# Evaluation of viral genome assembly and diversity estimation in deep metagenomes

**DOI:** 10.1186/1471-2164-15-989

**Published:** 2014-11-18

**Authors:** Daniel Aguirre de Cárcer, Florent E Angly, Antonio Alcamí

**Affiliations:** Centro de Biología Molecular Severo Ochoa, Consejo Superior de Investigaciones Científicas (CSIC)–Universidad Autónoma de Madrid, Madrid, Spain; Australian Centre for Ecogenomics, School of Chemistry and Molecular Biosciences, The University of Queensland, St Lucia, Brisbane, QLD 4072 Australia

**Keywords:** Assembly, Diversity, Metagenomics, Virome

## Abstract

**Background:**

Viruses have unique properties, small genome and regions of high similarity, whose effects on metagenomic assemblies have not been characterized so far. This study uses diverse *in silico* simulated viromes to evaluate how extensively genomes can be assembled using different sequencing platforms and assemblers. Further, it investigates the suitability of different methods to estimate viral diversity in metagenomes.

**Results:**

We created *in silico* metagenomes mimicking various platforms at different sequencing depths. The CLC assembler revealed subpar compared to IDBA_UD and CAMERA , which are metagenomic-specific. Up to a saturation point, Illumina platforms proved more capable of reconstructing large portions of viral genomes compared to 454. Read length was an important factor for limiting chimericity, while scaffolding marginally improved contig length and accuracy. The genome length of the various viruses in the metagenomes did not significantly affect genome reconstruction, but the co-existence of highly similar genomes was detrimental. When evaluating diversity estimation tools, we found that PHACCS results were more accurate than those from CatchAll and clustering, which were both orders of magnitude above expected.

**Conclusions:**

Assemblers designed specifically for the analysis of metagenomes should be used to facilitate the creation of high-quality long contigs. Despite the high coverage possible, scientists should not expect to always obtain complete genomes, because their reconstruction may be hindered by co-existing species bearing highly similar genomic regions. Further development of metagenomics-oriented assemblers may help bypass these limitations in future studies. Meanwhile, the lack of fully reconstructed communities keeps methods to estimate viral diversity relevant. While none of the three methods tested had absolute precision, only PHACCS was deemed suitable for comparative studies.

**Electronic supplementary material:**

The online version of this article (doi:10.1186/1471-2164-15-989) contains supplementary material, which is available to authorized users.

## Background

Several studies have demonstrated the potential for reconstructing genomes from viral metagenomes [1]. A major obstacle for metagenomic reconstruction is the existence of highly similar regions between coexisting genomes which can lead to fragmented assemblies, like repeats in single genome assemblies. In this regard, it has become important to analyze the performance of different sequencing platforms and assemblers in a metagenomic context, and assess their strengths and limitations. Initially, Mavromatis *et al*. [2] combined Sanger reads from sequenced bacterial isolates to form *in vitro* simulated communities of different complexities and benchmark assembly and other metagenomic processing methods. Pignatelli & Moya [3] derived short-read *in silico* simulated metagenomes from this work to explore facets of the assembly of high-throughput sequencing data. Charuvaha & Rangwala [4] evaluated the effects of k-mer size on the performance of the Bruijn-based assemblers. Later, Mende *et al*. [5] studied the effect of quality filtering on the assembly of Sanger, 454 and Illumina metagenomic datasets, while Luo *et al*. compared [6] the assembly of 454 and Illumina datasets from the same metagenome, and evaluated single bacterial genome reconstruction in a metagenomics setting [7].

The above-mentioned studies of metagenomic assembly all used bacterial communities and mainly focused on assessing functional and taxonomic annotations. While many of their results and findings can be applicable to the study of natural viral communities, viruses present unique properties. Viral genomes are usually smaller than those of *Bacteria* and it has become affordable to obtain high coverage of viral metagenomes using current high-throughput sequencing platforms and to attempt reconstructing environmental genomes. Viral genome reconstruction is an important step in the metagenomic analysis of viral communities. Viruses lack a universal phylogenetic marker gene that can be used as the ribosomal genes for cellular organisms. In this sense, both accurate phylogenetic annotation and putative host description rely heavily on the almost complete reconstruction of the viral genome. Additionally, the extent of intra-group variability among viruses is greater than in *Bacteria* due to their faster evolution rates, which poses increased difficulties to the assembler. Recently, Vázquez-Castellanos *et al*. [8] assessed the effects of different overlap-layout-consensus (OLC) assemblers for the functional and taxonomic annotation of an *in silico* simulated 454 viral metagenome, and Solonenko *et al.* [9] commented on how different library preparation choices bias the outcome of virome assembly.

Community diversity is an important ecological characteristic of natural communities, and its estimation usually complements taxonomic and functional analyses of viral metagenomes. There are currently three different approaches to estimate viral richness in metagenomic datasets; the use of clustering [10–12], PHACCS [13] and CatchAll [14, 15]. The latter two represent software tools which rely on assembly results, more precisely contig spectra for the fit of their diversity models. Unfortunately, none of these methods have been the subject of a comparative performance evaluation using viromes of known diversity.

In the present study, we investigate the ability of various sequencing Platform – Assembler – Depth (PAD) combinations to reconstruct the genomes from a high-throughput *in silico* simulated virome, and explore how genome relatedness impacts the success of genome reconstruction. Furthermore, we evaluate the applicability of three different methods to estimate viral community diversity. Collectively, our results should guide researchers undertaking deep viral metagenomic studies to adequate methods for genome reconstruction and diversity estimation, as well as understand their limitations.

## Results

### Metagenomic assembly

Using a single virtual viral community, composed of 300 genomes with different degrees of relatedness, from both ssDNA (*Microviridae*, *Circoviridae*, and *Nanoviridae*) and dsDNA (*Siphoviridae*, *Podoviridae*, and *Myoviridae*) viral families, we generated a large number of metagenomic reads mimicking Roche’s 454 and Illumina’s GAIIx sequencing platforms. Sequencing costs were kept similar for each technology (based on Reagent cost/Mb values), resulting in different sequencing depths. We also produced lower coverage datasets, containing 10% of the reads of these high coverage datasets, and complemented them with additional Illumina Miseq and Hiseq low coverage metagenomic libraries.

### Assembly statistics

We assessed assembly of these data using three de Bruijn k-mer-based assemblers, chosen either for their widespread use (CLC), or for their claimed performance in a metagenomic setting (CAMERA, IDBA_UD). The various platform-assembler-depth (PAD) combinations were evaluated based on contig length statistics, accuracy of the generated contigs, and comprehensiveness of the reconstructed genomes. There were some differences in the assembly statistics of different PADs (Table 1). Based on assembly results for the high-coverage datasets (Table 1a), CAMERA performed better than CLC with the 454 dataset with respect to the maximum contig size and N50 parameters. For the Illumina GAIIx dataset, and compared to the CLC assembler, IDBA_UD also had a much larger N50, even if it produced many more contigs (translating into an overall lower mean and median values). We compared the effect of scaffolding on these statistics by comparing IDBA_UD with and without scaffolding. For both high and low coverage datasets, the most striking difference was an increase in N50 when scaffolding.

**Table 1:** **Sequencing and assembly statistics**

	#reads	Read length	Read sum	#contigs	Contig sum	Contig mean	Contig max	N50
**a ) High-coverage mock**								
**454-CAMERA**	0.2 M	700	140 M	2,515	8.8 M	3,526	279 K	10.7 K
**454-CLC**	0.2 M	700	120 M	2,798	7.6 M	2,737	103 K	4.55 K
**GAIIx-CLC**	26.5 M	2x150	7950 M	2,359	8.0 M	3,392	280 K	9.86 K
**GAIIx-IDBA**	26.5 M	2x150	7950 M	4,002	9.9 M	2,476	280 K	25.6 K
GAIIx-IDBA*	26.5 M	2x150	7950 M	4,040	9.8 M	2,447	280 K	20.6 K
**b) Low-coverage mock**								
**454- CAMERA**	0.02 M	700	14 M	1,856	2.2 M	1,223	51 K	1.41 K
**GAIIx-IDBA**	2.65 M	2x150	795 M	3,406	10.1 M	2,973	280 K	32.3 K
GAIIx- IDBA*	2.65 M	2x150	795 M	3,476	10.1 M	2,912	280 K	27.7 K
**Miseq-IDBA**	1.65 M	2x300	990 M	3,859	9.9 M	2,565	280 K	27.0 K
**Hiseq -IDBA**	9.20 M	2x100	1840 M	2,674	9.5 M	3,568	280 K	32.3 K
**c) Empirical**								
**454- CAMERA**	0.023 M	220	5.1 M	147	0.17 M	1,177	8 K	1.38 K
**GAIIx-IDBA**	1.98 M	2x75	297 M	2,774	4.55 M	1,643	114 K	1.98 K

*No scaffolding.

We then compared sequencing platforms, focusing on the assembly statistics obtained with the best tested assembler for each platform (CAMERA for 454, and IDBA_UD for Illumina). For the high coverage datasets (Table 1a), Illumina GAIIx achieved a higher number of contigs, contig sum and N50 than 454. Similarly, for the low coverage datasets (Table 1b), Illumina GAIIx outperformed 454, and even surpassed the high coverage dataset by assembling the same amount of reads in fewer, larger contigs. This outcome indicates that the simulated community may have been oversequenced by the GAIIx high coverage dataset, and is consistent with the inability of Bruijn k-mer based assemblers to deal with large numbers of sequencing errors, resulting in more fragmented assemblies [16]. Illumina Miseq and Hiseq (low coverage) contigs (Table 1b) were also more numerous and longer than those of 454, and there were no major differences between them, other than Hiseq having a slightly larger N50 value. All Illumina platforms were able to completely recover the longest genome in the dataset (Pseudomonas_phage_phiKZ, 280 kbp).

We also analyzed the behavior of 454 and Illumina technologies using an empirical metagenomic data derived from a single Antarctic freshwater viral community (Table 1c). This dataset contains about 40% of the sequence information of the low coverage 454 and Illumina GAIIx datasets. In this case, it was possible to assemble more reads into contigs with Illumina than with 454, leading to much higher maximum contig length and N50 values.

### Contig correctness

Contig length statistics alone cannot indicate the degree to which assembly faithfully reconstructed the original community. To this end, we compared the contigs generated by each PAD combination to the original genomes, assessing their accuracy and chimericity. Contig accuracy represents how well a contig aligns to the genome its represents, while chimericity reflects how many genomes contributed to each contig. Overall, the contigs produced by all PAD combinations were accurate, with average accuracy and percentage of high-accuracy contigs usually over 98% (Table 2). The only exception was GAIIx-CLC with an accuracy of 94 ± 14%, and only 86% of contigs with high accuracy. We also noted that accuracy was more consistent (smaller standard deviation) at higher sequencing coverage and when scaffolding. No notable differences in chimericity could be attributed to the use of different assembly programs (Table 2). However, there were large differences between sequencing platforms, with Roche 454 producing both less chimeric contigs and a larger fraction of perfectly non-chimeric contigs. The Illumina Miseq dataset also had a much larger fraction of perfectly non-chimeric contigs when compared to that of Illumina Hiseq (21.9% and 5.2% respectively). These results seem to indicate that longer sequencing reads help prevent the formation of chimeras. Another remarkable result is that while the effect of scaffolding on chimericity for the high coverage GAIIx-IDBA dataset seems marginal, scaffolding reduced the fraction of perfectly non-chimeric contigs from 20.5% to 8.6% for its equivalent low coverage dataset.

**Table 2:** **Accuracy and chimericity statistics**

	Accuracy	Accuracy >90% ^a^	Chimericity	Non-chimeric ^b^
**a) High-coverage mock**				
**454- CAMERA**	99.8 ± 0.5	99.9%	0.93 ± 1.09	42.9%
**454-CLC**	99.1 ± 3.4	99.4%	0.90 ± 1.01	41.2%
**GAIIx-CLC**	94.0 ± 14.0	86.0%	1.30 ± 1.03	7.00%
**GAIIx-IDBA**	99.9 ± 1.1	99.7%	1.27 ± 0.94	6.10%
GAIIx-IDBA*	99.9 ± 1.4	99.7%	1.25 ± 0.95	6.20%
**b) Low-coverage mock**				
**454– CAMERA**	99.2 ± 3.4	99.9%	0.59 ± 0.87	53.8%
**GAIIx–IDBA**	99.6 ± 1.6	99.6%	1.27 ± 0.78	8.60%
GAIIx-IDBA*	99.5 ± 3.1	99.4%	1.26 ± 0.79	20.5%
**Miseq-IDBA**	99.5 ± 3.7	98.7%	1.32 ± 0.99	21.9%
**Hiseq-IDBA**	98.7 ± 3.9	98.2%	1.32 ± 0.87	5.20%

*No scaffolding. ± Represent SDs. ^a^Percentage of contigs with accuracy >90%. ^b^Percentage of non-chimeric contigs (Chimericity = 0).

### Contig coverage

**Figure 1:**
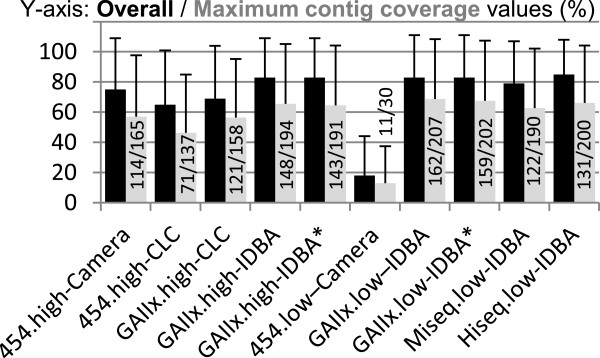
**Overall and maximum contig coverage statistics.** For each platform-assembler-depth combination black bars represent average overall contig coverage and gray bars average maximum contig coverage, with the error bars representing SDs. Within the gray bars, numbers represent the number of genomes showing *max contig* values above 95% and 50% respectively. High; high coverage datasets, low; low coverage datasets. *No scaffolding.

Next, we assessed how extensively the PAD combinations recovered the information contained within the original genomes by calculating overall contig coverage, i.e. the percentage of genome covered by its contigs, and the maximum contig coverage, i.e. the percentage of genome covered by its longest contig. CLC produced significantly lower overall contig coverage than both CAMERA (454 data) and IDBA (Illumina GAIIx data) (paired Mann–Whitney test; p < 0.05) (Figure 1, and Additional file 1: Tables S1 and S2). With regards to the sequencing technology, Illumina GAIIx outperformed 454 at high coverage. Differences became more pronounced at low coverage, with values of 18 ± 26 and 83 ± 28 for CAMERA-454 and GAIIx-IDBA respectively. Scaffolding produced marginal yet significantly higher overall contig coverage for the high but not low coverage dataset. No differences were observed between high and low coverage GAIIx values. Maximum contig coverage comparisons produced essentially the same results, with few exceptions. Mainly, scaffolding had a positive effect on both the high and low coverage datasets, and the low coverage GAIIx-IDBA produced larger maximum contig coverage values than its high coverage counterpart, again suggesting some detrimental effect associated with oversequencing. The number of genomes showing maximum contig coverage above 95%/50% followed a similar pattern than the above results.

### Genome and community characteristics effects on genome reconstruction

It is noteworthy that the best PAD combination for contig coverage (low-coverage GAIIx-IDBA) only yielded maximum contig coverage above 95% for 162 out of the 300 different genomes in the community, a figure that was not improved by increasing the sequencing effort 10-fold (Figure 1). This inability to obtain contigs spanning a large proportion of many original genomes, despite high sequencing coverage may be due to: i) limits to what the assembly algorithm can achieve given a particular community, either theoretically or due to imperfect design, ii) variable genome coverage along genomes (e.g. due to lower conservation or to GC bias), iii) large genome length, iv) the existence of repeats regions in the genome, and v) high community diversity.

Several of these factors were further studied using the Illumina Hiseq dataset showing best overall performance and likely future use, and while more contigs derived from this dataset were chimeric compared to its Miseq counterpart they were still highly accurate. Differential coverage along genomes (due for instance to %GC bias) was not studied as it was not modelled by the chosen metagenomic simulator, although some less versatile simulators include such feature [17]. First, we evaluated to which degree the existence of repeats regions within the genomes may have translated into low maximum contig coverage. We then analyzed the genomes in our evolved mock community for long repeats regions and found that the longest repeats region did not span more than 400 bp. Hence, the existence of repeats within the original genomes was not likely the cause of low maximum contig coverage.

**Figure 2:**
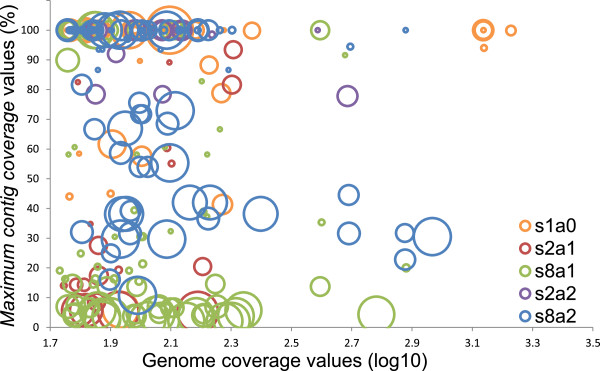
**Initial exploration of maximum contig coverage vs. genomic characteristics.** The diameter of each bubble positively correlates with genome length, and its color represent its complexity group; s1a0 represents the unmodified genomes, ‘s’ denotes number of siblings and ‘a’ the nucleotide transition rates employed (a1; α = 0.0025 intra-species. a2; α = 0.01 inter-species). Only genomes longer than 1700 nt are shown (see methods). Genome coverage; for each complexity group (color) no apparent trend of increasing maximum contig coverage (Y-axis) with genome coverage (X-axis) is observed. Genome size; for each complexity group (color) no apparent trend of increasing maximum contig coverage with decreasing bubble size (genome length) is observed. Number of sibling genomes; for each transition rate (a1, a2) the number of sibling genomes (s2, s8) seems to influence maximum contig coverage values attained (e.g. blue vs. purple). Degree of relatedness; transition rates employed had a profound effect on maximum contig coverage values obtained (e.g. green vs. blue).

The initially exploration of the results (Figure 2) indicated that the minimum coverages attained were sufficient to recover both large and small genomes. This result shows that the coverages attained were not a limiting factor for genome reconstruction, in line with recent results showing that a bacterial genome could be recovered from a complex community with as little as 20x coverage [7]. Moreover, genome length did not seem to have a strong influence on maximum contig coverage. On the other hand, grouping genomes by relatedness (unmodified genomes and genomes from groups of 2/8 siblings produced at α 0.0025/0.01 transition rates) revealed that the number of siblings per group and especially their degree of relatedness likely contributed to obtaining maximum contig coverage well below 95%.

Next, we studied whether or not we had retrieved at least one almost complete genome for the intra-species groups (α = 0.0025), which exhibit the lowest maximum contig coverage (Figure 2). For groups of two siblings, the largest maximum contig coverage corresponded to the genome with highest coverage (most abundant). However, only for one out of ten existing intra-species (α = 0.0025) sibling pairs did the maximum contig coverage value surpass the 95% threshold, and this group was characterized by the lowest *coverage by others* values (see below) of the ten groups. On the other hand, the groups with eight siblings showed a strikingly different behavior, with seven out of ten groups having at least one member surpassing the 95% maximum contig coverage threshold. For six of these groups, the genome showing greatest maximum contig coverage was not the most abundant, and maximum contig coverage for the other sibling were very low. The exception as before corresponded to a group with very low *coverage by others* values, where all 8 siblings surpassed the 95% threshold. Subsequent principal coordinates analyses based on pairwise nucleotide similarities of the six groups showing a similar behavior revealed that in five out of six cases the reconstructed genome represented a central genome within the group (Additional file 1: Figure S1).

**Figure 3:**
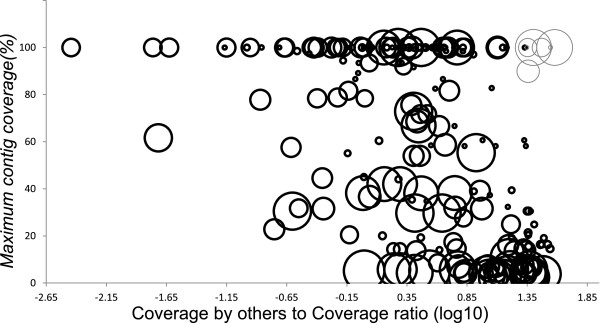
**Bubble chart of maximum contig coverage vs CbO/C ratio.** The diameter of each bubble positively correlates with genome length. Grey bubbles represent the single reconstructed genomes from the groups of eight highly similar siblings (intra-species).

We then aimed to evaluate the effect caused by interferences between similar genomes by refining the results obtained in Figure 2, studying the genomes’ *coverage by others* to *coverage* ratio (CbO/C). This new measure should serve as a proxy of the possible difficulties faced by the assembler to reconstruct each genome due to the existence within the community of other genomes with highly similar regions. Thirty eight genomes had no reads by others, and all but one of them had large maximum contig coverage (99.2 ± 2.6%).Plotting CbO/C against maximum contig coverage values revealed a particular phenomenon (Figure 3); while there is still the possibility of maximal reconstruction with high interference from other genomes, it diminished with growing ratio, and it seems to be unrelated from genome length. Hence, there is a tendency of diminishing maximum contig coverage with increasing CbO/C, but with noticeable dispersion. We further studied the main outlier group representing genomes with both very large CbO/C and maximum contig coverage (Figure 3, grey bubbles). Interestingly, these genomes belonged to the groups of 8 intra-species siblings (α = 0.0025) previously shown to produce a single reconstructed genome in detriment of its siblings’ maximum contig coverage. This indicates that the dispersion observed from the prominent tendency of diminishing maximum contig coverage with increasing CbO/C is due to foreign reads being assembled during he reconstruction of particular genomes to the detriment of the reconstruction of their original genomes (positive and negative dispersion along the Y-axis respectively).

### Estimating community diversity

We used PHACCS and CatchAll to estimate the number of viral species in the evolved simulated community using contig spectra derived from sub-samples of the Illumina GAIIx dataset. Since we observed that the assemblers were not able to resolve all cases between highly similar genomes we expected richness estimates *ca.* 200 – 300. However, both methods over-estimated the number of species (Additional file 1: Table S3), with CatchAll being always 1 to 3 orders of magnitudes off compared to PHACCS, and richness estimates increased with sequencing depth.

**Figure 4:**
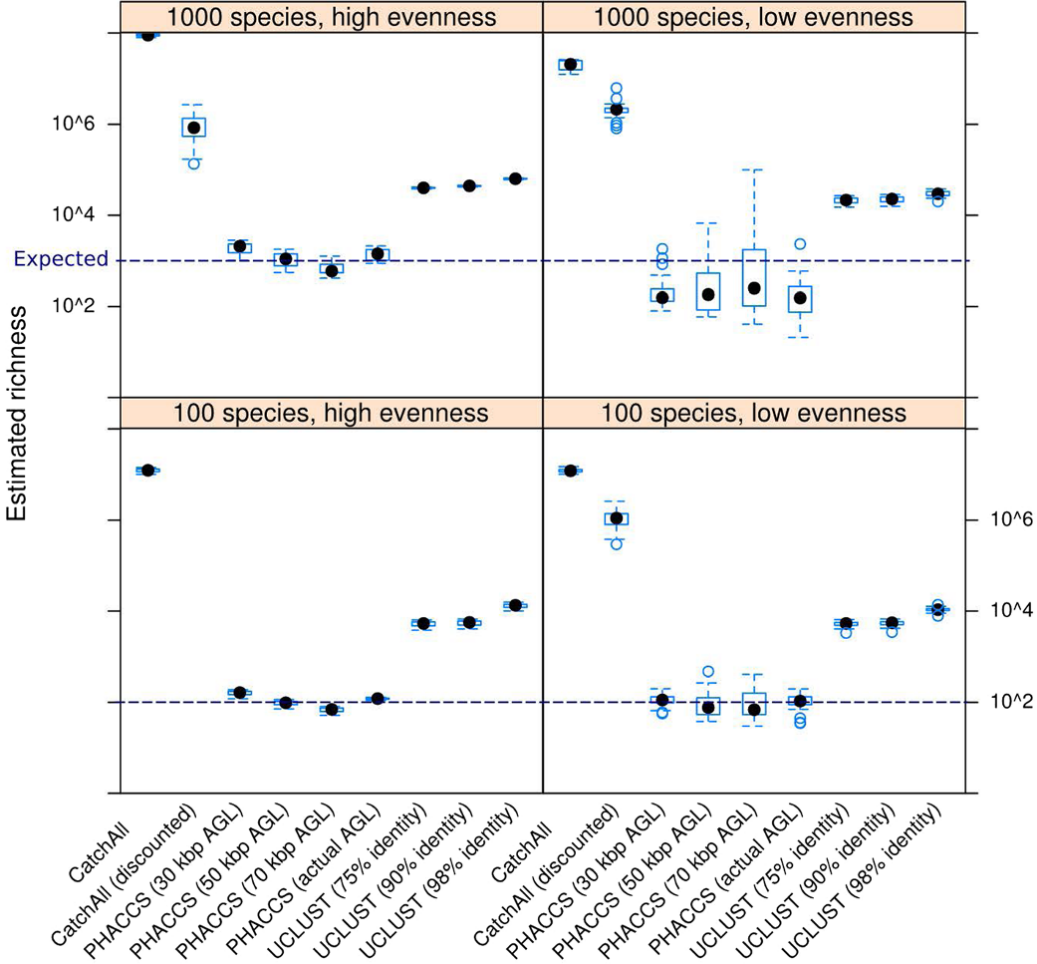
**Boxplots of richness estimates.** Boxplots of richness estimates (minimum, 25-th percentile, median, 75-th percentile and maximum) generated by UCLUST, PHACCS and CatchAll for four types of community structure. AGL: actual genome length.

To further investigate the accuracy of viral diversity estimation tools, we tested them on 100 simulated communities with different richness and evenness. For comparison, we complemented this analysis with richness estimates based on the number of clusters formed by UCLUST. The richness estimates of CatchAll and UCLUST were orders of magnitude higher than those obtained by PHACCS, which was the closest to the expected richness (Figure 4). While not absolutely accurate, the estimates produced by PHACCS were consistent with community richness and evenness (Additional file 1: Figure S2). When accounting for average genome length in the community (see Methods), UCLUST estimates improved dramatically, while CatchAll estimates were still at least one order of magnitude higher than expected (Additional file 1: Figure S3).

## Discussion

Our ability to accurately reconstruct the viral genomes within a deep metagenomic dataset represents a black box. Assembly success is usually described in terms of rather subjective proxies such as assembly statistics, or the number of apparently complete genomes recovered. However, as we remain ignorant of the richness, structure, and genetic diversity of the community it is not possible to work with more objective measures of success. This is especially true for viruses that have no ribosomal genes to help us estimate its composition, richness and structure. In this study, we have shed some light on this black box’s internal functions and mechanisms.

The assembly statistics derived from each PAD combination revealed that both CAMERA and IDBA_UD outperformed the popular commercial assembler CLC in terms of both total information in contigs (Sum) and N50, likely due to the fact that they have been specifically developed for metagenomic studies. For the high coverage datasets, both 454 and Illumina performed similarly. However, for the low coverage scenarios all Illumina platforms greatly outperformed 454, with Hiseq performing slightly better than Miseq. This result was apparent in the empirical metagenomic datasets, where Illumina was able to recover 26 times the amount of bases in contigs of 454.

Since single point errors in contigs can alter gene calling and predicted translated proteins, contig accuracy is important. The contigs and scaffolds produced by all PAD combinations were generally highly accurate, but CLC exhibited a lower accuracy that could substantially compromise annotation efforts. Concerning chimericity, no major differences between assemblers were observed. However, the 454 platform produced less chimeric contigs and a larger fraction of non-chimeric contigs than the Illumina platforms. Moreover, Miseq also produced a larger fraction of perfectly non-chimeric contigs compared to Hiseq. Both results indicate that read length correlates negatively with chimericity.

Overall, scaffolding marginally improved both contig length statistics and accuracy, with the tradeoff of increasing contig chimericity, which is consistent with previous results [5]. Both overall and maximum contig coverage derived from CLC assemblies were lower than for their CAMERA and IDBA_UD counterparts. 454 produced lower yet relatively similar values when compared to Illumina GAIIx for the high coverage datasets. However, its ability to reconstruct the original genomes (overall and maximum contig coverage) was much reduced in the low coverage dataset, when compared to all Illumina platforms.

Interestingly, while some large genomes were recovered completely as a single contig, we could not obtain contigs spanning a large proportion of many original genomes despite the high coverage values attained. This issue was further explored using the Illumina Hiseq dataset, which showed that genome length, sequencing coverage, or the existence of repeat regions had little effect on genome reconstruction. However, the co-existence of highly similar genomes within the community had a strong effect on genome reconstruction. It is noteworthy that usually, for pairs of highly similar genomes, the largest maximum contig coverage corresponded to the most abundant genome. However, in the more complex cases with eight intra-species genomes, a single genome was normally reconstructed. Instead of representing the most abundant species, the reconstructed genome tended to be that showing closest similarity to its siblings. In this sense, it seems that it is not possible to ascertain whether a retrieved genome corresponds to a single strain, or rather to a group of highly similar strains. One could take steps to study the intra-population structure of a given genome, e.g. by re-mapping onto it all metagenomic reads and then evaluating its nucleotide diversity, or using more sophisticated software [18, 19].

Despite advances in sequencing technologies and bioinformatic tools, the assembly of viral metagenomes thus remains incomplete. Since all genomes cannot be reconstructed, even with very high sequencing coverage, methods to estimate viral diversity in deep metagenomes remain highly relevant. PHACCS and CatchAll are sophisticated tools that model community diversity based on the assembly of metagenomes, while clustering is a simple method that provides a proxy for viral richness and has been used to generate rarefaction curves. Using 100 simulated viromes, we showed that CatchAll results are orders of magnitude higher than expected, due to its underlying assumption that each contig belongs to a different viral genome. The use of a discounted model was not sufficient to alleviate this fundamental limitation. By its nature, clustering creates many clusters for each genome and its richness estimates can thus be considered upper bounds of viral richness. However, correcting clustering results for genome length dramatically improved richness estimates, which suggest a new direction for modeling viral diversity in metagenomes. PHACCS was the most accurate of the tested tools, reaching a 44.5% ±33.0 relative error. Note that these results were obtained using the more thorough ‘cha’ mode and providing the exact average genome length in the community, which is rarely known with precision. We suggest that assuming an average for the viral genome length limits the absolute accuracy of PHACCS. However, PHACCS richness and evenness estimates were consistent with community structure, which makes PHACCS well suited for the estimation of viral diversity of communities in comparative studies.

## Conclusions

The amount of metagenomic information available for genome reconstruction had a profound effect on assembly success, as evidenced by the low performance demonstrated by the low coverage and empirical 454 datasets. However, despite the fact that all Illumina combinations tested likely presented per-genome coverage in excess of what seems to be needed for accurate genome reconstruction, they were unable to recover all genomes in the community, because of the presence within the community of genomes bearing highly similar regions. The assemblers were nevertheless generally able to recover at least a single genome from a highly similar group of genomes. Overall, we recommend the use of Illumina platforms such as HiSeq and MiSeq, bearing in mind that oversequencing may be detrimental,and a metagenomic-aware assembler such as IDBA-UD for the assembly of viral metagenomes; this PAD combinations provide good value for money, and yield long, accurate contigs.

Deep metagenomic studies can be complemented by analyses of community diversity, some of which are based on contig assembly. While our simulation results argue against the use of CatchAll for this purpose, PHACCS was shown to be well suited for comparative work. Clustering might also prove a worthy alternative in the future, provided average genome length in the community is taken into account.

## Methods

### The evolved mock community

To evaluate PAD combinations, we devised an *in silico* simulated community containing 300 genomes from six DNA virus families (both single and double-stranded) most commonly found in freshwater environments. Each family was given the same relative abundance and contained 40 evolved genomes (details below) (Additional file 1: Table S4).

The rank-abundance curve for the genomes of each family followed a power-law distribution (Additional file 1: Figure S4), with few abundant genomes and a long tail of low-abundance species as seen in many environmental viral communities [20] (Additional file 2). Within this distribution, we intercalated one original genome for each four evolved genomes to assess genome reconstruction without sibling noise.

We used the GemSIM simulator [21] to generate mock metagenomes with empirically derived, sequence-context based error mimicking the widespread sequencing platforms Roche 454 FLX + and Illumina GAIIx, Hiseq and Miseq. The GemSIM error models were derived from data obtained from our local sequencing center (454 FLX+, Hiseq, and Miseq) or published data (GAIIx) [22] (Additional file 3).

Sequencing depths were chosen based on: i) the amount of sequences being reported for environmental viromes, and ii) existing sequencing costs associated with each technology (Reagent cost/Mb) [23]. We thus produced high-coverage datasets containing 200,000 reads for Roche 454 FLX + (*ca.* 700 bp in length) and 26,500,000 read pairs for Illumina GAIIx (150 bp in length). In addition, we also produced low-coverage datasets representing 10% of their high-coverage counterparts, and complemented them with 1,657,913 MiSeq and 9,201,420 HiSeq read pairs (based on Reagent cost/Mb values provided by our local sequencing center).

### Evolved genomes

To simulate realistic communities, in which closely related viral species and strains co-exist, we used MetaSim’s population sampler, which produces evolved sequences based on a source genome (Additional file 4) and a given evolutionary tree [24]. We employed the default tree-simulation parameters but included two different nucleotide transition rates α (0.01 and 0.0025), and generated groups of two and eight sibling genomes for each transition rate. An initial exploration on the outcome of chosen transition rates on a single genome to produce 10 siblings resulted in average nucleotide identities (ANIs) ranging 0.786 – 0.947 (0.85 ± 0.04) and 0.935 – 0.985 (0.96 ± 0.01) for α 0.01 and 0.0025 respectively. The chosen α levels approximately correspond to intra and inter-species siblings based on the fact that 95% ANI can be considered a rough boundary for species in *Bacteria* [25]. Nevertheless, we acknowledge that this value may represent a rather artificial boundary with viruses. For each viral family we introduced five categories of genomes; 10 unmodified genomes, two pairs of sibling genomes produced using α at 0.01, two pairs of sibling genomes produced using α at 0.0025, two groups of eight sibling genomes produced using α at 0.01, and two groups of eight sibling genomes produced using α at 0.0025. Hence, we produced 40 evolved genomes from 8 original genomes for each family by generating duplicates of the four combinations (2 X α levels [0.01/0.0025] X group sizes [2/8]).

### The Limnopolar empirical community

We compared the assembly results derived from our simulated community to an actual viral metagenome obtained from Lake Limnopolar and composed mainly of unknown and ssDNA viruses [26]. We maintained the costs associated to each technology and compared 23,249 (average 220 bp in length) Roche 454 sequences against 1,989,155 (75 bp in length) Illumina GAIIx sequence pairs. The 454 virome was assembled with the CAMERA assembler and the Illumina version with IDBA_UD.

### Metagenomic assembly

A series of filtering and trimming steps were undertaken to remove low quality reads and bases using the prinseq-lite software [27] (*trim_qual_right* 28, *trim_qual_type* mean, *trim_qual_window* 5). Additionally, Lake Limnopolar 454 reads were dereplicated with prinseq and sequences shorter than 50 bp removed. The resulting reads were assembled into contigs using different assemblers; 454 reads were assembled using the CAMERA-assembler [28], and CLC Genomics Workbench 6.0 (CLC Inc, Aarhus, Denmark. Trial version). Illumina reads were assembled using CLC and IDBA_UD [29]. CAMERA and CLC were used with default settings, and IDBA_UD with recommended metagenomic settings (*mink* 20, *maxk* 120, *pre_correction*). In all cases, contigs shorter than 500 bp were removed from further analysis.

### Contig analysis

In order to evaluate the performance of the different PAD combinations we used previously developed analytical strategies for short read metagenomic assembly [4]. We calculated metrics reflecting the extent of genome reconstruction: overall contig coverage, the percentage of each genome covered by all its contigs, and maximum contig coverage, the percentage of each genome covered by its longest contig. First, contigs were aligned to the input genomes using nucmer (c 30, l 15) [30]. Then, the results were filtered allowing only ≥95% identity and ≥100 bp length alignments. For each contig, only the best-scoring alignments to a genome was allowed. Finally, a dedicated python script recorded the alignment position information for each contig, with the collection of all such positions for a given genome representing its contig coverage, expressed as a percentage of the total genome length. The same alignment file produced for the contig coverage calculations was parsed using an in-house script to obtain the proportion of the original genome’s length covered by the longest aligning contig (maximum contig coverage). To assess which particular PAD combination produced the best maximum and overall contig coverage, we conducted paired Mann–Whitney tests with R.

The accuracy of assemblies was established using a chimericity and *contig accuracy* metrics. Chimeric contigs are defined as contigs formed by reads derived from more than one genome. However, due to the short length of reads issued from high-throughput sequencing platforms and the existence of closely related viral genomes, chimericity does not necessarily mean lack of correspondence between a contig and its source genome. Reads were re-mapped to contigs using the bowtie2 read aligner [31] reporting only best hits at high stringency (score-min L,0,-0.2). For each contig, we used the counts of reads from each original genome to calculate chimericity, defined here as the entropy of the contigs:

1 $$\text{Entropy}=-\sum_{i}p_{i}.\text{log}\left(p_{i}\right)$$

Where *p*_*i*_ is the proportion of mapped reads arising from genome *i*.

The level to which each contig accurately represents the information contained within the original genomes was assessed using a *contig accuracy* score, defined as the identity of the local alignment multiplied by the ratio of alignment length to contig length. Contig accuracy values were also obtained by processing the filtered *nucmer* files with a dedicated script.

### Genome reconstruction

Both genome and community characteristics may impact our ability to assemble a particular genome from a complex community. We have used the PAD combination showing best overall performance (Hiseq) to assess the effect on maximum contig coverage caused by genome length, relative abundance, existence of closely-related genomes in the community, and repeats regions within the genome. Due to the interaction between the circular nature of many genomes and chosen alignment thresholds genomes shorter than 1700 nt were removed from further analysis as there is the possibility that their maximum contig coverage may have been slightly underestimated.

In most instances, the assembly only recovered one of the genomes (maximum contig coverage >95%) from the groups of eight intra-species genomes (α = 0.0025). We studied the possible effect of intra-group genetic similarity on genome recovery by obtaining pairwise nucleotide similarities between sibling genomes, which were then analyzed by principal coordinate analysis using the dudi.pco function of the ade4 package [32] in R.

The existence within a community of genomes bearing highly similar regions may also hamper the reconstruction of a genome. For instance, the reads originating from a particular genome might be used in the reconstruction of other genomes with OLC assemblers, or it may lead to graph structures not properly resolved with de Bruijn graph-based assemblers. To analyze this aspect, we mapped all metagenomic reads to each genome using *bowtie2* with default parameters but allowing all above-threshold hits. Then we recorded the number of metagenomic reads mapping to each genome minus the number of reads originating from each genome, and normalized for differing genome sizes dividing by genome length, obtaining a *coverage by others* parameter. Finally, we used the ratio of *coverage by others* to *coverage* as a proxy to assess possible genome reconstruction bias produced by this sort of interference.

### The hundred mock communities

We generated 100 *in silico* mock metagenomes with different community structures to benchmark the accuracy of viral diversity estimation methods more thoroughly. To this end, >2,200 complete genomes from the NCBI RefSeq database [33] were used as reference for the Grinder read simulator [34]. Each metagenome contained 200,000 reads designed to follow the length (~450 bp) and errors typical of 454 GS-FLX Ti pyrosequencing. The metagenomes followed a power law rank-abundance and were classified in four community structures, varying in richness (100 or 1,000 species) and evenness (most abundant genome at 2.0 or 25% relative abundance). We let Grinder automatically randomly generate 25 metagenomes of each type (total of 100 metagenomes) for statistical replication.

### Estimation of viral diversity

Using the GAIIx evolved mock metagenome, we determined the effect of metagenome size on estimated community viral diversity. We produced subsets of this metagenome containing 24,658, 248,525 and 2,485,933 reads. Their contig spectra was calculated with Circonspect [1] using the Minimo assembler [35] employing all reads and default parameters (98% identity, 35 bp overlap). Then, both PHACCS and CatchAll were employed with their default values to fit the contig spectra using all available models.

Using the hundred 454 mock metagenomes, we calculated the accuracy of viral diversity estimates obtained using PHACCS, CatchAll and UCLUST as a function of community structure. Contig spectra were generated with Circonspect at 3X fold coverage using Minimo (and default options). These contig spectra were provided to CatchAll and the estimated richness using the best model and the best discounted model were recorded. PHACCS was also given these contig spectra to estimate the viral richness and evenness, letting its optimization algorithm look for the best fit using the more exhaustive ‘cha’. For the clustering method, the entire metagenome was used as input to UCLUST (cluster_smallmem program, both strands, minimum identity of 98, 90 and 75%) and the number of resulting viral clusters was calculated. In an attempt to improve their accuracy, the UCLUST and CatchAll estimates were divided by the average genome length minus average read length.

## Electronic supplementary material

Additional file 1: **Tables and figures.** Supplementary tables and figures. (DOCX 490 KB)

Additional file 2: **Community Structure.** Simulated community members and structure. (XLSX 30 KB)

Additional file 3: **Error model statistics.** Error model statistics. (XLSX 73 KB)

Additional file 4: **Genome sequences.** 300 genomic sequences used for the evolved mock viromes, fasta format. (PDF 7 MB)
