# Supplementary material for: Evaluation of viral genome assembly and diversity estimation in deep metagenomes
Source: BMC Genomics. 2014 Nov 18;15(1):989. doi: 10.1186/1471-2164-15-989 (PMC4247695; doi:10.1186/1471-2164-15-989)
Supplement: Supplementary file 1 — Additional file 1: Tables and figures. Supplementary tables and figures. (DOCX 490 KB) [file 12864_2014_6692_MOESM1_ESM.docx]

**SUPPLEMENTARY INFORMATION**

**Evaluation of viral genome assembly and diversity estimation in deep metagenomes**

Daniel Aguirre de Cárcer, Florent E Angly & Antonio Alcamí

| **Suppl. Table 1. Maximum and overall contig coverage statistics.** | | | | | | | | |
| --- | --- | --- | --- | --- | --- | --- | --- | --- |
| High co. datasets | 454- CAMERA | 454- CLC | | GAIIx- CLC | | GAIIx- IDBA | | GAIIx- IDBA* |
| ***Overall*** | 74 ±34 | 65 ±36 | | 69 ±35 | | 83 ±26 | | 83 ±26 |
| ***Maximum*** | 57.0 ±40.7 | 46.3 ±38.7 | | 56.5 ±38.7 | | 65.61 ±39.6 | | 64.6 ±39.5 |
| ***Maximum* 95/50^a^** | 114/165 | 71/137 | | 121/158 | | 148/194 | | 143/191 |
|  | | | | | | | | |
| Low co. datasets | 454- CAMERA | GAIIx- IDBA | | GAIIx- IDBA* | | Miseq- IDBA | | Hiseq- IDBA |
| ***Overall*** | 18 ±26 | 83 ±28 | | 83 ±28 | | 79 ±28 | | 85 ±23 |
| ***Maximum*** | 12.9 ±24.6 | 68.7 ±39.6 | | 67.6 ±39.8 | | 62.7 ±39.4 | | 66.2 ±38.0 |
| ***Maximum* 95/50^a^** | 11/30 | 162/207 | | 159/202 | | 122/190 | | 131/200 |
| *No scaffolding. **^a^**Number of genomes with max contig >95% / 50%. | | | | | | | | |
| **Suppl. Table 2. Paired Mann-Whitney tests** | | | | | | |  |  |
|  | | | **Overall contig coverage** | | **Maximum contig coverage** | |  |  |
| 454- CAMERA **vs** 454- CAMERA ^S^ | | | *** | | *** | |  |  |
| 454- CAMERA **vs** 454- CLC | | | *** | | *** | |  |  |
| 454- CAMERA **vs** GAIIx- CLC | | | ** | | - | |  |  |
| 454- CAMERA **vs** GAIIx- IDBA | | | *** | | *** | |  |  |
| 454- CAMERA ^S^ **vs** GAIIx- IDBA ^S^ | | | *** | | *** | |  |  |
| 454- CAMERA ^S^ **vs** Miseq- IDBA ^S^ | | | *** | | *** | |  |  |
| 454- CAMERA ^S^ **vs** Hiseq- IDBA ^S^ | | | *** | | *** | |  |  |
| 454- CLC **vs** GAIIx- CLC | | | * | | *** | |  |  |
| 454- CLC **vs** GAIIx- IDBA | | | *** | | *** | |  |  |
| GAIIx- CLC **vs** GAIIx- IDBA | | | *** | | *** | |  |  |
| GAIIx- IDBA **vs** GAIIx- IDBA ^S^ | | | - | | *** | |  |  |
| GAIIx- IDBA **vs** GAIIx- IDBA**^a^** | | | ** | | *** | |  |  |
| GAIIx- IDBA **vs** GAIIx- IDBA ^S^ | | | - | | ** | |  |  |
| Miseq- IDBA ^S^ **vs** Hiseq- IDBA ^S^ | | | *** | | *** | |  |  |
| **^a^**No scaffolding. ^S^Low coverage dataset. *pval<0.05. **pval<0.005. ***pval<0.0005. | | | | | | |  |  |

| **Suppl. Table 3. Richness estimates.** Number of species estimated for sub-samples of the GAIIx dataset using dedicated software. | | |
| --- | --- | --- |
| **#Reads** | **PHACCS** | **CatchAll(Discount)^a^** |
| 2,485,933 | 11,959 | 1,417,908 |
| 248,525 | 1,874 | 239,535 |
| 24,658 | 481 | 7,612 |
| **^a^**Best Discounted model**.** | | |

| **Suppl. Table 4**. **Design of the simulated community;** including for each family relative abundance (%), total number of genomes (# genomes), and distribution of genomes into evolved siblings categories (Genome distribution). | | | |
| --- | --- | --- | --- |
| **Family** | **%** | **# genomes** | **Genome distribution** |
| *Microviridae* | 16.67% | 50 | 10 unmodified + (2 X α [0.01/0.0025] X groups [2/8]) |
| *Circoviridae* | 16.67% | 50 | 10 unmodified + (2 X α [0.01/0.0025] X groups [2/8]) |
| *Nanoviridae* | 16.67% | 50 | 10 unmodified + (2 X α [0.01/0.0025] X groups [2/8]) |
| *Myoviridae* | 16.67% | 50 | 10 unmodified + (2 X α [0.01/0.0025] X groups [2/8]) |
| *Siphoviridae* | 16.67% | 50 | 10 unmodified + (2 X α [0.01/0.0025] X groups [2/8]) |
| *Podoviridae* | 16.67% | 50 | 10 unmodified + (2 X α [0.01/0.0025] X groups [2/8]) |

**Suppl. Figure 1. Effect of intra-group genetic similarity on genome recovery**. The panels show the first two axis of principal coordinates analysis based on pairwise nucleotide similarities between sibling genomes. Only results for the intra-species groups of eight genomes where a single genome was reconstructed are shown. In the labels, the value after the letter represent maximum contig coverage values.

**
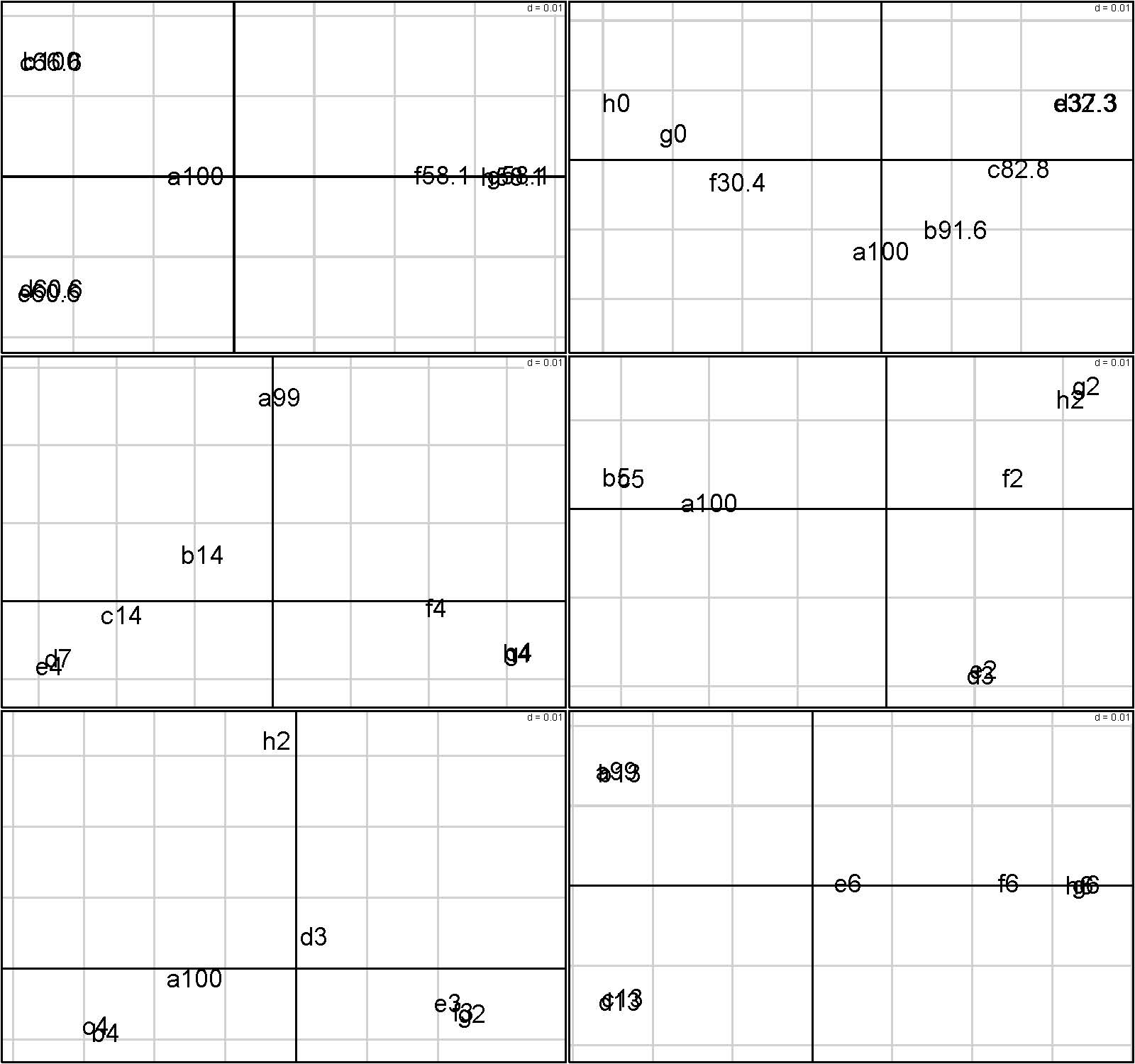
**

**Suppl. Figure 2. PHACCS evenness estimates.**

**
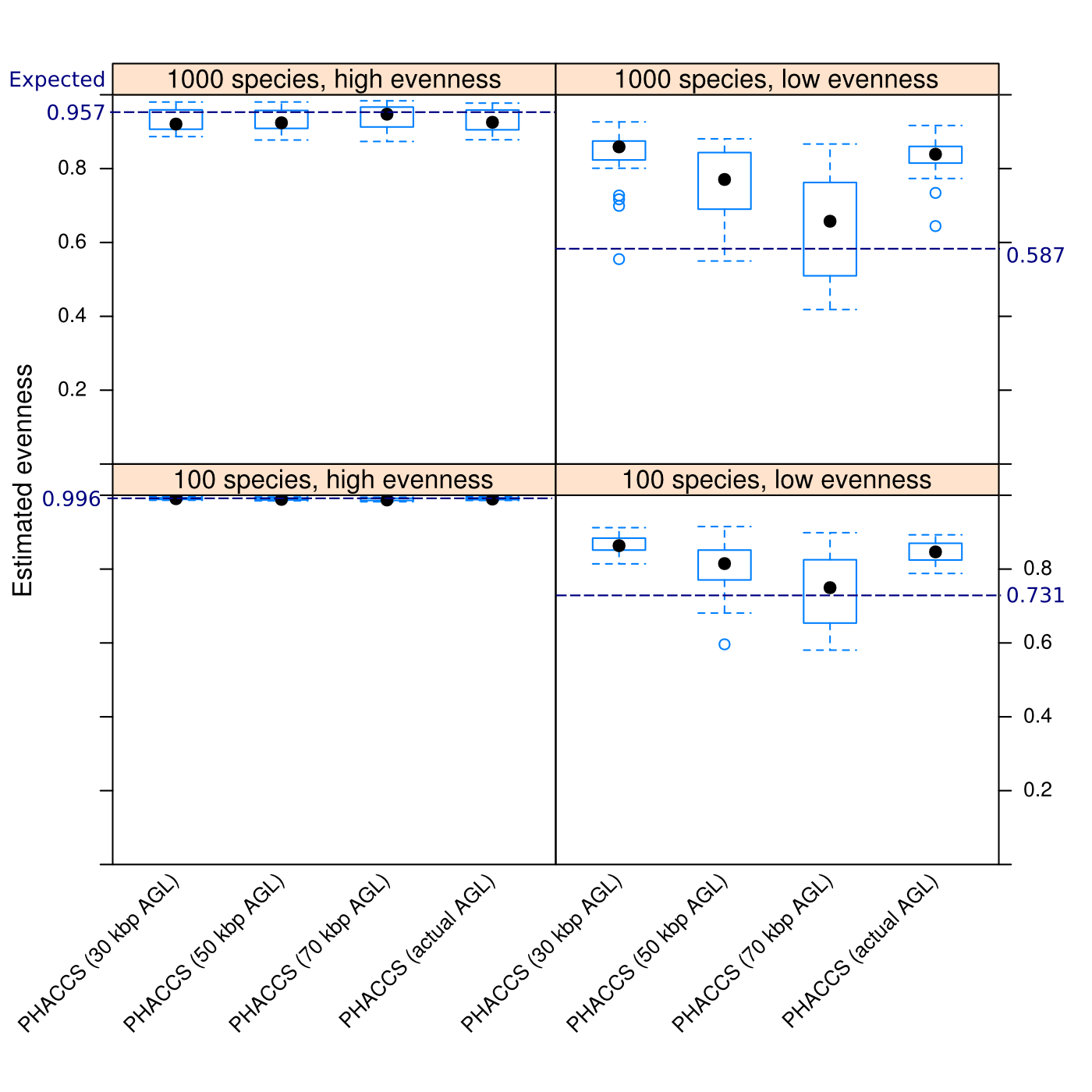
**

**Suppl. Figure 3. CatchAll and UCLUST richness estimates corrected for genome length.**


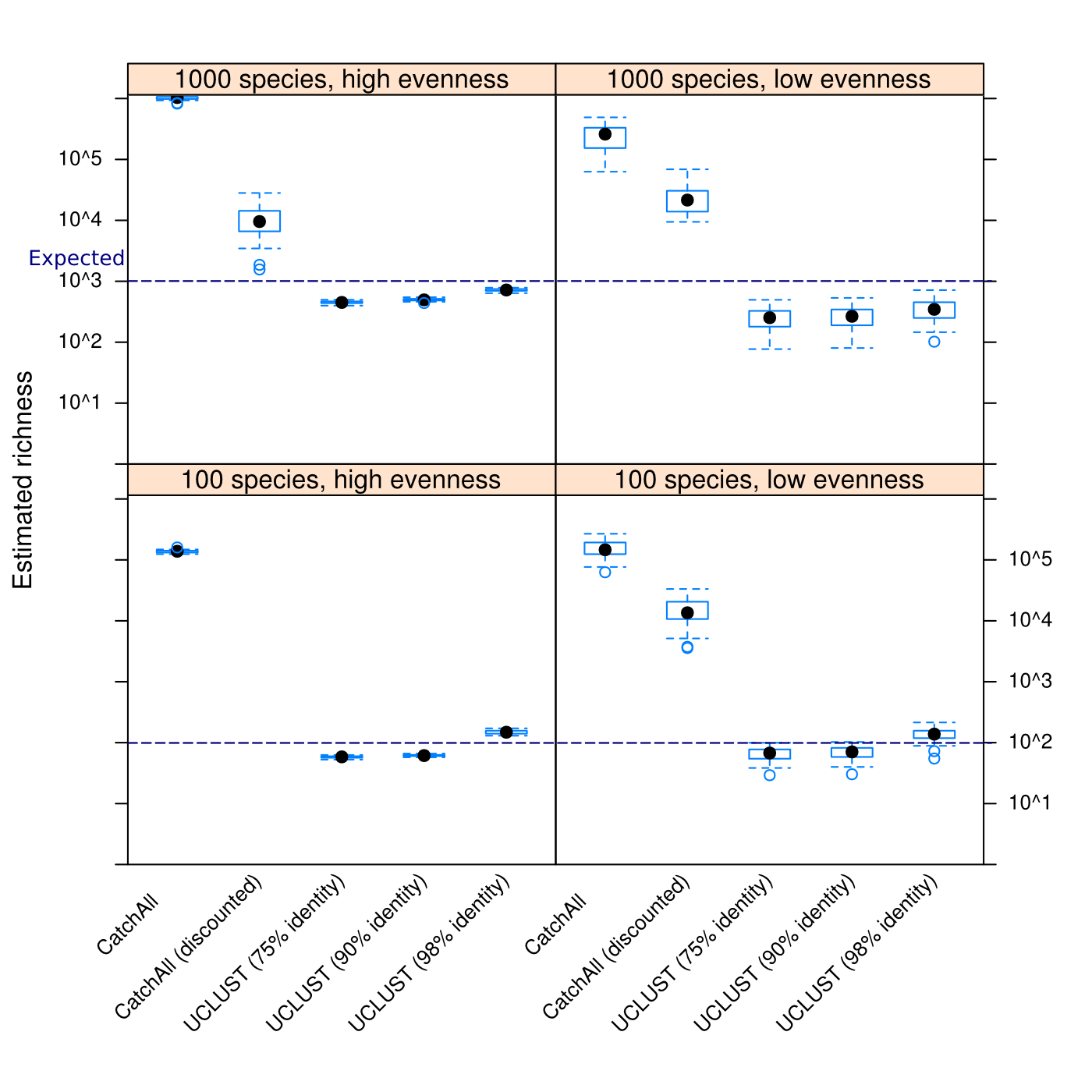


**Suppl. Figure 4. Shape of the Rank-abundance curve employed for each family.**
