# Supplementary material for: Evaluation of viral genome assembly and diversity estimation in deep metagenomes
Source: BMC Genomics. 2014 Nov 18;15(1):989. doi: 10.1186/1471-2164-15-989 (PMC4247695; doi:10.1186/1471-2164-15-989)
Supplement: Supplementary file 4 — Additional file 4: Genome sequences. 300 genomic sequences used for the evolved mock viromes, fasta format. (PDF 7 MB) [file 12864_2014_6692_MOESM4_ESM.pdf]

>NewGenomeName_1
ACCGGCCACTTGGAGCCACGGAGCCCCTTGCAGCTATGCCTCCGCAAAAGAGGGAAGCAG
CGGCTAGGAGGTTGTGCTTCACGCTGAATAACTACACAGATGAAGAGGTGTCCGCCGTGA
AGGCGTGGAACGCGAGTGAGTACCATTACGCTGTCGTTGGACGCGACAAGGGGGAGAATG
GTACGCCGCATTTGCAGGGCTACATTCATCTAAAGAAGAAGGCTCGGCTAAGCACTCTGA
AAAAATTACTTAGCCGTGCCCATTGGGAGAAAGGTCGCGGTTCGGACAGCGACAACGAGG
CGTACTGTACGAAAGATGGGGACGTGATACTTACCCTTGGCATGCCGGTGGAAGGGAACC
GTAGCGATCTCCCCGGAGCTGTTGCCGCCGTGAAAGCCGGAAGTCGAATGGTGGATATCG
CGCGAGAGTTCAGTGAAGTCTACGTCAAGTATGGGCGTGGCTTACGTGAACTGGCGCTTC
TGATTGGACAGAAACGGCAAGATTTCAAAACCGAATTTATCGTTGTCACCGGTCCGTCGG
GCGTGGGCAAGAGCCGTTATGCTAATGAGTATCCAGGCACTAAGTTTTATAAGATGAAAG
GGGACTGGTGGGACGGCTACTCTAACGAGGATGTTGTCGTTATTGACGACTTATATGGAT
GGATACCTTTTTGTGAGTTACTGCGTCTCACTGATAGGTATCCACATAAGGTTCCCGTGA
AAGGATCCTATGTGGAGTTTACGTCGAAGGTCGTCACAGTGACCAGCAATACGCACACCG
ACTCGTGGTACAATGAAGAGAAGTGCTATCTACCCGCGTTGTATCGGCGGATAAATAAGT
GGCTGACGTGGAATGCCATCCGGTTCGAGGATGCACCGGATTGTATGAAGAACTACCCTA
TAAACTATTGATTACGCCCCCTCCCTCATAGTGCCTTTCGAGGAAAGAGCAATCGCGGCC
AGCCAAGGCGGCCGCGTTCACGGGTACTTCGCGAGCGCACCGTCGCGAGCGAAGTTCCCT
AGTGAAGTTCCCCGAGGAAAACACTTGAGATGAGGCGATTTATAATTTTTACACTTAATA
AATAAAGACAGGAACCAGTATTGTGTGTCTGTACTTTATTTAGGGTTTGCGAGGTGGGTT
AGGTTCATTTGTCTGCACTCAACATATATCGTCACTTTTGCATGGAATAGTAAAGCATCG
TGGGGCTGGGGCCAACTGATGGCGAGGCCGTAGTGCTTTACTCTAGAGCCCGAGCTGGGT
AAGTTTTGGTCTCCTTTCTTCATCATAGGTATCCATGCGGATCTGGGGTTGGTTAGCCAT
TGCGCTGGAGTAACGTTTCCCGCGTCGGTGTCCGGAATGGTAATTTGGGGTCGGGGTCGG
AAAAGACGCTTGAATCCTTTGCGGAGGTCCCACTGTCTTGCGCCGTCCCAGTTTGCTAGC
GGGTCTGTCTCCAGGACTGGCTTCTTGTAGAAGTCTTCAAGGTGGTTGTCTTGAATGGGC
ACATTGTGCCCTATACCATGCCATGATCCCCAGTTCTCGTTTAGTGGACGCATTTCGAAT
TTTGCCAGCCGGATCCTGTAGTCTTCGAATGGTAGCCTCCAATTAATTGTTCCCCAGTCG
AGGAAGTCGTCCAGCATTATGATGAGGCCGTCGGTAGCCCATTTCAGGGTAGTGTCACCT
GAGCCGGCGCATTCGTGGCTGTACATGCGGACAAACTTGTATCGGTATACTCTGTTAGTT
CTGTGACCACACCAATTGGTGAGTCTGCGTCTGCGCCAGTAACGTCGTCGCCATCGCCGT
CTAATCGCCTGTGTCTGTCGGCGTCTCCTAGCAACCTGTATGAAAGAGCCAATCACGAGG
TGTCACGTAACCGTGGCTCCAAGTGGCCGTTGAGTATT
>NewGenomeName_2
ACCGGCGGGCCGGGGCCATGGGGCCATCTGGCCCCTGGCCAAGCATGCGCAGTAGTGGTG
CATGCTCGATGGCCGCGCAGCGGCCTTTATTTGCGGAGCATGCGCAGAAGGCCGCGCAGC
GGCCGCTATTTGCGAAACATCACGGCAGCACGGCGAGACAGCGGAGCCCGTCGCTGGTGC
TTTACTCTGAATAACTACACTCCCGAGGAAGAGGAGACCGCTCGCAATCTGATTCATGAT
GCAGATAAGTACGCGTTTGCGATAATCGGCAAAGAAGTTGGCGAAAGCGGTACTCCTCAT
CTGCAAGGCTTCATGCATTTTAAGCAAAAGCAGCGGCATACCGCTCTAAAGAAACTTAAT
CCTCGCGCGCATTTTGAGAAAGCTCGCGGCAGTGATCAGCAGAATGCTGATTATTGTGGT
AAAGACGGCGAAATACTTACCATGATCGGTACTCAGAGTGATACTAATTCGAGTGATCTT
GCGGGAGCTGTTGCCGCTGTGAAACGCGGAAGTCAAATAAGCGAAATCGCGCGAGAGTTC
AGTGAAGTCTACGTCAAGTATGGGCGGGGCCTCCGTGATCTCCAGTTCCTGATTGGTTGC
CCGCCCCGCGATTTCAAAACAGAAGTCATCGTTCTGATTGGCCCAGCTGGCTGTGGCAAG
TCAAAATTGGCCAATGAGATGGAAGGGTCTAAGTTCTACAAGATGAAAGGTGATTGGTGG
GATGGTTATGACAATCAAAATATTGTCATAATCGATGACTTCTACGGTTGGCTCCCGTAC
TGTGAGTGTCTACCTCTGTGTGACCGGAACCCTCACCGTGTGCCGGTGAAAGGTGACTAC
GTGGAATTCACCAGCAAGAAGATTGTGTTTACCAGCAATCGCCATGTGGATGGGTGGTGG
AAAGGTGAAATTGAGAAGAGTGCCTTCTACCGACGGATGAATGTGTACAAGTTTTATGAG
ACTGGTGAATTCAAGGACATGCCCGGACACATGCTCCCGCACCCAATCAATTATTCGGCG
CGAGATGAAAACACCCCCCTCGGGATGACAGTCGTTTTCATCTCGCGCCCCCGAGTCGCG
GAGTTCGCGAAGCGAACGGAGCGCGAGTGGCGCGCGAGATTGTCGACGACCGACGCGCGT
AGCGCGGAGGGAGGAGACCACTTGCTTTGAATTTAATATGCTATCTTTATTAAAGAGATC
CAGCAACATTCCTGTCTATGTCTTTTTCATTGTCAGTCATTGTTAAGCAGTCCCCGAACT
TTGACAGGTCTTCTTCAGATTTCTTCAACATTATCTGAGCGAACTGCCTGAATGTTACAT
ATATAGTTATTTCTGCATTATATCTCAGTGGCTGCCCAGGTGAAGGCCTAGAGAATGCCA

GCCCGTAGTGCACTACTCTTGTACCGTCCCTCTGGGGCCCTGCTGGCATTTTTAAATGTA
ACCAGCCTGATCTACTGCTATTCAGCCACATTGATGCTGAAAAGTTAGAGGCTGTCATAT
CTGATATGGTTAGTTGAGGCTTTGGGCGTACCAGCCTCTTAAACCCTCTGACCAATTTCC
ACGCCCTCGCCCCGTCCCAGTTATCCAGTGGGTCTTGCGTTTGATCCTGTAATTTTATAT
CTTTAACTCTGGCGTCCAGAACTGGGACTGTGTACCGCCAGCCCCTTGCAATTTTTGTAG
TGACACCTTCAGGCCTCAGCGCCACTTTTGCTAAATTAATCCTGTAATCCTCGAACGGCC
ATTCTGCCGGTATTCCCTCTTGACCTATAAAGTCATTCAGGTTGAAGGTGAAGAAATCTG
AGGCCCACTTTTGTGGGTCAGTCTTAGATGGGAAGTCCACTGTGAATTGCCGTCTAAATC
TGAACAAGTATATTCTGTTCAGTCTGTGCGCTCTTCGGTTCCTGGGCAACCTGTGACGTC
TGCGCACTCGTCTGTGAAACCGCCTTCCCCGGAAACCGCGAGTAACACGCCTCCTTCTGG
TTGCTGAGCGACGTACCTAAAAAAAGAATGGCCCCACGGCCCGCCGTATAGTATT
>NewGenomeName_3
CTAATAGTACATACAAAGTGTTTCCCCCCTATGGCCTTCTCATGCTCACCCCAAAGAGAG
CACCCCTATGGTGACTTTAGGTTTACTTTAGGTGTCCTTGAGGTGTACTTGAGGTGTACT
TTAGGTGTACTTAGTGCCTAACCTTATGTGTGACATTGGGTTTACTACTAGCTGTAGTGC
TTAGGGGGGACTTTAGGGGACTATATGTAGGCCTCCTTCTGTCTCTATCTGTAAGGGACC
CTCTAAGTATCTCCTGATGTCACCACCTTAAGTCCTCTCCTTATGTCACCACCTTATGTC
ACCACCTTAAGTCCTCTCCTTATGTCACCACCTTATGTCAGCCTCTATCCTTATGTTAAC
CTTCAGTCGGTGCCTTCAGTCTATAGACCTTATGTTACCACTCTTAGAGATACTGCTTAG
AGGCTACTTAGAGACTACATAGGGACTAGTTAGAGACTACTTAGCGACTCCTTATAGGCT
ACTTAGCGACTGCTTAAAGGCTACCTAGAGACTACTTACAGGCTACCTAGAGACTACTTA
TAGGCTACCTAGAGATACCCTTACAGTTAATACGACTCACTATTGGGAAGGTGTCTATTA
CTATGCCTTTCCTTATAGGTATCACTTAGAGTGCTAATTTAAAGAACATTCCAAGAACAC
TCAAAGAACACTTAAAGATGACTTTTTGTTACTCTAAGTATTGACTCTAAGATTAGATAC
TGTATGGTTCATCTCACATCAACCCAGTGAGTCGGCCTCTGAAACAACATGAGGTGTCCA
GGTGACAATCACTAGGTTGACAAGCAGTAACACTAAGACGTACAATGATTCACACAGATT
CACATAGATTCACCGCTCTTTAAAAACTTGGATAGTAACACTCTTGAGAATGACCAATAG
CGATTAACTCAAGGTGACTCGAAGAGAGAAGCCTTTATGATAATCACTAACTTATTAACT
AATGAGGTCATCACACTATGAACGAATACCCTGGCTAGTTCTTGACATATAACTCATTCT
CTAGGTACTTTGGGACACACCATTTGACAGGTCTAACGCCACTAGGTGTAGTGACTGCTG
CTCTCTTAGTCATCGCAACGTATAAGGCAATCAAGTATGGCTTTAACTACTATCGCGGGA
AGCAGCTCGCTAAGTCTCTAATTGTTCCTGAAGGGCCTCATAAAGGGTCTGAGGATACAC
AACGCTAACCTCTAGCACTTGAAGCATGGCCTGCTCTCTACAACTAACCTTCTACTATCT
TAAATAGGACACACTCTAATGAACTACACCGACATTCAGGCACGGCTAGCAATCAGCAAG
TCTCTCCCTATTAGAGAACAGGACAAGCGCCAACCTCTTCTGGTTGAAAGAGCTGCTGAC
ATTGTGAACGGTGAGACTAATAACGGTAACGATACAGATGTCTCTAAGGGTCTTGAGTAT
CAGGACTGGTGGCATAGCTTAGGGTCACTCATGGGTGATGCAGGGTTCAACCTGTTAGGT
AATGGTCACTTCAGTGCAGCTTACTCACACAAAATGTTACCCGGTCGAGTCATTAAGCTT
GGCTTTAAGAATGAAGACTCAGGAGCTGCATACACTGCGTTCTGTAGGATGCACCAAGGT
CGTGCTGGTATCCCTAACATCAATCACGTAGCACGGCACGCAGGCTGCTACACAGTTGTA
CTTGACCATTTGAGTGACTGTAAGCTAGACACTAATGAGACTCACGCTAACTATGCGTAT
GCTGCAAGGGCACTCATAGAATGTAACGATATCAATGTCGAAGCTTGTGCACATTTTGTT
AGCACTGAGTTCCTCGAAACGTGCAAGGTGATTCGTGAGTTCTTCTACGGGATTGCGTCT
TTCGATATGGACAGTGGGAACATTATGTTCGATAAGAACGATCCGGCTTATATAACTGAC
CCTGTTAGCTTCTCCCACGACCGTGAACGTGAGGATGGCTTCCCTTTGGATCCTGATGCA
CTGATTGCTGAGGTCGAGGCGATAGCTAACGAACGTATCATCGAACGTTGTCGGAACCGT
AAGGCAAAGCGTGACCCTAATGGTACATTCCGTGCTGAACATAAGGCTGCAATGAAACTC
CGTAAGCGTAACCGTAAGTTATCTGAATGTAGAGCTGAACGGGTAGCTAAGTGTGAAACT
AAGCTGGACCACTTACACTTAATGTCCGTCCAAAGGGATCACAAAGTAATCGTACGAAAC
GAACGTCGTGCTGAGATGCTCATGGGGTCTGAATGGCATGACTTCAGGTTACTCAATGGG
AACTCAACGATACACAAGGTTAACCAAGTGAACGACTTTAAGTGGCAAGTAGCTGACGGT
CTGGCTATTCCGGCAGGATGTTCACTAATGATTGATAAAACACTTGACGCACAACTGATG
GGATGATAAGGTGTATCTTCAAGGTCGGTACTAACTGCTCGCCTTTATAGATAAACTTTC
TTAACAACGTGAGGCAACAATGACTAACGTAATCATCGCACCAAAGAACGACTTCTCTGA
TATCGCTAATGCAATCCAGCCGTACAACATCCTGGCTGACCATTACGGTGCACAGCCTGC
AGCTACACAGCTTGAGTTGGAACATGAAGCGCACACTGAGGGCGGGAAGCGTTTCCTTAA
GGCAATGGAGCGTCAAATCAAAGCAGGTGAGTTCGGTGATAACACGGTAGCTAAACCGCT

CCTGTCATCCCTTGCACCAAAGTTTATCGAAGCGTGGAACACTTGGTTCATTGATGTAGA
AGCAAAGCGTGGTAAGCGTCCAGTGGCCTTCAATCGGGTACACAAGGTTGCACCTGAAGC
TGCTGCTTTCATCACTCTGAAAGTAACACTGGCTTGCTTGACGAAAGAAGAGTTCACCAA
CCTGCAGTCGGTAGCCACTTAGATTGGCCGTGGCATTGAGGACGAGTTACGCTTTGGGCG
CATCCGTGACGAAGAAGCGCAGCACTTTAAGAACCACGTACAGGAAGCACTGAACTAGCG
CGTTGGTATCGTCTACAGGAAAGCATTCATGCAAGCTGTAGAGGGCAGGATGCTCGATGC
CGGTGAACTGCAAACCAAATGGACTACTTGTACACATGAAGAAGTAATTCACGTTGGTGT
ACGTATGGTAGAGTTACTCATTGGGTCTACTGGTCTCGTTGAGTCGCACCGTCCGTTTGG
AGGCAACATTGAGAAAGACGGAGAGTACATCCAGTTAACCGAACAATATGTTGACCTACT
GTCTAAACGTGCTGGTGCACTCGCTGCAATTGCTCCAATGTAATAACCTTTTGTAGTCCC
TCCTAAGCCTTGGACTTCTCCAGTAGGTGGTGGCTATTGGGCTGCGGGTCGTAAGCCTTT
CCCTATGGTCCGCACTGGTTCCAAGAAGGGCTTAGCGCGTTACAACGATGTGTATATGCC
TGAAGTCTACAAGGCAGTGAACATCGCACAGAATACTCCTTGGAAGATTAACAAGAAGGT
TCTGGCATTGGTCAACGAAATTGTGAACTGGAAGCATTCCCGGGTTGCTGACGTACCTGC
TATGGAACGTGGTGAACTACCAGTCAAACCTGTAGACATCGATACGAACGAAGCGTCTCT
CAAAGCGTGGAAGAAAGCAGCATCTGCAATCTATCGTAAAGATAAGGCTCGTGTGTCTCG
TCCTATGAGCATGGAGGTCATGTTAGGTCAGGCCAATAAGTTCGCACAGTTTAAAGCTAT
CTGGTTCACTATGAACACGGACTGGCGTGGTCGTGTATACGCAGTACCAATGTTTAACCC
ACAAGGTAACGACATGACTAAAGGTCTGCTGACATTAGCTAAAGGTAAACCAATCGGTGT
TGATGGGTTCTACTGGTTGAAGATTCATGGCGCTAACACTGCTGATGTTGACAAGGTGGA
CTTCGCAGAACGCATCAAGTTCATTGAGGACAACAATGAGAACATCAAGAGCGTTGCAGC
TGACCCTATTGCTAACACTTGGTGGACTGAACAAGACTCACCGTTCTGCTTCCGCGCGTT
CTGATTTGAGTATGCAGGTGTGCAACATCACGGGTTGAACTACAACTGCTATATGCCTCT
GGCGTTCGATGGTAGCTGCTCTGGTATTCAACACTTCTCTGCAATGCTGTGTGACGAAGT
GGGTGGACGTGCTGTTAACCTGCTGCCAAGCAAAGAAGTACAAGACATCTATCGTATCGT
AGCTGAGTGTGTGAACGAGATTCTTAATCAAGATATATTCAACGGTACTGACAGCGAAGT
TGAATGACTGACCAATAAGGACACTGGAGAAATCACTGAGAAGCTCAAGCTGGGGACTAA
AGAACTGGCTGGTCAATGGTTGGCTTATGGTGTAACACGTAAGGTAACTACGCGTTCCGT
AATGACCTTGGCATATGGTTCCAAAGAGTATGGCTTCCGTGACCAAGTGTTTGAGGATAC
CATTCATCCCGCTATCCACGATGGCAAGGGCTTGATGTTCACTCAACCTAACCAAGCTGC
TGGATACATGGCTAAACTGATTTGGATTGCAGTAACAGTCACAGTGGTTGCAGCAGTTGA
GGCAATGAAGTGGTTGAAGTCCGCAGCTAAACTTCTGGCTGCTGAAGTTAAAGACAAGAA
GACTAAAGAGGTTCTTCGTAACCGTTGCGCGGTATAATGGGTAACTCCAGATGGCTTCCC
TGTATGGCAGGAGTATCGCAAGCCAGTACAGACCCGCTTGAACTTAATGTTCTTAGGTCA
GATTCGTCTACATCCTACTGTGGACACCAATAAGGATAGCGGAATTGATGCGCGTCAGTA
GGAGTCAGGCATCGCACCTAACTTTGTCCACTCAATGGATGGAAGTCACCTTCAAATGAC
TGTAGTTCGATCCTATGAGGTCTACGGTGTGGAATCCTTCGCACTGATTCATGACTCATT
TGGTACTATCCCGGCTGATACTGGTAACCTGTTCAAGGCTGTACGTGAAACTATGGTGAA
CACATACGAAGAAAACGATGTGCTTGCAGACTTCTATGACCAGTTTGCTGATCAATTACA
TGAATCACAGCTTGATAAGATGCCTGAGATGCCAGCTAAAGGGTCTCTAGATATCCAAGA
AATCCTCAAGTCAGACTTTGCGTTTGCCTAACGGAATTTAATACGACTCACTACTGGGAG
ACCCAAGGTTTTACCTACTTTAAGATACACTTTAAGATATACTCTAAGATGCACTTTAAG
TTTACTTTAAGAGGAAAGAACATCATGCGTACAAACTTTGAACCAATCACTAAGCATAAC
AACGTAATCAACGAACATGGTACTGAATGGCAGGAACGTAAAGACCACATGAAGAAACGC
CACAAGACACAGCGTGGTAACTCTGAGAAACGAAACTGGAAGGAGGGACTCCTATGAGTC
GCTTACATAGTGGGAACCTAAACGCCTTCAAGGCTGCTACTGACAGAATATCTAAGATGG
ACTTAGTGGTCGCTTGTGAGGAGTGGTATGAGTCACATGTAAGTCTGAACTTTATGTGGC
TACGCATAGAGGACAAGGAACGTAAACGGATTAACTCCATCACTTGGAGTCATCACGTCG
AGGACGTTCTATACAGTATGGGAACCGAGTGGTTGAACCGAATGTATGACCAGTTGAAAG
ACTGGAAGTAATACGACTCACTATTGCGAAAGAGTTAAACTCAAGGTCATCTAAAAGGTG
GCCTTTGTGATTAACTTTTTGATTAACTTTTGATTAAACCTTAACCAACACACACACATA
TACATCTATAGGAGTTCCACAATGGAAATCCCATTCAAGACTTGAACACATAAAGCTGTT
TCATTCGTTGAGTCTTCCGTAATGAAAGCTCTCGACAAAGCTGGCTACCTTATCTGCGAT
GTAAAGTATGATGGTATTCGTGGTAACATTGTAGTTGACAATACGGATGACAGCATGTGG
ATCTCCCGTGTCTCTAAGACCATCCCTGCCCTTGAGAACCTCAATGGGTTCGACTCTCGC
TGGCAGCGTCTTCTCAAAGATGACCGCTGTATCTTCCCTGATGGTTTCATGCTTGATTGT
GAACTGATGGTCAAAGACGAAGACTTCAACACAGAGTCTGGCCTTCTGCGTACCAAATGG

AGTGACCCTAAGAATTTCAGTACCAACTGTAACCCTTTGGCCTCTGAGTTCACCAAGAAG
TCTGCCAAGGTTCCCTACATGTTGTCCTCTGATAAGCTTAAGGTTGTTCTTTATGGTGTC
ATGCCGTTTGACTCTATGGTTTCTGGTGAGACCTACGAAGTGATGAACCTACTGATGCGT
GAGCACGTTAAGGCTTTGCTTCCGTTACTGAACGAGTACTTCCCTGAAATCGAATGGGAC
ATCTCTGAGAGCTATGAAGTCTACGACTATGTGGAACTGACAGCACTCTATGAGAAAGCT
CGAGCCGATGGTCAGGAAGGCCTAGTAGTGAAAGACCCTCTGGCATTCTATCAGCGTGGT
AAGAAGGCTGGCTTTTGGAAGCTAAAACCTGAGTGTGAAGCAGCTGGTATCATTCAGTCT
GTCAATTGGGGGACACCGGGTCTATCTAACGAAGGCTTGGTGATTGGCTTTAATGTTCTC
TTAGAGACTGGTCGCCATGTTGCAGCTAACAATATCTCACAGACTCTTATGGAAGAGCTC
ACAGCTAACGCTAAGGAACACGGTGAGGATTACTACAATGGATGGGCCTGTCAGGTAGCC
TACATGGGGGAGACGAGTGACGGGTCATTACGACATCCCTCATTCGTTATGTTCCGTGGC
TTTGAGTCAGACTCAATGGTCAAGGTCTGAATCACTCAATGGTCAGTCCTTAACAAGGGG
CTGGCCTTTTGCGTTTCTAATTAGACTCACTATGGAGGAACAACCACATGCTATTCCTAA
TTGTTGCTAGCATTGCTGCCACACTTGCCTTAGTTATCGTTCACGACAACTGCTGGCCTG
ATTGCCAATACGACTCACATATGGAGGACACACATATGCTACGATTACACCTCAATAAAT
GTAATGGTATCTACTCGGTTCGTGACTCTGGCCGCTCTCTGATCAAGGCTACCGAGAAAC
ATGCAGTCATCCTAAAAGTGCTGGCCGCTACCATTGAGTTAGCACCTCGTATTCACGCTG
TGATTACTACAGGCTTATTTAAAGAAGCGACAACTGGCTCCCGGCCTTTCGTTCCGGTTC
TCGTCACCCGTTTCCCTAAAGTCCGTCTGGTCATAGAGTGTATCAAGGAGGTGTTCTAAT
GTATGGTTGCACTGCATGTATAGAGAATGACGACCGCTACCCACATACCTGTGAGCCTGT
AGCTAAACCACAAGATATGGATGGTGTGCGCACTCCGAGCCACTATATGTTGTTTGATGA
CATTGAGGCTATCGAAGTGATTGCCCGATCGATGACCCGTGAACAGTTCAAAGGGTACTG
CTTAGGCAACATCCTTAAGTATCGTCTACGTGCTGGTAAGAAATCCGGACTGGCATCTTT
AGAGAAGGGCATGGCGAAAGCTGGGCTCTATGGAGAACTGTACGAGAAACATAAAGGAAT
ATGTTATGCTTAAACCTAGTGAATGGTGTGAGGGAAAATTCAACGAGACTGGCAATGTCG
ACTATCTCGAAATGTACAACCTGTGGAAAGGGAGAGGACAATGAGCATTTTAAACAAGGG
GTCACTGGTTAATGGTGACAAGAAGTTCTTCGCTACTGTAGAAGGCCAAGGCCAATCATT
CGACGTACCTGTGTTCGCTGCGTCTCTTGAAGAGGCCACCGAATTAGCTGAGTGGCAATA
TGACCCAGCTGGTTTTGCAGTGACACGCATGCGTCCAGACCGAAAGGTTTAATAGGACTC
ACTATCGGGAGACTAATAGTTTCCCACTTTTAACTTTCAGAAACTTAAGGAGAAACATCA
TGGCTTTCACCTAAAGTAAAGTATTCAATTCTGCTAGTGCTATTGCTGAACCGTACTGCT
ACCTGGCTAAGCCTGACTTTGGTAGTGGTGACTTCTAGAACGAACGTGGTGTCAATAACG
TATCACTAACTGTATCCAACGACGACCCTCGCTGTCAGAAAATGATTGACGAGATTGTCG
AAGCCCATGAGACGGATTATGCTGCACGTATGGAAGAGTACGCAGCTCACCCGCCGAAAG
TTGTTAGAGGTAAGAAACCGCTGAAGCCATATGTTGGCGACATGCCTTTCATGGATAACG
AAGATGGAACCACAACGTTCACCTTCAAGTGCTATGGTTCATACACTGACAGGAAGACTG
GGGAGCATAGAGCAATCGACTTGGCTATCGTGGACTCCAAAGGTAAGCGCATTCGTGGTG
AGCCTCCGGCTATCTCTGGTGGCTCCGAGCTGAAGATTAAATACACTCTGTTCCCTTATG
GGTGGTCTGCTGTGGCAGGTGCTTCCGTTAAGCTACAACTGGATTCCATTATGCTGATTA
AGCTGGTTGAGTTCGGTGGCAGTGGTGAGGATGACTGGGCTGATGAAATCGAAGAAGACG
GTTGTGTTGCTGTTGAAGCACAGACCCGTAAGCCTCAGAACGACTCTGGCTGGGATGAAG
AACCACAAGAGGACGATGAAGATGATGATGAGACCGGCGACTTTTAATATATGATAGGGA
GGTCAGAGGGCAGGTTATGGTGCAAAAGGAATCAAGAAGGTAGGTATCTGCCGAAGTGGA
TTCGAAGAGAAGAACATGAAACTCTTAGAGTCCCAGGGTGTTAAAGCTGAGTTCGAAATG
TGGAGAGTTCCTTATGTTGTTCCCGCAAGGAATCACCATTACGCACAAGACATTTTATTA
CCTAACGGTATCTTTGAGGAGACAAAAGGCTTATGGGAAAATGACGACCGCAACAAACAC
TTGCTGATTCGAGAACAGTGCCCCGACTTGGATATACGCCTGGTCTTCTCAAGCAGTCGC
ACCAAGATTTACAAGGGGTCTTCTAAAAGTTATGCGGAGTTCTGTGAGAAACATGGCATC
TTATTTGCTGACAAGCTGATTCCAGTCGCGTAGCTGAAGGAAGCATCCCGGCCTGTACCA
TTCGATAAGCTGAAGACCAAGAAGGAGAAGAATAAATGAGAAAGGTACAATTTAAACCAC
GCAGTTCTACAGAAGCGATCTTAGTACACTGCAGTGCACCAAAGCCGACTATGGACGTGG
GTGTCCGTGAAATCAGTCAGTGGCATAAAGAACAAGGTTGGTTAGCAATCGGCGATCATT
TTGTGATTCGCCGTGATGGGACCATTGAGGAAGGTCGCCCAGTAGAAGTCATTGGGTCTC
ACGTTAAGAGCTGGAACAGAAAGTCAGTTGGCGTGTGTCTCGTTGGTGGTATTGACGATA
GGGGCCACTTTGAGGCAAACTTTACGCCTGCTCAGATGGTCTCTCTTAAAGCGAAACTTG
CAGACCTGATGAACATGTATCCAGAGGCTGAGGTGAAAGCTCATCATGATGTTGCACCTA
AAGCGTACCCGTCATTCAAATTGTGCCACTGTCTTAAGACTGGAGAACTAGTCACAAGCG

ATGGGGGCTAATTAATAGAAGTCACTATGGGAGAGCCGAAAGGTTGCTCCGTTTGTTCGC
TAAAAATGATTAAGGAGTAACCACAATGTATAACCTTAAAGAGCATGTGTTCGTCATTTT
AATCTGTGTACTTATCATTGGCGAAGCCTGCCTGCGTCCGTTCTTATAGGATGTATCTAA
TGGATATGGAAGAACAGCAAGATAGCGTGTTCCTGTATCACCTGCCGTGTGAAAAGTGCG
GGTCGTCAGATGGTGACTCAATGTACTCCGATGGACACAGGCACTGCTTCGTGTGTAACA
ACTATGGGCCTCCCACGGATAAGACTCGTGACCAAGCGTCAACCAAGTGTCGTCGCTCAT
ATAACACTGGAGGTGACAAAATGTCTAACCTTTTAAACTTTGGGGATTCAGACGGTCGCT
ATGCGGACCTCAAAGCTCGTGGCCTTATGGAAGCTATCTCTCGTAAGTATGGATATTGCG
TAGCGATGGTCAAGGGAGAGATGCTTCAGGACGCGAACTACTATGACGTTGAGGGGAACC
TTGTGGGTCAGAAGGTGCGCGACAAGAATAAAGAGTTCTCTGCAAAGGGGAAACTCAAAG
CTGACCTATTGTTCGGTAATCAGATATGGAATGGTGGCAAGCAGATTGTAGTTACAGAAG
GTGAGATTAATTGCTTGACTGTAGCTCAACTTCAAGAAGGGAAGTATCCCGTTGTGAGTC
TCCCGATGGGTGCTCAAGCCGCAAAGAAAACATGCTCTGCCAACTATGAATACTTTGACC
CGTTCGACGAGATTATCCTTATGTTCGATATGGATGAACACGGTCGTAAGGCTATCGAAG
AGTGTGCTCCTGTCCCACCTAGTGGGAAAGTCGGAGTCGGTGTACTCCCTCTTAAGGATG
CCAATGAGTGTTGACTCACTGGACAAGCCAAAGCAGTAACCGACCAGATTTGGAATGCAC
AACCTTGGGTGCCTGATGGTGTAGTCTCTGCTGTGGCTCTTAAAGGACGAGTGCGTGAAG
CTATGGTCAAAGAGGAAACACCAGGTCTTCTATTTTCGGGTCAACCGAAACTAAATCATA
TGGCACTGGGTGCTCGTGGTGGTGAAGTCATCATGGTCACTTCACGTTCCGGTATGAGTA
AGTCCACGTTCGTTCGTCAACAGATGCTCTAGTGGGGCAAGGGTGGTGCGAAGGTAGGCT
TGGCTATGCTTGAAGAAGCCGTTGAGGAAACTGTTCAGGACTTAATGGGCTTGAACAATC
ATGTCCGTCTACTTCAATACAAAGAACTCAAGATGAAAACCTGAGAGTATGGACGCTTCG
ATGTATGGTATGGTGAACTGTTTAACACAGATATGTTCCACCTGTCTGATTCCTTTGCGG
AGTCACAGGAAGACCGTCTGTTTGCGAAGCTGGACTACATGGTCGATGGCTTAGACTGTA
ACGTCATCCTCTTAGACCATATCTCTACTGTGGTATCTGGGATGGAAGATAACTCTGATG
ATCGTAAGATGATTGACCGCTTGATGACCAAGCGGAAAGCCTTTGCGAAGACTAAACGTG
TGGTCGTTGTGGTAATCTGTCACCTCAAGAATCCTGAGAAGGGGAAAGCACATGAAGAAG
GTCGTCCAGTTAGCATTACTGACCTACGTGGCAGCGGTTCTCTGCGCCAACTATCAGATA
CTATCATTGCTCTCGAACGCAACCAGCAAGGGGTTTATCCAAACCTTGTTCAACTTAGGC
TACTTAAGTGTCAGTTTACTGGTGAGACTGGTGTGGCTGGGCATATGGCCTTCAAGAAGG
AAACCGGATGGCTTGAACCGACTGTCTCACCTGAAGATGAAGGAAGCAGAGATAGCGGCT
GGGAACCAGAAGAAGACGGTCAGAACTTCTGATAGTAAACCTATACAACTCACTAAAAGG
GCAGACAACCTGATCCCACTTATCAATCCTCTTAAGGAGAAGCACAATGTTTAACTTCAT
CGACACTCTCGGTAAGCTGGTAGTTAAGCTGTACTTCTGTGAAGCTAAACGTATTAACAA
AAAGGCTCGTAAAGGTGCTGATCGGGCACGGCGCTTGGCTAAAGATGCACGTATGCTATC
TGAAGCTGCAAGTGCTGGTGTAACGTCTGCTGCTTAGATTGCAGCTAAAGCAACCGACCT
CGACAAATTCTTCCTGTAAGGAGTAACATATCATGTCTATTAAATTCCCTGGCAATACCA
TTCGTCTATCTGACACCATTGACCACTATGCTCGTCGAGTGCACGTCAACGTTCGTAAGG
GAAAAGTCACTTTGGTCTACCGTTGGCAAGACCATAAGTCCACCAAGGCGCACACACAGC
GTGTAACTTTGGATGTCATGCAGGCGGGTCGTCTAATATCTTCTGTTGCTGTAGCTGCTA
CTGTGGCTGTAGGTGGGAATATTGTACGTCAGGCTATGCTAGGCAACACAATTGAAGCTG
AAGGCAACATGCTTGCTGAGAAGTGTTAGGAGTGGTAAGACTTAAACTAAAGGTCATCTC
CGGGTGGCCTTCGTGTTTAACTTTTGTATGAAAACTATTCAACTAACTAATGGAGTAAAC
ACAATGTTACTATCCGACTTAGAAGCAAATGGGTTGCTGCACAGCGTTAGTCAATTTCAC
TGTGCGGTGACTTACGACTACTCAACTGACCAGTACATCCGTTATCGACCTGCTGACTTT
GGGGCATACATTGATGCTCTCGAAGATGAGGTGGCTCGCGGTGTTCTCATTGTGTTTCAT
AATGGCCACAAGTATAACATCCCAGTAATCGAGTTGCTCGCCAAGAAACTGTTAGGACGT
GATGTCGAGTTCCCTAAAGAGAACGTCTTGGATACCCTAGTGCTGTCCCGCTTGGTTTAC
TCTAACATCAAAGACACCGATGCTGGGCTGCTTGGTGCAGGCAAGCTACCAGGTAAACGC
TTTGGTTCCCACGCTCTGGAGGCTTGGGGTTATCGCTTAGGTGAAACGAAGGGTGAGTCC
AAGGACGACTTCAAGGCCTCACTGGTTGAGTCTGGTGAAGACTACGTGGCTGGTCTGGAA
TGGTTGATGTTCAACGAAGACATGATGGAATATAACGTTCAAGACGTTGTAGTAACCAAA
GCTCTCCTCGAGAAACTTTGTAGTAACACATTTTATTTCGGTGAGGTGGAACCTAATGGA
AGCACTGTGGCAGAACGTTTCTGGGAGGGTAGCATTGAGGCTGTCAAGATGGAACACGAT
GCTGCATGGTTGTTAGCTAAGATGGAACGTAATGGTTTCCCTATTGACGTCAAGTCCTTA
GAGAATCTTTATGCTGAACTTGCTGGGCGCCGTGGTGAACTTCTGGTCGAGCTGACCAAC
ACTTTCGGCTCATGGTATCAGGTCAAGGGTGGAGTTGAAGCATTCCGTCACCCAAGGACT

GGTAAGCCTCTCACTAAGTATCCTCGTGTGAAATATCCGAAACAGGGTGGTATCTTTAAG
AATCCAAAGAACAAGAATCATCGCGAAGGTCTGGATCCTTGTGAAATGGACAATCGTGAT
TACGTAGAGGGCGCTCCATATATGCCTGTAGAGCACGTTGTGTTTAACCCTTCTAGTCGT
GACCACATCACTCGTGTGCTTACTAAAGCTGGCTGGGTGCCCGTTGAGTTCACTCCTAGT
GGTGCACCAAAGGTAGACGATGAGGTCTTAGAGCACGTTAAGGTAGACGACCCTGATGCA
CTTCGTTGCATTGAGTTGATTAAAGAATACCTTATGATTCAGAAGCGTGTCGGTCAGGTT
GCTCAAGGTGATAACGGTTGGCTCCGAATGATTGGAGAGGATGGTCGTATTCACGGTAGC
GTTAACCCGAACGGTGCAGTAACTGGACGTGCAACTCATAGTTTCCCTAACGTGGCACAC
GTGCCTTCTAGCCGTGCAGCTTATGGTGAACCTTGCCGTGCTGCTTTCGGTGCAGAGCAC
AACATGAAAGACGGTAAGCCACACCCTTGGATTCAAGTAGGTGTGGATGCGTCAGACCCT
GAGCTACGTTGCTTGGGTCACTTCATGAATCGATATGTCGAAAGTGAGTACTTTGAGACT
ATCCTTACTGGTTATATACATAGAAAGAACCAAATGGCCGCTGGGTTACCTACAAGAGAC
AACGCTAAGACGTTTAACACAATGGACGTCTATAAACCTATCTAATTCGATGGACATCCC
TATGGGACAATATCGAGCTAAATGATATTCGGTAGCCTTTAACTTAGGAGGCTCCATATG
TATTCACTAAACCATAACAAACCTAAATCCTCACAGACCGCGACTCCCAGCAAGTACACT
CAGGGGCACTTGAAGGATAAGAAGTGTAAGTGTTGCAATCTGGTATTTAACCCCAAATCT
CCTGATGAGTTATACTGCTCAGATAAGTGTAAGGATAGGGGAATCCAGACGGCATATCTT
ATGCGGAGCTACGGGATAACTCTTGGTGACTACGAAGAGATGTTGGAATCCCAGAAAGGT
CTATGCAAGATATGTGGCACCGGGGGTTTTGCAATGAACCCAAAAGTGCACAAGATAATA
CTTGTTGTTGACCACTGTCGCACCAAAGGTAATGTTAGAGGTTTATTGTGTCACAATTGC
AATCGTGCATTAGGGCTTCTTAAAGATAACGTGAAGTCCCTACAGCCTGCTATTGAATAT
TTAAATGTGTAACGACTATCCCGGAAGGGAGTAGGGCCAAGTGGCTCGAAACGGTAGGGC
GCTGAGGCGCAAGATATAGTCTGCTCTGCATGGCAACATGTAGCAGTTCATAAGCGAACG
GGCAGGGATTAACGACCCCTGTTGAACACTAAGGTATCTACGGATTTTTATATGGTCCTG
GTGCTGCAAAGATTGGCCAGATTGTTGGAGGTACGTCTGAGGATGGTAAGAGACGCATCA
AGAACTTCCTTGAGCAAACTCCGGCTATCGCTGCTTTACGTGAAACAATCACAGGGGCAT
TCGTCAAGGACTCCAAGTGGGTTGGTGGCGAACAGAAAGTAAGTTGGAGACGTCCTTGGG
TTCGTGGGTTGGATGGTCGTAAGGTACACGTCCGAAGTCCTCACGCTGCGTTCAACACCT
TGTTGCAGTCTGCTAGTGCTCTCATTTGTAAACTGTGGATTGTCAAGACAGAAGAGATGC
TTCTTGAGATGGGCTACGCGCACGGCTGGGATGGTGACTTCTGCCTAATGGGGTGGATAC
ACGATGAGCAACAGCTTGCTTGCCGTACTGAAGGGATTGCAAAAGTTGTAATCGAAGTGT
CTTAACAGGCAATTCGCTGGGTTGGAGACCACTTTAACTTCCGGTGCCAACTGGATACTG
AAGAAAAGATGGGACCGAATTGGGCTGTATGTCACTAATTAACAGGAGAAATATATCATG
GCTATTACTAGACGTTTTTAAGTATCATTCGAAGTAACCGCTGTAATCACCTCTGAGAAG
GAGAAACAATGAACTGATGCATTGATTCAGTTGGCTAAGAAGGCTGCCGCTGGTGAGAAT
CTTAGTCCCCTTGCCCGCGAATTTCTGGTTCAAGCATTGACCCACGGTGTCGAGGGTGCT
ATCTCCTTTTCTTCCAAACACGGTATGCGCGAGTTTATTCCCCAAGAGTTACCGGAAGCT
GGACTGACAATCTCTCCAGCAACTATTCGTGAGGTGAATTAATGGGTGAATATATACGTG
TACTGGCGGCTATCAAAAGCTGCCCTAAGACCTTTCAGTCAAACTATGTTCGTAACAACC
CGTCACTGGTAGCTGAGGCTGCAAGCCGTGGTCATATCTCTTGCCTATCAATCGATGGGC
GTAATGCTGATGCTTGGGAAGTAACAGGTGCTGGTCTCCGTTTCCTGAAGCAGGTGGGAG
GTTCTGTATGACTTTGCTTACTGACAACGTACAGGTGACTCGTGAAGCGTGGAATGACAT
GCAGATGTACATTCAGTCCCTTGAGAAAGACGAAGAGTTCCTGGATGCTCTGAGAGCCTG
TGGTGTGGACAATTGGGATGGCTACCTAGATGCTGTCGAAATGGTATGTAGGGAGGATGA
GTTATGAGTGCTATTACACTGGGGGAGTTCTATAAGTTACGCCAAGGTAGATCCGATAAG
GGAGTCTTGGTGATGGATGGCGTCTGGCTGGAATTCCAAGCGATGGCAGCAAGTGAAGTC
GAGACCCAATGGGATGATGACATTTGGAGTTTAGAGTGTGACCACGGGAAAGCGTGGAAC
ATCCTGCTTGACTCCGTTAAGTCCTATGCGAGCCGTAAGAAGGCTTGGCGTGAYGCACCT
ATCATGATGGCCTTCATTGACTCTGTGAACTGGCGTCAGGAATTAGTTGACCCAACCTAT
AAGGAGCACCGTAAGGCGTCTCGTAAGCCTGTAGGCTACCGTGCGTTCGTCAAGCGTGTT
CAAGCCTTTGAGGACTGGACGTCTATCCTTTAGCCTCGCTTAGAAGGTGATGACGTAATG
GGAATCATTGGGTCTGGTGCTGAAAACTTCGGGTTCAAGGAGGCTGTACTGGTCTCCTGT
GATAAGGACTTTAAGAACATCCCTAATTGTGACTTCTTGTGGTGCACTACTGGTAACATC
TTGACTTAAGCCCAGACTACCGCTGACTATTGGCACATCTATCAGACCATTAAAGGTGAC
TTGACTGATGGGTATGGTGGCATTCCGGGCTGGGGTGTGACAACTGCCGACTGTTTAGAG
AACCCGTATGCCTTTGAACAGGTTGAGAAAACCTTTAAGTCTGGTACTCGTAAGGGACAG
ACAGTTATCGAATGGAAGAAGGTGGGCTAGGGAGACAGAACCTTGTGGGAAGCTACTGTG

ACCCTTGGTGCCAAAGCTGGGATGCCTGAAGAGGAAATCATTAAACAAGATCAGATGGCT
CGCATCCTTCGTTTCGAAGAGTACAACTTTATCGACAAGGAGATTTCCCTATGGTCTCCT
GAACAGTTACATTCGTGAGGGCTACACTTATAGCTAAGAGTGTGTAACTCTAGGCATTCT
TCTACATGTTAAATTAATACGACTCACTATTGGAGAGGGGTGCCCATATGATACCTACTT
TAAGTTCTAATTGTAAGGAGAACATTAATTATGTTAACCCCTATCAAGAAGTACATGGAG
AACCCACAAGATATTACTAATGTTCCTCGGAGTGTCATGGAGTATCTTCCGGTTTAATAC
AATGCTGGATATGCTATTCAGTCTGGGCTTATCAACCGATTGAAACAGGCTGGGTGGTCT
GAAAGCTACATCGCTGGGTTCCTCGCGGGTCTCAACTATGCATCTCAAACCTTAGACGAT
ATGGAGCCATTCCGTAAGGAACAGGCAGAATCCTACTAACAGGAGGTAAGCTGATGTGTT
TTTCACTAAAGATTAAAATGCCAAAAGTAGATACCAATCAGGTACGTGCAATCGACCCTG
CGCCACTAACAGAGAAACCAAAAGGTATTCTGTTTGGTGGTGATGACACTGAAGCACCGA
AAGATGGCACAAGTCCTGAAGTGTCTACAGGTGGCAGGAAGTCCCTCAAAGTGAAACTTG
AAGACACCCCATCCAAGGCACTTAGGAGTGACAATTTGAAGAGACGGACCGGGGCCAAAG
CGTCTATCCGCAAGTCCGTATTCAACAACAACAAGTAATCAGGAGGTTTATCATGGGTTT
GGGCAAGAGCTTAAAGAAAGCTATCCAGAAAGTAACTAAGCCAATCGAAGGTATTACTAA
AGGTGCTCTAGGAGCTGCTGCTGGTATGCTGGATGGTGGTATGCCAGATGTCCCACAAGC
TGCACAGACCGTTGAGCCACCAAGCAAAGGTGACGTGAACACTGATGACAGTTCTCAGAC
TGAAAGTGGCAAGAAGAAAACACGTGCTAGCGGCAAGAAAGCACTGAGTGGCGCTCGCAG
TTCTGGCGCAGGCCTAAATATCTAACAAGGAGGCGCTAAATGGCAAGCTCACAGAAACGT
GAAGGCTTCGCTGAGAATGGTGCCAAGGCGGTGTATGATGCATTGAAGAACGACCGGAAC
TCCTATGAGACCCGTGCGGAGAACTGTGCTAAGTACACAATCCCCTCGTTGTTCCCGAAG
GACTCCAATAACGCCTCTACTGACTACACAACTCCGTGGCAAGCAGTGGGCGCTCGTGGT
CTGAACAACTTGGCCTCTAAGCTCATGCTTGCTCTGTTCCCTATGAAGACTTGGATGAAG
CTGATCATCTCTGAGTTCGAAGCGAAACAATTGGAAGCACAACCAGATGAACTGGCGAAG
GTAGAAGAAGGTCTCTCTATGGTCGAGCGAAGCTTGATGAACTACATCGAGTCAAACTCA
TACCGTGTGACACTCTTTGAGACCCTCAAGCAGTTGGTCGTAGCGGGTAACGCTCTGGTG
TGCATTCCTGAACCTCAAGGTACTTACAACCCCATGAAGTTATGCCGTCTGTCTTCTTAT
GTTGTCCAACGAGACGCATTCGGCACGGTACTACAGATTGTGACCTTGGATAAGACTGCC
TATGCAGCACTTCCCGAAGACGTAATGAACTCTATGGACTCTAGTCAGGAGCATAAGGGT
GACGAGATGATTGATGTGTACACTCACATATATCTCGATGAGGAATCTGGAGACTACCTG
AAGTACGAAGAGATTGATAGCGTTGAAGTCGATGGCACAGCTGCTTCTTATCCGGTGGAC
GATTGTCCATATATCCCGGTTCGCATGGTGCGCATTGATGGAGAGTCATATAGTCGTTCG
TACTGTGAGGAATACTACGGTGACTTACGTTCACTTGAGAACCTCCAGGAAGCAATCGTC
TAGATGTCTATGATTAGTGCAAAGGTCATCGGCTTGGTTAACCCAGCTGGTATCACACAG
GTTCGTCGCTTAACGAAAGCACAGACAGGTGACTTTGTGTCTGGTCGTCCTGAAGACATC
TCGTTCCTTCAGTTAGAAAAGGATGCTGACTTCTCTGTAGCGAGAGCTGTGAGTGAACAG
ATAGAAGGCCGCTTGTCCTATGCCTTTATGTTGAACTCAGTGGTACAGCGTACTGGAGAG
CGTGTGACAGCCGAAGAGATTCGCTATGTTGCATCTGAACTGGAAGATACCCTTGGTGGT
GTCTATTCTATTCTCTCACAGGAGCTCCAGTTACATATGGTTCGTGTACTACTGAAGCAA
CTTCAAGCAACCAATCAGATTCCTGAGTTACCTAAAGAGGCCGTTGAGCTAACTATCAGT
ACCGGGATGGAAGCATTGGGGCGCGGTCAAGAGCTCGATAGGCTGGAACGTTGTATTGCC
GCGTGGTCTGCTCTTGCTCCTATGCAGGGAGACCCGGACATCAACATTGCGAACATTAAG
CTGCGCATTGCGAACGCTATTTGTATCGACACTTCTGGCATCCTTAAGACACCTGAAGAG
AAGCAACAGGAAATGGCTGAGGCCGCTCAAGGTACTGCTATGCAGAATGTTGCTGCGCCT
GCTGGTGCGGGTGCTGGTACAATGGCTACTGCAAGTCCTGATAACATGAAAGCTGCTGCT
GCACAGGCTGGCATGGCACCTAATTAATACAACTCACTAATGGGCGAGCCACTATGTCCT
CCCTTTGAGTTATACATTTTATTATCAAAGGAGATAAAATATGTCCGGTGAATCTAATGC
TGATGTTTACGCATCTTTCGGTGTGAACTCTGCGGTTGTCGGTGGGTCTACTCCCACTGA
CCACGAACAGGCCATGCATGAACTGGATGTTGCTGCCCGTGATGGCGATGATGCAATCGA
GATAGTAGGCAATGATGACCCATACGGTAATCCAGACCCGTTTGGTGAAGAAGACGATAA
TCGTACTGAGATTCGTATCTCCACTGATGGTGAACCAGAAGAGACTGAAGAGGGTGAGAT
AGATTACTCTGAAAGCACTGAAGAAGAATCCTCAGCAGAGTTTGCACCTGTGGGTGAAGT
TCCAGAAGAACTGAGTGCAGCCTCACGTCAACTCGAAGAGCATCAAGCTGGCTTCGAAGA
GATGGTCAATCAGGCTGCTGGACGTGGTCTGTCTGAAGAAACTATCGTCCCTATCCAACA
GGAGTATGGTGAAGATGGTCTGTCTGAAGAGTCCTATGAAGAACTGGCTAAGGCTGGTTA
CTCTAAGTCCTTCGTTGACTCCTACATTCGTGGACAGGGAGCACTGGTCGAACAGTACGT
TCAGTCTGTTATGGAGTATGCTGGTGGTGAAGCACAGTTCCAAACACTCTACAATCACCT

CGAAGTGTCCAACCCGGATGCTGCCGAGTCTCTGGTTAGTGCTCAGGAAAACCGTGACCT
TGCGACCGTTAAGGCTATCGTGAATCCCGCTGGTGCAAGCCGTGCGAAGACTTTCGGTAC
ACCTGCTGCCCGAAGTATTCCTAAACGTGCCGTTCCTGCTGCACCTGTACGAGCCAAACA
GTCTGGCTTTGAGTCACAGGCTGAGATGAGTGCTGCAATGTCTGACCCTCGCTACCGCTC
GGACTCTAAGTTCCGTGCGGAAGTTGACCGTAAGATGATGTACAGTAACTTCTAAAATTA
ATACGACTCACTATTGGGAGAGACATCACGTTCCCCCACTTTGAGTGATACACAATGAGA
ACCAACTCGTTTCAAGTAGTACCTCACAATTTAAAACTTTCTAAACCAACAGAAGGAGAT
TCAACATGGCTAACATGAATGGTGGACAACAGATGGGTAAAGACCAAGGTAAAGGCATGA
GCGCTGAGGACAAACTGGCTCTGTTCCTGAAAGTCTTTGGTGGCGAAGTTCTGACAGCGT
TTACCCGTACCTCCGTAACTATGAACAAACACCGTGTTCGCTCTATCCAATCTGGGAAGT
CTGCTCAGTTCCCTGTGCTGGGTCGTACAAAAGCTGCTTACCTACAACCAGGTGAGAACC
TCGATGAGAAACGTAAAGACATGAAGCACACTGAGAAGACCATTAACATTGATGGGTTAC
TTACTGCTGACGTTCAGGTTTACGACATCGAAGACGCTATGAACCACTATGACGTTCGCT
CTGAGTACACTGCACAGTTGGGTGAATCTCTTGCAATGGCTGCTGAAGGTGCTGTACTGG
CTGAAATGGCTAAGTTATGTAACCTTACAACTGCTAATAATGAGAACATTGCTGGTCTTG
GTAAGGCTCACGTACTTGAAGTCGGCGACCAGGCTACCCTGCAAGGTGACCAAGTTAAGC
TAGGTCAGGCGATTATAGCACAGTTAACTCTGGCTCGTGCTAAACTGACTGGCAACTATG
TACCATCTAGTTTCCGTGTGTTCTACACTACTCCTGATAACTACTCTGCGATTCTGGCTC
CTCTGATGCCTAACGCTGCAAACTATCAGGCTCTGATCGACCCATCTACTGGTTCTATCC
GCAACGTAATGGGCTTTGAATTAATCGAAGTTCCTCACCTTACTGAGGGTGGTGCTGGTG
ATAACCGCGCTGAAGAAGGTGTAGCACCTACTAATCAGAAACACGCATTCCCTGATACTG
CTTCTGGTGATACTCGTGTTGCTTTGGATAACGTGGTTGGCCTGTTCAATCACCGTTCTG
CCGTTGGTACTATTAAGTTGAAAGATATGGCTCTGAAACGTGCACGTCGTGCTAACGTCC
AAGCAGACCAGATTATCGCTAAGTATGCAATGGGTCACGGTGCTCTACGCCCGGAAGCAT
GTGGTGCGCTGGTTTTCAAAAAGGCTTAACTTCTCGCAAACTCGTAGGTGACACTGAGTC
TCTTACTTAAGTTGAGCAAGAGCTAACGCCACAACAGAAAGCTGCCCGTACCCGTGCGGC
AAACAAGGCTCGTAAAGAAGCTGAGTTAGCTGCTACTAAGTGACAAGTATATGAAACCCC
TTGGGTGCCTTCGCGGGTGCTTGAGGGGTTTTGTCACTACAAGGAGGATACCCTTATGCG
TTCCTATGAAACTACCCTAGAGACAGTCGAAGAGTTGGCTGCTGTCAACGAGATGTTGGC
CTCTATTGGTGAACCCCCAGTATCCACTCTGGACCGTGACACTAATGCTGATGTGGATAA
CGCACGTCGAGTTCTCAATAAGATTAACCGACCGATTTAATCGAAAGGGTGGACGTTTAA
CAATGAGGGAGGACAACAGTTGCTACCTGATGTGTTCAACGGTATGATTCCCTACATGTC
AGACTACCTGTCTGTACTCTCGGAAGGTGGAGCAACTGCCTACGTTAACCGTGGTGGATA
TGTGTTTGATCGGATGACCGGAACTGATATCTTTGAGGGCCCAGTTACTGTGACCATCAT
CAAACTCCGAGAGTTCTATGAGATGCCTGAATGCTTCCGCTCATGGATTGTCACCAAGGC
AGCCCGTCAGTTTAACAACAGGTCCTTTGGTGCACCTGAGATTCACGCAGTGCTAGCTGA
AGAGGAACAGGAAGCCAAGATCCAGTGTCACGAGTATGAACTTGACTTTGGTAACTTCAA
CATGCTTGATGGTGATGCCTTCACAGGTGGTCTACTATCTCGTTTACGATAACATTTTAA
AGGAGGTCTCTAATGGCTCTTATTTCACAATCCGTCAAGAACCTGAAGGGCTGTATCAGT
CAAGAGCCGAACATCTTAAGGTTCCCCGAACAGGGTTCCGAGCAGATTAATGGTTGGTCC
TCGGAGACTTAGGGTCTTCAGAAGCGTCCACCTTTTGTCTTCACTAAGACCATTGGAGAC
CAGATTGCCCTTGGTGCCAAACCTCTAGTTCACCTCATCAACGGTGATAGTGCCGAACAG
TATTACGTAGGGTTTACCGGACAGGGTGTTCGTCTTATCGATCTAAACGGTAAAGAGTAT
GATGTAAAGGGTGATTTGTCCTACGTTAAGGTAGAGAACCCTCGAGATGACTTAAGGATG
GTCACTGTGGCTGACTACACATTTATCGTTAATCGCAATATGGTTGTGCGCCCAGATACT
ACCCCTTTGTATACCCTTAAGGAGAATGGAGACTGCTTGATTAACATCCGCGGGGGTATG
TATGGTCGTACGTTGGCATTCACTATTAACAACACAAAGATTGCATACGAGATTGCCCAT
GGGGACGCACCAGAACATTCTAAGCAGACTGCCGCACAGTGGCTCGTTAAGAAACTTGCT
GGTCTCGCGCGTCTGAATGTTGCCTTTAAGGGCTGGACGTTCACCGAAGGGCCTGGGTAC
ATCGATGTGATTGCTCCATGTAACAGTAAGATTAACTAACTGTCCACTGAAGATGGCTAC
GCCGACCAGTTGATGAACGCAGTGATGCACACCAGCCAGTCATTCAGTCGGTTGCCCGTT
GTGGCGCCCATTGGAAACACAGTGAAGATTGTTGGGGACACCTCTAAGACATCCGATATG
TTCTAAGTTCAGTTCGACAATATGAAGAAGGTATAGAAAGAGGTTGCTGGTTGGGGAGTA
CAGAAGGGACTTAATGGAGGCACGATGCCTCATGCTCTGGTACGTCAGGCTGATGGGTCA
TTCCAGATGCAAGTTCTACCGTGGACACAACGTACCTGTGGTGACATGGACACTCACCCT
TCGCCGTCTATTGTTGACCAGAAGATTAACGATGTGGTCTTCGTCCGTAACCGCTTAGGG
TTCCTCGCGGGTGACAACATTGTGATGTCCCGTACCTCTAAGTATTTTTCATTGTTCCCT

GCATCAGTAGCTAACCTGTCCGACGATGACCCTATCGACGTGGCTGTGTCTCACAACCGA
ATCTCTATCCTGAAGTACGCCGTACCGTTGTCTGAGGAGTTGCTCCTATGGTCAGACCAA
GCACAGTTCGTGTTGTCTGCTCAAGGTATCCACTCACCTAAGTAATTAGAGTTGGACCGC
ACGACCGAACTCGATGTGTCAGACCGAGCGAGACCTTTTGGCGTTGGGCGCGGTGTGTAC
TTTGCGTCACCTCGTGCTTCCTATACGTCACTTAACCGTTATTATGCGGTACAGGATGTT
AGCTCTGTGAAGTCTGCTGAGGATATGTGTGCTCACGTTGCTAACTACATTCCGAACGGT
GTGTTCTCTATACGAGGCTCCAGCACTGAGAACTTCATCTCTGTGTTGTCTTCAAATGCT
CCAAGCAGAATCTTCCTCTATCAGTTCCTCTACTTGAACGAAGAGATAGCACAACAGTCG
TGGTCACATTGGGATCTAGGAAGTAACATTACCGTACTGGCCGGTGACTCTATTGGTGCC
ACCATGTACATTGTGTTTCGCAATCAGTCACACACTTGGATGTGTCGTGCTCACTTTACG
AAGAACAGCATCGACTTCCCTGATGAGCCGTATCGTCTGTACATCGACAATAAGGCGAAG
TATGTGATTCCTGAAGGTTAATACAATGATGACACTTACGCTACCACTGTGAAACCTGTA
GACGTCTATGGGATGAAATACTGGACCGGTAAGTTCTACATTGTGGCCTCTGAAGGACTG
GTGTCATGGTTCGAGCCTCCTCGTGGTGGTTGGCCTAATGGTGTACCTATGTTAACAATG
AGTGGGAACCGTGAGAGTGAGACAATCTACGTTGGGTTGGCTATCAGCTTCCGCTATGTG
TTTTCGAAGTTCCTCATTAAGCAGACCGCTGATGATGGCTCTATAGCTACCGATGACATT
GGTCGTTTACAGTTACGCCGAGCATGGGTGAACTACGCGGACTCTGGTGCATTCACAGTG
GAAGTGGAGAACACCTCTCGACTGTTCAGCTATGATATGGCAGGTTCTCGCTTGGGTTCC
AATGTGTTACGTGCTGATGGACTTAATGTTGGTACGGGACAGTTCCGCTTACCGGTGACT
GGTAATGCACACTTGAATGAAGTACGTATTATCTCTGAACACACCACACCACTGAACGTT
ATCGGTTGTGGTTGGGAGGGTAATTAACTTCGTCGTTCTTCTGGTATCTAATTGAAACAC
ACCCTCTGACTCACGCAGTGAACAATACGACTCACTATTGGGGAAGGGGGTTTATACTAT
AGGAGAGAAACGCTATGATTATCCTACCTACAACAGAACGTGACTTTATGTGATTCAACC
TATCAGAAGAGGACATTGCTGAGGCCAAAGCCTACGGGATTGAACCTTCACTTCCACAAG
CCTCTGAGTGTGTGACCATGAGTTTACATGGGGTTCCTTAAGCTATTGGAGGCAACTGTG
GTGACCTAGTGTGGTTCGTAACGTCTGACGAAGTGTGGAGACTATCGAGACCTGCGAAAC
GGGAGGTCCGCAAGCTCATCCTAGAGTACCGCGATACGGTGCTAAAACAGTATCCAGTAA
TGTGGAAGTATGTCTGGATAGGCAACAAGTATAACATTAGGTTCTTGAAGTCTATCGGTG
CGGTATTCCACAATGTTTTTACTGGTGATAGTAATCAATTCCAACTATTCACAATAGGAG
GCTAAATTATGTGCTGGATGGCGGCTATCCCTCTCGCTATGCAAGGTACTTCCATGATTA
TGGGAGGTATGCAAGGTGAACTAGCAAAGGCTGCACAGATTGACCAAGGTCGTCGAAAAA
ATTGGCAGATGCTGAAAGAGATGAATTATACCGATGCTGACCTTAAGTTAGAATCCCGTT
ATCTCATCGAGTCAACTGTCCAATAGATGACCCAAGCCAATATGAACCGTGTGAGAAACA
TGGGGACTATCCGTGCGGCTATCGGTGAAGGACTGCTTGAAGGTAACTCTATGGACCGCG
TACAACGCGTGACCGAGGGTGACTTCATTCGAGAGTCTCAAGGTATCACCGAGAACTACC
AACGTGACTATAGTGTCATCTTAGGGAAGCGTTTAGCTAACCAGGAGAACACCGTTAGTC
AGATTAAAGAAATCAACGAGTGCGAGCCAAAACTAAAAGGTCGCTTAGAGCAAATCATCG
ACCCGTTAGGTATGGGTATGGGTCAACTGGCTGGTATGGCAACTTCTGGTTGACTCTTCG
ATGGCAAGTAGACTAAGAAGGCTACCACTAAGGGTAAGGCCCCGGGCGTTAAGTGAGCAG
ACCAAGATGGTAAATAAAGGAGGTACAGTTTATGAGTAAGTTAGCGTAAGCATTAGGTAG
CATGAACGCTCCGTCTACTAGTCCTCTGCGTGGCACTGGTCGCATGGAGGTCAAGGCTGC
AACAGTCTATGAAGACCCTAAATATGCCGAGAAGTCTAAGCTGATTGGCACTGTAGGTAA
ACGGGCTGAGATGGGTGCGGATGCGTATATGCAGTATGACCAGCGTCAGAAGGACAAGGC
AGACGAACGCTCTAACGAGATTATTCGTAAGCTGACTCCTGAGCAACGCAGAGAGGCCAT
TAAGGACGGAACGCTGTTATATCAGGGTGACCCATACGCAATGGAAGCACTCAAGCTTAA
GACTGGACGTAACGCTGCGTATCTCGTCGATGACGAAGTGGCACAGAAGGTGAAGAACGG
TGAGTTCCTCGCTCAGCAAGAGTTGGAAGAGTTCCTTCACAGTCGACTGCAAGAAGCCTC
TTAGAACTATGCGGTACAGTTAGGTATCAATGAGACTGATGAGTTCTACCAGAAGGGTTT
CAACTCCGACATTACTGAACGTAACATTGCCCTCTATGGTGCTCACGATAACTTCCTGAG
TGACCAAGCTAAGAAAGGTGCTGTCATCAACAGTCGCGTAGAGTTAAACTCTGTGTTGAA
CGACCCGGAAATCCTACGTTCTCCGTATGCTGGTGAGTTCTTTGTGAACTACTTCGGTGC
CGGATTGACGACTGGGAGTATCCCTGGTGATGACCAAGCGTTCACCATGATTAGCCAAGG
TCTGTCTGACGTGGTACACCGTGAAGGTGGCGGGCAGTTCCTGCAACAGATTGAGAACCG
TAAGGTGAAACTCCACGGAAAAGAGACAACCTACGAGGAACCGATGGGCGCCGAACGGTG
GAACAATCTGATGGTTAAGGCTCAACATAATGAGTTCCAGTTGAACGCTAAGAAGACCGA
GGCGTCCCAACTTAATGTGAACTCTGCGTTGAACCAAGAGAACATAATCACTGGTTGGGA
ACAGTTTCAATCTATCAAGGCTGAACTGGACACATTGCAACCGGGCGAAGAGATGACACC

TGAACGTCAGGCGTTAATCAATGCACAGACTCAGCTGCCGGACAGGATGAATCGGGAGAC
CGCTGAGTTAGCCAAGCAGATGGACAAACAACAGAATTCGATCAAGAAGATGAACGTCAT
AGAAACACAGTTCCAGAAGAGACTCAATGGTCAGTACGTGTCTACTGCGTATGGCGATAT
GCCTACCAACGATAACACTGGTGAGTTCACTCATAGTGACATGGTTAACTATGCGAACAA
GAAGCTGGCCGATATTGATGCTATGAATATTCCTGATGCGCAGAAAGACCGAATGAAGTT
GGACTTCCTCAAGGCTGGCTCCGAGAAGGCTGCTTTCCGTACTGCGGTTGGTGAACTTAT
TGGTGACTCTGAGAAGGAATGGACGTCCGCTGTGATTAACGGGAAGATGCCAGAAGATGG
TGCTGTGGCACGTAACGCCCTTCGACGTGTTCGTAATGCTGACCCCGACTTGTTCGCTGC
ATTGTATCCTGATAAGGCCGAAATGTTCCTCACTATGGATATGATGGACAACCAAGGGAT
TGACCCACAGATCTTACTGGATGCAGACAAGGCTCGGCAGTCTCTAACCAAAGAGATGCA
GTATGAGGATGATAAGTATTGGGCTTCTCTAATGAACAACTCGCAGCCCCCTGAGATTAA
GTACATGCCATCACTTCTCCAGAACGGTGCCAGTAAGATTTATGACTCTGTTAAGTACCG
CACAGGTAACCCTGATATGGCATTGCAACAGGTCGATAAGTATCTCACAGATAACACTAC
GACTCTTACAGGTGATGATGTTGAAGGGGATACCATTGGTGTCTTAACGAGAAACTCTCT
GCGTGTCACTGATGACCCGGACTCTTGGAAACAAGGTAAGGACATTATAGACACAGCCGC
TAAGAAACTCGCTGAGACTAACCCTTGGGTGACTCGCAAGCAACTCACTGTGTTTGAACG
TGGTGATTCTATCTACCTGATGAACACGATCGGACAGGTAAACGTTCGGTACGACAAGCA
GTTGCTCTCTAAGATGTACCAAGAGAACCAAGCGAAGCTGGATGAGGAAGCCCGTAATAA
GGCACTCAAAGATGCCAACAAGCGAACCCTACATACACGAGCTATGAACCGCAAGCGTGA
ACGTGAAGCTAAGAAGCCTAAACGCTCTGGCAGCATGTACGATAGCGTGAGTGGCAAAGG
TATTCTGGTTACACTTACTGGTAAAGACTAATAGACCGCGATAGGAGGTTCCAAGTGGAT
AAGTATAACCCGAACGAACCGCATGAATATGTTGCGTTATTTCAACAAGCTGCTGACACA
CATGGAGTCTCCTATGACCTTCTTCGGAAGGTCGGTTGGGTACAGTCCCGCTTTAAGCCC
ACGGCTCAATGACCAACAGGTCCAAGAGATGTCATGCAGTTCACTAAGGCAACAGGTCAG
GCTTAAGGTCTCCAGAACGATGAAGACTTCACTGACCCTGGTAAGTCAATTGATGCAAGT
GCTCGTTACCTTTCAGACCTCGTTAAGAAGTACGACGGTGATGAACTTACAGCTGCCTTA
GCGTACAACCAAGGTGAAGGACGTACTGGTAAGCCTCAGTTAGATGCCTACGATTCTGGG
AACTTTGCGAGCATTGGGGACGAAGGCCGTAACTATCTGCGTTCACTGCTGGATGTTACT
AAGTCTCCTAAGAGTGGAGACCTTGAGTCATTCGAGGGTATCACCACAAAGGCTAAAGGG
ATTCCGCTTGAAGCTGCTATGAGCGGTATAGGGAAGAAAGGAAGTGTGACAACTGAGCTC
CCTGAGTCTCACGACATGTCCTTTCGAGGTAAAGAACAGGCCGCTCCCAATCAGCCGTTC
GGAAAAGATTATTGGGAAGCGAAAGGTACAACTCTGGATGAAGCAAACAAGCCTTCAACC
TTCTTTGGATTCGGTAATGCTACTGAAGCAGAACTCTCCAACTCAACCTTGGGTGTTGCC
TTCCGTGCTGGTAAGCATGACGATGGTTTCGATGTTCTTACTGGCGTACTTCAACCGACC
CGGTTTAATAGCCCCGTTTGGACGCCTGAAGAACTTGAGAAGATTAGGACTGAAGTGAAG
AACCCTGTGTACATGAACGTTGTACTTGGGGGTTCCGCTGAGAACCTTGACGAACTAATC
AAGATGGCTAACGAGAACTATGAGACTGACGTAAAGACTGCAGACGCAGGCCTAGGTGCC
AAGCTGTCCGCTGGTCTTATTGGTGCTGGTATTGACCCTCAGAACGATGTTCCAATCGCT
GGGACTACCGCTAAGGGGTTAAAGTTGGTGAACAAAGCGTTAATGGTCGGTGCTCAAGCT
GGTGCACTTAATGTTGCCTCTGAGGGCCTACGTACTTCGATTGCTGGAGGTGAGGCCCAC
TATGCTGAAGCTGCCTTAGCTGGGATGCTACTTGCTGGTGGACTGACTGCAGTGGCAGAT
GCTGTAGCTGCTGGTCTCCGTCAGTCTGGTGCTGAACAGATTGAGAACCCGTTTGCGGCT
GCACAGATGCGCTTTGAGGCCCGTGAGACTGCACGCAACACTGGTGGTTATGACGCAAGC
CCTATGCCTCCTAGTGAAGACCGAGTGTTCTCACAGCACAATGGCATTGAGTATGCACCA
CTGGAAACCGAACCGGGTGCTGTAGTAGTGAAGGATGGTTCCATTATTAGTGATACCACT
CTAGCTAACCCTATGACAGCCAAAGAGTTTGCCGAGGCGGACCCTGAACGTGCTGCTTGG
GGATTGCCGATGCGTGGCCTGAGTGAAATAGGTATGAGAACACTACGTTCCAAACATGCT
GAAATCCGTGGTCTCGCCAAGCACTTAGTGCGCTCACCTACAGGTATGGAGTCTGGCTCA
CACGGTAAGTTTGGGGCCACTGCCGCCGATATCAAAGAGCGACTGCACAGTACCAACCAG
AGAACCCACAATGACCTCTATGGTGCAATGAAGGAAGCAATGGCTGACCCTGAGTGGTCT
GTGGGTGTGTTCAAGAGTGGTGCTCAAGGTGCTCGTCAAGAAATCTATCGTCTGGCAGCT
GTGGCTATCGAGCGTCCTGAACTTCAGGCTAACTTGACCAAGGCAGAGCGTAAGGTGATG
GACATCATGAAGGAACACTTTGACCTCAAGCGTGAGATGATGGAGAACCCGTCGATGTTC
GGCAACAAGGCCAACTCTAAATTCCCTAACAGTCGCCACAAAGGGACTTACGTTCCTCAT
GTGTACTCAAGGGAAGCCAAGCAGTTGTACTCACAGGCTCTGGGAGGTACCGATGGTCTA
CAGGAAGCAATTGCAGCAAGCTGGATGACATCCTATCGTTCACGACCTGAAGTGAAAGCT
CGTGTTGATGAACACTTAGCGGAAACCTTGGGTATTGACGTTAAGGCTGTTACTTAAGAG

ATGGTTATGAAACACGCTAGTGACAAGGGATATGGTATCGCCAAGACTGATCAGTTCAAC
TCTTCCAGTGTCCTTGATGATAACATTTAGGGTATTGTTGGTGTCGTGAACAACTCATTC
CTTGAGGCCCGTAACCTGTTCGACTCTGACATGCCGGTAACTCTCCCTAACGGACAACAG
TTCTCCGTGAATGACTTACGTGACTTCGATATGAAAAGCGTCATGCCAGCCTATGACCGC
CGTGTTGATGGTGATATTGCGATCAAGGGTGGTACAGGGAAGACTACTGCTGAACTCGTA
GCTGAAATCAGTACTCTCGATGAGAAGTTTGAAGGAAAAGGCACAGTGAAGGGTGAGGTA
GGAGAACTGAAAGATACCATTAAGATTCTCACTGGCCGTGCTCGGCGTAATCACGATACG
TTGGGAGACACTATGGTTAGCGCTCTGTCCGATATGTCCTTCTTTACGCAGAACGCATAC
ATGGGCTTGCAGAACCTAACTGAAATCTCTGGTATGTTGGCAAAGGGTAACATCGGTGCG
ATGCTCCATGGTGTCCCATCACTACGTGACTTAGCGTGCCGCAACAAGCCTGTCTCTGGT
AGTGAACTTAAAGAACTCCTCTCAATGGCGTTCGGCAGAGAGTTCGACCAGTTAATTCGA
CCGTCCCGTCTGGACATCATTCAGCGTCTCCGAGAGTCCACCGATACATCAGATATAGCC
GCTAAGGTTGCTGGTACTATCAAGCATACGACTCAAGAGTTAGCTGCCCGCTCACCTCTA
ACCAAGTACCTCAATGGTAAGTCAAACTACATTCTGGATATGGCTCGACAGGGTGTGATG
GGTGATGTGGTAACTCATGCTGTTACAGGCAAAGGTGCCAACAAGTGGATTAAGGGTGAC
ATGCTGAAGTCTGCGAGTATCTCTAAGGAACAGTGGGAAGGCATTCAGTCTCTCATTAGG
GAGAAGGTAACTCGTGGTGAAGACGGTAAGTTCACCTTCAAGGATAAGCGTAGGCTGGCT
AATGATCCTCGTGCCATGGATTTATGGCGTTTAGCTGATAAGGTTGCTGACGAAACGATG
CTCCGACCTCGTAAGATTTCCATACAGGATTCCCATGCGTTTGGGGCCACCTCTAAGTTA
GTCCTGCAGTTTAAGTCGTTTGTCATCAAGTCGATGAACTCTAAGTTTATCCGCACTGGT
AACGAAGCCTTTAAGAACCATCGCGCTATGGACATGGCGTTGACCTATGCTATCTCTGGT
GGTATTGCTGGATCCTACTATGTGGCACAGGCTCACTTGAAAGCTGCTGGCCTACCTAAA
GAGCAACAGAAGGAGTACTTGAAGAAATCCCTCGACCCTAAGATGATTGCCTACGCTGCA
GCCTCCAGAAGTTCACACTTAGGGGCTCCGCTGAGTATTGCTAACTTCGGTATGGGAGCT
GCTGGTTATGACCAAGGTCTTATGGTACGCTCTACGATTCTACCTAAAGGTGAGGACAAA
CGTGAGAGCAATAAGGCAGTATCTTCCCGTGACATGGGTGATTCCATTATGGGCGCTATT
GGTGAACAGGTGCCTGCTCTCGGATTCGCCGGTGCAACTTTTGCTGCTTGTCGGAATGCT
TATGGTGTCCTTACAGTTCCGAAAAAACTGACCGAGCGTGAGATGATGAGTGGCTTAATG
AATGCTCAACGAGAGTTGATACCTAACGACCCAATCTCTCAACAGATGCTCATTCAGTTC
TATGAGGCCAACGGTGTGGATATTAAGTCTAACAAGAAGTAAACAATACGACTCACTATT
GGGGAGGCTCTCATAGCTCCCCCTTTTTATTTACCCTTTATACCTCACCAAAAGGAGGAT
ACAGATGGCTAACAAAATTTCCACTATACGTACTTATCCGCTGAACGGTTCCGTGAACTT
CACGTTTACCTTTGAGTATCTATCACGTAAGTTCGTCCTAGTGACACTTATAGGGAAAGA
CCGTAAGGAGTTGGTTCTTAATCAGGACGACCGCTTTACCACCAAGACCGAAATCAAAAC
AGGCCGTACATGGACTGCTGCGGATGGCTATGAGATAATTGAGATTCGTAGATTCACTTC
TGCAACCGACCGTCTGGTTGACTTTGCTGATGGCTCAATCCTTCGGGCATATGACCTTAA
CATCTCACAGATTCAGACACTACACGTAGATGAGGAGGCACGAGGCCTTATTGCGGATAC
TATGGGTGTCAACAATGATGGCAACCTAGATGCCCGTGGTCGTAAGGTTATGAACCTAGC
GTTTGCTACTAGTGATTATGATGCTGTCCCTCTGAAGCAGATTACAGACCGTGAGTCCTC
TGTGTGGAACGCTGTGACCAAAGCTAGTGAACATGCTCACCGTTCTATCAAGGAAGCCAA
TCTCTCACGGGACGAAGCTGACCGTGCTAAAGGTGAAGCTGACCGCTCGACCCAACAGGC
AGGTGTCTCTGCGACACAATCAGTTGAGGCCAAGAAGCAAGCTGGCCGTTCCAACTCTGA
GGCCAACCGTTATAAAGGTTATGCTGATAGAATGACAGTCAGTGTTGAAGCTGCTAAGGG
CCATGCTGAGCGTGCCAGTAAGGAAGCTAATCGTTCCCGTGCTGAGGCTAATCGTGCTGC
TAATGAAGTTACTAAGGCCGCTGCTGAGGTTTCCAAAGCTGCTGCACATGTGGCTAGTGC
TAAGAATCAGGCCGACCGTTCCAGTACTGAGGCTAATCGTGCTAAGAGTGACGCAGACCG
TGCTAAGACTGAGGCCGATAAGTTAGGCAATATCAATGAGTTCGCTGGAACTCTCGAGAA
GGTGGTGGGAGTAACACCAACTTTCAAATCGGGGATTAAACTTCGTTCTGGTGAGCTCAA
TGCCGGGAGTGCTAGCTTCACTAAGAAAAGACAGGGTGGCGATTGGAGCCAGTGGACTGG
AGCTCTGACACCAGATGTACAACATGATATAGAAGGACAAGGGAACATCGCGTATCTCCT
TGAGCGAGCTACAACTTTGAGTAAAGAGCCCTTATATGCTAAGTGCCTATATAATAACCG
TATTGGCGAAAACTTCGTTATCAAGCAGTATCTTCGAAATGCACATTTTGACTTACGCGC
CGATGGAGCTCTTATCAGCTCTTCTGGTTGGAGTATCCCAGCCGATGGTAACTTATACAT
CGAGAAGTATGGGTCGAATCTTGATACATGGGTTAATAAGCGCCTAAGTGCACACGGCTA
CAGCAAGGGTGAAGTTAACAAGATGGTCGATGGACCACTCACTACCGAACAAGGTGATGC
TCGTTATGCACGCAAGAGCTCTGGTTGGAATGAGGCATGGCAAGGCTCTGCTGGTGGTGG
TGTTTCAGTAAGCCTCTCACAGGATTTCCGCTGGAGAACTATCTGGATTCTAGTTAATGA

TGGCATGTGTTTTGTTCAGATTGGAGCTGATGCTACTTACTTCGTGGTGGTCATGGGTGG
TTGGTTGAAGTTCACAATTTCCAACAACGGGAGAACTTTCCGTAACGACCAATATCGAAA
TACAGTACCTGAGCAAATCTTAGTTCGAAACTGATAAGGACTATACAGAATGACCATGGA
CGGTCTTTACCAATGAGGAGGACACCATGTTGTCTTTAGACTTTAACAATGAAGTAGTGA
AGGCCGCTCCGATTGTAGGGACAGGTGTGGCGGATGGTGCTGCCCGTCGGTTTTTCGGAC
TGTCGCTTAACGAGTGGTTCTACGTGGCCGCAATTGCGTGCACAGTGGCTCAAATTTGTG
CCAAGGTAGTCGATGTGATTCTCAAATGGAAGAAGAAGGGTAGAGGTGTCTAACGATAAG
TCTTTAATACAATTCCTTGAGATGCTGGACACTGAGATGGCACAACGTATGCTGAAAGAC
CTTCAGGATGATGAGAAGCGCACACCTCAACTGTACAACGCAATGGGGAAACTGTTAAAG
TGCCATAAGTTGCAAGTCTCCAAGTTACAGCCAGACCGTAACATCCTGGGTGGACTCGCA
GATGGACTCGAAGAGTACCAGATCCTATTGGGTGCTGATGGTCTCACAGAAGACGAGACG
TACACGCTGAACTGATGGAATAACTCAAGACCACTACATCTAGTGGTCTTTATGATATTT
CAACACAACTCACGGTGACGCTACGTGAAATCTGAAAATACTGGAGGGTCGTTATCCTCA
AGAATTTAAAGAGCTACGGCATAATCGCTGCGTTTGCCTTGGGGATTGCCCTTGCGTATA
ACCACGGATATGACAAAGCTAGCACTAAATGGGAACAGGAGGTGCACAATGAGTACGTCA
AGAAAGCTGAGGCGACTGCGGTTAAACAGGCTGCTATCAATGAAGTCTCACAGCAATACC
AAGAAGACCTTCCGGCGCTGGAGGGTAGCACTTATAGGGTCATTAATGATTTGCGTAACG
ATGGTAGGCGGATGCGTCTCAAACTATCAGCCACCTCACGAGAACCCCAAGCTAACGGTG
GATGCCTCGTTGATGGTCGAGCCGAACTTGACAAAGAGTTTAGTAGACGTCTTATCGGGG
CAACTCAAAGAGGGGATGCGGGGATTAAGGCGCTCCAAAATACAATCTAAGAAATGGGAC
ATAAGGAGAAACACAATAGCTAACGTAAGAATCAGAACCATATACACTCTCAGCGGACGT
GCACCAGATAGTCGTGACGTATGGGATAAGAACAGTGTGGGCCTTCTAGTAGGTCGTATT
GACCTTCCTGAGTCCTCTATGGTAACAGTAGAAGGATACACTAAGGTAGAATATCAGGTG
AAACTGTCTGATGGGGCTATGGAGGTGTACCCCAACTTCTCTGCATTCCTCGCTGCTTGG
AGCGGAACCATTGAGTCTAAAGTTGGCAGTGGATATATGACTGTGTTCCTTAATAGAGCT
AAAGGTGACACATTGGGTCTAGTACAGATTGACGTACATAAATAGTGGGATGACTCTACT
CCAGCCCCTGAGCCAGAACCGTCTCCTGAGATGGACGTAGCGGCTATCACTAAGTTCTTT
ACGACCTATGTGGATAACCCTGAGTACCTCTTTGTGTGGAACGAGATGACCACGGTAGGT
GGTTCAGAACGTAACCGTCGTCTGCCGCACGAGATGTTTGGTGTTGCTCTGGTTAACTCT
ATTAAGCATATGCGCCCACAGACCCTCAAGGCTCTTCATGATGCGCTAGGTATCACAGCA
ACCACCGAGTAACATTAGGAGGCCTCAATGTCTAATCAAGCAAGCAAGAATCCGCACATA
GTGGCGCAACTTAAGGGTGACTTCGTAGCTTTCTTGTTCGGCTTATGGAAGGCTCTGAAC
TTACCTAAACCAACCAAGTGTCAGATTGATATGGCACGTACAATCGCTGATGGTGACCAC
AAGAAGTTTATCCTTCAGGCGTTTCATGGTATCGGCAAGTCCTTTATCACTTGTGCGTTC
GTTGTGTGGGTGTTATGGAGGGACCCTCAACTTAAGGTTCTCATTGTGTCTGCCTCTAAG
GAACGTGCTGATGCTAACTTCATCTTCATCAAGAACATCATTGACCTGCTATCGTTCCTC
GCTGAGCTGAAACCTCGACCCGGACAGCGTGACTCTGTGATTAGCTTTGATGTTGGCTTA
GATAAACCTGACCATTCTCCCTCTGTGAAGTCTGTAGGTATTACTGGTCAGTAGACTGGT
AGCCAGGCTGACATTATCATTGCGGATGACGTTGAGGTGCCTGGTAACAGTTCTACAAGT
TCTGCTCGTGAGAAGCTGTGGTCGCTGGTAACTGAGTTTGCTGAATTACTTAAGCCATTA
CCAACCTCTCGTGTTATCTACCTAGGGACGCCTCAAACCGAGATGACTCTCTACAAGGAA
CTTGAGGATAACAAAGGGTACAGCACAGTAATCTGGCCCGCACAGTATCCAAGCAATGAT
GCTGAGGCTCTCTATTATAGGGACCGCTCGGCTCCTATGCTTAAGGCTGAATATGATGAA
GGCTTTGAGTTACTCCGTGGACAACCTACTGACCCTGTTCGATTCGATACGGATGACCTT
CGTGAACGTGAACTCGAATATGGCAAAGCTGGTTACACTCTTCAGTTCATGCTTAACCCT
AACCTTAGTGATGCCGAGAAGTACCTGTTACGTCTCCGTGACGCTATCGTGTGCGCTGTA
GACCCTGAACGTGCCCCATTATCATACCAGTGGTTGCCAAACGGTCAGAACCGCAATGAG
GAGCTTCCTAACGTGGGCCTGAAAGGTGATGACGTCCATAGTTTCCATACGTGCTCCTCT
AGAACCGTGGAGTACCAGTCTAAGATTTTTCACATTGACCCCAGTGGTCGTGGTAAGGAT
GAGACCGGGTTTGCAGTCCTCTATTCCCTGAATGGCTGCATCTACTTCATGGAAGTCGGT
CCGTTCCGTGGAGGCTACGGTGATGCTACCCTAGAGAAGCTCGCTAAGAAGGCCAAGCAG
TTGAAGGAACAAACAGTTGTCCACGAGTCCAACTTGGGTGACGGTATGTTCGGTAAGATT
TTCTCTACTGTTCTCCTTAAGCACCACGAGGCTGCACTCGAAGAGATTCGAGCTAAGGGC
ATCAAGGAGATGCGAATCTGCGATACCATTGAACCATTGATGGGCTCCCATAAGCTAATC
ATTCGTGATGAAGTTATCCGTGAAGACTATCAGACTTCTCGTGACCTAGATGGGAAACAC
GATGTTCGCTACAGTGCGTTCTATCAGATGACCCGTATGACTCGTGAGCGAGGCGCTCTA
GCACACGATCACCGACTTGATGCTATCGCATTGGGTATCGAATGGCTATGCGAGGGGATG

CTTGTAGATAGCAAGATAGGTGAAGAAGAGATGACCCTTGAGTTCCTTGAGGCACACATG
GAGAAACAGACAATTGGTGGTGACCAGATTCATAGCCTTGATGTTGGTGGCGTGGACATG
TATTATGAGGACGAGGAAGGAGGCTCTAGCTTCATCGACTGGTAACAGCTACACAAACAG
AAGGAGGTGTGAGTAATGAGAGTGGCAATTGATAGTATAACTGGTTGCCACATCTTCCTA
GGGTGTGACACTAAAGGTGGCTACGGTAGGAGTCGTGTTAATGGCGTACATTGGATGGAC
CATAGATACGCTCTGCACGTCCACCTGAAGCGTCCCTACAAGGAAGGATGTGTGGTAACG
CATTCGTGTGATAACCCTTCGTGTGTGAACCCAGAGCACTTATCTGAGGGAACACAGAAA
GAGGACATAGAGGAGTGTAGGTCGAAAGGTAGTATGGTCCATAGTGATTCTTCAGGTAGT
TCTTCAGGTAAGCATCAAAGTCACATAGATAAGATTCAAAAGGCATTCAATAGACGACTG
AAGGCTGTACTTGAAGCTGATGGTACGGATTCTGAGGTTGCTAAGATACTTGGCGTAAAC
ATCCAGTGGGTCAAGAAGGCTCGGTCTGGTACACTGAGTATGCACATAAAGTGACTTAAT
ATTCACAATGGGCACCGTATGGAGTGAGGTGAAAAAACCTAAGGAAATCAAGGGTGTCCC
TAATACGACTCACTATTGGGAAGGGCCCCCTAAGATAACTTAAAGACACTTAAAGATTGC
ATATGCATAGTGCATACTTAGTGACTACTCTTAGTGACTACTCTTAGTGACTACTCTTAG
TGACTACTCTTCGTGACTACTCTTAGTGACTACTCTTAGTGCCTACTCTTAGTGACTACT
CTTAGAGACCCTTACAGTGAGGGATGATAGTAATAGTAATTACATCCCCACTATCAGTGT
AACCATCAAGTATAACAAGGAGGACGCTCTATGCGCTTACTATACGTGCTCAAGACACTG
GCAACGCATCGAACGACTTATAAATTTCTCGTTGTACTTGCAGGTTCCATTGGTATTAGA
TCTGGTTCTGAACACATCGGTCAACTGGAAACTCTGCTTTGTCCTATACTCACTTGTGTT
CATTAGACTACTCATTGACGCTTAGTGAGCTCGTAGTGATTATGGTCGATGTACATCTTC
CCCTATAATCACTGTAAGAAGTCAACTCTAAGAGTAACCCTTATGTGCCCTCTGCGAGCC
TACAGGGTCTCTTAGAGTCTATCAAGGTCTCTTAGTACCTCTTAGTACCTCTCAGTGTCT
CTATGGGCCTCTAGGGTGCCCTCAGGACCACCTTATGATAGACACTCCAAGTGGTTAACC
TTATGAGACTCTTGGTGTGCACCCTTTAAGAATCCTACAGAAAAATCTGAGTGAGTATCT
AATAGTACATACAAAGTGTTCCCCCCCTATGGCCTCCTTATGCTCACCCCATAGAGAGCA
CGCCTATGGTGGCTTTAGGTTTACTTTAGGTGTACTTGAGGTGTACTTGAGGTGTACTTG
AGGTGTACTTAATGCCTAACCTTATGTGTGACATTGGGTTTACTACTAGATGTAGTGCTT
AGGGGGGACTTTAGGGTACTATATGTATGCCTCTTTCTGTCTCT
>NewGenomeName_4
CGATTATGTGGTTGAAATTGATTTGATTTGATTAGTTTTGAGGAGTGATTGAGTTGAAAT
ATTCACACCTTGCCGATCTCGTGAAAACTCTCCTGCAAAACAATCCTCCCTGGCTGGCGT
TTGTATATTTTCGTTCTCGACCGTTCACGAAACCATCCGGCGTAGTTCTCCGACGGTGTT
TTCATTTAGCAACACAAAAAGAACCAAATTATACCCCATATAATAATAATTAAAACAATA
GGTTAATAACATATATTAATGTAGGTGATCTTTTATGTATAGCGTGTTCCTTAGGGTATG
TGGTAGGGTGTGTTTGATTGATATTTCTATCTGTATATTATTTAGCCAATTGCGATTGAT
TTTGGTATTTGGCTTCAATAATGTATATATGTATTTAAACCCGTGAAATATCGTGAACAC
GTGAACAGCGACGGAACAAGGATTTATTTTCAGGAAAATCATCGACTTGAAATTTAATTT
TGTGTGCACGTGGGTATGGATCGTTAGCACACAAAAAACTACTTGACTAGAACAAACGGC
GGCGATACTATGCCTCCACCAACTAACCAAACAGGAGGATAACCAATCATGGCAACTACC
AAAGCGGTTTGCGACAGCTTGCGGATGCTGGCTGATAAGCTGTCAGACGGAGAGCCATAG
GCATTCGTCATCACTGACGCAACCATTACCGATAAACTGGATGAAGATGACCAGAAGAAG
CTTAAAGCTTCATATATGCGCACGATCATGAATCGTGTTCCAGAGGTGAAGGAGATCTGA
ACGGTTTCAATTACTCGCACTGAGCCGGTCGAGGAGGCGCCAGGTTTTAAGGTGGCAATC
AACCGCGAGGCAAAGCGGCAGGTACTCACCAAAAATGATATTCCGGCACTGGAAGAGCGG
TTCGGTGAGCGAATCGCCAAACGATAGCTAAACACAATGCCAAATATCACGGATCTGGAA
GGTGAACAACTCCAGGGCGCGGCAATCGGAATTAAGCGTTATCAGGATCTGATTAAGTCA
TTCGCAACGGGGGAATAAGCCATGAAATACGAACGCAAAGAGAAGATCGCAAAGGTTATT
GTTTATGAGGTTGGACGCGTCGAACTAACACAGGAACAATGGGAGGAAATGCAGGAGGCT
CACAGACACATGATTTCACCACGCGTGATTGTCAGATCTGGAGGCAGGGAATTTAAATAC
GTGGCTGATTCTCCAGCGCTTAAAACGGCTGTAAAAGTGTAGCTTAATAGTGGCGAAGAA
TTCCAAGTTGTCAAGGAGCGCGTAGATTGAAAGGATTCGCCTTTGACGCCGCAATGTGAT
GTGTTGTTATTGATAGTCCTGGCGTGGCGCTGGTTGTGATGGGTCTTATTTTATTGGGGG
TATTTGATGAATTAGAAGCTTTTAGATCTGGCCGTGGTGTTCTTTATCGCTGCTGCTGCA
TTTTCGCTGTTGTCAATTCCTTTTTCAATCCTTGCTTTCGCCGCGTCAATGCTGAAATGA
CGGATTACATCCTGATTGGTCAATACCACTTTTTGTTAAAACTGAATTGGGGTGGTTTGA
CATAATTACCCCCTTGACACGAAATGAGGAAAATAAAATGTTTGAACTGAAACGCGTAAC
AGTTGAGATCCTGAATGATGGCCCTTATAACGGCATGCAACACTTAACCTTCCCGATCCG

CATTTAGAATTGCATTGATTATGGGATTGAAGGTGGAACGGTTGACGTTCCGCATGATGC
ACTTATTGCGGCTGGCTATGACAAAGAGAAAGGCCAATGTTTCGATATACCGGATAGCTA
TGAAAGCGACACACATTTCCCGTTCACATATGGCTAATGCCGGATTGTTGATTGAGCTTA
TAAAATGAGCAGTTGGCACAATGAGCACGTAATGCAATTCTACCGGCACAGAATAAGGAT
ACTGCTAAATAACACGAATTGCTAAAACTAAATCAGGGGCATTTGTTATAGTGTCCCTAG
TTTAGTTAAGCAAAGGAAAATCTCTAATCTTAAGCATCGAGAAGATAAACTTCGCGATCA
CATTTAAATTCTATCAGCCTGACAAGGAAGTTACCGTCACCACTAAAACGCGTGGAGAGT
GGAAAGATGGTGGAAGTACAGGATTGAAACTTGAGTATTTCGTTAATATGCACAACAACA
AATATCCAGATCATATCCCTGGCAAAGATGGGTATTACATCATGACGGCTTGTGGCCAAG
AAGGAAAACACGAGGATAACGATCTGTTAGACGCTTTGAACTATTTAAATATCAAGCCTG
TCCAGGTCAATTTAAACGGTGTTGTATTCCCTGCGTCCGTAGATGCTAAAGATTACGATT
ACATTTGCAATGTTGAAAATATGCCACTTGGCGCAACCCTGTTAGGTGTTCATCGTGAAA
ATGGCGAATGGCACGAATAGGAATCTAAACCGCGCCGCCTTGAAGTACATGACGATGTTA
TTTGCTTGCTTTTTGGTTATGCTGGTGGCTACAAGAGCGGTAATAATTACGGCCCTGAAA
TCAACATTAAGATCTGTACTAGCGTAAAAATCAAAAACACCACGAATTGCTAAAACACAA
TTTTGGGCTTGCTGTATAGTAAGCTCATACCAAACAACGAAAGGGATCGAAAAATGGAAT
TAACACGTCACGCGGTTTATGGCTTACACGTCAAAGGCGGTAGAGGTTCAATATATGCGC
ACCCGTTTCATGTTAACGAGCGCGGCCAGGTTGATATCTCAATGCATGTTGGCCAACTGG
TTAAAGGTAGTGAGTCCGACAAATTCAGACCGGATTTAATTGAATATGATCACGCGTACC
GCCACGTCCACTGGCTATATGGTTATAAGTTTACAATGCTGGATAGCCATGTTATTGACA
CAAGCAAGTCAACAAAACGAATCTATAAGCGCATGGCTCGCAAAATCTTGAAGCAGAAAG
CGCAATAGCTCGAAATGTCAAGCGGTCATGTGTGCTGATTGGTACAATGACCGCATGAAC
CAACAAGAGAGGAGAGAAAAATGTCATAATAATCAATTAAGCAGTTTCTGAAATACGGCT
CTATGATGGTGCTCGCTACCGTTGTGGTGGCTGGCATCGCGTATAGCATTATTGAGTTTG
CGGGGTAATTGATTTTTACGATTAACTAGTGGGGTAGGGATTATGCTAATCCCCATGTTG
CGGCAAAACAGCGTTTCGTTATTTCCGACCTAGACGGCACATTGTCAAATGGTGAACTCT
GCTTGCACCTGACGCCACCAGTTGATTTGCATCTAACAGATAGCTGGAGTGAGTTTAATC
ACGCCGCAACGGATGATTTACCAGTCAAGGATAACATAGCCGTTCGCAATGCCATGTTTG
ATGCCGGATACCTAGTTATCATCCTAACTGGTCGGAGTGATGAAGTTGAGGCAGAAACGC
GAGCATGGTTAGCTGAAAACGGCGTTTCCTTCGATTGGCTGCTTATGCGCCGCGCGGATC
TCAACCGGGAAGATACCGTGATCAAAGAGGAAGTTTTTCGCACTATCGGACTTGATCGCA
TTTTCGCATGTTAGGATGACTCACCAAAGGTTATTTCACACCTGCGCGGCCTGGGCTTGA
ATGTTTACCAGGTGATTGTATACGGCGAGCCGTGACACGATCACTTAAAATCTGACGGGG
TGGAGGAATTGAGCAATTACAGAAAAATGAATTGAAGATGGGCATGGCGATACTGTGCTG
ATTTACAAAAAAGGTTAATCAGTGTGGATTGATTGCAAACATTGGGATGATACAAATTTG
CAGTTTCGCGGAATCTTTGACAAGAAAGGATCGTTGGAGTTAGCGGAGCCCATCAAGCAG
ATTGCGGAAGAATTACCAGAATAGAGGGTTTATTATGTTGACTTTTATCTTCATCGCTAT
CGTTTTACTTCACTATGTTGGCGGCGCTTACCTGATGCCAGCGCTACTAAAAGACGCTGA
CGCTGATGATAAAGATAGTTGGAAGCCTATTGTTTTGTGGCCTGGCTATATGATCGTGCC
GATTTACGAACTAATCCGCGATGGTGAGTTTAAGTGGTAAATAGCACTTTTTGCTAAAAC
TTGCTAAGGCACAGTTGTTAAAGTGTGCCTTGTCGAGACAAGCAAGGAGAATGACACATG
AAACATTAGAGGTGCGGCAGTCTTATTTATCGTGACGATGGGGTTAATATTATTGTTGCA
TATGATGACACGATGGAATTTTTTGAATCCATTTACACAAGGCGCGTTTTTAATTCAAAG
GTTGATGCGGGATTCATGAAAGAGTGCGAGCCACCAAAAGCGAAGCCGTTTTATAAAAAT
GTTGATTTCTGGCATAACGCCGTATTGATCACAATTCTGTTAATTCGATCTATGGTGTTT
ATTTTCTTATGATGAATGATAAGCTTAAAGAGACTGAAAAAAAGATCAGCTATCTGCGTG
TGCTTGCCGATAGACTGGAAAACGGCAAAGTTGGATTAAATGATATTACTATTGAAGCGC
GTAATATTTGTTGGATCGTTGAAGAAGAAACATTAACACTATAAACTGTTAAGGCGGTAA
ATAATGGCAAAAATTATCATCCTTAATGCGCCTCCTGGTGCTGGGAAAGTCACTATCGGC
TAGATGGTTTCTTTCTATTCCCCGGGTGATCAATGCCGAATCATTAGCTTTAAGGAGCCA
ATGTTTAATATTGCATTTGCTACTCTGGGTAAAGAGAACTATCAGCGTTTCATGTTGGCT
TATAGTGACAGAGGGAAAAAAGAAAGGCCGCAAACATTCCTGATGGGTATGTCACCGCGT
GAGTTCATGATCTGGATCAGCGAGGATGTAATCAAACCTAAATTCGGCAAGGATTATTTC
GGCGTAAGATTTAACTAGAAAGTAAAAGAGTGTGATGCTTCGGTTGTTTGCACTGATGTT
GGTTTTCCATATGAAATTATCGCTCTCATCAATTCCGGTAATGAGTTGAAGCTATGCCGC
TTGCATCGCGACGGGATCACTTTTGCAGGCGATAGCCGCAACTACATTCGAATCAACCCC
TATTACCATAAAAACGGATATAGGGAGCACGATTTCTATTTGACCGATAACAAGCCAATG

GGAACCGTTAACACGACTATCAGCATGTTCTTAAAATAGCACGAATTTCTAAAAATTGCG
CAAGGCCATTTGATATAGTGGCCTTATTCAAGAGAAGCAACCAATCAGTGGAATAAGCGA
TGCAGATTAAATGAACATAATCAAACACCATGAATCTGACAGTTGGCGCGATTTACCATA
GGGAAGAAGGTGTAAACGGGTTTACCCCAACTGTTACGCATGAATACGGCGGTCAATTCT
TCTTAAACGGTAAAGACGGATTAGCTGAAGATGTTGGCTCTTTCGAGCTTGCAGAGTTTG
AGATCATGGAGGAATAAAATGTTCTGGATTCATTCGCCGCAAGGATTGCTTTATGTGCCT
GGCGTTGAATTGGAAGAACGACCCGACGGCGAGACGGTAAACATCATTACGAAGGATACA
AAAGGCAATACGCTTAAAATGTACGTTTTTAACACTCCGGCTAAGGTTGATAAAGATGAT
TACGATGTTGTTAATATTTATCCTGCGTGTCATAATTGGCAATATTCTGATGGTCGCTGA
TTGTGTTACGTTTGGTGGCACTGTTTTCGGTATGACTGGTTATTACGTTGGGTATAAAAG
GCATAAATTAGATTAATGGAAAAATGCTGGTACTCGCATTCGTAAAGATTGCTAAAAGAT
GGATTGATTCAGCTAAAACTCCTTTCACTTCTAGCGTGTACGGTTACAAGAATGCTCGCG
CATGGGGTCGCATCATTGCTGATAGCTATGAATCCGAAATGCTGGAGGATTACGCATGCA
TGATTGGTACGACCCTGAATGTAGGGGGCCAGGCTGAATATGATTGTTGCGCAGAAGAAG
AAATTTATTGTTGGTAAGGAGAATTAACATGATTGCTAAAAATAACCTTCACAACCTTTT
AACTACTGGTAATGATTATCGGGTACTGAATAGCACAATAGACCGTTATTTTATAGAATG
TGATATTGGTGATCCAATGTGGTTGCGTAAGGATTTTTTCGAAATCGAACACCTAAGCAA
TGCAAAGCGGATCGCGGATGAAGCCAGCGCATTAGAAGAGCGTGAACGAATTCGTGAAAT
AGTAGCGGGGCAGGTTAGAAATTCTTGCTTTCCTGATGGCGATATCTTAAATTCAATGCG
CGGTGAAATGACGGTTGGAATTCAAGATCGCGTAATTGAAAAGTCAGCTGATTATGTAAT
TTAGAATGCGTAATTCACTGCGATGATTGACTCAGAGCATAACGAATACGTCAAAGTATT
AGAGCAACAGCTTGAAGTGCTGCGAGTGGCGGTAGGTGAAAATATTGTGACTCATGCTAA
GGTTGTAAGAGCGTTGGCTGATGCTTTCCTTGACGGTTACCCAGGAATGAAGTGCAAAAA
GCACTTTTTGTTAAAACTTCCGCGAGGGCATTTGTTAAGGTGTCCTCATTGAAGCGAAAC
ATGGAGATTTAGAAAATGCCTGTATACACTGGAAAGATGTTTGTAAGAGAAAACCCTATT
TACGGCGGCACTTATGTGCAAATCATTGATGAAGATCGCGTAATTCCGGCTGAAGATGAT
AACTAGGAATATTACGAAGTAGAAGCCGATAAACGGGATAACGACGTTATCTTTTTTGTT
GATATTAGCCGCATCAAGTTTTATGCAGTGGAGGAGGAAGAATGATCACAATTAACCTGT
CAGAAGAACAAGCGAAATCATTATTGCGCGGCCCTGGCTGGCGAATGGGTAGCGGTAGCG
AGGCGGTTGTTAACATAAAAGAGGCAGTAGCAAGAGAGCTTTCTGCATGGCTTGAGCATA
AACTGATGCCAGCCTCAACAAGCGCGGCGGAGTTGGCTGTGTGGAGGCATGAAAACGGCC
TTAAAGAAGTGGATGTTGAGAAGTGGAAGCGTAAAAACGAGGTGTTAGCATGATTTACAC
TCACTCTTACGGAGTTGGCAAGGTTGGCAACAATCAGATCCGAAAAATTCACGACAGGGT
CGATGAAGCCATTTCACAGCAACGCGTTTCAGGTGGAGGGGTTCTGGCGTTTGAGTGAGT
GGAAAACTTAGAAAGTGAATGGATGGATCGCGAGCATATAAAGGTTCTGATTGGTCATGA
GGTGTCATGCCTGATCGACGATCTCACAACGCACTCGCCAGGGTCGCCGAGAAACTATGA
GCCACGCTCAACGCTATGGGATTGCTGGAAGGTCAAAACGCTAAGCAACAGCCGTGATAT
TCTGGACGGAAAATTTAACGAAAATTGATAGCTTTTAAAGCGCCACAATCGCATTCTAAG
AGGCGCATTTTTTGGAAAGGTGATTCTAGGCCTGAATACATAAAAGCGTCGCAGTGGTTC
GCACAGAAGCTAGAGGAAGCGTTAGAGCACGGCGACACTGACACGGAGTTGCACTACTTT
GATATGTTTCAACTTTGGAAATCAAGAGGACCGTAAAATCTGAGGACTTAATGAGGCTGA
ATATAACATTGTTAAACGTGCTGCAAAAGAGTGCATGAAGGAACTGAAAGTTGAAGTGAC
AAGCGGCAACAAATACGACCATATTGCGGCTGCAATTATCACCAAACCTCACACGCCAAT
TTCTACCCCAATCACTCGAACAAAGTTTGTATGGCTTGCTGGCTATGTTGCTGGTCGATG
GGGTAAACCTGGCGAGTATGACTAATAAGCACTTGAATCAAAGAAGGTTGACGGCTTGTT
GTCTGATTGGTAGATTAAACGCCGTGTACATAAGAGACTCAATAGAGCAGAGCGTGGCGC
GTTAGCCGTAAATCCTAATCGGATGGGATTGCCTTGCCAATGACCGTCGCGGGAGCCGTA
ACCCGCAATCACTCAGGAGATTATTTTATGAAATCCACATTGCTTTTCAAAAAAAGAGTT
TTGCTCCCTCGTAGCGAACTTCTTGTTAATCGCGTGATGGTTTCAGAAGAAGATTTCGAT
ATTGATGTTCAAACGAATGGTGTCATAATCTCAAACAAAGAAGATACAAAGGCGTTTTCA
TTCCCCAAACACAACATCATCGTGATTATCGCAGTGGAAGTAAATAACGCACAATAAAGA
GATTGCAATGGTAACTATCATCATTGAACGTGACCGCAAGAAAACGCGATACAAAAAGCG
TTGTGCAAAAGGCGAGGCGGTATTAGGCAGGAGCGGTGATTTTATGGTGAAATTGCAAGA
GGATAAGCCGGATGTTGTTCAAGGTTAATTTAACTATCAGGCGCATGGGTCGCAATTGCG
GATCATGTAAGCAAGAATTTGAAACGGTTGTTACAGCATGCAGGGCTGAAATGGCTGTAA
GATTTGCTAATCAATATTCTGGAGCAGATTCAGAAACGCACGAATTTTCAATCTATTATG
TGAGGGCTATATAATGCGGATGTTTATTACTGGTTTTGGTTTTTGTGCGCTGCTTTGTTT

TGTTCTTTACCATTGCTTTTCGTGGTTTGCGCTTGGAAGAATTAAAGAAAACGATCTTGT
GATCGCGTCTTACAAAAACAAAAGCGATGTTTGGGAAATTACACGAAATTACGAAAAGAT
TGCTGATGAAATTGCATTCAGTCGCAAGTTTGGCAAGCCTGGAAGCATCAAGTTTATTGA
TTGATTCAACGCAAAAAGATAACCCGCCTTGTGCGGGTTTGTTTGTGCCTGCAATTTAGC
TATTATCACGCCTCAACAGGAGGATTTTATTATGGCTAAAAAAACAGAAGCGCCAGCGGC
GGAAAGTCTAAACTTTAAAACGTTATGAAATAAGCGTTCTTCCGATCTGGTAGGTAGTGA
GATTGAAAAACGCGCAACGCTAACGCCGGCACAAGTCATGAACCTGATTGTTCATTATTT
TGAGTGGGCGGAGCTAAACGCCATTAAAGCAGGTGAAACGGCATCATTTCAGGGTCGAGT
GTATCAGGATACGATTCACAAGCCGCGTATTTTTACATGGGAAGGGTTAAAGCTGTTTTG
CGGTTTTTCCGGCACAGCACTAGCAAAGTGGAAACTTAAACCAGGCTATGATTTTGTTAT
TAAGTTTGCGGCATCGGTAATAAAAGAACAAAAATACCAGTCAGCTGCAAACGGAATGAT
TAACAGCACCATGATCGCAAAGGAGCTTCGCATTGATAAAGGCGACACGATCAATAATAG
CGCCAACGCAAAAGATCCAGACAATACATAACAAGCGATGAGAAACGCAGTCGCAAACGT
CCTAAGCAAGATTTAAAGGTGAATGCTATGTTGATTTGGGAAGATTTGACAAAGCTTGAA
AAGCTGGCTACTAAGGAATTAAGCACACACGATTTTGATACTTTCGTTAAAAGCTGGTTC
CCGATCCAGCAGGGCGAAAAAATGATTCCTAACTGGCATCACCTTTATATTGCAAGGGCA
ATTGATGAAATTATTGAAGGTGTGCGCAAAGATACCATTTTTAACCTAACGCCAGGCTCA
GGCAAAACCGAATTGCGTTCGATCCACTTTCCGCCTTATTCATATTTGAAATTGAACAAC
GTGCGAAATGGGAATATTTCTTTTGCTGATACACTAGTTAAACGTAACTGCAAGCGTGTG
CGAGATCTGGTTAACTCGCGAGAATGGCAAGAGCTTCACCCCGCAAAAACTGGTACAAGC
AAGGATGATGAATTCCAGAACCTTAATGACGCTGGCAAAGTGCGCCTGGTAACGATTAGT
AATTCGATGGGAGGTCAGAATACTTGTAGCCGTGGCGGTTACATCACGTCTGGCGTTTAT
TCCGGTTGTGTGACGCTTGATGATCCAGAAAAGCCGGATGATATGTTTTTGAAGGTGAAG
CTTAAGTGCGGTCAAATGATCGCAAAGAACAACATGCGATCACGTCGCGCACATTCAGAA
ACGCCGATTATCGTTATTCAGCAGCGTCTACACGCGCAGGATATGATCTGGTTTCTGAAG
AATGGCGGAATGGGTATTGAATTTCGTCAGATATCAATTCCGGCGATGGTTACGGAGGAA
TACGGGATATCGCTTCCTGCTTGGTTACAGCCTCATTTTGAAGAAGATGTGCTTTCGTCT
GAATCCCTCGTTATTGACGGCGTGAAGTATTAGTCTTTTTGGCCTAGCAAGGAAAGCATT
CATGATCTTAAAACGCTGCGCGATGCCGATTTTTACAGGTTCCTTTCTCATTATCAGCAA
GAGCCAATTGCTCTTGGCGGTACCGCAATTAACGTTGGATCGCTCCAGTATTACGGATCC
GGAGAAAAATCAACGATGCCAAAACCGGATCGCCTTGATTATACGTTTATTACAGCAGCT
ACCGCTCAGAAAGCACGTGAGCTAAACGACTACAGCGTTTCATGTTATTGGGGCATGTTC
AAAGGCCGTATAAATTTTATTGACGCTGTGCGCGGAAGATGGGAAGCGCCAATGTTAGAG
AGGCAGTTTAAAGTGTTTGTTAAACAGTGCTGGAACCGAAAGAAAGAATGCGGAAACTTG
GGTGAAATTTACGTGGAACATAAAGTGAGCGGTACACGTTTGGTCCAGAATTGCCGCAAA
CCATTTCCGATAGAAATTACGCCAGTGCAACGCGGTAAAGACAAAGTGACGCGCTGCATG
GACGCACAGCCAGTGATCTAGAATGGTTATGTTGTATTGCCTGAATCGCATCACACGCTT
GCTGAATTTATGGCAGAGGCGGCGGCATTCACTTGTGATGATAGCCATCCTCATGTTGAT
ATTATGGGTAACCTATTTGATGCGTATAATATTGAGCTTAACCTTGCTGATAATGCGGTT
GATAGAATGAAGCGTTTACCTGGAATAGCGAAGCGTTAATGTATAAAATCAAAGGGTGGG
ATATTCCGGCCTTTTTATTATGGGGGTATTTATGAGCGACAAGGTAAAGGCCATTACTAA
AGATGATGGTTACGACGAAATTTTCGGATCTAACGATGGCACGTATAGGCCAAAGGCTTT
TTAAATGCAAAGATCGGCATTTAAAGCGTTATCGCAAGTTTATGAAGAAGATGGAATGGC
GCGGCGCATTGTCGATGTTATTCCCCAAGAAATGGTAACACTTGGTTTTGAGGTGGACGG
CGTGACAAATGAAAAGTCGTTTAAAACTTGTTGGGATAAATTGCGATTAAACGCTAAAAT
TATTGATGCGCTTTCATGATCGCGTTTATTTGTCGGTTCTGCAATGCTGGCTGTTGTTTC
TGATAACAAAATGCTGAATTCAACTGTAAAGCCTGGTGCACAACTTGAAGATATTCGCGT
ATATGATCGCTATCAGATCACGACTCATGCACGCAAGACTAATGCGTGCAGGGTTCGTTA
CGGTGAGCCAAAACTGTATAAGATCTCACCAGGCGGAGAGATACCGGAATTCTTTGATCA
CTACTCGCGTATTTGCATCATTGATGGTGAGCGCGTATCGAATGAAAAACGCAGTCAAAA
TCATGGTTGGATCGCAACTCTGTTAAACTAGCGTTTAATTGAGGCGATGGTTGATTAGAA
CCACTTCCACGTAATAGCAACGCAATTGCTACGCCGAAGACAACAAGAAGTTTTGAAAGC
GCGTGATCTGGCTTTGATGTGCGACGATGAAGAAAGACGCTATGCGGCGCGTTTGCGTTT
GGCTCAGGTTGATGGCGAAAGCGGAGTTGGTAAGGCAATTGATATTGAGGCCACCGATGA
GGAATATGAGGTTCTGAATTCTGACGGTTCCGGCGTTCCAGAGTTCTTACAGGAAAGGAT
TTATCGAATTGTCGCACTAACTGTCATTCATGACATCACCCTCAAAACTATGAACACAGG
CGGCGTTTGAGCGAGTCAAAACACGGCGCTAGAGACTTTCTATACGTTGATTGATCGCAA

GCGCGTAGAGTATTACAAGCCGATTCTTGAATTCCTTTTGCCGTTCATGAACTCAGAAAC
GGAATGGTCTATTGAATGCGAACCGTTAAGCGTTCAGAGTGATAAGGACAAAGCTGAAAT
TACGGATAAGAACGTTGAATCTGATGTTAAATTGAAAGCTGAACACGCAATTACTCTCAA
AGACACATGAGACACTTTGCGATCCATTTGTCCAGATCTTAAAATTATGGACAATGACAA
CATTGAATTGCCAGAACCGGAAGATTTAGATCCCGGGCCAGGGCAGGAGGGAGGATTGAA
TAAATGATGTTTCCTGATGTGACAGAGAGCGACGTTGAAAGGGCGCTTGATGATCTGGCA
GTTGATATCACTGAAAACAATGCTGATGTATAGGCCGCAATTGCCGGCTTTAGGTCGCTG
GTTTACGGGTTCGCCGTTAGTACATACAAATACCACACTCGTCAATTTATCAGAATGGCA
AAGCAAACAGGAGGAGCAAATAATCGATCCGTTATCATTCTGATTGGTGGCGTTCCCGGT
TGCATGAATGAACGTTGGTTCAATTCAAAATACAAAACCTGGCGATCACTTATTGAGGCA
TCCGTAAGGAAATTTGCGTATAATGTGCCTGATGATTGGAAAGTGCAACGGCGTTTAAAT
GAGTTACGGGAGCAAGCAGAAACAAGAGCGCAGAGCCGAAAGCGCCTAAGAAAATATAAG
GCGTGGGCTAAAGATAGGGCGACTGGTTATGTTGGTTTGTGGTATTCCGTGTTAATATAT
CAACGCGTGGTTGATACCGGAGTTACCCATTATATTTGGCGAACTCAGGAAGAGAAGAGG
GTTCGACCTGAACACGCTAAATTGGACATGAAACGGAACTCATTGGCAATAAAGCACATA
TTCCCAGGGCACGAATATAATTGTCGTTGTTGGGCGCTACCGGATTGGGAAACATAGGAA
GAGGTATATTTATGAAGAAGGTGCAACGTTTTGAAAGCGTTAGAGTAAAGGCACATTTTG
ATGCACACGGCTTTTTAGTGGATCGGCCTGTTGTGGCGCGAATTGGTTTTCAGATTTATC
AAACGCCGTAAGGCGAGCGTAGTGAAGTTAGGCGAGCAAGTGAATTGTTTAAAGCTGATA
GCCTGGCGACTTACGGAGGGAAGCCAATCACAGTAGGTCACGTCACAGTAACGCCAGAAA
ATGCGCGTGATGTTGTAGTTGGTACTTGTTTAGGCGCTGGCATTCCAAATGGGATCGGAG
TTGAATCACCTTTGAATATTTACGCAAAGGATGCAATTGAAAGCGCCAAGAAAAAACAAA
CGGCTGAAATTAGCGTTGGTTATACGTCGATTGATATTGGTAAGCCTGGTTATGGATCTA
ATCAAAATGGTGAATTCATCTTTGAAGAAGATCTAAAAGATGATGAACAGCCGCCTGAAG
ATTGGGTAAAATTTGATGCGCTGCATACAAGCATTAGTGTTAACCATATTGCGTTGGTGT
TTAAGGGTGCCTCTGGCATTGCGAAAGTTAATCGTCATAGCGAGCAAGAATTCCGGAATG
ATATCGACGTTACATTAAATAAAGAGGACGAGGTTATGACCGTTAAAATTAAACTTGATA
GTGCTGTTGTATTTGACGTACGAAAAGAAGTTGCTACTTTTGTCGAAACCTAGAAAACAG
ACGCTACAGCGGCAAAACCTAAAGCCGATAACCTGGAAGCAGAGCGCGATGCTCTAAAGG
CGAAAGTTGACGGCGTTGCGGCGCAGATTGAAGAAGCCGTGAAAAAAGCAACAACTGACG
CTGATGAACATGCCGCACTGGTAGCTACCGCTTCAGAAGTAGACGTAAAATGCGACGGCC
TAACAGCCAAAGAAATTTAGATTGCATATGCGAAAGAGGTCAGGGGTAATGACATTAGCG
CAAAGGCTGATGCATATATTGATACCGCTTTTGATATCGCAAGAAAATCTGATAAAATGG
CTACTCAACGTATCGCATTAGAAGGCGATGCAGCTAAAAACAATCAGGATACAGCGGAAT
CCGCTCTTGATCCTTCCGCACGTCTGAATAAAGCTAAGTAAGGAGAAAATATTATGGCTC
AGATTTCAGCAAGTTATCATGTTGCGATGGCTCGCGCCTTGCCTGGTCAAGTTTCTGACA
CCTCCGCGTATAACATCGCTGGCGCTTGTGTTCTTGATGGTGATGCCGGATCAAAAATTT
ATGTTGGAGTTGGTGTTCAACATGGCGGAGTTATCGCAAGCGGTGAGAAATTAATTAAGG
CTATGGCGGAGTCTGGTAATACTTATGGTGTGGCTATTCGATCGAACTTCCAGACCACAT
CTAAAGATGGTCGCATGGTTTATGAGGCTGGTAGCGGCATTAACGTAATGACTAAAGGGC
TCGGTTGGATGGTTTCTGTAGCATCAGAAGCGCCAGCATTTAAAACATCTATCAAAATTG
ATAATTCAGAACAGGTCGATTTAACGTCTGGATCAATTGAAACTACTTGGATCGCAACTG
GAGACTTTACGGAATCCTAAGGTCTAAAACTCGTTGAGGTTCTACTGGTATAATAATAAG
GCCGGATTATTCCGGCCCTTTATATATGGTGGTTTTATGTCTGATTACGGTTCTATAATA
GGTATAGTTGAAAATGGGTGCTTGGTAGATTCAAGCCTTTATAAGATAGTTGGCTCGCCT
TTTTTTGATGTTTCAGCCGTGATAGGTAAGGCCGTATGTGTTGATCGCATAGCTTGCGGT
TACAAAATAATCAGCCACAATTTTAATGAAAAATCAAAGCATTACGGAATCGTTACGCGT
GATTATATGGATTTTTGCAAAGAATCTTACGACGCCGGAGATCCGGTTAACGTAAGAACT
CAAGGTCGAATTGGGGTAATCACTCAGGACGAACTAGATATTTCATGGTATAAAGATCCT
GTTTTGGGTAAAGACAATGGCTATACCTCAACAAGTGGATTTCCTACCAGTTGGTTTGGT
ACTGGCGAATACGTAAGATATTCGCACGGATATATCTCACTATCAGTTCAGTTGTAGCGT
GCTAACACAAGAAACACTTGAACGTTTATCAAAAAATGCGATATTACAGCCATTATTATG
ACGGCTTTTCTATGAGGGGGAATATTGTGTCTAAAGAAATGAAATATGATGACTTTGAAG
CCAACGTGATCGACAACCATATGCAGTTACGCGGCGCTAAAAACGACGCATCTGATATGG
GTATTTGGACGTCTCAAGAGCGTCATAAACTTAAGGCTCAGGCCTATGAGAAAGTATAAC
CGACAGGTTCCGAGCCGCTTGTATTCCCTGTGAGGTCGGAACTTTTTGATACAGATAATA
CCTTTGAATATCATACCTTGGACAAAGTAGGACACGCAAAAATTATCGCTGATTCCACTG

ATGATCTGTCAACTCTTGACGCGCTGATGACTTCTGAATTCGGTAAAGTGTTCCGCCTTG
GCAATGCGTTCCTTATTTCCTTTGACGATACCAAAGCAGGTCAACGCACCGGCAAGATCC
TCCCAACCCGGAAGGCCAACGCCGCACAGACTGTGCGTGATCAGCTTGTAAATCACCTGG
TTTTCTAAGGTTCCAAACCGCATAAAATTATCAGCGTGCTTGAACTTCCAAACCTGACAA
CTTTCAACTCCGCAGGTTGGAACAACGCCGCAGGGCCTGGTAACAAGCTGGAAACCGCAC
AGGATGAATTAGAGCATGCGATTGAAAAAATCGAAACGTTGACAAACGAGCAGCACCGCG
CTAATATGATCCTGATTCCGCAGTCAATGCGTAAAGTTCTGATGGTTCGGATGCCGGAAA
CCACGATGAGCTATCTGGATTATGTTAAGCAGCAGAATGGCGGTGACACTATCGAATCCA
TGTCCGAGCTTGAGGATATAGACGGTGCAGGCACTAAAGCGGCGCTGGTTTACGAAAAAG
ATCCGATGTACATGAGCAATGAGATTCCAGAAGCGTTTAACATGCTTACCGCTTAACCTA
AAGACCTGCATTTCAAAGTGCCTTGCACCTTTCAATGTACTGGCCTGGCGATTTATCGTT
CGTTGACGCTTGTTCTGATTAAAGGTCTTGTAGTAGGCTGATAATTCAATTAATATAGGG
AATCGTTAAAGGTTCCCCTTTTGATATCTGGAGAAAAGCAAATGGCTAAAGATAAAGAAC
TTACACTGGTAAACACTGTCGTTGCACTGATTATTATAGATCGGGTTGACGTTATGCCAC
GTAAAGAAATTACTGTTTCGGAAAGCGTGTTAGAACGGCAGCGTCCAAAATCGCTGCGTG
CCCAGGGCAAGCTAACCGTAAAAGATAACAGCGAGCTAAACGATAAGATCATTGAAGCGT
TCAACGAAAACCGCAAGCCGAACCCATCCGAAGGCAAAAGCAAGGCACATCTTGAGGAAG
GCGGCGAGGATTAATTAGAAAGGGCGCTCATGTAGCGCCTTTTTTATTATGCAGAATACA
CCAGGAATGATATTGAGATCCTAGAGCAAATTTATAAAATCGAGACAGCATTCAAGAAAG
TAGATCCTGAATTGATTCATGCATGGATTGAACAGGCTAACGCTTTCGTTTGCGAAAAGC
ATTTGAAAGACAAATATTTCCGCGCTGGTGCTCTTGACACTTTGCACCTTATGACTCTTG
ACGGAGCTCTGAAACGGGAAAGTTAGAGCGTAGAAAGCGACTCGCACCTTATAGCGTCAT
TTTCCCTGACTGGTAAGATTATGCAAACGTTCAGCAAAGTTAGCGACCACACAAGCTGTA
ACACGTTGCGCCAAACTCCGTGCGGTAACATGTACGAAGTTCACAACAAGAAAGAAGAGG
GCGGATTTGGCCTTACAACTGCATTTCATCGGAGGTGCTCACGATGAATTATGAACAAAT
CAGATTTATGGCATCGGCTGGAATTAATTTTTTTAGCGATGGAATGGGTGAATTTGATTG
CATTACTCAACTTGGAAGCGTTGACATTGTTGGCGTTATTGAGCTTGAAAAACCCGAAAT
AAAGAGGAAGATTAAAGGTCTTGTGAGAGCACCACGGACGCGAGAAGTTGACGGAGAGCT
AATTAGAGTAACAGACAAGTTAGGCGTTTTTAATGCTGAGGTAGAGCTTAAAAACGGATA
TCAGATTGATAAAGACAGCGAGCGTTATGTTATGTTTGAAACAAGACCAATCATAACAAC
AAGCATTACCGTTGCTTACCGTCCAATAATGCGGATGGTTGCGGGTCATGGCTGATTATT
CGATCCGAGAATTTCTTTGAACTTTTGACAAATGGATCAAGTAAGTAGAAAGGGGATTGT
ATGACGTGATTCAGATTTATGGTGAAAAGGTTCACGGCGCATTAGTGGATATTGCTCCAG
TGGACACAGGACGATTAAAAGCCAATATGCCGATCACACCAAGCAAGCCGCCTCTTTTCG
CGCTCAATCAATATGATCCTGATGGTGAAAAAATAAAGGCGGAAGTTCGCAGGACGCTTT
ATGCTTAATTGCATGGCGGCGGCGCTATTAAATCAAGATACTGTTCAAATATGCTGATTT
ACGCCAATGCTCTTGAATATGGACATTCGAAGCAAGCGCCAGCGGGGGTATTTGGTATCG
TTGCGATCCGGTTGCGCTCTTACATGACGGAAGCGATAAGGGAGGCGAGAAAGAAAAATG
CACTATGAGTTATCTGTTGCGGCAAGAATGGCGCTGTCTCAAGAATATGAAAGTGCTTAT
ATGAACGATTATGAAAACGTTGAATTTACGCCCCCAAAAGTCGGTGGCATTTGGCTGTAA
TATAACTACAAAGAAGCAGATACAATAATACATGATCTTAAAAGGAAATGCATTAGCTAT
ATTGGAATGGTTCAGATCGGAATTGAATTCCCACCAGGATCAGGAATTGATAAGGCTCGC
AATCTGGCAAAAAAATATTGCTGATTTCTTTGAAGATGGTAAAATGCTTTAGAATGGCTAT
ATTTCAGAAGATCCAAAAGGGCATCAAGTTCAGAAATCAGAATCAAGCTGCTTTTTTCCA
GTGCGTTTTTATGTTCGTTATGATGGTTAATAAAGGGGGCTATTATGCACTTACCTATCG
GAGGACAGATTTATATTGAGAAAACTCGCGCACAGTCAGTAAGCGCAACCAACGTAAGCA
ATGCAACCAAACCCGAATTCACGCTTGAAAGCGGCGGCTATGCATTCAATAAAGGTGATT
ACATTATCGTTACCGCATCAAGTTGGGGTAAATTGCTTGATCGCGTTCGACGGGTGACGG
ATGCTGAATCAACCAAGGTTACTGTTGAATGTATCGACGCAACCGATACTAACGTTTTTC
CGGCCGGATCTGTAACTGCGAGCTTTGCAAAAATCACCGATTGGACAGAGATTCCTTGCA
TACAGGACATGGGGCAGGACGGCGGCGAGCAGCAGTATTACAATTACCAGTGCTTAAGCG
ATGACCAGGAGCAGCAGTTACGAACCTATAAAACTGCGGTATCACTGACTAACACATTTG
CTCATGTATACGATAACCAGATTTATCCGCTCTTGCGTAAGGCTGACGAATCCGGAGCCG
TTAAAGCATTCCGCATGTATGTACCAAAAGCGAAAGAAATGCGCCTTTGGGCTGGTGTGA
TTTCTTTCAACGAGAACCCACATGCGGCGGTAACCGTAATGGAAACCGTTTCTTTGTCGG
CATCCGTTAAAGGTCGCTTTACTTTCCTCCCTGCTAAGCAGGAATAATATGAAGGGGCGC
ATTGCGTCCCTTTTTTATCTGATGTAATAGCTCCTGAATATTTAACCACTCAGGAGATAT

TACAATGGCTGAGATGAAATCGACTCTTGCCCCGCTTCCAGATTTCAAACTTCCAGTTAA
ATTTGTATTGCCGGATGGTAACGTGCAGAAGATTGTGTTACCGTTTAAGCATAAAAAGGC
CAGCGAGATTCAAGAGCTTTACCAGAAAGAAGGTATTAAAGATCCTGAATTCCTCATGAA
TGTTGCCGTTGGTTGGGATCTGGAAGAAGAATTCACGGAAGAAAACGCTCAACTGTTAGT
TGATTATTATCCTGGCTCGGCGCTGGCTCTTATGGGTTCTGATTTAAGTGCGCTGGCAGG
GCAATGCGTAAAAGACTAAAACGGGCGGCTTATCTGTATTACCAGAAACCGCCGACAGAT
GCAGAATTGCAAGCCGTAGGATTCACCCGCGCGGACTATGAAGGAGAGGATCCGCCAGAG
GTCATATTTGATGAAAGTCTGATGCATTCATGGGATATATTTTGCGCGATGGGTACACAG
TGGCGTAGTGCTCGCGCTGGTGCTTACGGTTTTGATTATAATGTTTTGCCTATGTTACTC
AGAATCTATAAAATAGACGATGAAGAAATGGCCTTAAGTGACTTGCGCATCATGGAACAA
AAAGCACTGGGAATGATGCAAGCAAATAACAAATAAAGCGCCTACGGGCGCTTTTTTTAT
GAGGGTGATATATTGGGCTGAACTATTTGCAGGTTTAACACTTGGCGTTAACGTCTAGCA
TTTAAACAGGGCTGTAAAGTCATTGCATGACTTGTAAAAAAGCAAACGACGAAGCAATGA
AAGGCGTTGATAGCTTTGTTGATGAGGAAGAAATTGCAAAGCAAAGAGCAAAACAATTAG
CTGAAGAAGAGGCGACACAGAGCCGTGAACTTAAAAGCATTCAGGACACTGTTGATCCGA
CAGCGGCGCATATGCGCAAGCTAATCCAGGCCGCAAGTGATCTTGATAAGCTATGGCAAC
AGGGAACTGTTCCTGATGAAACGTTTTTCCAGTTGGGGAGTATGCAGGAAACTCAGATCA
GTAAGCTCGACCGTACTAAAAAGGCGTTAACGGAAGAAGGCCGCGCAGCGCTTCAGGAAT
CAAAAGCCAAAGAGAATGCGGCGATAGCCGGAAAAACTTTCCTTGCCGATCTGGAATCGC
AAGCCCCCGCTCTTGTAAAAACAAAAGCTGGCTTGCTGGAAATGAAAGCGGCGCAACTTG
GCGTAAGGGCTCAGGCAGCGCTATTTATCGCACAGCTTAAAGCGCAAGAAAAACAGATGA
AGTTAACCGGAATTAGCGCGGGGCAATATAACCAGGCCATGCGAATGCTTCCCGCTCAGA
TCACTTACGTAGTAACGTCGCTGGCTTCCGGTATGCCTGTTTGGTTGGTTGCAATCCAGC
AGGGTGGACAGATTAAAGATTCTTTCGGCCGTGTTGCTAATACGTTTAAAGCGCTGATGT
CGTTTCTGAATCCGGTAAACGTTGGCCTTCCCGCATTAGCCTATTCAATGGGTGCTTTTG
TTAAGGCTAGTTATGATGCGTATAAACCGCAACGTGACTTGCAAGAGGCGTTGGTGGTGA
CTGGTGGACATGCTGGCACAAGCGGCGCACAGTTCGATAGGCTGGCGCGGGACAGTAATT
ACAGCACTGATGCGCCGATAGGAAACATTCGATCCATCGCTACAGAGCTTGCAAAATCCG
GCCAGTTCACGATTAATCAGATTCAAGCAATCACGAACACTACCGCTCAGTGGTCAACTG
TAACTGGTGAATCAAGCGACAAGATAACCGCTTCCTTTAATAAGAGTGCTGGCGATCCGG
TTAAAGGTCCTGCAGTGCTATACGAGCAATTCAACTTTTTAAGCGAAGGGCAGTTAACTT
ATATTGCAGACCTGGGGAAAACGAAAGGCAAAACGGAAGCGGTAACGGCGGCGAACAAGT
TATCCGCTGATATTATGGATCAGAGATTAGCTAAAGTCGCAGACTGCGCAACGCCATTTG
AAAAGATGTGGGCGAACATTCAAAAAATCGCGTCAGATGGATGGGGTTGGGCTGGGGATC
ATACGATGGCGGCGCTTAACCTGATTATTGACGTTGGAGCCGGAACCAGTGAGCAGGTTC
GTTACTTGCTTAATCAGGGTGATATTCTCATTGGTGTGTTTATCGTGTCTGCAACTAAGC
CTAGGCAAAAAATACCTGGTCTTGATGGAGTTGGTCATTCAGTAATTGCACAGCAGCAAC
AGATCATTAATAAATCAATAGAGCAAAACAAGGAGTTATCAAAATCTATTGCCGAACGTA
ACGAGCGAGTCAGAAAAGGCGATCAAGGTTACATTGACATGATGAAAAACCGTGCGGCGG
TTGAACAGCAATATCCTACTAAAACATAGGAGAATATCAGGAAGGATGCCGAGGAATTAG
CCAAACGCGATAAAAAGCAAAAGGCTGAAAAAGTTATAGTATCGGCTGGCGATATGCTGG
AATAACAATACCAGCGTGATATCCCGGCTTAAGAAACTCAATTAAGAGTTTTGAAAGAGC
ATAAGACGATCACTGATACCATAAGCCAGCACCGTAAATCTTTATGGGCTGAACAAGCTA
ACATTCAGATTTTGCAAGTAGCCTCAACAAAGCGGCAACTTACTGATGAAGAAAAAAGCA
TTTTAGCCAACAAAGATAAGATCCTGGCGATGGCTGAACAAAAAGCCATTTTAGGCGATC
AAATTGTCGCACAAATTCGGCTTAATCAGTCGCAAGATAGCGCCATCAAGTTTATCCAGC
AGCAGAAAGCACCAACGGAAGCACAAGCAAAAACCAGAGGGTTAAGCGAGCGTGTGGCAG
CAAGAGAGCCGGCACGTGCACAGGTTGAATCTGATTATCTTGCTAAGGGAGGACAGAAAG
AAGATCCGCAACTTAAAGCCTTGATAGATGCCTTAGACAACCGATATAAAAAAGAAGATG
CTAAACGCGCTGATCGGTCATCTGGCGCTAAGGATGCCTTTGCGGAGTATGGTGAGGAAG
CAATGAACATGTATGATAATATCGCCAACATTGCGAGCCAGGCGCTAAACGGCCTATCGC
AGCAAATGGCTGACTTTTTGACTACTGGATAAGCCAACTTTGAAGACTTTGCAAAATCGA
TCATTAGCCTTATAGTTCAGATGATCACAAAGATGATTATCTTCAATAGCATTTCCGGCA
TGATGGGCGGCTCAACCTGGACGCGTGGAAGCCTTGTAAGCGGTGCTGGTTTTGCTCGGG
GCGGTTATACTGGCGACGGCGGCAAATATGATCCCGGTGGCGCTGTTCATAAAGGTGACT
GTGTTTCCCCCAAAGGGGCAACGCAAAGAATGGGAGCTAAAAACCTGTATCGCATGATGC
GTGGTTATGCAAACGGCGGTCCAGTAGGAAGCCTCACAACTGGCGGCGCTAGCATCAATC

GCGGCGCATCTCAATTTGCGTTTGGTGATATCAACGGTAACATTGACAACGGTTAATATC
CAAAAGGGATGGAAACTTGTATTAAGATGATTTTCACCGAAATGATTCAACGTTCATGCT
CGCAAGGTGGCGAGGTTTATTATTTTGTTCATGGGAGGGCTTAATGTTAGATGAATTTAC
TTGCTGTACGCAGATTCAGAACGGCGGCGGTGTAATGACAACCAACAACAACGACAGGGA
TGTGATTTTTGGTATCGGTTATAGCCAAAAGGCAACTTCCGGATTTAACACGGAGCGGAG
GGAGTTGTCTATTGCATACGCTGGCACTGATTATAAAAAAGTGAATGCCTTCATGACGGA
TCACAGATTAAAGCCTTTCTTGTGGAGGATGCCGGATGGTGATTTAGGGTTGTTTACGGT
TAAAGCAGGAACGGTAGGGCTAACGCCAATAAGCCCAACCGCTCAGGAAGTCAAAGCAAC
ATTTACCGCAGAATTTACATCCATGCGATAAATCAAAGCCGCCATTGTGCGGCTTTTTTA
TTGTGTCTATAATGGTGCTTTATTGGAGGGCATAGAACATGGCAGAAAAACAGAACAAAA
AGACTTTTGTCAATTGTCTGCAATCGCTTTTCCCTGGTGAAATAATTACGCTTGTTGTGG
TGGACGGCACACAATTTGGTGCGCAGGTTTACCGCTTTCCTGCTGAAAATATCGCGTACA
CTCCAGAGGAATTATTGCAAGCGCGTGAAACTGGAATATTGCCTCCGAAAGATATTAAAT
TTCGTGGCGAGGTTTACGGGGCGCGGACTTTTGGGATTAATGGAATTGGTTTTACAAGTG
ACGGAACGGCAGCAAAACCACAATTAGCGCTTTCGAATTTAGATAGCCGCTTATCTGCAG
TGATCAGATCACATAATGGAATGATGCAAGCCAAAGTGACAATTTGGGTTACATCAGGTG
ATTTAATTAATGAAGAAGGAAACGTGGAGGACGGCGCATATAGAAAGTTTGTTTATTATA
ATGAGCGTCCAAATTTTGTTAATCAGACAGTTGCACGTTTTGAATTAACATCACTTTTTG
CAATGGATGAAATTATGATCCCGCCACGACTCACGCAAAGCGTTTGTTATTGGGCGCAAT
GGGGATGGTATAGAAGCGGGAAAGGTTGCGGATATAACGGATCGGCAATGTTCTATAAAG
ACCATAATCCGGTAACAGATCCGGGCAAGGATAATTGCGCCGGCACAGTGACAGCCTGTA
CATTGCGCTCTGGCGATCAAAAAGAATTAGATTTCGGCGGTTGCGCGGTTGCATCAGTAT
TGAGGAAAAACCAATGATTAACGTAAGAATTAAACTTGAACTTCTGCGCCATGCCAATGA
CGTTTACCCTAATGAATGAGGCGGCCTGGTAACTCAAAAATCACGAGTACAGAAATATCT
ACGGATCGACAACGTAAGCAAGGAGCCGGAAAAACATTTTGAAATCGACGCTGAACAATA
TGCGGACGTTGAGGAAGGCGGCTCTGATATCATAGCGGTAGTTCACAGCCACACAGGGGA
AGGGGCAACAACAATCTCGAGCGCACACCATTTGTGGATGTGGTATGAAGCTTGTGTTTC
CTGGGTTATCGGTTCGATACCAGAAGGAGACATCAGAATCATAGAGCCACAATCACGGCC
TTTGATTGCTTGCCCCTGGTCGCTTGGCGCTTCTGATTGTGGGGGACTTATTATGGCCAG
GCATAAAGAGCAAGGCGTGATCCTGAATGATTTCACAAAGCCTTATGAATGGTGGAAACC
TGAATTTGGTGAAAACCTATATCAAGACAATTATTGGAAAGAGGGTTTTGTTGAAACTGG
CGAACAACCAAAACACGGAGATATGATCATAATGCAGCTTCAACCGCCAGTATGGAACCA
TGCCGGAATTTATTTAGGCAACCATCAACTATTACATCATGCATTCGGCAAGTGGTCGCG
GGTTGATTTATATTCAGGATGGTATCAGGCACTCTCAACAATGATTGGCAGAAATCAGGT
TCTTAAAAATGAATAATGTGATTAACGTAATATAATCTGGCTCATTGGGTCGCCGCTTTG
GTGTTTTCCATACTTATGCTGTTCATAGTTGGCCTGAATGCGCAAGGGCATTATCAAGCC
AAGTTGAAGGATTCAAAGAACTGATGCACAGTGAAATCGGATCACAGTTAAAATTTGCTA
TTTTCGTTGAAGGTAAAAATGTTGGACTGAATAATGATAGCGAATGGCGTTGCACTCGTG
AGGTTAGAATCGTTCCAGTTCCAACCGTTTTAAAATCTGGAGGCCTATGTCAAGTTGTAT
TAGGAGCTGTAATTATGGTTGCTGCATTCTATACTGGTGGCGCTTCACTTGTTGCCATGC
GGGCTATTTCTTCATCAGCTTTTATGATGGGCGGTGCTATGATGCTTGGCGGAGTAATGC
AGATGATTAGACCACAACAGTGATGCCTAAGTATGGAGAGTCAAAGCGCGGAAAATAAAC
CATCATATGCGTTTGGTGGTGCTGTTAATACTAGGGCGGCAGCTTATCGGGTATGTTTAC
CATATGGCTATAGAACGGTAGGCGGCGCTGTATTCAGAGATGGCTCATACGCAGAAGATA
AATTTTAACTATATAAACCCGCCGTTGCGCGGGTTTTTTGTTACCTGTACAATGGCAGCA
CGTTCAAATAGCACAAAAGCTTAAAAGCTATGATCAAAGATATGATAACTGGCAGTAAGG
GCGGTTCTCTAAAGCCTCATACACCTGTTGAAATCGAAGATAACCTCATTTCAATCAACA
GGATCAGAATTCTTTTAGCCGTTTCTGATGGCGAAGTTGATCCAACGTTTTCATTGAAAG
ATTTATATTTTGATGACCTTCCGGTTATGAATCCGGATGGTTCACTAAACTTTCAGTATT
TTGAGGCAGAATTCAGACCAGGGACGGAAACGCAGGATTATATTCAGGGCTTTACTGATA
CAGCCAGCGATGTTACTGCTGCTCGCGAACTGACAGCCGCAAAGCCTTATATTATGTCTG
TTATCAATAAGATCCTTTCTGCGAACCGTATTAAAATTTTAATGCCTCGCGGGGTTACTC
AGGAAGATAACGGAGATCTAACAGGTGTTCGCGTTGAATATGCTGTTGATATGGGTGTTG
ATGGTGCTGAATATAAAGAGGTTTTGCTTGATGTAACTGAAGGTAAAACAATGAGCGGTT
ACGAAAGAAGCCGCAGAATTGATTTACATGCTTTTAATGAGCGTATTTGATTGCGCGTCC
GTCGCCAGACAGATAGCCCGTCAGCAAGGGTAATGGATCTGATTTAAATGCAAAGCTATG
CGGAAGATGTAGACGCTAAATTTCGTTACCCACTGACTGGTTTAGTCTACGTTGAATTTG

ATTGCGAATTATTCCCTAATGCGTTATCGCATATGAGCATTAAAAAGAAATGGAACATCA
TAAATGTTACGTCGAATTACGATCCGATTAGCCGCATCTATTCAGGGGTATGGGATGGAC
CCTGGAAAAGAGCCTGGTCTAATAATCCGGCTTTTGTGCTGTATGATTTAATTACCAATC
AGCGTTACGGAGTCGATCAAAGAGAGTTCGGCATGGCGCTTGATAAATGGAGCATTTACG
CATGTGCGCAGTATTGCGATCAGATGGTTCCAGACGGTAACGGCGGAACCGAGCCGCGAT
ATGTTTGTCACGTTGTGATCCAGAGCTAAGTAGAAGCGTATCAGCTTGAGCGTGATATTT
GTTCAACCTTACGTGGCATGAGCTTTTGGAATGGTGAGAGCCTTTCAATCGTGATCGATA
AGCCTCGCGATGCGTCATACATCTTTACTGATGACAACGTGGTTAACGGTGAATTCACTA
ACACGTTTGCGAGGGAAAAAAGCATGTACACGCAATTCGACGTGACTTTCGACGACGAAC
AAAACATGTATCAACAGGATGTTGAGGGAGTTTTCGAAACAGAGGCGGCGTTACGCTTTG
GCTATAACCCAACGTCAATTACTGCGACTGGATGCACACGACGTAGAGACGCCAACCGCC
GTGGACTCTGGATTTTGACAGCCCACGTCAAGAGCACAACGGTAAACTTTGCTACAGGTC
TGGCAGGTATGATCCCAACCGTAAGTGATGTGGTTGTTGTATCTGATAACTTTTGGTCCA
GGTCTCTTACGCTGAATCTATCAGATCGTTTGATGGAGTTTAGCGGGTTTAAGGTGTTCA
CGCCGTTTAAGGTAGACGCAAGAGCAGGAGATCGCACTCTGGTAAATAAGCCGGATGGTA
AACCAGTTGGTCGAACCATTGCGCGTGTGAGAGATGACGGTAAAACGCTAACGCTAAACC
CGACATTTGGTTTTGACGTTCACACGGATACCAATTTTGGAATTGAGCGAACCGATAATG
CACAGCAACGATATATTGTAACCGGAATCACTAAGGGTGATGGTGATGAAGAATCTACCT
ACAACATAACGGCTGTTGAATACAATCCGAACAAATACGATGATATTGATTATGGCGTAA
ACATTGATGACCGTCCGACTTGAATCGTGCAGCCCGAAATTTTGCCAGCACCGCAAAACG
TCTAGATAGATTCTTATTCGCGTGTTGTTCAAGGCGCGAGCGTGGAAACAATGCATGTTT
CATGGGATAAGGTTGAATATGCAAGCCTTTTTTAAATGCAGTGGAGAAAAGACAATGGCA
ACTGGAACGGCACGCCGCGCACAGCCAACAAGGAAACGGAAGTTGAAGGAATTTATGCTG
CTAACTATCACGTAAGGGTTAGATCTGTTGCAGCGAATGGTTCAGCGTCTGGATGGCCTG
CGATCGTAAGTGCAGGATTAACCGGAAAAGATGGAGAGCCGGAAAAGCCAATTAACCTTA
CAGCGTCGGACAATGAAGCTTTCGGAATAGTCGTAAAGTAGGGTATGCCAGAAGGAAGCG
GAGACAGGGCCTAAATAGAGTTGCATCATGCGCCAAACGGTTCTGATGGTCATCCTATTG
TTGATGAAGGAACGCTCTTAAGGCTGGTACCGATCCCGCAATATGCGTATTGGCATTCAA
TACTGCCAGCGGGTCATGTTGTCTGTTACAAGGCAAGGGCATTTGACAAAATCGGTAACG
TTTCTGATTGGACTGATTTTGTACGCGGCATGGCTTCCGACGATACGAGCATTATGACGG
ATCAGATTAAGGTCGATATTGAACATTCTGATGGCTATAAGTGGTTTCAGGAAAACGCAA
TAAAGACCATTGATAAGATCCACAGCACAACAGGACCAGTGATTGAATACGCATTAGCTC
ATGCTAGAGATGTTCGACGTATGACGGTTTAAAACGGAAAGCGTAAAGCCAAGTTCTTGC
AATCGCTGAAACTCATTGCAGACAAATCAGAAGCCAGGGTAATACAGGTCACTCTAATGA
GTGCTCAATTTGACAAGAAATTAAGGGCTCAAACTAGCGAGTTGAGAGGAGTGATTGCTA
ACAGCACTGAGACCATTAGCCAAAGGATTAATCAGCTTACAGCTACGTTTGAGAGTGAAA
TTGATGGTTTTAAGCAGAATTTCAAAGCACAGATAACTGATGATAACCAGGCAATAACTA
ATGAAGCGGATGCGCGAGCGTCAGAGGATAGGGCGTTATCAACACAAATTGGAGATACGC
AATTAGCAGTGAATCAGAAACTTGATCCTTGGGTTAATACCGATAGCGTTGGTGCGCAGT
ACGGCGTTAAGTTGGGAATCAGGTATAACGGGCAGGAATATAGCGCAGGTTTGGCGCTTT
CTCTAGTCGCTGATGGTGGCGGAGTTAAATCACAATACCTGTTTGATGCAGGACGATTCG
CGACCATGAACAATGCACAAAGCGGAGCCTTTACATTGCTCTTTGTTGTTGAAAATAATC
AGGTGTTTATCAAAAGTTTGCTTGTTAAAAATGGTTCTATCGGTAACGCACGGCTTGCTA
ATTGCATCAACTATAACACCTGGCAGAGCGGGGTTGCAGGATGGGCTATTAACAAAGATG
GTTACGCAGAAGTTAACCAGATAACGGTAAGGGGTACTGTTTTCGCAACCGCCGGTTCAT
TCACTGGTAACGTCTACGCTACTGATGGTTGGTTTACGGGTAACGTTTACGCTGAAAAAA
TTGAGAGTGACGTTGCAAAGGCTGTTGTCGTTCCGTTTAACGGATGGGTTCTAATTCCGG
CGGGGAACTACAACAGACATCTTGTGACCCCTGATGTTGGTATTCAGGGGGACACATATT
CAGGTGGGACATGGGGCAGAGGTACTGTTTGGGTTGATTCAGCATATGGCGGGCGCCTTG
CTAACGTTCAGGCAACTGCAATGCATGGCTGTTCAAGTGGTTATGTTCTGTCGCCAGAAG
GTAACGCAACCACGCTCTCATATGGAGGAGATCTAAACCATGCAAACGCAGTACCGATCT
TTACATTTCTGCTCTTCAAAGCATAAAAGGAGGTAAATCAATGCCCTGGGTTAACGGTCG
GGGCATTTTGTTTTCTTTTCTTACACGATGTTCACAACCGAATGCGGCGTAAAATTGAAA
ATGTCTTGATAACAAAAAGAACAAATTCAAAATCTATCTAATACATACACTTAGAGTTAA
TTATTATTATATATATCTATTGGTATCCTTATTGTTTCCATTGTTCTATATGGTTTGTGG
TGGTTGTGTGTTTTGTATTCGTTGATGGTTATATATCACATGGTATGTATTTATATATGG
TATGCAGTGCAAATGCGTGAACAATGGAACACAAAAACAACAATTAGTAAAATCATAAAC

TTACGATGTGATTTTGTTTTCAATGCTAGTTGTAAACGTATTCAAAACAAAAAGAATACC
TAAATGTTATTGACTACATGAAACAAAGACATATAATGCAGCCATACCAACGAGAGGATG
ACAAAATGAGCGATTGTAAAGTTTACACCTTTGACCAGGTAAACAATTATGAATATCACT
ATCCTTAAACTTGGGCGGCTGAATACGTTAGCGGTTCAAGCCTGGCAGAAATCATTGCAA
GTTATCCGGCAGCTTGGAAAAACAGACCACGCGAGGAAAGCAAAGCGCTTGTTTTCGGTT
CGTAGTCCCATACAATCATTCAAAGAAGTGAGTTGTTCGCTAAAGAGTACCGACGAGCGC
CAGCGCCGGAAGATTAAGACAACTTAATCACAAGCCGAACGGGCTTAGCCAGCTAGTTAA
AATCTTTTGGCCTGACTGGTACAATCGGGAAAACTTATCCAGACCTGATAATAATGATGG
TTGATTGTGGTGAGGATTTTAAGGTACAGTGGCTTATTGAAATGATCGCAGACAGCCAGG
CGCGAGCGGATAACGTTGAATTGGTTCCGGCTAAAGATTACGACGCTTGCGTTATCATGC
GCCGCGTACTGGAAGACATACGGGAACACAACGCTTGCATGAATAGCTCAACCGCACAAC
GCGAGCGATCAATTTTCGGCAGTATCAAAGGCGTGAAAGTAAAAGTAAGGATTGACCATG
TAGATATTTCCCAGGATTTGCTTGCAACAGTGATTTCCGGCTATGATGGTGATGGTAATC
CGCGATATGAAACAACGCGATCTGATGAAGCGATTATGATCACGGATTATAAAACCACGT
GAAGCGGTAACCCGTCAGAGTTTGGAAGGCTGGCTTTCAATCATGGCTACTACTTGAAAA
TGACATTGCAGCATGACCTATTCAAGAAAGAGTATCCAGCAGAAACCCGCCCCGTAGTAG
TCAGATTGCTGTCGGAGGAAAAGAAGGAGCCTTATTTACCGATTGCATACCGAATGACGG
ATGAACAATTGAAGTTCGGACGGCTTCAATACATGAGTGCGAGAAACCAGTTTGCAGAAT
GCCAGGCAAATGACAGTTGGCCTTGATACGCAAACGGAGAGCCTGAAATTGATCTTCAAA
GGCCTGATTGGGTTCGTCGTCAATACAAAGGTTATGTATAAATTGCACTTTCTGCTAAAA
AAGCCATTCGATGCGTGGTATACTAGACGCATCAACCAACGGGAGAGAGAAAATAAAAAT
GGCACAGCAACTCAATGAACAAGAAAATCATGATGTGCGCGATCACGTAGGCGGCTTTGT
AAACGCATTAGGAATTATGCAACGCCACTTCGTGCGCGTACTTGAACCAATCGGATCACC
GGAAACTATTGAAAATGTTCGCAAGGGTGCATATTCGATTGATAACGTTGTGTTAACGGT
TCTTCAAATGGAAAACAATAAAGAACATCGCAAGATGATTAATCAGGCAAGTGAAATCAT
GATCCACAATTTGATTGATTATCACAACGAGAACGAAGAAGAGCATTATTTAACACGGCG
GCGCAAGCCGCCATTATTAAACCGGAGTCAATGAAATGAAATTTCCCGAACAAAACGCAA
AGGTACTTAAGGCATTGTTTGAGGTGCGTCAATTATTCAACAAAGTAAAAAAGGGTAAGC
AAAACGCGCACCTGAAAAATAAATATGCAACTCTTGACAGCGTGCTTGCAGCTATCATGC
CTGGCTTTACTGATAAGGATTTATTCCTTACTCAAGATCAGAAAGTGGGCGAAGCTTTGA
AATCAATGACGGATTTAACTCGCTTCATTCACGTTGAATCAAATGAATGGGTTGAATATA
GTTGCACGTAGCCAATGCAGAAATTACAACCACAAGGCGGCGGCTCAACAAACAGCTATG
CGCGACGTTATGCTCTTTGCACGGCCCTTAGCCTGGCAACGGCTGACGATGACGCAAATC
ATGCCACCAAAAACGCGCAGGATTGGAAAAAAGAATTTGATGCTAGCGATAACCTTACAG
ACTTGCAAGAGACGTTTAAAACTGCTTACAAGCAATCTGACGCAGCGAACCGCCGAAACA
TTAAAGAGCAATACGACAAGCTGAAAGCTGATATGGAAATCGGAAAGGCTCGCGGCTTTA
ATCCGGCTGAACAAGCGGCGAATGTAGCTAAGGATGAAAAGCTTGAAAAGGAACCGCCAC
AGGAAGTAAAATCACAGTCTATCACTGGATTTGATTGATAATTTGCCGGGCGTACTGCTC
CGAAAAGGAAAAAGAATGCACGTTATCACTGGTGAAATTCGCAAAGAGCCTTTTGTTAAG
CAAGGGCAAAACAGCACACTTTATATTGTTGAACTTTCAGAATCATAGAAAGATCGAGAG
GGTAACAGACAATACACAAATTACAAATTCTTCTTTAATGCTAAAAGCGAGGGAATGAAT
AATTGGTATCGTGAAGCATTCCAGCAGGGTAAAATGATTTCTGTGTCGTGCGACCAGTTG
CGCATTGATAGCAGCGAATATAGCGGGCAGATTTATAACACTCTAACAAGTGCCGGATTT
GCGAACTTGCAATTTAGCCAGCGTGGTGGAAGTCAAGCACCACAACAGCAACGACCACCT
CAGCAACAGCCACAACCGAAACAACAAGCATCACAAAACAACGAGCCTTCAATGGACCTG
GACGACGATATTCCATTCTAAAAATAAAGGGGCTTGCGCCCCTTTTCCACTTTCTTTGAT
ACCCGAAACAGCAATTACCTTAACTGAGATCCGGCTTGCAAGTTCAAGCTGGTGTGCGTT
AATCGGTCCTGTATTCATGATAGACAACTGTGCACACATAGGGCTAAGAGCGTTAAAATA
AGTAAAGGCTGATTGCATAATATCCATTTGTTGATGACCATCATTAACAATCGCTTGTGC
GCCTTATTCACCCAATAGAAATTGGTTCATTGTAAAGAATTGGTAAATAAAAAGAGGAGG
CGTTGCGCCTCCCTTTGTATTATGGAAGCTGTGGAATCGTTTCGGCATTACCTGCAGCCA
ATCCAGCAACCATTTTTTTTAATTGTTCAAAATCAGCTTTTAAACTTCAAATTTCAATTT
CTAACGCTTCATTTTTACCCTGCAACTCTTGAACAGCAACAATAAGATCAGAAATAACGG
CGCGATCATTGTCGTTCATGATTTCTAATTCATCACCGTTTTCATATGTAGCAGTGCCGC
CAAAGAAAACGTATGTGTCGACTATTTCAGCCATTTGTTGCGCAAGGACCCCACGCTGCA
GAACATGTTTTCCCTTATAATAAAACTCCATAACACAAATCTGTTTGACGCGATCCCATG
CGCCAGGCTTAGGAGCTTCAAATCTATCCTTAATGCGAACATCAGATCCTTGCATAGCGC

ATCGTCCCTCTGGGGCATCGAATCCATTATCGTTTCTCATTGAATACCATACGACATTAC
CGCCGAATGTGTTTAAAGACCAAACATATTGATACGCCTGCCCAGGAGTACCAACCAAAT
AATTTTGAATTCCGAACCATGAGTTCTCAATATACGTAAGAAGGTTTGAGCCATCGCTTG
ATTGCTGTGATTTCTCCTTTATTACAGCAGCAGTACAAGCTCCGCCTTGTGCGTAATTAG
CCTTAGCCCATACCGCATTAAATGTATCATCCTGATCAATTGCAACCCCTAAATTTCGTC
TAGCTTGAGCCACATTATCAGCAACAGGGCCACGAGATACGATTGGTAGCTCATTTCTTA
CTCCGGCGGAGCGATCAAAAACCCCCCAATCACCAGCGCTGTAAGCGAATAAATAAGTGT
TTTTATCAGGTGCCCAAAGTAACGTTTGTTCTCCAAGTCTATCAACCTGGTCAACAAGCA
GGTTTTCACGAGCTTTTGCAACAGTTGTTGCAACAGTACCACCTTGCTCAATACCTAGAG
CCGACCAACCTTTTAGATCGGGATCGAACATACCCCATGTGCCATCATTCCTTACAGATA
TCCTGTTTAAACCATCTGGAGAAAGCATTGACGTAAAAACAGAATCCTGAATTATTCTAT
TAACTTGCAAATTTATTTTTGCTTGCGTCACGTATTCAGCACAAGTTCCGCCAGCGCCAC
TAGAAAGTGCCGATGTGCTGTTGTTGTCATTACGTGCAACTCGCCATTCTCCATTGTTAG
CGATCCGCAATTCATAAGTCCCTGCAGGATTTCGGAAACAGTTGTAAGTGGCAGTATCTT
GATGTTTTACAGAATTATCAGCCTCAATTTTCAACGCTTCCCTTGCAAGGTCTTTGTTTG
CAAGATCATTAAGGTTTTGATCCTTATGCAGCAGATTTTCAGGTTGCACCTGTTGCGCCC
ATTGGCGAGCTTCATCACGCGCGTCTAACGCTTTATTTTTAATTACGGTTATCTCAGTAG
CGGAAGAATCCTTAATCTGTTGAGTTTGGTTTTTTATTGCGTCCGTGTCTGCCTTAATCT
GATTGGTTTCAGCAACGGCGGCATCTTTAATCCGTTGCGTTTGATTAACAGCGGTATCCT
TATTCTGTTGCGTTTCAGTAACAGCGTGATCCTTTATTGTTTGCGTGTCGTTTTTTAAAT
GTTCAGTCGCGGCTCGGTCTGCATCAGTTTTTTGCGCGGAAGCCTCTGCGCGAGCATCAA
AAGCCTGTATTGATTCTTAATCAAACTTCTTGAAAAATTCAATCGCTGCCTCAATAACAG
TCTTGTTACTTTGGTAATAACTCAGCGTGTCCGGTACATCTTGCGCCATTCCATCGATAG
TTAGCGAGTCATTTAACAGGATCACATAATTTCCGTTTTCTACTGGCTCGACGTCAGTCT
AGATCGCTTTCATTTCCGTGTCGCTTTCAATATCGCTATTCACAGCCAATTTACGAGGCG
ATGTGAGAGATACGATTGTTGCGCCAGTACGGATAAGAGAAAGAGGATCACGCCACTTTG
TGCCAGTACCAATAATAACGCCGTGCGCGTCCATTGCAGCCGTACCAGTTTGATAGAAAG
TCATAATTACACCTCATGTTATTCCTTGAAATAGTTTAGTGATTATTCGAAATTGCTCTG
TTACATGCAATAAAAACACCGCCTTAGCGGGGTTCGTCAACCACAACGAGAAGTCAATGA
AACAGATACATCGAAAAGTGTTAGTCGCAGGATTAATATTATAATAAGTCTCTTGCCTTG
TAAAGTCTCCGCTCTATTCCATTTTTGAATTTACCGTTAACTGGTAACATTAAGTCTAAT
TCGCGATCATCGGCAGCAATCAAAGTGTCGCGATCATTAACATGGCACAGAACGCGCATT
TTACTCTCTCCTTTGTATCTGTATACAACAATTTCAAAATTTGAAGGTGAGAAACACGCC
CATACTTCTTGCCCCGCCGCCAGCTGTAAATGTTGCGCATCAATCCAATCATGGCAAAGG
TAAATTGTTCTGTCAGGCTTGCCAGTTACCGCAACGCTTCCTCGCGTGTACTCTTTGGCG
TAAAAACTGCTTTTACCTTCCTCGTTGACGAAAAGACTATTGCACATATCATCATCCAAT
TCATCACTATGCACCAGGTGGCAAGGAATGGCGTGTATATGCACGCAATATCCATTGTCA
TGAGTTTTTACGCCAACTTGACACGATTCGCCGTCTAATGAATAAGAGGCCTCAAAAACG
CTTAGATTTATGATTCCCATGCGTTCAGCTTTATCCATCACCTCAACACAATTTTCATGT
GGTGCTTGCGATCCCAAATTGTAACCTGAATCACGTTTTGCCGCTTTGTCAGCCTTGTTG
ACAACCTCAACAGGAACCAAGTTTGACCATTCGCCTAGAGTGTCGATTGCTTCACTGTAA
TTTTCACCACGTATGCGCATGAATAATCCGATGACTGAATCATTCCCGCATTGGTTGCAA
ATGTTGTCGACGTCGCCTTTCTCGTTGATTATGTCAGTCCAACGAAAGCGATCTTTACCG
CCGCAATAAGGGCAAGTCTGATGTTTCCCGTTAAAGGTTAATTGAGGAATCTCGCAAAGT
TTCATTACTGCCGATTGCCACATGACGCGCATCTTTGGGATAACTTCTTCTTTTTGATAA
TGCATTTCTCAACCTCCGAATAAAAAACCTGGCACGATTATAAACCATGCCAGGCCGGAA
TTTGTAGCATGCAGTGCTATCGACGGAAATCACAACGGGTTTCATCTGATTGGCTTCCTT
TGCTTTGCGCCGTTCTTCTTTCGCCTTTTCCCGTTCAGCTTTGCGCCGCGCTTTCTCCTT
CTCGCGTTCTTCTGGACTCATAGAACGCAACGGATCGTAAGGCCGCACAAAACCTCTTGC
TTCCGTTTCCGCTTTGGTTACAAATCGCATCAGCTTTCTGTTGCTGCAACGATTCTCTAA
TGGCTTGCCGTTATTATCAAATTTGATATCTGGACGGCAATATGAAGCGCGGAAGCCGTT
AAAACCTTTGCGTCGGTAATCTTTCTGGATCAGTTGTGCGCCTTTAGTTAAAATCATTCC
GTGCTCTTTCCATCCCCATACTGTTTTAAGGTTAACGCCAATAGCACGAGCCAGGGCAGC
GCGACCGCCGTAAGCGTTGACAACTTCATCAAGCCGCGCTTTTAATCCTGCTCTTACTTC
TTCCTTTCATACAAGATAACCGCTAACAGCTGATCGACGTTTACGATCTTTACCACGTCT
TACGCCGTTGTTACCATTGATTGTGCGTTTATCGATTTCGCCAGTTGAGGAAGCAATTTT
GAATTCGGTCATATTTTCCCCTATAGCACTTTTTGCTAAAAAACTTGGTTAAGTTTGTGT

ATGATATACCGCAGATGGCATTTAGTCAAAAGGATAAAAAATGATTCCTAACATTGAAAA
GCAGATTGCAGATCTGGGAGAGGATAGAATCAAGGCAATCCAGAAGCGTTTCACGGGTGG
TGAGATTGTCCCGTATGAATACCAATGCGTTGCTTACGCTGAAATTTTAAAGCGCTTGAG
TAAGTATGTTAATCCGTTTTTCGTTAAAGCGGCGGTGTCGGCACGTAAAACAAACAGCTT
TGCTATGGTTGCGGCTCTGTGCACGAAAATGGGATTGAAAGCGTTATTTCTGGCGTGTCT
ATCTAAAATTGTGGATCAGGATACAAAAGAGATTAGCGCCTTTGGTGTCCCGAATCCCGT
TTATTGCGCTGGCCTGAATACCAAACGCGCTTATTTTCCGACTGTTGTCGGATCGGAAGG
TACTGTAATTGGCGGATTGCATAATGCGTTAGGTGATTACGTCGCATAGGTTTTAGGTAT
AGATGAACGTCATCAAGTCAACGGGAATGATATTGTTGAAGCCGAGCAAAACAGCGAAAG
CATAGAACAGATGATGACGCCGAAAGGTGAACCAGTTGAAGGCAATCCGTTGCTGTTAGG
TTCAGGCCGCGCACAATACTCCATTATCATTGCAGAAATGCGCCGCCGTTGTCTTGAGAC
TTACGGTCATCAGTTGCGTATATGTGGAATCACCGGATCGGAATATCGCGGAATTGAGCC
GATACTTGTCGAAGATAAAAGAATTCCTGGGTTTTGGCGTGAGCAGATCACAAACATTGA
TACTGATTACCGGGTAAAATTTGGTTCAGTTGTTCCCACTTATTTTGGCAATGTAGGTGA
TTTGGGTTACGACATATTAAAATTTACGCCAGTGCATGAATTTGGCGTTGATGATTATGA
CCGGAAGCAATTAAAGGCCATGAGTGACAAGATTCACCAATCAGTAACAATGACGCAGAC
AATATTGCAGATGGTTCACGAGGTGATGAAAACCCGCTTGTGGGCTCTTCTCACTTCCGC
TGATGATAGGCACTGCAAAGAGGCGGCGGCGCTTCCGTCTGGCACGAAATTTTACAT
TATTACCGGAAAAACTGGAGAGAGACAGCGGCGCGTATGGGTTGATGATTCCTATGAATG
GCGAGTGAAATATACTTTCATGGTTCCGGCGCTAACAACTGGCGTTAACTTTCCGCCGTG
GGATACCTCTGTTATTTTGCGCAAGATCGGAACTCTTATGCTATTAACTCAGCTTTTGGG
TCGTGGTATGCGTCAAGGAAAGACTTACCACAAAGAAGTTTTGGGAATGGACAAAACACA
TCATCTTGTTTTGGACTTTTCCGGCACGATGTTTGAAATGGGTAATTTGTATTTCGATCC
AATGCATGAGCAAGCGCAATTCCAGTTGCGTAAATCGCAGGATAAAGATCCGAAATTTTG
CCCGAATTGCGGAACGGAAAATTCATTCGAAGCGCGGCGTTGCATTAACGAGGATGAAAA
CGGAAATCGTTGTGAGCACTTTTGGACGTTCCAGGAATGTGAGGATCAGATTGATGAAAG
TCCAAAGAAAGTTATTGTAAACGGTTGCGGCGGAAAAAGTGACGTTGCTGCGCGTGTATG
CCGCTGCTGTAATGTTCAGTTACGAGATCCACATGATGACTTATGCGGCAAGGGGTCCCG
TAAAAACGACTGGTGCGACGTGTTAGATTTTAATGTGACGCTGACAAAAAACCAGGCTGG
CGTTAGATTCTGCTACACGCTGCGTGATAGTGGCGGCGTAGATTTCAGACCATAGGAAAA
ATGTTTCCCTAAATCAGAGCACAAGATATGCAAAACGCTTTGGACAAAGGCGGTTAGAAC
ACACGTTGTAGATCGACAGTCTGCGAACTATATCATCACTTGCCGCCACGCAATTAAGAT
CATGAGTTATGCGGCGCAAATATTGCCACCGAATCGCGTTACGCAACGCAAGAACACAAA
GAGAGAAGATTTAATAGCACAGCAGGAATTCTAATATGGTTACTGATAAAGGTGAGTATC
TGGAGTATTACGTCAAGTCAGATCCAGACGATAGAAAGGAGGAATCGCACCAGGGTGATT
CTTATTCATGGCTAACATATGAGTTTCCCATGTGGCTTGTTTGGCACACTAAAAACGAGG
GGAAGAAAACAATAGGCACAGCCGTAAAGGATCAGCAAGCGGGTGTGAAGAGGGGAGTTA
GCGACATTCTAATCCTGACTGGTTTTATGGGCTGCAAGTATTCATTTAAAGCCATTGAGC
TTAAACGCGCCAACAAGAAAGTCACGAAGGTAAGCGACGAACAAAAGGAATTCTTGCGAC
GTGTCAGAGAGTGCGGAGGATTTGCCGCCGTATGCTATGGAGTAGAGAAAGTAAAATTGG
CTATCGCAGACGCTACAAAATAGCACTTTGTGTTAATACAGGGGCAACGAAATGCGCCAT
GAGGCGCCCATCAGAACGAAACGGAGAATTAACCATGAAAAAGATGTTATGAGTAGTTGC
GTTATCACTTGGTCTTGTCGGCTGCATGGATAAACCAAAACCAAATATTTGCGGAACTGA
AGCGTGTGAGGTTACAAGCGACTATATTAAAGTTGTGCAAGGCAAAAGCAAAGGCGTTGT
AATGGATACTATCAGCAAAAATCAATATAAGTTGTTTACGCCAATTGGAACGGCTTTCTA
CACAGTTAATGAAAACACAAATGATATTAAAGTTGGCTTTTATCAGGACACTCTAACTTG
CGAGGTAAAATAAATTGGCTAAAGATATTCAGGACAAAGCCACAATTGACACTTTCGCAC
CGTAAATGGATTTTCATGAGCTGCAACACGGCAGATATTCAGATATTGCTTTGCGTTCAG
GTGGCCATTATCAGCCAGTTAGAGAAAACCCAATGTACAAAGTTAGCAGCAACCGTTATG
CAGGAAGTAAAACGACGGACGTTGTGCGCGACTTGTGGGAAACTGCAGATGAAATGGTTG
TATACATTGCTGAACGTTACGGCAAATATGATCTTGACACAGCAGCAACGGAAGATACTA
AAAAATGCGATAAATTTTGCAGCAAACAAACAAACTGCTTAAAGAGCTGGTGGGGTAGCA
AGAAGCACGTTTGGTTAAATCCACCATACAGCAACCCAGATCTATTTATCAAAAAAGCAA
CTGAGCAGATGGAACACGGTCACCAGATTGGTATTTTACTTCCGGCTGAAAATTCAACTG
CATGGTTTAGGGACGCTCAAAAACACGCGGCAGAAATTATCTGGATTACTGGTGAAACCT
GGAAGGATGAAGAAGGTCGCGAATATTCCCGAACTGGTCGAGTAGCGTTGATTTCTGGTA
TGACAGGTGAGCCAGTTAGCGGAAACAATAAAGGTAGTGTAATTTTTATCATGCGCCAAT

TGGAAGAAGGTGAGCAACAGGTAACGCACTATGTGAAGATTAGGCACATTTGCCCTTCCG
TAAAAAATAAACGAGCCAGAGCGCGGAGTATTTAAAATGAGCATTTGATTAGCCGATCAC
ATTAAATGGGATTTGTCTGTCGGTATGACAATTCGCTTACTTGATAAGCATGGTTATAAC
GCTGATAAAATGGAGTTGATAAACTGACTGGATATGACATTTGACGAAATTAAAGTTGCT
CCTTTATACATGTGGGAAAGAAAACTTGAGCACGACCTGATACAATACAAACGCAGAGAC
GGTGCACGAGTTTTTAGTGTGAAATAGCACTTTTTGTTAAGACCCGCTACGGCGGGCTTT
GTATTATGTTATTAACGGGAAACAAGAGGATTTCAAGAATGAAAATCGTAAACGTCGAAT
ACTTCAAAGATGCGCAATGGCTGGCTAAGGAATCATTATTTATGCTTGATCGCAAAAATT
AAAAGGTTATTGCACCAATACTATGGAATGACGCAATGGAGTTACTTAAAAAGGCTTACG
GTTATGAAAGTGACAAAAGGTAAAAAGGAAACTTGGGAGTTAGAGAAAAAAGGGGGTCCT
GATGAGGGGATCGCCAAAATAGCGCAATATTTCGAAATCAACGACGTGTGCGTTATTGTC
TGTAATGAAATGGCCTATGTGGAAGAAAAGCAGCGCAAGGTTCATCGCGTTCCGGCAATT
GCGTCAACAATCGACTGTAAAGAAGTTATTAACAAAACTAAAGAGCAAAAAAAAATATTTC
AAATGAGATACATTTACGTGACTATTTTTCCTCCTGTTATAGCTTGGGTTATTGTTCATT
TTTTAGGTATGGGGTTGTCATGAAATATAATGATTTATTTTCATATGAAAATGGAGCGCT
TACAACAAAGGTTGCGAAATCTAAATGATGTAGGATTGGCGATCCTGTTGGATTGATCAA
CAGAAATTGTTACTTTCAAACAAGTGGATCAGGTAAAAATGTTCTTGTTGATCGTATAAT
ATGGGAAATGCAAAACGGTAAAATTCCAAATGGAATGGAAATCGATCATGTAAATCACGA
TAGAACTGATAACAGGATTGAAAATCTAAGAATTGTTGATCACAAAGATAATCATAAAAA
CGTATCAATGCAATCAGATAATAATACTGGGGTTTGTGGGGTCAGGTTTATAGTAAGATG
GAATAAGTTCAGAGCATCAATAAAGGGAGACGGGGTAACTATATATTTGGGATACTTAAC
CAGCATTGATGATGCTAACAATGCAGGAAAGAAAGCAAATGATAGATTTAATTTTCACAA
AAACCACGGAATTTAATAAATGACTGCTAAAAAAATTACGGAAGAGCAATTGATTGCAGA
GCGCGGAGCGGGTTTAAAGTTGCGAGAGATTGCTGAAAAGTACGGAATGAGCTTACGCCA
GATGGGGCACCGTAACAGCTAATTGGCTAAGCGTGGCGAAATCGCAACTATCAAATCACC
TGGCTTTGCGGTAATTGGCGAATCGCACCTTATACGTGATCACGTTGAGGTTGTAATTAC
TTGGACCACAAGGCAGAAAGATAAAGAGAAATTAGAAGCAATTCTGCAAGCTGCTATGGA
TGCATATTCGGAAGAAGTGCCGCGCATTAAGCCGCAACCGGAGAAAATTACTGATTACTC
GCAAAAGCTGGCGCTATATCCGATTTTTGTTATTCAGATTGGCGCAATGGCTCATAAGCC
TGAAAGCGGTGAGCATTAGGACACGGCAACGGCGGAAAACGTCATGAATGAGTTTATCGA
TTATGCAGTTCATAAACCGCCCAATACCGAAAATGCTGTGTTGCTAATTGGTGGTGATAT
GATTCACAGCGACAGTCTGGAGGCTGTAACACCTGCAAGCGGTCACTTTGTGGATCAAGA
TAGCCGTTACGTAAAACTTGATTATGTTGCTATCAGAGCCACGCGTCGAGTAGTAAGCAG
AAACCTGGAAAAACATAAAGAGGTTGAATTGCAGGTCATTGAAGGTAATCACGACCAATC
AGGAATGATCTGGCTGCGTGCTGCAATGGCGGCGACGTATTAGAATGAGCCACGCGTAAA
GGATGATGTTTCGCTGCATATCGTGCATCACACGCAATATGGAATACCGTTCCTGGCTTA
TCATCATGGTCACACTAGATGCAAGCCAGAAACGCTTTTAATGATGTGTGCAGCGGATGG
GCGGCAAGGTCTTGGAAAGTCCAATTCAATGTATGCGCATGTAGGGCATTGGCATCACCA
GGCTGTTCCAGAAACGAAGCTCGGCATTGTTTAGGTGGATAGCACTATGGCGGCTAAAGA
TACTTATGCCGCGCGTGGCGGTTGGTGTTCACGCCGACGAGCGGCGGTAATTATCTATGA
TAAGGAATATGGTGAGATAGTGCCCTTTATGTTCTACGCAGAAATGATTGCATAATTAAA
TTATCACCTAAAAAGGCTAGCAAACTGCTAGCCTTAGTTTGTTTTTGTCGTTAACATAAT
CAGCCTATTTTTGACCTGGATAGTTTAATTAAACTGAGGATCACATCATGATGGACTTTC
TTTTAAGGGCGGAAAACTCTTTCGGAGGTGCAACCGTAGTTGGAACTTTTACTGGTGAGT
TTTTGCTTGCACATGCAGCGTTTATTTTGTACGCTATTTTCGGCTGTTGGGGCGCATGGC
TGAAATCGCGTTATAGTAAAGCGATCCGAGAGGCTCTATATTCAGGGGATTTATAAACGG
CGCTTAACCTAGGGCAGAAAGAGAGGTAGTAATGACAATCACGAAAGGTATAGCCGCCAC
CGTCACAGGGGCGGCTTTAATGTTTGCATCGCCTTTGACTAAAGAAATTGAAGGCGTAAA
GTATAAGCCTTACAAGGATATTGCTGGTATCTCGACTGTATGCCCCGGCATCACAGGAAA
TGATGTGATTCTGGGAAAGGAATATACCAGGCGAGAGTGTGCCGCACTATTAGCAAAGGA
TATGAAAGTAGGCGCTGACACTGTTGATATAGCTGTTAAGGTTGATATTCCTATTTCTAT
GCGAGCGGCTTTGTACTCATTCACATTCAACGCCGGAACAGGGGCATTCAGGAAATCAAC
CATATTGAAGAAGATCAATAATGGTGTTCTGAGTGGTTGTTGTGGCGAGCTTGGGAACTG
GACATATCACCGGAACCCGAAAACGGGCAAAAAGGAAAAATCAAAGGGATTGAAGAATTG
ACGCGCTGTTGAATATAAATATTGTGTAATGGATCTGAATAAATGAAAAGAAAATTACTG
GCTATTATGATGTGCATTGGACTTTCTTGCTGTTCAGCTTCAAGCGTGTTTCCTGGTGTG
ATTGGTAGCAAGCCTGATATAACGGCACAGGCTCGAGCGGAGAATGTGAAACAGACTGTT

GGTGTAACCGCAAAGCAGCATACATCAAGCAAGCAGGAAACCACACTCAAAGAATCGGAA
GTTGGTCATGATGACACATCCAACAAGAAGCAGGTGAGTACCTCAAGCATCAAGGCTGAT
AACATTAAGGCGGAAAGAATAGAAATTCGCAACAATGACGGCCTTGACGTCCCGGCAATC
GCAATGGGTATATTGATGTTCATTGCTTGTATGCTGGCAGGTAGAACGCTAACACGTAAT
AAGGCGGAATAAGCCGACTTTTCTTTTACTCAAATTTACTAACATCAATTAGCAATGCAC
CATTAGGATTTGTAAGTTGATGCTCTTGATCCAAAAATGCTCGCATATCGCTGTAAAGCA
ACAGCATTAAGGCATAGACAAAATTATCATGCGGAGTTTGCATTGCTGAACTTTCTTTCA
AAATAAGATCTATCAAATGCTAGCTTGCGGAATAATTCTACATCGCTCATTTTCTCAATG
TTCATGATCTCCCACGCATAACGATTGGAGTCACCTTCCAGGCAATGCAACTCAAAATTA
AACGGCCTCTTCATATTGTTTATGCACCAGTACACGCAATGACCGCCGTCAAGATATGCA
TAACCCATCACTCGCGCTAGAAACTCTTTGCTTGAGCTTGAGGCGAAATAGAGCGGCGTT
AACCCCGCATTTTAGGCGAGCCTTACACATTAACGGTGACGGAATATAAATCTTGCTAAC
TCCTGGCGGTTGAATTTCTAGCGAGACTCGCCAAAGCGATACAGCTCTAATAAGAACAAG
ATCACCCCATTAACGCAGGATTTACATAGACAAATTTACCAACGTTGCAAACATAATTCT
TTTCTTCAAGCATTGGTAATAATTGTTCCTCAAATCGTTTCATCACGCCTGATTGACCTG
TAAACGGCTTAACCTTACGCGCACTTTCATAGATAGCTCTTGCATTGGTAACGCTTTTAT
TTTGACGTGAGATCTTAATTATGCGTTCAATTAACTTGATCATTTCTGTATCATCGCCAG
CGAGACCGGAAGCATTCGCGGCGTTGATGTATGTTGTGCGTAATTCGCTGAACATCACCG
AAGCCTCTTGCATTGTCTCCACATAAATTTCTCGCGACTTTTGCGGCATACCGTTTGGAT
TAAACCAATTGCGGATCGTGTGCAATAGTGACGCAATGCGGATCCCTGGCTTATCCACTT
TAACCAAATTACCACGCAACATTGTGTGTGAATATTTCCCGCCGTCTGCTAATTATGGCT
CTAATTTCTGGCGAGCAATATTAAGAACACGCATTGGAGACCGTGACGGCTTCAATACAA
CATTTTGCTCAGACATGATGTTATGAATCAATTGGAACTATTGTGCCTTTAATTCTTTAT
CAACTGGCGTGTAATCAAGATCACCATTTTGATCAATAAAGACACGCTCGCCTAACATTG
TTTTTTCACGTACAAGCAAGAAACGTTCACTAACTCCAATACCCGGCGCCCCTGCATCCA
CGATCCCTTTTATCGTTTCATCCTGGACAATGACGCTCAGGCAGCCAAACTCAACAAAAC
TCATATTGTTTTCAGAGTTGGCGCGAGCTATTGAAACGTGTCCGGCGTCCCAAGCTTTTA
GAACCAATTCGCTGTTTGTCTAGCGATCACTATTAGCTAAAGTCAATCCGAGTAAGCTGT
TGATGCTTGTTGCTTCATCGGAAATGACGGCAAAGTTACCCTCCCGATTGTGGATCTTTC
CTAACCCTTCCGGCGTGGTGTCTGATACCTGAAAAACGATATCGCACATTTTTTCTAACT
TTTCCTCTAATTCCTCCTTTTCCTCAAACAACGCCGCTAACTAAGTGCAGGAATGTTCAG
CTTTCAATTCCTTTTCAAAGCCTTTTAATTTAGCTGCGATCTTCTTTCGCTCTTTCTTGC
GTTGTTCGTTGATTGGCCCTACTTCACAGCCCATTGGCGCAATAGCAAGGCTGTTGAGTG
CTGATTTACCAGTTGACGGTGGCTGACTGGTTACAACAGAAAGCGCAGTTGGTTGTTCAG
TGCCGTGGTACTCAACCTGGAAACGTCCAACCATAGCGGCGGAAACGCAACCAATGAAAT
GCATGTAGGCCGATGATTCAGGAAATTGAACAGATTGCGGAATCCTACGTGACAGTTTAC
CCACAACATCAACGTCATTCCCCAAAGAGATCACAGGATAGCGGTCATTAGCGCCGTTGA
TACCCTTTACGGCTTCCTAAAAGCTTGACGATTGGCGGTATCCGTTAGACTGAATGGTAA
CGCGAAGCGGTGATGTTTTCTGCGATTCTGCAATGGCTATAACTTGTTGAGGTGTCAATT
TGTTGGTGTCAAAATCAAACATCTAAAAATCTCTTTCTTGTTGCCGCTAATTATAACGGC
AACATTCTACAGCCTTTTAGCCAGTCGTGCTATTTGATCGGCTCAACCCTAGGTAATAAC
TATCCGTCTATAATGCTATGAAATTCAACAGTTTTAGGATCGAATGCAAGCGAACGATGG
AACGTATGACCAATCAGCAACATACCAGGCTTTTCCCTCACCTTGTAAAACTCATCGCCA
GGTTTAAACATTGCCGAGTCGCTGTAAACGACTTTATACCGGAATTCGTGCATCGCTTTT
GGGTAGTGCCTTGTATATTCAACGCCTTTAACCAATTGTAAAGTCCTTCAAACAGATAAG
CGGCACCAGAGGCACAGAAGCCGACTTTCTCACGATACAGCGTGCAACGGCCGCCGTTTT
CGTCGAACACATGTCCAGCAAGAGCACCTAACACTCGGCCTTGTTCGATCTGGTAACGCT
TATCAGCTTTGAAAGTTTTCTTGATGTGCATGGAATGGTTTTCCCATGTGCATTTAATGG
TTTTGGTCCCAACATTTCGGAATGTTAATCCGGTTAAGTTCGCGTGAATCGAGTTCCCAT
CTAACCTATCGAAGGTATCATCATTTTCGTCTACCCGCGCTTTGTATAGGCGGTTATCAG
ATCCGATTAGTTTGATTGTTTTGAATTTAGCAATTTGAATCATCTCCTTTTGGTTTCGTT
GAAAGCATTATGCCGGATACGTGATCCGGCGTTTTAACAAAAAGTCCTATTCTTGATTGG
CCAGGCTTACAGCAAGCGCAAAGCCGCGCGGAGTGAGTGAGCGGATCAGCTTTGTTCGCT
TATACTTACTTCCTAGCTTTTGCCAGCCCGGATTGTTGTTCCCTGTTGGCAATACTGCGT
TTGTATCCGGCATCACAAAACCGTTACCCGTCCAAAGGCAAGTTTTTTTGGGGTACGCAT
TGTGAGCAGGAATGATGTCAGGAAAGTGAGGATGTTTATCATCTTACGGCAACAGACCGC
CAAACGAGCAAGGATGAAATATAAAATCGGGCTGCCGCCACATCGATGACAGGACGCTAA

CAGGATGCTCGATCACGTATGGCACATTGAACCGATCCGCAATTTCAGCCGCAATCACGC
ATGTTGAAACTGCCTTATCCTGGAATAGCGGATCACGTTCACGCGTTGCGGAAAAGTGAC
GCGAACCGCCAACAGCTAAACCAGTGCAGGGAGGAAACACGAGCAATAAATCAGGCTTCC
CGAAAACCTCATTCCGAGCCTTAAAGCTGAAATCTGAATCAATCCAAACGTTCACAAACT
CAAGGCTGGGATGACTTATGCGCACTGAATGGTAATCACCATGATCGGCATCATCATAAT
TGAAACAGATTACTTTGTGACCACATTCAGCCCACGGCAACGCATCATAACCGAAACCGC
TAAAAAGAGAAAAAACTCACATACTTAAATCCTTTGTGTATGGCCTTGTGAGGCGTTTTG
TTTGTGGTGTCGCAACTGTATTGGCTACGGCCTGAATTCTAGCCATAATTCGAAACAGGG
CGCGATTTTTTTACTTACAGAATGGGCATGTAGATTTGTCCCCGCTTCCGCCGTGTTTTG
GACGGTAAATTTTATCAATAGGTGTCATTTTAAGTACTCCTTAGAATGGGAAATAACGCG
TGCAAGATGGATCGAAGTTACAACCGCAATCATTGATTATTGCGTTAGGATCAGAAAGCA
TCACGCTACGCTCTTGATCCATAGCCATACCGTCTAAAGCAACATCAAGCGTCATTTCAA
GATAGGAAACTTTAAGTGCCCATTCCTTGTTGAGTCCAGCCGCCTCCGCTTCATGTAGGC
GAGCAAATAAAGCATCTTGAACTATATCAACCCCACTTCTCAACAAAGAAGCTCATTTCG
TCAATCATCGCCTAAGCACCGCCTTCAGTCATGCGGCGTATTTTTCCCCCTGGGTCTTCG
CCAACAGCGAAACCAGTTTTTACGCGGGTAATCGTTAAGTGATCGAATCCAGAAGCGCGA
TTCGTTCACAAAACTACTTATACGTGAGCTTTGACAATGTTCATGGTTGATTCAGTTTTC
AGTTACATGTGTGATTCTGCATTTTGTTTATGTGAAATTATGATAGCAATCAGTGCGATA
TCGTTTTTAGCAATTCGTGCTCTTTACGGCGATAAACGGATATCCTGCTAAATCTGCATT
TCTATAACGTGCAGACCTAACCCAACAGCCGCCTTAGCTCATAATTCAACCGTTTTTATC
GCGTATGTTCACGCGGTACATTCTTGCGGTTTTTATGTTTTTGAAATATGTCATAATTTA
ACCTCATGATATTCTATGAATTCTTGCACATATGCGCATTCACGCGGCGACTCCTGGACT
TTTAGGACTCCACAGCCACCATTAGACAGATAGCAATGTTGGTAACGGTTGCCGCCTACG
TTTTCCTCAGCGATCTGAATTCCGGTACACAAACCGCCACCAAACCTATGAACGAAATGA
TAAGCGGAGCACCTGCAATTAATTTGCATACGCTTTCTTTTTGCCGTGGTTTTCATGAAA
ACCCCTGTTTATTTTTTCTCTCTGAATACCCATGCTAGCTAATTCAGAAGAATCCAAAAC
ACCAATATGGATTTCTCTCGCGTCTACTTTTAATCTTGCTCTCCATTTTTTAGCTGCGGA
ATGCCACGACACACCGCTAAACCCTGATTTATTTGCTTTGCAATATGATTGATTTTTCCC
GTTTTCATTTGCACTAACAAAGCGTAGATTTTCTATCCTGTTATCAGTCGTATCGTGGTT
TATATGATCAATCTGGATTCCATCTGGAATTTTGCCATTATGCATTTCCCAAAAGACTCG
ATGCGCAAGATAACTTTTATCATTCAGAGATATCTTTATGTAGCCATCATTTCTTATGCT
TCCGGCAACCTTCCCAGTCCATATTTTATTCCATGCAGAGTCCCTATATGATCTTATGTT
TGTGCGCGGCTTTCTTATAAGATTTCCGCAATCATATATAGAAAAGGCGTGCCGATTCAT
TTCAAATTCTCCGGTTCCTTTCGATGAATGTATTATGCCAAAATAAACCGGATCGCTTTT
TACAAAAAGTGCTATTTAGTCAGATTCCATTACGATTACTGGTTCCAAACGCTCTAGCAC
AACCTTGAAATCTGCAAAGGAAGTGGGACGTCGCGTAGTCAATTCCTCATAGTTATCAGT
TGCAATGCAAGCGCGATCACCTTCATAGCACACAACCCGTTTACTATATGCGCCACTAAT
TGCAACAACGCGATATGTATTCTTGATCATTTCATCACCTCAATTGCATAACCATGTTCA
ACGCCTGGCGCGTATACGCGATTTTTATCATCAGTAACGCAAAGGCAAAGAGGCAAGCGA
CGATTGTCTGTAATATGCAAGTGATTTTGTTTATCCATCAAAATTCCATATTCGCGATCT
TCATCTAAGCGCCAAACGCTCAACATACCATCGCCCAGCATTTTCATTTCTGCTTTCTTG
CCTACAAATTTTTGACCGATCAGTTATTTATCCTCCACAGTTTCAAATACGGTATTAAAT
GCCAGTAGTTTAAATTTCTTTCCAGGCGCAACCACTGCGATATTCCAGGTAATTCCGCTG
AGTTATGCAACCATATCATCAATATCGGCGGTGATTTGTGAGCCCTTCTTGAATAAGCCT
TTTTCATTGCTCGGATCGCGAACAGTAGCCACAGTTTCGCATTGCGTTTGTTGTTCAAAG
TTCATTTTCGATTCTCCTGATTGGTTGATGTGTGTATTATGCCGGAATAAGATCAGGCAC
GTTTAACAACAAGTGCTATTTCAAAACACAAAAGCCCTGGCGCTAACAGTATTATCTTTG
ACTATTTGCCGCGACTCCAGGCAACGAGCTATGCTGTGAAATTCTTGCGCTGTTACCGCC
TCACCTTGCATTGTTTGACGCGCAATAATTAGCACGTGCATCATTTTTCGTAACCTTCAT
TGTATGCAAGGTTAATGTGAATTGATGCACTTCCGAGAATTTCATCGGTTCCAATCTCAA
TCATGTATATCAATTCATGGCGGCTAAATGATGTGTATTCGTGGAATTCTATTGCAATGC
GCATAACCGGAAGTCCACTATATTTTGTGTTAGGCACTGCATATCCATTGCCAGTGCTCT
TAATGCGTATGAGAATCATTTTATTTTCGCGCGATCCCAAAGATAGAAATTATTGAGTTT
GTACGACTCCGGCCATACTTGCGAATAACAACAATTAAGTTTTCGCTTTGCCGATGCCCT
AACGTGATCGGCATTTAAGTACCATCATCTAATTCATCACCAACCTTGTTGGGCGTTTCT
CGCCACGCGCAAATGAAACCAAATTTATCACGCGTGAGATAGCGGTAGATGTTAGGAAGG
GGAAGCTTAACCCCTTCAAAGTCTAACGTTTGCGTGTAACTGCGGAAACTGAGCGTGGAA

TTCATAAATCACCTCTCCAAATTCTCCGATTACTCGCGACTTGTAATATCCTCCGCGATT
AGCTAACAAATACGCCTGGCTCAGTGTTTCAAGCGCGCGGTTACTGGCTTGCTGTTTTC
GTGTTCCCAGGTAATCAAGGTATTCATTGCTAAATCCTCAGTTCATTTCAATGAGGTAAT
CATAGAAATTCGTGGCTGATTGGTTTTAGCAAAAAATGCTATTCATGCGTTAAACCGAAT
GGGCTAGACCGATTGGTCTAATGAACACATCGTTTTCTGTATCGAACACATTCACGTTCC
TGTTACGTGATCGTTATATGATCAGTTGATGATTGATGTGTG
>NewGenomeName_5
TCCCACTACCGTGAATACCCGCGCGCCCCCATGCCGCTACCTGCGGGTGTTTTCGGGTGC
CTGCGCCGGGGACAGCTCGGGTCCGCTGTGCGTGCGTCGCGAATTTGCAGGTTCGTATGT
GCATCGGGAGGTTAGGGCACTCGGTAGGTGAAGAGGTTCTAGATCGGTTCGACCTTCTGG
GGCTTGGGAATGTCGTAGGCGGCCCCGTGGTTCTCTGGTGCCTCCCCTCCTCCCTTCTAT
GATAGGGGTCCGGTTCACTGGTGCCCCCTGGCGTCTCTAATGCTCCCTAGGTCTGCCGCG
CCACGTATGCCCTGCCCTGCCCTGGCGCTTTACCCGCTCATTGCCATGGTTGCACTGTGA
GCGTCCGGCTACCTTTACTGGTTTCCGTCTTCGCCGGCGGACTCGTGTAACAGCAGGGCC
TCGTGCGCTCGGTCTAGCCCCGCCGCCTTCGCTTCTCTCATTCTAGTGCGACAATTCTTT
TAAATCTAAGTACGACTATGTTAAATGAAGAATGGGGACCGCATGGGCGACAGCAGGTCT
GTCGAGTCCGCAGCCATGTTCAGGGCATGAGCCATACGCACTAACTAACGCAAAGCGAGG
TGGGGGTTCTGGCCTAACGACCAGGCGAGGCGGGCAATCATACACTCCAACTAGTACTCG
TCAATCACGGGACGCTGAAGGGTAGCCAGGCTTGACACGCTAAACGGATGGTGTAAGACG
CGAGTCGAAACTAAAGGTAGCCGCACAGCACAGGGCTCAGGCTGAGGGTTAACGTGCCAC
ACTGTTCACCCTTGGCTCTAGGCGTTCCGACCGTGCTTGGCTGTGGTCACGGTGAGCCGA
TGCTTGAAGATTCCTGCCATTGCGACGCACTCGTCGCGATGGTAAGGCGCCACGGAGGAC
GGTCCGGCCAGATAACGATGGTCGACACTTTCACAGAGCTTTAACTATTCGAGTCTGCTA
TGGAACGCCACTTAACCAGAGTTACGAATTTGCGCCACGAAGGCCAAGCTAACATCGCCT
TTGTCAAGAAACTCTAAAGCAAGTACGATACAGTACAAGGCATCATGTAAACGTGTCAGC
CTTTTGCCTTTCGTATTGCCGTGCTGACCGCCAGCCAGAGTAATTCGGGTATGGTATGGT
AAACGAACCTCCGTCGGGAGGGTACCATAGCCAAACTAACAAGCGAACGCCTGGTATTAA
CGATGGGTACCAAGCAAGACGCAAAAGGTGACCCGCAGTCAGCGGCCGGGCACCTGCAAA
CGAGTGAGTAGCTTGCTGATGAAGGTCTACGACTTATTCCGGTGCACAGGTACACTGAGC
GGATATTAATCTTACTCGATTATGCATCTCGAAGTGGAGTTGGACCGATGAGAGTAGACC
ATTCGCACCGCCGGGCTTAGTGATCCTTGCCATAAAGGCGGAAAGTGATAGTCCGTTGTG
AGTACCCTTACCTTGTTGGCGAACCTTGGGCACGGCAGGCTTATCCTGCATTGGATCCAG
GCAGCCCGGGAGTAATGCTCGGCCGCTGAAGATGCAGAAGCATGCCCGAGATCGAATAGG
AGGCACAGTCATGCAAGTGAATATTCCCGCGTTAGATCTCAAGCTGCGCCCCAAGGCGAA
TCGTCAGCAGCGCCGAACCGCTGCCCTCCCGCCTGACTTAGGCGCTATGCAGAAGCGAGC
CGAGCACGACCTACTCTGTCGACGCCAGCTCATCCGCGACTCTAACCATGCTGACACGTT
GAATGAGCCGCCCGCCACCGTGGAGGCTGAGCGCCAGTCCAACGACGTGTGCAATAATCG
TAATAGACGGAACCTGACCGGCTTCTTGACCCCGCAAGGATGATAGCCGACTGTCTGAGC
CAGCAGCCTTCAAGCGACAAGGTTTGCCCTTGTGCGTTGACGCATTGCCTCGCCCACGAG
CTCTATAAGGCTATGCGCCGCGTATACCACTTCGCGATAACACCATCAATCAACTGGGTA
GTGTAGCCTTTCCACAACAAAACCGGGCGGCTTGTTCCGCAGTCGGAGACCCACCGGGCG
TACTCAATAGTACATGCTGTCTGCCATGCAGCATTTTGATATCATTGTCAAGCGCATTGA
CTAATCCGTGTAACCGGTCGGCATGGGTCTGCGCCTTCACATTGGTGCTCACGTAGAAGT
CAGTCTGCCCGACAAGCTCCTGAAGACGCTGCCAAAGGGCGCCCGCGTCACGGCATTGGC
CGATTGATTCCAGAACCACGGCAAGACCACGGTGAGCCCAGTAAACTCCACCGCCAGGCA
ATTCCCGCTTGTATTCATCAAGGACGGCAGCACCGACCGAGTCGGCGCAGCTGCCACGGA
ATGGTACTAGTGCATCGTCGACGGAACCCTGGCCGTGGAGTTTGATTTCACCTCTCAATT
ATCGTAACTGCTGAAGCATGCCGCCGAAGCCGAAGCCCAGGGCTTTGACGTAAAGGGCGC
CGACGCCCTGTAGAAGTGCAAGGGCGTGATAGGGTTTGACCCCATTCCGGGATAATAACG
ACGTAGCAAGTACCTTCCGAGAATTAGCGATAGGGCCATTGTTCACGGTGAACTAACATA
AAATGCGCAAGCTCTGCAGCCGCAACACTGATAATTATTTTGTGGGTCGTTGGCTGTACG
TAGGCCGAGGCGAGCAAGTGCATCTATGCAAGCACGGGTCGTAACCTCGTCCCTGCTGCT
CAGCGCCCTACAGCCTCGCGAGCTGAAGGTAACGACCCACCACCGTACCGAGAACCGCCG
CATAACGCTTGCACACGGCGCTCGAAAAGCGTGAAGACGAGGGGAGTATGCGTACTGTAA
TCGAGAACATCCGTTCCGGCGCTCGCGGACCTACCGTAAACCGTGGCGTGTGGCGAAGTG
GATGGAACCGCACCGGGTACACCTACGTCCACAGTTGCGCAGTCATCCCACGATCTAATC
TCAGCAGCAAACGTAATCCGGGCATGTTCCGCAAGGTGGGCTAGTATGCAAAGCGTGCAA

GACATCTTCCGAGTCGTCATCGATCATAAGCACTACGCCGTTAGCAACTAGCGGTTGGAG
ATCACGTGTTGTGCAATTTGCTTCGCTCTGATGACACACTAAATCTGCCAGGACAAGTTC
TGCGCGGAGAGTCGTGCCGTCTAAGAGTGCGTTGAATAATGGGGTGCCCCGCCGGGTAGT
CGGATGTACGACGCCTTGCAGCGTCGCGGCGCTTGTCCTGAGGATATGAGTGCTTCCGAG
TAGGTTCCCTAGGCCGGTAAAGTGTTCTACTACTTCTAGGGCGGGCACTCACGAAGTGAA
AAGCAGCATGTCCGTAGACGTTCAAGAAGTTTTCAACAAAGGCATCGACGCCGGCCTGAA
CTGGGCGGGCGAGGAATTCGAGCAGTTCATGTGACACGCCCGATTGCGTGAATTGACTCT
GTACCTCATCAGCTACGACGATCGCAAGTCTGCCGGGAAAGGTATTGGGGCGTAGGTGCA
TAGCACCCTCCCATTTGAGAACTCACTCGTTGGGGCACGGGGCAGGAAGCTGCGGCAGGC
CCTGACCACGGCCGCCATGCGTGAAGTAATCCTAATAGAACTGTACCGGGACTGGGTGGA
CCGCGCGATACCGCTACTGCCGGACTCGCAGCCCGAAACCCTTCTAGGTGAAGAGACACC
GAGCATCTAGAACCTGAAATGCATTCTAGGACATGACGAGTAGGCCACCAACAAGCGCTA
CTTGGGTATGCCGAGTACTAAGGTCGTCCGCATCTTCACCGTAAGTAAGAAATCCGACCA
CTCTCAGACACTGCACGCCCAGCGGGAGTACGTCAAGTAGCACTGTAGACTCGACGTGGC
AATGGCGCACGGGCAGACTAGCATCTACTGCAAAGCCAAGGCCAAAGCATGGTTCCAGGC
GTTCAACAGCTTGGGGCCCCTCGTCTACTACCGAGAGATCACCTACGAGGAGGAATTGTC
GCTGGCGGTGACCCAGTTCGGCGAGCTTGTTCCCATCTTCAAAGAGGTGGGCGCCCCCAT
GAGCCTGTGCGAAAACCTGTAAGGGCTAATCCTGTCCGACACTCTTGGTCAGAGCCGCTT
CTTCTTGCCGACCGTGTGGATCCCGAACGAACAGGTGGTTTTTGCCGAAACCTTGCACGC
CGCACTGGACTGGGAAGTGGACGGTGCTGGGGTGGTGCGAACCGTGCATATACTTGTGAA
TAACGACCTGTTCCTGGAGTCGGTGTGATATATCTGTTCCTGTTCTCGCTAATTTGAAGC
CTCTCCACATGGGCGCTAATCAGCTTCCGTACAGATGTGCAACTACTGCGTAACCGCGAC
GACAGCGCTGTATTTCCAATTGTACTCACTAATAAGCTAGCCGAATAGCAATGCTGGGTC
GGACTACGTCGCAACTTCACGGAATACATCTATGCGTAAACCAGTGTCAAGCGTAGGCTA
GCGCTTACGCACCGTGCGCCGCCCCTGTGGCGCGCTCTATGTCGAACCCTGCTTCCTCGA
GCAAGACGATACGAGCGTCAGTGTACAGGCGTTCAAGAACTGCCAGCCTCCGTACGCACA
GGACTTTGGGGCACTGGGCTCGGATACCGTTGTCGCAGTAAGTCTGGAGGGACCGAGCCC
AGTATTGTCGAGCTCTCCCGTTCCTGGCGTTATCGTTCGCAATGCCGGGGAGCAGCACGC
CTGTCACTGCACTGACCAATCCTGTACGGCAGTACACGCGCCACGTAAGCGAGGAGTCGG
GTGTTAGCAACTCCGCTACCTTCGGCGCTATCAAACCAGGCATTATAAGCACGCGGCAGA
AGACCGAGCGCTTCGAGGGGTAGTCCCTCTCCGATAACAACGCAGCGAAGTAAGGAGTCG
TTAAGTACGACCAACCAAAGAGAGTCACCACCGTGTAGAGGGAGGCGCCCCGCCCCATCT
AGTTGCTCCTTTCGGTGACGCAGACGTACCACCTGTGAAAACCGATGCACGATGCCTTGT
CTCGCCGAGCCTTGGATGAGACCGCGCTGCGTGCAGTTTCCGAAGACCTCTTATCCGACT
TGGGGCTGGCTACTACATGTCTAGGTTATCAGCACGTATTCGACCGCCGTGGAGGGGAGG
TGGGCTATACTGTCGCGCCAGTTCGTAGCACCATAGAGCGCCTCGGGGAACTCGATGCTC
AAGGATGACGGTATAGTCGTCATGGCTTATAAATCCGGCCAGTCCAGGAGCGCGCTAGTC
GCTACCCGGAAACGTTCCGGCCTGGCGGGCGACTTCACAGGCGAGATGCGCTTCTAGCGC
CCCGGCAGTTAGGCAGGGGTGGCCGCCAAGGTGAACCTGTACCAGGACCCAGAGGCACTG
GAGCATGGCCCGTACGAAGCAGGTATCAACCGCGGTAGTTGCCAAGATGGACATGGCTGG
CAGTGCGTATGATGCAGCTAAGATACTAGGTCAACCCGGTGCAATCTTGGGTGGAGTATG
TGGTGGTCGGGCCTGTTCGTGACGCCCTGTACTGGGCGAATGATCGCCGCCGCGCGAGCG
AGTGTTTTGCGGTCAGAGACAACCGGCTCGTCCGCGGAGTGTGGCGGCGAATGGAGCTGG
ACCAGGTTCCCGTCGAATCAGCGTGGGTTGTGAGTACCCAAGCGCCAGATGGTCGCACCA
GTAACCGCTACAGCAATGAGGCAGAAGCCGGCAAACGCGCGTTTAAACTCGCCTGTCTCT
AAAAGGACGTGCCGTCCTACTTGGCCCGCGTCAACGAGACGAGCACCCACCCCAAGCCGG
GCCTGTCGGAACCCATAGTCGACAAATTGTAATGATCGCGTACGCTAACCTCGCTGAAGT
AGTGGCCACGGATGACGCACCGGGCTGGGCACTCCCACGCAGTACACGCTAAATCACAGG
TGCATGTGTTCTCCGAGACCCCAGCATCTTAGTCAGAATCGTTGTGGAAAAAGTAGACTT
CGATGGGAGTATAGCCATCTCTGTCGGCTAGGACATGAACGAGGGGCCTAGACTGACCTA
GGAAACCGCTGTCGAGCGGGGCGCCGCCCGTAAATCTGAAGTGGAGCCACTGTAGTGGCT
AAAGCATGGCGTAAAAGCAGCTGCGAGAACACAGGCATCCGCCGCGTCGCGATCTTGGCT
GGCAGAGCTTTCGGTGTGTACCCCGGTGTCAGTGCCCTCATGGCCTCGCTGCTGCGCCGG
ACTGGTGTGGGGCAGGCGTAGGAATACTCGCTGAAGTCCCAGGAGGGCGTATTAGTCGCA
AGAGATACACTATAGAACCGGTTAACGGTCGCGTGTTCTAGGAGAGTAAGATAGACGTTG
TGCAAAGCTAACGGCACTGGCAGTTGAAGCACATGCGGAAAGACGAACGCGGTCACAACG
TCGACCGAGACCTAGCCGCTGCTAAATAGAAGGGCATGGTCCTGGCGATTTAGGAGGTGC

CCGTGGAGAAGTAGCAGATACCGGGTTTTGCATAGAGTCCCGCTAAAGATCTTGCGTCCT
CGCCGCCTGGACCCCGGCGCTATCGCCAAGGCGCGGCAACATCTCCAACCATATCAAAAA
AGTTAGCGACCATTTGCTTACCGTCGAAGCCAAGGCCGCCGAGCTGATAGCTAAGGCCAA
AGCCCTTCGCGTGTAGGGGGTTTCCCAGGAGCGCATCCAGATGATCGCCTAGCGAGATGC
CATCCCTTTCGTCATTGGCCGCCAGGACGACAAGCCGATCATCACCGTCGTGGTACTGGG
CGAGGCTAAGACCGACGCCGGGAGGCAGATACGGGTCCTCGCTGAGGGCTGCTTCATCAG
CACTACCTACACTCTCAAGTCTAATCATACCGGGGACGAGTGGCGTCCGCAGGCTGGAGA
GACTACTGCGGTCGCTGAGCAGCCTGGCGAGTAGCATCGGGTCTTAGTCCAAGAGGATCG
CCGCATAACCCACCCCTTTTGCGGGTACCCGTAACCGCGGCACTAGCTGCTCAATTTCTG
TAACGACGAGGTGAGACCATGAAAGCTGTTAAGACACTGAACGGCGCTGCTCGCGTGGCT
GTCCAAGGTAAGCTGGATGCGGATGGCATGGAGCCGCGCATGTCCTTTGAGCAGGCGCAA
CAGCGTACCGAGCATCTGGGGCGGGTGCGCTAGACCGTGCAGCGGTACCCCGGGGAAAAC
GAGGGCTCGTGCAACGAGCTTGCCCACGCACAAAGCCAGATCTCATTGCTAGATTGCAGC
TGGAGTAAGCAACTCGAGACCATCCAGGCCCTGGAGGCCAAGCAAGAGGACCACGACGAG
TTTGTGATGAAGCTGGGACTGGATAAGGACATTGCCGAGGCTCCGATTCAGGACCACGAG
CCGCAAATTACTACTATGAAGCGGATTGCCGACGCCCAGTGCGGTGAGTTCGTATGATTG
CACTGGAAGCTCCCCTAGGAAACCGGTTAATTTGAAATTGTGATGTTAGCCTGAACGCGG
GCTGTAAGTCACGTTGGGTGTACAAGAGAATCTAAGCGATATCGCCGACGACGTGAGTCA
GATGCGTAGAGACTGGGTATAGTTTTCCCACCTCTAGGGGCCCGCTGATTTGCCAGGCCG
AATCGATGATTCCGACGCCGTGCACGCCGAGGCCGAGGTCCTGCGGGCTCGGAATGCAAA
ATGGCAAAGAGGCCATCAAGATATACATGAGCCAGCAGTATGTGAAGCCCTTGCGTAGTT
CCAAGCACCACGCACCGAGCTGGATGGGGCGCACGGCCTGCTGCGCAATATTGTCCCCGC
CGCGCGCATTAACCCCTCTAGCCAGCAGGGTACGGTAAATCCTTTACGCCGTGGCTACTG
CGTTGACTTGTTAAGTGACGCATTCCAGGCCTCCACCCAGGCGCCGGTAGGGCGGGGCTG
TATTAAGCGTCACCTCACAATGGCCGACTAGGGGGAGCGGCAGCAGGCAGTGGAGTGGGA
GTTAACGAATAGCTCCGACGAGATTGTCAGCTTTGCGCCGATTTATTACGCTGAAGAATG
CGCCATCAGTTGTCAAATGGCCGGACCCTTTTCCCTCTCGACACCACCCCTCATCCCGGC
CCTTACGTCCGAGTGCTGGTGGAGGCGATGGAAGGAATACTCGAGTATATACCATTGGGC
AATTCTGACGGCGAGTACTTCAACATCGTCAAAGCCAAGACAGCCTTATCCGCACATTGC
CACCCGCAATCGGTATGGCCATACCCGAGAACGTGTTTGTGCAAAAGGCGCAGCAGCTGG
CCATAGGGCAAACCTCGCGCACGTAGCACTGCGAGGACAAACGTGAAAGCCCCGAGAACG
GCAAAACCACCGAGCGTTGGTGGGCGTACAGCCACCGCTGTCACGAGCGCGGGCAGGTGC
TGAAGAAACCTGTTATCGTCGGTGCAGACAACCAGGCCCAGGAACGCTTTATGCCATGGA
CCGACGATGCTAAACGCGCAGTTAGCGACTAGCTGTCCGGCAGCGTGGACCGCAACCTGT
TTGACAAAGGCATCAATCTCGTTGGTATTCTGCCTGGGGTCCCCGTTTAGAGGAGCCGCC
GGCAGGGTAGCCTGATCCGCGCCATTGCGCACGGGTTGTTGGGTCGCGGCAAAGGCACAG
TGCAGCCGAAGTGAAGCGCGGACTCAAACCCAGGTCAGCACCCGGCAGAGTTCCCACCTC
ATCAGCACCATCATATTACGAGGCAGTGTGTGATCACTGAGGAATACTTCTCAGCGATTT
AGATAAGCTGGGCTTAGAATGATCCAGAAACTATTTCGAAAGCGGCACTCGGGAGTCGTG
TGCACATACGCCCGGCGGCTGCTCACCTCGGAGCAGATGAGTTGCTCTACGTCTTCGATG
GGGATCGAACCGGGTGTGACGGGTGGCCAGTAGCCCCCGGCCGCTTATGCGGGCTCGGCA
AGTCAGTGTGGATCTGCGACGCCCCTCTGGGTTTAGACCCCAAGGCTATCAAGACGCCAC
AGGTACAGGAGTTGAAAAATGGGTCGCCGCCCCAGTGCGGAGGAATTGGAGGCTATCGAA
CGTCCCCACGCTTCCGGGGTGAAGGAGCAGCGGGAGTACGGGCGGTACAACTGTCCCCCG
GGCATTTGGTACCGGATCGGGTCGGTGCGCGTTATGCAGTGATATCACTGCGGCTGGCAG
GACTTCCGGGATGTGCAATGAGAAACCGTGACCAACCGATCCACATGCTCAGGTACTGGA
TCAGTGATCATGGTATCGAGAGGCGGGCGGGGCATTACCCAAAGCTGACCGAGGACGACG
CCGCAATATGTGCGGCGTTGGAGCAACGTGCCCTGGTCGAGAGTGGGATGCATTCCGGGG
CGGATGACCTGCTGGACCGATCTGGCGACGTTGAAGATTATGGCCAGCAATATTTTGATC
CTTTAAGCGCCGCGGCAGAAGCAGACGTATCGACCGCTGATAGGCACCGTGCCCTCCGGG
GACCTTGGCACCGAGGCGAAGTTCCTATAGGCCTGGTATCCGTTAGCCCACCACGGGTAC
GCTGTTTACGAGTACCCGTCCCTCGGCGCAATTGGTTCCCAGGTGAAGCTCCTGTGCGGT
TACAGCCGTGAGCAGATGGCGAGGGTACGCAACGCCCTGCAGAACCGGCGCCAGCACGTT
GACCAGGATCCCATTACCGGCGTCTTGAATACTCTGCACGGGCGGGATTTCGCCGTTCGT
GACGGTGCTCTGCTCGCCCAGCACAACGCAGGGGGAGACGTTGATCTCATGTTCGAGTTC
ACTGGCATTGCGCAGGAGACGCATCTCGGCGTGCCCCAGGGGGCCAGCTCGGAGTGGTTA
AGGGACAGCAACGACCGCATCCCGAAAGGGGAGGAGAGGGACTTCGCCCTACTGTTCCCG

CTGCAAGTCTTGGAGGCGCACGTCTAAGGCCTCCTGGTAGGCGCCCCAGTGATTTTGACG
GGGCTCTCCGCGAATCGTAAGACGGGCCTCATTGGAACCATCCTAGCGCGCGTTGCGGGT
CAGGTAGACCGATACTCCGTCCAGGAGCGACCTATCCGCTAGCTGAACACCAGGGGTCAG
GGTCAGCGCTTCATCCCTCCGAACTAACAGCCCGGCCTCGGGGGGGTCATGGACGTGCTG
GTGGAGTTGAGCGACACCGTCACGCTGCGGGATGGGTACACCTGACAGGTTGGCAGTGTG
GATCGTGTCAGTTTACCGGATAGGCACTCTGCGTGATTCGCCGATATCGAGCAAGTCATC
GAAGCAATCAAGCACGGTATTGTCGTCTTGGATCTGACGTCGAACGTCCGGCTGGCGCAC
AGCGCCGGGCGTAGGAACAAGGTCGATGAGGTGGAGGAGAAGGAGCCATCCCCGCGTGCT
CGGTCGCTGCGCCACGCCCTGGGGAGCCTTGGGGCTGTCCAGATCAGCAACGAGGGGGAG
AACATTCTGTAGCCGGCGTACGCCGCGCTCAAAGACAGCAAGACCGGTAGCCAGGGCGAC
GTGGATCTCATGCTGACGATCGGGACGCTGGATGCACGTGCAATGCCGTGCCTACGTGAT
ATCAGCACGCTGAAAATCAGGGTCCAGCTGCCCAATGTACCAAGCCATATTACCTTGGAA
GTACTTTTGGGTGCAGCGAGATCTGAGTTCGACCAAGGTTCCTAAACAGTGAAAAGTAGG
CGTCTCAGACCATGCGTTATAGGCCGGGCACGGTCTCATCGTAAAGCAAGCGGGACACAA
GTGCTACCACGCGTCAGTCAACATAACCGACGCCAGACCCGCCTATTTCGAACAGCTGGA
GGACCGCATGAAAATCCAGATCAACAATGTTCTGTACGGACTCGGTAGCATACGTGGATG
GCCCAAGACCTCACCCTCGAGTGGAGCAGTATAGAGACTGACGAGGTGGACGGCGCACCT
GATCACCCATGACTCCTTCCCCAGCTGTCTCAGAGTGACACCTGGTGACAGGCTGTACTC
ACCCATTCCGACGCCGCGCGCCCGTCTGAGCATATCATCATGCACGTACCACCGGAGCAA
ACGAAAGATCACTGAACCTTTACGTTGGCCAGGTGCCTCGCCATATATTTACCCGATCGC
GATATACAATGATCTCTACTACATGTTGTGCTTGGATGAGACCCAAGTAGGCACGTACCA
GCAACTAACACTCGGCGGACGTTGGTTCACGTGCAATGTTGATGAGTAGCCTCCCTGGGA
CGAGTTCGTGGGGGTGGCATTCCTAAGGGGACAGTCGAACCTTAACCTCTACTGGAACGC
GACTCAAAACCTGCGTTGGTTGGCGCCAACTGGGCTGACTAAGGTATTGAACCTCACTCT
AAAAGCGAAGCGGGTTGGCTAGATTCGGGCACTCCGCCATGCAAGCCCCGACCAGGCGCG
GTCGATGCTGGAGCGTCCCCGTGACACAGGTGAAGCAAAGTACCAGGAGTTCTTGAATTA
GCACTAAGATGCCCCCCATGTGACATCCGACGGCGGACCATTCGGTGGACGGGCACCAGA
AGGCAGGCGAGATGCGAGTCGACCAAAGCCCGCTCCATGCATGTGAGATGGTGTCTGGGT
TCCGCCTACTGATCCACTTGACCTGCCAAGGGGAAGTGGCATTGACGTCACGCGAGGGCA
TTGAATGCAGGCACTTGTCTAGACTTGCGGTTCACGTTTCTTGCACCCTGTTCACGCCAG
TTACACCACTGCGTAGATACTACGACCTGGAGCTCTCGATCAACGGCATCCCGTATGAGA
AGATGACCGGAATACTACGCCTTATTACCCGCCTGCTCGGCCAGCACCTGAAGAACGTAC
AGGTCCTGATGTTCGACCTGGGAATATTAGGGGACTACGAAGGCCGGGGCGAGGATGGTG
TTCCGAATGTGCCGACCTTAGACGAACGTCCTCGACTCATTGGTTTCTAGGGGCGGGCCC
CATTCCTCCCGCTGATCGAGCGCCCGAGGTCCCTGTTGATCTAAGAGCAGTCTTGGACCG
TCTGCGCACTGGCTCTTATCCGGGCCCTGCCCCAGCCTGCCCGGGCTGCCGAGTGCGAGG
GCCTGATCCTCAAGGGGCCCCTCTTCTTACTCCGCAACGGCAAGGTCTCCGGGTGCTGGA
ATGTGAAGCATGGTTGCCGCGCCCCTTTGCCGCGTCTGTACTAGACGGATGGTACTGTCA
GCGACACCGTGTGGGGAGCCCCCGATGCGTCGGACGCCGATAAATTCATCGAATGCCTAG
TACGTATGGATCATGGGGCTGAAGTAAACGCAGCCGGTTTAACGCAGCACCACAGGTCCT
GAGACAACCAGAGTTACCACGCAAGCGCCTTTGAGGGTGGCATATCCCATACGCACTGCT
ATCGCCTCGCCTGGAATTTCCCCTATATGGGGCCCACTACACTTGGCAGTATCCGCCCCC
CCAAGTGCGACGGGCTCCGAGATATCCACAGGGCTGTAGGTTTGAAAGCGTGAAATAGTC
TTTTATGTTGCAGCTTGGCCCGACGCCGAAGCGCGGGGTGACACTAGGGATTGGCCCGGT
TTAATCCCGTCACTGGAAAGCGAGGTTCGACCGGCTCGATGGTGGCCAGTTGGGAGCCTG
GTCCCTCGGTGTGGGCGAGGTATGGTTATTCTGCTGTTGGCAGAAATGAAGGTACGGACA
ATAGATTTGGAGTACGATAACAAACCCTGGTCCGGGCAGCTCGCGTCCCCGCACAACCCT
GATAGCTACGTGGACTTGACCCGTTCATGTGGCCGCGGCCACGGCTTCACCCGTCGCCGC
GTACAGCATCTGCAATTCACCTCGCTGGGGAATGAACGCAGGTGAGACTGGTTCAAACTC
GACGGGGTAGATCCCCTCGAAGCACCCATCGCTGGGCACGAGGTGTCGTGGATCATAACC
CGCCACCTCAAAGAATTCGAGAAGTTCCGCTTACGTGCTGGCCGGGTGTTCTGTACCCAG
ATCGCCGAGTACATCCTTTCAAACGCCCCGCAAACGTACACCAGGTTGGAGGAGACAGCA
CCCCATTACGGTGGTCTTCACAAGATCGTTGCTGTAAAGGAAATGTGCAACGAGCGGCTC
CTCACCAGAAAGCTCGGACCGAAGTTCCGGGCCGAGTACGTCGCCGGTCCCAACCGTGAT
TTTAAACACACCCGCCGCATCTTCTGCGGGCTGTAGACGATGCTCCAGGAGCGCGTGCTG
TGAGGGCAGTTGGTGGAGCGCTGCGTAGACCCGCTGGCTTTCGCTTACTGCGAGTTCTAT
GGCTGGTACCTGAACACCGAGGGGACCGAGGCCAAACAGACCAAGGGGTAACCTGAGCTG

CCCCAGCTGCGAGCCGCAGTATAGCAGCTCACGACCGATCTGTCTAATCGGTTGAACTTC
AAGTGGGGCAATGACTACCATCGCAGCGCCGTTCTGTTCGGCAGTTCGGTGATATGCCGG
GCCCGCGGACGCCGCACCGGTGCCGGTGACCGCGCAGTTTACTAGAAGTATGAGTTTGCC
ATGCTCGGTGACAAATTCATCGCTGTGGTCTACCTCAACGCCTCGGTCGAGGCGGTGGTC
GCACATCTATGCCGGGGACACGACAACCCTCATCGCCATAAGGCCGGCGAGAACAAGGGC
GAGTTGAAGATTCATAAGGTTGAATCTGATCACAGGCCGATGAAGTGTGAGGATCCGGTG
TTGGTGTCCCTCGGAGGCTTATGGATTCTGCAGCTGCCCTCAGACGTCATGGACGCGTAC
GCGTAACACGGCACGGACTCGCGCGAAAACGGGGTTTGGACCGGCCGCCGCCTGCTGTGC
GACGTGAAGGCCGCGGTGTATCACACCAGCTCGCAGGTGCCCGAGGTACTGGCCTATCAC
GGCTTCGACGACGCCGATAAGCTTTAACGCACGGTATGCCTGGAGAAGGATAACGGCACT
TACGAACCCTCTGTGGAGATCGCCAAGGAGTGGAAGACCAAGATGGTCATAGGGATGCTC
CTGGACCACCAGACGGACGGGATCATCCACATCAGCTTGAACCTTCACGCAACGGTCTCT
GTTTGCCTGTCCCCCTCGTACCGGCTCCTCCAGAGCGTCACTCGAGCCGACAGCCGCTGG
GTCAAGGTGAGGTTCGAGTGTCTCTTCGGGACTGCGGGGCGGTTATTGGAAGTGGACTAC
TCAGTACTGTAAATGGTGATGTTGGCTACGCTCAGCTCCGACGAGAACCTGGTAGGGCTA
CCCGTCACCGGCTCACACTCACAATGCCTGCGTCTGGCCGCGAATCTCTGTGAACCCTAC
GAGCAGGTACTGCACAATGCAGTCGAGAACAAGCCGCATGCGGAGGACAAGGGGTACAAG
TAGATGCGGACGTACATCCAGCCCCGTAGCTTCGCTGCACGGTACGGGCCCTGTGCCGAG
GGTATCGCATACGCCACCGATTTATCTGTCGCGTACGCCAAGGATTTCCTGGAAACGGAT
GCCCGCCTGTTCCCGCTCAGCATCCTAATCAATGGGATAATGCTGGCTAAGGTCTAGCGG
AGCGGCGACCTGACCAAGGGCCGGGAACGCGAGCATGACGACGCCACTGGGGCGTGACGC
TTCCACAGCCGTGGTTACTGCCAGGCACCAGATGGTGCCCGTTACAGCTTGCGGGCGTGG
ACGCACCTGAACAAGGAGTTGGGCTATCAGGTTGTGGACTGAATCGACACCCGGATCGCC
AATTGCTGGTGCCAGAGCGACGCATTCTAACTCATGGCTGTATCGGCAGTATGAGTACTG
AGTTGGGTGCGTTCCGAGAATTTGCCATCGAACTAGGAGTTCCCGCAAGGTCGCGTGTGG
TTGATCAACAAAGCGCACGAATGGCTATAGTGCGACGTAGACAAATTGTTCGGACGGGTG
GTCGGGCTCCAGGTTCAGGGCATCATGCTAGACCAGTAGACGGAAAAGAGCGACCGCCTC
CGCTTCAAAAGCGCGCACTTCCCGTTCCCAGCATCCGCTCAGGCCGGGCTCTACATGGCA
GATGAGGAGCACATACACTGAGCAACTCCAATATCCCCACCAAGCACTGGCCCTAACGGC
TGACTGGCTGTACGCAATACCGCTGGACGCTCGCGAGTCTTGGGACAGGCCGACTTCGTA
TCGGGAAAGCGCAGGATAGGGTTACCATCATGACCCTGTCTGCGGAGGTCCTCTCTGTCC
CGCGGGGCAGCCATGCTCTGGGGTACAAGGCATGAACGTCCCCACTTATAACGTGTCGAT
GGTATGCCGCGAGGCGCCCGGCACCCCGCAACAGCCCCCCGAGCTGCCGACGAAATACGC
CTGCTGCTACCAAACGCAGCTTAAGCCACCTGGCTACGCCGTTCCCATGGAGGACCCCCA
GTCCGGGCGAAATTACTTCTTCAACCTGAACGTAGGTGAGTGCTTTGACGCCACCCCCGT
ACCGGAGAGAGTATGAGCCAGAAATTGCCAGAACTACTCGCCCTTGCCAACGTTGGCGCC
GAAACCGATTAGGTAGACCTAGGCTAAGTCTTCATGGGCAGAGACGATGGTAAGCACTAC
CCCGCAGCCTACGTTTTTGACAAATTCGTGCGGTAGATCGAGTTCGATATGCACGCATAG
GGGACGCAGGGAACGGCCAAGGACCCAGCCCTCCAGTACGACCTGGGCTTCACTCAGTTC
GGTGGGGTCTACCAGCACGTTTACGGCATCCCCGGCCTCTTTTCGACCTATCACATAAAG
ATCAGGGACGACGAAAAAACGGGCACGAAGATTATCTACGACAAGATGACCTGGATGGGC
CCGGCCAAGGCCTTCGTGCAAATACTCCGCGTCGCCGTCCTCGTGAAGATCGCGCGCAAG
GTCGTCCAGGAACAGCCCGGGAATCATCGGAGTGTCATGAATCTGGACGAGACCCAGCCG
CCACTCGACATGACCAGCAAGCACGCGTACAAGACTCCACAGATTCTCGACTACCCGCTC
GCCCTGTTCCTGTGATCACACCCGACTAAGGAAGCCTGGGACACCATGCACATGGAGGGC
ACCAATGAGACCACCTGCACGTACAAGAACTGCTTGAAAGAGATGATCGTCCTGGCTATC
GACTTGCCTGGCTCGGCCCGGGAGCAGCGCCTGTGCGGCACGGGGATCCTGCCCTCGCCA
GAGCAGCTCGACGCTTTGCCACCTGCGACGCCCGCAATAGCGCAAACGCCGGCCCCGCCT
AACATCCCGACAGCCCCGGCTATCCCCGCTGCCAGCGCGCCCGTCGCGCCGGCTGGGCCA
CAGGTCCCGGCAGTTCCTCAAGTGCCCGCTGCCGCACAGGTGCCGGTAGCGCCACTCCTC
CCGGACTTGCCCTGTACGCCGGGTGTCCCGGCAGGGCCGGAGGTACCGATGGAATGGGGA
TCATCCGAGACGTGGTGGTAACCGGGCACGATGACAAGTTCGAACGGATGGTGCCTAACC
GAGTACTGCTGACGGATGGCGTTGGGGCGCGGTACCGCCCTAGCGCTATTGTGAAGACTC
ATAATAATGATGATCGACGGGTCGTCAGGGAGCCTCTCCCCGACCAGTGCCACACCGGTC
CCCAGGAGTCCCGCGTACACCTCGCCGGCCAGGGTGGTCCGAAGGCCGTAGAGGGCCTGT
ATCGGACGAGGAATCCCTACCAGTGTACCCGTAAGGGTAAGCCTAAGACGGTGCTCTTGG
AGCCGCTGGGGTGGCTTCTGGCTACGGAGAGGCTATTCCATGACGGATTCCCCGAGAAGA

TCGGGGTCCACCTGCATCGATACAGGGAAGCCGGCGAGGCCCTGGTACAGGACGGCGTGC
CCTACGGCGTTCGCTCCGTCAGGAAGTCTGATGACCAGGACCTCCGGTCGACACCCGCCC
CGTTCTATGACTCGGAGAAGCGTAGGGTCGATGTCCTCCACGATGGCGACCGGATTGTCA
GGATCAACGCCGGGTTCACGTCATCGCTCTCGCTTTGTGTCACGGGGCATAGGAGGAAGT
TCTTCTGGCCACTGATGCTCATGGTGGAGACCGCGGATCACGGCCGTGGCCTCCAGCGCT
GGGAGGGGAAGAGCGTCGCAGACGCCGGTTCCCTGGACGTGCGAGGTCCTGTAGAGAGCC
ACAACGAGGCGGCCAACTGTATCTTTTGGGCCTACGGCAATATCGGCCAGGACGCTCTCG
CAGAGGCCCATGTTCTGTTACTGCGCCGCTCCGTGCCGGACTGCGCGTCCCGCTACCTCT
GTGAACTGGACCTTAACCCAAACCTGCGAATGTGGTTGTACTAGGTGAACGGCAACAACC
CACGAGCCTACGAGCAGAAGCTAGAGGAACGTGATGCGTAAGGAGAGTGGGACGAAGCTC
AGGGGCTGGCGGATCACCCACGTCGCGAAAGCGCTGGGATGGCGATGTCCACCCTGTGGT
ATCACGACTGTTTCGAGCCTCGCACGGCAAGCGGTGGTTGATAACCACCACAGTCTACGC
GAAAACCGGTACGGTCTCTACCGATCCTGTAATGCGGCGGGAGGCAAGGACAGCAAGGTC
CCCGGTCTGAGGTGTACCAAGAGGAGTAGCTATGTCGCCAACTTCGCGTTCCTGGAGAAT
GTGTTCCAGGACCTGAAAAGCTACGCTCCTGGGTTAATGTACCCAGGCCACAACCCACCC
GATCCAACCGCTGAGGCGATCGGGGTGACAAGCAGCCCGGCAGCAGCAGTGCGCCTGGCT
TCCGCCGAGGGCCGCAAGAACCCGCGTATGGAGACCCACCGGGTAACGACCTAGTGACAC
GAATTTGTTTCAAGGACAAGAACATACCCAATGCACTGTTGGTTGGCGGTGGTAACAAGC
CCCACGCCGGACATCACCTCAGCTATATGGGACGAGGGGTAGTAAGTCGGGAACTCTTGC
GACAATGTACACAACAGGGTAAACAGAAGGGTCGCACAAGCCAGAGTGAAACGACGGGGG
AGCCGGCAGGAAGTGCCACGAGTGCTGGGGGAAAGGGTACGCGCGCATTGGAACACGGTC
CATGAATTCTGAGCATCGACATCGCGAGCGGTACCAAGGTAGGTTGAGTGTGGGGACTCT
GGGAGTAGAACTTAGGACCAAACCAGTTCACCAACCTATGGAATATCCGCAGCTACTGTG
CGCACTAGATGCCTGATCCGATCGCCATCTACTCCGACCTCACCTAGGGAGAGAGCATTG
GAGAGGATTGGAAGCTAGTAGCTGAGCTGTACGAGCGGCAAAATGGAGCAGATCTTGTAG
TGGCACAGAACGGTACCCGATTCGATCGACCGAAGAATTAAGCCCAATTCGTGATGGATG
GTTACAAGCCAGCTCGCCCGTTCCGGGTAATCCACACCATGCTAATGCCGAATCAGCAGT
ACGGTTCCGCGAGAAAAAAGCTGGAGTGGATGAGCGACAAACTGTTCACGACCTAGAAAC
TCGAGCATTAGAAGTCCCCCGGTATGCCGTTGTGGCACCAGTTCTAGGCCGGCAAACGCG
GCGCCAGGCAGGAGATGCCGCACTACAAGATTGACGACTTAATCTCGTCAGAGGGGTTGT
AGTTTTGCTTACAACCCTTCTACCATGGCCATAAAAACGTGCCTGTCTTCAACGATGAGC
AGGGGATCGCCTGTCTGAAATGCGTCTCGCACGACCTAAAGCGCGAAGGGTGGACTGTCC
CCTAGTCCTGTAAGCACGAGGTTTATCATGGTAACGGTTGAGGCGGTTACAACCTTAAAC
GCAAAATCATACACTCGAAGCAGGTGCTTTATTCTCAGCCATCTAATTAAATACCCGAAG
GCATCCGAGCGCTATTTCTACACAATCCCCAGCAAACTAGGCCGAAGGTACGGGGATGAG
TATTGTAGGAAGGAAGGTTCTGCATGCGAGGGTGGGTACGTTCGGGCTGTACAAACGCCC
CTTGTGGGCCGGGAAAGGATCGACGCCGCCTACAACGCGGACACCGACAAGTCTGGCTGG
AACGTCCTTCTAAACTCCGCCAGCGGTTGTGGGCGAGCCGTGCGGGCCGTGGTTCATGTA
CTGAATATCCCTGTGCGGGATATCCGTGAAGGCGGAAAGGGGAACATCCCGCCTTCCTAG
CGCTAAGTACCGGACGCAGGAACGCGCTATGAGGATGACCCGCAACGCCGATTCGCCCAT
AACCGCGACGGTGAGACTCACGACGCCGAGTACGGCGAGTCGCTCTGGGGACAAGTTGAC
ACGCACGCACGGTTACTCAGGGTACAGGATCATCAGAAGCAGAAGACTGATGAGTGAGTG
CGTATCGTGCTTGCGCGGCGTTGACAGCTGCAAACCGGTGCCACGGAGAGTCGTCAAACC
AGAGGTACTCGACATCAACGAGCTGCGCCCGTACCACTCCAGGAGCCTAGAGCAACGCTT
TGCGCTCGCGGCTGAGGAGTTCAGCGAAAACAACATTGGTCGCGTGTGCCACAAGCCCTG
AGGTCCCGTGAGGTTGGACTCTCGCATAACGCAGGTCCAGTGCGGGAAGCGCCACAGCGC
TGAGCCTAATTCGCACCCCCTGCGCGACGTTCAAGAGGCCGCGCGAGCCGGCAGGGTCGA
TGAAATCCAAGCGGCCCGTCACTTTAGCGCCCGTGTGTCCGATCAAGTGCGGGATGCAAT
GGATCTGGTTATGCAGAACCAGGCACGCGGCCCAGGAACAGCGCTGTGTGGCTGGCCGCG
TCGGGGCCATACAGAGACCCTCGACGGTCTGAGCATCGGCATCGTACTGGGGCACGTCAT
GCGGGACACCGCCGCGTCACCAGCCAACCTACACTGCATCGGACGCGCCCTGGGGCGGGC
CATCGCGCTGGAGGCATTGGTGTAGGATGGATACCCTACGACAGGGCTATACCTCGACCT
GACTTCGCTATACGTGCGCACAGCGGGCACGCCCAGTAAACGGCACACCCAGACGACGTT
GGGTGCTGTGGTACACACAGTCCGGGTTGCTCAGCTAGGCGGGAACCTTACGCCCACTGA
GTACTTCCACATCGGCGATCACGGCCTACATGCGAGCATGGATACTGGGCGGGGGGAGCT
GCGGCGACAAGGGCAGGGTGCCGAGACGTATGCGAACTGCGTCCTCGCGGCTGAGGTGCG
GGCGGTCCTCGGTTTCGTGCCGCCTTAGATAGGCGGTGCCGCGCATATGATATTCGCTGA

GCCTATCGCATGGGACGGCGGGTCGGGCGGTTGGTCCTGCACAGGGCGTATGCAGCTCGA
CTTCCCACTGAGACGTTTGAACTGCCGTACTCGCGCGCCGTTGCGCCGCGACTTCCGGGA
TATCTTGTGGTAATGCGCCGATGTGATGGCCTGTGCGAACTAGCTTCAGGTCGAGGCGTT
CAGGATACACACCTCCACACGGGAGCTGATCAAGGAGGTGTGACCCAGCGACCGTGGTGT
CCTGGGCATACACTAGCACGGCCCACCTCCCGAGCTTGCGTTCGCATTACCAGCCAGCTG
GGACATGGACTCAGATCCCTAAATGTAGCTGGAGCGGTTTACGAGTTGGAAGCGTCATTT
GCTCGCCTGGCGCATGGGCAATATGGAAGGCGGCAAAGCTCAGCAATGCGTCGGCCAGGC
CCCCCATCTCGCCGCCGGCGCTTCAGCTGGAGAGTGGCTGCCGACGTTCATTGACAGCAG
TGGTGGGTACGAGTACCGTGGGGTGTTGAACCGGCGAGAGGACCAGATTTATAGGGAGCT
GTTGCACCTCGCCGAGAAGCGCCCGATGGGCCAGAGACGCCTCTAACGGATCCAGGTACT
TGTGCCAAATTTATCCGGCGAGGACAAGGCACGCTTCTATCCAAGGTCAGCGTAGGGTGT
TGAGCCCACGGATAATTTGTACGCCGCCCTGGAAGCTCCTGCAGACACGTGCTTTGCCGA
GGCCTACTCCTCGCTCGGGGCATCCGCTGCCTTGTGGCAATGGCGCGTGGCCGAGCGCTC
TTGAAACCCATCAACTAACGGCACTCGGCTGTGGATCCACAAGGAAGCGACATGCTCTGG
CAGCTAGCACATGTCAGCGATGCTCCACGACCCGGTGGGTGGGGAGTAAGTGGACCGCTA
TGACTGCGTGGGGGACTCCAAGACTGACTTCTAGCTCAAGATGGCCGAGGTGGAGGAGGC
CCCGCTCAACCGCGACATAGCCATCCCGGCCTATAAGGAACGGCACCTGGCCAAGCTCTA
GACAAAGTGGGTGTCACCCTGCATCCTGACTAACGAGCCCGTAATCACCTACGTATTTGG
AGCCAAACCGCGCGGTGTAGCGGAGGGCATTGCCGACCGGCTTGAAGACCGACCGGCGAT
CGTACCCGATAGTGTACGTATCTTCGACCCATAATACGACCTGCGCCGTGTGGTGTTCTC
TGCAATCGAGTATGCTGCGCCTGCCGCCGCCGCATCGATGCGTTGGGTGCGGGAGCAGGC
TCGCAGCGCCGCTGGCTTCTCACCGATGTTGTGGTTAAGCCCCTCTGGCCTACTGGGGGA
GCTCGACGACCGCGGCTCCGCGAAGCACCGGGTTAAGATCGGTCCGTGCGGTATCACAGA
CATCGTGGTGGCGGAGGCCCTCGACCTGACGCGAGACAATCGGCTGCAGAATGCCGCGCC
AGAGGACTTCGTACACGCCCTGGACGCACCGCATCTAACCTACACGGCCCGACTGCTAGA
GGTCGGTGGACATGCAGTGGTGGCGATCACCGACAGGTCTAGTACCCACGCCTCTAGCGT
GGATGAAATGCACACTGCTGCCGGGGAAGCGTTTGTTGGGCTGTACTCGGAGTTCGAATC
TAGCACTCTGTTCCTAGGTGGGATCGGTCACGAGAGTGTAGTACTACGGCCGAAAGGGTA
CCTGGAGTTGACGCTGTTCCGAAGCTCGCAGTCTGGGTTCTTTGTACGGTCGTACTATAA
TTACCAGGAGGAAATCGTCCCAAAGGAAAGCGAGGCAGAGGTTGAGTGCCCATCAGAACG
GATCTTGTAGTTGCTCACGACCTTCGGTTCCCAGCCCATCGGCCCTGGTTGGGATGTGCA
CGCAGTCATGTACAGGGCAGGTAGGGGCACCGTGATTGCTGCCTGTATCGCCAGTGTCTC
CACCCCGGTGACATTCCGCCTTGCCCACATTGATCTACCACTGGCGGCAGTCAGCGGAAC
GCACTAACGGAAGCTGGTACATGGATCAGGCGTAGGGGGAGCAGTCCGAGCAGCATTGTA
CACCCTTTCATGATGTTAAGGGACGCGCGCAGGTGGTAGTGTGCTCAATCGAGCGCCTGG
AGTCCTTGGCCACCATGGGCCGAAGGGCAGCCGGGATCGCTGTAGACGCAGGTGACGATG
GTCTGCCGGTAGGCGAGTGCCTCGCGTTGCAGTGGCCGTTCATCCTGCCTGAGTTCGGGA
ACGAGGGGGTGTCCCAATCGTCCGTTCGCAGAGAAAGGAATCTTGCCCGACAACTAAACA
TCACCACGAGATCCCTTACTCTCCGGATTGGTCCGAGGGATAGCCAGACAAGCTACAGGA
GGTTATATGGCCAAAACGATCTTAAAGGAAGTGGGCAGTGTCGTCAGAGGCTCAATTGGC
GGTGGGGAGAAGAGCGGTGCCGTCTTGCTTGGTGGCGGGGAGAAGGACTCCTCGAAATTA
GCACCGGCAGCGCCGCTGGAGGAGCAGTGCCGTGTAGCCCAGAACCCCCAGGTGGACCTG
AACGAAGAGAACATTCCCACGAATGACTCTGGTGGCACTGACGATTCACAACCAGAAGCC
GGCACTAAGGAGCGTGGTCGTCTTGCCGTAGTTGACGGAAACTCCCTCGGCATTAACGTC
GGACAGCGATAGGACGGCCAGCCCCTGCAATGTGGGCCGGCTACCAAGATTCGACTGCAA
TCCAGAAGGCCCAGGGCTTCAAACAGTTGACCATCGCCTCACTGATGGTGCCGACGATCG
ACAAGACGCACCAGTCGGAACTCGTTGAGTACGACTTGCCTTGTGCCCGCGCTTTCCTCC
TAAACAACTCGCCGGCCAAGGTGGCGGTGATCCTCTTCCCCCCGGGCCGAGACAGCTGCC
AGATCGACCTGGACGAGTCGCCACAAGATCTGGCAGCCGCTGACGGCATCGAGCAGTCCG
GACTGCACAATCGCACTGCCGACCTGGAGCGACTCGTTGCTCCGCTCCGGTTCGTAAACG
ATGGACTGTCGTAGCTGCACCGCCTGCTCATGCTTCTGATAGCCACAGGCAACGCCCGGT
GATACCGCTAGCCAGGCACAGGTATGATTCCCATCTGGCCCATGCAGCCGTACACGGTGA
TCAGCAGCTCTTACTGCAACCCCGCCGTGGTTGTCCCCCGCCAGCAGATGCCGATCCCAG
AGCTGACTCCCCGGATCCATGCAGATGCCCATGCGAAGCAGATTGCCAAACGCTACTCAG
ACAAGTGCGTCTTGTAAAGGTTGATCGAGGGACAAGCAACCCCGAACGGAGATGCATCG
CAGTATGGCACGAGCAAGAAGGTAAGTGCCTCGGCCCGGAATCGAGCTACCCTGAGCACG
TATACCCGTATGTTCCCGATGCGTGGATAGCATCCGACGGCGAGCAATTCGGTCGCGGCC

ACGTCGAAGAGTACAGTGGCAAACTCTCTCGTGTGTCCATCCTCCGCGGGCGCCTTGGCC
TGTCCGAGTTCGAGGCGGAGATTATGGTGAACCTCTTGGACGGGGCCAAGCTTGGCGGGG
TGGTCGTCTCTCGTGACACTCAGACCGGTGGCTTCATACCGGTACAGGTTAATTCGATTG
CGACGTAAGAGCACAGCGATTACAATCCGATCGCCCAGGCCGTGGCCAGCGTCGAGAGCA
TCGTGACGCGCCAGACTAGAGGGTTCAAGTACACCGAGCAAGTACGAGCCCCTGAACTTG
TGATGATCGAGGTGATTCGTAATGAAGCTGAGGAGGCACCGAACCTCCTGGACGGCGTCA
ACAATCTGCTGGATGAGTCGTTGCCGACGCCACTGGCCTACCTGACGATGCACGGGGCAT
CGCGTCGTAACGGAGGTATGCTCCTCGCTATCGCCCAGGGTGTGTACCGCCCGAGCATTA
GCATTGGTAACCCGGCGCAGACCAGGGCCCTAGAGACCACCAACATCCTCAGCGCAACAT
TGGAGACCAGAGAGAGCGTGCTGGCGCTGGACCAAATCACCAAGCGATTCGATCGTGAGA
AGCTCGTAGATCGTGTCTCAGCCTTCATCCCTGTTCATTTCCCTATGTTGTCGAAAGACA
CTGCCGTTATGGCTGCGGAAGCCGAGCAGGAATCTGCCTTTGGACAACAACCACTGGTTG
TAGCTAGCGGGGCGCTGGTTGCCGACACATCAGCAGGAGTCCTAACCTCAGGTGTGCCGA
GACACAAGCAGTGGTGTCGACCCCGGCCCCAAACGCTGAGCCCACGCCTTCGTCGGCGCC
TGCTCCTGTTGCTGGCCCGCCACCTTAGCTAGCTGCTGCTGCCTACGTTGCTCATTAGGT
TTCGAACCAACCTGCTGAGTTCACGGCAGCCCCACCCGAGCGTGGTGGCCTCCTCGTCCA
CATAGGCGAGGACGTGTTCAGTGATCCGACTGCATCCGTAAGTAGCGTTCTGGTAAACCT
CATCATGAAAGGTAAGCACCTGGATCTCGGACGCCCTTCTTGGAAGGCTATCGAGGAGGA
TGATCCATGCTTCATCGAAGAGCGCTATCTTATCGAGGTGCACGGCGAGCAGCAGACAAA
GTTGCTTCGCCGGGAAGCTGGTAGGTAGCTTTCGTCTTCCACCCTTACCGGCGACCGCCT
GCGTGATCAGATGCTCAAGGACATCGCTGGCAGTAAGTATACCCTGACTAAAGGGGGTTA
GGTGTTTTTCTCTGTCGCGGCCGAATCAATTCGGGCCGGGATCGCCGACCTTATCGAGAT
TGGCGAACTCAAGCAAATGAAGTTCGGCCGCAAGCAGATTAAGGACTTTGCGACGGCCTC
CGGGCGAGTCGTGGTGTACAACCAGCAACCTGGGGTCTCCTCGGGTGCGATGGAGTGGAT
TCTCCACGAGGCTTATAATGTGGACGTCAATGAGCGCAACCTCTCCCCGCAGAAGGATCA
GCTACTGCGTGCCCAGCTTAAGCCCGGCGTCGAGAGAGGTCCGTAAGTCGCTTGACCCAT
GACGTATTGCGCTGCTGAGACCCCCACTCGGTCGGGTTCGGGTCGTGCGAACGCGGATGT
GCGCTTGCACTTCGAGGAGTCCCTTGCACTGGTGGAATCGAGCTATATGTCCAGCTGGAA
GCTCGCCTCCAAGATGAACGGCCGTTCCCGGCCCGGCACCCTCAAAGTGCCCGTCGACCG
CGTATGTGCAAGCGCCATTGCAGGCCGGAAGGCCGGGGAGGGGGAGGTAGGCGAGAGGAG
CGCCAGCGACAAGCTGAACCGGACCTCGGACACTGTGGTATACGCCCGCCACTTCTTCGA
CAAGTCCGCCGGGAGGATCTCGAACCTGGATGTCCGCAGGTAAACCGCCCGGGTGGACCT
TGGCGCTATGACCCGCCAGTACGACCAAGCGTGAATCGTACCGCTCCAGAATTGCGGCTA
CCTACTAGCCCCGGCACATCTCAAGCCAGGCTTTGACAATGGTATACTGCTTCCGCTCTT
TCTTTCTGGCCTTGCCGGTGATGCCACACCCGATGCCGACGCCCTGGTAGCCGCTCACAA
CCAAGTCTTAGAGGCCATGATTTGCCTCGCGATGGACGCCCAGTTTCTGGTCTACGGTGT
CACCCTGCTGGACTTGATCATCTTCTCGGTCCTGGTGGAAAATGTGCGCCTGATCAACGT
CGACTTCGGCACCAACGAAGGTGCCAACTCATTAGGCGTTGGTCGTAGCGTCATGCTGAA
CGGCGCTCGTATGGCCCACACCCAGCGCTTCCAGTAGTCCGCGATCGCCTCGAACGCCGT
GCGTGCCGACTTCAACGTCACCGACGTCGAGGTTCCACGTAAGAAGATCACCTTCATCAC
GAGAATGGTTCTTCTCTCGGCCCGCGTGTACGCGTTAACCGCCCATCCCTAGTAGGAGAA
GAGAGACTTGCACCAGTACCAGGGCACCTGCCAGGCGTAGATCATTGGCCAGCGTCGCCC
CTAAGCTGTTGGGTTGCCCGACGGCACCGTCACCTTCCAGTAAGGGTTGCGGTATCAGCA
ATGACAAATCCCTCGGCGAAGGACCTTGTTTAGGTTCGTCTCGCTGAGAAGGCCAAACAA
AAGGAGATTCCCATGAATCTTACTTTTCTTGAGGCGGTGAACCTAGCCCTATGTGCCCTT
GGCGAGACCCCCCTGACCTAGGTAGCCGACACGTACCCGATACTGGCCTAGTGTCTAACC
GCTAAGGACGACGCTCGCCGACACACCCTTGCAGAGGGTTGGTGATTCCTCATCTTGGAC
GCCTTCACCCCAGTTCCCTCCCGGTCGCGTGTAGAGCTTCTATCCGAGGTGTCCTCCGCA
TTGTATCCACACAATGTGGCGAAGTTCGCCTGCGCGGGGCGTTACGTCGGTGTTGCTTGT
ACAGGGTCTAAGCTAGATGTCGCTCGGCTGAAGCGGCATGTCCTACTGGATATCCCCGAC
GACGAGCGCCTGGAGGGTCCGCGATACCTCGTAGTTTCCCGCTGCGCCTACCCGGTGTGT
CTAGCGGACCTCGGCGCGGTAACTACCGCCCAGGTCACTGTGAACTAGATGTCCGCAGCA
TACGAGCAGGTGCCGGTGGTCCGCATCAGGCAGGGGATGCTCACCCTGCGGTAGCGTACC
CCGGCCACCAGAGTCATCAAGCGAGGTACAACTAGTGAGTTACTGTGGGGGGCCGTTCGG
GCAGCTACTGTTTGGCGTAAGTCAACAGATTGCCAAGGGTGGCCTTAAAGGGCAGAGCAA
GTCGCACTTGAACATGCTGAGTGTTATGAAGACAGGTCCTCGCCGCCGTAGTCCGGTACA
ACTGAACGCTGACGCCATAGCCGCTACCGATGCCAACTGCCACGTGTACTTCCTGGCCAC

ATTGTCGGGGCACGAGGTGCTGCTCCTTGGGGCCACTCTAGACGGACGCTGGCTATCCA
CGGCGGTCCAAACGGCGATGGACAGTTCACGGGGTCAAACTGCTACTGTAACGCACGTAC
AGGGAGAAGCTCGGGATTCGCAACACTACATGACAGCATGTTCGTGGCGTATATCTAAGT
TATCTCGCTGACAAGGATATGGTCTGGTCCGAGTGCGCACCCGGACCCAGACCGAGCTGG
CTACCTGTACTGAGTTCACGGGGCGTTCTCGAAGCAGTACCGCCTATCCGTCATCAACCA
AGTCACGTGGCTCAGGAATTCCGTGGAAGTGTCCACATCTGCAACAGAGGCCATTCAGTA
TGCCGATGAATTCCTGATCACGTAGCTCCGCGCGGGGACAGATGCGGGTGTAACTATTGT
TACTGCCGCCGGTCTAGCGTACTACGATGAGAGTGCTGACCTGGTCCTTACCGCCCCAGA
AGCAATCGCGGCCAGTACCGACTAAGGCAGTAACTTCCATCGTGCACGTAATGCAGCTAG
TATCAGGGACCCCGCTGAGCTGCCTGCGAAGCTACCTGAAAGTGAGGACGGGTTGATAAT
CGCTATCGGCGCGGCTTACAACAAGAAACACAACCGCTGTGTCGGTCTTGTGCGGATATG
AGACGAGGATACGTCTTAGGGTTCACAAGCTGATCTGATGGACATGTCGGGACGGGTCAC
GTACTCGGCTCCGACCTTCAGACCGACTGCTTTGAACTACGAGCGCCGCGCATCTGGGGA
CGCTACGGCGAACCCTGCCTTTGAGTGCACCTAGACGGGAAACTCTGGAATAACGACCAC
TCAGTGGCCGTTGGCCTTGCTGGCTGGCGATTACGTGTGCATGTCCGCACGCGGGAACCC
CTTGCTTGGGTTCCGGGCCTGTGTCAGGGCCAACATTGACAACGATACTATCTAGGTCGA
GGCTACTAAAACAGTGGCCTCCCCGTATGAGTACGCCGTGGCCTTCAACAAGGACCTGGT
ACTCGATGCCCAAACCCGCAATGACCAGGTGGCGAGGACGAACCTACTCACGAATCGCAG
CGAGACAGCGGGGGTCATCAGATAGTACAGCTTCCAGTCCCCCGACTCGTCTGTGGTAGC
TGGGCCCACAGTGTTCCTGGCCAATCCCCGTAGCCGTTCAGCTAGCGCGGTGTGAGAGCT
GCTTGCAACCTAGTACGCTGATGCCCAAGTGGACCCGGGAGACAGTAAGTAGTACCTGCC
AAAGTACATTGCGAGGGCGATACGCTTCATGGCGATTTCCAGTACAACGAACATCGTGTT
CGTCCGTACGAGGTATCTCCACGAGATGGTTGCGCATGGATACCTCAGGCAGAGTGAGGA
GTAGTTTAACGGAGCGTTCCAAAAGTGGAGCCTCCTGCAGGACAGAACGCGTGCGTCCAT
CAGGACCGACCGCTACAAGATGCGATTCAAGGTCACGATTCGGGTGATCCTCGGGGAGCT
CTTCAATTAGCGACTTGGGGCTGCCCAGACTATCCCATGTGGACTCCAGGACCTGTAGCG
TGTCCGAGCTGCCAAATCGGGCTACGAAGCGGCAATCCAAGAGTTTCCTTCGGACGTGTA
CCCGGAGGATAGCGCCTTCGCGTACAAGCGCCCGGGCCAGCTGCAGTCACTCGGCCAGCA
CTGTGGGGATCGTCGTGTAGACTCGGTCCCGGTGTACAGCAAAGCTGACCGGACAAAGAC
GGGAGATCAGTACCGTATTCGGTATCGCCACCTCTCGTAGCTGGGGCCCACCCCCCCCAT
CCTCCGGGACCCCAACGACGTATCGAACACAACAACGCGCACGCAGTAGCATCGTGTAAC
ATGGTATCTCGGGAATACCGGGGTAGTAGCCTTCCGTGTGGCCGATCAGGCACGCAGGTC
GTCAACGTACACGAACACGGCGATTCGGCTCCACACCCGGGACCTTGGCGCGGGTTTGCC
AGAAAAGCCTACGGCAACCTTCGGCACCCCTGCTATGGTGGATATGTAGACGACACATTT
CTCACTTGGAAATGGCGACCGCTAGGAAGTCAAAATTACCGGCCTGGAGTACGGATTCCA
GTACAAGCAGCGCATTAGGAGGCAGTTATGGCATTACCAGCCGTACTCGCGTTCGGCAGG
TATTCGGCAGCCCAAGGTATCTGCGACGTATTGCCAAAACGCGAGCAGACTAAGGCACAG
CATATACTCACAACGGAAGCCAACCGATGGGACGTCCCACAGGCGTTCCAGGGCATATTG
GCAACCGACGTGCAGTGCGGGCACGTACGCTCACATACCGCCAATAAGTTTCAGTTCCCG
GGACCTGTGGCGGTCGAGGTGGGTGGAAGCCCACGCGCCGGCCAGGCGGCGTCGGGGGTG
AAGGGGGAAAGCGTTGGAGCCACCCAGGAGGAGATTGGCTCGCAGGGGGCCGAGGTATCT
GTTGAGCTACATCATCAGGACCGTGCGCAGGGGGGCGACTGGAGCTACTGTATCCGGGAG
CTGTTGCCCGGCACCCATGCATGCTTGGGCCAGCTCCAGGAGGTTCCGAGTACCCGGAGA
TAATTTCTCGGGGACCCTGTACAGGTTGGGCTGTCAGCTGGTTCCGCATACGGCTCTATG
ACGTTCTTGTTTGGTAGTGTCGGTAGCACATCAACCACGTGCGCCACTAACGAGCAAAGC
GGCGTAGCTGTTTCTGGTCCGGGTGGATTCCACCCGGGCGGAGGGCCCGACCGATAGGAG
AGACATATAGCTGAACGCGATCCGCAGCTCTTGGGTCAAGCTACTGGCAAACTGGTACAG
GTGGGTCGCTACAACCCGCATCGATTCCAGGCTGAGCGTGCGCAGGGCCAGGGCAACGGA
GCGCTCCTGGCGCGGAGTATCCTACTGCAGATTCGCGCGAGTATTGTTCAGGAGAAGTTC
GCTTCCGGCGTGATGAATGAGTACATACCGGGACAGCTGGCACTGATGCTCGGGCAGGCC
CTGACGGATGTGGGGGGCTATCCCCTCGCCCGACAGTTTGTTCGGGTCGTGTGCCAAGAT
TAAGACTACCGTAACCGCCAGGCGGACACTGGTCAGCGCGTGCACGCGTTCATCCAAACC
GACTGTCTGATATTGCCACCTGAGCAGTTCATACAGGACTTGTTGTGACAGTCCCGGGCG
ATCCTTGTGGGCATGGGTGACTGACTGTCTGATCAGGGCCCTGCCCAGGCCCTCGCCCCC
CAGAATCTTCTGGAGGATGCCATCGACGGTGCCCATTCTAAGGTCTGCGAGGCGAGGGGT
ATTGAGGAGGATAGGATGCGCAACTCTTCGTACGGCCACAGAATCCTTAAAAGCTACAGC
GAGCCCAAGCTGGGCGGGGACCTCGATACGCAGCAGTAGTACGACTAGCAGATGGCATGC

TTCCACTGCGACGTGGATGCCTTAGATTCCCCGCCCGTGGACATGCAGCAGCAGGTGGCT
GAGCAGTGCCTGATGAAAGCGCACAGTCAAGACCTCCGTGAGGTGGCAACAGCTATGAAG
TTCTGGGGCCTCCTGGACACCCTTGAGTTCAACATCCAGATAAAGGAGAATGAGGGGATA
CCAGTCTCGGAGGGCTGTACCAGCGCCCTGGACCGCGTGGGGACCAGCCGCAGAACTTCT
GAGTTCGAGGCCCTGGTCTGGCAAAGGGCCATCACGCCCGAGCAGATGCTACTTTATACG
GCGCAGGTGGATACTGGGGGGAGCAAGACCGCGAATCAGCTGAGGGCCCTCTTGGACAAG
TCGTACGCGGCGAGCTCGAGCAAGGAGGCTTTTGTGGATGTTTTGCAAGCCCAGCAAAGG
GGTGCTAAGCAGGACCTGGACATTTTCGGCTGGTCGGTTCCCGAGGCCCTTGTCGCTTGG
GCGGTGCAGGCATACCTCCAGCAAAAGACAGTGGCTACTCTGGCCGCTACGGCCCTGTGG
ATGGGGGCACGCCTCGGCACCCTCGCGAAACGCGCAGCCACTGAGCGAATCGCCGCCGTG
CGCCAGATCACCCTACACCCGGATGCTGCAGCCCCTGGTCCGGTTTAGCTTGCTCGGGCC
TTAGCGGACGCGGCCTATCTAATGACCACCACGCCCCCGGAAGGGGAGACAACAGCCCTT
AGCGGCCTGGAACGAGAGATTCCACGCAGTTTCAGCCAGATCACAGGGTACGTGACGAAG
GGCACTGCGCCGGTGAAGGTCCTACGCACTGCGCCCCTGCCCCAGCCAGCCTACGAACCG
CTGGATCGGCCGTCCCGGCGAGCTCAGTCCCGCAAGCTCGAGGACGTTGTCGAGGATGAG
ATGCAGACTGGCAAATTCTCCCAAATCCGGGGTCTTTCCACGGAAGGTAGCAGAACCCGG
GACGAGGGTTAAGGTTTCGAGACGCGGCGTGCGGAGCCGACTACGGAGGCTCGGGCCCCG
GCTCGACAGACATACTACGCCGGGTCGGCGCCCAGTGGGATTGCCCAACTGGCTGCCGCC
AGAGTCGAGCAACGCACGCTCCACGTCAGTCCGGAGGAGGGCAAGACTACGCGGGGAGTG
CTTCCAAGAAACGTTGCACTAGAACGCCTATTAGATAACCCGAGAGCCACAAAAGATCGG
GTGTCGCCTGCAATGTTGTCATTGTGCCCGGCAATTCCGACATGAGGCCGACGGGACTCC
TACTGGGACGGTGTAGGCGTGTGGGCGATGGGAGTGTATGTCGATGGCGGCCCCGCATCG
CAGCAGCTGGTGATTAGGCAGCACGTCAGTCTGCAGATCAACAAGAAGATCGACGAGACT
GTCAGCAAGGCCAATGGTTGTGGCGTCGGTCATCTCGTGACGCACGGCGAGGTCGAGCTG
AGTACCTAAGGCCACAAAAGGACGAGTAACCGACGGACGATCTGTCGCACTGGCGCCGC
GATTTTGTGCAGCAAGAAGGTATTCGACTTCATGCGTAATAGGATAGTAACGCCTAAGCA
ATAGGCGTCGGCGAGTAAGTCACTGGGAGCATGACGATCAGCAAAACGAGCACCCGCGAA
TAAGCCTCGGTGGGATTGCTCGTCAGTTCCGGGAGGGTACTGCTGGAAGGCGAGCGCAAT
GCACATGAGTTGGGCGTAACTACTGTGTGATCGCAGCCGGCCCTCGACGGCGCAGTGTAT
GAGCTCGGCCCACAAGGTGCCCGTGATATCGAAAAGACCTTAGAGGTAATCCCCAAAAAG
ATCTTCGGCACCCACTAGGAGCATGAGCGGAAGTCCAAGTTGTACAAGCAAACCCCAGTT
TGTGGTGAATACTTCATCACCAAGAAAGAGGGGCAATACTTCACAAACTTACGGCGGCTA
TATCACCAACCCAACAGCACCAAATATTCGACTCTCCCCGGAGGAGATCGTGCTATTGAT
CGCGTGTGCTGCTACTTCGGTTACCCAGGAAAGGCCCCCCGTGGACCTCGGCATGGCTTG
ATAGATAGCAGAGCAGCAGCAACTGGTTCGAGTTCCGCCGTTGAAAGTCGCGCGTACAGA
TGCTAATCGCAGCACTTGGGATACTCGGCCGGCCGGTCAGCAAGAGACCGAAACCTAGTA
CTTGCAACGAGCATACCTGGAGCATCAGCACGAGATGTTCCCTCTCCCCGACCCCTAATT
CGACTTCAAGCGGTAGTACGGCGACATTCTTCTTGCCCACAATATTCAGGATACCGACGA
CAATCTTACCACCACGGCGGATGTCAGGTTATCTGTGGAAGCGAGTTGGATCGCCACGGG
TATTGGCGATAAGGAGGCCCGCGAGGCTATCTTAGACCGCATCGGACTCGTCGCCTTCAC
GACAGGTAGGGGCGATCCACTGACGATCTATGTGGACAGCGCAATCTACGGTGTCACCCT
GCTGCTGCAGATGGGGCTCCTTTCCTGCGCCCTCGCTGGCGGGAGTCCTACCCCCGCGGT
GTCTTCCATGGCCTGCTACGTCGGTAAGGATTATGAGGCCCTGGACTACGCTATTAGCTG
CCGCCTGACCGGCGGGGCCTTCTCCCTGGTGGATGGTCGAGGCATGGACGGTCTGCGTTC
CAAAGCCGGGCATCCTGGGGGATCTGGGGGCATGTTCTGGGGTCTCACGAAATTCGTGTC
AGCTATCGACAACCGCATCCCCCCAGCCGTCGAGTCCGGGGAAGGGCTGGCCAATCCGGT
CGACGCTCCGGGGCGTGGTGCCGGTATCCTAACCAACTACAATGCGGCTCGTACCTTCCG
AATGAACTTAAACATGCTCGACGGCAACTTTACCATTTTGACGATGGGTCTGGGGAGGTC
TATCGCCCAGGTTTAACCTCTTCACACGTTCAGTATTAAAGTAGACGACAGTGGTTCAGA
CGGAGACGCGTGAGATGCTCGGGAGCGCAAGGTGGCAGGGGTGCTTGCCCGCCGGCGTGC
GGGCCGGACCCGCTTCGGTGCCTTCACGGATTGGCGCCTATACACGGACGTGAAGAGCCT
TGCCGACGTGGAGTTGGCGATCATTTCCCAGGGGGCGGAGCTGGCCAAGCGCCAGGGACG
AGCTGTGCTTGCGAATTGCACATCCAGCCGCGGTTATGGCCACCGCGCCTGGAGCCAGTT
GAAGAGGCGTGCCTTTGAGGACCTCTACGGTAGCAACACCGTTAAGAACCTGCTCACCGA
GAGCATCGTGCGGGGTGTAATCCTGACCAAGAGGGAGGCTGGGTCGAACTGTTCTGCGAA
TCTGACACGTACCCGATACAAGGCCGCCGGCCACCGCCCCGTTTTCATCGCTGGCCCGGG
CAAGACAGACACGGATCAAATAAGAGCTTTGCTCTGGGAGGCCGCTACAGACGACGCGAA

AATCAAGGGTCTCATGGGAAGGATCAAGGAGAAAGTTGATGAGGCCGGCATGTCCTAGGC
CAGCGAGTCTCGCTTACCGCTGGAAATGACCGTCGAGATGACCACACCAGACGGTCGCTC
AATTTCCGGGGTGCACATGACTGACACCGCTCTAAGTCGCCTGGCTGAGAACTACAACCA
GCAGCTGGCGGGTCGGTCAGCCCTCTCGGCAGCCGGTCTAGGCGGAGACTACGTAACTTT
CCAGGCCCACCGGTGCTGCTACCTCGACACATTGGAGAAGGCAAAGCTTATGTATGCAAA
CAGTGACGATCGTATGCTTCAGTTCAATCACCTAATGTAAGAACTGGCCGGTCACTGCCC
ACATGCCAGTGTGTTGTGTACAACTGCCCAACGGACTAAGAGCCTTGCGCCGGTCACAAA
GGTGACTGCCTCTGTGCTGTGGTAAGTAGCTGAATTCTGGACACTGGCGTTCCGTCACTG
GACCGATCGTACCATGGTAGAGCTCTTCTAACCGTCCCCATTCGTAGTCGGGTTGCACCC
TACGGCTGATCGCGATTCTGACTTCTGCTACCAGTGGTCTTCTATGGCTCGTATCGACCT
CGATCTCGAGGTTCGGGTAAGTGCCTGGCTTAGTCAGTACTAGGCTATCCTCCGCTTCCG
GGACAACTTGCTGGACCCCGAGCGTTATATGGGCAACCAGGCCACGCCGATCCTGGACGT
GATGAACTATGTCCATGCGCACCAGGAGCGCCTGAACTCCGACTTCACCATGACCACGAG
CGGCAGGGAGGGAAAGGGCTATCGTAACGCGTAGCAGATGCTACGCTAGTACGGCGTGGA
CGACTCGGCCGGGGGTAAGATCAAGAAAACTGAGGATGACAACGCCGAGTTCAACGGGAA
AAACGTAAGGAGTATGCACTGGGCCGGCTGGCCGGAGGATGCAGACGATGCCGTCATGAG
CGGAGTTTCACGGCTCATGGACTACACGTCCCTGTGCGGTAGGTCGGTTCAGGGCACTAG
TTTCGCGCGAAGCCCGCTCGGGCAGAGTCTGGGCCAGTTCCGTAGCTACGTGATCTTCGG
GCACAACAAGCTGATAAGCGGTACTATCCAGAACTCAGCTTAAACTGGACTGGTAGTCCC
CCTAACGTAGCAGTACCCACTCACCACGATGATTGTAGCTGGAAACGAGCTGGGCATAGG
CGAGCAGGGGGACCTGGAGTCAGAGGCGGGCCTCAGGAGCTTGGCAAGCACAGACTTTGG
CTAAGCTCCCGGTATCGGGTTTATCGATGACGCAGCAGGTTTCATCTGCCTGACTGGAGG
ACGTGGTGGGCCCTCTGCCCCTGTGACAGGCCTGGTCAACTCCCCTGGTGGTATTGCTCA
GCGAGCAGTGCACTTACTCTAAGGTGAGAGCTCTGAGCTCCATGACGATTGCATGAAGGC
TGCGCTTACCGCACTTTCTTTCGTGGGAGTGATGGCGGGTACTGCACGACTACAGAGCGA
ATTGAAGGAGCAACAACCATGTCGCAAACACGAAGTACATGGGACGACTACCTAGGGGAC
GGCGTAGAGGATACGGTCCACGACAAATTTCCGGACCGGAATCAGCAGAGGGAGTTTGTG
ACTTTGGGCGGGGATCCGGAACCTTACACATTCATCCCCGCAGGGGGGATTCAACTCACA
GCGGGCCCGGTAAATGGGGTCGCAATCCGTGTACGGCGCAGCGCTGAGGCATTCCACCCT
CGGCGCGACTCCGCCTACGGCGTGCCATTATGTCCGCGATCCATGGCGGAGAAAGATAAG
GTGTTTTTGTACTCTACAGAAGAGGCGCAGAATGAGACACATGACACTGGTCCCGAAGCT
CTGAGTGTCGCAGAAGAGGCCAGCGGTATTGCGTAGGCGCCATCGTACTAAGTGGTATCT
GCCGCCACTGACGCCGCCCGCCCGTTGCTTCTAGATCCCGCCGACCCGGGGCAGGGGCAA
GGGCTGGTAACCTACGACCGAGGCGTAAGTTAATCGGACGGGTCGGTCGATCAAAGCTTA
CTGTTTCTGGATATGGGCCGGGACACACCAGCGCAATTTCGCGCGGTTGGTGAAGTCGCC
AGGCACCCACTTTCCGCGCTATACGCGACCCTAGCGGAAGCTCAGATTGTCCATCCTCAT
GCTGTCGCGCTCTCCGACCACCTAGACTGGGCCGCCTTGCACGCTGTCGTGGATTCAGGG
GCTCCTGTACACACACCGTCAGGGGACTATCAGATACAGAGTGTGATTAGCGGAAGGGC
TCACTACAGATTTGGGGTGAGGGCGCTATATATATTTTAAGCCTGTCTGCACCGAACACT
GATAAATCCGTCCACAGTGGTGTGAGGGAATAAGCCGCCTCGCCGAGTATAGCCTTCGTG
TCGACTGGTTCCCCAACAACGGACTATGCCCCCACCCCTAATGTTGTGGCGGAGAATTTA
ACGATCATCCATAACCGGCGTGGCAGCAGCTTCTCGGGAGTTCGCGCGAGCTATCCGGCT
GGAGAGTTCTGTGAGGTCAGTGATCTTACAGGGACTACGGTGACAATCCGTTCAGCACCC
TCTGCCGTGAACTCAGGGGGTACGGTTGCGATCTAAAATGTACTCTTTGGTGTAGTTGAT
AAAGCTAGCAGTCACATCGTATGCAGGGCAGTACCAATGAATCGACTGCTAGTAGAGGTA
GTCGTTTTACCGCGTGTCGTTGACGTGACGATCACCCTTGTCAACGACACCAGTGTATCT
TTTGCCCACCGCTATGACCCTTAGCGCACTTTGGGGACAACATCGAACCTCGGCGACCGG
GGTTACGACATTTGAATAATCCTTGAGAACTCTCACGGCCGTGGGGCAGACATCTGTAAA
GTCTTCACTAGTCCACCTGACATCGACACTAGCGGCCATGCAGAAGTAGGCTGGGTTCCG
GTCCGTAAAGTGCGTATCTGTAGCTGCACACTTGGGAATGATATTGCCTTTCGGCAACAC
GGCGTGGACGTCCACGTGAACGTCGAGAAGTGCACCAAGGAAAACTGCACGGTCTGCGGT
GGTATATTTTGGCAGGGTGCGTATTTCCGCTACACACACTGTAGAATGACTAACGCGTTG
GGTGTCCCGACTCTTATCTCCGCTGAGATTCTTGGTGGTACATGGCTTGTAAACCACGGC
GCGTTGTACACTCCCGGCGATCCGCAACCTGGTTGCTGTGGGTTTATCGAGGCAGGTCGA
CGCTACCGAGTCCTGACTCCAAAAACAACGCAACCGTGTGAATTCCTTATACAAGGCGGC
TGTCTGCGTGCGCCCAGATTAAATAAGTCTCGTTACCTATTGGGCAAACGTCTTGATTGG
AGTACAGTTCCAGAAAAAATACAGTCAAGCCGTCAGGCTATTGATGCATGTTCTCTGTCC

AAGGTACTACTACGCGATATTGCCTCGTTCAGTGGCAATCGGGAGCACTTGATGGTGGAT
ATTTTAGCGGGGTTGGCAAGCTGCATCACGTAGCCGTCTACTACTGGCGGTTTCGCAAGT
GCCCCGAGGCGGTTGCCTGTGCTGCGTGGTAGGGTTTAAGTAACAAGGGCAACCAATGCG
AGTAGCGTTACTCCACCACTAACGTTCAGGTACCTTTATTCGAGGGGCACAACCGGCCAG
GTCAATGGGACTGACAGGAGATACGGCGGTAGAAGGATCGGCGGAGGTATCGCCAATACG
ACCGAAGCGCCCTGAGCGACGTAGGGTCTGTTCACGGAGCTCCCGACAAACTTGAGCTCA
GGCGTCCCTAAGCGGCTGAACTGGCAGGCGGGGAATTACGAGGTAACATTGTGCAAGCAA
CGAGCGATGGTCTCGGGGAATTCCAAAGCGCACTGGCGCAGCTTATGTTGTACGAGCTGC
GATGATCCCGAGAGCAGGAGCCTCCGCTTCCTTTGTCCACGGCTGTTAAGGCTGATACCC
TAAAATTCTTTAAGGATAACACCATTACGTGCGCTCCAACATGGGTCGATGACCTAGGGG
GGGGGCGCCAGCAGCGCCAGGGTAAACGAGCTGGGAGCTACACCCGCCCACGTACCCAGC
TAGAGCTTGCCAGGGGCTACGTCGCAACACTGTACGGAGGATATCCAATGGAGGTCCGAG
AACGGTTTGAACGGACGCAACGAGTGCGTATGATGTACCCGGAGTTCGTGGACTACTGCC
GCTGCGGAAGGGAGTACTTCCATTACAGCAAGACGTGTATGCAGGAAGACATTGCAGAGT
TGATGCACTTCGGGCCTGAACCCTCAATGGTCGCCGCCCAACGGGTCGAGCCCAAGCGTG
CCATTGCATGTCTGTTCGGCCTGTGGGACCTGGTGCAGGACCCCACGCATCTTGTGGTCC
CCGTCTGTAGCGGCTAGAATGAGGCTGAGGAGAAGCGCAATGTCATGCACCGGCTGATCC
AGAACTGGCCGCTGTTGCAGTACCTTGCCCCTACAAAGTACGCCGGGGACCGAACCTCTG
TGCGGGAGTTCCCCGTGGCCTGGGCGTTGATGGGTGTCGACACGTCCGCATTCGTGACCT
ATCTGGGTGCCAGTCGGTCCCTATAAGGGTCTCGTTGCAACCAGTTAAACCCAGGGGTTA
TCGCGAACACATAAAACGGGTCCACCGCCACCGCGCGTGCGAAGATCATCACGCACTCGG
AGGAGTTCACCTCCATTGTGGCCGACAGGAACCGATGCATCTTATACCCGGGCAGACCGC
AACCGCGTAAGTCTATTTCGTGCACCCGGACTCTCCGTGGCTTCATTGTACGCGTATGGC
CGGGACGATACACTGAAGTCCGCGAGCTTCCGAAGTGCCGCGATTCGCTGGAACCCGGGA
TTCTCGATAGGATTGGAGTACTCGGCGACCCCCGTCACTCTAGTCGTGACCCGGACAGTA
CTCGTCGTTGAAGGACCGGGCCGGAGCGGTTCACTGCGGAGGCGGTGTGTGCTAAACAGC
GGGACCAGGGGCGAGATAACTGAGAACTCCCGTTCCTGTTGAACACCGCCCTCGCAGACC
CTGCACGTCCGCATATGCTGCTCCGCGAGCTGATCGTGGCCGACATTTCACATGAGTCAT
GACCGGAGAACGGTTTCAGGGCCGCAGGCCTAAGAATCAAGGTCGAATTGCCCCTAGGGC
TACCGCTTAAGTCAGTGGAGATAGTCCGCCGAGCAAGAGTTAATGTAAGCTTCGCGCAGA
TCAAGGCGATCAGCCTGTTCCTCAACCCCGCCGGTAACGGCGGGGGTGAGCTCGCGTGTG
CCAAGGGCTGTGCAGTTGTGGTGTACATCCACGTCGTAGCCTTGTGCGGATCCAAGGACT
CCGTCTCTGATGCCATCCTCTACACCCTGGTTCAGTTGAGGAATGACTTCGGGGTGAAGG
TCGTCCTTGTAGGGAAAAACAAGTGCGGATGTACTGTGACCCATCTCTTCCGTAAACATT
TCGAGGGCTTTGGCCCTTAGGGTAAGCATAGCCCCCTAGGCGTAGGTGTAGACGTGCGGC
ATAAGACCGAGCTGAAGGAACTGCGAACCCTCAACACGATGCGTGCAGTGAGGCGGAAGA
ATCGCCCCGCACTGCATCGCAACGCAGTAGACAGGGACCTGGGGTTGCACTAGCAGTACC
CGTTGCAGGCCCCCGATGTGCGCTCGGGTCTGTACTAGAGGCACAAGATCACCTCAGCCG
GAGGCCCCTTAACCCAGGAAGCATGAATAGATGCTCTTGAGGGTGGGGTTGCGGAAGTCT
GTGTTGTCCTGGTGACCGACGCGATGAAGGAACAAGAGCGGCGCGATGCTGGAGTAGTGC
AGGAGTCTCTGCGTAAACCAATTGGCACGGGGCCGGATGTACGCCGCGCACTCCCGTCTT
GCCAGCGCGCTATTCGAGAGAGGTTGGGAATATGACAAAGAAAACTGATTTCCCGAGCAA
CGCCGCGCTGCAGGCTAGCATTAATGTACCTATCATCGGAGTGGCTGCAGCTACCTTTCT
AGAGTACACCGTGAGGGACTGTCTGGTGCTTCGTCCTGTGCTCTTTCAAGTAACGCCGCT
GGGGTGCAACGTCCGGAAGTAGGACCTGCTGGGGCGGGGTCAGGCCGCCGCGTAGTCTCA
AGGTTACATTGACCTCGGCCGCCCTTGCTCTCGTTCTGGGAACTGCCGGCGTGTACAACG
CAGCCGTGTCCTTCGGTCCAGAGGACGCCCTCCCCACCCTCCCGTACAGGGGCCACGGGG
GCGGCACTACCTCGTCAGATGGTCAGACTGTGGGAACCCCTAATGCCCGGTACACTGCGC
ACAAGTGCGCTGAGGCCCGGCTGACGGCTTTCCACGTGTACTGGGAAGGCATCAGCTAGT
ACGGGCCCCAGGAGGACCCAGAGAGCGTCAGAGCCGCGATGCTATCCGTGCCGTACAGCG
TGGGTGTGTACGGCCGGGTCTGGGTGCTAAAGGAGTGGGCTCGCAAGGTCCCCCCTCGCT
TCCGGGTAGCCCCGGCTTCACGCGACTATACAGCGACCTCCCACGCCATCCAGGCCCCCT
GGCAAGGAATCCACGGGGTAGCCTAAGGGTACAAGGTATCGGTGCAGAGTAAGCCTGTCC
GGGTGCTTGATTAGCGTCGATGAGTTGATGATGTACCGTGTATGGAGGACGTGAGATGAG
TATAAAGGTAGCCCTGCTCCTGGGCCTCGTTGTAGTGGTGGGAGTGGGCCTTTCCGCAAC
GCATAAGGCGCGGCAGTACCATCACACCCCCGATACCCTAGCGTCGCAGCTAGAGGCAGC
AGAGGCCCGAGCGGAGCGTTTTCAAGGAGGGGGACTCCCTGTCAGGACCAGGGCCACCGC

AGTAAAACGACAGGAGCGGGGTGCGTTGAATTAACAGCCTGCTTGGAGAGATTCTCCTGT
GCCTGCTGCCGGTCGGAGAGAACTGTGCGCGCATAACAACACCTGAACCTGTCCGCGGCC
AGCATCCCGCTGAGCAAGTGACGTCCACCTCTGGTGTGGTCCTCGGACTGCTAGCGTAGC
AACAGGCAGTAGCACTGTGCAATGCGCTGCACTGCTTCGTTGATTGAAAGAGATAGATAT
GGGCATTAGTTCAGCAACTGCTGGTCACTTCCTGACCGCCCGAGATAATGCAGCGACCAC
GCTGCCGTGTCGCCATGGCTTCACTCAGGGTGATTCAGACCCTCAGAATCTTGCCTTGGG
TGCCACCTGGGTACCCGAGGTGCATGCTGGCCTGGATCTTGCCTTGTGGACGTAGCGAAT
TGCCGGCGATGTACTGCTGGCCACGGGGATTGTCGTTGTTTATGGGCAGGCCCTGACCGG
TGACGCCTCCGCCGTCACCTACAGCACCACATTGACCTTCACAGTTTTATACGGCGCCAT
CACCGCTTTGGCCTTGAGTTAAGGATCCGGCGTGCTCAGGGTGCTCTTGTCTTAGCGGAC
GCCTTGGCTCCGGCTCGTGTCAGGGTGGAGTGATCTTACTGGCGGCTGGAATTCGCTGCC
ACGATCCGCCAGACCTCCCACGCCTGTGAAGTCCCGCGAGTCCCCACGGTCCTGAGTGCG
GGTTTCTTCGCTCCAGTGCTGGGGGTACAGTTCGCGTGCTATGTGCGGGCGTAGTTGCAT
TGCGGGTACCGACGTGCGTCGGGAGGTTTGGGCCCACGGCGCGTGGACAGGTTCTAGGGC
GGACCTCCCTTCTGCGGCCTAGTGACTTAATAGGCGGCCCAGTGGTTCACTGGTGCGTTC
CCTACTCCCTTCCCCGGTCCGGTATGGGTTCTCTGGCGCGCCCTGGTACCTCTCGTCCTG
CCTGGGGCTGCCT
>NewGenomeName_6
CTCATTCGCTCCCGCCTGCGGGCTTCACCAAGTAAATTTTTTGCAAAAGTGGATTACCGC
AAACCCATATGCCCAGACCGCGCCAGATAAAGGTTCCGCAAGGCATTGAGGATCTGAAAG
GATCGAAAGGAATTGCAACGTTGTACAGTTCGAAAGAGCCAGGGATGGCAGTAGAGAAAA
CGTAACCTGTCGAATCTATTGGGTACGTTATAATTTCCGTGGGCATTTGAATGAAGGGGG
ATGACCTGATTAGTTGCATTGCTACGGTTGGAATGCGTTTTGTTTCAGGTGGTTAGGCAT
GATGGGGCACCATCTCATAACTGAAATTTTGAAAACCGGCGACAGCCCCGACGCTTTGGC
TTCCCTCCAGCACGGAAAATCGGCTCTCCACGTAGAGGCCCCTTGAGTTTGGTTCTGATC
CGGACCTGTGGCAGACTGCCCGCTCAGACAAAGAGCAGATAAAGAGCGGGAGAATTCTAA
TGGTGCCCGGGGCCGGACTCGAACCGACTCGTCTTGCAACGGCGGATTTTGAAACCGATG
GGATACCTTTTAAATCAATAAGTTACTGATTTAAAAGGGGCGGAAAAAGGCAATATGGCT
AAATATGGCTAGATGTAACGGGCCTCGCTCGCCACGTTGTCGCTATTTTTTAGCGAGGTG
ATCGGGTTGAGACGCACCGCATCTTCCAGGTGGTCGGGGCCGAAGTGGGCATAGCGCGTG
GTCTGCTTGATGTCGGTGTGGCCGAGGATGCGCTGTAGCACCTGGATGTTGCCGCCGTTC
ATCATGAAGCGACTGGCGAATGTATGGCTCAGAACGTGGGTGCTCTGGCCGGCGTGCAAC
TTAATGCCAGCCCGTCTGGTCGCCTTCTCGTAGTCGGCATAACAATCATCGAACAGGCGG
CCAGTACGCCGGGGCAACATGGCCAGCATCCAGGCAGGAACGGCAACAGTCCGGTTTCGC
TTGCCCTTGGTCTTGGTGAAGGTGAGCCGACCCATGCCGATCTGGGAGCGGCTGACCTTT
TCTATCTCGGACCAACGGGGACCGATCGAGAGGCAGAGCAACACAATGAGCCACAGATCC
GACTGCTCTTGGCAGGCATCAAGCTGTTCCTCTATTTCATCCTGGGAGAGATAGGCCAGC
TCGCTTTCATGCACCTTGTACGGGCGCAGGTCGGCCAGCGGGTTACCGCCGCTCCACACC
CCCAGGCGTGCCGGCCCATTGAATACCGCCTACAGATAAAGCGGCTCCCGGATGATAGTG
GTCGGCGTCACCTGCTTGCGCTGCTCAGGCACATAGCGCTCACCACCCAGCTGGCGCTCG
CGGTATGCCGAAAAGAGCTCGGAGGTGAACACGGTCGCAATGGGGTTATCCAGTGCCTCG
CACAGCCACACCAACCTGTCCCGTCGCCTTTCACCGTCAGCCAGGGTCTGGCCGTGTCGT
CCGAACCAAGCGGCCACGAGGTCAGAAAGCCGCCGCTCATCGCCGGTAGCGACAACCGCC
ACTGTCTATTGCAAGGGCTTTACGGTGAGCATGTGGGATTCCCACGCCAGCGCCTCCCCC
TTGTTGGCAAAGCGCTTGCGCTTACGGGGCCCATCTCGGCCATCGGGGAATATTTCAGAC
AGCCAGGGTTTTGACTTGTGATCTGTTTTACGAACCGACATGACCACACACCGCCAGTGC
TTGTTTGTGGGATGTGGTGACCAGCGTCAGTAGTTCTGGTGCAGTCTGCACCGCACTGGC
GTCACCATTTCTGGCGAAGGTATAGAGCTGGCCGACAAACGCATCCGTGCGCTGCACCAG
GACAGCAGCCTGCCGGATAACCACATCATCATCCAGGTGGCAGGTACGCAAGCCGTTCAT
AGAACCACGAACGGCAAATATCATCGGATTGACAGTGCTTACCCGATACTCGGCGATCTG
GTGATAGTCAATCTGCTGGCCATCAAGCACGCCGCCAAAGAGCGCCAACACGCCATCAGT
GGTTTGGTTCATCTGCAGATAGATTTCCTGTTCCAGTCGACATTGCTCTGCGCGGGATAA
GACACGCAGGGCCTTGGCTGGTTGTTGCTCTCTCTGCACCATCCCGGTTTCCGTTGGCGG
CATAGGGGCATCAAACCCAGCCGGACGGAACAACCACGCGCCAGCGAGCGCTATTACCAA
CAGCAACCACACAACTGATCCGGCTTTATCACGGCGTACTGATGCAGCCCTCTGAGCCCG
TTCCTCTTTGCTCATCTTGAGTACAGACAGGGCATGACTGAAGAACAGCAGAATCCCATG
CACTGCAGCCAACACGGCCAACAGCACCAATCCGCCAGACATCTCGATTTTTCCTTCCGG

CACCATGCTGAGCAACGAAAAACCGAGGAATGCATAGAGCCCGATAGAAAGCGCATTCAG
CCGGCGCGTCTGTCCAAACTAAGTCGGTTTAAACAAACTGATCACCGGCATCAACAAGCA
TAAAAAGGCTGTCCAGATAGCCAACACAGAAAGCATGAGAACTCCTTAAAACTTCTGACC
ATCCCACACCACCCGGCCGACCAGGTCTAGCTCGGCCAGCTCGGCCTTGCTCAGGTCGCG
AGATTTATACAGAGGGTTGTCAAAGATGATGGGAACCCCGCCTAGGTCGAACTGCAAGCG
CTTGACGAACAGGCCGCCATCCAGGCGCAGGACATAGCGCCCATCGCGGGGTGCTTCCCC
GTTGTTCAGGCGAACTAGGATCACATCACCATCATTGATGGTCGGCTCGATCGAGTCGCC
CTTGGCCCGGATAACCGCCATCTTGGCCGGGTCAAACCCCTCTCGGCGCAGCCAGTCGGT
ACGCAGCGCTATCGGCTCGGCGAGTGGTTCATCTGTGATATTGGCGCCCTGCCCTGCGCT
AGCAAACACCTGATAGGCGGGAATCGTTGTGAACTCTTCGGTTACCTGAAAGCCTTGGAC
ATCAGAAGCGGGCGCAATAGGAGGATTTGTACGAGCGCTGGTCCCTGCCTCGCCGATAAC
AAACACCAGTCGGTCAAAGGTGACACCGCCTGCTCGCGCCAAGTTTAAAGCTCGATAAGT
AGGAGGAATGGTTCCCTCATACACATAGCGCTTGAGGCCGCTACCGCTCATGTCTGCTCA
TCGAGCGAAAGCGCGAATTGGTTCGTTGCCTATCACCTTAGTAAGTCGTTCGGAGAAGGA
TGACTGATCTAAATCAACCTTTAAAGCTTACTCCGTAGTCATAAAGCACCTTATAGAAAT
TTATCGGGCTATTACTACTCCCTCAGCTTGACCAAGCAACTCTTTAGATCAACATTTCAC
TTTGTGAGATTGAATTGAAGCATTAGCGCTATATCGAGCCGTGTTGAGCAACGTTTAAGG
GGAACTATGATGTCAGAGATTGCACTCAAGATCGACACACCAGTCAAAACCATCGAGCGG
TATAGTGCCGATACCGGCATCCCTGTTGGCACCGTCAAGAAAATGATCTCCAGTGGTGAT
CTGCAGATCATGCCAATACAAGGCCCAAAGCACCGCGGCCTGATCAACCTGGTCTCGCTC
TACCACAAAGCCGCAGCAGCAGCCTACGTCTCTGCCACCTCCTGATCACTACGCCACTCA
ATGGAGCGAGAACCATGTTTATCAGTAGCGACTGCAAACACACGCACCTCGAATCTGCAT
GCTGCAGATGTAAATCCAATCACGTGATCAGCCAGATCGCCCCCTGCGCTGGCATCGACG
CCCAGGTGCTGAGGAACAAGCTCAGTCCTGGCCAGCCGCACCAGCTGACCGTGGTCGATT
TGATCGCGCTCGACCACGCCACCGACGGGGACGAAACCCTGATCGACAGCATGCTGCTGG
TGTGTGGCCTGACGGACATCGCCATCCCCAGCGCCGACCGAGCGCCCTACCTACCCGACC
AGGCGATCCGCCGTAACGCTAACGTTGCCAGTATCGGCCTGCGTCCGGTGGAGCTGTCCG
AAGGGGGGCGGGTCACCCGCACCGAACGCAACACCATCAACAGTGTCGTCACCGCCACAA
TGTGGTCGCTAGCACTGCTCATCCACGACTTCGAGGCCCGCTTTCAAGCCGTTCCCGCCG
TCGCATGTGCATTAGACATCCTGATGCAAACCGTGACCATGTAATGGGAAACCCATGCAA
CAGACACAAATCAAACACGACGAACGCAATCTCGCGGGCCTGACTCCGCTAGAGCAAGTA
GCCATGAACACCGCCGGCTGCCAGATCCTGCTGAAGTTGTTCGGCAAAACGCGCTACAGA
CTGGATACCGACTGGCTGGCCCTGAAGCCGGCCAAAAAGGCGGCCTTCTGTCCCATCGCC
AGACAGCCACAGGGCGAGCTGATGACCCCGACCATGTCAGGCCTGTCACATGCACAACGC
GAGGCGGTAAGGCTTGCGGTGATCGCGCTCGAGTACCAGGGGGTGTTTCGCGGAGGCTCT
TACGCCAAGGTGTGGTACCCGGCGCCAGTGGCCAGGCCAATCGGGGATATCGAGAGAGAG
AAGAAGGAGAGAGCCGACAAGCTTCGTATGAAGCGCGCCGTCATGGCAGCCAGCCAGATG
ACACAAAGCGACCGGCTAGCAATCGGGCAAGAAAAAACCCCGCAAAGGTGGTGGAACACC
GCGGGGCTTTCATCAGTGACTTATCGACTAGGAAAATCGACATGCCAACTCTAGCCATTC
TCGACACAGTGCGCAACCTGCGCCTGCAAAACCGCAAGCTGGCCCTCAAGGGTCAACGCT
ACAAGTTCAGCCCTGACCTGATCCATGCAGTAGAACGCTCGGCCGCCATGGAGTGACGTG
CGGTCTGGTGGTGCGTCAACAGCCGTGGGGGGATCTGATCATAGCCGCCGTTATCGCTTG
TCACACCGAACCGACCATCAAGGCCGCCAGCGCCTATCTGGTCAGCCGCAGTTACATCAA
CTGCGCCACAACCTGGCTACGCGGCAAGAACGGGTACGCCCGGATGGAGCACCTGACCTC
ATGCTCGATTCGCATCATCGACGGGGTGGCATAAAAAAGCTGTGCCACCCCATCACCCGG
CAGGCCGACCTGGCCGATTTGGCCGATCTGCCGCACCGCATCAGGGCTAAGACCTACACC
ACCAGGCGCGGCCCCGGGGGCGGCAATCTGCGTGAGCAGGCCGGGCTGCAACTGCACTGG
CACCAGCTATTCCACACCATGATTCGGAGGGCCCAAGCATGAGCATAGACGCTATTCATA
TCGCCAAGCGGGCAGAGCGGCCCGTCCTGCCGCTGTTCTCACACCTGCTGGCCACCGGCG
AGCAGGGATACCGCATCGCGCTGGGCGAGCTCTAGTCCGGGGACGAGTTCATCCAGTTGC
TACTGGTCGTGACCAGGCGCCCTGCCGAGCTGCTTTAGGACGACTCAGTGATGGGGGATG
AGGGATGACAGACCTGTTTGAACTGGAGGCCCCGCTGGACGGCCAGGGCACCACAGTGGC
GGGCCTCGCCCATATGCAACCGCCGGTGACAGTCAGCCCGATGGCCAAGGATTGGCAAGC
GGCACAGGATGTGTTCAGCGCCTCGGGCAGCGACGCAACGAGAAACAGAAACAACACCCA
GGAGCGGCTGGCCCTGGGCCCTATCCGCGACGTGTACTTGCTGGCGCTGGGTAGCAGTGA
CGTCTCCCTGGCGAGAGAAATTGCCGAGTGGTGGGCCGACTGCGCACCGATTCACGGGCT
GGGGTAGACCATCAAATGAGCCACCGCCTGATCTCCGACCTGCAGACCCGCGTGGATAGA

TTGTTCGACACCATGATGGACGACGAGGCCCGCCTGCGCAGCTGCCAGCGCGAACTGCTG
GCAATGCGTCGGCTTTCACCTCGCCCACGCTGCACGGTATCCCTAACCCTGCGCCAGTGT
GCCGCAGCCCGAAAGTTGGTGGGGCATGCCCGTCGCACCCCGGACTCCTTCCGGAACAAC
ATCAAAGAGCCGTGGGGTAGAAATCACCAATGACCAACGAACAAATAACAGGGCCAGCCG
CCGAGGCTGACCAATTCGTTTTTTCCATCAGCCGTCTGCCGCCGCCGAAACGGAACCAGC
GACCGCTGTCGAAAAAGACCCTCAAGGCGCGTGATTATGCGCTCGCTAACGCCGTGCCGG
GCACCAAGCTCGAAGCCGCATTTGTAGTGGCCCCTGATGAATCCGATCTGGTCTGGGCGG
TGCAACTGCTCGATGGTCTCACCACGCATTTCAGCCAGGCTCTGTTCAAGCAGTATGTGC
GCCGCCGCAAAGATGGCACCACCCGCAACTGCCGCAGCGCAAACATCTGGCTTTGCGAAC
GGGTTAAGTGGGTCCGCTCCCTGGTGACGGCGCTGCCGGTCGATGCCCAGCACCTGCGCG
ACGAAGAAGGCCGCTAGCGTGTCACCCACCAGTTCGCCAACCAGACCGCAGCCATCTGGA
ACAACATCGAGCAGAACAGCACCGCCGTTGAGCTTGCTCTGATGGAGACATGGGAAGCCA
TCAAGCAGCCAGCCGACCAATGGGCATTCATAGGCAAGATGCCGAAATTCAAAACCAAAG
AGGCAAGGGATAACTGGATCCGGAGCGTGATGGTGCACCTGCTCTCAGCCAAGTGGTGCG
AGAAGCGCATCAACCGCTGCTGGGTCAGGCTGCAAGAACACATCGCCATTCTGCTAGGCA
AGGGGCTCAAGGGGGTCTCCGCCTGGGTCTTGAACGCCACCATGAAGGTGGTGCGCGCGC
GCAAGCGGGCCATGATGCGCTGGCTGGCCGAGTCGGAGGTGATGAACGAGCAGCACGACC
TTGTTGTCTAGATGAAGGATTGCTGGGAGGCCTGCAATGCCAACCAAGTCATCCGCCTAG
CCGAGATGATGACCCCAATGCGTGGCTTTGAAGACTAGGCCGAGGAACAGGGACATGTGA
GGGTGTTCTTTACCTGGACAGCCCCGAGCCGCTTCCATGCCTGGCAGACGGGCCGCAACG
GCAAGACCATAGAGAACGACAAGTACCAGTGCGGCACCCCGCGCGACACCTGCGCTTATC
TGGCCCAGCTCTGGATCCGGGACCGGTCATACCTGAAGCGCTGGGGCATGCCTATCTATG
GCTTCCGGGTTTGTGAGCCGCACCACGATGTCACGCCTCACTGGCACATGCTGCTGTATC
TGCGCAAATGCGACCGGAATGGGGTGATCAGCACCATGCAGCGTTACGCCCTGACGGATG
ATCGGCAGGAGCTGGAGCGCAACAACCTGGGCATTCCCTTCACCGACTTTACGCCGCGCT
TGGACTGTAAGGAGATCGGCCCGTCCAAGGGGGACGCCTCCGGCTACATCGGCGCCTATG
TCGCCAAGAACATCGACGGCGAGCATGTTGATGGTGATGACGAGGCAGGTACCAAGGCTG
ACCAGGGCGCACAACATGCATGCGCCTTGGCCAGTTGGTGGGGGATCCGCACGTTCCAGC
AGATCGGAGCCACACCGGTTGGCGTATGGCGCGAGCTGCGCCGCATCAGTAACGCCAGGA
AGAATGGCGATCTAGTGGAGCCACCCAAGCCGGTCTTGCAAGACCCACGCTTTGCGGCCG
CGCGCTATGCCGCCGATAACGCCATCTTTCTCTGCTACCTCGAAGCCATGGTCGGGGCAC
TGGCTACCAGAGCCGAGCACCCCATCAAGCTGGCCCACCTCATCGAAGATCATGCCAACA
GCTACGGAGAAGACATCAAGCGCCTGATGGGGCTGCACATCGCTCATCTGGGTATCAAGA
CTCGCCTGCAAGGGTGGGAAGTGGTACCAGCAGGCACCTTTGAGGACACCAAGGCCTCCG
GGGGTTCGGTTGGGGGTGTTGTGGTTAAGTCGGGCGAAAGCCGGGCTCCTTGGAGCTCTG
ACAATAACTGTACGCAGCAGGATCTAGAGGCGTTCGCGGATCGTTTGATGGCAGAGCAAT
GCGGTTTATCTCCCTTCTCTATCGGGCATGTGCGGGCAGGTGCCAGCGTGACTACGGACG
GCTACACCCTCCGGCTTGAGAACGGCCAGGTTCAATCGAGCCGGTCGGAACCAAGCGAAC
CGAACTGGGTGCCAGAGGGCCAGTCGCCAGCAGAGCCGAACCAGCAGGCTGAGTTCGCGG
TACCGGAAGGCGATAAGGACTGGCCCATACTGGTTGAACTGTCCGGTAAGGTTTACCAGG
CACAGGGCCACGCCGGTAAGCGCTGCTGGATAGAGATGCTGTCGGAACCATATCAGTCAG
AGATGTGGCGGGTATGGGAAGGGCTAGATGCACCGGAGTGGATGCAGGAACAGGACTACT
ACAGCGCGGAGTGGGCATGAGCATGAGTAGCAAACGGACCGTCAGCCGCGAAGAGTACCG
CCGCCTGGATAGCCGGGTGACCTGCATTCTCCAGCAGCGCTGGCCAGCCAACGAGATCAG
CCGGTGGGTGGGGCTGCTCAAGGGTAAACAGCAGGCTGTGGAGTGCGCCATTCTGCGCCG
CCGCCACCCTCGCCCAACATCGCAGGCCCTGCCGACCATCGTTGGCGACAAACCGAACCC
ATTTCAGGCCAAGGCCAACCGCTCCACCGCGTCGGTGCTGACAGCTGGTGGCCGCGCGGT
TGGCCGGCGCCATATCGTGGACGGACTGACCCCCGGGGCCATCGACCAGAGCGGCACCTT
CCGGTGCGCCGTTACTGGCCGCCCCCTATTGATCGCACCGGGCAGCGCCATCGACCGCGC
CAACCCGGCTGCCGCCGAACAGCTCAACGCAACCTACCGGCCGGCACCGCACCAGGTACT
GGCTGACCTCTGTCAACATGTAGAAGCCGGGGAGTAAACATGAAGCATATCAACTGGAAT
CAGGCGAGCGAACTGGGCCTTATCGTTCGCATCAACCGCGAGGTTCTTCACCCAGAGGGA
CTGGCCATGACCAGAAATCCGGAGAACCGGGTATCGGATACGCTGCTGGTGGGTCCAGAT
GGGATCTGGGAGTACGAACACCAGCTGATGACCAATGCACCAATAGTCAGCGAAGATGAA
GCACGGGCAAAAATTGCAGAGTGGACGAAGGAGCAGCAGGCATGACCGACCCCCGCTAAC
ATCCAGTGCCGGACGACGTACTAACCCGCTTGTGTACCACCAGGGAAGTGACCGACATCA
AGTGCAACAACCGTGGCACAGTCACCGATGTGGTGTTCGGCGATCCATCCACCGATGCCC

TGGCGAAGGAGGCAACCATCTCTAACTGGCGGGCATGGGCCAAACGGGATGTTGTGGTGG
TCAGAGAGGGAGCAGCATGGATCTGACCATCCCAATCAGCCCCATGGTTCGAGTTCAGTA
GCTCCCACCCGAGTGAATTGAGATTGATATTCATAATACAGCCGTGCCTGAAGGCTGGTA
ACTTGTGCCAGCGGAGCCTATGAGAGCCATGTGGCGAAGCCTTTGATAAGCCCATAAAGA
GTGGGAGAGCGGTAACGAATGGTAGCTCGTTCGTCCTAACCACCAACGGAAAGCCATGTT
GGCTAGGGCACCAAGGCCATAAGGGGAAGAAGGATGACCCCAGCCACGCAAGCAGGTCCA
GTAGGCTAGACAGACAGCTATAGGTATCAAGCCGGTGGCGGTGCCGCTCGCCGAGCGAGA
GCGCCAGAAGATCGCAACGCTGCGTATGGCCCGGGCTGGTCGCTGTTAGCCCTACTCGAA
CGATGGGTACTTTAGTACCCTGATCTAGCGGCACTGGAAACGCTGGCTGGATATGCTGAG
CCAGCAAGACAGGGAAGCATTCAAGCCGTGGCCTGGCTGGCCTCGCGCTTTATGAGCCTC
ACCCAGGAGCTGGAGAATTTGGGCCAGTGCTCGCGCATCTATCACGCCCGCCAACGCGCC
AACCGTAAGATCTCCAGCTATAAGTTACTGACTCGCCAGAGGCTAGAACTCCCAGGCTCA
TCGTTCCCCATCGTTTACTAGTTGTCGCTCGTGATGGCTCTTAGTGGTTTTAAATAAACA
TGTATACTTGCAAGAATTAAATAAATGCGTATATTGACGGTCAGGCGCGTTGTGCAAGCT
ACGCCCTAATTCCGAGGCCCAGCCATTTGATTGTTGGGCTTTCGTGTATCTGAATCACGG
AGGCTAAAATTGCTACACAGCGCCTATCTTGACGAGATTGGCAACGCAGGCCCCTATCTT
GGTAATGACCATCCAAAGCACCATACCCATTCAGTGTTTGGTATTGGCGGCTTTGTGCTA
CCGTCCTACAAGGTACTGGCATTCTCCTCTTTCTTCTTCCAGTTCAAAAATCATCTCCTA
AGTACTGATCTTGGCGATACTCAAGCCAAGGCGATGACGGATCAGAAACCCTTCCTTCCC
GCCAAGTGTGAGAAAAAAGGGACATCCGTCCTGACTGAGAAGAATCTCACCCGCTATCGT
GAAACCAGGAGGGCAATTGACCGAGTCTTGAATTGTATCAGAGACGATGAGCGCTTCGCC
TTCTATGTGGGCCAGCAGAAAAGACAGGGGCTAGAACTCGACTCAACCAAGCTTTATCTC
GATGTGATGAGAGAAGTGATCAAACGGCTCGATCAGTAATGCAGCACACATAAGAGCCAG
TTGATGATCATCATCGCCCAGGACACTGAACGGAAAAAAATTGTCGAGACAGCTTCAATC
GAGATGTTCGTTGAGCAAGGAAGACGCTGTCTACTCGAGGCGCCACCGCAGGTCGAACGT
CACCTGTATCAAACTCTCCAGTGTGCTGACTGGCGATGTGCTCTCTACGGCCGCTTGAGT
CACCATAAATGTGAACCAGAAGGCGAGGCTGGGTTTCACGTCTTCGAAAAGTACTCCGGT
GAGCGGTTAAGGCAAGTTCATAAAAGAAGCCGGATTCGGTCATTTGGCGGGCCAGCGAGC
ACAGCCAGACTGCAACTGATGGCCTATAGGTACTCAGCGGTCGGATGAGTCAAGGGGCGC
TATTGGGCCCCTAGTCCTTTCAGTATCAAGTGCCGCCCCTCTGGCGTCAGTGATCCAAGT
AGCCCCAGCACCCACCGGTTCGTCGTCCTGGCCGACGGACTCTGCGTATGAGTGAACGAC
AGTGTGGGTACCCAGCTATGACCACACTCCGTATCAGTGCACTGGCAATAGAGATCGGAG
ACCTCATCACCCAGCCGATTTGACTTGGTAATATGGCCTCGATGTTAACACTCTTTGCAA
AAAACCCGCATCACGCCTCCCGATAATTCGAGATCTCAATCACCAGGTTGCATCTTACAT
CAATCTGACGGTGTTTTTATACAGCGGCACCTTTCGTTTCTCGAAACGTGACCCACAGCG
CCCGGGGCAGGCCAGCGCTGTGGATGGCGTCCTGCACCAGTTCACACAGCGGCAGCACAT
CGTTTCTGGCATACGTGGCGTCGTCCTTCTCGGGATCCCTGAGCCCGCCCCCGCCATTGG
TGGGGATAATGCCGGCCAGCGCTGGCGGGTACCGATGCGCCGTCAGCACATCCTGGTCAG
TGATCCCCCTGATAGCCGCGAATTCGTCCTTGGTTGCGATGTGCCCCGCCGGGATGAGCT
TGATGCCATCGGGCTTGCCATCGGGGATGTTCACGAACATCGAGCGGAAGTTCCCTACCC
CTTTGGAGTTCGCGATCATCTCCTTTATCTCCTCATCGGTGTATTCGTCCTTGTTGGGGT
CCGTGGCGTCGAAGATGAACCCCATGTGGGTGCCGTTGAGGAAGTAGCGGCGTCGGAACA
GGGTGGCGTCGTGTTTGAGCAGCGCAGACTCCAGGCCGCCAAGGTAACCGGGCATGCCAT
AGACCTGCTTCGCGGGGTCATACTGGGGCAGCCAAAGCACGTCATCCGGGCGGTAAATCA
GGTTTGGATTGCCCTGCTGCAGGTAGACAAAGTAGCCATCCTCGCGACGGCGCAGCTAGA
CGCTAGTGAGCGGGTGCAACCCCACCGCCTGGCCGAAACTGTTGCGCAGCTTCAGCAAGC
CACCATCACCAAACTGCAGGTAGTTGTGTACACACGCCGTGATGGTGGCGCGTTGGTTGG
TGAAGCGCCCCGCAACCATGTACCGTCTCGCCATCAGGATGGCCACATGGTGGGCATTGG
CCCGGGCTACCTTAGCCAGCCCCTTGCGGTTGATGGGAGGCTGGTAGTATTCCCCATAGG
GGTTGTAAAACACCCCGGTGTAATCGGTCGTCCAGGCCGTGGGGTCGATGGCCTGCCGCA
TGCTGAACACCACCGAGGGCCGGGGGGATGAGGCGGCCGCCTGGGCCGGTTGCTGCTGTC
GTTTGGTCATGCGAGGGCACTCTCCGGAGAGTCTTTGAATTGGCACATGAAGAGGTAATT
GAACTCCTCCTGCGGGTACTCGTATTTGAGCTCATCAATGTCCATGAGATGGCAGCCGAG
TCGAACGGCATCCTCAATGGTGTTGACATAGCGCCCCTGACGTTCAGGGCAAAGCTGGCC
GCCATCTCTCAACGATGAAGCGCTAGGGAAAGTTATCGCGTCTCTGCCAGGGTAGAGGTT
CTTGCAGTGCACCCCACACCGGAAGTTTCGCGCCATACCGCGGCACGCGGAGAGCGTTGA
ACAGTAGGTCCTGTGCCCACGATTGTGCTTAGCCATAGCGCCGGCCAAGTGCAGCGCTTT

TCCAAAATCTGGCATCCAGATATACTCATCAACGTAGGCACGGCCCGCTTTTTCAGCAAA
CGTCAGATCATCAAGAGAATGAAAATGGATATGGGCACCGTTCGACAGGACGAAGGCGCC
ACCGACCAGGGTTACGCCGATGAAATTCTTGGCTATATAACAAACATGGGTGCGGTAGAC
ATCTGCCATATCTGGGGTGTCTGCCAAGAAAATCTGATTGTCACCAGTCAATAATGCATC
TTCTAAAGCCTCCGCGGCGAACTAATAGGACATGACGATCTGATGAGCTTTCAAGATGTT
GCGGGTGCGCGCGAGGGACGGGTCTATCTGGGAGATTCGGCAGCGCAGTTGGTAGCCAAT
CAAGGTGCTGAGCCATACGTGAAAGTGATCTGCTGTCGAATGATTGATATCGTTCTTCAT
TCTGCGTTTTTCTCCTGGCCTGTTGCCCAGGTGGATTTGCGTTTGCGGTGGGTATCGAGG
GGCTCATTGGCCACACCGTGGGCGATGGCAAAAAACACGTCGGCGTGAGCGGTCACGTTG
TCACGCGCATCCCGCGAAGTCTTTTGGCCGCCGCCGGTGGTGCTGCGCTCGGTGGCGAGG
GAGGCGAAGGGGATTTACCGCTCCGAGCTGTCCCACTCGATGCGGTATGCCACCAGCACA
TCGATCATCTTGAGGACCAGCCGTGACTTGCTCTCGATGCCTTAGTTGATGGGGTGGCGC
ACCCCTTTGAACCCGGGCTTCAAGAGGCCATATACCCCGGCGCCGGTGCCGGAGACATCG
ACTCCCAGATATGTGACCCGGATCTTCTTGGCGATGCGCTCTATCTCCTGCGCCTGATAC
AGTAATTTCAACCCGCCCCAGTAGTGCTTTTCCAGCACTCGGAAGCGCTCGCCAGCCTCC
GTGGACGGGGCGACCACCACCAGGGTCGCATTGTCGCGGGTACGGCTCGAGTCATAACCC
ATCCACACCTCGTGCCTCCCGAACGGGTCAGGACGCCCGGGCGTGAAGTTCTCCCAACGG
GTGGGGTCCACCCCTGCCAGTTCCATGTCCTGGAACTTGTGCACAGACAGGGCATCGTCG
ATAAAGCGGCACATGTAGAGGCGGTCGAACACCTCCTCCGGGTATTCGTCCTTGAGCTCC
TCTTTGTTGATGAGGTGGCGATCGATGCGAATGGCCTCCTCGATGGTGATGACGTAGCGC
CACTACCTGTCAGGGCTGATGAGGCCGCCGTCGCGTAGGTCATCTTCACCAGGTAAGTCG
ATGGCCACCCGGCTGAGCCGCTGTCCCTTCCAGTGATCCCCGGTACAGAACCGGTACGCC
TCGTGCAGCTTGCTCGACGGGGTCGAGATGTAGGTCTTGCGCCAGTGGCTCTGGGTCGCC
ATGGCACTGGCCACATCAGAGAGCTTCTCGCAGTTGGGGATCCAGAAGTATTCGTCCATG
AAGACGTTGCCGGAGCGCGACTGGGCAGTGTTGGAGTTGGTGGAGGAGAAGTGCAGCTTG
GCCCCGTTCGACAGCACGATGGCGTTGCCGGTCAGGGTGACGCCGAGGAAGGTCTGGGCG
ATCTTGCAGAGATAAGAGCGGAACACCTCCGCCTGGAGGCGGGTGGCGGACAGGAAAAAC
TCGCTGCCACACGTCAGAATGGCATCCTCCCTCGCCTCGCCGACGAAATAGTAAGTACTC
CCGATCTGACGGGACTCGAGAATGTTACGGGTACGCGGGAGCGCCGGGTCGTTCTTGGCC
TCCCACACACGCAACTGATAGCCTAACAGGGTGCCCAGCCACTCGGTTAAGTCATCGTCC
GACAGGGGCCCGATCTCGTTCTTGGCCTTCTTGCCACCCTTGCGGTCGCCACCTCCCTGG
CTGTTGTGCTCACGCTTGCCACGGCTGGGCCCTGGCTCACTTCCCTCCGCCCGCTCGGCC
TTGAGGTCCTGCACGCGCCCGGCCCACTTGAGCGCCTTCTGTTTGAGGCTGCCATGATGG
CCGATGAGCCTGTCCCGCTCCCCCTGCTCGCCCGGGTTTTCTTCTCACGGTGCAGCAAC
ACCTGCACCCGGCGAGTGATGGCATCCTCCACAGCCTCTTCGGTCAGCAGGTCACGCCAG
CCGAGCTTGTCGGCCCAGTAGTAGATGATGCGATACGAATTGAGCCCCATGTTGTCCTTG
ATATCCTGGGGTGTCCATCGTTTAAGGTAGAGACCCTTCGCCGCATTGCGGATCTCTTCG
AGGTACGCCACGGCGCCTCCATCCGGTGAATGATGCCCCCATCATAGCCAGCCCCTTCCC
CCCACTTATCCCACTGATGTTCTGAGCAATCCGGATTTCCCGTTGGATCCGAATCCATCA
GAACGCCGTAGGGTGAAACCCCCATGCCGGGCCGATAGCCTGAGCCCGCTTCAATTGGGA
GTAGGCATGAACACATCAACCTTGAGAACTGGCTGGGTCTGTATCGCCACCGAAGGGACC
ACCGTCGATGGCAGTGAGATCACCGCCGCTTGGCTGACTGATATGGCCGAGGCCTACGAC
CCGGAATAATACACAGCACTGCTATGGCCCGAGCACGACCGCAGGGCAAGCTTCGGCTAT
GTGCAGGAGATCAAGACCGACGTCGTAGAGGGCAATCTCAAGCTGTCCGCCATCCTGAGT
CCGAATCGCGATCTTGTTTATTACAACCAGGTTGGCCAGTACCAGTTCTGTTCCATCGAA
CCACAAGCGCAGTTTGCCGATCTGGGCCGCACTTATCTGCTCGGCCTGGGGGTCACAGAC
CTGCCAGCCAGCAAAGCCACCACCCACCCCAAGTTCAACAAGAAGGGCGAGTCTCGCTTG
ATCGGTACCAGCGAACCGCTAGTTCTCTCCATATTCAAGCTACCCAAGCACAGAAAGGCC
GATGGCCTGATCGCCAAGTTTTTCACCTTCATGGCCAGCCACGGCGAGACAGCTATCCAA
CCTACCCCCTGCCAACCCGAGGTTCAGGAACTGACCAAAGAACAGTTCGATCAGATGCTG
GGGGCCCTCAATGGCCTTGGCAGCAAGATCGATGGCTTCAGCACCAAGCTTGAAGCCAAA
CCGGAAACCACCGCCCCGGTGACCGAACCAGCGAAACAAGAGAAGGCCGGCATCAGCGAC
GAGCAGTTCAGCAAACTGGAAGACTCCCTGAACGGTCTGGCCAGCACCGTCGGCGAGCTG
AAAGGCCAGGGCGACAAATTCGCCCACAAAGTGCCGGGGCACCGCCCGGGCGCCATCGGC
GGTGACGATACCACCTACCAAGTTTGCTAAGGGGCCTTCAGTGAGCCAAGCCCTGACCGT
TTAGGCCGAACAGCGCCTGAACAAATACTGCGATGCGCTGGCCAAAGCCTACGGCATCGA
CATCAGCAAGCTGGACAAGCAGCTCCGTGTCTCCGGCCCGGTGAAAACCACCCTCCGATC

TGCCCTGCTCGCCTCTGTCGAGTTCCGTGGCCTCATCACCTGCCTGGACGTGGACCACGT
CAAGGGCCAGGTCGTGCAGGTTGGCGTGGGTCAGCTCTCCACCGGTCGTAACAAAGGTGG
TAGCTTCAAGGGCAAAGTCGGCGTGGATGGCAACACCTACGATCTGACCGAGACCCACTC
CTGCGCCTCGCTAGATTGGGCGACCCTCTGCACCTGGGCCAACGCCGGTAGCGAGGGCGA
GTTCATCAAGCTGGTAGGCAAGTTCGACAACAAGGCGGTTGCTCTCGACATGCTTCGGGT
CGGCTGGAATGGCGTGTCTGCCGCCGATGACACCGATCCGACGGCGAACCCGCTGGTTCA
AGACGTCAATAAGGGCTGGCACCAACTCGCACGCGAGTCGAAAGGTGGTAGCCAGATCAT
CAAGGCCGCGGCAGGCGAGAAGATGTACTTCGACCCGGACGCCAAGGTCGAGGACAAGAC
CCTGGACGAGATGGCCTCCGACCTCATCAACACCACCATTGACCCACTGTTCCAGCAGGA
CCCCCGTCTGGTGGTGCGGGTCGGCACCGATCTGGCCGCAAGCGCTCAGGCCAAGCTCTA
CAGCGAAGCCGCCAAGCCGAGCGAGCAGATCGCCGCTCAGCAATTGGCCAAGTCCATCGC
CGGTCGCAAGGCCTATATCCTGTCCTTCTTCCCGGGCAAGCGGATGGTGGACACCACCCT
GGACAACCTGCACTGCTACACCCAGCGCAGCACCCGCAAGCGCAAGGCGGACGACAACCA
GAACAGCAAGAGCTTCGGCAACCAGTACTGGCGCATGGAGGGCTATGCCCTGGGCGAGCA
CAAGGCCTACGGCGGCTTCGAAGCGGCCGAGATCGGGAGCGGTGCTGATCCGGCAGTCCT
CGCCGTCGAAGCCGCCTCTCAATCGGGCCAAGGCTAACCCATGAGCTCATCCGGTCAATG
CCACAAACAGCGGGCACTCGCCAGGCAGGGGGCCGAGCAGGCCGCCTGCTCCGGCATCGC
CACCGGCGCGGTGGCCGACAGCGTGCACCTGCAACTAATTGCCCTGGAGCAGGACATGGT
TCGCTTGCGCAAGCTGGCCCGCATTGGGGACCGGGTTAACATGAAACGCGATGAGCTGAT
GCCCAAGTACCGCCCCTATTAGGAGCGCTAGCTGGCCGCTGTCGCTGAGTGCGGCCAGCC
CTATCAGAACCAGCTGGCTCAGCGCATCATCATTTTGGGTTTCGATGTGGGCGACTTCGA
CGACGGCATCGCCTGGGCCGATCTCGCCATCGCCCAGGGCCAGCGCACCCCGGCGAACAT
CATGCGCGACTGGTCCAACTTCGTGACCGACACCGTGCTGGAGTGGGCCCAGAAGCAGGC
GGCCGAGGGGCACTCCGTCGCACCCTGCTTCTCCCGGGTATTCGACAAGGTGCTCAATGA
CTGGCGCTTCAACGAGCGGCCGACCGCCAAGTGGTTCAAGGTGGCCGGTTGCCTGCTGTT
GCGCGACCACGACGGTAATTCCCGCCCCAGCTCCGTTGGGGATAGCGCCACCCTGGAGCA
GGCAGACCACTGGTAGGCCCAGGCCGACAAACTGCTCGGCAAGGTGGGCGTCGACACCTT
GCGCCAGAAGATTGACATGCGCCTGCGGGCGCTGAGTCCGGAGTAATCAAATCTCCGCGC
CGTCGCACCCCGGCTGGGAGGATAAGCGAGCCGCGAGGTTCGCCCCGAATCCTGCGATCC
GTGGCTACAGGGGTGCACCTTTTCTCGCCGCGCCATCGGCGAATCGGGCAACAGGGGCAG
TGGTGTTCACATTGGCAAGAATCAACAAAGGGCCAGACATGTTTGTAGGCTAGGACATCG
ACTACACCGACGCCACTATCCGCAATGACGGGTTCTGGCCGGATGTGGCCGTCGCCGACT
TCGAGCGCCGCCGCGCCCTGCCTGCCGATCTGGGCCAGCTGACCCCCGGCGCCGCCTTGC
TGGCCGGCGTCCCTGAAACAAACCGTCAGCTCGCCACCCTCCAGGCCCCCCTGCAGGCCC
AGGGTTATGCCAGCGCCGCGGCTGTACCGGCGCCCAGCCTGGAAGATGGTACCAATGCCC
TGACCGAGCAGTACCTGGCAGACATCTTCGCCCGGGCCAAGGCGGCCTGGCTGCCAACGA
TCGCCACCGTCACCGAGCGGGCGACCGCCAACAACAAGGTAAAGCGTTCGCCAGACCAAC
GCAACCAACTGCTGGCAGAGAGTCCGCAGTTGGTCCGCAGCATCAAGGGCAAGCGCCGTG
CCGGGGTATCGCAGGTATGAGTAACGACATGGACGAGCCGCAGGCCCAGGGCTACTTACT
GCATGCGCTCCACGCCGAGCAGCTGCGGGTACTGCCGGCTAAGTGCCACAAGACGCTCGA
TAGCTGGATGGAGAACGGCACCATCAAGTTGGAACCAAAGAAGGTGGGGCCCACCGGGGT
GGAAGTGGCTTGGCTCACCTATCAGGCGGTGTTCACCATCGAGCAACTGCCGTTTCGAGA
GCTGGATCAGGCCATCCTGCTGGCGGCCGTCGCCGCCTCGGAGCAGGAGCACGACGAGGT
GCGCGAGCAGCTCGGGCTTCCCGATCACGATTACGCCGTCACCCCGAACGAGGCGCAGAC
GGCCGACCTCGAGACCCAGCTCCCTTTAGCCGAGCCTCTTCGTCTTATCAAGCACCCGCA
AGGGCCCATCAACTGCCTCGGCAAGCGCTGGAACGCGGCCCCCTATGACATCTGGGTAGC
CGAGCAGATTGACCTGAACGTGGGTGATTCCGGCGCTCACCAGGTAGGCGGCCTCTCATG
ATCACCATCACCCTGGACACCCGTCGCGGCAAAGACCAGCTCAACCTGCTGGCCCTGCCA
CCCAAGAAGCGCAAGCGGCTGGTATGGCGAGCCGACAAGGAGATGAAAAAGCTGGCTACC
CGTAACGTGCGCCAGCAGCAAGCCCCAAATTGCAATGGTTGGACACCGCGCAAGCGGGGC
AAGCGCGAGATGTAGCGTGGCCTGCCCAAGCCGTTGCAGATTCGCCAGCCTCGCCAGGAC
ACGGCCGAGCTGGGGTTCACCACGGGCATCATGAGCGCCCACGCAGCGGGCATCGCCAAG
ACCCACCACAAGGTCCACACCTACAAGGTGAAGGCCGCCAGCCGGCGCCGCATTGCCCCC
AGTGAAGTGGGCAAATACAAGGAGGCCAGCAAGGCACAGGCGCGCAAGCTGCGCGAGCTT
CGGTTCAAGCGTCCTGGCAAGCGCAAGCGAGCATGCCGCTCGGCATCGCTGGGTAGGATC
ACCGCCAACCTCATCTACGCCCAGGCGGGGTTGCTAATCAAGAAGCTCAAGGACGAACCG
GTGAAAGAGAGCTGGGAGATCCAGCTACCAGCCCGCCCGTTCCTGGGCGCAAACGCCAGG

CATCGGCAGCAGGCCTTCGCCCTGCGCCCGGAGAGCATCAATTACGGATGGGACCTCAAC
AAGCAAGACATGAAGCGGAAATAACGGCATGTGGCCTTACGTACAGATCTACAACTTGAA
CCGGATGCAGGGGCGCGTGACGGAAGTCGAGCGCCACCTGCTGTTCATCGGCAGCGCCGC
CAGCAACACCGGTAAGCTGGTCTCCCTCAACGCCCAATCCGCCTTTGATCCGTTGCTCGG
GGCCGCGGGCGGCGAGCTCAAGGCCAACCTGCTGGGCGCCCGTGATAACGCCGGCCAGAA
CTGGTCGGCGGCGGCCTATGTCATTCACACCGACAAGCCCTGGCTGGACGCGGCCCGCGA
CTCGCAGCAGACCCCGCCGTTAGAGGGGGTCGTGGTGCTCGGTCAAGAGTGTCACCAGGC
GGCCATCAACCACGCCCAAGCCCTCAACCAGGAGCTGATCGCAAAATGGGGGCGATGGCA
GTTCATGCTGCTGGCCGTGCCGGCAATCGCCGACGAACAGGACTGGACCGCCTTCGAGGC
CGAACTGGCCACCCTGCAGGACGGTATCGCGGCAAGCTCGGTTTCCCTGATCCCGCAGCT
TTGGCCGACCCTGGTTGGGGCCTACGCGGGCCGCCTCTGCAAGCGGGCAGTGAGCATCGC
CGACAGCCCCTGCCGGGTGAAGACCGGCTCCCTAGTGGGCCTTGGCAAGAAGCCGGTGGG
CAAGGAAGGGATTTCGCAGCCACTGGCCACCCTGCAGACCCTGGAGGCCAACCGCTACTC
GGTGCCGATGTGGTACCCGGACTGTGACTGGATCTACTGGGCCGATGGCCGCAGGCTGGC
GGACGAGGGCGGCGACTACCAGGTGATCGAGAACCTGCGCATTGCCTACAAGGTGGCGCG
CCGGATGCGCCTGCGCTCCATCGCCCGCATCGGGGGTCGCTCGTCGAACTCCAGCCCGTG
CAGAACCGCCGGCGCCATCACCTACTTCGGCGAAGATCTGTGTGAGATGGCCAAGTCGAC
CACCAGCAACGGCCAGCCGTTCCCGGGCGAAATCGCCTCCCCCCAGGATGGCGACATCCG
CATCCAGTGGGTCGCCAAGAACCTGGTCTCGGTTTTTGTGGTGGTGCGCACCGTGGACTG
CCCCAAGGGGATCACCGTCAATATCATGCTCGATTTGAGCCTAAACAATGGGGACGGCTA
ACCCATGTCCCGTCGTATCTCCGGTGCCATCGTTGACACCACCCTCTTGGGGGCCGTGGT
CCACGTCGAAAAGGCCAGCCTCTCCATCACCGACAATAGCGCCGTGGCGCAATCCCGGGG
GATCGCGGACGGCTTCGTCGATGGCGATGTCGCCGCCGAGCTGGAGTTCGCTTTCGACAC
CAAGAATCTCTCGCAGTTGGCAGATGCCGCCTAACGCGCGGGGAGCTGGTGCGGAATGGA
GCCGGACTATGTGCTGTTCTACGCCTACACCGTCACCGAAACCATGAAGGGGGAGGCCTT
CGCGGTGAAGCTCCTTGTCTCCGACCTGCTCGACATCGACTCCAAGGGCGGCAGCAGGAC
CGTGCACAAGGTGAAAGGCTTTGTGACCTCCCCCGACTTCGTTCACCTCAACGGCGTGCC
TTACCTCTCCAAGGAAGACACCCGCCACCTGTTGGGATAAGGGGGATGATTGGACGACAT
CGACCGCGCCACCCGCCACGCCGCCCGCATGCTGGCGGTCCAACTGGACAACCAGGTGGG
CAAGGGGCACTAGCAGGGGGAGAGCCTGCCCCAGTGCGAAGAGTGCGGGGACGACATCCC
CGAGGGACGACGCCGCCACGTACCCGGGGAGCGCCTTAGCGTCCCCTGCCACACCAGCCT
TGAACGGTTGGCCCGCTAAACAGAGCAACGGACATGAGCATCATGCTCCATATAGAACCC
ACTCTCTGGGCCCTGCTCTTGGGCTGGTTGATGGACAACATGCCCACCGTATATGGCACT
GCATTAGCAGTAATAACGGCCTGGCTGCGCATCCAGTATGGCGCAGGCAGTGGCCGTCGT
AGGCTCCTGGAGTCCGTCTTATGCGGAGCCTTTACCTTGGCATTTATCAGTGCCTTTGGC
TGGTTCGGGATGCCAGGAGAGGCTGCTGGATTTGTCGGCGGCATGGTGGGCCTTATCGGT
GTTGAGACCATTCGCGGCTTAGCCGAACGGTAGCTCACCACCAAATTACCGAAGGAGTGA
ACAGTCGTGAGTACTGTTCAAATGCATATCCATAAACTTCACGAGTGCATCAAGCTGGTC
AATGCAAATTCGTTGGCCTACCTGAAAACTATGCCATCTCACAGCTTGGATCTTGTGGCT
GTTGATCCGCCCTCCTTCCGAGTGAAAGACCTGGTCTGCGGTAACCAATGGCCCGACGTC
ACCGCCTACTTAGATTGGTTGGAAATATGTGTGATCGAGTTTGCCCGCATCCTCAAGCCA
AACGGGCCACTATATCTGTATTGTGGCCCCGGGCTCAATGCCGAAATCGAACTGTTAGTG
CGCAAGCAGTTGAAGGTTCTGAGCCACATCGTCTGGTCAAAACCCTCAGGCGTGTGGAAC
CGGCCGGACAAGTCTACCTTGCGCTATTTCTGGCCCGCGAGCGAACGCATTATTTTTGCG
GGGCAACTGGACACCCATGGTGTGGCGCAACACAGCGGCTATGGCCAAGCCCGTCAGGCG
CTTCGTAGCCAGGTGATGCAGCCGCTGATCGACTACTTCCAGCAGGCCAAAGCTGCCTGT
GGCATCTCCAACAAGGGGATCGACCAGGCAATGGAGTGCCATATGGCTGGGCATTGGTTC
GGTCGCAGCCAGTGGAGTCTGCCAACTGTAGCACGGTACGCCCGCTTGCAAGCCCTGTTC
GCCAAGAAGACTGACGCCCTTTGCCAGCCCCATCATCAGCTCAAAGAAGCCTATTGGGGA
TTACATCGCACCTACCAAACCGTCGTGGCCAGTTATGACGAACTGAAGGCCGACTAGGAG
CGACTGTGCCGCCCGTTCAGCGTCACCAAAGAGGAGCCCTTTACCTAGGTCTGGACCTAC
CCCTCGGTGCAGGCTTACCCGGGCAAGCACCCTTGTGAAAAGCCGGCTGCCATACTGGAG
CACATCGTGCGCACCAGCAGCCTGCCAGCTGATGTTGTTGGAGACTTCTTTATGGGGTCT
GGTGTCACAGGAAAAGCAGCCTGGCGGCTTGGCCGCTGATTCAGTGGTGAGGAGCTGGAA
CCCCCGTGGTTTTCCCTAACATGCCAGGATTTTCAGACCCTAGTCAGTCAGGAGGAAAAG
CACCATGCCGCGCAGTGTCTGCCACCCCCAGATTGCCGCCTTTCTCGACCAGCTCGCCTA
GGCCGGGGGCACCAAGGGGCTGGGCGAGGACGATTACAACGAGCTGATCATCCCGGCCGG

GTTCTTCCAGGACTACCGCGAACACCCCGATGTGTTTGTGCGGGTCAACCCGGCCCTTCA
CAGCACCGCTGCCGGACGCTATCAATTCCTGTCCTGTCATTGGGGCCACTACCAGGCGGC
GCCCGGCCTGCCGGACTTATGCCCCGTCTCACAGGACACCTGGGCTATCCAGCTTTTCCG
CGACCGCAAGGCGCTTGACGTCGTCATCATGGGGCGCATCCTCCAGGCGATCAGCAAGTG
CGCCAACATGTGGGCCAGCCTGCCCGGCGCCGGCTACGGGCAGCGCGAGCACAAGCTGGC
GGATCTGCTGGCCAAGTTCACCGAGTTTGGCGGTGTGCTCGCATGATCACGGTCATCCGG
TTGTTGCCGTCCCTGATGGGGTTGGTAATCGGGAGCTTGCTGTGTGCTCAGGGGGAGCGG
CTCACCCAGCGCACCCGGGAGCTCGCCACCGCCAACGACACCATTGCCACCCTGCGAGAG
GCCAAGGACCAGACGGGCGATGCGCTCAAGATGCTGCGAGAAGAAGACAGCGCCCTGCGC
CATCTGCTCTTCCATCCGAACGCGTCGTTGGCCGAACTCGACACCTAGAAGAGGAAGACA
GCCAATGACCTGTAACAAACCCTGGCCATACCGCCGTTGGGCCGCCCGGATTGCGCTCGC
GAGCCTTTGCCTGCTGGCCCTCTCCGCCTGCTCCAGCCAGCCCACAACCGTGGTGCAAAC
CCGGGTCGTCACGCGGATGCCGCCGCCGGGACTGGTGCCCCACTGCCCGGAGCCTGAAAT
CACGGGGAGCACCTTCGGCGACACCGTGCGGTTTATCCCCACCGTGCAGATGGCGCTCCG
CCGCTGCCCAACCCATATCACCACCCAGAACAAGGGGATACCGCAAGGGGACACAACCCC
ATGAGAAAGACCCTGACCCTGACTGTGGCCGGTACCGACATCAGCTTTTGACCGACCATG
ACCGCCTACAACGGCTTCATCAACGACATGATGCCGAGCGAAAAGGTGGCACCGGCCCAC
AACTACCTCAAGAAGATCGTCTGCCAGGAGAGTCAGGCAGAGCTCGACGAGCTGCTCAAG
CGGCCGGGCGCAGCCCTGCAGTTGGCGGGGGCCATTAACTAGCTGTTCGCCCCCACCCTG
GAAATCACCGTAAAAAACTGACCGCGCGTGCCGAGGCCATCGAGCGCAATCCCCTGTAGC
AGGTGCTGGCCCGGCGGCGCTACTACCTGCCCCATGAAGATGACTACCTCGACAGCCTGG
CTCGCGCCATCTGGTTAGACAAACAGCACTGAGAGTCCAACGCCGCCGCCGTGGCCGAGG
GCATTGCCAAAGCACTGAACGGGTCACGACTGATGGCCTGGATGGAAAAATTGATGATGC
AGGTGGCCCTGGTTGACCAGGTCACCAAGCCCCTTGCCGGCATCAAAGCCCAGATGGACA
AGGTCAGCAAGGCGGGCCGCCAGGGGTGGAGCAACATTGCCATGGGGGCCACCACCCTAG
GCGCCGGCGTGCCCGCGGTGCTGGCGGCCCACGGCCCCGCCATCGAGATGGACTGGGTCC
TCTCCGAGGTGGCCTCCCTCGATGTGCAAAAGGACGTGCTCGGCGCGCTCGGCCGTGAGG
CCCTCAAGCTGTCCGTGCAGTACAGTAACTCGGCCACCGAGATTGTCCGCTCCTCCTACG
AGATCCCATCCGCCATCGCAGGGCTGGAGGGCAACGAGCTGGCCGCCTTCACCCGCGCCT
CAACCGTCCTGGCCAAGGCCACCAAGGCCGACACCGCCACCATCACCAACTACATGGGCA
CCATGTACGGCATCGACGAGCAGCAGGCCAAGAGAATGGGCAAGGCCAACTGGGTGGAAG
ATGCGACGGGCAAGACGGGGCTGGCGGTACAGATGTTCAAGACCTCCGGCCAGGGCATGG
CCGATGCCTTCGGGGCCATCGGTGCCAACGCCACGGCGGCCGGGGTGTCGATGGATGAAC
AGTTCGCCGTGCTGGGGCAACTGCAGGCCACCATGGGCGGCGGTGAGGCCGGGACCAAGT
TCAAGGCGTTCCTGGCAGGGGTGGGCAGTGCCCTGACGGCGCTCGGCGTGAAGTTCACGG
ACTCAGCGGGCAACATGCTGCCGGTGGTCAACATGCTCGATAAACTCAAGGCGCGCTATG
GCGGGACCCTGAGTGTGGCCCAGGGAGACGAGATAAAGGACTCGTTTGGCTCGGATGAGG
CGGTGGCCATCGTAAAACTCCTGATGACCAACACCAAGGCCCTTTCCACCAACATCAACG
CCCTGGCCAACACCCACGGTATGGCCAAGGCAGAGCAGATGGCGGCGGCCATGACCCACC
CTTGGGAACGGGTAACCCAGGCCTGGTTCGCCATCCCGGCAGCCGCCTTCGGGGTGGTGC
TGCCGGCCACCGACCAGGTGTTGGGGGTCTTTCCCGACGGGACCGACACCGTGCTGCGTT
GGACCCACCTATTCCCGAACCTCACCCGGGCGGTGAGCTATGCCCTGCTCGCCATCGTGG
GCCTTGGCATGGTGTCCGGTGCCTGGATGCTGGTCGCTGGCGTGGCCAAGCTGGCCACCC
TGGGGCTTGGCATCGCCTGGTCGCTACTGCTCGCCCCGCTAAACCTGCTCAAGGCCGGGA
TGGTTGCCTTTCTTGCCATCCTGCTGGCCGTCAACATCATGATGGACGCGAACCCATCCT
TGCTACTGGCCAATGTGGTCGGCGGCCTGCTCGTCGGTGCTATTGGGGTGGCGAACTACT
ACTGGGACGATCTCAAGAAGACCCTGGCGGATGGGGGCGTGTTTGAGGCAGTCAAGGCGA
TGATCGACGGGGCCGCCGCTGGCTGGGCCAGTTCCATGCAGCTGCTCGGCAACCTGAGCC
CCTTCCAGTTGCTGGGGCGGGCGGTGGACTGGCTAATCGAGAAGCTCAACTTGATCCCGG
GGGTCAATATCGAGCTCGGGAGCTTGCCGGACCTGCAGATGCCCGCGCTCACCCCGCTGA
CCCTCGCGGTCATGCCTGGGACGGTGAAGGCCCCCATGGTGGCAGAGCCGCAACAGGCCT
CGATCATCGCTCCCCTCCCCCCCTATAGCCAGCCGGAGCAGAGCAAGGTGCCGGCCGGAG
GACTGGGCAAGCAACTGATCCAGGCCAACGCGGCCGCGTGCTCTGTCAACCGGAAGCCGG
CCAAGTCCCTGCACATCGGCGAGGTGCACATCACACACCAGAACCCCCTGACCCCGGAGC
AACTGGCTGAAAAAATCTGGCTGGAGACCAAGTGATGAACGAACCCAAGTACATCGACGT
CCTGGTGGTGAACGGTGCCTGGCAGCCCGTTGCAGGGGGCCAGCCCCGCACCACCCAGGA
CCGCCACAGCATCGTTCAGGACATCAAGCACCGCATCCTGGAATCCGGGATCGCCCGGAA

GCTCATCGGCGTGCGTAGCCCGACCTTGAGCAGCGCCTTGGTGTCCGAGATTGAGCTGCT
GGTGGAAGACGACGAGCGGCTGGTGCCTGGCACCATCTTCATCAGCGAAGAGGCCCCCGA
CCGGGTGCTGGTCACGGCACGGAACTATGAATTCGACGAACTGGAGGTAACCCTGGGAGT
CTGCGCCCGACCGTGGACTTTACGACCCTGTTGGCCGAGGCTGGCGGACCGACCACCGAG
GCGGCCATGTAGGCCGAGCTCAAGAAGGAAGTGGTGGCCGCCGGTTCCCTCATCACCAAC
GGCAGCGATGTGAGCCCCTTCTGGCGGCTGGTGGGCGGCGTGGTCATCACCCCGGCCCTC
CGGCTTATCCGCACCCTGCTGGCGAGCCATGTGCTGCCTAACACCGTCGGGGCCACCGCC
ACCGATGCCTATCTGGACCTCAAGGCCTGGGATGTGGACCTTACTCGCAAGGCGGCCCAG
AAAACCCGGGGCCTGGTCAATTTCGTCAAAGCCAACCCGAGCGACGCCGTCACCATTCGG
GCCGAGATCTGGATCACCAACGAGCGCATCGACGGCACCATCTAGCGCCTGCGGCCCATG
CAGGCGGTGGTGAGCCCCGCCGGGGAAGCGATAGCCCGGGTCGTGTGCGAGGCCGAGCTC
ACCGGAGAAGCCTGGAACCAGGCTTCGGGGTATTACAACCTGCTGAGCAAATAGGTGACC
GGCATTCTCTCTGCCCGCAACGATGACCAGGAGTGGATCACCACCCAGGGCAGCGACGTC
GAGAGCAACGATGCACTCGGCCTGCGCATCCAGATCCAGTTTTCTGCGGTTGGGTGCTAC
CTCATCGACGCGATTTACCGCTCCATGCTGGCGAGAGTCGCGGGGATCCGCGTCGATCTC
ATCTTCTTCGAGCACGAAACCCCGCGGGGGCCCGGCACCGCCAACTCTTACATTCTGCTG
GACGTGGGGGCCACCCCGGCCGGCCTCATCAATCCGCTCAACGACTACGTGGGGCGCCTG
GGCAACCATGGCCATGTGGATGACCTGTTCGTAATGAGCATCCCCGTGACCCAGCACAGC
TTCACACTGGCGCTCTGGCCCCAGGCCAACCTCAGCGACGAGCTGCGGGCTGCGTTCAAA
GCGGGTACCGACTTCCTGGCCCGGGCAGGGTTTCGCCAGTCGGCAGATTTCCCGGCCGTC
ACCCGCACCTGGCCGCGCTCCCGCTCCTCGCTCTCCCAGCTTGCCCGCGAGCTGCACAGC
CAGTTCCCGCAACTCCTGAGCCTACGCCTTGGCGAGAGTGACAACGTTGCAGGACTCGCC
ATCCCTCGCCTGAGTACGCTGGAGGTGACCCTCCATGCCCGACCCGACGCCCCTTGAGCA
CGAGCGGCAGGCGCCGCTGCTAGCGGATGCCTGCGCCCCCTGGTGGGAAGATGGCTACAC
CATCAGCCCCTCCCATGCCGAGCCCGGGTTCCTGGGCAAGGGCATCAACGCCTTCTGGCA
ACGCCTCAAGGGCTGGCTGGTGCTGCCGTTGGCCCGGCAAGACCCGATGACTTGCTCGGA
GTCCTGGCTGCCCCTGCTGGCCTGGGAGCGAGACATCACCCGTTTCAATGGCGAGCCGCT
GCTGCTCTTTGGCAAGCGGGTCATATTCGCCTTCATGAACGCCCGGGATGCCGGTGAAGT
GGCAGGCTTTAAACCCATCTTAGAGCGCACGGGCATCGGCTGGTGCGACATGCACGAACG
CCCGGCCGGCACGCCCTGCGACGTAATCACCATCCAGGTCACCGATAGCGCTATCGAGAG
CAACCAGCAGATGATGGAAACCCTCATCCAACACTATGGCCGTACCTGCTGCCGCTACCG
CATCCAGGTGGTTTACCCGGTCACCGCCACCCTGTGCCCCGGTCGCATCGACATGGGTTA
GCAGGTGTTCGGCGCGACACTCAAGAGGACCCCATGAGCCAGATCATTACCAGCGCTTTC
ACCAGCTACCTGCAGGCGAGCCTCGCCAACCAGACGCCGGTGGTGCTCGATGAGTTCGTG
CTGGCCAACGTGCCCGACCTAGACCCCGAGGCGGCTATTGAACCGGACATCGGCCAGCCG
CCGGCGGGCCAGATTGTGCATCGTCACACAGTGGATCAACGCGGTCGCATCAACAACGAC
CCGGTGGACTACACCACCGTGATGGACACCACGTTCGGCGATTTCAGCTTCAACGCCCTG
TACCTCATCAACACAGCCAGTGGCATGGTGGGGATGGTCGTACATAAGGGGCTGGAGACC
AAACTCAAGACCAATGAGACCATCGGCCAGACCGGTAACAGCCTGGTCAAGTCCATGCTG
ATGGAGTACGACCGGGCAAGCCAGGCCACCACCACCCACATGGATGCCAGCACCTGGCAG
TTTGACTATGCGGCCCCGTTGCGCGGGATGGACGACGACCTGCGCCTGCAGGACCTGCGG
TTCTTCGGGCCGGCCACCTTCCACGGAGACGGCTTCAAGCTAGTAAGCGAATCTGGTGTC
TATAAGGTGCAACCCCAGGTGGCCTATGTGGACGGCCTGCGCTACCAGCTCGACGAAATG
AGGAAGGTCACCGCCGGCTCCAAGCCGGTGGGGCTCTGGCACGACATCTACCGGGCGGGC
TCCCTGCTCAACGCCTGGGTGAATCACTTCTCCCTCACTCTTAGCGTGCCAGACATGCTG
GATGACGTGGACAGCAACGGCTCTATTCACCACGTCGCCAAGGTAGCAATCGTCAATAGC
AAAGGCAGCATCACCGATGTGCGCCACAAGCGCACCATCGAGCTGACGGGGGTCGTGACA
GGAAAGGGCATTGTGGAATATGCCACGAGCGTTTCGATCTCTGTGGAGATCAAAGACGGC
AGTCACCGCCACAAGTGGGATGAACTCTAGCAGGTGCCTGAGACCGCCAGCCGCTGGCCC
AGCTACAACGAGGTGACCAATAAGCCGAATTTAGCCGCGGCCAATCATAGCCATATGGGC
ACCCTGACCAATCCGAACCAGCTGTCCAACGAATTCCTGAATACTATCATGAGCCCCGGC
ATCTATCGGCAGGACTGGAACGGCAATGCGACAGCCGGGCTCAATTACCCAGAGACACAT
GCGGGGTGCCTGATCGTGACAGTTGGCGCCGGCGGACAGCAGCTTTACCACGTATATAAC
TCGAGTCGGGTCTATAGTCGCGCCCAGTACCACCCGGGGGCTTTCACTGCCTGGGCCAGG
GATTACAACAGCCAGAACAAACCGAGCGCCGCCGATGTGGGGCTAGGGAACCTTGTGAAT
AAGGGTGGGAATTATGTCGCCATAGCAGACAAGTATGCCGTTCGCGAGACTTCCGGCGAC
CTTAATGCTAGCCTGTTCAGGGCGACTTACCCCTACGCGGCAACTTGCTGTGGAGCCTTG

GCCTTCCGGGTAAACAACGACAATAACTCTTACACCCGATACTGCAACAACCCGGCAGCG
GTGCGAGCCTGGCAGAATTAACATAGAACATCGTGGGATATGGGGTGGCGCGCATACATC
GAGGCTCCCAGCAACCCGATGACCGAAAACCACATTCCAGGTACGTACGCGTTAATTACC
TAACCGACCGGCGACGGTACCTATTTGATTGCCTCATCCAACGGCGGGGGTGGTGCAATA
GTACAGCGTCTGCGCCTCGATACAAACGGCAACCTCAATGCCACCGGCGGCGTGCCTGAT
ATGGGGCATCGGGTATACAGCCCGGTCAACCCGCCCCACAACTCTCATAATCACACGGCC
GCCCAAGGCAACTCAGACATTGTCGGCGGTGGCTGGGGGCTGGTCGGCACTCACATCCTT
GCTTCCAACGCCTACGGCACCACCATGGGCTTAGGCTCAGTAGTCTCGGCTTCCAGCGTT
GTGCCAGCCTCGGCAGGGGAACGGCACCATGACGGGGTCAGACTGCCAGGCACCTCGAAA
TGTCTCGGCTATTCCGTTGGTTCGCTTGGCCCCTTCGATACCCGCATCACTCTCTGGATA
AGGATAGCCTGGTGGGGATCATCACCCCCAAACAAGGCATCGCCTACACCAACCGACCGG
ACGCCCTGGACATGGTGGTTGCCTTCGACCACCTGCCCGGGCAGTTTGTCTCCTTTACCG
CCAGAAAGGACGTCTCGGCCGAACATGGGCGCGAGCTCTATGTGCGCGCCATGTTTGGCG
AGTTCGGCAAGATCCGCACGCAGGCCCAATCCGAGCACCTCCCGAGCGAAGCCGAGCACC
AGCGCAAGCTCGATGCCCTGCAGGCCGAGGTCACTCTGCACATCGCTCCCCTGCAGGACG
TCAAAGAGCTGAAGCTGGCCACCCCGCAAGAACTCGACAAGCTGGAAGCTCTGCAACGCT
CTCGCATCGCCCTGATGCGCCTGCCGCAAAGCGAGGGCTGGCCAAGCTCAGTAACATGGC
CGGAGATGCCCCAATGAGCTGGGCCCAGGGTGCGCTGCGCTGGCCAGCAAGCGTTGACAC
CCTGCACACCCGCGCCCGGGAGGTGCTGGGGCAGCCTCCGGAGAGCAAGGCGAGCGCCAT
GGATCGAATCCTGGGATTGGCCGCGCGGGCCCATTATCGGCGCCATCCGCTTAGCGAGGC
GGCCGCGGGCTCGGCCGCTCTTCGGACCGAGTTCGACCCGTTGCTGGTGATTGGCCACTG
CCTCACCATCACCCCCTATCAGCAAGGAGTGGGACAACTGAAGGGCACCCAGCACCATCT
GTCCGCCCACTACTCGGTGGCGGCACTGCCCGCCATGCTGCAGGATGGCGCCGACCGACG
GTAGCCCGGCGGGCAACAGCATGCCCTCGCCTGGGTGCTGACAGGCAACAGCGCCGATGA
CCTAGCCCGGCAGTTGGCGCTCCTCTGCGCCCTGCTGCCGTTGCCGGAGTGGTGCGCCAC
CTTGGGCCGACTCACGGCCAGCAACGTCGCCATGCACCAGCCCAGCGCCCCCAGGGTGCC
GCGCTGGCGCGCGGGGGAGCCCCTTTGATGGGCACCGCTGCGCCCGGGCCTGGCGGCACT
TGGCGCGAAGCTGTCCCAACTGGAGAGCCTCGCCCGGGACAGCCAGAGCCCCATCGACAA
GCTGCAGGGGCTCGCCGTGCGCCGCACCGCCCGCCTTGATGCGCTGGCCACCGCTCTGGC
CCAACTTGGTAAGCTGTCCGGCACGCTCTGGCACTGGCATGGCCAGGGGGACGCGGCGAG
CCTCGGCACCCAACTCTAGCAGAGCAGGCCCCCCGACCACAGCATGCGCATGACGGTCGC
TCCCCTGCTGCTCACCCCTTCCCCGCTCACCTTCTGGCAGGAGTTGACCCCATGAGCCAA
GCCATGCAGACCCTGGATGGCGAACCCATCATCATGAAATCGATGCCGGTGTCCGCATCG
AGGCAGTTTAAGAACAAGGACCAGAGCGGCCAGACCAGCGCGACCAGCAGTGCCGAGCAA
GGCGCCAAGGGCAAGGAGCTCGACGTCTCGGGCCTCATCGCGTTCATGGAGGAGCGCATG
TTGAGCCGGCTGTTTGAGCTGGCCGATGCCAAATGTAGCGGCGGCAAGCGCCACATCTAC
CGGGTCGGGTCGATGCTGGCCAACGCGGTGAAGGTGCGCCAGGCGAGGTTCGCCGGGCGC
ATCACCGAGAGCGACCAGGAGGGGCTGCTTTCGTGGCAGGTGCAGTTCACCTTGAAGGAG
TTCAACTCGGTACCGGAGAAGCGCGAACAGCGTTTGCCCAAGCAAGCCCCGCCCATGGGC
CAGAGTATCTCCAAAAACAGCGCCGCCAAGCCCGGCGACAAACGAACGAAGAAGGATTAA
AAAGGCCTCAGCAGTTTCGAGCGCTATGTCCTACAACCGATGGGTGACATGCTGGCATGA
AACTGACCACCTCTCTGACCCTCGCGGGTCAACCGGTGTACCTCATCGACCATGATCTGG
TGCTGGATCTCAACGCTGGAGGCCGCGCCGCGCTCACCAGCGAGGGCACCGCCCGCAAGG
GGCAGCCCTTCACCCTGGACACCGGCTATAACGGCGACCTGCGGCGCTGGTTCACGGGCT
ACGTGTACGACGTGCACCCCGCCGCCAACGATGCCAGCAAGCTGCTGTGGCGGGAGTTCG
CCGGTGCTTTGGGATCCCGCCTCCCCGACAGTCAGCAGCATGCCACCCTGCGTGGCCTGC
TCGCCTGGCTGACCGACCGCGGCGGGCTGTCCTTCCTGCTGCCCCAGGGGGCGGACTACA
CTGACCGACCGAACCCAAACTTCACCAGCGCCGGCACCGGCTATCAGTTGCTCGATAACG
CCGGGCGCGCCTTCGAGGTACCGGACATTGACTAGTACCAGCAACCGGATGCCTCCATCT
ACGCGGGGAGCCTCGCCCATAGCCGCTGGCATGGCAAGGAGGCGACGCTCGATCCCGCCT
GGTCAGGGCGCCAAGCGGGTGACACCCTCAGTCTGTCGCCGGTGCCGGCCATCAGGCCCG
GGGCCATCATCAACGGCAAGCGGGTAATGCGGGTACGCTCCAGGGGAGACGAGATGACCC
TCACCACCGTTACCCCCGGCAAACCGGCGAAGTCGCCGGAGCGGCGCAAAATAGAGAGCG
AGTTCCCGAAGCTGGCGTACAAGATGCACCTACCCAAGTTCCGGCAGGTCGAGGAGGTGA
GCGATCAGGCCACTGCTGGCCAGCTCAATGATCCCTTTCGTCCCCGCTATGCGGTGGATG
TGCAACTGCTGGGCGAGGATGGCGAGCCGGACAAGGCGGCCCGGCTGTATCCGGCGGTGC
CGCTGCCGGTGCTGTTCGGCGAGCTGGAGCAGGGCCTGCTGCAGTTCCCCATTGAGGGGA

CTTTGGTTGAGCTGGGGTTCGCCTTCGGCCGGGCCGACCGGCCCTTCATTCGCACCGTGC
TCGGCAGCGCCTGGGCCTTGCCCAACATCACCCCGGGCGTGCAACTGCAGCAGCAACGGG
CTGAGGTGTTCAGTCGCACCGATATCGTGGGGGACCTCTGCCACCACACCGACCGTCGCG
TGAACGACCAGGCCCTGCAGATGCAGCACCGAACCGATCACTACCTGGGGGATCATGGCC
AGCATCGGCTGCAGGTGGCGCAGCGCAGCGTCGAGGAGGTGGGCGGGTTCCAGCTCATTG
CAGCACTGGGAGCCATCGAGCTGCTGACCGGCGACGCTCTCAGCCTGGGGAGCCTGGGCA
ACCTGAACCAGACAACGGCCGGGGATCTGGTCGACGTGATGGGACAACAGCGCCGGGCGT
TCGCCGGCGAACTGCAGCACTTGGAGGCGCCACGCTCCTGGGTGGGGACAGATAGCGTGA
ACATCCTCCGGCTGCTGCTGCAACTGATGAACGTCGTGGAGCAACTGGCCTCCGCCACGG
CTGGCCATACCCACGGCAGCGGACCAGCCCCAGGGAACACCGCAGCCATGACGGATCAGG
GCCAGCAAACCAAGCACCTGGCCAACTCCCTCTCAGCCATCATCGAGTAACCTGCCAAAC
CAAGAAGGCCCCAGAGTGTGGGGCCTTCTTCTTATGAGCCTGACAGCGCCTGAACCTGGC
CAGGGAAATGTGCCGCATTAAGACGCGCCACCGGGCAGCGCTACAACATGCGTGAAGCCT
GCGCACGCTCACAGCCTCCTGACGAGTCACGGAAACACCCGCGGCGGCGTCTGCGCCACG
GAATCCGCGCTCATGCGCTCCCGCC
>NewGenomeName_7
ACGAGGGGGGCTTATTATGACGCCCCCTGCTGGGCCGCGAGATCCTATTAGTAATGGGCT
TAATACATAAATTGGCCGCTAAGTAACCGTACAGTTCAGTATCTTCGGTTTGGGCCTCGG
CCCAAAATTAACAGCACGTGTGCGCTTGCTTGGGTTGGCCCGTAGGTCCGGTTCGAAGGC
GGAATATTCCTTGCTTCTATAGGAAGAAACAGCGAAATAAAATAAATCGCTGTGACACGT
GTCAGCGGACTATTAGTTCGCAGAGTTTAAGCATACGCTTCAATTTGATGGGTGTGGTGC
GCTCTCTCTTATGTCATACGTTGTGCCATGAGGAAGCCCTCCGGGGCGAAAGAAGGGCGG
AGGTGGAGGCGCCGCCCCGCCCGTCCCTCCGACGAAGGATACGAAGTCGTAGGTATGTGA
GGGTATTTATATAGGCCTCGTGTACGAAACGCGCCTGTATCCTCTGTGTATATGTGTATT
AAAATATTTAAATACTAAACCTTAACGAATAAAATATATGTTTAATAAACTAAGACATGA
CAATAAAAGAAAACAATGACACAATAATAAAACTGTATTATGCCAACGGTGTATAATTAA
GGTCTGTTTAATAAACCTATATATATGTAGATTACAGTCTTGTTTAAAATAAGTGACTTG
GAAGGAAATAATAATATTAATGATAAGCAATAAAATATACCGACTACATAAAAAGGGGAA
AATGCAATTAAGACCTGTCACGTTTTCACATTTCGAACAAATTAAGGGCCGAAGGCCCCT
CAATCTGCAGGTATATGGGTGATTGCCTCGTGACGAAGCGAGGGTATTATTGTGAAAAAG
AAAGCATCTGTCAGCTATAATAGTGGTCCCCTATACAGCTGTCATATGACAGCTGGCAAA
GGACCATTCGGGGGACTCCAAATATATACTAAATATAATATATTAAATGTATAAGGTATA
GATACTATTCTTAATTAAAAAAGGCGGGAACGAGGACACGTGCGCCGCACGCCATTTTAG
TGGTGGGACCGATGTCCCGCGTTAGTGCGCTTACGTC
>NewGenomeName_8
ACCGGCCACTTGGAGCCACGGAGCCACCACAATGGCACCTGTTAGATCTGCTGCTGCAAA
GAGATGGTGCTTTACGTTGAATAACTATACGGCAGAAGAAGAAGCTAAAGTGAGGGCCCT
GTTCCCTGGTGAGTTCCACTTTGCAATATGTGGGAAGGAACGGGGGGAGCAGGAAACTCC
TCATTTGCAGGGCTTTCTACATTTTAAAAAGAAGCAGCGACTGAGTGCCCTGAGGAAACT
AGTTGCCCGAGCTCACTGGGAAAAGGCCCGTGGCTCAGATCATGATAACGAGGAGTATTG
TAGTAAGGAAAACGACGTGATACTCACCATCGGGGAGCAAGTTCAAGGCAACCGGAGTGA
CCTTGCCGGAGCTGCTGCCGCCGTGAAGGCCGGAAGGAGAATGGTGGATTTCGCGCGAGA
GTTCAGCGAGATCTACCTCAAATATGGGCGTGGTCTGAGGGATTTGGCCCTGATGATTGG
ACAGAAACCCCGTGACTTCAAGACGGAAGTCGTCGTTATCACGGGGCCTTCCGGGGTGGG
CTAGTCGAGACTTGCCTCTGAAATGGTAGGATCGAAGTTCTACAAGATGACAGGTGACTG
GTGGTATGGGTATTCCAATGAGGATATAGTCATCACGGATGATTTCTATGGGTGGCTGCC
ATTCTGTGAAATGTTGAGATTAATGGACAGGTATCCACATAAAGTTCCCGTGAAGAGGTC
TTATGTGGAGTTCATGTCGAAGAAGATTGTCATAACGAGCAACACGCACCTGGAAAGTTG
GTACTGTTCCGATAAGTGCTATCTGCCTGCTTTGTTCAGGAGAATCAATAAGTGGATGTA
CTGGGATGGACTGAGATTCGAGTATGTGCCGGATGCTCTGAAGAAGCACCTGATCAACTA
TTAACACTCAGCCCCGAGAAAAAGAGCGTCTTGCAGGTGATTAATATAAAATGCGCGCGC
AGCGCGCGGCGAAGGCCGAAGGCCGAAGCCGCCTGAGACCGCGGGAAGCTCGGGCAGGCG
CGAGCGACAGCGCGCGGGCAGACCCCGGGTCCCCCCGGGGTCGCCCCCGTCGAGGGGCCT
AAGGCCCCGAAGAAAGCTATTTGTACCCCCCATCAAATAAAAAGACTTCAGACAAATAA
ATGTTTGGTGTCTCTATTTTGGAGTTGCCAAGTGAGTTAAGTTCATTTGCCGGAATTCAC
AGTAGATGGTGACCTTGACCTGGTGGTCCACCTGGTTGGTGGTGGGTTCTGGCCAACTGA
AGGCTAACCCGAAGTGTTTGACCCTGGGTCCGGAGCTGGGAAGGTTCTGGTCACGTTTCT

TCATGATCAGTATCCAGGGGGATTTGGGATTGTTGAGCCACAGGGCCGCCGTGACGTGAG
CAGCCTCAGTGTCGGTCACGCTTAGTTGTGGTCGTGGTTTAAATAGCCTTTTGAAGACTT
TTCTGATATCCCATTTACGGCCCCGTCCCAGTTGGCTACGGGGTCGGCATCAAGTCTTG
TCTTTTTGAAGAAGTCCTCTAGGTGATGGTCTTGGATAGGTACATTGTGCCCGAACCCTT
TCCACTCCTCCCAGCTTTCATTGAGGGGTCTCATTTCCACTTTTGCTAACCTGATTCTAT
AATATTCAAAAGGTAACCTCCAATTGATAGTACCCCAATCAACAAAGTCATCCAGTATAA
TGTTGATTCCATCAGTTCCAAATGACAGGTGAGACCCCCCTGTCCCTATTTGTAGTACCT
GACCGAACTCCCTATCAAATCTGACTCTATAGACCCTGTTGGTTCGGTGGCCCCTCCTGT
TGGCAGGGATCCTTCTCCTCCTCCYCCAGTCACGACGCCTCCATCTCCTCCTCCTGGGAG
CCAAAGGTCGTCGTCTCCTAGCAACCTGGTTAGAGGTTACGCACATCCGGGTTAATAATT
AACCAGTGGGYCCAAGAGGCCGTGRCAGTATT
>NewGenomeName_9
TCGTTTTGAATACTTATTTTAGGCTTGTTATTACGCATTTTATAAACTCCTTAATTGTTA
TTTGATACCAATTTGATACCGTTTAATCAAATATGCTCATAGCTTGATGTTTTTTATCAG
TATATAAATGAGAGTACGTTTGAATTGTTTCTGTAATGTTAGAATGCCTCATTAATTCCA
TTAATAAATTCATATCTACACCATTGTGAATAAAATAGCTAGCGTACGAGTGTCTTAAAT
GGTGTATTTTTAGATTCGGGAATACAGATTTAAAATGATACGAATAAGTAACGTATCTAA
TAGGTTCTAAACCCCCGAATATAAAATAGTTTTCGTCAAAATATTTATATCTTTTAGAAG
ATTCATTATACATGTTTTTAAGCATCTCTCTAATTAAATTTGGTACAGGTATTATCCCTT
TAGAGCTTTCTTTTTTTAGATTATATTCAATTTTTCTATTACTTAAATTGATTTTCTTAT
TTACGTCAATTTCGCCTTTTATTTTATCGTAATCTTTCCACTGCAAAGCTAAAGCTTCTC
CTATTCTAAGACCAGAATAAAATAACAGTCTAGTTAGCTGACGAGAAGTATCATTTGTGA
TTTGTTCTACTTTTTCATCAAATTCTTCACGAGTGATAAATTTAGCTTGTGGTTTTGTTC
CGGGAATAGGAGTTACCGATAATGTGGGGTCGTATAAGAGCTTGTAATGCTTTTTGGCGT
AATTGATAACTGCTTTAAAACCTGCCCACACAGATCGTGCATAGTCAACAGAAAGACCTG
CATCGTTTAACAAATAATTCCTGAAAGCAGTACATTGCGTAGTAGTGATTTTGCCAATAG
GGATATTTCCGAACCTTTCTTTTATGTGAGTATTATATTCTGTAGTTCGCTTTTCTATTG
AGCGTGCAGAAAGATTTTCATTTTTTAAACGATCAAAAAATATATATTCAAAGGGTTGAT
TGTCCGAGTATCCATATTTAACATTTTGTATAAATTCGCTTTCAGCTAGTTTGACATCTT
TCTTACGTTCAAACCCACGCTTCATTTTTTGTTTGTTATTACCGTATACATCTTTATATC
TAATGGAAAAATACCATTTACCTGTATTACCATCCTTATATACTGGCATTTTACTTCTCC
CTCCTCAAAATTGGCAAAAAATAATAAGGGTAGGCGGGCTACCCGTGAAAATTGTATAAA
AAAAGAGAGAGCGCAGATGCACCCTCTCATGTCGCAAATATTTCAGCGACTTGTCTAATT
TGAAGCTTGCCGCAAATATTTCAGCGGCTTGTTTTGTATATATGTAATATACCATCAAAG
AGAGTGTAGTTCAAGCGATTTAACTAAGAAATCTAATTTTTATCCTATTTTCAATTTTAT
CTACTGTTTCTTTTGAATATGATATTTCTCCGGCAGGGTCATACCTATTAATTTTCGATA
TTCTATCCTTGCTGATTGTAGTGATATTTAAAACGTTAGCATAGGTCTTTTTATACTTGA
ATCGCTCATATCTTTTGCGAACCTTCGAATATTTTTTGAAGTCGTCATTCAGCGATTTGT
TTTCATCAAGTAATTTTTGATCGTATGGGTTTTCTGCTTTTGACACCTTTTCAAGATTGT
TCATGATTTTTTTAGCTAAATCCTTACCCGTTACGTCCATTTTTTCCAATCCTAAAGGTA
ACAAATCTTCTTCGATATGCACATTGAATTTACTTCTGGAAGATGTAAGTGGCACTACCG
TTAATATTGGATTTTTATTTGAATCGTGATTATTAAGTACCATACAAAAATGGTTTCCAG
AAAACTCTCTGCCAACATTAACACCTAACTTTACATAAATTATAGTGCCTTTTTTATATC
TGGTGTAACTTTTGTTTTCTGTTAACAATCTAACTTCATCCAATAAAAACTCTGAATATT
CAAGACACCATGAATTCATATATTTAAATTTGTAAATCTCGCTATTTTGAATCTTTTTAA
AATGATTAACTGCTGTTTCTAAAGGTGCGTTCTCTTCCATCCCTCATCCTCCTCACGCCA
CACAAGCGCTATTAATCAATATCCAATAATTGTTGTTTTTTCTTATCGAACTCTTCCTGA
GAAATTACTCCGACATCTAATAATTCTTTATATTTTATTATTTCATCAGCAACAGAAAAA
CTCATTTTTTCAGAATTGGATGGTTTCATAGAACTTTCTCGAATAGAGATTTGATCTTGT
ATTGTTTCCGCCATTCTAGATACAGTGTTTTTTGATATGCTTCCTATAGCGATACTTGAT
GAACCGTGATGTATAATTATTTCGCCAAAAAGAAGTCCTTTTTTATACGAAACAGAATTG
ATTTTCTCGAATGGAAATTCATGAAATTTCAAACCATATATCATACCTTTACCTAAGAAT
AACAATCTTAGATAAGTACATACTATTAAGTAGGTATTATTATTGTACAATCCCGAAGTT
ACATACATTATGTTTTCATTATCTTTTAAAATCATAGGTAGTTCTTTCACTTCTTTTTTT
GTACCAAACAAATCCTCTCCACCTATTTCGCAAAATCTTTGGTAGATTTTAGATAAGTTT
TCGTCAGATTTATTGATTTCACTTTCAAATTTCACTTCTTTTCTTGGTTTACTTTGGTAT
TCTTTTAAAATTTCTCTTTTGTCTTCAACAGATAGTTGCTTGTATTGTTTCTTTTCTTCT

TTTGTTTTAGTTGCTAAATATTGACTCTCAATCATACTTTCTTTGAACGTTAATCTGCTC
TTAGGTAATTCTTTCATGTTCATTTCTCCTTTATTTATATTTCTTTATATTTAAAAACTC
TCAACGGCTCTAATGTAATCGAATACTCGCCATAGTGAGATCCAATACCATATATCTTTT
TATATTGTTCTATTGCTTCTAATATGTATTCTTCGCTTAATTGTAGATACTCAGACAACT
CATACAAGTTGCGTACGCCATAATTATAAGCTTCTCCAATTTCGCGTAGCGGTACAGCTG
AGATAAAGCCGTGTCGTCTTGCGTAATTTTCGAACTTGCGATTGTTGAATTTCGATTGAT
CTAAAATGTTGCCATACGTCAACTTGTGGTGGCAAGTTCTTCATATAATACTTCTAAGT
TGTTCCTTTCGGATAAGGAAGGTCTAATAAAAATTTCTCCTTCTTGATACCAACCATCGA
ATCCTCGAGGTACTCTTTGTGTTTCTTTCACTTCAACTTCACATTTCATAAGCAATTCTT
CGTATTTTCCCATGCGCCAAACCCCTTTGGTGTCTTATTTCTTTCTATCTCTAACCCATT
GCATAAAATTTTCGATTTCTTCCCATTCTTCGGGAGTAAATTCATCTTTATTTGCATGAC
CGGCTATAGTTTCTTGATGAATACTTCTTTCTTCTGTAATTCTCGATTTAGGTACATTAA
AGTAATCTGCTAATTGTTGGACTTTTGATATTCTAGGATATTTAAGTTCTTTAAGCCAGT
TAGTGATTTTTGATTGACTTACCCCGATTGCTTCAGACAATTCTACTTTAGTAATGTTGT
TCTCTTTCATAAGTTGCTCTAAGTTCTCTGATAAAATTTTTCTAGCACTCTTATATTCCA
TAATTTTCTCCTTTAGTATTACTTAATGTAATACTAATTTACCATAAGTAATATCACTTT
TCAATACAAAATATTACTTTTTTGAAATAAATATCACTTTAGGTGTTGACATATTACTTT
AAGTGATAGTATAGTTGTAAATGTCAACGGGAGGTGATACGAAATGCCAGAAAATTTTAA
AGAGTTCTCTGTAAAGGTCTGGAGAACTAATTCGAATATGACACAACAAGATGTCGCTGA
TAAATTAGGCGTTACTAAACAATCTGTAATAAGATGGGAAAAAGATGACGCAGAATTAAA
AGGCTTACAATTGTATGCTTTAGCCAAATTATTCAACACAGAAGTTGATTATATAAAGGC
TAAAAAAATTTAACATTAATATCACTTTAAGTGATAAAGGAGGAACTCAGATGCAAGCAT
TACTAACATTTAATTTTGAAGAATTACCAGTAAGAACATTAACAGTAAATGAGGAACCGT
ATTTTGTAGGGAAAGATGTAGCAGATATTCTAGGTTACAAAAATGGCAGTCGTGATATTA
ACGCTCATGTTGATGCAGAACATAAGCTGACGTACCAAATCAGTACCGCAGGTCAAAGAC
GAAATCAAACAATCATCAACGAATCGGGTTTATACAGCCTAATCTTCTCATCAAAACTAG
AATCAGCTAAACGATTCAAACGTTGGGTAACTTCAGACGTCCTACCCGCTATTCGAAAAC
ACGGTAACTACGCAACAGACAATGTAATTGAACAAACATTAAAAGATCCAGACTACATCA
TTAAAGTGTTGACTGAGTATAAGAAAGAAAAAGAGCGAAACTTATTTTTACAACAAGAAA
TTGGAGAGCTAAAACCCAAAGCAGACTATGTCGATGAAATCTTAAAGTCAACTGGCACAT
TAGCCACAACTCAAATCGCGGCAGACTACGGTATATCAGCACAAAAGTTAAACAAACTAC
TACACGAAGCTAGATTACAACGAAAAGTGAATAAACAGTGGGTGCTTTACTCAGAACACA
TGGGCAAGAGTTACACAGAATCAGACACTATACCAATTGTACGCTCTGACGGTAGAGAAG
ACACAGTTTTACAAACTAGATGGACACAAAAAGGCAGATTGAAAATACATGAAATCATGA
CTGAATTCGGTTATGAAGCTAATTTAGGGGGAGCGTAAATGACACCAGAACAAAAAGAAA
AGCTAAACAATATAGTATTAACACTTTATGCAGTTAAAGAAAACAAAAGTCAAACATACA
CACACAAAGATACTCTTACTGTGACATATGCAGGCGAGATTGAGCACACTTACGAAGTCG
ACAGAGAGAAACACCTTGAATCAATGATTGAGTGGGCAATTGACCAAATCGAACAGCACT
TTGATTTAGACGAAGAAGAATAACACACAATTTAACAAACAACTTAATAGGAGCAATTAC
AAATGAATGCACTATACAAAACAACCCTCCTCACCACAATGGCAGTTGTGACGTGGAAGG
TTGTAAAGATTGAGAAAAACACAAGATTTAAACTTAGAAATTTTGATTATCCAAAAATTA
ATAATGCTCAGAGCAAATCATTGTTGGAGATTGCTAGTCACGATCTAAAAGATATTTAAC
TGTATTCAAAATTTTCATATCTTGTTGAGCTTTTAAGCATTCGTAGAAAGCTATTGAATA
AATAATTTCGTAAGATACGTTTTCAGGAGCATCTTCTTTCAACTTATTTATTCTATCCCT
AAAAAAGTCACTGTCACCACCGAATTCTTTTTCGGCTTGATTACTAAGTTCACCAAAGAA
ATTTTGAAAATCATTAAATTCCATACTTATCACCTCCTTTCACTAGGAGATAACTAAATT
ATACACAACACAAAAATAAAAAGGAGGAATAGATACGATAAAAAATAGTTTGCAAGCTAA
AGAACTTGCAGTAATTTTATCTGTTTCTAAATCCAAAGCAGGACAAATAATAAGAGAACT
GAATAAAGAGCTTGAAGACGAAGGTTACATTGCGATTCGAGGCAGAATACCAGTCCAATT
AGCTAGAGAAAAATTCCCTTATCACGGCTTGTCAGACGAGAGAATAATGGAGGCGTTGAA
AAAAGAAAATGAGTAACATTTATAAAAGCTACCTATTAGCAGTATTGTGCTTCACAGTCT
TAGCGATTGTACTCATGCCGTTTCTATACTTCACTACAGCATGGTCAATTGCGGGATTCG
CAAGTATCGCAACATTCATATTTCATAAAGAATACTTCTATGACAACAAAGGAGAATGAA
AAGATGTCAGATAAAGACTTGATGGAAAAAGTTGACAAGAGAAGGAAAGAAAAAGATTTA
ACGGTGAGAGAAATAGGATATTTGCTAGGTTTCTCTGATACTTATTTTATTAAGTTAAGA
AATGGTTCAAGAAGAATTACCGATCGGAAGAGAGATAGAATTAATCGTTATTTAAACGGT
GAATACGACAATGTAAAAATTCCTAAATATTCAAGAGATTCTGAACAAGTAGCATATGAC

AAGGGATATAAACAAGCTTTAAAAGATTTAGAAGAATTTGTAAATAAGAAAAAAACTGCT
ACTTGCGACAACAAGTAACAGTGACAAACACTTAAGAAAAAATTCATGTTCAATATAAAA
CGAAATAAAGGAAGTGTCAACAATGTACAACAAAATTGGCGATGTATGTCAAAAAGTAAT
TAATGTAGACCGATTCCATTTTAAAGTAGCAGTTAAGAAACAAGATTACAGCATTCTAGT
GAATGTCTTAGATTTAGAAGATAGATTTATCGACGGTATAAATATAACAGATGAGAATGA
TCTATACACAGCATTAGACATATTAAATCAATTTATTTATGAATGGATTGAAGAGAACAC
AGACGAAAGAGACAGGCTAATTAACTTAGTCATGAGATGGTAGGAGGTCGCTATGAAGCA
GACTGTAACTTACATCATCCGTCATAGGGATATGCCAATTTATATAACTAACAAACCAAC
TGATAATAATTCAGATGTTAGTTACTCCACAAATAGAAATCGAGCTAGGGAATTTAACGG
TATGGAAGAAGCGAGTATCAATATGGAATATCACAAAGCAATCAAGAAAACAGTGACAGA
AACTATTGAGTACGAGGAGGTAGAACATGACTGAACAAACATTATTTGAACAGTTGAACA
GTAAAAACGTGAATGATCATACAGAACAAAAAAATGGATTAACTTATCTAGCATGGTCAT
ATGCACACCAAGAGCTGAAAAAGATTGACCCAAACTACACAGTAAAAGTACACGAGTTTC
CACATCCAGATATTAACACAGAAAATTATTTTGTACCTTATTTGGCTACACCAGAAGGCT
ATTTTGTACAGGTATCTGTGACTGTGAAAGATAGTACAGAGACTGAGTGGCTTCCAGTAT
TGGACTTTAGAAATAAATCGCTTGCTAAAGGTAGTGCAACAACATTCGATATTAACAAAG
CGCAAAAACGATGTTTTGTTAAAGCTTCGGCTTTACACGGTTTAGGCTTATATATCTACA
ACGGCGAGGAACTACCAAGTGCAAGTGACCACGATATTACAGAATTAGAAGAGCGTATCA
ATAAGTTCGTGAACTTATCTCAAGAAAAAGGGCGAGATGCAACTATCGATAAAACGATGA
GATGGCTAAAAATATCTAACATTAATAAATTAAGTCAAAAACAAATCGCAGAAGCACACC
AAAAATTAGATGCGGGATTAAAACAATTGGATAGTGTGGAGAAACAATAATGTTAAACAG
AGCAGTATTAGTAGGACGCTTAACAAAAGACCCAGAATTAAGAAGCGCGCCAAATGCCGT
AAATGTAGGTACATTCACATTGGCAGTAAACAGAACATTCACGAATGCTCAAGGCGAGCG
TGAAGCAGATTTTATAAACGTAGTAGTGTTCAAGAAACAAGCTGAAAATGTTAAAAACTA
CCTTTCTAAAGGGTCGCTGGCAGGTGTAGACGGGCGACTACAAACACGTAGCTACGAAAA
AAAAGTCGGGCAACGTGTATTTGTGACAGAAGTAGTAGCGGACAGTGTTCAATTCTTAGA
ACCGAAGAATAACAACCAACAACCAAACAACAATTATCACCAACAAAGACAAACTCAAAC
TGGTAATAATCCTTTCGAAAATACCACTCCGATTACTGATGATGACTTACCGTTCTGATT
GGAATGATTAAATGCCGAAAATTACTAGTTATATCACTCAAGACGACGGCACAACAACAG
TTGTCATCTCTGATGTTGAATTAGGTAATAAAGAAACATTACTACTTGATAACGGGTTTG
ATGTAGAAGTCGATGTAAGCGTCATAGATCCATTTCGAATTACCGGCAAGCAACGCAAAT
TGATATTCGCATTGTGTAACGATATAGAAGCTCATACAGGACAACCTCGAGATTATATGA
GGCAAATGTTCCAAGATTATGTGAAGTTTCTGTATGGCTATGAAGAACGCATATCTTTAC
CAAATTGTTCTCGAACTATAACTAAGCAAATTATAGAAGCGATGTTTGAGTGGATTTTTA
CAAATGCGATTCCATTAAATTATAAAACAAGCAAATTAATGAAAGAAGATAAAAATTATC
TTTATTGGGCAACTGTGACGCGTCATTGCATTATATGCGGAAAGCCTCACGCTGACTTAG
CACATTATGAAGCAGTCGGTAGAGGCATGAACAGAAACAAGATGAATCACTATGACAAAC
ATGTATTAGCGTTATGTCGCGAACATCACAACGAGCAACATGCAATTGGTGTTAAGTCGT
TTGATGATAAATATCACTTGCATGACTCGTGGATAAAAGTTGATGAGAGGCTCAATAAAC
TGTTGAAAGGAGAGAAAAAGGAATGAATAGACTAAGAATATTAAAAATAGCACTCCTAAT
CGTCATCTTGGCGGAAGAGATTAGAAATGCTATGCATGCTGTAAAAGTGGAGAAAATTTT
AAAATCTCCGTTTAGTTAATACAGGTTTTTACAAAAGCTTTACCATAGGCGGACAAACTA
ATTGAGCCTTTTTTGATGTCTATTACCCAGGGGCTGTAATGTAACTTTAATACTTCAAAT
TCAATGCCAGAAAGTTTACTTATTGTTTCTAGGTTGTGTCCTGACTTTAACATTCTTTTA
ACAAATTCTAATCCCGAAACAAATCTTTGTTTTTCTATAATCTTATTAAAGTGATTTAAA
AACTGAGGAGCATAAAACTTATTATAAATTCCTTTTTTTGTTAAGTAAGACATGTCAAAA
GTTTCATTTAAAACCCCTAACCTTACTAGGTTATTAATTGAAATTTCGGTTGATTCTATA
TCTAACGGAGAGTCTTTTATTAGCGTGTCCGATATATTCATACCGTCATTCTTTGGGTTT
AAAACCGCTCTATATTTAACGGCAGGATGTACTTCGTGATTCTTTAAATGTTTTAAAAGA
ATAGCATCATTTGGGGATAATTGTTTCATTATTTCAACAAATGAATGGTGGGTTAATGAG
TTTTTTCTGTCATCCATAGATGATGCTACTAGTTTTGCGAACATATTACTTAAAGTTTTT
TCACTAATGTAAAACTTTGAAGCTTCTAGAGCAGGACCTAGAAGAGAAAAGTGTGGTTCT
TGTAAATTATTTTCAGGTACAGAAGATATTTCTTTTTTATATTGTTCTTTGAATTTTTCA
AATTCTACTTCTCTTTGATAAATAACTTTATCCACATAAAGCTGGAATTTCCCAAAGACA
AGTTCCCAAGTTTTAGAGAATGTTTCTACAGGCCCTTTTGATGCGCCTTCAATAATTTTA
TCAATACCTTTACCTAAAATAGGATCCATAATTATTCACCCCCAATCTAACGCAATAGCG
ATAATAAAATTAAACCAGAAAGGAGATAACGAAATGGCAACATTTAGAACGATAAAAGAA

AGTGGCGATTTTGTAACTGTGCATAAATCTTTTGTGTTCGATAGTAATTTAAGTGCTAAA
GCTAAAGGGATATTATTGTATTTCCTAAGTCGTCCTGGCAATTGGCAAATATACACGTCA
GAAGTAGTTAAACATATGAATGATGGACAAAAATCAATCAATAGTGGCGTTCAAGAACTT
ATGGACAATAAATATGTTCACAGAATACAAAAAAGAGCTGAAAACGGTGTGTTTAAAGGT
TTTGAATATTTAGTATACGAAAAACCAACCGAAATGCCATTTTCGGAAAACGGATTATCG
GCAAACGGGTTTTCGGAAAACGGAAAAACGGAAAACCAAAAAGGGCGTACTACTAATAAT
AATAGTACTAATAATGATTTAACTAATAATAACAATACTAATAATGATGGAAGTATATTG
TCGGGCAACCCGACTGTGTATTCCATTCCCTATAAAGAAATTATCGAATACTTAAACAAA
AAAGCAGGAAAGCATTTTAAACATAATACAGCTAAAACAAAAGATTTTATTAAAGCAAGA
TGGAATCAAGATTTTAGGTTGGTGGATTTTAAAAAGGTGATTGATATCAAAACAGCTGAA
TGGTTAAACACGGATAGCGATAAATACCTTAGACCAGAAACACTTTTTGGCAGTAAATTT
GAGGGGTACCTCAATCAAAAAATACAACCAACTGGCACGAATCAATTGGAACGCATGAAG
TACGACGAAAGTTATTGGGATTAGGGGGATATTATGAAACCACTATTCAGCGAAAAGATA
AACGAAAGCTTGAAAAAATATCAACCTACTCATGTCGAAAAAGGATTGAAATGTGAGAGA
TGTGGAAGTGAATACGACTTATATAAGTTTGCTCCGACTAAAAAACACCCGAATGGTTAC
GAGTATAAAGACGGTTGCAAATGTGAAATCTATGAGGAATATAAGCGAAACAAGCAACGG
AAGATAAACAACATATTCAATCAATCAAACGTTAATCCGTCTTTAAGAGATGCAACAGTC
AAAAACTACAAGCCACAAAATGAAAAACAAGTACACGCTAAACAAACAGCAATAGAGTAC
GTTCAAGGCTTCTCTACAAAAGAGCCAAAATCATTAATATTGCAAGGTTCATACGGAACT
GGTAAAAGCCACCTAGCATACGCTATCGCAAAAGCAGTTAAAGCTAAAGGGCATACGGTT
GCTTTTATGCACATACCAATGTTGATGGATCGTATCAAAGCGACATACAACAAAAATGCA
GTAGAGACTACAGACGAGCTAGTCAGATTGCTAAGTGATATTGATTTACTTGTACTAGAT
GATATGGGTGTAGAAAACACAGAGCACACTTTAAATAAACTTTTCAGCATTGTTGATAAC
AGAGTAGGTAAAAACAACATCTTTACAACTAACTTTAGTGATAAAGAACTAAATCAAAAT
ATAAACTGGCAACGTATCAATTCAAGAATGAAACACAATGCAAGAAAAGTAAGAGTAATC
GGAGACGATTTCAGGGAGCGAGACGCATGGTAACAAAAGAATTTTTGAAAATTAAACTTG
AGTGTTCAGATATGTACGCTCAGAAACTCATAGACGAGGCACAGGGCGATGAAAATAAGT
TATATGACCTATTTATCCAAAAACTTGCAGAGCGTCATACACGCCCCGCTATCGTCGAAT
ATTAAGGAGTGTTAAAAACGCCGAAAGAAAAATATTACTTATACCGAGAAGATGGCACAG
AAGATATTAAGGTCATCAAGTATAAAGAGAATGAGAATGAAGTGTAATCGCTCACAGGAG
CCCATTTCAGCGACGAAAAGAAAATTATGACTGATAGTGACCTAAAACGATTTAAAGGCG
CTCACGGACTTCTATATGAGCAAGAGCTAGGTTTACAAGCAACGATATTTGATATTTAGA
GGTGGACGATGAGTAAATACAACGCTAAGAAAGTTGAGTACAAAGGAATTGTATTTGATA
GCAAAGTAGAGTGTGAATATTACCAATATTTAGAAAGTAATATGAATGGCACTAACTATG
ATCGTATCGAACTACAACCTAAATTCGAACTACAACCTAAATTTGGGAAGCAAAGACCGA
TTACGTATATAGCCGATTTCTCTTTGTGGAAGGAAGGGAAACTGGTTAAAGTTATAGACG
TTAAAGGTAAGGCGACTGAAGTTGCCAACATCAAAGCTAAGATATTCAGATATCAGTATA
GAGATGTGAATTTAACGTGGATATGTAAAGCGCCTAAATACACAGGTCAAGAATGGATGG
TATATGAGGACTTAGTGAAAGTCAGACGTAAAAGAAAAAGAGAAATGAAGTGATCTAATG
CAACAACAAGCATATATAAATGCAACGATTGATATAAGAATACCTACCGAAGTTGAATAT
CAGCATTTTGATGATGTGGATAAAGAAAAAGAAACGCTGGCAAAGCGCTTAGATAACAAT
CCGGACGAATTACTAAAATATGACAGCATAACAATAAGACATGCATATATAGAGGTGGAA
TAAATGAGTATCGTAAAGATTAACGGTAAACCGTATAAATTTACCGAACATGAAAATGAA
TTGATAAAAAAGAATGGTTTAACTCCAGGAATGGTTGCAAAAAGAGTACGAGGTGGCTGG
GCGTTGTTAGAAGCCGTAAACGCACCTTATGGCATGCGCTTAGCTGAGTATAAAGAAATT
GTGTTATCCAGAATTATGCAACGAGAGGCTAGAGAACGTGAAATAGCTAGACAACGACGT
AAATAGGCTGAATTACGTAAGAAGAAGCCACATTTGTTTAATGTACCTCAGAAACATTCA
CGTGATCCGTACTGGTTTGATAATACTTATAACCAAATGTTCAAGAAATGGAGTGAAGCA
TAATGAGCATAATCAGTAACAGAAAAGTAGATATGAACAAAACGCGAGACAATGTTAAGC
AACCGGCGCATTACACATACGGCAACATTGAAATTATAGATTTTATTGAACAAGTTACGG
CGCAGTACCCACCACAATTAGCATTCGCAATAGGTAATGCAATCAAATACTTGTCTAGAG
CACCGTTAAAGAATGGTCATGAGGATTTAGCAAAAGCGAAGTTTTATGTCGATAGAGTAT
TTGATTTGTGGGAGGGGTAGCGATGGCAACGCAAAAACAAGTTGATTACGTAATGTCATT
ACAGGAGCAATTGGAATTAGAAGACTGCGAAAAATACACAGACGAACAAGTTAAAGCAAT
GAGTCATAAAGAAGTTAGCAATGTGATTGAAAACTATAAGGCAAGCATAAGTAATGAAGA
GCTATACGAGGAATGCATGTCGTTTGGTCTGCCTAATTGTTAAAAGGAGGGATGACCATG
ACAGATAGCGCGCGTAAAGAATACCTAAATCAATTCTTCGGATCTACGAGATATCTGTAT

CAGGATAACGAACGAGTGGCACATATCCATGTAGTAAACGGCACTTATTACTTTCACGGT
CATATCGGGCCAGGTTGGCAAGGCGTGAAAAAGACATTTGATACAGCGGAAGAGCTTGAA
ACATATATAAAGCAACATGGTTTGGAATACGAGTAACAGAAGCAACTAACTTTATTTTAA
AAGGGCGGAAACAATGAAAATCAAAATTGAAAAAGAAATGAATTTACCTGAACTTATCCA
ATGGGCTTGGGATAACCCCAAGTTATCAGGTAATAAAAGATTCTATTCAAATGATGTTGA
ACGCAACTGTTGTGTGACTTTTGATGTTGATAGCATCTTATGTAATGTGACTGGATACGT
ATCAATTAACGATAAATTTACTGTTCAAGAGGAGATATAACAATGAAAATCAAAGTTAAA
AAAGAAATGAGATTAGATGAATTAATTAAATGGGCGCGAGAAAATCAGGATCTATCACAA
GGAACAACATTTTTTTCAACAGGATTTAGTGATGGATTCGTTCGCTTTCATCCAAATACA
AATAAGTGTTCGACGTCAAGTTTTATTTCAATTGATATCCCCTTCATAGTTGATATTGAA
AAAGAAGTAACGGAAGAGACTAAGGTTGATAGGTTGATTGAATTATTCGAGATTCAAGAA
GGAGACTATAACTCTACATTATATGAGAACGCTAGTATAAAAGAATGTTTATATGGCAGA
TGTGTGCCTACCAAAGCATTCTACATCTTAAACGATGACCTAACTATGACGTTAATCTGG
AAAGATGGGGAGTTCCTAGTATGATGTTGAAATTTAAAGCTTGGGATAAAGATAAAAAAG
TTATGAGTATTATTGACGAAATCGATTTTAATAGTGGGTACATTTTGATTTCAACAGGTT
ATAAAAGTTTCAATGAAGTAAAACTATTACAATACACAGGATTTAAAGATGTGCACGGTG
TGGAGATTTATGAAGGGGATATTGTTCAAGATTGTTATTCGAGAGAAGTAAGTTTTATCG
AGTTTAAAGAAGGAGCTTTTTATATAACTTTTAGCAATGTAACTGAATTACTAAGTGAAA
ATGACGATATTATTGAAATTGTTGGAAATATTTTTGAAAATGAGATGCTATTGGAGGTTA
TGAGATGACGTTCACCTTATCAGTTGAACAATATAAAAATCTTTGTACTAACTCTAACAA
GTTATTAGATAAACTTCACAAAGCATTAAAAGATCGTGAAGAGTACAAGAAGCAACGAGA
TGAGCTTATTGGGGATATAGCGAAGTTACGAGATTGTAACAAAGAACTGGAGAAGAAAGC
AAGCGCATGGGATAGGTATTGCAAGAGCGTTGAAAAAGATTTAATAAACGAATTCGGTAA
CGATGATGAAAGAGTTAAATTCGGAATGGAATTAAACAATAAAATTTTTATGGAGGATGA
CACTAATAAATAACCGTGAACAAATAGAACAATCCGTTATAAGTGCTAGTGCATATAACC
GTAATGACACAGAGGGATTACTAAAAGAGATTGAGGACGTATATAAGAAAGCGCAAGCGT
TTGATGAAATACTTAAGGGTTTACCTAATGCTATGCAAGATGCACTCAAAGAAGATATTG
GTCTTGATGAGGCAGTAGGGATTATGACGGGTCAAGTGGTCTATAAATATGAGGAGGAGC
AGGAAAATGACTAACACATTAACAATTGATCAGTTACAAGAGTTATTACAAATACAAAAG
GACTTTGACGATAGAATACCAACACTAAATTTACGAGATAGCAAGATTGCGTATGTGGTT
GAATTCTTTGAATGGTTTAACACATTGGAAACGTTCAAGAATTGGAAGAAGAAACCAGGT
AAACCGTTAGACGTACAGCTAGACCAGTTATCAGACATGTTAGCGTTTGGATTGAGTATT
GCTAATCAACAAGCAGATAACATGGAAGAAATTTTGGGTTATTTAGATGACGGAGATTTT
AACGACTATATAGAACGAGTTGAAATCGATTTTATCGATAGTGATGTAGTAGATGAATTT
ATGTCAACTATAGATGAAATGTATGAAAGTCCATATCGTAGCAACTTATTTTTACCGTTT
GCATTAGCGAACAACTACTACACTATCGATCAACTCATTGACGCATACAAAAAGAAAATG
AAAAGGAACCACGAAAGACAAGATGGAACAGCAGACGCAGGGAAAGGATACGTATAAAGA
CATATTAGATCGGGTCAAGGAGGTTTTGGGGAAGTGACACAATACTTAGTCACAACACTC
AAAGATTCAACAGGACAACCACATGAACATTTTACTACTGCTAGAGATAATCAGACGTTT
ACAGTTGTTGAGGCAGAGAGTAAAGAAGAAGCGAAAGAAAAGTACGAGGCACAAGTTAAA
AGAGATGCAATTATTAAATTAGGTCAGTGGTTTGAAAATATAAGGGAGTGTCGGAAATGA
CGGATGTTAAAATTAAAACTATTTCAGGTGGAGTTTATTTTGTAAAAACAGCTGAATCTT
TTGAAAAATATGTTGAAAGAATGACGAGTTTTAATGGTTATATTTACGCAAGTACTATAA
TCAAGCAACCAACGTATATTAAAACAGATACGATTGAATCAATCACACTTATTGAGGAGC
GTGGGAAATGAATCAGCTGAGAATTTTATTACATGACGGTAGTAGTTTGGTATTACATGA
AGATGAATTATTTAACGATATAGTATTTTTTTTGGATGATTTTAGAAATGATGATGAGTA
TTTAACGATAGAAAAAGATTATGGCAGAGAACTTGTATTGAACAAAGGTTATATAGTTGG
GATTAATGTTGAGGAGGCAGACGATGACTAAACAAATATTAAGACTATTATTCTTACTAG
CGATGTATGAGCTAGGTAAGTATGTAACGGAGCAAGTATATATTATGATGACGGCTAATG
ATGATGTAGAGATGCCGAGTGACTTCGCAAAGTTGAGCGATCAGTCTGATTTGATGAGGG
CGGAGGTGTCAGAGTAGATGATGTGGTTAGTCATAGCAATTATATTACTAGTCATCTTAT
TGTTTGGTGTGATGTTGCAAGCTGAACAGTTAAAAGGCGATGTGAAAGTTAAAGAGCGGG
AGATAGAGATATTAAGAAGTAGATTGAGACATTTTGAAGATTAAACATATTTGTACGGAG
GGTATTCATGACTAAAAAGAAATACGGATTAAAATTATCAACAGTTCGGAAATTAGAAGA
CGAGTTGTGCGATTATCCTAATTATCATAAACAACTTGAAGATTTAAGAAGTGAAATAAT
GACACCGTGGATTCCAACAGATACAAATATAGGCGGGGAGTTTGTACCATCTAATACATC
AAAAACAGAAATGGCAGTAACTAATTATCTTTGTAGTATACGAAGAGGTAAAATTCTTGA

GTTTAAGAGTGCGATTGAACGTATAATCAACACATCAAGTAGGAAAGAACGCGAATTCAT
TCAAGAGTATTATTTTAATAAAAAGACTTTGATTGCGGTTTGTTATGACATACACATCTC
TGAAAGTACAGCGCATAGAGTCAAGAAGAAAATAGTGTCTAAACTAGCCGAAGAATTAGG
AGAATACTAAATTTGACAGTAAAATGACAGTTTTTGACACCTATAACGAGATATTATGAT
AGTGTAGGATATTGACTATCTTACTGCGTTTCCCTGATCGCAATTAGGAATAAAGGATCT
ATGTGGGTTGGCTGATTATAGCCAATCCCTTTTTTAATTTTAAAAAGCGTATAGCGCGAG
ACTTGGTGGTAAATGAAATGAACAAATTAACTAAAAAGCAACGTTTGTTTGCAGAAGTAT
ATACAATACCTGGTACTGAATGTTATGGCAATGCTACTAAGTCAGCTGTGCATGCCGGAT
ATAGCGAAAAGACGGCGTACTCACAAGGACAGCGTATGTTGAAGAATGTTGAAATTCAGA
ATTATAACAAGGAGGTTGAAACAAAACTCTTTGACGAGAATATTATGTCAGGGAAAGAAG
TGTTGTATAGGCTAACTAGAACAGCTAGAGGAGAACACACGGAAGTTGAAGCTGTCGTAA
CAAAAACTGGAGACTATAAAGGGAATCCGGATACTGGCAAAATGCAATTAGTATACGATG
AACACATACAACTTGTTACTAAGTCCCCTAAAATAAGTGACCAAAACAAAGCCTGAGAGA
TGTTAGGTAGACATCACAAATTATTTACAGACAAACAAGAAGTCGACCACAAAATACCGA
TGTTTGTTGATAATATTCCGGAAGATGATTAGTCATGTATGAAATACTTGATCTAAAAAA
TAAAATCGGTGGTGGCTACAATAAGTTTTGGCACAACAAAATCTTTTACCGTGTTGTTAA
AGGTTCAAGGGGTAGCAAGAAAAGTAAAACTACCGCTATTAATCTCATTTATCGAATAAT
GAAATATGATTGGGCAAATATACTTGTAGTCAGAAGATTTAGCAACACTAACAAACAATC
AACGTATACAGATTTAAAGTGGGCAACTAACCAATTAGGCGTTGCTCACTTATTTAAATT
CAAGGAAAGTTTGCCGGAAATAACGTATAAACCTACTGGACAAAAAATACTGTTTAGAGG
TTTAGACGACCCATTGAAAATAACATCGATTACTGTTGATACAGGCATTTTGTGTTGGGC
TTGGTTTGAAGAGGCTTATCAAATAGAAACATTCTCTAAGTTTAGCACTGTTGTTGAGTC
AATACGTGGTAGCTACGATAGTCCGGAATTTTTCAAGCAAATCACAGTCACTTTTAACCC
GTGGTCGGAAAGACATTGGTTGAAGCCTACATTTTTTGATGAAGAAACAAAATTAAACAA
TACTTTTTCAGATACAACAACTTATAGAGTTAATGAATGGCTAGATAAAGTCGATATTGT
ACGATATGAAGATTTGTTTATAAAGAATCCTAGACGTGCAAGAATCGTTTGTGATGGAGA
TTGGGGTGTTGCAGAGGGGCTTGTATTCGATAATTTTAAAGTGGAAGACTTTGATTGGTT
TGAGGAGTTTAAAAGAACGCAAGAAATAACTCACGGAATGGATTTTGGATTTAGTCAAGA
CCATACAACAGTTGTTAGTACGGTTGTAGATTTAAAAAACAAAAAGTTATTCATCTATGA
TGAACACTATAAAAAAAGCGATGTTAACTGATGATATAAAACAAATGCTTATTAAAAAAGG
ATTAGGTGATGTAGATATTGCAGCTGATTATGGGGCTGGTGGAGATAGAGTGATCAGTGA
ATTGAAATCTACAGGGATTAAAGGTATAAGAAAAGCGTTGAAAGGCGCTAATACTATTTT
ACCAGGCATTCAATTCATTCAAGGCTTTGAAGTTATTATACACCCATCATGTGAACACGC
TATTGAAGAGTTCAACACTTATACATTTGACCAAGATAATGATGGTAAGTGGTTGAACAA
GCCTATAGATGCTAATAACCATATTATCGATGTATTGCGGTATAGTCTTGGGAAATATCA
TATCGTCCGTAAAAAACGTAAAAAGAATATAGAAAGCAAAACAAAAGTAATTAAATTTCT
AGGATTATAGGAGGGAACAAATGTTAAAGGCAAACGAATTTGAAACGGATACTGATTTAC
GAGAAAACAGAAATTACTTGTTTAACGATGAAGCTAATGTTGTTTACACATATGACGGGA
CAGAGTCTGATTTATTACAAAACATTAATGAAGTAAGTAAATACATTGACCATCACATGG
ATTACCAACGACCTAGATTAAAAGTGTTAAGTGATTATTACGAAGGTAAAACTAAGAATC
TGGTTGAGTTAACACGACGCAAAGAAGAGTACATGGCAGATAACCGTGTAGCCCATGATT
ACGCATCTTATATTAGCGATTTTATTAACGGTTATTTCTTAGGTAATCCGATTCAATGTC
AAGATGATGATAAAGATGTATTAGAAGCTATTGAGGCGTTCAATGATTTAAATGATGTTG
AGTCACACAATAGATCTTTAGGATTAGATTTGTCAATTTATGGCAAAGCTTATTAGTTAA
TGATTAGAAACCAAGATGATGAAACGCGTGTATACAAGAGTGATGCAATGAGTACTTTTG
TCATATACGACAATACAATTGAACGTAATAGTATCGCAGGCGTTAGATATTTAAGAACTA
AACCAATAGACAAGACTGACGAAGATGAAGTGTTTACAGTTGATTTATTTACTTCTCACG
GTGTTTATAGATATCTTACCAGTAGAACAAATGGATTGAAGCTCACACCACGTGAAAACG
GTTTTGAATCACACTCTTTCGAACGTATGCCTATTACAGAATTTAGCAACAACGAAAGAA
GAAAAGGAGATTATGAGAAAGTAATCACTTTGATTGATTTGTATGGTAATGCAGAATCAG
ATACTGCTAACTATATGAGTGATTTAAACGACGCTATGTTATTGATTAAAGGTAACTGAT
CAATAGATCCAGATGAAGTAAAAAAACAAAAAGAAGCCAACGTGTTATTTTTAGAGCCAA
CCGTTTATGAGAATAGGGATACAGGTATCGAAACAGAAGGTTCAGTTGACGGCGGTTATA
TTTATAAGCAATACGATGTACAAGGTACCGAAGCTTATAAAGACCGTTTGAACAGTGATA
TACACATGTTTACCAACACGCCTAACATGAAAGATGATAACTTTAGTGGCACTCAATCGG
GCGAGGCAATGAAATATCAATTGTTCGGATTAGAACAACGTACTAAAACTAAAGAAGGAT
TGTTTACTAAAGGGTTAAGACGTCGTGCTAAGTTGTTAGGGACAATACTTAAAAATACAC

GGTCGATTGACGCTAACAAAGATTTCAATACTGTTAGATACGTATACAACAGAAACTTAC
CTAAATCATTAATCGAAGAATTAAAAGCTTATATTGATTCTGGCGGGAAGATTAGTCAAA
CAACTTTAATGTCTCTATTCTCGTTCTTCCAAGACCCTGAATTGGAAATCAAGAAAATAG
AAGAAGATGAGAAAGAATCTATTAAAAAAGCGCAAAAAGGTATTTATAAAGACCCTAGAG
ACATCAATGATGACGAACAAGATGATGATACAAAAGATACTGTTGATAAAAAGGAATGAT
TGTAATTGCCTAACAAAAACACTCAAGAATATTGGGAAGAGCGCGGACGCAAAGCAATCG
AGAATGAGTTGAAGCGTGATAAAACTAAAGCTGAAGAAATAGAACGTATATTGAATATGA
TGATTAAGCGCATTGAAAAAGAAATCAATGCGTTTATTGTTAAGTACGGAGATTTTGCAG
GCGTTACATTACAAGAAGCACAAAAGATTATTGATGAGTTCGATGTAAAAGCGTTTCAAG
AAGAAGCAAAAAGATTGGTCGAAAACAAGGACTTTAGCGATAGAGCAAATGAAGAATTAA
AGAAGTATAACACTAAGATGTATGTATCAAGAGAACAGATGTTAAAGATTCAAATAGAAT
TCTAAATTGCTTATGCAACAGCTCAAACAGAATTATCGATGAGGGAATATTTCGAATCAA
CAGCTTATCGTGTGTTCAGTGATCAAGCGGGTATTTTAGGTGAAGGTGTACAAGTAGCTA
AAGAAGTTATAGATACAATCGTTGATACACAATTTCATTGTGTCGTTTGGTCAGAGCGAT
TATGGACTAATACTGAAGCGATGAAACAAGAAGTAGAAGAAATAATTGCTAATGTGGTTA
TTAGAGGTCGACATCCTAATGAATATGTTAAAGATATGCGCAAGCACCTAAACAAATTCG
AAGGCACAGCAAGACAAAAGACTGCAGCAATTAAATCATTGCTTTATACGGAATCGGCAC
GTGTTCACGCACAATCAAGTATTGACAGCATGAAAGAAATTTCACCGGAAGGATATTATA
TGTATATTGCAAAAAATGATAGTAGAACAACTAAAGTATGCAAGGGGCTTAATGGAGAAA
TATTCAAAGTTAAAGACGCTAAAATTGGTGCTAATTTCTACCCTATGCATATCAATTGTC
GTTCAGATTGTGCATTACTACCTAAATCTATGTGGCCGAAAAAACCAAACAAGAAACGAA
AAACAAAATACTTCGGAGGAAAAGTGAAAAGCGATGATTGATTTAAAAGTAAAAGTTTTT
AAAGGCAAGTTAGCATTGTATGATAGTAAATTAAGTGTTTGGAGGATATTGGTATGAGCA
ATACTGACAAATACTTTAGAGACATAGCAAGAGAGTTAAAAGGTATACGTAAAGAGTTAC
AAAAGCGAAATGAAACAGTTATTATTGAGGCAAACTTAGACAGCGTAAGGTCGGCTGTAT
TAGCCAATAAAGAAAAACCGAAATATAACGAACCACTCTTTTAATAGCTAGCACTTAATT
GTGTTGGCTATTTTTTATGTCCAAAATGTGCTGATGACATAAAAAGCACGCATGGAAAAA
CAGTCGACAGACTATAAATGGAGGTATATCTCATGGAAGAAAATAAACTTAAGTTTAATT
TGCAATTTTTTGCAGACCAATCAGATGATCCGGACGAACCAGGCGGAGATGGTAAAAAAG
GAAATCCTGATAAGAAAGAATATGACGAAGGTACTGAAATAACTTTCACGCCAGAGCAAC
TAAAGAAAGTTGATGAAATACTTGAACGTCGTGTAGCCCACGAAAAGAAAAAAGCTGATG
AGCATGCAAAAGAAAAAGCAGAAGAAGCCGCTAAAGAAGCTGCTAAATTAGCGAAAATGA
ACAAGGATCAAAAAGATGAATATGAACGCAAACAATTGGAAAAAGAGCTGGAGCAATTAC
GCTCAGAAAAACAATTAAATGAAATGCGTTCAGAAGCAAGGAAAATGTTAAGTGAAGCAG
ACGTTGATTCATCAGATGAGGTTGTTAATTTAGTTGTAACAGATACTGCTGAACAAACTA
AATTGAATGTTGAAGCTTTTTCTAATGCAGTAAAAAAAGCGGTTAATGAAGCGGTTAAGA
TTAACGCTAGACAATCGCCATTGACTGGTGGAGATTCATTTAATCACTCGACTAAAAATA
AACCGCAAAACTTAGCTGAAATAGCTAGACAAAAAAGAAGTATTAAAAATTAACGGAGGC
ATTTAAATGGAACAAACACAAAAATTAAAATTAAATTTGCAACATTTTGCGAGTAACAAT
GTTAAACCGCAAGTATTTAACCCTGATAATGTAATGATGCACGAAAAGAAAGATGGCACG
TTGATGAATGAATTCACAACGCCCATCTTACAAGAGGTTATGGAAAACTCTAAAATTATG
CAATTAGGTAAGTAGGAACCAAGGGAAGGTACTGAGAAGAAGTTTACTTTTTGGCCTGAT
AAACCAGGTGCTTACTGGGTAGGTGAAGGTCAAAAAATCGAAACATCTAAAGCTACATGG
GTTAATGCTACTATGAGAGCGTTTAAATTAGGGGTTATCTTACATGTAACAAAAGAGTTT
TTGAATTACACTTATTCACAATTCTTTGAAGAAATGAAGCCTATGATTGCTGAAGCATTC
TATAAAAAGTTTGATGAAGCGGGTATTTTGAATCAAGGTAACAATCCATTCGGTAAATCA
ATTGCACAATCAATTGAAAAAACTAATAAGGTTATTAAAGGTGACTTCACACAAGATAAC
ATTATTGATTTAGAGGCATTACTTGAAGATGACGAATTAGAAGCAAATGCGTTTATCTCA
AAAACGCAAAACAGGAGCTTATTACGTAAAATTGTAGATCCTGAAACAAAAGAACGCATT
TATGACCGTAACAGTGATTCATTAGACGGTTTACCTGTGGTTAATCTTAAATCAAGTAAC
TTAAAACGTGGTGAATTAATCACTGGTGATTTCGATAAGTTGATTTACGGTATCCCTCAA
TTAATTGAATACAAAATCGATGAAACAGCACAATTATCTACAGTTAAAAACGAAGATGGC
ACACCTGTAAACTTATTCGAACAAGATATGGTGGCCATTACGTGCAACTATGCATGTAGC
ATTGCATATCGCTGATGATAAAGCATTTGCTAAGTTAGTTCCTGCTGATGCAAAACCATC
TTCAAATCCAGGAGAAGTTTAATAAATAATTAGGAGTGGTAACATGCCCGAAATCATTGG
AATTGTTAAAGTAGATTTTACGGAGTTAGAAGATAACAGACATGACTATATGAAAGGGCA
TGTCTACCCTCGCAAAGGTTATGATCCTACAAATGAACGTATAAAATCTTTAGCCAGCGT

TGAAAATAAACGCAATGAGCAAATGATTTACATTGTAAATGACAAATTAACCAAAAAAGA
ACTTGTCGAAATAGCAAGTGTTGTTGGCTTACAAGTTGATGAAAAACAAACAAAAGCTGA
AATTATCAACACTTTTGAGTCGCTAGAGTAGGTGGTTATATGACTACGCTAGCTGATGTA
AAAAAACGTATTGGTCTTAAAGATGAAAAGCAAGATGAACAATTAGAGGAAATTATAAAA
AGTTGTGAAAGCCAGTTGTTATCAATGTTACCTATTGAAGTTGAACAAATACCGGAAAGG
TTTAGTTACATGATTAAAGAAGGTGCAGTTAAACGCTACAACAGGATTGGTGCTGAAGGT
ATGACATCAGAAGCGGTTGACGGACGTAGCAATGCGTATGAATTGAACGATTTCAAGGAG
TATGAAGCTATTATTGATAATTACTTTAATGCTAGAACGAGAACTTAAAAAGGAAGGGCT
GTGTTCTTTTGAGATATGAAGATAGAGTTATTTTTCAATTAGAACAATTAGCAACTTACA
ATCCTAAAACTAGCAAAAAAGAAAACGCACTAATCACTTATGATGCGATACCATGCAATA
TTAACCCCATTTCTAGAGCAAGAAAGCAACTTGAATTTGGCGATGTAAAAAACGATGTAA
GTGTCCTGAGGGTAAAAGAATCAATATCTTACCCTGTTAGCCACGTGTTGGTTAATGGCA
TTCGCTACAAGATAGTTGATACAAGGATATACAGACACGAAACGTCATATTATATCGAAG
AGGTCAATTGATGAATATAGATGGATTAGACGCACTGTTAAACCAATTTCACGATATGAA
AAACAACATCGATGATGATGTAGATGATACTTTACAAGAAAACGCCAAAGAATATGTAGT
ACGAGCTAAATTGAAAGCTAGAGAAGTAATGAATAAGGGTTATTGGACTGGTAATCTATC
ACGCAATATCAGATATAAAAAAACTCGCGATTTGCAATACACTATCACATCGCATGCAGC
TTATAGTGGTTTCTTAGAGTTTGGTACTCGATACATGGAGGCAGAACCTTTTATGTGGCC
AGTATATGAGGTAATAAGAAAATCGACTGTAGAAGAGTTGAAAGCGTTGTTTGAATAGGA
GATAAAAGCATGACACCAAACTTACAACTTTATAATAATGCGTTTGAAACGCTACAAGGA
TATGGATTCCCTGTTATTTCTCGTAAAGAGATGCAACAAGAGGTTCCGTATCCTTTTTTT
GGAATAAAAATGCCGGAGTCAAACAGAAGTAAATACACGTTTGATAGTTATTCTGGTGAC
ACGAATTTAGTTATTGATATTTGGAGTGTAAGTGATGATCTAGGACATCATGACGGACTT
GTTAAAAGATGTATTGATGATTTAACACCTAGCGTTAAAACAAACGATTATGACTTTGAA
GAAGATGATACTAACATCACACAGTTAGTTGATGAGACTACCAATCAAGAATTGCTACAC
ACATCAGTAACGATATCTTACAAAACATTTTAAAAAACGGAGGAATATTGAATGGCAAAT
ATGAAAAATAGTAATGATCGTATTATTTTATTTAGAAAAGCTGGCGAAAAAGTAGATGCT
ACTAAAATGCTTTTTTTAACTGAATACGGCTTATCACATGAAGCTGATACAGATACAGAG
GATACAATGGACGGTTCTTATAACACTGGTGGTTCTGTTGATTCAACAATGTCTGGTACT
GCTAAAATGTTTTATGGCGACGATTTTGCAGATGAAATTGAAGATGCAGTTGTAGATCGC
GTATTGTATGAGGCTTGGGAAGTTGAAAGTAGAATACCAGGCAAAAATGGAGATGCCACT
AAATTTAAAGCGAAATATTTCCAAGGTTTCCACAATAAATTTGAATTAAAAGCAGAAGCT
AACGGTATTGATGAATATGAATATGAATATGGAGTGAATGGTCGTTTCCAACGTGGATTT
GCAACACTACCTGAGGCTGTAACAAAGAAACTTAAGGCGACTGGATACAGATTCCATGAC
ACTACAAAAGCAGATGCGTTAACTGGCGAAGATTTAACAGCAATTCCACAACCTAAGGTA
GATTCATCAACGGTTACACCAGGAGAGGTATAAAAATAGGGCGTTAAGCCCTATTTATTT
TGTTTAAATTAATCATGAATGGAGATTTTAAGTTATGAATGTAGAAATTAAAGGAAAGTC
ATTAGAATTAAGTTTTGGTTTTAAATTTTTAAGAGAAATGGATAACCGATTAGGTTTAAA
AGTTGAGCAAGCTTCTATCGGTCAAGGTGTATCAATGTTGCCGGTAGGTTTAGAGAGTGG
AAATCCTGTTGTGATTGGCGAAGTTTTAATTGCAGCTACATCTCACTTGAAAAAACAAGC
AATTACTATTAATAACATTGATGAAGCGCTAGATGAAATCGCAGAAAATGTCGGACTAGA
AGAATTCGGTTCAGATATTTTAACGGAGTTGGGAAAGCGACCTATGACCCGAAACCTAGT
CGAAGTAGTGGAAGCGGAAGAGAAACCAGCGGAAGCGTAATAACTTACGACAGAATCGTT
ATAACTTGTATGTCAACACTTGGTATTACGGATTTGAACGTTATTGAGCAAATGACATTA
ACAGAATATAACTATCGAATGTATGCGAAAGAGTATGAAATGCTAACCCAAGAATTCGAA
CGTTACAAACTTGCGTTTGCTATTCGTGATGCTGCAGCTACTAAAAATGTTGGGACAGAA
AATAAACCTAAAGAGGAATATGTTTTTAACAACGCAAACGACGTATTGCCTTATGAAGAA
AATATCCAACGGCTTAACGAAGGTAAAGATATAAGATTTAGTAGCGAACGTGATGAATAC
GAACCACAAAATAATGAATTCTTTAAAGTTATAGCAGAATTTAACAAGCAATAGAAAGAG
AGGTGTTAATGTGACGGAATATAAAATTAAAGCGACTATTGAAGCTAGTGTAGCCAAATT
CAAAAGGCAAATCGATAGTGCGGTTAAGTCTGTGCAAAGATTTAAACGAGTAGCAGATCA
AACTAAAGATGTCGAATTAAATGCTGATGATAAAAAATTACAAAAAACTATCAAAGTTGC
TAAAAAGTCTTTAGATGCCTTTAGTAACAAAAAAGTAAAAGCTAAATTAGATGCTAGTAT
ACAAGACTTGCAACAAAAGGTACTAGAATCGAATTTTGAACTAGACAAACTAAACTCTAA
AGAAGTTACTCCAGAGATTAAATTACAAAAACAAGAATTGACTAAAGATATCGCTGAAGC
AGAAGCTAAGTTATCCGAACTAGAAAAGAAGCGTGTCAATATTGACATCAATGCTGATAA
CAGTAAATTCAATCGAGTGTTAAAAGTATCTAAAGCTAGTCTTGAAGCTTTAAATAGGTC

TAAAGCCAAAGCTATTTTAGATGTAGACAACAGTGTTGCTAATTCTAAAGCCAAACGTAC
TAAAGAAGAGCTTAAAAGTATTCCAAATAAAACTAGATCTCGACTTGATGTAGATACAAG
GCTTTCTATACCAACTATTTATGCATTTAAAAAATCATTAGACGCATTGCCAAACAAAAA
AACAACAAAGGTAGATGTCGATACTAATGGTTTAAAGAAAGCTTATGCCTACATAATAAA
AGCAAACGACAATATCCAAAGACAGATGGGGAATTTAGCTAATATGTTCCGTGTGTTCGG
TACTGTAGGTTCTAATATGGTTGGTGGATTACTAACGTCATCTTTTAGCATTTTAATACC
TGTAATAGCGAGCGTAGTACCTGTAGTGTTTGCACTATTAAACGCTATCAAAGTGTTAAC
TGGTGGTGTACTTGCTTTAGGTGGTGCTGTAGCAATAGCTGGTGCTGGCTTTGTAGCATT
TGGCGCAATGGCTATCAGCGCTATAAAGATGCTTAGTGACGGCACGTTACAAGCTAGCTC
AGCAACAAACGAATACAAAAAAGCTTTAGATGGCGTAAAGTCAGCATGGACTGATATTAT
AAAGCAAAATCAATCCGCTATCTTCACAACTCTTGCAAATGGTTTAAATACTCTTAAAAC
TGCAATGCAGAGCTTACAACCGTTTTTTAGTGGTATTTCAAGAGGAATGGAAGAGGCGTC
TCAAAGTGTACTTAAATGGGCTCAAAATAGCGGTGTAGCATCAAGGTTCTTCAACATGAT
CAATACAACTGGTGTTTCGGTATTTAACAAGCTATTAAGTGCTGCAGGCGGTTTCGGTGA
CGGTTTAGTCAATGTATTCACACAATTAGCACCACTGTTTCAATGGTCGGCTGATTGGTT
GGATAGATTAGGTCAATCTTTCTCTAACTGGGCTAATAGTGCAGCTGGAGAAAATTCGAT
TACTCGTTTTATTGAATACACAAAAACAAATTTACCTATCATTGGTACTATTTTTAAAAA
TGTTTTCGCTGGAATTAACAATTTGATGAATGCATTTAGTGGATCGTCAACTGGTATATT
CCAGTCTCTCGAACAGATGACAGCTAAATTTAGGGAATGGTCTGAACAAGTAGGACAATC
TCAAGGGTTCAAAGGCTTTGTCAGTTATATACAAACAAATGGACCACTAATAATGCAATT
AATTGGGAACATTGCAAGAGGATTAGTTGCATTCGCAACAGCGATGGCTCCTATAGCTAG
TGCAGTATTACGCGTTGCAGTAGCAATAACTGGTTGGATAGCTAACTTGTTTGAGGCGCA
TCCAGCTACAGCACAATTAGTTGGCGTCATTATAACTTTGGTTGGTGCATTTAGATTTTT
AATTGCTCCAATATTAGCGGTAATGGATTTTCTGGGGCCATTAGCAGCAAGATTAGTCGC
ATTAGTAACTAAGTTGGGTTGGGCTAAAACAGGAACTTTAGTATTAAGTAAGGCAATGAC
ATCGTTAAAAGGTCCAATAAAATTAGTTACAGCTATATTCCAATTGTTATTCGGTAAGAT
TGGATTAATTAGAAATGCTATCACAGGACTAGTAACTGTGTTTGGTATTTTAGGCGGTCC
AATAACAATGGTAATTGGTGTAATTGCTGCATTAATAGCTATATTCGTTTTATTGTGGAA
TAAAAATGAAGGATTCAGAAACTTTATAATAAATGCTTGGAATGCGATAAAAACGTTTAT
GGTTAATGTTTGGAATGTATTAAAAGCTGTAGCTTCGGTTGTATGGAATGCTATTTTAAC
AGCTATCACTACAGCAGTATCGAATGTTTACAATTTTATAATGATTGTTTGGAATCAAAT
AGTCGCTTATTTACAAGGGCTATGGAATGGAATTATCGCTATTGCAACAACAGTAGGGAA
CCTTTTAGTTACAATCATTACAACTGTTTTCACGACGATAATGACAATAGTTATGACGAT
ATGGACAGCTATTTGGACGTTCTTAAGTACAATCTGGAATACGATAATTACAATCGCTAC
AACGATTTGGAATTTGTTGGTCACTGTAATAACTACTGTGTTTACAACAATCATGACTAT
CGCAAAGACAATTTGGAACGCTATTTGGACGTTCTTACAACCGTTGTGGAACACTATAGT
TACTGTGGCAACTAAGGTTTAGAACGCTATCACTACAGCTATCTCTACTGCGTTACAAGC
GGCATGGAGTTTCATTTCTAATATCTGGAATACGATTTGGAGTTTCTTATCTGGTATATT
AACGACGATTTGGAATAAAGTTGTAAGTATATTCACACAAGTTGTATCAACTATATCAGA
CAAAATGTCTCAAGCTTGGAACTTCATTGTCACTAAAGGTATGCAATGGGTATCTACTAT
AACAAGTACGCTAATTAACTTTGTTAATAGAGTTATTCAAGGATTCGTTAATGTTGTAAA
CAAAGTTAGTCAAGGTATGACAAATGCAGTAAATAAAATAAAAAGTTTTATAGGAGATTT
TGTGTCTGCAGGTGCTGATATGCTCCGTGGTTTAATTAGAGGTATTGGACAAATGGCTGG
CCAATTAGTAGATGCACCTAAAAATGTTGCTAAGAAAGCTTTAGATGCAGCTAAAAGTGC
TTTGGGTATTCACTCACCTTCACGTGAATTCATGGATGTTGGTATGTATTCAATGCTAGG
TTTTGTTAAAGGTATAGATAATCATTCAAGTAAAGTTATCCGTAATGTTTCTAATGTTGC
AGATAAAGTAGTTTATGCATTTCAACCTACATTAAACGCACCTGACATTTCTAGTATTAC
AGGAAACTTAAGTAATTTAGGTGGAAATATAAATGCGCAAGTACAACACACACATTCTAT
TGAAACATCACCGAACATGAAAACTGTTAAAGTTGAATTCGATGTTAATAACGATGCGCT
TACTAGTATTGTTAACGCCAGAAATGCTAAACGCAATTCGGAGTATTACTTATAAAGGAG
GTTACAAATGGACATAGAATTAACAAAAAAAGATGGTACTGTAATCAAATTAAGTGAATA
CGGGTTTATCGTTAACGATATATTAATTGATAGCATGCAAATCAACACAAAGTATCAAGA
CAAAGAAAATATGAACGGTCGCATATTAATGGGGAGCAATTATATCAGTAGAGATATAGT
TGTTCCTTGTTTTTGTAAAGTAAAAAATCGTTCAGACAGTGCTTATATGCGAGATATGTT
GTATTCGTTAACTACTGATATAGAACCAATGTATTTACGTGGAATCAGAAGAAAAGAAGA
GTTGAATTACAGGTTTACTCAACCAACTTCTGATGATTACGTGAAATCAGATAAAAACAA
CTTCCCGGATTATAAATCTTCAAGACACGATCAACAAATTTATGTAAACGGTAAACAGTA

TAAAGTTATTTTAGCGGAGTTATAAATCCTAAACAAAAAGGTAACAAAGTTGCTTTCGA
ATTGAAGTTTGAAACTACAGAATTACCATACGGCGAAAGTATTGGAACAAGCCTAGAGTT
AGAAGAAAACAAAAAGGTTGGATTGTGGTCGTTTGATTTTAATATTGATTGGCATGCAGG
TGGGGATAAGCGCCAGTATACATTTGAAAATGTTAGCAAAGATACAGTTTACTATCATGG
TAGTGCTCCTAACGACCAATTCAACATGTATAAAAAGATAACAATTATTTTGGGCGAAGA
TACAGAATCGTTTGAATGGAATTTAACGCATGCTGAAATAATGAAAATCGAAGGTATCAA
ACTAAAAGCTGGAGACAAAATTGTTGATGATAGCTTCCGAGTTTATAAAAACGGTGTTGA
AATAAGTACCGAAACGAATATAGCCCAACCAAAATTTAAATACGGAGCTAATAAATTTGA
GTTTAATCAAACGGTACAAAAAGTTCAGTTTGATTTGAAATTTTATTATAAGTAGGTGTC
AGAATGACAATAATTGTAAGACCACCTAGAGGTAATGGCGTACCTGTACCAGTAGAAACA
ACTTTAGTGAAAAAAAGTTAATGCTGATGGTGTATTAACTTTTGATATTCTAGAAAATAAA
TATACTTATGAAGTTATTAACGCTATAGGGAAAAGATGGATTGTTAGTCATGTCGAAGGT
GAAAACGACAAGAAAGAATATGTAATAACTGTCATCGATAGGAAATCAGAAGGCGACAGA
CGACTGGTTGAATGTACTGATAGAGAGATTCCCATAGACAAGTTAATGATTGATAGAATT
TATGTTAATGTAACAGGATCTTTTACAGTAGAAAGATATTTTAACATTGTGTTTCAAGGT
ACTGGAATGCTTTTTGAAGTCGAGGGCAAAGTTAAATCTTCAAAGTTTGAAAACGGCGGT
GAAGGCGACACAAGGTTAGAAATGTTAAAAAAAGGTTTGGAACATTTTGGATTAGAATAT
AAGATCACATATGACAAAAAGAAAGACAGATATAAGTTTGTATTGACGCCTTTTGCAAAT
CAAAAAGCGTCTTATTTTATTTCTGACGAAGTCAACGCCAACGCTATAAAACTCGAGGAA
GATGCAAGTGATTTCGCCACCTTCATTAGGGGATATGGTAATTATTCAGGAGAAGAAACA
TTCGAACACGCTGGGCTCGTAATGGGAGCTAGAAGTGCATTAGCTGAAATATACGGCGAC
ATCCACGCAGAACCACGTAAAGATGGTAAAGTGACTGACCAAGAAACTATGGATAAAGAA
TTACAATCGAGATTGAAAAAGTCGTTAAAACAATCTTTGTCTTTGGACTTTTTGGTGTTA
AGAGAATCATATCCAGAAGCAGACCCACAACCCGGAGACATAGTACAAATAAAATCTACC
AAACTAGGTTTGAATGATTTATTCCGTATAGTACAAGTTAAAACGATTAGGGGTATAAAC
AATGTAATTGTTAAGCAAGATGTAACGCTTGGTGAGTTTAATCGAGAACAACGATATATG
AAAAAAGTTACTACTGCAGCTAACTATGTTTCTGGATTAAATGATGTTAACCTTTCTAAT
CCTAGTAAAGCGGCAGAAAACTTGAAGTCTAAAGTAGCGTCAATAGCTAAATCAACACTC
GATTTGATGAGTAGAACTGATATAATTGAAGACAAACAACAGAAAGTGAGTTCTAAGACT
GTAACTACATCAGATGGCACTATCGTTCATGATTTTATAGATCAATCAAACATTAAAGAT
GTAAAAACAAGTGGAACGATTGGCGATTCTGTAGCTAGAGGATCACATGCGAAAACTAAT
TTCACAGAAATGTTAGGCAAGAAGTTAAAAGCTAAAACGAGCAACCTTGCAATAGGTGGC
GCAACAATGGCAACAGTTCCAATAGGTAAAGAAGCGGTAGAAAACCGCATTTATTGACAA
GCAGAGCAAATAAGAGGAGACCTAATCATATTACAAGGTACAGATGATGACTGGTTACAT
GGTTATTGGGCAGGCGTACCGATAGGCACTGATAAAACCGACACTGAAACGTTTTACGGC
GCCTTTTGTTCTGCAATTGAAGTTATCAGGAAAAATAATCCAGCTTCAAAAATACTTGTA
ATGACAGCTACTAGGCAATGCCCTATGAGTGGTACAATGATACGCCGTAAAGATACGGAC
AAAAACAAACTAGGGTTAACGTTAGAGGATTATGTCAATGCTCAGATATTGGCTTGTAGT
GAATTGGATGTACCAGTATATGATGCCTATCATACAGATTATTTTAAGCCATATAATCCA
GCGTTCAGAAAATCAAGTATGCCAGACGGATTGCATCCGAACGAGAGGGGTCATGAAGTT
ATTATGTACGAACTTATTAAAAATTATTACCAGTTTTACGGATAGAAAAGGAGGAAGACA
TGGATAACAAATTAATTACAGACTTAAGTAGAGTCTTTGACTACAGATATGTAGATGAAA
ATGAGTATAACTTTAAACTTATTTCAGACATGCTGACGGATTTTAATTTCTCTCTTGAAT
ACCATAGAAATAAAGAGGTATTTGCACATAATGGAGAGCAAATAAAGTATGAGCATTTAA
ATGTCACAAGTAGCGTCTCTGATTTTTTAACGTATCTAAACGGCCGTTTCAGCAATATGG
TACTAGGTCATAACGGCGACGGTATCAACGAAGTAAAAGACGCGCGCGTTGATAATACAG
GTTATGGTCATAAGACATTGCAAGATCGTTTGTATCATGATTATTCAACACTAGATGCTT
TCACTAAAAAGGTTGAGAAAGCTGTAGATGAACACTATAAAGAATATCGAGCGACAGAAT
ACCGATTCGAACCAAAAGAGCAAGAACCGGAATTCATCACAGATTTATCGCCATATACTA
ACGCAGTAATGCAATCATTTTGGGTAGACCCTAGAACGAAAATTATTTATATGACGCAAG
CTCGTCCAGGTAATCATTACATGTTATCTAGATTGAAGCCCAACGGACAATTTATTGATA
GATTGCTTGTTAAAAACGGCGGTCACGGTACACACAATGCGTATAGATACATTGATGGAG
AATTATGGATTTATTCAGCTGTATTGGACAGTAACAAAAACAACAAGTTTGTACGTTTCC
AATATAGAACTGGAGAAATAACTTATGGTAATGAAATGCAAGATGTCATGCCGAATATAT
TTAACGACAGATATACGTCAGCGATTTATAATCCGGTAGAAAATTTAATGATTTTTAGAC
GTGAATATAAACCCACTGAAAGACAACTTAAGAATTCGTTGAACTTTGTTGAGGTTAGAA
GTGCTGACGATATTGATAAAGTTATAGACAAAGTATTGTATCAAATGGATATACCTATGG

AATACACTTCAGATACACAACCTATGCAAGGTATCACTTATGATGCAGGTATCTTATATT
GGTATACAGGTGATTCGAATACAGCCAACCCTAACTACTTACAAGGTTTCGATATAAAAA
CAAAAGAATTGTTATTTAAACGACGTATCGATATTGGCGGTGTGAATAATAACTTTAAAG
GAGACTTCCAAGAAGCTGAGGGTCTAGATATGTATTACGATCTAGAAACAGGACGCAAAG
CGCTTTTAATAGGGGTAACTATTGGACCTGGTAACAACAGACATCACTCAATTTATTCCA
TCGGCCAAAGAGGTGTTAACCAATTCTTAAAAAACATTGCACCTCAAGTATCGATGACTG
ATTCAGGCGGACGTGTTAAACCGTTACCAATACAGAACCCAGCATATCTAAGTGATATTA
CGGAAGTTGGTCATTAGTATATCTATACGCAAGACACACAAAATGCATTAGATTTCCGGT
TACCGAAAGCGTTTAGAGATGCAGGTTGGTTCTTTGATGTACTGCCTGGACACTATAATG
GTGCTCTAAGACAAGTACTTACCAGAAATAGCACAGGTAGCAATATGCTTAAATTCGAAC
GTGTCATTGACATTTTCAATAAGAAAAACAACGGAGCATGGAATTTCTGTCCGCAAAACG
CCGGTTATTGGGAACATATCCCTAAGAGTATTACAAAATTATCAGATTTAAAAATCGTTG
GTTTAGATTTCTATATCACTACTGAAGAATCAAAACGATTTACTGATTTTCCTAAAGACT
TTAAAGGTATTGCAGGTTGGATATTAGAAGTAAAATCGAATACACCAGGTAACACAACAC
AAGTATTAAGACGTAATAACTTCCCGTCTGCACATCAATTTTTAGTTAGAAACTTTGGTT
CTGGTGGCGTTGGTAAATGGAGTTTATTCGAAGGAAAGGTGGTTGAATAATGGTAGTAGA
TAATTTTTCGAAAGATGATAACTTAATCGAGTTACAAACAACATCACAATATAATCCGGT
TATTGACTCAAACATCAGTTTCTATGAATCAGATAGAGGAACTGGTGTTTTAAATTTTGC
AGTAACTAAGAATAACAGACCGTTATCTATAAGTTCTGAACATGTTAAAACATCTATCGT
GTTAAAAACCGATGATCATAACGTAGATAGAGGCGCTTATATTTCAGACGAATTAACGAT
AGTAGACGCGATTAATGGGCGTTTGCAGTATGTGATACCGAATGAATTTTTAAAACATTC
AGGCAAGGTGCATGCTCAGGCATTCTTTACACAAAACGGGAGTAATAATGTTGTTGTTGA
ACGTCAATTTAGCTTCAATATTGAAAATGATTTAGTTAGTGGGTTTGATGGTATAACAAA
GCTTGTTTATATCAAATCTATTCAAGATACTATCGAAGCTGTCGGTAAAGACTTTAACCA
ATTAAAGCAAAATATGGCTGATACACAAACGTTAATAGCAAAAGTGAATGATAGTGCGAC
AAAAGGCATTCAACAAATCGAAATCAAGGAAAACGAAGCTATACAAGCTATTACTGCGAC
GCAAACTAGTGCAACACAAGCTGTTACAGCTGAATTCGATAAAATAGTTGATAAAGAGCA
AGCGATTTTTGAACGTGTTAACGAAGTTGAACAACAAATCAATGGCGCTGACCTTGTTAA
AGGTAATTCAACAACAAATTGGCAAAGGTCTAAACTTACTGATGGTTACGGTATAGCAAT
TGAATCGTCTGAGCAGTCCATAGATAGCGTTTTAAGCGCAATTAACACATCTAGGATTAT
TCATATCACTAGCGCAACAGATGCGCCAACATTTAAAGATATAGGCACTTTAGAGACGCC
TAAGGAAGATGGCGTTGATGATGGTTCTGAAGTTTCAGCAACTACGAATACTTTAGGGAA
ATCAGGCTTGTTAGTTGGTTATGTTGTTGATGACAGTACAGCTCGTGCTACATGGTATCC
AGACGATTCAAATGATGAGTACACAAAATATAAAATCGGTGGCACATGGTATCAGTTCTA
TAAAAAAGTTGACGAAGAATTAACGAAGAAATTTGTTGAAGAAACGGCTAACAACGCTTT
AAATCAAGCTAAGCAGTATGTAGATGATAAATTCGGAACAACGAGCTGGCAACAACATAA
GATGACAGAGGCGAATGGTCAATCAATTCAAGTTAACTTAAATAATGCGCAAGGCGATTT
GGGATATTTAACTGCTGGTAATTACTATGCAACAAGAGTGCCGGATTTACCAGGTAGCGT
TGAAAGTTATGAGGGTTATTTATCGGTATTCGTTAAAGATGATACAAACAAGCTATTTAA
CTTCACACCTTATAACTCTAAAAAGATTTACACACGATCAATCACAAACGGCAGACTTGA
GCAACAGTGGACAGTTCCTAATGAACATAAATCAACGGTATTGTCCGACGGTGGCGCAAA
TGGTGTAGGTACAACAATCAATCTAACTGAACCGTACACAAACTATTCTATTTTGTTGGT
AAGTGGAACTTATCCAGGTGGCGTTATTGAGGGATTCGGACTAACCGCATTACCTAACGC
GATTCAATTGAGTAAAGCGAATGTAGTTGACTCAGACGGCAACGGTGGCGGTATTTATGA
GTGCTTACTATCCAAAACAAGTAGCACTACTTTAAGAATAGATAACGATGTGTACTTTGA
TTTAGGTAAAACATCAGGTTCTGGAGCGAATGCCAACAAATTTACTATAACTAAAATTAT
GGGGTGGAAATAATGAAAATCACAGTAAACGATAAAAACGTAGTTATCGGATTCGTTAAT
ACTGGCGGTTTACGCAATGGTTTAGATGTAGATGATAACAATGTGCCTATTAAATTTAAA
GAAGAGTTCGAACCTAGAAAGTTTGTTTTCACTAACGGCGAAATTAAATACAATAGCAAT
TTCGAAAAAGAAGACGTACCGAATGCATCAAACCAACAAAGTGCGTCAGATTTAAGTGAT
GAGGAACTTCGCGGAATGGTTGCGAGTATGCAAATGCAGGTGGCACAAGTAAACGTATTA
ACAATGGAATTAGCTCAACAAAACGCTATGTTAACACAACAGTTGACTGAACTGAAAATT
AACAAAACAAGTACTGAGGGGGACGTTTAAATAATGAAGATGATTTATCCAACTTTCAAG
GACATTAAAACTTTTTATGTGTGGGGTTGCTATAAAAATGACCAAATTAAGTGGTACGTA
GACATGGGTGTAATCGACAAAGAAGAATATGCATTGATCACTGGAGAAAAATATCCAGAA
ACAAAAGATGAAAAGTCACAGGTGTAATGCTTGTGGCTTTTTAATTTAACACAAAGTAGG
TGGCGTAATGTTTGGATGTACCAAACGGCACGAACATGAATGGCGAATTAGAAGATTAGA

AGAGAATGATAAAACATTGCTTAGCACTCTCAATGAGATTCAATTAGTTCAAAAAACTCA
AGAGAAAGTTAACATTAAATTAGATAAAACTTTAGATGCTATCCAGAGGGAAAGACAGAT
AGACGAAAAAAATAAGAAAGAAAACGACAAAAATATACGCTATATGAAAAGGTGGATTCT
CGGTTTGTTAGGGACTATCTTCAGTACGATTGTCATAGCTTTACTAAGAACTATTTTTGG
TATTTAAAGGAGGTGATTACCATGCTTAAAGGGATTTTAGGATATAGCTTCTGGGCGTGC
TTCTGGTTTGGTAAATGTAAATAACAGTTAAGAGTCAGTGCTTCGGCACTGGCTTTTTAT
TTTGATTGAAATGAGGTGCATACATGGGATTACCTAATCCAAAGACTCGAAAACCTACAG
CTAGTGAAGTAGTAGAGTGGGCGTTGTATATTGCTAAAAAGAAAATAGCTATTGATGTAC
CTGGTTCTGGAATGGGGGCGCAATGTTGGGACTTGCCTAATTACTTGCTTGATAAATATT
GGGGGTTTAGAACATGGGGAAATGCTGATGCTATGGCTCAGAAATCTAATTATAGAGGTA
GAGATTTCAAGATAATTAGAAATACAAAAGATTTTGTACCACAACCAGGCGACTGGGGTG
TTTGGACTGGTGGTTGGGCAGGACATGTAAACATTGTAGTGGGAGCATGCACAAAAGACT
ATTGGTATGGTGTGGATCAAAACTGGTATACAAATAATGCAACAGGAAGTCCGCCGTATA
AAATCAAACACTCTTATCATGATGGACCAGGTGGAGGAGTTAAATATTTTGTTAGACCAC
CATATCATCCGGAGAAATCTACGCCGGCACCTAAACCCGAAGACAACAGTGATAATAACG
AAAAAAATAATAAAAAAGTTCCGATTTGGAAAGATGTAACAACTATAAAGTACACTATTT
CTAGCCAAGAGGTTAATTATCCAGAATATATTTATCACTTTATAGTAGAAGGTAATCGAC
GACTCGAAAAACCTAAGGGAATAATGATTAGAAACGCACAAACGATGAGCTCGGTAGAAA
GTTTATATAACAGTAGGAAGAAATACAAACAGGATGTAGAATATCCCCACTTTTATGTTG
ACAGACATAATATTTGGGCACCTAGTAGAGCTGTATTTGAAGTTCCTAATGAACCTGATT
ATATAGTTATAGACGTATGTGAAGATTATAGTGCGAGTAAAAATGAATTTATTTTTAATG
AGATTCACTCAATGGTTGTAGCTGTAGATATGATGGCCAAATATGAGATACCTCTAAGTA
TTGAAAATTTAAAAGTAGACGACAGCATTTGGCGTTCGATGTTGGAACATGTTAATTGGA
ATATGATTGACGGCGGTGTTCCCCCTAAAGATAAATACGAAGCATTAGAAAAGGCATTAC
TTAATATATTTAAAAACAGAGAAAAATTATTAAATTCTATAACTAAACCAACAGTAACAA
AATCTAGAATAAAAGTTATGGTAGATAATAAAAACGCTGATATAGCGAATGTAAGAGACT
CATCACCAACAGCTAATAATGGCTCGGCATCTAAACAACCGCAGATCATAACAGAAACGA
GTCCTTATACATTCAAACAAGCACTGGATAAACAAATGGCAAGAGGTAACCCGAAAAAAT
CTAATGCTTGGGGTTGGGCTAACGCTACACGAGCACAAACGAGTTCAGCAATGAATGTTA
AACGAATATGGGAAAGTAACACGCAGTGCTACCAAATGCTTAATTTAGGCAAGTATCAAG
GCGTTTCAGTTAGTTCGCTTAATAAGATACTTAAAGGTAAGGGGACATTGAATAATGAAG
GTAAAGCGTTCGCAGAAGCTTGTAAAAAGCACAACATTAATGAAATTTATTTAATCGCGC
ATGCTTTCTTAGAAAGTGGATATGGAACAAGCAACTTCGCTAACGGAAAAGATGGGGTAT
ACAACTACTTCGGCATCGGCGCTTACGACAACAATCCTAACTACGCAATGACGTTTGCTA
GGAATAAAGGTTGGACATCTCCAGCAAAAGCAATCATGGGCGGTGCTAGCTTCGTAAGAA
AGGATTACATCAATAAAGGTCAAAACACGTTGTACCGAATTAGATGGAATCCTAAGAATC
CAGCTACCCACCAATACGCTACTGCTATAGAGTGGTGTCAACATCGAGCAAGTACAATCG
CTAAGTGATATAAACAAATCGGCTTAAAAGGTATCTACTTCACAAGGGATAAATATAAAT
AAAGAGGTGTATAAATGTACAAAATAAAAGATGTTGAAACGAGAATAAAAAATGATGGTG
TTGACTTAGGTGACATTGGCTGTCGATTTTACACTGAAGATGAAAATACAGCATCTATAA
GAATAGGTATCAATGACAAACAAGGTCGTATCGATCTAAAAGCACATGGCTTAACACCTA
GATTACATTTGTTTATGGAAGATGGCTCTATATTCAAAAATGAGCCCCTTATTATCGACG
ATGTTGTAAAAGGGTTCCTTACCTACAAAATACCTAAAAAGGTTATCAAACACGCTGGTT
ATGTTCGCTGTAAGCTGTTTTTAGAGAAAGAAGAAGAAAAAATACATGTCGCAAACTTTT
CTTTCAATATCGTTGATAGTGGTATTGAATCTGCTGTAGCAAAAGAAATCGATGTTAAAT
TGGTAGATGATGCTATTACGAGAATTTTAAAAGATAACGCGACAGATTTATTGAGCAAAG
ACTTTAAAGAGAAAATAGATAAAGATGTCATTTCTTACATCGAAAAGAATGAAAGTAGAT
TTAAAGGTGCGAAAGGTGATAAAGGCGAACCGGGACAACCTGGTGCGAAAGGTGATACAG
GTAAAAAAGGAGAACAAGGCGCACCCGGTAAAAACGGTACTGTAGTATCAATCAATCCTG
ACACTAAAATGTGGCAAATTGACGGTAAAGATACAGATATCAAAGCAGAACCTGAGTTAT
TGGATAAAATCAATATCGCAAATGTTGAAGGGTTAGAAGATAAATTGCAAGAAGTTAAAA
AAATCAAAGATACAACTCTCAACGACTCTAAAACGTATACGGATTCAAAGATTGCTGAAC
TAGTTGATAGCGCGCCTGAATCTATGAATACATTAAGAGAATTAGCAGAAGCAATACAAA
ACAACTCTATTTCAGAAAGTGTATTGCAACAGATTGGCTCAAAAGTTAGTACAGAAGACT
TTGAGGAATTCAAACAAACACTAAATGATTTATATGCTCCAAAAAATCATAATCATGACG
AGCGGTATGTTTTGTCATCTCAAGCTTTTACTAAACAACAAGCGGATAATTTATATCAAC
TAAAAAGCGCATCTCAACCGACGGTTAAAATTTGGACAGGAACAGAAAATGAATATAACT

ATATATATCAAAAAGACCCTAATACACTTTACTTAATTAAGGGGTGATTTTTATGGAAGG
TAATTTTAAAAATGTAAAGAAGTTTATTTACGAAGGTGAAGAATATACAAAAGTATATGC
TGGAAATATCCAAGTATGGAAAAAGCCTTCATCTTTTGTAATAAAACCCTTACCTAAAAA
TAAATATCCGGATAGCATAGAAGATTCAACAGCAAAATGGACAATAAATGGAGTTGAACC
TAATAAAAGTTATCAGGTGACAATAGAAAATGTACGTAGCGGTATAATGAGGATTTCGCA
AACTAATTTAGGGTCAAGTGATTTAGGAATATCAGGAGTCAATAGCGGAGTGGCAAGTAA
AAATATCAACTTTAGTAATCCTTCAGGGATGTTGTATGTCACTATAAGTGATGTTTATTC
AGGATCTCCGACATTGACCATTGAATAATTTTAAACGACTAATTTATCAGTCGTTTTTTA
TTTTGGATAAAAGGAGCAAACAAATGGATATTAACTGGAAATTGAGATTTAAAAATAAAG
CAGTATTAACAGGTTTAGTGGGTGCGTTGTTGTTATTTATCAAACAAATCACAGATTTAT
TCGGATTCGATTTATCAACTCAATTAAATCAAGCCAGCGCCATCATAGGTGCTATCCTCA
CGCTACTTACAGGGATTGGCGTTATTACTGACCCAACGTCAAAAGGCGTCTCAGATTCAT
CTATAGCACAGACATATCAAGCGCCTAGAGATAGCAATAAAGAAGAACATCAAGTTACGT
GGAAATCATCACAAGACAGTAGTTTAACGCCAGAATTAAGCGCGAAAGCACCAAAAGAAT
ATGATACATCACAACCTTTCACAGACGCCTGTAACGATGTTGGCTTTGATGTGAATGAGT
ATCATCATGGAGGTGGAGACAATGCAAGCAAAATTAACTAAAAAAGAGTTTATAGAGTGG
TTGAAAACTTCTGAAGAAAAACAATTCAATGTGGACTTATGGTATGGATTTCAATGCTTT
GATTATGCCAATGCTGCTTGGAAAGTTTTGTTTGGATTACTTCTGAAAGGTTTAGGTGCA
AAAGATATACCATTTGCAAACAATTTTGATGGACTAGCCACTGTATACCAAAATACGTCG
GTCTTTTTGGCAAAACCCGACGATATGGTTGTGTTCGGTAGTAATTACGGTGCAGGATAC
GGACACGTAGCATGGGTAATTGAAGCAACTTTAGATTATATCATTGTATATGAGCAGAAT
TGGCTAGGCGGTGGCTGCACTGACGGAATCGAACAACCCGGCTGGGGTTGGGAAAAAGTT
ACAAGACGACAACATGCTTATGATTTCCCTATGTGGTTTATCCGTCCGAATTTTAAAAGT
GAGACAGCGCCACAATCAGTTCAATCTCCTACACAAGCACCTAAAAAAGAAACAGCTAAG
CCACAACGTAAAGCAGTAGAACTTAAAATCATCAAAGATGTGGTTAAAGGTTATGACCTA
CCTAAGCGTGGTAGTAACCCTAAAGGTATAGTTATACACAACGACGCAGGGAGCAAAGGG
GCGACTGCTGAAGCATATCGTAACGGATTAGTAAACGCACCTTTATCAAGATTAGAAGCG
GGCATTGCTCATAGTTACGTATCAGGCAACACAGTTTGGCAAGCCTTAGATGAATCACAA
GTAGGATGGCATACCGCTAATCAAATAGGTAATAAATATTATTACGGTATTGAAGTATGT
CAATCAATGGGCGCATATAACGCGACATTCTTAAAAAATGAACAGGCAACTTTCCAAGAA
TGCGCTAGATTGTTGAAAAAATGGGGATTACCAGCAAACAGAAATACAATCAGATTGCAC
AATGAATTTACTTCAACATCATGCCCTCATAGAGGTTCGGTTTTACACACTGGTTTTGAC
CCAGTAACTCGCGGTCTATTGCCGGAAGATAAACAATTACAACTTAAAGACTACTTTATC
AAGCAAATCAGAGTGTATATGGACGGTAAGATACCAGTTGCCACTGTCTCTAATGAGTCA
AGCGCTTCAAGTAATACAGTTAAACCAGTTGCGAGTGCATGGAATCGTAATAAATATGGT
ACTTACTACATGGAAGAAAGTGCTAGATTCACAAACGGTAATCAACCAATCATTGTAAGA
AAAATAGGACCATTCTTATCATGCCCGGTAGCTTACCAATTCCAACCTGGTGGATATTGT
GATTATACAGAAGTGATGTTACAAGATGGTCATGTTTGGGTAGGATATACATGGGAGGGG
CAACGTTATTACTTGCCTATTAGAACATGGAATGGTTCAGCCCCACCTAATCAGATATTA
CGTGACTTATGGGGAGAAATCAGTTAGAATGACATAGTCATGTCTATTTAAGCAGGTGCG
TTACATACCTGCTTTCTATTTACATTTAAAGATAAAATGTGCTATTATTTTACTAGAACT
TTTTAACATTTCTCTCAAGATTTAAATGTAGACAACAGGCAGGTACTACGGTACTTGCCT
ATTTTTTATGTTCTAATGTAATTACATTACCAGTAACCAATCTGGCTTAAAACCACATTT
CCCGTAGCCAATCCGGCTATGCAGAGGACTTACTTGCGTAAAGTAGTAAGAAGCTGACTG
CATATTTAAACCACCCATACTAGTTACTGGGTGGTTGTTTTTTTTTCGCCATTATGTTCTG
TCTACATCTTTTTGCGCAAGTTGAGTATTATGTATATATAGTATAAATAAGGGATATTTT
AAATGGAAGATAATAAAAATTTAGCAAAAATGTATTCATTCAAAGCTTTATATTTTTTTA
CTGCAGCTTTTGTAAATATATTGACTATTATAACAACTAAAAATTTTGAGGTCGCTATGT
TAGGTGTAACACTTTATTTTATGCCTATTGTTCTAGAAAATTACATGAAGAATGTTTACA
ATAAAGCAACTAAGATTTTGAGAAGAATAGGATATATATTTCCTAGTTTATTCATATTTA
TAAACATTTTAATATTTATTTTATGTTCTTATATGCCTACACATAAGTTTATCATTAGTA
CAAATTGGCATGTCGGTGCTATTATAATAACAACAATACCTTTAGTTTTTATTAGTATAT
TAGATATTTTTCTTTATGGCTTTAACAACGAGGAAATCAAAGCTAGTAATGATACTAAAG
AGTATTTGAGAAAGAACAGAGAACAAAGTAAAGAACTAAAAATAAAGCAAGAGAAAGTAC
TGAAAGAGGAAGAACGTCAATTTAAAATAGAACGTTCTAGAAGAAAGGGATGATAGTAAT
GGTTATAACAATTATAAGCGTTTTTGTTATAAGTGCTATCTTATTATTTTATTTGTCAGC
GGTAACTTTTACCGCAGTTAGATCTTATGATTATAATATTAAATTTAGTGTACCAACGCA

ATTTGCAATACCATTAATTTTGGTCAGTACACATATAAAAATGTACAATACTTACAAATT
GTCTGACAGAAAAAAAGCCTTCAGAATGCTTTTATTTATATTTATAAAATATCCGGTAGC
AATAGCTATGTTTTTAACTATTATTGCTACAAATATCGCTGAACAAAATGTTAAATTATC
AAATGGTGCAACTTATAAGAAAACTGAAAATTTACAGCAGAAGAAACGTAAAAAAATGAT
TAACAAGGTTCTTCAAAGTAATACTTATGGAGAAATGCTTTTGGGAATATAAAGAAGTTA
CTTAGTTTTTCTTATGATTTTAATATAATAGATTTATTATCTTTTCTTGATGAAATTATA
ACCGTACCCTTTAAGATGCGGTTATTTTTATTCCTTTACAATCAACAAAACCACAACACC
TATTAATTTAGGAGTGTGGTTTTTTATATGTGAAGCTAAAATAACTACAAATGATACCAT
TTTTGATACCATTTTGTTGTAAAACAGAAAAAATAAGGAAAATAAAAAAGGCAAAAAACG
CATTAAATCAACGTTTATTGTCTCATGAAATTTAAATGTATATAAATTTCACTTCCCATG
GG
>NewGenomeName_10
AGCGCTGGGGACTATTATTACCCCTAGCGCTCGGCACGGGACATGGGCTAATGGATTGTG
GATATAGGGCCCAAAGGGCCCGTTTAGATGGGTTTTGGGCTCATGGGCTTTATCCAGAAG
ACCAAAAACAGGCGGGAACCGTCCCAAATTCAAACTTCGATTGCTTGCCCTGCAACGCAT
CTAGAAGTCTATAAATACCAGTGTCTAGATAGATGTTCAGACCAGAAATGGCTAGGTATC
CGAAGAAATCCATCAAGAAGAGGCGGGTTGGGCGCCGGAAGTATGGCAGCAAGGCGGCCA
CGAGCCACGACTACTCGTCGTCAGGGTCAATATTAGTTCCTGAAAACACCGTCAAGGTAT
TTCGGATTGAGCCTACTGATAAAACATTACCCAGATATTTTATCTGGAAAATGTTTATAC
TTCTTGTGTGCAAGGTGAAGCCCGAAAGAATACTTCATTGGGCTATGATCAAGAGCTCTT
GGGAAATCAACCATCCGACAACCTGTCTGGAAGCCCCAGGTTTATTTATTAAACCTGAAC
ACAGCCATCTGGTTAAACTGGTATGTAGTGGGGAACTTGAAGCAGGAGTCGCAACAGGAA
CATCAGATGTTGAATGTCTTTTGAGGAAGACAACCGTGTTGAGGAAGAATGTAACAGAGG
TGGATTATTTATATTTGGCATTCTATTGTAGTTCTGGAGTAAGTATAAACTACCAGAACA
GAATTACATATCATGTTTGATATATTTATGTAAACATAAACTATTGTATGGAATGAAATC
CAAATAACATACAACACGCTATGAAATACAAGACGCTATGACAAAAGTACTGGTATATGA
TTAGGTATCCTAACGAACTAGGGCCGAAGGCCCGTGAGCAATATGCGTCGAAATAATGTT
TGACAAACAAATATACATGATACGGATAGTTGAATACATAAACAACGAGATATACAATAC
AACAAACTATAGTAAAGAAATAAAAAAATAAGAAGAGAGAGTATATTTGTGTCGGATCAGC
ATCAGACCCACCACTTTAGTGGTGGGCCAGATGTCCCGAGTTAGTGCGCCACGTA
>NewGenomeName_11
GGGGCGGGTCATAGTATAACCCCCGCCCCGGGTTCAGAGTCACGTACGGAGTTACTTATG
GCCTTTAGATTATGATACACGTGGACGATCAGGATCTGTGATTCGTGAAGCGAATCTGAC
GGAAGATCGTCTGCAGCTTCGTGGTAGGTCCCAATTGTTGCTTTAGTTTCCTTTTTTATA
AGTAAAGTAAGATGCTGTCTCTAACTTTATTCCCTTTGAGTGGAATACAGCTGTCTTTAC
TTCCTCACGAAGCAAAGCATTATTTCTCTCTCTATAAAAAGCTGCGTTCGTCTTCGTTGT
TCTTACAACGAAGATGGGTCTGAAATATTTCTCTCATCTTCCTGAAATGCTTAGAGAGAA
GATTGTTCACGATCATCTACAACAAGAGCGAAAGAAAGAGTTTCTTGAGAAGGCAATAGA
AGATAGTTGCAGAAGGCATGTGTCGTTATTAAAGTCAGACCCAGCTCCTTCATAGATGTA
TTCTCTGAGTACGTTTTTAGATTCATTGGGTGATTACGTTGGTAGACAATTTAATACACG
TTGTCTGATTAAATGGAGGAAGGATCTTCCTGCTAATATCTAGTTTCAAGTTATGGAAGA
GCAACACTTGAGGTTATATGGTTTTCTTGATATGGACGACTTATCATGTCGTGAATTACT
GCCGCCTGAAGAGGACGACGATATTACATACGAAGAGGGTATGATTGTCAACTGCAGTGA
ATTGGATAAATTATTTGCGGCTCTAGGCATTAGAGTTGTTTATATTACTGTCAGTAATAA
TTGTATTTGTACTCCTTTGAACAAGGATATTGTAATTAGTTGATTCAATGTAATTAAATG
ATATATTATTGTTAATGTTACTATCAAATACATATTTATTGTTTTTTAATACTCTGCGAA
GCAGTATGTTAAGGCCCAATAGGCCCAATAGCACTAAGGCCCAATAAAATAATCTGTTAA
ATGAGTCAGCTGACGTCAGCTGACTCCTTGATGACGTAG
>NewGenomeName_12
ATGGGTCGGGGCTTAGTATGACCCCGGCCCCAGGATCACTCAAGTCACCACGTGAGAAGC
ACATGACATCAAATGTTATCAGTCATAGCAAGTGGATCTTGGTCCCTACCACGGCACTTT
AAAAGGCTGCACAGAATATATGCTTTGCTTCGTTAGTACGCAAAGGTAACGAAAATACAT
GACTTCTGATTGCTTTATTCCATGTGCAGAGACTTTAAATGAATGGATCAGACTTAATCG
CACATATATATCTTCCAATGGAAGATTGGTTATCCCACTATAAATATAGGAGACCTGTGG
AGCTATTTTTTGGGTATTCGGAATTCCATAGTTGTTGTTCTTCGTCTGAAACAAGATGCC
GTTGATTAGGCGTTTCAAGCTTTCTATGAGAGAATTGCATAAGCTGAAAGAACAACGAGA
CTCTTTCTGGAAATCATAGCATGAGTATCTTGGAGCTAATGAAGAAGCGTTAGGAGAGTT

TTGTAGATACCATGGAAGGAGAATATTAGCTTATCCAAATATATCCAGTTATGCTCCAAC
AAGGTGGTTGTTGAAAACTCGGCCTTTCTATGACATCAGAGTTGTGGATTGTGAGGAATG
CCAGAAACACGAATAATGTAGAAGTCGTTTCTATGTCAGAAATACAGAAGGCCTGCAGGA
CTTGGATCATTACGGTAATTACCGATTTCAAGTGTATTATGGTAATCATAATGATGTATT
AAGATATTGTGTTAATAAACGACACCTATCATTGGAATATATTAAGATATGTTATAAAAG
TTTATTATGGTTTGTGTTGTTGGGCCTTGGGGCTATTGGACCTATTGGTCCGGTAGTTAT
TCAGTTCGTGTATTCCTATATACTAAACTGTAACATGATTGTATCCATTGTTGTGCCATA
AAGAATAAATATAAATGTTAACTTATATTTGCTATGATATTGATATAGTTTTTATGACC
TTAACGAAGCGATATGTATTTAATTGTATTAAAAAAGACGTCGCTAGATCCCTTGCGGAG
>NewGenomeName_13
ACGAGGTGGGTTCTAGTATTACCTCGCCTCGTTACACTACCCCGTTACCTTTTCTCTCTC
TAGAGTGAATGGCATCTAAGCGTTGGCGCTTTACGGTGAACTATAAAACAGCGTTAGAGA
GAGAAACATTCATTTCTCTCTTCCCTCGCGACGAATTGAACTAGTACGTTTGTGGAGACG
AATTCGCTTCCACTACTGGGCAGAAACATCTCAAAGGATCTGTTTCGATGAAGAAATTAA
TTCGTGTGGGTGGATTGAAGAAAATATGTGAATCTTTTGCTCATCGCGAGATCGCTAAGG
GAGACGATTTTCAGTGTCGCGATTAATGTACGAAAGAAACGCAAATTGCAGAAATTGGGG
CTCTGGTTAAGAAAGGCTCTAAGCGAAGGAAGATACTGGAGATATACGAAGAAGATCCAG
AAGAAATGAAATTGCGAGATCCGGTTACTGCCCTTCGCTGTAAGGCGAAGAAATTAAGAG
AAGAGTATTGTTCTGTGGTTTCGGTGTTTTCTCTTCGACCACGGCAAATCGAGCTACGCA
GAGCGTTAATGGAGGTTCCTGACGAGAGAACAATTATTTGGGCGTAGGGCCCAGATGGAG
CCGAAGCTAAATCTACCTTCGCGAAAGAATTAATCAAATATGGTTGGTTTTATACTGCTG
GAGGAAAGACCCAGGACATACTGTATAGGTATCCGCAAGATCCTGAAAGAAATATTGAGT
GCGATGTACCCAAGTGTTCTTCTCATATGATGAACTATCAAGCATTGGAAATGATGAAGA
ATAGAGTATTTGCAAGTACAAAATATAGGCCTGTAGATCTTTGTATAAGAAAGAAGGTGC
ATTTAATTGTATTTGCCAACGTGGCACCCGAACTCACAAAATTAAGTGAGGACAGAATTG
TTATTATCAATTGTTGATTAAATGAATCATCTATTATTTTGTGGAACTCCTTAGCGCCAG
CTGTCCTTTTTTTGGAGAGTGTCCAAACAAACCTCGTTACACTATATAT
>NewGenomeName_14
ACGTAGGGGCGGGGCTTAGTATTACCCCCGCCCCAGGATCAGCGGAGTCATTTACACTCG
CTATAGGCCGTTAGATGTGTATACACGTGGACGATCACGATCTGTGATTCGTGAAGCGAA
TCTGACGGAAGATCGTCCGAAGCTTCGTGGTAGGGCCCCCATGTTGCTCTATCTTTACTC
TAATAAAGTAAAGTAAGATGCTGTCCCTTGCTTTATTCGTTTGCTAGTTGTTTACAGCTG
TCTTTGCTTCGCCGCGAAGCAAAGGACATTCTCATCGTCTATAAAAGCTGTGTTCTCCGT
GTGTAATTCGTCGGAATTATCGAGCGAAAGTCTCGTTCTTGTTCTATGGAAGCTTTCAAG
CAACCAAAGCTCTCTTATGGAGAGATAGTTCAAATGAAGGAAGAACAAGATGCTTTCTGG
AGTTGTTATCATGAGTTTCTCCGTCGCAATGAAGATGTTCTTGGAGAGATGTGCAGACGC
CATGGAAGGAAATTGCCTGCATATCCAAAGCTCCCTACATATGCCCCGATACGGTGGGTT
CTTAAGACTAAGGCAATTTATGATGTCAGAGTAGATGAATGTAAGTCATGTTCGCATGAG
GAAATTAGTAGAAGGGATTATAATCCCATTAAGAAAGAAGGTTTAAAAGACCTGTATGAT
TCAGGTAATTATAGATATCAAGTGTATTATTCTTCATCGTGTAATAGAGATAAATCAGAT
TAATAGATAAATAATTACAATTGTATTGTTTGTGCTATTGTTTATGTTTCATTAATAATT
TATGGTTTGCAAAACCAGAGCTATTCAGAGGTAGAAGACAATTACAATTGATACCATATA
CATTACAATGATATTTGAGAAATATATTATTTTTAATAACTCTGCGAAGCTATATGTCTT
GGCCCAATAGGCCCAAATGCTCTGAAGCCCAATGAAATTAACACTGAAGTGAGTCAGCTG
ACGTCAGCTGACTCCTTGATG
>NewGenomeName_15
TAGTATTACCCCCGTGCCGGGATCAGAGACATTTGACCAATAGTAGACTAGTATAATAGC
CCTTGGATTAAATGACACGTGGACGCTCAGCATCTGTGATGCTAGTGAAGCGCTTAAGCT
AAACGAATCTGACGGAAGAGCTTTCACACTTAGATCTAGTTAGCGTACTTAGTACGCGTT
GTCTTGGGTCTATAAATAGAGTGCTACTGAACAGATTGTTCAGAAATTCATAGCGAGATG
GATTCAGGTGATGGCTACGATACATACTCATATGAAGAAGGTGCTGGAGATGCGAAGAAG
GAAGTTTTATATAAAATAGGTATTATTATGTTATGTATTGTAGGGATTGTAGTTTTATGG
GTTTTAATTATATTATGATGTGCTGTTCCTCGCTATGCTAAATCAACGATGCACGCTTGG
TTATCTGCGTCTTCCATTATGAAGTGGAAGATGGCTTCAAGGATTACTGGTACTCCGTTT
GAAGAAACTGGTCCTCATCGTGAAAGAAGATGGGCTGAAAGAAGAACTGAAGCGACGAAC
CAGAATAATAATGATAATGTAAATAGATTTAGTTGATATGTTGTTATTTTATATGGATTA
ATGAGAATTATTATTATTCTGTTCTTCGTCTGTGTTTTTTAAGCTTTTTCTGTGTTTTAA

TGGCGTCTGGAGAGAGAAAGGAATAATTGTAAGGTAGACGACGATGTAGTCGATTACAGT
TGTCTTTACTTCGCCTCGAAGAAAGACGCATTTCAAGTTGTGAGTGTTATTGCTTTTGAG
GAAGCTTCCTCGAAGCAGCGTATAACTTTAATTTGAATTTGGTTTTGGCGCGTTAGTGAA
ATTGCGGCTGTAAACGTGTCAAGTTGTGAGTGGCTGAAATAAGATAATAGATATATTATT
ATTGTTTTAATTTAATTCCGCGAAGCGATATGTCAAGTGATAAATGAAACGAAGCGTTTT
GATGACGTCATATGACTCCGTGCCTACGTCAGCACGGGGCT
>NewGenomeName_16
ATGACTGAGCCTGGAGCATCGGAGTTCAGGTGGGGACCGGGAATGCATACGCATCCTGAC
TTGGACTACGACGCGGGGAAGTCTCTCGCGCCCAACAATGTGGAGACGCTGCACGATCAG
ATTCGCGATCTGCAGGCGAGCAAGCGGAGCAACGCCGCACGCGCTGGAGAGACGATGCGG
GACATGTACTTTGCACTCGGGAATCCGGATCGCTACCGGCCCAAGCCCGCGCTTGTGCAA
GGCGATGGCAAGCCGGAGCCTAGCCCCACGCCCGAGGAGTCACCGCAGACATTCGAACGC
AAGCCGTAATCGTTTGTGCAATAACAGATGTGCAGTCGGTCGGATTCGTGGCCAGGGAGG
CCACTGCCGGCCGACAGTATTATTGAGGGACGAATCCGAGCCTGACAAAAGGGAATTGCA
ATGCCACGAGGTGATACCACCAAGGAGCTGACCGACCGCACCGTGGGGAAGCCCGACGGG
CTAGAGGAGTTCCGCCCCGAGTTTGGAATCAAGGTCGGAACGTTCACAATGCGTCGCAAG
GATTTGGACCATCCCAACTATCGCAGCATGCCGTGTCTGATGTTCCATGTGCCGTTGCAG
TTCCGCGGACACTATCGTGTGGAACGTCTCGCGAACTTCTCGGGCGACCCGGACGACTAT
CGAATCGACATCCACGGCTACCGGCTGTGTGAGGCTCAGACGCCGCGACGGCCCACGGGT
TGCCTCAAGCGTGCAAGCAACCGACAGCCGTACTTCGAGCTGCATGGCGCGCGCCTACAC
CCGCTGGACAAGGTGCGAGTTAATCCCAGGGATCCCAGCACCATGACGCGCATGGAGCTG
TTGACGCACGGGTACATTGATGTGGAAGACCTCACCGACGACGAACTCAAAAATGGGCTT
GTGGCTCCGGGGCGTGGTGGTAATGCAATGATCCACTTGCCCAAGGACGTGTACACCAAG
ATCGTCAACAGGCACTTTGACCGTGTCAAGGAGCTGATGGTTGATGGGATGATCCCGGCC
ATCAAGGTGCTCAGAGACATTGCAGAGAACAAGCACGCCATCTACGAAGCCAAAGACCGC
GTCAAGGCCGCCACCTACATTGTGGACAGAGTCCTTGGTAAGCAAACAGATACGGCACTG
GTGCTGGGTGGTGCTGATGGCGGGGCCCAGGGGCCCTGGCACCAGATTGTGGCCTGGATC
ATGGACAACACGCGTGCGGAATCCCGTGCGGCGCGCGCGCTCCCCTCTGGCGGAGAAGGC
GCTACGCTCAACCACAACGGTGAGGCCGAGGAGGTTCTCGATCTTGAGGTTGTGGAAGAG
TCGGCGGCAGATCATGATTCAGCGACGCCGCGAACGCCAGACGACACGGGACCTGATGCG
AGGGCTCTCATCGCAGCCGTAGCTCCCAATGCAAAGCCCAAGCGCGCCAAGGCCAAGGCG
TCTGCTGGCACGAGTGGCACGAGTGGCACGAGTGGCACGAGTGGAAAGCCCGCCAAGCTG
CGGGTCAAGCGGCCGAAGCGCGACGCCAGGTAAGCTTCGCGGTGCGGGGTGGACGGCAGG
CCGCCAAGCAACCGGAGTGGACCCGGGATTCGCGCAGATTCCCTTCTGTCCCGGGTTCAC
TCCCAAGAACTGCAGGACTGGCAAGGAGCTGAGCATGGCGAAGCAGGGTGTGCGTCGGGA
CATCAACGGTCCCACCAAGCAGGCACCGTCTGTCACCAAGGCTGTGCAGCCCGCGCCGAA
GACCAAGTTGCACCGGCCCAAGACAGCCTAGGAGGGCACCATGGCCAAGGGCGTGAAGAA
GCTTCCAAAGCGCAAGGGCACCAACCCCATTCCGCGTGACAAGTGGAACTCTGACGACAT
CGCTCGGCGTCAGCTCGAGCAGGACCAGAAGCTCCACCTCACCACCAAGGGTTCGCACAC
CGGGACCAACGACAGCTTCAAGTAGCACCTCGCCATGGACAAGCTGCCCGTGGCGGTTAC
CGGGAGTGGCACTGTCCAGGTGGCTGGGCGCGGTCCCGCTGGCGAGGCTGTGGTGCTAGA
GACGACGCTCCAGCTCTGGCGGGCAGCCAACAACTAAGTCGAGCGCGTGGCCCATGCCCT
CGGGTTACCGGCGCGAACCGACCCGACAGAGTAGGATCACGTGCATGCCCAAGAAGAAGG
TCACCGGAGCCAAGCTCAAATGTGGTAGCGCTGCCCAGAAGCGTGCTCAGCGAAAGGCTG
CAGAAGCCAGTGCCCGGAAACGGCGCAAACGGGGCCTGCAGGACATTTTGAAGCGCAAAA
GGCGCGCAGCCGGTAGCTTCTCCGAGGTTCGTAAGAGCTCGGTCAAGGTGGCCAGGGAGC
ACGACCTGTCGGGCACCGGGTACGGGGCCAACAAGGAGGCCTACCGTGCCAGTGCAGCCG
AGAGCCGCAAGATCAAGGCCGCTGGCGGGACGAGCTCGGCCGACCGCGTCTACAGCACTG
CCCGCAAGCTCAAGTACACCGCGCCACGCGGTAAGGCAGGCAACCAAGGCAAGGTGGGCC
TGAGCTCGCGTAACAAGGGTTCAGGCTCGTCGTCGGACAAGTTCAAGCAATGCGATACTG
TCTATGCGGACGGCAAGTCCTGGACAATAAGGAGTTTCCGCGAGGCCAATGGCAATACCT
ATGTCGATGCCAAGGCCGACAACGGAACCGATCTCTCCCTCGTCATCAAGGGCACCGGCC
TGGACAAGAAGACCATCCGGACGGCTGTGGGCGATCGCCCGCGTTCCGAGTCCAAGACTG
TCAAGCCTGGCGACAGCGCTGCTGCACATCGCGCCAAGAGCTTCTCTCAGAATGCGCGTG
GCGTGTCGATGGGTGGCCCACGATCGAGCTCCAGAGCGCCCAAGGACCTGAGCAGCATGT
CCGACCGCGACTTGCAGAACGGCATTCTGAACGCTCGGACCGCAGCCGACAAGAAGGCGT
ACTCCAACGAGTTGGCCAAGCGCAATCTCGACAATCACTTTCCCAAGGGCAGCAAGAACG

CCTCGACAGAAGAGCTGCAGAACAAGGTGAAGGACGGCACCACTCCGTCCTCGGTAAAGA
CCGCCATCGCCGCCGAGCTGGACCGCCGAGCCAAGGCTAAGGCACCCGCGAAGGCCCGTG
GTGGGCGTCGACCTGAGAAGCCGATCGCCCAGGTGATGAAGCCGCCCAAGGACGCGGTGC
CGCTGGTTGACAAGAATGGCAATACGACCCCAGAGGCGATCCGCCGATACGGGAACGGCA
AGAAGGAGTCTGGCTATCCCGACGCCGATGATCGTGCGGCCTGGAACAAGCTCGTGGGTC
GCGAAACATACCGCGACTTGAGAGACGACATGACACCGCGCCAGCGCGCCAACTACCGCA
AGGAGCTGCAGCGTAAGCTGGACAACCCGCGCATCAGCGACACTGTCAAGAAGCAGGCCC
GCGATGAGATGGCTCTGCTGGATGCGCTCGACAAGGGGTCTGGCAGAGGATCCGCGAATT
CGGGGGAGGCCAGGGCACAGCGCGTACTTGATCTGCACGACCACGAGCTGCAGGACCTGG
CCAGCGAGATGGCCCGTCTGGACTTGTACAAGCGGGATCTCAAGGGCGAAGGACACTTCG
ACCGGAAGCCTTCCGAAATCTCCAAGGAAGAGCGCGTTCGCAATGCCATTCAGGGTCTGA
TGCGTTACGTCGAAGAAGATCTTGGGTACGAGGTCGATTGGGACGAGCAGCGGCTCGACA
ACGCCCTCAACAACGACCCGCGCATGAAGAAGCTGCGAGCCATCCTGGCCAAGCTGGGAA
AATTGACAGGGCCGACCGAATCCCGGTCGGCCAATCGCACAGAAATCAAGGGTAAGACTT
CGGACGATGTGGATACGGCCATTGATAACCTCAAGGCTGCACAAGACAGCAACAATTCCG
ACGCCAAGGCTAAGGTCGCTCTGAAACGTACTCGGCACTACGACAGCCCCGCCGACTTCA
AGCCTCCTCGATCTGCACAAGACAACAAACGCCAGAGTCTAGCCTCGTACCGGGATGTGT
CCTCGGCGATCAACGGCTCACTGCGCTCTGGCGAGAATATCTCCAGGAACGCCTGGAAGC
GTGGCGTTGTTGAGGATCTGGACAGCATGATCAGCGAGTCCACCCTCAAGCGGCCGATCA
CCGTGCGCCGCTTCATCGGCCCGAGCGCTGGCGGAAGTGGTATTCGTGGCGACTATGCAA
GGGTCAAGAATCAGCTAGAGTCTGGCACCTTCAAGGACAAAGGCTATATGTCCACAGAGG
CCGTTACGCCGGGATACTCGGTCGGCCAGATGGGACGGCTGTGGTCCGACCGAGAGAAGC
CCGGTGAGAACGATTTCGTGCTGGAAATGGATCTTCCCAAAGGTTTCAATGCGCTTGATC
TCAGGAGGGGAGACCTCAACGACAAGCATCTCAGCGAGATCCTCCTGCCTCGGAACACCG
AGTGGGAGGTGACCGGTACGCGCACCGAGGACGGCATCACCGTCTACAAGCTCCGGCCCA
GCACTAAGCCCACGTATGACGAGGTTCCTTCCAAGCGACGCAAGAAGTCATGACCAGCAC
AGCCAATCCTGCTGCTGCACAACTTGATCGCTGGCAGATTTACGACACCGAGATCGAGGT
CACCACCGAGCGCGGCCGAGAGCGCCGCAAGGTCTGGGACCCACACGCTGGTCAGCTCGC
CATTGAGGAAAGCCCTGCGCGTAACAAGGTGGCCTCCTGTGGCCGACGCTTCGGCAAATC
GGACCTGGGTGGCAAGCGGCTGGTTCCGCCTGCATTCCTGGCGTACTACCGACAGGACCT
GCTCCGCACCACCAACAAGGCGATGATCTACTGGATTGTGGGGCCGGAGTACTCGGACGC
CGAGAAGGAGTTCCGCGTTCTGTGGAACACCTTGGTATCATTGGGTGTACCCTTCGACAA
GCCTGGCAGCTACTACGATGCCGTTGGCGGAAACATGCACCTGAGCCTTTGCCAGGGTGC
ATACCAGGTGCATGCCAGGAGCGCTAAGTATCCCGACACGCTCGTGGGTGAGGGCTTGAG
TGGCGTGATCATGGCCGAGGCCGCGAGGCAAAAGCCGTCGGTGTGGACTAAGCATGTGCG
TCCGACGCTGGGAGACTTCACGGGCTGATCGCTTCCTACGTCAACTCCTGAGGGCAAGAA
CCACTTCCACGACAAGTTCCAGATGGGCCAGGATCCTACCAATCCGGAGTGGGAGAGCTG
GCGCATGCCCAGCTGGGCGAATCCGTACGTGTACACGCGCACGGGCCGTCTGGTCGCGAT
GGCCAAGCTTCCTCCCGAGACGCCCAAACCCGACCACGAGTACACGCTCGACCGACACCT
CACCATCATGCAGCAGCTGATGCGAGAGAACCCGACGGTGTCGCCATTCAAAATCGTTGC
GGACCATAAGCTTCGGGTTGACCAAGAGGTGATTGAGCGCGCAGCCGACATGAGCATCGA
GAGCTTCAACCAGGAGATCGGCGCGGACTTCACCGAGTACGTGGGCAAGGTGTTCAAGGA
CTGGGACGAGGAATACCATGTTGCAGACCTGGTTGACTACAACGTCAAGCATTTCGGAAC
CTACTTCAACCCCAATTACGAGACCTATGCAGCTGCGGACTACGGTTACACAAACCCAAA
TGTGTGGCTAGTCATACAGATTGGCAAATGGGGAGAGGTCAACGTCCTCCGCGAGATCTA
CATGCCGGGGCTGAGGGCCGATGCGTTCGCTGATGAGATTCGGCGTCAGAGGCGCAACCC
GCCAAACCTTCGAATCTTCTATCCGGACCCCGCTGACCCTAGGTCGTCAGAGACGTTGAG
CCAAAAGCTCGGCATCCGCTCCGCTGGCGGAACAGGTGGTGAGAACCGCATACGCCTCAA
CCTGATTCGACAGTTCCTGAAGCGTGGGCGTAACGACCCCACCGACGTCACGGGTATGGG
ATGGCGTCCGCAGCTGCTGTTTGATCGGAGCTGTGCGAATGCGCGCCGTGAGATGGAGGC
GTACCGCTACCCTGACAGCAAGGACAAGCCGAATCCAAGCTCGCTGGTCTATGAGGAGCC
GCTCAAGAAGGATGACCATGTGCCCGAGGCGCTGGGTCGATTCTTCGTGGGGCACTACGG
CGAGAAGGCGCTGGTGAACGAGCGTGCCGTAACACGCATTTCCAAGGCCAACATCGAACA
CCAGCGGACAAACCGCGAGCGCCGTAGCACTAACGCGCCCAAGGACATCAAGTACCAGCG
CCTAACGGATGGTGAACCTCCTGCATACCAGTACAAGAATCCCGACGTGGGTTGGAGTAA
AGAGCTGCTCGACTGATTCGGAGTTGGATAAACACCAATGGGCCAGGACAAACGCCAATA
CGGCAGTTCGCAGCAGGTGCGTGCAGGAGAAGCCATTTTCCCCAACGCCGGAACGGATTT

CGACAAGGCGCGGCTGGCCAGTTACCGGCTGTACGTGGACATGTACCTCACCAACACGTC
CGACTACCAGGTGATCTTGCGTGGTGGCGACGAAGGTGACCAGCGGCCCATCTACGTGCC
CAACGGCGAGAAGCTCATCGAGGCGAAGATGCGCTTCCTCGGACAGGGGCTGCAGTGGGA
GTTCTCCAAGAAGGATGCCAAAGTCGACGACGCCATCAGAGCCCTGTTCGACCGGGAGAA
CTGGGAGCAGAAGTTCGAAAGTCTCAAGCGCTGGACCGAAATCCGCGGTGACTATGTGCT
GCTGCTGATCGGCGACGACAAGAAGGACGAGGGATCCAGGCTCTCCTTGCACGAGGTGGA
CCCCAGCACGTATTTCGCGTACGAGGATCCGTGTGACCCTGGCCAGGTGCTGGGCGTGTA
CTTGGTGGATGAGTATCCGCACCCGCATTCCGATAAGAAGAACGAGAAGTGTGCGCGCGT
CCAGAAGTACATGAAGACGCTCGACGACGACGGGAAGCCAGTCCCCGGTGGCGCGATCAA
ATACACAGAGGAGCTGTACGAGCCAGGAAAGTGGGACGACCGTCCCGAGTCACCTCTCGA
GCCCGACGACATCAAGAAGCTGAGCACCCTCACTGAGGAAGAGCCTCTGCCCGAACAGAA
CACGACACTGCCGGTGTGCCATTTCCGCGGACACCCGATTATGAACGCGATGTTCGGACG
CTCTAGCCTGGCGGGGCTCGAGTCGTTGATTGCATCTGTCAACCAGACCATGACGGTCGA
GGATCTGATCATGGTGTTTGGGGGACTGGGCTTCTACGCAACAGGCTCAGCTCCGCCGCG
TGATTCGCGCGGCAGTATGGTGCCGTGGACAATCAGCCCGCTGGGCATGGTCGAGCATGC
CCAGAACAACAAGATCTACCGAGTCAATGGTGTTGCAAGCCTTGAGCCTAGCCAGACTCA
CATGAACAAGGCCGAAGAGGCCATGCAACAAACCAAGGGCATCCCCGACAGCGCTGTAGG
CGTGGTGGACGCTGCGGTCGCCGAGAGCCGCATTGCCCTGGATCTCCAGCTCAGCGCAAT
CCTCAGCAGTTGCGCAGAGCTGGAGCTAGAGCTGAAGTCAGTGCTCAAGCAATTCTTCTA
TAATCTGGTGACGCAGTGGCTTCCGGCGTATGAGGGTGTGGGGATAGACGACGCTGACAA
GAAGCTCACCGTCACCATAACCTTCCGAGATCCCAAGCCGGTCAACAACGAGAAGCGATT
CGGACAACTGTTGGAGTTGTGGGAGGCTGGGCTGATCCCTGCGAAGAAGCTCAGCGAGGA
GCTCAGCAAGATCATGGGCTTCGAGCTGACCGAGGAAGACTTCAGGCAGGCCACCGAGGA
CAAGAAGACCCAGGGCATTGGCCAAGCGGAAGCCGCCGACCCGTTCGGGGCGCAAATGGC
AGCAGAACAGGGTTTCCCCGACGAGGAGGACGACCAAGCCCTGAACGGACAACCGTTGTG
AGGAGCTAGCCATGGCCAAGGGTAGGCGCAGCAGCAAGAAGCGCAAGACATATGCCTGCT
TCAAGTCAAAGAGGTCGTGGCGCTGGGCTTTTGCGACCAAGCAGCCCTGGGCACGCGAGA
AGTCCCACAAGACCAAGGGAGGACCCAAGGGCCGTTACCGTCGACTTCCAGAGAGCAAAC
ACTCTGGTGCAGGCAAGAAGGTCTACAGAAGGAAGAAGCGTTGACGATCGAGCTGTAACG
TCCTGAGGACGAGCAGGTTTGGACACGAGTTCCGCATGAGCTTCCCCAGGACGCCGTGGC
TTGCCTGGTGCATATGTGTCAGCAGAGCCCCAAGGCCGAGATCTGCGGCTTTATCACCGC
GAGCCATGGCATCACCCCGATTCCAAATGTATCATCAACGCCGGAACGCGAATTCGTCAT
GGACCAGGGTCAGATGATGCAGCCGCTGTTTCGAGAGAAGGTGATCGGCACCTACCATTC
CCACCCGACGGGTCGACCCTGGCCAAGCGCTTACGATACTGATCACATTGGTTACCTGTA
CCAGCAGGGCTGCCCGTGGGATTACTACATAGTGACGAATGCAGGAGTGTTCCGATAGGA
GCACAGGGACAGGCGCGTGGCGGACGTCGTTGAATCCAGAGATGATGATAGTTCATGTGA
TGCCGGTGGACGACCTGATTGAGCATGTAGAATCGCCGGACTGCCCGTGCGATCCGGACC
TCAAGCTGATCGAGCGTACCGACGACCATTTTGGACAGATGTACAGCCACAATGCTGCCG
ATGGTCGGAATGATGAGGCTGAGGAAGAGGATCTGTTTTGACCTCTCCACTCGAAGAGCC
GTCAGTATCACCTCAGCGGCCTCGCAAAAACTGGCTCCTGAGATACCTTGGAGTACAGGA
GAAATACGATGCAATCATCTCAACAGCCCTGCTTGACGCTGCGGAAGATGCGGAACAAGG
CATACGCCAAAGGCTTGGCGACGGCCGACCTGGATCCTCGACTCGAAGATATCAACTGCA
GCTCGCGCAGTCTGCTGCACGTGACTCACTCAAGCTGCTTTTCCGAAACGTGCATTCAGC
GATCACAGCGGGTCAATCGGAGGCAGCGGTCGCGGCGGTCGAAGCAGGATTCCACGACGA
TCGCCGCAATCTGAATCGGCTGTTCCCGAACCCCGCTCGGCGCGCCAAGTACGAAGACTC
CATCCGGCAGAGCGCATCCCGTGGCGTTCAGGCCATGATGACGCGTGTGCTACAGAGCAA
CCGACCCCTTAACCAGCGAGTTTACCACAAGCAAGCCCTGGCCAACGGCACGGTCGACCG
AGTGATCAACTCAGCACTGGTGAAGGGTGATGGTGCGGCCGCAATCGCCAAGGCCGTGCG
CCAGAGTATCCGCCCGGACGTGCCTGGCGGAGTCAGCTACGCTGCAATGCGCTTGGGGCG
CACCGAGAGTAACAATGCATTCCATGCTCAGGCGATGAACGACATCAACACCAAGCCATG
GGTGGATCATGTCGAGTGGCACCTGAGCAAAACACACAAGGAGCAGGGCTGTCGGTGCGA
AGTGAATGCTCGCATCAAACGCTTTCCAAAGGAGGCGATTCCAGACAAGCCCCATCCGCA
ATGCCTCTGCTATATCACGGCTTCGCTGACACCCTGGGAGGAATTCGAAAACAACTTTCT
GATGGGAATGTACGACAGCTACATCGACAACAGTTTGCGGTAATAGGGTATGGTAATCCG
CAGGAAGGAACTCTCATGACTGACACATTCCTCGCATGGCTCGCCGCTACCGGCTTGACG
CTGTCTGGCCACGACGACGATGGCGATGGTGACTTCGATGGTTACGACGACGATTCGGAT
GATTCGGACGACGGTGCATCGGATCGAGGATCGTCTCGAAAGTCCGACGACGGTGATGAT

CGCGACAGTGGCGATGACGCCAATGATGCTTACGACGACGATCTTCGAGCAGATCGCAAG
GCGAAGGACAAGAAGTTCGCCAAGCTCGAGCGGACCTCGAAGACGCCCTCGAGATGATC
CAGGCGGCAGAAACCAAGCGCCGCAGGCGAGGTGATGATGACTACGATGACGACGACACC
AAGTACCACGAGCTGCGCGACGAGAACGACAAGCTCCGCGCTCTGCTCAATGGTCCGTAC
ATCCGTTCGCAGATCTCGAGCTTCCGCGGCACGGACGGGAATCCCAAATGGGACAGGCAG
GACCCGGAAACCGCGTTCGCGCTTCTGAAGACGGCAGACCTCGAGGTCGACGTCGAGACG
GGCGAGATCGACGGTCTGGAGGAGCAGCTGCGAGATCTTGCACGGAACAAGCCGTCCCTG
TTGCGGCAGTGCAGCTCTCGTCGTGGCGCTTCGGGTAAAGGTCCCGGTGGCTACAGCGAC
AAGAAGAAACACAGTCGGGAAGACCTCGCGTCCGACTTCTCCGCATTCAACACCATGATC
TGATTGAGAGGTTGACCAATGGCGAGGTATGCCAAGTACAACACCTACGGCGGAGGCTTT
CGAGCCCCGCTGGCAGCCGACTGGACTGACGCCTACGCTGGCAAGCTGTATGCCGTTGGC
ATCAACAACGTCGGGGCTGTCGTGAAGGGCGCAGGCCAGTCCGGCGTGGCTGGCGTGCTG
GTGCTCACCAAGGGGGCCAAGGCCGGATCCATCGTGGACGTCATGAAGGTTGGCGAGCTC
GTCGAATTCGGTCCCACCTCGGGCACTCCCGGCACTGCCTTCGGCGCTGATGGTACGGCG
TACTACGCCGACACCAGCACCGGCGCAATCAACTCCACGTCCGGCGAGGCCAAGGTCAAG
GTCGGACACACCGTCGGTGCTCAGCGACTGATCGTCGCCGTGGCCGATGGCGTTGTCGAA
CCTTCGCCGAACGAGGAGTGAGGCAGCCCGATGAAAACTGCAACAGAAGGAAGGGTCATT
AGGGGGAGTTTCCTCGAGATCATCGCTGCGGTGGGCCTCTCTGGTCATGACAACCAGGGT
GGCTTCAACACTGCCGGTGACATCAAGTACAAGAACGCCGATGGCGTGTCGTACGACAGC
CTGTGGAACCTGTTCAGCAAGGTAACGGACGAGTGGAACAAGCACAAGTCCAAGATGGTT
CAGCTGATGACGTTCCCGGTCATCAACCAGACCGAGAGGGTGCCGCGCATCGGCCAGTTC
GTCTTCGAGAAGGCCTCGGAGTTCGGCGTCCCCGAGAGCAAGCGGACCGAGCTGAGCTTC
TACCAGCTCGCGTACGACTTCGAGGACTACGACCGGGCCTTCCGATACACCTGGAAGTTC
CTGCGTGACGCTCCGTCGTCGCAGATCAAGGCGTACCACAACCAGGGTCTCCAGGCCGAC
GCCAAGCTCATCCACCGCAAGGTGATGGAGGCGATCTTCGACAACCGCCAGCGCGAAGCG
GACATCGAAGGCCTGCCCTACAAGGTCTATCCACTCTACAACGGCGACAACATGATCCCG
CCGGAGTACAACGGCACGACCTACACCACCGCCCATAACCACTACCTGGTCTCCGGTGGG
ACGAAGATCGACTCCGCCCATGTGGAGATGGCTGCCGACCACATCCGTGAGCACGGCTAC
ACGGAGGAAAACGGCACCCAGCTCATCGCCTTCGCACACAAGGCCGAGATCCAGGAAGTC
CGTCGGTTCCGCTTCGGACAGACGAACAACAACTCGGCCGTGGCGAACTCCGACTTCGTG
CAGTCGCAGGGCGAATCGCCCCTGTACCTGCCGAACGCGGACGGCCTGCTCGGCAAGCAG
CCGCAGTCGATGTGGAAGGTCTTGCGCCTCAAGGGATCCTACGACGACGTGCTCTGGATT
GAAGAGCCGACCATGCCGGCAGGCTGCGATCTGTTCGTGGCCACTGGCGGAACGCTCGCC
CAGCAGGACCTCGTCGGCTTGCGCGAGCACGAGGACGCGGCGTGGCGCGGTCTGCGTCAG
ATCCCCGGGAACCAGACCCGGTACCCGCTGATCGACAGCTTCTTCCAGCAGTCGTTCCGT
ACCGGTATCCGTCAGCGTGGTGGTGCACTTGTCCTGCAGGTCAAGGCCTCTGGCACTTAC
GACATCCCGACCAAGTGGACGAACGGTGGTGGATTCGAATGAGCCGTGTAATCCCAGAGG
ACCGTCCGCTGTCCGATGAGGACCGCTCATGGCTCAACAAGCGCAGCATGGGGTCGAAGG
TCGAGCTGATCGACCGCGCCTACCCTCCGGGGTCGGACAAGGTCGAGGATGACACCTCGA
CCAAACCGTCCGAGCCGCAGGGCGATGACGACGCGGAGGTGGAGGTCGACAAGGACTTCT
CGAACTACGTCGGAGACCTCCCCAACAAGGACGCCGTCAAGGAGCGCCTGATCCTGGAGG
AGGTGGAGTTCGAAGACGATGCGAAGCGTGAGGAACTCAATGGCCTCCTCGCGATCCACC
TGCAGGAACAGCGGAACGCCGGCAAGGACGTCGAGCTCGGAGACGGATTGTGGCCTCCGA
AGCAGATGTCCGGCTGTTTAAGACGCAGCTGTCGGGCGTCGATGGTATTTGGGAAGATTC
CGAGATCTCAGATACCATCGACGCCCTCGGGTCTGTCGGCAAGGCAGTGCGTGCTTACTG
GTCCAAGGGGGTCAACACCACATACCTGCTGACCGACGTGAGCGAGAGTGGCAGCTCGCG
TAACGCAACCATCATCTACCGCAATGCCATGGGGCAGTTGCAACGGTGGGATGAGATCAT
CAAGGAAGAGGACGCCAAACAGGAAGCCGAGACCGTCGGTAATGTGAGGTTCAACCGAAT
TTCGAGGAATTTCTCGTCATGACCGTGTCAGCCAGTGCGCAAAGGGGATTGGAATTCCTG
CGTGCTGGCACACGCGCATTCATCGACGATGATCCGACCACGATCGTCCTCAATCGTGGC
CAAGCAACCCGCGTCGAGAAGCCTGGCGGCGGATACGATTTCACCCCAGGGGCTCCACGA
ACTGCACAGATCTTCAAGGTCATCAACCAAACTGGCGACGGGTCGGCCCTAACCGAGGCC
CAAGACAGCATCCAGACGCTGGTACGCGAGTACATCCTGCTGGGGGCTCATGATTCTGTG
GCCGAGGTTGGCGACTGGTGGGTCGACGGCAACAATCGGTACACAGTGACCGAATTGCTC
GTGGCAAATGGCTACGAGCGCAAATGGCGTGTCCGCAGCGTGGGGCTGGAGCCCAATTAT
GGCTAAGATCGAGTTCAAGTTCAAGGACTTCGAGCTACGCCAGGGGGTGGAAGATATGGA
AGCCAAGGTCGACCGCGCCATGAAAGCCACCAGCAATTACCATGCGGTCGAGGGCACGGC

CCACATGAAGGAGCATGCTCCCTGGACTGAGCGGACGGGTGCTGCGCGTGCTGGTTTGCA
CGCTGTTGCCAGCACTCCACAGCCCGACCGCTACGAAATCGTGTTTGCCCACACGGTGCA
CTATGGCATCTGGCTCGAGATCGCAAACAGCGGACGGTAAGAGATCATCATGCCCACGGT
CCATCATGAGGGCAAGCTCATGGCTCAGCGTCTTCGCGGCCTCCTGGGGAGACTCCGATG
AGCAGAGCCGCAGTGCTAGATGCGCTCCGGGCCGATGTGGCGCTCGGGCAAATTCTCGTT
CCGGGCAACATTCTGACGAATTACTCCAAAGAGGGTCCGCCCAACCATTTGGCTCCAGGG
CCATTTGCAGTGATTCGTTGGGGTGGCAAAACGATTGACCCTGCGGTGAATCGCGGACCC
AGAGACGTGAACATCTGGGTTCACATCCCGCAACGTCAAAGCACTGACTACACGCGGATC
GATCGAATCCTCAAGCGCACCAAAGAGATCATGCTGTCTCTGGAAGACGTTGCTGGCGCT
GATGGGGCCCACTTGGTCAGCACGCCCTTCCTGGCGGAGAGCGATGATCTCGTTGACCCG
GGATTCGAAACGATCACGCGCTACGCGACATTCAGCGTGCTCAGCAGAAGCACCTGAAGT
CAAGTACGATAACCAACAGGAAATGATGCTCTCATGGCGAATCAGATCACCCCTCCGCCC
TCGGATGTCAAGCCCGCAGCGGCCTCTGCACCCGAGGCCCAGGATTCGTGCTCGGGCAAG
CGGAAAGATCTCAAGTTCGTTGGCACGAAGAAGATCTCACCTTCGCATCGCGCCGGCAGT
CACTACCACATCCCCGAGGCACAGCTGGTGGTAGCCGGTGTGCCGCGCGAAAGGTGCTTC
AAGTGCAAGGGCTCTGGCAAGGTTGCGCTCGCGGTGACCGAGGTCACCTTCGGCGCCGAA
ACTCAGCACAAGGTGCCGCTCGACGCCTTGTACAAGGAGGCCGCAGACCGGCTGTTACTG
AAGCCGGACATCATACTCGTTGAGGAGTAGGGAACGCGTGAGTGCAATACCGATGCAAGG
GCAAGGTTCACGGGGAGCTGTCGGAAGACGGTCTCCTCGTGTTCATGTGCTCCCACATCG
CTTGCACACGAGGGAAGAAGCGATCGTTCGTCCTTCACTACATGGACCCGAACACCGGCA
CCATCAAATACACCTCGCGACCATACCGGAACCCATTGAAAGGTAAGGAAAATGGCAATT
GACACCGCATTGCCCTATGGTCTGAGGGACGTGAAGATCACACCCTATGCACACGAGGGT
GGCACCGTTCTCGGCGATACGAGCTACGACCTGCCGGACGCACAGACGTTCAGCTTCAAC
GAAACTGAGGAGTTAACCGAGCTTCGTGGCGATGACCGGCTGGTAGCGACGCCCGGCAAT
GGCGCACAGGTCGACTGGTCGCTAGAGGCCGGTGGCATCAGTCTGGTCATCTGGACGATC
CTGACGGGTGGCCAGTTGATCCAGTCCGGGCTGACCCCGAATCGCAAGGAGCTCATGCGG
AAGCGCGGCACCGACGTGCGTCCTTACTTCCGCGTGGACGGCTAGGTCTTGTCGGAGTCC
GGCGGCGACATTGTGGCGCGCGTGTACCGCTGCCGCTGCAATGACAACATCGGCGGTGAC
TTCGGCGACGTTACTTTCCACATCACCAGCTGCTCGGGTGTCGGTCTGCCCCTGCTGGAT
GAGGCGAACGACCTGCTGCACGACATCATCCGTAACGAGACCAAGACGTCTCTGACGCTC
ACCCCTGAGCCCAACCCGCTGCAGTCGCCGCAGAACCTGTCGGCCGGCACGACCACCGCC
ACCAGCGTCGTCCTGTCGTGGACGGAAGTCGTTGGCGCGGACGAGTATCGGGTGCAGAAT
TCTCCGGACGCCGGTGTCACCTGGAGCCTCGTCGACGACGTGGAGGACCCCACCATCACA
GTGAGCGATCCCACCGCCAGCTCGGCTTACCAGTTCCGTGTGGCGTCCAAGGTCGGCAGT
GCGCTGCCGGTGTTCTCGGCTCCTTTGCCGGGCACCACGCCTGCCCAAACCTAACCCAAG
GCTCTCTGGGGCGCAGGGTGTGGCAACTCGACTGCGCCCCAGAGAATTCCATCTAGAGCC
CAAGGGGGCCATGCTATGCCGGAAATCAGCGAGCAAGAGTTCGAAGAGCTCAGACGGATC
AGAGAACAGACTCTGGCACAGGAGAATGCCCGTATGTCTTCAGGAATCGAAGAATAGGCC
AAAAAGGCCGCTCTTGATGCCCTGGCAGAGGCCGAGGAAGACAAGTTCCCCGTCAAGGGC
TGGGGCAAGGATGTCGTCCGTAAGACATTCGACATCCAATGTCCCAGCGGACAGTGGTGC
CACGCCAAGACTTTGACCATCGAGGACGCGATGTCGCTCGGTTTGTTGGACAGCCCTTAT
CTGTTCACCAGTACGCTCATGGCTCCGATCATGGCCAACGATGAGGACAAGGAGCGCGCC
GAAGAAGAGCGGAATGCTGGTCTCCTCAACAGCCTCAAGGCTTCTGGCAAGCGTGCCAGC
GTCTTCGGCACGGTGAACCGTGTGACGACGCATTGCGTGCTGAAGCCGCGCGTGGTCCTC
GAGGACGACGGCAACCTCCCCGAAGGCACTGTCTTCGCCGACGACATCCCGTTCGCTGAC
AAGATGCACATCTTCCGGAATGTGTTCGGGGCAATGGGGAATTCCACGATGAACACGTTT
CGCGAAGGACCGGAAGGCAGCGTGGCGGCTGTACCAGATGGCGCTCCTGTACAGGACGCG
TCCGAGTGACCTAGTCGGTTTGGAGGATCCGTTGCTGCGATACTCCTCTGATCGAGGGCT
GCGGATCTTCGGCGTAACGGTGCAGAACAGGGTCGATGATGCTGGCCATGCTGCGGGCCG
TGGCGCCAAGAACAACCGAAATAGGGAAGCGCTTCCAAACGGTGCGCGCAAGCGCACCTT
GGCCAAGCTGCTGGGCGAAGACATGGTGAGTAGCTAAGCTGGCTTCCGAGATCCGGCGAA
GGCGGTGTGACGTGCCAGATTACGACCTTGGGCGTGCCCATGGCAAGATCGTCTTCGACG
TTGACAAGAGTGGTGTTCGACGCGCCGAAGGCGCGATGGACCGCATGGAGGAGTCGGCCC
ACAGGGTAGCCGAAGGATTCGACGAGGCTGAGAAGTCAACCTCTCCTTGGGAGAAGACCA
ATCGCAAGGCTGTGGGCGCCACTGAGGCTCAGGCGCGAGCGGTCGATCGGTACAGCCGCA
TTCAGGAGGAAGCCGAGCGCGCTGCCATTTAGCGTCGCAAGGCCGAGAAGGAGCTCGAGG
AAATCCTCGATGCCGGTAAGGCTACCGAGGAGGAGACCCAGAAGGCCTTCGAGAAGACCA

ACAAGGCCAAGGGCAAAGCGCTACAGCTCGCCAAGGAGCGCAAGAAGGCTGAGCGCGATG
TCGAGCGAGCTGCAGCTGGCTTGCCCACCGAGCACGAGACCAAGCTCAAGATCAACACCA
AGGACGCCGATCGGACGTCGATCGTTTCTTCGACAATGTTGATCGTCGTGCTGAACGTA
GTGCGCTCGTCATCGCCAAGCTGGGCACAGGAACCATCAAGGCTGGTGCCGCGACGGCTG
GCGTAGGTGCGCTGGGCGGGTTGCTGGGTGTAGCGGGGTCGGGTGCTACTGGCGTCGCAA
GCATCGGCATCTTTCAGCTTGGCGCGGCGATCGCTGATCTGTCTGGTGCACTGGGTGTTC
TGCCAGGCGTCGTGGGTGCAGGTGCCATCGCGATGGGCACGATGCAGCTTGCAACCAACC
GATTCGGCGAGGCTCTTAAGGACCTCGGAACCGACGACTTCGCCGACGCGATCAAGGAGC
TAGCTCCGGCTGCACAGTATGTGGCCGTTGAGCTCAACACGCTTCTGCCCGTGTTTAAGA
ACTTCCAGAAGAACGCCGAACAGGCGCTCTTCGTGCCGCTCAAGGGTGTGACCACTGAGC
TGACGACGACCTTCCTGCCGACGATCCAGGGTGTAACTAACCAGATCGCCAACATGTTCG
GTCGCACCGGCGCTCGCCTGGCGGAATGGCACATGAAGCCAGAACAGCAGCGCGACATCC
AGAACTTCCTGTCGAACACCGTCGAGGGCTTCGAGCGTCTGTCGGATGCTGCTCAGCCAC
TGCTGCAGGCATTCACCGACATCATGACGGTAAGCGGCGAGTTCCTGCCCCAGATCGGTG
GTGAGATCGCTGAGGCTGCAAAGGAATTCGCTGGCTGGATCCGCACCATGCGGGAGAATG
GCGGACCCGCCGCGTGGATCCGCGATGGCATCGAGGAGCTGAAGACCTTCCTTGGTGCGC
TCAAGAACTTTGGGCTGGGCCTGGTGTACAACGGCCGCATCGCCAACGAGTTCGGCAGCG
GTTTCATCGCTCTGTTCGAGAAACTTAGCCAGACTTTCCTTTCCTGGACGCAAAGTGTTG
AGGGACAGGAAACCCTCACCAAGTTCTTCATCGCGACGAGTGAGGCCGCGCGTGTGTTCA
TGCCGCTGCTTGAGAAGCTGGGCACGCTGTTGTTCGGCACCATCGGAGCAGGATTGGCGC
GCTTGGGAATTGAGCTCGCTCCGGCGTTCAACTTCCTGTTCGAGACGCTGAATGTCGGCT
TCCATGATCTGATCCAGGTGCTGATCAACAGCGCACCCCAGATCACCCAGTTCCTCACGA
CTCTGGGAAGTCTTTTCGTAGACATCATCAGACAAGTCGCACCGCACTTGCAGGCATTGC
TCGGGACTATTGTAGATTTGTTCTACAAGCTCGGGCTGGCCATTAGTCCGCTAGCGAGCG
CGCTGGTTCAGGGTGTCAACCTGATCATCAACGCTTTCAATGATAAGTGGGACACAATCG
CCAACAGCCTCAATGGCTTTGGGGCTTCTGTTGCGAAGGCGATTGAGACTGGTGATTGGT
CTGATGTCGTTGATCGCATCACGAACCTGTTCCAGCCCTTGGGCGATGCTGCGATGCAGG
CTCTGCTCGACGCTATCAATCGTTGGACCGAGGATATTGCGGAAGCCGGTCACAAAGTCA
TAATCGCCTTGGCCGACGAGATCTCCCCCTGGATCGAGATCAATGCCCCGACATGGGGCG
AGAAGCTCGTCAAGGGGCTGCTCGACGGCATGGTCGGAGCTATCCCGGTCATCGGACCGG
TGGCCAAGAAGGTCCTTGGGGCGCTGAGCGATTGTTTCCCAAGTTCTCCGGCCAAGAAGG
GGCCGTTTTCGGGTACCGGCTGGACGCCGTACCGTGGCAAGGCGTTGATGGAGGGCTTCG
CCGAAGGCATCCTGGCTGGCGGAGCAAGCGCACAGAAGGCCGTGGGATCGGCCGTGGGAG
GCGCGAGCGCCGGGATGAACTCCGGCCTCAGCGACCTCGTCAGGGACCTCACCGAGCTCA
CGAGCTTTGGTCGGAGTCTGCCCAGCTTTGTCCAGGGCATCACCGACGTCGTGTTCCAGA
CGATTAAGTTCGCCACCACAGACTTCAAGACGGGCGAGAGCACCCTGCCAAAGCGCTTCG
TGCGCAATGTGAGCGATGAAGAGCTCGCCAAGCGTCGTGAGGACAAGGCGTATCGCGACA
ACCTCAAGGAGCAGAAGGAGAGCGGCACCGGTCCCACGGGCGCTCTGTCGCCGGAACTTC
AGCGCCTGCTTGAGCAGTCGAATCCTGGTGGAACAGGTGAAGCCGCAGTTCCGTTGATCC
AGTAGGAAGACGGCACCAGGACTTCTCCGAACAAGGAGTGGGCCAAGCTCATTCAGCGTG
AGTCGAGTGGCAACGCCACAATCACCCAAGGCATTCAGGGCGCCAACTCCGGCGGGAATG
AGGCCGAGGGGCTGTTCCAGATCACGCCGAGCACTTGGAAGGCGTACGGGGCCACAGACT
TTGCTCCGAACGCGAAGGCCGCCACTCCGCAGCAGCAGGCGATCATCGTTGCGCGGATCA
TCCAAAAGAATCCGAGCGGGTCTGACTGGGGTGCGAAACTTCCGGAGCGTGAGGACCCCG
AAGAGCTGCTGAGAGGCTTGACGACTGCCAGCGCAACTAGCACGACGAGCAATCCATCGG
CCAAGTCGAAGCCCAAGGCTGCAGGCAGCGAGGAAACGCTCAAGCCTGCGACTGGCCTCC
AGATGGAGGAGTTCGGAGACGGTTTCTTCAAGGACAAGAAGACCGAGGCCGTCTATCGAG
AGATCGATGGATCTTATTACAACGTCACCGATTTGGCCGTCGGCAAGGACGGCAAGCCGC
TCCTCAACGAGGACGGCAAGCCCATCGCCCGCACGAGCGCCAACAAGAGCGCGTCTCGGC
CATCTGCGCCAAAGGCACGGGAGCCGTATGGCCTCAAGCCTGGCACCGATACGCATGGCT
ACGGGAATGGCAATGCCGATGTGTTCCCGGAGTGGGTCATGGATCTAGACGATCAGTTCG
GCATCAAGGCCAGCACCTACAAGGGTCATCAGGAGTCTGACCGCAACGAGCCTGGCTATG
CACCAAATCCGCAGCGCCTCAACCGAGGGGTTGACTGGGTCGGTCCGACTAAGAACCTGC
AGGCGTTCGCCGAATATCTCGAGACGATACCGGACGCCCTGGAGCAGGAGATCTGGAAGA
ACCCCGACACCGGCCGCAAGACCGGTACGGCTGGCGGAAAGATCAACCCTGGATACTATC
CGCAGGACACCTATGATGTCCACGGCGGCAATGATCCTAGCAACGTCCACGTTCACACCC
GGCAGTCGATGTCGATCCCGTTGCCGGGCGAGGATTAGGTGCCTGGTGAGACGCCGACCA

GCACGGGTGATTCGATCCTGGACGAACTCCAGAAGATCACGGCGAACACCGAGGAAAGCA
GCAGCACCGAGGACAAGACGCTGCGCCAATTCCTCGATCAGAATCCGATCCTGAACGAAC
TCGTCACGGCCGCACAGGATCCCAGCAGCTCGGATGAACAGGTACAGGCTGCCCTGGGCG
GGCTGCAGAAAGCCATCGACGCTCTAAACCAAATGGACACGCCTGCCAGTCGGTATCTTG
CCGATCAGCTGAGTGGCATGCAGAGCCAGATCTTGACAGATCGTGGCTTCGGCGTGGCCG
ACAATCCTGTTGATGCGGCTCAGGCCATCGCCAATGGGGCGATGGGCCTGGTTGCCGACG
TCTTCAAGATCATCGACGACACGCTCAAGTCCATTGAGTCGGCAAGCGAGATCAGCTCCA
CTCTTGTTCGCGGTATTGAGAATACCGAGGACATCATGAAGATCATCGACAACGTACAGA
GCTTCATCCAGCTGGCGGAACCATCGCCCAGACGGAGACAGACGGCCCGAACTTCGCGA
GCTCGATCGCGAGCACTGCCGGCGGAGCACCCGGTGGCCAGGGTGCCGTGGGCGGGCTTC
AGGCGGCAGCGGCGATCTCGGGAGTCATCACGTCGGTGATCCAATCAGTCAATGCCGCAA
TTGATCTGGGCCAGGAAGCGTACCGAATCACCAGCAAATACTACGGCAAGTTCCTCAGCT
ACCTGCTGGGAGCTGGCGAAGGTTCTCTGATGGGCGACATCAGATTCCATCTGGTCAAGA
ACGATTGGACGCTCAAGGCGTGGTCCGAGGACAATCCCGTGGACAAGCGCAGCCATCAGG
TTCCGGCCTGGCTGCGGGATGAGCGGAGCGTCGCGGAGCAGGGCGGCAAGATCCGAGACC
TCAACATGTACATCGGGCCTGGCACAGACGCGAATGAGGCCATGAACGAGGGCTCGTGGC
GCGTGATGACTGACCAAGGAGGGGTGTTCACCAGGGAGTATTAGTCTCGAACCTGGCCAG
TACCAGATCGGAGATCTGGTGTTCGTCCGTGGCACCATGTATCCTGTGACAAGCATTAAG
ATCCAGAGCTCCCAAGTGCAGGCACAGGATTTCCAGGTGATTCGCAGCGACGAGGTGCAA
TTCGGGCAGGGCAGCCTGCTGCCCGGCCCAATCGTGTTCGAGCTCGGTGTGTATGATTTC
AAACCAATCGCCAATGTGGAGCCGTACACAGGCCGTGTCTTCAACGAGGAGCCCTTCAAG
AAGCTCCTCAACAAGGTCGCAGCCGAGTGGCGGTGTGACGACGTCCGCCAGAACTGGGGT
GCGATGAAGGCGCTGAAATGCTGCGAGCGTGATGGTAAGGTACTTGTGTGGTACGGACGT
CCCGGCAAGTTTCAGACCACCAAGCGATCACGGAAATCGTCCTTCTTCACGGTGACTGCA
GAGTTTCGTAGTGCAGACACGCTCATGTACTCCGACAAGGAGTCTGGCGACAAGGTGCTC
AAGGGTGATGTCCCGACAATCATCAATCGGGCTGACGGCGAGGCCAAGAGATGGTTGCGC
GTGGTGGGCTATGGGCCGCTGACGCACCCGGTCATCACCATCGGAGACCAGCAGGTCGAG
CATGACGTTGATTTGGCAGATGGAGAACCCTTCGAGGTCAGCTCGTATCCGTGGGAGCGT
CGTGCTATCAACAATTCTGGACAGAACATCAGTTCGAAGCTGATCGGCGACTCCCAGTAC
CTCGACCGTCTGCAACTCCTTCCAGCCGTAGTTCCGACGCCGGTACGGTGGACCTCCGAC
GAGATCAACACATGGGTCCGCGAGCTGGGCAATCAGTCCTGGATGGAGACGATCGAAGGG
GTGAACAACTTCAATTTTCCCAACACCTTCAAGACGATCCATGGCCGGGTGGTCCTGCGA
TTTGACCTGTTCAATCCGGAGTTCGACGAGAAGTTCATCGGCTCGGGCATGCTTGGCGGA
ATCAGCGCCTGCCTGTACACTAAGAAGACCTTCGCCACGCGGAGCCAGTACTGCGAAGCC
AAAATCGTTGAGCCGTGGGGTGGTCGGAGCGGCATCGTCATCATGAGCAATGCCACGATG
ACCAACTTCGCACTGCTCGAGGTGACTTCAGGACCCAACAACGACTACCTCTAGATCCGT
ACTGGCAGCGCCTACAACACATACTCGAACGTCCGGAACAGGTGGAAGAATCCGAATAGC
TGGAAAGTGACGGACGTGGTGGGAATCGGCTCCTCGTACGATGAGGACACCGAGAGCACC
ACCTACACAACGTTTTTCAATGGCGATCCCGTCGCTGAGTGGTCCGACGAGGAAAATGTC
GTCAGCACAGCTGCGACCAATCGGTCACAGGGATTCATCTTCGACATGGACAGTCAGCTT
CTGACCATCGGCACAGGGTTCAAGGACATTCTGGCTTACTACACTGGGACGGTGCAGGCT
CCTACCGGCAGAGTGTTCGTGCTCTGGCGTGATGCATACACGGTGGCCTCATGAAAGCCC
ATGACCGCAACCGCTTCATCATCGAGGAAGCGCGCACGGGCCGAATCCTCACCCGCGATC
TTCACGTACAGAATCCGAAGATCCTCAAGAAGCTCAGTGGCCCTTGCGTTATTGAGTTTG
ATGTCGATCACCGACACCCCTCGGTCCAACACCCCGACGGCACAGGCCCCATCCTGTTCA
AGCCCTGGGGCCACTGGTGCCATGTTGAGCGCGAGATTCGGGGCAAGCGCGTCATCATCG
CCTCAGCCCTATTCCAGCCCAGAGAGGTCGACCAGGAGACAGACCTGCTGAAGGCGCAGT
GGCAGGGCTTCTCGGGATATCCCAAAGACCTTCCCTGGTTACAGAACTGGAATCCGATCG
CGGTCGATCCATTCGAGATCGTGCACCGCATCTGGACGCACCTCCAGAGCTATAGCAATG
GCAATCTCGGCGTACAGGTGTACTCTCCTGGCCCTGGCGGAACACGCGTCACTCCACCAA
ACAGCAACACCGAGTTGCTTCCTGGATTCTCCTTCGATAGCCAGCAGTTCATCCAAGACT
TCTTCGCCATCTTCATTCGGCGCGTTGAGTTCACCGACTGCGGCGATTACATCAACAAGC
TGGCTCGCGACATTCAGTTCGACTACTTCGAGGAGTCGGAATGGAACGAGGATCACACAG
CCATCGACAAATTCATTCGGCTCGAGTATCCGCACCGGGGTGTATTCCAGGATAATCTGA
GCTTCCGGATGAACGAGAATGTGATCCGGGGCAAGAGATAGCTCGAGACAGAGATTGAGT
GGACCAGCGACATTGGCATTCGCGTCTGGTTCCCCGGCAAGGTCTACAGCTCTCAGATCA
GCAACGCCGACCCAAACCGCTACAGACGCTTCATCCTTGAAGAAGATGCTCCCAACAACA

GCACCGAGCGGTCTGAAGCCTGGGCGCATCGGCAGCTGACCAGACGTCAATTCCCCTCGT
ACTGGGAAACAATCACCATTAATTTGCATCGTCCGAATGCTCCGTTCGGTACGTGGGATG
TCAGCGACCAGATCAGAGTACAGGGGCTGATGCCGTGGGGCGGATATGTTGACCAGACGC
ACAAGATCATTGCGTGGTCCCTTGACGAAACGTCGGCGACATTCGAATTGACACTTCGAG
CTGAAGGCGCATTCAACTACGATCCGATCTTCTTCGAAGGCAAGCTTCCGAACCTTCTGG
CGAATCCGAGTTTCACCGCCAGCATGTTCAACTGGACTGCCGCTCTGGGCAGCTGGTCGC
GTGAAAGCCTGCGAGGCATGGATTCTCGCGGAGCGGCTCGTGTCACGTGTAACGGCCAAG
AGAAGCTGCTCCTGAGCGAAGCCGTACCAGTTGCGGCAGGCGATGATGTAACGCTGAGCA
CGTGGGTGTATTGGCAGAATCTCGTGGCCACCGGCGAGCCGCTCGAGGTGATCCTCAACA
CCTACAACTCGGATGGCGAACTGATCAACACCGTTGTGTTCGATAGCACATCGGGCTCTG
GAGAGTCGCCGGGCTGGGTACAGCTCAGCAACGCATATACGATTCCCTCAGGAGTCGCGT
CGATGCGGGCTGGTGCACGAGTCACCGAGGACGCCATGACCGGGTCTGTATGGTATGACG
AGTTCTACTTCACGAACGACGACTGATGGCCTACGAGTGCGAGAGCAAGGAATCTCGGGC
TCTGCGGTCTATCGAGAGCAAGAACTACCCTGACACCAACAAGGATTTCGTCAAGAATCT
GAGTCGGCTCAACAGTTCTGTTGATTACATCTCGTCCTACATGATCATCATGCAAAAGGG
CATTGATGACGCCAATAAGAACTTCATCGAGCAGATCCAAAGTTTCATCAATGATCTAAT
CGTTCTGTTTGCTGGCGGAGAGCCCACGGGCATCGAGCTCGGCGACCTCAAGTACATCAT
CCAGGCTCTGGGCGCGCTGTTCGGCTTCAACGGGCCATTCCCGATGAGCTTGATCAACGC
GGTGCAGCACTTCTTCCTCGGGTATGTTGTTTCGCTGCCACAGTTTACAGACGTCATCAC
CGACACCATCATCGCCTGGGCCGAAGAGCGCGGGCTGGACCCAGAGTTCGTTGATGCGCT
GCGCGAGCGCGCCGACGCCATCAATGATCTTGGCATGAGCATTGGCGATTTGTTCACCAA
TATCATGAGTATCTTCAACATCTTTCAGATTCCAGAGCTCGGTACAGGACCTGTCGCCGA
ACCATGGGATCTGCTGGCAGGCATTTTCGATGGAACCAACGGGAATGCGCTGCGGCCGGT
ATTCCTCGCCATCAGCAACTGGACCATTCCGTTCATCGAAGGTCTCGCTTCACTGGTCAA
CTATCTGGACGACCTGGTCGACGGCCTTGCCAACGGAAAGTTCCTCAACATAAACTCACC
GCTGAATGCGGCAAATCTATTCGGTGCTATCGGATCTAATCTTATCAAGGTCGTTCCTGC
TGGTGCACTGACCAATCGTCGCGTTGAGCTAATCGACAATCCGCACTTCACCGAGGATGC
CATCGCTCCGGGCAACGAGTGGGCCGTGGACATGCTGTCGTCCCGCACCGACAATGATGG
CACAGGTAGCGCGACGGTGATCGCCAACGGTCAGCCCCACGCACTCAACACCGGGGCAAA
CAAGGACGACAAGATTCCTTTCGGCCCCGGACAGCGTATTCCTTTGGGCATCTACATAAG
TCATGACGGCGCTGTCGCTACAGGACCGGCGATCATTCTGCAGGTTCGGCGATTCAATGG
CAACATCCCTATCGTCGGCCTGTACGAAATTGGATCGTACAATCCTCCTGTGGAAGACCT
GCCGTGGCCTGGCCACAAGCTCGAGGGTGTCTACACAGTCGAGGAAGATGTCACCCACAT
CCAGGTGCGCGTGTACGTCACCGAAGCCGCCCTGGCGGGTCGGTATCGCCTTGACGATGT
GGATTTCGCCAAGGTCACCGACCTTGCCTTGATCCCTAGTCTGACACAAGCCTTGGAGGC
GCTCGAGTCTGGCCAGCGTGCTCTGCAGCACGCAATCGCCAACGCGCTCAGCAACATCCC
GATCATCGGCGATGGGCTCGAGGATCTGATCAATGCGCTACAGAACTTCAATCCGGCCAA
TATCGCTGGAGCTCTGGGCAGTTCACTGCTGAAGGGCGAACTGTTCGGGATCATCCGACA
CATTATCGCTGCGGCGCGTGGTGTTCCGGTTTCGGCGGTAGCCGAGGATGCCACGCTCGC
AGACCTGTACTACGCGATGAATGCGGCGCTCAACAATGTCGCCGGATCCGACGAGACAAC
CTTCGTCGATACCACGACATTCGTTCCGGTGAGTGCGGCTGGTACCCACTACATTGATGT
CATCGGCGTGGGCAAGGGCAAGGACGGCATGGACGGCCCGACGGTGCCGCCGATCCCCGG
TGTGTTCGGACAGGGTGGGCACCTGGGAAAGGTGCTCGCCACCACATGGGTCAAAGCTGT
CCACTACGACAATGATCTCACCGGCGTCCAGGTCACCTTCAACAGCAATGGCTCTGTGAC
CTTCTCGATTCCCGGTTACAGCCTCACCTGTCCTCCTGCAAGCGGACCTACGCGCCAATT
TCAATTCGGAACAGGCTGGCACGGCAAGGGTCCGGGCACATATGTCTACAACGACCGCCC
ATACGCTGCTGGCGGAACACAAAACCACCCTGGAGGCAACGGAATTGGACCGCGTGGCTC
TGGCGCTGGTGGTCGCGTGCTTCAGAGCGGTGGCCGAGGCGGACCTGCCGGCGGATGGGT
GCGTCGGCGTGCTGCTGAAGCAGAGAATCCGTCTACTGGATCAGACACGACCCCTCCGAC
CCCTCCGAAAGTTAGCGTTGTCAATGCGACGAATGACACTCTGTATCTGCTCCCGAGCGG
AAGCGTGGACCAGTAATGGCTAATCATGTCAGCGGCTTGGCCGGATATCTGTTCTACCTT
GAGGACTCAACGGCACCCCTCAACCCAGAGCAGTTTCTGCCCAGCTCTGGTCTGCCGTAT
CCCATACGAGGCCTTGACGCAAACACTGACTATAGCAATCGCATCTACGCCACGGCGATC
GACAATGCTGACAATGAGACCGACCCCATCTCGCTGGAGGAAGCTCTCGACGAGCTGAGC
CCATTCCCCGCCAGCACGCTCAGTCCCACGCCAGAACAATCGCCCATTAACTCAACTCGC
ACCACTGCCATCGACAACATCATCAAGCGATGCATCGCGGCTGGTTCGGGTCTTGGTGTG
ACGGTGGGGATTACGAGCCCGTGGGGATACTACCTTGGCAGCTTCGGTGAGGGCACCGGC

GTCGACCGACACTTCCTCATGGGCAGCCAAACCAAGAGCTTCCTCATCTGGGCTGTGATG
ATGGCCATTGACGAAGGCCGTCTCTCCCTCGACACAACAATCAACGACATTCTCGACACG
CCGTGGAGCACCAATCCGACCATTCAGCAGCTGATGATGATGCGGTCGGGTATGTACAAC
TACCAATCGGACTAGGCATTCGGTCTGCAGGTGGCGATGAACCCTGCGATGTCGTACAGC
CTGCAGCAAATGATCGCCAAGAGTCGCAACGGCGCCAAGCACTTTGAGCCTGGCGAGAAG
TATGACTACAACAACGGAAACTCGGTCGCTCTAGCATTGATGCTCGAGGAAACGGACCCC
AAAGGCCGCAAGATCCAGGAAATCATCAAGCAGGACATCCTTGATCCGCTGGATCTCGTC
AACACGAATTTCCCTACTGTCACCAGAGGACCTCCGGCACCTTCTGCCATCCTGTTCTCG
TGGAACTTCTTGTTGTCGCTCATCGGCATCAAGCTCCGCCAGAACGTGACCATGCAGACC
CCCAACATCACATGGGCGGCTGGCTGTATGACCAGTGTCGTTGGCGACATGCTGAAGTGG
GGGAAGGAGCTTCGCGACTGCACGCTGCTGTCACCAGAATCAGCCGATCTGATCCACACA
ACTCACATCATGTATCCCAGTGCACCCTATGGCCTCAACAAGGACGGTGCAACAGAATTC
GGATACGGATTGCACGGCCATATCAAAGTTGGCTCCTGGCGGGGATCCCACCGCAGTTGG
ATCGGCTGCGACTCTGCGACAATGTACGAGCCCACCACCGGCACCATCATCACGGTGTAC
GAGAATTTTCAGACACCGGGATTGCTTTCCTTGGCCACAGTTTGGCACGAATGCGCGGAG
TATCTGATGCCGGGGTCGGCGACGTATCCGGGCTACCAGACCGGCGAGAATGCGGCAGGT
ACGGTCGGCACATCGGTGAAGAAGCTTGGCACGTTGTCCGAGGGTGCGGTGTTTGCTCCT
GGCACCGAAGGCGTTGAATTCGACAATGCGAGTGCGGTCAGCACGAATCGGGACGAAACC
GCAGCATTCAACATCGCGCCCGATGCAAAGGCATTGGTGGCCTGGGTTTCTGCACAAGGT
AGCACGGACTGGGGAGGCGCAGCCGTCAAGGCCAAGATTGATGGCATTGAAATGGAGAAG
GTTGTGTCGACAACCGATGGCACCACCTCCGGGAATGGCCTGGCATGCTTCCGCCTCCTC
AATCCGCCGACCGGGGCGAACAAGAAGGTGACATTCGAAGGGATGGGATCTCTTGCACAC
TATTATGCCAATGGCGCAGCCTCGTACAAGAATGTCGGGTCCTTCGGAGCGCCTGTGAAG
AACAGGGGTTCGGGGTCGTCTGCCAGCCTTACTGTCGGCGGCACAACATCTTTGGATATG
GTTGTGGGCGGATACTATTTCCAGGGTGCGCACAACTCCTTCAACAAGAATGTTCGTGGT
GTCAGAGGTCAGGCAGCATTCGTCAACCAGGGCCTGTTGTCCGGAGACTCTTCCGCTGGC
GGAGACGTCACCTTGACCGAGACATTCACGAGCTCGATTCAGTGGTCAGGGACGGCGTTC
GTTCTTCGGGCTGGGGAGGGCTGATGGCTGTCGCATGGAAGGCTGAGAGCTTCTCGATAA
TAGAGACCGAGCTTAACCTGCTCGTCAATGAATCTGTCGGTGACCAGAGCCAGTATGGCC
TTGCTACCGTTGAACTCCAGAAGCTTCGAACTGAAATGGAGGCAATCGCTTACGACTATC
TAGTGTCGTCGGCGACTGAGTTCAAGAAACTCTCCACGAGCGCCTCTGCTGGACAAGTGT
ATTTCGCCAACATCCAGACAGCCGTGAAGAAGCTCGTGACCCAAGCGCTTGCCGGAGTCA
CAGCTCCGGTAACATGGCAGGGCACAGGCCCGCTCCCGACAGGCGTCGGCATCCCTGTCC
AGAACAACTGGACAGCATCGGCTGGAACCGATGTGTACATCGCAGCCGTCATGGACCGCT
CGGGCAGGTCCACCGGCGCGACATTCGGTTGCGAAGCGGGCACTCTGATGGCAGAAGTGT
TGCACAACAACAACGCTAGCACAGGACGTACGGCGTTGTGGCGTTTCGCAGGCAAGGGCG
ACGGCACCAACAAGACCTTCTCCATCACAGGTAGCGGCTATGGTTGGTTCGGTGTGTGCA
TCTTTGCCTTCTCTGGCGTTGTGTCGGTCGGAACACCCGTGACCACGTACGGATCGGGCA
CTTCGCCTTCGCAGGTGGTCACGGAACCAGGGGTGCAGATACTCGGACGAGGCGCTGGCG
GAGCTGGTGTCAGCACACCGAGTGCCTTCTCTGGTGTGACCAACAGACTCAACCAGAACA
TCAACGGTTCGAGCCTCGCAGTCAATCAAGTTGAGGTGCCAGGAACAAAAGGGGCAACCA
CGCAGAATTACCCTTATGGGTACATCTTCCTGCCCCTCCGGAATACATAGGAGTGCGAGT
TGAATACAGAGTTGCAGGCGATCAAGGACGCAATAAATACCCTTGACTTCGATACTGCGC
GTGATCTGGCCAAGTTGTATGTCGAGAACCATCCGGAGGAGTTCGTCGACTATTACCAGA
GTGTGGAGGAGTCGGCCAACGAGGAAGAAGCGATCGCCAAGGTCGTGAAGTCCATTGAGG
TGTTGCGAGCTGCAGGCCTCACCCAGCAGCAGTATCGTGGTGAGACCTTCCATCTGTTCC
GCTGGGAGCCGCAGACCATTGGCGCCACAACGGAACCTGTGGTGCGCAATCCAATCCCGC
CGACTGTGAGGAATGACTGAACACGGCGAAGGCATATTCTGTCACGCTGGATGTGGCCCA
GGGAATGCTCGGTGGTAGCACCAGCTACGCCGAAGCTCTTGGGTCCTCGCCAAAGATCAA
GATTTACTCAGGCACTCCACCGGTCAATGCTGGGGCTGCGCTGAGCGGCAACCCGCTACT
GGCCACTCTCACATGCGCAAGCACGCCTATCGCCAGCCAGAGCGATACTGGCACTGCCGC
GCGTGCGACGTGGGCCACGTTCGCGTCTGCGGTGGCCGCAGCCGCAGGTACGGCCACCTT
CTTCCGCACCACCACGTCGTCCGACACAGTGAGCGACCAGGGTTCCATCGATACTACTGG
GGCAGTCCTCAACATGTCGACGACGTCATTGACCGCTGGGGCCACCATCGCAATGAGCTC
GAGGACAACAGACTTTCCTTACGGCCCATAGGAGACAGCATGGCATTCATCCAAAAGCAG
GGCTACTGGAAATCCGAGAATGGATGGCGCATGCGTGACACTGCCGAATTGGACTACAGG
GCTGTGCCGGGGACCAGCTTCAAGCTCGGCGTTCTCAAGGGATCACCCAACATCATTCTC

AAAGCACTGATCTGGCGTCTTGACAAGATCGAACCCATGATCACGTAGCAAATCGGTTGC
TACACAGCCGAAAACTCGATGGCGAACAGCAACCACAACTCTGCGACGGCGATCGACTAC
AACTGGAACAAGCACCCCTACCAGAAATGGGGGACATGGCCAAATCGTGCGGCCGTCGAT
AAGATCGTCGACGATTTCCGCGGAATCATCGAATTCGGGGGTGACTGGACCTCGCCGCGT
GACGAGATTCACTTTGAGCTGCACTTCGCCGAGGGGCATGCTGGCACCGAGGCGCTCGCC
AGGGATTTGGCCAATGGCCTATGGGTTATCTGGAAGCCCGGAGCAGCCCCAGCCCCCACC
CCCGCGCCTGGCGGAGGGAACGACGGCATTCTGCGGATCGGCTCCGAGGGGCCGGAGGTG
CTCAAGATGCAGCGCGGTATGAACTCTGTGTTCAAGAATTACCGCGCCATGCCGCTGTTG
GAAGACGGCATCTTCGGGGCCGAGACCAAGGAAGCTGTCGTCGAGTTCCAGCAGCGTGCA
CTCATTGATGTGGACGGTGAAGTTGGCCCGCAGACCAAGGCGAAGCTCGCCGAGTACGGC
ATCGTGCTGACCGGAGCGACTGCTCCGACCGCTCCGACAGCCATCGTGCCGCAGAAGGTC
TGGCCTCAGACCGCCGGTGACCGTGAGCTTCTCGAGTACATTGCGGCGCAGCTCGGGCCT
GGCGACCCGTCGTGGCCGATCAAGCTCGGCAACAACGAGAAGGGCGATCCGCTCACGGTT
CGCGATCGACTTGGCTTGACGACCAAGGACATTGAAGAGATCAAGGGAAGGCTGTCGCAG
TGAAGAATCTTTCGAATTACTGGAAGGCTGGCATTGCGCTGGTGGGTACGGCTGGCACAG
CCGTGGCTACCCTCGCAGCTGACGAGAATGTTCGCACCGCCGTGGGGGAATCCGGTGTCA
CCTGGCTGGCTGTTGCCGGTGTCGCCCTCACGACCGCGCTGACCTGGCTCAAGCGTAACG
AGCCCACGGTAACTGAAGCAGAAGAGATCCTGCGCCGTGCAAAGGAACGCGCATCCTCGT
CCTCCTCTGCATAGGAGGATGGGCGCAGTGGCTATGGTGCTGCTACTCGTATTGACCTGT
GTGTCGTGTCCGCTGGCTGAGTGCCCAGCCGACGGCAATGACCAGCACCATAGCCAGCTG
TGCAGGGATGAGGAATACGCCAAAAAGCATACGGTCGAGTGTGGACGGGTGCAGAGTGGC
CCCATATCAATCACGCCTCCGACTGGCGGACACGGTGGGGGAGCTCCGCGCCGAGGTTTG
ATCGGGAGGATCCTGGACAAAGTAGGGCTGGGAGGGCTCCTCTGATGTTCCGTCCAACAG
ACTCACGACGCTTGCGCAGCATATTCCGCAAGCCTCGTCGGATTGACCACGGCAGAATGT
ACGTGTCGTGTATGATGGCGCTCTAGTTGTGGTCTCTGTTATTGCTGGTGTTCGGACCTG
TTCGGAACTCAACCATCGACGAGCTCATAGACTATGTCCAAAACATCCTTGCTTCATGTA
TCTTCATCGGCTCGTTCGTGTGTCTGTGCGGCATCGCCCTCGGCACTTAGTACGTCCACC
CCAAAGCGGATATCCGCTTGTGATACCGCTTCTCGTTGTGGGGAATCCCCGCCTTGGCAG
GGAGCGTCGGAACGTACGCATGGGCCATCGCCCAAAATACTGGCTCATTTTGGGTATTGG
CGTATGCAGCTTCCATCGGAACATTCATCTGCCTCGGAATCGTCTGGAATGGGATAGATT
TGCTCTTCGAGATTGCTCGACTGAATGAGGAGATCAATTACCTCAAATACGGTGCGGGCT
CTGAAGAGCGGCTAGAGGACAGGGACGATGAACGGAAATGTTGATGTTGGCTGGATCCTC
ACATTCCTGGCCATCGTGTCTGCTAGCGGGCCAGTGCTTTCCGTTGTCCTTGACTATCGC
AGCCGCGAGCGTCAGGGAGAGGCAGAGGCGAACAACAAAGATGCCGATATCGGGATAAAG
AAGGTTTCTGAGCAGGAACTTTCGCAACGCGTTCCGTCAATGAATGATGAGCGCTGGATC
ACGCGTGAGAAGCGATGGCAGGAGCGCGAAGACGCTCTCGAGCGCAAAATTCAAGAGCTG
CGAAAAGAGATGGATAATTTGCACTCTGACATGAATGCCTAGCTTGAATTCATCGCCAAT
GATGAGCAGTGGCATTTCTATGACCGGATGCACCGCATCCAGCACGATCTCCCTGCGCCA
CAGGATCGGATCACATTCCAGCAGTTCATGGAGTCGCGCCGAGAGGCGGCGGGGAGGATG
TAACATGGAACTCAAGGTTGGCTCAAGCGACGCGAACACTGATGGTGAGGTAACTCGCTG
GCAGAACTGGGTAAAGCGGTATGCCGCAAGATAGTCCGACATCATGGGCCCTATCGATGA
GTACAATGGCTGCCCGGATGCCAACTTCACCCGTGAGCTACAGCGCCATCTGGGGCTGCC
GATAACCGTCGTGTTCGATGATGTGACAGACCTGCACCCAAAGGTCAATTACCGCTGGAA
GGGGCAAGGCGCCGGGCAACGAGCCGCACGCCGGAAGATCTCGTTCTACTCCAATCCCGG
TTCGGTCGCAAACGAATTCGTCGGCCCATCGTTCGAGGTCGGCGAATTCTGCAAGAACGT
CCTGAAAATCAACCACCAGCCGGTGCCTTCGGCCAACGGCGGATATCTCGTACTGATGGG
TGGCGACCCCAAGGTCAGCTACAACGACGTCATCTATGACCAGTACAAGTCGCTGGAGTG
GTTGCTGGACAACAATCCTGACACCAAGGACCCAGACGTCGAGTTCTGGTTCTCGGGCTA
CTCGCAGAAGGCCGACGGCCTCGAAGACGCACTCGAGATCTTGTTCGGCGATGGGGGATT
CCCCATCCCCAAGACGGGCGAAACCGAGGGTCCGGGTAAGTACCGGCACCTGCGGCCAAG
AATCAACGGCACCATACAGTTTGGCAGTCGCAGAACGCAACCCGGCCCCACGCGCGTCGG
GAATCGCCCACCCGGAAGCGGCATCTCCCGCAAGAAGCGGCCGCAGTGGCTCAGCATGCT
CACCTGGGACATCGTCACCACTTCCCCAGGGGCACCGGACTTCTACGCTGCCTGCGACGA
TGACATCCGACAGTTGTACTACGAGTGGTTCATCAAGGCCGACACGGAACTGCCCTTCGT
GGTGTACACGCCGCAGATCATCATCCCGGCGCTCCTGAATCTTCTGGCTCCATTCTTGGG
AGGCTTCGGAGGGGTGACGAGCCCGCTGGCTGACGGGATTCTGGCGAGCGCTACCGTCTT
GCCGATGAACCTGCTCCACGGCCTGCTCTGCGGGGTGGCTGCAGCCGACAATGCTCCGAA

CCCCAAGCTCATCGAACTCCTCAGCGTGCGTGGTGTTCTGACGAACATCCCGCAGCTCAT
CAAGCTGCTGACCAACATCTCCGGCGTCCAGACGCACGGCGAATACCACCTTCCGAAGCC
AGAGTTCAACGGCCGCAGCGGAATCCAGGTCGGCTGCGACATCGTTGCGGGCTGCCGGCG
GTAAGGAACACTCATGGAACGCGTGATAGTTTACCAAGACATCAACAACGAATGGCGCTG
GCGGTTCAAAGCCAACAATGGCCGGGTGATGGCTGACAGCGGCGAGGGTTACCAGAACAA
GCAGGACGCCCTCACAGCGATCGATGCTGTGTTTGGCGGGCTTCCGCCAGAGGTGGTGGA
TGAGGCTGGCTAGCAGCTCCGGGTAGTGCCGAACGACCCAGCGCTCGAGGCCGCGCTGAC
GCTCTTGACGCTGATGACAGGGGACGACTGGGACCGCCCGAAGCTCGTGAAGTGCTCAGC
GAGCTTGCGCCAGTACGCTTGCGCAACGCTCGTGAAGCAAGGTATTGGCGTCGCACAAGC
AGAGGCTATCGTGGGCGAGCGGTGGGCTGAATAAGGGAGCCCCGAACCAGCTGAGCATAC
TCCTGTAGCTGCAACGGCCGATCGCTCGGCAGAAGCCAACGTAGCACCCAAAATGGATCT
GTCTCCGGGAACGCCTCCCACCGGCGAGGAGCTTCGGCTCAAGGCGCGCAGCCGTATCGA
CAGATGGCGCAAAGTAAGAGCCGAAGCCCCTGAGGACTCCGGCTCCGATACTTAGCCAGA
CTAGATTTCCAGCGCGGGCTGCTCGTCCTCCTTCTTGGAGGCCTGGAGCTCCTTGCGAGC
TGCCTTGACGGTGAACTTGAAGGCTTCGCCGAACTCGCAATTTCTGTAGACCGGGCCGAT
GCCCAGGTAGACGCTCAGCTCCTCGGTCAACGTTCGGGTGCAGACGCAGCAGATTCCGTA
GATTGCGCCGAACTCCTTCATCTCCTCGTAGCTCATCCGCTGCTCGTCGGTCAGGCCTCG
GAGCGGCCGCTTGCCCTCGAACTCGAAGTGAGCGGTGAAGACGGTGCCAGCAGTCTCGTC
GTGGCGGTTTTCCTCGACGACGACCAAGCGCTTGGCGACCTGGTGGCCGCGCTGGGTTTT
GTAGACCTTGTAGATGGTACCGTCGGCGACGCGGTACATGCCATCTTCCAGTTCGTCGGT
GGCAGGCTTCGCTTTCTCGGCGACCGGGGTGATGTGGGTTCCCTGGATTTCGACCGTCTC
ACCAGCCATCTCGGTGGTCCGGATGACCGGATCTGCGCCCTTGATGGGCATGCTCTTCAG
CCAATCGATCAGGTTGCTGGCATCCTTCGACCTGAAGGACTTGTAGCAGAACTCGTAGCC
GTCCGATGCACCGTGGTTGTCGACGACATAAGCCAGCGCAGCGTTCACGCGTTCGCCGAG
CAGGTCGCTACTCATGGCCTTCCTGATCTCATCCATGACATACGGGGTGCCGTCCACACC
GTTGTCGTCGCGCTTGATGGCCCAGTCGATCATCAGGCGGATGGTCGAAGCGCGATACAC
AGCCTTGTGGCGGTAGTCGCTGGCCCACGCACGCTGCTCGAGCAGGTCCTTGATCCAGTT
GATCTGCTTGTTGGTCGCGGGCTTGACGGTCGCGGTCGCGTTCATTTTGGATTCCCTTCT
CCGGTGTCCGAGGCGCTTTGGGTAGCCTCGCTGACAAGACGAACGTTACGGCAGAAATTG
CAGTTTCGCTACTCCTTTCAAAAGTCAGCGCTCACGGCGCGTGGGTCCGGCTGGCCTACC
AGGACAAACCTTCTGCAGTTGCACAAGACGCTCCTCGTCGTCTCCCAGCAAGCCGCATGG
CCGCCACCGCGCGGCTGGTAGTGCGAGGACCACGGGTGTCCGCCGGCGCACTGGTCTACG
GCGTACCGGGTGATGCGGAGAAGCTGCATTCCCCGCATCACCCACTGCCAACGCTCCATC
AGGCGATGGCCCGGATCAGCGCCCGCACCGCGCGGCGCATGGCGTTCTCCGGCATGCCGG
TGATGACCACGACAGTCTTGCCGTCGCGATTGACGACCCACTTGCGGCTATCGTCTTCGC
CGACTGCGAGGGTGTCTGCCACCTGGCGGTCGTCACGGTATGCCGTCACGCTATCGCGCA
GCGTACGGACGGTGAGATCGTCCTCATCGGTGTCATTGCCGGCCAACCCGGGAACGCTCA
GCGCTTTCACGATGCTGTCCACCTTGTACATGAAGTTCAGGCTGTGGTCCGCGCTCCGGA
CTGCCCCGTCAACCACCTTGTCCAGGTAGTTGGAGCGGACCAGAACTTGCTTGGCCTGCC
CGCGCTCGAAGGTGATCGCACTGGCCACCGTTTTGTCGGCGATCACCTGCTCGCGGGACG
TCAATCCGAAATTAGCGGCAACTTCGGCGATCTGCATCCGCTGGTTGACGTTGGTGATGG
GCATGAGGCCTCCTGGGCTCGTGGGGATGTTTGCGCCCCGAACGTTACGCCACCGCGCCG
ATTCGGGCTACTGCCTTCTACATGCGTCGTCGTAGGGGTGGCAACAATATTCGGCCCGGA
GTAGCGCGAATCGGGTCGACTAGAGTAACGTTCCTCCTGTCCGCCTCCCCACATCCCCAG
GAGCCCCAATGAGCATCACATTCCAGCCCGGTCAGCAGGTTGAGGTCTACTCCGGCGACA
ACGTGCTGCACCCCGAGACGCTGAAGCCCTTCAGGTATCGCATGGTCGTCACAGCCGGAC
CCTTCGAGGTGAATACCGGCCACGAGGGGTACCGCGTCCTCGGTCCGGGGAAGCCGGAGC
CGTCATTCGACCAGTATTGCAAGGCTGCGAATGGGATGCGTCCCGTCGCCGACGTCGATC
CCGAGCTCGCTGCCCGGTACGACGCGTTCCGGAAGTGGGTCGGCAAGTGATCCGGCTGGC
ACGCCGCGCAGGTTACTGGGTCTGCAAGCGGTGCTGGCAGGGCAACCTTGGCGGAGAGAA
GTGCCTGTACTGCGGGAGGGCCAAGTGAGCGAGCTGTGCGCCCTCTGCGGGCACGAGTTC
GAGGAGCACTACTCGCAGGGAGACTCGAGCGGAAGGCTGGTGCAGGGTTGTGAGGCCTAT
ATCAGGACACCCTGCCCAGCCTACAACCACCGCGACCCGGAGCGTTGCGAGTGCGACTAC
GTTCCCTGCGATTGCCCGGGATTCGAGAGCGAACCCGGCACCGACCCCGACATTACCGAA
ATCGACATGTACGACCCGGAAGGACGCTGATGGAGTACATCGACGTGCTAATCACCACGC
ATAGCGCAGACGAAAGCAAGGATCTGGACCAGATCATCATGCCGACTCATATCGCCAAAG
ATCTTGAGGCTGCTGGCTGGACTGTCCGCAGCACAATCGTTCTGGATCACAATACGGGGC

TGACGGGCAACTATCGCAACGAGAAGGTTCAGCACCGCATCGAGAAGGAAGCAGGACACT
GAAATGGCCAAGGAAAGCATCATCAACCGAGTGCAGAAGCTGCTGAACCTCGCGATGGAC
CAGAGCGCCACCGAGGACGAGCGCAAGCTCGCCCAGGAACGCGCGGACGCGCTGATGGCG
CAGCACATGATTGACCAGATGGACCTCAAGCCCGAGGACCCCGGTCGTTCGCGCGTCACC
AGCACAAAGTGGGAGTTCTTCTTCGAGTTCGAGTTCGCAGTGCAGCTAAAGGACTTGCTC
GCAGCCGTCGTCCGGCACGCTACCTGCCGTGCCACGAGCACGCGCAGGATCGCCAAGGTC
GGCGATCCCAACATGGTGGTGACCATTGTTGGTACGGCGGAGAACATCGTCTACGCCGAG
CGCCTGTGGATGGTCGTTTTCTCCCAGCTCACCAGGAACATGTTCCCGAAGATGGACCCC
AACGAGAGCTTCGACAGAAATGTGGACAACTTCGTGAAGGCTGGCTTTAAGTGGCAGGAG
ATCCATGAGCTGCTCTGGCGGCACAAGGACGATCCGCATTGGATGGAATTCCCGTTGAAC
GACCCGTATCCGCCAGAGAAGCAGAGCGAATATAGCTGGCATGCCAAGGTGTATGGTGGC
GACGGCGGACGTCTGAAGCGTGCCTACACTCGTAAGGTCAAGCGTCTCGGCGAAAGCAGA
GCGCACCACACCACCCGACATGGCGTGTACCGCCGGAGCTATGTGCAGAGCTACTCGAGC
ATAATCTCATTGCGGCTGCACGACATGCGGAGCAAGTGCGAGGACGCTGTGAGCGATCGC
GACAAGTTCGCCCTGGCGGTGCGCAGCTCGCAGGACGAAGCTGGCGCCGAGTTCTATCCG
CTGTTCCCGCAGTTCGACCCGGAGAACATCCGCAAGCGGCGTGAGGAGATGCTCCGCCAG
GAGCAGATCCGTTGGCAGAACAGGACTCCCGAGGAGCGGGCTCGCGCTGAGAAGGAGCGC
CTCAAACGTGAAGAACGCGCTCGCAAGCACTGGGAGAAGACGCGGGACACGAGCTACGAC
CAGGCTGGCTGGGCGCGCGGCAGTGCTGTGGCCATGAAGGTCAATCTCAACGACGACACG
CAGATCCACCACAGCAAGAAGGAGATTGGGTGATGTGGATTGACGACTGGCTCGAGGCAG
CCTACGAGGAGCGCACCGAAGCTCCCGACGATCTGGACAACGACGAGTGGTGGCCGGAGG
ACGATGGGGATGAGTGACCAAACGCTCAAGCTCGCAAGCGAACTCAACGAGGCAGCGATC
GCCTTCCGGGACGGCTACGTCTACGGCAAGAGCCTCACCCGAGCTCTCGACAAGGCCATC
CTCATGGCCGATGCGGCCGACCGGCTGAACGGCCATCTCCTCGACGTCATCAACGATCAG
CAGTAGCAACACACCTGACCATCAGCAGCTAGGCTACGCCTTACCTCGTCTCAGAAGGGA
ATCTGACACATGAGCAAGACCACCTCAGCCATCGCCACAGGCAAGACCGCCGCAGTCTCC
GCTCTGGCGGAGAAGCACGACTGGACGGCCGAAACCAAGGAAGACGTCAAGAACGACATC
CCCACCCTCAAGCTTATACGCGGCGATGAGTGGGTTGAGATCCGCTGGGACGGAACTCC
TGCAAGGAGATGCCGACGGTCTGCCACGAATGGCAGCTCAGGTACGTCCGGAACGTTGCA
GCTGCGAAGCGCGTCCTCGAGGCTACTGCCGAGCAGAATGCGGAGCCTTTCCAGAAGCGG
GCTGTGGCCAAGCAAACTGCGCGCAAGACCACGCCTGCCAAGCGGCCTCGCCGGAAGGAC
AAGCTCGAGGAGGCGCTTCCCGACAGCGACGACTAGCAGATCAAGGAAGCCCTCCTCGGC
CGGAAGATCCTCTGGCGTAACAGCACGAGCGGCGAGTTTGAGGAGGATGTGGTGTTGGCC
GGCAAGAATCCGAATGGCCAGTACTATGTGTCCCACGAGGGTGAGAAACGCCAGATCAAC
TTCATCGGCGCATATGGTTTTCGAGCGGTGAAGATTTCTGCGATTGCGCAGGTCAGATAG
GCAAAACGAGTAGCCCGAATCGGATCGACTAAGGTACGATTGTCTTATCCAATCGGAAGC
AAGAAGTGAATCTCAAAATGGACGTTCGTACCCTCACCTACAGGGATCGCAAGGTTGCCG
CCCAGCTGGCCCAGCTGGCCTCTGACGACCAGCTGACCTACCGGATAGCCGAAGGTGCGC
GTCGGGCCAAGATCGGCCGTACCGTGTGGTACGAGATGGCTGCAACTCGTGTGTCTGACG
GTAGGCAGTACCCCTATTGGCTTGCCAAAGACTCGGGTAGCTTCCGTGGCTCATTCCTGA
ACGGCTTCGACCTGTAGTAGCGCGAATCGGGCCGATGGGGTAACGTTCGGTCTGTCAACG
AGGAAGGGAATCCAATGAGCGGGCTCAATGTAGGCGATCGAGCGCGAATCATCAGGGTCA
GCGATCTGGACAACGACCGCTAGGTCATCACCGACCCCGCCGGGGCAGCTGCGCTGTTCG
GAGGGTACGGACCTGGAATTGGCGATGTCGTGGTCATCACCGCGAAGACCCTCGGCGAAC
TGTACGACTACGAAGCCGAATGGGAGATGGAGAAGGGCTACTAGACCTTCAGCTTTCTGG
GGGAAGAGCTGGAGCCAGTCAAGTGAGTAAGCGCGAGTGGAAGATATGGTTGGAGGACGG
CAAGATCGTCCTGCTGACCGACGGGTACGATACGGCGGTCGTCGAGAACATCAAGCTGTA
CTTGCCGACGCCGCGCTCGTTCCGGCGTACCACCAAGACGTGGCACGTCCCCCAGATGTG
GGAGACTTGTGTCGCCCTCCGCCACCTCGCCAATCGGCATCGAGCCGACCTGAAGATGAG
CGACGAGCTCCGGCTGTGGGCTATGGCCGAGAAGCTCCGGCTGGCGAGCATCCCCGACGT
CAACGAACACAACGTGCTGCAGCCCATCCCGAACATTGAGCGCGAGCTGACAGCCATGGC
GGAGGCGCTGAAGGCACGCCCCTTCCAGACAGTGGGCGTTCGGTTCGGCGCCACAGCGCG
GAACGTTCTGATCGCCGACCACCCCGGTCTGGGCAAGACGCTGCAAACGATCGGCGCTGT
GGCCGAGTGCGGCCTGAAAGGACCCGTCCCGGTAGTTGCCGCGCCGAAGTCGGCGGCCGT
CGTTACCTGGCCCAACGAGCTGCGGCAGTGGGTGCCGGACGACGTCGTGATCACGATCTC
GGGGAGCATGAAGCCTGCAGAAAGCGCGGAAGCTGTCGAGCGCGCCAAGGCTTACGCCGA
GCACGGCGACATGCGCGTCTGGGTCGTCACGAGCCCCAACTACGTTCGCATGCGGGCTGA

GCTCGACACCTACGGCAACTATGTGAACGAGGGTGGCCAGAAGATTGTGCGGCCGGTACG
CGAGGCTCTGCAAGGGCTGGTCGACTTCGAGTGGTCAGCGATCGTCGTGGACGAGTCGCA
CAAGACGCTCGCCTGCGGTACGGGTAACCGCAAGAAGTGGTCTGCACAGCGCCTGGGCCT
AGAGCTGCTCCGGGTGAAGAAGGATGGGCTGCGGATAGCCCTGAGCGGCACACCCTTCCG
GGGCAAGGAAGACAACGCCTACGGCACCTTGCAGTGGCTGCGACCCGACCTGTACACCGG
ATTCTGGAAGTGGGGTGAGAAGCACTTCCAGATCTACGGCGATGGTTCCGGACAGATCGT
GGGTGGGGTCAATGACTCAGAGGAGTTCTACAACAGCCTCAAGCCAGTCATGGTGCGGCG
AACCAAGGCCGAGGTCGTCAAGGACCTGCCTCCGAAGATCTATGCCGGTGAGCATCTGGA
CCACGAGGACCCGCATTCCTGAGTGGCAATTTGGCTTCAGATGGAGGGGAAGCAGCTCAC
GGCGTATCAGGCTATGGTCAAGGATGCAGCCGTCAAGCTGGAGAGCGGTGTCCTTTCCTC
CAACGGCGTTCTGGCGGAGATGAAGCGTCTGAAGCAGATGGCCTGCTCCTGTGGCGATCT
GGCTATTGCTCCGACAGGCGAGCAGACGTTCCGTCCGTCGTTGCCGAGCAACAAGTTTGA
TTACCGTGTAGACCTTCTGAGCGATCGTGGCATCGGTAGTGGCGAGTTCGACCCTACCGG
GAATGTGATTGTCGCCAGCCAGTTCACCAAGCTGCTGAATCTCTTCTACGCCGAGCTGAA
CAAGCTGGGCATCAAGTGCCACCTGCTCACCGGCGCGACTTCGGAGAAGGAGAGGGTCCG
TATGGCAGACGCCTTCCAGAACACCAAGATTGGCGACGTCACGCCGCACGTGTTTCTGCT
GAATACCATCGCTGGTGGCGCGTCTATCACTCTGGACGCTGCGGATGATGTTGTGACGCT
TGACGAAACCTGGAACAAGGACGACCAGGAGCAGGTCGAAGATCGCGCTCACCGCATCAG
CCGTACCGACCACACCGTAACCATCCACAACCTTCGCAGTAGGGGCAGCATCGAGGAGCG
AATCGCCACGAGTGCCTTTGCTCAGGATCGAAACATCAAGTCCATCATCGACGGAGCGCG
CGGTGTCGAGTTCGCACTCGAGCTCCTGGAAGGAGTCAAGGCATGAGGACCCTCAGCGCA
TGCTTCATCACGGCCATGCTCAGCGGGTCATCAATGTATTTGCTGGCCTGGCAGATTCAC
GGCAAGCCTCTCACGAGAGAGGAGATGCGGGATTACATGGAGCGGTATGTACACCGGGAG
AAGGCAGAGCTGGCAGACCGCCGTCTCCAGGACCAGCTCTACAACGCTCGGCGTCGGGCT
GTGCCGCATCCGGACGCGGTTGCACCCGTGCGGATCACGAGGTTGCACGTGTCTAGAAAG
GGGAGAGGCGAGCGAGAGGAGTAAAGTCAAATAAAAGTCGGAAAAATTACCGATTCCGAC
CAAGAACCAGTAAGCTTCATTACACGCCCAAAAACTTCATAATCCCAACGAACAGAAGGA
AAAACGATGTCCCCGAAGACCGCGTCCAAAGAGACCAGCACCACCCGCGAAGGCAAGGTG
CGCTTCGATCCGTTCGCCAATCCGGGCACCCTCGCCGAGCAGGAGGCCGCGTGGCTGAAC
GAGCAGTTCCCCGGCCTGAACGTCAAGGCCAGCCACGTCCGTGCGGTCATCAGCAACCAC
ACGCGCTTCCAGAAGTCCGACCTTCGCCGCCAAGCGCGGGACGCCGAGCGAGCCAAGCTC
GCCCAGGATCGCGAGGCACGGAAGGCTGCCGCCGAGCAGCGCAAGGCCGACGCCGAACAG
CGCAAGGCAGAGCGTGAGCAGCGCAAGGGAGAGCGTGCGGCCGAGGCAGCAGCCAAGGCT
GCCGAGAAGCCCGAAGCCAAGCCTGCTGCCAAGAAGGCCACTCCGGCCAAAGCCGCGCCC
ACGCCTGCTCCCAAGAAGCGCGTCTCGCGTCCCCGGAAGCGCACCGGTGACGTGCCCGCG
GAGGACGCATTCTGATCCACCCCACCCCCTCGGGGTGGCTGAGCCTCTGGGACATGGGGA
GGGCCCAGAGTCTCGGTGGAGGTCGAGCAAGTGCGCCTCTGAGCATCGGATCACCATCAC
AGGACTCCAGTCAAATCCAGGAATTACTCAGCCTCCTCCAAGACAAAAGCTCACTGACAG
AAGGGAATCTGCAAAGTGAAACGCATCAAGAAAGCCGCGAGCGCCGGTGTGGTGGTTACC
CAAGTCGGCCTCGGAATCCTCAGCGCCGCACCGGCGAACGCCGACTATGTGATGGGGTGT
GAGCGAGTCCGCTGGGGCTTCCTGTTCAGTGGCTATCGCACCATCTGTGACGGCCCCAAG
CGTCCGGACGGCAGCTGGGACCGGACGCGTCGTGAATGGACCCCAGCGCACATGGCTGGC
GGATACTGCTCGGGCAGCAGGTATTACGTCAGCTGCACCCCGAGCTACTGGTTCAACGAG
AGCACACGACGCCTCGAGACCTATCCGGTCACTGATGCCACTGTGTTGCCTGGCGAACCC
GGCTGGCTCCCTCCGGGCACGAACAGGATCCTCTGATCCATGGCACCCAAAAGCCCACGA
ACATAAGGGAATCCATGAAACTCAAGGTGTTGACATTCATTGCCTGCGAGCAGTGCGACA
CCCACTTCGGTGGGCCAGAAACTGGCGAATACGACTACCGAAATCTTCCTGGCAAGATGA
CGAGTGCCGAATATCTCGTCGAAGAAGCTCTGGCAAGTATGTGGATTCGTGTCATCTGGG
GAGCCGACGACGTAATTCTGTGTTACCCTGATTGCGTCAAGAGCTGGCTTGATGAGGTGG
TGTCGCGTGGCAACTAGAATCCCGCTGCCCCTGCTACTTACGAGCGAACGCACCGTGTAC
AAGAGGTGCCCGTGGAAGTGGTACCAGAGCGTTGTGCGCGGCCTCGTGCCTAACAATCCC
CGCCAGGACGCGCGGTGGTTCGGTACTGGCATTCACCTGTGCAAGGTCGAGTGGTACGTC
CCCGGACGCAAGCGTGGCCGCGACATGCACGAGACCTGGGACGAGTTCTCACAGGGTACA
TTCACCATAGTCAGCTCGCAAGGGCTGGTGAACGACGAATGGGAGAGCGAGTGGGTTGAC
GCAAACAAGCTGGCGCACGCCATGATTGACACCCACCTACAGGCGTATGGGAACGACGAC
AACTGGGAGGAGATCGCTGCTGAGCATCCATTTTCGGTCCTGATCCCGCACCCCGACAAG
ACAATCAACCGTGCGGTGTGCCGGTGCGTCGGCACCATCGACCGGATCATCCGCGAGCTG

GACACCGGCGAGATTTGGCTGGACGACACCAAGACCGCCGCGCATCTATACCCACCAC
CTGACACTGTTGGAGCAGCCAGCTGCGTAGGTAGCTGTGGGCACGCACGTCCTCCGGGAG
CAGGAGATGATTGGCCCCAAGGAGCGGGTCAAAAGCATCATCTTCGACTTCATGCGGAAG
GGCTTTCCGGACGATCGACATCGCAACGAGCGCGGCGAGTATGGCAACAAGCCGGTCAAG
AAGCACTTTGTCGAAGCCATCCTGAATTGTCGGGGTTATCAAGGTCCTCTGAACACCGAG
CGAGATCTGCTTCGGCTTAAGCTCGACGAACTGCAACACATCGCCGATGAGCTCAACCTA
ACGGTTCTGGGCGATGTCTCAGCCAACCAGGGTGCGCCCCTCCTGTTGCGAGAGAAGATC
GTGAAGAGCCCGAAGGAGTGCTCACGGCAGCTCCGCAGGATCTGCGAGGAGGTGCTACAC
ATGAACGCTGTGCGCGCCGGTAAGCTCCCGATCCTCAAGACGCCGCAGGAGGACTGCAAC
TTCTGCGATTACTTCGATCTCTGCGAGCTCGACGATTCTGGCGGAGACACGGAGTATTTC
GAGTCGCAGGCATTTGTGGTTCGTGATGCCTACGCGGTTCATCGCGAAGGCGCTGAGAAT
TCGAAGATCAGCGTCAAGAACAAATATAAGACAGGAGTGCGATGAGGTGGCCGGTAGCCT
GAAAGATATGGGCCTCAAGGAGGTGGCAGCCCTCAAGAAACGCGTGAACAGCCAGTACGC
CTTCAATCGCATCAACGGTGGTGACCGAGACAACCTAATCGAGCTCATCAACGAGGTTCA
GGCGTACATCATCAAAATGCCTGAAGGCGAACGAAGCATCCCCGATTTCTACTGAGAAAG
GAAGGCTGATGGTCCAATCCAAGATCATCAAAGTCGCCGACGACGAGGACTACGTGAATC
TCCTCGTGTACGGTGACTCCGGGGGGGGCAAGACGGTGTTCTGCGGCAGTGACGACAAGG
TGTTGTTCGTTGCGCCGGAAGACAAATCAGACGGCCTGCTCTCCGCCAAGCTGGCGGGTA
CCACGGCGGACAAGTGGCCCATCCGGGACTGGGGTGATCTCGTTGAGGCGTACAACTATC
TCGACGAGCTCGAAGAGATTCCATACAACTGGATCGTGGTCGACTCGCTGACCGAGATGC
AGCTCATGGCCATGCGGGACATCCTCGATCGTGCCGTCGAGGACAATCCGAGCCGTGATC
CGGACATCCCGCAGATCCAGGACTGGCAGAAGTGTTACGAAATGGTGAAGCGGATGATCA
AGTGATTCAACGCTTTGCCGGTCAACTTTCTGTACACGGCGCTGTCGCGGCAGACCGAGG
ACGAGGAAGGTACCGAGTACCTTCTCCCCGATCTGCAGGGCAAGAAGGACAACTACGCCC
AGCAGGTGGTCAGCTGGATGACCAGCTTCGGTTGCATGCAGATCAAGCGCGTCCGTGTCA
AGACGGACGACGCCATCGCCAAGAAGGTGAAGGAGGTGCGCCGAATCACCTGGAAGGACA
CAGGTTTGGTGACTGGCAAGGACCGCACCAACGCTCTCACCCCGTACACCGACATCCGCG
ATGTCACCGATCCTGAGGATGATGGACTCACCCTCAAGGACATCAGGCTCCGCATCGATC
GGAAGAAGTCCGGCTCTGCCAAGTCGTCAACCACCAAACGTCCGGCCAGAAAAACTGCGT
CGGCTCGTACTCAGAAGGAGAGTGCATAAATGCCCAAGCTCAAGCCATTCGGCATCGGCG
ATGTCGAGGACATGATCAACGCCGAGAACTCCTTCAACACGGAGGTCGTGCCCAAGGGCG
TGTACCAGGCGAAGGTCAAGCGCATGAAGCACGCCACCATCCAGAAGGAAGGTCCGAATA
AGGGCGAGAGCCGCTTCAGGATCTTGATGGAGGTCGTCGGCCCGAAGTCGACGCCGTAGA
AGTACATCGGTGCCTCAATCTCGCACAATATCAGCGTGATCGAGTCCGGCGCTGGTTTCT
GCAACGACTTACTCAACGCGCTGGCGGGACCGGAGCCCGCCAAGCAGGCCGCGCTGAAGC
GTGCGCTCTGGGCAGCCGACATCGTCGTCGATGCGGACGGGCACGTCATGAAGATCGGCA
AGAAGCGTGTCAATGGCGACGGCAATGGCGAGCTCGTTGTCACCGTCAAGACCAAGCTCG
GCAAGGACACTCGTTCGGGCGATCCGCAGGCACAGGTTGGCAAGTTCCTGGTCCCGCAGG
CTAACGACGACGACGATGACGACGACCTCGATGAGGACCTCGAGGACGACAATGATCTCG
CCGTTGTCGACGACGAGGCTTCGGACGACGATGATTCGGACGACGAGGATTCGGACGACG
AAGACGACGAAGACGACGAAGACGACGAAGACGACGAAGACGACGAAGACGACGAAGACG
ACGAGGACGACGAGGACGAGGACGAGGACGAAGACGACGAGGACGACGAGGACGAGGACG
AGGACGAGGATGGTGAATCCGACTCGCGCCGTGCGGAACTCGAAGCCCTGACCATCGCCA
AGCTCCGCGTCGAGGCCACGAGGGTCGGTGTACCGCCAGCAACGATCAATGGCCAGTCCA
AGGACGAGCTCATCGATACCATTAGCGACGCCGAGGCTGACTCGGACGGCGAGGACGACC
CGTTCTGATCGGGTAGTACATCGGGTACCTGACAGACGAAACCGTCAAGCTAAGTCGTGG
TAGGGACGTCCACAGCCCGACCCCAATAACTTTCAGCAAAGGAGCCATCATGGCCGATGA
GCGTCGGTGCGCCTGCGGATGTATCATGGTCTGGTCGAATGAAAACCACACATGGCTCTG
CATCCACTGCGACCTCAACATCGAACCTCAGGAGTACGCCTCGTGAAATCTGTTGCCATC
CTTGGTCGTGGGCCTTCTGGTCTCCTCGTGGCTCATGCAGCCGCCATGCTCGGCTGGGAC
TTCCGAATCTACAGCAAGAAGCGCATGAGCTTCTCGATTGGCAGCCAATACCTACACGAG
GCCATCCCCGGCATGACAGAACCAGTTGCGGCGATCGTGGCATACAACCTTGTGGGTACG
CCAGAAGGCTATCGGCAGAAGGTGTATGGCGCTGGCTACGATGGCAGCGTGTCGCCAGAA
GACCTCGAGCACGATCACTACCCCTGGAACATCCGCACCACCTGCGACAAGCTGTGGAAT
GCCTATCACGACGAGATTGTCCACGTGGTGATCGACAAGCGCTGGGATTCGGCCGAATTC
TTCCGGAATGGCAGCACTGACTTCCGCCCCGACCTGATCATCAGCACGGTCCCTCGCAAG
ATCTGGGCCAAAATTGGCGATGTGTTCGCAAGCCAGAAGGTGTGGACGATCGGGGATGCG

CCCGAGCTAGGACAGACGGTTCCGTTCGAGCCCAAACAGGACAACACGATCATTTGCGAC
GGCACAGCCGATGTCGGCTGGTACCGGCTGAGAAAGGTGTTCGGCTACACCACCATAGAG
TGGCCACACGACCGCAAACCTCGTATCCCGAACATGGCTGAGGTCGAGAAGCCGCTCTGC
CACAACAGTTCTGGAGCCAGCGACTTCATTCACATCGGTCGGTACGGCGCCTGGGAGAAG
GGCGTGGTGACGACATACGCCTTCCACCAGGCCATGAAGTACCTCGGACAGGACAAGTTG
GGCTGAGCGATGATGGGCAAACCTGTTGTTGGACTTGACATTGATGGGACACTATTCGAC
TACCATGGGGACTTCTTGCGCTTTGCAGATGATTGGTATGGCCGACCAATGCCGGACCCG
CAGGACATCAACCCAGGCCTGCCCCTTCACAAGTTTATGCGTACGTCGAAAGCTACGTAC
CGGCAGTGTAAGCTGGCATTTCGCCAGGGAGGCCTTGAGCGCAGCATGCCGGTGTATGAT
GGGGCGTCGGAGCTGACCCGAGCTATCCGCCGCGCTGGCGGAGAAGTGTGGATCTGTACG
ACCCGTCCATATCTCAAGCTCGACACCCAAGCGCCAAACACCATACACTTGTTGCGGCGC
AACAGAATCCAATTCGATCACATGCTCAGCGGGCCACACAAGTATCGCGACCTCGTGAAT
CAGGTGGGTGCGGACCGAATTGTCGGAGTGCTTGACGACCTGTCAGAGGTGTACGAGCAA
GCAGAAATGTTGGGGCTCAACCCCATACTTCGGGACCAGCCGTACAACCGACACATCACC
GCAAACCGCATCAAACATCTGGACCTCACAAAGATCTCATTCCTCGCGAGGATCGAAGTG
TACAAAGAGAAACAGAAGGGAAGCTGATGGGCTCGACATATTCAACAGACAATCCGTTGA
GCTTCGCGCTGTTCGGGCTGTCCCGCCAGGCAGAGTGGCCGGTCTGGCGGAGGCTGGACT
GGGGATACCCCATCCTGCACCTCGGTCCTGGCGACAAGATAGTCCGGGGCGCTGAGGAAC
TGGACTTCCCCGCATTCGATTTCGGTGACCCAGAGATCAGCTTGCCGTACGATGACGGGT
CAGTCGGCGGTGTCATCGCCACGCATGTGCTGGAGCATCTGGCTGATCCACGCCCAATCA
TCCGGGAGGTGGCGCGTGTGTTGAACTTCGGGTGCCCATTCAACATCCTGGTGCCACACG
GTCAGTCGCCGATGTATTTGCAGGACCTCGACCATAAGACACCGTTCGTGCTTGACACGT
GGAAGAACTTCCTGCACAACGGACTCTATGACAACATTTACGGCGAGGGCGGATTGCCGC
TCTGTATCGGCGCCATCTTCAAGTTTGCCCTCAAGGAGGGCAACGAAACCATCATCACCC
AGCTCATCAAGGAGTGACAGTGTCCAACGAAAACAAGGGTGTTGCAGTCATTCTGGAAGA
GCTCACCTCCGAGTAGGCCAGTCCGGTGGATGACTTGATCGCCAAACATCTTCCGCGCGT
GCCAGAGCTGTTCCGGAGCAAGGCGCGCGACTACAGCGACCGCTCCGGCATCTTCGCGGC
CGAGTTGCTCGGTGCGCCGGGGCAGTTCGCCGAGATCTGGCGCAAGATTCCCAAGCTCAA
AAAGGGCATGTGGGACCAGGACGCACTCGAGAACGAGACCGTCTAAGAGATCCTGTTCGA
TCTTATCGGGCATTGCCTGCTCGCGTTGGACATGATCCCAGACAGAATGCGGATCGGCCA
GGCTCCCAGCGGACACTCATGGATACGAGGGTACGAGATCGGCGAAGTCAGGCGCGTTCC
TTCTGGCACCGAACGCTTCACTGAATCAGACAAGGGTCCGATAACGGGCAAGCGCGATGC
TAACGGTGGCGACTTCCGTCTCTAGGGCATGGGTGCTCGGTGGCACGTCCGGCGTCGGGA
AGGCTTGCGCCGAGCGGCTCGGCGAATACATGGTGGCCACGAGCACCGGTCTACGACAAG
GGGATTGGGACGTCCGCAGCGATTTGCGATCCATCTTCATACACGATGAGGTCGATACTC
CGACCCACATCGTGTATGGTGCAGGTATCAACCATCTCGACTGGATCGGGAATGGTGCGC
CGAGCCATGTCGAAGTTATCGACACCAACCTGAATGGATTCATTCGTCTGATGGACGGCC
TGGTGCGCTTTGGCTACTACAGCGCTGGCAAGCTGGGAGGGTATCCGTACCACATAAAGC
CCAGCATCGTCGTGATCAGCAGTGATGCGGCTGAGCGACCGCTCCGAACCTCTATCGGGT
ACTGCGCAAGCAAGGAAGGGCTCAACATGGCCGTCAAGGTGGCTGCCCGAGAACTCGGTC
CGCAAGCCTGGCGGATCAATGCCGACTCGCCGGGGATGACTGCGCCCACGGGCATGAGTG
ATTACATCGACAAACGTGTTCCGGAAGCGCGAGGTTGGACTCCGATCGAAGCTGCGCAGT
ACGAGGCACAGCAGGAGGTCGTCCCTGGACGCATCGATCCTCGAGAGGTGGCAGAAGTGG
TGTTCGATGTGCTCACCGGACCTGAGCACCTCCACGGGTCCATCATCACCATCAATGGCG
GGCGCTGACCAGTAGGAATTCGGCCTCCCCGAGACGGGAGGCTGAATTTTCCTCCGAAAC
AACCCGATTCGCGCCGGTGTAGTAGGCTTCGGCCCAGTGTCTAGAAGGGAATATTGAGTT
GAAGGACCTTGAGCTCAGCCAGCCAGACCCGATCCGCGAGAAGGGAATTGGTGAGGAGCT
CTCTTTTTCCAAAACCAGCAATGGCTTGTCGGTGAAACCGATGAAGTTCGTATCGCTTCA
CACCCACTCAACGTTCTCCTACGGCGATGGCTACGGACCAGTCAAGGAGCACGTCCAGCG
AGTTGCGGAGCTGGGCATGGGCGCTCTGGCGCTCACCGAGCACGGCATCCTGAGCAGCCA
CGCACAACTCGAGAAGGCCTGCAACGAAATTGGAATCAAGCCCATCTTCGGGTGCGAGCT
GTACGTTGCTCCGCCAGGCGAGAACCGCAAGTGCCATCAGACGGTCCTGGCAATGAACGA
GGTCGGGCTCCAGAATCTGAACCGCATCGTCACCAAGAGCTGGAAGAACTTCTACCGCAG
GCCCACGACCAGCTGGAAGATGTTGGAGGAACACAATGAGGGGCTCATCGTACTTTCAGG
ATGCGCCGATAACGTGCTATCATGCACGCTGCTGGGTGGTAAGCTTTACGGTGACAAGCG
ACTCACCGCAAGCAAGCGCGACATCGCTCGTGCCGCTGGGCTTATTGCACGGTACCAAGA
GATATTTGGAGATCGGTACTACCTTGAAGCCCAGAGATTTCCAGGACTTGATCGCACTTG

CGCTCTCATTCCGCTCCTCGCACGGATATCGGAACTCACCGGAGCTCGTCTTGTTGGAAC
GGCTGATGTTCATTATCCACGTGTATCCGACAATGATATGCAGAAGATTCTTCACGCGGC
TCATCGCGGGTCTCCTGTGGAGCCTACTGATGCGTCCTGGGAGTATGACATTCTCCTCAC
TTACCCGGAATACGACCGAGAAATCTATATGGACCTGGTGGGTACGGGGCTGACCAAAGC
CCAAGCGCGCGAAGCGGTTCTGGAGTCTGGCCGAAACGCTGAGCGCTGTAACGTGATTCT
GCCGAAGAACCAGCCGATCCGTTTCCCCAAGCCTACGAAGGAAGTTGACGGCACGCAGGT
GCCGATGAGCGCCAACGAGCTGACCCGTAAGTGGCTCAACGAGGGCTGGCACTTCCGTAT
GGCGACCAGCCCGAACATGCGGAAGAACCGCCAGGCATACAAAGCACGCGTCGAGCATGA
GCTTGGCATGATGGAGGAGAAGGCCTTCCTTGATTACTTCTTGGTACTCAGCGATGCGGT
CCGTTGGGCCAAAGACCACAAGATTCGGGTTGGGCCCGCTCGTGGTAGTGCTGCGGCGTC
GCTTGTGTGCTACGGGCTTCGGATCACTGAAGTCGATCCGATGGTCTTCCCGCACATGCT
GTTTGAACGTTTCCTTGATCCCTCACGTACAGCGCTGCCCGACGTCGACCTTGACTTCTC
AGACGGCCGGCGAATCGAAGTCAAGCGCTACCTAGAAGAGAAGTACGGTGCGGACCGTGT
TGGTAACATCGGAAACTTCACGCGTTATCGTGGCAAGGACTCGATCGACGACGTGGCTCG
CGTGTACAACATCCCCGTCTGGGAAACGGAAATCGTCAAGAACCTCATCATCGAGCGCTC
TGGCGGTGACAGTCGTATCAGTGATTCTCTCGAGGATACATTCAACATGTTTCCCAAGGC
CGCAGCAATTCTGGAGCGTCACCCCGAGCTCGCCAACGCGATTCGCCTGGAGGGAAATTA
CCGGGGGATGGGAGTTCATGCTACGGGCATCGTCATTAGTAACACTCCCATCACCGATAC
CTGCGCAGTATACGAGCGGGAATCGGCCGGCAAGTCGACGCAGGTCATCGCATACGACAA
GACGGACGGCGAGTATGTGGGCATGATCAAGGCAGACTTCCTCGGTCTGTAGAAGATGGG
CATGATCGGTATCGCCCTTGACATGATTGGGATGGACCTTGAAGAGCTATATCGAATCCC
TCTTGATGACCAGAAGGTGCTGCAGGCATTCCGGCGTGGAGACGTCACCGGCATCTTTCA
GTTTGAAGGACGAGCTACCCGGATCGTGTGCAGTGACGTTGTGCCAGACCACTTCCAACA
CCTGGCGGATATCAACGCACTCAGCCGCCCGGGTCCGCTCTTCTCAGGAATGACTGCGGC
TTACTGCGAGGTCAAGCACGGCCGCAAGACTCCAGAGCGGTACCATCCCATTCTGGACAA
TCTGACTGACTGGACATATGGTCAGGTGGTGTAGCAGGAGCAGGTTCTCAGCACCATCAG
GGAACTCGGAGGCTTCCCGATGAACGAGGTCCACAATATCCGTAAGATCATCTCCCAAAA
GCTCGGCGAGGCCCAGTTCGAGGCCAAATACAAGATGTTCGACGACAATGCTTGCCAGAA
CCACGGCTGGACCCCGGAGCAGGCGCGGAAGATCTGGCGCTTCCTGGCGACATCAGCCAC
CTACTCATTCAACACCGCGCACTGCATCAGCTACAGCATGCTCACCTACTGGCTGATGTG
GATCGAGCAGCACCACCCCACCGCGTTCTATGCGGCCCAGCTGCGCAAGGTCAACGACGA
GAAGCTCTGCAAGTTGATGCGGGATGCGGTGCGGCACAACTTATCTATCCTCCCGCCAAG
CATCAACGAGTCGATGGCGAGCTGGTCGGCTCTCATGGAAGGTGTTGTGCGCGCAGGTTT
CCTGCAGATACCGGGAGTGGGTCCCAAGACATCGGAGGTGATAGTCGAATGGCGCAATCA
GAAGATCAGGAAGGGACAGAAGGCAATCGCAGCCAGTGCCCCGGACCTCATGTGGTCGGA
CCTGCTTGAGGTAAAGGGGATTGGCCCCAAGTCCATCGAGAAGATCGTGGCATTCTGAGA
GGCAGAGGACCCTTTCGGGCTCAACAGGGTTCAAGACCTGTTCAAGTCTCTGCGGCGAAT
CATCAAGCCCGGCAAAGGGTATGGGGTGGCAGCGCCTACGCATAACACCGAGAGCATCCC
GCGTACGGGCGACCACGACGGCATTGTCTGGATGGGCATCCCCAAGGCCAAGAACTATCA
GGACTATATCGAGAACCAGCGGTCTCGGTACGGCAAGGAAGAGGCCGAGATTCTCGCTGA
GATGAAGGATCCGCTTCTGGTCAAGAGCTGTGTGGTGCAGTGCTTTGACGACTATGACGA
CGACGTGTACTGCCGGTGGAATCGCTGGCACTTCACCAAGTTTGAGAAGATGCTGGAATC
GCTGGCCGTTGATGGCAGCAACGTCCTCATTGTGCGCGGTCGCAAGCGTGAGGACTTCGG
AGTGTCACTCCACGTGACGGATGCGTGGATGCTTGAGTGGGACGAGGACGATAGGCAGAA
GTATGGGCAAGAAGAAGAAGCGTGTGGGCAAGCCCGGCCCCAAGCACAACCCGCTGAAGC
TGGTCGGTGGTGGCGAGGACTCGGCCGCACGCGTCACCAAGGTCATCCAGAACATGCTCA
AGCCGCCAGAGACGGTGCTCGCGCACATCCAGCGCGTAGGGGCTCCGTTCTGGGTAGCAC
CATTTGGAGTTTCACAGGAGGGCGAGCAGTACGCGCGTCCGTGCCTGGTGGTGCCGTACG
ACGACCAGATTTCGGCCGAGCTGGCCCATCTGGCTGCTGGCGGGGAGCTTGCCGAGACGT
ACACGGAGGAAGCCCGCAATGCGAGCCGCGCAGCTGCGCGTAGTGTCGCAGGCACACAGC
CCAAGAGCGACCTGGACCCCACAGCCATTCTGGCCCAGGTCTAGGAGATGCTGCAGCAGG
TTCACGGCGCACAGTGGCGGGTTGCTGACACAGCATCAAAGATCGAACCCACCGAGCTTT
CGGACGAGGAGCGCGACGAGCTCCTCCGCAAGCTGCGCGGTTAATTCCAACAACAAGAAG
GGAATCACATCATGACCGGGAAAGAGATCGCCAACTACGCCGACGTGGCGATGTTCAAGG
CCGAGAAGATCGACCGCACCAAGGGACCGCAGGTGTTCCTGCTGAGCTGCAACAACGATC
CTCTTGGGAGCATCGCGGCTGCTGCCAAGGCGTACAAGGGCGAGTTCGTTGAGTCACTGG
CCCAGATCACCGACGACGAGCGCCGGTACTACCTCAAGGAGGTGCAGAAGTCGGTGCTTG

AAATGCCTCTGGAGGCTGTCCAGTTCCACTTCCGCATCACCGGTGTGACTCGCGGGTTCA
CCCACCAGATGGTCCGGCAGCGCAACGCAGCCTACTCCCAGGAGAGCACGCGCTTCGCCG
TCAAGGAAGACGTCCCAGACGGATTGCCACCCAGCCTGCAAGACACGATGCCATGGAACG
AATGGGTCGAGGAATGTCCGATGGAGATCTATCCAGTTCGCAGCCAACGGCGTGAGTATC
TTCCGGACACGCGTGACCAGGCTGAAGAGTTCGCCGAAACGATGGCCGACAAGGATCAGC
TCTGCCGTCGTCGGTGGGATAGGGCTATCGGCGTCATCGAGGAGGAGTACAACGCTCTGG
TCAACGAGGGCATGCCAGCCGAGGACGCGCGTGGATTGCTGCCGACGCAGCTTCTGACCC
AGATGAACTACATCACCGATCTGCGTGGCCTCAAAGGCCATGCAGGCGTGCGGCTCTGCA
CGCATGCGCAATTCGAGTGGCGTCAGGTCTGTGCCCAGATCGTCCGGGCGATCCGTGAGT
ACGGGAAGACCCAGACGTACATGACGAACGACAATGTCCATGACAATCCGGAGAACCAGA
AGTCGTCGCAGTGGCAGTTCGATGCCTTGGCGGACATCTTCCGGCCGATCTGCTCCTACC
GGGGGAGCAGCCAGGTCCAGGCAGACTTCGACCGGCACTGCATAATCCGTGATCGCGTCG
AGGAGAATGCCGGTATCAATCGGCCGAGCACCGACTGGGACAATGAATGGGTCGGACCGA
TTCCTCTAAGCCCCACTGGCGCTGAGGGTTACATCGGACCGATCTTCCCGGTTGAGTGGC
TCCTGGATCCCTCAGCGGCAAGGACGAAGTGACCAAGGAGACCGGCGCATGCCGACTGTG
TGGGAGGGTGGTCGATCGGGCCGCCCTCACCCAACACTGGCTACACAGCTTCATCTGCGC
GCGATGCAGTGAAGGATTGAAACGATTGAGCGCGAAGCTATTTCCCACGACAGGAGGGGA
TTCAGTTGGGAATCATCGTCATTGATTTGGCCGAAGGGAGTTCGCACCGTGCCGCTATGC
GTAATAAGAAGCGCAACAGAGACAGCGAAGCCGGCATCAACTATTCCACAGCACCGGTCA
TCGCTGTTGATCCGGGCGGAACGACCGGCTGGTGCTTCATGTGTGTGGAGCCATTGGCTC
TGCACGACCCGCAGGAAATGGTACTCGAGAACGTCATTACTCATCAGCACGGCGAGCTCA
GCAGCACTGTCGGATGCAATGTCGCAGAAGGCGAAAACCTCTGCGTCGCAGACCTGTATG
AATTCATCTCGGCTTGGCCCCAGGCCGCTGTCCTGGTGGAGGACTTCGTTATCCGAAGCA
ACAATCGCAGTCGCGTATTCCTTTCGCCCGTGCGGATTACCGCCGCACTCAAGCACCTGC
TATGGGCCGACAACCGATACATCATCCGATAGACACCAGCAAACGCCAAGATCACTGCGA
GCGATGACCGGCTGAAGAGCTGGGGCTTGTTCGACGAGCATGGCAGTCTTGGGCACGCAC
GCGATGCCGATCGGCACGCAATTACATTCCTGCGCAAGGCCAAAAGCAAACCGAAGCTCC
GGGCGATGGCTTGGCCGTACCTATTCGGAGAGGAGGGTATTTATGCAGTCGAAGACGACG
GACAAAGCTGGTGCTGATGTGCCGCCAGCGTGGCTGGTTCGGGTGCTGGGCGAACACCAG
CCGCAGATGACTGGGCTGTGGTCCACATGCTCTTGGAAGAGCACCGACGCCTTCGGCGCT
CCGAAGCACTACACAACCTTCTCTGCGGAACATCAAGCGGCCGAAGTACATCGCGCTCTG
AGTGATGGCGTCGCAGCCCTTTTCTAGGAGGTTCGGTGAGCAAATCGGACGAGTTCTATG
CCAAGGCGTTGGTCGCGCTAGGTAATGCAGAAGCTGCGCAAGCTCACCTCAAGGAAAGCT
TCGACGACAATCACAACACCTTCTGGTCTAAGCGCGTACTGGCGCACTGTGCCTTGGCGA
ATGCCTATCTCAAGGTCGCAGAGTGGGAGCTCGGCGTCGAGGCCTGGAACAGGAAGGAAG
AGGACCAGGTCTCGGTGCTCACCTTCGGCACAGACGAGGTGGTCGCGACGTTCCCCGACG
ACATCGAGGAGCGCCGATGAGGCGCGCGGGCATGAACGACCTCTATCCGACAGTCGTCAA
GAGCCAGAACGACGACCAATACAAGAATCCAAATCCCAAGGACGCCTTCACATTCGGGCG
CACGTGCCCGGACTACGCTGGCGGGCTACAGGTCAAGTGGGACCGGATGAAGTTCCGGCC
CAAGCGCAAGGTATGGATCTGTCCTGCCTCCTACGAGATTGAACTGGAAGGGCTGCCACC
AATCAACACGGTCGGCCCCGCATCGGAGGAGGAAGAATGCCCCGTGGAGCAGCGATGACT
AAGGAGCAAATCGGAAGGGCGGAGGAGCTTTTGAACGAAGGCTGCTCTTACCTCGACGTC
TCCCGAACCATCGGCATCGCCGAGTTCAGCATTCGCCTCAAGTTCCCCGGTCGGGGCTGG
ACACAAGAGCAAAAAGCCGAGCACAAACGTCTCAATGATCTGTTCAAGCATCTCGACGCC
ATCTGGGGCTTGGAGAGGGCACAGAGAGGCGAGAGGCGCGCATCATAGGTTCAGTAGCCC
GCATCGGCCCGAACCAGTACCATCAAAACCGTAACAAGGAGGAACCATGAAGCTCACCGG
AACTGCCCCGCGAACGTCGCGGGTGAGCGGGTGGGTCGAGCCAGAACTCGACCAAGCGCT
CAAGGAGTATGCCCGCGAGCTCGGCACCAAGACCTCACCAGCCTTGCGCCGCGTCATCAT
CCTTGGGTTGAGCCAAGAGGGCTTCCAGATCGACAGCGAAATGGTTCCCATGCCCGACAG
ACCATCACACAGGAAGGATTGACCGATGAGCCGAGTGGAGGTGGCCCTAGCGAGCGTAGC
GCTGTCTGCGGCGATGCTCCTGAGCCAGGCCGCTACTGTGGGCGCAGAGCCCGAGCAGCT
GTCCGGGAAGGACCAGTTGTACCTCACACCCCTAGTCATTGGCGAGGTGCCATACGGATC
GGCTGCAAATGCCCTCTCCATTGCACGTCGTGTATGCCAGGCTACCACTGGCGGAGACTG
GGACGCCACCTCTCTCGGGAAGCTCAGCCAAGAGAGCATCCGCAACAACCCGAAGTTCGA
CGCCGACTCGGCAGCGAACATCATGGTGTCGAGCATGACACTGTACTGCCCCGAGAACAT
CCCCGACTGGTTCCACGCCGAGAGGGCCAAGTATCTCGCCGAGGACGCCGGTGTGGCTGT
CCCTGTGGCGACGAATCCTGGTCCCTACAAACCCCTCCCACGCTAGGAGCGTTTGCCATG

GAAACAGCGCCTTTTGGCGTATCTACCGCTGACTTCATCGTCTGGGGGATCACCTTCGCG
CTCACGGCTCTCACAGCTTGGCTGCTGAAGCATGGTTGGCGCGTGAAGACTCCGCTAGAG
CTTGTGCATCGCACCTTCAACAAGCACGGCATGCCCACCAGCGGTATCTGCCCACATGGC
TTTGATCGTACGGCGCACCACTACGCGCTCCGAGGCTATGACCAGGTCGAAACCCAGGGG
TGCGGCGAATGCCTGAAGGAGCTGACCCCAACTCAGTTTACCCACATCACTAGCTGTCCC
AGCTGCTGGTTCGTCGGGGCGCTACCGTTCGGCCCGATAGAAGCATACTTCAATCTCCCA
CCCGAGCAGACGATCTGGGTCGAGAATAACGATGGCAGCGTCCATCTCCGCAAAGCCTTG
GGAGAGAACTGGGCCGACACCAAATACAAGGTCTGGGGAGAATGGCAGCGAGAATGCAAG
ACCAAGGACCTGAATCTGATCCGGGAATTTCGGGTGAAGCCTGTGGTGTACCGCATATGC
CCGAGTTGCGCGTACACACGGTTTGAGCACTAACCGAAGGGAATACATCATGATAATGAA
AAACAGTTCGGAGGACGCACTGCGGAAGGCTGCAGCTGCATTTGCTCTGCGTCAAATTCG
TGAGGTCAAGGAGCGCGCTGACTACATGCTCGTCCACGGCCTGACCGAGGCCGACGTGTA
CGGAAAGCATGCTCGTGAGGGCTGGGAGCAGCGCGAGAATCTTGGTCGCTGGATCATCAC
GCCCAAAGGACTCAAGAGGGTAGTCCCAGACCCGGACGACGTCGTCGGCTGCGTGCCGGT
TCTCGTCCGAGACCCGGACGACATTGGCGAGCACCGAGAGCGTAACGAAACGTTGCGCCG
GTACTACGATCCGCGCTTCGGCAAGGATTGGTCGGGCTTCGTCGGCAAGGTTCCCTACAA
GCAGAGGTATCGGGAACACAAGGAGCCACAGCATCTACGGCACAAGCCCTATGGGTATCC
GAGCCCGTCCTGGGAGCTGAACTACTTCCGCGTCAACACCTTCGGTTGGACGCACTATGT
CTGCCGAACCAAGCAGGACGAAAGCTGCTGGGGCACGAAGAGCCCAGAGGAGGTGATGGA
CTGATGGACTGGAATCTCCTGATCCGCGTTGCGATGGGCGCTGCGATGGGCGTGACGATC
TATTTGGTCGGATGCATGGTCGGATACATCGTCGGATACAGAGCAGACAAAAGGAGGCCC
CACCAATGAGAGGCGTGAAGGGCTCTGGCCAGGGCACATGGGCAGCGCACTACGACAGCG
TGTGCTCGGTGTGCCCGCACTGAATCTTCAAGGGTGATCCAGTTCACAAGATGCACAACA
AGGTAGGCCACGAGGAGTGCCATACGCTAGATGAGCGTGGCGGGTTCGGACTTGATGTCA
GCGGCATTGAGCAAGAGCGCATCGACGGCCGATACGGCAAAGGCTACGAGGACCGGCCGA
GCCACACAGTCCGGGGCCGACGCAAGCACGAACGCAAATGTGGCGAGTGTTTCCAGATAC
ATGCAGGAGAATGCCCATGAAACTCGAAGACACACACGGCGCATACGCCGGTAAGAGCTT
GGCCGATAAGATGCAGGAGAAACTCGACTCTGCTTACAAGGCGTGGCGCGATGCCGGTGG
CAGTGATCATCGACAGAACTCGCAACAGGGACGGCACAAGAGGAACTTCGACCGGGTTGA
GGGTTGTGCGGCGCAACTCTGAATCCTCAGGGGCACAAGTACAAAACATGAGATCGAGGA
AGCGGAAAGGCGAGACAGATGAGGAATCCCTTCAAGTACGGTAGCCCGCAGCGGCTTGTG
TTGGCGTACTGGCTGATGCGGTGGGCGTACCGGATCGTTGAGAGCATTGATCGCGGTCCC
GTCCCGAAGATCAGCATGTGGACCTTCTCCTTTGAGCGTGGGAAGGGTGCGGTAGTTCGC
TAGGACGATCGGGCAGAGAACAAGGGTTGCAGACTCGTGTACCTCAATGATCGTGAGTAT
GACCGTGCCTGGTCGGATAGTGCGTCGTTCGGCAAAGACGAGGACGAGGACCAAGACGCC
TTCCTCACCCTGGATCTGCCCGATAAGCGGTACAGCCAAGTACGACAAGAAGATCGGCAT
CAATGCCGACTGGCGGAAGACCACGACGCGCGGGCCACGAGGAGCGTACGGCAGTCGCCC
AGACAGTCCAGGAGACCCAGAAGCACCCAGTTGTAAGGGATTCGGGTCACGCTGGCTGCC
AGACGGCACTTGCCCCGGCTGCGGCAGGCACTGGACCTGCGTGTACCAGACGCGCGACGC
AAAACGCAATCCCTTCGGCACCAACGGAAAACTCACCATACCTTCTCACAAGACCAAACC
ATTGGAGGGACAATGAACGATCCAGGCGCCGAAGCCGGACAACGGGCGATGAGGAAGGTG
CACGACTACCCAAGGCCCAACCATGACCTGATCGTCGCCGCCCGTGAGGCGTTGAAGCCG
ATCCGCGAACTGCACTACCCACAACCTGATGACCGAGCCCCCTTCTGCCCGGAATGCCTT
GTCGAGTGGCACTGCGACACCGCCAAGCTGGTCTACACCACCGAGGAGCTGGAGCGATGA
TCTTATGGCATGCTCGGCGTGAACTCGAGCGCTCTCGGAAACATCGTTTCTACAATCGGC
CGGTGGACAAGAGTGTCCAAGCTATGTACACCTGGTGGGAGTACATGGACGAGATCGACT
TGGTGAATGGCGAGAACGACACCATACGCATCACGGTCATGGAAGAAGAGACTCACCCTC
CTCCAGGAGATCTGCTCCCTGAAAAGCCCAAGGTCATTCGCGCACTTCATCAGAGGTTGA
CACCGATAATTAGCCTCGAGTACCAGATCAACACAGCGTACGTAGCCCAAAGCACCATCA
GCCCGCTCGAGGAGATCAGGGCCGACGGGTGGATGGCGCTCGGCCACTATAAGATCCATC
GGTACTGCATCCCAGGCCGCATCAGGCCTGGCGGAGACGGTTACGAGCCATATCGCATTA
TCATCCTGGCGGGTGAGGTCAGCCCTCCGGGCATATGGTATCCGTGGTCAGAACAGCCCG
AGGACGTGCCGAACGAGGTACTTGACGGAACTTACCACGGGATGTTTATGGCAGATAAAT
AGCCCATCCCCAGAAACAAATACCATCCCCTCACAGAAAGACAGCATATATGTTCTGTCG
TAGGAATCGCGCGGCCGCAAAAGAGAACGCGCGCATGTACAATCTCGGCAAGGTCTATGG
CTACCTCGACGCTCTCAAGGAGCTCACCACAGCCATACAAGTGGAAACTCAACTGCGCGA
AGAGAAGGCGCATGTGGTGTCTCCGAGCGAGCCGCCGTGGACCCTCCACAGTTTTACCTG

CTGGTTAGAGGGCGAGGTGCTTCGAATGAGCAAGACAAAACAGATTCCGTCAGGCGAGTG
GGTCAAGGAGGTCGAGCGCGTACAGTCACGCCTAGACCGTCTCACGCGTCGTCGGAGGTG
AACGACATGGATGTCGACCTCATGATGGCAGTACGTAAACAGCGGGCCACATGGCCTCGA
CGAGGTGAGTGGAAGCCGCAACCCACCGACAAGCCCAAGGCTGAGCCTGTTAAACGGTAA
GCTATCTAGCTGCGGTAATAGGTCGACCTTGCGGGTCTAGGCCAGAGAATAAGAGACACA
TCTTATCATGTCAACAGCTGGAAATATCAACAGCGCCAAGCCATTCCGACGCGCGGTAGA
AGGCTACCGTGAACGGGGATGGGCGGGAACGCTACCGCTGTCGCCTCGGAGCAAGAAGAA
GCCTCCAACAGGTTACACCGGGCACACAGCGCCGTACCCGAGCGACGATGACATCGCGAG
CTGGCTGCGAATGCCCAAGTACGCCCAGGGCAATATCTGTCTGCACCTGGGTCAGGTAGC
GACGCGTGGGCCGCACGGAGAACGCCTGGAGCTCATCGGGATTGACGTCGATGACTATGA
GGAAGGTGACAACGACAAGGAGGGCGGCAAGCAGCTCGCCGCGCTCGAAGCCGAACTCGG
GCCACTCCCCAAGACGTATACCAGCAGCTCGCGCACAGGCGTGGGCGGGAACCGCTTCTT
CCTGGTCCCCGCCCAGCACGCATGGATGGGGAAGGCCGCGCCGCACATCGACATTGTCTG
CAAGACATACCGTTACGCCATCGTATTACCAAGTTGGCACCCAGGCGAGGTCGATGAGGA
GACGGGGAAGGTCATCACCGAAGGTGGCCAGTACAAGTGGCCCGATCCGCAGGGGCAGTT
GCTGGATGGGCTAGAGGACATCCCTGACGTTAAGGATCTGCCCCTCCTGCCCGATGCGTG
GGTAGAGCGGCTGAGCCGTGGCGGCATGAAGCTCTCGGACGAGGACGCCGAGATCGACAT
GGATCTGAACGTTGACGAGATGTTCCGGTGGGCCGTGGGTACGTTGCCGGGCAGTGACGA
GGACGCCCAGCCCTGCTCCAAAATGAAAGCGGCAGTTAAGTACTGGCAGGGGCAGATTGA
GGAGGACGCCTCTACCCACGACAAGATCACCGGCTCGCACTGGAATCTGGCCTGCTTGGC
GACCGAGGGGCACACCGGCTAGCGGCAGGCCATGGCAGCTGTGGATGCGAGCGTCGCCGA
AGACACCATCAAGAACCGTGGGAAGCGCGAAATCAGCGAGCCGCGCTCAGAAATCACGCA
TAGCAAGGTGAATGCACTCCGAAAGCTCAAAGCCCAGATAGACAAGGGCAGGCGCAACGT
ACTCACGGTGTGCACGTGCTACGAGCCCAGCGCAGACGAGCTCGACGCGTTCACCGCCAG
CCTCAACAGCGCAAAGGCAGCTGCCAGCGTGCCTACCGAGGAGCGCGCGGTCGCCAGCGG
CGAGGGGCGAGACGGATATTGGCGGGATGGCATGCTCAATGGCACCCCGCAGGACCCCGG
CGACTACGAGCAAAGCGACCAGGGCAACGTCGATCACTGGATCAACCTGCACAAGGACAA
TGCGTTCTACGTTCCGGCGCTGGGACAGTGGATCATGTGGACAGGGAAGAGCTGGATCGT
GGGGGAAGGCTGTGCCGAGCGGAGCTACCGACGCGTGAAGGCTCGCCAGAAGCGGAATGC
CGAACAGCTTATGCGGCGCGCGGCTGAGCTCAAGGCTGCGCAGGATCCTACGGCCAACGC
GGCTGTGGCGATGGCCAAGAGCTGGCGTAGCTAGGCCACCCGCAGCGGCGATGTGGGGCT
GATTGAGCGTGCCCTCAAGGCCGCCAGCATGGAGCTCGGCATCTAAGAAGGCGAGCACAA
CTCTAATCCGGCGCTGATCTGCTGCGGTAATGGCGTGCTCGAACTCGATCTGTCGCCGCT
CGACTCCCTGGCGGACGACTCCCAGAGCGCTGGGCGCAACGGGGATAGAGGTAGGCGCAA
CGGACGCAACGGCACTGCAAATGTGGCTAGCTTGGCCGAGGACGACGAAGAGTTTGATCT
TGACTACGAGGCTGTGGCCCGCAATAGCCATGTGCGCCTGCGTGAGATCCGGCGCGAAGA
CTTGCTGACGCTGAGTACTGGGACTAACTACCTGCCCTGGCAGGAGCTGGTGGCCGCGGA
GTTCGGCAAACAGGAAGCGCTGTATGCGAGCACGTGGGCCCGCGCAGTAGAGATGTATCT
GCCTGATGAGGAGGTGCGGATGTTCCTGCAGAAACTGCTAGGGTACAGCCGGCTCGTCGA
CAACCGAGAGCGCATCGTCGTGTTTCCGCACGGGCCAACGGGCAGTGGTAAATCCACCTT
CCTCAACGCCACGCTCAACGCGCTCGGCGAATATGCGGATGTGGTTGATCTGGGCATCTT
CAAGGGCGACCGGCAGACCAACCCAGCTCAGGCCTACGCCCTCCCCAAGCGCATTGTGAC
GTGCTCCGAGGCTAGCCAGCGAAACGTGCTGCACGCCGACATGTTCTAGCGAATTACAGG
CGGTGACCCGTTGAGTGCAGAACTGAAGTATAGCAACGAATCTGTCAAGCGGAAGCCCGC
ATTTGTGCCGTGGAACGCCACCAACACACCACCCAGCATCCCTGGGGCCGATGCTGCGGT
GGTTGATCGGACGGTGGTGGTGGGATTCAATGAGCAGATCCGTAAACAAGACGTCGGCAT
GAATGCGATGCTGTCTAGTCCGAGGGCCAAAACGGCTGTGCTCGCGTGGGCGGTCGAGGG
CTGGGGAATGAACCGGCGCGAGGGGATGAGGCGCGCTGATTTCCCGACTGCAGTCAAGCG
TGAGAGCATTGAATTCACCAACCAGATCTCCGATGTGAGCGAGTTCCTGAGTGACTGCGT
GGAGGAGGCTCCGATGAGCCTCCGGCGGAAGGCTGAGAGGCGCGCGTGGCGCGTGTGCGA
CTGGCCCGAGGAATGGCACATAACGGTCAGCGAGGTGTACGACGTGTACGTCACCTGGTG
CCAGGAGAACCGCGTTGCGGATCGTAACATCATGAAGAAAAATGGGTTCTCGCGCCAACT
GAAGGACTATGGATATCGGGCCGAAGTTGTCAAGAAAGATGGCTTCACTTCGAGAACTTA
CGCTGGCTTCAAGCTCAACTCTCCAAATGCACGCGTTTTGAAATTGCAAAAAGACAGCTG
ATTATTGGACAAGAATGGCTCTGAGACGGGGCTGTTACGCAATGCGTATCCCAATCAGTA
ACTACATTTGACCAGCGGAAACACATCATTTTGGTTACTGGTTGGGGTTCGTTTTCGCTT
AGGGCTATACCTACTCTCGTTCTTATACCTCTACCCTGTTTTCCCTACTACCTATTATCT

AAAAAAGCGAAACGGGTAACTCAGGAGGGTATGAAAATGCTGGTAGAGCAATGGATTCGC
ATTTTTGAGAGACCCGGAGTGCGCTACCGAATAGGTAACCCGAACACAGGCTACATGCGG
AAACATGTTCGTTTCGCATAATTTCGGTGTGACGGATAGCCTGGGAGGGTATCAAGGAGC
GTAAATTTGTGGGATCAGAGATGGTTTGGACAGCAGCAATCAAGACTGATGGAATTGGAG
GAATGGC
>NewGenomeName_17
CTGAAGGACGGTCTTTCTTAATCACCAAAACGACAAGCATACGAGTTAGTAAAGTGCGAG
ATATCAAAGAAAAGGTTAAACAAGATTACATAAAAGGGATGAAACAAAAGGAAATTTCAA
CAAAGTATGACAGTAGTTTAAACACTTTAAAGACATGGATAAAAACAGAAAATTGGGGTA
GCGAGAAAACAATGGGTGCACCTAGAGATAAAAGAGGTCCACCCATAGGTAATAAAAATC
CCACTTGTCCTCCTGGAACTAAAAATGCTGAAAAGTTTGGATTCTTCTTAAAATATCTAC
CAGTAGAAGCTAGGGAATCAATACAAGAGATGTCGATAAAAGATAAATTTGTTATTCTTT
GGGAACAAATATCAACTCAATATGCAGAAATAATAAGAGCACAAAGTATAATGAATGTTT
AAGACATGGAAGAAATAATTAAGGAATTAAAGAAACATGAAAGCACAGAAAATGGTGAGA
AGACAGCGTATGAGTTTCAATTTGCATGGGATCGGCAAGCATTGTTTCTTGCTGCACAGA
GTCGGGCTATAAGTAAGTTAGGGAGTTTAATTAAACAGTATGATGAAATGATGCATATGG
ATTGGAACGTAGCTACAGAGGAGCAGAAAAATAGAGCTGAAAGGTTAAAATGTGAAGTTC
TTAACCTTAAGAAAAGTGATACTGGAGATGATTCAAAAATTTGGGTTGAAGCTATACAAA
ATATTGCAATGAAACGTTGTGTTACCAATGGCTAAAGCTTTATTGTCGCTATTAGATTGT
TATTGGGATAATCCTGTTTGGTTTGCAAAGGGTATGTTAAATGTTAGAGCTGACAAGTAG
CCGTCTGATGATCTGATGTCTTTAGCCCAAACCCCACAAGTATCTACTAGAAGTGGTCAG
GGAGTAGGTAAAACTGGATTACAAAGAATCGCTACTGTGTGGTATGTAAGCACTTGACCT
TTCCCTAAAGCAGTTGTTACAGTTCCAACACGACAACAATTATCTGACCTACTATGGGCT
GAAATAGCTAAATGGCTAAGTAATAGCAAGGTTGAGAAGCTACTTGAGAGGACTAAAACA
AATGTGTATATGAATGGCTTTGATGAATGATGGTGGGCTACACCTAGAACAGCAGTAAAT
CCCTAGAATATGCAATGTTTCAGTGATGATGATGTGTTATTTGTTGTTGATGAATCTCCG
GGAGTTGCGGACCCCATTATTGAAGCTAGATTGGGAACATTATCAGATGCAGAAAGTCAA
CGTCTTTTGTGCGGAAACCCATCTCGAACGAGGGGAACGTTTTACGATAGCCATAATAGA
GACAGAGGTTTATATAATACAGTTAAAGTATCTTCTTTAGACAGGCCTAGAACAGCGATA
GAAAATGTTGATATACGCAAAAGAAAGTACCTTGAAGGTTCTGACCCTTGGAGTGTCAGA
GTACTTGGAGAAGTTCCAGAAGGTGAAAGTGATTCTTTAATATCTTTAGAAGCTGTTGGA
ACAAGCGCAAAATGAGAAGTGAATCTATTTAATGACTATATATTCAATATTTCTGCGAAT
TTAGCAAGATATGGTGATGATGAACCCATAATAGTTCCTAGAATAGGCGTTAACGTTTTT
GACTTATTAACTTATTCAACCAAAGATACAATGGAAACCGGAGGAACTAGACTACGAGCA
GTTGCTAAATTTAAAAATATCTATCATCGAATTAACAGAGTAAAAATAAAAACGGATGAT
GATGGCTTAGTTGCAGGTGTAATAGATAGATTAAAAGAAGTTATAAGACATGAAAGACTT
AAATATGAAGTAATACATATTTAAAATGGTCGTAGTTCTATAGAAAAAGATAAGTACTAT
AAAAAAGCTTCTGAAATGTGGAATAAAATGAGGGAGGAATTAGACGCAAATTTACGTAGT
TTTATACTAAATAAATAAGCTATAATACCGCCTCCTAATGATGATAAACTTATTAAACAA
CTATCAAATATAAAATATCCAGTAGATTCAAAAGGGAAAATACAACGAGATAGTAAAAAG
GAAAGGAGAAAAAGAATTGGAGAACCACCCGATAGCGCTGATGCAGTAATATATTAGTTT
GCAGAAAGTAACAATACCGATTTATCTTTACTTAAAGGGGGTAGGGTATGGGGATAATAT
CTTATTTAAAAAAGCTAGTTAAAACAGCTGCAGGCGCGATTATGCGTATCTCTACTGGTA
AAATTGGAGGATATAAATTAGACGATACTACAATTGATTATGAGTTAGCAAAAGAAATGT
ATCAAAATAAAAATCCTAATTACAAGTTAGGTTCGAGTTTTGTTAGACCGATTGTCAAGT
CAACAACTCGTTTTATGTGTGGACCTCATTTTCAAATAGAAGATGAAGAAGCACAATATA
TAATAGATGAATTTGTTTTATATAACACATCTAAAATGTTAAAAGCACATACAAATAGTT
TAAAGCAAGGTGATTGTTAAATTTGGATAACTAGAGAAGAAAGAGAAAATCCCTTATCTA
CCGCTTAAAAAGTTAGATTAATATATAACTTCATATCACCCGAAGAAGTCAAAGTAATAA
TATTATACCCCACAACAAAAGAGCCTATAGCTGATATATTAGAAAGTCAAAATGAATGGA
CTGACTCAGGAGAAAACGAGAGAATGGCTCACGTTGATCAAATAAGAATTGCTGAGAGTA
GAATTGTTGAGGTTGAAGGTGATAAGATAGAAGGTTTAGAAGAAGGGGAAACGCCTAATG
TCTGGTGTTTTATACCAAAAATACATTTTAAAAATGAAGCTGGTGAAACATTGAAATATG
GGCAAAGTGAAATGGCACCAATAGAAGCTCTTTTAAAAGCTGATCATGATGTTATGTGAC
ATGCGTAAAAAGGTAGCAAAATACCCTCTCCTCCAAAGCTAAAGTTGAAATTCACTGCTG
TTGCTAGTTTTTTAGCACACAATTTTGGTGTTGAAGACTCAGTTAAATTTGCCTAAGAAG
GTGGAGAGATAAATCTTGATGGGCATGAAATACTATTCTTAAACCAAGATGAAGAAGCTC

AGTTTGTAGAAGTAAAATCAGCCATACGAGATGCTAAGGAGCATTTACAGCTTCTTTTTT
ACTGCATAGTAGACGTATCTGCAACACCCGAGTTTAAATTTGGAGTACATACACTTAGTG
CTTTATCTTCTGTAAAAGAACAAATGCCTATTATGGTAAATAAGATAAGTAGAAAGAGAG
AACAATTTACAAATAGCTGGCAATTACTTGCAAGCATGGTTTTAATAATGAGTACTAATT
CTAGTGGTATGGAATATTCATCTTATGATGTGACTATAGGTTAGGATGAAGTAAATCCAC
GAGATGCTAAACAATTAGCTGACACGCTAGAAAAAGTAACTAGTGCATTAGATAAAGCTT
TACAGGGTGGCTTTATTAGTGAAGAATCAACAGTGAACTCTTTAGCACAGTATATATATA
AAATGAGCTATTATATAAGTGATGACCATGAAATAGTTGGAAGAAGAGAAAAGATAATAA
AAACCACGATGTTAAAAAACAGATTAGATAATTCTCATGGTTTAAATGATGAGCCAAAAG
AATTTGAGAACGAAAGAAATATAATAAAGGATTATAAAGGGAATGAATAAATGTAATTCG
GAATTAATAACTGTTGCAGGGGAGTACAAGAAATGGGCATTAGTAGCTAGAAAAAAATTT
ATAGATTAAACGCTCAAGCAAGAGGATGAAATAAGAGCAATGTGTCTTAACATAACAAGA
AATAATACAAAAGAAATAAGAAAAGGAAATCTTTCAGACTTTAACAGAGTTTGGTTAAAT
CAGATACTAAAACAATTACCACAAGCAATAAAAATATTAAATCAACAACTAGTATTTAAT
TTTGTTGAACACTTAAATAATAATGTTGAAATAGCTATTAATCACTCTAAAAATAATTTA
ATTAATGCATTTGAGACAGCTGAAATAACTAAGGTAACTAAAACAGTGATACAAAAAGCT
TTCTATGATATTAATATAAGAACTCTTGAAGCTTATTATTCACGGGTTATGGATGGTTTA
TTTTTCTCTGATAGAATTTAGTCTAAGTGTAAGAAGTACAGAGCAGATATGCAAGTTAGA
TTACAAACAGCAGTAACAGAAGGTCGAGCCTGTGTTGAAACAGCTAAAATGGTAGACAAA
TATGATCTAAAAGGTAGGAAAACTTTAGTTGATGGCCATCCAAATATGATAAAACGAATA
GGAAATAGAGTACCTCAAAATATAAGTTATGACGCTTTATGATTTGTAAGAACTGAAATG
ACTTCAGATTATGGTGTCGGAGTTTTAGCTTCTGCAATGATTAACCCTGCAAGCATAGGT
AGGCAGTTTATGATTTCCATGGAACATCCTCCCACAGATATATGAGACGAAATATGTGGA
GAGGATAATTTTGTTTTGGGTAAAGGTGTTATTCCTATAAATGAAGCTCCTGTGTATCCA
TTCCACCCTCATTGTTTGTCTTTTATGCTTAATGTAGTCCAACCTTTAGACAGATTAGTT
GGAAGGTAGAAATATTGGATTAAAGATCCTATGAATAATGTACCTCTTGAAATATGGTAT
CAAGAGGTGGATGGTAATTTGAATTTTTGAACAGAAAGGTGGTGATACAATGAATGTAAT
AACTGGAGAAGTGGACTCAATGAATGCGTTAATAACTAGTATAACGCCTTCTGATTTTCC
TTAAGCTAAAGATATAGCCATAGACGCTTTAAAATCTATAGATGTAGAACCTCTTGAGGT
AGTTGCTTAGATACCAGCTACAAAATCTAGATGGATGTGGAATTATACTGCTAAAAGCTT
GAAAGATATCGTAGATAACACTAATGAAAATAATCTTAATGGCTTTTTAGGACCACAAAA
AGCTGAAATTATATCGAATGAATTTGAACTACCTGTAAAGCATTGGATAGGTGCAGAACC
GAAAGGGGATAAGGCTTTGATCAGAGGGCTGAGTGATGCTGAGGCAACAAATTTAAAAGG
ATGGACTAGGACTAAATGGATAAACGAATGTAGTATATTTGGTTGCTCCAAACTTAAAAA
GAGTGCTAAAGGCGAAATGAATGTTATAGGATATGAGCCACTATCTATTGATTGGACTGC
TCTACATAGACCAGGTAAGACAACAAGTATCGTAGGTAGGGAAATGAGCCCTAATGGCGA
ACAGTAAGATGCAACTTTTGAAGCTTTAAGAATAGATTTATGAGAAGCTTTAAAAGCCAA
GTTTTCTATAAATCATAATAATTCATATCTCGATATACAAAACTTAAGATATAAAACCAA
TACTGTGAGATATGACTTAGACCAAAATGTATTATGCAAGCTTTATGGTATACCATTCAG
TATATTTGAAAATAAAGTAAATCTAGGTGAAGGAATAGAAGTAATCAAGAATATAAGCTA
TGAACCTAAAGGAGAAATGGAAGTAGCGGAAAACAAATTGGAAGGAAAAGAGTTAATACA
AAATGTCAAAGGACTACTGCAAACTAGTCACCTATCATATTCAGAGGTCATACAACGAAT
AGGCTTAACTAAGGAAATTTCGACAGGAGAGATGGAAGACGTAAAAAGATCATAAATAGC
AGAAAAATAATTAGGAGAAGTGAAAAAAGTACTAGGAATAGTAGGAGACATGGACACAGT
TGAAGTGGCAAAAACGGCTTCATACGCTTTAAAAAATGAGAAAAAGGAAGCGTGGAACTG
TATGGTTAATAAAGCAATTAAAGATAAAGTGCCTGGTGTAATAGGTCAAACATTAGTTAG
AGAAGTGTTAAATCATCAGGAAGGCTCATGTTAAGGAGTAATAACAGGAGAAAGACAAAA
TGTATTAAATTATGTGTTTGTAAAATATACAATGTCTAATAGGTATAAAGATAATGCAAC
AACAACAGGTCTGTTAAACTCTAGCAATGCTGGAAGTTTAGCATCTAAGAAAAATAGATT
ATCAAGGAGTGATGTTTATATGTCATTTAAAGGTCAACCAACGCCAAGCACAGTAACACA
GAAAACAAGAGTAAAATTAAGTGATGGGAACTCTGTAAGAGTTATTCTTTCAGACGGTGC
AAGGACTAAAACACAACAATAGAATCCTATAAATGGATTCTTTGGCGACGCTATGCAAAA
GGGAGAAAAAGGCGAAGAAATTATTTTGCAAAAAGCGCCAGCTGAACACGAAACGGCTAA
TATTTTTACATCAGAAGCTTTTGAGGCAGGGAAATTGACTTATAAGGATAATACAGCGCA
GAAATTTACTACTACATCTGCACGTAATAGGCTAGTTGGTAGAGTAAAAGTTAGGTAAAA
CAGTAATAATGTAATTTGCTTGATATTATTACCTCAACTAAACATAAGGAGTGATAAATT
TATGGAATTTAAAGTAATTAGTCAGGAAATTTTGCTGGAACTAAAAAGAAAGGAAACTTT

AAAAGAAGATATACCATTTATAGTATATGGTGAAATGGAATATGTAACAAAGAAAAAATC
AAATGGAGAAATGGAAGCCTTGGACTTAAATGAGCCACTCGGTGACATGATGACTTTTAG
CACGACTTCAAGTTTAAAAGAGTTATTAAGAAAAGTCGTATTCGATGTTGAACTAGGCAG
AGAGAAAGTACGACTATTATATTAACCACGCTATGACAGTATAGCAGTTTCTAATCTACC
ACAAGTTATGGATGCTAAGTGGGCTTTACAAGTTATCTGTGTATTCCTGGAGCATATTGA
AGGTGAAGAAATTAAATTCGGTACAATAAATGCAGAAAATGGTCCAGTTGCAAGGATACA
AACTTACGCAACTCGTTTTGCGTATACTAAAGAAATGAAGGATTTTAACCAACCATTTAG
TGTTGAGATATTAGGTACATCAATTGGTGAGAGTTACAATGCCTTGTTAAGCTTCATACA
TCTAAGCCCAAGATTATAATTTAATTATAAAGCTTCAAATAAGACAGCTTTCAAAGGTGA
TACTAATGACCCAATGTGGCTAGGAATTTGGAGAATACTAACAAAAGCACAAGATGATAC
AGTTATTGCAAAAGGACAAGGTAACACATTAATGGGTTCTAGTGCTGAACAAATTGAACT
AGAAATGGCGTTAAATGGAGGACATTTATTAATCGGAAGCATGTATCCATCTATAAAATT
TATAGCAACAGTAATTTATTATGATGTGTGGGAGGTTACTGTTGGTAAAAAAACATATTC
TTACAAAGGTTTTACACCAGGCAAAGGATATTCGATAAGACATCAGCGGGGATTCAAAGA
GTTTATAAAGAGAGATTAAACAACACAGGTTGGAAATGCTGACTTATGTAAGTGAGTAGG
AAATCAAATGGTAGGTCATTGTTATGGAGCTCCTATGGCAGCAGTAGAATAAAATGTACA
AGAAATAAGTTTTAGATCAAACACTCATAATAGTGTTATTTTTATGAGGTGATAATTTAT
GACATCATCAAGTAATTTGATAGAAAATTTAAGACTATTATTAAATGATCATGATAAAAA
TTCATTTACAGATGAAGAATTAAACTTGTTTTTAGAAGAAGCAGACTATTTTTACTGTGC
ATCTTCTCAAGAATGGGCATTAAAATTTCTTCAATATGAATATACAGTAGGGGAAAAGTA
TGAGTATAAAGTGGGTCAAGAAACATATAAAAGCTCTAGTGTAAATGACCTAGTATCTGT
AGCTTTTCAAAATGCAGAGAAATTCAAGGATCTGGGTACTAACAAAAAAGTAAACGGTAG
TCTTATGTTAGGAATAAGCTCAGAATTTGAAATATGCTAAATATTGAAAGAAGAAAAAAA
GACATAATAAGATCTATTAATATGAACCCTACAGATATTACTATAACTAATATTAAAAAA
ACTGAAATAGAAGGAGCTTTAGCAGACACTGAAACAGAGATAAAATGTGTTCTTAGAAAA
TTTAATGAAAAGACAGCAGAGAAACAAATATCAAGTGAACAGCAAGGTACAATTAGTTCT
ATTAGAACATATGGAATGTTAGTAAGTGGTGATGTTGTCTTAGATGTTAACAGTAGAGAT
CCTTTGGAGTTTCAGCGCATATATGGCCGAATGAAAATAGTCAATGTATATCCTCTAATT
GTAAAAGGTGAACATTGTGGGTATCCAGGTTCACTCGAAAGGATTGATTAAAATGAGTGC
TATCACAAATGCAATAGATGATATAAATAGAAAAAAAGCAGGTATGTTTGTACGTTGTAT
GAGTGCAAGTGCAATGATAGAAACTAAAGCTAAAGCAAATGCAAGTTGGACACACAGAAC
ATCACAAGAAAGAAAAAGTTGAAATGCAAAAACTCTTGGAGGAGGAAATAATTTCATTAT
TAGATTATCGCATGGTGCAGAATATGGAGGAATACTTGAAGAAGGTTCAGAACCACATGT
TATTACTCCAAAATCAGCTTAAGCTTTATAGTGGAGAGGTGCTTCAGATACTGTAAAAGC
AGTTCACCATCCTGGTACAAAAGCAAGGCCTATCATAAAACCAACTAGTGATAAAAATAT
AGAAAAAATAGGTAATATGATTTTTAGATATTGGGGTGATTAAATGAGGGCAGGAATAAG
AAAAGGTTTAATAGATAATATAAAAGTATTGAAAGATTATTATGAACCTAATGTCCCAAA
CCAAGCTACAAATAGGCCTTATCTGGTAGGTGGACAAGGGCAAGACAATGACAAGGGAGA
TTCGATAGGTTTTGAAATAACTATAGAAGACTGGATTTATGAAGGTAGAACTACATTTAA
GAAGTTAGATACATTAACTAACCAACTTATTGTAAGCTTCGACATGAATATTATAGTTGA
TGAATGTGAAAATGAAGCTTTCACTTGCATTTATAAAGTGACAAATGAAAATGATATTGT
GGGTCAGGAATGGGATGCGATAGCAAGATGCATAAGTTTTAGTGTTGTAGCTTCAGCAGA
TTAAGAAGATGAAACTAGCGTTAGAGGGGTAGAAGCTTTATCGAAGTACACAAAAGATTA
CTTAGAAGTTGATAGTTATAAACATATTTGGATGAAAAACTTTATAGATCCTTGCGCATT
ATGGCGTACTACAAATGCTGAAAACAAAAGAATAAATTATCATTTGATTGAGATTACCAG
AACTACGAAATGCCATCTTGTAAGCAAAAAGAAGGATGAAATCGTCACGGTTCGTGACGC
ATAAGAAACAAGTTTAATAATAGATAAAGGAGTAAGACTAAGAGAGGATAAGAATAGATG
TTTAACTCTTGTTAGAGTAGTTGAGGATAGGGACTCAGTCATGTTGACAACAGGACCATT
AACAGTTGTTTTCAAAATGATAGGTAAGATAAAAAGAGTAGGACCTGTTATGGACGAAAT
TTATAGTAATGGAAGTTTACAATGGGAGGTGTAAGGGTTGGCAGAAACAACTAATAAAAA
GACTAATGTAAGTAAGCAGAAAGAAAAATATTTGAAAATTGCTTTCTTGAAAAATAGTAA
AGAACTTGTCTACGTAAAAATGGTAGTTGCAGGTGCTTTATTTAATTGTAAGAAAGAAGA
ACTTACAAAATCAGAGTTTATGAAATTAATAAAAGATTTTTTAGCAAGAGAGGTGAAGTA
AAAACGCTACTGGTACTTGGATTGAAAAAGACAAAAAGAAGATACCGGGCTTTGATAACA
GATTCAAAACACAAGCAGAAAAGTCTGCAAACACACGATTAAAGGGTCGATTAGCAATCC
CAGTTAAGGGTAATTGCGGAGAAGATGGCAAGTTTTTAACAATAAAAAATGACTTGAGAC
AGCTTAAAACTTTGTTTGGTGGTGTTTTGCACTAGTCAGCGTATAAGTTACGTAAATTAG

CTTTCTTCTGAAATGTAAAAGAGGTGTTATTATATCGGCTTGTTGATGGAAAAGAAATGA
AGGGTAAATTAACACTACAGGATACTACAGAGAATAATGCAAAAGATGAAATTAAGCTAG
AGGCTAATTACCCAACAGCTAGAAACTTTAATGTAACAATAAAATCTAATTTAGTAGATG
CAGATAAATAGGACTTTATATACCTTGAAAATACTAAACAGTTATTTAGGTCAAGTTTTA
ATGGCACTATAGATGAGATAGAACTAGAAATAAACTCTAATTTAGATAATGAATAAGTAA
TTGCAACTAAAGTAGCTGATAGTGTTACAACACTAGCAACCTTAGTAAATGTCGCACTTG
AACGTGGTAATGATGGTTGCGTATCTATTACTAATGAGTTTTATTTAAAAGCTTTAGAAG
ATTCTGAAAGATAAAGTTTTGATAGCGTTGCACTTGACGGTGTGGCTGATGAGGCATTGC
AGGAAACTCCAAAAGCTTGGGTAGCTTAAGATAAAGAATTAGGAAAAGATATTCTACTTT
TTCTAGGTGGAAAAAACTGAGGATTATATAAACCAAATTAATGACAAATCGAAAGGTTTTA
ATGATGGAAAGATAATCAATGTTGGGAGCTCAGCTTATTACGAGGAAACAAAGTATACAC
CTAGTGAAGTAGCTGTTTATATAGGAGCATGGGCGGTAAGCAAAGGTATAACAGGAAGGA
TACGTATTGCTAAGAATATATTTGAAGAAGTAGAACCACGATTAAGCCATTCAGAAGTTA
GAGAGTGTTTGAAAACTGGTACAGTCGTCTTAGATTTTGATGATGGACACGTGGTTAAAG
TTGATCCTGTGACTACATTTAAAAAATATGTACATGATAAAAACGAAGCGACGGGAAATA
GCTCTAATATCATGTTTATTAGTACTATAAATCAACATACTTCATTAAAAAGAAAAGAGT
TTCTAGGTAAAATATCTAATGATTCAACCGGTCAAACAACTGTTATATGTGCATTGTAGA
ATCATTTTGAAGAATTAATGAGTCAAGGTATTATATCAGAGTTTAATGTTGATATAGATA
CAGAAATTCAAGCAACTACCAAAGCAGGTTAATTTTAATGGAAAAGGGATGGTGTTAAGG
TTGATGTAATAAAGAAAATTTATGGTACTGGATACCTAGGATAGGTAGGTTGTAAGGTAA
GGGAAAATATGATAAAAATATTATAGATGCTACAAGTGTTGTCGATGGTTCAAATGCTAG
AATAATAAATGATGTAGAAGAAGAAGGATAGGGAACAGAAGTTACAGGTGCAGTTGGAAA
TGATAAGAAAACTTTTAGATTAAGTTGTTGTAAATGGGAACTAAACAAGGCATCCACTCA
AAAAGGTACTTTTTCTTTACTAGTACTTAAAACTACATCTAAGTGGATTAAAAAAGGATT
TAATAAATTTGAAATAATTACAGAAATAGTTATTCCTGGGCTAGTTGGATCTGAGAGAAT
TAGATATAAAAATTGTATGGTCGAGAAAATACAACTAGCAAGCATAAAATCTGATGAAAA
TCTAGAAATACAAGTAGATGGAACTTATAAAGGATTTGAACTTTTAGATGAAATAACATA
ATAACTAAATTAAACTTGCACGTAATTAATTTTATGTGTAGCTTTTTATAAACCTATAAC
ATTGGAGGAAGTTAAAAATCGAAAAATTAGATAAGGAATTTTGCATTGAAAGAATAGAAG
AAGAAAGTGAGCTTACTAAAGATGAAATAACAAAGCAACAAGGATGTAATATAATTATGA
CATTGACGGAGGATGCTATATTACCTGAAAAAAATATTTTTTTACAAAAATTAGATGTAC
CACTTACGCTTAGGGCCTTAACGGAAAAAGAGCCAAGTGCATTGCAAAAAAAATATACCA
AAGCTACTCAGGTAAGAGGTAGTAGGGAAAGCAAACAAATGGAAGATGAATTTAATATAG
CTCTAATAGAAGAGGCTACAATATTTCCTAATTTCAGTGATGCAAGACTTCTTAATTCCC
TGAATGTATCTAAAGGTGTTGAATTTATAAGAAAAAAGTTCTTAGCAGGAGAAATCGCAT
TAATTTGTGATGAGGTACTAGAATTGTCTGGATTTTATGAAGAATTAAGTGATGATGAAA
TAAAAAGCTAATAAAGAGAGCTGGGAAGATTACTATTTTATACAACGCATAGGTTAAACA
TAGTGTTCTTCCAGAAGATATTCTGAAAAGCGAGAAAACTCCTCAACAGCTCTTTAAAGT
TTTTACACAGTATGAAATAGAGCAGGATAATAAAGCTATGAAAAATAAATAAAATTCAAA
CTGGAAGCGAAGTGAGAGAAATAGCTAAAAATGAGATGTATCACATTGATGTTATCATTG
ATGTTACAGAACATGAACAAACTAAAAACATATTAAGTGCTATGGTAAGATACACGACAC
AGACAGAAAAAACAATATAAGCACTTAATAGGACAAACGCTAATCCAGTTATACACACTC
GACTAAAAACAGCTAGTGTTGTAACTATAATTAGCAACAACTTGAACAGAGTGAGTATAA
CTATATCTACCACTATAAATGCAAAAGCTAAAGCAACTCGTGTTGTAAATCGAGTGCAAA
ATAAAGTAAATAGTGTGCTTACACGTAGACAAAGAGAAGTTCTATTTAGGGCTAGAGACA
GAGCTAGTCAAGTTGTAGATAAAGTAAAAGCTGAGTTACAAAATATGACTGCGGCTACTA
TAATTAGTTTGAAAATGAGAGCCGACCCAGCATTAACAGTTATTTCTCAAACTAAAAGGA
AATTAGGAGAACTCAAGAATAATACAATATCAAATATTAAAGCAAAAGGTGAAGAAGCTT
TGAATACTATTTCTCGTACTAAGAGTAAAGTACAAGATTTTTCTAATAGAACTTACCAAG
CAATTGTAAAACTAAAAGATGAAGCTAGTCCAAGATTGAGTGGCTTAGATGTCAAGATAA
GTACTTTTCTAAGTAGTACTATTAGTGAGTTTACACAATCAGCGGTAACAGCCACAGCGT
TAATTGGTGGGGTTGGGGTAGGAAGTGCTCTAAAAGGATTTGCAGACTTAGAACAACCAA
TGAAAAACGCACAAACTGTATTAGGTGCAGATTCAAAAGAAATTGCAGAAATGACTGCAA
TGCCAAGGGTAATGGGCCGTACAACTAGCTTTACAACTAAAGTTGAAGCGAATGCTATGT
ATTTTATGGGGATGGCTGGCTGGAAAAGCATAGAAATGATTGCGGGTCTACTTGGTATAT
TAAATTTGGCAGCAACAGGTCAGTCAGATTGAGCCCTAACAGGAGATATTGGGACTGATG
GTTTAACTGCTTCACGGCTAACTGCAAAGGCTACAGGAATGTTTGTTGATGCCATGGCAG

CAATAGTGGTAAACTATAATACAGACATAGATAGAATGGGTGAAACTTTGAAGTATATGT
GAAGTGTTGTAGGAGCATTAGGTGTTTCCATGAAAGATTTGAGTTTAGCAGATGGCTTAA
CGGCTAGTGCATGTGTTAAAGGGAGCATGGCAGGTACTGCACTTAGAGGTGGTTTAGTAA
GATGAATAAAATCCCCAGCTGAGGTACAGAAAGCTATGAATAAATTTGGAATATAAGCAA
ATAAAACAAAAGATGGAAATTTAGATGTAGCGAGCAAAATCGTTGGCCTTAGAGAAAAAT
TAGGCGGACTTGGAGGAGTACAATAAGGTGGTGCAATAAGTCGTTTGTTTGGGCTTACAG
CCATGGTAGGTTGGGCGGCTGTAGTAAACGCAAGCGAAAAGCATTTTAATAAATTCACAA
CAGCGATTGCAGAGAGTTAAGGAGAAGCTAAGAGAATTTCTGACATGAAATTAGATACTC
TATCGGGACAATTTACTATTTTAAAAAGTGCTATTGATGATGTTAGAATATCAGTAGGAC
AGAAGCTTGGGAAAGGTACAAGAGGTTCTGTTAAGCAAGTTACAAAAGATATGCCTAAAA
TAGGAGATAAAGTAGCTGGCTTTGTAAGCGGTTTTATTAAAAATTTTGACAAAATCAAAC
GTGTTATACAAGGGGTAATTTTTATTATTGGTGGTGTTGTTGCTGGATTTATGGCTTTGA
AGTCTTTTACAGTAATTTCTAGCGTTATAAGTAGAATTACAAAGAAAGCCCCAGCATCAA
GATTAATAGGAGGAATCGTTGGGGTTCTTGGACCTACTGGTGTAATAGTTTTCGCTATAG
GTATGTTGGCAGGAGCGTTTCCATTTGCTAAGCAAAAATCCGAAATTTTTAAAATTTGTA
GTACGAATATAGGGACATCAATGTCTAATATTTTAAAACCTTTAAATACTTTTATAGTAC
GAATGAAAAGTAAATTTAAAGAACTTATGAATGTGGTAGAACCTTTGTTTTTAGCTGTTT
CTGAATTAGCCTCCATGATTATAAGCGCCTTATAACCACTTATATAATTTTTGGCGACAG
TTTTCATAATAAAATATGTATATACGTTTAAGGTAATTGTTATCAAGGTAAAAGCTGTAG
TAAATCCAATAACAGGAGTAGTCAATGGTTTAGTAGAGATATTTAAAGGAATAGTTGATG
TAGTTAAAGGCTTTATAAATGGAGATGTAAGCCAAGTAGCTAACGGGCCTAAATCTATTT
TTAAGGGGATTATAGGTATTGTTACGTCGTTGTGGAGTTTGTTAGTAGATTTTGTGACAT
CACCAATTCAAACTGTAGTCGATATTTTGGATACTAGGTTTGGTAAAAAAGTGGAAGACA
TAAAGAAAAAAATGGAATGAATTAAAGGACCTTTTAACAAATCCTGCAGAAGCAGTTCTAA
TGGTTCAGCAAGTTATTTTATATAGCGAGAAAGCATCAAACGAGTTACAAGCCTCTTCAA
ACGGAGCAAAAGCATATATAAGCTCATTAAGTCAAAAAATAGGGAAAGGTATCGGAATGA
GTAAAGAGAGATTTGGAGAGCACAAAACATCTGCGACAGAAGGATGTAATAATATAGTAA
CTTTTAGAGGCGGCAGAGCAGCTGAATTCAAAGACAAACTTTTGGAAGGCATAACACCTG
CTTTAGATGCATTTAAAGAAGCTTTTTCTAATCTTAGAGAAACTTTCGGGGGCAATTTGG
ACCGTATAAAAGCAGCTTTTGGCAGTTAAAAAACTGTATTTAATGAATATAATTAAACAA
CTTTTGAAAATTTAAAACAAAAAGTTTTAGTAACAAAAGAGAGTTTAAAAGCAGTTTTTG
ATAATTTAAAAACTAGTTCTGCAGAGATAGGAAAAGCTCTTGAACCAAAAAAAGAGGACT
TTAGTGGGATAAAACAATATTTTTCAAATCTATATAAACGAATTGAAGATGATGGGGCAA
CTAAGCCTAGAAAAACTAATATGGATAAGTTAAAACAATCAACACAAAGTGCAGGAAGGT
CTTTCTCAGAGCTAGGGAATGCTTATAATCAATTAAAAGAAGTAGCAAAACCCTTTGTAG
ATTAATTAAAACGAATAAAAGATGCTTTAGCATCTACTTTCCGAGATATGGGAGGAGAAT
TACTAAAAGGTGTAGCAACTTGTATAGTTTTAGTTATAACTTCTGTTATTAATGCGAGTG
CATCTATTAAAAACGCTGTAGCAGGTGCTATAAAAGGTGTAATTGACATAAGGCAAGTAA
TATTCGAAATCATAGGTGGGATAATGTGCGGTTATGGCGAAAACATAAAACAAGGATTTT
CTGATATTTTCTAAGGAAATGGGGAAGTAGTTAAATCATTGTGGGAAGGTATAAAAGGTG
TTTTAGGAGCACCAATTAAATCTGTTGTAGATTTTGTAACTAATGGTTTTTCGGAAAAAG
TAGGTCAGGTAAAACAATGGTGGACCGATTTAAAAACTAATGTCGGTCAAAATATAAGGG
GATTTGTTAGTTTTGTAAGCAATGGTTTTCAGCAAAAAGTTCCACGAGTTGGTGTATGGT
GGCAAGGATTAAAGACTAAATAATCTGGTAAAATAAGTGGATTTGTAAGTCTTGCAGAAA
ACGGATTTAAAAGTAATGTAGATTCAATTGAATCTGCTTGGGCTTCTATTAAAATGCAAC
TATCAACCAAAATAAGTGGGTTTGTAAGTCTAGTAAAAACTGGAATACGTATTGTATTAG
ACCGTTTTGCTGAGGGTGGAGATGCAAGTAAAACAAGCATTTGTGGAGAAGCAGGCCCCG
AAATGGTTATTCCTCTTTCTGATAGCAAGAGAAGTAGAGCGCTAAGTTTGTATGAACAAG
CAGGACAGATGCTTCGAACTCAAACAATTAATAATATTATTCCAATCTCTCAAAAATTAG
GAAATAGTTTTAATTCTACAACTAGAATCCAACATAGTAATACTAGTATTAGTAATAATG
TTAGGCAATTTCCTGCCAAAAAAGATGAATTTAAAAATACCGAAAATAGAATTTCCCAAG
AATATCAACCCCAAAACATAATTACTAGTGGAAGTATTGCAAGTAATGTTGGTGGACCAT
CTATAATTATTCAAGGTAGCAATTACAAAGAAGAAATGATGCAAGGAATATTGTCTCAAG
TAGAAAGTGAATTAAGACAAGCAATAGCAAACATTGCATCATTGTAGAATTATTGTTGAA
AAAATCCTCCTTATATATGCTATAATTTAATAATAAATCATGTGAGGGGGATTTTTACTA
ATGTGGGAAAAGTGTAGAAGTCTAAACGTTTTTTTAAAGATACTTATAGTAATTATAGCT
GTTGGTATGTAACCAGCAACATTATTTCTTATGTCAATAATTCTTTTTGTTACACTTTTT

AAAAAGAAAAAAAAAGATTGCATGCATAATGTCACTTATTTTAGTTGCTTTAACATTTAAC
TTGGCACATTCTTGGTATTTGATGGATACAACTGTTCCTGCCCGTAATACAAAATCGGTA
CAAATAAAAAAAACTCATAAAAAGAAATGGGAAGTGAAAAAAAGTACAGGGGAAGCACAT
AAGAAGTAAGAACAAGGAGAACAAAAAAATACAAGAAGAAGTAAAAAAATGCAGATGAACAG
AATAAACATGCAGAAAAGAAAGTACCAACTGAACAACACAAAGAAGATGAAGAAGCAAAA
AACACAGGAGGTGATAAGTAAAAACAAGTAGATGCTCACAGAAAAGCACAAGCAGAACAA
ACCAAAAATAATGAACTCGTGAAAATCCTAGCAACATATGAAAAATTATATTAAATGATT
CCTGCTAGTGTAGAAGTAACATCAGCTGCAAATAATGTACTTGCGATTCAAAAAACATTT
GCAACGGGAAGAGATGTCGGTTCAAAATATTGGAAAGAGCTAGATGATTTAAAAGATAAA
TATAGTAGTGAATCAAATGAGTATAAAGCTATACAGAATTTGCAGTTCGCATGTTTTGTA
GTAAAAGAGGCATGTAAAAATGGAACTAAATATTTACACTAAAATGAATATATATATTAC
GAGAAACATGAAAAAAAATTGTAATGAAGCGTGTGCTTGGTGTGGTGATTTTGGAACTTTA
AAAGAGAATATTAAATAAAAAAATTAAAATCAATATCAAAACACCTACTAATTAGTGGGT
GTTTTTAATATGCACTGAAATAAGTGAGGTGGTAACTTGGTAATAGGCAAATATCTAAAA
AACGAAAAAGAAAAAATAGATTTCCATTTGCCAGTTAAACCACAAGATTCCCTATCTATT
GAAAAAGAAAAAAGGTTTCGAACTCTAGACATATTAAATTTAGGTGAATTTGACATTAAA
AAAGATGTGGAGAAGATAATAGAAATATCATTCAAAACCTTCTGACCCAACTTTTATGAC
GCTTTTTATTGCAGATACAGCGAGTTAAAAAATCCAATCGAAATAGTGTCAGTGATTGAT
AAATGGGTAGACCAAGCCGAACCTTTAATACTTAATATCGCTGGTTTTGTTTTGAATGGA
TTAGTCACAATATCTAATTTTAGCAATACTCAAACAGCAGGAAGAGAAGGAGATAGAGAC
ATTGAGATAACATTTAGTACATACACAGATCTGAAGATGGAGACATTGAAAAAAGAAACT
AAAAGTACTACTAAAACAAATTCAATGGATAATAGACCTAATACCCAAACTATATCCAAA
ATATATACAGTTACATCAACAGACACATTATGGAGCATTGCAAAGAAATTTTTAGGTAAA
GGCTCAAGGTGGACAGAGAATTACAATATCCCCGAAAACAAAAAAGTCATTGGTAAAAAT
CCTATTGTGATTAAAAAAGGACAAAAGTTGGTGACACCAAGCAAGTGAAAATAATACTAA
ATGGAAAATATGATATTATAAATTTTAACCAGGGAATAACGTTACGTGAAGCTATAGACG
GGGTTGCATATAAAATGGATGTATCTATAGTAGGACCTAAACAACTTCAAGATACCGGAA
TTAAAAAGGGTGAGAATATAGTTCTAATTTACATTGCATATGAGAGTAAAAAAGAAGATA
TGGTATTTGGTGATGTCAAATGGGATACTAGGAGAAGTGAAAAGATTAAGAAACTGACAC
TATCTTGTACAGAGAGAACGGTTTACATGGAAGAATCAGAGGAACAATATTCGTTTAAAG
AAAATACAGCAACACAGAGGATTGAGTCCTACAGTATACAATGGAATATATCTTACTTCA
ATCTAGCAAATACAAGGAAGAAACTTGCTAACGTAATACATAAGACTAATATGTTAGATA
TGATTAAAAAGGAATTAAAAGCAAATGTAACAAAAGGTGGAGACTTATTTAGAGTAAGAA
TGGCTAATAAGCTTAAATTATTTAAAATTGGAACTAATGCAAATGTATACAAACTGCATA
GTATATTAGAAGATGCAAACTGGACAAGTCGTTATAATGATGCAGTTACATCTGTAAAGG
TTTTAGGAAAGAGTAAAGACAAAAATACAAAAGCACCTATTATCGGAACATATAAGAGAG
TCTCAGATAAGTCTGGAACACTACAAAAGATTAAACAAAATGAAAAGATAAAAGATGCTA
AAGTAGCTAAGAAATTAGCCGAAGAAATGTTTAATTCGGGGGAAGAAACAATAAGTGAAG
CTTGCATGGTAGATAGAAAGAGAATAAGGGCAGGTGACAAGGTAAGTATAAAAAGTAAAG
AATATTCTGTTATAGATGTCACTCATACACTGGATTGGAGACCAGAAATTAAGCTCAAGA
TAGGGACTTTACAATATATAAGGAGGAAGTTCTATACAATTGGCTGATGCTAGATTTAAT
GGAGTTGCTACAACATTGACAGAAAAAATGAATAAAAGAGTTAATGATGTAGTTTTTGGA
ATAGTGTGCGAACTTGCAGGAATTACAATAAAAGGTCTTAAGGTGGTCGGTTATTAAGAT
GAAAAACAGGATTCTTTAAAATGATAGAATTGGTCATTAAAAGAAGATTATTTTACTTTT
TCAGATTCAGCTTTAAGTGGAGCATCTCGGCATAAGCATAAAATAGAAACTATAAAAGAA
TTAAAATCATTGAACATAGGTGATAAGGTGTAAGTAGCCGTCATGGGTGCTGAGTTTGTT
GTCAGTGGGAGGGTTGTAAATGGCAAACCTATTTCCAGTTAATGAAACCTTTGAAATTGT
AGAATTAAACAGTAAAAACGAGAATGAACTGGACCTAAAGGGTTCTTGGTTATTTGACTT
AAGGAAAGGTGAATATGTTAAAAATGCAGATGGAGCATTAAAATGATGTGATAAAGTTCA
AGCATGTATACAATGGTGTCAAAAGGCTCTATTAACACCTAGATACAAAAAAGCCGCGTA
TACAAACATTTCTGCAAGTGACATAAAAGACTTAATTGCCAGTAACTTATCTCAAAGCGC
AAAAGAGCGTGAATTAACTAGATTAATAAAAGAAACTATTTTAGTGCTTCCATTTACAAA
AGAAGTCAGTAATTTTAAATTTTTATGGCTTGAAAACAGTAGACTTGTTAATTATGAATA
TGATGTACTAACAATAGATGATGAAAACATAACCATAGATAGAAATATAAAATGGGAGGT
GAATACATGGAAAAAGAGGTACCGTCACCAGTTTTTTTGTCAGAAGATGAAGACACTATA
CATGGAAGGATGTTAAGCAAGTTTCAAGATGTTTCTACATTAGAAGGTGAGTTTATCTAT
GAGGCAACAAGACCTACAGCTGATCAAATAAACGAATTAAAACGACTATGATTACAAAAT

AATTTAAAGATAGCATTACATCAAACTTGATATAGAACTTACTTTGAATTGCTTGGTTAA
TGTAAGGAAGTATTTAACAATCAACCTACGAAAGCAATGGGAACAATTACATATACAGGT
GTACAAGGAACTGTCATTACTCAAGGAACTATAGTAACTACTATTGCAACTGAGGAAAAA
CAAACCATAGAATTTGAGCTTCCTGAAACTAAAACTATAGGAGAAAATGAAACAGTAGCT
ATTAAAGCAGAAAGTAGGATTGTACGAACGACAGGGAATTTGTCTAAAGGTAGTATATCC
GTTTTACTAGGTTCTATTAGTGGTGTTAAATCAGTTAATAATAAAGAAGATTTCAGAGGT
GGAACAAATATAGAAGATGAAGGACATATTAGAGAAAGAGTTCTTGTAGCGGAACAAGAA
GATAAACTTAGTGGAGCTAGTTCAGATTATATTCGTTGGGCTCAAGAAGTTAATGGAGTT
GGATATGCTTATGTAGTTCCCGAATGGGCTGCAGCAGGTACAGTAAAAGTATTATTACTA
CATAAAAATAGATAAGCAGCAACCCAGGAATTGATAAAGAATGTTCAAGAATATATATAT
CCAAATGTGGAAGAGGGTCAAAAACGAGATTCAAAAGCTCATGAAGGTTCTATTGTGGCT
ATAGCCACTCCACTAACATTATTAATTAATGTAAAAGCTAGTTTTGTTTTTTCAAATGGA
TTTAGTCAAGAAACTGTGACAAATATCTTAAAAAATAGAATTGACAAATATCTGGATTAA
ATAGATATTGGAGGTACTGTTTCTTATAGTTCAATTTATTCACTAGTAGGTTCTATGATG
CTTGCAGATGAAGGAATACATGATTTTCCTAACTTAACAATAAATGATGGAACAACAAAG
ATTATTTTAAATTACCAAGTTGTTGGCATAGGTGAAATAATCCATGAGGTGATAGCGTGA
TAACTTCTAAAAAAGGTAGAGAAATGCTTCTAACATTATCTCCTATCTATGACCAATCTA
TCATAATGCAAAGGTTATATGAAGCTATAGGAAGCGAATTTGATAATCTAGAGTTGTTGT
ATGAAGAATTAGAATTACAATTATCCCCTCAGAGCGCACAATGGGGACTTGAATTTTGGG
AAAATAGGGTAGGTTTATATACTAATATAGATGAAGGTATAGAAGTTAGAAGAGGAAAGG
TCATTGCTAAGCGACAAATGAAATATATTGTTAACCCTGACACGTTAGCGACTATAATAA
AAAGCTATACTGGTGCAGACGTATATCGAAAAGTAAATATAGCTCCATACGTTTTTAAAG
TTACTGCTAATGGAGGTGATGTTGTTAATTCTGAAGATTTAAAATTTAGAACAAATACAA
TTAAAACAAATCATCTTCATTGGATGCCTTCCTTTGCACTCAAATACACAGATATAGAAA
AATTTCAGGTAAAGATGATTAACCGAATATTTAGAGATTTTAGAGGGAATATAAGAAATT
TCTTAGATGCTATGTGGTTATTACATGGAGTTAAAAACTTACGTGCTTCTATATTTTAAA
ATGAGTCAATAAGCTTAAATATGAAAAATAAGTTATTTGTAAAAGAAGGTGAATTATTTA
CATATCTTCAACTAAAAATTAAAAAGAACTTATATTACCTGAATGTGATACAAATGCTAA
ATGGAAATAAACTGCTTAATACAGAAGTAAGAGAGGAAGTGTTATGAGTGGCAAGTGCAG
GAACAACAGAAATTGCAAGACAAAAAATGTGTAAACATAGAGCAGTGGATGTACCATTGC
CTCCGATAGTTGGTATGGTGTTTGGAGATGGGGGGGTTGAGAATGATGGAACTATAATAG
CTCCACTTTCAAGTGAGACAGCGCTAAAAAATGAAGTTGTTAGGAAAGATATAGGAAGTT
ACGTATATCCAAGCCCAGCTAATTGCAGATATTCAACTACTCTATTAAAATATGAAGCAG
AAGGAAAAAACATAAATGAAATAGGTTTAATAGATTCGGTTGCTGACTTAATAGCAATAA
AAACTCTTGGAAATAAGTATAAAGACAGTGATATGGAAATGGTTTTTCAGATAGATGACG
AGTTCTAGGAGATGAATAAATGACTAATGAAATACCTTTTAATAATGAAATAGAAGAATA
TTTAATAACTACACCAGCTCATGCGAATGAGTTTAATAATCGACAACAAAAGTGGATAGG
CAATCATAAATATTTAGATAATAAAATTGCTACAACTAAAAAAGAGTTAAACACTAGCAT
TGACACAGAAAATGGTAAACACAACATTAAAATTGACCAATTAATTGCAGGTGGTTCAAG
TGCTGGATGCACTCAAAGGGTCGCAATTGATGATTGGGTGGAGGATGGGGAAAATGGATT
CAAAGCAACTGTAACACATAGTTTGTGAACACAGAGAATAGTTGTAAATATTATAGATGC
TACTACAAAAGAAAATGGAGATACAAACTTTAAAATTATAGATGATAATTCTATTCAAAT
TAGAAGTGGAACTAGGTTAGAATTAAACGTATCTGTGATCAATGGAAATACAGAAACTCA
TTTTATTAATCCAACTGTAGATGAAAACAGAGAGTCTGACATGACTACTTATTCATCTAA
GAAAATAGAAGACAGCTTTGTTCATATAGAGCAAAAAGTAAATGGTGTTTTATGTAATAT
TGCAACAAGTGTAAATGAGTTGATTACTTATTGTTAGAGAGGAGAGTGAGAAAATGCAGA
CTGAATGGAATTTTAATTATGCTAATTATGTACAAAATGTTTCATTGCAACCTGGGCGAT
ATAAATATGAATGTTGGGGTGCTTGTGGTGGTCTTGTCGATACCAGCGATTGGACTGATT
GTGCAAAAAGTGGTTATTCAAAAGGTGAGATTGTATTTAAAAAAAGAACTTATCTACAGA
TTTGTGTCGCTGACTCTGGTTATTAGAAAGATTCTGAAGGCTCAAGACTTACTAGAAGAG
GTTTTAACGGTGCAGGCGCTGCTGGCAAAGTTACTACTGGTAGCTTTGCTTATTTTAAAT
ACGGTGATGGAGCAACTGATATAAGGCTTTATGATCCGAGTGCAACTAGGGGTAACACCG
AAAGTTTGCTTTCCCGCATATTTGTTGCAGGCGGTGGCGGAGGTAAGAAGAATAATTTCA
CTTCTGCTCGTTCTATTGGTCAAGGTGGGGGTTATGTAGGCCTTAATGGAGTTGGTCGTG
ACCGAGATTTTTGTGGCGGTGGTTCTCAATTCTAAGGCGGCACAAGTTACGAGACCGAAG
AGCACCATGGCTTATCAGGAAAAGGAGGTTATGGTAACATAGGAATAGGTGGTGGAGGGG
GTTGGTACGGTGGTGCTGGTTCTTATTCTATTGAATGTGGAGGACGTGGAAGTGGTTACG

CAATAAATAAAGATAATTATAAGGCACCCGGATATACACCAACACCTGAATATTATCTTG
AAAATATAGTCATGACAATTGGAGGTAATACTACTAAAGCAGATGGTGATGCTAAGATAA
CGTTACTACAGGCCTTACCATTTTTAACAGGATCTTCCTATAATTCCATTACAGCAACAT
TTAAACCTGACCACACAGACCCTACATTGCTTACGGAAATAGAATATTTTATAGATGATG
TGTCAAAAGAAACTAGAACAAGCGATTTAACTCTTGAAGAAACAATTAACTATACATTAG
AATATAATGCACTATACACACTTAAGATAGTTGTTACAGCCAGTAATAATGCTACAGCAG
AAATAGTGTTATGTATAAGTAAGGATATAATGCCACTGCCCGAAAATGTAAATTAGCACG
ATATAGCTTCTAAACTAATTGAAGTAAACGAAGGATTTACAACTGATAAAACAAGTATTA
TAAATACTTTGGCATTAAAGAATAGACAAGCAAGTTAGAATAATACACTTGATGAGTTGT
CCGTGAACATAAAATCAAGTTTTGATAGTTCCGATGCTAGTGTACAGGATTTGATGAATC
TGTTAAAACGAGCTACTAATACCATAACACAGTTAAATTCCTAATTTCAAGTAGCAGGTG
TTACTTCTGTTGCAAAAAGAAGCGACGGAACTACACTTACTGATGAGTATAAAACGTCTA
CAAGTTCTAAATTTGCTGGGTGGCTCAAAATTAATGGATCAGCTTTTAAACCTACCTTTT
TTGTAGCTGAATGCGAATATTATGTCAGCGATTATAGAGTTGACTATAAATTTTTTACAT
TTGCTTGTTGTGGCGTTCCTACAAGTGGTAAACAAGATTTTGTTGCTGTAGCAATTTAAG
GTCGCTCATGTGGTATAGATAATTACACTGATGACGGTTTTGTTTATAACAATAATGAAG
TGGATTTAGGGTTTAATGCTAGTGGCGTTTAAGTTCCTGCTTATTTACCAGGTGGTGGTG
AAAGTACGACTTATGCTTGGAGATCTGTCAAAGTTATGAATACTTTATAGAGGTGGTAAA
GTAGATAGAGCAAATAGAATTAGATGTGACCAAACAGGTAAGATACTCTTGCCAACTGGA
GAAGCAACAGGGGATGTATTACCACACAATAAAATAACAGAATTACGTTTTATTGATCTT
CCCTATGGAAGTATAGATTATATGAAATATAGAATTGTACGGATAAATAAAGAAACAAAA
CAGCCAATTTTGGCAGATATACCAGTAGATGTAAGAGAAGCAGAAAGAGAAAAATAAGAT
TTAGAAGATCAATTACTGTCATTAACACATCAAGAAATCCGAGGAGGTACTTTATAATGA
ATACCAATAATGTAGTGGTAAGAATATTAGCAGAGAGAATATAAAATGGAGGGTTAAACC
GTTTGAAAAATAGAGAGTTTCAACTTGAGGTTGTAACTAACACAGAATATAGAAAAGCAG
TAGAGGATTATATTATAAAAAATAGTGTAGTAGTAGAAGGAATAGAACCAGCAGCGCAGT
AGGGCCTTTTTTTATTGAAAGAAGGTGACTAAATGGCCTTTAAAGTGTTAGTTAATAAA
GTTAGAAATCTTGTATTAGAAGCAAATAATGTAACTATAGAAGATACAGAGAATAATTTT
ACAAGTCTTAATATAGAAGGAGCATTAAAAGAGGTTTTTCAACGTGGAGTTAACGCTAAG
AATAATATAGTAACAGCATAAAACTCCCAAGGTCCAGAGGTTACTACAAGCGATACATGG
GAAGAAATAAAGAATTAAATTGATATAAAAGGGGGGCGATTAGATTTAATGGAAACAACA
CTTGAAGATAACTATCCGTATTTAGTAACAAATGGAGCTATGAAATATATTGAAAGATGT
AGTTGGAACTTAAGGACATTTGAATATGACGAACCATATCTTTATGTAATTAAAGTAACT
CATCTAATTAAAATTAATGTTATTGCTGAAACAGTAGTGGTTGACATTACTTTAGCTAAT
GCTAACTTCTCATGAATCTGTGTTACACAAGTGTATTTATTGATATCAGACAATACTATA
TTATATAAAATACATAAGACACCCGGAATTGAAGTGAAGTCAATAGAAGGTGCTTATTAT
AAGTTTTGTACTTAAGGGGAATTTATTCGTGTAGTATATGCAGATGAGACTTCTTCTATA
CTTCATCAAATGTGAATATCTGATACGCATTTAATCTTAACTATGAATTTAGTTTGTAAT
GGTATTTACAAGTAGAAAGGAGGAAAGTTTGTTTGCAATAGGAATGCTATTAATACTGCA
ATAAAACACTCAAATTCTAGTAGGATTACAACATGTCATTTATCTAAAATAAATTTTGAT
TGTGCCATTGCTAAAGATTTTGGAATTGGAGGATATTTGCATATGAAAAACATTAAGATT
TTAAATGATTTTGTTATTGCATCTGATGCAGCAGGAGGTATAGAAATTGATGGCAAAGAA
AAAAGTGCTTTAGTAGAATCTGACGTAAATTTAAATTTAAATGTCAATTCAGACGATAGC
CAATATGACAATTTTCATATATATAATGGATTTATATAAGCTATATAATCGCTATCTAGT
AGTCATTTCGGAAAAATGAGTTTAAATGCTCTAAAAGGTAACGAAAGCCACTGAAAACTT
ACTGAAACATACCGAAGTTCTAGTATGTTTGTAATAAATGATATAGTTTTCCTTATCAGT
AGTGGAATTTTTAGAAATATATCGTCGAAGAAGGTATATTCGGAAGAGCAAGGAGAATCA
TTATAGTTTATCTATGAAATTTAATGGGTACAGAAGAACTAAACATAAAATATGTTGCTA
TGATACACTATGGACCGAATGTGTTATCGGAGGAAAACTTAAAAGAAGGTATTTCGATAG
AGGAATTACGAACACCAAAATACACTCTAATTAAAGAAACAAAGTTATTTATAAAGATAG
ATACTAAAGAGGTTTTCTATAGAGATACACATATTAAAAGTAGCATAGAAGACAAAGAAA
ATTCTACACAACGAACAATAGCAGATTTAACACTTCAATTAATGTCAAATGGGGTGTTAT
AAATGAATTGGTTCAAGATAATAACAGATCTCTATTATAATGGTAATTGGACTAAAGAGC
AAGATAAAACGGCACTAGAAAAGAATAAGATAACAGCCCGTGAATATAAAGACCTTGTAC
GAGAGGACTATATAGCTTAGTCTTTTTGAATTCAAAATTTAGGAGGTTTTCATGAGAGAA
GCACTTTTCGATGCAGATTTAAAAATACATGAAACCAGAATAAATAATCATGGAGAAGAA
ATAGACGCAACAAAAATAGCAAATATAGAGTCTAAAGGAGAGTTAAAAGCAATGTGTGTG

AATCTAAACTCACTTGCATGTATGCTCAAAAGGCTAATTGTTACAATGAGTACAACACTT
GTAGGTTTCTTTCTATTTGCAGATCAAAGTGGTATATTTTAATTAATTCGGGGGATAAGC
GATGGGTAATTTAATAAGTTTTATACCAGAGGAGTTGCTATTTTTAGTAGCTGCTCTCTC
TATCATAGGTCAGGGCTGTAAGATGTATAAGCAACTAGATAACAAATATATTCCAGTAGG
GTTATTACTACTTGGTATAGGATTTTCTATTTGGAAGTTAGGACTAAGTCCTGTTGCAGG
CTTACAAGGCGTGATTTGTTGGGGTATATAAATAGGTATAAACCAAACTTACAAACAGTT
GAAGGGGGAAAATAAATAATGAAAATATGTATACCAGTAGGGCACTGTATGTTAAAAAGT
GTAGCATGTACTTCTGCTGATGGAGTAGTTGACCAGTATCAATACAACAAATCTCTTGCA
CCAGTATTAGCACATACATTTAGAAAAGAAGGGCATTGGCTAGATGTAATAATATGCCCA
GAAAAGCAGTTTAAAACTAAGAATGAAGAAAATTCTGGAAAAATACCTAGAGTTACTAGT
GGAGGATATGTTTTACTTATAGAGTTACATTCAAGTGCAACTAACGGTCAAGGTGAAGGT
TCAGCATTTCTATACTATAGTAATTACGGCTTAGAATACACAGCTAGAGTATGTGCTAAA
CTAGGTACAGTATTTAAAAATAGCGGTGCTAAATTAGATAAAAGATTATATATCTTATAC
AGGTCAAAGCCTACCGCAGTATTAATTGAAAGTTTCTCCTGTGATAATAAAGAGGATTAT
GATAATGCTAAGAAACTAGGACATCAAGATATTGCTGAGTTAATTGTAGAAGGTGTATTA
AGTAAAAATATAAATTATGAGGGAGTTAAACAGATGTACAAACATACGATTGTTTATGAT
GGAGAAGTTGACAACCTATCTGCAACTGTAGTTGGTTGTGGTTATAATGATGGGAAAATA
CTGATTTGTGATATAAAAGATTACGTGCCAGGTTAGACGCAAAATCTTTATGTTGTAGGA
GGTGGCGCATGTGAAAAGATAAGTCCTATTACTAAAGAAAAGTTTATTATGATAAAAGGT
AATGATAGATTGGATACACTTTATAAAGCATTGGCTTTTACTAAAAGACACATATAAGTT
ATCAACAAGAAGTGGTTGTTTGTGGTGATAACTCTATTATTGTAATATATTGTCAATATA
TTATTGTGATAATGGAGGATTTATGTATGTATGCATTGGTTTATGAGAGATTTGTATGAG
CAACTTTTAGTATTAAACTTAATACCTTGATTAATGGAAGCGAGGATTTAGGTGGATTGG
ATGAAGTTGATATCTTGAGAGCAGCAAATAATATACATGAATTAAATGAGATAAAAATAG
GTACTGGGTATGCTATTGCAATCTTTAATAAGGAGATATTATAGTCTTTTAATGTTTCTT
ATTATGTTAGCAATATAATAATAGAACTATTTGATAGAAGCTTAATTGCAAATTCAAGGG
AGGATATATTGGGTATAATAAGAGAATATGACACTTATAGAGTACGACACTTCACTTTCA
ATTGGAAGAGATAGAATGCTTGAGTTAGGAAGTAGTTATTTTAGTTAGTTATTACTTTCT
TTCACATCCTTTTATTATTTCAGTAAGAGTATTTCCAAATAATAATTTATTCAATAATAC
TTAGTAGAAAATAAAATTTTGAATCATTATAAAATTAAATGAAATGAGGTAGTAAAATAT
GGGTGAAAAATATAGACGTAACGCAGTTGAGAGTAAAGAGAATTGATTACTATATTGAAA
AATCAGATAATAAAGCGAGTCTTTTTCTATTATTAAGTAGTGCAATTATATGTTTTTTAT
TTTCAGGGAAGGACAAAATAATGAATCACTTAAGTATAAGAAATATGAGATGTTTAGAGA
TGCTATTTGACATTGTTATATAATATACATTTGTTGTTTCGAATGATTTTTCGATTATGG
TTTTAAAGCCAAGGAAATCTAAAATACAATATAAATATACATCGATTTTTTATAATAAAG
AGATAGCATCTTTAAATAATGAACAGTACAAAGAAGCATTTGAAAATGCATTTAAAGACA
AAGAAAACTTGATCAATGCAGCACTTGTTCAAATAAAAGAATTATCTTTAATCAGTGATA
AGAAAATGTATTATGTGAAAAAGTCAGTAGAAGCTTTTAGTTTAGGTGGAATTATATTGT
TTATATATATACTAGTAGTAGTTTTTAATGCTATTTAGAAAATGTTTTATCTGGTTAGTA
TTCCTCTATGAAGATAGAATCAAACAATAGAAGAATACCATTTACATATTTGTAGATTCT
TGTCATTGATTTTAAGTTAATTTTTTAAATGCTAGTTTTACATTTTCTTTAAAATCAGGT
ACTGTGTTTTCATTAAAATCTTCAAGTTTATCCTCTCTTATCCATATAAGTTCTGTATGT
TTTGATGTCGGTTCAAACTAAGAAATGTCATATTCATTTATTATTTCTGCAAGAGTTATA
ATCCCTTTGTGTAAACCATCACTTGTTTTAACTTGATAAATTGCTAGAGGTATAGATTGG
CAATCATCTTTTCTTGTGCATTCACTAATATGATCAATGTTTATAGTAAAATGTTTTTCA
TATTCACGTTTAATAGTATTTATAATTGAAGTTTCTAGACTTGCATTAACACAACCAAAT
ACCCATGTACTCGCATTATTATTTCTGTTATCACGTCTTTTCCCAATTAATATTTTGGAA
GTTGATTCATAATAACAAACCGCAACACAGTGTCATTCCATTAAATCTACTTTTATATAG
TCTTTATCTATTGTATTTCTATCGTAATGGGGGTCTGTTTTAGAAATCACGTCACCTATT
TTTTCACTCTTAACACTAAGATTTCTGTGGATACGTATTTTATCCAAAGCCTACGCGACA
GAATTAAATATAAAAGAGTCAATTAGTAATTTACTTTCTCCTTTTTTATAAAAAAAATAT
GTTCGAACCAATTAATTTTCTTCTATTTAATCAACAGAAAAACTATCATCAAATTATCTA
TCATTATTTATTCCTTTTATATGATACCAAATGATAGGGTATGGTTTATATTTCCAGATA
GCCTTTAATTTTTTATATGTAATTATATGTAATTTTGATAAAATATCAGCTTTTCTTGAT
CATATATAACCTTGTTACAAACTCAGAAAAAGTCTTTCTGGGCATGTTGGCTTTGATATT
CTTAAACCAGCATCAATATCATTTCTTAAGAACTCAAGTATTTTATAATTATTGGAGAAA
TCGTACATAGCAGAAATAGTTTCATATTGTTCATTATTTTCTAAAGCATTAAAATTAGGA

ATGCATGATACTATAGCAATCCAGGCCGCTCCTTTTAATAAAATAAGATTTTGAAGGTTC
ATTAAGTTTTTTTCAGATTCAGAAAACCGTTCTAGTGTAGAGAATATATTTACAATTTTT
TTATCTTTAGCAGTGCTTGTTAACATGTTAAAGTTCATATCTATGTATTTTTAAATTTCA
TCATGATTTTTTCGTACGATAATGGAAATTACCTCATCACCAAGAACTCTCCAAAGTTGT
GCTTAAAAGTTTTCATAAACTCTATATTGTAACTTACGAGGTATATGTAATAATACCTTA
GCCCATGCGCTATGATGTATATTTTTATATCATGATGAATTAGCTATATCAAAATAAAGG
AACAAAATCACTTGTGGATGCTTAGGTTCTTTTTCTTTACCATAAATTTCAGTATTAATA
CTTGCCTGATGCGTTTTCTTATACTTTTTAAGATTAAAAGCGTTTTTGTTTTTAGCTGCA
CTATAAACATTATGTACATATTGAGCTTTATATTCTTTATCATCCACCATTGTAAATATC
CTAACCCTTTCCCTCTATTAGATGCTCCATAAGAGGAAACCTTTAAGTTATCTGCACTTT
TTGAAGTTTAGACCTTATTCCCTACTGTGTTTTCATCCATAATTCTTTCATATTACGGTC
TAGGCATCAACAAGGCTGGTGCGAATTCATTGGATTGATACTCCTTGTTGGTATTGCTAC
CTCTAAAATACTGACTTCCAGCTTGACTATTCCATAGTCCATCATCAATGCCATATCCCA
TGTGTAAAAATACATGTCCTAGTTCATGCTATATGGTAAGATTCTTTCTGGTATCTGGGT
GATCAGGAGATGCCACTATTACAAATGAATCTTCAACTTTTCTAAAAAATCCTTCAGAGT
ATCCACATAAAGAACTATCTGCTATTACCTTTACTCCTAAAGCATCCACAACTTGATTTA
TATTTCGTATTGGAAGTTGGATAGTGTATGTTTGGTTATTATCTTGGGTTATATCATTAA
TTAGCTCTCTGATTTTGGAATCCATTAAATCGCATTCGTATATAATATTTCATTTAAATA
ATTATTTATTGTAATATTCATTATATCAAAAACTATTTTTTTTGTTACATTTAATATTAA
TTATTTTCATTAACCTTTTGTATTATACAATATAATTATACCATATATCAACAGTTTTTT
GTCGAATGTATATTTGAAAAAATGAAAATTAGTAAATTATCTTAAACATAATTAGATATA
CTTTGCAAGAAATAACAATCTTAACAATAAGATAAATCGTTGAAATACCAATTTGTGTTA
CATATACACAATAAACAAAAAATAAAATTTTAGATTTCAGAATATGAAAGGACCAAAAAA
CGATTAACTAGTAATCTTAATGAAATTCATTTAGTTTATGTTAAAGTTCGAAAGCTACAG
TAGAAGTTGTAATAATAAAACGAACGAAAATTATGGTACTAAAACATAACACATGCTGTA
ATTGTATTAGAGAACTGATTGAATATATACCACAAGCAATCTTTATATAACAGTGCTTCT
TGTTTCGAAATTCATCAACATATAAACTATCAAGAATATTACTCAACACACCTTCAAATT
GTTTTAAATTCTTTTGCATACACAAAGTTATAATATACAACAAAAGAGTACAGAAGTAAC
CCCAACACATCTATCCTAATTCAACCAATATTATATAGAGCATTCTTCATTATGGAGAGT
GCTTTTTTATCTCTTTGAATAATCATGTTGATTCTTTATAATATTTCGATTTAACTTATC
TTTTTCTATCAAATTTAATACACAATTAATACAAATGTGAGTTTTCATATGACCTTTGTT
ATGAAAAGTAATAGACTTATTATCAACTATATCTATTCCTTTATTACTGTAGTCACATAA
AATAGTCCTAGTCATATTTTTATTCCGTTTATTTTATATTTGGCATAATTAAGTTTAACA
TATTTCGTATATCCCTATGTCTATAGAGTTAAAACAAAGTTTTTTATCGCAACATACAAT
ATATTGTATAAATAAAAAAAATGGAGAAGAGATATTTGATAATTGTAAAATAATGTTTTAA
TTAAAAAGCAAGAATAATCTTGTGGAACTACAATCTAAGAGTGGAGATTCATTTTCTACA
TTCCATCCTTTAAAAGGAAGGAGGTGGATAAAAGAGCGAGTTTTTACCAGGAGTGTTAGC
TAGTTTAAGAGCTAGCTTTATTACATATACTATTACCAGTAAAGTAAAAAGCCACCCTGG
CAGGAGTGACTCTGAGCTTGATGGATAAATCAAGATTAATAAAAAACGACATTAATATTT
AGTTCATGAAACTTCGCTCTCGGCTAAGGGTTTATAGTTTCATTGTTTGTTTTTGATACG
AATTTACATCTTTATTAAATCTCCTTTTGCGAAAAAATATAACTATGAAGATTAGAACTC
TGTTTTATTTTATCTCTTAGTATATATATTATTAGCCACCTAAAAAACTTTTCTAAAAAC
AAGAAGAACAATATCTTTTTGGCAACTGGCTGGCATCTTTTACAAGAATTTTATAAATCT
TACTCACTGTATCTTTGCGTCACTAATTTTCTCTAGTGTTGCCGATTTAGTTTTGTTATA
CAACTAAAATAATACAATAGAATTAGTTAATGTTCAACAAGGTTGTATGAAAACTAAAGA
AATTTATATGTATGAATAGTGTTTATTCCTGAAGAGGCACAGAGTATTTAAAGGTCAAGG
CAAGTTAGAATAATATTGTGAAGTAACATTTGATTATGCGAAATTCTATGAAACAGGGAC
CCTGTGACTTTAGTCACGGGAGGTTCAGTGTATTTCGCTTATTTCAATTGATATAAGTAT
TCTATTTATTTGGGCTGACTAATAATAGCCAAGTGGATACTATATGTTAGGCCAAATAAT
TATATAAGATTAATAGTGATGTTTTTGTTATTTTCTGTTCCTCTATAGTCTATTATAGAT
AACCAAAAACGTCTGCGTTCGACCCTGCTTAAATCACTAGACATCTCATTAATATCAGTA
TCTAAGAGTTTTTTTATATTAGATGTATCAATAATTTGACTCTCCCGTTCTTCAGTTATA
TTATCCAGTTGAGATTGTAATTTTTCATAATCTTTTCTATAAGTCTCTTTATCGATCAAA
TCCTCTAAATATAAATCTTTGATTTTATCTAATTTTTACATGATTTTCTTCTTTTCATCT
TTTTTGTTAGATTTCCTATTTCGTCTGATGTCTTCATTTTGTGCTTCTAATTTCACCATA
TGTTCATATAATTGTGGCTTTAAGTTAGTCAAATGGTAGTTTCCTATATGTTTTTCATTA
TGTGTTACCTTGTGTACACAGTTTTTATTTATATAGAATGTAGAACATCTATAAGATAAA

TAAACTTTTTCTCCAACAATACTAGAATTACCCTTTAATGTATAACCACACTCCTTACAT
TCTAAAAGACCAGAGAAAATATGATAGTGTATGTTTTTATTAGGTGGAGCTTCTTTATTT
TTTTCTAATATTCTTTCGATTTGTTTAAATTGTTTTTTCGATATAATAGGTTCATAATAA
TCTTCAACATAAAATCCTTGTTTATTATATATTCCTATATAAAGTTCATTTCTTAGAATA
TCCTTTATTTTATTTTCACTCTATTATTCGCCATGAAGATTAACAATTGTTTCTATTAAA
GATCTAACAGAACCTGATTTCTCGTATTCGTCAAGTATCGCTTTAACTATATATTTTTTT
TCTTTATCAATAACTTGATGCTTATTTTCTATTTTGTAACCTATGGGAATTGTACCACTT
ATAACTTGATAGCGTCTCAATTTATTCTGAAAATCAAATTTTATTCTTTCAGATGTTTGA
GCTTCTTCATTTTCTGCTACAGAAAAGATAATGTTTATACGTAATCTACCAGCAGCAGTA
CTAGAATCGTGGGTTTCTAAAATGGTTTGCCAATCACACTTATGTTTTTCCAGGGTTTCA
ATGTTTTTATACTAGATTTTAACGCCACGACTTCGTCTATCAATTTTAGACATTAAAACT
ACGACTGCATAAGCGTTTTCCACATCTCTTAATAATCGCTGTCAGTTAGGTTTTTGTAAA
TTAGTTGCAGAGCAACCTTCATCAATCTATTTACCAACTATCTTAAAATCATTATCTCTA
GGATACTGCCCGAAAGCCTCTATTTGTGTCGGTATGCTGTCTGCATTTAAATATTGTTCT
TCTGTGGTAACAAGTATATAAAGTGCTACTCTTAACATTTTACATTCCCCCTTATTGTAA
ATTAAAGTAGAGCTGGACGGCTCTTAAGTTTACTATGAATTGTTACAAAAAAGTCGCATT
CATAATACTAATTCTTGGCTCAAAAAATATCACATACTTATCTAGAATAGATGAAAAACC
ATATTAACTTTTATAACACTCTAAAGCTTCATTTAGAAACTTTACTGTTACATCAAGGTA
TTCTGCCATTTGGGTTATAGTCTTACAAGCAGCATTAAAACTATCTATTATAGGTTTAAG
ACCAATTAATTTGTTGCAAGCCATCAATCTACCTGTATATTCTTGTTTGCTATTTTCAAC
TTTATTTAGGTCTAATATATTTCCATTTGAAGTATAATGATATACTAGTTCTTCTGTTGA
CGCACAAGCTTTTTCGCTTATATGATTAAGCGTAGTATTATTTAAAGGTTTCCCATTATT
TTCATATAATCCATGAGAATTCGAAATAGAAGGAACTTCTCTAACAATAATATTCTTATC
ATCTGCTTTTGAGAGTAACTTTTCATAATTATTCATAAGTTCACCTACCTATTTTACCAA
TTATCCATATCATCTAAATCTTGATATATTTTTTCAATTTCTCCTTCTTTATGTAAATTT
ACATTGTGTGCCGCTATTGTGTCTCTGTGATTGTTATTTACATATTTACCTATTTGAGTA
AGTTCATCTAATCTTTCTATTGCTTCGTTTTGTCCTATATCGTTTAAGGCTTCAAAGCTT
GACAAAAGCTTATTTACTTTAATTTTAATTTGATGGCCGTCTTCGTTCCACCCCATTCTA
AAAGCAGGAGATTCATCTAATGCATGTGCTAAAATTTCTAGCTTATCAAGAGGAATATTT
TTTAGAGCTCCCGTTTCATAGCTCTGTAAAGTGGCCTTTCTTTGACAAGTCTTGTTAACT
AAATATTGATACGAGTTATTAATTTCTAATCGTTTATGTTTCGTTCAATTTGTAATCTCC
AACATTTTTAAATCATTTTCAGTAGTCAAATTTATCACGTCTATAATATATATTATAAAG
TATTTTTTCATAAATGCAACACATATATTGGAAAATATAAAAAAATGTTGCATTTTTGAC
TTGACAATATAAAATGGATGTATTAATTTTGAATAAGCGCAAATAGGCGACGAGGTGATG
TAATGAATATAAGTAAATTAAAAGGAAAAATGGTCGAAAAGAAATACACTCAAAAGAGAT
TAGCAGAAGAGTAGGGTGTGACTGTTCAAGCTTTAAATGCGAAATTAAAAATTCGTTCAT
ACTTCAAGATTGACGAAGCAGTTAAGATACCAGATCTTTTGCAAACCGAAGAGCCTGGTT
AGATTTTTTTTGAGAATTACAGCCCAAAATTGCAACGAAATTAAATAAGAAAACCTAACA
GATAAAAATTAAAACTAGAGAGGTAGAAATTATGAGTAATAATTTACAAGTAATGGATAG
AAACAACAAAATAGTTCTAACTACACACCGACTAGCAGATGTAGATGAATCTGACTCAAT
GAATATAAGTAACAATTTCAACAATAACAAAGATAGGTTTATTCGAGGTTAACATTATTT
GTTATTACACGGTGTTGATTTAAGGAATTTTAAAGGTATTCATACGGACTATCAAGTGAT
TTAAGTAAGAATTAAGTTAAATAAACCAAGTACCTTGAAAACTGAATACAGAATATTCTA
AAGAAGTGATTAGATCCAAATAGATCAAACAACAATAGGCCTGCCTATATCAGGCGAATA
AGCGCAAAAGAGGACTTATTCTTATGTTTCTTTGCATTTTTCAGCATTTAATTTTTCTAA
ACCTAATTTGACTAATTTAATAGTAGCTTGAGTTCTATTTGGAAACCTTTTATCAAACGT
AAAATCATCAATTTGTTTAAAAAGTTCATCATCAACAGTTATTGTGAAACTTGGTTTTTG
AGTAGCCATTATATCACCTCACCTTAAGTGTATTATACACCAGTGTTGAAACGGTGTGAA
TATAAAAAATAATAATATAATAAAAAGGTGTTGACATGGTTTACCAGTTCATCTATAATA
AAAATTTAAAGACCTTCACCACTGGTGACAAAAGGAGGATGAAAATGCAAATTAAATCTA
AAAGCATTTCAATAACTATATTTCGAGAATTCGAAATAGATATAGATGCTTTAAAACAGG
AAAAGTTTTATAAAGAATCTCACAGTCCAATGCTAAGATATCTTCTTAAACTTGGGCTTC
AAGTAAATAAAGAAAAGGTCTATAAAAAGGAATAAAAGAGGAGGTATAAGCTTGAATGAT
TTGACAATAGTCTAGCAAAATAATAAATTCTTAATTGAGAGTAGAGAAGTCGCAGAGTTA
ACAGGAAAGAAACGTGATAATTTCTTAAGAGAGATAAGTGGATACAAGAAAATTTTAGAG
GGCTCATTAAATTTGAGGATTCATGATTTTTACATAGAAAGTACATAGCTAAATACTCAT
AATGAAATTCAGCCTTGTTTCTTATTAACTAAATAAGGTAGTGACATGGTAGCTAATAAG

ATGACAGGTGAAAAGGGAATTATATTGACAGACATTTGTATAACTAAGTTTGAAGAAATG
GAACGAGAGATAAAATAACAACAACCTAAACTAATAACTCCATACAAGGAAGCATTGCAA
CAGTTATTAATAGATGTTGACGAAAAAGAACAATGACAATTAGAAAATCAAGAAATAGAT
AGGGTAATTCAGTAACAGCAACCAAAAGTACTGCTTGCTGATTCAGTAGTATCTTCAGAC
ACTTCGATCTTAGTTGGAGAATTAGCAAAATAGCTTAGACAAAATTGAATTGATACAGGA
CAAAATAGATTATTTGACTGGTTAAGAAATAATGGTTACTTAATAGATCGTAAAGGCGAG
GATTACAATAAACCAACTCAAAAAAGTGTAGAATGAGGAGTCATAGAAACAAGAGAAGGT
ACAAGAGTACATGCAGATGGTCAGACAAGTATCACTAAAACACCCAAAATTGAAGGTAAG
GGAGAAATATACTTTATTAATAAGTTTAAAAGGAACAATCGGATATCAATGTTAAGTTAA
ATCATAACAGCACTTTGAAAACTAAATACGGAATATTTTGAAAGTAGCGGGGTGATTTAA
ATGGGCAAAAAAAGTTAATACACAGTAACAACAGTTGGTGAACCAAATATACATGCGTAC
GGCGAACCTTAAGCTAAAAGGCTTACAGAGAAATTTGGGGTACTAATAACTCATACATAC
ATTCAGAACTATGATTTTGATAAAGAGAGAGGGGTTGATGTAGATGGAGCTATTGAATGT
GAACTTTTATCTAGGGAAATTAAAAAATAGTCAATGTAGTGAGTGTAGGTTCTAAGAGGT
TTGGGTTAAATATTAATTGTATTGTAGTAGTAGCAACTGACCAGGGCCTAAATTTAGCTT
ATTATCGCAAAGATAAGTGATCTATTGAGTATGACGCACTGATACCAGAGGATATAAGAT
ATGGTGATTATATTCTTGAAAGATTAGGTTAGGAGGAAATAAAAGGGCAGCTTTAATAAT
AGTAGGCTTATTTGCAATCTGTTTAGTAGGATTAGTTCAAAATAGAGATTAATTAAAAGG
GGGGTTTGATTATGGAAAGTTTAAAAATAGTTTGACAAAAAGTTGAAGGACAGGTCAATT
TTTCTAGGTAAGAAATCGACCGAAATAAGGAGACTTATGAGATTCTCAAAAATAAATCTT
GCAGGGATAGGTCAGAGGACATAAATTTAACTGATGGATTTACAGAAGTAATAGTAAACG
AACGATTAAAAATATTTGCTAAGTCATTACTAGAGTTGTAAGATACACAAATCAAAATAA
AAGAGAGTGAAGAAAGTGAAGACTATGAAAGACTTTGTATGGGCGTAGACGAATTTGGAA
GAGATAGACCTATAGATGTCCAGATATAAAAAAAGAGCCATTAGGAGTGGCTCGATTCAG
AAAATGTTTAAAAATTAAAATATGACTAAATTATAGCATAAAAGGAGGGTAATTATGAGT
ACTTTATATGATTTAACTACAGATTTATTAGAAATCGAAGGAGGTGTAACACAGGTAACA
GGAAATGAACCTTAAAAACTAGAGAAAATAAAAGAATTAAGAAAACAAGGGATACAAAAT
AGAAGCACTCGGATCGTTTCATTGATAATAGATATTGATACTGATATAAACTCTTTAGAC
GTAGAGATTAAGAGAAAGCAAGAGTTAAAGATGAGTAAGAAGAATAGTCTTGATATATTA
AAAAGTAAGAGAAAGGAATGTATGGAACCGCTTGGTATTATAAAGGTAGAAACATGTTTA
GGAAATATAAGTATGTGAAAATCAGCAGGTAGCTTAGTCATAGAAGATGTAGGAAAGATT
CTTGCTATATTTAAAACAGTAGAGCAAGCTGTAAAAGTAGATAAAATTACCATTAAAGAC
TTTATCAGAAAAGGTCATGAGGCTGAAGGGTGTAGGATTGAATATGGAACTACACTAACA
ATTCCGAAAGCTAAAAAAGAGTAGGAGAGGACCATGGAAACTAATAATGTTTATATAAGA
CTTGTAAATATCCCGAGTACATTCAAAGCTCGTAAAAGTTAATTTAATAGCTTTGGGAAA
TACAACTATAGTAGTTGCGAGGATATACTAGAAGGTTTGAAGCCTATTCCAAAAGAAGAA
AAAGCACTAGTTATATTAGATGATAAAATTATTCAGATATGCACTAGATTCTATGTAGAA
GCTACAGCAACTTTAATAGATGCAGAAACAGAAGAAAAAGTATCTGCAAATGCATTAGCG
AGCGAAGATGAAACTAAAAAAGGCATGGACTTAGCACAAGTGACTGGAAGTGTATCAAGT
TATGCAAGAAAATATGCTTGAAATGGACTATACTGCATTGAGGATACAAAGGATAGTGAT
CCAACAAATACACTTGTAAAAGAATAAAAAGAAACACAAGTTAGTGAAGATGAATTAAGT
ACACTATATTTTTTAGGTGAGTCTATAGATAAGGATATGAAGAGAGTCGATAGTGAGGTT
TATAAGAAGTTTAGTAAAATAGCAATAGATTTGACGGAGCTAGAGTATGAAAAGGTTCTA
AATTGATACAAGCGCTTTTTAGATAAGCAAAAGCAAGAGTAGGTGATATATTGAATTTTA
ATAAGGAAACATACTTTCACATAAACTTTGATGACCCATTTACCAGAGTACCTAATACAA
TCCTTTATAGTGAAAAACTTAGTTATTAAACTATAGGAGTAGTTACTCAGATGTTAAGGT
TTCAAAGGTCGCGTAGTCATAAAGTTTATGCAAAATCATTATTAAGCTATAGAAAAGATA
GAAAGCCAAAAGTAAGTACTGCTTTAAAGGAGCTTATGCAGGAAGGTTTTGTTATTAGGA
GACAATTAAGGGATGAAAAAGCTCAAATGAAAGGTTATAAATATGATATTTTTGATACAC
CTCAAACTGTAAATTCTGAAAGTGTTGAAAGGACTGAACCTCACCCGTGTGCCGTTTTCC
CAACACCGGTAAAACCGGAAGCTGGTAAAACCGAAGTGGGTGACGCCGGGAGCCGGCACA
ACTGAGGTCGGGAAATCGGCAACATACAACAAAATAGTTTGAAAAAGAAAATAGGTTTAC
GAGAAAATGATTGTATTACCACTGTTATTTCTAAACAATCTGAAAAAAATAAGTTTGTTT
ACATATAAAAATATTATGAATCTTAGATAGCTGTGATTACTCCAAATTATTTTCTACAAC
TACTCACTTATTTATATGATGGAAGGGTAGCTGATGTAATAATAAGAGATGTTGATGAAG
CTGTAGACAGTGTAGAAAAGAGTTATAAGTAGGTAAAGACAATTTTAAATAATTGGATAG
AAGCAGGTTTAAAAACTGTTTTATTACTTCCAGAATAGCAGAATGCATTTGATAGAAAGA

AAAAGATTAAGCTGGAAAAGAAGCAGTCTAATAGTAAAACTGTGAATACTAAGAATGCGA
ATAAAAATAAGTCTGCTAACTTCAATCAAACTTTCCCTCAATATGCAGAAACAGAGCTAT
ATGATATAATACAAAAGAGTCAGAAGGAAAAATGTAAATACAATTAAACTTGTAGGGAGC
AAATATCAATATATTGCTTCTTAGAAAAGGGGCGGTATAAAATGGCGAGAATATATACAC
AAAGAATTGCTTCTTTAAATGAACAAGATAGATTGAAGTTATTAAGATTACTAGGGAAAG
CTGGACATAGACTAAAGCTGGCTAGAGCGAAGCAAAATAGCAAGACAACTCATAGTTACA
TTGTTGATTATACAGAAGAACAGGAGGAAAAATAGAGGGGGGCTACTCGAATGAAGACAA
TAGCCTTAGTTGGAAGATTAGTTCCAGAGGCAGAATTGAAGTACCTTCAAAATTCAGGTA
CTCCAAAAGTAACCTTTTCAATGGCAGTAGAAAGAAGGTTTAAAGATAAATATGGAAATA
TAATACCTGATTCTATTCCATGCGAGCAATTAGTAACACATGCAGATTATATAGTACAAT
ATCTTGTTAAAGGTAAGCCTATATATGCTAATAGAGAGTTAAATATAAATAATTACAAAG
ATAAAAATGGTTACTGGATATCTATTACTAAGGTTAATGTAAATGCTTTAGAACTACTTT
CTAGTAAAAATTATAATAATGCTAAACAAGAATGTGTCCCACCAGGATTAGACCCATAAG
GTTTTCAAGCAATAGATGATTTCGATCTACCTTTTTAATTAAGTTAAATAATCTAGGGAG
AAATGATGCAATATTACTTTCTCGGAGTTAAAAAATTGGAGGGATGGTAGATGGAGGAAG
AGTATAGAAAATTTTTTTAATGCGTAATGGTGAGAAAATTGAGATCGTACAAAATTGCG
AAGGAGCAATAAAGATAAGCCTAAATAATATAAAAACAGATAATTCGCAAGGACCTTGGA
ATTTAAAGGCAGAAGGAACAGCTACAGTGCCGCTTTACCTAGGTAAACTGCTTTTTGAAG
TAAAGCTGTGGCTTAAAATATAATATCTGTTAGAAGTTATAGAAGAAAAACTGATGATGG
GGGGATTCGGAGTGATAATAATTACATGTCAAAATAAATTAGATTTAATGAGCGTTAATC
GAGTTGAAATAGATAGTAAATACGTATATGCAGTGTTTGAAGCTGAATCAAATGTTAGAG
AACTAGGTAGGTATAAATCGGAAGAAAGAGCTCTTGAAGTATTAAACAGAATACAGGAGG
CTATTATAGCAAGAACTAAGTTTTACATTATATATAAATACGGGGTTAGATGCAACAAAG
AACAACTGTTGGGAACGCCAGTTGAATAAGGATGGGGCTGAAATGTTAAAGGTTGATAGA
TATTTTAATGGTTCTGTTGCAGATAGTATATTTGAAGATGATTTGACTCTTAAGAATTAT
TAAGCACTATATTGCTGTGTATATGGAGTAAACAAAAATGGAGAACCTGTATATCCAACA
CGTGGAAAAATCCTAAAAGCGTTTAATGTTGATAGGGATAGAAAAAAGAGAAAGACAGCT
TCAAAAGTAAAATTATTTAAACTCAAAACTGGTGAAGAAAAAATGTTTGATTCTATGGAT
AGTGCAGCATTTTTTTTAAGACTACACAGTCAGGCAGTTTACCAAACGATTAAAAAGAAA
ACTAAAACAAGAGGAGGCTGGAAAGCTGAATATATTGAGCAGGAATAATGGAAGTTTCAA
GCACTGAATACACAATTAAATGAGCAAAAGAGTTATATGATAATGGAGGGGATATATTTA
TTGCTATAGCTAAGGCTAGAGAAGAATATGAGGTGTAGGTTAAAAGTGAATATCTTAGCT
AGTGTGATATTAGAAATAGGGAGTTTTATAGATGGTAGAGTTCATGAGTATAGATTGGAT
TTAAATTAAAATGATGAAGCTGATTCAAAAGTACTTTTAGATGTTGTTAATGAAATTAGT
GTGTTAAGAGAAGAATATGGAAGTCTTAAAGTAAAGTGACAAGAGTAAGATGTACTGTTC
ATCAATAGATTAAAAAATTTTTTACATGATTAAAAGATATCCGAATGTTGAATTTTTGAC
TGTAAGATTGATGATATTGAATATGAGTGTGATTATTGTATCAAAAAGTGGCTTGCTAGC
GAAGAACGTATATTAGAAAACAAGAAATATCTAATTAAAACAATTTAGAGAGTTGCAAGA
TGTTTTTTACTATAATTATCACTGAGATGTTTTGCACCTCTCAAAATTGAAGATAAGGAG
GCGTTGTATTGCTTGCATTTTTAGATTTACTCGCAGGGATAGGTGGCTTTAGGCTACGGT
TGGAAACATCCTGACAAACATGTTTGGGACATTGCGAATATGATAACTTGGCAGATTTAA
GTTATAATGCCATGCACGATCCGAAGGACGATGAATGGTTTGAAAGAGATATTGGAGATA
TTAGAACCGAAAATATCACGAGAGCAGGTGTCTGGTGTTTTGGATTCTCATGTCAAGACA
TTTCTGTTTCAGGGAAACAACTTGGATTCAGCGCAGAACGTTAAAGTTTATTTTTTACAG
TTACTAAACTCATTAGAGAACTCAAAGAACAAGACAGACCCAAGTATTTACTTATTGAAA
ACTTTAAAAATCTACCCATTATTAATGGAGGATTTGATTGCCTCGAAGTTCTGGTGGAGA
TGGCTGAAATCGGCTATGCTGCGGACTGGCAAGTACTTAATTCTAAAAACTGCGGAGTAA
CCTAAAATATTGAACAAATATGCATTGTTTGACATGTTAGAGGACGAAGTACAAGAAAAG
TATTTCCCATCGAAAGAAAAAGTAGAAAAAATCTTGAGCTACTAAATACTCCAACTCACA
GGACAAATACAATTTATGATTCAGTTGGAATTGCTAGATGTATTAGAAGTCAGGCAGTAG
GTGGAGGTGCTAAAACAGCACTATACTTTATAGACTTAAATAAAAACTCTAAAGTAACAA
TAAATGCTACTTGCCTTAAAGCAACATCTTATGCAGGTGTGACAAATAGAAATTGTGATA
ATAGTGGAGTTTTAGCTACTGCAGTTTGAAGGCCCGATTGGCTTGAGAAGGAGAAGTAAA
TAATAAGAGCCTGCAAATTAAAACTAGTTAGGAGGAATAACTTATGAAGATTTATTTATT
GACTATACTGCTAATAATTATTTGTATATAAGCAAATTATGTGAGAAATCGCACATATAA
AAAATCTATAAATAATCTATAATATAAATATTCTGTAGCGGAAAAGATTATATATCATCA
AATAGCCTGTTTCTATAACCGAATGGTTGGTTGTGAAATCTTAGAAAAAATGTTTTAGTAG

GAAATTTAGATAAAGAAATACCCCGCTTTATAAAATAAAAGCCTATGTAGGTGATAACGG
TAAAATATGGGTTATACCAGAGTGGAGAATTGAATGTCTTGCTACGAGTTATGGAGAATT
TCCAAAACATTAAATAATAGAAATTAGCTGGAGAAGGAGATTGTAAATTACGTTTTAATT
CTATAAGGTGCAAATAAAGACAAAAAGCACATTGGAGCAGGTAAGAACTCAAAGCTTAGA
CTTTGAGTATTAAGAAAAAGAATTAAAAAATACTCTGAAATACTAAAACCTGATTGAGGA
TCTAAAAGTGTTAATTGTTAAATGTGGAGATGAATATTGTCTAGCTAATTACAATGAACA
AGATAGTAAAATAATCATTGAAGCACATTATGTTTTAGAGCAGGTTGAATATACTGGATC
TTATATAAATGAATATGAACGATTTAAAAAAGATTGGTACAATAGTAATCGTGATGGTGA
AGCCTCTATTTTATTTTCAGATGATGGAATTGAGATAATTGAGAAGATAAGCGAGGCTTA
AATATGAATAAAAGAATTAAAATGAAAAAAAGATTAATGCATAAAAAGTTTTATGAAAGA
TGTGTCAACAATGACTTTGTAATCAGCAACAACCTTAGAACTTATAAAGTGTGTATAGGA
TACATATACAAAGAAAATGTGGATAAAGTATGTCAAGAGAACTATAAGAAATTAAGAAGT
AAAAATAATAAAATAGTCAAGGTAAGTTGGTGAATGAAACTAGAATGTTATAGGCTTGAC
TTATAAAAGGAGTGCGTTAAATGGCTAAGAGATATTGTTAAAATTATTATTGTAAAAACT
ACTTTGAAGATATGTGTATACTTGAAAGGATTGAAATTAATAACATCAAAGAATGCGAAA
GGTATCTTGAAGGTAAAGATGTGCTATATGAATTAGAAAGACGATATCCTTTACATCCTA
AAGATTTGAAAAAGGCGAAAAGTAAAGATTATTCCGTTGAAGTTACCCATATCCCAACTG
GTGTTACAGTAAAATGCCGTTCTACAAATAGTATTTTTAAAAATTAAAAGAAGCGTTTGG
AAATTCTAGAAGAAAAACTAATAAAAATAAACTCTCACTTAGAGCTAGAGGATTTACGGT
AGACAGGAAGTGATTTAAAGAAACGAAGAAGATGCAGTTCTTGCGGAAAATTATTTTATC
TCGTAGAAAAATCTAAGGATGTTTTTTGCCGTAAAGAATGTAGGAAGAACGCTAAGATGC
TCAAAAAATGAGAGCCTTTCCTCAAATAGATGGAGAACCAGTTCCTAAAGAAAGACCTAG
ATTTAACTTAGCTACTAAAAGGACCTATACACCAAAGAAAACTAAGGACTCAGAAGAATT
AACAAAATGGTAATTCCAATCTAATGTTAGACTTTATTTGACTGGATACATAAAGATGAC
TTTAAGATGTAATTACTCTATTGCAAAAAGTGACAGTGAAAAGATTAAAGAGCCGAAAAG
AAAAAATATGTTAAGCTACAATAAAAAACCAGATATCGATGATGTCGTCAAGATTATTGC
TGATTCACTCACTGAGATAGCTGATAAAAATGATACGCAGATTGTTGAGGTTGTAGCTAG
TAAATATTACAATGATGAACCAAGAGTTCATGTTATTTTAGAGGATGTTATTAGACCAAC
GGAAAAATCCGTTCGTTAGATTAGGTACTCAGGTTTTCATAAAAATGTGAAAAAGCTGAC
TAGAGAATATATCAAATGGTAAATGAGAGATAAATTATGAATGAAAATATAATTAAAGAA
ATAACAGTACTTGGAACTCTAAAAATCGACGGAATGAAATTTCATAACATGGAGAGTAGA
TTTGGAGAAGATAACCAAGCAATGCTAGTAAAAGTTATTGCTGACATACATAATAGTGAA
TCTATACAGATTCAGGAGCTTATAAATAAGAATAGAAAAAGATTAAAAGATGGAAAAGAC
ATATAAGATTTGTTAGGTGTTGGTTTGGACGAAACCAAAACAATAGAATTAGGATTTACT
CAGCAATCAATACATTCATATTGAGGGTTAAAAAACAAAGCGTTACTATCTGGGATATAT
ATATTAGCTGAAAGAAGTTATGCAACATTACCAAAAATATTAGAAGATGTTATAACTTGC
GAAATATATGAGAAGTTAGTTGATGGATATTTCTCTATGAGAAAAGAACTAAATAATCCT
CTTTTAAGTGCATCAAAGGAGTACCAACCTATATTTATGCTAGATAAGAAACAAGAAGGC
TTAGAAACTAAAATAGATAATGTTAATGAGAAGTTAGAAAACTTTATGGATGATGCACCA
TTATTAAATATCGAGTGTGAGTGTATTGGTAAAGACGTTAAGAGAATAGCTACAAAATCA
CTTGGTGGACATGGAAGTAAGGCTTATCAAAATAAATCTCTAAGAGGAAAAGTTTATGGT
GATATATACCATCCGATTAAACGACAATTTGGAGTAGATAGTTATAAGTCTAAAAAGCGT
TGCCAGTTAGATAAAGTATTAGAGATTATAAATAGTTTTAAGTTACCTATAGTGTTTGAA
GAAGAAATAATACTTACGAATAGTCAATTATCAATTGTGAGTTAAGACAAGAATAGACTG
TTCTCCTTTAAAGGGGAAAACCTGCACTAGTCATTTAAAAGAAAAAAGGAGTGCTTTCAC
ACTCCACTTGTCATAAATATGAAACTTTTATATACAAATATAATCATAACATAAAAAACT
GCTAGGAGTGTGGAAGAATGAATAAAAAGACACTTTTTCAAGAGGTTGAAGGTAGTATAT
ATAACTATAAAAAACAAGACAGTCGAATAAGAATAAAACATATATATATTAAAAAATTAG
AGAATGAATCTGACGGATGCAAGGCTCAAAGTTATGAAGAAAGAACAGGAACTACCTATA
ATATAAGATAAAGTGTTGAAAATGAACTGGTTTAAAGAGAAGAAGATTAAAATAAATCAA
ATGAAGATAAGAAAACATTAGAAATTGAAAAAGAAACTATAGAACGTGCATTAAAAAGTT
AGAACAGTTTTGAAACCGAGTTCTTCAATAAAATGTGTACGCATAATGAAAAGATAAATC
TGGATTATATGTGTAATGCTACGCCCATAGATAGAACTCATTGCTTTAGAATAAGAAAAA
GGATAGTTTGTCAGATTATGGAAATGTTATATCCAAAGATAAAGGAGTTTGAATTACCCA
TTTTTTCATAGCAAGCTTACAAATAAGACTATTTTGTGACTTTTTCGAGACTATCATGAG
ACTTTTTATTGGTAAAAACATGAGACTATCATACTGTGGAAATGAAGATTACCCTGTCAA
AATTAAATATAAATAAGGCTAGAGTTAAGGCATTCTCTTTGCTCTAGCCAAAGTAAATAC

TTTATATAATTAAGGGCATCATCTAAAAAGGTGAAAAATTTTCAAATGACGCTTTAAATA
TAAATAATAAATATATTTCTCTTTAGGTATTGACACTTTTACATAAGGCGCCTACAATAA
ATATATAAAAATATATAGGAGATGCATGCCTGAGCGTCAAATAAACTAAGATTAGAAGTT
CCCAATGAAATAGATTATAAGATTGTGCAATGCCTTAATAAGGCAAACGAAGAAATTAAA
GAATATATAGGAAAACAAAACCTTACATCTAAAAATGGATTGCCATTTCAGTTTTGGGAT
TTGTTAAATGATGCAACTGTTACTGCATGTAATAGTTTTAATTTTAAAGCGTATAAACCT
AAACGTGGAGGTTGGAGAATGATTTTTATATATGATAAAAATGGCAACTACCTTTATGTG
TTAATGAAAGAGACAAGATATAAAAAACTTCATAATAATGACAAAAAAAGAGAAGAAATG
CATTATGTTGATATTTTAACTAAAGTATTAAATCGAGATTTAACAGCCCCTATTGAACAA
CCAACAGATTTTTCATTAGAATTTGATAATGAAGATAAAATTGAGATATATTTACAGAAA
TTAATTGATACCATAAAGACCGATGGTCGTATCATTGAACACCATGTATTATTGATATTT
AATCAAACATCCGGATAGGATTTCACGTCTATTCGTTCTCTGATCGTTGACTCAATTTGA
AATATTGTCTCAGAACAAAATTTGAGGTCTTATATATCAATAGAAGAAAGTACAGTTGTA
GAAAACGTTGATTAGACTAATTCTGTTGCAAATAATCCTACTCCTGGATTGAACCTAACT
GCAAAAGCAGATGCAAGAAAGAAAACAGATCCTTTAAAAAAGGCGAAAAAGGAGGAAGAT
GCCAAGTAGGCTCTTTTTGGTTAATAGATGGAGGAAATAATTATGGAAATTAATTTTAAT
GGAGAGCGTTTAAATTAATCTCGTATATATAAGGGAATGACAGTATCTGAAATAGCTGAG
ATAATAGTCTGTCAAAGACTGACTGTTTCAGTGTATGTGTATAATAAATCGAAACGAAAA
GATAATATTGTAGTGAAACGAATAGCAAAGGGGTTAGGTTTCCGAGTTAAATTCATTTTA
GAAACAGGAAATAATATTGCAATAGGTTCAACATATTTCAGAGCATTATTGACTACAAAT
TAAAAAAATAGAGCAGAGCAAATTCAAAAAATGGAATTCCTAGCAGAAATATATTTCTTC
TTACAAGATTATATTGCATTCCCAACATAAAATTTACCAGATTGTTCTGGGAAAACACCA
GAAGAAGCTTCTTTAGTTTTAATAGAAACTTGGGGATTAGAATTAAAACCTGTTGACCAT
ATCATATATGAAGTAGAACAACACGGAATACATGTAACATATTTCTCGACTTCTACATAT
GGTATAGCTGCATTCAGTCAGCTCGTAGATATGTCGGGTGAGTCATTTTGTCTGATTGGA
TATTCTAGTAATAAGACTTCTGCTTCTAGAATTCATTTTGATATAGCCCATGAATTTGGG
CATATATGATTGGATGAGTGGCGCGAGGATGTAGAAGCGTTAGAAAAACAAGAATTTAAT
GACAGAGAATCAGAAGCTAATAGATTTGCATCCGTATTCTTATTACTAAAATAGACTTTT
AAAATTGAGGCTAAGAGAACTCCTTTACGTATTCGAAACTATACAGAATTGAAACGTAAA
TGGAAAGTTTCCATTCAAGGACTGATTCGACGTTCATATTGTTTAGGAATCATCAGCATG
GATGAGTATCATTCTATGATTCGTACTTTACAACGTCGGGGACTCAGAAAATCAGAACCA
TTAGATGATCAATTATTAACTTCTCTACCAGTATTGTCAAAAACAGCAGTTTTGACGTTA
TTAAAATACCACGTATTTACTCCAAAAGAGGTTATGGATGAACTTTCATTTTCCTATAAC
TTTAGTCTAGAGCCAGAAGAAGTCGAATATCTATTAAGTTTACCTAAAAATACTCTAACT
TCTGGTAAATTTGTATAATTTCCTGATTTACAACTTAAAAAATATGGTGAAATCATATTT
TAAATAGAGCGAGGACTTGTGACCTCCCTTATATTCTATGAAATGGGGGGAAATATGAAA
TGGGAGTATAAGAAGGTGCAAATAAAATAAGAGAGATAGCCAAAAAGATGGCTATAGAAA
AAGGAATAACAGTTCAAGAGGCATGGAAGGATGCTATTAACGAGTACAAGGAAAAATATC
AATTTAGTTAAGACCAATGAATGGTGTTTTTTTTATTACTATGAACAGACTAGGAAGGGC
GTGAGGACCCACTTAGTTCGATCCTAACTATGTTCATATATTAATCATCGTATACACTAA
AAGTAGAGAAATTGAGGGCAAAATCTTATATTTTGTATCTTAAGTCGGAGGTCTAAAAAA
CGAGTGGGGCTTGGTAACCTCACTCACCACGCAAGTACTGGTTTAGTTTAGGTTCCATTC
TTGGAACTTGCTCCCTTTAATAATTTGTATCCCCCATTAAAAAGGCTTAGATTACCTTCT
AGGTCTTTTTTAATACAACAATTTATTATAAATGCACGGCATGCACTGTTCAGATATGCA
TGTCATGCTTGGCTAAATTCGAATAGTTAATATTTTCAATGGCTCGAATTGAGGGGTCGA
AAAAAAATACAGGAGGTAGCAGTATGTTGAAAATTTTACAAGAGAAAAACGTAAAAGTAA
TGTGGTCCAAAAATGGAGAAGAAGTTTGGTTTAAAGTAAATGACGGAGGAGAGGCACTAG
ACATAGTAAATATTCGTAATACATTAAGAAGTTTAGAGAGTGAATATAATAAGAAATTTA
ATGAGTCTACTGTAGGAGAGTCCTACACTAGAAACTTTAAAGATAAAATGCCTAACTTAG
GTACTACTTTTGTTACAGAAGAAGCTGTGTACAATATGTCATTTAGAAGTAAAAAACCAG
AAGCAAAGTTATTCACAAAATGGGTTACAAAAACCCTTAAACCAATTAGAATACATCGTT
ATGATATTGTTATAGAAAAAGACCAGCAATGGCCGGATATAAGCACAGAAGGCAAAAAAG
TAAAAAAATATTTTACAGATGAAATACAAGAGTTTGTATATTATGTTACTAGTCAAGGTA
GCAATCAACCTCAGATGGATTATAAACATTGTACCGAACTTGTAGGAGAAAAATTAGGTA
GTCAAAAAGGTGGGCAAAGAGATGAGTTAAATCAAAGCGAACTATTTGATATAAAAGCAC
TTGCAAGAATTATATCTATGCAATTCCCTAAGTTATTATATAAAGATATGAATTATAAAG
AGGAATATAAAAAGCTTAAGCAATTATTAGAAATGATTTAAGGGACTGTC

>NewGenomeName_18
TCCCGTCTCTGATCAGCCCACCCTTCCCCCGTAGGCCCTCCGCATGGTGGGCCATCCTCG
CCTTGCCCGGGTGTTGCCTGGGTTATCCCTGGGAGCTTCCGGCTTCGCCGGTGGTCCGGT
GGGCTTCCCTGCCCTGTCGCCAGCCATTCTACACGGCTCAGCGGGAGTGTCAACCCTTGG
GATAGCGCTGTCGCTCTACCGGGCTTGGCCGTAGTACCTGGAGCGCTCCATCACGTCGCG
GTGCATGGCCCTGGCTACCTCGTACTCCTGGGATGCCTGCCGATTCCGCCCGGGTGGTGC
GTAGTGCCGGTGGTCCTGCGCTGCGCACTGTGCTAGCTGCCTCTGCCATGCCGCGTCCTG
CTGGTACCAGGCACGCTCACCTCTGTTCTGTCTGCTCAGGATCCTCTGTGCCTCCTGTGG
TCCTGTCGGGTGGTGGTGCGGGAGTGGCTGGCCTTGCCTCTCAGCGTAGCCTTGCCTCTT
GTCTTGCCATGGCTCTTCCTGGCTACTCCCTGGGTATTCACTGGGGTTGCCTGGGTGGTC
CTGGCCACTCCCTCTAGGCCACGTACTCTACTCGCCTCTACCTGTCCTGTCTAGTCCTGC
TCCCTGTCGCTCCCTGGGTGCTGGTCTGTCTCCCTGGCGCTGCACTGGTCCAGCTGTCTT
ACCTCGCCGCTCCCTCGCCTGCTCGGTCGCACCTGCGGCGCTGATGGACTCCTATTTGTC
CATTGTGTGTGAGAGAACCGCAGCCTTAGTGCCATGCGGGTTCCAGGGCGATGGCCTAGC
TGGCTGGGTGGACCCTGGCTGTTCCCTGGGAACGATAATCCTGGCTAGCTAGACTGAGCC
TCGCATGCGTGGGTCTCTATACGTGCGTTGAGGCTCCAGGCCGGGCCTAGGCGGGGTGTT
GACAAGACAGGTAGGCGACTGTACTATGCGCCCTGTCTCCACGCAACACCGCCTCTACAG
GCAGCCTGGAGACAGGGTTGACACACTACCGGGACATCGGTAGCGTGCGTAGACAGAGGG
ACGGAGCAACGCAACCTTCCAAGCGCGGCACATGCCATAGGCGCAAGGGACAAGGCGAGG
TTCCAGGGCAAGACCGACTACGCGTAAGGCCGGCAGGCATCGCAGAGGGGATTGACAGTA
ACACGCTCAGTCGGTACGATGGGCACCACTCGGCAAGCAACGCAACATCAGCTACCGGGT
TGACACGACAAGGCCGAGTCGCTAACATGGCCACCAGCAACAAAGCGAACCGGCGGATAA
CATGGTCCGGCAAGCTCCAGCAAACGGGCTGGGCTGGACGGGTAAGACGAGTGAAGCTGA
CAGCGTAGGCCCTTCGTAGAAGGCAGCCGAGGTGGATCACGCACCAGAAGGTGAACTGTA
GGAATCGGGCAGCGCAGTAGGACTGCCAGGGGGTCAAGGCTAACCCCGCAAAAGAAACAG
CCGAGCGTTAGGATCACGTCCCGGAGCAGTGTCCCGGGCAGTATCTTGTACAACCGACAG
TAGTCGGATGCCTGCAAGCCGAATCAACGGCTGGCCTACGGGCTGGGCATCCCCTCGCAA
GTCGGTTGTATCTGGCAAGAGCCATCGGGTTGGCGGATGGGCTGCGAATGGGCAGCGATG
GCTCTTGACCAGATACAACCAACCGGAGTAACCATGAGCAAGAACGCCTCTGGTGCAGCC
AAGGCCGCCAAGCTACGGGCCGATATGAAACGCTATCTCGCTGGCCACCGCGTCACGAAG
GTAGCCGAACTGATCGCCATCGGCGTCAGCTCCGCACGGGCTGAACTGCGCAAGATGCGC
GATGTTCGGTACAGCACGGACAAGGCAACCACTCCCGGACACTTCTTCAATCCCAAGGGC
AAGCACTTTGCCCGTGGTTCCGAAGGCAAGGCACGCAACATCCTCAAGGGCTTCAACGCC
CGCAAATCCCAAGCAAAAGGACTCTGACCATGACCAACCTACTGACCAAAACCGTCATCG
CTTTCGTCGGTCCCGCTGCCATCACCAACGCCATCGAAGCCATCAGCGTTCGTGGCAAGG
AACTCGACGAGGCCATCCAACTGACCGGCCTGTCCATCCTGAACCACGTCGATCTCCACG
GCGACATGACCGTAGTCAAGGCGCTGTACGAAGCCATGCCGCAGGGTAGTCGCTGCAATG
CGCTGGTCGAGTGGCTGACCATGTTCGGCCAGGTACAGGTGAACACCGACAAGAAAACCA
ACAAGGAGCATCCGTTCCTGTACAACAAGTTTGGCAAGACCGATCTCGTCGGCGCAACCA
ACGAACCGTGGTACGGGTTCAAGCCGGAGAAATCGCTCGATCAGGAATTCAACCTCGCCG
CCGCACTCGCCGCTGTCCGCAAGCAAGTGCTCCAGGCGCAGACCAAAGGCAAGGTGATCG
TCGGCGCTGAACTGCTGGCCGACCTGGAAGTCCTGGCCGCCAAGGCCGCACCCATGGTCG
AGAAAGCCGAGCGCGCTACTGCCGACTAACTAGCTCAAGTCGAGCGCCTGCGAGGCGGGC
GCTCCGCTGGATCTAGTACAACTGGAGACACCACGATGACTTTCAAGCAACGTCTGCAAC
GTCAAATCGCTCTGGCTCAGTATAGCCGGCCCGCCCACTATCCCTACGGCGAGCAGGCCG
TACAGGAGGCAAAGTAATGGATATCTGGATCGCTCTTCCCTTCTTCGAATTCGGCCTGAA
CGTTAGCGAGTGGGCCGTGCAATTCACGCTGGGCATGATCGCAACCGCCCTGTTCATCCA
TCTGCTGCTGAATCGGTGACTCGAGTCATGGCCCTGGCGGGCGACCCTCGCCTACTCTGG
GGCCATCGCTGGACCCATCACAAGACAGAACTAAACAATGCAAGCTTTGAATACTCTGTA
GATGGCAATTCCCAAGGACCCGCCAGGCCGATTCAACGCAGCCGACAAGGCGCTGTGCGC
CCATGGCTTCCTTATGGGTGACCTGAACCGCGCCCACGTCCTGACCCCTGGCGGGTTCTC
CGTGGTAGGCGCCGGCGTGACCGTCAACCGCTACGACGAAGCGTATCGCTTGAGCCGCGA
TATCGACTCCGCAGGCTTTGACGTACTGCTGGTCCAGGGCAGCCCGCTGTCCGGGCACGT
CACCTGCCAGGCGTACGGCTGGATCAACGCTGAGTACCACAAGGGCTGCGCAAACGGGCG
CCCGATCTTCGACATTGCAGGAACCACGTACCATGTCCTCGCGCGATCCCTACCGTATCG
GCCACCGCGTGGGGCTGGTGAACTACAGCGACCGCTACCTGGGTGCCGACGCGGCAGGCA
CCCGGGGCATCATTGGAGCCATAACCCGACCGACCCGCTGTATGACGATCTACCACGTAC

GCTGCGAGCGGACCCTGCGCCTTATCGAGGCCGAGGCCCGTAGCGTGGGGTTCATCAGAC
AGCTACCGACCCGCAAATGACGTGGCTCATCATCGGTGTCAGCCCGTCGGGCGGTTGCGC
CTTCGTGTGGAGTCGCAAGCGACCGGTGCGTCCATTGCGATTCTACTCACACAAGGCAGC
TAAACGCTGGCTTCGCAAGCATCGCCGCAAGGCCTTGCTTGGGAGCCGATTCCTCATAGT
GAATTGGAGCAAACGCATATGGATCCTACTTACGTCGTCCGTCTCAAGGACGGAACCACC
CGCCAGGTGGCCGCCGACACCATCACCACCGGCCACAACTTCGTCGAACTCTGGTTCCTC
GGTGGCGTGGTCGCTATCTACCCGGCCTGTGAAGTACAAGAGGTGCATCGCACTGACCTC
GTGAAGGAGGGCGAGTAATGACCGTCGAGACCGTGTATGTCAAGCCTGAAGACCCGGCGC
CACCAGTCTTGTCCGTAGGACGCCTGTCACCAGGTGAACTCTACAAGGTGGTGACGCCTA
GCTCGGCAGAGGGCGCCGTTATGCTGGCGACCAAGCAGACGCCTGCCCTCGCTCAGGCAG
TCATCGTACTGCACAGCATGAACCCCGCGCAGTAGGCGGTGGGCACGGCCATACTAAACA
TAGACTGGCGGTGCCGCCGCCTGGGTTCCGGCGAGTACATCAAGCTCGTTCAAGGGGAGG
CCGACCAATGATCGCTGCCCTGTTCGTACTCGACCTGTACTGCTGCCTCGGCCTGCTTGC
AGGCGACTGCTACTGCATCGCAGTGGCGCCGCCGTTCGAGAACCCTACGGAAGTGCAGTT
CCAGGACGCCTGCCTGATCGGGTGCCTCTGGCCCTTCGTGCTCGTAATCATGGTCTACCT
AGGGGTGCGATCCTTCTTCTGTGGCGCTCTGGGTGGCTTGCGCCGCCATCGGCAACGTCT
TCACAACAAGCGATAAGGAACTGCGATGATCCGTACCCATATCCACGGCACCAACCGTAA
GCGACTCCGTCTTCACAAGGACACTGAATTAGAGGCGGGCGAGCTGTACCGAGTCGTGCA
GCCCGAGTCCCGTCGGGGCACGCTGGTACTCGGCGTGGGGAGCACGACCGACCGTGGCGT
CCGCACGGTGGTGCGTGCAGTCATCCACGATGAAGTTGAGGGGAACGTGTCTACTCCGAG
TCCTACCGGGCTGCGTAACGACACCTGGCGCATGGTGCTCGTGAACAAGGGCACCCAACT
GACACTCACCGCCGAGTGACCAAGGCGAAGGCTGGCGCGCCAGCCTTCCACCGTGGCCAT
TCCTTGCCGCGAACCAACTCAACTGAGGAGCTACAGCATGACCAACGTCAACACCGCCAC
CGAGACCACCACCGCTGCCGTCCTGGGCGCCAAGATCGTCAAGAAGCCGGCTACCGTGGA
AGACTTCCGCAACAACGTGGTCTTCCACTACACCGCCCTGGCCAAGCTGACCGAGGCCTA
CAACGCGGCGGTCGCTGCCCTGCAAACCGCCGAGCGCCTGTCCAGCCTCGTCGCCGGTGA
CGTTATCACCTTCGACCACGGCAAGGGCGAGAAGGCCGAAGTGCTGAGCGGCGAAGTCAT
CAGCGTGGTCGCCGGCGACTATCAGGTGCTGGTGCGTATCAGCGACAGCGACCCGGCCAA
GCTGCTAGACGTGAAGGCTGGCGCCATCCGCGCCGTCCAAGCTCCTGCTGCTCCGGCTGA
ATCCCTGGACGAAGCCATCGCCCAGGGCGAGTAAGGCCCGCACGTAATAGGTCTGGCCCT
GCCGGGCCTATTGCGAGCTAGCCATACCATAGGAGGAGTCAACGTGAGCAAGCACAACCC
CGAACACATCAGCGGCCCGGTACGCAGCGTGAGCGTAGCAAAGTCTCCCGTCGTCCAGGA
CCAGGAAGACCGCCTGGAATCCGCCCTGGGCGTGTGCCAGGAACGCGCCCAGGATATCGA
CACCCTGAGCCGCCGGCTCCAGGCTGCCGGGCGGACACGCCGCTGGGACATCGACGAGAT
TCGCAACCGTCAGGCGACGATCCGTCTACTCTAGAACGACTTGAACGCAGCGCATGATGC
CCACGAGGCACAAGAGCGCCGTGCACGCAAGGCAACCATCATGGCCTGGGTATGCCTGCT
GACCGCATGTTTGGCCGTCACCCCGAAACTGGCAGGAGCGTGACCATGCAGTGCAAAGAC
CTTTACACGAACCTCGCACCGGACATGTTCAGCGTACCGTGCTCCCAGGTGACCCCGGAG
ATGCGCCGCGTGGCCAAGAGCCGGGCATTCGCCCACGCCTATACGCCCAAGAAACAGGCT
TCTGGCGGGACTTACACCGCCCGTGTGAGCGGCGTCACCTTTGACGGTGGTAAGGTGGAG
GTGCGCCTGGATAACGTGGAGCGCGTCAGCACCTGTGACTATGCCGATCTAGAGGCGCTG
GTAGCGGCCAGCTTGTGCCAGGCCGATGCGAAGCGCGCCGACGAGTACGAAAAGTTGCTG
CTCAAGGCATTCCCGCTGGTATCGAAGAAGGCCGGCCCGCTGTCTGCCAAGGACTTCGAG
GTACGTCTGCACGATTTGCTCGTAACCAAGCTGGCGGTAAACCGTGCCCTGCGCGATGCC
GGAATAGAGATGGACGGACCGCTGCGCAGCCGGGTACGGAAGCTTGCGGACCGGAATAAC
GAGATGGGCGCCAAACTGTTCAGCCTCCAGCAGGGCTTGACCCGGCTGGTGGAGGTAGGT
CAACGGGCCGGCCTGTATTGGGACGACGCGGAGACTCAGCGATTGCTGACGGTGGCCCCG
ACCAAGGGCGTCTGTCGGCTCATCAGCGACCTGACCGGCGTGCGGTATGCCTCCCGCGCT
TCCGTGGCGAAGGCTGAGGCCGAGGCTCGTGAGCGGGCGAAGGCGGCGGCTAAGCACACC
TGGCAGGCAGCAACCTTCGCATCCACAATCGCCGGCGGCGTCGTGGGCAGCGTGCTGACG
TACCTGCTCGCCTAAGCGACCGGGGCCTACTCCGGGGTCAAATCCGAGGGCGTTCCTAGA
GCGCCCTCTCGTGTGCGTCTGGAGGATCACGAACATGCAATACCACTTCACGCATTACAA
CGGATACTGCTTCGGCGTCGAGCTGGAGGACGAAGCCGTCTTCCCGTGCATCAACGGAAA
GCGGGCTACCTGGGACAAGGTGGCGGCGTGTGCCGGTAGCCTTGTGCATTACATGGCGCA
GGACCTGATGGACTTCGGCCAGCGCAAGTTGAGAGAGATCGAAGATCAGCAAGACGAGTC
TGTACCCGCTGACCCTGAATCCCGGCCTGATACAGATCAAGACGATCCACGTATTCAGCA
TCCAGGTGTCGAAGGACGAGCCGAACTGGTGGCAGTGGTTCCTGTGGCAGCGGAAGTACC

ACCCACTCCGCGACAGCCTGGACACGGCGGGGAACTGAGTGCGAGTATCGCCGAGTATG
TCCACCGCCTCCGCCGGAATGGATGTCGAGATAGGGACATCTGGAGCAACAAGGGCGGCG
TGCTGGCCCTCGGTGCCTTCAGCCTCGCCGGCGAGATGATTGGCTCCTGCCTCGTGGTGG
ACAACGAACTGCGCGCCCTGTGCGTGGACGACCGGGACAAGGGGCAGGGTATTGGCGCTG
AGCTGGTGCGCGCTGCCGAGCTGGCAGGTGCCGACCACCTGAGGTGCTTCGAGTTCCTGG
AACCGTTCTACGCCGGCTTGGGCTGGCGCACCGAGCGCCGCGCGGCAAACTTGACTGATG
GTGAGCCGGACGTGCTGCACATGAGGGCGCCCGGTTATGACGCATGAGGTGATGACATGG
CTTATCGCGAACAAACCGCTGGTCATCGGAGTCGCCTTCAGTCTGGTGGCCCTGGGCGTT
CTGATAACGCAGGACAACGGCGGCCCGCCTACGGCACCCGCATGACCTGGGACCTCCAGG
ACATGTTCGAGGCCCGAGGCGGGCTGCGTCCCCTGTGGGAGGGGTGGTATCAATGGCATT
GCGCCGCGACTCCTGGGTGAAGCAGGCGCAGGCGCTCGCCGTAGACCAGACTGGCAGGGT
CCGGCACGTCCTGGGATGCAAGAGCATGAGCCGGGGAGGGACCAACATGACCGTCAAAAA
TCTGCCGGACCGTTGGGTAGCGTATTGCTACCCCTGCCAGGAAGGTGGCGTGGTCGAGAA
AACGCATGTGCGGAGGGTGCAATGCGCGGATCAAGAACGCTTTATGCCCTGGCCCGAAGA
TGCCTCGGACTGGACGCGGGCCGCCTGCTATCAGTCGCTCTAGGGTTTGCTGCTGTCCAA
GGGCATCGACTACAACGTGATGACCCCAGGGCTGCCGCTGCTGTACAGCGGAAGGCAGCA
TCGGCCTATCTTCCCTACCGACGCGGGCTGGATTGGGCGCGCTACTGCCGACCAAAATCC
CAAGTGGGTGGGCTACGTGTATCCTGCCCCGGATTACCATGGATGGCCCCAGGACTTATC
AAAGGGCAGGGCATGGGTGCTGACGGAAGACTACTTGTCGGCGCTGAAGGTGCGGTGGGC
CTGTCTCGAAGTCTCTGCTGTCGGTCTGAACGGTACAAGGCTGCGCGACAGGCTGGCGGC
GATCATTTTGCAGCAGACCTGCAAGCGCGCCTTCATCTTCTTGGATGGCGACCCGGCAGG
TGTCCGTGGTAGTGCACGCGTGATGCGTCGGCTCCGGTCCCTGCGTATCGAAGGCCAAGT
AATACCTACGCCGGGCGGGTTCGACCCCAAGGACCTGACCCGCGAGCAGATAAGGAGCCT
AGTAATTGGACGTATTGACGCTACACGCACTGAGTGACAGGGACCGCTTCCGCACGTTGC
GGTGTGTGGTGCCTGAAGGGATGATGGGGCCGGAGACGTGCTTTGTCATCGACTGGATCG
ACCAATACTGGCAGGTCTACCCCGCGCATCAGAAGGCGGACCCGCAGGCGCTGCGCGAAC
TGATCAAGCTGCGAGGTGGCTACCAAGCGGAACAACTGGCGGTAGTCCTGAACCTCGTCA
ACCAACTGGACAAGCCGGTAGACCCGGACTCGCTACAGGGCGTCGTGTCCCAGCTCAACG
AGCTGTATTTTTCAGGGCGGGTGGATGCCCTCCTGGCGCAGTACAACCAGGGCGAGGACA
TCGACCTAGCGGATGAGCCGCGCAGGCTGAGCGACGAGGCCCTGCGCCGCGAAGGGGTCA
GCAAGCCGACTGACTACGTGACGGACGACGTGTTCGATATCCTGGCCGAGGAGCAGGCTG
ACCTCGGCATCAAGCTGCCGGGGCTTGTGCTACCAGCGTATATGAAGGGCCTCCACGCCG
GGGCCTCGGTGCTGGTGGCTGCGCCACCGGATGCGGGCATGCCCTCGTTCATGGCCTGGA
TCGCCGTACACATTGCACCGCAGCTCAAGCGGTACTTCGACCCAGGCCGGCCCATCCTAT
GGCTGAATAACGAGGGCAAGGGGCGGCGGATCAAGCCGCGCCTGTACTCGGCGGCCTTGG
GCATGACCGTGGGCGAGATCCTTGCCATGGACCCGGAAGTGGTACGCAAGCAGTACGCCG
ACATGATCGCCGGCGACTCCGAGCTGATTCGCATCAAGGCCTTCCATGGTGGGTCCCTGG
CCCAGGCCGAGCAGGTCATTGACGCGATGAAGCCGGCGGTGGTGTTTTGGGACATGATGG
GTCACGTCAAGGATGGCCCGCGCAAGGACCAGAACCGCACCGACGAGATGGAGTATAAGG
TGGCCGAGGTCCGCGAGATGGCGGTGCGCCACGACTTCATCAGCTTTATGACGTGGCAGG
TCAGTAACGATGGCCACGACCAGTTGTTCCCGCCGCAGTCCTGCCTCAAGGATTCGAAGA
CAGCGGTACAGGGCGCCGTGGATGTGCAAATCCACCTGGGCCGTCGCAACGGTGCGGATC
AACAGGTCATGCGTGGCCTGTCCCTGCCGAAGAACAAGTTCCAGATGGACGGGAAGCCTA
CGAACGTGGAGGCGATGATTAACTTCGACACCGCTCGGTGTCGTTTCTTGGAGAGTGTAA
ACCATGCAAGCTAAGCATAGCCGGGTGCTCGAAGGCACCAAAGAAATTCCACTGGGTTCC
ATTGCCCTGCCGACCGCGGCCAAGGCCCTGTTGCTGCGCCTGTACTCTGATGCACGGCCC
AACGAGGGCGTCGCCCTAGCCGGTGGGTTCCCTCGCGACCTCATGCATGGCGCCACTCCA
AAGGACGGGGACATCGCCCTGTACGGCATGACGCAGTATCAGGCTGAAGTGCTCATCAAC
AGCGTGTTTCCTACCCTGGACCCGCGCTTCGTCCGGGACGGTGGCTGGAGTACCGAGTAC
GCCAACGCAGGTGAGGGCCGTATCTTCAAGGGCGTGCTGTCCCTCGTAGGCTCCCGTGGC
CTGGAGGGCATGGACGTGGATTTTAACTACTACGACGCCGACAGCCTTGTCCGGGTCATG
GAGTCGTTCGACTTCACCATCAACCAGGTAGGCATTGCGTACTACCGGCCCGACCCCGAA
GGCGGCCCGCGCCTGGCTGCGTACCTGCACAAGGACGTTACCTGGGGCGTGAACAAGGAA
GTCGGTGCAGGCTCACGTCTGCCGGAACGATGCGAGAAAATGCGAGCCAAGGCCGCGTGC
TACGGATGGGGTAACGTATGATGAGCAAGCGCGACGTGGTACTGGATATCGAGAAAGGCA
TCTGGCGTGGTGTTGACCAGAACGACAAGGCCGTCGAGGCCATCATCAAGAAGAACGGGT
AAGTGATCCTCGAGCCTACGATCGACGGGTGCCGTGCCATCGTCGGTGCGCATGGCGTGG

TGTCCCGCAGCGGGCACCGCTTCCCTGCCCTGGACGGCCTGGAGGATCGCATCCTCGCGA
AACTGTCCCGGTCCGGCCTGGACTCCGGTCTGGTGCTGGACTGTGAGATGTACCTGGAAG
GCATGCCCTTCAGTGAGGCGACAGGGCGTATGGCCGGCAAGACCCCGCTGACCGAGGCCG
AGCTGAAGTGCCTTCATTTCGCCGTATTTGACGCCACCCATATCGGCGTGCTCCGCAAGT
CGCGCAAGTCCCACCTCGTGTAGGTTGAGCGCCGCGCCATGGTCCGCAGCCTCATGGAAG
ATTGCCCGCGGGGCGACACCTCGTACTTCTTCCAGTTAGCCGCACAATCCTGCCGTGCTA
TGGAGGCGGTACTATGCTGGTACGGCTACCACCGCTCCATGGGCTTCGAGGGGTCGATGG
AGAAAGACCCGAGCCTGACCTACCGGAACGGCAAGGTTGCAGGCTGCTACAAGCGCAAGC
CGGAGATCACCGTGGACGGGCGTATCGTCGGGTACGTGATGGGCAAGACTGGCAAGAACG
TGGGCCGCGTCGTGGGGTACCGTGTGGAGCTGGAAGATGGTTCCGGCACCGTGGCCGCCA
CCGGCCTGAGCGAGGAGCACATCCAGCTCCTCACCTGCTCCCACGTCAACGCCCACATCG
ACGAGGCCATGCCGAACTACGGTGGTATCGTCGAGGTCTCCGCGATGGAGCGCTCGGCCA
ACACCCTCCGCCATCCCAGCTTTAGCCGCTTCCGCGACCTGGCCAGTAACCCTGGAGTGA
AGGTATGAAGATTCGAAAGTCCCATAACCGCAACTACCCGGAAGATATGGTATACCAAGC
CACCAACCGGGATTCGCTGCTGTATCCGAAGTACGTCATGGGTTCCGTGTTCAGCAGCCA
GGACGGAACATTCCGCATCTGCGTCATGGCAGGGACTTGGGACCACGTTGGGTCCGAAGT
TCGGCATCAGGCGCGGGACATCAAATCCCTCGGCGCCGGTCGCCGGAAGTTGCACCGCGT
CATGCGACGGCTGCGTCGCAATCTGCGACAGGTAGCAGTCAAAGTATGAGAATACCCCCC
GCGGAAGAACGTATGATCCGCTGCTTGCTGGCGTATATCCACGAACCCCTGGGCCTGCTG
TTCCCCGGCCTCCGCACCAAGGCCCACGTGGACCCTCAAGCGGAAGAGCTGTCGATTCGA
ATTGACTACGACCTTGCGAAGCTGGGCCGTATGGGATTCTGCCACGCGGTCTCCCTGTAC
CAACTGACCAGATGGGGACGTGAAGGAATGGACCGCTACCTGATGCAGGAGATTCCCCGC
CGTGTGCTGGAAGGTCTGCTGGTCAAGGCGCAGCAGTACAGTCAAAGCAACTGGTACAGC
AAATGACGACTATCCGAATCCTCGACCTCGAAACCGAGAGCTACGAGCACAAGGGTCGCA
AGGCGTCGCCCTTTGACCCCCGCAACTACATCGTCATGGCCGGCTGGCGGGACGATGTTG
ACGGCAAGGTCGGCCAGAAGGTGGAACATCGCTTCCGCAGCCGGGCCGAAGCCGAAGACC
CGAACAACCGCTGGTTCAACCTCGACGGCGTGGACGTGATCGGAGCTCACAATGCCATGT
TCGAGCCGAACTGGATCTTCACCCGCTACCGTGACGAGTACCTGGCCTTCCTGCGACGCG
GGGGCCGGGTCTGGTGTACCCAGCAGGCCGAGTATCTGCTGAGTCATCAGACGTGGCTGT
ACCCATCACTCGACGAGCTGGCTCCGAAGTACGGCGGCACCCACAAGGTGGACGGCATCA
AGATGGTGTGGGACCAGGGAGTGCTCACCTCGGAGATGGGCCAGGACCGGCTGAGCGAGT
ACCTGTCCGGCCCCTGCGGTGATATCGAGAACACCGCCCTCGTTTTCTACGGCCAGTTGT
TGAAGCTTCAGGCCCGTTGTATGTCGGCAGGTTACCTGGATCGCTGCGAGGCCCTGATCG
GTTTCTCGGCGATGGAGTGCGCCGGCCTGAAGGTGGACCTCGAAGTCGCCAAGGTGAACC
ACGCCAAGCAACTGGAAGAGGTGGCCGGGATCGAGGCTGAGCTGAAGAAGCTCATGCCCG
ACTTCCCGGAATACTTCGAGTTCAAGTATACCAGCCTGTATCACATGAGCGCATGGCTCT
ACGGCGGCGAAGTGCGGTACAAGGGCCGGGTGCCCTACGGAGATGGCCGGATGGAGAAAG
CCGACTTCGTGCGCTTCGGTACGGCCAAGCGGGGGACACCAATCAAGAGTACCTCGGTGC
GGGTCCCGCTCAGCGAGGTGATCCTGGACGACGACCAATGGAACTGGGCGGCCATCACCG
AGCTGGCGACCAAGTACGGCCCGGTCATCACGTTCTCCGCCGGCAAGAACAAGGGCAGCA
TCAAGGTGTTCCGCGAGGACACGGACATTCCGGCGAACAAGTGGGATGACGACCAGCAAT
TCCGGTTCCCCGGCCTGATCAACCTGCCCAACCAGCCGGAAGTAGTGCGTGAGACAGTCC
TGGGCAAACGCCCGGAGTTCCAGTGCGCCCTCACCCTTCCGGATGGATCGCCCGTGTTCA
GCACCAGCGGTGAAGCCCTCAAGGCTCTGGAGAAACAGGGCTTCGAGGCGGCCAAGCTGT
TGATGCGCCTGGCCGAGCTCCACAAGGACAACTCCTTGTTCTACATCACCCATACCTACA
ATAAGGATGGGACGATTAAGGACACGAAGGGGATGCTCCAGTACGTGGACGACGACGGTA
TCATCCACCATTCACTGAATACGACGGCGACGGCGACAACGCGCCTGTCGTCCAGCCGCC
CGTACCTCCAGCAGCTCCCGTCGAAGGACGAGGACGACCCGGAAGCCGGCAGCCGCGTGA
AGGAGATGTTCGTGTCTCGCTTCGGCGCGGACGGGATGATCGGCGAGACCGACTATACCG
CCCTGGAGGTGGTGATATTGGCGGCCCTGTCGAAGGATCGGAACCTCCTGGCGAAACTGA
TGGCCGGCACTGACATGCACTTGTACCGCCTGGCAGGGAAACACAACAACTGGAACGGGT
TCGACTACGACCAGCTCGTGGCCATCAAGAATGACCCCGACCAACCGTGGCACGGTCGCA
TGATGCAGGCTCGAAAGCACATCAAGCCCAAGGCATTCTCGGCGCAGTACGGCGCGAGTG
CGGCTGGTATCGCATTCAACACCGGCTGTACCGTGGAAGAGGCCCAGGAATTCCTGGACA
ACGAGGCGGCCCTGTTCCCAGAGTCCATCGCATTCCGGCAGATCGTCCGCGACAGTGCAG
AGGCCACCAGCCTCGTCATGTACAAGGCCGAGGACCAGATGCCGGCAGGCGCCTTCAGCG
AGATGGGGCCGGATGGCAACTGGCGCCAGTACCGCCGGGGATTCTGGCAAGCGCCGGGTG

GCACCTGCTACAGCTTCCGCCAACACGAGCGCTGTGACAAGGAACAGCGCAAGACGGTAA
TGGACTGCAAGGACACGCAGATCGCCAACTACTGGAACCAGGGCGAGGCTGGGTTCATGA
TGACCGTGAGCCTAGGGCGCATCTTCCGTTGGATGCTGCATCGCCCAGGATTCATGGTTA
CCGATTTCCTGATCAACGACATACACGATGCCGTGTACACCGACTGCCACAAGGACACCG
CCGCCGAGGTCAACAATGGGGTGCGCGACATCATGGCCGACGCCGCCCGCTACATGAGCG
AGCGCCTGGGCTACGACATCGCCGACGTTCCCTTCCCGGCAGTGGCTGAGATGGGGCCGA
ACATGTTCAATATGGAGGTGATCCAGTGAAAGATCTGCACCCGCTGCACACGCCTGAGTT
CGTCAAGACATTCCTGGACCAGACCGGGTGCCTGCCGGGAGTACGCCGTACGGGCCGCAC
CACCGGCAGTGCTCTACAGGCCATTGGCATGGCGCTGTCCCATCCGAGGGAAACCCTGAC
GTTCGTGGACCACCCGGACGGCAGCGCGGCAGCACTGGTGGCCAGCATTGAAACCATACT
GGCGACCTTGGGCTACGAGAACGTCCTCGTTCGACCCACAACCCGTGCGGATGGGCGCAG
CGTGAGAATCGTCTTCAAGACGCTGCCGAACGCCTGATGACCCTTCTCTACTCCGGCCTT
AAATCTTCATCCGACACGAGAGAGACCACGCATGACTCAACGACTCAACGCTCTGCAAGC
CGCCCTCGCCCTGGCCAACAAGGCTGCCGAGACCGCAACCATCGACATGTCCGAAACCTC
CACCGGGGGTGGCGACGGTCGCATCTTTCCGGCGGGCACCGCCCTGGGCCGCTTCTTCAT
CTACATCGAGCTGGGGGACCACGCCAAGGAATTCCAGGGCAAGCTCAAGAATACGGCCCC
TCAAATCCGCCTGGGCTTCGCATTGTGGGGCTACGTGAACCCGCAGGCCGGTAACCCGCA
GAGCCGCCCGGACGACCTGTTCCACACCTACGAGGCCGACGGCTCGATCAAGCCCGGCCT
GTTCCGTACCTTCGAGATGACCCTCGGCAACAACGAAAAGTCCAAGACCAAGCTGGCCTT
CGACAAGATGAACTTGAGCGGGCAGCATACCCACTTCGCTCAGAGGCTCGGCCAGGCGTT
CATCATCCCGATAAAGCGCTCCAAGATCACCAACGGCAACAACGCCGGTAAGGAACGCAA
CGACATCGATTGGGGCGGCATCATGAAGCCCTATAACCCGGTCGATGGCAGCCCGTATAA
CGTGCCGGAACTGCCGACGGAACTGTTGCAGTATTTCTTCTTCCACGCGCCGACCAAGGA
GACCTGGGACGCCCTGTATATCGAGGGCACCTCGGACAACGGCAAGTCCAAGAACTTCCT
GCAAGAGACCATTCGCTCGGCCACCAACTTCCCCGGCTCGGCCCTGCACCTCATGTTGGG
CGGGGGCGACGATCTGATCATCAAGCCTACGAGCCAGGCCGCAGGCAGTAACCTGCCGGC
AGTGCCCAACGTGGCCGCCGATGCAAGCGTAGCAGCAGCCCCCGCCGTCCCGGCAGTCCC
GCAGGCAGTTGCTCAGGCGGCCCCCAGCGTGCCCCAGGTGGCGAATGTGGCTGCCCCTGT
GGTCGGTACTGCCGAGGCGCAGAACGTGCTGCCTGACGTCCCGCAGGTGGCCCAGGTGGC
GGCTCCGGCAGCAGTCGAAGTGCCGTCGGTCCCGGTAGTCCCGGAAGTACCGCAGGGTTA
AAGCGGCTGCCATCGGAAGAGTTCCTGTCAGGACTATCCGAGCAGTTCGACCGTACCAGG
GCAGGCGGGACGTTGGTGTGTGACACCGACGGTCCCGCTTACGTGGCTTCGGCTACTGCA
AAGACCCTGTACACTGCGCTCCGGCGTTTCTGGAAGATCATACTGGAGCAGCAGTTCCTG
GCGCACTGTACAGGGACACGGGTCCACCTCACGGCAGCAGGTGGGGCTAAGGCGTACCGC
GACGTGTATCCGACCATGAAACCCTACCAGGGCCAGCGCAAGGGGAAGGCAAAGCCCGCC
CTGCTGGAGCCGCTCCGGCGGGCTGTGGCCGACGTGCATGAACGAAGCGGGGCGCCTGAA
GGGATCGCCGTTATCCTGCACACGTTCTTCGAGGCGGACGACGGCATGATGATGGATGCC
TACGCCATGCAGGACAAGGCCATCATCCAATCCGACGACAAGGACTTACGGATGACGATC
TACCCGTATTGGGAGATCGATACAGCGTGTGTGAGCAGGATCGACGGGGGCTTCGGCTAC
CTCAAGGAGGCGTATACGCCGTCCGGTCAGTTCCAGCTCAAAGGGCACGGACGCAAGTTG
TTCCTGGCGCAGTGGCTCGGCGGGGATACCGCTGACAACATCCGAGGAATCGATCGATTC
AACGGTAAGCTCTGCGGGATGAAGACGGCCTTCGATATCCTCCATCTGATCACGGACGAG
GACGAGGCCATCGACATGATCCTGGAGGCGTACGCCAAGGTCAAGCAAAACCCGCTGGCA
GAGGCCGAGGTGCCGTGGATGCGCCGAAGGCCTACCGACAATGCAGCGCCGTACCTGTTA
AGCCGAGACCTTCGTCCGGCCTTCCGCCAGTGGATCATCGAGCTGGACGCCTACCACGAG
GCGCCGCTCCAGAAGCGGAGGGAGAGTGACTATGACGAGTGATCCGAAGGTCTATCAGAT
ACCGCGCAGCCAACAGCGCACTTTCCCCCTGAAGCTGTGGGCCGAGCAGGGCAAGCTGTG
GCCGCTCTGCGGTAAGCCCATCGATATCAGCGTGAAGGGCGAGGCGGTGATGGACCACGA
CCACGAGGCGGGGCTGGTGCGGGGCGTCCTGCATCGATTCTGTAACACCGCAGAAGGCAA
GATCACGAACGCGGCGGGTTCCTGGGGATGCAAGTCGATGAAATACACCGACATCATCCC
CTACCTTCGTGCCCTCCTGACGTACCTGGAGGGGCCGAAGCATCCGCTGATCTACCCCCT
GCACAAGACCGACGAGGAGAAACCCGAAGCGAAGCTGGCCACGCGCCGGCAGGCAGTGGC
CAAACGCAAGGCGGCGATGGCCGTCGCAAAGCACAACGCGAGGAACGTATGAGCAAACTC
CGCAAGCAATTCACCAATGAGTACCTGCGCAACGTCTATGTCGAGCTGGGTCTCAAGACG
GGCGACCAGCATCTAACCAGGCATTCGCGCTTCGGCGAAGTGAGCCGCCAGTGCTTCCGC
AACTGGTGCATCAAGCTGGCCTTCCACGACAGCAGGACACGCGGCACGTACGCCAAGAAG
GGCGCTCAGCACTGGCTGGACCGAAAGGCCGCCGAGGTAGTGCGCAAGTTCCCTGGCGCC

GTGGGCAACGTTGTTGGCAAGGGTCCGAAGGTACTGAGCCTGGACATCGAGACCTCGCCT
ATCGAGGGTTGGGTCTGGTCGCTCTGGAAGCAGAACGTTGGCCTCAACCAGATCAAGCGG
GACTGGACCATTCTGGCGTTCTGCGCGAAGTGGAAGCACGGCGACGAGGTGATCTACATG
GAGTGCCAAGGTGATCCCTTGGACGACATGCACCTGCTTGTCGCCCTGCATAAGCTGCTG
GACGAGGCCGACATCATCATCGTCCAGAACGGCAAGCGCTTCGACGTGCCCAAGATCAAC
GCCCTGTTCTTCCTGAACAAAATGCCGCCGCCGCGACCGTTCAAGGTGATCGACACCTTG
ATCATCGCCAAGCAGCAATTCGCGTTCACCAGCAGCAAGCTGGAGTACATGACCCACAAG
GCGTGCGCCATCAAGAAACGCCTCCACGGCAAGTTCCTCGGATTCGACCTGTGGGCAGCC
GGCCTCCAGGACAACCCGGATGCCTGGGAGGAGATGCGCCTGTACAACATTGACGACGTA
CGGTCGATGGAGGAGCTGTACATCCTGATGCGCCCGTGGTTCGTCGGTCATCCCAACGTG
GCCGTGTACTTCAACGACGCCGAGCCGACCATCCGATGCCCGACGTGTGGCGACACGGAT
GTCAAGCAAGAAGACTGGGTGCATACGCAGACCGGCAAGTACGAGCACTATCACAGCGGC
GGCTGCGGTGGCTGGAGCCGAGGGCGCTACACCCGCAACACCTCGGAACGGCGCAAGGCC
CTGCTGAGCAACTAAGGAGACACCATGATCGTCGACACGTCGTATGAGGTCGCGCTAGCT
GAGCGCTACTAGAAGGGCGCCGCCCTGGACGAACGCAAGGTCGGCAAACTCCCGATGCAC
CTGGTGGTCGATGGCTTCCCGCTGCTCAAGCGGGAGCTTGCCTGCATGATGCAGTGGGCC
GCCGAGGTCAAGGGCTACAAGCCGCACGACTGGAAGAAGATGTCGGTTGGCGAGTTCAAG
GCCGCACAGCGTCGGCACGAGTCCAAGCGCCTGCTTGAAGGGCCCCTGGATTACGAGTCG
GGCCTGATGCACCTCGAGCATGAGGCATACAACACAATGGCTGCCGCCGAGGTGGCCCTG
ATGGATCGGGAGAAAGGCAATGAGTAAAATCTGTTGGTGTACCCGACCGCACGAGACCGA
TGAAGGTGTTCGGGTCATCTGGGCCTTCAACGAGCGGGGCATCGGGGTCAACTACGTCAC
AGCGTACATCACGCCGGCGATGGTCAGCCATCGGGACTGGAGCGATGTCATACTCCCGGA
CATTCTCCGGGTGATGGCGGAGCGCCTGGAGCGGGAAGTGAAGCTGGTGGAACTGCGCTG
GTTCCGCGCTGAGATTCTGAGCTGCGGGGAATGGCGTGACTACCGAGCGATGACGCTGGA
TGGGGCGGTTAGCCTGGCCGAGGCCGAGTGGGGTCCCGAGGATATCGGGCGCGTAATCGA
AAGACGATAGGAGACGGAATCGACCTGATACAACAGCAGCTCGCCCACGAAGAGGCCCTG
GTCGGGGCGGCGCAGAATGACGCCCGCATTGCCTTGGAAAAGGCGATTGCCCAAGGGTCC
ATCGACCGCATCCCGAGGGCGCGCATCATATTGATGCGGATGCTCCCCATCGTGACCGAA
CCGATCTACGCCCACCAGGAAGCGAAGGCGGCGGGGCAGGCAGCGAAGCTACGGCACCTG
CTGCGGATCATCGACGCCCAGGACCTCGCGGTCATGGCGCTGCGGGCTGGGCTGTCGATG
CTCATCAACTACCCAACGATCACTGCGACGAAGTATTACACCCACATGGGCAAGATGCTC
TGTCGCGAGATCGAAGTGCGGTTGGCCTTCAAGGTCAACCAACCCTCTTACGACCGGACG
CTGGACTACCTCAAGACCAGCAGGACTCGCAGCGTCCGGCACATCCAGTAGACGATGGAC
GCTCTTCTGGACGCGGTGCTGCCCGAAGAGGCGCGCATCGACCTGCCCGATGGCGACTAT
CTGCGCCTGGGCAAGTTCATCGGCGATCCGCTCATCCAGTGCGGCCTACTCGAGCCGAAC
CGGTTTGTAGGGCGAGGCGGCACGAGTGTCCACCTGGAGACGTCGCCGGAGGCCAAGGAG
TTCCTACAGGACCCCTCGGCGGCAATGACGTGGGGCGGCCCAGGGCGTAGCGTGATGCTG
GCCCCGCCAAGACCCTGGCAGGACTGGTGCGATGGAGGCTACTACAGCGCCAAGGGGCAG
AAGCACCACGTACTGGTGCGACGCACCAAGCACCAGACCAAGCGGGCACGGCAGATGCAG
CTCCGCCACCTGGGCCGGGACAAGATGCCCCGAGTCTACGAGGCGGTCAACGTGCTGCAA
TCGGTAGCCTACGAGATCAACCACGACGTGTACGAGATCATCGAGCGCGTCGTCACCTCC
GGTGGCGGCGTGCTGGGCATCCCCCAGCGCACGTACCCGGACAAGCCTGAGCTCCCGCTC
GGCGACGAGTGGGCCAAGGAGAATGCCAGTGAGCAAGAGCTGGAGGCCTTCAACCGTTGG
AAACGCACCGTCCACCGGTAGTACACCGAGGAGCGAGAGCATACCGCCAAGCTTCGCGAA
TTTGCTCCACTCTACCTAGTTGTTCGAGAGCATCATGGCAAGGCAGTGTACTTCCCCATG
CACGTTGACTCCCGTGGCCGCATGTACTATTGGGGCACACCGAATCCCCAAGGGTCCGAC
ATCGTCAAGGCCGGCCTGCGCTTCCACGAGAAGCGAGTGCTCGGCAAGCGCGGACTGTAC
TGGCTCAAGGTCCACGTCGCCAACTCCCTCGGATGCGATAAGGTGTACTTCGACGACCGG
GCAGCCTGGGTCGAAGAGCGATGGGATGACTTCCAGCGAGAGCTCGACGAGGGGCCGGAG
AACTATGCGAGTCTCTTTCCCGAAGCCGAGTCGCCCCTGTGCGCCATCGCAGGTCTGCCG
GAACTTCGGGCGGCTTACGCCTCCGGCAACCCCGAAGGGTACGCCAGCGGCTTCATCGTC
CACATGGACGCCACCTGCTCCGGTCTCCAGCACTACTCGGCTATTCTCCGAGATGAGAGT
GGCGGAGTCTACGTCAACCTCCTACCTCCTGGACTTGCCAAAGCTGACATCTACTCCCGA
GTGCTCGGACTCGATAATGAGGCTCTGGAGAGAGACCGAGCGGAAGGCGCGGATGGCGAG
GCGCGGGGTTATGCCCTTCTATAGGATAAAGCTGGTCTGACGTGGAACCTGACCAAGAAT
CCCTGCATGACGCTGGTGTAAGGCACCACGTTCAAGGGCGTCGTGGCCCACTGCCTGGAC
TACCTCGACGAGTCCGGCGTGGAGATTCCCGAGGGTGCCCCGTCATACCGCCTGGGAAGC

TACATTGCGACGCTCAAACTGGACGCAATCCGCGAGACAGTACCATCGGCAGTCTTCGCC
ATGGAGTGGCTCCAGCGGCTTGCTAAGGCCCTTCCTGACGCATCCAAGGGTTTGCACTGG
ACAACGCCGCTCGGCATGCAGGTCTTCCAGTCGTACCCGAAGACCGAGGAGGTGCGGCTA
CGGCTGCGAGGCGAGGCTGTCGAGTACGTGACCCTGTACGAGGCCAACGACGAGCTAGAC
CCGCTGCGCAACGCCACCGGCATCGCTCCGAACTTCGTGCATGGCCTCGACAGCAGCCAC
CTGGGACTGACGGCCCTGGCCTGTGCGGCAGAGGGAATCCCGATCCAAGCCATCCACGAC
AGCATGGGGACCTACGCTGGCGACGTGGACCGGATGCACGTCCACATCAGGGAGCAGGTC
GTCACCATGTACAGCGGCCCCTGCGTGCTCGTAGAGCTGGCTCGACAGCTTGGGGTAGAG
GCTACCCCGCCCCGTCGGGGATCGTTGAATCTGGAGGCTGTGCGGGACTCCTGGGCGTTC
TTCTGCTGAGTAGATTATGTCACCCACATAGGAGCAAGTGCATCCGTCCAGAGGCCTCGT
AGAGGAAGAGGGCTCAGGAAAGACCCAAGAGGAGGAGAAGTGAGAGAAGACGACTACCAA
GGATTCTAGATAGAGTAGAATAACCAGCATAGGAGATATGATAGATGGTTACTATGAAGA
GTAAAGGGCCAAGGATTATGTCACCCACAGTGGAAGGATCGAGAACAGGCAAGGGTAAGG
CCCACCCTGTGACGTTCACCTCTCAGCAGATCGAGTGGCTAGAACAGACCTTCCCCGAAC
ATCAGATCACTCCTGGAACCACGATGGAAGACATCCAGTTTCAGGCTGGCCGCCGCGATG
TGGTGCGGGCTATCCGCCTGCGTCGCAGCGATGCAATCGCAGTGGAGCTTACAGGATGAA
CAAGTCACTCTGGCGAGTCCACGCAAAAGCCGGCGCCCCTTCCGAACTGATGGGGCTGTG
CTGGCTGGCAGTACAGGAGCTGGAAGAGTTCACCCTCTTCCTCTCGAACGAAGAAGCCCT
GGAAGCGATGCTCGTCAGTATCGAGGGCAATGATCGGACCGAGCTGCTGGTATTCCGTGA
TGGCCAACTGGCCGGCGGAGCCTGCATTGTGTTCGAGGACGATCCCCACATCGGCCCGTG
CGTCACCGCGCAGTGGCAGTACGTTTGGCCACGCTCCCGCAATACAGGCGTGGCGCGAGA
GTTCATCCGCGAACTGCACCGACAGGCCGGCTGGGGTCAAATCCCCCTCGTGTGCTGGAG
CCTTCGCGAGAGCGATAGCCGGTATACGATCCACTACCGGAGAGCCAAGCCTTATGGGCA
AGAAAGTGAAAAAGGTACTGGGCAAGACGATGATCGGCAAATTCGCTGATGGCCTGCTCG
GCACTGACCTGATCGGCGCACAATCCGATGCCCGCAAGATGGAAGAGCCGAACCGCCTCA
TGCAACAGCAGGCGGTCCAGCTCGCAAGAAACCAGCAGGTTGACCTAACCACCGAGAACG
TGGCGCAGGTTGACCTAGGAGCGATGGCCGATGCCACTGGCACCGGCAATCGAAGACGCC
GGAATCAGGCAGGCACTGGCGTATCGCAAACCCTCGGTATCAACTACTGACGAGGTACGC
CATGAAGTCTACCGCAGCAATGCTGTGGGAAAAGCTTCGGGATGGGAGCGTGGAGCAGCG
AGCTATCGAGTTCGACAAGACCACGCTACCCTACCTGATGGTCGATCCCATGTCCGGCAT
CCGAGGAGTCGTAGAGCACGACTTCCAAGCTGCCGATGCCCTGCTCGTGAACAACCTCGC
CGCAAAGTTCGCGCGGTCGCTGTTCCCGACGGGGATTCCGTTCTTACGTTCCGAACTTAC
CGATGCGATCCGGCGCGAAGCCGACAGCCGGGACACTGACATCACCGAAGTGACTGCTTC
CCTGGCCCGCGTGGATCGCAAAGCAACACAGCGCCTGTTCCAGAACGCCTCCCTGGCGGA
CCTGACGCAGGTGATCAAGCTACTGATCGTGACTGGCAACGCCCCGCTATACCGCAACAG
CGACGAGGCCACGGTAGTCGCATGGTCGCTCCGCTACTACGCGGTGCGCCGAGATGCGAC
CGGCCGATGGATGGACGTCGTGCTCAAGCAGCGCTACAAGTCCATGGACCTGGAGGAGGC
GTACAAGCAGGACCTGATGCGTGCGGGTCGCAACCTGTCCGGTTCGGGCAGTGTTTACCT
CTACACGCATGTTCAGCGCAAGAATGGCACGGCGATGGAATACGGCGAGCTGTACCACGA
GATCGACGGCGTGCGTGTGGGCGAGGAAGGCAGCTGGCCTATTCACCTCTGCCCGTACAT
CGTACCGACCTGGAACCTCGCCCCCGGCGAGCACTACAGTCGAGGCCACGTCGAGGCCTA
CATCGGCGACTTCGCCAAGCTGTCCCTGCTGAGCGAGAATCTCGGCCTGTACGAGCTGGA
GTCGCTGGAGGTCCTCAACCTCGTGGACGAGGCCAAGGGCGCGGTGGTCGATTACTACCA
GGACGCTGAGATGGGCGACTACGTGCCGGGTGGCGCGGAGGCCGTCCGGGCCTATGAGCG
AGGCGACTACAACAAGATGGCTGCTATCCAGCAGGGTCTCCAGGCTGTAGTCGTCCGGCT
GAACCAAGCGTTCATGTACGGCGCCAACCAGCGCGACGCCGAGGGCGTCACCGCCGAAGA
GGTCCGCATCACTGCGGAGGAGGCGGAGAACACGCTGGGTGGCACCTACTCGCTGCTCAC
CGAGAATCTCCAGTCGCCCCTGGCGTACGTCTGCCTCTCGGAGGTGGATGATGCGCCCCT
CCAGGGCTTGATCACCAAGCAGCACAAGCCGGCTATCGAGACGGGCCTCCCAGCCCTGTC
CCGGTCCGCCGCCGTGCAGAGCATGCTCAACGCATCCCAGGTCATCGCAGGCTTGGCCCC
GATTGCTCAGCTCGACCCACTCATCTCGCTACCGAAGATGATGGACACGATTTGGGCCGC
TTTCAGCGTCGACACCTCGCAGTTCTACAAGAGCGAGGAAGAGCTACAAGCCGAGGCAGA
ACAGCGGCGCCAGCAGGCCGCGCAGGCCCAGGCCGCACAGGAGACCTTGCTGTAAGGCGC
TTCCGACATGACCAACGCACTCGCTGGCGTCTGATAGATGCAACAGAACGAACAGCAACT
GCCACCAGGCCTCGCTAACCTGGTTGCCAACGTCCCGCCCGTCGCCGCACCGACCCCGAG
TCATGTGCAGGTGATGCCGAATCCCGTCATCCAGCCGCAGCCCCAGTATGCGGCGCAGCC
GGGACAGGCAGGGTCGCCGCAGCAACTGGCTATCCCGACCCAGCAGCCCCAACCCGTTCC

GACCAGCGCCATGACGCCGCACTACCAGCCGGTAGCGGTGCCCGCCGCTTGCCAACCCGT
TGTACCGCAAGCGACCGCACAGCCGGCTCCGGTAGCTCCTCCGGCTCCGGGCGCAGTTCC
TCCCGAGAACCTGGAGGTTCCGCCGCCGCCCGCATTCACTCCCAACGGAGAGATCGTAGG
CACCCTGGCGGGGAATCTGGAAGGCGACCCGCAACTGGCGCCGTTCATCAGCTATCTGGA
AGCGTTCACCGACAAGATCGACACCGTTCGGGCCTCCGGTAAGGCCGCCGAGCACCGCGT
TCCCCGCTTCATCGACGAGCACTATCTGAAAGACGCCCTGGGCGAGGCCCAAGCGCAGCA
CGTCATCAACGTGGCGAAGGGCGTTCTGACCTACGTGGATGCTCAGACCCAGGCAGTCCT
GAACCAGACCTATGCCGCCGTCGGCGGTGAGGCTCTGCTCAAGTAGGCCGCCAGCGTCTT
CAACCAACACGCTGACCCGGCTACCAAGACCGCCATCGGTCGCCTGATGGACTCGGGCGA
TGCTCAGTCCATGCAATACCCCGCGAAGCTGATCGTTGCCTTCGCTCAAGGCTCCGGTGC
CGTGGTGAAGGCAACCGGCCAACCCCTTGGCGCAGCGGCGCCTGCCCTGGCGGCCCTGTC
CGCTGAGCAGTACCGTGCCGAGGTCTCGAAGCTCCCGTTGAACGCATCGGAAGCCGACAT
AGCCGCTCTGCGTGAGCGCCGCAAGGCAGGTATGGCGCATGGTCACTAACGACCTTGCCC
TACGCCGGCCTTAAACCAACATCCAAAAGAGAGAGAATCTCATGAGCTTTCTGAACGACT
TGACCCGTCCGAACTACGCTGGCAAGAACGCGGACGTTGAAATCCACCTGGAAGAGCACC
TCGGCATCGTCGACAAGCACTTCGCCTACACCTCCAAGTTCGCGCCGCTGATGAACATCC
GCGACCTGCGCGGCTCCAACGTCGTCCGCCTGGATCGCCTGGGTAACGAGCAAGTCAAGG
GCCGTCGTGCCGGCGAAGAGCTGGAGCGCAGCCGAGTCGTGAACGACAAGTGGAACCTGA
CCGTCGACACCCCGCTGTACCCCCGCCACCAGTTCGATCACCAGGGCGAGTGGACCCAAT
CCTTCGACATGCGCAAGGAAGTCGCCGTGCTGGACGGCCAGGAACTGGCCCGTAAGTTCG
ACCAAGCCTGCCTGATCCATGTGATCAAGGCCGCAGCGATGGATGCTCCGGTGGACCTGG
AAGATTCCTTCTCGCCGGGCGTGCTGGAGAAGCTGGACCTCACCGGCGTCACCTCCTCCA
CGGAAGCCGCCAACAAGATCGTCCGCATGCACCGCAAGGTGGTCGAGTCCTTCATCAACC
GCGACCTCGGCGATGCCGTCTACTCCGAGGGCCTGACTCCGATGTCGCCTCGTGTGTTCA
GCCTCCTGCAGGAGCACGACAAGCTGATGAACGTCGAGAACCAAGCCACCGGCGCGACCA
ACGACTACGTGAAGTCGCGTGTGGCCATCCTCAATGGCGTGAAGGTGCTGGAGACTCCGC
GCTTCGCCACCGAGGCCATCGCAGCACATCCGCTGGGCCGCCACTTCAACGTGAGCGCCG
AGGAAGCCGAGCGCCAGATCGCCCTGTTCCTCCCGAGCAACACCCTGATCACCGCCCAGG
GGGCGCCGGCTCAGGCCAAGCTGTGGGAAGACCACGATCAGTTCTCGTGGGTCCTGGATA
CCTTGCAGATGTACAACATCGGTGCCCGTCGCCCGGATACCGCTGGCGCCATCGAACTGA
AAGGCATCAACGCCTTCGACATCACCGAGTGATGCCGCGAAACCCCGCACTTCGGTGTGG
GGTTTCTTCAAAGCCTAACGACCCGCGCAGATTCCCTGCGTGGGTTTTTGCGCTTTAGGA
GGAACCCTATGCTACTACTCGACTCAGTGAATGTCATCCTGGGCAAGATCGGCGAGTTGC
CGATCCCGAGCATGGATGAAACGTACCCAACCAGGGCCATCGCCCTCCCCGAGTTGGAGG
ACCAGCGCATCCAGTTGCTGACGCAAGGCTGGTGGTTCAACACCTGGTGGAAGCACAAGC
TGACACCCGACCCGCAGGGCTGTATCAACCTGCCAAAGGACACCTTGGCGTTCTACCCGG
ATTCCCCCCAACTCCAGTGGGACGGCCTGGGAGTGCGCGACGCCAACACCGGCGACGACC
GTATCGGCAAGGTAGTAGAGGGCTGGCTCGTGCTGTCCCGTGAGTGGGACCGTATCCCCG
AGATTGCCCAGCGCGTCATAGCGCACCAAGCCGCCCTGGCCGTGTACACCCACGAGATCG
GGCCGGACGAGACGGCGCAGGTCATCGCCCAGGAGTTGCAGGCGTACCAGAACGAACTGA
GCCGCATGCACACCCGATCCCGCCCGCTCAACACCCAGGCCAAGCGCAGCTCTAGCCGGT
GGCGGCGCAGCTTGAGGACCTGACCATGAGCTACAAGCAATCCGCGTACCCCAACCTGCT
GATGGGCGTGAGCCAGCAGGTGCCGTTCGAGCGCCTGCCAGGGCAGCTCAGCGAGCAGAT
CAACATGGAGTCGGACCCCGTGTCCGGCCTTCGCCGCCGTAGCGGTATCGAGCTGATGGC
GCACCTGCTGCACACTGACCAGCCTTGGCCGAGGCCGTTCCTGTACCACACCAACCTGGG
CGGTCGCAGCATTGCGATGCTGGTGGCGCAGCACCGGGGCGAGCTAAACCTGTTCGACGA
GCGGGATGGCCGCATGCTGATGGGGCAGCCCCTGGTCCACGACTACCTCAAAGCCGCCGA
CTTCCGGCAGCTTCGCGCCGCCACGGTGGCTGATGACCTGTTCATCGCCAACCTGAGCGT
GAAGCCGGAAGCCGACCGCACTGACGTGAAGGGCGTGGACCCCAATAAGGCGGGCTGGCT
CTACATCAAGGCGGGTCAGTATTCGAAGGCGTTCTCCATGACCATCATGGTCAAGGACAA
TGCCACCGGTACGACCTACAGCCACAGGGCCACCTACGTGACGCCGGACAACGCCAGCAC
CAACCCCAACCTCGCCGAGGCCCCGTTCCAGACGAGCGTGGGCTACATCGCGTGGCAGCT
CTACGGCAAGTTCTTCGGCGCCCCGGAGTACACCCTGCCCGACTCCACGAAGAGGTACCC
CAAGGTTGACCCCGAAACGGCTGCGGCTACGGTAGCCGGCTATCTCAACCAGCGGGGCGT
GCAGGACGGCTACATCGCCTTCCGGGGCGACGGTGACATCGTGGTCGAGGTGTCTACCAA
TATGGGCAACAACTACGGCATCGCCTCGGGTGGCATGAGCCTCAATGCCACCGCAGACCT
ACCGGCCCTACTGCCGGGTGCGGGTACGCCGGGCACTGGCGCGTAGTTCATGGACGGGGC

CGTCATGGCCAGTGGCTCGACCAAGGCGCCAGTCTACTTCGAGTGGGATTCGGCGAACCG
CCGCTGGGCGGAGCGCGCAGCCTACGGGACCGATTGGGTCTTGAAGAAGATGCCCTTGGC
CCTACGCTGGGACGAGGCGACCCACACCTACAGCTTGAACGAGCAGAACTACGACCGGCG
CGGCTCCGGCGACGAGCACACGAACCCCACGTTCAACTTCGTCACCCGGGCCATCACCGG
CATGACGACCTTCCAGGGCCAGCTGGTCCTCCTGTCTCAGGAGTACGTCTGTATGTCGGC
GAGCAACATCCCGCACCGCTGGTTCAAGAAGTCGGCGGCGGCGCTCAACGACGATGACCC
CATCGAGATCGCGGCCCAGGGCAGCCTGACCGAGCCGTACGAGCACGAAGTCACCTTCAA
CAAGGACTTGATCGTCTTCGCCAAGAAGTATCAGGCCGTGGTCCCTGGCGGCGGTATCGT
GACCCGGAGAACGGCGGTCATCAGCATTACCACGCAGAACGACCTCGATACCCGAGCGGC
ACCCGCCGTCACAGGCCGTAGCGTGTTCTTCGCCGCTGAGCGTGCCCTGGGCTTCATGGG
CCTGCACGAGATGGCCCCATCGCCATCCACGGACAGCCACTACGTCGCTGAGGACGTGAC
CAGCCACATCCCGAGCTACATGCCAGGGCCGGCAGAGTACATCCAAGCGGCGGCCTCAAG
CGGCTACCTCGTGTTCGGCACCAGTACGGCGGACGAGATGATTTGCGACCAGTACCTGTG
GCAGGGCTACGAGAAAGTGCAGAACGCATTCCACCGCTGGACGCTACGGCACCAGATCAT
CGGGACCTACTTCACCTGGGACAACCTGATGGTCCTGATCCAGAAGCGTCAGGAGATAGC
CCGGGGCCGGATGCACGTGAACAGCCTGCCTGCACGGGAGGGCCTGCAATATCCGAAGTA
CGACGACTGGCGGCGTATCGAAGCGACTGTCGAGGGCGGGCTGGAGCTGACCAAGCAGCA
TTGGGACCTGATCAAGGACGCCCCTGCCGTGTACCAGCTACAGCGCGTGGCCGGCGCATT
CATGGAGCGCTACCAGCTCGGCGTGAAGCGCGAGACCAACACCAATGTGTGCCTCGACGT
TCCCGAGGCCGTTGTCGGCTCCGTCTACGTGGTTGGCTGCGAGTTCTGGTCCAAGGTGGA
GTTCACCCCGCCAGTGCGGCGGGACCACAACGGCCTGCCGATGACCGCGGCCCGTGCAGT
GCTGCACCGCTACAACGTCAACTTCGGATGGACCGGAGAGTTCCTGTGGCGCATCAGCGA
CACGGCCCGGCCCAACCAGCCGGGGTACGACACGACGCCTCTACGGCTGTTCAGCCGGCA
ACTCAACGCCGGCGAGCCGCTGGTGGACAGCGCCGTGGTGCCGCTGCCGGCCCGGGTCGC
TATGGCGACCTCCAAGTTCGAGTTGAGCTGCCATAGCCCTTACGATATGAACGTGCGGGC
CGTGGAGTACAACTTCAAGTCCAACCAAACCTACAGGAGGGTGTGATGGCATTCTGGCTA
CCACTACTGGCCGCTGGCGGCATGTCGGCTCTCCAGCAGGGCCTATCCAACAAGGAAGAA
CGCCGAACGATCAAGGCCGAGAACAAGGCCCGCCTCAAGACGGACCTCGACAACCTGGGC
GCCGCTGCCTGCGACATCGCCAACCTGGGCGTCATGGCTGCAAGCTACCGCAAGCAGGCC
GTGGCCTCCCAGCTCGAGGCCAAGCGGCATGGCATGCTCGCCGGCGGGTGCGCCTCGGCA
CAGGCAGGGGCCTTCGGGGTCAAGGGTGCATCCGTAGATGCGGTGGCCCTGGACATCGAG
CGCGAGGTGGGCGAGGCCCTGATCCAGATTGACGATAACCTGGACAACCAGATGTGGAAC
CTCGCAGAGGAGGCACACTCCAGCCAGGCCCAGGCTAAGGCCGGCCTGCTGGGGCAGAAG
AGCACCACAGCGGGGCAACGCTCCCCGCTGGTCGCCGGCCTGATGTCTGCGGGTTCCCTA
TACGCAAGCCAGTACTTCAAGTTCGTCGCCACGCCTAAAGGAGGCAACTGATGGCGGAAT
CGCAACGTGCCTCCCAGGAGCTTAGGATCAACGTCGGACAGACTCAACTCCAGCCGGGCC
AGAGCGCCCGGCGCGGGGTGCGAGACTCCGAGGTCAACCATAGCGGCCCCAGCGTGGGTT
CGCAGATTCTCGACGGCATACTTGGTGCAGGTCATCAGATCGCTGGCAAGTGGTTCGAGC
ACAACGTGCAGCAGGAAGTGCTTCGCGGCGAGCGTGCCCGCATGGCCGGGGAGGCCGAGG
ACGCCGTAGACAGCAACATCCTGGCCAAGCCCTTCGTGAAGGGCGGCTGGCGCAAGCAGG
ACTACCGGATCGCCCAGGCTGACTTCAGTATCAAGATGCAGCAGTTTATCGCCACCAAGA
GCCGGGAGATGACTCCCGACGAGTTCCGAAAGCACCTGTCGCAGGAAGCGGCCCACGTGC
TGGACTCCACCGAGGGCATGAACCCCAACGACGCCCTACAGGCGCTGGCCCAGCAGCAGA
AGGCCGAAGAACAACTCTTCGGCATGCAGGCCTAGGCCTACATGGACTGGTCCATCGACC
AAGCCGCCCGAGGCTTCCGCACCCAGGGTAACAGCATCCTGGCCAAGGCCGTGCAGACCC
AGGCCACAGGCGACGAGCTGTCCCGGCAGTTGAGCCTGGAAGAGGCCGGCCTGTTCTACA
CCAACATCATGACCTCCGAGGACATCCCGCTGGAGGTGCGCGACAAGGTGGGCATGCAGT
TCCTGGCCGCCAGCCCGGATATGAACCAGCGGGGCATCTACGAGAGTCTGCGCGACGCCG
GCTTCCTCGACGGCATGGCCTTCGACGACCGACGTGCGCTTAATGGCCTCTACGAGAAGT
CGAAGGCACAGACCCTGGCCAAGGACTCGATGGCCACGCTACGGGCCTACGCGGACTTCC
AGCAGCGGGTGGCCAACGGCGCCATCACCGACCTTGCAGAGGTCGAGGCGTATTCTCGGG
GAATGGTGGCGGAAGGCCGGTGGAGCGACGCACAGGCCATCTCCTTCATGACCAAGGCTA
TGACCGGCCTGGGCAACGCACAGCGCATGCAGGGCATCATGGCGGCCCTGGAGGCCGGCG
ACATCAACGCACTGCACACCCTCGGGACCAACGTTACCGAGGCCCTGGGGCAGTGGGATA
AGATGCAGGCGGCCAACTGCTCCAGTCTCTCCGATCGGTTGGTACAGGGCACGCAACTCG
GCCTACGCCTGGGGACCTTCCCGAAGACCTACGGCGAGTCCGTTGGCAGCGCGGTCCGCA
TGATCCAGGCCGCCAAGGAAGGCGAGGCCAACCCGGAGTTGGTCTACACGCTGAACAGCA

TCTTCGAGCAGGTGGCCTCCGTGCAGGAGATCAACCCATCTGCCGGCAACGTGATGCTCT
CGGGTATCCCGGAGGGCGAGCAGGCCGCCGTAGCCTGGGCACTCACGCAGATGAGGATGG
TCATCGCCCCGGCGCAGGCCCTGCGGGAGTTCAGCGCCAACGCGGAAGTCGTCAAGGAGA
TGGACGCGTTTGAGAAGGGCCAGAACACCAAGCCCTTCAAGGACCACCTCGGTAAGCAGA
TCAACGACAAGTTCGTGAACAACATCTTCGGTCGGGCCTGGAACATGCTGACCGGACAGA
GCGACCTCAGCAACAACGCGGCCGTCCTAAGCATGTACCGCCGTGCAACCATCGACGAGG
CCAACTGGCTGGCAAGCGACCGCAAGCACGCAGGACTCCTCACCAGCGACACAGGCCGGG
AGGCTCTGCTGGAGATCGCCGCCGCCAACGTCCGCAACCGGACTATCCAGGTCGGTGAGT
CCCCCAACCTCAAGGAGGGGGAGCTGTTCAGCCGCCGCGACAGCGCGCCGCTGATCCTGG
CGAGAGGCACTACCGCCGAGCAGCTATTCGGCACCCACGACACCGAGACCACCGGCGCAG
TCCTGGCCGAGCAGCACAAGCCGCAGGTTGAGGGCCTCCTGGGCTACAAATCCGTGGTGG
CGTTTGAGTTCGACCGCACCAGTGGCAGCCTACTCGGCGTCGAGTACGCCGAGAGCGGGG
TGGCCCTGGACCGTACGCGGGTCCATCCATAGGCCGTGGGTAACGAAGTGCTCAAGCGCA
ACTTCGACAAGCTGAACGCTGTAAGGGGCGCGGAGTATGGGGCCAACGTCAAGGTCAGCG
GCACGGACATTCGCATGAACGGCGGCAACAGCGCCGGCATATTGAAGCAGGATGTGTTCA
ACTGGCGTAAGGAGCTGGCACAGTTCGAGGCCTATCGAGGGGAGGCGTACAAGGATGCAG
ACGGTTATAGCGTGGGCCTGGGTCCCTACCTAGGCAGTGGCAATGCTGGGGCAGGAACTA
CGGTCACGCCTGAGCAGGCCGCGCAGTGGTTCGCCGAGGACACCGACCGAGCGCTCGACC
AGGGCGTGAGGCTGGCCGACGAGCTGGGCGTTACGAACAATGCTTCGATCCTGGGCTTGG
CCTGTATGGCCTTCCAGATGGGCGAAGGACGTGCCCGGCAGTTCCGCAACACCTTCCAGG
CGATCCGGGATCGCAACAAGGAGGCCTTCGAGGCCGGTGTCAGGAACAGCAAGTGGTACA
CGCAGACGCCCAACCGGGCAGAGGCATTCATCAAGCGCATGGCGCCCCACTTCGATACCC
CGAGTCAAGTCGGGGTCGACTGGTACAGCGCCGCAACAGCGGAGTAAGATATGGCAAAGC
AATTCAAGGGCCGTATGACGCCCAAGTATCCCCTCGACCAAGCAGTCCTCGACGAGGCCC
AAGTACAGGGTCTGCTCGATGCATTGTCCACCGTGGAGTTCGACGCCCTGACGGGCCGCG
AGATCGGAGAACGGAATGTGGCGGGAGGCCAACGTGCCAACGCTCGGGAACTGGAGCGCA
TCGTCGCAGACCACAACCTGCCGGCCCTTGACCGTGCTTCTGCACTCTGGAATCAGTCCA
CTCTCGTCGGCCGCTGGGACGATTCGCTCCAACACGACGCAGACCTTGCGGCGAACAGCA
CCGGCGAGGTGGACCCCAACTTCGACGCCGGGACCTACGGGGTCCAGGCGCTCCAGGCGG
CTGGCATCCAGCCGACTGACAACTACCTCCAGATCATGGCCCGCGCCGGCAACGCCGAGG
ACGCGGCCTACCTACTGTCGAGGATTCAACGGTATGAGCAAGACGAGCAAATCGTGCGGG
ACAACCCGTACTGGAACTTCGCAGCGGGCATGCTGGACCCGGCTGCGCCGGCAGTCGATG
CGGTTACTTTCGGTGCTGGCCGCGTTCTACGGCTCGGTCGTGCTGGCATGGCTGCTGCTG
GCGGCGCTGGCCAAGTCGGGTAAGTTGCTTGGCTGGATGCCGCAGGTGCCGACGTGGATG
CCGGAACCTACATCGTCGCTGGCGCTCTGGGTGCTGGCGTGGGTGCTCTGCTGGGGTCTG
GTGCTGGACGCAGTGCCGCCGAAGCCCCAACGCAACCGCATGTGCCCGAAGTATCGGCCC
CTACTGTCGGGCTGCCAGGAGAAGCGATGACCGCCGAGGAAGCCGCCGCTCGTGGCTTCA
AGGCAGGCGATGTGGTGGACCTGCTGGACGAGGGCACTGTCCTGTCCCGGGTAAGCTACC
GAGTGGAGCAGGCCGAGATACCGGCCATCCCGCGCCTGGACACTGCCTTCGGTGACGAGC
TGCACGGCCTGTCGGGCCGCAAGCTGTCGGAGGTGGTGGACCACCTCAAGACCCACGCCG
AGGTGCCCAAGCCGTTGCACGGCGTGGCCGCCAAGGTGGCCGAGACGTTGAAGACCCTGG
AGGGCCTGGGGCAGCGCACCATGTTCCGCGTGGTGCAGGGCGGGGATACCGCTAGCTCCG
CCTTCCTCAAGCCAAGCACGGCTGGTCTGCATTCGACGCAGGGATTGGACACTCTGGTCC
AGGTGCGTGGCAGCACGGCGCCGGGCCGCGTGAGAACCAACCCTGTCACGGTCCTCCACG
AGGCGGTTCACGCCGCCATGGTAGGCGTGATGAATACCGCCCTGCGGAATCCTGGCGCGT
TGACTCCCAAGGTGGCCCAGGCAATGCAGACCCTGGAGAACGTCCGGGGTAACGTGCTCA
ACGCCTTGAAGCAGGACCGGGCCGCCGGACGGCAACTTTCCGAGTTCGAGGAAACCCTGC
TGGCGGGCAACTCCAATACCCTGGCGAACGTCAAGGAGCTGGTGGCCTGGGGCCTGACGG
ACACCCGCTGCCAGAAGACCCTGAACCGGCTGCGCTACGGCGACGGCGGGAAGGGCCTCT
GGTCCCGGTTCGTCGAGGGCATCCGCAGTCTGCTGGGCCTGCGGCCTGATGCGGACACGG
CGCTGAGCCGCATTCTGGCGGCCTCAGAGACAATCATGGACTCCATGCCGGGGTACACCA
AGGCCCAGGCCAAGTGGGCCAACAAGGGCGCGCCAGTGACCGAGGAGGCCAGCCTGGAGA
CCATCGTGCGATCCACCCGGGAACGTGCCCGAGAGGGTGCCGGCTTCGTGAACCGATTCT
ATAGCGAGGCCGACCTCCTGGCACAGCCGGGAGAGGGTGCGCGGCTACTCCTGAGCCGTC
TTATCGATGACCCGGTACGTCGGGATGGGTAGAGCACCAAAGACAACGCAGCGAGCTATT
TGCGCCGCTACCGCAACGAGTTCGAGGGCTACGTCAAGTCCAACGACGAGATGATGGCGA
AGGCGATGGCTGAGCAGGGCGTGGGCCTGACCGCTCGGGCACTCAACTCCCGCCGCGCTA

TGGCCGTCCGGGATCAGCTCAACGAGCAGGTCACCCGGGAGGTGCTGCGCTGGGACCGGG
AGTGGACCGCCTATGGCAGCGTCCGCGTAGACCCGAACCTACCGGGCACCATCAAGGCGC
TGGCTGACCACTCCGACGAGATTCACGGACTGATGGGCCAGCGTGCACGGGAGGCCGGCG
TGCGTGGGTTCGAGAACTTCGCACCACGCCCCGGGTACTTCCACCGCTCGTGGAACTGGT
CGAAGATGGCGCAGCTTGACGAGGCCGCCCCAGGCTTGGCTCGCCGCGCTATCAGCGACG
CCGTATTCCGGGGCATCCCTGGATTGGAACGTGCGGATGCTGACACCATCGCACAGGCCA
TCGTATAGCGGGCACGAGACCGGGCGAGCGGTATCCGCTCCGAGTTCATGGGCGCGATGG
GCGTGGCTGATACGGCCTTCATCCGGCAGGCGCTGGAAGAGGCCAGCGTGTCCCAGGCCA
AGTTCGACAGCATCATGGCGAAGATCGAGCAGAAGCAGTCCGAGCAGGGCACCTTCAAGT
ACGGCAAGGGCCGGCTGTCGCGGGACATGACCGCCGAGCTCAACCACAACGGCACCGTGT
ACCGCGTACAGGACCTGATCGACCGGGACCTCGACCGACTGATGGAGAGCTATTAAGGCA
GTATGTCGGGCCTCTCGGCGCTGGCTCGGGCAGGTATGCCGGGCGACTCCGAGGTCGAGG
CGTACATCCGTGAGTACCAGCGGTAGGCCGCGCACCTGGGCACCGACAAGGTGCAGGAGC
TGACGGGCCAACTGCGGGGCATCTTCGGGGACTTCACCGGCAACGTGCCGCGCGAGCACC
AGCTCGGCCCCGTCGCTCAGCGGGCCAGCGGGCTGACCAGTGCCACCATGGTGGGGTTCT
CCGGCGTGTACCAGCTTGCAGAGCTGGCCACGATAGACCACCGCCATGGCGTCTTCAACG
TCATGAAGGCCATAATGAACTCCCGCCTCGGGGACTTCGTAGGCGCCATGCGCCGTAACC
CGGACCTCGCCGACGAGATGCAGACCGTCCTCGGCCTGAACCTCGCCAACGACATCCGCA
TGAAGGCCTGGAAGCGGCAGTTCGACACCTTCCCCGCCAGCCAGGATACCTTCATGGATC
GCTTCCTGCACGCAGGTGAGCAGGCTATCCCTGTCCTCAACGGCATGAAGTTTATCCACA
ACTGGCAATCCCGCATGAACGCCAACCTCACCTTGAACAAGGTGGCGCGGGCCGCGCAGG
GGGATGAAGCAGCCCTTCGAGTGCTCCAGCAGTACGGGAAGGACTTGGACTGGATGCCAG
TGCTGGCGCGGGTTCGCGGTTATGTCACATACAGAGGAAGGAACGCCCAATCTATGAATT
GGGGCGTCTGGAGCCAAGCAGACGTGAACACCGTCATGAACACCGCACTGCGGATCATGG
ACGACTCGCTGCTGTATGGTAGGGTCGGTCAGAACTCGGGCTCTGCCCGGTCCCCTGTCG
GTCAGATCCTGGGCCAGTTCCGCAGCTTCGTGGCCTTCGCGCACAACAAGCTCCTCCGGG
GAACCTACGAGAACTCCGGCGTGCTTGGCGTGGCCTCTCTCCTCGCATTCCAGTATCCGC
TCACAGCGCTGATGATGGGTGCCAAGGCAGCGATCAACGGCAAGTTCGACACCTCTGATG
AAGGCATCCGCAAGATGGCCATCGACGTCATCGGCTACACTGCGGGCCTCGGCTCCACCG
CCGACATGTGGGGCGTAGTCACCGGGCACTCCCCGCGGTCCGCCCCGGTCTTCGGGCTGG
CGGAGCACTCCAACGAGGTGTTCCGTGGCGTCAAGGACCTAGTAACCGGCGACGACCCCG
CAGCCGCCACCGGCGATATCGTCAGCGGCGCCGCAGCGGCACTGCCTTTCGTCAACGTAT
TCCCGGCGACCAAGTTGCTGCTGGAATCCATCAAAGGGGAATAACGTGGCTCGGTTCAAG
TATCCCGAGACCCTCCACGTTGCAGATGGGGTCGAGGCTGTCTTCAGTCTCGACTTCCCG
TTCCTGCGGCGTGAGGACGTATTAGTCCAGGTCGATAAGATACTCGTCACCGACTATACG
TGGGTAGACGACACCAACTTCCAATTGGGGGTGGTGACGAAGAAGGACCAAGAGGTCCGC
ATCTTCCGCGACGCGCCCCCCCAGGTCCGGGACACACACTTCAGCCAGGGCATCCCGTTC
CTGCCTCGATACATCGACGCGAACAACAAGCAGCTCCTGTACGCTGTGCTGGAACGCATC
AACACCGCGATCCTCGCTCTCGCTGGCGTACTCGACGCGATCCGTATCGCCGAGGAGGCT
CGTCGCCTGGCGCAGGAAGGACTCGACGCCGCCAATGCGGCGCTTCGCCGTGCCCTGGGC
TTCGCTGAGATTCGCACCGTGTCCGAGGACTCGGCTATTGATCCGAGCTGGCGGGGTTAC
TGGAACCGCTGCATCACTGCCGACAAGCCTCTGACCTTGACCATGCAGATGGAAGACCCG
GATGCACCGTGGGTCGAGTTCATCGAGGTTCACTTCGAGCAGGCCGGTGTGCGTGACCTA
AACATCGTAGCCGGTCCTGGCGTTACCATCAACCGTTTGCAGAACACCACCACGCAGCTC
TACGGCGAGAATGGCGTGTGTACTCTCAAGCGGCTGGGCGCTAACCACTGGATCGTGTTC
GGGGCCATGGAGGACGAATAAAGCACGGCATTATCGCGGGCATCATGGCCTCCCAAATTC
GACGGCCCAAGCCCATCCTGGCGACCTACCCGTATCCCATCATGCAGGCGGATAATCGCT
GGGCTGCTCGGCCCAATATGGTGGCAGCTCTGACCAGGGACACTCTGAAGGAGGTCCGGC
CAGAAGACACGCTGGAGCACTACAGTGCAGCCACCGCTGTACTGGACGCCAGCATGCGCA
GCCTGACACAAACGGGCTACGGCGGGGCCTGGCCGTACCAGCTCCTAACAGGTGTGGCAG
ATACCACCCTCCGGTCGCTGGTGAAATCTACTACGGTCGAGGCTCAGCCCTATCTAGCCA
CGCCGGCGATCCACTCGGCGGACCTGCGGGTAGTGCTCATTATCTCGAATTACGAGGTAG
AGCCGTTCCATTACACCCTGACCAACAGCCTTGTACAGGCGGAGCTGAAAAATGTTTAAG
ACCGAAGTAAAGAGACGTTACACCCTGATTCGCCGCAAGGCGGACGGCACTCCTGTGGAG
ACTCTGGAGTTCGACAACATCATTACGAATGCGGGCCTGGATTGGATCGCCGCTATGGAT
ACCGACCTCATGGGCGAACCCGTAGCGGTCAGCACTTCTACAGCCGATCCCAACCCGAGC
GCACCCGCCATCCGGGAGGTTGTGCAACGCACGTCCGCATCTGCCCCTGGTGGAGGTACT

ACATCGGGCCTGGATGGCGAGTGGCTGTTCTGGCGGAAGCGTTGGAGATTCCCGCAGGGC
ACCCTAGCTGGTCAAGTCCTGGCCACCGTGGGCCTCATCTGCAACTCGGATCGTCGCTTC
GAGAATAACACGGGTGAGCTGATCCCGAAGGATACCCCGCTGTCGTACACTCGCATCAAG
GACGCCGCCGGGCAGCCTACTACTCTGGTGGTGGCCGCTGACGAGATTCTGGATGTCCAG
TACGAGTTCCGCAGCCGGCCCGTAGGAACGGCTGAGGCCAAGTTCGTGATCTCCGGCGTG
GAACGCACCTTCCGGCTGATCCCACAGCCATTTGCGGACCGTGCTAATCTCTCCGGGGAA
CGCTACATCTTCTACAACACCAACCCCTACATCAACGGAAAGGACGCCTCCGGCGGCAAT
GTCCTAGACGGTCAGTGGCAGAAGAAATATCCCAAGTACGTGCGCGCCTCTTACAAGGCG
CAGATCACGCTGCTGACCCAGGTCCAAAACGGCAATATGGCTGGCGGCATCACCGGCACC
GAGGAACTCAAGATTTACAGTGGACGTAACTATGTGCTCGATATCAACCCGCCTGTTGTG
AAGAACAATACCCAGGAGTTCACCGTGACCCCGGAGTGTACGGTGGCGAGGGCATAACCT
ATGGCACTGATCTACGACTTCAACCCGGACCTTGATCCGAAGGCTAAGTCCAAGTTCGTA
GGTGCGCGTGGCCGTAGGGACTTCAGCGACGTGCTGGACTTCTGCGAGGGGGGTCTGGCT
ATCCAGGACCCGTCGCAGGGCATGATGGTCCGCGTGTGGCGAACAGACCTTCGCCAGGAC
GGGACCTACCTGGGTCACGAGGACGCCTCGAACGAGATTCGCATCCGCGGAGGTATCGAA
GAAGGTATCTCCCAGATGTCCCTCGACTTCGACAGCAACATGAACTACGTGTGTGCTTTC
GTACGAGCCGACCGGACTGGTGCAATGTCCTACTCCAACGTGCAGCAGCGCCGCCGGCTC
CTCGTGGAGCTTGGGCAGGTTGACTATGCCAAGGTGGCTCTGGACGACAATCGTCCGGGG
GCTACCGCCTTGGAGCAGGTTATCGTGCCCTACACACGCAACGGGAACCTCTACGTCCGC
ACGCAAAACGAGAACTACACCGAAGAGCACCTGGAGGTGGATACCGGCAAGGTATTCTGG
CCTCTGGTGAAATGCGGTATGGGCACCAACCTCCGCTTCCAAGTCCAATTCAGAGGGCAC
ATGTAATGAGTAAAAAGCAGACCGCGAGTGCTGAGCGGCTGGGCCTGCTTCACGAGCTGG
TCTGCACCGCTATCGAGCGCAACTTCAAGTGGTACATGGACAAAGACATCCCGATCCCCG
CATCGGATATCGCTGCCGCCACCAAGTTCCTCAAGGACAACGAGATCACCTGTGATCCGT
CCGACACCATCAACATCGACCGTCTCCGCGAGGAGATGCGGCAGGCGCAGGCCGAGAATC
GCCGAATTGCCTTGGAGGGCTTCATCGCTGGCGAGACCGACGACGAGATGGAACGCCTGT
ACACCCACTAAGGAGGCAGCATGACGCCGCAAGAACGATTCCAGATAGCCCACGAAGTGC
GGGATATGTACCCGCGCTTCCGGGACTTCTGCCTGGACGCCATGCTGTTCCTCGGCTTCA
AGATGACCTGGATGCAGCTCGACATCGCCGACTTCATGCAGGACTCGCCCAACAAGGCGA
TGGTTGCTGCCCAGCGTGGCGAAGCTAAGTCCACCATCGCCTGTATCTACGTGGTCTGGG
GCATCGCGCAGAACCCGGCCACCCGTGTCATGCTGGTCTCCGGCTCCGGGGACAAGGCCG
AAGAAGACAGCCACTTGATCACGAAGCTGATCATGCATTGGGACCTGTTGGCGTACCTGC
GCCCGGACGCCCGCATGGGCGACCGTACCTCGGCTACCAGCTTCGATGAGAACTGGGCGT
TGAAGGGCGTCGAGAAGTCGGCCTCCATCAACTGCATCCGGATCACCGCCGCCCTCCAGG
GCTACCGGGCTGACATCCTGATCCCTGACGACATCGAGACCACGAAGAACGGCCTGACCG
CAACCGAGCGGGCCAAGCTGACGCGGCAGTCGCAGGAGTTCACCTCCCTCTGTACCCACG
GTAAGATTCTGTGCCTGGGCACTCCGCAGTCGCGGGAATCCATCTACAACGGCCTGCCTG
CGCGGGGCTTTCTCATGCGCATCTGGCCGGGCCCGTGCCCGACCCTGGACGAGCAGAAGC
GCTACGGCGAGTGGCTGGCCCCCTCTATCGTGGACCGTATCGCCCGCCTGGAAGAGAAGG
ACCACAACCCCCGCAAAGGCAAGGGCCTGGACGGCACCCGTGGCTGGGCCGATGACCCGC
AGCGCTACAACGAAGAGGACCTGCTCGACAAGGAACTGGACCAGGGTGCCGAGGGCTTCC
AGCTCCAGTACATGCTGGACACAAGCCTCGCCGACGAGCAGCGCATGCAGCTGAAGGTTC
GTGACCTGCTGTTCATCGACGCGACGCACGAGAGCGTACCCGAGCAGGTGGCCTGGGCTG
CCGACGAGCGCTTCAAGCTCAAGTTCGACGCCCACCGATTCCCGGTCATCAAGCCTGAGC
TGTACCTGCCGGCGCTGATGGCTGGCGGCTGGGCCTCGCTCCAGCAAATGACGATGTTCG
TGGACCCTGCCGGCGACGGTGGCGACGAGCTGTCGTATGCCGTGGGCGGGACTCTTGGCC
CGTACATCCACGTCGTGAGCATCGGCGGCAGGAAGGGCGGCTTTGCCGAGGAGAACCTGG
AGAAATGTATTGCCCTGGCTGCGCGCTATGGCGTCAAGGTGATCTATGTCGAGAAAAACA
TCGGCGCTGGTGCAGTTGGCCAGCTCTCCCGCCACCACATGCGATCCATCGACCCGGACA
CCAACAAGCCCCGCTATGAGGGGATCGGCGTAGAAGACCGCCAGAAGTCCGGACAGAAAG
AGCGTCGGATCATCGACACCCTGCGGCCCATCATGCAGCGGCACCGTCTGATCTTCCACG
TATCGGCGATGGATTCCGACCAAGTGGCCTGTAAGCAGTACCCAGCGGACAAGCGCAATG
AGCGCTCCGTGTTCCACCAGATTCACAACATCACCACGGACTGAGGCTCACTGCCTAAGG
ACGACCGGATCGATGCCCTTGAGGGCCTTGTCCGCGAGCTGACACCATCGCTCGTAAAGG
ACTACGAAGCCGCAACCCGCGCTCGTGAAGAGGCTGCCAAGAAGGAATGGCTGAACAACC
CGATGGGTTACACTAAGTCTGTCCTTCGGTCTCTCGGCATGGGGCGGGATCGTCGCAAGG
GCCGTCCAAAAGGACGAAGACTATGATGATTGATACCGCTACCGAGGCGGGCAAAGGCAC

CCTCGCCGTCTCCGGCGTGGGATCGCCGTTTACTCGCCCTATGAGATCGCCAGCCTCTG
TGCTGCGGTACTCACCGCGCTCTACGTAGGCGCCCAGCTCATCACCCTGCTCCCGAAGAT
GCTCGATAGCCTCGCGGAGTTTCGCCGGAGGTTCAAGAAGTGAACAAGCCCCTGCGCGGC
GCAGCCCTTGCGGCTGCCCTCGGCGGCCTTGTCGCCCTGGAAGGCAGTGAGACCACCGCC
TACCGAGACATCGCCGGCGTCCCCACCATCTGCTCCGGAACTACTGCCGGGGTCAAAATG
GGCGACAAAGCCACACCAGAGCAGTGCTACCAGATGACGCTCAAGGACTACCAACGCTTC
GAGTGCATCGTCCTGGACGCCATCAAGGTGTCGCTGAACGTCAACGAGCAGACCGCCCTG
ACGTTCTTCTGCTAAAACGTGGTTCTAGTCTGTACAACCAGCACAGCGTTCAAGCGCTTC
AACCAAGGCCGCGCCACTGAGGGCTGCCAAGCCCTGGCCATGTGGAGCAAGGTCACGATC
AACGGCCAGAAGGTCGTATCCAAGGGCCTCGTGAATCGCCGCAACGCGGAGATCAAACAA
TGCCTCGAACCATCGTCGCAATACTCGTCCTTGCTGTGGTAGCCCTGGGAGCCTCGTACG
GCTTCGTCCAGGGCTACCGGGCCTTGGGTATCGCCCAGGAGGAGATCAAGCGGCAGACGG
CCCGTGCGGAGGCCCTGGAGGTGCGCTACGCCACCTTGCAGCGCCACGTCCAGGAGGTCT
CTGCCAGGACCAACACCCAGCGCCAGGAGGTTGACCATGCCCTGGACCAGAACCGTCCGT
GGGCTGGCCGTCCTGTGCCTGCTGCTGTTGGTGGCAGCCTGTGTAACCGCCCCGGCGCCC
GCTGTGCTGTGCGAACACCCACTGATTGACCCTACCACCCAGGCTAGCCTGATCCGCGCT
GTAGCGGCCTATCAGGACGCCCTGGACCTATGCAACGCCCTAAATCAAGGAGATTGACAA
GGCGAACACCCGCGAGCGGTACCTCGCTGGCCGGGTCACCGGCCTGACCTTCTACCAGGT
GTGCCAGCCCGGCACGAACAACCGCATCGCCCTACACGACATGGACGAGGCCGATGTCAG
GGCCAAGGCCACCGCTGTGATCGCAGCAGCCACTGCCCTGGGCGGCGAGGGCGGCGCTAC
CCCACCGGACCCGCTCACCGCCTACAAGGTGAAGAACGGTAACACCCTGCCCGTGGACGG
CGGTGGTTCCGTGAAGGTGACCATAGCCAACGGCGCCATCACCAAGGTCGTGTACACCGC
ACCGGCGGGCTGAGCTTCAGCCCGTCCAACCTGACTCCATCCGTAACACAAGGAACTGAA
CCATGGCAACCTTCGCCGGTGCAACTCAGAAAGACCTCCGCGCCTTCGCCGGCACCATCA
AGAACCTGATCCGTCCGCTGGAAGAAGCGGCCCTGGGTTCTGGCTACAACGAGACCAAGG
TGCGAGCTGAGCTGGTCGCACACCTCGACGAGCTGATGGCTGCTGTCGAGTCTGACAAGG
TCACGGTCTACGCGTAGTAGGTCCAGCCGAGCACCTGCATAGTCGGGTGCTCCACTGGAA
CTACTGGAATTTTTATTGAGGGTGTGTGTGGGCTGGGTTGGCTGGTGTGGGCGGGAGTAG
TTACTCCGGGTCTAATTTTGGTATCGTCGTGTGAGAACCCTCCCGACTCTGATCAGCCCA
CCCTTCCCCCGTAGGCCCTCGGCCTGGTGGGCCATCCTCGCCTTGCGTGGGTGTCGCCTG
GGTCATCCCTGGGAGCTTCCGGCTTCGCCGGTGGTGCGGTGGGCTTCCCTGCGCTGTCGC
CAGCCATTCTACCCGGCTCAGCGGGAGTGTCATCCCTTGGGCTAGCGCTGTCGCTCTACC
GGGCTTGGCCGTAGTACCTGGTGCGCTCCAGCGCGTCGCGGTGCATGGCCCTGGCTACCT
CGTACTCCTGGGATCCCTGCCGCTTCCGCCAGGGTGCTGCGTAGTGCCTGTGGTCCTGCG
CTGCCCACTGTGCTAGCTGCCTCTGCCATGCCGCGTCCTGCTGGTACCAGGCACGCTTAC
CTGTGTGCTGTCTGCTCAGCATCCTCTGTGCCTCCTGTGGTCCTGTCGGGTGGTGGTGCG
GGAGTGGCTGGC
>NewGenomeName_19
TTAAAATTTAACATTCTTAACCAGTTTCATAAAGTCTTATGCGCGTTTGTCGTTATGCAG
AATACGATACCAGTTAGCTACAATATCATACAGTAACGGGCTATTCTCTTTAAACTCCGC
ACAAACTGCTTTAATTCGATCGTTTGCTGCCGTGAATTTTTCATCACAGATCTTTTTAGT
TTCTTCGTCCATAATCCAACGAGTATGAAGGAGTAACTTGGAAACCTCGTTATTATAATG
ACGACCGTACAGTCTGTCAATAAATTCTGGAATACAGCGTTCTAGTTCATCGCCATGAAA
CAACATAGTGATCACACCCAGATATTCGCCATACCCCAGATGATTGGTCTTAACCTTCTT
CGCCAGACCAGCCAGCTTACCGCGAACAAATTCTAGCGCACATTCCAATTGTTCGTCAAC
GAGATATTTTTGTGTCTCCTTTCGAGTAATAATATACTTTGTTACCCCGAAGTAGTCAAG
CGCCGTATGTAATGTATTTTCACTATAGATCGATTTACGACTACGGCTTTCGAATGCATC
ACCTACGCGGAACATGTGCAAGCCGGATAACTCGCGAATCTCTACCGCTTTCATGTGATG
GCTGGTTTTATGCCACCTGTCACCAATCTGTTCCATGATATAAACGTTAGGCAGCTTGGG
AGTCGCTTCACGTTGTGTACGCTCGGTCTTCTCCGGCTAAACTGCTTTAGCACGCAGATC
ATCGGCCTCAGAAACTTTAATCACGGTCATTTCAGAATCATCATAATACTGTTTGATGTA
CTCGAAATTCTCCATATGTTTTTCGTTGCGCTGACGGAACACAAGTACCTTTTTATTGAT
GCCGTATTCCTGGACCATGCCGTTAAGAGTCAGTGCACGCAGTTTCGGTTTATCGGTATC
ATCAATAAGACACATAATATCTTCTTTATGCATCCATACAAAATGCGTAATGTTGTATTG
ACTGCTAACGAAAGCAGGGCGCAATTTACTGATTGTGAGACGAGTACGATACAGAGTATA
CACCTGACCTACTACGTCATTAGGCGAATCTTTACCCGCATTCACAAACTTATTATAGAT
TGCTACCGGATCGTCCCATTGTCCGATCTTACCCATCTTGCTAATAATATTGCGTTGCGT

CGATGAATACTCGCTCATAAATTGAGCAAGTTTACGCTCTGTTTTAATGTTGGCGATCTT
TTCTTTAACCTTTTCCAACATCACTTCATTCATTTCTGCAATGCGTTTTTCGATGTTCGC
TTTAGTCTAAATTGACAAGTGAACTTCTTCACGCGATGGCATCAGGTCCAGATCACCCAA
CTCAAAATTAATGTAAATGGTCTGGTGCTGGCGGTTTACAGCTTCTAACCATGTTCCGCG
AACATATTCATACGGGATCGGATACTGGATCTTACCCATGATTGCATATACTACATAGGA
ATCGTTATCACGCCCATTACAAACATAATAATTATCAAAGCTAGGGAAATAACTAATTCA
GATTGCATTGATATTCGGTTTGTGATCCCCAAAATGACGCAGTACAAATTGCGCTTCTCG
ATGCCCTGTCTGAATGTCTTTTTCTTTCACCGGAATAGTGATTTTCAGACCATTGTCTTC
ACAGTTCGGTTCGTCGAAAATCTGCACCATTTTCGGCATAGAGCTTTGCACAAATGCCGT
AAAGCCATAAGCGCGACCATTGTAATAGTTTACGATTGTAAGGGTGTCGGTATAGCTATA
AGCAGACTTCGCACCTAAACCAAAAGATCCGATATAATCATTAGTCTGGTCTTTTGTTGA
TTCAAAGTAAACCGTAAACAGTTGTTCTAATTCTTTCTTAGACGGAATACCAGTACCATA
ATCACGCACAGAAAAAATACGGTTCGATTGCAGTCGGTAAATGAATATCAACCGGACGATC
TAATGTTCCGGCTTCTTTGTGAGTGTGGGTAGCATTACATGAATACTCACGGACGCAAGC
ACGCTCACAATATTGATAAGTGTTCTTAGAAAGCACCATAAAAGCCTTGCTACTTGCTTT
CAGTGAAAAATCAACCGCACCGCTCAAGTCACTGCCAAATACTTCTTCATTATCAATATG
CATTTTCATAATATAATCCTCACTTAATTAAAATCTTCTTTGTCCATCACTGCGTAGATT
ACATCCCACGCCCATAAATCAATAAACGCATTATATGTCACGGTGCAATCGTTGTCAATC
ACTTCTTTCGCATATTTCTTGATGTTTTCGTAGGAAACTTGCGGATCAATTCCGTGTTTA
CGAAGGTGTGCATATTGATACCTCAAACATTTCATAAACAGGTCCATCGATTTCTTTTCT
CGATGGCACTTGGCGATCAGAAAATCAAAACTTTGACTCATTTTCAACTCCTTATACATT
TTCAATCACCCATTGCAGATGGCCTTCTGCGTCAACCAGTTCAGCGCGGCTTTTCTCTAA
GCACTTGCTGTATTCTTTACGTTCCCACTCGGCAATTTCGGTTTTCAGGACGTCTTCATA
GTAGGCAATTACGCCTTTCAGGTCTGAAACTTTACGGCTTGCCTATTCGATGTAATGGTT
ACGCATTTTGATTCTCCTTAGTGGTATCTCGTTTCGATGGGGACATTATGATATATCCCC
GATCCAGTGTCAACAGATTTTTAATCTTTTTTCAATGCATCCATTACTCGCTCTGCGATC
AGTTCTCGTAAGCACTAATAACCATGATATTTGATGGCAATGTCACGCAGATCATTAACC
AGTTCCCGTACTTCATGGTCTTTCAGATCGTCTCTGTATTTCGATTTAACCGTGTCTTTA
GGTGGATTGTGGATAACCTGAAACCCTTGTTCGGCAATCACTGCGGCTTCGCGCTGGCGG
TCATGCTGTACTGTATTTTTGATCAGGTCTGCTTCCATGAACTTCGCCATACTCATCATC
ATGTTATTCTTTTTGCTCATTATTAAATCCTCAGAAAAGAAAAGGGAGACTCCCATTATA
GAGAATCTCCCTTGTATGTCAACTAAAATTTAAAGATGCAAACTCCCCGTGGTGAATTTT
TGCATATTCATCATATGCTCTAGCGGCTTCCTCCTGAGTAGCAAACGAACCTATGTATTT
TCTGTATCCGTTTATCCTTATTTGTGCTTGCCACGGTCTTTTGCTCGATCCTTTGCGATG
AGTAACACCATTATATATGCTCGTACACCCGTCCATTTTCTTTTTATTGTACATGTTTTG
TTGTTGTGTTGCCTCCCGTAAATTTGGTGGATTGTTGTTTTCTGGATTACCGTCTATGTG
GTCTATTACCTTAACCTGTGTTCGATATTCAAGAAACCAGACAATTCTATGTACAAGATA
TGTAGATGTTCTCTTTTTAACCTTATTGTAAACATCTATTGCTAAATGACCGTAATTTAA
CTTTTTTGTTTTTTCTTTATCCCCGACTTGGTAGAAATATCCCTCTGGTAGACCATTAAA
AGACGGACTATTTATTTTGTGATATAGTACACCTTCCGTGTGTCCAAATCTTCGTTTTAA
TTCCTCTTGAATTTCGCTATTCCAAAATTTCGCACTGTTCATATTATGACGCCATCTATG
CCTTCCTCATGTCTGAATCTTCACTATAGAGCAAATCGAATAGCTCTTTATAGTTTTCCG
GTAACGTGATGATCTCCATGACAGGTTCGTTGATCACGCGATGATAGTCTGATTCTGTGA
GCGATGCTAAACCTTTGATATATCGATTGTTGTAACCTTTGATATTATCTCGGTCGTTCT
CGAAATCTTCTAGGTCATAATACCATCAAACATCATCACCTTTCTTTGGTTCAGCAATGA
TAATAGGTGTTTTGATAAACCGGATTCGTCCATCTTCGAACAATTCAGGCCAACGTGCAA
AGAAAGAGATCAGACTAGGTTTAATAGACCCGATCCCGTCAGTATCAGCGTCGGTCATAA
TCGCAATATTTTTGTATTACATAACTTCCAATGCATCAACACCAAATTGCAAGCCAGTGA
TCGCCATGATGTTAAAAATTTCGACGTTCTTAACAATCTTTGCCTCCGGTTGTTCCCACG
TTGTAAGAGTTTTGCCTCGTAACGGGAAACCTCCATGTAAATCTTGATCACGTACAGAGA
TCAGATAGATGATCGCGGAATCACCTTCCGTCAAAAACAGGGTAGTTTCTGCGTCAGTTC
CAATAAGTTTAGCTTTAACGTGTTTTGCTACCTTAGCCCGTTTTGCTGCCTTTGCTGCCT
TAGTCGCTGCCGCTTTATCTGCTGCCTCTTTGCGTGCCAGGACTACAGCAATAATAGGCA
TGATAATGTTTTCTTTCTTCGCAATGACTTTAGCCAGCTTTTTAAAATCAAGATCAATAT
GACGGTTGATATCACCAGTTGGGTTAGTCAAGCGTTCTTTTGTCTGTGAATCAAAACGAA
GGTTAGGCAATTCACGAACAATCAAAATACAGGTCAATCCAGACTTAACCGCAGCTTTAG
TAACATCAACCTTATATTCTTTCTTGATTCGTTTTACAATCTATTCATAAAGATCATCTG

GCACGTAGTCTACGTGTGTTCCCCCGTTTTTGGTAAACAGACCGTTAGCAAATGATACTG
ATTTAAAACCTTCTCCCTCGACCGCCGGGACTAGGGCAAGTGAAAAGTATTTATCTTCAA
TGACAAACGGATCATCATTGTATAGGTTAGCATAATCCTTGAACTTACTAACAATACGTT
TACCGTTAAACTTAAACGTGATCTTAGGAAATATTACGCTCAATGATTGCAGACGATCAT
GAACAATATCTAAAACAGAGTTGTCCATCATATAACCTTCGAAGTGGTCGAAGTCTGGGC
AAAATGTAACCTTTGTTCCCTTCAATTTTGATGGTTACGATGACCATGTGATATTTTTAG
CACCGTTTGTACATTGAACGGTCATTTCTGTTTCGCCGTCACAGGTCACACCAGTAAACC
AATCTCTAAAGAAATTTGATAACGACGACCCAACACCGTTCATCCCTTGCGTTAATCGAT
TGGTATCATCAAAGTTAGACCCTGCTTGTGTTCGTGTCCATGCGGCTACAGGTTGCGGAA
TCTGCGTACCCTCTGGGGTAGTAACCATATGTTGAGGAATACCGCGACCGTTGTCTGTTA
CCGTAACAATATTGTTTTGGATATCGACGCTAATCACATTGGCAAATTCAAAATTAGTGC
GAATCGCCTCGTCTACAGAGTTGTCGATGATCTCATCAATGATTTTCACCCGCCCAGGAA
CATATGAAATTTTTGTCCATTTACCGAACAGGAAACGTTGATGTTCTTCGTAAGTTGTTG
ATCCTATATACATTCCTGCACGCTTAAGGACATGCTCACGATCAGATAACATGCGAAACT
CACTCATTATTAAACCTCTTCATAACATAACCATAACTTACTGCAACCGCCACAAAGACG
GCTGCAACAATCATATTAAAAATTGAGTGATTGTCAAGTATATGACTCATGACACGCGCA
CATTTTTATCATAGTACCCGCCATTAAGACCGCGCGGGGGAGTTAATCCCTGTTTCCATT
CTTTACCACATTCAGGGCATTATGCCTTTTCACGTTAGGAAATTTTGCGGATTTTCTGAA
ATTGACCGTGTTCTTCACAAGAATAATCATATAATGGCATATTATCCCCTTTGTTTCTTA
ACTGAATTTGGATCTGGATTTTCTGGCAAGTCTTCCAGACCCATAGCATAACGTTGTGCA
TAAATTAGCATAAGAATATCGCGAGCGCAATCATGCATTGCGTCATGCGCGATAAATGCG
TCTAGCGTACTCATTGGTAACGGGCATTCACACATACCACGATGCATCAATGTACGCTCA
ATGGCGGTGCGAACATCTCGGATATTCCAGAATCGCACAGGTTCAAGGCTGAACGTCTCG
CGTGTATTGTGTGCACGGCGGATCATATCAACTAGTAACGGAATATCAAACGCCTTACCA
CGTGTGCACATTACGGATTTAACCGGATCTACTCCTACGGCTTCACAGAATGCGTTAAAA
CGCGCTAAACCTTCTTTAGTTGATACTTCCGTACCATTTGTGATTAATTGCTTTCTCATG
CCTTCTGAGGCGGTATTCTTCCACCATTCAACCACACCATGATCAAACACCCTTACACCT
TTCTGGGAAGGAATATCAAATTTTATCAGAAAGGAATTGTCAACCAGTTCTTTAAATGTA
GGTGGATTGTGCGGATCTTCTTTAAATCCGATTGCCGCCATATTGATAACCGCCCCATCA
GGCGGATAACCTAATCTTTCAAAGTCAATCATTAAATCCACTGGAAGCATTTTAAATAAC
CTCCGCGATATGCCGGAACAACTCCAGATCGTTTTTATCCAGAATGAGACCGCTACAATC
AATAATGCCGTTACCGTAATTATCGGTTAACAGTCGTTCCGTTGCCACGTATGCGCGTAC
TTTGTTGTTAAAATTCATTACATGTTGTGCATAGTCACAATGAATCTTTTGACGGTTCCA
CGCGACAATTGATTGTAGATGTATTACATTAGCCACATTATTAATAACAACCATATCAAA
AACCTCGCTTAGTGAAATGCTCGATCATTTTATTGATACATGCAGCTTCACTATCGCCGG
ACACGTTCCAGCCGTATAGACCCCATCTAAAGCACATGGTAACAGTTCCATTGTGACGGC
TTACCGTCACATCATTAAGTTCTGGGATACTTCGCATAATCATAATAATTTCCTCAAATA
TAGTCTACTCGATACCGCGCGCGGGTAACACCAACATATAATAACTGCATTGCCAATTCA
AAGTCAACATTGTCTGTACACATGCAAGGAGTATACAGGAACACGCGGTCTACGCTGATC
CCTTGTGACTTATGAATGATGCAAGCTGGTAATGCATTTACATTCAAGAACATACGGTTC
ATCGTCCAGAATGAATCCCAATCTGGTTTAAATTTTGGATTCTCTTTCTTCATTTCGCGA
TATTGTCCGGCGACCGTATTCAGATAGAAAGAGAAAATGTTCTTCTGACGTTGTTCTGTC
AGAATGTTCATTTCTACTAAATCCGGTTTATCTGTAGAGATTCCGACTGCATCTAAACCG
ACTTTCTGATTTAGGATCTATTTGTCTTCGTCATGATCGACGCTTTCCACTGTCAGAGTA
TATCCTAAAACTGGTTGCGGATCTACCCCCGTACAACGTAATGTAAATTCGCGCGGTATA
ACCTCTTTAACTCGCATCATCTCACCATTGTTAAAAATAACACTAGGAATCTTCTTCCCG
CGTTCATCTCGCAGATATTCGATCAATGGTTCCTGAGTAACCAGAATTTCATCTACAATC
ACTGGTTCATCGGTGTTATAAACGCGCTTACGGACAATTTCATTGAGTTTTTCGACGCTC
TTGTTAGTGTAGGCCAGAATACGGTTTTCTCGAAGGTCTTCCGGCGTTTTAACGTTTTCG
AATTAATGCTTCATAAACTCCGGCAGAGTTGAGCTATGGAATACACCCTCGTTACCGCAC
TGCTTTTCGAATCCCCATTTACCATTACGGACAGCGGTCGCCACATCAATTATTGGCGCA
TCAGATCGTTTAATCTCTGTAAGTTCTACATTCTGAAACCTTTCATCCGTGAAGAAAGGA
GAGATCGACGTTGCACCGCTCGGATCAACTGGTCGTAACTGGTGCGGATCGCCAACTGCA
ATAATAACAATTCGACCGTTGATACTCTTCATGAGAATATCAAATAGTTCCTTATCGACC
ATACTCGCCTCATCAACAATGATGATCTGAACATCATCTAGCTTAGGAGTATCTTTTTGC
TCAAATAGTTGAGTATCTTCATAGTTGGTCGGGTTTAGTCGCAGTAGTGCATGTAAAGTA
AATGCTTGACGACCTACCGCTTTACTCAAAACTTTTTTAGCCTGGTGGGTAGGCGTTGCG

AAAACCACACCAGTTACACCACGGCGGACCATTTCCTGAACAATGAATTTCATCATTGCG
GTTTTACCAGTACCCGCCGCACCTCGGACAGTTGTATGCTTCTTTTGTTCAATGCGTTTC
ATTAGAACATCAAAAGCATGTTTTTGCCCGTCTGTTAACATATCGAAACTTAATACTCTG
CTCATTATCCTAAAATCCTCACCTTGCGAACACAGATAGTATTGAGTTCTCGCCGTACCC
GTTCTTTTTCATATTCAGGAATTTTATCATCGATACCATCTCGAACAATTTCAGAAGACC
GGATTATGCCAAATTTTCGTTTAGGTACAACCTTGTATTACGGTTCACCCGCCAAAAGAC
TCTCACAATAATCTATCTGATCACACATTGACTCCAACGCAGCATCATCTGTTTCGCGAG
TTAGGACGCGACCCTTCGCGTCCACTGAGTATTGAAAGAATCCGTCTTTATCACGATAAC
ATACAGCAAATACATGCATGGTAAACCTACCTTCGCTTTCATCAATTCAATCCACCACGC
ATCAATCGGAAGCCCGTATTTAACGCGTTCTTTTTGTGCTGGCGGTAAGTTCTCATACTT
AGAAAGAGATTCAATCAAAACGCTGGTATATTTTAGCACGACTTCGGTATTGTTTGCAAT
AGCCTTAAGTTTTTCTTCTTTCTTGTTGATTTCGCTTAGGGCTTGCTTGAGATCTGCAAT
TTTCTGATAACGTTCTTCCATACGGAAGAGAGTATTTTTAATAGCTCGCTCGACTGCCTC
TGCTTTCTACGGTTTGTTGTGCTTTTTGTGGCACTCGATTGCCAATTTCTTTTCTTCGAT
TGCTATTTCCTGATTTTTGATGCGATTCTCGTTAAGTTCGATCCACTCTATGTACATCGC
ACGTTGGGCAATTTCATCATCAGAAAGTTTACCGTTAGTTCCTAGTTCATGGATCTCTTT
AAGAGATTCGATAGTGTGATTGCCACGGAAAGAATTGTATTTCAGTTTAACCGGCATTAC
ATTGCCATCAACATATCCCAGATTGCAATCAATGCGCTCTAAGGAGAGTCCATCATCACC
TTCACCGAAAGCCTCACCGGAATAGGCGCAATGAGTCTGTCGGTAAATGTTACGCACAGT
GACTAAGCTCATAGAGAAGTCTTTGTTGCGACGTGACGCACCAGCCATGATAGAGTTCAG
ACGGAAAACAACCAGCGCCAATTCTTCGTTAGTTTTAAAAGCAAACAGTTTCATAATTGA
TTCTCCTTAGTTGGTATGTGTGTATTATTGTCTATCTGGATTGTGCTGTCAACACTTAAA
TTAAAGAAAATTTGTCAATCTCAATCAGATCGCCATAACACGCGTAAACTTCCTCGATCT
CATCAAGGATCATGGTCGTCCCTGTCTTGGATATGCATTCGCGCTCATCTGCCAACATAG
CATACAAAGACAGTCGCGTAGTCGGATTGTCATACTCTTTAGACACCACTCGGTCAATAG
CATGTTCGTAATAATTCATGCAAGGCTTGGTAGGTTTTATTGCAATAATCTTTCCTGTTG
CATAAACGTCTGTTGCAAAGTCCTCTGCTGTTGATTGCAGTGGCGTAAAGCTCATAGGAC
GCTCTACAACGACAGAATCACCTACAATTAAGGTATCCAGCTTACGGCCTTCTCTTTTGC
TCACACCGCGATACAAGGCTTCATGATGCACTAACCGCTCAGTCTTTTCAATCAATCCAT
TAAGGATCGACATATCGATATTTTCTTTAGAGTTCATCACTTCATCAATGATGTTAAGTT
CTTCTGGTGTAAATTGCATAAAAAAAAAGCCTCCAATCTTTCGACGGGAGGCATAATAAA
ACCATTGATTGAGTTTGTCAATCACAAAGTGTATGTGATCTATAAATAAGTTTGTTAAAA
CTAAAGGAGATCACAAAATGAACTATAAACGTATTTATGATAACCTGATTGAGCGTGCTA
AGACTAGAAATCTCGACGGATACACAGAAAAGCATCACATCATACCACGTTGGATGCATG
GAAGCGATCACCCCACCAACCTAGTAAGACTTACACCTGAAGAACATTATATGGCACATC
TTTTGTTAGGAAAGATATATCCAGATAATAATAAACTGATATATGCCGCTTACATGATGT
GCGTTTCTTGTCATGGACAAAGACAAACAAATAAGCGTCACGGATGATTGCGTAGAAAAA
CGACGGAAGCGCATAGTAAAAGAATACGCGATAGAATGGAGAACGATCCAGAATATAGAT
CTAAGGTTTCGGAATGGGCTAGAAAAATGGGCAAAGCCATGAAAGGCGTTAAGCGAGGAC
CGGAATTTAGCAGAAAGTTGGCAGTAAGAAACAGAAATACCGTGATGAGAGAAGAAACTA
AACGTAAGATCGGGGAAGCCAATAAAGGAAAGAAACGTACAGAAGAATATAAAAGAAAAC
ATAGTGAAAGGTATAACGGTGAAGGTAATCCGATGTATGGTAAACGAGGAAAGGACCATC
CGGCATATGGTACTAAACCGTGGAACAACCAAACATCATTTGTTGAACAGAAAGCATGGT
GACGGTTCGCTGGCATGTTTTATGACTGGTATCGGAGAGATATTGACCGTGGAACACATA
AACGCCACGGTCATAAGAGAATGTTGGATGAGTTAGGTTTCGTTGGTTGGCGAGAAAAAG
AGCCAGCCGCAGCAAGACGATCAATTGAGATGTTCGCTAAAGGCTGGAATCCTTATGAAG
ACCAAGAATGGTTAGATTATGTTAATACCCGCTGTCCAAACGACGATAGTTTTCCGCGTT
CTTCAACATATACAAGACAAACAGATCCTTCGCAGTAAGCCCCAACGCCAAGAAGTTACA
CATAATGAAATGGAGTTGGTCGACGGCTTCTAGGTATGTTTCCAAACGATCCGCCTCAGA
TAAATCACTAAAGAGGCGGTTGCGTGCTTCCATATGATTGGCTTTCCAGTGTTTCCACAC
CGCGCTAGCGTCTTTCCCGTTAGACATACCACCCAACGCAGTGTACAGTTCTCGCGTTTC
GTCGTCAAGCGCATTATCATTAGCTCGAATAGAATCTAAAATCTCTCCACATGTTTTCAA
TTCATCAATTTTTGCTACCCACGGACGGTCATTAGATACCTGGTCTTGTAGTGCTTTCTG
CATGTCCATCATGTGTTGAAATGGGTTATCACCGTTTGCGATAATCTCTTTATATTTTTC
TTGTGCAAAATTCAGAGTTTCCGCCTTTACTAAGTGTGCACAAGAATTAAATTCTGACAT
ATTCACTTCATTAATTAAAATTTACAATATTTCATAAACCGCATTTTAGCCATCAGACCT
TGTACGGTGTTCTCTTTGATAAATTCCATGATCTGGTCGCGTGTAGCGCCCTCGTCCTGA

ATCATGGCATTAACGTCTTTAGAAAGCCGCGGGCAACGATCCCACAATACAATCTTTTCC
CCTGCATTGATAAACTTTTCCAGTCGGCGCATTGTATCGGCAGATCGTGGTTCCGCATCA
AGTACAAACACTCGCTGGTCTGGGAAAGGTATTTCATCTAATCCCAAAGTACCGCCAGTA
ATTGCACACCCGTTATCAATAAACAATGAATCGATCGGACCTTCTAAGAAATACACTGGT
TTACTCGGATCGGCGCGTTCAAGACCATAGACCTTACTCGCTAACACATGAGTTTTAATC
GTCATATATTTGGTCTTTCCGTAATCCTTTCTCAATGCTCGCCCCTGAATGGATTCTATC
TTGTTCTCTCGGTTAAAGATAGCAATCACTAAACGCGGCTCAGGTTGTGGATTCTTGAAA
GTATCAGCCTTAATGTGATTTGCTACCTTTTGCCATTCTCTCGTGAAATACAGAAGATCC
CATTTATCGTGGGGAATGCAACGATCACTGACATACTTTCTTATAGGATGATTAGTCGGA
AGAGTATCTAATCTCTCGCAGAAAGGCAATGTTTGCACTTTCTTAATCTCCGGCTTTTCT
ACCTTTTTTAGTGGCTTAAACGTGGTGTTCTTGTGGTCGCCTTGTTCCTTCCGTCGTTCC
ATAAGCCATTCCCTGAATAAGTCCGGCTTATACTGTTTGAGAAATTCACCTCACGGCTGA
TTGTAGTTACAGTTGAAACAACCACAACGCAAACTATCCTTATAGGTATAGATACGGAAT
CGCGCTTGAGTATGATTTTTCTTTGAGTCACCGCAGATCGGACAACTACAGACCACCGTA
AATGAGCTAGTATGCAACCGTTTAAAATTTGGTTCGCCGTATATGGCTTTTTCCGCTATT
TCATAATCTACGTAGTAAATCATACTAACCTCTTATAGCTCAAATTTCATGAAAGTTACC
ACACTTTCATTTTCATTCTCTCCGTTTTCATCTAAGCAAAATTTGTAGACACTACCATCT
GGCGATATGTAAGAGAATACTCGAAAACGGCCTACCGGGTCAAGTTCTGTAAATTCATAT
TCTTTTCCGGCTTCAAAAAGTGCATATGCGCTTTTAAGACATTTCATCGTAATCATTTAA
GCGCCACCGATGAGAACATGATAATAATACCAGAATACATAACAGTCTCAGAAATTTTTT
CTGTTTTTGTCTTTTATCCAGACAACCCGAAAAAGACAACACCCGAAATAGCCGTCACAA
GCCCGATAATGAATACAATAAAACTAACCATTGTCTTTCTCCTTTTTGCGTTTATCTTTA
CCCGCTACACTAGGTCCACGATTGGTAATAGATCCGCTCGTTTCCCCTGCTGCGATCTTC
TCAGGATTGCCGCCAGCATCGCCAGCGGTCATATCTTCTTTGAGTTTATTTTCTTCAACA
AACGGTTTAAAACCAATCATAGAATAATCTCTTTCAGTAAGTCAACAATCTGGTCTTTCG
TCAGTTTATCCTGATTCTCTACAATCTTGATAACACAAGCGTTAACAACTTTGCGAGTCA
CGCGGGACATCGTTTTAATACGATGAAACGGTTCGTAGAAATATACTGCTTCATTATAGG
TCGCAGCAATTTTTTGACGCATTTGATCTTTATCGGCCATAAAAACAATTGCAATTTTCG
GATCTACACCAGCCACATCATAAAGTTCGGCGACTCCCCAGCGTCCTCCCTGTTCAATCA
TCCCAGCGTGTTTACCGTTAATAAAGATAAGAATGTCTCCGTTATTTGCGGTTTAAAATG
CTACATCTTCCGGCGTACTCATATTCTTCATAATATCGTCTCCTTAGAGTTTCATTATAG
ATCAAGAGAACGTAGAAAGCTAATGTTTGATTTCTTTCAGTTGTCGTTAAAGGTCTTTGC
GTTTCTCTTTCAACTGTCGTTTAGCATTGTTTCGTTGTTTTTCAACATCTTTATCAATGT
GCTTAGTACAATTATCAATATCAGCATAAGTTGGCATACCCAATTCTAGACCTAATGCGG
TTGGATGCAGGGTAACAGTCCATAAACCGCTAATCTTCTTAAATAGGTCTGGGTGTAGTG
TACTATCTACATCGTCCGGTGTCAACTCTTTTTCTTCTTTCTGTGGTGGGATCTCGTCAT
ATGGTTCCAATGCATTTACTTTAATATCAGATACCTCATCAAAGAATCCGCGTACATCCG
CAGGAAATACACAAGCAATTTCCCAACCGTGATCACGCAGAGAAGTAATATTTTTCACTC
CCCCACCATGTGTGATGATTGATGTGACATTGCCATCGAGGTCCACCGAATTTACGAGAA
ATGGCTTATCGTCTGTCAGATCATTATAAAACTGTGCTTCGGCTGTAGTCATCTCCAGAA
ACAGATCTCGGATCAGACGACCATCAGGTGCGTTACCTTCCTTGAGAGTATAAAAGCGAC
TTACGATCCATTTTGGCGGTAAAGCATTAGCGATCATGTTATATCTCCTATCAAATCATT
CACCAGCGCCCCGCAGGGCGCATTGTATTAGAAAGTCATGTTGTTTGCTAGCTCGTCGAG
TTTTTCTCGTTTCTCGGCTTGCTGTGCGCTTTGTGGTTTAACTTGTGCATTCTCCTGATC
TGCGATCTGGTTTGGTACTTCATACCAACGCTGATTGCCTTTCTTCACACCAACGTAAAA
CCGTGAATAATAGTTTTTGTCACCGAAAAAAGACTTGATCTGCTTCATGAGTTGGACGCC
CATTTTAGCCGGGTCTTCGGTTTCGATAACACCCAACATAAAGTCGGCAGTAGCAGGAAG
ACCTGCTGATTCAGCGGCATCACTCATGTTAAGGTCGGAAGCATCCCAACCCGCGCGGGT
AGTCTGTGCCGCAGTCCAACCAACGACATCCCACTTAACAAAAAATCCTCGCAGTTCTTC
CGCAATCGCCTTAACTAGTGTGTAACTATTTTCAGAGTATACACGCAAGCGAGACGATGC
GCAAATCCCCAGATAGTCAACAATCACAACATCAGGTTTAAACTGTTTCTTGAGTCGCAA
TTCATTCATTAACGCCTCAAAGTCATTGGAGTTCGCGCCACCAGTAGGATACTGCTTGAT
CATCAATTTTCCAACCGGTCGTTCTCCGACCTTCTTATTCTTAACACGCTTCATGCGTGC
GGAATATTCCGCAAAAGAAACATTACCATTATCCAATTCATCTAATGTAATGTCAAGCAA
GTTTGCGTCGATACGTTTGGCTACGACATGCTCTGCCATCTCCATTGAGATATACAAGAC
ATCATAACCACATTCCATATAATCCGCAGCAAGAGAACACAAGCCAAGCGATTTACCAAC
GTTTACACCAGCAAGCAACACATTTAGCGTTTTTCTTTCCGCCCCTCCTTTCGTGATTTT

ATTCAACATCGGCATCTTAAACGGGATCTTATTCGCCTTAGTCTGATATAGCAAGAATCG
TTTATCAAAGTCATTGAACCAGTCATGACCTACATCAGAATCAAACGTAATGGATAGTGC
TTCCTGCATGATCTCAGGAATAGCACCCTCACGAGGCAATTTCTTATTCTGGTTCTCTTT
TGGTAGTTGTGCGTTCGCCTGAATCTCTAGCGCCCGTGACATTGCATTATACATGGCACG
GTCACGAATAAATTTTTCGGTTTCCTTCATTAACCAATCAAGGTCTTCCGGTGTATTCTC
AAGCGAATCAAGAAGAGTTTTAGCCCCTTCATATGTTGTCTGATTGCCTCTCTCCTTGTC
TAAAGCAATCTGTAATGCAGTTGGTGTAGGAATATTGGAATATTCGTCTACGTGCTTTTT
AATCAGCTTATAAATCAGTTTTTCTTCGTCTTCGGTGAAGTAGTCAGCTTTAAGGTGAGG
CCATACATTCGTAAAGAATGCGCCATTGTAAATCAACTGTGTAAAGATAGTTTTAATCAA
ACATATCCCCCAACTACACGCATAACAAAGGTCTTGAGTTTATACCAGAAAGTTTCTTTT
TTCAACTTTTCTTCTGCTTCTTTGATCTGCGCGGTTAACGCCTTAATGACGTGTGGATAT
ACCACATCACGCTCTGACGGCGTGGAGTAGTCGACCACAATACCTTTCGGGTCTGGATCG
TATTCAAGATTATGAATATAGACAATATGATGTTCTCCCTCTTCATCAACAAATATCAAT
TCCTGAATAACTGTCTTCATTGCTTCTTGAATCAAGCCGTGTGCTTCTTCTAAACTTGTT
GGTTTCTTATCCACAATATAATCCTCTCTAGATATGAAAAAGGGGCACGTAGCCACTTTA
CTACCATTGTTATGCTAACATATCTTCGATTTCTTTGTCAACTGCTTCGTCGATGATCAC
TGGCCCCAGCTTATAATGATCGTTGATTGCTTCGTTAAACTTATCGTTTGTCAGAAGTGG
TTTCCAGAACTCAATGCTATTGGTTTCTTTAGCACAGAACTTGCGTTCTTCGGTTTCCAT
CTCTCCGGTCTCAGTATTCAGGATTGAACGAGAATACCAGCCGTTTGACGGTTTCACTAC
CCAGCCTAACTCTACAGCCAACTCCAGCAAACCGGAATAAGGATCGATACCACCGCTATA
TGTTACGCAAATCGGGAACTTAGATTTTTCTTTAACGGTTCGTCATTTTTCCGCATTCAG
GATAAAATCATAACCAGCAATTTCGGCTCCTTCTTTCTGCTGACGACGACCAATAATGAA
AACTTCATTGGCTGAATACATCGCGCCCGTACCGCCGGACATAACGGTCTTACTAAACAT
TTCTTGCGTTTCGTAACTGTGGTTCATAGCGATGCACGGGATATCATTCATAGTCAGATA
CGGCGTAACCATACGGAACAAGCCTTTAAGTGCTTTCGCTCGCGTCATATCCTGTGCGGA
TTTCTCATTAATCGCGTCTTCAACTTCTTTTTTACTTGCTAAGTTACCGATTGAGTCAAT
AAAGATGATCACTTTATCTTCACGGGTAATTTGCTCAAGCTGGTTCATGATTTCAAATTT
AAGTTCTTCGATGTTCTTGATTGGAACATGCACCACGCGATCAGGATCTACACCCATAGA
TTAAAGATAAGCGGGTGTAATACCGAACTCAGAATCAAAGAACAAACACGCCGCATCAGG
ATTCTGTTTCATGTATGCACTAACACAAGTTAATCCCATGTTTGATTTAAAGTGCTTAGA
CGGACCAGCAAAGAAAATCAGACCAGATTGAAAGCCTTTTTTAAGATCACCTGAAAACGC
AATATTCAGCATCGGGATCTTAGTGCGAGTATTTGTTTTTTCGTTAAAGAGGCTGGATTT
AGACAGTACCGCAGTAGTTTTCAGGGTAGAGTTCTTTTTCAGTTTTTCCAGTACAGACAT
TATTAATACCTCTATTCAGTTAATCCGTAATACTTTAATAGCTTCATTGTTATTCGATCT
TTGTAGTGCTCTTGTATTAACTTTTTCATCGCATCAACATTGATCGTCACAAATACTTCG
TTGCTGTGATTTCCGTGAACTTTCTTGAAATGCTCTATGCCTTCCTCACGTTCACGTTTA
ATCTTTTCAATGTCTCTCATAGTATATTTCTCACGTTGGCGGGTATCGTCACCCAACACC
CGCCATTATACACACTTTATCTTAAATGTCAAACAGGTCCGTCAGTGAAGTAACTTTTTC
ATATTGCAGCTTCGCAGCCGTGGTAATGTTCTCCAGTGGTTTGACGAAAGTCTTATTAAA
CAACAAAGGCCAATCCAACCACTTCAACACCTCTGATTCAAATTCTGGCGGAATACGAGT
ACCCGACAACCAACCAAAACAGATTTCTCCAAACGGATTTCGGTCTTTAAGCGGTACAAT
CATAACCTTCTCGCCTTCACGAATCGGATCTAGTCCGAATTTTCGAGCATTACGATTATA
ACACAATACACTCTTAACCGCATACGGACAACCCTTAATCGGATACCCGTTTGAATCGCT
GTATTTCTGTAGGTTGTTTGCAGATGATACCGCAGCAACATCAAGATAGTCAAGTTTTCT
AAATTCTTGCTCAAAAATACCGTAGTATTCGTGCAATGACTCTTCACCTTCCTGCAACAT
ACGGCGAATACATTCTTTCAGTGATTTCTGGCATGCTTTAGGGGTGGAGCTACGTTGCGT
TTCCAAGCCCATGAATTTTAGCTTAAGTTCAGCAAAGCGCGTTCCTTGCATATACCAAAC
ATTAAGAGCATACCGCTTTTTAGCAGTCCAGAATCCACCGATACCCGCAGATTTCAACGG
AGGACCAGCAATTGCCTCACGGTCCATAAACATCAAGTGCTCATAGTTGTTCATGTACTC
GCATAACTCGCGATATCCCTTGTCGATAATAGGTTCCATTCGATGTTTACCGAAATTATC
AAGGAAGTCAACAAGTTTTTCGGTATCGTCGAAACGGTCTTCCCCGCCAACCTTATCAAT
AACATTGTTAACCAGCACATAAACGGAGTCAGTATCACAATAACGAACATATGCAAAATC
AGTCGTTCCGCAAAGCTCGTTTAAGTATTCGTTAATCCTACGCTCAATCCACTGAATTGC
AAGCTGACCGAACAGTGTGATCGCACTAGCGTTACGGAGATCATAAAAACGGAATTTAGG
ATTCCCCAACGCACCATTAAGGGAGTTGATCAAAACTTTACGAGCAAGCTGGTTTGAATT
GCACAAGATCGACTCGTCTTTACATTCTTCCAGTAATTTCTCTAATGCATCTTTACTCAA
TTCAGAAAGCATCGATTTAAACGCTTCATCGAAGTCGTGACGCACTTCAACGCTATAGCT

AATGGTTCCCCCATAAACATGATTTTCATCGTTTAGAATCGCTTTACAGAGTTCAGCATT
GCGGTCACATGCCAACATCATGTTTTTGTACTGCTTACGCTGATCGAATACCTTTTTAAT
CTCAATCGGTACGATACCGTGAATATCTTTTCTATACATCCAACCGTTAGGCGAGCAAGA
ATACTCATCACTCGGACGTGGTGCAGTTTTATTGATATACTCGTGCATCGGTGCAGCATC
AACAGATCCGGCGATTGTTGCCGGCGAAATATTAACCTGACGGATGATTGACGGATACCA
TTATGTTCAGCGTGGTTCGCTACTCCCGCGCCCGTTCTCTTGCGAACTGCTACATGTCAC
CATGTAGATCAGACTATATCACATGCTCATAGAGAATCCTGCCATTTCGAATCGTTTGAT
TCTACTCTACTCGGTTCGTTACCCTTTCGATAGTCGTTGAACAACTGCATCTTTAAGCTC
ATTTTGATCAAACGGAGTTCTCTTACAGTATCCGCTATAATTCTTCACCCTGATACGGAA
TAGACACCAGTTTAATGTAGTCGTTTAATGCTTCCTCGTTATGTATTTCGTACTTTCTTA
AAATGTCTTTCATCTCGCGCCTCCAATGTATATCAATATCTATATACAGTATAAGACACG
TTAGCTCAAAATGCAACTACTGCTGATTGTCTCCTTAGAGATTTCCCAGCAATTAAGCAG
GTTATTCGATCACTGTCACCAGTGAAAGCCCCATATCAGTTCAGGGAAGTAAGGTCAAAG
CTCATCATCCAACGGTAAGCATTCGGCACAGGTTCTTTTACTAATGCTCCCGGATAAGTC
TGCTTAGGCTGTATCTTGTTCTCTGGTACTACTTTACCCTCAGCTTTCAGAGATCCGAAG
ATAATAGCATCCCATACTTTCATCGGGTTATAAACATGGTTAAATTTAATCTTTGCATAG
AAACATAGGCTATACACCAGATCGATAAATCCGCGCTTTTTATCAATCGCCAACACACGG
TCAACGTCGCCGATGTTATAATCGATGTAAAGTTGATGGTTGTCATCACGCAATTTGTTC
AACGGACCATCATAATTAACCATACCGATTCCAGTCTCCTATTCAGCAACATAATCTAAC
GCATATGTTGGTTGAGTCGTAAAGCTAACTTTCTTGTACAGGTCAATATAATCAAGGCTT
TCGATCCCCATGATCGTAAATGCTTCAAGCTCGTTGCCAAAATCATCCTGTGAGATTCGG
GATTTAATCTTACCGCACGGGCTTAGACGGTTAGCGGTTCTACTTCCGAACTTATTACTG
ATACGTTTAACGATATATGGAATATCGAATGTTTCTGTGTTCCAGCCAGTGAAACCAACG
GGTCGGTTTTCCTCAAAGAATCGGATGTATTCTAACAGCAATTCATATTCTGTGTCATAG
ACGTTATAAACAACGCGCTCTACAATATCAGGTCGAAGAATAGACTTGCTAGATTCCCAG
ATTTCACCGTTTTTTCCTTTCAAATCAAATACAAAGAAACGATCATGTACGGAAACATAA
TGCGTAATTGCACCGATAGGATATTTCGCTTCTTCTGGTTTAGGAAATTCTGGGGCAGTG
ACCTCGATATCAACCCATGCTACACGGATTTTATTTCGGTCATATCGAATCTCGCCTTTA
TACGTATCAGACAGATAGGAGGTGATAAAGTCATCCATCCCTAGCGCCTCCTGCCCCATT
TCTCCTATCTTTTTACGCCGTTTGTTGGCCTCGTTCATAGATGGGAATTTTTTAGCAATG
CAAGGCTTGGCATAGATATCTTTATAATTCGTAACCATACCAGGCGCGGCATGATGAAAT
AAAGTTGGCGTATATTCAACGTAACGCTCTTGTTCTTGTCCAGCATCAACAATATAACGC
TCGTAGATCTTATCACCCACTCGCACACCTGACAGAAAATACATAATATAAACTCCTTCA
CTTACGAAAGACACTCATTATAGGGGAATCGACTTAGTTTGTCAATACCCCTATCCCATC
AGTATCTTAGCGTCCGATCTGATATTTCTCTATCAGCGTCCACTCTTCTTTCTGTTTGAA
AGACAGTACACGGAAATTATTAACTTCGGTAGTTTCAAAATCACTATCAATCAGGGTACA
CAGATTCCACTGCGCAAGAAGTTTTGCAATGATATTACGGCGTAATTCATCTTCCCACGT
CATTTTAACACTACGACCATCTAATTTCAACAACTCCTTGAAATGAACGATGTAATAGCG
CCGTTTCTTCTGGAGAATATGACAAGACTGATACAGTGTCTTGTCTCGATTGTTGGCAAT
GCCGCAGCGGGTTAAAGTTTCCTTAATTTTAAGGAAGTTTTCAGGACTAATCAAATTAAT
TTCAATCATTTTACCACCTTTCTATAATCTTTTTAAGTCGTTTCCTTTCTGTCTGATTCT
TAGTAACACCCGCCAAAAGTACATCGGTCGCATACGGTTTATTGTGCTTCAATAACTTGT
CCATATATCCCTTTTCATTAGCCAAATCGAGATAAAACCGGGCGTCATCCATATTTACAC
AATATGCTTTCGCCACTGCCATTAAAACCAATTTTTGGTCCAATTCTTCTGTTAGTTTAG
CCCGTTTACCGTATCGTTTCCCCTTAGAAATACTCTGTAAAAGATAATCAAAATGCATTT
GATATGGGAGGCCATGACCGTTTAAATTCATCACATACGCGCTATATAGGCAATCTGCGT
GCTGGGCAAGAGCGGTGTTAATCCATCTCTGATTATATGCAGAATTGTTTCCCACATTAA
CACGCTGTTTATCTAAGGTGATATGATCCATGAGAGCAAATAATGCATTGTCGGTTTTCT
CGGTGAATGTGACAGCTAATGCAGACACCTTTTCCCAATCACGAGAGCGCCATGCAATTT
CATGCTCGTTTAATTGTGTATCATCTTCACCGAAAAATTGACCTAACATTATACGAACTC
CAATTCAACCATTAATTGCATTAACAAAAAGACAATGAGACTCTCTTGATCAGCCGCGTG
CGCAAACATCTGATTATTTTCACCGATAATCTCAGTGAGACGAATAATAGAGGCTGGCTT
AACTTGAGTATACAACTCAGTATAAAGTTTCTTGACAAATTGAGCGTATGTATTGGTATA
GCGTGGTGCGAGTTTACGCACATTAGCAAACTGTTTAGTTTTAAGTGCTTCCACCACATC
GGTTACTTCGGTATTGCTACTTTCGATAACCATGCTCAATACGCCAGCATGAATTTTACC
CTGTTTAGCGTAGCGGTCCAAAAGTTTCATTACGCGCCGGAAGTCTGGGAAGTTCTGCTT
AACCAGTTCCGCTAATACGCGCATTTCTTCTACTGGCACACCTTCTTTATCAAGAATTGC

TTTACACCGGACGATCATTTCTTTCAACATGCGAAGCTGATCATCTCTAGTCGGAGATCC
AAACGTGATGGGGTCAAAACGAGATCGTAACGCTGGGATAATACCTTCTAGATCGTTTGC
TGTGATAATGAATGTCACATTGTTTGAGTATGCCTCAGAGAAACTACGCAAGTGTCTTTG
AGCGTCAGCCAACCCCGCTCGGTCATACTCATCGATAATAACAACTTTGCCCTTGTCTCG
TTTTTCTTCATCGAGTGATACACTCTGCGCAAAGCGTGTAATCTCATCGCGGATAAAATC
AATCTTACAATCTGCGCCGTTAACAAACAGATAGTCGACACCCACATCTTCGCAAAGCGC
CTTAGCAACGGTTGTTTTACCAGTACCCGGCGAGGGGCTATGCAGGATCATAGAAGGGAT
TCGGCCTTGCTTAACAATCTGCGCGAAGATCTTTTTATCTGCTTCCGGCAGAATACATTC
AGAAATTGATTTCGGACGGTATTTCTGTTCCCACTGAAACTCATTTTCATTAACACTCAG
CATATAATCACCTCTCACAAATTCCATCATTAAAATGGGGAGTATTTACACTCCCCACAA
ACATTATATTAGTCGGTGTAGCTGGCGTCAAGGTCACACGCTATCATGTAAGATATGGTT
TCACTTGCCAGCCATACAGCCTTACGGCGTGTGAAGCTCAGTTTGTAATCGCCGAAGTCC
GTAGCAACCAGTTTCATGTTATCTTTACGAATAAAGAAATGGAAGTCTGCGGTTCCGGTA
TATTCGCCCATATCAACACAAAACATCTCATTAGGATTATTTTTGTCAATCTCTTCCCAA
CCTTTGATCATCAGACGACCATCAATCGGTAGAAATTCAAGGCGATCCACTTTAAGCTGA
TTGCTCATTTTCATAAGCTGTCTCAGATCGTCGTTGCTCAGTTCGTAATCTACATCGCTT
GCAGGTGGAGTAACTCGGCCTTTCGGTGATACGATAGCAGCAGCACTAGCACCAGGATAA
TTAATTTCCAGACGATCATTAGACACACGAATGTTTCCGGTGCGGTTATCAACCGTAATA
TCTGCTTCATCGCCCACCAGATTCAGAATGCCCAGTAACGAGTTAAGATCATAGATCCCG
ATTACGTGTTCCAGTTCTTGATTCAGAGTCGCTTCACCATATACAACGATTGTCATGTTT
TTGGTGATTAGGACGTTTCCTTTTTTCAGGATGATACCTGGGTTAATAGCGGTAAAGTTT
TTCAGGGTAGCAATGGTTTCTTTAGTCAGTTTCATCAAATCACCTTTATCATGTTAATCA
ATTAATTACGGGTTTACTTCGTGCTCATTTTGAATCTTTAGCATTCTTTCGATAAATTTT
TTCTCGACTTCCTTATCGGATGAGTGAGTATACTTCAATCCAATGTAAGCTGTCAAGTAT
TGTTGCAGAGCATAGACAGTAACAGGATTCTTTGCGGTCTTTTTTCCGTCGAGATATACC
AGATCACCACGAACATCAAACGCAGGTTTACGACGAATCACATCATCAACAAATTTATCT
AGGACAAAATACCACTCAATACACTCTATCCCGTGACGGTAGTTCATTACATCATTATCT
TTGATATAGTCAAACGGAATAGGACATAATTTACTATTTTGTAGGATGAATAGCATTTGT
TCTTTTAAGCTCATACGTTTCATTCTATCACTTCCCATCAGCAATTGCGATAAGACGAGT
TACTACTACGCTAGCGGCTTCAATCAGCAGTTCATCAGTGTCAATCTGATAACCCTTTTC
GAGAATATCTTTACCTCGAATAGTTGTAGCGCCTTTAGCAATTGAATCATCGTAATACTT
GCGCATTGCCGCGATCCATTCTTTTTGCACCATATCATCAATATGCGCGTACAGTTCAAA
GCGTGCTTGCTGGGGGATAACCTTCATTTTAGGTTTCTCGCTTTCTGGCATTGGTAGTAC
CCAAGCTTTACGGATCTTGCTCATTGCTCGGTCAAATTCGAGACTGGTTCTCTTGTTCTG
AATGTCTGCTACATTAATTTTAGCCAGGTTTGCGGAAGTAGTCATAATATTAATCTCCAA
AATAAAATCAGTATATTACGTTCTAAAGTTTGTTGCAACCCCAGCATCAGGGTAATCAAG
TTTATCAAAGTTGGGGACCAGCATAAACTCATGATGTCGACAAGACAACATTTTTGTTAT
GTCCGGATCGCTGTATATGCTGTTTTTCATATCCCGCATACTAACTACATATTCTTCACC
GGAATATAGATTGATTAATGTATACAATTCTAACTCCGGCTGCGTTACTATGTCAAATGT
TAGCATTCATTTCAGTGAATCGCCCACGTTTAACCATCTTGAGATGACGGCTAAAATCAT
CAGGGTTATGGTCACGATGGCTGATAATATACACGTTATCATCTAACTTGTCAATGATGG
AATCAAGTTCTTTTACGCCCTGAACGTCCGTAGCCCCATCATAAATTTCGTCGAGTACCA
ACAAGGAAAAATTAGTACCAGATACTTTACCAGCAACATCACGCCATGTAAAGAGCATCG
CAAGGTCAATACGTGCCTTTTCGCCTTGACTGAATGATGTATACGAAAACGATTCACGCC
CCCGTGATTTGATTGTTTCGATAAACTCTTCATCTAGCGTAAAGATATCATCCGCTTCCA
TGATATCAAGATAATACGCAATCCGATCATTGAAATATGGAATATATTTCTTGACGATAG
ACGCCTTAACACCGGAATCCTTCAACATTTCTGTTACAAATCCACGGTGATATTTTTCAA
GAACTAGCATTGATTTTTCACTAGTAATCTCTACCAGTTCGTGTTCCAGTTTAGCAAGTT
CTTCGGAATTGTCAACCCTTTCTTTTTGCAGTTCCTGCATTGCTTTCTGGATCTGTTTAG
CCCGACCTACAGATGCCGTTAAATTCGCTCTCTGTGACTCTATAGCGCGTTTAATTTCCA
AGACCTTAGTTTGTGCTGTCTGGAAAGAATTGGCCTTCTCTCCGTATTCTACGCGTTTCT
CGTCAATCAGAGACAGTTTATGTTCTGCTTGTGCTTTTTGTTCGCGTACAGCTTGATCAT
TACCTTGAATGATCGGTTGTGTGCACGTAGGACAGATCCCGCCTTTCTCGTGCATATGGA
TAACACGGTCAAAGTTCTGGATCTGCGCACCAATGTGTGCGATAGCCGTATTAATTTTAG
CCAATGCCGCGCTAGGATCTTCCGGCATAACAACAGCCAATAGCTCATCGGTCAAGCGCG
TTTCGTCGGCTTTAACTGCTTTAGCCGATTCTACCTCTTTATCATACATATTCTGCAAAC
GCTGTAGATTCTCGGCACTAGCCTTGCTCGCGTCCTCTATGTACTTTTTATGAGTGTTGA

TTTGTTGATGAACGTGATTAGCTTTCATTTCCTGGATTTGGATATTCTGGGTAATCTCGC
GCACATACGCTTTATTCAGTTTATCCATTTCGCCTAGCGTAGCAATATCAAGCAAATCCT
CGACCAGTTTACGGCGACCCGCTGCCGGACGACTCATAAACGGAACATAACCAGCAGTGC
CTAAAACAACGATCTGTTTAAAGCTGGCTAGGTTCATTCCTATGATACCTTCAAAATAAT
CCTGAAAGTCTTTGGTGCTTGCGCTCTCGTCGATCTTTTGACCGTCTCGATGCACTTCGA
ATACTTCCGGTTTTTGACCACGTTTGATATAAAACTCTTTCGCGTCCAGCTCAAACCAGA
CTTCGACTAACAGTTTCTTTTTGTTGACGCTGTTAATCAATTGGCCTTTCTTGATACTAC
GCAACGGTTTACCGAACAACACAAAGCAAAGCGCCTCGATCATGGTCGATTTACCAGCAC
CGTTAACACCAGTGATTACTGTCTTTTTGAATCCGTCTAGCTGAATCTCAACAGGTTCAG
CACCCACGCTCATTATATTTTGATAGCGCAAAGTTTTAAAATTTCACTTTGCCATTAAGC
CACTCCGCAATCACACATTCCATAATATTAACTGCTAACACATCTTCATCAATGTTGTAA
ACACTTGCTGCCTTCACTTTAACTAATGTGTCTCTGCGATCAGGCAGCATGATCACATAA
TCCAGTGTCGCAGGATCATATGTCATAAAGGGATCAACATAAATCCCTTTATTCAATGCC
AGATATTCGCGAAGACATTCTATAACCCTGTGTCTAAGGTAGGTCATCGATAGAATCATT
TTAGTTTATGTTCCTCTAAAAATTCTTCAATTTCACCTTGCAGGTCTTCCGCTTGCTTTT
CCAGAATATCGCGAAATTCACCTACAGTCAAGCGGTAATCACATTTCACCAGTCGATGCG
GGAGAATATACCCTTTATACATTGTCTCTGAGGCTTCTAGGAATACCCCACTATGCATGA
CAGAGGTCCAACCTGTTAACGTCAATGGAGTATTCGGAAAATAATACTTGCTGGATTGTT
TCCACTGGACGAAAGCACCATATACAATCGGATTATATTCACCAACCACACTCTTAATAA
TGGATTCTACTTCGTCTTTCACAAAGTCGCCAATGCCCATAGTCACCATCAATTCTTGTG
GGTGAATCACTTTAAGGTAACGACGGTAGCCGTCATCATACCACAGCTTAACCACTGGCT
GACCCCACATTTTAGCAATACGCGCTTTCAGATAACCATAGATCGGATTCTTGTAATATG
ATTGTTTTAGTGTGTACGCGTGCATCAATTCAATAAAATATTCCATTGCTATCGAATCAG
GATCAGCCGCGACCATAGTCGTATTCATAATATGTTCCTCGTTCAATTTCATATTGTTTC
AGGTCTGCTAAAAGTTCATCCACTGCTTGTTTCAGCACATGACGCAGCGTAAAACAATAG
CTGTCATCAATTTCTATTTCCGCGCGAGAAATAGATCGAATCTTACACTCAGCAGTAGTA
TCGGTGAGTCGCTTTTCGACCATCATCCGAACTTCTTCATGCTCTTTGGAATACCATACA
TCAACGGAGATTCCCTTGCGTCGAGCAAACATAGGCAATGCCGCATTAAATTCCATTACC
TTTTGTGCAAGTGTTTTCATCATACAGACTCCATAGACCAACGAGCAATAACATTCTGGG
TAATATCAGCAACCTTACTGGATACGATATTTGTTAGCCCCTCTTTGGTGATATTTTCAC
GATCAAATGGGATTTCGTCATGAGTCCACAAACCATAGTTAGACCGAAATGATAGCATAT
ACTGACCATCTCGCACAACCTCAAATACATGGATCTTAGTGAATTTCGACAAACCTTCGT
TGATTTCTTTCAGTGAATTAGCAATCATAACAACCTCCAATCAATTTTTAGAGAGTATAA
CGGGGATTTCTCCCCGCGTCAACTAACTTTTTGCGTTATTTACTTCGGCTAAAAGTTCAT
CAGAAATACGCTTGACCGCCGCAAGTTCTTCCGGTTCTAGGTTTTGTTCTTCCAGATATT
CATAGACCATCTGAATAGTGCTTTTAACTTCAATAACACCTGTTTCCTGTGATGCGCTAC
TCTCCAGTTTATTGACCACCTTTAGCTTGTGTAAAAAGGCCTCTAACGCGCTTTCTAACT
TAGTTAACCCATTATCAGCGACCGTTGCGATAAGACGCACACTAAGCCCTTTAAAGCGTT
CTACATCGATTTTATCGTCTGACGGGTAAGTGCTACGCACATGATAAGTTTAGTCATTCT
GAATAAACTCTTTTTTGTGTGTATCGGTATCGAATACCCAAAAACCACGCGGACCGTTTA
CGTCACCCGCCGTTAGTGTGTATGGTGTACCAATATACGTTACGTTACCGCCGTCACTGA
TAGTATGAAAGTGTCTCGAAATCACTTCACGGTATTTCTTGAGGAAGTCCGCGCTATAAC
CTTCGGATTTTAGCCCTTTATAGAAATAGTATCCAGACAATTCCCAATGGCCTACAGAAT
ATTCGCTGAATGAGTTTTGAATAAACTCTAAAATCTCTGATTTATTGTCATCACAAAACC
ACGGGAACAGGTCAAATGATACACCATCAAAGGTAACTGGTTCCGGTTTATCGATAACCT
TAATGCAATCATACTGGCTTAACACTTCACGCGGTGCGTTAGGCTGTATCTTATGACGTT
TGAATAGATCATGGTTCCCTACGGTCACATAACAGGTGATTCCAGATTCTTCCAGTAACG
GGATGACCTTAGTACGGTTACAACTCATCGTCGCATGAGTAATAGCTTTTCGGCAATCAA
ACCAATCCCCTGTTTGAATCCATGTCTTAATATTATGCTCTTTGGAGTATTCAACCGCTT
TCTTGATGGTATCAAGAATATACTCATCCACCCACGGATCATCGCCACCGATACCAGTAT
GCAGATCACCTAAAAGAAAAATTTTCTTAGTCTCTTCCTCTCTGCGTTTTCGATGCTCTC
ACTATATCACAGAAAAATAACAAGGGAAACCCCTTTCAGAGGCTCCCGTATAAAAGCCAT
TACAGCATACTTAATATCGTTTGCCGTGTACTGACCCACATAGCAGAAGTTAGTTCTGGC
GTGTGTGGCTGTTTGTAGATATCACGTAATTTTCCGATAGCGTAGATCTACGCATCACGA
TTATCATCGATGATAGCTTGTTCAGCCCACCGCTTAAGTCGTGCAATCTCTCGCGAGTTC
TTACGCAAGATCTTTTCCGCTTTCTTAGCGGCTTTAGCCTCTAGTTCTGCTTGTACTTTG
TCTACTTCATTCACTGCAATCTTGTCATGCTGTTCATCGTAAGTCATGTTATTTTACCTC

TGGGATATAATCAATTATTAAAGAAAATTCATCTTCTGATACATTATAGCTATGTGAGAT
TGTACCCGTCAAGACCTCAAGATCCGGTGATTTGATATTGCACGGATAGATAATCACCGG
AACGCCGCAATCTCTTAAAAATAATTTGGCGAAATACGGCACGAGGTCAGTTACAAACTC
TGCTTCTTCCAGCAAACGGGAAATGTTAATTTTCAATCTCATAAAATTGGTCCAGGCTAG
GCAATTCCTCAGTCAATACATCTTCAACCTTTGGCGCTTTCGGTATATTGACCGATTCCT
CGTAGTTGTTGATTTTATCGTAAATGTCCTGTATGAACGCCTCATCAGCAATCTGGCACA
TATCAGCGTCGCGTACGTCATATACATGATGCACAAAATATTTGTATTTTGGGGCTGTCG
CTGTTCTCTCTTTTTTAATGCGCTGTACAAAAGCATTAAAGCACGCTTGCGTGATGTAAG
CATGAGGATTTTTATACTTTGTCTCGTCAAAATTATTCAAACCCTTGACCGCCGCTTCGA
TTCCGTCGTCGATCATTTCTTCGCGCCATGTTTGACTATAACCAGAAAAGTTAAAACGAC
GTGATAGATTTTTAGACATTTGCATAATTGCTAGGCCGATTTGATCGCTCATTGGAACTT
GCCGACCTTCTGCTGCAATTTGTTGTTTCCAACGGCAAATTTCTTCGTAAAGCCCTTTAT
TGTTTACATAATCAGACACTTCGCACCCTCGCTATTATTTAGGAAATAACCCTTTCGGGC
TATTCCCCATTGCCATTATACTTTCTTTGCTTGGTTATCCACACTCTAACCATCTCTACT
GATTCACCGTCAGACGGATCAAGAGTTTTTCCTTTTTACTTCATGCGATACTTAAATCCG
TTAAATCTTGCATTCTCTTACTCGTTAAAGTCGAACATCTTTTTAAATGATTTTCGCCCT
TTACGCTCACCACATTCTAGCGGAATCATTCGGTCTGTTATTACATTGGCATTAGGTAGA
AACGTTTTTAGATAATCTACCGACGCCCAATCAGGAACATATAGATTAACATTCTCCTTG
CTCTCGTTGTCTCTCAGGTTCCAACGCAAACATAATTGAAATGCTGGTTCCAGATAATCA
GACACATGACGCGCTTTCGAAAACTCGTTTGTCGGGAGATCGCAACTATTAGCAAGACCT
TTTAAAATATCAATCTCCCATCGTTGAGGGTTAAAGCTGAACATCAGAGTAACATTTTTA
TGATGAATGTAAGAGTTAAGACCATAAGATCCGTAAGGTATCCGCTCACCAATACCATTT
GTGCACGCTTCTCGGTCCCGATTCACGGTATAAATGAAGTCATCGCCACATGCATTTTTA
ACCAAATCAACAAGACGTTCAAAGGTATTGCTTGTTTCTGACTGGTGACGCGACCAGCGT
TCGAAGTCAGTCATTAGGATGATGTTAACTATATCTGTATTTTTAAACTCGTTGATATAT
AGTCTGGATTCTAGCGCACTCGGAACAAAATCGCAATTTGCATAGTATCGTAGATACTTC
CCGCTATATGTATCAAAGAGATTTGCACATGCTATCGTAACCTTTTTAAAGCCTCCCCAC
GTACCGTGTTTGTTATCGTTCAAGAAAACAATTTTCGACCCATTCTTCCGGTGTCCTTCG
TTATGTTTTACCATAACGTTGATTTTCTTCTCTAATAACGCCTCATAAAGCCCTCGAAGA
GAAGAATCGATCATATCAGTACCATCCTTTGCACTTTCTATCTTTTCTTTGATAGCGGCC
TTCTCAGAGTCTTTTAGAACTAAGTTCTGTAGGCAGTCCGATTCGTCGACGATTGGTTCG
CAATGCTTCGTGATAAGTTCAAGATTAGACTTAAACGGATACTCATTAAATTCAACCAAT
TGAGGAAATTCATCAATGTAAAGAGCATAATCACCAAACATTTTTGAACGTTCCATATTA
GATAACGCTGTATGTGTAATGAATATCACATCAGCACCGTCTTTAACTGATTGCATTATC
GACGCTTGCACTGTTTTAAGGCTTTTTAGTCTTGTGCTATCGATCAAGACAGCACGACCG
CCCATTCTTTTAAACTTATTGTAAGATTGTTCACTTAAAACACGAGATGGCGAAGCAATA
ACCGCTTTTTCTTTTGTCTCTAGGACGTGGGAAAGTATAGCATCAGTTTTCCCCGCCACT
GGCAATGCGATTAATGTTTCAATTTTCATTAGAAACCCTTACGTCGTGCTTTCTGGCATT
TCTTGATCATTTTATCAGAAATTGCGCGCTTAGGTGCAATCTGTTTCACACCGAAAGCAC
TAATGCCAAATTCAGAGAGAATATATTCAACTAACAATTCACGCACACGCGCTTTACCGA
TATTCACGCCGTTATTTTTCAGATATGTCTGGCCTTCGACTTGCATCAGATCTAAATCTG
CGTCGGTAATATTGATTTCTTCCATGCGCCCAAATCGAGAATAAACCATGTGTGCGATTG
CATTGTGTACTAACATAATATATTCCTCACTCAAGAATTACTTAATGATACTTACTTTAC
CAGATCCGTGACAATGAGCACAACACCTATCACCACGCAATCCTGTTCCGTTACAGTGTT
TACACTTCTCCAACGGTAAAGCCATTATAGCAAACAAAGCAATAATGAAGATCGAAACTA
AATTCTCAATAAGCATAATTCTCCCTAAGTTCTTTATAGCCGCCAATGTGCTCGTTGTCA
ATGAATATCTGGGGGTATACAAATCGGAGGCTCTTTGAGTTCAGCCTCCCCGCCAGTTCT
TCTATCATATCCCGGGCGGGAATAATGTCAACCCAAACCTGCTTGAATACTGGTTTAAGT
GTATACGGAATACCCAGATCATCCAGTAGCGTCATTGCACTAATACAACCATAACAATGA
TTAGCCTCTTTCGGTATGCCGTATATCTCGATCATTTCTCAGCCTTTAATCGTTCTAGTT
CTTCTTCCAGTTCCTTGATCCGTTGGTCACGTTCAAAGTCACGCTGCAACTTAGTGGCGA
ATACTCGTTTAAACTGATTGTAATCATCGATTTTAAACACTTCTTCTTCGCCATAGCGAC
GTACAGAAACTTCGAAGTTATTGAAGAATTGTTGCATCATTGCTTCGGATGATAGTTCTG
CCGATACTACTTTAAAATATTGATCTTCTGTAGGCGAAAACCAATTGCAAGCATAAAGAT
AAACCTTTTCATTGTCTGGAGACTGGTAAGCCTTACCATCGATTTCAATCACAGAAACAT
ATTCAGGTATAGTAGATGAATCAATAGCTAAGACTTTAAACGAACGGTCCCCGATAAACT
CGGAGATCACACGATTCGCATCACTGGTTGCAAATACTTTACGCGCTTATTCGTTTTTCC

ACTGATAGGTCATACCAACAACAAACATATTACCAACCTCGCTCCTTACCAATCAAAAAT
TCAACTTCACGACGTTTAATGTCTTCTTGAAGGTCTTTAATCTTCTGGCGATCTTGCATT
ATTTCGTCCATCTTGTCAAGTGCTGCGATGAAATCATCGAAACGTTCGAAGGTTTTACCT
TCATTTGCTTCGCCAAATTTAACCATTGTCAGGCCGTAACGACTACTAAACAGACGTTTC
AACTGTCCAACGATTTTAGAATCAGTGGTTATAACTTCCAATTCTTCTACTTCACGAAAG
AATTTTCTATCAGAATCAATAAACCAGCTAGTTCCCTTATGGTCGCTATCTTCTGTTGCG
CTCAGTAGAGTATGGTCTTTACCATCAATTTCAATTACATCAATTTGCCCACATGGACTA
ACATTTTTAACAGTAAAAGGTCTATAATTTATTGCTCGAGCAATAACATAATTAAGGTTA
GATACGTTTTCAGCATAATGGCAGAAGTCTGTCATAGCATTCGCATCTTTCCAAGTATAA
GTTTTACCAACTTCCCATTTCTGGGGAACTTCTTCAACTTCACGAAAGAATTTTCTATCA
AATGGACTAAAGAAATATGTTGCAGTCCAAATGTCTTTTCCTGTAGCAGTTTTCGCCGTA
TGATAAACACCGTCTAGCTCTATTGCATCAATCCAACCGTTACCCTCGATCTCGATGATG
GTAAATGGCTTGTCACCAATAGCTGCACTAATATCGCTGTTAAGTGGAGAAAGAACACCT
TCTCCGTTAGTAATAAACTCAGTTTTAGCGTTTTCGTCTTTCCAGGTGTAAGTTTTACCA
ATTTCCCATTTCATAATATATTCCCCTTAACCGATTTTATCAAGTTCGTTTAATACTTTC
TCTGCTTCCTGTATAGCTTGCACCCTTTCCGGCATTTTGTCAAGAAAATCTTTCAACTCT
TCAAAACTGGAGATATGACGCAAGGCGTTAGCACCGATAGCCGCATGTGATTTGCATTCA
AAGCGGATTTTATAATTATCCATCGCCAATTTGAGAAGATCAATTAATTTAGATTCTTCT
GGAATAATAATTTCTTCAAATCTGCTACGGTCATGATCCAGAAACCAACAAGATAGACCT
ACATCGGTTGAACTCATATAAGTTCCATCATGAAGATAAACAGACCGCACGTATCCTTTT
TTCGCATCAACCGACGCAACTTTAAACGGTAATCTCAGATATTTCAAATAATCAACATCA
TACCATTTAAAAAATTGGTTTGCATCTTTTACATCTTTCCACGCATAGGTCTTACCAACT
TCCCATTTCATAATATCATCTCTCATTTTTGATACGTGCTTCTAATTGGATCATATGGTC
GCGCGTTTGCTTGTATTCGCGGATAGCACCGATCCGGCTAGGCATACCATCAAGCACGCT
CTTAATACCTTCCAGGGTAGTAATTTTGGCAATGCTATTATGTAACCCCATCTTATCGAT
CAACTGAATGTCGTAGGCTTCCATAAGCAGCCCCAGGCGTTCTAAAGTTAGGTCTTCTTC
TTTTTCTTCCGATTCTGGAATCTTAGGTTCAATAGTAACTTCGGTAAAGAAACGTCGCTC
ACCCGACCAAAACCACACACCGGAACAGCCAATAGGGGATTCTTTTAACGTCATAGAGTC
GAAATTACTACCAATAATAGTAATTTCTGTAACATCACCATCATCGTCAAGGTCTTCTAC
CCTAAACGGGCGAGCAGCCACGAAGTCCACAATACATCTGTTAATAGACGACGAAAATTT
AGACAGTTCGGCTTTCGACGCTTCGCATTCAATCTTGTAAGTTTTACCAACTTCCCATTC
CATAATAATCACCTCAAATAATTTTCTCTGGAATACTATTAACAATCTTGTATTGTACTT
GATTGTCAGACCCCCGCCACGGCTTCTTCGTTGGCATATCTTTAATATAACGCCCATCGA
TGAACATGTCAACTGATTCTACAATTTTTCTTTGCTCATCATTCAATTGGTCAAGAGTAT
AACCGGACCATAGCCAAATATCTTTATTTGGCATCCATGATTTTACTTCATCGATGATCC
GCTGTACACCCTTTAAACTGCGTTTATACAACGGATCACCACCTAAGAATGTTAGCCCCT
GCATAAAATCAGGTCTAAGCAGATCCAGAATCAATCCCATTGTATCATCGGTGAATGGTT
CCCCGTTCTTAGGATTGAGTAGGCTAGCATTAAAGCAACCATCACATGCGTGTGAACATC
CGGTAACGTAAAGGGAGACTCTCACTCCCTCACCGTTAACCATTTCACAATCTTTAATAT
CGGCATAGCGTAGTTTGCCGTATGTATCAGAACTCATATTCAATTACCCCGAACTCGTTT
ACAATCTGCGCTTTATAGTAACCACCGCTAGATGCTAATAGAGAAGCTGGGCTTAGGTAT
TCGTATTCACGGATTTCTACTTGTGCGTTTTCAGTTTCCCAAATCAGTAAAGTCCACATA
TTTGATTCTCCTGTAAAGTTGTATCTCGTCTTGATGAATATAGTATAGGGGATCTCGCGA
TCCCCGTCAAGCATTTATTTAAATTTTTCGAGAGATTTCAACCACAGGCGGCCTGGTAGG
TACTCTCCGGCAAACATGAAATTGTCATCTGGATAAGAACGCAGCATATTCAAAATAAAC
TTCCGGCACTCTTCGTAATCTTTACGTTTAAGATAAATCACATTCTCGCCAGTACCGATC
ACACCTTTTCTATATCCATGCTCTTTACGCACAGACCATTGTACAGTATCTTTTTTAACC
CGAATCACAACAGGTTCCCAATACCCAGCGCATCCGGCTACACCACACGAACAGGTGAGG
AAGTATTGACCTTTTGCTTTGTTTTCCTCCCACCCGATATTAACCGGATGCCATTCGTGA
TACTAGGCTAAAGCATCGTGTGTCGCTTCGATCTGTTTATTGTCAATTCTTACCGTAACC
ATCCATACAGGGATCTTATTTGTTGGTTCATCATCATAACATGAATCATCATCAATATGT
TCTAATGTCATGTCAACATCAATTGATTTTGCGTGTTCCATGTATCGTTGTGCTTTACGC
TTACGATTCAATAATTTAGACATATTCACCTCACATTATAAACGAAATTGTACGCCATCA
AAATGAGAACTTGCATTACAGCTATTATAGATAATAAATCCTGTATCTGGCGCTTGTACT
ACAAATTGACTTGCATTAACAGCGGCAACAGTAAAAATACGATCCGTGAGACTATGTTTA
ATTTCTGTCCCAACTTCCAATGGGATGTACTCTTCCTCTTCGGCCCCAACACCAAAACAG
CCTTCTTCATGATACGCCTGAATCTTTTTATCACAATTACACGGTTTAGCAAAATCAAGC

GTATACAATTCACGAGAGTTGTCGGTATAAACCAACACAGATTGATTATGATAGCGAATC
ACATACAGGTTATTATCTTCGTCAACGACACAAAAACTACGGATTTGTGTGCCTTCGCCT
TTACTCCAGCAGATATTAACAACCGTTCCCATAATAAAGAAACTTTCTTTTGTACCAAAG
CTAGCCAGTGTGCTAGCGTGCATTTTAACAACTACACCATCACCAATTTCTAACTTGCGA
ATTTTAATTTCTTTATTCAATATACAAAACATAATGTTGTCCTCTCTTGTTTTCGATGTG
AGCATTATACACGAGCTAATCCGGTTGTCAACACCCCAAAACGAAAAAGCCGGGCCTTTC
GGATCGACAAACAATTACATGTGCTTAACGCGGTTTACCATCTCGGAGTGTTTCCCGTGG
TTACATGGACGCAGATCAACAGATCCAAGATATCCACTAACGCGACGGATAACATTCATC
TTTTCCAGATTGTCATTTCCGCAATCAGGGCAAACAAAACCAGACTCGGTCGCTCTCATT
TCCTTATTAGATCCGAATACATGACATACATCAGAAGGAGTATTAACACCGAAGTAGTGC
AATTTATCCATTGCATAGTCTACTACAGTTTCAAGACCTTTCAGGTTGTTTTTCATGTCT
GGGAACTCAACATAAGAGATCATGCCACCGGACGCCAGCCAGTGATAATCTTTCTCAAAG
TCGATTTTCTCAAATGGTGTAACTTTCCGGTCTACGTCAAGGTGGAATGAGTTGGTGTAC
CAGTCTTTTTCGGTAGCACCCTTAACAATACCAAATTGCTCTCTGTCCAGTCGATTAACT
CGATAGCAAAGGCTCTCGGCTGGCGTACCGTAAAGACTAAACCCAGGTCCGGTTTCGTCC
TTCCATTTTTCAGTATAATGACGCGAGATCTTAAGAATATGTATACCGTATCGCTGCGCA
TCTTCCGAATCAAACGGATGAACACCAAACAGGACGCTCAGTACCTCATGTAAACCAATA
TATCCAAGAGAGATACTAGCGCGACCATCTTCCAGCACTTCCAAAATTTCATCGTTTTGA
TGAATACGCAAAAATGCACCTTCTGTATACAGTACAGGAGCAACATTGGCTTTAGCCCCT
TTTAAAGCGTCTACGCGACTCATAAGCGCATCAAAGCAAATAGGCATACGATTAGCTAGC
TCTTGTTCGAATAGCTCAATTCTGCGCATTATAGACGCTTTACCAGCACGACGCGCGGCA
ATAAGAGCAATACGCGGAAGATTCAGGGTCACAACACCCAAGTTATTACGTCCGTCTAAT
ACTTCCTCGCCTTTGTCGTTATGCCACACACCCAGGAAACTTCTACACATTATTTGTTCA
GGGTAGGTCGCTAGTCTACCCCCGCCTTTCGGCTGCTACATGTCGCCATGCAGATCAGAC
TATATCTTCTATCTTTCGATAGTCTACCATTTCGGATCACTTGATCCTACGCCGCTACAT
TCATCACGGCTAGTCGTTAGGCATTTCATAGCGGAACTCATATTCATAAGTATTGGTTTT
TTTACCATCAAGAATTGCAGATACATTTTTACGATGCAAGCCCAATGGTTCAGAAAGACT
TCTCACGCTTTTAAATGTTTGCTCTTGTCCGGTTTCTTTATGGATCGCAGTAACCTTATA
TGAACGACGTTTTAACTTATAGCACCCGTGTTCGTAACCTTCTTTCGTGTTTTCGCTGTT
AGTCCCCCACTCAAGGTTAGATGCATCGTTTATAGTTTTATCGTCGTTTTTATGTTTCAC
ATATCGATAGCCGTTAGGATTCGGAATCCAACATTCAGCCACAATAACATGAACACGCAA
ACATAAGGGTTTCTTGTCTCTCCTTACCCTTACTAATGTATATCCGGTATTGTTTTCTAT
CCAGGTACTCAACTGTTTACCTTTAGGAGAAAACACCCGTCCATCTTTCGCTACCATATA
TTCGGTATCACGAATTTTTCTAAGTTCCATAATATATCCTCTTATACACATTGGCGTAAA
GAGAATATGACCGCTATGTTTAGCACGGGATTGTCTTCTAGAGAGTTCCCCCGTTAAGGC
AGATTTTAGATGAGCTGTTTAAAGTTTACCCATCGGGCTAACCGGAACACTAGATCCAGC
GATGCTACGGTTATTCTTCGCAGAGATATAATCAGGGTAAAGACGCTTACTAGGACATTC
CAATGCATGTTGCTTAATGTCGTAGTTCGGATCTTCCGGTTTAAGGTTTAATCCTTCTTC
TAAGAACATCACCAGCTTAGGAAACACCGGAGTTCTACCGGCTTTACCTAAACCTTTAGT
TCGAACTTTCAGGATCGCTTTCTGAATCAGTTTTTCTTCCCAGCTAGTACCCATACCAAA
GGTTAAAGTCACAAAAGGTTTTTGACCGTTGGTAGAGAACAACGTGTTGATCTCACATTC
CAACGCCTGATATCCGTCTAACGTTTCTTTTTCTGTTTTCTCTTGTGCATAGAGAGTAAC
AGCTTTGATTCCGTAGGTTTCGGCATCAGCAAGGTGTTTATGATATGTTTTCTTAACATA
CGGCGCTAACACCTGGTCGATGTTTGCAATTGTGGTCCCGCCATATTGATGACTTACCAC
CTGTGCGATAATTTGAGCGGTTACAGCACATGCAACACCGATCGATTTAGGACTTTCGAT
TTGTGCGCTACCCAATTTAAAACCATTCTCTAACATGTCTTTGAGATTAACAAGACAGCA
GTTAGTAAAAGGCATAGCTGGTGCATAATCCATATCATGATAATGAATATCACCGTCTAC
ATGCGCTTTATAGATGTGCTCAGGTAACCCGGTTTTAGCAATATGTTTAGACACAGCGCC
AGAAATCAGATCTCGACGTGTCGGGAATGTTCGCGCGTCTTTGTTCGCGTTCTCTTTGCT
TACTTCGTTGTCTTTACCCGTTGCGATCTCTGTAATGTCTCTGATTAGTTTTTCCATTGT
ATCACCTATGAAAAAGCGGGATTTCTCACGCTCGTTGATAGTTATTTTACCAGTTCTGCT
AATTCTTTTTCTTTAGCTTCTAGTTCAGCTTTAAGCTGTTTAATCTCTTGTCGCTTCTTA
TTAGCGGCTTCCTGCTTACTATTTCGGATAACCTGATCTAGATACAACTCCCAACCCTGA
ATATTTTTGATAATGTATCCGTCAACATTAATTGGTCGGAGATTCTGTACAGCTTCCAGT
TTAGTTGCAAAAACTTTAATTGCTGGGGTGAAGTATTCATATTCGTTATCTTCAAACCAA
TAACCTAGACTTGCATTCTCAATACGAAGATGAAGTTTAAGATCAAGGTCTTTATCATCG
GAATATAGGTCTTTTACACACCCCATAGAATTAACAACAACTTTAAAACGAATTTTATCT

ACACCACCAAAAACATCTTCAAGAATATCAGCAATTGAGCTATTAGCGCAAGAATGGTCG
CTGAATGCGTCAAGGTTTTCGTTAGTTACAGTATACCAAACATCTTGTACCCACATAATA
TAATTCTCTCGTTGTTAAGTGGGAGTATTATAGCATACTCCCTTCGCATGTCAAGCACTC
ACTTTTTCCTTTACTGCTTTCACCTTCTGGCGAATTACTCGAATCTCTTCGATCTCTTTT
TCAGTCTCTTTAACAATTTCTTTGCGATTCTGAATATCTGTATCGAGATTATCATCAAGT
TTAGGCAGCATGTCAATCAAGAACTTTTTAAAGTCTTTCAGGTTTCGGATAACAGCACCT
TCACCCATTACCGCCACACCTACACCGTCCGGCAGATCACCAAACAGTTTAACCAGTTCA
GACAGATGATCATGGTCTTCTAATGCTTCCTCCGTTTCAGACGGCAGTTCAACAAAGTTA
TAACGTTCATCATTGCTGATCAAGTGTTACCAGCAAAGCCCGAAAGATTTCGGTGTATAT
TCTACACCATCAATTTCAACCGCGTCAACACTTCCATAATCATCTACTTTAGTTACCTTA
AATCCTTGTCCCAGCTTTTGAACAATTTCAACAACATGACTTGATACAGTTTCAGCGGTA
AACCAATATGGCAAATTATGCTGATTTGGCTTGTAGCGTTCACCTACTTTCAATCTCTGA
CCATCCGCTTGCATAGCATGAAATTGTTGTTTCGTGATTTCCGCGAAGTATTCAAATTCG
CCACCATCAAACACACAAGCCCATTGATCACAAAAACGATCTGCCTTAACAGTTTCACCT
TTCGCGTTGATCATGGTAGCTGATCCGTGCAATTCTTCTTCAACTAAATAGCCATATTCG
ACAATAACAACAAGTTTCATAGCTTCTTTGTGATCTGGATCATGCTCTCCCCAGAATCGC
TTAAATTCTCGCAAACCTTCTGGTGTAAACGAATAACATTTACCTTATTCAAAGTAAGTC
GGCATTGCATCCTCTTTCTTGTTCATCGCTTCGTATGCCTCTTTTGAGATGCGTTTAAAG
TATTTACGCTCATCGTGCATAATCATAGTCGCCCAATAATAAACGATATCCTCAGCGGAA
TAAACTACCTCTCCAGCTTCAATAGTAATAACATGACCGTTGTCATCTTTTTCTAATACT
TTAAAGCCCTTTTCGACGATTTCAGCAACCCGAATATGACCACGGCGCGAAGCATTGGTC
AGAATAATTGAATAGCGAGAAAAGTCTAGAAACTCATCTTTACCACCTTCACGGAATGCA
TAGTAATGACCTTCAAAGAATTTAGCCATAATATAATCCTCTCGTTGTTTAAATGGGAGT
ATCTTACTACTCCCGTTTCATCATGTCAAGCGTTAAATGACTTTTTGGATCTCTTTCAGT
TCAGCTACTTTATCGGCGCTGAATTTAGCGTAGGTATCATGTCGAATAATTTCACTTTCA
AGAAAGGTTTCCAGCCGAGATCTATTTTCAATAAGGAACTCTTTCAGATCTTCCGCGTTC
TTGATAAAGATTTTATTGTCAGTAATGTCATCTTCAATCGCAAAGGAGATAGAGCGAACT
TTACCAAACAAGTTAATCAGATCTTCCAGCGATTCTTCTTTCTTAACTGGTTTCATTTCT
TCAAACTGCTTTTTAGTGATGCGTTTAAAGTATTTGCACTCGCTTGCATCAATCATGATT
AACGCACCCTCATAATCATGAGTTACTACCAAATACACTTTACCCATAACATTAACCGTA
AGAACTTTAAAGCCCCTGTGAAGCACACCATACAGGAACTTGTTATGTTGTGGATAGCGT
TTAACAAATTCCTCTGCGGACTCAGCATTACGGAAGGTGTAATAACGACCTCGTTCAAAA
ACAGCATCATTTGAAGACTTGGCTGGTTACAATTTATACGTTTTCTGTACCATAATATAA
TCCTCTATAATTTCACTGATAGAACAGCACGAGACAGAATGCGGTCACACGGAAGAAGCA
TAAATTCCCAACGCGGATCAAAGTCTTTAGCGGTATATGTTTTCCCATCAACCGAGATTT
CACTTACATAACCAATAGGTGACACTTTAAGAACAGTAAACCCATTCTTGATGACTTCGT
AGGTATCTTTCAATCCTTCGTAGTCATCCTCTAGGAAGTCTTTACGTGCTTGCTCAGATT
TAAAAACATAGGCCACACCGGATTTAAACATCTTACGCATTGTTGATTCTCCTTCTTGAT
TAGATGGGAGTATTTTACTACTCCCATTTCTTATTGTCAACTACTATCGTAATCTTTGTA
TAAATTGTTTCTTATACTTCTTCGTGATCTCTTGACTTGTATCAGCGTCACAGTAATCGA
AACCTTCTGCGTCCATGATAGCAACCATTTCCGCGCGTGTCAAGCGTGAAAATCGTTTTA
CTTTATCAACAGCATATTGAGGATGTAAATGATTGTTGGTGTAGTCTCCTTTGATGTAAT
TAACATAGTTTGTAGCCCAAACAACGAAATCAGCACCTAAAGCACCAACACCAAACCGTT
TAAAGTCTCCGGTCAACGATCCTTCTAGGCGGTTGCAACACGAATGAAGCAATCCCCTTA
CACGGCCTTGCCCTTTCGCATCTAGATCGTGGTCGTGGTCAAGATGGTTTTTGCTCATAT
CCTCAAAAAGTGGAAATTGACAAAGGGGGCAAATACCCCCCTGTTTTTCGAATAGTTCTT
GTTTGTACTCTTTAGCACTCTTAGCAGTCAGTAACATAAATTCACCCTCCAAAGAAGGTA
TTTAACCATGAATCTTGTTTTTGACTACAAAGGATCGGAACTCATCAAAGCCACCAATAG
AATCACCATCATAGAAAATCTGCGGCATGGTGGCAATATCTTCTACCCCTGCGCGATCCT
GAATGTCGGCAATGATAGAATCAATCTTTACAGGGCGACCGTTTTCGTCCACACCATCAT
TGACAGGAATATAGATGTAAGGGATCTTACGCGATTCATAAAGGCGCTTTGCATTCTGGC
AATACTTACAGTTGTAACCCTCAAAACCAAAAATTTCTACCATGATGTACCTCAATCTAC
GTTAGTTACATCAACTTCTTGTGTATCAGAAGACTTACCACCTGAACGATCTAGGCAAGA
GGTCTTATTCAACAGGACGCAGTTTAAATCATTCCGCTTGATAAAGTCAAGGAGATGATC
ATTTACCTTTCCCTTATGATGTGTGACATTACTGATACCAAACCGAAAACCACGCTCCAT
AATAGATTCTACCCACTGATAGAATTTAATCTCTTCCTCGTCACTCCAGAATTTATTGTA
TTCCGCAGAAGTGATAAGGTACGGCGGGTCCAAATAAACGAAATCACCTGGCAAGATCTC

GATCTCGTCATAGCGACCAGACGTAAAACTGATTCGACCGTCTTTGTTTTCTTCCTTAAA
ATTCAGGACACGCTCAATCGCGTTTTCGTTAAAAGTCCGGCGACCAAAATTAGCATTAAA
TTCTCCCTTCCTGTTATGGCGGTTAATGTTGCTAAAACTGTGCTGAATCAGAAGAAACAG
AGATAGCGGATCTGGATTCTCATTATACTTCTTACGGAATGCCATGTAAGCGTCTTTATC
TTTGCGAGACAAACCATTCTCGGCAATAATCCGCTTGATCGTAGAAACCGGGTCAGGACA
CTCGTCAATCGCTTTGTACGCGTCAATTAAGCGGCGATCATAGTCATTAGCCAGCACATC
CCCTTCGACGTACAGAGACACGCTCAAGCCGCCACAAAAGGCATCAACAAAGCGGTTATA
GTGCTTAGGGAAATACTTACTAACACCAGGCAATAACTTTTCCTTATTGCCAGTATAGGG
AATTAAACCTAATGCTTTCTTCATTATCGCTTCCTCAATTAAGTTCTCTCGATCTCTACC
AGTTCGAAAAATTCTTGCCAATCACACGGAAAATTGCAGTTTCCGAAATGAACCTCTTTG
TAAAGTTCTTTACCGGATGGCAGCTTAATATCTGTTACGCTGTAGTATCCGCAACAATCG
CAATCACCATCTGTATATTCTGTTTTAGTCGGAATATCCATCATTGCCAGATACAAAGGT
ACATCGTCTTGCTCAAAGTTCTTGTAACCTTCATGGTCGCATGAAACAAAGTTATCATCA
ACACCGAAAGGGATATAATGGTCATAATCGGTTACAGGGTGACCGAGTAATTCCCATTCT
ACAATTTTAACATTACCACTAAGCTTTATACCCATAATTTCAACAAGAAAATGAGTATCG
TCAATGTAAATCTTACTAGGGGTTGGATAAACCATAATATAATCCTCATACCTCAGAGAA
TAGAATCTCTGTTAGCTTCACTGACTGCTCTCAGAATGTTAGCGTATACTCGAAAGGAAA
GTCAATACCCAACTTCAAAAGAGAATAGCCTACCGCCTCTTTATGCTTATCGGTTAGGGC
GATCCTAACCAATTCCCGTTGAATCTCTTTAGGCGGTGAATGGATAGTAACAGGGGTATC
ATGCAATGTCCAGTCACCATTACGAATCAAGATTAACGGTTTATCGTCATTGAAGATCAC
CGACCCCATTCCGGTCGGTGAGTGTTTTATACTAATCATCAAATTCTAAGAAATATTCTG
GAACGATATAACCGTTAAAGTCATTACACTTATAAAAATGGGGTGCATTTAAGGCTGGAT
GAGCATTAGTAATGAGTTTACCCTCTAGAAACAGAATGTAATTAATAGGTCTAATACCTA
CAGAATCAGGGTCTAAACCTTTTTCTTTCAGTTCAGCCAGCGGTAAAACTCGTCCAGATC
GTAACTTATCCATGATTTACCCCCAATTCATCAAGGTTATGTCGCAGCATTTCAGAGTAT
TTTTCTACTTCCTTAATCTTAGTGTGGATCTCCACGATCTCATCTTCACTATCAGAAAGT
TTAATGGCAATAGCGGTACTTCCTTGACCTGTCTTGATCGCTTCTTTCAGTTCAACCAAC
ATGTCCGCGTGTTTCAATAGCAATTCTTCACGTCGAACATAAGCGACGGTCAAAGCCTCA
TCAGCGATTTCTAAAGCATTTTCAGCAACACCAACAACCATTTTACGAGTAGACAGCATA
AATTCACCTCACATTATTTCACGTTAACGCCGTCATCAATCCAGCCGACGCTATTTGGTT
TAACAGTTTTACCATAGGATGCATACTTAATCAAGCAGGGTTTAACAGCATTATCAAATC
TTCCTAAACGTACTCCCAAATCAGGAATATCCATGTATGCGGCTTTATATCCCCATGTAC
CGTATTCAGAATCAATCTCAACAGGACGGCCTTTAAAAGCATACACTACACCGTGACGGT
CAACGATAACATAGCGATAGCCAGCCGGAAGATACACAGTTTCGCCGTAAACTTTAACTG
GAGTAGCAGAAGAGATAAACATAATATAATCCTCACATCGTTCATTTGATGTGGTCATTA
TATGATAACCGCATTTTCATGTGAACAGTTTTTTAAAAATCTTCTTCCGGTTTATATCGA
CCGTCATCGACTGGTTTTGGACTGAATCCTTTATGCGTGCGCGGCTTCGCTTCCGGTTTA
CCAGCAGTCATCAAACCAAAACATTTCAGCCCCTTATAACACATAACATTATCAACCACG
GTCCAAGTTATAGGCCATACGATTGCAGCAATACAAAATGCTCCTGTATCGACCTCTTTG
GTGTCCTTGTCGAATCTGGCGTACAAGCAGAATACTAGATACGAGCTCATTGCACCAGTA
GTCCAGATATAAAGCACATTAACAATATCTTGTGTTGTCACATTATCTTCCTTACATTTC
TTGCAGTGCATAAAAGCATTTTTCTTCATAGTAATAGAAAATGTCAAAGTATGCTTTTTC
TACATACTCGCCACTAATCCAAGCGGCAGTAACAATCAGAGTGGTCCAGTTAGATCCGCT
CTCAACGGTAAAGGTTTTAATACCCAGCTTCACTGCCAAGTCGAACGCAAACCGGACGCA
ACCACGGCAGGAAGTGATATTGGAAGTTTTGATTTTAAAGCCACCGCGAGTTTTAGAAAT
TTCTACTTTAGCGAAAGACAGGTTGCTCATTTTGAGTCTCCTTAAGAAAGTGTGTTTAGC
TTTGATGTGTGTAGTATAGGGGTTACGTGATCCCCTGTCAACAAATTATTTATGATTTTA
GTGAATTAACCACTACATCAAAATGATGGACAGAGCACACCTTATTAACCACATACTCAT
CGTGTTTAGGAATAGCGCCTAACCGCTTTATTTCGCTCGCTGACACGATAAACCGTTTAT
CAGGTGCATTTACCTTACATGCTTCAACAACGCACGGGAAACGCGTTTCAGAGAGTTCTG
GACAGTTTTGTGAATCCATCAGGACAATGAATACCCTGTTACATTGATTGTAGAATCCTG
TTTTTGATTTCATCAAGTTTTCTTCTCTGTACAATTCGTGCAATCTTCCTTTCATGATTT
CGTTGGTTCTTGTCTTCACAGTTTACCCATTCAACAAGATCTTTACCCAACTCGAAAGCC
CATTCTCGGTCTTCCTTTACGTTTTGTAGGTATGGACTTTCTGACTCTCTACGAAGTGAT
GTTGTCGTAACAAGAATGATCCCAGGAATGGTGGGATGTTCCTTTCCATATACAATAGGA
CGCGAGTCACTATCAATACCTTGTAAGAAGGTGTATCCTGAATCACTTAGAATCATATAC
TCTTTCATTTCACACATCACAGTTTCCTCAACCAGTTATTAAAGCGAGCAAACCATGACG

CTTTTCGTGCTTCTTTTACTTTATGTGCACGAAAACCACTGAAACACCATTCATAATCAT
CACCGAAACATGATTCAACAGGAACACCCGCATTTCGTAGTTCTCTGGTCTTTATTATAA
ATCCTTGACATAAGGGTCGACCTTCAACAATGATAGGAAGTTTAACTTTTTCTATGAAAG
TATACCCAGCACGGTCAATAATTTCAATTTTCATTTTACATTATCTCCATAGATCTTTCC
AGCAATATCAATTTCACCAGCGCGAACATCTAGGAAAAGATGCGGTAGGTGTATGCAGTT
TTCCCACCCCACTCGCTTCATATCTGAAACCGTGACGTAAGCCCATCCGTTCGCTGTAAT
AAAGTCTACTTCAACCTTGAGAGGAAAGCAAGACTTTTCTATGTCTTCAAATCCGATAAC
GTGTTTTGTCGTTGAATATGGAATTTATCCGCTGATCATTTTGTGATCTCCTTATACTCA
ACACACCATAAGCAGGTAGCCCATGATAAACGCTCATAACGCTGGCAGAAATCGGACCAT
AGACATTGTGCACCGTTCGGCATAGTTGAAATATGCGTTTTCTCTCCAACTTTCACGAAA
TAAGTATTCGATTTTGTGATCTCGACCGGAGTATATCCGAAAGCGGGTTCCAACTCAACA
TCTGAACTAAATGCCAATTTAACAGAATTTGTTTTCCTGACCGCTGTCCGGTTTCCTTCT
GGGTACAGCACAGCAGGTTTAGCGCCTAAGCGGATAAACTCAGTATTTTTAATGAACAGA
ATAGCATGATAACCGTCATATTCCGACACTTCCATAGTAGCAGGAAAATCGACATGTGCT
AAACCTGGTAGATCTCGCATATCATGAAAGGTCGCCATCAACTTTTTCTTTCTATATTCC
TCACTCTTCTGTTTCATTTCTTTTATATGGTAATATAACAGGATTAATACAGCAAGCACC
AATTACGTTATGAGCCCCACAAGTTAAACATTTATAGTCATGGAACTTATTAAGGAATCG
ACCAAACTCATAACCGACTAGCGCCGCGTGTACTTGAGTCTCGATCTCATCCATATCAGC
CAATGTTTTAACCTTCATAGTATAATCCTCCAATGGTAGTTTTGCGAAGATGGTAATTAC
ATGCTTTTAATGCATCAGTCGCGGTGTCATAGCGAGTATTATCATAGCACCAGTAACCAT
CTGTCAACTGATAAGTCAATCCGTCCTTTTTAGCTTCAATGAAAGACAATTCTTTGAAAG
TTTCTACTTTCTTTGCTGATACTCCACGGTCACGGCTTTTATAGAGTTTAGCTAAAATGC
GTTCAGCTTGTTCCTCTTTATTGCCAGACAGATCCCAGATGATGTTTCTTATATGCTTGA
TTAATTGTTCAGTGGTCATTTTTGATTCTCCTTAGTTGGTATGCGTGCATTATAGCATTG
TGATTATCTTTGTCAACACCCAACAACAAAAAAGGCTATCCCGCAGGATAGCCCATAAAA
TTACACGCGAGCACCGTAGATCGTGCCTACTTTATCCCAGCGTGGAGCATTACCAGTAAC
GGCTTTACCAGCAGCACCACCGTCATATTCTGTACCGTTCCCCTGAGTATTACATCTTCC
TCCAGCAGCACCAACGTTACCGCCCGCACCGCCTGTATATGCACTAAGAGACCCCTCACC
AACAGATCCTTTACCAGGAGCGGAAATATTACCAGCAGTTGCACCGGAACTCATATGAGA
GGAAGACCCACCAGCACCGAATGGGCGACCACCGCCACCACCAAATACTAGTCGACCCCT
GTTACCGCCGCCACCGCCGCCACCGCCGCCAGCGATAGCACCGTTATTTGTAATTCGTAG
ACGTGTACCGATAGCGTTATTGATTGCGTTTCCACCAGCAGCGCAAGCAGCGTTACTACC
ACCATTACCCCCACGTCCGTAGACAGTCACACCGGACTGAATGATCAATGTTACATATTC
GTTTGTTAACGAGCAAGGGAAGTCAAGACAAGGAATGCCAGTAGATTGGGAAACAATATC
ACCAGTGATAGTAATAACTCCAGGAGTAGATCCTTGTGCTTTCATCCAGTTAATAAGAGT
GTCTTGATTGTAAGAGTTACTAGCACCAATGCTATAATGAATCTCTTTAGAGCGTCCGAC
CATTGCCGACATATACGCCGGACGACCTAAGCGTACAGCTTCCATTGCGGCACTCATCCA
CCGTTGACCAGTCTCGGACACAGCAGATGATCCCACCCATCCAGAAGATATTGCCATAAA
ATTTCCTCACAAGAAAAGCCCCATTTAAGGGGCATTGATTGATAATATTACTTAGATGAT
CTCTGCTTCACCATCCAAGAAGAAAGACCATGTTTTGTTAGGATAGCGAAGCGCCCATGA
TTCGGCAGTTTTAACACCAGTTTCGAACAACTGACGGCTGGTCACTTTAGCCATACCATC
TTCGATGGCAACGTTTTCAATAATCATCGGGTACTTAGCGCCACGCATGAAACCGAAGCC
AGCGGAAGATTTGATAATTTTAACAGTTGCCATTTTTGATTCTCCTAGAAAGTGTCGTGT
TTCGATGGGTGTATTATGCAATAACACCGGATCGCTGTCAACACTTTCTAAGAAAAATTT
TTAAACTAATCGAATATAGCCCAGAATTTCAGCACCTTTAGCCAGTCCTAAAACTTCCCA
TTCAAGGTCGCCGTAGTCATAATTTGCAACCATCAACTGACCATTTTTCATGCAGCAATC
ATGAGAGAAAATTTGCCCCGCATAAACTACGATAGTAATAAGCTTTCCTGATTCTGGCGG
TGTGGAAGACAAAGCGACCATATGACCATTCAGCTTAGTAACCAGTGATGGAACAATAAA
ATTCGGGCTAACCTTAATACCGTTAAATTCGTTTGTGTCTTTCATGCCAGACCTCGCAAA
ACACTAATAGCATAATCATGCTGGTTATTCATAATATCAATTGCATTGTGATAACGAGCT
AAACCATCAAGGATAAGTTCTTGTTTATCCTGTTTCAGTTTGGTATCACGCGCAGCCATA
AGAGCATCAAGAACGGGTCCAAAGTGATTATCTAAATCATCTTCGGTTGCAATGTTTCCT
ATTGCCTCGTCTATCCGTGATTCTAATGCTTGCTTTTCGTTGATCACATGCTGGGTATAT
TCCAACACATCAAGACTATCGCGGGTAGCGTCGCCATGATAAGGTATTCCCGCATGAAGA
TGATCAATTAGTCGTTGAATGTCAATGTTCATTTGTAACCCTCCTCTGAAAGCACATCCC
AGATCGTTGTGTCAAGGTGCATTAAATTCCACATAATAGAGTCATAACTACCGAAGCCAG
AAGTAAAAATTACATCACACAGGCGATATGTCTTATGAAAATCGGTGGCTGTCTCTGCAT

CAATAGGCATATCGTCACCTGACAGCACCAGACATTTAAGAATCATAACTCTGTTAGCCG
TTGGCGCTTGCTTAGTCTTTACAGCATATTTTTCGATGATCCGTCTTGCCTTTTCTTCTT
TGATACGTCCAGACCAACTCCGGTTTCGGTATTGATTCATAATAATTTCAATCACATCAT
TTTGCATTCTTCACGGTCTCCATAATGGTTTTGTAAGCCAGTTTAAGACGATCACCGAAA
AACTTTGCGGTGTTACCCTTCGGATCGCGGAGCATGGTTTCTACGATCTCATCGGCAGTA
ACAATTTCTCCTTTCTTTGCTGCATGTTCTACCATATACTTCAAAGATTCTTCGGAAGCA
AGTGCTGCGGCTACTGGATTAATCATTTTTGATTCTCCTTTGTTGTCGATGTGTGCATTA
TGCTATACCCTATTCCGGTTGTCAACACCTAAAAACGAAAAAGCCACCCTTTCGGATGGC
TTTTTGATCATATTTTATTCAGGTCCATTTGTATAGTAGTGTTTGAATCGACCTTTGCTA
AACTAGTTAAACATAGTAACCAATTCACCATCCCTTTCCAATGATAACCCTTCCGCGTCT
TAGACGGGTCTTCCGATTCGGTAGTAATTGAACACGCGAAAGTATGAGGAAGCCAAATAG
TTGAGTTAATGCTAGTATTCTTTTACTTCTGGTAGGCATCCCACGGTGACTAACATTAAG
CCAGTCGAGTTAGTTTATAAATTGTTTTAAAGCATACAGCTTTGATATCGTCTAAAACGC
TCTGTAACGAGCTATCAACATCATTATAAATCTCACTAGCCATTTGCGTTAACTCTTGCA
AATAGGCAATTTCGCTAATCTCCTTGCGGACGACCAACTTTGGCTGATAACGACCAGTGA
TCCCAATATTGGTTTCAGTGAAATTATCAACCAATTCTTGCACTTCAAGATAGAACTCTT
CTAGAGCCTTATGTTTTGCATAAGATCCAGGACCAGTTACCCCAAAATGCTCGGCATGCA
TATAAGCAACACTTATCAGACATTCACCGATAAAGAGGTCGACTTTATTAGCCTTTCCCT
CTTCGGACGTCTTTAGAAAAGCACTGAATTTCATGTTCTAGATTCTCCATATTATGCGAC
AGAACAAATTGTTGTGCGGCATATCCCCCCATAATAAAACACTGCCTAGAATCCATACAT
TTACTACGAGCATACATCTTCCTAATATGGGCATGAAATTTCTGGCTGGTTTCAATGGAA
GGAGATATGTTGTTTACATATACTATCATTGCGCCGTCGAAATAATCAGTAAAGTCACCA
GTTGGATCATATTGATCAGCACCGACACTTCCGACCGCAGTCAAGCATATTGCCGCTGTT
AGAGTTTTACGAATAAATGACATAAGTCATTCCCCCAGATTGAAAATATTCAGTTATATT
TATAATCTGGGGCTTTATTTATGATTAACTCTTATTTGGTAGTACCGTCAATAAAATGTT
TACTACACACAGAAACATATTTATCCTCTGCACCAACTTCTATTTGAGCACCATCGCGAA
TAACATTACCCTCTTTATCTACACGAAGGACCATTGTTGCTTTCTTGCCACAATGACAAA
TACCCTTCATTTCGACCAGTTTATCAGCAAATGCCATTAATGCCGCAGATCCTTCAAAAA
GATTGCCCTGAAAGTCAGTGCGAAGCCCATATCACAGTACAGGGATATTAGCATCATCAA
CAAGCGCACACAAGCCTAAAACATGCGCTTTAGTCATAAATTGGGCTTCATCTACAAATA
CAGCATCAGGACGCCAGCGATCCGGTCTACGCTTACCTAACGATTCACGAGAAACCAAAT
CGAAAAGATCATCCGATTTAGTGAAGGTGATCGCTGGGGCACTTAGGCCAATTCGGGAAA
CGACTTCACTTTATGAATCTCGATTATCAATCGCTGGCTTAAGAATTAATACTCGCATAT
CACGTTCTGTATAGTTATAAGCACTTTGTAACAGAGAAGTTGATTTGCCAGCGTTCATAG
ATGCATAGTAGAAATATAATTGCGCCATTAATTAATCTACAAAAATCCAACAAGGCCTAA
ACCGATATAAGTCCAGATACCAATAAAAAAGAAGTTAATGCTCATGGCAAGGATAAACCC
TATCAGCGTTTTGGGATACCCGACCCAATATTCCCGACCATCTTCAAACCGGAACAGTTT
ATTGCCTTGAGAGTCCATATCAACCACAACATGTTTATTTCGAGATTGCAATAGATATTG
TTTTACAATCTCAACACCCGCCATAATAACAATAACGAAAATAAAAGTCCACACAACAAG
ATTAATGATAGTCTCAAGCATTTTCTATCACCTCCACCCAATCAGGTGTTTCCTGAAAGA
CTCCTTCTCCTTCATGATGGTCGCAACCTTTATTGCTACATTACATCCACCAATCCCACA
TTCCCCACGGAAAACGCTGCATATCGTCGTTACAGCAAGTGAAGATAATAGGCCAACCAC
ATCTAACACATCGGCAGTTTTCTTTCATTTGAGCGGTATTCGGCTTCTTCATAGATGACT
CCAATATAATCGACATTTACATTTAGGGCAATGCGGTTCTTCAATATAATACCCGTCTGT
CTTTGGAACACTAACAGGAGCGGTTTTTATATTGTATTCAGTGAAACAACGAAAACAAGT
TACGTTTTCTACAGACTGACGTTTCACCCAATCACCGGAATATAGATCATTTTTATTTGT
CATATTCAACCACCACAATGTTAATGTCTGGAGTTGCAATATTAATGATCTCCTCTACAC
ATTCCCACTCAAGGCCACCGATACCGCAACCGATTTTAGGAATATAGAAAGGCTGTATAG
CACCTTCAAAAACATCATTGATAGTCTCAAATCCTTTCAGGATAGCACCATATTCAGCTT
TTGGACCCGGTCGGAATTGGGTATATAGATTCACCCCGCACCAGTTATAACCATTAACTT
GATGCGTCCAGACACTAAACCCACCCAGCTTTTCTACCTTACCGCGAGAAGTTTGCTTGT
CAATCTCTAACGCTTGCGGGAACTCTTTCGCGATCTTTGCTGCAATACCAGCACCCATAG
TATGAAAACGATTACAACCATGAATTAAATGATCGTTTCCTTGTTTAAACATGGCGATGA
GATCGCCCTTTACAGTATCACTAATCATAATATAAACCTCTCATTAGTCACGGGAAGACA
ACCCAGCCATAAAACGGCTTAACAGTGTGTCGCGATAAGCAAAGCACAATTGCTTATCAG
GGGCAGTATCACAAAGGTTGCGTTTTGTTTTAGCAAAGCTGGCAATACTCAGATCCCACG
CTCGATTTTTTCGTTTTTCGTCTCGACCTTGTTTGTATGCTTCGTCGAGTTCAAGTGCGC

TAAAATCACTACACATGTTATCATCATCACAGAAAGTTTTAATGATCTTTTTGTTGATCT
CAAATCCTTTGTATTCGAAATTTGGATAAGGGCGCTCATAACTAGCAAGCGCACCAGCCG
AGAACATCATAGCAATCATAGCAATTATGGTCAACTTTTTCATATATTCCTTAATAGCGT
TTAGTCTTAAGGATGCCTTTACTCTTTAGATGGTTAGCCTTTTTTAGTACACCATCAGCA
TAACCTAACCCGGCTTTATAGTTCCATCCAGCATTGTAAGAAGCTAGCGCGAGTCGGAAA
TTACCGTTTCGAACATCCAGCCAGTATGCTAACTCATCCATCGCGTAGAATGCAGAAACC
TCGCGATTCATGATAAAGTTGATAGTCTGGCGATCACTCATTTTAACACCGCGCTTTGCA
TTTTTCGCTTTCACCGTTTTAAGGTAATTTTGAAATAATCCATCAGCATGATGATTTTTC
TTTGGTGCTAAGTTTGCACCCGCGCTTGATTCTTGCCATGCGATACCCGCCAGGCTGTGA
CCTAAACCCTTTCCGTTATAGCGAATATTCGGATGGTCCTTTTTCAACTTACCGCTTTTC
TCGAATTGCTCGCCGTATTGATAGGCGAAATGTAAATTATCTAGTTGGTGTGCTGTAAAG
TTGTCTATCGGCGCCTTTTTGATCGCTGCGCTAGTCGATGCGTGTGTAGCACCAACGGCT
AGTGACAGACTCAATACTATCGCTTTTAGAACTTTTAACATTAATTTCTCCCGTTTGGCT
TAACTTTCTGATTCCATAATATAGCCCTATCACCAGAAAGTCAAGCATTATTTTTAACCA
ATTTCCATTATTGGTCTACCTTCAAAACAACCGCCGACATTTTACCCGCCAGTCGTTTTG
GATTCTCTATAATCATTCGGCAGCATAGACCGTGATATGGCTTACCCCCTTCGATTAGAT
CTACTGTCATCCCGATCCACATGTTACCGTCAGTAATTTCTAATCGATTCGGTCGGTCTT
CCATTTTCAGGAAGTCGAAGACCTCCGGCAAACGAGTTTCTATCTTATTGAATAGTTCCA
TTGTCTTATGTTCATCGAAAAGACGATCGGTTAGACGGTCGATCATATGCGGACTAAAGC
GAATGAAAATATACTCACCGTAACCATCATTCAAGCACTTTTCTTTGATCTGCGCATTGG
TTTTGCGCATTTCGGTTTCGAAGAATCTACGCAGCTTACTACGGCGAACATGTACGTTAA
TTGTTTCCATTATATTATCGTTTCTTAAACAAATTGCGAATCCAGTATACAAACGGGATT
TCTTTAAACTTGAATTTCTCTATAGTTAATTCTTTAGTAGCAAGTTGAATACCATAAGCA
TCATGTGTAACTATATGCCATTTTCTATAGCCCAAATGTGAACAACCGTCTGGATCTAGG
TTAATAAGAGTTTCTCGCTCACTAGGATTATTTTTCCACAATTTTGAAATAGGATCGCAC
ATAATTCGACCTAGCGTACCATGAATAATCTCACAACGGTAAAGTTTTCCAGCAGTGAGA
TAGGCGGACCCGTCATAGTCGGTATAAACAAACACTTCTTTAACACTCATTTTAATACTC
CTTTGTTGTTTCGATGGGGGTATATTGCTACCCCCGATCATGTGTGTCAACTACTTTTTC
AAGATTTTTTTTGATTGCATTCCAAAGACCTTTATTTTCAGGTTGTTTTGGTTCTTCGAT
CAGTGTCCATGTTCGACCGTCTATGTGTGCACAATTTTTAAGGCAAATAAAAATCTGGTG
TCCCTTATCAGCACGGATATAACCACTATTCGCAAATCTTTCATGCGGTGTATATTCATA
CAGTGTATTAGGTGTAATATACTTGCGGTTACTATAAGGTGTCATCACATACATCATGGT
ACTAACTCCCAACGATCACCGCGAAGATGAGAACATCCACCATCAAGAAGTATCGTAATA
CGATAACCTATATCATCTCTGATGTAACCGATTGTCGGTGGATTTTCGTCGTCTCGTGCC
GGGGTAAAATCATACATCTTTCCGGGCGTAATATCTTGTCCTTTATTGGTATTGGTATAA
CCTTGCATTTTCTTAATCTCCTATATTAACTAACCACGGGTATAGTGTTCCAGAACAACG
GTCAGATGAATATATTCTAATAGATCTTTCCAAAAGTCAACATCTGGTCCTTCCGGCATA
CCGTTTTTCTTTGCCTCTTCACGCGCGAGTTTAACACGCTCGTAGAGATCTTTCAATTCT
TCCCGCACATTATCAAAGGTTCTTTCCCCTAACTTACACGCTAAAATAGAAGAACGTCGC
GCGAGCGGGAATTGTAGATCACCTGTCTGATAGATTTCAAGTAGCTGATAACCAACATGC
AGAGCATGAGAAAGATCTTTCCAGTTACTGAAATACTTTTCAGCATTGGCTACCTGCTTT
TTGAGATAGCCAGACAAACCCCACATACCAACATGATAGAATTTATCTCGATGTGTTCGG
ATGTATTCCCATTCTCTGGAGGATTCAACCAACATGTTACTAGGCGCGTGCACCATATCA
ATAGCGCCGATATCGCCTTTACATGCCAGATTGATAAACCGTTTTAGACTGAACATATTA
ACGTCAATGTCGTCTTTGGTGTCCTTGATCCCTTGACCGCTAGTGCTTTGATCATAGTGT
TCTTTCATCGATCCTAAGATAATTTCTTTCGCAGTCGGAACAAAGATACCACGATAATCT
TTATCGGAATTTTCAGTATCAAGGCCGTATAAGTGAGATCCGAAGACCATTTTAACCATT
GTATGCATAATTAAACCCTCATTAGTATTTTACGTTACGTCGAACGCCTTTTCGAATCTG
ACAAATAGCAGACGGTGAAACATTATATTTTCTTGCAAGTTCTACACCCTTCATATGGGG
AGAAGATAAAATCTCTTCCACTTGGTCGTCGGTAAGTTGTGCGTTTCCGTGGTTCTCTCC
CCAATTTTTGTTTAATCCTTCTTCCTTCGCATGAACAAGATTTCGGGTAGCTGTCGTCCA
TTCAAGATTATAAAGGTCATAGTTTTGTTTGTTTCCATCCTTATGATTAACTACACGGTA
GTTATTTGGATTCGGAATAAACGCAACCGCCATAGCTTTATGGATGTTCACGGTTTTTCT
AAATCCATCCGGCTTATAACATACTGTAAATTTAGAATAACCAGTTTTACCATAAACACT
AATATAATTTCCGGTCTCCGTATTTCTAACCCTACCATGAGAGCTAATCTCAAATTGAGT
GTAGGGCAACAGCGCATCAATTACTTTCCATTCTTCGACAGGATGGTCATCACAATATCT
TTTAATCATCATATCACCTCATAAAAATAAAGCCAACCCTTTCGGATTGGCTTAAAAATT

TACATTGCTTCCGTAAAGGTGCGCGAAATAACGTCACGTTGTTGGTCTGGAGTCAGACTA
TTGAATCTTACGGCATATCCGCGAACGCGAATGGTTAGTTGCGGATACTTATCAGGATTA
GCAACCGCATCGAGAAGAAGATCGCGACTCGTAACATTGAAATTAGCGTGCTCACCACCT
TCGACAACCGGAGCACCATTATCTACAATCACCGGACGGAAGCTAGTTGTGTGCAGTGCC
AGAGAATCGACATCTTCTGCGGCGATCACATGGTCTTCTGCGAATGTTTTACTAACAACT
ACTTTAGCGCCGATCAGTGCTATCGGTTCGGATACCAGAATCATAGGCGCGAAGTGGCCA
CCAATAATAGTACCTTTATGCTCACCTTTCATGATTTGAATTGCTTTAAATTCCATAATT
CACCTCTTATAGATCAGGACCTTCGTCATTAATTTTACCAACCAAAACACCAAAGAGTTT
ATCAGGTTGGACTTTACGATTCTTTTTAATTGTGGCACGGAATCGACGAGCTACGCTATC
TTTATTTTTCATTAGTTGATACCTAAGCCAATGAAAATAGGTTGGTCTTCTACATTACCC
ATCCCAACACTAAGTAGATTCAATTCTAAAAACTGCAATACGCTGTCAGTAAATCCGCGC
ATGGTAGTATCTAGGCTAAAAATTGTTTTGTCAAGACTACGACCTCGCCATGTGATCGGA
TTCAGGAAAGTTCGGATAGATGTACAATCTACATCTGAAATATTTACATTTTCACCGCGA
CCGCGATCTCGGATCGTTTTGGCATGTTCCCGCGTCGTTGAAACGATAAAGACACTATAG
CCTTTTCGCGTATAATGGTCATATAACTTGATTGTAGCGTCTGTATGCCCTGTCTGACGG
CCTAAATTGATTGAGACGATGGAATCTAGGTCATCAGCAAAATTGCGTCGCTCACGGCGT
TCTATGATGCCATTTACGGGAGATACACACGCAGCATAAATCAAATCTTTACTTTCTAAT
TTTACATCAATATTCATGATAAAGTCTCCAGTGAAGGGGGAGAGATCCTTAGATTGTCGC
CCCGTTGATGGTAAATATTTTATTTTGGATCTGGGAAATTCAATAGAGCATATTGTCCAT
AAAATTTCCTAGCTGCATTATCATAAGCGATTGCAGCTTCGATTTCTGTGTCATATTGTC
CAAGAACATGTCTTTGTAGGTTGACCATTATACTAGCTCTCCATTTCTTACGCAAATCCT
TCCTGTTACACTTACTAACACCCTTATACACACTAGAAAACTGTTTAGACTTCTTCTTTC
TATTATGTGGATTTTGCGCGTCGGTTGCCTCCCTAAGATTTGGAGGATTGTTATTAGCAG
GATTACCGTCAATATGATCAATTAATCTAACTTGCGTCCCATGTTCTAGAAACCAAACAA
CCCTATGCACGAATAGATGAACTCTAGGAAGACCGTCATCTCTACTATGCCAAAATCAAA
GATGACCATCTTTATTTGTTCTGAGTTTAACTAAATCACCGTGTTTATATCTACTCCCTA
ATGGTCGTCCTTTGATAGCTTTACTATCGACTCTATAATATAAGATACCCTCTATATGAG
CATATCTAAGTTTTATCTCGTTTTGTAGGGATTCATCCCAATATTTAAAAGCGATTTTAT
AATCCTCACAAAGCCAGCGATTAAGCTGGCTTTATTTTAGTTATACCGGGTCTGGATAGA
CCTTTTTATCGAGTTCCATATGCGGTCCGTCTTTAAAAGTAACCCAATCAACACCGCAGA
TAACAGGAACACCCAATTCTCGACCAGCTTGTTTAAATGCATCAGCGATCATACGGTAGT
ATTTCATGTTCCATGTATCCTTATCTTTCGGATACGCTACAACGTCAACCGCGAAACCGT
GAAGATGACGAGATCTCATAGTCTTAGATTTACCTTCCGCAACATATTTCTTTTGCGTTT
CTAGACTACGTACACCTTCCGTAATACCAAAGTCGACCGGAGTCAGTTCTAACGCACGAT
GAACGACTTTTACCAGGTCTGGATGCACACCAACAAGCCGATCTTTGCTTCTTTGAGATA
ATTTAAGCATTATTCACCCCCTTTGTGAGTGATAATATTTAGATCCCGGTGATCCTGCAT
AGAGTTCTTGTATTAGCACCAATAAGGATAGCATTAACGGCAACCATTCCCCAATCAACT
CACTGATTATAAGAAATGCTAAATCCGTTTGCTAGGCCAAATACAAGCAATCCGGTACAC
ATACTCAGAGCAATAATCAACATCACGGTAAAACCAAATGCAAATCTCATATTATTTTCC
TTTTCTCTGTATACGTTTATAAAAGTAATACTTTACCTCATCGCGTCCGGTGATCCAACG
TGCTAGGGCGGTAAACAGGTTTACAATTCCCAACACACCTACTGACGCGGCGCTGTCAAC
AGACATAGGAACAGTAAAGGCCATAGCTAACATAGCAACGCCACAAATGACATTAAGCAC
CATTATAATAAGACCGAAAAAAACCATTGTCAATCCTCCGACACAAGAAAACCAACAATC
ACGCCAGTGATAAGACATGATGCGCCGCTAATCAGACAACCCGCACCACGTGCTTCCCAC
CAGATCAGAGAAGCGACAATAACACAGAATCCCCATACTACCGCAATCACAACATAGGCA
AGGGTCGCCCAAAACATATATTTGTTCATAATAAATCCCATTCACCAAACAAATAAGAAA
AAAGCCATATCAACAGATACGAGGCAATTGACGCGGCAAGCAAAAGGAGATTAGAGAATC
CGCCGTCTATCCAGGCGACAAAACAGAATACGCCAGTATAGAAGCATAAACCAATTACAA
AGCATATTATAAGCAACCATAGATAAAACGCCATAGAAAGCCCTCCATCGAGATAAATTA
GTGCGGGTAGTACATTACCCTACCCGCATTGTTAGTCATCGCAGAGCGGGTTCTAGGACG
ATTCCACACCAACCGCTAACCACACCCAAACACACAGCAACAAGCAGATTCATTCGGTCT
TTTGGAATCATCGCAAACAGAACAACACCCACACAGAAGGCCATTACCATTGAAGTTAAC
ATTTGTTCAACCATTATCACACCTTTAAAGTCATAGACGCATAAACATTAATGTCAGGCC
AGTTAGGTTCGCAATTATACTCAACATCCCACAAATAAAGCGGTTTAGATGTATCAGCCA
AGTTTTTAAAATCTTCTTCCCACTTTTCAAGGCTATATGATGGATAAACTAGGCCATCAT
ATCCGCCGTAACGTTGGCTTACTGGTCGGTCAGCAAAAACTACCTCAAGATCCGGTAATT
CTTTTTCAAGGATAACCGCCAGCGCATAAATCAGACGGTCTTCCTTGTTACGCTGCCGAA

GGACCGTTGTCAGTGGGTTTTTCTGGTTCACTAGCAAGCACCTCCACATTATCACAGAAT
GAAATTTTCACAGACACAATTTTACCATAAGTGAAGAATGAATATACATTATCGAGATAT
TCAACGTCACCTACAATCACATCAAACTGCGGATCAGCAACCGCCGCAAAATATTCTACC
AGTTCATCAAGAGAATGCAACTTAATTTCATCAATTCGGCTGTATTTGGTGGCATCTTTG
ATTTTAAAACCACGTTCTTTCAGCTTATCATTTAGAATTTCACGCGGAGTTTCAAACAGT
TTCCTCATAATATAATCCTCACATAGTTAATCAATGGTCAATAATCTTTTACATTATAAC
AGAAAGAACAAAGACCGCAACCCACATATAGATCCCATCAATCAATGCAATTTCAATATC
ACAATATCCAAGATATACAACAGGAATAAAAACAAACATACAGACCACCGCAAAGAAACT
AGCAAAGACGCTAGAAAATCCGATAGCAGTATTAGTAATATTCATATTAACAATCCTCGC
GGCTTAAGTCAACAGCAGTTGCATACAGCATAATGAATATGCTGGCCGATCCGAGAATCA
ATGCACCCTCACATTTTGAAAGTCGCTCAACAAAATGTAACATTATCCATCCGAGGATGT
TAAAGGCGCAGACCGCGCCTATGTTCGCTAACCAATTAATCTTTTTCATAATATATCTTA
GAAAGAAACAGTTTTCAAGAAATCCAGATTTTTAGTACCGAAAACAGAAAAGTAGTGTTT
TCCTTTTTCGGTTTTAGAGAAAGCGATTGTCTTCTCTACATGGTGCCGGAAGTCAGACAG
ACGATCACCCAGGTCAAGGCGGCAAGCGTAAGTAGTGCCGTTTTCGAAAGTAACCTCGAT
TTGAGTTTTGTCATATTCGCTTTCATAGGCTTTTGCTGCCGCTTCCTGTGCTTTACGCTC
GAAGTCTTCGAAGTGGAATACTTCGTTTGCGTTAGCGAATACAGAAGATTCAGAGAAGAA
AACTTTAACAGATACTGGTTACATTTTGATACTCCTTAGTAGTTCGTGTTTCGTTTCGAT
GGGTGTATTATGCAATACACCCTTTCTCGTGTCAACAACTTTTTAGTCTTTTTTCATAAT
TTCTAGAGCAGTTTCTACAGCACCTTTCCAACCTTCCCACATAATGGACATAGTAAGGAA
GTTAAAGCACCCGCAGGAATCAACCTTTTAAGAGATAAACCATTCTACAAACGCTTCGCT
CATACCATGCTCTTTAGCGGATTCGCGTAATTGTTCAATTTGGTCTTCCATAGTATCACC
TCAACACTTAACGTTATTCATACTCAAACGGTCTATTCATCCTATTGTAAAAGTACATAT
AACAAGCGTAGTTATGTTTTGGTTGTACCGTATCAGATAGACACGCTTTTACAGCCTCAC
GGTCCGCTGGGTCTACACCCTCAGCTTTAAGTAATGCTAAAATCTGGTTATAGCGTACTC
TTAGTTGTACTAATGGTTTATCCCCCATATTACCCCCTTATAGTTGATCAGCGATGTAAG
CGTGACAAGCGCGGAGTTTCATGAATGCACTACCTTTCACTTCGCCTACGATGTTCATAA
CTTCGAAGCGGTCGGATGGATTCATCTTAGAGCACCAGATCTGGTTAGCCAATTCCAGTT
TACTCATATTTGGTTCCATCTCAAAGAGTTCACGCTCAAAGTCGATAATTTCTTTTGCTG
CGTCTTTAAAATTAATCATTTTGATTCTCCCGTTTCGTTGATGGTTGTATTGTAGGGGAG
TTATCCTCCCCTGTCAACACTTTTATCAGAAATATGCAATATAGTTTGCCAGTCCGACAG
AGTCATCATTCGGCATTGCTATTACTGCCAGTTCAAACCCTGCTTCAACTTTCTATTTAG
CGGATTCTTCGTTATAACCCCAGATTACCAGAATAGCTACTACGTTTTCTTTGCTTACTG
CTTTCAGTGCTTTAGTGATGCGACGTGCCATAATTTGATTCTCCGTTTAAGAAAGTGTGT
TTCTTTTCGATGGGTGAATTATGCTATAGGCCGGATCTGGTGTAAACAACTTTTTTTAAA
AAAAATTTTTAGGCAATAAAAAAGCCACTCCGAAGAGTGGCTTATAGGGAAGGTAGCGAA
TCTTCCTTGTCTCCAACCTGCTAGCGGCCTAGCAAGTGAATGCTTTAGCGTACCGGATCG
CCCATACGCTGCAATAAATGCGGCTAGGTGGCGTCCAAATCATTCTAACCGCGCTGTCTT
TAGTTCACTATTAGTTATATCGCTTTCTCTCGTGACGTATTTGATTTTCTTCCTTGATAG
AGTTAAATGCATCAAAAAGACACATGACACTAATAATAAACATAACCGTATTTTCTACGA
TATCCCAGGGCTTCGCATCAGTACAGATCCCCCAAACGGTAAGAAATGCAAATAGTGCAG
AAGAGATCACAAGCATGATAGTTGCTAAGATATAAAAAGTCATTTTCATGGTTTTACCTC
AATTCTTCGATATTCATCACACATCCACGCAGTAAGCAAGAATCCTAACACATAAGCAAC
AAAATACGTTAATGCTTCTTCTGGTTCTGTGCTGTGTATCATCATTAAACCAGCTAGAAC
GGTCTTAACAATTACATTTAAAATAATTAGCCACGCAAATAATTTCATCGTTTGCCCCAT
TTCGGTCGAGTCTTTTCAAACTTGTACATTCCGTAACAATCTCGAATTATCCACGTAACA
AGAGCACCAGTCAACACAGACAATACACAGAAGAAGTAAAACGGTCCAGTTGCATTATCA
ACATGAGCAAGCTATGTAAAGAAAACACATGCTAAGAATGTAAAGAACATCATAAAACAC
AAGAAAGCAAAATATTTTATGCTACTCATAGCGCCACCATACAACAGATCATAAGAAAAC
CCAACGCAATAAGAATTGCATCAAATTTAGTGAACTCAGGTCCACCTATCAACGTCACTC
GTTCCTGTTCATTCATAGATCCCAGAGCCATCTTACAGCACCACATAAAGCGATCAAAAG
ACTAGGTATAAACGAGATTACAACAAGTTTACAAAAATCATCTAAGGTAACACCGGAATA
GCTACCTACTGTAACAACACCGATCATCAGACACAGAAAGAAAACAAATGCAATCAGAAA
CAAAGTACTGCTAAACAGTTTAGTGATCATTAGACACCCCCACGACGATCACTTTCAAAA
ACTTTTTTACCAACAACGAAGCATGTCACAACTGCCTCGTTGACTTCCGCAAATTGTTGT
GTCACCCACTTAAGGTCAGAGCGAAACAATGTTCTATTAATAACAAATGTTTTACCGCTC
TTATCCTTACCCATAAATTTTGCTTTAACCATCTCCAATACCATATTATCACCTTATTTG

CTAATCGCTAAACCCAATCCGAGGAGAGCACCCGATGCTACAGTATACCCGAAAGTTTCA
AATGCAGTAAAGATATCTAAGCCACCAGCTAACAGAGTTGCAGTAAACAGAGTACCAGCT
ACGAACATAAAAGTTTTACCGATAAATTTCATTTTGATTCTCCTTTCGTTTATTTGTGGG
GGTATCTTACTACCCCCGTTTCATCTTGTCAACTGTTTTTAAGAAAATTTTACTTCGTCT
ACAACGTCTTTCATGACATTGATTTCCATTCCGGTCAGAACACGTTGGAACATGTGCTGC
ACATACGCTTTCAGGTATTCATTTGATGGCATGATATCGAAAGTCTTCTTAGTGTAGTAG
TCATCACCGAAAGCTACTGTAATTACATTTTCGTCGTCGGTCCAAGCGATATAAGCAAAT
TTGCCGTTATCCCAGAAGCGAGCACTAATTGCTACTTTTGTTGAATCGAATCCTACTTCG
CTGTGAGATTCAAGAACTACTTGCGTACCAGTTTCTTTATGAATGTAGTAGCGGGATACT
AAGTCAAAGTTACGGGTGTAGTGACATTCGAATTTATCTTTAAACATCATCATTTTGATT
CTCCAAAGTGTGTTTCGTTTCGATATGTGCATTGTGCTATAGTCGTTTATGGTTGTCAAC
ACCTAACAACAAAAAACCCCGAAAAATTTTCGGGGTATTAAATTACAGTTTGGTCGTCCA
CTCCAGGTCTAGATCTTCGACATACTCATGAGATCGGACAATCTCTAAATCGAACTCTAG
GTCAGTGCCAAATTTACGCGCACCAACATCATAAGAAAGCGTGATATGCGGTACATATTC
AGGATAATCCCACGTCGCGCCTAAAGCAGTGCCGATATCGTGTCTGAATTGCATGTAGTC
GCTTTCAAATGCCAACACAAGCGAATTAGTACCGGATTGAGTTTCAAACACTTTCAGATG
GCATAGAGAGCCAAAAAACTTTTCACCCTCTTCTGGGATAAACGGAACATAGACACGAGA
ATATAAGATAGTGCTATGCAGTTTATCACGAGGAACGGCATTAGGCAGATCGTAGAACTC
CTGCAATTTTTCAATGCGGTCTACTGTATCCTCAGTGCATTTAAGAGCCGCATATGTACC
TTTAACGGATTCTCGAAACGGGGTCATAATCAAGCCTCCGTGATCAGTTCTACTTCCTGT
TCTGCCTCTTCCGCTGCTTCGTCTTTAGGGAGAAGTGCTTTCACTTCTTCGACAATAGCA
TCAAGGGAAACAGTCCCGTTTTCGTCTGCCTGAATACCCAGAATGCGTACCAGTTCCATC
AATACACGCTCACGATCTTTCAGTTGGCCTTCTAAGAATGCGGCCTTTTCTTGAGTGGCA
AACAGACGTACTTTCAGGACATTAATCATTTGTTCATGCTGATTGGACATATTCACATCT
CCTACTTGTTGATAACTTTTTATTAAATGTTCGCGAGAATCGCGCTTTCAAATTCTTCTA
ATGTACCATTATGTATGATAATGGTTTCACCGTCAATAGGCAAAATTCCTTTTTCGGTGG
AGTGAGTCGATACAATACCAGAATCACGCTGAATATGCAACACCTTAGCACCCATAGCAC
GCACGAGAGCAATTTCAATAGGCTGAAGCACATCAACCACTACAAAATAACCTTCTTCAT
TTTTGCGCTTCATAAACTCATTCACGAATAGATTACACCAATAAAGCGGATTGATCAAGT
TAACCATTATATCAGTACCGAGAGTGGTTAACATGAGACGAATAGACCAGACGTTATTAT
GTTGTTCGCCAATCTCATTAACGACCTTGTAAATCTCAGCCAGACGATCACAAGTAAAGG
AACTACCGCCATACTTCGAATCTAGCATAAGCAGACACTGAATAAACCACTTATGCGCGG
TATAGGTATCAATACCTAGATCGGCTTCTCTGTCACGCCCTAACCCGTCAAAGTCAAGAT
AAGTCAGACCTAAGTCTGGTTAACATTCGCACAATATTTCTTTGATCGGTGCTGCCAGTT
GATAACTATGTCCATCAATGATTAACGCGGCAGTATCTTTTCCTACTCGCTTTTTACCAC
AAAGTGCAATCAATTTCATGATATAAGCCTCTTAGTACTTTCTAAAAGCAGGGTGCATAG
CTTTAATAACTTCCACCCTCTGACGATCCGCCGCCTGAATTGCTGGGCGAGGAACGATCA
GTTGACCATCACGTTCCATGCTCATTGTTTTAAAGGGAAGAATTGCAGTACACACCAGAG
AATCATGCATATCATCGGTGTATGTTAACTCAAGTGCCCCCAATTGTGAACACCAACAAC
CATAAAAGTTAAAAGTGCACACAATGTTACGTCGTTCGTTGTCCATCATGTGAGCTTGAA
CGGCTTCCGGCATTGAATCCTTCCACGCAGTGGAGTTACCGTTATTGTAGTCTACTATAG
AAAGCATCCATGTGTAAAGGTTTAGATAGCTTTCCAAATGCTCATCGACAATAAACCGGA
CTACCAACGGGTCAAACTCCACATGACTAGACGGATAATCACCGACCGCCAAACCATATT
GACCAGCAACTTGCTTAGTCGGTGGAATGGTTATAGCCGGAAGTGTAACACCTTGGGCAT
TTAAAGTAAAGCCTTTTGTTAGTTGTCCATCTGGAATATTGATCTGCCAGTTGCTGGGGT
TAGCATAGTTAAAGAGATTAGACATTACTGATTCCTCAAATGCAAATATTTATTAACAAT
TGTCGCTTTGCGACGTGCCGGAGGCACTGACTACTTTTTAACCGAAACAAATTTTCTATT
ATTCTAAAGATCTTTCTTCGTTTTGGTTATGTTTTAACCAATAACTATCTTTTTATACAG
TTATAAATAAAACGGGTACTATACGAGGACTTCTTATGACAGAAGACATTTTGTTAAAAC
TCTATGATCGTCTGGATAACGAATTAGCAACATTGCGGGGTGAAGTTCTCTATTTGTCTA
AATGCAATACAGATCTGCTTAAGCAGATTGCAGAAAGGGATGATCAAATAAAATCCCTTT
CAGGTGCATTACGTGATCGCGACAAGGCATTAGAGCTTTTACAGCTTGTTACCAATAGGG
AAGAATTAAAAGACTTCCTAAATGGTTATGCTCATGCCATCGATCTATAATAAAAAAGCC
CGTTATTGACGGGCTTAATGTTAACTATTGATCATATCAATGACGTTTTGTAGCTCTGAT
ATGGATAGCGTTTCTGTATATCCATCTGGCACATCTACTTTACTAACATAGAAACCTTTA
TACTTTTTCTTTATTGCACATTCCATAGAACGAGCATCATCACCGTAATCGTATTTTTTA
TAAACTGTCAACTCATGACGAAATATAGATTTTGAAGATTGGTTTTGTATTCTTTTTATT

AATCTGTTGTTGGTTATACCAAATTTTATAGCAACCGCAACATCTTCTTTGTATAAAGTT
TGTACATAAATGTATGCAGGTTTATTCCTCTTAAATCCATGATTGCTACACATAGGGCAA
CCGCTATTTTTTGATACGCAGTCTCCTAAGTTTGGCGTAAATTCATGTCCGCTACTACAA
CGCATTACCAATTTTGTTTTTAGGATTCCTCTGTACTCACCGCCAACAAAATCAACAAAT
GTATCACGTTTATCAATCGCCTTATCTGTTATTTCCTTTACTACTTGCTCAGTTGACTTC
TTGTTTTTACCTAAACATAGTTTACATCTAGATCCATTAACTAAAAACGAGTGACCGGAT
GCAGTATATTCATGTTCTTTAGGGCATTTAACTAAGATATTTCTTTCATAAAAATCTTTA
TAATCAGACACATAGCCAATGGTTTCGTGGTCTTTTTGGTTATTCCTCATCTCTGTTAGC
AACTCTTCGCTATCCCAGCGTTTACCCTTAACGCAGAGACAACCATGACCTTGATAAAGA
TATATAAATTTCTTAGTAAAGTTTTTGTTGCAATCAGGACAAAGCATTAGTAATTCTGTG
ATTTGACCTGTATATCCTTTAGGAATATCGATCAATGTGTATTTTCCGCATATTGTCGTT
AATGCATCATCTATTCTTTCTTTGGTCCAGCGTAAGCGACCAGTGCATTCATGACATTTT
ACACCCAGTCTAAGAGTATCTAAACTAAATATAGTTGACGTATGTCCTTTATCACATTTT
AACTTAAATCTAGTATCCTTTGCGTTGGTGTATTCTCCGACTAGACCATACCATTCATAA
CAACATGGGATATGTTCTTTTACCTTCTCTAGATATACACCGAACGGTCTAGGAACTGAA
TTGGGTTTTCTTTCTTTAGTCATATTTTTATATCCTGTAAATTAAAGCGCCGTCACCCGC
GGCGCTATTTTTATAATCATTAAAACAATTTCCACACCTTACGAGCATCATATTTCTTGT
CTTTGCTCATAAACTGGTAGATCGGAAGATACACAACCGTGCTCCAATCTTTTGGTGCGA
TCTCAATCAGTTTTGTTTGGATGTGTCCAGGCAAATATGCTTTGATCATGTGGTCTGCGC
CTCTCATATCTTTAACATCACTCCATTTAATTTTTAAACGAGTTTTATTACTAAGAGTAG
GTGTACTCGCGTATTGTCGCAACAACGCTTCTAAGAACTGCTGGCGAGCTTTAGGTGGCG
CATAGTGCAAGTTCAAACCTAGCATAATCTCGTTTCCCGCTTTAGATGTTCCAAATCCTA
GACAGATAATCAAGGGGTATCGGTCAAAGAATGGCCAAGAATCCCGATATTTTGGGTTAT
ACGCATAAGTCTAGATCCGTCCCACTTGTGGTATTACTACCTGATGACCTTTTACACCAT
CCTTGATGGTCTTCATAAACCACTCCTGTGATTTTTCTTTAGGACGACTCTTTGATACAG
GACGCATAGACGCACGAAAATCATTAATCACCATCATTTCGCGTTGCTGCTTATTGAGAC
CCTTTGCACCTTTCCCTTTTAGTTTTTCAACTTCTATTGCTGTTTTAATTTCGCTGGTAT
AACGAGACATACTTTTAGTGAAAGTAGCATAGGCTATTCCCTTAGAATCTGCAAATGCTT
TAACTGTCATGCCTTTGGCTTTTGCTTTCGCAAATTCTACACCCAATTCGACCCACTGTT
TAGACATTCGTTTTTGGGGAAGACGGCTTTTCGTGTTCTGTGATAATTTCAAATACGCTC
ATAATATTATCCTCTATTTGTTAAGCCCTTTAAAGCCAAACACTTTCTTTAATGCATGTT
CGGTGATGATCTTAAACTCCATGTTATGCTTTTGGCATAATTTCACGGCAGCTAACCATT
TATCAGTATTTTTTGTGTATGTTCATACTTCATGCATATATCGTTGCTTTGTCTTCGCGG
TCATTCTTTTTGGCTTTTCCGGTGGCCTTGTCTCTTTAGCTGGTTTAACTTCCCATAAAT
GCTCCTTGCCATCATCAATCTTAATGTAAAAATCCATAAAGTATATGTGTCTTTTTCCGT
CTGTTGATGACTCATAAGGAATTACTACGGTCTCAGATCCCCATTTTGCAACTCTAGGAT
TGCGGTCTAGCCACTGAATGATCCATCTTTCCCAGCTTGAACGGTATGTGATCTTACGTA
TATCCCCTTTATACTTTTCCTTATTCTCAGGAACATACTTGCCCTTATAAGTGTTAAATT
CTCCCATTTTACCACCCTTTATTATAAATACATAATACTATTTATCAATTGAGGGTGATA
AAATGCTCTTTTCTTTCTTCCCTGCCATTGAATACAACGGCAAGATGATTACTGACATTG
TGCGCAATTACCGCGCATATTTCAACGCTATAATTTCTGATTATAACATACAAACATATT
ACATTCAAGGTAGTCCACGACCGGTACAAGTTGCATATGAAGTATACGGAAACCAACAAC
TTTACTGCATCTTGCTGATGCTTAATGACGTATACGATCCGTTCCATGACTGGATCACCG
ATCAAGAGACAGCATATGAATCAGCCGAACAACGATATGCAAATGTCGGAGCAAATCAAG
TATTATACCACGTTGATATTGACGGAAACAAGTATCGGAATCTTACCGAATATCCGAAAG
GATCAAGCAATTGGTATGATAAAGGGGATATTCATCACCGTCATTTGCAGTTTCATGTTG
CATTGTCGGCAGTGGACACACTGGAAGATGCGATCCTCCGTAATGAGGAACGACACAAGA
TCAAGATCATAGCTCCTACACAAATTAAACAATTTATTAACGCATTCATTCGAGAAATGG
AAAAGGCTGATGTGTAAGGTGATTAATTATGCTAATGTTTAATAACTCCGTTAAATGGTT
TACCGGAATTGTAGAAAACAGGGAAGACCCATTAATGCTGGGGCGTGTTCAGGTTAGGAT
TCATGGTATACATCCCTCGCTATCCGCGCACGGTGACATTCAAGCTCTTCCAACAGAAGA
TCTATTATGGATTACGCCAGTACAAGATATTACAAGCGCGGCGGTCTCCGGTATAGGTCA
AAGCCCTACTGGTATTGTTGAAGGTTCAATCGTATTTGGTTTGGCTTTAGATGAACGTTA
TTTATACTGCGTTATTCTGGGTACTATTGCCGGACAATATACAGAGAAACCACTCACTAC
AGAAGGTTTTTGCGACCCTTCTGGTGCATATCCTCGTTATATTGGTAATGATGTTAATAT
TCTTGCTCGTGGCGGTATTAACCCAGAAACCGGACAAACAACAAAACCTGATAGTGTCTA
TATTCGAGTTGAAAACACTGGAGTTGCAGTAAATCCAGACGACGAGCCATTAGACCAAAT

TCCGGTTGATGATAATCCTGATTTTACAATAGAAAAAATGCTGAGGGGCGATGAGGGATA
TCGCGAGAAATGGTATCTTGATTCAGAAGGATATCCGACAATAGGCATCGGGCATTTGAT
CATTTATAAGAAAACCAGTGATCTGGGTATTATCAATAATGAACTTTCTAAGCTGGTAGG
GCGTGAAGTTACAAACGGAAGACTTACCGCCGAAGAAGTTAGTAAAGTATTTGCTGACGA
TATAGAAAGGACTCGTCGTGATATGCGCAAACATCCAGGAATTGCACCTGTCTATAATAA
ATGTAATGCCTCTCGTCGTAGGGCATTAGAAAATATGGCGTTTCAAATGGGTGTTGGTGG
ATTGGGTAAATTTAAGAATAGTCTTGCTGCGATGTTAGCGGAAGAATGGAAACAAGCCTA
TGATGGATTACGTCAATCTGTATGGGCTAATCAAACTCCAGGACGGTCAAGCCGTGTTTC
TAAAATTATTCTTACTGGTAACTTAGAATCATATGGCGTAATTGCTCCAAAGAAAGAGGA
ATCTGTTTCTGATGATCCGCGAGCACGTTGGAGAAATGCCCGTATTGCTGCCTATAAGAC
GCAGAGAGCAGAAGAAGACCCAGAAGCACCGTTTACCCCACAAGACACACGAATCATGTT
TAAAGAGCCTAAGAGTTCGTATTCTGCGAGGTATCCTTATAACCATGTGTACGAGTCAGA
GAGCGGTCATATTATCGAGATTGATGATACTCCATCTCATGAGCGTTATCACCGCAAACA
TCCTTCTGGTACATTTGAAGAAACCAGACCGGACGGAACGAGGGTAGAAAAGATTGAAGG
TGATGATTATCTAATCGTTAAACAAGGCCGGAAAGTAAACGTTAAGGGTAATTTGCAAGT
TGTTGTTGAAGGTGACGCGCAAGTTTATTATATGGGTAATGTGATGCAAACCGTTGATGG
TAACGTCACTGAATTTATTCGTGGCAATGTTAATCAGACCGTTGAAGGTACGGCTAACAT
GCATGTTGTAGGTGATGTTACCGCACAACTTGACGCTAATTTAACGGCTAATGTCAAGGG
TAATGCTGAATTAATGATAGAGGGTAACACTACCGAAACAATTAAAGGTAACTATGATTT
AACCGTTGAGGGTAATTTTAATATGACCGTCAATGGCACTAAATCAGATCTGGTTAATGG
TGACTGGTCCCTTAATTGTGGTGCAAGCGTTAAAGATATTGCATCTGGTACAGTTCAAAT
TGATGGTAGCCGTATTGATTTTGGGTGAGGTAACTAATGGTTGAAATTCTTCCGATGAAT
TCAAAATTGGAGGATATCAGCGAGCGAGAAATCTTTGAGGTGGTGTTCTCCTATAGAGCA
ACGCCACCGGATGCAAATACCACATACACAATTAAAGAGTTTAAGATCAGCGAGTATCGC
CCTAATAAGGGCGTTACCGTTGAAGAAACTAAAATATCTGGTGGGTATCGGGATAGTTTT
TCGCTTGGCGCGGATTCTCTTAAATATATAACGAAAGATAGAATTAAAAAATCTGCCGGA
AGTTTTGATGATTTACCAGATCCAACTACCGCAGATCTATATTTTTTTAACGCTCCTACA
CGTCTACAGGAGGTTTATACTTATAAAGTAACCCTAACCTATATCCAATCTGTTTCAGAG
TCTTCTGGTGGTGCTGGTGAAGGAAATAGTGGTGGTTCCAATAGCCGAGAGGGTGCTGGT
GAAACTAATCCTCCTCCGGTAGTAACCGAACACACAATAGAGAAAGTTTATACTCAAACC
GTTTTTGGAAATTGGGATGTGTGGGAGCAGCAACTAAGAGTTTATGTACATAGGGGTGTT
TAATGGCTGGATTATCCTACGATAAAGCTAAAACTACAGGGCACGGAAACTACCCACCTA
CGGTAATTAATGCAACTCAATCTAAGGTATTTGTTGAGGGTATCCCTGTATTAGTCGAAG
GAGACCCAATTACACCACACACAAAAACAACAAAGCCTTATGATACACACGGGGGAACTA
CGGTCGCCAGTACCCCGAAAGTACTTGTTACCGGAAAGATAGCAATTCAAATGGCAGATC
GTAGTAGCTGTGGCGATACCGTCGCTATGGCATCACCGAAGGTATTCATAAAACGATAAA
GCAAAAGATTACACAAATTCCTGATATTTTTAATGCTGCTACATTCGATGAAATTAAAAG
CGCATTAACAGACTGGATGAGAAACCAGGAAGAATTTAAAGATTTTGATTTTAATGGTGC
TGGTATTAACGTATTGATGGACTTGCTCGCATATAACACTCTGTATATTCAGCAATTCGC
TAACGCCGCGCTATATGAATCTTTTATCAGAACGGCCATTAAGCGTAGTTCAGTTGTTCA
AGCCGCGCAAGATATAGGATATTTGCCAGCAAGTAAAACTAGTGCATCGACTACAATTAT
GTTGCACGTAACACATCCTCGAAATCCGGCAAGTATTCGAATCCCACGAGGAACTAAATT
TATTGCAAGTGTTGATCGCCAGACGTCTTATCCGTTTGTGACTACAGAAGACGTTTTGAG
CGTTTTAGGTTCAAATGGTTTATATACGCCTATGCTTTAAGTCGCACAAGGTCGTATTGT
TCGTACAGAAATGTTATTTAATCCGAAGAAACAAATTCTGATCCGCGATCCTGATATTGA
CCGCGCTCAGGGTAAATTGTGGGTAGACGGTGCTGAATGGAAAGACTGGACACGAGAAAG
CATTGTTAACGCAACTGGTGCAAGCAATATTTTCTATATGCGTGAAACCGTAGACGGTCA
TACAGAAATCTTCTTTGGTAAAGGTGAATCAACTTTATCTGCTGCTGGCGGTGCACTGGA
AGCAAATTATATTGGTGGACTTAAGCCGTCAACTGGTGCTAATATTGTTATCGAATATAT
CAGCACTAAAGGCGCGGAAGCTAACGGATCTGTCAATTTCACATACGCGGATACATTGGC
ATCTATCTCTATTGTTAAAATTAATGAGAATCCGACAAATGATCGTGATTATGTCGGTGC
AGATGGTGGTGGTGATCCTGAATCAATCGAACGTATGCGTGAACTTGCTCCGGTAATGCG
TGAATCTCAGAGTCGTTGTGTTACTGCTTCTGACTATGAGGCTTTTGTATCTAGCCGTTT
TGGTAGTATTGTTCAGGCGGTACAATGTTTCACCGACCGAGAAAAACCGGGTAATGCTTT
TGTTGCTGTTAAGCCTAAATCTGGCTTGCGTTTGACAACTGTTCAAAAAGAAGATATGCG
CGATTATCTGGCAAAATATAATCTTGCCCCTATCACGCTGAGCATCATTGACCCAAACTA
TCTGTATATTAAACAGAATATTAAAGTTTCCTATTCAATCAATGACTTGTCAGAATCAGA

GCAGTGGTTGCGCGGTGCGGTTATTGATCAGATTGATGCATATTATACCGACGTAGTGGA
AATTTTTAACCAGACCTTTAGTAAGTCTAAAATGCTTGCTCGTATTGACAAAGCTGATGT
ATCTGTGATAGGTTCTAGTGCAGAAATCGAGATGGTGCGAGAGATCGACAACTTCTATAA
AGCACCTATGGCTGGAATCAAGTTTTTAAACCAGTATAAGAGCGGAATCAAGTCTAGTTC
ATTCAACTTAGAAAAGGACGACAAGCAATATCCGATCCATTATGTAAGTACAGAAATGAA
CAAAGAATCCGGTTTAGCTAAATTGCTGGTTGGACCTTTCCCTGTTGGTTCTGCAATCGC
TTTACCGCACTATTCCGGCACTGATTTTGATAAAGAGGTTATCGACGGTGCGGACCTATA
CTTTGAGGTTGGAGCGGTTCAGCATTATGACGACCTTATCTCATGGGATCTGGGTAAACT
GAATATCAATAGCGATCGCTTTACTGGTTCATATATTGAGTTGACAGCTAAACCGATTGG
TGATAACATCTTTAATAAAGACGGTTCTTTGATCGTGTTTGAAAAGGATCTCCGTCCTCA
GTACACAACTATTAGTCTGGAGGCAATTGCTCAATGATTAAAGCGCCTAGTGTAACTAGC
TTGACAATAGAAAAATTGTCTGCTAATCAGGCGTATATCACATGGGATGACATTGGCGAA
AACTTCTTTTATTTTGTGGAGTTAGCCGAAACCAGAACACGAGACGGGGAGATCATCCCC
GTCGATGAGTTAAGCTGGGTAAACCTGGGATATACTGCCGCACAAAACTGGTTTGAGGAT
AAAAACCTTTTTCCAGAATATTATTACAAATTTCGTGTAGCCGTAGCCGCAGAAGGATTT
GAACAAAGTAAATGGATTGAAACCGAAGAGTTTCAGACATTTAAAACAAACGCATTTAAT
TTGTCTATGATGCGAGAGTTCAACCTTGCAAACGAGTTTGTTAAACAAAAGTGTACATAT
GATAATCGTGATTATATTAAATTCAATAGTGATTTAATTCAAGCATCATTGATGAAGGAA
GATTTTGTCTTTAGTCCTGAATATGCAAACATGACAAATATCTCAAACTACATTGTAGTT
GATGAGAACTTTCATGAAATACAGGATCACATAGAAAACGTTTGTATTGATATAAACCGC
ACTATGTTAGCGGAAATGAACGGTGTTTTGTACATTTTCGAACGGTTCCAGCCGATTGTG
AAAGTGTCAAATGATAAAGGTCAATCATGGAAAGCATACAAGGCTTTTAATGACCGTGTA
GGCAATCCGGTTTCCCGTACATGCGTTTATCAGTCTAAAACCACTACTTACGTTTTAGGA
TATGACCGAATCTTTTACGGACGACAAGCTAGAGATACTCGATGGAGCGCGGACGATGTT
CGATTTAGTGATGATACGATCACTTTTGCAAAGGTTGGTGACGAGAAGGATTTAGGCTTT
GCCGTTGAGGTCTTTGGTGTATTCTCCAGATTGACAGGTGACACCACAAAATACGCCGAA
GCTATGGCGTGCAGTGCCGATTATTTGTGGGTTGGTGCTAAAGATGTTATTCGCCGGATT
CGTCTTAAGAATACTCCTGTTGATACCGTACAGGGTTCGCCTACGTTTGGAGAAAAAATA
TTCGATCCTGATGTTATCCGAGTAACAGGAAATGATAAAGCTGTCATTCATAAGATGGAT
GTGATCGACGGTAAATTGCTTGTATTTGTTACGGGGGAAGTTAAACAGCGTTATCAAGAT
CCAACTAAACCAGAAAACGTTGTTCCGTCGGATGATGCGGGTGTATACTTAATAGGGAGA
GATGGTCTGTCTTTTCAGAGAGTATTTGGCAACACTCCGCAGGAAAGGGAATATATTCAG
CATGGGTTCAGTAATATGTCAACCAATGGGGATGAGGTGTTCATAAGCAATTATAATCAT
CACTTTGAAAGTATTTTACCTGATCCAGAAACTGCCGCGAAATATGAATTAGGCGAAGCT
TTGAAATATTCTCAGGAGCCAACTTATCTAACTGATAAGAAAATACATCAAACTACTTTC
AGAGCAATTTATGATGATTTGCGTATTTGGGAGTTGGGACCGCAAGCGTATTATAATGAG
GCTGATTATTCATGGATGGGTCGAAACGGTGTCCGTGTGTGGATCACAAATGATAATCGC
CCGTTGGTTGTTTATCCAGAGGTTATCTATACTCAAGAAGTTGATACATCTGGCATTCTT
GACCAAAATAGGGTCAACCGAGAAGTTTATGATAAAAGGACCGTGGCCTGTTATCTTAAC
AATGTTAAATTTGAAGGATTCAAACAATATGCATCCGGTGTAATGTTCCATAAAAACACG
GGAGAGATCATAGGATTCTATCAATTTAATTATCCTGTTGGTGACCAAGTTGGTGTATAT
TGGAAACCTACATATGTCGCATTTGTTGCTGAATTGCAAAATCAGGTTCGGGCGGTTCCC
TGGGTTCCTGTTCATTATGACAGAGAGCAAGATCCAGAAATTCGTCCTTTACTGAATAAG
ATGACGCCTGATAGCTTTTCACTTGAGAAGGAAAATTTCAGAAAATTCTCTGAATATTAT
CTGCAATTCTTAAGTGATGGTTCAGGAACATATTATAATAAACTAAAAAATCTGATTAAA
AACAAATATCCACGCGAGCAAGATGCATATGAGTATCTTTGGTCAGAGATGAACAAACGA
AACATCTATGCAGATAAAAACGAGCGAGATGCGGTCGTAAGATTCTTTGAATCTCGCAAA
TCAGATTTTTATTCCACTAAGGGCGTGGAGGCTTCTTACAAGTTCTAGTTTAAGTTACTT
GATAATGAAGGTGTGGAAATTGAGGTTGAATCACAAGCCGGACTAGAGTATGATATTGTT
GTTAGTTCAACGAACATTGACGAGGATATCGTAGGACGCACGATCTATACTCCAACCGGA
CGGGCTAACGTGACTTATCTTGAAAGGGAATATGAAGGTGGACGTCTACGCTGGCGAGTA
ACCATTCATAACCTGATCGGGCGGTTTGACGAAGGTCAAGTGATCAAATCAGAGAAAACA
GCGTTTGAGGGGATGATCCTTGTTGGTGTACGTGGTAAAGAGTTGTTGAGTAGTTCAATC
GACTATATCAACCGCTCACGAAGCCAATACACCATGAAGATTAAAAGTGCATTGCCAGTT
TCGCGCTATGAAAATGATGTAATGCGATTCGTTCATCCTGTCGGCTTTGGTTTCATAGGC
GTTACTCTTCTTACAATGTTTATTAATAGCGGGTTAAGTATGAGACACGTAGAAACTATA
ATTCAATCTCTGAAAAATTATAAGTTTGACAGCGGATTGCCCAGCGTGTATCCTGATCAT

GTTGCTGATCTTGATGTTGATGGAAATATTAAGATTGACCCACTAACAGGGAAACCATTA
TACTTAACGTCACCGACAATCGGCGAAGAGTTCCTTGTTCCTCCTGAATATGATCACGAG
AACAGCGAATTGTTCCACGGACTTAAAGCAAGCGAACGAAGATTTGCAATGAGTCCACTG
TTTGACCAGTCGGCGGTAACATTCTCAATGTTCCGTCAACTTGTAGATAAACGGTTGAAG
GATAATATAGGGAATCCACGCGATCCGAAGAATCCGACACAGAGGAAAATTAATGGCTAA
TGAAAATAGTGTGATCTATCGCTCACTCATTACTAGCAAATTCAGAACAGAAAAACTTCT
GAACTTCTATAATTCTGTAGGCGATGGAGCAAATCAAATTACAATTTATGGAACGTTTGG
ACGTTCGGAGCCGTGGTCAGAAAACGAAAAAGAGGTGGGCTTTGCCCCACCTTATCCGAC
CGATTCCACGGCTGGTGTTGTTGACATGTGGACCCATATGATGGGGGCGGTTAAGATTAA
CAAATCACTTCTTGATGCGGTTATCCCTCGTAAAGATTGGGGTGATACCGGACAGGATAA
CCCGCGCACATTCTTTATCAATGACATTGTTGTTGTTAACTCAGCACCATATAACCGAAC
TGATGTTGGTAGCGGCTGGATGGTGTATCGCGTAACCGATGTTCCTGAACGCGGTTACTG
CTCGGTTTCAAGCATTGACGCTAAGGTAGAGTGTTTGAAGTTGGGCGGAAAGTGGACCCA
CACTCATGAATCAATTGCGCCACCTGTAGGAAAGGGCGATTCTTTTGATATGGGTGATGG
GTATCGTTGGGAATATCTGTACACAATTCCACCTGATGCGGCGATCAACCGTTGCACCAA
TGAACATATTGTTGTACCGTTCCCCGATGAACTGCGACAAGATCCGGCGCGTTGGGGCTA
TGACAATGTGATCACGTGGTATAATGACTATAATCTTCTTTATCGGCTGAAAGTAGTAAC
TTTGCGTTTTCAGGCTTATATGGACTCTGCGCGATTCCCAACTACCGCGTTAGTGGGTAA
TACTGGCTTCCGTCAGTTAGGCATTATCCTTAACCCATTGGAAAAGAAAGCACGCCCTAG
CGATCCAGATGTTAAAGCGGTCAAAGATAAGTACCGACCGCAAGACTTAGAGCCCCAGTC
AGGCGAAATGATCTACATGGAAAACCGACAACCTATCGTTCGGTCATTAGGTCAAACAGA
ACTTGTTTCTGTATTGTTTGAGTTCTAAGTGTATGATTTTAAAAGCCAATTCGAGAGAGT
TGGCTTTTCCCATTTTTGGAGTTGACGATCTTATTTGGTAAGGTATAATACACACATCTA
AACAACTGAGGATAAATTATGGCACGTCGTAAATTTACAGATACTTGTGAAGAAATTCAG
GTATCCCCTTGCGGTTGGGATGTGATCGCGAACGGTGAAAAGATCGGCGAGATCATCGAT
CATACTCATTCATCACTAGCCGATTACACCGATGAACTATGGGAGGGTATCAACTTCATG
AATGACACTAAACGATTCAAGACTATCGACGAAGCTATCTAATTCGTGTATAATCCAACT
TTCGGTATGTGGGGAACTAGAAAATGGCACGCGTAAATATTAAATTGCATTACACATTGA
ATAAAGCTAAGTTCCCAATGCAAAAGCATTACTATGGTCTACGTGCACAAGATCCTAAAC
TCTATGGTACTCATTGGTTTTGTAAACATGATAACCAATGGCACACATACGAAGAAATGC
ATGAGATGATGAAACAGGGTATTGAATTTAGTTGTTCTAATTATGACCATCGTATAACTT
GCATTCGAAAATTGAAACGATGGATTCGTAAGAATCCGCAATTTAAAAACTTTGTGTTCT
TTATGTCTCATGTTTATTTGGGACATAGCGTTACCGTCACAGCAAAGGTTAAATACTGGC
ACGACTTTCTAAACAAGAACAAAAATTACACGATCGGGTGATGGAGTTGATTTATTCAGA
TCGCCCATTGACATATGAAGAAAAAGAATTTTGCTATAATAACTATCATGGCGATGGTCT
AGGCGGTTCAGGTGCATTGTTTACCCCAGAAGGGCTTGCATGGGATTTTACCATCGATGC
ACATTGCACAGGAAAAGTATTAGATCTTTGTGCTGGGATCGGTCGCTTATCTTTTTGTAT
GTTACATCGTTGTAAGCCAGAAAAGATCGTATGCGTCGAACTCAATCAAGAATTTGCGCG
AATTGGCAAGAGACTATTACCGGCGGCAGAATGGATCACCAGCGATGTATTCGAATTTGA
GTCAAACGAAAACTTCGATGTGGTGATCAGCAATCCACCTTTCGGGAAAATCAATACAAC
CGACACAAAGGATGTATTTGAGTACACTGGCGGCGAATTTGAATTTAAGGTTATGACATT
AGGCCAGAAGTTTGCCGATGTAGGTTATTTTATTGTTCCTGCTGGTTCCGCTGGTTTTGC
TTATTCCGGCAGACCTTATTATGACGGGAGCATGAAGTATAATAAATATCTAAAATGGAG
CAAACAAACTGGTTTGGTGACTTACGCCGGATGGGGGATCGATACATCAATCTATCGCGA
TGAATGGCATAGTACCAACATTTTAACAGAAGTGGTTGAGGTTCGGTATTACGAGAAAGA
ATCTGTAGGAGCGCCTAAATCACGTCCTAACGAACAAATAAAAGAAAGTAAGGAGATTAC
CTTACCGAAACAGGAAATCGAGTCTGGGACGCTCTCAGATTTGTTTGACATTTAATTATA
AGCCGTCCTTGTGGGCGGCTTTTTTGTATGTATTCTATAAATAATACATCATAACATGAG
GAATTAAACAATGGCACAAGTTCCAAAGAAAAGAATAGACACAGGTACTGTCGGCAATCC
CAGCACCGGGGACCTCCTCTATGACGGCGGTAATAAAATTAACGATAACTTTGATTCAAT
ATATGACGCATTTGCTGATCAACGGTTTAAAGACGTAGACCACGGCGTAGGAAGAATGGT
TATTCATGCAACCGGATACTATCAGAAACATCCACGTCAAACCTACTCTGGTTCCGCTGT
TGAGATTGGGAGTCTACACGTTATCGATACAACTCAAGGCGCACTTATTGCTATTCTACC
TAAAGCAAAGGTCGGTGAGGGTGTTGTATTCATAAATAGTAACGGTAGCCTTTCAACGAC
TACACCGCTCAATATTCGAACTCAAGCAGGTGATTCTATTAGCGGGTATTCCGGTGATGC
TACAATAACACAACCATTTTCAAAAGTCACTGTTTGGTGTACTGCCGTTGAAGGTAGTCG
CGTAACATGGCGATTGGGTATCGAAAGTATGTTTACCGATACAACAACGCCATTAGATAA

AACAGTTAACGTAACTGGAGTAGCAACAAACGTTGCAATTGCTGCAAAAACAGAATACGT
TTCCATCAAATTATTAACTACCGCTAGCAATACATCCGGTTCAAAAATGCGTTCAAGTGA
AACCCTACTGATGATCGATTCTAAAGCCGGAAAAGTTTATGCGACGGAATATGCAGTTTT
GCCGAAGACAGAAGACGAATTATACACGGTTCGTTATTTCATCGGTTCCGGCGATAAAGT
ATATGCAGAGTATAAGGCTACTGGCGAAAATATTCGTTTAGAAGTTAAAGCCGTAGACAC
AATTAAAATCGGGGTTGCTACATGAAACAATTAATTAATATCGGTATCGTTGTTGATGAC
GGTACAGGTGATTATCTAAGAAAAGGCGGTCAAAAGATTAATGACAACTTTTCTGAACTT
TATGCTAAGCAGGGGGATGGTGATATCCCTCATGCATCCGGCGCGTGGAAAACTCACACA
CAACCAGTATTAAAACCAAATTTTGGTGATGCATGGGCGATTAATACAACGAACAACGCT
GTTTCTGTTGAATTGGCTAAAGGTGGAGTTGGTGATTATAACAAGACAATTAAACTTCGT
GATGTTTGGCGCTCGTGGGCGTCTCATAACGTAACACTTAAGCCAGCACAAGGTGATACC
ATTAAGGGATCTCCAATCGACCGTGAGTTGTATAAAGATTTTATGGATGTTGAACTGGTT
TATTGTGCGCCTGGACGCTGGGAATATATCGAAAACAAGCGAGTTGACACGATCAGCACG
TCTGACCTAAGCACAGTAGCTAAGGAGGTTTATGTTGCCACATAAGGGCAAACAGATTTC
CCTAATGTATTTGGTTATAACACATATAACACTCGAAATGTTGAAGTATATTATTGAGGA
AACCTTTTATATTATGGCAAGTCTGTTAGTAATGACTCTAACTACGGTTCAATTGGTGAG
GATGGATCACTTGTTGAACTTGATGGCAAAAGCATAAAACTTCGTTTTCCTTGTTCGGCT
GGGGATACCGTCCAAATTATTACATATATGGACGGAATTGCATCATTCCGATCTAGTTAC
ATCACACACACAATTAGAGTGTATGATACCGGAATGACAGCACTAGAAACTATTCCTGGT
GAAGTGTGGGTAGGTGATTTATCGACGAAGAAAGAATTTACCAATGCTGATTTGGGAGTT
AGTAAACGTGATATAATTAACCCAAATTCATTTGAGTTATTAATAAATGGTACTCAAATG
ATTAAAGCTGGTGATGCGGATCTTCCTTCGTTTGTTTGTGAAGGTGATGACTGAGAAACC
GAAGAGAGTTGCGCACTTGCGGGCGGTCAGTGGGTACAGTCTGGAGAAGATTATTCACTG
GTATTTGATGAGAATAGCATTGTAACAGGGATTAAATTATCTAAAGGGTTGGACAATAGG
GATACTATAACAATTCGTTGGTTTAATAACGATATCGGAACTCTATTAGAATGGGATGGT
GTCGGCGGAATTAAAGAAAAAGCAGACAAGATTTATCTCAATAACGAAGACGAAGTTAAT
TTAACAAAACAAATTGAATATACCGACTTCCAGAATCCTAGCCAGAAAAACATTCGTCCG
GAGGAAGAGTATTCAAGTCGAATTATGGATATATATGCACTGTTTGATATTTTCCATCCG
ATCGGCACGATATATGAAAACGCACATAACCAAGCTAACCCAGCGACATATATGGGTATT
GGTATCTGGGTTCGGTATGCGGAAGGAGAATTACTAGCTGGTTGGTCTTCCGATGCCAGC
GACCAGAATTTTGCATTAAACAATAACGATCTTGATATTCATGGACAACCTACACACACC
GCTGGTGGTACTGTTGGTGAAGTTGCCTATCAATTCAAGAAAGATATGATTCCTGAATTA
CAATCCAGCGATCGTGTTTTGGTGAAAGACGATACAGGGTCAATTATCATTGGTGGTTGT
CAAGTTGACCCTGATGCGGCAGGTCCAGCGTTTACGAAATATAGAGAGGATATCGTAAAA
GTTAATACTGGAAATGCTGATCCATCAATGATGGGTATTCTTCCTCCATATCACACCGTT
CATCGCTGGTGAAGGGTAGGTTAATATGATTTCTCAAACGCGTTCTAAAGCAAGCGTATT
TTCTAGCGACGCAGCATTCATTTCATACGATCCAGGGTCTAAAGACCCCATTATTGGTGA
CATTCGCCCAATTGGTGGATTAAACGTAGACCAATCTCGCAAAGGTTCTTTTATTAACAA
CGTACAATCAGCCATTGATGATTTGTATAGCATTTCTATGCTTAATGTCGGTGATGTATT
GGTAACTACAAATTCAACTCCACCGGAAGCGGCGGGACAAATAGAAACTCTTTCTTTCAC
TGGAACGGTAACAAATAAACACGATCCAGCCGCGAAAAAAGTTGTGATTGAAGTTTTCGG
TTATCCATTTGTTGTTAATGTTGGAACATCGAATGTTGCATTATGCGAAATGGTCACAAC
AAAATTTACTGAATTGGAAGACAAAAGCATCCTGTTCTCTAAAGTTCAGAGGAAAGGCAC
TGGCAACGATCAAATAGAGATTCAGTATATTGATTCAAACGCACACGAAGCAACGGTCGT
AGAAAAATATGGTATTAGCGTTCATGGGGAAATAAATTCCCCTGCTTGTTATGGTTATGG
TGCATGGGTCAGATTGGGAACGGAAGAAAAATTTGATTCAACACTATTTTATTTTAAGCG
TATTGCGTAAGGTCTAGAATTATGAATAACACTATTAATCACGTAAAAGACGATGCACAG
TATGTTAAGTTTGACCCTACCGGAAATTGGCCTAGCAATATAAAAAACGTACAAGCGGCA
TTAGCCGCTATTCAGGGATTTGCTATTGCAGGTCTTCCAACGGCAACCGAAGAAACAGCG
GGTATTGCTGAAATTGCTACTCAACAAGAAGTAGATGATGGGTTGGATGGAACTAAAATT
GTTACCCCCAAAACATTGGCTGTTAAGCTGTCAAGACCGGACGCAACAAAATTTGTCAAA
GGGATCACTCGCTATGCTAATAACGATGAAGCTATGGGCGATACTCCAAATATTGCAATT
GGTCCTGATACATTAACCCATGTATTCGAGAATAAGAAAGCAACGGAAACTGTACAGGGT
ACAATTAAAATCTGTTCTATGGATGCGGCAAAAATCGGTAGTGATGATACTATGGCGGTC
ACGCCTAAAAAGATGCACGCTGCTATTGCTCAGAATGTTACTGGGTTGATTCCAGAACAA
AACACGGCGACCGAGTCCCGTGAGGGTTTAGTTCGACTGGCGACAAGCCACCAAACGCTT
GCTGGTACTATCAGAGAAGGATTTGCCGTTAGCCCATATGCATTTTCCAACGCAAGAGCC

AATGAAAATCAGGCGGGTACTGTTAAAATTGCGTCATCCCAACAAATGATTGACGGTAAT
GACAACACAGTAGTAGTATCAGCACAAAAATTTGCGAACACTAAAGCAACCACTGGACAA
TTCGGTGTTGTTAAGCTAACAGATAACCCAGGATCAGGCGAGGCAAACACAGCACTGTCG
GCAAACGCTCGTGTATCGCCTCTAAGTGATGGTGTCGTTACTGGTGATGTGTATCGTCAT
AGTGGAGGCGACGGAAACCAGTTTATCATTAAAAATGAACTGGATGGTCTTTGTATGCCC
ATTGGTGGCATCATTCTTACAGCATTTAACAGTTCCGACCACGCACACTTCAAAATCTGC
AACGGTCAATGATTAAATAAACACCAATATCCAGTATTATTTTCACGAATCGGTTTCACT
TACGGCGGAGATGGCGGAGACAACTTTGCTCTACCCGATATGCGAGGGCTTGTTGCTAGA
GGATGCGACCACGGTCGAGGACTAGATCCAGGTCGTGGATTTGGTACTTATCAAGACGAC
ACCATGCAACATATGACAGGTAACTTCCCTGTAACCAACCGTTGGCGAGGTTGGACTGGT
GGTGTATTTGCCATCACAGGCGGTTAATGGAGCACGAACTATAAAAACGGTGGTGGTGAT
GATTGGGGTAGTATTGTTAACTTTGACAGCGCAAGACAGGTTAGAACGTCTGGCGAAACT
CGCGTTAAGAGCCTTGCATTAAACTACATGATAAGGGTACAATAATGATCGAACAGTTAA
ACATTCGAAAGATCCCTAACGTCGATGGTGTCCCTGGAGAGGGACAGCATTCTATTAGCT
GGATTAAAAATGGTGAATGTCTAAGCGCAGGAACCACAGAAACAGGACACGAGGGGACGC
TTAACCGCGCCCCTTATGCGATTCAAGAAAATATTGTTCGTCTAGATGAAAATTCTAAGA
CAACGGTAACAAAAGTTAACGAAGTTGATACAGCAGTTAATGATATTAAAACTACTTTAG
GCGCGGTTGCTGATGGTAGTATTATTGATATTGTCAATCAAACTATTGCAAAGGTTGATT
CTGTAGAAGAAAATATAGGAGAAACTTCAAAGATATTACAAACCACAGTAGATGAATTGG
CAGAGACAGGAAAGAAAATTGGTGTTCGTCCGGCAGCAGCTCCGAGTACACGAACAATAT
ATAATGATATTTCTTTCATCAAACAAGAATTGGGCGCTTTCCCTAACTTCAATATCAACG
GTTCTCCAGATACGGGTAGTTCCGGTTCCGGTATCAAATATCGAGTTATCCAGAATACGG
AAGCATTAAACAACCACGGTCAGCGAATCCGCCAACTAGAAAACGACTGGCAAGATTCTG
ACGTTGGAGAGCTAACAAAACAAGTTAACAAGATGCGAAGCGAGCTAGGCGATACATCAC
TAGCAACACAGGACAATGTTTATCTTCGTCTTCGCACAAATACTCGCAATATCGCTAGTA
TATCAAACGAAGTTAAAGAAGTAAAGAAAGCGATTCTATTTGAATAAATTCCTGATATCG
GGACTAAAGTAACTAAGCTACAATCTGATTATACACAGCTAAATGATGAAGTCAACGGCG
TTGACGCTGGTATCCGTCCTCGTTTAACTCTTCTGGAAAATAATAATGGCAATCCTCAAG
CCGCTGGAAGTATTGATTATCGATTGACTAATCAAGATCGGGAATTTGTAGATCTTAAAT
CTGTTGTCGGTGTTAGTGGTGCTGTCGGTCTACGTGGTGAGGTTGCACGAATCTCTAACA
CAGTTGGTACTGATAGTGAGCGTCATACTATCGCAGGTCGTCTAAAAATCGTCGAAAACA
CACAGGGTCGACTAGAAGCTAGCGTAAAGGATATCGAGCAAATGATCGGCGATCGAGATT
CCGGTCTAGCCGCTGGGGTTATTAAGCTGTCAAGCGCCATGTACGGCAATCCAGAAGGAA
GCACGCAGCTTGAACAAGATGGGATCGTGAAAACTGTTAAGGAATTGAGCACATCAGATG
CAAGCAAGGTAACAGACGTTCCTGATGATGGTTTCCATTGTCTACGTAAGCACGGGGAAT
GGGTTCAGGTCGCATGGGCGGCTGGTGCATTTAAAAACACCGCTCAGATTGATATTACTC
TCGACGGACAAGATCAATACAAAGGAATCCCGCTAAACGATATGACCGAAGAAGTTCCTG
CGCGTGGAATGGTTAAGAGCGAATCACTTGTTACGGTTAACGACAAAGGCTTATTCCGTT
GTGATTTCCGTTGTTCTGTAAATGCGGCACATAACGCTACTTTCGTAGTCGGTATTCCTA
AAAATGGTCTGATTGTACATGAGGTACGGAATGGTCATTATGGACCTGATGATTTAATTA
GCTATTCTACCAGTGTTTTCCTAAATATTGAAAAGGGCGACCGGATCGATATTCGCATTA
AGGGCGTATCCGAAGAAGCATTAGCGCAACAGCTACAAATTAAAGAGTTCCTCTTTGGAA
TTTCTCCGGTATAAGAAATTAAAGGGGCGTTAAGCCCCTTTTTCGTTTATAGGTGATATT
ATGTCAGGATATGTAACCAATAACCCAAGAGAATTAAAAGACGCTATTCTGCGTCGTTTA
GGCGCACCAGTAATTAACGTAGAAGTAACAGAAGACCAGATTTACGATTGTATTCAGAGA
GCATTAGAACTCTATGGGGAATATCATTTCGATGGTGTGAATAAAGGATATAAAGTATTT
CGTGTTAGTGCAGAGCAAGCGCAACACGGGGTATTTGATTTAAGCGATTCTGGAGTATTC
GCAGTAACTAAAATAATTCGAACAAACGTAGGTAGCGCGGCTACGATGGATGGTACTGCC
GTTTATCCGTGGTTTACTGACTTTCTGTTGGGTTAAGCGGGTGTAAATGGCGGTTTAGGA
TCTGGCTGTAGTAAATTTTATGGACCAAATGCAATGGGCGGTAATCTAGGATATTTCGAA
CAGCATATGAGCTATCGAGCCACTATGCAAAATATGCTTAATCCGCTGCCAGATTATTGG
TATAATAGCGCAAACGGACAACTTAAGATAACAGGAAACTTCCGAGAAGGTGATCTCATT
GTTGTTGAATTATATGTTAAAAGTTATCTTGATGTACCTGATATGATCGGCGCGGTTGCT
GGTTATGCTCATGCGGGAGGATGTTCTACCGCTGGCGGGATTAGCGCCGTTTATGATAAC
CCCACTGCTGGTTTAAGCGGGATTGTTGCTGCCATGGGACCGATCCGTGATAACAAGCAG
GGCGCATATAATAACCGTTGGGTTAAAGACTGCGCGACTGCGTTTACTCGTTAGCTATGG
GGGCAGATCTTAGCTAAACATCAAGGGTTGCAATTACCAGGTGGTGGCACTGTTGATGGG

GTCGGAATCATCGAAGAAGCACGCGCGGATATTGAGAAATTGCGACAAGAACTACTTGAC
TTAGATCCTCCGTTCGGCATCCTCATGATTTGATTTATTGGGGGAGGATTCCCCCATTTG
GAGTAACATATGTTTAACAAGACTCTTTTTGCTCGCCTTGAATCGCAAAAGGTTTATGAG
AATACACTACAAACCGAGATTCTCAATCCTTACGTAAATTTCAACCGTCATACAAACACC
CAGACGTTAGCCGACGTTATGGTCGCCGAAGCTATTCAGATGCGAGGTGTAGAATTATAT
TACATCCCTCGTGAATTTCTCAAACCAGATATGATCTTTGGTGAGGATGTTCAAAGCAAA
TTTACTAAAGCATGGAAATTTGCTGCGTATATAAACTCTTTCGACGGGTATGAAGGTGCA
GGAAACTTTTTTCAATCTATGGGTTACAAGGCAAACGACGAATTAACGTTTACGGTGAAT
CCTAACCTATTCAAGCATCAAGTAGATGATCAAGAGCCAAAATCAGGTGGCTTGATTTAT
ATTCCAATGAGTAATGATCTCTTTGAAATTAATTACGTCGCTCCATATCAGCCGTTTTTT
CAGGCTGGCAAAAATGCCATGCGTAAAATTATTGCAGAGAAATTTGTGTATAGCGGTGAG
GAGTTGCGACCAGAATTACAACGCAATGAGGGAATTAATGTTGATGAGTTTGCGGATTTG
GATCTTGCACCGATCACTAATCTGGACGCGTTAACAGATATCAACTTAGATCAGTACAAA
GAAGATAAGCAATTCAGAAGCGAAGGACAAGATTTTATTGATCCGTTTGATCCTATTAAT
GGCAAGGGTTCACCTTTCTCCGATTTTTGATAAATACTTGTATTGATATAATATTAGGAT
CTAATGACTTATGAAAAACTATTATCTATACGAAATAAAGAACAATATAAATGGTAAGAT
ATATGTTGGCATTCGTGGGTATGATGGTGACATAAGTCAAGATCCTTATACGGGTAGTGG
ATTGACTCTTAAGAGGTCTATTAAAAAGAACGGGATAGAAAACTTCACAAAAACAATTCT
GTAAACGTTTGACTCATGGAAAGAGGCAAGGAAAGCAGAAAGAGAAATAGTTACAGAGGA
ATTTGTAAAAAGAGAAGAGACCTATAATGTTTCGGTTGGTGGACATGGTGGAAATACTCA
CGCTGGGATCAGCGAAGAACGAAGAAAACTTGCAAGCGAAAGACACGCTGAAGCAAACCG
AAATAGCGTATGGAAGCAAGAATCTAGAGATAAGGTTAGCGCAAGATTCAAAGGTCATAA
GCTATCACAGAAGCAGATAGACGCGCTAAAGAAGGCAAACACAGGAAGAAAGTTCACAGA
AGAAAACAAGTTCAAAATAGGTAAAACGTGGCGAGGAAAGAAACGAACAGATATAGGAGA
GAAGGCTGCGGCAAAATTAACAGGGAGAAAACTACCCCCAGAACATGTGGCAGCAATAAC
GCAAAATACACCGAGAGGAAAACAACACTTTAGAGCAAAAGGATTCTTCATCGACGATAT
GGAATTTTATATTTTGAGTAATGCAGCTATAATGTTTGGTATAAGTCATAAAACGGTTAG
AGCTAGACTTCTCTCAGATAAGTTTCCTACATGGAGATATGAATAAATGTTTTCACATTT
TTACAATTCGTCAATTAGAAGGTATATTGTATTGCTGGGTAATTTATTCACCGGCGTACA
AATTAAAAGGGTGCGTAGTGACGGTGAGCGTTTTATTAAAGTTCCTATCACCTACGCAAA
GAAAGAAAATATCATGATGAGTCCGGACAAATATAACAATATCTTAAGCGAGGAAGACGT
CGCAAAGGTAGAAACTATTTTACCTCGTATGTGTCTTTCTTTGATTGATATGACATACGC
CCAGAACTTTAAAACCAATATTGCAAATAAGACAGTACCAAAATATACGGCTGATGGAAT
CAGGAGCGTAACACAATATAATCCTGGACCATATAAGTTTACTTTCGAGTTAGGGATTTA
TACGCGTAACGAAGATGATATGTTTCAGATAATTAAACAGATTCTTCCTTACTTCCAGCC
TCATTTTAACTGTAAGATTAAAGAACTACACGGTAATGATGTATGGATTGATCGAGATAT
TCAGGTCACAATTAGTTCTATTACTCCAGATGAACAGGTAGGCGAAGATAAGTTTTCTCG
TCGTCGTCTTGAGTGGACCATTATGTTTGACTTCATCGGGTTTATCTATCCTCCAGCCAC
CGACGTTAAGGGGGAAATCAGAACGATTTATCTTGATTTTCATGCTAATTCTCGTAAATT
AAATCCTAGCGGTAATTTTGAATCAGTGGATCATGAAGTTGTTAAACGTGATGTTGAGCA
AGAAGAATGGGACGGAACTATCAAAGAATCATATTCTAGTGATATTCCAATACCAGTTGC
ACCGACTCCACCGGGTCCACGTATTGTAAGAGAATTTTAAGAGGTAATTTATTATGTCAG
ATCTAGGCTTAGATATGAAGCAATTAATGAATTTCGAAAGTCTAGGTCTTCCGGGGTCAT
TCGACGAGGAAGAAGATCGAGTGAGCTATGATCCGCTCGTCTTGACTCCGGTCGAAAGCC
ATCCAGAAGATCGTAATATTGACTTACAACGAGATTATAAGGAGGCACGTCAGAATATTC
ATTTTCAGAATCAGATGATGATGGACGCCGAAAAGATTTATCTTGAACTTGCTAAAAATA
GTGAGTCTCCAAGGTTCCTACAGGCATTTAACGGCCTCATGCAGCAAATGAACAACAACA
ATAAAGAATTGCTGAACATCCATAAGGATATGAAGAAAATCACCGAGCAAGCTGGAGAGA
AAAAGAAAGATAATACCCCAGCCGCGCCAGTTAATATCCAGAATGCGACCGTCTTTATGG
GTTCTCCTAGTGATTTAATGGATGAAATCGAAGACGAGGAAGCGCGAGTTATTGAAGGCG
AGACAGTATGAGCGTGATTGAAGGGATTAACGCAATGGCGACGGATGAACACCCGCTGCA
TTTAGCTCATCCGTCTCCACTAGAAACTAAAATTGATTCAAATGGTATTGAATGGATTTT
ATCAATGCATGATGATAAATGGTATCCTAAAAAGTTTTCTGACTATCTGAAATTAAACAG
ACCGCAGAAAATAAGGATGCAAAGCACTGATCCAACAAATTATAAGTTCTATAAAGATAG
TGACAATATCCGCACGCGGTATATGCGGTTAAAGAACTTGCGAAGGGCGAATATTAAAAC
TCAGTATACCCCTGAAATGATCGCGGAGTGGAAACAATGCCGTAAAGATATTGTATATTT
CGCAGAAACCTATTGCGCAATTACACACATCGACTACGGTACAATCAAAGTACAATTGCG

TGATTATCAGAAAGATATGCTTAAGATCATGCATGAAAACCGCATGAGCGCCCATAAACT
CAGTCGTCAGCTAGGGAAAACTACCGCTGTAGCAATATTCCTTGCTCACTACGTTTGCTT
TAATAAAGACAAAGCGGTAGGTATACTGGCACACAAAGGATCAATGGCAGTTGAGGTTCT
AGAACGTACTAATCAAGCAATTGAACTATTGCCGGACTTCTTCCAGCCTTGTATTGTAGA
ATGGAACAAGAAATCTATTGTGTAAGAAAACGGGTCTAGTATCGGCGCTTATGCATCGTC
ACCAGATGCGGTTCGCGGAAACTCCTTTAGTTTCATATACATAGACGAGGCAGCCTTTAT
TAGTGCGTGGCAGGACGCATGGTTAGCTATTCAACCAGTTATCTCATCCGGTCGTGAATC
TAAGCTGCACATGCCAACAACTCCGAACGGGCTTAATCACTTTTATGACATTTGGCAATC
AGCCATTGATGGTAAAAGCGGTTATGTACCTTATGAGGCGGTTTGGCATTCTGTTAAAGA
ACGTCTTTACAAAAAAGCTGATATATTCGATGATGGTTATGAGTGGTCATCTCAGGCGAT
GGCCGGATCTAGTATTGAGCAATTCTTACAGGAACATAACGCTGAATTTTCCGGTAGTTC
CGGTACTCTGATCAGGGCAACCACACTATCAAGATTATCATTTATTGACGTTGTTAATGA
AAACGGGTTTAATCAGTTTGAGAAACCGAAAGAAGGTAGAAAATATGTTGCTACACTAGA
TTGCTCAGAAGGTCGAGGACAGGACTATCATGCAGTGCAAATCATTGATATTACAGAATT
TCCTTATAAGCAGGTTGCAGTATATCACAGCAATACTACGTCCCATTTTATATTACCGGA
TATTGTCTTTAAATATCTCATGATGTATAACGAATGTCCAGTTTATATCGAGCTAAATAG
TACGGGTGTATCTATTGCTAAGTCTCTTGCGATGGATCTTGAATATGATAATACTATTTG
CGACTCATTCATAGATCTAGGGATGAAACAATCCAAAAGATCTAAAGCAATGGGATGCTC
GGCACTAAAAGACTTAATAGAAAAAGATAAGCTGATCATTAATCATAAAGGAACTATTCA
GGAGTTGCGCACATGCTCCGAGAAGGGAGTAAGCTGGGCGGCAGAAGAAGGTTTTCACGA
TGATTTGGTCATGCGTCTGGTAATATTTCGTTGGTTAACAACTCAAGAAAAATTCGCCGA
ATACGCAGGAAAAGACGAAATGCGGATCGCCTCCGAAATATTCCGAAAGAAATTAGATGA
ATTGGGGGAAGAATACGCGCCCGTGGGAATATATGACGGTGCAAACGGCATCGAAGAATA
TAGGCCGCGTGAAGGTCTAACAATGATCTAAATACGAGAATGCATAAAAAGATGAGGAAA
TTCTGATGACTCTATTATCACCGGGTTTCGAAACGAAAGAAACTACTCTCTCCACTACCA
TTGTGCAATCTGCGACTGGTCGCGCGGCGCTGGTAGGTAAATTTCAATGGGCTCCAGCTT
TCCAGATTGTTCAAGTAACTAATGAAGTTGAATTAGTTAATAAACTTGGTCAGCCGGATA
ATAAAACCGCAGACTATTTTATGAGCGGGGCTAACTTCTTCCAATATGGCAATGACCTGC
GTGTTGTTCGTGTACTCAATCAAGAAAAAGCAAAAAACGCAACTGCCCTAGCTGGTAACG
TAGAGTTTGAAATCACAAACGAAGGTTCAAACTATGAAGTATGCGATACCATCAAGATTA
AACATAACCGTCAAGATATTGAAACGGCAGGTAAAGTAACCAAAGTTGATGGTGATGGTA
AAGTTAAGGGCGTATTTATCCCAACTGGTAAAATTATCGCTCACGCTAAAGCAATCGGCG
TATATCCAGAACTTGATGGCGGTTGGACGGCAGAGTTCACTTCTTCGTCAGGTAACGGTA
GTGCGGCGTTGTCAGTAACTAAAATTGTTACCGATTCCGGTCTTCTCTTAACGGATCTGG
AAACTTCCCGAGCCAATATCACCAATCAAGATTTTTTGACAAAGCTCAAAAAGTATGATA
TGCCAGCGGTAAATGCAATCTATGCTGGTGAGATCGGCAACTCTCTGGAAGTTGAAATTC
TGGCACGTAGCTCGTTCAGTGGCGCGGTAGCACCTGAATTAACTATGTATCCGTTCGGCG
GAACTCGTGCCGCAGCACGCAACCTAATTCCATATGGACCGCAGAATGATAATCAATATA
CGTTTATTGTTCGTCGAGATGGTGTTGTGGTAGAATCCTATGTACTGTCTACTCTGAAAG
GGGATAAAGACGTATACGGCAATTCTATCTACATGGACGACTTCTTTGGTCGTGGTTCTA
GCCAGTACATCTATGCAACCGCTCAAGGGTGGGTAGATGGTTTCAGTGGTATTATCTCTC
TTGCTGGGGGTGTGTCTGCTAACGAAGCCAGTACGGGCGACCGTGGAAATGATCCGTTCA
TTGGTGCGATGATGCAAGGTTGGGATCTGTTTGCTGAACGAGAATCTATTCACGTAAACC
TGTTGATTGCTGGTGCATGTGCTGGCGAAGGCGATGCGTTCTCTACCGTACAGAAACATG
CTGTTTCCATCGGTGATGAGCGTCAGGATTGCTTAGTAAGGGTTTCTCCGCCGCGTAGCA
CTGTTGTTAATATTCCGGTCACTACCGCAATCGATAATCTGATCGCATGCCGAGAAGGAA
ATAGCAATTATTCAGACAACAACATGAACATTAATACCACTTACGCGGTTATTGATGGTA
ACTACAAATATCAGTATGACAAATACAACGACGTACACCGATGGGTTCCGTTGGCTGCTG
ATATTGCTGGTTTGTGCGCTCGTACTTATGCTGTATCCCATCCGTGGATGAGTCCGGCAG
TTTATAACCGTGGTCAGATCATGAACGTGGTTAAACTGGCTATTGAGCCTCGCAAGGCAC
ACCGTGATCGTCTGTATCAGGCTGCAATTAACCCGGTGATCGGTGCTGGTGGTGAAGGGT
TTATCCTGATGGGTGATAAAACCGCTACGACTGTTCCTAGCCCGTTTGACCGCATTAACG
TTCGTCGTCTGTTTAACATGCTGAAAAAGAATACCGGCGATTCAAGCAAATACAAACTAT
TTGTAAACAACGATAACTTTACTCGCGCTTCCTTCCGTATGGAGGTTTCGCAATATCTCA
GCACGATTCGCTCTCTTGGTGGCATTTATGACTTCCGAGTACAATGCGACACCACGAACA
ACACGCCGGATGTAATGGATCGTAACGGATTTGTTGATGGTATGTTCATCAAACCAGCAA
AATCGATCAACTATATCATGCTCAATTTCACTGCTGTTGCAACTGGTGCTGATTTTGACG

AAATTATCGGTCCAGCTAACCAGGCATAATCAGTAAATTAGCATAGATTAAAAGTTAAAT
CAAGCGGGGATTTTCTCCCCGCTCTATTAAGGAATATCAATTATGGCACTCGATTTGAAT
GATATTACACGCGGTTTCGAGTCTGACGATTTTGCTCGTCCTAACCTGTTTGAAGTTGAG
ATCCCGTATCTGGGGCAAAACTTTAAGCTGATGTGTAAGGTTGCGACTGTCTCGGCTGCT
ACCGTAGAAAAAATTGCTGTAGGCTATCAAAACCGTAAAATTAACATCGCTCGCGACCGT
GTATACGACGATTGGACCATCACTGTATTCAACGATGACGCACACAAGATCCGTGATGCT
ATCGTTGCATGGAGCGATCAGACACATGGCACAGGTTCGGCGATCACTGGTGCAACTCCT
GCACAGTATAAGAAACAAGCTATTGTTCGTCGACTTAACCGTAACACCGAATCAACTAGC
GAAAAAACATTGTACGGTATTTTCCCGACCAACGTTGGTGAAGTAACCCCCGATTTGGGA
CAAAACAACGAAGTTGAAACGTTTGAAGTTACTTTTGCTATCGATTGGGTACAATAAATA
TATTATCATGCGGGGAGAAGTTCCCCGCTTAATTTCTCCCAATTCGAGGTGAATAATGTT
TAGTAGATTAAAAATGCTTGCGCGTTGGGCTGATTTTGACAACGACAAATACGAAGATCA
AATTAAAGATAAAGCAGAGTCTATCGCCGCACCTAAAAACAATGATGGTGCTACGGAAGT
AGAAATTAATGATAATTCACCTTCTAGCGCGTGGCACTCTATTACCCAGCAATTTTCCAG
CACCGACCAGAAAATCAGCACAACTAAGCAATTAGTTAACACATATCGCGGGTTGATGAA
TAACAATGAAGTCGAAAACGCTGTACAAAATATCGTTAATGATGCGATTGTATTTGAAGA
AGGTCATGAAGTTGTTTCATTAAATCTTGAGGCGACTGGCTTTAGTGAATCTTTAAAAGA
GCGCATTCATGAAGAATTTAAAGATCTTTTAAACACTATCCAGTTTGATAAGCGTGGTCA
GGATATGTTCCGTCGCTGGTATGTCGATTCGCGTATTTTCTTCCAAAAAATCATCGGCAA
AAATCGAAAAGATGGTATCGTAGAATTACGCCAGCTTGACCCACGAAATCTTGAATACGT
TCGAGAGATCGTTACAGAAGACACACCGGAAGCTAAAATTTATAAAGCAATAAAAGAATA
TTTCATTTACACTGTAGGCAATAGCTCTTATTGGGCAGGTGGTCAAGTATTCTCTCCTAA
TAGTCGCGTTACAATCCCGCGTAGTGCTATTACATACGCTCACAGCGGCTTGATGGATTG
CGATGATAAATATATCATTGGTTATTTGCATCGGGCTGTTAAGCCAGCAAACCAGCTTAA
ATTGTTGGAAGACGCAATGGTCGTTTATCGTATCACGCGTGCACCGGAACGCCGAGTGTT
CTTTATTGATACCGGAAACATGAACAACCGCAAGGCTGCACAGCACATGAACTCAGTGGC
ACAGTCTTTTAAAAACCGCGTTGTTTATGATGCATCGACTGGTAAACTTAAAAACCAGCA
AGCAAATCTATCAATGACAGAGGACTACTGGTTACAGCGTCGAGATGGTAAAGCAATCAC
CGACGTTACTACTTTCCCGAGTGCATCCGGTATGAGTGATATCGATGATATTCGCTACTT
TAACCGTAAATTGTATGAAGCGTTGCGTGTGCCATTGTCACGTAGCAATCTCTCCGATGC
TAATATGGTGATCGGTGGTGATGGTTCAGAAATTACTCGCGATGAGTTGGAATTTTCGAA
ATTCATTAGAACTCTGCAATCACAGTTTTCGGAAGCATTGCGCGATCCTCTGAAATATAA
TCTGATTCTCAAAAATGTACTTACGGAGGATGATTGGGATCGTGAAATAAATAATATCAA
GGTGGTTTTCCATCGGGACTCTTACTATACAGAAGTTAAAGACGCGGAAATTTTAGAACG
TCGAATCGGTCTAATCGAACGTATTACACCTTACAATGGTAAATATTTCTCTAACCAGAC
AGTGCTGCGTGACATTCTGAAATATACCGATGACCAGATGGACACCGAGAAGAAACAGAT
CGAAGAAGAAGCGAACGATCCGCGCTTTAAACAAACTCCCGATGAAATAGAGGATTTTTA
AATGAAACAATTTATCGAAGCAATTAAAACTCGCGATCTAACGTTGATCGAAAAAGGATT
TGATAAGATCATGGAGGCTAAAAAAGCCCAGATCATCGAATCCCCGCGTACCGAAATCGC
ATCCAGTATCATGATCGAAGGTGAAGAAGAAAAAGACCGCGGTGATGAAGAAGATATGGA
AGATGAAGATCAGGACGTCGAAGATCGCAAAGACGATAAAAAAGACAAAGAAGACGACGA
ATAATAGGAAGCCAACATGATTAAAGATTTTACACCTAAAGCTGACTTAGAAACTTTTCT
GCCGGAAGCACAGGAAAAATTGGACATGTTGGCTATGTTTGAGTCAGAAGAACTATTGAA
AATCGTTGAAGGTATCTCCGAGGATGAGCCGGAACTGGCGGTAGCAATGCTGTCTATCAT
CGACGGAATTACTGTAAATGAAGCAATGGTTAAACATGTAAACGCACGCGGGGAAGTCGA
ACGTAAGAAAGATCGCAAAACCCGTGAGCGTAATGCTTACATGACTACTGGCCTAAGCAA
AGCTAAACGCCGTCAAATTGCCCGTCGTGCTGCAAAAACCAAACGTGCAAATCCACCTAT
CGAACGTCATGCGGAACGTAAGCGCAAAAAAGCAATGCGTAAACGTAAGGCGTTAGGATT
ATAACAGGAAACAACATGGAAAAGAACGAATTACTGATCGAACAATGGGGCGTTCCTTGT
GCTCGCGGTAGTTCGTCATTGCTTGAATCATTTGATGATAAGGGTACAGGAAATCTCTAT
ATTCAAGGGATTTTCCTGCAAGCCGAAACGGTAAACCGTAATAAACGCTGGTATCCTCGC
AGTGTTCTAGAAAATGCGGTTACTAAATACATACATGAACAAGTTGAGACACATCAAGCT
CTAGGCGAACTTAATCACCCAGCAAGAGCAATGCCAGATCCATCTAATGCTTGCATCATG
ATTGAAAAATTGTGGTGGGAAGGTAATAACGTGATGGGTAAAGCCCGTGTTATTGATGGG
GATAAAGGCGCAGGTGATAAACTTGCTGCACTGATTCGCGCTGGCTGGATTCCTGGCGTT
TCCTCCCGTGGATTGGGTCGGTTGAAAGACTCTGGTCGCGGATACAATATTGTACAGGAA
GGTTTTGTGTTGACGGTTGGTGTTGATGTTGTTTGGGGTCCGACCGCACCGGATGCGTGG

GTAACTCCTGTGGTTGAGTCAACGGATAGTGATTCTAGCGCGTAAGACGCACCAGAAAGC
GATAAAACCGAAAAAGTGCCTAACCCTACAGCGTCACAACAAAACATCGTCGAATCGAAT
AATATCGCGTTTATGGCGCTCGCAGAAGCGTTATCTAAACGCTATAAATAATAACGGCGT
TATCAACAAGAAGGTTATAACCATGCTTAAAGAAATGCTGCTCGAAGAAGCAAAACAGAT
CACCGTAGACGTTGCTCTCGATGACCAGTTTGAATCCGTCGAACTGTCGCCGGAAGTAAA
AGAAAATTTTGGTTCTATCTACGCTCAGGCGGTAAAAGCTAATGCTGTTGCTCTGGCAGA
AAGCCACATTGAAAAAATCGCCGCTAAAGCCGATGAACTGGTAGAATCAAAGGTCGAAGA
AGCACGCACCGAGATTGAAACCAAACTGTACGAAGATGCAGATAAATTCCTGAATCACTT
AGGCGCTCAATGGCTGGCAGAAAATAAAGAAGCCGTTCCCCGTAATATTAAAGCGGATCT
GTGCGAATCCCTGATTGGTTCTCTGAAAGATGTTTTCGTTACTCACAATGTTGTAGTGCC
GGAAGAATCTGTAGACGTTGTTGCTGAACTTGACGAAGCACTCAAAGAAGAAAAAGAAAA
AACCTCCGAACTCTTTGATGCCAAACTGCAACTTGAAAGCGAAATTCGTGGTATGAAGCG
CGAACAAGCGATCAATGAAAGCACTCGTGATCTTTCCGATACTCAGAAAGAAAAAGTAAC
CGCGCTGATTGAAGGTCTGGAATAGAGCGAAACTTTCGATAAGAAATTAACTGCAATTGT
CGAAATGGTGGCTAAGAAAGAAGACAAACCAGCGAAGATTGAAGAATCACTAAATAATGA
TGCCGACAAACTGAATGTTATTGCAGAAGCGGTCGAAGAATCAGAGAAAAAAGCATCTGT
AGATTCCGGCGTATCTCGCTACCTGAATTTCATCGAATGCAATTCAAATCTACTAATAAA
TACTTTAGGTTAACAACAAACAAGGTTTAAAGAACATGAAAACTACCAAAGAACTGATGG
AAAAATGGTCCCCGCTGCTGGAGAACGAAAAACTGCCGGAAATCGCAACTGCGTCTAAGC
AGAAACTGGTTGCTAAAATTCTGGAATCTCAGGAAGCGGACTTTGCTGTAGATCCGATCT
ATAAGGATGAAAAAGTTGTTGAAGCATTCGGTGGCTTTATCGCAGAAGCCGAAGTTGCTG
GCGACCACGGTTATAACGCATCTAATATCGCGTCTGGTCAGACTACTGGTGCAATCACCA
ACGTAGGTCCGGCTGTTATCGGCATGGTTCGCCGTGCTATTCCTAACCTGAACGCATTTG
ATATTTGTGCTGTTCAGCCGATGAGCACTCCGACTAGCCAGATCTTTGCAATCCGTTCTG
TATACGGGGGCGACCCGCTGGCTGAACACGCTAAAGAAGCATTCCATCCGATGTACTCTC
CGAATGCGTTCCATAGCTCTCTGGCTGCTAAAGATGCAACGACTGTTTCTCCTACTGGCA
CTGCGTTCCAGAAACGCACTCTCAGTACTCCGATTGCCGCAGGTGATATTGTACACCACA
CCTTCGCAGAAACTGGCATCGCATATCTGCAAAACGTTACCGCAGAACAAGTAACCCCGA
CCAAAGCTGACAGCGAGTCAGATGATGAAGTTGTCATGAAGCTGATGGAAGAAGGTAAAC
TGGCTGAAATCGCTGTCGGTATGGCTACTTCCATCGCTGAATTGCATCAAGGGTTTAATG
GTTCTCAGAATAACCCGTGGAACGAAATGTCAATGCGTATTGACAAACAGATTGTTGAAG
CGAAATCTCGCCAACTGAAAGCCCGTTATTCTATCGAAGTTGCGCAGGATCTGCGTGCTG
TTCACGGTATGGGTGCTGATGCTGAACTGAATGCTATCCTGGCGAACGAAGTTCTGCTGG
AAATCAACCGTGATATCGTTGACGTGTTCAACTTTACCGCTCAGGTTGGGAAAACTGGTA
TGGCTCAGACCGTTGGTTCTAAACCTGGTGTATTTGACCTGCAAGACCCGATTGATACTC
GCGGTGCTCGTGGGGCTGGTTAATCCTTCAAGTCTCTGATCTATCAGATCGACAAAGAAG
CTGCTGAAATCGCTCGTCAGACTGGTCGCGGTGCTGGTAACTTTGTTATCGCTTCCCGTA
ACGTTGTAAACATTCTGGCTTCTGCTGATCAGGGTTTCAGTCTTGCTATGCAGGGTGCTG
CACAGGGTCTGAATACCGACACCACTAAAGCTGTTTTCGCTGGTGTTCTGGCTGGTAAGT
ACAAAGTATTCATCGACCAGTATGCTCGTCAGGACTACCTCACCTTAGGTTACAAAGGCG
ATAACGAAATGGATGCTGGTATCTACTATGCTCCGTATGTTGCTCTGACTCCGCTGCGTG
CAACTGATCCGCAGTCTTTCCACCCGGTTCAGGGCTTCAAGACTCGCTACGGCATCGGCA
TCAACCCGCTTGCTGACAGCGAATCCCAGGCGCCGTCCGCTCGCATCACCTCCAGTATGT
TGAGCAGAGACAGCGTAGGTAAAAACGCATACTTCCGTCGTGTATGGGTCAAAGGTTGCT
AATATAAGCCATTCCTGAAAACTCCGGTCGGGTGGAATACCCGACTTATCATAAACCTTA
TGACAGTGTAAAGTGTTAATTCCAAAGGGAAGACTCGAAAGGGTCTTCCCTTTTTGCGTA
TTATAACCCGTCATAAATTAGGTTGAAAGCCTATACTCTATTGGGAGCTAAAATAGATAT
ACACAACAAACAATTCGACCAATTTTCGTAGTAACTGCATGGCAAAGGGACCAATGAATG
AAATGAACGTTCATGTTCATATTGGGTCGGATAGATTTGGTAGATATACAGATCTGGTGT
ATGCCGACCTTGTTATGACATTCCAGCGTGACGCTAGCGGTACTTTTGGTCTTGGTACGC
TGTTCCCTGAATTAACCCCAAATTCTCCATAAGGAGGAAATGCATGAGTAAGAAAGAAAA
CAGCTATGTTATGACTCGTAAGGAATACAACGAATTGATCTTAGCATTAGCTTATGCAAT
TATTCGTTCTGATCATGCAATCAATCTGGTAAACAAAGTTAACCGTAGCGTTGATAATGT
TTCTAGGAAGAAGTAAGTTTATTCTGGAATCCTTCGGATTCCTTATTCTTTTTGTATAAA
TATGATATATCCATTAAGGCAAATACAGGATTCATAAACATGACTCAAATTTTTACTGAA
CTGTTGCGGGAATCTACTTCCTCAGTAGCTAACCAGACAGCGCGACCGCAACTATTATCA
ATAACTCGCGCGGTAAACAACCTCATTTTCTCTGATCTGGTAGCAATCCAGCCAACGGAT

CAACCAGTACCTGCATTGTACGGCCTACGTTATCTTAATCGTGATGGTCAGATGACTTTC
CGAAATGCTACAACCTACGGTGGCGCGGCTGGTGATCGCACAGAGATTGAAGAGTTTTCA
AAAGAAAAATCTTATGACGAAGGCGCACTGTTCAAATCAGACAATGTTTTATACGAAGTT
GTGACCGCTGGTACAGTCGGTGCAGATGCCGAAACTCCAGAAGCTGCAATCTTTAAAGGC
ATAATGACAAACAATATTCGTTTCTATAGCGATTGTGCATCGGTAGAATACTTTGAAGAT
AAAAACACCGAAATTTCGTCTACCTCTATGCAGTTTGATAAATGGCAGGTTAATGTAGGT
TCTCGTAAACTGAAAACCTCATTGACGACCGAATTGATGCAGGATCTTGAGGCAAGCCAA
ATCAACTCTGAAAACTCTGTAATTGATCTTCTGGCTACGATCGCTTCAGAAGAGATCAAC
AAAGATATTATTCAGAAGTTGATCACTGTATCATCTCGCTATAGAATTAAAGGGATCACC
CCTGACGGCGTACTTTCTCTCGTTAGCGTTCAGGATGCACCAGCACAAGCGCGTGAACTT
TACCGCTATGCTTGCGAAATGACCTATCAAATGTTGCGGACTTCCTCCTTTTCTGGTACA
TACGTTTTGGCGTCTTCTCGCGTGGTTGGCCTCCTACAATCTACCGGATGGATGGAAGAA
ACCGACAATGCTCTAAGCGAAGGTCGTCTACGTTGCGGTCTGGAAGTGTACGCAGACACG
ACAGCACCTTTCGATTATATGCTCGTTGGTTGTAAGCACATGATCGGTGATATGGAGAGT
GTAGGTTCTCTGTTCCACTCCCCATATACAGAAGCCGACGGCGCTGGCGCGTATAAGAGT
GTAATTGACCCTAATAGCTTCCAGCATCATGTAGCTATTATGAATCGTTATTCACTCTCT
GTCAACCCATATACATCAAAAGTTGATCAGGAAGAACACTAGGTCATCAAGGGTGACGAT
TGGGGTAAAATGGCTGGACGTTCAGAAATGTCCTACATTTTAGGAATCGAACTGCCACCG
CTTGAGATTGACAACGCTTAACTAAATATCTAACCAAGACAAGTCAGATCCGCCCAAGTT
TTTTTCGACGGCGGGTCTATTTTCTTGATGATATAAGTTAAAACATGGTATCCCATGTCT
ACGGGCAACATAAACAGCAATTCTAGTTCCTCCCGAAACGATTCCATTAACTTCTTTCGC
CCAGAATATCACACAATCTACAGGATCATCAAGTGTCTCACCTAAGACTTGAGCAACATT
TCTAGAAAACAGATTTTGCATGTATAACGGTATAGAACGAGGATTATCATAAACGTGTTC
TAATTCATCCATCGCCAAATTTTTTTGTTCTAAGGTTTGCCATGCAATAATATCATGACC
GTTATGCTTGTGGTCATAAAACTTCCAGTTTGGAATGAAAATTTTTCTATAATCTGGATC
GTAATACTTCATGAAAGCATTGTCCATACCGTAAGCCCCTCCAGAATGCGCTACAACGCG
ATTTTCTGACAATACCCTCCCAATGGTACAAGCCATTATCAATATGTCGCTAGGCCCTTT
TCTAGAGCCTATTAAAGCACATCTACGAAGTGGTTTTACTCCAAATAGATCTTGTAACTT
GTCAAACATAATATTTCCTCCATAGAAAGCAATATCATACTACAAGTTAATAACAAATAC
AAGTATCCGCGTAGCGGAAGCGAGCACGGCGAGCAATAAAACACTTCCAGTAATTGATTA
ATAAATGGTCGAAACAAATTTTTATTATTCTATAAGCTAAGTCTTCGTTTTGATCATTAT
TTAATCACACATGTAAAAACTACTCAAAAGGATATCGCTTCGCGAACTCCTACGGAGTAT
AACATTTGTGTTACAATATGTAGTGATTAATAATTAACCAAAGATACATTTTCTATGTTC
TAAAGATCTTTCTTCGTTTTGATCATAATTTAATCAAACAGTATTAATATTGTGCACATG
TAAACTAAAATTATGTTATTAACAGACAACAAAAAACGCCGACCCGGAGGACGGCTAATG
TTAATTATTTGTTGATAACGATCTGATTTCGGCGAATGGTAACATTACTCTTAGAGATGA
TCAGATCATCCGGCACTACGTTAGTTTTGTATAGCTGTAAGCGAACAAGCATCTGATTGC
CTTTAGACCCTTCTTTAATCTTGATTTCCATCTGGATATTGTTGCCTACGTGAGCAACCG
CAATTTCACCTTTATTCTCATAGACCCACTTGCTGGTAACGTCTCCTGCAAAAGCCTTAA
TAACTTGCAATGCTTTGTCGTATGCGTTCGAGCTGTGAACTTTCAGAAGGCCAGCCGTAG
CAATACGGGAGCAGGTGATCGCCGGAGATTGCCAAACATCAACTTTAATTTGCTCAATCA
GAGTAACACCGCCCTGATCAACTGCTTTGAATGCTTAGAACATTGCCTTTTTCATTGCTA
CGTGGTTGTTACGGAATTTCATAATTAGTCACCTCTATTGATTAGTTATTCAGAATATTA
ATTACAAATCAGGACATTGCCCGTGTGAATGCGTCCATTATAACAGTGTTATTTGGCTTG
TCGACACGTTTTTGAACTTTATTTTTAAATTTTTTGTTGATGTGGTGTTTACGATAAACA
ACCTTTTCACGGCTGGCGTAAGCCTCGCGTGCATCTTTTACCATCAATCATGCCTTAGCC
GCATCTTGTCCGCTGATCCCGCCAATCTTCTTAGCAATACCAACAAAATTAAGACCGTGG
TCTTCGCGTAGCATGTAAACCATTAACGCTGTTTTCTTGTCCATCACTTCGCCCCATTGA
CGATTAACAGGATAGTTATCAGTACCGGAAACTGGATCATGACAGTACCAATAAACCGGA
TAAACGCATTTTCTGACTTCCACACATGACATGCGACTAGCTGATAGCCCATTGTTGCAA
CCAAAGCCAGAAAATACATCTCGATTGGTGCTTCTTTGATCAATCGTACAATCTCATTCA
TACTACACCTCATACATTCATAAACACAACAACGAATAATACAGTAACAGTTATCAACGC
CATTTTCAACCCCATATTCATAATATTTTCTTCGCTTCACTATTGACAGTAGCATGTCGA
TGCGTGCCATTATAGGCATCTAAATCGCTCTGTCAAGCACAAAAACAAAAAAGCCACCCC
GAAGGATGGCTGATAGATTTCTTTAAGCAGGATGCGTTTCAGGAATTCGTAGAACTCGCT
CTAACGTATCTCGGTCCGGTTGATGTAAATCTTTGGAATGATACATGAAACCATTGCGAG
ATTCCATGCAAGTGCAATCACTATATTGTTCTTCCGCTGCCGCGATTGTTTCCAGATCTT

TGGCGTACTTGCTGGTTGAATAAACTTCTTGATCACGCCACTCTAACCCAGCCTCATTGA
ATTTAACAATCTCGTCAAATAACCACCATCCAATGTAGATAAATGAAGTTTTGCGCCACG
GGATCGGGTGTCCGTAACGAAACATTTCAACAACTTCCATTGAACATTTTCGGCTTGTAC
TTGCTGCAACTTCTACTGTTTCATCTTCGATCTGTTGATTTAGTTTAGCAACACACCAGA
ATTTGGCTGTGTCATCGTAAGTAGCACTCTCTTTGAAAAGATGACCGTCTGGCATGATAG
AAAATCCTTCCAGTGCTTCGCCGTCCTCACTAACGATTAGAGTCGGTGACAGTTGCCCTT
CGCGCTGCAAATACCAGTTATGTTTGTTCGTCAATTCAGGCATACCGCTAGAATCGCCTA
CAATGTCCTCTGACACAACATAATTAAGTAAGATCTCATTACCTAGTGCCACGGTCTTTG
ATTCCACCACATCCTGTTCTACGTTCATTGTTGCCAGCATGATTTTAGTCGATGCGCTAT
CAGCTGAATTTTCCGACTCACCCCACACTTTTACAGTATACACGCCGTTTTTATCTGCTG
TAAATTTTTTGATTGTAGTATTCTGTTTTGTGTCTATAACTTCTTCGCGCTTACTCCAAC
TATAATGTAGTTCATAGTTGTCTGCTGGAGTCTTCCCACCCGAAAAATTTACTGTTGCAT
GTAATTCTACATCTTTTTGTGATGATACGTAGAAATGCGGCGGTTCGGTGATATTGATGA
TTAATTCCGCTTCTTCCATGAGGAAAACTTCAGATTCTACTGATTCCATCGTATCAGTAT
CTTGTACCTTGACTTTATAGCTACCCGCATTTTCACTTGTTGCGCCTTCTACAGTTAGCG
TAGCTGTGTTAGTTTGATGCTGTTGGTCGTCTTTTGTCCATGTATATGTCAAATTGCTTG
GTAATGGTTCGTCACCCGCTAAAGTCGCCATTAAGGTGAAACCATCTCCAGCAAGAAAAC
ATCTTTTGTTAGATTGTATGGATAGTGTGAAAGCCATATAAAGCCACTCCCATAGAAAAT
CCGAATTTAATTAATTGAAATTGTTGATTGCCACCAACCAGATAACCATTGCGATATTCC
AATACATGAGTATTAGAATAGTGAGGCAACGAGGCCGAAATAGTCTCGATCTCGTCTGGG
TATTTGTTCTGTTTGATGTAATACATTGATTTTGTTAGGCATGTAGTATCCTCTTTTTAA
TTGCATAACGGAACTACAATTAAAATTATAACGAATATTTCACAACTTTTATCACCCAAA
AATTACTTGAGTGTGTGGAATTGGCTATGTTTAGTCTCTTGCACCTTGACAGACCCATCG
CGTTTGATATTATAGATCAGTGACTTGAGGGTGTCACGTACATACGAGTGCGGGAATCGT
TCTGGATACTTCTTCCAATCTGGAGTATTAACGATCCAGTCGAAAATCCACCACGGGCAG
GAGTAGTAACCACGACCGCCACCGATCAAGCAAATGGTCTGACGGAAGCCAGTAAGATCG
CAAAATGAATCAGTGTCATGTGCTAGGGAACGATGCTCTGCTTCGTCAGAGAAAATGTTT
GCTACCGGAGATTTCACTTTAACTTCCGGTGCTTCTTCAACATCTGACTTAACTTCTTAG
ACCACTGGTTCAACTGCTGGAGATTCTTCTTTTGTTGCTTCAATGACGATCTCAGGTTCT
TTCATTGCTTCAACCAGAGACGGATCAACTTCGTCGAATACTATTTTATCATCTGCGGTG
TCTGCTGCCAGAATAAGATCGTTAATACTGGTTCCTTCTGCCGGAGTTTCTTCTTCCATT
TCGGAAGCCAGTTTAGTTAATTCGGTCTCTACGAACTCGATCATGTTCTCTAGTGATAAA
GTCTTTTTGACCTTAATATCAAATTGTTTTGCGTAGTCAGCTAATTCTGTTTTAGCTGTT
TTCTTTGCTTCTTTGTCGTCGGACGCCGCAATAGCGCGGAGTTCTTCGAAATATTCTTTA
TCAAACATGTTATTCTCCTGATTGGGGGTGTTTGTATAAATCTTTGTATAATGTATTTAT
TGAGTGAGGCTATTCGCTATGCAAGATATTAAAATTCTCTTTCTGAACTATTCCCATGAT
CAAATTGAATGTAGCGATTCAATTTTAATAGAAATACGAGATTATTTTAGTTTTGAAGTC
GAGGGAGCGCGGTTTCAAAAACGAGTAAAATACGGAGGCTGGGATGGGCGTATTCGTCTT
TTAGAGTACAACGGAAAGCTGCCAATCGGCCTAACTAAAACCGTGGGAATCTTCGCTAAA
AATATGGGCTATAGTGTATGGGTAGATCCTCGCTTATTCGAAAAAGAACAACTTACAGAA
GAAGAAATTACAAAATGGTGTAATGCCTTAGAATTATATTCCGGTGCTAACAGGATCACC
TCGCATTGGTATCAATCTTAGGCTGTCTTTGAAGGTATCCATCATAGACGCCGTATGTTA
GTTTTGCCTACGTCCGCTGGTAAATCTGCGATTGCTTGTATGTTGTCTCGCTGGTACTTA
GAGAACTACGAAGGGAAAGTGCTGATCATCGTTCCTACGACGTTGCTAGTGCTGCAAATG
CGTGATGACTTTGTTGATTATCGCCAGTTTCCTTATGAAGCAATTCATACTATCATGAGC
GGTACTAGTAAACACGTAGGTGATCGCCTGATCGTTGTTAGTACGTGGCAATCTGCATGT
AAACAACCTCGTGAGTGGTTCCAGCAATTTGGGATGGTTATTGTTGACGAATGTCACAAA
GCCACTGCTAAAAACCTGCTAAACATCGTTACTGATATGGATCATTGCCAGTTTAAGATC
GGCATGACTGGTTCCCCTCGTGACGGGCAAGCTAACATGATGCAGTATGTCGGGCTTTTC
GGTGATATTTCTAAGATCGTGAGTATTGACCGCCTGATGGAAGACGGGCAGGTGACAAAA
CAGAAAATTAACTGTCTTTTCCTTCGTTATACCGATGAGGAATGTAGCGCGGTTAAGGGG
CGAGAATATGCGGAAGAGATCAAGTATATTACATCTAACCCACGACGAAATAAGTTTGCT
TGTAATTTGGCGCTAAAACTCGCTAGGAAGGGCGAAAACGTATTCCTGATGTTCCGCAAC
ACTAAGCACGGAAAACTTATGTATGACGCGTTACAGAAGGTACACGACAAGGTTTATTAT
ATTGACGGTGGAGTTAAGACAGAAGAACGAGACGAGTTCAAAAAATTAGCGGAAGGGGAA
ACAGGGATCATTTGTGTTGCGTCTTATGGTGTATTCTCTACTGGTGTTTCAATTAGGAAT
TTGCATCATGTTATTTTCGGTCATCCAGTGAAGGAATCAACTATCGTTCGTCAGAGCATC

GGTCGTGCATTGCGTAAACATGGTTCTAAGGATATTGCAACGGTCTGGGATGTTATCTAT
CATTTGGCAGCTAAGACAAAGAGCAAGAATGCTAAGAAACAGTTTAGCCACCTAAATTAT
GCCCTTAAACATGCGCTTGAAAGAATAAAAATTTACAACACCGATCGATTCGAATATGTC
ACGAAAACAATCGTGATCTAATAAACAGGGGCTTTAATGTCCCTTTATTCTACGGAGATT
AAAAATGATTAGCTTTAAACGTTTTTTGGTTGAGGCCGCTATTGACGGTTTCATGAGTAA
GATTTATCCTTGTAATCCTATCGACGGATTAAATGAACTTGAGGCATATTATGAGAAGCG
CAAGAAAGAATCAGAATTGAAACCTGCTGACGACATTTCGATCCGTGATGCTATCGCTGG
ACGCCGTAAGCATCTGGAAGCGGACAACGAGCCAGAAGAGGAGCCGGAAGAGGATTTTTA
AGATATAAAAAAGCCCCATCAATGATGGGGCAAAATAAACCAATTAAAATCGGTTATCAA
CAAATTACATTAACAGCATTGTTTGCTCTACGAGTTGTTCTTGTTCTTCGCCGTTCGCAT
CTTCACTAATCGAACGTTCTGTTACTAGCTGTAAGCAGTAGCCAGTGTGCACAACGCCGT
TTTCTGTCGGGACTGATAACGCATCCTCGACAGGTTGTTTACAACAAATACAGATTTTCA
TAAAAATTCCTCCTGCATGTATTTAGCGACCAGATTCCAGCATACGCAATTCTATAGCAT
TCTTGATCGCAAATCCACGAGATTTAACGATATCAAGTGCGCGACCAGCAAAATCCATTA
CCATCTGGTAATATGCAATTTTTGTATCAAGCTGTAAGATCTCGTCGTCTGCTGCCATGA
CAACTTTCAATTCTGATTTCTCATATTCAGCACGACACCAGTCATCACTACGGTTAGTGT
AAAAGTCAAGACGATTTTTCATAGCCTTTTTCTTTTTAGCCTCAAGAGCAATCGTCCCTC
TTTTCGCCTCGCTGTAAATCCTTAACCACTTTGAGTATACTACCGGATTATTCAGCGCCT
CCCATTGCAACTTAGTCGCATCGATCTTCGTGTCAGCTTCTAATTGTTCTTTCAAATCGT
TTAGGTCCATTATATCCCCTTATTCACACTAAGACCTATATTATATCACACACAAAATCA
CTTTGCAAGTCTTATGCGTAGTTGTTCAAGATCATCTGGGTTATCTATTACTGAAAAGTA
GATTGATACTATGATTGAGTTATCGTCGTACTGCGGAGTTACTTCTACTGTCAGGTTTCG
GACCCGTGGTTCATAGTTTCGGATCGCCTCATTGATGTTTTTTGATACGGTATCCACTGT
CATTGGGTTCATGTTCTCAAACAGTGATATATTGAGATCGCATCCAAACAGTGGATCAAA
TGGACGAGATCCCTTGCGAGTAGTCACGATCAGTAGAATAGAATCTTTAACCGCTTTAAT
CCCTATGTTCTTTTTAACGTCGTTATTCCACGATCTGGATATATTGACATCCAAATCAGT
ATACATCTGATTAATAGTTGCCATGTTATAGCTCAAATAATTTTAAGGAAACTGGATAGA
CCTGATAATGAATATACACCAGATTCCCCGCATTCGCACTTGATCGGTACTGCCAGCGTG
ATTTGATTTCTTGTTAGCTCGTCGATCACTTCTTCCACTTTATCCGTGGTAAGCATATCC
AGCACGCGGTCAAAGTCCTGTGCGTTCAGGTGGTCTATATTGAATTGCTGATCGCCGATC
ACGATATATTCGATACATGCAATTACCATCTGCATAATATCAACATCTTCTGTAAACTTA
GGTTGACTAAACTTGATTTTTACTTGTCCTAAGTCCAGAATATACGGTTCAACTTCTGGT
ATCTGTGTGCATGAAACATCAAGAGGGTATACTTTCTCTTTATTGCATACACCACACACC
CAAGTAGCATCAATTTTCATCTTATTGAGGCTGTGACACCATAAGAGAAGAAAAATATAT
TCTGCTTCGCATTTCTCTAGCTGTAATGCATTGATCCCCGTACATTCATGTATTATCTCT
ATAACAACCGCTGACAGCTTGCCAGTCTCTTTTGCTGCTACCAGTTTTTGAAATTCGCCG
AAGGTGAAAGCACGGGCGTGTATCAACTTTTCGCCTAATTGGATCTTGAAAAAGTATTGC
ATAATAAAACACCCTTTATAAACACATAGAATTATTGAGTATTTTATAAATACCCGAAAG
GGGGATTATACCATGAATAGTATCCGAATAAAACTGCCGGAAGATATGCAGAGGTTTAAA
CCATTCACGGTAAAAGATTATAGAGATTTCCTTCTGGTGTCAACTTAAATTAAGATGAAT
CCAGAAGAAGAACAAACGATTCTGGATGAGCTATTAGAAGAAATATGTCGAGAAGTAGAT
CCGGCATTTCGTGAAAATATTTTCCTGAATGTCTTTACTAGCTCAATTGGTAAAACAAAG
ATACCGCCTTGTTTTAGTTGCCCGACTTGCAACAAAGAAAGAAAAAATGTTGCTTAACTTA
CAGGTTGAAGCACTAAAACCAATCGCTCTAGAGGCCGCAGGGCTTAAAATAACTTTCCGG
TATGTTAAACCATCAACCGACTATGCAAAAACGTTTCTGGATGCGATAGGGCGTGTATCT
GATGGTATTAATGATTATCTATGGACTGAATTACCGGAAGAAGTTAGAGAACAGGTTATC
GATTCAATCTCATTTGCTGAATTTGAAGAAGTTGTTAAGCAGATGCACACAATCCAGATC
GAACAAAAGATATGTTGTTGTGAAAGTCATGACCTGAAATACATAGGTCTTCTTCCATTA
TTCAAATTGTTGTTGAATCCAGATGAGTTATTCATCTTTTATCGAGTAAACCATCTATTG
GCAAAATCAAATTATTCTATCAGTGATCTTATGGATATGTTACCGATTGAGAGAAACATT
GCACTGACACTGGTCGAGAAAGACATAAAAGAGGCAAATAATGCAAAGAACGGGGTTTCC
TAATGTCTAAATAAAAGCTTATCAGGATTATGAGGCATTTCTAAATCATCGCTTTCTAGA
GTTGGGTGCTACCTTCGTTACAATGACTCTGCGTGATTCGATTCGTGGGGTGAATGAAGG
ATTGTTGCAATTCTATGATAATAAGGTGATGCATACAAAATTAAACGGGAGAGAGATCAT
TCAGATTTCGCTATCCACTGCTAATACAGCTGAAGTGTTTAACCGAATATATGGGATCTC
TCATAGTAATGTAACAATTGACCAAAAATCCGACAACATTTTAACATACCAGTTAAAGAT
CTATCACGAAACCCTAAACCTTAAATTTTCTCGCTCATTGACAAATAACGCGGTTAATAA

CGTAACTAGCATGATTGATGCCATCTATAAGTAAGTTCCTTTATGGAAACCTACTATAAA
GGGTGATAACGTTGTTATTCCTGGTTGTCCGTGGGTTAGTACCATAAATGACTATATGGA
TTTTGTTAGGGCATACGGGCAAAGCGTAGAAAGCGAGTCTTTCGTGTATTGCTGGGAAGA
TTTTGATAGTATAACGATTGCTGGATACGATACAATATTAAAAAATGAGCCAATACCAGC
TATAATCTGTGAGCCTAGACTCATGGGGCAATTTATTGATATGTCGCCTAATCTGATGGT
TTTAAATTTTGAGTGGCAGACTAAGAGCAATGCTAAAGTTAAAAAATTCATTTAGCAATG
TGACATACACCACTATTGATTTTGTTACTAAGCAGTATAATAAAATTATTGTTGGTAATG
GGGATAATATTGCACACTTTGCTCATGGTGGCGCTTATGGCGATTTAACTTATAAGAATG
CGTTTTTGGAAGGTTCTAAACTTGCCACAATAGAACAATATGATTCATATGCTACCGCCG
TGTGTCACGGTAATTTTTCATTGCGCCCCGGTATGGTTATTCGATTCTTTGATGAGATGC
AACAAGCACGCACTGATTTTATTGTTGACGAAGTGGTGCATGAAATTAGCCGAGAGCAAT
CAATAACTCACCTGTATATGATCGGTAACAGCGAACAATTACAGGAAGTCAAATTTGAAG
GTAAGATATAATGACAGAAACTAAACCTATGTTAAAATTTGTTGTTCCTGTACAAAAATT
AGAGATAAACGGGAAACATATTAAGATCCCAAAAATGGGGATTAAGCAACATCGATTATT
AAAAGATGTTCGTAGCTGTGATGAAACATTAAAGATTCTGCTTGATTCAATCTGTCCTGG
GTTAAACGCAGCCGAGACTGAGTTGATTATGCTTAACCTTTGTGCGTTTAATGGTAAGTG
TCTGGAAGAAAAAGATGGTCTTAAGTTGTCTGATGTATATATTTGTACAGAGACTGAATT
TACTTTAACTGGTAAAGCCTTTAAATTTAAAAATCCAATGTATACTTCTGACCGCATTAT
CGATGACGCTGATTTCGTGAGTCATCATTATCACGATCCTAGCGTAGACTTCCATGATTT
CCCAGCTTTTATTATGGATTGGGCTACAGGATTAAGAAAAATTATTGCACTGGATACACC
GGAAGGTACTATCTATGGTGGAATTAACATATTGGATCGCCTATCATGAATGACGCATTA
ACATCATTTCGCGGGATGCGGAAGGAAGCGGAAGAAAACCCGATTGATAAACTTAACAAA
TTAGATAAACTTAATTCTACTGATAATTTGCAAGCCGTTACCGAATTGGTGGCAGAGACA
GTAGAGCAAAAATCTAATGAAGTTGTTGGTGCGGTCGAGGATAACACCGCAGCCAATGAA
TTGACGGCAGAAAATACACAATCTACCGCTGGCAATACCCAAAAAACTTATGAAGAATTG
CAAAAACACAATAATTACTCTTCTCAGATGAATGAGAAATTACGTCGTTTTGGTGTTATG
ATGGAACGTAGATTTGGTGTTGTCAGTAAAATGGCTAGCGGTATTGGTGCCATTGAAGAA
GCATTGAAAAAACCGGAACAACCGCAAACAATGCCATCACCTCAGCCAGTTTTGCCTACT
GTACCGGAACAGCCTAACAATGACAACTATCAAGGTCTTCCAAAGAAAAAACCAGATGCT
GATGACCAAAAGAAAAAGAACGCCACAGATAAGCGTAATGCTGATTCTATGGAAAATCTT
CTTACAGTTGTTCGTGGAGGATTTAAAGAAACCATAGGTATCTCTAATAAGGTCTTAGGG
ATGTTATTTAAGATCACTTTGACCGCTATGGCAGAAGCCGCAAAATGGGGTGCTATTTTG
ATGGGTATTGTTTTTGGCATTGATACCCTGATGGTTCATTTCAGATATTGGAGTGATTTA
TTCGAAACAAAATTCAATGAATTTATGGATAAGGCCGGAGGATGGGCTGGTCCTATTAGC
GATATTTTAACTACTGTTCGTCAGGTTCGAGATTATTGGTCTAAAGGCGAATATGGAGAG
TTAATTAAATCTCTTGTTATGGGTATTGGTGATGCATTCTATAAAACCTTTATCCAACTT
GATCGAATTATCACTACGGGTATTGCTAAAATTCTTCGTATGATCCCTGGCATGGGTGAT
TATGCCGATAAGTTAGAATATGGTGCTTTAAAATCTGCTGTCGCTCAAGGCTATACGCCA
AATGAAAGAGAACTCGAATTAATGGATAAGGTCGAGTCTGAACACGACGAAGACAAATAT
GGAGAGCGCACAGGGTGGACCGGAAAAGCCCGTGATATTGGCGAGGCCATAGGCGAATCT
ATTAAGGTTAAAGTTAACGAAGGTCTTGTTAGTCTGGGCTGGAGAGAACAAAAGGAGGTT
GACGCAGAGAAACGACAAGAAGAATTAAAAGGCGGAGAATATAAGTCTGTTTCTGCTGAA
CAACGATCTGCTAGTCGTAAACTTAGAATTAAAAGCGAGGGCGCGATCAATAATATTAAT
GAAGTTATGGAGAATTTGAGCGGTGACTATGACAAAGAAAGAATGGGAGATTTGAAAAAG
GATATTGATGTTTACCGTGAGCAAGTTCAAGATCCTACATTAGTTGAAAGTGATCCTTCT
CAGTTAGAGCGTTTTATTGAAAAATTTGATGAGATGTATGCTGATAAAACTAAGGGTGTG
GTTCCAACTAAATCAGTTCCGGCAACTGAAACGGAAACAGCAAAACAAGCGGAACGTACT
GAACAAATGCAGAAACAAGCTACAATCCAGCAACAAACGACAAATCAGACCTCAAACGTA
AATAATACACAAATTGTAACGAACAATAGAACTATTAAACAGGGTGCACCAACAACTAGA
ATTGATGCCCCAGGTACAATTAGTATGGGGAACTTTTAATGAAAATTAGTGTTATCAATG
ATGCCGTTGATAGCTTTAAAGCGGGGGTTAAAACCTCCGCTGGTTTTACCTCAAAAAATA
AAGGGAAAACATTAACCGCTCAATTCCCCGCTGAACGTGCTAGCGGTAATGATGCTAGCG
GTTATTATATCAACGACTTATATAACGATGGTCTGTTATTCACTGCATACGATTACACAT
CCCGTACTAGTGGATCGTTGCGTGATTTCAGGAAAAAGAAAAATGTTGCTAGCGGTTTTG
GCGGTAGCGTTAATATCGCTGGTTTTGATTTAAAGTTGGGTGGAGAAATGCTGCATTTG
ACCGAGAGGCAATTGCTAATATTCTTCTTCCTCGTAGTCAATCTGACGTTGACGCTGCCA
GCCATAAGTGTAATGATGTTGGTGAATCTGTTATTTCTCGCGGCGGTGGTACTTTAGGCG

GTGCTCTTTCTAATATGCCATCTACTGCTGTTTTTGGTGGTATCGAAAGTATTACTGGTG
GATACCTTGCCGATCACGGTGAACAGATTTATAATACTGCGCGAAGCATGTATGCGGGTG
CTGACGCACGAACGAAAAACTACGTATGGCATCTAACCCCACGTAGTATTGAAGACCTTC
TCAATATTCTGATCATATACGAAACGTTTTTAGAGTTGTCTTATGATTCTAGTGGTATTT
CCAGCACCGCAAAAGAATTAAAAGCGGAAGTTGATGCGTGGTATAAAAATACACTATTGA
GCAAATCCACTCCAGCTGAGGCAAAGAGAAATGATACGCTGTTGGAAGGGATCACCGATT
TTCTATCCAACGTAATTACTGTATCTAACCCGACAATATGGATGATCTCTAACTTCGGCA
AGCGTCCATCGTTTGAAGGGCGTAGCGATGTAGTTGGTCCCGCTCAGATCTCAAGTGTAC
GTCTGGACAAGTCGCCTGATGGTAAGTTTAATGGCCTTGCTATTTCTCCTAACTTGCCGA
GTACCTTTGTTCTGGAAGTATCATTTAGAGAGATCTTGACACTTTCCCGTGGTACTATAT
TTGGGAGTGCGGCATAATGTTTGATTTAAATGACTTTAACTAACAAGCCGCTAACCTTGA
CTTTCAGCGATCTAGCTTATTATGCGTGGCATTTGCCACCACTCCAAGTAATAAGACACA
AGCGATCCTAGAAAGTATGGGTGGTGCGGTATATGATATTATACCAAATGCATTAAATGA
CTATTTCGGGGTTACTCGTGGTGATTATACTTACGCATTAACAAATCTTGCTGTACAGGG
TGTTAGGCGTGCTGTTGATTCATCTGGTGTCAAAAAGTATCTTTTAGGTGCAATGTCATC
CCGCGTAGTTCAATCTCTTCTGGGGCAATTTGATGTTGGTACATATGCGCTTGACTGGTT
CAATATGGCATATAAGACAAGCGGTCTTCTGGTGTATGCTGTTAAGGATCCAGAAAACAG
ATTGAATTATGAAATGGACCGTAACCACAATGCGCCAAACATTCGAATTACCGGACGTGA
TTTTGATCCGCTTGTATTGAGCTTTAGAATGGACTCTAGCGGATCAAACTATAGAGCAAT
GCAGGACTGGGTTAACTCAGTAGAAGACCCCGTTACAGGGCTTCGTGCGCTTCCTGTTGA
CGTTGAAGCAGATATTCAGGTCAACTTACATAACCGAATGGGAGCTCCTCACACGATCAT
GATGTTTAATGGTTGTGTTCCTGTTGGTGTGAGTGCACTGGAATTAAACTATGAAAACAA
CAACGAAATCACAACATTCGATGTTACTTTCGCATATCGTTCTATGCAAACTGGTGCTGT
AGGAGAGCATGCCGCGCGAGAATGGATCGAGGATAAGGCAATAAACGCAATAACAAACAC
TTTCGGTAATAATTTACTTGATAGTGGGATATCTGCTGCTGGTAATGCTTTATCGCGTCT
AAATGGCGTTGGTGGGCGTGTGGTGAATACTGTCACTAACTGGTTCTGATAATCGAAAGG
CGGGGATAATCCCCGCCTTTTTATTTTGCTAAATCATTGCAATATTTTAGTGCCTTAGTT
TGAAATTCTGCGCTGTTAAGTTCTTCATAACGGTCACAATCACCGTTTAATGCTGCCTTT
CTTGCTTCTTGCTCTATCTGCATGTCAGACATGATAATACAGCGATCCCGTGCTTTCATC
TTCTCAAGTTGTATAGACTCTTCTTTCTGTTTGCGCAATTCTTCTTGACGCTTCTTTTCT
TCTCGTGCTTTCTCTTCCCGCGCTTCTTTGATTGCATCCTTGAACATCTGCGTTTGTGGT
GCTAAACCAACTTTAACGCGATCCACGTCTCGACGTTCCATTTTAGAAATTAAGCATGAT
TCGATCTGATCATCACTTGCATATGCTGCTTTACATTCACGAAGCAAGAAAACCGGATCT
GATTTAGCATTCATCATAGCTTCCATGTTAGCACTACCAGTAAGAATGAATGCAGTCGCA
AGACCAATCAGTTTGATCATTTTCATTTAGTCACCTCACCGAAAATTGTAAATTGTCTAC
GATTCTCATCGCCGAAAGATTCTACTACGTCGTTTACCTTGACGCAAGTCCCGCGCGGTA
AAATTACTTCACATTCGCAAGGGTTCCATGCAATGGACCCAACAACAAAACCTTTATGAT
CTTTTACATCAATCAACCATGATACATTACAAGAATGTTGCATCTTTCCGGTCATTGACT
CATATCGATTCTCACAATAAAGGCTAGTGCTGTTTGATAGCCATCCTCCAAAGATGATCG
GTGTCATGCTGGTCGATACAAAGTTTTTAAAGGTAAACTCCTTATCTTCAATGATGCGCT
CTATTGTCTGAATGTCAAGATGTTGACCGCGATAAAGAGTACCCTTTAACTTTTCGGTTT
TATCCATGACGCAATCCATCAAATCAACAATCTCTGATATCTCATCGGTCATGTCTTCTT
CGCCTAAGAGAGCACCATTTACCCATTCGTATTGTTCAGACACATAATCACAGATTGCAG
TATATTCTTCTGGGGTTATGTTGTTATGTTTGTACTGTTTGAATACATTATCTCCAAAGT
CTTCGGAGTGTACTTCATACATCACATTAATGATTTTCTTGACAAATCCATAATCTTGTG
TCTCACGCTCAAGGAAGCGGATAACCTTCACATAATCATCAAAGTTAATCTGATCGCTGA
TCATCTCATTGATCATGTAACCATTATTCTCCTCAATGTCATATGTTGCGATGTAATTAT
CCATACGTTCCATCATTGATTCGCGTGATTCTTCCGAAAGACGAAAATCCTGTAATTGTT
TGAACATTTTAAACCTCCCATATAACAGAAAACGGGGCTAGTATAAAACTAGTCCCCGTT
TGAGTCAACCATTATTTCTTAAAAATTACATTTTAAATTTGTCTGGCATTGCGTCTACAG
ATACCAGATCAGCGATAAGCTGACGTAGAGTTGCCTTATCTGCTTTCTTACGTTCGGTTT
TAGCTTCGCTAATGAAGCTGGAGAAAGACAGGTCAGAAGCAGGTTTAACAACACCTTCAC
GGATGAATGCATCACCGTCTGCGATCATCTCGCCAGATTCTTGTAATGCTGCCAGTTCAT
TTTTGTTCATAATATGTGCCTCAATGTAATGAGTTTCTTGTGAATCCGTCGATGCCGTAG
CACGAGTTACCGTATGAAAGGATAACACAACATCGCGAGGAAGTAAAATCTCGCATTCTT
TGTAAGCAAATGCTTTGTTACCTACTGGCAATACAGGGATGTTTTCCGCGCCAGCAATGG
AGAAAGCAACATTAGCGCCACCTTCACCGCGAGAGCCAGCCATAGAATCAACACCAGCAA

CGGCACTAGCCACGTTTGAATTCCAGCCGCCAAAAATGTTAGGTGCAAGGGAAGTGCTCA
CATAGTTTTAAAGTAAAACGCTTTTTGCTTACGTGCAGTTTCCCACATTTGCGACCGGA
AGTCCATACCACGATACTGAATAGTACCCGGATCAAGTTTAACGCCATAGTTGGAGAAGG
CATTATCCATTGCATCGCACATCTTGACCAATTCTTCTGGATCTGAGATTCCTTCAAACT
CTTCCAGCTTACGGTCGGGTAGGAAGTTGTTCATATTGATGTATGCTGCATCGGCATATT
CGCTCAACACAAAACCTTCCGCGCCTGGCAGATCAAGATCAATATTTGCCATCATTTCAC
CCATGTTGCTAGCAATTGCTTCAAACATACCGGAATCAACATTACGTAGGGCATCGGTTT
TAATCTGGGTGATAATACTACGCAGCATTTCAGCTTTTACATCTGCTGGCATAGACTGGT
CTACTCGATCCATTGCCAGTGCGGTGATCTTGTCTTCGAATACTTCCTCGCTCATGATTT
CTATTAAGCGTTTGTTTAATGCTGCGATAACTTCCGCAGACAACGGATCATATTGATCCT
GAACTTTTACCAGTTCGCTTACTGCGTATTTTGCATCGGCTTCGCTTTCAAAAATAGTAC
GCATGAAACCATAGCGAGTGATAATATCACTTGTCCAGTCATTCAGAGAATGGGCGGTTG
AACGTTCTAAGGCTTGACGATCAATCAGAGTATTAAACTGTTTCTTAGGTTCTGCTTCGG
TGATATCTTCTTCCGGTTTAAGATTAATTTTTTCTGGATGATTCACGTAGTCGCTTTTAT
AGGTCGCTTGAGTTTTCAGCATTTCTTCTTTAGTCGAACGAGATTTAGCCGCTACGCGTT
GTTCCGCTTCCGCGTTTCGTTCTTCTTCTGACACAAGACTAGCCGCATACGCTTCATCTT
TAGTCAGAGATTCACCAGTAGCCTTAGATACCCAGCGATCAGCTAAATCACTTTCTACTT
TAGTGAACAAGGAATCATCAAGTTTAATCCCTTTAATCATATCAATCGCCATACCGCGAC
GATTGACAACAACTAAAGCGATCTTTTTGCTGGTCATTACTTCCTGCTGGAATACTTCAA
ACTTCGGTGTACGACGCACCAAACGCTCTATAACGCGCTTAACCAGTTCGGTCTTACCTT
TCATGCGGGATACGTTAAAACGGAACATCACAGCGTCTAGTTGCGCTTTACGGGCTGTTG
CCATAACCACGTCAAAAGCACTTGACATAACTGCAATCGGGATATCGCCTAGACCATTTT
TAAGCTGTACAATACGACCGCTTTTGTTCATTTCGCACAGTACAACATGTGCGGTCTTGT
CCCCCATACGAATGATTTTAGGTGTTTTGTTCGTGAAGTTACAAAGACGGACAATCACAT
CTTTATCGCGTGTCTGTACTGCAAAAAGCTGTTTAGGATTCTGCTTAGGCTTTCAGCACT
GTACCGGGGCAATACTACCAAAGTTTTCTTCCTCTTTCGTCAACAGCCATTTCTCGAAAG
ATTCAATCATTAGATCGTCGATTTCTAAGTTAAACATTTTATATCCTCTAGTTGAGAACC
AATAAACCTATATTTATGAAAACGGGGCTTAACGCCCCTTTTTATTATAATGCACCATCG
AGCAATTGCTCTAAGGTGTATCCTGAATAGTCTTTTTCAATCAGGATATACTTTCCTACT
CGTTTAATACAACCGCGCATTTCAACGTATTCTGCGTAGTTGATGGTACGAGTTGTTCCA
TCTTCCAGTAATGCAGTACCGAGTTTGTTAATGCGGTCATAAGTTACGTCGGTAATGATG
ATTTCGATGTTAGCCATTTGATACTCCTTAGTAGTTCGTTTCTGTATGGGTGTATTATGA
TATACACCCTCTGTTATGTCAACAACTTTTAGAACATTTTTGAAATTAATTCTGGTTTGA
TTTCGCAGAGAACATCTAAAGATTTCTCGTCCCCCGCTTTTGCTGCTTCTGCTTGCATTT
TTGAAATGGCTTCAATCTCAGTGCAATTATAAGCCGCTTGAACTTTAGCGATTGCGATGA
TTGCCGATTCGATGGTTAAGCGTTTCATGTTTGATTCTCCTTTCGTTTCGGTATGTGTGT
ATTATAGGGGATCGATGATCTCCTGTCAACAACGTTTTTAGTTATTTTTTAATTGCTCAT
CTTGCAATAGGGCCATCACATGCTGGCGATGTTCTTCCGATTGAGGCACACCGAAAAAGT
TTGTTTTGTAGAAATACTCTATTCGGTATTCTCGTGAGCTCTCTTCCATCATGATCGCGG
GTAGTCCGAGATTTCGGAAACGTCGCTTTTCTGCTGGCGTAAAATCACTGTAATACAACA
TAGGATCATCATTACCCGCAATGCTAAACTCATCCTTCATAAAGGAGAATATTGCGTTTA
AACGCTGTTTACCGTCCACGATTTCGATCTCATAGCGAGAGCCATCATCAATCTTAGCCA
TTGCGATAGCACCAACCGGAAAATCATTAATCAGGTTTTCGATAAACATACGTTTGTTTT
CTGTGTCCCATACTAAAGGACGCTGATAATCTGGGTTCATGTTGATATAGCCATCTTCTA
ACCATGCATCATAAACAGAGATGACAACGTTTTTAGTATTGGTATAGTAACGGCGTTTAG
GCGTTAAACGGTTCATGTTATAGTCTCCTGAATGGATCATCACGTAAATCAATATCTTTT
AAGAACTCATCCCATGAATGCCGGATATTGTTCTTAATTCTCCAGATAAGCGCAGGGATT
GAACGCTCTCCCATCTGGAAGTAAGGATCGTATGATCCGTATGCCCAATATGCTACTACA
ATTCCACAATCAGCGCAATAGTATTCTATTTCACTTGCTGGCGACCAAAGTCCATCAATA
TGATCGGTGATCTTGCTTTCGATGTTATGGCTATGACAACCAGGGCAACGGATGCAATGA
CCTTGTTTATCGTAATTTGACGTTAGCGAATACTTAAACAATCTCATTATAGATCCTTAT
TTGCTTCAAATACTGCCTTTGCGAATCCTCGCGGGGTGAGTGACCTTATAGTCTTTCTTT
TCGTTCCTTTCCCGCCTCGCTTAGAATGCTGTTTACTGTACCCTTTATTAATCTGCAACG
GTTTCTTTTCTGGCATTATAAAGCCTTGTCCGGACCATAAGCAAGTCTTTTTCGGGTACA
TATCACGGGCGGCAATATACTCAGGAAACGTAGGATGCTTATCATCTTCCGGCAAATAAC
CGCCATATTCCCACGGATGGAACACATAATCAGGTTTACGCCACATGCTAGAGATAACGC
TTACAGGATTCTCTATCATGTAGGGAACTCCTAGCCATTCACCCAAATAAGCGGTTATTT

TAGCTGCCCGTACATCATGAACTTGAATCAATGGTCCTTGCTCTCGTTTCTATCGTTCAA
AGTGTGCAGCGCCCCTAACAGCTAAATTGGTGCACGGTGGGACTGCAAAGATAATATCCG
GTTTCCCCCATTCGAGGTCCATTGCTCTTTCCAGCCAGTTCAAATCAATCCAGGTGTTTA
CATAGGTAATGTTTGGATGTTCAAATCTGGTATTATACTTTTTGTATGTACCGTGGTCGG
CGTCATCCTCATTAAAGCAGACTACCTGATGACCTGCTTTAGCCCATTCTAGCCCCATTA
TCCCCGATCCGTCAAAGTTGCAGAATATTAATTTTTGTTTCACGCTGCCCCCATTGGTTT
GATAGTAACAGAATCTAAAAGACCCTTGTTAGCCAGTTCGCGGCAAGTTTTCAGATCCGC
AACTTCCCAGCCAGCCGCAATCAGGTTATCAATGAGGCTGTTAGCAACTTCTTCGCTCAT
GAAAGAGCACTGTTTGCCGATGGTGTAGCGTACACGCTGTAATTTCAGTTTACGGAAGCC
TCTAACGTTCAGGTCTTTAGCGATTTTCTTTGCGGTTTCGATGTTAGCCATTGTTTGATT
CTCCTAAGTGGTATCTAGTCTTTATGGTTGTATTGTAGGGGAGTTATCCTCCCCTGTCAA
CACTTTTTATAAAATTATTTGTTAATTTCTGGTGCGTTGCTAGGATAGTAACCTTCTTTA
ACTCCGAGATATTTCGCAACCATCCCTACAATCTTCTGTGCTACTACAATGTCAAATTTC
AGATAATCTTCGCCGTGGGTTTCAATGATATATGCTTCACATGCTGCTTTAGTCTCGAAC
ATTTTGATTCTCCTATTTCGTTTCGGTATGGGTGTATTATAGGGGATCGGTGATCCCCTT
TCAACAACTTTTAGCGACGGCGACGATGTTTTTTGCGAGTTGCCTTTTTGTATGCTTTAT
ATGCCTTTTTACCGTTTTTATATGCTTTTTTGGTATATGATTGATTTTTCTTATTGTTTT
TGTATGTTTCCCGGTCAGTATTAATTTCGTTACATTCCACGAAACGGTGTCGATGTTCTT
GTGGTATGATCAAACATTTATCATAATTAACTATTTCGTTTTTCGTTGTTTGTGGTTTCT
TTGGTGTGTTGTTTGCACTAACAGACCCACCAAAGAGAATGAGTATAGCGCTCATTAACG
AAAACATTGTTTTCTTAATCATAATATTTTCCTTTAGTTTCTGCGGGAGTGTGAATATAA
CACTCCCTTAATATTTTGTATTATTCGTGATTAGTTTCGATGTTGTGCATTTTGCACCAG
TTCAGCCAATCGCCCAGATCTTGATAGTTAATGCTTTTATCTGCTGCCATCGTTTTTGTC
TTGATACCCATATGTGCGTTATGAGTAAGCATTTTTACAGTGAACTCGATTTTATCGTCT
TTAGCTTTAACATCAACCCGTTTGGTCCACTGGCTACCAGCAACGCGAATGTTCATACCA
ACGATGTTTCCGATGAATGCTTCTTTGTTTTTCAGGGTTTCGGTTGCTTTGATGAATTTC
ATTTTTGATTCTTCAGTTCGTTTCGGTATGGGTGTATTATGCTATGTTCTTACTTTGGTG
TCAACAACTTTTTTTAGATATTTTCAGAAATAAATTTTAAAACTTGACGTAAATGTTTCT
CATCTTTGATTGTAGCCGGAAGTTCTTCTTTTACTTCCTGTGCAAGATAGATATTTCCGT
TCCAAGTGAAAGATCCGTCTTCGTTTACTCGATAGCTGATCATCCACTCGCAATCATTTT
CGATTTCATATACACACAGAAGGGTGTAATCGTTAAAGCTATTGATTTCAGGGTCGAAGC
CGTGACGTTTTGCAAAGTTGGTAGTGGTTTTAGTCAGTACCATTGTTTGATTCTCCTAAG
TGGTATCTCGTCTTGATGGGTGTATTATGCTATACACAGGATCTGGTGTCAATGCCTTTT
TTAAAATTTTATGCAATAAAAAAGCCGACGCTAAGGTCGGCTAATAAAATCATTTAGACA
AATAATCATTCAGTGCAATCCGGTCGGCGTATGCTGGTCTTACGTCAAGCGCAATCACTT
TAACGCGTGCTTTCTTGCGACCTGGGTTTTCTCGTAACCAGTGCTCATGACCGTGAACAT
TTTATACTGCGATGCTTCTTCGAAAGTACGGACCGGACAAGGGTAATCAGCGTAAAGGAT
CTCTAGCTCTTCGGTGATGTAAAAATATACATCATAATTACTGGTCTCTAGTTTAAGCAG
AGACTTTCGGAACATTGGGAGATTTTCGCGGGTAAATTCAAAGATATTTTCTTTCATAGT
ATTATCCTCAATAGTTGGATGGAAAGGGGGGCTTAATGCCCCATATATTAAAACAGTCCT
TCCTGTACACCGTAGTAGAGTTCATCGATGATAATTTCATTAATGCGACGGTGTGCATAT
TCCTGATTTGCGAAGTTGTCAGCAAGTTCTACCTTAGTTTCGCCATCATGAGTCCATTTA
GTGATAACTTTACCATCGGCAAATTTGAAGTTGAACTCTTTAGCTACCGCATCGCGTACC
AGACGTGCAAACTTATTGTTGCGGATTGCATTGTCGGTTTCGTGTGCAGCGTGAGCGAAT
TTGTTAACTTTCAGAGTGATAGTAATGTATGCCATAATTTGATTCTCCTAGTTAGAAAGT
GTATCTCGTTTCTACGGGTGTATTATGCTATACACCCGATCCATTGTCAACGCCTTTTTA
TAAAATAATTTTCGATATCTTTATGATGTTTTTCTCGTGCTTGGTCTGCCTCGGCTGGCG
ATACGTTGGAAGCGATAATCTTCGCTACTGTTGCACCTTCTTCGGTGTCCGACACCCAAT
GATTATGGAAACCGCTATTATTAAACACTTGATAATAAAATGATTTAATGTTAGGGAAAT
TATAAGTGTAATAACGTACCCTTAATTCTGAATCTACGTTTAGATAGATCAACGCACCTA
GATAGACCGTCTCACTCGTTCCGTCAACTAACAGTAGAGTATTTCGCAATTCTTCTAATT
TGTCTTTTGTAAGAAAAATCATAGTATAATCCTCAATTAAACCAAATTAACTTTGGTGTT
GTGGTGCTTTTCTCGTGCGTCGTCCGCTTCTTCCGGTGTCACGTTACGAGCGACTACAAT
AACCCTTGCTGCCTCTCCGTGATGATAGATCTCTACCCAATGGTTATGATTTGCGAATCC
GTGTTTTATTGCGTAAGCGAACGGCTTAGACGTCCAGTATTAACTATAATATCGTACATT
AAGATCGCTATCAATGTACAACTATGTGCTGTCGTTTACATTGCCACTAACAACAAAAAC
AAGACAATTGCGAAGATCTGACAATATTTTGTCTGTCAATTTTATAATGTGACTGTTCAT

AATTAACACCACTCAAGAGTTTTTGAAAACACATCTATCCATTGTTGAGTATATGCGGTT
TCTTTCCATGACACAATACCAGTCGAATAATGGTTTTTGTTTCCAGAAAAGGACGGGCAT
TTTGATGCATCTTCCATCATCTTTTCGGCGAACTCTGGTTCGTATACCTGCAGAATCTGT
TGATATCCTTTGTATTCTTTAAATTTGCTCATAATCATTCACTACCTTATCAGCTTCTTT
TGTTGTTTTTACATCAACTGCGATAACCCGTAAAGGTTCGTCATGGTTCCACCAATGATC
GTGAATATCGGCTTGTGATTTATCACCAAAGGAATCACGAATATCATTTTTACATCTATA
GCTAATGTGACCGTCATAGTCAACATAAAGGTAATTACCGAAAATCGGCGTTTTTAAGAT
CATACCGAAAAATCTTTCTTCGTGTGTCTTATCCAATGTTGCAGTAATTCGCATCATAAT
ATATTCCTCTTAGAAAGGGATGGTCATTTCTTGAGCTTGTACATAGTCGTTAGGATTAAA
CTTCATCCAACCGATATTGTCTACCAGCATATGCACATTTTTATCATATCCTACGTTGGT
AGTCAATACTGTATGTGCTTTACCTTCAACGATGATTTCCACGTACTTCTGAACTTCGAT
AGCTTTCATTTTGATTCTCCGTTTGTTGTCTTGATGGGAGTATCTTACTACTCCCTTTAA
TCATTGTCAACTATTATTTGGGAATTTCCGGTGCGTTGCTCGGATAGTAGCCTTCTTTAA
CACCCAGATATTTTGCAACCATTCCGGTGATCTTCTGTGCTACTACGATATCGAATTTCA
GGTAGTCTTCACCGTGGTTTGCTACAATGTATGCTTCGCATTCTGCTTTAGTCTGGAACA
TTTTTGATTCTCCTATATCTCGTTGGTATGTGTGCATTATAGGGGATCACCGGTCCCCTG
TCAACAACTTTATAATGCTTTATATGCGTATTTCATAAATTTGTCTGCCACTTCATTGGC
ATGTTCAGATTCTACGCCTGATTCGAGAACAATAGCTAGGTCGGTTTCGTCATTCCCGCC
GTACCAATGATTTGATGTTCGATTTTTTTCAATTGGAAATTGGTCACTTTTATCTGTGCG
ACAAAATACATCTAACTGCTCATCGATGTAAAAGATCCAATCTTCATAAAGAATAACGGG
TATTAGCAAGATTTTTATTGGCTTCAACGAAAGGTTCGGCTACAAACAAATTAGGATGTT
CCATAGTAATTTCCTCAAAATTATACAGCTTCGTAAACTTCCCAATGTCCAACACCAGCA
CGCTTAACGAAAAGGTTTTCTTTGATGTTATTCATAAACGTGTGGATGGATTTAATGCGG
TTTGAAGGTTTCCATTATTCGTTGGAATTGCGGCGATACATAATAACATTACCAGTTCGA
TTATATTCAGCAACCATTTTAAGACAACACATAATAGTATCCTCAATTTGTGGGAGAGTA
TTACCCCTCCCGATGTTGTTATATTACATTACACAAAATGGCGTGTCAATGCTTCTTTTG
CGGTTCCGCTAAGACTTTCGATCTCGCCTGGGATAACATCAACCGCCAGAACTTCTACAA
CATCCTCGGACCCTAACCAGTGAGTATGGCCTTTGTAGAACTTGATAACCAGACTACCTT
CCTCGTCCCATTTAACGCTATCGCTAGCCCACCACACATCACCGTCTATAAAGTTGATGT
AAAGCACACTACGCCACATTGAATTAATTACTTTCAATACTGCGCCTTTCATCTCTTCCA
CGGTATGGTCTTCCATTTTGAAGACCTCACCTACCTGAACTTTTGCCATGTTACATGCTC
CGAATATGTTTAGCTGCATCTTCCAGTTCGTCGATACTGACACTGTGTGCCAGTAAGTGT
ACTGGTTGTGGTTCTTCACTTTCCATAGTATTGATCAGATGACCGTTTCCTGGGTGGAAT
GTATTGTTGCCGTTCCAAATAGGATACACATCATCTTCTGTTACGTAGATACTAAGTCCA
ATCGGTGATTCTGGTAATTGAAAAATTTTACCCACTGCATCTTTGCGATTGTTTACGATA
TCTTTTACTGTCTTCATTTAGTTACCTCTCGTTAAATTACATCACAATCACGCCAGCCGT
TGAAAGTTGATGTTTCAACTGGGCATTTTCTGCTACTGTCAAATCAATGATTTCTTGTGA
CGCTGCAATCATATAGCCATTGGTGATATTGTTTTGCATCAGTTAAATGGTTTCTTCGTT
GTAGCGGATTTTAGCGATGATTTCGTTGAAGTTCATGTTTGATTCTCCTAGTTCGTTTCG
GTATGTGTGTATTATAGGGGAGTTTAACCTCCCCTGTCAAGAACTTTTTTGAAGTTTTTC
CATGATAACGGTCATAATTACATCTTCTGCGCGAGCGGTCAGATTATCAGCGTCAGCATG
TGGGCGGTTGGTTGTTACAGAGATAACACCGTTGCGCACTGGTTTGATATTGATATCAAG
ATGATCCGCGAAACGTTCGCCAGTTACAGAATGAATTTTGGTGTTAAATTCTTTTGCCAG
TGCCTTGCGAACTTCACGAATCAGGATAGCGTTTTTCAGTTGGTTCTCCGCGCTCATGGT
CTTCTGGGTGTTCATGGTTCCGTCGAAGTCAATCATTGGCAGCATTACGATGTATTTCAT
TTTTGATTCTCCGTTTAAGAAAGTGTGTTTCGTTTTGATGTGTGTAGTATAGGGGATACG
TGATCCCCTGTCAACAACTTTTGTGAATTATTGTTTCATGAGCTTAATGGCTTCTTCCAT
AGCCAGCACATTTCGATAGTTAGGGTATCGTGTTAGATCGTCATACATTCGCTTAAGGGT
GGAAACTTCCTCAAACTCTGGGTTTTCTAGATACAGTTGAACACATTGTTTCTCTACCAT
CTCGGCTTTTAGTTTGTCTGCTTGTTCCTGTTCAGATGATTTCGTTGGACCCAACACCAA
CAAAATGATAAAGGTGATCAACGGGCTAATGATCAATGATAGGATAACATAACCGATAAC
GCTACGGTTTAAACGGCTTGCGTACATGCCTACCAGGAAAACAGAAAGGATATAGATACA
CAGAAAGAACATATAATAGTTACTCCTTATTTTCTCGCTTGTACTTTTCGAAATAAAATT
CTACTGGCTGGTCATGAATTGACAGCAAGCCAAAGCGTTTAGCCGCTCGCAATCCCATAT
TGCGCTGAATATACTTCTGGAAGTTCTCGCGTATACACTTACGCCAGTATTCTAAGCCCA
TACAGCTTTTATAAAGATTGCATTGTACACACGCCGGATTCATGTTATCAAGTATATCAT
GTTCCGGTTTATGCATTCCTACGACCTGATACTTCCACGATCCATCATCATTACGGGCAA

TTCGTCCATTTTCATCATAGACTACCTTTGAGCGTCGCAATACTGGTTTGATGTGGTCTG
CGTGCCAGCGATCCCCTAGTTCCTCGCCACAATAGGCACAACGTCCACCATATTTCATTT
TCAGGATCTGGCGTTGCTCTTTCTTTGACAATTTTTTAGGTTTAGGGGCTTTCGCACCTT
CATTATCACACATCGGATACCTCTTTTACCGTATTTGCAGTGGTCTTGTCGTAGCGAATA
CGTTTGATGATTGGGAGAAATAAAGAGAACTCCGGTTCATCTTCTTTACGTGATTTGCTG
CGGATATACCCGTTGCATGTCATCTCAATGACTTTTCCAATCAGGTTATGACGGTCAGCC
CATAACATTTCTCGGTCGAGTTCATTTCGACGATCGTACGGAATTACACGTTCAGTACCA
TCATCACCTTTTTCGGTTGTAGTGTCGATAAATCCCGATCCGGTGTTAGTCATCTTTCGT
CCGCACGCACTACGGACAGTAAAACCGCCTACCTTATTGGGGTCTTTACTATGCGGATAT
ACATCAACAATTTCCGCGGCGAAGTCAATCACTTCTTTAAATTTAACTTGATCTTTAGTA
CGGCGATCGTCAAATACACCATGTAAATTTTTAAGGGTAATACCTTCTAAGCCCATGTTT
ACATAGCGATGATATACTGCGCGAGCTTCATCTATATTGTTAACTACAGTGTTTTCGATT
ATCTGAACACGAGGATCGTTAAGTTCTTCAACAATCTGTTTTAGTTCTTCGAATCGATCT
TTATATTGCTTCGTTGCTTTCTCGTTTCCGTAATACACATCACGGTCGATAACATCCCAG
ACAGTGTAAACAATACATTGGGCTTCTTCTTTTGGAATAGTTCCTTTCAGTGATTTGTTG
AGGATACCATTTCCGGTCTGACGATATGCAACAACGGATTCTTTTACTGCTTCCGCTACT
GGCGTTAATGGCATTTCCTCTTCTTCAAATATCCATGACAGAGTACCATCATAAACGCTT
TCAACGAAGTTCTGGGCTGGTGTTTGCGAAAGGGTAGGCATATATACCAATTCCCCATCT
AGAACAATTCCCCGGCCTCGCAGACGCTCTAAAACAGCATACAAATCATCCAGCCCCTGA
TAGGTATTACCTGAACGGGTATAGATTGCTAATGTTTCCCCGTCAATATCAGCCATCCCA
CCAGCGCCATCGCTTTTAAGTTGTGCATACGCCAGATACTTGATATTAGCAATAGTCTTA
TCAGAACATGACGATGCAAGGTGACACGGTTGCTGGGGGATCAGTTTCTTCCATAGCTTG
TTTGCAGTGGTAGCTGAGCAACCGAATTCCAGATCACGATTCAGGACCATCATTGCAACT
TTAGCATCATACTCTGACAAGCATGTCAGAGTATCGGTAACTAGCTGTAATGCTGCATTA
CCAGTTACTTGACGCGTGGCAAGTTTATTTTTAAAGACCTGTAATGCACCGGATAAATAC
AATACATACGAATAACGCGATGGTTTATCGTATTTCTTTACGCCAAACGTAACCTGTTTG
CAATATGTCGGATAAAAACATTCTTTCAGTGTTTCATTGCTTGCAGATTCTTTCAGGATC
TTTTCTTGCAGCGATGTTTTGTCTGTTGCTGCAAACTGGTTAATGATATTGAGAATTGAC
ATTTTCACCTCGTTTTGTTAAACGTTCACGAATATCATTGAGGATACTACAAAATTCCGA
TTCAGTCAATATAGTCTTTAACGGATCAGTTCCACCTATGATATGACGTAATTTCTTATT
TCCTGTAGCGAGTCATGCTTTACAAAATTCTGGGTTATTCCGTGCCATCGTAACAAATGC
CAGATGTACCAGTCTGTAATAATGCTCACTCATTCGATGAATCGGGCGAACCTGAAAATA
TAACATGTGATTCTCAATCTTTTCAGTTCTTCCGGTTCTTTTTGCTTTGCCACCGTGCAT
CAACAAGATTTCTCTTTGTCTCTGAGGGTTCTTAAATCTTAGCCCCTGTAAAAATCCTTC
TGTGCTGTTAATATCGCAACCAAATAGACGGAATGGTCTTTTATTGATATTACTCAATTC
ACCCGATGGATAAGGTCCACCCGCATAGAGATCAATTTCAGAAGTCATTGAAACTAATCT
CGCTAATCAGAGAGAACGGATCATAATCGTCATTAGGAATATCGATAGTAATCGAATGAG
GATTTACCCATACACCAAACTGGTCTTCTTCGGTGAATACTTCGGTATGGATTTCGTGGA
TGTTTACATTCATAGTTTTCATACTAAAACTCCTTAATAATCAATAACTTACAAAATCAT
ATAGCCAAAATATAGGCGGGATTATTTCCCGCCGTTTAAAATCATTAACCGTAAACTACC
ACAATATTCTGTTTGTGTGTTACAGTCCATTTCAGGTCGATCTCTTCTTACTTCTTAACG
CCGTTAATAACGTCTGGGTGAGGAACTTTATTCATCTTGCCATGTGGCAGTAGAACAGTT
TTACCCAGCAATTCTTTCTCTACAGTCTCGCCAATATCAATGATCTTACCTACCAGCGGA
ATCTCGCCCATAGTGCGCTTTTGCTCAATAATGATCCCCATAGAGCTTTTTACTTCGGTC
CCTGCGCTTTCTGCTTTAGCTTCGATAATTACATAATCACCAAGTGCTATAGGAGTTTTA
ACGGTCATAATTTTATTACGTCTTCAAATCAATACAGTATAATGATGAATTATTTATAAA
TTTCGACGATCTGCGCGGGAGTCAGCCCATCTACATTAATCAGACTATCAAGCCCAAAAC
TACGGAAACCTTCAACTGCAATTTCATATACTGGCAAAGATGTTTCTGATTCTTTACGCG
GTTTTTCAACGTTCGGAACTAACCACTTTTCGTATGTTTCGAACGGCATCAGTGCCGGAT
CTCGCGTGGCGATCATCTCAGGGACAGTACCATCTTTCTTAGTAAACTGCAAAGAGTAAG
TACCGACCGAAAACATTTTCTTTAGTGCTTCACGAAGTGCGATATTATCAATTTGTTCAG
TCATTATCAATACCCTTCAAATCAGTCAGTAATGCTTTTGGAATACGCGCGATCGTAATA
TCTGCATTATTCAGAATATCAGCCCAATCACGCCCACCAGTTCGATCGTATGTGTCGCAA
TACTATAGATGTTTAATTCCACTGTTTGCAATTGCTTTAGCGCAATCTGGACACGGTGAA
GCAGTACACCACATACCAGCGCCTTCAATCGATACACCGCTACGGGCAGCAAACAAAATA
GCATTTAATTCAGCGTGGATCTCGTTTTTCTGTGACCATGCACTATGCTCTGGTCGTTTT
TCTGGGATCATTTTGCCGTCATCATCCAGCCAACCGTTTTTATGTGCATGACAGCTACAA

TTTACTTGCCCTGCTGGAGTTCCGTTAAATCCAGTAGAAATGATTCGCCCGTCTTTTTCG
ATCAGTGCGCATACCTTCCACGAAACACATTTAGAGTTCTTTACGATTAGATAGTCGATC
TGCATGTAAGTTTGTGACAACATAATATAATCCTCACATATTAATTAACCGTATACGCGA
AAGTATTCTACCGATCCGTCGCTATACTGTTTTACATTGAATGTATTCACACCATGATCA
TTGCTCATAATGACATATAAACTCCGACGCTCATCACCAAACGGGTTAACTAGTGCGGGT
TCAATTACAACATTAAAACCACGTTTAGCAAGTGCTTCGCCTAATTGCTTGAGTTTATCG
GTAAACATTAAAAGTCTCCGTAGTTAACCTGAAAACATTCTACACCAATTGAGCGGTACA
TGTCAACCACTTGGTCACGGTCATCTACTGCCAAAACAACATGATAACGCGGTGCAATTT
TCGAGAAGAAGATCTCTTTCTTCACTTCATGGTCTGGACGTCGATCATTAGTTTCACGCT
GGAAGTGATCGCTAGGTTCAATACCCTGTTCACGCAGCCATTGCAGAGTATCTTCTTCGC
AAACATCCTCACGCCCTGAGCATGTAATGATACGATAACCATTATCACGATACAGTTTAA
GCATTTCGATCACTTGCGGACGCGGTTTATCTTCTTTGACCTTTGTCCAGTCAAACGGAT
GACGACCGCCCTTATCCGCTAATGGCCCATCAATACCAAAGATCACCGCTTTCGGTAGCA
TGGTATTCGGTTTGTATACTTCTCGACCCATATATTCCTGCATAAGATAAAATTGGTTGC
GCAGATGAGAAAGTGGAGTTGCTTTCTCGCCGCGATAGAAGTTACGTTTCTTTAGTGTCT
CAAATAATTCATTGAACACTTGTTCAGTATACACCGCGTTAAATTCTTTTGCAAGTTCTT
TGTAGAATACACGATGTTTTTCGTTCAGGGTGGTATCAGAGATCATGATACTCAATCCGC
GTGATAGAGTCTCGCGAGCAATCATTCTCTGAATAGACTCTACCAGCTTTTCTTTTTCCT
TAGTATACCTGTGTTTCAAAAGTGAGTCAACACCAAACAACATAAGTCGAAGATCATCAC
GATTCACATTACGATAATTATTAAACCGTTTACATGTTTCCATAGCCCATCTAGTTTTAC
CGGACACAGAACAACCAACGGTTAAAATCACTTCACATTTCGGCATTATTTCTCCTTCGG
TTTGCACCGTTCCTCATTAAGTTCTTTGCGATAATAACACATGATCAACTTACTTTCAAG
TGTATATCTCTTAATATCAGCCAACCAGACTCTAAATTTTTGGGAGTCCTCCCACGCCAT
AGAAACTTTAGGTTTACCGTATATTGTATCCACTTTCAGGTCAATGTCAACTTTTTCTAT
TGGTGCTGGTAGTGGAGGATGTAATTTCGGTTCCTGTGAATAATGGTCCATTGTACACGA
AGAAAGAGCAAATACAATAGGTACAGCGAAGACATGGATTATGGCGGTCTGAAAATTCTT
ATGTGCTTTCTTAAACAACCAGTGTATCACGAAAATTGAAAACACCATAAGGGTAATAAT
GCTTGCACCTATCCCCAGTTAATCAACCAGATTAAAATTGCTTATCTGCTCTTGCGTAGG
CAATATTTCTATGCTCATTTAGTAGCCTCCTTTAATTCTTCGGTAAACTCCTTAAATGAA
TCATTAAGCTGTTTCTCTACCAACTTGGCTTTTGGTGCTACTACATAGCCTCGTGTAGCG
TCTTTCATTGCTTTGTTTTGTTCTTCTGCAAACTTAGTTCTTATTCCGTACATCTCCTTT
TGTAGTGAGTCTACGACCTTCATCTTTTCATCTGTCTTTTTCTTATACTCTGTAAAAGCC
TGATTGACTCGGTCCACCTCCTTTTTATTTTCCCACCACATATTGCCACCTACCCCCAGC
AACACAGATATGATAACAATGTATATCAGCTTTGACATATTACCCCCAACGACAAAAGGC
GGGGAACTAGCCCCGGTTATTATTACCAATCAATCTTATCAGCCGGAATCAGTTTCTTAT
AATTCCGTAGATAGTAAGTTTTCATTGAAGCCAGAATACGGTCATAATCAAGGCCATCAA
ATGCAATCATCAGACACATGAAAATATATGATTGTGCATCACCATGTGCACGTGAGTCGA
TAGTCGCATTGGATGCATATGTCTTTCTGTCTTCTCCCGGGTGTGCGTTATAAAATTCTG
TTGCTGTCGTAAAGGCATTGCGAAGAATGGACCTGAATGCTTCCTCGAATAGCTCAATTC
GTTCCAGTGCTTCCGGTTCGCCTTCATATGCAGCACGCAAATCATCGGTTTCGCTTTCTA
CGACATAGTAGATCAGGTTATTAGGAAACATCGATGTGTTTTTAATGTTTTGCACACCAA
TATACCATTCACTCTTCACCTTGAACATTTGACCGTCTTTAGTGCGTGCAACACTTCCTT
CGATACCTTCTGCGGTCCGAACTTCATCAAGAATATTGCTAAAACGCGCTGAATCAACCT
GTAACGCATTTACCAGATGAGGACGTAGGATTGCGTCAGCAAATAGCATTTGATGCGGTA
CGTACTCTCCTGTGACGTTATGGCGTACATTCAGAAGTGTTAGCGACGGTTCAGAATATT
CTACAACAATTCGGTTGCTGGGTGCTGTATATTCAAAGTTGAAATTATAATCAGACCCAA
TATCGGCGATCTTAGCGCGTAGATCCGCATACTTCTCTGAATACAGAAGACCGTTTGCTC
TCTCTGCCTGGCTTGAGTACAAGGCCGCTTTAGACTTCAACTGAACATAGCCGTTATCAA
TGTATGTGCTTACTAGACTACCCTCCATTTTGTCCATGACCATTTCCACATCTTCCGGCT
TAAAGCGAACGCGATCATTCTCACCCAAATTAAAAAACTTCTCCATAGGACGAGATGCAA
TGCGGACAGGTCGGTCTTTATAATCGATTTCAAACATGATCCCGCAACACTCTAACGCAT
CTGGTTTATTCCACATATCCGGTTTGCTCAAGTACAGATAACTAAAAATGCGCATACGGG
TTTGCATCGCCGTAATTTCATCTTTGTAATAGAACGTATCAGATCCGTCACATAGTGCCA
TCAGATTTTTGAACAATGCTAAAACTTTTTTTGTTGTTTTCTTTTGTTGACATAATTTGT
GTTCCATTCTGGTTTAAATTTGGTGATCAAAACAGGTTCTTCGATATCGGCAGTGCTTAT
AATATCTTTACCCGTTGGATTCTCGATGTACAACTCAAAACATTGGCGGTAATAGAATGT
TACTGTCTTACCTTCCTCTAGCGCCTTATGAATATTCTTACTCTTGCGAGTATCCGCTGT

TGGTGTTTGTCTGTTAATGCTCGTCCGGTAATAATTGATCCGCTTTCTGAGATTAACAGT
CTGACCAATGTATACCAGTTCATCATCAACCAGAATAGCGTAAAGAACATTTAAACGGTT
TTTCTGAGGCGGTAGAATCACATCACCATCTTTACACTCTAAACATGAATATTTAACGAA
TCCATAGTTTTCTATTAGCTCATTAATCATTATCCTTTCCTCACGTCACAACAAAGGCGG
GATTTCTCCCGCCCACCTTATTAACTTTCCAGAATATCGCGAGCTTCGTTACGCTCCTTG
ATGATTTTCAGGACAAATTCCAGTTTTTCTTTGTTCAGTGTTCCGTTTTCCATTGCTTCT
TTGACGGTAAACACTCCGCCACCTTGCCCGTATACAGTGACAGTGATTTCACCTAACATC
GTGGTATGAAAACCGCCAATTTTCGGTGATTCGAAAGAAATAATCACCTCACGGATAGTA
TTGCCATCGTTGATTTCTTCCAGCGTAATATCGCTTTTGTAAGCGGTTGCTTTAACCATA
CGGATCAGCATAGTCTGATCTAGGGGGATGGTCATTTTGTCCATTGCATGTTGCAATGCA
GCGTAACCGAAAACTTTAACGACGTCCAGAACGTCTTTGCGAGTTTGAAATTTGATCATC
TTGATACTCCTTAGTAAGTTAGTTTCGTTTCGATGGGGTATCTTACTACCCCCGGTTCAG
GCTGTCAACTACTTTTTAATGTCAAGTTCGAATTTTGCTACCTTACGTCCTCGTTTATTG
AAAGCAACCCACGGACGGGTTTCAATGTAGAACAATTCTTCGCCAATAATGAATACTGGC
TGATTGTAAAGGCGACCCATCAGGTCACGCTGGCGAATGCTAGTAGATTCGTATACTTCG
CCTACTACAAAGTGTTTGCTGATAGAGTCGGTGCAAGTAAATTTCATTTTGATTCTCCTG
TGTTTCGTTTCGATGGGGGTATCTTACTACCCCCCGCTATTCAGTGTCAACTACTTTTTA
CTTGTTGTATTCATTCCACCATTGAGTGAGCTTATCAATTATTTTTGTGTTTCTATCCAA
CTTATCCCACCGTTTTCGAGGGTTTATCAACCGGAATAGGTAAGGTGTACTTGTTAAGTG
AACTCGGTCACTGTAGCGATTGATGTACTGCTTTTCAATTCTAAGTTTCATCTTTTTACG
GTGAAAATATGCAATGACTTGTTCTTCTGTTTCCATATGTTCCTCCGTTTCGATGTGCGT
AATATGCTATGTTAAGATCCGATTGTCAACACCCAATAACAAAAACAGTCGCCACTATGG
GCGACTTTGAACAAACATTAATCATGACCGTGATTTGCAATATCCGCTCGTGTCGTGTCT
TTGTGATTACCTACGTCAAAGACACGATAACCATTTTCACGAAGAACTTTAAACACTTCT
GGGTTATCCTCAAAGACTCCGACAACTTCATCTTTATCAAAGGATTTAATTACCTCTAAT
TTGATGTGATAGTCTTTACGGGAATCATTGCGACCTCTCATAAAGATTTTATCGAAACAC
ACATCATTAGCGATCAACCATTCCACCGTTTCCATGTAAGCATGTGAACGTCGTCCGGTG
AGGATGTAAATCAAATGCCCGTTCTCGGAAAGAGCATTGATAACACGGATTACATCGTAA
ATGGGGGAATCTCCCCCACATGCCTTGTTATATTCGATCCACGACTCGACTTTATCAAGA
TTTTCGGTTGGCATCTTTGCTAGTCGATGTGTTGGATCTGTGATCGTAAAATCTAAATCG
ACAAGATATATCATAGATATTTCCCGAATCGGTTGTAGTCATCGTCTGTTAAATCGGTAT
TTACCTGAGACACCAGATACATAATCTCTACTTCCTGCGCTGCATTCTGCACTTCGTCAG
AGTTGAACGACTTACGCATCCACGGTAAAGGATGACGACGAACACTAAACGGGCTTTTCA
GTCCGGCAGCACGCATACGTGAATCTGCCAGGTATTCTACATAGGTTTTCAGAATTTCAG
GTGACAGGTTAGGAATTGCCCCCATTGCGAATAGGTGGTCTACCCATTCTTTTTCTTGCT
CGACAACATCCAGAAAGATTTTCGTTGCTTCGCCTTCAAGTTCTACCGCAATCTGTTTCC
ACTCATCACCATCAACCCCCGTTTGCCATTGACGAATAATGTATTGTGTGCCATTTAAGT
GAAGTTGTTCATCACGCGCAATAATTTTCATGATCTTCGCGTTACCTTCCAGTAAACCAC
GTTCGGCAAAGCTGAAAGTACATGCAAAGGACACATAAAAACGAATTGCATCCAGTGCGT
TGACGCAGTGTGCACACAGATAAATCGAAGCCATCAGATCTCGTTTTGTTTCTTCAAAAA
ATCTTCTTGCATAATCTAATGCTGTCTGGTCGTCTCGTTCTTCCCAATAGCGGAGATCTT
CTTTTGCATTCTCATACTTACGGATCCAATAAAGAACATCATCATAATATTTCCCGATAG
ATTCCGCTCGCTTCATGATAGAAGGGTTATCGATGATCTGGTCGAACTCACTAGCCGGAT
CAATGAAACAGTTACGCATAATGTGTGTATAGCTTCGGCTATGAATCGTTTCGCTGAATG
TCCATGTTTGCAACCATGTATCGAGAGTTGGATCAGATACAATTGGACCAAATGCTGCGG
ACGGTGCGCGACCTTGAATAGAATCAAGTAATGTCTGATACTTCAAGTTTTCTGTAAAGA
AACGCTTTTCAACGTCACCCATATTAAGATATTGAATGCGGTGGGTTGCAAGTGAAATTT
CTTCTGGACGCCAGAAAAATGACAGAGATTTTTCAATTAATTCTTCAAATACTTGATGAC
GCTGAATGTCATAACGAGCGATACCTAAACCGCTACCCAGAAACATCGGTTCATTCATAA
TATCAACGTGTTCACGATTAAATACCGTATTTCCCATTCACATATTCCTCATTTTACAGT
TATCAAAGTGGCATCGTAGAGCATTGCCTTTATTGGTAATTTTCCCGCAATGCGGACAAG
GATCTGCCATTGTTGCTTCCTTTGTCCGTTGTGAAATCAGCTTTCTCATTTCCTCGCTAT
TTGGTATTCCTTTAGTTGGCGATGGTTTCCCAAACATAGGATTTTTACTTCCCATTCTAG
CTTTTGAAATGTTTAACGCGTGCTCCTTTGTTTTTGGCTTACGCATTTTAGCTTTTGTCT
CTTCTGATTTCAATACACCTTTCGAAGATTCGCTAATCTTTCTTCGCGTTTCTTCTTTCA
TCGGCTTGCCCTTTCTTTGTTCAGAGATCAGTACCCTAGCTTGTTCATATACCCTAGACC
AAACAATATACTCTCTTTTATTGGTCTTCGCATTTTTTGCTAAACACATCATACCAAATG

CTTGTGCGATAGGTCCACCATGAATTTTGGCCAGCAGATGATGAATCACGAAATGTTCTT
TTGCTGTCAACTCAATCAGGTTATCAACTTAATCACCGCCACCCATAGATCGCGGTAAAA
TATGATGTGTTTCCTTATATCCTTCAACGGTTCCTCTGATCTTTGCTTTTTCTATGATTG
CGTCATGTAGTTTTTGTAAGTTCATGAATTAATCCTCTTTTCTGTATACCATTATTTATC
AGGTATCCAGAAGAAGGATTAATTGAACTCACTATTATCCGACAACCTCAAAACATTTAT
CATCAGGAAGAATAGCCCATTCTACGCCGTATTCATCCTCAACGTAGTAGCAAGTCCCAT
CATGTCGAGCATCATATTCATCACCGACAACAAAGCTATCAGCTAGCTCAAAATCTACAG
CAACACATTTTAATTTGGTCATAATAAAATCCTCACTTGTTTTTGATGGTTGTCTTATAG
CGGGGATTTCTCCCCGCATCAACACTTATTTTACAGCTTGCAAGATGAGCACTCGCTAGC
GTCTTCCGTACCATCTGCACCGTCGCGAACGTGCGCATAATACAGGGTTTTCAGACCGAA
ATATTGAGCAAACAACATATCATCTAACACAGCAGCAATATCGACGTTACCAGTCTTATA
AGTGCTTGGATCGTATGATGTATTGGAACTAATCGCCTGATCCACAAACTTCTGCATGAT
CGCTGCCAGTGTGAGATAAGGCTTGTTACCTTTCTTGATCAGTGTCCAAAGCAGGTCAAT
AACATCAGCATTATTCTCTACGTTCGGAATAACCTGATTAAATTGCCCTTCTTTCGATCC
TTTAACCGATACAATCCCGCGAGGCGGTTCAATACCGTTGGTGCTGTTAGATACCTGAGA
AGACGACTCACAAGGCATCAGCGCCGACAGTGTACTGTTACGAATGCCATATTGTTTCAG
GTGTTCGCGCAGTTCTTCCCAATCCATCTCATACACCGGATCCACCAGTTCATCTACTGT
CTTTTTGTACCAGTCGATAGGAAGTTCACCGCGACCATATCGTGTTTCGTGCATCAGTTC
GCAAGCACCCTTCTCTTTAGCCAGTCGCACACTAGCCTTAATCAGAGCATATTGCAAGCG
TTCAAACGTTTTATGCGTAAACTCGTTAGCGTCTTCATATGTGAAGAAATTATCTGCTAG
ATATGATGCAAAGTTAGTCACACCAACACCCAGAGAGCGACGTTTTTGGCTGTCAGCGC
TTGTTCTGCAGGATAATCCTGATAATCTAGCAGGTTGTCAAGAGCACGCACCATAACCAT
AGCAATGCGTTCTACTTCTTCCATATTTTCGATATCGAAATTACCTAAAACATATGCTGC
CAAAGTACATAGGCTAATTTCTTAATCATCACGACCCATCGGTTTTGTAGGCAGAGCAAT
TTCACAACACAGGTTAGATTGTTTAATCGGTGCTACATTACGAATAAAGGCCCCGTGATT
GTTCATGTTGTCTGTGAACGTTGGGTAGATTCGACCAGTGTTAAAACGTTCAATTGCCAC
CGCTAAAAACAGGTCTTCGGCATTAACACGAGTTTTACGAACACTCGGATCTTTATCCAG
TATTACGTAAAGCTGGCGGAAACCTTCCTCGTCTCGGAAGTAGAGATCCCACAACAAGCC
ACCCGCAACATCTGGACTAAACAGAGTAATGTAATCCCGTTTAAGGAATCGCTCTACCAT
CAGGTTATTAATCTGTACGCCGTAATCAAGATGACGAATGCGGTTATCTTCCACCCCCTT
ATTGTTCTTTAACACAATCAGACTTTCAAACTCTAAATGCCATATTGGGTAATAGAGAGT
CGCCGCCCCGCCAGGAATGCCGCCTTGTGAGCATGACTTAACGGCAGTCTGAAAATGTTT
CCAGAATGGGATAACGCCAATATGACGAACTTCCCCATGCCCGATCTTAGATCCTTCCGC
GCGAATCATGCCAGCGTTGATACCGATACCAGCACGCTTGCTAATATACTTGATGATTGC
ATTTGCTGTTTCGTTGATGCCCTGCAATGAGTCATCACTTTCGATCACCACACACGAACT
AAATTGGCGTGTTGGGGTACGCACACCTGCCATAATCGGAGTAGGTAGAGAGATTTGCTT
ATTCGCAACAGCATTATAAAAATCAATCACATTTTCAAGTCGATTTTCTGTCTCTTCTTG
ATGCAAGCTCATACCGATCAGCATATACGCAAATGGTGGTGTTTCGATGATTTTCTTAGT
GCTGCGATCCTGAACAAGATATTTCTCTTTTAACTGCATCGCACCAGCATTGGTCATTTT
AAAATCAAGTTTATGGTCGATGAATTTCTCCAGATATTCCACTTCTTCATGGGAATAGTT
TTGCAGAATTTCGCGATCATATTTTCCGGTTGCAATATTACGCTCAATATGGCTGTAAAA
ATCAATCGGTTGGAATTGACCGTATAGTGATTTGCGAAGCGCGAACATTGCAAGGTTACT
TGCTACATGTTGATAATCGGGTGTTTCTACAGTAATGTTATCTGCTGCGACCTTAATCAA
TTCTTTGTGGATATCGTTGGTAGACATACCTTCTGTCAGAAGTGGTTCAACCATATCAAA
CAATTCATATGGATCAACGTTATGACCTTCGGCTGCCGCTTCCAGAATTTTCATTACTTT
TTCTTTTTCAAACGGAACAGTCAGACCGGAAGACTTAACAACATTTTTAATCATATTACC
TCAGTTCAAATTTTAGCAGGAACGAGTTTATCCTCCATATTGGGGGCCTTGGCCCCCTTT
GTTATACTGCCATTTGAGCGGTGATCGCTGGATGGCTTTGATAGTTGTGAATTTTAAAAT
CTTTCGGAGTCTTTGTCAATACCTCATCTAAAGATTTAAAATCCGGCAACTCAATTATTG
GTTTATCAAACGGAACACGGCTTAACTGGTCCATAACTTGATCAAAATGGTTTTTGTAGA
TGTGGCAATCCCCACCCTGATAAACCAGTTCGCCAACTTCTAATCCGCAAATCTTCGCGA
TGATATGCGTTAACAGTGCATAGCTCGCTACGTTGAACGGATTCCCTAAAAAGGGATCAT
TCGAACGCATGAAAAATAAACAACTCAATTTGTTGTCGTTGGCAACATAAAACTGTACAA
TCCGATGACATACACCTAACACAGCATTATGCGTTTCCTGTACGTTCCATGAATCAATGA
TAATTCGACGACTATATGGATCATTCTTGATCATGTCAATAACTTTTTGTAACTGGTCTT
CCCAAATCTTCGGCCGATAGACATATTCTTCATCGTCTTTAGTGCTTGATGTGTCTACGT
CCATACTAACAAACATCGTGGTATCCGGTGCTATAATATCCCCACCCAGATAATCACTAT

CAAGCAGATAACCGAAATAACCATCATCTACCCATTCCTGGAATCCCCACAAGCAATATG
CATCATTAACAAAATTAGATTGAGTCTTCCAGTGGTCTGCTACCAGAATATAATCAAACT
CTTCATAACGCATACCACGCCAGATAGCACATAAACGGTCTTCTGTTTCATTGGTTGGTG
TAAACTATTCCACCCACGGGTGCTTAAATGTATGTGTATATTTGCTCTTAACGCGCTTAA
TTTTGATTTCACCGTGTGGCTAAATCGGTTTTCGTCGCCATGCCATGCCATACATATTAC
CGACATTATAATCATTGAATCGCAAGGGAGTCTTTTCTTTCATATCAAGACAATTGCCGA
ACCAAATATCAAAGGCTTCCTCGTCAAACTTACCGCGTGTAATTTCCCGTAAGCGACGGC
GGTCAAGTGATCCCTCCATAAACCATAGCAATTCGCCAATTACAGCGGCTGTAGCCATCT
TTTTAGTGGTCAGTAAGGGGAAACCCTCGCTTAAATCAATTCGCATTTCCGCACCGAATT
TAGAGAGCGTACCTACCCCAGTTCGGTCTTCACGAAGCCAGCCATTATCAAGAACATCAC
GCAATAGTTTCAAATATTCTTTCATCTCACACCACCTTTTTCTTAGTGATTGCTTTCTTA
CGCAGTTTTGCTACTGTCTCGTCAATGTCTATAAAGATAACACCACGGTCGAATACAGTT
GCAGGTCTTGAGATATCGCTATTTTCTTGCACAGGAAGCCATGATGCGCTCGCTTCATCA
TAGAGATATTGTAAACCGTAATAATCTACGGCTACTGCGTTGTTTTCACTGGTAGCCGTC
CATAATTGAATAATACCGACTTTCATTATTGTTCCCGTCCACAATGTAAACACCGACTAC
CAGCAAACATCCAAGATGCACCACAATGTTTGCACTCATAATCTGTCAGAATTTTCCAGT
TACGCCTTCTTACGTTCTAAAATTTGTTCGCTGATCAGGTAATCAGCAAGGAAGTAATTA
TGTGCTTCTTTTGTTGCAAAATCAAAATATAATTCCTGAATCATTTCCTTAGAGATACCA
ACATCATATTCGAACGGATCATTAATTCCTGCTCGTTTAACATATGTTAACACAATACGG
TCCGCAAAGTCAACCGCTTTTTCAATCAGGGAAGGTCCACCAATAACGCTTACCAAACCT
TGCAATTTCTCTATGACTTCTAATAATTCCTTCAATCCACCATCATTAGTAATCGCGGAA
TACACATCTGGTTGGTCGCCGTTACTTGCTTTCGGTGCTGGCTTACTGGTATCGGTCCTA
ACAACCACATTCACGCGACCATCCAGTGCTTTAGGAAGACTTTCAAAGGTATTACGTCCC
ATTACCACCGCCGCAAGATCAACATCTCCCATAGTGCGCATTTTAAAGTTTTCCATGTCC
TTTTTGATTGGACGACCCCACGGTAAACCGGCTTTAAAACCAAAAGGCCAATTTTCATCC
GACATTTTAGTGTCACCAATCGCAAATACACATTGAATCATAATATAAATCTCCTATCAA
ACCAGCTTGAGATAGTTTTCGTTAACGTTTTCTTCTGTGATGTCGGTTTTAAACTCAATA
CCAACACCAGGAGAAACATAATAGCGTTCGGAATAATCACGCCACATTTTCAGTAGGCTG
ACACCAACATTAACACCATTTTCCCAGAAACGGAAGATAATTTTATTTGCGGTGTGGGGG
TAATCGCTTTGAATCAAGCTGCCATCACCGCGACGTGATTCATAAACCGTAATACATTCA
CGGTTAGCGGCTTTAAGACATTCTTCAATAGTACCGCATGAGATAATTTCGGTTGAGTCG
TTTACGATTTGTTCTACAGTCCACATAATATAATCCTCTCTTTGTTTCGATGTGTGTATT
ATACATTACCACATAAGCATGTCAACACCTTTTTCATAAATTTTTAGGCAATAAAAAAGC
CTCCCCGAAGGAAGGCTTTAAAATTCATTACATCCCTAACAGAGCGTCAAGATCATCCAG
ATCAGAAGCGGAGGAAGTACCGCCACCCAGATCCAGCGATGCCATCAGGTCAACAGCGGA
AGAATCTAACGCCGTGCTAGCTGCGTTAATACCAACTACAGTGCTAGCTGCGTTAATACC
AGCATCGAATGGATTCATCTGTGCGTCAAAAACGTCAAGCTGGCTGTTAATCGCTGCCGC
AGCACTGGAAGCCGAAGCACCAGCGACCATAGAAACGCCCATAGTTTCTTAAATTTGGT
TTCCAGTTCATCGAACGGATTAAACTGGCTAGGCGCTACAATCTCACTCAGGTCAACCAT
GTGTTCCATCAGATATTTCTGATATGCTTCGTCTTCAATTTTCGGAAGCTGTGATTGCTG
CATGAAACGAGAATCATCATAGTTCTGGTGTTTATCAACCTTCTTAGCTTTCAGGAGGAA
GTTAGCACCGGAGAACACACAAGTTACATCTACCGGAACTTCGCCCATATTGGTATTCCC
GTTCACCATTGCAACGATCTTATCATAAATTTTCTGACCGAAGCGGAATTTGAATACCTT
ACCTTCATTTTCTGGTGCTTGAGGATCTTTCAGTACCAGAATATTTGCGTAATAAGAGGC
TTTACGCTTAATCAGTTGCCATTCGGTTTTATCGGTATTGTACAGATCTTTTTCGTTAAT
ATATTTGCACACAGGGCAAGAATCAAAATCACCGTGGGTGCTGGAGCAGTTTTCACAATA
CCATTTGCCATTAACTTTGAAGGAATGGATTACCAGTTTCACAATCGGCAGACCGTCTTC
TGTCTTAGACGGCAGGAAGCGAATCAGGGCTTCACCTTTACCTGCTGCATCAGTCTTAAT
CTTCCACTCTTTCGGATCTTCTTTGGAGAAACCGTTGCCACCTTTCAGTGCGGCCAGTTG
GGCTTGCAGTTTGGAAGAATCTTGGCGTTTAAACATATTCACTATTCCTTCTTTTGATTA
ATTTACTAGACTATTATACAGATTTAAATTTACTTGTCAAACATTTGTTTCCGTTTTTAA
TTGAGATATTTCTTTACACTCTTTAACTACTTTAACAAATAATGCTCTTGCTTTTGCGGC
GTCAATTACATGTAGTTTCTTGTACGCCTTCATTTTAACACTATAAGTTTGCCAAATCAA
GTCTTTGGCTACCTTGTCGTGTTCGTCGGTTATATTTAGAAAGCTATCTAACAACATAAA
CGACTCAAACGATATAATCCGATTTTGCAGTAGTTTAAAGATACCGCTAGTTTCGGTTGC
GGGATTGTATGTAATTAGCTCTTTAAGAGTAACCCCGCGTTTCTCGGAGAAGTATACCAG
ACTTTTAATGTCATCGTCAAATCGTGTTGCTGCATCGGTTATCTTTCCTATGTATTCCAT

ATAAAAGCCGTATGCGTCAACATCAGTGATTTCACCTACCCATGCATCGGTATTAGCTAC
CAGATTGCTTAGGAATAGGAAGTATAATTCTCTTAGATTGTACTTTTCCGCTAATTTTGC
GAAATAGTACGTATCTCTGCGTTTATTGAATGCGGCATCAGACAACCGCATCCTCAAATT
GTATTTTACGCAATCATAACGTCCGTTCATGTGAGCTTTCATCATGCAATATAACATATA
CACAGATTTAGCATTAATTCGCATATCTGGATCTGGAGGAAGACGGTTTCTGATCATACA
TACCCCTTAAATATCGAGAGTGTTGGTCGGTTCGTCGATCACTCCCTTCCTGAGCGTTCT
ATTTACTATACATTCTTGTGTGATCTTGTCAATAATGGACATCGGAATATACTTCTGATA
GTGACCATATTCGATAGAGTTTTCCTCTACAAACTGCGTTGTCGCTTCAAGATAAGTCAA
TCCCTCTTCAACAACCAGTTTTTCGATTGCAAGACCGTTTGCTGTTTTGTTCGCGGCCTT
TACCGCATGTTCATTAAGCATTGAACACCCGATCATAGAGTTCTACCGCTTCGTCGCGTT
CTGCTTCGAACGTTTCGCGCGGTTGCTGGTGATACAGTTTAAACAGAATGCCCCACATTT
TACCATCGACGCCTAATTCTTCTTTAGCACGCAGTTTGATCTCTTTGATCTTATCGCCGT
ATGCTTCTTGAATGGTTTTCTGATCGCTGGCTTCTTTAATCAGTTGTGCCAGGGTATTTC
CGTGCACTGCTTCGTCAAATACTACTTTTTCTTTTTTCTGTTCAGTCAAAATATTAACCC
TCTTTCGTCATTAATTGAAAACCACGTTCAGCTTTTGAAATTTCGCTAGGACTTAAATCG
TCACCAACATATTCTAGCATCATTTCTCGGATAATGTCTAGTGCTTTTTCATCACTTGAT
TTTAACTCGAACTCAAGTTCATTGTGAGCATCTCCCATTCTTTCGCCGACTTTGCAATAA
TAGAAAGTGTCTTTGCACATTTCCACTATAGCACCCTCAAGGTCAAGTGTCAATGACTTG
CGGTATGTCATTACTTGCAATAGAGGAACGATAAGGTCGGAGTTTACGCCTAGACTATCA
ACATAATGATAGAAATCATCCATTGTTGAAAAAGTGATTTCTTCTCGGTTGAATAGTCCA
GATTCACTAACACCTATTTTGACCATAATCAATGATCTATTAACGTCAAGTCGAGCATAA
ATTCCTTTTCTACGCATTGCATGATCTTCGGTATCATAATAAAATTCGTGTTTTAGAGTG
TTAGCACGATCTACTAGATTGTACCCGTCATACATTGCTACCCACAAATCTCAGATTGTT
TCGACGCCAATTTGTGAGTGTGCATAGTATCTTAATTCAAGTCCTTTCATATGTCAACCC
TTAGAACTCACCAATTTTCTGCAACATTTTAACTAACGCATTGGTTACAAAGTAGTTATA
AATTTTACCGCGTGGTGCTGGTTATGATTTCTCCCATGCTAACAAAATTTCATATTCGAT
GTTATCAGGAATAAATTCAAAATCAATAAGTTCGCGGTTTTCGTCAAAGCGTAGTGCTTC
TTCCTCTGTCATCAAAGACTTTGGATCTTCGGCATCGAAAATTTGCTCTAACCATTTAGT
TGCAATTGGTTTAGCTCGTTCCCCTTCCAGTCGTGAGATAATGAAATCACTACGACACTT
GATAGGAGCTACACCGTCCATTGCGTCACCCTTAACACATTTTACCATAAGGTCTTTGTA
AGGGCTAGCATATTTGCATTTCTTCTATGCTTTCTGTACTGGCGCCCACTGTTTCACGTT
TTTGTATTTGTGCAACTGTGTGTAGTCACCATCGCCGGAGTTGATTAGCACCTGGCGACC
TTCTACAGAAAACTTTTTAGTTAACACAGCGATTATATCATCTGCTTCAACTTTGTCAAC
CTTAATAACGTGATAAGGCATGTGCTCTTTAATCTCTTCGGTGATCTTATTGATCGCAGC
AAAGATCATCTCCCAATCACGCGGATCTGCGTCTCGTTTTTCCTTACGGTTGCGTTTATA
GTAAGGGGCGATATCACGTGGCCAGTAACCTTTACTTGAATCAAAGGCAAGTACCGTAAT
TGGGTATTGCTCTTTGAATTTTGTCATATTATAACGCATGGTGTCAAGGATAAGATGACG
AATCATGCCTTCATTAATAGGATCTTCCGGTTTAAAGGAATGCGAGATAGTAGCAATCAT
AAGCTGCGACACATCAATCAAATGCACACCTTCCGGCAGTTTATCTTCTGGATCATCGAA
GAAATCATTAAGTGTAGCGGTAGTTTTTGTAAACGGGTTATACGTCATAATTAAGTTCTC
TCCACTATCACAACAACATATTTGCCAAACGAGTTAGATACTTCACGATCATACCCGTAT
TTCTCCATAACTCGCAGAATTGCATTGTTTAATTCGGCACGGAAATAATTATAATCCTTA
TCAGGGATTCTGTCAACTACCCATTTTGCACCTTCTGACAAATCAACTTTAACCATAATA
CATCATCCTCTTTTGTTTCTATCAAGCCTCATTTTATAAATATGAACATACTATAAAAGG
AGACCCAATAATGGCAGATTTTTCTCAACATTTCAAGGCCACATTTGGCTTTGACGCTGG
TAATGAAAAGGTCGTCAATGTAGCATACGCAGTTAAAAACACATCAACGGATGCGGTCAA
CGTTGAATTTTCCAATTACGATAATGGTATTACTCAGTATGACACAACTCGCGGTTATGA
TCAATACGGAACTACAATCTATCAAAACCGTCCGTTTTATGCAAAAGAAGATATCACAAA
GCCAGCAGGTGATTTTGACCCGACTAAATGGCAAGCACTGCGCACGGACCCACGTTGGGA
TTATATTGCAGCAACTTCCAGTGATACTCCATTAAAGTCTGGTGATTATATCGCAGCAGA
TGGTCAATTTGCAAACTTGACTTTCTCTATGCCAACTATACCAGCAGAGGGAGACACTAT
AACAGTAAAAGATATCGGTGGCAAGTGTGGTGTTAACGAATTATCGTTCTTGAGTTCATG
ACATGAGTTTTACCATAACGGCAATGTGTATAACCGAAAATGGTATTGCGCTACCCCGTA
TGCAATGAACTATTTCATCTTTGTTCGGAATCGTTGGCATGTTAATCAGACGGGAACAGA
GCCGCGCGGTGTATACGCACAGCCAGCGATTGATGCTATTCAAATGCGAAATGGTGATCA
GGTATATCGTCGTAGTTCGTTGGGTAATATTACCTTAGTGCTTCCTAAGTTTGCCAACAA
TGGCGACTTGATCCAGACTACAGACCTTGACGGATTAACCGCAACCAACCACGTTACGGT

TAAAGTTCATGAGCACGCAGAAGAACAATCAATTGGCACGTCCGGTCTAAAAGAGATTAT
CGGTAAGCGTAGCGGTCATGGTCTATTCATCTTTGACAAGACGGAAAAATTATGGCGTTT
GTGGGATGGTGATCAGTCTGTTCGTCTTAAACCGATCATCGATGACACTCGCTTGCGACC
TAATAGCTATGTTGCTGTATTTGGTTGTCGTGATGTTACTCTGACGCTGCCGACCAATGT
CGAGCCGGGTGATCGTATTCAAGTTTCTATGCAGTATATGCAAGGAAACCAGAATTGCCG
TATTATCACCGCCACAGAGGATACAAAAATCCTTATGCAGAAAAACATGGTTCAGTTTCC
TAAGCGTAGCGAGTACCCATTCAATGGGACTGATACATGGACTGGAGTCTCTGAATTGTC
TTTCCACGCTGCAAATGATTATATACCTTATCTGGAGTTCTCTTATTCAGAAGGTGATGA
TGGTAAGAAATACTGGTTGGTGGCACATGCTCACCCGATTGTAGAACGAGTAGATCCGAC
TCGTAAAGATCGTGTTGGTGTAATTGCACTGGCTACCCAAACTGAAACGAATAAAAACCA
TGAAGAGAATCCTACCGACGAAGTTGCAGTAACACCAAAAATGCTGGCTAATAAAACCGC
CAACGAAACTCGACGCGGTATTGCTCGCATTGCGACTCAAGCGGAAACGCATCAAGATAC
TGGTTCCAACTTCCTGGATGATGTTATCGATACGCCTAAAAAGTTAACTGACTGTGTTCC
TACCGAAGCCCGTCGTGTTGTGATGGAAATTGCAACCCAGCACGAAACCAATGAAGTTTT
AGACGATACCCGCGCTATTACTCCTAAGAAATTAGAAGAACGTCGCGCTTCCGAAGATCT
CGCTGGTATTGCTGAAATTGTTCAGGCTGGCGGCAGTGCTGGCGCTCGACGTGGCGAAGC
TGGTACAGGGATCTATAACATCAATGACCATGCTAAAATTGTTACGCCAAAAAACATCAA
CGAAGTCAAGGCGACCGAAACTTCCCGTGGTGTTGGCTATCTGGCAACCGACGCGGAAGT
GCAAGGGGCTACAGAATCAACCCCGCAAGATGCTTTACTCATCACGACTCGCACGCTAAC
GAAACGTACTGCTACCGAATCGCGCACTGGTATCGCTGAAACTGCAACTCAGGAAGAAAC
AAACTTAGGTCAAAGTGATAACCATATCATCACGCCTAAAAAGTTGCATACTCGCCGCGC
AACGGAAACTTTACACGGTTTAGCCGAAATTGCAACTCAGCCGGAATTTGACGCAGGTCT
TGACGATGCTCGCATTTCTACCCCATTGAAGATTAAAACCTTCTTTGAGAATGCACAGCG
TTTGAAAGTCGATCCGACTCAGGGGCTTAACCTTACTGGTGATTTGTGGCAAGGTATCAC
GATTCGCGGTTTGGATGCAACCGAAGATACAAAAGGTGTCGCTAAAGTTGCTACTACTCA
GCTTACGGATGCTGGTCAAGATGATACTACAATCATCACGCCTAAAAAGCTGCAAGCCAA
AAAGGCCACCGAAGGTAAAGAAGGTATTATCCGAGTTGGTACTCAAGCCGAAACCGTGGC
TGGTGCACTGGCTAATGTAGCAATTTCTCCGAAGAACTTCAAATATGTTGTTCAGACAGA
GGATACATGGAAAGCAACCGAAGCACGTCGTGGTTTCTTGAAAGTTGCTACTCAGGAAAA
CTGTTTCGTTGGTGATAATCTGCAGGGTTCTACTCAAGAACTAGGAAACTACCAGCATGA
CGGAATTGCTGTTACCCCTAAAGGATTAAACTACGCACTTGCTAATTTCTTACCTATGAT
GGCAACCGCTCAGAATAGTTTGAAATTGGGTAACGTAGAGGCCGCTAAATGGGCGCGTAG
AGACATTGACCAGACCATAGAGAGCAATTACACGTTTAATAAAAACGTTAACGTGAAGGG
CGATTTAGAGTGCTTAAAATCAGGTTCCTTTGAAACTCTGTCTGTGACTAAGAACTCAAC
CGACGATCCGAGTAATGGTCATCTGGTGTTGGGTGAGCGTGGCGTTGACGGTCACACCGG
AATTACTCTGCATAGCACAACCGCATCGGAAGGCATGAAAAACTCGTGGTCTATTATCGT
TGGTGGTACTGGAACAGCGCAAGTTGTTGATAGTGATGCTATCGCTTTCGGTAAAATCAA
TGATGGTGGAGTCGTCGAGCATTACGCCTTTGCTATGGAACACAATGGCGACGCGACAGC
ATATAAAGACTTTATTGCAGGTCGTAACCTGTATGCGAAACAAGGCGGTTTATACATCGT
TGACCAGTCTAATCCGGCAATGACTCGTTCCCCAGATGCTATGCTGAATATCGGTCACGG
TGGAAGTGTTAACATCAAAGCAGGTCATCAAACGTTAACTACAGAGATCGCCGGACAGAA
ATATCAGATCGTACACGCAGGAAACGCTGATGAAGTTCTTAACCGTCAGTTTGTTAAGAA
TGCTGGCGATACGATGGTCGGTAAGCTGACTATGGATAATGCGCCTATTGGTTCTGTTAA
GCATGAAGCTAGCGCGGCAACCGCTCCGGCAATCAGCAACATCGGTTTCTGGAATATGCG
CGTTACTATCCAGAACATCAAAGAAACTTATCCAGAGAAGAAAAACGGTACTCTGATGCA
ATGGGGTACTGACGCGGACGGGCTTACCCAGCTTTGGTCCCCAGATGGTACTCATAAACA
TTATATCCGTTCCGGTACTGGTGGTCAATGGACCGCATGGGGTGAAACTCACACCAAACA
GAATAAGCCTACCGCTCAGGAAATCGGGGCAGTAGTTGCAGAAGGTGGTTTAATGAACTC
TATGACGGTGCGTGACTGGATCAAAGTTGGTAATGTTAAAATCATCGCCAATAATCTGAC
TCGTACCGTAGACTTTATCTGGGAAGACTAATTAAAGGGGGCGAAGGCCCCCTATGGGGT
TATTATGACACAAGATATAGAACTACGCAACACTGGAATTAGGTTTGCGCATTTCGACGA
AGACTCTGCGACATTTACCAGCATTTCAGAAAATAACTCTGTGATGTATAAGCTGGAAGT
GCGCGGATCTAATGCGGATTCGACCAAACCAGCAAGGATTAGATTTAATGGAGTGGAGAT
CGTTGGCACTGGCTATGAAAATGGCCTAAATGTTAAAGTTCTTACTCCAACTGGACAACT
ACACGAATAAAAGGAGTTCTATGGTCGCGGTGCTGTTTTGGCAATGCGTGATTATCTTTA
TTTGCTGAAAGGTGACTATATCATTGCGATGGCAACACATGGAGAATTGTTTGCAGATCC
GATAAGTGATGATATATTTTCTAAGCTGGGATCTGTATCATTCCCGAATCACATTTTGTT

TCAGCAAATGCCTCGTGTATCCTATGCAGCAATTTATTCTACTAAAATGGGTAAAATTGT
GTGCGAAGGGATGCAAGCAACGCACGGAGAAGGTCAAGATTCGTCTATACAAATTGAGAA
AGTTTATGATACTATAGACGATCTTGCAATAACAGGAACACCACAACGATTTTTAGATTA
TCCGGTTGAATATGTTAGCGAAGATGCCGAACATTTCGAGCTAATACAATGGCCTCATGA
TGAGATCAGCGCACCACTTGAATATTTTAACATAAAGGCAGGTGACAAACTATCAATATC
CTTTGAATTGTTCCGTGATAGTGCCGCAGCCGCAGCCAACGTAACAGCGCGTTTCTATAA
CAACTACTTCACTGATGGACAATATCAAACTGGCGTTCGTTATAATACCAGCCAGAAGGA
TCAATGGGAAAAGTTTGAAGCCGTCTATACCGTACCGGAAGGAGTTGATAGTGTTACCAC
TGGTTGTATCAGATATCCAACCAACTCAAATGAAGGTATTGTTAAAATTAGAAACATACT
AATAACGCCGATCAGCGGAGTAGACAAAACTACTGGTCCGACATCATTTGGTGTTAATGG
TGTTAAAAAAACTCATATTCAGGATAACGGTCATTTTACTAACCCTGTAATGAGTCTTCT
TAAACTGCCTAGAGTTAATAAACACATCACATCCAACAACTTTAAAGAGTTCGATGTTGA
TTAACATTTTTAACAAGCCGATCCTCGTGGTCGGCTTTATTTTTATACAGTTGGTTAAAA
CATAACCGAAACGAAGAAAGATCTTTAGAATAATAGAAAATTTGTTTCGGTTAAAAAATA
GTCAGTGCCTACGGCACGTCGCGAAGCGATATTTTGTTAACATTCTATAAATACTCGGAA
CTATAACTAATTTTTGAGGGTGCTTTATGGCAGATTTGAAACATGGATCTACTGTAGGCG
GATCTCCCATTTGGACGCAAGGAAACTTGACGAGCCAGCCAGCGGGGGATCAGCTTTTCT
ACAAAGGTCATAAAATCTATACCGCTTTTGATAAACCTTCGGCTGCCGATTTTGATGCTG
TTAGTGCTAACGACGGTGGCACATTTCAAAAGGAGGTCCAGTTTGAGGAAGGTTTAAGCG
TAGGTAGCGCATCGGCTGGAGAAACCAAAAAGAACGGGATCTTTAAAGGAAATAGTGATG
CGGCTAACTTTGATGGTGTAAGTTGGGGTTTGCATTCGTGGAAATCTATTGGTTTTGTTA
ATGCCCGTGATGGCGTTATCATGGCTTATATTGACACTACCACAGGTGATTTTAAAACTA
AAGGGACTATCGAAGGGACAACCATTAAAGACACAGGTCAACGAGTCTATAGCCCTGTTA
ATAAACCAACGAATAATGATTTAGATTTGGTGTCTCGTCGCGGTGATACCCTCACAGGAA
CATATAATTTAAACACTGTCACTGTTAAACTATCGGTTGATTCGAAATTAATTGCAGGGG
AACGTGATTTAATTAATTTTGATGCGACTAAGTTCTATTATGGTAACGTTCTTGATACGT
CGGTCTTCCGAAGTAAAGATGAGCCTACCATTTTTGTTAATGGCAGAGAAGGTCGTTTTT
ATCACACCAACAATAAACCAACTAAAGCCGATGTTGGGCTAGCCAATGTCACCAATGATG
CACAGGTTAAACGTGCTGGTGATATTATTGAAGGTAACTTGCAAGCGCCTAGAATGTTGG
CAACAAACGATCCGCCTGGTCCTAATGAATTGGTGCGTTTGAGCTACTTTGAGAGAAAAT
CAATGGTAGCAAATCCAACCATCATTGGCAACGTTGATTGGAACACTCTTATTAATCGCG
GTATCTATCGTGTGGAAAATGCTGGGTCAGGTACTAATAAACCGTCTGATAGCTATTATA
ACGGCGTTTTGATGGTATATCGCCCAGAAGATGCGGTCGGTACTCGTCGAATTGTTCAGG
TGTATTATCCTGAATCTATTGACCATCCGATGTGCTGGCGTTCGTGTTCTAATGAATCAT
GGACTGCATGGAACTATGTAGATCACCGTAAATTAGGCGATATTCGCTATGTTAACGTAA
CTGGCGATACCATGACAGGACCGTTAACTGTTCCGGCTGCAAACGGTGTTAGAACAGCTA
GAGGTGCATATAATGGTGATAACTACTATGCTGGGTTAAATAGTGCCGATGGCACGGCTA
TGATTCACCGTATCAGCGATATTAAAGCCACTGAAAAATTGGGTAATACACATGATTCGA
AAGCTGTTTTCGTTAAATCGCGTGGAAATACTCAAGCCACAGACCGCTATAAACTTTACC
ACGAAGGGCGTAAACCTACCCCTGCCGAAGTTGGCGCGGTTCCACTTAATGCGGTGATTG
ATTTTGGTACTTTCTAATTTTAGCGGGGATTATCTCCCCGCTTTTCTAAATAATATTATT
CTAATAACCGTTTAAGGATTTTATAAAATGGCAGATTTAAACAGAATCCAATTTAAACGT
ACTAGCACGAAAGGTTGTAAACCTGATGCTACTACAATGACGCAAGGTGAACTTGCTATC
AACCTGCATGATTATTATTTGTTTACAAAAAATGACTCTGGTTCTATTATTAACTTAAGC
TGTCCTCCGATCTATGACCGCGATGTTACCATGGCTGGTAAAGTAAAGGGAGATTATTAC
ATTTTAGGTAAAACCGCCAATTATCTTGAAGATCAGACAGCGGGAGACCTTAACCGTTTT
GGAGCATTCCGTACCAACGGTCTAAATGGATTTTTAGATTTGACCTTGCATGTTCCTCAT
TCTGCTGGCGTTCAACATGGTCGAGGATTTACTTTCCATTATGCGACTGGCGGATCTCGT
GTTGAAACATATGGGTATAATGCACAGGGACTAAAAGCATTTAGCTAGAAAATGTATCAT
GAAGGTGATAAACCAACTCCAGGAGAACTGAACGTTTATAGCAAACAAGAAGTAGATAGA
ATGTGTGTTAAAAACGTTATACTTTCTACAGGTTCTGGTGATATCGTTGACGGTTATTTC
AAATTAGCGACTGCGATGATTCCACAAGACGGTCGTAGTGTATTTTTCCGTATTCGTGGT
GGTAACGGATATAACATTACTCCATACGATCAAGTTGATATTGTAAAAATTGTTATTCGC
AGTGGAAATAATCGTCCTAAAGGTGTTAACGTTATTGCATACCGCCGAAATACAAACAAA
GCATTTGATGTTTTGGCTGTTAATACTTCTGGTGATAACTATCATATCTACGTGAAATAT
CAGCGTTACACTTATAACGTTATTGTTGAATTTGGTAAAAGTGTTGATGTTAATCTGGTA
GTCCATGACGTTCCAGACTTTGTTGTTGATCGTCCTGTTGGCGATAATGTTATTGGCGGT

CGCGCGGTAACTCTTTTCAACACCGAAAACAAACGAGGTATGTTGAGTTTTGACGACAAT
ACACAAAACAGCTATGATATTGTTCACATGAGTAATGATAAAGGTACTGGACGAAAATAT
ATTCCTAAATTCCGTCGTAACTATAATGAAATGATCTGGCATGAGACGGTTCAAGGTAAC
GTATATCGTTTAGCTACTGGAACTACCGACGCAAACGAAGTTCTCAAAATTACCGACCGC
ACGTTGCAAACTGGCAAGGCTGTTTTTGACGGTGGTAATAATAATGTTCGTTTACATCGT
AAACCCGGAAAAAGTAACTATCTGGAATATTATGATGCCCGTGACGGAGCAAATAAACGT
CAGGGATATATTGGGTATGGCGATGCCACAACTAACCAGTTGTCAATTATTAACGAACTC
TCCGAAGGTAATAACCCGATTTTCTTTAACGAGACAGGTCAAATTTCTCTTAACGTAGGA
AAGACAAAAATTGTTCATAGCAACGGACAATATATTTCTACCAACGCCGATAACTATCGT
ATAATTTATGGTAATTATGGAACATTCTGGCGAAATGATAGCGTTAAACTTTATCTCATG
TCCACTGCCGAAAATGATAAATGGGGTACTTGGAATAGCTATCGACCATTCATTTACAAC
CTAACCAGCGGCGATGTTCAATTGGGTGGTGATGGTAGCGAATCGGCATTAACGTTAGAA
TGTGCTTCTCGTGCCGCTCGCTTTAGTAATGACGTTTACATTAAGAAAGGGCTTTTGACT
TTCGACGCTGGGCGATTAGGATCTCGCGATTATATTAAATTTAATCATTGGAGCGATAGC
AATAACGGTCGCGATAACATCCTACAGAGTGAAGACAGTCAGGGCGCACATTTTACCACT
CAACGTGCTATGGGAACTGGTAACATAACAGCACGTTTTAAACGCTTTGTTCGTGTTGAC
TCTGGTGAGATTGCATTTGATGCTAATCGGGGGTCGCAGTCTCAAGTTACCTTACACACA
TGGGGTAACGAGCAACGCGAACACGTTTTTGAATGTAAGGATGCTACAAGTTATCACTGG
TATACTGAACGTACTCAGGGTGGCACTGGACCTATTCTGTTCGTTATGGCGGGTAGTCTA
AACGTTGGTAGCAATATCACAACAACTGGTCCGGATATTACGTTTAAACGCGCTGGCAAT
AAGCACATCTGGTTTAGAGATCCAAACGGCTTAGAGTTAGGCTTGATGTACTGCGATGAT
GCTGGTGCTATTCGCTTCCGTGGACAGAAACAAGCCCAGGCGTGGAAATTTGCAGCTAAA
ATGATCCAGTTGGAATCTGGCACTGTATCCGGTGGCGGTAATGGCCTGATTCGTGGTGAA
GTTGCTGGCGGTAGTGGGGCTAGCTGGCGTGACCGTGCTGCTGGCCTTATGGTTGGGTGT
CCTCAATCAACCAATTCCGCTCATAACATCTGGAAAGCTACTCATTGGGGACAATATCAC
ATTGCAGCAACCGGTGTACATGTTCCTAACGGTACTATCGGTGATGCTATAGCTCGCCTA
AACGTTCATGACGCCAACTTTGACTTTAGCGCCTCCGGTGACATGCCGGCAGGGCGTAAC
GGTTCGTTTAACGATGTTTGTATTCGTTCTGATGCTCGCCTTAAAATCAATAAGGAAGAG
TATAAGGAGATTGCCACTGATAAAGTTAATCGCTTGACTGTGTACACCTACGATAAGGTT
AAATCTTTAACTGACCGTAGTGTTATCGCCCATGAAGTTGGTATTATTGCTCAGGATCTT
GAGAAAGAATTGCCGGGAGCAGTAACAACTTCTAAGGTCGGCGACCCAGATAAACCAGAA
GAGATCTTAACAATTTCTAACTCTGCTGTCAACGCTCGTTTAATTAAGGCGTTTCAGGAA
ATGAGCGAAGAAATGAAAGCCGTTAAAGCTGAACTAGCGGAACTTAAAAGGAATTAAGGA
AACTCCCAGGGAAGGGAACTACTAAGGAGAAACGGCTATGGTCTATAATATCGTCATTGG
TGCTTTTTCTAATTTCGGAATGAAATTATTATCAAAGTTAATTAGTGAAAAGATGCTCAC
TAAAATTTTCTTCTATTGCGCTCGTCGTCTTGCGGCTTATACCAATACGCCAATTGATGA
TCGTTTTGTGGAAGAACATTATAAGGAGTTTAATAAAGATCTGTCAGAAGATGGTAACGA
GCAAGATAAACCTAGTGATAGTCAACTACCTAAATAAGAGTACACGGTCATAGTTATCAT
AGGGCATTATATGCAAGTTTCTGAAAAAGGGAAAGACTTTGCTATCTCAAATGTCCTGCG
CGCTATTTTCACAACTAAAAGCACGGAGTTGCTCGTACTTCGTGGTTTTGCTGCCGTCGT
GTTGTCCATAGTGGCATTTGTCGTTTATAGTAAAAACGAGTTATTTGCGCTTTATAAAGA
GCCTCGTTATGAGACTTATGCTCACATTCTCCAGGTAGAGAAAGACCGCAATTTTGATAA
TGCAGCACAGGAGCAGTTACAGATAGTTCATGTATCGGCTGATGACGATTTTTCTGCTGT
TTTTAGTTTTCGTCCGAAAAACCTAAACTATTTTGTTGATTTGGTGGCATATGAAGGAAA
GCTACCGCATACAATCGATGAAAAGAACTTAGGCGGATTCCCTACCAATAAAACGAGTGA
AGTATATCGTAGGCATCTTTTAGGTAAATCATATTTTACCGATAAAGATTTCCAGTATAT
TCCATCAAGAGAAAAGAAATTAGAGAGTATCGATATAGGGGTTATGTATTCTTGTCCTAT
ATTCAATTTCGACAATGTCTATGCTGGTAGTATCTCCATTGCATGGAAGAATAAACCGGA
TATTGACATTGAGAATTTAGATACTCTTTGCAATCAATCTGCCAGAATCCTCGGAAGGAT
TAGATACCTTAAAGGGGAAGCCTTTAGGCCTCCCCTTCTTTTTATTGGCGCATAGAAGCC
CCTGAATCACATTTTGTCGCTGTATGATGAAAACAGGTAGCGTACCAGTCATCGGCTTGT
TCGTCTTCGTCAGAAAACGCGTAATTGCGTTGTATACCGTTTTCTACTTTGACCAGTGCA
ATAGCTGTTTCTGGAAGATCAATCAATCGGACAAAATCACCATCTTCCCACTCTACACGA
GCAATATCATAGCCCATCTCAAAGTATACAATCGCTTGTTCTAATTTCATTCTGGAAACT
TCCCGTAAACATTCTCTGCCAACTTACGTGCATCTTTTACAATATCTTACATGTAATGAC
TATAATTTTTAGTTAGATCAATGTTGCAATCAACAAAGAAGTAATAGCGTCGCAGTGCTA
CCAGCACAACATCTTCAAGATGACCATTTTCTCCAATCGCTGAAATGTGTTTAAATTCCT

TTCGGATCGCATCCATCACTACATTAACTTCTTTTATAATATCTTTCGTATTATATGCAC
CTCGGCTAATCAAAATTTTGGTTAGGTCTTCTGCTTTTTCTTGAAAGTTGTCAATTTCAA
ACTCAATCATAATCACCTCTCATAATTTCACGCAGTTGTTTCAACATTTTTTCGTGGTTT
GCTAATTCTATACCTTGTAATAGCATAACCAGTGCAAGTTTCAATGATTCATCATCTTTC
ATAATCAAAATTTACCGAAAATGTTCTCTGTCATAATTCTTGCTTCGTCGATAATTTTCG
TCATGTTCATGGTGTAGTTATGATCATTTAGTGCTTTAATAACAACGAGCAAGGAATCTA
AAAACACTCGATACCGGGTATATTCTACGCTATTTGTGAATACCGATGTTTTCTCCACTT
TATCGATCAGATAGCCTAAAACCGATAGCGCCTCGTTTACTTCCCTTGTTACTTTATCGG
CGCTAAACAACACCGGATAAAGACGCGGTGTGATTAACCGCGCTGCCTCTTTCAATTCGA
TCGCTTTCCATGATTCATATTTGTCATAGTTAAACATTATATAATCCTCTTACAGATGAA
AACCTGTTACGATCACCGCATCGATCATACCGCTAGGAATAAACGGTTCGCCCTCTTTTA
TTTCAACTTTGAATTGATTCCATTTCGCTAATACTTCTTCCGGTACACCCAGCGATTCGG
CGTCTGCATTAGTTTCATCCATAATCCAATTAGGCTGCATGAGCGCATAGTTTACATAGT
CCATTTCTGGGGTCATGTCATCAATGTTAATATATTTGCAGTTGACGCGTTTCCGAATTT
CAAATTCTTTAATATCAACCTGTACCATGTATAATTCAGTAGTTTCTGGGATCAGTTCCC
ACACAATCAGTACATTTTTCATAGTTCACCTCACTTAACAAAGATTTTCTTTAATGCTTT
CAGGATCGTTGCTTCCGGTGCTTCTTCCGGTGGAGGAAGCAATTCTACCACTTCCTCTTC
CAACTCGATATAGTCTACCAGATATTCTACATAGAATGCAAGTGCTTCCATGAAATTAGA
TGCTACATCCGATTCACCACAAGCGCCTACATGCATTGTATAAACATCACTGCGATCAAG
TTTGTGGAGGCTTCCCAGATAGTTTCGGTTCTACCAGACTTCCCACACTTTCAGCTTTCC
GGTTTTGGTTGCTGTTTTATAATCAGTTTCGTAATGATGCAATTGACGTAGCAAATAATA
GATAATAACACATTTTTCGTAATTGTTTTGAATTTCCATGATGTAATCCTCACTTATCGT
TAAGAAGTTCTAAAACTTTCTGTCGTACTTTGTCAATATTAGTTGATTCAAAAACTGTAC
GGTATCCCCAGCTTTGACCGTATGATCGCGAGCAATCCCCATATCCCCATTGTTCAACCT
GATAGTATTCAACGAATGGGTGCTGCCGAACTATGACACCATCGCGATTAAAAATTACCA
TAATATAATCCTCACGTCAACAACTTATTTTCGGCTGTACTTCTGCCACTTTCGGTAATA
CTGTTTGTAAAATTGTTTTACTTTTTCACCATCTTTACTATACATGTTATCTACCATCAA
CTCAATAAGACCTCCCGGTGTCGGGTAGTTCCCCACTCGGATAAGTTCTTTAATTAAAGT
GTCCTGATGATCTTTGTATACTACGTTAATGATGGTCTTTTCTGCTTTCTTCGTCCCGAC
CATCCGGTGATTAGCTTTCATCGCATCCCACCAATCTTTTGTGATCTTCATGTTATTAAA
TTTCATCACTTAACCTCTTCGCAAGTATATCTCCACTCAATACGTTTTTCGTTTCCACCG
CGCTCAGCATCATAGATATGACGGAATGTCAATTTGCTTTCATCATCAACAAGTTTATTT
CCTTCGAATTTAGCGCCATGTTTTGCAGAGAATATCAATCCGATTTTATAATCTTCGCTG
TTGATCATAGTTCCGTTATCTGTGATTACAAAATCCATAACTACAGGGTCTTGCATTGTG
CTGGAGATCATGATTTTAGCAATGTCACCTCTCTTAGAAACTTTAATTTTTGCATTTCGT
TTCATAATATCATCATCTTCTACACTCATGCCATTGATTGTAGTAATTTTAACAACTTTC
GGGCAAATGTAGGAAAACGTGGTTTTCTTCGGATTGATTACTTCCGGCTGTTTCTCTTCA
ACCTTCGGTTCTTCCACCACTTTTTCTTCCTGTTTCGGTTGTTCTTCTTCAACCTTCGCA
GAAGTCTCTGAATCGCTTTCTGGTGCTGTTTCTTCTTTAGGTAATACATTCATCATGTTT
TGAATTTGAGCGTTATATACAGCCTCCAGTGGCTGAATCTGCCCGATATTGATCATTGTA
TCACGAACAGTTTTATTCCAGTTGCGTTGCTCTTGTTTCAGAGACGGATCAATCTTAACA
GCCTTAACATACACCTCATGAAGTTTAGAGTCCAGTGCCGAGATTGACTCATTAGCACAA
ATAAACTTCTCTGATTTGTTAGTAGCTTTAGCGCAATCAAAGGACGCAGCCTTAGCACCG
AAAGCAGTAGCAGAAAGAACGATAGCAGCGATAATGTTTTTCATAATATATTCCTCAGTG
TTTTGTCTTGATGGGAGTATCTTACTACTCCCTTAATCCGGTGTCAACTACTTTTTTAAA
TTTCTTCTACAGTAAAATCATCAATGTTGTTGTGCTCGGCGAACGTTGCAAGACCAGCAT
TCCATGCATCATCCTCAGAGAAGAATACACCGTAACGTTCATATCCTTTAGCGCCTTCAC
ATTTAACCATTAATTTGTGAGTATGTTTCATTTTGATTCTCCTTCGTTGTCTTGATGAGT
TTAGTATAGGGGATCGCCGATCCCCTGTCAACAACTACTTTTTAATTTTTACCCGCTCGT
GCATTAATACGCATTTTCTGCATAGCCATGATCTGGCTTACTACTTCTTCTTTACGTTCT
TGCGTGATAACGTGACCAGATTCCGCAAACTCTCCCGCAACTTCTTCTACGATCTCGCGA
ACTTGAGAACGTCCAAAGATGCGGTTTCCTTCGTAGCTCATAAATTCTGTTACTACTTGC
TCGATAGATTTCATGTGATTCTCGTTTCGTTCGGTATGGGGGTATAATACTACTCCCATT
TCTTTACGTCAACAACTATTTTAAATTATTTTCGACCTTCACCCATTCCGCGTATTCTTG
CGCGGCTACTTTCGGATTCAGGCCGTCTTCGTACATTGCTTCAAGTAATTCTTCGTCGGT
GGGCTTATACCCTAGATAAGCCTCCACCTGATTTTTCCAAATTTTATACCAGTTCATATT
ATCACCAGTCAAGAGCGCGTGTCTCTGCGTCTTCCCATGCTTGATCCATAACAGCCTTGA

AAACATATTCTAGAGTATCATCTTTAGGGAATCCGAAGACTTTAGACGGAATATCTTCAA
TTGCTCCTCCAGCAACTTCTGCATTACGCGCTTGGCAATAATGCAACTCTAAGTTATCGA
GATAGCGACCGCCTAATTTCACCATATCATCCCAGCATTTCCAGTAACCTACTTTATTTG
CAATTACTAACACTTTGCTGTTTTCGTTGCTCATCTTTGATTCTCCTTTTGTTTGGTATG
TGTGTATTATGCGAGTCTGGATTCGTGAAGTCAACAACTTTTTAAAATAAAAAAGCCGAC
CACAAGGATCGGCTTAATAATCAGTTACTTACTAAATTTCTTTTTGGCTTCGTCTACCGC
ATGAGATGCCAGTACCGCAGCCGCATGTGCACTTTTATCTGTGTTGCAGAGAACGCCCAG
ATAAACATCACGCCAAAGGTTAATCATCTCGTAGATCATATCAATATCATTTTCTTGTTT
GTTGGCGATGCTATTCGTCGTTATCTGGTAAGGTAACGGCTTGATCTGTACTGGTTCCGC
CATCGGGCTAATCGGTTTCGGATCAGTCAAAATAGGTGCTGTTGTGGGTGGTGTTTTCCA
TACCCCATTATTCCACGGTTTAATCGTTGTGTAGTAAAGTTTATAAGGATTTTTGTCTGG
GTCAACCATCCATGCATGATGAGTACCCAAACCATTTGAAGTGATGCGTCGTTCAGTCAT
AATTTATTTCCTCTCTGAAAATTCTTTCATCAAGCTGTCGATCTTTGCTCTGGGTTCTGG
TGTGTTTGTCATCTCTGCTAATTTGCTCAGAGATTCACTGGTGATCCGGTTGGATTCTTC
CAGTGTTTCAACGACCATTGGCACAATCTTTTTATTTTTGTACACAAATGCAATATAACC
GCCTTCTGGATATTCTTTCATAATCAACCCTTCTTAAGTTCTTTAAGATCTTTCTTATAT
TCCTTCATTGCGGTCGTCTTTGTCCAATACTCATATTCACTATGATGTTTATCTGCATCT
TTGACCAGTCGATCATACTCTTCTTGAGTAACGCTATACAATGGCATAGAAACTAGCTTA
TCGGCGTATGGACGCAGTTTTTCATGTCCATTGATGGATTGCATTACCTCGGCCTTTTTC
TTGCCCTGTAGCTTGATTTCGCCCTTAATTACGCGATCGATAAACTCGGCTTTAGCCATT
TCATAATTGTATGCTTCCAGCGTTTCAGCTTGCATCAATTCAATGCGTTTATCGACATAC
CCTAAGCGGATTTCGACGAATCGACGGATCAGATCGCTCGCTTTCTCGAATACTTCTACT
ATACCACGTTCATTAACCACTACTATATTCTGGGCTACTAACTGTGTTAGTTTAAAATCC
TTCATGATTTGCTCATGTCGAGCGGATTAATCTTCGCTTTTGAAATAATCGCGTTTCAGT
GTTACTTCAAATCTGAATCCATCCTTAGAGCAACGGTCATTATACGATACAATCAGATCT
TTTTCTTCCAGTGCATCCAGTACCGCTACATATTCTTCACGGTCGAACTTATAAGGAACT
TCTGATATAGTCAGCTTAGTCTTGCCGTTTAGTTCATATGTACCCTCAAGAATCACACCA
TCACGGTCATCACGAGGGATAACTTTACCCGCGAATTTCGAGAATTGAACACTAGGTTCT
TTATCCAGTTTTCCGGCAAGTGCTAGCTCTGTGCATTCAATAAGGCTTTCCAGTGAATGC
GGTAAAATGTCAGTTTTATACCCTGTAGCAATACCACTAATACCATTAGCCAATACCATT
GGCACAACTGGCAAGTAGAACGCTGGCGGGATGTGTTCCAGATCTTCGTGCTTAGGTGCG
CAATTCATATCTTTATAGATTTTAAAGAAGTTTTCATGCACACGAGCAAAGATATAACCT
GCCGCACCTGCTTTCTGGACTAAACGCGATCCGAAGTTACCTTGACCGTCAAGCAGTGGA
GTATTATTGCTCCATGTATTCGCCATGAGTGATCCGGCTTCTACGGCATTAGTTTCCCCG
TGGTGATAACCCAACTCAGCAACACCACATCCAATATCTGCCATCTTACGGAATTTCTTA
CGGTCGCCTTTACTCTTTTGAACTGCGCACTCCAGAACAAAGCGTTGCACTGGTTTAAGT
CCATCCACCAATTGAGGAATCCGGCGATCCTCGGCAGAATAGATCGCATATTCCTTAGCT
TCATTATGTACAATGCTTTCAAGGCTGCGGTAAGTAAATGTGTTTACAATTTGACTAATT
ATATCCATCTTCACCTCATACACGCTTACAAAACACATTCTTTACTTTATCAGACTTATC
AGCCCAATCCAATAGCCATTTTAACGGAAGGATTGACACAATAAACGGGGCAGCAATCAA
AGCAAACATGATACACCTGATAATCAGGAAAACGCAAGTGACAAAACTAGCGATAGTGAT
CCCGACAAAACAAAATACCATAGAAAACAGAGTAATGAGATAGAAAGGCCAGAATACCCA
CTCCCGCAGCTGAACGCCTTTGTATGCTCTGAATTGCTTAATTTCTACAAACAACTCTCG
GATATACGCGTACAGGACCGAAAAATCATCTTTCATTGCTCTGAATGAGCAATCTTCTAT
TTTAGAATGACCTAGCATTATTAACCGCCTTTCAACATTGATACAGGAATTGTTTTCTGA
TTTGTGTATAATTGGCCTGCTTCAATTTTTTCTTGAATCACCACGACGTTACAATATCCA
CTGTCTTTAAGTTTTCTGACAATGACTCGCTTGTGTTCGTCGGTGATAGCATTGACTGTT
CTCACCAATTCACCGCAAATAATAGCACCATCTTTACAATTGCCAGTAACTTTCATGCTA
CGACCGTGAACAACAACCACATCACCGTAAATCACATTTTCGTCGTCGTATTTGGTAATA
ACCGTGACCGTCATAATGTATTCCCTCTGAGAAAGGACGCGCAAAGCGGCCTATGTTGAT
TATTGTTCTACAGTATAACCGATGTTTTCAAGCCATGCAATAACAATCTTTTCGCCAATT
TCATCGAGAATTTCATCAGCGCCGTATTCTTTAATGAAAGTAGATGGATCATTATTTCTT
ACTACGTGCTGGATCACAATATGTTCAGGAGTTGCGTCTAGCAGTTCACTATGATCATTC
TCATTAACGATATCTTCGACACTAACATTATTACCCAAATCAACATCATCCATCGTCACA
GTAATGCGACCGGACGGACGCAACGAATATTGGCTACTTTCAACTTCAATATGTCCACTT
TCGCAAGTAACTTTCATATCAATCATAATTAATTCCTCTGTTAGTAATGGCTAAACCCTT
CGGTCACATCTTCAACCACATAGTAACGATGACAGCGCATTTTGGCATTCGCATGATCTT

TCGGGATACTAACAACGTGACGCGGATCAACTTTACATTTTATGATTCGGCCTCGTCCAC
CGCCATAGTGCGGAATATAGTGTTTCGCTGCAACGTGGAGGCCATTAGAGCAAGTGCGAT
CATCATCTGGGTCCACGTCTTTACGATTCATTTAACCGTGCGCCCTGGGCTATTATCAA
ACGTATGAGTAAAGAAATCTTTATAATCAACATCAACACGTTTCCATGCATAGAAACAAC
CATCTTCCCCGATCTCAATGTCGTTGTGCTCTAAGAAGTCAAATAGACGATCTACCGCCT
TACGGCTAGGGTTTTTCTTGCACTTCTCAAAGAAATTCAGATAGTGCGTATATGGCTTAT
TCTCTTAAATCGACTCAACAACTCGACGAGTCAGATTAATATCAAGAACAATATTATGAT
AATACACAATAACATTTTCGAAACGGAAAGCACCATGATAATATACTTCAACCGCTTTCT
TAATGTCAATCAGCGTAATAGCTTTGATGATATCATTATGTTTAATCGCTTCCAGTGCTT
CATTAAAGTTAACAAGCGACGGATCAGCAGTATAAACGATGTCACCACATGTAATAGTAA
TAAACTTAGTTGAGGCTATTGCTACGATTTTTGAGGAAGTAGTCACGGGCTCTTTTGCGC
GTTCACCGCCTTTTCCTTTACTGGTTTTACGGCGTGCTTTACGTTTTTCTAGTGCCTTGC
GACCTTCCGCTACAGCGTCTTTCTGAATCAGTAATACACGGGAGGCAGTACTACGAGATA
CCCCAAATGCTGCCGCAAGTGCTTCGCGGGTAGTAAACTTACCTTCGCCCCACTGTATAG
CCAGTTTGATTTGTTTCGCAGAATCCAGGCATTGATCGTTACGGTCAGAGACGGTAATTT
TTGCTTTAGACATAGTATTCATTCCTCTATTAGTTGGGCGACTATCGCTGCCCGATTCAC
ATCATTATACCAGAATTATTTTGCTTCTACAACCTTGCGGATTGTGCGGTCTGATACATT
ATAGATTCGTGCCAGTTCGCCTTTAGTCAAGCCGCGAGCGTATTGTTTACGGATGGTTTC
TTTCTGGATAGCGGTCAGTTTAGTCATAATATCACCTC
>NewGenomeName_20
GGTAAATGTTCTATAGCTAATTAAACCTAACAACTGTGGTTTCCCATACAACACGAATAT
CGTATACGGTATTACCAGATTTTTCCACCCATTTTCAAGTTTCACCTCTTTGTCATATA
GTCTGTAATTTCTGGAGAACAAATTTCTTTGCATTAACACCTCTGACCACATCCAATTAT
TGTTAATTATGCGTGGTATTAACTCTCTCATTAAAGGATGCTTTATTACTATGTTTTCAT
TTATTGATGCATACGGTTCTGTGCCAATGAATTTTATATTTTTCTTGTCTCTTCCAAATC
CAAGATGATCAATGTCTTGAGATATTCTATTTACAATGCTTTCCTCAAGCTGAAACTGTG
CATTTACGGCATTGTAAGCACCATAAGAAAATATTGTTGATATTAAAAGAATAAAAGAAA
AATATATTCTTGATATTAACTGCTTATCTTCAAAAGCATGGAATACGCATAGGCATCAAA
AAAACATAAAGCCACCCATACCAATCAATACCCTCGGTGCGTATATTGGTGATTTTAGAA
AAATCATTGGTCCGATGATGAAAAACATTGATGCCAATAAAATTAAAACTACTAGCAAGA
ACTTTGTTTTCTTAATTTCATCTCTTTTGATTACTTTTAAAACTATGACTATCAAAGAAC
TGATTAGCGCAAAGAACAGCGAGTAGTAGATTAAGTAATTATCGCCATTGAAGATCGTGC
TAAACATTCTATAAAATGATAAGACGTTAGAAATTATCCCTTCAAATAAACTTGAGTTTA
TCTCTATAATCTTACTATGTTCGATATTGTAAGAACCTGTTACAAGTCTTTTTGCCATAA
AGTAAGAATTGGCAAAATATCCTACTGTTAAACCAGCGACAGAAGACGCTGTATTTTTTG
TGATATTTGAAATTGAGTTTTTCTTAACCACATCTGAAATTATAAAGGCCAACAAGAATA
TTGCGTAAGTATTCAGCGCAGCCTGATAAAGACTAAGGAATGCAATGGTTAAAATGGATG
ATATTATGATATTTATAGGCTTGTATTGATAAGCGACATACGATGAGATAATAGATATTG
TCACACTCATGCACATTGTTAATGAATCATATCTATATGATAGATTTTCAATAAAGAATG
GGTTTGCCAAAATCATCATAAAACAAAGAGATGCTGTGATGTAGTCATCTCCAAACAGCT
TTTCCCTGATGCAGGATAGTGCCAATGCTAAAATAACTATCCCTAGCATTAAAGGTAGCG
GAGAAGCATCTATAATTGGGGTTCCAAAATTAATGATAGAGAAAATAAAGTCGGAAAGTG
GGCGACCATTGCCTGACCAACCCAACCCGCCATATAAAGACCTAGCTAAGTCATCAACGA
AAAATGATTGATGTGTCAATAAAGGAAATGTATATATAATCGCCAATCCAAGAAAGATTG
ATATAAATATCCTATCATTACTATTAAATTTCACTTTTAAAACCCTGGCGCTTTAATGTG
TATTTAGGCCGCTGTTTGGTTTCTATGTAAATTCTACCAATATATTCTCCAAGAATACCT
ATTCCTATCAATTGAACGCCACCCAGGAAAAGAACAGAAACAAGAAGAGACGGGTAGCCA
GGAACATTATTTCCAAATATTAATTCATCAATAATCATCCATGCACCGAAAAGGAATGAC
ATACCTGCAATAAACAATCCAATGTAAGTCCATATGCGGAGCGGAAATGTTGAGAAAGAA
GTTATTCCCTCCAGCGCCAGGTTCCATAATTTCCAGCCGTTGAAGTTCGAATCACCGGCC
ACGCGTTCGGCACGGGCATATTTAAGAACATCCGTTTTTCCGCGAACCCAACTGAGCACA
CCCTTCATAAACAAGTTGCGTTCTGGCATTTGTTTGATGTTCTCGACAACCGCACGGCTC
ATAAACCGAAAGACGCCAACATTTTCTTCGATTTTTGGATTGCTGATTTTATTGAGCAGC
TAATAAAACCACTCAGCTGTCGTACGCTTCAACCTCCCATCTGTTGAGCGGTCTGAGCGC
TTATCCAGCACCATATCCGCGCCAGCCTGCCACTTCTCAATGAGATGAGGGATAACTTCT
ATCGGATCCTCTAAATCGACATTAATAGGAATGACCGCATCTCCGGTTGCATGGTCGAGA
CCCGCGAAAAGAGCAGGTTCTTTACCGAAGTTTCGCGTAAACGAAAGCGGAATAACGAGC

GGATCAGATGCGGCTATTTTGTTAATTATTGATTCAGTCGCATCTTTACTACCATCATTA
ATAAAAACGATCTCAATTTCATATTCTTTTAGCTCATTAAACTCACGTACCGTTTTATAG
AAAATCGGTATCGTGTCTTCTTCGTTAAAAACTGGAACGACAAGAGAGATTTTCATCTTA
TATCCCTGAAAACAATGAATCTGGAATAGATGAACCCGCATACCAGGCTAATTGCCGAGA
AAGTGATAAGGGTAATCAATGGTGGCAAGGAACATTGGTCAGCCATCCAGTCAACAACAG
CGCTCAGTGTTCCCATGAATCCCACATACATCATGTAGCGAAGCGTGGTGGTGGTGGCAT
TAAAGGTGACACGCGCATTGGCATAGAAGCTGAACGATACGGCGATAACAAAACCGGAAA
AGTTCGCCAGCGCCTGATGCGTATGCATCCCATACACACAAAAAGCAAATACGCCCCAAT
GAATAAGCGTGTTAAGAACACCGATCGATGTGTACTTAGCGAATAACTTCAACATTATGA
AAATCAGCGGATTCGGAAAGGTCTGGAGTGTAGCACTACAAATTTCTTTGATAGATATAA
ACGATCAATAATGTCAACTTTGATAGTTTAAAGTTATTGTTTGCTCGTTAATTGATCGTT
GTTACCGATCAATTTTTATTGCTGATTGCTAAGTGGTTTGGGACAAAAACGGGACACACA
AAGCTTTGCATCGGCTTGCCAGGCTTTGCATGTTTTTCGAAGATGGGACGTGTGAGCGCA
GGTATGACGCGGTATGTTGTTGACTTAAAAGGTAGTTCTTGTAATTCGTAATGCGAAGGT
CGTAGGTTCGACTCCTATTATCGGCACCAGTTAAATCAAATACTTACGTATTATTCGTGC
CTTCCTTATTTTTACTGTGGGACATATTAGGGACAGAAGTACCAAAAATCGAGTCAACTT
GTCGAGCATGTTCAGTCATGTGATTTGGTGCCAGATGAGCATATCTGTGAACCATTTCGA
TAGACTCCCAGCCACCCATTTCCTGCAATACCGAAATCGGAACGCCAGCCTGAACTAACC
AGCTTGCCCACGTGTGCCTCAGGTCATGAAAACGGAAGTCTTCAATGCCTGCTCGTTTTA
ATGCTTACCACCATGCAGTATTAGCGTCATAGCGCATCTTCCTCACTACAGGTGATTTAG
TTCCGTCTGGCTTGGTGCTGCTTTCCTTGTAGACGAACACCCATTTGTGATGGTTGCCTA
TTTGCTTTTTCAGCACCCGGCAAGCAGTATCATTCAGCGCCACGCCAATGGCCTGATTTG
ACTTACTTTGTTCCGGGTGTAACCATGCCACCTTTCGCTGCATGTCTATATGCTGCCACT
CCAGATTGATAATGTTAGACCGCCTTAAGCCAGTAGAAAGCGCAAACTCTACGACTGACT
TTAGCGGTTCCGGGCATTCATCAATCAACCTTTTTGCCTCGTGAGGCTCAAGCCAGCGGA
TACGCTTATTTTTCGGCTGAGGAACTTTGATGATCGGAGCCTTATCCAGCATCTTCCATT
CGCGTTCAGCAGCCCGGAGGAGTGCCTTAATGAATGAAAGGTGAGTTGCTTTTGTGGCTA
ATGCTGCCGGCTTAGGCTTGAATACTGGAGGTTTCTTCCCATTCTTCCTGCAAGCTTCAT
CCATTAACTTCCAGTTTTCCTCATGCCGCCGATTAGTCATCTTCTGGATGGCGGAGTAAA
TCTTCGTCTCGGTAATATCCTTCAACTGCATCCCTGCAAAATGCTGGAACCAGAATCCTA
TCCGACTCTTGTCGTCATCCAGCGACTTCTTATGCGCCTTCTCCTCTAACCACCTGACAC
AGGCCTCCTCAAAAGTCATGTCAGGCGTCTCTCCTAATTTACTTACCCTCCATGGTTCTG
CCTTCAGCTTGTCATGAAGCTCTGTGGCCTGCCTTTTGTCCTTTGTCCTAAGAGACTGCT
TAAGTCTTTTGCCCTCCGGCAATGTGAAACTGGCGTACCAGGTATCACCTCTTTGCAATA
GTGACATTTCAGTTCCTCTGTTATGTCATCACCCGCGCTCACCTGGACAGTATGCAGCGG
AGACTGAAGTGCCGCAATGCAGGCTTGTCGTGTGGTGAGGTAAGGGGATTTCGGTTTGGT
GGGGTCTTTACGTGTTGCCTGTAGTCGGCCTGTGCGAATCCAGTCGGTGGCGGTAGGTCT
GGATATCTTGAGAAATGCACAGGCCTAATCGAGTGTGAGACTGTGTGATTCCATGATTAC
TCCCGAGCTTCTTCTTCAACTTCATTCGCGATGTCGTAGAATTTCCTGTAAGTTATTTTC
TTGAAGCTGTCAGGGATAACAACTTCGCCAGGCCTCTCTTCTTTGTTATTTGGTATTGCA
AAAATAAGACAATCATTGCGCTTCGGGTGCTTACCGCCATAAGTTGATAACATAACGAAA
CCAAGTCCACGGCCCGATTGACCACCAATTCCTGAACGCATAATCCCGTAGTGGTTGGTT
ATGTAGTAATTGCATTCAGGCAAGGATTTTAGCTTGGCGTTAGCGTTATGTATGATTGCA
TCCAGCTCTTTGTTGTATGCGCGGCCTTCCTTTGTGTTTCCCTTTCCTCGCGCTATCACA
ACTCTCTTCCCGTCCCAAAAATCCTCGTGTTTGATTGTTATCTGGCATGGGAATTCATAT
CCTTTTTCCCAAACGAAGCTTTGTAGAAGTCCGCCTTCTCCACCCCAGCTACTGGTTGTA
GTCCATGGGTTTGCGCCAACCTTTTCAGCGGCTGTGGTTAGGATTGAATTTCGTTGGTCG
TTAATGGTGTCGTGTGACTGGATAAGCTCCTTAACATCCTCACCCTCTACCATGTAGTAG
TCGTAATATTTGCTCTGGTCTGACATTTCTTGTCTCCAATAAAAAACCGCCATCAGGCGG
CTTGGTGTTCTTTGAGTTCTTCAATTCGAATATTGGTTACATTGTTTTCATATATGAATA
AATAAATTAGCTTTTTTCGTTGCCTTTGCGTTCCTTATTAATTCTGACAAACTCGTTTTT
ACCACGCTCTCCAAATGCGTCTTTAGAGTCGTTGTTTCCGCAATCGCAGCACACATAATC
ATCAGACCATCCACGCATTGTTTTTTCTTTTGCAATATTTCCAGAACCGCATTTTTGACA
AGACATGTCACTACCTCCACAGCATGAGTGAGATGACAACGTAACATTGATTGGAGATTA
ACAATAGATTGCTGATGTAAAAGATATGGATAAGCTTCGCTATCAAAGGGGAGGATCTGG
TAGCTGCATCCAGTGGCTTACACCGATACTTTCCATACCCTCCCATTAGTCAAAGAACCC
ATCATCGTCGTATGTAGCAACGAACATCCCCTGACCCAGACATTTTCCGGTAAAAATTGC

GATGGGTTTAGATTCATCATTATCTGGCATTCGCTCACTACACCTTATCCAGCCACCCGG
AATTACCGGAGAGTTGCCATTCACATCGAAATTTGGTTCAGCGTCCTGAACCAGACGAAT
GTAGACATTCTTTGCTGTGTCCAGTTCTGATACCTCGGTGACAGTACCGAAATAGCGATT
CTCGGCATCAGCATCACAAGTGCTGACATCAATGGACACCTCCATGCCTTCGATTAATTC
TGGCAACTCGTAAGTTTGGCTTACAGGTTGGTTTAGTTTTTCTAAATCATGATGCAGGGC
GGCGCGAACTGTACGCGCAATTCGTTCACGTAACTGTTGTGTGCCGTGATACTCAATAGC
AATATCATGCAGCTCGTTTACAATCTCCCGGATTAGATTATCTTTCACGATTGACCTCCG
TTGAGCATGGCATTGCGGCAGGCGTTATAGCCTTGCGCAAAGCTCTTTTGATAAAGATTC
ATGTCATCAGATGTCGCCATTTCATCAGGCACTACCGGCGCAGGCTGCTCTTTGATATGC
AGCCGCGGCTCGCCGTCTTTCGGCTCCGGCCACTGGCGGGCCTTGTTTATCGCTAGCTTT
TCCACCATCGCCATTGTAATCTTCTCGTCAGTAACACCAGCACCGCGTTGAGCATCCCAT
AGCAGGAACTGCATATCAGCCCATTCAAGCGGATCGGATGGGTCGGCAGCGGCCTCCAAT
GCTTTTTTCGAGAGATGTTTCAGCGGACCGACAGGGCCAACATCGCCGAATGTCTTATCT
GACCACTCGGCGTGCTCACGGCGTATCCGGTTGCGTTCTGGTGCAGGCTGCTCTTTGCCA
ACCAGACGCGTTATTTCTGATTCCAGGAGTGAGCCTAAAACTGTACTCGTGCAGTGCTCA
GCCCGTTCGTTTTTTTTCATCAGGCCGATGATATTGAGCACATCATTGTAAACGCCAGTG
TCCTGCACTGGCGGGGCGGCGTAGAGCGGTATATACACGGCAACATCATCAGCAGCATTT
GGCTGCCGCTCTAACGTCCCGCATGTACCGGAAAATTTATTCAGGTATCGCACAGACTCA
GCATACAGCGATGCCGGCGCACGCTTCAGCACAATAAGAATTTTGGCGTCATCATCGTCG
AGGCCAAACGGAATATCGTCGCGAGTGTTTTGAAATTCAGCGATAGTTTGCTGTAGCCAT
TCTTTGGTAATAGTGGTCATGGGATAGCCCTCAGCCTGCCGTGCTTTCAACTGGAGATGG
GAAATCAATAGCCCCTTCAATATGCGCACCTGCTACACGGCACGCCGGGGACCAGTCTGC
CGCTTTGCCAGTGTCCAGCGCCTTTTCAACTTCGTTGTATTTGCGCAGGTCATACAGGTG
AATATTCGAATCGCCGATGGTGTAAAAACCGATTTTTTTCGGTGACGGGCAACGGGCCAG
CACCGCGTTAACCTCATCAAGCCAGGCTTGTTCTTTCTTCGTTAATTTTGCCATTTGTTT
TTAGCCTCCACATCAATGAATGCACCGCAGCAGCCGCATTCATATTCGTTGATGAATCGA
CCGTTTTCTGTCTAGTCGTTAGACTTGCATATGTTGCAGCGAATCTCATAATTACCAAGT
CGTGCTATATCAGTGGCGCTTACCCACTGACCATCATCAAACCTAAATCGGGCTATCTCA
TGCGGCACATAGCGGATAACCATTCGCCCCGCGTCGTTCTCACATTCCGTGGGATAGCGT
AAAAAAATCCGAGCCATTCTGTTTACGCCCTTGTGGGTCGTCTCCGTTAATTAGGTCGTC
CAGTACGTTAATTCTTCAGCTACGTGATAATTGGCGTCATCCTGGTCCTCAAGTGGCGGA
TCTGTATTGAGGTGTTCTTCAGCTGTGATTGCCGCATTCTCCCGACAGAATGCTTTCCAC
GCTTTACGGCCACCTTTCCAGCATTGCCCCTGATGCCATGCCAAAGCCTCTGTTTCGGTA
CGCCATGCCCGGTTTGCTAGCTGCATTTGTGTCTTAGCCATGCGCACCCCTTAACCTTGA
TGCCAGGGGTGCGTGTTTCGTGTATCGCATTATCATTACCAGCACACCAACCCTCGGCAT
AATCCCGGCTGAATCCGCTCATGTGCATGACTTCTCCAACGCTGAGTTTTGACAGGTTGA
ACTTCCGCGCCTCCAGTTCTGCCACCCGGCGAGCCTCACGGGTTGCAATTCCCGCGTATT
CAATCAGGCGTTGGTCCAGTTCGGCTATGAGGCGTTCTGCTGTATCCAAATCCTCGCCTA
ACTTCTGCTCCATCCGAAACCAATTGGCGCGCTGTTCCTCTTTGGTTTCCAGCGCATCCA
GCAGCGCCAGCATAGTGGCGGGGTTGGTTTGCGGCGATAAATTCCGCGTCACGTTTCTCA
ACAGTATGGGCCAACGTCACTTCTTCACCGCAAAGAGAAAATGGCGTTACCGTGATGCCA
TTGAACAGTGATGAGGTGCGGCGCCAATTCCCCGGCGTAGCCCTCTCAGCCACTTCACGA
AGCGCCCGTTTGTCGATGTTGCTCATTGGGCGGCCTCCTGGCGAAGTTGGGTTTCCCATT
CTTCTATCGCCTTCTCCGCGTATTCACCTGACAAACCGTCATCCGCGGGTAGTGGGTCAT
TGGCTAAATCCTCTTTCGCCGACAAAATCATGCGTGTCACGTCGAGAACTTCTGATACAG
GTTTATCGAGGAATCCCTGATTGAATGCGGCGGCGAGGCGGCTGGCGGCATAGTTGATAC
CTTCATTACGCGCACTTGCCAGCACCTCAGCCAGCGCCGCGTATTTATCCTCAAGCTCCG
CATAATCACTATGACGCACCATGTCAGTACAGAATGATTCTCCTGTTATTGGTGGTGATA
ACTGGTCACTGACAATCGTGTATATTTTCACTTCTTTCATTTCTTCCCACTCCGCAACAT
TGCATTCAGATATTTGTTTTCATTAACAGAACCGAAACTATTTCGCTTAAGCATTTCTTC
GCGTGGAATATCGTTGATGGGTTTGGAGCGGTGCCGAATAATCATTTCCGATGGAAGGAT
GCCGGGGTCGTAGGACAAACCTCTCATGATGAATTCCTCAGTTATTGCTGATAGCGCCGT
AACGCGAACGGTAATTTTTAAGGCGCGGGTCTATTTCAATGAATTTGGTGTATGTGGCTT
TGCGGAATGGCCGGATGGATGTCTGGTAAATTCGCTCGCGTTCTTCTTTCTCTGCAAGCC
ATATACAGTGGCGAAATTCCTTTTCCTCTTTCGTTTCCTGCGGTAGCGACATTATCAGGT
CGTAGTTCTTTCTGAATTTTTCCAGCATCTCCGATACAGAATTGCCGGAACAGCGGCGCG
TGTCATCCGCACCATATAGAGGCGCTGGCATAATTTACTCCAGGTAGGTTATCCGAATA

ATGTGGTACGTATAGGGTTATTTCGTTCGTAAACGTGATAGCCTGCTTACTACCGACTCT
TCACTTCGCCCGAGAATTTTTGCTACATTTCTTTGTGTATAGCCTGATGAGATAATCGCC
TGCATCTTTTTGTCTTCGTCGTCGCTCCATCTTGGCTTAACGAATGCCGTTTTTAATGAC
AGTTTTTTAGCTATGTAATAAAACTGATTTATGTTTAGGCCCCGTTTTTCTGCTGCACGG
CAAGCTACCATGCGACCGCAAACTGACTCCATCTCAGCTGGAGTTATGTTTAATCTTCTC
ATTAAGCCACCTGTTTAAGCTCATTTATTCTGATATTCATTACCTGAACGCATCTTGTCT
GCGCATCATCGTGACCAGCCAATAATTGCCAGTCATGCTGGTATCTCTCAATTAGTTTTT
TCTTGTCCGTTTCTGTTGCTGCATAATCGCTGAAGTCTTTCAGGATTTGTTCGCAGTCAA
CCGATGGAGATTTCTGGTTGGTATTTTCTGGTGATGGTTGATTGCATGATGCTGGCATGG
CTCAGTCCGGCAGCGATGGAGGGAGCCAGTAAAATCGTGTTCCATCCTTCAGTTTGGCCC
TGTGCCATCCTTGTTTCTTATCACTGGATATCTGCGCAAAACCTTCCTCAAGGTTATATA
GATACCGACCAATTCCCCACTGAACGGCAGCACGCTTCATTGCGCCGGAGCGACCACCTT
TGACGGCTTCTACCTGTGTGTTTTCAGCAGCATCCCATTTAGTTACCCATTCGGAATCAA
TCTTGATTGATATGCCGCATTCAACGCCACCGTTGTTGGGAATATCGCGGTATTCATTGC
GCCATCATTCTTTGCCGCAAACATCGTCCAGGCGTTTCATGATTGCCCTGTCCGTGACAT
AAGCCAGCACCATAGCCCACACTTTGCCATCGCGTGTTTTACCGCTTTGCTGTATTCGCC
ATTCGATATCTTCAGGATTGAATGGGGCGTCGAATTTATTCAAATCTATAATTCACCTCA
GAATGGTAATTCGGAAGGATTAGCCAGAAATTCACCTATGTTTATTCTCTCGTTTTTGGC
TAATGAAAGGCAATTTCGTTTCATCGATTTATTACCTGACTTGCGCCAGTACATTGCCTC
TGTCAGGTGATACTGACGTTTTAACCTGCTCAACTCCGGTGTCCTTGCTAAATCTACTGG
TATCATTTCAACCTCCATTCGCGAAAGGCTTCTAAAGCTTCGCGATACATTATTTTGTCA
CCAAGTTAAACAGCAATTGCGAATTTAGACTGAATAGCCATAAGTGATTTATCCATTACA
CGGCACTCCTGGTTGATTCAGGATATCGACCAGACGTTTCCATCCGGCCCGTAATTTTCT
GGAGATACGCTCTAAAAGTGATTCATTAAGGTGTGCGATACCCATGACGGCACCGCCCGC
GATAGCAAATGTCATCGTGGGATTCTCCATTTTGATTTATTGGCATAGCTAAAACGCCTC
GATATGAAGCGCTGTGGATATGCGATAAAACAGCCGCACTCAGGCGGCGGCTGTTGTTTC
TTCTTTCAGGCTTTCGATATATTCACGAGGGTCGTCGTAACACTGGCATTCGCTATACCA
ATCCACCCAGCGATCCGTAAGCTCCATTTCATCCAAATCCTGGTCAGTAAGGCTCTCATC
CCACATCTCAAGGCCGTTAGCGTTGCTGTAGTCAGGTTTGATGTTGTTGTCATACTGAAA
TGCGTCATAATCAGCCAGTGCATCCATCACTGGCACACCCTCTTCAACACTTGCTACTTC
TACAATGAATGGCTTAATAGGAACTTGCGGGATATGCCAGACACGTAATTTCATATTTCC
TCCAGGTAAAAATAATGCCACCCATATAGAGCGGCAAATAACATCAAGGGATGATTTTTC
GATTAACCAGAACGAGTCGTCGTCCTCGCTTGGTTACGAGCGATATTGCTCACATAGCAG
ACTCCTAAATCTGCTATAGGTCCTTATTCGCAGCCAAAAATACGCTTACCCAGTTACTTC
ATCTGCATATTCTTTACTTGTTAACCAATCCGGGCGTTCACCTTTACCAATATAGAAATC
GATAATGTCCAGAAGACGTGGATAAAATTTAAGAGCTTTACGACCATCCATCTCAGCAAT
GTCCTGCTTACTATATTTTCTCCATTCCTCAAATGTGTGGTTCTGGCATCCTGCTCGTAC
ATATTCAACGTTCGTTATACTTATGAAGTATTTCTCACCCAGAATTACGAAAGTGTGATC
AGGCAGGTCGGCATCGCACAGGTTGGCACCGCGCAGGTCGGCACCGCGCAGGTCGGCACC
GCGCAGGTTGGTACCGCACAGCTCGGCACCGCGCAGGTTGGTACCGCACAGGTCGGCACC
GCGCAGGTCGGCACCGCGCAGGTCGGCACCGCGAAGGTCGGCACCGCGCAGGTCCGCACC
GCGCAGGTCGACACCGCGCAGGTTGGTACCGCACAGGTCGGCTCTAGATCCGCTCTCACG
CACTGAGGTAATCCTCACTTTGTGTTCTTCAAGAATCTTCGATACATCTGCTGAATTCAT
GTTGTTATTCCTGAAATTTTGGCAATAAAAAAGGCCGCATTGCGACCTGATTAGATATTT
GAAGTGAGATAAAAGAAGACCAACTATGTAGCCTTTAGTTTTTCCAGCTCTCTGGCAATC
ATTGCGGTGGTCCTGATTGCCCATTTATCGACAATCGTTCCATCTTCTCTCACCAGAGCC
ATTTCCTCAGGCTTCACCATGCATTCAGCATCAATCTTGCAGCCTTTGCATTTCACAAAG
CGACTACACCATTGGTTGGTATCAATAGTCGTAGTCATATTGGTACTCCTGGTATTGTTC
CATCACATCCTGAGGATGCTCTTCGAACTCTTCAAATTCGTCTTTCATATCTCATCTCAA
TCGTAATAAGCCGGAATTGATTTTCCGCGCTGCTTCTGTACGGCGTGTATTTTATTTCCC
AGCGGGTTAACGTCCCGGTAGTAAATGCGGTTCTTCTTAACCGCTGTTACTTCAACTTTC
TTCTGACGCGTTCGGGCAAGCGAAATGGCTTTGGTAACGCGGTCAATTCTTTTGGCTTTG
ACCTCATGAGAAGCATCAGGAACATCGCAGCCAAAAATTGAATCGATGATATTGCAGATG
GTGTCTCGCTCTATGGCTAGCGTTCTGCGCCGCTCATGACGGCGAGTTTTAGCATTGCCT
GCAAACGTTGACTTCCCGTAGGTGATAACCGTAATGATTTATCCCTAAAGAAATTTTATT
GATTGAAGTTAAAAGAAAGACGACGTACCAGGAGTGGTGCCCGGTAAAATGGTTTATCTA
AAAACGAATATTTGTGAAACCAAACACGACCTCGCTCGTCTCCATTTCTGTTGCCTTCAG

GGCGACGCTTTAAGGTGAAATGAATAATTGTTTTTGTAGTGGTTTTTGGCTTACGCATCT
GTTTACCCTCATGTGAAATGGCTTTGGTGTTACAGATAGCCAGGCGTCTAACCCTGACAG
CGTACTCATTGCCGAGCGCCTCCGCCGAAGAGGTTGGCTTCTACCTGCAACCCAAACCCA
TCTCGTTTGGTATTTGTTCGCGCTTTGTCAGCGCATCATCGAAGTTAAAGAGCGTTGCCT
TTCCGTTTGGCTACCAGCGTCCTGCTGATGGCTAAACAATACAAAATTTACTTAATATCG
TCAATACAAAATGTACTTAAAATTGATAAATAAATAATATGTGTATGAAACTGAATGGAA
AAAATATTTTGGTATTAAAAAACCCGCATAGGCGGGCTAGGGGAGGGAATTGTTAGAGGC
CTTGCCATTTTGCTTCAATGACAACACCGATAATGCGGCAATTTCCGTTTATGGGGATCA
TGTGATAGCTGGGGTTTAACGGTTTAAGATATTTCTGTCCAGCGTCAACAATATATTTCT
TGAAGGTTGCCTCATTTTCAGACTCAAGCTTTGCCACCACGAGTCTACCATTAGTCGGTT
CGATAGCCGGATCAACAAGAATTTGCATTCCTTCCGGTATGCTTAATCCTGTAGGAGATG
TCATAGAGTCGCCACGAACGGTTAGCCAGAATGACCTTTCGCTTGCATGTGCAGTTGTCT
CAGGCCACACCTCTATTTCTCGGAGTTGATAAGGTTCAACAGCCTCACGCCAGTTACCTA
CGCTCACCAAGCTAATCAGGGGAAATCTTCTTATTTCTGTGTGTGGACGAGGACTTGAAA
CATTGCTCAGGCTGGAGTCTGGATAATCAACCATCCCATCAGAGCTTAATACAAGCTCCT
TCAATCCTAGCTGTTTCATGATCGCTGCAATATCTTCAATACTTGGTTCACGGCGGCCAT
TAAGCCAATGACCTGTCGCCCCCTGAGTCTTACCGAGGGCTTCAGCAAGTTTATCCTGGG
TTAGGCCTATTTGTTTCATTCTGGCTTTCGCCAGCTCATTCCACGGTGTTTTCATGCGCA
GATTATTACGAGATGTATTGACTGTGACAACACACATATTGTATTAATTACCTTGCTTTT
ATCTAGTACGAAATGTATTATTGAGTTACGTACCATCCTGACGAGATATACCGATGAGCA
ATCTTCGGGACATCCGGGAAACCATGAAGGTATCCCAGGCCGTTCTGGCCGAAAAGATTG
GGTGTACTCAGGGAGCGATTGGTCATTACGAATCAGGGCGACGCCATCCGGTTTTGAGAA
TGTGCCGCCAGCTCGTAGAGGCGCTCAACAGTTTTGGCGCGAATGTTCAGCTAGACGATG
TGTTCCCACCTGAACTTAATGCTGCCTAAGTAGTACCGCTCTTTACCAATCTGAACCGCC
GACAACGCGGTAAATCTATCCAACGGATTTGCGTGTATTTGCGAATCCAACTCTATCTAA
TTTCTAAGGAATATTTTGAATGAACGTAGTTGCAACTAAAAGCAAGAAGGCGGCTCGCAT
TGAGTCCACTTTACTCAACAAGTTAGCCATGATGGGCCAGAAGACATTCGCTAAAGCTAT
GGGTGTTCCTGAATACCAGGTAAGCCGATGGAAGAACGGTTTCTTCTCTCAGGTCAGCAT
GTTGCTTGCTGTTCTGGAGTATGGAATCGAAGACGAGGAAATGGCAGAGCTCACCAGGCG
ACTTGCTACCTACCTGACAAAAGAAAAAGCCCCGAAGAACGGCGAATTCTTCGAGGCCTG
ATGTAGAAAGACTGGATCAATCCACAGGAGTAATTATGCCAAAACAACTCAGTCCTGACC
AGGACAAATTACACAAAAACATACTACGTGATCGGTTCCTATCCAGCTTCAAACAGCCTG
GTCGATTTCGGGCTGAGTTGGAGAAAGTGAAGCTAATACTGAAGAGGAAAGGTCATGAGT
AACATATCCAATCTAGCCGAAGCCAGAGAGGCCAGAAGGATCCAGAAGCCGCGTACAAAT
GGCGGCAAGGGGTTTGCCTTGATTCACCGCCAATTCATGGATAGCAAGCTATAAAAGGAT
TCTCAGGCTGTACATCTTTTCCTGCATCTGATACTGAAAGCCAATCACACTGCGGCCGTC
GTAACTACCGACATTGGTGAGATGTTGGTTGAGCGAGGACAGCTAATTACCGGACGGCCA
AAACTGGTACGTGAAACATTCATCCCGGATAACAAAGTGAAAAGTTTGCTTCGTTCTTTT
GAATGGAATGGAATGATTTGTATCGAGTCGAAAGGGAGAAAATTCAGCCAGAGAACAGTG
TTGAAATATGATGATTTTCAGGCTCCAAATTGTGCAACGGATGTCCAACGGATGTCCAAC
GCAAACACCCGTAATGACGCGGCTCACAGCAAATGTTGTCCAACGGATGTCCAACGGTTG
TCCATAAACAATAATATAAATAATATCTCTAATACTAACGTATTAGAAACTACCGCAGCA
GACGAAAATCCTGACAAGAAAAAATCTGCTCCCAGTTGTCAGGATGGTGTCGATGATTAC
CACGAATTACTTCCTGAAGCTTCCAGGGTTCGCGCACTGAATGACAAACGTAAAAACCAG
ATCCGAGCTTTCTGGCGAAAAGCCGAAGTGATAACACGCCAACTTGACGGGCATGGGTTC
ACGATGCAGGACTGGAGAAATTATCTGAGCTACGTAGGCGAAAATTGCCGATGGATGTTC
GAAGAGCGCCAAAACCATCAACGCGGAACCGTCTGGCACAAAAAGGGATTTGATTTCCTG
CTTAACGATAATACCTACCTGAAAGTTCGTGAGGGTGAACACGATGACCGATAATTTTTA
CGCGCCGCCACATAGCATCGAGGCGGAGCAGGCGGTGATTGGTGGATTGCTTCTGGATGA
TGACAGCAGTGAGCGCGTCCAGAAAGTTCTGGCGACGCTGAAGCCTGATTCATTTTACAG
CCGACCACACAAAATCCTTTTCGAAGAAATAACCGGAATGCACCGGGAGCAAAAGCCAGT
AGATGGCCTGACGCTTTTCGATGAACTGGTGCGTAAATCGTTAACGGTGTCAGTTGGCGG
TTTTGCTTATATCGCTGATATCGCAAAGAACACGCCAAGCGCAGCAAACATCGTTGCCTA
TGCAATGCAGGTTCGCGAAACCGCAATGGAACGCTACGCCATCAACCGCATGTCTGAAGC
GCCGGAATTGCTCTATTCCTGCAACGGAATGACTGCAACGCAGAAGTACGAAGCTATTCA
GGCGATCTTCACGCAACTGACAGACCATGCAAAAACCGGATCACGTCGCGGCCTTCGCTC
ATTTGGTGAGGTCATGGAGGACTGGGTTGGTGACCTTGAAAAGCGATTTGACCCGTCAGG

CGAACAACGAGGAATGAGCACAGGGATCCCATCGTTGGACAGGATGCTGTCACCTAAAGG
TCTGGTAAAAGGCTCTCTGTTCGTCATTGGCGCTCGCCCTAAGATGGGGAAAACTACGCT
ATACAGCCAGATGGCAATCAACTGCGCAGTGCATGAGAAAAAACCTGCCCTGATGTTCAG
CCTTGAAATGCCAGGCGACCAGATACTGGAAAAACTGGTAGGGCAAAAGTCAGGTGTTAA
CCCGAATATTTTTTACCTTCCGGAGACAAATGACGCTGATGACGGCTATCAGGGTGATTA
CGATGGTGACTTCAACAAGGCGATCGAAACAGCCAATCGCTTGAGTGAAATTGACATGCT
TTACATCGACGACACGCCGGGATTATCTCTGGCTCAAATCGTCAGCGAAAGCCGTCGAAT
CAAGCGAGAAAAAGGATGTATTGGCATGATTCTGGTCGATTACCTGACACTAATGACCGC
TGAAAAGGCCGATCGCAACGACCTTGCCTACGGCATGATCACCAAAGGACTGAAGAACCT
TGCCAAAGAGCTTGATTGCGTTGTTGTGCTTCTGATGCAGCTTAACCGCGCACTGGAAAA
CCGAACCAATAAACGCCCATTACCAAGTGGCTCACGAGATACAGGGCAGATTGAACAGGA
TTGCGATTATTGGGTTGGGATCCATCGTGAAGGTGCTTATGATGACAGTGTTCCACCTGG
TGAAACCGAACTAATCCTTCGTCTCAATCGTCATGGCAATACCGGCACGGTGTATTGCAT
TCAGGCAAATGGCGCTATCTATGACACAGACCGACAGTCTGCTGAAATGCGCTGCCGTGA
ACGCGAGGAACCGCAGTCCAAGAAGAAAGGAGGGTTCTGATGACCATCTACATCACTGAG
CTAATAACAGGCCTGCTGGTAATCGCAGGTCTTTTTATTTGGGGGAGAGGGTAAATGAAG
GATTTATTAGTAACGCTAAATGTCGGTTTAAGCCTTCTTGGTTACGCCTACATTATGTTC
AAAACAGGCCAGTGGATTATTACAAATGCACTTAAGCAGTGGGATAAGCGTAGAAAAGTG
TCAGCCAAGCACAAGGCGGGTGATGCGCTATATGAAGCATACGAACTGGATAAGGTAAGC
GAAGGAGATACTGTAAAAGTGGCGACAAAAGAAGGTCTGGTAATCATGATTTGCAGACAT
GAAAAGACTAACACCCCAGCACACTGATGGAGAGGAATGATGAGTACATTGGCTCAATTC
ATTAATGCCGACCTTGAAGAGTCAGGAGCACGGCATTATCGCTATTGGAAAGCTTCGGGA
CTTCCGACTAGGGAGCGATACAAGCGTAGGCCAAAACCAAAGAGCAGCTAGCGAGACAGG
GTACTTAAGCGCCTAATGCAGATAAACATGTCGCAGTTTACTGATTTCACCTGGTTCAAG
CGGTGATGGAGAGGAATATGGACGAATCAAGAAAGGCTTTCGAGCAATGGTTCCAGAGCA
AATACAAATGCACTATGGGAACGATGAAGGTTATGCAAATCAAAGTCGAACTTGCTTGGG
AGGCATGGCAGGCCAGCCGTGAAGCTATCGAGATAAAGCTCGATGACAAAGTAATGGTTG
AGGATGAGTTCGACAAAGGCCACAACTGCGCAATCGACTATTGCGCTGATGTCATCCGCG
CCGCCGGAATCAAAGTGAAGGAGTGAGTATGAGCAAAGTATCAAGAGGAATGAAAATATA
GCTTATTTTCATCCTTAATCCGCATCGTATCTTTTTGGCTTCAGCAGTATGGCTGTCAGA
TTTTGTTTATTGGTTAGCAGATAAATTGGATGATTTTGCGAGATGGCTTGAGAGTTCTGC
GAATGCGAGGTTTTGGTTATGGCCGCTTATCGGAGAGAGGATGTCTGACGAATTAAACCG
GTATTACGCGGATAAGCGCAAGGAGAAGAGCAGGAGGGCAAGTGAAGCAATTATTCCTGC
TTCGCAACGTAGCAGTCAGAAATAACGCCATAGACGCGATTCTCTCACTACTCATCGACG
ACAAGTCACCCCACGAAGTCCACGTTAAAGCACCCAAGCGAACCAAAGCACAGAACGACC
GTATGTGGCCGATGCTTCATGATGTCTCCCGTCAGGTGCTTTGGCATGGTCAACGACTGT
CTCCGGAAGACTGGAAAGACATATTCACTGCGCTGTGGCTCAAGACTAAAAAGCTGGAGC
AAAGAAGCGTACCAAGTATTGATGGCGGTGTTGTTCTTCTTGGGGTACGTACCAGCAAGA
TGAGGAAGGCGAGCATGACAGAACTTATCGAAATCACGTTCTGGTTCGGATCAGAACGTA
ACGTGCGATGGAGTGATGATTCCCGGAGAGAGTGCGAATGGTCACAACTAACAGGGAGAG
TTGCATGAAACGATGTTACCGATGTGGAGAAAGCAAAGACGATTATCGATTCCGGCCAAA
TCAACCTTATTGGCAACAATGGTGTATCAGATGTGATCGGTCGCCAGTAGGTAATTTCCC
GCTGCCAGAGACGAAGGAGGACGTATGGCACGACAGCGACGAAGTATCACCGACATAATC
TGCGAAAACTGCAAATACCTACCAACGAAACGCTCCAGAAATAAACCCAAGCCAATCCCC
AAAGAATCTGACGTAAAAACCTTCAATTACACGGCTCACCTGTCGGATATCCGGTGGCTT
ATAGAACGTGCGAGGAAATGACAATGGAGTATTCACAGTTAAGAGATTTTGAAATTAACC
GAATGGTAGGAGACATAATTTTTAAAGCCCTTTGGGCATGTAAGCCGGAAACGTCAGGGA
ATAACACCAACAAATGGTATTACGGAAACGCTGATACAACTTTTGAGCCATTAAACCATT
TACCTGACTACTGCAATGATCCGAGTGCCTCATGGCCGATTATTGAGAAACACAGGATTT
CTATCTTAGACCAGTAAACTGAATGGTGTGTTGATGCAAAAGGCGTAAGCCCAATATTTG
ATACCAGACCTCTCCGCGCCGCCACGATTGTCTTTCTCATGATGCAGGACGCCAATAATG
CTTAGCCCATCCCAATCCCTTCAATACCAGAAAGAAAGCGTTGATCGGGCTTTAACGTGC
GCTAACTGCGGTCAGAAGCTGCATGTGCTGGAAGTTCACGTGTGCTCCGATTGCTGCGCA
GAGTTAATGGCAGACCCTAACGGACAAATGCTGGAGGAAGATGATGAGTGAGTTACGCGC
AGGTGGCATCGCAATAGTCATTTTTTCAGAAAAGAAACCCGAAATTGGCAGATGCGTTGA
GTTAATCGAAAAAGTAACAAACGGATATGTATTTAATTTTCCTGGTGCAGGTACGCATGG
GTGGCGTGAGGATGCCCCTGCGTGGTTAGTTAAAGGCGATGTATCGATTTATACAAACAA

GCCTTCAGGTGGTTTCTCTTATTTTTACAGTGATGAACTCATGCCAATCGACGGAGAAGA
CTTCTCTCACGAAGATGAGCAACAGAAGGAGCTGGCAAATGGCTAATCTACACAAAGAAG
CGCGCGGCAGAGAATGCCAGGTACGTATTTATGGCGTATGTACTGGCAATCCTGAAATTA
CAGTTCTGGCACGTTACCGGATGGCTGGAATTTGCGGAACGGGGATGAAGCCTGACGACC
TGATAGGTGCATGGGCTTGTAGCGCGTGTCACGATGAAATCGATCGACGAACCCATATTC
TCGACAACAAAGACGCCAGACTTTACCACCTCGAAGGCGTGATCAGAACGCAGGCGATAC
TGCTGAAGGAGGGGAAGATTAAGGCATGAAAACATACCGAATAAAATTGCCGTGGCCTCC
TTCAAACAACCGATATGGGCGACACTCAAGAGGGGTCCACTACATCAGCGATTGGGGAAA
GCGATACCGGCGAGAAGTAATCGAAATAATTCAGCAACAACAGCTAGACCTTAAAATCAC
ACCCCGCAACAGGATCACCATTCTCGCAGGACCTCCCGATAACCGCAAACGCGACCTGGA
CAATCTACCAAAAGCCGTTTTTGACGCACTCACTAGTGCGGGCTTCTGGCTGGATGACGG
CCAGATAGACGATATGCGCATCAAGCGCTGTCAGGCGATTAAAGGCGGAATGCTTGTGCT
GGTAGTGACTGAGACGTGCGGGAATTTGCCAATGATTACGGAACTACTGGAGGCCGCATG
ACACACACTGTCAAAACCATTCCAGATATGCCCATAGAGACATACGGAAACCAGACAGAA
GTAGCCAGGCGATTGTCGTGCCATCGAAACACAGTCAGGCGCTATCTGTACGACAAAGAA
GCTAGGTATTACGCCATCGTTAACGGCGTTTTAATGATTCATCAGGGCGTGAGAGGTGTT
TATGACCGTAACCAGCATTAACCAGGCGAAACAGCAGCGTGAGCGTGACGAACCCGAATT
GCGCAGCGTCAGTGAGATGACGGAGCAACACCAGAGGGCAATGAAATATCTGCATGAGCG
AGAGCGCGAACTGGTGAACCGGCTTGGATTGAACAAGCCAGCGGTAGGCGATGCTGCATG
AGTATACGAGAATTGAACCTCACTAAAGAGCAGCATGACTGGCTTAATGGGTGGCTTGAG
CCATGGGGGGCATGGGTTTATTCGGGTCGTCTGGAAAAGCGCATGAGCAGCGTAATAGCG
AAGTTCATGGAGAGCGTAGAGCCGGGAAGAGTTATGACAAGACCAATGTGCAATGATGAT
GATGGAATGTTGATTTCTCAGGTCGTCGATTCCGTCATGTACATTGACAAGAAAGCCTTT
GGCATCCTCCTCAGCTACTTCGCTCATGGTTCATCCAAGCGAGCAATTGCATCCTACTGT
CACGCGACTGCAAAGCCACGCAAGATGTGTGGACGGGGTGGCGATGGATTGAGAAAACCT
TCACAGGCAACCTGTAGAAATGAAATTGACGAAATCCTGAAAGCGTCATTATTTGTTTTA
TACCAGCCAATGCAAAATGCTTTCAAAATGCGTAAACGTGTTGAGAAAGTTAAGCATGTT
GCTGTTAAAAGTCTTGACATGCAATTATCCATTTAGCCATAATTAGAGGGTAAGCTGCCG
TTAGTGACTCTTAAGTTGCAACGGTGGCTTTTTTATTTGGGTCAGTCGTATAAAGGTCAT
TACGGAAGGCTGTTAACCTTCTTATCGTGGTTCGAGTCCACGCTGTCCCGCCAAACATGC
TGGTTTAGCTCAATGGTTGGAGCGCCTTCCTAAGAAGGGAGTTGCGGTTCAGTTCCGTCA
ATCAGCACCAACCAATCCCGCTGCCAGGGATTGCAGGCCGTAGAGTATACGTGCCTTACC
CTCTTATCTTCTTTCCCACCTAGCCGTGGGCTTTTCATTCAGGCCGCCGACAATCACCCT
CATAAGCAACGTAGCTATCATGTCGGACGGCCTTTCCCCACTACAAATACAGCACCCCGT
TTCTTCGGAGGTGGTATGGCAAAACGTATGAATGACGACCATAAAGTTGTAGGCCTGTCC
TGGCTAATTCTGCTTGGAATTGCCTGCTGGGGCGGCTTGGTTCGCTACCTGATTGACGTA
ACGCAGAATAAAGCAACATGGAGTTGGATAAACGCTTTCGCACAGATCGCGGTATCTGGC
TTTACCGGCCTGATTGGCGGGTTGATAAGCGTTGAGGGTGGGCTTAGTTTTCACATGATC
CTGGTAAAGTCGGGAATTAGCGGGGCAATGGGTTCCGTTGCGCTAACCTACTTCTGCGAG
CGTCTTACAGGGATGAAAAATGCAAACCAATAAGTTTAAATTTTCTCAGCGTAGCGAGAA
GAACCTGAACGGAGTTAATCCTGACCTGGTCAAAGTGATCCGCCGAGCACTGGAAATTAC
ACCAGTAGACTTCATCGTTATCGAAGGGGTGAGAACACAAGCCAGGCAGAAAGATATGGT
TGCTTCTGGGAAGTCACAGACGATGAACAGCCGCCACCTGAGTGGTAATGCTGTCGACAT
CATCCCGGTTAACACTACCTGGCAGATTGAAGAGTTCAAGCCGTTGCTCAAGGCGGTTAA
ACAGGCAGCTTATGAACAGGGCATGAATCTACGATTCGGCACTGACTGGAAGCATGATCC
GTCACTGCTCATTAAAACCAAATTCATCGATGCCCCCCACGTTGAGATACCCGCATGAAA
ATCAGCCTGAAGTCGCTGATTGTGCCTGTTGTAATGCTGTTGCTAACGGCAACGGCATAC
ATCTATCACGGCAAATATCAGGATGAATTGGCGCGAGCGGAACCAGCCGAAAGCAACCTG
GTGCTGGCCAATCTAACAATAGCTGACATGCAGAAGCGTCAACGCGACGTAGCGAAGCAC
GATGCCAGATACACAAAGGAGCTTGCTGATGCTAACGCGACTATCGAAAGTCTCCGTGCT
GATGTATCTGCTGGTCGTAAGCGCCTGCAAGTCGCCGCCACCTGTGCAAAGTCAACGACC
GGAGCCGGCGGCATGGTCGATGGAGAAAGCCCAAGACTTACAGCAAATGCTGTACTCAAT
TATTACCGTCTCCGAAGTGGAATCGACAGCATAACCGCGCAGGTTAACTACCTGCAGGAG
TATGTAAGGACTCAGTGCCTGAAATGATTTGTCAACCAAAGCGGAAGCAAAAGCGAAGTA
GCCATAACAAAGCCCATCTACGGGTGGGCTTGATAATGAAACCGGAGTTAATTTCTGGTC
ACCAATTAACGGCAGTACCACGTAGCAACCCAAGCCAGTAAGTGGGGAAATAACACTGGC
AGCCACTGAAAGATGAACCTCCTGCCTTATGGCAAAAAAGATTCTTTGTGGTGTCGGACT

GATGGAAAGACATCGGTTATTGCAGGGGCTATTCAATGAGTGGTCTCGACAATGGCTTAT
CCCAACAACCGGAGCCAACACAATCGCAGAGATTACAGCATTGACACAATTACAGCAGAT
GAACCTCGATATCCTCCGTTTAGTTCAAAGCGACACCGCAGCAGCAGAGAAAGCGATCGC
ATTCGTTGCTGGAAGTAAGCTGAACTTCGAACTGTTCAAAGACCAACTGGTTTTGGCGCA
GGGTGTAGGAACGGCATTAGCTCGCGCAGAAAAGTCTATTCTTGAGGCAAAAGAAGCGTT
AGACCTGTTCACTGCCGGAGCATAACGAATGGCAAAGACGAAGTGGCCTAAACTTCCCCG
GTTCTTCGTGCCATTGTTCCATAGCGCCTATGTCTACCTGTGTCGTTCAAAGGAAGAGTG
GGATCAGGCTTGCATTCATCTTGGAGTTGGTAGCGGCGGGAATGAGATGCTGGCGGGGGC
AACACAGTCATATTGCAATACCGAAACAGGCGAGAATCTTTACCTGCTTGGTGTATTCAA
TGGTGAGGCGGCCACATTGGTTCATGAATGCGCTCACGTTGCATTTTATGTCTGCCGAGA
TGTTGGTGTAACCACTTATCCTGGCGACGCAAACGAAACCTACTGCTACATGCTTGACAG
AATGTTCAGTCACTTCCGGCCGTTCTTTCATGAACCATAAAAAGAAGGAGCCAAGTAATG
GCAAACCCAAACTTCACGCCAGCATGGCCTCTATACAAAGATGGTGACGGTGTATATGTG
TCTGCGCTTCCGATTAAAGCTATCAAATACGCTAATGACGGAAGTGCAAACGCAGAATTC
GACGGCCCGTAGGCTGACCAGTACATGTCAGCGCAAACAGTAGCCGTATTCAAGCCGGAG
GTTGGCGGATATCTGTTCCGGAGCCAGTACGGCGAGCTGCTCTATATGAGCAAGACAGCA
TTTGAAGCTAACTACACTTCTGCAAGCGGTTCAGTAGCTAATGCAGAGACGGCGGATAAG
TTATCTACTGCCCGCACTATCACACTAACCGGAGGGGTCACAGGTTCAGCGTCCTTTGAT
GGTTCGGCTAACGTGACTATCGAAACAACATCAGGAAGTTAACTTATGGCAGCACCAAAG
GGCAACCGATTCTGGGAGGCCCGCAGTAGCCATGGGCGTAACCCGAAATTCGAGTCGCCT
GAGGCGCTGTGGGCTGCTTGTTGTGAATGCTTCGAGTGGGTGGAGGCTAACCCACGATGG
GAGATGAAGGCTTTCTCATATCAAGGAGAAGTTACACAACAGCCTATTGCCAAGATGAGG
GCGATGACCATCACTGGGCTAACGCTATTCCTCGATGTGACGCTTGAGAGATGGCGACAA
TACAGGGTGAGAGAACACTTATCTGAGGTCGTTACGCGAGCAGAGCAAATCATCTACGAC
CAGAAATTCTGCGGCGCAGCCGCTGATCTTCTCAACGCTAACATCATCGCCCGCGATTTG
GGCCTCAAAGAGCAGTCGCAATTTGAAGACGTGACACCTGATAAGGGAGATCGCGATAAC
CGCCGCTCTCGTATCAAGGAGCTACTCAACCGTGGAACTGGACGCGATTCTTGATAACCT
GAGCGACGAAGAGCAAATCGAATTGCTCGAGCCACTCGAAGAAGAAGAGAACTACCGAAA
TACACACTTGCTATATGAGTTTACGCCATACAGCAAACAGCGTGAGTTGATCGACGCAGG
TCATGATTATCCAGAGCGATGTTTTAAGGCTGGTAACCAGCTTGGTCAGTCATTTACTGG
CGCTGCTGAAGTCGCGTTCCACCTTACCGGGCGATACCCGGGAACGAAAGGTTATCCGGC
TGATGGTAAATATGGCGGAGAGTGGAAAGGTAAGCGTTTCTATGAGCCAGTTGTCTTCTG
GGTTGGCGGTGAAACAAACGAGACTGTAACCAAAACGACTCAACGCATCCTGTGCGGGCG
TATCGAAGAGAATGATGAACCTGGCTATGGGTCAATCCCGAAAGAGGACATCATTAGCTG
GAAGAAGTCTCCGTTCTTCCCTAATCTTGTTGATCACCTTCTTGTTAAGCACCACACGCC
AGAAGGCGTCGAAGATGGCATCTCAATATGCTACTTTAAGCCTTACTCACAGGGCCGCGC
CCGCTGGCAGGGCGACACAATTCACGGCGTCTGGTTTGACGAAGAGCCGCCATATAGCAT
CTATGGCGAAGGTCTTACCCGTACAAAAAAATACGGGCAATTCTCAATTCTGACGTTTAC
CCCGCTGATGGAGATGTCTGACGTTGTTACGAAGTTCCTGAAGAATCCCAGTAAGTCGCA
GAAAGTGGTCAACATGACCATCTATGATGCTGAGCACTACACCGACGAGCAGAAAGAGCA
AATCATAGCATCCTATCCTGAGCATGAGAGAGAAGCACGTGCTCGTGGTAGTCCTACGAT
GGGTAGCGGTCGAATATTCCAGATACCGGAAGAGACGATTAAGTGCCAGCCGTTTGAGTG
TCCCGATCACTTCTATGTTATCGACGCTCAGGACTTCGGATGGAACCACCCGCAAGCTCA
CATCCAGCTTTGGTGGGACAAAGACGCAGATGTTTTCTATCTGGCGCGTGTGTGGAAGAA
ATCAGAGAACACCGCAGTTCAGGCATGGGGTGCTGTTAAGTCGTGGGCTAACAAAATACC
TGTCGCGTGGCCTCATGACGGTCACCAACACGAAAAGGGCGGTGGTGAGCAACTTAAAAC
CCAATATGCGGACGCCGGGTTCTCTATGCTTCCCGAACACGCAACGTTCCCGGATGGCGG
TAACTCAGTAGAGTCAGGCATTGGTGAACTTCGTGACCTGATGCTTGAAGGAAGATTCAA
AGTCTTCAACACATGCGAACCATTTTTTGAAGAGTTCCGTCTATATCATCGCGACCAGAA
CGGCAAGATTGTCAAGACCAACGATGATGTGCTCGATGCTACTCGCTACGGCTACATGAT
GCGCCGCTTCGCCAGGATGATGCGCGATATCAGAAAGCCGCGAGAAAAGAAAATCCCCGC
ACCGATTAGACCAGTACGCAGAGGACGATAATGGCCGACAATGAAAACAGGCTGGAGAGC
ATCCTGTCGCGCTTTGATGCGGACTGGACAGCCAGCGATGACGCCAGACGCGAGGCAAAG
AACGACCTGTTCTTTAGTCGGATCAGCCAATGGGATGACTGGCTATCACAATACACAACC
CTGCAGTATCGCGGGCAGTTCGATGTTGTACGACCAGTGGTGCGCAAACTCGTTTCTGAG
ATGCGTCAGAACCCTATTGATGTTCTGTATCGCCCAAAGGATGGAGCAAGTCCTGACGCC
GCTGATGTGCTTATGGGTATGTATCGTACAGACATGCGGCACAACACGGCAAAAATCGCG

GTCAATGTGGCTGATGGTGAGCAGATTGAATCTGGCGTAGGTGCGTGGGGTCTGGTCACT
GACTACGAAGGTCAAAGTCCAACGAGCAACAATCAGGTTATCCGTCGAGAGCCTATCCAT
AGTGCCTGCTCCCATGTTATCTGGGACAGTAACAGCAAGCTGATGGACAAGTCCGACTCC
CGTCACTGCACAGTTATCCACTCAATGAGCCAGAATGGTTGGGAGGATTTCGCTGAAAAA
TACGACCTCGATGCTGATGAAATTCCATCATTCCAGAACCCCAACGATTGGGTATTTCCA
TGGCTGACGCAGGACACAATTCAGATCGCTGAGTTTTACGAAGTGGTCGAGAAGAAAGAG
ACGGCGTTTATCTACCAAGACCCGGTTACGGGTGAGCCGGTAAGCTACTTTAAGCGCGAT
ATTAAAGACGTCATCTACGACCTGGCTGATAGTGGATTTATCAAAATTGCAGAGCGCCAG
TTTAAGCGTCGCCGGGTATACAAATCGATTATCACCTGCACCGCTATACTCAAAGACTAG
CAGCTCATTGCTGGCGAGCATATCCCAATTGTTCCGGTATTCGGAGAGTGGGGCTTCGTT
GAAGATAAAGAGGTGTATGAGGGCGTCGTCCGCCTGACAAAAGACGGTCAGCGTCTACGC
AACATAATTATGTCGTTCAACGCCGACATCGTGGCCCGCACCCCGAAGAAGAAGCCTTTG
TTCTGGCCTGAGCAGATTGCAGGGTTTGAGCATATGTATGACGGTAACGACGATTACCCG
TATTACCTGCTCAATCGCACGGATGAGCACAATGGAGAAACGCCAACTCAGCCGCTGGCA
TATTACGAAAACACTGAGGTACCGCAAGCCAACGCCTACATGCTGGAAGCAGCCACCGCA
GCAGTGAAAGAGGTAGCGACGCTCGGCGTTGATGCAGAAGCAGTAAACGGTGGACAGGTA
GCCTACGACACTGTTAACCAGCTAAACATGCGCGCTGACCTTGAGACATATGTGTTTCAG
GAGAATCTGGCTACCGCTATGCGCCGTGACGGTGAGATTTACCAGTCGATAGTTAATGAC
ATCTACGATGTTCCTCGCAACGTGACAATCACCCTTGAGGATGGCAGTCAAAAAGAGGTT
CAGCTAATGGGTGAGGTTGTTGACCTTGCCACTGGTGAACGGCAGTTACTGAACGATATC
AGGGGGCGCTATGAATGCTACACGGATGTTGGACCATCATTCCAGTCCATGAAGCAGCAA
AACCGCGCAGAAATTCTTGAGTCGCTCGGCAAGACGCCACAGGGAACGCCAGAATATCAA
CTGCTGCTGCTTCAGTACTTCACCCTGCTTGATGGTAAAGGTGTTGAGATGATGCGTGAC
TATGCCAACAAGCAGCTTATACAGATGGGCGTTAAGAAGCCAGAAACGCCTGAAGAGCAG
CAATGGTTAGTAGAGGCGCAACAAGCCAAAGAAGGTCAACAAGACCCGGCAATGGTTCAG
GCTCAGGGCGTACTCCTGCAGGGGTAGGCTGAACTGGCTAAAGCTCAGAACCCGACGCTG
TCCCTGCAAATCGATGCAGCTAAAGTCGAAGCGCAGAACCAGCTTAACGCGGTCAGAATC
GCACAAATCTTCAACAACATGGACCTCAGTAAACAATCTGAGTTTAGAGAGTTCCTTAAA
ACCGTTGCTTCATTCCAGCAGGACCGCAGCGAAGACGCTCGCGCAAATGCTGAGTTACTC
CTTAATGTCAATGAACAGACGCACAAGCAGCGAATGGACATTGCCAACATCCTGCAATCG
CAGAGACAAAATCAACCTTCCGGCAGTGTAGCCGAGACACCTCAATAAGAGAGAGTTAAT
CATGGGACCAACCACCGAAATTCAGGCAACTGAAGACTTAACCCTGTCCGGCGATCATGC
AGCGGCATCTGCTGATAGCTTAGTTGTCGATAATGCCAACGACAATGCAGGTCAGGAAGA
GGGCTTTGAGATTGTCCTGAAGTACGATGAGAAAGCACCAAAACAAGACCCGGCAAAGAA
CGCAGAATTCGCCCGCCGCCGCATCGAGCGCAAACGACAGCGCGAGCTTGAGCAGCAGAT
GGAAGCAGTTAAACGCGGAGAATTGCCGGAGAGTTTACGAGTAAACCCTGACCTTCCACC
TCAGCCGGATATTAATGCCTATCTGTCAGAAGAAGGCCTGGCCAAATATGACTATGACAA
CAGCCGTGCGCTTGCCGCTTTCAATGCTGCTCATACCGAATGGCTAATGAAAGCGCAGGA
CGACCGCAGCAATGCCGTAGCAGAACAGGGCCGCAAGACTCAGGAGTTTACCCAGAAATC
AGCGCAATACGTCGAAGCTGCACGCAAACACTATGACGCGGCGGAAAAGCTCAATATCCC
TGACTATCAGGAGAAAGGAGATGCATTTATGCATCTGGTTCCGCCTACGGTTGGGGCCGA
CATTATGCGCCTGTTCCCGGAGAAGTCCGCCGCGCTCATGTATCACCTGGGTGCAAACCC
GGAGAAAGCCCGCCAGTTACTGGCGATGGATGGGCAGTCCGCGCTGAATGAACTCACTCG
ACTATCCGAACGCTTAACTCTCAAGCCTCGCGGTAAACAAATCTCTTCCGCTCCCCCAGC
TGACCAGCCGATTACCGGTGATGTCAGCGCAGCAAATAAAGATGCCATTCGTAAACAGAT
GGACGCAGCTGCGAGCAAGGCAGATGTGGAAACCTACCGCAAGCTAAAGGCAAAACTTAA
AGGAATCCGATAATGGCTTTGAACGAAGGTCAAATTGTTACACTGGCGGTGGATGAGATT
ATTGAAACCATCTCCGCAATCACTCCAATGGCGCAGAAAGCCAAGAAATACACCCCGCCT
GCTGCTTCCATGCAGCGCTCCAGCAATACCATCTGGATGCCTGTAGAGCAGGAGTCCCCC
ACTCAGGAGGGTTGGGATTTAACTGATAAAGCGACAGGGTTACTGGAGCTTAACGTCGCG
GTAAACATGGGAGAGCCAGATAACGACTTCTTCCAGTTACGCGCCGATGATTTGCGTGAT
GAGACAGCGTATCCTCACCGAATCCAGTCCGCAGCCCGCAAACTGGCTAACAACGTTGAG
CTGAAAGTCGCAAACATGGCCGCCGAGATGGGGTCATTGGTTATCACTTCGCCGGACGCT
ATCGGCACTAACACCGCACACGCATGGAACTTTGTGGCCGATGCAGAAGAACTGATGTTC
TCCCGGGAACTTAACCGCGACATGGGGACATCGTACTTCTTCAACCCACAGGACTACAAA
AAGGCGGGTTATTACCTGACTAAACGCGATATCTTCGGGCGCCTTCCTGTAGAAGCGTAC
CGCGATGGCACTATCCAGCGTCAGGTTGCTGGCTTCGATGATGTCCTGCGCTCTCCGAAA

CTTCCTGTGCTGACCAAATCTACTGCAACTGGCATCACTTTATCCGGTGCGCAGTCCTTC
AAGCCTGTCGCATGGCTACTGGATAACGATGGCATCAAAGTTAACGTTGATAACCGTATT
ACTACCGTCACCCTGTCTGCAACTACCGGCCTGAAACGAGGCGACAAAATTTCGTTTACT
GGCGTGAAGTTCCTTGGTCAGATGGCTAAGAACCTACTGGCGCAGGACGCAACTTTCTCC
GTAGTTCGCGTTGTTGATGGTACTCACGTTGAAATAACGCCGAAGCCTGTAGCACTGGAT
GATGTTTCTCTTTCTCCTGAGCAACGCGCCTACGCCAACGTTAACACCTCACTGGCTGAT
GCAATGGCCGTGAACATCCTGAACGTTAAGGATGCCCGTACTAACGTGTTCTGGGCTGAT
TACGCCATCCGTATTGTGTCTCAGCCGATTCCTGCTAACCACGAATTGTTTGCAGGTATG
TAAACTACCTCATTCAGCATCCCGGATGTCGGCCTTAACGGTATCTTCGCTACACAGGGT
GATATTTCCACCCTGTCCGGCCTGTGCCGTATTGCGCTGTGGTACGGCGTAAACGCGACA
CGACCGGAAACAATCGGTGTTGGTCTGCCTGGTCAGACTGCGTAACTAACAGGGGCTTCG
GCCCCTTTCTTATTTGAGGTGACATATGGGCGTAATGCTATATAAGCAGGGTCGTGGAAC
GAAGGTATGGGGCAAGGAAGTTCAGGCTAAAGTTGTCGACGACAGCGATGTCGAAGATCA
CCTTGACGATGGTTGGGTTAAGCACCCAAATGAGGGGCCGGAGACTAATGACGAACCAAT
CGGCGAGTCAGGAGTGGCCAAGAAAGACATGGGTGAAGTATCTGATGGATACCACACCTT
TAACGAACTCTATGCACATCGCGTGCGCCTGTTTTCAACACTAATGAATGCCTTCCCAGA
AAGCGCATGGTGGAGCTTCCAGCATCATGACGGCCAGCAATGGGATGGATGGGTGTTAGC
TGGCATCGACACCCCAAAAGGCGCGGTAACATACCACCCCCCAGAGAGTGAAATTGAACA
TCTGCCTAAAAGCACAGAAATTGAGTTTGGCAAGGAATGGGACGGGCACACAGCAGATGA
TGTGTTGAATCGTCTGCTAAGCCTGCGACCGAAAGAACCGGCAACCAAAGAACGCAAAAA
GCCAGGACCAAAGCCTAAGGCGGAAAGCGATGCAGATAAAGACTAAAGGCGATCTGGCCA
GGGTGGCGCTGCGTTAGCTTGGTGTAGCATCAGATGCAACTCTCACTGATATCGAACCTC
AGTCTATGCAGGATGCTGTTGACGACCTTGAAGCGATGATGGCTGAGTGGTATCAGGACG
GAAAGGAAATCATCACCGGATATATATTCTCAGATGATGATAATCCTCCCGCTGAAGGTG
ATGATCACCGTCTTCGCTCAAGCGCAGTCAGCGCAGTATTCCACAATCTGGCCTGCAGAA
TTGCTCCGGATTATGCGCTTGAGGCTACCGCCAAAATTATCGCAACCGCTAAATATGGGA
AGGAACTTCTCTATAAGCAGACCGCCATCGCCAGAGCTAAACGAGCGCCTTACCCGTCAC
GTATGCCAACTGGCAGTGGAAACAGATTCGCCAATCTGAACGAATGGCATTATTTCCCCC
GAGAGCAGAATGCCGATTCAACAACTCCCCATGATGAAGGGAATCGGTAAGGACTTCAAG
AATGCCGACTAAATTGATTACCTACCAATCAACATGTTGGCCACACCAAAATAAGTCCTT
GACTCATCGGGTTATTTACGCTCATTCCCGGGCATAGCGAAGCGCAACGATGTAAATGGA
GTATCGCGCGGAGATGAGTATAACACCGCTCAGAACGCTGTATATCGTGTTTGTGGCGGC
AAGCTCTACAAAGGTGAAGCCGTAGTCGGTGATGTTGCCGGAAGTGGTCGCGTATCAATG
GCACATGATCGCACATCACAGGCGGTAGGCGTTAATGGTCAGCTCATCGAGTATCGCTAT
GATGGCGCGGTTAAAACCGTCTCAAACTGGCCTGCAGACAGCGGATTCACGCAGTATGAG
TTAGGCTCAGTCCGTGACATTACTCGCTTACGTGGGCGTTATGCATGGTCAAAAGACGGT
ACAGATTCATGGTTTATCACTGACCTTGAAGATGATTCGCATCCTGACCGCTACAGTGCA
GAATATCGCGCAGAATCGCAGCCTGACGGGATAATTGGCATAGGTTCATGGCGAGATTTC
ATCGTCTGCTTTGGCTCGTCGACGATAGAGTATTTCTCCCTGACAGGCGCATCCACCGTT
GGCGCTGCGTTGGATGTCGCGCAGCCATCATTAATGGTACAGAAGGGGATTGCCGGAACA
TACTGTAAAACGCCATTCGCTGATTCATATGCATTCATCAGTCACCCGGCTACTGGCGCC
CCTTCCGTCTACATCATCGGGTCAGGGCAGGCTTCACCAATTGCGACGGCCAGTATTGAG
AAAATTATCCGCTCATACACGGCTGATGAACTGGCAACCGGGGTGATGGAGGCGTTGAGG
TTCGATTCGCATGAACTGCTGATTATCCATCTCCCGCGTCATTTGCTGGTTTACGATGCC
TCATCAAGCCAGAACGGGCCGCAATGGTGCGTACTGAAAACCGGTTTATACGACGATGTT
TATCGCGCCATCGATTTCATGTACGAAGGCAACCAGATTGCGTGTGGCGACAAGTCAGAA
GCGGTGACAGGGCAGTTGCAATTCGACATTAGTAGTCAGTACGACAAGCAGCAAGAACAT
CTGCTGTTTACTCCCATCTTCAAGGCTGATCACGCCAGATGCTTCGATCTGGAAGTTGAA
TCCTCGACAGGCGTTGCGCAGTACGCTGACCGCCTGTTCCTGTCTGCGACCACAGACGGA
ATCAATTACGGCCGCGAACAAATGATTGAGCAGAATGAGCCGTTTGTGTGCGACAAGAGA
GCTTTATGGAAGCGTGTAGGTCGTATTCGTCGATTAATCGCATTCAAACTGCGTGTAATC
ACCAAATCACCAGTAACACTATCCGGGTGTCAAATGCGTCTGGAGTAACATATCGCAGAC
CCGTCACTTAATAAGCCTGTCGTGGTTCAGGCTACACGCATGGATGCATCTATTCTCCCT
CGCAACATATTCAGTCAGTTTTACCTTCTGTATGTCATAAATCAGGGTAATGATGTTGGC
TCCATTGCAGAAAAGGCAAATCAGGCAGGAGGCGGTGCTTATGATGCGCAGGTCAGAAAT
GATGAGCAGGATTTAATTCTTGATGAGCACGAAAAAAGAATTGCAAAAACTGAAGAGGAT
ATTTCAGGAATAAAAGTAAAGCTTCTTGAAATAGAGAATGATGTTAATGGCCTGAAAATA

AAAGTTCAGGATTTCGAGGGTAAGGTATCAGAGATAATCGTCGATTATGTTTCACTCAGC
AGAACAGGAACTCAAACACTTTCCTCATCCCTTAGCGTATCAGGAAATCATTCTGTTAAC
GGTACAAAAGTTGTTGGCGCTCGCCAGACTGGATGGACCGCGGCAACAGGTACGGCGAAT
AAAGGCGTATTCAATGCTGACCTGACATTCACCGTTAGCGATACTTACACGCAATCTGAA
ATACAGGCTATAGCCAATGCTCTAATTGCTGAGCGTCGGCGCACTAAGGCTTTGGAAGAC
ACCTTGCGTGCACATGGGTTGTTTGATTAATGATTACATTTACTCCAACACGCAACATCG
ACCTGATAGAAACTGTCGGCAACCATCCTGACATTATTGCCGGGAGCAACAACGGTGACG
GGTACGACTCCAAGCCTGAGTGCCGCTATTTCGAAGTGAACGTACATGGTCAGTTGGGTG
GCATCGTGTATTACAACGAGATTCAGCCGCTGACCTTTGACTGCCACGCCATGTATCTGC
CTGAGATTAGAGGATTCAGTAAGGAAATCGGGCTGACGTGATGGCGATACATCCTCGCCA
ACACCACCGTTCAGTGCGTTACATCATTTGCTGCACGCAAATTTCGCCACGGTCAAATGT
ACTGCGCAATGATTGGCCTTAAGCGTGTAGGAACCATCAAGTAATACTTCAAAGGCGTGG
ATGACGTGACCTTTTACAGCGCCACACGCGAAGAACTAATCGACTTCCTGAATCACGGGA
GATAGCCATGTTATATGCATTGAAGCTGGGCAGGAAACTGCGCGGTGAGGAACCTCTTTA
CCCTGAAAAAGCCGGTAAAGGCGGCTCATCAAGCAGCGGAGCAAAAGAGGCCGCAAGAGC
AACACAGTACGCCGCAGACCTGCAAAACCAACAATTCAATCGCGTGATGGAACAGTTGGC
ACCTTACGCCGCCGCAGGTTTGCCGGCTCTCCAGCAGATTCAGCAGCTATCAACGCTGGA
GGGTCAGAACAGCGCTCTCAATCAGTATTACAACTCAGACCAGTATAAACAGTTGGATGA
TCAGGCTCGCTATCAAAGCCTGAATGCAGCGGAAGCCACCGGAGGTCTTGGCTCTACAGC
AACATCAAACCAAATTGCATCCATTGCACCAACGCTCGGGCAGAACTGGTTGTCAGGACA
GATGCAAAGCTATGGCGACCTGTTAAACGTTGGTCAGTCTGCGGCAGCAGGCCAGGCATC
GGCAGGACAGAACTATGCAAATAACGCAGGTAATCTTGCGCAACAGATGGCGGCGATCCG
CTCTCAGGGTTCTGGTCAATCAACGTTTGGAAGTGCCATTAGCGGTGGTACAAGTGGTGC
TCCTGCAGGAGCTGGTCTTGCCGGGATGCTCGGTGCATCGACGCCATGGGGTGCTGGTAT
TGGCGCAGGTATCGGATTGCTTGGCTCACTCTTCTAAGGAGTTATCGTGGCTACATTTCC
ACTTGCTGGTTTGCCATCAATGCAGGTGGCGAACCAGAACGCTCCCGGGCAGCCATCACT
ATCAAACTACGACTTTAGCCAGCGACCAAACGTTGGAGTTCAACTTGCTCAGGGTCTTGG
TGCAGTTGGTCAGGCAATGAGGCTTTCTGACTTTCAAAAACCTTTCGGTCAGGCTTATGC
GGCAGGTGACCGGGATGCCTTGCGTCAACTTGGGGCCACCAATCCAGGCCAGATTGAAAC
AATTCGTCAGGGCATTGGGTTTGTTGATGCTGATCGCAATCAGGCAATGGGAGATATGTC
TGCACGATTGAACATTGCCGCCGCTCAGGGGCCTGAAGCGGTGATGCGAGAGCTTGCCAC
GCACCACAATACACTGCAGCAAATTGGCGTATCTCCTGAACAGGCGTGGCAGACATATCA
ACAAAGCCCTGAAGGCTTCACGCAGTTAACAGACCTTATTGGAATGCACGCGGTAGGACC
AGCAAAGTATTTAGATATTCAGGACAAGTTGACAGGTCGCGAGATTGATCGAGGTCGACT
TGCTGAAACAATCCGCAGCAATAAAGCAGGGGAAGGACTTCAGGCTCGCGGGCAAAATAT
TACTATGCGCGGACAAGACATGTCAGCCTCTACAGCCCGCCGCGGTCAGGATTTGGCAAT
GCAAAGGGCAAACGCCAGAACGATCTCAGGAGTTGAGGGGAATCGGGTCGTTCAGCTTGC
AGATGGTAGAACAGTCAACATTGACGGAAAACTTCACGGCGCAGGGGCTAATGCATTTTA
CGAAGGTATTGACGATAACAGCAATATGGTTCGTGTCCCAGCAAGTGCTATTGCAGCGCC
TCCAACGTCTGCAGCAAGCGCACAGACCTACGCCATGAAGAAAGACATTGACGCAATCGC
AAATGCACATGCTTCTGCTCTCGATTTCATGACTGGCATGACTGGAGGAGCAGGAAATCC
GGCAATTGGTGCAGATGTTCGCAGCCGACTCACAGGCAAAGAACAACGACAGTTATATAA
CTCCGCACAACGTATTCAGGGAAGAATGCAGAATCAGGGCGTGGCAGCAGCAAGAGATAT
GGGTGCTAGCGGTATCAACACCATTGCAGAAGCAAAGATGTATTTTCAGGGTATGCCGCA
GGTTGACTACACAAGCCCGGAGGCTATGCAGCAGTCGATTCGTGAGATTCAGGAATACAC
CAACAATTATAACAAGCAGTACAACGTTATTGTTGGTAAATGGCAGTATCAGCAATCTCA
ACCTGTACAGGAATCACAGCCTGCATCCAACAGCAACTTTTCTTCACAATGGGGTGATTA
ATGGCTAAGGCATGGAAAGACGTTATTGCCTCTCAAAAGTACCAGGCATTAGCACCAGAG
CAGAAAGCACAGGCGCAGGAGCAATACTTCAATGTAGTAGTAGCACCGCAAGCCGGAAAC
GATGCCGAACAGTCTAAACAGGCTTTCTGTGCTGCTTATCCACCTCCAACGGCTCAACAA
CCAGCAAATCAACCACATGGACCGGCGCAGCCACAGCAACAAGGTGGCTTCATGTCTGAC
CTTAGCAATGGTGCTGCGGAGACGGGGCGTGGATTGCTTCAGGCTGGCGTTAATCTGGCA
AATATCCCGGCATCAATGGCTGATGCAGTCGCCAGCGCCGGGGCATGGGCTGGTCAGAAG
CTTGGCATTGGTGACGGAACTTATCAGCCATCGCCTCGCGTCACGGCACAAGGACTTGAG
CAGGACTTTGGCTTGCAACAAGGTGCGCTTACTCCACAGACGACAGAAGGCAAAATCTTC
TCTGAAGCACTGCCATATTTGACTCCTGTTGGGGCCGAGAGAATTGCAGCGCAGGCATCA
TCTATTGCCGGTCGAGTTGCTCAGTGTGCATCACGCTTGTTGGCGGAGAACGCTGTTGGT

TCATTGGCTGCAAACAGTGAGCGTGATGATCCAGGAGCACTGGCAACAGACTTAGGAACT
GGTGTTGCATTAGGCGGGGCAATGAATCAGTTAGGCCGTGCCGCTGGTGCTGCTTATCGT
GGGATTCGCGGGACGATCGCACCAGAAGCGCAGCAGGCTATTCAGTTCGCTAATGCTGCT
GATGTTCCCTTGCATACAACTGACGTTTTGCAGCCAAATTCCCGCGTCGGGCGCATGGCA
CAGACCACCGCTGAAAACATCCCATTTGCTGGAACAAGCACTATGCGAGCTAATCAGCAA
GAAGCGCGCAGCCAGTTGGTAGAGGAATTTGCATCACGGTCTGGTGAGTAAGATCCGTCA
ATTGTTATTGGCAGCCTGAAGGCAAAAACATCAGGAATTCGGAAAGCAGCAGGGAACCGT
CTTGAGCAAGTTCAGAGCGCAATGACAGGAGTCAACATTCAGCCAACGCGAGCAATTCAG
CAGATAGATGATGAGATTGGAAAACTGCAAAAATTAGGACAAGTTGCCGACACGGATACA
ATTAGCAAACTTCAGGCATACAGGAATGAATTGGCTAAAGGTGATGTTAACCTGGAACAG
TTAAGCAGACTGAGAACGCACTTTAGGATGGATGTCAGTGGAGAAAGGACACAAATGCCA
CCGCCAGCTGAGGCGGCAGTGCAGCGTGTATACAGGGCAATGACAGGAGACATTGATAAC
TCCATTGGCCAGAACCTTGGAAACGACACTCTGCGCAGATACAAGCAGGCCAATGCGGTA
TACGCAGATGAGGCTAGTAAGCTCCAGAATACCCGCTTGAAGAACGTTCTGATGAAAGGG
GATCTAACTCCTGACGTTGTCAACAACATGTTGTTCAGCAAGAATAAATCAGAAGTTCAG
AATCTGTACCGGTCAGTCGGTCAGGTGGACGCGCTCAGATGCGTAACGGCATCATCGGA
AAGGCTATGGAGAAATCAGGCGGTTCTCCGGATCAGTTCCTGCGCCAGGTTAATTTAATG
TCTACCCAGACGGGAATCGCTTTTAAAGGACGAGATGCTGCGTATCTGAAAGGACTGAAG
AACTATCTTGAGTCAACCAAGCGTGCTGGTCAGGCAGGAGTAACAACGCCTACAGGTCAG
CAAACTATACCGTTCATCCTAGGTATTGGAACGGTAACTACCCCTGCGCGGGTAGGTGTT
GGTGGCGGGTATGGTTTGCTGGCAAGAATGGATGAGAGTGAACCAGCACGTAATGCAATG
CTTCGCCTGGCTAATACTCCACGTGGTTCTACCGCATTCGAGAAAGCGTTAGCCGAAGTT
GAGCGGGCTGTTAACTCTGTTGCTCAAGGTGCTAAATCAGATGCATTAAGCGAATAGCAG
TCTACCAACTACGATGCCGAAGATAAGGAATGCAAAGTTCAATAAGTCTCTGTTCATAAA
TCATCGTAGGAACCAATAGAGATCATTCTTTGATCTATATATTATCTGAATCCCTTACTT
AATTGGGTGATGATAATGAAAAAAGGTGTGATGGTTGGCTGTTTTTGTGTATTTCTCGCT
GGGTGCGCCACAGCAACAAAAACGTATGCTCCAGATGGAAGAGAGGCATATACGATAGAA
TGCTCTGGAGTAGGGGGTTCATGGGCTATGTGTCAGGCCGAGGCCGGAGATCTTTGTGGT
TCCAAAGGCTATGACCTGTTTAGCACTGGTAGTGATCAGGGAGCTATTGCAAACATTGAC
GCAAGTACTGGCAACGCATTTGCAGCAAACACCATATCAAGAAGCATGTATATAGCTTGC
AAAAAATGAGTAAAGCCTGGTTCGCCGGGCTATTTTTTTCGATAGATATCTTTCAACTTT
TCGAATACTAATTCTTGAATTTCTCTGGAAACGATGTCTGCTTCGAGTTCAGCATTATAC
CGGTATCCGATTACAGGCGTAGGCTTTTCAAGTGAATCAGCCACTATCTGCACTAGCCCT
GCACTTAGTGACCTTCCGTTTGCCTTTGCCCTGTGCTTAACCTTTTCTTTCAGCTCGTAG
GGTAGCCTGAGGTTAAATTGCGGGTCATCTCTTCCCATTTCTGATGCCCCACTTTGGTAA
GTGGATCGGCATCATATGATCTACTGGTTATATCCACAATAAGACCACCGTGGTCTTAAT
GACGCACTGCCGTAGCCACGCTGCGGCGATTCCTTGCCTCTGGAGCACATTAAATGACAG
ATATCACTGCCAACGTAGTTGTTTCTAACCCTCGCCCAATCTTTACTGAATCCCGTTCGT
TTAAAGCTGTTGCTAATGGGAAAATTTACATTGGTCAGATTGATACCGATCCGGTTAATC
CTGCCAATCAGATACCCGTATACATTGAAAATGAGGATGGCTCTCACGTCCAGATTGCTC
ACCAGCTAATTATCAACGCAGCCGGTAAAATCGTATACAACGGCCAACTGGTGAAAATTG
TCACCGTTCAGGGTCATAGCATGGCAATCTATGATGCCAGTGGTTCTCAGGTTGACTATA
TTGCTAACCTATTGAAGTACGATCCAGATCAATATTCAATAGAAGCTGATAAAAAATTTA
AGTATTCAGTAAAATTATCAGATTATCCAACATTGCAGGATGCAGCATCTGCTGCGGTTG
ATGGCCTTCTTATCGATGTTGATTATCATTTTTATAATGGAGAGAAAGTTGAGTTTGGTG
GTAAGGTTCTGACTATAGAATGTAAAGCTAAGTTTATAGGAGATGGAAATCTTATTTTTA
CGAAATTAGGCAAAGGTGCCCGCATTGCCGGGGTTTTTATGGAAAGCACTACAACACCAT
GGGTTATCAAACCTTGGACGGATGACAATCAGTGGCCAACGGATGCCGCAGCGGTCGTTG
ACACTTTAAAACAATCGAAAACCGATGGGTATCAGCCAACCGTAAGCGATTACGTCAAAT
TCCCAGGAATAGAAACGTTACTCCCACCTAATGCAAAAGGGCAAAACATAACGTCTACGT
TAGAAATTAGAGAATGTATAGGGGTCGAAGTTCATCGGGCTAGCGGTCTAATGGCTGGTT
TTTTGTTTAGAGGGTGTCACTTCTGCAAGATGGTAGACGCCAATAATCCAAGCGGAGGTA
AAGATGGCATTATAACCTTCGAAAACCTTAGCGGCGATTGGGGTAAGGGTAACTATGTCA
TTGGCGTACGAACCAGCTATGGATCAGTAAGTAGCGCCCAATTTTTACGTGATAATGGCG
GCTTTGAACGTGATGGTGGAGTTATTGGGTTTACTTCACATCGCGTTGGGGAGAGTGGTG
TCAAACCTTGGCAAGGTACTGTGGGCTCGACAACCTCTCGCAACTATAATCTGCAATTCC
GCGACTCGGTCGTTATTTACCCCGTATGGACGGATTCGATTTAGGTGCTGACACTGACA

TGAATCCGGAGTTGGACAGGCCTGGGGACTACCCTATAACCCAATACCCACTGCATCAGT
TACCCCTAAATCACCTGATTGATAATCTTCTGGTTCGCGGGGCGTTAGGTGTAGGTTTTG
GTATGGATGGTAAGGGCATGTATGCGTCTAATATTACCGTAGAAGATTGCGCTGGCTCTG
GCGCGTACCTACTAACCCATGAATCAGTATTTACCAATATAGCCATAATTGATACCAATA
CTAAGCATTTCCCGGCTAGTCAGATATATATATCTGGGGCTTGCCGTGTGAACGGTTTAC
GTTTAATTGGGATCCGCTCAACCGATGGGCAGGGTCTAACCATAGACGCGCCTAACTCTA
CCGTAAGCGGTATCACCGGGATGGTAGACCCCTCTAGAATTAATGTTGCTAATTTGGCAG
AAGAAGGGTTAGGTAATATCCGCGCTAATAGTTTCGGGTATGATAGCGCAGCGAGTAAAC
TTCGGATTCATAAGTTATCAAAGACCTTAGATAGCGGAGCATTGTACTCCCACATTAACG
TGGGGCCCGGTTCTGGCTCAGCGTGGACTCAACTTACTGCTCTTTCAGGTAACACACTTG
AGGCTGTATCATTAAAAGTTAACCACAAAGATTGCAGGGGGGCAGAGAGACCATTTGTCT
CTGACATCGCGTCAGATGATTTTATAAAGGATTCCTCATGTTTTTTGCCATATTGGGAAA
ATAATTCTACTTCTTTAAAGGCTTTAGTGAAAAAACTCAATGGAGAATTAGTTAGATTAA
CCTTAGGAACACTTTAGATATGTAATAAAAATGGGTGTAAACACCCATTTTTATTTTAT
>NewGenomeName_21
ACCCCCGCCCCAGGTTCAGCGGAGTCATTGAGACTCCATCGCATCCGTTCATTGAATAGA
CACGTGTGCAATGAGGATCTGTAATTCATGACGCGCATCTGACGTAAGATTGTACACACT
AACGTTGGTGGCCGACAATGCTCTTCTGTACTTTTTAAAAGTAAAGAAAGATTCTTTTTA
TTATTATATTATATGTAATATATTTTATCATTGCTTCTTCATGAAGCACAGAACTTTATC
TCTCTATAAATACCAGTTCTGTTTTCGTTGTTCATACAACGAAAAATGGTGAGCAATTGG
AATTGGTCAGGCAAGAAGGGTAGAAGAACACCTCGTCGTGGTTACACCAGGCCTTTCAAG
TCACCTCTTCCTACAATCAGAGTCGTCGTTCATCAGTCAGATGTATTAAAGAAAGATGAC
GTGTCTAGTAGTTAAATTAAGCCTGAAGGTGATGTTGCGCGTTACAAAATTCGTAAGGTG
ATGTTGAGTTGGACCCTGAGGATGTGTCAGGGAGAGTTGGTGAATTATCTGATCATCAAG
TGTAGTTCACCTATCGTTAATTCGTCTGAAGCTTTCCCAGCACCTGCTCTAATGGTGAAA
GAGAGTTGACAAGATATGATTACTATCATATGTAAAGGTAAAGTTGAATCGAATGGCGTA
GCTTGATCAGATTGTACTAAATCATTCAATAAGTTTATCAGATTAGGAGCGGGTATTAGT
CTAACACAACATTTGTACGTTGTAATGTATACCAGTGAAGCTGTTAAGACTGTATTAGAA
CATAGAGTCTATATTGAAGTTTAATTCCAGTGTGTAACGAAGAACATATGGGTAATGAAT
AAATATTGCACTGGATCTATTTACTCCGCGTAGCGGTATGTTTTGTTGTCTTAGTTTCCA
TAGATACCCTACGTTAATGAACGGGTAATGTCTATTTTGCCCTTATGTGACGTCATTTGA
TCCCGTGCTGAGCTGGGGCGCGGCTTAGTATT
>NewGenomeName_22
TTGGCGGTCTGATTATCTTGGCTGAATTAAATACCAGAAAATAAATTAACCCTGGCTTCG
GTCAGGGCTTTTTCTTTACAAAAAGAACGGAAAGTTATATAATGTTCCTGTTCCAAAAAT
ACTTTTTTCATAAAGTTTATCCTAAGCGTCCCTCTCCTAACTGGGGCGCTTTTTTTATGC
TATAATATAGTCGGGATGTTTGTTAGATTTCATCCTATTCCTAGAGTCAAGCCATTCTTC
GGAGTGGCTTTTTTCATAGCTTCTTTTACTTCATCAGTTAGAGGTCTGCCATCAAATGAA
ACCCACTTGTCCCAATCAACTTTGCTATCATCTACTAAATCGGCAAGGTCAATACCTTGT
TTTTGTTGTTCAGTTTTAATAGAAACAATTTTAGATTTCGGCTCTTCTTGTTCTTTTAAT
TGAACATTTGCAGTATCTAAAACTATTTTTTGACTTGGCTCTTCCAATTTCGTCATAACT
GTAATAGTTTCTTCTATAGTTTCAGAATCATTTTCTGATTCTCGCGGTTCAATCATCGGA
AGGTCATAACCCATTAACCACGGCTCACTAACTCCCAAAGTTTGAGATAATAATATCAAT
TTTTGTTTATCGAGATTAGATTTTCCATTTATATAATTAGATAAATGAGTTTTTGGCATT
TTTTTACCCAATTTATCTTGAAAAGGTTTTGATTTTTCAAGTATATCAACCAGTCTTAAT
CCTCGGTCGTTCATTATTTTCTTTAATCTCATTTGAGAGTTTTCTTTTTTCATGAACTTA
TTATAAAAATATTTATAAAAAAGTTCTAACTTTTTATACTTTTTCCTTGACAATTATTTT
CCAAATGATATAATTAAATCATAAAGTTCAAAACATTTGAACTTTATATCAAACAAGCGA
ACGATCATGGAGCATTCAGTACGGCAGACGGAACAGGCTCAAATCAAGGTACACGACGTA
TCCACCGCGACGTAAGTAGCAAGTTTGGCAAATAAAAAGCCCCAGAGGGGCAGAAAGGAT
GTATAATGACTATTGATTATTCTAAATTGAAAGGTCGCATTAAAGAAAAGTATGGTAGTC
AGCAAGATGTTGCAAAGGCTATAGGTTTATCAGAAAAAATTATTTCCGATAAGCGTAATA
ATAAATCATACTGGAAACAATCAGATATCGATGCCGCTACAGAACTTCTTGGTATTGAAA
AAGAAGATATTGGTATTTACTTTTTTAATAAAAAAATCCAAAAAATTTGAACTTTATAAA
AAGAAAGGATTCGAAAATGAATCAATTAATTACAATCACACAAAACGAAAACAACGAACA
AGTAGTAAGCGGTCGTGAACTACATCAATTTTTAGGAGTAAAAACTCGATATAATGACTG
GTTTGAAGATATGGTCAAGTACGGGTTTACAGAGAACGTTGATTTTATAGGTTTTACTGA

AAAAAGAGTAAAAGCTCGAGGCGGTCGTCCAAGCGTTGACCACGCTCTTAAACTTGACAT
GGCAAAAGAAATTTCTATGATTCAACGTAACGAAAAAGGAAAACAAGCTCGTCAATATTT
CATTGAAGTTGAAAAAGAACCCAAACAACAGCTTTTACCGCAAACTCCTGAACAACAAAT
TGCATTACTCGCTCAAGGTAACGTGAACTTGAATAAAAAAGTTGAGCAAATCGAAAATTC
AGTTCTTGATTTGACTGACGGATTCGGGCTTCCTTCAAATAAAGCTAACGTTTTGCAAAA
GAAAGTAGCAAGCAAAGTTTATATGTTTACTGGCGTTAAGTATTCAAATGCTCATCAGAA
ATTAGGAGCTAAGGTATTCAGAGAGTTCTATAAAGATTTGAACAATCGCTTCGATGTTGT
TAAATATAGCGATATTCCACTAAGCCGTTATGACGAAGCAACAGAATATCTTGACATGTG
GCAACCATCTTTCGATACAACGCTTGAAATTCGTGGATTGAACTCACAAACTAACTTTGA
CTTTGAAGAATAGAGAGGAAAACACATGGAACAAACACTTGAAGTACAAGCAACTATTTC
AGTTTTAATTCCAGAAGAAAAGATTCTTGTAGATAAAGTTGAATATCAAGAGCTTAAAGA
AAAAGACTTTGACGGTTGGGTTGGTATGGATGTATTCATAAAAAAATCAAACCGTAGTAT
TCCAACAGTTTCCAAGGTTTTAACAAAACCTGATTTAAGAAAAAGAATATCAGTTGAAAA
CGGAGGTTGGGTATATTCCCCAACTGGTAAAGGAGATAACTGGTCGTTTAGGTTTAAAGA
AATGATGGATTTTATAAACAAAGAATTTTATCAAAGTTTTCAGGAGGGAGTAAATAAAA
TGTTCGGATTTAAAACAAAAGAAGAAAGTGCGATTCTTGCAGACCATAATAATACGGTTA
GAGATATTAAAGAGATGATGGCATTGATTGTCCAAATGTCAGCTACAATCGCAACACAAG
CTCAAATGATTGGTACAAGAGATCAATTGGTCGATGAAGCATATTTAAAACTTGAATCAG
CCGAAACAGAATTAATTATTCGGCGCAAAAATGATGAATATCGTCAAAAGTTGGCGGTGA
TGAAATTAAAAATCAAGAAAAAACAATTAATCATCTTGGTCAAGTAGTTTATCAAGAGTC
AGTCGAATTTTATAAAGAAAAACTTTCTGTTTACTCAAAAGATTTTCTTAAAAATTCGCT
CCTCCCACAGCTTTATGAATGGTCAAATGCTTATAAAGCAGCGGTTGAACTGACAAAATA
AAAAAGCCCGCACTGACTTGCGGACTCGGTAGAAAATTTACGAGAATTTCTACCTCTAGT
ATAACAACTTGGAGGAAAAATGACAATTAATCAAGGGGGGGATATGGAAATGAAATCAAT
TCCAGACTATCCTAACTATGCTGTGACTCGAGATGGGAGAGTTTGGTCCTATATAAGTAA
TAAATTTATAAGACCAAGGGTATCTAAAGAGAATTCCGTCATAGTAAATTTGAATTTTGA
AGGAATAAGGTTCAGAAGGAATGTTGCTAGGCTAGTATTTATAGTTTTAAAAGGATATGA
GCCTGAAATGGTACGTCATATTGGTGGTAATCCTCTTAATAATAATATTGATAGCCTCAA
AGCAATGATTAGAAATGAACACTTGAAAAGTTTAGGTAACCCTAGCAATTTCAAAAATTA
TAATCGTAACCTTAAAATGATTAAAGTTGATGTTGAATCAGAAAGTAAGCAGGTTGTTGC
ATACCCATACAGGTCAACTGAATATGATTCAGCTTTACTATGTTGCAATTTAAAAAGATT
CACTTACAAAGGAGCTTTGTACTTTTGGGAGAATCGGAAAAATGAATTAATTGAGGAATT
GAAAGTTAGAATTATGCTAAATAATTTACGTTTGAATTCTGATATAAGGGAATTTCAGCA
GATTGGAATGTATAAAAGAAATATAAAAAAACAACAAAAATATCTTGAAATACTAGAAAA
AATTTAGAGGAGAAAAAATAAATGCTTGACAATTGCCTATGGGTTCTAATGGCAGAACGT
CAAATAAAAATTTCTAGTATTAGTCATGATACGGGTATATCTAGGACAACACTAACTAGC
CTAAAATATCGTCGAGAAAGCAGAATTGACTTTAAAACCTTAGATATACTATGCCAATAC
TTGCGTATTACTCCAGGAGAATTTTTTGAATATAAACCAGAAAAGAACGGATTTTAAATA
ATGGCAGAAGAAAAGCCAAGATTTAGTTTTTCTGATATTAGAACTTTTCAGGAATGTCCT
GTTAGATTAAGGGAAAGAAAAGCAAAACGATATAAGGAATCTCCTACAGAAGCCATGCTA
GTTGGCTCTTATGCTCATGCAATGCTTGAGGGAGATAAAAGCACCGATGATTTTATCCAA
GAGCATTATACGGATACGATGGGCAATATTGGTAAGAAAAACCAAGGCATTAAAAAAGTT
TTTAAAGATATTGTGATCGCGGTTAATGAAGTCAAAAATACTAAAACTTATCAGTCTTTC
GATACTTTACATACTCATAAAGAACGTTATATCAGAGCAGATTATGATGATTTTGTGATT
AGTGGAAGAATTGATGTCTTAAGGTTTGACCACGAAAATAAGATCAGTGATATAATTGAC
TGGAAAACTGCCGCAAGCTTTGAAGATATATTCGATAAAAATATCCGAGCTGATTTGGAA
TGGTACTTCCATTATAGAGAGCAATTAGCTTTATACGCATGGTTAGTTGCTCAAGAATTC
GGTGATTATACAAAACTAGATTATAAAGTGGTAGGTAAAATCGTAGGTTTTACCAAGAAA
CTTCCAGTAAACATTAAGACAATTACAATGGATTTTGGAAAACTTGCTGATATTTCAGAT
AAAATTCTTGTTCAAACTGGGTTATCTGAGTTAGATAATATCGCTCATAATATTGAGCAT
GAGGGAATGGATGGATACTTTTGTCATAATTGCGACTGATGTATCCAAAATAAAAAATAC
GAAGAATGAGAGGTAGAAGTATGTTAATGCAAATTAAACCTGCAGGGACTAGAAGCCCTA
AACTGACACGAGTCCTTATTTCAGGAGGCGGGCTTTCAGGAAAAACGACACTAGCGGCGA
AGTTTGCCAGTAGTAATGATAGAGCCTTATTTATCACTACTGACGGGAATGCATCCAAAC
AAGGGTATCAAGCGATAGATTTTGAGTTTTCACAAAAGGCCGAACAAATCATCACAAACT
TTACTCAAGCATTAAATATGGCAGAACAAAATGCTGAAAGCTGGGATGTCCTTGTAATTG
ATTTGATTGAAGACTTTGACGAACGGGCCCAAACCTTATTGCGTGGCGCACTTAATGATT

file:///E|/Curro/Simulación/paper/submission/Additional%20file%204.txt[27/06/2014 19:27:32]

TTAAATCGACAATGAAAGCATGGGGTAAAATTAACAGTTTGTATAAGGATATGCAAAGTT
TAATGATGAGCAAGTTCCATGAGAAGACAATTGTTTGCTTAGTCGAGATGTTGAAGAAA
TTGGCCAAAAATCAGGCGAAGTCATTGGATATAGGCCTGCTTTAAGAAAATCTCTCAAAA
ATATCATTCTAAAAGACCAAGATGTTAAAATCAGAGCTTATTTTGGTAGAAGCGGTAATC
GTCAATTTGATATTTCTAATTTAAGGTATGAAGAAATGAAAGGGATGCTTCAACAGATTA
TTGCTAAACCGTTTGAGATTCCTCAACCTGCCATTGACCCAAAAGAGCAACAAGAATCAA
AGGAAAAAGCTAATAAATTAAAAGCACAATATGATAAAGCTTTTGCGGCTGCAGCATCTC
ATAAAGCAAGTGATAAAGATATTGAGTATTGGAAAAATATGGAACCGTCAGAAGCGATTA
TGTCTATTGCAGATTGGATTCGAATTAAAGAATCTGCTCAGTCAGGCGTTGATGAAGAAG
AACTAATTGTGGATGAATTATTTCCAGTAGGTCAAAACTAAACCTATGAGCAAACCGCAG
TCCTCACTAATCCTGTGCAGTAGAATTAGAAATAATTCAACTTTAGCAAAGCCACCTTGG
GCGGTCGTTTCGTATCTAGGCAAAGCTGGAGGGTGGCGTAACGTCCGTAAAATCCATGAG
TATTCAGTGCCTGCACATAAACACTCATTGCCAGCTTTTAATTTGAAAAATAAAACTTGA
AATAAATACTCCATAGAAAGGGGAAGTGTGGCAAAAACAAGCAAAAAGAAAATATATTTC
TGGTTAAAACTGGATGAAAACTTTTTCAAAAATATATTAACAAAAAAAGCAGGAGAATCT
GCTGGTGGAGATACAATGGTGCTCATGTATCAGCGCATGATGCTGCAGTCGTTAAGTAGT
GAAGGTGTTCTTTATTATGAAGGAGCACTTGATAATCTTTCTGAAGATATATCGCTTAGC
TTGGATGAAGATGTCGAAAAAATATTAATGACTTTAGCTTTCCTTTCAAAATATGGATTG
ATACAAATTGATGAAGAACAAAATGCCGAAATGTTACAAGTTCATGCGATTGTAGACCAA
GAAACAGACTGGGCTAGATATAAGAGAAATCAAAGATCCAGCCAAAAATTGGACAATGTC
CAATCAGTGTCCAATACTTGTCCAACAGAGACAGAGTTAGAGACAGAGTTAGAGACAGAG
TTAGAGAAAGAGAAAGAGAAAGAGAAAGATATAGAGTTAAAGAAAGATATAGCGATTAGA
GATATAGATTCAAACTCTTTACTTTCCAAATTTCTAGATACTCTCATTAATTTTTCAAGT
AAAAACCGTTCAAAACGTGATGTAGCAACTGCTGAGTTTATAAAACTCCCTTCTTTTCAA
AGAGAACAGGCAGTTATTGGGGCCATGAACTACATACAGTCTTATAAAAACTAGCATCCT
GAGGATGAAACTGCTCGATATAGTGTAAATGCAGCTAATTTCTTGTCCAACATGATGTTC
ATGGATTATCAAGAAAAAGTGAAAGCAGATACTGGATATGATGAGGATTTGGGCTTTACG
AAGGAATACATGCAGTCAATGGCAGATGGAATCCGAGAGTTCCATAAAATGAGAGAAGTT
AAAACAGGTCTATTTTGTGAAGAACATCAACAAACCGAACTGGTTAGATATAAACATCCA
GTAAGTATAGGTTATGATCGGTTGATTCATGGCGAGTTAGTTGATGGTTTGCAGGTAACC
TCAATGAGTTATTGTGCAGAATGTGCTAAAGAGGGAATATACCAACATGACCAACAATCA
AGTGCTTAAATAAAAGCAAATGAAGATTTCTTGAATAAAAGTAAGTATGGAAAATATAGC
TTGCTTAAAACACAAAGCCTTGTAGGCAAGAAATCGCTCTGGTTTGCACGTTTCAACACT
TTTAAGGCAAATAGCTCAGAAGAGCAAAACTTGTTAAATCAAGCCCAAAGAATAGCCAGA
GAGTACACTCAAGGTCAAATGTTTAACAGTGTATTCGCTGGTGGAGCTGGTAAAGGGAAA
TCTCACTTAGCAATGGCTATTTTGCAAGAAGTAAATGAAAATCTTAAAGATAATAAATTC
TCAACATTGTTTATCAATATTAGTGAATTAATTCGAGAAATTAAAAATAGTTGGAATTAC
TCTGTTACCAAACCAGAAGAAGAACGACTGACAACATTAATGCGAACAGTTGATTTGCTT
GTTATTGACGATTTGGGAACTGAAAGCACGTTTTCTAAAGATAATAGCTGGGTATAAGGT
GTTATTTACAATATTTACAATGCAAGAGAAGACAATACGATTATCACATCAAATGTAACT
GGTAAAGAAATGCGTTCATCTTATGACGATCTAATAGTTTCTCGAATCATGGAAGCCTCA
AAAAATAGTGTTGGTAAATTTGAGGGCATTACTGATAAGAGGAAAAGTAAATGAAAACAA
TAATCATTGAGCAGTGGGAAAACGAGCATTACCCACTCGGAAGAATTAAAAAGCAGAAGC
TGGCTGAGAAATCTGAGCATGAGATTATTTTTATTCTTAAGCGCATTGCTCAGATGCCTG
CAATTGCTAGATTTGGGGAAGCAAGTGAACTTTGTTGATTTATTTGCCGGGATTGGTGGA
TTTAGACTTGGCCTTGAGCAAGCCGGACATGAATGTGTAGGGTTTTGCGAAATCGATAAA
TTTGCCCGGCAGAGTTATAAAGCCATTCACAATACAGAAGGAGAACGAGAATATCATGAC
ATTACAACAGTCAGCAATGAAGAGTGGCGAACCTTACGCGGAACAGTTTAGCTTATTTGC
GGAGGATTCCCTTGCCAAGCTTTCTCCATTGCAGGTAAAAGAAAAGGATTCCTTGATGAA
ACTCGTGGAACGTTATTCTTCGAGATTGCCAGAGCGGCTGAACAAATCAAACCACGGACT
TTATTCCTTGAAAACGTTAGAGGGCTTTTATCTCCAGACAAAGGGAGAACTTTTAGAACT
ATCATATCCACCCTTGATGAATTGGGGTACGATGCAGAATGGCAGATACTTGACAGCAAA
TATTTCGGTGTTCCACAAAACCGAGAGAGGGTGTTCATTATCAGACATCTTAGAGAAGAA
CGTGGACGAGAAGTATTACCTATCACCAGAGAAAGCAAATGAAATGGAAAGATTTATACA
AAACACTTGAAGAATTGACCAAAGATAAACAAATAGACCTGTAATAGCTCTAATTCATGA
AAATTACGGTTACATTGAGCGCTTAAACCATTTCATGGACAATTGATCACGAACAAGCTA
AAAGCGCTTAGAAGCTAAAATATGAGGTAGTAATATGTTCAGCAAAAATGAAATAAGGCG

TGGAGATAAGATATGCTTCCGCGACACAAAATTCTTAAAAGTTATCGAAGTTACTGACAA
ATACATAACGGTTGAAAAAGACCAGTTCACTAAAAAATCGGTTAAGCGTGAGGTTTTTAG
AATTGTGAAAATCAATGAAAGATACCATGCATGTGAACTCTTTGACACAGTTGTGAAGTG
AGGGATGAGATGAAGTGTAAAAAATGCGGTAACAAAATAGATTGTGATTGTATGGGATGC
CATGAGTGCCAACCAGAATATACTTGTGAAACGTGCGGAATTTGCCATATTGACGGTTGG
GAAGCTGGGGCATGTTGGAGTTTAGCCAATGACCCTGATTATGACCGATTTGACATTTAA
AGGAGATATGAAATGACAAATTTGTATGATGCAACAGTCACGATTATAGAAAGCCACGAT
AAAAAAATTGCCGATATTGAATATATTGGTAGTTCAGAGACAAAAATTAATACAAGTAAA
GCGCTCGAACTGATGAAAAAGACAAAGTATTATAGCGGTTATGGCGGTAAAGAAATAGCA
GAAAACCTAATGATTAAAGGGAATGGTTTCATCATGACACGAGGGGAATATGATGGCTCT
GAATGGTGGGATTATATGCAAACAGACCCGTCTTTACCGCAAGTAGAGAGAGATGTTAAA
AGTTTTAAAGCAAACATAGGCTGGGACAGCTCAGCGGGAATTAATAGCTTGGAGGACACG
AAAAATGACTAAGTTTGAAGAAGAATTTAACTACTTGATTGAATTATCAGGAAAACTATT
ACTTGGACAAGTAGAAGCTGAAACATTTAAAAAAAACAGAATTGCATTTTTTTAAAAGTA
TGAAACAGACCAACAGCAAGACCTACCAGTCCTGCCTGAAGATGTTGATAAAGCGATCAA
ATACTTGAAAACTCAGAATAATTTTTCCACACTTACTGATTTGAAGAATCTTGATATTTT
GACAGAAAAAGGCTTTTGGTGGCTGAATGATTTCCAATTTTAAGATAGACGATTTGGTTT
TGGAGGTCTAAATAAGAAGTTATTTATCCTTTCTCATTTAGCTATTACAGGCTATCAAGT
AGAAAAACCGCAGCTGTTCTATATTGACTTACCAAAATATTTTGGATTAAGCGATTCAAC
TAGCGATTCAACCTTCGTATCAAAAGCGGAAAGTGGAATAATCCTAGAATTTACAAACGG
GAAAGATTATGCATTAAAATTAAAAGAACAAGAAATCAAGTCAATTGATGAGCGTTACTG
GCAGTTTGCTGTGCCTGTGGAGGTGCAAGAATGAGCGAGAAAAAATATTATGTGTTTCTT
AGTCCGTTAAATAATGGGAACAAGCCATTTTTTCAACTAGTAAGTTTTCGGTTTATGGCA
GAATTATTTGGTTTTGCTAAATGTAATACCAAAAATAAAAACGGTAGATATGAGCACAAA
TACTCTAAATTCACAAAATCAGAACGTGCAGAAATCATGGGCGGTGCTTTGTATAAACAA
ACAGATTCTTTACCATTTGAATGGCTTTATTCGTTTGAATCGTTAAAAGAAAAGCTTGGG
TGGGGATTTCACGAAAATATTGAAAAATGGGAGTATTATAATCCAATAATTGAGCTTGTG
CCTGTGGAGGACCGAGAATGACAATTACTGAGCAACAATTCTATGACATGCTCAATGTTG
ATGAACATATGAATTTCACAAATCGAATTCGAGAACTTGTTTTTGATAAAAAAGGACGTG
AAGAATTTTACTCTAAAATCTTAAATATCCACCATGACATGAGTGTTGATTTTTTCAGAG
ATTATTTTATGACTCACTCGGCTGTTTCGTCAAAAGGGCAGCATTATACACCAGATGCAA
TTGGTAAACTCACAGCATTGCTTGTAGGTGGTTCTGGAGGGGCTGATTTAACTGGAGCAG
GAACAGGAACTCTAATTATTCAAAAATGGCAAGATGACCGATTGAATGCAGACTTTTTTA
ACTATTTGCCGAGTAACTATTGGTACCAGGCATTAGAATTATCGGATGAAGCTATTTCAT
TCTTGATTCATGCTTTTGGAATTAGAGGAATGAATGGCGTGATTATTCACTGTAATGCAT
TGGAAGTGGCCGTGAAACAAGTTTATTTCATTCAAAACAGTGCTAATAATCCAATTGGTT
TCTCATAGATAAATGTTATCCCTCACGGCAAAAATGCAATGCAATTTATAGGGATTCATG
AATTGACGGAACAGCCAATTGAACATATTGAAAGTAAATTTCCTGACTGGATTCCACTAA
TAGAATAAAATAAGGAAAATTAAAATGAAAGTATATGTCTTAAGCGCAGATACCTATGGT
GAACGTAATGAAACAACACCGTAAATCACGGTACTTTAGTGCCGTGAGTGTTCAAGGAGA
ATATCAAATGAAAATTACAAAAATTAGCCAGCTTATCAAAGCTCTTGAATACACGAAAGA
AAATCTACAATCAGATGATTTATCAGAAGTCATGTCTGTTTATGTTTCAAATCAAGCACG
AGAAGACGGAGTTCAAATCACAGACCCAGCATCTTTCTCAGAAGATGATGGAGATCCATC
AATTTCAATTAATATGGTTGCGGTTGATGCATTGTCCTATATTTTAAAAGATGATGAACC
GTCACGGATGGGAAGGGAGTGCTATTAATGAAACTTTTGTGCAAGCTGTACGGGCATAAG
TGGAGATTCAATCCCATGGATGATATTCGTCCATATTGTGCTAGATGTTGGATTGAAGAA
ACTTCACTCGATAAAGAGATAGACCAACGAATTAAATCTTGGAACCGCTCAGACCTTGAC
GTGTCTGAGAACGTGTTCCTGAAAAATGGCTTGATAAGCATATGGATTGAGGTGGAGAAT
GCGTAAAAAAATAAGAGATTACTTGACAAAATTTGTTATATTCGTAGTTGTTTTCATGAC
TACTTCTAATATGATTAGTATGTTTTCTAAAAATGCAACACCTAGCGAATGGACTTTTAC
TATTGTTATGTCTTTAATCGCAGCATATCTATATCTGGATTGAGGTGGATATGAAAAAAT
TTGAGTTATATAGCGCTGCCATCTGCAAGCCAGAAGGAATTGCATTTGAAAAAAATACAG
TTAAAGCTGATAGTTTTGCTGATGTTATTGAGTATATCGAAAGCAACGCAGGTTGGTACA
CTGCTATCAACTGAGCTTTCAAAGTCGCCTATATCGAGGAGGTTGTGGAATGAAATTACT
TAAATCGTTATTGATAGCAATTGGCATTGTAACTTTAGGTTTTACCATTGTTATAGGAGA
AGTATTAATATTTAAAGTTTTTGGAACAGTCGCTTTCCTTTTAGTAATGGCTCTGCTCTT
AGTCATTGCTTATACGACTTTGTTTTACTTAACGGAAAAGGATAATTGAACGCGAAAAAA

GCCCAAGCTGACCAAGCTTGAGCGTTTATTGTAAAAATTATGGAACAACACCGAAAAACT
CGGTACTTTAGTGCCGTGAGTGTTCAAATGTTAAATTAATTAATTAGTAGAAAGAGGAAT
AAATAATGAAAGACATTAAACAAGTACCATTGATTCAAAGAGTGCTATTAGAAGTTGGAC
AATTTAAAAAACATAAGCTAAATGAATTTTTAGTAGGGGAAACGGACTGGTGGTATTCTC
CTATCAATGATATTGTAATGGCGATGACTAATGGAGAAAAACCTGGACCTTTTAAATATG
CTGAAGTAGAAACAAACGATTGGCTATGGGTTATGTCAACAATACACGGAAATCTTGTAT
TGGAGGGAAAAGGCGGAATAGGAATCGAGGTTGAATAGAATGTTTAATAAATATAAAGAC
TTAGAATATCGAATTAACGACTTGCAAAAGGCAAATGAAAAGTTATGTTAGATTTAAAGA
CTGGTAAAACAATTGATTTTGTTCAATAAAAGGAGAAAAAATGAAGCAACGGACAGATGA
GTTATCTCAAAAGCTTAGATATAAACGTTCTGATTTAAAACTGAATCGCGGTAAGCTTTG
TAAAATATTACAAATAGGTAGCCATACATTAAAAACTTTAGAGGAAGGTTCTTGCGAAAT
TAAACAATCAACTTATATAAAATTGCTTGTATGGTTAGTTGGTTAAATAAAACAAAAAGC
CCGCTGGGAACGGGCTTCGGCATGATTGTATCTAATATCATTATACCATAGACGGAGGAA
TCTTTTAAACGGCGGATAGATTAGATTTGTTATTAAGTGACTACATGACTGGAATGCTTC
AAGTTAGAATTAATTCAAGAGAACGATGGATCACTCGTGAGAAACATGAGGAAAGAATCG
GAAGTGGTGGGAGTAGTTGAAACACTGCACCACAAGAGCGCAAATATTTGATTAAAGAAG
CTGACAAAGAACTTGGAAGACTTAATGACCAGAAACAAACGCTTGATGAATTAATGGAAG
TTATACAAGGTACAATTGCAAAAGATATTAGTATTGCTAGATTTAAGCACAGAATTTCTT
GGCATAACGTGGCTATCAGAGTTTGCTTAGAAGAGAGTACAGCTAGAAAGCAGTATGATT
CATTTAAAAAAACTTTGAGAGATGGATTATGGAGAGAAACTTTAGACTAATCTCACTTTC
CTTTTTATTCACGTTTTATTCACGTTTATATCCTAATTTAAGTGCGATAATGGTAGCATG
AAGTTATCAGCGAAAGCAAACAAAATGTAATTCGTTCGGTTGGATATAGTTCTAACGTTG
CTGGACGACAAAACCAGCGTAGCAAGTAAAGCACCACTGGAGAGTGCGCAGGCTTCGACT
CCCTGACTTGCTATTATAATTTATTACAGGTTGTCCAATGGGCAGCCTTTTATTGTTGAT
GAGAGGAGATTCTATCTTGCCGATGACTGGACGTTGTCGTGAGCCCAACTGCCACGCTGT
AGTTATTAGACCACTACACTATTGTACTAAGCACGCTGATAAAGAAGCTGCATATCAAGC
AAGCTGAGAGCGATGGACTAATCGTAATGATAATACAAAAAGATATAAGGACTATACCAA
ACGCAAGCGTGAGTATAGCCACATTAAAGTAGAACAGAATAAGTTCTACCAAAGCAAGCA
ATGGAAGTCTATACGTGATGTAGTAAGACGTAGAGACAACTTCCTTTGTCAGTACTGTAA
AGCGCATAATAGAGTAAGAACTGGTAAGATAGTGGACCACATCGTGCCAGTGGAGTTTGA
CTTGAATGGTAAGACCGTGATGGATAACTTGGCTTTCTGTTGTAGCAAATACCACACAAG
GAAGACTAAGTGGGAACAAATTTATTATGGAACTGGGTACGGAAATAAAACTAAAAATCT
AATCCCCATAAAAAATGTAAAAGATGTCCCTGATTTTCAAAAAAATGAACGATGATTTTT
TACAACCCTCCCCCCTATCTTTTTAAAGGGAAAGCACACACATAGGTTTAGAAACGTTTT
AAAGTCCAATTTTGAAAATTTTTATATAGGGGGGGTCAAAACACTAAAAGAAAGGAGAAA
AAATGACAGCTAAGAAGTTCAGAGACAGTAATGACGGCAAGTTGTCCTATCGTGCACCTA
AGCACCTTTCTCCTCTCGCAAGTGCTTGTTGGCGCAAAACTGTTGCCTTGCTTCAGGAAC
AAAAGCCAGTTGATAAGATTGATTCGTTTTTAGTTGAAATGTACTGTACTCAGTATGAAA
TTTATAGAAATTCATATGAACATCTCAAAAAACATGGTGAGGTTCAAGAAATTTATAAAC
CAGTTCAAGATATGACTGGTGAAATTATTGACAGACAATTTCAAGGTTTCAAACGTAATC
CAATGACTCAAATTTACTCGGATGCTATAAAAAATCTTAAAAAGATTGGTTCTGAATTAG
GGTTATCTCCAAAATCTCGTTCTGAATTGATGGAACTTAATATGCAAACAAACGAAAATG
AAGATGAGGGAATGGGGGATTTCTTCGATGCAGATTGATTTAACTCAAACCCATGATATT
ATCGGTACATATCATTAGCTAAATTATGAAGATATTAGAGAAGAATATCGAGACCATGCT
ACAAAATATGCTTTTGATGTCTTAGATGAAAAGTACACAACAGGATATTTAATGCAATTA
GCATGTTTTAGGCATTTACAGGACTTAAAAAGGATAGGAAATGAAGAATTTCCTTTTAAT
TACGAAGTAAAACATGTAGAAAGGTTAATGAAATTCTCTAAAATGGCTCCAAACGTAGAT
ACTATGGAACCGACTAAATTAATGGAGTGGCAGAAGTTTATGCTATCTTTATTAATAGGT
TGGAGAAACAAAGAAGGTGGTAAACTTTTCAGTCGTGCAATTATATCTGTAGGACGCGGT
CAAGGGAAAACTTATATGTTAGCCATATTAATGGCCTATTCATTTTTTGTAGAAAGTCGT
GGTTTAAGTAACCAGGACTTTTTAGTTTCATCCATTAATGCAAAACAAACAGGTAAATTA
TATGGCTATTTGAAATCGATGATTAATGTCCTTAGAACAATTAATCCATGTAGAAAAATA
GCTGATAAAACCGACCTAAGCTTACAAGCTGACAAAATTATTATGAAAAACCATAATAAT
GTGATTCGCCCAATTTCTCATGAAGCTGGACAGTATGATTCATATCACTTTACAACCGCT
ATCTTTGATCAAATAGGCGAAGTAAAAAGCCGTGAGAACATTACTAGGATTGTGTCAGGG
CAAGTTAAAGTTCCTAACCGTCAATTTGTTCAAATTTCAACAGCATATCCTGACCCTACA
GTTCCCTTTCATGAAGATGAGATGATGCTGCAACAAGCAATGGAACAAGACTTTTTAGGA

GATGCTGATACTTATCCATGTTTAATTTGGAGTAATGACAGTTTAGATGAAACTTATAAG
CCAGAAACTTGGGTAAAATCAAATCCTTTATTAGATTTAGCTTCAGAACATGATAATCTC
ATGCAAGGACTACTTGATACGCGTGATAACGATGTACTTACTGGTGCTGTTCATGACTTC
CAATGTAAGAATCTTAACATGTGGCTTTCAACAGATATAGACAGTTATTTAAAGCTAGCA
GATGTTGAAAAAGCGATTGTTCCTGAATTTAATATCTATGGTCAACGCTGTTATGTTAGT
GTTGACTATTCTATGTCATCAGATAATACAGCAGTTGCTTTTGTTTACCCTTATGTAAGC
GAAGAAGGGCAGGCAAAATGGCATGTTGAACAACATTCGTTTATTCCTTTTCAAGCTGCA
GGGTCAATTGAAGCCAAAGAAAAGCAAGATGGTATTAACTATAGAGAACTTGAAACCAAA
GGATTCTGTACAATCACAAGTCATCAACGGGGATTAATCAATGATGATGAATTCTATGAG
TGGATTGTAAATTCTATAGAAGAAAATTCACTTAACGTTTTATTTTTTGGATATGATGCA
ATGGGAGTGACTAAAGTAATTCAAATGCTGCTTTATAATACTGGATTTAATTTGCAACCT
ATTAGGCAACGGACAGGAGAACAGGCAAAGCCCACTAAATTCTTACAAAAGATATTTGTT
GAAGGAACAATTAGCCGTCTGGATGACAAAAAAATGGAAAAAGCATTATTAACTGCTGTA
TTGCGTGAAGATTCAGTTGGTATTCAAGTAGATTAGCGAAAAGCTACACTTAAAATTGAC
GTTGTTGATGCAATTATAGATGCTCTATACCAAGGTATGAATCATTTTGAAGATTATGGT
ATGGCTAATGATAGAAGTTGGCAAGTTGAGCATATGACACCAGAACAAGTAAAAGAATGG
GTTACTAGCCAAGAATCTGGCTAATTAGACCTTGAGGATGAAATAGATGATGATTGGGGA
TTCGATGAAGATTTTTAAAGCGTTTTTTAAAAAAATATGGGATGTTTTTGATGTTCTATG
CTTCTCTTTAGCAGCTATTACGTTAAATATCACGATTTTTCTAATGAACTTATTTGCTGG
TGGAATTACATTGACAGTAACATTTATTATATTTGGAGTTAGTTCTTGGTTTATTAGTTC
CAAAATTACGAAGGGAGGTGATTGAATTTGCCAATATTAAACTTTATCAACCAAACAAAT
GATGCGCCAGAAGCCGGTAGTGTTCAAAGCTATTTTCCAGATGGAAATGATGCTCAAATA
ATGGAAAGTTTGCTTGGAGATAATAATGGATGGGTTTCAGCTCGTGCAGCATTAGGAAAT
TCAGACCTATTTTCTATTATTTTGCAACTATATAGTGATTTAGCAATAGTTAAGATCAAC
GCTGAAAAGAAAAAGAATCGAGGAATCATTCATAATCCAAGTACTAATGCTAATAAACAT
GGGTTTTGGCAATCAATGTTTACACAGTTGCTTTTAGGAGGCGAAGCATTCGCTTATCGT
TGGAGAAATGCTAACGGCGCTGATATGAAATGGGAATATTTAAGACCATCTCAAGTGAAT
ACTTATTATTTTGAGTATGAAAATGGAATGTATTACAACATCACTTTTGATGAACCTAAG
TTAGAACCTATTTTACGAGCACCACAGAGCGATTTGATTCATATGAAACTACTATCAATC
GATGGTGGTAAAACTGGAATTAGTCCACTTCACTCTTTGAGGCGTGAATCAAAAATCCAA
AGAGCCTCTGATAGATTAACAATTACTTCATTGAATAGTTGATTAAATGTTCCTGGTGTA
CTTACTGTTAAAGGTGGTGGGCTTCTTAGTGATAAAGATAAAGCATCTCGTTCTCGTTCG
TTGATGAAACGTTCAAGAAGTGGTGGCCCTGTAGTATTAGATGACCTTGCAGAATTTACT
GCACTAGAAATGAAATCAAATGTAGCTCAATTATTATCACAAACAGATTGGACTTCTAAG
CAATATGCTAAAGTATATGGGCTTCCTGACAGCTATATTGGTGGACAAGGTGACCAACAA
TCTTCAATCCAACAAATAAGTGGAATGTACGCAAGTGCATTAAATCGCTATTTAAGACCT
GCTATAAGTGAATTAGAGTATAAGTTTAGCGACCACATAAGCGTTAACATGAGACCAGCT
ATTGACCCTCTTGGTGATAATTACTTATCTACTATTAGTACTGCTACAAGATGGGGTGCA
TTGGCTGACAATCAAGCTACGTTTGTCTTGCAAGAAGCAGGATATATTACAAAAGACCTA
CCGGCTCCTGAAAATACAAATAAAAAGACAACTGGCCAAAGTAATGAGCCAGTGCCATAG
GAAAGGAGGTGGTCATGGTGATTATTCTTAGAAAGGAGGTAAATGATGACAGTAATCGAC
ATTAAAGGAGATGTACTTGATAATAGTTACGGAATGATGTATGACTGGTTTGGAATCGAT
TATACAAGTCCATCTAAAGTTAATGATGCCTTGGTAAATGCTGATGATGAAGAAATTGTT
TTAAATATCGCTTCTAATGGCGGAGATGTATTTGCAGCTTCTGAGATTTATACTGCTATT
AAGATGAATGGTAAACCTGTAACTGTAAATATTCAAGGGTTGGCAGCATCTGCAGCATCG
GTAATTGCAATGGCTGGCGATACGGCAAATATCTCTCCAACAGCCCAATTGATGATTCAT
AAGGCTATGAGTGGTAGCCAAGGAAATGCTGATGACTTTGAGCAAGAAGCTAAAGTTTTA
AATGGCGTTGACCAATCTATTGCTGCAGCTTATGAATTAAACACTAGTATGAAACAATCT
GACTTATTGCAGTTGATGTCTAACGAAACATGGATGACAGCTCCAGATGCAGTGGATAAA
GGATTTGCAGATAATATTATGTTTGTAGATGCTAATAAACCAGTATTTTCTAACTCAATC
GGCAATATTCCAACTGCTGATAAACTTACTGAATTTATGAATGTCATGAATTTCAAAAAT
CGGAATAACCCTCCGAAAGAAGAAACAATTATAAAAAACAAACAAGCTGATTTACGTTCT
CGTAAGTTGGCTATTTTATTAGAAAGATAAAGGAGACTCAAATGGGAGTTAAATTAACAG
TAAATCAATTGAACGAAGCATGGATTGCTTCAGGAGATAAAGTCACAGACTTTAATGACC
AAATTAACATGGCTCTTAATGATGATAATTTTTCAGCAGAGGCTATGTCAGAATTAAAAA
ATAAACGTGATAATGAAAAAGTTCGCCGCGACGCATTGAGAGAACAACTTGTTGAAGCTC
AACGTGAGCAAGTAGTTAATATGCGTGAAGAAGAAAAAGGTCCATTGAACAAAAGCGAAA

ATGAACTCAAAGACAAATTTGTTAAGGACTTCGAGAATATGGTTCGTAACCCTATGGCAT
TTCTCAATACCGTTTCATCTAAAACTGAAACTAGCTAAAGTGATAGTGCTGCTGGACTTA
CTATTCCGCAAGATATCCGTACTATGATTAACACATTGGTTCGCCAATATGACTCGCTAC
AACAATATGCACGTGTTGAGAGTGTTTCTACTTGAAGCGGTAGTCGTGTATAGGAAAAAT
GGACTGAAGTTACTCCGTTGAAAGCAATGGATGAAGACGATGGAAAAATTCCTGATCTTG
ATAATCCACTTTTGACAATTATTAAATACTTGATTAAACGTTGTTCGGGAATCATCACTG
CAACTAATACATTGCTTAAAGATACAGGAGAAAATATTCTTGCATGGTTATCAAGCTGGA
TTGCTAAGAAAGTGGTTGTGACTTGTAACCAAGCGATTATTGCAGCAATGGGTACAGTTC
CTAAAAAACCAACAATCGCTAAGTGTGACGATGTTATTACTATGATTAATACATCTGTTG
ATCCTGCGATTATCGCTAGTTCAAGTCTTTTGACTAACCAGTCAGGGTTGAATAAACTTG
CTTTGGTTAAAACTGCTGAAGGTAAATATTTGCTCGAACCAGACCCAACAAAACCTAATT
CATATCTAATTAAAGGTAAACAAGTTATTGTTGTTGCAGATCGCTGGCTTCCAAATAGTG
GATCAACAGTTTATCCACTTTTCTATGGAGATATGTCTCAAGCTATTACATTGTTTGACC
GTGAAAACATGTCATTGCTTCCAACAAATATTGGTGCTGGTGCATTGGAAACTGATACTA
CTAAAATTCGTGTAATCGATCGCTTCGATGTAAAAGCGACTGACTCAGAAGCTTTAGTTA
CTGGTTCATTTACTGCAATTGCAGACCAAGTAGGTAACTTCAAAACTACAACGTCTACTG
CCGTATAATCAGGAGGTATTTAAATGAGTCTAACTGTTGATAACTTACTAGATCAGTTAT
CAGAAGATGATGATCGCAAACCACAACTTCAAATTTATTTTGATACAGCTACAGCATATG
TGAAAAATGCAGTGAGTGCTGATACAGTTGATGCTCCATTTTTCAATGTAGAAAACGTTT
CTCCAATTTATGATGTAGCTGTTCTTAGCTATTCGATGGATTTGTTGATTAATCGTTCTA
CAACTATGCCTCCTACTACGGCTGTAGATCACATAGTCGGTCAGTTGAGAGGTCTTTATT
CTTCGTGGGAGGAGGAGCAAGCTGGCCAAAACTTACAAACCGAATGATTTTCACAGAAAA
TGTAAGATTGGAGTTACTAAAACAGTAACTACTCCAACTGGAGGTAAGATTGAAATAATT
GACCCAGCTACGGATTTAAATGTTCGATTTGCGGCTAAAATGAGATCACTTGCGCTTCAA
ATTCAGATAATTGGTACAACTACGGCTGATACTTTCGACATTGCAATTAGACAGAATAAG
CTAGTTACAAAGAAAATGTTTGTTCAAATAGATGACGTTCTTTACAACATTATTAATATT
TCTTCAGATGAATCTGCAAAGCTTATTAAATTTGATATTTTGACTCTTCAAGCGAAGAAG
AAAGGAGCATAATATGGCTACTTTTGAGGAAGCAATGCAATTAATGATTAATCAAGCAGA
ATCATTAAGCACAAAGATGACTGTAGAAGATAAAGCCGTAGTTACAAAGGCGAGCGCTAA
AGTTTTTGAGCAAGCATTAGCTTATGAAGTTAGAAATAGACACTACCGTCATCGTGATAC
TGGAGAAGACCCACATTTAGCTGATAGTATTGTTATGAAGAATAAGAATATTGACGGTGT
TAAAGATGGCCAAAGTGTTGTCGGATGGGAAAGAAGTACGGAAAAACGTACTCATACAAA
AGGTTATATCGCTAATATTATTAATAACGGTAGTCGTTTTCCTCAGTACACAACACGTTC
AGGAAGAAAGTACAAAAAGCCTGGTGAAGTTGCAGTTCATGCAGAGCATTTTATTGAAGA
AACAAGAATGACCCTCATTGTTCAGCAAGGAATATTAAAAGCTGAAGCTGAGGCAATGAG
GAAAATAATTAATAGAAAAAAGAAGGAGAATAACTTATGAAAAGACCAGTTGAAATTGTT
CAAGACATAATTGCAGCGAGTGACTTTCCGCATGATGAAATCTTTCTTGATTTTATTCCT
AGTGAAAAATTAGATTCTAGTAATGAAACTCAGGTTTTACTTACAGAATCAGAGAATGGT
CCTAATGATTATTGTAACTCAGATTTTATTTCAGTCATGTATGGTGTTTATATTCAAATC
TTTTACACGAACGCTGAAGATTCGGATATAAATATTATTCAAAGCGAAATTAATCTGATG
AAATCATTTATACAAAATGATTGGCTTATTGCGCAATCAAAAAGTCACTATATAGACCCT
GATACACGGCAAATTATTAAAAATTTATCGGTGCAACGCATCATGACATTAAGCGAGATA
GCAAATAGCTAACTGATTTTTTATTTAAGAAAGGAATTTAAAATGGCAACAAAAGGTTTA
AAAATGGTTACACTTGCTCTATTGGATGAAAAAGGAGTGATCGTTAAAGGAGACAGTGGT
TTATCTACCAATGGAGTCTTCCCAATTACTGATGAAATGTTAGGTACAAAAACTGCAAAC
ATCACTAATTTATCAAGCGCTCCAACAATGATTTATGGTAATGATGGTCAAGTAGATGCA
GATATTGCAACAGGAACTCCTTCAGTAGCTTTTGCATTTAATGGTCTACCAGTAGATATT
AAAAACAAATTGCCTGGTCGTGTAAACGATACTAACGGTGGTTATACACAAGGAAGTATT
CCAAAAGTAGCAGTCTTGATTCAAACAACTACAATCGGTACTGCGAAGCCACAATATGTT
GCTTTTGCTGCTGGTAAAATGAACGAAACAGCAATGAATTTGCAAACAAATACTAATGCA
GTTGTTCGTGTTGATGACGCATTGACATTTACTGCATTCTCTGTAAGTCGTTGGGGCGGA
GAATCTGTCAAATTCTTTGATGATGGAGATTCCAAATTTAATGAAGACGTGATGATGGAA
GATGTATTTAATGGTTATGCTGGAGATGAAGTTTAATAAATTATACAACGACTATAACTA
GACATTAATAATTAAAAGGCGGAGTAATCCGCTTTTTATGTGGGATAGATAGATGGTCTA
TTATATTAGGTTCGATACCTGACTATTCCTTTACACAAAGTAAAATAGAGGAGATATACA
ATGAAATTATCATTACCAGAAATTAGAGAAGAATCATTTGAAGTTAAAACTTCAATTAAG
AACATTAAAAAAATGCATGCCTACCAATTGGAACTAGCAAAAAGCGAAGAAAAACTTGCT

GCAGTTCAGGATGGAACACTAGAAGAACTAACCAAAGCAATCGCTCTTGATGATATGTCA
GTAATTAATAATGCTTAAAAATTTATTACTGAAATTCTAGGTTTAAATAAAAAAGAAGTA
GAGAAATTACAAGAATTTGACCGTGGGCAATTTATGATTTTACAATCTAAACTTGTTCTT
TCACTTCAAGGGTATGATGATGATCAAATCGATACTATGTTTACTGAGGAGGTTGACTCT
GCCGAAAAAAAAGTTCAAGCATTGAAGAACGAAAAGGCTACCACCAGAACCAATTAATAG
CTTTACAACTATTTAAAAAAAATATTATCGAAAATTGGCACTGGACATTAGAGCAAGTAG
ATAATCATGATTATTATGACTTAATTGATGTGTTTAAAGCAAATGAAGATAATATAATGG
CTTCATTTGATGATTTGAAGAAAATGTTTGGACAATAATAAATATTATCTTGTAATATGA
ACTATATAGGGGAATAATTTACTTATTAAAAATCAGGAGAGATTCATGAAATATTGCCCT
AATTGTGGAAAAGAAATAATAAGTTCATCTAATTTTTGCTCAAATTGTGCTTTTAATTTT
AACCATAAAACTGATATTAAACAAGAATCTGGTGCTATTAATGTACCTAAAAAAAGTGTT
GAAGAATTCAAAAATGCTTACTTAGCCAATATTATTTTAGCTGTGATAACTGGGATTTAC
GTTGTTTTAAAATTTTTACAAATTTTTGGAACATATACTATTAACTTGAAAATTTTCCCA
TTTATAATTTTAGCTTGTGCTATCTATGCAATTGTTATTAATAAAAAGTTCAAATTAACT
TATACAGCTTCAATTATTATTTATATTTTTAGTATATTTTCAATATTTTTCTCAAGATTT
CTATCTCTTGGACTTATGTTCTATTTTGCTTTAATATTGATAGTAGTACCATATGGAGCA
TCGGCTGTCCTAAGTGCAACTTTAGCATATGGAATAAAGCAGAACATTTTAAAGTGATAA
AAGACTCAATTCAATAAAAATAAAACGTCTAGATGAATTAGGCGTTTTTTTATACTCAAA
AATTAGAAAGGAGTAAAAATGGCATATATAATGGTTGATTCAGTCACCACAGGGATTGAC
TTTAATGAGACAAAGGCTGTTGAGGCTATCAACCGCTTAAAATCAGCATTTAAAGATAGT
ACTCGTGAATGACAGATAAATGAAGCACAGGCTAAATCTGCTGAAGATGCTGTTTCTGCA
TCAAAATATCGCTATGAAGGTCTTAGTGAAGCAATGGAAAAGCAAAAAGCTTATATTGCT
AACCTTTCAGAAGGTATGAAGACAATCAATAGAGATACTGATGCTGGTGAAAAGGCTTAT
CAAAAATATAATGCTCAGTTAACCACGGCAAAACGTTCTCTTGCCTCAATGACAGTGCAA
CTAAACCGTACGAAATCAGCTTATGAATATCAACAAACAGGAATTGAGGATTTAAATAAA
TCTCTCAGTCGTAATGATAAACTCATGCAGTCTCAAATTGATTTATGTGAGAAAACTCGT
AATAAAATGGGAGCTGCCAAAGCTGAAGTTTCTGGTCTATCTACTTCATACGCAAAGCAA
ACTGAGATTTATAGAGCCCAAGTAACTGAGCTTAAACGTTTAGAATCTGCTGAGGGCACA
AGTTCAGAAACTGTTGTCAAACAAAAAAACAAGAGTAAATGAAGCTGCGTCGTCATTATTG
AACTACAGAAATAAACTTTTAGAAGCTAACTTGGCAGTTACAAAGATGCAACCGTTTAAT
TCTGAGTCTCTCATTGTTAAAGGTTTAGATACTGTTTATCAAACAACTGAGAACGCTACC
GATGTAATGACAGCAGGATATCAGAAAGTAAAAAATGCAGCATATCAAAGCGCTTTTGGT
ATTGCAGCAATTCGAGCGGCAGCTATAAAAGGCTCTCAAATGGCAGCTGATTTGCAGGAT
AGTTATATTAGAACATCCAATCTTGCTGTAGCTGGTGGAGAAAAAGTTGCGGAAGTAACG
AAAAATGTTGCTCAAATGCAAAAAGATGGGGCTCAATACTCTGTAGAATATGGTAAATCG
CAAAAAGAAATTGCTGAAGGTTATCAAGAACTTATTAAACGTGGATATACTTCTACTGAA
GCTTTGGGTGCTATGCGTTCAGAATTCCAAGCCTCTGTTGCTTCTGGCGATGATTTTAAT
AATGTATTGACCGTAACCTCTCAAGCTGTCGATGCTTACGGCATGCGTACTGATAATGCA
GCACAAATGACTCAAAATACAAAAGATGTAGTTAAACAATTAGCTTATGCAGCAGATATG
ACAGCGACAGATTTTGCAAGTATGGGTAAAGCTATGGAGTATGTTGGTGATTCATCTCAT
TCAGCAGGTTTTCAATTATCAGAGGCATCTTCAGCAATTGGTATTCTTTCGAACCACGGT
TTAGAAGCTGATAAAGCTGGTACTGGACTTCGTAGAGTAATTAACGCAATTACTGGTGCA
TTAAGTGTTCAGTCAGCTAGCCAAGATGATGCTGCTAAATCAGAGTCTGCACTTAATGAA
AAAATTGCAGAGCACCAAAAGAAACATGAACAAGCTCAAGCGGTTGTTAATTCTGGTACA
GGGAATACTAAAGCAGCAACTAAAGCTATTGAAAAGCAAAAAGAAGCAATAGGGAAGCTT
CAAGATAAAGTAGACCAATTGCAATCTGGTGGTGCTGCCAAAGATATGCTTGCTAAACTT
GGAATTAAAAATAGTGATTTAGTAGATGCTAATGGTATCCTAAAGGATTTAACAACTATT
ATGTCCATAGTAAATGATCATACAAAAAATATGGGAACGGCTGAAAAAAACTCTGTTTTT
CAAAAATTATTTGGAACAACAGGGCAACAAGCTGGTATTATTCTCGCTCAAAATAATAAA
GAACTTGAAGAGCTAAATAATAAAGTAAAAAACTCTGCTGATGGTCAAGCATATGTTGCA
AATCTTGCAAAGAAAAATATGGGATCGGTTAAGTCGGAGATAAAACAATTCAAGGCAGCT
GGTGAGGCTCTTTTAATTATGATTGGTCAAAAGTTCTAGCCAGTTTAATCTGATGCAGCT
ACTTCAATGGCTAAGGCTTTTAATTCTAAAGAAGGTAAGCAAGGGCTTGAAGAAATAGGT
AGTTGGATTGCAAAGATTTTCCAAGGTATTGTTGATACTGTCAAAGTCATTGGAACTCAT
AAAGATGAAGTAGTAACCTTTGGTAAAATCTTTGCTGGAATTTGGGCCACTAAGAAAATT
GGAGATGTTATTGTATGGCTTGAAAAATTGAAAAAATCTTTACTTGAAATTGAAGCTATT
GATGCATTATGAGGAGGTTTAGGAACAGGAGGCATTAAATCTTGTGTAGGTAAAGGTGTC

GCTGCTGAAGCTGGAACAGTTGCTTCAACAGTAACTAAAAGAGGCGTAGTTGCTGAAGGC
GAAGCGCTTGTTGCCTCTGGCGGTTTATCAAAAGCTACTTCCTTAATTCCAAGACTATTA
GGAATTATTGGTTCTGTAGGCGGAAGTACAGTCTTGTCTGGCGGAATAAATGCAGGAGCT
GAATTACTCAGTAAAGATAGTACAGCTCAGAAAACTGGTGGAGTTGCTGGCTCACTCGGT
GGAGCAGCAGCAGGTGCAGCTATTGGTTCTCTTACCGATCCTGGTATTGGTACAGCAATT
GGTGCAGCGATTGGCGGAATGGGTGGTAAAAACTTAGGTAAAAAGCTTGGGGATTTGATT
AATTATGGATTAAAAGAATCTTCATTAAAAAGCGAAAAGCTACCAGTTGTTAAGTTCGAC
CCAAAAGCACCAACAAAAGATATGAAAGAGTTCTCCAAGGACTACCAAGGTTTCTTGGAT
AAAATTAACAAGGCATCAAATGTTGATATTGTCGATGAGAAGTCACTTGAAAAAGCTAAG
AAAGCAACTGCTGATGCTTATGCACAAATGTCTAAAGATGTCGATAAATTCGACCAAAAA
CAAGAAAAGGATTCTAAAAAGCAAATTTACATTCTAGTTAAAAATGGTGTAATTACTCAA
GCTCAGGCTGACCAATTAAACAAAGGTCAAAAAGACTCAGACGATAAGCAGAAGGCAGCT
CAGAAAAAGAATCTTGATGAGATGAAGAAGAATACTGATAAATATTATTCCGAGGTGGCC
AAATCTCAAAAAAGTTATGACACACAATCTCAAAAGGATGCTAGTAACCATGCTGCAGCA
CTTAAAAAAATTAAGTCAGGCAATACCGATGCGCTCTTAGCTATTGAAAAAAAATATGGT
AAAAATTCACCTCAATATCAAAAAGAGATGAATAAAGAAATTGCTAAAGAAAATAGTGAT
TTCAATAAAGCTGAAAAAGCTGCTAAGAAGAAACATAATGAAGCGATGGATAAGCTTGAA
AAAGCTTATGCAAAAAATCAAACCAAAGCCGAAGAGCAGATGAATAGTCAAATCAACACT
GCTACCAAAATCGCCCAAAACAAGCAAATGGATCTTTTAGAAGATTTAAAAAATAAAAAA
GGTAAATTAAATCAGAAACAATTTATTGATACTATTGAAAAAGCTGACTATGAATATAAA
GGAGTTAAGGATAAGGCTCAAAAGCAAAAAGATGAAGCTGTTAAAGCAGCTAACGAAAAA
TACAAGAAGACAGTAGCAGCAGCGGAAAAAGAACGTGCAGAGAATGGATCAATGTCTAAA
GCCCAATATGATGAAATTGTTAAAAATGCTAAAAAGCAACGAGATGATACAATTTCATCA
GTCAAAAAGCAACAAGAAGATGTTACTGATAAAGCACAAAAAACCCATGATAAAACAGTT
GAATTAGCGAATAGTAAAGCCGATAAAAATGTGAAAGCTGCAGCTAAAGAGCAAGGAGAG
ACTGTCGAACAATATACAAAAGGATTTAAGGATTCTAGAAATTTAATCAATTCATTCATT
GATGGAATTAACGGAGTTCTTAACTTCTTACATAAAGCTTGGGGGAATATTGGGCATGTA
AGCCTCAAAGGTTTTGCGACAGGTACTAGTGGTTTAGCTCAAGATGAAACAGCTTTAGTT
GGTGAAGATGGATTTGAACTTGCTCACCATCCAAACCGAGGTATTTTTGCGGTTGGTCAA
CAAGGCCCTGAAATTCGTAGTTTGAAAGCTGGAACTTCAATTCTTCCCCACTCAATGTCA
AAAGAGTTTCTTTCATTAACAGCAAATTTACCAACTCATGCGGACGGTGTATCTGGCTTC
CTAGCAGATGCGCTTGGATGGGTTAAATCAAGATATAAAGATGTTACTAGCGTTATTTCA
AAAGGTCCTAAAGGTGTTGTAGAAGCTATTTACAACGGATTAGGATTAGATGATTTAGAA
AATGACTTTCCGCCAGGTGTAACTAGGATGGCAAAAGGTTCTGCCCAAACAGCACAAGAT
AATTTTGTAAAATTCTTACAATCATTTTTCAAAAAAGCTGAATCAGATGCCGGAGGTTCA
CAAGGCTCGCCATCTGGTTCCGGTGTTCAACGTTGGGCTGGACACGTTAAACAGGCGCTT
GCAGCTAACGGCTTAAGCACAAGCCAAGACATGATTGACCGTGTGCTCCGTCAAATTTCT
TCTGAATCAAGCGGAAATGAAAAAGCCGTTCAAGGGAACATCGGAGATATTAACAAATTC
ACTGGTGACATTGCTAAAGGGTTGATGCAAACCATTTCCTCAACTTTCAACGCCGATAAA
TTCCCTGGTCACGGTGATATTTTTATTGGTTACGATAACTTATTAGTTGCTCTTAAGTAT
GCTAAAAAAAACTATGGTCCAAGTTTGTCATTCCTTGGGAATGGACATGGCTATGAAAAT
GGTGAAATCATAAATGCTCATGGTTTCTATGATATTCCTGAAGGAAACCGTCCTGAGATG
GTTATCCCCCTTGACCCACAGAAGAAATCAAGGGCTAATCAGCTATTAAACCAAGCTAAA
CAAACAATTCATTCAAATTCATCACATCAAAGTAATTCTTTAGGAGATGGTGGACAGTTA
GCTGCTTTAGTTTCAAGTCTTTTAACACTATTAAAAAACGGTCAAGCAGATCCTATTATC
CAACTTATGTTAGATAAAAACGGTTTTGCACAAGAATTGGCAGAGCCTATGAACAAACAA
TTAAGTGTTTTAAATAGCCGCAATAAAATAATAAATGCAGGAAGGACAACATAATGACAT
TTTCAGTTAATTAGAATGGTGTTGAACTATCTGAATTAGTAAGTGGATTCACAGCAATTG
ATAGAGGGTTTGGTTCAGTCTGGAATAATACACTATCAAATCAAGTAGCTAGATATGGTC
AATCATTTGTAAAGAATACATTAAGCGCAAAAAAAATTACTATTACCATTAGAAAAAGCG
GAATTCCAAGTGATTGGATAGGCTTAAAAAAGGAATTAGCAAAGGTGCTGGACGTCAATG
AACCAGCGCCTTTAATTTTTGGTGATTAGCCTAATAAAGTTTGGTATGCAGTAGCAGATG
GAACTCAAGCCGTATCAGAAGATATCTCTAACTTAATTGCTACTGGAACTCTTACATTTA
TAGTCCAGGAAGGGCACGCTTATTCGACATATACTAATGTTTTAAACGCAGATAATTCAG
GTGGCGTCAATGGTTCTAACACTCCTAATGCTGATGGTTCGGTTGATATCACAATTAATA
ATCAAGGAACATTACCGACTTGGATTGATTTAAAGCTTACCAATAATCATGACAATGGGT
ATTTTGGTATTGCTGCGGTTAACGGAAGCTGAGAATTAGGTAATCGAGAAGAACTCGATG

GTGTTACTATCAAGCATAGCGATATTCTTTATGATAGTAAAACTGATCCTAAATTTTCTA
AATTTGTATCAGCCGCAGGTCAACCCCATCCAGAATGGGCTGGTGCAGGAACAAATGGAA
CAATTAGGTATCAAGAATATCATTATAAGTCTTCCAATGGGCAACAGAAAACAATGAAGG
CTGTTAAGTTGATTGATCCTGGAAGTCAGACAGGATTTCGTGGGGGCATGACAGAGCTTG
TGTTACCACCAGATTCACATGGGGATTCTGGTGCAGTTAATTTCTATGCTAGGTTCAGAT
TATTTACTTGGACAACTGTTCTCGGCCAAACTGGGGCGATGCAAATTCTAGTCACTGATG
AAAATGATAATTTTGTGGCTGGTTATGGGACATTAAAGGATGATAAAACAGGAAATATTC
GGACTGTTGGATTTTGGATTGGCGGAGCTAACAAAGGGCAATGGAAACATATTCCTTATG
TCGCAAATAATGGAGAACAAGCTGGATTAAAAGATAATAATACGATGCTTAATGATAATC
GAGGGCAATTAGAGTTCGCTAAACAAGGAGCAACTATTGGTTTTTATTAGAAAGGTGGCC
ATCAAACAATTGTAGTTCCAGTACTTCAAGATGTCGCAATTCATAAGGTTAAGTTCTTCT
TTGGGAACTGGTCAACTAACCGAAGAGGAACGATGACACATATGGTTATTCGCGACTTTT
GGTGCCGCAAAGACTTTGTTAATACATGGAATGACGTACCAAATCGCTATAATAATGGCT
CAGTCGTTGAAATTGATATGGCTAGTGGTAATGTATCCAATGATGGAATATCAGCCAATA
CAGAGGTAGTAAATGGAACACAACCTTTCAATATTCCATCTGGAATTAGTCAGACCAAAA
TCATTCAATCATTTTGGAATAATACACCACCAGACGTTGAAATATCATGGAAAGAAAGGA
TTTTATAATGCTCATTAATGTTTTAAATGGTTCATTAAAAAAAGTAGCGATCTTAAGTAA
TGAACTTCCCAATGCACCTAGTTATTATAACGATGAGTTTCATGACTATTTAGAACAAGG
AGCAACTACATTTAGTTTTACAGTTGCTAAAGTGATTAATAATCAGCTACAGGATTATAC
ACATTTCTTAAGTGATCAAAGTTATTTTAGCTACAGGAAAACTGGTCGCGACTACTTGAT
GACTCCTTCATCCGAGCAATCAGTTCATGAAACAAGTACTGAAATTTCATTTTTCTGCGT
TTCTTTAGACCGAGAACTTATTAATGAGCAAGCAAATTCATTAGCGAATACTGGAAGCCA
TATTATTCAATGGTATTTTGTCCGAATGGGTTTAATCTCTAGGACTAAAATTACTATTGG
AATTAACGAAGTTTCAAATCTAACACGAGTGATTAATTATGATGCGCAAGAATCTACATT
ATCAAGACTTATTTCGCTAATCAATAATTTTAACGCTGAATTTGATTTTGTAACGACTTT
AAATAATGATGGTTCTCTTGGAACTGTTGTTCTACATATCTATAAAGAAAATGACGGTCT
AAATATTCAAGGTGTGGGAACAAAACGTGATGATGTCAGACTAACATTTGGCAAAAACAT
CGAGGATCTCGAAGTAGATGTTAGTGATGACGGATTTTTTAACGCCGCTTATGTGACTGG
TGCTGACGGTTTTAGTTGGAAAAATTATTATTTCAGTTCTTAAAATTCAGACGGTGTAGA
AGAGTTCTATAAAAGAAAGGGTTCTGAAATAGCTCATGCTCCATTATCTGCCAAAATGTT
CCCTCCTCAATTGAAAACAGACACAGGAGACATTTGGACAAACTCAGACCGAACTACTGA
ATATAAAAATGACAATGATATGTGGGGCTATATTGTCAGTCAATTTAAGCAATATGCCTA
CAAACAAATCACATATACCGTTACTCCTTCATCTACTTTAGTTAATCAACTTATTGGAGA
TGGAAGACCTCTCCAAAAGTGAGATACAGTTATTATCCAAGATGATAACTACATGGATAT
TGATGGCAATGTGGGTCTAATTTTATCTGCAAGAGTCTCTGAAATCATGACATCAGATAC
AAACCCTGCTAATAATAAAATTGTTTTTTCAAATTATAAAAAAGTAAAGAGCGATGCTTC
TTCTGATATCAAAGCGATTGTCAGTCAGTTAGTCAATGATGCTACACCGTATTTCGCAGT
CATAGCAACTTCAAATGGAGTTCAGTTCAAAAACGGCACTGGTTCAACAACTTTATCAGC
TCATATCTATAAAGGCTCTGCAACGACTGAAGCAACCGCAGACAGCTACGAATGGTCGAA
GGATGGAACTATTGTCGCTCCAACTCAGACTATTACAGTTGATGCCAGCGGAGTTGCGGA
TAAGGAAGTTTATAGTTTTAAAGCAACGGTTGCGGGTAAAGTGGTCGCTAGTCAGTCGGT
GACTTTCACTAATGTGAATGATGGAACAAATGGACGTTCTGTTACAAACGTTTCTCAAAA
GTGGCGTTTGACAACGACTACTGCAACACCAACGCAAGCGTGGTCAGACCCAGGTTGGCT
CACTACTCAACCAACAACGACAGCTACTGATAAATATCTATGGTCTATCACTCTAACAAC
TTTCAATATAGCACCTTTAACGCAAGATGTTATTGAACAAAAAGCAGTTTATGGTGATAA
AGGCGATAAGGGAGATACTGGAAAAGATGGAAGAGCAGGTAAGGACGGTGTTGGAATTAA
TTCAACTACAATTACGTACGCTATATCTTCAAGCGGTACAGTTACCCCAACGGCTGGATG
GAATTCGCAAGTCCCTACTTTAGTCAAAGGGCAATATCTCTGGACGAAAACAGTTTGGAA
TTACTCAGACGGAACGAGTGAATCGGCATATACAGTCTCTTACATTGCGAAAGACGGGAA
CAACGGTAATGATGGAATTGCTGGTAAAGATGGTACTGGTATCAAAACTACGACCATTAC
CTATGCAGGCTCAACAAGTGGAACAACAGCACCAACTAGCGGTTGGACTTCTACAGTTCC
GACAGTTGCAGCAGGTAGTTATCTGTGGACTAAGACCGTTTGGGCTTATACGGACAGTAC
CAGTGAAACAGGGTATTCAGTTGCGATGATGGGAGTTAAAGGCGAAGAGGGAGATCAAGG
TATTCAAGGTATTCAAGGTGTTGATGGACGTCAAGGTATTCCTGGACCTAAAGGCGCTGA
CGGAAAAACACAATATACACATATCGCTTACGCAAATAGTGCCGATGGTAGCAAAGACTT
TTCAACTTCTGATTCTAATCCTACCTATATCGGGATGTCTGTTGATTTTAACGTCAATGA
TTCAACCACTCCGAGTGATTACTCATGGACACTCGTAAAAGGAGCGGATGGAACGCAAGG

GACACCGGGTAAACCTCGGACAGACGGTATGACACCGTATTTTCACACAGCATGGACTTA
CAGCGCATACGGTACTGATGGTTTCACGACTGTTTATCCGAATTTGAATCTAGGAGATAA
TACCAAAACTTTTGTCGGCTCAGAGTATGAGTTAAGTAATATAAGAGGTTCTATTAGAAT
AGACCCATCAACTCAAAAAACACAGGATGGAGATTTTTATCATTTAACCGTTAAAGCACC
AAGAGATAATTATCATTGGTTTAGATTCTATTTAAGACCAAAGTCAACAACGCCTAATAT
GACGAAAGTCGCGATTAAACCAAATACATAATATACATTTAGTGTTATGCTAAAAGGTAC
TGGACAACATACGATTTATGCATACCAAAATTGGACTGCTCCCAACACGCCTTGGACTTT
ACAAATTAATCTAACTAGTGAATGGAAGATATATACATTTACAGTAACATCATCAAATGT
TATTCCAGATAGAGATGTTCAATTCTTTATAAGAAGTAACAATGGAACAGAAATTAATCT
TAAATACCCTAAAGTAGAAGAAGGCTCAACTGCTACTCCATAGATGCAATCAGCTAGCGA
AGTCACAACTGCTGATTATCCAAGCTACATCGGTCAGTACACAGATTTTGCGCAAGCTGA
CAGAACTAATCCATCCGCCTATACTTGGAGTCTGATACGGGGGAATGACGGGGAAAGAGG
TCTATAAGGATTAAAAGGTGAGCCTGGCGCAACTGGTATCCCCGGACAACCCGGAGCTGA
CGGAAAAACAAGCTATCTGCACATCGCCTATGCCACGAACTCAACTGGTACGGCTGGATT
TGATGTATCAAATGCGACTGGTAAAACTTATATTGGACAATATACAGACTTTACGAGTGC
TGATTCTACAGACCCAAGTAAGTACACATGGAGCTTGATTAAAGGTGATAAAGGCGAAAA
GGGAGATACTGGAAAGGATGGGAGAGCAGGTAAGCACGGTGTTGGAATTAATTCAACTAC
AATTACGTACGCTATATCTTCAAGCGGAACAGTTACCCCAACGGCTGGATGGAATTCACA
AGTCCCTACTTTAGTCAAAGGGCAATATCTCTGGACGAAAACAGTTTGGAATTACTCAGA
CGGAACGAGTGATTCGGGATATACAGTCTCTTACATTGCGAACGACGGGAACAACGGTAA
TGATGGAATTGCTGCTAAAGATGGTACTGGCATCAAAACTACGACCATTACCTATGCAGG
CTCAACAAGTGGACCAACAGCACCAAGGATCGGTTGGACTTCTACAGTTCCGACAGTTGC
AGCAGGTAGTTATCTGTGGTCTAAGACAGTTTGGGCTTATACGGACAATACCAGTGAAAC
AGGGTATTCAGTTGCGATGCTGGGAGTTAACGGCGATAAGGGAGAGCGAGGCATCAAAGG
TCCTGCTGGAAACAACGGTGACCATGGTAAAATCGTTTCCGATACTGGGCTAAATACACG
CTTCAAAGGCTTGACTTGGAAGTATTTAGGTATGTCTGACCTTACAGCTAGTGATGGAAC
AGTTATATTATCTGGCACCGAATATTATTGGCCGGGAACTAAGTGGATATTAAACGAAAT
AAATGCACATAATATCAATGGTGATAATCTAAGTGTTACAAACGGAACTTTTACCGACGG
GAAAATAAAAAGTACATGGAACTCAGCTACAACATCGTGTAGCACGCAAATTGAAAATAG
TCACATCAACATGAGTGCAACAAATTCATCATGTAAAGTTACAAATAGTTTAGGACTGGA
TATCCAACAAGGTTTTGGGATGCGTTGGACTGATCCAGCTTCTAATCAATCAAGATCAGT
TTCACTTGATTATGGAAACTTAGCTTTTGATAGAAATGGAGTTGCAGCAGGAATAAACTT
TGATGGTACCAATTTATCAAGTTCTATGCCTATTCAAGCCCAGAACACAATTGTTAAGTC
AACTCAGTTTAGCGTTAGTGGGATAGGAATCCGTTTAGACCGAATGATGTCATTTGTTGC
AGTTCCTTTTTCAGGGCAAGCTTCTTCAACGATTAACAACTCTCCAACAATTATCAATGT
TCTTCCAGAAGGATTTAGACCAATTAGTCCATACTCAATAGATTATATGGTACCTGGTAA
TTCAGCTAATAATGGTTATATCTATTTTAATCCAGATGGTTCAATTAGTATTATGGCAAC
AATTAATAGTGGTTATTACATTAGGGGAACAAGATTTTATATCACTAATGATGTTTATCC
CTCATAAAAAAGAAAGGAATTTATTATGACAGTAAAAGCAACGGGCGAGTCTATGAATCG
CGAATTTAGAAATGAAAATGGAGAAGTAATTGTTTCATCATCAGCCAATGTTGTAATAAA
GACCATCGGATCAATGACCCTTAGATTATTAGATGCTCAAAAAATAAAAGATTCAGAAAC
AATTGTAGAAGAAATAAAATTTTTGATTGATGATGTATTGGCAATGTCCGCTAAATACTT
GAATTAGAAAGCAGGGGTTATGGAATTAAAACAGCTCGTAGAGCAGCATGAGACTAAACT
CAACCAACATGATAAGGAACTGTCTCGACTCAACGACATGTCGGTTGAAATGCAAAAACA
AATGAGTGAAGGACTTGCTCGTGTGGATGAATCCAATCGCTTTTTAAGAGAACAGAATAT
TCGACAATCTGAACAAAATGCGCAAATTTTGCAAGCCGTCATCAAAGGCAATGAAAGTTC
AGATGCACGGCACTTTCAGTTGAAGCTGCTTGATAAAACTAACTTATGGAAATAGATTTT
CGGAATTGGCGGAGCTTCGGCGGCCATATATACGATTCTCATCGCAGTTATAAAATTAAT
TAAATAAAAGAGGAAAAATAAAATGGATCAGAATTTAATGATAATCTTTAGTGGTATTTT
AATAGTAGTCGGCTCTGTAGTCTCTTACTTCATTTCACAGACTGCTAAAAAACATAGCAA
TGTGAAAAACATTGATGCTTTAGCAAAATTGGCTAATCAAGGGGTAAGTTGGGCACAGAA
ATACTTCAATGAGAATCCTGAAAAGTTATCTGAAGCTATTAATTATGTGACACAAGAAGC
TAAGAAACTTAAAATTAAGACTAATCCAGCTCAGATTGAAGCTCAGATTGAAGCTTCTTT
GGCTCAATTGAAAAAGAACTTTACTGCTGACCCTGCTAAAACAATTAAAGATGTTGCACA
TGCTACATCTGAACTTGCTCAATCAGTATCTAAAACAGCAGATACTATGTCTACTAACGC
TGAAGAACTGTCTAATCTTGTAGACCCAATTATTAATGGAATAGAAGGACTCACTGAAGA
ATAGGGGGGTATTATGAATGGAATTGACATTTCCAGCTATCAAGCAGAATTGAATGCTGG

AATTGTTCCTTCTGACTTTGTATTTATTAAAGCAACGGAGGGAACAAAATATATTAATAC
AACTTGGAGAGAACAAGCTGGTCAAATCACTCAGGCAAATAAGCTTCTAGGTTTCTATCA
TTTCGCTAGTACTGGCAATCCAATTGCGGAGGCAGACTTCTTTATCAGCGTTGTTAAAGA
CTATATTAGTAAAGCAGTTCTGGTCTTAGACTTTGAAGCTGGGGCAATTAATGCATGGGG
AAATGTAGGTGCTCGTCAATTTTTGAATCGTGTAAAAGAAAATACTGGCATCAATCCAAT
GATTTACATGTCAGCAGAAGTTACTCGTCAGTTTAATTGGAGTACTATTTCAAATAGTAA
TGCTTTGTGGGTTGCTCAGTATGCTTCTACAAGTCCTACTGGTTATCAATCAGAACCTTG
GTCCGATGGCAAAGGGTATGGCGCTTGGAGTTCTGCTGCGATTCATCAGTGCAGTTCGTC
AGGGACTTTAATGAACTGGAACGGATACCTTGATTTAAACTTGGCTTATATTGATGCTAA
TCAATGGAAACAGCTAGCTAGTGGAAGTTCTACATCGCAAAATAACAACAACTCAAATAC
TAATTCAAAATTGGCGGATGATGACCTTATGAAATTTACATATCGAATTATTGATGCAAA
AACAGGAAACACGCAAGGAACTATTTACTACTATGATGGAAATAAAGTAGTTGCTTTATC
TCATGAAGATCAACTTAAAATTATCCGTCAAATCTATAAAGAGACGACAGGAAAAGACTT
GAAACAGTATAGTTGGAGAACTGATGCTCCTTGGTACGTTCATTTCATGCAAGCAATCAA
CCAAAAAACAGTAGAAGTTGCTTGGAAATAAACTATGGTAGAATTTTTCCAAAGAATTAA
AGAATTGCCCGACCTGAAACTATTTTTAGACTACTGGTGGGTTTGGCTGATTATAG
>NewGenomeName_23
TTCCCGCGAAGCCGCCTGTGCGGTGCGGTGGTGGACGAAACCGGATAAAACAACCGCATT
GTGCAGATATCGATTGAATTTGGTGCTGCTGAGTGATATCTGAACAATCACAGCGGTCAG
TCTTCATGAGTTTTTAACACGGGGCCTCAGAACGTGACATGGCAAAGCTGGAATGGTAAA
AGCTGGAGCAGGCATTCCGATACCAACATGCGGAAACGGGAATAACACTACTATAATGGT
GCCGGAAGAAAAAGATTAATTACAACACCGGCAGAACCCGTATAGAAATGGGCAGAATCG
ATCATGAAAGTGATCATAAGACCGCACATGAAATAGATCATGACATCTCAGATGAAGGAC
CCAGCAATGACGCGGGTTCCGGCGATGAAAAATATGAAAAAAACTCTGAAAAAAACAGCG
CAAATTCGGCAGAAACGAAACGGATTGGTAGTTCCCGGCTTTTGCCACCTTCAAACGCTT
TATCTCAGAGAAACCCCCACGCCGTAAGAAACCGTGGATATCCGAAGTATCTTGAGGCAG
AAAACCTCATGGATGGTGCGTCCGACAAGCTAATATTCGATGGACTGGTGTTCACCGGGG
CCCGCGCACTTTCAGTAACTAAGGCCCTTAAAGGGATGTGCCCCGACTTGGACGAGGTAG
ATGACGTGTGAACCCGTGTTGCTCTTTACGGCAAATTACTCACACCTGAACAGGCCCTTG
ACCGAAATATTGACCGTATCGAGTCAACTGTACGCTCAATGCTGACGCAGGACGTCTTGG
GTGAGCCAGCAGCATAACTTCGTGGTGACCGGGAAGGAATCCACGCTGCCATAGATAAAC
TCACCGCTGAAAACAATATTCTGACCAACCATCGTCGGGGCATTGTTACGCCTCTCAGTG
ACACCGTGTCACCGCTTCCTGAAATGAGTAATTCGGGGAGACTGTACGACATTCCGGAAG
AATGAACCGCGATGTGATGAGCCGTCAGAAATTACCGAGGCTGAACTACGTCGGTTCATC
ATAACAAAACTGAACAATCCCAGGTGGCGGCTCAATCACCTCTACAACATACTGAACGAA
ATAGGTGAACTGGGCACCTTCAGTACGCGATCGACGCAGCGCCAAGTGTTCCGGAGCATG
CACAATAAAAATATTATCTTGAAAGCACGCCGGCCGGGATTTGCCACGGCCATTGATATT
TATCTTCTCTACCACCCATTATTCATCCCGCTTCTCAAATGCGGGATCGTCGCTCAGGAT
TACCAGGCTGCCAATGAAATTCTCCGCACAGAAATTGCAGTACCGATTGATCATCTCCGT
GATTGGCTAAGAGCCTCATTCACCATCGTTGAACGTCGCAGCGGTGCCAGCGGTGGCTAT
AGCATGTTTGGTCACGGCTCTAGTGTCAAGTGGGCAACCTCATTCCGTTCAAGAACGGTG
CAGCGCCTCCATATCTCAGATCAAGGTAAAATTTGCGCGAAATATCCGGCTAAGGCGGAA
GAACTGCGAGTCAGTACTCTTAATGCCGTCTCTGATGCAGGCATTATTTTTGATGGGTCA
ATACCAGAAGGCGTGGGTGGTGATATTAACGAGATGAGTAACCGAGCACAGGAGATCACT
GCATCAGGCTCATTGTTGACGGCACGGGATTATATATTCCATGTTTACATCTGGTGGCAG
GATCCTCAGTACAGCACCAAAGTGCCGGATAGCGGGCTGAAGCTGTCAGGCGAATAAAGG
AGGTTTTTTGCTACGGCTGAGAACGCAATGAACATCAAGCTGACTGACGAACCGAAGCAT
TGGAACATCAATAAGGAAACGGAATAACGTGAGGAAACGAAGCAGGCGATTCCCTCAACG
CCACAGGACGCGTGTCTGACGTCCGGACTACGTGTTGTCAGTGCCGAAAGTATATTGCAG
GCCGAATCATTCGGATCCCCACCGATGATTGTTTACAACATTGACAATGTTACAGGAACG
AGGACTAAAGCTCAGTCTCCACGTGAAGGAAATAAAAACTAGTTGCAGCGTACGCTGATG
AGTGATCTGCTGGTATGGGAACTTCCGGATGCGGAGGAAGCGTATGTTTGTGGAGCAGAT
ACTGCCGAAGGGCTGGAACACTGAGACCGCTCATCGCTGTATGTTGTGAAATGCAGTAGT
GGCGAGCAGGTTGGTCAGTGGTTCGGGCATTTCGCTGCTTGACTTTTTCCTCATCTCATT
TCGCAGGTCAGTCGTATGTTTATCAAAGCGTATTTGGGGCCGGAGCGGAATACTCACGGT
TATGCAGTTAACCTGAAACTCCGGGAGCTCTATCCGACACGTCATATATACAACGAATTG
CATCTTGAGCAGCCATCCGACGACGATACGCCCCGCCCTGGCTGGCTGACAGCCCATCAG

AGGAAACCTGTCCTGACGCAAGTAATGAAAACGCTTCTGAATAATGGAATATCAGGGATC
CGCTGGGCAGGCACATTATCGGAAATGGACACCTACGATGATTACGCGAAAGGCTGCAAG
AATGCACAAGACGGATGCTTCGATGATCAGCTCATGAGGTTCATAATTGCCCCGGTGATG
CTCGCCAGAATGCCGGGGGGGGTAAAACCGACAACGGATAAACGCAGAACCACACACTGG
ATGCCTCACTGATGTAAAATGAAACTAACCCCATGGGGCCGAAAAACGACAATGGAGCCA
CGCCGCGTTTTTCTCAGCGCCAGTTTCAGGTGCTTTGTTCTGCCATTGACCGCCAGCCTA
AATGGTGTGATGCCCCAAACAAGGTCTGTGCGTATTACGATGGCTATCAGCTGCTACGGG
CAGTTCTCCAGGTATTGAAGGATCGGGGTCGTCCGATGACTATCCATAACCTCATCGCGC
GTACCGTCAATGGCGTTCTCGGCATGGAGGCCAAAACACGGACTGATCGGGCGCTTATGA
CAAACGAGCCAGACGATGACAATGAAAAATTGGCTGAACCTATGAATGCTGAACTTGCGG
AGGCATGTAGCCTTGGCAGTATGAATAAAGCCCGCTCTCATGCCTATGCGGAACTAAACA
AAGCGGGCCTCAGTTGTAAGGAGGTCTGTCGAAACAGCGATCCGGCAGAGCCTGGATATT
AAGTGCCTACTGTCAGCCGGAATGAGGTTTTCTGGGACGGGCTGAGCGGGGAGGCTGATT
TAAGTGACTGCCGATGGCTGATGCGTCGCGTCTGGATGGATACCGTTGAGGCAGAAGCAA
CATTCCTTGGAATGGCACAGGTGATCGATTAAGCCATTGACGAATGGCGTGGTATTGTCT
ATACCACGGTTGCTGAAGGCCAGCCCAGTCCGTTTATGAGTGCATGACTCGAGTATAAAT
CATGGGATCGAAAGCCGAACGAATGTCCTCAGCGTGAACGCCGACGTGTGCTGCTTGATG
TACTTTATTACCGTACAATCGAGCGTCTTTCGGTGATTGAAGTCAGTAAGGGACGGGTGG
TGGCCTTTGCTAAAGCAAACCTTACGTAGGCGGCAGCCGTGGGGTCCGCGTGGGTTCAGG
CGAAAGCTAGGCGGGAAAGACGTATTCGTCAAGCCTGGTTTATCGGCCCACACTTTATTG
TGGATCGCCTCTGTACTGCTCCGCATGGGATGTTTCCGCTGTTCCCTTTAGGGGGCTACC
GAAAGGATAAAACCGGGGAGCCAGACGGGCTAATTTCCCGCGCCATTCCGGCACAGGACG
TGGTGAACTTTCGTCGTATCCAGCTGACCTGGTTGCTTCAGGCCGAATGCGCGATTATGG
ACGAGGATGCCACGACGTTGTCAGACAACCACCCGATGGAGCATATCGAACGTCGGGATG
GCACTCTTATACTGAACCCGGTCCGAAAAAATCACCAGAGTGTTGCAGATGTTTTTCGGA
TTGAGCACGATTTTCAGGTTGCCAACCAGCTGTTTCAGGTCATGCAGGAACCGTATAAAC
ATATCCAGGAAACCACGCGAGTTTATTCCACATTTCTTGGGCAGGATTCATGTGCGACGT
CAGGCATTTCTATCAGTAAACTGGTGAAACAGGGGGCGACAACCCTTTCGCAAATCAACC
CTAACTACCAGTTTGCATGCAAGCAGGTGGGTCGACAGTTGCTGCCTTATTTTCTCGATG
ACCTGCAAAAACGCCGTAGTCATGCAGTAGTGATTCCTCGCGAAGATCGCCAGCGTCGCC
TGACCATTGTCCGCAATGCTGAAGGTGATAATGGTGAACTGACCAATGATAGTTGAAGGT
TATATACACATATTGCCCTGGCGCGTGTTCAGCAGACACCAGCGTTTAATGCATAGCGTG
CGCCGCGAATGTCAGTGGTTATTCAGGGGCAGACGCCCCCGGTGCAGGCTGTTGTGCTGG
ACCTGGGGGTTAATCGTCTGGATGTGCCACAGAAACAGGAGTTTATTGAGCGTATTCGTG
CCGCGCTGGCGCCACCCTAAGCACCGGAGGAAAGGACGCAGGAAGAACAGGAAGTAGCGG
CCCAACAACAGACACTTCATCTACAACACGCGGAACTGCAGATGCGCGAGATGGCTGGGA
TAGTGGCTAAACAGGAAGCTAACGCCGCCGGGGCACATGCCGCTGCACACCGGGATAATG
CCAGTGTCCAGCGCGAAGTCGCACTGAAACAGGGGCACCGTTATGTGGATGGGCTTAAGC
AGGCACATACCGCAGAAATTATTACGGGCGTACAGAATATGGAACAGGAGGTGTACGATC
CTCAGTAACGGATGCTGTATACGTTACAACAGGGGACGACGGTAATGTCGCTCTGAAAAT
TCTGGCTTCAATTGAACCCCGTCATAGTACGGGGTTTTTTGTGTCCGGAGGTAATCGTTC
CGGAAGCGGTGCGTTTCTTCGCGGGGGCTGCGATAAGCCTTATTTACTCACTCATCCCTA
CCTGTCTCATACATCGACCATGGGGAGTTATTTATGAATTTTGAGCTTACGGGCGAAGCA
AGCCCGGAACAACTAGAAACACTGCTGGCCGGACTTAGGGATGTCGATATAGGCACTCTC
GCACAGGACGTCGTCACGGAAGCTACCACGGAAAAAGATGCGGATGAGGAACTTCAGACT
CAGACGGGCGATAACACTGTTGCACCGACGCCCGATGCCAATGTGGAGCAGACGCAGGAC
GTGAAGGTGTCGGAAGTGAAGGGGGGGCTCACCCGCGACGGTAAACACGTCATTCCCTAT
GATGTCCTAGAGGCTGAACGTGACGGTAAGCAACAGGACGAACAGGAAGCCGCACTTCTC
CGTGGGCGGCCAGCTGGAGAACAACGCAGGGCGGAACCGCTGACATCTCAGATCCACCAT
GCCGGTAGGAACCCCACTCCGTTACCGGAAAACGAAAAAATTTCCGATAAGCAGATTGCC
CGTATCAGGGAGATGTAGCGGGAAATTTGTGACGCGATAGCCTCTCTCATCCATAATAAT
ATCTATCTCCAGGCCCGTGTTCAGCATTCAGCACCGTATGCAACAGGGAATGGTGATGAG
GATTTATCAGCCGTTCTTAATTCGATGAATGCCGTGCGGGTGCTGAAAACGTGGCAGGAG
TCCGATCCATATCGCTTTTCGGTTGCTGTATCCATCGACGGGAAGCTCCAGAATGACCAC
TCATGGAAAGACAATACGCACACTGAACGTTTCGCTGAAGTAGCCGGTCATACGCATGTG
GCTTTCGGAGAAACCAGTGAATCGTCGGCTGACAACAAGGAAAACAAAAGGGATATCCGG
AAAACGCCGGAAGAGAAAGTGAAGACCGCTGAAGAGGTGCAGGCAGTACCTGCTTCCCCC

TCAGATTTAGGCACGACGGCTTACGGCGGAACCGGTAATAATTTTGAACGGTTACTTTGC
GCTTCGCATTCAGAGGCAGAGGCGATTATGCGCGGTGTGACGAATGCTGAGATAGACGCG
ATTCTGGAGAAGCTCGGGAAACTTGCTGAAGAAGAACTGAAATAATGACGAATGTAACAT
CAGCCCAGTCGAATAAGCAGCATCAGGTGGCGCTTTTTATCGCTCGCAACCGCAACCGAT
CGATGGTAAATATCCTCATTGAACAGCAGGACGCGACAGAAGCGGTTTCGCCGGACAAGA
CAATCACGAAGTAGTCCGGCGGGAGTGCGCGGGTTGCCCGTGTCACAAACCTTAACAAAC
TGGGCCGTGCTGAAGTGACCATCAGCGTCAAGCACCAACTCTCTAAACGTCCGACTCTGG
GAGATGAGCGTGTTGAGGGTAGAGGTGAGGATCTCAGCCATGCGGACTACTCCCTGAAAA
TCCATCACGGACGTAACCTGGTGTATGCAGCCGGACTGATGAGGCAGCAGCGCTCGAAGT
TTAACCTGGCATCCTCAGCCAGAACGTTTCTGGGGACGTACTTCAATGACCTGCAGGAAC
GTAGTGCGATAGTGCATCTTGCTGTAGCTCGTCCTGATTATGATGCTGGCTCAGTATTCT
GCCTACGCCGGAGCACGCTGAAATCAAAAAAAGCTTGATTAACGATGTACTGCCACCGAC
ACATGACCGTCCCTCTTTTGGCGGTGATGCGACAGGCTTTGAGCATATTGAAGCGGCAGA
TATTTTTTCTATTGGCCTGGTAGACAATCTCACCCTGTTCATTGACAAAATGCCGCATGC
GGAAAAGCCGGTTCCTTTGTCCGGTGATGAACTTTACGGAGAAGAGCCATATTACGTCCT
GTCCGTCACGCCGCGTCAGGGGAATGAGTGGTACACCTCGAGGTCCGGTAAGGACTGGGA
CCGTATGATGGTTCGTGCCGTGAAGCCTGCAAAAGGTTTTAATCATGCGCTGTTCAAAGG
TGAATGTGATATGAGGTGCAAAATCCTGGATCGTAAGCGAGCGGCAATGCCGAACCATTT
CTATCAGGGGTCAAAGGTTCTAGTATCAAAGACTAACCTGACGGCAACCGCGTAAGAGGT
CGCTATTGCAACCAATCTTGACGGCGCCATGTTAATGGGGGCTCACGGGCTGGCCAATGC
TTACGATGAGAAGGCGGGCGGTTACTTCAGCATGGTTGAGAAGAAAACGACTATGGATAT
CCGTACTGAGATAGCAATCATCTTGATCAACAGTCTGAAAAAAATCCGTTTGCCCCAGAG
GAGCGTCAAGATGCAGGGTCCCGGCGTGATTGGCGTTGATTCAGTCGTGAAGCTCTGATT
ACTTCCTTTCCCTATGCCGGGGTTTCGCGCCGCTTTTTCATGAGTCATTAATTATGGCAA
AGACTATCCTTGCCCCGTCCCTGAGTGAACGTGTGTATACGGGTACACAGGGTAATGAGT
CAGTGTCAGAAGGCGTAATTACGGTGAATGCTGCGGAAGCGGAAAGTGTTATTCATCTTA
TTTCACTACCAGTGGGCATCCGTATCAACCCGCTCCAACTGGTCTCGACGGGTGGTCTGG
CACCTGCAACCTTCAGCATTCTGTCCGGTGAGCATGCTCTCTTAGATATCAGCGATGCTA
TTTCTGCAAAATTTGCGAGAGATGTGCCAGTGGAACCGAATAACACACTGCGTGACGGGG
AGCTGGTTACTGTCACGCTTAAGACTGCCGCTTCAACCGGCATCCTGAATGTTGTGCTGC
GTTATACAGTGGTGGGATACTGATTAAAACCGTCCGGCCCGCGTCATGCGCGCTTTTTGC
CCGGGAAATTATATGAGTGAGAAAATTGCCGTTTTCTATATCGGTCCAAAACCCGCGGAA
AAGGACACCATTACCGGAAGTCGAATGCTGTTCCCACGTCTCGAGCCGGTGTATGTTGAC
AGCGCGATAGCCTCGCAACAGCTGCGGTTTCCGGTTTTCTGGTTTCGTCGTGAAGAGCAT
GGTGATGCTCTGAAAAAGCAACAAAATAATGATCAGTGCCGGCAGGCACAGTAGGCGCAT
GAAAGAGTGCTTGCTGCGCTGGCAGATGCGCAGAACAGTTTTGTTGTTTCTGTTGACGGC
CCGGAGGGGGATTAAAGTGAGCTCACCTCAGCACGGCTGGCGACGCAGTGTCAGGCAGAA
GAGCTGGATATTCACAAAGACCCGAAACAAACGGCAGAGGCATTCCGTATCCGGGTGCGT
GAGGCATTTCGCCGTCGTGTTGCGGAGACTGACCAGCAGGGCGGAACAGATGGCCTCTGT
ACCGTATGTCCGTCGTCATATGATCCGTCCACTGAACATTATGATTACGGATGCACTGTC
AATGACTGCCGTGGCATTCAGCAGCCAGTCGTTGGTGTGCCGTCGGGTGATTACTCTCGT
ACCAGTAGCAGAAAAAGAACGCGTGCTTCCGTATGACAAAGACGCTGAGGAGTGCGATCA
TAGCATCCGTATCTCGGGAGATAATCATGAGCTTTTTGTGGCTCGGGATGTGTATATCAT
CACCGGGCACTCCCTGCGATTGGCCTGTTCTCCCGGTGAGGTGAGCGTGCTTCATGGCGT
CGCTCCTAAAGCCGGACGCAGCCAGATACCGGATGAACTCCTCACATGGCCACAAGAAGT
CGCTGCGGGGGCGCTTGGGCGTTTGTTCATGCAGTCTGGTGTTTCATGGTCACATCCGTT
ACGCGCAGAGTATTTTTCTGTGCAGTTTTCTGCGGGGATTTGTCGGGCATTTTGTCATAC
AATGGCGCCAAGCCCCTACTCTTGATACCGCAACCCTGTAATCAGGCAGAGGTTGTTCTG
ATGACGTCGGTTATAGAAATCATCGGACGTGTAAATACAAAGCAGGTTGACCCGGTGTTG
GTTTACTGGCCCCTGCACGAATTGTGCGATGATTACAATGATGCTGTGAGGACAGGGATT
CTGGCGAGACTGGATGGTGGCGGAAGCCTGAAAACAATTAGTGGTGTGCCGGGCGCCCGT
CTGGTTGTGCCCGATGTTGTAATACACCCTCTTGACGTGATATGCCTCAGGGACGGTAGT
GCAGTGAGACCATTATCACGAGATGTGCTGGATGCACAGTATCGTGAGTGGCCCACAATG
AAAGGTATTCCTGAATGTTTTATCAGCAACCACTTGTCCCCGCGTACATTCAGGCTGATT
CCTGCGCCTGACAAAAAAAAAAGTAATGATGCAGTGGTGAGCCTCATACAGGAGGCAGTG
TATGTTCTGATGCAGGCAGATGATACGCCAGTACCACTGGAAGAAGCTTATTTTGACCCA
CTGCTGGAGTGGAGGTTGTTTCGTGCTTTCAGTTATAATGCGGCCGGTGGCGAAGAATCG

GGGATGGCTGCGCTGCATTACCAGAGTTTTGTTGAGCAACTTGGGATCCAGCAGGGGGCA
GAAAGTGCACTGTATACCCGTAAGAAAGTGTTTAACGTAGGGTCAGAATGAGCGTTGTTG
TTCCGGGGACGCAAAAATCTCCTGCTGGTGTGGCGATATCAGGAGCAAATACTACCCTGT
CGGCCCTGCCAGTTTCACCGGATACGCTAAGCGGCACCAGTGGGTCGGCAGCGCCTAGTG
AAGCTGGATATTACGGAATGTCGATGTATCCGGGGGAGTAGGCTGTTTCGGTGACGGTGA
AAGGGAAGACTGCTGTCTACGGACGTGTGCGTATTGTAGACCCCGAAAGTACGGTGACGC
TGAATATGATGTCATGACACAGTATTGTTGAGGTTAGCATACCCGGAGAACTGGCGACAG
ATTTCCGGCAGATATAGAGTTACGTGGCTGATGACCTTGCCACTATTCTTCGCCTGAATG
AAGACACGTGGACGAAAAACACAGAGGACACACAGGAAAAAAGAAGTGAAGCATCCCGTG
CTAAGTGTGCATTTGACAGTGCAAAGAGGGCAACCAGCAGGGCGGCTGCAGCAGGACAAA
AAGCCACTGATGTCACTGAGGCTGGGACCCGTGCAGTCACAGCAACGGGGAATGCAGATG
GAAGCTCGACCCGTGCCGGATAGTCTAAAAAAGCTGCCGCCGCTGATGCAAAAAAAGCCA
GACCGGCTGCTCAAAAGGCCAGGCTGGCGAAGGAGAGCGCCGGAGAGATCGCTAAGCGGG
CAGAGCCTGGCACTGTCAGGGCTGAAGAGGACAGACGTATGGCTGAGAATGCACGGGGGC
CTCGGGGGCCTCAGGGAGAAACTGGTCCGTAGGGGGATGTCGGTCCTAAAGGCGATAACG
GTCCAGTGGGCCCTCAAGGGCCCGCAGGGCCGAAAGGAGAGCGTGGTGACGTTGGTGCTC
AGCGGGCTGTAGGGCCTGCTGGGCCGCATGGTGAAAACGGTGGACATGGGGGGTGAGGAC
CGCAGGGAATCTCAGGCTTGAAGGGGCATACCGGAGAGCGGGGGCCTTAAGGGGACCAGG
GGTATATGGAGCCAAAAGGCGAAAGAGGTGATCCGGGGGGTCCTGCAGGCCCGCAAGGTC
CTAAAGGGGAACGAGCAGAAGCCGGACCACAGGGACCGATGTAAGCACGGGGTCAGCCGG
AGGAGACTGGCCCCCGAGGTGAACCTGGTCCTACTGGTCCGAGAGGCGAACGCGGAGAGC
CCGGACCTCAGGGACCACGTGGAGAGCCATGTCCGGCAGGCATCGCTGCATATGTGGGTG
ATGCAACGACGGCACACAAGGGAATTGTGGAGTTAAGCAGCGCACCGGTCGGTTATGATG
GCATGAAGGCTGCCACCCCGAAAGCGGTGCATGCGGCAATGGATGTGGCAAATTAAGCGA
AAACAAAGGCAGAAGAGGCTGCAGCAGGAGGTGGAGTTCCCAGTCCGAAAGAAGATAAAG
TGGACACGGGGCCAGCATGTCCGGCTGGGTCGGAGGCTGATAAGGGAGAGGGCGGTGCCA
CCGGCCCTGTCGGGGCACCCGGCAAACGGGGACCGGCAGATCTTACTTGTCCGGCAGGCC
CGCAGGGGCCGCATGGTGACAGGGGAGAGCGGGGAGAGACCGGTCTGACGGGAAATGCCG
GTCCACAGGGTCCAAAGGGAGATACCGGTGCGGCAGCCCCGGCAGTCCCACATGGACTAA
AAAGAGAAACAGGTGCGGCAGGCCCGGTAGGGGCGACCGGACCTCAGGGGCCGAAGGGCG
ACCCGGGGGATACGCAAAGACGGTTCGCTCTGGGGCTGGGAAACATTATTGAGACAAAAA
GCCATAGCTGGTTCCCGGATACATATGGCGCACTCATCACGTGAGTGACCTTTCTTGACC
CCCAAGATGCCACCCGGGTTCTGGGTTTTTTCCAGCATTTGCAAGTCAGGCTTCGTGACG
GGCCGTGCCAGGATGTCAGGGGGCTGGGTGAAGTGGACAGAGATACAGGCAGAACAGGAG
AATGACTTGAATATTTTGAGAAAGCTTATGAAGAGTCTGTGTGGTTGTGGATAGCATGAG
GACTGTGAGGACAGGCAGTCGCTTACAGTAGAACTGCGATTGAGACCTGGAGAGATTCTG
TCGTCAGATGAGAAGGGCATAGTTCCGGAGCAGGTCGGGGTAATCACACAGGTGCTGATA
CTGAGTTCTGATAAAAATCAGACACAGTGTGTGGTAAGACCGCTGCAAATCTTGAGTGCT
GAAGGGTCGTGGGAAAATAGTGGCGGGATGAAATAGCCGACAGCTTCAGAAAAACCGTAG
CCCGGCTCGGGTTTTTGTTGTCATGTATAGGCGGGGGTTACTAGAGAGTGAAGTAATAAA
TATGTTAATAGGATGGTGTGAAGGTTGCCGTGTAATTCTGGTTCAAGAGTTTTTTATGCC
GGGAAATCGCATGACTATTGTGGATAGTATAGAATCCTGGTTAATAATCTGTGATAGTCA
CTTGGGCCATTAAATGTGCAGTATGTATCAGGGACGCCGTTTTATTCAGCTGAATCGGGA
AACATTGAAAGGGGTACATTGTGTGGCCTTGCGCGTGAATTGGGAATTCACACGAACACA
GTGAATAGCTTTCCATATACGGGAAAGGCGAAAAATGGACGGTATGGTGTGAGTGAGTAC
CATCTAGCGTGTGCGGAGTGATGCGTCTCGTTGCCACAGCGGCAAAGATAATGCTATGCA
AAGAGGGTAGGAATATGCTCACCCAAACCGAAAGAAAATTGCAATGATCAATGAAGTTGT
TTAGTCATCATCAGAATTTCATGCAAGACATTTTGTTACAGTGATGCTGCTCGCGATTTT
AGCGAAGTCCCTCATATATCCCCAGACTTAAAAGCCTGAAGCAGTCAAAGCACTCTCTAT
GCCCTGTATCGATATCACCAAGATGCGTGGGATGATGACGCGCGTTGTGACATCCATGCT
GCTCGAGCATTCCGCTGTACTGGCGGAAGACTGCCATTTCCAGTTTGGTGGTATTACACG
AGACTGTCAGATATTCGGGGTAGAGAAAACATTCACAATGAAGCCAATAACCATTTTTCA
TTAGCGTGCCGATTTCTTGTGTGCATGGCCGGATGTGGTGGATGAGATCCGCACAACGAT
CGCTCAGGACCCCCACGAGCGTATTTACGACACTGACGGGCGTATTCCTAAAGTGGCTGA
TGCGACTATTGCCACAAAAGGAGATGTGAATCACCCGACATCAGAGTATGGTATGGCGAT
CTCGGCGCCTACGACCACTCCTGTCTGAACTGTTCGGCAGGGCGTCGATGTTTCCGTCGA
TAACCCGGAGGATGATGAAGCCCGGTCTTATACGGGAGCCTTTGTCCCAGATTATGGTGA

AGAAGGTACGCCAGGTCTGGCGTCTCTGGAGGTGGCACTCCGTACTCGAGCTACTGCGGT
ACACATGACGCTGGATCCGGTGCCATTGGTGAATTCCAGTATTAAACGTCGCCGGCTTTA
TCGCTCGGCATCAGGTGGGGGGCAGGCGGTTTTTACTTGTGGCTGAGTTGTATGCATC
CGTGTTCAGTTACACGGACGAGGTACCGGCGATAAACCTCGCACCTTCCTTGGCGACATG
GGATCACCTACCGCCACCAGTGAATATGACAGGCCTTTGCCTGATGGCTAACGGTATCGC
CGCCGGGTTTGCCGGTAATGAAGTGATGTTTTCGGAAGCGTATCTGCCGTATCCATCGCC
GGAAGTGAATCGAGACACAACCGCAGACGATATTGTAGCTATCTGTAAGCTGGGAACGTT
ACTGGTGGTGGCGACAAATGGGGAGCCTTATTAGTTCAGTGTGGCATCACTGTCCACTAT
TTCTGGTTCCAAAATCCCTTCAATGCAGGCGTGTGTGAGCAGGGGGAGTATGGCTGCGAT
GGAGGGGTTTGTGCTGTATGAAGGAACATATGGCCTGGTGTCTGTTAAAGCAAACGGGAA
TGTCGCGCTGGCGAAGGAACAGATTGTTTCACCGGAACACTGGCAGAGTCAGTTTAATCC
GGCCTCCATTGTGGCTTATCCCTGGCGTGGTGAATACGTTGCCTGTTACCAGAAACCGGA
TGTTAAGCAGGATGTGGTTGATTTCCGTCCGGTGTACATGGATATCCGTTATCACAGTAC
ACCGTTTGAGTGCGCAGGACTTAATCTCGCGAAAGATATGATGCGTGTGGTGACAGGAGA
CCAAAGGTCAGTGCTTGCCGGGGAGGCTCTGCCCTCCAGGATAAGGTGGAATCCAAAAAT
TTTTTGATACCCTGAAAGAATCTCTTCTTCCTCTATCAGGGTGAAATCTCCAGCGCCTGA
GGGGCTCTGGATGACCATTAGGGCTGATGATGTTACTGTGATTCATTTTGCGCCGGGTAC
GTTTAAGGGAAGTGTGGTGAGTCGTCCGGCAGCAACCGGGCAAAACTGGCACGGGCCAGC
ATCCGGATTCGGGCAGCTTCAACAAATAACCCTGACTACATCAATGTCGGAGATGACGAT
ATGTCCATAAAACCGTGGCGTACGGGGAAGGATTTATCCACTGTTGTGGAGAATATGGAT
ATTGGCACCGGGCAGCGCGGTGGAGGACGTCATGCATTTGTAACCCGTGGGGAACTGATT
GGTCTTAAACTGGCCCGGCGACGAACATTGGGTGGTGGCTCATATGCACCGAATCCGCGT
TTTAAGATTGACAGTACTTTAATGACTGTTGATTTTCTAACAAAACCGCTGAATTTTAAG
GCGACCGGTGGATTTGCCTCGATTATTGTTGAATTAGATATGCCTAATTAGCGCAGACAT
TCAGTGACTGAAATCTAGCGGGGTACGGAGCATGTCCTTCCTGATGCAGTGCTGGTTCCC
ACGACGCCGGGGCAGGTTTACGGCGATCCGATTGACCCTGGCTGGTCGGGATTTTGCTGG
ATACGTTTTGTTAACGAGGCAGAACTGAAAGGTCCATGGAATACGGAAAAAGGCACTCCG
GCACAAACACAGATGGGCGTGAAGGCCATTAGTGACCAGATCCGAGATGAGGCTGCACAG
TCGACGATTGTGTCGGAGCTGCGTAAAGAAATCACAAATGCGCAGGAGCAGGCGGTAAAG
GATGCTGCAATTGAGACAACCGAAGTTGTAGGGACTCTAAGGCAAGAAACGACCAGAACG
ATTGGTGGTATTGAAACCCGCATTAGCACACCGGATTCGTCAACCAGTGGACCGCTTAAT
GAGGTGCAAAAGCGCATCACTAAATTGGATAAAGAACGCGGTGAAGCTTTTCTCGCAATG
TGGTCAAACAAAGCTGGATTTGATGGTATCTCAACGGGAATCGGGATTCGAGCCGGAAAA
GACAGTGAAGGGAGACCGGTATGTCACGGTACACTTTCTGCGTCGAAGTTATTTGTCTTT
TATCCGAATCTTCAGGATAACAGAGCCTATCTGTTTGCGGTATCAGGTTGCAAGGTAGTG
ATCCCGAAAGCGATGATCTATGACGTGGTGATTGAAACGCTGGGGTCGCGGAAGGTTTTA
GCATATCAGGTTAAAGCCGGGTTAAGTATCACTTCACCCGTTAGCCGGTGTGCCGTTATA
CAGAACGGGAACTTTCAGGTTGATTCTCAGGGTACCAAGAATATTTGAAGCCTTTTATGT
GTTACTTCACAAGGGCAACTGACCATTCGTTACTCTAATCAGAATGTAGGACAGGTGATC
CGCAATGATAAAATTGAGCTTTAAGACCAGAATGGAGGACTTGCTGTACGAACAGTCAGC
TTACGCTGATCAGTAGGTGAGTATTGGAATGCGGTTGTGCCATGTATAACAGAAATAATG
TCAATGTTACGGGCCTGCTAACTCCAGCAATTTTCCTGGACAGATTTACAGCGGAGGCTG
ACTCAAAGCCGTACTCTAATAAACCCGACGGGAAATCATTGCTAGCCGTATGTTGTTTAT
TTCCTTGGAATAATGTATTTGCGAATCGGAAAGTTCCGAAGATGACCCATAAGGACAATA
CGGTGACGTGGTCGAATCTTGAGCAGGGTTTAGGCTCTTACATTTATACATTTCGGGGAT
AAGTATTCTGTACGGTTTGAGGGTTATGAAGGCGGGTGGTAGCGTATGGTTAAGTCCAGG
TTTTGCGCCGCAGTGTCTGATCAACAAAGGCACCTTGCCGGCGACTGAAAAGTCCTTCTT
GAAAACATCAAACACGTCAAGCAAAAGTTGTTTCTTCTTTATCAAAACAGAGTAGAAGGC
GGAAGTCATGTAGAAGTATGGTCAGATCGAAGGATATCAAGCACTAAGGTTTCAAGTAAT
TGTCAGGGGAACGAACCCTGGTTTCACGACGGCTTATGTGTACTCGGATATGGTTACTCC
ACCTTCTGAGTAGGGGATCGCCCTGTAGAACCCGGACGGTGAGATGATTTATCAAGGCGA
ACTGATGCTGCTGGACGCGAAGTTAATACCGGCTGGTAGCAGTATTGAAAAGCACCTTGG
ATATCAATACGCAATAATACCCGCACTAGTCGGGTATTATAACTGGAGAAGAACTCCTTA
AGATCGACCGAATAATATGTCATCCTCTGGTGCTGCAAGAAATAGATTATATTCCTGTGA
GCAATGTTCCGGTGGTGCAACATGGGATATTCGAAAGCCGTATATAGATGAGGTCCTGGT
TATTAAAACGTCAGTATATGATTAGTTGCAGCGAGTCTTTAATGTTCATTGAAAATGTCT
ATAGAGATGTATTATTAAAAAGTTTAGCGTGTTGTCTGAATACAGGATGTCTTAAATGAA

GAGTATAGCAACACTGGTTGATTGTGCAATCTCCGGGATGGCCTGTGTAAATTTACCTGC
ACATGCAGCAGAACGAGAGCATACAATTTCTCTGGGCTATGGGCACTTTCAGTTTCCGGG
ACTAGAGAATTTGGTCGATGATGCGACTGCTCATAAGAGGGAGACATTTAGTCATCTCGT
CAACAGAAACTACTTTTCATCATTGGGCGAATATACAGACGGTAATGTCAGTAGATATGA
AGGCAAGGATATAAGTCCACAGGGAATTGATATCAGGTCTAGCTACGAAATAACGGATGA
TTTTGGCGTTAGCTGCTCTTTTACATGGGCACCTTCTCTCACTAACTGAGAGACATTTAT
TGATGTTCAGTCAGCCGATCATTCCAGGAAGATTAAGAAACCGGCAGCTTCTGCCAGCAC
GGATCTCAGGGCGAATTACTCGAGTCTGTTAGCGGGGCCTTCGTGCCGTGTTAATCAGTA
CATGAGTTTGTAAGCGATGGCATGGATGGGCGTTGCTAAAGTTAGCGCTTACCAGAAAAT
TAATGCCAATACTAACAGGAGAGGCGGATTTTCTGAAAGCAACCGGACGAAGACAACCTC
CCTTGCGTGTGCTGCAGGTGCACGGTTTAAACTGAATGAGAGTGTGACACTGGATGTGGC
TTTCTAAGATTGCGGCTCTAGAGACTGGCGCACGCGCGGCGTTACCGCTGGCATTCGCCT
GAACTTCTGACCTGTATCCGGTAACCCTTTACTCCCCGCTGTGATGGCGGGTTTTTTACT
GCCCGAACAGGGAAACAACTGTGTATTATGTGAGGGGGCTTTTCGGCTGTTGACTGGAGG
GTGAGCCTGATGGCCTGATGTGAAAGGGCCCCGATACGAGTTAAGGTTGACCCGAGACCC
CAACCCTAATTACCTTAAGCAAGTAGATTGTTAGCGCCTCTGTGTAAAAGGAGTCAAGCG
CTATGTCGCAAAAAGCGCTTATGACCGTCTCGATTTGCATGACGGTTATCTTCACCATCT
GGATGTTGCACGGTTCACAGTGTGAATTCCGGCTGAATTCGACGGGAGCGGAGATTGCGG
GGTTCTTACAGAGTAAGCAGTAGGAAAAGCGCGACGGGGACGAAAGTCCCCGTCAACTGG
TTGCTGAGGTTCAGCCGATATGCCACCCGTTTCCGGTGAGAGAATGAACGATAAAATACT
CGGGTATAGGCATCGTGTTGTGAGCAATTCCCGCAACTATGAATTTATGAATGAACTGAA
AGAGGCCGGCCTTAAAAAGCAGGCAGTTTGTTTTGAGGTACCTGATGGCTCTTAGGCGCT
GCGTTCTGTGGACTGTCCTGATCGTATCCCTTACGTTCTAGTGTCGTTGGGCGTGTGTAC
GGGGAGAAACAGCGTTGGGCGTTACCAGCCGGATGGGAAGACATTAACCCATCTGGCTGG
CGGACACTGGGATGAGTTCCATACCGCAAGCCGGTGATTTATCCGGCTGGGAAACCGAGT
GGGCTTTGATCGAATGCCGGATGAAGACGATGGCTTTATGGTCTTGAGGATAGCGGACTG
ACGGCTAAAGTTTTCTTCATTGTGTTTAATCCAACATTGTAAGTCACATTCAGACCCTGC
TCCGGCAGGGTTTTTTGATATCCAGGGGGCCATGATGGGTGGAAGTGAAGGCGGTGGTGA
TACTACAGTAAAACCAACTGCAGCGCAAATATCACAGGAAGAGGTGGCCTCGAAAGGGTG
GCTGGATTACAAAAATATCCTCCAACCGGCTGCGGATAACTACATGGTAAAGGTGGATGA
CCTGAACACTGAGCAGCATTACGACAATACCGCGGGCACCACAAAGCTGGGGTATCAGAA
ACAGTTTGGAGAGGCACGGCAGGTCCTTGCTAGTGATCTTGCTCAGTCCGGCGTTGACCC
ATCGAGTGGTCGTTTTAATGCGGTAATCCATGCCAACCAGAGTGACCAGGTAACCGGGCA
GATAGACACACCCACACTGGGGCAGATATCGCAGGCGGATAAGTATGTTGCCCGTCTACG
GGATGTGGCTGCTCTCGGTGCCGGTCAGCAGGCGGATGCGTTACAGAGGTCTAACTCTCT
GCCAGACAGCAGTCTAGCAAAAGCAAAATGGGCTGCACAGGCGACGTTTACAAAACAGCA
GGGGCGAGCCTCTCTTGTTGGCGCTGGTCTGGGTGCGGCGGGTGCATCTGCGATGCATGA
GCCTGGCGGTAGGGGAGTAAGTGGCGGTCCTTGGTCACCCGGCACCAGCGCTAATGCCTT
TCTTGATCAGGCTCAGAATTGGACACTGTAATTATGGAGTATGGCAAATACGAAACTCTC
GCATGGGCCGGTTATTCAGGAGCAGCCCACCCCCAGGGTTACTGGCAGACGTCAGCAACG
CTGACACCCTAACAACACGACGAATGGCGAATCAGATATTTGCCCCGCGTCGCAAGGCAA
GATGAACTTGGGGAGAACAACAGCCTGATGAATGCACGGCTTGCACGGGTGGGAGGACTT
GCAACTTCCGGTCTCCGGACAGCTCAGCTGGCGCAGGATAACCAGATAGTGAGAAATGGG
GTAAACCGCCTGGATAATCCCGACAGGAGTACGCTGGGTTTACGTAATGCACTGGCAATT
GCTGGCGCGAAGCATGGTATACGTGAAGCCGAACAGGATCGACAGCTGACTATTCTGACC
GGGGCCTCTGCACAGGCAAGACAGAAACTGAGTGTTGGCGGCCGACTGGTGGAAGCCTAA
GGCGGCGATATGGGATACGCAGTAGTGGATATTGCAAATCTGTCGCGGCGAGAGGCATTA
CAAGGAATAAGTGACGGAGACAGACGACGTGAAGAAATTGAGGCTGCGTACAAAAAGATG
GCGACGCAACACATAGCGTAGAACAAGCAGAATATCGGTACTGGCATTAGTACGGGGGCG
GCTATTGGCGCATCCGGTGTTGGTCCTGTTGGTGCTGTTCTTGGAACAGTCATTGGCGGC
GTTGCTGGTTCATTGTTTTAAGGTGTGGTGCATGTTCGGAGTAGCACAGGGGTTACTTGC
CGGATTCAGCAGCGTCGACCAGGCAAGGACCCTTCGTAAGGAGCTTGGTCTGCGTTAAGC
ACAGCTTGCCCGGCTCCAGAAAAATAACGAGCGCGATTTTGCCTTCACGGAATCTCAGTT
TGAACACAATAAAGACGGTGATCAGCGGAACTTTGATTACAGAGCAAAAGATGACGATCG
TTATCATGCACTAAAGGAGAGTGAGTTTAACGCTAACCAGGATTACCGGCACGCGTCACT
GCGTATGGCGCAGCAGCGACTCCAGTTGCAGAAACACAACTAGCGACGGCTTGAGTATAA
CGATATAATTGCCCATAGCGAGGCACTAATGGACGCGCTAAGAAAGGCAATTGAGGATGT

CGATAAGCACGCTGTAACGCTTAAGTTAGGGCAGCTGCCAAAGGACATCCATTAATTCT
TATGTCGAACGGAGACTACGCATCTAAAGTGGGAGAGGCCGTGTGCAACCTGCAAAAAAT
ATTTGGTGATAAACCATACATGGCGATCGATTCATGGAATAGCCCTGAAAATCTCGATGT
GCTTTCAGGCGTGTTTGCCCCGCGACTACAACAGCGTATCGGCATGCCTGATGCAACCGG
TGACCAAACCATACAAGAGGCCACGATTGGCCCTATCGTACCAGCGCAGCGGTAAGGCAA
CGTACCTATCGGCCTAGATTTCACATACAGCGATGCCTCCACCGCGCATAAAGCTTTAAC
GGAGTACGGCAGTGCGCGCCCTGATGATCAAACCGTCTTGGCGCTACCCGTTGATAAGGC
TATCGCTCTGGTCAGAGCTTGCAGCAAATTTGCAGAGATATCGAAGAATTATGGGTATCT
TATGCCGACGCAGCAGGGACTTCATCCGAAAGAGCTTTAGAAGGGCGACAGCAACGTAGC
GTCGGACGCGATCAAGAATGGCGGAACGGCCCCGGCTGCGGGGGATGAATATTATGCTGC
GCCGGGGTCTCCACCGCATCAACAGAAAATTGAGCAACAACAACTGCAGCAGCAGATTAT
TAACTGGGCGGGAAATGATCATGATAAGCTTTCGTTGGCCAGTAATGTAGCGGCCCGTCA
GCCTGAAATGCTGGAACCTCAGAATAAGAGATTGCTGGGGAACGGGCATACGAATCTTTT
CCGTACTCAAAAGGCCAGGGGGGAACAGGCCAGAGATGAAATAGCTTCATCTGCATCTCA
GTTTATACGTGGACTGAAACAGAATTACGCCCAGTAATTCACGATATTCCATTAATACCA
ATTGCTGAAGACCGGCCAATGTGCCGGGTTTTTTTATGGTGTCTGTATGATCGATGCAGC
GGTACAGCTTCCTGAGGCGCAACTTGGTAACCAAAATCGTAAAAGGCAGAACATTCTGCA
ACCCGCCGAAACTAACAGCAATAAAGCATTTGTCTCTGAACCGAATGGCTGGAAGAAGAA
CACTACGTCGCGCAGCCTGGGTGATGCATTGCCAACAATGGGTAAAGGTTTCGCCCAGTC
CGTCCGGGGAATATGGGAAATAACCCGTGGACTCGGTCATGCGATGATTCAGAGCTCGGT
ATAAACAGGGGCGCGGATTGTAAATGAGTTCAGCCGAATGGGTATGCCGGGTGTTCCAAC
TGTGAAGGATATTTTTGCCGGTGGCAGCAGGTGGACTGATGATGTCATCGATACCCTCCG
TAATGGCAAAAACGCGGTTACTGATACTATCGGTAAAGGTCTGAGGGTAAACGGTAAGGC
TGTCAGTGCTGGTGCCAATGCCACTGATGAATGACTGACTGGTAAGATGTCGCCGGGTGC
AGTTCGCGCGCTGAATGCGCCGATGACCGAAGGCTCTAATGAATCTGCGGTATGGGAGGC
GAAGGCTGTAAACCTGATTGGTGCGCTTTTACCTGATATGATTGCTGTCGGTGTGGCTAG
AAAGGTGGGTGAGGGCCCACTGCGTAATGTGCTAAGCGCCGGGCTGGAGTAAAAATGCCT
CGCGGAAGGGGTGCAACTGGCAAGAGCCACAGCACTGTCAGCAGAAGCTGTCGATAAAAA
AATGCCGGATTTATTCAAGGCGGGAGTGATCACCCATTCCACTGTGAGTGCATAGGACCG
GCGTGCAATAGCGGCATCAGATGCTGTTCTTAATGCAGATTGCCCTGAGCTGGCGCAGTC
ACGGAAAGTTCAGCAGACGTTTATGTCCATTGAAGCCGACCCGCAGCGCGCAAAGCTAAC
CGAACGCCAGAACATGGATCTAGCAAAAGATCGTGTTGACGATAAGTAGCGTACGCCGCT
GGCAACCGATCCTGAATAGAGGGCTGTGAGTGCCATAGCGGGAAAACTGTTAGGCGCACT
ACTGTTTAATCTGGTGACACGAGGCACAGTGAAGACCGGTAAAAGCCGCAGTGTCAGGAA
TGCCGCGGAACAGGGGCCGATTATTGCGGCCCAGATCGGCTATTCACGCTATCAAGAAAA
CACGGCATTGCGTGGGACCGCCCGAAAGGGTGTGTCACCGCGGGAAGGCGTGGCTGATGC
AACGATCCAAGGTGCAGCCTTTGGTGATGCGATAGGGGCTCCGTTCGGTGAGCTTGCCGA
ATATCGTGGCTGACGTCAGGCCGCAGAAGCAACCGCCATGCGTGATACTGAATCGGTGCC
GCAGGGCTTCGCAGCCTCCCAACCCGAATCTGTTCCTCCGGTTGCGCAGCAGCGTGAACG
CATTAAGGGGATGAATCGCGAGCAGCTTCTGGAGCAGTATGCTGATCCGGATGTGGGGAC
AGTGGCTGACGTATCTGCGGCTCAGCGCCAGGACGCTACCAACCAGTGGTGGAATGAACT
GGACGACCAGACGAAGCGACAGGCTGCGATGAATGAGCTCAAGGCTAGGCCGCTTTCTGA
ACTGCGTGTGGCATACGGCAGGCTCAGCCAGAATGAGGGGCGCACCCAGACTGAAGACAA
ACAGTTTCCGGGAATAGGCGGAGTCATTCGCCCGGAACATGAAGTAACGGCGGAAGCACA
GTCACAGCCTGAAAATGCGGAGGATCGTAGCGGGAGAATTTACCGGACGGTGCGCTTCCG
GGATCCGAAGGAAGTCCGCAATGATATTCCCGGGATTGGAGCGTCCAGACCAGCCCAACG
CATTGAGAAGGTGCGCCCGGACAACCGTTATTTTACGGATGAGAGAAGCGCCATGGGGAT
TGATGTTTTCCGTAATGCCACCGCCACCGGCCTGAAAGCGTCTGTAGTGAAGAAAGGCGA
GAATGAGTAGGGCGTGGAAATGGAGCAGCCTGCGTTCTCTGAAGATGTGGCAACGGAAAC
CTTAAACACCCTTCCTGGCGACAAGCGTATTGCTGATGGTGACCCGATGGAGCAGCCTCC
GTTGATGCGTAACCAGCGATTCGGAAGTTTCACGGGGGATGATACGGACGTACAGGCATG
TCTTGTCCGTGGCGACGCGCCGACGGCAGAGGACCTTGTAGGTTCACAGATGGTTGAAGA
TGATGCCGGTCCGCAAGCACATGAGTTAACAGAGAGTCCACGCCTGCCCGCTCATGGCGA
TATTCCTTCCGGACAGGGATATCCTTTCCCGGCAGAAGTGGCGCATACGCCGGATTAAAA
TCAAGCCGGACGTGCTGGTCGTTTTATCAAAACCGGTGAGGTTCAGGGCCAGAGATTCCA
GAAAGAACAAGCTCGGGCCCCGGAAAACGTCGCTGGTCGACCGGGGGAAACACTCGAGCG
TGACCTGGTTTGTGGGGGTCTGCCGTCACCGGATTCGCAGAACGCGACAGCACCGGTACG

TGAAGGACTACCGGCTCCGGACATTGCGCGTAATGTTCGTATGCCTCATTCTGAATCGCT
TCCCCGCACTGTACGGGACTCACTTCCTGAGCTTGCACAGCAGGCAGAAGTACGCCGACA
GGCCTGAGGAAATCGTGAAATCCCGCAGCCTGAGCCAATCACACCTGAATCTAAACCAAC
TGTCTCTACTGATAGGGATACTACAGTGCGCGGAAGTGAAGTTAGGGGCAAAAAAATTGA
AGACTTTGGTGAAGATATTAAGGGCGCGGCAAAACACCGTTATGCCCAGTCTGCTGAAAC
ACTGGGTAAAACGCTGGAAGACAGGGATTATGTCACGCAGCCGCCCAGCAAACTGTTCCC
GAAACCGGACTACGTAAAACTGGCGAAGAAAGGTGCTGATGGTGCTACCCTGAGTATGAT
ACCGCTGTATCGTAACGAGATTGCGGCGAAGAGGAAACACAATACGGCAGGATGGGGGG
GAGCATAAAAAGAGTACGCGACAGTATATCGGGAATTATGAACGTAACGGTCAACGCGAA
ACGGCTCGCAGAATGGATGGAAGGCAGAAGGCCCTCCCGTTGCGCGGATACCTGGCAACT
GTTACGCACTCTTCCGCTCTCACAGATAGAGCGGGCTTCTGCTTATCTGGTGGGAACGGG
TGTGTATCAGGCGGGACGAGGGCAGCGTTTCGATTCGACACAGACACCTTATTCACTGCG
CGATAAGGACAGTAAGGGGCGTAACCTCTTCTTCCCGGAATGCAGGGATGAATCACTAGT
AAACGCGAGATTCTGGTTTGACGAGCAGGAGGAAAAATCACAGGCGAAAGGTGGTGAAGA
AACAGCACCGTCACCGGGTGACAAAATGAGCTTTAACGTTTTCCCGAATACCCGCAGTGG
CGTCATTTTTATCGCTTAAGTTAAAAAGAGAATGAGGGTGAGAGGTGGCTTTAAGTCAGC
CAGTGAGGCGCGTATGTACATTGATCCACATCGTGATGAGCTTGTTCGTGATGTTAAGGA
GATGCGGGAGATTTCGCGAGAGGAGCAGCGAAACCCCCCGAACCGCTACCGTACCGGACC
AGAACGCTGTAAGGGGAATGTTTCGCCGGAGCAGTTCAGTGTTGCGTTCGGTTTCCGTGG
TTTGCAGTTTTGTAACTACGGGGTATGTTCGCGTCGTTAGGCTGATTTTAACCGGCATTA
TGACTCGCTGCATAACCTTACGGGAGTACTGAATGTACCGACAAAAGCGTTTTCCCTGAT
CGGTCGACTTGGACTGGCATTTGGTGTCCGTGGTAAGGGTAAAGCCTCGCCACACTAGGA
ATCGGGTGACGTGGCATTCAACCTGACAAAAAGTAACGGACCGGGTGCGCTGGCGCACAA
AAGGTTCCATTCTCTTGATAATTATTTAGGCCGTTATGACGTTTCCACTTTCGGGAAAAT
TACGTCAGGTCGCCACTCGATGACGGCAGCACAGCGTCTCCGGCGCATATCTAAAGACGG
CAGGTATGTTGGTGCCGAATAGCCGGTGCGTCAGAAGGTTTACGACGCCTTTAAAGGTGT
GATTCCGGCCATTAAAAACAGTGACATGTCGCGTCGTTCAGCGGTTCTCGATGAGGTGCG
CACAAAAACTTACTGGTCAACGGAGGTTGAAATCGCGGCACGTTCCTTGAAGCGTTATGT
TCAGGAACAGGCGCGTATGGCTGGCGTGGAGAATGAATATCTGGTCTATATCCGTAACGT
ACCTGAGTACAACACAGAGAACACCGACGCTTATACGACGAATCCGGAACTGGATGGCGG
TAGTCGTGAGGCATTCGATCACCTGTTCCGCACCCTGAAAATCCCTGAGCCGGACAAGGG
CGATTCGTATTATTCACGTAAGGGCGTTACCCGCACACCTGAAAGTAATCCCATTACGGA
TATTAACCGTAGTGCGGGAGCCGAAGGCCGCCCGGTGCCGCAGGTTGGAGAGGTTGCCCG
TGGCGTGACGAGCAGCATTGTGGACAGTCACCTGAAGGTCCGCGTGGTGACGTCAAAGCA
AGAGGCTGAAGCGCTGGCGCGTGACTTGTTTAATGGTTACGGCAGGGGGCGTTCATTCGA
TCGTCCGGACAAACGAGAAATTGTCCTTGTGGCGGATAACATCCCTGACGGGCCTACCGC
TCGCGAGAAGCTGCGTCAGGAGGTCATTCGTCAAGCCGTGGAGCATGTTGTCACAGCAGC
GGAACATCAGGCGATTTTCAATACCGTGCTTAAAACCCGCGACAGTGATAACGTAGCCCT
GCGTGAAGCCTGGCGTAAGATTGATGATTCCTAAGGTCAGGAATCACCGGAAGTACAGGT
AGGTGAATTTTTGGCACATATGGCGGATATATTGCCGGAAAAATTCGTGGCGGCATGGGA
GCGTGTTGTTACCCTGGTTAATGAGGTTCTGCTTCGTACGGGGTTACTTAAGCCGACTAA
ACTGAATAATATCGGACTTGTTCCCGCGGCTATGCGTACGTTAGGCCAGCGTGTGAGGCA
AGGTTACACGCCGCGTGAGAATGGCGCGGGCGCATCGATTAAATCCTACTGTCGTTGTAA
ACGTGATCCGTTCAAAGTGCAGGCAGATGAGGGCGAGCGTTATCGTGATGAGCTTGCAAG
AATGATGAAATCTCAGCGCACCAAAGATTTCACGGTCAACATCGGGCGTATGCCACCGTT
ATTGCGTGACCTTAGTGTACCGGACTTGACTTTGGTAATTTCCCGCGATACTGTGCTGAA
GGTCCCCAATGGTGTGAAAAATGGGGTGCCGATGGAATTTATCGAGAGACTACCGGAACT
AATGCACGATCCGGATGCAATTCACGGCTCAGCGCCAAAAAGCAATGCAGTTGTGATGCT
GCTCGATGACGTGGAGAAAAATGGTGATCCGGTGGAGTCAGTGACACACATGAAGGGTGT
ACGGTCTCGTCTGGAAACCAACTAGGTAGCTTTTGTTTTCGGTACAGAGTATGGCTAAAA
ACTGAAGAGTATAGGAATGACAGGGTCAACGTTGTACCGGAGCGAAGAATTAAGCCGCGA
TAACCTTCTGCACAGAGGGCTCCAATTGCCCAAAGGGGCACATTCTTATAGCGGCTCTGC
GGATAAGATACTCTATCCAGAAGACATTCGGAAGGGGCCGTATGACTCCCGAACCAGCAC
TCTGACACAGGAAGAGAAAATTGCCTCGCGTTTTGTACGCCAGAACCAGGATAAAGTCCA
GGCGCTGAATGCCGTTCAGGATAAGATCCGAAAAACTTGCGGCAAAGTGGCCGACAGTCA
CAACGCTAAGATGGCGGAAGACCTCTTCCACGGAAAGGCGGAAAACGACCTTAACGTGAT
GAAGGTGCGCTACGTGCAGTCACTGGCTAAACTTCTGGCGGAGTACAAAATTGCGCAGGC

CGATCTTGAGGGGTACCTCTACCCCCGTCACGCGCCGGAACGCAACGCGCATAACGCGAA
ATTCAACCCGAAAATGCCGGACGGCACTTCCGAGATGCCCACCGACGACGCGGCGGAAAT
CATACAGCGTGGATGTAACAGTGGCAAACAGGCACAGTGTGTCCGTCCGGCAGGGATTAT
TGACGATATGCTGGCCCATCGCCGTGAGGTTATCCGTTAGCAGGGACTTGAAGAGACCGT
TGTGGTGGATGACTGGCGGAACGCCTACCGTTACTACGTTCCCCTGTAAGGTCAGGATGT
TGACGGTGTGGTGTCACTGCCACGCACAGGTAAGGGCTTCAGCATCTGCATACGTGAAAG
CAGTCAGGCTACGTGGCGTGCATCCCGGGCACTGTCTCCGTCCACTCAGGCGATACAGGA
CCTGAGCGAATCGGTGAACCGTTATCGCAAAAACGAAGTGGGGGACGCCTTCCTGAAACC
GGTGCAGGATAATCCCGACAAGGATTACTGCCAGGTATTCACCGATGACAGACCGGATAA
CATCCGAACGGTTCTAGAGCGCAACGAGCAGGCAACTGGTGAAAACATTCGCTAGGTTGT
AGGACGACCGGTACCGATGGCCATGATGGCAGACCCGTACTTCACCACTGAAAAGAACGG
CAAAATGTACTACATCAAACTCCATGATCCGCGCCTGATCCGTTCGATGAAGAGTATGGG
ACCGGAGACCATCAATGCCTTTGGTAGTATGCGGGGGAAAGATAACCGCTGCCTGGCAAC
GGTAAACACGTCGTATAACCCGGAATTGCTGGTCAGTAACTTCATCCGTGACGTACTGAA
GTCCGTGATGAATCTGAAGGCGGAGCAGGGAATGAGCGACGGTAAACTGAAATGGCTGCA
TAACTTATCCGCCCGGGCTGAGGTGATAGGCAGCCGTTCTTCCAAGTCAGCCATATAAGC
CAGTCTGCGTAGTAAAACCCTCACGGGAACAGGTGCACAGTGGCACAAAGTGTGGAAGGA
GTTTGTTGAGGACGGAGGGAAAACCGGCTGGTTCTACACGGGTGGCCTTGAAGGCCCGAA
GAAGGAAATGGATCGCCTTGTCTCACTGGCGAAGGGGGGATAGAAAGGCCAGAGAATCGG
GGCGTGGAATTCGTTCCTTAACCGTGGCGCGGATGCCAACGGTGCGTTTGAAAACGCTCT
GCCTCTTTCTGCCTATAAGCACGCGCGTGATGCCTCATTGTCACGGCAGCAGACGGCGTC
CCTTGACACAAACATGACGGAGGACTTTGACCGTAGTGGTGAGCAGGGCGCGCTGATGAA
TTCGCTGCACATGTTCGCCGAGGCGAACATTCCGGGAACGGCAAATCTGGTGAGAACGCT
CGGACACCCTAATAGTGAGGGACCTTTAGTGGAGCCCCTTCCCTTGAAGTATCTGAACGT
TCTGGAGAAAATCGCGCTTGCCGCTGTGGGGGCAGGTTACCTGTTTGGCTCGCTGCACCG
CAGCATGGCAGGTGAGGATGACGATGGGAATAACTGGTATGAAAAGGTGCCCACTCATGA
CAAAGAGCGTAACCTCGTCAATATGAAATCTGAGTTCGGGGGAAATGCCGGAGAGAACTA
GAATATTCCTCTGTCTTACGGGTACAACGTTTTCCTCCTGCTCGGGCATACTACTGAAGG
TGTGGCGGCGGGTGACCTGACGGCGTCCCGTGTTGCCTGCAATGGTGTTCGTGGTGTCCT
CGGTGCACTCAGCCCGATGGGCAGCGAGACGGCGGAAACACTGTCCGGGGCACTGCTGAA
CAATGCAGCGCCGTACATTCTGTGTCAGTTTTCTAACCTTGCCATGAATGAAGTTGTCAT
GGGGAAGTAGGTTTACCTGGAGAACATGCCTTTTGGTATACCAAAATCTGGCAACCAGCT
GGGGAGACGTTCAACGCCAGAAGCATACATGGCCTTTGCATCCTGGCTGAATGCGTTCTC
CGGTGGCAGCCAGTACCGTCCCGGGGCGGTGGATATCACACCGGAATGGCTGAAATTCTG
CATTGATTACACCCCCGGCGGGACAGGGCGCTTCATTTCCATAACTACGGTTGCGGCGGC
TAAAGCGCTGACCGGTATTGATATCCCGGAACAGCAGGTGCCCTTCCTGATGAAACCTTC
GCAAGAGGTGATGCCGTATGCAGACCAGGCGAATATGTACGACCGGATGGCGGATCTAGC
GCAGTCTCACGCAGAGCTGAGAAGTCTGGCCGTTGCAGAAAGGACAGCGTGCATTGCCTA
GTGCAACGGAAGATCGTCAATGAACGGGCTTATGTATGATACCCGGCAGAGTCTGAAAGA
TTTGCGTAATCAGCGTGATGCTATTTACGCCGACCGAACTCTAAGTCTGTCGCAACAGTC
GGCGAAGGTGATATCGCTAGTGCGGGATATGCAAATTGCCGTGCATCGGTTTAACCGCGA
GTACAACAAAAAAGTGGGACTGGATTAACAAAAATGGCACCGGAGGGAAGTGTGGGGCTG
ATTAAGAAATCAACACTCATTGACCTGTAATTACCGAAGGGTTTAACTCATAGTCAGATC
AGCATTTCATGTGATGCACAGAGCCGATTTATTTTTAATGATGAAAAAGTTGCGGAAATG
GCAGTATATTTGCTGAAAGAGCATGGCGGATGTATGTGTTTGATTAAGCTTAAGCAGGAG
CGGTAATGGCGAATGGCAGTTATGAGATAAATAATCTTGATATGTAAGTAAGCGTACTGA
TGCAGGCGAAATGATGTTCACCCCTTATCGCCAGGAAACAATACCGGCAAGCAGAATTGC
GGATGGAACTATTCAGGCCCACGAGGAGATCGATGAGGAGTTTTTCACCCAGCATTGGA
TGGCTTTCTTATATCCAAAGATAGGCCCTTTGACATCCTCCACGACCTTAATGACGGAGT
TTTAGACCGCAGCGGACAACACTCGGTGTTTTTTTGGAAAAACCTGTGATAACTCACAGA
AACCCCGTCAGTAAGACGGGCTTAGCAAGCTGGGACGGTTACTATAATAATTTCAGTGCC
TTTATATCCACTTTAACACTGCTCAAGCATTTAGCTATTTCACCCTCAATTCTTACTTTG
TTTTTCGGAGAGATAATCTGCGAGGCCCATATGCTGTCATCGAAATACGTGACAATTGTA
CCGCTATTGTCGCGGAACTCATAACGTTCATCACCCACTTTTTAACGATTCTCCCTTTA
AGGATACCCCATGGATCATCCTTCAGTTCTTTTGCCTGCGCTACTGTAGAACGCTCTGCT
TCAGGCCCTTGGAAACCGCCCTGCTGTGCAAAAGCGCCAAAAGACACAGAATAAATAGAG
GCTGCAACCAATATCTTTTTCATTCATAGTCCTCTTTCTTAGATGAACATTCAAACAGCA

TTTTCAGTACGGTAATGCGCGGGTGGGTTGACGATGCCTGAAACATCAGTGGTGATGGTA
GATTACTCCCCTGCTAGGTCTCTTAGTTGTCAGAGTCGGAGTCTACGAAGACAGCGACCT
CCGTCGGACCGCTTCGGACTCGCACCTGACAGACGTCTTTCTTCGGTGCCAGTGCCGTCA
ATATGACGGTCAACCAGATGACAAGCAGGGCGATTAACATCGCCCATTGCTGCTTTATAG
CCTGCTTCTCCTTCCCTTTCGGCACGTAAGAGACTTATCTACCTGTATTCAGCATGGATT
GAGCCTCAGATTAATGTTAAGCGTCTTGTCGGACCCGTCATGTAAACTGGGGCTTTTCTA
TATCTGCCCTAGGTGTTCATGCCCGAGGAAGATGGCCTCAAGCACCCGCAGCAATTCTCC
TTAACTCTCTTTTTCCGGCAAGACGTTTTTATCTCCAGCGAAAAAGCGAATAAACAACAA
ACACCTCTGCCATTACTGCTCCTACTTCTGCGAATCCTACAGGCCGGGTCATTGATTCAC
CTTCTTGTCAATATTTTTAATGTCATTGTCCGCATACCGTATTTCAGTTCTTCCTTCCCG
CAACCGTGCTTTGGTGTTCTGTTCTTCACGCTCGAGACTGGCAACAGATTCTCGGAGCTG
ATCGTGTCTGTTATGAAACTGTCGATCGTCTCTCACCACAGCCTCACCATCGTTCGAACA
CCGCAGAACTTACCCAAATGCGACCACAGCACATCCACACTGGAAGCCCAGCAAAACGCG
ATCCTGTTCACCAACCTCAATGGCGCAACTTTTAAAGCTCTGGTGTGTGTAGTTTTTTCT
GCCATTTATTGCAATGTTCAGCAGCTTCTCTTCATCGCGCTTCGCCTGCACCAGTGTGAT
GATATTATCGTCTTCATTATCGATCAGTTCACATACTGCGGCGGTTGTGATAGCGGCAAC
CAGTCTTACGGTTTCCACGTCGCACCCGGAATTCATCACCCGTCATTAGCGTCAGGATGC
CCCGGCATGAAGGTGGCCCCCTTCACTCGCCAGTCACCTTTCCTGTCAAACTCCACGGCA
ACAAGAACGGCTGTCATGGTATTCAACATTCGCTCACTTCGACTTATCCAACCATCCCCA
GTTAGCGGAGAGTTGTCAAACACCGCATTCTGCAACCGTAACAGGTAAACGTATTCTTGA
ACTCTGTTTCCGTCGCACGCCTGAAGCCATTGCTCAGCCTTTACCGCATCAATGTGAAAG
GCACAAGTTCCACCGCCACCAAATTGCATTTCGCAGAGGTCCGCAATCTGTTCAAACCGC
GTGTGTGGCAACTTGTAAGTTTGGCTTACGAGTTCGGCTTCCTGTTCGGATATGCGCTTC
TCTGATGCTCCAACTAACGCATGCTTATCGCGTAGCGCTTCTTCCGGTTCGGCAACATGG
CATTCACTATCATTAAGGTCGTTCTATGCTGCTCCCACCTCACCTCTCAGCTTCCCATAC
GTAAGCGCAATCTCCTCGTTGTCCTGGTCGTGGCGTTCGATGTATTGCTGCTTTCTTTCC
CGTTCATCCAGCAGTTCCAGCACGGTAGCCTGGTTAGTGTCTGCTATGAATTCAGCGTTT
CATAAGCCTGCGCATCTGTTTCACTCATGCTATTAACATGACATTCTGCAATCACACCAC
CGTGTTCTCGTTTCCATTTTTGGGCAACAAACACTCCTGTTAAATAGCCGTGCTGGTTAA
CAGATGTACGCCCTACGATGTAGCTTCCTTCAGTTGCTTTCTCTGCCCTATAACGGAGTG
CCTGATAGGCAATCTTGCTCATTTTACTCTTCACTCGGGTATACATGGATTACTACGCCC
CCTCATCTAATTGCGCAAGCTGGATACCCTGGTTCCATGCCGTCAAAACCTAAGGTTTCG
AAAAATGCATCTATTTACTTACGGCGTTGCTCCTGCTCTCGGCGTTTCGTCCATTTTTTC
ACGGACAACATTGACAGCCCACCTCCTCTGCAGAACGTGATGTAGAAAGAACCCAGGAGA
GCTAGGCCGGTGTTCAGGGCTGTAGTGATCGTTGTCGTGGTGTTTATGTTCATGTTACAC
CTCTCTGTTTGTTGATAAGTTCATTATCCTGCTAGCAACTGGCGCAAGCTCTGCATCCAC
GAACAGCCAGGCGTCGCCGCTCCTCTATCGGAGCCCCACACTCGCAAAAATGAATAGAAG
ATGGGGCATTTCTGACGGATTTGTATTTTCGAAGGGAGAGATTGCGCTGAGATTCTTCGA
TTTCATCGGCGTTGTCGACGCAATCTGCCGATTTCCTTTCCTTCAGGCATGAAACAAGGA
GCCGAAGCTCCTTTGTTTTTAGTATTCGAATTGCCTTGCGCTCAGGCTTTTCAACTTTGG
TCTGGCCCGCTGAAATACGAAACTCGACTGGTCAAGCCGTGCAGCCTCTCTCAGTATCAC
AAATCTGTTCTTCGTCACCATGGAGAGGGTCGCATACCCAATGTCATACAACTTGTTCGT
GTAGGAGGAGTTCAGCTCTTTCACGATCGGATACAGGTGATTGCTGAGGTCCTGGTCTTT
TTCCACCCAGAGTTGCTTGTAGCAGAGGAGGATCATTTCCTCGTCTGTGAGTTGTTGCTG
TAATTATGATTGTGCTGACTCCGTGTTGCGCAGTTTGTTTTCGCACTCGATGTAGTATCT
GCGTATCTGTTGGCCTATTTCGTTACGATCTAACGCCGCCGTTTCTTTGACTGTATCGAG
GGTAGGGAGATGACCATTTTTCCCTCGCCCATAACCTATTTCCCGGTTCTGGGAAATAGC
TATATAGTCCTGGTTTACTACGAATCCGCACTATTCAATACGTTCTGTAATCCGGTTTAC
CAAGCGTTTTCCTTCTCCAAGAATAGTATGTAAATCACGGGCATTAACCAGAAGGGTGTT
TTAGTTGGCGATATTCCCGTTGAATACGGGGATTAGTTGCCTGGTCATGATGATCACTTT
TTATGTTTAGTTTATAACCTCCCGTTAGTATCTGGCGGTCGGGTGTCAACTGAGCCAAAT
AAGAAGCTCAGGGCATATTCCCCTTGCAGGGGTTGTATTACGCCTCTCCACCCCACCTTT
GTACGGCTGTAACTATGCCAAATTCCAGGCATAAAAAGGACGCAATGCTACGGGGTGCGG
AGCACGCTTATTTTTTCAGAGCTCTAAGTATGCGATAGCTGTGGAGGATTTGTCAATCGG
CGGAATCTGGATGTACGCGTAACGGTAAATGCCTCATCCAGTAAATTAACGCAAATTCTC
ATCTCAAACTTGCGTGAATTATCGGAAATATAAAGTTAATTTCCCTTAAATGTATGGGGC
ACTATTGGGGCTAAATGTGGTGGTTTGGGGCATATTTGGGGCAAAAAACAACGTATAAAG

AAGGGTGTAACATTGATTTGTTCATGGTCAAAATGAGTTGTAACAGATTGAATAAGATAT
GCTCTTGGACGAGGTTCGGTAATTGTGTGTTTTTAATGCTTCTTGGTTTGAACACGTCTA
AGGATATGGTTTATCGTTTTGAAAAAAATAATTTTGTTTCATCCTCCTGGTGACTTAGGG
TCACCTCTGGGGCACGGGCATGACGGACATTATCCAGCCTGGCATCTTGACTCACGCTCC
GTTCAGGCATCCATGCACCATAAACATTGTAGACAATGCTAGAGCTGGAGTGCCTCATCT
GTGTTTCAATAAACGTCGGGTCTGCTCGGTAAGATAAAGCCCAGCACGCATAGGTATGGC
GTGTCAGCTAGGCTTTACGGGATTAGATACCCGCTCTTGTTATTGATGAATCCCATGTGG
CTCCGATGGGGCTTATCGCGTAGGTAATAGCCGCCGTGTGATTCTTGCGAACGATTTGAG
GACAGAAAACAAAAGTGCACTCGTGCACAGTTGTTCATCCGTACTCGCGTAAGTGAACAG
TGATCTGATGCTGCGTGCTACGACGAGGAGGCATCGCCTGGTTTTTAAGCGCTTCACTTG
CTGGTGCCAGAAGATGTATGCCCCGGTTGGTGCCTGAGTCGGTCATTGGTAGGGTAAAAT
CGCCTATTTTTGTAAAATTTCGTCGCACTGTTATCGGGACGGCTTTCAGGTCCATAGCCT
CCCATGTAAGTCCAGCAATTTGACCGTGTCGCAACCTTGTAAAAACAGCCGCTGCCCAGC
GGTTTTTGGTCTGTTGATGATGGCAGGCATGAATGAAACGGCTAAAGTCATCTCTGGTCA
GTGGTGCCGGCTCTGGTTTTAATTTCCACAGCGGTGTTCTTGAAGTAAACGAGTTTTTCT
CCGCATGCCAGTTTTCGGCAGCAAAGCTGAACATTCCGGCTGTTGTTGTCATGTAACAGT
TCACTGTGGGTACGGTTCATCCTTTTCGGGACGTGCTGGTTTGCCAGGTTCCCTCTTCGC
CGGGCTGTAAATCTTTCCTGATACACAACAGCTCTTCTTATGTAATCGCTGACGCCAGCC
TGCCAGGACCCAGCCTCGGTAGCATATTTTTCATCACTGATTGATAATGATTTAAGGCGT
TACTACTGATTACCCTTGCTTTCAGAGTAAGCTATTTCTGTGCCAGTTCACCCACGTTGA
TATCTTTTTTTTCCAGTTCAAATAGCTTGAGGTCAGGTGAGACGGGAAATCGATCGGCAT
AATTAAACGTGCTTGTTCTGATTCCAGAGTAGACCGTAGCCCTAAACTCACCAGCGATCT
TTCTGTTTTGCAGTGTATCCGGCACGCGAAGATTTTCGCGCACTCGTTTACCCCTGTACT
CAAACGTTATTTGGAGTTTTCCTCCGTGATTGTCAACGCCGGTTGGATAAGCTGAATTCG
CCATTGTTCCTCCTGCGTCCAAGAACATGCTCAGGTTACACTCAGATGGATAAGATTTAA
ACACCCTGAGATAGTAAGCGTTAGTTTTTGATCCAAGTGTTGGTGGTACGAAGATGTTAG
AGAGATTCGCAGTTTTTCTTTGGCGTTCCGTCTGGGGCTATTTGTTTATATTCTCTGCCG
TTCATCCTCGCACTGTCCCTGACGCGTGATAGTGTCCCTTTTCTCAGGCCTGATACCGAA
ACTATGAGCTCCACTGTTACCCAATCATTCGCATTTATCCGTACATCAGGTGTGATGACT
GGCTGAATGCCATGCTTTTGTAGGTTTCATCACTCACCCATAAGTTGCCTCTGCATGTGT
AAGAAAAACCCGTCCTCGGGGGGCTTGGTGTTCTTTTAGTCTTTCAATTCGAATCTTGGT
TACGTCTTCTTCGATACGAACTCCTTGTTTTTCGTCTTTTGATAGTGCTAAGTCATAAAT
TTGCGCGGCGCTTTACCCATCTCGCATCCATGAATTTAAGGCGCGAACAGCCTCGCTACG
CTTTTTATCTTCTCTCGCATTTTTGATATTGACGAGGACATCAACGCAAGTATGGAAAAT
GTGGATTTTGACTTTACATGCGAGCATGACGGCTTCGCCATCATTTCGGCCACACAGTGA
GTACAAATCTTCAGGGTCTGGCTGGTATTTCTATAACGTTAGAGGGTTGAATGGTGAACA
AGCCATAATCATCCCCATAAAACAAAACTCGCCGTAGGGAGTTCAGATAAAAGAAATCCT
CGCGAGTGCGAGGAATGTTATACAGTGGCGATATTCATCTCTATAGCGATTCCCTTTGAC
GGTTTATCACCGAAGTGCGGATGTGTGATTGTCTTGATTTCATATCCCTCTTACGGAACT
TTAATTCTTCGGCTGGAATCGTTGCGCTTCGGGTATCACTTTGTTGTTATCATGCGGTCA
TACTCGCGGAACATAAATCGCTTATTCCAGTAGTTATTAAACAGGCGATACTCTACCGTT
TTCTGTCCGGGAGTAATGGCATCCAAGTATTCACCTTTAACTGCAAGTGGCAGGTTAGTC
ACGGTTAGCCTCCTGCGGCGGTTCTGGTAGCGGCATCCCGTGAGTTGCTTGCTCAATACC
ATCTCCCGGCTTAACCGTTGCATCTCCGCGCCGAAAGGTGCTTGCGGAGTACCGTGCGTA
CCATATTAGCGCTTCAACCAGACAGCGATCGAGATTCACCGAAATAAGCACGTTCTGACC
TTTTTTAGGCAAGCGACCACTACAGCTTATCCAACTATTCGGAGTTACCGGAGATTTACC
TGCCTCATCAGCGGATTTCATCCAGTGCGTAAGCGTTGAGTTGCTTACACATACCCAATC
AACGTTTATGTTTTCTTTTTTTTCTGACAACCATTCTTGGAATGGACGCTTGGCAGTCCG
GCTTTCTGCATCAATTCGTGGTTGGCAGATATATAGTGGTACATTTCCCGGCTTCATCGT
ATTGTCTCGACAAATAAACGTGATACAACCATATTTAACGACCTCAATTCCCACTGTGTA
ATTAGGGGCGAATGGTTTAGTGGTCAATGCAGTCAACGCCATTTCTGAAGCACGGCGCTC
ACTATTATCTCGCAAGTCGAGGCTGCCGATTCGCTCTTTGGTTTCTTTAGTCATTTCTTT
CTCGGTGTACATTGTCATGTGTTAGTACTTCTCTACTTCGAGGCCATCTTTCAGCGTGAA
GCCGTGGTAATCATCAGCCAAACTGGTTAACCCAGGCGCATGAAGGGTAGGAATATAGAC
GCTTGCAGTGGGGTAGCCCTTATCGTTATTAATGCTGGCAACGTGCTCGCCGTTGTATGC
GCTCAGCGTGTCTAGGACACAATAAAAATTTCCTACGGCTGCCCTGAAATGCTTTACTGC
CTTCACAAGTGGTTTCCACGCTTTTTCCTCTTCTGGCGTCAGGACGATTAATTCCTCCAA

AGATGCCATTTCAGTCTTCGTAATATGGGTTGCTTTTAATTTGCTGTGTGTTGAGCGACG
CCCATACCACGTAATTATACAACTCATTAACTAGATCAATGATTTTCCGGTTTGCCCAGA
CAAAAATTTGCGGGGATATCGGTATCAGCAGCCCGATAAACAGAATGAGAAAGAAAAATC
CTGTCGCATTAATTTTTCCCGGCTACTCTTTTCAGGATAATGTAGGCACATCATTCTCCT
TTCTTGCTCCTCAAAATTTTATGCCCTGGCGCATAAGCACGCGTTCTGTCTTTGCTTATT
CGACAGCGATTCTTGCACGCCTCTTTTGTACATCAAGCCCATGACCGACCGATAGAGTCA
CCGAAGCCTGGCCACTTATCCTTGCCATCTGTACAGTGGAGGCAATCACTATAGAGATGC
ATGGTTTAACTTGCAGCAAAAGCCATATCACTCTCTTTTAGTGCGCAAGTGGTTTTACCT
GCGGTTTTGCTCCGCGCTGGGCTTTTTGCAAAAACCACAATCCCTCATCCCGTAATATTT
CATCAACCCCATGCGTCGGTTGTTGAGTCACCCCCACTGCCATACGCCAGGAGCGTTTCT
ACGAACTAACAGAATCTTTGCATTACGGTATTTCATCGGTTTGCTCTCCTGCATCTCTTT
GGTGCTCGGCGTGCCGATGTAATCCCGGTAGGGCGGCGCTTTGGTGTCGGGATGATGTTC
TTTGCCATTAGCGCAGAAGCCCGAAAACGAGACGGATACAGTAACAAGCAGATACATGCC
ACACGCACTATTCACCTCTTTCGATGGGACTGCCAGCGGCGCGCTCGGCTACACTTTGTC
CCCAGAACCACATGTGAAGCGCCATAAGCTTGTCGTCAATCGATGCATATTTGCGATTAG
AGTAGGCCTGAGCATCATTCTCAGGTTCGCCAGGTAATTCGCCCGGGCCAAACACTTTGT
TATAAATCCATATTAGTCCGTTGTTAGCGTCGCCAGTTAGCTGCCATTCGGTAATCGTAG
CCTGCATGACCATAATGCTAAGCCCGATTAATAGGTTCAGATTTTTGTACCGTGTGCGGA
TGTTTGCATTCTCGCTTTGTACTTTTACGTTGCGCTTTTTTGAGCCTTCATGTAACGCCT
GCTTATCGCGTAGAGCTTCTACCAGTTCAGCAACATGGCATTCACTCTCAATAAGGTTGT
TTTCTGCTGCTTCAAGCTCAACACGCAGCTTCCCAACCGTACGCTCAATCTCCGCGTTCT
CCTGGTCGCGGCGTTTGATGCATGGCAGGTATCTTTCCTGTTCATCGCGCAGTACCAGCT
TGGAAGCCGGGTTAGTCTAAGCTATGAGTCCAGCGTTAGCATAAGCGTGATCTTCTGTGT
CAATCAGGCGTTTAACATGACATTCGGCAATTACGCCACCGGGTTATCCTTTCCATTTAT
GGCAAACAACAACTACTGTTAAATGGCCGCGCTGGTTAACGGACGCATGCCCTACGAGGT
AGCTTCCTTTGGATGGTTTCTCTGCCTTCTCACGCAGTCCCTGATAATTAATTTCGCTCA
CTTCGAACCTCTCTGTTTACTGATCAGCTACAGCTCCTCCTGACAACTTGCACAAGTCCG
ACAACCCTAAACGGACAAGCGTCTCCGCTCATCGAGGGAATTGCCACACTCACAACACTG
ATTGGCAGAAATTGCCTGGTGGTTCAGGCGGCGCAATTTTATTGCTGTGTTGCGCTGTAA
TTCTTCAGTTTCCGATGTTGAATCAATGAGGTCTGCGATCTTTCATTAATCACTAAATTG
TTAGGGAAGACGCTTGAGGGTGAACGCGAATAATAAAAAAGGAGCCTGTAGCTCCATGGT
GATTTTGCTTTTCATCTACTCGGTTTCTTATAGACGCCGTTTAAAATGCCGATCGCCAGA
CCTAAATGAGTCGGAGTGAATCCCATTAGCGTTACCGACTCGCGGAGGTTCTTCACAACA
CGTCGGCAAACGTGATCGTAGTTTTTATCTGGAAACCGCTGTCTGGCTTTTTTGATTTCA
GTATTTGCCGGACGAGCAATGCTGCGAAGGGCGTTTTCCTGCTCAGGTGTCATTCAACAA
GTCCCGTGTCGGCAAACCTAAGCAAACAGAATAAGAAGCCTGCTGCCAGAAAAATGCATT
CTGTTGTTGTCAAGCCGGGTCTCTCTAGTTTGCTTCTGCTTTCGCAGCCATCATTTCTAG
CTTTTGTAAAAGGAATGTGACTGACGTATGAAATTCTTCGTCTGTTTCTACTGGTATTGG
CACAAACCTGACTGCAATTTTAGCGAGGCTATGTGCCCACGTGAAACTCGTTCTTATCTC
AACGGGAGATGCTGTATGCGGACAGATGCCCGTTTATTATTTATCTCCTAAGCCAGCTGT
TGTGCTTTCAGGTTATTTCTGATAACACAAAGGCCGGGAAATACCCCGCCTAGCTTTGTA
ACGGAGTAGACGAAAGCGCTCTAACCTACCCGGATATTATCGTGAGGATACTTCATCGGC
ATTACTCACCAAATACAAAACCACTTTCAGCCAGTGCCTCGTCCAATTTGTCAATGAACT
ACGGCAGCATCTCGTCAAAACTCGCCATGTACTTGTCATCCCGCTCAACCACAACATAAT
GCAGGCCTCCAGGCTTTATACCTGGGTACTAGTTGGCAAAAGACAAGGCATCTTTTCGTG
TCACCCACATGCTGGACTGCACCTGGGCCCTGTAAGCCGGCTTTATGGCCTCGAAACCAC
CGAGTCGGAACTTCATGAAATCCCGGTAGGTAATCGGGAATTTCAGTTCAATGCCATTTG
AGGCACTGCATAAAGCATCGGGAGAGCAGGCGGTACGCATACTCTCATCGCGATAGATGA
TGGGGGATTCAATAACAGTCACTCCGGAGGTGAACTCAAACAGGGTTCTGACATCGATCT
CGTACTGTTTTCCCCAGTCGAGTGCTTTAGCGTTAACTTCCGTAGCCACACCGGTGCAAA
CTTCGGCAAGCAGGTTGTCGAAGTAGTAAATTTTCATGTCAGGCCAGATCTTTCCTCAGC
GTGGCAGTGCTATCACGTTGTACACTTCTGAAGTGGTGATGACGCCGAGCCGTAATTTGA
GCCGTGTATCAACCCCCTTTTCGACAGCTCTGTCGTCAGTTCCGGTAAGCTGCAGGATAA
TGTGCGGTGTCATGCAGCCACCTTCTGCTCAGAGGCTTTCTGTTTCAGGAACGCAAGAAC
TTCCCCTGCTTCGGCCTGTGTCAGTTCTGACGATGCGTGAATGTCGAAGCGAAATATCTG
GGAACAAAGCGACGATAAGTCGTCATCCCATGTTTTATCCAGGGCGAACAGCAGTGTGTT
AATCTCCTGCACGGTTTCATTGTTAACCGGAGTGATGTTGCGTTCCGCCTGGCGTGCTGC

AGTCTAGGCAGTATTTGCGACAATGCGCTCGGCTTCATCCTTGTCAGAGATACGAGCCAA
TCCGAAGGCTAGACGGGCACACTGAATCATGGCTTTATGCCGTATCATCAGGTTGGGATG
CGACTGCCACGGGCCTGTGATTTCTCTGGCTTTGCGGGTTTTGAATGGCTCGCTGCGGCA
TTCATCCATCCACTCCGTAACGCAGATCGGATGATTACGGTCCATGCGGTAAATCCGGGA
TGTAGAGCATTCATTGTGCCGCTCAAAGCCCTTGCCATCAAACTGCCGGTTTTAATTGAT
GACGCGGGGCCACCCACCAACGCCCATCACCGGAACGATGCCGTTCTGCTTATCAGGGAA
GGCGTAAATTTCATTCGTCCACCCATTATGGCAGTACTGGTGGGCGACGATCAACAAGGC
GATGAACTGCGAATCTATGGCATCACCTATAAATGCCGTCTGGCGATGAGTGGTGAACAG
TTCCTCTCGATCGACTGAATCCATGAAGACACGTTCAGACAGGTTCCCTGCCAGCGTTTC
GACTGCTGTACTCTTCCGGTTTATACCTCAGAATCAATATCAACCTGCGGGCGGCCACTT
GTTTCAACCATGTCCTGGATGTGCTCTACCTTGCGTTCCTTAAACTCAAAATCGTCATTA
AACGCATGGGTAATAGCTTTTTTGCCGGCCCCGAGGCGTTGCTAATGATCCCTGCAGAGT
GATTCAAACAGTTGCTGGGACAGACCTATTCCCAAGTCGTCTGCCAGCTCTGCGTCTTTC
TTTTCACGGGCGATCTGCTGGTAGTGTCGCGTCCAGCGCTGAGCCTCAAGACAATCCTGA
AAGGTATTAGCGTTCATGGTTGAACTACTGATGTAGCGCAGAAAATATCGCCGGCGAAAA
GGCGGGCTGATTGGGAAAACAGGAAAGGGGGTTAGTGAATGCTTTAGCTTGATCTCAGTT
CCAGTATTACTATCCATCTATTATAAGCGTCGACGGCCTCAGGAAACATCTTTTCATCGT
TAACAAAAGTGGCGGTAGTGAATTTAGTCGGGATAGCCATAAGTGTTGGATCCATTTTTG
GGGACTCTTGGCTGATTTAGTCTGTCGATAAGGCGTTTCCATCCTTCACGTAATTTACGG
CTGATTCGTTCAAGTAAAGATTCGGAAGGGCAGCCAGCAACAGGCTACCTCGCAATGGCA
CAGTACATGGTGTGCTCCTTATTTATACATGAGGAAATCCGCCTCGAATGAAGCGTTATG
GGTATGCGGTAACGCCGCGCTCAGACGGCTTTGCTTGTCACATCATCTGAATCAAATAGA
AATGAAGTATCGAGATCGGTAATTCTTATTCCTTCGCTACCATCCATTGGGGGCCATGCT
TCTTGACCATTTCTATCATTCCAGTCGAACTCACACAAAACACCATATGCCTTTAAGTAG
CTTGAAAAAGCCATAAGCAGAGCACCTCGCGCAAGCCTGATCAATACACCATTTAATAAA
GAGCCGTGTTTATTGAGTGGGTATTCAGAGTCTGACCAGAAATTATTAACTTGGTGAAGT
TTTTCCTCTATCATTACGTCATGGTCGTTTGAAATTTCTATAGATTCTTTTCAGTCGTAA
TCAATGTTTTATATTTTGATGTTTGGCATCTGTTCATATCCTCCCTGATAAAAAATCACC
CTCACATTGGATGGCGAAGAAGATTACCAATAATCAGGTCAAGTCGGCCACTGTTTAGTT
ACGAGCGTGATTGCTCCGTGTTGTCACTCGTGGGTATCAATACACAGTGCTCTGTTTATT
CTGTTGTTTATGCTAGAAATAAAGGCCACCATCAGCTAGCCTTGTTGTAATTGTTGCAGG
TACAAAGTAAGTGATTAGATGGAGCGCCATACATTATCAATTCATGCTATGTCGGGTTCA
TCTCCACCTCTTGTGGTATGGGCATTCTTGCGTTAGTGTCGTAAGCGGAGAAACATAAAA
CAGCCCAGGCACCGATTGTTTTAAAAAGAACAGCAATGGGCTGTGGTTTTGCTGCATTTG
CGGTAACGCGACAATCACGAATCGCGGTTTCATGAAATTCCATATATCACCTAAAATTAG
TGATTTGCTGCCTAATTTCACTTTCTGGCAACCAACACAAGTCATCTTGCTGTCAGTTGT
TTGGATTTCCGCCAGCCTGCCGCTTAAAGAGCTACATTTGGAAGACGAGTTGAGTCTTCA
TATTTTCTGATCAAAGTTGTCGGTGATATTTCTTCTGCTCTGATCGCTGGTTTGCGTTCG
CTTTGTAAGACCACTCGTGATGGGCTTGGGCTGTGTGGTTTGAGGGAGACAATCGCCTCG
TGACTTTGCATGTTTGCGAGACGAGCTCTACGGCGAGAAGCTGCGGCGCCTTTACATTCT
GTTTTTCGGGACATAGATTCATCTCGAATAATCGTTCTCGATGCCATCTCGAACCTCCTC
CTGAGTCGGGCGCTTCTCCATAGCACTCCACAGCTTATGCGGTTGGGTGATTTGGTTTTT
CAGCCACGTAGTCGAGAGTCCACGTTGTTTAAAGAGCCTGCCACTATGTTCCACTTGGCT
TGCAGCGTCCTGCTGATGGTTAAAGGGTAAGATATGCAATTCACTTGGTTAATACAATTT
GTTCTAAAACGAGGCGTCTTTTTGCAACGCTTTGTATTTAATAGTATTGTTTTTTATCGT
GTGTGTATTGCGTCGGCGATGTAAGGAAAGATCAGAATTGCGTTGTTGAGTGAGTTGTAT
CACTTTCCTTGCCAATAAATATAATTGTCTATGTGGCTTTTTGGGCGAGAAGAAGAGAAA
ACCCTGAATGGAGGCTGGGTTGATGATTACCCGTGTGGCCTTGAGCGAGAATGCTCAGAC
ACATGTTCAAGATCTCTATGCCATGATCGTTCACAGGCATTCGCTTGAACTGCTCGATTG
AATTGGTCTTTCAATTTGTATGTAGACATCTGTATTTTCTCATATTGGCCGCAGCCGAAC
AACCGCATGAAAAAAGGTACCGGACTCGCCAAGGTTACATCGCAAATACTGTGTGCCTTT
ACAATGAATCCTTTTGGAGGCTCTGGTTAAACTGCATCATTGGCGGACTTGGGAGTTGGA
CATTGTGCAGAATGCTTTTGCATTATTGAGGCAGTACGTAAACTTTCAGCATTGAAGAGT
TGCGTTCGGCTTCTTCGTTTGTGAGATCGTTAGGAAGGCCGTTAATCGTAACTGTAACCG
CTGATTCGGGTTGAAGAAGTACTGGAAGATCATAGCTATGATGTGCCTTAGCCTCGCCTT
CGACTTTTGTTGGTTCGCCAGTCAAATCTTTTTCCTCACTGGATTCTTTGTTAATCGGAG
CGACTGGGATTTCTTCACCTCCGTGGGCAGCTTTAAACTTTTTGATTTCCTTTTGCATGC

GGCTTTTATATGCAGTCATGCTGCTGTCGGTAGTCTTCTGCTCAGATCCATTGATGTATC
GCTCGGCACGCTCATTCACATCAAGTTGAGTAACATCACACATTTCCTCTTCTTGGACGA
CCGTTAAGAGGGGATCGAATGAGTCTTTTAAATTTCGAGCGGTGTCATCCTTCATTATGT
TCAGTGACAGAAGATCTTCAAGAAATTGCTTGAATGCCTGCACGCTAACGTATGAATTAG
CCATTTGCTCTCTCTCTGAGTTATTGACTCAGATCGTGTTTATCCAGGAATTGATTTGCA
TGAAAGACTCAAGTCTTCAGAGTTTCCCTTCGAGATCCACTCTCAGTAAAAAAGGCCGCA
GCTCTGCGACCTTTCATCTCCTAAATATCTTTTCTCCTACCTATAGAAACAGCAGGGTCG
GTAAGCCCTAACAACTTCAAGCGCATCGTTCAGAGAAAGCTCCATGCTATATAGGTCTTA
TTTCATATCTTTTTGCAGCGAAGAAATTTTCCCTCTGTGCAGAGAGCGTATGCCCTTGAT
TCTGGTTGATGGTATGACGCTTTTTCCCCAGCCTCACCCTGCCATACAGGACGCTGCTCA
TCGATATTTATCGGCCGGTCATTGATCTTAGAGACAATATCAAGCTCACGACGGATGTAC
TCAGATGGCCTTCTGCTTTCAATGAATAGCTGAACTTCTTTTTTTCCGCTTAATACTCAT
AGCCATTAAAGTCCAGCTATCCTCTTTACCCATACGTCTCTTCAGGGCATTGGCTGACGA
TAGCTTTCCACCCAACGGAACAACTCTCAGTGCATGGGATCATTGGATATTGCGGGTTTA
GTGGTTGTAGAAAGAGCAGACCGCTATCCCTGATCAGTTTGTTGACCGTAAATCCATCAC
CACCTAGTCTGGCGATGTAGAAATCGCCGGGCTAAACAGCTTCCTCAGGGTCAACCAGAA
TTAACATCCCGTCATCAGAACGAGGTTTGGAACCAGTTGGTGCGGTCATTGACTTACCTT
CGACCCCAAGCTGGACGGCAGAGTCACTGGCCCTTTTGGTTGTGCGTACTAATCTCTCCG
CATCACCTTTGGTAAAGGTTCCGAGTTCTGGAGAGAACATCCCAGGCTAAATATGAGAAA
AGACAGTGTACTCATATTGTTTCTTAATTGGGGCCGACGACTATTCGCCACCAGGTGAAA
ATGTCCCGTCGTGGTTAAATGATATGTTATCAATACCTAAGTATTTCAACACCACACCAG
AATCACTAAGAGATGGATGACGAGACCCTCGCAACCACTGTCCTATTCCACCGTGCGGCA
TACCTAGCCTCTCGGCGCACTTCGCTTGAGTTATGCCGAGCTCTTTAATTCCGAATCTAG
CTAGTTCATACCATTTCATTTTCATGCCCTTATTATTACGCTCGGTACAGGAACCACCCA
TGCACAATGTGTATTTTTACTTGTATTTGAGAAGTATATATTGTATTCTTTTATTAATGG
TTACTATGGAGGGCATATGAGCATCCTACGAAAATATCTAGGGTCACTGAATATCTCTCC
TACAACACTTGCTAAGGCAGTTTGATGCACACAGGGAACTATCGGACATTGCGAAGCTGG
TCGTCGCTTCCCATACCTTAACACATGCCGTGCCCTTCTTGCCTGTCTAAATAACTTAGC
CGCAAAAGAAAGTCTTGATGACGTGTTCTCGCTGGAGCAAAAAACGGCTTAATAAGCAGT
GCAGCTCTTTGTAACAACGTACATTCGTTCTATGTCGCTTGAAGTCGAGTCCCAAGATAT
CTGACCAACTAAGGCCATATGCGTTTCCACGTATACCTCTCAACTAGCAATTCACTATCT
GAAATCTTTAGAAATGGAACCAGCAAGTTACAGCAAACTATCACAGCCAGAGATTGAACG
CGCTGAAACTGATTTACTCATCAACCTGTCAACGCTTACCCGGCGCGGTCTGGCAAAGAT
GATTGGGCGTCAGGAAGCGAAGACAAGGAGATCGGACTGGAGAGTGATTGCTTCGGTCTT
GTGTGGTTTCGGAATGGCAGCAGATATCAGTCCGATTAGCAGGGCGTTTAAGTACGCGCT
TCAAGAAATCACACGGAAAAAATCCCCGGCCGCCACCGAGGTTTTAAAGAAAATTTATTT
GCAATTCTGAGGGAATTACTGGATCAATCCACAGGAGCAATTATCACAGAACAACTCAGT
GCTGGCCAGGACAAAAGTCACAAACACGTCCTACGTGTTCGCTTACTGTCCAGCTTCAAT
CAGGCTGGTCGATTCTGGGCTTAGTTGGAAAAAGTGAAGCTGATGCAGAAGGAGAAGGGT
CATGAGTGACACACCTAATCTAGCGGACGCAAGGGAGGCAGGAACGCTACAACAACCGTA
TCAAAACAGCGGTAAGGGGTATGCCTTGCGTCACCGTAAATTTATGGATGTGCCGTTTTA
CAAGTCCGCAGAAACAGCGCATTTGTGGGTCCGCTTAATCCTCAACGCAAAACATACGCC
TGAGTATGTAATGACTGGCGCAGGGGAAGTTCTGGTAGGCAGAGGGAAGCTACGTGGCGG
AAGAAACTCTCTGGCGCATGGAACAGGACTCAAACCAGTCCGCTTTCAGTACCTGCGCAG
AAAGGTCAAAAGTCTCGCCGTGATTGGCTGGGTTTCACACGGTAAATTCTCAGTTTTCTC
GGTAGAGAAATATGTCGATTATCATTCATATTTTGTACCAGCAGATTAACAGCAAATTAC
CACCGCAAAGCCAGCAATACGAATGCCTGCAAGCAATACTGTACCAGCAGATTACCAGCA
AATTACCACAGATAAAGAATATGAGCATATTATCTATAAGACTAACGTATTAGAGGGTGC
CACAGAAGACACAAAGTCTGTCAAGAAAAACCCTTCCGTTAGCTGTCAGGATATTGTCGT
TGCATACCACGAGATACTTCCTGAAGCGCCAAACAGCCGCGCACTGAATGACAAGCGTAA
AATCGCGATCCGTACGTTCTGGCGCAAGGCCGAAGATATAACCAGCCGACTTGACTGGCC
TGCGCTCACGATACAGGACTGGATAAATTATTCGAGCTACGTAGGCCAAAATTGCCGATG
TACGCTCCAAGAGCGCCCAAACAATCACCCCGGAACCGGCTCGCACAAAAAGGAGTTTTA
GTTCCCGCTTAACGATTAGACCGACTTGAACGTTCGTGAGGGTGAACACGATGACCGATA
ATTTTTTTTTGCCGCCCCATAGCATCGAGGGAGAGCAGGCAGTGATGGGTGGATTCCTGC
TGGATGATGACAGTGGTGAGCGCGTCCACAAAGGTCTGGCGGTGCTGCAGCCTGATTGAT
AATACAGCCGAACACACAAAATCATTTTCGACGAAATAACCAGAATGCACCGGGAGTAAA

AGATAGTCAATGGCCTGAAGCTTTTCGATGAACTCGAGCATAAATCGTTAACGGCGACTG
TTAGCGGTATAGCGTATATCGCTGAGATCACTAAGAACACGCCAAGCGCAGGTAACCTAG
TTGCCTATGCGATGGAGCTTTGTGAAACCGTAATGGAACGCTACGCCATCAACCGGAAGC
CTGAAGCGAGGGAATTGCATTCTTCCCGTAACAGAATGAGTGCAACGCAAAACTACGCAG
CTATCCAGGCGATTTTCACGCAACTGACAGACCATCCAACATCACGATCACGTTGCGGCT
TTCGGTTATGCGGCGAGGTCATGGAAGACTGGGTTAGGGATCTTGAGAAGCGATTTGACC
CGTCAGGCGAACAACGAGAAATGACCACAGTGATCCCATCGGTGGACAGAATGCTATCAC
CGTAAGGTCTGGTGAAAGGTTGTATCTTTGTCCGGGGCGCTCGCCCTAAGATAGGGAAAA
TGACGCTATACAGCCAGATGGCAATCTTTTTCGCAGAGCATGGGCAAAAGCCCGCTCAGA
CGTTCTGCCGTGAAATGCCAGGTGATTAGATACTGGCAAAACTGGTAGGACAGAAGTCAG
GTGTTAACCCGAATATTAGTTACCTTCCGGCGACAAACGACCGCGATAACGGCTACCAGG
GTTCTAACGATGGCTACTGCAACAGGGCGATCGAAACAGCTAATCGCTTGAGTGAAATCG
ACTTGCTTTACATCGACGACACGTCGGGATTATCTCTGGATCGAATGATCAGCGAAAGCA
GTCGAATCAAACGAGAAAAAGGATGCGTTGGCATGATTCTGGTCGATTAAGTAACACTAG
TGACCGCTGAGACAGCAGATCGCAAGAACCTTGGTTACGGCGTGATCACCAAAGGACTGA
AGAACCTTGCCACAGAGCTTGATCGCGTTGTTGTGCTTCTGACGCATCTTGACCACGTAC
TGCAAAGCCGACCCAATAATCCCACACTACCAAGTGACTGAAGAAATACAGGCGAGATTG
AACAGGATTGGGATTATTAGGAGAGGATCCATCGTGAAGGTGCTTTTTATGACAGTGTTC
CACCTGGTGAAACCGAACTAATCCTCCGTCTCACTCGTCATGGCAATTCCGACACTGTGT
AATGCATCCAGGCACGTGGCGCTATTTGTGACCCAGACCAGGAGTCTGCTGAAATGCGCC
GAGGCGAACGCTAGGAACCGCCGGCCGAGAAGAAAGGAGGATTCTGATGAATAAAAAAAA
ATTTGCCATTGTGGAAAAGGCATGGGATGCACAAATATCCTTAGCTTTGAAAGAACAGGC
ACTACAGGTAATCCAGACCGAATCGAAAGTCGCCAGGCAGTTCTGCGATGGCGGATTCCT
GAGCGAAATTTAAATAACGCGCCAGATGATGACGTTTAAAGGGAATGATGTAGATCATCA
AGGTAGAGCGGCGTATTGCTCCCATCTGCCCGATGACGTGGACGTTGATGAAATGGTTAG
GGAGAACAAGCATTGACCATCTACATCCCTGAGCTAATAACAGGCCTGCCGGTAATCGCA
GGCCTCTTAACTTGTGGGAGAGGGAAGTCAACGATAGCTGCCGTCAGTTTGAAAACTGGG
GGTTATAAGAGAAAGGCCAGTTCACGGGATATGATGAATTAAAAGAGTTTACCTGGGTGA
TATGGTAGGCATCGCGCTCTGCTATTGAACTTGATATCGACTCGCCCGAATCGAATGACG
ACTTTTGGTAGGATGGTGAGGAAGGTGCTTATGCGATGGGCTATGAGTATGGGCGGCACA
AAACGGTAATTGCAGTAATCAAAGCCATCAGCGCCGCAGGAATCAAAGAAAAGAATTTCG
ATTAAGCAAATAAGAAGTCAATAAATCACTTTTTAGGGATCACAAACGCTCTGTAGCGAG
GTAAACGCGGGCAAGGCATGCCAATAAGCAGCGAGAATGCAAGAGGCGTGAGAATGTGTG
TGAGGAGGTGTTAAGAAGTGAGTACGAGAGCTGAGCTTGTCAGGGCTAAGTTTCGCTAAG
AGTAGGTGCGTACGTATCTGTATAGTTCATCGTCCAGTTTGCCGTATGATGAGTATTATG
AGCATTATCCTGCCGCACGACGCTATCCGCGTGACCGTGTTTTTCGACGGTTGGTCAAAC
TCAACATTGAGTTTAAGCGCAACACGATTATCCGGAGTCAGGATTAAGTGTGAGGGACTT
AGCATGAGCGACCGATCCTTAACCCACCCAACGCTAAAAGAATTTCCGTTTTGCGGCGGT
GATGCTGGTCTGTGGGTTGAAGCCGGAATAAATAATGAGGTGTGGAGCTATGCAGAAGGT
GACCTCTGTGAAGGCTGAGTGTCAAGGGCACCAACAGTTGCTTTGGCGGCTAAAAAATGT
AACCGGAGAGCCGGAGATGAAGCAAACATTTCTGCTTCGAAACGAATCAATCAGAAATAA
CGCCATAGACGCCATTCTCTCACTACCCATAGAAGACAACTCACCCGGCGAAGTCCCTGT
TAAAGAACCCAAGCGCAGCAAAGCCCAAAATGACGGTATGTGGCCGATGCTGAACGATGT
TTCTCGTCAGGTACTGTGGCATGGTCAACAGCTGGCGCCGGAATACTGGAAAGACCTGTT
CACTGCCCTGTGCTTAAAGCCCAAAAAACTGGAGGAACGAAGTGTACCTGGTATCGACTG
TGGCGTTGTCATGCTTGGCGTGCGTATCAGCAAATTGCGCAAGGCCAGCATGACTGCGCT
TATCGAAGTCATGTTCTGGCTCGGCTCACAGCACAACGTACGGTGGATTGATTACTACCG
GCGAAAGTATGAATGCTCACAACTAAAAAGTAGGGCTGTATGATTATCAAAGCAAATACG
CCACCAGACGACGAGGACTGCTAGCTAACGCCGCTTTGGCTTTTTGATGCACTGGATATT
GGGATTAGATTCTGGCTGGAATCAGCTGCGAGCGACAAAAACGCTCGGTGTGCGCCCTGG
CGAACTGGGGCTCACGACGCATCAATTTCTACGGGGATAAGTCACGATGCTGTCTGGAAT
AACCCATCGTCCAGCAATATCAGGCCGTGGGTGGATAAAGCCGCAGAGCAGTGCATACAA
AACCCACAGACGGTAGTGATGAATCTCCCAGAGGATATGTCAGTAGGATGGTTCAGCATG
GCTCTGGAGAGTCTGGACGACGTTCGCATCATCGCTGATGGACGGATTAATTCTATGGAC
CCGCCGACTGGGCTGCAGATGAAGGGAAACAGCTAAGGCTGAATGTTGCTGATTTGTCGA
CCGTTCATCAGTTCTCGACGGATGTTTACTATCGTATACAAAGCGGCATTGACGGCGAGC
GGGCTGGGCGTAAGGAGGGCTGCATGAGGCCACAGCGACAAAGTATCACAGACATCATCT

CCGAAAACTGCATATAACTTCCAACGAAACGATCCGGAATTAAACGCAAGCCAATCCCAA
AAGAATCTGACGTCAAAACCTTCAATTACACCGCTCACCCGTCGGATATCCGGTGGCTAA
GATATCGCGCGACATAATGACAATGCTGTCAATGCAACCTGGATTTGGAGTTAAAATCAA
AAAAGGCCTCATGTTTGTCGACAAAGAGTCACAACGAAAAATGGCGTCTATCCGATCGCC
CTTTATCAGTCTTTGTTGGCTCAACATGGAGGCAACAAATTATTGGTATACATGCGCCAG
AGCCGCATTTAATGACCCAGACTGGTTTGTGAAAAACCACCGCGCGGTTCGTTAGGCAAA
GAGAACGGCCAATATCACATACATGAAGGCTTAGCAAAAAGCATGGAAAGGACACCGCGA
TCGATACCATCAAGACATGGGAAAGCTTAAATCAGAAAACATGGAACTAAGATGAAAGCT
CGGTGACGCAATACGAAACATTGATGCCTACAATCGACGTGTAAATGGTGACAGCAATGC
TTAGCCCATCCTAATCCCTTCAATACCAGAAAGAAAGCGTCGAGAGGGCTTTAAGGTGCG
CTAACAGCAGTCATAAGCTGCATGTGCTTGAAGTTCACATGTCTGGGTACTGCTGTGCTC
AACTGAGGAGCGATCAGGATACCTCAATGTACGAGAAAGAACACGATGGACAAACCTGCG
CGATGGAAATCCAATATATGCAAGGAGTGGTGTGACCCGCCATTCTCAAATTAGCGGTGG
TACTGCCCGGAGCACGGAACTCAGTTAGCACTCAAACTAGAAAGTAAACAGCGAGAAAAA
GTGGAAAACGCAGCAGCGGAGAAAGGCCGACGAGAGGGCCAGAAACAGAAAGATAGAATG
GAGATTAGAAAACTCGCCTTAAAGGCCCGCAGTTACTGGAATAAACAAGCCAAACTCGCC
GGAAACGCCCGCATCAGAAACTGAGCCCGCGACTTACGATGTATCGGGTGCGGAACGCTA
ATGTCTGCTCAGTGGGATGCCGGACATTACCGGACAACTGCTGCGGCGCCTCAATTCTGA
TTTAATGAAGGCAACATTCACAAGCAGTGCGTGGAGTGAAACCAGCATAAAAGCGGAATT
CTCATTCCCTATCGCGTCGAACTGATGAACCGAATCGGAAAGGAAGCTGTATACGAAATC
GAAGCAAACCATAACCTCCACCACTGGACTATAGAAGAGTGCAGGGCCATCACGGCAAAG
TAACAGCAGAAACTCAAAGACCTGCGGATCAGCAGAATTGATGCGGCATGACGTTCTCAG
GAAAAACCATTCCTGACATGCTCGTTGAGGCAAATGAAAAGCAGACCGAGGTACCCAGAA
TACTGAACTTTAGTCGCAACTCTACCAGTAAATAAACGGGCGATAAAGAAGGAAAAAGAC
TCGCTATGGCCATCGGTGTTCTTATAGTACATCGCGGATAGGGTAAAGCTACCGATGCGT
GATATCCGGCAGGATCTTGAGTGCTGGGGGGTACGAGCGGCAAATAACCATGAGGCTGTG
ACCTGGTCACCCATTGCCGCCGGATTTAAGGGACTGATCCCCGAAAAAGTAAAATCTCGC
CCGCAGTGCTTTGACGATGATGAGATGGTGATATGCGGCTGTATAGCCCGCCTTTACCGG
AACAATCGCGATCCGCATGTCTTGCTGGTTGATTATTACGTGCTGGGGAGGACGTTCATG
GCGCTGGCACCGAGGCATGGGTACTCTGACACCAAGATAGCTAAACGCCTTCACAAAGCG
GAGGGGATCGTTGAAGGCCTGCTGATGATGCTGGGAGTGAGGCTTGACGTGGATCGGCAT
GTTGAGTGTGAATAGCCGTGAGGGATAACCTCTGTATTTTGTCAGCGAAAAAATAGTTGA
CGTTCGTAAAAGTCTGCATATCATGATAAGAGTGGTAACATTGCCACGCTGCTTCACCCC
CCGATGCGCGGGATTTTTGGTAATCATAATCCTGTCAGCTAGTCGAAAAGTATACAGAAA
GGTAGGTGCGACCACAATTATTACCACAATCTTAAAAATTGCACATGGCACTATTAGTTT
TCTATATATTGTGTATTTTTTGTATTGCAGGGTGATCGTGTAACGAATTTTCCGTAAAAG
CATTTTGCTCCACGTGTTTGCCAGCCTCCCCCAATGGATGGCTTTTTTATGCCCGTAACA
TCCTGGGTATCAATAATTATTGTTGTCTACGTACGTCAATCCGTCGCATTTGATCTCACC
TGATATGTGAAGGTTGCAGCGCTCCTTGAATAGAATTATCATTTTTATTACGTTACTGTT
TCGTTAATCCGGAGCGCCGTAAAAGGCCGTCCTTCAGCGGATGGAGGATGTCAAGAATAT
AGGTATTGTATGGTTCTCAAGGAGTATTGTGTATCATGAAAATAATTATTTTGAGAGTGC
TCGATTTTTCCTTTGTTATCTTTTCTGTTAATGTGGTTGCGAGGGAACTTACCTTAGACT
TCTCGACCGCAAAGACGTATGTAGATTCGCTGTATGTCATTCGCTCCGCAACAGGTACAC
CATTACAGACTAGTACACCAGGAGATACGTCTTTACCGATGAAGGATAGTGGCTCAGGGG
ATAATCTGTTTGCAGTCGGTGTCAGAGGGATCAATCCAGAGGAAGGGCGATTTCATAATC
AATAGCTTATTGTTGATCGAAATAATTTATATGTGGCAGGGTTTGTAAACAGGACTTATA
ATGGTTTTTCTCGGTTTACTGATGTTTTACAGGTTAGCTTTCTAGGTACAACAGCGGTTA
CAATGTCTGGTGACAGCAGCTATACCACTTTACAGCGTGTTGCAGGTATCAGGCGTACGG
GGATACAGATAAATCGCCTTTCGTAGACAACCTCATATCTGGTTTTTATGTCAAATATTG
GAACCTCACTGACGCATTCTGTGGCAAGAGCGATGTCTACGTCTGTTACTGTGACAGCTG
AAGCTTTAGGTTTTCGGCAAATACCGAGGGGATTTCGTACGACACTGGAGGATCTCAGTG
GGCGGTCTTATGGAATGACTGCTAAACATGTTGATCTTACAGGTAACTGGCGAAGGTTGA
GTAGTGTCCTGCCTGATTTTGATGGACAAGACTCTGTTCGTGTAGGAAGACTTACTTTTG
GAAGCATTAATGCAATTCAGGGAAGCGTGGCATTAATACTGAATCGTCATCATCAGGCAT
CGCGAGTTGCCAGAATGGAGTCTGATGAGTTTCCTTCTATGTGGCCGGCAGATGGAAGAG
TCCGTGGCATAACGCACAATAATATCTTGTGGGATGCATACGCTCTGGGGGCAATTCTGA
TGCGCATTACCAGTAGCAGGTGAGGAGGTTAAATGAAAAAAACATTATTGATAGCTGCAT

CGCTTTCATTACTTTCAGCAAGTGCGCTAGCGGCACCTGATTGCGTCACTGGAAAGGTGG
AGTATACAAAATATAATGCTGCCGATACCGTTACAGTCAACGTGGGTGATAAAGAATTAT
ATACCAAAAGATGGAATCTTCGGTCTCTGCTTCTCGGTGCGCAAATTACTGGGCTGACTG
TAGCCATTAAAGCTGATGCCTGTCATAATGGAGGGGGATTTAGCGAAGTTATTTTTCGTT
GACTTACAATAGCTCAGTGAAAATAGCAGGGTGAGATTCAAAAATGTTAAATACATCTCA
AGTCAGTCAGTTCTTGCCGGTCTGTTAATGGATGCCTTAGAGATTTTCTGCATGCGGAAT
CCCCCTGTGCGGAGGGGCGACATGTGAACGGTAAGATCTCTTTGATGAGCGTAAGCGAAA
ATACGCGGGTTTGGTGGCCCCGGGCCGTGCTCGCCGGGAGGCACCCGGCACTTTGCAATG
GCACAGAGCGCTACCCTCCAACCCCTCTCCGGAGGGGCTTTCTTTTTTACAGCAGAAGCC
CGCACAGGGAGACGCTGCCGGGGACGAATAAACAACCAAACGTGAAGTAGTATTTCAGCT
TGCGCCTAATATGCGACAGTAATCACTCTGCGCAATACCGCGGCCGATTTCGTATTGCGA
GATGTTGTCTATCTTCTCCCGTTCTCCTATAGCTTCCGCATTTCAGCACGCTCATCATTT
TACACACAATATTATCCCGGCCGGGACGATTCATGGCATTGCCACACTACGATGCGGTCA
GGGCGGCATCGCCGTCAGACCTTGCTGATGCCCTTGCGCAAAAAATCTGTCAAGGATGGC
AACGATTCCGTGTGCCGTTGTCTTCGTTTACGGATGATCGCGCGCCACTGATTCAGGCGA
TTGTCACAGAAGGTGATGTGAGCACACCTGTTGTGGTGAAGCTGAGAGGTGGTTAAGCTG
CAGTAACCAGCGCCACCAGGGACCCCGGGTATTACTTTGTTGTCGTTCTGCCAGGGCAGA
CAAACGCCCTGTCCTATGGTGCAGGTCTTCTGTTTCAGGAGACATACGACCGTCCGGACC
CGCCCATAAAGTAGTCGGCGCGTCGCAGTACGATGACACCGGACGGTGTCGCCTGTAAAT
ATAACGACATCATTCCGGCGGACCATTTTCTGCATGATATGCAGGACATTATCCGCCTTA
GCCATCCGAAAGCGGACCTTTTAAAGGGGCAGTAAGGAACCGTGGGGACGGGGCTACATA
TCGCCAAAAAATCGCAGCCGTTTATACCGGAGAATCCGGGCATTCTGTTGGTCCCGTACT
GTCGGGGTGGTTAAGCGTTCACCACCGGGGGCGAGGGCACATACAGTGCCGCGAGCGGTG
CCTCGAAGAATTCAACCCGCTGAGGTGTGGACAAGCCGCTGTATAAGGACCTTATCGGTC
TAACAAAAGCAGCAATCAAGAAGTATCCGGAATATGTTCTGATTCCAGTGGTGAGGACGC
AGGGGGAATTTTATTTGGGCGGTACGCCGGCAAATCACGCAGCATAGTTTGGTGAGGTCG
TTGATAAATTCCGTGTAGACCTGGCGGAAATGACAGGTCAGTGTGTCGTTTGCTCTCCTG
AGGTTGTTCCCTGGATATGCGGGTACAGGACGTATTTCTGGAAGCAGAATAACGAATCCT
CGTACCAGACGGTTTATGGCGGCTGCTAAAACAAAACGGAAAAGCATATCCATTTCGTAC
CGTCCATGACGGATCAGAATTGGGTGAATGTGCCGACGAACAAACCGGATGATGACCCGG
ACATTCGGGGTATCGGCTATTGCGGTTCGAAATGGCGTGACAGCTCAGCCACCTGGACGT
CACAGGACAGGGCGACCCATTTCAGCGCCTAGGCAAGCCGTCGGATTATTTCCCACCGTC
TGGCAACGGCGATTTTGCGCCATTCCGGAAGATTGGCGCTAATCGCGGGGGCATCATCGA
CAGTATCAGAGGTGGGCCCGACATCGCCTTCCGGTGCAGAACCCACAGCCGTCACAACAC
TGCTCTCTTACCTTGGCAGCGAGACAGAGGGAAGCCTGAAAGTACAGGGATGGTCAGCAC
GTGGCGGGAGGGGTCCAGTCGTCGGCTAGACGGAGGGATCCGGAGGTTAGGCAGGGAAGC
TGACCAAGGAAGCCGGTAGAAGGAGCTGGGTGTTGGTGTACGCCGCGGGCAATGGTGCGG
ATCTGTTCCAGATAGGGTGGCAGATTCGCTGCCAGTTTTAGCTTTCGGGAGTGCTAGCTG
CGAACCAGTATGTTTTGCCGTTTTACTGGGCCGTATCTTCGCTGGCACAGGGGGTTGCGC
TGACCAGAGACGGGTCGAATAACCAGGTGGCTGCGTTCTACATCTAGACAGACGCGATAG
AACTGAATGTGATGTACCACAATGCGAAAGTGGCGATAAACAACTTGATACAGGGAACTT
GTGGTGGATTTGCTAACGTATCGCATACGGTTGCCTTCCCCCTCGCCGGGAATAACAGCC
TTCAGGTGACGCCGCTTGTCGATGTTCAGGATGGCATTCCGTTCCCGCTGAAGCAGTCAC
CGGTCAGTGGAATTACGGCGGATCAACCGCATGTGACAAACATAACCAAAGGTGCTACTT
ACCCGGTGCTGATGGACAGCATTGCAGTGGAATTGTACAACACGGACACCCCGGCATGAT
AAAAAAAACCGTCAGCGACAGGAATCCAAGCTGGCCGTGGTAATACCTATGGAGAAAAAA
TAAACGAGCGATACATTCGTGGTATGTACTCTTAAATGAACACAGTTCTTAGTGTCAACC
ATAACGGAAAGAAATTTTAACATTTATGTACCAGTTAAAGCTGTACTTCTGTAGGGTGGT
CTGTGGGCTTTATCTCCTATCAGGTGGGTACTCGGCCATGCGTGACTTGTGGCACAGACG
GATTGCCAAAATGGCCGCTTAGAAAAGCAGTGCCAGTCACTCAGCCGTAAACAAGCCCGA
AGTGCCGCACATTTAGCGGCAACTTTCTTAATCACATCTTTCGACGACAAAATCCCCTGT
CAGGTATTACATCAGTGGTGACTGCTTAAGCACTGAAGGAAGACCTGGGCTCTGAAGGAG
TCCGGAGTGCCCTGAAACAGATACTTCGGCAAAACCTGGAAACGCGTCTTGAAGCAGAGG
TTGATGCCATTCTGCAAGAGCTCCTGGCCGCACCGGCAGCTCCGGAGCCGGAAGGCATCT
CGGGTGAGGGGAGTACTTCGGAATGCGGTTACCCCACACCGGACAGCGACATGATGATGT
GAGCATGCGTCATGGACCATCGGTGTGCGCCGTTGGTCTTTTTCCTGCTGTGTCCCTCC
GGATTGCGAGAAAGGGGGTATGTACTAGATGGAAAAAATCAAAACCGGTGTGGCGTACAC

CACGTAAGCGGAGGGAATGGGCTATTGGTTCCTGCAGTTGCTGGAGAGGGTTGCCCCGTC
TCAGTGGGCGACCATAGGCGTGCTGGGGATGCTGCTGTTTGGGATGCTGACATATCTGAC
GAACCTGTATATAAAGATTAAATTAGACCGCCATATGGAGGTTCCGGGAGAGTTAGCTCA
TGAGCAGGAAACTCCGCTATGGTTTATCGCCTGCCGTTCTAGCGCTGATTGCCGCAGGTG
CTTCTGCGCCTGCAATCCTTGACCAGTATCTGGATGAAAAACAAGGTGACCACAACACAG
CATCCCGTGATAGTGCGGGTAACTAGACCATCTGGCGTGGAGCGACCCGGGTGATTGGTA
AGCGTGTTATTTCTGTAATGAAGCTCTCGAAGGATAAAAGCGACCGGGTTAACGCCATTG
ACCGCGCTAAGGCGCTGGCATGGCTGGAGAAAAACATCAAAGTGCCGCTGACCGAATACC
AGAAAGCGGGGATCGGGTCATTTTGGCCGTGCAACATTGGACCCGGTAAGTGTTCCCCTT
CCACGTTATACAGAGGAATTAAGGCAGGTGATTGCAATGGTGCATCCGAAGCCATTCGCT
GGTGGATTAAGGACGGTAGCAGAGACTGCCGTCTTCGTTCAAACAAATGTTACGGTCAGG
TATCCCATCGTGACCAGCAGAGCGCGCCGGCGTGCCCGGGATTCGACAGATACGATGAAT
ATTCTGCTGGGAAATGATGATTGTTTACAGGTACGGTTAACACGAAATGCCGCAAATCGG
CAAAATGTAAGTGCATAAAGTCAAAACAGTTGTATAACACTTAGGCACCGTACCCAAGCC
TTTGTCAATTATGCGTATCTCACGCGCATCTCCCAACACAGATCGTTTCAGGATGACCCT
TCAGGATACCGGTTTAGCTGTCGGTGCCTTTCTGTGGGCTGGATTCCAGTGGGACAACGT
TCGTCACTACAAGGAAGTAACCGATGAATACGATGGCCATGCCGTTTCACGGCAACTCTC
TTTATGTACTTAACCATAATGGCCAACCATACATTCCCATAAAATTTCTCGTTGCGGGGA
TGGGGGTAGCCTCGAAATCCCAGGTGGGTAAGTTAAGATTGCGTTTTGCGTCAACTATAA
CGGAAATCGTTATGGCTGCTGAGGATGAGAAACAACGCCATAGGGTATCCATGCCACTTC
GAAGACTTGCCAGCTGCCTACAAACCATAAATCCCAATAGAGTAAAACCCGAAATTCGCG
AGAAGGTCATCCAGGATCAGGAAGAGTGCAACGATGGTCATTACGCGTACTGGACGTAGG
GTTTTGGCGTTACTCACCGTAAAATGAGCGTGATGGAAGAACTCAACCAGCATTGTGCGG
GCATGAAACGGGATAAAAGCATTGCCGGTGTGTTTGCTGCCGGGCTGAATGAGTGGAAAC
AGGTACGTGCCTCGCATGTATCAAAACTCCTTACGCTGGTAGGTGAAGCCAATATGCTGA
TTGATTTTGTCCTGGGTGATACAGGCAAAGGGAAAATAAAAAAGGAGGATTAATGGGGTG
GTCAATGATATCACATGAACTCATAACGCCGGTGAAGAGCCTCTATCTACTTGTCTGCAT
ATCATACTTAGTCCTGCAGGTTGCCATTTTCTTTTCCACCGCCTGTCGAGTCCTGACGTT
ATCGGGACTGGTGGGGTCAAACCGAGATGAACCGTGTTCGGTGGGTGGCGATTCTTGTCC
TGGCGGTTGGCTATGGTGCGCTATGGCTGGCAACAAGCCATTACGGTGACAACTCGCTCA
CCTATAAAGCTCAGGTCGATACAAAAGCCAGACAGCTGGAACAGGCTAATGCCAGCGTTA
CTGACATGAAGGTGCGCCAACGTGCTTTGGCTGCGCTCGATGAAAATTGATCGAGGGAGT
TAGTCGATGCGAGTGCTGAAAATGATACTCTGCGCGCTGAAGTTGCCTCTGGTCTTAAGC
GCCTGCGGATCAACGCAAACTGCGCCGGTACCGTGCATGTAGCCACCCGCACCTCCCTCG
TGGATAATGCAACCGGCCCGCCACGGGCAGAAAACGCTGAACGGGATTATTGGGTCCTCG
GAGGGAGGCTCGTCACTATTCAAAAACCACTGGAAGGAACCGAGAAGTATATTAATGAGC
TGTTCAGATAGAGTTGCACATATCGATGGGCAATTCATGCAATTATTGTCAGCAATAGAC
CCGCGCTTTCAGCGGAGTATAAATTACTATAGTAATAAAACCGTGCAATCCATATAAGAT
TATTTGCTGGGTTTTTGTCTTAACAACCTTGTTAGTGCCGCGACAATGTTTGGCTGTACC
TACAGTTTTCTGCTGCCCATTTCCAGAAACGCAGAACTGATGGGTGATGGTTGTCTATTG
TGCAATTGCTGTCTGTTTCTTTTGAACAGTAAACGTCTGTTGAGCCCTTCGTGTAATAAG
AAGGGCCAGCCCAGTAGTGAGTCGCATTTTCTTCACGGCGATAATCACGATGCTCTTTGA
AGTTCGCAGAATCGTATGTGTGGAAAATTAAACGAACCCTAAACAATGAGTTGAAATTTT
ATATTGTTAATATTTATTAATGAAGGTGAGGTGCGATGAAAAGACATTGTTTCCCCGGGT
TAACTATGTCCACAGCCCTGACGGGGCACTTCCCTGCGGGAGTGTACGGCGATAATCAAG
ACCGATGCACACCGGGTTTTCTCACTTTTCACGAGATGGGAGCGAT
>NewGenomeName_24
CAGTCGGTGAAGCTGCAGATATTATGAACTGTGTTTGTGACATCCTGTATTTGCAATACA
AGGCAGATTATCCTGAACTCAGTGAAACTCAGATTGTGGATTTGATTAAAACCGAATTGA
ATTTCCAACTCAACTTAAAAGCTGAAAAATGGATGATGAAAGCATGTACCAAGTAATTTT
TATCGAAATTGTCATAGTCGTCCTTATTGCGTGTCACATGTCCATGTGGGATGACTATTG
GAACGGTAAATGCAAAAACGAAATCACTGAAATTAACGCATGGCTCAAAAAGTATGATAT
GACACTGTGGCCAAAAGACCGGAAAGCTGGTCCATTTCCAAAGTGGGTAATTTGTTAGAT
GACACTTTGAAAGACATCAAAATAAGCCAATGCCAGGGTATCAGTTTCCGAAAATTGTAT
AAAGCTCTAGCTGAAATTCGCAAAGACGATATTGCTAATCCTGATTTCGATCAAACTGGA
AATTTGTTGTCTTTGCTTTACAGATCGCAAATGAAAGATGTTAAAAAAAATCCATTTGGG
AGGACTAAATGTCAACTGCAGCTGTCATTGCGAGTATTGTGCTCATGTTCGCAAAAACAC

AAAGTAGTGATATAATGGCAACTACTCTATCAATTCATTTTGAGGAATATTAATGTCTGA
TGAATTCAAAGTTCTAACTCCTCGCGAACACGTAATTCTTGGCCCAAATATGTATGTAGG
TTCTGTCGCATATGAACCACATGAGCGATTCTTGTTTGGTAAGTATCAATCCGTTTCCTA
TGTTCCGGGCCTGGTAAAAATCAACGATGAAATCATCGATAACTCGGTTGACGAAGGTAT
TCGTACCGATTTTAAATTTGCAAATAAGATTGATATTCGCTTTGATTTGCTTGAAGGTTC
TCTGACGGTAGCGGATAACGGCCGAGGTATTCCTCAATCACTGGTAAACACTCCAGACGG
AGAAATCCCTAAGCCTGTTGCTGCCTGGACACAAACTATGGCAGGCTCAAACTTTGATGA
TACCAACCGCGTATCAATGGGGATGAATGGCGTAGGTTCTTCTCTGACAAACTCCTGCTC
CAAGACTTTTATCGGTGAAACGTGCGATGGCAAAAACTTTCTCACTGTCACTTGCTCTGA
TGGTGCATCTCAAGTCAATTGGGAAACCAAGCCGGGTAATATCAAAGGAACGTCGGTATA
TTGCATCCTTGATGAAAAGCAGTTTGAAGGAAAAACGAAGGATCCGCAGATAGTAGATAT
CATTTCAGATCGAATCACGGCATTGTCTGTGGTGTTCCCGAAGATTAAATTCACCATCAA
CGGTGAAGTGGGTTCAATGAAGTTTGCAGCATACGCTAAAATGGTCGGTGATGAATCTCT
CATTCTCGAAAATGATAATTTTAGCATCGCAATCTGCAATTCTCCTGAAGGATTCCGTCA
AGTCAGTTATGTCAATGGTTAGAATATCAAGAATGGTGGTTCCCACATCGATTACTTGAT
AGATGGCATTGGCGACGAACTTATTCCATCAATCAAACGAAAGTATAAAATTGAAATAAG
CAAAGCTCGAATTAAAGAATGCTTGACAATGATTGTGGTTGCTCGTGGAATGAGTAATCT
TCGGTTTGATTCTCAGACAAAGGAACGTTTGACCAATCCGCTTGGCGAAGTGAACTCACA
CATTGAGCTGGATTTCAAAAAGTTAGGAAAGCAAATCATGAACAATGATGCTATCCTGAT
GCCAATCATCGAGGCTGCTCTTGCTCGTCATCTGGCTTCAGAAAAAGCAGCATTGACGAA
AGCCCAAAAAGCAGCAGCAAAAACCAAAGTCGCTAAGCATATAAAGGCCGGTTCCTGGGG
TGACATTTCCAAAGAAACAACTCTGTTCCTGACAGAAGGTGATTCGGCCATTGGTTATTT
GTTGTGTGTTCGTGACAGCGAGTTGCATGGTGGATATGCTCATCGAGGAAAGGTTATGAA
CACTTGGGGAATGAAAGGTTCTGACGTGCTAAAGAACCGAGTATTGGCCGATATCTGTGC
AATCATGGGTCTCGATTTAGCTAATCCGGAAGATGTCGAAAATCGCAAATACAAAAATGT
TGCAATCATGACCGACGCCGATCCAGATGGCCAAGGCTCAATTTACCCATGTCGACTGGC
GTTCCTTAGCCGTTTGCCAAAGTTGTATGATCTCGGTTGCGTACGATTTGTAAAAACTCC
GGTAGTCATTCTTTCTAAAGGAAAGCAGACCAAGTGGTTTTAAACCATAACTGAGTTTGA
TGCGCAAAAGTAAAAGTACAAAGGTTGGGAACTTCGATATATCAAAGGATTGGGTTCACT
TCGCGAACATGAATATTCCGAAGTTGTTAATAACCCTCAGTATGATATCGTCGATCTTGG
ACCCGATTGGGAAGAATTGTTCGAAATGCTGCTTGGCGATGAAGCAGAACTTCGAAAACA
AGGGATGTCACAATGAAAAGGGCTCTTCGGAGCCCTTTGTTGTTTTTGAAAAACAGTGTA
CAACGCTTAATGTGAATGGTATTATGGCTCCATCAAATCAACCAAGGAGTACATCATGAA
ATTCGTTCGAGTTAAATTCAAATCCCCGGAACATCGTAAAGCTTTTGCTAAATCTGCCTC
GTACAAAGCCGAATTTGAGAAATTCATGCCAGACGAATTTTTGGCTAGGTTTGACCCACA
CAAAAATGTTCAATACCGTTTGGTCGATTCTGATGGTGTGATGATCGAGCTGCCGGAATC
AACTGGTGGTGGCGGAATCATTTTAGACGACGAAGAACTTCATTATTTCATGGAAATTAT
TGAATAATTTCACAACAAAGCAGTTTACAATCGTGAGGCTATGGGATACTATAGCCTCAA
GTTAAACGAACTAGCAAAAACATGGAGTAATTTAAAATCATCAACTTCACCGAAGCATTC
GGCAACGTTAAATCCCATGCGATGTTCTTCTCCCGGTATCTTGATACCTTTATCAGTGTT
TACGGTGTTGAAACTGATTCTCCGTCAGTCTTCATGTACAACAAAGGCACAAAGAAAGCA
TAGCGTCTCGGCGGCAAGAAAGGTGCTTCATACATCGAATACGTTCAAATTGAATTGGCG
ATAGGAGAATAAAATGAAAGCACGTTTCAAATCTGAAGAAGCACGTCAGCAGTTCATCGA
CTACGGCGAAAAGCGTTGGGCTATTGGCCCTCTGGGCGACACAAATAACAACCGTCGCAT
CGCTCGTCATTTAGGAATGAAAGAGTTCGATGTTGTCAAAATCGATTTTCCATTTGCTGG
CTGGTTCGACATCGTCATTGATGACATGGTGTTCGAGTCTATCGCTAGTGACGAAGTTTG
CTTCTTCGAGTTCATTGATTGATTAACTGGCCGGGCAGCCGCCCGGCTTTTTTGTATCAA
AACAGTTTGAGGCTTTGCCTCTTCGAGCGAAGCTCGAATTTATCCTTTTAGTTATAATAT
CTATTAAGCCATAAAGGATAAAAATATCTAGTTCGACTGGGTGGAACCAGGTAAACCTGG
CTGAACTCAATAGTTTAGTCAATAATACATTCACATAAGAATAAGGTATTATCCAGGTTC
AACCCAGTTAATCCAAAAATTATTTTAACTTTAACTGTGTCATTGTAATGTTTACTGTGT
TAGGATAACACCAGTAGAAAAACAAAGGAGGTCAATATGACCCTGCATGAAGAAATATCT
GAAGCTCTGGTTCGAATTTGTCGCATCAACGTACGATTCATGGGTGATGAAGAAACAGCC
GATGAACTGAGCGACGAAGTTAACCCAATTATAGCTAATATCAAAGCTGGTATTGCAACC
CGCAAAGAACTCGGCCGGCTGTGTCATTTGGGTCTTGTGCACGAAGCTAATCTTTATGGT
TCCGATTGGGGCAAAATTGTTTCCAAAATGGAACACCGCATGAAAAAGGAACATAGTTAT
GCAAATCACCTTTAAATCCGAAGAACATCGCAAAGAATTTGCACGTATTGCCAAATGCAA

TGAATGGATTGCTCACACTCTCGGTATGGACACATGGGAATGCCGAGAAGAACCGGCAGA
TGAATATGACAAAACCCCAGTATTCGCTGTTCTTGACGGTTTCGGCGATCAGGTCGCCGG
AGTGTTTGTGTATGACGGCGGTCAGTGTGAAGATCATGCATGGATTTTCTGGGGTGAGCG
CAAATATTTCGATATTACTGAATAAAGGAAACACATAATGATTATCGATTCTAACGAACA
AGGTGTGATGATTGGTAATGCACCTTCTAAACGGACCGGGTTTAAAATCCAGGCTGGGCG
GAAGGCCTTCCAAGTACTTTCTGAAAAGCTTTATAAAGACAAAGCTGAAGCAGTTTTACG
TAAGCTCTCGTGCAACGCGGTTGATATCCATTGCGAACTTGGAAAAGGACATATTCCGTT
CAACGTCACTCTCCCAACTTCAAGTTACCCATGGTTCGTAATCCGTGGTTTTGGAGCTGG
GCTGACGCAAGAGCAAGTTGATGAAATTTTCACTGTCTATTTTGCTTCCACCAAAGATGG
TTCCGATGATTCAATCGGTGGTTTCGGCCTTGGTTGCAAGTCTCGATTTGCATACAGCGA
GTCGGGTGGCGGTTTCACTGTAAAAAGCTATCAAAATGGCCTGTGTACTGTCTACAACAT
GTATATGGATAATGGAGAACCATTTGCTACACCGATGTCGACTACTCCGACGCCCGAAGA
AGATGGTCTCGAGATTCTCGTTCCTATCCCTGAGCACGAACATGCTCGCTGGGAAGTACT
AGCTCGCAAAGTGTACCGGGCATTTGATCGCTGCAAACCTTACATCACAAATATGGATGC
AAGCAGCATTGAAGAATTTCCGAATCAAGATTCGTTCTTCAATACTGACGAATTCGCCCG
TCCAGGAGAAGTGTATGCTGTAATCGGTGGAGTAGTGTACCCGATCCCTCAACGTCTCCT
GGAATCCGATATGGTATTCCGATATGCTGGTAAAGCTGCATATACCAAGTGCCCTATTGG
CTCAGACGACATTGCTCCTTCTCGGGAAGAACTGTCGTTGACCAAAGAAGGCGAAGATTA
CATTATTGGTCGACTTCGTGATTTTTCTGATAACTTTGCCGAATCGATAAAGAAGGAATT
TGACGGAATAACCGATAAACGAAAAGCCGTAATTAAAGCCAGTGGATACAACAACTATGT
TCGTCAAGCTATTTTAGATTTGACCATTGATGGCGAGCGAATTAACGATCTTATTGATCG
ATATACGGATATGTCAAAGCTTAGTGACATTACCGTTTGCGTATATCGTGGCGGTAGTTT
GCGCTAGCATATTACGCGTTCGGGATATTCGTGGAGAAGTTCCAATATAGATGCAAAAGA
AGTCTTTGGCATCAGTCGGGGTAAAGTGCATGTTCTCATCAATGATACAGGTGAAGGTAT
TCCGAATATGGTTCGGGCGTATCATCCTCATATTGGTGATACCATTCCAGTCGTTGTGTC
GAACAAAACTCGCAAACTAAAGAAGAGTGAGTACGCCCGATATGGACGAACCATGAAAGC
TATTTGCGAAAAATTTAGCGTTGACGAACGCTCAATTCTCAAATTTAGCGAAATGAATGA
TATTCGAAAAGCATTCACCATACATAAGCGCAAAACATCTACTTCTCGCGATGCTAAACC
AAACGTTCAACGTCATTACATCAATGACTCCGACCTTCGTCACGTTGAATTAATTTACAT
GACATCCGATGAAATAAAAGCTCTTGAAGGAATTGCGGTTATTTCGTATAGCGATACATT
TGACAGAATTGCGCTTAAAGCTTCGCAGTATACAATGCGTAGTGCCCCTAGCAAATTGAT
AGAGCTTGGAGCAACCGAAGTTTACGTCATCGATAATGGAAAGCGTTCTGCCCGCTGTGA
AGGCTCCTGGTTTGAAATGCGGTTTTAAGTGGGCATATGACCAGTACAAAGTGTCGAAAG
ATGCTTTTGATATTCTTAGTGCCGAGTGCCCTAATATCGGAGACTATGAAGAAGCAATTC
GTCTTAAGCAAATGGGCATTCTTGGAATTGATATAACCGAAAAGTTTGACTAAAACAATG
ATGATTTCCTGAATGATGCATCCCAATGGGTTGAAATCGGAAATCCCGATTATGCCGATT
TTGCTGAAATCAAAGAATATGTTGATGAACAGAATATCAAGTATTACGAAATGTTCTATG
ATCATAGGGAGTCATTGAAAATTAAACACCCGCTATTGTTTGCAGTTGCCGTTAGTGGTA
TATAATTAAGTCCTGAAATCATGAAAGATATCGAAATTCTCCGGGCTCAGTAAGAGCCCT
TAAAACAAAGAGGTATGATATGGCTAAGGTTAAAATTGTTAAGTGTTTTGATAATGAAAC
CAAGCAACAGATTTTTGACAAATATTCTCTTGGGAATGTGTCTCAAGCTTATTTGGCAAG
TGAATACTGCGTATCGTTAGATACAATTGCTCGGGCGCTGAAAGAAGTCCGAAATTGGGA
TATCTCTGCTGCAACGTCAGAGGATTCACCTGCCGAGGAAGAAATTGCTGATAAGCTGGA
ATGTACCTGGATGGCCAATTCCAAGTTCCTTTCTGTGACCATCGGCGCCGAAACTTATAA
CGCTTCTGGCGATCACCCACAGTTCATGGAAATCATCAGTCTTCTGCGCGAAGAAAAATA
CGATGAAGCAATGGAAATGTTGAATATCAAGCGTGGTCTTGCGCGATATGTCTCTGGCGA
TCTGGTTATCGAAGGCGGCACACTGAAATGCAAAGATCTGATCATTGATTCTGGTCTTAC
TCGTCGCATCGTCGGAAAGATGCAAAACGGCGAACCTTTCGAGCCGCTCGTTGCATTCTT
TGAAAATCTGATGGCTAACCCGAGTCGTCGAGCCGTATATCAGCTGTACGACTTCCTGGA
ACACAACGACATTGAAATCACCGATGATGGTTATTTCATTGCTTGAAAGCGTGTAAACAG
CAATTTCAAAGACATGGACACTGGCAAATTCGATAACTCTCCTGGAACAGTGGTTAAGGT
TAATCGCTTCCAGATTGACGAAAACCCTAACGTTACCTGCTCTCACGGTCTTCACGTTGC
TGCCAAGTCTTATATCCCTCATTATGGTGGTGGTTCTGGAAAAGTGATTGCATGCAAGGT
CAACCCAGCAGATGTCGTCGCTATTCCAACCGACTACAACAATGCCAAAATGCGTTGTGC
CGGGTATGAAGTTCTTTACGAAGTAACTTCCGACTTCAAAGAAATGAAGCACTACTAATT
TTCATAAGCCCTCTTCGGAGGGCTTGTTTGTAAGGGGTTGTATGAGCTCACCATTTAGCA
TTATATTTTACAAAGACGATGACAGTGTTGGTTCGGGTTATGGAAATCATAGTTGGATTT

CGCCGAATGACTTACAGTAAGCGAAGAACATAATTGCAATTGTTGAAGGAACATCTTGGC
AGTTAGGACCATTCGAAAAAGAATGGACTCATTTCATTGCTGCTGGCCGAGTGTTTTATG
CTCAAGATTACGAGAAAATGAAACAAATCATTTCTCTTATAGAAAGCTTTGATTTATCCG
ATTTTTCATTGTCATATTCTGAAATTGTGAGGAAATTGTAATGACGGTACCGATGAACGT
ATCCCATGACGAATTTATCGCCCTTCGTCATATCCAGCCGTTTGACGCGGATATTATTCG
TCTTCAGATCTGCGACCAACTCAAAAAGCATCCTGACACGAGGGATGAGCTTTTTGAAAA
ACGGTAAAGGAGATATGATGATACCATCCAATACGCTCTCGATGAAGCGGCTGGGAATGA
TATTCCTCGGCAAGAGGTCATTGATAGAACGGTTCGTTCACTGAATGCCGAAATGGGTGT
CGCATATATCCTGGGTGGTGTCTGGGGGGTGTTTGACTGGTTAGACAATGACAATATCTC
CTTTGATGTGCCAACACCTGGGTTCAATCTGAAAATAAAGACTTCTTTTGCGAAATGGCC
AACAATGCATACCGCTGGTGCTAAACCGTATGGCAAATCAGAAGGGCTGTGCTTGTACGC
TGGTTTGCGCGGAGATGCTGATGTTTACTTGGTATTGAACATCAATGAATCTGCTGGCCG
TATCACATATAGGCCGAAATACTTCTTAACAAAGCGAGCTATGGAAAATTATCCGTATGT
GATCTACGTTCCCAAGCGCGGCGGTGGAGCATACATCAATAGACAGCAGAACAAATTTGT
TTATTTTTATAACAAGTGGTGTACACTCTGATTGTGCGTGATAAAATGCTCCTATCAACA
ACACTGGAGCATTTTGCATCTAGGAAATGAAAATGAGAACTCACGATTTTCTGAAAAGAC
GCGCCAGGCCAGGCTGTGCCGAGCAAAAGAACGAGCTGATAGCTGAGTACAGAATGCTTT
GCTCAATGATCGATCATCACATCATCATGTGCTATGACCGCGAGGACTCACCTGAGCTCA
TTCAAGCCAAGAAAGACCGCGATGCAATCGAAAAACTCCTAATGGAGATTACTAAATGAA
AACTAATACACATCACGTCACAGCTATTGATGCCCCGTGTGTTGGCACTTTTGAACTTGA
ACCGGCAAAACTTGCGGATTCAAAGTACACTTTCGAAGAAGTGATGCATAGAATCGGTTA
TACGATGGTTGATTCGTTAGCAACGCTTGAAAGCTCCCTCGAAGGTATTGAAGCACTTGT
TTATGAAAACATGATCACCTTCCCCTGCTGGGTAAAACGGGTTGGGAATCACTTCGATGA
AATGTATGACGTGAACTGGGTTTTCTTTGATTTTGTTCCACTTTCTGAATAAAACAAGTT
TACAAACTCGTGGCTATAGGTTACTATAGCCTCAAGTTAAACAAATAGATAAACACGGAG
TATTTTTATGATCAACTATACTTTCAAATGCGAAACTTCTACCGAAGCCTTCAATTATGT
TGTCGGCACCAAGCGTGGTAAAGCCTGGGCAAATAAAACTCTTCGCGAGTATCTGGATGA
AACTGGCGATAAGGATGCCACTATCACCCTCATGAAAGAAGAAAAACTGTAAATGGAGTA
AGTTATGAAAGGCCCTAAAACATTCGACAGCCAATTCAGGCCTCGCGGCGGTTTTGCGCA
AGACATCAGCAACGGCGAAATCAAGAACGAGCCAATGTTTTTCAACTCAGACTTGGAATT
CGCGTACGAGAACGGCGGCCCTATCACTCGCAGTTTTATCGAGAATCTCCCGCTCGATTG
GAAAAGCATTGTGATTTTCGATTCGCGTGTTCACATGCTTATGCCTGGGTGGTACCCAGC
GATTCCCGGATATCACCATGATGACGTGCCGCGCCCGCCGATTCCGGTAGGCCAGCACTT
TGCAACTGCAGGACAGCCAGACTATGACAATCCTCGTTATCTGTCTCGCCATATCATTGG
TTTGGTTAACGGCGATATCTGTCCTAGTCATTTTCCAGTAGGACGATGGGAATTCAGCCA
AATTCCTGATGGTGAACTTATCTATCGGCAATGGCAACAGGAAGTCATGGCTAAACTCGA
AACTGGCGAGTTAAGTAAGCATATTGCTCCTGATCGAACTCTTGTAGAGTTTGACTGGCA
AACCTGGCACACTGGGTCAAAAGCGGTTAGAAACGGCTGGCGTTGGTTTGGTCGAGTTTC
TCGCGACACTGATCGAGTTCTGACACCGTCGAATGAAATTCGACGTCAAGTTCAAGTTTA
CATGGAATTCCCGATGGAGGGTTGGTGATGGTTAAGTATCGTTTTGTTAGTGAAGAGCAA
TTCAACGCATGGGCCAAGCAAAATTATGCACAGGAATTCGCATTTGCTTTTGATTCATGG
GATACAATCTTCGAAATGGAAGACATCGGGCGTAAAGTTTGCTCTGACACTACCAAAATC
GACGGCGAAATTTACATGAGTACTCAATGGTACCCATTCAATATAACAGACCGTGGCAAT
CGCATCATCGAGGTGAAAGAATGAAACTCTACTTCTTGCAGCACATTGAAATCAAATAGC
TGGCATGACTTCTGCGAATTTGAAATCGGAAGTTATGGTGCAAAAGATTATCACGTTGTT
TGCTTAACAGCTGTGTTAGTTTAAAATACAAGTCAACCAATTGAATAGAGAAAAATATCA
TGAAAACTAATCTTACTGCTCAACGTATTGTAGTTACTGGTGCCCAACGTGTTGGTGCTT
TTGAATTTGACGCAGGAAAATTCCTGGATGTGAAATTCACTTTTGCGCCTGGCAAAAATC
GTGAAAGCATTAGCAAGCCCGGCATTTATGTTATCGCTAAAGATCGTGTTGCTGTAGCTC
GTACTTTTGCTGGCCAACAAGAAAAGAGTGTTGGTTTTAACGAGACTATTTCGCGAGTAA
TTAGCTGCTGTTGGAAATATACTGACGGTCCTCCGCGGAAGATTTACCGCTAAGTCACAG
GTCAAAACAAGACACATACGGTTTACCATATCCCGATGTCCTTGGTGCCAAGACTGTTGA
ATATGCAACAACTTGAGCTGTTTGAAGATAACACCACCAAATTTCAGTCGCTCACTGCTG
TTTCGCGTCAACTGTTTAAGGTATATAATTTCGAATACCAAAAACGATAAGGAATACCCA
TGGCATTTAGTTTAGATGATTTTTTTGATCAAGAGCCGATTTTGCCGGTTCCAAAACCGG
CATCTAATCCCGAAACTACGCCTTGGAATGAAAGCAAAGATGATGAATTGTTTCCTCCGG
CAGGAATTACCATCAACCAGGTCACCGATGAAACGGTAATCGAAACTGAAGTACTCGATT

CTTCGGTTGTTAAAGAAGTCGATGAAACTGAAGTACTTGAAGACCAAATTCGGCAATGCC
AATCTGAAATTGATTCAGATATTCCGGAATGGAAGAAAGCCGTATTGCAAAGTCATTTAA
CTAATCTGCATAATCGACATCTGCAATCGATCATCAACAATGAAGCTCTTGAATTTGCGA
TGTATACACTTGAAGAACGTGCAATCCCATCTATCATCGATGGCTTTAAACCGGTTCAAC
GCTTTTTCATGTATCGCGCATTGGAATATGCGAATCAAAATAAGACCAGGTTTAACAAAG
TTGCAGCGCTTGCAGGCGGTGTTTCTGAGGCAGCTTATCACCATGGCGAAGGTTCTGCCG
CAAGTGCTGGACAACTTATTGCAAATACATGGAACAACAACATTCCAGTATTGGAAGGAG
ACGGAAACCTTGGTTCTCGTCTTGTTCAACAAGCAGCTGCACCACGAAATGTGTTTTGTC
GTGTCCATCAGAATTTCTGGGACATCTATAAAGACATTTCTCTTTCTCCGGTGCATCCAG
ACCCTGAGCATTTGCCGCCGCGTTTTTATCTGCCTGTTATTCCTATGGTTCTCGCGAATG
GCGTAAAAGGTATTGCCACTGGCTATGCTACTACAATTTTCCCGCATTCTTACGAGAGTC
TAGTGAAGTGCACACTGGCTGCAGTAAAAGGCGAAGAAATCCCCGAGCCGGAAGTGCAGT
TCCCATGCTTTAAAGGTATTGTTCGTCGAATCAATTCAAAATCTATCGAGATCGAAGGTC
TATTTGAGCTAGTTGGACAAACCAAACTCATTATTACCGAAGTTCCAGTTCGATATGATC
GTCTTGAGCACGTCAGTATTCTCGACAAGCTCGAAGAACAGGGAAAAATCGTAGGTTACA
AGGAACAAACAAGTGATGGAATCCGGTTTGAAGTAACTCTCAAACGTCAGGATAAACTTG
GCGAATTGATGTAAAAAAATCCGGAAGTTGCACATGAGCGTATCATCATAATGTTCAACC
TTCGCCAAAATCTTTCGCAAAACATCACCGTTCTCGGCGAGAATGGAAAATTGGCAGAGT
ATCAATGTGGCGCCGATCTGATTCGAGACTTTGTGGAGTATCGCAAAAAATACACCAATC
AGCGTGTTATCTTCGAAAAAGAAAAGGCAGAATATTCGCGTGATTTGGCTTGGGCCAAAT
TTGCCTTTGTTAATGAAGTGATTGAAGCAAAAATTGTTCTTCGTGGAAAATCAAAAGCGC
AACTTATTGCCGAAGTAAAGTCCAAGCCGAATATGGCAGGATTTGAAAACGAGCTAGTTG
GCATGAGCATTTACCGTCTAACTGAAGACGAAACTCATAAACTTCAGGAAGAAGCTCCGA
AACAGACCAAAGAATACGACTACTGGTCGAGCACTACTCCAGAAAACGAATATGTTAAAG
ACCTCGAAACTTTAACAAAATAAAAACAAAGGCCTCTTCGGAGGCCTTTGTTGTTTCTAG
ACAAAAGTGTGTACAAATGCTTTAGATTGTATAGAATAGCCTCAAGTTAAACTAACTGTC
ATGGAGCATATTATGATTTCACAAGCGCTGAAAAACAAAATCAGCAGGTATTATGAGGAA
CGCGTACAGTGGATGCGCGAAAATCATATCGTTGACGTTACAACTCCAACTTATACTCTC
AAGTTTATAAATCACAAGGCTTATGTTGGTCAATGTCATTACTCTAACCGTGAAAACAGT
ATTTCAGAAAAACAAAATCAATATGCAACATGGGAGTCCATTAAAGACACAATCAACCAT
GTGCTGGCTCACTGGGCAACCATAGGTCATGGCCACGATGAAGCATGGCAGCAAATGGCC
GTTCGTCTTGGAGCAACTCCTGCGAAAACTTGCACCCTTGGCCATCTGCAAAAACCGTAT
GTCAAAATGTGCAAAGGCAAAGTTGTAGGATATTCTGAGGTATTGCGAGTTGGTTCAGAC
GCTGCCACACGATACATCAAAGGAAAAAAGAAACAAACTCTTGGATTTTTGGAGTATAAG
GTAAATCCAGAATTCATCGATAACTCCCACTGGAGCGAACCAAGTGTCGAAATGCCAACA
CGAATCAAAAAGACAAACAGCATTATCGCCGATTTTCTCAGCGATCTTTGAGGAAAAGAA
TGAAATTTTCAAAATAATGCAAAAACTAGTGTACTCCGTTTGGTATGGTGATACTCTAGC
ATCATACCAAACAAATTGATTAAAAACAACGGAGTATATCAAATGTTCAAAATCACCAAA
ATCCAAGTAATGCAGATCAACCTCATGAAGAAAGCTGTTGAAAATTTCCAAGGTTTTGAA
ATCAACGCAGTAATCATGAATCCGCTCAACAAAATGATGATCAACGAAGTTCTCGATAAG
CGTGAAATGAATCGCTGCGAACAGTTCTTCAACGACATTATTCAAGACGATCTGATGTGG
CAAAATGCTGATTGCGCTAAACTCGCAAGATCCATGAAGCGTAAACTTGCTGCAGCGATG
AAAAAAGCTTAAGTTATTTCAACAAAGCGGTTTACAACGCTAAACCGCTTGATTAAAATA
GCTACAAGTTAAACAAACTACCACAAAGCAAACGGAGTATTTAAAATGACCATGACCAAA
ATCGAAGCTATCGCAAAAGTTCTGAACAACTCCAGCATCTCTGAAAACGCTACTTCTGTC
TTCATTCAAGTTGCAAAGAAAGCTTTCGTTACTGCTGTTGAAATTGCGGAAGCCACTAAA
ATGAACAAAAACGCAGTTTACTCCAATATCGGCGTGATGATGAAAAAGGGTTTGATCGAA
AAATCTGGCGACGGCTACGTAACCACCGAAAGTGGTGATGCCATTCTCATCGAAGCTGCC
GAAATGTGGAAAGCTGCCCAACCTGAAGTTGAAGCACCTAAAGTAGAAAAGAAAAAAGGA
ACTCGTAAAGCTCGCGAAATCACAGTCGAAATGACTAAAAATATCGAGCTCGTCGAGCGG
CTGCTGGCTAACCTCGGGATCGAGCACAAGCAAGCAGTCATCAATCGCCAAAACTACGAA
ATCACTCTGAACAAACGCCACGCTGATGGATACCGCAAATTCGAAATTCTCAACCGCGGA
ATCTTCCGTATCGTTGGTTACAAAGTATCTGAAGAAATCATCAACAACTTCAAAACTTTG
GGTTGTGAAATCAAGCAAGGTCCGGCAAACTGCTACATCGACATCACCGCGAATGAAAAC
AACATCGTTCGTCTGATGGAAGAAATTCAAGCCGCTAACTAATGCGACTAAATACTAGCG
ACTAAGAAAACTATCAGCCATCGCATGTTATAATGTGATGGTTGATTTCATTTGAGGAAA
AATATGAAAATTTTAATTGTTGGTGCTGGTTTGACTGGAGCAATTATTGCGCGTTGTCTC

GCTGAACAAGGGCATGACATCACAGTCATTGACAAACGGAAACACATCGGCGGAAACTGC
TATGACGAAGTTCGAATTGGTACACTAGTACGTAAGTACGGACCACATCTTTTGCATCAT
AGCGACTTGGGTGTGCACAATTTCCTTTCACGCTGGACTTCCTGGATTCCCTATGAGCAC
AAGGTAAAAGTCGAATATAGAGATACATTAATCTCTTTCCCCGTGAATCTGAAAACACTC
CAAGAGCTCTATGGTATTGAAACGGAGCAAGAGGCCATCAAGTATTTTGAAAAGGTTCGA
GACACGAAATCTCCCACCAACTGTGATGAACTTTTCGATCATTCCGTAGGAAAAGACCTT
GCCGATAATTTCTTTAGACCATACACGAAGAAAATGTGGGGCACTGATCCTAAAAACATT
GAGTGTGCGGTTGGCGCAAGGATTCCCGTGAGCACCTCTGATGATGACCGATATTTTAGT
GATAAGTACCAATATATGCCCGAAGGCGGTTACACTAAGATGTTTGAAAGAATGCTTGAC
CATGAACGAATTGAGGTCAAAATTCGCACGCCATTCGAAAATGAAATGGAAGCTCACTAT
GACTTCATTTTCAACTCGATGCCTATCGATGCGTACTATGACTTCAAATTTGGCAAATTG
CCGTACCGTTCTTTGCGCATGGAAGTAAAAGAAGAGAAGCATGATAATGGAATTATTACC
GTGAATAACTCTGCTTCTGAAGAAGATCATACCCGTTACACCGTTTGGAAGAATATTCCC
GGCCATGGCGATGGTGACATGGTGACTTACGAGTACCCGTGCGACTACGATGGCACAAAC
GAGCCGTATTATCCAGTGACTCACAGAAAAGAACTCTATGCTAAGTATGCCGAGCTTGCA
AAGAGTGATAAAATGATGTTTATCGGCCGTATGGGTGCATATCAGTATATCGATAGGTGG
AAAGCAGTTCGCACTGGCCTCGATATCTTTTCAGATTTCAAAGCATAACTCATAGCCGGC
CGTTGGCCGGCTTTTCAAAAGGAGATAAAATGAAAATTCTTGTAACTGGCACTAATAACT
ATCATGCTCGCCCCAACTGGCATCTGGAAAAACGTTCTCGCTATCGTCTCGTGACTGCGA
CTACCGGTCTCGTCGAAGCTCTCAAAGATATGGGCCACGATGTTGATCAACGCTTCGTTA
CCCCAGGCGAAGACCTGAGTGGCTACGACAAAGTCTTCACTGCTTATTTCGATTCTACTG
GCGGTATGCAACCAGGTGGTTATTGCGTTTACTGGCTTCTGGCAAATCGTCCCGACGCGA
TTATTGTCATTGATGACTGGCACACTCCCGAACGTTTGATGAAAGGCTTCAAAAAGTCTG
CCACTCGCACCCGTGAACAATTTGAAGAAGTTCTGTTCCGCGAATTTATGCTGAACGACC
AGCACAAAGAAGCTACTGATGCAATCACTGCTGATCCTTCGTTGAAAGAAGATATCGTCC
GCGGTTATGCTAAGAACGTCTTCGATTCTCACCGAATTCTGCATTGCGCATTCGACAAAT
TCGAAGTTTCCAAGATTTTCGATTATCCACATGAATCTTATTCGTTCTGTCCCGAATCCT
ATGTTCCTAGCCCAGATGATCGGCTGCGCGAAATGGGTCTGGCACGTCAAGATATTTTGC
GCGAACGTAAATGGATTATCGCCGGTATCGGTGGTCTTCCTACAAAGAAAGCATGTAAGT
TCATAGACAACTTCACTTGGGATGTAGAAGTATTTGGTCGTGCAACTATCGACGTTGATG
CCAAACTTGCAAAAGAGCCCGAAGTAATTCAAGCCTACTCGGGTGCCCGCGCTGTTTTCT
ACCCAGACCAAAACAACTTCGGCTCCAACTGGTGTCGCAATCGACCACGCCAATGCGCTG
ATTTGGGTGCTGTTCTCTTTATGATGTCTGATCAAGAAGCTTCATTGTTTGGCCCATCTT
ATATGGGGCTTTCGATCGAGAAGTTAGAAGCCATGACTGACGAAGAGCTCGATGATCTTG
CTGCTCGTCAGAAGGCCGATTATTACAAGTCAAATCCTCTGGACAAAGAAAAAGTTAAAT
CTTGTCTTGATTTGATTATGGCGTGATGAGTTTTACCAAAGCGGCTTCGGCCGCTTTTTC
GGGAATTTTATGAAGCAAATTATCGTAAGGCTGTTCAGCCCGATTTCACGCAACTAGAAA
TCACACAAAATGGCATGGGCACGAGTGTGGCAAGCTGCTATCGAACACCACACTGGGTTT
CCATGTGAGTTGATCCATGAGGCTAAACCATGGTCAGAATATGATCGGGTCTATGTCTAT
TTCAATCTCGAGCCGTATGACAGATTGAGTTAGTTTGGCGGTCCTCAGCCACAATACGCT
GAGTTTATTAAATGTGCTGAAGGATTCACCGGTGAAGTTGTTGCATTAGACAAACCTTTG
GCCCGTTTTGGTGAATTCTGCAAAGCACGTTGTTTCAACGAACATGGTGAACCCAACATG
AAAGTTCCCGAGCTATGGAGAGGAATTGATTGGGATTCCATTCAAGCGCAATTTGACAAA
GCCACCTGTTTAACGATGGCTTCTTTGCCGCTTGAAGATGTTACACTCGGTGATTCTCAC
AGTATTTCAGTGTGGCAGCCAGGCTATACGGCTTATCGCTGCGACGCGCAAACATTACGT
GGAGCTTTGAAAAGGGGATTGTCAAGTATCCTTCCTGATAAAGTTCGGAATTTGACAATT
TATTTTGGAAACATCGACGTTCGGTATCATTTCGGTTTACACAATAACCCGATTTCGGAT
GTACTTGAAAAGCTCGACGAGTATGAAAAACAACTAGTCGGACTAAAGGAGTCTGGACAA
TTTACCGGAGAAATCGAACTTGTGCACCTGCTTCCGATCGAAGATGAGTCAAGAAAAATC
CCAACAAACAAGATGCTTAACGGAAAATCGGTTAACGGCAGTTGGGCTTAAAGAAATTCA
TGGATGAGCATTTTCTATAATGGATTATCGGAAATAGCATTCAAACACGGTTTCAAGGTT
CATACTTGGCCAAACAGTTGGTATGTTGAAACGTATAAAGACCCAAAATCATTCTTTACT
CGCTTGGAAGCCATGAAAGTTTTGCATCTTGCCTTACCATTCTATCGGTATGAAGGGTTT
AAAGATAGCAACACTCTAGAGTTGTTTGACATCTAAAGGCCTTCGGGCCTTTTTTGGGAT
AAAAGTGTGTACATGTGTTTTTATGCTTGATATTATGGCATTGCTTGATAAAGGAGAATT
GAAAATGTCAAACGACCAACATGTTAAAGCGACAATTAAACGGCTCGAGTACAAAAGTGC
GCTGTATCGCTGTCGTGCCATGGGGCTAAAGTTTCCGGGAACCGAAATTTGTGAATTTAT

CAGTCTTGAAGTATACAACGACTGGCTGAATCGTGAAGATGACGATGGCCTAATGAAATA
TGACGGCAAATTCGATCCGACCCACCCGTTCGTCGTAACAGTCAAGCAGGCTGGTTCTCG
GAAAATCTATGTTGAGATCATCGGCTAAGAAAGTCAACCTTTGGTAGATTGCGAGTACTA
TGCCTTTGTTCCTGAAGATTTCAGACTCATTCGAAAAGTATAAGGACACAGTAGGCTTCA
GAAAATGCGATAAAGAGTGAAAATAAATGTGTACTCACAATGAGGCTATTGATACTATAG
CCTCATCAAATAAACGAATACACAAACACGGAGTATATAAAATGCAAGTAACCATCAAAA
ACGGTCTTTACTTTGGTAATGTTGTTAACGGTACTTTTGGAACTAACGGAAACACCTGGT
GGGATGCCAGCGAGAACGATCCCCGCGAAGGTAAAGTTAACGCCGTTCTGAAAGGCAAAT
CTCGGATGGTTTGGGTAAATCGCAATGACATTGTGATTACCGAAAGTTTTGATGGTGTTG
CTCAAACTGATGTCGCTGAAGAAACTGTTGACGAAATGCGTTCTCGAGTCGCAAAACGCT
CCGATGTAATGGACAAAATGACCTTAGGACTTGTTAAAGGCAGCATTCGTTCTCTGATTA
TCTCTGGTGCTCCTGGAATCGGCAAGACCTTCAGCTTGGAGAAAAAGCTGAAACATGCTG
ATGGAATTGGCGACATCTCTTTCTCTTCCATCAAAGGAAAGTGCTCTCCCATCGGCCTCT
ACATCCACCTCTGGGAAAACCGTGATGAAGACTGTGTTGTGCTGCTGGATGACGTCGATG
TGTTCTCCAACGAAGACACTCTCAACGTACTGAAACCTGCTCTTGACACTGGCGAAGAAC
GCATTATGACATGGGGTACTGCCAGCAACTACTTGGCAGAACGCGATATCCCCAACAACT
TCGAGTTCAAAGGTTCCATCGTCTTCATCACTAACGCAGATATCGACGCTGAAATCGATC
GTGGCACTAAACTTGCACCGCACCTCGATGCTCTGCAGTCTCGCTCCATCTACCTAGACC
TTGGGGTTCACACTAGCCGCGAAATCATGGTTCGAGTGGAATACGTAATCACTAAAACTG
ATATGCTGCAAAAACGCGGACTGTCTGAAGCACAAATTGTAAATGCGTTGGAATGGATGA
AAGAGAACGTGGACCGCCTCCGCTCTGTATCTCTGCGAACTGCCCTCTACCTCGGCGATT
TCATCAACACTGATAGTGATTGGGCCGATGTTGCTGAAGTTACACTCCTGAAGTAATACG
GATGAGCTGGAGGAATTCCTCCAGCTCCTTAATATTGTTATTGATCTTTGTAAAAACAAT
CAAGCTGGTTCACACTGGTTAACCCAAATGGAGAAATGAAATGAATCCGAAAAAATGGTC
TTATCATGAAATATTTTGGGAGTGCAGAAGCAAAGCCCCAAATTCCTAGGGTTGAAGGTA
TTCGAGCAAATGCTCTTGAAATTTTCAAGCAACTCGAAGATCGAATCGCTGCTGATTCGC
AGACTCTTGACCAAATTGCCACTCGCCGGCAAAACGAACCGCAACCGCATGAACTTGCCA
TCAAGAATTTGATGGAACGTCATTCGGCCATAGTGAACAATTTGTCTGCTGAGCAAACTC
AAGTGAGTGACTCCATCAAAACTGCTCAAGTTATGAAGGCTAATATTGCTCAAATTTGCT
AATAACCAGAGGGCTCCTGAGGAGCCCTCGTTTTTTGGAATCAATATGCAATACAGTTAT
GGCCAAATCACGTTTTTGGTATTTGCTCTATTATTCATTATGTGGCTTTTGTATAAAGCT
CGACATTTCATATTGGATATTTTGGATGCTGTTTGTGGCACGTTTGATTAGGAGAGATTT
TGAATCCAGTTGCTAAACATGACTTCAACAAAGGTGGAGCTCATGTAGACAAGAAACGAC
GCTTAAATGAAAAGCGCAAACTAAAACATAAGAAGGTGTACAATGATGGAACTAATTATT
GATGTTTGGAAAATTGTAAGCGTCGCAATCGGCGCATTTATCATCATGCGATTGATCGTA
ATTGCTTTAGCTAGCAAAGGCTTTATTTCTGCAAGCGACCAAATCAACGAAATTTTAGAC
AAATGGCCAAGTTTGAAATCTTCGGGTCAAGAGTTGTTTGGTAATTACAGCATTGTTGTC
ACCCTTTTGGTCATTGCTCTCCTTCTTGTTTTGATCTCTCTGTTTTGGCCAATTCTGTTG
CTGTATATTATCGCAAAATAAAAAGGTATGTACTTTTTGAAAAGATTTTTGTAGAATGTA
ACATATCAAATTTTGCCGTATTGATAAGAGAAACAACATGTCATACAATGAAACAATAAA
AGAAATCATTTCAATTGCGTCAGTTCTGATCAAATTTGGCTGTGAAGACATTCCGAACAA
TCAAGAACTTTTCGTATCGTTCTTGAACGAATTGGCTTTAAAATCACCAAGTGGGGAAGA
GTTCACTCGAGCTGGTTTTAGGACAAATGATGAAGCGTCTACCAGCTGATCAGCGTGAAG
AATTAGTAGAAATGTTCAACCAAGGTCATCGCGACATCAATCACCAAATGATCATGTATA
CAAACAGCAACAATGGAAATATTATGGAAATCGAAATCGATAAAAAGTACCGTGTAAGCG
ACGCCTCTCGTTTTGAAAACGAGCACGGCATCAAAAATGGCCATGAATTCTTCTTGGATT
CGCTAGACGAGGATGGAGATATTAGGTCTCGCGGAATTGCTTGGAATGGAGTAGACGGCG
ACAGCAACGATGCTCCATACTCCGGTTGGGCATTGCTGATGAAAGGCTATCAAGATCATT
GTCCAGAAACACCAGCGGATTACTCAGGCGCTATTGAACAAGTATGAAAAAAGGGAACCG
TAAGGTTCCCTTTGTTTTATCTAGATCTACTCTAAAAGTCTTTCGGCTTGATTGCAAATA
ACGAAAAGATTGTTTTCATTAATTACTGGCTTTTTACGCCAAAACATCGATACTGCCCCA
GCATAAACGTTGTCTACGTTCACAATCGGGCAACAGTACATGTATTCAAACTCACTGTGC
CCCGCAGGAAGAAACGCACAATCCTTGTAATTGCTAAAATGTCTTCCTGAAAAATGGACC
ATATATTCATTAGATGTTTTGTTTATTGGATACCCGCCCTTTTCCATATCTACAAGTTCC
AACGGCGTTTTCCCCTCCGTGGCGACCATGTCAACGAAATAGTTCAAGCTTCTAGGTCTA
AAAGAAAATATAACTGAAAAATCTGCTCGACTTGAAACATGTGCTATTTCAAGCTGTTCT
TGTGTCGCTACCTGAAATTTTCTATCGCGTTCGGCTTGAAGAATTTCCTGGTAATTTTGA

AAACGAGTATCTCGATAAACATTGAAGATTTCGTCCTTGTTGTACCAACTGAAACCAAGT
ATGAATACAAGGATCAGCAAAATTGCCCGATAAAATACAACTTCGCCGGTGACAGCGTCT
TTGAACAATCGGTCCAGAATTTCCATCAGAATCCCCGCACGACCAGGGGTTTGAGCTCCC
TGCGTTTGGTTTCCTGTACTCATAAAGTTGTTACTCCTGTATAACAGCTCTATTTAATGC
TTTCGGCACCTAACGGTGCCGAAATGTCATTTAAAAGAATCCATTGTGCCAGTTAGTACA
ACTTTTGCAACTCGCATTGCCCTGTTGGGGGTTTGCTTTGCCCAAGATGAATTAAGCAAT
TCAGAGTATGCTTTAGACCAGTCCTTATTGGCTATGAATGCGAGTGATATTTTGAATCCA
GATAGACGAGGACCTCCCATTTGGAACATTAAGTTACAAATAGCATTTTTTCTAATAGGG
TCACAAGGATCATATGCTAATTTGATATTAGGATATTTCTTGATTTCACTTTTGACTGTT
TGAACATCAGATCTAAACAGGGCACTGCATTCACTCTCAGAGATAACCCCGTTCACCTGG
CGACCAACCTGTTTACTCAATGTATGGTTTATTAAGGCCATATCGGTTGTCTTCTGGGGT
ATAATCAGGTGACCTATCCCAACAGTAGGGTACCCTAGATGGTCCCAATACACTTTCAAA
ACTCTTCCCTCATCGTAGATGAGCATGTCTTCTAACGTCATATGCTTTCTCCATAATTAT
TTTCCGAATATTGACGTGTTTGGTGGCCAGAAAATGATTTAAACAGCTCGCCGTTATACG
CCGCAAGATGCCCGGCACCCGGCAAAGCCATATTCAAATATGGCGACCATGACGAATTTT
GACCTCTATCACCCACATCATGTTGATCAAAATCTCCATAGTTTCCAACTACTTCAAATT
GTATTCGGCCTCGGCCCGTTGGATGCGTGAAGTTGAAAGTACGTGAGCCATACCAATCGG
AAACATATTGAGCTGGTGGCGTGTATACTCGAGTTTCTTGCTACGGCTGGTTATCCCATC
TCCATCGTAGATTAACGTATCCGAGATCTCCGCCGCGAAGAGACGCCCGGCCAGTTCCGA
TGTATCCGATTTTTATGGTTGCACGTTGTACTTGAGTGAATTCCGTGGAGTAACTGAAAT
TGTGATTTCTGTTGTACGGATATCTAGAGCCAGACCTCATGTAGCACCGCGATGGCGATC
TGCTTTTATTGAAGTAACTCGATACCCACCAATTTATAGGTGACGCACCTTGTCCATAAT
AAAACGCCGAAAGTTGTGGGTCTGCCGCGTTGCCTGCGGCAGATAGTCCGGCAAATCGCC
GGACGTTGGTAAACCAATTTGGCTTTTTGATATCACCGGTATAACCAATCGCGTTATACC
CAATGCTCCAAAATCGCCCATGTCGTTGAGACATTTTGCCTCCTAATGTTATATAACGTT
CAACGCAGCGTTCACAGTATAAACTATTGTTCCGCCTTCAGTGGCAGCGAACTCGTAAGT
TTGAGATCGAACTGTTCCCTTGTTTGGATTTAATACCCCGCGCTGCACACCATCTACTTG
ATACCATCGAGCCTCTGTGCAGTTGATATATGTGATTCCACCATTTCGTATACGCACGCA
TGCCCTGCCGTCGAAAATAGGGTAAGATTTAACGGTTGTGCCATCTATATAAGTGTAGAT
TGTCTGACGGGTTGATGCATTATAGCTAAACGTCGCATATGGCGGTGTATTGTTTCCAGT
GACAACGGATTGTATAGATGCAATCTGCTTAAGACTGGCCATACCAGCCAACTGCCCGAG
TAAACCGGTTGTGCCAAGACCGGCTTTACTCAGAATGTTGGAGCCCCATCCATTGGCCTG
GAACTCGGTGTTTGCCTGAGACAACCAGAAGGGAACGTTAGGTACGGCCATATTCCAACT
CCTTCACCTTCTCGGAGAGTTCCTTGACTGCCCCCACCAGTAGCGCAATCAATGCGGAGT
TTGATAGCGTCATGATTTCGGTCCCGTCTTTGTTCCTTGAATCCACTACACGAGTAACAG
CTTCGGGTAGCACCTGCTCGACGTCTTGTGCAATCAATCCGACCTCTTTTTTGTCGTACT
CAGTGGCTTCAAGCGTCTGCTTCTTGTCGTAGGAGTAGGCGGTCAGTGCTGACACTTTCC
GTAGCGCGTCACGTACTTCCACAAAGTTCTTCTTAAGTCGCCGGTATGACCGGATGTAAA
CGTCATTCGCGTTCACATTCCCTGTCATAATCCCTGCATACGAAGCGAGTCCTTGTGTGT
GGAAGCGGAAGTCCCCTCCCTCCCACAAGTGTAACGTACTGGCAAAGCGGCCTGCCATGT
GGAACCCGACGCCCGGGCGACTGTCGATAGCGGGGTTACCGTTCACCTCGATGTCGCACT
GGTAGTAGTCGTTTCCCACGGCATTATTTGCCACAAACCTGTCGCCTTGTTTCACCAGCT
CGAAAGGCCCTTTCTGGGCACCGGCCTCCTGTCCGCCAATCCACGCTTTAGTTCCAGCAA
AGTTGATAGCCAAGTACCCGAAGAATACTGCTGCGTTTCCGTGAGCTTGGTTGAAGAGAT
ATGTCCATTCAGTATTCGGAATGCCCGCAATGTTGGGTCCTTGGAAGAATCCAGATTGAC
GACGGAGATTACAATCGCCCAAAACTGTGGAAATGCCAGTTCCGTACGTCCCAAAAGCAA
AACCTGCCGGAGCCGCACCAACATCCGAGGCAGTCGGCTTGTTCCCTTCATGGTAAATCT
TGCACATAGCCCCACGTGCGCCACTACGCCATGTGAGAGAGTTATCAGCCCCTATCGTGC
CCCCAATCCACAGGCGTCCTCGCTCAGTGCCATCAGGTGCGCTCCACACGATGTCTGCCA
TGTCACTAGCAGAGTGCCCTACAAGGTCAAGTATCCCTGTGACTCCTAGCATCCCGGATA
GAGTCCCACCAGCAACAGGCAGAGCCCCGATTTCAGACAGCGACCAAGACACGTCACCCG
CACCGTTAACGGACTTAGCAGCGTTGCCGATAGTCAGGGTGCGGGCTGTACCCCAGTTCG
CCGTGGTGATGTTGCCGGGAACGTCAAAGTTGGTGCCGTTAATCTGGCGAGCCGTAGCCA
GCTTGGTTGCCGTGGCCGCATTGCCAGCAAGAGCGCCCGTGCATGTCGGAGCGGTCACCC
CAGCAGCCACGACAAGCCCTGTAGGCTTTAGTGACATCCAATCGGTAGTGTTCGCTCCGT
TTACACTCTGCCACTTGAAGTATTCGTTACCGTTATCCCCGGTTTTGAACCACATGTAGC
TGTCGGTGTCGTCGTCAGAGGCATTCTTGAAACAAATCTTCGCGTAATCAGAATTGAAGA

CCCAGCCCAGCTCGCAGGCAGCATCGACGAACGTAAGCCCACCGGTCATTGCATCGCCAG
CTTTAGCAACCGCACCAACGTTAGTTGCAGAAATCGAAATATTCGATGTGCCATCAAAAG
GAACACCTGCAATAAGTCTAGCTGTCTGTAATCTAGAAGCCGTAACGGTATTTCCATTGA
CTGGAAGAGCTCCTACTGCTTCTGGAGTTGGGTTATATCCGTTATCGTAAAAACGATTGC
TTCCGTTATAAGTACCAGAGCCCTTATCAAAATTCGCAAGAACAATGGTTTTTGTATTTC
CACTTACTTGGCCATTAACCGCAAAGATAGATGTTCCATATGTAACATCAGTTGCACAGG
CGGTGACGGCTGTTTCGATATCGACTCTAACTCATATCGGGAAAGCACGTGTTTCAACGT
AAACAACAATCCCATCTACGGCCTCAGATGGTCCGTGGATTGAGTGAATTGCTCTTTCTG
ATGTAGAATAAATCGTAAACTGGCCTACGGCATAACTTGCACCGTCACCCCATCCACTGG
CGCGAACATTACCGGTAAATGAACAATTGTTCATTGGAAGTGTTGTATGGCCAGATAAAG
TATCGATATAGACAAATGAGTTGGATGCACCATTTGTTTCAAAAAGAACTGGAATATATC
CTGAAGCAGGTGCTCCAGCAGGAAGTGTGATCGTCATATCTTTCACACCTTTACCTGAAG
CAGTAACAGTCGATTCAACAAAATCACGACGTGCCACAGAATTAGCTTCGGTGCCTTGCG
CGGCAGACAAAAGAACTTTTGGAACAGTCAAATTGCCAGACATAGTGTCGCCGATTTTTT
TAACGTAATCGGAGCGTGTATGGCTGTCGTTTGTTACTGTCACTGCTAGAGAAGCATTTG
CACTACCGTCGAACGATACTGAGCCAGTTGCGCCTCCGGTCAAACTGATAGTTCTTGCGC
TTTGCAACTTTGATGCAGATACAGCAGTTGCACCGGCAGCTAATGCACCAATTTCTGCAG
GAGTAGGTTTATCAGCTTCGGGATAAATCTTTTGGTTTGCTAATTTAGTGTCTGCCGAGT
CGTATGCGTATGTGTACATACCATAACCGATACTTCCGTAATCGAAAGTAATACTACGGG
CATTCGCACCGTTACTGGCCGATGGATGAGAAACGTTAATAGTCAATCCACTGAATCCGC
CCAGACTGTTATGTCTGAAGAATCCGGAATAGCCCATTGACAAGTTATGAGGTGTTCCCA
TATCAGACGATTTGCTCAGCAAGTATCCATTTTTTCGCAGTATAGGTGTTCAGTCTGATA
AACCGTGCATCAGCCTCGCTCTGGTTATAGTATGGATTATCCATAGCAGCGCGTTGGATA
AGACCGGTGTCGCTAATCAAAAGTCCGCCCGTCGTATTGTATACGGCGGAATCAAAGCGA
ATTTTAGTGGTAGTCATTCCATCTTGGGCATAACCGAGTACTAAATCAGTTCCACCAGGT
GCGCCAATATACAGGTTTTTAGACGTGTTTGCTCGAATGAACTGGACACTTCCATACTTT
ATATCAAAGTTTCCAGTATTAGCGGATACCAACGCTCCTATCAGAGAAACATCACCATTT
AGATTTAGCGATTTTAAGTCCTTCAAATGCCCATGCGCCGTTGGTGTCCGTCATTGCCAC
TTTAAAGTCACCAGCCGCATCAGTTCCAACATATGACTGAGGTGTATTTCCCTTTGGAAA
ACGAATATAAGCAGGACCACTAACGCTTGACTTAAGCGCTAATGGGTAATCAGTGGAAGT
TTCGATGTACAGAACACCCGAGATAGGATCGCCAGCACGAGATACTACTTGGAGTTCATT
TGCACTTGGTTTATCGTTTTCCGAATAAATTCTTGCACCCTTGTACAGGATAGCATTACC
GCTTGGTACCAGCGCAAGATTGCCTTGGTGCCAAACAGGTGAACAAACGATCGTGGTTCC
AACTTTTAAATCTGCCATATGGGCTCCCTTGTTTAACGAATGATTGGTGATTTCTTATTA
TATTTAAAGCACTCAAATTAAAAGCAATCCTGGCTCCAGGTTCAAGCTTGCTTGAACTTA
TCTTTTTATCCTTTTAGTTATAATATCAATTTAGCCATAAAGGATAAGTTTTGGCAGGGC
CAAAACCTGGAAGCCAGGATCTTATATTCTATTTAAAGCAAACAAAGGGCAAAGAGGCCC
TTATCTTTCGTTAAAATCGTTTGAAGTAATGATATTCTTTTGTTTCAGATTCAAAAGCTG
TAATACAGGATTTGTGTAATTACCGTTATTGCTTATGATATTAGCTCTAATACCATTCAC
ACCAATTGCTGCATCCGCCACTTTATCATCATCGTCCCTTGTAACCCGCGTTATAGTGAC
ATTTCGCACACCTGCTGTTCCAGCACTGACGCTTGACGGATATCGGTATGCTGCAGAAGC
AAAAATCTTCGCCCCTGAGCTTTGCGTTGGCAGTTTGAAATAACCTTCAAATTTTGTCTA
CACACCTGGGGCCGGTGTGTGCGTTCCTAGCCATATACTGGTTTTCCATATGCCTGAAGA
GTCTTGAGTGCATACATAAAAAAAACATCTTCCACCGGCGGTAACTAGTGTCTAGTCGCT
GTGCAGCTCGCATTGGATATGATATATTGATTCCCATTGCGTTGTTATATTTTCAGACAA
ATAACGCTTAAATTCGTATTGATTCCCCACTGAGAAATATTCATTTATGTCATCGACATT
TCTTGACGGTAATCCACACGCTCCAATATCGACACCGTCGTCAAAAACAACTTGCAAATA
GGCATTTGTGTCTTCTGCGAAACTGTTAGGATTATTCCCAACAAAATTCTCGTAACAAAA
ACGCTTTAATGTTGTATTGAAAATTGCAACGTACGAACTTCTATATGCTCTCGGAGTAAA
AAGAGCATCGGATTGTCGATTTAGATAACTAGAAACAGGCCACGACATAGCTCCCTATTG
TTTCATGAATGTGTCAAAATCCTATGACGATCTGATAGCATCATGCGAAACTACTACTAC
TATATTGTCAGATGGTGCAGACATTAGATATGTTCTTAAAGTGTCAAGATGCTCGCGTCT
AGCATATATGTCCAACGTCGCAAAATTAGATGCTACTCCGGTTGGAGAAATAGTGATAGC
GTTCAAACCCCTCCCGTACGAAGGCAAAACCGAAACACCATTGATTTTGACATACGGCAC
TCTGACACTGGAGTCATCATTGACTCCAAGTGCCTGTATCATATACTTTATTGCGTTATT
TTCACCTAAGTGATTTGATTCAACGTAAGATGGCCCAAGATGGGCCATAAATTGTTCTGC
CATAACGATTCCTTATTCTTCCCAGATAAACTCAACTGTCTTGGTCAAGTTGTTGGCAAC

GATTTTCACATTACCAATTTTGTTCCAATCTCTAATCGTTATATTATTGGAAGATGAACC
TGTAATATCAACTGCACCTACGTCTTGTGCAGTAGGTTTGTTACCCTTGTGATAAATCTC
GTACTCAAAGTTTCCGATCTTTACGATTGGATTCTGATTCGTATCAAGACACACACGGCC
TCTCGCTGTATCGGTATTTCCGAAAACGAAGTCATTTCCACGAGAGGTAATGTAACGATT
TGAGTTTTTGAATTGCAAATACTTAACATAGTCACCAGTGTTTGACAGAAGCATTGCTAC
AGTCATAGTCGATGTACCATCACGACGAAGATATGTTCGGTTGAGCTTTGTCTGAGCGTT
GGCAGTATTAATTACCGGTGAGCCATTAATTGTGAAGGCATCAGTAGAGCTTGCCAATAC
AGCCATTGTTGAACTAGCATTAACAACTATGAATCCGCCTGCAACAGATTGTGCAAATTG
ATTCGCCGCTAATTCGGTTACACCCAGATAAATTCTCGCACCTATGTTTTCCATAACAAG
GAAACCAGCAGTTGATTTTATATGTTGTGCAATTCGTACATCACCGGCTCTATTAACTTT
CAGCTTACTAGAGAAAGTTGAACCGTCTTTAGTTGCAATAGCAATTTCACTGCCAGAAAC
TTCAGAACCACCAACGCCACCTGCAATAAATGCCCAATCGTTTATTGCAGCATCACTTCC
AGCCATTATGGTGATATCATTGGCTCCATCAGCAGCTCTAGTTTTAGCTATTTGAACAGA
GTTTGTCGACGTATTATCGTTAATCACTCTTGCGCTAGTTGTCGTAGCATTGGTAATATC
TGCAGCTACCATTGCGGCAGTAAAAGTCATTCCGCCTGTTACGGTTTGAGCAACAGTTCG
ACGGACGCAATCGTTAGCAACAACACCACCAAGTTTCAGTGAGTTCTGGGCGGTTGCCAT
TTTCGGCATATAGTTTTGCAACGCATAGTTCAGACTCAGTGGACTAACGGCCAGCCCATC
ATTTGAATATGATGTGTACGCTTGGGTTGAACCGACAGTATCGTGTCCAACGAAAGTTGA
CTCTACTGTTGCCGCTTTGACAAACCCACGGCTTGTTTCATTTCCTGACCATGTCGGTTC
AGTTTGCGCTATATATATGAAGTCTTCTGGACTAACAGCCAAATTAGTTACTGTTCCAGT
TCTAACTCCAGCGGTTGTCGCAACCCGGATAATACCGTCCAGAGTTCTTGTCGCTTTTCT
TCCATCGAGTTTCTTTGGAGTTACGATGGTATTGTCTGAACTAGATCCATTCGCCTCAAG
TTGGGTTGAAACACGCAATGTCCCACGTTGAGCTTCTGTAGCAAGACTTATTCCAACAGA
AACAGTGCTCCAAATGGTTCCAGATTGGGTCAAACCATCAGTCGTGTTTACCGAAATTCT
TGCCGTATTGTTGAAACGATTCTTCAATTTCAATGGAGTGACGATAGTTAAATCGTTTGT
CGCAGTATCTATTTCAGACGTCGTGGCAATACGGGCAATACCTGTGAGAGTTTCTGTTGA
AGTGCGATCATTCAACTTTTTGGGAGTAATAGCACGGGTATCATCAGTTCCGGCATTTGT
TTCAGTCTGGGTTGCAATCTCAATCAGATCATGATTTGATTCTGTCGCTCGTCGAGCTCC
CAATCTTTCGGCTGTTACAAGCAAAGGCCCAAGTGGATCAGATGAACCCCCGTCGACTTC
CGCCTGTGTGACAATATAACCTATACCCTTAGCTGTTTCGGTAGCCTTGAATTCATCCAG
GGTTAACGGTGTGACAGCTTTAGACTGATCGGCTTTGTTATAAACAACAGTCCCAGCAGC
TGAGCGAGTTGTACCAGCTGTTCCATTTTTAGAAACCACGGCTAAGATACTGGTCAGAGT
TTCTGACGCAATACGATTATGCAACTTTTTGGGAGTCACAATTGTTATGTCGCCAGTTGA
GCCGTTTGCTTCTGCTTGAGTTGCAATCTCCGCTAAACCACACCGAGGTTCGGTAGCAGT
TCTTTCATTTAACTTTTTCGGAGTGACGATAATGTCATCCAAATAAGTTGAAGTGCTTAA
CTGATTGACTTCAGCGGTTGTTGCCAAACGGGCAATGCCACGACGAGTTTAAGTAGCAGT
CTTATTAGCTAATGATTTTGGAGTGACAATAGTTTCGTCATTCGGGTTATCTTCGTGATT
TTTGTTTACTTCTGCTTGATCCGCGAGAGAAGCCACGCCCAGACGATCACGACGAGTTGC
ATCAACACGTTCAACTTTTGGTTGAACATTTCCGATCATCCATCCGTATTGCGAAGAAGT
AACACCCACCAGGGACGGGTCTAAGTGTGCGTACATTAGCTCGACATATGGAACGTAATC
TGTGTCAGCACTTATAGAAATAGATGAAACCAATTCCCACCCATTTGTCATCCGGTATTC
CGAACGACGCTGGAACTGCATTTGAGCTTTCGACCAAACAATCAGCTCATTTGAACCTGG
ATTTACTCGGATTGTACAAGTTTGACCTTTTCTCAGATAATCCATAGTGATTTGAACACG
GTCACCTATGGCTACTTCGTCTGGAAGAGTCAGCACGGTGGTCTGACTTGGCAAATTACT
GTTGCCAGTCACCAGAATGTGTTCAATCGGCAGCAGTGCTGTGTCAGTTTTGGTAACACG
AATGCGTGTCCTTTGGTCTCCATCCCATACTTTCCAAATCAAAGAAGGAAAATCGACCAC
GAATGATCCGTAACCAGATGTGCGCGATTCGATTTTTGTAACACCGGTTTCATTAATATT
GTGACCTGAACCTGGGTAAATCTAAAGAGTCGAGTGGTTAATCGGGTTCATCCCATCAAT
ATCATGGGTTGTAATTACATCGCCATGCCTAGCATGTTTTGGCTGAACAAGACGAATTGT
TCCTGCAGCAGTACGTCTCCAGGTAACCATCCCTGACTGCAACTGGAATCCTTGAATCGA
TGGCTCAACAAAATAACTGTTCATTGCTGTAGCTGCTATATCTACTTGCCATCTGTTTCC
AGCTGCTTTATAGACGAACATATATTGGCAGAATGGGACAGTTGCACGGAAGCGAGCGCC
GACACTGGCTGCCTTATATTCGATGACTGATGTTCCTATAGATACAACTTCCAGATCGTT
AACACCTGGCTGACCGCCTACGTCCTTTATTACTAAAGTATCGCCATCGACTGGTGATGT
TGGCAAGTTGAAAACCAGATTGTTGAATCTATTGTCAGCAGTGATATATTGACCAGAACT
CAACTGAAGTGCGGGCCCAGTCGAGTTAATGAACTGCCATGTTGGGTCAACACCGAGACC
AGTCCATTTTGTGACGTCGAAAGCTCGAGCCGGGGAAGCAATATCTGCTTTTGCGTAATA

AATTCGACGCTCGAAAATTACAGCATGTTTTGCATCATACCCACTGGTGCTGTCATATTC
GTAAATTGTGTTATATTTGTGGAAAAATCCAACGCTAACGCCATCGGACAAAATTGTCTT
ATCGGCCCTTGCAACGTTGATCAGTTTTTCGCCAGCTGCATCAAGGCCGCTTGTGGCGCG
AAAGGCTTGTCCTAACATTTTTGAAGCTCTCCTGTTACTAACTTAGTTATTTATGTTAGT
ATGTCTAGGTGTTCAATAAGAGAAATTGATATGAACTTAAATGATTTCATGGGTGAAACC
ACAAAGGATGCTGTCAATATTATTGACGCATCTCAATTAATTATCGCGACAATAATGGAT
AACTTTACTCCTGAAGATGTAAATGAGCGAATGCTCAGACATTTAATTCTGGATACGGTT
CGAAACAAAGTCAAAAAGTTCAAAAACGAATATCCCGAAACTATTATCACATTTGACGAT
TCTGCCAATGGTTATTGGAGACGTGACATTGCTTGGTATTACAAATTAAACCGAAAGGCT
AATAAAGAAGAATCTCCATCGGATCGGGAACTTCTCTTTAGTATTATCAATAAAGTGGTC
AACGAAATGCTTAATATCTACCCAGGTGTTAAGATGATCAAACTTGATAAAACTGAGGCT
GACGATATTATTGCAGTTTTGGTCAAAAAGTTCACCCAAGAAGGTCGATGTGTTATGATT
TTTTCTTCTGACTCTGACTTTACTCAATTGCACAAATACAAGGGTGTCAAGCAATATTCA
CCTGCTCAGAAGAAAGCAGTAAAGCCAAAATATGGTAGTCCAAAGCATGACCTGTTTGTT
AAACTGATTAAGGGTGATGCAAAAGATGGTGTTTCGGGAATAAAAGTTGGTGATACATTT
GTCCTTGATCGTGTCGAAGGTGAACGTGCTCCACCTTGTTCTACCAAGTGGATTAATTCA
ATCGTTGAATCTGAAGACCCTCGAGCTGCATTGGAAAATGAAGAATGGCAAAAGCGTTGG
GATATGAATGTTCGGTTGCTTGATTTGGATAAAATTCCAGCACATGTTTCGGATCGAATC
ATTCAAGCTTATAATTCTTCAGTAGTCAATCCTCGTGGTAAATTGTACAGCTATTTTGTT
AAAAATGGCTTGGTTAAACTTCTCCATAAAGTAAATCAATTCTAAGGAAATAACATGAAA
GAACCGAAGAAAAAGAAAGAAACAGTACGAATTCAACCCGGCTGTTCATGGCGAACGACT
CCAAGAAATTATTACCGAAGCATCAAATCTGCGCACCATGCAAGAATCTTATGCTGAAAA
GGTAAAAGAGCAGCGCAACTTTGCCATTACTGAATGCGGTGTTTACGGCAAACTGTTCAA
TCAGCTGTTCCCGATTTACCACAAGCAGGAACGTGAGCAGTTTGAAGATGCTGCCGATGA
AGTTACTTCAGTGTTTGACCAAACCTTTAAAGGCAAATGATGTCAAATCCGAACAATCTG
AATGATAAAACCGCAAACGGATAGAGATAGAAAATCTGGTTAAAACTGGATTGACATAT
CTGGAAGCATGTGTTCAGTGGTTGGAAGAAAATTCTTTAGAAATTAACTCGTGCCACAAA
TATATCCCGCGAGCGGTAATTGACAAACTTTCAAAAGAATGTGTCGATTCCGACATGCTT
AGACCTTCCATTGCAAAATCGATGACACGAAATAGTTTGGACTTTTTGATGTGAGATTTG
TATGATCGCTCTCCGATATCCGCCTAGACCTGATAGATTTATTAGTGCAAAGGGCGCGTT
TATGCTTTATTTGATGATGAAACAACATATGGCTGGAAAATATGACATAATCAAATATAG
TTGGAATATGAAAGTTACTGATGCTGCGTTTAATAAACGCAGAGATAAGTACTTCTTTGA
TAAGACGAGCGATAAATTTCACCTTAAAGACTTGACAGAGATATTTCTGGCAACGTTTTT
GGAAAATTCAAATGGCTGGGCCGGAGATCTTGTTTCTGAAGATGCAATGGCAGCCCATCG
AGAATTGTTGGGAAGATACATCAGATTCCCTGAAAGATACAGGGAAGACCTGAAAAGCCT
GGTGTTATTTTTCCAATAAAGTCAATGTTCCGATAGGTAAACTATTGGAGTATAATGAAA
AACAACAAACGAGCCCAATTTTCAAATTACTCCAATCTGGCCTGATCAAAACAGAAACAT
TTTTGGTTATTGATTCATTCCTTCGTATAATCGACAAAATCGATGAGAAAATGTCAGCTG
ATATAATCTTGCAGAACTGGCATACCAAATTGGTCAATTACCGGAAATTGGTAGGAATAA
ATAATGACATAGCTAAAGCCAAGTTTATTCAACACGTAAAAGAGTTTAAAGCTGAAGTTT
AGCTATATCAAAATTGATGTAAAATGTAAAAACGCAAAAACAACTGTCAAAAATAAGATA
GGAAAATAAATATCTTCAAGCGCAGCAATCCTTCTCAACTTCAAGCTCAACTGGCCGCTC
TGAAAGGTAATGCAGGTGGTTTCGGTGGAGATAAGAATGAATGGAAACTGAAAACTGATG
CAGCTGGTAATGGTCAAGCAGTAATTCGTTTCGTTCCCGGTAAAGGTGATGAAGGTCTTC
CGTTTGTTAAGCTGATTAACCACGGCTTCAAGAAAAATAACAAATGGTATATCGAAAATT
GTTCTTCTACCCACGGTGATTTTGACAACTGCCCGGTTTGCCAACATCTCAGCCGCAATG
ATTCTTACAGCACCAACGTTGAAGAATATAAGCTGCTGCAGCGTAAGACTTCCTATTGGG
AAAACATTCTGGTAATCAAAGACCCTGCCACTCCTGAAAATGAAGGCAAGGTGATGAAGA
TTCGCATTGGCGCAAAGGTAATGGAAAAAATTACTGCCATGATCAACGTCGACCCGGAAA
TGGGCGAAACCCCAGTAGACGTAACTTGCGTTTTCGAAGGTGCAAACTTCGTTTACAAAA
CCAAGAAAGTTGGCGGTTTTACCAACTATGATGACTGCAAATTCTTGGCTCAATCCGAAA
TTGCAAAAATCCACGATCCCGAATTCCAAAAGTTCCTCACTGATGGCATGGAAGATTTGA
GCAAAAATCGTTGCTCCTTCCGAATTCAAGCCTCTTTAAGAACTGGAGAAGAAATTCAAG
CAAGTAATGGGTACTTCCATCGCTGCTGGCTCTGCCGCTTCTGCCGCATCTTCTCTGTCT
AGCGAACTGAATGACTTCGACGAACAGCTTACCTCGTTCGACACTGATGTAGTTCAGCCA
TCCGTGCCTAATGCTTCTTCTGATCTCGATGACGATCTGGATGCTCTTCTCGGCTAATAT
AGAAAAGCAACCTTCGGGTCCTTTTTTTGCATCAAAATCTTTCAAAAAGTAGTGTACAAC

TATGCCATACAGTGTAGAATGTCTCCATCAAATCAACCAAGGAGTAATAAAAATGAAAGA
CATTCTCGATTATCCAGTTGACACCAAGGGTTCGGATTTGCCACAAGAAATTCTCGCTTT
GCACGTAAGTCGTTGTGAAAAGACGGTTGCCGCGTTTGGAGGTCAAGATGAATTTTTCGA
ACTCATCAACACTAATGCATGTGGCGGTTGGGTTGTGGACTATAAGGCCAAGTTTGAAGA
AGTAAATGGCGTCAAATTCCCCAGCAATATCAGATTCGAATTATAAGGAAACTTGAAATG
TCATCGAACAAAGTTCTCATCAAATTCAAATCTCCTAAAGCTCGTGCTAAATTTGCAGCT
TCCTCTGCAGCTAATAGCGCAATTGCTGAATATATGCAGCATTACGAGCTGACTGCTGTC
AATAAAGGGAATGGCGATATTGTCATTCTCGGAGATGGCAAAGAATTTGAGCTCGATGGA
TACTGGACTCCCGCGATTTTTATGTCAGAGCGTCGATATTTTGACATCGACGTGATTTAA
CTCAAAACATTGTTCAAAAGGAAATATTATGAAAGTTGACACTAAATCTTGGCACTATCG
TTTTCTGAATTCTCTCAACACAAATATCCCGAATTCTTTATGCCCGTATTTGTGGAAAGT
CGTGTTTACGATCGCATGGATCACGGGCTCGATTTTACTGCTCATTGCTTTTTTCACATC
TATTGGTGCCAGAATCTTCGAAGGACTTGGCTTTGCCCTCGATAATATGGTATTGTACTG
GAGCCTTGCCGCTGTAGTAGGTACTGTCACCGTCACATCTGCTATTGTGCTGGTTGTCGC
AGTAATATTCGGATTTGTTTGGGCCAAAGAAAATTTCAAGCCACCATACAAAGAAGAACC
GTTGATTATCACCTTTGTCAAGAACAAAAAGTCTAAAATTTGCCCGCGTCTAGAATTTGA
AGACATGAGCAAAAAGACTGAAGAATAAGTTCGCAACCAGTGAGGTCATGATAAAATGAC
CTCACTAAAATAAAACAATCTCCGGCTTATAATGTTTCACTAAGTAAGGAATGATTAATA
TGATTATTAATAATGAACGTCAACTGGCTTCTATTCGCAAAATTGCCGATCTTCAGCCGA
TCCCGGGTGCTGATGCTATCGAATGCGCAACCATCGACGGCTGGGAAATAGTCGTGAAGA
AAGGCGAATTCGCAATTGATGATGATTGCGTATATTTCGAAATTGATTCCATGCTACCGA
CAGATAATCCTGCTTTCAAATTCTTGGAATCCCGTGCCAGGATGTACGAAAGCAAAATGC
GAGCTCGAGTGAAGACCATTAAACTTCGCGGCCAACTTTCTCAAGGTATAGCTTTGCCGG
TTAACGCATTTACCATCGGAGAACTGCTCGGTGTTTATGAAATGGACAAAGGTCTTGACC
AAATTCTCGGTATTATCAAATATGAGCCGGCTCAAGACGGTTCTGGCTGTAACCCGGCCG
GTAGTTTCCCTGTTTTCATTCCGAAAACTGATGAAGAACGCTGCCAAAACATTTTCAGCA
AATACAGCGCTCGTTACAAAGATGTTGTGTTCCAAAAGTCTCTGAAGCTGGATGGTAGTT
CTATCACCATGGCATGGGTAACAGATCCTGACCTGTTCCTCGAGCTTGGAACCGAAGATG
AACCATATGCTCACGATTACGACGACGCTCAATTGATCCTAGCTTCTCGTAATCCGGTTC
TCCGCTATAACCCCGAAAGCAAGTGGTGGATAGGTGTTGAAAACTACCAAATCATCGAAC
AACTGAAGAAGATGGGCAAATCTGTCGCAATTCAGGGTGAACTGATGGGTCCTGGTATCC
AGAAAACCCGCGAAAACTTCGACAAGTACCGGATTTTCGCATTCCGTGCCTGGTTTATCG
AAGAGCAACGTTTTGCTACCGACGAAGCGTTCCACGACCTGTGCCGTGCTCTCGGCATGG
AAATGGTGCCGCAGCTTGGTCGTTGCAAGCCGTTTGAACAATTTGAAACTGTCACAGAAA
TGTTGGCTGATGCGGATGTTCCGTCCATCAACCACACGATTGCTGACGGTGTCGTTTATA
AGTCTGTTGACCTTGTAGATGGCGAGATGATTCACTTCAAAGCAATCAACAATAAATTCT
TGCTGAAATGTGAGGACTAACATGAAAATTAAGAAATTGCAACCTTTACAAGTCTTGTGT
CCGAAATGCTGGATAATTGAAGATTATTGCTACTGCTATTGAGAATTCTATGTCTAACAG
ATTGGGGCTTAAATGCCCCTTTTCTCTTGAAGAGATGAAACAGATATCAAGTCATTTAAC
CGAATCCAACACGGAGGTTCAGTGTGATTAAAGCCGTATTTGCTTTAGGTGTACCGTCGG
ATTTTCATAAGCAGGGAGTTTTTGTATTTGGTAGAGATGGCGATTTACCATGGCCATCGA
TCCCCGAAGACTTTAAACTTTTCAGGGAAAAAACCGATGGGACTATCTGTGTTATGGGTG
CAAATACATTCCAGTCGCTCCCAAAGAATCTTCAGGGAAGGGTAATGGTTGTTATTGGCG
ATCCTAAGCGTTAGTGCCGGAACAAATCAGGTGAACTTCCTCATAATTTCGTCAATCTTG
ATATGGACTGGATTTCTGTGGTCAAAGAACTATATCCAGAAAATGATATATCGATTATCG
GTGGCCTTGGTCTGATTCTGTCAGTCGCCCACAAGTGTGATGAACTCCATATCAATTTTC
TTATGCTTACTTCTGACTATGATACAATTGACCCAACAGTTTATATCACTGATACCGCAC
TAGGAGTTTTCCTGTGTAAACGGAAAATGAATGCTATCGCGTATCGAGTTGATCGTGAAC
ATTGCACCAACATTAAAGCAGAAATTTGGAAATGATTCAATAAACCGATTTAGTATGCGA
TATTATCACCAATGGGCGACAGAAGACTGATCGCACTGGAGTAGGCACCCGTTCGGTATT
CGGTGGAATGCTTCGCTTTGACCTCAGCAAAGGCTTCCCGGCCACCACATGCAAACGTCT
AGCATTTGTTCCAGTTGTGGTTAAATTGCTGTGGTTTATGCAAGGTTCAACCAATGTCGA
ACATCTGCGCAAATTGACCTATGGCGAAAACTCTGATCGTTAAACCATCTGGGATGATAA
CTATAACGAGCAAGCCGTTGATCTTGGATATTCTGATGGATACCTTGGTCCCGTTTATGG
CAAGCAATGGCGTATGTTCGGTAATAAAAACCCGGTCGAACAACTGCAAAATGTGATCGA
TCAAATCAAAACAAACCCCGACAGTCGTCGTCGTTTGGTTGTTGCATGAAATCCTGAAGA
TATCGAAAAATGGCTTTGCCTCCATGTCACTTTGCGCTCCAGTTTGAAGTATATGAAGGC

AAACTCAGTTTGATGTGGAGCCAACGCTCCGTCGATGTTGGTCTTGGTCTGCCATTCAAT
ATTGCGTCCTATGCGCTACTTGTCCATATTGTAGCTCGAATTTGCAATTTGGAAGTTGGT
GAATTGGTATTCTCTGGTGCAGATGTCCACATTTACGAAAATCACCTGAGTCATCTCGGC
GGTATGCTTTCTCGTGAAGAACGCCCTTTGCCTACGTTGAGTTTCCCGGATATTGACAGT
TTGAGTGATCTGCGTTTACTGGAAGAAGCCGATTTCCGTCTGGACGGGTATGACCGACAT
CCAACAATTAAACTACCGATGGCTGTGTAAATGACTATATGCACCGTTGTAAACGTCAAG
CATGAAAGATGTGACGTTCGTATACAGCGAGGCACTATATGGGGTAATCCAGACGGAGAA
CCCCATTGGAAACGGCAGGATTATCTTGACAATTACAAAAAGCATTTAAAACACCAAATA
AAAACTGGGGTTATTACTCTTGCCGATTTAGAATCACTACGAGGCAAGAAATTGGGGTGT
GGTTGCAAACCATTGCCATGCCACGGAGATATTCTTGCTGATCTAGTTAATCGCATGTTT
AAGGATGTGTCAAGCGTAGATGATTTCATATGACATTAAAAGAACACGGATTCATTCAGA
TAGCAGAATTGACCATAGGTAAAAACGGACGTATATCCAGAGATTTTTGCTAAAGACCCA
TCACTAAAACACATCATTTATTTGTTTACAATTGATGATGAAGTTGTGTATATTGGTCAA
ACTAAGAATTTGCACAAACGAATGGACTCATATGCTAATGGCAAGTATTGGAAAAATACG
AACAAGTCACATATCCAAAAATCCAATTTTCTGGAAGAAGCAGTAAACTCGGGTAAGAGA
GTTTGTGTTTGGATAAAGCATTGCTTCCAAATCGTTATAACCACTCCTGCTGGGTCAAAC
GTTATAGCTGACCTAGACGCGGAAGAGAAAAATTTATCGGGATTTTTAATCCGGTTCTAA
ATTCAAAACTGAAGGTTAAATTATGAGCAGAACAACTGAACTTTTTAATAACTTGATGTC
TGTTGTGGAAAAATCCGAAAAGGGCAATTTCTACTTCAAGGACGTCATTACGAGTATGGG
GACAAAAGCTCGAATTTTCAGCTATTTTATCGCTTCATATACCGATTGGCTGCAAGATGA
TGCTTTGGAATGTGGAGGCATTACGTTTGAGCTAAATGATAAAAACGAGCCAGTTCGAAT
CATGGCTCGCCCAATGCAGAAGTTCTTCAATCAGAAAGAACACCCCATGACCATTGGGCT
CGATTAAACTAAGATGATCGGTTTGATGGAAAAAGCGGATGGGTCGCTGATTAGCAGTTA
TCATGATCGAGGCTACGTTTAACTGAAATCTAAAGCTGCGGTTTTCTCCGATCAAGCCAA
TAAAGCAATGGTGATGTTGAATTCGCCTGCTTATGAAAAATTGCGCGATGCAATTGTACG
AGCCGGTCCCGATTCTACTTTCAATATGGAATATGTTGGACCAAGCAATCGGGTTGTCCT
TCCATACGAGGAAGAAGAATTGATTGTTCTCAACGTACGTCACAATGAAACAGGTCAATA
TGTAGAATTTTCTACTCTGCTGGATGACCCATTGATTCGTCATCGTATGATCGGGGTTTA
TCCTTGCCCGGATTGGAGTAAAGTTACTCCCGAAGAATGGGAAGCTGAGACTCGTGCCGA
GACCGATATTGAAGGAGTTATCGGTATTATGCCAGATGGCCAGCTGTTCAAACTGAAGAC
CGACTGGTACTCCTCTTTGCATCGTACCAAAGACTCGATCAATAACAATAAGGCATTGTT
CCAAGCCATCAAAGAACGTGCTTCCGACGATCTTCGTGGCCTGTTTTCCGATGATAATGT
CGTTTTGGCAAAAATCGAGGCCTTTGAATCCGCTTATATTGATACTGTTGCTAAATACCA
CAAAATTTGCGCAGAAGTGTTTTACGATTTGCGAGGTTTTGACCGTCGAAGTTTTGCTAT
CGAAGCACAAGCGAGAATGAAAGACTTTCGGTATCTTTTTAACGTAGTTATGCAACAGTA
CGGCCGCGATTGGGACGGCGAACTCGCTGACGAGAAAATCGAAGAGCACATCATCAAAGA
ATACGCAACATACGTTCCAATGGCGTACCGTTAAATAAAGTTGCAAAAACTGTGTACATT
TGAGGTTGTAATGCTTAGAATAAATCCATAATCAACATGGAGTTAACCGAATGAATCATT
ACAATTACACCGTCGAAATTCTCATCAACACGTACGCTCTCGGCGGCAGTTCGGATGGAA
TTCGAATTTTCGACAAAAACGGTAAAGTTGTAGGGTATATTACTGATTTGCGCAAAGCTG
CTCATTCTGCAGCCGAAAAGAAAGAAAAATCCTAATCGTACTCTCGGGAATACTATCAAC
AAGCTAAAACTAACATTGGCCCAATGGTCAATGAAGTTGTGAACGAGTTGAAAAATGTTT
TTGCAGGCACACCCGCAAAGGTGTTTATTAACGAAACGATGCCGAATGTCAATTTCATGG
GAGCCAAGTTCTATTTTATCTTGTCTCCACTGACTGACAAAATTCGTGTATACATCACTC
ATAAGGATGCATGCCGCATGAAGTATGTTTTCCCGCAACATGCCAAACTTGATATTCGTC
AAGGCGGAAAAAGCATTTTGATTGATGGTTTGACACAGAGCGAAGGCATCGCTCTTGTCA
AAGGATTCTGCGGAACTGTTGTTAACTTTGAGTTCTCTGCTCAGTTGGTTAAAAAGTAAT
GCGTTATATCTCATCATAATGGTCTTAATGGCCATTATGATGAAAAATCAACACGACGAA
ATCTCTACGATAAAAAATAGTTTAGAATCGATGAAATCATTCCAAACTAAGTCGTATGAG
AATGCAAAACCCGTCACTGAGGCCCTGCAGAAATCTCCAAAAGCTACAAAGCAAATGGAA
AAAATTGCGGAAAAGAAACCGCAACTATTAGAAAAACGAATGAATATGGGCTTCCAGAAG
TTAGCTGATCAACTTCAGGAGAGTACGAAATGAGGTATATCATCATTCATCTCGGATGTC
TCCTGATCACATCCTGTGCATCTAAGCCCATGTTGCCAAAACCATCGCCGATACATAAAC
TCGATATAACATGGAAAGCAACCGAAAATGGAGCTTTACTAAGTTTTGAGGATTACAATA
AGTTGGGTGTATGGTTAACCGATGTCAACAGATATATCAAAGCTCAAAAAGAAATAATTC
AAATATGTGAGGATACAATTAATGACTGAACGTTTCGCATCTCAACAACTGATTTTGACT
GTTGGTCCTACGTGTTCCGGAAAAAGCACCTGGGCAAATGAGCAAGTTCGGGCAAATCCT

ACGAAATACGTTAATTTGACTCGTGATGATTTTCGTTTTAGCCTTTTTGGCTGCCGGACT
TGGTCTGACTACAAGTTCAATAAGCATAATGAGCAACTGGTAACGAATGCTATCCAAAAA
GCAGCGATTGAAGCTATTGCCATGAGCAAGACTATCATTATTTCCGACACCAATCTGAAT
AAGACGACTCAAGGAACTTGGAAAATTTCGGAGATACTCATAACGTTGAGTTTGTGCTGA
AATATTTTGCGCCGGGCCTTCTGTCTACTCTCATGGAGCGTAATCGCTAGAGGCGAGCAG
GCTTTGCCTGAATTCGTTGTGAAGCGTCAATATGTCCAATATATGGTTGATTTTGGAGGC
CAGCGGGTTTATACTCCCGACTTTGCAAATCCTCGGGCAATCATTTTTGACCTGGATGGC
ACCCTGTTTGATAACAGCCAACGGCGTGCCTTCGATTGGTCGATAGTTCTGGATGACACT
CCCCGTGACTCTGTCGTAGAACTTTTCAAAATGTACGTTGATCGCGGTTATGGTTGCATT
ACCGTTTCTGGCCGTGATGGTGTTTCTGAAGCAGATTCTATCAAGGCTCTGAATAACGCC
GGTCTTTTCCCTGCGGCCCATTACCAACGCGCTGCAGGCGATTCTCGTTCTGATGATATC
GTAAGAGAAGAAATTTTCTGGCAACAGATCGAGCCCAATTGGTGTGCTGTTCGTGCAATC
GATGATCGTCAAAAGGTTGTTGATATGTGGCGTCGTATCGGGCTGGAATGCTGGCAAGTT
CAACCTGCTGATTTCTGATTCAAAGGCCCTCTTCGGAGCGCATTTTTGTAAGAAATCAAT
AAAAAGTAGTGTACAGGTCGGTTTAGTGTCATAATATAGTCACATGAACAAAATGGAGTA
AAAAAATGTTTTTTGACACCTACGGCACTGCGCTGGCAGAGTTGGAAAAGCGCTGCGGCG
AATTCCAGAACAAAGTTAAAGCTCGCGACTAAAATGCGATGGTTTCAATAACTCAAATTA
GCATCAACGAATTTGAGTTGATCGTCGCCAATGGTGCAGGCGGGCGTGACAATTATTTGT
TTGCAATTTCGTTGGCCCAACCTCCTGTGCCGAAGTTTATCATCGGCTGCTCTCGGGTAA
TCGCATGAAAGATACAAGTGTATTACAACATGCCTATATCGTTGCACAAGAATCTCATTG
TGTTTCGTGGAAAGTAGGTGCTATCATTTCGAAAGATGGTCGAATTATTTAGACCGGTTA
TAACGGAACTCCAGCTGGTTGCAACCATGCCAATTGTGATGACCATGCGAAGAAAAGTGG
TTGGCTTGATTCTTACGGTAAGTTGCGAGGAATGTATCGGCAAGCACACAGTGAATGGAC
AAAGGCAAATGAAATTCACGCAGAATTGAACGCCATTCTGTATGCCGCGAAAACCGGGCA
AAGTATCGATGGTGCTGAAATGCACGTTACTGTTTCTCCGTGTCCAGATTGTGCAAAAGC
CATAGCTCATTCGGGGATTAAGCGAGTCGTCTATAATGAGTTATATGATCGTAATCCGTC
TGGCTGGGATGACATACTCATTAAGTCTGGAGTAGAAGTAGTGAAAAACGAACAGCCTCT
CAACATTATCAAGAAAATTTACACCACCAGCAACCAAAATTGAGGAATTGATTATGATCG
TTGAAGTATCTCCTAACGCCGAAGCACTTGAAGTCAAATTCTTTGAAGTTAATAGCGTCG
CAGAAGCTGCCGTTCTGGGAATCGATCCCGAATGGGTTGACGATGTCAAAGCCGTCGTCA
AAGTTGACAAAAACGAACCGCTGTTTGGTTATTATCCGTGCCTTATCGGTTTTGTCACTG
GTCATCCGGATTTTGCGGATGGTAAAGTGATTCGCACTTCTACTGTAGATATGGTAATTC
ATACTCGTGGTGAGTTCGAAGAACGTACTATCGTTTTCACTCGCAATTCTCGTTATGAGG
TGATTAAATGAAAATTCAGCTCGATACTAAAGCACTCGAATCACTTTTCCCCGAAGGTTC
GGAGGCTCGTGTAGAACTGCAAAATGCAGTTATTGCTAACTTCGCAAAGAAAATGCAAGA
AAAGCATCTTGAAGATGTAACACGGGACCAGATTCGTACAGCTGTTCAAGAGGCTGGTGT
TTCGATGGATTCATATTCCATTGTTCGTAACATGCTGAACAAAACGATCACCAACAACGG
TTGGGATGCATCAAGTGCGCAATTGAAGACCAATTCCAGTTTGGCTCGTGCTATCCATAA
CTATGCCATTCAGCAAGACCTGAAATTGATGAAGGGTATGGAAGCATGGGCCAATGAAAT
GGTTAGTAAAGAACTTGGAAAGCGCGAAGAAGCTGGCAAAGGTTACGTTGAGCATTACAT
ACAAGAAGCTGAAATGAATAGCGCAAAAGCAAAGAACCAAGCTTTTGCGGAAAGCGAATC
TCGGATTGTTGCTGCCTGTAAGCAAAATATGCCGGAAATCATTCGTCGTGAAATGGCGAA
TTATCTGAACAAACCAAATACACTGTAAGGAAATAAAATGTCTAAGCAATATGTACTTCA
ACAAATGCTGTTCTCTATTTTGGCTGACGGCGAACATGAACTGATTTTGCGAAAAGCAGA
CGATTCGCATCGGGTAATGCTGGCTACTCGTGACACTGTCCTGATTGAGGTTGCTGAAAA
AGGTGACAAAGCTTTTACGGCAGCAGAACCTGAAGAAAAGAAAGAACGCAAAGAATGTAA
TATTTTGAACGTCGTCGTTTACGATTTGGAAGCAAAAGCTTGGCGATCGTTCCGAGTCGA
TCGACTCGTATCTATCAACGGTACTGGTATGGCCACGCTGGCATCTCTTATCGGTGCTCA
ACATCGCAATCAACTGAAAATGGGCCACATCGAAGAGCGTTACGTTTCTACCGAAAGCAA
GGGTTACGTGAATTTTAAATCCGTTGACCCCGATGAGTTGAGCGATTTGGCCGAAAACTC
TTCGCGATTTGAACTTTTGTCCGACGAACACGGTACCAATACATGGTTCCTTGTAGAAGA
CGAATCCAATTTTTAATCTGTGCTGAAGTTTGCAATACGATGGGATAACTTATAATTATC
CCAGCAATAAAACAGTTAAAGAGAAAATCATGACTAACGTAACACCTATTGCTCAATTTG
AAAGGATTATTCTTGAATCAAAAAATTTGGCATCCGCAGGTGATGAAATCATTTCAGATT
CTGGATTTGTTCTTGGAAAACGCGATCTTCCTGAAGTTCCTCTCACTGCAGAGATTATCT
CAGTCGGTAAAGACGTACCGAACCATTTGAAAGTAGGTATGCAAGTTTTGATTCCAAATG
CCCGTATGCACAATTTACCCGATCCTCGTTATCTGTCCGGCGAAATCAGTGCGAACAATA

GTCGAAAGCTGTCAGCATGCCACTGGAAAGATATTCAAGTCCTGTATGCTCAATTGATTG
TCAATATAAGATTAAACAGATTAAACAAAAGGAAATGAAAATGGCTAAGAAAAAGAAAGC
AGTTGAGAAAAAAGTAGAAGCTCCGAAAACTGGCCGTCAAGGTTACAAGCGTAAATCCAA
CAGCCGTGTTGATCAAGTTGGTGATGCTCTGGTAGCTCGTGTTATGCGCCAAGTCAAACA
TGTTGATAAGTTTGGTCAGCCTAAGCACGAAGGTCGCAAGCGCCAGAAGTGATTAATGGG
ACCTTCGGGTCCCATTTTATTTTGAAAAGCGGTTTAAAAAGGAAATATAATGCAATACGA
AAGACTGATTATTTTGACTGATGTCGACGGGGTTTTGGTTCAATGGACATCTGGCCTCCC
ATATTTCGCTCAGAAAAATGGCATTCGGACCGACGAAATTTTGAAAACACTGGTCGACGA
ACGATTCCGCACTGGTGAAGAATTGTTCGGTGTCAACGCGCGTATTGCAAATATTTTGAT
GCGCGAGTATATCAACTCAGATTTCATCAAATATCTTTCTGGATATGTCGATGCAATCGA
TGTTGTAAATCGAATGAAAGACCCATATGACTTTATTGCCATTACTGCGCGAGGTGATTC
TAACCAAGCTCTGCTCAACCGGTGTTGCAACCTGAACACACTTTTCCCGGGCGCGTTCAA
AGATATTCTGTGTGTTGACATCGGCGAAACCAAAATGCCACATTACATTTCCGTAAAGCA
AAAATATAAAAATCGTTTGGTTTGTTTTGTTGATGATTTGCTAACTAATCTCCAAGATTG
TCATGATGCGATGTCGCAATTGCCTCAAATTCATATGCTTCGAACTGAAGGCGCGCGTGA
AGTAAAACATGATATCAACAAATACCACACTGCTAAAAATTGGTACGATGTTGAAAAGCT
ATTAGATCAACTTGATCCAAAACCAAATACAATCGATTTGTCACATCATTCGACTTGGCA
AGATAAACCTGCCGTCTCTGAGGTAAAATGAAATGCATATTTTAGACATTCTGAACGAAA
TTTCCTCAACTGATTCGTCAAAAGAAAAAGAAAAAAATCCTGAATCGCAACAAAGAAAAT
AACCTTCTCAAAACTGTTTTCACCCTTGCGTATAACCGTCAGTTAACGTACGGCATTACA
CCAAAAGGTTTGAATTTCGGCGGTTCGGTTGGACCTATGAGTTTGGAAAATTTCTGCGAC
GAGCTTGTCAAAAAAATATTCCACCCGAAATTTGACAGGTAACGCAGGTATTCACTACATG
CAACAGATGCTTGATAGTATGTCAAGTTCTAGTTGTGAAGTTGCCAGACGTATTCTTTCA
AGGGACCTCAACTGTGGAGCATCTGTTGCTCTTGGAAACAAAACATGGCCCGGCATTATT
CCTGAACAACCTCAGTTTCTTGCAGCACCATACAGGGAAAAGAATTTGAAGAAAATCCGT
TGGCCTGCTATTCCTCAATTGAAAGCCGATGGTGCCCGTTGCATGGCTCTCGTATCTGCG
GATGGCTCACAGGTATCACTGGTGTCACGGAACGGTAATGAATATCTGAAGTTGTATGAC
ATTACATCACAGTTGAAGGAACTCGCTCAGGTTGTCAGAGAGACTCTGCAACATTATGGT
GATTTTGTTATAGACAGCGAATTGATTCATCTAGCTCCATTCTCTGCATCCATTAAAACG
GCTAAACAACATACACTGGATGATCTGTTTGGAATTCCTGAGACAAATGATGAAGAAGAA
ATCGAATCAGATCGAAACAAGTCCTATGGTTTAAGTAATAAAGCTCTAAATGGAACAATT
AGCGACGTTGAACAATCAGAAATGATTTTTAACGTATGGGATATTATTCCGCAGGAAGTT
TATTACGAGCGCGAAAAGAGCACATCGTACTACGAGAATCGATTTCCTACTCTTGCTTGC
CTGACTGAAGCTGTCGATGGTACCCAAAAATATCAAAACATTCAACTCATTGAATCCGAA
GAAGTTGAGACATACGAGCAAGCTATAAAAATTTATCAGAGATATGTTGATCAAGACCGA
GAAGGTACCATTCTTAAAAATAAAGATGGCCTGTGGGCAAACGCTAGAACAACTGACCAA
ATCAAGTTCAAGGTAGAAGTAGAAATTGTTTTGAAGATTGTCGAAGCTTATCCTCATGGA
AAAGATGCGTCGAAGGTAGGTGGCTTTGTTCTGAAAGATTCTTCAGGTCAAATCAGGGTT
CGTTGCGGTTCCGGCTTAAAAGACACAACCCATAAAAAGGTAAATGGCGCTAAAATCGAA
ATTCCTATCAGCGAAAGGCATGAATATGACCGCGAAGCGCTTATGCTGATAAAAAAATGAG
TTGATCGGCCAGATTGTTCAATGCAAATGCAATGGATTGCAAGAGAGGAAGGGGCGTAAG
CCGAATGAAGCAAAATTCAAGCTGTTTCTTCCTATTTTCCAACTCATCCGACGTGGCAAA
TCAAAACCAAATCATACCCATGATGTTTTCCCACATGCTATTCTGGACTGAAAAAGGCCT
TCGGCCCTTTTTTCGTTTCTGTTGTGTACATGTTGATTCGGCTTTGATAGTATGAATACG
TACACAACTATTGGAGAAATTCACATGATAGTTTAAGTTCGATACAATTTTGAAGAACAC
ACTGGCGAAGCCGACGACGCTCAAACCGCTTTTGAGAAAGCAACTGAAGTTATGCTGTCA
CAGCTTCCCCAAAAACCAGAAAACACGTTTTACGCCACTGTTCCAAATGCAAGCCGTTTT
GGCAGCGGTGATATTCGTGCAATGAAAGTTATCCGTATGACCCGTGTTGACCTCATGGCT
GTTCGGGCGGACGACTTCGAATTTCCGTTCTTTGTAAAAGTAGGTGAATAAGCTCCTTCG
GGAGCTTATTGGAGAAAATTATGCTAAACCCTTCAACTGTTGTACCTGTGGCTATTGAAA
AGCCCATATTTTTCAAATCACATCTGTCAATCGGCGGATCTACCGCGGACACAATAAATT
CCCCAATAACTTTTGAAGAATTAGAGTGGGCATGTAAAAACGTAGAATACGCTCATGATG
TTAGCAGCCACGGAAACATGCCATGGATGAATTTGGAATTGGGGTCATATGAGCAAGTGC
GGCTAATTTTTGACTATCATGGTAAAAACTCCCCAGATATAATCAGAATGTGCAATCGTA
TAGCCGAGCAACGATACACCGACAGTTGCATGTGTATTTTGAAAAAAATATGACGACTACG
TGTCTAGCTATTGCGATGAAGAATTTTCAAACGTTTCTGCTGCTTTGCGGTCTGGAATAT
TCGACGATGGAAATAAAAAATTCGTAACCGAGTGCATACGACGGATGGATTCACATATGG

CAGAATCTGAAAATTTTAATAAATCTGTATGGAGAGGAATGTCTCTCAATAAGGCAGATT
ATATGAAATTTAAAGATAGTGGGTCTATTCTCTTTAAAAATCCCGTATCGACATCATTTT
GCCCGCTTTTTTTCAATTACGGCATTGCGCAATGCAGACATCTCGATTTTAACGAAATCA
CACGCGAAATTGTTATCGATGGCGACCCACATCGACCTGTCACTATAAACATGCACATTG
ACTGTCATAATGTGAAACACCTGATTCCGGGAAACCTTAGTCCCTACCCGGAGGAGTGCG
AAGTAATTTTGGACAAAAACACTGTACTCGTAATATATGAGATTTTCGAATATGTGTCTC
ACGTCGACAGTTTTTGTTTTGTTAAAGCGAAAGCGGTTCCGTTAAGTCAATTCACTGGAA
CAACATTAGTAGTGTAAATAACATTTTAGACCTATAAGGAAAACAAAATGATGCTATTTG
AGATGACAGATGAGCAATTGGACGAGGTATTCGACGGTGGAGTTGGAAAATACCGCGTGG
TCAATCTTTTGCCAAAGACAAAAGTTCCGTATGTATTAAACATCCAAGTTCCTGGTTCAG
AAAATCTGGCTTTGCGATTCTGTAATTTTACTTCAAAGGGTGATTCCGCGAAGCAGATCA
AACCTGGCGACAAATTCATGCAAACACAGGTCATCCAGATTTCAGAAAAAGGAACACCAC
AGATTCTGAGAGATGGTCTCGGTAGTGACCCCATTGGCGCAATGAATATGATAACTGACG
AAATCGTTAATATGCTTGTCAGGTATAGAATGGATGCAACGCTTCTGATGTTTCCGACAA
AGGCACTGAAAGGCCAAGCTAAAAATATTGCGCGAATTACATCCCGGCTTTTGCAAAAAC
GATCGGGTGGAAAGTTTATTGTTTTGCCAGAATTGTTGGAAAGAGGCAAAAAATATACAT
ACATTATGATTTACCGAAAATCAACAGGAAGCACTAGTGTCAAAGGATTGGAAACTGATC
CTGAGCTTTATACGGTTTCATCTACCAGTGTAGGCGATGTCGTCATTGATGTGAAAACTG
GTGAAAAAGTCGCAAAGGCAGAAGCTATTGCTGGGTCAATTGAAAAGAGAGAAGAAAAAC
GCTCTACTAAGTCAATGACAAACTTCAAGATACCACGTGGAGATTTGATCAAGGCATTCA
CAACCAGTATTGCCTCTTCGGACAAGGAAAACAATTACTACGACCACTTTGGTGTTGGCC
CAGTCGAGAAAAAACCGCCAACTAGCGATGCTGAGGAAGCAGATATCAATGCTTATAACA
CGATAACCAGAGAACAAATACGGAAATCGCATTATTACAAATTCTCTAATTTAGCCGCTG
CTAATTGTCTTGACAAAATTGACTTCGACAAATATGATGCGTCAAGCCATGAATTGCTTG
CTCTCTATGCGGAGACGGTTCGTCAAACGTCAGAGAAAGCCGCACAGAAATGGGTAGATG
AAAATATTCCGGATGAACATCCGGGTGTGAAGAAAGGAAAAGTCGATTTCATGGTTTATA
AGGAGGTGAGACGCGCTATTATGGAATGGGCGAATTATCTTGGTAACAAATTTGATGATA
CTGTCGGCAGTGTTAATATGAGAAAAAGATTGCCACCTGCATGTGTTCATGCTATACAAA
CTTACACTTCGAATGACTATCGTAAGATTAATAATGTTTTCATCGCCAAGGTAAAACCAT
CAGCTAAAACCGTAAATGATATACAGAATCTCGACAGAGCTGTTGACATTGCAGGAATTA
AACTTCCTGAGAATCTCACCCTTTGGCGGGGTATAACATGCCCAAATTATCCAGTTAAAG
TAGCTTTGGAAAATAAAATGTTTTATTTTTCTAACTACGTCTCATGTTCTGCTGTTCCTA
TTGTATTTTTAGGTGGGTCTATTGCCGAGTATGACATTGACATTCTTAATGCTAAAGAGA
ATGATGACGCGCAGGTTGAAACAGCCAAAAAAACACGGGGCTCATTTCGCATTTGCTATC
AAAAATGTCCAGGTCCCAGCTCTTATAGTTAACGGGATGTCGAATTATACGAAAGAACGC
GAAATTGTTCTTCCACGAGGAACAATTTTTGAATTTAAAGAGGTCAGTAATGTTGGTGAA
TTTACTTCAGTAAAAAGGAAATAGCTGATAGATTGTTCTGCCGTTTTCAAAAGTCAAATC
GACGAATCAACAGAGATTTACGACGGTGATTTGCTAATGCAAACAGGCGAAGTCAAACTG
ATTGAAGGTTTTTCTGGATTCATGAAATCTCGCGAAGATGAAGAAAAAGAAAGATTCAAA
GCTGAAAGGATACAAAGAGACAATTATTTCTCAACTCTCATCGACTTAGGTGGAGAAACC
AAATTTGTCCAGGTAGTAGAATAAAAAAGGGAGCCGTATGGCTCCCTTTTTGTTATCCAA
ATGAACCAAATTTTGACAACCATCCACCAGCTTGTTTGCCGAGTTCAGGTAATAAATCAC
CCTTAAGCCAGTCTTTAAGCATTGCTTGATTAACCGCACCAGTGTTCATGACTCGATACG
CGAATGTAACGTCAAACGTTGTAATAGCATTGTTATCCTCGTAGCTAAGTTCAGGTGCTG
ATACGCTGACAGGTATGCATCCATTAAACATCGTCACAGTGTGGGGTATACCGTCTCTGG
AGTGCAAATTCATTTGTATATCTTCTTCAACATCACCAGGCAGTGAACGCAGTCCGGTGA
CAGGGTCTTCGACCGCATTTACCCAATCCTGCATAGCCCGGTCATTGGCAGCCTAATGGT
CCATTCTGAAACTAATTATGAGAGGATCATACTCGCGCCCCATCAATTTGATGTTTGGAG
CGTTGTGAAATTTGTCCATTTCATAATTTAGCCGGTTTTCTGGCATTTTTACAGAATAAA
CCGTTAAACCAGTATGTGTATTGCCAGCATTGAAAAAGTCAAGCAAACACGTACCGACTT
CAAATTGACCAAGTAGGGACTGTATAGTTCGAGACGTCATTGCGCCGCTCAAGTATCTCG
AAATACCTGATTTGCGTATGATACTTTTGTGTGCCTTGAACAATGACTTTATTCAAGGTG
CTCGAAAGCATACCTTGTGTCAACCCGGCAAAATCTTTCCCAAATGGAAGATTGTTATAG
AGATATGCACCGAATCCATCAAGCAAATCGGTTTGTTTTAGATGATGGGGTTGTTGCGAA
AACAACAGAGAACATATTTGTTCTCTGAAAGTCTTTATTCAATCCTTGTTGAAATTCATC
TAATGTATACATGATTATTCCTCATCATATAGATTAGATCAATTGCGGGCAAGAATCTCT
CTGAAAGTGACTTCAAGTACAAAACTTGAAGGCAAATTATGTGCTATTGCTAAACCCCCG

AAGTGACCGTCAGGGCTCTTATCGAACCGTAGACTTTGAATTTGCGCTGGCCCAAATAAA
TCAGACCGCATATCAAATGAACTTGTTTCACCGAAATTTCTAATAGTCCAAATAATTGGA
TTAGAAACAACGTTAACATTACTCAAAAATGATGTTATTCTTTCAAACATCGTAGTTTGA
CCATGAGTTGCTTCTGGTGTGACTGCGTTAATGAATGTTTCCCTGTATGCTTCATCAAGA
GTGCTTTTTATTTGACTCGCCGTTTTTGACTTTTCTAGTGCTCCGTACGAGTAATACGAG
AGCATTTCGTAAATTATCAAAATTTGCATTAGGTCGTATACATTACGAGGCGTTAATTGC
CAGGTATATGCTTTTGTACGATTCTCTGCGCCAGCGTACAGTGCCCTGGATGCGATGTAT
ACTTGCTCACCACGGTCAGCAAATGCTCCCTGGGTAATAGACTCCAATCCGCCATACAAT
GCATGAGAAGCCATCGAGCTCAGTGCCCCGGTCTCAGAACTGTCGCCTCGTTCAACCAGT
GATTGCTGGACATCATTGAATTTATGGCTAATAGTATCTACATCTGTCTGTCCCCGAGGC
ATCAGGATGTTAGCTATGGCTTCCTTGACAACACCCTTACGATAATCGCCAATGTTATTT
TGACCTGATCTGAACGAACGCAAATCCCCAGTCTGTCTGCCGGCATAGTTAAATGCTGAC
AATATCAAACCGTTTTTATACAGATCATTAACATTGAACTCTTGCGCACAGTCATTCGCT
GAATTTCTCTCGGCAGGGAATTGGGCTATGTCAATTTTGCTAATAGGCCTGCGAGGTGAA
GAACCCGTTGCCGGGTTCTCTCCTTTCAATATTCCGCCTACCACATCCTGAACCCCGCCG
TCTAAAAGTTCAGTTACTCTCATAAGATGTTATTTCCTATACCAGGTCCAGGCGAAGATT
TTCTTGGAGCCTGGACAAATGTTTGATTCTGATTTTTGACAATGTTAGCCTTTGTCTGAT
CGTTGTTTTGACTGGCTGCCTCTTGCTTCTTGCTTGCAACTCTCTTCACTTCTTTGTCGA
TAGATTTAACAGTCGAAACCTCTGGTTCAGGAGCAGGTTTTATTGCTGCTGTCGGCACAA
CCAATGTTTTCTCATGCTCGGCCAAGAATTTAGTCTGTTTGTCTATTTCCACTCTAAGTT
GTTCATAAGATTCCTTAGTCAACTTACCTTCTTGATAAGACTGTTCAACTTCATTCGAAC
GATCATCGAGCAACTCGCGGTGTTTGTTCAATAATTCAGGAGAAGTTTCATTCTTTTTAG
CAGTAACGCCAACATGGTTTATTTTCCCGCTCAGAACGTCATATCTGCCAAATTGCTCTG
CTGTAGTATTTTTGGCAATTACCATGCGTTTTTCACGCTTTTCTTTCTCGTCAGGGGTCT
CTTCATCTTCCCCATTCACTTTACGCCATTGCTTTTTAAACCAATTTGCTTCGGTGTTAT
TTGCGTCTTCAGCTTCGCGTCTTCTAACACGGCCGATTACTTCTTCCTCTTCTTTTGAAG
GAGTATATCCGACTTCTGATGCAGCAACTGATATTGCAGGAGCTTCGAGCGAATCGGCTT
TTTCATCTGCACCGAGAGCTCTTAATATAGTTGCGCCAAGTTTGGCCAAACCGACCATCA
TCATATTGACCATATATTTCATTTGGTCAAAGGCACCTTCGGCAAGACGAACAAACATTT
TTGTATATTCATCACTCTTGAAATAATTCATCAGGTCTGTTATAACTCCATGAACATTTT
CAAATGGTGTTGCTAAAGAGCCCAATTGATTTTTAAATTCGGCGTAATTCTCTTCAAACA
TTTTAGTGCAATGAGTAAAATGACGAGATAAAACGTCAAATGTCATAATCAAGGAAAGCA
CGAGTGCAGCCATTTTGGCAAAATTGAGAACGGCAGTCAAAGTGTATTTGAACAACGTTG
AACTTATTGAATCGGCAACGCTAATAGTTTTCTTAAAACGATTATCGACTGACGACACAA
TACGATCTCCGATTCCGTCAATCGCACGTTCATCATTTTCTTCTTTGTCTGGTTTAACTT
TATCCTCTTTTTCTTCTTCCTTTGGCTTATCGTTTTTTAGGAATTTGATATCTTCAGAAA
TAGTTTTCAAATACCCTAAAACAGGGTTATTTTTGGGTGTTTGTTCTTCTTCCTCTACTA
TCTTTTTATCTGTTTGGTCACTAGTGTTTGTTGCCGTAATTGGATTTTTTTCACCATCCG
CTGAGAAATTATTTTTTAGCTTTTCAGCAAATTCACTTAGTTTGCTAAATGTTTTCTTGG
AAGTATCTGAAATCTCTTTCAAGTGCTGATTAGAAATTTCAGTGGCTTCCGCGGAAAGTT
CTGTTGCAGCAGCAATATTTTCGGTTTCAGAAGTGTTGTTAACTATTTTACGAAGTTCGG
AATTACCTTGTTCAACTGTTTCAGCTATAAGCTCAGATGAAGCTAAAAGATCGCCAGTGT
TGTCTGGCGGATTTTCCAAAGCAGTTTTGATATTAGATTGCTCAGAATCTCTTTGCCTAT
CTCTTCTCAAGTCTCTTAGTGTCTTTTGAGTAGCCATTAAATTATTCCAATAATGTTTGA
GCCACCATTTATTTTGCCAAATGGTGTATCAAGTGAAATAGTAGAGACTATAAAATCTGC
CCAATCCAATACAAGCGCAGGTGCATCTTCGAAATTAAAGTCAAACCCGCTTTTTTCTTT
GTCGTATTGCTTGGTCAGTATGTCCAATTTCGATACAAACGTATCAGTTATCATGGGAGG
TTTGAAATACAGAATATTTCCGTTTAGCTCGAATTCATATTTTGCGCTAATATAAGCTTT
GTCAATATGAACGTCAAAACCGTTAATGGTTTGAACGGTCGGCAACTTTTTGTTGAATGC
CAGCAGATGCAAAATTACCATATCTCGTTCAGCGGCAGTCAAACCCGAGCGAATAGAATC
AAGCAATTCGATCATACATGAGTCTACACCCTGCATATCGTTCAGCAATTTGATATGCCT
GAAACCAAGTTTTGGAATTGTGATCTCTTTTCCATTGACTTTTATGGTCTTAACAGCAGG
GATGATGTTCAATTTTGGTTTCATGTTATTTGCCTTTCGTTAAAATCTGTATGAGCTTTC
GAGTTGTTGAAAAGATACATATTTGTTATAGATTGCTCTCTGCTTATTTCATGTATAACT
TCATCAACGAAATACTCGCCTCTAAATTGATTCTTTTGATCGTAGAATTCCAAACGCATG
CCTGGTACGAGACCAAATTCTCCGTATGTTTTTGCTGTGCAATACGCATCGGATTCTGAA
AATATTAGAGACCTATTGTACTCTGCGGCACCAGGGCGGCTTATTTGGTCATAATATGCA

CCAGAACGGCTAATACAAACTGAATTTAGCCCTTCGCCAGTAACAACCCGAGTTTGCATT
TTGTTTATGAATGAGTGAGCATAAAACGAGGTATTCTCGTATGGGTTACGGGTATACGCG
TTTGCTTTTGTCAACCATTCAAAATCAAAGCATATTTTTTATCTACCATCGCAATAGTT
TCACCAAACAAACGCGGCTCCGTCACGACGTATTGTATTGGATCATTTTTGCTAATAGAT
TCAAAACCTGACATATGAATGCCTAACATATCTTCCCAGACGAATGGAACACTTTCGTAT
CCAGTCGTTTGGCCATGTCGGCTAACAAACTTGTAGTATGCATTTATGTCCAACACCCAA
TTTGTCATTGGCACTCTAGTGCCGATCGAATTGATTGTCGGCGCGAGTTTGGGAGTTCCA
GAATACCAATAATCAATCATCTCGTTCACGGATGCTACAGCATCATTTGTAAACATTCTG
CTAAACTTGAGATTACGTACCGAATGTAATGAACCCAACTGGACTGTTATGATGTTGTCG
CCTTTCATATCTACGTTTGGAGAAAGATGTTTGATGCCATACAACCGGTTATAAGTTATT
TCAGTGTTCGCTGTTTTGAGAGATGTTTGGATTATTTCATCACCAGTCAAACGTGTCTGT
AAACCGTTTAAGTCATAAATTTGCAGCAGCCCTTCATTTATACCATTGGTTAGACCATCT
CTCATGGTCATGGTAATAAAAGTTGCTGCTAATTCAATAAACCTATTCTCCATCCAGGCA
TCATAATTCTGGTAAAGTTTTATGGAGACATTGGGATAACCAGCGCGTTGTAACATTAAT
TTTTCTCCTTTAATTCTTTTTCCACCAGGCTGAGAACAATGGACCTTTCGACTGGTAGCA
TATTAATTATGTCGGCCAGAGAATAGTCATGTCTTACTAGCGCTCTGTTTATCTTGTAAA
AATTGAAAATTTGATCAGGACTAACCAAAACCTCAGACAAATGGAGCATATTAGTAAAAT
TGATGTCATAATCTTTACAACATTTGATTTTTTGATTTACTAAAATCGGGTTGAATGATT
TTGATAGTTCAACAAATTATTCGTTTGAAATCATATCAACCAATTGACTGCGCTCATCTT
CACCCAGTTCATCCCAGGTGTATTCGTTGTGCCCGTCATATATCCTATCAACAGTCTCAT
AGAAGAGTTCGTCCGGTGAACCTGATTTACGAGGCATACGAAATTTGAACGTTATGTTGT
TTACCTGCAACTCTGGAGTTTTTAGTGGCTCTTGTGTCAACTTCAATCTAAGATTGTGCA
TCGACTTGCAGTGCGGGCATGTGAAAGTTGCGTCCACAAGTGATTTGCCCAAACTTCCAG
AAAAAACTATCAGAAAAATATGTTCACGTTCAAACGGCGTATAATCCGGATACATTTCTT
CGAGCAAATCGGAAAATATCTCAGATTTCTCTAAAGGCTTACTTCTCATCTCATTTCTGA
TCAATAATAAATCACGATATTCTGTAACTGTTATCGGTTTAAATCTGTGTGTGCCGCTCG
GAAGCTTTATGCGGATTATATTCATAATAGATACACTCCTTTTAGGTTATTTAAATACAT
TCAGGAGGACTGTATGGACATTAAAATCACAGTTGGAAATAAGCAAGTAGAAACTACCAT
GTTGTCGGTGCGCGAATATCTGTCTATCGTAAATGACGCAGATGAAATGATTCCGAAAAT
CATTTTCAATAAAATAGGTCAGCCGTTACCGAAACATCTAACTGAGCAAGCTCTCATCAA
ATTGATAGCGATCTAAAAGAAAAAAGTCATCAAAATGAAATCAGAGTGCACGCTATGTCA
AAAAGAAAATAAGTTTGAAGTGCATCCCGATAATGTCTGTGTTTCTGACGGATTGGGGCT
ATCGCATGTCGCTGGCGGAATGGTTATCAATATGAGATACCCGATGATGTTCGAAGATCG
GGATATGTTTGAGATGATAGACAGATGTATCGTGTCATTTGAATATGCAGATCAGCTATT
CAAGTGGGAAGATTCAACAGAAAACGAAAAAGACATTTTATTTAAGCATTTGACGTTTTC
AGATATTGAAGCTATCGTAGAAAAACTTCAATCTCCACAGGTATATGCTGCAGTTCCAAT
CGAGTGTGAATGTGGAAATCAGTACCCAGCGGCTATATATGGTGCTAGTAAATTGTCAGA
TTCACTGGGGGTAAAATGAATATAAATCTGCTTTATACAGATCTTGACCCAGATTTCAAC
AAATCGTGGGACAATGATGTGTTAATGACACGAGGAGCTCGTGCCGTAAAAAATTCTATC
ATAGGGATAGTTTCTACTCCCATTGGTGCGAGACCATTTAACCCATACTTTGGCTGCGAC
TTACCAGACCAGCTATTTGAAAACATTACGCCGTTGGTGATAGACACGGTTAGGCGAAAT
ATTCAAACGGCTATACGAAACTTTGAGCCACGTGTATACAATCTTCAATTCGACTCAACT
GGAAATTACGATAATAACGAACTAATTGTCACAATTCGATTTTCAATTATCGATAATCCA
GCAGTAATAGACGAAATAAAAATGAAACTTTCAAATTAATTTAGCCCTCTTCGGAGGGCT
TTTTTGTACGAAAATGCAAGAAAAACGTCACTTGGGAAACAGCATAAATATAAAGTCAGT
TGAATTGCTGAAAAGAATAAGATACTCCAGGAAACGGGATGTAAAAATCAATACTGGAGC
CCATGGCTCCAACGAGCTTGCTCGTTAATTTTTATCCTTTTAGTTATAATATCTATTAAG
CCATAAAGGATAAGTTTTGGCAAGGCCAAAACCTGGAAGCCAGATTTTACTGTTCAACTA
TCGATTTATAGTGTTATACTGGCCTCAACACTAAAGGAGAGTAAAACATGATCATGAATA
AAATTCAAGAAGTTCGTATGTCTTTTCGCAAGCTTGGCTCTGATGGCGCTAAAGCTGCAA
ATCTTCTCGGTCTAGTAATTGGCCAGGTCCAGCAAACTCGAGATAATTCCGACGCTGCAG
TTATCAAAGTAATCAATTCCCTTTTGAAGGGAATTCGCGAGCGTATTGCTCCAAAACACA
ATCAGCACGACCTCGGCAATGCATATCGAGAAGAAGCACTTCTTTCTCAGTTTATTCCTC
AAGCCATTACTGACGAACAATATAACTCGGTCGTCAATGGTAAAGCTGTGCTAGACCTTT
ATCGGGTGCATGGCCCAAATACCAATCTTATCACTGGAAAACTTATGGCACAGCTGTCAG
CCGGTGTGAAAGATGGAAATTGGTCTATCCCTGACGCTGCTTCGTTGAAAAAACAGATCG
AAGTATACATCCTTATCGCTTTGGACGTAATTGGAGAATAAAATGAACGATGAAATTTGC

GTAGTATGTAAACAGTTAGTTCCTAAAGCACTTGCTGTTTACACATCTTTTGGACCAGCG
CATCCTGGTCCATGTGCCCATTATGTTGAAACTTTACCTCTTTCAGAATCTACCGGAATT
CTTGAAGAGACCGAACTTCTGGTGTAAAATAAAAAGGCCTGCATACGCAGGCCTTTGTTT
TATCAGAAGTCTTCAGGCGGTTCGCTTTCTTCTCTGGCATTTTCGAATTCACTTCTACGG
CCAGCGATCGCATCTCGCACCGAAATATCATCGGTGGGTGTCAATTCAACTTCTTTAACT
CGCTTGTTGTAATATTTTTCTAGCTCATCTAGTCCTTCTGCTGTTTGACACGCGGCAATT
TTAGACATAAATGAATCCACTGTTGATTCTGCTATAAATTGCTGAAACATTCCCATTGCA
TCCCTCCTATAACGAAATTTTATGCAATGTGTAATTGCATTTTTCTGACACATATCTTTC
AATACGTTCTATTGCGTGTGTCAGGGCGTAATTTTTCTTTGTGTATCTCCCATGAACATT
GATTATCCCAATATCGTCAATAATGTCCCATACTGTAGCAATACTCTTGGACGAGTGCTT
TCTCAAAACTCGCCCAATAGTTTGGAGAACAGTTACCTTTGATTTAACAGGCTGTGCAAA
AATGATATGATGCAAATTTTTGATTGACACACCAGTAGAGAAAACACCATATGATGCTAC
GAAAACATGACCAGTTCCACCTTCGGCGATGATTTTCACTTGATCACGGATTTCGGTGTC
TGTATCTCCAGATATAAAATGAACTTTACCTGGGTCATGAATCTTTCTAAGCGTATTGTA
CAAAGCCATACCATGCGCTTTCAGGCGAAACATAACAAATGCATTTTCATTTTTGTCTGC
GATTTTTTTAGCCATCGAACAAACAACTTTATTTCTTTTCGTATGATTCGTGATAAATTT
TATTTCTTCATCAGAAGAAAATTTTCTGGCAGAAGTTCGATCTTCGCTCGAATAACCAAG
CTGAATTGCTTTAACTTGCAAATCACATACTTGGCCCTCTTCCATTAGCTGTTTTGTTGT
TACTGGTCTAAATATATCCCCAAACATGCCAACATATTGCAAAATGTTTGCTTTTCCATC
TCGCAGAGAACCAGACAAAGCGTATTAAAACGGTATATGATCGACGGGTTCGATGAGTTT
CTTTATTGATGTCCCAGTTGATAGATGACACTCGTCGTTCATAACACAACCGAATTGGTC
AAACCATTCTTTTTTCTGCTTAATGGCAGATTGCCAAGTAGAAACATATATCAAAGCATT
CGACTCTTTTACGGTCCCACTTTTTATCCCAAGCATAGCTTCTCGAGGGAAAAGACGGTA
ATCGACAAAGTCATCAATCATTTGGTTGACAAGACATGTCGTTGGAACATTGACAAGAAC
TTTGCCTTTGTAATTTTCCAAATACGACAGAGACAGCAATGCTAGAATCAGCGATTTACC
AGCAGATGTAGGCAAATTTAGCAATTTTCTTTTTGCGCATATCCCTTCATACACAGCATC
AGCCTGATACCAGTGAGGAGTGATTTTGTTGTTACCAGAATAAATATCTTTGCTGTTTAC
CCATGCATCAAATTCGGAACGTGAAATATTTTGTCTTTCCGTGATCAACGGGTCAACAAC
TATTTTATAATCGTTCTGCTCGGCAAACTTTATTGCTTGAGTGACTAGTCCATAAGGAAG
CAAATGATCATATCCTAGCAGGAATATGTCACCAGACCAACCTCCACACTTGAAACGCGG
AGAAAATTCATAGCCATCTGCTTTAAATGAAAAATACTCCCGCATTTCATAGAAGATCGA
GTCTTCGCAAACAACCCTGGCAAAACTGTAGTTTTCTAATACAATATTTATCAGTAAACG
TCTCTTAAATAAGTCATATAGTTTTATTTATACAAAGGAATCACCATGAAACAGAAACTT
TTTAGTCGCTCTGATATCGGATCTCAGTTTTGTTCTCTTTCTGCCGCAGAAGGCAAGAAA
AAACTAGCAGATTATGCTCACAAGAATTACGACATTAGACTCGTCAGAAACAAGTCGTTT
GAAAATATGATGAACGATTTTGAATCTGCTGTCATTACCAAATTCGAGGCCGCTCGTGCG
GAAGCTGGAGATGCTGAAGAAACTATCGATATGGGAGAACGTGTTGACCTATCAAAGGGG
ATTGAGCTTGACCCTGATAAAATGGATATTGAAGATGAAATATCTGAAACAACAGAGCAG
CCTGTTGTTGCTGAACCATCGGTCGAGATTGTCGATCAAGACCAAGTAGAAGAAAAACCG
GCCATCCCATTTTCTGAGGTGAAAATTGAAGCCCCTACAGCAACTGAGACAACTACCCAA
GTAGAAGAAAAACCGGTTTCTGATTTTGTTTTGTCATTCCCTGAAGACTATGCGCCAAAA
TATCGGCTGGTCGGACCAGAAGGCCGAGCATTTATTAATGTTCCGTATTGGATACACGAT
TGGATGCAGGGAAGAAATTGGAAAATTGAAATTGCAGAATGTCCATATGACAAAGAGCTG
TTGAAGTCATTGGCATTTTACATTGAGCGAGATGGTTCTGTTGCTATTCGCGAATCACGA
AATAGCAAATTCATCGTTATTTCATAAACCGGGGTCTATGACCCCTTCATTTGTTTAAGG
AAAAAACAATATGGCAATTACTGCTTCTATCTCTCCACTTGATCCATCCGTTTCGGAAGG
TAGTATTGTTGACTTTACCGTTACTTTTAGCGGGGATGAGACGGTTAAGGAAGTTGTTGG
TTACGAATGGCTTGTTGACGGCGTTGCTCAAAGCGGAGAAACATCAACTACTTTTAGCAA
AACCTTTGATTCGGCCGGTTCGTTTACTGTAAAATGCAATGTGACATATGCTCTTGTTGC
TGACGACGGTGCTGAGCCAGTTGTTCTTGCAGCCGAGAGTGCTGTGACCGTCGAAGAAGT
ACCCGCACTTGATGGAAAGCATTGGTTCGTTCACCCCTTGGCTTTCAAAAACTCGAGTTT
CTCGTGGGTTCCGTACTGGGTGCACGACTGGATGGTAGCGAATCCAACATGGCGTCCAAC
ACCGGCAGAAAGTCCTTGGCCAATCATTACTTTCGCGATTGACCAAGCGGTGGAGGAATA
CAGCGAATGCTTGATGCAAGAATCCCGTAATGGGTACATTTACAAAGCATCTGCTTATAC
CAAGTAAAAGGAGCCTTCGGGCTCCTTTTTTGTTTACATTTCTAGAAAAACGAATTATGA
TGGCTTTGCCAACTTTGGGCAATTCACAAGGTTCCGAATAATTAAGGAATCTTCCTAATC
GAAAGTGAGTAAATTATGCTGAAATCGGCATTAGAAAATGTGATTTTGGAAAATAGTCAA

GCCATCGAAGGCAAAGTGCTTGTCACCGATGCCAAGAAAATGTTCACTGACCCGAATTCG
CCAATTCATCACAGAGCCAAGAACCGTGATGAGCGGTTTCAAATCCAATTCCGTGATTTG
ATGTACACTGCAACAACTTATCCAGTTGCAATTGACGGCCCGATATGGGAATCGTCGTAT
TCCCTGATGTATCCGATGATTCTGCGTAACCGTTTTGGTGATTTTAACCTCAATGGCCAT
AATTTACGTGACGTTATTGATACTCTGCTTGAACTCAGGCGTGAATTCAAAAAGAAAATG
GCTCTTGCGCATCTTGCAAAACTGCGTGACTGTCCAGATGACGGTGTCGATTACTACAAA
GCACAGTGGTACACTAAAGCTATTTTATGTTTGATGTATGCTCGATTTGCTGATGATCGA
GAATTCGACATCGAAAAAGTTGTAAAAATTGCAAAAAGCGAATACGTTCGAGCCGCAATT
TCCATAATCTCATCCGGTGGGATTTTGCTCAATGCCTCAGTCGATTCATATGTTTATGCT
TCAGCATTACCGATTACTTTTTCTGAGACACAACATAACGACAAATATTCAAAGATAGTG
ATTTTTGAACACGGATACGTCGCTGACCCGGGCAAAATGCATCGAGTTCGTCTGCTTACA
GAAACAATCAAAGATCGGCTGATCAAAAACCAGCGTCAACAATTCATGAAATAGTTGTAC
AGTGATGGGCTATGTGTAATGATAGTCCCATCAAATCTACCAAGGAGCATATCATGAGAA
CAATCCACTGCAAAGACGACTATGTCGAGTTTTATCATGGCTCTTGTACACTCGGCCTTA
AAGGTGATATGCTTCTTCCGCCTGAAGCAACAGACACGATTTCCGAAAAGGGCCGGATAA
AGAACTTGCACCGTGTGTTTTTCACCAAAGACCGCGGTCTAGCCAAGGTTTATGCTAGAC
GTGCTGCTCTTTCTATTGGCGGCGAGCCAACTCTATTTCGAGTTACTGCACCGGTTGACA
CGGTCTTTATGTCTGAAACGCCGGGCGCGACGGTTTACCATGCTGCCTGGGCTTTTGTTG
AACAGATTCCATTTGGTAAATTGAGCGACTAGAAAGATGAAATTCACGACAACTTACTTC
AATTTCAAAACAAACCGTCCGGACCATATCATCAGCGGTGCGATAGTGCCAAAGACAAAA
GAAGAAATTATCGAAGGCATCAAAGCTTACGTTGATTCTATTTGTCCGTTAGAAGATCGA
ACCGATGAAAACGGAAATGACACTCTCAAAATCGAAGAGGGTGCCCGACTCCCAGAGTCG
ATGCGTTCGCGGCCAATTCAAGTCACTATGAGGTAATCGTAATGAGATATACTCAAAAGG
ATCTCGCAGATGCCATTAGCTTTGCTGTGGGCGTTCACATTGATCAATTTGACAAAGGCG
GAAATCCGTATATTTTGCACCCATTGAAATTGCTGCATTGGGCCAGACATGCCAATGAAG
ACGTTGATGTTCAAATTCTGTGCATGCTTCATGATTGTGCAGAGGATAGTTCGCTGGGTG
TTGCAGAGACACTGGATTTGATCGAGATCACTTTTGGCACCGAAATGCGTCAACTCGTGG
ATAATCTGACGAAGCGTGATGGTGAAAAATATCAGGAATATCTTGATCGAATCAAATTGA
ACGTCAGATCCGCGAAAGTGAAGTGCTTTGATCTTAGACACAATTCTGATCTGCGCCGAC
TGAAATGTATTACAGTCAAAGATCAAGAACGTACCTTGAAATACCAAGTCGCTTATGCCA
AACTGAATATTTGGATTCAAGAGCTAAAGGAAGAAGAAAATGCGTAATATTTCTGAGCTT
TCGTCACCCGAAAAGGTACAAGTGCATTTCATGTTTCGTGAAATGAAGCATGGATTCGTA
CAAATCGCCAAAATGTATGACATTCGCGCGACAAATGTCATCGATGTCGTCAAAGAAGTT
GATGACATGCGCGACAATCATCTGGAAGCTCTTACCCGAGAGCCGGTAGTGTATCGGAAG
CACTTTGTGAATCCCAAACGGCATCAAAATGATGGTCGCTTCAAAGCTCGGAAGCGCGCT
AAAGGCGAAACTTTTGAGACTTTGCCATCAACATTGACTCCCGAGAAATCGCTTGAGATT
TCTCAAGCCTCGGCCAACATGCCCGTCAGGCCAACAAGAGAAGAGCTTGAAGCTTTGTTG
AACAAATTCAACACAAACTGATGTACATTCTCCGCGATTTTGGTTAAAACTCTCCCATAA
TGAAAAACCGAAATCACGGAGTATTAAAATGTCATCTAGAATGGATATGCTTATACGCTA
CAATGCCATTGCTTCGGCCTCAGTCAAAGTTCCTGAACCTGTCAAAAAGCATCATTTTGA
AATTCTTCGAGAGAAGCGCAGCACCGAAATTGAAGAAAAAATTCAAGCTGAGCGAGATGA
AGTTGCTCGTCAAATTGAATACATGGCTTTATTCAGCGCAAATAAGCGTTACTTGCAACC
GTATCAAGTTAAAGTAAAAAGCTGCGAAGAGCGCGGCTTAGAGTGGCGTATAACTTTTCA
ACAATTTGTTGAATTGATGAAAGTCGAAAAATGTGCATACACTGGTACTGTTTTCCCGAA
AGACCATGGCCGTTCTATTGAGCGCATAAACCCCAAAGTCGGATACACGATGGAAAACGT
GGTAATCGTCACTGCTCGTGCAAACAGCCAAAAGGCTCATCTTGACCAATTTGTTCACGA
AGAGCATATCCCCGCTGATATGAAAGTCAAACTTTTTGCGCAAAGCAATTTATCAATTGG
AGAAAAATACAAAATGAATTGGATTAAACTTTTTGAAAATGCTATAGTACATGACGAGTA
TATGGTATTTTCCGAGAACTGGACAAATCAAAGTGATTTTGCTGCCAAAATCATCGAATT
GTTCAATGAAAATACTAAACATGGTGGATCCGCTCCAACAGTTGTGCCAATGGGAAATTG
GCCATCTAAGAGCTATCTTAGCAACATGCCTCGTTTGTTTATGTTGAAACGCTATGGATG
GGAACTGGCGCACGATTATCGTCAATGGTGCAAAGATAATCCTGGCAAAATTTGGACTGT
CGAAGTATTCGGCAGCCCTGAAGCGAAATATCCTACATCATTTATGATGAAATACGAAAA
AGGATTTAAATTCAAATTGTTTTGATAAAAGAAAAGCCCCGACGAGTCGGGGCTTTGTTG
TATTTGCAATTTGTAAATTGATCCGATTACACCGGAATGATTCTGGGTAATTTCACACCT
AACATATATGACATATCAGATTGACCTACCAAGTTATCCCAATCATCACCTGTTACATTA
CGTCCACCGGATTGACCCATACGTGTAGTGTACTGGTTAATGGAGAAACCATACCGAAGC

ATAATGCCAAAAGATGGTTGCAGTGATGTTGAGTCATTGATAATTTTATAAGCACCCAAA
TCATCTGCTTCAATATACGGTGAATAGAACAACGAACCTACGTGTTCAATATCATTGACG
TAATGCTTACAGCCAGTGATCAGATAGTCAAATGACGATACCGTATCAACATACACTTTC
AGTCCGTTGCGCAGAGTTCCTGCTGAAAGAGGATCTTCGTTCGCATACATTCATCCGGAT
GCTTCAAGCATAGCAGAAACGCGAGTTGTACAGAGAACATAGGTCGCTTCATATGATGTG
TTTCTCAGCATCTAATTTTTCAGATCGCATGCTTGTTGATACAGAGTTCTTCCAATAGCC
GGGGCGTCAGTGCTAGCGGTTAAATCCAGAATCCCATTTGGCGAAATATATTCTACCTTA
TAGCGGCGCGAAACAGTTATAAGAGTCTGAATAATGTCTTTGTTAATATCATCAGATATC
ATAGTTGACAGGGCATCATCGACTACGCCAACACCATCCATTTTATTTGCTTCCAAATCC
TACATCAACTCAACACTTATATTTGTCTTAAGTTTGCGTGTGCGAACTGGAACTGTCCAC
CGATCAAACTTGAAATCAGTTTCGACAAATGATGATGCACCTTCAAATTCTTCTGCTAAT
GCTGCTTCAGACTTGATTCTCAGGTTGCCGAGCATTACAGCATCACCGATTGCATCGGCA
ATGTTTCCAGTTGACGATGTCAAAGTCAGTGGGTTTGTGGCAGTGTAAGCCACATCATTT
GAATCTTTGAAAACATACCCCTTAGCCATGGGAGTGTCTAGTTGCGCAAGAGGAATCGAA
TCCCGATATCCGACTGACCCCATGTAAGTTGCAGCGCTGGAGAATGATTGATTACCGGCA
GGGTTCATATATTTCACACCGTACAGTGTGGAGGTTGGCATGTTAGTTTCTTGAATTGCT
ACCATATCGGTAAAAATCAAGTTGTTTGCGGCCCGGGTCAAGCCGACAAGAGCAGGACGG
CTCGGACTATTTGCCCCGGTAGTTGTTGCTTCAGAAATCAGATCGTTAATGCTGCTCATA
TTTTTCTCTCATTGTTCTGCGATTATACGAACTCGTGTAATCGTGAAGTTTGACGTAGAT
GAAACCAATTCCAAAGCACCACCATTCGTGGCAAAATTTCCGTTTTTATCAACACTTATG
AATGAGTTGCAGTTTATTGTCTGCGAAGCCTGCCCAGATATTCTGACGACAGTCATTGTA
TCAATATTTACGCCTGCATTAACGGTGAGTTCCAACGAGTTTGCAGGTTCTGCATCACCA
AATGAGCCAGTGATTGATATTTTATATTTAAGTGACCTGACGCGCCATTTGCAGCTATTT
GGCATGTCACTGATTCCATTTTGGAAGATTCATGCGTAGAGGACATAACTGGAATATTTG
ATTTTATTAGCAGCATATATTTTTCCTTATGCAAAAAAGGGTGCCCGAAGCCTCCCTTAA
CCATTCCTTGGTTGTGGAACCAAGTCCTTTTGAATTACAGACCTTTAACCCATACACGGC
GGAAGTAGGCATTCTTACCAACGCTGTGAGCACCAGGCATACCATTGCTGATTCGACTGG
TAGGAGATTGGGTGCGGGATTCAGCGAACGGGTTAACACCGATGGCGTAACGAGTCTTGA
AGCCCATGACCGGTTGGAAGTTCTTCGGATCAGAACCACGCAGCGGGGTCAGTGCAAAGT
ATGGGCAGTAGTAGATACCGGCATCCAGGTAGTTGGCACCGCGGTAACCCATGGTGAAGT
AATCTTGACGGGCGTATTGGTCTGTGTAGAACTTGTAACGACCACCCAGAACACCAGCGA
ACACACCATTGGTAGTATCACCGTTCAGGCCAGAAGCCATACCTTGAGCAGCTCGGGTGA
TACCAGTATCAACCAGAGCCAGGGCAGAAACTACGTTACGAGAAGCGATGATGAAGTTAC
CTGCACCACGACCAGTTTGACGAGCAATTTCGTTAGCTTCCTTGTCGATTTGGATCAGCA
GAGCTTTGTAGCTTTCACCAGCCCAACGAGCACCACGGACATCGATCGGGTCTTGGAAGT
CAAACACACCGGCAGCAGAACCTACAGTTTGAGTCCAGCCAGATTTACCAACTTGAGCGG
TGTAGTTAATCTAATCGATAACTTCACGGTTGATTTCCAGCATAACTTCGTTAGCCAGGA
TTCCGTTCAGCTCGCTGTCAGCATCCATACCATGAACAGCGCGCAGGTCTTGGGCCAGTT
CGATAGAGTATTGAGCTCTCAGTTGACGAGATTTCGCTTCGACGGTTTGCTTGTCGATAC
GGACGCTCATTTCGTTCCAAGGGTTATCGGTGGTTCCGTTAAATCCTTGACGAAGTTCGG
CGATAGAGGTAGCCATACCTTCAGCAATTTCAGCCAGTTCGGCAGCAGCAATCTTGGCAG
TAACCAGGGCATCCAGAGCAGCACCAGTTTCGTTGGTACCGACAGTCACGCTTCCGCCAG
TCACCTTCTGCAGATAAGCTGAACCAGACTCGTAGAAGAAGTGATAAACGATATCACCGG
TAGCAACAGCTTGACCAGCAGTCAGCGTAGCAAACGGAGTGCCATCGTCTGCACTGCTAG
CACCCTTGGCACCCAGACCTGAATGCCAAGCATCAGGAGCATACATCGGGTGGAAAGCCT
CCTTACCACCAGAAGCCAGAGCGTCTTTGCCGTAAACAGAACGCAGGGCAAATACTTGGC
CAGTAGGACCAGTCATCGGCTAAACACCAGCGATATCGAAGGCGATCAGGGATGGAATTG
CGCGTCGTACCATGCCGATAACAGCAGGGCCGATGTTTGTAATTGCTCCTGAACTGTGGC
CAGCAGCAATATTGGTAGGATAGTAACCGTGATCACCAGCAACTTCGGCTTCCATCAGAG
ACTGACCGAATGCTTCAATCAGTTTGTCATCGCGATACACCGGATCGGTTTTGGAATCTT
TTTCCTGAGCTTCGAGAACTTGGGCTACAAGAGCTTGCTTGTTTTTACCAGCAATTTCAG
GCAGACCTTCACCTTCCAGCAGCGGAGTCCATTTGTTCAGAATTTCTTTGGTTTTCAGAG
ATATTGTTATAACCTTTATTTAATTAAGAAGACATACGGGAAGCAGCGGCCAGATAGGCG
GCCCTGGAATTATTTGCCTGCTTGTTATCATTTTGCTCAACAATCACTTCGGGTTCGAAG
TTTACCGCAGCTGCAGATTCATCAATATTTAATGCTTGTTCCTGGATCGGTTTTTGTTCC
TTTCGACCAGAATTAGCAACCATTTCGACAATCGCAGACAGTTTTGGTGATTAACTATCA
CCATATTCCAGGCCTTCAACAAGACTCAGAACTTTTTCTTTCTGTGACTCGGTCAAACCT

GCAGTAGCTTCTGAAATAGCTTTTTCTTTCTTCAGTGAAGAAATTTGCTCTTCCAGAGCC
AAATTTTATCAACAGCGATTTAATTTCAGCTGTAGATTCATCCAGCTCAGCATTCAGTTC
GTCAATAACATATACTTGCTCTTCTGGTACAGAGACGTTATTTTCAATGAACAGTTCTGT
GAGACCGGTTGCAAAGGATTCGAACAGCTCAGACTTGATGTTTGTCTTCACTTGCAGCGC
ATTTTCTTTCAGCTATTCTTTGCCCAGATGAGTTAAAAATTTATCAGCATCTTCGTAAAG
TTTTGTTTCAACTTCTTCACGGGCTGCTTCAATTCCTTCGGCCAGTTGAGTTTCGGCTGA
AGAAGCAATATCGGCAATGTGAGATTCAGCAAGCGGGATTACAGCCGATCGTACAGCTTG
CTCAAACGCAGTACTGACATTTTCTTTTACGGCTTCGTCCAGCTGAACAGACTCGAAAAT
ACTGTCCAGTTCAACGGCTACCGCGCCCAGTTTCTCGCTGGATTCAGCCAGCAGTTTATC
TTTAAGGGACATTTGGTTTTTCCTTTAGTTCAACATTGAAGCCTATGTTTTTAGCCGATT
GGCTGAACGCTTCATTAAGTGTACTTACATTGTTATTTATAGCATCTGTATGAGCTTCAG
ATTCTTGTTCTTTGCTCTCTCTTAGTGGTTTAACGTACGCTTGTGGAGCAGATGGCCCCC
AAACAACATCAACCGCTACAGTGAGGCGATAACCCTCCTGAACAAAGCAACCTTTAACTT
GCCCGAGACCACGTACTGATACTCCCGGAATCCAACCAGCTCGTATATTGGCGGCAAGTT
TATCACCTGGACCAAAATCACCTTCAATTACCCTGGCAGGACCCATTACATTATCGCCTT
CCCACCATAGTTTTTCTACTATGATTGAAGACAAATTGGGGGCAGGCATCGGCCTTGCAG
GATGATTAGTTTCGCCGAGTGATTGTTTAGAATTTATTTGGGTAGCAATATAATCGTCGA
CAGCTTTGGCAAGAACAGCTTTAGGATAAATTCTCCCGTTTCTATTCTTAACGTTAGCTT
GCATAAAGATGCCTTCAATATACAAAGCACCTTTACCATCATCCACTGATTCGACTACAA
GTTCAGAACAGGTGCCGCCGGGAAGTCCGCATACTACAATTAGCAGCTCGTCTTCCATCT
TATAACCCCAAAGCTTTACGGCGTTTCAAGGCACGTTTGTTTTGCGCTGCGCCTTTATCC
CAATGATTGGTTGAGAGCGCTTAGTTTTTCGCCGCCTTACGAGCAATTTGTCTACAAGTA
GCAGCAGTTAGCCCAGTAGTTTTAAATGCATTGCGCTTCCGTGTTTCTCGATCTTTGGTA
CGAGTTACTTCACCTTTCGGATTAACATGTTTGACAATTGCTTCTGAAATGGAAATATTT
TCAACTGCAGCAATCATTGCCAATGACATCTCTGTTTCGTCAAGTGAATGTTTGGAAATA
ACACCGATAATTTCATCGGTGCTAAATCCGGCTTCTACGAAGGATTTAACACGAATCACT
GCTTGCGCAAACTGAGATTCAATGACTTGTTGATTTGCTGAATAGCCGGATGGAATAAGC
ATTTATTCCTCCTCTTCATCTTCATCATCTTCGTCGTCATTCTCATCATCTTCGTCGTCC
TCGGGTTTTTCACCTTCGGTAACAACTGCGCGAGCAATGGCCTGTTTTTCTTCGGCGTAT
ACTGACTCTTTCACAAGTGCCATTTTCGCATTAAATTCACGTTTTGCTTCATTCAAATCA
CCTGATTTGATAGCATCGATCAATTTTTCCATTTAGAAATCATCCTCGTTTTCAGGGTTC
TGGAATCGTTTCACATTGGCCTCTTTTTCTATTTGCTTTTCCTCTTGAGCAATTTGCTCA
TCAGTCATCTGCAAAATGTTTTTCATGACATAATCATGCGAGACATATTTACCTACATAC
GGCTCGATTTGCGAAAGAGCGTCAACTCGTTGTTGAAGAGTTTCAATATCTTTCAATTCG
GTATAATAGGAATCTTGATGGAAATTAACCTTTATGTTATTTATTTGCTCTTCCCATTCG
CTTTCTAACATGATTTTCTTGTAAATCAAATTGGTTTTGAGCGGGTCAAGGAAAATTTCT
TCAAAATTGTGTTGAAGCTGAACAATAAATTTTCTGAAATCTAATTCATCGCGGGTGATA
GCCATATCTTGGCCACCAATCACCATTCCGCCATCGTCGCGAGGCATCCGGGACAAAGGA
ATTCTCAGAGCTTCACAGAGCTTTTTATTGAACCAGCGAACATGATCCATTTCACCCATT
GTTTGGGCGCCTGGAAGAGAAGTAACTTCGGTAACAGATTTACCATCTCTCCGCATCAAC
CAATAATCTTCAGTCATTGAAAGATTGCGTTTTTGATTTTTCACAGTACCTGTATTCGAA
TCGTATACAACACGATTTTTCAGGCTCTGCATGATGCCATTCACGTATTCAGTTGCTTTG
CGATTAGGCATATTTCCTACGTCAATGTAGAAAACACGTCTTTCCGGAGCACGCGTAATA
CGATATATTACCAAAGCATCTTCTAAGAGTTTTAATTGGTTTGCAGGCTTAACAGCATTA
TGCAAATAACCGACAATCCCACGATCAGAACAATCCTGAAGGCCTGAATGCGCATATACA
ATGGCACTTCTCGGAATTTTAATGCGTGTGTTCGGTTCAGACAGTCTGCCGTTATAAGCA
TAACCTTCGCTGCCGGCGGTATAAACAAAAAATTCGCGATAGCCTTTTACTATAGAAGTA
CCACCGACGTCAGCCGTAACGATTTCACGATAATATTCGACATGACGTGGGTCTAACCTG
CGTAATTCAACAATGCCTTCTTTAGGATTAGGCATTATTTTATGCAAGAATATGCGGGAG
TCAACATACCATCTGCAGAACAACGTGTCGGGTTTGCGAGATACATTTAGCAATCTGCAA
ATTTCATCAAACTCTTCTAAAATTTTGTCTTTGATAGATGAACTAAATTCAGTATCATCC
AAATCAAGCGATACTACTTTCTGACCCTTTTCATAAACAACAGCTTCATTTCCGATATTT
GCTACAGCTCGCTCAACTTCAGGATTATTCATGAGTTGGCGATATGTGTTTATGAGGTTC
TTGGTACCGGAAATGTTGTTATCGATATCAAAAAATTGTTGCATTAGAGCGTTATATGAT
GATTCTCCGTCGCGAGCTTCAATCTCGGTGGCACCATCATCTTCTTTAGGAGCCGCAATC
GATTCGTGGCCTTGTCTGAGCCGCTCGTCATATTCATTTTGATCGACACGAGCCCAAAAT
TTAAACAAATCTAAAAACTTCATACAAACTCCTAATCAGGGAGCATTACTGCTGCCTGTA

TTTATTATTCCCACCAGTCGAGTGCAAACGTAGTTTCAAATGTAGAAACTGTGTTGTTAT
CATCCCAGTCGAGAGTAACTTCACCAACGTTAGTTGGGAAAAGACCGACAATGGTATATT
CCTTAGTTGTATTTCCAGTACGATCTTTTTGACGAACAATGCCAGGCCTTTTATATGCTT
CTAGAATATCGCCAGTGATTTCATTACCCATTCCATGAGTCAAGGCATTCCAATCGACAA
TTGCCTGTCGAGTGTTATGGGCCGAATCGTTATAAATCGTAATCGTCCAGTCGTCAAAAA
TACGGTCACCTGCAACATTAAGTTTGCGGTTCATGTATCCAACCGGGATTTTTTCAACAG
TGGCTGCCGGCATTGTACCTGCTTTAGCTTTAAATTTGGAATTTTGGCCCAGGTATGGGA
TTTCAATTTCAAACAAGTTAGGGCGGGCGAAATCACCGGACTCAAAAGCCCTGAGGAGAT
CAGTTACGTCGATTATCAACCTCTATGTTTATTTATGCGCCCATATTTGGGCCCAACGCT
GAATTATGCTAATTGGGCATGGCCAATTAACTCGTCAAAGTTAGCACCAGTTGATGTCGC
TACAAAGTTCAGAGTGATGTAGTTAATTGAACGAGGAGCTTTGATATAAATCGTAGCTAC
AAATTTATTACTGTCGATAACTTGCGGAGTGTTGTGTGTTGTATCACAGACAACGCGGAA
GTCATAAACACCACCCAGTGACCTGATGTTGTCGAGATATTGCGAAACTTCCATACGGAA
CTCTGACGAGTAAATGCATCGTTATTTCAAACAATTCGTATTTAGAAGTATCACCAATAT
TTTTCTTCAACATGTTGAAGAGGCGACGAACGTTAATTCGGTCAAATGGAGATGGAACCT
GAGTTGCCATTTTGTCTCCAAAGAGAACAAATCCGTCACCGCCGGCAAAACCTGTAACCG
GGTTAATTGACACTTGTTACATGGTATCACGCAGCGATTGCTTAGGTTCAATTGCCAGTT
TGATGGTGTTGCGAAGTTGGCCGCGACGATATCCAGCAGGGGACATCCATGGATGTCCTA
CTTGATCGGTGTAGGCACACAATCCGGCAATATCGGCAGAAAGAGGAACCCGCCGGTTAA
TATCATTGTATTTGTCATACTGATATTTATAGTTGCCTGAAATAAAAGCATATGTTGAGC
TAATATTCATGTTATTGTCGACAACTTCACCACCAAATGTCACACCATTTCGCCATTCGA
CAATATTCTTAACCGCTTGAGTGGTAGGAACACCAACAAGTAATTCTGACGGAGGATTGA
CGCATGCTACACAGTCTTGGCGGTCATCTGCGAGAGCAACGACATGTTTCTGGACAGTAG
ATGCAACAGCAGCACCGTCAGATGCACATGCTCCAGCAATCATCAGGTTGAAATGCAGAG
CTTCTCTGTCGGCGAACAGATCCCAACCAGCAATCAATTCATCTGAACCAACAGCATCAT
TTGCCGAAGCGCCTCCACCGAGCCGGATGAGACCGGCAAAGCCAGCATGCCAATTTACAC
TAGAAGCATAAATGAAATTACTGGAACCATTACGGAAATAGTCGTCCATGAAATGTATTT
CCATAAACATCTCGGTCTCCGCGACGAGTGGACAACACAGTAGATTCGACTACGATGCCG
TCTCGACGAACAATAATAGCAAATTGGTCATGAGTCATCGGGCCGTATTGAATGACGCTT
GCGCTTTGCTAGTGCGAGTACCACCAAACAGGTAAATTGGCTGGGCTGCACCACTTTGGA
ATGCGGCCTTAGAAATTATTTCAACTTCGACAGTTGAACCGATTTCCCCGGGATAGACGG
CTGAAATCAGAGGCATTCCATATTTGGCAAAATTAGCCAGCACTTTGGTAGAAGTCATAA
CATCTGGAGCTTCTTCCGAATTACCAAAAGTAACTCCAGAATCAACTACGATATTACCCA
AGGTTAAAGCAGCAGCAGAACCGCCAGATGCGCCCGATACTTCTGCGCGCCAGTTATTTC
CTAAGACAGGATATGTTCCCAGCTGTTTGGCTTTTGCAATTACTGCAGCTGAAGGAACAA
GCAAAGCCTTAATTTTACCGTCAGCCACAACTTTAGTTACTTTTCCTTCTTCTGTAACAG
TTGTGGTGTTATGCTTAATAGAAACGGTATCACCTACGGTATAACCTTGTCCTTCGGTAG
TAATTGTTACTTCGATTTGGTTAAACAATGGAGATGCGTTTTTAGCTTGCTCCATGTCAA
TTACGCGCACGAGACGAAGATCATTACCATACTGCAAGAAGTTCATAGCACTCATGAAAT
ACGGAGCAGTGTCATTGTCTGGGCTACCAAACATATCAACCAATTCGACTCCATTTGTTA
CTTGACGAATTTCATAGGCAGGACCCCATGCGAATTTGCCGACTAATGCGGCACGGCCAA
GAGCTGACCGAACGACAGTTGAATTGATGGAAGTTTCCTTCATTTCAATGCCGGGGCTCA
GAAGTTCCATATTTTGGTTCCTTGGAAGTTAGTTTAACACATGTTTATTTATTGCTTTAT
ATTTACACGAACGACATACCATTACTGGCAACTTCGAAAGTTTCTTCTCCGCTGTCAACT
ATAACGACAGTCGTATAGTCGTCATATAAATTTTCCATTTCCTGACGAAATATTTCATTT
GCCAATCGCATGTCATCTCTCTCGGTAAAATCAGAAAATCTCGGTTGGGTAGTAAGCCAA
GCAAATATTACCAATGATATCACCAAGTCATCATGGAAACCGTCTTCGGCTGCGCATGAA
ACACCTTTTTCTGAAAATGTTCTCAGTTCCATTATCGTGCTTTTATGGTAAAGCACTAAT
TTTTCTTTTTCGATTAAATCCTTGAGAGTAGAACAACCTAGGGCTTTTGACCGTTTAGTT
TGCTTCATCCCGAGGTCGTTGTACGAGTCACATATAATGTTTTCGTATTCCAGTCCGGAA
TACAACGACTTAGCTATGGATACACCCGTTGCATTCAGCTCAATATAGATGTATGGCTGA
TAATACATATTTAAGTACTTGAGCAACACATCAGGGAGTATCAGGTGTGACGTGGTGTTG
CTGTGATATACTGCGACCTGTTCGTACGGGAATTCTGTTATGTCGTATATGTGCATAGCA
TGAGAATCTTGCCCACGACCATCAGCAGAGTCTAGTGTTGCGATATATTTATGACCTTCA
ATTGGTTCCTTAAACATCGTGAAATTGTCTGATGCTGGAACTTCTTTCCACGTCATTTTT
GACAGTTTGAACCCGTTGATCAACGTTCCAGATGTTCCCTGGAAAGCAGTATTAAGCTCC
TGTTGAAATGCCTCAAGGCTAGAACCGGCGATTGTCTTGGCTGACCAAGAATATCCGTCG

TTAAAATATGAATCGATGCCATCTGTTTTAGAACCATCCGAATACATGCGCTCTTTTACG
GACGACCATGTTGCGGTATACGGAACAAAACCAGATTTGCTTCCACTTCCATCCGAATTA
GGGGTAATTGCAGCAGTCCAGATATCGTACCAATGATTAAGCCCATTCGGCGTTGTTGTC
ATCAAAATCTTCGAATGACGACCTGATGATATAACTGGCTGGATTGCTAACCACGCGTCG
TTGAAGTTGGGAATAAAAGCAACTTCGTCGATGTAAATCAGCGCAAACGAGTTACTCCGC
ACAGCATCAGGGGAACCGGAAAATGCACCAATCGCACAACCATTTCCAAAAGTGATAGAC
CCTTTATTCCATTCAACTATACCAGGCTGCAGAAAATCTGGCAATAGCTCCAATGCCTGC
TTTGTTCTATGAAGAACTTCCGCTGACATGGATGCTTTATGCACAAGGATACCAACGTTT
TTAGCCGAATTGAAGCAAACAAAATGTGCCAAGAAAATAGCTACTACTGTCGTTTTTCCG
AGCTGACGAGACAAATTTGCTGCCATCAGACGATTGCCAGCCATTATTCTAAGCATATCT
TTTTGGTAGTCACGCAATTGGACCCGGATAATGCCATAGTCAATATGCTCAATGCAGCAA
TAGTTTTCGGCGAAATAAACGATATCTTCTTTGCATCTCTTTCGCTCGGCTAACATCTCC
TTTGTACATTTTATTTTAATGTTAGCACGCTTTAGATTCGGCAAAACATTATATCTAGAA
CGCTTGTTTGTTTTATCTTTAAACGTTCTAAACATTGACGGGTCGTCTGCTTGGATTTTT
ACCCTCTTTACTCCCTGTGATTTCAGATATCTATCAAATGTCGAGGGATACCACTTATCA
TCGTGATTTGATAAAATCCATTCCCCATCTTCGCGCATTTCTCTCTTTAACGTGGATGGA
TAGGTCATTTGCAATGGGTAATCACTTTCGAAAATATTCATACTTCATCACCCAATTCCA
GTTCAAGCAATTCATCAGGAGAACCTTCGAATTCTATAATTTCTCCGCTTGCATCATTTT
GAACTGTGCCGAGTATGGATGGTTCTGACGATGTTAGTTCTTTCATTTCCTTGTGCATAT
TAATGATTTCTTTATTGGTTGAAGTCATTTGACCCATTAGCTGAGAAAATACTTCAACCA
TACGTGGACTATCGGCATTTTTGGCATTTTCCAATGCTATCTTTGCCATGTCCATCATCA
TTTGAGTTTGAAAATGCATGTTGCGTCTAACAAGCGTATAGTCATCTTCCTGGTCAATTT
TTCTATTGTTTGGATTTGATGTCGGTTCTTGTAAGACCAACGGGGCGTATACCTGCACGT
CTTCCCCGTTGAGTCCTTCGATACCAGACAAATCCTTGAGACTAGTGAAGTCTAAAAGAT
CATTTATAGAGTATTTCCTATTGTGGTCGAGGCGGTGTATTCTGATCGGTATTGTTTGTC
CATACTTCCATCGAACTCCCGTCCCAATCTTGAGCTGGCACTGTTTTTGGATCAACTTGG
TGATCAACTGATTCAAAATGAGTATCTTTGTTTATTTCTCTTTCATTGCCAAAGAAATCA
AGATAAATCGTTCTAATTTCACCTGCAATATTTTTGGTGTTTGGATATAACCAACCAGTG
AAACTAAACATGATTGACCATTCAATACGGCGTCGTTGTTCGCCAGGGCCATCTAGCTGC
TCATCAGGCGTTATGGATGTGATAGTTATATTGATAACCCGCCCTTTAATAATTATTTCG
TTGTCATACAGCTCGGTTATTGTGCATGAAAAATGTGGTCGAAAGTATGGTAAAATTTGT
TCAACTATTTGAAATGCATCATCTTCATGTCGGGTCCATATTCCTAGTTCAAACATGAAT
GTGTATGACACAGGGTTAGATTGCGCCAACGCTTTCCCATTGGGTTTATTTATCCATTTA
TTTTGAGTATTTCCAGTTTTTACCATTGAGTTATAATGCATATCGATCAAATGCAAATTC
ATTCTAGGAAGAATTGCTTCGACTTTTGCAATGTTGAATTCGGAAGTTGGCATCGTAATA
TCATCAATAGACATTACCAATTTTTCTTTACTTGCGTAGGTGATAGGAACATCTTGAAAA
TAATGCTGGCCATCTCGAAAACGAGAAACTTGAATATGACCAAAAAGTTCCATTAGCATC
AATACATATCGGTGTATTGAGCTGTTATAAAAATGATTGCCAAACATTACTGTCCCTCAT
GCCGCCCAAATGGACTATTAAAGCTGTTCACTGGGTTTGTACCAATTGGATCAAATGGCT
CAACAAATGGTCTAGCTTCATATTGCCCCTGAACACTCTCACCAAACTGAGCGATTTTTT
GATCAATTAAAACCATCTAAATTCAATATTGGCTCCAGGTCTAAACCATTATACGGATCT
TCAATTTCAGGTTTTTCCTGGAACTGAGGAGCCAATTTTTTCACCAGAGTATATGAATTT
CTGGGCAACAATTACCCTGATTGGATTTTTTCCCATCTGATAAAACGGCGTGTAAGGTTC
TACCCATGTTATCTAAAACAGGCTGTTATCCATTGGCAAATAAATCAGATCACCTAGTTT
AGGTTCTTTTCCATTTACTTGATGCTTGAAAAGGCCTGGGTTGATATAAATTCTGACTTC
GTCATTTTGTTGATGGCCAAATTTAGAAAAGAAACTCTGTTGTCCCTCATTTGACTCGAA
GCTATTGAGCCATGCCGCAAATTTCCATGCTTTTTCAAATCTGCTATTTGGGTCTTCTCC
AAACACAACGTCCAAATTTGTATATTCTCTTTCGAGATAATAAAATTCCAAGCCTCTCAT
TTGAATAGATTCTGCAACTAGAGTGTCGTGGAGGGCCTGAGAGCACTCATACCGATTGAA
TTTGACATACGGGTTAAGAATATTTTGCTCATTCATTTGTTGATATCCGCTTCTATATTC
TTAAGTAGCGAATAGTGACTTATCCATCATAATTTACCCCATGATAATAGGAGGAGGCCC
ATCCAGCAATTCGAGTTCTTGTCTAAGCTTATCGATATCTATTCTAGCTTCTTCAATTAG
GCGTATTCCATCGAGTGTTACACCACCTGGAAGCTGCATACCTCGATGTTTCGCTAAAAC
TTGGCCGTTTAGTTCTTTCACTTTTTGCTTTTGCCATGTCTTTTACCCAGCGATTATTGT
ATGACTTATCTTTTGTCATACCATTGGGTTGGCCTGCATAATTTTGGCCATGTGACATAT
TAGGATATGCGTATTTTTGCGCCACTGACGGCACACTATCGTCATCTGACATGGCAAACC
CATATCCAGCCGAAGTGCCTGAAAGCATTGAGTCAACATTCGCATACGATTTGCAGTAAA

CCTCAAATACCAGCAAATCTCCTTTTTGCAATTTGCCAGCAACTCTCATCATTTCGGTAG
TACTGTTGAAAAAGAAATCAGGCAAAGAAGAAAGCATGTCTCCCATCATGTCTCGATATG
ACATCAATTGAGTGAAATATCCGAGATCTGCGCCAAAAGCATTAGGCCCATACCATCGGC
ATTGGCCGCCAATTGAAGCTCCAGTCATACCCATAACAAAAACAGTAAACCACGGATAAA
CAGCCGTGCCGTCCATGGTTATAATTGACCCAATATTGGTTCGAATTACCTGAGTAACTG
CTAAATCATTGCCACCTGCAAAACTAATTACACCAGTTTTAGCTTGTTCTTCGGTTAATA
CTAATGTTGCATATGTTTTGTGAAATCCATCGTAGTGATATTCATCGTACAATTCAAGAG
CTCTAGATATGCAATCAAATACTTGTTGCTCTGTTACTTCGACGTGCACGATAGGTGCAC
CTAGACGTCTAAGTATAGCGTCCTTTAATTCTAACGGGTTATTAGTGGTATATGACATAA
GTGTCTCCTTTATGCTTTTTAAGCAAATTATGGCGGAGCCTTTCGAGCTAGCTCGAAAAT
GCTATCCTTTTAGTTCTAATATCTATTAAGCCATAAAGGATAAGTTGTATTTACAATAAA
GGCTCCCGAAGGAGCCTTGAGATTATGCTGGGACAATAACAGCAGACATTTTGCCGATAG
TTATCGATTTAGCTGAACTTGAGTCAAGTGCTTTGACATAAATTGCGATAGTATCATCCA
AAATTACATTGTACAGCCAGTTGTGTGCGTAGAGTCTACCGTTTGATCTCAGGGTAAATT
CTTGCAAATTGGTGTATGATGTAAGTCCGCCGCTTGTATGAGCTATTGAGATATCCAGAT
CATCTGTTTCTAACAAACCAGATGTTTCAATTTCGATGCTTGCATTAACTAAACCGCCAT
CAGTGAATTTGATTTGTCCGGATTCAAATATGCAACCGTTTGCTACGGTCTGATCAAAAT
CAACAAATGGAATAATCTTCGACGTTTCAGCTGTAGTTATTTGAAAATCTTTCTTTTCAA
CAGCAACCATGATGTTAGTTGCTTTAACCCAAACACCGTTTTCGCAATACCAGCGTCCTA
CTGCAAGTGGCTTATCTAACTTAAGTTGAAGAGTTTCGCTGCTTTCTTTGACAGTTTTCT
TAATACCATCTCTTTCAAGTTGTGTAATTCCCGCCGCATTTCCATACAAGTCGGTTGACA
TCAGAATGTTTGCAGAAACAAGACCTGAATTTTCATCACCCACACGTGAAATAACGTCAT
TCAACGATAAAGACATTTGGTTGTGATTTGTCTGTAAAATGTACAGTCGTCCATTTATAG
GTGATGGTTGTCCGATAGAGCCAATAGCGGCATTGAGATCTGAAATATCCCCCTGAATTC
CACTGGAACTGTTTGCCCCTAAAATCTGCGTAATTGTTGCAATATTAGAGGTATTTCGAT
TGATGTCGTGCAAAATTGTATTTTGCGTATTAACATCACCGATTTTCTGCTCTACCGCTC
CAAGACGAGATAACGCACCTGTCACAGGGGTATTCATCGTGTTCGCGATAGAACTCAACG
CACTTTCAATCAACTCCACCCTTGACGCTATCGACACAGGGTTGGAAAAATCGATTTTTA
TCTTGATTTGATCAACGTCATCATTACCACCACCTAAAGTATTTTCGATAGCGGTCAATC
TGGTATCAATCGGAATTCCTGTACCAAGCTTAGTATTGCCGACTTCCATCCTCAAATCAT
ATAAATCAGACGTCAATTGACCTACATCTGACTGCGCCCATGAATTTTCAAGCGCGGGTA
TGCGCTGGGTGTTCAGAGACACGGTTTGGTTTACTTGACTAAACCGATACTTTAATCCCG
AACCTATCGAAGAAGGAACAGAATTACCGTTTATGTCGAAGCCTGGATATGCACCTAATT
CGGTTTTTACCCATTACAAATCACCATATACAGATCGAGTTGTGTCGTCTTTGAGATATC
GATGGCCAAGCTTTTCCGAAGTTGATTCTGAAAGAGAAAGAACACCTGCTAAATTAGTTT
CAACCTCGCTAATATCTTGCTTGATAGCTAAAATATCATTAGAATTAGCATTGATTTGGT
CAATTACCGTATTATCACCGATTACACCCAAGTTATCTTGGATAATATTTACCGCATCGA
CAATCTCATTGATTTTTAGGAATGACTGGGCTTGGTCTTCGTCTAGCTGAACAACGTTCT
GTTGAATATTAAGTGGAGCCCTGTTGTAAACACCGTCGCTGGCTGTTTGGGTGGAAGCGG
CCGAAAGCCGCTCTCCGTTTTTAATCCAACCAATTCGAAGTTGACCATCACCTGGAGTAG
TATCAACATAAGGAATAACTCCTTTCGTGATTTTGTCTATCATATTTTTTCCTCGAGTTT
TTCTAATCTGGATTTAAGCTCTTTATTAGCCTCAATCAGTAATGCAATGAGAGACATTGG
AGAAACAGTAAGAGTTCCGTCGTCAAGTGTTTTTACGCCTTCTGGTAAAACTTTTTGCAA
TTGCTGTGCAAAAATACCTGCCTCTTTGCCTATCACGATTTTGTCAGTCAAAGATTTATG
TTTTGTGTAAATCCCAGCGTCAAGGCTATCTATTTTTACGAGTGCATCTTTGATAGGCTG
AATATCAGTTTTTAACCTGGTATCGCAACGAACATACATATCGTTAATGTCGATGTTTCC
GGCTAGCCATAAATGACCACCTGGCCTAAGCGTAGCAATTGTGGTATCTACGTCATTGGT
GCGAGTTGTCCTTCTAAAATACTCGTTGTTATTGTCTCCAACAGCAAACAGCAAATACGA
ATTCGTGTCAACGTCACTATCGTTTTTGAATGTTATAGCAGCCAAATCGGTATTTCTATT
CCACAAAATTCCTTGATTGTTTTCTGTGAAATTGATATTACCAGTCATTGTGCCACCTGA
GCGATACAACACCTGGGCATTAGCAGAAAGAGCCGTATTCGCCTGTGAGCTTTCAGTATT
AAGAAGGCGAACTAATCCTTTTTGAGTGCTAGTAGCATTCAATATGGAAAGTTTAGCTGG
ATTAATAACGACAGTGTTATCAGCTAATGCTAATGTTTGCGAATTTGTTGCTACTTTAAG
CAATCCAGCTGAGCTTTCCGTCCCTGCCATTCTTGATAAAGTATATGGACTAACAGCATA
GCCATCTCTAAGAAGTCCTGCTAAAGCTTGAGCCAATGTGGCCAAACGGACAACTCCGTC
TGAGCTTTCTGTTGCAGTTGCCCATGATTCTGAGAAATGCGCAATTGCCTGCCTGGTCTT
AAGAGGAGTCATAGCAGTGGTATCGTCGGTGCCAGATATTGCCTGGGCAGCAGTCGATAT

file:///E|/Curro/Simulación/paper/submission/Additional%20file%204.txt[27/06/2014 19:27:32]

AAGGATTGTTCCTCTTGCCGCTTCGGTAGAACCTTTATTTGCAATGACATACCCCAGTTT
CGAAGGTGTTATTGCAGCGGTGTTAATAGATAAGGCCAAAGCTTCGTCATTTGTTGCTTG
AGGAGTATTACCAATAATGGCGTCGGTAGCCTGCGGCGTCTGCATCGCAATCCGCAGAAG
ATTAGGAGTAATAACAGTATTGTCACTCATGCCAGCTGCGGCTTCAGCTTCAGTAGCAAT
TCTAATGATGCCAGGTACAACCTCAGTTGCTATCGGCAGGCCATTAACAGTTAATGCCCA
TGGCGCTATTTCATTCAATGCGTCTGATACGGTTTCAACTGAACTGGGCCAATCTGGCCC
AGAGAAAAGTACCGAATCCGCTGAATCCGAAATATGATATTTGATATTGTTATTCATTAA
TCAATCCTCTTGAAGTAGTAAAGATCAAATGAACTGGTTACTGACCCACCAGTAAGAGTT
TTTGACTCTTTACCCATATATTCCCAGTTACCATATCCAGGCTTTCCAGGAACAGTAATT
TCACGTTGAACAACAATTCCCCAACTGCTAAAACTTTCGATATTCCCATGCTCAGCCGAA
TCAATGATTTGCACTTCTATGATGGGAGTTGTTGTGCCAGATTGTCTTTGAACAGAATAA
AATAAAATGCCGGCATCTCGCATTTCTTCAAATTTTGCCAAAACTATGTCGCATTCTGCA
TCAGCGTTAGTTCCAGCGGCAATAACCAAAGGTATACCGTAAACTGTAATGACTGATTTG
TTTAATTCAGAAGGAAGAACACTTCCGCTAAATGTAAGTATCTCAGCTTGACGAAGACCT
TCTGGGGAAACTTGAACATCTGAAGTATATATCGCGTTAATAGGCAACATTGTCAGTGTT
TGGAGTTCGTCGATAGCAGATTGCACGTTTGGATAGTTCACACCGTCAATAAATGAGTCT
ACCGTCGGCCCACCTATATTTCGGGCACTCATTACTTGGGTGCCCGGTTTGGCCAGATAC
TCGATATGAGTTGCTTTTCTAAAGATGTTGTGTGCACTGTCTAATGTTTTTGAAATCATT
TTCCCACCCTTACCCATTTGTATGCTGTAATTATCGGAGGAAGAATATTGATGTCTCCTG
GCGATGTATTTCCTTTATTTACAGTAAGAACTGATTCTACGTATTTTGTATAGCCAGGAC
CAGATTCATCAGGGTCAACTTGGCAGCCGCCAATGATGATATTTCCATCGTTATTTGCGA
CTAAAACTTTATTGCTTGAAACAAGCTCAGGCACATTTTGAGGAATTAGTTCAACAGATT
TTGAACCGTATGTTCCACCTGCGGTATGGCTTGCATTACCCTGATTGTCGAGGTCGTTAT
TGTATAAACCAAATTCGACATCAGCCTGATTCGATGTCCATCCTGCAACCAAACGCCCTT
CAGCATATCTTTTCCATACACCAAACCCCCTGTACTCACGTGGGTTGGCAGGGTTGTTGG
CATTCTCATATATTGTCCCAATCGGATAGAAAGCATCGAACAACTGTTGGATATTTTCAA
ATCTCCCGCTGAAAGTGCCTGGAATAATTTGTGACGTACGCTGGCCAGGATTGTCAAAAT
CGGTGTATTCGATTTGATTTTGGATTATTAATTCTTGCTCGGTGTTGAGGTAAACAGAAT
CAGCTTTTTCTTTTATACCATTTGTACCTTTCCAATCCAACAGAGTACCAATATCCTTAT
TGAACCATCTGATGGTGATCATGTCACCGGATTCAAATGTTCTTCCAAACTTAATATCGG
TAATATTGCCGTTGTCATCCAATACGATATAATAATCAATAGATGACGGAACCCAGTTTC
CACCATTATCCCAACACATTTGTTGAGTATCGCCATTTGCATTCTCGCAGAAGAACATCG
GCAAATCGCCCGCCCCTGCTCGATTAAGCATCGTGCCATTTAGAAGAACTTCAAGCGAAT
TTGGATTTATTCTATTGTTTGGCTGAATATCGAATTCGTCAATAGTGAAATCAGTTTTTC
TCGAAATATCTCCAACCCAGATTTCACCAGGCGATGACGTTTTTTGTGTTGTTGATGCAT
CAAAAATCTGAATATTCTTAAGGTTTTATGAACTCCGGTAAGAAGCGATTCCATCCATGT
AGGTGACACTTGTTACAGTATCACCTTTTTCGATGTCAATATCACGCATTCGAATACTTC
TTCCATCTAATGGCAGCACTGATGTCGAATTGGCTGGGTCGATAGATCCATAGTTCGAGT
TTTCGTTTAAAGTGTCGCCGTAATACAGCAAGTTACCGCGTAGATAGACTTCGAGGTTTG
CAATATTGTAATTTGGTGTCCCGAACACATTAGGAAAATCCGTTTGACCATCAACAGTCA
CAACTATTTCTTTTTTGGCAACAGTACTCAAATCGGATGTTGTAATTTTATCGACACGCT
TGTTCTCTACGTATTCCCATCTGCCTGGCGAACAATATACAAGCTCAACGTCCATCAAAT
TTTTGTCAAGATTACGAGGATTTGGTGAACCCTTTAACGTATCTCCCGTAGCAACAACCA
AAATTACGTTTTGACTTGCCCATGTGCCCCACACATCCCGCAATTTTATTGCTTTATTGT
AGTCGGAAACAGATCATTTAGGCAAATTGACAGTAATAGAACCAGCGGTGGTGTTTATTG
CCCATGAATCTCCGAATAATGGAGAAAGAACTCCTACCGAATGATTTTTCCACGCACCTG
GAGCGAATGGGCGATCTCCGTCGCCCAGTTGTGAATAGAGTTCGGTAAAGTAGTTATTGA
CTTTTTGCCCACCACGGCGGAGAGAATCGCCTTGGCCATCATCAACAACGTTTCCGATTT
TGATTATTTCCATTATGATATTGCCCCAATATTAATTGTGTCAATGGCTTTTATTGAAAA
ACGTATTTGCCCACTGAGAGATTTTACTTCAACGTAGACGTTATCGCCCGCGCCAATAAA
GAAACGAATTGTGTATAATTCGTTAGAGGTATTGTTAAGAACAGCATACTCAGTATTTAA
AACCGCATTTGTAACCGTATCTACTGATATCAAGTATTCCGCATTTTCAAAAACTGTGCC
AGCAGTGTTTCGGGCAGCAACCATCAACTTTATCGCAGAATATTCGTTTTTACCAGCAAT
ACGAATATTAGTTGCAGTAGTTGTGGCCAAAATAGTTTTTTCAACTGGCATAATAGAATT
TCCAAACATTGGGGTTATTCCGTATTCCCAATATTTTTCTGATCCTTCGTTCTTTGTGCA
CCAGACAAAAACTCTGGCGTATGGAGACGTTATAATAAGAGAGCCAGATGTTCCTAAGAT
ACTTTCGCCCGCTTGAGTGCGAATTATGAGAGGATTTGACGGAGAAATTGATCTATTACT

ATTTATAAAGATGCAACCTTCCCCAAACTTTGCATTTGGTAGAGTCATGGTCAACGCGCC
GCCTGACGTGTCTGCGTCATGCATAGAACCAATATCTATCGCATTCCCGGCATAATATTG
TCGCGATAATTTCTGGTAATATCCAGTGTCGTAAAGAATTTGAGAGTCAGCTCCTTCAGA
AGCTGACGAATAAAGTCTATAATCACCAAATGTGTTATAAAGCGCATCAATAATTTGGTT
GAGTTTGACACCACCGTCATAGAGTATATCACCAGTTGAAGGATTACCTATAACGCCTAC
ATCAATTCTCTTTTTTGGTAGATTGTAACATTTGTATTCCTTAAAATGAGAAAATGATAT
TGATTTCTTCTGTCTGATCGAGAGCTCGAGTGATAGGCTGTCTATTTTCCATGTATATCA
TTTCTCCTGAATATTTTTCCAATTCTTCAGGTTTACATGCTATCAGGGTCGCTTTAACAT
CTGGATCGGACGGCAATGATTTTTTAACTAACGGGTTGATGATTACGCTCATCTGGCGAA
AACCAGTGTTGCCTGGGAGTGATGCCTCAGGGAAGTATATTGAGTCCATATACGCTCTGA
ACCGGAGTGTGTCTACTTTAATCCGGTAAATGAGGTCTAACTTATCCGGATACCATGGAA
TAGCATTTTCATACCCCGATCTGGCAGGATCGTCAGAAAGTTCATCCAGGAAAGGGACAA
CAATATGTTCATTTGTGCATCTGTTTATTGATACATCGGGCGGAATAGTGTACAGATAGT
CCCACACACAGCCATCACTCATGTCGATTGCCATAGCAGTCCCGCGAGGTGGTTCAACTG
TTTCATGAGTTGCAGTCCATTTTCCACCTATAGTGATACACTCTATGTTGTCATCTATTC
CCGAGATAGAACACGAACCAATAGAAGGAACATCAACACAGCGGTAAATCATCCAACCAA
CCGAACTGTCGGTACGATTGTAGGTAGCCGAGTTGACTACGACGATATCGTCGATAAAAA
CTGTCTTTGGATTGTCATATCTGATATCTCCCCAATCTTTACGCGGAATTATTGCGTCCA
GTAGAGATTTATTAATTTTTACTGCACCTAGCATGTTTGTCCACAGGCCAACAACGCCAT
CAACCGAATCATTTGGATATGGAGGTGCAAAGCCGACATCGGTTTCATTCGTTGACCACG
GAGTATCACGTCCAAATGTGGCGTATATTGTGTTTTTGGTGGGCTCGTCGCCCACCATGT
CGTAAAAGTTTAGCAAATTTTTTGTTCTAAATTTACTACTAACAATAGCACGATAGAAAT
TACTCATCTATTTTCACCTGTGTTGGGTTTTGTGGATCACGAGGATTTCCAACGTCATCT
TTCAGTCGTTTTTCAACTAGTCGTCTAAATTGTGAAAACGTCGCAGCTGACTGATCAAAC
AAAGGACTCATTGCAAATCTTCGTTCTGATGGAAGTTTTCCTAACCATGATTCTTCTTCA
GTGTCATATTCAGGAGGAGGAACAAATGGTTCGCCAGCATTTGGCCCAACCAAGTATAAA
GCTTCTCCGGTGGCAGAGTCATGCTCAATATTTCCGTCAACGTCTAATTTGGCTACACGA
TCGTATTGCTCAGTTGGGTAACCAGCATCCCATCGATACGTTTTCTAAATTATCGATGAT
AGTTTCTACTTGTTTCATCGATAAACCAGAATTGATGAACATCGTGAGAAGTGTTATTCC
AACAAATCCAAAACCAACTGGATGAACAAAACGGAGAACATCTTGCGCATATCTAGACGC
CGGAAGAGTTGACTTTATAGTCATGGTGTAATATGATCTACTTCGATTGATATAGTCAAT
CGAATCGCTCATAAGCTCCTTTCCTCTAACCCCTACCAATATTTCACCTACGAACGAACT
AGTTTCGGATTTTATTTGCTGGCCTTCAAAGCATTTACGAATCGTATTGTGAATGGTTAT
TCTCCACTTCAATTGGCCTTCAACATACTCACGATCCACGTATGAGACGTTTGCACGTCC
TGTAGGAGTATATATGGTTCTTCCAACAATGTCTTGGCTTATGTTGGTTGAATAAACTAC
GATGTTATATTCTATTCCATGCTTTGACTCGATGTCGATTTCAACATCTTCGTTATAAAG
CAATTTGAAAAGGAATTTATATGATTGTTCAACCCCTTTCGTGGAATTAAAATGGTAGGC
CCGAGATTCAAAAAATCTTGCGACAGAATCTCGCTTAGCTTCATCCAAATAGATGTTTCT
TTTTAAAACTTCTGACCAAAGATATTCATACGCATATTGCTCGCGAGGGTATTTGTTTTT
TATCAAATTGAGCAATTTGTTGTAATACGTCCCGTTTCTGTCCGACAAGAATTGCAGATA
ATATTTCGCAAATGCACCAAAATTGGTATCTTCGTCCAAACAACTTTCAGGCATCATTTT
AGTAACAAGTGGCTGAAGGTCAGGATCCTTTAATCCAACAAATGGAGCAGGTGTCCACGG
AACTTCACGCTCTTGATTGCGCAGCTCAGCTACCATCATGACGTTGGCCGGCTTCCAGAA
AATGTATATAGTATTTCTTACCCGATATGGGAATTCATAATACCCAATTATTTCGCCAGT
ATTTTTATGGAAAAGTACCCCATTGGCATATTGTTTGAAAGTGGTAAAGTATACGTTATT
TGCATACAGTGTTACAACGCCTTTGTTGTAAGTTTCTCTGTTTACCCTTTTATCACTTTC
TGTTCCTTCGTCGTCGATTGAGAACGAATATTCGATTTTTGGGTAAACAACAAGAGCTCT
GTCGTTGTGTGTTATCCAACACCTTGTGCTAGAACGAGCCATCCATGTGAACGCTGCTTC
GTTATAGTATTGCTGAGGGCCAAGTTTCCACTCTGAATCATCGGCATTGATTCTGAATGT
TCCCATATGAATTGGGATATCGGAATTATAACCTGGAGTCAATTCGTATTTTACTGCTTC
AAATACGTTGTCATATTTGTTTGGTAAATCTAGATCTGGCAAAGTACCTGGATATTTGAA
GTTTATCTGAGAAACAAATATCTCTTCTCCATTTGTTGACATATTGCTATATTCATGCGA
TATAAAAGTATGGTCTTCGCCAATACCAAAAACTTTTCGCCAAGAAGTACCATCGAACTC
ATAAACTCCAGCAATTGGGTCATGGACAACATTTTTGGGGATTGTTGGGTCTAGACCTGC
CTCAGTAACTACGCCAGTAATCAAAGCATAAAGCTTGCCGTTGAATGTATCAAGCTTTTT
CGTAACAGCCCGTGCGTTTGCTGTTATATTGAAAGAGGTTGGGTCACAAAGTTTCTCACC
AAATGTCGGACTACCTGGATCAGTATCAATCGGGGCATTAGATGTTTTGATTCTGCGCAA

CTTATTTCTAGCTGCTACATATACCCAATCGTCATCGGCAGCATTATTTTTCTCCCTTCC
TAAAAATATCACCCGGAAGCCGAGCGTATGAGTTGAATAATTCAACATCAAAACCAAGAT
TCAGTTCATCGCCAGTTTTTGAGAATGTCAAACTATCTGAGCTAAATCTGACGTCATCAG
CTGACCATCTGACATCATTTGCCCGACGACCGTAATAGATGTTTTAATATCCAAGAAGAT
ATGTAGTAGTGCTAGTTTGAGATGTTACTGTTCTACTAACTGGGTTGCCGACACGCCCGT
TGAAAGCTTTGTAATATTTCCAGTTTTGCCCTTTGTCATTCGACACTTTGGCCATTGGCT
GAAAACGCTAAAACAGATACAACACGTCGTCAATTTCAGCTAGCATTGTTCTATTTTTGT
CAACACACACTTGCTCAATAAAGCCTTGTATTTCGTGATGGTTTTCATCTGAAACTATTT
TGTCATAAAGATTCGTCGCATCAGTAATTTGATTGCTGAACACGAAATCTTCGCTCATTA
GCGAAGCCATAATTACATCAGTATTGAAATTGACGTAATTTCCGTTCTTTTTAGAGAACT
TTTCTCTCAAAAAGGAAGCGGCAGGTGTGAATTCTTTCATGGCAGAAATGTAGTAGGCAT
TTTCCGAAAAGGTTTGAAACTCCTCAGTTTCTACCCAATCAGACATCTCAAATCCGTTTC
CAGAAACGCGAACTCGCATTTTGTAATATGTGCCTGCTTGTATAGATGTGTCTTCAAACC
ATTCTTCGACCGATGTTACACCTAATTGACCCCAACTGCTTCCGGACCCTGCTCTAGTCT
GAAGAAGTTCGGCAATATAGTAAAAATTTGAACCTACATCGTCCCAAGTCAAATATACTT
GATTCGCCGATAAAATTTTGATTTTAAGGCTCGTTACGGCCGGTGCTTTTATGATCATAA
AATCTCCAAAGGGCCGTTAGGCCCTTATTGTGAAATTGCTTCAAGAGTAATAGCAGTGTA
CTGTTGACGTAAATCATTCTCAAATACGATAGGAGTTCCATCTTTCGCACAGATAGTGTC
ATCAACTGGTAATGCTTCTATTACTATATATGCTCCAGCAAATGATTCTGCCGGTACAGG
TAAAATACCAAGGTCAAAGTTTATAACACTGAGAGCATAGTCAACTGTTACAACTTCATA
TTACTTGTTTCTTCCACCCACTGTTGTTTTTGTGAAATCATTACAAGAGGACTCTGATGC
AGGAATATCGCCATCAGCAAATGGCCCAATCAAAATTTTACCAGTTTGTGACGTAGAAAC
GTAATTAACTTCATACGTTACGTTATTCTTGGTGAATTTGATTGTAGAACTATTGACTGA
CCCCTTTTTTATTAGCATTATGGAAAGATATTCCAGCCATCGGAGTTTTGAAGAAATTAT
CTGCTTCCCTGACAAGAGATATTTCAGCAGAAGAGCCGATAATAGAATCATCCGACGAAT
CTACACGTGTCAACATCTTAGATTTAGAGAAGTTTTTGTTGAAAATTTCAACTTCATCAG
TGTAATAGCGGTCAATAGAATCAATTATTTGCCCGCGAAGCAATTCTTCAGAGTTATACA
ATTCTGAAAGGTTATATGTCACTTCGATTTTTGACTTGATGTACATGTAATTTGGGTCTA
TTACTGCAGGGGTAATAGGGCGCGAGATTATATTGTGACAAATAATTTTCAATGTCTTCT
TTTTGAACAGTTGTTAATCTAAGCCCTTGTTTTGGCTTAATAGCGATGAACGCGTATCCT
CTTTTCTCACTGTCAGTGAAGCACTGAACGGCTTGAACAACTGAACCAAACTTGTGAGAG
ACAAATGACTCGTAGTCGGTTGTAGTAACACATCTGCGCTGAGATTCTCTCATAACCGGG
GAACAGTTCGCGGATTCTTTCGATGTCTTCGGGATCACCACCACCCTGAGTACCCACATA
ATCGGCGTCATCAGTCGGATTCTCAATCAGATCAGTTATGACGATATTTTGAAGAGTATC
GGTGTATGCGAAATCTCTTGACCCATTTGCTGGCTCACCACGAGTGCTCAAATATTCGAT
CACGATTGTTGACCCGATATTCGGTTTGAGCCCGCCAATGTAATTTGCTACAAGAGCCCC
GCCAGCGATTTCGGTGCTAGCTTCACCTTCGCCAAAGAATATCTCAGTCGTCTGGTCAAC
GGTCTCACGCATATAAAATACGTTAGAAGCACCCGAAATATCAACCATCGGATTATTGGT
CCAATCGACGCAAGGTGAACCATCTACCCATACACGAACTTTAGATCTGTCGATATTGGG
GTCACGAATCAAAATTTGACTAATTCCATCAAATTTCAACTCAGTTCGAACTATTCGTCC
TTGATACACGTTTACAAGGGATTCGTAGGTATTATTCTGACCACGAATTATTGTAACATC
GTCAAGAGTAACGAATGGGTAGTAATCAGTACGTTCCACCGTACCAACGAATTTAGTTCC
TCTCGGAATACGAATGCTGGTAGGATTCAACGGGTTTGTCGCTTTGAGCAGTAAAGTCGT
ACTAGAAGCAGTACGAAAATCTGGCATATATCCCATGTCCTGAGCATGTTGAACAACTGA
ACTTCTTAATACAGCGGTTCGGATAAAACTCTCGAATAATGCTGAATTTGAAAATTGCTG
AATATAGAGTGTGCAATATGCCAGAAAATCGGTTAAAACGTTTAATCTTGAACCAGTAAA
GTCATAATCTTTAAATTCATCCTGGTTACCTAACCAGTCAATTAAGTCGCTTTTGATTTC
GTCAGAGGTAGCGCCTTTAAAAAATATCTGGGATATTTGTTTTCAATTTCATCGTATAAA
CACCTTACTAGATGATTGCGCAATTGTGTCTCCGCAAGCTATTGGATCACCAATTGATAG
TGCGGGTTTTCCACCAACAAAAACCTTCGCCGTAGTGGGAATAACGTAGCCGTCATGAAC
ATCATAAGGCTCAACAGTGTTTCGATGAGGTATTATTCTGTCGCCTTCAACGACGACCTT
TATACCGTCAACAAAAACTTTTCCCTGCGTGGCTTGTATTTGTGTAGGAGGATATGTGTC
ATGGCCAGTTGTCCAAGCCATATCGTATGTCAATGCAGGCATAAAAGGCCCTCTTATGTG
ATTCTATTTAAGAGCTCCCGGGAGGCATTAAATAAAATAATCAGGAGTGAAACTATATGA
TTTTTTCATTTTTTGTACCCAATAGATTATCAGGGTAAATCTACTACTGATATTTTTAAG
AATTACAGAGCGTATTTTAAACGTGTTATCGTAAAATACAAGCCTGAAGTGTATTGGATA
AATGGTGCAACAAGACCTGAAATGTTGGCACATGAGCTTTATGGCAATCAACAGCTATAT

TGGGTTTTGCTAATGCTAAACGATGTATATGACCCTTTTTGCGGATGGATAACAACACAA
GATGCTGCATATGATTACGCGCAGCAACAATATCCAGAAAATAAGGTCGTATATCATGTT
GATGCCATTGGTGAAAAGTATTGGAATTTGGTAGAAGACCCAGATTTCCCGAAACATTGG
TATGATGCTGGAGATACATCTAGATCACATATTCAATATGTTGGAGCACTTCGGGCCGTT
GATTCCCTTGAAGCTCAATCTGCTGTCAATGAGCAGAAACGAAAAATTCTGATATTTAGC
GAAGCTGATATCAACTCGTTCCTAAACGACATGATACGTGAAATGGAAAAGGTTTAAGGT
GCATAATATTAAAAGGTATTGATAGATTTTTTACTGGCGTCGTAGAAGATCGTCAGGATC
CGTACATGTTAGGAAGGGTGAGAGTGCGTGTCCACGGTATTCACCCAGAACAAAAGGTTA
GAGCAGACAACTATGGTTTAGCAACCGAGGATTTGCTGTGGATGTCAGTAACCATGCCTG
TTACATCAGCGTCAGTTTCGGGTGTAGGCCAGGCTCCAGTAGGCATCGTGACCGGTACTC
ACGTATACGGAATTTTCTTGGATGAATTTTATCAAAATGGCCTAGTTCTCGGGACATATC
CAGGCATATATCCAATGATGCCTGTTTTCAATAAAGGTTTTTCTGACCCATCAAGAGAAT
ATCCTCGGTACGTCGGTTCAGATGTGAATATTTTGGCTAGAGGTGGAAAAGAAATACAAA
TTTCACCAGGCCAAATTGTAAAAGACAACCAAATTGTTCCAGTTGTCGTAGAAGATCAAA
ATCTCAAGCGAGATGTCGCACAAGGTGCAGATAGAACACCAAATGACGAAATACACCCAA
ATCCAGATCCAGACGCAACAATAGAGGATATGTTACGTTATGACGAAGTTATTCGTGTAT
CGGTTTATTGGGACAGTGAAGGATATCCAACTGTTGGAATAGGTCATTTGATAGTTCCCG
AAAAAACTAGAAATATGACAAGAATAAACCAAATGCTCAGTCAACAAGTAGGCAGGCAGG
TTAATGGGAGAATAACCGAAGAAGAATGCTCTATGCTGTTCGAACGCCATCTTTCCAGCG
TCTATTCTAGCGTATCAAGCAATTACAAGGTCGGCCCGGTATATTCGATGCTAGACGTTA
CTCGTAAAATGGCTATCGTCAATATGACGTTCCAAATGGGTGTTGGTGGCGTAGCAAATT
TCAATATGATGCTTGGATACTTGGCACTAGGCCAATACGATAATGCGGCTGATGCTGCTC
TCGATTCTTTATGGGCTAGACAAACTCCATCACGAGCTCGTCGTGAGTCAGAAGTCATAA
GAAATGGAAACTTGGTGCCATACGGTGGGTCACAAGGATCTGCGCCTTCATTGCGGACGA
TGAGAATGTTCAAAACGGCAAGTGTATCATCAACCGCCCAAGCTCCCTCGTTCACCGAGC
CTGAATCACCTTACTCGGCTGTATATCCATATAACAAAGTGTATGAAAGTGAGTCTGGTC
AAATCCAGGAATTTGATGATACACCCGGGCATGAACGCATAAGAACATATCATCGGACGG
GTACGTTAGAAGAAATATAACCTGATGGAACAAAAGTCACGAAAATTAATGGCGTTGATT
TCTACATTGTCAAGAACGATGGGAATGTTCATATCGGCGGAAAACTCTAGATCGTTGTCG
ACGGGGATGCTGATATTTTTGTTCTAGGCGATTCTAATAGCACAATATCTGGAAATGCTA
CCCAATTCGTTCGTGGTAATGTTGACGAGCGAGTAGAAGGTAATGTCAATCAGTATGTCA
CTGGAAATTCATCGGCGTTAGTAAATGGAAATCTAAATGCAGATGTTGATGGAAACGCCG
ATATTAAAGTTATGGGTGACTGTTTTAACACGATTGATGGAAAGTACACTCTAAAAATTG
GAGGAAATTTTTCTACCGAAGTTGGAGGAAGTAGGACAGATGATGTTTCTGGTAATTGGA
ATAGACAATCGGCCCAAGTATCCGATGTTGCACAAGGAACATTCTCAATTGATGGTTCTA
GAGTAAACTTGGGGTAATTCATGTCAAATATATTACCAGCATCAGCCGTTTTGCCTGTTA
AAAGGGAAAACGAAATAGTATCGCAGACATTTAGCGCAAACCTAACTCAATCACGGGCAA
CCCTGGTTTCTTTGGAAATTAGTGCTATAGATCAAAATCAAGGAATAGTTATTGTCAACA
ATGGATTTTCAGGACAATATACTGGAGTTTTTACCCTTGACGGTGGTTTATCATATCGTT
TAAAAACCGGAGAAAGTGAAACAGCAAATAGATGGGACGATTTGCCTGATCCACAAACAA
CGGATTTGTATTCTTTTCTTGCGCCAAGAGTAATGGAAAAAGAATATTCATACCTTGTTG
TTTTAACATACGACGTTGTTACGCCAACTAGAGCCAGGTAATCCTCCAAGACTAACCAGT
TAGATATGTGATAAGCAAGACGTATACACAAAAAGTAGTTGGTGACTGGAACACATGGGC
TAACAATATGCGAGATTATGTATATAGGGGTAGATAATGGCATATTTTGGAAATTTAGGC
CAAAAACATCGAAAAATACAAGGGCAATGCAGATAAAATCACGTATAGGTCCAGTTGGGG
AGCGTGGGTCATGAAATTTTGCGATCAGTCGCCTTCAGTTTTAGCATGGAATTCTGAAGA
AGGAATAATCCCGTGCTTTTCGAATGCTGACGGAAAAAGACGAAGATATTTCATGGATTT
TTGGGTCAAAATGTCAAACGGCCAAGTATTCCTGTTCGAAGTAAAACCCGAAAAGGAATG
CAGACCTCCCCAGATGAAGTCTACGAATACTGCGAAAGCCAAAAAGCAATTTATGGCAGA
AATGTACACATGGCAAGTGAATACAGATAAGTGGAAAGCTGCTAATGCCTTGTGTATGGA
AAAAGGTTGGATTTTCAAGATAATAACTGAAAATTCTCTTAGAACTCATTTTGGATGGAG
AGGATAATGATTTTTGACTATATCAATGAAGCTCCACAGAAAAAGTCAAGAAACCAATGG
GTAACATTGGGAATAGAATGGAGAGAAGCAAAATCCAAAGGTGTATCGGGAAAGGATTTT
GCAAAATCAAAGCGTCTAAATTATGACACGTTTAGACGGACTATGCTTAAATTCGCAAAG
CAAGTTGATATTGCCATAGAAGTCGAAGGTCTAAAAAATAAAGCAAAATTAAATGCACGC
GAAAAAGCATTAGTCATGATAAATGACTTTAGAAGTCAATTACGTTCTAGAGCAGCTGAC
ACTGGAGCAGCAAACACTAATAAATCTCTAAAATGGTTCAATGATACTGTCAAAAAATCA

ATTCGTGGTCACATGGTATCAAACCCGAAACCAGGGAGAATTTACACTTTTGCATATGAT
GCAAAGCACAAAGATACTCTCGAATATTGGGATAGATTCCCGCTAATTTTCTTTTTGGGA
ATAGGCACATCTTCATCAGGCCCTTAATGTATGGCTTAAACTTGCATTATATTCCTCCAA
AAGCTAGACAAAGCTTTTTAGAAGAATTGCTAAAAAATTATGCAAGCACCGAACGACTAT
CCAATAAAACGACTCTGAAAATAAATTGGTCAAATGTTAAAGTAATGAATGGTTCCGACG
TCATGATAAAAGCATACCTACCAGCTCATTTAAAAGGTTCAATGCTAGAAGTGAAACTTA
GCGACTGGGTGAAGCTCATTTACATGCCGCTGCAGCAGTTTACGTCGAAAGGTAAACGAT
ACTCTGCCAAAAAGGTATGGGCACGTGCTTAATGGTAATGGCCTGGACTTCGTCTGGGCC
ATTTTATTTTATAGATTAATAAACCATATATCTCGACGTAATGAGTTTAGCAGGGTGATC
CCGGTTAAACCCTGTATGCATCCAGTTATATAAAGGTTACTGGCTTGCCAGATTCAAGCT
TGCTTGAATCATTCATTATTATCCTTTCTGGCTTAATAGATATTATAACTAAAAGGATAA
AAAGATAAGTTTGAGCAAGTTCAAACCTGTAAACAGGTTTTAATTTTTATGGAGTTTTAC
AATGGCTTTAAAAAATCAATCGAATATTACTAACTTTACGCTTGATATACCGTCATCTGC
TGGTATTAAGACCATGCTCATGAACGTGCAATCAGCATCAATACCAGGTTTTCGTATACC
TCCGACAGAAATGCCATTAAATTCTCAGGGAACTGCTAGACAAAACGTGCCCAGTACAAC
AACCGAATTTGATCCGTTGATTGTTCGAGTGTTGCTTGATGAGGATTTTGAAGCATATAA
CGAAATGTACAGATGGATGCTTTCATTGAATGACTATGCTCGGCAAACTCCCACTAAGTG
GGGAACGGTTAATCAACCGCCAGCAATAACGCTTCATGTTCGTAATAACTCTAAGCGTGA
TATAATTGCCTCATTCAATTATCATGGTGCTTGGCCATCTGAAATCGGTGAAATAGAGTA
TTCTTACCCTGAAGATGGTGATGTTTCTGTGACATTTACAATTACCTTTTTCTTCAAATA
TTTTGAAATTGAAAAAGATGGTGAATTAATACGACCTTTGAGAGTATAACAATGATAATT
GCGTTATCGGCGAAAAAGAGATGTGGAAAAGACACAGTAGGCGATCTATTGGAAAAAGAA
GGTTTTCATAAGTACGCTTTAGCAAAACCTATCAAGATTTTCCTGTACCGTGCAATCCGA
GATGACAATCGGTTGCCAATGTTTGTCCAATATTTTGATATGAATGATTTTAACGGTCTT
GGTTATGACCGTGAAGAATATTTGCCCATTTCTAATTCGGATGTTTTTAGAATTATGCGA
TCTGCATGGATGCTTGTGTGTGATGACCAACAAATCGATTACACGTATGCGCATACCGGA
ATAATTTCAGAAGCAGTTCATGGCAATAAGAAAGCATGGTCAATTCGTCGACTGATGCAA
ACATTTGGAACTGATATTGGTTGCGCAATAAATAAACGTATATGGTTGCAATATCTAGAA
AATTTCCTATTGAATCACGAAAATGTCGTTGTTGCTGACTGTCGGCAAGACCATGAAATG
TCAGAAATGCGAGAAATGGGAGCAGTCGTTGCTCATATTATTCGTCGTACTGATTCAGTA
GATACTCATTCCACCGAAGCAGGTCTTCCTATCCGGTCTGGTGATCATATAAATGATAAT
ACTGGAACGCTTGAAGATTTGCATGAATGCGTTAACAATTTCGTAAAAACACTAAACCCT
AAGGATATAAAATGAACGCAGAAGAACAAACCATCGCTTACCTGAAAATTCGAGTTTTTG
ATCTGATCGAGCAACGTGTTACTACTCAACAACAAGCAGAAGAATTTTTAGATGCGCTCA
CCAAAATCGTCCACCTTTTGGGTGTGACCCCGGCCGATGGTGAAGATAGCATTGCCCTTT
CTTCTATTGTAGAAGCAGTAGAAGCTCTGGTTCCTTCTCAAGAAGTTGAAGTGGTAGAAG
AATAATGTTTACAGACAGCTTCGTAGGAACATACGTTGCTTGTAAATTTAGTGATGAAAC
GCTGGATTTTATCCAGCGTATTCAAGAAGAACTTAGATTGCCAAATCCTGTTCCTCGAGA
CGAACGTCATTCTACTATCGTTTATAGCAGAGTATATGTTCCATTTATCCCAGACGATTC
GCCTGAACATTTGGCCGATAGTTGCTATCTGAGAATTTTTGAAACTCCAGAAAAGAATTT
TCTTGTTCTGGCGTATGATTCTCCGTACATGCAAAAACGACATGCGTACGGCCAGGTCCT
TGGCTCGTCAGCTCATGATTTTGACGAATACAATCCTCACATCACGATTGCAAAAGACAT
TGGTCCTCTTAAGTATGAAGGATCATACGACTTCCCTATCGTAACAACACACGAATATGT
GGAAGAATTACGTGAAGATTAAAGCAAAAAGCAGTTAATGTTCAAAGCTGCGAAAATTGA
TGTACTATGAAATTCATGAACAAAACATTGTTCAGAAATGAAAACTAAGCAACCTTAAGG
AAAATAAAATGCAAAAGCAAGTAATTCTGTCCACTGGTTCTACCCTGACTGTATCCGGCC
GTGCTGTTTCTGTTGAAGTAGGCCCTGTTTTTACTGCCCTTGTCAAAGGTGAAGGTGAAA
AACGCGTAGTAGTTGCTGAAGTTCGCAGTGAACTGGCTGATGTTATTGTTCATGAACCTC
GCCGCCTGGGTGAAGCTTTAGAAGCTGCTGAATAATTTTCAAATGCCCTCTTCGGAGGGC
ATTTTTGTAACTATAGGAGAAGTTATGGAAATCTTTTTGTTTTATTTTGTGATCACATAT
TTGGCAGCATGTACAGTTTTGGCTATTAACGTGATAAAGTATACAACATCAACATTCAGA
TGGAATTTTGTCTGGACAACTGGCCAAAAATGCGGAGTTGTATTGACAACTGCACTATGC
AGCTACATTATTCCGTTTCGCGTTTATGCAGATACAATTGGAAAAATAAATGACAGATCA
GCAAGAAGAATTTTTACATACGAAAACATCCGAATTAGTTAAAGTCTGGTGTGAATATGA
CATAAATGGCAGTTTTGGAGGTAACAACAACGAAGAAGTTTGTCGTATCATGTTTCCATC
AGATATGCCGCCAGATGATTATTCTGCTCATATTAGCATCATGGTTATGAAACATATGAA
AGAGCAATCTGGCTTAGATGAAGACGATCTTGATGGTCCTATTGGATGGGACTACTTTAT

GCCTTTGGAATTGTGATGGAAAACGTTGGCATTGCAAGAGTTGTCAGAACAATTGAGTTC
GATTAAGATAAAGCATAGACTTGAGGTATAGAAAATGAAATTTGCACCCGGAACTGTAAT
TCCAGAAGGATAGGTCATTAAAATCACTTCCTGGGAAAATGATGGTGAAGATTACGATCA
TGTCATTCATACCGGCAGGCCAATGAAAGATATCGAATTCTTTGAGAAAATCAAACCGAT
TTTCAGTTCATACCACGACGAAACAAATAAAGGCTATGGCAACAAAGATTATAATCCTGG
GATGGCCGCCGATTTGTTTGAGATTTTTGTAGAAAATAGGCATTTGTCTTTGCAATTCAA
ACGCTATCTAGGGTTGACACCAGATATGTATGATCCACTTGATGGCGATTTATACAAATT
CATTCTGCCATGACAATTTCTGCAAAATGCTGAAAGAACACATCACCGGTTATGCCAAAC
AGTATTACTTCGATTTAATTTGTGTGGTCGAGTCGATTGAAATCGAGTATAACCAGTCTG
AATACGTTTTTCCAAAAATTACACCTCGTTAAAATTAAGGTAATCTAATGCAATTTTTAT
TTGAATTATTTGCAGCTAGCGTTCAATACACTGCTGTTGCACTGATTGTATATGTTTTGC
TACATGGTTATGAATTCTTTGACGAATGGCGGAAAGGAAAGCCCGACTCAAAGTTTCCAG
CAAAAAGCCTATCAACCAGGCAGATTTATGGAGCCATTGCAGCAGCATGGCTACCTGTAG
CTCTTTTCTTGCTATGGAAGCTAATTGAATTCATTTACGTCAAAGTAGTAGTCGATAAAA
TTTACAAAAAGTAGTTTACATTGATGGGACTATGTTTTATGATAGTCCCATCAAATCAAC
ACGGAGTAATTTAAATGAAAATCTATCACACGCAACATATCGCGAAAGTTAACGGTATTA
CAAAGGATGACATGATTCGCGGCCATAAAACTCAAGGATTTCATTGCTATATCATGTTTA
AAGATGGCAAGGTCATTGATTGCACCTTCAAAAATGTCACGGCACAATTCCGCAATATTG
AAACGATTGCTCGTGAAGTAGCAGCAGAACATCAATACACTCTGTGAGAAACAACACAGT
TCTGACAATGGGTTGACTATAATACCCATCGAAATTGGCCGTGGAGGGAATCAATAACGC
GTGCTGAAATACTAATACCGAAGGAGGTAAAATGAAAAAAATTGTTTGGTTTTGGCTCTT
GATTTTTCATCTTCTTATGCGAGCGCAGATTCAACCAATTTGATAATTGGTGGTTTTTTC
AAAACATTACGAAAATGATGAAATGAATGAAAAACATCCGGCGATAGGGGTCGAAATTGA
CAACTTTTCAATAGTATATACGTCGAAAAACTCGATTAATAAACCTTCGGTTCAGTTGGC
TTATTCTGACACATTTTACACTTATGGAATTTTTGATATTGGTTAGCGCGTTGGTTTTAT
GACCGGTTATCGATATGAAACAAAATATGCCAATATCCGATGGTATAATGGTGTTGATTT
GGGTGCCGGGGTAGCCCCGCTCACCGCCAATGATATTTCGGTAGATACCCCTATTCCGAG
ACTCAAATTTGTTACAAGCGTAACACCAGTTGTTACGATGTTCGGATTAAAGATTGAACT
TTGGTAAATTTGAGAACGCAAAATGAAACTATTTCACCGACTGGTAAAGAAAAAACTTTC
AAATCGTGGAAAAGCCTGAAAGAGTGGCTGTACGCTAATTGTGATTCGTGGGATGCTGAT
ATATTAGATATTCCATTTAACTCGACAGTACATTGGGGTCTGTGCCCATTTGATCAAGAT
ATCTACAATGGAAAAGAAACGTTTGTTTGGAATTTCTGCAAAACCCTTTTAGAAAAGGAA
CTCGAGAGCCGAGCTGAGGAGATTTGAATTGGAAACCTTCGTTGTAAAACTGCCCGCTCA
TCTAAAAGAATGGTTCATTTCTTATATGTGCGATTCGGGTGAACAGCATCTCATGGAAGC
ATGGCATGATGAAGGCATCGAAGGCGATCGCGTCGATTTCAGATATCCATATTATTCTCG
AATGCGTGGCGAAGAAGTTGACGAGACCGCTGATATCGTAGTTACTGAGTATAAGTGAAT
TATTGCGAAACGTTTTTAAGTTATCGCTGAAATCGAGCAAAAATTCACATACCAGGCACC
CATAAATAGATATACCAAATAACAAAAGGTCGAGCGCGATGCGGTAAGGAAGCGAAGCGG
TTACATCCTTAATACTGAACCGATTAAGTTACGGCGGATGGACTGGGCTATCGGTAAACG
GGGGAGCATAGACCCGAGCACCTGGCGTTATTATGCAAGTATGGTGGAATTGGTATACAC
TGGAGGCTTAAAACCTCCCGCCTTCGAGATTGTGGGCTCGAGTTCCACTACTTGCACCAA
ATTCAAATTTCTAAAGCATATTGTTTACACGCTTTGGAAATAAGATATGATTAACTTATC
GAATCAAAACACGCATTCAGTCTTGAATGGGATAAATTGTGAAAAGCCATGGTTTCCTG
GAGCAAGCCTTAAGTGGACAGAACTCTCATCCTAGTGACGCAGGAAGTGTTTTGTTTTGA
AAAGAATTCTGGAAGATTGGCCCGAGTTGGTTTAAGGCACCGGTCTTGAAAACCGGCGAT
CGTAGGAATACGGTCTATCCGTTCGAATCGGATATCTTCCTCCAAATTGAGGTGCTGTGA
GCCGAGCGGCTTAAGGCGGTCCTAATGCAGGACATACGAAAGCGTATCGTAGGTTCGAAT
CCTACCAGCACCCATTAATTCTGAAGCATATTGTTCAGAGTTGCTGGAAATGAGATATTA
TTGTCTCTTCGATTAAAACAAGTTTATTTTGGGGTGATGTAGCATAACGGTCATGCGGCC
GGCTGTTAACCGGTGAGGAAACTCTACGTTGGTTCGACTCCAACCATCACCGCCAAAATC
AATTTCATGGAACGTTCGGGTAACGGGATCCCAAGCGGCTGTAACCCGCTCGCCTCTGCC
ATTCTTGGTTCGAGTCCAAGGCGTTCCACCAATTTAACATGAGAAATATATTTTATGAAG
CAAGATCTATTTGATAGATACGGATCCTTCTGATTGAAAGAAGGAATACACGGTGATTTT
GAAATAACTGATGATTTAGTTCATTCGATATTTTTCAATCATATCTATCAGACGACCAAA
TTGCCATTTATATGAAAAACGGCAGCTGGGTTAAGAGCATTTGCGCCCAGATGAATTACG
TTAGAGTATCTCTTCTCAATTTGAAATATTATCGATCTGGAAAAGCGTCGGGAATAAATG
AAGGTTTTGTTTATCTGATAACAAATCCAGCTTGGCCACATCATTTTAAAATTGGAAGTA

AAATATCAATACAATCTCGATTATCATTGTACCATAGTTATTCACCGAATCGCGATTTTG
ATATCGTCTCTTATTTCTTCTCTAAATTTCGTTTTGCGGATGAAAAAGCTTTACACAATT
TGTTTGAAGCTGATGACGAGTGGATTTATGACAAAACGGGAGATAAATTGAAAGAAGCTC
TGCAATTCTTCAATCATAAGCGCAAAATAGATAAACAAGTTTTACGAGCATCTCTAACTC
AATTGGTAGAGTAGCGGACCTCTAATCCGTCAAGTCTGAGTTCGAGTCTCAGGGGATGCA
CCAAATAACGGAGAGGTATTGCGGTAAAGAGCCGTCCAGGCATGTAAAACCGGTACGTTA
CGAGTCTTGTTGGAATCGAAACCAACCCTCTCCACCAGTTTTGCAGTAAAGAAGTTGTAA
TGCGTTGTCATCAGGCACTTATGCGGAGTAACGTTCGAACCATAACGGAATGAGTCCAGG
GTGCTACAATTTGAGCGGCGAGTGTATGGCCGAGCACACCACCTTTTAGGCGCCGGAAGA
CGGGTTCGAGTCCCGTACCGCTCACTGTAATTAATTTCAAGTAATCATTGGTGTGAACTT
AGTAGTAATGGTAGCTATCCTGGATGTGACCCAGGGAGTACGGGTTCGAACCCCGTAGGT
CACCCCAATGATTATTTTCGCGGGTAAGGTGTTTATGGATACATGCTAGCCTTCCAAGCT
TGAGTAGACCGGTTCGATACCGGCTACCCGCTCCAAACACAAGTCACAGGGCTCTAGATT
CCTAGTCAGTTTCCCACCCCTGGTTGATGCACTGTCCATTATTGCGCCGTGACTGGAGCC
GGCGAGTTGGACAACCGTATTGTCTCCGGCACTTAGATGACCGGCTTTAACAAGATAAAT
CGGGAGACGTCCCGTAAAATCCTCGGACAAAACTTCAGTAGTCACTCCCGTCATTTTGCG
CGGTGAAACGAGGAAGTATAGACGGGTAACGAATTCAATCTCAGCGTAGGCAAGTGGTGT
GTCGTTCCGTTTGGGACGGAATTTTCGGGTGTTCGATTCACCCCGCTGAGACCAAATTAC
GTAAGCGGATACTTGGTTGCAAACAAGTCCGTAAAGTCGTCGAATGTGGTAACATCCTCT
CCTGGCACTATCCCAGTGATGCGGTTATCCAGAACGGCGCAAACTGAAGACTTGAGTCAG
ATCCGTAAGGCCCTGACTCCAATTCTTCAAACACTCTTGTTTATGTGGCGGTCGAGACAT
CCCCACGTTCAAAACTGAAATCTCGATGTCCCGAGCTGCAGCGTTAGGGAAGAGTGTTTG
AAGAATTGAACTAACTCTGAGGAACATGTTATGAATGTTGTCAAAATTACAACCACGCGC
GATGTCTGTTGTGATCAAAACCAATTCATTGGTTCACATGTATTCTGCTATGGCCCATGT
GATGCTGGTGATGGTTGGATTGCTAAATGTGCGGTTTGTCAAAAAGAATGGGAAATCGAT
AGGAGCTGTATTACCTACGTCGTTAAGACTTAGCGTGAAATTGAAGTTGACACAAACAAA
ATTTCGCGTGACCTGACCGTTGGTGATTTGGTTCACATTTTAAGCAGATTTGATTTCCAT
ACTCCAATTGTTAGCATTCGATTGAAAAGTTCATCTAATCTTGTATTTGCTTCATCCAAC
GGAGATGGCGTAAATATACATGATTAATTGCCGGTTTAGCTCATCTGGTAGAGCACAGCC
TTTGTAACGCTGGGGTGGTCCGTTCGAGTCGGACAATCGGCACCGAATTTTTAAATGAGG
AAAATATTATGTTATTTGCTTATCAAGTTATTGCTGTTATCTTCTTTATTTGCTCTTTCT
TGGGCGATTATAAGCGATATTCGGATTCCCCTTCCGAAATTGCGGGTAATTTGATTGGCT
CTGCTTTTGCAGGTGGTTTGTGGTCATTGACGGTACTTATCCATATTTTTTGCATCGCAA
ACCGCAATCGCTAAATAAATACAAAGGAACGGACTTCTTCGGACCCGTTCGTTAAGTAAA
AGGGAAGACCATCTGGATATCACGAGGGCGTCGGCCTGTATCATATCTTGCTGGCCCTTG
GCCTCCGACGAGGTTTTGTCTGGAAATGGTGCGCCCAGCTCGTTTCAATATGACATTGGT
GTTAGCGGTAGCATGCCGGTCTCCAAAACAGTGCGGCCAGGGTTCGAATCCTTGATGTCA
TGCCAATAGTGGATTAGCTCAGTAGGTAGAGCACTCGACCGATAATCGAGAGCGCATTGG
TTCGATCCCAATATCCACTACCAAATTTTTAGGAGAAGTGGATGAGCGGCTGGAATCGCT
TCCCTGCTAAGGAAGTAAACCGAAAGGTTTCGAGAGTTCAAATCTCTCCTTCTCCGCCAA
ATGTATCCTGAAGCATATTGTTTCAAATTTCTCAATGAGTGATATTATTAACTCATACCA
AAACAGAGTTTAGATCGGAGCCATAGTTTATTTGGTTAAAATAGTCCCCTGTCACGGGAC
ACCACCGAGTTCGATCCTCGGTGGCTCCGCTCTAACTTTTGATGCGATGTAGAGGAGAGG
TCGTCCTCGTCGGGCTCATATCCCGAAAATCAGCGGATCGAATCCGCTCATCGCTTCCAA
CTTCAATCGCTTGTTGAACGTGGGAAAATAGCAAAATCTGGGAAAGGGTCGCGAACCGGA
ACCTTAAAGTAGCTTTCTAAGTACTTGTTGTCTGGCAGTTCACACATACCTATATTTGTG
ATGGGTTCGAGACCCTAACAAGCGCCCGAATAACAACGCCGGCGACCAATCGAATGATCG
TTATCGGCTTTTTCATCTGACGGCCCTGTAGCTAGACGGTTCAAGCAGGCGGCTCATAAC
CGCTCGTAGTACGTTCGATTCCTACCAGGGTCACCAATTTGGGTTCTTCGTTTAATGGGA
AGACCGTAGGTTGCTTTGCATCTTGATGATGCTATTTCGATACTAGCAGAACCCACCAAT
TTGAGGAAAATACTATGGATATCGGATCCGGCTCGCCTTGGCCATCGTGTGCATTGAGTA
ATTTTGCTCCGCATGAATTCGTTATCGATGGGATTAAATGTTCTTCAATGGAAGGATTCC
TTCAGTCTCTCAAATTCAAATCGCCTGAAATGCAAGAACACGTTTGCAGTCTGGTTGGTA
AAGCCGCCAAATTAAAAGGCACAAAGAAAAACTGGTGGAGAGCTCAAACTCTGTACTGGA
AAGGTAAACCGATGCGGCGGCAAAGCGATGCTTATTCTGATTTAATCTACCGTGCGTACG
ACCAAATTGCGAAGAATTCCGGATTTCAACGAGCAATCCTAGCCACCCGGAATTCTTCGT
TAACCCATTCAATGGGTAAAAGTAAACAGAATGAAACATTGTTAACTGAACAAGAATTTT

GTTCAAATCTGTATCGTATTCGAGATAAACTCCAAGCCGCTTGATTGCGGCTTTTTAGTC
ATATGGTCTCTTAGCTCAGTTGGATAGAGCAGCGGCCTTCTAAGCTGCGGGTCATTGGTT
CAAATCCAATAGGGACTGCCAAATTAAAGGTGATGTCATGGCGATGATGCACTAAAAATC
TCATGCCCGAGAGGGAAGGGGCAGTGGAAATGCACGAAGCGAGAATTGGTATAATTCTCC
ATTGTGGAAAAACACCGGCCCAAATAGAAATCCCGGAGTAGTAAAAGTACAAATTCCAGA
AGATCAATTAACTGATGAACAGAAGCTTCGCAGGCGTCAACAGGAATTGCTCAGATCAAT
TTATTAAGGTAATTAATATGTCTCATTTACAACGTATGAAAGATGAGCTGAGTGAGTTGG
GTGTTCGACTGAATAAGCTCAACGATTTCATTATCACGTCTGATATTTACAAGACTCTTG
ATCCTGATGAGAAGTTTCTCATGGAACAACAGTATATCGCGATGGACAGGTATTACGATA
TGCTAAAACGGCGAATTAAGCGTCAATCATAATTAGGGCTCCTACGGGAGCCCTTTTCGT
ATTCTCTCGACGAGGTTTCAACATGAACAACTATTTCGATTATAACTCTTTCTTCGTAAA
TGCAATGCGTGTTGGTTCGGTCAATTACCCACCGGGGTGCGAGATGGAGGTATTGAAAGA
TCGATCAGATTTTTTAGAATCCAATAGAACAATATCCATAAATGTTGGTCGGCAAACCGG
TAAAACAACTTCATTTATCAGGCTTGGTAACGAATTAAGCAATCATGCCAAAGTCGCAAT
GGTAGTCATGAACACCCAAAATAAGAAAATGACCAAAAGTATGGGAGCTAAATTCGATAT
TCTGACTTTTTAATGATATTAAGAGACAATTGCAGATTCTGGATTTAGAAAAAATTCAAT
TCATCATGGTTGACGAGAGCGAATTTATTCTCTTTGATGAGAACACGGTGCACGATTTTT
ACAAATGGGCAAAATTAGTAAAGGCCGAATTTGTACTAAAAACTTGATGCAGTCTGTTAA
ATTATTCAAGCCTAGTGGTATTATTGCCATATCAACTAAACACACATTACAAGTATTAAA
AATGTCTAAAGCTACTCACACTATCGATTTCGGTTTTGGTAAAATCGTTGCTCAGAAATC
TGGTCATTATTTCAATGTTATTGAGAGTCATGACCCAATGATTCCCACAAATACCAAATT
TCCAGTCAATGACACTTTGGTTCTGGAAGCAAAAGTAATCGAATTGGAGTAATCGGCCTT
AACGCTTATTTTTCACTATTCTAAAAGACATAATGAGTGAGCACTACAATCGCGATAGTG
CAGATGACCCAATCAAAGTTGTTAAAACATCGGGCGTAATTTATACGGCCAGTTTGATGC
ATAAATTATCTTACGATCAACTACCTGGAGGAGCGCTGATCGTAGCAGACAGAGATATTG
CAAATGACATATGGCATGCATGTGATAAACCGAAAATGTTAGATACTTTGTCGTTTTGCA
AAGATAAAATGCATTTTGAGCTTTTGCATAGGCCTCCAGTAATCATATTTGTAACTTCTG
CCAATGTAGAGAGCATACGCACTGCTTTATTTTCAAAGACTGGGTTTTCAAAATCTACTG
AATTTACGGCGTATTACCTCGAACAATGCGACGGTTATCTCCAAAAATTGAGAAATCTTA
TAGGATTTTAAATGTACTAACTTTACAAAACCCTTCCGATATATCAAAGCCAAGAAGGTT
GGATTATCAAATACCCTTAGTTTGCCAAATTGGCAGTTGAGCAAATGCATGCTTTTTGGC
CATGGGATGAACCAGTTGTTGATAATGATGCTCAAGATCTGCGCACCAAATTGACACCTG
GCGAGTTGAACGGTATTACGACCGTTCTTAAACTGTTTGCAATTTACGAGCGCAAAGTCG
GTGAAGATTATTGGTCAGCACGAATCGCTCGAACATTCGGTCGCCCTGAAATCAAACGCA
TGGCAACTCTATTTGCCGCCGCAGAAGGTAACTCGCATGCACCTTTTTACAATAAAGTTA
ATGAAGTTCTTTATCTGGATTACGAAGCTTTCTACACTTCTTGGAAAGAAAGCGACGAAT
TATCTCGTCGCATTCAGTTTGTCGGTAAATCGGTATCAGACCCCGATGATGCCAAATCTT
TTGCAGCTTTCACTTTCATTGAAGGTGCCGTACTTTATTCCAGCTTTGCATTCCTCAAAC
ACTTCCAAGCCCAGGAATGTGGCAAAGACTTGATGCGAAATATTTGCCGTGGTGTTGACC
TTTCAGTTGCAGATGAACATCACCGCTCAATTGGTGGAGCAATGTTGTTCCAAACACTTT
GCCGTGAAATGAAAGAAAAATATGGTGAAGATGTCCGGGCAAAACTGAAAAATGACATTA
TCAACATGGCATATCAAGTGTTTGATCACGAGTCTGGCATCATCGATCTCGTTTTCGCTG
AACCAATTGATGGTATAACTGCTGACGAGCTACGAGTTTTTGTCATATCTCGAATTAATC
TTTGCCGTGAAAATCTTGGTTATGAGCCCATTTTCGAAATCGAAAATAACCCTATCGCTG
ATTGGTTCTATCGCACCATTAATGCCAAGAAATTCCATGTTTTCTTCACTGGCTCAGGTT
CGGAATACAACATCAATTGGAACCGAGAAGGTTTCTCTGATTGCTGGTAAAATAATTCTG
CCCTGGTAAACCAGGGCAATCTTTTTAAAATCATGGTGCTTTAATGTCTAAAATTTCAAA
ATTCGAATCTTTGTCAGCCGAACGCAAACATATGCACTCAATTGGCGAAATGCCAGAGTG
GTTTACAACGCAGGGCTATTCGATGTTCCGGAAAAATTATCAGTACAAAGGAGAAACCGT
TCGTGGTGCATTCCGGCGAGTCGCCTCAACACTTGCAAAGCATGATATACGGCCAGAAGC
TGAAGAGCGCTATTTCGACCTTCTCTGGTCCGGTAAGCTGGCGATGGCTACCCCAGTTTT
CTGTAACACCGGCACAGATCGTGGAATGCCTGTTTCCTGCGCAGGAAGTTATGTTGGGGA
CAGTGTCCTTGACTTCTACGAAGGCCAAACGGAAGTCGCAATGCTCGCTAAAAACGGTTT
CGGAACTGCGTCATATCTTGGAGACATCCGACCTCGTGGGTCCGATATCTCGACCAGCCA
CAACACAGCAGACGGACTCGTTCCTGTATTTGACAACTTCGTTAACACCAAAACAAAGGT
TGCAGCTGCTGGCCGACGTGGTGAATGGGCCGGTTACGTTGACTTCTCGCATGGTGATTT
CTGGGAACTTTAGGGGTATGTGTTGAAAAACCCGGCTACTGCACACGTTGGTTGGGTATT

TGAGAAAGAAGATTACGAAAAGCTTCTGGCTCGCGATCCTGAATATATGGCACGCTGGAA
TGAACTGCTTTAGATGCGCGCTCGTACTGGTAAGGGCTATATGTGGAAGAACTGGATCGC
TAACGATCTTGCTCCACAAGCAATCAAAAATTCAGGTATCCGTATTCGTTCAAGCCAGCT
GTGCACCGAAATCGCATTGCCATCTGACAATATGCATACTTTTACCTGTATCCTGAGCTC
ATTGAATTTGTCTCGTTGGGATGAAATTAGTGCTGAAGATATCAAATGGGCTGTTCGTTT
CCTTGATTCGGTATGCTCAGAATTCTTGGAAAAAGCGAAAAAACTCCCGTATGACACTCT
TCATGAAGCTATCAGATTTACCGAAAAAGCACGTGCGCTCGGTCTTGGTACTTTGGGTTT
CCATACCTATCTCCAGATGAATGATACCGCGTTCGAATCTGGCGCCGCCCCCATTCTGAA
CAATAGCAATTATTCGCAAATCAAGGTTTATGCAGAAGCTGCTTCTCGGGAATTAGCAGA
AGAGCATGGTGAACCAGAATGGTGTGTTGGAACAGGTATCCGTAATGCTACATTGATCAC
TATTGCTCCAAATATGAGTTCTGCTCTATTGGCTGGTGGTGTATCTCAGGGTGTTGAGCC
TCTCGCATGTAACAGCTTCATCCAACAGTCAAATGCTGGTGATTTTGTTCGCTCGAACCC
GGTATTGGCTAAAGTATTGTCCCACCGTCTTTCTGATTAAGAAATCAAAGACCCCATGGA
AAATATTGCCACCTATCATAATGGTTCTGTTCAACATCTGGATATTTTGACAGACAATGA
GAAAATGGTGTTCAAAACAGCTTATGAGATTGACCAACATGCAATCATACGTCTTGCTTC
AGCTCGTCAACGGCACATTGACCAAGCGCAATCAATCAATTTGTTCTTCGCTGCTGATGA
AAAACAATCAGTCGTTGCGTCCGTTCATAGAGCGTTTATGGATGACCCTCGTCTGAAATC
TCTTTATTATTTGCGTTCCGAGCGTGGAGTAAAAGCTTCAACTGGTGAGGCGGAGTGTCT
AGGATGCCAGGGCTAACTGCAAGGGGCGAAAGCCCCTTTTATAGAACATATGTGACAAAA
TTTGAAACATCATCAGGTGAGAAAATTTATTGTGGTAAGCATAAAAGTACGCATGCTAAT
CCTTTATCGGACCCCTATTGTGGGTCTGGACTAAGTTTAAGAAATGCCATATCAAAATAC
GGTGTTTCTTGTATAAAATCGGTTGAATGGTTTGAACATTCAACCGAAGAAGAGATGATT
GAAGCGGAAATTTTATTAATTTCAAGTATGGTTGATAACCCGAATTGTATCAATATCGCA
TACGGTGGTTTTGGTGGAAATGCTTTACGGTATGCTTCCGATGAGAGAAAAGAAGCATTT
CGCGAAACATCACGCCAGAAAATGATCAAGCGATATAAAGACAGTGATGAAATTAAAAAG
CACAGCAACCGCGTGCGAGTTGCATGTAATCGACCGGAAGAACGGCAAATACGAGCTGAG
CGACATGCTGAACTTTATAGCCGCCCTGGTTATTCCTAGCGTATGTCGGCAATACAAAAA
GAAACGCAAAGCCGCCCTGACGTCAAGGCCAAAAATGCTGAAGGTGTAAGAGCGGCGAGA
AAGGCAATAAAATTCTTAAAACAAATTAATATGTCGGACGTAATAACTATTATAGATGGG
CGATTTGTTGATAAATTAAAACTCGCGGATGAGTTGTCAACAAAATATGATATCAGATAA
ACCCAAATATTTTGAGAATTATCGACCTGATATTAATTCATTGCTGGGATAAATGTGGCT
CTACGAATTTTATGCGCAATTTTTGTCTATGCCAATCCGCTTTTTGGATTTGGGTATATA
ATTCACGTATTACTTTCAACTGTGATTTCTTTCTTTGTTTTGGAAGGAATTACTGCAATA
GCTTATTATACAGGAAAAATAAATGATTTATCTTTTCGGCGCTGACTGGTGTGCAAACTG
CAAACACGTAAAACCTATGCTTGCCAATGTTGACTATAAATATATTGATGTCGATTCTGA
TGAAGGCATAGCTCTAACAGCAAAATACGCAATTCGAGGTTTGCCTACAAAGATTAATGT
AGAAACAACCGATCGTTTTACGGGAGTTCCGAAAAACGTTGCGGAATTGAAGCAAAAAAT
GGGAATTTAACAATGGGCTCTTCGGAGCCCATTTTCATTTTCATTTTATGGTTCACATCG
CGAAAAATCCATGTTAGAATAAGTTTGAATCAATAACATATGGAGTATTATCATGAATCG
CTACAAACTTCGGACAATCCTCGAAGCAGAGTTTAAAGAAATCAACAGAAAAATCGAAGA
AGCTAGGTTGGCTTCTGGTCATGGCCACCGTTTTCATCTGGAATACACTTATCATTTCAT
AGATGACCTGTTGCTTCGCAATATCAATATCGATGATACGCTGAGCTTGATCAAAGGTTT
GAAGGAAAAAATACACGAAGTTGACGCATACATGTCTCTTCCGTATCCTCCGCATCTTGA
CGAAAAACGAGTCGAAGGTGTAGAATATCGACCAACTCTCTTAGAAATCACTAATGGATC
ACTTTGGAGTGGAATTACTCCTTCCAAAATTCCGCCATCATCAGAATACAGTTCTTCTTT
GACATGTCGAATGGCAATAGTCAATTCACGACGTTTAACTTCAAACGTAACTACAAAAGT
AATTAAAATGGAGGGGTTATGAGGCTATTGATGATTCTGATGATCGTATTTGCCACACAT
GTTTATGCCGGTCCGTCGGTAACAGATTTTACGGACACTCAAAAGTACAATTTGTCGTAT
GCTTACTACTATGGAAAAGCTTATGACTTGACGGGAGAATTGCGAGATGTCAATAATCCG
AATTACTGGAACGATCGTCATCTTGGCACATTATTCGCTGCTATCGCATGGGAAGAATCC
TCAGCTGGAATAAACACTGGTAGATATAAAACGGGTCATCATGCGTATGGGATGTTTCAA
AATCTTTACACAACGGTTACATCTAAACTCGACAATAGAGGAATTCCGTACAGACATTGG
TATCTGAAAAGGGATTTGGAGCGTCAAAGTTCTTCTGCCGAATGGGCTATGGATGAACTC
TCATACTGGCTCAAGGTTCGAAAAGGTAACATTCGAATTGCTCTGGCTTCATACAACGCA
GGTTGGAATTACCATGCTGGTTTGAGCTATGCCAATCGTGTTTTGGCAAAAAATCAAGAG
CTCAAACAATCGGACTTTATTTACAAATAAATGTTTACATTTTTGAGGTTATGGATACT
ATAACCTCAGTTAAACAAACCAATCAAATGTGGAGTAAGTTATGGACTTCATCATCAATA

AGTTCTGTATCGCGTTTATTGTGGCTGTTGAAGTTGATCGCGCTAAATTGGAAGGTTGCC
GATTTTTCAATTCACTTTACGATATGAACGTAGCTATGCCAGAAGAATCTATCGGATACG
AGTTTTTGATTACTCGCGATTCCCACGATTCCCAAATTCTGATTCACACCGATTCCCGAA
TTATGATTCACACCGATTTTAACTCGTCTGAAGTCCGCATAGAAACTGAAAGATTCATTC
GGGAATACTGTTTGTGAGGTAAAGCGCAATATCATCTTTTGTTATTGCTGGGCGTCCATA
CGTGGACGCCGTAGATATCGAATTCAATCGTATCCATTACAGCAATGGAAATGACCGTTA
TGGCTTGGCTCATTCGGGTGGAGTCGGCTTCCAGAGTTATGATTTCAATGATGTTCTCGC
ACACGAGGGTGAAATATGAAAAAGTCAATAGCAGCTGTTTTAGCACTGTCAATTTATGGT
TGCGGCGCGGCCGCTCCTGCAGTAGCAAACATGAACGAGACGTTCTGGTTTCCTGAAAAA
TCTGGAGTAATCAAAGACTTCACCGATGAAAATGGCACGTATGAATCTGCTCTGTCCGTT
TCATGTGATATGCAATACGCACAGAAACGTGACGATTGCCTTGGGATTATTTTCAATAGT
GTTTATTGGATAATTGACTCTGCACGATCTGACGACAGTATTTTATCTGGGCCTGAGCGA
GTTATGATTAGCTTCTACGGAGTTGTTCAAATCGTTGACTATGTTCGGACTGGGTCCGCT
ATCGTTATTTGGCGTAAAGATAATCCCGATTTCAACCCGATAGGCAAAGTAATGTCAATT
ACTACACCTTCCGGCAAACGAGAGTTCACATTATGATCGTTAACACAGTCAAAGGTAATG
CAATCAGTCTGTTTCTGAACGGCGAATATGACATGTTTGCGCATGGTTGTGACATTTTTA
ACCGAATGGGTTCTGGCATTGCTAAAGAAGTTCGTGAACGCCTCCCTGAGCTTTGGGAAC
TCGATCAAAAGACAGTCGAAGGTGATCGTTCCAAGCTTGGAACGATCTCGGCCACAAGTT
ACCCCTATGCTGGCCTTGCCTTCAACTTCTATACTCCGGCGACTTACTGGGACCCTAGCG
ATATGTTGTCCTAAGATGCGGCCCGTTCATGCTTTGAAACCTTGAACACTATGTGTGAAG
AAACTGTAGATTTTGGCCTCGGTAAGCCGACTATGTGTATTCCTAAAATAGGTGCTGGTC
TTGCCCGAGGTTATTGGGGCAAAATTGAAGCAATCATCAACGAAGTAACTCCAAATATTG
ATATTACAGTGGTGGAATTCGATGGCAGTCGGTAAATGGCAACAGTGTGGTCCGGTGATG
AACCGTATCTTCGAATGTGCAATAGTCGAATGACTTGGCGAAGAATGTAAAGGTATTATA
TTCCACTCATATCGTGACGCCGGCGATGTAGTTCTCGTTAATTACGAGTTGATGTACCAC
GGCCGAGTGTTTATTGGTCAAACCTCAATGGTGCCGTAATGAAATTTCCAAAAGAATTTT
TGATTGAACTCATTGGCGAATCGCTGGAAATCATCCCAGGAGAAGGTCTTCCATCAGAAC
TGTGTGGCAAATATGAAACTTTAGTAAACGAAGTTGTCGACCGCGGGCGCTGGACTGTTT
ATTACGACTTTGTTTTCTCCTTTGAAAAGGATGGAGTAAAGCGCTATTTCCGCGAACCAT
ATGAAGTCGGTGCAACAGAAGAGCAGGACAGCCCGATTTGGGAAAATGAACCTGATCTGA
TGGATGTCAGTGAAGCCGAACCGTATGAAGTGACTACAATTGCATACAAGTGGAAGAACA
ATGGCTAAAGAAATAGAACGTAAATGGCTTGTTAAATTTGTTCCATACAAGAATTTGGAG
CAGTTTCCTCATAAGCAAATTGCACAATTCTACTTTGATGGTAAACGCTATCGAAGTGTG
ATGGAAACTGGCGTTCGTTAAAAATTCATAGAAACAGTGAAAACTGGCGCCGGTTTGGTT
CGCGAAGAGGTCGAAAACGAAATCGACTAGAATACTGCATGGGAAGCCCATGATGCTGCC
GGTTTACCAACTTGTGTTGTTAAAAACTCGGTACGAAATCCCGCATGGTCATGGATTAAC
GATCGAACTCGATATGTTTGCTTGTGGGGAACAGTATATCGAGATCGAATTTGATAACGT
CGAAGATGTCAATGCATTCATTCCACCGTGCTGGTTCGGCGAAGAAGTTACCAATGATGA
ATATCACACCAACTATGCGCTTTTCAAGCGGCCCAACGAGGTCTAAATGGCAAGTCTTCA
TTTTCACTGTGCTGCAATGAATTCTGGCAAAAGCACACATTTGCTTCAAGTAGCACACAA
TTACGAAGAACGTGACATGCGTTCTATTATCATAAACCCTGCGATTGATACGCGTGATGG
TTCAACCGTATCCTCAAGAATTGGTATATCGCGGCCTGCTATTATGGTGAGGCAAAGCGA
TAGTTTGACCGCCTTAATCAGCAAAGAGCATAATGGGCAGCACATTAGTTGTGTTTTGGT
AGATGAAGCGCAATTTCTGCTTCCAGAACAAGTCAGCCAGCTTGCGCCTGTTGTAGACGA
ACTGGACATTCCAGTAATGTGCTATGGATTGTTGTCTGATTCAAATGGTAACATATTTCC
GGCAGCAGAACGACTGATGGTTTTGGCAGAAGAAAAGGTTGAGCACAAAACCATATGTTG
GTGTGGCACCAAAGCTACAATGAACATGAGAATCGATGAGAATGGCAAGAAAGTCTGGGG
GCCAATTGTTTCGATAGGTGGCAATGATCGCTATATTTCAGTATGTCGTAAACACTGGAC
ATTGGGGAAATCTTCAAAATAATTTCAAAAAGATGTTTACAACAAGGTTGTTTTGTATAG
AATATCCTCAAGTTAAACGAAATGAGAAACACATTATGAAAAGTTCTGATGTTTTTGCCC
AAGTTCAACTTGCTCAAGCTTTTGAAACTGAAGAGTTGAAACGCAGATTAGCTCCGGTTG
GAAAATTCGTTGCCGACGAAATGATTTCAAAACGAGTCTTTACAATCACCTTCGCGGGCG
ATAAATTCCCTGGAATTTACACCAGAGATATCGTCGACTATCTAAGAACTCTTGGATTTG
AGATTCGCACCGAATCTCATCGTAATGAAAGCTATATCATCGTAACTGCAAAACCCTAAT
TCAATCAAAAGGAAATATAATGCGTACTATGTCCAAAACCCCGAAAATTACCAAGAAAAT
GGGCCTTTCTATCGCAGCCGGTCTGCTGACCGCAATTACTGCCCTGAATATCTTTACTGT
GGTCGGTGATGGTTCCGTTGCAACCACCACTTTCCTGGGTAAAGTTAGCCCGAATATCAT

GCAGCCCGGTCTGAATATCATCAACCCGCGGGCATCAGTTGATACCTATTCAACTCGTGA
TCTGAAGATGGAATTCTCAAACGTACAAGTTCCTTCTCAAGATAAACTGAAAACTTCTGT
CGATATCACTCTGATGCTGCGATTCGACGGTGATAAAGCCCAGATGGTTCGCATCAACGG
CGGTACCGAACGTCAGGCCATTGATAAGTGCGTTGCGAAGAAATTCGAGAGTACTGTTCG
TGAATCCGGCAAAAACATCAAGAAAGCGCAAGATCTGTTTGGCGACGCTACCACTCAGTC
CATGCTGCAAGACATGATCAAAACTGAAGTCAACGACTATTCCAAGCCGTTTGGTTATGA
GGTAACCGAAGTGTTCCTGCAAGAGATTACTCTGCCGAAGCTGATTCAAGACCAGGTTGA
GCAAACCAAAATTCGTGAAGAAGCCGTAAACCAAGCCCAAGCGGATCTGGATAAAGCCGA
AAAAGTGGCACAACAACAGGTTAAGACCGCCGAAGCTGCGCCTGAAGCTCGTGAACAAAA
CGCGGTTGCTAACGAGCGTGATGCTGATGCTAAACTGTACGCCGCAGGTAAAGAAGCTGA
AGCTAACGCTCTGCTGCAGAAAACCATTACTCCTGAAATGATCAAATGGCGCCAGCTGGA
AGTCGAAATGATTCATACCAACAAATATCAAGGTGGTGTACCGCAGACTGTGGTCGGCGC
TGGTTATGATGGCCAAATGCTGATGGACATGCGTTCCAAGTAATTTAACTCGGGCTCCTT
CGGGAGCCCATTTTGATAAGATAACTTGTTAAATTGGAATATAATATGTTTAGCCGTGGT
TTTACTTTAATCGAATTGCTGGTATTAGTGGCGATTGTTATCATTCTGTTAGCCATAGCA
ATACCGGCTTTTTCGGATAACGAAATTTATAATCCGATCAATTCAGTATGCCCTCATGGG
TTAACAACCGCAATTACGCCAAATGGCGAAGAAATTCTTGTTTGCCGAAAAAATCAAGTG
ATCAATAAACAAACACTAACAATCGGAGAATAAATGCAATAGCAACTCAAAATGGGCATC
ATGGCGGCATTCGTAGCAGCAGCACTTTCTGGTTGTGGCGAGCAACAGTCTGATACATTT
TCTTATGATGGTCTCGGTGGAGAACCTAGGCATACCGTCACACCGACTTCGGCAGAGCAA
TGTCAAGAACTGGGTGGTGGTTCGACGGAACAATGCCAACTAGCATTTAAGCAAGCGAGG
GATGCACACACCTCAGCTGCACCGAAGTTCAATGACCAAACATCATGTGAATCTGGCACA
GGTGCTGTATGCACCCAAACACAAATTCAGAATTCCGATGGTAGTTTCAGCGACGTGTTC
GTTCCAGCTACGGCTGGTATGATTGTTGGCCAATTGATGTCAAGCAATGCTCGCCCGATG
CCAGTATATGCGCCAAGTCGACCCGAAGATCGTCGAAATGGCTTCGTCACTGCGAAGGGT
TCCTACGTTCCTCCTGGAAAAGGTTCTATCGGTGCAAACATTTTCAATACTTCCTTTGGC
AAGGGAAGTTTTGCCAAACCGGTAGCATCTTCAAAACCTGTTGTGAGAATGTCATCAGGC
GGTTTCTGAAAATCCGGTGGCTTCGGCTCTTCAGGTGGTTAATTTCAAAAAGCGGTTTAC
AGTGATGGGAGTATGATTTACTATAGTCCCATCAAATCATTAAGGAGTACATCATGATTT
ATGCTGGCCAAAAATATAGAATCACCAGACCTGTAAGCGAACAGCACATTGTTGGTGTTG
GTGATATTATAACAATCTCCCAAATCACTAGAAAAAGGCATTCGTTCTATGGATATTTCT
GTTGACGATGATGGAATTCCATATGAACGAATTCTAATGTTTGAAGGCCCGTACAATATC
CAGGAGTACTGGGAAAAAGTCGAAATGTGCATGGAGGCCGTTTAAATGATTGACGGAATT
TATCGGATTATTGGCGAAATCTCTATGAATCATCGTTGCATTAGCCAAGGTGACGAAATT
TATTTGAAAGAAGATGAATACGGAAATATCCGCGGTCCATCAGTTGACTGGGACGGGCAT
GAAACTGAAATTGGTTACATTTTGTGCCCAGACGCAGTGGAAGAAATTCCCGGCAAAACT
TACAACGAAGCTTACCGAATTTGGATTGACACTGTTTTGGAGAAAGTAGAATGAAACGCG
TATCTATTGAAGCTCGTAAAGATTGGTTGCCACAGTTGACTTCTGAAGGTGTTTTGTGGA
CCACTACTGACGAAGGTCCTTATTGGGCCGAAGCAATGGTTCAGCCCAAGTATAACTCCT
TCACTCGCAAAGAGCAAGAGCAACTCGAGAAAGCCGCAAACAAAATCCATGAAATGTGTC
TTGAGACAATGGAATGGCTGTTTACTAGCGACGCCAAAAAGCAAAACGAAATTTTCAACA
TGTTCAATATCCCATACGATTCTCGTGATCAAATCATATGCAGTTGGGAAGATGAAGAAT
TGGGCCTTTATGGACGGTTTGATTTCCTCATGACCGAATACGGACCCAAGCTTCTCGAAT
ATAATGCCGATACCCCCACCATTCTCATTGAATCTGCAATTTCTCAATGTAATTGGTTCA
ACGATCAACGCGAAAAATTCCATCAAGAAGCTTTTCAATTTAACGAAATTCATGAAGCAT
TGGTTAATCACTGGCGTGACATGAGCACTTATAACGCAATTGGCGATAAAATCAGCTTTG
TAGCTACTACGCAAGTTGAAGATTTTGCTACAATCTCATACATGGCTGAAACTGCTTCAG
AAGCTGGACTGAATGTAAAACTGTTCGATATCGCCGAAATTCAGCTAGGTGATGACGGAA
ATTTTTATGACATTGAAGGCCAGCATATCGACACTTGCTTCAAACTGTATCCATGGGAAT
GGCTGTATGAAGACGATTTTGGCAAAGCCATCGATGGTTGCAACACTCGGTTTATTGAAC
CAACCTGGAAAATGCTGCTTTCCAACAAAGCACTTTTGGTTCTTCTTTATGAGCGTTATC
CTGATTGCGAGTGGTTGGTCCCAGCGTATACAGAATATGATTTTGACCGATTGACTCTGT
TCACATATGAAAATCCGAAATGGGTGTCAAAGCCTATTGTTTCTCGCGAAGGATGTAATG
TTCATATTTTTGAATATGGCGGAGAAAGTGAATTCACCGATGGTAATTACGCAGAAGAAC
CGCGTATCGTCCAGCAATATATCGAATGGAAAGATTTCGATGGCTGCTACCCAATGCTTG
GTGTATGGATGGTAGGTAGCGATGCCGTGGGTTTGGGTATCAGAGAAGATGATAGCAGAA
TTACCGGTAATAACAGCCGACTTATTCCGCACGTCGTAGAAAATTGACAAACAGCAGATG

ATTTAAACCCTCTGTAAGGCGATAAATATGATAGGAGTATAAAGCCATGGATTGGCCAGA
TGCCTTGTCATATTGGTTAAACCAGTATGCGGTAAAGACAAAGGCATCAAATAGGCTTTG
CCTTTGTTTAATACCATCAAGGAGGGGCCAATGGCCCATTTGTCGTATGTTAAAAACATT
ATCAATAATCACATCGGTTTTAGCTTCAATGCACTTCGTAGAGGCGGACCCTGAAAATGG
GTCTTATGATGAGTACATGCGTGGCGCGTTAATAGTGTATAGCAACGAAATAGGGCCATC
TCTAGATAATTCGGTTGAATTTCTTAAGCATTTGAACGATAAATGGAAATCTGCTAAATG
CTCTTCGAGTTGTTATCAAACTGGATATCTTGAAGCAAAGACATTCATAGCAGAAAGAGA
AGGTCATGGAAGTAAGATTCGGTGATGTTGTAACCGAGAAAACAAGCATTTTTGATGAGT
TTATTGGTGCGTGTTTAATGTCAAGCACATATGCGCATTCCGCGCACTTTTATGACAAAG
TCGTATGAAACGCATATAGCATACGAATATTTCTATGACAATATGCCAGGGCTGATTGAT
ATTTTCGCTGAAGCGTATATGGGTTCAGGATTTACATATCGGCCGCGACTGTCCATTTAT
AATGGAACCTTTGAGTCATTTGTCAAGCAACTTGCCCAGATGGCGGAAAAGGTATCTGCC
AGTGCACCTAATACGGTTCTCAAAAACACCTCAGATGATATCCAAGAGCTTTGTCGTATG
ACAATGTATAAACTATCATTGCACTAATTCAAATGCCCTGGTGTGAACCAGGGCATTTTT
GTATCCGTTTGAAATAAAATTCAAAATGGTTGTACTCCTACGTTAAACGAGTTTCGAATG
TTATCATAATCAACCAACGGAGACAACCGATGAAACGTGTTAATGAAATGCCCAAAAATG
GTCAGTTTGTAGTTGTCTGGGTATATGACGGCAAACTCTGGTCTGAAACCTGGCGCTTGA
ACAACGGGGTGGTTGAAATCCTCATGGATGATGACACTGCTGATGAATGGCGTACAAACG
ACTGTTATTACGATGATGGTTATGAAGATCTCGAACTCAACTATATTGTGATTTAAAAAT
GTCTATACTTCAGGAAATCGACAGGCTGTCGAAACAAATAGGTGTGGAAGCATCAGAATT
CAAGATTTCGCCTTTCTGGAAAATTGTGCGCAAACCTGAGTGCTCCTATGGGCTTTCATT
TGAGCGCGAATTCACTGTTTACGAACATAAAGAAACGGGCTATACTATCGCAGTTCATTC
TGAGCGGCATAGAGCTCAATTTGTCAACGAAAACAACGAAGAGGCTATGGGTTTCGCTGA
AATGATCGTTTCGGTGAGCGAAGTAGAACCTCACAAAAGCAAACATGGCACACGTTGGCA
ATTTGTGTAACGCTTTAGTCATTTTAGTGTATATTACCAGTACAATGGTTTAGACTAAAA
AGACTGGAAATAAAAACTCGAGCGAAGCTCGAACGCGAAGCGATAACTAATTGGTGATAA
TATGCAAATTATCCTTCAGCCTGCAGGCGAAGGTAAAACCGCCGAAACAATCAAATCTGC
CTATATGGCAGCAATGAATCACCCTGTTCTTATCGTTTGTTCTGTAGAGTATCCAGCTGT
ACTGGAGCATCGAGTTAACATGTTCAGTATTCAGAATCCTGCTATCAAAAACCGCTATGA
CATCGTTACTTGTTTGGAACCTGCAACTCCCGAAATGTTCAAAGATCTTGGCTATTTCGA
CCGTTTTGGCACCATTATTCTGGATGTCAGCTTCGCAATGAGTCGCGGCCCTTGGATTAC
AGTGTGTGCCGAACTCGAAGCATATGGTTATGAAGTAATTGCTACTCAAGAACTTGTTAA
ACCTGCGCAAAAATCGCACAAAGTGTATCACTGATATGATCGTGGCCCCTTAAATGGGGC
CATTTGGAGGAATCATGAAAAGATATCGTTTTAAATCTGATGAAGCTCGACATGAATTTG
CTGCCATAAGCGACATCAATGCTGCTATTGCTAATAGCGTATGCGATCGTGTGTTTTACG
AAGCTATGCCACGAAATACTATCGATGAGATTTTTTTCGAAATCGACGGTATTACTTTGT
TTCCCGAGTATTTTCCTGTTTTCTTAGCTAACGAAGTAAAAGAATATTTCGAAGAAGTAA
GCGCTGGGGATGTTGCTCACGAGATATCCCAGAAGATATTGGAAAATAACGATGTTGAGC
AATTGCTTGCAGAAAACGCGGCCCTGCGGGAACGCATGTCTAAACTTGAAAATCGTCTTG
AACGTCTTGAACGGTTGGTATAAATGAAATCTACAGCTTTAGCGTATGTTCTTTGGTTTT
TTCTGGGTGGGTTTGGTAGTCACCGTTTCTACACCAGAAATTATGTCACCGGAGTTATTT
GGTTTTTTACTTGCGGTCTGTTTGGTCTTGGCTGGTTTGTCGACTTTTTTATCACTGCAG
GATTAGTTCGACGCGCCAACATTGAATGGCGCCAAAATCAGATAGAAATTAATACAATGA
TTCGTCGAGGTTATTGACATGGTTTATAATTATGATCGTGCTGTTGTTATTGGCCGCTTT
TTCCAGCTTTTTCCACCGTGTACATGAAATGCTGATTTACAAAGCGCTTCAAACTGCTGA
AACTGTTGCTGTTGTCTTCGGGTCATCCCATTGTAATCCAAACACAGTCAACCCACTTCC
AACACTGGTTCGAATCAGAATGTTCAAAGAATGGGTGAGTAACAATCTCACTCCTAGCGA
ACAGGCCAGAATTGTTTTCGGTAATGTTCCTGACTACCGCTATAACGAAGATCGCTGGTA
AACAGAAGTTCGTGAGGCCGCGCAAGAGCAACCGGGCGAGCGTGTTGCGATGATTGCATA
CGATAAGGATGAAGAATCTTATTGGATCAAAACCTTTGGATGGGACCATATTCAATGCAT
GGGCGTTCAGAATCACGGGAAAGACATCTCAAATACCCCAATGCGCGATGAATATCTTCG
AACCGGTTCTATCCACGACGATTTCCCTATCAATCCGTCAGTGCGGTCATTTCTTTTGAA
ATTTTGGAATACTGAAGGTTCCGAAATACGCAGCCGGTTGACGGAAGAACGTGCGATGTG
GGACAATGAACATGCAAAGGTTAAAGATAATCCATACGCTGATGCTCTCAGCTGCAATAC
TGCTGATGCCGTAGTAACGTGCAACAACCATATTCTGGTTATTAGCCGCAAACATGCACC
AGGCAAAGGTGCTCTTGCTCTTCCAGGTGGTCACAAGAATTCCAATGAGACCTTCCGCGA
TTGTGCAATTCGCGAATTGTATGAAGAAGTTCGGATTGATGTCGATCGTCAAAAAATCAT

TGGGTCTATAAAAGCACACGAAGTATTTGATCACCCAAAGAGAACGGCAGAGTTCTGCAA
ACCGACGGTTGCTTATTATATCGTACTTCAACCCAAAGACGACGGTAGTTTGCCTGAAGT
TCGTGCTGATGATGATGCAAAAGCAGCATTTTGGATGCCAATGCATGAATACAAGAGAAA
TCGGGCACGTATGTTTGACGATCATGCTGAAATCATTTCACGCTTCACCGGTATGTAGGA
TACAATTACGGAAAATGATGTAACATCACTAAAGGAGAATAAAAATGAAAGCAATCTGCA
TTCGCAAGAAAATCAAAAAGCAAAAATTTGAGCGCCCTACACCCGGTGATGACGTGCGAT
ACCAAAATGCTCGTTGGGAAGTTGCAACACTTTGTTGCATCACTGAAAATCCAATGATTA
TTTCACGCAAAAAGATGAGTCGAATTCATGTTATTATCGAACTTGCCACTGACTGCAGCG
AAATGTTTTCCCAGTTTAAAGAAATTTCTCCCGAAGGTATTACCAGCTGGGGTGAAACGT
CTGAAACTTCCGAAATCAAATTCGTCAGTGGTCATTTCGGCGTGATGAAAGAAAACGAAG
AAACTGTTTACACCCCGATTCAAGTCGGCGACGAATTCATCCATCGTAATCATGATAAAC
GATACGTGGTTAAATTCATCGCAGAAGACGGTTCTCTAATTTCTTGGGATTCTGATTATC
ATAGGTCAGTTTTCTTTAACCGCAACGATCGTGATCAACTTAAAGCATATGACATGAAAT
TATCTTAAGGTGGAAATTATGTAAAAACTAGTTTTTGAGGGCCTTCACGGCTCTCATCTT
TACGGTCTCAATACTCCTGAATCTGGCCTGGATATGAAAGGCATTTTCATCCCCGATCCC
AAAGATATTCTGCTTGGCCGAGCCAAAAGAAGCTATTCCATCACAACTGGCAACGACAAG
TCCAAGAACAGCAAGGATGATGTTGATATCGAAATGTTTTCGCTCCAGCAATTCATCCTG
GATGCGGCCAAGGGTGAAACCTATGCGCTGGATATGCTGCATTCTCCAAAAGACAAAATC
ATCACCGACTCGTCTGTTTGGCAATATATCCATCAAAACCGGTCTGCTTTCTACACGACC
AATATGACTTCCTATATGGGTTATGTTATGAAGCAAGCGGCAAAATATGGTGTTAAAGGT
TCTCGTTTAGCCGCATTACGAGAAGTAAAACAGTTCATGGATGCTCTTCAGATTCACGAA
AACCCAAGACTGCGAGACCTAAAAGATCATCTGCCACAAAACGAGTTCTGCTTCTGGACT
CACGTTGAAGAAAATCCCGACACCATGTCGTATTACACCATGATGGGCCGTAGGTACATT
GCTGGTGTTAAGATTGCTGAATTCTGCGGGGCAGTTGACAAGCTCTGGGCCGAATATGGC
GATCGAGCTCGCAAAGCCGAAGCAAATGAAGGTATCGACTGGAAAGCTCTAAGCCATGCG
TTGCGAGGCGGTCTTCAATTGCTTGAAATTTACAAAACTGGCGGTTTGAAGTATCCATTG
GCCGATGCTGAAGAAATCAAACTGGTTAAAGCTGGCAAACTGTCTTTAGCTGTAGTTCAA
GAACGACTGGAAGAAATCGTTGAAGCTGTTGATACGGCCGCAAAAGAGGCATCTAAGAAC
GGAATGCCCGATAAAGTGGACATTGGACCATGGGAAGCGTTTGTCGAACAGGTTTACGCT
GAAGCAATGAAAGAATATCTGGAGAGCAAATAATGAAATTGATTCGCTTTAAATTCAAAA
GTAAGGAAATGCTGACCGAGTATACTACTCGGTTCAGACGCCATATTTGGGCAGTAGGTT
TCAGCGCAAACACAGTTTTTATTGCCAGAATCAACAAACGTGGCCAAGTCAAAAGTATTT
TTGATGGTAAAACTCTTAAAGAAATTGTTTCTTCGAAAGAAGGTTGGCTTCCTTTTGCCT
CCTTTGAGTTTGAAAAGTTTTGTGAAAAAGTAAGCTAAACGGTTTACATTGATGGGGCTA
TGATTTACTATAGTCCCATCAAATCAACAAGGAGTATGTCATGAAAATCACTAAATCAAC
TAACCTGCAATCCATGGATTTCGTTGGCCGTGTCGTTAAGGCAGCGACAGAAACCAAAGA
GGTTAAAAATTTACCCCGATTCGAAACCATTGCTGCCGGTATTACCGTAGGAATCGTTGG
CGCTTGGGCTGCAATGTGCTTTATTGGCGATATCGATATGGCTCGTAAAATCTTCATTGC
TGGTATTATCGCTGGCGCAACTTTCCTCGTTATGAAAAAGGGACTGATGAAGTGATTAAA
GCTGCTGAAATGGCAAAAATGGTTGCACAGAACTCCGCAGAAATCGTCAATTCCGTATCT
GTGCATTTTGATGATTTCTTCAATGATAATATGAAAGACACAATGATTGTCAAAGGCGGC
TATATGTGTGTTGACGCGGGCCAATTCTTGCGTTACCTACGTGAAAAGCAGCCTTTTACT
CATTTGTATAATGATGACATTATCAAACGTTTTATTGCAGAACTGCGGGGATTGGGCTAT
AAAGCAGAACTTGTTTGTCCACCTATGAGCCCTGATTACATTTCAGTAGCGTTGGTAACA
AAATGATCGTTTAGGGGCATTAAATGGAGCTGAACATCGTTAAAGTATACTTGGAAAGCA
CACTCCAAGTTGAATCGCTCATCGGTAATAAAGTCGGTGAACTTGAGATTTTCACTAAAG
AATATTGGCTGGTGAAAGACGGATTTATCTTTCAGCATGACGGGCCAGGTATTTCCAAAA
ATGATTGTTCGCTCTGTTATGAAATTCTCCGGAAATGTCACTTTTCTGTTGAAATCACGG
TGTTTATGGTAGACGACTATGTTTCCAAAAGCGCCAAGGCTATTGTAGTTATTACTGATA
AGGAAGACAAATCATGAGTGGATAAATCTACCCTTCGTTCTCAACGTAAACACCAGCGCT
GACGTCACAACGACAGTGCTCATGTTCGCAATTTTTTGAACACCTTACAATCCGAGCAGC
ATGATCGCGATTGGCTCAATCTGTTTTGCGAAATGTTCAATGTTAAAACAATGGTGAAAT
GGGATAATACGTATCAAAAAACAGCCAGGATTCCGGCGTGGTTTTGGTACAAAATTGACC
GAATTATGAGAGAAAACCATATTCCATGGTGTAAAGCCAACACTGGTATAGCCATATACA
TGCATGAGAGAGGCGTCTCGCGAGTTGGCGAGTTGGCGTTTTCTACCGACAAAGGCGTAT
TGATGATCGCACGGCTTCTTGAAATTGAAGTTCATTCCTCTGGTATGAATTTAGTTTTGC
CGGTGTTGAATCGCTATCGGAACCGTCGGAATACCGAACTTCGCACGAAAATGGTTCATC

CTAAGCCTGAAAACATTCATTATGCGACTACCGAAGAGATTGATAGCATGAGAGATACTA
TTAGCATTGATAAAATGTTAAAAGAAATCCCAGCAATCGAGTCACGAATCAGCGCTCTTG
CTCGCGAACTTGCCCAATTGCAAGATAAACTCCAAAATACACGACAAGATGTCCGCGATT
ATCTTTCGAAAAAGTATGGTATTTGATTGAAAAGTTAGTGTACAGTGATGGGAACCTTGG
TTATAATGTTCCCATCAATAAACGAACGGAGTAATTTAAATGTCCATCAAAACTGCTGCA
ATCAACATTGGGTACATAGCGTCACTTGCATTGTTTTATTCGATGTATATCGGAAATGCT
TATGCCGTTCTCGGATGTGCAAGGGCAGATATCGGTTCAACCATTATTGTGAGAATTTGC
GAATGAATTATTATGCGACAGTTAATAACTTTTTGGAAAATCGTCGGGATACTTCCAATA
ATCACTTTAAAACCTATAAGCCTGTAGCTGAACGGTTGGCAAATTATCACCTCTTCATGA
AGTTGGTAAATGATATTTTGCCGAATCTGATGCCCGCATCGCCTCTCGGTAAACACAATG
GCATCTCCAAAATCAAGCCAAACTACTTTAATGTTGATGCCGAAAACAACGTCACGGTTA
AAGGAATTATTTGGCTCAACACAGCGGTTGAAGCAGATTGTTTTGCAGCGAGACTGGAAC
TGCTTAGGCATTATGGGGCTGCCGTAAAAATTGATATTTCTGAAAATGGCAGAAATCAAA
TAGTTAGATTTAGCATCATCTTGAGCGAAATTAAACGTGTCAAGACTGTTGCTGAAGATG
CAAACATTGTCGAAGAGGCACGATCGGCAGAACCTGTTAATCGCGAAACTGAAAAAGAAA
TTTGCGAAAGCATCTTCTTTTCTGAAGCGATCGGAAAAGCTATCAACACTGTTCGAGGTG
CACTCCACTTTGGCTCTAAACGGTTGAACGAAATTGACAGTGAAGTCGCCAAGTTGCAAG
CACTACGTAAAGAAATCGAAGACAACATGAAAATCATCAAAAACTCAATTGGGGCTTAAT
TAAGAATATGATCCGTCCACACATCGGCGTGAAAGGTATCCCTCTGATCGCAATGACTGA
TTATTACAAAATCACCCATTGGAACCAGATGCCTGAAGGTACCGAAGAAGCAGTTTTCTA
CGTCGAAGCTCGTGGTGGCAAATTCGATGAAATCGTTATCGGTGGCGTGGAATATCTGCT
GACCAAGTTTGCAGAAATCATCACCAAAGATGATATTGACGAAATGGATGCCATGAGAAA
GGCTCACTTTGGCCAAGACGTATTCAACCGTGAAGGTTGGGAAGCAGTCCGCTAATATCA
AGAACAGCATGGCTGTCTGCCCATCCAAATCGATGCTGTTCTGGATGGGACTGTTGTCCC
AGTCAAGCATGTTGTGCTGACTGTTCGTGTCAAAGCACCATTCGTTTGGCTGGCGGGTTG
GTTTGACCCAATCATTTTGAGAGCTGTCTGGTACACTTCTACTGTTGCCACGCTGAGCCG
CGAATGTATCAAATCTATTCGCCGCCACCTGATTGAAACTTCTGACCTGGAAGTAGGCAG
TCCTGATTTCAACTTTGTGCTGATGACCCGACTACATGATTGTGGTTCTCGCGGCGTTAG
TTCTGGTGAATCTGCTGCAATCGGCGGTTCTGCTCATCTGTACAACTCCATGGGTACTGA
CACTGTCGAGTCGCTGTATTTCGCAAAACAGATGTTTGGCGCTGGAATGGCAGGCATCTC
TGTACCAGCTCGTGAGCACTCCACCGTAACGTCTTGGGGTGCCGATCGCGAAAAAGACGC
CTACCTCAACTCGGTTGAACTCTTCGGTGCGGGTGCATATTCCATTGTGTATGACTCTAC
CGATTTCATGAAGGCAAACCGTGAAATCGAGAGCTATAAGGACAAAGTTATCGCCGAGGG
TGGTACTCTGATCGCTCGACCTGACAGTGGTGATATGATCGACAATATCAGTTTTGCTCT
GGAAACTCTGGGCGAAATCTTCGGTTATGAAACCAACAGCAAGGGCTACAAGGTTCTGAA
TAAGCATTGTCGTATTATTCAAGGTGATGGCATCGACGGTCCCGAAGTTATCAACCGCGA
TCTGACTGTTATGAAAGCTCGCGGTTGGGCTGCAGAGAATATTGCGTTCGGTATGGGTGG
CGGTCTTCTGCAAATGGTAAATCGTGACACCTGCAAGTGGGCCATGAGAATGTCTGCCAT
CAAAATCAACGGTGTATGGAAAGACGTATTCAAATGCCCAAAAGGCGCTGAATGGAAAGC
TTCCAAGAACGGTCAGCTTAAGCTGCGAATCATGGATGACGGCCGCTATTACACAGAAGG
AACTATTGAAGGTGGTCAAATGCTGTACGGCGTAAGCACCAAAGACGGTTTTGTTGAACT
TATGTCGAAAGATGCATTCGTCCGGTATTATGACGCTGAAAAGGGTCAAAAATTCTAGAG
CAATCTGGCTGAAATCCGCCAGCGTACTGCAATTAAATAATCATTCAAATGCCCTCCGAA
GAGGGCATTTGTCATTGGAGAAATCAGGATCAACAAAATTATCAAGTGGTATGAGCAAGA
ATGGCTGTACAAAAAGAAAATAATCTATGAATGGGAAAATTGATACCTGGTAAAATATTT
TTCGATTGACGGAGAATTCGGACATATTCCATCATATTGGGGCCAAATGTATGACGGATA
TAAGTTAAATCGTTTACGACTCTTTATTGTTTCTATTGAATGGGGTCATATTGTTATGTC
AAAAGGGGAGTGGGAAAGTAATGCAAATCAAACTGACAAGAAATGAATTCAAAAAAGCTC
TAGAAAATAATGACCAATACGAATATCCTCTGCACAATGAAACAATCAACCAATTGCGTT
CAGATTTTTCTGTCCCACAAGTTGAATATGTTTATTTGTCATTTCAAGAATATCAATCCA
TTTTACCCATAATCCCTCATGACGCGAGTATGTATGCGCATACATAATCATGCCAAAGAA
TTCACCAGGGTGATCGGTCGCAATATCAGAGTTAATCTTTTGTCGACAGATCATGAACAA
ATCAAAAACACGATATATTGGGCATTTCCTGATATAGAAGAAGTTGAAATTGGAAATTCG
AAATATTTTGCCCAAGACGGTAAAATATATAACGCAACAATAGACTACAACACCAAACAG
GTTATAATGGTGGCAAAATGACCCTCAAGTTTAGAAAATTCAAAGCAAAAGACGAACGTA
ATATGTTTAAAGTTACCACCAACTTCAGCGATAAGTTTGATGATAAAACGATTCATTGGG
AACTGCCAGCTGTTAATTTAAACGAATCTTCTATTGAAATTGAGCGTCTTGCCATTTTCC

TGGAAAAATGCAAAAATCGATACCCAAACGGTAAGGGTGGTCTGGACGATTTCACCGACA
TTCCCGCCTACTATCGCTATTTCTGCGAAGAGGAAGAAAGCATAGAAATCTGGGAAAAAT
ACGGTGTTGAAACGCTATGGTGGCCACGCCATGATGGTGAATATCCAAAAAACCTTGCCA
GCTATGAAATCGTATATACTGATAACGATAACGTGAAGTACAGTGTAACCGTGTAATTAA
ATAAGAGCATATGGAGGAAACTTTATGCTCTTAAATTCCAAGTCTGCTGATGAATTAAAA
CAGAAATTGTTTGAAGAACAAAATAAGTGTTGTAAATTGTCTGGAAACGAGTTTGACGGT
CCATACAATAAACAGCAATTGGATCACGATCACGTATTGCAAGGCCCGAATGCCGGAAAG
ATTCGAGGTCTACTTTGCAGTAGATGCAATCTACTTGAAGGCATAATGAAACACAAATTT
ATACGGTCAGGATTAAAGGCAAAGGATGTTGATTACATCCAATGGATGAGATCGCTATTA
CAATATGTAGATTCAGATCATTCAAAAAATCCAATACACCCGAATTACGTTTCCGACATG
ACCAAAAATTTCTCGCGGAAAAACTTGAGTGAAATGAAGGCCGAACTACGCTAACGAGGA
TCTGACTTTGATGAAAAAGATCAAAAAGCTGTTTTAGTCAAAAAATGCAGAAAGCAATTT
AAGAAGGCCATTCAATGACTACCCGATCACTTGTAAAGCAATTAGTAGAAGGCCAATCTG
GCCTGCACCACGAAAATGCGAATAAAAACGCTGACTTTATCCCGACCATGCGAGATATGA
TGGCATCTATCTCCTTATAGGATTACGCTAAAAGCATTTTGCCATCTCATTTAATCGAAG
CACATTATAGTGGAGATATTCATATTCACGATCTAGATTATGCTTTTACTCTTTCAATGA
CAAATTGTTGCTTGGTTAATCTCAAAGAAATGTTTAAAAATGGCTTCAACATGGGTGATG
CCCAAATTGAAGAGCCTAAATCCTTTGGTGTAGCTTCAGCCATTATGGCTCAAATTACCG
CACAAGTTTCTTCCCACCAATATGGCGGGACTACTCTTGCCAACGTTGACCAAGTCCTTG
CGCCATACGCCATAATGTCATGGAATAAGCATTTATCTGTCGACTGCAGCTTCATCAGCG
ACAATGATAAAATTGAAAAATATGCATACGAGATGACTGCTAAAGAAATCTATGACGGTA
TGCAAGCTTTCGAATACGAAGTCAACACGATGTTTACCACAAATGGCCAAACACCGTTCG
TGACAATTACATTTGGGATGGGAACTAACTGGTTCGAAAAAGAAATTCAAAAAGCTATTT
TGAAAGTACGAATCAAGGGTCTTGGAAAGAATGGCATCACACCAGTATTTCCAAAAACTC
GTTATGTTCATGGAAGCTGGTATAAACTTAAATCCTGGAGATCCAGACTATGAAATTAAA
CACCTTGCTCTGGAATGTTCAGCCAAGCGAATGTACCCTGACATTATCAGCGCGAAAAAC
AACCGATCCATCACTGGTAGTAAGACTCCCGTTAGTCCAATGGGTTGCCGGTCTTTCCTG
GCTGGTATAAAGTCTGGCGAACTAGATGGCAGAAACAATTTAGGTGTTGTCTCTGTCAAT
ATTCCCCGGATTGCTATCGAAGCAAACGGATCACAAGAATCGTTCTTTAAACTACTTCAA
GAAGGTTTTGAATTGTCAGTTGAAGCATCTCTTGAACGAATTAAACACCTAGAAGGTGCT
AAGACAAAAGACCGCCCCAATTCTCTACACTGAAGGGGCCTTTGGTCTATATCTTAATCC
GGAAGAAGAGTTTTTTGCCGCATTTGAAAGAACGAACTTCGATTTCAATTGGTTATATTG
GCCTTTATGAAGCTGCTCTGATTATGACTGGCAAATCAACAGCCAATGATCTAGAAGCAC
GCAATTTTGCGCTTTCGATTGCAACATATCTCAATTTTTCATGCGCAAAAGCAAAACTCA
ACACCGGATGGAACTTTAGTCTTTATTCTACTCCGTCTGAATCTCTGTGCGATAGATTCT
GCCAGCTGGATCGCAAGAAATATGGTGTTATCGAAGGAATTACGGACAAAGATTGGTATA
CGAATAGCTTCCATATTCCTGTTGATGCCCAGTTGAACCCGTATGACAAAATAGATGCCG
AAAAAGAATTTCATTGGATTGCATCGGGTGGACATATCAGCTATGTCGAGCTACCCAATA
TGCGAAACAATCTCGAGGCCCTTGAGCAAATTTGGGATTACGCGATGGAAAATCTTTTCC
TATTTTTGGAACAAACACACCTGTTGATAAATGCTACGAATGCGGTTTTGATGGTGAATT
CAAATGCACCTCGAAAGGATTTAGTTGCCCGAGCTGCGGTAACCACGAGTCGGCAAAGAT
GTCAGTTATCCGAAGAGTTTGTGGGTATCCTGGTGCTCCGTTGGCGAGAGGATTCTATAA
AAGCAAACAAGCCGAGGTTTCATCACGTGTCAAGCATGTGAAATAATTTTTAAAAACTTG
TTTACATTTGTGCAGATGTGTGTTAATATACATCTGCACAAACTAAACAGCCCAAATAAG
GTTATTATGTCTGATATTAAGCAAAGTATTGAAACCTTTGAAAAGAAATTCCAGGATATT
AAGTCCATCCTGGAAACCCCAATGGATAAAGATGAAGCTCAACAAGCAATCCACAAACTG
AATTGTATGTCTGAGGAACTTGAATCAATCGCTCAAGCTGTTGCTGATCGATTCTGCATT
TCCTTCTCGACTGGTGATTATGGATATGGCCGTACCTATTATCCTAAAGGTAACCGTTTT
TGGTCAGAAAGCAACGCCGAAGAGTATGGCCAAGGAACCGAGAATGGCTACTGGCTCAGT
TCCTCTGATATGTGTTAAGGAGAAAAGAAATGGCTCGTTCTAATGAATTGGTAGAAAAGG
CAGTTCAACTTCAAAAACTTCTTGTTGAAGTTGAAGCACTGGCAACAGAACATGCTTACG
GCATGGAAATCAATGGCAATGAAGTTGATTTCGACGACTAGCTGTCAAGCAGTTGTTATG
GCGAAGGTTCTGAAGCATTTGGCATTAATGCAGATGGCTCCGTGTGGATGGAAAGTAGTT
GCTAATTAAGGGGCTCTTCGGAGCCCCAATTTGAGGGTTTTGTTACGATTATTGTTGGCT
CTCGTGCTTTTCGATCTAGGCTAACTCCGTTTGTTGCTCCTACAGTAAATATGGACCATG
ACTTCATTGCATACGAAGAAGAATGGAACGAATATCAAGTCAGAATGCAAGGTCGGACTA
TTGAAGTAAAAAGCCCGAATGTTCGAGCTTTCGTCGTCGGTGATGGTAAATCTCGTTGCT

GTTATCACGAAGCATATATCGTTCAACCTGGCTCGTCCGATGAAATGATTTACAAACTCA
TGGATGCTGTTCCCGGTAAAGACGTATATGCCACTCCCGAAATTCTTCTCGCCATGAAAA
TGGCTCATCGGTTCAAAAAGAACACTCGCAATTTCATCAAAACCATGATGCACATTCGTG
AATTGCGTGAATTTGGTATAGTCATGGGGCCTGAAGACTTGGAAATTTTCAAAAAGCGTG
AACAAGAATCTTTGAATTATAGCCACCCGAAGCTTAACGTCTGTAAAGGCGCATTTTTCA
AAGACGACATCTACACCTATGATCATGACACAATTCACGAAGCAGTGGCGGTTGAAGGTC
TTCTAGCATATCGATCATACATGGTTGATGGCGCTGAAGTAATGACGTCAAAAGAAAAAT
TCTTTAATTGCTCTCGTCGTATTCAGTTGATGGGTGTTTATGAAGAATCTTGTGTTTTGG
CTCTGGAACGTTGTCTTATTCCTTTCCCAGGAAAAGTTCAACCCTATAAAGCTTTTCAAA
TGGCCCTTGAGAAAGTATGCACTTCTATTACATCTGGCTGGTTCCGTGAATTTGCGTGGG
AAAATTATTGTGACGTTATGAAAATTTACGCCGATCAAGTCAGGGCTGGCGACTCATACG
ACGAATTGTTCAAACGAGACCAGCATCTCCTGAAAATTCACAGTAAATAAATATGGATTT
TCACATAGGAGGTATACTGTGGAAGGACACATAGTCCAACAAAAGTTCGTCAGAACTGAC
ATCCCGGAACCAGAACCGGAAAAAGAGGATGAATCCGAAAATCCTAAAACAGACGAATAG
AAGCTGTTAATAGTTTATGCCCAGGTGTTATAATGACCCTGGGCATTTTTGTAATTGGAG
AATGTAGTTGATATTGAAATGCATATTGAAGCACAAAAACAGATTCGAGAAGCTGAATGG
TCTGTTCGCACAGCAAAACACGCTCGAATGCGCGAACTGCGAAACGCATATGGAGAAGAA
AAAGAAGCTGGCCGAGTCGAATCTGAACTTATTTCAGAAGATCATGTTGCTAAAGCTATT
TCGAGTTATTTGAAGTCGAATGAATATGACGTAATGCTTGACCGTAGAAAAGAAGTCGCA
AAACAAAAATTTCGTTGTCCATGTTGCGATACCGCTCAAATCCAACTTGTTTCATGGGTT
AATAAAGTAGTTGATCTTCGATGACGCAAGTGCAACCGGAAATTTATTTGAGAGTCTCCG
ATATGATGTTTGGAGAAGCTAAAGAGTTAATAAAACGCCCTCCTAAACTTTTCTTTGGTA
AAGAATATGATGGGACTCATGTAGGCAGTATTTTGGAAAAAATACAGCAATACAATGGTC
CAGGAGATGCTTATCGAGTGGAGTATCGGGTACTGCTAGACGGCGGTGATGTCGTAACTG
GAGTAGGAACAGCCAATTATAACTGGATAACAAAAGTTGGCTACAGGCCTGATGTTGAAA
TAACAAGAATCAAACCCACAGAGGCGACGTTAGACCGATGGAGCTGTATCTATTCGGAGA
AATGGCATGATTGAAATATACGGAATTGTTTCAGAATCGCACAAATTCCCGGCATGCGAC
TTTATTGTCAAACGTCTTGAAGAAGATGGCATAGAATATTCCATAAAAAACTGTTTAGCG
GGATGCTAAAACTGATCTCGGGTTTGAGTACCAGCGAGACGTCATCGATGAGCTCAGGCG
TCGTATAGGGCAACCCCAAGGTTCCCTGGAGCTTCCCAAGGTTTTGGTTAATGGGATATA
CATCGGCGGTCCCAAAGCTCTCCTGGATTATCTGGATTCATAGGCCTCTTCGGAGGCCTT
TTTCGTAAAAGTAATGTACAAGGTGTTTTTGCGATGATAAGATAAGTCACAAGTCAAGTG
AATGAGATTTACATTATGATCGATATACCGAATATTATTGAAAATATGAACAAAATGTTT
GCGATTCTTGGGTATAAGCCCAATAACGGCAAAGCGTGGACAGGCTATGTTGACGCAGAA
GGCAAAGGCCGCCAACCAATCAATTGGCAAACTGCTAAAATGGTAGGCTTGAACATCTGG
TCGCCGCTGTGCCAGCACGAGAAACTGTTTGGAATTACTGCAACTCCGCAGCAACTTGAC
GAAGTTCGCAAGTTTGCAAAAGACAACCAACGAACAATTGGTTGGTCAAATATGCTGGTG
TATGACAAAAAGAACAAACTAAATAGAGCAATGTGTTTGATTGCTAATCCTATGTCAACT
ATGTGAGAAAATACTTTGTCTATGAAAAATATTGCCGAATTTATTCGTACAAAAATCAAC
TCGTTTGTCCAGAAAAATACTTCTGTCGAAGACCAATATGCGAAAGCAGCAGAACAAGTG
ATTAGCCAAATCACCAGGCTGGAAACGGCACATGTGAAATCCGTTTCAGAAGAAAAACGG
TTGAGTCAATATGCAAAAGATAAGCAAGTTGAGGGGCGGCGAAAAGATAATGAAATTCGT
ATGCTCATGGATTAAAATGTTCCCGTCGAAACTCATGTTAAACTTGCTCTTCTGTACCGT
CGTACGGCAGATGCGCTGAATGCCAAAGCTGATGATTTGGAAAAAATGCGGGACGAGATT
AAGCTGGCGGTTGTTGAACTCGACAATTCTCGTTTAGACTTGGCTGCCAAACTTGAGTTT
ATCCGCGAAACTCGCAATGCAGAAGCGCTTGGTATTTCTTGTTCCGAAGATGTCATCGAA
CTGGCTGGTCTCACCAAAGTCGATGTCAACGATGTTATGATGCGTATCGAAACCTTCAAC
GGCGATTAACAAACAGCTGTAACAATTAGCGAAATTGAAAATTATATCAATGGTCTGAAG
TAATTAAAAAGGGCCTCCTTGGAGGCCCTTTGAAAATGGAGTATGTTAATGATTTGCTCA
ACATTAGTAACTCTAGTCGATCCAAATGGAATTCGAACTAACTATCGGTATAATCCGGTG
ACAATGGTATATACCAACACGAAAGGCGAATCTATTGACGCATACACCGCAGATTCTATG
GGATATACTATCAGCCGAACCGAAGAAGCCAAAGAAACGATCGATTCGTTTCTCAAACTG
TCTAATGCCGAAATGAAAAAGAATTATCGCGAAATGATAGGTAAAGATCGTCAGATTGAA
CGAGAAGAGCATGATGGCAGTGCTAAAATGGATTACGAAACTTTGGCTAGAGCCACTGAA
ACTGCTGATAGTCTTATACTCAATAAGATTAAAACGAGTGATCTTTTGAATTTGAAGGCG
CAGTATATTTCATCTGTTTCTGGTATGTCGGCTGCTGCGGCTGCCGTTGAACAAATCTCC
GGTGGTATTCAAATTGGAGTAGAGCGGCGATGGAAGCGGACTTCGGAAGCCAGCCACGGT

GAAAAGTATATCGCGCATTATAGCATTTTGCCTGTAGCAGATTTCAGAGCCGTTGGTGAC
GCAATCAAATTCGTCAAACTCCTGCGGTCTGCACATCCTCAGTTGTCACATATCGGTTTT
ACAAAATTGGCTTACACTAAACTGGCTTTAAACAACCGCGAAAGTGAAGTAAAATTTGTC
AAAGAATGGATTAGCCCATTTACCGAGGACGTGCTTATCAATATCATGCTGGAAAAAGGC
TATAACATGTCGATCAGCTGCCCAAATCAAATATTGCTGTTGGGTGAGTTTTCGAATTAT
TCCATTCCTGAAATTGCGCGAATTGAAATGTTTGTAGACTATTGCCGCCGAACTGCTCCG
AAGAGAGACTGAAACAGTCTACAATGACCGTCAATCGTGATAAGATAATATAGTTGAAAC
AAGCATTCAAATTTTCGAGAGGAATTCAAATATGTCTAATGCATTCATTGCGGTTCGTAA
CTTTGCTGTACACACTGCTGCCGCCGAATTCACCGCTGACAATATTCTTGGTCGTGAAAT
CACCGAGCAAAAGATCGACAAAGTTGAAAACACAGTATGGGATGCTCTGATGGAAGCGGG
TGTTACCGGCATTGGACGCCGAGCGATTCGCAGTCTTATTGTGCAATATATCGCGGATGA
ATTCGGTACCCAGCCGCTTGGCCACACTCCTACACACGCAGGTTACAAGCAAGTCGCCGA
AGACGCGTACAAGAAATATCAAGGAAAAGCTCGCAAAATCCGCCAAGAAATGTACGGCAG
CAAGCGATAATTTTCTTAGCTCCAAATAAAGTATCTGAAGGCTTTTCGCAGTCTTCCGAA
ATAGGAATATCACAATGGAATTCAAAATTATTGTTTCAAGCCGTCATGCTGGTCCGATTG
ATCGCTATATTATAGATTTGAAAAATATCGACCGCGTTTCTCCGGGTGGTTGCGAAATGC
ATATTTTACGTACTACTTCTGGTGCTGATAACATTTTCCATGTTGACGCTTATGACCCCG
AATTTTCCAACGAAGTTCACCGGCACGTTCATTACATTCTGAATGCCATTCGTGTGCCGG
CTGGTGCACACGTAGTCGGAGTTATATAACGAGAAATTATAGCCGCCAGGCCGAGAAAGC
TGTTAGCGAATTTATCTACATGGAATAATTAAAATGAATTTCAAGGGCTTTTATGCTGGT
GTAGGGAGTCGCGAGACTCCCGAACATATTTGTCGTTTGATGAAAGATATCGCGGAGGTT
GCTGCGATCATAGGATATACCGGACGTACTGGTGGTGCCGATAAAGCAGATGACGCATTT
GAAACTGAATTTTTGGCAGTTGCTGATAGTTTAGTCATCGACAATATGGCTGAATTTGAT
GTTTATCTGCCATGGTAACGATTCAATGGCCGTTTTGCACCAAAACATGCTCGTCACAAC
CTCATTTTACCAAGCGCCAATGAGTGGGACGCCGAGCAAATCATGAAAACGGTCCACCCA
ATTTACTCCAAAGGAGGTAAATTGACTGGTGGTGCTCTTGCACTTCATACTCGAAATGTG
AATCAAGTTCTAGGTTATAATCTGAAAACACCTTCAGAATTCTTGGTGTGTTATTCGGTT
CCAACGGATAACGGAGTTTCTGGAGGAACAAATACTGTGTGGCAGCTTGCAATGTGTCAT
GGTGTAGAGTGTTATAATCTTTACAACCAGTTTGACATTGAGCGGCTTAAGGAATTTTTG
GATCTGTGAAACTTTACACTCTCTCCGCTAAACCAGCTGCAGGAAAAACTGCAGCTATTA
TAAATCATATTGTTGAAAATAACGAAAAGGCAAGTATAGCCAGCATATCTAGACAATTGT
CGAAGCAATCTTTCGAATACTTTGTCGGTCTGGGCGGAGAAGGCGACTTAGTTGACACCG
ATAACCGTCAAGGCCAAAAGTCGGTACACGAAGCAGTTATCAAAGCATCCGAAGAATTTG
ATGTGATTTTTATAACGCACGCGGCTTTAATGGGTCTTCGAGATATTGATGCTTTAAAAG
ATTTTAATCTGTATATCGATGAGGTTCCAGAATTAACAAGCTTCGAGCGATTTACTTTCA
CAACATCAGTCCAAAGTCATATACTCGAATTGTGCAAAATAGATGAAACTGGACTATTAG
AGCTGCGAGATGAGCATCGAAAAACATTCCAAAAAATGGCAATAGATGGCTTTAGTGGAA
ATGATGACATTTATTCAACTCTTTTTCCGTTGTATAAAGCACTCCTGACAGGTGTCCCTG
TCAGGCTGTCAGTAGAGGGAGGTGTGACGCAATGTTATTAAGTGAATGACGCGTCAAATG
ATGATTGGGCAAAATTCAAGTTAATAACTGTCTGTTCTGCAAATTTTGAGCAGACTTTTA
CTGGCATGATTATGAAGCATTTTAACGGATGGGAATTTGAAGAGTCTCCGCTAGTTGAGC
GGTTAGCTTTCCGAGATTATCCTAAAACTGCTCGCGTAAAAATTCATGTAATGTTTGATG
ATGATTGGTCTCGTTACACAGCCGACAAGGAAGTTGATGGGATTTCGAATTACACTCGCA
TCGAAAATACTCTCGACACAATCATAAATGGCGATTTCATTTACAATCGAAATTCTTATC
GGCCAAGAATGCCAAAGGGGACGGAAGTTCCTGATAACCCGCACGGTCTAAACAAATATT
CGGATTTTACCAATGTGGTTGTGATGTTTTCGTATAATCCGGCGCCTTGGCAAGTTCCAC
TTTTGAAAGAACTCGCAAAGTCGGTAGGCTTAGATGAAAGTGCAATGGTTGACGCGTACA
TAGTTTCAAAGTATCTTGAACCCGCATTTCAACTTTGCACAAGATGTGATATCCGAAATG
AACATTCGCTAAAACCCATCAACCTGTATGTTCCTGATATGAAATTAGCTCAATATATGT
TGAGATATTTGCCGCACGCTGAGATATGTGACGGTAATTCAATCGAAAATCACAAACAGG
TAAAAAGAAATCGAGAATCGCATCAAGCGCTATACCAAATGACCGAGAAAGAAAGGTTCA
AATACAAGTATCCGCTAAGAAAACTTGGGCGAATACTCGATATCAATTCTGCTAAAGACC
AGGAAATTATCCAGGACTGGATAATCACAACGCGAGAAAAAGCCAAAAAGAAGGCCTCTA
CATCAGGCCTTTGTCGTATGCACATGATAACATAGGGATCACTCTGGGGTTATCAGGAAT
TTTTGATGATAGGTTATTAGTCTAATGACCTTGTTTAAACCTGGTTGATTATAGAAATCC
TGGCTCCAGGTTCAAGCTTGCTTGAACTTATCTTTTTTATCCTTTTAGTTATAATATCTA
TTAAGCCATAAAGGATAAGTTTTGGCAGGGCCAAAACCTGCAGGCAGTAAAGTTAAATAA

GTTTAGCAAGCTTATTAGTATGATATACTCAATCATAGGAGGAATTGCCCTATGAAATAT
GTTGACAATGAAGTTCTATACCTGAATATTTGCAAATGGAAACAAGATATGCGTGATGCA
GGGCTTCACATCAAGATGCCAGATGCAATTGGTATAGACATTATGAAGATTGCAAAAGGT
TTTTCCGGATATTACAAATTTGCTGGATATACGCAAAATTGGAAAGACGGAATGAGATCT
GATGCAGTCTAAGCTGTTGTAAAGGGATCAGTAAATTCCGATGAAGAAAACTACAAAAAC
CCCCATGCTTATATCACACAGGCTAGTTAGCGCGCCTTTATTGGACGCATCAAGTACGAA
AAACACGAAATGGCTATCAAATACCGGTATTTTTTGACCAACGTGTATGACGAGCATGAT
GAGGATATGTCACGATTGGTTGATGAAAATTTCATCCAAGACATCCATGGCAAACTAGTA
GCCTATGAATCATCCCTTAAATCTAAAAAGAAAGATAAAGAGGATGTTCCTAATTTGGAT
TCATTCTATGAAGACGACCCTGAAGATTAATCTTTTACCCATCATTGATTCAACATGCGG
AGAAATAGAATTATTAAGCGTATTTCGTAAAACTCCAATTATTGGATTATAATATTCCTG
TAGTTTTAAAATCCTGCCAATATTTCATGGCAGCCGTTCGAAGTAGAAACTAGGCCAACT
CGATTGGCATATTGATGTAGACACAATGACAATGGAGATTTCGTATGTCGCCTGATTACG
ATGTTGACCTTAGCACTGGTGCAGAAGCTCTGAGTGAAGTTTATGACCATAATCTCATGG
ATTTACAAGAAGATCTACGAAAAACAAGCTGAAGAAAAAAGCCGCAAAGCATCTTAAAAC
TCATGGGAGAGAAATTAAGCGGCTGAAGAAACATGCTGAACGAGCTCTATTCGCAAACAA
TAAAGAACAGTATGCGTATGCTATCAGTAAACTTCGTACCCTGTACAAGCAAAAGCAACT
TCCGCATTCTGCAATGATCACTATGTTTGAAACTAGTCGTCAACAAATTGTTGACATGGC
TAAAGCATATGCTACTGCCATCCAACCGGCAGAAAAATAATCAAGCAAAATCGCAAACTG
TTCCAGTTTGTTCTATTTGATCTATTATAGGCTCCACGGAGCCTATTTTTGTATTAGGAT
TTAAACATGAAACTTTTGCATCTCGGTGATATGCACAATGGCGTAAAAGATGATGACCCT
TGGCATGAAAACATCGCTGAACACATAATTGACCAGGCTGTTGAATATCCTAAAGCAAAT
GGGATAACTCAATGGCTTCAGAGTGACGACTTTTTTGACGTTAGAAAGGCTGTAACACAA
CGTACAATGGAATTCGTCAGGACTCGAATTACGCCCAAATTGAAAGAAGCTGGTATTATA
TGCTATGTGATCTTGGGAAATCACGCCATGAATAAGAAGGATAAAATACATCCAAATAGC
GTAACCGAAACTCTGGGTAAAGATGAAACGTATGTCGTGATTGATAAACCGACGACGGTC
AATTTCGAAGGTCTTGATTACGACCTTATTCCGTGGTTGTGTAAAGAAAATGGATCAGAC
ATTTTCGAGTTTGTCAAAAAGTCAGAATCCGCATCGTGTCTTGGTCATTGGGAACTAAGC
GGATTCTATTTCTACAAAAATATTCCGAGTTCGGGATACAGTGGAGATTTTTTGAAAAAG
TATGAAAAAGTTGTCTCTGGACATTTTCATACTCAATCAGACGGTGAAAATATTCACTAC
ATCGGCACTACTTATACGATTACAGCCGGTGATGAAGATGACCCTCGCGGTTTCTGGGTT
CTTGACACAGAAACCCGTGAATTTGAATTTATTCCGATTTCCAAAACATGGCACAAACGA
CTGACTTATCCTGGTGTTACAATAGAAGAAATTCGAGAGTGCTCCGGTTGTTCTGTCCGT
TTGATTACTAATGAAGTTGATGAAGGCTTGACAAAAGTTGAATGTCTTCTGGCTAAAATT
GTTCATGATCTGAGAACCATCAACAAATCTGCCAAAATTGAAGTTGATTCTTCATCTGAT
ATAACCGATGACGGCGAAACTCTTACTGTTTCATCTATGGATACTTCTGGTAGTTCGATG
ACCATAATGGGTTTATTCGCTGAAAGTGTCGCAAATTCCAGCATCGGTGCGGAACATAAA
GAAGCAATCGTAATTATGGCAAATGAACTTTATGCTGAGGCTATTGCATCATGATTGAAA
TTAAAAAGAAGCAGCTTGGATTTATTCGAGTTCGCTACAAAAACATCATGTCCGTAGGCG
ATGATCCTATCGCTATGTCGCTTAACAAATATGCTAAAACATGGTGCACTGGCAAAAATG
GTGCCGGAAAATCGACAATGTTGGAGGCAATTTATTTTGCGCTATACGATAAACCTTTTC
GAGATATTACAAAAAACCAACTGCTGAATGAAAACACTGGCAAAAATTTGTTAGTGGAAC
TTTGGTTGGAATACGAAGAAAAGGTTTTTCACATTACACGTGGAATTATGCCTAATAAGT
TCTCGATAACTTGTGACGGCGTGGCTATAGATGCTGCAGCATCAGTTAAAGATTTTCAGT
CATATTTCGAAGAAATGATTGGAATGAATTCTACCAGTTTTAAACAGATAGTTGTTTTGG
GCACTGCTGAATATACGCCGTTTATGCAACTAAAAAGCGCCGGAAAGGCGCAAACTGGTG
GAAGATTTGCTGGAAGTATCCGTCTTAGCTCAAATGGATAAAATCAATAAAAGCCACGGA
TTAAAGAGTTAAACCAGGATATCGCTTCAGTTGATATGGAAATAAACCATATAAACTCAC
AGCTGAAAACATACCAGGATCACCAGAATAACTCTAATGCATCCAATGCTGCTGCCATAT
CCGACCATCAGGCATTATATGACACTGCTCGCGAAAAAGCAAAAGAAATCAAGTCGGCTA
TTGATTTGCTCACAACGCAAATTACAGAATTCGTGATGACTGAATCCCCTGCATCCGATC
TTGGCAAGTTTACAATGGCTAGAGCAAAAGCCGCAGCGCAACATGAACAATTTTCCGGTA
TTGCTCATATGCATGAAGATGGTGGAACGTGCCCAACATGCATGCAGCAATTTGACGATC
CTGAACTGCTAGTGCAATTGCGTGATAAAATGTCGCAGATCCAAGGTCAACTTGACACAG
CCGATATTCGCATAAATGAACTCAACCAAAAAATGATAGAGTTCAATGAAGCAGCTGTTA
ATTTCCGTGATCTTCAGAATAAGTTAAACAACGAAAAACAGTCTAATGATCTTTATGTTA
ACCAAGCTAAAGCTGCAAAAGCTGCAATCGAGCGTCTGCAAGATGAAAGAACAGATTACA

CATCGGAAATCGACCAACTCGGGAATAACCTACGTCTAAAAATTAGTGCTAAGTCTGATT
TAGAAAAAGAGAAGCACCGTAGGTCTTTGATAGCTGATCTTTTGAAGGATTCGGGAATTA
AAGCCGTCATCATGGCCCGCTATATTCCGTATTTCAATAATCGCATCTCTCATTATCTTG
ATATAATGGATGCAGATTTTGGATTTACTCTTGATTCCGAATTTAAAGAAGTAATAAAAT
CTTCAGGAAGAAGCACATTCAGTTATAACAGTTTTTCTCAAGGCGAAAAAGCTCGTATTG
AGATGGCTGTCATGTTTGCTTGGCGTGATTTAGCATCTGAAGTTTCTGGTGTAGATGTTA
ATGCGCTGTTTATGGATGAAGTATTTGACGGTCCTTATGATGATGATGCACAACGCTCAG
TGTTTTCAATCATAGAAAACATGAAAGCCAACGTGTTTATCATTTCGCATGGAGAACACG
ATCCTCAGAAATTTGACAGACACATTGTAATGTCAAAGCTAGGGAGAACTAGCGTAATGA
ATGAGGTAATTTTTGATGTTTTATAATCTTCCAACAACTGCTCCTGAAATAAGAAGTTAT
AATTTAACTGATGAAAATGGTCATGTTAATTTGATTGTCACTGATGAAGACCGGAAACAG
GTCTTCAGCGAAGAAGATATCCGCAAGTTTAAATCAAATCGCCATGCTTATTACAATTTA
GTTGAAATTTTCGACTAATGATTTGCGTGAATAGAAATTATATTAGAATGTTCGTTATAA
ACACACTGAGGAATATGTATGCGTAATCAAGCCGTTCTTTTTGTACCAGCCGATATCCGC
CCAATGAGCGATAACGTGTTTGAAAATTCAGTTAAAATCCGAAAAGCTTGGGTATCATTT
ATCAGTGATATTCGTGCAAACCAATTGCAAGCTCTTGAGCAGAAAATTCGGTTCGAACTA
TATGCCGAAATTGATAAAATGGTTCGAGACATGTATATCGAATTGCTCGTACGCCGCCGA
GAAAACATTCTGGCCAATGGCGGATATCATGTTAAAGGAATGAACGACAAAAAAGTACTG
GCCAAACAATTCCAATACGACACTGACGAACTAATTATTCAAGCTGCTCAGACTGTCATG
GCAGATTACTAATAAGCTATATCGTAGTTGTGTTATAATATTTCAAATACTGAAAGGAAT
ATGAAATGAAACTTTCAAAAGAAACACTGTCCGTAATGAAGAATTTTGCAGCTATCAATC
CATCTCTGCGACTTACCCCTGGCAATTTCATCATGACCAAGTCGGTAAATGGTGTTGCAT
ACGCTGAAGCTACAATCGCTGATGAAATTGATTCCGAGCGGAACATTTATGACCTGCCTA
ACTTCCTGTCAATTCTTGATCAACTGGGTGAAGGTTCTGAAATCAATCTCTCAAACGGGG
AAATTGTCATCCAGAATGGTCGTGCAAAAGTAAATCTGCCTGATGCTGAATCTTTAGTCA
TCGTGGTTCCGAAGCAACGGCTGCGTATGCCACCGGCTGATGTAGAGTTTGACCTGAAAG
CTGAAGATTTAGCAGAAATTCTCAAAATCTCTCGAGCAGTTGGTGCAGACCGCATCGCGA
TTACTAATCGCAATGATCATATCGTTATTGATGCGTTTGCCGTCGAAGACGGCGATAATG
CACGAACGCGATATTCTTTGACCGTCTGTCCTTACGAAGGCACCAACAACTTCAGTTTCG
TTATCAACCTTGAAAACGTCAGTGTCGTCGTTGCCGATTACAAAATCAATATTTCTTCCA
AGGGCGCTGCTCAATTCCAGGGAATTAATACCGCATATGTATTTGTCCTTGAAACATCTT
AAAAGCACGACTTCTAATAAATCCTGGTGTGATATCATGCGGGCGTAAAGGCCCGCTTTC
TTTTGAGAGGAATTTTAATATGTTGACAGTTAATGCCGGCGAATTCATGTTTGAAAACAA
ATATCGCCCAGGAACAATCGATGAATGCATTTTGCCAGAATATGACAAAAAGATTTTCAA
ATCTATCGTTAAATCTGGTCGTATCCCCAACATTATTCTTCATTCTCCTTCCCCAGGGAC
AGGTAAAACTACCGTCGCCAAAGCTCTGGTATCAGAAGTTGAAGCCGAGTTTCTGTTCGT
TAACGGCGCAGACGCAAAAATTGACTTTGTTCGCGATGTTATGACTCCATTTGCAAGCTC
TAAAACTATGGCAGCAGGTGCAAAAGTAATTATCATCGATGAATTTGATCGAGCAGGTCT
TGCAGATTCTCAGCGTCATCTTCGCTCATTCATGGAAGCTCATTCCAAAAATTGCACATA
TATCATTACTGCGAATGATTTGGGTGGAATTATTCCAGCACTTCAATCACGGTGTCGTGT
TATTCAATTTGGTAAAGCCGATCAATCTGATAAAGTCAATATAATGAAACAGATGATTGT
TCGTGCCGAAGCAATTTGTGATGCTGAAAATATCGAAGTTGAAGATCGCAAAGTTATTGC
CGCTTTGGTTAAAAAGAACTTCCCTGATTTCCGTAAAACTCTCAACGAGATGGATTTGTA
TGCATCCGAAGGTAAAATTGACAGCGGTATTCTGCGTCTGGTTCTCAATACCCGCAACGA
TATCGAACAAGTCATTGATGCACTTAAAGCTAAAGATTTGAAATCGCTTCGTGCAATGGC
TGGTCAATACACGACTGATTACGCTGGTTTTGTTGAACGTCTATGTAATGCACTGTACCC
GCTCGTTAATGGTCCGGCAAAAATTCGCATGTACGAAATTATCGGTGAAAACAACCAACT
GTTTGGCAAAGCCGCAAATATCGAGTTTCATGTTCAATATATGTTCGTTCAGCTTGCCGT
AGAATTCCATGGTAACTGGCTGTAAGGAGTTCGATATGAATTTGAATTTCCTTATGGGTG
ATGATGAGCAACTCAATGAGCACGAAATTGCTTGGAAATCGAAAGATTGGGATGCCGTAT
CAGCATTAGCTGATTCGTTCAAAGAACCTTCCGAAAACAATGCATTCGCATTCTTGGATG
ATATCACTTAAACCAAAACGGAAAGATGTGTATCAAATAGTGATTATCCGGCATGGTTTA
TCAATAATGCTCTTTCGATGCATGCCGAAACACTGTATTCGGCCTACGTCATGAATCTTC
TGTCAGCATTGCCGGCCCAAGCGCAATACAATTACCTGGTGTCATCTGTAAGAAAAGGCA
AGATTTACGGCAAATGGCCTAAACTAGATGAATCAATCATCGAAAAGCTGTATATTGCCG
TTATACAAGCAGCGAATAACGTTGATTTGCATACCGCAAAGATGTATATGCAAATCATGC
AAAGGCGAAACACTTTGCATGATTTTCTGAAAAATCACAAATTTATTGTAACTCCAGCAT

TTGTTAAAACTGTTGCGAAAACAGCAAAAGATCAAACGCAGATGCTTAAGTTGGCTAAAA
AATTCTAAGGTTTTCTTATGATCCAAATCGATATCAATCATCCTGATGATTTTCTGAAAA
TTGCTGAAACACTTACACGAATTGGCATTGCATCTAATAAAGAAAAGAAACTTTATCAAA
GCTGCCATATTCTTCAAAAGCAAGGCAAATATTTCATCGTTCATTTCAAGGAAATGCTGA
AACTAGATGGTCTACCAGTATCAGTTTCCGAAGAAGATATTTCTAGACGAAATAACATTC
CTTCATTGCTTCAGTCATGGAATTTGTGTAAAATACTAACTCCTATTGAATTGTCATCTC
ACAATAATTTTAGAGTTATTAGCCACAAGCAGAAGGGCGAATGGCAACTATTCGCGAAGT
ATCAATGTGGTTAATGAAATGGCCTCAACAGAGGCCATTTAGAAGGACATATTATGCAGC
ATTACACGAACATCGAAGTTATTGGAAATACCATTTTTGAGCGTTACATTAAAGACGGTG
TAAGACTATCGCGAAAAACAAATTCTCAGCCAACACTCTTTCATCATGCCGCCCCAGGTG
TAGAGACGAAGCACAAAGACATCTATGGGCGTTTTTGCCTACCAAAGAAGCATGAAAATA
TTTGGGAAGCAAAGCAATGGCAAAAACGAATGGCCGAAATTGGCCAAGATGCCATGGGTA
TGGACGACTATTGTATTTCCTACATCTCCGATAATTATCGCGGAGTTATTGATTTTGATC
GTGACAATCTCATTATCGTTGTAGTCGATATCGAAGTAACAGCTCCAGAATTCCCTGATC
CCAAATTTGCAAAATATGAAATTGATATGATTTCCCATGTGCGTTTGCACGAGGGCAAAA
AGACATATTACATTTTTGACCTTGTTAAAGATGTAGGCCATTGGGACCCATCTAAGTCTG
TTCTAGACAGATATATTCTTGACAATGTCGTTTACATGCCATTCGACACTGAAATTGATC
TTCTTCTCAACTATATTCAACTATGGAAATCATCTACCCCTGATTTGGTGTTTGGTTGGA
ACAGCGAAGGTTTCGATATTCCGTACATTATCACTCGAATTATCAACATCTTGGGTGAAA
GTGCGGTTAACCAAATAAGTCCGTACGGCAAGATCGGCTCAATAACAATTACAAACATGT
ACGGTGAAAAAACGATCTATCGTATCCATGGTGTTGCACTGATGGATTATATGGATGTTT
TCAAAAAGTTCATTTTCACTGTTATGCCTGACTACAAGCTAGGTAACGTTGGATATCGCG
AAGTTAAAGCAGACAAATTGGAATAAGACGGCCCAAGTAATAAGTTCCGAGCAGCAGATC
ACCAACGATATGTCGATTACTGTGTGCGAGATACGGATATTATCCTGTTGATCGATGGCC
GTCGATGCTTTATTGATCTTATTCTGTCTCTTTGATATTACGCGAAAATTCGTTTCGAGG
ACGTATTACGAACTATTACAGTTTGGGATTCTATCATTTTCAATTGGCTTGTCGAGAAAA
ATGTAGTTATTCCGATGATGAAAGCTAGCCCAAAGCAAAGTTTCCCTGGGGCATACGTAA
AAGAACCTGTTCCTGGTGCGTATCGATATGCCATGAGCTTTTACTTAACATCTCTGTATC
CGAGTATTCTACGTCTTCTCAATATCAGCCCAGAGATGATTGCCGGAATGTTTGCACCTG
CACATCTTGACGACTATATCAATAAGACTGCTCCTAAACCATCTGAAGTTTATTGTTGTG
CCCCAAATGGCATGATGTACAAACGGGGTGAAGTTGATGTACTGCCAGCTGAAACTGAGA
AAGTATTCTTGCAACGTAAAGCCGAAAAGAAAATGATGCTTGCTGCTATACTCAATCAGG
AAGCAATTAAGAAAAATTCTGGCTTCCAGGGGCGAAAAGGTAGACCACTAATGTTCCGCT
TTCGTATGGGTGTTATATTATGCACCCATCTTGGAGGAATTACATTATGTGGTATATTTA
CAAAATAACGAATGAGCTAAACGGCAAAATTTACGTCGGGGTTCACAAATCCGCAGACAT
GAAAAACGACCCTTACATGGGTTCAGGCGTAGGTGTTAAACGAGCAATCGCCAAATATGG
TGTTGAAAATTTCACTCGGACGACGTTGTACGAATTTGCGACAGCCGAAGAAGCTTACAA
TATGGAAGCCGCCATAGTGACTGAAGAGTTTTTCAGCCGTCCAGACGTCTACAACGCAAT
GGTCGGCGGTAAAGGTGGTTGGGCCCATATCACAAAGCCCGGGCAGCATATGAAAGACCC
CGTCATGGCTGCTTACATTGGGTCTTGCGTTCGCCGCGCATATGAGCAAGACCCGAATAA
AAAGCTTATTAGCATCGCCAATTTCAAAAAAGCGTCGGAAAGGGACCGTGGCAAGAAACA
TTCAGAGGAGACTCGCCAAAAGCGGTCCGACTCGCTAAACGATTTCTACGCGAATAACAC
ATCTGTTTTCACTGGTATCCCAAAGTCAGCTCAGCATCGCAACAAAATGAAAGCCGCCTG
GACTGAAGAACGCCGCGCAAGCCAAGCAGAGCGTCGAAAGGCCCTAGTAGCCTCAGGTAA
TGACGCATTCGCTAAAAACAGGCTGGGCAAAACTAATAGCGAAGAAACGAAGGCTAAGAT
GTCCGAAGCGAAGAAAAAATACTGGGCTGAAAAGCCAAAAGTTATTGTAAAATGTCCACA
CTGCGGAAAAGAAGGCATCAAACATGCCATGCTCAGATGGCATTTCGACAATTGCAAACA
CAAAGAGGCATAATTATGGAACTCGAATTATTCTGCGACTTCAATGAAGATGAACTCGCG
TATTTGAATACTCTTTCAACCGATGAACTTCATACCATGATCGGTCGTTGTGAAAAAGAA
GAACAAAAACGCAACACCAACCAATTGAACCGCAAAATTTTGATCAACTCGCTTTACGGA
GCTTTTTAACACTTCATTAACTTGGAGCTCCTTAAACCCGGTGAACTCAGGGAACCTCTC
AATGAGACAATCCTGAGCCAAGTCTCGAAAGAGAAAGGTGCATCGACTATCGAACGGCGG
GTAATACCGAACCGAGTAGAGTACACCCAAGTGGGTGGAAGCGCCGGGGGTGCAGCAATG
CATATGATATAGTCTGCTCTGCATAGCGATATGCAGCTGTGAGTAAAGGTCGCGACGACA
TATTCCACGGTCTTGGATTAACGACCCAAGGCGAACACCTGGTAGGTAACAACCTCTTCC
GATTCTACGATTTGCGAAATGCTGAAGCTATTACAACTTACGGTCAATTGGCTATTCGGT
GGATTGAGCGTAAACTGAACGAATGTATCAACAATCTTGTCAAACCAACTAATGTTGATT

ATGTGTGTTATATTGATACTGACTCGGTGTATCTCAACATGGAAGCAGTGGTTAATAAAG
TAGGGATTGACAAATTCCGGGACACCAACCATCTGATCGATTTCCTTGATAATCTCGGAC
ATAAAAAGCTCGAGCCATTTATCGACGAGTCATACAAAGAACACGAGGAGTATATGAACA
ATGAGCACCATCCCTTGCTCATGGACCGCGAAGCCATTTTTGGAGCTCCATTGGGTTCTG
ACGGAATTGGTGGTTTCTGGACAGGTAAAAAGCGATACGCTTTGAACGTTTACGATATGG
AAGGCACTCGATATGCTGAACCGCATCTGAAAATCATGGGTCTAGAAACTCAACGATCAT
CCACACCTTTGGCTTGTCAAAAATCGTTAAAAGAGTCAATTCGTCGGTTGTTGCAAGAAG
GTGAATCTTCTTTGCAAAATTATTTCATTCAATTCAAGAATCAATTCAAAACAATCGATT
ATCAAGAGATTGCCGCAGTGTCTTCTGCGAATAATATCGCCAAAAACGCGGATTCGGCAG
GATATCCAACTAAAAGAGCTCCGTATCATGTGAAAGGAGCCCTTGCATATAACCGGATTG
CAAAAACTGACCAAGCAATTCCAGAGATTGTTGAAGGCGAAAAGGTAATGGTTATTCCTC
TTCGTGATCGCAATCCATTCAATGAATCGTGTTTTGCTTGGCCGTCGGGAACACAAATTC
CACCTGTCGTAGAAGCCGATGTTTTGAAGTTTATAGATATAAATAAGCTTTTTGATAAAA
CATTCGTTAAACCACTCGCTTCAATTTGCGAAGCTACGAAAACAGATTATGAATTTAGGA
ATAATTTGTTTGACATGTTTGATATTTGAGGAAATAGTATGTGTTATATGACAGTTCAAG
ATTTTCGTGACATCATGGTGTCAAAATACAAATCAAATGAATTTGTTACTGACAAATCCG
GGGTTAAAACTGTCGAAATTGCCCCAGTTATGTTTAAGGCCGATGAAGATCTAATTTTCG
GTAAACTTAATCACGATTATATTGAGCGAGAGTTGTCTTGGTATTTGAGTCAATCTCGTT
ATGTTGCTGATATTCCAGGTAAAACCCCAGCAATTTGGGAAGCTGTTGCAGATTGTAACG
GTATGATCAACTCTAATTATGGCTGCGCTATTTTCAGCGAAGAAAATGGAAATCAGTTTG
CAAATGTTGTCAAACATCGAATTGAAAATCCATACACTCGTCGTGCCAACATGATTTATA
CTCGACCGTCTATGCATACTGATTACGATGCAAATGGTATGTCCGATTTCATGTGCACCA
ATAACGTCCAATACCAGATTCGGGATGGAAAACTGAATGCAGTCGTTCATATGAGGTCAA
ATGACGCTATTTTCGGTTATCGGAATGATTGGGCGTGGCAGGATTATGTTTTGTCTTGAC
TGGTTGATACTCTTCAATCCCATGAAATCATGGTAGAAAAGGGTGATATCTACTGGTGTG
CTGATTCACTCCATGTGTATGAAAGACATTTTGGCCTGATTGAACAATATATTGCTCAAT
AATTCTCAATTTCAGCATAGCGAGCATAAGATTGCTCGCTATGTTTAGAATCTTTATAGG
GTTGGTATAATGTCACCACACAAAGGGAACATTTATGGCATGTACCACTGAAGATGAAAC
TCCCAAAAACTTAAATGACGCTCATGCTCTTATACAGCATGCTATGGAAAACGTTAAACA
AGAGCTGCTGATAATCGATAAATCAGGCAAATCACACCCTGTTATATTTTTGATGCTCAT
ATACATGATGGAAAACTGCATGTAGATTACAAAGCAGTTAATGAAAATGATGAAATTCAT
CAACTTGTTCATGCAGCTATGAATGCACAAGTCAAACAAATTATGAAAGATAAAGAGGCA
CAAAATTGGTTGAAACAATCCTTGAAAACCTTCCTCTTCAACAATACGTACTTTATTTCG
GCGTACCCGTACATAAAAGAAGATTATTTCGACCGTGGGCCTTACCGCGAGATTTTTAAA
CTCATCCAAAAACACGTGAATGAGTACAATACAATCCCAACCAAAACTGCACTTGCAATC
GCACTTGACAAAAAGTCTATCGATCAAGTAACTCATGACGGTGTGAAGGACGTTTTGGCT
CTTCTGTCTTCTAAACCAGAAGATCATGCCTGGTTAATTAAAGAAACTGAATCTTACTGC
AAAGACCAAGCGATGTATAACGCGTTGTCTAGGGCAATCGAGATACAGGAAAATGCGGCC
AAACCATTTGAAGAAGGACATAAGAAATTGCCGGATGTTGGCGCAATCGAAGATTTGATG
AAAGAATCATTGGCTATTTCGTTTGATGGCTCAGTCGGCCATGATTGGTTTGAAGATTAT
GAACGACGTTATATGCTCTATCAATCCAAAGCTAATAAAGTCCCGTTCATCTCAAACGTT
CTGAATAAAATAACCAAGGGTGGTGCAGAACTTGGCACGCTAAATGTTATCATGGCGGGT
GTCAACGTTGGTAAATTTCTTGGTCTTTGCTCTTTGGCCGCAGATTATCTCCAAACTGGC
AAAAACGTCGTTTATTTCTCGATGGAGATGGCTGAACATGTTGTTGCGAAACGTATAGAT
GCCAACTTGCTTGATGTCACGCTTGACGAAATAGATGATGGCAACATGAGCTTGGCTGAG
TATAAAGCTCGGATGGAGCGTCTGAAATCCAGAAATATGGGTCGTTTGATTATCAAGCAA
TACCCAACTTCTGGTGCAAATGCTAATCATTTCAACGCGTTTTTGAACGAGCTGAAGCTG
AAGAAAAACTTCAAAGCAGACGGTGTTATTGTGGACTATCTGGGTATCTGTGCATCCACA
CGAATTCGTGGAGGTTATGAGAACTCGTATACTTTGGTAAAAGCTATTGCCGAAGAATTA
CGTGGCCTGGCTGTCCAACATCAAGTTGTTCTATGGACTGGTGCTCAAACCACTCGATCT
GCATGGGATTCGACCGACATCGACATGTCAGATGTAGCCGAATCTGCTGGCTTACCAGCA
ACTGCTGATTTTATGCTTGCAGTGATGGAAACTGAAGAGTTAGCTCAAATGGGTCTCCAA
CTGATGAAGCAAATAAAATCGCGGTATGGAGATAAAAATTGGATTAACAAATTCAAAATA
GGGCGTTAACAAGGGCAACCAGAGATGGTACGATGTAGAAGAAGAACAATCAAGGCCAGG
AACGCCAAACACACCTTCTCCAAGACAAGCTGACAATACGAAGGGCAATAACTCAAGACA
GCATCTTGACGAACTTGCCGCGACAATGCATTTCTGATGGCCTAACGGCCATCTACATAG
GAAACTGATGATGAAAACATTTCTAGAGTGTTCTGGTAAACAGAAAGATCATGTCAATAT

GGATATACCGGTTGTCGAAGACGTTGTGGCTGGTGATTCTGCAGGAGACCCCGTCAACAT
CGCAGCTGGTAAAACGTCTGGCGCAGTCATTTCCCCTGGGCCTAAACTCCTTCCTAAGAA
GGAAAAGAAGAAGAAATGATTACGTTCGAAGCCGCACAAGAAGCGTGCGAGGCTTTAGGT
CATGTAGCCTTAAAGAATCTTAGAAATCAACCTCATCTTGATATTCTGAAAACAATCTAC
ACGAATTCCTTTCCAAGCATGATGAAGGATGTCGTGTATGAGATGGCTGTTAATGCTGCA
GAAATTGATTCGTACTTGGTTACGCAAGGAAAGCCATTGTCTCGTTTAGAATATGCTCAA
CACCGTGAAATTCTGTTGGAAGCATGTCGGCAATTGGATGCTAATGGATTTTTGTCGTAG
TATAATGGGGCCAAGAGCCCCATTTGGAGTTATTATGTTTGCTGATTTTTTGTTTGCCGA
ACGAGCAATGATGCATTTGCCAAAATTCAAACGAAAGAAGGGCAAGTTCAATTCAAGATG
TCCAATATGCGGTGATTCCATGACGGACATGAACAAAGACCGCTTTTGGATGTATGAGAA
GAAGGGCGAGTGGCAAGTTCATTGCTTTAACTGTGGTTATCATAGCAATTTGGGCGGGTA
TTTAAAGGAGCGAGAAGAAGTACTTTATCGTGGATGGCTATTAGAAAGAAGGAAAGAAAG
CACTGTCTTCAAGGCAGAAGCAAAACCAGTAGAAAATCTCTTCACCAAAAAGATGCCGGT
GATTGAAAAGTTAAATTTTTGTGATCGTCTAGAATTGCTACCAGCGAACCACCCTATAGA
AAAATATGTCGCAAAAAGAAAAATACCAGAATCAGCATATAAGCGGTTATGGTTTACTCG
CGAATGGCAGAAATTAGTAAATTCTATTTCACCAGATACGTATACGCGCGAATCACGGGA
AAATAGATTGGTTCTACCGATATTTGACAAAAACGGTGAAATCCAGTCTTTTCAGGGAAG
AGCTCTATCAGCTAGTCCAAACAAATACATCACCATAAAGGCTCATGAAGATGCATCAAA
AATTTCGGAATGGATACAATTGATGGGAACCGAACTGTATGGTTTATGGAAGGACCGATT
GAGTCCCTTTTCATTCCGAATTCTGGAGCTATCACTGGCGGTAGTCTCGCATTAAGTGAA
GTTCCATTCAAAAGCACTCGAGTATGGGTTCTTGATAATGAACCATTTCATCCTGATACG
TGCAAGCGACTGTCTAGACTAATTGATGCAGGTGAACGAGTTGTGATGTGGGACAGGTGT
GCTTGGGCGTCCAAAGATGTAAACGATATGATAATGAATGAAAATGCAACTGCCGAAGAT
ATTGTCCAGTATTGCAAGGACAACGTCGTGTCGGGTTTGCAAGCAAAACTGCGATGTGGC
AAATGGAAGCGTTGCTAAGGCCTCTTCGGAGGCCTTAATTGTTTTTATTGAACGATGACG
AACAAAACAGTGTACACTATCTCTAAAGATGATACTATATCATCATACCAAACAAACTGA
TTAAAACAACGGAGTATATCATGACAAGTATAAATCTCGAACATCCTTCTCTATTGTGCA
ATGCTCACCTCATAGGCGAATGGTATGAGAATCCTCGGGTTGCGAGCATTTTGATCAAGA
AAAACGGAAAGACTGGTAAAATTCCATCAAAATTTACCGTTCGCACAAATAAAAACCCTC
TCGGCGGTCGAGGGCATGTAGCTTTCTTTTACAACAAATTGGCTTGGGTACGCGATCGAT
ATCTGATGCTAATGGAAGAAATGACAAAACGCGGCTATTCGGCGACTGACAATTGGAAAG
TTGCAATTTTCTTCCCTGAATACGCCCATTTGTTTGGTGAATGGGAACCGTCAGACGATG
ACATCTTGCTGAGTCGCACAAGAATTTCTGAGATGATTCCCGAAAAGCACAAGCTAACTG
AAATGCAAATTCAACAACTTAAGCAGATTGACGAATATAAGGAAAAAATGAACGAAATCT
GAGCTTTATAAACGTGGATACAAGTGGGCTTTTGACTTTAATGAATTTGATCGCGGCGCA
TTGGAGGCTGTCCGCGATATGCAAAATACGGAGAACAAAACATGAAAGCTCTGACTATTG
CTCTATTATTCGCTGCTTCTACAGCTTCTGCTGCTAGCTTCGATTGTCAAGTAAAAAATG
TGAATGCCATCGAGAAAGCTATTTGCGCTGTTCCAGAAACTTCTTTGCTGGATGATCAAG
TGGCAGAAGCATTAAAGGCAGTATCCCATATTCCTGAAGTTAAGGCTGACCAAAGAGCTT
ACTTCAAAGAGCGCAACGCATCAACGTCTTTGTCTTCGCTTAAAGACAATATGACTGACC
GCATCATTGAGTTGCAGATTATTGCCGAATTGGAAGGTGTTCAGAAAAAACCGCATTCTG
AAATTGTCCGAACCGATTCTGCTAAAGCTACAAGTAATCGACCCGTCGAAAAAGACACGG
TGGCAGATCTCACTTGGCAAGCAAAGCAAGGGGATGAACTTAGCTTGCGTGGAAAAAACG
AGCTACACAGATTACCGTGACAAAAATGCTCCAATTTGGATGCGCGACTGTGCGTACAGG
ATAGTTCGCAACGATGTGATGGGCAGATGGGTAGCTCAAGCTCGTTCTGCTGGTCTAATT
AATGAATTCAAATCTGTAGAGTATAAAATCGAAACCGGTTTTTTCAATATGACTATTGAT
AATTTGAAAATGAATATTCAGCAGGGTCGTCAAGTGTGCGAACCTATGGCAATAGGACCA
ATGAGGTAATACAAAATGTGGATTCTTATTCTCACCATAGTTGGAACAGGGATGTCTTCT
GTTCATTCTATCCCGTTTAACACGTATGATTCTTGTGAAAAAGCTCGAATTGTGTGAATG
AAAATACTCCTGATTCTAGGAAATATACTGCTGTCTGTGTCGAAAAGTGATATAATGACT
ACAACTAACATAGCAGAACAAAATGACACCCCAATTTAATGAATGCGCACAACTAACTGA
TAATATGGATAAGGCAGTAAATGCACTGTATGATTGCCTTATTGCCAACGATAATCCTCT
CAATCGAATGATTGACATGCAACGTCAACTTCAAATTGAGCTCGCACAACGGCATCCAAA
ATACAATCGCGACCCACGAGAGCTTAAAACTTGTGGCGAAATTCTTGACTGGTGTCAAGC
TCAGGACGATTATATTGCCGACGAAATTCGCGAGCATTATACTGCTCTAGGTGGAATGAG
TAACCCCAAGCCGAATGCGATCTGGAAACCATGGCGCGCTGAACATGCCGAATATCGCAA
TCGACTATTTTCTGAATTGTCGCCTGAAGACCAACTCGAAGCAAAATTCGAGTTGATTGA

CCAAATTCACTTTGTTCTTAACAAGATCATTGCGATGGGCATGGATGGCGATGAAATCTT
CAAACTCTATTATTTGAAAAATGCCGAAAACTTTGCACGGAAGGAGAATGGATACTAATG
CTTGAACAAGATGAAATTGATGAAATCAATCGCTTGCGCAAACTAGATGTTGAAGAAGGT
AGGGCGGATATTTACGCCCCATATATTCAATTTCAAACAATAAAATATGAGGATTTTTAT
TATGTATGAAGTCAAATGGATTGAAATTAACAGAGACAATCATGGCGTAACATTTGCTAT
GAATACACCCGACGGCGTTCTAATCGGTGTTAATTACACTTTTAAAGCGGAAAAGTTTGA
TATGACATTCATTCCTGATATAAAAGTTGTCAATAATCCCAATGGAAATGGATATATTTT
CTGCCACGGGTGATTCATGAAAAAATATGTAATCCAATGGAAAGACGTCGAAGGATTCTG
TCATTTTCTGGATGGCAATTTGTATGGCTCATCTAGTGGTGTCGTATTCCATAATTCATG
GGAAGATGCCAAAACTAATATGCAGAAAGCGAAAGAAGCCATAACAGAGTATTTGAAAGG
AACAAAAGTCGTCAAAAAACGACTATTTGGGCCTCCAACGATAGTCACAATCAACAAGCC
GTCGGAATATTTGGCCAATGCGCATAAACGAATGTGTGATACAATGCATATCGTTGATAT
TAATGTGATGCCAAAACAAATGAGGTAAAAATGGTAACATATGATGATTTGACTGTTGGC
CAAAAAGATGCTATTGAAAAAGCACTCCAAGAAATGAGAACTAAACGTCATATTACCATT
CGAGGGCCTGCGGGCTCTGGTAAAACTACTATGACCCGTTTTCTGCTTGAGCGTCTTTTC
CAGACGGGCCAACAAGGTATTGTTCTCACAGCACCTACGCACCAAGCAAAGAAAGAATTG
TCCAAACATGCCTTGCGCAAGTCATATACAATTCAGTCAGTCTTGAAGATTAACCCAAGC
ACTCTCGAAGAAAATCAAATTTTTGAGCAGAAAGGTACTCCTGACTTCTCCAAAACACGT
GTGCTTATCTGCGACGAAGTATCGTTTTATACCCGCAAATTGTTTGACATTCTGATGCGA
AATGTTCCATCTCATTGTGTTGTTATCGGCATTGGAGAGAAGGAGCAAATTCGGGGAGTA
TCAGAAGATGATACGCATGAACTCAGTCCATTCTTCACAGACAAGCGAGTTGAACAAGTT
GAATTGACGGAAGTAGAACGTCACCAAGGTCCAATCATTGAAGTTGCAACAGATATCCGA
AATGGCAAATGGATTTATGAAAAGCTTGACGATTCTGTTAATGGCGTATAGCAATTTCAT
ACGGTCAAAGATTTTCTTTCAAAATATTTCGAAAGAACAAAAACGCCCAATGACCTACTT
GAAAATCGCATAATGGCTTACACAAACAATTCTGATGATAAGCTAAATTCCGTTATCCGC
AAACAGCTATATGGAGGAAACGCTGCGCCATTCCTGCCGGATGAAATATTGGTCATGCAA
GAACCGCTCATGTTTGACATAGACGTTGGTGGCCAAACTCTAAAAGAAGTCATTTTCAAC
AATGGGCAAAACGTACGAGGTATAAACGTTAAACCTTCTCGCAAAACGCTTAAAGCAAAA
GGTGTCGGCGAAATTGAAGTCGAATGCACAATGCTCGAGTGTGAATCTTATGTAGAAGAT
GAAGATGACTATAGACGAGCGTGGTTTACGGTTGTTCATGACCAAAATACTCAATATGCA
ATAAACGAATTTTTGTCTATTATTGCTGAAAAATATAGAAGTCGTGAAGTTTTCCCCAAC
TGGAAAGACTTCTGAGCAATACGCAATACTTTCACCAAAGTTCGACCCTTGGGTGCAATG
ACATTTCATAAATCACAGGGTTCAACATTCGATAATGCATATCTTTTTACTCCGTGTTTG
CATCAATATTGCAGAGACCCGGATGTTGCACAGGAATTGATTTATGTCGGAAATACTCGT
GCCCGCAAAAATGTCTGTTTCGTATAAGTATCCTGAAAAAGCGATTTATAGACCAGGTCA
ACTGGTCTATTTTATCAAAAAACGGGTATCGCTATAGCGGAACCGTATTACAATACAAAT
TCGGAGGTTATCGCATATTGAGTGATGCTTTTGATATGGTATTGCACATTAAACCAAACC
AAGTGGAGAGTAGATTATTATGAGCAAAGTGACAGCTTTTATTATTGACTGGGAAACAAT
GGTCAATGCTCCTGATGGTGCAGTAGTTGACCTTGCTGTTTTGGTGTTTGAGCATGACCC
GTACAAACTGCCTACGTTTGAAGATCTGTGTAAACGCGGGCGTCGATTCAAATTCAATCT
CCAAGAACAAAAAGGCATGAGATTCTTTGACCAAGGAACAATCGATTGGTGGAAAAAACA
AGATGTCGAAGCTCAAATGCATCTTAAACCATCGAGCGAAGATGTCAGCGTCATGGAATT
TTATGAAAGCTTCCTTGATTTTCTTGCAGCACAAGGGTTTGACAAGAAAACAGGTCATGG
CTGGTGTCGGGGTCAAAGCTTCGATTTTCCTATCATGATCGATGTTATCCGCAAAATTTA
CAACACTCGTGAAACATTCAAATACGAACCATGTTTCTTCTGGCAGCAACGTGATGTTCG
TACTACTGTAGAAGCTACTCTGCTGATGCGTGATATGACCATGTGTCCTATGCCGAAGGG
TGTTTTGGATGGATTTGTTGCTCATAACGCAATTCACGATTGTGCCAAGGCTGCGCTAGA
ATTGCTTTATTCCAAGCGATATGCTCTCGGTCTCGACGATATTCCCACCGGTGATGATGT
TGACCCGCGGAGTGTCCGCAAATGATAGTTGCTTTTGGAGAGGGGTTGAAAAAACCCCTT
CTTTTATCGCACATCACCTGGCGAGATGACAAAATTCAAAGTGCTTAGGTTATTAACGGG
GCATGGGAATTTAACAGGGTTAATGGAGTTTTGTGTGTCGAAGGTGATGTTAATTCTCCA
ATGGAAGAATTACAAATTGCAATAGATGTTCCTCAGGCGTTTGTTAGTGGTGATTACAAT
GTAATCATCGAATGGGCTAAAAATGAGGTATCAGAATGATTTACTAGCATTACAACGCAG
ATAGCTTCTGGGGTACTACGATCCAAACAGCAATGGAAATCCGAAATCGGTTGAATTCAA
CGATGTTTCTGCGGTTTGTCAAACGAAAAGATTATAATGCGCGATATGAAGCGTTGATTA
AATCTATCGGTGAAATGCTTGGGCAAAAGCATCCGACGGCCAAAACTTGGGTAGCAAAAC
CAAATCCTTTCAGGGATGGTGTACTAGTGGAGATTCGAGAGTGAAACAAAATTACGCTTG

GCTAGCATATCACTTCGGCTTGGTTCCAGCAAATGCTTGCAAATATGGCACTCGCGGATG
TACGTTTGCTAACAATAACCCGCACTTTGCATAATTCATTTCAACTGAAGATTTTTGTGC
TATTTTGCGAGTCGCACGACACTGCTTCCTTAAGGGCCGCGAAGTTGCCGTTCATGATCA
TGACAGATTGATTTACGGCTGCTTGTCTGGGGCGGATGAGCGTTTTATCAAAAGTCGAGA
CACTCGAAAGCTGAACTTTCAGTTTTGGGTATCAAAATCAGCGACTCATCATGTTGTGAA
CCGTGTCATCAACACTGATTTCCGATGGATTCAACGAACCAAATGAGGCCCGAAATGGAT
CAGCAAGTAAATGAAGAAGTAGTTGTTTCTCTGGATATTGACGGTGTTCCCAATACGCAG
GATGATCACATGGAATGGAGAACTGACAAGAACAGTCACCTGTGTCGTGTTTCGCCATAC
AATCGTGGCGATATTGTCAGCAACCATAGACTCAGTTTGCTGAGGGAATGGCTAAAGAAA
CATGATGCTAAAGTGGTTGTAATTAGCTCGTGGGTCTTTTTGCGTGATACTGGAAAAATG
ATATGTGATTTTCTCGAACTTCCGTATCATTCCGAAGCATACGATACTGGCGGTGGAAAA
TCTCGTGGCACTGGTGTTCTTCGTCATGTAGCCGATCATAATATCCATAGATGGGTGGTC
TTGGATGACGCCAAGTGCATGCATGATCACGATGAGGTCCTACTTAGGCACCTGGTTCAT
ATCAAAGGTGGTCTCAATGAATCATACTTGGAAGAGGCCGACTTCATTCCGTGACACCTG
GTGGTCCCGCACCGAGAGTCGCCTGGGAAACAGCATAAATAAAAAGTCAGTTGAATTGAT
AAAACAACTGAGTGACTAACTGGAATAAAAAGAACCGGGCTTCGCCCGTCGTGCTTGCAC
GAATCCAGTTTGTCGTTTCATCTGATTTATATAGGGGCTCTTCGGAGCCCCTTTTTTGTT
TTCAAAACTAGTGTACAAGATAGTGCTTCATGATAAAATGACTTCAGGTTGAACAACAAG
GAGTACTCTTATGATTTACGCGATTAACAAATTCGGTGTAGCATTTGCGATAGAAGCCAA
CATTTGTGCTTATTCGATTGCTTCTAGAGGCTGCCAAGTTTTCTCGTGTGCTGACGATAT
GTATGCTGCCATAGATCCTCAGGCTGTCGGTTACGAGTATCACGTCAAGCAAGATGGCGA
TGCTATCATTTGCGAACTCGAACAGACCAACGTTCGCGTAGAAAAAGATTTTGCTGAATT
TATCTCTGAGTACGAAGTTTAAGTGTACAACTGATGTGGCTGTTTCATAATTGCCACATC
AAACAAACATGGAGTAATGAAAATGCGTATTTCCAAAGATTTCTTCCTCGACGTAATGTT
TTAAAACAAGGATTCTGTTGTTGAGAAATCCCTCGAAGAGCATCTCTGCTATACCACTGT
TCTGTATGTTTTCGAAGGCAATACTCGAATTGCTGTTCTCGAACGTGTTATCACCGCAGA
AGGTACCAATTGGGTTCTGAAAGAAGGTATCTACGAAGAGCTGAAGGGTAAAATCTGATG
GGAGCTCCGAATCATAGATTGCTCCTATGCGAAGAAAAAAGTCATGGAGTTGAAAAGGAA
TTGTATGACCTTTTCTTCGAAATGAACTCTATTGGTGCAAACAATCAAGACACTTGGGGC
AAACTCTTTAAGACTAAGGCATACAATGAGTCTCGAGCTCAAATGGCCGAGTATAAAGAG
AAAATTCGCAAAATCATTGCGACTAATTTTCAGCAATCTGTCACGTACGCGTACGAGCTG
ATTGGTTTGAGGGGAATTGTGGATGTTTCCATGAAACGTGGCCAATGAATTTTAGTGAAA
CTTCAATACGAGTAATAAAAATGAGAATTCGTCTTGTTGATAAGAAAGGTTTCATAGATG
CAAACCGCCAAGACAGTCAATCCTACAGATTGGCAAGGACCATTGTTGAATTTGCAGAAT
GGAGGCAACTCTGTGTTATAGGCAATGATGGTCCCGAATGGCTTCTCAATGTCTACGGTC
CATGCTGGCGCGGTAGAATTTCTGCAAACTACAAGCAATATTTCGAGGTTCAAAATGAAA
CCTAAATTGAAAATCCCTCTCTCCGAATATCAACAACGTTTTTGCAAAACGGAACGGCAG
AGGTTGTGGGCAAGTACGCTTTAACAGAAATCCAAATCTGGAGAACTTGAAGTTCTCGAA
ACAATGCCGGACGGGACATTGAGAACAAATGCTACTTTCCTTGGATACCACATTGGAATT
CCACTCGAAGAACAACGATATTTTGAATTTGAGGAGCAAAAATGATTGATGACGTTAAAA
TCGATATGACTAGCAGCTTTAACGCTAGCATGGCTGTTCCTCCAAGATCAATGGGTTTTG
TGCTGGCCAAGCTGATGGAAGAAGTAGGTGAATTTGCAAAGTCGCTGAACCAGCCAGAAC
GTTGCGATGAACGCC
>NewGenomeName_25
CTCTATCGCGTCTTAATACACTATTCAAATCAAATGCCTGTTAAATGGGTTTTACGTTTC
AATAAAATAGCTAATTCCTATCAATGGAGGACACTGTGGATGATGTAGTACGAATAGTGT
GAGGGCTTTTACTCGGTGGTTTGTCTATCTGGATTTTGAGAGGTCATAACAATGGCAAAC
AATAAATACGATGTACTAATCCAAGACAACTACTTGGAAGGTATTACGGTCACAACCGAC
GTGCACAGCGCTTAGGTAGCGTTTAGTGTTCGTAATCGTGCATTACAACATTTGATTAAG
AAAGCTCTAATGGTAGAAAATCGCGGGCACAAAGAGGTGGTTGAGGACTGCCAAGACATT
ATTGATTCGACTGTACGAGCCAAAGAACTAGATGCCCAGCTGCAATACATTGAATGGGCA
CATGCACGAGATAAGGAACTAAACATGGAGGCTATCCCCAATGGGTGATAACCTAGCAGC
TGTGTGGATTATGATTATGTTCCTGTTTATCATAAAAAAGACTGGTCCGATTAAGGCCCT
TAACTTATTCTCGTTTACACCACTTCGGAAGCGGTATTAGCCAGCGCAATACGGCTACTG
CTGTTATCAAAAAGAAGCGTAAGACTCCCACAATGAACCACGATGTTGGAACATTGGTCT
GGTTGATTAGTTGGGCTTTATTACGAACGTTATCCCTACCTACTACATTCTCAACATTTT
CCGCTTTCACCTCAAGATTAGAACCCATAGCGTACTCTTTATCTCCTATCTGAGTATCTA

CTTGTTAAGCAGCATTCTGGTCAGGAAGTAAAACACCCTTAGCAATCTCTACTGGAGACA
GAGAACTACACCCATTAATAGATAGGACCAGCAGTAAACCTGTCGAGACGAGTAGGGATT
TCATCCTACTTTTTACGTAGCTCCTCAATAACCCTCCGGTGATATCTCATCTCAGTCTGG
ATAATTACTAAGTTCTCATTCCGGTGTCGAAGTTCGTTAACTAACGCCCTGTTCTCTGAT
TGTTGGCTTTGCAACTGACGGACCCCTGTTTGAAGCTGACCAACCTTATAAGATCAAGAT
AGAGTAGCACCACCAATAGCCACTCCAACAGAGAGGAACGTATTGATTATAGGAGACTTC
CAGTCGTTTAGCATAACTACCTCCCATACCGTCCAAATATGCTGCAACAATATTTAGGCG
CATAGCGTAAGTATAGGAATTATCTACAATGCCTGAGCTATCGTAGACCTGGAGAATACG
ATCTCGCGGTATCCCTTTATATTCAAGAGAAAAGATAGAAGTGTAGACCTTGTGGTCATA
ACAGCGGATAAGAGAGAGGCCCAAGGTAGGGCCATCTCTAATCTGGAACGGTGCTAGTAA
GAACAGAAACATCACCAAGAGTAATATAGCACGTTTCATACTTAAACTCTATAGTATTGT
TCATACTTACCTCGCAGATCCGAAGGGTCCATTAAATCTGGAGCACCTTCGTCTATCTTA
CCAGATTGCTGATCGTTGTATCGAAGTCCTGCAAAGACGGTTTTCAACGCGTCTGGAGTA
ATAACTCGTTGTCTAAATGCCTCGAAGGCTTTATCTTCTTCCTTTGACAGCATCATGGTC
CTTATTGGTGCACCACCTCGTACTGGAGCATTCTCTAGAGCGTCTGCCTTCTGGCGTTTG
ACCTCAAGAGGTTGTTTCCAGTCGATAGGACCTTTGATTAACAGTTCTAAGTACTGCTCT
GGAGAAGCAGCAAAGTTCTGCCAAATATCAGCATTTTCTTTCAATACGTTGTACGTATCA
ATCAATCGAGACTTACCATAAGTATCCTCCAAACCATTAATAACCACTTTCTCTTTACCA
GCAGAGCGAGTGTACATACCACCACGGGCTCGCTCGGGGCGGTATTGTGAACCATAGTCG
AGCTTATCGGGCTCAGTATTCACTAGAATCTTAATGTCATCTAGTTTGTCCATTGTTGCT
TCCATACGTCCACGTAACGCACTGGCAACTGCATATAGCGCGTGGTCGATAGTGGTTTCC
ATCCACTCTTTATCTCGTGCAAAGGTAGCTTTGTACTGAGGAGTTAACCGTCGAGTCATG
TTCTCTAGTAAAGACCGCAAAGCTCTGTCGATAACAGGTTGCGCAGTAGCAATATTATTA
CGAATCATATCTGGGTTATATACCGCCCGACCTTGCTTGTTGTTTAGAGCTAGGGTAGTA
GGAACAACTTTCTCAGTCTCTCCCACCTTTTCACTCTCTTTCGCTTCTTCAGGGTTGGCA
GTAATACCCACCATTTCGTTAGTAACCGCACCATAAGCCCGCTGTAAGCTTTATCCCAAG
TTCTGAGATTTAGTCATTAGGTCTGAGAGGGATGCGTTGCTCTTAATCAGTGCCTTAGCA
CGTTCAACACCAGCCATAGACCGGAAGATGGACTGTTTCATTGAGTCAGGAATAGGGTTG
TTCTTGGCTAAGTAAGTTAGGACACCATAGTTCTGGTTGCCCCTCTGTATAATCATTTCC
GCAGGCAAATCATTCTTAGCTTTAACGCATTTAATTGCTGTCAGGTCACTAACCATCGTC
TTCATGTTTTGTTTGAACTGTTTAACTCGTAATTCATTAGTTTCAATCGTGTCCTTAGTA
ATCGCACCAGGGTTAGTAGCAGTCTGCATTTATGTTTTCTGTAACAGGACAGCACTCAGT
TCTAGTCCAATCTCTAGGGCACGTTGTTGTTCCAATCCAAATGTAGCAGCTGGAGAGTTG
AACATGGTTCCGAAAATATCATCTTGAGCACGAAAAGTCGCGTCATTAACCGCGCCATAA
ATATCATCTGTGTAGTTCTCAGCATTGAGTTTACGAATCTCACTCTGACGCTGTGATGGA
ATCAAGCTGATTGCAGTTTGTTGAGATACACCGAACTCTAACTGAGTCAGTGTTACCCTC
TCACGATATTCATTCAGGGCTTTCTCTTCTGTAGATAGTTCCATAACTACCTTACGCAAA
CTCGAACTAGATGAACCGATGCCAAACAAAGTAGCACCACGTTCACGAGTTGGCATCGCA
AGGAATGGATATGAGTTGATAGCTTCTGTCATGATGGCACGGGCTGTAACCTGTACCTCA
CGGCGTTGCATACCAGCTTTAACCTTATCTGCTAATCCACCTATACGCCCTAGGTACGGT
TGAATCTCAGTAGGGATTCCTATATCTAGTGGGTTATCTAGAAAGCTTCGAGTATCGTTA
ACAACACTCTGAGAAACTACATCACCACCAATCTTGTGCCCAAGGCTCCCGACCGCCTGA
ACGAGTCGTGCTAGACTATCATCATGAATACCAGCTTGTATTGGAGTTTGATTAGAAGCT
ACTGCTTGGATAGAGTCGCTTGTGTTAAAGATAATAGTTCCCGTATAACTTTATTCTCCT
TGTAGTGTATCAATTCCATCTACATTCTAACGGTTTGATGAACTGTGGAAGGAATAACCT
GAGCGAACCTGTTTAATAATCATGTTGCGACTGAACTCTCCTTTCGGTTCGCTACCACTT
AGCAGCTAATCAACTGCAGCTTGACGTAATTGGTATAATCCTGCGGGGCCATCTGCAAGT
GAGCTATCAATCGAGTTCATAATTAAACCAGTTGCTCTCACTCCAGCCACGTCGTTAATA
TCCCACCCCATGCCCACTTTTTTAATAAGCTTCACAGACAACTGCACGTCCGTACGTTTT
AGGACGCTACGTGCTATCACCGAATATTGTGCCTCATAGTCTTCACCCACACACTGTGGA
TTAAAGCCCAATGCCTTTGTTAAGGCTTGAGCCACACTAACTTCTTGCGGCCACAATGGT
TGTCCGTTAGCTTTGAAGATTGCTTGTCCGTTATACATCACACACGCACCGATTAAGTTA
CGAGTTGATGTAGGAATCTCTAGCAATGATTCGCCCCTAAATTGCACCATGTTAACGGCA
ACTGTCAGTGATAGATCATCCGCAGATAACGCCACTTTGCCCATAAACATCAAGTTCTGC
TAGAACCCAATCTGGTGACTACCGGGGCCAAAGACACGTCCGTACACACGGCCTGCAGCA
CATGCCGCTAATTCTATTAGTGAGTAATCCCCACTAATGTATCTAGTTACACTCTCCTCT
ACAGCATTCAAGTTTCCGAAGCGAGTATCCACGTCTGCATCCACAACAAAGCCTCTTATA

GCTAATCCAGTGGAGCCTCCTTTCAAAGACACTATCACGATTGGGTTTAGGTCTCGTGGC
TCTATACCTGCCCACGATAAAGCAGAATCAACTAACAAGTTACCGTAAGGAATACCACTA
GGTCCGAAGAGCATTAGCTGGCTTGTCACTAGGTTAATTTTGTCTTGGGTGGATAGGGTA
GTGCCTAACTTAGCCTCGATACATTTAGTAGCAACCTGTTGGAAATGGAACGGAATTGAT
TGCCAACCTTTCTGGAACCACGCACGGTTAGATTGTGACATATTCAAACGGAATGCTTCG
GTACGAGCAACCACTTGTTTCAATGTCATATCGTCAAACCTAGCTCCCTTGATACCTGCC
GTACAGTCGTCCTAAGCGATAACAAGCGAGATACGAGAAGTAACCAACTCACCTTGTTCA
AAGACGAATCGCCCTTTCTCTATGAATCGGCTCAACATGGATTGTGTTACTGGAATGCAA
CTAGCCGCAATACGGTAGTCACCTTGACCAGTAACAGCAGAATCGTATAAGCCTGCCTTC
TTCCATCCTTCGTAGAATTCACGAATATCCGGTAAATTATTGTCACTACCTTGTAAGTGA
CTCTCAACGATACCTGGATCTTTCGCTAAGTCTAAGAAGTGTCACTTAGTCATGCGATCA
AACGCTCTCAGTGCGGATTGATTACTGATTCCAGCTGCTAGAGTAGAACCCATAGCGTGT
ACCAAGAAGTGAGCAGGAGACAACAAACCTAGCAGTAAGATAAACGCAGCTCCTTTGAGA
TTCTGAACTGGGTCGGATTGGTTCATACGATATAAACCTTCTGCCATTCCAGGCTTGTCC
TTGCGGTCTAGATACTCTGCTAGAGAATTAGCCATGTTTGACATATCACTTTGTTTCTTA
CTCATAATACTCATCATCTGAGTTACTTGTTCATGAGAAATCAATAGTTTGTCACGAACT
AGGGCATCTTCTAGTTTTTCAGCGTCAATACGGCGAGAAGCTTGGAAATTCTGGGTACCA
ATATCTTTTCTTTGATGTTTACTAGCTTGGTTCATCCAACCGTCGTGTAGCGCCTGTTTC
AATTGGCTAATCGGGTAACGAGTTGAGATGTTACCGAAGTATTGGCCTCACGCTTCGATT
GGAGAAATGAACTCAGCCTCTATGCTTTCTGGACTAAACCTGATACGTTCCACAGCACCA
GCACTACGGAACTTACCTCCGTACACGTTGATAATATCTGTATCACGTTCAGCAGCATCC
ATCTCAGGGTCACGTAGAACATTGTTATTCTTGTCAGGGTTACGTGCTTTAAGATTCTCA
GCATATCGTTCAGCATCCACTACATTATCAAAGTAACAGCTTGTCTTGTTATATGCCCGA
CTCTTACCATCTAGATTGATTGGAACTTCCTCTTTAACAAAGTGGTAAGCCTCCTTGTAC
ATAAGAGGCATATAGCCCTTACGACTATTTAGAACCAGTGGGTCAAGGTCACGGATATAT
TCACGTCTAACGATAGCGAATGTCCAACCCTGAATACCTTTCATCCAGAACTCGTTTGGA
GTATCTAGATAAGTCAGTTGATAACGTGTCTGATAGAAATCTTCCCACATTTCACCTGTC
ATGACAGTGATGATTCGGGCATTAGGGGGGTCATACACACAGATCTCTTTACGAGCTTGG
AAACATTGTCTAGCACTGGTAGCGACTTCCTTAGCTTTAGAATACGCAGACTGTTCACCA
ATCTGTTCAGTTTTGTTCCCATAGCACTGAAGCTGTCTATATAAGGCGTCATTCTATACC
GGCCACCCTTGGTCAGCAGTTTGCCGCATTGTGTCGTATGCTTTGAACTCAGAATCATGA
AGTTGAGGTAAATCAGGTCTGACACCTTTCACCATATCACGGTATGAATATACCTCATCA
ATCAGTGTGCCTTTCTTTAGAATCAGATCTAGCTTTTGTAATCCCTCAGTATTAACACCT
ATCCAACCTTCATTCATAGCTTTTTGGAATCCACTACGAATCATAGCACGTTCACCCGAT
ACACGAGTGAATGTACCCACAAAGAATGCACGGTCACGGTCTAGGGAAGTGTTTGGACTC
ACTACCTTAGCCTTGGTCCTAGCCATTATGCTAATCTTGTCTGTGTAGCAGCCACCACGT
AATGCATCTTGGTTGTACTCGATTATCACTATAGTTACGTTACGTACTTGTGTTGCACCA
GTCATAGGGCTAACAATGTCACACTTGAACTTCACACCCTTGTCCCTAGTTTCCAATGTC
AGGTTAGATACTTCGTGTCGCTCCCCAATACGCGCAGCAATATTCTCAGCAAATGCTTGT
TGCTCTTGTGGAGTCAGTGGAACACATTCACGGGCCACGTCCTTAGTTTCAGCAAAGATA
CCATGAACAGTTGACCAACGTTTCTGAACTTCGTGTTCAATCTCTGGAGCAGCACCTTCT
AGGATAACTTCCATATCTTGAATAGGAGCAGCAGTCCTAGCTGCATCTAAACGAGACATT
GATACCGCACAAGCGGCATCCTCCGAACGCGAGGCAACGTCTACAACGGTAGCGGCCACA
TCTTGATCGTGGACTACATTTCGAGCAGTAGACGATAGACGTTTTAAAGACGCCACCGCC
TTGATACCAGTTAATCCTAGAATAAATTTATCTACCTTATCTAGAATAGAAGCTAGTTGA
GTGTTGGATTCAGTCTCTACGGCAGTAGCAGAAATTAGGAATACCTTCTGTAGGAACTCT
TTATCTTCTGCTTCTTGTGAAGCTTCTCGCAACTCTTTAAATTCACCAACACGTTCTTTA
GGAGGCGATCCCATAAATCGTTTTCCTAGGTCTGCTCGGCCACCACCGGAAGTCATAACC
TCAGTGAACCAGTTAACGGCTTCTGGAGTGACTACCTGTAGGGAATCCGCAGATGGAGCA
CTGTGTCAACCCGGTAAGAACATAGCAGTTAGGGCATCTATAGAAGTCATCGTTCCTCAC
ACCCGTTCATCCCGCACCCCACCGATTTGCCGGAGAACCCATAATTAAGTTGCAGCATCT
TCCCGTTCTTCTGGAGTTAGATAAGTGTTCAATGAGTCTACGTAGGCCAGTTCAGCACCT
AGCTCACGGGCATAATCTACATACGCCTCGTTCTCACTCTTAGTCAGTTCTGCAGCAGAG
TTAGCTAATACTTGTGCATTATCTCCCGCAAGCTCATCAATAGCCATTGCTGTACCAGTT
ATAGCCGCTTCCATAGCTTATTTTTGAGAGTTAGATAGACGTACAATTGGGTCAGCATTG
TACGCTTCCATATAAGTTTTCATAAGGTCGTGATCACCAGTACGGAACTTACTAGTGGAT
TCACGAATAGCTACCTCTTTCTTAGATCAAATGCGTACAAATTGTTGCTCTTGTCCCATT

TCTAGTGGATTGGAATAGACAATTCCTTATCCAATTGGCATCGCTTATCTCCTTATATAA
TTCTTTAACGAACTGACCAAAGTGAGCATCTTGCATTCCAATGTTAGCAATAGCTCCAGC
AGTATTAGCGCGGCTAGAAGCATTAGCAGCGGTTTGAGAATAACCACTAGCTTCTTTACA
TATCTGACGGGTTTGGGCTCAGAAACTCAGGTTCGCATTAGTGTGTGAGCTAATGGACGC
TACCGCACGTGCTTCACAGGAGCTACCGGACGTACCGGTTTGAGCAGCATTATTCTCAAT
TCTTGCTCGTTGGATTCGGGCTTGTTGTAAAGCAAGGTCCCGTCTTCGGGTTTGCTCAAC
ATCTGCAATCCGAGATTGGACATCTTGCTGTATTCGTTGCTCTTGAGATGCCTTGCTAGA
TTGGGCAATGAATACGGCTGTAATAGCGACATCTGCACCAGCAGCAACAACCTGAGCAGC
AGCTGCACCAGCGGCAGTTGCCATATCACACCTCCAATAGTTTACTGTAAACGATGATAT
CTACGTGATACCCAAATCGCTCCATAGAGGAGGTAGTGGGAGTGAAGCCAATGTGGAGAA
CTGTTGCCCTGTACCGGTGTGCGGAACTCTCAGCAACATCGAACATTCTCTTGAACCCCA
TGCCCCCGCGATAATCTGGTTTTAGGTACAAACAAATAGTAGATGCGACACATCCTTTGC
TGAATACGGCTTTAGGAAGTACATTGTAAATCACGTATCCAATTGGAGTTTCCTCGTCTC
TGCGCACAATGATTTTGATCATACCCGCCGCAAGGAGATTATATCAGCTTTCTAGATTCA
ACTCGAAGAGTGATTTAGTTGAACGAGCGTCTGCCTCAAGAAAATTATCCTCAAACAGAC
CACTAATATACAATTCCTCACGAATTTCCGGTTTACTTTCCATGTAAATGTTTAACAGAC
CTTAGTACCACCCCTGACGGTTACTGCCCAACATTACAACTTACAATCCTTGCTTGGCTT
TGTTCTGAACACAAACTGGAATGCCGTTCCTGAGCCTCTAAGTCGCGCTGTGGCCGTGTT
TACTTCATAAGGGTACACATTGACACCCAGTTTGAGATTCATCGCTCTACGGCAACGCTA
GCACTGACTGTGACGAGTAGTCTCAGTGCACTTCTTGCCCGTAGGATAGTCGGTAACGCC
CCAACGAATACGTAACAACATGCTAGATTCGTTTTCTCTCATAGCTTCTCCATCCACATC
TTTGCTCCAAGTATCAGTTCCCATCATGTGAGTTGCTGGGCTTGTATTTTATGCACGGGC
TTGAGTATCGTGTGATTTCTGTGGAGCACTTAACAGGTAAGCCTATGCATGCACTAGGCA
GAAATCTTTAAAGTAATCTTGGTTAAACGAATAGAATCCAAACGAAACATCATCACTTTT
GATTGTTAGATACTTAGTATCTCTCAACTCTTCATCCCCGATTCTACGTCCCAATGGGAA
TGTAACATCAGAAGACTCTAGTTTAACTCTATCTATACCAGCAAAGGTACAGTAATCACT
AACTACCTTGTTACCAATAACGTTACGCTCCGGAACATATCCGGTAACTCTTTACGCCAT
GGTAATGTCACTCTTGAACCGGTTAATGAATAATGCAACATAACGTACGCTTAAGGTAAT
CTCTAGGTCAAAGTTACTTGTATTGGCAATTTCACTAACTAGCCAGCGAACATATTTACC
TGTACGTTTTACAGTAGCTATCGACTTAACCTTACTGGTAATAGTTATCTCATTATATAA
CGATTGAATCGTTAGATCTGTTAGTTTCGGTGCAGAGTGTTATCCAGTTGTTTGGTCTGG
AGATAGGACAATGATTCCTTCCTCTGCCCACTAGAAAACGGTATAACCATATGCAACAAC
AGAACATGGTGACACACCACCGTAGACAGTAAACTTGTCTACGTGATAACCAGTAGCGGA
GAACAGACCATCTGACGTACCACCCAACAACCACACTCCATTTTCAGCAATGATAAACAG
ACCGCTGGAAACGTCTTCCAGTCCAACAATCTTGCGAGTTGCGTTAATTGTGATGAAAAC
ACCGTCTGTATCAACAAGATTACTGTCCACTTCACTTGTGGGGTCAGCTAACTGATAACA
CTTGTTAATCTCTGCGTTAGATTTAACTAACTGAGAGAAGAATACGTGGTTACGGAAATC
AGGACGTACATCATCAATTTTTCTTGGGGTTGCTTGGAACCCTCCATAGAATACTCGACC
TGCCAATGTTTGAATGCAAGAAAGTTTACCACTAGCTAACAGTTGTAATGACGCTACCGT
CGAACCACGAGTTGCAGGAGTGTTTTGACGTTCCTTACCGATACCGGTAGTTGTGCTGAC
TTTCATCCGAATTGGAGTAAACTTTCCGATTGCGTTTGCGTCTGTACCAGCAGTTGTTTT
AAATAACCACCAAATCTCGTAGCCACTTGGATAAGCACCGATTGTCGCAAAAGTTGAGTT
AATCTTTTTACTATCCCCACCTTGGTTAATTAGCTCGGACCGATAATCTGTCGATAGGGT
TTTTGGCCGTTCTCTACTTGGAGCCGAACTTCCCCCCACATCACGAGTTTCAACGAACAC
AACCTTTCTACCTACTACGATTTAACGACGTGCAGTGATAGAGTCTGTTTGAGTATCCAT
AATCTGGAGTGATATAGGGTCTGCATCCTGAGTTGCAAATACACTGATACCCTCTACCGA
AGCGCCACTCATAAGATCCTCTGTAGTACCTTCTGTGGATAAAATTTATTGGTTGTTCAT
TTACAATTTCCCGTTAGATAATGGGTCAACAGAAGTATCAACGAATTTTAATTTATCACC
GAGCTGTACTACTAGAACTTCTGGAATGGCGACATAGTCAGTTTGCCATACGTTAACACT
AGATACTAAAATTTGGTCTGAGAAGTCTACGTAGGTAGGATACAAAGTACCTTAAACTTC
CAAATCCAAACCATAGCGTCTTGCCCAAGCGTTAGCTTTTGAGAGGATGATGTTCTCACC
CTCTCTGACAGAATCCATAGGCATATTTAGAATGTTCCCTCCTGTCATTAGACCAGACTG
AAACTGATTGTATTCTAGATTAGCCTGTCATCTTGCCAGTTGCTTTTGCTCCTCGTATTT
CAGTAGCTAGGTATTGTTTGATGCGTTGCCAGGCGAACGCTCTTGAGGGGTATTGCCCTT
TAAGAAACTCTGGTACTTGACCACCGCCGTCTAACTAAATCTCGTATACAGTACCTTCGT
TATCTGTAATCTCTACTTTAAAAGCCTTTGTTACTGCCATTAAAATCTCCTTGATAAGAT
GTTTCGAAATATGGACTACGACGATTTGGGCAAGTTGAAGATGTTGGTCCGCCATAGTTG

GGGAATCGAATACCACCTTTGGCTTGCCAACTCTTGCGGCTCATATGATGACGATGGATT
GTAGCCCATTGTTCGTCTTTAGCTGCGTGCATTTGTCTAATCTTGGCACTGAATGCTGGC
TTTGCTTCACTGATTAGGTATGGGAGCATGGGAGCTGGTAAATCAATAATCCAAGCATTC
TCCAGCACAAACTTAGGACTAATATAAGCGATGCCTTGAATTTTATCTTTGTGGATTGTA
GATTCTACGTCTGTGTTGTAGCTATCGTATACAAGCTACTCATCATCGAATGAAGTCCAG
AATTGTGGAGCTTAATCGTGTTGGATGAAGTACACAATGCCGTTGTCATCACGGACCTTC
TGTACGTTGGACTGACCTGAGTTGATAGTAGCTAGTCGAGCTTCGAACTCATCTGGATAC
ACGTACTTTAACTCAGTGTAAGATACTGGACGCTCTTTCATTGAGGTATCTTATGGCAAG
TACGTCATCTCTTGAATGTTCTCAGGTAGATTTAAGTAGTTTGGTTTATCCACATCACTT
ACTCCATCAAACTGTACTCCACGGCGAAAGTAATCAATCGTTGAGTTGGTGATTAGGTTG
TCATACGAAGAACGCAGGATGTACGCCGCCTGTATCGACTCAGTGGTATCATCAATAGAG
TTGACGTTACCACCATCCGAATCACTCAGCATGTCTTGAACAAGATGAATAACAGTATAT
TTCATTATCGTTTACTATATCTACCACCACGAGCCTTTCCAGCTCTGTCACCATATTTAC
CACCATCGGAACGGTTGGCACTTCGAGAACGTACTCGTAAATTACCATCTCCATTACCGC
CACCGTGTTCCAAAGTTCTTTTGTGATCTACGTCTTTCCCGTAGCCCTTTCGAACTTTTC
CCTCACGTCCCATCTTCCGACGAACCGCATTCCCTTTTGCCGGATTCTTCTTTTGTTCTG
GAGTACCCTGGTACTTGTCGTACTCCTGTCGGTCAGTACGCTTCATTGCCATTACTGAAT
CTACGAGATTTAAAGAATTAGCGTTTCATCTTGACTAACAACGCTGAATTGACCGGCTAC
ATATTTCATTTTACCACGAACGGACAACATATAGTCAGTACCGACAGGAATGATTGAGCC
ACGGAAATCCATGATATCATTCTGGTATTCAGACGGCCCACCTACTGAGGATTTGAACAG
AGTGAAGATGTATTTACTACCTCCGATTGGCGTATATACAGCAGATTTGCCATAAGGGTC
AATCGAAATTCCTGTTGGAGTCGCAAATGACTCATTGTTGAGTGGGACAAGCACAGTAGT
GCCATCACCTTTGGCACGTAAGAATGTGGTAATTGGAGCATTAACAGCTCCTTATACTAC
ACGTAAGTATTCATCTGGAATATCCTGATGTTGTATATCAGCCACTGGAATGACTCCTTG
TTACGAGATACTGATGTAATTAAGTAGAATTCCTTAATGTATCGTGTTATCCATTCCTGA
ATTAACATACTCGTCTTCGCCACTACCTGTCAAGTCACCCTAGACCAATTAGTTTTGAAG
CAAAGTGTACACGAAAATATGCAGGTAAAAGAAAGCCCTCCGCCGAGGGCCTTGAGATTA
GTGTTGCAATCGGGATCCGCCTAAAGTGACACCAGCATTTGTTGGGATTCTAGCAATACT
ATGAGGACTACTGATAATTCCAATCTTTTTGGACCTGTCGGATGTGTACTCTTTGAATCG
TACTATCTCACCAGCTACGACACTTATGGGAACAGTGTAATGAATGAGTTTAACATCACC
GGAATCACCACCAGCCAGTATCAGCAATCTAGTAGTTCCACAGACAGGACTCCATGAGAT
ACCATCCATTGATGCTTCTCCATACATTCCCCACAATACCTCTCCAGCACCTACGGTTTC
TCTAATGACCTGTACTGACATATTGGACATTGCTTTCGCACCATCGGCGAAAGTTAGTAC
TCAAGATGTTAGGTCTGTTTGTACAAAATCACTGGCAATCTATGTAACGTTGAAAGTAAT
TAGAGAACCAATGATTGATGTTCTCAACAGTTGTTTAACCGGTAAGGACTTAGGAGTAGT
GTAGCTGATTCTTATAATGTGCTCTTTAAACTCCGGTAAGGCTTGGTTTTCCCACCGAGT
TTCTGTGAGGTTATAAATCAACACTTCACCATCCGTTTGGATCGAACAAACCATTAGCAC
AATAACTATTGACTCTATAGTGTCTGGAATAAACTCTAAAGCACTCTCAGATTCGTTACC
CGCCACTAGTTGGTTACCCTGCCCTACGTAAGTGTTTGGCCAATCTAACAACTCGAGGAA
AGTCTGTTTACCTTGAGGCTGTCCATCAACATACTCAACAGTTGCTAGTTCCTTATCTAA
CCAAGGACGCATTCATAGAGAGTAGGCTGGGACACCATCACTGCTGCCTTTAACGTACAA
GTCTGGTCCGATTCCGTAAGCTATTGCAACTAGAACTGCACCTTGTCGTAAGTTAATAGG
CGTCGGTAGAGTGAAGGTAGTTATTACTACAGATTGGCTGCTACCCATATCTCCATCAAA
CACGATAGAGTTGTTGTAAGTTAGTCTGAAATGTAAGTGAGGAACCTCGTTAGTGCAATA
CAATACACTTTTAAACACAGTCTCACTGTATGGAACAGTTACGATAAATTCACGGTTGAT
TACAACCTCAGATACGTTTGTAGTTCTAACCACGTATTCGAGAGGGCCATGCACTCGTCT
AACAGCGGTAAAAGTATCACTGTCAGAAATACCCACACCCTGCCCAGATGGGAAATAAGA
TTTTCCTGTGGCCTTGTTTAAGAACCCTACTTTCTCTCCGAAACTACCGATTTTATGTAC
ACCCTCTGACACTGAGTTAGATGAAACATCGAGTTCACCCTCGGAATTGGAGAATACACG
AGTGTCCACGAATTTCGAGGTATCCACATCCCACTTAATCATGTGATTGTAAGAAACTCC
TCCATCAATTACAGATTCTATATTATTGTCTATGCTATCAGCAATAACACACTTCGCCTT
GATAACACCAGCATTGATACTATCATTTGAGTTCATATCTAGGTCACTTTCAATTTGGTT
TGGTTAACCTTCCGGGTTGTCCCTGAATGGAGCCACATTATTGTGTATGTCCTTAATCTT
CTGGAAATTACGATTAATTGGCACAGAAGTAGCGGAGGTAACATCACTCAGAACATTCTT
AGCCATACACTATCTCCTTAGTAGTATAATTTTTGATCTATAAAACAAAAGGGAGAGCCT
AAGCCCTCCCTTGCATTCGTTCCACCTATCTTAGATTGAACCTTGACCTTTTGCACCAGC
AGCGGCATCAGGATTCCTACCTCCTTTCACATAACGACCAGTTAGACGAACTTTACCTTC

ATCAGTCACAGTTTGCGAAGTACCAGCGGCTTTGGAGGTAACTTCGCTATCAGTAGTTAA
AGCACATGCCCAAGTACTTTCTGGAGTTGGTTTAACTACGCCCACTCCTTCCTCTTGTGC
TTCTAGCGGTGGAACACCGTTGGTACCTTCTGAGCCTTTAGGACCTACTGCAATAACAGG
GTCAGTACCACCAAGAGAAAACACCTCGGATACGTGAGCAGAAGCATCAACAACTGAAGC
ACCTTTAGGCAGAAGTACTTTGTTGTATTTAGAATAGTCTTTCTAGAAAGAAGATCCCCA
AATATCAAAGGTCATTTCCATAATGGAACGTTCTGTCTTAATGCCGCTTGTTGGTGACCG
TGTACCACGCGCTCCATATAAGTCGTGAGTACCAAGACCAGCATAACTTTCAAAACCCAT
AATAAGCTCCTAAGATTAAATCTAGCAATGAACCGGAGGAGGGGCACTCCCTCGCTCGGG
TCATTTTACTTACTTGTCAGCAGTCTCAGAAGCGAGCAATGCAAACAGAGTGTCGTTACG
TTGGATAACAAGACCATAACGCCGACGAGCAACGAACTCATTACGGGCAAGGTCTTTGTA
ACGTTCAGACTCAACACGAGGAGCCTGACGTTCTGCGTACATTATAGGTTTGGAGTTGTA
ATCTGCGACACACATACCGCTACACGCAACACCGTTGGTGATTGTCACACCACCAACCTG
TTCCCTCACGATACGTGGTAGACGGTTAGAAGTTATCAAGTGCCAGCCGTACAAGTTGGA
AATGAACTTCTGCTCATGAGCACGACCGCCTTTCTACACATCCATCGAGAATGGGTTAGA
GTCGATAGCACTTGCAGCGATTTGGAACTTGGTATTCAGAGTTTCCTCAATGAATGGGTC
AACAATCTGAATACCACCACCTAGTGGAAAGTTCGCCTTGTCAGCAGCAAGTTTCAGGTA
AATCAAGTCTTGTCGTTCGATGGTCTTGTTAGTACCTGTATCGATACGCTTGTGAGCTAA
GCCGTTAATCTCGGTAGCCTCAGCAGCACTTAGACCTGCGTAAGCTACTTGCAGATAGCG
GGTTTCAAAGTCTTCTTGCATCGTACGAGAAGACTCTTAAGCACGCATTGCCATCTGAGT
TTCGCTTTGAGCACCGTCTTGACGTAATTTGTCAGTAACAGACCAATCATCGCCGTTGCA
GTTAGTAAAACGCCTCTTTAGGTTCCCAGTCACGCTTAGTTTGTAGAGCAGTGGAGTTGC
TACTTCCACATCCTGAATAGTTACCGTATCCACAGTTTTAATGTTTAGCACTTCACCCGC
GAGAAAATCTAAGACGTTACGATACATCATTGCTGGCAAGGAACCATCGTGTAGGTTTTC
CACAATGAATTTTGGATATTGTTGAGCCTCAATAAAATGGCGTGAGTTGCCAGTATTTTG
TGAACCAGTAGCACCAGAAATATGTAAATCTCCTTAAAAGTTAGTAAGCTTGTGAAACTT
CTTTACCACCTTTGACCGCGAAACCACATGCACGGAAGGCAGCGACTAAGTCAGAAGTAG
AACCTGTTGAACCCATTTTGAAGTTGAGTTGTGGCTCTGGCGGGCTCTGAAATTACGCCG
TGGTATGGGTACCTTTCGAAGTAACAGTCGAAGTAGTTGGTTTAGATTCGACATTAAAAT
AACTTGGAACTGGTTTAGGTGATTTGGCAGCGAGGTCTTTCAAAAAATAGAAACCTACTC
CTAATCTAGGACCCTTGGACTCGAGAGCCTTGGTCGCCCTATTGCTAGCGCCAAATTCAC
CAATCAGGGCATTAGTTACCGCTACTTGGTTAGCTTGTGCTTGAGCTCGGGCATCACGTT
TGTTAAGTAATTCCAACACCGCCTGTTCATCTAGACCTGCTTGCACTTGTGGTGCAGTTG
GGAGGTGACTTACAGCAAGAGACGCTACTACGTCCTCCAGATTGTTAGCGTTCGCCAGGT
CAGCCTTCAATGTCTCATTCTCTTGCTTTATACGTTGGTTTTCAGCTTCGAGATTAGCAA
GGAGCGATTGAGCGTGTGGAACACTAGCTAAAGCTGCTTGAAGGTCTGCGTATGTACGAC
CTTCACCCACATAAGCCGCAAGTTCTTGAGCCAGTTGAGGAGCCGCAGGCGCAGGTCGTT
GTACGGGTTGCTATGGTTCTGGAGCTTGTGTTTCTTGAGCCTGTGCGAATAAATATGATT
GGTCAGCCATATAATTCTCCCTATTCAAATTCCTAAACGTGATTACGAAATAATTCTAAT
AACTTAAGGTTACAAGTCAATAACTTCTAGTCAAGTTTTTTCTTGACTATTTTATGCAGC
CTGTTTGAAGCCCCAATTAGGACAGTCGAAACCATCCAGTTTGATTTGTTATTCTCGATT
CTCAGCGATACGCTGTCTTAGAATTTCCACCAGTCTTTCCCTGACTATCTGACTTCCAGC
GAAATCGTTGGCGAACTGTACCTTTTGTTCACCTTTCATTCCTTGCGTACAGACAGTTTT
TATCCTTATCTCCTTATCCTAACGGTACTTGTGCTCCTTCTTCGATTTGTGCTTGAGATT
GGTTAACAAGTGCAGGGGTTGTCTGTGCTCCCATAACTGCTACGTTAGGTTTGAAAATAT
CCCATCCACCTAAGCTTAAGGTGTGTTCCAACATTTTAGCCAAGTTCTCAGTAGACACAT
GAGGTTTAATCTCTTGCCAAATTGGAGTATTAGCAAAGCCCATTAGGCTTTGTACAACTT
GCGCTTGTTCTGCAAAGTGTCGAGCGCCTCCAGGACGTAGACGACATTTTGCTGGGATAC
CGTCTTTGTTAACGTTCATGTAGGTTGCCACTTTATCCTCTGTGTTAAACACACTAATGG
CATAAGCTACATCTAAATTACGACGAGAAATCTCTAGCATCGCATTCAGAACCTTAGCCA
CCAACATTAGTTCAAAGTTCATAATCTTCTCTTGGAACATACGCCCAGCGGCGGTTTGAA
GCTGTTGGGCCTCGAAAGGTGTGTACTCACGGGGAGCACGAATACCCATACCTTAACGAG
GAACTCCTGCAAACTCTTGCATCTTAGCTTCAAGAATCTGAATCTGAATATCTGCTTGTA
GAGCTTGGGTATAGGGAGCCATCATCTATACATCACCATCACCATTGATGTAAATCTGTT
CTATAGGTCCCCAAACAAACTGCTCAACATCTCCTATAACATTCATTGAAGGAAATGCGA
TTAGGTCAACCACGGCAGCTTTCAAGTTCTCAAGGTGGTCGATACTATATTGCATACCTA
CTAGGTTATCTAGTGGTCCCATACAGTACAGGTTATGTTGACGAATACGCCAACAACAAT
GGAAGATTGGAGCTTGTGCAAAGCATGATAGATTCTCTTTATCCTCGATTACAAACATGC

GGTCAATGATTGTGACTTTCATATTACGCTTAAATGTACCTGACTGAGTATCGTGGTAGC
CACCATAGAATGTCAGCACCTCAATTTAAAGACTTTGGGAGTAGTCGTAAAGGATGCCAA
ATCCGTCCATCGAGCCGCCTACAGCCTTTTCAGAGTCTTCCCTAGTGTAAGTGTCTAGCC
CACGACTCATCTCTCGTCTACGAGCGACTGAGGAAGCGGGAGAGTCGTTCTCTGGCTGGT
CTAGTTCCATTTGTGGTTACCCTCCCTCATTCAACACCGTACGGATGATTTTGGAGAGT
GAGCAAAGTCTACAGCGACGGGATTAAACACACTATCTTTTGGGTCGTTGAGAACTGCAC
GAGGACCAAAATACGTATCAAGTGTAGCGCCAGATTCCTCATCCTAAGTGGTTTCCTTCT
CATACTGGACGGTGGCAAAACAGTTGCCGTAATAAATGTACTCGAATAGGAGTTGACTGA
TGATTGTGCCGAAGTCCGATTCCTTAACCTTGTTATCCATGTATTGCTGAATAGCGTCCC
GTTTGGCCTCATCGTGAAGGCTCTTACCTTCCCGCATCAACCACTGTTCATTAGGAAGCA
GTGCACTAATGTAGCTGCAGTGAAAGCAATCTCGAATCTGACACAACTTAGGTAAAGTAG
TTTTGTTCTTCCACAGTAACGTGCGGTTAGTCGTCTTACAGGTATCAGTAGCAGAGATGT
AGTTACGTAGTTCACTCTTTTGAGATAACTACTCATGCCGCTGACTGTTCCAGTTCTGCC
AAGTATCAGCAATCTGTTTAGCCAGATCATCACGAGTGTCGGTGGGCATCTGTTGTAGTT
ATAGAACCTTCCCTTTCATTAATTAATTACTCCAAATCTAGGGATAAATTGAATGACGTT
GCTTTCTCGTTTAGCGCTTGCAGAAAGTTGCAGTGGTTTTACCGCAATCTCTACAGCATA
AGCTAGAGGGTCTTTCAAGTCATCATGAGGTGGACGAGCCATCACAAGTTCTTCCTCTAG
CATTGGAGTGTATCCACCTTTTCTATGCCACATAATACCTTGCTCATCCCGTGGTTCCAA
TACAGCAACCGTACGCTCTTCCTTAGAACCAAGCGCGTTAGTTGGTCGAAACCCGTCGAT
ACTAATAGTCATGCCTACCTTGCAGAACTTCTCTTTAGTATCGTTTACAATGATTTGCTG
TGCCACAGTACCCTCAGCACGAAGTTTCCTATATTCCCACTTACTATGCAAAGCAGAGCT
AGGGTCATTGTAATCGTTAATCTTATCTGTTTTGAATCGGTCAATATCCGAAACGTAGAT
GTAACCCTGTGAATCAATCCCAATAACAACGATTGATGTAAGGTTGCCAATCTTAGAGAA
ACTAACTGCAAAGTCCACAGCAGCTCAGATATTCAAAGGTCTGTCCTTAAATCAGTGTCT
ACCTTCCCGATAGGTAGCGAATATCTTATAGTAGTATTGGAAAGTGCTATAGTTCATACG
TGCTGCTTCCGGGTCGTTTGGGACATTGTAGTACTGAGCAAAGAACTGAACACGATCAAC
GTATTTAGCCTTCTTCTTAGCCAGAACACGGTTCTCAAATCCAAACTCCTTACCATCAGG
ACCACGATGAGTACCAGGCAACAAGCATACACCATCTTCTTCTACTACACGCTGAAAAAT
ATCCCAAACAGGCCTTTCCTCTCCAGTCTCTTCTCCATATTCGTCGAATACGGGTTGATG
CTGATTCTTCCATGTGTCGTAAATATCAGCAGGAAGATACCGAGTACCACAAGTTTTAAC
GATACCATCAGCGTTCAGGATAGATTCCATCTGAGAGTTAGCAGCAGCTACCTTGGGTCT
ACCATCCTCCAGATATTCGTTATCTGGAACTACAACTTCATTAGAAGTAATAACGTCAGC
GTGCCGACCAGGGGTGTTAGGGGTGAGGCCAGCAGTCTGAATAGTAAAGTCCCGGATGCC
ATCCATCTCACGTTTTGGCTGGTCAACTGCGATTACACTGTCAGTACATTTCTCACGTTT
ACCCTCCTCCTCATTCATCATATCCGACCAGTCCCGGCGATAGATTTTGGAAGTCATGAT
GTTCTTACTATCCTATAACTGCATTTCAGCAAGTTCACTGGTAGCAGAGATATACAGAGT
GGTTGGCTCGGGATGATTAGTCACACACCAAGTTACCCCAAGAGCCATCATGTGAGGTTT
TATGTGACCACGAGGTGACAGTAGAAGTTGGTCGTACTCATTAATCTCCATCCACTTTGC
CACTTCCTTGTGGACCGCACCGTAAACGTATTGTGGATTAAGCAATCGTCAAAACATCCA
GAAGTGAGATTCAGCAGCTTCTCTCATTTCGTCAATCTTACACACTCAAACCTCGTAATG
GTCGTTCATAGTCTTCCTCTAATGCATTCTTAACCCGTGATGCAATCCATGACTCACGAA
CTATTTCTTCCTTGCTTGGGCGTCCTGCCCCACGTTCGTCCTATCCTACTTCGGCAAGCC
ATTTGGCAGCTGCAAAAGACTTACCATCTCGTGCCATTTCCATTGCACTGAGTACACCGA
TAGAGCGATCTTGAATCTCGGCTTCTTCATGCCATTCCTCGAACACAGCGTAAAATTCTT
GGTTAGCCTTAATCTTCTTCCAGTGTTGCCACCCACCTAAGCATTCCCTCGCATATTTAA
TCTCATTTGGGTCTTCGATGTACGTCTCGACAAACAGCCGTTTAATGCTTAGGTACTTTT
CACCTTTGAACATACGGTCTTCATCCGATATCGTGTAAATTCAGCTTGGCTTGAATCCCT
TCCGTAAAAATAACCACTGAGTAATCCATCTACCCATACAGGCCTTCAATTCCTTCTTCA
TTCGACCCCCTTAGTATGGCTTATTATAGGGGTAATATAAAGTAAATACTTTACTACTCC
AAAGGGTATTTTAATTAATTGGCCTTAACAGCAGCCCATCAAGCCATCTAGGCTTGGCGA
GCCATGTCAATCTGCCACCCCATAAATAATGGGTCACTAACTACTCGATATGCCGCTTGT
ATACGTATTAGATTATCTTCATTTATATCTACTGGAAGAATTTCTAATGTGTTTCCAAGT
AGTTCTTCTTAATTCACAAATGTAGAACAAGCTTCATCCCATACCCATTTACCGGAGGCA
AGTTCCTCCGGTGTTAATCTTAGTTTACTCTTAGAATTTCCTTTGTTGATTCGACATCCT
AACAGTATACCACTATTTACAGAGTTAACGTTAGACGTACCATCAACGGCAGCCCCAGCA
GCACCTCCTGCCTGCCCACCGAGTCCAGGAGTCTGCATTCCTGATGCACCGGTGGCTCCA
CTTACACCTGCGACACCAAACTTACCACCATTACCTCCGTTACCACCTCGACCTTCGTCA

GTCCCACCGTTAACACCTGCAAGACCGTTTGTCGCTGCTTGCGAGACTCCTTGACCTTTT
CCGCCATTACCAGCGGAACCACCGGTAGTGTATGTAGTATTGGGCACTGTTATAGTTCCC
TACATTGCGTAAGCCTTCAAAACACCCACAGCTTCACCGTCGAATTTGGATGCCTAGTAC
GTCCACCCTCCCACGGCTATAGTGATACCGTAGGTAACACCAGTGCCAAAGTCTGGACCA
GACCAACGATATATCTCGGAGTGACACATACGGAGGAGACAGTTACGAGCACGAAATTCG
CTTTGGTTCCCTGTAGCGTATGTCCGAGCTCCAGATGATGGGTCACGGAGTGTAGATTCA
GTAGTAAAGAAACCGTTACCACCTTGTCCTCCTTTACGACCACCACAGCCTCCACCTGCG
ATTGTGCCGTTGTTGGTTAAGTTAATGTTTTGTGAAGTCTGTATTCGTAAGGCAGGGCCA
CCAGCTTGCCCTACTCCAGTTCTCCCAACACCAATTTGTCCCTAGATATTACCATTCTTG
ATGATGTTCATTGTCTTGACTACAAAGACGTCTGGAAACACAATTGCTGGAGTGCTGACG
TTGGTTGAGTACACGCTCACACCGGAGTTAATCACTAGATTATTGATAACCTCTTCTCTA
CTACTTAACGCCGTAGCTAGGTTGAAACTTGCATTACTCTGACTGCTTGTGTAAGCAAAG
TCGGGGTTTTTGAATTTCTTCCATTCCCCGTTGTCACGAACCAAACCAAGCGGATATTTC
AAAGCTCCACCGGCATTAACTTTCATTGTAGCCTGTCTGTAAGTACCAGAAACATTTACT
CCAACTGGCATGGTCCCTGCTTATACGGACCGTAGCCAACTATCTCCATCGGCACCACCA
TAAGGTGCGCTAGAAGATACAGTAATCTGTCGCTTTCTATCTGCGGCTACATGAGGAGAA
GAACGGGCATCAGTTGCATCATCATATGCGGCTTTTACACCGGCAGTTGCAGCATAAGTA
GTGGTACTTGTTGTGTTAGTAGGACTAGATGCTCCCCCATCGTTTACGTTGCTTAAACTA
ACCTCAGCTGTAGTAGTCCAACCATTTGCGAACTCCGGAGTGCCTGTAATCCCCGACCAT
GCTGGAACCTAACCAATAGGTTGGTATGTCGCCCCACTCTCACCTTTAGAATAGGCAGTA
CCTGCTAACTCCAATCCAGATCTAGCAACTGGAATAACTTGGACCTTAGTCCTTCCTTCC
AACTCCTCTGAACGTACGGCCGTAGGGGTAGCACCCAGAGCACCTCTATCCGTTGGGGTG
GGTTTAAACCCTGTGTGGTAGATTTTACGCCATGGGCGAGATTCCCAATTTGTCCTATGA
CCTGATTTGAAATATAAAATCTCACTATTAATTCCATACCCAGTTAATAATTGGAATGCC
CTATCTGCTGGAGGTGATAAAAATGACCCTAAGTTACCGTAATTAGTTCCCTCTTGCATA
TTTGGACTGTTGTTACCCAGGCGAACAACACCAGTTAGATGTGGATACTGAGTCAAATCG
AGAGTCTTAGACGCCGCAGCTAACATCACACGGTTTCCCACATCAACAGACGGAACAGCT
CCAACAACTGAGGCAGTGGGTGTATAACTTGGAGAGTATTCCCTAACCCTACCCTCATAT
AGTGAGGTAGATGACACCACTTTATCTCGCAGTATTGTTACTGATGAGGTTGCAGCCTCA
TGTATTCTTCCAGTTCAAACAACTACCTTATGATCTATACCCTGACCAGCATTGTTAATA
TACACACTGCCTCTATTATCCATCACTCTGTACCCATCGAACTTTGCAACGCCTTGGGTT
GGAGTGGTATTTACAGAATCGGAGTCGATGAGCAGCTCAGCAGTCATTGTAGCTCCAGCT
ATATTTACGGCTCCTAACTTTGCGTGAGTTGGTTTGTTCGTATCCGAGTAGAACTTAATA
TCCGCGTCTGATTTAGAGTACACAGTGTTGGCTTCGGCTAGGCCGGTGACGATACCTACT
CGACCATTAACGGACTGTACTCGAGTACCACTCTCTACAATACCCCAAGAGGAACCAGTC
CAAACAATCTGGTCACTGTCGTTCACTGTTAGTGTTTCAGCTGGGATTTGTGTAGACACG
CCATTTGGGTTGTTAGTAACTTCAACATAGGCTGCACCTTCTGGTGCTGGGTCAGTTGGA
GACATACCTTGGTTGATTGTTGGGTCGTCTGGGTCTCCCATCACAAAGATGCTTGTTGGA
GTGGTAAATACATTGTCCACTTGTGCCTCACCCTTAATACTGATAGAACCGGCACTACCG
GCCAGCAAGTCACTTACCAGCAGTTTCTCTAACTCTTTTGACGAGTCATTTTGAATCATG
AGGTATTCAAAGTTACTGATAGTCCCCTTTGCAGGGGCCGAATCCGCGTCGATTACTTTG
GTTTGAATCTTGTCATCCATCGAAATCTCCTCAGGCAATCACAGATACTCCCCTAAATTG
AGAACCAGTAGTGGTTACTCTGCTTAGTTTGACGTTAATTACCGGAGAAGTTACGTATCC
ATTGAGGATAAGTTCTATAGTGTTCCACTCTCTAGGAGAAGGAATAGTAAACGACCAAGT
GACTCCAGAAAATCCATAACAAGTTTTGTTATTTGCGCTAACATAGGCACGTCTGGAAGA
CTCATACACTACGGAACCGTTTACTCGAATACTCCAAGTGATTTGAACATCTGCCAAGAA
TGATGGACTGAACGAGGACAACAAGACTAGTAAATCCTCCAAACTCGCCATTGCAGCAGC
CGCAAACGGGGTACTAATCACTCTGAGACGAAGACTTAATGAACGTGCGTGGTTAAGTGG
AGTATCCATTCCAACGAACGTTGTACTTGACAGTGCAGTAGCTGACCCATTGTTTAACTT
GGTACCACTTACACCATTCATCACTTTGATTGTGAAACCTTGAACGAATTCCACCTGTTC
CCACTCTCCAGAACCACGATACACGCGAGATTCTGAATACCCTTGAGATTTGCTGTACTG
GATACCAGCATCAGATACAACAGGGTTTGGGTTAATAGCGTTAATCAATGTTTAGCTCCC
CCTGGTGCCTGTGGCTTCAATATACCACTCGAGAGAACCACGTTCACCCTGGTCCCCTTG
TGGAACATTGTCACCTGTTCGAGCCTGTGGTCCCATTGGACCTCGTGCGCCTTCAGGCCC
TTGTGGCCCGCTAGGGCATTGAGCACCTGTTAGTCCTACTGGACCTGGCGGACCAATAAT
ACCTTGAGGACTCCGGACCCCTTGTGGGCCTGTTAAACCCCTATCCCCTTGAAGGCCTTT
CTCAACCTGTGGGCCACGTTCCCCTTGGGGACCTTGGATACCTTGAGGCCATCGGTCGCC

TTGCACTCCCGTCTCGCCTTGCGGACCCCGAGCACCTTGTGGACCTGTAGAGCCGATGGG
TCCTTGCTTACCTTGAGGTCCTTGTGGACCTAGACCAAACGGAATTGCTGGAGACCAATC
ACCAGACGCTGCGATCGACTTTCTGAAGATCGCACCCTTAGTCACAAATGGAGACTGGAG
ACGTACTGTGAAGCGGACTCCAGTTTTAGGAGCTTCACCACACGTTAGAACGTTACCCGC
TCTCTAAAAAGTATCTAGGGACTGGTATGAGTATCCTTGCCAAACATATCGGTTACTCTT
CAAGTTTGGAGTGAACAACAAAGTGAAACTCTTAGTAGACGCATCTCCAACAAATTCCTG
TTCTTGAAATGTAGAATCAAATGAATAGTCTGTTGCAAAGTAGATAAATGGTGGACCTTC
ATCATCGTATGCACACAAACCACTAAACAAACCTTGAGCATCGAAGAACAGAGAGTCGCC
CTTATCACCTTTTCGGCCAACTGGACGAGCAGCACCGGTATGGCCTGGGTGACCCGTTAG
GTCTTCGTAGTTACCCTCCGTCCCGTCAGGGTTGCGGAATCGTAGGTTCGCACCATCCCA
GTCATGCTCTGGAGATAAACCTTGTGGGCCTGCAAGAACAACAACGACCTGCGGACCTTG
AATGTTTACCCAAGGACCCCCCTCAATAACTACACCAGTAACTGGGTCAATCTTGTTGTC
GAATCGAATTGATGTGGTAGATTGAGTAATGCTGTCACCAAATTGGTGGATTGGAGTACG
ACCTACTACGCCTTGTGGTCCCTGCGGACCCAGAATATCTTGTTGACATTGAATACGCTG
TGGACATTGTTATCCAGTAGGGCCTCGCGGACCCTCTCGCCCAGCCGGGGCAGGCGGACC
AAATACTTCAAGTGAAGCATATGAGTAAATGTCCACCTTGTCTGTAGACGTTGCGACGTA
GTTAATGAATTGTACCTGTGTGCCCACTGTAGTGTATTCCACTTCTGGAAATAGACGGAT
ACCGTTTTTATACACTTTGGTAGAGAAAGGACCTGCCTCTACCTTAGTACCGCTGTCGAC
GTCAAAAGAAAATCCATTACCAGTATGTCGCATCACCGTTGGAACAGTACCAGATACATC
ACCTTCTAAGGTTTCAAGGTCTTCCACTCGAAGAGGTTAGGTAGGGTTAATTCGAGCAGG
TCGGTTAATTAGACGGTTGGATTTAACAACAAACTGATGTTCCAGTTGTTTGGAACCATT
CTTAACTGAGATAAGATTGGAGTTTAGAGCCTCTCCAAACTTATCAAACTTGGAGTTTAT
TGAGGGGGTGTTAAAGCCTCCTCTTACTTCAGTAAGAGTAATAGTCATAGTTATTTAAGT
TCCATAAGATTTTTAGAAATAGTTAATATGTCAAGTATCTAGACTATAATAATGATTATG
TATTAATACATTCCGATATAGAAACATATCAATACTTAAAAATACCCCCGTAGGTTACGC
AACTACGTTGACCGGGTAGATGCTATTCCTTTCATTACTAAGTCTCCGCTGGCATCAACT
ACGCAAGTATTCAACATACCCAACTCTTTAGTGGCTACTCATTCATGAAAGATACAATAC
CAACAATCTATCGGGCTGTGGTGGGGCCGCGACTTGTTTACTATAGTGTATCGCCCCGTA
GAGTGAAGCCGTATAGACGTCTAGAGCACAAATATTATCAAATTTGCTAGCTCTTATAAA
TGACAAATAAGAAATCTGGAACGCCATGATTTCTGTGGGAAATTTTTATGTTGCTGTGCT
AACCAAAATAGCACGCCTCGTGTCCCCCTCTACCCATCTCCGAGGATGTGCATGTACTCC
TTTTTATCTTTATGTGCGCATTTAGCCGTACGGAGACATTGTTGATGGCTTGGATGGTTG
AGCATTAACGTTCAGCAATTTTCTCTACAATTAGCAATCTCCCGACACTTGCACGATTAA
GTGTCCGCACAGCGATTCGGTGCGTTAACATATTGAATTATCTATTATTGGGATGAGCTG
AGTGTTCTGTATATAGCTGTACATGATACTGAACGCTAGCTATCTACCAACTAGGTGATA
ATAGGTTGTTTATTTGTACCTGATACACGTATATACTGAGCGTATTCCCGATTCATATCC
ATGTTGAAAAATGTGATGTATGGCACTTTTAGTCCAGTTAGGCTTGGTGTTAATTTTCAC
CCCGTCGGTAAGGCAACAGACTAACGATATAGAGATAGATAGTTACAGTGTTTATCCCTG
CTCTTTAAAAATGTGGTTGACAATGATTACTCATTTAAGGTAATCTTCACACCGTCAAAT
GTTTACGGTGATACGTAGTCCTGTGTTGCTCTGGGTTGATTGAGGTAAGCGGCGTTACCG
ACTACTATCAAATACCGTTCTAGATGTACCGAACAGGAAGCTAGCCAACAACGCTTGACG
TGGGGAGGATGATTCAGTTAACATGGTCACCACGCTCTGAGTAGCATTCGAGTTAACCGA
ACGGTGCTCTAAACGTTCTCTAGTAATTCGGATATTGACACTGATTTAACATCGGTAATT
AGGTTGATGATGCGAAGTCTGGCGTAGCTGTGAGGAGTTAGACTCTGAAGGGTATAGAGC
TACGGTGCGCTCGGTTCCGAGTCTGTAACGCTTTATCTATAAATCGTCCAGCTAAACTAA
CGGTTATGTATATAACTACAGTGCGGTTGACGTTGATATGGTTGAATTTTGAATGCTGAG
TGTTGATAAGCCGAGACGAAATGCGTGTCCACCGTGAAACCAAAAGGTATGCGTCAGATC
TAATCAGCACGCCAAGCAGTCCGCCGTGATTAATTGTAATAGGACTGTATCTTTAATGGC
ATGAGATACCTAATTAATATCAATCTATGAGTTAATTAGTAATATCTGGTAGAGCTAAAG
GTTGTTTAGTGTCCTAAAAAATTTATGGTGAGGGGTTAAGTAATCTAATGTGTATAAAGG
ATTAATACTGCTACCCAATGTGATTGAGTTCTATGTGCTCCGTGTTCAATGTTTACTCAC
CAGAGGGCTCGTTAAATGTTGACTAGCCTTTATTGTATTCACTAACACGCGAGGGGTAAC
AATGCCAACTTGAATTACAACTAACGTTTTACCAGACAAGGTAAACCGAATCAATAGCCT
GTTAGGTATTGGAATTACTGCGGTAGGTCAGTCTGAAGTGGAAGCGAACCAACAAGGTTA
TAAATTGGTTCAAGTTATGTGTGAAGCATTCTCTATTATTCCACTCTCTGACTCCCTTAC
CAGTAAAGAATTAAACTTGTTCCTGACTAGACTAATCACTCGTCTAACTCTACCATCCAA
CTAATCTCATGGGTGATTAGCATGGCCCTACAACTAACTGAATCTAACTTCAAAGCTAAA

CTTAACAATTGGGTAAAAGGCAGCGAGTCTAAGCGTGATGAACTGCAAGAATTTATTGTG
TTCGGTGTACTACATACCCCACAAAACGACAAGGCCTTTACCAAACTGTCTGAAGTAATG
TTTGCTTGTGAAGATGCGAAGGCATTCACTAACCAGAATGTATCTGACTACAGCAAGAAA
GTAATGAACGCTGTAGTATTGAATGTTAAGGGTAAAGTGTTCAAGAAAGCTAACAAGAAA
GCGTCTATTGAATATAACATTGGCTACCTAGAATCTGAGGCCTTCTACGGGTTTAGCAAG
GCCACGAAAGCAGCACAAGTAATGGATATTATCAATGCGGATGCACGTTGGGCTAAACAA
CTGCGTGAGAACACTGATAAAGGTTCGTTTGAAGACGGTCTACCAGATAACGCCGAAGAC
CTACTGAATACACTGCGTCAGTTTGTAGGGTAGTAAACTAGCAGCCTTTCCAAATGAGAG
GCTGTTATCGTTATTGTCCATATACTTGATGAACGAGACTATTCTGTCTATTAAGATGCG
TCCATGTGCTTTGTCCTCATTTCAATTTACTGATCCTCCGTTTGCTGGATACGTGGGTGC
TCACAAGTACATTAAGACTGAGGATAAATGGATTGTGACTGCGATGGTTCTGTGACTCTT
AGAGGCGGTACTGTGGGTAAATTGTACACTGCGAATGACGCTAACGGTGTTAAATGGTCA
TTTGTCGTGTTCAACTATAACGTTGAAACATACGTAACCGGTGGGCTAGATTGGTTTGCT
CGTGAGTTCGAGCAGTCCGATTTTGAAAACTTGTGTGACCCAACACCCACCATCGACAAT
ACTCCGGCCAAACTAGCTGAAAGCTTCCAGCATTTGCTATGACACTGAGTGATTTACTAA
AGGCTATCTGATTAGGTAGCCTTTGTTAGTTTATTTCACGCAACCATTGGTGAATATAAT
GCGTATTTTAAATATTGTCAGCAACGCCGTAGTAACTAACGCAGACGAAGTAGCACAGGA
AGATAGCGTATAGATCAAAGGTACTACCTATGTTCCATGTTTGATTCTGGGTCATTGGGT
TATTGTTCGTAGCGATAAGGTGGTTGATGCGAAGACAGCAGACGTGCCAACAACCTTCGG
GATGTTAGCTCTGATTGTCTAAATGCTAGGTCGAAAAGTACTATCATCATCATTCCCTCA
GCGATATGGAGACAGCAACATGAAACAACCCAATCCAAACTGGGCCCATAGTCCAGTGTG
TTTGTTGTTACACAAGCAAATAGCAGACGTTGGACTAATCAAAGTGGACAATATAATCTC
TCGTGCAATTGGTGGTGCGGTGAATGGTGGTCGCCTTGGTTTTATCTGTGCGGTTATTAA
ACAGGCCGGTGCAAACAACCGTAGTCTGTCAACGTGGGAAGCATAAGGTCAAGTGTATGA
CTTATTAACCACATTGGCTCTTGTCCACTAACTGATTAGGTATCCAATCATGTTCCATAC
AATTACTCTGAGTGTAGCCGAGCTGGTTATGGGATTGGCCATTCTCTTAAATAGTCAAGT
GCTAATCCGCTCTATCTTAAAGCCCAAGTCACTAGGCCAATATGTACAGATTATTACACT
AATGATTGACATTTCCACGATTGTGGCGTGGTTCTTTAAGATGCCACTCGGAGCATGATT
GATGCTTTTCGGTAAATTTGTGAACTTAATCCTGCTTATTTACCTACTTTTACGGAGAAA
ATACACTATTTGTAGTCTAGTTCCTGGTCGTATGGATGCGCAAGAACGTTTACAAAAGGC
TTACGTTGGTGGTACATCTCGTAGCGCTGTTATCTTCCCGAAGAAACGTAACTGGATGGG
TACAACGCCCCGTGTTAAGTCTGGAAGTACTAAGACTTATCGAACAGCGACACCTCCTCG
TGGCTTTAAGCACTTAAACTGAATAAATATTAGGAGTCCATTATGGTACGTACGATCGCA
TCAGGCGCTTTTTCTTTCGTTGCTACTCATCGTGAAGATGATCTATCCGTATCAAATATC
TTTGATGGTCGTGAACCAGACGCGACTCAGTTGAATCCAAAGCGTGTCGAGCCTATTGCA
GACCTTTTGGCTAGTCAAATTCGTGAAGAAGAGTCATTTTTGGCAGCAAAAACCCTGCGT
GAAACAGAATTTATGGTCTGTATCTGTGCAGTAGTATTGGATGAACCAGTACGTGACGCC
TTCTTTGAAGCGGCAGAGGAAGCAGTCGAGCAATTGATTGTCGAAGGTGAATCACTCCAA
GCAACTAAACATTAATCCCGCTAACCAGAAAGAACTACCATCACGTTCCCAATAGAAGGA
ATCATAATTATGTCTATGATCTCTAAAACTTTCCCAGTATTTGGCTCAACTGTGGTTCAC
CTAATGAATACTCAAACCCTCTTTGGCTTTACCATTGTAGACGGTGAAGAAATGGTCGAA
GGTACTTTGAAAGACCCACTCTTGGAAGGGGCAATGGCTGCATTCGAGGACTTCAGCGAA
AACCACGCAAAAGCTGCGGCTCTACTATTCAAAGAAGCTAAGTTCTTCCACCAGAAAATG
ACCACCAAGATATCATCGGACTGCTTGGGCGGTAACATGAACGTTGCAATGGTTGCTCCA
TGTGAATCAAGCCGTGAGGTAGGTTACGTTGTCTCCAAGACTCGTGACGAGGATGGTCAT
TGGGAAGTTGTTCACCATGAAATGCTCAACCTAGCAGACGATAGCAGTGTTGCAGACATG
GTGCACAGCTGGAACCACGGTGCCGCGGAACATTTAGCAGTGTCTCTAATGCTGACTTGT
TCGCTAGGCTTAACTAGGTCTCAACAATCAAATCGGTCCCCTACTGTGGTGGTGGGAGCC
CATCCACCCACTCAACTTGAAAGAGAGAAATACCATGAAAGGCATTCGTTTAAATCAACA
AATCCGTTCTGGAAGCGTAAGAAACGTGCTCTAAGCTTGGGCTATTGAGAACCCAAAACC
TGAGTGTGTGGGTAAACCAAGCAAAAACACTTTGTTAGATGCCGCATACGCCCCATGTTA
GAAAGGCTTCCAACTGGATATTCAATTTGGTTGTGGTTTGAAAGACGAAATACTAAACAA
AGAGTGCTACGTAGCTCTTCGTGTCGATGGTGAACTCGGCACTGAAATCATCTACGAAGG
TATGTACTTCAAAAAATGCAGGAGGTAAGAAAGAGCGCCGACCCTTCCTACCAACTTGTGC
TCTACACTTAACTACCTCTTCACCGGTGGACATCGCATGGAAGAAGCAACATGATGAGTT
CACTCTTCAACATGAGCAAATGAAGAAGTGCAAACAAGCTCGTGCTGACAGAGCCACTGC
AATCCGTGCTGTGCTACACAGTGTGAACACCACCAAGCAATTGCTTGAAGCATGGGCAGA

GCTTGAACGCTTTATGCCGAGTGGTTATGTTGACCCAAGCAAAGTACAACTACCAGCAAT
CCTTCCAACCTTACCAACTGGAGAGTTAAATATGAATTCCTTTCTACTGAGTGTATTCTT
GATTCTAACTTTGTGCGTATCGCTTCCCTCAGAAGCGGAACCAAGTTCTAATGTTACAAG
AGCCACGTTTGTAGAATACAATTGCAGTTGTATCACTGATGGATACATTGCAGCGGCTAC
GCTTAACGGTAAATTCTGTGTAGACTTCATCTTAGCAACTAAGAAGTCACAGAATTATTA
CGATAGACTCGACCGCGCAGAAAAACGATTGATTTCAGTTTATCTCTGGGAATGAAAATC
TAAATAGATTCGTGAAAAAGGTTGCAGACTAGCTCTAGATGCCATGCAATAAGGCTCAGA
TGAAATGGCTGCCGCTTTGGTTTATGTAGTAGGTTTGTAGTGAATAAGTCAAGGGTATCT
AATTGGATGCCCTTTATCGTGTTCCTCAATCAGTTGGCAACTGTTATGAAATACTATATT
GAATCCCGTTTAACCCACTATCTTAGTATCTTTCCTAATGTGGTTGCCGTAGTAGAACCT
TTGTTGGCCGTTCTGGGTAAAGGTGAGGATATGTACAACAAAGGATGCATCCGGGGCAAT
TAGCAATATGGGGAACCGTATAACTTTGGTGAGCCTGTCCCCTTCACTCATATCTATTCA
TGGTCAACTATAGACGACGTTCAACCCTTTCGTAAGTTTACTGGAAGCTGTGATGTTGGC
TTTAAAGACGCTGCCCAATACTTCCTGCCTTGTTTAAGGTTCTCTGCAGACTAGATTGAA
GGTCTCAAGCCTTGGAAAGATAATCTTGATGTTGTCAAAAGTGATCTACTCAATACGCTA
ACAATGACTGACGAGTATGAATCTCCCGATGATACCGATAAATTCTTAACCAAGTTAGAT
GGAGCCAAGATTACTAAAGAGCATGGCCGATGTGACTGAACAAAAATGCAACCAAATTCA
GTTAGAAAAGTGTGGTCCCTGGATCTTCAATGGTCAGGGTGCCCTGAGCTTGCAGAGAAA
GAAGTGCGTGAAAGTTGGTTTGGGTTTAGCCTAGGTAATGATCACTACTTCTGTAAAACA
GAGGTAGATGATGATCTATTCGACCGTTACCTAAATATTTACTTATGGTTGAAACACAAA
GGCGTAACGGAGGGTGAACAGTTTCTAGTTCATTGGTGAAGGTAATTTTACTGGATAAGT
AGACAGCAGTTTCTAGTGCTTTCAATCATGTTCATTGACTTATTAGGAGAAAATATCATG
AAACGTATTGCTATTGCTTACAGTGCACAAGAAGGCGGTAACACTAAAGTTAAAGTTTTC
TGGTCGAACGCAGAAGACAAAGAGATTGCTATGTTCAATTTCTAATAATACCGCGATGCG
CAAACCGACCAACAGACCACCAATTGGTACTAGATGCACCAATTCACTAACTAACAACGG
TACTATCATCATGAGCACACTTCATAGAAGGCTAACACTGGAAAACAAAACCGATGCTCC
CAACGGACAAAGAGTCAAAGTATTGACCCACAACAATAAGGATTATGATGTAGAGCTGTT
ACCTAACGAATCAGATAGTTTTGGCTCTTAAGCAATCAGTAGACAGATTGAAGACGAATC
AAACTTGTGTTTTGTAGTCACCCACTCTAGTTCGAGTACGTGTGCTTATGTGAGGTTCAG
TAAACGGGAGCTATTCAGTTTGCTTGAAAAATGTTTAACTAATACTGGTAAATAACATGC
ACCTTGAAATAGATGCAGGGCGATTACTTGCCCGTGTTATTGCTGGAGATTTTGGCATCA
AGCTAGTCATGCGAGAGGACTCTACGGTTCCAATGACTGACGGTAAGACAATCACAATGC
CAGTGCTAGGTCCCGATTGGGATGTTCACAGTGCCAAGGTGCAGCGTTGGTGTGCAGGAC
TTATTCACGAGTGTTATCACCATAACGGAACTATCGCTTTTGATTTTAAGTTACTGCGTG
AATTGATAATCGACTCCAACAAATTCTTTGGTATGGTACTCAACATGGTTGTTGACCATA
ACATTGAATACAAAGAGTATGGGAAACTCGCGGGTGCTGATGAATGGATGGATGCTTTCT
ATTCTAAAAGTTACATGAAAATCAGCGGTAAGTTTGGTAGTAATCCAGAAGAATTTAAGG
AAGCGTGTGCATTAAAAGCCCTTGTTGCCTTTGACCACACAATGCGCTCTCAATGGAGTG
GCATTGTTAATCTTCACTTAGCGGAGGGACTCACTACTGAACTTGCCCGCACTATGTTCC
AAACTATGATGGACGAAGCCCAAGACCTAGACTTAACCAAAAGTGTTGGTGGTTTGCCTA
GCCTACCAGTCTGCAACCTGTTGATGGACTTGTTAGACCTGAAAGAAGAAAAAGGTGGAG
CCGAGTCTAAACCAGAGGGTTCATCCGACTCTAAATCAGTACAAGGTGAATGTAAGCCCA
GCGTCGGCGAAGGCGAAGTTGACGAGGGAGAAGGCGCAGGAAGTGACTCAGGACCACCTA
GAGAGGGGGAAGGTGAAGCAGGTGAAGGGGAAGTACCTCAAGAACTAATTGAGATGGTCT
ACGGGGATTCTGGGCGTACTCCCGACGATTGTAAGCGTGCTCCTGGTACTAACGTTAAGC
TAACTTACGATTGGGACGAACGAGATACGTCTGGCTATTGTGTGGTAACTGCTGCAGAGT
TACGACCTAAACCCGTTGCAGGCCGCCGCGGTAATATTTGCGTAGATAATGTGCTGTCTC
ACTTCCATTTTACTCTGAGTGATAAGGTCAGTAACATCCTCAAAGTTATGTCCCGAGTCA
AAAGGCAGCGTGGACACAAGCGAGGCAAGTTACATAGCAAAGCTGTGGCTAAATTAACGA
CTGGCAGTGTTGTTATATGCCCTCAAAAGGAACCGAAGGTTGTATTAGATACGCCTGTAA
CTGCTCTGTTAGATAGCTCAGGCTCTATGTCTGGACGTAGCTAGTACTTCCACGGTCTGT
TGGCGTGTTGTGTGTTGAATGATTCCTTGAACAAAGTCGGCATGCGGATTGAAGTTCGAG
GCTTTACTCAAACTTACGAAGGCGGCAATTCGTACAACCAGCTTCTCATTCAGACACCGT
ACGGTCGTCGTGATACGGCTCGTGACTTGTGAGAGTCAATGGATGGAGTTGAATTAGCCA
ACAATGAGGATTGTGCTGCTATTATGTGGGTACACTCTCGTCTAATTCGCCACAAAGCTG
CACACAGAGTCTTCATTGTGTTAAGTGATGGTAGCCCTGCATGTTTGCAAGATAACTCGT
ACGCATTAAATAAGCAAGGGGTCGAGGAGATTGAAAAGGAATCTCCAGTAGAAGTTTACG

GCATCGGCATCATGGACGATACATTTAAACGCATCTACACACAAAGCGAAGTTATCAGAC
GACCAGAGCAACTAGAATAAGCTCTACTTAATGTCGTTAAATCTATAGTTCTCGGTTAAA
ACGGAAAACATATCATGGCAACAAGGACAACAGCAACATCAGAAGCTCTACGTTCTCGTG
TACCGGAGGCACTGGCAGCAGGTATGGGACGTGAGGCTCGTAAAGATGAGTACGTGACTA
CGGCTACACAACCAGAACCTCCAAAGAAACCTACTCTCTTTCAAGATGAGTTTCCACAAC
CATCAGGTGATGGTGTGAAAGAGTATGCTGAAATCATTTGGCATCCTCATCCAAACGGTG
AGTTTTATATCCACACCTGCAGGAAGGAAGATTGGACGGAAGAACTACAATCTCTGATTC
CAGAAATTGATACCAACATGGAGTTTGATGATGACCTCGCTCTTGCTATTCTTATGGGGA
TTGAATACGACAAACCTGTATTCGCTTATAGCGCACCCGGAGCTGGTAAGTCAGTGACTC
CAGAACAAATCTGTGCTCGTATCGGGTATCCTGTCATGTTCTGTGCTGGTATGGGTGGTA
CCGAGCCTAGCGACTACGTGGGTTCTCCTTGGGAAAGTGACGGCAGTATGGAAGGGAAAG
ACGGCCCGATGTCTTACCCTGTACGTAAATGTGTATTCCTTCTGTACGATGAGCCAGTCA
ACGCGTCTGCACAAACTAACATGTGTATCCAATCACTGCTAGGTACTCGTCGTACTCTGA
AATTGTACGGTCATCCTCACCCTGTTAATGGTAACTTACGTGCTAATGGTAGTTTTCGTA
TCATGCGTGCTGGTAACGTCCGTGGTTCTGGCGACCAGATGGATTAGATTGCGGCAGAGA
ATCAAGACCAATCTACTCTGAACCGTATGGAGTACAAAATTCATGTGCCATACCCAACTG
AGAAGGCTGATAAAGAAATTCTACGCCGTAAGATCCCACAACTAACTGAGCGTTTGGCTA
TTAAGATTGTTTCCTTAGCTAAAATGAGTCGTAAAGGATGGTAGCGAGGGACTATCAACA
TGCCTTACTCTGTACGTGATTCCCAAGATATGGCTAAACAATCACTGCGTTTACGTAACC
CTGAGTAGGCTTTCAAAATTACCTACTTCGATTCTGTCAATGCCGAAGTTGAGAAGAAAG
AGCTTGAGAAAATGTGGAACCTGGTTAAGTTCGTCGAGTACAAACTCGAAGGAGTTATCA
TGGCCTATTATGGTCGTCCGGTCTTTGTGTTTAGCACAGACCCCACTAAACTAGCCTATC
CTCGTGATTCGTTTGAGGAAGAGTATGACCTCAAACACGTCGAAAGAGCTAGTGATTCAA
CTAACTTCGTCATAGGCGGTAGAAAATGTTTGAGAGACACCCTATCCGACGGCCACGAGA
GGGAAGCAAGTGATGCTCTAGTGTGGATGTTTAAATCAGACTTGTTAAAGGGCTGTGGGT
AAGTTTATCCACTATTCATCAACTTAGATGAGGAGGTTAGCGATGGGTACTACTTTGTGA
AGTGGTCGTGGACTAGCACAATATTCCACTCTAATCGTTGATGTGTGGCACCTGTCTGAA
CAAGTTAAAGAAGAGGCTATTAAGGCTATTCTTGAAGAGTTTGGAATCAAGACACCTCGT
ACATACAGCTTCACAAAAGATAGCTGTGGATATCGGATCTTACGAGACATTAATCCATCT
CACCACCAGTTAACTCAAACTGCAATGACAGAGATGGCTGAGTGGAGGAATCATAGAATA
TGTTGATTCCAAACCACGAACCAGAGACTACATTCTCGTTAAGATAGAGGAGTTATTAGT
ATGATTGATTCTCCTTGGTTACGCTGTAAAGCCTGTGATGAAGAAATTCAACCACATAAT
TTTCGTGTTCTCTGTGAGCATGATGAAATTTTAGAGGTGTGGGAAAACCTGTGTCCTGAG
TGTCGTGCTAAAGTCTCACATTGGAACTCTGGCACTGAACAATGGAACAATGATTCAACA
GGGATTCGATTCACTAACCCCAGTATGGGAGATTCACTTGGTGAGTTGATGCACTCATTA
CATAACGGAGCTTCTTCTGGTGGCAGTTTTCATTCCGAATACCGAGATTAAAGCCGTCGC
AGACGACAACCCAGAGTGGGATATCGGTGAAACGAAACATTTCTTTAACTGTAACGAAGG
CCACAACAACAACCGATTCTATGTGACCCACGTAGAGAACGGTGTTTACCTTGGCTACTG
TCATGCTTGCGGTGGTTAGGGTATCTATCGTGAGAAGGATGCCCCTGTTTAAAATAAATT
AGCAGGTAGTAGTCCTAAGGAAATCCACCGTGATTATAATCTTCCAAGGACTATTACCAC
TAACATGTCAGAGTGGTCGTCAAAGGCTAAAGTGTGGATGTGAAAAGCCATGCCCACGGA
TGATGAAATTAAAGATAGAGGTTTCTGTTACGCTACCGAATCAGGTCGTGTGTTACTTCC
TACGTATTTTGATGGTGAGTATCAGGGATACCTCTCTCGAAGAGTGGAGGATCACGATGA
AGCGAAATACTTGGCAATGTCTAAATACCGAGAAAAGTTCGCGCTGTATTCGAACCAAGG
CACTAGCGACTGTATTGTGGTGGAAGATGCTCTATCTTTTATCCCCATCAGTAGACACAC
CAGTTGCGTCTGACTTTTTGGAACATCTATATCAGACAATGTTCTAAATCTTATACGCGG
TAATCACAATAAGTACAACATCTGGTTTGATAATGAAAACCGACAGGTCAAAATCAAGCT
GCTCGAGCTTAAAGCTAAGTTGGAGTGGTTTGGTTCCGTCCGAATGATTAACAGAGACAA
AGACCCTAAAGAACATTCTGACCCTGAACTCAAGGAGGTACAATGCCTTTAAAATTTACA
TTCGGGTATACGGTTAAAGGTGTAGATAACGTTCCGATCTACACATCACATTCTATGGAT
GGTGCAAGATACCCTGCGTGGGTGAATGGCTCTAGTCCGAGACGCCTCACTAAATACGAA
ATCATTAGAGTGCATCAAGATGCAACTACAAGAATTGGTGTTTATAATATCTACGCGGTT
CAAAATGCGAGTGGTCAAGTGTACCTATGCGGTGAGCGAGGTCTGGAACTAGATACACCT
ATTATCAGTGCTCCAGTATCTGCAATCGCACCAACACCGGTTTCGTCTGTTTCAGAGTCA
TGGATGCGTAATGATGGGTATGAACACGATACAATGCGTGGCCGTGTAGTCGATGCTCGG
AAGATAGGCAACAGCATTAAGGGAGCGTGATGGCATGTGGGTGATGACCACCGTGACGGT
TTGGGGGGGACCTGAAATGAAGCATTCCGCAAGTTAGACCCGTTAGATGCGGCAGCAATG

TCACTCAACTTATCAGACATTACATCTGGAGTGTAATCATGGGTGAAGAGATTAATGTTG
GTTCTTGGGTTCGCTATGTTACTTCTGGTGGGCCTGACTTAACTGACAACAAAGTGTACC
TAATTATTCAGGTCTACAGGTCTGATGCAACTTACGGAAGGATTCGGCTTTTGCATGATG
TGGGTGATGTTCACGACTTGAAGGAATATGAGTATGAGTGGTTAGATACTCGTAATCCTG
ACTGGATTCTGGTGTTCCAGAAAGGTGATAGTACTCCAAACTGAGGAGAGTGGTCAAGCC
TCACAGTCTTCGACGATGAAGGCCAATGGTACTATATCCGAACTATTTAAGGGGAGCGAA
GGGTTGTGAGTGACTCAACTGCTACAAAAGATCCGTTAGAAGCAGCTCGTGCCGCGGCAC
AGTTATTAGTCTTTGCTGATGAGGAGGATAAATTGTGAGTTTAGACTTACTGAAAACTAT
GGAGAGCCGTGAGAAGTACGAAAGTTACTCTCCATTAGTTCGTGAACATACGGTAGTCCC
TTCCGTCTGGAAGGGAATCCAAGCGATTAAAACTTGGTTTAAAGAGTCTGAAGAACGTGA
AGACATTGATGCGGGTTCTTTCTCGACGTGGTTCTGTGCTATTAATTATGGTCATTTATC
TAGTGACAAGCTTGATATATATCGTGCTATCTTTGATAAATTACCGGCTCATGTTGTTGA
TGCTACCTTGGAACGCAGTATCGGTGAGAACTTTATCACTCGCGGTTACTGTACTGCATT
GTTGGATAAAGCTCTCCGAGGTGCGGAAGGTGAGCCGGTCACGCTTTATGACCTTCAAAA
AATCGTAGATGATTTGACCAACGTGTTAGCTTGAGTTGATGTACTTGATTTGTTTGCTCT
CGAAGACTCTATCGAGGATATAGTTAAGCGTATCACCACCGGTGGCTACGATTGGCGTAT
ACAATTCATAAACAAGTCAGTTGGTCGTCTGGCAGAGAAAGGTAAGCTGGTTGTGATTGT
AGTTCGTCCTAACACTGGTAAAACAACGCGCATGGCTTATAACGCTTCTGGTTTTCCTTC
TAGAATGGCAGAAGATAAAGTTGTGTTGCGGTTCAAGAACGAGCAAGATGGCGGTCAGGT
CATTTTACGTACTCTCCAAGCTGCTATCAACTGGGCAAGTGCTGACATTGAGCTTGCTCC
GTCTCGTGCATTGTCCGTTTATGTGGATTATGTTGGTAACAAAGATAAGATTAAGATTAT
CGACAAGGCTGACACTAGTGTCGACGATGTAGAACAATACATTGAGAAGTACAATGTAGG
CTTAATTGTTATTGACCAGTTGTGGAAACACCACTGGTTTGAACGTGAGACAGCGATCGA
AGTAGCTCGTCAAACTAAAATCTTTAACTGGGCGCGTGAGAGTCCAAAGAAATATGCCAG
TGTTATGGGTACTCTACCAGCTGATGCGCAAGCGCAAGGTCAGAAATGGTTGGATACGTC
CATGATGTACCGCAGGAAAACTGCTTCACAACGTGTGGCAGACACAAGCATCATGATGGG
ACGTACTGATGAGGAGTGTGAGAAAGCCGTTCGATTCCTCACCATTGCTAAAAACATGAA
TGCGGTAAGACGTGAAGTAGACCCTGCACTACTTGAAGGCCGTGCCGAGTTACGTATCGT
TCGAGATACTGCTAAATCTAAGGAGATCTAAATGTGGTTAGACCATGGCCATGACCCTAC
TGAGATTTTTGCTACACCGGTGTCATTCGACTGTGAAACAACCATACGTAGTCAGGGTAT
TGGGAAGTCACAAGCTTCTCCTTACCATCCTGCAAAGAAGATGTTGTGCTTTGGTTGCAA
AACGCTAGACGCTGAACCTGTAGTACTTCGTCCATAACATGCTGACTTCCCAACAATCAC
TCGTGAGAATGATGTGTTTTTTTTGGTAGGCCACAACATTAAGTTCGACTTGCTGTGGAT
GATGCGAACGTATCCTGTTTTCTGTGATATGTTACCTAACCTATGTAATTCGGATACCCA
ACTTGCTGAGTACTTGTTAACTGGACAAGACCACAAGATGGCGTCTCTTAACGGGTTGGC
TATGGAGTATGGAGGTACATAAAAAGATGACCGTATCGCCGAATTTTGGAAAGCTGGAGT
TGATGCTTCTGACATTGCTTTCCCGATGCTCAAAGAGTATCGGACACAAGACGTAATCAA
CACTCAGATACTTTTCCTAGAATAAGTGGACCAAATCATGAAGGCAGGTCTTCTTAATCT
TACTATGCCTCAGATGTACGCTATAATGGCTACCACTGAGATGCTATGGCATGTTATGAA
GGTCGACAAAGAACTTGGCTTGGATTTAGATAAACCTCTCCAAGCTGAGACAGGAGATAT
TAAGGGTGCTGTATCTTCTGTGCTGGAAGCTGTAGGTTTTCCTAAAGATTCAATCAACGT
GAGCAGTAACTCACAGATTAGTGCTCTTATCTTTGGGGGTGTTGACAAGTACAAACAGAA
AAATTACTTCGACAACGACGATGGTGCGTTTGCTACATTCAAAGGGGGTGCTCGTGCTGG
CCAACCTCAGACCAAAATCTTTGAACGCTCCAAGCCTCTGGAAGGTATGTGTTAACCAAT
CAATCCGAAAGGTAAGAACGGTTCGTTTCCAGTAGGTGACGACTCTCTATCCAAAGTCAA
AGACAAATACAAAGGGGACGCTGTTGTTGAACAGTTATTCGGTGACATTCTTAAATTCCG
CCAATTGGAAAAGGATGTTAACACTTACTATACTGGCTACGCTTCTCAATTGTTCCCTGA
TGGTTGCCTACATGGTAACTATAACCAAACTAAAACGGCTAATGGTCGATTGTCTTCTAT
TTCGCCAAATATCCAAAAGATCAGCAACTAATGTTTTGTAAGTTATATGGTTCGTGGTAT
ATTCGGTGTAAAGCCTCTACGAGGTACGTTGTGGGTAATTGTGTTGACAATAGATTACAT
CGAATATACTGGGCGATGAAGGGCCGTTGTGAGTATCCTAAGAATGTCCGCTACTCTCGA
TATGGTGGGAGAGAGATTTCAGTAGAGTTCCAATGAAATCCAGAGTTCAGAGAGTGGGCA
TACCAAAATGGGGACTCGCCGACATTAACAAAAGATAGAATAGACAAGGACAAGAGCTCC
AGTCCCGAAAATTGTAGCTGGATAAGTCACTATGATAAGACGCAACTTGGTTCCTTGAAG
TATTGGAATCACATTGAAAGGGTAGATGGCTCTATAGGGTATGTAACTAACAATCCTACA
GAATTCTGTAGGGAGCATTCCTGAACTTAGAGTTCTTAAAGTAGAGTGGGAAAGACCTAC
AGAGGAAAAACAACAAAACAATACAAAGGTTTCAGATGTCAGATACCAAGCGATGTCTAA

TCTCCCGATTTCCCAATGGAAAATTCATGGATTGTGATTTCTCTCAACTTGAGGTAGTTG
GTGGTGCGGTGTTCACTGGCGACCCAATGATAATGAAAGATATTATTGAGGGTGTTGATA
CTCACTCTATGTCTGCTTCTTGGGCAAATCCAAAATACTCGTCTGAGGCAATCTTCGCAG
GGTACAAGGCAGAAGACCCTTTCTTCACCGAACGAAGTTAGAATGCCATGCCTGTTTACT
TTGCTTTGCAATACGGTGCTGGTGCTGAGGCTGCTGTGGAGAACGCAGGTATCGAAGGAG
AACAAGCTCAAGCAATCATTGATAACTACTACAATCGTTATTGCGTGTTGAAGCAGTTCC
ATGATGATATAATCGAGCAAGTTAACAAGAGTCGTCAACCCTCTAGTCGTCGTACTGAAA
TCTGTATGCCTGTTGGTCGTGGTGAGTACCGTAATCCATCAGGACGAATGTTTACTTTCT
ATGCTTACGATTCTCCTGAGTCGCTTCGTCGTCGTGTACAGATGAACTCATTCTCCCCCA
CACAATGTAAGAATGACCCGAGCCAATCTTTTGCACATGGTGATATTGTTCCTATGATGG
TGGGACAGATCTACAAACTACTGATGACAGACAAAACTGTACGGGATAAGTGCCTCCTAA
TTGGTACAGTACATGATTCTGTAGTGTTGGATGTTCATCCCGACATTCTGTATTATGCAG
CAAAGGCCGTTCGTGGGTTGTTGTTACGTACGCCTGCATATGTGGAACGTTACCTTAAAG
TTCCATTTGATGTCCCTTTGGCTTGTGACGTTGACATCGGCGAGGGCGCATGGTTCAATA
TGCATGGATACAAGCGTTAGGAGGTTCCATGCAAACAGTATCACGGAAAAGATGGTAAGG
CGAAAACGAAGAGCACCACCGTGATAAGGGAAGAAAGTGGCTATGGAAACTCCAGAGTAC
GCTTCTCTAATTAAGCTACGCCACAGATTAAGGAAGAACTCCGGCATGACACTAGAGGAA
TACAACCGTATGGTTGAATGCTGTAATAACCAATGCCAAATTTGTCTGCAGACTCCTGAT
GGTAGTAAGTAAGACACTCTTCTTTGTATAGACGACAACCATGAAACTGGAGAACTACGA
GTATTGATTTGTTGACGATGTAACCTAATGTTAGGCTATACTATAGATGATCCAGAGATA
CAAACCAATGCAAGTAAATACCTGCTAAAATAACTTAGCAAACTTCCGTAAATCATAATA
ACAATAGGAGATATTTCTATGACGACTATGACTGGTACAATCACTCCTAAAGCTCGCACG
GGTAAATCTATTAACCTAGGTGACGGTAACTGGTACTCAGCATTCGCTGCTTGGCAGTTA
CCGGCTAAACTTAAAGCAGGTGTCTCAGTAACTTTTGAATATAAAGAGAATGGCCGTTAC
CGTAACATTCAAGGTGACCTTAAGAAAGCAGGTGGTTCTGCTCTTGCTGCGAGAGGAGGT
AAAAGTGCTCCAGCTTCCGTTGGTCGCGGTTATCGTGCTAACGGTCAAGAAGGTGGTTTC
CCAATCCATCCACTAGCGTATGATCGTGCTCATGACCTCCGTAACGCAATCGCTGCGGCA
AGTACTATCATTGCTTGTCAGATTGCACATGGCACTGAGTTTGAAGATATTGCCGATGCT
ACACTCTAACTAGCAATAACCTTCGAGGCAAAATCAGCTGCTGATATGGGACGTGTGGCT
ATGGCTGATATCACTTCTGGCGACTCTGAAGAAGTTTGATTGTAAAATCCTTTGGCTCCT
TCGGGCGCCTTACAATTCTTTGGAGGCAAAATGTTAGAGAAACATGCTCGGTTACTTCGT
ATGCTTCTTGGTTGTGTTACTCTGGCTTATGTTGACCTCTTCTTTGACAAGACCAGACAA
CAATACGTTAAGGTTCCACGGAAAGTTTTGAAGGATTAGAACAACGAAGATGAGTTGAGT
CGCCAAGCTCGAGAACGTCAGAAACGTGGAGAAGAACGTCAACGTTTGGCAGAGAACCGC
ATCGCTAGAATCAACGACCTGTTAGAGGAATAATGATGAGTTTAGATGAACTATTATATA
AGTTTGTAGTCGAGAATGCTGAGAAAACAATCCAGTTGGCTGGACCAAAATTCACCGATG
TAGGGAAGTTAGAAGCCTATCGCAGTGTTTTAGGGTCCATCGAACAAGGATGCAGTCTAG
AACACCTAGCTCAGAATCTTCATAAGGGTATCGACAGGGTGGAAACTCGGTTAGGGCTAC
ACAATGAAGATTGAGACAACTCTCCCACCTCCTGAAGTGGCTCCTTGGTACAAATGCAGT
AAGGAACAATGCACCAACTTATGGCAACCCTATCAAGGTAACGTCGGCTGCCTATGTTCT
GAGTGCGACGATTAAACCCCTGACTATGTAAAACACACGTATTTGGAGACAAGAATCATG
GCTACAATCAAATTATCTAAAGATGTAACCGCTGAAATCGTTTTTAGTAACACTGTTAAA
GTTACAACCCACCAACGTGTTCGCGGTGGTGGTTCTTGTAACCTCATCTGTTGGGCATTA
ACTGCTTCTAAGTTGGATATCATAACTCTTCTGATTGAGATTAACCCACTCGCTTCTTGT
GATGTTGCAGAAGATATTCGTAAAGCAATACGTAATGACAATCCAATGATTCATCACCTA
GGTTTAGAGGTACATCAGGCTTAATATGACGACAGTTGCAGTAATTGGTGAAGACACTCC
AACGTTCGCTCCGGCAATTAGCTTTGAAGATTTTCACTATAAAACACCGGAGGGTGATTG
CTATCAACGAAAGAGTGATGCTCCTAAGTCGCTACAAGACTCTCTAATTAAAGTATGGGA
GGCCACAGCGCCTCGACGATTTGCTGTATCTCGTTTTGAGAAATTCATCCAAGATATGCT
AGATGATGGTCAGTGCGACGATTATGTGTTGTTTATTGGTGGCGGGAATAAATACCGATA
AGATTTTCCACATCCTAAAGGATACCCTTACAAGGGCAATCGCACCAACATGCACAAACC
TTTCTATCTTGATGAGTGCAAAAACTTTCTAATGGAGTGCCTTCCAACGATTGTTTTCGA
AGGCTACGAAGCTGACGATGGTTGCGGCATTTTGATGAGTGGAGGTATGGAAGGTTACGA
TGAACTTCCGTTATGTCATATTGATAAAGATATGGATATGATTGAAGGTAATCACATGCG
TTGGAGGCGTAACATCAATGGAAAAGACTTCCCAGCTAAGTTCTACACGGTGAACAAAGT
AGCAGCTCTACGACACCTCTATCTCCAACCGCTTTCTGGTGACCCGACTGATAATATTCT
GGTCTATTATTAATTCACTGGTAAGAAATTAGTTGCTGGGATCAAGTCTGGATTGCAAAC

GCTGTCTACTGAATTAGATATGTGGTCTCCCGTTCGCCAAGTCTATGCTAAAGCAGGTAT
GCGTGACGAGAACATCCTTATCGAAATTGGTCGCTTGCTTTGGATTCAACGTAAAGAAGG
TGAAATGTGGAACACTCCTACGAACGTTTTACGGTACTAGCACTACCTGCCGAATAGCAA
CAGTACACTATAACCTATAAATTTGTAGCTCATATTATTGTCACACTAACCCACAATGAT
AGGTATGCTGTAGCTCATGTACCTACAGCCCTATTTGAGTCTCTTGAATATGAAGACTTG
GCAGCATATATCGCTGATAATCCGTGTACAGACCTTAAAGCAGGTTACGGAGTATTTAAA
GATGCGTAACATTGAACACAATAATCGTCAAAAGCACAAAGCAACTGTGCTAGAAAAAGC
TCGTCGACGCCAACGTGCTAAGTACCATCGTCGCAAAGAGGAGGACAAACTGCGGGAAGA
TTACGACGCGGAAGTGTCGTGTGTCTAAACGTTACGAGACCCGCAAGTCGGGCTTCCGTA
GCGGATTCAAGCATAACTTTGCTAAAAACTGTCCAGTACCTTATGAGTATGAGACAGAGT
AATTACCATATCCAGTTACGTTAACTAAGCATTAAATGCCTGCTTTTGTCTTGATTAAGA
CAGACGGAAGTAAGCTGTATCTGGAATTGAAGGGTCGGTGTACTGCTTCAGGCCGTGTTA
AAATGAAGCGAATCAAGAATTGTTGCCCGACTTTAGAAATTAATATGGCGTTCCAGATGG
GCACTAACCGGATTACTAAAGTTGCCAAAACAACTTGTATCCAATGGTGTGCTAAAGAAG
GTATCCTTGCATAGGATTGAAAGGGTAAACCAGCACACATTCCAACTGAGTGGTTAGAGG
AGTTGAGTTGAGCTAGTATACTGAGCCACATCCAGTACCTTGGACTGGTAAAGACCTTAA
AGCTGCTTGGCAATTCACAATCAGAGTTAATTGTGTCCGTATGCTCCGCGATGAAGCAGG
AAACCTAATGTGACGTAAAGGTAAGCCGTTAGATAACTTAAAAAATGTAGGGGAGGAAAT
CTCCGATGCATAAATTTAATGTGGGAGTTGGGAAGCGTCTATATCTCACTGTTGTTCCTG
TGGCACTTCAGTTGGCTCCGTTGATGCTGCGAATGTTTATTCTCTTTTTCCCGTTGTTGT
TAAACGTATAGATTTAGGTGTCGATATCAATCCAACTTCAGAGGATATTAATACTTCATT
GAGTCTTGTTTTAGCTGAGGTACACGAGGGTTGAGTTCCCCGTGAAATTAATGGCAAAGG
TTAACTCTACAAAGTGAAACCTAACGAGACTTACGATGTTCAAGTCATTGGTAGGGTAGA
GGGTACTGGTAAACATAAAGGGCGGATTGGAGCACTACTTACTCCAATGGGTAATATTGG
TACTGGATTCACTGATAAAGAACGTGCGTCGTGGTGGTCACAATACCCCAGGTTTGAAAG
TACACCACGTAAAATTATTGAAGTTGAATGTTGGGAATTGACTAAATCTGGTCAGTTCCG
TCACCCTCGCAATATTCGTTTACGGGAGGATAAATAATTGCCTTTGGACCTAAAGGAGTG
GCTCCGGCGTACATAGCAATGAATGAGATAAAATAGTACACACTTGGTGATGCTAGTGCT
GCATTTGAACAGAATAAGTCCACCATGTACAGTGTTCCTCTTGATGATATATTAGTATTA
CATAGCCTATCACACGCGACTTGTATGAAACCAAGAGATTTTATCGGAGTACTAGAAGAA
GCAATTCTTCCAATGTTGGAGTCGAGCACACAATTATGATTAATACAACAAGCAAAAATG
ATTACTTACCTTCAACTGTTCAAAACATTACGTTATGTCATTATGAAAAAGATTTTGTTC
ACAACGGTGATGAGAACGTCCAGCTTACGAAGCTGGAAGAGGAGTTGTGAGAGGTTCTCG
AAGTGTTCTGTGAGGGTCACAGTCACGAACACTGTAAGGAAGAAATCGGTGATAGGATTG
TGGTCTTAATCAACCTGTGTGAACGCGGTGGTTACAACCTTGCTGAATGTGCTAACACTG
CATTCCAGAAGATTAACAAGCGCAAGGGTAAGAGCATTAATGTTGGTTTTTTTAAGTATG
AGGATTTGAAGTAATGGGTAGTTCGTACTTACTAGAACCGGAAATTAACTTGGATGGTAC
AAACATCATCCTAACTACTGGAGTTCTGGGAGCCGGTAAAACGGCTGCCCTAATTAACGC
AGTAGAACAATCGGAAAAGAAAGGTCCGGCTAACTGTTTGATTATTAAACCAGAGGTGTA
TGCTCGTGATGAAGGTGTAGGTGCACGGGATCGACGCACTCGACCGTATACCCCAATTGG
AACCGGAGAATAAGTTGTTCGGGTAAGTTCTTTGTCTGGCAGAATAGTTTTTTTGCTCGT
TGATGAGGCACAATTCTTTAGTCCCGAGTTCATTAAGCCAATCTGCCTTGCAGTAAATCT
CGACAACATCCCCAACGTAGCATTCTACAGTTTAAAGACTGATTATCTCGATAATACGTT
TGAGTAATGTAAAGAGATTGCCAAGTACGTAAACCGGATGTGTGAGTTACAACACACTTG
TGAAATGAGTGGTAATCAAAATCCGGTACACAAAATGCGTATTTCTGGTGGTGATTCTTT
GATTGAAGTTGGTACTGACAATTATCGAGCTGTTTGTTCTGTATGTTGGAGTAAGAAGAA
ACGAGTAAGATGTTCAAATGTGAACACTGTGCTGCGGAGCCTGAAGGTATTAAAATCGTA
GACAGCCGAACTAAGTTTGAGTTAGGTGATGACTATCTCATGCAAATCAAACGTTGTTCT
GAATGCGGTGGTTGCTCCAAGTCTATCAGAGTAGATGAGGGTAGTTGCGATTATTTTCGA
GAAGATCCTGACTGTGCACGCGGCGCTTGCAAAATCTGAGATTAGTCCCTGCTGTCCAAC
CCTGTGCGATAAGCGCGGGGTAAATGATTACATCACCCACCTTGAGAATGTCTTTAAAAA
TCAACGAGAAGAAATTGAGATGCTTAGGGCGAGGATTAATGAGTACAGTAGTGAGTGACT
GTTCAGCAGTTATTGGTAGTAAAGCTGTCTCTAACCTACTACGTAACAACGGTAATAATC
ATGCCAACTGTATACACGTTATGCAGAAGTTACACACATTTCGGGTAAGCAAGAGGAATT
ACTTCACCCTCACATTGATGTATTGTGCACTCTTTCCCAAACGATCTGTCTTGCCTGTTG
TGGTACTCACTGTGTAATCGATTATTCTCATGCCTGTAACTCTCATATCGATGCAGTAGC
AACTGACCAAGGCTTTACTTGTCCTAACTGTGGCTGTCTCGATAGCCGGAGGATGTTGAC

TAAGCGAATCTGTTGTTATCGAGGTAATCCAACAGGACGCGGGTTCGATAATGGGAGGCA
GGATGAGTTGATTCGGGGAGTTAAACCTTGTTGATTGGATTTACGGAGGAGCTCGTCTTA
TGGCCTCTGTGCTTGCTTTTTGCGCTGATGACATGGAAGAAGTAGACCATGATGCTTTCG
TAGAAACTATGAAGGAGTTAGATAATGAGTGATGTAATTGGTATCGGAAACGCTGCGGTC
AATCCGGATAACGAGTTGGGGATTGACAATGGTAATTATGAATCGGTAATTGTTCTTGGT
TCCTAGAAAGATGGCTACTTAGGTGGTCGGCCCAGTACTAACCTAACACGCGAGAAAATC
TTATGGTTGCGTGAGCAATTCAATCACAAATACGTACTTAGTAGGTCGAACGAGGTGGTG
TGATGGGAGAGAAATTAAGGGGAGTAGCTTTCCGAGTTAGGCCAGACTTCTCAGTAGCTC
TTGCTCTAGATATTCTGGCTTATGCCGCAGGTGTAGCTATGGCCAGTCCTACAGTCACCT
CTGTCGGATTTACCTGACAGACTTTTATCAAGGGGTGGGGTTACAGTGAGGATTACTATC
TCATATACAACGCTAAAGAAGATGGTTGGTATATGAAGCACAGAGTAGCAGTTGACATAA
TGGGTTTAGATGTGATTGATGTGGAAGCTATGGAAAACACCACAAAAGAAGAACCAGTTT
GGAGTTATCTATGATTGTTTTTCTTGGAAATGAAGTACAGGCTGGAGATGAGGTAGTTGC
TATGGAGCGTCCTGGCGAACGTTCCTAGGAACTAATCAGAGGATTCGTTATTAAGATTAC
TCCTAAGAGAGTTGAGTTATATCAACTGGGTATCGGAGAAAGCGTCATTGATATTAGTAC
AATCAATAGTGTGAAAGTAAAGCAAGTAGAACATGAAAAAGTTGTTGAAGTGACTACAGA
TAAAATTGAATTTGTGTGATTAACTCTCTCTATCTACTTAATATTCTATTACAAT
>NewGenomeName_26
ATGAATCTATCACTCAAACAACACTTCACTAAACCAGATTACGATTACTACAAATATAAC
GGAAAGGTACCTGCGAACCAAAAGTCATTTGAGCAATGGAAAGACCGTTATTTTTTTAAA
AACGTAGCGACAAAACATACAGGAGATAAACTCTTAAATTATTTTGTCGCTAATTTTGTA
GACAATCCTAAAGGATATCTCAGATCATTTAGTGAGGATACCTATAATAAATGGTGAATA
CATCAAGAGTCTTTCACTTATAAATTTAAACAAGATGTAAATCTTTTACTCGATGATTAT
GAGTATCCATATCAGGAAGCATTTGATAGAATGTTCATAGTCACTTCTGGTCAGCATCCA
AAAATCATGAGACACTATCTATCTGGAGAGATCTCATTAGAAACATTGGTTGTATTAGAA
ACATGTTTAGGGTGCGTTGAAAATTTTGATAAAACTTTGTCAGATCCTATCTGGAAAGAT
ACCCGAATGAAGGTTATGGAGTATAAACCATTTCTAAAATTAAATTGTCAAGATTATAAG
TCGGTAATCTTAGATACTATTAAAACAAAGTTATGAGTAATTTTTTCGAGTCCCAACAAG
TCCAAGAAGATCTACAATCAATCTTTAATTTTTATAAAAAAATTTCAGCAGAGACAGTAC
CTTTAGGAATCATGGATAAAGAAGAGAAACTAGAACACATTGAAGATTGTAAAGCACATT
TTGCTAAACAAAAGACTTGCTATACAAGGTTGTGTCTAGCATCGAAAGAAGATAAAGAAG
CAGCAGATATGAAGAACAGAGTCAATGATTTAGCTTGTGCGTTTGGATATTAGGACCTAA
TCGAGTGTATGAATGCAATGGTTGTTACACTAGAGGAAGCAGCAAAGAGGGATGATCTAT
GAAAATTAATATGATATCTGATGCACTCAAGATAGAATTAAAAGATCGTATCAATGAGGT
TTTAGATAAGAGAGAACTTCAGAAAAAACTAGACGGTCCTTGTGACTTTCCAGAAGATAC
TGATTGGCAACCAGATGATGATGGTCTTGATTATGAAGTAAATTACTATGGATAAGAAGT
TTAAAGATTTTATAGAGAAATGGAAGAAGCGTTTACGTTTCCCCAAAATGCCACCACCTC
GAAGTTGCCCCGCTTGACAACACCTATATACTATGCTACGATTACACACTAGTATACAAA
CAACTTACACACTAAAACGGAGAATACGATTATGTCTTTCGCAGCACCTAAGAAAGCATC
TAGCACGGGAAATACTTTTGCTAGATTGACTAAGGAGATCGAGAAACTAAACCAACCTGC
TCAAGGTAACAGTGGCGCAGACGACCGTCTTTGGAAACCAGAATTGGACAAGAACGGTAA
CGGTTATCCTGTTATGAGATTCTTGCCCGCACCTGATGGAGAGGAACTTCCTTTCGCTAA
GATCTGGTCGCATGCCTTCAAAGGTCCTGGTGGTCAATGGTATATTGAAAACTCATTGTC
AACTCTTGGTAAACAAGACCCTGTTTCAGCATACAATACTGAACTCTGGAACGCTGCAGG
TGAGGGTTCTTCTGAACGTGCACAAGCACGAGCACAGAAGAGAAAACTTTCTTACTACTC
TAACATTTACGTTGTGAGTGATCCTGCACACCCTGAGAATGAGGGTAAGGTCTTCCTATA
CAAATACGCTAAAAAGATTTTTGATACACTCGTTGAAGGAATGCAACCTGCATTTGCAGA
CGAGACTCCAATCGATCCTTTCAATTTCTGGAAGGGTGCTAACTTCCAATTGAAGATCCG
CAAGGTCGATGGTTACTGGAACTATGATAAGCCTGAATTTGCCCCACCTAATACACTAGG
TGATTATGATGACAAACGTCTAGAACAAATTTGGAAAGAGGCATACTCTCTTGCTGAGTT
TGAAGACCCTAAGAACTTTAAGTCTTATGACCAACTTAAGGGACGTCTTAGTTTAATCTT
GGGTAACACTACTCCAGTAGCACGTCTTGATGAATCTTGTGAAGATGTTAAACCACAGAC
TCCAGATTGGGGATCAGAAGTTACAGATTTTCGAGAGAAAGAAGTTGCCTCTGTTCCTGC
CGAGGATGACGTAGAACTTTCCTACTTTGCAAAACTTGCTGAAGAGGACTGATTATAAAC
TGGCACAAGGAGGGTTGTAATGACCCTCCTTTTCTAGTATAATTAAAACATACACAAGCA
ATACAATGAAAACTGGCCTCGCTGCAATCATGTTACTTGCATCTGTACCTGTAAATGCTG
AAAGCATTGGAGACAGGAGTAATCGTCAAGCGTATGACTCCTCTGGAGGATATGCATACG

AACAAAATTGCTATCGTTATGAATATCGGGAAGAGTACGTTCCTGGCACTTCTAGATCGC
CTGGTTACGTCAGATCTAACCGAGAAAACATTGCAACACCATGTTTCTATGGTGATCATA
GCAGATCTTCAACTGCCCGTTCAAGAGTTTACAGAACTCCATCACCTGATGGTAATGAGT
GTAGCGAAGGAGCAATTCTTGGAGGAATCTTAGGAGGAGGTGCAGGTGCAGCATTATCAC
ACGGAGATGGTCGTTGGTGGGCAATACCTTTGGGAATTGTCGGTGGATCTATGATCGGTT
GTGATATTGATGGAGGTTAGTGATGTCAAAAACTAAAACTCCTATAAGCAAAGAAGAAGT
CGAAGATATGATTGAGTTTGCTATCAACAGAAACAATCGTAATGCAGGACTCATCAGTAT
GGTTTTAGGATTTCTATTCATCGCACTCTTTGCAGACGGATTATCTAGAGTTATCGGAGA
GATACCTCCATTTATGGGTATCGATGTTGATATACTCAAAGAGATAGTGGAAAGAACAAA
AGATGAAATATTAAAAGCACTTTCAAGATCATGAACAACATAGGATTAGAAGTTGTCTTC
TGGACAATATTGTCAGTTTATTTACTAGCAAAAAATCGGAGTCTTTCAAAAGATATGAATG
ATGTCGCACTAGTAATATACTTGATTCTATTTGTTATGTTATTCGCAGCAACCGTTGCAT
ACATGTTTAGGTTGATGGGCTCTACGTTGGATTCTTTTAACAAGGCATCAATTGGACAGC
CTGAGTTGCGAGGGGTTAGAGAAGGCGAACAGTTGTTAGGAGTTACTTTCAAGGGATATG
AAGAACTATCACTAGATGAAGTAGAAAAATATCCAGTAAGTGCACAAAGAGACCATGAGC
CCATTGAACTCTTTCGCGATTTACAAGATCGTATTGATGAACTTGATGATGAAGACGATG
ATGATGATGACGATGGTGATATCCCTGCTAAACCGTACGTCGGTTCTGGAATCTGAAATT
GACTTTTAGTTCCCAAGAAACCGCAAGAAAATTTCCACCAAAAATTTGACCCCTAGAGAT
TTTGATGAGTGACATACATTTTAAAAAACACCGAGTGTTCCGAGATACAGACGGTGTTAT
TTTTTATGACATACCAGTAGAGGAATCTAACGCTTCTGACTTAGTTGTTCATGAAGGTCC
TGCTGAATCTCCTCCACCTGATTGTGTAGGAGGGAAACAATTCTATATACATTGCTTTCT
AGATGATTACAACAGAGTTGTATCAGGCACAAGAATGTTTGAATTAGTTAATTATGATTG
GAGATTCCCATGTCATATAGTGCACCTTGATGTGCATAGTGGTGCATTAATTATTCCTCG
TGGAACATTTCATAGATCTCAATCAGGAGAACATGGATCTATTGTAATCAATCAAGCAAA
GAGATACGATTGATTTGATGCTAGTCCAGAGTTTTACCCTGTATCTGGTGCAGAGAACGT
TCAACTATATAATGTATTAAAGAATGAGAAACCAGTAATTCATCGCTTAGGAGAATGACT
CAAAGTTATCACATTTATTTTCAAGAAAAAGTCCTCTTTAAAAATTTGACAATAGAGGCA
TTTACTTTAATATGGGACAAACGCTATACTTCCTATTGGAAAGATGACATAACGTATTCT
ATATGTTATGATGAAGCATGTGAACTAGAACCCTCTTTTTAATTATGATTCCATTTTTAC
TTACTAGTGCAGCATTTCTTAACTTTGCATTTTATATCTATGCAATTGGATTTGTAATTG
CACTCGCCTTAGAGCAGTATCTCAAGGCCAGACCCCTGTCTGCTGACTCATCGATGAATG
AGAGAAATAATTATATTGTGCAGACTAATAGAAAATATTGTTGGCAACAGGAATGGGTAG
TAAATCTTTTTTGGTTCCTATGTAATATAGGATTGTTTGTTTTATCAAGGAACATGCAAA
CACCTCAAGATACTTTCTGGAACGGAATATGAAGAAGGAATCAAAAGAAAGAGAGTATGC
TTAGGATAGAATGGAATACTTCAGAGAGTTCCATCGAGTCATTGCACCAGTAGTTGTAAT
GAAAGTGGAGGGTAAAGATGAATAAAATTTATGCAACTTGTCTTATTGGTGCAGTAGCAT
GGTGCACTGCAGCACAAGCTTGTAGTCCTCGTTTGGATGGGGGAGAAACTTATTGGCCTC
CAATTGATGATGTATTCTTACCAAAAGAAGAGATGAAGGGTAAAATTGATGTTTCCGATC
TTAATCACTGGGCAGCTATACAAGGTATGTTTATCAGAAACCAAAGACGAGAACAGATAG
AAGAAAATGCGACTCATCCTACTGACGCAATAGATAAGGCATTACTTGATTTTTGGAATG
ATGACAGAGTATGAAAGACGAGCAGAAGATCCCTGTTGGCAGCATAAACAAAAGTGTATC
GCAATGTTCACCCTCGATTCACACAACACTAGCTACATATATCGTAGAGAAGATGGAACA
TATTACTGGCAGCATCGTAGAAAGGAAGCGGAAGACGACCTCTTCGTCGATGCTGATGGG
TTGCAACTAGAACTTCTAGGTGACCCTGTGCTATCTAAAGAATTTATTTTTAAAGCAATA
TTTTACTAATATCCGCCACCATATCCAGATCCAGAAGAGCCGCTAGAACTTGGACTAGAA
CTGCTAGAACTAGAGGAGGAACTAGAAGAAGAACTACTACTGGAACTCGAACTGGTTGAA
CCGGTCGAACTGGTTGATGAGTATGCTTGTTGGTTACTGACATCGCCTACGTTGCTAGTG
GTGGTTGTGCTAGTTATTGCTGCTGTTGTCGAATTTATTACACCTGTGGTTGCACTGGTT
GCAGAAGGTCCGTTATCAAATGATCTCACAGCAGTATTAACTGAAGATTAAGAAGCACTA
CCAATAACATAACCAACAGCATCAAAGAATGTAGAAGCAATACTGAGAGGTGACTTCTTA
TTATTTACATCATCTAATTCTGAATGAGGAGCGTACGCAACGAGAGTTTCAAACTCTTGA
ATCATTCTATCTACAATTCCTCTATTGGGAACTTTAATTATTCTTTTTAATTTATTTAAG
TAATATTCATGCTCATAATTAGATACTGGATATATTGAATCACTCTTACCTTTTGTAGTT
CCGTCAGGTAAAACTGTTCTGTAAGTTGAATTTACTTGAATTCCTTCTTTGACTAAAACT
ATTCCATTCTATAAAACCTCGTTTGTTTCATAGTGATGCGCAACTTCTGGATCGCGATAC
TTTTTAACAACCATATCTTGTAGATCTGCTTCACTTCTTGGCCACTGCTCATAAAAATCA
GTAATATTGTTTAACATTAATACAACCCATTCTAAAAATGGATCTCCTGATGCTACTTCG

GCAATCATTGAGGGAGTTTCGCCTTCTGCTAACGAATATGATGCAAAGAAGATTGTATAT
TTTTCTAGGTCTTCTCTTACTATAATTCTACGAGATATATTTATTGCTAAACGATATTTG
AAGGGTTCGTCATTCTTGACACCCTCGCCAACATATACGTTTGGTAGAAAATTAAAATAT
GCCATTAGTATCCGTTTATGATGTCTGATTGACTGAGGAGACGAACCTCAGTAAATTGTA
CTTGTAGTTCAACAGCAGGAACTTGAATCATGCTCCCGTCAATTCTTTTTATTGAAGTAT
ACTGATTGTCTAGTGTGTAATTAACAGATAACCCAGAACTTACACATGTATCCATTTTAA
AATGTAAACTGATGTTAGTTTCATGTTCAGCAGTGCTTGAAGTGCCTAATGGATCACTAA
ATTCACCTGACTTTGGAGTCATTCTCATAAATGCCAGTTCATATTAATCAGGAATACCAA
AAAATCTATACCCTGACATCAGACCACCATTATCTCTGACAAATTTTTTAACATTATATG
CCGTTTGATCCTTGTCACCTGTAGTATTTTCAAGTTTTTTAGAATCTTTTATATCTGTTA
AATTTAATAAATCACTTTCTTGACTTACATTGGCACCTTCATTAGGGTGAGCACCAACTT
TTACATATTGAATTATTTTTCTGATTTCTTGTGCTTCTTTATCGTTTCTTGCAAACATTT
TAAAAGCGAATGAAAGATTTCCAAGATTCATTTGACTAAATATCTGCTCAACATAAGGAT
TGAATACATTTCCTTCTGTTAAACCTGCTAGAGAGTTCATCTTTAAATTCCCTCGTACGC
CTAGGAAACCAGATAAAGCATTAGCACCTGATGCAATAGTAGAAGCAAAAAACTCTGGAA
GCACAGCTCTTGCAGGGTCTTGAAGTGTACCTGCTACGTTTTCAAAAGATGTTCCTCTTT
GAATTGCATTGATCGCAGCCGCACCTCCAATACCAAAATTTGTTTGATTATATACTGGTG
AAGACTGAGCATTAATACCACCAGGAATCGCAAGATATGCTATTGAAGAATGAAAATTTG
TTCTACCCAAGTTTCTAGGATAATTATTCTGCCCGTAATAATCTTTTCCTCCATCATGAT
ATTCAGTTTTCTTTCTACGTATTCTTAGGTAATCTGTCTTTCCAGTAAGACCATCAACTG
AATCTCCATTAAAGTCTGAGTCCACTACAGGGGGTTGCAACGGATAACGTAGGGTTGATT
TACCGTAATCTCCGTCGTCTAAAAATTCAGTTGCCAAGATTTCTTCCTAAATACTAACAT
GGTCTTTATGTATTTATGAGGTATCAAGGAATATATCGACCAAACGTTCCAAGAAAGTAT
AAAGGTGACCCCAATAACGTTATTTATAGATCGTCTTGGGAGTATAAGTTCATGAAATGG
CCTGATATAACCTCCACGATTCAAGAATGGGGTAGTGAAGAGATTATTATTCCCTATATT
TCGCCTGTTGATGGTAAGAGGCACAGATACTTCCCAGATTTCTTTGTAAAGATAGAAGGT
AGAAGGTATTTGGTTGAAGTTAAACGATTGAAACAAACAAAAGAACCTAAAACTCAGAAA
CGAATGACAAAACGTTATATTAATGAAGTCGTGACTTGGAGTGTCAAGCAAGCAAAATGG
AAAGCAGCACCTGAGTTTTGTAAAGATCACCACTGGGAATTCAAGTTGATCACCGAAAAG
GAACTTAAAGTATAATGGCAATCATTAACACAGAACAAGCACAGTATCCTTCATATCAAG
AGTTCCTAGCGTTCTCGAGGAAAGAAGATCATCATCCTAGTTTTACTAACCTGTTCACGG
TGCAGTTTGCTGCGCCTAGATTATTACAAAGTAATTTGGGACCTTCAGTAGGAGGAAGTA
GAACAAAAAGATTACATCCAGAGACTCCTCAACTCCGCACTCTCTTAAATTACTATGCTA
ATTCTATAAACCTTCCCAGTAAACAGATGACTACTGGTTCTTTCCTAAGTGTAGGAACAG
CAATTAAGTATGCGACTAACCAAGCGTACAGTCAGATGAATATATCTTTCATTATGCCAC
GTTCACAGTATACTAGAGTATTTTTTGAAGAGTGGGCTACAACCATTTCCGCTGATTCAA
ATCAATATGTAGAATTTTATGATGATTATGTTTGTTCATCACTTAGAGTATATAAATGGG
AGAGAGGTGGAGGAAAAGATGTAGTGCAAGATGAGAAAATGATCAAAGCATTGAAGGATG
CAGGTGAACCTTATCTAATTGCGAAACAATATAGAGTAACTGCGGTATGGGGGATCCGAA
ATGTATTTCCCTTTAATATTGGATCAATTCAATTAAACAATGACACCTCTAGAGCAATGA
CTCTGACAGTAGGACTCTTATTTGAACGCTATCGTCTTACAGTTGCGTATGACTTCCGTG
ATCCTGGCACATATCAGTTTCCTGGTGATAGTAATCCATTTACATCAAACATGCCTCAAC
TTGATAGATATAATAGTGGAGTCTTCTAAAACCAAAATCGATTTTCAGCTCCCAGATAAC
CGCAAAAAAAATCCCGCCAAAAATTCGACCCCTAGCGTTTTTGACTAAATAACTATACTG
AATTGAACTTCTACGGCATTACCTAAGTTAAATGTACCAAAGTACAAAATGAAACTACCG
TCAGATGGTAGAACGGTGAATTATAGACCATTCCGCGTTAAAGAAGAGAAACTTTTACTT
CTCGCAACTCAATCTCAAGACCAAGAACAGTTAATCGTTGCTATTAAAGATATCATGGCT
GCTTGCACAGATATCAAGGATATTGATAAATTGGCAACTTTTGACATTGAATACCTTTTC
TTACATATTAGTACTAAATCTGTTGGTGAAACTGTAAAGGTCTCTTTGACCTGCCCTGAT
GATGGTGAAACTACTGTTGGAGTAGATATTCCTCTTGATGAAATCAAAGTCAAAAAGACT
AGAGGTCACAAAACTGATCTTAAAATTACTGATGAATTCACGGTTACTATGGGGTATCCA
AGTTTAGATACTTTTGTTAGTATGAACTTTGTTGGTGATGGTTCTGAAGTCGGTGTTGAT
CAAGTCTTTGAAATGGCAGGAAGTTGTATGAAGACAATTTCTGATCGAGAACAAGTTTAT
GACTGTCAGGACGTACCTAAAAAAGAAATTCAAGACTTCTTTGAAGGTATGAACACTAAA
CAGTTTCAAATGGTTCAAGCCTTTTTTGAAACTATGCCGAAACTAACTCACACAGTTAAA
GTTACGAATCCAAACACAGGTGTTGAGAGTGATGTAATTCTGGAGGGATTTGCAAGTTTT
TTCGGCTAGCCCTAATGCACATGGACTTGAAGACATATTATAAGAGCAATTTTGCTTTAA

TTCACCATCACAAGTGGAAGATTGAATATATTGACAATCTCATGCCATGGGAAAAGGAAA
TCTATATTAATCTTCAAATTAACTTCTTAAAAGAAGAAGAAAAACTTATGAAGGAGCAAA
AAGCACAAGGTGGCTAAGATACAAGCCTACAAGCTTATAAATCCTGGAGTGGCGACTATC
AAAACTCCATCGTTTGTTCCTGCGAGACAGACAATACCTGCTCAAAATAGACTTGGTAAG
ACATTCGAGGGCGTTGGACAAACAGTCCTTGACTTAGAAAAAATCACCAACTTACGTCTA
GGAATACAAAAGAAAGCGGATGAAGCGGAAAGAAGCCAGGACCAGAGAGAAAGAGATGCT
GAAGCTGAAGAGTTAGCAGAGAAAGGTTTATCAAAATATTTCAATAAAGAGGGAAAAGAG
GCAAAAAAATTTAAACCAGATAATAAACTAAAGAGTGCATTTAGTAAAATGTTTGGATGG
GTAGGTCCTCTCCTATCTCCATTTGTAGACCTAGCAAGAAAACGATTTGGTTTATTTCTA
ATTAAAGAATTATTAGAGTATACTGCAGATGAAGAAAACTTAAAAAAAATAGAGACCTTC
CTAATGAAGACAGATTTTGTCTTTAGGAAGATATATGGTTTTGGTAAATCGTTAATTAAG
GATAATATGGTAGATCGTATTGAGCAATTATTTGGTGATGATACAACATTATTAGGTAGA
CTTGGTGGTCTCGGTAAGTTGATGACAGGTATCATTGGGTTGAAATACCTCATGAACGCA
TTCAGTATTATTACTGATATTATCTTTCTAGCAGATCTTATTAGTGCAACTAGTTTCATT
CCAGGAAATGGTAGATGTTTACCGAATATTAAAAATTCCAAATCTAATTTAAAAAATAAT
AAGGTAAACTTGAGAAGGACACTAAGCGGTGGGAAACAGATGAATCCTGGTCCTCTCAGT
GGAGTAAGAGAGTGGCTTAGAAAATTTAGAACGACTGCAGGTAATAAAATTACTCAAAGC
ACTACGACACGTGAAAACCTTCTTCAAAGACTTACCGATAGTGTAAAGAAAGCTGTTACA
CCAAGCAAAGTTACTCAATCAGGCACTGGTAATATTTTTACTAGAGCACTTGATGGCATA
AAGAGTAAATTTACAAAACCTAAGGTTTCAGGTAATGTAAAACCCAATCTCAATTTAACG
GTATAACCAAACAATATTATATAACCCAAAAATATCAAGGTAAAACCTAAAGCGGGTGGA
GGACCACTGGGCACCCTGTCAGCGATTGCTCTTGATATAGGAATTCAAGCGGGATTTGGT
TCTATAGAACAGAAAAAATTTGAAAATTACCTCATAAAGTTTGAGGCAATGAGGAAGGTG
GAACAATTAAAAGAAATTCAAAGGAGAATAAAATTAAGAGATTATGCTAAATCAAAAACT
TCTGGTGCTTACGGTGTTTTTCAAGAAATTATTACTGGAGGTGGATCGTTAGGAACGAAT
TATGATAAACAAACGTTGGAGAGGGAGACTAATTATCTGGAGGCAATGCATCAGATAATG
GGAACTGTTGACAGTTCTGCTATGAATGATAATGAGTATAAGCAGTTCTATGAGAACATA
AAACCTGAGAACAAGGATACGAGTAAATTTGATGATCTGAATCCTAAAAATGATAAAAAA
TGGTGGAACCCAACAACATGGTTTAAAGCAGATGGTGGTAAGTTACCAGAGTTCCTTATC
GGTGGTTTATTTAAATCAGGTAAAAACGTAGTTAGTGGTGTAGTTAACACTGTTACTAAC
GTTGCAAAGACTGTATGGAATGGAGTGGGTAAGGTTGCTTCAAACCCACTCGGATTTACT
GTAGCGTCATTTATTCCTGGTGCTAATATTAACGTACCTGCTATCAAGGCAGTAAATGCA
CTTAGTTCTGGTGATATCATGGGTGCTGCAATGAATGCACTTGATGGTATCAGTAATTTA
GCTAATTTCAATACAGTTAATGCTATTAATCAACCTACAGGGATGCAAAACTTGAGATTT
AGTAAGTTTGGACAAGGTGTCTCAAACATGTATCATGGTGCACTCAAGGCAGTATCTAAT
ATTAGTAGTAGAGTTAATGGTTTCTTTGATATGGCAAGAAATAGGACTATTGGTAAGATT
GGGATGAAAGTTTTTAATGGTAATATTGGTGGTGCTATTGGAACTCTAGTAGGCCAAATG
CCTGGTTTCTCAGGAGGTATTGAGAATTTTGGTAATTGGTTGAAGAAAAATAAATTAGAG
GGTATCTTAGGAGCGGTTCCTGGTCTTGGAGGTTTAGCAAGTAAAGTTCCTAATATATTG
TCAATTCCAGGCATGGAATCTATCCTAGGTATACCAGGCGAGGGTTTTAGTGCCTTAGGT
GCTATTGGTAATATTGCTGACAAAGTAGGTATGAAAGGTGTATATCCAGCTATCATGAGT
GGTGTTCAATCTGGAAAGTTTATTGAAGGATTACCAGAACTTGCTGCAGAAATAGGTGTT
GATCCGAGAGTTTTAGGTGTTCTCGACAGAGGACGGGATTTATTACAGAATAATCAGTTC
AAAGCAGAGTATGCAAAGCAGACTGCTATTGAATCTTTACCTGTTCCGTTGACCGTAGAA
AAAATCGTTGCATCACCTACCCCTGTGCCAATAAATAGCGTTGATACCTACTTAGTAGCA
CCATCCTCTACAACTAACAGATAATGGCAACAGTAAACAAAGGTGCGAAACTAGATTTTT
ATAAGTTTGTTTACCCTAATGCGGGAGCAAGCACTACTTCTAGAGCTAATGCAAAAGGAG
GGAATAGAGAATTAACTACTGTTATAAAGCAAAATACAAGAGCAATTAATAGTATGGGTA
GAGTTGTCAATACTATTGGCAGCACTATAGTGTCTATAAAAGACGTGCAGATGAGGTTTT
TGAAGATAGATGAAGAGAGATTAAAGAAACCATCATTCGTACCCCAATATACTAAATAAC
AACCTCCTCGTAAAATGAAAGCATTTGATAGTTTATTCAAGGGGAAAATACCAGGTTTCT
TTGAATCACTTGCAAAATTAGCAAGAGCGTTGATAAAGTTTTTCTCAGTTCTTCCTGCTC
TTAAATGGTTATCTAATCCTGAGTAGCAAGATAAGGTTGTTAAAGGACTTGAAGTTCTTG
CGAAAGTTTTTAAATTTATTGCATCTGTTGCTAATTTTGCCTGTGTTAATACTATAGACG
GACTGTACGATCTTTTAAAAGAAGATGCTACATGGATGGAAAGAATAAGTGGTTTTACGA
GAGCACTTGCAGGATTGGGAACCGCATTCTTAGCATTAAATTTTCTTACAAATCCAATGG
GGGTTATCAAAACCTTCTCAAGTGTGCTATTATTCTTTCTCAAGGGTCTCCTTGCTGCCT

TTGCCATACTAGCTACCCATCCTTTGATTGCAGGTGCAGCACTGTTTCTTCTTCCAAAAT
ATCCTGATCAAATTCCTGGTTTAGTCAGTAAAAATGAGGGAGCGTTTGCAGAGGCTTTAG
AAACAACAACAAATGAATCTGGTGATACTATCATTGCAGAATCTGATGAAAAAGCACAAA
GAATCGCAGATCTTAAAAAACAAAAAGAAGATCTTTCCCTTCTTGATAGGTTGTTTGGAA
AAGGTAAAGAAATTCAAGAGTTAATTTATTACTTAGAAACTGGTAAAACTAAAAGTTATG
GATTATTTGAACATGGAGGTTATCTAGATGGTTATCCTAAGGGTGGTTGGATTTCTGCTC
ATCAGTCAGGGTATTCCGTGTCACTAGATGGTAATAAACCTGATTTTATTGGACATGGAA
CTGAATATGTTGCACAGAAGTCGGATGGTGGTGCATTTATTGTTCCGTTTGATACTCCTG
CAACTAAAAAGGATCCTGATCGAACATCTCGAAGAATGACTGAAGCAAGACTTCTAGGAT
TTTTTGAAGAAGGTGGTGGGTACGATACGTTTGCTAAGAGAGTGTTTAAGATTCATGAGG
GTTTAAGTCCAACAGCAATACCTGAACCTAATGGTGGAATGTCTATTGGATTTGGTCATT
ATATTAAACCTTCTGATAATTTCCCTCCCACTATTAGTCGGGCGTTTGCAAATCAACTGT
TCAAACTAGATTATAACGATCATAAAAACGCTGCTATGAAAATACCTGGATTCGGTAAAT
CTAGTCATCAACAAAAAGCAGCATTGGTAGCTCTAACCTTTAATCAAGGTGCAGGTTGGC
ATACAGGATTCCCCAAGTTCATGGCAGCGTTCATCAAAGGTCATTATGAAATCGCAGGAG
ATGAACTAAAAGATAGTCTTTGGTTTAATCAAGTTGGACGTAGATGACCTACTATTGTCA
ATTTGATGAAGAATAAAGGTTTGGGAGACGGTATTGGACAATACCTTTTAGATAGAGGTT
TAGTAGTCCCTATGGACGAGTCGAAATCAAGTAAAGGATATGATTGGACATTTGGATTAG
CACAATTTTTTGCTGGAGCACCTGCTGCAGCTGCAACATTAGATTCTAATATCAATGAGA
ATAGACTTGATGGTATGGAGACTAATACTAAAAGGGATTCATATAGAGTCGTTCCTACTT
CACATGCAGATACAGGTTCTGGACGGGGTATTGAGGGAGTTACCGATAAATTTGGTCGCC
CTTTGGAATTCTCTCAACCTGCTGCTCAAATGTTTGCTAAGATGATGCAAGCATCAAATG
GAATGGTAAAAGGATCTGATGTTGCAAGCAGTGGTAGATCACCATCAAAAAATAAAGAAG
TTGACGGTCACAAAAACTCAGTTCATTTATATGGAGAAGGTATAGACATCTCTGGGTCTT
CAAATGCATGGATGAAAAACAATGCTTCTAGATTTGGTTGGAATTATGGATACAGTGACG
GACCTGGTAGTGGTCATTATGATTATGAGGGTGGAGGTGCAGGTAAGACTCCTATCTTAG
GATAAGCTGGATCTCCTTCATATTCGTTCATGGATTTACAGAAAGAAAACGCATGAAGAA
AATTACTTGCTTCAAGTAGTCTATCTGGATTAAGTTTATTTGGTAATAATAATGATCAAC
CAGGTTCTGGATGACCTTTTACTGACATGTTCTCGGAGTGAACATTTGGTAACATGGGAG
ATTTCTTCACAACCCAAGGGTATCAACCAAACATATTTAATTCTCAGAATAGAACGAGGT
TTGCTCAAAATAATAGTGAACAAATCAGAGTTAAGAAAGTAACAGAACAAAGAAACCAAG
CAAGAACAGAAGTCAATGCAAAGACTTCGGAGATTGTGCAGATGGCGTTGGCTGCTGTTG
AAGCGCAAAGTGGTTCCAATAGGCAATTCATTTCGACGGCTGAGTCGGCAATTAGGAGTT
TATTAGGAGCTCAACAAGGTGGAGGTACGTTCGTTAATGTTGGTGGAACAACAGGAACAG
TATTACGAACTGCTGTTGCTGTCTTAAATTCATTTAATAATCCTTTAAGAGGTATTTTCC
GATGAGTAAAAATTCTTCTAAAGAAGGGTCATTAGTTAGAGAACAGGCAGGTCAGAGAGA
GGTAACTCTATCCATTTACAGAAATGGAAAAAGTGTGGAAACTCCAGATGGAGCATATGA
TTTAGTTACATTTTTAAGAGGATTTGAAATTTATGAAAGTATTGCAAATCCCTGCATGGA
GTGTCGCATTATTTTAGAAGATGCAGGAGGACTTATAGGATCTTTAACAGGAAATGAGGC
ATTTACTATTCAAGTTAGAACCTCTATCAAAGATAGAATTTACAATTTCAGATCCAATCA
AATTCAATCAAGAATTAGGACTAGACAAACTAATGACACATTTTTAGTTAACTGTGTTTC
TGATGAATATCTAATCAACGAAACTACAAATGTATTTGGTAATTCTGAAGTCATTTTCAA
GAATGAAACAGAAGCTGGTGCTATCGTGAAAAAATTATTAGGCAAAGAATATATAAAATC
TGAGAAAAAATTATATATTGAAAATACTGTTAACAAACAATCTGTTATATCACCAAACTG
GCGAGTATTTGATTTAATTTCTTGGATGTCACAAAGAAGTATTCGTAAATCGAGTAAAAA
GGGTGTTATGCCAAATGGGTTTGCTTTTTATGAAAATGCACTTGGATTCAATTTTAAATC
AATTGAGTCTATGATTGAGGATATTGGTAATCAAGGAGAAACATCTGAGACAGATATAGC
ATCTGGTAAACCAGAACTATACACATATCATTACACACCCAAAAGAATCGGTGAAGCAGC
TGATGTTCAATTTAATATAGATCGTATTGCATTTCCTGATGAGAAAAACTTTTTGATGGG
TCTAAGACATGGTTCTTGGTCAGGATATAGTGTCGGTTTTGATTCTGTATTCATCACCAG
ATCTAGAATGGGAACTAGCACAGATTTATCTGCAGACGCTTATCGCTATTCGTACGCTGA
ATTATGGAAAAGAATGGCAAATCTGGATGGTGGTAATGCTGTTAATCCTCAACAGAAAAT
GGATAATTTTTCTAAAAATGCTGGTAATTTTCCTAATCGAGTTAGATACTCAATGATTCC
TAATCAAATTTTTGATGCTAAGTTTCAAAACAACCCTCAAAGAAATTATCAACAGCTCGT
TGAATTACAAGCATATCAATGGATGCGTATTGAATCATTAAAACAAAATCAACTGACTAT
CATCATGCCTGGCAATTTAGATTTATATGCGGGTTCGGGGGTTAACTTAAATATCCCATC
GACTCATATAGAGGGTGACACACCTGAAAGAGATCCTAAGTAGAGTGGTGGATGGTTGAT

TGCTGCAGTAGCACATAAGGCAGTAGGTTTAAACTTACAAACAGAACTTGCTCGAATGAA
AGTCTCGGATATCCCGAATCTCTCACAAAACCGAGAGGTAGGCATACCATAACTTGACAT
CCACACTCACATTTTTTATAATATAAGTATAAATAATTATGTATCAACGAGGTACACAAA
TGGACACTATAGAACAACACATTCAAAAAGATAAAGATCTAATCGAAAATCCTCTCACAT
CACCTTCTGCTCGTAGACACTTTAAACAAGAACTACAAGAACTAGAAGAGTATGTAGAGC
ATCACAAAACTGAAATTAAAGCAGGAGATCATCACGATCCAAACGCACTTGAACTATTTT
GTGATCAAAACCCAGAGGAACCAGAATGCCTAGTTTATGACGATTAATTATGACAGCTAA
TATACAGGACTTTGTGACTTTTCCTTATAGTGATTTGAAATTAAAAGATTTCATTGGTAG
ATGGGAGAAGAAAGTTCCAGATGAATTATGTGATAGAACCGTAGATTAGATTGAGTCTCA
ACCACTTGATAAAAATATTGGTAATGATAACGCAGTGAGAGAGGATCGAAATACGTTTCT
TATCGAAACTAAACCAGAGCATAAAGAATGGTTTAGAGAACTTGACGATTACCTAACCCC
TACAGCAACAGAATATGCAAATACATGGGGTGCTTTAAAGGGTGCGTTTTTAACGAACAG
TGAGATTAAATTACAAAAAACATTGCCTTGTCAGGGTTATCATGTTTGGCATTGTGAAAG
AAATGCAATGCAATTTGTAATGAGAGAATTGGTATGGTCTATCTACTTAAATGATATGCC
TGAGGGAGAAGGAGAAACAGAATTTTTATTTCAAAAATATAGATATCAACCACAGAAAGG
GGACATCCTAATCTTCCCTGCATCTTTTACTCATACTCATAGAGGTAATCCTCCATATAC
AAGAACGAAATATATTGCAACTGGTTGGTATCTTTATACACCACCTGGTGGTATTCATAT
GCAAGAAACTGCAGAGACAGCGGGTATACCAGTTACTACACTTATTGATCGAGATTGCTA
TACTACACCTGGTGGTGTTATACAAGAAAAAGACGTATAAATACTTTCAAAGTCTAGAAT
ATTCTCATGTCAACGATTAATGTAGGATCCGTTCAACGTAGTCCAACGGTAACAGGTGAT
TTAACTGTCAGTGGAACAATTAGATCGTCTGGCGTACGACACCCATCTGCTAACACAGAT
GCAATTTCTGCTGCTGCGGACGGAACGGAGACTGTGGCAGGTGTTGTTAAGACAGCTGCA
ATTCAAGATTTATCAGCTAATACGCTTGGTGGTGGTCAAGTTTTACAAGTTGTTCAAAGG
GTTAGTAACTCATATGGTGAGTTTGGTAACTCAACTTATCAGAACAACATTAATAACTTA
ACTGCTATTGGTGATTGGTATATTGACAGTACGACTACAAAAGCAAATTCTAGGATTTTG
TGTCAGTGGAAAACTAAGATGTATGGTCCTAACACTCAGCACCAATGCGTTGACTTAAGA
AGAAGAATCGGTAGTGGTGGTTTTACTTCATTAGTTGAAGCTTATAGATCAAAGAGCACT
ATTCATACATTTGCAGGAATTCACTGGCAAAATGGTAACGGTGCTTTTGAAGGAGACTAC
TTCTCAGCATTCATTGTTACACCAAACCAACCTGCAGGTACGTCACTTCGTTACCAACAA
CACCTTGGAGGTTGGGCAGGAGGAACTATCGACTTCGGTGGTTGGGATGCTAACAACAAC
CAGAACGCTCGTGGTATGATTGTTATGATGTGCTATGAACTTGCACCTTAATTTTTAATT
ACAATGACACTTCCAGTTCAAAACGTTAAAACTCCATCTTACGGTTCTGCAATTTCTAAG
ATAAGACCTGATTGTGTATGGATTATCTATGAAAATGATCTGTCAACTCTTGAGTGGTCT
TCTGAAAATACATTGCCTAGACCTAATGATGCTGAAATCATTGCCGAACTTGACAGTCAA
ATGACTGCTTTCAATGCTAAACAATTTCAACGTGATAGACAACCTGAGTATCCAGATTTG
AAATCACAGTTAGACATGTTGTATCATGGCATCAAGGACGGTAAGTTAGATAGAGGCACA
TGGATTGCAGCAGTTGAAGCAGTAAAAGCAAAACATCCTAAACCTTGATGATTGACGATT
TTATTATAGGACACTGGACAAATAGATATCAAGCACAATCAGCACCTCACCATTTCTCTA
CTGTTGAATCTATATGGGAAAAGGTAGAGGGTGGTTATCATTCAGAGAATTTTTATAGAA
GCGACGGTGCTAATAAACCGTATAGAGAAAGATATCACAAGGTTAATGCAATATCCTTTG
ATAAATTACTTTTTGAAAATTATAACTTACACTGGACAAGATCAGAGAATTGTGATATGA
TATTCACATTCGATGGTGAAGCATCGCATGGACACCTAGTAGGGGATAAATGCGCTGGTG
ACAAAGGATATATGATTGTATCTGAGATCACTCTTTACGGGAATCGACTGCATAGTATGG
ATCAAGGATATGATGATAAAGGTAACATGGTATGGGGTAGTGAGGGCATTTATAAATTCA
CTTGCTGTATCGACCCTAAATAAAGTTGTAGCAATCTCAAAAATATGCAGACCATTGATG
GTATTGTAAATGAACCTACGGTCAATTTTGTCGGAAAAGATGGATTTTTCTGGTGGGTTG
GTGAAGTAGAAGATAACGAAGATCCTATGGAATTAGGTAGGGTCAAACTTCGTGAGCTTG
GATATCATACTAACGTACGAGGCGGAACAAGTACAGCACTTCCTACAGTAAATCTACCGT
GGGCGACAAGTTTGCAACATACTGGCCAAGGAGGTAATGACGGACAAAGTGTAAGTTCTG
GACAACTTCAACCTGGTGCAATCGTCATGGGATTCTTTATGGATGGCGATCAGGCACAGA
TGCCTATTGTCATGGGAGTTCTTCGTGTTCAGAAATCAAACGCTACAAAAATACAACAAC
CATTTGCTTTTTCTGGTGAAGCAATGGAGCCAGGTCTTAGTGTTAATGCATCTGCATTAC
ACCCCGCAAAACCAAACTCTAGATTGGCAGGAACTAAAGAAGAGAGTTTCCTGAGACAGG
GTGCACATAATACAGTTTGCGTTCCTGGCATGAAAACATGTGAGGAAGGAGGTCCTGGTT
CTCCTAAGAAACTTGGAAATGATGTAGGTATTATTGGTAGTAAACGTAATACTGTAAAAC
CTAGAGATCCAGAAAAACCAATACACACTGCTAATGGTGTTGGTGGTCCTTGGAGATCAC
TCGTCTATAAACTTTCATATTTGATTGCAAATATTGCTGATCATGCAGGAACTCTCGTAA

AAAAAGAAGACGGGGACTTCCTCGATTTAATGACAGGAAAAATTGTAACCGCAAAAGAGT
TAACTGCTAAGGTGGAAAATTTCTTGAGTGCTATATTTATTCAAGTCGTAAGTGCTATCA
AGCAATCTTTGGCAAACCTTGCAGAACAATTAAATGTTATAACTTAGCTTGCAGGTGCAA
CTGGTATTCCATTTGTCACTTTTACTGCTGTTACTGCTGCAGTTCAAAAGATTTTGAGTG
CTCTTTGTATTGTTGATAAAGATCTTTTAAGTCTCATTCAATCTCCTATTGATATTCTTC
TTAGCATTGTTGAAAACTTCCTTGAGGGTATCATCTCTCAAGCACAAATGTTCATGAACA
GTGTTCAGAAAGTCATTGATGATATTGTTTGCTCTATTCAAACGATTCTTAATAAAGCAC
TCGGCATTGTAAATGATGTAAAATCTATTGTCTCTGGAATCGAAAAGGCACAAGAGCTCA
TTAAAACATGGGAACAAGGAACACAAATTTTCAACTATCTTCAAAGTTTCTTTAAAAATG
GCATAACCAACTTAACTGGATTGATGGCATTATTTCTTAAGTTTGCAGGAAGTAACTTTA
ATCGTAAACTTTCTGGTGCTGAAAATGATAATGGTTGGTATCCTATATTCAGTACAACTA
ATTGCATACCTGAGGAACTCTCTAATAACAATAGCATTCGAGGAAGAAATTGAGGTGGTT
GTGGCGAAAATGATAAAGGTGGCGGATTACTTGATAATATTCTTTCGCAAGCAGACCCTT
ATTTGTCTGCTGCTAAAAACTTTGTAAATGGTGCATCTGAATTATATCTTGGCACACCTG
GTCGTTTAGCAACCATTATTCGTAAAGAAAATGGAACAACACTTACATCAGTTAAAATAA
GTCAAACATCGTACGCTGAATATACTTTCAAGAAAGCATAGCGTAAGGAAAAACCAGATA
TATCTGATGATGAAATAGAAAAACAATTAGTTGTTTTCAGAAAGAGACAAAAAGAAAAGA
AAGATGATGTTCTGGTTGCAGATCACGCAACGTACGTTGGTAATAGCACTAAAGAAGTTC
ATGGTGATCAATCTGAAGCAATTGATGGTTCACAGGTTACTAATATTGAAGGTGACTATC
ATTTAGACACTACAGGTGATTGTCATATTTCTGTTGCTGGATGCTTTATGCTTAATGCTC
AAGGTGCACCTAAACAGGTAAAACCAGATTGAACTCCATCTGATGAAAAAGACATTAAAA
AACATTCATTAACATATGGTTCTGATGTTGATATGAATATCTCAGGTGCTGCATTTACTT
TTGACGGTTCAGAATTTAGTATGGGTGCGGTCACTACAAAAATTACTGGATCTAACTGTG
AAATTTCATCTACAACAACTAGCATTGCTAGTGGTGAAATTTTATTGACTGCAGAAAACT
CAGTTGATATTACTACACCATCTTTAGTAGAATTAATTAACTTCCCTCCTTCACCCATAC
CTAAGGTTAAAACTGGTATTCTTAGAAAGGTTGGTGGTTCTATGGAAACATATATGACAC
CGGGTCTAGCTTCAGCAGATGCTATTCGTAGACATATCGTTGCAAACCCTGGAGGATATG
CTTTTCATCAAAATGGTTTGGCATACACTAACTTAGTTATGACAGGACCATGGCTCGCAA
CTGCTACAGCAGGTCTTGCTAGTTTAACAGCAGGTGCAGTAGTAAATATCACTGCAGGTT
CTGCCATGAACATCACTGCAACAGCATCAGTAAACGTAAAAGGGTTGACGATTTTCCTAA
AATAGTGCTATACTATGAGTGTATCCGCGGATGCCCCACTTGGTTTGGTGCACAACACCT
CCGCAGAATGACAGGAGACCCCATGCAATACACTCAAGTTGAGCAAATTTTTGTTAATTT
CTCTAGGAGATCTCTAAAAATCCTAGATATTGAAGGGTACCACAAAACAATAGAATGGGA
ATGGAATGAAGAGGGAGCGGAAGGTTTCTCAGTAACTATTTCTCACCTTTGTGATGTCCT
TGACACTGACCTTATTACCTATTGTTTTGCAGAAACAGAATGATTATTCCACATTTCAAT
TTTGATCCCAACATTACATTTCCGATTTCAATTGCAGTAGTTACTGTATTGTTTATCTTC
TATGGTATCTACAGAGGTTTCTTTGCAAATGAAGGATTGACAGACCCATTTGACTACCAC
GACGAGTAAAACTATGATTGGACCTATTGGCAGTACATTAGATCAAGCAGGTGAAAACTT
TCATTTCATGATGGATCTAACAGCAAACCAAAGACTTTGCTGGAAAATTTCAACACCTAA
GGGATCTGTTATGTTAGTTCCTGTAAATGAAATTGCTCCAGTATCTGATGAAATTCCAGA
TCAAGTAGAGGAGTTTCGGAAAAGTTTTATGGAGAAAGATGCGGCCTGAAACTAGACATG
CCATGGAAATGCTTTTTCATTCAAAATGGAACTTGCCAAAAGCAGCAAAACATGCTAATC
TAACTAATAAGGAAATGAAGATTACTTTTGAAGAATACTGTAATTTCCATGAAACTTCCG
AACTGGCAACACCACTCAAAAAAGGAGAAAAAGAGACATCTTAAACCTGAAGCACTTCGT
GCTGCTAAAAAAAGATTACAAGCATTAAAGAAAACAGTGGGAGTGTGGCGGAATCGGTAG
ACGCACCAGACTTAAAATCTGTTGGGCATGTGCCCGTGAGAGTTCAAGTCTCTCTACTCC
TACTGTCGGGGGAATACAAAAGATCTCTATCTAGAAAGAGTGCCCCCCTTCAAAACATAC
TAAAATAACATGACAGTATTAATCATCATAGTTGTGCTCATTGTAGTAGCAGGAGCACTT
ATAAGGTACTACGATCCTCATTAACTGAAAGTTGACTCTAATACGGAACCCTTTCCCTTT
TTGTTTATTGAGGATTTGTATACAGAGGAAGAATTGCGTCTAATATGGTGTGAGTTAGAT
TATTATCAATCTAATAAGTATATACTAGATGCAAATACTAATCCTTCTGTAACAGATGAT
GGAAAACCAAGAACTAGAAAACAAGGAAACTTCGTTGATAATGTTTTCCAATTGAGAGAG
TATTCTAATATTTTAAATCTTTCTAGAAAAGTATTAGAACCTGGTTTGATATGTCGTCGT
GACCATCTTATTCAGTGGAAATATTTCCAACCTGATGTAGATCACTCTTTACTTTCATAT
TATGATGATGGTGGATCTTATCTACCTCACCACGATAACACCTTAGTTAGTGTGATATCA
TGGTTATGGAAAGAACCTAAATGTTTTGAAGGCGGGGATTTTGTATTTGAATAATATAGA
ATTACTGTTAAATTTACAAACAAGAGTGCAGTTGCATTTCCTGGCACTATCAGACATGGA

GTCACTCCTATCAAAATGGAAGATCAACATAAAGATCTAGGTTTAGGTAGGTATTCTTTA
TCACATTTTTTGAATTTTAGCTAATGGGGTACTTAGTTCATCCTTTACCACCTAGAAAAG
TTTGGGTCAAAAAAGAATATCTTTATGATGTAGAAAAAGGTCATGGAGAACTTACACCTG
GCACTTGGATCTCAGTAAGGAGTATCCAAGCAAAGGCATTATATTTTGAGACATTGCTAG
CAGATTATGGTGCACTCTTTGATATGTTACCACTCAGTGCATTTGTATGGAAACCAGATA
TTGATTGGGATGATCAGTTGCCATTAGATGTATTAGAACTGTGGGATTGTTTTGCTTATA
TTAGTACAGTTGTAGAAAAAAACTATACTAGGTAGATGTCAGTTCTTTGGTAAGGACAAGA
AGACGCACGCAGGAGAGTATGAGTTTACTATTGATACTTCACACCCTGACTTATCTGTAT
TAGATGTAAACTTTTCAGAGCATGATCCAGAACATAAGACCTGTAACATCATTGCACTAG
ACAATGGACAGTTTGCAGCACAACCAAATAACAGATGTCAGTTCCTCGATAATAGTTTGG
TAGATAATGATAATCTCAAGAACCCTGACTTCAAAGTATGCACACAAAACTATGCTGTAG
AAACCCTACCTAAGTGGTGGTCTGTGGGTCACACAGCTGAGTGGGCATATAAGACAGGGG
AAGAAGAGGAAGAAGAAATAGACCGAACAGGCGGTCGACTTTCTTTATAAATAGACCTGT
AGCAAATAGTGTGGTTATCTGTGGGAACCCGTAAAATATCTTAGATGGATACAATATCAG
ATGCTAACCTAACGGGTGAAGCAATTCTCCCCGTGGTCGTGTCTGACCCATTGATTCCTA
ACGGAAAAGCAAAAGTAAATCAACTCTTCCGTGGAGTTTCACAAGGAACTAAGGCATCGC
CTGGTCTTTGTTTTGATTTAGACAGAGACAGTGGACTATACCAATCAGCGTACGATCAAA
TCGGTGTTGCATTTGGTGATGGTAGTTTATATTTTAGTAGAGTCAGCAATACTGTAACTA
GTTCTTCGCTATTTGTAACTGCTGTTGATGATACTGCTACTAATACGGACATTGTTTTCG
CACCGAAAGGAACGGGAACTGTCAGAGTTACCGGTCTATTTACTATTGATGACGGTTCTG
TGATCTTAGAAGATGCACAAGCACCTAAGGCAAGATTTGAGGTAAGTAATGTTGAAACGG
GAACTAATACTCGCATCTTTACGCTACCTGCCATTACATCTGGTAATGGAAGAGTCTTGA
TTGGTGATGATACACAGCAAACACTAAGAAATAAAACCATTCTTATTGGCGAGGATAATC
TCTTTATTACTGATGGCACTGAAGAAGCAATATAACAAATTAACTGGGTTGATACATCAA
ACGCTAGAAGATCATATTTTCTTCCTGGTGCGGGAACTGTAACAAATACAGTAGAACCAA
CTGCGACTACAACTACATTGTTAGATACAAAGGCAGAACAAACAGCATTTAGTAAGACTC
TTGTTAATCTTAAATTAGCAAAAGACGCTGAAGTTGCAACCCATCGGGCACAGTTCAACA
CTAATGCTCTATCTGCTAATAGAACTCTAACTGTTCCTGATTTATCAGGTAATCTTGCTT
TCACTGATGCTACTCAAGTATTACAGAACAAAACTGTTGAAAATTTAATTCTTCAAGGTC
CAACGGATACTTCAAAGGAGATTACATTTTCAGTTGCTAACTCAAATACTTTTACCAACG
TGACTGTTCAAATACCTCCTACTGCAAACCTAAATAACACAGGAGTTCCTAATACACTTG
CTACAGAGGCTGTTACACAGGAGCTGTCAAATAAATCATTGACTAACCCTGTGCTGAAGG
GTGCAGGATCAAAAACTGTAGGTACTGTTACTCTTTCTATAGATAATATGACAGCTAACA
GAACTATCAGATTCCCTGGTTTTGATGCAACTCTGTTATCTACTGAAAACGTTACATTTG
ATGACGTTACATTTGGTGCAGGTATTAGTGCAGCAAACTTAACGGGTCGAACAAGACAAC
AACAATTTTTCTACGCAGGATTTTAATTAAAAATGGCTAATCAAGGTTTACTTGCACAAC
ACAAACCCGCAGCGAACACGAATACGTTGTTCTATTCTGCTCATGTTGATAAATCTGCAA
GCACGATGATCAACGTCGCTAATGACGGAAAAGCATCAGACTACTCTGTTGCTCTTAAGA
ATTTTGGTCAGAAGTTAGTAGTAAATGGTTCTGCAAACGCATACAAATTACATGAGTATG
ACGTCATCACTGCATATAAGATGACAGTTGACACTGCCTTTGACCCTGCAGCACAGGGTT
TTACTGGCGGTCTTCAAGTTACGAGTGCTGATAATGAATCAAAGTTTGGATATGAATCTG
CCATTATTCCTAATTATGTAGAACTTTTTGTAAAAACTTTTGCTATCAGACAGATCACGA
TTCAAAGTGTTACTGGTACGTTCTCTGTTGGTAATACTATTACCAAAGGTTCTGGTAGTG
ATGCAACTACTGCAGTAATCTATGGTAATAATGGAACTATTCTTCATGTAGGTCCTTCTA
CTATTAACGGATCTGGTGCAGAATTTGCTGCAGGTGACAGTATTTCAAACGGTGCGGGTG
CATCAGCTACAGTTGCAACTGGTGGTGTAGGATCTGCATCTAATAAATTTGTATTCTCTA
GCACATCAGGCGGAGCTTATGACCTTCGTTTGATTTCTGCAGGAAACGGTTTTGAACTAT
TCAACGATAGATCATACAGATTTAATCTTGCTGACTCTACTAACTCAGGTCACGTTTTTC
CATTATCAACAACTATTAATGGTGAGTGGGGTTCTGACGGAACAACAGGTAACTCTGATG
ATGGAACTGAATATACTACTGGTAAAACTACTAATGGCACCATTGGTTCTAGTGGTGCAT
ACATCCAATATGCTTTTACTAGCACTTCACCTACTTTGTTATCCTGGTATAACTCTGTTA
CTGGAACAGCTGCTAACAGTAGTTTTGGTGGATCAGAGGCATATCTTACAACAACTTCTA
CATCTACATTCAATGTATTTTACATCTATGATGTAGAGTGATCTTGGACTAACTCTACAT
CTACCTTTGTTCAGAACAGTATCACATATACTGTTACTACTCAAACTTCTGCAGCGTACG
GTTATTTTCGTACTTACAGTGGTAATAACTTATATGTTATTAAAGGACTTAATTCAGCAG
ACTTTGCAGGTTCAGATACTTTCTTATATAACCCCAAGTTATCTACTGCAACAAGATCTA
CTGTGACTGTAAATAGTGTTGCTGTTGTAACTACTGCTGTGGAAAATAATTACATTATTC

AAGGTGCAACTAACGCTGGTCATGCAGTCGCAAAGAATTCTTCTATTGTTGTTGGACATG
GTGAAAGATTGATTATCAACAGCACTACACAAAACAACGCCTCCTCCCTTATTGGGTTTG
AAGATTCCTCAAGTGCGTTCACTCCCCGAACATTCGGCAGTTGAATAAATACAAACAAAG
CGGATAGGTAATGTCACTAACTAGACTAAAGAATATTATTACGTCCAGTATTGGACGTAT
CATATATGTAAACCCTGACGATTTCTATGCATCAGATGCTATTGATAACAGGGGAAACTC
TGCTTTGCATCCTTTTAAAAGTTTGCAAAGAGCATTCTTAGAAGTAGCAAGATTTTCATA
TAGAGTTGGTTTGAGTAATGACGAGTTTGATGCTTTTAGTATCATGCACCACCCTGTTGA
ATATTTTGTAGACAATAGACCAGGTGATGTTTTATACACGAACGTTGCACCTATTGATGC
AAACTCAGACTTAGACTTACCTTCTCCTGACCATGTTCTATACAAAGATAGTTCAGTAGA
AGGTGGTATTAGCGTTCCTAGAGGTTGTTCTTTAGTTGGAACTGACCTTCGTCGTACAAA
AATTATTCCAAAATATGTTCCATATCCTACAACGTATGCTGCAAAAGGTATTAACACAGA
GGATCAAGTTCCTCCAAGAACAGCAATCTTCAAAGTTACTGGTGGTACTTATTTCTGACA
ATTCTCTTTCTTCGACGGTGCTGAAGAGGGTGTATACTTCGAACCTGATTCCACTGAAAC
ATTAGCACCTAAGTTTTCACATCATAGACTGACTTGTTTTGAGTTTGCTGATGGTCTTAA
TCCATTATCAACTCTTATTTCACAAGGAACCGTTCCCTCTGCTGATTACTCTGCTGTATC
TAATATCCTGCAGAGAAATGATTTAGAGATGTATTATCAGAAAGTGTCGTAAGCTTTCGC
AACTATCCCAGATACATCTGGGGATCCTGCACCTGACCAGATTCAAGCAAGGGTAGAAGA
AAACCGTATTGTAGGTCCTATCTCCGATGAATACCGAGTCCTTCAGATCACAACAAATGT
ACAGACAGCTACGGCTGTCACCGTTGACGAGTTTGATAACCCCAGAGACCACGGATTTTC
TGTGGGTGTTAACATCGACGTTAGTGGTGTTACAGGATCAACTGGATCGCAATCCGATCT
TGAAGCAGGAGTTTATAACGGGTCTTTCTCCGTCACTTCGGCCTCGGGTAACGTCTTCAC
TTACCAGATGCAATCAGTCCCCTCAGGAAACGGAGCCGGATCAAACATAACTGTTAAGAC
TGAGACTGATACTGTTGACTCAGCATCACCGTATGCATTTAACCTATCACTTAGAACAGT
GTGGGGTAATAATGGTATGCACGCAAACGGTGCAAAAGCAACTGTTTTCAAATCAATGGT
TGTGGCACCGTTTACTGGACTGTCACAACAGAAAGACGATAGAGCATTTGTAAGATATAA
CGCATCAACAGGTAACTATGATGTAGCAACAGCAGGTGATGGTGCACATATAGATGGTTT
CGCTGAATATAGAAATGGATGGCGTCATGAACACATCAAGTGTAGTAGCGACTCATTCAT
ACAGGCAGTTTCCGTGTTCGCTGTGGGATACCAAGTTCACTTAACTGCACTAAGCGGTGG
TGTCATGTCCATCACCAACTCTAACTCTAACTTTGGTAACACTGCTCTCAGATCAGCAGT
ATTCAAAGCAAAAGCATTTTTAAAAGATAAAGCGGGTATGATTACACATATCTTTCCCCC
AAAAGCACTCAATACAATTTCAACAACTGCAAGAGGAACTAATGGTTCTTTAAATATTAC
TCTTGCTAATGATGGATCAGTGAATGGTCTGATTCAAGGTATGACTGTATCTGGAACAAA
TATTGCTACTGGTGCAATCGTTAGTTCAATTAACACAAATACTAGAGTAATCACACTTTC
TGCTGCAGATACGGGAACTGTTACCGGGCACGTAATCTTTGGTGAAGAGACATCTGTTAA
CTGGGTAAACATTGATACTCAAAGAACTAAAGTAATTAATACTGCACTTTCTGGACAAGG
TGGAACACCAGGCACAAGACTATACTTATATGGTTATACTGTTGAAGCGTATCCTCCAAC
TAGTAGAGTTCAAGGTTTTACAGTCGGTGCAAGACAAGACTGAACTGGTAATAGTGCTAT
AGCAGATAAAATTAACTGCTTGTTAGGTGCTCAAGCTGCATCTTCTGCAACTGTACAGTC
AGCAAGCATATCACCTTATGGTCCTAGTGTATCTGGTCTTACAGCTGGTGTAATTGGATA
TCCATTACAATATGATAGTAATACATATACTATCAATGGTGTTGCAGGTTCAGTCGGTGG
TTGGTATCTAAGTGTTTCTTCTACTTATAACAGTATTTACGAAGCGTTAGCAAATAATAC
ACAATACAATACAGTCAGTTTTACTCCTACTACATTCCTCAAGAGAATCCCTGATTCAAG
AGACTTAGAAGATAGAACATATCGTGTACGTTATGTAAATGATACGGATAAGACTAATCC
ACTACCTAGAGATCCTATCTCTGTTTTCGTATTACAACCTCATAACAGTGATACGACAAC
ATATAACTTTCAACGTGCATTTTATATTTACGATATCGAGACTGTTCAAAAGTTTGTAAG
AGGTGTTAGTGATGGAATATATTATATAACATTACTATGCGGTTCTATTGCACCTACTAC
ATGTAACTTTGATGATAGGAAGTTCTCACAGAACGTTAACGAAGTTTATCCTACATTCGA
CAGAGATAATCCAGTTGCTGATCCTCCTGCTGCAGTCACTGAAGCTTCCAATGTTACCCT
AGGTCTTGTTATTTCTACTGATGGTGCAACTCCAACTGCTGCACTTGACCCCCAGAGAAT
TATTACTAAAGAAGCAATTATATTCTTACTGCCTGATACTGGTTGGCAGAATCCAGGTTC
AACTCCTGCGTACGATTCAGTTAACGGACGTCTTGCAGGTATTGAACTGATTGCACGGGC
AGGAGACGAAGAAACCAGTAAGATTAATATTAGTCAAGATAACTCTGGAGTGGTTGCACC
AATCAACGTAGAGTTTAGACGATACTCAATCATGCGTTCTGGTAACCATACGTTTGAATA
TCTTGGTTTTGGTCCAGGTAACTACTCAACTGCGTTCCCTCAAACTCAGGGAGAAACATT
ATCACCTGAGCAGATCAGATTCTCACAGTCTATTAAAGAAGAAGGAGGAGTTGCATTTTA
CTCTGGTTTAAACAGTAATGGTGATTTAATCATTGGTAACCAGGTGATTAACCCTGTTAT
GGGTCAGATCACTATCGAAGATATTGCACAGTTGAATGTTTTTGGTGAAGAGAAAACAAC

CATTGAAACGTTCTTTGAACTAGTATTGACTGATAAACTGACTGTTATTTGTGGTGCATC
TAACGCACTTGAATCAATCTTTGCAGGTCCTGTCACATTCCAAGGTCAATCTACATATAC
AAACAACATAACCGCTAAGAAATTTACCTATAATAACCAAGATGGTACAGTTATTAAACA
AACCTTACTAGCACCAGAAAATGCGAGTGGACTCCCCGATTTTAGTAATATTACAGGATA
CGATACGCCTGGTGATGGTGATATTGTTTACAACATCAATTGGTCACCTGGTAAGTCTCT
TGGTTGGATTTTTTACGGAGCTGCGTCGTATGAGTTTGGTATCACGGATACTGGTCAAAT
CAATGTTATTATAGACTCTGGTGTCACGAGGATTGGTCTTGGTGTTGCTCCTACGTCTCC
TTACAGGGCAAACATCAACGGTTCACTAACGATTGATGGAGACTTAGTTGTTACTGGAAC
AGGTGGTGTAACAGCTGCCAAATATAAAAAGAATCAATACAAAGGAGACGGTAACCAATT
AACTTTCGCTGTTAGCACATAGACTGGTGGCAGTCAACACACTGAGAACTCTTTAATAGT
ATTCTTAAATGGTGTTGCACAAATTCCAGGCACTAACTACACTGTTGATTCAAATGGTGC
AAACGTGGTGTTCAGTGGTGGAGACGCACCTCTTTCAACAGATATTGTGCATATTCTCGA
ATTACCTATCTAAATAACAAGGAGGGTCGCAACAGAACATGGCGATTACAAGAATTAGTG
GAAATCAAATTTCCGACAGCACAAGTGCAATCATCACTACATTAAGTTTCTTAAGCACTA
ACTCAGTGTTTAGATTACCTGCAGGTTCCACAGCAAACAGACCTACAGGTGTATCTGTTG
GAACTTTACGTTTTAATACTTCATAAGATTCTGCAGAAATTTATAAGGATGATGATCGAA
CTGGATCCGCAGGGTGGTTACCTGTTGCAGGTGGTGGTCCTTCTCTCGGAACGGACAGTA
TTTTCAGAACTAACCCTACAACTATTCCAGAAGTTATTACAGTCGGACCTTCAGCAGGAA
ATGAGTTTGCAAACGGAATGAGTGCAGGACCAATAACAATAGGAAATGGTTCTACAGTGA
CAGTAGAGTCAGGGGGGTCATGGAGTGTAGTATAAATGAAACTCAACGTTGGGGAAATAA
GAGGATTACTAGCAAACAGTGCTAATATAACTGTTCCTACAGAGACTTCAATTGGTTTTG
AAACTGCAGCTCAATTAACGCATACTACTTTAGGAACCAGTCACATGGTAGTTACTTATG
GCACTACTACAGAGTGGGATAATCAAGTAAGAGTTCATCAAAAAGATTTTTTGAATGGAC
AAATGCGATGGAATACAACAGACCAACAACTCCAATTTTATTACAATGGTTGGATGAAAA
TGGTGTCTGGTATTGCTGTAGGAGATATCGGAAGTCAAACAAACCCTGCAGTAGATGGAA
ATGCAATATATAGAGCAGGTAAATCAAGTGGTATATATTGGATTCAACCAGAAGGGCAGT
CATCATATCAAATGTATGTTAATAATGATGATAATGGAGGTGGTTGGGTATTATGTGTTC
ATGCAAGGACATCAACCTGTCAAGATCATATGACTACAGGATCAGTCAGAATTAGCGGTA
CTACAGGTCCTAGAACAAATAATACATCTACAACTAAGATGGCGGACAGTTGGATTCAAG
CATTACGCAATAGTTCCAGTTATACTGGCACTACTGGATATTGGATGCACGCATTAGATT
TCGGTCCTAAGAACGATTTTATTTCAAGTGCAGCAACTGTAAATTTGAATAATTGTGATT
CTGAAGAGAATCCTAGAGCTAGAATCTCTACTACATATCAAGGGGGTCTCTCTGACAGAG
GTCCTAATACAGGAACTAAAGGTTAAGGAGATCACCATACTTCTGGTGGAACATATTTTG
CATACGGTAGACACCCTGAGTCAGGTAACAACTGTGGATTCAGAGAAGATACTAACGGTG
CATCAAACGGATACTTATGGGTAAAGTAAAATGAGTAAAGTAACTCTTGTAGCATTAGGT
GGTATTTTAAACACTATTGGACAGGTAACTGTACCTGCAGGAAATACTTTGACAGTTGAA
GGAGACATATATGATCATCAAAATACTGGTGCATTGAAAGTGCCTACAGGTGCGACTGCA
CAGAGACCAAATAGTCCAGCTGCAGGTGTAGTGAGATTTAATACTGGTGAAAATTACTTA
GAGGTTTAGACTGGTAGTGCACGGAATAATGTCATTGGTCCTGATGCTGCTGCAACTTCT
AATTTAGGAACTGAATCAAACCCTGCTGTTAGTGGTATGGCACTCAAGGCAGAGGTATTA
CCTTCTGGTCTTTATTGGATTAAACCTAATGGAAGTGTAACTAATTATAAAATGTATGTT
GATAATGATCGTAATGGTGGTAGTTGGGTTTTAGTTGCACATGTTAGGATATCAACCTGT
CAAGATCACATGACTAATAGTGCTGTTCGTATTAGTAATAGTATAGGTCCTAGATTTGGT
AACACAAGAACAACTAAAGTTGAAGACTCATGGATGAACGCCATGAGAACTGCATCAACA
TACTCTGGTAGCACTCGATGGTTGTTGGAAGCACATGATTTTGGTACCCCTGGTAAGAAT
ATGATTTTTGATAGTGCTACTACTGCAGATTTACCATCAAGTGCTAGTAATCAGAACGCA
AGATAACGAGTCTCTACTACCTATGAAGGTAGTATCTCTGACAGAGGACCTAACACAGGA
ACTAGAGGTTTAGGTGATCATCATACTTCTGGGGGTACCTATATTGCTTATGGTAGACAC
CCAGAACAAGGTAACAACTGCGGGTTTAGGTCTGACAATTCGGGTGCATCACACGGATAC
TTATGGTTAAAATATAATGAGCATTTTAAACGTCGGCGAATTAAATGGATCTACGGAGAA
CAACCGACAGATTATAATGGAGACCAGTAACAATCTTGTTATCACTGGAACTCTTACTCA
AAATCGTTTAAGTAGATTTACATTTCCTATTGGAACGACTGCAGAAAGAACAAGTAGTCC
AGCGGCCGGTCATGTGAGGTATAGCACAACTTTAGGGTATGCTGAGGTTTATAATGGTAG
TGCATGGTATAGAGCAAGTTCTGGTATTCTCGGAACTGTAGGAACTGAGGGAAGTCCCGC
AACAAATTCTCCACAACTCGCAGGACTACCTTCTGGTGTCTATTGGTTCCAACCAAGTAG
TCAAACAAAATATCAAATGTATGTTGATAACAGTAGACTTGGTGGAGGTTGGGTCTTGAT
GGCAAGTGTTAGAACATCTACATGTCAAGAGCATATGGATCAAGGTGCTGTTCGTATTAG

TGGTACGACTGGTCCTAGACTAGATGATACATCAACATCTAAGATGGCAGACGCATGGAT
CAATGGTTTTGTATCTCGTTCTAACTATACTGGTTCTACTAGATATTGGATGGAATCAAC
TGGATTCAATAAAAATGTGTTCAATGATTCTAATGCTACTGTAGATTTACTCAGTTCTGC
AAGTAATCAGAACGCAAGAACAAGAATATCTAACTCCTATGAAGGAGGTTTAGATGATAG
AGGTCCTAATACAGTAACTAGAGGTTTTGGAGATCACCATACCTCTGCCGTACCGTACTT
CGCTTATGTTAGACACCCTGAATCAGGTAACAACTGCGGAGTTAGAGCAGACTCTCTATG
TGCACCTAATGGTTACCTATGGGTAAGATGATAAATAGAAACATACAGAATTTTTCAGAC
ATGTCGGATATTAAAGTAGATAAAGTTAAAGGTAGACAGGCACCCGCAAGTCGTCCTGAC
GACACCTTTGATTCGTCTGGAAACATATCTTTTGCAGGTAATATTTCATCTATTGGTGAT
GTCCCTGCTGATGATGTTACAGTCAACTTCTGACTTCTTATTCCCAGATATGCAACGAGT
GCTTTACCTAGTAGTGCAACTACAGGAAGTATTGCATGGGATACAGATGATGAAGTAATT
AAAGCTTGGGACGGAACGGAATGGAGAAATGTTGGAAAGGGAGCAGGAATATCAGGTAGT
GGTGGGTCAGTTTCAACTGCAGGTGGATATACTATCCATACATTTCCAAGTGGTGGAGCA
TTAACTATTGATGGAGAAGGAACTGTTGATGTACTTTTAGTTGGTCGTGGTGCAGGTGGT
GGAACTCGTAATGCTGGTCCTAACTCAGGAGGAACTGACGGAGGTTCTGGTGGAGGAGCA
GGTGGTTGGGTTCAAGTAGCAGGTATCCCTCTCACATCTGGAAGTTATCCTGTTAGTGTT
GGTGGCGGTGGTAATGGTTATCAGTCTGGACAAAATCCTGGTCAAAATGGTTCGCCCTCT
ACATTCAATGGTCTTGTAGCATACGGTGGAGGATACGGTGCCTCAGGTCCTGGTAATAGG
CCAAGTGGTACTGGTGGATCTGGCGGAGGAGCAGGAGGAGGTGGAGGTTCACCTGGTCAA
GGTGGTTCTGCGCTACAACCTCGTGCACCTGGTCAGTCTGGTTCTAATGGGCATGGAAAT
CCTGGTGGTCCTAATCCTACCCAAGCATGTTACTCTGGTACTGCTGGAGGAGGAGCAGGA
GGATCTGGTTCGACTGGTGGAAATGGTCGTCAGGCACCTGTTGGAGATGGAAGATCAAGT
GTATAGTCTGGAAGTAAGACAACCTACGCAGGTGGCGGAGGAGGTGGCGGTAGTCATCCA
GGTTCCTGTGGTGGAGGTAACGGAGGTTCTGGTGGCGGTGGTCACGGTGGTGTTGCTCCT
AACCGTCAGAATAATGGTTATGGAGGTGCAGGTTCACCTGGTCAAGGCGGCGGTCGCGGA
GGAGGTGCAGGAAATCCTTGGCCAAATGGGTCAGGTGGTAATGGTGGTAGTGGTGTTGTT
ATTGTTAGATACCAGACACCCTAATTCTGCAAAGTATATCGCCTCAATCTTAGATCGCTT
CATAGTTTCAATAGCATCAACAGGAGTTTCAACAATACATTCACCCGCAAGGTTAAAAGA
TGTATTGAACAAGATAGGAACTCCTGTTTTTTCATGGAAACATGTAATTAGTTGATAGTA
ATGATAATTTTGATTGTCAGTCACCGTCTGAACTCTACAAGTGTAGTCAACATGAGTGAC
ACCAGGTATAGTATCTGATGTAACATTTACCGCATACATCATATAAGGACTCTCATCTAA
TCCTCTCATATCAAAGTAATCATTAGCACGTTCTTTCATAACTGTTGCAGCGAAAGGTCT
AAACCCTTCACGTCTCTTGACTCGATTGACTATTTCTTTTGCTGCACCGTTACGAGGATC
AAACAAGATGGATCAATTTCCTAATGCTCTAGGTCCTGCTTCGGATCTACCTTGATAGAT
TGCTATTATTTTTACGTCAGCGATCATATCTGCTACTTCAAATGGTGTTGTAGGTCTAGT
GCTTTCCATATACAACCGAGTAACGTTATCGTGGACAGGTCCGAGATATAAACTATTCAA
TGGTCGTTTTGTTTCATCACCAGTAAGTTCATGGTGAATAAGTTTTGCTGCTCCAATAGA
CGTACCTGCATCACTTGATACAGGTTCAATGTATAAGTTTACATCACTAGGTAACACACT
CAAATAGTAAGAGTTTGCAACGCAATTCAGAAAAAATCCCCCAGATAAACAAACGTTCTT
ACACCCAGTTTGGTCAATTAGATTGAGAATATCTTGTGCAACGTGTTCTTGTGTTTGTTT
TTGTAAGGTGAAAGCAAAATCCGCTTTTGAATTAAAATCACCAAGCAAAAAATTTAATCC
AGTATCATGTAAATCTTTACCGATATAAAATAATTGTTTGTCAATTAATCCATTATCATA
TATTGATAAGGGTAGTTCCTCTCCATAAGATGCTATCCCCATAACTTTACCTGCATCTAG
TTCATGAAACCCAAATCGCATTGATGTCTATTGAAATGCCATACCCTCACCAAGGTTGTT
AGAGATATGAGCCACACCAAATTTATGATTAGATTCAAAGGGGACAGCAACGTGTCTATC
TCCAACGGTAAAAACCGAAGGATCTGTTGCCCAAAAGGTCGTAGATAATTCTCTACCATA
GGTGCCTGATTGAAACATATCTCCTACTAGAGGTACGTCGGATCCCATCCCGTCTTTTAC
AATACAGATTGCTTCATCAAATCCTGAGTTGTAAAAAGCGTGTGCTGCATGAAGTTTATG
ATCTGATAGTGATAGGTCATGAACCTCAGTGTCATACTTATTTTCTTTCGTCTTTACATA
TAACCTATCTGGGTCATCATCAATAAAACAGTCAGCAGATGTCAATTTGCCTACACCTGC
AATACAAATATTACCAAGGTTTAAGGTGTCAAGGTCAGTCAAACATTGAAATGGAAAGGC
ATCAGACGTTCTATTAGATAATCTTTCATTCTCAAGGTGGTAGACAATTCCGCCATTTTG
TAGTAAAGTAGTATAGGAATTATTAACTCTACTGATACCTAAATTTCTCATAGGATTTCT
TGATTCCTGATACATATATTATAACATGATTTTTAAATTATGACATTTAAAACAATGTGG
TATGAGACCGATATTCCATGGGAATTTGTAAGTCTCATAGAGAGAGAATGCACACCGTAT
GATGATATAGTTAAGGCAGCGAGTGTAAGAGAAGGAGAGATATTTGCTACAAGAGATAGT
GAAACATCTTGGATTCCTGCAGATAATTGGGTCGGAGGATTCTGTATGTCTTCTGTATTG

AAGTCAAACAGAGATAACTTTATGTATGATATAGAGGGTATTGATGGCAATGAAATTCAA
TATACAGTATATGAGCAAGGTCAGTTTTATAATTGGCATCAAGATGCAGATCATACTGCT
GCAGACGAAAATGGTAAGTTGAGAAAACTGTCATTTAATTTACAACTCTCGTCTCCTGAT
GACTATCAAGGTGGTAATATTGAAATGAAAAACACTGATGATGATGTATATCTAGTGCCT
AGACGTAGGGGTACATTTATTGTGTTTGATAGTATAACTGCTCATAGAGTCACAGAGGTT
ACTGGTGGTATTCGTAAGACTCTGGTAGGGTGGGTAATTGGACCGATGTGACGATGATTC
ATTACAAAGATTGGAGTCTTATTCAACTCAAGCATTTGGCGACTGCTCCCCCGTTAGTTG
AAGAACCCCCTCTTGAACGAGGAAATTTTGGATATGATAAAAATGGCAGAATGGAAAATA
TGTCTGAAGAAGGACAAGTTCCAAATAGTCTTGCAAGATATAATCACCCTAAGTATAAAC
AATTATACAAAGGTGTTCAATCAAAAATTGAAACTATCTTAGGGGAAACGTTATATCCAA
CATATTACTTTGATAGATTTTATTTCAAGGGTCTAGAATTAAAGAAAGATCATGATAGAC
CTGCATGTGAAATCAGTGTACCAACGAATATCACCACTAACGCAACTACACCATGGCCAA
TATATTTTGAACTTCCTAACGGAGATGTTAAGGAGCTTTACACAAATCATGGTGATGCTG
TATTATATAAGGGTATGGAGTTAGAACATTGGCGAAAATGATTAGAAGGTACGCCAAAAG
TTTACTATCACCAAATTTTCATGCACTTTGTAAGGGCAGACGGATATCACGTCGAATATG
CTTATGACACCAAGTGCTAAATAACTAAAGCACAACCTAACTGATTAAGATGGATGGCAC
ATTACGCTAATATAAATGATGATAATGTTGTCGAGAGAGTCGAAAAACTTGACGACTTTT
ATGAATGGACGGACACAGGTGAACTCGATGAACAACGTGCGATCACCACTATGAGTAAGT
TTTTTGGAGCAACAACTGATTGGGTGACGACCTCATCCAATGCCAAGATTCGTGGTATGT
TTGCAGGTGTTGGAGATATATCTCGTCCCGATTTACATAAGTTTGTATCTGCAAAACCGG
CAGGTATGGACTCATGGGTTTTAAATGAGGAAACCCTACAGTGGGAACCCTCAATACCAA
TGTCCCCTGCTAACGAAGATCGTACTTGGGAGTGGAATGAAACAACTAAAGCATGGGACG
AAGAAAAACATTGAAAAAACTTTATGAATTGGTCTCTTTTGATGTAGCAATTAATTTATT
GAGACCTCGTGATAAATGGACTCTAGATCATGGCAAGTTTAATTGGAAAGACCCTAGACC
TTGCCCAACTATGGAAGAAGTTGAAGATTGTCTAAACAAAATAAAAGAAGTTGAAGAGAG
TCTTCCTTATATTCTTTAAACAGAACAACAAGAAGCATAAGCAGATACATGTCAAGATCC
CGAATTTATCGCTTGCAATTATAAGCACGATCTTGAAGAGAGAGGTGTACATGTAGGAGG
AGGAAATGAGCAAGGAGAAGGGATAGGTGGTAAAGGTGGTGTAGCTCCAGACTAATTTAT
GAGTGATTATTTTTATCATTACAGATTGAATGACATACAAAAATTCAAATCTGATGTTGA
CAATGCAGTTAATGATTGTGGGTTAGATTGCCTCAACACTCATATGTTTGATATATCTAC
CAGTTTACAAGGTAGGCAGTATCATTTGATGGAAGAGAGAACACTTCCATATCTTAAAGG
CATCATAGATCAAGTTAAAGAAATGATAGTAGATGCTGTAGATGCAAAAAATCTCAAGTT
AGCATCTGCATGGACAGTATATGGGGAAGAAGGAACATATCATACAATGCATCAACACAA
CACTAACAATGATAATTGTAGTGTGATATACTTAGATGTAGAGGAAGAAATTTTCCCTGC
AAAAAATGGATCTTTTTATTATTTCTTGGACGGAGTAAAACTAGTTCCGCCAGGTGTAGG
AGACGTTTTGATATTTCCTGCAACTCTTTGGCATGGTGCATACCCTCAGAAAACTAAAAT
GTGACATGTATTAAATCTTGATTTTACACATGAAACAAATTTTTAGTAATCAATTCCATT
ATCAATATAGAATGCCTGGCTTTGATATTCTTAAAGAAAGGTTAGATACAGTTGAAAAAT
TTGATGATTGAGATTTCACATGGGGAGACCTATGTAAGATTGAACGTGACTCATACAATG
TAAATGATTTCTTTGATATTCTAGTCAAACCTCTTGGTTTGATGTCTGGTGATCTTAATG
TTAAATTCAATGCTAAGATTTTAAATCCTTGGTTGAACAGATATAATCGTAGTGGATTTC
AAGAGATACATTATCACGATGATTGAGACATTGCAGGTGTGGTATTTTTAAATGATGGTG
AAGATTTCTCAAAATTTTATTTCTGGGACGCTCACCATACATCATTTACTAAACCTTGGA
TTAAGATACTCACTCAGATGAAGTTGTCTAACATATATTATCCAGAGGTGAAAGCGGGTG
ATGTTTTGTTATTTCCCTCACACATGTTACATGGTGTTTCCCCTCATAATTCTGATACAA
TTAGAGAGACATTTTCTTTTAATGTCGTAGTGACAAATGTTGAATGAAGTGTATCTAACT
GATAATTTTTTGAGTGATGACATATGTAATTGGTTCATGTCATTTCATCAAACTATGTTT
CCTTTATATGGATAAGAGTTTGAGAATAGAAGAATTATTAATTTAACAGAACTAAATCAT
GTTCTCTTTAATAATAATGGACCTTATGAGGTAAGAGATTATCTAAAAATAGTCCAAGCA
AACTTGACTACAGAGGTTAGGAAGTACGACCCTAAAGCATTTCCTAATTATATTCATTGC
ACAGTGTGTACAGCACCACACTATCAACCAATACACACAGATTTTGATGAACACGTTTGG
ACATCTATTCTATACTTGAATGATAACTTCACAGGTGGTAATACTATTATTGAAGGTGAA
AAGCTACCGCCAAAAAAAGGAACTTGTAGCACCTTTAAAGGAGAGTTAAAACATGGAGTT
GAAGAAGTGACAAAGGGTAATCGTTATACTATTTCGATATGGTATAAAAATCACGTAGGA
GTAAACAAATACAGATGATAATAGATTTAATCCCAACATCAATTTACATGGATAGTTTTG
AATTATCCCCCGAAGATCATGCAAATTTATCTCAAGTAAAACTTACTAGAAACAGAGATC
AATGTGCATGGGTCAGTTCACCTATACATTTACTTGATTGGTATCAAAGTATTGAAGTTA

AAGTTAGAAAGCACGTTGAACAGTAAGTTTAGAATGACATTGGTTTGAGTAGAGGATACA
ATTTGCAAGGTCATGGTGCATGGTTGAACAGACATGATGAAGGAGATTACACAGAGATAC
ATCATCATTCTAATTCACTTCTAAGTGGTGTATATTATTTGTCGGTCAATGACCAACAAG
GACGTATACAATTCTATGATGATAAAGATGGATTGTTTCGTAGATATTTCACAGTATTAA
ATTACACTGAACCTAACAATAGAAATTCTCATAGAGCAAACGTAGAATGTAAGAATGGAA
CGAATGTGTCATTTCCCTCACGTCTGAAGCATACCGTTGCACCCAATCTATTCGAAGAAC
CTAGATTCTCACTAGCATTTGACTACACGTGAGAAGGTGTATGTGATGCAATGGTGATCA
AGGTGAACTATGTGCCAGTTAAATGAACTGTCCACAGGCTCGCCATTTCAAAAAAATTGT
GTTATACTAATAGGGTAACGCAAACAGAACAATGCCCCACTTCACTCTCATCTGCACAGA
CGAAGATGAGACCCTCACAAAAAAAGAGCTTGATGCCGACATTCTTGATGATGTTGTCGA
TAAAACTGCCGACTTCCTACATGGAGTAGGTTATTGTTTCCAGGATCTTTCAACGCAAGT
GTATCCAACTCAGGAAACCTGATACATACTACAAGTAGTTTACAATTTTACACACTCAAA
ATGGGTGAGACATTTCGGCGAGGTGGTAGCGATCAAGGTTACTACTCTTATGGTAAAGCA
ATCCGGGACAAGCGACAAACAAAAATGGTAATTAAGAAAATCGAACATGAAAACAAAAGG
GAATTAAAAAATGAAAGATCAAAACCCAATACGTCTGAATGAAAGTTCTGATGTAAAATA
TCAGAGAGCATTAGACCTTTTTACCGAGTCAGTTATGAAACCAGACCCAGATTCGCGTGG
GTGTGCCCATAATCAGGATTGTTTCACTGAACTGATGGAGATAGGAGAACACGCTTTGAA
ATATCTTTCAACGTTGAAATCAACACAAAACTTTGAAAACCCAGATGAATCCGATACAAT
AGAACAAGAGGCCTTGGAACACACTTCCCCTTTATCTAAATGGAGATAAATGTATCTTAA
ATCTGCTATATTAACTAAAATGCAAAAAGAACTATTGAAAGATGCTCTGTTGGCATACGT
TTCAAATTTAAAGAAACGATACTATGGGGATAAAATTATTGATGAATCTTTTTATCTAAG
TAAGATGAAAGAAATTCAGAATATTGTTGATGAACTACATGTACAGGAGTTATACACATA
ATGTTTCGTAAAGAAATTAAACTATTGAAGCACGCTATCATGAAAGGTCAGGAAGACCCA
TTTCTATACTGCGAAGAGGAGTTTCATAAACTCAAGAAGAAACTTAGGCAATTCAGGGAG
TGGAAGAAATCGACTATTATAGCACAGAAGGGTGGATTTGGATACGAAGTGTGACAGCTA
AGTTTGTGTCACAACCCTATTGACATTACTTGTAATATCGTTTATATTAAGAATGTCGAA
ACAAACAAACGTAAAACTTTCAAGGTAACGTATACCCAGAGGAATACGTTTTTAAGACGA
ACTTAAGTAGTTGAGTTTTGTTTCGACCCACCTATTATTTCTTGACATGGAAGTCCAAGC
ACATGGTAACAAATTTGAAGACATTGTAACTCGTGTGAGAACTGGGCTATCAAAGAAAGA
ATATGATGCTCTAAAGAAGAATGGTTATGCCTCATCATTTGATTTACCAAAAGGTCTTAA
GGTAGAGTATGATGCTAGTATTAAAACTACTGGCAACAATACTATTTTCTGCTCTGATAT
TCTCAGAATGATGAGACATAATGATTATAGACTAATTGTTGGGTGCTACGATCAAGTAGG
AGATCAAAAAATATTTCACGCACAATATGAGTTTTCTATCCAACCAAAAGACTATCTAAC
TTTATGGGTAGACATGGATTTTCATAGAGTACAATTCTTTCTAAACTATGATAAGAGTAT
TCCAGTGGGAGCAAAAGCAAGAGACGATTCTAAATTAGTGAGAGATAATTTGCAAGAAAG
TGTATCATGCGATAAAGCATTATACACTATCAATCCAAAGGTAGATAGTAAGAAACAAAG
AAGAGTTCAATGTTCATTAAAACTTGACGAACTAATTCCAATTGGTGTAGAATATGAAAA
GAAAGATATATCTTTAATCCTAGAATCTAAAAAACGTACGTTTAATAAATGAGAGCATTT
TGGCCACCAACAAATATACCTGAGAAAGATATAGTGATGACCCCAGAGTATCTTGCTAAG
GAGATCATTAATCATTTCAATCCTACTGGTAGAATTCTTGACCCTAGTAGAGGAGAAGGT
GCATTTTATCATAATTATGATACGGACAATAAAGATTGGTGTGAACTAGTAGAGGGAAGC
GATTTTTTACAATATCAAAAGAAAGTTGATTGGATTATTACTAATCCGCCTTGGTCTATG
ATGCAACAATTTCTAGTGCATGGCATGGAAGTGGCGGACAATATTGTTCATTTAACAACG
ATCAATCATTATACTACTAAACGTAGAATAAGAGATATGAGAGAATATAACTTTGCAATT
AAAGAGATATATAATGTTCCCACACCTACAAAACCTTGGCCACAACTAGGGTTTCAACTA
GCTGCTGTTCATACACAGAGAGATTATAAAGGAAATATTAAGTTTTCTTATTCCCCTGAT
CGTTCCAGATATAATATGACTACATACTGGTGTCAATCCTACGATTGATATTTACCGCCT
AATTAGGACGTTACAGATTAACTATTATGAATAGAAACCACAGACGTAAGCAACGTGTAA
ATGTTGCTGATTTATTTGATCTTGAGAAAATTATTTCAATAGAAGACTGGAATGACGAGT
GGGAAGTGTGCTCAATACAAAGAGACCACGAAGAGAGAGCAAGAAAACCTAAGCACAGAA
ATAAGTTATCTACACTAGTATCATCACACTTATAAGTAGATGGTGCAGTATTAACAAAAG
ATTGTTACGACCCAGAGACGAAAAACACATACAAAGCAGGAACTAGATTTAAAACTAACG
GACATACTAGAGACGCACACTGGTGGTCAGATGATGCTGACGATTGGCAACCCTCACACG
TTCGTGAAAAGTATAAAGAACACGAAAATATTAATTCCATATAAAAAGAATACTTAATGC
ACGACAACCTAGATGACGCAGAGATTGCATCTGATAGGGTAGATGGTGCGTATCGTGCTG
TATTTGGAGAGAGGTATATTGTAATTAAAGATGGTACGTTGCGTAATGTTGAACCATTAC
AACACGCAGCTTTACTATGTTTTTCTAACAAGTATAACGTCAAGATGAAAACAAATACTG

TTAACATCAAGATGTGGGTTAGTGATATTGAGGATGCTGTTTTATTGGTTCGTAAAGTAT
ATCTTGATTCTGAATTTAATAGTTGAAAGCAAAAAACTCTGCCACACTATAACCGATTTA
CTTGGGCATATCTTGTTTCTTATATGAAGTCTAAGGAAGTTCCAGAAGCACTAGAAAAAC
TAAAGGAATTGATATACAGAGCATCTAATTATGAAGTGGTAGTTGACCCCTCAAATGGAC
GTGAAGATAGATTTAGTCCTCTAAATGTATTATTGAGAGAGTGGTAACAACTCAAAGCAG
GTACGTCAAGGTATGTTCAGACCTCAGCAATGAACGGAAGTTATCCCTCTAACAATATGA
AATCATTTACACTATTGTGTATTGATAAATTTATTGAAGGTAAGTATCTTAGTAAAGATG
GTAGCATGAGAGGTGTTGACTGGAAAGCATACTTAGAAGAGTGGGAACTTGCTTGGAAAG
TAGCACATGGTATGACAGCACCTCACGTTGAGTATGGGACATTACCACTAGAGATGTTCC
CCGAAGACGAAGACTATACCAGTTAGGATAGTGTCACAACCCCTCGACAAAGGGGTTTTT
TTATGCTATACTGATTGTATCGAAAGGTTATTATCCAACTACGTCCACACCAAACAAGAG
CATTCAACTCTATGCAAGAGAGTAACAAGGGTCAAATCATTGTTCCTACTGGTGGTGGTT
AAACATACATCATGATTGCTGATACTCTTAAGAGATTCAAGTTACCAGTTGCACAGACTA
CAGTTGTAGTTGCACCTCGTATCTTACTTGCTAATCAATTGTGTGCCGAGTTCCTAGAGC
ATAATCTAGATGGATATTACAATCAAGGTGTTGATGTTGCTCATGTTCACTCAGGAGAGA
CTCATCACTTCAGCACAACAAATCAATTTGAATTAAATACTTGGGTCAACAACAGCAAAA
AGCACATTTTGATATTGACCACATATCATTCACTTAAAAAGGTGGTCAATGCAGTTGATG
TAGAAGTTGATACTATTTACTTTGATGAAGCACATAATGGTTGTGGTAAGCACTCCCATA
TTGCTCTATCTCTAATAGTCCAGTATGCAAAACGGAGTGACTTCTTTACAGCATCACGTC
GCATGGGTCGTGGTGTATCACTTGACACAGGTATGAACAACTCCTCAGTATATGGTGGTG
TATTGGAGAGTGTTCTCGCACAGGAACTGATCAAGTCAGGTGCTATTGTTCCCCCTAAGA
TTGTTCCCTTTGAGACTCGTAACTCAACCCCTCGTGACAAGTATAATGCTCATGAGATTG
ATGCTGATAACCTCAGAGATATTATCGATACATTTGATGATAGTCAGAACAACAAGATTC
TAGTTGCAGCACCTAGTTCAAGAGTCTTGGGGAATACGCTCGGACATACTACCATTGTTA
AGTATTTCAAGCACAATGGATATGACGTTATGCACATCACATCAAAGTTTGGTACTATCC
TCAATGGCACAAAAGTTGGTAGAGAAGAGTTTTTTGACACACTCACAAAGTGGGGTCAGG
ACGATAGTGAAAGATTTGTTATTTTCCACTATTCAATATTGTCTGAATGTATCAATGTTC
CAGGTTTGACTCATACAGTTTTGTTGAGAAACCTACCTATCACTGAAATGGCACAAACTA
CTGGTCGTGTCATTCGAGTTCATTCTGATGATCGTAAATCAGTTGCTGATGGTCTCATTC
CTGCAGGTGCATTTCACTTATACAAAAAACAATTTGGACAAGTAAGTGTTCCTACTGGAC
AAAAACGTGGAGACGCAATTGGTAAAAGATTGCAGAATGTAGTCAACCAAATTTTTGTTG
ATGGTGTTCCCCCTATCGCTTATTGCCAATGTAATTAAGACCTCAAAAAGATACAATTCT
ATATGGGGATTGTAGGAATACAATTCCCACTATACATGAGAGAGTCCAGATGTGTGTAAC
TTCCCACCCTTATTATGGACTAAGGGATTATGGTGGATAGTCATCACAAATAGGACAAGA
ACAAACGCCCGAAGAATATATTGAAGAACTTGTAAATGTATTCCGAGAAGTTAAGAATGT
TTTAGCAGATCATGGCACTCTATGGTTCAACATAGGGGATAGTTGTTATAACCATAGACC
TGGTAAAGGTCAATCATATCCTAAACAAACAGTATCTAAAACTAAACAAGATTTAGCTGA
TAAATGTAAGAAACGAGGTAACAAATTAGAAGGATTAAAAGAGAAAAACCTTATTGGTAT
TCCTTGGTTGTTAGCGTTTGCCTTCCGCAAAGATGGGTGTTATCTTAGGCAAGATATTAT
TTGGCATAAACCTAATCCAATGCCAGAAAGTGTAAAAGATAGATGCACAAAAGCACATGA
ATATATTTTCTTATTCTCTAAGAACAAGAAATATTATTATGACAATAAAGAAATTAAAGA
ACCTGCACAACATTGGGGAACTAGAGATAGAACTAAAGGAATGTATCACAATAAAGGAAC
TAGATTAAGTCCTCATACTGGACTCAATAAGAGTTATCCTACAAAGAATAAAAGATCATT
ATGGTCAGTAACTAATAAATCATATAAGGGAATTAACTTAGCAGTATTTCCCCCTGACTT
AATTGAACTTTGTATCCTAGCAGCGAGTAAGAAAGGAGAATTAATTCTTGACCCATTTAT
GGGTAGTGGCACAACAGCAATGGTATCTAAACAATTAGACAGGCATTATATTGGTTGTGA
ATTGCATGAAGAATATAAATCACTGATTGATTCAAGATTACCAAACACAGCACTTACTGA
CTTGTTGTAGTGTGACAGTTAACGTACTGCACACTATCGGTTGCATATGACCCTATAGTC
ACTTATAGTATATTCATACAACAGATTGTTATTATGTCAACAAATGCAAGAAGCGCACTC
AAACTTTCCGATAAAACTTTTGTTTCAGTTAATCATCATTGGGACGGTTATCTTGAGTGG
TTGGGTGTAGTCCTAACTGAACAGTATAACAAACTAGAGTCTTTTATTGAACTTTTCTCA
GGTGGAGACATAAGTTCATGTTGGTCAGATAATGAGTATGATTTCGAGAAAAAGGAATTG
GTAAAACGTTACCCAAAACCTACTTACTACTCAGAGAGAGGAGAGACAGCACCCCCTAGA
ATCGCACACTCAATTACAGAGTTCCTAGATCATTGTGACCAGTGTGGTGGAGAATTTGGT
TATGTCTATGATAAGGGAGAGTGGTTCGCTTATGAGACTACAGACCGCACTTTAGTTGAC
ATTCCAGAGGAGATAGCAGAATGAAATTTATAGTAAAAGACATTGAACTCTAGTTAAGTG
ATGTTCGAGAGGGAGACCCCGATTTACAATTCACAGATCGAGAAGAGAATGTAATGCACC

AAAGATGTCTAGGTTGATGGACAGCAAGAAATGAAAAACATTAAAAAGAAAGGATTTTTG
ATTTCATAGGTTATCATGTAGAGTTTGTTGACTACGAGGTAAAAGCATGAACACCTACCC
ACTTCCGAAGGTGCTACAAAGACATGGTATTGAAGTTATGCTGATGAATGAACATCAAAG
AAGAATCTGGATAGAGGACGTTAAAAAGATACACCCAGATTTCCCGTTTGACAGCATGGT
GTATGACACTCACACTAGTGGCACATAGTTCCCCCATTCCCCTCTATCATACCCTATACT
AAAAATATTTCCAGAAAGAAATGCAAAACATTCCATTCTACCACTTCCCCAAATCCCCTA
TTCTCATCCTAGGGTTATTTGGAATTGTAGTCGCACTCGTCACACTTGACGTTGTTAATG
TAAAGTATTTCAATAGTCCATTCAACGAGGATAACAACTAATGCTAGATTTTTTATCAGA
TGTCTTAGAGGATTTTTGCTCTAAGTATAATCTACCACTCATGAGTGCAGATGACCTATT
ATATGCACCTGCATGGTCAGAAATCAATCTTTCACAACCTCAAAAAATTTGGTTAACCAA
TTATATGGAAGTCTGGGATATTATCCAAGAAAATTCTTAAGGAGAACTTTATCATGACAA
CTCAAGAACGTGACAATGCAATTACTCAAATGTCTAAGCACTTGTTTAGCGTGATGCAGA
CCAGAGTCAAAGAGGACAGGCATCTAGATGCCCTATCAATTTGCGAGAAGTGGGTAGTAA
ATGGCAAAGACCCTCAGGACGAGGATACAGAGTTCATTTTTATTCCTAACACAACTTTTG
GCGATTGGACATGGGAGGTGTGACAGTACATTTAGCGTCACATACTTTCCCTATTCATAT
CCATTACTCACTATAATAAAGATATGAACAAAACAAAACCAAAAGATAAAACCATTACCA
AACTTATGGAAATGGTTATTGACACACTTAAGTATTGTGATGATCTTGATGACCCTGCAT
TTGCAATGTATTATGTCATGAGAGACGCAGTTGAAATAGAAGTTCGCTATCCTATGGAGT
TTTAACTAATGAATCAAAACGACAAGAACGAACTTGAAAACTCTGGTATGACTCAAAATG
AGTTGTATGAACTAAGATACCATTATGTTGACAGATATGTTGAAAATATGTCAACTAAGG
ATTTAGTCCAGTATGTCTTTGCTGATTTGCTCAATTATGTTCATAATCAACCAGATGCAG
AATTTGTTGATGAAGCACAAAACTATTGGGAAGATCTCTATGATGATGTTGTTACTGACA
GCAAAGAGTATGCAAACAGCGATTTTAATAAGCCCTTAGAGGAGAGCAGAAATAATGACT
AGATCAGAACTAGAAAACAAAATTATCAATTTTGTTTTAGAGAATAAGCTTGATGCTTCA
TTTGTGCAACAAAAAGAGGGATTAACTTATGTTCGCTTCGCAGTAGAAGAGGATTTTGAA
GAATGATAAAAATTGAACTAACAAAAGGACAATTTCAAGAAATCTATGATGCAGTTAGGG
ATTTACCTGACGAGATCAGAGAAGACTTAACACTTGATGGGGAAGAACCTGATACAGCAC
ATTTAGATTTGTTTAGTGCAGTTGATGAACTCGCAAGAATTGATAAGAGACTCAACAAAC
AATTTAAGCAAGTAAAACTCAATTCGCCAGATTTGGAGTGACAGTTAATTATGTATCACA
CACTACCACGCATAGGTTACAAAATACCCTATAATACGAATAAGCGAACAAAGAACAAAA
AATGATTCCAGAAACATTGCCAGTTCACTTGAGTGGAGATCAACTCTACAATATGGTAAA
ACTCTAGGATCAACTCAGTGACATGAGTTTTGAACTCATACCCGAACAGATCGAAGTGTT
CGAGAATATTCAAGATGCAGAAGTCAACGGAGGATTTTTCTAATGAAATCATTTCCATCA
AAAGTTACGATCGTTTTTGATATTAATAATCCTGAAGCGAACTCTAAAGAAGAGTATATC
CAAAAGTTAAAAGATCAGTATGAAGAATGTTATGGAATTTACCTACACGATCATGAGATC
ACAATGATCGAAGAGGAAGAACCAATTAATCTTTATCAGGGAGCATAATGGAAAGTTACT
TAAATCTATTTGAGCATATCCCAGAGGACGAGCATTCACATATTTCTAACAAGATATGGG
AAGCACTTGATCGGGCAGGGATTGAACTAAGACAAGATGCAGAATTATCAATCCGCATTT
ATGATGACAACTATGAAGGAGAATATGATGGGGAAGAGTATGACATTTAAACAAGTGTCA
CGTACGTTTACCATTCCACTCTATTATCCTTTATAATAATTGTAATTAAACATTTTTATT
ATGATACAGTATTTTATTGAAATCCCAAATACACCGATCAGAGAACCAGTGACCGGTTTT
TGTTATGACATACTATATGATATGGCACAACAGTATGTTCATGCAGAATAAGTATGGTCT
GCTAATTATGGCACTAGAATGATACAGGGCATCTATACAGATAAAGAGTAATCAATTCAT
TCAAAGGAGATTTTTAAAATGGGTGCTACACGTGACGTGTGTGCTAATTTTGATGAGACT
TATAGGCAAGAAATGGAGGTCCCTAAAGATTCCGATCATTGCATTCAAGATTATCAACCC
GATCTTAATTACTACTGGGATAGTCCAATAGAAGAGGATTATAATTGGAGAGACGAATTT
CCTCATGCTGATTGTATGTGAGAAATCTGTTTTGATATAGCAAACCCAGAAAAGAAAATC
AAATGGAATTGTGCCAGATGTTAAAGTGTCACACAGTTTCCCTATTCCTATACATTTACC
TTTATAATATTAGTATATTCAACAAACATCATGCAGAACTTTAAAGAATTTCAAGACTAT
TGTGAATCATTCTACGACCCATCACACCCAGATGTTTTATATCCTATTGATGGATTAACT
CGCGAAGAGTTAGCACTTGCTACACTCACATACCTAGACTTATGTGCATCATCTGATGGT
CAAGTAAACTGGGGAGACGGAGTTAGTTTAGATCGTGAGAGAGTCAGAGATTTTGTTTAC
GCAAGGAGGTCAGTAGCATGAGGAAATTTATAATTCTAGAGAGATTTGTTGGTTATAATG
AAATAACGATCGAAGCGAATACCGAAGAGGAAGCGATCGAACTTTATAATCGTGGACATT
TTCCCGATAGTGCGGTAGATCAAGATGATACGTTTTACGATTTTGAATTTAGAGATATAT
GTCCAGAATGATGGAAGAATTAACAGAAAAACAAAAGTTGCTCATTCACTTGAATGAAGT
TAAAAACATATTGAACAATTGCACATCACTTGATGATGAACTAATTGGATTATACCAGAA

TTTTAACACGATCACAGACGTGGACATTTTTAAACCATTAATGGACAGTTCAAATACTGT
CACATAGTTTGACCATTCACACTATTATGGACTATAATAGTAGTATAAACAAAGAGGATT
TATGAACAACACTTATCTATTCACTGATGCACAGATGAGAGTCATCAATGTAATGCTCAC
ATATGTTGACGAGTTGGGAATCAATGCAAAATACAAAACCGATTTTGAAAGTCTTTATGA
CTATATTCCAACAGGCTGAGAACTATTCTTAGGAGAGTAAAATGTCAGTATCACATCACG
AGAGCATTCTCGAAACTTGCTATGATGAAGCATGAATAGACTACGCAAAAGAGCATGGTC
TAACATACGATCAACTTACAACACTTGAGCAAAATTCTGACTACGGTATTCTACCAGGGA
TCGAAGCAGAAGCAAGAAACAGATTTGAGGACTTGTGCCAGTAGGAATACTTTCACCTAG
TTCCCCCATTCAACCCACCTATCCTTTATAATAAGAGTAACAACAAACAAATCTATGAGG
ATGACTAATAAGCAAGCAGTTCAGCAATTTAGATGGGATTGGTCAGACTTCCTAAAATCT
AACCCCAGTGGGAGGGGAGACAAAATTGCAAAGAGGTATGCTTTTAATGATTTTGTTGAT
GCTCTCAATAAAGATGGTCTAGTCTCACATTATCAAGCATATAACTGGTCAAACCCATTC
TAAGGACATTTTTAAAAATGACAACTATTGTACTTTTAGAAGAGTCACTCAAGCAGTTAA
AGATTATTCAACTTGAGAATTTGAAGAGAGAACCGAACCACCCCAGAAACAAATTTGACT
AAACCGTTGTAGTTCCAGATACTGATCTAGGTTATCATGAACACTATACAATGGATTTAG
AAGTTGCAAAGAAAAGTGAAATTGAGTGGGCAAGAGACTACGGTCGGGGATCTGTAGAAG
ATCGTAACCTAAAAACAGTATTCGCAGTGCGGTAGTGTGACAGTTAGATTCGTGGCACAT
GGTAACCCCGTTGTCACTAGTTTACCTTTATAGTAATCGTATAAGAAACAAACTTCTAAA
AAACATGACAATCTCAGAATACAAAAAACTTCAAAAAAACGACACTCTAATGATCGGTCT
TGATGTATCTGAAGAGAAAACAAACAGAGTAATCAACAGAATCCTCGAAGTTGACAATTT
TCAAAATGTTGCTTGCTATTGTGCAGACTTCTCAGAATTTGTGGAAGCCTTGGCAGAGTG
GGGTGTTGATGGGTGTTCTAAGGTCGATTTTGATGACCCTGATCTAGACATTGCTAAACT
AGACAGATTTATCAAATCTGAAAACGGAAACATTAGGGGAGAGTAATGGAACTAACCCCT
AAACAACTAGAGTATGTCCTCGAATGTCTCAATTTTCAGTACGCAGAGAATGAGCATATT
AAAAAGGACATTATCGAACTTAATGCTAGTATATGCAGACTAGTAGCAAACCAACTACAG
GAGATTTACAGAAATGACTAGAGCAGAATACGAGTTAGTTTTTCAAGCATTTAAAAACTA
TCGTCTTTATATGACAGATGAGCAAGAAGTTTTAAGCGAGAAAATTCTAGACGATCTTTT
TTACCCCGAATTCGATAAACTAAAAGGAACCGAAGAGACCGCAGGAGATTTACCAGTTTT
AGAAATTCCCGAACATCTCGGAGAGGACACATCAGAAATTAAATCACTTAATTTTCGATA
AGTCAACGAACCTTGTGACACTTTGATTAATGGCATAGGGTTTCCCCATTTGCGGGTCGC
CCGCCCTGTAATTATAGTATAACATACAAAGAAACCTTTTTATTATGAGAAAAGTCGAAG
CAACAAAGAACTCAATGATTCGCAACGGTGTAGCATGGTCAGATGGTAATACTGCCACTA
CTTACGATCATGATGGAAATTGCCTAGTTTCCCTACACGGCAATGATATCGCAACTGTTG
GCGACTCATTCATGGCATTCTTTGACGGTGGTTATCAATCTAACACAAGTAAGTCTAGAC
TCAACGCATTGCGACAAGAATTCAGACCACACACAAAAGTGTTCGCTAAAAACTTGCAAT
GGTTTATCAAAGCACATAATACAGTAGTTCCATTTATCTCAGGGAGTCTAGTATAATGAC
AGTATTGAATGAATATGAAAAAATGTTTAATACCGTCCAGTCTCTACACTTTGGGAGAAC
ATTCTGGATAGATGACGAAAATAACCTATGTTCAGCACCAACTTATAAAGATGGTTCTCC
ACAATGGGAACAATGGGATTATGTTAGGGTGTGGACAGACTTAGAAGGGGTTAACCTCGT
TAAATGACTTGATGTTCGTAAAACTTGTCTAGAGTAAAAAAACTATGGTGCGGAGGATTT
TACCTAGTGCATCATGTTCTTTACGACTGGTCGGAATTAGGAGAAATTGATAAGCACGTT
ACCCAATTAGAAAAGGGAATCGAAGCAATCAATCAAGCAATTAAATCATGTAACCTAACA
GAGAACAAAGATTATAAAGAACCACTATATAGAGTTAGATCGGAATTGAATAAACAACTC
AATGACATTCTAGCACATTTCCCAGATTATTCAAAACAATGCAAATAGGGGATATAATCA
GATTTAAAGGAGTGCAGGGTTTTATCACTTTTAAATGTAAGGATTATATTACCTATTGCA
TTCACCAGTATAAAAAAACAGATGAAGAAAGAAAACACGCCCGAAGAGAATACACACAGG
TCAATGTTCTCATCTACCGACATGAGTGGGGTATCATTAAAGCAACAGTTGAAGTATGTA
AGGATAACGAGAAGAGAATTGACGTTAATGTTATGGGATAATTATCGGAAGTATTAGCTA
AAACAGCGAAGAGACAGAAATGCTGCAAATAAATAAATATTGAAATAAATCTAAGTGTGT
GTTTTATCTCTCGGTAAAACTCTCAGAGTAATGCACCCTTAGCACGCATCTCAACAGTTG
TCAAGTCACTCGAGGATATGCACACATTTCCAGTAAGGGCTTGACATTATTGGGAGATAT
GCCTTATAATAACACTGTAAGGGTTTAAGAAAACAACTGTATGCTTTCAACTAAGTCTAA
AATGATCTACTCACTCTATAACGAAGAGGATGAATTACTAGGACAGTTCTCTAGTATATA
CGCAATGCAGATATATGATCATGTTCTCAGAGTTGAGAGAGACGAATGGAGGGTACCGAA
GAACCAGGATATAAGCGTATTTGATTATATCAAATCAATCGGTTATACTTGGGATTGTGT
CCCTAGTTCTCAGGTAGGAGAGAGTTGACAATGTAATTACCCTATGCTATAATATGAGTA
AATCGGAGGTTATCCAATTGTAACACTGAGAACAGTTACTGATTAATTAGAAGGCAATCT

TATAAGCCCCCTTAATGTAAAAATCGGCCACTACCCTAAGCTACAAAGGTTCCCCAGAGC
ACTTGATATATTATTCATAATTTCATTTACATCAGTATTAAAAAAATTTTGGAGAACAAA
AATGCCCCCAGAGGACGATCTAAAAACAAACAAAGAATTTAGTGGTAGACAAGAGCGAAG
AGTATGGGCGATTGAACAACTAATTAGACTGGAAGGGTGTTTAGATAACAGAATGTATGA
AGGTGCAGATCATGCTGATTCTATCTACAATGTAAAGAATAAGGATTCACTATATACACT
ATGGACGGATTGGAAAAGGAATCATCCCACTACATATCCACCTAATAACAGACTATAGAT
GAAGATTTATGTCCCACAGAATTACACAAATACTAGAGGAAGATGATTATGGAGACTTAC
TACTCACTATCCCCTATGAATTGTGTCAAGAGATGGTTTGGAACGGAGGAACTGAACTAC
AGTATGAACTCAATGATGATGGAACAGGTTTTATTTTAAAGAAAGTGAATGACTGAAAAA
GATCAAGACGAAATTTCTACAGCATTGAATGCTATTAATGAATGTTTACTTGCTCTTAAT
ACACGATTAGAGGCATTAGAGAAGCATGTTTCCGAACTACCAACACCAGACAAAACATAT
TTCAAACCAGAAGGTGAAGAACACTACCTAAATATCAAAGGTAACTATGACCTGATATAT
AAACGTCTTAAGAAACTAGAACATGGGATGTAAAATCACTGCGAGAGGACAGAACCCTAT
CTTTAACTCTAATTCCCTTAACGATAATCACTGTTATGCGTACGATCCTGAGGCAACAGG
GCCGGTATCGATCACTTACAGTGAATATCCTACTGCAACAATTCGAGATTATACCCAATA
TTACATACCAGCAAGAGAACATAACTTCGTCATGTATAATGCTTCGGAAGTATGTCGTAA
CACTTGTCAATCATATTTTCAAACAGCAGGTGCATGTGGTAAGATATTTAAGGATAATAA
TTGTGTCAATAATAAAACTTGTCTATTAACCTTTGATTATACCCCTTTAGAACTTTGTTT
TGACTTTGCATACTCTGACGTATGGTTCTCATACTTATATGATACGTCTGTTAAAGCGGG
TCATGTAGGAACTCCTGCGTATTATACTGAAGATGAGATCAAAACTACGAGTGGAGGAAC
TGGTTCTGGGTATGGTCGTGATAATCAAAACACTTCATCCAATCGTTTCCCTTGCACGAG
TCAATTTACCTGCACCGCAGCGACCACAGACCTTAAGTATACCGCTCCCACAGATTATAC
AGGCGACCCCGACTGCCCTCACCCCACTCTATTCGGGTTTGGAACCACATCTAACAAGAT
CGCTTTCAAGTATGATGCGCTATCCACACAGATACCAAATGGGACGGTGGACTTTGAGGT
GGCAGAAATGGGTTCATCTACCTTTACTGATATATGGAATGAGAACCAACTACAAGGTAC
GCCATTCCTTTCCACTGCAAATAACAACTGGGTAGTTGATGGTGAAGCAGGTTTCGAGGA
TTTTTAGAAATATGACCTTAACGGAGCACAGATGACCTCTGGTTCTCCTAACTATAACGA
TAATGCAACTACAGGAGTTAGGTTAAAGTTTCGTATTGAACCTAATCTTCTTGCTAATGG
CACCATTAACGGAACTAATTGGGAACTAGCGGAGCTTATGAGCAATGGATCAGGATATTC
AGTAGGTGATACATTCGTGATATCCTATGCCAATACTCATACTAACGGAAGCACAACTAC
ATTTCAATTAAAAGTTAGGGTAAAAACTGTAGGACAAATCAATTCAATAGGTAGTTCATC
AGGTTTTGACCTACTGAGATCGAATGATACTATCAATGGACATACCATTACCCGCGTTTT
CCACACAGATCAAGAAAACTTCGAGTATCATGTCGCATACTTAGACGTAAACGGTAATAA
TTTTGCAAAAGTTAGTCAATATACATCAAATAGGGCGCACCAAATCACCACAATAGCAGG
AAAAGGCATTCCCGACCGCGCAATCTTGATTGGAAAGTATGAATTTATCGATAAATCTGT
GCAATTTGTCACCGCAGACGTAGATTTGGACGCACCAGACATTTATAACTCCATAAAACA
ACCAGATGTAACACTTACAATCACAAATGGGCGCGTAACAGGCACTACAATTGTGGATGG
AGGGACTGGTTGGAACCAATTGAAGGAAGTACCTGACCTTGTTGTCACTGCACCACTCAT
TGAGAGTGGAAAAAACGCGGAATTGAAGGGTACGTTCTCTAATGGAGTGCTCACGGCGGT
AGAAATTACAAATGCAGGTAGCGGATATGACGCAGATAATGCTCCAAAGGCATGGATTCG
CAATATTTACAAGGAAGTTACCACAACTCAAGAGAATTTTCCCGATGATGACTATACTCC
CAACACTTCTAAGCAAAAAGTTATCCAAACTCTGAATAATTTACCTAAAGCGGGGGTAGT
TCCAGTCCATGTAGACTCATTTGCGACTGATCAACCCGAAACTTACCAAAGTTTGATTCA
ATTAGGTCTTACTGATAGTGAAATTAGAGCAGTTCTTACTGAAAATGAGGTAAAACGTTC
ACAAATTGACCCTTCTACGTCTCCAAAAGGGACACAAACCGAATTTGCGTCTCTAGACGC
GATTTTTGTCTTCGAAGAAAGGCAAAATTTTACTGAAAAAACAACTCATATCAATATTAA
GTCAGATCCTGACCGTAGAAGAGTTCAAAAACTAGGACAAAGAGGGTATTCCGCTAGTTC
TTTGGATACTCTTCGTGATGCTACACAAACTGATCAGTATAGTTTGAACTTTTTGGACCA
TTCTGAACTTCCCGACAAGGTAAAAACGTTTTTGAAAGATGATAAAGAGCAAAGGCTTGC
AAATAGAGATGCGGTTCTTGATGAAATGACACAAAAAGTGAATCCTGAGTATACAAACTA
TATAGAAAACTTAGTTGAGACTGTTCAAGGTCCGTTAGTCAATCTTCCAGAGGCATCCAC
GGGGACTAAATATATGATGACACAGTATCGTGCGGATCCTACGTCAAAAGTGAACTTAAA
TGTTACAGTTTCTATGACACTAGTAAATTCGGGGAATTCACATTTTACTTGTAACCCTCC
TACACCTTCAACAGGTGGAACAGTCACAGGCGGAAATGGGGAAACGATTGTAACTTCTTA
TCAAATTTCTAGTTTACTCGGACCTGTTTCTCAAGCGTGGAGTTGTAGCGGAACATTATC
TGTTTACCATGAACTTGGTCGAGCAGCAGATAATGCTGCCCGCGCAGGTGCAGCGTACGG
AAATCCTTTCACAATAACCTAATGGCATTCATTCCAACAGCATCAGGAGCACTTGGTTCT

GCATTATTCATGGGAACATGTACTGGACACGGAGCGTGAACAGGATATCCACATCATCCT
GGTCTTGGTGGAGGAGTATTGAAAGGTTCTTCTTGTCCACACGCACCTCTTGCTCCAAGA
GTTTCTCCCAGACCAGTAGATGCGATGGATGTTACAACCATATGGCCTCCGCATCCGCAA
TTACCTTTTGCTGCATTATTGCGGAATGTGATTGTTAATGGTATAATGCCTCTTATCGAT
TTCGTTCTTCTTATTACTCACCCTACTCCAACCTCTCATCCTCACACAGTTGTTACACCG
CTTCCAAATGGTGTATGTGTTCATACTACATCTTCTCCTGCGTGGCACTGTACAGTAGGA
ACTGCGGGTGGTAGACAAGCAGCACCAGGTCATGCTAGAAAATTACTCACTACTAGTAAG
AACGTATTCATCAATGGTCGACGAGCAGGAATGTTTGGTGATCCTCTAGGTGATGGTACC
GTAGCGTTTCCGTCTAACTCGCGTGTTGCGGGTTGGAGACCAAATGTATTCATAGGAGCT
TAATTATGGCAATGAGATTTCACTTGGGTAACACCATTGAGACAACCCCAAAGAAAACAA
GACAAGGTAGAGGACAGCATACTAAGTATAGTGCTACCTCTCGTAACACAGCAAAAAAGA
GGTATCGTCGTCTAGGCAAATAGAATTGTAGACGGTAAACGTAATGCTAGCATTCCTGTA
GACATGTCAGATCATTTTTATGATCATGGAAATGAATATTGTCGTTATTGAATTACTGAT
CCACGTTCTGATAGATCAATTAGGAAAAAATCTGAAAAAGACGCTAAATAATACTGGCAC
TTCTCGTATTAGATGGCGTTAAAGAAAATATCAGGTAAAGATCAAAACAGATCACGTTCA
TTTACGGATCTCTCATTTGGTATGGTGAAAAATGCGAATACACAGGATGATGCTGTCGGG
AAAAATGACAATGCAATCAGACAGGCGATCAAAAATCTAATTATGTCCACGCCTGGTGAA
AAACCATTTCAACCTCTAGTAGGATCTAATATATCAAAATTCTTATTTGAACCTCGAGAC
GATTTTACAAGTGATGCTATCAAACAAGAGATCATAAATACGATTAATCGGTTTGAACCT
CGCGTACGTATGACAGGAGTTAGAGTACAACCTCGTTATGATAGAAATACATTCAATGTA
ACGATGGTGTATAAAATAGCTGGAATCCCAATCAACGAAACTATCGAGTTCGTGTTACAG
AGACCTGCATAATGCAACCAAATAACCTCACAGAATTAGACTTAGAACATATCAAAGCAT
CGATCAAATCCTATCTAAGAAGTCGGAACGAATTTACGGATTATGACTTTGAAGGATCTT
CTTTATCGTATCTTATCGACACATTAGCGTATAATTCATATTATACTGCATTTAATGCTA
ATATGGCGATGAACGAGGCATTTTTGCCTTCTGCTACTATTTGAGATAATGTCGTTAACG
TTGCAAAACTTTTAAAATACGTTCCACGTTCAATTACTTCATCTAGAGCATGCTTATAAC
TAGATGTACAAACAACACAGACTGCAGGGGCATATCCTAGCAGTATAACATTAAAAAAAG
GTGCTATTGCATCTGGTGGAAATCATGTTTTTAACATTTTAGAAGATATTACAAGTACGG
TCAGTTCTACCACAGGTGTTGGCACTTTTGACAAAGTAATGATCATGGAAGGTAGTATAG
TTACCTTCCAATACATTGTTAATACCTTTGCAACTCAGGTTTATAAAGTCCCTTCCGAAG
ATGCAGACATTTCTACTCTTAGTGTAAGAGTAAAACCTAACGAATCCTCTACAACATCAG
ACTTATACAGTTTGACTGATACAATCACTGATTTGACCGCAACTACTCGTGCATACTTGC
TCAGTGAAGGTGAAGATATGCGTTATGAGGTAAAATCTGGTGATGATACTGCAGGTAGAG
CGTGAAAAGATGTAGAAGTCGTACTTTTTGAGTATTTGGTTACTTCTGGAACAGAAGCAA
ATGAAATTACAACATTTATGTTCATAGGACAGATTACAGACAATATTGGAGGTGCCTATA
GTGGCACATCAGTCACTTTGAGCATGAAAGAGAAGTCACAACTCGGTGCTGCAGCAGAAA
CTTTAGAATCTATTAAATATAATGCACCAAGATACTACTCTGCACAATACAGAGCAGTAA
CAGCACAAGATTATGCATTAATTGCGAAAAAAGTGTATTCTAACGCCGATTCTGTTGTTG
CATATGGTGGGGACTCTTTAAATCCCCCAATTTACGGAAAAGTCTTTATTGTAATCAAAA
CCAAAACTGGTTCTTTACTTAGCCACGCAACTAAAAAGTCAATTTCTGCAGATTTAAGAA
AATATGCTATGGCATCAATTGATCCTGTAGTTATTGACCCAGAACAGATGTATTTGTATT
TGAAAGACTTTGCACAATACGATCCTGGCACTGCATCTAATACTTCCGATATCAAAACTA
ATATTCAGAACGGAATTAGCGATTGGGCAACACAAACTCAGATTAATAACTTTAATGCTA
CATTCAGAGCACAAGCATTTGAAAAAGCGGTGACTTTATCCGATAATAGGTTGAGTGATG
TGTCTTTACAACTATCTATATTAAAATACATCTATCCAAACACAAATCAGACTAATACTT
ACTGTATTGCTACTGGTGCGGATCTATATGATAGTGGTCCAAGCAATTCCGATGGAACTA
CTTGTAGAAAGGAACCTAGATTGCTTTCAGGACCTTTTAGAACAGCAGATAGACCAGGAA
TTGATCAACAGTTTGAAGATGATGGTTTTGGCAACTTAAGAACATTTTATAACACTGGTA
ATAAGAAAGTATATACAAATAATGCTGCAGGACCTGTAAATTATGCTACAGGTCAAATTT
GTTTTGGTCCTGTTAATATAATTGGTTCAGGTGTCAATACACCTCTAACTGGATCTATAA
CAATTACAGATTCAACAACTGGTGCAGGTTCCGTAACAAATGCAGATGTACTTCCTACAG
GTCTTCAGATTCCTGTTCTGTTTATACCTGCAAACGTTGCATCTATTAATGCTGCAACAC
CAGGAACTATTAACAATATTATAAACCCAGAGGTTACAGTTGTCCCAGTAGGTACGATTC
CTCCTCCAACAATACCTCTAAATAGTTTGACGCCATCAGTGTTTAATACGGTTCCCACAA
CTATTACTGTTGCTGAAATCTCAAACGCAGGTGATCTAACATCCTCTTCTTGCTTCTAGG
TGTAGATGAACATTAATAAGGTCTCTCAGTCAGTTGTTTCACAGACTCCAGATTTTATTG
GAAACGATCATCCATTATTCAATAAATTTATTGAATATTACTATCGTTCTCAAGAAAAAA

CGGGTTTAGGGCAGAATATTGTCAATGAGTTTTTACAATATCTTGATATTGATAAACTTA
ACATCGATATTCTTGATGGTGCTACAAAGTTGGTTTAATCGACAACTACAACTGCTGATG
AGATACTAGTTGAAAGTATTGACAGTTATTTAACTTAAGATGTTTCTGTTTAAATTGGTA
ATGAAGGAGTATACTTTGAATCTACTACAGCATCACCAAACATTGCTCTCAGTCCTGGTA
TTTCTTACGAGCACCTAAAATTAAAGTAGACAACTCTTGCAAGTCTTCTAGATTCCTTTG
ATGGCACTACAGTTAGCTTTAATTTAGCATCTCAGGATAGTCCAGTAGTAGCACCTTCGG
CACAACACTTAATTGTTTCTATTTTCGGAAAAATATTAACACCTGGTATTGATTACACAG
TTGATGGCACTGCTATTGTCTTTACTACTGCTACTAGGAGAAGAATTCCTGCAGATGACT
CAACTTCAACTTATATTAAATATCAATGTGGATTTGTTGATAATACAATTGTTGAAATAG
ATAATTTATCTAGTTCTTTAGGTGAAGGTAAGAGACAGTTTACCATGACAAGAAATGGTT
CTCCTTAAGAACCTGTTGTAGGTGAATATATCTTAGCAATTTACGATCAACGTCCATTAG
TTTCTAAAGTAGACTTCTTTTATGATAAAGAACCGTTTATTTTTCTTACAGCACCTCTCA
ATGGAAGATTTCTCTCACTATGCTCAATTGAAGCACCTATTCATTCATTTGGATCTGGTG
CTCTGGGGTACTCTCGTCTTGACGATAAAGGAATTTTAACTAGCGTTTCTGTAAAAGAAA
ATGGTAGTAATTATCGTTTTGAATACCCTCCTAAAGTCACTATCAACTGTGCTGAAGGTA
GCGGTGCTTCTGTAACTGCACTTGTAAATGGTATTAAGACTCTATGACTCTTAGAAGGTG
GTAAAGGATACAGCGATACTAACCCTCCTGTAGTTCAAATTCAATCAGCATCAAAACCTG
GTTCTGTAGCAGCAACTCTTAAAGCAACTGTAACTAACGGTAGTGTTTCTGGGATAGAAG
TTATTAGTTCTGGTAGTGGATATACATTTACACCTAGAATCACCTTCAAGCAACCTGGCG
GTGCTATACTAGGCACCTGTCCTATTGTAGGGGGTCAGGTATCTGGAACTATTCCTATTA
CTGATAGTGGTTTTGGATATACTACAGTCCCCACAATTTACATTGATGAACCAACGGGAA
CTAATCCTATTAGAGCAACTCTAACTCCTGTATTGACTGATGGCGTAATAATTTCTATTA
CAATTACTAATGCAGGTCAAGGATATATCACTACTCCTAGAATATCTGTAGTTGATCCAG
TTGGTGCACAGGTCTTGTCTACAACTGTTGATTCAAATGGGCTTCTTGTAGGAATTGAAT
TATTAGATGGTGGTAGCGGATATGATGACGTACCTTCTGTTTATGTCGTAGATAATAGAG
TAGATGCGACAGGAGCTTAAGCGGGCGGAACTGGTGCTACTGCAACTGCTGCTATATTCA
ACGGTAGAATTACTGATATTAATATTACTGCTTTTGGTTCTGGTTCTATTGCTGCAAATC
CTCCTGAGATAGTAATTCAAGCACCTACTCAGGCAAAAGCATCTGCAACAGTTGGTCTTA
ATGCAGTTACTGGATTTAAAGTAAATCAGTCAGGTAAAGGAGATTCAAAATCAAAACTAC
TTGGTTGTGCAAGAGCAGCAAGTGGTATTACTTCTTATACTGAAGATGGTAACGCTGTAT
TTACAAATAACACTACAGCAGCAGCACATACGATTAATTCTGATGTAAAATGTCTAGATG
CTTTATTTGTAAAACGTTTACTAGACAAATACACTGAACAGTTTTTACCTGATGTTCAAG
AACTACATTATAAAAAAATTGATGTTAGAACAGCAATCAAGACAATTAAAGATTTTTATA
CTGCAAAAGGAACATCTTTCAGTATTAGTTACCTCTTTAAACTGCTATATGGTTAACAGG
TAAGTATTAATTATCCAAAAGATCAAATTATCAAACCCTCTGCAGCAACATGGTCTATTG
ACACTATTTTGCGTGCAACTTTGGTAAGTGGAGATCCTACCAATATTAGAGACGGTTTAT
TAACTCAGGGTGCTGATATTGCTGATGTTAACGTGGCAGCAGCGAGTGCTTTGATTGAAA
GCTATATTTCTATTAAAACTTCTGACGTAGAAATTTTTGAACTTGTTCTTTCGGAAGAAA
CTATTACAGGTTCTTTTGCAGTTCCTTATAAAACAAAACTTGCTGAACCTTTAGGCACTG
AAGACAGTATTATCACTGTTGACTCTACAATTGGTTGGCCAGAGAGAAACGGTGAGTTTG
TAATCGGATATAGTGAAATTGTAAGATATAAAGACAAATCACTCAACCAGTTTATTGAGT
GTACACGTTCTGTTAATAAGATTGTTGAAGCTTGGGATTCTGCTACCGAGGTTGATTCTA
ACTTCTCAGTTTTCGTTAACAAAGGCACTCCTCTGGAAGTTCTCATGAAAATTGTTGGTA
TTGTTGATGCTCAACAAACAACTCTTACTGATACTGGTTCATATTACTTAGCTGGTGATA
AATTAACAGTGTCTGAGTTGGGTGGGACAGGACCTAATGCTGAACTTACAACTTGTTTGT
ATAACGTTAAAAAATTAATTAATATCAGTAGTATTACGTTTGGTGGTGTTGAAAATCAGT
CCGCAACTGTAACTTGTGCTAATAATCATGGTCTCTTAATTGGAGATCAGGTTACAATCT
ATGGTGTTAACCCAATCATCTATAATGGAACTTTCTTAGTTACATCTAGAGATAGTAATA
CAGTGTTCCAATATCAATTACCACTACCTCCTACAGTCGTACCTCAAGGTAATATTCGTG
TATCTGTTGACCTCAATAAAGGTAAGACTGCTGATACAGCTGTTTTCAATTCTATCGGAC
CTTACACAACTAATATTCAAAACTCATTCTTCAATAAAAACGACGTTTACGTTGCTTCTA
CAGGTATACCCAACTATAAGATCGGTCTGTTTCCTGGTTCTGCTCTTTTGCCAGATAACC
AACGTAAATTGAAGCGTTTCCCATTAGCGGGAACTACAATTTCTACAAAGAATAATATTT
CACCTGGTCCGATTGGAACATGGGTAAATGGTGTTTCTATCTGGTCTTATAAGTCTACAA
TCAAGAAAACTTTTGGTGCTGTTACTGATATCACTATTTCTAACGCAGGTTCTGGATATG
ATGCTGCTTCTCCTCCAAATATCACAATTTCTGGTGGTGGAGGAACTGGTGCCACATCTA
GCGTAACTGTTAATGGATCTGTTAATGAAATTACTGTAACTGCAGGTGGATCTGGATATA

CTTCGTTTCCTCTAGTTTCTATCGCAGGTGGAGGTGGATCAGGTGCTTCTGCAACTGCAA
TCATCACTAAAGGTGTTGTATCTAGAGTTTTGATTAAGGATGGTGGAACAGGATATACAT
CTCAACCTACTATTACTATTGTTGGTGGTGGAGTAACTGGTGCTACTGGAACTGCATCTA
TTCGAGGTCCTATTCAAGCTGTAACTGTATCCAATGGTGCTGCGTGTTATACTTCAAATC
CTACTGTCACACTAAGTTCTGGTAGCGGTGCTGTTGCTCAAGCAATCGTTCAGAATGGTC
GTATTATCTCTATCGCTAATATTTCGGCAGGTTCTGGATATACTACTGCTCCTACAATTG
ATATTCAAGGAGTAGGTTTCGGTACTGTTGCTCGTGCAACGCTTGATGTTGATGGTGATA
TTGCAGGTAGAGTTACTGGTATCGAAATTGTCAATAGAGGTATTGGATATATTCAAGGTA
CGACTTTAATTAATTTAAACTCTGTTGGTTCTGGTGCAACATTTACAGCAATCGTATTCC
CATGGACTTATAACTTACAAGAAACATCTACTCTTGATGTTGCAAAGGGCGGAGTATTTG
AAGGATTTAATAATCAATATGGTGGTGAATATGCTGACGTATCAAACCCACAAAGATTAA
GATATCTTCTTGGTGCTAATTTATATGAAAATAATGCAGGATTAGTCAAAGAGCAAGCAA
CTCCATTAGCACACTCACCGATTATTGGTTGGGCATTTGATGGTAACCCATTTTATGGTC
CATATGGTCACTCAGATCGTACTGATCAGTACTCTGCTATTGCTAAATTAAACACATCTT
ACCAACTTAAAGCAGAACTTGTATATAATACTGTCACAAACCAAAATCCTGTTAGAACAC
CAGGACCTCGTCTTTCTGAAGAATCTGCAGGTAGGTTTGTTGAAGCTTATCAAGATGTTT
TTGGTCGGGGTGATTTAGATCAATATAATGGTCGTTTCCGTAAGACACCTGATTATCCTG
TTGGTAGATATTGCTATTTTGTTACTATTGATTCTTCTAAAGATGGTAATCCTCTATTCC
CATATGTTCTTGGTCCGAGTTTCAACTCTGTCGTAGATACTTGGATTTTAAGTGCTGGTG
CTGTTCAACAAAATATTCCTACTGGTGTTGTTAGATAACGTGCTCCATATGATACCGTTG
ATATTGACGTTGAGAGGGCAGCAAATGCCTCTACAAACGCTATAACCCTAGAGAATGGTG
ATATACTACTATTTGTAGTGGAAGACGAAGATATAAGTGGTATTATTGCTCAGGATGAAA
CTGATGATCTAGATCAGGTTTTTGAAGAATCTCCATTACAATTATTTGATTATTTCCCTA
AAGTATAACTTGATTCTAAAGTTGACACTGAAGTTGAGACAATTTCTAAATTTGAAGATG
CATCTGTAACAGGATTCACTATTGAAAATGCAGGTGTTAGTTATTAGGTAAATGATAGAT
TAGTATTTGATAATACTGATACAGATGGTTCTGGAGTTTCTGCTCGTGTTTCTAGAATCA
AAGGTGAATCAGTATTAACTTATGGTTTTGAGAGTACTAGTGGTATAAATTATGGTCTTC
TTAAAACTACAAAACCTCATAATTTAGTTGCAGGTGATAGAGTATTTGTTGAGTATTCTC
CTGTAACGGACAATACTAATAAGTCTTATATCGTACGTCAATATAAAGGTATTGAGGAGA
TTCAAATTGATCTTACTGGATCTGGTTACAACACAGATATTCCCCCAACAATTATTATTG
ATGGTAATGGATCTGACGGTAGTCTTGAAGCAGTTGTTACTAGTGTAGGTTCTATTGACA
CTGATAATATTCTTAATTCTGGTTCTGGATATACTTCCAATCCTAGAGTTATCTTATCTC
ACCCACAGGTATTCAAAAAAGCAGATTACTATGTTTCATTGATTGAAAATCAAAATTATG
CATCTGTAGATGATGTATATGTGAATGATAATAAGGAAGTCTACATTTGTGGTAAAACAC
ACGACATATATGATAATCTTTTTGCTTTTGTAGCAAAACTTTCTGCAACTGGTATCAAGG
AATGGGAAGCAACTTTAGAACTAACTGGTGGTATAGATGATGCAGAATTCCAAAAGTTAT
ATGTTGATGGTAAGGATATTAGGGTTGCAGGAAGAAACTATCCTAACTCTTCTCTATTAA
ATGCATACAATCCAGATATTATCTTAGTTAAGTATGTTGAGGCAGAAAATGGTCTTAGTG
CAAAACTGAATTTCGAAAGAGCATATGCAGGTATTTCTGGATCTACTCGTTCAGATGGAA
TTACTGCACTTAAAAAATTCTCTGATTCTGGATTTGTTATTGGTGGATATACATATACTC
ACTCTGCTAATCATTACGACGCTTTTGTTGCTCTAGTTGATACTACTGGAACTTTTGCTA
TTAAGAGAGAACTTACATCAGCAAATAGTTCTGAAAAGATTACTGATATTGTAGTTAATG
GAAGTGATGTATATTCATCTATGGAGATTGCATCTAGTGTTTCTGCAACTGCAGTTGATG
TTGGTTATGGTAAAGCAACTATTGGAACTAGTGCAATTTCAATTCCTTATATCAAACAGA
TTAGTAATACAGTGTATTCTTTCTGGAATACTAGTATTGCAATGGATGAATTTGATGAGG
CATATATTAGTGCTACAACTAGACGCAAATCTGATAATACAACTAAAACTGGTTTCTGGG
TAGGTAAATTTAATACAACTGGCGATGTAATTTACATTAGGAGATATGCTGTTGCAGGTG
GATCTATTACTGCAGCAAGTAAGTGTGTTATTGATATATTTGGTGATCTTAACGTAGCAG
TTAGTAAAATTGCTACAACTGATTCAGTGCGAACTGCAGAGTGTATTAAAATCGGAACTA
ATGGTATAATTAAGAGTCATACAACTCAACAGTTTAATCTCAATAATATTGAAGGTATTA
CTGCAAATTCTCTTGCTGTTGATAACTCTGGAGATGTTCATCTATTTGGTCGAACTTCTT
GGAATAGGAATGAATTTATTTACCCATTGACATCTGGATCTAACGCTGATACAACTGGTC
ATTATACTTTCACATCTACATCAACTAATAACTCTATCACTTATGCTGATAATGTTGCTA
AGATTTATGGTTACACCCCTGCAGGATCTAACTCTACTTGGGTAAATTCACAACTTAAAA
TTACTTCGGCTCAGTTAGGAACAAAATTAAATGAGAATTGGACTATTGAATTTGGACTTT
ATAAAGACGCAAGTTCTTCTCAAACATTATCTCAAACACAACAAACATTAGTTTCTATTG
GTGATGCTGAAGACGCTACAGGTGGTCTTTGGTTATACTATGATGTTTCTAGTGGAAAAC

TAGAATTAGTAGTTACAAATAATACTACTAGATTAAATTCTGCAGGATCTGCACTTCAGT
CTACATTAACTACATTGTTTGCTGATAATACATGGCAAAACATTGGACTTAAGAAAGAAG
GTAATCAGTTTACTGCATATGTAAATGGAATTCAAGCATTTACAGGAAGGGTTTCTAATA
CAGCATTAAACGGTAAAGATCTTCTCTTTGGTCAAATTACTGGTAGAGATGGAACTGCAG
GAACTATTCGTAAGAATGAGCAAGGTCAATACTTTATTGATCATCTCAGACTTAGAATTA
GAGCAATCACTCCTAGCGTACCTAGTGATGTTACTGCATTCCCAACTACAGGTGCTTCTG
CCTTTGCATATACTTGGGTTGATAGTGGATGGTTTAATACTAACCTTTCTAAGTCTGATT
ATATTGATTACATTGGTTGGGGTCTTAAGATGGATAGAAATGCTGAAGCTGCAAGAATTG
GAACTCTAACTGTTCAAACGAATACTCATGTTGGATTTGTTAGAACAAGTGTAAGTGCTG
TAACAGGTGTAGATCTTACTGTCACTAATACTGGTTTTGCTTTAGGTGATGCAGGATTCC
AAAATCTAGACTTTGATGATTCAGCAGCCACTATGAATGCAGGCACTGAGTCACTTACTT
ATAAGCAGGATATCTGGAGTTCTAGAACTGCAACTGTTCCTTCAACAGGTTCTAGAAAAC
TTAAGGTTACTGCTATTGTTAGAGACAGATATTACTTTAAAGTAACTCCAAAATCTAAGA
TTGATAATATTCAAGAACTTACCAATAATCAATCATTTAACTTTACTGTAGGTACAAAAT
TAAACTTAGTAAATGCTACTTCTGGGGCATTTATTAATAGTGGTTACATTATTTCTACTG
AAAATGTTAATAATAAAATATTCCTTTCTGTTAATAATAATGGTTGGTCTGATGATTTGA
ATCAGGGTCAATTAGTCACAGAGCAATTTAGTGAACAGTCTACTTATAGCATTGTAGGTC
CTATTCCTGCAGATGTTAATGAAATTGAAGGATATAATTTTGCACAAGTAAATAACACAA
CACCAGGCACATTTGATATTGATCTTGACAAGGATAATTTAGATGGAATTTATAATGCTG
CAGGTGGACAGAATTTAGATAGTTTTGCAAAATTCAAACCACGTAGTGATATTGCTTATG
CGATTAGAATTGATGAATTTTCTCCATCCGGATCTCCGTTTATTGTTGGATCTGTAGTCA
CAATCACTTCCTCAGATATTAGTTTTAATACTGCATACTCTACAGCACAAGTTACCAACC
TAACTGGTGTTTTAAAGATCACTGTGATTGCAAACTGCACTAAGATTGTACAAGTTACTG
CAGTTACAAATACTGATCAAGTTTATGTAATTACACGAACTAATCATTACCTATCTACTG
GATCAATGATTTATGTTGATGGTAATCCTACTCAAACTATTGGTTCTACCACATATGATG
AATATGATGGATCCTTTGCAGTTTCTCCAATTGTTAGTGCCAGAGAATTTGTATAAAAAC
TTCCTAGTGCTGCATTAACTTCACCTGCTACCACAGCAGCAAATGTTAGCATCTACGTTA
AGTCTCCCGTTCTTAAAATGTATTATGGGCATCAATCCTCGTTTGATGTCAGTCATTCCT
CAATGAGCGGTGGTAACTTATCTATATCTAAAGATCCTTTAAACAAATGAGAATATTCCT
TCAACTCTATTGAACGTGTTGGAACACCTGGTCTTACAGGAGCAGGAAATCCTACTCCTA
CTGTAAAACTTAAAGTTGATTACGGTATTATTACAAATATTTCTTATTACTTTGATCCCT
CTAGAACTGGTGCTGATTCTCCTGTTTCAGATGTAAGTTATCGTGATGTTACTGACTCTC
CTTACAAGGGGTCGTTCTCAGTTCAGTCTGTTCAAGGTGCTACTATTACTCGTGGTGCTG
ATATTAGTAGATTTCCTCATCTCAACGAACCAGAAGCAGCTGCTGATGTAAACCAAGCAT
CTTATTCAACAAGTTCTCTTAAGGCAGTTGGATCTATTGGTGCTATTCGTATTGTTAATC
CAGGTGGTTTCTATACAAGATTACCTGTTGTTACTGGAATTCAATCTACTAGACAAATTG
AAAGAGTTCCTATTAACGCACCAGGCACAGAATATGCTGTAGGAACTTATACTGGTGTTC
CTATTGGTGGTGATGGTGAAGGTGGATTTGTAGAAATTACTGTTGCTGACGGAGCTTAGG
CTAACGGAGTAACTATTCCTGGTCAAATTCAATCAGTTGTTGTTACATCACCTGGTAAAA
ATTATACTTCAGCACCTATTGATGTTGAGGCAATTACTGGTATTCTAGGATCTGGTGTAA
CTGGTTCAGGTGCAGATCTAGAAGTTGTTATTCCTGCTTCTGGAACTGGTGCATCTATTT
TCACTCAAGGTGATAAAGTTGGTAAGATTAAGGAGCTTAAGAACAATAAGTTTGGTTATG
ATTATCCTCATGACTATACACTACGTCCTGAGATTACATTCCCAATAAATGTTCAGCTAA
CATCTACCTGTATCCTAGACAGTATTACAGTTACCGGTCCAGGTTCTGGATATACACAGG
CACCTGCATTGGTAATTACTGGTGGTGGCGGAACAGGTGCTGTTGGCGAAGCAACAATTA
AGAATGGTCGTTTAGATTTAATTATTGTTTAAGATCCAGGCGCAGGTTATTCTTCTACTC
CTGCAGTTGCTCTTAGATCTTCTTTCAATTATGTTGTAGATCTTGACTTAGGTCTTTTAC
AGTTTGCATTCCCTCATGATATTGCTAACGGTTCTGCGGTTAAACTTAATGTTGTTGATA
CTGGTGAAGGTGCAGACTTCCCGCTTGCTGCAGGTGCGATCGATCGTTTGAAAGGAACTA
CAACTTATTACGCAATTACTGGTTCTGCAAACTCTCTTGAACCAGATCAATTAAAACTTG
CTATTACTGCATCAAACGCAAACTTAGGTGATGCTATTTCCTATGTCAATGCAGGATCAG
GTCGTCAACAATTAGTCACAGAATCTTTCGGTGGTGCTGCTAAAGCAAACGTTATTTCAT
CTACCTTCTTAGAAGGAGAACTTGTTTATCAAAGTGATTCTTTAGACAACTCAACTGCTC
AAGGTTATGTTTCTACAAATAATGGTTGGCAGATAGGACCTAGAATTCTTAAGATTGTAG
ATTATACTGGTGACTTTATTGAAGGACAAAGAGTAACTGGTGTTATATCCAAATCTTCGG
GTACGATCAGTGACCTGAATATTGCTAAAGGTGTTCTTGAGATTGGTTCTCTTACCAAGA
CTACAGGTCAGTTTATTGATGATGTTGGTAAACCTTCTGAAATTATCGAAAAGATTCAAG

ACTCTTACTATTATCAAGATTTCTCTTATACTATTAAGTCTGCTGTTTCTATCGGTGAGT
GGAAAGACATTCTTATCAAAAACGTTCACCATGCACCATTCAAGGTATTTGGTGAATTAA
TTCTTTGTGATTACGGATTTGTTCCTAACAAAGAGAATGATTTCCAACTTACTAAATCTG
TTGAACTTGCTAGAGATGCGATTGTTCATAATATTGAAAGTTTTGCTTTAGTAGAACCAG
TTTATTCAGAATTCAATAATACAGAAATACTTTTCCGTCAGAAGAGACTTACATCTTCAG
AAAAAATTCTGACTTCTGTTGTTCAACGTATTGATGATATTTCTACTTTATCTGATGGTG
AAAGGATTGCATTCCCACTGACAGTTGGTGGAACTAATATTGTTGCTAATGCTAATCAAT
TGATGATTGTTTTAAATGGTGTTGTTCAGAATTCTGGCACATCTTTCAATCTTCAAGGAG
ATTCGATTGTATTCAACGTACCACCACAACCTCCTGCAAGTATCAAATACGTTATCGTTA
CTATTCAAAATATTCCTACTAAGAAAATTTCATTTGATGATATCGGTGGTATTTTCCCAA
ATCCTGGTAATGAACTGGTTGGAATAGCATCAGGAAATCGTCTGACTGTTACCAGTGTTG
TAGGTAATGATGTATTTGGTTTCTTCACTCATGGAACTGCATTTACTCCTAGTGAACTTG
TTACAGTTGGTGCAACAGGCTTTGCTTCTAATGTTGCTGCAAGTAATGGTGTAACTGATG
TTCAGAATATCGGTCTATTTACTTTTGGTGAGAAAGTTACTGACTTCCAAGGTCATACTG
CTAGAGTTGAATCAATCAACGTACACAAAGGTCGAGAAACACCTGTAGCACAATTAAGAT
ATACTATCGGTCCTTCTACTACCTCATTTGAAGTAGTTGCATATAAGACTGATAATAGTG
TTGCTGATGCACCTGTTGCTGACGGAACATTTACTACTGGTCAAAACTATCAGTTTGGCT
CTGAAATTTTCTTGCTTAATAGTATTACACAGGATTCTCAATCTACAACTTTTGGAGTTA
CAAGAGCAGAGTCTTGAACCACGGCTGTATCACAGCAAGAAGATAATCCTGTTTATGGAA
CTCAAATTTCTGTCAATAATCAACTAACTTTAAGTAAAACAACTGGAACATATCAATCTA
CACCTGGTCTTTACTACATTCAATTAAATGAAGTTATTATTGGTGCGCAATCTGGTGTAG
ATGCTCGTGTTACTGCAACTTCTACATATCAAGATCCTGTAACTCAAGAATTTATTAGTC
AGGTTAATATCTCTGAAGGTTCTTCTTTCTTTGGTCTATTATTCAACAGGATTACTTCTC
AGACTTCTCCAAACGTTGTTCTTGATGAGATTTCTAAATCTCACGTAAGAATTGTTGATT
TTACAGATAATCAAACAGATTACAATACTAAATTCCCTTCTAATGAAGCACTTAACAATT
ATGTTATTCCTTACGATAATGCTTCGGGCACTCTACAAAATGAGACTATCCGTAACTACA
AATTAGAGTATGGTAATAATGATGGTGAATTTGCTACTGGTGAAGGTGCTGTAGTTCGTA
AATTAACTTATAGAGATACAGTTGGAATTGGTTTATTCGTTCCTGGTCAAATTATCAGAA
CTCGTGATACGAAGGCAGAAGTTATTGGTGCAAACCAAGCAAGAAAAACTGTATACGTTG
GTAAGGTTGGTAGATGTCTAAGAAACGGTGGAGATTATCATACATTCACATTTAATAATG
CATATCTCAACGCAACCAATCAGAAGTTAGGTGATGCTTGTTTAGGTCTTTCACCGGGAA
CCGAAGCACATACTTTTGTAAGTGGTGTTGCTGGTTCAATCACTGCAGGTGGCGGTGCAA
CAGGTACGTTTACTGCTGCAACAGGTACTACCTATAATGGATTGACAGGTGATCTAGTAA
TTGAAATTGGAAATCACTCTTTAGCAACAAGTAACAAAGTTCTTATCGCTGATAACAGTG
TTGGATTTATTTGTGCACAAGATGGTAATACCTCTACTAAAGCATATCCTCGTAGCACTG
ACCCTGCATCAGGATCTGCTCGTAATATAGCTGCAGGTACATCAACTACGATAACAGTTA
ACGTGCGTGCAGTTGCAATTGATGACAGTTCTGCAGTCGCGGCTTTTGACGAATTTGGGT
TTGGCACTGCAGCGTTTACAATCGAGTGTTGGATTAGACCAATTTCTGTTGGTGCAGGAA
CAAGATCTATCTTTGATTTCAGAACTGCTGCAACTGAAGTTGCTCCTTACTTATATCTTG
ATGGTGCAAATCTAGACTATTATGTTAATGGTTCCCTAGTAATTCAAGGAACAGTAAAAA
TATTTGCAAATCAATGGACACACGTTGCTATTAGTAAATCAGGTTCTGTAGCTAGACTGT
TTGTTAATGGTGTTCAAGATGGTAGTAATTACAGCGATACCAATCACTACGGAACAAACA
AACCAATTAGAATTGGTGAGGATACTGCAGGTAATGATCACTTTATTGGATATATTGATG
AGTTAAGAGTTTCTACAACTGGAGGTTATACCGCAACACTTACTGGAGCTACAGGTATCT
TCCAACGTGATTCAAACACTGTATTACTCATTCATTTTGATGGTGTTGAAGGACAACAGC
ATACAGATGATTGGTCTGGAACTGTATCATTTACTAAGGATGAATATTTCAGTAATGATG
CAATTCTAGAAACGTCTAGAAAGACTGGTGCACCTGCAGGATTTGCGGGTAGATCCCACA
GATACTATAACGCTGCAGATGATATCGGAGGTAACATCGACTTTATCAAGAAAGAGGTTG
TATATCAAATGAGACAGAGATATCCAGAACTTGTTATTCTAGTAACTCGTTTCACTCCAA
CTAATGCAACATATGATGCTGTAACTGGTCTATTGTCAATGACAGTGACTGGAAACACAT
TTACTAATGGTGGACAATTTACACCTCAGTCTGCATCATACAATCCTTCGACTGGTATAT
TGACTATAACTAAGATTAATTACGGTGTAACAAATGGTAAGAGAATCAATATTAAAGTTG
GTGGTATCACATTTACATGTGCAGAAGATATTAACGCTCCAAGTCATCCATATCCTCGTT
CTACAGATCTTGTTGCAGGTAAGTGGTTGACAGTATCTAATGCGACTGCAGATACATTTA
CAGTTGATGTTGGAACCTCATCTAATACTACAACTCATACTTTCGTAAGTGCCTTAAGTA
ATGCAATTACTGTTGAGAAAGATAGACTTAAGATTTTAGATAATTCCTTGCCATTCACTT
GTGCGATGGATGGTAATACTGCTAATAAAACTTATCCTCGTCCAACAGTTCCTGCATCTA

AAGATGTTGCTCTTCCAATCGTAGCTTCCAGTTCTACCAATTTAACAGTTAATGTAGGAC
CTTCACCTTTAGTTAATTTCCAACCAACTAACGCAACGTACGATCCTGCAACTGGTGCAT
TCGTCATGACGATTGCTGATTATACAATCAATGTTGGAACACAAATTAGATTAACTGCAA
ATTCATTTACATTTACTTGCACACAAGATGGTAACAGTGCACAGAAAACTTATCCTCGTG
CAACTGCGGGCGATGGTCAACCTGATCCTGCATATAATACTGCTCTAGATGTAACAGCAG
TCGGAACAACTACACAATATATTAGTAGTGCAGCGTATGTTCCTACAATAGGTATTTTAA
CGGTCAATACAAGTGGTAACCATGGATTATCTACTGGCAATAGAATTCAAATTGATGATC
ATTCCTTAACATTCACATGTGCTTATGATAGTAATGCTCCAAACCACACGTATCCAAGAC
AGACTGATCCTATCAGAGGTGAGTGGGTTGAAGTAACCGTTTTTGATGCTGATACATTTA
CTAATGATATTGGAACATCTAGTGATACTTCTGCACATTCATTTGTTTCTGCGACTGCAG
GTGCACTAATTAAACAAACTGGAACTGTAACTGTTCACGTTGGTGTTTCTGCTCAGGCAG
ATCAATATGCTCATACTTTTGTATCTGCTGCAACAAACGCAGTTGTTACAGGTGGTAATT
ACTTACATACATTTGTTTCTGCTACAAATAATGCAGTCATTATTGACGAAGGAATTAATT
GTGAAGATGATATTCGTGATACATTGCATGCGATCATTCAAGATCTTCGTAATGGTTCTA
ACAATCATGTTTGCGATGCTGCATCTTACTATGTTAATAGAACTGTTAACCCAGTTGAGA
TTGCACAGATTGAACCTGCTGTTAAAGAAACATTATTTGCTTATGGAAAAGTTGATGATG
TGCTTCAGTATGTCATTACTAATGCTCTTTGGCCAGTTCAAGGTGATGATGGTTTAACTC
AATTTACTGACGTTTCAATTACTGATTCTTCTTACACTACATTGAATCAGTTTACACCTA
CTAATGTAACTTATAATCCTGCTACTGGTGATATTGTATTGACTCTTGAATCTCATTCAA
TGACCACATCATCAATAGGTAAAATTGCAGTCGGTGGAATCACATTTACTTGTTACAAAG
ATGGCAATGATCGTCCTACTGCATATCCACGGAAGACTCTTCCTGCTGCTAAGGCAGTTC
TAGCTGTCACTGCTGTCGGTGGTGGTAACACAACTATCACAATTAACGTAGGTAAATCAT
CGGCAAGAGATCAATATACTCATACATTTGTATCTGCAGTAACTAATTGTGTCACTGAAT
TAAATTATACTACTGGTGACTGTGCTGATGTCTATGCAACTGTTGGTAACTTAATTAACA
TCATTACAGATACAATTACAAATGCTTCTGCATCATCACCTGTTGATCATTTGGCAAGTG
TTACTAAAGTTGAACCTGCATATGAGTTCGTTGGTGGAACAGTTGACTGATATCTAGAAA
CAACTTTAGCAGTTGATTATCCTAATGCAGGAACTGATTTATCATATACAAATCAGATTG
GTCGTGATACGCAATATCGTTTCAGACATGCTGCAAACTTAATCAGACTTAATCGCACAG
CAATCGTTGATAAAGCAGCGTACGATATGTTACAACGTTATCCTACTTTGGCACAAACAA
TGCCTAGAAACCAAGGTGGTGCAAGCACTGACGGAACTATTGGTTGTAAGACTGACTTAG
GTAATTTCTTAGATGCTATCGCAAATGATTTACAAGAAGGTGGTAATCTTGAAACTACAA
CAGGAACTGGATTCTATCTCTTCCAAAATGGAGGACTACGTTATATTCGTCTTCAAGTAT
TGCAGTCAGTATATGCTCATGGAAGACTTGCTGCGTATATGAAACAAGCAGTTTTAGGAA
CCTTAACTGACGCCAATACTAACAGTATTGTTGTTGGGGACTGGGGAGTTACTGGTTCAG
GTGTTCCTGGTTACTTTAATGCTGTCACTGCTAGAATTGATGAACTCATTGCAACTATTA
ATAATTTTATTGCTCCAACAGGTTCAGACTTTGAAACTGCTGCTGATAGATTATTCTTTA
ACAAGCAATTTATAAAGGTAGAAATTAACGCTCTTGTTCTACAGTTTCTTTCAGATGTCT
TAAACGGTGTCACCTATAATGCAGGTGCTTGGAATGATGTATTAGATGTTGAAGATTTAA
TTGTTGATCTTATTTCTGATTTACAAGCGGGTGCAACAGATAGCACAATTCAAGAAGCAA
GCACTTATCTAAATCCTGCTCTTAATATTCAAACTCTTGACAATGTTCTCCCTGCAGCAT
TATATGCTATTGAACAAGTTGGAATCCTTGGTGCGTATGCGATTGAAAATGGATTATTTG
CTCAAGCAGAAGCCGGAAGTGCAAACAGTTCTACTCACTATGAAGCGCAATATACTGATG
AATCAGGATATAGAAATTCTATTTCAAATTCAGCAATCAATGGTGTTGTATATGTATGGC
GAGACTTAATTGAGATTGTTAAGGATATATTCTCACCTGGCGGAAAAGAAGCAAGATCTG
CTGCTAAACAATTAGAGTATAATGAAAATTATTACACTAATGAATTAAATAACGCAGTTA
CCAATCAATTTGGATCTGGTGCATGGTCTACTGATCAGGAAAACTTTATAGAAGGTATTG
TTGGAGACATTGTTCATGATTTTATGACAACTGATACTTACGATAAATCCACAGCATATA
CAATTACATTAAGTGCAATTTCGGGTACGTTTGGTGTTGGAGAACTTGTTACTGCCAGTA
ATGGTGGTGAAGCAACTGTTCTTGAATACGACGATGTAAATAAAATCCTTTATGTCGGTG
CATTTGGTGGAAGTGCATGGGTGGCAACTAATACATTATCTGCTCCTAGCGGTGCTACAG
GAACCATTGCATCTGGTGGCGTAAGTTCTGCATACAATTGGTATAATAATCCAACTAACG
TTAAGATTATTGATAGAGCAAGAATCCTTACATCTAATATTAAAGGTCTTGTTTCAACTA
CAAATATTTTCCCTGATCCCGAAAATCTTAATGCTACAGGTTGGACTGCATCAGAAACAA
CAACATCAGTAGATGTTACTGCTGCTCCTGACGACACTCAAACTGCTGATAAGTTAATTG
CTACAACAAATAATACTGAGCCCGACAAGTATAAGAATTTCACTCTAAATTCCTTTGAGT
CATTTGATAGTGATAGTACTAAATTTGACTCTACTACTTAAACATTCGATACTGGTTCTG
CAGGTGATGCAACTACTCAAACATTTACATATTCAATCTTTGTTAAGGCAGCTGAGTATA

CTAAGACTCGTATATACCTAGCACTTGATGACTCTCTTGCAACACGTCAAAGAGCATTCT
TTGATCTTGATTTATCAAATGGAACTAAAGGTTCTATCTTTCTACCTGATGTTGCTAAAA
CTGTAGATGCCTTTGGAGTAATTCCTCATGGTAATGGATGGTATCGTGCTTATATTGCAG
CAACATTTGGATTTGGATTCTCTCAACTTCGCACACAAGTTTATACTCGAGGTTCTAACG
GAGCACTCGTCCATACAGGTAATGGATCTGATGGATTATTTGTTTGGGGTCATAAACTCA
ATAGTGGTGCCCTTGATCTTTATACTGCAGCATCTGGTACATTATTCTATTCAAACACTG
TTTATAATGTCAAGACATTTGCTTTGGATAATCTTGAAGACTTTATGGGTAAGGCACTTG
ACAATACTTAAACATCTCCTTCACCTTTTGCATCTTTCTAAAAGTTCTATGATGCTTCTC
TTGCATCAGATTACAACAAATCTACTATTCAAAGGTTTATTCGTTATCATCTAAATGTTA
TTAGAAATCAGTTAGCACAGTCAACTTATTATACAACTCTTACTAACAACAATGCGATCA
CTGCTCCTACAATCAAATATGGTAATCCTGAGGTTCCAATTGGACTTAGTGGTGGTGTTA
AACCTGCTGATTACTTCTATGGTTTACTTAGTTACGCATCAGCCGAAGTTGAAACTGTTA
CACAGAATACTGGTGAAGTAGTTCAAGTTTATAATAGAATTAGAATTGACGGTAACATAA
CTGATTTTCCTTTCACAATGGGTGAAACTGTTGCTAAAAAGGGTGCAGGTGGTGTTACAG
GAGTTGTATACGGTTTCTACGAAGATGCAAACTATAAGTATCTTGATGTTCGGGTAACCG
CAGGGGTTTGGGCAGTTACTGACAATATTGTAGGTGTAGCAAACTCTACCTCTGCTCAAA
TTAGTGCTATTGAAGATCGTCTTCATATTATCGATCTTCAAGGATTGTTTGAGAATGATA
TCCCATTCAAAGGATATACATCTGGTCAGACAGCACAACCAACAGGATTCTATCAAAATA
ATGCAGCGATTACAGATAACACTGGAGGTACTTTAACAGTTGATAGTGCAAGTCTAACTG
GAACATGTGAAACCACTGCAGTGGTATATCCTCAGACCTCTAGGAAGTTTATTGATGTCA
AGAAGATTGATGGTTTAGATGTTAAGGTTGGTGATGTAGTCGCTTACACAGGTAATATTC
GTCTTGCTATTAATATCCTTAATAACTTGAATGTATTTACCGAAGGTAATAGACTGTGTT
CAATAGTTGCAGGTCAGCAGCAAACCAAATATGTAGTTATTTCTGGGATAGATCTTGCTA
ATAATTACCTATACGTCACTCCTGTTGGCGGATTTACACCTTCAATTGGTGGTGTTCTTG
GTGATTATGGAACAGGAACTGCATTATCTGTGGGACAAATTCAGCTTACAACTGTTGTTA
CCAATCCAGGTCAGGGTTCTGCTAGAGTTCAAGATATTGAAAATATTGGTTCGAATAAGA
GATTATCTCTCTCTTCTGTTGTTGGTTCATTTACTTACAATTATGGTATTAAAGGTCCTG
ATAATTATGCTTCAGCGATTCTTAATGTTGAAGATGTTAAAGCTCGTGTAAAACGTTCCT
TCAGAGGATTTGATGGAACACAAACATCATTCAAACTCACTACAGATAATGGAACTCAAT
ATCTGCCTGACCCTGCAGGACACATGCTCATCTTTGTTGATGGTATCCTACAACCACCTG
GTGCAACTAATGCATACACAGCGTTCTCTGGTACTATTCAGTTCACTGAAGCACCTGATT
TGGGAGCATCCTTTACAGGATTCTACGTTGGTAAACTAAGACAACTTGATGATATTAATA
TCGAGTGTGATTCATTAAGTCAATCATTCAATTTGAAACGTAATGATGTGTTCTATTCAT
TGACTCTAACTGATGGTGTGCAGTCAAGCACAATTAGACCCGAAAACAATATTATCGTTT
CAGTTAATGGTATGATTCACGAACCTGGTATTGGTTTTGAGATAGTTTGTTCTAGAATCA
TCTTTACTGAAGTTCCTAGATTTGGTTCTACATTCGTGGCATTCTCTTATGTTGGTTCTG
AGGCAGACGTTGATGCTGCTGAGGTTGTTCCTCCAATTGAACCAGGTGACTTTATTGACA
TTCAAGGTGAGACTGCAGATAGAGAAGTTGCTGTTATTGAATCATCAAACTCTCTAATTA
CATTTGATTATCTTGGATCTGTATTCGAACAGAAAGCTGTCGGAACTGCAGCTCTAACAA
CTGGATTTATTAAGAATGTTCAGGTAACTGGTGGTGGTTCAGGATACACTTCCACACCTA
CTGTTCGTGTTGATTCAATCGCAGGTTTTGAGGGTAATATCCGTGCACTAGTTGGTGTTG
CGGGTGTTGAACTCAGTGCAACTGGTTCTGGATATCAAAATCCATCAGTTGCAGTTGAGA
CCACTGTCCCTGATGATTATGTCGCACCTAATCTTGCAGATTATGGTGAAGAACTAATTG
ATCCAGAGATCCCATAAATAACTAAAAATCGTAGCGAGTAATGGCTAAACAAGCACTAGG
TCTAGGTTAAGCAGCAAATGATAACTCAGGGGATACCCCGAGCGCTGGTGGTGACAAGAT
CAATGACAACTTTACTGAAATATACGGTGCCATTGTTAATGGAACTACACTGACTGTTAG
TGTTACAAACCCTGCTGTTGGTCAGGTTCTTAGATATAACGGAAGTACATTTTTGACTTC
TGACTATACTCAACTTACTTCAGCATTAGATGTAAATGGTATCAATATTGTATCCTCTTC
TAACGGTAATATCGTTATTGATCATAACGGAACTGGCAACGTAACTATTTCTAATGGAAG
TGTCACAAACACTTTTAATGGAACTACTGCAATAGTAGACTTTCCTACAAAAATTCAGTA
TAAGAATGAGTTTACATCTCTTGGTGCTGCTCCTGCTGCAGCAACATATACTGGTTATTT
CTTCTCTGTAGATGGTAATGATAATACATCTGTGAACATTAATATAACAGCAGGTGGAGC
GGGCGTAATAAGAGCAAAGCTTGTAACTGAATATTTGAGTATTGGATTACTTTCAGATGT
TGATATTACTACTGCAGCACCAACTAATAACCAAGTCTTAAAATGGAATGGAACTAAGTT
CGTTCCGGCTGATAATATTCAGGGTACTTCAGGAGGTGCCACTCAAACCCTCTTCGCTAC
TATCTTTGGTGACAGTGGCAGCACTACAGCGAATACAACAACAGATTCTTTAACAATTGC
AGGTGGAACTAACATCACTACTGCAGTTGTTGGTGACACTTTAACAGTGAACTTCTGTGG

AACGTTGACAAGCACCATATCAGCGTTAACTGACACTGATATGTCAGGACTTACTCAAGG
TGATTCATTATTCTATAATGGAACTAATTGGGCTCCTACTAGATCACCTATCACATGGTG
GGAACTTGGTGCCACTGGTAATGCACAGAACTTTACGTTCAATGGTCATGGTTTCTCTGC
AGCAACAAATGATCCTCAGATCTATGTTCAAAGAGGAGCAACGTATGCTTTTGATAACTC
TACTAATGGTGGTGCACATCCATTTAGAATTCTGAGTTCAGCAGGTTTAAGTGGCACTCC
ATACACCACTGGTCAAAGTGGTAGTGGAACTGCAGTTCTTTATTGGACCGATCCTATGGA
TGCGCCTGGCACAATTTATTATGAATGCACAATCCATTCACATGTGAATGGGACTATCAC
CGTTGTAAGTTAATAAATGACAAGAACTGTTCCTGGAACTGGAGCTGTAATCGAACCAAT
ATATGATGGAATATTTGGTGTTCGTGCAGTAAAAGTATTAAATGGAGGATCTGGATTCGT
TCCTAAGGATCTCCCACGGCTTACTGTGAGTGGTTGCGGAACTCCTGATGAAGCAGCGTT
ATTATATCCAATCATTGATGCTCATTCTGGAAGAATCACTGATGTAAGAGTATTGAATAG
AGGAAAGGGACATGATCCTCTAAGATTGCAAATTATTCCTGAGCAGGATACACTAAACGT
CGTTACTTCATTTGATGTCAATAGAGTTTGCCAAAGTCATCCTAACTCTCCTACAACAGG
TACCTTTACATCTGTTAGACTAAGATTAGTTAGTGATAATCACCCTAAACCAACTCAGCA
TTTCTTAACTGAAAGACAACCTGGCGGTTCTACAGTTGTCGTTGATAGAAGTTGTGATGA
TACGATAATCTACCGTGGTGGAAAGGATGTTCCTTATGTTGGTCAAAGAGATTTTCAGAA
AAACAAAGCACTTGGCATGATGGCCAATGGTGTCTTACTTCATACTCCAGAATGGGGAAC
ATCAGCAGGAACTCCTACAAACTTCTCTGCTGATACTGCAAAATACGGTTATGTAAAAAA
TACTAATACTTATGATGCCGTAAATGATAGTGGCGTTTATTATTATCAATCAAGTAAAAT
TGTAAATGAGTATGCTCCTGATAATGGTGTATTTGATTGGGGTGATATACAACTCTACAC
TTGGAATATTTAAGTAGAAACAGGAAACCTTGTTGTTGGCGTATCTAACTTAGATGAAAC
ACTTGGGTCTATTGAAGTTGGTAGAATTGTAGATGAAGTCTCAGGAACTGGTAGAGGTGA
AATTGCTAAGATTGTTAGAGATAATCAGAATAATGTTACTAGAGTTTAAATTAGAGCATT
AACAGGTGATGCTTTCCAAGACAATGATCTTTTGTTAGGTTCTAATGGATTTGAATTTAG
ACTCAGTGTTGATCCAATATCAATCAATCCTTATTATATTGATTTTGGATCTGACGCAGC
AAAGTTCGGTCCTTTCACACCTGGCCAATTTTATATCGCTCCCGAAGATATTCAAGTAAG
AGCAAATTATTTAATTAAATTTAATCAATCTGACGCATCTAATCAGGCAGGTACAGGACA
TCCTATCAGATTTAGCGCAACTCCTGAGTGTACGTTGAATAACGGAACATTATACTACAA
TAGCACTGGTGTATCTCAAGCAACTGCTACAGATTATGAATCAATTTATGCTCCATTATT
CTTGATGAATAGTGATGAACAAACAAGGATTTACTTCCATTGTGCTTATTACAGATATAT
GGGAGGATATGCAGGTGATGAAGGTTACATGACCCTCAATTCATCTGCAGAAACATATAC
TCCTACTAACAATTATTATGCTAGGGACTTTTATCAAAGTAATAGCAGTGATCCCAACAC
TATAGATAAGTCTAGATATATTGATGGACACTCAAAAATTCTTGGTGTTTCTTTTGATGG
TTATGCAATTTACGGACCTTGGGGATATAATTCTAGTGGTGCAGTAGCAAGAGAAACCTC
TTCTTATAGACTTAGAACCACTGCTGAATTAGCGGGTGCTCGTCCTCAGGTAAATACAGT
ATCAACGGTAACATATGCTGTTACCATTTGTAATGTACAGTTCCTTTTTGGAGGTTCTCG
CCCAAACTTTTTAGAATTGGGTCGCGGTAAAACTTACAATTTCAATCAAAATGACGCATC
TAATGACAGTCAACACTTACTAATCTCAACTGCGGACGATGGATGGCATGGGCAAAATCC
TGTTATCATTGGGAATACTGCAAATTTGTATTCAGGCAATGGAATTAAATATTATATTGA
CGGGAGCGAAGTAACCTATCAAGCATATTTGTCAGGTTTTAAATTGGCAACATCTCGTGA
GGAACTATTTACAGTTCCTGTGAATGCACCTACTGCGTTATATTTGTTTGCATATACCAC
TGCAGGTCATGGAATTAGAACTGTTCAAGAAGGATATGTTCTAGGAGATTTGGTAGAAGA
TTGTATTTACGATTCTTCTGTAGGAACTCTTGATGAATTTAACTGTAAATTTGCTGTTAC
ACCACAGTATCCTAACGGAACCTATGCATACTTCATGAGTGAAGATAGTAGTGGTAATCC
TGCATATCCATATGCTGTCGGTCCTAAGTTTTACGGAACACCATTATTTGAAGGAGATAT
TCCACCCGCGAAACCAACTGTATTCCCAACAGATGCAGAAGGTGATGTAGTTCTTGCTGA
GGATGGTAGTGTTTATTATATCAAGATGACGAAGAAAGGTGATAATTTCTTCGGACCTGC
AAAAGCCAAAATTCTTTGTGGGGAAGGAACTGGTGCGGTTGGAACTCCTACTGTTCAAAT
TGTTACTGGTCTAGCACTACTCGATGGTGGTAGAAGTTATGCTACTCCTCCAACACTTAT
TTTTGAAGGTGTTGGTGGACAAGGTGCAGAAGGTGAGCCTGAAATTGATACTCTAGGTAA
AGTTACATCTATTGCAATTACAAATCCAGGTGAATTCTACCAAGAACCTCAATTTATTTT
AATCACAGGTGGTGGCGATATTGGTGCCAAGGCGGAAGCAACTATCGATCAAGGTGTTAT
AACAGCAATTAATATTACAGATCCTGGTAATGGATATACTTCAATTCCAAACGTTATCTT
TACAAAATTAGTAACACTTAAGAGAAAAACTAGAGCACGTGAGGCATTTAACTAATCAAA
CATTTATTTGACTGGTCTTGTTAACAATGTTACGTCGAATGATACAGAAATATATGTTAA
TTCTACAGATGCATTTCCTGGCTCTGGTCAAATCATCCTTAATTATGAAACAATCTCTTA
TACCTCTAAATCAGCAGGTAAGTTTGGTGGTCTGACAAGAGGTGTAAACTTTAATTATGA

TCAACGGGTTATTCATAACGCAGGTCAAAATGATTCAAATGGTGTTTCAACCTATAAGTT
TAAGGTTGGTTATAGAGTTATTCGTAAATTTGAAAATGCTAATAACAAAGTTGCAAAAGT
ATATGACTGGAATCCTGTTACCAGAGAACTTCTAGTTGCTTTTCTAGTTGATGAATTAGC
ATTTATTGACGGTGGGATTCCTTCTACTGAAGATCCTATCGTACAGTTTGATGCAGGAGT
TGCTTCTAGTGCACCTTTTGGATTCCAACCTCATACTGTTCTTACAACTACTGGATATAA
TATTTTCTTATTGACAGATCCTCCTTCTCTTTTGGCAGACAGATCTTTCCAAGATATTCT
ACCAACAGCAGCAGGTGATGGAATTGCTGACCTAGATAAGACTGGCACTGATTATGCTAA
TCAAATTAGTCTTGATGGTGGTATCTATCAGTCTTTATATGGTATAGAAGAAACTCAAGG
TGGAACAAATACAAGACTATTCCAACTTGGTGATAATGTAAAAGATGGAACTATTCCATT
GAAGTATGCAACAATTATGACTGTAGGTGCTTTGAGTGATGGTGTTGAACACACTGCACT
TCTAACTATTAAACTTGATGCAGCAAATGGTAATGGTATAAATCATAGCGTTAATGAAGT
TGTTACAGGAGCAACTTCTGGAGTGATTGTAACTGTTGTTTCTTATGATCCAACAACGAC
AACAGTAGTTGTAGGTCGCATTGTCCCATTTAATACTGGTTATATCAATGTTGGTGTTGG
TGGATATCTTTATGAGTTCTCTGATAGTGGAACTGTAGTTGATGTTATAATTCAAAATCC
TGGCAGAAACTATACTGCTGCACCAACAGTAACGTTGGAAAATATTGGTGCCATTCAAGC
GACAGGAACTGCTGTTCTCACAGCTGCAGGAGACAAAGTTGCATCTATTACTATCAATAT
TGGTGGATATGGTATTCAACAATCTGTTGATAACACATATAACCTACACCCCACAGTAAC
ATTCACTAATGCAACTGGTGATACCACTGGTGCGGGTGCAGTTGCACAAGCAATTCGAGG
AGGTGAGGAGCTCGTTGTGAACGGTGGTGCACGATATAGAATCAATAGTATAGAATATCA
AGCAACTGTTCGCTCCGCACCCTCATAAATAAACCGGAGAACAATAGTATCCCAAAATGG
CAGCCCTATTAACTGATCAATTTAGAATATTTTCTGCTAAGAAATTTATAAAAGCACTGG
AAGGTCCTGACGCAACTCAGAGTGACGATGCAGCGGGAACTTCCCGTGATCGTTTGTATG
TTTTCAGTGGTAGACCCCAAACTTGGGATAATGAAACCTCGCCCCCACAGGCAGTAGACT
TATTCTCAGAATTTTCTGGATCGTACGATGAAATGATGTCGATGAAGAGAGTCCTCGCTT
CTGACACGATTCAGGTCGTTCGCAGAATTGACTGGGTTTCCCCAGAACAAACTACAGGTG
GACTAGGTTTTACCTATGACATGTATAGACTTGACTATTCTCCTACCAAAACTGCTGATT
CAGGTGCTAGCAAATTATATGACTCAGACTTTTATGTTGTGAATTCACAATATCAATGTT
ATAAAGTCATCTATAATGGAACTTCTCCTTCAGACCCAAATGGAAAACCTTCTACAGTTG
AACCTACAGGTACTTCTACTTCAATTATCACTACTGGTGATGGTTATCTTTGGAAATACA
TGTATACCATCCCCGTAGCATCGGGTCTTAAATTTTTTTCAAATGACTACATCCCTGTCT
CCACCAATACATCGGTAAAGACAAATGCAGTTGTAGGTGAAATTGATACAGTTGTTATCA
ACTCTGCAGGTTCTGGTTACAACAACGGAACTTATGCTAATGTTGCAATTAATGGTGATG
GAACTGGTGGTCGTGTTTCTATCGTTGTAGACGGTGGTAAGATTATTTCTGCTACTGATA
CTTCTGGGGGACCAGGATATACATTTGGTAAAATAAGCGTTAACAACATCACTGGTATTC
GAACAGGAACTTGTGTCATAGTTGCTGTTATCATTCCTCCTCCAGGCGGACATGGATCAG
ACGCAGTTGTTGAACTGGGTGCTTTCCGTGTAACGATCAACGCGAAACTATCATGTGATG
AAGGTGCAGGTGACTTCCCAATCGATACCGATTATCGTGGTATTGGTCTTGTTACTAATC
CTTTAAATTTTGGAACTTCTGAATTGATTTCTGATTTGACGGTTTCTGCTACGAAGGCAG
CAATCTTCTCCCCTACATTCCAAGGCAACTATGTGGCTGATGAAATTATTACTCAAACAC
GAGTTGTTGGTGGAACCAGCGTTACTGCTCGTGGTCGTGTTATTTCTTGGAATGCCACAA
CTAAAGTTTAGAAATATTATCAGAACGCTATTGATGGAATCTTCCCCGAAGTTACAGGGG
CACAAAATGAGTTTGACGGATCTAACGTCATCAATGGTGCAACTTCTCGTGCAGCTGGTC
ATGGTCAGCCAGATGTTAATTTCCCTGCTGTTCCCAACTCTTCTTCTAGAACAATCAACA
ACACAGAATATGATTTGGGTATGTAGTTTAACAATGGTTATTCCCAACCAGAGTTAGCGT
CAAATAGCGGTCAGGTAGTTTATATAGATAATAGATGAGCAATCAGTCGTGCTAACGACC
AAGTAGATGACATCAAAATCGCAATCGAACTCTAATGGCACAAAATACCAACCGAAACGT
CACAACTTATTACGACGACTTTGATACGAGCAAAAACTTCTAACGAATTTTGTTACGTCC
TGGTTTCCCTATTCAAGCGAGGGAACTTACAACGATGCAAAGCATCATGCAAAACCGAGT
GGAGAATGGTGGTGCTCACTTGTTTAAAGATGGTGCGATGGTTATTCCAGGTCAAGTTGG
TTATGACCTGACTGTTGATTGCATCATGCTCCAAGAATCTTTCTTAGGTGCTGATGTTGA
ACTTTATAGAACTCAATTAAATGACAAGGTCGTCACTGGTCTTACTACAGGTGCAAAGGC
AAAAGTTCTTTACAGTATTTCTTCTACAGAATCAGATAAAGGTTATATCACTCTATATGT
AAAGTAGATTGAGTCTGGTGGAGCTAACAGCACTCAAGCAACTTTCGCAAATAATGAACA
ATTAATTACAGATACTGAAATCACTTTCGGAACCACTTTGATTGAAGTTGGTTCTCCTTT
CGCACTATTACTTCCTACTGGTGCTCTTCAAACAGGTTCTGTTGCTTATGTACAATCTGG
TGTATATTTCATCAGAGGATTCTTTGTTGATGTCCCATACCAATACATGCTTTTAGATCA
ATATCGAAGCAACCCCTCCTATCGTATCAGACTTGATATTCAAGAATCAATCGTTACCCC

AGAAGATGACCTTAGCCTCAACGATAATGCTGCAGGAACATCTAACTATGCAGCTCCTGG
TTCTCATAGATCTGGAATCACCACAAACTTAATTAAGAAAATTCTTACTGATGATGCCGA
TAAAAACTTTATCGAACTTCTTCGTATCAATGCATCAAAGGTAGAGAAACTTCTTGATAG
AACTGCGTATGATGAATTAGAGAAGAGTATTGCACTTAGAACTTTTGAGGAGTCTGGAGA
TTTCGTTGTTAAAGACTTTGAGGTCACTGTTAGAGAAAACCTTGATGATGGTTTTAACAA
TCGTGTATATGCTGCGGGTGCTACACCTTCATCTGGTGCAGTTTCTGCAGGATCAAAATA
CTCTGTTGAATTGGGTGCAGGTGTAGCGTACGTTAGAGGATATAGAATTAAGAGATTAGC
ACCAACCTACGTTGAGTTAGACAAACCTCGTGAAGTTAAGGCAGCACAGAACACGATTGT
TCCGTTTGAAACGGGCAACTTTACATCTGTAAAAAATGTTTATGGATTCCCTAATGTTGC
AGGTTCTACAATCACTAATGCATATCAAACTGTCGAATTAAGACATGCATTTAGAACTAC
ACCAGGTGATGCAAATGGTGTTGTTATTGGGTTTGCTCGTGTTTCCTCTATGGAGCATGT
TGGTGATCCTGATAATACTTTTGGAAATGCTGATGATCAATATAAGTTGAATATCTTCGA
TGTTCAAATGTTCACAGTTTTAGAACTCGCAACTGCTCAAACTATTGCAGCAGCTTCACT
AGTTATTGGTGCAACCTCAGGTTATAGAGCATATTTAGTCAATGCTATTTCTAGTGATGG
TACTATCATTGTATATCAAGTAGAAGGTACTTTTGTAGAAGGTGAGATGGTCACCGTTGA
TGGAGAAGGAGTTGATACTATCTCTGCATTACATAGTTACACATATTCTGAGACTCGTCA
GTATGTCTCCAGAAATGAAAGCACAAATGCTGTTGAATTAACTGCAGATGCAATCTTAAA
AGATGTTTTACCGGTTGAGGGTGAAACCTTTACATACAGCACTACTGCAGGTAATGTTTT
AACTACAGATACTGTCAGTGCTGCTGATGGGTCTAGAACTGCAGGAACTTATATCGTAGG
TGCATCTGACTACACTACTGATGCAAATGGAACAGGAGCAACAATCTCTATCGAAATTGA
CGGTTCTGGTGCTGCAACAGTTACTATTCTTAAGGGAGGTTTTGGGCACGTTGTAGATGC
AACATTTACTATAGCAGATGCTAAACTTGGAGGAGGCGGTGGTGCTGCACTCACATTGGA
TGTTGCTACAGTTGGTCCTTCTATTGAGGGTTTACAATCAAACTTTGCTTTAGATCTAAG
ACCTGGTGATCAAGTATATATGAGTTCTACAGAATTTGTTACTGTTGACACTGTAACTCA
AACAGCAATCGCTACTACTAATACATCAAACATCTTTAATTATGGAAGACAACTTGTAAA
TGTAACAGCTGGTTCTGCTGCTCCTTCTGCAGACACTTATAATGCTGCAGTTAGATTAAG
AACTAAATTGAATGGCATCAATAATGCTTATCTTCTTAGCGAAATGCCTAAGGAATACGT
TAAGAGTATTCCTGATGAATCAATGATGGTTAGAAGAACTTTTGATGCTCAGACTGTTGC
TTCTAACTCTATCTCTATTACTTTACCTGAGAACGAACAGTTCCAATCAATTAGTGATGA
GAACTATACATTTACAGTTCTTGCAGGATCTAACTCTACTAACCCAGTTGGTGATCAGAT
CACTATCAATACTACTAACTCAAGTGCTGTTTGATATACCACATTCTCAACTTCTGAAAG
AACGACTTTACAGATTCCTAACTTAACAAATATTACTTCTATTAAAGTAACATCGTCTAT
TTCTAAGAACGTTACTACTAGAAAAACAAAATCTGGTAATCATATGTTTGTTCTTAAAGT
TAATTAAACAACTTAAAATCTTGATAAGCTAAATTATAATTTAACATATTCAAATCTATA
CGGAACAAGTATACAAGATAGAGACATCTCTCTTGGTTTAGTCGACTGCTATCGTCATCA
TGCTGTACATGAATCTCTAGATGATAATGATCCTGTTATTCCATCTGTAACTTTACTCGA
ACCTACTTTCTTTGCTACTGGAACTATCGTAACTGGTAGAACTTCAAAAGCAAGGGCAAA
AGTTGTTGCTTTCTCTTCAGGAACCCTAAAACTTAATGTTGTTTATCTATCTGGTATATT
CCAACCAGGTGAAATTCTTGATGGTTTTGACAGTAATAGTACTGCAATCAATGCAATTAT
TAATGATAGTGCAGGATCTGTTATTGTTGGCTCAAAAGTTGTAACTGATAGTTACTGCAT
GGAATCAAATCAAACAGGTTTTTTGTATGATCTTTCTAAAATTGTTCGTAAGAAAGGTAT
CGTTGCTCCTGTTAGAAAATTAAAAACTATTATTGACTACTACAGTCATTCTGCAACTGG
AGATTATTTTGGTGGTCAATCTTATCTCGATACATCATACAGCGATATTGCGTTCTTTAA
GGTTAAATTCCTTGCTGATTACCTAGACTTTAGACCAGGTGCAAAGAACTTGTTTAGTGG
ACCAGGTACGGTAGCATCTCCTGCATTTGTAAATTGTTCTACATTTGACTTTAAGTCAAG
AGTGTTTATTGTATCTGGAACACCTAATGCTACAGTCTTTGATATTCCAAAATTATATAG
TAATTTCCGTTGTGATTTTGATTGGTATCTACCTAGAGTTGATAAATGTTTTGTCACTCC
TGATGGTGAGTTCCAAATTATTAAAGGTAAATCTTCTGAAAGACCAAAAGAACCTGATTC
TCTAAAAGATGGTATGCTTATAGCAACCATATCACATAAACCATATGGTTTCGATCCACT
TGCAGATGTAGTAATTAAACGTTCTGATAAGAAACGTTATACCATGCGTGATATCGGTGG
TTTAGAACGTAGATTAGATCAGGTTTAATATTATACATCACTCAACATGCTTGAGACTGA
TACAGTCAATAATCAAACTCTTGATGCTAGTGGTAAGAATCGTTTGAAGAATGCATTTAT
TGTTGATGATTTTGGTGATCATAGTAAGTCTGATGTTGGACGTGAAGATTTTTCTGCATC
GCTTGACTTTAGACAAGGGTATGCTCGTCCCTCTCATTACACAACTAACGTTCCTATAGT
AATTAATACAACTTTATCCACTAATATTCAACAAACTGGTCCTCTAATTACGTTACCATA
TACTGAAGAGGCTATTGTTAATCAACCATATGCTTCTCGTGTTGAGAATATTAACCGATT
TAACGTCTCTACCTATATCGGTCGTATTACTTTAACACCTGGTTCTGATGATAGGTTGTA

TACTAATAGACTTCCTGCATCAGTTCAACAGATTGAAGGTGATTTCTTACAAGTTTCTTC
TGAATGAAATGTTGATCAGAATGGTTTTGCTCCAATTGAATGGGGAGCATGGAAACTAC
ATGGACAGGTGAAGAAGTTAGATCATCTGAGGTAACTAGACACTCTCATTGGTTAGAAGA
AGATATCGGTAGATCACCTAGACCTGATGTTTGGGATGGTCGTGGTATGCGTCGTGGTAA
TCAGACTGAGACAATCGAAGTTACTGAAGATCAAGTAAGATCTGGTGTTGGAACTCAAGT
TATTCCTAGAATTGATTGTCAATCTTTGGGTGATGCTATTCTTTCTAGCACATCTATTCC
TTGGATTCGTTCTAGAAACGTTGAAGTAGATGATGCACGTCTAAAAGCAAGAACTCGTTT
CTATTCATTCTTTGATGGAAAGGCAACTATTGATTATCAGATTCCAAAGATTATTGAAAT
TATTAAAGATCCTACGGTTGATAGTAGAACAAACTCTACTCCCTTTGTTATTGGTGAAAC
CGTAACAGGTGAGATTAGTGGTTGTAAGTTTAAGGATGCTGCACCTGATGATTTTTATAA
GTTCAATCCTTATGATAATACAGCACTTCCTACTTCATATGCATCTACAACTGCATTCTT
GAACATTGATACTACTGAACTTTCTAAGCAAGCAATCGGTGCCTTCTATGGTAATATGCA
GGTTCAAGAAGTATTAGTTGGAACTTCTGGTGCTCGTGCTGTTGTTAAAGACCGTCGTAT
AATTTCCGATCGTTTTGGTAAATTAAAAGCATCATTCTTTATTCCTAATCCTGGTGTAGG
TTCAAACCCAAGATGGGCAACAGGAACTAGAACTTTAAGACTTTCTACATCTGATGTTGA
TTCACGTCTTTCTGGTGCAGTTGCATCTTCCGCTCAAACTTCATATGAAGCAAGAGATAC
GTTAAACACAGTTCAAGAAAGTATGTTGGCATTTCGTAACGCAGAAATAGTTACTGATAC
TGTAACTCAAGAACAAACAATTCAGACAACTAGAACTGAGAGTAGACAGATTGGTTGGTA
TGACCCTCTCGCACAATCATTCATTGTTGATGAAGTCGGTGGTCGTTTCCTTACTTCAGT
TGATGTTTACTTCTCTAAAAAGGATGCAAACATTCCAGTATCTATGCAAATCAGAACAAT
GGAAACTGGTAATCCTACAACAACCATTCTACCTTTCTCTGATGTTACTCTAGAACCTGA
TCAGGTTCAGTTATCAGAATCTGCTGCAGTTGCAACTAGATTCACATTTGATGCCCCTGT
ATACATTCCTCAATCACAAGAACATTGTTTTGTTCTCCCATGTGACTCTAACTCATATCA
GATTTGGATTTCTAGAATGGGTGAGATTGATATCTCTGGTGACAGAACAATTTCCGAACA
ACCTTATGCAGTTGTTCTATTCAAATCTCAGAACGCTACTACATGGACTACAGATCAAAA
TGAAGATCTTAAGTTTATTATCAACGCTGCATCATTTAGTAATACTGGACAAACTTAACT
TGTATTGAATAATGCTGAACTCGGTAGAGGAAATGGTGGTAAGTTAAATCTTCGTTCTGA
TGCTATACAAACATTCCAACCAGAATTAGTTCTTTCACTTAATTCCACAACCTTACCTTA
CACAGTTGGTTCTCGTATCTATCAACAGACTACTTTGGCAGAGGGTACGATTACTGCAAG
AACTGTAACAGCAGGTGGTGTTCAATTAACTATTAATGATATTAGTGGTAACTGGGCAGC
AGGATCTAATACTGGTAGAGTAATTGCTAACCGTGTTGTTTCTTCTAAGACATTAGCAAC
TATGGTTGTTAGTGGTGCTTCTGGTGACTTTACAGTCGGTGACACAATTACAGGTAACTC
CTCTACTGCTCCTACTGCAGAAGTTGTAAGTTGGACTACTGCAACAAATACACTGACACT
TGAATATGTGTCTTCTGCGTTTGCTCCATCATCAGAAACTATTACTGGTGGAACTTCAAC
TAAAACTGCAACAGCCAGCAGTATTACTTACTCTGGAGACCCTGTTGAGTCTGCTGTAAT
CCAACCTTCCTTCCCAACAGGAACACCAACCTATTATACAAATCAAAGAAAGATTCGTGT
TTATCATAGTAATCACTGTATGCACAGTGCATCTAACAATGTGATTATTGAAGGTGCTAA
ATCTGAGGTTTCTGATACTGCTTTGACTGCAGCTATATCTGCTACAGATATTAGTATTGC
TGTAGGAGACGCTACTGCATTCCACAAGATTATTAACGGTGCTGCTATTGGTACAAGTAA
CGTTGGTTACATTAAGATTAATGATGAAATTATTTCTTATAGTGCAATCTCTAGTGATAA
CAAAACTATTACTGCTCATGAGCGTGGTCTTGGAGGAACAACCGCAGCATCACATACAGA
TGAGTCTGTAGTTGAGTATTATAACTTATATGGTATTCCTCTAATCGAAATTAATAAACC
TCATGCAGGAATTGGAGATGTTACTTTTGATTACTATGAAATTTCCACATCATCAATTGC
TAGATTAGGAATTCTTGGTGGTGGAGCAGGTATCATACCATAACAAAATATTGAATACAG
CACTCTTGTTCCTCATATTCAGAAGATGTTGCTTCCTAAGACTTACATTACTGCTAGAAT
TAACACTATTAGTTGTACGTCTATCAATGATGGATCTACTTTATCGGAAGCATCATTCTC
AAATGATGGAGTATTCTCTGATATTATTTTAAGTCAGGATAATGATTTGATGACACCACA
ATTAGTTTGTTCTGCAATCTATGAATCTTCTGAACTTAGCGGTGCTAAGTCATTCAGAAT
GGATCTTACAATGACTACTGATAAGACTACAATTTCTCCTGTTATTGATACAGATAGAAT
GTCTCTCACTTTGGTCTCTAACCGTATCAATAGACCTTCAGATCCTAATACTGCAAAACT
ATCAGTTGGTGATGGACATGATGCAGTTTATATTACTAAGACTGCAACTCTAACTAATCC
TTCTGGTTCTATCAAAGTTTATTTTGCGGGATATCGCCGACCCAACAGTGAGATTAAGGT
GCTATATAGAGTGCGTCCTGTTGGATCTTCTGTTCCTATAGAACAACTGGGATTTTCCTT
ATTCCCAGCTGCAGTTTCTAAGGTTCCAGCAACTACAGAAAGAGAAATATATTCATCATA
TGAATATGAAGTATCTGGCTTGAGTTTTGATCAATATCAAATCAAAGTAGTGTTTGTTTC
TCCTAATCAGGCATTAGTGCCGATTATCAAAGATCTAACAGCAATCGCTCTTGCTGTATA
ATGCAAATTCCTGTAAAAGACAATGCGAATTGGTTCAGGGACTCCTATGCGGGTTCCGTC

CAGTGTGCTGATAAATCTCTGAATGAGAAATATATGGCAGCAGATAAAGCCCGACAAGTC
AAGAAAAAGGAATTGACTACTTTACAAAATGAAGTTTCTGAGTTAACATCAGATATGAGT
GAAATTAAATCACTCCTACTAACGTTAGTCCAAAACAAAACATCATAATGACTGTTGAAA
AAGTATCACAAGAAGAGATGCATTCTCAATTCAAGGAGAGATATGCAAAGTTGATTGAAG
AAAATCAACACTTGTCTGCGAAAATTAAAGAAAATGAGGTGACTGCTCTTAAATTATTGG
GTGCTATTGAAACACTAGAATATTATACATAGGAAGAGGAAGAGACCGCTTCTACTCCTC
CTGATTCAGTTCCATCAGAATAATTTGAAGGGGGCACAGTCCCCCTCTTTTTTTAGCATA
AATAATAAATGAAGCATAATCTAATCGAGTTGTCGTAACAAATGGCAAATAGATTTCAAT
TAAGAAGAGGTGGCGAACAGGAATGGGCGAACTCTAACCCGACTCTTGCACAAGGTGAAT
TTGGTATCGAACTTGACACGGGTCGTTTTAAGATCGGTGATGCTGTATCTGCATGGAACA
CCTTAAGGTACGAACGTCCTGTAGAGTCTACACCTAATACTGCAAATACTTTAGTCCAAC
GGGATGCTGATGGTAACTTCTCTGCAGGTGCCATTACTTCTACTGTTATTGGTAGCTCGT
CTACTGCGTCTAGACTTGCTTCATCTAGACAAATCCAACTCTCTAATGAGGTTACTGCTT
CTGGTGTTTTTGATGGTTCTCAAAACTAAAATCTATCTGCTGAATTAGCACTTGTCTCTA
CTTTACCACATTACGATGGTACATCATCTCCCTCAGCAACTTACAATAAGGTAACTGTTG
ATGCTAAAGTTAGAGTAACAAACGCACAAGATTTTACTGCTTCTAACAACGGTACTCTTG
CTGATTATGGTCTTGATGGAACCAATGAAGGAACATCTGCACAACCATACGATTTAGACT
TAGTTGCTTTAGCAGGTTTGACCACTACTGGTATGATCTCTAGAACTGCTTCTAATACTG
TAGCAACTAGAGCAATTACTGGAACTGCCACAAGAATCTCTGTAAGTGAGCGTGCAGGTA
TTTCTGGTAACCCAACTATTGATATGATTACCACTGCTGTTACAGCAGGTAACTATAATA
CGGAATCCCTGACATCTGTATCTGGTGCAGGTGGAAATGGCGGACCCTTTGGTACCGCTA
CAGTTAACGCTACTCAGTTTACTGTTGATGCCTATGGTAGATTGACAAGTGCAACAAATG
TGCCTTTCTCTACTGCTGAAGAAGGTAGTCAGTATGCTAGCTATAATGCAGGTAGTACTT
ACGTTAGATATGACATCATTGCTAACGCATCTAAAGTTTACCAAGCCATCACTGGCATTG
CTGCAGGTGCAGGTGCACCTACTCATAGTAGCGGTGACTCTGGCGGGTGGAGATAACTCG
CTGCCGAAGCAACAGAGCAGAAGGGATTGGCTAGTTTTGCACAAGAAGATTTCGACGTTG
ACGCGAACGCGCACGTCACGATCGCCACCGCAGGAATAGATAATACACAACTACAACATA
ATAAACTTATTTTCACAGATGGAAATGCTGTTGAAAATTTTGAATTAGATAACGAACTTA
CAACTGCTACCGCCTACACTGGTTTTAATGAATTAAACTTTATTAAAATCAATAATACTT
CTGGCGGTTTATTATTCGGTGCTAATAATACTGGTGATAGTGGTGCAGGTGAGATCGATG
TAAACGTACGTTCTTACTTTAGCGATCCTGATATTACTTTAGATGGTTTAGTTGCACAAA
CTCTAAACAAAACTGGTGATGGTAATCTTACTTTCACAACTACTCAAAACTCATCATCTC
CAAGATCTTTAGCAGTTTCAGCATCAAACTCTGGTTCTGGAACTAGGACAGTTACAATTA
CTGCAGAAGATGTAGTTGATATTGATGCCTCAGATTCGAACGGAAAAGCTCATGTCGAAG
ATTCTAAATTCCAAGATAACTACATTGCTACGTCTAATGCCACTATGCACCTTGATCCTG
GTGATGATCGTGCTACTAGTGGGTTAGTTCGTGTTCATGGAGATTTACAGGTAGATGGTA
CCACTACGACAATTAATTCAACAGTTACACCAATTGATGATCCCACTATTACTCTTGGTG
GCGATACTGCTCCAGGTAGTGATGACAATAAAGATCGTGGAGTTGAATTCAGATACTACG
ACAATCAAGCAAGAATTCGCTTCTTTGGTTACGATGATTCTTACACTGACCGCGGGGGAC
ACGTCGGAGGATATACATTTTTACACAACGCCACAAATACATTAGAGGTCTTTAGTGGAA
CTGCCTCAGGTATAGCTGCAGGTAATTTAAAACTTACAACAAATACTAACTTAACATCTA
ATACTGCAGGAGACTTAGTAGTTGCAGGTGGTGTTGGTACTGGAGATGATGTTAATATTG
GTGGATTGCTAGATGTCGATGGCACATACCGTGCTAATAGCACATCTAGATTTGATGGTA
ACATTGTATTCCAAGGTGCTTCTAAGACATTATCATTGAATAATGGTAGTGGCACAACTA
AAATAGAACTTCACACTACTACAGGTAATGTAGAAATTTGTGGTATCATAACAAATACTG
GTGCTATTGATGCAAACAGCACTTTAAATGTTGCATCCACAGTACGTTTTGAAGACACAG
AAGAGCCAACTGTAGCACAGAATGGTGGAACTGGTATTTATGAATTTCAATCAAAAGATG
ATGGTGCATTCAAATTTGATGGTGGTGGATATGTTGAAGGTGATACAGTATTCAATAGTG
ATGTTTTTATTAACGGTGTTCAAAACCAAAGAGACAGTGCTACAGAATCATTCGGTAAAA
GAAACTATCTAAACATCACATATAAATTACGTTCTGGTTCAAGTGCAGCGTATACTCCTT
CATATGCATCTGATAATACTTCTAACTTAAGAATTTTTGGTGGTGCAGATATTCAAACTG
ACCTCCATATTGGTGATGATCTTTATATTGGTTAATTAAACTCTGGTGACAGTGTTTTAT
TCCAAGTATTAGGAGAGTCAGGTAATAAAACTACTCAAGGAACATTAACAGTAGAAGGTC
AAACAAATATTAATGACTCCCTAATTGGTAATGCTTCTAACGAAAACTTTAAGATTCAAA
ATGGACCTGCTGCAGATAAATTTACAGTTGATACTGATAATGGTAATACATTAATTGAAG
GAACACTTAATGTAAACGGTGTAACTGATATTGACGCAGACTTCGCAGTTAGAAACGGAA
CGACTGATAAATGTTTTGTCGCATCTGCCTCAGGTAATACCCAAATTAAAGGCAATCTGA

CTGCTGATGGTCATACTGAATTAAATTCAACACTTAATGTTGACAGATCTACAACACTTG
GTGCACAACTTACAGTAACTGGTAACTCAGAGTTCAACGGAACTCTAGATGTTGATGCAA
ACTTTGCAGTCAGATCAGGAACTACTGATAAGATGACTGTTGCATCTTCTACAGATAACA
TAGCAACTGATGGAACTCTAGTTGTTGCAGGTCAAACAACTATTAATGATTCTCTAATTG
TTCAAAGTGATAACGAAGTAGTTAATATAAAAAATGGTTCTGGCACAACTAAGTTCAGCA
TTGATACTGATAACGGTAATACAAATATTATCCGAACATTGACAGTTGCAGATGCTACTC
AAATTAATAATACTCTTGGTGTTTCTAACGTTGTAACCTTAACAAGAAACACTCAGCATA
CTCTGACTGGATCTTATTCTGCTGATGGTGCATTCAAACTAACTGGTGTTGCAGGTATCG
GTAAAAACCTTGGTGTTGGTGAAGGTCTAAGAGTATATGGAGGCACTGAACTTACTGGTG
CCCTTCAGCTTAATAATAGTGCTGATATATCTGGTGCTTTAGTAACTCATTATGATGTTA
CTCTCACTGCAGATAATAAGATGTTCAAGGTGCAGAATGCTTCTGCAGCGAACAAGTTTA
CAGTCGACACCGATAATGGTAACACTGATATTCAAGGAACATTGGATGTTCCAGGTGATG
TAACTGCATCATCTGACCTTACAGTCACAGGAAATCTTACAGTCAATGGAACGACAACGA
CTGTCTATTATAAGGTCACAACAATCGATGACCCTGTTATTACTGTTGGTGGTGATACAG
TACCCGCGTCTAGTGATGGAAAGGATCGTGGTATTGAATTTCGTTATTATGATGGTTCTG
CTAAAATCGGTTTCTTCGGTTTTGATAGATCATCCTCCCAATTCCAATTCTTAACAAGTG
CAACAAATACTTCTTAAGTTCTCTCAGGAACAGATGGTGCTCTAAGAGCAGGTAGCTTAA
ATCTTACTGGTGCGGGCACATCTCTTGATGTTGATGCTAATGCAAACATTGATGGCACAC
TGACTGTAGATGGTCAGATTATTTCACAAGTATCTTCACGTCCTGCTCTTGTTACCCCAA
CAACAGACAAGATTAATAATCATTACGCAGACTTACTAGATGGTGCTACAACCGCAACTG
CTGCAACCGCATCCACAGTTGTTCTTCGTGATTCATCTGGTGATTTTGCTGCCAATCGAA
TCACTGCTGCTAGTGCTGCAGGATCCGGTGCAGGTTTCTTAGTAAACGCATCCACTGCTG
ACGCATGGAAGAGTGCTAGAACACTCACCATTAGCGGTGTTGTATCTGGTTTTGTAGTCA
TCGATGGAAGTGAAAATGAAACACTTACAACAACTTATGTTGATGCAGACATCACTGCTC
TAGCAGCGATGAGTGGAACGGGTTATGTCGTACGAACTGCTGCTAACACTTTTGCCCAAA
AAACCCTCCAAGTCACCGCATCGTCTGGAATCACTCTTACTAAAGCTGATGGCGTTTCTG
GTAATACTACAATTAACGTTGCTAGTGCATCTTCTAATTCTGCAAACAACCTCGTCCTAA
GAGACGGATCTGGTAACTATGTTGCAGGAACTATTACTGCTGCCTTAACTGGTAATGGCA
CAGGCAGTGTAACTGGTAATGTAACTGGTCACGTATCTGATATCTCAAACCATGATTCTG
GTGATCTTACTGAGGGCACTAACCTCTACTACACTAATGAAAGAGTTGACGATCGAGTCA
TTGCTCTTATTAATGCAGGAACTGGTATTAGTAAGGTTTACGATGCCGCTGCAAATACAT
ATACACTGACTGTAACTCAGTCAGATATCAAAACTGATAATGTAACAGAGGGATCAACAG
ATCTATTCACAACTGCTGCTAGAACTAGAACTCATTTCACATATGGAACTGGTATCACAC
ACAGTTCTGGAACTTTATCAGTTACTCAGTCTGATATTAATACTGACAACGTAACTGAAG
GGTCAACTAACCTATTTACTACTGCTGCTAGAACATGAACACACTTTACATACGGAACTG
GTATTACACACAGTTCTGGAACTTTATCAGTTACACAATAAGATATCAACACTGATAATA
TTACTGAAGGTTCTACAAACCTATTCATTACTAATGAACGTGTTGATGATAGAGTCAACG
CTGTAACTGTTGCAGGAACTGGTGTAAGTAAGACATATGATGACGCTACGGGAACTCTAA
CTTTAGCAGTTGACTTTAGTGAGTTTAATACAGGCAATATTACGGAGGGTTCTAACCTTT
ACATTACAAACACTCGTGCTGATGCAAGGGCAGACTTGAAAGTTGCTGCTGCAACTGGTT
CAAACCTTGATCTTTCTTCTAAGTCTACGTCCGCTCTTTCTGAAGGAACTAATCAATACT
ATACAGAAGCAAGAGTTCGAGCAAAACTTGACAATGGTTATGAGCAACTAAGAGCAATGT
TAACTAACCTTGCAACTAGCACCACTCTAAATCTTAACTTGTCTGGTGAGCCTACACCTG
GCGCAGTTGGTATCACTGGTGTTTGTGTTGGTGGTGGCGGTGGATTTACTGCAGGAACAG
CGATTGCTACATTTGGTTCAGCAACTGGATCTGGATTGACTGTTGATACTACGGTTGAGA
GCGATGGAAACATCACTGCTGCTGCGGTAAATGCAGGTGGTTCTGGATTTCTAATTACTG
ATACTGTCACAGTCACTAACCCCAACGCAGGTAAGATTCTTACATTGAACTTGGCAACAT
TATCAGGTGGAACAGGTTATTCATCTGCTACTTGAGTTGCAATCAATGGGGGTGACGGTT
CTTCTGCGACTGTTGACATTACTGCATCTGCAGGTGCTATCACTAACGTAACAGTTAATA
ACGGTGGAACAGGATTCGCTGCGGGTAATACTATAAATATTGCGAATGCTAATGCTACTG
AAGGTAAGAATCTTGGATCTATTACTCCTGCAGGAGCTGGATATTCAACAGGAACTGCCA
TTGCAACATCAGCGTCCGCATCTGGATCTGGAGCAACATTAAATATCACTTCGGTCAATG
CTAGTGGTGGTATCACTGGTGTTGCAATCAACGATGATGGATCTGGTTACGCTGCATCTG
AAGTCTTGACAATTACTAATGCTAACGCATCTGGTATCCCAACGGTAGGAAACATTGGTG
CTGCTGATGCATCAAGAACTGCAGGAACCTATAACTTAGGCACATCTGATTATGGAACTG
AAGCATCTGGTGCCAACGCAACATTCACTGTTGTTGTTGCTTCAAATGGTGCTGCTTCTA
TTACCGTAACAGATGATGGTAGCGGATTTATCGCCAATGAAACTATCACAGTTGCTGATG

CTCAACTTGGTGGTCGCGGTGGTGCTGCTCTTACATTCGATGTAACAGCAATCCATGGTA
GCACTGCTACAGTTCCAGTATCTGCGATTCATGGAAACGGTACAAGAGTTAATGTCGCTA
CCGTTGCAACAACTGCAACTCTAACTCTAACTGACATCACACCGATGGAAGTTGGAGCAA
CAGTTACAGGTGCTACTCCTGGCACCACAGGTGTCATCACTGCTCTGGGAACTAACCAGA
TCACTGTCAATACCGTTGACGGATTCTTCAAGAAAGGAGGAGTCGTCAGTTCTAATGATG
TTACTACACTTACTATCTCCTCATTAAATTAATAAGTTATGTCTGCCACCAGACCCGTAA
ATAAAAGTGAACTACGAGACTATGCTCTCCGTCGTTTAGGATTTCCTACTATAGACATCA
ACGTTCCTACTGAGCACTTAGATGATTTGATAGAAGAAGCGATTGATTTCTATCAAGAGT
TTCACTATGAAGGTAGTTACAAATCCTTCATGTTTATTGAAGTTGCAGATGCTATTAAAA
CAGCAGCAAAAAGTTCAACTCAACAAGGATGTACCAATTGGTATGAGAACAATAACTATG
TTTCATTACCACCTAACATCTTAGGAGTCAATCAGTTATATACACAGCTTGGTGCTTATG
GTATTGTGCCTGGGAACACGTTCAATATTAAGTATCAAATCTTTTTAAATGATATATACG
CTATGACCCATGGTCATATTCTTCATTACTTTATGACATCTCAATATCTTGAGACACCTG
CTTTTGTAACAGACTCTAGTAGGAGTAGAAGAGTAAAATATAACGAGAAACAAGGTCGAT
TGTATCTCGATATGGATCGGGATTCATTGGGAACAGGAGATAAGATTTTAGTCGAAGTTC
TCGTACGTCAAGATCCAGAAACTTATACAGCAATGTATAATGATAACTGGTTAAAAGGTT
ATGTTGAAGCATTATTTCAACAACAATGGGGAAGAACCCTAAGTAAGTATGATGGCATTC
AAATGTTAGGTGACATCACACTTCATGGTCGTCAAATTCTTGAGGACACAAGTCGATTCA
AGAAAGACCTTGAAGATAAAATTCGTGATACATATGAACTTCCTCCTTTAGACTTAGTGG
GGTAATCTCTAATGGTCCTTCGCAACTCTCCTGCACAAGATTATGTTCAACCGGATTTTT
CTAACGCAGGACGCTTCAAAGCAAATGGTTCTACACAGGAACAAAAATTTATTGAAAATC
TATTAGTTGAAAGCATTGAAATCTATGGGCAAGATATTTACTACGTTCCGAGAACGATTG
TCAACACAGATACGGTCTTTGGAGAGGACTCGGATTCGCGATTCGATACTGCGAAACCGA
TTAGAGCATATGTCAATAGTGCTGAAGGATGGGAAGGACAAGGTGAGTTACTTAGCAAAT
TTGGAGTCCGTATCGAAGTCAAGACAATTTTTATATTCTCCCGTGAGAAATTTGAAGAGC
ATGTGGACGATGGTGTGACGCTTAACGTCGAAGGGTGACCAAACGAAGGGGACTTAATCT
GGTTTCCAACAGCAAAGCATGTATTTGAAATACAATTCGTAGAAGCAGAAAAACCATTTT
ATCAATTAGGAAAAGGATATGTTTGGGAATGTCAGTGTGAACTCTTTGAATACAGTGACG
AAGATCTTGACACAGGAATCGCAGAGATCGATGCAATCGAAGCTGGCTTTGCTAATGCTA
TTACAGTTAACTTTGCTACTGGAAGGTCTAGCGATTTCACAGTTGGTGAAATTGTTGCAG
GAGGTACATCTAAGGTGACTGCTGAGGTTCAAGCATGGGATTCTACAACTAGACAGTTAC
AGGTCTTTAATCGTTCAGGTGTCTTTACGATACCTGAAACGGTTACAGGACAGACCTCAG
GTGCTGCTTGGACAACTGCATCATACAACACACTAAATAATGTTAATACCGCTAACTCTA
TTGATCGAAACAACGACTTTGAAACTTTGGATAACGATATTATTGACTGTACCGAAACTA
ATCCATTCGGTTCAATTGGTTCCATTACTGACGCTACAAGCTAATGTTAGGCACTTATTC
ATATCACGAAATTTTTAGGAAGACTATCGTTGCTTTTGGAACGTTATTCAAGAATATTGA
ATTACGTCGTAATGATGAAGTTATGACAGTACCTTTAGCGTACGGTCCTAAACAAAAATT
CTTAGCAAGGTTAGATCAAGTTCCTGATCCAACCAACAAAAGAGTTGAACTTACTCTACC
CAGAATCTCTTTTTAAATTAACGGGGTATCATATGATAGTGCAAGAAAACTTGCACCCAC
ACAAATGATCAAGATGGCAAGCAGTTCAACTACAAATAAGAACGTTTTTATGCCTGTTCC
TTATAATCTTCGTTTTGAACTAGCAATCATCTCTAAAAATCAAGAAGATGGTTCACAAAT
TTTAGAACATATTCTACCAATATTCCAACCTCATTTCAATATGGCAGTAAAACTATTGCC
TGAGATGACTGAGATTAGAGACATTCCCGTAGTCCTCAATGACGTCGAATATGAAGATGA
CTATGAGGGTAACTTTACTACAAGAAGAGCGATCATTTATACCTTACAATTTACTGCAAA
AACTCATCTATACGGTCCTGTATCGGAACCAAAAGTTATCAAAAAGGCAATTACAGATTA
CTATACAGATACCAATACTACATCTGCACCAAGAGAAGTACGGTATACTGTTGTTCCTAA
TCCTCTCACAGCAGATGCTGATGATGATTTCGGATTCGGAGAGACTACTGCAGTCTTTAC
AGATGACAAGAAACGTAATCCTACTAGTGGTGCTGACGAGGCAATTTAATCATGTCAAAT
CCTTTCGATGGTCTCAACGATGCTTTTGGAACTGAACCTACAGAACTTCAAAAGCATGTT
GAAAAAATAAAACCTACTCTGAAAAAAACAGAGACTGAAGATGTGAAGCAAGACTACACG
ATATCTCGTGCTGCGTTACATAGTTTAGTGATGAAAGGACAAAAAGCAGTGGATGGTATA
TTGGAAGTAGCACAAGCATCAGATCATCCTCGTGCCTATGAAGTTGCTGCAACAACGATT
AGGAACGTAGCAGATACTGCTGATAAACTAATTGATCTTCAGAAGAAGATGAAAGAGTTT
GATGCTGAGGATATAAAGAATGCACCTACTACAGTAAAGAATACTATGTTTGTTGGTAGC
ATTGCAGAGTTACAGAAAATGTTAAAGCAGCAAAAAGAGATAAATAAAAAGGACGAGAAA
TAACAACACACGACATGTCAGTATTAAATGTTCTTAGCACTAATACAGTTGCTGCAGGTG
CAACTGAATATCAAGTAATTCAAACAGGTTATAACAGAATTCGATCTACTTCAGGTGCAG

CAACAGTATCACTTAATGGTGGTCCTGCAATCACACTTGTTCAGAATGAATTCATTCTTC
TCAAAGGTGGTAAACCTGGTCTAGCACGAATTGTAAAAGCAGTATCTGATTCTACGGGTG
ACTATATCCTAGGTCAGTATATTCATTCTACAGGTGATGCACATCCATACACATCTGGTC
CTTTTATCGCAGTTGAAGATGATAGCACATCTCCTGCTATTGATAGTAACTTCCTTTCTG
CAGGAACTGCAGGTAAAAAGATTACTGCTGTCACAGGAAAGACAATCGCTACTGATATTG
ATTCAGCAGGTGCATCAGCAGATTACACTTTTGCCTTCTCAGGTCCTCAAGCAGTTGTTA
AAAGATGCATTAAAATCGTTGCTGCTACAAGTGCAGTAATCATATAAGAACTTCAAGTGG
ATGGTGGTTAAGATGCCCGCTGTAAATCAAGAGGCAGAGAGAATAGTTAAAGGGATGAAG
AAGAATCATCATCGCTTTAAGAAACTCTATGGAGATCGAGAGAAAGAAGTTATGTACGCT
ACTGCTAATTAGTTAACACAAAAAGAACAATTGAAAGATATGTAATATCAAGATGTCATT
AAACTCGTCGAAGGTAATCCTACTCCAAGGATGCTATCGAAAGCCAAGTCATAGACTACT
GGAAACATTTCTGCTGATCATGGAACTGACGAAAAGAAGAATAGAGTAATTAGAAAGTCT
CTAGAAAAAGATCTTGAGAAGAAAGGTATAGGTTATAAAAAAGGTGTAGGGGAGTATAAA
TATTCCTCAGGAGAGGGAACAGGACGTGAGGTTTCATACCAAACAAGTCTTGGCAAAGTA
ATGTCTAAGAGACGTTTTGGTAAGGTCATGCGTCGTCTAGGACGAAGGCATGGTCAAGAA
TCAGTTATCACTACCAAGTCAGGTAAACCTGCTACACTACATGATACTGAGTCTAAAAAA
CCATCTAAGTCTTTGAACTTAGGTAAAGCAAAACCAGGAAAAAATCCTGGTGGTTTCGGA
GAAACTTCTGGTACTAAAGTCAGAAAAGGTAAATTAGGTAAAACTAACAAACCCGCGATG
CACTATGGCTACTAATGAAATTATCGAAAAGAACAAAAGGGGTGACAGTTCTTTGCACGA
CTGGTTTTGTAAGAGTAAGTCTTCTGATGGGAAGCCTGGTTGGGTTCAGCTCGGTGGTAA
ATACGCAGGTAATCCCTGTGCCAAACAACCTGGACAAACATCCAAACCCGAGTGCGGTTC
CAGTAAGATGAAAAGAAACCTAAATAAAAAAGAGGAAGATGCTGCCTTTCGACGCAAAAA
CAAGCAAGATCCTAATCCAAACAGAAAAGGAAAAGCAATCAACGTGAAAACAGAATCTAC
ACAAATACACGAAGGCGAAAAAGATGCATGTTACCATAAGGTAAAATCTCGCTATTCAGT
TTGGCCAAGTCCTTATGCAAGCGGTGCACTTGTCAAGTGCCGAAAAGTTTGTGCAACAAA
TTGGGGGAACAAAAGTAAGAAAAACGAATTTGATATGAAGGTAAAAGGATTCCAAAGTTT
TCAATCAGAGTGTTGGAAGACTCATAAAAAAGTTGGTATGAAAATGAAGGGTGGTAAGTT
AGTAAACGATTGTCGTCCAAAGAACGAAGCAAAGGAATACACAGGACCTGACAAAGCGGA
CAGAAAACTTATCAACAAAATGGATAACCCTATTTATGCTTAAAAGTTAGCAGACTATGA
AAAGAACATGGATCATAAGAAGCGTCAAGCACTTAAGGATAAAGCGACTAAGGGTATGAA
GTTTACTCATGAAGAAACTACCAACGAAGCTAAGAAATGTTGGCCAGGTTATGAGAAAAA
AGGAACCCAAAAACTAGTCGGCAAAACGTACAACCGCTGTGTAAAAAAGGAAGACTTGGA
GTATGTCAGAGAAAAAGCAGCAGCATGGACTAGGAAACCAGGAAAAAACCAATCAGGCGG
ACTTAACGAAAAAGGCAGAAAAAGTTTCGAACGCGAAAATCCTGGATCTGACCTTAAAGC
ACCTAGCAAAAAAGTTGGAAACCCCCGCAGGGCATCCTTCTGCGCTAGAATGAAAGGAAT
GAAAAAGAAATTAACTTCAAAGAAAACAGCCAGCGATCCTGATTCAAGTATTAACAAAAG
CTTGCGTGCTTGGAATTGTTGATATATAATAGACGGTAGTCTGTACAATTAAAATGAAAA
TCTGTCCGAAGTGTAGTGCTACATGGATCGGGGAACAGTTATATTGGTCTACAGAAAAAG
AGGGTTGCCCTCATGATCTTGCAGGACTACTATGTAATAATCATGGAGATGAAACTTGTA
TTAATCCATGTAAAGGATCCACTAGTGGTCAAACATGGGAATATCGTCGTGGAATGATTG
ATGGTTTGACGGAGGGGTATGGTAAAGATAGTTCGTAATGTATGTCATCAATCTTTAATA
GAGTTTATATTTGAAACAGCAGAAAGAAGAGAAGCGTGGCATTGGAACTTCCCTATGGGA
GCAGCGTTCGATAAGACGCATGCAAAATTAGACATTGTATAAGATGGTAAGATCAAAGAT
GCTTTTCTAGCAGGTCTTGCATCTTCTGTTTTGTTGCAAGTATGGGAAGCAAGTAATAGA
GAACTATTCATACCTGAGATATTTTATGCGGGTGTGGCAATAAAAGATAAGCATAGAAAA
GATAACGTACCTGTAGATGACGCTAACAGAAATGATACCATTAAAATTTTGGGTATCTTG
AATAGTGACTGGGATCCGAACACGATGGGTGGTGGATTTATGCATGGTTGTGTCATACAT
AAATTGAACCCTACCGACTTTTGTATTTTCGATCCAAGGACTCCTCATGCAGCAGAGGCT
GTAATCTGTGATCACAAAAGATTTGCCATGGATTTCTCTGTTAAGAAAACGTAACCAGCA
TGCAACTTTAAAATCTATCAACTATAATATAGTGTAGACTTTAAAAGGAGAACGATGTTT
TTTGCATCACACCCGTCTGTTTATACTTTACCTGGCACATGGGGACCCCAACCAGTTATA
TTGTATAACCCGACACTTCTTATTTCTCTTTAATCAACAGCATTTGCTATTGGTGGGATT
GTATCGATTTTAGCGGTTAGGAGAAAGAGAAAACGCATCTAATAAATGACTGACCTAAAC
ACAATTATGTTAAACACAACCGTTGCCATTATTGATTTTTTGTATAAAGATAGAGACTTC
CAAAGATTTTGGGTGCTTGAGGAGATAGCTCGAGCACCCTATTTTGCTTTTATGAGTGTC
CTACATTTCTGAGAATCAATGGGGTTACGAGGACCAGAACACATTTATCTAATGGAGGAG
CATTTTGGTCAAACGCTTAACGAAACAGAACATCTTGAATATCTTGAAAGTCGGGGTGGT

AACGCTTATTGGATTGAACGTGCTTTCGCCAAACACCTCGTCCTTATCTCCTATTGGTTC
ATGGTGGTTTATTATGTGTTATTTCCTGTATCTGCATACGACCTAAATGAAAAAGTAGAG
TGGCATGCTGCACATACATATGAAGAATACCTAACACGTTTTCCAGATGACGAGGACATC
ACTCGAATCAAAGATGATAAAATTAAACATGCAAATGAATTATCCAAAGCAATAGAGTTA
ATTAAATGAGAACCCTACCCAATAACGAGTATAAACTAGTATTCGATGCTCTTTGGAAAC
GTCAGCGATGCTTTAGTGCAGGTCATAGAATGTTCAAAGAATACGAGAAACTTATTGAGG
AGTTTGAAAATGCCAGTCTATCGTGACAATGAGATTAGACTTAATCTCAATGAATTAATT
GAACAAAGGATTCCAACCTATGACTTGCTGCATCCAGACCACTGCTTATCTGAAGCACAA
GTGGCACAGCTAGCACATGATATTAATATGGATTTAGCCTTGCACCCAATGTATCACCAG
ATTGATGATCATATCATGAGATAAGTAAAGGCAGCAGGTATTGATAACTCAGAGCAATGG
GTTGAGAAAAAACTAAATGATTTAGATTTCTGAAAAGAATGAAAAATACAGAATCTTCTG
AACAACTTATTCAACGTTTTACCAAACGTACGATGCAGTTGACAGCAAGGAAACAAGAAC
TTCGATCTGCATATGATGACTATGTAAAATTAGAAAGAGATCTGACTAGACTTGATGGTT
CTGTGCAAGCAATTGAATGTGTTGCTTTCGGCAAATTACCAGGACATGGTAACCATACTG
GTATGAAAGATCACTCTCCCAACTAAATAAGGGTGGCGAGGATCGATCTCGACTTAGCCG
ATTTATGAGTTCGGTGCATTCACCAGATTGCTACACCCTCAATGGAGTTGAAACTATCAT
GTCCCATTAAACTGTAGGTTATCACGATAAGCAAAATCAACATTATGAAATATGTGAGTC
TGCTGAAGATGTTTATCATGATATCAAACAAGCATGCGAAGATTTAGAAGGATTTAATAA
TCCACACGCAGCAGAATATTGTATCAAAGAAAATTAAATGGTTGTCAGGGGTGTCATATG
GATGGTCTTTATACTCGTTGTAATCGTCTCTTGGTATATCTACTATATACTTAGAATGGC
CTACAAGGAGATGGACGATGGCAGCGATGCAACCACCGAGCAGGAAAAGCTGCTATAACT
TTAGAGTAACGGAGATTAATCGTGTTGTTGACGGGGATACTATTGATGTCACCATTGATT
TGGGGTTTTATCTATACAAGAAAGAAAGAGTTAGAATTGCAGGAGTTGATACGCCAGAGA
AAAGAACCAGAGATCTGGATGAGAAGGCATTGGGAATAGATGCTACAAACTGGATGAAAA
AAAATTTAGAGGATGCTATTAATGGAGATGATGAGCTCACTATACGAACTGAACTTAAAG
GTGGGGTTGGCAAGTATGGTAGGTTGCTTGTTTGGTTATACGATGCCGATGATGATGTAT
CACTCTACGAAAAAATGATTGTGCAAGGATATGCATGGTCGTACGACGGTGGAACAAAAC
AAAAAAACTTCGATGAACTCCGTGAAATTAGACGAAGCTTCGGAACTTTGCTCTAGTCTA
GCATCGCTGATTAGAACTAAGATTTCAAGGTTATCACATTTTGAAGAACTTGCTTCAGAT
TATGATGAGGTGCATACAGGTGATGTATCCATCTATAGTTGCATGTGGACTTCTAAGGGC
TTAAGAAAGTGACATATAGAAAGAGCACTTACAAATAAAGGGATAGAGATACTACACTGT
GTATTATTTCCAGATCCAGAGTTGCCTATTCCTAACTTTGGATGTGATATCGTAGAGGCA
GGTGGTAAAGTAACTGCTGCTATAGTTGATGTGTCACCAGTGCATAAGGTAGATTAAAGT
TTACGTAATATAACATATAATTTTAAAAAGAATAGGCATCTACCAGAGTGGGGTGAGATA
TTTTCACCATGGTGTAAGTTTGTAAGATTAGAAAAGGATGAGTATGATGACTTTTTATTA
TTGTGTAGTGAATACTTAGAGGTATTCTGTCACATAGTAAGAACAGCAGAAAGAGAAACA
GACTGGAGAAATACTATGAGGAGATATGACGATCAGTTATGCTATTGCACATCGCAGATG
AAGAACAAGAAAACTGAGGCGGTGCTATCCCCATGGTTCGATAATCCCTGGGCATCTATA
TATCTACAGAACATACTATTTGACAAACCCAAACTATGAGAAGAGAAAAGTTGGAAGCTC
TGAAGGCACCTGCCATTGGCAACATCAAGAAAGCAAAGATGAACGTTTAGGTTTACCTTA
AGAATCCTGTTGGAATTGGTTAACACCCAGATGTTCTTGGTGCTATTCAAGAACAAATTG
ATTTGATTGCGAAAGAAGAGGAACGTTTGGAAGTAATCGAAGAGTATTTTGAAGTAGGAA
TGGATCTTTATGGTGGAACTGAATGAGTGATCAGTATCTCGGCAACCCAAATCTAAAAAA
AGTTAACACATCAATTGACTTCAAACCAGAAGAAGTTGCTGAGGTATTGAAGTGCCAAGA
GGATCCCATATACTTTATGAAAAATTATATCAAGATCGTTTCTCTAGATGACGGTCTAGT
TCCATTTAACATGTATCATTTCCAAGAGGAAATGGTATCTAAGTTTCATGATCATAGATT
CAATATCGCTAAACTACCACGAGAGAGTGGTCAGTCTACCATTGTTACTGCATACTTACT
TTGGTATGTGTTATTCAATGCAAATGTCAACGTAGCAATCCTTGGAAACAAATCAGCAAC
TGCTAGGGAGATGCTTCAAAGATTACAATTAAGTTATGAAAAACTCCCAAACTGGATGCA
ACAAGGAATCTTCCAATGGAACAGCGGTTCTCTGGAACTTGAAAACGGCAGTAAAATCAT
GGCTGCTTCTACTTCCGCTTGTGCTGTCAGGGGTATGTCATTTAACGTTATATTTCTGGA
TGAATTCGCGTTCATTCCGAATCATATCGCTGATCAGTTTTATAGTTCTGTTTATCCAAC
TATCTCTTAAGGTAAGTCTACGAAAGTTATTATCATATCTACTCCTCACGGAATGAATAT
GTTCTACAAACTCTGGCATGATGGAGAGCCTGGAACGAATGAATTTGTGCCTACGGAAGT
ACACTGGTCAGAAGTGCCAGGTAGAGATGATGTATGGAAAGAGCAAACTATTAAGAACAC
ATCAGAATCTCAGTTTCGTGTTGAGATTGAGTGTGAGTTCTTAGGATCTGTTGATACATT
GATTGCTCCTAGTAAGTTGAGAATCATGCCATATCATGATCCAATTACATCTAATCGTGG

ACTTGCAGTATCTGAACAAGTAGTTCCAGAACATAATAATATTATCACTGTTGACGTATC
AACAGGTGTTGGTAATGATTACTCAGCATTTTGTGTAATAGATACCACAACTATTCCTTA
TAAGATGGTTACCAGATATAAGAACAATGAAATTAAACCCATCGTTCTACCAAATATTAT
CGTAGACATTGCAAAGAATTATAATAAAGCATACATCTTATGTGAAGTAAATGATATTGG
TGGACAGGTAGCAGATATTATTCCGTTAGATTTGGAGTATGAGAAGTTGTTGATGGCTGT
AATGAGAGGTCGTGCAGGTCAACAACTAGGTCAAGGTTTTTCTGGTAAGAAAACACAACT
TGGTGTTAAGATGTCCACAGCAACAAAACAAGTTGGATGTTCTAGCCTTAAAGCATTAAT
TGAAGTAGATAAATTGCTTATAAATGATTATGACACGATTGCGGAAGTAACAACGTTCAT
TGCGAAAGGTCAAAGATTCCAAGCGGAAGAAGGTGGTAATGACGACCTTGCTATGTGTTT
AGTGATAATTGGTTGGATGGCAATGCAACCATACTTTAAAGAGATGCCCGATAACGACGT
ACGTCAACGCATCCATGAACAACAGAACGATATGATTGAACAAGACATGGCACCATTTGG
TTTTGTAACTGACGGAATGGAAGATGATTATTTTGCAGACGTTCAGGGCGATGTGTGGAA
ACTCGCGGAATACGGGGTTAAATCCTACATGTGGGAGTTTCAGTAAGGTTTCAAAAATAT
GAATAATTCTAGACATCTGATGTTGGAATTACCCTAGGAGAAAATTAACCATGGCAGCCA
ATCAATTATCGCCAGGTGTAGTGGTGCAGGAGAGAGACCTTACTACTATTACCACATTAT
CGACCGCTAACGTTGGTGTGCTCGCTGCACCTTTTGAGCTCGGTCCGGTTGAAGAAGTCG
TCGAAATCTCCAGTGAAAGGGATCTTGCAGAGCAATTTGGAAAACCGAATGAGTATAACT
ACGAGTATTGGTATACTGCAGCACAATTTCTTTCCTATGGCGGACTACTAAAAACAGTTC
GTGTTGACTCGACATGTCTCAAAAACGCAGTTAGTAATGGAACTGCAGTTAAAGTCAAGA
ATTTAAGTGAGTATGAAACCACTTACGTAGGTGCTACTAACACACGGAAGGGGGCAGCAA
GAACTTCTGGAACTACAGGAAACACTATCGGTGTATTTGTAACTGACGCAGGTGCAGATC
GAATCGCTGTTGTTCCTGCTCCTGGCTCAGGTGACGATCATGAATTCGTTGCTGATGAAG
CACTTTCCGCAACTTCTGGTGCTGCAGGTAAAGTATTCAAATACAGCCTAGTATTAACAG
TCACAACTGTTGTTGGCGACTTCGTTCCTAGCACTACTACAACTGTTTCAATCTCTGGTA
CTAACCAGACAGTTACAGTGCTTGCTTGGGATCCTAGTAACAAGAAACTTGAAATCGGTC
TTCCTTCTGGTGGTATCACTGGTATCCTTGCTGATGGTCAGACTGTAACTCAAGGATCTA
ATAATTGTGTGATTCCATCATCCGGTATTGAAAGAAGAGTTAATATTGGATTAAACAAAG
GTAGCATTGAATTCGCTGCTGCTGAGAGTATCGCTGATACTAACTCAACTGCTGTTTCAA
TCAGTTCTGTTCGTTCTGAGTATGCAGAGCGTGAGTATCTACCTGGTGCAAAATGGATTA
ACGTTGCTGCTCGTCCTTCTACGTCACTCTTTACAAATAATGCAGGTGGATCTAGAGATG
TACTTCACGTTCTAGTTATTTATATCGATGGTGGTAACACTGGAACAACTGGTGCTGTTC
TTGAGCGTTTCATTGGTCTTTCAAAAGCATCGGACGCTAAGACATCTGTTGGTGAAGCAA
ACTACAACAAAGAAGTAATCAAGCAAAAGTCTGTATATATCTACTGGGGTTTACATGAAG
AAGGAGTATTCAATGCAACTGCTTCTGCTGCTGCAGGAAACTGGGGTTTAGCTGCTGCTT
CTAGACAGTTAAACTTACTACGTTCTGCAGATGGTTCTACTGGTTATCCTGAGGGCCGCG
CAACTGTAGGGTATAAGAACAATGCAACATACTACTACAGATTAACTGGGGGTGTTGACT
ACGGTATTTCTGGTGGACTTTACAGTGTAACAAACACATCTCTTGCAACTGCATATTGTC
TTGCAGAAGTTCCTGAGTCACAAACAATTGACTTCATTCTTACTGGTCCTTCTGGTGCAG
AGGATGCTTCTGCAATCGCTAAAGCAACTTCTCTAGTTTACATTGCTGAAGAGAGAAGAG
ATTGCTTAGTATTCGTTTCACCTGGTAGAGGAAACGTAATTGGAGTTACTAACTCTACTA
CTGCAACTGATCATATCGTTAACTTCTTCGATCTATTACCGAGTTCTTCTTACATGGTAT
CTGATTCTGGATACAAGTATACGTACGATAAGTATAACGATTTATACAGATACGTTCCAA
CCAACGGTGACATCGCAGGTTTATGTTTACAGACAACTAAGGTTGCAGAACCATGGTTCT
CACCTGCAGGTTTCCAACGTGGTGTTTTAAGAAATGCTATCAAACTAGCATACACACCAA
CTAAGACACAAAGAGATCGCTTGTATGCAAACAGAATGAACCCTGTCGTATCATTCCCTG
GTCAAGGCGTCGTCCTCTTTGGTGACAAGACTGAACTCGGATTTGCATCCGCGTTTGATA
GAATCAACGTACGTCGTCTATTCCTCACCATCGAACGTGTTATCTCAGGTGCTGCTAAGG
CACAACTCTTTGAGCAAAACGATGAAGCACAGAGATCACTCTTCTTGAATATCATCGAAC
CTTATCTAAGGGATGTTCAAGGTCGTCGTGGTGTAACTGACTTCTTAGTTAAGTGTGATT
CATATAACAACCCACCAGAGGCAGTTGATCGTGGAGAGTTCTACGCGGAGATTTTCGTGA
AACCAACACGCACAATCAACTACATTACCTTGACACTTGTAGCAAATAGAACTGGTGTTG
CCTTCAGCGAAGTAGCAAATTAAATTAAATTCATACTTTTTTCAGACGGTTTCCGCATCG
GTACCCTCTGAAAACTTTTATTCGTCCAAATATAACTGACTGAGACACCTAGAAAAATGG
CAAAAAGAGGAACGATTGACGATTTTAAGGCAAATGTAGCATCAGACTTTGCTCGTCCTA
ACCTATTCCAAGTTGATTTAGCATTTCACTCTGGAATCATTAATAATTCTAATTTGGTAA
ACCTTGCTAAGTTCACTGTTCGTGCAGCGAATCTTCCTTCTTCCCAGATTGGTGTAATTG
AAGATCTTTTCAGAGGACGTACGCTAAAGATCGCAGGAGACAGAACGTTTGAACCTTGGA

CAATCACTGTTATGAATGACAGTAAGTTTGTTCTAAGATCCGCATTTGAATTGTGGGCAT
CAAGCATTCAAGCATACAACGAAAACTTCACTTCCGCAGCAGGTCGTGGAGACACAGATG
ATGCAACTGGATAATTTGCTGATATGAAGGTACATCAATGAGCAAGAGATATTAGATCAG
GAGAGAAACCAAAAGGTCTCAAATCATATAAGTTCTATAACTTCTTCCCTAGTGCAATCG
CAACGATTGATCTAGATTACGGAAACAACGATGCAATCGAAGAATTCACCGTTGAGATGC
AGGTTCAGTATTGGACTCCTCTAGCAGACGCCATCGACTAACCCGCTAAATAGAACAGGA
CCAATAATTAAAACATTTAATAATGGCAAATCAGCTCTTCGGTTTTTCGCTTGAGAGAGC
GAAGAATGTGCCTAAGGGACCTTCTTTTGTTCAAAAGGATAGTATGGATGGATCGAAACC
TATTGTAGGGGGCGGTTACTATGGATATTCCCTCGATTTTGATGGCACGGTTCGCAATGA
GTATGAACTAATCACTCGTTACAGAGAGATGTTTCTACAACCAGAGTGCGATAGTGCGGT
TGATGACATCGTGAATGAAACTATTTGTGGCAACTTTGACGACGTTCCCGTCGAGGTCAA
GTTGTCTAACTTGCAACAATCCGACAAAATTAAAAAATTAATCAGGGAAGAGTTCCAAAC
AATTCTTCGCTTGCTTGATTTCGGTAATAGATCGTACGAAATTTTCCGTCGCTGGTATGT
CGATACAAGATTATTTTATCATAAGGTTATCGATCCAGATAACCCTAGAAATGGTCTTGC
TGAGTTACGTTATATTGACGCTCGTAAGATTCGTAAGGTTACAGAGTATGACCAGAAACG
TCCAAACGAACTTCGTGGACTAGATCTGAATGCTCAACTTACACAAAAATATGCAGATTA
TTATCTATATAATCCTAAGGGTTTGAAGAACTCTGGATATAAGCAAGGAATTAAAATTGC
TGCGGATTCAATCACTTATTGCCATTCTGGTATACTGGATCTCAATAAGAATATGACATT
AAGGCATCTACATAAAGCAATCAAAGCGGTTAACCAACTCCGTCTGATTGAAGACTCATT
GGAAATCTATAGATCATCAAGAGCACCAGAAAGAAGAATTTTCTACATCCATGTTGGTAA
CCTTCCTAAGAACAAAGCGGAACAATACGTCCGCAAAGTTATAGGAAGGTATCGTAATAA
ATTGGTTTATGATGCAAACACTGGTGAGATTAAAGATGACAAGAAGTTCATGTCCATGTT
GGAAGACTTCTGGTCACCGTGTCGTGAAGGCGGTAGAGGAACTGGGATATCCACTCTTCC
TGGCGGACAAAACCTTGGTGAACTAGAAGAGGTTAAGTCTTTCCAGAAAAAGTTATACAA
AGCGTTGAACGTACCGTCATCAAGACTTGAGACTGAGCCTACCTTTAACATAGGTCGTGC
TGCTGAAATTACTAGGGACGAAGTAAAGTTCCATAAATTTATTGCACGTCTCCGCAAGAG
ATTCTCAGAACTGTTTATGGATCTCCTTAAAACTCAACTCGTTCTGAAGGGCACAATGAC
TCTGGAGAATTGGGATGATATCAAAGAACACATTCAATTTGACTTTATTGCTGACGACTA
CTTCACTGAACTAAAGGAAATTGAAGTACGCAATGAGCGTATGAATCAAGTGAACACAAT
GGATCCTTACGTTGGTACATACTTCTCTATTGAGTATATGCGTCGTCAGGTTCTAAAACA
AACTGAACAGCAGATTAAGGAAATTGACAAACAAATGGATTCTGAACGAGAAGCAGGTCT
TATACTCGATCCAGAGCAAGCAATGCATCCCGCTACGGATCCTGGCGCTGCCCCAAATGG
GGAAGTAGCTCCACAAGAAACTCCTCAAGTAGACGCGGGTGACACGAAACGGGGAGAAAT
CTGAACTATAAATAATAAATGTGAAAGGAAAATTATGCCTACTGAAATTGCAAACCAAAT
AGTTCAACAAATCTTCGGAGACGATAAATCGAAAGCAGTTGGCTCAGTGAATGATGCATT
GGCTGCAACTGCATATGATGCCATTCAAGCAAGAAAGGTTGAGTTCGATCAAAGTATGGA
GTTTGAACTAGATGATACCGCACAGGATGCTGCAGATGAAATTACAGATAACCTACCTGA
CGGAACTGACGAACCTGAAACTGTTGAAGTTGATGGTCGCAAACCTGAAGATCCACCAGA
AGCACCAGAAGGACCCGTTGCTGACGGAACACCTTTTTCTGTAGAAGAACCAATCGAGGA
ACCAACCAATGAAACTAATAGCTGAAGAAATTACAAACGTTGATTCTATCTGCGAAGAAA
AAGAAGGCAAGAAAACTTACCTCATTGAAGGTGTCTTTCTACAAGCGGAATTAAAAAACC
GCAACAATAGAATGTATCCCTTGAAGACTTTCTCCAAAGAAGTCGCTAAATATGATGAGA
ACTACATTCAACAAGGGCGTGCCCTTGGAGAATTAGGTCACCCTGATGGTCCGTCAATTA
ACCTTGACCGCGTTTCCCATAAGATTCGTTCTCTAAAGGAAGATGGAAACAACTTCATAG
GTAGAGCAAAACGGCTCGACACACCTATGGGTGGAATCGTAAAGAACCTCTTAGATGAGG
GTGTCAAACTAGGTGTTTCATCTAGAGGCATGGGTTCAATTCGTAAAGAAGAGAACTGTA
ATGTTGTTATGGACGACTTTATGCTCGCAACTGCAGCAGAAATAGACGCCGATCCTTCCG
CTCCTGATGCTTTTGTCAATGGAATTATGGAAGGAAAGGAGTGGGTTTGGGACAATGGAG
TCCTAAAAGAAGCTGATGTGGCAGAAATTAAACAAGAAATTGATCAAGCAACTCTAATTA
ATCTCCAAGAACGTAAGGTTTCCGCGTTTGAGGCGTTTTTAAAGAGTTTGTGATTTATAA
ATAAATACAGACAACGCTAATGCATAACGGAGTTTAAACAAATGGCTGAGACCCACGATA
AAGAGCTAGATAACATGGAGCAAGTGGACGAAGGCGCTGATCCTATCACCAAGAACGCAA
AACCTGGTGAGAAGATGCACACTTCCAAAGCAGGAAGTCCAAAAAAATGTCGTGAACGTAG
AAGGACCTGTAGGTGCTTCAATGGAAGGCGCAAAAGGAACTAAGAACGCAGGTAGTTCTG
CTGCAGGTGCAGTGAACTATGAAGGTTCTAAGTCTCTGAGCACAAAACCAAGTGCTGCAT
CCGCAAAACAAGAGGAGGTAGACACGGGAGATGGCGAAAAAGAAGAAATCGCTGAAACCA
AGTACGAATTTACTCAGGATGTTGACGCTCTTGTCGCAGGTGAAGAACTATCAGAAGAGT

TCCGAGTAAAAGCAGCTACTATTTTTGAAGCAACTGTTACTGCCAAAGTTAACGACGAAG
TTAAAGCGTTGCAAGAAGCGTTTGAAACTACGCCGACTGAAGAAGTCGAAAAAGTTCAAA
CAGAATTGGCCGAGAAGGTTGACGATTAATTCTCTTATGCTGCCGAGCAATGGATGAAAG
AAAACTCATTGCGAATCGAGCATGGCATCAAGACAGAGATGGCAGAATCGTTCTTCAAAG
GTCTAAAAGGTCTCTTCTTAGAGCACAACTTTACCGTGGCTGAGGAGAAGTTCAACCTGC
TAGATGGAATGGCAGGAGAACTAGTTGATATGGAAGCTAAACTCAACGAGCAAATCGACA
CTAATGTTGCTTTGAACAAGCGCATTGGTGAGTTTGTAAAAATGGAAATTGTGAACGACG
CAGCTACTGGACTAGCAGATACCCAAAAGGAGAAGTTACCATCATTAGCAGAAGGTGTTG
AGTTTGAAAATGAAGAAGATTTTCGCAAGAAGGTCCAAACTATCAAGGAATCCTACTTTA
CTAAAAAGGCTGAAGTCGCTACAAAAGCAACTGAACCCACTGAAGAAAGTTCCGCTCCTT
TGGTAGAAGATACTAACAGCAGCACAATGTCGAAGTACGTTGATGCACTCGCTCGTTGGT
CCAAATAATTAACTACTAATCCCAAGAGGTAAATTTAAAATGTCTTTAAATCAACTTCAG
GAGAAGTGGGCACCCGTTCTAAATCACGAATCTCTACCTGAGATTGATGATGCACATAAG
CGTGGCGTCGTAGCACAACTCCTAGAGAACCAAGAAAAAGCATTAGTCGAAGAAGGACAA
ATCCTTACAGAGACTCTACAAACCGCAGGTACAGGTGGTTTCGGTGGCGGTGCCACTGCA
ACTGGTCCTGTGGCAGGTTTTGACCCAGTATTGATCAGTCTTATCAGACGTTCAATGCCA
CAATTAATTGCATATGATATTGCAGGTGTCCAGCCAACGAATGGTCTTACAGGTCTTATC
TTTGCAATGAGAACACAGTACGGTACTGAAAGAGATCCTTCCAGTGGCGATTACGGAGAA
GCATTCTTCAATGAGCCTAACGCATGTTTCTCTGGTGCTAATGGGAACCGTCTTGCTGAC
AACGATCCAACTGCATCTGGGAGTTCTGCCATGAACGATGCTGAAGGTGCTAACCCAGGT
CTTCTTAATGATTCCCCTGCAGGAACATATGAGTTGACAGGTGACGCTCAAGGAATGAAC
ACCACTGCTCTTGAAGCAATTACAGATGCTGCTGCAGCAACTGCCTTCATAGAAATGGGT
TTCTCAAGCGAGCAAGTTACTGTTACTGCTAAAGCTCGCGCTTTAAAGGCAGAGTACAGT
ATTGAGCTTGCTGAAGACTTGAAAGCAATTCATGGTCTTGATGCCGAGCAGGAGGTAGCA
AACATTCTTTCAACCGAAATCCTTGCTGAAATCAACAGGGAAGTTGTTAGAACAATCGAT
GTTAACGCTGTTGCAGGTGCTCAAAACAACACTGCTAACGCAGGTATCTTCTACCTTGAC
GTTGACTCAAATGGTAGATGGTCTGTTGAGAAATTCAAAGGACTTCTGTTCCAGATTGAA
AGCGACGCAAACGCAATCGGTCATCAGACACGTCGCGGAAAGGGTAATATCATCATCGCA
TCTGCTGATGTTGTATCTGCTCTTGGTATGGCTGGCGTGCTTGATTACGCTCCTGGTCTT
CAAGGTAACAATGGTCTTGTTCCTGATGACACATCTTCAAGTCTTGTTGGAACTCTTAAC
GGAAGAATCAAGGTTTACGTTGACCCATATTCAGCAAACTTAAGTGACAAGCACTACTAC
GTTGCAGGCTACAAAGGAACTTCTCCTTATGACGCCGGATTATTCTACTGCCCATATGCA
CCTTTACAGCAAGTCAGAGCAAGCAACCCTGACACTCTCCAGCCAAAAATTGGATTTAAG
ACTAGACAAGGTATGGTTTCTAACCCATTCGCTAAAGGTCATACCCAAGGTTCTGGAGCA
CTTGCTGCTAATACTAACTAGTATTACAGAAGAGTTCAGGTTGCTAACTTAATGTAATAA
GTAGTCTTACTTAACTTTCTAGAGGGTGCTTGACACCCTCTTTTTTTATGCTATAGTATA
TTTGTTGGACGCAACACTGGGAGTGACTGAATAAACTTGCTGGCATATAGCTGGTTAAGG
TGATGCGACAGAGGTGGTGCTCGCTGTCAGGAATGGCAGAACTACAAACCAAGTAGGTCG
TAGGCAGAGTGGTAATTCTAAACTGTAGAAATGCCCTGCTCTTGTTGGTATACAGGAATC
CAACCTCCCCCATTAATTTTTTAGTAGAAGGGTAAAGTACCTAGTGAAATTGTCAGGTTT
GCATTATAAGTAGTAATAGAATTAGGGTTAACAAGATGCACTAAACTTTCCTTGTTAATC
ACTTCAGAAATGGAGAGAGATTGATGCACAACCTAGTATCATATAATCAATTAACAGGTT
CTTACTAGGAAGAACACGACACACGGCTAACAGAATACTACGAATGTTTAATCGAATGTG
ACGACCAACAACAGATCTGTAAAAGAATTTGCAGAGAGGTTCTTATGTAACACATATTCG
GGAAGACTTAGAAAGGGGGCAGAGATGCTCCCTTTTTTATGTGCTAAATAGCTCTATCAA
AATGGAATTATATTATGGATTATAATCCTTATTCTCCTGAGTGGCAGCGAAAAACGTATC
TATAGGAAGCACTCTTTAAATTCCTTGTTGACTACGTTGAGAACGATATCATTAGAAATG
ATTTCAAAGTTATCCTGCCCAGTAGATCTGATGAAGCGTACAAAGAGTATACTAAACTCA
ATCAACTATCCGCCAAACGCAGTAAAGAATAACAAATGCTTTCAACCCAATACCGACTCC
GACTGGAGTCTATCTGTAAACAGATCGTAGATGGTGAAAATGTCAAACTAGAAGACATGA
TTTGGGCGCAAAAACTAGCAAAAAGAAATACCACTGCTGCTACTTGGATGCGACAAGCAC
GACAGAAAGCAGCAAATCCTGACATGAAAAATGGAGGGACGGACGATTTTCTGAACAAAA
TGGGGTTAGGAGAACCCGACCCATCGGATTATAGAAGCGGGTTCAATAGTGCACATGACA
TTGGTGTATGGTTTAACAGAGAGAAACCTGACGACTGGAGACAACGAGACTAATGGCAAT
CTGGTTCCAAGACCAACTAACAAATAGAAACTTTCTTTCCCCTATAGGATATCTTTTCCT
GTTAGATCAGGCAAAGAAGGTGTCTTTTTTATGTCAACAAGGAGAGATTCCATCAATAAA
TTTAGGTGAAGTTCTTATTCCTACTGCAGGTTTAGTCCCTTTATCGAGAGAAGGAAACAT

ACGCTATGGTGATTTGACCGTTGAGTTTATGGTAGATGAAGATCAAAGAAACTACATGGA
AATCCACAACTGGATGAGTGCTTTAGGAACTCCACAATATTATGATGAGGGTGTTGCATG
GGAAACCAAGTATCAAGACGCACCATCACAAGACGTACGTTTCTCAGACGCTACTCTTCA
AGTATTAAACAAGAATAACTTAGCAAACTGTGAAGTAGTCTTAAAAGATTTGTTTCCTAC
AGAACTATCAACACTATCATTTGATGTTTCACAAGGTGAAAATTCATTCATGACTGCACA
GGCAACTTTTAAACATTCTCGATACGAAATCAGAAACGTAAATTCGAGAACTAGACGATG
AAGAAGTTCACTCTATTCAAGTTTGAGCATACATGTCGTGGTAAAGAGAACTGGTATACT
AAATCCACTAGGTGGGCGGAGAAGCAACCCTTCCCTTTAAACCATTTAATTAAAGGTTGT
ATCGAATGGTTGCATATTCAGTGGAATGATGGTATAATTAATATGGCTATGGACGACGTT
GATCGTCAGGTAGATAAAATCCAATCCCAGTGGGAGGAAAATGAGCAACCAACAAGACAC
ACAATCGTGGAGACAGGAGTATTTGGAGATGAAGGCTGGTCTCTCGAAATTACAAATCCG
ATTGTTGAAAGAGGGTCCTCATCAACTAGAACAGGCATGGTTACTCCAAGCGATGCACAA
CGACTACAAGAAGATGAAGGGGATCAAGGAACCACCTAGTCGTGAGTCTGGATATCAGAT
ATCAATGTAGGAGTGGTTTCAACGTGAAAAGGATCAGGGTGTATGAATCTTGAGCAATTT
CAAGAGATGTGGGAAAAGGATAGTGTAATTTACGGAGATTTTGACTGCGAAGAATCTACA
AAAATGCCACAACTTCATATGAAATATATGGAGTATCACAATACTTTCTCTCTTATGAAA
AAAGAAAGAGAGTTAGGAATGAAACGTCTCATAAGAGAAAAATGGTTAAACTATGTAGGT
AAAGCACCCTCTTCAGTATACAAATAAACACCATTTGATTTTAAACTTACTACATAAGAA
GAGATCAATATGTTTATTGAAGCAGATGATGCTATCTGGAAACTACAATTTAAGATAGAC
TACATAGAACAAGTTCTACTGTATCTTGATAGTATCTTACGAATGATAAACAATCGTATT
TGTCAAATTAAGGATGCAATTGAATGGGAAAAATTTAAGAATGGTTTTTAATGAATTACG
GATTGCCCTACAAAGAAGTTATCTTCAATACAAAGGCACAGGATATAGTCAACAAGGTAA
TTTCGTATAGACATAATTGGACTTGAGGACAAATTCGTAGGGAAGTTCAATCAAATAGAA
GTTCCGAGATAGCATGGTTAGGAGATAGAGAACTCCTGTCCATGATTCTTCGTATGATTA
AGAAAATAAATGAAGATGCTCGTTGCAATCTAAAGATTACAGGTGTCGAACCAGTTCAGT
ATGGTAGATATGGTGAAGATGATTTTTATGATTGGCACATAGATCAACACCCTCAACCAG
TAAAGGGATTAGTAAGAAGGGTTAGTATGACACTCTTTCTAAATGAAGATTACGAAGGAG
GCGAGTTTGATTTGGAGATATATAAACCAGAGACAGATCCCAGAAATAAAACTTTCAAGT
TAAATGCAGGATCTGCTATATTTTTCCAAAGTGATCGATGGCACAGGGTACGTCCTAGCA
CATCTGGAACTCGTCAATCTTTAGTAGCATGGTTTTATGGACCTCCTTATTCGTAAAAAG
AATGAAGTCTATCTTAAGGTTGAAGCGCAACCTCACATTAATTATGAGTTAGCAGCTTTC
TTTACCTTCGAGGTAGAGTCTGCAAAATTTATGCAGAAGACTAGAAGGTATACAGGATGG
GATTGTAAGATTAGATTGTTTTCTCCTGCAACAGGAGAGATATATCGTGGTCTTGTAGAT
TACTTAACAGACTGGGCGGAAGAAAAGGTATTTGAATATCAAATAGAAGATGATGAATAC
TTTGGTCATCCTATCACAGAGAATGAACTTATCACTCCCAAGTCGGTTGTAGGGTTTGTA
AAATCACTGCGTCTGCCCCCGACTCTACAGGTAAGGGATTATCAGTATATGGCAATTTAC
GAAGCACTAAAATACAACAGACGACTGTTGCTGTCCCCCACAGCGTCAGGATAAGCTTTG
ATGATTTATGCATTGGTTAGATTTCGGTTAAACGTTAGTCGTAATGTTTTAATTGTAGTC
CCCACTACCTCTCTTGTCGAACAAATGTATAAGGACATTGAAACATATGGTTGGATGGCA
AAGAAAGATTGCCATAAGATATATGCAGGGCAAGACAAACATACAGATCACAATGTAATA
ATCACGACTTGGCAGTCAGTATATAAAGAACCAAAGAAATCGTTTGATAGATTCGACTGC
GTGATTGGTGATGAAGCACATCAGTTCAAAGTAAAATCTCTAAGTACATTGATGGGTAAG
TTGCATGATTGTAAATACCGTATTGGTTTTACAGGAACACTAGATGGTGCCAATGTCAAT
CAACTTGTATTAGAAGGTTTGTTTGGTAGATGTTCTCAAGTTACTAGAACTAATCAATTC
ATGAAGGCAGGGCACATTGCTAAATTAAAAGTCAAGGTAGTGTTTTTAAAACATGAAAAA
AAACTATTTGAAGGATATCAAGAGGAAATTGATTATCTAGTTGAACACGAAGGAAGGAAT
AAATTTATCCGTAATCTTGCGTGTGATCTCAAAGGAACTACCCTTGTGCTTTTCAATTAT
GTAGAACGACATGGTAAATCTCTTTACACATTGATAAATAGTTACACAGACAGACCCGTG
TTCTTTGTTCATGGGGGTGTAGATGTCGATGATAGCGAAGAAATTCGCATGCTAACAGAG
GCATCAGACAAGGCAATCATTATTGCATCGTATGGTACGTTCTCTAATGCCATTAATATT
AAAAACTTACATAATGATATTTTCGCTTCTCCTTCTAGATCTCGGATTCGTAATTTACAG
TCTATTGGACGAGTATTGCGGAAAGGAGACAATAAATCAAAAGCCACTTTATATGATATT
GCTGATGATATTACAACAGACAGGGGCAATAACTACACGTGGAATAACCTGATGGAGAGA
GTCAAGATTCATAATGAAGAGAAATTTCAGTATGGGATCATAGATGTAAAAGTAAAGGCT
TATGATTAACTACGGAAAACACGACGAAGAATTTCACGGTATATTCAAACTGGTTAGTGG
GTAGGAAATCCTTGCTAAGGCAGTTATGACAGAAGACCGTGGAGAGAGTTTAATGTTCAT
GTCTGATCATGTTAGCGCATTGGCAATCACTAAGGACGTCGGCGAGCAAAAAAATATTAAG

AGGTATGGGTTTCAGTAAATGGATTGCAATGTCTGACGAAGATTTTTTATTTTAAGCGA
GAAGGATATCATGACCATGGCAACAATGAGTAGACCTGTCAAACTCATGTACGATGCATA
CATCATCGGCGAAGATGCACATGGAAAGCAAATGAAAGAACGTCAAGTTCCGCTCTCGAT
TGCCGAAGGATATCTAGGAAACACAAAAGACATCCGTGCCTTGCTACAAAAATTGTAAAA
GAAGTAAGTTATATTTCCTCTGACCCCTTACAGTGTTAGTAGACTTGTCCTTGACAGGTT
TGTCAAGTGTTGTTATAATTAAAACAAAGAAAAATTTGTATGAAGATATCTTCCCCCAAA
AAGCGACAGCATTATGTAGATAATCAAGAGTTTCTTGCGGCTATTATCAAGTATAAAGTG
AAAGTAGACCATGCAAAAGAAAAGGGTCTACCAAAACCTCGTGTCAATAATTACATTGGT
GGTTGCTTTTTAAAGATTGCTACTCATCTATCATATAGATCAAACTTTATTAATTACATG
TATAAAGATGATATGGTATGTGATGGTATCGAGAATTGTATACAGTATATTGATAACTTT
GATCCTGCTAAGAGTAGAACCCCCTTTGCCTATTTCACTCAAATCGTATACTTTGCTTTC
CTAAGAAGTATGGCTAAAGAGAAAAGACAAATGGATATTAAAGAAAGAATTATAAAAAAA
TCTGTATACGATCATGTGTTCACAGTTGACGGAGACGCAAGTTCAGAGTATAATCAAATT
AAGTCCCGTGTCGAAATGAATTCTAAACGATGAAGATTCTCTTAACAATAGATCAACACT
TTGGCGTTCGAAATGACAACCAACATTTTATCCATCATTACAAACAGTTTTACAATAATA
TTATAATTCCTTTTATAAAAGCATCTGGCATTAAACAGGTCATTGCTCTTGGTGATACCT
TTGATCGCCGTAGGTTCATTAACTTTATGTCTCTATCCGAATCAAAAGATATGTGGTTTG
ATCCATTGAGAGATATGGGTATCCCAATGACCATGCTTGTTTGCAACCATGACATATACT
AAAAAAATACTCTTCGAGTTAATGCCCCAAATGAATTACTCAGAGGGTACGACAACATCA
CAATCATTGATGACCACGATACTGTAACTTTTGATGGTCTACCTATTCTTCTCCTTTCTT
GGATTTGTGATGACAATCGCGAACGATTTCTCAAAGTTGTCGAAGGATCTGATGCCCCTG
TCGGTATGGGTCATTTAGAGCTTAACGGTTTTGAGGCTCATCCAGGTCATGTGATGGAAG
GTGGGATGGATCCCAATGTCTTTAGTAAATTTGAAAGAGTATTCTCTGGACACTATCATC
AGAAATCTACTAGAGGTAAAATCAGATACTTAGGTAATCCTTATGAAGTTTATTGGAATG
ACTACGGAGAGAAAAGAGGTTTTCACGTCTTTGATACAGGCACTCTTAAAACTACTTTTT
ATATGAATCCCTTTGATGTTGTTTATAAACTGTATTATAATTCTGGAGTGTTTCACCCAG
ACGAATCAGAAATGAGAGGATCATTCGTCAAATTAATTGTAGAAGATAAAGGAGACTATC
AGAAGTTCGACTACAATGTAAAACGCATACAGGATATGGGTTGTGCAGATCTTAAGATTG
TTGAAGATCTTAGTGTGGAACTGGAGAATGGAAGTGAAGTGCTAGAAACCGAAGACACAA
TGATGTTGTTAGACAAATATATAGATGAGATAGATTTAAGAGTCATTAGACCTAATGTTA
AGTCTGTCATGCGATCACTATGTGTAGAGGAATCAGAACTATAATGTTGGTTCTTACTGA
TAAAAAAATTGGTGGTGTTTATGCTAACTCTAAGTCAGGCGACGAACAGGGTAAGAAGAA
CGTGCTTGTTTTTGCAGAGAAAGACGATGCAGAAAGATATATCGTACTTCTAGAGGCAGA
TGATTTTAATCAGGAACTTGACATTCTGGAAGTAGATGCAGAAATTGTTGCCATGAATTG
TGGTAATTATGGGTATTCTTATGCTATAATAGAACCTACTGACTTACTCATACCAAAAAT
CAAATTTGATAAATGATTGTTTTTGAATATATCAAGTGGAAGAATTTTCTTTCCAGTGGT
GATCAATGGACAGAAATTAGTTTGAATGACTCTCCATCAACTCTTATTGTTGGTGCAAAC
GGTGCAGGTAAATCTACTCTCTTAGATGCATTGTGTTTTGTTTTATTCAATAAACCTTTG
AGAAACATTAGTAGAGGACAGTTAGTAAATAGTATCAATGAAAAAGGTCTTAAGGTTGAA
GTATTATTCTCTATTGGTGTTGATGAGTATCGCGTCTTTCGTGGAGCGTAACCTAATATA
TTCGAGGTCTACAAAAACAATAAAATGGTGGACCAAGATGCTGCTGCCAAAGACACGCAG
AAGTATCTCGAACAATCCGTTCTCAAACTCAACTTTAAATCCTTTACACAGGTCGTCATC
GTGGGTTCATCCACCTTTGTCCCCTTCAGGCAACTCAACGCACCTGTCAGGCGAGAAGTT
ATTGAAGATTTACTTGACATCAACATCTTCTCCCAAATGAAGACGATCCTAAAAGATCGT
GTTAGACATGCAATGTCACAACAACGTGATTGTGACCTTCGCCTTCATATCGCAGGGGAA
CGTGTAAACAATCAAGAAGGATTTATTAGCCCTCTTGCTGATGTGAGTGCAACTCGTCAG
AAAGATAAGAAAAGTAAAATTAAAAACAATAATGAGAAAATTAAATCAGAAGAAAAGCAA
CGTGATCTACCGAAAGAAGAAGTAGAAACTCTTACTATAGGTTTGAAGAATGTTGATAAT
CATAAAGCAGTATTAGATGACCTTCGTCAAGAACAATCTGATATCAATTCAGAGTTAAAA
TCTGCTGCCAAACAACTAAAGTTTTTTAAATCACATGATGAATGTCCTACCTGTTCTCAT
CAGATTGATAAGGAATTCAAGACAGCAGTGATTGGTAATTTAGAAACTAAAGGTCAGAAT
CTTACAAAAGATTTTAAAGGTTTGACTAATAAAATTGCTGATGCTGTTAGTGTTGTAGAG
AAAATGGAGATTCTTTCTAGAAATATCATGGAAACTCGAAGTAATGTATCCTCTGCAGAA
AGAGAGATCGTACGTTTAAAGACAGAGAACCTTAAAATAAATGAAGAGATATTAGAACTA
CAAACAAACACTCCTAAAATTAGAGAAGAAAAACTATCGTTAGTTAAGTTTCAAAAAGAA
TTAGAAGAAACACAAAAAGATTGTGGAAAGAGTCATCAGACTCTTGATGAATTTAAAGTC
ATAGGTAATTTGTTAAAGGACTCTGGTATTAAGAGTCAAATAATTAAGGAGTATGTTCCT

ATTTTTAATAATATAATCAATAAATAGCTGCACAGCATGGACTTCTTTGATAACTTCACA
CTTGATGAAGAATTTAAAGAAGTAATTAAAAGTAGATTTAGAGATGAATTTTCATATCCA
TCTTTTTCTGAAGGAGAAAAAGCAAAAATTGATTTAGCATTGCTATTTACATGGAGAGAC
GTTGCTCGTATGAAAAATAGTGTTGCTACTAATTTAATCATACTTGATGAAGTATTCGAT
AGTTCTCTGGACGCAGAAGGAATAAATGAACTCTTGAAGATTCTTCATGCTTTGGGTATT
TCAAGTAACATATTTGTTATCTCACACAAGGGTGATGTCTTATTAGACAAATTCCTAAGG
ACACTTAGATTTTAAAAGATTAATGATTTCTCGCGGATGTCAGACGACTCGTAAATTTTG
GAGAGTATGGGCAAAAGCACTTGGAGACAAGTCTGGAAACTCAGATAAAGAAGCGGATTG
GATTGCTTTAATCCGAACATTTATTTTCAGTCGGTGAGTAGTTACTAACTGTTTTATCGT
AGCAGGTAACATAAGACACTGGAACGATCATCACGTTCCGCCATCATATCAGCGTGTGCC
AATTAAAAAAGTGGCACAATAGCTAGCGCATTCTTTATTGGTCTGCTATAATAGGTATAT
ACGAAACACAGACACATGATCAATCAAGAAGTAAAAGGAACACTTGCTAAACTACTAGCA
ACAGAAAACCTCACAGTTGAGCACCGTCAGGTTACAACTGCATACTTCGATGTTGAGAAT
CGTGTTCTTTGCCTCCCGATCTGGAAGTCTGCCTCAAACACTGTTTACGATCTACTTGTA
GGTCACGAGCTAGGACATACACTTTATACTCCTGCAGATGATTGGGATAAAAGTGTTCCT
ATAGCATTCATCAATGTTCTCGAAGATGCTCGTATTGAAAAACTAATGAAGCGTACCTAT
CCTGGTCTTCGTAAGTCTTTCTTTGACGGATACAAAGAACTATGGAATCAAGATTTCTTC
GGAGTCAAACATGAGGATCATGAGATTCTATCACTGATTGATCGCATCAATCTTTACTTC
AAAGGTAATCTATATCTTCCTTTCTCAGATGAAGAGAAAGCATGGGTAAACAGAACTGGA
AAAACTGAAACAATTCAAGAGGTAGTAGACCTTGCCAGAGATCTATACGGTTGGGCAAAA
GAAAAGCAAGCAGAGAACGAGCAAGAATATATGGAAACTGATACTGAGATTGGTAATGGC
GATGGTAATATGAGTGGTGGAGTTTCTGCTACAGATATCGAAGTCAAAGAGAAAGATTAT
TCTGAAGGAGAAAAATCTGCACCTGCGATTGATCCTAATCAACCATGGGATAGTTCAGAG
AATCCTCTTGAGGATCTATCAGAAGGTCTCAATAAATACTCAGCACCTATCGGTGGCGAT
GGATCTGATCTATTCAATGACTTTGATCAAGTAGAAGAAACTGAGAGTATTACAGATAAA
GCATTAGAAGAATCACTAGAAGACTTTGTTGATGATGATGCTAAGGAGTGTGTTTGCCTT
GGTCTTCCTAAAGTAGAAATTGACAAACTTCTAGTTTCTCACTCTACATTTCAAAGTGAT
CTTGAAGGATACTTCTCTTTTTACAAAAATGGTGACGAAGATGCAGAAAACTATAAGAGA
GAGTGTCTTCAGTATGCAGATGATCATTACAACAAATTCCAGAACGATGCACAGAAATCT
GTAAACAATCTTGTCAAACATTTTGAGATGAAAAAGTCTGCTGATGAAAACAAACGTGCA
GCAGTATCAAAGTCAGGTGTTATCAACACTAATACTCTTTACAAATACAAACTAACTGAT
GACATCTTCATGAAAGTTTCAACTGTTCCTGAGGGTAAGAATCGTGGTCTTATACTACAT
CTTGATTGGTCTGGTTCCATGCAGTATCAACTCTTGGATACTGTAACGGAAGTTTTCAAC
TTGGTATGGTTCTGTCGTAAAGCAGGTATTCCTTTCCGTGTCAACGCATTCCAGAGATCA
TACTATAGTTGTGATCATAGTAATTGCAAACAATGGAATCCTGGTGTTGGACCTAAGGAA
GGTAATCTTGGTATTGCCGATGATTTCAGACTTCTTGAATTACTATCTTCTAAGCAAAAT
GCTAAGTCACTTGAGAATTCACTCAGACAAATTTACAGACAAGTATTTGCTATCGGTGGA
TACCGTATGTCTGCTATGCCCACATACACTCTTGGTGGAACTCCACTTGCTGAAGCAGTT
TTCTGCACTCGTCAGATGGTAAAAGATCTCAAGAAGGTAGAAAATGTTCAGAAAGTAAAT
GTTGTATGCTTAACTGATGGCGAAGCAAATCCAATGTCATACTGGGCAATAAGTCCTTAT
AGCACTTACATGCAGGAGATGCGTGAGGAAAAATTCAGACTTGCTCAAATGTGTCACTCA
AGAAATAGAGTATTCATTCCTCGTGATTCTCACACTGGTTATCAACGCAAACTTAATGGT
AGTCCATATGAAACTACAAAAGAGATCGGAAGTTACATGATAGATGTTACTGATTACAAC
TGGATTGGTATCAGACTCTGCAGTAAAGGCGAGGTATCAGGATTGATTCGTATCCTTGGT
ATGGACATACGACGTCAAGACAAACTTGACAAGGAATGGAAGATAGAAAGATTTGCTTCT
ATCAAAGATGAAGTTGGATTCTCTCAATCTATCTATATGCCTGACAGGGGCAATGGTGAA
GGAACTCAAGACCTGACTGTAAAACAGAAAGGCGAGGTTGCTACCAAAGCAGAACTACAA
CGTGCTTTCAAAAAGCACATGGGTTCTAAGGCCACACACTAAACTCTCCTCAACGCATTC
ATTGAACAAATCACATGAAGTGTAAAGTAACTCTATTTAAAGCGGGAACAATCTTTGAAG
AAACTGTTATCACTGTGAATTATGATGATTCAAAAAAAGTTGCATTAGCAAGAAATCCTA
ATTCTACCGTAATGAGTGTGACAGCAGTGTTCGGATAAATAACTGTCCACATGCGGTAGC
ACTCAGCAGATATACCTGCTATAATAAATGTATAAACACACAAAGACTATAACTTTTGAA
CCAAACCCTGTGACAACTGAACAACTTGTTCAACACCTTACTGATAATGTTGTAACTGAA
GTCGGATGCAAAGACATTCGTGAGTCAGCAAAGATACTTAAACTATCTTTCACAACTGCA
AGTAAAAGACATAAGTCTTATAAATCAGGTATAGGCAAGTGGAATCTAACCATTCAAGAA
ATTCAACGTGCGTAGGAAGCACCTGCTGCTAAGAAAAAAGCAAACTATGTTCCTGATAAA
GATAAGACCTATGTTCCTTTTGGTAACTTCACTTCTGTAAAGAAAGTTATCTCATCGAAG

AAGTTCAATCCTCTATTCATTACAGTTCTATCTGGAAACGGTAAGACTCGATCAGTTGAA
CAGGCGTGTGCTGCAACTAATAGGGAGTTAATCCGGGTCAACATCACAATCGAAACTGAC
GAGGACGATCTTATTGGTGGGTTTCGTCTTGTTAATGGCGACACTGTTTGGCACAACGGA
CCTGCGGTTGAGGCTTTGGAAAGGGTAGCAGTATTACTTCTAGATGAGATCGACCTAGCA
TCTAACAAAATTCTTTGTTTGCAGTCTGTCCTTGAAGGCAAAGGTGTATTCCTTAAGAAG
ATTGGTAGGTATGTAAAACCTGCTCAAGGATTTACTTTTAGCGCTACTGCTAACACTAAA
CGTAAAGGATCTGATGATGGTAGATTCGTAGGAACATATATTGTCGACGAAGCCTTCTTT
GAGAGATTCCCAATCACATTTGAGCAAGAGTATCCTTCTGCTACTATCGAGACTAAGATC
TTGATCAATCAGGGATGTGACAAAGACTTTGCAGAGAACCTAATCAAGTGGGCAGGTGTA
ATCCGTAAGACATTCTTTGATGGTGGTGTTGATGAAGTTATCACAACTCGTCGTTTGGTT
CACATTGTTTCAGCATATCAGATCTTTGGAGATAAGTTGACAGCAATCAGATATTGTGTA
AACCGTTTTGACGATGATACAAAATCATCTTTCCTAGATCTTAATACAAAAGTTGACGCA
GGAGAAGATTCAGAGTATACTGATAATAACGAAGTTCAATCATTCAGTTAATTATGAAGT
ATCGTGAAGAGGAGTTCCTACAGTAACTCCGACATTACATCGAGAGCACTTACTCTCAAC
ACTATGCTAGTGATAAGATTCAGACTCTTGATGTAATCGATGAGTGTCGCGATGCTACAG
CATTTTGTAGAAGTAACATTTTAAAGTACGCTTCTCGCTTCGATAGAAAAGGTAGTGCTA
GACAAGATCTCTTAAAGGTGCTACACTACGGAGTGTTACTCATGCACTTTTATACAAGAG
ATCAAGCAAACTACGCAGCAGCAACTTTTGCAGCACAAGAAGACTAATTCATTATGACAG
TAATTTCCAAACCCACCATTGAAGTCCTTAAAAACTTCTGTTCAATCAACAAGTCTATTG
TTATTAAACCTGGCAATAAGATTTCTACGCTCAGTATTAATAAGAATATTCTCGCTATTG
CCGAAGTCGAAGAACAATTTGATTCGCAAATTTCTATCTATGATTTGGGAGTTTTCCAGG
GAGGGTTGTCTCTGTTTGACTCACCAAAAATCGATACTACTGATACCTACTACGTCACAG
TAAGTGACCTACAAGGTAAATCATAGACACGTTATTTCTATGCTGATCCTGATATCATTA
CTCAGTCACCAGAAAAAGAAATCAACTTGCCATCAGAGGATGTTGAATTTAAACTTGAAT
CTAAGATTCTGATGCAATTACAACGTGCTGCTATGGTATCTCAACTCCCTGTTCTTTTTT
GGTATGGGGATGGAGAAACTATGCAGTTATGTGGGACAGATAAAAAGAATGATACTTCCA
ATAATTATTCAGTAGTAGTTGGAACTACTACAGATGAGTGTTGTTTTTGTTTCAAAGTTG
AAAACTTAAAACTTTTATCTGGGGATTATGATGTAGTATTGAGTAAGACAAATGTTGCTT
TGTTCAAAGGTGACGGAATCAAATACTACATTGCTCTAGAACCTAACACATGAGACGTTG
GCATTCTGACACCGAACTAATCTGGGTAGGTGACAAGGATGGTCCTTATGCAGAGGAGTA
TAGTTTCGTAACTAATCTCCACTGCCCTAAGTGCGATTCTTACGTTGAAGTTTATTATCC
TAAAAAAGATGAAAGAATTTGATTATGAACTCGATTATGAGAGACTTGACTTTACAGACG
AGGAGACTCGTAAACTATATCGCATTGGAAGAGGGGACCAAGGGGTTCTACTGGTTCGCC
CTTATACTAACGATATATGTGCTCATTAGAGATTCAAAACTCCCACTGAGGCAGTAAAAT
CTTCCAACCATATCTTTGGTATGGATCTTGATTAACGTGATGAAATAGATTTCATCGGTA
TGGATATGTGTCGTAAGTTTCTTGAAATGGGTTTTACCAGATCGAGGAGATATGCCATTT
ATAGAGATGGTAAAAAGTATGATAAAGAAGGTAATGTCATACCCCAAGAACCAGATCAGG
CTACCTGTCATTTTGCTGAGTCTACTAAAATTTTTAAGAAGGTTCGAGATATTGTTGCGA
AGAATAAAACATATGTTACACTGAGGAAAGAATGGAGGGCATCTGCATGAACATTTTTGT
AACTGATCCTTCCCCAACTAAGTCTGCTCAGGTATTACCTGACAAGCACATCGTCAAGAT
GCCACTAGAGACCTGTCAAATGCTTTCTATTGTTGCATCTGATAAATGGGGTCACGGTTC
TGGTTCTCTACCTAAACTAGATGGCACACCATACAAAACAAATAAGGGTGCCTTTTGTAA
TCATCCCTGCACTGTATGGGCACAGGATAATTGGACATGCTGGATCGTGCATGATCTTGC
CTTATGCTTTGAATATACTCTTAGATACGGTAAGATACATAGTTGTCAATCGACTATCGA
ACATTCTGAAAAAATATTTCCTCCTCAGGATACTGATCCAACACATTTTGTTTTCGCAGG
TCCTGATCAGTTTAAGCATGATAAGACTATTGACATTTTTACTGCATACAAGTATTATAT
CAATAGTAAACCAAGGGTATCAGATAATTATCTAAGGTTACCTGATAAAAAACCTAACTG
GGTTAATTAATTATTATGAATGATTTTTTGTGGGTCGAGAAGAATCGTCCTAAAAACGTA
GAACAATGCATCTTGCCCGAATCTTTGAAGACTACCTTCAAGAGTTTTATACAGCAGGGT
GAGATTCCTATTCTCCTTCTATCTGGGACAGCGGGGGTAGGTAAAACAACTATTGCTAAA
GCACTTTGTCATGAACTAGGAGCCGACTATTATGTTATCATTGGATCCGATTAAGGACGA
TTCCTCGACACCGTACGAAACTAAGCAAAGAACTTTGCTGCAACTATATCAATTACAGCA
GAAGCAAAGCACGAAGTTCTTATCGTCGACGAAGCAGACAACACTATACCAGATGTTCAA
CTCTTACTTCGTGCATCGATAGAAGAATTTCAAAAGAATTGTAGATTTATATTTACCTGT
CATTTTAAAAACAAAATTATTGAACCTCTACACAGTAGAACAACTGTCGTAGAATTCAAC
GATCGTGGGAAAACTAAACAACAACTTGCTGCAGAATTCTTTGCTAGATGTAAAGGTATT
CTGATTGCTGAGGAAGTTACCTTCTCAGAAAAAGTTGTAGCAGACGTTGTTCAGAAGTAT

TTTCCAGACTTCCGAAGGACTCTTAATGAGTTGCAACGATATGCAAGCACAGGAACTTTT
GATACTGTAATCCTAGCAGCACTAGGAGACGCAAATATCGATACTCTAGTCGAATCATTG
ATAGCAAAACGATTCAATGATGTAAAGAAATGGGTTACACAAAACCTAGATTCTGATTCT
GTCTCTATCATGAGGAAACTCAATGATAATGCTTATAATGTAATGACAGGTCCTAGTATT
GCAGCAGCAGTTTTAATCATTGCTGAATATCAATACAAGTCTGCATTCGTTCTAGATCAG
GAGATCAATCTCCTTGCATGCTTAACTCAATTGATGCTTGGATGCGAATTCAAGTAACAC
CCAGTGACCTTTACAAAGAAGTAAAGGAACTTGTCTTAAGTCCTGATTTTCCTTGGCATT
GGCATGACAAAGCATACAATGATGATGAGGTTACTGAAGGTAGAACTAACTTTGGTTACT
TGTCACATGTAGTCTTAGAGAGACCTGGTTATACATACCTAACTCCTAAGATAAATCCTG
ATCACTTTCCATTGTTTCATGATTTATTTGTTGAGATCTGTTGAGACAATAACATTGACC
CTAAAGTAATTTACAGGATCAATGCTAACCTGACTACTGAATCACACCATAACAAATATG
GTCCTGACCACACAGACCATGACTATCCTCATAAGAATATGTTAATATACTTAACAGGAA
CTCATGGAGGTCTTACTAAAGTTGGAAACACCAACTACTCTTGTATGCTTAATGAAGCAA
TTGTTTTTGAAGGTGTTCACGCACACATAGAACCTACGTTTGGAAGACGAGTAGTTCTAG
TTTACACATTTTTATGATTATGACTATCAAATTTATTACAAAACGTGAATAGGTTAAAGC
ACAGGTAAAGTCCAGATTCTACTACTGGTTCTGGGGTGCTATGGCAGGTGCTGTTGTAGG
TGGTCAACTATATGTTGGATCTTCATACAGACAAATGGCAAGATCAATGGACAGATGGTT
TGAAGAGACCTTTGAGGTGATACAAATTCCACTAAAACCTCCAACGGGACGCATGATGCC
TGTTCCAATGCCTAATCCAGATTTCTATGACGATCCTATGATTATCAGATGACACCGTTA
AAAACACCACTAAGATATCCTGGCGGTAAGTCTCGTGCTACTAAAAAAATGGCACAATAT
TTTCCAGACTTAAGAGACTATCCTGAATTCCGTGAACCTTTTCATGGAGGTGGATCTGTA
GCGTTATACGTTACACAGATGTATCCTCATTTGGAAATCTGGGTCAATGATCTATACGAA
CCTCTTTATACATTTTGGACACAACTGTAAAATCATAGTGAAGAACTTAGGAACCAACTT
GTTCAACTCAAGCAAAAATATCCTGATCCGTCTTCGGCAAAAAATCTTTTCTTATAATCC
AAAAAGTATCCTACTAGACACCTAAGAGATTGCGATGTTAAGGCTCGTGCTGTCGCTTTC
TATGTTGTTAACAAGTGCTCATTATCTGGTCTCACTGCGTCCTCATCGTTCAGCTAACAA
GCGTCTGATTCAAACTTTTCAATGCGAGGAATTGAAAAACTACCATACTACGGAAGACTT
ATCAAAAGGTGGGAAATCACTAATCTGCCATACGAAAAATTATTAACAGATGATACTAAT
GCATTCCTATACTTAGATCCTCCGTACGATATCAAGGCAAACTTGTAAGGAAAGAGAGGA
ACAATGCATATTGGATTCGACCATGATCCTTTTGCAAGAAGGTGTGATAGAGTCGAGTGT
GATCAAATGAAATCTTAAAATTCTTCTAATTTAGTAAAGCAAATATTCCAAGGTTGGAGA
GCACAAGAATACGATCATACTTATACTATGAGATCTGTCGGTGACTACATGTAAGATCAA
GAACAACGCAAAGGACTTCTCCTTTTAAATTATGGCATATGATGAAAGATAACCTCTAAA
GGATTATCTAAACTTAAGCAACTTCAGTAAAGATTATTTGATGGATGAGGATCCTGCATG
GGAGAAAAACTATCCTTCCTATGTAATCAATAAATACATGTCTCAACATTTGGATACGAT
CATGTATGCTAATGAAATGAACATACATTCTCATATAGAGAAACGTTTGCAATATGCTTT
CTTTATAAATATCGTGAGACCCCGAAAGAGATTTACTCCCTGGGGTAACAAACAGAAAGT
GAAAGATCTTGACCTTGTTACAAAATACTATGGTTATAGTATTTATAAAGCATATCAAGA
CTTAGAGATCTTAACTCCATCCCAACTTAATTACATTAAGGATCAACTGAACAAATGAGG
TAAGACCAGATGAATGAAGTTAAAGAAGTCAAGTGGACTAAGAATGATATGGTTGAAGTG
AATTTAAAAGAACCAGACGATTTTCTAACGGTTCGTGAAACACTTACTCGTATCGGTGTA
GCATCTCGTAAAGAGAAAAAGTTATACCAGTCCTGTCATATTCTACTTAAGAAAGGACAA
TAGTAAATCGTGCATCTCATAGAATTATTTGCTCTTGATGGTAAGAAAGCATATCTATCT
GAGATTGATGTGCAACGTAGAAACAGAATTATTAACTTACTATCTGACTGGGGACTAGTT
GAAATTGTAACTGTATCAGCAATCTCAGACGCTGCTCCATTAAGTCAAATTAAAGTAATT
GCATACAAAGAGAAAGGAGTTTGGACGCTTGAGTCCAAGTATAAAATCGGGAAAAAACGT
CAAGTCACAGAATGATATATAGTTTAGTGTTATCATACCAGCATAACGGCAGCCGCAAAG
ACAGTCGAGGATAAACCAAATGGTCCTCTTGGAAAAATCAAAGAGTTCAATGAAGATAAA
GAAGAACAACTTGCTCTCCTAAGTACATTCGTACGTGTGGGAATTTTGATTTGGAGTGGT
GGTATCTTAACTTTAAATTACGTTACGATACCTGGTTGAGAACTAGATAAAATCGATCCA
ACTTTTATAGCGTCGGTCTTCACAGGAGTCACAGCTACTGTCGGCCATCAAGCGGGAGGT
AAGAAAACGAATGGAGACAGTGGTGGTGGTGCAAACATCTCTAAGAAAGACATGGAGATG
CTTATAGAAAAAGCAACACAGGCAGCACCAACTCAAACTATTAACTTAGAAGTTCCTACA
GTTAAAATTACTAGTTAAAATCATGCAAAAAATTATCAACGGAATCGCTATCTTCTCAGG
TGTTGTGCCACTTGGTGTAGTAGGACTAGGTGGATATGAATACTTCCGTAAGGATGCTAT
CATTGAAGACATCAAAGAGAAAGCAATCAATGCAGCACCTGGAGGAGTAAGTGACTCTCG
ACCTGACATGGTAGATACACAACTACCTCCTGTAAATGGTGCACCAGTTCCCTTATCTAC

ATTACCATGAACTTTAAATGGATATCAATCGGTGTAGTTGGTAGTATATTCGCAGTATCT
CATCTGGGTATGATAGGTTATATTGCCAGTCGGAAAACCGAGAGTCAATTACCTAGGATA
GATGTTCCTGTAGGTGACTATACTTCATATGCCATCTCAGCTGATAAGGATGGATATAGA
ATTAGTTACTCAGCAAACGATCCTAAGACAGCATTTATTACTAAGGACATCAAAGAGAAA
GGTGGTTTCTTAGGACTAGCAAATGAAACTACTAAGATCACTGAAGAATACTGCTTAGAT
GGTAAGACTAATCAAGGCGGTGCTGTATCTAACAAGAGATCTTGGCTTGATCAACCACCT
GGTGTGACTGCAGAGCAATCAGCAGAAATAGTCGCTGCTCGAAAAAGTGAAGCCTGTATC
AAAGCAATCGGATCAGCAGAGGGTACAGGACGTCTCGTTGGCACATCAATTGGTGCTGCG
GCTGCTCCTTCTCTCAGTAGAATCCCCTTTGTAGGTTGGGTCGCTGCTGGCTGGGTGGCA
ATGTTTGGTGGCAATCAGGGTGCTGATATAGGTGGAATTATGGCAGAGGATATGAGTAAG
GGTTGTTAATTCATTTTCTTCTTAGGACGTTTGAATGGAGGTAATCCTTTCTTCTCACGA
TACTTATTACACTGTAACTCATTACGACTTAACTTAGGTGGTTCTTCATCGGGCTTCTTT
TGCATGATCGTAGTGAGTTTTTTAATGAGTGCTTTTATAACTCTCAACAGTAATGGTGTT
GCAGCAGCAGATGCTGTAGCTACCACTGCGATAGATGCTGATACGCTGACTTGATTTGTG
CTTGGTAGAAATCTTTGAATTTCTGTAGTATCTTCATACAACACTACACATTGACCAGCT
CTAACTTCATGACCTGTAACTCTTTCTTCACCATTCTGAGTTAGATCACCTACTCTAGGT
TGATTAGGTGCAGGACACTCAATCTCTTTATCAGTTTTTGGTATCCGTGGAACCTCTGTT
GTATCTACTTCTGGTGTAGGTGGTGGTTCAACGACAGGAGGTGGTGGTGCCTCTATGACC
ATCTGTAATTGATCAGGTTGATAATCCATCGCATCATATGATGGCACACCTGCATCACAG
AATACTAGTACTTTATCTTCATCGTCTTCAATTATTTTATTATTCTTGTCACTCTGCTCG
TGTGCTTCCACACAACCAGGCATCTGAATGATGGGTTAACCTATGTTTACAGTAGCAGGA
GGTACGAAAGGTATTGAGTGAACTGGTTGTTTAGTTAACCAATCTGGGATATCACTAACA
AATATTCTCGTTCTTCCTATGTCTCGAACACTTGCGTTATTGACTTCTATCTTTGGTATA
TCCATACATCATGGGTTTCCTATTGATATATCTTCAATGTTACTTGCATTTGTGCTAGGA
ATCTCATGAGCAAGAGGAGCAATGATATTAACATTCTGCGCAACAACGTCTGCACGTATA
CTATAGTATGGTGACTTAGGATGGTATGAAATTCCGTCTTTTATCAAATTTCCCCATTTT
TTCAAACGAGCTATCTCAAAGTCAAGTCTCTTATTCGCTACGGTTTGATTCATTAATGCG
CTATTTGCTGCTGCTGCCTCTTTACATTGCACTTGAAGTTTCTTATCTAATGGTCTAGAC
CATGCTGCAGATACACCAACTGAGAAATTATAATCTTCTTTCTGTGCTGTTCTTGTAGGA
ACATGGTATTAAATATTACCTGGATTATCTAATACACCGTCATCATTCAAATCTGACATA
TCATACATAGGATCCATATAGTATGGTTCGTACGGTTTTTGTGCTGAAGCCGTCCCTGTG
ATGTACGGAGTAATATTCTTGGGAGCACCTTGACATAGAATGCCATTACCATATGTGTTT
GTTATATACGGACCTTGTAAAACTTGTATTGCCTGGTTCGTCACTGAGCCTGAAGAGGTG
GTGATTAGACTTGCAGCCGGACTGACACCACCAACTGTCTCTGCGTTAACAATAGGAACA
ATGGGCATGAATCCAGTAATGGAAAGTAATGCTATTGACTGAAGATACTTGTTGTATCTG
TTACCGACGTTACGGTTGTTTCTCTTTGTATTACCGTGTGGTTTGAAAGACCTGGTCCCA
TGTAGGTTTCTGTGTATTGAAACGCTGCACCTGGCGTCTGTATTGTGAAGTTGGGTTTGT
CGCTTATACCCGTCCATGTCGAATTCAATCCTTCAATAGTATTAACTTGGGTTGCATTAG
TGGCTGATAAATTATCATCTGTAGTTACAACGTGCCCACTGACCGACCATTGATAGCCTG
TGTTATAGTCCATCGAAGGTATGGTCTCCACCACAGTGGAAGTCGTTTCCGTGTGGGTGC
TCATCGAGCCCTGTTGGAAATTTGGCACCACAGGCACGGCTATGACTGGGTTTATAGCAC
CTCCCATAGCTATTAGTAGGAATAACTTATATGTATTCCTCATGACTATTAGTCAAAAAT
AGTAATTTCGCTAACAAACCGACCTGTCAATGAATTACCTGCACCGAGAGATCCTGCTGA
ACTTGTGCCCTGATTAATCGTGATAGTCTGACCATTTGTGATTGTTCCTGGTGCACCTGT
TCCTGCTGTTCCTGCACTTTGTGTGGTGATATCAACCCAACTAGCAGCTGCTGCAGAAGA
ATTGTCACACTGTTGCCATGTTTGGGCTAAGGAGAACGATCCTGCAAATGCTAGGTCATC
ATCTGCAGATGATCCTGTGCATCCATTTTGACCACCAGTAATTGTTCCAACTGTTCCAAC
ACCTGTAGTAGCACTATAAGTGATAATACCGATACCACCATCTACAGCACTACCATTGGC
AGGTGTATGTGTTGTGATCACGTTATTTCCAGAGATCAAATAGCTATTCCCAATTCGTGT
CTGAGTCACGATTTGAGGTTCAACAGTTAGTTGTAAACTAGATTGGTGTCTAGTCGATAG
TCCGCCCGCATCTGCTGCTGTGCCGAACGTCAAAAGCATAATGATAGGAATCAATTTCTT
CATTATTTTACTCATAGGGAATCCTGCACTATATAGGTGTGCAAAACCTTACACATGTGT
TCGGCATGTACCAATTCCTGACATCTCAGTCTATGGTTAAATAGTAGTGTCGCCTTCGGG
GACATAAACTAACACTCGCTTATTTAAGGAGCAACATGACTAACTTATCAAAGTGGAAAT
CGAAGGATGTCGATGCAATTTTTAACGCTGCAAATCGTTACAGCGTAGGATTCGATGATC
TGTTCGACAGACTACACGCATACGGAACAGGATCACCTACTGGACAATACCCTCCATATA
ATATTGTCAAAGAATCCGGAGACAAATGGAGACTAGAACTAGCACTCGCAGGATGGACAG

CAGACCAAGTAGAAGTATCTACAGAGCAGAACGTACTACTAGTCAAATCAAAACAGCAAG
ATCCAAATACGAAAGAAGTACAATACGTTCATAGAGGTGTAGCATCCAGAACATTTGCTA
GGGGATTTAATCTATCAGATGACGTAGAACTTTGCAAAGTATCCTTTGTTAATGGAATGC
TAACAATTGAGTTACAAAAGGTAATTCCAGAACATCAGAAAAGACGAGTCTATGAAATAG
GTTAACTAACACTTTCGCAGTTCTTAACCTGTAGTTAAAGATACAAAACACTCACTTATG
CTATAATAAGTGGGTGTTATTTTTTTTAAACAAATGAAAGCATTAGCAATTGCATTGCTT
CCTTTACTATGCACAAGTGGATGTGCAAATTCAAGAACAAGGTTGAGTGGTGCAGGTGCA
TCATGCCCATCTAAAATGTATACTCGTTGGTTCTCAGACGTAGCAAAATCTGGTGGAGCA
AAAGTAAACTATCAGGCAGTTGGTTCTGGTAGTGGAAGAAAAGCATTTATTGACGAGACC
GTAAACTTCGGTGCCTCTGACGATCCTATGAAACAAAAGGATATAGACAAAGTAACAAGA
GGCTTAGTTTAGATACCTATGACAAGTGGAACTATTGCATTTGGATATAACAATCCTGAT
TGTGATCTAAAACTTACACAACAACAGCCAGGTGAAGTTGCTATGGGTATCATCACTAAC
TGGAAAGAAGTTGGATGTGATGATCAGAGGATGACTTCGGCACATCGGTCAGTGGGTACT
GGAACAACCAAGGCATTTACAAGTAGCATGCAAGCATTCTCTCCTACATGGACACTTGGC
ACAGGGAAATCTGTAGCGTGGCCAACAGGTGTGGGTGGTAAAGGTAACGCAGGAGTCGCA
GGAGTAATTAAAAATACACCTGGTTCAATAGGATATGTGAATCAATCTTACATTGATGAT
GTTGTAAGACCCGCAGCATTACAAAACAAGTGGGGTGACTTTGTAAAACCATCAGTTGAC
GCAGGATCAAAGGCACTTAACGGTATCACTCCTGATGAGAACCTTGCAGGAACAAATACA
GATCCAGCAGCCGAAGGAGCGTATCCGATTGCCACTATGACTTGGATACTTGCATATGAA
ACAGGTAACGGTAAGAATACAGATGCAATCAAGACAACATTATCAAATCTCTTGAGTGAT
GAGTATCAAGATAAAGCAGGTAAGTTAGGATTCGTTCCTCTTAAAGGTGACATTCTTGAG
AAGGCAAGAGCAGCAGTTGACAAGATCGGTAACTAGTTTTATAATATAAACGTATCGTAA
TTATTTTATGAGTGTAAGAATCGTACGAACCAGAAACGGTGACGATGTTTTCTGTGATCT
TTATGAGGTCACAACTAACGAAGATCCAAGTAAAGCAGGAGCCTTTCACCTTAACAATCC
ATATATTATATGGTTGCAAGGAAAGAAAACTGAAGAACCTAAAATTCTTGTTGAAGTTGA
TGCAGGTGACATTGTAAGTAAAATCAAAGATCCCGATATCTACTTCCAACCTTGGATTCC
TCTGTCTTCTAACAAACAAATTCTTTTGAAGTTAGAGGAAGTGGTAACCGCATATGAGAC
CTATCCAGAGGTCATCACTAAATACAACACATTAATCGAGGCAGATGGAGGAAAACCAAA
TCAAACCCCTGTTGATGAATCAATGGAGTGAATACCTTATTGGAAAGGTAAAAGAGTTAG
ATGAGGAACCTAGTATCCTTATCGAAAAATGCTATGAAGAGACGGGCGAGGATAGGATGA
TCCCGTTTCCAAAATACAGTTCACAACGTGATGTCTTCTTGACATCTGAAAACATCTTGA
GTATACTAGATCCGACACCAAAACTGTTGGAAAGCGACAATAAATTATGAGTTCTTTCTA
CACCAACATTCAACTAGCTGGTGACACTATACTTTACAGAGGGTATGAGAATGGAGAAGC
GGTTCAATATCGCACATCCTTTTCTCCTACCCTTTATGTTTTGTCTAAGAAAAAAGAAAA
GTTTACAACACTAGATGGGAGATATGTGTCTCCTGTCAACTTTCATAATGCAAGAGAAGC
CAGAGAGTTTATCAAACAGTATGAACATGTAGAGAATTTTGAAGTTCATGGTTACGAAAG
ATTTGTTTATCAATTCATTCGTCAGCAGTATCCAGATGATGTTGACTACCACATTGATCA
GATGAAAATCTATGCATTGGACATCGAGGTTCAATGTGAAAATGGATTCCCCGATGCAGA
AGCAGCAGCAGAAGAAATGCTATCAATCACCATTAACGATATGGTGACTAAAAAATATTA
CAGGTCGACAACTAGAGAGTTTGATGCACCAGATAATCAGGAGTTGAATGTCTCTTGGAC
AGAACAAGAGATACTTACAAACTTTATTACCTGGTGGGCACAAAACACTCCAGACATTCT
AACTAGTTGGAACGTTAACTTGTATTACGTCCCTTACATTGCTCGTCGAGTCAATCGTAT
TCTCGGTGAGAAATGGATGAAGAGTTTATCCCCATGGAATCGTGCAAATGCAAGAGAAGT
ATACGTCCAAGGTAGGGGGAATTACGCTTATGATATCTCTGGTGTTAATATCCTTGATTA
CCTCGACCTATATCGTAAGTTTACATACTCAAATCAGGAGTCTTATAGACTCGATCACAT
TGCCTTTGTCGAACTCGGACAAAGAAAAATAAATCATGATGAGTATGAAAACGTCAAAGA
CTTCTATACATCTGATTGGCAGAAGTTTATGGAATACAACATTCATGACGTTGAGTTGAT
TGACCGTCTTGAAGATAAGATGAAGTTGCTAGAACTAGCAATTCCTATGTCTTATGATGC
CAAAGCAAACTTTGAAGATGTGTATAGTCAGGTGCGCATGTGGGATACGATGATATATAA
GTATCTTACAGATAGAAACGTTGTTGTTCCCCCTCGAAAAGGAGCGAAGAAAGATGAAGA
ACACGCAGGAGCGTATGTCAAAGAACCGAAACCAGGATGCTATGATTGGGTTGTCAGTTT
TGACCTCAATAGCCTGTATCCTCATCTTATTATGCAATACAATATCTCGCCCGAAACACT
CAGGGAGACTAGACATCACAGTGCAAGCGTTGAGAGAATCCTCAATCAGGAGTTGGAGAT
AAAGGAAGACTATTGTGTATGTGCAAACGGTGCCATGTACCGAAAAGATATTCATTGTTC
CCTGCCAGAGATGATGCAGAAGATCTACGATGAACGTACAATCTACAAAAAGAAAATGCT
TGCTGCAAAACAGCAACACGAAAACTCACCATCTAACAAACTAGTTGCTGACATTTCAAA
GTTCAACAACATTCAAATGGCAAGAAAGATTCAACTTAACTCTGCCTATGGTGCTATCGG

CAATCAATACTTCCGTTACTACAATCTGGCAAATGATGAGGCAATTACTGTGTCATGGCA
ACTTTCAATCCGTTGGACCGGAAACAAAATGAACGAGTATCTCAACAAGATACTCAAAAC
TACAGGAGAAGATTATGTTATTGCTCATGATACTGATAGTATCTACCTCAATTTGGGTCC
TCTGGTCGAGGCTGTATACAAGGGGAGAGAGGCAACTGATTCGAGCATCGCCTCTTTCCT
CAATAGTGTGTGTGAGGTGGAACTTGAAAAATATATTACTAGTTCTTATGAACCGTTGGC
CACACATGTAAACGCATACGAACAGAAGATGGTCATGAAGCGTGAGATTATTGCCAACAA
AGGTATCTGGACTGCCAAGAAAAGATATATTCTCAACGTTTGGGATAGTGAGGGTGTTCG
CTATGAGAGACCTAAACGTAAGTTCATGGGTATCGAAGAAATTAAATCTTCTACACCTGC
AGCATGTCGAACATCTATTCGTGATTGCCTAACAGTTATCAGGAATGAAGATGATTAGTC
TGCACAGGGGTTTATTGCTGACTTCAAAAATCATTTTTCTTCTTTACCTATTGAAGACAT
CTCATTTCCTAGAGGTTGTAACAATCTAAATAAGTGGTCGAACCCTACAACGATCTATAG
CAAAGGGACACCCATACATGTGAGAGGGGCACTGTTATATAACTTTTATAATAAGAAAAA
CAAACTTACTCACAAGTAGCCCTTAATTCAAGATGGAGAGACGATCATGTTCGTCTATTT
GAAGACTCAAAACAAAATCAACGAGAACATTATCAGTTATCTTAATACGTTCCCGAAAGA
GTTAGGACTTGACCAACAGGTGGATTATGATCTACAATTTGGGAAGAGTTGCTTAGAACC
CATCAAAGTGATTATGGACGTCATTGGTTGGCAACCAGAAAAAATCGCATCATTGGAGTT
TCTATTTTCATGAAATTTGTTGTTGTATACCAAAAAACATTTGGTATCCCAGACAAAAGA
GAACAAGTATTTGACGATGAGTCAGAAGCAAAATGGTTTGAACGTGCTATGAAACGTACG
AACTTTATTACTAAAATTACGGAGGTTGACGAGTGAGTTTTCTGAAGACAGTTGTATCAG
AAATCGGAAATGAATATGCAGGACTAGTATCAGACGGAGTTTATGCAGGTGATACAAAAT
ATTATATTGATACTGGTTCTTATGTCTTTAATGCCTTAGTTTCAGGAACAATTTATGGCG
GTGTGCCAAGCAATAAAATCACAGCGATTGCAGGTGAATCATCAACTGGTAAAACTTTCT
TTTGCCTTGGTATTGGGCAACACTTTCCAGATTCTGATCCTGATGCAGGTGTAATTTATT
TTGAATCTGAGTCTGCTATTAGCAAACAAATGATTGTTGATAGAGGTATTGATGCAGATA
GAATGATGATCGTTCCTGTGTCTACTATCGTAGAGTTTAGAACTCAATCCTGCAGGATTC
TTGACAAATATATGGAACAGAATGAGTCTGATCGCAAACCTACGATGTTTGTATTAGATT
CTCTTGGTATGCTTGCGAGTAATAAAGAAGTTGAAGACGTTGCTAATGATAAGAATGTCA
GGGATATGACAAGAACTCAACTAATTAAAGGTGCGTTTCGTGTTCTTGCGCTAAAATTAG
GTAAAGCAAACGTTCCTATGCTCGTAAGGAATCATACATATGATGTTATAGGAAGTCATA
TTCCAACGAAAGAAATGGGAGGTGGAAGTGGACTCAAATACGCTTCATCAACAATTGTAT
ATCTATCAAAGAAGAAGGAAAAAGATGGTACGGAGGTTGTTGGAAAAATTATCAAATGTA
AAGCCCAAAAACCCCGGCTAACAAAAGCGAATTCTATTGTAGAGACACGGTTATATTATG
ATCGTGGACTTGATAGGTATTATGGATTACTAGAATTAGGTGAAAAGCATGGAGTATTCA
CACGCAAAGGTAATCGTATTATGGTTGGTGAAACTTCTGTATATCCTATATCCATTCTTG
CAGATCCCTAAAAGTATTTTACTGATGAAGTAATGGAAGCATTAGACGAGGCAGCAAAGA
AGGAGTTTCAATATGGTCAGTAATCTTACTGACTACATTAAATTCTATGATCATATGTTT
GAAGAGGATTTTTGTAAATCTGTAATTCAAACATTCACTGAATCATAAAGAGATGTTACT
CGTATAGATAGAGAACAAAGACCAACCTTTAATGAGTTGAATATCTCACAAAGGTTTATG
GCAAAAGATCCTGCATGGTTGAGTACTCAGAAACATATACAGACAGTCTTTATTGATATT
GTCAAAGTTTATATGAATGCTTTACAGTGTGACCCAGATTTTCCTGCAAAATATCCTTTG
GAAGAGGTTAGAATTAAACAATATAATAATGATGGAATAGATCAATTTAAAGATCATGTA
GATGTAGGAGACTATAATTCTGCTCGTAGATTTTTAGTGATGTTTTTATATTTGAAGGAC
TTTGATGATGGTGGACAAACTAATTTTCCTAGATTGAACCATGCAGTTTCACCTAAACGT
GGTAGAATATTATTATTCCCTGCCACATGGCAGTATCGTCACTCAGGATTGCCACCTGAG
TCTGACAAAAAGTACATTGTTGGAACTTAATTACACTACCTATGATTCTCGAAGTCACGA
TTGTATGTGATCTCTTGTATAACGAGAAGTATGCAATGAAGGTTCTTCCTTTCTTGTAGG
TAGATTATTTTACCGTACGTTCTTACAAGATTATTATTCTTGAACTACATGAAAACATTA
GTAAGTATGATGCATTGCCATCTCTGAGCGCACTTAGTATAGAATGTCAAGAAAGAAATG
ATCTTACAGAAGAACAATTTAAAGAAATTGTTGAGCTTTTAAATGTCCTTTCCGATGATC
CCAAAGACCACGACTGGCTCGTGGATTCTACAGAAAAGTGGTGTCAAGAGCGTGCGATCT
ACTTATCGCTTATGGAATCTGTTAAGATTGCTGACGGTCAAGATACCAAACGCGATAAGG
GTGCTATTCCGCAGATTCTTTCAGAAGCATTAGGAGTATCATTTGATCAACATGTCGGTC
ACGATTATGTTGAGATCGCTACTGATCGTTTTGAATGTTATCATAGAAAGGAAGACAAGA
TTCCTTTAGATCTGGAGTTTTTTAATAAGACCACAAAGGGTGGTTTAGTAAAAAAATCAC
TAAACATCCTTCTTGCAGGAACAGGTGTTGGTAAATCTTTGGTCATGTGTCACTTTGCTG
CATCAACTTTATTGCAAGGTAAGAATGTTTTTTACATTACTTGTGAAATGGCAGAAGTAA
AGATTGCAGAACCTATTGATGCAAACCTTTTAAATGTTCCTATTCACTAACTCGCTGATC

TACCACATCCAATCTTTGAAAGGATGATTAAAACACTAGCAAAGAAAACTCAGGGTAAAC
TTATGATCAAAGACTATCCTACAGCGTCAGCACATGTAGGTCATTTTAAATCTCTTATCA
ATGATCTTGCATTGAAGAGAAGTATTAGACCTGATATTATCTTTGTAGATTATCTAAACA
TCTGTGCTTCCCAGATATACAAAGGAATTATTGTTAATTCTTATACCTATGTTAAAGCAA
CTGCTGAAGAACATAGAGGACTTGCGGTGGAATGTAACGTCCCGATTGTCTCCGCGACAA
AAACGACACGCTCTGGTTTTGGGTCTACTGAAATTGATCTCACTGATACGATTGAATACT
TTGGTCTCCCTGCTACTGCTGACCTCATGTCTGCTCTTATCTCGACTGAGGAACTTGAGG
GGATGAATCAAATTATGGTCAAACAAATTAAGAATCGCTATGATGATCCTACTATGAATA
AAAGATTTTGTGTCGGTATTGACCGAGCGAAGATGAGGTTGGATGATGTAGAGGAGTCTG
CTCAATATGACCTTGTTGATTCTGGTCAAGAAGAAGAGAAGGTTGATCTTGTCAGACGAT
TTACATCAAAGAAAACTTTTAGTTAACTAAAGTATGATTGACTTTAAGAAGTATACTGAA
TTTGTAGATGCTGTTACATCTGAATAGAGTAAGCATAGTGGTCATTTCCAAGATCGTTTA
AGAGAATTAGATTATAAAGACTTCTCTTCACATAGAGCATTAACTGCTGCACTCGGGCTA
TGTGCTGAGTCAGGAGAATTTACTGAGATAGTAAAGAAGATATTATTTGAAGGTAAACCA
GTTACTGAAGAGAATCTATTTAATATGAAACGTGAGCTAGGTGATATCATGTGGTATTTT
ATACAGGCTTGTATAGTATTAGATGTATCACCAGAAGAGGTTATAGAAATGAATGTAGAT
AAGTTGAAGAGCATATATCCTGGTGGAGAGTTTGATCCCCACTATTCTGTAAATCGTAAA
CAAGTAGATGTATAATGGTAGGACCTGGTGAATACATGGTAGTTGTTAGTGATCTTGCTA
ACGCATACAATACTATTGAATGGAAAGATGCTATTCCATATTTGATTTGTATTATTGGAC
TTTATTGGATTAAAGTTAAAATCGATACAAGTTTAGGACTTGGTAAAAAGCAATCAAGAG
AACTAAAAAAAATTATTGTTGATGCAATAGTTGAAGGTCCTCAGAAAGCACATAGATCCT
GAAGTTAGGTAAACTTGATCCAGAAGAAAGGGTTCTATCTGAAAAAAAGATAGAACCTAT
CCAATTGACAGCAGAGTTAATTGCTGAGATTAATTGTGCTATGGCACATACAAAGAAAGA
TGGCACATATAATTGGTTGCCTACTGATGAGTATGAAGTTCAAATAGCAGGTCCGTTTGC
TGCCGATAGATTTATTGTTATGAAAAACACAACAAAGAATCCAGTAGTCTATGCTGAACC
TCATCCTTACTTTGACTATGAGAAAAAAGTCTTTACCAAAGATGGTAGAAAGGAGTATAT
GAAAGAGTTTAAAATAAATAAAGATAAAGATAAAAAAGAAGATGGAGACAAACGCTAGAG
AAACTGCTAAACAAGAGAATGGTTCTAGAGTTTTTTTCAAGTCTGTAATCGAAAGAAACA
AAGAACCATCCTTAAAAGAAATGGTAAAAGTCTATGAAGGATACGGTCCTGAATGGAGAT
CAACATATCTTAAACAAACTGCAGCATTAAGAAAGTTTATAGGATCTAATAAAGGGTTTG
AATATTCCAGAGATAAAGGAATCATGCCTTACATTAATCACATTGCTAAGAAAGAGTGTG
GTGTACCAGTAAATGATCGTTGGAATCCTATGGATATTGTATTGGTTAAGAAGAGTGCAA
AGAGAGTTGTAGAAGGAACTATAAAAGAAATTACTAACATTGATGGAAGGAGTAAAGATG
CAAAACTTACTCTACTCAAGGCGTATATGCGCGAAGCATTGAAGTCTAAAATTTTAATTG
GTGTATCACTTAATGCAATTGCAGCAAAGAAAAACACTGCTAACGCAGAGCTTGCAAATG
CAGGTAGAACAACTGGTTAACCTACCATGGTTAACATGGTTCCTAAATATTTGAAGTGTA
CGTTAACTTTAGGAAAGAAAAAGAATTACCTATTTGATACTTGTGAACTTGGTTTTGATA
TAGTTACAGTGAAAGGTGGTAAAATACGTGGTCAATCAAGAAGTCTTCAATATTCTAAGG
AAAGAAATTTTGTTCAAACAGAGCTCACTCCCAAAGGTAAAGTCGCAGGAGGAAAACTTG
GTAAAGTTTCTAGTGTTGCATTAGATAAATTTCTAGGAGATTTAAGATTAACTCGTCCAA
CTTCTACAGCAAAACATAATCATATTCCTAGAGTTGGTGGTTGGACAGATAGAGATAAAA
AATACTGGATTGACTTGTATAAAAAATTAGTTACATCTCGTATGATTGATTTTGGTGAAG
TTGCTGTGTGTGAAAATAACCAAAAGGTCGCTGAAGCTATCGAAGCAGTTATAGACTATG
CAATTTCATACGAAACTCGTCAGGCAGATAGAAGTTCTGCAGGTAGATTCTCTTCTAAGT
TGATTGCTATGGAATGGGCACATACTTGGAGGCAGATTGCTAAGATGAAGAAAATGCAAC
AGTGGTGCACAGCGCGATACTTTGGTAGAAAAGAAGAAGTCGGTGATTCTAACGGTCCGT
TCCTAAAGATATACTAGTCCAGCTTATAGAGTGTCCACTATCACTCGCATTTCACCCTCA
GCGTGCTATAATATTAGTATACAGACAGAGGACTCCTTGCCTAACCAACACCTTCAACAC
CTTGAGGATCTGATCTTTTCTGGTCGTAAGGAAGCGTTGGATGCAGTATGGTCTGCACTT
AACAAACCAGAACTGAGTGTTAAGTGGGATGGTGCTCCTGCTATCATCTTTGGCACAAAC
CCTGCAAATGGCAAATTCTTCGATGGACCAAAATTCGTTTTTAACAAACACAAGATCAAG
ATCTGTTATGATCAAACAGATATTGACGAACTTTACTCTGGCATCCTTGCGGACATTCTG
CGCTTATGTCTTTGGAAGCTTCCTCGTATCAGTGGAATTGTCCAGGCTGATTTCATCGGT
GTCGGAGGGGGCCGTGTTTATCGCCCTAATACTTTTGAGTATCGTCTTCCCTCTCTGATC
CTTAGCGATATTATCCGTGCTCCACATACTTCTTATACCGAAGTTTCTTCAAATGCTGTT
GGCAGCATTCGCCACGATCTACCTTCTACATTATCTACTTCTTACTTTATAGTTAAGAAA
GAGGCCGATGCATCTCTTGCTAAGACTCCTTTCTTCAATTGGGTCAAGTTTCTCAGCAGG

TTGCCTTGATGTAAAGTGCCTAGCGAGAAGGTACGTCCACATATTCAGAAACATATCAAC
AGGTTTTTCCGTTGTGATTTACCAATTCCTTGAGCAGAATTTTTGTATATTACTTTACCT
GATAAATAGAAGTATGAAGTTAATGTTACCACATTCAAAGTGTGGCATATGTTATTTCAA
TTGAACCATAGTTTATTGAAAAACATCGTTGTCGTTGGAACTGTGAAATGCTACATAGAT
GGCCAACCTTCAGAACATGAGGGTTTTGTCACTGTTTCAGATTCTCCCTACAAAATTGTA
GAGAGATTGACTTTGAGCAAAGCGAGCTTGAACCTTAGTAAAAATTGGACGAATGAAAAA
ATTTAATGCTTTTCTAATAGAAGCACAGAGATCGTTTGCTGCGAAAGCAGCGGAAAAATT
AAATCTTAACCATGTAGGTTACGGTAAATACGCAGACCCATCTGGGAATGTGACTCACAT
GTCTAAGGATGGTAAGCTTGTAAAAATTACAGCAAAAGATGCAGCGAGGACACAACAAAA
TGGAGGAGATGAAACGGAATGTGGCGAAGGTCTGGTCGATCAAGGCGTAATCTCTATTAC
ATTTGGAAGATTTCATCCACCGACTATTGGGCACGAGAAACTTCTTAAGAAAGTAGAACA
AGAAGCAAAGTGTGGTGGAGGAGAGTATAGAATATATCCTTCAAGAACTGAAGATCCTTA
GAAGAATCCTCTTGATTCTGGAACTAAGATCAGGTTTATGAGACAAGCATATCCAGACCA
TGTAAATGCGATCATTGATAATGAGGACATGAGAACTATCTTTGATGTTCTTACTGCTCT
TGACAATGATGGGTATAGTTCAGATAATATTGTAGTGGGTGGTGATAGAGTATGTGAATT
TAATTCATTCGCAACGAAGTATAACGGTGACTTATATACATTTGATGAGATTAAGGCAAC
ATCAGCAGGAGGAAGAGACCCTGACTCTGTAGGTGTAGAGGGTATGTCAGGATCTAAGAT
GAGAAAGGCTGCGATGGAAGGTGGTCAAGATTCTTTCAATAAAGGTATACCTAAAGCGAT
GTATAAGAAAGATAAAGAAGCGATGTATCTAGCACTCAGACAATCAATGAATGTGCAGGA
AACATTTGTTGACTTCGCTGAAGCATCATATCATCATTATGAGATCCCTCCTAAGTTAGA
TCCTCAAGGTTTAAGGGAAGCATACTATGATACTGGAATGTTTGAAGTACGTACTTTTGT
TGAAAATATAAACACAGGTATTGCAGGTAAGGTCGTTAGTCGTGGTAGTAACTACATCAT
TTACATTGATGAGCATGATAATATCTTCCGCTCATGGTTGAAAGACCTTGTTGAGACTAA
GAGTTCTGATTATGGATTCGAGTTTACACCTGCAGGTGAGATAGGAACCCACGAATTAGC
TGCATATTATCGTAGAATGACGCCTGGTGTGTTCATAAAGAAGATAAATAAAAAAGTAAA
GGTGACTTAGTAAGATGGATTTAAAGAACTTACCTGACATGATTGCTGCATACCAAGAGG
TGCAGGGAAAAGCAAAGAAGCTAGATCCCGTTGGAAAAGAAGGCGGTGATATAGATAATC
ACGGTGATAAAGATTCATCTGATAAGTATCTTATGAAGCGTCGTAAGGCAATTTCCAAAG
CAATGAAGGTCTAACATCATCAAAAAGATGAGAATCGTAAAGTCATCGAGCATGATGTTG
AAGAGGACGAAGTAAAAGAAGCATGCACAGTAACGAATGCTGATAAGAAAGGTCACACCA
AAGTATATCAGAACTTTAAAGCAGGTATGAAGGGTAAAGATGGTAAACCTTTATACAAAG
CAGCAGATCACATGAAAGAGAATCAAGATATTCATCCTGATGACAACGTTTTATCAACAG
AAGAACTAGAGAAAGTAGCAGAACTCACTAGACAGTGGGATGCTAAGCTGGAAGAAGGTA
ATGGTATGATGAAAGGGTATCCTAAAGGTGGAGAGGTAAAGGCACCAAGAATGCAGAAAG
GTGCTATGGCATATGATGGTCCTAACACGGCAGCAAGTGAAGCAAAGGATAGAATCCTTG
CTAAAACTAAGGAGAAACGCGAAGAAATGAAAAAGTAAGGTTATCTTTCAAACACCTATC
TGAAAAAAAAACTAAAATTAAACTAAATCCTAAGAAGGAGGATGTCCTGGAGGGAGATAA
AACTCTCAACCATGGCGAAGATTGCGGGTGCATGAAATGCGACAAAAAACGTCGCAAGGG
TGAACTTGGCGATGAAAAATCCGTATCTACCGAAGAAAAAATTTAAGACAGTAACGAATA
AGTCTCAGAAGAAAGCACAGAAAGCAATGCTCAAAGCGATAACTATGAAACGAACTTGTT
AACCTTCAATCAATTTCATGAAGGATCATCTTATGGCTTATATAAAGGATCAGGTAAAGC
ATCGGGTCCTATGGCTCCCTTTGCCAAACCAACTAAACCTAACCCTAAAGCAAAAACAAA
GAGAGTGAAGTATGTAGGTGTAGGTGAAGAGACTAAGTATGAAGTCATGAGTAAAGAAAA
GTATAAGAATACTCATAAAGACTTCAAGGGTAGCACTAAGAAAGAACCTAGGGTATCAGT
TTACGATCCCGGAAAGAAAGCAACAGTATCAAGATTGGTTAAATTCTCTGAAGCAAAGTA
TGAGAGAGGTGCGTCAAACTATGGTAAGATGTCTATCAGAAATAAAAGAGCAGTAGGTTA
CGGTGTCAACGCAGCACCTCCAGAGGAGAGTAGGAAGGCACATGATGAGAGAATGAAGAA
GCATAAAGCATCTCAAATGAAAGAAGAAAAGCATAGAGATGCCGCACTTGATACTGTAGA
GAAATCAAACATTGCCCAGTATGGTAAAGGTGCTATCATGAGAAAGGGTAGCGATCAACA
GAACAAAGTCCGAGGTGCTAAGTCTATTGCAGGAACTGGTAAGTATTAGAAGGTGGCAGA
TCAGAAAAAGCAAACTGCTTCAGACGCGAAGAAGCGTGGGTTTAAGTCTGTTCAGAACTA
CACTGATGCTATGGCACGTTATGGTGATAGAGATAATTACGACAAGGGTAGAGGATTAGG
GTCATGAGCACACCTCTTGACCTATCTCATTGTCCCGATGGTCAGTATTATTGCTTTGAT
GATAGAAAATGCAAACCAATACCCAAAGGTATGTCAGTAGGAAAGAATGGTATGTTGGTC
AAAGAAACGACAGAGTTAAAAAACGAAATAATTGCGAAGGCACAGAAAAAACATACTATG
GCAGAGTCAAAAAAATTCAAGGATGTCATGGCAAAAGGAAAAGAAGCAAAAGATAAGTTA
TATAAGACTACTAGAGAAAAAGGCGTACGTTTCTATGACAAGAAAGGTTCTGGTTACATG

AAGGACGGTAAGAAAAAATACGATTGATAGCCTATATATTGTGTAATTACATATTAAGAT
CATGATTGGTAATTTTTTAATGCCACTGGCATACAAGGTAATCTATTCTGCTGTCAAAAA
AATCCCTGATGATGCAGAACTCGGAGAGAAACTAATCGATATTTGTTAATTGATTATTGG
CAAGGCAGTGAAACTCACTAAGACGACCGCTGACGACGCTCTATTTGAAAAAGTAAAGGA
AGCACTTGCTGCTAAATAAGTAGCGTTTGCGACCCTTTTAGAGGGGTCTCAGAGACCCCT
TTTTTTATAAATAACTACAGGAAAAAACAGAATTTTAGGAGCATAACCATGGCACTTTAC
GGTGTAACTGATGCAGACGAATCTATGCCAAAGTGGGCTGTGAGAGGGAGCGCTGTAGAC
CCCCAAAATATTTTTGCAACCACTGATTGTTGGGTTCTTTGTCATTACAAGAATGCCGCG
AAGACAAAGTTTTGGGATGAAATCTTAGTCTCAGTTGATGGTCTAGTAGGTGCAGGTTCT
CGTGGAACCAATACCTTTGGTGGTGCAGATATTGCTGCAGTATTCTTTGAAGAGACAGGT
TATGCAGGTGGAGCAACAGGTTGTGTTGTTGTCAGATATAACGAACAGGTTGACCTCACA
AACGGTGCAACTTTAGTCGTTAGAAATACTACTAACAGTGCCAAAATCACTGCAATTGCT
GCAGCACAAACGGGTGTAAACCGTGTTGAGTTTACATTCACTGCGGCTGCAACTGGTAAA
GCACATGCTATCAAAGCACAAACAATCTCTGGAACAATCGTTGACTCCAGTGGTGGTGCA
GCATCCGATAAGGTATTTGTTTCTGGTGGTGTAGTTGGTGCAGATGGAAGTGGTTCTACC
GCAACATTTACTGCAAGTTAACTAACATATGAAATTTGACGAACTAAATGAGGATACATG
CCGCGTGTTCGCCATTGAGCATTATGAAAATCCTCACTGTGTGACTAGAGAGGATTTTGT
TGAAGATTTAAAAGGCTTCAAGTATCTTAAAAGACTCATGAAGCGTTATGTGAGAAGAGG
TCCGTTAAGGATTCATCTCGTCATCAATCATATAATCATCTTATATAAAGATTTTGGTGA
AGCAGCAACTCCTCTTCTCTTCTTTAAATTAGAAAGAGAATATTGGAGTTTAATAAAGAC
TCTACTGATCTATTTGAGTAAATATCCAGTAGGGAAGCTTCCAGACTTGGAATGATACCC
CGATTTAGAAGAAGAACTTAAAAAGGTATAGGATATGTTGACAGCAGGAACAGGCGGATT
TAGTGGTAGCGTAGCTGCCAAAGGTCCTGTTGCGGTTTTTGATCCTGTCATGAAGATGAG
AGGTAAACTTAAACAAATTAAGGCACAACAATCTAGTTGCTGTGAAGAAACAGATAGCGT
AAACCCTCAAGGTCCTTCTAGATTATTCCAATACAAAATTAACGTACCTGAGGTTGGTGA
AACAATCGTGTTTGCTAATAGTCCTGCTGAACTCAGGATGAAATTAAGGATGGCAATCAT
GCCTAAGTATAAGTCTGGTATTAATATTGAAAGAATAATGCCTGGTGGTGCTGCTAAATT
CTGCATGGACAAGAGAATGAAGCACATGAAGAATGTGTAGGAAGAGTCTGAACTAAAGAA
AGCAAATATAAACTATAAGACTACGAAGAGCGGTGGTAAGAAAACTCGTCATGTCAATAA
GAATGACGTGGCAGATGCACAAGCAGCGATGAAGAATGATCCAAAATACATTCTTGGTAA
GACTAGAGTAAAACCTGTTAAAGAAGCTGCAGGGGATTAGCAGATGTACAATCAAATGAA
TCAGCAAAAGATTCAGAACCAGAAGAAGAAAGTCATGTTAAAAAAGCAAGAATTACAAAA
ACAATTACAACTAAAAACACAACAACTTAAAAAACAAGCAAGGACTGGAGTAGAACAAGA
CGCAACAAGGTAATGTCTGACCTTAACACCGCTATAATAGAGACACTCGAAAAACTAGTT
GATTCATTGCAGGAGTATTCTGTAAAAATGGGTCAATTTCGTGCTGTGCATAATGAGAAG
TTAGATAAACAAGACAAGATCGATCGAGTTTTGTTTGAAAAGGTTGATAGATTGCATGGA
GATATTAATAGAGAGACAGAACAGATAAAGAAAGGTTGTGAAAGAGACATCCGTAAGATA
GATGATCGTCTAAGGTTGATGGAAAAGAAAATGTGGACTATATTTGGAGCAGTTTCAATC
GTAACGTTCCCTGTTTCAGCACCAGGAGAGGCAGTTTTGAAGAAGTTGACAAACAATCAA
AATACTAGTATTATAACGACAGAGATTATTAGGTCTACTTGATTGACAATTTTTATGCGA
ACCTACTCTCTGCTCGTCTTGACAGATTTAAGCAGGTCAAGAACGGGACGTACAACTTTA
GGTGTCCCTATTGCGGTGACTCCCAAAAACGTAAGAATAAAGCAAGAGGTTACTTCTTCA
CTAAGAAGAGTGGTCTCGTTTTTAAGTGCCATAACTGCGGTGTAGCCAGATCGTTTGGTA
ACTTTCTAAAGGAACAAGCGAGTGACCTTCATGATGAATACATCATCGAGAGATACAAGG
CAGGTCTTACTGGAAAAGGTACGAATGTAGCAGACCCTGAGTTTAATTATGAAAAACCAA
TATTCAAAAAGAAGGGAGAAGTACAAAGTATTTCAGAGCTAAATAGTTCGCACTCAGCAT
TAGCATACTTAACCAAACGTGGTATACCGAGGAAACATTTCTCGAATTTTTACTATGTCG
ATGAGTTTTGCACATGGGTTAACACACAGAAACCTACCTTCCGAAATGTCACCAAGGATC
ACCCAAGGATTATTATTCCTTTTATTGATGAAAACGGAGAATGGTTTGGATTTCAAGGGA
GGTCTCTGAATCCCAACGACAAACTTAGGTATATAACTATCATGTTGGATGAAGATCGAA
TTAAGGTTTATGGTGTTGATCGTGTCAACTTCAACAAGACCGTGTATATTACTGAAGGTC
TTATCGATAGTCTTTTTATCGACAATGCAATTGCTACAGCAGGTGCTGATGTTGATTGGA
AACTAATAGATCGAAAAGAGGCAGTCTTTGTTTACGATAATGAAAAACGTAGTAAGGAAA
TTGTTAACCGTTCTGAAAAAGCAATTGATAAAGGATATGAAGTAGTAATCTGGCCTAGTA
GCCTACAAGAGAAAGATCTAAATGACATGTTCTCTTCTGGACACGACGTGCAATCTATTG
TAGAATTTGATACTTACCAAGGATTAGAAACAACAATTAAATTAACCGAATGGAAAAAGG
TCTGATTAGTCCAAGGACACCCAAACCAATCAACGTCACAAAAAGAGAAGGAGAGAAGAC

ACCCTTAGACCTTAATAAGGTTCATAAGATGGTAGAACTTGCCTGTGTAGGTCTTGCAGG
GGTCTCTGAATCTGCTGTAGAGATTAACAGTGGTCTTCAATTTTTTGACCGAATTAAGAC
AGCAGACATTCAAGAAATTCTTATTCGTTCAGCGAATGATCTGATCTCTCTTGATCATCC
TAACTATCAGTATCTTGCTGCATGATTGCTGCTGTACGGTCTTAGAAAGGCAGTATATAA
CGGTCACCCTGAAGGTCATCCTCCTTTACTTCAGCATGTCAAGAAGTGTATAGACAAAGG
TGTGCATGATAGCAGTATTGTTTCCAAATATACTGAAGAAGAATGGGAAAAACTGACTAG
TTATATTGATCACGATAGAGATTATTTGTTTACATATGCAGGTATTCGTCAAGCAGTCGC
TAAATATCTCGTACAAGATCGTACATCAGGAGAAGTATACGAAACTTCACAGTTCATGTA
TATGATGGTGGCGATTACTCTCTTGCAAGACGATGAAAAATTTTATCACTTGGAGTATGT
AAGAAAATACTATGACGCAATCTCAAAACACAGACTCAACATTCCAACGGCGATTATGGG
AGGAGTGCGTACGCCACTTAGACAATTTGCAAGCTGTGTTCTGGTTGAGGTTGATGACAC
CCGCGATAGCATTTTTAGCAGTGACATGGCTATTGGCTAGTATGTTGCACAAAGGCCGGG
TATCGGTAACAACGCGGGTAGAATCCGTGGCATCAACAGCAAAATCCGTAGCGCAGAAGT
ACAACACACAGGTGTTGTACCTTTCCTCAAAAAGTCTGAAAGTACTGTCAGATGCTGCAC
TCAGAATGGCATTATAGGGGGATCAGCAACTTTCCACTTCCCAATCTGGCACCAAGAACG
AGAAGATATTATTGTTCTAACAAATAATAAAGGAACAGCAGACAACAGAGTAAGGTAACT
AGACTATAGTATCCAGATTAGTCAGTTCTTTTATCGAAGATTCATTGATGGTCGCAGCAT
CTCTTTGTTTAGTCCACATGATGTGCCAGGTTTATACGAAGCATTTGGAACTCCTGAGTT
TGATGATCTATATGTAGAGTATGAAAATGATCCTGCTATTCCTAGAAAGACTATGAAGGC
ACAGACACTCATCCTTGATCTATTGAAGGAGAGTGCAGAAACAGGTCGTCTTTATATAAT
GAACATTGATCACTGTAATAGTCACAGTTCATTCAAAGTTCAAGAAAACATGAGGAACCT
CTGTCAGGAGATTACTCTTCCAACAGCTCCTATTCAACATATAGATGGATCAGGTGAGAT
TGCTTTGTGTATCCTATCTGCTATCAACGTAGGTAAGTTAAAGAACCTTGAAGGACTTGA
TGAACTATGTGAACTTGCTGTTCGTGGTCTTGATGCATTGATTGATTATCAACAGTATCC
TGTCAATGCTGCAAGGCAAAGCACATTGAATAGTAGATCACTTGATTTTGGTTACATTGG
ACTAGCACATTATACTGCTAAGAATGGTCCTAAGTAGGATTGTCAGAAAGCATTTGATTT
AGTTCAGAAACTTACTGAAAGATTCCAGTATGCTCTTCTAACAGCATCGAATCGTATGGC
AATGGAGAAAGGTCCTTGCGGTTATTTCGGTAAGACAAAGTATGCTGAGGGAATTCTTCC
TATCGATACATATAAGAGTGAAGTAGATGAGATTTTTCCGCATGACCTATCATGTGATTG
GGAGTTTCTCAGGGCCAGGGTCAAGGAGTATGGACTCAGGCACAGCAGTTTGTCCGCACA
GATGCCTTCTGAGAGCAGTTCCGTTGTGTCAAATGCCACAAACGGAATCGAACCTCCTAG
AGACTACCTGTCCGTTAAGAAACCAAAGAAAGGACCTCTTAAGCAGATTGTTCCGTCTTA
TAACTATCTAAAAGCAAACTATACCCTGCTATGGGATATGCATAATAATGATGGTTACAT
CAAAGTAACTGCAGTTATGCAGAAATTCTACGATCAGGCAACGAGTGGTAACTGGAGTTA
CAATCCAGAGAATTATCCTGACAATGAAGTGCCTGTATCTGTGATGGCAAAAGATCTTTT
GACCACATACAAATATGGTTGGAAGACTTCATATTAACAAAACACTTATGATGCTAAAAA
AGATGGTGACGACGAAACAAACGTCGATAAATTAATCAACGACATACTCACCTCGGAGGA
AGAAGACGGTGACAGTTGCAAGGTCTTATGCAGAAGTAAAATGAATGACAGTATTTAACA
ATAACAAAGTAAACACAAAGAAACAACCAATGTTTTTTGGACAACCATTAGGAGTCCACA
GATATGATGAATATAAGTATCCAGTATTTGATAGACTTACACAATCACAACTAGTATATT
TCTGGAGACCGGAAGAGGTGTCTCTACAGAAAGATAGATCAGACTATCAAACTATTACAC
CAGAACAGAAGCACATCTTTACTTCTAACTTGAAGTATCAGATCATGCTTGACTCAGTTC
AAGGTAGAGGACCAGGTATGGCATTCATTCCCTACTGCTCTCTTCCTTAGTTGGAAGCAT
GCATGACGGTATGGGACATTATGGAAATGATCCATAGTCGTTCCTATACATACATCATCA
AGAATGTATATCCAGATCCTTCAGAGGCATTTGATACTATCTTAGATGATGAGAATGTAA
TGTCTCATGCATCATCTGTGACTAAACCGTACGATGAATTTATTAATTCAGCACACGAGT
TTGATAATTGTCAAACATGGGATCTGGCAAGACAAGGACATTTAACTGGAACCTATGAAA
GAAAAGAACTCAAGAGAAAACTCTATAGGGCAATTGTAAACGTTAACATTCTTGAAGGAA
TTCGTTTCTATGTCTCCTTTGCGTGTTCGTTTGCTTTTGGCGAAAATAAACTTATGGGAG
GCTCAGCTAAGATTCTCTCTCTTATCGCTAGAGATGAAAGTCAACACTTGGTTATCACAT
AAAATATCCTCCAGAACTGGGCAAAAGGTGACGACTCCGAAATGGCAGAAATCGCAAACG
AAGAAAAACCATTCGACGTAGATATGTTCAAGAGAACTGTTGAAGAGGAGATGACTTGGG
CAATCTATTTGTTTAGAGAAGGTAGTATGATTGGATTGAATGATCGCTTACTACATAATT
ATGTTGAGTGGATTGCCAATCGTAGAATGAAAGCAATCGGATTAGATCCTGTGTTTGATC
AAGTTGCTAGAAACAATCCTTTGCCATGGACTCAACACTGGTTAAATAGTAAGGGTCAAC
AGAACGCACCACAAGAAAGGGGGATTGAAAGTTATGTCGTCGGAGGAATCAAACAAGACG
TCAAAGGAGACTCCTTTGCAGGATTCTCTCTCTAGCACAGAGTGGTTAGATAAAACTTAT

AATGATCTAGTGGAGGCAGGAAATGACTACGGTCCAGACATTACTGACATGATCTGGACT
GCTGCTATGAAAGAAGCAACTAAGAGGTTGCAGGAAGACTTAAGCAAAGCTAAAGATAAG
GATAAAGTGTGTAAAAGTGTATCATAGTGACCTACCAATGTCAGGAATCTATGATATAAA
TATAATGTACAGGTAACAGTTGTTACCAAACGTTCATCCAATGTACGGACTCGCACTACT
GTTGTTGATGTTTGCGGAACACGATGGTTCCCACTGGGAAGTATCATGTAACGAATGGAA
TCAATCAAGGATTGAGATACTCAGCGATGAGAATCTTAGTTCAGATGCTCATGAGTATCT
TATAGATTACTTCTATACCAAAGTATCAGAACCAAATTGCGAAGCTTGCCAACTTAGACG
CAAGTAATTGGCGGAAAGGAGCGTTCATCCCATGATCCCTATTTTAATTGCTACTGCAGT
GACATGTGCTGATATTCAACCATTAATCGAGCGTGCTCGAATTTATGAGGGAATTGATGA
ACAATCAAGGCAAGAGATCATTGAAATTTATCAAGTAGATTTTACCAATGCTGTAGGACT
TGAGTGTGATTGGGACGCAAACGAATAAAGGAACGGACCTTAAACAAGTCACTAACTTTA
GGAGTAAAATCATGACTACAATTATTTACAGAGGCGTCAAGTATGACGCTGAAGCATACA
AAGCAAAGGTTCTTGCAGAGCAAGCAAGTAACAACCGTCACGATCTGATGTATCGTGGAC
TGAAGGTCGAGCGTAACTTCGCATCCCAGTCTTAATCGTGACAGAACTACAAACCATTGG
GATGGTATCCCTAGGGTCTGTTGTTTTCATGATTATGATCTATCTGGAACTGCGTCTTTT
GCCTCTAAAGAGACCAACAGTTGCTAAAGATTAATCTTGAGTGGGATTATGGTCTTCCCG
AATATGATGCCACTAAACACGATCCAGTCAAGACCTTCGCTTTTTTAACTTATCGTGGAG
TACACTATCCTAAGTGGGTTTACCTCAAGGTAGACTTCGGTGGTGCACAACCGTGGAACA
TAACATCTAAATTATGTTAAGATAAACACTTTAACCCTAGTCCTTTACGACTGTGGTTTT
TTATGTTACAATAAATATTATGAATTATGCGAAGGAGCACCACGAAAATTTGTCTGGATA
GTTCTAATGCCGAAACGATCTCTAATGCTTTTGAGAACGATTTAATTGACGGTGTAACTA
CCAACCCATCTCTTATGTTAAAAGCAGGACAAGATGCTATGGAAGTCATAACGGAAATTT
CAAATGTATTTCTTTTCCATGCTTCAATCTCTGCCGAAGTGGTAGGTGATACTGCCACTG
AAATGTTAGATATGGCAGAGAAGTATAGCGAGTTTGGACCAAACATCACAATTACAGTTC
CATGCACACCAGAAGCTCTTAAGTCATGCAGAGTCCTCACTAACGATGAAGTTAGTGTAA
ATATCACATTAGTATTCTCAACAGCACAAGCAATTCTAGCATCTAAAGCAGGTGCAACGT
ACGTTTCTCCTTTTCTAGGAAGAGTATACGATAACTCTTTGGATGGAATCCACTTGAGTG
AGGAGATTGCAGACGTATTTGCAACTCATGGACGAGACACAAAGGTTCTCGCTGCTTCAG
TTAGGGAAGTTCAACAGGTATCATCTGCCTTTAGAGTAGGAGCAGAAATATGCACCCTCC
CTGTTCCTGTTTTCAATAAGATGTATAAACACATTCTTACAGATAAAGGATTAGAACTGT
TTGATAAAGACTGGCAGGAATTGCAAAGATGTCTGAAGAAATATATCGTAGATATCCTCC
CTCTGGGAGAGGTCATATGAGAAAGATTGATATCGCACCCAGAATGTTTAGACTCATACA
CGAACTCTATAATGAACATGGAGGTGCAAGTAATGACTGGAAAGCAGGTGCACACTATTC
CCTCGATAGAGTTCTACAAATCCTACAAGAATATTACTCATGAAGAAAAGAAATTTAAAA
ACGTTGATACATGACCTTGAGGTTGCAGTAGCAGAGTTAAAATCTGAGGTCTATTCAGAC
ACTGGAGCATATCGTATAAGTAGTGATACTGACAAGCACCCTACTTATCGTGACAGCAAC
GACGAAGTCGGACTCTGCGATTGATTATGAAAATCCCCGGCTATATGAAGGTTCAACTTT
CACTTCTAACGATATTGGCGATTTCTTCGGTTTCGTCTACCGTGTTACACATAAGGTTAA
TGGGCGAATCTACATCGGGAGAAAGTATTTTTATGCCTTTAGAACACCAAAAGGAAAGAA
AAGAAAACGAAAACAAGAATCCGACTGGAAGAAGTATTATGGATCTTGTCCAGAATTAAA
AGAAGATATATAATTGTATGGAAAGGAACAGTGTAAACGAGCGATTATAAGCCTACATAA
CCCGAAGGGTCAATGCACTTATGAGGAGACCCGACAACTGTTTTTCAATAATGTACTTAC
GGAGGCAACAAAAGAGGGAACCCCTGCATTTTACAACTCGAACATACTTGGTAGGTACAT
GCGTAAAGATTATTTCAATCCTTGACAACTGCTGATAACTCTTCTATAATGTCAAGGTAG
TCAAGGGGGTTCTCTTATGAACGAAGAATTCAACATTTCAGATGCGATGATCGACATCAT
GATCGATCAGTTGCATAAATGTGCTGAGCTCGAAGAGGAGGAACTTGAAAACCACTGGGA
CAGTAGCTCAGCGGAAAGAGCAACTGCCTTCTAAGCAGTCGGTCGTAGGTTCAATTCCTA
GCTGTCTCGTTGCCTTCGGGCAAATCGGTCAATACAGGTTAAAAAAATGACTACAGCACA
ACGTTTCTCGTCTTGTCTCGATATCCTTACCGATGCAGTTGACAGACAGATTATACTTGA
CACCGAACATCCTATCATATATAATGAACTAGTAAAACACTACGAGGATAAAGGTGTTCA
ACTCTACGGTGATGTAGATGAGGAGTACGAAATCCATTTATCCAAACTTGAAATCGATCT
TTATTATGAAACTTGAAACCATTTTAGAACGGTATCCATACAGTTATGTGTCGGTTGGAA
CTCTTCCAAACGGTCATCCAGACTATCGTATCCAGAAGTTTCACGAATGAACACAACGCT
ACAGAGTCATGTATCTACTTGACAATTCAGGTCAACTGGATTATGTTATTGAAGACTTTG
AATACACAAAATGGTTAGACCCTGATCCTGAGGTCACTGCGTAAGGCAGAAAACGGGATG
TAGTTTCCGCATCTAATTAATGAGTTCCGAATTGTTATTGAGGATCTACAGAGCAGTGAC
TAAAAAACCTGTCTCTTCTTCACGAAAACATTATAATATCCTAACATACGGATAAATCCC

GTCTTTGCCAATAGACGTTAAACTAGATGGTTTTAGGCGCGAAAGCAACTCATAGTCCTA
GTCCTAATCAGCACCCTTCGGGGTGCTTTTAATTTTCTTATATGTTGAATCAGAAATCTT
AATATTTCAAACGCTTGACAAATGTTAAAACATTATAAATAATTGTAACAGTTCTTAATA
ATACTACACAAAATGACAACAACAACTGAATCAGGCGGAAGACAATATATGTTTCCAACT
GAAACTCGTCCTTACCTTGAGGAGAACTATCAATAATACGGAAAGAACGCTGAACTACTC
ATTGGTGGCTTGGCGATGCTTGGTTTAGTTGCGGGTCTCGTCTCTTACGTTACCACAGGT
AACTTCTTCTTCGGTGGTCTTCTTTGTTTCTAACCATGGAACTTATCGAATCAATTATAT
ACACACAATCAACACGCAACAGGAACACCATCATGACTCCAGAAGTAGAAAGATTTAATG
GATGGGCAGCATTGCTCGGTTTCGTAGCAGCAGTTGGTGCTTACGTTACAACTGGTCAAA
TCATTCCAGGTATTTTCTAATGTATCCATCAAATACAAAAGAAGTAGAAGCACAGAAAGT
AGTTGCTGAGAAAGTTAACGGTAGATTAGCCATGCTTGGTATCATCGCAGGAATCGGTGC
TTACTTAACAACAGGTCAACTCATTCCAGGTTTCGTATAATGAAACATTGGATTTTTGCA
GAGGAATTAAACGGTAGACTAGCGATGATTGGTCTACTCGCAGCAGTAGTGAACTATGGT
TTCGCAGGTTGGATTGCACCAGGTATTTTCTAATGAACAATCACGACGTCATGGAAGCGT
ATAAGAGACCTATGTCTGTACGTTACATTCCATTCTTCTACTGTATTATCGCCACAGGTT
TAAGAGCAACTGTTGGATTACCTGTATACGCTCACGGATTAATCTAATGTATATTGATCA
ACAAACGTAGATTCATACAATGTTGTTTCCGTTTATGCCTGTCATAACGCTCTTCATTGT
TTGTTTTGTCATGCTAGGTGATCTTCCTTGGGACGATGATGACGATGATGATGATGGTGG
CGGGGGCGTAATGACTCCAGTTTATAACCATACACCTCAAGGAGCATAAATAAATTATAT
TATTAGCATCAGGTGTTGACGATGCCAAATCCTAAAGCACTCTATGAAGATATGGAGACC
CTTAACATGCTCTATGAAGAGTTACTATGGGATCCTGATGCAGAGTTAGATTTTAAAGCA
GATTACAGTAACAACAGAATAGTTATACAACCTAAAGAGGACGCTTGACTTCCTCTTTTT
TTATAAATAATCTTATGATAACTTGTAATTAATCATGGCAGCAGATCCACACAACACTTC
CGTATGGGTAGCAACCCGAAGGATTGACGATAAAGTAATGGAATTCTTAGTTTCACATAC
TGCATGGAACCCCGACAAGAGATTCGCAAAAATTTTTGACACTCAAGCAGGTGCCAGAAA
GTTACTTAAAGAAGGGGGTATGAAAGGTACCGTACGAAAACATTCATGACAATTCCCGCA
TCAGATAAATTACCGTATGTTGGATGGTTTGATGACAACCTTTTAAAAGGAACAAAATAC
ATAGACAAACCTGTCTACGCATCATGTGATATTTCTATACACCAACAGATGTATGACATG
TGCACAAGACATCTGAGTAGAATAGGTGGATCAGAAAATTAGTATTAATACTCATCGTTT
TCACTTGACTTAGTCGTAAAGATATATTACTATAAATAAATCAACTGGGTGAGGAAATCC
TCACCTTTTCTATGTGCACCCGTTTAACCGAGACCTATGGGTGGGTAAATTACGTCTCTC
ATATCCATCAGTGAAGGGATTGATGGAAATATAGTATCGCTGTACCCTTTGAGCCCTACT
TAACAACGTCCTAATGATAACTCTTTCAAGACAAGGCAGACAAGGTGGACTCCTACAAGG
ATGGCCAGAGTTCTGCGAATGGGTAACAAGCACTAACAACAGACTTTATGTTGGTTGGTT
CGGTGTCTTAATGATCCCATGTTTATTAACAGCAGCAGCATGTTTCATTGTTGCATTTAT
TGCTGCACCACCTGTCGATATCGACGGAATCAGAGAACCTGTAGCAGGTGCTCTAATGTA
TGGAAACAACATCATCTCAGGTGCAGTTGTTCCATCTTCAAACGCAATCGGTCTTCACTT
CTACCCAATTTGGGAAGCAGCTACAATCGATGAGTGGTTATATAATGGTGGTCCTTACCA
GTTGGTATTCTTCCATTTCCTAATCGGAGTCTCTGCCTACATGGGTAGACAGTGGGAATT
ATCATACAGACTAGGTATGAGACCTTGGATCTGTGTTGCTTATACAGCACCAGTATCTGC
AGCATTTGCAGTATTCCTTGTATACCCATTTGGTCAGGGTTGTTTCTCTGATGGTATGCC
TCTAGGAATTTCTGGGACATTCAACTTCATGTTCGTATTCCAAGCAGAGCACAGCATTCT
TATGCATCCCTTCCAGATGGAAGGAGTAGCCGGTATGTTCGGTGGATCTATATTCTCCGC
TATGCATGGTTCACTTGTAACATCTTCTCTAATCAGAGAGACTACTGAGGAAGAATCTCA
GAACTATGGTTACAAGTTCGGTCAAGAAGAAGAAACCTATAATATTGTTGCAGCACATGG
TTACTTCGGTAGGTTAATCTTCCAGTATGCAAGTTTCAGCAACTCAAGAAGTCTTCACTT
CTTCCTAGCAGTGTTCCCAGTTGTATGTGTATGGTTAACCTCTATGGGTATTTGTACAAT
GCCAATCAACTTGAACGGAATTAACTTTAACCAATCAGTTGTAGATGTTAACGGTAAGAT
CATTCCTACATGGGGTGACGTACTTAACAGAGCAAACCTTGGTATGGAAGTAATGCATGA
GCGTAACGCTCACAACTTCCCACTAAACTTAGCATCTGCTGAAACTACAGAGGTTGCTTT
AACTGCACCAACAATCGGATAAATTAATGCTGAGGAGCACAAGCACAAATGACTCAATTT
CTCGTAAAATACGGAGGATACTTTTCAATATTTGAATTCATATTCTTTATGGCAATAGGT
ATCACGTTACAACCTGACCTAACCTAATCGAAATAGAGGGGTAAGACCCCTCTATTTTTA
TGGCATGTTATTATCCAGACGGTAGTTTTGGTACTGTATGTGATCTACCTCCCACTGATG
CTGAGATAGCACAGAGGCCACGATTTGCTGTGTCTGATACTTCGCGTTTAGGAGTAGACT
CTGAACGTGTTGTTACTCTTGATTTGACAGGTTAATAAGATAGTATGCAAGAAGTTATTG
GAGAAGTGACCTCCTTCTTAAAGCGTCAAAGATGTAAACAAAGAACGTTACCTGATGGCA

CTATAGAAAATTACGATTGTGTATGGGAATATCAAGGTGACCTTGGAGAGAATTATGATA
GTGGTTGGAATTATGCCGAGGCAAATAATGAAGTTAGTTTAGGTAATTCTTTTCCTCAAC
CAACATTATCTCCAGCAAGATGTTCACAATATCAACCAGACATTAATATTAGACCTCTAA
CTTTCTTCAATGCTAAATGAACTTTAGGTATAAAAACTGCTAATGAAGGATCTTCACCTG
TAACATTTCCAGTTGAATCTGAGTATCTATGGTCCTCTGAAGCAAATGAATATGGTGTAT
GGACTAACGCAGGAGAGTGTACGTTACCACTCGCACCTCAATCAGTAACATATAAAATTA
ATATCACAGTAGCAGGAACTTAGGGATTTACTTTTGCTGCTGACGATACTGGATCTGTTA
CTATTGGATCAGAGACTAGTGCGTTCTTCACAAATAGTGGTGGTAGGACAACCACTGGAA
CTCCCACTACTGCTACAAGATCATTGTCAGTAGGTGTTTTATTTGTGACAGTTATATACA
GGAATGAAGTCGCTAATCCCCCAACGATAACTGATGATGAATATCTTTGGGGAAATAATC
CTCGTGGATGGTATCTCAAGATATGTAAGGGTGGAGCATGTGGAAGTGCAACTAGTATGT
CTTGGGTAACCTCAGGTCCTCATCCTCTATGGTCAGCATTCATGAATACTTACGCAGTAT
TCCCAAGTGATAATGATCCTTTACTAGAGGCTGGTCAAACAGCAACGTATAATATAAATA
TTCCTACAGCAGGTAATTATGATTTTGAATGTCAGGCAGATAACACTGCTACATTCACAC
TTGATGGAACACAGATTGCGACGTCCAGTTCCTATACAACAAGTAGCACTGCTACACTTT
CTAACTTGAGTGCAGGAATGCATACTTGAGTTGTCTCAGTTACTAACACTACTACATCTG
GTAACGTCGCTAAAACTTGGACAGATAATCCTGGTGGTGCGGCATGGACAATTTCTCAAT
CTGGTGCTATAATACCTTCATCATTACATCTTTCTACATCTGGTGACGGAAATCTTTTCT
GGCACACTAGAAAGGCAACAGGATATACATACTCAATCACATAATGGAACTCCCCAAAAT
CCCAAACAATCAACTACGTAAAGAGATAAAAGAAATTGTAGGTGATGCTGATGCACACTT
TGATTTGGTTGTAGATCCTACGGAAATCATAGACGTACCCTTAGACACAGATGAATACTT
TGAAGGGAAATATCAAGTGCAAAAAATGTTAATTAAAAACCGTAAACGAACACACGAATA
GCATGAATCCAAAAGAAACAATCAAAGCAGCAAAAAGAATCATTAAGGAACGCAAGATTA
ATAAAAAATTGTGCACTAAAGAAGATGCTCTCTTTGCCAAATTAAGCAAAAGAAGAGCAA
AGAAAAAATTGGCAGAAGAAGACACTGGACAGATCCCAAAGAAAGTGTTAGTATAAATAC
CCGAAGCGAAGGACGCTTCGCGAACAAAGATTTCCGAATGGTTCAATTACTCCCGCTAGG
TCTCTACAGTTCAAATCCTAACGAGAACACGTCGAGTTCTCTAACATCTGCAGGTATAAC
TCTGCAAGAAAATAAGAAAAACAAATGATTAAATCAACAATCGCTGCAGTCGCTGCAGCC
CCTCTTCTAGTTTGGGGTGCAGCTTTAGCTGGTCCATATGTTAACTTAGAAGCAACTGGA
TCATATTCTGATGGAACATACAGATCTGGTGGATGAGAAGCAGTTGTAGGATACGAAGGA
GAAACACCAGGTAGTATCGGTTGGTAAGTATCTGGTGGTCCTACTGTAACTCATACAGAA
TCAACTGATGAGTTCGGAGACGTTGAATTGGGAGGATACCTTGGTGGATCTTACGATAAG
TTCTACGGAGAAATCTCTGGTGTAACTACATCTGATGACGACATTGACTTCCCTGCTAAG
GCAGGTGTTAAGTTCGATTTCTAAACGCATCTATATAGGATGAATCCCAGGGACTCTATG
AGTCCCTTTTTTATTCAACTAAACTATTATGAATTTTACTGTTTACACACGAGACGGTTG
TCCTTACTGCACTCAAATCAAACAAGTATTAGAAGGAAAACAATATAATTATCGGAAATA
CAGATTAGGGGTTGACTTTCAAGGAGAAGCATTCTATAATCAATTTGGAGAGGGATCAAC
TTTCCCTCCAGTTGTATTAGGTGGCACCAAGCTTGGTGGTTGCACTGATACAGTTCCATA
CCTTCGGGAAAACAACCTTATCTGATGGTAGAATTTTACGGTCTTGTTGAATCTGCTATT
GATGCTGCGTTGGAAAAGAATATGTTTCTCTTCAAACCGTATAATTATTTGACTCACAGC
AAGATTAATCGTAAAGAAATACAAGAGTTCATTGATAGCACAACTGCTAAGGAATTAGCA
TTGACTATTTCTGGTCTCGATGCTTATGTCAAGGGTGGATCTGACTCTTTTCATCAGTTA
CTTCGTGAAGCATATGGGCATCTTGGTAAACCAAAAGCAAGGATAATCTCAAAGTACTTG
TCACAGATTTTGTTACATGCTCGGCAATACGAATGGTATAAGAGACCAGGTCGTAGTAAA
ACCTCTAAATAAAAATAGCTAAGGAGAGTCCTATGGAAATTGCACTTGTAGTATTAACAA
CAATCGGTGCTTTTATTCTTGGTATAACCGTCTCTTGGTTAGCGACAGGGTACGTTGAAG
ATTACATCGAAAACGCTGCCTATGCAAAATCGGTCACACATCCTTAGATGTTTGATGAAG
ACGGTGACATGATTCATGATGAACTAATCTATATTCGACCAGACTCTAGGTTCTTACAAC
ATCAAGATGACGATGACGATGATGATTAACAAATTATGCCTACACGATCAATTGAAAACA
CTAACTCTAGGTTACTAATTAGTGAGATCCTAAGAAAGGTCTCAGATGCTAAAACTAAAA
AAGAAAAATTTGACTTACTGATGAAACACAACACTCCCGCACTACGTCAACTAATGATCA
TCAACTTTGATGAGAGTATTGTATCAGAACTCCCCGTAGGTGAGTTTCCATACACTCCTA
ACGATACCCCTGTAGGGACAGATCATACTAGACTTGAACAAGAATACAGAGTACTATACA
GAGTCTTCAAAGGAGGAGACAATCGTCTCACGTCTTTGAAGAGAGAGTCCATGTTTGTGC
AACTTCTAGAAGGATTGTCTGCTGAGGAAGCAGAACTTTTGGTTCTATGTAAGGACGGAA
AGATGGTAGATAAGTATAGGAGATTCACTAAGGCAGTAGTTTCTGAAGCATTTCCATCTA
TTGAATGGGGAGGCAGAAGTTCAAAGGAGTTCGAGTTTTCAAAGAAAAATGCACTCCCGA

TGATGCAAAAGATAAATCATTACCTTACACCGCCTATTTGGTTGAGTATAACGTAGATGA
TAAACCTGCGTTTGATATTGCAATCGCAAGTAAAGCAGTAGATCTTTTTGATTATTACTA
TGATCTTTATAAAAAGCACTTTGTAAAGTTTACACAATCAGAAGGTAGAATTAACCCTAA
ATTATGGAACGATCCAACACAGAAGAAAGGCAAGCGAGACAAAAGATGAGCGTCTACTTT
GACCGTAGAAAAGCACTCGATGCGGAGGAAAATAAAAAGAATGAAGAAGCAGCAAAAGCA
GTTGCTAGAGTAGTTCAATTCTTTGTTCAACCTGCGATTGTTATGTTACTATGGAAGTGG
TTGATGCCAGGTTTGTTTGGTCTTGCCACTATTGGATATCTAAAAGCGTTGGGTTTGTCT
CTGATGTCGAAAATTTTATTTGGAAAGTATGAGTAAAGTATTTTTGATATCTGTAACTCC
TGATGCAGAAAAAACTATCGGGTATGTTGCTAGGGTGAGTAATCCTAAAAAACAAGAGAA
CCCTGACGTCGGGGGTTTACTTAAGTATTGTATTAAGCATGGTCATTGGAGTGTCTTTGA
ACAAGCGTTCATGACTCTAGAGATTAACACGACTCGTGCTATTGCTGCTCAAGTCCTAAG
GCATCGGTCGTACACATATCAAGAATTTTCACAGTGGTACGCTGACTCTTCTCTACTTGC
AGATAGCATCCCTCTTCCAGAACTACGTCGTCAAGACGATAAGATTAGACAGAATTGTAT
TGATTATGTAGATCCATTTATCAAACAGGATTACGAGTTAAGAATGTAAAGGCATTTTGT
AGATGCAATGAAACTCTATAAAGAAATGCTTCACTCAGGTATTGCAAAAGGATCTGCTAG
CATGGTATTGCCTCTAGCAACACCAACTAAAATGTATATGAGTCGATCACTTCGCTCATG
GATTCATTACATTGACCTGCGTTCTGCTAATGGAACACAGAAAGAACATATGAAAATTGC
AGAAGCAGGCAAACAACATTTTATATGTCAGTTCCCAATCATCTACAAGGCACTAGATTG
GTGTCCTGATGATGATTGTAAATGTTCTGATGAAGACTACTACAATGACCTACAGCCCTG
TTTGAGGATAGATTAATGCCTAGTTACCCAGTGATAAACAAAAAAACTGAAGAGAAAAAA
GAGCTCCACATGACCATGAAAGAATATGATCAGTGGAGAAAAGATAGTCCCGATTGGGAT
AAAGACTGGCAAGCAGGTGTTGGAGGTATGACCTATGGTCACCCTAAGATGGCGGACGGA
TTCAAAGAAGTCATGTCCAAAGTCCAATCAGCACATCCTAAAGCGAATCTATCTCGTTTC
ACATAATGGAAAAAGCAAGAAAAAGAACTAACTCTCCTAAAACTTTCCCCAATTGTATGT
CGAGGAAACACATGAAACGTAAGAAACCTATCGACTCATCATACATGACAGAGGATAAAC
CTCTGACAGATAATCAGTCACTAGCATTTGCTCAGTATGCTGAAGGTAAGAACTTGTTAC
TGCATGGTGTTGCAGGAACAGGTAAAACTTTTATTACTTTATATATTGCTTTGCAAGAAG
TGCTTGACGAAAACACAGCGTATCATAAAATATACATTGTAAGGTCTCTACTTCCTACTA
GAGAGATTGGTTTCCTACCAGGTGACCATGAAGATAAATCTGCACTAAATCAGATTCCAT
ACAAAAATATGGTAAGGTATATGTTTAGTATGCCTGATGACAACGCCTTTGAAATGTTAT
ATGATAAATTGCGAGCACAAGGAACTATTTCCTTTTGATCTACTTCTTTTATTCTTGGAG
TCACTCTTGATAATGCTATTGTTATGGACGATGAGTTCAGTAACTTAAACTTTCATGAGT
TGGATTCTATGATTACTCGCATAGGTGAAGACAGTAAGATTATGTTGTGTGGTGACATTA
CCCAGTCTGATCTTACACGAGAGAATGATAAGTCTGGTATCTCAGACTTTATAAAAATTC
TGCAGGAGATGAATGAGTTTTGATGCATTGAGTTTGATATCAATGATCTCGTTCGCTCAG
GACTTCTTAAGTCTTATCTCTTGTCAAAATATAATCTTGGTCTCTAATGTTTAAATTCGT
TGATGTTGGTAATATTTCAGTTGAAGTAGAACCAGTAAGTAAAGATGGAGTACGTTTCTA
GCCTATTCCTGGTGCGGATAAGTATTATCCAAGCGTAACCTCAATCACATCGTTTAAGAA
CGCACAGTTTTTCAAAGAGTGGAGAACCCGAATAGGTGAGGACGAGGCGAATCGCATTAC
TGCTAGATCAACTCAACGAGGAACAGCAGTTCATAACATTGCGGAAGATTATTTCAAAGG
TGAATTAAACCTTGACAGATACTTGGAAAATACTCCATTGCCTGTTAGAATGTTTCAATC
TGCAAAGTCCACACTAAACCGGATCAATAACATTCATTGTCTAGAGACATTTATCTATTC
ACACTATCTTGGTTTAGCAGGTCGAGTAGATTGCATTGCTGAGTTCGATGGCGAGTAGGC
ACTGATCGATTTCAAAACGTCTACTAAAGAAAAAAAGGATGATCATATCGAGAACTATTT
TGTTCAAGAAACTGCATACGCATCGATGTTCCTAGAACGTTCAGGAATTGAGGTCAAGAA
AATTGTCACACTTACCGCCACTGAAGAAGGATCTATTCAAGTATTTGAGAAGTACAATCT
TGATGACTATTTACAGTTGCTGAAAACTTACATCGAAGAATTTGTTAGGGGAAGACATGC
CTAAAGAAAAACTTGAGGATAAATTTTTAACAGCTACCAAATGCTCACAAGAGATTGTAA
GGTTAGTTAAGAAAAGTAATGGTCTCATTACATATATTGTAGCAGTACTTACATACTGCC
AAGAGAATGAGATCGAGGTAGAGACAGTTCCCAAACTGATTTCTAAACCGTTGAAAGAAC
GTTTGCGTCACGAAGCACAACCTTTGAATTATATGAAAGCATCTTCTAAAGGAGTATTAC
CACTGTGACAGGGTTTGAAGTGTATAAG
>NewGenomeName_27
ACCGGGGCTTGTACTCAAGACTCCGCGGCCAACGGAATAGTAACTCCAATGGCGAAGAGC
CGCAACTACTTATACAAGAGGTGAATCTTCACCCGTAATAACCCCACCTTTGAAGACTTT
GTTCATGTGCTAGATTTTTGCGCGCTCGACAATTGCAAGTTTACTATCGCCGGCGAGGCG
AACCTCGTGAATGCTACGCCTCACGTTCTGTGATTCCTGCACCTGCGAAGTAATGCTCGA

GCTGCTGCACTGGAGGAGTCGCTTGGAGGAAGAGCCTGGCTCTCTCGTGCCCGGGGATCT
GTTGAAGATGATGAAGAGTATTGCGCAAAGGAGTCGACATACCTTCAAGTTGGTGAGCCA
TTCTCTGAGGGTCGATCCTCTGATCTAGCCGAAGCGACATTCACTGTGATGGCTGGCGTA
CCGATGACTGAGGTGGCCAGGAAGTTGCCCACTACTCATGTCGTCTTTCGGCGTGGCCTG
GAACGCCTCCGGCACCTGTTCGTCCTGACGCAACGTGATTGGAAGTCCGAAGTTATCGTG
CTGATTGGTCCTCCTGTCAGTGAAAAGAGCCGTTATGCATTTGATTTTCCCGCCGAAAGC
AAGTATTACCAGCCTCGCGGGAAGTGGTGGCACGGTCACTCGGTAAATGACGTACTCGTC
ATGGACGACTTGTACTGTTCTTTTCCGTATGCTGCTTCGTTGAGAATTACCAACTTTTAC
CCGTTACGGGTTGAATTCAAAGTCGGTATGACTCATTTTGTGGCTAAGACTCTGATCATT
GCAAGCAACCGCGAGCCTCGTGATTGGTACAAGAGCGAGTTTGAGCTTTCAGATCTGTAG
CGCAGAATTTGCAGGTATCTTGTGTATAACACTAACAAGTATGAACCCGCCCAGTCATGT
ACCCTCCCGTTCCCAATAAACTACTGAGACGAATTTGCCGTGAGTCGTTTATTAAGGTAA
ACATTTGGCAGCGATTAAGCAGTTGGGGAGATCGGTTAAGGTTAGGCACTTGGCAGGGAG
TAAACACTTGGGCAGCTCGGTTAAGTTAAGGCACTTGGGACGTATTAAACACTTGTGCAC
CTCGGTTAAGGAAAGGAACGTGATACGGTTTAAACACTTGGGCACCTCGGTTAAGGTTAA
AACGAGATCTCGTTTTTGCCTAGATGCCCGTGATGTGTCCAGATTTAATGTCGAATGTGT
ATTGCGCCTGTATGTCGTACTGCGCGTTCATTCGTGTAGTGTTGTCCGGTTTACGAGGAC
TCATGCCCATGCCGTAACGCCCCACTTAGTCTTCAGTGCAGTTGATCCGTGTAAAGTTAC
GTCCCCCGAGGAAGAAGGTGGAGTATTTCGTCGATTGGCACGCCCCTTGTACGAGGGGCT
TAGGTACGAAGTAGCTTCTGTCTACTCTGGAGGGTTCCCCAGTTCTCGTGGGGCTCGTTG
AGCCGTAAGGGTCTATAGACCATCCGGTGGTTGCCGTCTTAACTGTCTGCCCGTATTTGT
CTATCACGGCTGTTCTCTGTGTCTTGCTCTGCTGATATATGTTGTACGCTGGTCTGAGAG
TGATAGCTAGACCCATGATCATGTATTAATCGTATGGGTAGTGGTAGTTAAGAATTGAGC
GCTCCATGTAGCTGTTCGCGTCTCCGAACTTCGCACATCTCATGTGTAAGCCGCATATAT
TGTTGCCACGGGCCGGTGGTTCTGAGTACTTCGCGCACTCTTCTAAGCTTAAGCTCTGCC
ATTTGCCAGCCGCTGTCGGCGCTATTTGTGAGCCGAAAAGGCCGAATTCGGTGTTCCTTG
TGACCCAATATGTCACTACGGAAACCCGTCGTCGGGGGCTTGCGATTATTAGCCTTCGTC
TTCTGAATCTCCTGAGTAGACCCCGCCTCTACCTCCGACCACGAGATGCACGCCGTCAGG
AGCGAGCCCGCATTGGCACCGGCGGCAGTGACTTCCTGTGAGTTATAAACGACCGCACAG
GCGCCGTTATATTATT
>NewGenomeName_28
ATGATAATCCAACGTGCCGAAGAGAAGCTCCCAGTATCCGGTAGTGTACAGCGCTATCAG
ACTGGGATCGCGATTAATGCTGAGACTTGCAGTATTCTTATCGACGGTATTTATAACGAC
AAAGTTCTGGAGGCGGTGCGTGAAACGCTGTTCAACGCTGTTGACTCACACACGGAAACT
GGTTGCCGTGATAAGCCGATTATCATCCACACCCCAACCGGCCTGGAGCCTTGGTGCTAC
GTAAGGGATGGTGGTTTAGGTATGGACTTCGACATGGTTACACAGACCTTCGTGATGTTG
GGGACCTCCACTAAGCGTGGCTCCAATGAATTGATTGGGGCCAAAGGCATCGGCTCAAAG
GCTCCGTTCACAGTGACCGGCTTGTTTACGGTCACCTCCGTCAAAAACGGTATGAAAACT
GTTTACTCGGTGCATAAGAATGAGGGTATTCCAGAGGTTGTTCCTCTGCGCGAGTCGAAG
ACTACAGAGGAAACTGGCGTTGAAATCAAATTCAACGTAGATCATTCTGAGACTTCCAAA
TATCGCCAGGCCATCGTGAAGTGCCTGCGGTTTGCAAAATTCCCGTATGAGATCAATGAA
CCGTTTGCGACCTCTGAGATCTATGACCGAACCTACCCGGTTCAGTATCGTTATGAGGAC
AAAGAAACAGGTTGGATGCTGGAAATGTACGGTTCCGTATCCCGCAATGCAGACAGCGTG
GTTGTGATGGTCCAGCAACCTTATCTGTCGAACTATCTGTCCAGTAATAAGAACTGGCCT
ATGATGATGGTTTCTATTCCTATCGGGGATTGTGATGTGAACCCTGGTCGTGAATGGACC
GTCGAAGGTAAGAATGACCGTGGTTTCCGTGATCGCCTCGAAAAGTTTGTTACTGGGGCT
ATTGAAAAACGTGGTCAGGAAGTCGTGGAAGCGCTTAAGCAGCTACCTACACTGAAAGAA
GTGCTGGAGTATATGAAGCGTGTCGGTGCCTGCTTCTCAACCCGTTACGGGTCGGGGTTT
ATCGCAGACAAGTTCCATGACCACTTTGGGACTTTCAGTATTGACAAGTGCATAACTTAT
GGTGAGAAGGGTACTCGCCGTGAAACTGACAAGACTTACGGTTATGCTGACCTTATGAAC
GGTTCGCCACTGGTTTACAACGATGACGGTAAGTGCATCCGCACCACATGTAACTGGCTG
TCTGAGAACTGCTTCAAACGTGTCTACGTTACGGAACGTATTGAGGCGCTGGAGATCCTG
AAAGACCCGTTCTTCGTTGGAATGGTGCATAAACTTTCCGACCTTGAGAAGCGTCCTGTC
GTGAAGGGTGAGAAGAAGTACGGTCGTTATGGCTTGTACGAACCTGGTCACAAAGTGTGG
GTTATCGACCAAGATGGTTCTATCAAGAGCGACCGTATTTCTCGCGCCGACTTTGATGAT
ATCGAAGCCGCCAGGATCTACAGTGATGGAGAGGTGACTGGTAATTGTGATCTTGGTGCT
GTTAAGTATCTGCACCGTACACAACAGCCAGAGACTTTCCTGAAGGACTTGGGGGTCGAG

GGCAAGATGTATATTGTGCCTCTTAATCGCAGTTGCTGGTTACCTGATGAAACAAGGATC
GTTACCAAAGATGACCCTTATGAGCTTGCGAAACAGAATCTCATGGAGTATCATCTGAAC
CAGCGAACCACTTGCTCATCAACTTATCGCCAGTTGGTGAAGGAGCTTGGTATTTTTGGG
GTTTAAGTTGAATCCGACCCTGAGTATAAGCCTAAAGCGAACATGTCTTATTCGCTTTCG
AACATCAAGGGATATTACGCTCTGGAGAAAAACGCTGAAAGGATTGTCGATGCACGTATG
CGTATTGGTAAAAAGCTTTAGGAAGTTAAAAAATCTCGATACCCGCTGCTTAAGTATGTT
GACCTGAAGCACTTCAACACACCAGAGATGATTGAGTATCGACAACTTATTGATAGCAAA
GGAGGAACTAAATGAGTCAGTTATTTGTATCCCGTAGCGCAGCGCGTAAGTTTGCAGCAG
ACAACGGCATGAAGGTCGTGGACCGTGGTTTGTCTCCGGCTGTACTCGCAACCGGGCGCT
GGATGGTTGTACCAAAAGACAGCCAAGGGGCATCTAAGCCCCCAGAGGTAGAAATGAGTT
TCACCTCGGAAGCCAATCCTGCTCAGGTTTCACAGTTCTTCGCAAGCCGTGGTGATGCCC
GTGATTTTGCAAATGGGAAAAAGAATGAGAAGATCATTGATCATGCGAAAACATCCGATG
GTCGTGATAATCAAGAGGGTGTGGTTAAAGACCTCGGTCAGCAGGGTAAGAGCTGAGAAG
TCGTCTTTGACGTTACCGATCTGCATATCGAGGCCACTGTAACCCTGTTGCCTGAACCAC
ACAAGGTTCCTGTGACTGTGGTTGTTGAAGAGTCCGAGCCGAAGTCGTACTTCGACGAAG
AGACTCTTGAGAAGAAAGACCCTGTGGTCATCATGACTCCCGGCAATGTTTGCATCTCGA
TGCCTGATGGTACTCAGCACACCATCAACCGTGACAACGATATCTTCCCTGATGTGGCTC
TACTGCTGATTGAGGGTGATGTCGAAGGGGCTATCGCGCTGATCGAAGCAGGTAAATAAG
CCAAGGCTGAAAAGTTTGTCGATCTCGGCCCTGACCTTAAATTGCTGGATGGCGTTCTGT
ACTGGCACGGTCAGAAACAGGAAAGCACCCTGGCTGATCGCATTCTTAACGACATTAAGA
ATGATACGTTCGATGACCGTTATGTAAAGTTCATGCAGAAGCTAATGCCCAACCCGTCTT
ATGCATCTGTGAAAATGCTGTATGACTTGATTCAGCACAACGATCTTGAGATTCTTGAGA
ACGGTGATGTGAACGCATATAAATAGATCACCATGATTACCGATGGACCTCGCGATAGCT
ACACAAGAAAGGTTCCTAACTATGAAGGGCAAATCATTTCCATGCCTCGAAACATGGTGG
AAGAAAACCCTTACCAAACTTGTAGCCAGGGTCTGCATATCGCCTCTCTAGAGTATGCGG
GTCGGTTCGGTGGCAACGGACTGATTGAGGTTGCGGTTAACCCTGCTGATGTGGTCAGTG
TACCTTACGACTACAAGCACCAGAAGTGTGGTTGCTGCCGTTATGAAGTCCTGACAGGTG
CAGAAAAACCCGTTCGTGCGCCGGATGTGCTGGTTATCAGGACGATGGGGGAAATTCTGG
AAGAAATTTACCTGGAAGAAGAAACTAAGGAGGATTGATGTCACATTTTGGTGTGGGCGT
TGAAGCTGCCCTTGGTGCTGTCATGTCGGGGGATAACGTATTCGTTACGGGACCCGGTGG
TAGCGGCAAAACTTACACCATAAAGACCATCCAATCGCTCTACGCAGGTTCTGTCCTCAC
AGTAGCCCCTACGGGGGCTGCTGCCATTAACGTCAATCAGATGACTGCACACCGTGCATT
TGGGCTTACAATGGGGGTTGCTACCAAAAAGGATACTGAAGAGATTAAGCCGAAAGTGAA
ACGGCTATTGAAGTGTAAAGCGCTAAAGATCATCATCATTGATGAGATCAGTATGTTCAG
GGCTGATAAGTTGTGGGAAATGGATATGAAATGCCGACTCGCAAGAAAGCAACCAAATAA
GCCGTTTGGTGGTTTCCAGATCTGCATGTTTGGTGACTTCTTCCAGAACCCGCCCGTCCT
TACCGATACAGAAAAGGAGACGTATTTCCAGTTCAATTACACAGAATTGGCTTGTTTCTC
AGATACTTGGAAGGAACTGAATCCTTATCCAATACTACTTGAAAAAGTTTATCGCCAGAG
CAGTGTCCATTTCTCAACGATGCTTAATTGTCTGCGTTGTGGACAGCGGATTCCAGAAAT
TGTTAAATTCAAGAACACGCATTGTTATGATCATGGCAAGACTCTTGATGCCATAACGAT
CACATCTACACATGCTGCTGCCGATAAAGTAAACAAGAAGCGTTTTGATGAGGTCCCTGG
GTTGCCTACGTTGTACACTGCGAAGAAATCTGGTGAGTTTACTCACAAACCTGGGCCTGA
AGAGCTTTACCTGAAGGAAGGAGCACAGGTAATGATTACAGTAAACGACCCTAAAGGCTT
TGATGAGCTAGAGTATGTCAATGGTTCCCGTGGAGAGATATTCGAACTCGGTAAGGATAC
TGTGAAGGTTCGTTTGGACTGTGGGAAAGTCGTTGATGTAGATGCCTATGTGTGGGAGAA
TGTGGAATATAACCCAGTCAAGAGCAGGAATTCTGATGGTACAACAACCGAAGAGATTGA
GAAGATCACTATCGGTGACTACCGTGCTCTCCCTATCCGTTTGGGCTGTGCTGTAACAAT
TCATAAAGCGCAGCGCTTGACTTTACCTGAACTTAATATAGACTTTGGTTATGGCGCTTT
TGCACCCGGTATGGCTTATGTTGCTTTCAACAGGCCTACATCAGCTAAAGGATTAAGACT
ATTCAAAAAGGTCAAAGAAAAGAATATTATTATTGACCCACGTATAGTAAGATTTTATGA
AGAGACTTTCCCAGGTAAATAGGAGATAAAATTACGGCATCATACTCTCGAAAGCAGGTA
GTCAAGAAGTTCCAAAAGGAACTTGAAGATTGGAACAACGCCCCAGCCGCAAATGCGTTG
AAGCCACCGAAGCATACTACAAATATCCAAATGGACTCTCTTGATGAGATGATGTCACCA
TACGGCTACGTTCATCGTCGTGTTCCACCATATCCGAACATTACAGATGGTCGTTTTGGT
TACCAGTCCCTTTCTGGTAATGATTTTATCTCCCTGGAAGATGCGGTGTCATACCACAAT
AATGCGATGTTCCGTGGTAACCCTTTCAGAAAGATTCCTGATCGTTTAAGCTTCGCTCTT
CAAAACCGTATTGTGGAGCAGGGGTCTGTACAGCGTGATAAATCCCGCTATGCAGGTGGT

TCAGGTATTAAATCTCACAAATTATGGATGAAATTTGATGACATTTGAAGTAGAACATTT
TTCTGATCTGAAGAAAGTTTCAACAGAAAAACTGTTGAAACTTCGAGATGTGGCTAATGA
TAATTATAACGAGGCGCATCGTCACATGACAAAAGCCTTGTTGCATTTGTGGTCAATTAA
AGACGAACTGCAGTTGCGTAAAAATGCTGAAAAAACTATTTAACTGGCTGTTTGCCAAGA
AGGAAGAACCTGAGCGTGAAGTTAATGTGTTTGATTTCACAAAACGTGGAGCAGGGCATG
ATATTACTCTTCGCATGATTGATGATGGGGAGTATGCAGAGGCAATGGTTTTCCTTGCTT
TTGATCAACAGGCACCGGAGGTTGGCGACTTTATTGTTGCCAATCTTGAACAATCATCTA
TGCAGGGAGAGGTGGATTACCACATGACTTTTGTGGTTGAATATGTGACTTGTATTGCAC
TGGGTATTTCCCAGCTAACCCTAACCCGCTACGAAGGTGCAGAAGATGAGTAAGTCCCGT
AAAGTCGAGATTGAATTTGAGTACGGGCGCCCAACCGAAGGTGTGGTTGCAGTAGAAGCA
CTTTATACTGTTCAGAAGCCGGACTTTTACAGCAAAGAGAGCGATTGGGATTACAATGGT
TTTCAGGACTTAGAACATTATGCTGTATTCAAAAACGGCACCCAGATCCTCGTTGACATT
CCATATGATGTCATGTACCATCACCTACGTGAACATCTGCGTAATGCAGAGATCGATGGT
TGTTTCCATCGTGAGGAGGAATTGTAATGGCAAAAAGAGAAGTTAAACTGGTTGAATTCA
ATATGAAGCAGACCAAGAAAGAAGAGGCTGAAGAAATTCTGGCAGGTCTTATTGATGAAG
ATTTTGTCCTCATGGGGCAGCACGAAGCCGAAAGTTGGATGAATTTTACAATGGTAAAAT
CCGATAATTCACTAAACGTTGTCCATAATACGGATTGCGGCCTTACACCTGCTCTCCAGT
TCCAGGTGGTCAATGGTGCAGCAGAGCGAGGTGTTCATTGAGGTCCGTATGAATAAAGTG
GTGTGGAGCCTTTTCGATCGCCCCGGCATCATGGGGTGGGAGTATTCTAAAAATGGTTAT
AAGGTGTACTGCTTCAACTTTGATGCTGGGGGTCATGGTGGCTATGAGTTGGTAAGGGTT
AAGCATCCTAATATTCAGTATGTCAATTCTTGGATCTATGAGGGTTTTGATCCAGAGAAA
GATGGGATTCCTAAGCCAGATATTATCGTTGCATTTCCGCCATGTACCGATATGGCAGTT
AGCGGTGCTGCTCATTTTGCCTCAAAAGCAGCCAAAGACCCTCTTCTTCAGTACAAAGCC
GTGAATACAGCTAAGGTTGCAGCAATGTTAGGAGATAAGTACGGGTGTCCATACATGATA
GAGGACCCTGTGAGTGTTCTGTCCTCCAAGAGGAGGAGACCGAACCACATGTTCCATCCG
TATGAATATGGGGGTTATGTACCCCATGATGATGTTCATCCGTTCTTCCCTGAGTATATT
AATCCGCGGGACTCATACCCTAAAAAGACGTGTCTTTGGACGGGTAATGGTTTTAAAATC
GCACCCGCAAAGCCCGTTGCGGTAGCGAAGGGTTACTCAGAACAGCATAAAAAGCTGGGA
GGAAAGTCTGCCAAAACAAAACGGATCCGTTCTTTAACACCCCGTGGTTTTGCTGCTGCG
GTTTTTTATTCCAACCAGGAAGTGAGGCTAAGTGAAAGAGTTATTATTAGCTGGTGGTCG
AGCAGGGCCATCAGATGGAGATACACATATGCTGCTACAGGCTGACAAAGTGAGTGGCAT
CTACCCCATAGATCAAGCAAACCCGTCGCGAGCATTCACAACTTACGGGACCCCTGCTGG
ACAAAGTACAGTGCAGGCTAAGTTTGGTCAATATTCGTTTGACTGCCGCAATGGTGGTTT
TCAAATGTCTGGAACAGTAAAACCTGATCTTGCATTTGCAAGAGGTGATCCATTCACGAT
TGAGTTTTGGTCGTACAACACGGCAGTCGATAGTGCAACCTGGTAGATGTACTTTAACAT
AGGAACTTCTTCCTGTTTAAAAACCTATCAGTCAACTAGTTATTTGCAAGATAACTCTGC
TTCTGTACAGGTTGGTCCACTGTCTCGCCTACCGACGAACCAGTGGAACCACATTGCGAT
TGTTTACGATGGGACAAATCTCCAGTTCTATGTGAACGGAAACCGCATTGTATCGCAAGC
ATCTGCTGGCGGCTGGAGTACAGCTTCATCATTTGCAAGGGTGATGGGTGGTGAGGGAAC
AGCGAAGTGTTTCATGGATCAGTTTCGAATTTCGGGTGTTGCGAGGTATTCAGGTACTTC
ATTCACTGTACCCACAGCACCTTTTGAGATGGATTAGGAGGTAAGTTGAAAGATGTGGTG
ATCGCAAAAGCTTCAACCCTACAAAAGTTGGAGGATAATTTGAATTTCTTTTTTGAGAAG
GGGTATCTCATCCCAGGTGGTATTTCCTTCCAGGATGGATTTTATATTGCTGTTGTTGTT
AGGCCGTTTTAAGGAGCGATGATGGGGAATGGTCCGGATTGGGGCAGCATTGTGGAGTAC
CGAGACGGAAAGCTGTACTGGAAAGTAAACACCTTCTCAGGTAGACACCACAATCATCCA
GCGGCAGTTATTGGCAAGGAAGCTGGATCAATATCTGGAAAGGGATATTGCGCTATACGC
TTTCAAGGGGACAGGTGTTATAGGCATGTTATCGTGTGGGGGCTTCACCACGGACCAATC
CCAAAAGGATTGCAGGTTGACCATATCAAATCGTTAAGGGAGACAGGCGGTGTTGCCGAT
GACCGAATAACTAATTTACAACTCCTTACAAGAGGTACAAATACTCGTAAAGGTGGTGGT
AACATGCCTAATTCTTCCAACAAGTCTTCTGCAAGAAGAGGGGTTTCTTGGGATAGAGGT
TATCCATCAATGCCTTGGAGGGCAAGGAAGACCGTAGACGGAAAAGTTCTCCTTAAAATT
TTTGCGAGTTTTAATGACGCCTCTCAGCAAAGACAGTTGTTGGAAAAAGAGTATTGCTAT
GGAGGACTATGAGTAATTTTGATTTTTCGGGTTATTGACAAGGTGGACAGGAAAACCCGA
AAGCACAAAGATTTGGCAATAACGGTAATCGTGGAGGAAGAGTGAGTTATCAGCAGCGCG
AAGAAGAGACCCTGGAATCTGTAGTCAATGGCACCCGTATTCTGGCAGTTCCTGAAATGA
GTCTCTCGCTTGATTCAGCGAAGTATTTTAAAATCCGGTCTCCTGTTTCAGCAAGCGACG
GTGTAACCATTACAGAAAACTACTTGCCATATTATGAAAAGTACGGGAAGCTTACTGGTT

ATAACAAACGTGACTGGACACTACCAAAACAGAAGAAGGGTCACTTCTCTGTGGTCGGCA
CTGTAAAGGCAGCATCTTAGTTCTTTGGTCAGCATGAAGCTGCTATGGGCGCTGCGCGTA
AACAGATTTTTGTTGTTGAGGGTGAAGGTGATGTGACTGCTGGGTGGCAGGCGGGTTTTG
ATTTTGTCAAAGCTTTAGCTTGCAGCCCCAATGCCCCGAAAGGGGTGAAGGATTGGGCGG
CAAGTGTTCTGGAAGGTATCAGTGCCATTCAGAAAGGTGAGGAAGTTGCAGGTATTCCGT
CACTGCCTGTTGTCGGATTAAACTGTGGCACAGTTAACGCAGTTGATACCTTCGCTAACA
ATGAGAAGTTTATCCTTGGTTACGAAAATGTAGTTCTTGGTTTTGATAACCATGAAGCAA
CAGCGCTGGAAAAGGAACGTAAAATCAAGAAAGGTAAGGAAGCGACAGATTATGTTCCAG
CATTCTTGGTGTCAGACAATATCTGTGTAGCTCGTTACCCTAATGTACGTAACGATCCGG
CTGGTGTTAAAGATATTCGAGATATGTACGATGCTGGGAAGGGTCGTGAAATCTGGAACA
CGTTTTCCAAGGCAGAAGTCAGGTATGTGCCTTACAAACTGATCAGTCTGAGCAATATCA
CTATTGAGAACCTTCGTAAGAAGAAGAAAGATGGTGTTCCATTACCAGGTATGCCTCGAC
TTTATGAGTTGACAAAAGGCCCACGTACTGGAGAGCCTTGGACACTGACTGGACCAAGTG
GCGGGGGTTGACAATCTAGCGCCTTCATGCAGTAATGTATGAAGAAAACCTTGTGAATTC
GGTGAAAACCCTAACGAGAAGACGAGGGCAATACCGAGCGAAGCCGAAGAGTCTCTCCTT
TTATAAGGAGGGATTATGTGGAAAAGTATAGAAAATTTTGATGGATACGAAGTTTCTGAC
AAAGGTGAAGTAGGAGGGAAGAGAGGCTCGAATATAAAACGTGATACCAGTGGCAGAAAC
TCCCGTGTTAGCCTGTGCGGAGAAGCGATAGTTACTCGGTGGATGGTTCACCGTCTTGTA
GCTTTTGCATTTTTGGACCCCATAGAGGGTAAACCTTATGTGAATCACAAAAACGTTAAT
AAAACGGATAACTCAGTTGACAATCTTGAATGGGTAACCCAACGGGAAAATCAAGACCAT
GCTGTTTTAACAAGCTTGTGTCCTCGTGGGGAGGAAATATGGACATCTAAAGTCACAGAG
AGTGTAGTTCTTCAGATATCGGAAACTTTGCAACTTGGACTTCCAGTATCAGAAGTGGCG
AGAAGATTCTCCGTTCCTTTTCGGAATGTAAAAAGAATTAAATACAGAGAAACTTAGGAA
TGGTTAACATCTGAATACGAGTTCTTTTCAGGAAGACCCAAACTAACCCTGCTTTCGGGA
GAGCGGCAACAGATATACGACCTGTTGAAGTATGGCTTCCCCAGCAGAGAGGTTGCCAAG
ATGTTTGGTGATTCTAAAACGACTGTATTAAACATCAAGAGAGACTTTTCGGAACGTGTA
ACGACTAGCCGTGATGAGTGTAACGGTGTACATCCAAGTGGGCGGAAGCGCAAGGCATCC
TGAATGGATGATGATATAGCCTGCTCTGCATGGAAACATGCAGCAGACCCCGATACGAGA
TAGGTGTCGGGCAAGGAATAACGAACCTTGCTGAACTTAAGGAAAAGTACCATTGCTCGC
AAGATCGAGTATGCAATTGTGGATTATCTTCGCAATATGGATATCCCTCGTCTTGACGGC
TGGACGGAGCACGAGGAGGTTGCAATCATCCGTCTTGAAGAGGATCAGGAGGAGTCAGTA
AACAGCCTGTACGCTGAAGAGTTGAAGGTTGACCCTAAAGCATTTGCTGCAGATCCTAAG
CAGTTCCTAACTGAGGAAGAGCACCTTGCCATTCACCAACGGTGGATTTCTGAGGACAAG
ATTAAGATCTTTGACCACTTCGGTTCTATCCCGACAGATCAGTTGATTCAGAAGCTGAAA
CAGATGGTGTTCCTTGATGGTTGTATGTGGATTATCCTTGACCACCTGTCGATGGTGATC
TCCGGTCTTAAGTCAAACAATGAACGCCGAGATTTGGATAATATCATGACCGAGCTTGCG
GCATTCTGTAAGCAGTATGAGGTGTTCATTCTCAGTATTAGCCACATGAATCGTAAGGCG
CTTCAGTTATCGTAGGATAAAGACGGTAATATGTTGCCTATCTGGTATCCTGTACGTAAA
GAAGACCTGCGTGGTTCTGCTGCACTGGAACAGCTTTCCTGGATTGTCCTGGGTGTTGAG
CCAGAAGAACTACCAGACCGCTCTCGTGGTCGTGTGCGTGTGGTTGTCATGAAGAACCGC
CCAGACAAAAAACTCGGTGTCGCAGACCCCATGATCATGGACGAAAACGGACAATTCACG
GATGCGTCTGGTTGGGTAAGGGAAGATGGGATGTTCAAGCTGGACGGGGAAGTCATGCTT
CGCCCTGAAGGTATGGTTCAGACCCTACGACTGGAGACTCCGGTGGGTAAGGTCAATGTA
CCAGCACCTGTCGTGGAGAATCCGGTGCAACTCCCATCGCCAGCAGACATAGATGAGGAT
ACGCCGTTTTGATGATATATTAACCAAATGAGAGGTGGGTGTCGATCCAGACCAACCCAC
CTTGCATAGGAGGAGCATGAAAGGTTTGTTTATAAGGGACAACGAAGCTGTTGGTCTGTT
AGAGGAAGTAACTAAGTACCACTGTACCCTTCTGAAGGAATACGGTGTTAACAACTGGAA
TCTGTTTTTAGATCCTGCACACCCACAGTATGAGAGCGCCGTAACATATGCTCGCAGCAA
GACAGAAGCCAATCTCACGATCCGCTCATATGACGAGCTTGAGGCTTTCCTGAAAACTTG
CCGTGGTATTGCGTGTCACAACCTTTTTGGTTATGATCTACGCCTCTGGAAGAATCTGTC
AGGCATTGAGTACGACATGTTCAAAGACCCTAAGTGTCTGGGGACGATTGGGGACACCCA
AGTCAACCTGTACGATACTCTGTCTATGAGCCGTGTACTTTTTCCTGACCGACCAGTACC
AAATGGCTGTCCTGCTGCAGTTTTGAACCCGGTAACCCGGAAACGTGACCGTGTTGGTCC
ACATGGCCTTATGGCTTGGGGCTATCATGTCGCGAAGAAGAAAGTTCAGATTGATGACTG
GCGTAACCAGCCGCTCTGGGAGTACGTTAACCGTGTGTGGGAAGACGTTCTGATCGACGA
ACTGACGTGGAAAGCATTGATTGATGAATCCACTGGTGCTCGTTGGCCCGATGACAAGCA
GTTCATGTACACGGATAAACCCGAAGGTATTCGCCAGATTAACTGGAAAAACGCTCTTCG

TCGCCGTATGCTGACGGATTACCTGATGATTGAGCAAGAGATTCAGGGTGTTCCGGTCAT
CAAGCATGAGGCGGAAAAACTTCGAGATCGCCTTGATGTTATGATGAAAGAGATCGAAGA
AGAGGTTGAACTGTTACTTCCTATGAAGGAGATGACAAAATCTCAACAACCTAAGTTCCC
AGCTAACCCGTTTAATGGTGTTGTTAATATCTCCCACCACGGATGGAACTGGTTACAGTA
CCAGTTTCGGTATCCGGGTAACCGCGAGGGGCTGGAGTTTAAGGGTTCACCAAAATCCGC
TTTCAAAGGTTATGGTGAGGTCAGTGCGGCAGGGGAAAGATACTCTCTCCAGAACGGTGT
TGAAGACCCTATGCACTTCGGGGATTTTATCCGGGAACAAAGGAATAAAGAGAATACCCT
GGTGCCTCTGCCATCTGACCTGATGGAGAAGGCCAAAGAGGATTTGCGGAATGGTAAGAT
GCCTGACCTGATGGTCCCGATGAAGATCTCGAACCAGGACGACATCAAGAAGTACCCGAT
CCGCGATGCTGACTGGAAGCCTAGCCTGTGGCGTGTAAAAGATGTGACCAAAGATCAGCT
CAAGAGGGCCAGAACTGACCCAGAGATTGACGAATTAGTCAGAAAGTACATTGAGGAGGT
TGGTGAATCAGAGTATAAGTCTTTGATTATTGAGCACTTAAATAACACAGACGACAAATT
CAACACCTCAGAGCGCAAGTTTGATCATCGTTATGCGTCTGACAAGGCGTATGCGGAAAT
ATTCAAGAAGTTCCGGCGCAAAGCTCGTCAGTTACCAACTTCACCACAGGTGAAGGATAA
CTTCGGCAAGCTATGTCCGAACCTCGAACGTATTGATGTTCACTTGGCAAAACAGATCGT
TAAGTGGTTGTCACTGCGTAACCGTCGCTCAGTCCTCGACCCGATTGATGAGGATAAGAA
TGACACAGGTTTGCTCAACCACCCTCGCCTGTCGATTGATGGGAAGCTTCCTGCCCGTTT
TTCTGGAATCACCAACACCGGGCGTTGCAGGCACACCATTTGTGCAAACATGCGTAAGCC
AGATCCGAAGGTGTTGCTCGGAGAAGAAATGCATGGATTGTGGGGTGTTACTGACGGGTA
TTATCATGTCGGTATCGACGGCTCCAACCTAGAGGGCATGATCGCCGCATGGGGTTCATA
CCAGTTCGATGGGGGTGAAAACTTGCGGCTAATGGACAGCGGAGACGCTCACGCACGAAA
CGCAGAAGCGTACACCAAAGCTTCAGGAACCCTCGTTACACGTAGCGGCGGTAAAGGTGT
AACCTACGGTATCGTGTACGGTGCTCAGGCGGCGCAGATTGCAGGGATTTTGAACATCTC
CCTTGACAAAGCTCAGGCGGTAATTGATGCTTTCTGGGATAGGAACTTCGGTTTGAAAGG
TCGTAGAGAATGGCTGGAAAACTTCTGGGAAGCCACAGGGAAGCGTTATATTCCTGGATT
GGATGGTCGTAAGATTTGGACCCGTTCTAAGCACTCGTTACTGAATGCTTACCAGCAGAA
CGGTGGTGCATCACTGTTTGACCTTGGTGGCATCCTGCTACACTGGGAAATGGTGAAGCT
TGGGTGGTATGATGACGATGTACGTCGCCTGATCTACTACCATAAGTAACATAGTGGCTT
TGTGAGGTGACTCACATCGAATAACTCTTTTAATTGTCGGGAAGGCTAAGGTTGGCAGTT
TTGGTAGACTGTTATACTATGCTAATCCGCAGCGAAGCTTGTCAAGAATTTTAGAGACAA
GAACGTTCAGAGACTAATTCAATTAAAATACTCCAGCTAAATGCGAGGAGATACATGAAA
ACTACCAAAAAGGATCGAGACATGCTGATTGCCATTACACTTGGCGATGGATGGTTATCC
AAGCATGGGCATCTCGAAGTGTGGCACTCTATCCACCAGAAAGAGTACACATTGATGAAA
TATGAGAAGATTAAGAAATTTTGTCCAATTGTTATTTTATACCGGAAAAACAGCTTGGGT
AAGGAACAGGTGGGTTTTAGAACTAAAAGATTACCTTTCCTAAAATTACTTCGTCGTATA
TTGTATCCTGAAGGTAAAAAAGTTTTCAGTGGTAGATTACTAAATAGACTTGGACCAGAG
CACTTGGCGATATTGTGGATGGACGATGGAAGTTTAACGACCCTGAAGAATAAGAAGGGT
GTTAAGTCATCCTCCAGATATACGTTAAGTCTTTGTACCAGCAGAGAGCAATGTTTGCTA
GTTGCTGACTCGATAAAATCTCTTACTGGCGTATGCCCTTGGATAAGAAGTGTCAGGGGT
GGTAAATATTTTTCTCTTTGTTTTACAACAGGGGAGGGGAGGATTTTTAGCCAAGTAATA
CGACCATTCATGTGTAAAAGCATGATGTAGAAGGTGAGCCAAGATTAATTCGTGGGGAAG
CGACCAAGTGGCGTTGCAAAAGAGAGCATCCCTTGTGGATGGTTAGAAAGTCGGATCCTA
CGCGAAAACGTATGGCAACACGCAATGGTGTCGGCGACATTAACGACATCGCTAAACTTC
TGGATGAATACCAGCTCCAGGTTCCTAAGAAATACTTGAGGAAGTGGGAGTTTGATACCC
TGCAAGATGCAAAAGCTTTCGTAAAAGAGTGGGAAACAAAAGGCCGCGTATTGGACGGGC
ATGAGTGGAGAAAGGCTGTAATAGACGAGAATGGTGAAAAAGTCAAGGATGCCGAAGGTA
ATACGGTGTATTAGGGCGTCTTTGGTGAAGTTAGGAAGATACACATCCGATATTGCCCTG
TTGGTGAGATGGTGGTGAAATGTGTTGAGAAAGCGGCTCGTATGATGGGTTCTCCGGTGC
ATATCACAGGTGCCTACTTAACGGGTAAAAACTGGGCTGAGTGTCACTAAATTTAAAGGG
GCAGACGCCCCTGTTCTTGGAGACTATAAATGTTTGAGATGATGCTTGCTACAAGTGAAA
CAAAGAAATCGATTCCTGTTAGTAATGTGGAATTTACCAACATATCTTCCTCCCCACAAT
CATGGACTGTCCCTGCCAATGTCGAGTAGGTCAGTGTACTTTGTGTTGGTCAAGGAGGGG
CCGCACCAACTAATCCACTTCCAAGACGTGGAGGTGCGGGTGGAGACTTGGTGTGGAGAA
ACACCATCCCAGTTTTTCGTGGAGAGATTTTAGAAGTGTTTACACAAAATAATGCAGATG
GGACGACGCTTGTAGATTTGTTTCGTGGTTCTGTACCACTGGTGACTGCTGGTTTCGGGT
CAGGACCTGAACCGTACACGGGGCAATACCCAGATATGGGTGATGCAGTGATAAATAAAG
GCGGCGCGGGTGGAATAATACCTCAGCCCATGATATAAAACGTTGCAGTGGCGCAGGTG

GTGCCGCAGGGTATATTGGGAAAGGTGGTGATGGCGGTTATGCATACCCAGCATCAACAA
TGTACTCTCAAGGCAAGGATGGTCAGGGAGGCGGTGGCGGTGGCTCATGTGGTTATTACT
TCTCTGGTTCCACATCTGGGGAGAACTCCGGTGGCGGTGGCGCTGTCAAAATCTATGCCG
TTGGGCTCAATGGTCATGGAGGTAGAGGTGGATATTCACCAACAATAGCAAGAGGCGGGT
CGGGTGGGGCAGACCCTACTGGTCGCCAGGGTGGTAAATTTGGGGGTGGGGATGAACACG
GATCTTCAAGCTCACCTGGTTCTTCAGCGGTGCGGATAATCTGGGGAAATACAGAAAAGA
GAGAGTTCCCAAACAATGCCCGAACGGTATAAATAAATAAGAGGAGGATGTATGATTAAA
GAAGGTCAGTTGGTTAGATTGAAATCAGGTGGCCGTGGCATGGTTGTTGAGCGTATCGTT
GAAAGACCGCTGACCTCCAACGGGGTAATCAGTATCGTTAAATATGCTGCCTGCTCATGG
ATGAATGAAAGCAATGTGAAACAGGTGGAAGACTGTGCTTTTCCTGCACTTCAAATTATC
GGAGAGTGATATGAAAGTAAAAGGCACACAAACCACGGTGCAAACCGTGGCTGTGGAACT
AAGCGATGACGCCATCAAGTTGGTTGTTTATGGTCAGACGATTGATTATCTTGCACATGC
TGTGGAGGAAAAACTTTTGTACGGCTACCTCCACAGCTTGCCACCGGAGTTTAACGGTAA
AAGGACGATCAGGGAAAGTTACCGATCTGGGAATAAAGGTAAACTAGCGCTGGTGCATGT
GGATGCTGCCTGGAATTACCACAACAATGTTGGAGAGGACAATGAAGTTCGTTTATTGAC
GGAAGAGGAATGGGAAGGTTACAAGGAGCTTTGTAATATCTCCAAGGTAATTAAAGACTT
GCCAAAACACCGTAAAAGCCCCTTCACTTCATGGTTGAGGGGCTTTATTTTTAGGGGAGT
TAAATAACATGGAGGGTGGTGTATGTTAGAGGCAATCGATGATTTCTTTTTAGTGCTGGC
GGCAGTGACATGTGTGTGGTGTGGCTTTGGCGCTGTCTTTCCAAAAAATGAAACACACCG
TGCGGTGTACGTGGTTTGTGCAGTAATCTATGCTTCCTTTAGGGTGCATGTCTGGAGTAT
CGCCTTATAAAAGGTCACATGCCTTGGATTAAACCCTTGGTGATTGTCGGAATAATTGTT
ATTGCGGTGTTTATTGTTGTTGACATCGCAGATACCGTCACCCTTAATTGTAAGCCTAAC
GGTGAACAACGCCTCGCAAACAGTGCTGATGGAGTTGTCGTTGAGAACAAACTGATCTGC
GACAGTGGTGGCGTGAAGTGGTCTCGTTACTAACCTTGAATAAATAATATCAAGAGGAGA
TTCGAATGAAAAAATTAACGATTGCTCTGCTGGCTATGGCTTTTGTTGCACCCGCCCATG
CGGAAGAAGATGATGATTTCCAGACCGAACCTATGAGAAAGGTTGCGGTGTGCAACAAGA
TCGAGCGTTTCTCTGTGAGCATTTGGTCTCATAGGAAGGATAGTATGGGAGAGACCTGTA
TCGACACGGTGATCCCTAAAAAGCATGCTCAGGTGGGGAATGTTCTGAGCGAAGAGTCGC
GCTGGTATCAGGGGTCATCTATCAACCCAACCAAAAAATCTGTGACCCGTGTGTCACAAG
TCTACCAATGACAGAAGGGGTGATCATGGAAAAGATTATCCTTGCCACCCTGCTCGCGCT
TTCTTGCTCTGTACAAGCAACAGAGTTCTATACCGATACCTTGGCAAAAAGGATCACAGA
TGCAGGCTATGCTGATATGCAGGATTCTTGCAAACAGGGTCTGGAAAGAGCAAAGGCTAA
AAAAGATGGGGCAGAAATTGAAGTAAAAATTGCAGAAGTGCAAGTGGCAGGTTCCAAAAT
GGAAAAGCAGATGATTATCCTCACAAACGCTGTTGAAGAAATGGAGAAAAGCAAATTACT
ATGACTCGTGGCCTTATCTTCCGAAAATTTGCACCGCTGACCAACGGGCATGTGGCATTT
ATTCGCCAGGCGGCTTCGCAGGTGAGTTGCCTGTATCTGTTCCTGTCGCATGACCATAAA
TTTGTTGACGCACAACGAGAGTGGATTCGTCCAAAGTTGGGTCTTGCTGATCGCTATCGT
TACTTGCTGGATGTTATTGCCGATGAGGGTCTGGATAACGTGAAGGTTGACTACGTTGAT
GAGTCGAATATACGCGGATACCCTGAAGCAGGTGCTGCTTATGCAAAACTGATCCGTGTA
AAGCAACCTTTCGTTAAATACGACTTTGCTTTCTGTTCTGAGCCGGAGTACGAGTCTTAC
TCCAGTGAGTTCTTCCCTGAAGCGAAGCATGTTGTTATCGACGCAGAATGTAAAGCTGTT
CCTATATCTGCAACGATGATTCGTAACGACCCTTACAACAATTTCTATAACCTGGCATGG
CCTGCACGTAAACGCTTCATGAAAAAGGTCGCCATTGTTGGTGTTGAGAGCACTGGTAAA
ACGACCCTGACACAACACTTGGCACACACATTCTGTGCAGCTTGGATTCCTGAAATTGGA
AGACTGATCTGTGAGCGTGAGTATCACTCCAGTGAGCTTGCGATGAATCGTGAGGATTAC
CTGCGTGTTGCAATGGAGCATCGCCTGAAGGAGATGCAGGTGGCTGGTTGGGGTGACCGT
GGGGTAATGTTCTCCGACACCACAACTCTGATCACACACTTCTCTGGTGTTTGTGCAGAT
AAGATCGACTACTCCGACCTGTTGTTCCGGACACTGAGCCGTGAAGAGAGCTACAACTTC
TACGATCTGTTCCTGTTCCTCACACCCGAGGTTCCGTGGGTTGCTGATCCTCTTCGTTTG
CAGGATACACCAGAAAAGCGTAAAGAAACGCATTCTTTGCTTGACACAATGATCAGGGCA
TCTTATGATACAAGCAAGGTGGGGGTCATCTCCGGCAATGATTAAGAAGAGCGTACTGAA
AAACCCGAAGAAGCTGTCCGTAAATTGCTGAAGATCAAAGGAGAATAAGATGAACGTAAC
TAATTCAACCATGAAGCAGGCTTTTGGTGTTCTGAACGTTATTTTGGAAAAGGCCCCTGA
GTTAGCTGAAGGTCTTCAGGCTTATGCTAACTCTCGTGAGCAAGGCTTCTCTATAACCAA
GTAGAATCACAAGTGTGATAATGACTTTTCGAGGCAAGTTTCTTTCTCCGAGTGTCGTAA
CAGTGATCAGATCAATGTGTACTGGGGCCGACCAGGAAACTTCAATTACCAGACGAATGT
GCCTGACGACGACCCGTATTTTTCTCGTCGCAAATACTTTGGTTACGAGGAGTATGAAGA

CGCGGCAGACTGGAGCATCGCGTACCTGAAAGGTGACTTAGATGGATCAAAATAATCAGG
TTTTGGTCTGGAAAACCCAAGAAGGTAATCTTGACCGGATCACCCATATGTCTGACGAGC
ATTTACGTAATGCAATCATCTGGCTACTGACAGACTCTTGTCTTGATGACAAGACGGAAG
GTGTGCCGATAACGTCGTGGGTAGAAGCAATGGCAGTAGAGTTAAACAGCCGGATGGTTG
GTAATGTCTAAGGTTCTTAACATTTACCATATTGGTAAGGTTATCCCTGAAGGTGCCGTC
TACATGGGCCGCACCATGAAGAAATTTAACCTGGTGCGTCATAAGCTAGCCAACCCATTC
CCTCTTGAGAAAGGTGGTGGGAATCGTGATGAGGTCATAGAAACCTACCATGGGTGGTTG
TGGGAGAAGATCCAGAAAGGTGACATTACTTTGCAAGATCTTCTTGGCTTGGACAGGAAA
GTCCTTGTATGCTTTTGTTCACCGAAGAGATGTCACTGTGATGTAAACATGGCGGCAATA
GAATGGGCCAAGGGGAGGGTTACAAAATGAGTTTCAAAAAAGATCACACTTATAGCGAGT
TATATAAGAACATCGCTGGAGGTCACTCCCTTGTGCTCTCTGAGGATGCCGGAGGTTATC
GTCTGGCAGGTACAAAGATAACTGGTCAACCACTAGCGCGTTTTGAAATTTCTGTTGTAG
AACTGATGATCGCAGTAAATAATCACCAAGGAGAATAACATGGTTTATGGTAGGGGTATA
AACGACTCAGATTCTCCCTGTCAGATCTTCAAGTGGGTAGATGGTAGACAGGTTTTGGAA
TGGAGATGCCCTTACTATGAACTTTGGATAGGGAACTTGTACAGATGTTATTCACAGCAG
TTCCACAAAAGACAACCTACCTATGCCGGATGCCAAGTGGCGGATGACTGGTTAAGGTTT
TCTGTCTGCAAAGAGTGGGTTTCTCAACAACCCCTGCACGCACAGTGGTTGAATAAGGAA
GGTGATCTTCAATTTGACAAAGACATTCTTTTGGAGGGGAACAAAAATTACACTCCTGAA
GCATGTGCGTTCGTTCCAAGATACTTAAACACCGCATTAGACTATAGTAAGAATAGCGGC
CTACCTATGGGTGTAAACAAAAAAGAAAACAGGAAGGTCTAAGCGGCGGGGTGCAGGGTG
GACGGGAAAAGAAAATACTTGGGCGTGCGAGCAACCCCCCTTGCTGCTCATGCATTATGG
CAAAAGCAAAAGGCTGAAAATCTTCCATCTGTGATCGAGGTATACACCCCATCTGACTTT
TATAATGAGCTTATTGCGAAAGCCCTGCACAAAAGAGTTGACAGAATACTAAATGACCTC
CAAAATGGAGAGGAAACAACTTACTTATGAGGAGAAAAGATTACATGATGAATCTAGACG
TAAAAGCAACCTGGACCGCCTTTTATGGTTGGTCGAAACTTGAATATCTGTGGCTGTTCA
TCTGTTCAGCCTCCATCGCTCTCGTGTCGATCATCTTGGGCGGTCGGGTTATTGAATTTA
TCTTCTCTGTGACAGGTAGTATCTGAGCGATCCTTGTTGCAAAAGGTAAACTTTCCAGTT
ATTATTGGGGCTTTGTGGCGACAGTGTTGTACTCATACATCTCCTTCACGTACAAATTGT
ACGGTGAGACGATTATGTACCCTCTGCTATTTACTCTGATGCACGTGATAGGTGTGATTA
TCTGGGCGAAAAAGCTGACTGTCTCTGAAGACGGTGAACGTGCGGATGTTATCAAAAAGT
ACCTCACCACAAAGCAGCGGTGGTCGGTTGGGATTGGAACGTTGGTTGTGATCGCCTTGC
ATGCAGAATTTGTAAGCCCTCTGAAAGGAAGTATGCCTGGACTGGATTCCGCAACAGCGA
TCCTCTCTGTTCTGGCAACCTATCTGATGATGGTACGTTATGCAGTACAGTGGTACGTGT
GGATCATTGTGAACTTAGTCGCAGTACCTTTGTGGATTCAGACAGCGCTCCACCTTGAAA
CTCAGGGGTGGGCGGTACTTGCAATGTGGATCACTTTCCTGTTAAACTCTATTCATGGTG
CCTACAAGTGGCGTAAAAACGAAGTAGCTATGTAATGATTGGATTTTGGGAATGGTTGAC
GATGCACAATAACTGGGTGCCAGGTACCGCCCTTTATATTACGATTTTTGTTTGGGCCAC
TGTCTTATCTTTCAGGGAAACACGGAAGTATAAAGTTATGGCATATTTAATGCTGGTTGT
GGTAGTTGGTACGCTATTTAATGCCTTGTGAACTGATAGTGATGGTTATCGCATGTATAA
AAATCACTGGACCAGTGGTGCAAAACCTGAAGCTGTGGCATCATTCTATGTGTTCGATGC
ACAGAAAGAACGGTGCTTTAAACCAGTAGTGGGGTTTGCACAAGTTGATGATATGCAGGT
GAAGACCCTAACACCAAAACAGATGGAGACAGGCGAATGGTTATGAAATCTGAGCGAACA
GGTCAAAAATTTGTAAAAATGAACGAAGCCAGTTTAGGTGAGAATACAAATCTGGAAGAG
CAGTTGGTGCTGGAATACTTCAAGAAGAATGTTGAATATCTTCCTGTAGATTATGATAAT
CCAACCAGTTGCCGTAAAGCCTTCCGTCAGGTCGATTTCTGCAAGAAGGGTTATCAAGAT
ATGGTGATCTGTAGCCTAATATCGGGTGGTGAATTGAACACAATCTGGTCTGTACTGGAG
GAGTTTTATGCGTAAATTCTTTAAGTGGCTACTGAGCCTGGTTAGTTCAAACTCTGTAGG
GACCATTGGCAGTGGTAACACCACCACAGTGACGCAGACCTCAACTGTGCATGTTCTGAA
GGGACGTGACCCAGAGGGGGATTAGAAGTACAAGCTTAAACCCCTGACTGAAAATGAGAT
CAGGGATATTGCTAAAAAGGCAGTGGCAGATGTAGCAGCTAAAAAGCTGGTAACAGAGGG
CCGAGAAGAGCGGCTGAAAGAGATAATGGAAGAGTTGGATTGCCAGCGTAGGAACCGTCG
CCCACCACCGCCTCCTTCACCCCCACGTCGCCGTGTGGTTGTGGATGATGATTCTCTCGC
ACGATAGAGACGCAGTGAAACGAGTTACAGTCGTAACGATGACGATAGCATTGTAACCAG
AGCTTTTGTTGCCTCTGCTGTTTATCACTCGTCAAGTAGTGACGATGGTGGAAGTTATAG
CAGTGGCCGTTGTGACTCCGGCAGTTCTTCAGGAGGATGTGATTGAAACTAACGAAAACG
TTAGTTTGTCCAAACGGTCATTCCACTACCTCAGTCGTAGAGCACTTTGTATCCACGATT
GACTGTGCCAAATGTGGTGTAAAAGCCACATATGTAATGGCACTGGAGGATGGTAATATG

GTCGCAGGAACAACATCTTGTAGCTTCCCATCGTGCCTGGATGATCCGTTTATTAAACCT
ATGAATGTAAACAAGGAGTTTATGTAATGAAATTTTCATCTGCACTGGCGCTTTTATTCA
TCGCCTTGAAACTGTGCGGTGTTATTGCATGGTCATGGTGGTGGGTCTTGGCACCACTCT
TCTTCCCGTTCGCGATTTTTGTGGGGATCATAATTTTGTTTTTCGCCAGTGTCTTTGGTG
TTGCAGGGGCGGCTGCTCTTCTGGACAAAATCAAATTAAGTAAGGAAAGATCTCGATAAC
TATTGACATACAGAGTTGAAAGGGGTAATATACCCCTTCACTAACAAACGGAGGATTGAT
GATTTAGGCAGTAACCCCTATCGAAACCCAATCAGAAAAAGAAAAACTGGTCTTCCTCGA
ACTCTACGTAGGTCAGTTTGGAACAGTTGTTCTTCTTTCCAGTGCCGGAGCACAGATGAA
TCAGTTCATCGGCTCAGAGCCTATGCGTGATGAGATGCTGAAGGTTATCCAAAGCGGCAA
CATCACTACTGTGGAAGAAGCTCTTGCATGGGTGCGTGGACACCGCAGTAACCTTCTGCA
ATTAAATGGTGCATCGTCTTTTGCCCTGAATCTACTGAGTCAGGTTGAAGATCTGGTGAT
CGATAGACTTTTGGGCGTTAACAGTGATACCCAACAAGAAGAAACTAAGTGATAGGAGAG
AGAATGAGCGTAATTAACGAATACAAAGATCCGATCTCCGGCACGTTAAACCACTTCTCA
AAGGGCAGCATCAAATTCATCAGCATCATACCTGTTAAGAATGGAGATCCTGATGGTGTG
TAACGTACCCATATCCCAGCGCGAAATGGTGAGCCTGCAAAGGTTTTCGAATCAACACAT
ACCATAAGCTTCTTGATGCAAGGGGTGGACGACAATAACAACGTTATTGATCCTCAAAGC
CAGGGTGAGTGGGTCGGCATGGGGGAGAAGAAGCTACATGCCAGCCACACCGACAAAGTG
CAGGTTAAGCTGGACTCTGGTTACAAAGACATTCTGCCTGGTATGGTTGCATCTTTCCCG
CTAAAAGTGAGCACGAACGGCGATGAGACCTATGTCAACGGCTCTCTGAGTGGCAAGACT
TTCAACATTCTGGACGAAAGCAAAGCAGGTCAACCTGCACCACGTCAGCAGCCGCAGCAA
AGTGCTCAAGGTCAACAGTCTGGTGGTGGGGTGAAAGTTTACGGCGAGATAACCGCTATT
GCAGGGAATCTGGCGACTGTAAATGATGAGAAGAACGGCCCTGATAATGTTGTTCTGTCT
GATGAGCAACTGGCACAGGTACAGGTTGGTGGTCGCCTGACAGCTTTTGTAAATCTTGCT
GATGGCACAATCCTGAACGGCTTTAAAGCTTACGGCCCAGTAGGTCAATGAACTGGCTCA
CGTGGTAAGAAGTCTGGTGGGAATTATGACCCTATTGGTGTAGCTTGTGGTCACGGTATC
AACGGCCTTAAGGAATTGATGGGTGCAGGTTACAAAGTGAAGGATGAACTGGAAACAGTA
AAAGCCATTCACCGTGCAACTGTAGACATGAAGGCTTTCGTTGCGGAGCGTACTGGTAAG
GATGTTGAATCAAACGCTGTTGGAGCAATTGCAGGTAATGCAATCCTTGTTGCATGTAGC
CGATTCAATGCCAAATCGGAAGTAACTGAGTCAACGATCCTGGAAAGCGCCAAGAACGTG
TATGTTACGCTCTCTGAGCCGTTTTATGAGTTCTTGACTAACGATGGTAAGCCTGCGAAT
CAAAGGCAACCACAGAGTTCTCAGGAGCCTCTTAGCGGCACTGTTGATGTACCACCTATG
GATGCTTATGAGCCTCCGATGGACTTTGCCGACGATATGCACTTTGCCCCAATCAGTCTG
CAATATGGTAGCAACTTCATCCATTGCATTTAATAAAAAGAAAAGGAGCCTTTCGGCTCC
TTTTTGTTTTATTGCGGTTGAGAGGGTACGCCTGGTTCGCCGGGATCCACAGGATCTGGT
GGGGCTGTTAAGTCGGAAACACTGACATACGAATGATCAAATGCCTCAACCTGACCTTCC
CTGTAAATAAGACGAACACCACAACGAGCATCACCAACTGCAATACAGGAAAGGAATCCA
CCTTCATCAATCATCACCACATCTGGATTTGTAGTTGTTAATTCTCCGTCTATGAAGTTA
GTATCAACATTGGCTGGAGAAATTTCAATTCCGACAACGTTCGGACCTGGTGTATAGTCA
TGACTGTTTTTATCCATGAACACGAACACATCACCAACTTCCTTTTGTGGGATGGAATCG
GTAGTCACAGAGAAGATGGAAACATAACCGCCAGAAGTATCTGTGTGTGTTTTAGCATCC
GCGCCATACCCTCTTTTAGCAGTCATTGTGATGTCAAATCCACCCTCTTCAAGGAAGGTC
ATAATACCATTTGAGTCTACAGTTGCGATATTAGGAGCACTCGATGTGAACGAATTATCA
GAAATCTCAGCATCTGCTGGCTCAACAGTCCACGTAAGTTGACTTCTCGTCCCTTTGATA
GCGTATGGGATAGACCCTGTGGTGATAGCATCCGCAGTTGTCCCCCCAAAGGATGCTTCT
GGGATGTTTGTGAACTCACCAGTGCTATCGGTTGACCCAAATAAACCATAAAGGTTACCA
GCGTAGTAAAGGTTCCCGTCATACACCATCCAATGGCTGATAGAGTTCCCCTGGTTAAGG
ACATCTGTCTTCATTTCTACAATGTATGTCAGCTTCGAAAGATCCCAATCTCTTCCTGTA
GGAACTGGAACAAGGGTCGGTTCTATCACCATTGTTGATGGTGTGCCAAGACCAAGTTGA
GCAGTTAGGTCACCAAGACCGTAAAGCTTGTTATCAGCAAGCAGAAAATGAGACTGACCA
CCGTCGATAACCCTGAATGTCTCGTAAGGACCAATACCCTCCTTCTTAGAGTATTGGGCT
GTCCCGATGCCCCTCGTATATAAATAATGCTCTGTATCATCAACACCTGTAAAGGCGATA
GCCATTAAGTTTGTTTCGTTCGCTAACCACTCTTTAACATAGACGGTTTCAGCCACAGAG
GCAAAACTAACATCGATAAAGTTTTGTGTTTGTGTTGTGGAAGTACAGTCTATTTGATGG
GTGGCCCCACAATCACGGGGAAGTCCAATATTGTTCAGGTATGTCACGCCAGCATTTGAG
CTGTAAGCTATCACAGAGGATGCACTAATACTTCTTGGGATCGATATTACGGTAGTATTA
CCAGAGCCAAGGCATCCATTTGTGTTTGACCCGCTTCCATAAAGTCTTCCCTCTACCATC
TGCAATAGTGTGTTACCGAGAGCACAAGAAACACTTTGTAAATTTGGGATTGGTACTGTG

CCTGTTATTGATAAAGGGGAGCTTGTCCAAGAATAATAGATAGACCCACTACCAGTGAAT
TGACAGGTGTCCCCACAATACTGCCAGGCACCATTGTTGTATTTGACAATAAAGGCTCTA
CCACCACCAAAAACATCAGCTACACCATGAGCCGCCAAGTGCCAATGATCCTTAAACGGG
CTTATGTTCCCGTTTGCGCATTCAGACCACGAATTATCCCCTTGGGTATAGAGTCTCCCA
TCAATCAGGAGGTTCGCCATTGACCTTTTGGTAGCACCACCAACCAGACCACGGGTAAAG
GCCACCTTCTTAAGACTACCTGGTGGTAGTTGTTCTGTTCCTCCTCCAGATAATGTCAAG
AAGAGACATGGAATAGGATACATTAAAACGTCCTAAAAGAACGACTGTCAGAAGACAGCC
TTGAATCATTACGGACGACGAACAATTACAGTGTCGATAACACCGCCTTCAACACCATTA
TAGGTTAATTGAAGAATAGCCACCGAACCAGCCGTCTCGTTAATTGTATCGCTGTTTAAA
ACCAAGTATTTAGGATCCAAAGTCACAGTGTGACCGCCAGTACCATCTTGAGTCAGGTAT
ATGACAGCAGAAAAAGCATCTGTCGGTTCAGGCCAAGCACCAATTGTTAAATCCGCCTCC
ACTGTTACGTTGTAGATGTTGGAATCTCCACTTGGGGACGATGCCGCTATAGAGCTATCA
ATATCCACAGCAATAAGTGTCAGGAATTCAACACTTGCCTTGGTTAAGATTGCCGAATCC
TGTACCGTAAGGCTATTGGCAGTTACTGCACCAGTAGAGGAAAGAGATCCTACGGTGATG
TTCTGACCTGTCAAGTCAACAGAAAGACCTGTAACCGTACCCTGAATAACAAGGTTGTTA
ACAGTCGTTGTTCCTGTTGCGTTGTTAAGGTTGACATCAACGTTTGCGGTCAGGTTATCC
TGGACTGTAACATCGGCAAGTACGCTAAGATCCTCAATAAACTCAGACGTTCCTGTAACA
GAGAGTTTTCCGCCAATTGTACCATCTGCTGTTCCGGTTGTAAATCCACCATTGAGACTG
GTTAACCCACCCACTGTGAATGGACCAGTGGTGTTTCCGTTACCCGCAGAGAAATCTTCG
AGTGTTGTATTACCGTTTACGGTAAGATCACTTGTAATATCAAGGCCACCGCTTGTTGCG
TTTCCAAGGTTGGTAACACCACTGACCTGTAAGTCACCACCTACAGTTTCACCGTCTGCG
GTTGTAACAGAGTTTAGTGAAATATCAAGTCCGTTGACACTGAAGGTGATACCTGTTACG
TTACCTGTAATATTCAGGTTTTCTACCTCAGTTATACATGAGACCTTTAATCTCGGTGCT
GGAGGATCTTTACTTGCGTTACCGCGGATATACACATAATCGAAAACGTTATTACCCACA
TCTGTCAGAGACATGGCTGTATCATCTTCAGCCACTGTTCCGTTGTCAATAATGTCCGTC
TGGATGCTGTGGGTTTGGATCGCATATGGGTCAAGGCGCGTCTTAAAGAATGTCGGATTA
TCTGCGTCTGTAAATACGAAGGTAAGACTTGACAACTCTTTGGTCAGGATCAGGTTCTGG
AAACGACCTGTTGAGTTATCTGCGTTTACAGGGCCATTAAGCTGGATTGTCCCACCAACC
ACCAAACTATTGAGTGTTGTAGCACATGTTACAGACAGGTTTGCAAAGGTAACACTACCA
CCTGAGATGCTTGACAAGGCGCTTGCTATAGCGGTTGTAACAAAGGCAGTTGTGGCAATT
GTTGTACTGTTGCTGTTAGCCGCAGGGGTTGGAGCCAGTGGGTTTACTGTCAGGATCGGG
CTGTTGATTGGTGCATAGATAGATGCCAGCGCCGCTGCGTCGTTGTACAATCTCCAGTTA
ACACTGTTAGGCGGGTTTTGTGGTAAAACGACAGGGGCGCTTGGCGTTGCGGTTGGTGCA
TACCACCATTGAACCCCATTTGTGCCATTAAACGCATAAAGCTGGTTGAATACTGTGGTC
TGAGTACCCGCGATCCAAGGCATAGGCGGTGTTTTAAAGAACAGGTTGTCAAGAAGAGCT
TTACGCACCGTTGGAATGGTGCTACCATCTTCAGTAACAACAGTTTCGGATGCCGTTCCG
TTGACAACCTTGTGGAGGCGTTCAGAGTCTTCAACAACCTGATCGACAGCCTGTTCAAAT
TGAGTCATGTCTGCCACAAGAGTCTTCTTATTGGTCTGACTTTGGTTCTGTACGTTGGGC
TTCTGTTACTCCCCAGCGTAGAGATATAAGCTTACTGATTAGTTGAGGTGCAGCAACTGT
GGGTGCATAAATCAACATGTACCACTCGGTCAACGTTCCATTGAATCCCCAGTAAAGGAA
ATCTGCGGTCATTGAAGCCAAGCCGATATTGCTCCAAAATTTTGTGTGAGAAACCCTCTC
AGGGTCTACAGGTGATCTCACAAGATCTTTAATAAACTTCACTAGTGATTTTAACATATT
TCCTCAAGATTCTCAATGAACTAACATGGGTAGGAACCTTGCAAGAACCACGAGGCTTAC
CACAGTAGCCGTTTTTAGCCACTCTTTAACCGTATTGTATTTAAATGATGCACAGAGGAT
CAATACTTCCATAACGGTCTGATATGAAGATTTGATGACAAAACTCAATGCAAGTGGCAA
AAGATATGAAAATTGCATGACTATTATCTGGCAGAAGAGTACAGGCACTGATACGGAAAT
GATCAGCTTCTTCTTCCATCCCGACAATACACACGCTGTGGCTAACAGGAGGGTTATGTC
GAGCAACCAACATAATGACATATATAGAGACAGGTCAAAGAACTGGATATGAACAGAAAA
CCACGAGTACAGAATGTTCTCCAGAAGGATATGGATCATACCACAAATAGACATGATCCT
TATACCCTTGTCTCCATTAAGCAGCAGCACTGCAAAGCATAAGAAATACAGTGCTGAACT
TATCATCGACGTTGATTCTCCATGAGTAGCTTGAACTGATTGCTGAGGTCAGCACGAAGA
TCGCCCACGTCTTCTTTAATGTCCTTACGCAAGTCTGAAACGTCATCACGAACCGTTTTG
AGTTTCTTATCCAGAATCTCGTCAAGTTTTTCCTTAGTGATCATCTCTGCCTTAATCCTT
ATGATGTCTCTATCAAGTTCATCAAGGCGCTCTTGCGTTTTGTTGTAATCTGAGTTTGTG
ATTCTCAGGACACCAATTAAAACAATCGTGATAACGCCCCAAACAGCTTTGAGGATAGCA
AATAAGTCAATAGACATTATAGAATACTTTTGCTCTTGGCTGGTCTTTCAGGCCAATTGA
TATTAGGAGCATCCTCCGGGTATATTGCTAAAAGATCAATACGGTACGTCTTTAGCTTTT

CGAGATACGCTTTCTGGGAATCCAAAGTAGGTCCTCTGGAAATCAAAACTTCAAGGGCTA
AAATCTGGTTTGTAGCCCAAGACATTTCACTGTCCAGAAGAACTTCGGCCTGTGCAATAG
CCTCTTTATTGTAAGCTTTAAACTCCCCAGAGATGAATTGCACCGCCCCTAAACGGGATT
GAATACCATCAGGTACTTCATCAATAACAGCAACCTTCATTTGGTCTGGGAAGTATGCTG
ATGGATCAGAAGACACACACAACACGACATCACCGGAAGACAGAAGAAGGAACTTGGTCC
CCTCTTTCAATTTTGTCAACTCTTTCACTATGACGTACCAATCTTCCCCGTCTTTATCTT
TCAGTAAAACTGCACCTGACTCCAGGAATGTGGCGTTCTCGTCCGTCTTTGTTGGGGTGT
ATAATGAAAATTCACCATATACCTTTGTCATAAATCCTCCTTAGTCCAGGTTGGAAACAG
TTACCCATTGTCCGTTAATGAGATACTGTAGTGGTGCAGTGTAGTAAGTTGCGCCCCTCA
ATCCTTTCTTGTTACCGAAAGCCCGCCCGGTCATGACCTGACCTGCATAAAATCTGGATT
CACTCACACCTGTGTCAAACCTATTACCTAAACGGATCTGCTGAACAAGTCCAGACAGGT
TTAGTGTGACGTTACCGTTGCCGTCTGCCGTCGCACCATTGATTGCTCTCACAACGTTTC
GTCCACCAACTGTAGCGGAGTTGTTGTTGACAACAAGGGAGTTAACGTTACACTGTCCGT
TTGCGCTGGCTTATGGGGTTGTTAAAGACCTTCTTGCGACAACGGTGAACCCTTGAACGT
TTCCTGTTGCCGTAAAGGTCCCTGTCCTGCTTGTTGTGGACTGAATAGTTAGAGCATTCT
GCGTACCGCTTAAGGTAGTGTTTGTGAAGTTTGCAGTGGTTGCAGTGACGCTTGCCCCGC
TTATACTTCCGTTTGCGATAATGTTTCCAGTTCACCTACACAGTACAGCAGTGCCACTGA
TCGCATTCAACTCCATCATTGTGAATTTTGAGCCACCAGAAGGGACGAAGCGGAAACGCA
CACTATCGATGCTCGACGTACCTGGGGCATTACCGATATTATCACCAACTACAAAATCAA
ACAATGTGGTGTCTGCTGCTGGGTTTGTTGTCGTGCTGTATGCGTAATCTGATTCGGCTG
GTTGGAGGCTCAGGCGTCGAACAGATAATGCACCAGTCATGGTGTCGCCTGAAGTGTTCA
CACATCTGGTATCAGACTCTGTTTTACTCCAAACATCGAGGTTTGTTCTGGCTGCGGCCT
TGTCTGCAAGGTCTGCAAGGGTCTGATTCACACGTAAGAAACGACTGTCGAGACCAGGGA
TCTGCTCACCAGTGATATACTTGGCCCATGCAGTCAGCATATTTAAAACGGAGTTAAATT
CTTCGACTGTTGGCTTTTCACCAGGGTCCCAACCTTTGTTCCACAGGTCATCGATAGGAC
GTCCTTTGTTTAATTCCTGGGTGTTAGGAAGAACAATATCTCCATAAGCCCAAATCTCAA
TGGGTACTGTTGGCGCTGCCATTGTAATCTCCCTTAAATCAAGGTATTCTCTTCATCAGA
CACATACGTCAGGTGAGCAAGACCACCCTCTCCTTTTCGGCTTCCTGAGTGGATAGAACC
GAATCCGAGTGCATGTTTATCGCCTGCGAGTCCAAAAGCCGTACCATTTGCCTCTGTTAG
TCTCAGGTGTGTTGGCATTGGCAACATGACTTGAATCTCCTGGAGAGCAGTCTCCAATTC
GAAACATGTTTTGGAAATGTTAATATCGAAACGGTAGTTATCACCCGTGTATGTGGTAAA
GTTACCTTCCCCGAACAACTGGTTAAGGGTATCGATAACTTCAGGTCTTGTCCCGTGCTT
CGCAGCACTTGCCAAAAGGATGATAATGATGGCACGATAGTTCATATCGCTCACGACATC
ACGCTCAATCCCGACCTACTTCCCTATCTCATCAAGGCTGACACCTGTCGCATTTTTCAG
TAATCTGCCCTCAGACATGTTGACCCACATTTCATCAAGCTTTTTGAGGCGTTCCAGATA
AACCGTCATAAATTTGACAAGGTTTTCTTTCTCTTTCAGAAAATCGCCAGGGAGATATTC
AATACCTCCCTCAATGAAATCTGGCAGAGTATGGATGTGGTTAACATCCTTTAAATAGTC
TGCCATACTTACCTCATTAGATGATTTGCTGTAATGTACTGTCGTCTGTATCTAAAGCAA
AGACTTCTGTCATAGCTGCAACAACTTCGCTGTTAGAATAAGCACTGTCAGGTTGGTCAG
TGTTCTTAACATCCACCAACAATTGCGTGAAGCGCCCGGGTGAAATAGAGGAACCAACTG
CGGATACCAGCTGGATGTTCTAAAGTGTATCTGCAATGTCGAGAGGATCAACAACGGCTT
TCAATGCATCTTTGATTGTGTTTTGCTCTGTCACGGACAGGAGCTTTCCTTTATAACGAA
CACGGACTGCAAGCTCACGAGCCTGTGCTTTGCTGTGATAGATACGTTCCGTCTGATCAT
CCTCTGTGGTAACATCATAAAAAACATTACAATAGGTGGCGTTGTACAATGCGATTGTGT
TATAAAGTGCTTCACTGATTTCTTCTGTGGAACCACCATAAACTACAGTCTCAAGCTTAT
ATGACGGAAGACCAAACTGGTCTGTTTCCCCTGTGTTGTTTGAGAATACACGAACCTTGC
TTACGCCTTCGACGATTACAACGGCAGAAATAACGGCAGGTTTAGTGGCTGCAGCACCTC
CTGCGGTGCTTTGTGAAGCACGAACCTCGTAATCAGTGTCTGTCTCAACATCGGAACCGT
CATTAAAGGCGGTCATGTTGGTCATGCTGAGAAAACCGCTTGGTGTCGGTGTGAAATTCG
TTTCAGTATTTGCTTCACGAGAAATAGGCCCTGCTTCAGCAGCAATTACTGCAATGCTAA
TTGTTCTCTGGCCCACTACGGGTGAGGAACGGAAATCAACACGACTGTTCAGTCCGATCA
TTTTCTTGTTAGCATCAGACCCGATATACATCGCGCCACCCGCACAGTCAATGAAGATCC
TGTCTTCGTTTAACTGTGTGGAGTTGTCAACAATGAAATCCTTAGTCGATGACATGAACG
CATTTAACTGAGGGCTGTTAGGCACCTTATTACTTAGCGTCAAGTTCATTGATTTTGTTG
AACCATCGTTCTGATTAATCATCTGGAACGTTTAGTTACCAAGAACCCAATCTTGGTTAA
GGATTTGTTGCGCCAGGATATTCCCTGCGACAGGTGTGTCCTGCGTGAGTACGAAGTTAC
CACTGTCAATGCTATATGTTGAAGAACTGTAAATCATGTTGTACGGGACGGTGTTATTAA

CAACCATCTGGATAGTACCTGATCCTCTGGTTTTACCACGACGATAAATACCACAACGAC
CAAGCAGGTCATCATGATAAATACCTTCTGCCCCTGCAAGTGTCTGTGCAGAATACACAG
CCGCAGCCAGAAGGATCAACTGATATTCTCTTTCATTCAAAATTGTGGTAAGTTTGTCAA
CAACAGTGTTAGCCTGAGTGTTGAAGTTTTCACCGAAAGTTCGGATCAGCGACTGCTTGG
TAACCGCGATTAAATCATCGAGGGATGGGATCGCGAAGCCGTAGTCATTTAATCCATATG
GAGCAGCCACGAACGCCTCCTCTTGCTTGATTTTTGCTCTAAGATGTTGCTATTTTATCA
TATTGTGATTTGATTATAAAGGCTTATAGTAAGAAAAGGGGCTGAAGCCCCTTGTTATTT
AAGTCTTTTCGCGGTATACTACGGGATTTGTCGAAACAAGAGCCGTGTTATCAGGTGTGG
CTGTCTGGATTGGATCTGCAATGTCCTGATATAACGGTGTGATCCAAGCACCTTTTTGTT
ATGGCGCTCTTATAACCGTTGTCAGGAGATCCGCACCACCGGGGCCTGCGCCTGGGATGG
TGAGTTGCAACCAAGCAGATTTAAATGCACGAGTAGCCCAAGGTTGTCGGATACTTGAAT
CTGGTGCACACTCAATCGTATTTGAGCCAACCACAGTTGCCTTGATGAAATCATTAGGTT
CAATGTAAGAACCCCCTGTTTCAACAGCAAACATACCACCTGTCTTCTTCAGAATGATGT
CCCCTTTAACGAATTCCTTTCCTTGAGGAAAATCATCATTATCAGAGATATAGATGTTAC
CAGCATTAAGCAGTATGACACCTCTGTTTCCGCTTGTCTTTAAGTGCTCAACCAAATGGA
AGTTACAACCGTATTTTGTTAAGTGGTCAAAGCCTACCTGACCGAACTTACCGTTAGCGA
CACCATTATCATCAAGGCTATTGATATTACTGATCTCAGCAGAACACCAAGCCCAACGCT
TCATCGCACCGCCTGCCTCACCCTGTACAGCTTCATAGTCGCCAATACCATCAAACTGTT
TACAGCGGATGACAGCAAGACGAAGTTTTAAGACAAGACCGCGATATTTGAAAGTGTGGC
ACTCAATGTCCTCAACCTTCACATCGTTGGTCCAGTCATCCATAGCACTAACTGGAATAG
CTACCAGTCCCGTATGAGTACGTACATTTCGCAGTGTAAAGTCACGGCACGGAGCGGCAA
AGGTTCCTTGGTTAACACCCCACCCAAAATAAAAGAATCGAGTAGCACCGTTGACCATTT
CGCCACGAACACCATCAATGATATAGTCTTTGTAACCATAACATACGACACCACCGGATG
CCAGCAGTGTTCCGTTAGAAACGGATGCATCAGGATAAACCTCAACGTTCTCGACCTTAA
AGTCACGGCACAGCTCGAAGTGGATACATTGACGAACTCGACGCATCTTAACGTTACGAA
TGATGACCCCACTAACATATTGGTCATCAGAGGCGTTAGCACCATACGGTCCCTGGCCCG
CAACACCGATACCAATACCCCAGTTAGTAGAGTTATCTGTATTGTTGAAGTTGTCGATGT
CAATATCTTCGATTGTCAAACCACCCGCTTTATAGTGTCTCTGTACCACGTTACACTCAA
TAGCATCGCCTGTCAAATCGTTAAAGTTGAGACGGGCAAAACGTGCGCGAGTAACAAGAG
CGCCTGTACCTTGCTGAAGGATACCATATTTACAACCTCTGAACTCACTATCCAGGATGT
CAATTGTAGCTTCTATAGGGAGGGCTTGTAGTAACCTCGCTGCGGTGTTGGTATTGTTAA
CCCATGAGGAGTCTGTAATGATCAGACGCCCACCATGATGGCGAGTACAAACACCTGTGA
AGCTCACCCAGCGGAATGTGCTGTTTGATTAATACATGTATCTGTTGCCGCCAGTCTGGT
TAAAGCTTCCACCATACCATTCAACACCGTCGATATACACACTTGTCTCAAGAACAAGGT
TCCATCCCCATGCAAGATCAGCTTTTTTGTTCTCTTTTGCGTATGCAGCTAACGCTGTTA
CAGCCTCTGTCGTTGTTGGATATTTGCCCATGAATTCTGCAGGGGTAACAAAACCATCGA
GGGATTGAGCCACTGTTCCCGCTGGTCCACCGATAAAGGTTGAGCCATTAGCACCTGCAA
GGTCTGCACGTAGAGATGGGTCGTTAGCTGACCACGCCCCTTCACCAACACCACCTGTAG
TCTCCGGTGTGGAGTCGGCAGGAACAAGTTTCGGGTATGCCCCGGTCCATTGATAGATCC
TGTTGGTTGATCTGTCGATCACCTGATCTTTGGGGGAGACAAGCGTCCCCCTTCTTGGA
AGGAAAACAGGCGTATCAACCCGAAACTGGAGAACATATCTGACCATGTTGATTTACGGG
TTGCATCCGCCTGTTTAACAACCAGGTAGTCTCCCTCGAAAGATCCCGTCGCAGTCGGTA
AGTCTGAGACCTTGACGGTAGGTAATAGATCTGCCATTCAATCCTCTTATCTGGTAATGT
TTGTGTACATACCATACAGACCAAATCCTCTTAACAATTCCCCGGCGTGAAAGCCGAAGA
ACATCCCATTGTCAGTATCTGCACGAGGAGCAACAGGCCAACACTCATTCATCGGAGGAT
TCGGAGTCTGAATGATGGAGTTATCGTGGATAAGACCGAAACAACGTTCTGCTAGCAAGT
CGATGTTAGGGATACGATCACGAATTCCTGCCATCCCTAGCATAGAACACCCACCCATGA
ACAAGCCTGACATGTGGCTGGTAAAGTCATCTTCAGGTGCAAGCACCGTTACATCAGGAT
TAAAGATGGTAGGAGTTCTTCCCTCGTTGTTGTCTTGGAACCATTTGAGATAATCAACAC
AGTTCTTCGCAACAGTGAATAGCTTACTTGGAACAGCTTCTCGACGCTGAGACAATTCAT
AAACGCAACGAAATACACAGAAGAATGCACGGGCTTCATATACATCCCAAGCCGTTTCGT
TCCAGTGCTTCATTGTGAAAGCGTTTGGCTCACCGTACTTCTTCGCATCCCAACGATCCC
ATACAAATTCCTGCGCCATTGGTCCAGGCATGACAGGATTGAAAGAGTTATAAAACCAGA
TCTGCAAATCATAAAGGAAGTCAATGGTGTTATTCAATCTTGTCCAGTTGATGTGATGAC
CTTTGAAACAATACAGCGTAGGGTACTGATACCCAGGGTACGGAAGGCCACGCCAACCAT
CGTACAGCGCCGTGTTCGGGTCACTGATGTTGGAGAACGGGATGAGTCCTGGGGTATAGT
GTAGGCTGTTTGTCTTATAATCAATGATCGTACAGTCGCCAAGCTTCGCACTGTATGGGT

TGTCTCCGGACACAGTGATACTGAAATACCTAGTATAATCGTCACCACCTGTGGAAGTGT
ACAACTCAGGACCAGCATTCACACAGTACCAATCGATCCGTGCGCTGACACGATCAACAG
GGTCGGTGTCAGGTGCCAAGTTAATCTGCTTCAGATTATGGAGATCGATTTGGTTTGGTC
GTCGATCAGTTTCCGGATGATCTGGTTGATAACTACTTCGTACAAACCTGGTTGTCGTAA
TCTGAAGTGTAGTCCAATCACCGTTTGTTGCCGGGATCATGGCCCACCATCTCCAGTTGT
TTACATCCGTAATCGTAATGTTTAAGTTATCAGCGTAAGAGCGATATGTAAACGCAGAAA
GCTCTGCTGTTTTGTTATCTATCAGCCAGAAACCAGTCGTACAACCACCATCCGCATCTG
TGATGAGGGTGCAAGCTTGGTCATTAATCCTTCCAAGAATACCAGTCTGGTACTCAAAGT
CAACAACTGTGTTGTCACCTCAATCCACAACTATACGAGGGTCTGCAACAATGGAGTTAC
CACCACCCGGAGGTGTTAAACGAACAAAGTTTGACAGGGGGATGTCCATTGATACAATCG
AGTCAGTACCGCGAGGAAGATCACAACGGTATGTCACCCGGCCTGTTTCAGACTTCGTTT
TATTGAGGTCTAGTTCAGGTCTGAACAATAGCCAATTGCCAAGGTCGTCCTTACCTCCAA
ACTGAACACGCAACTTGGAGTTACCGTCTACACGGAATCAGATCGGCTGCTCTTCAAGGG
TTGTTTGAGCAGATACTGCCTGACGAATACCAATATACCTTTCCGGGTCACGACTATACT
CAGGGCCCGCTGTTGAGGGGTAAGGGTAAACGTAGCTGATCCCGTCAGTGAACGGAATGA
TGGAAAAGGTCGATTTGCGGAAAAACTTATCGAAACGGTCAATGTCAGTATAATTGACAC
AAACGAGCAAAGACGACAGCCAGCAAAGCCAGTATTTTCTTTCTCCGGTGATCTGATACA
TAACATAGTAGGCATCACACCACCAAGTTTCAGCATAAGAGGCGTTGTCCTGAAATCCCA
TCTCAATAGGTACGTTGACTGGTCGGTTATGCCACATCGTGTTCCGATCCATCCAGTACC
CACCATGCTCTACAGGGTTGCAAGTTGCATAGTTGAATTTGAAATTTCCTGTTACCGTGG
TATCTTTGAGTTGAACAGTCCCCCACTCCGCCTCTGGGAAACCCTCTTCAAGAATATTAC
CGTCCCAATCAACTTTTCTGCCGAGTCTGTGAATGATCCAATCGACATCGCACTGAGACC
CATACTGATCCCAATTTGTCGTACCATCTGCGTTAGCAGCATATACTGTGGCGTTAACGG
TATTCCACCCAAGATTACCATCAAAGGCGAATCAAGCCTTGTCAAGGTATTCTCCCCAAT
GTGGAGCACCTTGAGGAATTAACGTCTTACCATTTGTCCATTACATAACGCTACCCTTAT
ACCCACCCTGGGTTGGGTATCCATCGTCTGTCGGTGGGTAATGTGCAAGCCTTGGCTCTT
TCACGTCAATGATCCAGTTCGGGCGATACACTGCCGGAGGATCTGAGATTGGCTGACCCG
CGAAGAACCAGTCGATATATGCTTGCCAAAAATCTTCTGCAATTTCCAGATATTTAGGGT
CGTTGGTTGCAAGATATACATATGCGCAACCGAGGATCAAGACAGACTGACCCTCGGTAG
TTGCATCACCGTTAGGCTCGGCTTCTTGAGCCGTCCAGTTGATAAAGATGCGGTTGCTAG
CCAAAACTTTCTGTAAGTTCAGGATGTAGTGCTGAACTGTCGGGTCAACGGTCTCTCCCT
TGTTTCTTTCAAGGAACTGTATGTGACCATCGATCAATTGAAAAACATTATGAATACTGT
GGCGTAACTGTGCCATTACTCAACTCCTGCCATAATAAGGGAGCCGAACCAACAATTCCC
ACCATCATAAGTGACGAACCGAAAGATGTCTCGGACCCCGGTGTTATAAGAAAGAACTGG
AACACGCCCATAATTCCATTTAATATTACTGGGCCATGAAACCAAATTCGCCCCTATACC
CTGTGTTAAGGATAAGGTAAACGACTGAGATTCATCCTGATCCGTTGATGGATTGGTAAT
TACGACAACGCACTGACCACCTGTCAAGTTGGCAACAAAAGAAACACCCCTGTTAAGGTT
ACATGTTGCTGTCCATTGCACATTGCCAAGATTGACAACCTTACCTGTTGAACCGCCGCC
ACCACTGGCACCGAACACTTGCCAGGCGTTCTCACTGATGCCGCCTGTGGTTGACGGGGA
AGATGACGTTGGAATTGTTTCGGGTAAACACCATTCCATGTGTAGTACTTACCATTCGT
ATCAAAGAATGCAAGGTCTTTCTTGGATTCAAGGAACCCACCCTCAGAAAAGAAGACAAC
GTTCGTAATACCCATACGAGTCATGATGGTGTCAAGAGAAGCCTTCCTGGTTGTGTCTGC
CTGATCCATAACTACATAGTCACCACCAGATACGGTAACGGCGGAAGGAAGGTCAGAGAC
CTTCACCGTTTGAAAAGAAACATCTGCCATTATTCACCCCTTAGTGGCTACGTTTGATGG
ACTTGCCATCCTCTGTGGTGATAATCTTATTCTTATCATCAAGTAAACTACCAACCGGAA
TAGGGTTATCCATATCAGCACCAGACCAAGTATTCCACCATGTAGCATCGCCAGTGCGAG
GAAGACGGAAGTTGATCAAGTAATAAAGTTTGTTAGCCCAGGTGATCCAACCATCGTCAT
CACAAAGAACGGTTGCACCTTCATCAGGGGTTGGGTAATAATACTCGTCTTGACCTACAA
AAGCGAGTCTTACCACTTCCTGTTCTTTGGTGGTGACTTCATAAAAGGCTTGATAACTAT
GGGTTGTTCTATCCATAGTTGAATAGCAAGAGTTGATTTCAAGAACGTCAGGTTCAAGAC
GTGTTACCTCCTTGATCTTATTGTCAAGGACAGTCTTCATTACTTTTTTGCTTAAATATG
CTCGGTATGGGAAACCAAATGTCTCATCGAAATACCAGTTACAAGCCCAAATGTTGAATC
TTAGCCACAGGCAGTAACGAAGGCTGGTCTGGTTTGACTCGATAAGCTTAAGCCCCGAGG
AGACATCAAGGTTTCCTGTAACTGGGTCCATAAGGAGATCTAAGTAAAGAGTTGCCATTT
AAAACTCTTATTTGTTGGGTGGGTTGGTATTACCAGAACCACCATCATCTGTCCATGTGT
AGAAGTGATTATGACTGACAAACCAATCATAGAAATCATTCATATTCACACCACGGGCTG
TAATAAAGTTGCCATCTGGTGTGACTGTTGCCCCGTTCATTTCAATATTCCCATCCAGCT

TTGCAGTCAGTGTTGCTGCACCATTGGTGAAGGACATTTGACCAGTTTTATCAACAATGA
ACGTTCCACCCGGACCAGTTAATGAGAAATCCCCTTCAGGCGTCATGGAGAAGTGAACTT
GGTCGTTCCACAACTCAACATTGTTTGGGTCTATAGTCACGGCGTTGCCATCACTATGAA
GACCGATAATGGACCACCCGGGAAAAAGTCCGTGGGTGGTCGTATCATTAGCGTGACCTT
CATTGCGCTCAGAGAAGGAGAGACCAACAACGTCACCAGGCTTCACAGGAGGCGTCAGGC
GAGCTTTTCCTCCATTAGCTGAAGGCATGGATAGCGGACATCGAAAACCGCAGGATATG
CGTCTACGGTGCCATCGTCGAAGTTTGTTGAGGCTAAGGGTTGGACAGTGGCAGAAGGGA
TTGCGTAATCCACATCAACCACTCTTGCCCTTAGTCCTGTGTGGATGTCGCGAGCTTGCC
TTGCGAAGCATTGGTTAAACGCAGCATACATACGTTGAATTGATGCCATTATACCTCCAC
TACTCCACCACGGGTCTCTGCAAGACCGATCTCAGACACCCATTAACCACCCTCAAGTTG
CCCTTTGTGGGCCAGGTACACAACTTTATAAAACCCTGTGTATTTCTGACTTTTCACGTA
AATCGTGCTTTCAGGAATGATCGCACCATTAAGCTCTGTCACCACAACAAGACCTGCGTC
TTCTCTGATCTCGGCACGACTTGGACCTTTGTTGGTTTTTCGTTTCTTTTTGGATTTACT
TTTCATTTCCAGATTACGCTTTGCTGGTTCAGGGTTTTGCGGGGTTGGTCAATCAGGCAT
ACCTGACTCTGCACTGATCTCAAAAACAGCCGTCTTAAACCTCTTGCCTTGTGTTGTCCA
GTAAACTGCACCATCTTGAACACTGAATGTTGATCCAGTATTCTTCGCCACCCTTTCAAG
ATTGTTCGCAGCGTTACCTGTGAAAGCAATTGACTGCTGGAGAGTCTGACCACGGAACTT
AACAACCTGCGCAATTGGTAATTTCAAGTCTGAGATACGGTCGTTAAGAACTGTATCAAG
AAGAGTCCCCTTCTTATAAGACCTAGCGGTCTTTGCAGTCATAATGTTGGTCGTTCCATC
ACCCAGGATCATCTTTGGTTGTCTGGTGTGTCCATCCCATTTATCCTGGACAAACTCAAC
AGTCCCTGAAAAAATCATCTGGTTTTCACCATCAAACCCCGCCTCAAGAACAATGGCAAT
AGACTCTCTCAGGTTAGCATCAAGATAACTAACTGTTGCGTCACTCAGGTTATATAGCGT
GACATAACCTTTATTCGGGTCTTTGGAGTTATCCTTCTTCACCTCAAACTCGATGTTTGC
AGCGCCTGTTTCGTTACTAACAACATACGTGTTTTTTGTATCTTGGGTGGTATAATCAGA
GATGTTCGTTGGTTTCTGTCCAATATCAACAGGTTTGCCAATGGTGAGTCTGAATCTACG
TGTTCTGTAAGTCCGCATCTGTTAATCCTCGTCTTCAGTGACCTCGTCGTCAGGAGACGA
ATAAAGGAGTTGCAAGTCAGACAAGAACCCGATATTGTATCTCCCCACACGTTTGGTTGA
CTGGGTGAATGAAAAGACACGCAGTTGCCCTGAAGGAATGTTTTCCAAATATTTAAAAGG
GTCACAGATGTCGAAAATTGTTGTTCATCTGAAAGAGATGGTTGGATCAGAGCCAACATC
CCCAAATGAAACGAGCCAAGATTCATCTCTCTCCTTCCACTGGAATCTCATCTCGTATGT
TTGACTGTCCAATACCACACGCATAGTTTGGTCAGCAAAACCATCAATATCCCATAAGAA
TGTGATCTTTGACAATATTTACCTACCGCCTCATCGTAATCAATAATGGAGCCTTCTTGA
GTCCTAATCTTACCAACTGGGGTCATCACACCAACAACTTGGTCTTGCTTGGTAAGGTTT
CCAGTATCCATTGAAGCAGCAATTGCTTGCCATCCATCTTTGTTTGGGCCAGCAAATTTG
GTTCGACGAGCAGTGACTTCAACCTCATTGTCGGTAGCTGAAAACTGCACAGACCCTTTC
TGCTTGGTTTAACCACCAGACTTTTTAGGGTCCGAATAGATTGTCGCCATCGCAGTTTTG
CCAAGGATGAAGGTGCGGAACTCCGTGAACTCAAGCTGGAAAACCAATGCAGCGCCGTCA
GAGTTGCTACGAGAAGCCTCCATCGAGTTTAAAATGTAATTCTCGATGATCTTATCTTCA
GTCACCAAGGTAACGATCTGACGATCAGTGATAAGTCTTTCGAGGACCTCAAGTGCCTTT
TCTGGGCGGCGTGAGGCAACCGGGTTTTGCTTGTCTGTATCTGTATCAATGTAGTTGTTT
TGGATAATATAAACAGGAAACGAGTTCACCCTTGCACTGAAAGAAAATTTCCCATCTTCG
ATGACACCGTGGTCACTGAACTTTACCTTTTCTTCAACAGCGTAGGCTGTCTTATCCACC
GTCCTTGTGTAAGAGCGTTTTTCAACAACGTCGAAAATGATCGCGAGGTCGTTGGTGTAG
TTCTTGTCCTTGAGTTCTACGTGTCTACCGCCGTAGTTCAAACCACTGGCGAAGAGCGTA
TATTGCACCTCAGCGCTGGCTTCTTGGGCTGTGTTGACCTTTATCTCTACGTTAGCCGTG
GCCTTACCCTGTTGCGGTTTGGACTGGTTGTTGGTTAAAAATGCCATGAGTCCTCCAATT
ATACATGTTACGATTGTATCATAAATCTTGAGATAAAACAATAAAAAAGGGGCCGAAGCC
CCTTTAAATTATTGCGGGACACCAAGGATTAAGTTGATGTTCCCCATGTTTGCAGCTTCG
ATCTGCTGGTCGATCATGTTCTTCATCTCACCTGTATCAATCTTGATTGTGATCTCGCCA
TTGACAGGTTCGGTTGGAATGACCATAGGAGCATTTCCTGTCGGGCCAGCCCCAGGCATC
ATCGAGTTCTGGTTCAGGTAGTTGAGTCTATACTGATCAGTCATTGCCCTCTGACCAGTA
GTAAAGTCCCAAGTTTTACCCATCCACGAGTTCTTGTAAAGGTTACCACCTGCGGTTGTA
TCTGTGCCGTATGATGATTCACTGTAATCTTGAGCTTGGTCGAAGACATCATCCCCGGGA
AGCATACTTTCTCCAGCAATCAACCCATACAGACCAAGCCAACGTGCAGCTTTACCCATC
TTACCGAAGCTTGGACCATTTCTTTTCTTCTTACCGCCGCCCTCGCCACCTTCATCATCC
CCACCGCCGAAGATCGTTGTTAGGAATTTTAAAGCTCCGGAGAGTCCAGCAATCTTCTAA
AGAATACCGAACACACGGATCAGAGCAGAAGCAAAGAACAGAGCACCAACACCCCACCCT

ACCCATTTCCATGCAGGGGATAACTCCTCGCCATTTTTACGGAAGATTGGAATGTAACGT
TCCATGATGCCTTGGATCAGCACAAAGGTATTGTAAACGGTATAGACCATCTCCATGAAC
CCGGCACTGAACCCAGCAATGAATTGACCCAGATTTTGAGCCAACTCCCCGTTGTAGTCA
AGGATTTTTGCAAGTTCGTTGAACGCGCTTGTTAACTCATCCCCAAATCCGCTCTGGAAA
ACCTTGTTCTGAAAGTCCATCCAGGTTAAGGTCAGTCTCTGCATCGCAACACGGTTGCCC
TGCAATGCCTTTTGTAATGCACCCCCTTTATTTGCAGACTCGGAGTAATACTTGGCTACA
AAAGGTATGACCTTCTCAGCTATCAACTCGCCCTTCTGCAACATATTGTGCATCTTGGTT
ACGTCGATGGTGGAATCATTGAAAGCCTCTTTGGAAGCCTTGATGAACAACTGTAAAGAT
CCCGGAATACCTTCAGCAAGTTGCTGCTTCAGTTCTTCCGCCATGATCTGTCCTTTACCC
ATCATCTGCTGGATTGCGGTAATACCAAGTTGGTATTTGACCGGATCAACCTGTAAGGCA
GTAGAGTATTCAGAGAACGCCTTGAAAAGTTCATCGTTCTGTGATTTACTAAGAGCACGA
TCCGCTGCGATTGCCATTTGGGTGTAACCCTGCGTTGTAATCTTGAGGACAACACCCAAG
CGGTAAGACTGGTCACCAACAAACTTCATGCGTTTCCCAGCTTCTTCGGAGCTATCAGAA
ACCATTAGCATGGTGGCTTCTATGCCCTGGAAGAACTGACCAGCTTTAACAACGCTTTCA
GCTGCATTGAATGCACCGTATGCTGTGCTAACGCTGATTAAAGTGCTACGAAGGTTTTTC
AACCCGTCTGTCAGGGTAAGAGTACTCGCATTTGCCTTCTTGAGTCCAGTCTCCATTGCT
GCAAGTTCAGCACGGTAATTTGAAGACTTCATGGTATTACTGTCAAGGAAGCGGTCTTTC
CGCAAATCGTAACCTTTCAATTTTGAGGCATAATCGTCACCATATTTAGCAAGGAGACGA
ACACCTGCGTTTGCGACCGTAGATTCCTTACCTGCAAGATAACGGGAATAACGCTGATCC
TCAGCTTCCATTTTCTTACGGGCGTGCTCACGGACGCGGAGTTGCTCTTTGGTGACTTCC
TTTGCAACTCTTGTTTAACGTTGGATCTGACGCACCTGTGCAGCGTGTAGTTCTGGGTCG
TAAGCTTTGTTTGAGTCACCAACCATGCCTGTAGCATTTTCGGCGTTCGAACGTTTTGAT
AGTTGCTCTTGGAGAGCGGCTTGACGTGCAGCGGAAATTGCTGGATTTGTGCCGTAACCA
CCACCTGTCTTTTCCATCTCACGGAGACGAGCCTTTAGGCCATTGTCATCCGCTTGAGAT
TCTGGAGATCTTTTTGCGGTCAGTTGACCAACAGCCTGCTTTCTACGGGCCGCTTGAATT
TTGCCGATTGCTTCACGGGCGAGAGCTTCCTTCTTAGCCATTGCAATAGCATGGGCTGTA
GTTTTCGCTTGCTCTGCACGTTCCGTTTGTTTTAATCTTTTGTAGACAGGACGTGCCTGC
ATTGCAGATCGGCTTAATGTGGCTGCTGGATCAGTCTTACTGGAAGAGAACGCCCTGCCA
GCTTGTTCCCATTCACTCTTCAAAGACTTCACAGCTTTCATGGCATGGCCGAAAGAAGTC
CTGTCCACGTCCCATGTCACTTTGTTGACTGTCTGGGTTACGATAATATTCGCCATTGAT
TCCCTCTCAATAAAAATCTCAGGCTCCCGAAAAGGAACCCGGCTTGAGATTAACCTACTC
TACCACGGCGCGGCCAGTTTGCAGCTTGTTTAGCCCTTGCTTTACTTTCTTCCTCTCCTT
CTTGGCCATCAGAAACGTAGTCTTACATATCGAGGTATTCGTTCGGCTTGAGCAGATAGG
CCATATCAGCAGATTCAAGGGAGCTATAGGATTCCCCCTTGAAGTTTTTCAGAGCACTAC
AACATAAGTAATCGAACTATGTCAGTGAGCTATTTTTCTTTGCATAATCCATAGCTCTCA
GGACGTCTTTCGAAATTTTGGACGGTGTTACTCCGCTGCCACTTCAATTGCCTGATCCAC
CGAGTTCACTTGATGGGTCTGTCCCGCGCGTCCCAGAAGTCCCTGAAGGTCTACGAAACC
GTCTTTCGTGAAAAAACAGCCGTGGTTAATCTCCAGAACTTTAGCAACCAGCTTGATGAG
GTCTAACATTTTGTCTTGGAACAGAGCGTCAATATCTACTTTACCCGCCATACCATAAAC
GGTTACATCGTCAAGAATCAATGCGCAAAGTTTTTCGAGATCAGCTTGCTCCATCTGTTC
AAAGAGGTATAGAATAGCTGTAGGGAGGGCATCAGAAAGGTTCTGGCCTCCAGTCATCAT
GGAACCAGGAATAGTTGCCATTGGGACTGCTACATAACGTCCGATCTTCGGAAGGTTTTT
CATGACCTTCGTTGGAGACCAGTGCGTGATAATGAAGTTGTGACCTGCGAGGTCGATTTC
AGTTGTCGGTCTGATATTCATGTACGCACCTGCGTCTTCGTGGTTGAGTTATCCATGCGT
GTATAAAGGCCACGTCCATTTGGCAAACGAAACACTTAAACGATACCAGTCAGGCCAGCA
AGACCGAAGCCTGCGCAAGACAGAGTATCTGGGCTTAACCAAGCATCAAGGATGCCGATT
GCCCAGTCCAACTGGCCCACTTCTGTTCCGTAGGTCGGGTCTGGTTGTTTCTGAATCCAA
GAGATGGTGCTGAGTCCCATACCCTGAGAACCTTCAAGAAGGACAGGGAAGAAAATCAGT
CCGGTGATAGAGGCTTGTTTCTGCCAGTTTGCAAAGTTTCCGTTCCAAGAAGCGGTGTTC
TGAAGGGAAACCGTCATGACACCAGATTGGTTACGTGACAGTGCGGCGGATAACTCACCA
TCGACCCCCATGTGGGGGATGATGTTATCTTCGTTACGGGCAACGACGATTTTAGTATCT
GCGGCGAAACCAGACACACGCTGGGTCATCAGGTACAGTTTGACTATTTTAGGGTCATAC
GCATACGGCGTTAGGATAGCCGTATCCATTTATTACAGTCGTGCTGGTACAGTCTGACCG
TTTCCGGAATTAGACCCTGTTTTATCTAACAGAACGGTCATGGGGTTCTTAACGAAGTGC
AGTGGCGTGTTGTACACAAGCTCCACTTTCACGTTGTTCAGAGTACGAGCAGCCAGATCG
TTAGTCGGGATGGTCGCACGAAGCGGGATTGTGATCACCGGATCGTAGAACACTTTGTTA
ACGTCATCGTATCCAGTAAGGATGGACCCGTTCGAAATACCAACGTTGACCGGGTTGTTC

ATCATGACAGATTTCACGTTAGGAAGATCGTCATCACTCATCTTCTTGGAGAGACCCAGG
TTAGAACGGCGAGACATGTATCCAAACACACTTTCTTCTGTACGGAATTAAAGCCAGTGA
CCGAAACGAACAACGTCAACGTAGTTACCAGAAGCACACTTACCTTCGTCGAAGGAAACG
ACACCGGTAATCAGACGGTAGAAGTTCAGATTCTGGTCCCAATTGGTCATGCGATCACTC
ACACTCAGAGTTGGAGCGATAACACCTTCCACCGTCTTCAGGTGGATTGAGTCACCATAA
GAAGGGTCGTTGGAAGCCATTGGACCGATAATTCCACCCTCTGGGAATGCGGTATCAGCA
AGAGGATCGTACATGCCGATGGTTGTATCGTACTGGAGTGCTTTCAGTTGGTTAGCAATG
GAGTTGCTACTAGCACTCTTAGCCAGTTCGTCAGCAGTTGAATAAACGTGCAGCTTGTAG
TTTGCAGCAGCGAAAGCAGCAGCACCAAGGATTGAAGCGTTGGTGTGAGATTCAGTGCTC
AGGAAGTACCAGTTCTCAGTTGCAGTCAGGACTTGTGGCAGAACAGACGACACATGTTCA
CTTGAGGAGGTTGTGATTGTCATGTTACCGCTGTCACGACCAATCCTGAAAGTACCATCA
ACTTTTGGAGCGATGGTTACAACGCCTGCGCTTGCCGTACCAGTCAGAACAGTTGTCAGA
GTTGCATCACCATTAAGGGCTGCTGCCAGGGCAGTTGCCAGAGAGCTTGGGGTAGCTGCA
CCAGTCACTGAAAGAATAACGCTGGTCTGATAAGCTCCGGAAACTACGTTCAGAGCCACC
GGGTTTTCGGTTTCAGTGTTTGTTTGTCCTGTGAAGTCCACGATGGTAGTGGTAAACGCT
TGACGACCAATCATCAAGTACTGAGGACGGAATGTACCAGCAAACGCTTTTGTTGCACAG
GTGTAAGCTGGGGAACCTGGCGCGAATCCATCATCTACAAGCTGATCGAGTTCGGTATAA
GCGCGAAAACGTTCAGGGATGACGTTGTGGATAGCCAGGAACAGCGGGGTTTCAAACCCT
TTGATGTCGATTGGTTGAGTACCAAGCGTGACTGTAACGTCAACCACCTTGTCATTATAA
GCCATCGGTTTCCTCTTCTTTATTTCTGTAAATACAGTTATTGACCAGCACCTCTTTGTG
CTGTATTCTGGGGGCTTGCGCCCCCTATACGATCACTAATAAGCCAGTGCTTAACAATAA
CCGAGTGGTTTTCTTTAGCCATCTGCTGGGTAGTGATAAACCAGTTGAGCGTTGATCTTC
TCCAGTTCTTCCAAAGCTCCGGTCTTTGTTTCGATGAAGCACACGTTAAATGTGATCAAG
ACAACATCACGGGTTTCAAACTTCTGCATATTCAATGGAACTCTTAAACGAGAAATTGTC
GAAGAGGACGAGTATGCAAAAGGAGAGTTGGTTGGGAAATATTTGTCATAATTGAACGGT
AGGTTCATTGCTTGCAATACCCTGCTTAAGGCGTTGGGAGCTTTACCACTGTAGGCTGTG
ACGGCGTAAACAACCTCCTAATTATGCACCGTCATGGCAGTGCCTTGAGTATCAACACCT
TCGTCAGACTGCCCGTCAATCTGGTCAACTGCCGTCAGGTCAACAAGAATGAATTCACCA
TCCACCTTGGGAATGTCATCTTCACCTGCCAATAAAACTATTCTTCCTGTGGTTTCTTTA
CAGAATGCTCCCACTGTATTGGTAAGGGCGTCAAAGAATTCGTCCGAAATGGTGAATCCA
GCCTTTAAATTCCTCCGTTCGTCCCTTCAGGGACGGATACTACGTAAGCTCTGAAACGGG
CGACACCTGGAGTCTGATAAGCATCAGATTTAAACACAGTGAACCACATCATTTCCCCAC
GGGAATTCATCAGCTTGATTTGGCCAGCAAGCTCGTTTGAGCCTTCAACAGGACCTTTGA
GCATCACAGAGGAATAAACGGTGAAGGGGTCATACTGACTACCACCTTCCTGCATCAATT
GTGATGTATAATCTCTTGCAGCCTTACCTGTTGATGGTTGGACAACCTACTCAAGAACAT
CAAACTCTTCATACTCAACTTCAACACCTTGGTTTTCGTATCCATTGCTTGCGTCAACTT
TTGAATATTGACTGCGACGCCTTGTAAAGGTTGGGCGAGGTATTAATCTATTTTTTCCAA
TAAGTCTGTAACCAGCCATTATGCCCTCGACCCTCTGCTGTTGGATACTCTGAATGTTGT
GGACTCTTTAAGGTCACCATAGTGGTACATCGCCTCACTGAATCCCTTGGCATCGGCCCA
CGCATCGGATACTCTGTTATGAGGAAACAGACCGGAGTCGATAACGAACTTTATCTTTCT
TGCACCTGCTTTGCCGATCGTGTTTAGCATCGGTGTCGGATTACGACCGCCTGAAGCAAG
GGATCCGAAAAGTTCCTTCTGGAGTTTTTCCAGTTCCTTATTGAATGCAACTGCTGCGCT
TGTCATAAAGGTACGGGCAGGAAGACCATTCCAACCTTCTTGATGGATTGCAGCGAGCGC
TGCAGTATTAAGATCTGAGTAGTGAGGGTCGTCATAATAACCATATGAAACCTGACGCGA
ATTAAGTTGTTGGGTTGCTCTGAAAAAGTTCTCAAACTGGCTCAGATCTTGCTTACTCGT
GAAGTTTGGTCTCATAGCCACATCCGTTTAGGTAATTGACCACATTATCTTAGCAAAATC
AACGACCAAGCGAAGGATAATTAACGAGGCCGGCGGTGAGGGTATCTGCGAGGAATTCCT
GGTACACCTGTCTGGGGTACAAAACCTGCAACCTTGAAGGGATCTCTTGAGTTCGGGTTC
CCTTTCACACGCCAGTGTTCATCTTGTCGGACACCACCAATGATGAATAAACTGGAAACC
GCGCTGAGACAACCGTCCACATAATCAGGATTGGCCTGTAACCAGGTCAGGAAATCTCAC
CAGTTCTGGTATGTAGAACCACCTTTCACCTGGACTGTTTCGTCACCGATCTTTTCCAAG
CGTTCTGTGATAGATGCCTCTCCAGAGTTTACTTCTCGAAGAATCAACCAACGTACCACA
TCAACGAGCGTGTTATAAACAACTCGCCACATGTCGCAGCTTGTTGATTGAGGGTTGGGC
CAGTTTGCCTTAACCTTCTCAATAGCAAGAAATGCCAGAATTCTTTCGTCTGGTGAAACC
TCTGGGGGAATGGACCCAAGGAGCAGTCGAATACATGCAATAATATCTTGGTCTTTCATT
TTCGCTCCTGTTGGATTGCTCTTCTATTAATCAATATTTTATCACAAAATATTCTATTTG
TCATTAAAAGCACTTGAAACGACGTTAAGTTCTGTTATCTTTAAGTCATCAAACAAAACT

GGAGAACATTTTATGAAACATTCTAGGGCCGTTATCATTGGAATTATCGCTGGAGCACTT
ACCGGGGCGGGAGCTTTTACGTATGTAATGAAGCATCCTGATGTTAATAACATAAAAGCA
ATTGAATCACGTTGGTTGTCACCTGAAGAGAAAACTGCCAGCGTCCATCGACTTGTTCGT
CTCGGTTGCGACCAACGCTTCAGGAAAGCATTCAAACCTGGCGTCTCAGTTGTTCAGACA
GGTGAGATGGATGAGATAACAGAACAGGCTGAAGCATACCAGTTTTTGAATGATGTTGCG
ATCCTTTATGGAACTGAAGGATTCAAAGGGGCGTACATCTGCACCTATTATAAAGATGGA
AAAGACCCCGCGGTCAATGTGAATATGGAAGCAGGTCTACGTAAGCTTCTAAAAAAAAAG
GCCCCGAATGGCCTTTTGTTACACCATAATCCCAGCGGATTTAAGCTTCGCAAGCAGTCC
GTTAAAGTCTGCTACCAGAGTTTCAACAACGGTTGCTACAGAATCTGCCTGTCCTGAAGC
TTTCATAACACCACCGAGCGTGGTAGTTGTTGCTGCTGGAAGTGTGTAACCTGCACCACC
TTCTACGAGGTCAGCAACAAAGATTGATTGACCAGTACCAACAGCGATAAGTTTGGTTGC
ATAAACAAGACCAGCTACACCAGAAGTTGCCATATTTTATTTCTCCTTATAGGCATGAGG
AAGGTAATTGCGGAATATTTTCAACAGAGGTTGCCTGATCCACACTATCAATGTAAGTCC
AGTAAGACTTCATCTACTCCTAATGAGATCTGGATACGCTGTTGATTAAAGCTGCCATTT
CTCTATGGGTGAGATCAGCCTTGTAACCATGGAGGAACTGGAATGGTTAGACATTTGTGG
TTTCAGAACCATCTTCAACCAAAACATTCTTGATGGTTAATAAAGTTGCAAGATCAAAAC
TCTTGACGCGAACTTGGTACTCCCTCTCATCGTAAGGATAAGGGATACCGATGTTAATGT
CCCCGCTAAGAACAGTTTGTGCATCAGCCTTTCTCTGATCTTTTGCAGAGACAAGGTTGG
CCGAAATTTCTTCTTCAGTGAAATCTCGTACATCCCAACTTTGTTCCCATTCTTGGGTTT
GGTCATTGAAATGTGGAGCGCCTTCAGTGAACACATCCCGTTCAGGCATTTCTTTCGGAA
TTACCGGGAAGTAACCTAACTCGACAAGGTTTTCAGACTAGATCCAAGAACCGGCGCTCA
CGTTCGGATTATCCGCTCTGAAGTCAGAAAGATATACCGGATATTTCCCATCAGACGTTC
TGATAAGAGGTGTATATGCGTAAACTACCATTTACTCTCCTTGAGAAGAGCGCCGAAGCC
CCTTTATTAATCGACAGTCAAAGACTTTGTACCTGTCAGGTTTGTCCCGTCCCAGAGTAT
AGTAACAACCGTGCGGGTAGTTCCCAGAGTAACCGCTGTGCCGTTTGACCATGCTAGGTT
GCCGGGCCACGTCAGTGACCCACCGGAACCAGAGAACACGGTAACGATTGTCATTGCTCT
GTTTGCAGGTAGGTTAGTGAAGCTCATGGTTTTGTTTGCTGTACCGTCAACCTTGAAGAC
TTGAGACACGCCAACATGCATAGCCCCCGTTGTAGAAGTAACCAGAAGATCCTAACGATC
AAGTTTCTTCCACGCATAGTCTACACTGACATAAGAGCCTGTATCTGCTGGTGCTTCAAG
TACCGGAACATCAACCCACCCAGCATTTGTTCGAGCTTGCGGAATGGTAGAGGCTGAATC
ATGAACGATTCCTCCACCCATGTACCCCAGAGAAGCCCATGCGTTAGCAGATGTTTTCTT
GACGGATTCCAATGTGGATTTATTGATGAAGTAATCACCAATTCGCTCATCAGCAGGACC
TGGGTTACGGGAGAATTTTCACCAAAGTGTTCCTTGGTCTCCTTTAATTCCCTGGATACC
CTGAATACCTTGTGGACCCTGTGGACCTTGTGGTCCAACATCAGCTTGAGGCCCTTGGAT
AGGTCCCAAGTTCTCATAAGTGGTTCCAGTCCATCCCCAGAAATCTCCCTCAAGCAAGAA
TCCCTGACCAAGCTCTCCAGTAGAAGGAAGTTCAGTGGTATTGTCGAGTTTGCCAAGGAT
TTCTACCCCAGCACCCACCTGACCCTCTGGTCCTTGTGGTCCTTGAGGGCCTTCTGGACC
TGTCAAGTCACCCATCCGTTCCCATGCGGTCCCGCTGTAAACATAAGGGTAACCTTTGTT
CCCACCGGAAGTTACTGAATACATCCAGCATTGTTCTGGGTTTTGAACATCAAGCAGGTC
CTCATAAGTCGCAATAACACCTTTAGCTACGCTACTTGCACCCTTCGGTCCTTGGTCACC
TTGGATACCAGGATCACCCTGTGGGGCACGCATGACTCCAAGATTTACCCAGTTAGTACC
ATCGAAAGCAAAAGCTTCTCCAGTGTCCTCTGTGGTGTAAGGATCTCCAGTTGCTGCGCC
GGACGGCAAAGCTGCTTGATTGGCTACTGTACCTCTGGCAGACAGTGAAACGCCAATTGG
ACCCTGTGGTCCTGTAGCACCTGCGGGACCTTGAGGTCCTGCATCAGCCTGATCGCCTTT
AGGCCCCTGTGGGCCATAAGCCCCAGTATCACCTTTAGGACCGACATCCCCTGTCGCCCC
GGTGTCACCCTTGTCGCCTTTTGGACCAACGTAAGGCCCTACGTTTATCCATTGAGCATC
AGCCTGATTCCAGACGTAAAGGTTTGGAACTATAATCCAAGCATCGCCTGGTGTTCCTGT
TGCAGCAAGATCTGAAACAGCGCCTTTTTTCCCAAGAACACGAGCACCATAACCAGTTTC
ACCACGATCACCCTTCGGACCTTGAGGTCCTTGTGGACCGACAGGGCCAGTTAAACCAGT
ATCGCCCTTATCTCCCTTTGTACCGATAGGCCCTTGAATACCCTGTGGACCTTCTGCTCC
AGCAGGACCCTCAGCCCTCGCAGGACCTTCTGGGCCTTGGTCGCCCTTTCGTCCTAGGGG
CCCTTGTGGGCCGACATCAACTTGCTCGCCTTCAGGTCCTTGAACTGGACCAATATCTTC
CCAAGTGTCGTCAGATGTCCAAACCCAAACGTGTTTGTCCGTTTGACTAGCCCATGCATC
AGCCACAGCATTACCAAAATCAGGCAATTCGTCATCTGTTGCTTTTGTTCCCTTCAGTTT
TACAATTGCGGCATCGTTACCTTTATCGCCCTTGTCACCTTTAGGTCCTTGAGGCCCTGT
TAAGCCCTGAATCCCTTGAATACCTTGGTCACCTTTCTCTCCACGTTCACCTTTATCGCC
TTTAGGCCCCTGCGGTCCTGTGGGACCAGTAGCCCCGTGTGGACCAGCAGGCCCTACTTG

CCCTACAGGTTGCCATCCGTCTGCATCGCCAGTCCAAACCCACATCTGGTCTTGGATGAT
GTAGGTAACGCCTTCCGTATTTCCTTGAGTCGGAAGGAGAGATGTGTCAGAAAATGCACC
CCTGATCACCCATCCTATACCATCTTGCCCTTGAGGTCCAGCGAAATCGCCAAGATCAGC
CCAAGTGCCTTTCACAACTGTCCAGTAGTGAGTTCCAATTACGTAAGTATCTCCCTCTTG
CATGGTTTCATCAGGAGGAAGTCGGGATGGGTCTCCAACAATTCCTTGGATGTAAATAAC
AACACCCGGATCACCTTGATCACCCTTGTCGCCCTTATCGCCTTTTTCTCCGGGAGGGAC
AGAAAGACCTCCTTCACCAGGGTCGGTCACTTCAACAAGAACGTTTCCGGTCTTGGTTGA
AATAGGCACAACGATCTGAGGAGGAGCAACATACAGCGTTCCTCCTGCCAATACAAGCAG
GTTATCAGTGAGAGGGCTAACCCCGACAGAAAGTCCGTCAGGGCCAGCTACAAGTGCGTT
ACCCGAAGCAGGATCTACAACAACAGCGGCCTTCAGCGGAGTATCGGAGTTACCATTACC
GAGAAGTGTTACTGTGGTCGTATCTTCTGTGTGGAATTGTCCATCGTCCACAGCGGTTCC
ACCGCCAGCACCTTCCGGAGGGATAGGTTCAGCGTTGACGACCGTATCATTGTAATCTGC
CATTTTTACCCCTTATTTTACAGGCTCTAATTCTTCAATCGCAGCCTTGTAGTCAGCCAT
CATGTTTTCGAAAGACTTGTTTTTGGCAAGGGTAATGCCTTTGGTTGCAGCGAGGTCAGC
AAGTTTAGCTTTGCTCCCTTTGTTGTCAGCTTCGTCGTTCAGTGCTTCTGCTTCAGCCAG
AATTGCTTCGTAACCAGCAGACTTCACTTTCACCTCTTCAGTCTTGACTTCTTCACCTTT
AGGGCCTACAATTGCCTCCACTGCCTTACCGCGACAATTCGGGGTTGGAAGAGGACTGTT
GAAGAATACGATGTATGTACCCATGCGGAGGGTAGAGTGTTCTGCATCCACATCAAGTCC
AGAGATCTCGGCAAGCGCCTTGAAGAACTCAACAGAGTTGCGGAAAGCCCTTTGTTCAGT
ACCGACACAAAAGCCGTTCAGAGGAAGACGGGCAGAACTACCAACATTCAGATCCGCTGC
GTAAGTACCGAAGAAATTGAACAATTCCACCTTGTTAGGGAAAATACGATAGTTTTGATT
AGCCATAAAATCTCCATTTGGTTACCTTAGTAAAAGCCACTTGACAGCGTTGATGAAGTG
GCTATTATTAAGGCAGAAAAATAAAAAGGGAGAGGTGAAGAATCACCTCCCCCTCTTAGA
GGTAGCAGTTACGCTATCTTATGCGAAAGTACCAATAGAGCGAACTACCTGTTCCGGACG
GATGTTAACAGTCGGGAAGGAAGTTTCGGACTCAACTTTTTATTCACGCAGGTATGCAGA
AGATTGGTACCACAGGTACAGTTCCTGTGCAGGAGTGTTCGCTTCACGAACGTCATCTGC
TGGCGCATAGTGAATCTGGAACCTGTCGTCGATTCCTTGCGGAAGAATGTAAGCTTCACC
ATCAGGGATGTAGTTGGAGATATCCTCGATGTAAGTCACGTTCTTGTGAACGAACATACG
GTTGTTGGCGTTCTCTGCTCCCTGACCCAGACGACAACGAAGCGGCTCTTGAGTAGAAGA
GTAGTATTGATAAGCGTTCATTACCAGCGGATGAGCGATCGGCGCTGAGAACCACTTACG
AGAAGCCAGAACTACGATACCGTAGTTGTTGCCATTGTCACCCGCATTGTCGATGATGTA
AGCACGAGCTTCAGCTTCGTGAACGTCGGTCGGGTCGATTGCAACATTGGTGAAGTCAAT
GTTTGCAGTGTGCTGAGTAACACGCTACTCGTTGAAGTAGTTGTACTGAGCAGTCGGGTG
TTGTGGTGCCCAAGATTTACCCATGATCGCCTGAAGCATTGCTTTTTGTTTCAGTTGTAC
ATGAGAGATGCGGATACGACGAACCACCCGAGCAACAACATCTTCTACAGTCTTCGGAGC
ATCAGTAGTGAAATACTTACTGAAGTTCTGTACGTCTGCCGGAGTGATCTGACGATCAAG
TGGGAAGAACGGGATGTTGATGTTTTTCAGTTGGGCCTTTTCAGTACCAACGTAGTTACG
TTCACCCAGACGACGACGAGCCGGGAATTCGGTTACAACCTCGTCAACACGTTCAATCTG
AGCGACAGTCGAAACACCGTGGTACGCTGTGAACAGGTCCATGTAGGTGATAAGACGGTA
CTGACGTGGGACGATTTCCAGAGTTGCGCCAAGATCGACGATCGGGAAGTTATTAGAGCG
TACTACTGCCATTGTTATTCTCCTTGGTGTAGATTAGATAGCCGTTACTGGGATCTCAAC
AGTGCCGTCGTAATATTTATCGGTGATCTTCAGACCTTTGGCCTCAAGAGCAGCGATAGC
GACAGACTTGATTGCAGTAGTACCGTCAGCAAAAAACAGTTTGAATTTGTTCAGGGTCAG
ACCACGAACCGCCACAACGAAGTTGTAAGTAGTACCAACCACGAAGTCACGAGGAGCATC
GTAAGCAGGAACCAGATCCCGGTCAGTGATTACACCGAATGCGTCAGCAGCAGCGGAAGC
GGCAATTGGTGCGCCAGCGGCGTTCACAATCATACCTTCTTTCAGACCAGCTACGAACTC
AATCGGCATTTCCACTAAGCTGTAGCCCTGGTCAGAAGAGTCCACTTTACCCAGAACGAT
GTCAGAGTAGTACCCCTGGATTAGATTAGCCATTTATTTAAATCTCCTCGATTAGCCGTC
TAGTGCAGCAGCTTCACGAGCGCGAGCTTTCCCTTTCTCGATGTGAGCCTGAAGGATGTC
TTCGGCAGACTGGTTGATGTCATCAACAGTTGCTTCACCGTCTTTTCCGTGTTCAGCAGT
GCCGACCTCTTTCTTCACGGTTTCAACTTCTGTTTCCAGTTCGCTGATACGAGATTGGGC
TTTTTCCAGAAGAGCTACTACCGGAGCGTTAGCTTTTTCGATGATGAAATCTGCCACAGA
AGCGTGGTCAGCTTCAGCAGCAAAGGTGAAACCTTGTGCGCTAGTCAGAGCGGCTTTCTT
TTCTACTTCAACAGCGGCTTTGATGATTTCATAAGCAGCAGCATCTTTAGCTTTGGCATC
TTCCAGTTGCCTCACCAGGTCAGGGTATTTCTGAGACTTGGTTACATCAGTAACATTATC
GGACACTGAGGTCTCCTTATTTTCTTTGGATGTATCAATTACGGGGTCAGTACCTGCTTC
TTTGACGACCCCAGTCGCCTGGAAATCTTCGGACTCACCTTCGGAGAAGGTCAGGGCTAA

TTCGCTAACTCCCTTTTCAATCAGTTGCTGAAGCTTCAGAAGTTCATACTGTTTAACAAG
GTCGATTTCTTCACCGCTGTGCGCTGCCTTGGCGATAGAGATTCACTCCATACGCCCTTC
CAGCCATTCCTGGTGCTGTGCGTTCCAAGCTTGGACCCACTCATGATCTGGGTTTTCAGC
GGCCTCATTTTCAAGTTCAGTTTCAAAGCCCAGCAGTTTGACAAGCAGTTCTGCATCGTC
GCTCCACATATAGAAGAACTTACCCAGGAACTCTTCGAAACTCATGGAAACGGTAACCTG
TTGCAGCGCCTTGATATCGTCTTCGGTAATCTTTTCCGGTTTCAGGTCAGACTTCATCAG
AAGTGATACTGGACGGAGGTTTGCAGAAAACCCTTGACTCTCATGGCAGAGTGCGATACC
TTTTTCCTTAATGGCCTTGGCTTTTGCAATTGTCATTCGCCATCGTCCTCAACATACTCG
ACACCGAAATCAGCGTTGGTGATTACTCCTGTTGCTTTGTCGATTGATCCGGAACACTGA
ATGCTCAGGCCACTAACAACGTTGGACTTTTTAAGGTCCCACAGCACAGGGTCGTTATAT
TTAACTTTAGCTACCCAGGTGCCAGCCTTGATTACTTGCTCGGTCCCGATAACCACCACA
TCAAATTCTTCCTGAATCCAAGTCTTCGCGATTGTGAACGAGTCAATCTCAATCATGTGG
AAGAGGTTCTCTTTAACAATGCCAGCTACGTTTGCCTTATCAAAAGACTCCTTGGCCTTA
GCGATTGTCTCAGCAGACATCCATTCGCCGTGAACGTCCTTGGCATCTGGTTCGTAGATA
ACTTCGTAAGAAATCATCTGCTCATCAGCATCTTTCTGGATGCTCACAACAGAGGTTGAC
TTGTTGATTTCAGTCTTAACATTCATTTTGAGAATGTCTTTGGAAGCCTTCTCGATAGCG
GCTTCAGGAGATTGGCCTGAAGCAATCAGGCCATTTGCAATACTCAGTACCTTGCTCTTC
TGAACGAGTGACAGATCAGATTCCGATGCAGGAAGGTCAGCAATACTTGTGTATTTCATC
ATTTTCCCCGATGATAGTTATTTTATCACCGTTCATTTTCTTTTGCAAGCTATTTCAGTA
AGTTGAAATTAATTATCCATATTGGATACGGAATTGTCGCTTGTGGAAGAGATTTTTCCT
GTTCCATTACCGTTAGAGCCTGCTGTCATACCATCACCGGAGCGACTGGTATCCTGACCA
ACGAGTTTCAGAAGTTCATCAGTTGATATGACTTCCGGGATCTCTTCATCAAAGCCACCA
ACTTCAAGGATCTTGTTGATAACTGTAGGAGTTTTAGGAAGATACCCAACACCACCGATA
CGCTGAACGAACTTGGAGAAGTCTTCCATTTCGACTTCTTGGATAAGGCCTGGTTTCAGT
TTTGGCAGGTCTTCGTCTGAAAGACGGATGTCATTCAGTTCCAGAAGCTGATGGATCAGG
TTCTTGTTGCAAGCCTCAACAAGGATGTCGATGTCACGCTGAACGAAATGCCCGTGGATG
GACTGCTTCGATTCGGATAAGTTGTAAGACCCCTGACCATCGTTACCAAGGTTGATGAAT
CCAGCGCCAAATCTGTCGAGGATTGCCTTCTTACGAGAGTTCACCAGTTCCTCTGTACTG
TACTGCTTACCTGCACCGTCAATCCCTCTATGAGACATCTTGTACTGTTCACCACCCTGA
GCGTTCATATCTGAAGGAAGGACGAAGTAGCCTTGCTCGCCTCCGTGGGCGTTTGCAGCA
TCCGCCATCAATCCTTGAACCATTTCTGATTCTGGTGATTTCGGGTCAATCGCTGCCTTG
TTCAAGATCTGAGAAGGGATCTTGAGATCGATGATACCACCAAGATCTTTGGAAGCACCA
ATTGTTTCGAGGTTCTCAATCAGAATCTTTTCACGGAAAGCACGGTAACACCCAACAAGA
GGAGAAACACCAGCAGGGTTTGACTCTGTACCACCAAGACTCATCACCATAAGTTTACTG
ATAGGAATGAACACTTCATCCGCAGAGGAGGTCAGATTAGTAACCAGGCTCATAGCACTG
GAGATTTGCGTCAGACCGTTCTGGAAGTTGGCAAAGGCCATCTTACTCTGGTAACTCCCT
TTCAGGGTGCGCCCATCCTCATCAAAAACCCAAGGTTTTGAGCGAGAAAGACTCGATTGT
GGCCTAAAGCCAATCTTATCAATGGTGATGTACCCAGCATATTTAGATGGGGCAGAGTCA
GTACGGTACACCTTCTCATAGATGGAGAATCCATACTCATTAAAGGTTGCGGCACTACGT
GCAATATCGCGAGGAGTTTGCTGGTTGGCAAGGTTCTTCACCGCGTATTCTACAAACTCG
GCTGCATCTTTGGATGCCTTACTGTCACGATTATACAGGACTTTGAAATCATTGAAAGCT
TTCGTTACGAAAACATACTTCGTATCAAGCGCCGTGGAGAGAGTATCGTCCTGCTTCATA
TCCTCGACGGTTGCCAGAAAACAAGGCCAGCGTAACTCTTCCACCTTCATAACTTCGGAT
TCTGCACGAAGCTGTGAAAGATCACCAGAACCTAGTTCCCCGGTACGGAGACGACTAACA
GCCAGATATTCGTTCCCGGCTTTAACCACCTCCGATGGTTGTGCAAATCTTGTTGACACG
AGTTATCCCCTCTGTTATATTCTCTTGTATAAGGTCGAAGCGACAATTGTCGGGATAACA
ACAGGTCTGGCGATCTCGTTCTTCTCCAAGTAATTAATACTGGAGGCAACGGCATCCGCC
CAGTCATCCTTACGGATTGTTGTGCTTCGCTCACCATTAAACTTTTCAAGTGCAGTCATT
AAGGCTTCATATGTCTTAGCATCGAAACTGCTTCTGACAATCCTCACCATTCCTGACTGA
GCAAGTTTTGCAAAAGGAGTGAAGCGTGTAAGTTTTGATTTATTCCCTCGCGTCGGGTCT
CTCTCAACCACAAACCCTTCAGCCAGAAGCTCACGAGAGCTGGTTGTGAACTCCCTAATG
CCTGCTTGACCGGGGTCAACCCTGAAGATGATAACAACATCATCCCCATGGTAATGGGCT
AGCTCTTTAATGATCACATCACGTTCACCAGCTTTCTTACAGAAACGGCCTAGCGTTTGA
GTGGAGTATTGACCATCATCAACAAACTCAGGGCAATAATCTCCTGAAAGATAATAAAAC
CCATCAGGGTCTTTACTTACCTTCACAGATGCAGTAAAGTCAGAGTATCTGGTACCAGAT
GTTCGTTCTCTACCTGCCTTGTCGTATGGTCTAACCGACGTAACGCCCAGTGGTAGGCTA
GGAACTTCCTTGCGATAGGATCGCGGACATAGTTCGCACCTACTGGACGAACTTTCCAG

TTTCCGTGGAGTAGCTGTGCCTTTTCAATTTCCGGAAGACCCTCAAGGAAAGGAAGATAA
TCTGGGTTGTTGTCAAGCATCGGTGGGTTATCGAAGATAGTCGCACTGATGAATGAGAAG
GAAAGTGGTTTTGGCTTCTTACGAATGTCCACACCGTCCCAATACTGCTCAATAAGTTCC
TCTGCTGTATCTCCCCAGATGAACACACCGTCACGACGAATGAACCAACGGATAATCCCG
TCTCGCTCCGCAATGGGGAAACCATCATCGTCTAGATACCACTCAATCAACCCTTTAATC
TTGTGGTCCGGGTCCGGGTTACATGAGATGACCATACGGCTCGGGTATTTGGATTCAGAT
CGAAGACGAGACATGAGGTATTCGATCTAAGACCATTCAAACTGTGTTCCTTCGTCAAAA
CCAACAAGGGTGTACTGTAGACCCTGGTGAGAATACTTATCTTTCTCGTATTCCTTATGG
GACCATTTTACTTTAGCACCAGAAGGGAAAATTGCTTCCAGGTCTTTCTCCCTGAAGCGT
GGTTTCCATTCCGGATCAAGCTGTGTATAAATGCCTCGTGCTGTATCATACAAACCACCA
CCACCCTTCCACTGAGGGGTGTTTCTACGGTACATGATGCAGGTTGTTCGTGGATCGTCG
ATGATATTTAACGGCATTAATTGAAGCAGGTAGGATTTACCTGACCCCGCCGCACCCCCA
ATGATGAGGATTTGTGCTTTGTTTTCCAGTATCATTTTCTGCTTAGGGCTGGCAGGTCCG
AACGTTTTACTCAAAAGACCTCCATATTAAAGGGGTAATTTGGCCTATAGGAGGTAGTTT
ACCACGAAACTTGATAAAAATAAAGCCCTTATCCCGAATTAAGGGCCTTGTCAATCACTT
TTGCGGATTAAAAATGTTACGCACGGATTGTTGCAGAGTCCTTAACTTGCTGCGGTCGTT
GGCAGCCTTCAGGTTGTTGGTCTTCATGATCGCGTTGTTCTCTGAGTTTGAAGAACCATA
CTTCAGGGCAGCTGGCTCCACAGGATCAACCATGGTAGCAGCACGTGGTTGTGCAAGAAC
CTTTGCAGGATCAAACTTCTCAACTACCGGAACAACCTCCGGCTTCGCCTGACAACCGAC
CAGTAGGGTTACTGCCATCAGCACTGCCATTAATGCTTTATTCATTTCACCCCCAAGCTT
TGGTTGTAGATGTCAACAAACTGGTTGTCAAGCCCTAGCTTCTTGCCTGCATCAGACCTG
CTGTAAGCGTCTTACTTTGCGTTAAGCTGTGCCTGCAATTCTGCATTTTTTGCCTGCTCT
TCTGCCAGTTCCCTTTCAAGGATATCGCTTGCGATCTGTTGTTCATCCGTGTAAGACTGA
TTCTCATCCTTGTTTTTCTGTTGCAGATCCTTCCATTTTCGATCTGTATCACTCACCCCT
GAATTGTAGGACTGCCTGATCTGCCCGTTATACCAGTGCTTTGCATACAATCCCCCGCCA
ACGAGGACAGCAAGCAGGATGAGCACTGCGATTACCTTTAGATTGCTTTTCAGAAACTTC
AGAGCCATTTCAATTTGTATCATGAGATTTCCCCATTGGTATCAATTACCCGCTCGATTT
TGAGCAGAGTTGTATCTTAATCTTCGATCTCTATTGCAGCATCATTGTAGGCAATCTTGG
TAGGGTCGCTCACAGACACCTCAAGAAGGACAGCGCCAGGTAAAGAGGATTTTTCTCCCC
ATCTACCAGTTCCGCCTTTCTCACCCCAACTCTTGTATACGTTCAAGGCTTCCTGCTCGT
CTACATAAAGGTTCATCACATCAAGGAAATTTTGTGCTGGTTTCTCCTCCAGAAGACCAA
CTTTGTGCCTTTGATCTACAGCTTGCATCAGGTAGAAGTTGTCAGAAGGACAAACTCCCC
ACAGAGTTTTAAAATATGGCCTTCCAGTATTCTTTCGGAGGCGTTGGTACATGGTAAAGA
TATTTTCCTCTTTAAGACCTGCCCAACCACCTTTACCACCCTTCTCAGAGTCAGTTGCAA
TAGCGCTGATGATTTTATCTCCAGCACTATCCATTTTATCAAATAATAGCTCAAATGAAA
ATGGCCTTTCTTCATCGAAAAGCTCTTCAACCTTCGTACCATACATACCACTCAGGTGGG
CCTAGGCATATTCCCAAGTATCAATGCTCAAACTAGAAGCTACAATGCCAAGCTTCATGG
TCTCCCGGCGAGAGATTGTATATCCGCGAGCGCGGTACTTATCAGCACGAAGCATACTAA
TAATCGGGTAATCTGTCTTGGGATTCAATACCAAAACCTTCTGAGCGATGTGCTTCATGA
ACTTTTTGACAAACGCCGACTGCTCTTTTGCAAACCTGAATGACCCCATCTTAATCGTAA
AGTCAAACTTATCAAATATCTTCTGTGCATCAGGGAAAACATCAAAATCGATACATTGCA
GAACCGGGAGCTTTTTTATTGGTAAACATGACACTTTTATCGGTCATACAGATATTGTCA
ATGTTGTCAATCTTACTCTCTTTAACCGCTTGGGTGAATGCCTTAAAATCCTCAGCAGAC
ATATCTTTCCAGATTTTTGCCAAGGCAACAACTCACAGGCGTGTCATCGGTTTATCTTCA
GATTTGATCACCAGGGTATGGACATAATCTTTAATGCTTCCCGGATTGTCAAGTTCCCTG
AAATATTTGAATGACCGTTTTGAATTGCCCCAATGATTAGTCGCCTCTTTCAGGTCTGAT
TGACCTTTCAAGAGGCTACGATCAGTCATGTACGCAAGGAAAACTTCCAGGTTCTCCCAT
GAAGGGAAGTAAAGGTCTATGTCGTTTACTTCTCGGTTGGAGAATAAGGATGTCAGTGTA
CCACCCGCGACACGGACTTCGAGGCGTTTAAGGATCTCCATCAACCCGTTTGGAATAAGG
CTACGGAGGATCAGGTATTCACGGTGGTATTCTTCTTTTGGAAAGATATGAAGCATGGTT
TTCTCCTTTAGTAATTAAACCATTGGAGTGTTCCAGAAAAAGAAAAGCCCCGCAATAGCG
GGGCGAGGCAAAGGAGAGGAGATTGAATATTTATTATTATTAGAATAAGAAAAGTGGCGT
AGGAACCTTTACTCGTCGAGGGTGTCTTACCGTTTCCACAGCCGACACCTTCCTTAGTGT
CCACTTAGTAAGTTGGATGCGTGAGCGGGAGTCGAACCCGCAATCTTCGGCTTATGAGGC
CAACATGATCTATATATCCGTTTCACTACCACGCAATTGTTTGGTGGGGCCGGGTGGACT
CGAACCACCGACCATCTGCTCTTCAGGCAGGCCCTCTACCAACTGAGTTACAACCCCAAG
TAATTGGTACTTCCTGATGGTATGGAACCCCCGACGCTTGCCGTGTAAAGACAACGTTCT

ACCCTTGAACTAAGGAAGCATCAAAGTGGTGGGCGACAGGGGACTCGAACCCCCAAAATC
CTACTTCTAAGTACGGCACTTATACCAATTCCATCAATCGCCCAAATAGGTGGAAGCCCT
ACTGTCTTCCTGATCAGCCAGAATCCTTATCCAGCATTGTCATCGGCCCTGTAGATCCTA
TCATGAACCAGGGAGTCGATGCCTTTATTTGGTGGAGAATAAGAGATTCGAACTCTTCTG
ATATCCTCGGTGCAAGCGAGGTGTCCACTCCAAGCAGACCCATTCCCCAATTGGTGCAAG
TACAGGGATTCGAAGCCCGATTCTCAGCTTCGTAGGATGATGCTCTGTCCAGTTGAACTA
TACCTGCATTAATAATATAATGTTCTCAACCCTTCAATGAAGCCAAGAACCCATGCCCTC
TTTTGCTGCCACTCAAAGTCTGAGTAAGTACAGTCGTCGATGGTCATGCGACCTGACATT
GCGGCATCATAACCACGACAATAGATTTGATCAAGTGTCCTGATTTGTAGTCTTCCAATA
TCAGATATTTAAAATGGTCATCCAGGCAGGACTCGAACCTGCGACCCGAAGCATCCAAGG
CTCCCACGCTACCAACTGCGCTACGTACTGATTGTGTAAGTTTTACTTGCCCCAGAATCT
TACAACTGGTATCGCTGGCTAGGTAATAAGTTCTCCAAGCAAAGGTTTAATTTGCAAGGA
GGATGTTTGGCGAGGTCGAAGGGGATCGAACCCTCATTTTCCGCATAGACAGTGCGGCAC
CATTACCTTGTCAGCCACGGCCCCATAAATTTGGCGACGAGTGAAGGATTTGAACCTTCC
TTGAGTTTTCACTCACGGCTTAACAGGCCGTCGCCCAACCACTAGGCCAACTCGTCATTT
AACTACTTTATCAGCCAAAAAAACCGTTTGCAAGAATTATTTTCACTAATTTAAAAATGG
TGGGTCTGGAGGGATTCGAACCCCCGATCCTCCGATTAAAAGTCGGATGCCTTCGACTTG
CGTCACCACTTGGCTACAGACCCGGTATTTGGAGGAGAGTGGAGGAATCGAACCCCTACC
GTTTCCAGCACCACAAGGTTCAAACCTGCTTGTCCATTGTTGGACGGCAATCTCCTTGGA
GGAAGATGTCAGACTTGAACTGACACACCCCTTTTGGGGCTACTGTCTCTTTAGCACAGA
GATCCCTTACCTGATTTGGGTTAATCTTCCAGATGTGGCGGAGAAAGAGGGAATCGAACT
CTCAAGCCGTTTTACCAGTCAGCACCTTACCAAGGTGTTTTCGTCACCTGTCGATTTGCT
TCTCCAATTTGGGGTGACCTGTGGGTATCGCGCCCACTTGCTCAACGTCACAGGTTGAAC
ATCGTCTCATAAATGCTTAGGCCACACATGAGCAGTCTCAAAAGAAAATGTTCAAATTTG
GCAGGGGGTGGAGGATTCGAACCTCCGATGTCGAGTTCAAAGCCCGATGCATTAGGCCAG
CCATGCTAACCCCCTATTGGCGTGAAGGTGAGGAATCGAACCCCTTACGACTGGTTTTGG
AGGCCAGCTTGCAGCCACTGCAAGTATGTTACCCGCACAATGTTTGGCGTCCTGTGCGGG
GATCGAACCCGCAAAACCCACCTTGAAAGGGTGGTGACTTTACCAGTTTGTCTAACAGGA
CATAGTTAAGCGTCATATCGGATTCGAACCGGTGCCACCGACTTGGAAGGACGGGATGCT
GGACCGCCAACACTAATGACGCATAATTTTGGTGGGAGTAGCAGGCTTTGCTCCTGCGAC
CTCTCGATTATCGGTCGAGCGCTCTACTAACTGAGCTATACTCCCTTTGTTTTGGTGGCC
CACCCTGGATTCGAACCTGGAACCCCGAAGGGCACGGAATTAGAATCCGCTGCAATAACC
GTTCTGCCAATTAGCCAAAATCTTTATAGCGGTGGACGACGATCTGCAAAGAAGCTGATG
ATCCAAACCACCATCGCTACGATAAATACAACTTCTGCAGCCCAAGCTGCTGTTCCTGCC
ATTGCCCCAAATCCCTGAAATGCCGCGATTAATGCGATAACAAGAAACATCAAACCCCAA
TTCAACATATTAGTATCCTCATATTAAGATATTTTGGTTAACCATTTTGTTTGAAAGGAA
CGATGTCGGTAGTGCAAGGTTTTGATGGAGCCTTCTCCGGAACCTTATTACCGTCATCAA
CGTTTTTAGTTTCTTCGTCACTTTCCTTTTCTTCATCGAAGTAATCAGGCATCATTACTT
CGCCTTTATCGATGACGAAACGTTCAATGTTCTCTCTGATGTTGTCCTTGGTGCTGGAAT
TCATCATCAGGTCACGTAAAACCTGGTGTGCGTTGGGAATTATGGCGATCATCTTTGTCT
TCAGGTCAAGAAGATTCTTCTCAAGCTGAATTGCATTTGACTCCTCTTTTAAAGGACGCC
CCTTTCCACGACGACGCTTTGTAAGCTGTTGGTTTTCTGCCATAATTAACCCCACTCATT
CATATTATCAGAGATGTTTTTCTCTGTCAAGGGTTATTTTAACATATTTTACTTTAAATT
TAAAGCTTTGGAGCACTGTGAGGGAATCGAACCCACGACATTGGTTTTGCAGACGAAGCT
CTTACCACTCCAGCAACAGTGCTTGGTCGAGGCAGCAGGATTCGAACCCGCGTAAAGCAG
TTTTGAAGACTCCAGCCTATCCGCTAGACTATGCCCCGTAAATTCTGGTGAAGGTGACGA
GACTCGAACTCGTAATCATTGAAGCGGGAGATTTTAAGTCTCCTGTGTATCTCCATTTCA
ACCACACCTGCAATCATCGTTCCTGAAATATGACACAACAAAAACTTTTCCAAGAGCAGG
TCCACCGCTTTGTATATGCTTACCATATTGCCCTGAGCAAGACCTCGAACAAAAAGGGCC
AGATTTTTTATCAGATCTGTTCTTCCTTTGTGATTTTGCCAATTTAAATGTATTACCACA
CCAAAGACAGTTGCAATATATTTTCTTTGCTCGTAGAGCGCTCTTATCTGCGTTAGCCAT
TCTTGTTAAGTCTTGCAGATTAGAGATGTTGTTGTTATTCACATCCCTGTCTATATGGTC
AACTGTTTCTAAATCTGGATCAAGCTCTCTTCCAAGATGCATCTCCATAAGATAGTTTGG
GTCGCTTCCTTTCATTTGCATACCGTCATAAAGATAGATGATCACATGTTGCCTGCCATC
ATCCCTAACATACGGACCTAAAACTTTTCTAATCTTATCGAGATACATATGCCTCTACCA
GATTGGACCAAGGACGCAAAACATATTTTCAGCTGCATGGTGACTCCACCGACCTCCGTT
CTCCGCGAAGGGCGCTCTACGACCCTGAGACTTCTGTTCCAACAACGTGGCCTTTGTCGG

TGGAACTCCATTTCGTTAAGCTCATTCTCCGAACAGATGATCAGTCTTTGAATCAAATAA
TCTGAGCTAACGCACTCTAAAACACGCTAAACATCTTATGTACGGGCTGAAGTCCTGAAT
ATCGCCATATTCCCCACACAAGTACCAAGCGTCTGCGAGACACTCCGCCTGTACCATTCT
GTAATAATCGGTTCTCGTGATAACCAATCTACGGAATCACACCCCAGTCGGTGCAGCAAG
GTTTTAAGATTCTCAAACCCGTACATAAGATGTTTGGAGCCGGGTGACGGAATCGAACCG
CATATGTATTCTCCTCCCACACCACGCTGGAGGGTATTTTCAGGCCTGGATGGACCTCGA
CCTTCCCAAAGGTCTGACCCGGCATTGGCTCCTCGTACAGGAATCGAACCTGTGCTTATC
CCGAATCTTGGGACTGCTCTACCAACGTGAGCTAACTAGGAATTGTTTGGTGCTGCCAGT
AGGAATCGAACCCACTTCTTCGGCTTACAAGGCCGCTGCATCGCCACTTATGCTTTAGGA
GCAAGAATGCTGTTTCCGATTAACTGCCCGGAGGTGTGGTTAGCCATATCTTGTGAGCTA
ATTACCTTTGACCCACGATTTCAATAATCAACGAAATTTTATCTTTGTGTTTTGAAAGTT
TCAAGTTTAAACGTTGACCTTTGAACTTTAATCTTTGAGTTTTGAACTTTGAACTATTCG
ATCTCGCGATTCGAAGTTTGTTTCGTTCGGAGTTGTAGTCCGAAGAACATACGTCGAGGT
ATCATCCTTTATGATTAAGTTCGAGGCAGTTTAACCTCCGGCCTCGGTAGAGGCAAGATT
TTTCCAACTTCGCTAACCAAAAACAGCATTTAAACACTTTACCAGTTAAAAACAGGAATT
ACAAGAACTTTTTCATAATTCCCGCAATTAATTTTAGAGTGATCCCTCAATCTCTACTTC
AGTCTTCGCATTGATCTCTGACAGTGCAAAGTAAACGTTGGAAGTGAAGTCTTCGATCTC
TGCTTCCATAGAGCGGATGGTGTCTGACAGACCCAGAGGGTCAACGATAGACGGGGTGCT
GATCATGCGGGTCGGACCTTCTACGACCTTCAGCTGTTAAGCACCCGGAGACTTCTCACG
ACCGATGTACGATGCGCGATTCTGTTCCAGTTTTGCTTCGAACCCCATAGTCTCCTTGTT
CAGGCGAGCCTGAGCAAGGTTGTACTGGGCTTTCAGCTTGGCAAGCAGAAGCTCTTTGTA
CTGAATGGTGCGGTTGCGTTCAATTGCCTGAGCAACTGTCATCTCCTGGTCACCAACGGT
AACCTTGGTGGTTGCGTTGCACTGCACAACGGCAGCTTTCAGTTCGTCACGAACTTTCAG
GAGGTCTTGCAGGCCCTGATAGTCTGCTTTCGCAGCTTTCTCAAAGGCATCAAAACTGAT
CAGTGAACCAGGGATCTGACGGTTACCGTCAATACCTTTTGCAATGTGAACAGTGTTAAG
CTGGTCAACACGTTTCTCGATTTTACCCTCGATCTTGGTCAGAGTAGCCAAGGCGCGGGT
TACAGAAATCTTAGTAGATATATTATTCTCCTCTCATTTAATTTTGAATTTTAAAGTCAC
TGCAAATTATTAGCCGACTTCCTTGTCGGCACAGCATATTAGTTTCTGCCAGTGTTGACT
ACAACGCCTTGGCGACCAGCATCTGCCTGAGCAATAGCGAGACGGGTTTCACGGGCAGCT
TTCAGGTCAGCACGAGCAGCTTCAAGCTGTTCAGTAAGCATTGCGATACGGTCATCTTTC
GTTTTACTATTAGCAGTCAGTTCTGCCGATTCAACTTTGTGTGCAGATTCCAGGTTGGAC
TTCGCTGCGTTCTGTTCGATTGCAGCACTGCGCTTAACATCAGCAACAGCGGCATTGATC
GCGTCTTCGTTATTGTCCTGTGCAACAGCCAGTTCATTACGCAGTTCTTCAACAACAGAA
GGATCGATTGCAACCAGACCGCGAGATTTCAGCAGGTTTGACAGAACGCCTTCTTCGTTT
TCCAGAGCCTTGATCTTCAGCTTGGATTTTTCTTCAGCCAGCTTTTTGTTAAACTGAACT
TCAATCTGTCCCAGTTCCTGTTGTTTGAACTGAATCTCCTGACTGATCTGCTCAGAGCCT
TCCGCCAGGGTTTGAAGATCGGCAACTGCCTTGCAAAGGTTGGTTGCAGCAGCGGTTACA
GCTTTGGTGGCACGTTCAGTTACGGAAAGTACAGTTTTAGAATCAGACATTAAGTGCTCC
TTTGAGTTAAATTAGGTTTTAAATACTACACTTACAGATTGTGTTTGTCAAGCGAATCTT
TGATCATTTCTTCGGTTGTCGGGTTGTTTTCGAACCACGACCAGTAATCCCAAAGAATGA
TAATCGCACAAATGACAAGCATTGCCAGAAAAATCATGCCGCGGCTCGTCGTACAACGCC
GTTAATAGAATCACAAACTTCACCAACAACGTCTGCGGTCGGAGATACGATCATCTCATC
GGCAGGGTGAACATCCTCTACTGCGACATCCACAAAAGGTTCTGCAACAGTTGCAGTAAC
AGCAGCAGCAGTACCAACAACATCACCAGCCATGCTTAACAGAGATCCGAACATTTATTT
GCTCCTTTATAAAGTTGGTGGGGCATGAAGGATTCGAACCTTCGACCTTCGGCTTAAGAG
GCCGCTACACTAAACGCTGTGTTAATACCCCGAATCTGGCCCGTGTTTTTCAAGACGTTG
CACGGGATCGTCACGGTTGCCACGGCCCTTTATTTTAAGACGCATCGCCGCAGCCAGCTT
CGTATATCCCCATGCCGGAGGAATTGCTTCCTCTTAGCTTTCGGCGCTACGGCTTCAGCA
ACCTTAGCGTCTTTAGCTTAACCAGCGCGTTCACGCTTGGTCTTGCCAGTGCGAACACGA
TGTGAGGCAGAAATGACCTGACCACCTTCAACGCGGGAAACTGATTCCCAGATTGTTACA
ACGTTGCCATCTTCATCACGACGCTTCTTACGAATAGAAACCGTCTCTGTTACACCGCCC
AGACGAACATCAAGAGGGGCAGAGTGGGTCTGCGTAGACTTGGACTCAAACTGGAACGGC
TGCTTGATTGCGTCGGTTGCAGAAACTTTCTTTTTTGCGTTTTGCTTAGCCATGTTTTAT
TTCGCCTCTATTTGCTAATGTCTCGTTGACGTTGGTAATACTAAACTCTCTCACTTCCTT
TGACAATCGTCTTTTAAATCTTTTTTCACTTCTTGATGAAGACCGAGTGAGAGGTCATAA
CGATCATATGTATGAACCGAGGATGCCTTGATAGAATCAAATCGGCTTTTCAATGATTCT
GACATACGTGATTTTTCAATGATACCAAAACACTAATATACACGTTTCATTGCATCTCCG

GTTTGTTAGTTCTGGCAGGAATTGAACCTGCGACCTCTTCCTTATGAGGGAAGCGCTCTA
ACCAACTAAGCTACAGAACCATTCCGTCTTGGTGAGATGAATACTAATGGTTAAAGAAAC
CATTGTCAACACCTAAAATGAAAAAAAGCCCAGTATTTTTCTGTCCGGTGCTTCTGGTAT
GACTTGCGATATGAAGTCGCCTGGTTGTCAAGACGCTGGTTAACTCGTGCAGCGTCACCG
CGAACAAACAGAACATCCTGCTTCATGACATCAACGTCAGATTGCAGTTGATCAACTTTC
TGGTTGAGAGATGCAACATCGCCTGACAGTTTTTCAAGACGCTCAATTTTGTGGCTGGTG
CAACCAGTTACTGCCAGTGTCCCTATTTGAACCGCGCCAAGAATAGCCTTTTTAAACATT
TTATTTCTCCTGGGATATGTGCAGCAGTAGTATCTGCATGATCTATTTTACTGCAAAAGG
TCCCAATTTACTTCAGGAAATGTCCGAAAGAGAAACATTCTGTTTACAAGCATTGCAGGT
GGTTACCTTCTTTGGACTCATGAACGGAAGGCCGCACACATCCCATTCAGTGTGGTAATA
AAAATTACGGAATGTTTCAGTGGGCCAGTCTCTGTCGCTCAATTCAAAGATTGAGTCAAA
GGTTTGGTCTTTCATCAGAACAAATACCCTCTCCGAAACCTACGAAAATATCTACGTCTT
CAGCCTGGCGAATGCCTTCACCGTAACCAATAAATCCGTCAAATTCGTCTTTCATCTTTT
TACTCCTTTTCGCTTTGAACGTGTAATCACCGCTTCAGCACGGCGCTGCTTCCGGTTTTT
ATACTGTCCGGAAGATTCGGAGAGCGTACTTGATTATAGAATCACCGTCAACGGGTTAGA
AATAGTATAAGCCGCCTTCTTCTCCGAAGGATGCAACGTATTGCCATGCCTCTATGCCAA
AGCAAGCCGTAGAAAAAGCGATGTCATTAGCTTCTTCAGTCGCTGCGTTAAGGGTTGGGT
GGTGTGATTCGTGCTCAATGTGCTCTCCATTATCTGATTTATGTACAAGCTTTAATTTAA
ACATTATGACCTAGCGTTAAGGTGTTAAGAATTTCGAGTTGATTCGATAGTTTAGAACTT
ATCAGTGGTCACTCTTTTTGTCAATCAAACAATCCCTGATGCCTTGACACCACGACGATA
TACTGCTAGTGCTAACGTCTTGGGGTGAAGCTCTTCCTCATCTTCCTTCGGATTATCAAT
CTCGAGGAAATGTTCCTCCAGACCAACAAGGATCTCCGGCTCCCACGCCTACTTCTGGTC
TGTGTGTACAGATGCACGATTGAAATCATGACGACTAACTGCATTTCGCACTCAATATCT
CCTTTTCTCTATCTCGTCAAAATATTGCATCATCAAGGTTCCTGAATGTTTTATTTCGTT
TTTCAGTTCCCCATTTTATTGTCACAACATAATGATTTCGTTTTTCAACCCACGTTATCT
TAACATGTCCAGTGTTTGAAGATGCAGTGACATTCCTATTATTCATGTCGCGTGTTGCTT
TCTTCAGGTTTGCCCACGCATTGTTGGTCCTTATCCTCTCATCATGGTCAGCGAATTCAG
GCATTTATCCAGTCATATAGAGATATGCAAGTCTATGAGCCAAGTATAGTTTTCCATCAA
TACGTATTACAACATAACCGTGCTTTCCAACGCAAGAACACTCTGTTCCACCAAGGCGGG
TAGCGTCTTCCTTTCTTGAAGAACCCTCTTTTAACCATGTGAAAATGCCTGTCTGCGGAT
CGTAATGCAAACGGGTTTTGAGATACTCTTGCGTTAGACTACGCACAGGATTCCTCTTCC
AAATCAATTTTTCCTGTTTTAGTGTTTACCCACACTGTTTTATCCTTGCTGACGTTCATC
TGATGACGGCAGCCTAAGGACACGATGCAAACGCCTGGGCCGTGGGCCTCAAGATTAGCT
TCAAATTCCTATTTACAGACTTTGCACTCCATCATTACTGGTCCAAACATCTTAGGCTCC
TCAATAAAATACGGGTTCGTTGGTGTGTTATTACACGTAAGGCAATCTCGTACTGGGACT
TCATGCCACTTGCATGGATTTTCCATAGCCTACCTATCAGAAAAGCCCCCGAAGGGGCTG
ATAATTTAAAGCTGAGAGGCTGCCCGACCACGAATCTGTGTCAGGTCTTCGTCAATCATC
AACTGACCGTCTCGGAGGACACAACCGAGTTCGTTATCACGAGACTGTACCTCATGCACC
TCCAGATTGTCAACCAGTTTTAACTGATTGTCTTCATCACGAATAACCTTCAGGAAACCT
TTTGCAGATTTCTTAGCGAGGTCAGTCTTCGGGTCTTTGACAACCATGATCTGCTTGCCG
TCGATAACTGCACCCGTTGCTTTCACAGCCATACCCAGTGAATCACGAGTTGTGTACTGG
GCTGTGTAAGAACCAACACCGTACACGATGTTACTCGATGCAAGGCCACGATCTTTCAGT
CGTTCAAAGATATCTTTTGCACGAGCAACAGTGATATAGTCACCGTAGATCAGGCCGACA
TGACTATCAAGAACTTTGTACCATCTGGAGTTAACTTCTCCACCAAAGATTTCCCACAAA
CATTCTACTGCACCTTTTACCTCATTATCTTCAAGACCTGACGCTTGGAAATCAAAGAAT
GCATTATCTTCATCATCTACCAGAATGCGATAATATTTACCCTCAATCTTAGCAACTTCA
GAGTTTGAATTATCCTGTAATGCTGTGTCCGCATCTGACCAGTCATGGAACTCACCCAAG
CAGCGATACCCGGCAAAAATATGCTCAGGCATACCTGAGTCAGGACGAAATACAACACGA
CCTTCACGAGAAAGAGTTTCATGTTTCAAGATTGCTGCTGCTTTCGTGATAATCGCCAAG
AGGCCATAGCTATCTGCAACGTAGGAAACAAAACCTTTCGGAAAAATTTCCGTGATGTAG
CGCTTCAGGAACTCGACTTCTGCAAGATAACGTTTAAGATCAAGGCTCACGAATTCTTGA
TTTGCTGTGTTTTCTTCCATCTCATTAACGATGAAACCGATATTGGTTGTTGCAACATTG
TGTTCAGATGCAGGGACAGACCCAGAAAGGAACATACATTTCATGTTGTAATAACGATTT
GCATACACAATCGCGGGAATGGTATCAGTACCCATAGAACTGATCAGTTGTCCAAATCCA
CTCTGTGCTGCGTCATGAGTCGCACCTTCACCACGATATGCAAAACAGTGGTTCTGGAAG
GGAACATGCAGATTATTATCACATGTTTCAGCAGCCCATTTCTGAGACAGTTTACGATAT
TGCCAGAATGTTGTTGCAGTTGTGACTGTTTTCCAGGTTGCTGCTGACATCACAGTTTAC

AGGAAGTTCGGTAACCAGTAAAAATCCGGATGGGTATTCTGGATAGTGAACATTGGAACC
TGCACCGGAGCCAGAGTGCCTTCTGGTAAAGCACGGATTTCCAGAGGTAGATAACCAAGG
TCGTGTAGCTTCTCAAAACCCCACATTTCCACCATACCCGGTCCAATACTCAGGTCGCAA
ATTGCTTTGTAATCTGAAATCGCTTCTTCCTTTGAGCGTTTGAAAAAGTTTTAAGTAAAC
ATATCGATCAGGAACCACTTGATGAATCCCTGAATACCAGCAAACATCACACGGTGGTCA
TTGTCTGCGAAGTGGATAAAGTGCTTTGCGTTACGGGCGTTCATATTGGCCTGAACAATG
TCAGAACGTTTGTCATACATCGAGATGTGACTCGGTTTGTAAAAATCAGTGAAAGTAATC
GGGCTTTCTTTATGCAGCATGTGTGTCTCTCCTCTGTGAATTTTGTGGGAGCATTCTTGG
ACCCGTTTTATACTATGTCAAGAAATTTTAGAAGTTTTCTATCCAGTCGTGATATGCATA
AATTTCATCTACACCATCATCAAGAATGACCTGCTTGCCGAATGAGAAAATACCGTGTGT
GACATAAAGGATAACACGTTTTGCACCCTTCTCCATCAACGCTTGTGCAAGGGCCATCAA
TGTGCGACCACCATCACAAATGTCATCAACGATGACACATTCACGACCAGTAACATCCTC
GAAGACAGCAGTACCTGTGATCTCGTGAGTTTTTACATCACGGACCTTACCTGCTTCAAC
AAGCGGAAGGCGGAGATGTTCAGAAACCTTCTGCGCTTTTTTACGTGCTCCGGCATCTGG
GGCGATAATCGTAACATCCCTACCAGCAAGTACATCACTATAGGATGCTGCGAGTTCATG
TTGGTGCCGAATACGAACATTGTTCAGAAGTGCAGCAGAAACATCACTGTGCGGGTCATC
TACGATAACCTTATCAAACCCAAGTGACTTAACGATGTGTGCAAAAACCTTGATGGACAA
TGCCTCGCCAGGCTGCTTAACACGAGCTTGACGCGCATGGGGGATGTACGGCAGGTGAAG
GAGATACTGTGCTTGAATATCAATGTTATCAAGTCGAGAGCAAGCATCTGCAAGCAGAGC
AAGAGCCATAACATCCGCAGATGTCTTGATTTTTGCTGTGATGTCAACCAAACCAATGTG
ACCTTTTGGTGCATAAATTTAATCGATCTTAACCTGTTCTTCACCACCCTTGAATGTAAG
GCGATCAACAGGGCCTATCAGGTTGTTCAGACGAAGTTTTAAACTCATTTTATTTCTCCT
CAATTATTTGATTAGTCTTTAAAATATCTTGCTTGTGGATTGTCTCTGTGAAGCCGTCAG
GTAACCAAGCTTTTTCACAAACAAATTGTGGGATCGGACGCAACACAATCTGCTCAACTT
TGGCAGCTTCACCATAACTTTTGAACAGCATGGAGTACATCAGTTTGTGCTGAAACAAAC
TTTTCTTTGAATGTTGGCGCATTCTTTCGAAAACATAAAATGCTATACCAACGTTTCCGA
TTGTTAAGCCTGGGCCTGCAAGCTCTTGAACATAAATGTAGCAGGTTCTTTCTAATTTTG
GATTTGTAGAATCGCCAAACGAAATACTGTACTGACTACTCTCCGTTCCACTGTCAGAGA
TGGCGGTTTTCTTCTCGATCCAACCCTTTTCATACAGAGAAGAGAGTGACCTACCAACAC
TGGCTCTACTCAACCCTGTGGCGTTTATGAAAACTTGGTAAGTATAAACCCCAATACCAT
TCAACCCCATAAACATCAAAAGAACCTTTTCAGAAGCACTTATTTCTTTCGATTTTATAA
CTTCATGCATAACATCCACTATCTCTTAACAATTACGTAATGCCTCGGTTTTGCATTCCC
TTTAGAATCTTGAAAACCAGAGCGTATGCGGCGCACGTATCCAAGATACTCTATCTTTGT
CAAGAACTCTGATACTTTCGGTTGTCACTTCTCACTCAACCCAAAAAGATGACACAAAGA
TTTTTGGGATGGAAAGAATATCTCATCACCACCATCAGGGATTAAACTCTTGAAGTAATC
ATACCTCCACCACAATTGTATCAACCTTTGCAAATCTTGTGGTGAGAGACGGTCATCAAA
AAGCCAGTGTGTCGGGATAGTGACGTAAGAGCCATCCCTTATGTTGACAGCGTTATCACC
AATCTTTATCTCCACCACCATCTCCTATTTATTCCAGAACAGATATAATTAGAATGTACA
CCAACGTAACGCAGATGTCAACATAATTATTCCCAATCGGGAATGTCTAATTATTCCCAA
AAAGGAATCCAATACTAACCTATCAATTAAACTAACTTATGATCTTTTAAAATCAAAACA
ATAAAACATAAGATCTATATAAAAGATCTTTAAGTATGATCTATAGATTCTACAGTCATA
GAGCCAGTGGCTCAGTTGGGTCAGTTGGGTCAGTATGGATGAACAAAAATCTGTTGTCAA
GAGTTGACTTCTCGTTTTGTTCATGTACAAAGCTTCTATCGTTTTAGAGAAGAGTGTGAA
TGTTGTCAAGTAAACAAGCTAAAGCTGTTGGAGATATGGTGTGTGGTAAGAGTCGTCGTA
AGGCGATGCCTACAGATGAAGAAACCACACGATGGATGATGGTATGTGAAGTCTTTCATG
AATGCAATAAGGAACAGAAAGATTGTGTGGTGTTACCAGATGGTCAATCTGCACATGAGG
TGTTTGGAAATTACGACGACTTTGTTCGTGGTCGTCTACGCAGACCAAATGTGGTGGTGA
CTCAGATGAAAGACTTGACAGTGGAGCACATCGAAGGTAATAATTGTGTGGTGGATCTGA
GTGAGATTCTCATAACGCTCTGAGAGCCTCTCTGAGACGTTTTGCTGGTGTGGTGGTACA
CTTGGGTTCCTCACACCATTTTAATTCAATACAGGGCCGCTCAGAGGCTCTGAGGGCTAT
TGTGGAAGATTATTTTCAAGCTTAGTCACATATCTGGTGAAAAATCTGTATGCAGAGAGG
CTTTTATGGCTCTCTTGAACATCATGGCTTTCGCTGAAAAGCGGTTCAATACAAAACTGT
ATCTCGACAGCCGTGTGGATATTGGTAAGTCGTGGTTGGAGTCATTTGTGTACATTCATG
GTGATCGAAATGTCGTCAACTTGGTGTGCTTGAAGCTTATTACTTCGCAGCACAACAATC
TGCCCCGTGGAAACCAATACCTTCTTTACGCATACCCTCCGGAGGACATGGACAAATGAA
AAAAGACATCAAGAAACCTGTAATGGAAAAATTCGATACAACAGCTTTTGTTTTCAATAC
AAAGGTAGAGATCATCAAAGAAGCCATTCAACGCTTGCACGATCAAGAGGCAGATCCTAG

CGCAGAACCACTTACAAAAGATGAAATTTCGTGGCTGGGGTGGTGGGAACTAAACATGCC
AGAACTGGAAAGGTGTGGACTCACAGAGGTTATGACGTGCGCCCAAGAGAACGCCGCTTT
AGGGCTGTATAAGAAAACAACGAAAAGGCAGAAAAAGAAATGACCAATATCGATCATGTA
ACATCAGATACAAATTGGTGGCATACCAATATTCTTGGTTTCCCATCCTTTGATAACTTC
CTTAAAAACCTGTACGGACCGAAAGAAGATTCAGCGAGGGTTGGCAAGATGAATGACGAC
ATGGTTAAAACATGGAACAAACATGTGAAACCTGATGATGTTGTTGCACATTTAGGTGAT
TTTGTGATCGCGTATGGTAAGCATATGGAGCCGAAGGTTCGTGACATTCTTAGCCGAATC
AACGGAAAGATCATTTTGGTTGGTGGCAACCACGACAACCACACTACAAAGCGGGTATTT
CGCGAGTTTGGTCACGAAGTTGTTGACTATAAAGAATTTGATTTCAGCACAGGAAAAGAA
AAAGTTCGGGTGTGTATGTCGCACTGCCCGTTTGCATCTTGGAATAAAGCACACCACGGA
TCAGTCATGCTCCACGGACATAGGCACGGCTCATATAAGGCACCTGGTGGACGAATCCTT
GATGTGGGTCGGGACGTCCACGGTCGACCTTTGACGATGCTTGAGGCCGTTTCTGAGGGA
CTTAAAAAGCCTATTTACGATATTCCTCACTGAGGAGATGGTGTGCACAACCAAAAACGC
AATTTCTTGCCTAAGAAAACATCAAAATCTTTTTACGCCACAATACATATTGCTGGCAGT
TACAACCAGGCGGAAAATCTTTGCCGACACTGGGTGAGGAGGGGAGCATGTGTGCAGATT
TCCCCTTGTACTTCTGTTTATACTGGTGGTGTGGAAGACGGTATGACTGTGCGTATCATG
CAGTATCCTCGTTTTGAGCGACCAGGACATGAAATTCTTGACATGGCTGTTGAACTTGGG
ACATCCTTGGCTCAGGAATTATGCCAGGTAAGCTTCAGTGTTGATACACCGTATAATAAA
ACGTATTATCAGGCAGATTGTTATGAAAAAAGATCTTAGGTCCCCTCTTGGTATCAATGA
CTCTGAGATAATTACACAGAGTGTTGTTGATGGAAAAAGGATTTTCCATCCTGTATATCT
TAAGTGGCGAAATATGTTGCGAAGATGTCTTGTAAAGCACAATAAACACCAATATTATGA
AAGAGTATCAGTATGCGATGAATGGTATTACCTTTCCAATTTCATTTCGTGGGTTGGTGA
TACGGATGTTGCTGGCCTCTACTTGGATAAGGATATCCTCGGTGACGGAAAAATATACTC
ACAAGAGTCTTGTGCATTCGTAACACAATAATGTAATCAATTCTTTTCATCTGTGAAGAA
AAATGGCAAGTATATGATAGGGGTTTCCAAAGGAACCCGCCTTGCGGACACATTATACTG
CCACTACAAAGGTAGGTATATTGGTAGTTTTAGAACAGAAACCGATGCTCATAAAGCTTG
GCACATTGAAAAGAGAAATCATGCAAAATCTCTTGCAATCTCAGAACAGGATGTTAGAAT
AAGACAAAAGCTGCAAGATATTAGTGCAAAAATTGAATATGACATCCTAATGGGATAAGA
AACATGCTCTTTAACGTAGACGATCTGGTCGCTAAATAGCTGGTCACCAAGAAGACTGAC
ACTGATAGCCCGTTCGCTGGCCTATCAGTTCTGAAGTACAAGAACAATGTCTTCTGGGAT
AATCTGTGGAACACAGATCCACGTCTCTTGGAATGCCGTGGCATGGAGGTTGATTCGGAT
GACAATGTGGTTATCTGGCCTTTCACCAAGATTTTCAATCACTTCGAAAGTGGCACAGAC
TTTCCGGCAGACAAGGTGGTTGATGCAGTTCGTAAAGTTAAGGGATTTATGGCATCCGCT
GGTGTGTATAAGGGCCAGCTTGTGGTGTCAACTACAGGAACTCTGGAATCAGACTTTGCT
GTTCTGGCGAAGACTCACATCATTGCTGAATGCAATGACATTGAGCACGTCATTAAGTGG
ACCTAAAGTGCAAAGGCGACATTCATCTTCGATATCTGTGATTCATGTGATCCGCATATT
GGTGATGATGAGCGTGGGGCGTACCTGATTGGTGTTCGAATCCAGATCCGTGATGGCACA
AGTTTTATGCTGCCTGAAGGTGACCTTGACGGAATCGCGAATGTTACTAAATTCACGAAT
CCTGAAGTCCACCAGATGGTTAAATTTTCAGTTGTTGTTGAAATGTCAAAACATTGCAAC
CACGAAGGTTATGTTGTGCGTGTGCCAAACTATCCGTTCAGCTTACTGTAGAAAATCAAA
TCCCCTCATTATCTGGCGAAGAAGTTCTTGATGCGTGGTGGTGATAACATGTGGGATATG
ATCTGGGATCATCCAGACAACGCAAAGCAGCGTATTGATGAAGAGTATTACGAATTGCTT
GATCATATTCCTGAATACTACACCAAAGATGCTTGGTCAGCTATGGATTCACAGCAGCAT
CGTAAGATAGTTGAAGATTATTTTACGATAGAAGATCTCTTTAACCGTGGGAGTCGTTTT
TATGTTGGTGTGGCTCGATGACTTAAGGGACCCCGTTAATTACGGGTATCCAGATGCCTT
GTGGATAAAAAATTCGCAGGATTTTACTCAGTTTCTCGAAAATCGGTCAAAACCATATCG
TCGTGTCACGGAGTGGCACTTCGATAATGATCTTGGAGAAGATTCTGATCATGATGGGTA
TTGGCGTTTCCTGGCGCTTGAGGAAAAGATTGTCGTCGTAAAGATGCTGTTTGGCCCAGT
TAAGCTGTTCGTTCACACATCGGACCCTTCACCAGGGCATAAGTTTATGCTTTCAAAAGA
TAGTTTGGCACGGTCTCGTGTAACCATTCTGAGAAACAATTACTGAGGAGAGGGTATGGA
AATGACATTAATCATCATTCGTGGGGTTGCTGGTGCGGGTAAATCAACCGTTGCAGAACT
CCTGAAGAATGACAGCAATGTTGCACATTACGACGCGGATATGTTCGACTATGTTGATGG
TGAGGACCGGTGGAAGCAGGAAAACCAGCACAAGGCGGACGAGTGGTGCCAGAAAAACGT
TCGCCTGGCAATGGATATCGGTTATGATCGCGTGCTCGTTTCAAATACCTCAACCAGCGA
GAAAGAAATTAAACCGTAGCTTGACTTGGCTAATGATTATGGATATCAACTAGTTTGCTT
GGTGGTTGAAAACCGTCATGGTAACGATGCTATTCACAGCGTTCCGCAAGAAACACGAGA
TGCTCAAGAGCGTCGTCTGCGTAACTCGTTGAAATTACAATAGGAGAAATAAGTGAGACG

TTTGATTGTTATTTCTGGTGCTGGTTTCTCTGCTGGGAGTGGTGTTCGAACTTTCCGTAC
TGATGAGTCAACAGGTAAGGCGTTGTGGGATGAATACGATCTGGAAGAGGTTTGCAATAT
CCACGCATTCCGTGGAAACTTCTACGATAAAACCCACATGTTTTACAATAAGCGTCGTGC
TGAATTGCTGACTGTACATCCGAACCTGGCTCACCTTCACGTTGCAGAGCGGTTCCAGCG
TTATCCAGGGCAGGTTGTTAAATTGACCACCAACGTTGATGATCTTTTGGAACGTGATGG
TGTGACGAAAGAGGATACGGTCTACATTCATGGATACCTGACGGAAATCATTGTCAAGGA
CAACCCGAAAGGAAAGCCGAAGATTATGGATGTAGGCTATAACGAGGTTGATCCTGTTCA
GTATAAATGGTGTAAGCCCAATGTTGTGTTCTTTGGTGAATTTGCACCAGAATACAGCAG
GATGTATGACATCCTTGACGCCCTTACCAATGACGATATGGTGATCGTTGTCGGTTGCTC
AAATACAGGCATCAACTTCAACTGGGAGTTGTTTCCTGCTCTCCGTAGAGAAACAAAGGT
GCTTGTGGTAAACCCACGTATCAACTATCTTGAGCATGAGCAGTATGATGCTCATGGTGT
TATGCAATTCCGTTGTGGTGCTGTAGAGGTCTTCAGCAACAACAATTTCATCAAGATTGT
GGGAGATCATCTTGAATGTAAAACAGCATTACAAGGTAAGGAGAAATAATGTGGATTGCT
TTACTTATCCCAACACTGTACGCCATTAACAAGGACGGATCTTATCAGGACTGGAATGTT
AGAACAGAGGGCGATGAGGTAGTTGTTGAGTCTGGTACACAAAATGGAAAGGTACAGCAA
AAACGTACCAAATGTGAAATGAAAAATCGTGGTCGTGCTAATCAAACAACTGCTGCATTT
CAGGCTGTGCTTGAAGCGCGATCAATATGGGAGAAGCAAGTACGCCTTGGTTATCGTGAG
ACAAAAGAGGCGTTACAGACTGAGGAGAACTTCTCTCCCATGCTCGCACACGATGCAATT
AAACGTTCAAAAGCATTAGTTTACCCTGCATATGAGCAGCCTAAGCTTGATGGGGTACGC
GCCCTTGTAACTCTTGACAAAGATGTTATCCCAGTATTTAATAGTCGCGGAAACAAGACG
TACCCTGTTCAAGGTGCATTGTTGCAGCAGACTTCAGAACTTGCAGAGCATACTGGGTTT
GACAAATTTGATGGTGAACTATATATTCATGGTCTGAGCTTACAGAAGATTGTTGCTCTG
GCTAAGAAGTGGCGTAAAACTGAGCAGATCGAGGTTGAGATTGACAAGGATTACCAATCA
GATCTGAAACGCTATCAGAAAGCTATTGACAACGGTGAGCAGGTATGGAAAGATTTTGAT
GGTGATGAATACGACGTAAGTATTGAACCGGTTAAGGATGTTGACCGATATGGCGGTTCC
ACAAGTTTGGACCTTGAATATCACATTTTCGATATTCCTGTAAACGCAAACAGCCCGTGG
CATTCAGAGAGCCATGATAAAAACCGACTATCTGATCTTGTTACCGTGATGTATTCACTT
GATAGTGAGGTCAGTGCACCAAAAATAAATGTTGTTCGTGGTGAGTTTGTCCTGAATGAA
GATGCGGTTAAGCGGTTCATTGGGCGATACATGCAAGAGGGTTATGAAGGTGTTATTATC
CGCAACTCCAAAGGCGTGTATGAATTCGGTCCGCGATCTTCCGACCTTCAGAAATGGAAG
CTGTTCCAGGATGGTGAAGCGAAGGTGTTGGACTCTGTGGAGCGCACAGACGGTGAGCGC
GTTCTCCTCTGCGAAGAAAAAGACGTAACCAGATTTACCCGTAAGATGAAGGGGACGCGT
GAAGAACGTTCTCAAGCGAGAAAGTTACTTCTGGTCGGTAAGTTCATCAACTTCACATTC
CAAGCAAGGACTGAGGACGGGGTTCCTCGGTTCCCTGTAGGGCCGCGTGTACGAGTAGTT
GATTCATTAACATGGGAGCCTGTATACTGATGGCATTTTATAAGAGAAAATATCAAGAAG
TGGAAGCGCATGTGTTCAATGGTTCAAGCACAGGTGTTGGTCAAGTGATAAAGTGGATGG
AGACTGGTGTCTGGAAAGATTCAGAGATTCACACACGAGACATCCATAACACGGATGTGA
ATGGGACCCAAGCTTTTCCTGGTGATTATATCGTTAAGGTTGGCGACCAATTCCGTGCAA
TGTCACCACAGGATTTTAGCGACCTGTATGAGCCTGTGACCGATGATGATCGTCTACATG
GTGATGACAAGGCTAAGAACGTGGTGATGGGTGATAAGGTTGAGATGTGGGGCAAAGAAT
ATCTCGTCGAAGGGGTCAAGGTAAGTGGTGATGACACCATGCTTGGACTCGACGATGGGT
CTAATGTCCAGATCCGTGGTTAAACTGCTGTAAAAGTCACTGAGGTACGTGCATAATGAT
CTGGTATGGACTGGCGTTGCTTGGTGCAACAATACTCACTGATGGCGGGATTTATCTCGC
CTACCGATTCCGTAAAGATAAGCTCGATGTTTTTATCGACTGGACTCAGGGAAAGATTAA
GCGTTTACTGATTGTCTGGTCGGTTGTATTGGGTCTTGTGACGGTGGCCCTTATCAAACC
GTTGTGGAACACAATGACATGCCAGATTGATGGCATGGCATACAAGGTTAATACCTCCTA
TTCGTGGTATAAGAACGGGAGTTATGGCGATAAGTGTCTTTTCGTTACAAAGAACGGAGC
GCTGCTTCCATTAAGAATTATTCGTGATCAGCCTGATGGTGATCATGCTTCAGCTGTTGT
TAATTAAGGAGAATAAATCAACTTCGGTGTAACTTATGAAACGCTACTGTCAATAACCAA
ACCGAAAGATATGGTTGAGTATGGTCGCCATGTAGCTCAATGGAACCACATAGCGCGTGT
TGCTCGTGGCGGGAAGGCAACTCCGGTTGATCTGCAATTCTCTTCTGTTGTTGAAGAGTT
CTAAGAGCTTATGAAGGCTCTCAGCGAAGGTGACCGAGTTGAGGGTGTTGATGGGGCCTC
TGATCTGTTCGTGGTCTCATCCTATGCTTTGTGGCTGCTTGAATCTTCTGGTGTTCGCTG
GGAGAAGTGTCTGAAGCCAGCAGAGGGTGCTCAGTTCAACAACGGTGAGATTGTAGAGCT
TATTTTCAACACCCCCTTGTCTGTGGAATCAGTTGCTGATATCTTGCAGCAGTCGGTGGC
ATTATGTTTCCGCTTAGATATCAACTTGGATTACAACATGCGTCAGGTGGTATCCTCTAA
TGACTCTAAGTATCCGACGATGACACAACTAAGAGAGGCTCACCCTACTCTGAGCTTATA

TAGTGACGATGAGTTGCTTATTGCCGAATGCAAAGCTATTGAGAAACGATCCAACGGTCG
CTACTCTTCTGTGAACGCTGTACGTAGTGGTGAAAACATCGTGTTCTTCGATGGAAAGGG
TAAGATCATGAAGCCGAGCACTTTCGTAGAACCGAAAATTATTGCTAGACAATAAAGAAG
GGGTCTGTATAATGGACCCCATCAAGACAGAGAGGAGAAATAAATTGGAAAAAGATTTTG
ACGAAGTTTGCAGGGAACTGTTAGAATTGTCAAAACGTCTTAATGTTCAAATCGTTATCG
GCAACAGGCCGACATTCAGGCCGAAGCAACATATGATCCGCTTCACAATGAAGCGTCGTT
GGGGCGGTCTCAAGAAGTACGCTTATGAATGCACTCTCGAATATCTGAAGACGTATGTAG
AGCAAATCGACAAAGCTGGCGGAAAGATTATCGATATCGAATATAAGTGTTTGACAAAAA
GATTTAAATAGCTATACTGAACTCACTAAACAAACAGAGGAGAAACAAATCTATGCCACG
TATGCCTAATATCAAAGTAGAACTGACAAACACCCTGCCTTCAGACGCTGTAGAGCGTGA
GCGCCTGAAAAAAGTTGTTCAGGAAGGTTTTGATGCGAAGATCGCCCAAAAAGATGCGGC
GAGCCAGTTCAAAGACATCGTTGCGGTTGATAATGACAGCCATAACTACGACCCGAAGTT
CATCAAGACCCTGATCAATGCTGAATTTGACCGCCAGTATCAGGCAGGTAAAAAACGTAA
AGATCTCGAAGAGCAAGTTGAGCAGATGACCGAAGCTAATATTCTGTTTGGTCGCGTGAG
CAACTAATGAAATGTTACCTGCATGGTGTTGGACTTTTTGTTTTCTTGTGCTTCATCTTC
GGGTTCGCAGTACCGACTCTGGTTTCAGCAAAGAATACACTTTTAGTCATGGGTGGTTTC
GCCCTTGTATTCTCCGCCCCAGTTGTTGTATGGTACTGGCTCCGAAAGGTGTTTATTAAA
CGTGGAGGGGTAAATGCTGCTAAATCTTAAAGCGTCTACGTGGGAAAAGTTGCAGGCTCT
GGCCCGTTCCAAGAATAACTCTGCTGCTTCTTACCTAACAAAGATTGTTGATTAGTACGT
TGACGAAAACGTAAAAACTGAATACAAAATCAAAGAGGAGAACGAAACTAATACTTAAGA
ATATCAAACGTGTACTGTTTGCTGGCCTGATCGCTTTGGGGGCAATTAGCCTGACTGGTT
GCGAACGTGCCACCGTGCCTGCGGGTTACGTTGGCGTAAAGGTTGACCTGTACGGTGACG
ACAATGGTGTCTAGCAGCAAGAAGTTGGTGTTGGTAAGTATTGGTTGACCTGGAACGGAG
AGATTTATCAGTTCCCGACATTCAACCAGTTGAATAACTACGAGCAGCCGTTCAACTTCC
AGACCAAGGATTCGATGACGGTATCCGCTAAAATCGGTGTTGAATACTTTGTTGATCGCT
CAGAGGCCACGAAGATTTTCCAGACCTACCGCAAAAGTGTTCAGGAAATCACTGATGTAA
ACCTTCGACAGAATATCTCGGATGCCTTGATCAAAGAAGCTGGCTCAATGGACATTGGTA
CGCTGCCTGGTGATTGTAACAGTCAGTTGCTTGAACGTGTTACCAAGCAGCTTAAGTCCA
GGCTTGATCCGATTGGTATTGTGATTGTAAAACTGTCATATACCGATGAGCTGATTTACC
CCCCGCAGGTAACAGCGTCAATCAACGCGAAGATCGAAGCAACTCAGAAATCTCTGCTGC
GTGAGAACGAAATCCAGCAGTCCAAGGCTGAAGCTCAGAAACAGATCGAAGCCGCTCGCG
GCGAAGCTGAATCTAAGCGTCTGCAAGCTCAGGGCGAGTCCGATGCTAAAGTGGCACTGG
CAAAAGGTGAAGCAGAATCCATTCAGATGCGTGGTGATGCTCTCCGTAAAAACCCGGGGA
TCATGCAACTGGAAGCCATTAACAAGTGGAATGGCGTTACCCCGGTATATATGACCAGTG
GAGCACAAGCCCCGTTCGTTACTCTGCCAGCAGGAAAGTAACAGTTGACAAAGGGCCCGG
TGATGATTATCATCGGGTCTCAACATATAAAAAGGAGATACAAAATGATCCGAGTAAAAT
TCTTCATAGCAGTATTACTGCTCTCTTCCCCGGCATGCGCCACAGTCAACACCCTCACCT
TGGTGGACGAATACACCGATGGAAGCTCCAAAGTCTGCATTTACAGTGATGGTAATCGCA
CAGAATCCTGTGTTAAGGATGGCCCTAGTTCTTGTCCTTCCAAAAAGACATTCCACTGAG
GTGATTTTATGAAAAAGTTCTTTGGTCTGCTTCTGTTTTATCCTTTGACGATCTTGTTGG
GGTGGTATACTGGCACCCTCCTGTTCGCATTTTTCACATGGACGGCCCCGGTGTTGGAAC
TCCCCGGATGGGCTGTAAGGGTTAGATTCTTATGGGTTCTCGGTATAACCGGGTTCATGA
CATGGAGGGGCAGAAATGAGAGTAACGGATAAGTTTGTTATCTTTTTCACCCAAGACGAT
ATCTTCAGTAACCATTATCGCTGTGCCAATCCTCTCTGGATTCCAAAGCACCAAGCGGTT
CGTGCAAAGTTCTCGACTGTAGATCACTTCATGATGTATGAAAAGGCTATGCTGTTTGGC
GATACAAATATCGCGGTTGAAATTTCTAATGTTTATCACCCACAACAGGCTAAGAAACCC
AGTCGGCGGGTGAAGAACTTCGACAACAGCAAATGGGTAGCGTTTCGTGAAAATATCGTT
GTGAATGGTCTTTATGCCAAGATGATGACTAATCCCACCTTTAAGGCTGCGGCTATCAAC
CTTCGTAAAGAAGGCAGGGTCTTTGTTGAGGCATCTCCCTACGATGCAATCTGGGTAATC
AAGATGGCTGAGAACGACTCAGGAGTAGAGGACCCAACTAACTGGAAAGGTCTTAACCTT
CTCGGAAATTTCGGGCACAAAGCGATTGACCGATTAATTGACACTCTTGAGTTGCATTGA
AATTAGAGATCGAACTGGTTCCTAAAACAGCTTGGTTCACTAACTTACGTTCTCACCTCT
CTAAGTCCAATTGGGATGTTGTAAGGAAGAGATGTTATGCAAAAGCCAATTATATATGTG
AGATTTGCGGGGGAAAAGGAACCAAACACCCCGTAGAGAGTCACGAAATCTGGGACTTTG
GCAATGCCAAGATCACGCTAAAAGGTCTGATCGCACTTTGTCCTTCCTGCCACGAAGTCA
AACATATTGGATTGGCGGGTATTCGTGGTAGGGGGGAAATCGCTCTTCGTCACTTCATGA
AGGTCAATGGTGTCTCCAGACAAATTGCAGACCAGTACGTGAAAGTGGCTTTCGCTTTAT

ATCACGAACTCAGTAAACAGGAAAAATGGGAAATGGATGTGCGGTATTTGGACGAGTATT
TGTGTAACGATTGAGAGGGCTAGTGGGATACCCGTATGATAAAGATTGGAAAAATAAAGT
CACGGATGACTGTGTGATCGCTTTAGACCTTGACCAGACTTGCTTTGTGTCTGCCGCTGG
CGCTGAGAACCGTACAATAAAGGCAAGTGACATCGCTTCTGGTCGGTCAAAGATCATTAA
AAACAGAACAGAGTTCTGGGGCTCCACCAAGAAGGTGCTTGGCGGATGGTTGAAGGACCA
GAACGCCATTATGGAAGTGAAGGCGATAGCTGAAGACCGTGAGTTCACTCCTTGGGGAAG
AGATGACTTCTTAATCGAAGATATTCAAACCCCTGAACCAGAGGAACACTGCCTGCATAT
ACTGAAGACAAAGATTAAGGCTATCTTTGAAGATTTGGGAATGGACGGCAGCAATGGCCT
TGGTGTGCTCGGCGGGGACAACAGCTTCCGTCTGTTGTTACCAGCACCGGAACGTTACAA
AGGAAACCGTGAAGACACCTTTCGTCCTTTGCTGTTGAAGGAAACCCGCGAGTATGGCAA
GAAGAAGTATAATGCCAAGGTCATTGATGGTGTTGAGGCAGATGATTACCCGTCTGTTCT
GATGTATACCGGATGGGAACACTTCCAGGCGACTGGTAAGTTCAACTACATGGTTGCGTC
GTTCGACAAGGACCAGAAAGGAACACCGGGTTTACTTCTTGATACGATGCGTGATAGCGA
GGAGAAAAACTGGAAACACCCGATCCCGATGATTATCGACGATTCTATGGGTGAGATCTG
GATGGAAGGAAACAAGGTCAAGGGATGGGGACGTAAGTTCTTTGGCTATCAGATGCTTTG
CGGTGATAGTTCAGACAATATCAAGCCTTAGCAATGCTTCGATATCTTTGGGCGTTTTGG
AGATAATGCTGCATTCAACCTGATCCGGCATCTTCAGAACGAGAAGGAAATGTGGACGGC
AACCGTTGACCAGTACAAGACTTGGTTCCCTAATGGGGTTGAGTTCACCTCTTGGGATGG
TTTACAGCGCAAGATGACCGCAGGGCAATGGGCTTCAATCATCATCCAGATGGTTTACAT
GAAGCGAACTGCCAACGACCATACTACGCTCACAACTGAACATCGTCGCGTGGGGGTTAT
ATGATTGATAAGATGATCATGGATGCTGTAATGAGGGACACCCAGGCCCTCTCGGTGTCC
TCTCTGTAGCCAAAATTCATGGTTCGAACAGGGCTGTGGAGAATGCTGGAAACAGATTAT
GGGGACTATGTTGTCTTCACGGATGACTTCCTTGAGCCTGATAAGGCATTCACTGTACTT
CTTCTGGAGGATTGTGAGCTTGTGGGGGGGGAGAGATTAGTTCTCCTGACAAGCGTGGCT
TACGGAGACCTTGAGTGTTCTCTGGTGTTGTTCAAGGACCAAGCTGTAGGAGACTCCGAT
GATGTTGATGTTGGTTGACCAAAGCCTAAGTCACTGTGCTGTCGTAATATGGGAACATGG
TGATCCCGTTTACAGGGAAATGATCAGGACAGGCTCAACACACAGTAAAGGGAAGAGGCA
GAAGGATGTTGTTTATTTTGACAACGTTTATGTGCAAATCCGTTATATTGTGAACAAGAT
AACAACCTTGGCAGAGGAGCACAGAATTGCCCACTACGTCATGGAAGCCCTCAGTCTTGG
TTCTATCGGTAGTGCAACTCGTCATTTGGCTGGCCTTTTTTACTGCGTTGTGTACGCATT
GTACCAGTTTGGTATTTTAGGGTATGGTTCAATCCACACTGTTGCACCAACATCTGTGAA
ATCTTTTTCGAGAGGTCTTTTACCGGAACAAGAGCGCACTTTTGTGAAAGTAAATGTTGA
CACAAAGCCAGGTAAGAAGGTATACTCAACGGGCCAAGTGAAGATGGAAAAATCGCATAT
GGTCAAGGCTGTTGAGAGCGACATGCCTGGCTGGTTAGACGGTCTTACGCTCGCGGCTGG
TAAGGCGGATTATGCGGATGCGTATTTGATCGGGAAGAAGTTCATGGAGGGAGTATCCGT
TGAAAAAGGATAAGGAGTCCTGCATCAAAGCCCAAGGCAAGGAAGAAACCTGTCGTAGTG
CCATTGACGCCAGGACTTTGGGTAAAAGATGCAAGACAAATCGCTGCATTCAGGGAGAAA
TTAATCCTTGAGCAGGGGGGTCTTGATCCTGTGCTTGGGGAGCCACTTCACAAACCTTGC
TTGGACCATGACGACTTCGATGGTAAATGTCGTGGAGTCTTGAGTCAGTGAGTGAACACC
TTCGAATGATATGTGCTGAAGGCATGGATGAAGTACGTGTCTGCGTACACAGATACGTCT
TTGTCCACAGCGCTCCGAAATCTTGCTGACTATTTGGAACAGGATTTTAGCGGGTTCCCT
ATCCATGCAGCGCACAAAGACGATATCCAGAAGTTCTTGAGGAGGTGTACCAATGACTAC
ATCATTGAAAGGGCGGAGACTGACCTCGGCCTGATCATCCCCAAAGGTACTCAGCAGCAT
GATAGCAAAACGCTTTACCATACAGAGTTTGTCAGCCTAACGGAGGATAGATTTGCTGGA
CAATTTGAGCAAGCACCTGAAGTTCTATTACAAGAACTGGCGGGGGGAATACGGTTATCG
AACCGGGATGGACCCGATTATGTGGTTCGGTTCCACAGAGTATCACAAAGAGCCGCAGTG
GATGATCAAAGCATACGATGTTGAGAAAGATGCCATCCGTGACTTTGCTGTGAATGACAT
TGTTGAATTTATACGAGAGGTGTAATGCTGAGTGCAGAAGAAATCCTTGAATTAAAAGCG
AAAGGTATGAACAACCGTGAAGTTGCTCGTCGTTATCTGGGGATGGAGAGTAAGGAATCT
CATATCCGTCTGATCCTGAAGGCGCACCAGTATGATGCAGAGGCTGCTCAGGTGATCACC
GAGACAGTTGTTCGCAATCCGGGGGCGAAGATCTTCCTTGGTTACGTAGAGGTATCACCA
ACGCTCGCTTGGGTCTCTAACCGATTCAAGGCGTTTGTAACACCTTCCCACGTAGAACAT
TAGCCTTATATGTACACATGGGCGGGTAAGTGGTTGGATAACCCTGCGATTATCAGTCGC
AAGCTGCCGGATTATGAGACGTTCAAAACCGATATCCACGACGACCGTGAACTGGTTGAA
GAACTGTGGCATAGCCTTGATGAGTGTGATATTTTCAGTGCACACAACGCACGTTTCGAC
AAAGGATGGGCTAACTAACGGTTTGCTTTCCACGGTGTGAAACCGCCGTCACCGTACATC
GTGATCGATACACTTGCAGAACTGAAGTCGGCTTTCTCCCTGCCGTCCAACGCTCTGGAT

GCCGCTTGTGATTACTTCCAGCTTGATGCCCGTAAGCGCCATCACGAAGGGATCACCTTG
TGGATTCGTTGCTTCCACGGTGAGGTTGCAGCATTCGAAGAAATGGAGTTTTACAACATC
GGGGATATTCCGACGCTTGAAGGTATCTACTTGAAAGTTAGACCTTTCATGAAGAACCAC
CCGAACGTGAAGCTTTATAGCGAAGAAGACGACACCACTGTCCTTCGCTGCGTTCGTTGT
AACTCTGATAAGGTACTGCCTATGGAAGGTAAGGTGGGGACAACCTACCTGTCAAAATTC
CAGGCATATCGCTGTGAATGCTGTGGTTCTGTTATGCGTGACCGTCGCAACATCCGTACC
AAGGAAGAGATGGCTAACACGCTTGTGAATATCATTTGAGGGCTTCGGCCCTTTTGCAAA
GGAGAGATGAATGAGTAGAGTTATCAGCTTCAAAGTCCCTGACTCGTTTGATAATGACTG
GTGGATAAAGCAGCTTCGTGATCTTGACATGCGTCTGGAGGATTTGGTGGAATCGGAATC
TGATGAAGAGCGCTTGAATAAGATCGGGGCCTGTCTGGACATCGTTAACGCTATGCAAGA
ATATGCGGGGTACTGATGGATACTATCCTTCTGAACGCACCGCCGTTGAGCGGTAAAGAT
GACATCTCCACCTATCTGTGCGATGGTCACCCAAACCTGCACCACGAGGCAGTTAAGGAA
GTTCTGTTTGAAATGGCTGTAAGGACTGCTGGCATCTCTCGCGACCTGTGGGATGCTATG
TACACTCGTCGTTACAAGGATGTCCCCAGTCCCTACCTGATGATTGAGGGTGTGAATGTA
TCTCCTCGTCAGTGGATGATTCACTGCTCTGAGAAGGTGATCAAACCACTCTTCGGCAAG
TCTGCGTTTGGCAAGGCAGCGGTTGAAAATCTGAAGCAGAATTATGCGAACGATGAGGTT
ATTGTTTTTTCTGATGGTGTTATCCCGGAAGAATTCCGAGAGCTTTCTGATCACGCTTAT
GCAACTGGTGGGGAGTTTTTCCTTGCACGTATTCACCGGAAAGGCTATGATCGGGGTAAC
GACAGCAGAAACTGGTTATACCTTGACGGTATTCGAGGTCATGAAAAAGACTTCGATAAC
AAAGAGGGGAAGTTGACTGACTGCGCCGAATCTGTCCTTGACTGGGCGAGATCATTCAGC
TATGGAGAAGAGAATGCGTAAGCCACTTAACATCGGTATTGATGTAGACCTAACGTTCGT
AGATTCATGCACACCCTGGCTTCAATGGATGGAGCAGGTATACGGTGTAAAGGCAGATTA
TAACCTGTCGCATATAAACCCGCAGGGGATGGATTATTATAATCTGTCAAAACACTTCCC
TCCTGCGAAGGTAAATCAGTTACCTCCGTATGAGTTTTGGGAAGATCCGTACCTGTACGA
CAAACTGCGTCCCCGCGCCGGGGCTGCTGATGCACTTGCAGGTTGGGCGGCTGCTGGACA
CTTCAACCATTTCGTGTCTTACTGCAAGAAAGGCCATTTCTCGTCCAAGGTAAGATCCCT
GAAACGAGTGACAGAGGAGTTTTTCAGCATTGAACCTGGAAGTGGTCACGGTTTTTATGC
AACAAAAAACAAAGCTGGTATTAAGATTGATGTCATCATTGATGACCGTAATGAGTTCCT
GAACCAGTTTGGGCCAGAGGTGATTAAGATCAAGTTCAAGACACCTTATGACCAATCTGA
AGAACTTCGCGTCTCTCTCGATCTTGAGAGCGATGACTGGAACGTCATCAAAGACTTCAT
GCTGAATATTGCTTAATGAAGAGATGCCCCACAGGAGACTTCGTTGTCCACTAGGAGGAT
TGTGTGGAGGTATTCACCGAGGACGGAACCTTCCTCATATCCTTCAAAGCAACAAGTGAC
ATGTGTCAGTACATACTGACACGATATCTGGTGTTTAGACCCGGGATGGACCCGGAAACA
CCTGGCCGAATCAAGAGGAGAGGCCAATGAGTCTTAAACCAGAATCTTTCGACATCATCA
AAAACGAACTTCTGCGTCGTGCTGAAAAAATCCCACAAGTGCTCGGCCTTGATGCTATAG
GGACCAATGCAGCAGACCTTGGGATCACCGAAGATTTCGTTTCCCGTCTGATCACTGAGG
CATACCTTGGAATATTTACGACCGACGAGGTTGAGGAAATGGTAGCCTTCCAGCAAAAAT
TCGCAGATCGCAATCTCATTCTTGAGAAAGTTATTGAGCAAGCATCTGCTAAAGTTTTAA
AAGAACACAAAGAGTCGCTGTATAAGAAACTGGAAGGAGCACAATAATGAAAAACGCTGT
AGTTGCCCGTGTTGTCGCGGATAGTATTACACCGAAGGGTCGCCGTATTACAACTTTTGA
GCTTGAGTACCCTCGTATCATTCACAGCGAACTGATGACTCACCGTCTGTTCAGTCGGAA
CGCTAAGAGCAGCGGTGCAATCCCGGTCAAAAAGATGATCTAGCAGGTTCAGACAGACCC
TGCAATGCCAGTTAAGTTTGGAAAGAATCAACCCGGAATTCAGGATGCTTGTGACCATGA
CGCACAGCTTGGTGATGGGAATTCGGCTGAAGATTGGTGGAAACTCGCAGGATTAAGTGC
GGCACGCTTTGCAGCAGAATTCGCAGATGCTGGTTACCACAAACAGATCGCGAACCGCCT
GCTTGAGCCTTTCCAGCGAATGAAATCGGTCTTGACAGCAAGTGACTTTGAGAAATTCTG
GTGGTTCAGGGTACCTAAGGATGCCGATAAAACCATCTACGCTTTAGCAGAGGCCATGAA
AAAGGAATTTGACGCATCAGTACCTGAATATCTGAAGCCAGGGCAATGGCACACCCCTTA
TGTTGATCACCTATACTCTGCAGGAGAGCAGGCAAGTGAAGACACATTTGATTACTGTGT
TCTTGACGAGAACAACAAACCAGTGATCTTGACAGTGGATGAAACTAAGGCTATTTCTGC
ATCTTGTTGTGCTCAGGTGTCTTATCGTGTGCTTAACAACACGAAAGAGAAAGCTTTAGA
CATTTATGGGAAACTTTTAAGCGGCAATAAGGTACACGCTTCTCCTTTTGAACATGTTGC
AACCCCGATGAGAGGTGAGTTCAGCCAGTACGGTTATGACACAAGCATCAATTTGTCGGA
ATACCCAGTATCTTGGGAAGAAGGCATTACCCATGTTGATCGCAAAGGTCGACTATGGAG
TGGGAGCTATCGAGGCTGTATTCAGTTGCGTCTATTACTGCCTAATAACTATGTTGAAGG
TTGATGTAGCTGGATATGAAGGGATTTATCAGGTAGATACTGATGGTAATGTTTGGTCTC
TTGAAAGGTGGGTTGAGAAAACTCGCCACGGAAAAAGAACAGCGCCACAGTTGGTCCCTG

CAAAGAAAAGAAAGTGTTCTGAGCATGTCACAGTCTATCTGCCTGTTAGATTGGCAAACA
ACGGTGTTGTAAAAACTTTCAGGGTGCATAGGTTGACTGCTGAGGCTCTCCTACCTAACC
CACACAACAAGCCATATGTGAATCATAAAGATGGTGATAAGAGAAACAACAAGGTCTCAA
ATCTTGCATGGGTAACCGAGAAAGAGAACACTGAACATGCTATAGAAACAGGGCTGAAGC
CTGAAAATCTGAGAGATCGCATGACTGGGCGTTACGTGAGCGAACAACTGGAAATAACGT
AGTTTCTGGTTGACAAAGTCTCCAGTAAAACGTATCTTGGATGTATGAATTGCGGGGAGA
AAATACTCCCCGATTATAGGCAGAATGGAGGTTACAAGATTAAAACAAAACGCAAACCAA
CCCAACATATTTCGGTCACTTTGGACTTTCTGAGAACCGTACCCGGATTCGATGTAACCC
AAGATAGTGGACTCCGTGCTGCCCTTTATGCAGTAGGCTTCCAAGTCACCGATAGTGAGG
GGAATCCTAAGCAAGTAACAATGCTTAACATGAAAAATGTACGATGTGCCAATTTGCCCT
ATACGTACCGTAAAACAACAATCTTTGTTGGTGACATGCGTCCGGATTATCCGTATGCAA
AAATTTATAACGGTGTTGATATTCTCGACGTAGGGATTTACTCCGAGCGCGACATGGAGT
TCGTTCTTGATCTCCCATACGATATTCCAGTGAATGAGAAGGTTAATACCCGTAAGTACA
CCAAACGTGGAGATCGTCCAGATTGGTTTGATATTACGTTCGATAGTGAAGATGAAGCAC
GTCTCAGTGACGCATTTGGTCTGGGGGAATAATGAGTAAAGAGAAGACCAAGTAGAAAGA
CCGTGACTTCGATGAAAAGCAATACTACGCCGGGTGGCTCTACTTCAATGATTTAACCGA
TGACAGCAAGATGTACGACGATGTTTACTATGATGAGTATTCTGGTCAAATAGTGAACGT
CACTAGGCCGAGTCAGGATGATAAACCAGACGAAGACGTTGACGAAGCTGATAAGTATCT
TGCTCAATTCGAAGAAAAATAAGGAGCAAGTTTGATCAATACATTTGTAAAGAGTGATGG
AACCAAGGTGCCGTTTGAACCAGAACGCCTTAACAAGGCAGCGATCTTTTGTGATGACGG
TAATGGTAACTGGTCACATATCTCTATGGACGCCTACAAGCGCCTGTATGATGGTTGCAC
CACTCGAGAAGTTAACCAAGCATTGATCGATGCCTGCGTGAGCCCGAGAGACGAAGCCCA
TTCAAGGATGGCTGGGCGAGTTCTGATCGGGCAGATCTATAAAGAAGCTTATGGCGGTTT
CAGCAAGATCACTGCTCGTTATAATTTCTATCACGAGATGATTGAGAAGGGTTACTGGGA
AGACACGGGTTACGGTCAGATGGAGTTCGTGGAACTTGAGAGTGTCATTAATCATTCCAA
GGACCTTGACTACGGCTACGCCGTCCTGAAGCAATTCCGTGACAAGTACAGTATCAAAGA
CAACATCGACGATGTTCTTTTTGAGTCACCGCAGATGATGTTTATGGGTATTGCGATGGC
GGTAATGACGCGTATGCCTCGTCATCGTCGTATCAAGGATGTCATTAAGCTTTATACGTA
TCTGTCGGATCTGAAGATCAATGCTCCGACACCGTATCTGAACGGTCTCCGTACCGGACG
AACTGGATATGCGTCCTGCTGCATTATTAAGGCTGACGATACAGCGAAAAGCATTAGTGT
TGCTCGTGAAGTTACGTATACGATGACAGTTGCACAGGCAGATATTAGCTACTACCTCTC
CTCCCGCTCAATTGGTGACTGCGTTCGCGGCAATACGATCAAACACATGGGTAAACTTCC
ATATTACCGTGGGATTGATGCCGGGGTGAAAGAAAACCGTCAGTCCACCCGTGGTGGTTC
TGCTACTATCGCTTTCCTGGCCCTTGACCCGCAGATCGAAGTACTGTTGCGCCTTAAGTA
CCCGCAAACAGTTTCTTCAAAACGTATTAACAAAATGGATTACTCCATTGGTATTAACCA
GTCGTTCATGAACCGGGTGGCGAAGAACCTTGACTGGAGGCTGGTATCTTACAAGGACGC
ACCTCAACTACACGAAGGTATGGTCAAGATGACCATGCAGGAATTTGATGCAGATGTTGC
TCGTGTGGCAGCGGATACTAAGATCCCTAAAACTTGGGTGGAGGCTCGCGATCTGGCACT
AGAGATCCTTACCCAACGAGCAGAAACAGGTCGCCCGCACGTTTACTGGCCTGACGAGAT
GAACCGGCACACGCCTTTCCTGGAGACGATTTATTCCTCCAACCTGTGCCAAGAAATCTG
TCTGCCTACCAAGGGATACAAAGATATGCGTAACATCTTTGCTACCTTTTCAGACGATGG
TGAGGTCGCTCTTTGCTTCCTGTCATCTCTGGATGCCGGGCGTGTTTCTCCGGAAGAGTA
CGAGGATGTTGCTTACTACACCGTTCTTATGATCGACAACGTTATGGATATCATGGATTA
TCCGTATGAGAATATGGAACACACCGCCAATTCTGGTCGTTCAACCGGTGTTGGTGTGAC
AAACCTTGCTCATTACATTGCGAAACACAAGGTTGCTTACGGCTCTGCTGAGAGCAAGCA
GTTGTTTCATGACCTGGCAGAACTGCACAGCTTCAGCTTACATAAGGCAAGCCTGGGTCT
TGCAAAAGAGCGTGGTGTAGCTCCGTGGATGGACAAGACCAAGTACCCACACGGGTGGTT
GCCAATAGACACCTACAACAAGGCAGTTGATGGAGTTGTTAAGAATCCAAGTCTCAAGCA
GTATTGGGAAACTTTACGACAAGTGAATATCGAGGTTGCTGGTATCCACAATAGTGTGTT
GGAAGCTTATATGCTGAACGAAATTTCTTCTCTGGCGTCGAAAACTACTAACGGGCTTTA
CCCGGTGCGAGATCACATTATCTTCAAGAAATCACCGCAACGGTCTGTGCTGTTTATCGT
GCCTGAGTATGAAGAACTGAAGGATTACTACACCTCTTCGTGGGATATTAACACCAACGA
CCTTATTGACATTTACGCCGTCATTCAGAAGTTCGCAGGGCAGGCGATTAGTGTTGACCT
CTACATCGACTATACCCAGTTGAAAGACGGAAAGGTCTCGATGAAGGACCAGATCGGTTA
CCTGATCCGTGCAACGAAGATGGGGATGAAGACTTGGTACTACTTGGACTCGAAGGTTGG
TGATGGCGACTCCCTCACCGCAGAACTTCAGGCGAAGAAGCAAGCAGAGGAAGAGACCCG
CAAGGATTATCCACTGTTTGCCGACACCTTCGTTGAAGACCCGTAATGTGAAGGTTGTGC

ATTGTAATCCTTATGGGGCTTCGGCCCCTATTTTTCCGGAGGCGAATTAGTGGCAGTATT
CAACGGTGAGAACACCGCTCATAAAACTGGTGATTATCCGCTGTTCCTCGGACAGCAAAT
GGGTCTGTATGACTCTATCTACAAAAAATACCCAGCCTTGTTTGACCTGTACAAGGCTCA
AAAGGAACAGGATTGGTCTGAAGACGAAGTTGAGCTAAATCAGTCAATCACTGACTTTGC
AACCTGTAGCACATCCACGTATGATGTCCTGGTTCAGACCCTGATGTGGCTGTGTGAGGC
AGATAGTGCTGCAGCACAGGCGATCATCTGTCTGTTTGCACCTTTCATCACAAACAGTGA
ACTTTTCGCCATGTTGATGCAGCAATCATAGATCGAAGTTCTCCACGCCCTGACTTATTC
TGATATCGTCCGTCAGTGTTTGCCAAACTCTCGGCAAATTAATGAAGATATTCAAAACAA
TCAGGCAGTTCTCGAACGTTCTGGTGTGATCGTCAAATACATGCGTGAACTTGAAGTTCT
TGGAGCACATTATCGTCTCGATCGTGAGTCTGTAGACAAAGAACAGGTTCGACGTTGAAT
CCTACGTGCATTGTTCGCGCTGATCGGTCTGGAAGGGATTGAATTTATCTCCAGCTTCGC
GTGCACCTTTGCCTTGGCAGAACAGGGTGTGTTTGTTCAGGTCGGTCAGCTTGTCCAGAA
AATCATGCTGGATGAAATGCTGCACACCAAGATGGATTTCGAGATTATTGATATCCTTCT
GAAAGGCCCGGTGTGGAAGTCTAGTTTTAATGCCATTAAGCACGAGATCAAGGAAATCCT
TGACGAAGTTCGAACAACAGAGTATGGTTGGGGTCATTACCTGTTCAGTGATGGTCGTGC
AATCATCGGATTGAATGCGCCGCTCCTGAAAGACTGGACTGACTGGAACTGTGCTCCGAT
TTATGACTACTACGGTATTCCAAAAGATTTCGAAGCTCCTAAGCGCGATCCTCTGCCGTT
TATGGATATCTGGATGAATCCAAGCCGTCAGCAGAATGCAAACTAGGAGCAGATAAACAC
GGTTTATCGTTTAAATGCGGCAGTCAACGATACAGACCGTGAAGTCTGGGACTTTTGAGG
AGTAACATTGGACAATTCGATTTTTGAGTTCGGGGTTGCTTACGACCCCGCCTAGATTCA
TGTCACTTTCGATGGTGAGCGTATTATGGGCCTGGATGGTGATGTCAAGGTTTCATGCTC
TCGCGGTCTGAATGGTGTGGCCTGTGCAAAGGTCTTTATTCAAGGTCAAAGCCCTTGGGT
TATCCGACTGAAAAAGCTTCTGGGTGAGCAAGGGCGATTGCAACTTGATTACACCGGAAG
TGGACCATTCCACGACAGTTGTAACTTTGTGGCTGATATGGTTGTGAAAGGTTATGACGT
TGATTATACTGGCAACATCCCAGTTTTTGTTTTTACCTTGGAAACGGAGAGATCATGAGT
ATCAAAGACAACATTTTCATTATCTACGGGAAGGACAATTTCCTACACTGCGTCCGGGCA
AAAGAGTTCGCTAAATACAAAGGTCAGGAGTTTATGTATCTGACCCTGGAAAAAAACTAG
ACCAAAGAGGAACTGGTGGCAAAATGCTCTCCAGTGATCCCGACAACCGTACCCCGTATC
TTTATGGATGATGAAACCAGCACCCGGTACATCGGAACAGGCGATGAGTTCATTGCATAC
GTCAATGCAAACATCTGAGGGGGTAGAGGAGAGAAAGTAGTAAAACGCGATGTGAGTGTT
GAAGACTTCTCTCTGGAAAAAGTTAAACGAGCGGTTGAGGCCGCTCTGTTAGCAAAAGGT
ATGGACCCAGAAGCAGCGCATCACACTGCGGTCCGTATCGGTCAGAGGACTTTCAAGCTA
TTCATGACGACACCTGTGAGACATGTTGAAGATTTCCATCGTGAAGTGGAAAATCTGTTG
ATGGAAGAACCGCTACTCCGTGATGCTTCTCGCGCCTATATTGAGTATCGCCATGATCAT
GACAAAGCGAGTGAAGGAAAGGAAAACTTTATGCAGATATCACCGGATTCGGGGACCAA
TCCACAGAAGAGTTCACCCGTGAGAATGCAAACATACCAAGCACGGTCGTAAACAGGCAC
AGGGACCTCTTAGCCGGGATTTCGAGTAAACATGTGGCGTTGACCCAGATACTGCCTAAA
AACCTCGCAGAATGGCATACAGAGGGTTTTGGACATATTCACGACCTTGATTACCTGGTA
AGCTCTCTGACAAAATGTTGCTTGGTAAATTTTCGGGACATGCTTGAAAATGGTTTCAAG
ATCGGCAATGCTAAGATTTAGAAGCCGAAATCAATTGGTGTGGCAACCACTGTATTGACA
CAAATCATCCACGCTGTAGCGTCAAGCCAGTATGGTGGACAGACTTGTGCTAACATCGAT
GAAGGTCTGAAGCAATACGTTGAAATGAGCTTCATGAAGAATGTGAAGCAGTCCATGAGC
CACAGGAACGCCGTTGGGCCGACCAACAAAATGGTTTATGATGCAATGCAGACACTGCTT
TATCAGGTCAATACCCAGATGAGCGTAAATGGTCAGACTCCGTTCATTACGATCAGCCTT
GGTTTGGATACATCCCTGTTCGGTAGGATGATAAGTTATAATTAACTAAAGGTCCACAAA
GAGGGCCTCGGCGCTGATAAGGTGAGACCAGTGTTCCCGAAAGTTGTGTTCTTCCTGAAG
GAAGGTGTAAACATGAATCCTGGCGATCCGATTTATGATCTGAAGTTGCTGGCTATGGAG
TGTTGTGCTGAGAGAATTTACCCCGACTTCGTCTCAGTACCGTTGAACGTGCAAGTGACC
GGATCTTCGGACGGCAAGGTGCCGAGCATGGGTTGTCGAAGCTTTTTATCTCACTTTGCA
ATCAAAAATGGAGAAAAGTACGATGGGCGTATCAATCTTGGCGTGGTATCATTAAACTTA
CCCATGATTGCAGCCGAAGCAAAAGCCGGAAAAGGTTTCTTCACAGACCTGCTTGAGAAA
CATATGGAAATGGCTTATGAGGCACACATGCTTCGTGTTGGCCGTCTGATGGATGTTACG
GCATCACAGAACCCGACATTGGTCATGGAATGTGCTCTGGCAAGGCTCGGGCCTGATGAG
AAGATCAGTAAACTCTTCTATGACGGGTACGGCTCCATTAGTATCGGGTTTGTCTACCTT
TATGAGGCTGTGGATATCCCTTATGATGAGCCAGTACGCAAAGAGATGGCGATGAATATC
CTGAAGTACATGAAGGATATCTGTGCACAGTTCAAGGAGCGCAGTGGGTTAGGTTTCAGC
CTGTATGGAACACCTGCGGAAAGCCTGTGTTATCGCTTTGCAAGCAAGTTACAGGAGAAA

CCTCCTGGTTTGACTGAAAGAGATTACCTGACAAACCCTTTCTACCAACCTGTGTGGCTC
AACAGCGACCCATTCAGTAAGTGGGAGTACGAATCAGGTTTCGCAGAGATCTGTAACGGC
GGTAATATCGGATACGTGGAAACCCCGAACCTGAAGCATAATATGCAGGCTCTTGAAGCG
CTTATTAACTATGGGTACGAGAACATTCATTATTTCGGTATAAATCAGCCAGTGGATCAG
TGCTTTAAATGCGGATTCAGCGGCGAGTTCAAGGCGACTGCAAAAGGATTCGAATGTCCT
TCTTGCGGGAACCATGAAGAAGGAACGATTTGTGTGATTCGTCGAGTTTCCGGATATCTG
TCAGCACCAAACTCACGACCCTACAACAAGGGTAAGATGGAAGAGGTGCTGTAAAGCGTG
AAACACGTATGAATATCTGTGGTTATGAACCAACAGACCTCGTGAACGGAGAGGGGGTCA
GGTGCTCCCTTTGGGTTAGCGGTTGCAGTCACGGATGTCGTGGCTGCTTTAACCAAAAAG
CATGGTCGAATAACTACGGTCAGCCTTTCACCAATGCGGATGTTCTCAGGATCATCAAAG
ATATGGGAAGGCCATTTATCAAGGGCTTGTCGATACTCGGAGGTGAACCACTTGACCCGC
AAAATGTTGATGAGGTCGTAAAAGTTATCCTGCGTGGCCTTGCAGTATTCGGAGATAGCA
GGGATATCATGGTGCGGACTGGATCCACCTTCGAAGAAGTCCCTGAAAGGGTTCAAAAAC
TTGTTGACATTATAATGGACGGCAAGTATGATGAGTGCAATCCTACAACCAAAAGGTTCA
GAGGTTCTGACAACCAGAGGATGTGGGTAGAAATATACGGTTTATGGATAGAGGCGAAGT
AGATGAGTAAGAAGATGATGCGTAAGGCATACCAGAAAGAACTGGCACTGGAACTCCGTG
GCAAAAAGCGTAAGAACAAGCGCACCACTGAAGACGAACTGATGCAACTGGTAGCTGACG
CTGCTGTCCGTGGCAACTACTGAGTTACTCCATGACTGACGTTGTCAACGAAGAACAGAC
CTTTGTGCCACAATCGCAACTGGAAACGCTGCTACTCGCAGATTCGGAGGAACTGGCCCG
TGAGTTTGTGGGCCTTGTTTTCCCAAGACTGATCAATCAGTTTACGGAACAAACTCAGTA
TCAAATTGAAGCCCTAAAGCAAATGAAGGCTGACGCACGTAAAGGCCAGGATGCTGCGAT
GGAGAAAGAGCTTAATGCTGCTCTGGTTAAGCTGAAGAAAGCTCTTTCTGAAGAGGTTAT
GCAGAAGGTGATGGATACGATGGTTGACCTTCTGAAAGAAGCGCCGACACCGCACGAGCT
TGCTTACGCGAACTACCAAGAACGTATCACCATCAAGATTTCTGGGACTGCCGCCCGACT
TGAACCGGCTTTTGAAGAGGCGCTCGGAGAGGGCTAATCCGAAAGGAGGTCTTCAATGAA
TGCTGCAATTGCGGAGATTCAGCGTATGCTGATCGAAGGTGGGTTTAGCGTCGGCAAGTC
TGGTGCTGATGGATTGTACGGACCCGCTACAAAAGCCGGACTGCAAAAGTGCATTGCACA
GGCTACCAGTGGAAACAATAAAGGAGGTACTTTGAAACTCACCCAAGCACAACTGGACAA
AATCTTCCCCGTTGGTGCCAGTTCTGGGAGGAATGGAAAATTCCTGATGCCGCTCAATGA
CATGTTTGAAAAGACAGAGATTAATACTGTAAATCGGGTTGCAGGATTCCTGTCTCAGAT
TGGTGTGGAGTAGGCGGAGTTCCGGTATGTACGTGAACTCGGGAACGATGCCTACTTTGA
CAAGTACGACACTGGTCCTATTGCAGAATGACTTGGAAAAACACCCCAGAAAGATGGGGA
TGGTGCCAAGTACAAGCGGAGAGGTCTGATTCAGGTGACCGGACGCGCAAACTACAAGGC
TTGCGCTAAAGCACTCGGTCTAGACCTCGTTAACCACCCTGAGTTGCTTGAACAGCCTGA
GTATGCAGTTGCCAGCGTTCGTTGGTATTGGGACACGAGAAACATCAACGCCGCTTGCGA
TGCTGATGATATCGTGTAAATTACCAAGCTGGTAAACGGTGGTACAAATCACCTTGCCGA
ACGCACAGCCTGTTACAAAAAAGCAAAATCTGTTCTAACCTCGTAAGGAGAACCAATATG
GGAAGAAGCAAAGCCGCTCGTGCATTAAAACTTTCGGCTCGTCACCAACAAGGTGCAGAA
AGACAAGCAAAGCACCACCCGAAATTCGATGAGGAACGTAACAGCGTCCCGGCTCTGGTT
ACTCTGAATGAAAAGCAGCAGGATTACCTTCACAAATTGCAAACTTGCAATATCATCATT
GCGAAAGGTATCTTCGGGACAGGTAAAACCTACCTGGCATCCGCCCACGCTGCCGACTTG
CTACGTAGAAACGAACTCGACAAGATCATTGTCGCTCGGCCAGGCGTGCAGACGGGTACA
AGCTCCGGCTTCAAACCGGGTACTTCACTGGAAAAGCTGTTCCCCTATGTTCGTAACATG
TTGGATAGCATCCGCAAACGCATGGGCGACGGGGCATATTACAATGCTCTGAAAGAGGGT
TTAAATGGGCGTATCGAAGTTCAGGAATTGGAAAGCATCCGTGGTCGATCATTCGACGAA
AAGGGCTTCCTGCTGATCAACGAAGCCCAGCAGAGTACCCCAGAAGAAATGCTGAGTATC
ATTACTCGTATTTCCGATACCTGTACACTCGTTGTGATGGGGGATGCCAGTCAGAAAGAC
ATTTATGATCAGTCTGGCCTGGAGTTACTGATTGAGTTTACCCAGCGACACAACCTTGCA
GGTGTGGGTACTGTGTCATTTGATGACCCGAATGAGGATATCGTTCGTGGTGGTATGGTT
CGTGAGATCGCTCTTGGCCTGATGGCTGACCGTGAGGCAAAACTGTACACGCCGCTGGCT
TCTTAACAGGAGGAAAATGGCATATTATACTGGGGTAGGATCACGCGAAACACCCCCAGA
GGTTATCAGCGTGATGGAAGACGCAGCTTTGCGTCTCGCCAGGCTCGGATTCACTCTTCG
AAGTGGGAAAGCTGGAGGAGCGGATGCTACATCTCAGAGAGTGATGTAAAAAAATGACGA
AACTCTTCACAATGGAAAGAAGAAAGAGTATCGTACACGCTTGGCAGAGATTTACATGCC
TTGGGACGGCTTTGCTTCAGACAATGAAGACCTTTGGGACTGCTGGGTTTATCCCTTGAG
TTACCTTGATAATCTCATGACTGAACAAAAGCATAAAAGGGATCGGTTGGTTGAGGAAAT
CCATCCGAATTATGAGGCTCAGAAAAGGAAAAGAGGTGCTTTTGCACTCCATTCCCGTAA

TGTGCATCAAGTTCTTGGAGCAAACATGCTTGATCCAAAGCCCTCTGCCTTCTGCCTTTA
CTATCCTTCGGAAGATAAGCATGGCAACCCAAAAGGCGGAACTGTAACGGCTGTCAATGT
CGCCAAAAAGGATGGCGTGAGGACGCTTAACCTAAACACTCCGGAGCGCTTGGCCTTGCA
TGAGCAGTTCCTGACCAGTCTTGAGAGGAAACTAGGTGTCCAAGTTTAGAGAGAAGCTGA
ATCAGTCCTTCTGCCTTGTGAAGGTTGAAAGAAAGGGCTGGGTTCTGGATAAAAAGAGAG
GAAAACGGATTTATGTTACCGAAGCAGGTGCGGAGATTCGTTATGTTTGCACCACAGATG
ACACGAATCAGTTCAAACATCTGACTGAATCTCTAGAGTACGAGTCCAAGAAAACTCTTT
TGGTCGGAGATACAGTAAAGCTGGACAATTTCGGGCTGTTTTACATTGTGGATGCAGGAT
ACCGTGGAGATGACGGTGAAGTTAAGTACCTGATCGCTCGATAGGAGGAGTATGACAGTT
AGTATTCATGGGGTTGTTTCCCATCGAAAACTTATGGAACTGAATAATTGTGGAGTTGTC
GAGCCTGAATTTTCCGAGGAAGAAGTAACGAAGCGTTTGACCTTTTACCGTAAGGTAATA
TCACAGAGTTTGTACGAGATGAATGATGGGTTTCTTACTCGTATGATCTACAGCGAAAAG
GTGGCTGAACGTGATGCTCGTTGGATGTTTCCGGACAGACGTGATCTACTTAATCATCTG
ATTTATCTGGATCATCAGGGTCGTGAATCGAGTTATGTGAATATCAGTCCGAAGGAACTG
AATGAGTGCACATTGCAGCACACAGCAGCGTTAGCCTCTGAGAGGGCAAGAAAAGTTCTC
GTGACATTCAAGTACAGCGATCAAGTGCTTGTGGAGGACGAGACAGGGTACTACATTGAC
TGGCTGATGAAGCATGCTTACATTCTGAAAGGCTACATTGCAAACGCTCGCGAGGAGGAT
TAACATGGAACAGAACAAAAACTTTGTCGGTATGCTGCCGTGGATGAGTGGGAACATTTA
TAGCTACCCGGTCCACTCTCACGATCACGTCATCTACATTGATGATCTGTCATGGCTGGA
AGCCCATCAGGATCGTCTGCAAGTTATCCGCCAGGCAAGCCCTGATGCCACGATCCGGGT
GGTGATTTACACTCCTAGTGGGGTAGTTTCTATCGCTATGGCGTATGTCAGTGCGATCCG
TGAGAGTCAGGCCCAGATCGACACCCATGCCGAAGGCAACGTTTGTTCTGCCGGGACAGT
TTTGTGGCTGGCCTGTAAGTATCGCACAGTGTCACCACTGACAGAGTTCGGGTTCGATAA
TTCGCAGGGCGGGACTTTCGGTGATGGTGCAAACATGCACGCTCAGATTGCATTCGAGAA
GCATTAATTTGATCGTCTGATTGCCACCTTCTACACCGGGGTTCTGACCGATACTGAGAT
TGCAACCATCAAAGGTGGTGGTCAGGTGTGGATGGATGAGATCGAGATCGTTAAGCGTAC
CACTGCGGTGCTGTTGGATGAGAAAAACATCAAACGTACGCAGCAAGGTCAGAACCCAAT
CATTTCCGGGGAGAAGAAGGCTAAAGAGGAGAAAAAGGTCGTTGAGAATGTTGACCTGAA
CAACCTGCCAGAAGGTGAGCCTGAGAGCGTGTTTCTGAAGATTGATGTTGACCGGGAAAT
GTTCAAGTTCGATGTATCCACGCTGAGTGCAAAAGATTTTGATGCCTTCAATGTGGAAGA
ACTGCAATCCATCCTGTCTCAGATCGGTGCGCTGGCAGCAGGCTCTGAAGAACTTCTGAA
GGTTACCACTCGAGATCGCCAGGAACTGAACGGTGAAATCCTCAAAGCCTCTGAGGAAAT
CGTTGCAAGTATTCTGGAGCAAATGGCGAGCTGATGTCTGCACCTTTAAAAAAGAAGGAA
GAGCGCAGGGCATACGATGTCTACGAAACCCCGACTTGGGCTGTTGAGGCCCTCTTAAAG
GTGAGCCCGATTGATTACACCAAAACTTACTTGGAGCCTTGCAGAGGCTCCGGTCGGATC
TACAAATTCCTGCCTTTGGGAAGCGCGTGGGGTGAAATCCGTCAGGGCGTAGATTCTCTT
AACTCCGAGTACAACCACGTTGATATGGTTGTGACCAACCCACCATACTCTTTGGCGCAA
GAGTTCGTCACCAAGGCACTTGGATAGGCGGATGTTGTGATCATGTTGTTGAGCATAGGA
TTCCTTGAGAGCATGCAGCGTTGGGAGTGGTGGCAGGAGAACCCAATCAGTTCTCTTTTG
GTTCTTTCTCAAAGGCCATCATTTACAGACGATGGAAAAACCGATGGCAGTGGTTATGCC
TGGTTTGTTTGGGACAAGAAAAACAAGCTCGGGCTGAAGCCATTTTATTTCCTGGAGGGA
CAGAGTGACGTATGTCGCAAACATGATGCGAGAGATCGCAAACAAAGGAGAAAAGCAAAC
CCCGGAGTCACTGAAGGAACTGTACGATCAGATAGTGGACGGGATAGCGCAGTGTGCGAG
GAAGGGGATGTAGGGGATGGGACTTTCTCTCCAGTTCCCGGAGGAGATGAATGATTATCT
GCCCCATATTGTTGGCGATCTTCGTGGTGGCGGGTTCACAGTCGATATCATCAGCTACCA
ACCAACAAAACAAGGCACTGCAATAAACTTTTTCGTAACATGGTAAATAAACCCGCTTCG
GCGGATTTTTTCGTTTTTGTACTTGACAAGGAGTAAATAAACACTTAAGCTGTTATCCAA
CAGAGGAGAACAACATGCGATTTTACATTTACTACCGTCCTTTAGAGACCATCCGCTTCG
GAGAATACATGGGTGACCTTATCCATATCTCGACAGAAGAGAAGGATTACTGCCCTGACC
GGAAGATTTGGAAAGTTAAACGTTGCAATCGCCCTGAGAGTGTGTATACTCGATTAATCA
AGATATTGAAACACGGCGGGATGGAGGACAATATGGTTTCTCCACTCGACGGAAATAGAT
CACTAAAAGATCTGGTCAAATAGATGACCCAATGGGAGAACAACAAATGAAACAAAGTCT
TACCAAAGAACAGGCCCTGATCATCACCGGGTTCACAGGAATCCTTTGCTGTCCGTTCAA
CCTGTTTCACGACGATGTCGAAAAGCGCCTGGGCCGATCCTTGTAGACGCACGAGTTGGG
CAGTAAAGAGTTCTCCGCAGAACTGAAGGTTGTTTATCGTGGAGACTTCATGGCGATGAT
TTACAAAGGGGAATGATGATGACCGAACTCAATGCCCGTGAAAAGCTTTTGGCGATAGCT
GATTGGCTGGGATGGCAAAACGAAAACCTTTCATTTGGCTTGAAATCCCCAGACGACGCT

GTTAAGCTGTACAACTACTGGCAGGAACATGCAGACAGTCTCCCGGAAATGGCAGACGAC
GAGCAGGACGATGAGGGACGCTGGTTGTTTATTTTCCGTAAGGCAGCACTGGGATATGAC
CCGATGGGCCACGACGCTTCTATTTTGGAAGCCCTTGGGGAAGAAAAGTACGACGCTGAA
AATAAAGACACCGACATCGGCTTTTACGACATCCGACAAACAGTGCGTATAATGGACATC
ATCGATGCAGAATGGAAGTTACAAAAGCAGTACGGCCTTGTCTAAACTGAAACAAAACAG
GAGAAAATAAATGATTAATAAAACATAAACCGTTGTTAAAGACCTGTGCAATGAGAACAT
GGGGACACCCTCCACAGTGGGTCAGGTGGTAACCACAATTGTGGAAGTTCCTGCCGATAC
CGCTGTAGTTGTGTTCGACCAAGAAGGCCAGTTTTGTTTCACCTCTACAGTCCGTAGCGA
TGTATTTCTTACCAAACGTGGAAAATCCAGAAGCAGAGGTGGGTTTGGTGGTGGAATCCT
AAACCGAATTAATGCTTTCCGTATCAACGGTGTTTGGTATGAAGGCAACGATGTGTACTC
CGCAGAAGAACGGGGGATCACCGAATGAATCTCTATGCAAAATTGACTGACCCCCAGAAT
GGTTGGAATGACGACATGGAAAAGGCCCGTGAATTTTTAAAAACTCACGATCAGGAGGAA
ATTCTTCTTGTTACTGAAGTTGCTATTGACAGGTCGAGCACCGAAGTGGTATTGTCTAAT
CACGGGCATGGTTGGAACAGTGTACAATTTACCTTCTTCATTGAGGGTGAGATCGGTCTT
GAAGAGTATGACATCTTCAGCAACAAGAAATCATTAATACAAATTTATAAAACATATATT
CACGAAGATTTCTAAGGTGAAAAGATGAAGGTATATTTGGTAGGTGGCGCAGTACGTGAC
GGTTTGCTGGGCCGTGAAGTCCACGACTGTGACTCCGTGGTTGTGGGTGCAACACACGAA
CAAATGATTGAAAACGGGTTCATCCATGTGGGCGCTTCCTTCCCGGTATACCTCCGACCG
GAGACCAAGGAAGAATACGCGCTCGCTCGAACTGAACGTAAGGTTGGAAGCGGTCACAAA
GGTTTTGAGACCTTTCTCAGCCCTGATGTAACACTGGCAGAAGACCTGTCTCGTCAGGAT
CTGACTATTAATGCGATGGCTCAGGATCTGGATACCGGAGAAATCATTTACCCGTTCGGT
GGTCAATATGATATCGAAAAGAAGGTCCTGCGACACACAACGTTGGCGTTTCTTGATGAT
CTTCTGCGTATTCTGAGAGCTTACAGATTCAAAGCGCAGTTGGGTAAGGAACGGGATCTC
CATCGGGAGACTAAATCTCTTTGTTACCAGAACCGTCACGAACTTGAAAGTATCAGTGGC
GAGCGTAAGTGGAAAGAGATGGAAAAGGCCCTAGGGTCTGATAACTTCCGTTCATATGCT
GAAGCAATGGCAGAATTGGGCGAGTTGGGAGAACTGGAATCGCTGCGTGGTGTAGAGCAA
CCTCCGGAACACCATCCGGAAGGTGACGCATTCATACACACAGTGCTGTGCCTAGATATC
GCATACCAGTACGAGGCAAACCCATACACGAAGTTTGCAGTCCTGTGTAACGACTTTGGC
AAGGCCGTGACATTCTCCAAGTATGGGAACCTGCTCGGTCATGAAGAGGCAGGGCTTGGT
CCGGTTGATGCACTGTGTGAACGTATTCGTGTGCCAAACGATTTCCGTGACGTTGCTCGC
TACGTGACTGAGCACCATACGCGTGTTCATTGCATCGCCCCTCGGGGTGGAAACAAGGGT
GCAAAACCGCGATCACTAATGAAGCTTTTCGAAGCTGCTGGTAACGTAAAGCGCATCATG
ACAGCTATGAAGGTCAGCGCCCTGGCTGACGCATGTTTCTGTGATGCAAAAGGCCGTGGC
CCGACAAAAGCAGCAGAACCTTACCCACAGGGCTACGTTGTGATGGATGCTTTCCGGTGC
ATGGTGGCGGTAGATACCAAAGTAATCTCCGCTGAGAGTTTGAGCAAGGGCAAAACGGGG
ATTGAGATCGGGGAAATGATTAGGGTGGCACGTATCGACGCAATCCGCAGCGTCACACCG
CATCAAATTGCCTGGTAAGGCGTTCTGTATGATGAAATCGGATAAGGAAGAGTACACAGA
CGAGAAACCTCACTTTAACGCTGACAAACACGCAGGGAAGCAGGTTTGTGCATGATGCGG
CCTCGTGGCCTTAAGGAACAAGGCAATCGACTGGTGCGTGGATAAGGGATGCAATTACGC
ATTGCATCCATCGTACAGAAGCGCCATGTTTAAGTTCACACAGTTTTTCAAATAGGGGAA
AGATAATACCTATTGAGCTTGTCATTTATTTGTGCATTGGTATACTAGCTATGTTGACGA
TTCTGACGTTGGTTTGGTTTTGTATGGATACGATAATTTCATCATCCATCGGCATCGGGC
TGCTGATGATCGTCTTTTGGCCTCTCGGACTTTACTGTTTGGTGGGTGGCACCATTGCCG
CCATTTTTGAAGAAGATTTTGTTATTGCAAGGAGAAAGAAATGAAGACACGTATGTACTG
CGCGGTAAATCAATACATTCCTGGCATTCACGCAGGTATCCAGTCAGCACACGCGATGTC
TGAGATGTTCTTGGATTACTCTCAACGCCGGAACAATGCCGGAAACCTGTTGTGGGATAG
GGCTGACCGCGTTAAGGCCGTGATCGTCCTCAATGGTGGATACCAGTCGTCATTGCAAGA
ACTGTGTGAGAAGTTGAAACCTCTGAGCAGCACTTATCCGTGGGCCTCTTTCTGTGAAGA
ACAGGATGCACTGAACGGCGCTATGAAAGCAGTAGCTGTGGTATTGCCACAGTAAATGTA
TGACCCCCAGTATTCAGATGTGGTCAATCTGTACTAACCCGGTAAGCACATCGCCTCTGT
GGTAGCCAAACAGTACCGCGATGAAATGGGAAATGTGGTGCATAAGTACACACAACCTGC
AAAAGACCTGATCACAATGATCAAATCATTCCGACTGAAAGGAGAATAAGATGAAAGTAA
GTGAACTTGACCGAAGTGTGATTATCGAGATGATCCGTTCAGCTACGCTGATCGCTACTA
CTGAGATCCGAACTATCGGGTCAGATTACAAAAAGAATACGAATTTCTTTGACCTCGACC
TGCGGGATGTGGTAAAAGGTGTCGAACAACAACTGTTAAAGGACGCGGAATAATGATCTA
CACAATTTATACCCTATTCACCCTGCTTGGTGTGGTATTGCTGACCCGTCACTGGATGAT
CGGGGAAGATTTCAAGATGGAGGCAAGAAGTTTCCCCATGTTCGTCTGCCTTGCAGTTAT

GGAGGGCTTGATGATGGTAACAACCCTGATCCCACCTTCTGCTGGGCTGTTCGCAGGAAT
CTTGTTTGGGCTTTGGATGTGGAGACATATTTTGTCTGCCCGTGCAATCTTGCAGGAGAA
AGTAATCAAGAACGAAAAGATGCTGGTATCCATCGTCCTGAATGTTCTTTTCTTTGTGCT
TGTATCGGTGAGCTACTTGAGGTGAGGTGGATAAATGACAGACGTTCGTGGAGAGGACTT
GAAGATTGGTGATGAGGTTTATATCTACTGGGGAGGCAATGAATTGCGCCCTGGTAAGGT
AAACCAAATCAAGGGAAATAAGGCGAAGATCCTTGTTGATTCTTGGCCTAATCACCCAGA
TCCAGCACACCGTTATTCCATGTCGAAATGGAAGTCGGGCGAGTGCATGATCAAGGCAGA
AGAGCCAACAGGCTGGTAGCCGGACGAGGTAGTTTTCTTGAATCAGGAGATCATTGCTTT
GAGAGACGAGGCGAAATTCTTGGAGGAGAAGACCAAGTACCAAGATCTCTTGAATTTCTG
GGATAAAAACCGACCATCCAATCTTGTTTGCTCTTAAGGAGAAATATGAAAACTGTAGAC
TTGTTGCGTTGCTACTCTGGCAGCATCGCTTATGGGACTAACCTCCCAACATCGGATACC
GATATCCGAGGGCTTTTCTGTGCAGAACCGAAGTTCATCCGCACACCATTCATCAATATC
AAGGAGCAAACCCTTGCTGACAGAGAAGATGGGAAGATTTATGAACTGACAAACTTCATG
AAGCTTTTCGTGGAGATGAACCCCAACATCATCGAGCTTATGTTCGTGAATGAGAAGGAT
ATTCTGCAAAGCTCTGATGCGTATGACTATCAACGTACAATGGCACCAGAACTGCTATCG
AGCAAGGTGGCCTTTAGCTTCTCAGGCTATGCAATAGCACAGCTTAAGCGTATTCGTGGT
CACGATAAGTGGATCAGCAATCCACAACCTGAAGCAAAGCCAATCCAGAAGGAGTTTTTC
CGCTTGGTGCATAACTACACCGAGCACCCGTCACTGAAACATGAGGATTTCATGCAGGCA
CTGGACGGCCTGAACATCTTCTGCATCCTCGTACCTTACGGTGACAACGTTTACGGTGTG
ATGGAGAACTTTAATAGTTCTGGCCGATTCAATGCTGACGGATCGAGTCGTAATGTCGAT
TACCAGCAACTGACCGATACGGATAAAAAGGGTAAGCCTTCGTTTATCGTAAAATATCTG
GCAGAAGAGCACAAGCTGGCGAAAGAGAAGCATCGGAACTACTGGACGTGGAAGGACTAT
CGCAATGAGGCTCGTCATGAACTCGACGTCCAGTTCGGATACGATACCAAACATGCGCTG
CACCTCGTGCGCCTGATGCGTATGGCGGAAGAGATTCTTACTGACGGTAAAGTTCTGGTT
AAGCGGCCTTACGCTCAGGAACTGCTTTACATCCGTGGTGGCAAGTGGACCTTTGAAGAG
CTTCTTGCTTGGGCTGACGAGAAAGATCGTTATATCCGAGAAGACCTGTACAAAAAGACA
GATCTCCCGAAAACAACCGATCTTGAACTCGCCGCTCGTGTACTTAGTACCGCTCAAGAT
ATCTGCTGGGCCAAGATCGATGCTTGAAGCAGTAATCGGTATACTTGGGGTTGCATATGC
TGTTATTGCTGTGGTATGCTCCATAAAGATGGAGAACGATAAGGTTCTCCGTCAGATTTA
CAAGGCCAAGACAGACCATGATTGGTCTGAGATCTGCTTCGCAGTCGGGTTCGCCTGGCC
GCTTGGGGGACTGGTCCATTATGTTATGAGGAGAAGAAATGAGACAGCTTGAATTTATGG
GAAGAATGTTCGAGTTGGTTGCGAAACGGCAAAGTGAATACAAAATGCACCGTAACATCT
TTGATGAACCAGAAAACTGTCCCGATATTCGAGACCAGGAGGACTGGCTTGGTGATTTTT
TTGTGTGGTTCCAGTGTAAGGGGTACACATTATGAGTTAAGGTTTGTTAACGCCAGAGCA
GGCGAAGATCTTACGTAAGCGTTTCCTCTTGCAAGAAGCAACAGGCAACGACCTCAAGGA
TATTGCAAAGAACGGTGGTGTTGGTTGTATGAACGATGCCTGCCCGAATTGCGGTGGCAC
AGGTATTCGTAAAGGTGGCCTGGGTATGTGTGTTCACGGCATCTCCTGCCCCTGCCCGAA
GTGTTCTTTCACATGTTGATAGGCTGTAATTGCAATCACTTAGGATTTTGAACCTTTGTG
CGATTGAGCCTTCCTCCCATATAAAATTTGCCACAACCATTATAAAATGGAGGTTAATAT
TATGATAGGAGGTAGTATGTTTCAGAAATTGGTTCGTGAACACCCTACCCTACAAGAACA
CTGGCAAACCCTGCTGGAAAAGAGTTTTAATCATCAGGGATGTTAAAAATAGCATCCTTT
ACACCAATCGTGTAGGGCGATTGGAGGTAACTCGTGGTGTTGACACTCGAAACCCAATGA
CATGCACGTTCCGTGTTGGAGATATGGGTAAGCTCGATCCTGATGACTTGGAAGAGGCTT
TCGACCAGTACGAGGGTTTTGAGACGGCGATCCGGGACATGTATAGAACAATCGACATCA
TCATCAATGAGCACAAGAAGTCTCTGGGATGGGGATAGAGAATCAAAGACTGGTTCAGGA
ACCCTCCTAACAAGGTTTTCTTTGTCGTTTTCATTATCTACGCAATAATTAGCGTGGTTG
TGTATTTTTATCCATTATTTTGAGGAGATTGCATGAGGGACAGAGGAGATTATAACAAAC
AGTATGCCGCGCGGAGGCGTGAGATAGACCGTCAGCGTCGATCCACACCAGAAGGAAAAG
CAACACAATTGGAAATCCGCCTTGACAGAATTGAGGCGGAGAAACGTGCAACACTCAATA
GTGAGTGGGACATCTTCGTTCGTGAAGAGGCCAACCTTCTTTGCAACGAGAGGATGCGTG
GCACAGGCATTCCTTGGGAGTCTGACCACATGCTGCCCCTCCGTGCGAAGATTGTATCTG
GCCTAAACTGTGGTGATAATATCCAATTTATCCCCGCAAAAATGAACCGTAGTAAGAACA
AATACATGATCTACACCAACCGACTGGATTCGATTAAGGATTTGGAGACCCTATGCAGGA
CGAATTAATAAAGCTTTTTGTAAAGGATACAAATCACAGCGTGATCAGTAAACTGCCGGT
AATATCCGGAGCAGTGGCAAAAGTCAAATCCGGATCAGAATGGTCAATCGTTTTCGTTGT
GAAGGCTAAACACAATGCCTATTCTGATGGAGTGCTTTATCCAAAGCTTACAATAAAGAA
GAGCGCAGGAAAAAACGAGCATATCTTTTTGATAACAACGAACAAGGATGAGGGAATCTG

GATTTATCCTGATCGTGTGAGCATCGGTGAGGGCGAGGTATACAACCGAGAAATGTTTTT
AGACTCATCATCCCCAACAGATGAAGAAGCCCAAATGCTAAGAATGAGCAAGGGTGCGTA
TCTTGATATCCTTATGCAGAAATTCGGAAGAGTACCCGAAAGTATCTTGGAAAGGGTGAT
GTCTGGAATGTTTGTTGTGGAGTATGTCTCGGAAAGCAAAGAAAGAGTGATTGTTTTAAT
AAGTTAAACGGCCATTGGCGGGTTTTCTTTCATCAATCCTTTGTCATCCTTTCAATGACC
AAACTGTCCGAATCATCAAGAACTGACAGGTGCTCAATAGGAATGTTCATCGTAGTAGGC
ATCTCCCGGTCATGACATTCGAACCAAATATCGTCCACCGGGTAAGGCATTCGGTTTGGA
CCAATCTTGGATCGTCCAAGAACATAACACACCCAGCCGTTTAGCTGGAATGTGAGCGGG
TTCTTGGGGCTGAATCCGTATAAAAGGCAGATTGTATCCGGGCGCACACGTTGGTCTGGC
TCATTACGGTCCGGGCGACCGCCCTTATCTTCTAACAGCAGCATGGTGTCTCCTTATTGG
GTTGTTTAAGACATCATATTTGATAATTGTGCGTTAGTCGATGGTTGACAAAATATTTAT
TTCTGCCATAATGGGGAATAACTTCGGATAGGAGATTGATATGTGTGAAAAGTATGGTGA
TGCAAGGGTAGAACTTGGGAGAATGATCGAAAGGAAGGAGATTCTTTAAGAAATCCTGAG
TGATATCGACCTGTGTATCCAAATCAGGAATGGTGCTGAGAAAGGCTCCCAGGAAAGGGC
AAACTGCTCGCTTCTTATCGACGCATTAATACACACTGCACAGAAGATCCAGAAAAGAGG
TCTTCAATGACGTACAGTATCTACGGCAGAATGCCTATGGCCCCATGCTCCCGCGAGGGA
GCGTGGTACTGCCTGAAAAGTAAAGTACCGTTTGACAAGACGGTAGAAGTAATGCAACAT
TACAGAAAAACTTGGCGATAAGTTCAGAGGAGACTTGAATGTTTGGATGGTGGAAAAGGC
GCAAGCTTAACCTGATCGGAACCTCCACTTGGACGGTCAACTGGACCGACACTGGAGAAA
GAGATCGTGAACAATGGTTGTTCTATGAGTCTGAGAGTGGTCGCAGGTACTTCGAAAGTA
CTCATGTACCGTGGTTACTGAGTAAAGAAAACCGACTTCCCGGTTACGCCGGGTGCAAGG
CGTGGGCCAGTGGTGGGCGACTTCCAGATACTTTCACACCAGTGAAGGTGATTGAATGAT
CGACCGAATCGTAAATAATGTGATCCATGCACCTGCGTTTTGCGTAGTGATGTTCGGGTT
ATATGCTGGGCTGGATATGAAGATTTATCCGGTCATGTACGCATGTGCTGCGTGTGCGTT
GGTTGCAGGCTGGCTTCTAACTAAGCACATCATGGGAGATGAAAGATTCTCAGAAATGCT
TTTCCGTTTTTCACACCTCATCTGGAGGACATAATGCAAAGCATACACTACGAGCAGCTT
CTACGAGAGATCCACAAAGATTCAATTGATCAGTGGTTCCATTCTGAAGAGGAACACCAA
CAACACGAGGAGAAAGAGATGGGCGGCGATACAATTTTTGATTTCATCAAGCGGGAAGTG
TGGCGGGATAGCGATACAGGAAAGTGGCGCTTCAAGAACAAGGAGTTCAAGCGAGAGGGG
CAAGCAGAAAAGGCCGCTGTGCAGTATTTCAAGAAGAAAAACTTTTGATTTTATGGGAAA
CTAGAGTTTTACCCAAAGAGAAGACAATATGTTTGAAATGCTCTTGGGCGGTAAAGCCCC
CATCTCCGCCGGAAGCATCAGAAAGGCAATGGCTGGGGGTGCGGATCTACTTGCTCTATC
TGAAAGCGATAAGCTGTACGGTATTTGTCGGACGTACCTAAGCGGCACTGGTGTTGCCAC
AACGACTTGGGCGAAGATTCTCGATGATGTTTAGGATTTTTGGTCATGATTCAGGCAATG
CCTTGCAAAAATGAAAGATGCGAGATGGTTATTTTGGGGGCCAAACTCCGTGGGAGCGAT
CCTTATTGTACGGGCTGGAATGCAGCCGTCGAGTTTATCCTGAAACAGGACAACCACAAA
GATTCCCCCCACACTAATTGGTAATACGGGCGGTATATGCTGTTGTTGCCATCATGCTGC
TTCTCACTCTGTAACCGACCTTTACCTGAAGTGACCCACCACACAAGGGGAGCCTGAATT
GGCTCCTCTAGTCCTGCCTGAAGAGTCCCACCAAGTTTGTTTAAAGAGGCACATCAATTT
CCTCTTGACGTAAACTGTCAGCCCTGCTACTATTACCTAAAGAGTCACAGGTGCTGAAGG
TCTGCACGAAACAAACACCACGGGTGCCTTGCACCTGAGAATTGAATAATTCTCATTCAA
CAATCTTTACGGACGTTGGATGTCCTGATTGTTGAACTGTACCACCGCTCCCGCGTGAAA
GGTTGTACAGTTCATTGACACACAATGCCCGTTGCACGGGTGAACACGTTCATTGCTCTG
CAAGACAACATGTTTAATGAATTGCATATCTTCGACACATGTAGCCTGAAGTGTCGATAC
TGTAAACATGTCACAGGGCGATTAGCCACAGGCTAAACCGTATACGGGTAAGCATATCAA
AACCCACCCCTCCCCCGCCCGGAGGGAAATAAAAGAGGCCCTGTCTTGTATGTCTTGGGG
ACGAAAAATATCCGTTGGCTCTATGTCTGATGTGATAGAAGGACAATCACAGCCCCCGAA
GGTTGTCAAGGATCTTTACAAAATATCTGAGTGGTCCGATGTCGAGACCCTTGCGAGTTT
TTGGCACGATGATTGCATGATGATTACTTGTGTGACTAATGATTGCCTAATGAATCTATG
ATGATTTGCTTGATCAGTCAGCTTACAGAACGATTTGCCCTTGACTTGCTTACTTTTGTG
TGGGTTGGTCTATTTAACATAACACACGTTATACGCACCACAGATCGGTCTGTTGCATGG
GTGATAGTTGCACTATGTTACCATTGCATCAATGAACCATTACTATGACGATGATTAGCC
TGCTATATGTTATCACTGTGACAGTTTACACCTGTTATTATTCATTATGTGGGGTGTATT
GTAGATGATAATGCTTTTCATAATCATTATGATTTCGATTTTCATTTCAATTTCTAAATG
AAAATCATTCCGCAAATAAAAATATTTTGTAAATTTCGCACAAAACACTTGACAGGGAGG
GAAAATTGTGTTTAGCAATACTCTGCTTTTGAGCATTTTGCCACATGATAACAATTCTCG
TTTAGCACTGTGATGGACCGGATACCCGGGAATGCTAATGCATTGAAATAGTTACTTATT

GTAACTATTGTCTGTATTGCTACACAAGGGATTAACAGACTTTGCCTATCAAGAAAAAGC
TTGACACTTGAGGCATCTTTTATGCTCAAAAACAAGCCTTTCAGCAGGCCAGCGGCCTGA
TTTACAGATCGATTAATCATGGTGTGTCAACCATTGACGGCGATTTATTGCTAATAAAAA
CATCTTGACTTTTATCACCTCGCGAGACTATAACACAAAGCGCTTACGGGTTGTTTTCGC
CACGGAAAACAATGTCCAGATTGACCAAAGGGGTTTTGTGAATCCCCTTTATCTGGACAC
TATAACACGAGAGCTATCAACGTTAGTTGATCGGTGTTATCGTGGCGAGTGTGATAATTT
CATTGTGTAACTATTCACTGTAAATGAATAATAATGTAAATGATTAGCCGTTGCTTAATC
ACGTATGTGATTATATCGTAGCGTCTACGAGCCACGCTAACGCGAACGAAAATGATAAGT
ATTATCATTTGAGTTACTCTCGTGTTATAGTGTCGCTCGCTACGCTCGCGCCACTCACCA
CGATCACGCGAACGATTATTTTATTTGACAAGCTTTTATTTTTATGCTCAAAAAAGACAG
TACACACAATGGCTAAGATGAGAAAAGTTATCAAATGAGAGTTATTATCACGTATTCACA
TGAAACGCTTAAACATGCGTGTTTTATTCACTTGATAGCATGCGTTAGCAATTTTGGGGT
GTTAGTTGGTCAAAGGCGATAAGCAATACCCTTTTAAACGCAATATAACGAGTTTTGAGC
GTCTTTTCTCGTGAGCCTTGTCGTTATATTCCTGTCCTGGGATCTCTTTAATTTCACTTT
TTTGCATAATTGCCAAAGGGGTTGGTGAGAATAATTCTCAGTTGCGATATTGCAACCAGC
GTTAAAGCGGGTAAAGATTTGGTGTTGTGTTTGGGGTGTTTGAGTGATATGCAAAAGCCG
GGACAATTCCCGGCCTTACTTACAACGTGACAGCCAGTGATCCGGCAATGATGAACGGTA
GATCATAACGGTCGAACCGTTTAAATCTGTGATATTTCACCACACAATAAAACGCCAATT
GCAAGCCGTCGATCATGGTGTTAACTGTCTGGGCCGCTGCGTCACTGAGTACAGGGCAGG
CAATCCAGCCATCAAAAACACTTGACAAAACAGCCATAAAGCAAGCCCTGGTTTTATAAA
GATTTTGGCCTTATCGGTAACGGTGCATTGATTGACTGTAAACCGATCCGCCGTCGAAAC
TTTCCGCCTTCCTCAATGCGTCCACCATTTCGCACATTTTGCGAAACACATAAGCACAAT
GATCGTTAAAGTCAAGCGCTTTTCTAATTATCTCTTCTTTTGTCTCCCCGAAGATAAAGA
CCAGGTGATCAAGGGTGTTGTAGTGATTGAACCACGCTCTCACCTGCTCACATTCCATGG
GGCGATCCCTCTCGCTCAAACTTGACAGGCCGAAAGATTCAAACGTGGTTACATTGTCCA
TCAATTCACTGTAGGCGGTGTCCCAGCCACTAGCCCAGAACTCTACAGGATCGGTGTGAA
AATCAGGATCGTCCGGATGAATAAAGCAGATAGAAAACAGTTGCTGATTTTCACGCTTTG
TAAGGGTGTATCGTGCGCTATCCAGGCCGCGCCCTAACATATCTTTCGGGGTTATTTCAT
ATTTCATGATCATTAGTCCTGGTTGTGTGGGTATGCGCCGTAAAGTCCATCACTATTAAA
ATCATCGTCAAAATCGGCCAGGTATTCGAAAAATTCAAATTCCATAGCTACAACATGCTT
GACAAGCTCACTATTTTTATACTCTTCAGGAGACAGGCGGATCGGCGCGTCACTATACAG
ATCCGTTAGTTCGATATACTCCCCGACCTTTAGAGCGGTAACGGTGTTTTTCTTGTTGTC
AAAATTTACTTTTTCCATGATATGCGCTCGACAATAATGATCGCTTGTGGGATAGAATCC
GGGGCAACTAACAGGGGTTTTGATTGGCGGATCTTAAAGCCTAATTTCTCTTTGCAAACG
GTGGCGGTCATTTTCCGCACTTCCGGATCGACGTTTTGATCCTTACAATACCACACAGAT
TTTTCATCTGCAATAAGGTTGGGTAAAAATTCAGTTTGACCAGTTGCATTTTTAATCATT
TTAATTTACCTTTCAGATTATGCCGGGGATGTCCCCGGCCTTGTTTTGTTTAGTATTCGC
TAGCATACATTGCTAACGGGTCATCAAAGGCGCTTTGAGCATCAGCACGATCCCAGAATA
AGTCTTGATCTTGCTGCTCTCCAATGTCTAACTGATTATACGCCGCTTGCGCTTGATATT
CCATAGCCAAAGATAAACGGCTATCAAATTCATTAATAACGCTTTGAGATAAACATAATT
GCATACTATCCTTTAACATTTCGAGCATGTACGCCGCCGTGCTTTCCTCCTGGTCGTGGC
AATATGCAGACCAGATAGCGGGCTTAACATTTTTCAAATGTGCATAAAGATCTTTATCGA
TAAACGGCGAAACATCGTTTAAGATACAGATCAAAGATTGCGGTTTGTCATTATTGCTGA
TAGTGATGAAATTTTGCATTTTCGTTTACCTTTCCGGCTTGCCGGGGAACCATACCCCGG
CCTCAAAACGATATTACCCGATCCGCGCCTGGCTTGCAAGCTTTTTTTTTATTCATCCTC
ACCGTAATCATCGCCAACGTTGAACATGTCGAGCATATCACCAGCGGCGATCCATAGCAG
GTACATTTCCAGGGTGTGCCGATCCATATTGTCGATCCCGTCTACCTCTTCACGCTCGAT
CCCTGTCTCGGCCAGGTAATCGCGGATCATTTGGTCGCTGTATGCGCTAAACTGTAAGCC
GTCCCGGAAATAAGGCAGGGCCTCCACCGTTGACGCTTCACAGTCCCCAGAGTGGGCGAT
CTCTTCTACCTGCTCACGGTCCCGAAAGATGAGACAAAGGCGACCGCATGAGGAGGACCA
ATAAACATATTTATTTTTCATAAATCTTTACCTTCCCATTATGACCGGGGGCGATCCCCG
ACCTTTAAGAATACTTTAGCCGATCTGGTTTTCAGAGTCAACCGAAAAAACAGCATTCAC
AATCGCAACAACCAAGGCGAACACGTCCACAGATTGACGGATCGGCAAACTGTCAGCCTG
CTTTACGTAGCCTTTCGCGCCTTCAATCTGATAAGACACGTGATGATCTTTTACTGCCTG
TCGTGATGCGTGATCAATGGTTGTTTCCGTCCGGATCATAATGTCCAGACGATCCCCGTT
CAGAGCGTCATTAACATGGCAAAAACGCATTACGGTTTTATCATCGAAACGACGAAAATC
ACATTTTGCATTATCGGAAACGCCAGCCAGGAAAAAGAATTTATTTGCAATTTCCATTTT

TAGAACCTCGTTTTAGATGAAAGATAATAACAGCAGGAAGATCACGAAAGACCACACCAG
GCGACACATAAAACGCGCCTCAATGTAAAACGTGTTTTCTGTAGTGTATTCTGCTTTGTA
ACCGTTCATGATTCACCTCTACCAGGTGACCGGGTACAGCTCCCGGCCTTGTCTGGTAAT
TATTGATCAACGGATCGCCGTTGTCAAATGTTTTTATGGCCTTGGTTCCACTTCTGGCGA
AGGAATACAGGTCGGATTGATATAATCAGGATCACGGCGTAAACCACTAGCGGCGATCTC
GCAAGATAGCGGATGATCGTATAATTCAATAACAAAATCATCACATTGCATCATCATAGA
TGCGCCATAAAAACAGATACCCGCGAAAACTTCAAACATTTGATTAACCTTATTTTTTCA
GGTAAAGAATTTGGGTTTTGTCGCCTTTTTTAGCGCCATCTTTCAACTCCTCACAAAGGC
CGAATTGCTCGCCTTTCAGAAGATTATAGCTGTTTGCGTACACGTTGCAAGCCTTTTCTT
CTGTAAAAGTTTCCTTTACAATCCAATTAAACTCTAATTTCATGGACGTAAGGGAAATGT
GAAAAGCCATTACTATAAATCCGAACATGATCAATTATCCTCTAAAATTATATAGAAAGC
AACAACGGCAAGCATTGCGACCAAAGAAGATACCTGCGCCACCACACACCAAGAATTAGT
ATACTCTAATATTTTGCTTACCCTCGTTTGTGGTGGCACCGTTGCCGCCTATGTAGAATC
ATAGCATAAGCGGCGCGGGTGTCAAGCGTTAATTCTTGATCTGGTTTGTGAGGTTGTAAT
CCTCATAACGTACACGGGAAACCGTGACCGATTGAAGCAAGAGGCAACGAACACGATCCG
CCCGGTTGTCATATTTTTTGATATGCTCAGGGGTAATGTGGCGGGTAAACTTAACACGCA
AACGTAATTTAAGCGGATCACGGTCATCAATGCCAGTAACCACACCGAAACCGATTTGCC
CGTAATTGTGGTAAACCATTTTACCCAGGAATTGAGATTCAAGCGCCGCGATCTGAGATT
TTACGTTTGGCATTTTATTTACCTTTGGTTTGTGTGGTTGGCACCATTGCCGCCCTACGC
ATTAAAGATAACATAACCACAGCAGGACGCAAGCACTATTTTAAATTATTTTTGTTGAGA
ATTGTTCTCATCTGCAGCGATTTTGCATGAACAGCGACAATTATGCAGATAGATTCACTG
CGTTACTAAATTTAAGGGCCTTTGAGCGTGGCGGGGTTATGTCATACCCTGCAAAAAGCT
CTAAAACGCTTTCTAAGCGCTTCCAGGCTTGACCCTGGCAAGTGCTTCACCTCGCGGAGA
TCTCCTTAAATTCACTTTTCTGCATAGAAAAAGCCGGGAATTATCCCGGCCTTTCGTTTA
ATAATCTGGTACGGCTTGCCACTGTAAAGCGAGCTTTCTTTCGGCTAACCGCTTGTTAAG
ATTTGGATCGTAGCGGTCCGGCGTCCAGGTTTTGGCGATGCTCTCCGGCTTGTGTAGTTT
GTCGTTTCGCTGGCGTCGCTCGTTTACTTCCCCATATGCCAGGCGATCACGCTTGCTTGT
CATCTTTTCAATGTTGCGCAGATTTTAAACCCTCGTTTTATTTTCGGTTGTTGTTTGCAT
GTTCAAAGATCCGGTCTTGTTTCTCGGCTAATTCATTAGCTCGCCATTCGATGATCTCCG
CGATCTGCTCCTTTGTGCCTTCTTTGTGATACGTGACTACTCCGCCCCGCTTTTTCACTT
CACACAATAAGCCGTTACCCTCAAGATCATTTAACGGACCAGATCGCCGGGTGTGGTAGT
GGTTCCTGTGAAATCTTTATTCAAAATAATGGTACACTTTGCCCATCCTGTATTCATAAT
ATACCCACAGCAGGCCCCGCCATAACCTGGCGGGGATCTTCCAGTAGACAGCAGCAGCAG
CCACCAAAAATAGGGTTTTAAACACTGTTAAAAATCCTCTAACATCTCGCGGAGTGTCTC
CAGGTCTTCACCTGTAAACACTTTGCCGAGTTCCTCGCCCGTCTTCTGACAAGCAAGATA
AATCTCTAAACCTTTGCGGCTATCCTCATCATAGCCCATATTAGCGCAAGAACCAGCATG
AGATTCAGATCCCAGATCAATATCTGACAGCAGGCAGCAATGCACGTTTGCGTCGCTAGG
TGTCGCAACATAAACACAGTTACCGAAAGACATTGCTTTATTGTCGTTGCGCTTGTAATC
GTCGCGTGTCCAGTTATTTCGATCACCGATGGAAACCGTAGCATCATCTTTAACTAATTC
TCTTGCGTCGGTTGGGGTTAAGCCAGTGTGACGGCCTACACGGTGACCCGTTCCCGTTTT
AAATTCAGAGCGGGAAATCTACCCGCCTTTTTCGAAACTCACGAAAAACAAATCAGCTTG
CCATTCTGGATCGTTGTTAACGCCACCATATACCGCTTTAAAATCAATCCCTTTTTCTTT
CAGGAAGTTGAAAACGGCAACATCTTGATCGTTGAAAGTGATTTTGTTATTCATGATGAT
TTACCTTTTGTTTGGTTTGGTATTATTGCCGGGTTATTTCCGTTACTTGGTCGGGCTTAT
CTTAAAGCCCCACCTATTAAGAGTCAACAATTATTTAATGATTCCTTTCCCGAAATAATC
GAAAGCGGCGATCACTTTCAAGGTATCATCTAACAGCTTACCGGGGATCTTTAGATTGTC
GATCCGTTGGCTTGCCTCTTGCTTTACGTCCTGAATTGTGCGGTTTGCAGGATTAAAGAT
CGTTAAAACTTGACCGTTAGCCATGCTCACGACCTTTTCACACCGCCTTTAAATAATTGT
TAGTAACCCGTTACACATGGCGTTAAGCGCGCTTTTTAGTGTGGGGCGCTTGGGTCATTG
ATTCATAAGCCAGATCATAACCGATTTTTTCAAGCCGGTAAAGCGCTGCGCTTTTCCCTT
CGTTGTGTGATTGTTCCCGGCGACGGGGCGACTCCAGGACCAGCACACCGAAACCAGTGT
AACCATTTTGCACGGATACCAGATCCCCACGGTGAAGCGTGGCGCGGTATTTGTTGATCG
TGTTTCCGTTAACATCGCGACAAAAATCAAAGGAATCAACAACAACGAAAAAATCCCCGG
TTTGGTTCGTGCGTGACAATGAGGCCGGGCGGTTTAGAGCGTCATACACATTGATAGATG
TTTTAAACTTTGCCACTTTCTTTACCTTTTCAGTCTGGCGGGAACCTCTCCTGCCTTGAT
TAATAATGTAACCGATCCGCCGATGGTTTGCAAGCTATGCGATGAAGTTTTTTAAATCTT
TTTTGCGCTTGTCTGTTTCTTGGGTGACGTTGATTTTTCAGCCGTTCTCACTAGCCCACC

GATCCAGGTGGCCCGATACTGATTTTATCCCGGTCATAGTGGAAAGATCTTTCATTGCAA
CTTGTAAAGCGTGGGAACCGTAACCATAACGCAGATCCCGCGACGTGTACAGCGTCGGCC
AGGTGTCGATCTCCGGGGTAGTGAACGAAAGCTCATAAACAAAATATGTATTACCGTACA
TATCTGTAAAGTCAAGAGAATGAAAATTTAAAGTTGCGGTTTTGTTTTCTAATGCTTTTT
TGAAAATCTCGATCATGTTTTCCATTTCGTTTACCTGCTTTCTTTTGGTCTGGGTATTAT
GCGCCAGTCTCCCGGCGCTTGTCAAATTACTTTTTATCGGGCCATATGCGCCGCAAAACG
TGCGGCGATCTGGGATCGCTGATGGGTTACCGGGATCGGCTTGCCGTTTGCGTCGTGTAC
ATAGGAGACCCGCGCCGCCTGGCGGTTACGGTAACCCGCTTTAATTTCCAGAGTAACAAC
CAGGGCCGGGGCGCTTTCCATTGCAGCAGGTAGATCGGCGGTGTCCTGGATCGCTTCCAC
CATAACGTCGGGGATGACCTGCCAGCGTTTACCAGCAGGCGCGGCGGTGCCGTTGTCGAT
CACTTTTCCGCCGTTGGCTTTTGCAGCAGTACGGGCAAGGGCGCGAGAATTGAAATATTT
TTTCATGGTGTAACCTCGTTTGTGTGGTTGATTTGTTTCGCTTGTCAATGTGTGGAGATT
ATAACCCCACACATTAGCCAGCGTCAACAATTATCTTACAGTTATTTTTTGACCTTTCAC
AATTAGATAAGTGTGTCCGGCGTTACTATATTGATCAACGTATACCCGACGATCACGCCC
CTGGTAATTAACTTTCCATTGTGTAGGGATCTTGGTCCCATACCCGGATCGGGATTTAGT
TAGCCCCTTTTGTTGCCACCACAAAGGCGCGTCAATCGCCGTAACTTCCGCCAACAACGA
CCCGTCATTGTCAATCAATAATGTTTCCATTTTGTTTACCTCTCGTTAGTGCCGGGGCGC
GTCCCCGGCTTGTTAGTAAGATAACCGATCCGGACTTGGTGATCAAGCTTTTTCGAAAAT
ATTTTTCAGGCCGTCAAAGTTTGCGATGATCTCCGCGCCTGCCTTTTTTGCAGCGTCCAG
CGCTTCACCGTTGATCCCGTTGCCGTTGTAAACTTCTTTTGCTTCGGTCAACAAATCGCT
TTCGTCCTGGTATGAATAGGTAAAACTATAACCAATAATCTCATTATCCAGGAGGGTGAT
CCCGTTCGCGGCGGCCGTCACATTGAAACCATAATGACTGGCGTTTAAATCGCGCTCCAA
AGCGTCCTGTGCGGCCTCGTAAGCGTGGCGCGATGTGTTATCATCACCACGGGCGATGAG
GTCTGCGATCCGCTCTTTCAGGCTGTATTGTAACGGGATAGCATACTTATAACTAGTGCG
ATCCGCGTTAGGATTCTTTACAGTAACCCCGCCCTGGTGATCGTCGGTCAATGCCCACTC
ATCGCCAAATGTATCATTTGTATTATAGCATACAACGTCAACGCTAAAATCAATGCCGTT
TTCGGAATAATTGAAAGTTTCGATGGTGTCCATTTGCTTTACCTTTTCAGTGTGGGGCGT
CGGTGTGTCGCCCTGATGTGATACATAGTAAACGGCTTACTAACTCCCTTTAATACCCCA
GGACATAAATTTTTAAAAAAGTTTTTCCTTTATACTTGTTGCATCCTGGCGGTGATCGTG
TACTATATAGGGACTGAGACAACAACGGCGGGTAAAGAAAATGGATAACGTAAACGTAGT
AACAAGCGAAACAATGGTTACCCTTTTCACCTGGGAAACATCAGCAAATCATTGTCAGTC
TAGGTCGTTGCCGTTGCCTCATGCCTTGCGTATGCTTAACGGCGGATTCTACCGGGCGAA
AGGGGTAGCGGAAGACCTGGAAGTTTTAGCGAATACCCTGTAAAAAATATTTGGTCCTGG
GGCTTGCAATCGGCGCGGCCCTGGGTTATAGTTAGTTCTAAGAAGAGTAAACGCCACCCC
ACTGCGACAAGGCCGGGCTAACGTACCGGGCGCGGGTGCCAGGTCGGAAGGTGGATCGCC
GGTAGTTCGACCTGATGTCATTTGTAAAGATTTGTAAAGTAGCTTGATCCGTGGAGTGGT
TATGCTATTATTTACACATGGCGAGGGGTAACGGGAATCGCGAGACAAACACCGAAGCTG
ACCGCTATTCTGCAAATCAGAATGATTCTCATTCAACAAAAAATTTCTCTTGACATCGCG
TGAGCGTGTGTGAGAAAATTACCGATCCCTAAATAATTTTTTCGCTCTATAGAAAAAATA
CGTTTTCATAACTGAAAACTCGATCGAGAACCGCGCGGAAAAACTTTTGCGATAGACAGA
AAAAATACGTTTTCGGTTTTGAAAAACCGACCGAGTACCCAGTGTTATCCAGAAAAACTT
TTTCAGCGATAAATAAAAAATACGATTTTGAAATCGAAAACTTGATCGAGTACGCGGACT
TAAAACCCAACAAAAGGGATATAACATTTCTTGAATTTTGCCACAATTATCGTACCGGGG
CATGGAAATTCCATTAGTAATCATTATGTTGCATGGGTGGGGTTTAGGATAAGTTGCTGT
ACTTTCCAAAATCTTAAATCACAATAAAATGGATTTACAACCCGGCTATGAGGGAAAACC
ATTTCAAAAACCATTTCGAATTTCATTTTGATTTCCAGTTCAAAATTCCCCGACCAATAT
CAAGGGCAAACAAATATCATTTTGAAAAACCGTACCCTTTCAAAACAAGTTCCTGAAGAG
AAATAGCAAGTGATTTCAGGTCGTTATCAAATATATTCTCAGATTCCCATGCAGCATGTG
CCTCTTGCTCTGTGTCAAAATATCCTATATACCTTCCCCTTGGGTCATTTTTAGATCTCT
GTGCGTTGAACTTACCAAGTTTTTTGTCAAATGTTACACCTCGTGCCTGTTTTCCATTTC
GAGAAATATAACTGTTCAACCACACAGGGATGTAAATACAAGTATCAGGGCCGTAAGCCC
TGTTCCTTTCTACCAGCAAAACTTTGTCAAGTGCATATCCTTGTTGATAATTGTTGCACC
ACCATGTATGAAAGTTCATAAAATAATGCCACTTATCGACCACAACAACCCCAAGATAAG
AGGGGTATTTACCGCTGTAGCTTCTGATTATCATATTGTGCCAATCCTTGTAGGCCGTAT
GGTGATGGAGTTTTCCATCTTTCCGCATACTCGTGATAAACGGTGCATCCTTTATGCCAA
CACCACATACTAAACTACGATGTTTTGTAGATGGTTTCGATTCCATTTCCAAATCTAAAC
TCATACCTTCCTCCTAAATATCACACGGTTTTCATTTTGGTTTCTTTCCGAAAGTCTCTC

TCCACCCACAAATATCGTACTCTGCATGGTGCGACATTTTATACATCAGGATTTTATCAT
CAGTTGGATTCATCCCGTCCATGAAAAGATCCTTCATCTCTGAGAGCCTGAAGCGATAGA
CCTTTTTATCCTTCGTCAAGATAATATCAATGCTCCCATTGACAGCATCTATTCTTAAGG
TATCATCACCTTCACATAAACACAGTGTCTTACCGCAAAGAGTGATCCAGTCCTGAACAC
CAACAACCATATTGCTTGCAGTGCTCCCATGCAGCATAAGCTCAACAACAATAGAACCTG
CGTAACCAGTTCGCTCTTCCTGAATGATGTTGAAAACAAAACCACGCTGAAAAATATTGC
AACCGCGAGACTCAAGAGCGCTGTAAAGGCTGGAGTCTTTGTTAAATTCCCTTACCGCTT
CTCCCGACAGATACAAAAACTCATCCGAGATTTATGCACGACCCATCTTGTTATCAACAG
AGAAAATATCTTTACTCAGGGTATCTTCCCGTTCGCCAACAGAGATCAGGGAGTCTGTGA
GAGTAACGACAAGCGCTTCCAGTTGGGTGACATAATTCTTATAGTCATTAAGCTCTGAAC
CCTTCGCTATACCCTTCCGTTCAGAAAGCTCAGACTCTACATTCCCGTGTCGTTTGTTAA
ACTCTGCCTCATCATCTTCATCAGGTGAAAATTCAAGGTAAAATAGCTCGTGCTCTGTGC
GCTCAAATATCTTGAGTAACTCGGATTCAAGATCCGGGTGAGGGTATTCGTTCTGTGTAT
GATCGAGAATCTCTGTGGTGTGAAAAACGGTTGTCATCTTAAACTCTCATCTATCGCTTT
GTGAGGCGTTTTAATAAATAGCTGTACGTTCGCCTAACCTGTATGGTAAAAACCCCCCAG
AAGCGTTTCTACGCAACATACGGGGCATTGTGGTGACATATCTTACTGACCGTGCTTCTC
GCTCATCTGATACTTCATGTATGCCATGTTCATTTTATGCTTTACATCATAGGGGATTGG
TAAGTTTCCGAACATGAAGTCGCTCAGCTCATACATCTTCTCCCAGAACTTGAAGGTGTA
GTAAGGGATCTTCATCAACGCACCTCCACAGGCTCAATGAAGTAGTTCTGGCCATGCTTC
GTCTGACAGAAAGATCCTGTGTACCAAACCCCACGCCCAACTAGGCTTGTCTTACGCATG
AGGTCGAAGTTGGTCAACTTTTCTTCACGCAGTAGGGCTCCTGATGCATCGACCAGTTTC
ATTAGTAAGTCGAAACCACACATCGAGATGATCTACTTGTTGCCATCGCTGTCAACAATG
TCAATGTTTGCAGCAGACCGCCCACGGCTGAATCCGGTATACCGGATTTCAAGGTGACGG
ATCTGGTGATGTTCTGATCTTTCGACTTTTCGTTCAATACCGTAATCGTTCTCATACCCT
GTATACCATGCCCCGGTGTCTTTGTTATACAGCGCGGGGTATTTCCTCTTTCCGAGCATC
GCCGTTCAATTTCTCCTCATTTTTCTCTATCCAAAGGATCATACCCTGGAGTTGTTGTTT
CATGGGGATCAATACAGGGAAATCATCGATCCAGATATCCGGTATCCACCCCAGCTTTGC
ACAGACAGCGGGCTTTTGCACTCCGCCGCAGTAAATGATGCCTATTCCAAGTTCTTCAGC
CCAGTATTTGATATCATCGTTCCTGTCAGTTTCGAATCGGAATGTCACGAACTTGACAGT
GCAGTCTGAATCTCTTGAACAGAAGAGGTGGACGAACTGCGACCACCTTTCCGGATCAAG
AGTGAATGTGTCATCGAAATCAAGCGCTATACGCTTTTTCTCAAATGATTTCACTCAGCC
CCCATCTTCTCTACGATACTGTCCCACTTATTGCACTGCTTGTCATCATAATGTTCAAGA
CGTCGCTGGACGGTTTCTGCGATCTCTTTCCGATCAGAATCGCGAGGGTTTATACAGTCC
TGAAGCTCAAGCATGTAAGCGAGGTCGTTTGCACAGTTGATCACATCGGCAATCTCTGCG
ATTGCAGGTTCATCACAACGATGAGGGCGATTCAGTGCTGGTGCCAGTTCTCCAACTTCC
TCTGCCAGATACATCATGACAGAAACAGTAGACCGGGCTTGCGCCCGGACCGAGGACTGG
AGTACGCGTTCAGCCATCCCGGTCATTTAGACTCCTTCTCTTTTGCCTTCTAAGCTTCAG
CGGCTTTTGCAGCAGAATCCAGGTCTTCGGTGTACTTCTGAATCTGCGCATCAAGGTACT
TTATGGTGCTGTCAGCCAGTTGCTGAGTACGTGGTGATTCTACCACATTCTGACCGACAT
ACGCCGCCAGCATTTTGTAAGCTGTTTCTTTCTGAGGCATGAAGTTTGCATATACAATCC
CCAGGAAACATACCACCATACCGAACACAGCAATCTTACGAGGCCCTTTTGTCAGGTCGC
GCTTAGGCACAGGTTGGCGCTCTGCTTCACGTTTACGAGGTGCTGTTACCTCCCCCAAAA
CTTCGAATCGCAGCCAACCAGAAGTATCACCATTATGATAAGGTACCATCATTGTACCAT
CATTATCAATCCTGGTGACAACTAACACATCTCCGGAATTATAATTGGACCAGTCCTAAG
CTAGGACAAGGTTCTGCCCCAGAAAGAATCCAAACCGTTCCTTCTTACCATCCGTCTCTT
CGTAAGTCTTCTGAATTGCATACATGATAACATATGCAACCGCTGAAATGAAGAGCAGGA
AGCCCCAACCGCCGTAAGTACCATCAGGAGTGAGTCCATCGATGAGGTAAATCAGAAAAG
GCCATGACATCAGTTATTCTCCTATTTATAAAAATTTGGCTCAATTACAGAACGACCATC
TTTACAATGAACACTTGAGGACAGTGCCTCAAAAGCCATGAAGGAATCAATCTGGGTCTT
ACCACCAGAGAACTCTATCGCCCAGTCATATTCTTTGGCGTTATCCCTTTTCTCTGGCAG
ATAACACCCCGTAAAAAGAATGGATAACAAAAGTGGTATGAAGACTTTTTTCATTTCTCA
TTAAGCTCCGCAATCAGTGCGTCAGCACGTTTGTTGTTCATGCCATTGATATTAGCAGGT
TGGCCTGCCATGTCAAGACGATTTCGCAAAGTTTCTCCTGAATCGTCTTCACCCATCGCC
CAGAATGGCTCCAGACCAAGGAAAGAACGCCAGTATGCTTTTCCCCACGAGTCCACCGCT
TTAGGGCACCTATCGTCTGTGTAGGTCCAGTGTGCAGCGCCGTAACCTGTCAGGCCATCC
ACAGTCAAGTACAGGCTTGGCATACAATCGCAACTGTCATCAACATGACAAGAGCAACTT
GAGCTACTGGTAATCACAAGGTACTTCGGAACCAGACGTTGAATAGAACGTTGAATATTA

ACCATTTTTCGGCCTCCGCGCCGCCCCGGAGTAGATCGGGTAAACAAGTTTGCCCCTGTC
GCCAATCTGCTCCGTTAACATTGAACTTCCACACACATCTGAAGTGCTTCGCTATCGCTC
CATCTCTTCCTCTTGAAACCAACTATACACAAACAAAAGCATAAATCAAGTCTTTTAGAA
AAGATCTTTTGTTTTTTGTTTTTAAAGACCTTATGTGTATTAATTTATATATGTCTTCTT
GTGCAAATTTTGCAGGACACAAGGTGCAAATTTTGCATAACAGCGAGGTGTATTCGTATG
TGCTCATGCCTCTCCTTTCCACAGATAAGTAGCCGTTTGTGACCAAGTTCTTCATAGAGG
TACTGATGGACCTTTCAGAAACACCAGTGGCCTCGCTTATCGTCTTGTAACTTGCGAAAT
ATTGCTTTGTTTTTGTCTCTAAGTATCCGGCTTTGTATTTTATATGCAGCAGGACACGAA
GATCTGTACAGGACAAATTCTTGTCATGTAACTCCTTCTCATCACAAGGTACCCACCTCT
CGCTCTTTCTGGACAACTGTAACCTCTCCACTATAGACAGATGGTAAAAATCCTTCGACA
ATACCTACAGCCTTTTCAAAATACTTGCTTTCAAAACACTCTGTGCCACCAGGTATCCTC
AAATCCATCATTGGAATCTGCCACTTTATCATCCTTTCTAAATGCCCTGCCAGGCCCCCA
TTCTCAAAAGTATACACAAATTCCATTGTGGAAACACTACCATATTGAGAAAGTTACTGT
TCCCGTTCATTAGGGTTTTGCGTAATACCTACTGTACGAACACCATCACAAAACCTGCCA
ATATACAAAGACTTATTTTTACCACCAGGGCTATATAAGTAAGCATAATCTGCCATAGTA
ACTGTTTTATTTTCTCGATCCAAAAGCAAGAAAACTCTGTTCTCCACATTTCTCAGGCAT
TGACTCAGCAACCCCCATTCCATGTTAGTATCTAAGAAAAGAAGTTCTACAGGGTAATTT
TTAATATCTTTCCGAGAATCGAAGCATCTTTTGACCTTGCAATAGCAAAGCACATCATCC
CCAGTCAATTTGTCAACCTGAGTCAAGCGATCAGGTGTACACATTTGCTATGACTTTAAC
ATTTTCATTCTCACCACTCCATATAACCTCTTAAGAATTCAATAACATACTCCTTCGCAT
ATTCTTCATCAGCATCATTATGCCACATGCGGGTAATTACACCCGCCATAGTGTACAAAG
ACCTCTCAAATGGTGAAAGTCCAGGTAAAATACTAACCATAAACTCCTCCTCTGTGGTGA
TCTCCAGAGGATTTACCGTGTAATCTGGTGGGATAATCTTCTCCCACTCTCCTCCCTTGA
AGTTCTTAAGGGTCAAGGTTCTCATGTTGGAAGTTGTGAGGTACTCACAATACTCACAAC
CATCCTTTTTGAAGATTATTCGCACAATTGCCCCGGTGTACATGAAATCCACATCCAGAC
CGGGACCAACGGCGACGATTTTATTTTGAGTGCTCATCTATATCTACCGCCCTTCCGAAA
GGTGGATTAAACTTCGCACGGTTTCTACCGCAAAGGACCCTCAGTGTTTCCCATTTACGA
CCCCAGTCTTTTTTCCGATCACTGAGATCCTCAATATATCCATCAGTGAAGATTATCACT
TCTGTTGCCTTCGGAATGTTACCTTCGATTTACTGGAAAGCCACACCTGCCATAGTACCG
CCTGTGGAGGTTACATTGTAGTCCGGCATCTCTTTAATGTTCTCATGTGTGTACACTATC
ACATCACCAACCTTCGTTGACCAGCAAAATAATGTCACACGGAATGTTTTATAAAGCAGA
CACAAACGAATAATCTCGTTGAAGATCTTGGTGAGCGTCGTCTGACATGTAGAACCTGAC
ACATCAAAACCGATAACGATGTCGATTGTATCCTGTTGCTTCCTTCCTGGCAAGATGATA
CTTGGCTTGTTGGTGATAGCAACGTAGTTTCTTAACACTTGCGTAACAGACCCCGATCTA
CGAGCAGGTTTGCGGAACGAAAGATGCGATCTCACACGAGAAATCATTCGTTGTTTGACG
ATCTGCATGTAATCGATAGGTGGTTTACGCATTCTGGCTACCAGTTCTCGTGCCTCTTTT
GGTCCTTCACCACCCGCAGCCTTTAATGCAGCCGCAATAAGACCCGCTGACCATGCAAAG
TTCTCATCTTTTGCTACCGGACCTTGACACGGGTGTGGATCAGTGTACCCAAGAATGTCA
CGATCTGGGTCATAATCCCCGATATGAGTACCCATTCGGTGACCTGAGACATCCTTCCCT
TTGTGAATGGCGCTGTAAATTTCTTCCGCCGTCATCCCGTGGAAAGAGAAATCACAGTAA
CAGTATGCCATAAATCCGAATTCTTTATTTTTATCAAAGACTGTTTGCGCCCCACCAGGG
AAAAATCGCATACCCTGAGAACGTGGTCCAAGATCGCTTATCACAATATCTGTGTTGATA
TACTGCTCCGCAGCAATATTGTACAGTTTTGAATCGAAGAACTTACCACGAGCCATATGA
TCATTGGTTATATGACATGCCTCATGGATGAGAATAACCACCACTTCCCTTGGTGTCTTT
TTACGATAGAATACATTGATATATTCTTCGTAGTCATCCTTCTGTTCCTGAGTCATGTGG
GGGTGTTTATCAATCCGAGAAAACACCTTCTTCTTCCGCTCTTCCGGCATACCCATGATG
AACTCAGGGTTATAATACAGGTTGCGCTGGTCAGTCGCCGCAGTAGGCAACCAACGCCAA
TCTGCGATGATCGGCAAAGAACTCAGCAAAGTCCCGTAAAATGGGCGCTGTTGTAACAAT
GCAATACGTGCAGATTGAATGACCTTCAGAGCATCTTTCTGAAGCTTTTCAATCATTTCG
CTTTCGTATTTATACATATCTCTCTCCAAATAAAAAAGGCCATCAATCAAGATGGCCTCA
GTGTATCTCATTTTTGAATGCGGATCAACTCTTTTTCTTCAACGGAATATTCTCTTCCTT
CCACCCTTAATCCCCATGCCAGTTTTGGCTTTGGCTCATACGGGGTTGGAGGAACAGCAA
ACAGTACAATTGCCTCCACGTTTCCAGAATGGGCTTCGTCGATAATCATAACCCCCTCAC
CAACTGCAATCGCAGGCTTGAAACCATGATCCAGCATCTTTTGTTCAGGGGCCGTTGCTG
GTCGGCCCAAGATGTGTTCGTACATTATACCACGTTATTCAGCATCGCGTTGATCTTCGG
CAGACTGAGACCTGTATGGATCATCAATTGGGTCATTGCTGTTTCCATTTTCTTGGATAG
AATGATGAAATTATCTTTCGCAAAGCGAAGTTTAAGGTCGAGGTTCCGGAGTGACTTGGG

TTCACCTGTCAACTTCTCCCCTGTCAACGAGCAGGTGTGACGGACATATTTTGCCTTGAA
CTGTGCATATGTCACACTTACATTCATCCCTACAGCAGAGAAGGTTTTGCAGTAGTTTGC
ACACGCCAGACCTACCGCTATGTCATCCGGCAGTTTTGCTGGCTCCGGTGCTTTTTCTAC
GACCTCTTCCACAGCCTTGGTTACTTCCTCAGTAACTTCTCCCATGTGCATATGCTTTTC
TGCTTCCTGGTTCAGTTTCGCTTCGATAGCAATCATCTAGTTCTCCTGTTGTGGGATATA
TTTAGTTTTCAGCATTTCCATGTGGTCTTCTGACATGGTCAGCATCATTGCCTGAACAAT
CTCGCGATCAAGCGGTTCAATTATAGTGATTGCGGTCTTCTTGGCAACCAAACAATCTTT
CAATTCGTTGGCACGACGCATTACAACACTGACGTTGCCACGCACATTACCTTTTTATC
GTCGATACGTTCAACTGAAGGGTATTTTGGGTTATAACCCGCCACGGTTCCCATAGGAAA
CGGCATGTTGGTGTAGTCGCAAGTACCCATACCGAGTAACTTCTTACCGATCATGAACCA
ATCATCGAGGGTGAGTGAGAACTCGTTACCACGTTTTCTTGTACACTCAATCTTTGTGTG
GTAACGCTCTTCGAGATTTTTCATCTCGTTTTGTGTGAATTCTTTTTTCCAGTCAATCAT
GTTCTATTCCTGGGTTGTTCCCGTTCTTGTGGGGTGAATTATCTGGCATTCAATCCGCGC
TGTCAACACTTGATTTCAAATTCTTTACAGTGTTTGCTTTGTGTACTTTTGTTTGAAGGA
AGGATAAAAATGACGAAATTCAACGTTGATGTTACACAAGTAAATGCCCCCAAGGTTACA
GCAGATATCGACTGGAGCTTATTAAAAGTTGACCAGACCACTCGCGCCCTGATCGAGCAC
GGGCAAAGAGAGCACGAGTATCGTCAAATCGCAATAATGGGTGTCTGGGATACGCCAAGA
ATGAATACCGATGATTTTCTTTCAGGGGTGAACCCAAGGGTGATGGAAGACCATCACCTG
GCTGAACGACTCAATGGCCTGCATGAAAGGGTGGATAGGGGAGAAGGACCATTTTCTTCG
TTCTGGGGTTATGACTGGGATGGTTATCTGCGGGAGAGCCTTGATAAGAGGTTTTTGGCC
CGACTAGAGGTAGATGTCAGAGACAGGGCCTTTAACTCAAGCCCGTTCGAGCGGGAAGGG
GTCTCAAGAGGGAATGTCTCTGCAAATATCCGGGGGCATGGGGATGGGGATGCCTTTACA
AAGAGTTCTGCTTGGAGGTTTTCAATTCGTACAAGGGAGTTTCTCAGTTTGTTCAAGCCC
CTGATCAGACAAATGGGGCGTTGTGAACCAGACCTTGTAATCAATGCAGAGTTCCGCAGA
TACGGCTTTGGTTCAAAATTCACGCAGAGGTCAAGAAACGTTATTGATATTATTGGGATC
TCCTTGTCTATCGGAGATCTTCTTATATTTTGCGACTTCAAGGACAGGGATAACTTCGAT
GTGCAGTGCAACCACATTGGTCACAGCGTCTTCCACTTGGGTGATCGCGGCGTTAAGATC
AAGAATGCCCGTGAAGGGTTGTTGACTTGCAATAAGAATGAGACATTATTCACCTTCCGT
TGGGAAGCAAGAATCAAAAATATGATGGCAGGGGAAACACCCGGCCCGGAAGAGTAAATG
ATGATGGAAATGGCTGGATATAAAGATGAGTTCTGGCTTGACAACATTGATGGTTTTGAT
ACACTCAAGATCAAACATTTTAATTTGATGGAGCAACAAAGATGGTACGAACTTTCAATT
CCACGATTCCAGTTTTAACCCCGGCGCAAGAACTTGCTCTGGCTATCAAGATCGCAGCAG
AGGACCACCTGAATCAGAGAGACAAGGGCGGCAATCCTTATATCCTTCATCCCCTGAAGG
AAATGCACTACCTGAAGACTGATGATTTTCAACTCATGCCGATTGCAGTCCTGCACGCTG
TTTTGGAAGATACCGATGTAACGGCTGCTGATCTGGTCATTCTGGGCTTCTCCAACCGTG
TGGAAGATGCTGTGGTTCTTCTGACAAAGACCCCCAATCAAACACCGGAGGAAAATGTCC
AGGGGATTGCAAGCAACTATGATGCTGTTCGTGTGAAACTTGCAGACCTACGCCATAACT
CTGATGTTCGTCGCCTGAAAGGTCTTACAGATAAGGATTTGTTGCGTGTCCGTAAATACC
ACGGCATGTATCTTCGTCTGACAAAGATGAAAGAACATCATGAAGCAATTAACCTGTTGT
CACAATTATGAATCTCTTGACAGTGCTTCGCACAAACAAGTATACTCAGAAACAGATGTG
CTACCTGCTGGGGATGCCCTCGTGGAGAGTGAGTAAGCTCGTTCTCAAGTTGGAGAAACG
TGGTTACATTCAGGTAAGTCGCAGATACAAATTGAGCGGGCCGGTGTCAAGACCCTTTTG
TGTTTACGATCTAAATCTTTACAAAGTGACTCTATCCACGAGCAAAGAATGTCCAGAAAT
CAAGGCTACACCCCCAAAGGCACAATCAAAAAACCCCGCCGCGGCTGGGCGTGGACTGAG
AAAGTTCGCGAAACATCTCGTTACTGGATGTCTTACCAGCATTCAGTAACAGGAAAAGCC
GTGAAGTATCGTAAAACTGACGGGAAACTGGTGGGTGGGGGTGACTACCTTGACCTTGAA
TCCATTAAGGAGCTTTCATATGAACAAATCTTTACAAGAGGCTATCGAGATAAGGCGTAG
TAAAGGGCAGAGCGATGCGGAAATTGTGGACTACCTCTTGAGGCGCTCTGATCGCAAAGA
AGAACACCTTACCTACTATAAAAATCGTGCCTCTGAACTGAAATCAGCTTTGATTCCGGC
ACAGTTTTAAAGTGGCCGTCGGGTAAAAGTCACGGTTGACAACATCATCAGAACATGTTG
GGGAAATTACGTGTATCATAAAAACACGCGTTACGTTTAGGATGGTAAGTTGAAGGCATG
GGTTAAAGACCCTAGCAAAAACAGTTTGTTAAAGCACAATATCAAACACCTGCCAGGCAA
ACTTCATGTGGAGGTTATTCTTCCTGACGAAGAAATGCCCCGGAAATGGGAGGGGGTTGC
TGTTGGTAATCTGTTTGGTAAACGCCTGCTTGCAGTAGAAAAAGACGGGATGTGGATCAA
CTCTGATGATACATTCATTATCACTAAGAAGGAGAAGAACAATGCATAACTGGAGAACCC
GGTTTGATTTGCGTGATGTGTGGGGTCAACGTACTGGTGAGTGCAGTCCAGAAGAGTGGA
CAGATAAGACTGTTCATGAACTAGCAAAAGAGATCGCACGTCGAATAAAACGTAAGTTTC

CACAACACATGATTGATGGACACGACTGGGACCCTCGTTTGACAGAGATTCAAGACATGT
TCGAATCAGTACCGACTCTTAAAGAGCCCCGTGACATGCTGGCCCGTCTCATTGCAAACG
AAAATGTTTGCGATGATGAGACCTGCGAAATATCCAAAGATACCCCGATGGCGCAGTTCT
ACGAGGCGATGTACCTGTTTTATGATAGGTGTGATAGTAGTTCTGTGTGGGAGGATAAGT
GAGAGCTTAAGATTTTTCATGTGCCGCAGTCATGCTGCTTCCTGGTCACCGCTTGACTCG
TGGTGCATGGTATGCAGTCCTTGGTGCCCAAAAGCTTGAGCCAGATGAAAAAATGTTCTC
TGAGGATCTTGCAACGAGGATTCATACCCTATACAATGATATTGCCCCTGAAGAAATTGA
GGGAGTAATTGCAAATTATCTTGAAGAAAGGAGACACACGAATGACGAGTCGTAATATTT
TTGATGATAACCGGATCACCCTGCGTGACATCCCGGATTTAATGCGCTTCTGTCAGCTCC
GCGGGCGCTCGCTGATGATGTTCGGTGGTGCTGGCCTAGGGAAACGTCAGGCAATGAAGC
AGGTCGCAAAGATGCTGTTTGGCGAACATGACGCCAACCTGATCGATTTCCGTCTTGCGG
ATAAAGAGCCGTCAGATGTTGTGGGCGTACAGATCCCGGTCACAGACGACAAGTGTGTTA
CCCGGACCGGTTGTGCGATCCCTAACTTCTGGCCTGAAGATCCTAACTGGAAAGGTATCC
TGTTCCTGGATGAGCTTCTTCATGCGGAGCCTTACCTTCAGAAGGTTGCATACCAGATCA
TGCTGGATCACCGTAATGGCACCTACATGTTCCCGAAAGGAGCGGTGTTTGCAGGTGCTG
GCAACCGTCCCGGCGATGGTACTGCGGTAAGTATGCTGGAAGCACCTCTGGCTAACCGTA
TGATCCTGGTCGAACTCGATTACGGCCCGACTGTTTGGCTGCATGATTATGCTATGCACA
ACGATGTTCACCACAACGTTGTTGCGTTTATCGATGCAAACCCAGGTAAACTGGAAAACT
ATGAGGAGATGTTGGAAATCAACTCCCCTTCATACGCCACACCCCGTACTTGGGTTATCG
CCAGCGATATCCTGTACGATTATGATGCAGGTATCTTGTCCACCCGCCTGGCACGAGCCG
CTCTACAAGGATCTATCGGCAAAGCATTGACCGATGAGTTGTGGTATTATCACACCCGTA
TTGCAAATATGGTTCCTTTCGAGGGGGTTATGGACGGATCGATCAAGAAGAACACCGGGT
CAGACACCTCAGATGTGTTATGGACCCTAGGCTCTCAGGGTTCAATCTGGTTGCGTAAGC
CAATTCAGAACAAAGAGTACACAGATGATCAGGTTATTGACTTTGCTGCGAGCTTCTTGC
AATACATGTATGATAATTTTGGTCAGCATGATACGTCATCGAACCGTGATTTCGTGAGTT
CTGTGTTGATGTCCTTCATCCAAGAGAACTCTTTTGCAAAAGCAATCCTGTTGTCGGGTG
ATCGTGAGCGCTTACCTGCCACATTGTTGGGTACATACCCGGTAGTAATGGAGAGCATCA
CCGAATACCGCAAGAAATTTGCTGAAGGTGTTGATTCAATCGTAAATTAATCGTTGACAG
CCCCAAGTGGGGCTGTTATTCTTTGCTCACACAAACAAAAGCGAGGACAAACCCAATGAC
TAAGTTAAACTGGAAAGAAGCACAGCAGGCTATGAGTGAAGGTAACAAGGTACGCAACCA
ATACTTTACTCCAGAGGAGTTCTTCGAGATGAAGAACGGGCAAATCGTGTGCGAAATGGG
ATACAACATGGCTGGTTGGTATCTTGGCGAGGCATGGCAGGATGAGGGATGGAGCGTGAT
TGATGCGTAGACCTAGCTTCGGGTATATTTATATCCTTGATAAAGGTTGTGATGCACGTT
TTGTTTGTTGAGGCTCTCTTGACATAGAATATGTGAAGGATCACTACGGCCCAGACGCAA
AGAACTGCCACTTCCGTGAAGACAATGGTTTTTGTCAATCTTCCTGGATGGCTGGAGAGA
GTGCTGGGGTTATACTAGCCAAGATAGCGGAGCACGACAGAGAATATTTTCACTTTGTCA
GGGATTTCGCACAACAGAACAGAATTGTTCTCATGAAGGGGGAACTATGGTAAAAGAGTA
ACTTCGTCCAATTCCTAATTATGGCGATGTGATGGATGTACATTCATGGGATTGCCGTGT
AAAATGCGGTGCCTTCATTCCTGATGGTGGAAATGGGTACTGGTGTCCTGATGAAAATTA
TGAGTCCGACATCAGTCGCTGGAGTCCACGTCCTGAATGGGCGACCCACGTAGCGTGGTA
CAATCGCTATTGGCAACGTTCAAATTTCCGCTGAAAGAGATCAACGAGGTTTGGTGGATT
GATCGCTTTGACATGGTGATGGCTGGTTACACATACCAGCGTCAGCGTATTTTTGTTGTC
TGTGCTAATACGAAGGGTGAAGCTCGTGAAAGCATGGACGCTTTTAAAGTGAGGAATTGT
GCATGAAAGTAAAAATGAACTGCAACTACAGCGACCGCTTAGACCATCCCCGTGCTGGAG
ATGTCCTCGATGTTTTGGAAGAGTTTTATAACGGGACTTTCATTGAATATTATCGTTGTG
AATGGAAAGGTGGTGACCTTGACGTTTACCCATACGAGTGTGGTGAGGTGAAGAAATAAC
ATGAAGGAGAGTGAAGAGGAATTGCAGGCGCTTAAATCTTCCTTCTCATATGACCCTGAG
ACAGGTTTGTTTACGTGTTTAAACCCTATCGCTCGTCGTCATTTTGGAAAGGTTGCTGGT
ACAAAAAGAAAAGACGGTTACGTAGCGCTTGGTGTTAAAATTGAAGGTAAAGGTTTTTAC
TTCTTGGCACACAGGGTTGCTTGGGCTTTTTATTACGGGGTATGGCCCACTTACTTTATT
GACCACAAGGATCGACAAAAACATAACAACCGCATTACAAACCTAAGAGATGTTCAGAAA
TTTGTAAACGTTTATAATTATAGCAGGGGTAAAAAGAACCAAACAGGTTTCCGTGGTATA
TTCGTAGATAAAAGGTGGACAAGACTCAGATTCCATGCCGTGTATAGCAAAACCCAACTT
GGAACATTCGACTCTGCGGAGGAAGCCAGTGAATGATATCAAGCATACGTTAAAACGATT
CATGACATTGTAGTGGAGGCCTGACGCTTAAGTATAAAGTAGGTGACCTGATTGAGGCTG
TGAAATCTGGGGAGGTCAATGTATTCGCGCATGGCTGCAATTGTTATTGCACGATGGGAA
GCGGGATCGCTCCTCTAATCAAAGAGGCGTTCCCTAAAATGTTCGCTGCCGACCTGAAAA

CTGAGAAGGGCGACAAGACCAAGCTGGGAACCTGCACAGTGGCCTTTTATAATGATGGGT
CTCTCGCAGGATTTAATCTTTACTTACAATACTGCTATAATCGTCGTAAACAGGGTCTCA
GGCACCTCGATTACAATGCCCTGTACGATTCGATGGTTGAAATCAAAAAGCTCTTACAGA
GCTATACAGACGGCCCTAGGGACCTCTATCGAATCACGTTCCCTAAACTAGGTGCTGGGC
TTGCAGGGGGTGACTGGAATGTCATCGAAGCAATGATCAAATCAATCTTCCACGATTGTG
ATGTAACAGTGTATATTTTACCGGAGAAAAAATGAGGGTGGAAATTATCTATCTTTTGAT
AGCATTCTTTGGATTCTACATCTTCATTAAGAGGACGCAGAAAGATTTCGGGAGAGTCGT
TAGTGTAGATGTCTTTGCTGCTATATGCATGGGCTTGCTCGGACCTGTGTACTTCACTAA
TCTTGTGGTGCTGCGAATTATCAACGGAGACTGGAAATGATTGAACTATTTCCTGGAATG
AAGGTCCATGCTAAAATTGTGGATGTATATGCAACAGATCGAACTGTATTTACATAGAGT
GCTCCTCGTGGCAAAAAAATATGCCCTCATCTTGATGACAGATGAAGATCCCAGAGATGA
TGTTGACAATCTCTTAAAGGCTTATGAGTGGATTGAATTCCTTGGGTGGAAACACGAGGG
GGATGACAGATCGAAGAAATTTACGTTATCATTGATACCCACAATGCAACCTTCTGGAAG
TCTGCTAACGACAAGACATTATGGCCTTCTGCGCGGACAGCGAAGTCTGCTTTTGGTTCA
AGTCGATACAGTCCGCTGCTTTGTCTGTACTCCCAGCAGGACCTATTTAAGATTGCGAAG
GTAACCACCGCAGGGGTGATTTATGAGTAAGGACAGAGTTTACGTCATCTATGATAAAGA
GGATGACTGCCTGTGGCACCATATAGCAAAGATCGGTTGGATAAGTTCCGGCGCTGCAAA
AAACGCCTGGAACCTTGGCAAGAAAATAAAATTTGACAACCAGAATCGTTAAGTTGTCGT
CTGTGTTAACGAACTGCTGGTTAAGTCTTTAATTGAGCAGAAGAGAAATGACAATGAGTA
AATCTGTATCAGGTATCGGTCAGACCGAACAGGAAGCGGTATACATCGGTGGTAAGCGAT
TTGCTGGCGCACTTACCAATGCCCGTCCTTTTTACGGCTTTGGTGACACGAAGGATGTTC
GGTGCATCAAAGGCTTTTGTTATCGTGATCTGAAAAATCGCAGCTTCGACAATGGCGATA
TCCGAACCTCTGTTGTAAAAGAACTGATCATTCATGAGCGTGACATCTACGCCATCACGA
TGAACAGTGCTTATCGACTGGACAACGTGTGCCTGTTGGCATTCGTTGCTTCCGGTGTGG
CGTGATGACGTCCTTAAACTGCTGGCAGATGCAACGAAAGATGCTTATCCGGAAGAAGGG
GTTCTGTCCGTAGAGGCTAATCTGACCTATCGTGTGACCCCAGAATATTTCCAGGAGGCC
ACAAAACGTTTATCAGCCGGCGAATGGGTTGTCACCCAAGCAGATTCTGTGAAGCGCACC
CTGAAAGTCGGTAACGAAGTGTATGTGGAGGAGAAATGATTGACTAAAGCTATCAAATAC
CCAAGCACTAACCAGTTCCGTCAGGTGATCCGTTCTGTCCACGACCGTCTTACCTTGGAC
GGGATTGATGAAAACGGCTGCGTAAAACGCAAGGTTGTTAACCCGACTGAGTTTCTCCTG
CCCTACGTGGGGCCTGTGAAGATCCACGGGACCAACGGGAGCATTGTGTTCTACTCCGAA
GATGAAGTTGTGTTTCAGTCCAAAGCACGTGTTCTGTCTCTTGAGCACGATAACAGCGGC
TTCATGGCGTTCATGTTGCGTAAAGATACTGGAGCACTGCTGGACCAGGTAAAACGCATC
TGCGAAATCAACGAAGTCCCGTTCGAGTTCCCGGTTGAAATCGCTGGTGAATGTGCTGGT
CGCGGTCTTCAGAAAGGTGTCGCAGTCAGTGAGGTTGAGCAGTTCTTTGCCATCTTCCGT
GTTGCAATCGGTCAGAAAGAAACCTCACTGAAATGGTTGCCACCAAGCGCAATCTTCGGC
ATGGGCCTCCCGGAACAGCGGATCTTCAACGTGCTGGGTTTCGGTTACTGGTACGTGCAT
ATCCCGTTCAACGAACCGGAAATGGTACAGAACGATCTGACAACGCTGACCTTGCAGGTG
TAAGCTCAATGCCCTGCTGGTAAGTGCTTCGGTGTTGAGGGTGTCGGTGAAGGGATCGTG
TGGTCTCTAAAGAGCACTGAACTGGCACAAGTTTCCGGTCTGTGGTTCAAAGTGAAAAGC
GAGAAACATTCTGTCTCCAAGGTCAAGACCCTGGTAGAAGTTGATCCGGTACGCCTACCG
AATATCCGTGAGTTTGTCGAGTATGAGGTCACTGAAAACCGTCTGTAGCAGGGTCTGGGT
GAAGTGGGTCTCGACCAGACCCAGATCGGGGAGTTCAACGGTTGGGTTAGCCGTGACATC
AACAAGGAAGAAGGTGATGTTCTGGAAGCGAATGCCCTAACCATGAAGGATGTTGCGGAA
TTTATCAGCAACAAATCTAGTGGTTGGTATATGTAAAAACTGAAAGAGTGGCTGTAATGG
CCCTTATCGTATCCACAACCCGTGAGGTTAAAGACAAGCCTTTGAAGGCGAAGTGTGAAA
CTTTAATCTTCGAGGATGAAGAACAGGGTAGACAATATGTGGAAGAACATGCTACCTTTG
TCTTCTCAGTTCAAAAAGCGAGGATTGTGAAGAATCGAAAAACCGAAACCCAAGTTTGAG
GTGGGACAGAAAGGAAAGGATCTTCTTAGCGGCAGGATACTACGCATACCTGGTCGCTGT
TACTTTAGTGGTTGGGTATACTCTTTCGGTTCTGTGGGTATGTATTTGTACCCAGAGCAA
GATCTGGAGGCTGTAAATGGAACTGAGTGAGTGGTTAAATGTGAAGAGCAGACAAATGGA
GGATAACGTGGTAAACTCTATAAGTATCAATGGCAATACCATCATCAATGGTAACGTTGT
TGGTGGTGATATGAACATTACTTCCCGTGGTGATAACATTTACATCAACGGCGAGTTAGT
TCACACCACGCAAGAGAAGAACATCACTGCGGTTATTCATGGTGACACCAAGGGTTTTTC
CACTGTTGCTGGTGATGTCAATGTTTACGGCGTAAATGCTGGCAATATCAAGACAACTTC
TGGTGATGTTCATGTTGAGCACGGCGCACTGGGGGACGTAACCACAGTGTCCGGGGATGT
TTATGCAGAAAAGATCGAAGGTAATGTGAAAACTGTCTCAGGGGACATCTCCCGCCGTTA

ATTGATAGCCCCTTTCTTGGGGCTTTTTAATTTGGAGCAAGATTTGAAAATAAAAGCAAT
AATCCTGGCAACCGCCTGTGCATTTTCTCTGACTACCACACCAGTCTTAGCCAAAGAAAA
CGCAAAGAAGCCCAAGGTGATTCATCTTTGTAAAAAAGATGATACAGCGGTAAACATCTT
GGCATGTAATATGTATCGTGAAGCCCGTGGTGAAAGCGATCATTGTCTCATGTCAAAAGC
TTTTGTGACGCTTAACCGAAAAGACAATGATAAGTCTCCTCGGACGGTGAAGAAGATTGT
CTACCAACCGGGGCAGTTTTCCTGGACATCCTACAGTACATGCTTCAAAGTGTATTAGAA
AGATCGTTGGGAAACGGCCAAGGAGTTCGCAGAGGTATTGACCAAGATCCACAACACTAA
CAAAGTTGTTTATGTTGCCATTGACATCACCAAGGGACCTACGTATTATCATTCAAAGAA
GGTTGAGCCTTACTGGACCAAGGCGATGATCCGCACGGTCAGTATTGATAACCACATCTC
CTACAAAGAAAAGCCGGACCCCCAGGGAGCCTGAACTTATGAAGAATTTTTTTGCTGCTT
TGTGTTTGCATTATCCCGTATCTTGTGATACTGTCTATTACTCAATGGATGGTCTGGGAA
CAAGTGTCAGGAGCGTATCTTCTTGGATTACTGGCAGGCATCTACCTAGTGCGGAAAGCT
CGCAAAAGGAACCCCATAAATAACTGCGAATAGAGGAGAGAAGG
>NewGenomeName_29
AGGACACGTTCATTCGATCGGCTCGTACTCGCGATTGATGTCGTCGGCGGTCCAGTCGAC
TCCGACGTTCTTCCACACGGTGATGATGACGTTGTCGCCAGCGAGGTGACTGGTGCGCGG
TTCCAGGTCTTCGACGATGCGCCGGACGGTGACGAGCGGGTAGTCCGCCAGATCCTCCGG
GAACTGCAGTCGGGCAATGCAGTACGCGAGATCCATCGTCATGACATCGACCCGGTCCTC
GTAGTGGTGCTCGACGGCAGCCGCGTCGTACACCGTGGGCAGGCGGGCGGCGTTCTGCTT
GGGGCTGATCACGCTACCGTCGAGAACGGCATGGATGTCATCCACAGTGACGGTGAAGCC
CTTCTCGGACATGGGCAATGACCTTTCAGAGGGCGCTGGTGATGATGGCGAAGACGAACA
TGAACAGGAACAGGCCGACCATCTTGACCAGCCCCCAGCCGACGAGGCGGCCGAGGACCA
TCCACGGTGACGGGATCGGCTCCCGGCGTGGAGTGTAGGCGTTGTGGATTCCCATGGCTG
GGGTCTACTCTCAGAACGGCGGTTCGTCGGGGATCTCGTAGACGACCGGCTGGGTTTGCG
GGTGCGGCGGCGGATCGGCTGCGTCGCAACGATGCTCGCGGATGATGTACCCCAGCTTGG
GCACCGGCTCCCGGCGGTTCATGGAGGTGGCGCGGTGCGCGAAGCTGGGGTGGTGAGCGT
AGAAGGTGCGCAGGCCCTGCAGTAGGGCGTCTACTTCGCCGACCTGACTGAGCTGGTACG
GGTCGAACTTGGTGTCGATGCCGTGGACCGCGCCCGCCAGCACGACACTCCCGCACCGAC
CGCATTTGCCCTGTCGCGGGTAGTCGGTGGTGAGGTGAGAGTTGTGCAGACGCTGCCACT
TCTGGGCCTTCTTGCGCGGCGGCACGGCTACGCCTCTTCGAGCATGCCGACGTTCTTGCA
GTAGGTCACCTAGCGGCGGTGGTCGACGTGGAACGAGGTGCGCGGTTCCAGGTCGGTGAT
GATCTGCTGGAGGTCGTAGAAGATGGTGCCGTTCCGGACCTAGGTCCCGACGTCGCCCTT
GTACAGGGACGTCGCCAGCTCGTACGTCAGCAGCTCAGGCCGGACCTCGCTGCTGTGCAG
CAGGCCGACGTAGCCGCCGTCGTACTGCAGCGGCTGCGGCTCACCCAGCTCGAACTCGGC
CACGATCGCCAGCAGGTCCCCGGCTTTTACGTAGACGTCGTATCCATTCATGGTGTTTCA
CCTTTTCCTCAGTCCCACAATCTCTCTCAACGCCCTCTCGGCGTCTGGCTCGTACTGCTC
CTTGCCGACTACTTCGCGGGTCTGCTGGTTCACGATGGTCCAGCCGATCCCGGAGATGTA
GAGGGCGGTAGCGATGTGCGCCCCGGTGGAATCAAAGGCCTTGATGGTCCACTTCTCGGG
GCGCAGGACCATAGGTTCCCCGATTCCCGTGGGGCCTAGTGCGAATACTCGCTTGTCCAT
TCGGTCACACCTCCTGCCCTCCTTCATCCTCGATCAGCGGGCGGAGCTTGTCGACCCAGC
CGTTGAGTGTCTCCACGAAGCCTGGTGCTAGGCGACGGGTGACGCCCTCATCGTCGGCAC
CGGCCGTGAAGACGACGTGGCCGTACCGCTCCTGTCCGAGGACGCTCTGGGTGACGGCGT
GGACGATCGCCCCGGCGTACCGGTTCGCGCCGCGCTCGCCGACCTGGACCTGGCCGTCGT
CGTCGACCGTGAACTCCTGCGACAGCAGGGCCTCGTCGTCGACCCACAGGGTCAGGTTCG
GTGCGAGCTTGATCGCGTTCACGGCCGAGCAGCCGATCGCGTCGTACAGCGCCCGCAGTG
CAGCCTGGTCGGAGCCTCCGAGGTCGATGCGCTCGGTGCTGCCATCGGTCTTCAGCAGGT
CGGCCAGTCGTGTCATGAATCTCTCCTCCGTGAGTGCATCGTCTTAGTTCGCGTGAGTGT
ATCTACGGGGTCGGACTACCCCCTGGCTTTGACGCTGGCGGCCTCATCCTGGTCCGGCAG
GGTGCAGAGGCCAGCTTCGACCAGGCGGACGCCAGAACGGCCGTAGGCCCCCTGTAAGTG
CCACACCAGGCGGGTGTCGATCAATTCCTGGAAGGGCGAGGTGGTCTGCTCGTCGTCCAG
TTCGTCCTGTTCGAAGGCGATGATGCGGTCGATCACGTCACTCATCGGTCTATCCTCTCC
TTGTGCGGGTCTTCTTCAGTAGTTCGATCCCCGCCAGCGCCGGCAGCCTGGTGATGGCGA
CCGGAGTGCTGGGGGTGTGGTGCGCCCGGAACAGGGCCTCGGCCTCGTCCTGCGCCTTCT
CGTGGACGACGATGTGCTGGGCTGCGTCCACCGCGGCCTTGCTCGGCCGGGGGCCGGTGG
CCTTGCGGTAGCCCCGCAGTGCGGGGTCGTGGGCCAGGTACCGGCGGCGGATGTAGTCGG
CCTTCTCGAGCACCTCCTTCGGGTACCGCTTCGCTGGCGGGGCCGGGATCTCGTACTCGA
CCTCGGTGCCCTTGGTGACGTCCACGACGACCGGGTGGCGGTTGGGATGACGCAGGACCC

TGACGGTGCCGTGCAGCGGACCGGGGGACCATACCTCGGCTTCCTCCATGTGGCCGCTGA
GCGCGACGTACTCCCTCACCCCACCTCCGCGTACAGGTCGATGGCGGCCTCGCTGGCGAC
GATCTCCTCGAGCATCTGCCGGTGGCTGTGCGGCACGACGGGGATGTCGCCCGGGACGAT
CCAGCCGCCGCGGAACGTGCGCCAGGCGATCGGGATGCTGGACGTGCGGATGACGTAGGT
GAGCGTGTCGCGGTGTGCGGCATGGAAGTCGGCGTGGTGAGACTCCGGCAGGTCGCCGAA
TCTCGCCTCACCGATGGCGTAGCCACCCTGCAGGCGGCCGGTCCGGAACTCCTCGCGGCG
CTCGATGGCCGCTGGCACCTCCTCGGCGCGGAGGCGGCTCTGGCGAACTCTCATTAGGTC
ACCCCCACAGGTCGATTTAGGCGATGGTGAGGCCGACGGCATCCGGCCGGACGGTGATCT
TCGTCCGCTCGGTGATGTATGCCGTCTCGCCGTAGCAGGACAGGTGATCGGCCCAGCAGG
TGAGGACCGAGTAGAAGCCCTCGCCGGTCGGCGTCGCGGTGTACGTGCCGGGCGCGATGT
CGGCGGGGACGCGCAGGGTGCCGGTGTCGCCGTCGCACATCCCGGGCGGGGTGTCGGCGT
CGGGGGTGTGCTGCGACGTGCTGAACGGGTCGAAGGGCGGGGTGTCGACGGTGCTCTTTC
CGCCGTCCCCGACCGACAGGGCCGCGCACCCGGAGAAGGCGAGCGTTCCGGCGGCGGCGA
CCGCGACGAGGGTGTAGGGCCAGAGCCGCTGGGGGGACATTGTGAATCTCCTTGGGACTG
TTCAGGAATTGGCGTAGAAGTAGCCCGCGCAGAAGCCGGTGTAGCACTTCTGATCGGGGT
CAAATCGGTCGTCTCCCCAGATGGCTCCGAGGGTCGTGCCGAGGGAGGACGGGGTGCTCG
GATCGAAGCGAGAGAACTCCTCCTGCGTATATCGGTCGAAGATGACGACGTCGCCGTCGA
TCTGCTGGATGAGCAGGGTGCCGTCGGGCGGTAGCTGTGCCACTGGTCTCTCCTCTCAGC
GGAACCAGAAGTCGCGGGGGTCTCGGTAACCTCCGCGCTGGCGCTTAGCGATGGCGGCCT
CGCGCTCGCGGGCGTAGTGCCGCTTGATGCGCGCCTTGAGGGACTTCGGGTGCGGGCCAC
TCTTGCCTCCGTGGAAGATGTATCCGGCGGCCAGGCTGCTCAGGAATCCAATCATCAGAT
CCCCCTCTCGTCGGCTTCTGCTTTGTCGATCAGCAGGTCGATGTGGACGATCAGCTCGCG
TCGGATGTCGAGGCGGAGTAGGTCGTTCGGGGTGGCCAGGTCTAGGTCGTCGTAGAAGTA
GGCGCGGCGGGTGTCGGACAAGCGGGCAGCCTCGGCGAGGATGTCGGACCGGAGCTGGTC
GTAGGTGTCGGCCATCGGAATCACTTCCAGTTGGTGGTCGGATTGTCGGACAGGATCAGG
CGGCAGGTGCTGCAGGAGACCGCCTTGAGAGACTTGCTGGCGAGGGCTTTCTCGACGTGC
GGGAGCGGTTTTCCGCAGACGCTGCGGGGTCCTCGGAAGGCGTGGCGGACGGGGTGCGGG
TCGTCGGGCTTGGCGACGCGGATCGGCTGGCTCATGGGTTCGGCTCCTGCAGGTAGTGGT
CGATGGCGCCGTACTGGACGCGCCGGTCGAAGATGGTGGCCATCAGGGCGGCGAGGGCCT
CGGGGTCAACGGTGAGTCGCGCCTCGGCCGCCAGGTCGACAGCTGAGGCGATCATCAGGT
CACGGGTGAACTGGCGGCTGAGCATTTTGATCAGATCCTTTGCGGCACAACGTAGAGGGT
AGTCAGGCAGGCGGTGCAGAATGCCGAGCCGGTGAGCGAGGGGTAGCCCTTCCCGCAGGC
CTTGCAGTGGTGGCGGCGCTCGGCCAGCATGGTGATCTTTGGGAAGTCGACGAGGGTGGC
GGGGTCAACGATCGGTGCGGCCAGGGCAATCACGTCCAAGATGGAGTTGGAGTAGTTCTC
GGTGACCCAGACTGCGAGCCTGCGCATCTCGGGTGTCATCGGCGACCCAGCTCGCTGTCG
GCCTTGGAGTCATACTTGCACTCGCCGGTCACGGGGTAGGCTAGGGTGTCGACCAGCTCA
CCGATCAGGGCCGCGATGTGCCGGACGGGTACCTCGGCGATGGTCTCGACCCGCCGCTCG
CCCTGCGACCACTGGACGGATCCGGCCTTGGTGGCCAGGTAGACGACGGTGACGCGCTTG
AAGTCGACGACCTCGTAGTGCTCGGGGTCGAGGAACCTTGGCTGGACGCCGAAGCTGGTT
TCGGTGCGGGTAGCCATGACGGCCTCCTTCGGTCTGTGGCGGTGGTTTGCGGTGGTGCCC
TGCCCCGGACTCGGAACCGGGGGCGTGACCGTTCTGCAGGGCTGTGGAGCATCAGGCCAG
CACCTCCCGGAGGACGGTGTGGACCTTGTTGTCGACGGCCGTGAACTGGCCGGTGATGGC
ACCCTCTGCGTTGCGCTCGGCGCGGGTAGCGCCCTTCACGGTGGACTGGTGATGTGCCCA
GGTGTTGACGGCCTGGATGACGCCGAACGCGGTGCCGTTCCAGGTGTTGGCTCGGGCGTC
GTGCCGGTACATCGTCGTCAGCTCGTCGCGCTGGCGGGTGGCGATGTCCGGCGCCCGGCC
CTTCTCCTCCGGCACCGGCGGCCAGGCGGCCAGGAACTGAAACCACTGCCGGTCGGTGAC
CGTCGTCTGGACCAGCTCGTGGAGGGTGTCGGTGAACTGGTCCGCCGTCTGCTCGATCAG
CTTGAGCGCGGAGCGGGACTCGTCGATGCGGAGGTTCGAGGACCGGCTGTGGCGGATCTT
GACCTGTGCAGCCTTGGCCTGCCCGAGAGCGATGGCCATCGTGTTGTCGCAGACCGTGGC
GGTGATCGTCCGGCCGTACGTGGTCGAGAGGCTGCCGTCGAGCGAGGTGGCGGCCAGCAG
GTTGGGCCGGTAGGCGAAGCCGGCGACCTCGGCGTGGAGCGTCTCAGGGACGGACACCTC
GACCCAGCGCTGGCCGACCTGCCGGAGGAGACCGGCGCTGGTGATCTTCAGGCTGTCGCC
GAGGATGTTGCTGACCGCTCCGAGCAGCCACTCGCGGTACTGGTGCGGCTCGTACCCGTC
GGTGAAGAGCTTGAAGACGTGGTGGTTGCCGGAGCGGGCGATGGCGGCGAGTCCGGGGAC
CGGCCGGGCGGCGACGGGGATGCCGTCAGCGTTGAGGCCAGTGGCCTCGGTGAGGTCGGC
GGGGAACTCAGCGAGGACGGGGACCTTGACGGCCTCCCAGTCGTAGAGGCGCTCATCGAC
GTCACTGACCGTGATGGCCCCCTCGTAGTGGTTCGAGCGTGCGGCCTGGTCCTCTGCGCG

GTAGTGCCAGGCGTGGCCCCGGTTGGCGGTCATGCCGATCAGGGTGTTGGTGTTCAGCCA
AAGAGAGGTTTCAGCGGACATTGTTCTCTATCTTTCGGTTTCAGACTTCGATGAGTTCGA
GTACGCGGGTGAGCTTGTTGCCGGTGGTGTTTCCACCCCGGACGATGACCCCGGTGCCGT
TCGTTGCGGTGAAGCGGGCGACGGAGCCGGTGCGGGTGAACCAGACGCTGAGCTGCTCGA
CGTCGCGCTCGAAGCGGACGAGGCTACTGGTCTCGCTCGGGGGGTGGAGCTAGGACCAGC
CGTGCTTGGTGGCGGCGGTCGCGACCTGCTGTCGTGCGGTCATGGCGGGGACTCCCTAGT
AGTCGAATCGCTGTGCGAGGCGGGCGTCCTGGGCTTCCACCTCGAGGTTGGAGCCGTCGG
TGTCGATGGAGCGCTCGGCGTGGCGGAGGATCCAGGCGATCTCGCGGGTGACGTACTCCC
GTCGGCGGAAGGCCCCCCAGGTGGCGAGGCCGTGCCCGCAGACCCAGGTCCAGGGGTGGT
GCCCCTCTATCGCGCCGAGGTCGTTGACGTAGTCGTTGGCGAAGCCGCTGGCCCCGTGGG
CAAAGGTGAGTGCGACCGCCTCCTCGATGTCCGTGGGGTAAACGTTCAGGACGGAGAAGG
CGGCGCGGGTGGCGGTGGTCATGGCCGGTGCCTTTCAGATGTCGTAGGTGGCGGTGAGGC
GGACCCGGCGTCCCTTGACGTAGCCGAGGATCAGGATGGCCTCGGTGCCGTTCTTGTGCT
CGGTGCGCTTCCAGTAGCTTACGTCCATGGACTGGCGGACGATGTCGAACTCGGTCCAGG
TGGTCTTGGTGGCTCGCTTGCCGAAGTAGGAGATCAGCGGGTTGTTCATCGTGAACGCTC
CTTATCGGGGTTCTGGATGCCTCGCTCCGCTCAGCCCCAGGGATCGTTGATGTCGGTGCG
GGCATTGGGGATTGAGATCGGGGTGCGGGTTGGCTGGTGGTCCCGCCATCCACGGCATCC
GTAGTCGTGGCGGTTTTCCCAAGTGCCGCAGTCGCAAACATTCATTGCCTGTTCCTTTGC
GCTGCAGTAGAAGTGGGTGGCGGGCCGGCCAGCGGAGGCTGGTCTCGCCGGTCGGCCCGT
CTCGTGTCGCCGCGAGAGCTTGCATCTGTGCCGCCCCTGGTGAATGGGCGATGCGGGGAC
CTACTAGTTGGTCACTCTGTGGAGTTCTCTGACTTCTTATCCGGAGCCGGGTCCTGGCCG
ATGATCCGTGCGGTGGTGTCCGCGTCTACCCGCTCTCGCTGGCGGGTCTCCTGGTCGCCG
ACCCTTTCGGGGCAGGATTGGCGCTCTGTCGATTCTCTCGGTGGGTTGTGGGGGCTTTCG
GTGTTGCTGTTGTGATGACCACCTTGACCCTGGACCCCAGAAGTAGGCTAGGGGTTTGAG
GACGTTCGTTATCAATCCGTTATAGGGTTGGTCGTGCCAGTCCCTTGATCAACAGGCCTT
TGGGCAGGTCAGGCTGTACTTCCGGCGTCGGGAGACCTTGTGTCCATTTCGGGCAGGTTC
TGGGGAAAGTGACCGTGAATCTCCGTACCAACTTCTGGCGGTCGTACGCTGGTCGTCATG
AGTAGCCGTTATGAGGGCCGCCGGGCGGTCATCTACACCCGAGTGTCGAAGGACCGCGCT
GGCGGCCGGTTTGTGGCCGAGCAGGAGTCCGAGTGCCGGATCGTCTGCCAGCGTGAGGGC
TGGTCCGGGGCCGACGTGCTGTCCGACAACGACGGGGGCGCGTTCCGCTGGTCGAAGGGG
GACCGGCCGATCTACGACCGGCTGAGCCGGATCCTGCAGCCCGGCGACGTCCTGGTCACC
TGGGAGGGCTCCCGAGCGCAGCGCGACCTCGCGGCCTACAACGACCTGCGCCACCTCTGC
ACCGAGCGCGACGTCCTGTGGTGCTACTCCGACCGCGTCTTCGACCTCACGCGCTCCGAC
GACCGGTTCACGACGCTGCTCAACATCGGCCTGGCCGACAAGGAGGTCGACGTGACCCGC
GAGCGGATCATGCGGACCCTGCGGGCGAACCTGCAGGCCGGGAAGCCCCACGGGATCCGG
CCGTACGGGTACCGATCCATCCGCGACCCGGACACCGGCAAGCCGATGGGCCGGGAGCCG
GACCCTGTCGAGGGGCCGATCGTGCGCGGCATCGTGGACCGCTTCCTGTCAGGTGTACCG
TTGCGGCGAATCGCAATTGACCTGAACGCGTACGGCGTCAAGCCGCAGCGGGCCGAGGAA
TGGACGGCCAGCCTGCTCGGGAAGATGCTGCAGAAGCGCGCGTACGCCGGTCTGCTGACG
CACCACGGGGAGATCGTCGGCCCCGGCACCTGGCCAGCGATCATCACCGAGGACGAGCAC
TACCGGATCATGGCGATCCTGACGGACCCGGCCCGCCGGACGCAGCGCGGCACCTCGCCG
AAGTCCCTCGTCACGAACATCGCGTTCTGCGGGAAGTGCGGCACGACGGTGCGCCGGAAC
ACGAAGGCCGGGCGGACCTTGAACGACTGCCGGGACATCGGCTGCGTGACCATGACGGCG
TCCGACGTGGACGAGTACGTCGAGGAGTGGATGTTCAAGCTGCTGTCCGACCCGCGCGTA
CACAGGCGCGTACGCCAGCAGGGCGACAGCAAGGCCTCGGCGGACGCCCTGGCCAAGATC
GACGAGATCCGCGCCCGCAGGGACGAGTACCCCGTAGAGGCCGCCAAGGGGGACATCAGC
CCGCGAGCCTTCGCTCTCATGGAGAAGCAGTGGCTGACCGAGATCGCGGAGCTGGATCAG
GTGGCGAACCAGGCCCTGATCCACCCGAGACTGGCCGACCTCACCGGCCCGTACGTCGAG
GCGCTCTGGCCGGACATGGACCTGGACCTGCAGCCCGACATCTTGCGGTCCATCCTGGAA
GTCACCCTATACCCGCAGAAGCTGCAACGCGAGCAAGACAAGCCGCCGATCGAAGTCCTT
TGGCGCACATAGCAAAAGCCCCTGCCTGGAGTGGATGCCGGTCGGGAGCTTTGCTTTTCG
AGCTAGACGACGGCTGAGAGCGTGATCTCGTAGGCGAGCCTGTTCAGCGCAGTGAGCTCG
TCCAGCAGGTCCTTCGGGTCGTCGGTCTGTGCTGCGCTGGCGAGCAGGCCCGTGCTTAGG
TTCACGAGCTGGCGAAAGGTGATCGCGGTCGACGCTGACGTGCGATGGGGTGGCGATGCG
GGGCGGCGTCGGCTGCTGCTGTTGCTGGACCGGCTGCGGGGTGGGTCGCAGCGGTTCAAG
GGCGGTGGGTTCCGGCTTGGCATCCTCATCGACGACCGGGTCGGCTCCCGAGGTCAATGT
GGCCTTCGAACTACCGAGGCACCACCCAAGCGGGGCGTCGATCGACTCGAGGAAGCGCTC

CGTCGGCCGGTCACCTCCGCGCCCAACTTCGTACTGTCGCAAGGTTTCCGAAGATCTGCC
GCCGAGCTGCGCGAGTGCTTCCTGGGTTAGATCCAGCTCGGCGCGGCGTTGCTGGATGGC
CTCGCCGAGGAGGCGCTTGCGCTTGGTGACCGTATCCGGGTCGAGGTTCTTGTAACGGTT
CATGTTGATCATCCTTTCATGTTTGGCCAACATCTTGGTCGGTTTCTTCGCACCTTCCAA
CTTGCCGGGTGTAACGCCCTGAGTTGTCTCGCGATGCTGCTATTGTGCCCCGCCCAAACA
TGGGTGTAGAGAGAAGGGCCAAAGAATCATGCCCGGTCCTGTATACATACGGTAACAGAC
ACATCAATTTATGCTCTGACCTGCGGGTATGCCACTGGGGACAATTTGCGATGTGTTTGC
AATGGCCAAGGGCTTGCCCAAACTTTTGGGGTTGGTATTTATTGGGTTCCAGTAAGCCAG
CCGGTGATCATCACCACCGACGTCCAGCAGGAACCGACCGCATAAGTGAGCAAGATCACC
GGACGGACGTCCAACACGCCCGACATGAAAGTGATCTTGGTCACACAAAAAGACCACCGG
TTCTTACTGCAGTCCCAGAAGTTGCACCAAGTCCTTCGTCTGGAGGCAGCTTCGCACCAC
CAGCGAGGCGCAGAGCGAATAGGAGACCTCTGCGATCTCCACCGCCGAGCAAACCCATCG
CCGCACCTACCTCACCCCCGAGGAGTGCGCGGCGGAGAACGAGAAGGGTTTCGTCCACCT
GTGCCGCGAGGTCAGGCGGATGGCCCGCCCATGGAACATTACCGTCACCTCGAACGTGGC
CAAGCTGGTCGCCCGGACCGTCTTCGTCCAGCGCCAGCTCGTAGCCGACGGGGAGGCCGG
AGCCAGCCTGGCGCACTCGGACGTGACCGGCGAGCTGGCCGTCTACCGGCAACTGCAGGT
CATCGCGACCGAGCACCGGTCCTAAAGCTCCACCCCAAAACCACCCGGCAGCCCAGCCAC
CACCTATCCCGAGGGTCAACCAGTCCGATACCGCCCTCACCCCGGCCGGGCTGCCGGGTT
TTTCGCACCCACCCACCACCCGCTTTCAACAGGCGGGGGGCACTTCGTCATGTCCGCATA
CAGGCAGGACTACCCGCTCGAGCCGGTTTACGGCGACGAGCTCGACGACATCGTCAACTG
GAAACTCGCCGGAGGAGGACAGAAACGTGGCATCTACGGCGACCATGCGCGGCCGGAAGT
CTGCAGCAACCCCAAGCACCCGCACGCACGCTTGCACGGCGGCCTGGAGGACATCCGCAG
CCGGGTCAACGGGCACGTCGAAGACCAGTGCACCGTATCCTGGCACTTCCGCCAAGGCGG
TAAGCGTCTAAATACCTGACCCACCACAGGAAGTCTTCGACCGCCTGTTCCGAAACCTCT
CAGCGGCCACCACCGCCGCATTCTCCCGAGGAGCTTGATCATCATGGCGCAGTACACCTT
CAACAACCCCGACTTCCCGAACGAGAACCGCGACGCCCTCGGTTCGATCCTGATCGGGGC
CATGGGCTTTAACCTCGAGTTCGCTGAGGCGAGCGGCAAGCCGACCTCCGCCCAGCATGT
CGCGAAGGACGTCGCCTGGGTCGCTGAGCACGTCGGCTGGCACCGCGATCCCGAGAAGGC
CACCCGCACGTTCAACGTCGAGAACTGCACCTGCGGCGAAGACCACGACGGCATCGAACA
CCTGGTGAAGGGTACGCTGCTGGCGTACGACATCGGCGCGATCCAGACCGCCGACCAGCT
CACCCGGATGTTCGAGCGGGTCGCCTCCGAGACCGGGTGGGTGATGGCATGAGCACCGAT
GAGACCGGCCGCATCCTGCGCGAGGCCGCCGTCCTGATCCAGACCAAGGGCTGGGCACGG
TTCCTCTTCGCCAGCACCCACGGCGAGCACTGCGCGCTCGGAGCGATCCGGGAGGCCGCC
GACGGCAGCATCTTCGAGACCGACAAGTCCAGGGCAGCAACCGCACGACTCGCCCAGACC
CTGCGCGATCGGGGCGTCGAGGGCGACCCGGAGAGCGACTCCTCCTGGCTCGGCGACAGC
GCCTACCGCAAGGAGTCCGAGAACTTCGTCCTGGTGACCGACCGGAACGACGCCGACGAC
CGCACAGCGGCCGAGGTGATCCAGGCCCTCCGAGAGGCAAGCCACGGATGACCGGCATCA
GGCTCTATGGGATCAGCATCCCAACCGTCCACGACCAGCCCTGGCGGCAGAAGGCGAACT
GTCTCGGCCAGAACTGGAAGGAGTACGTGGTCGAGGAGATGCTGAAGGGCCGCAACGCTC
CCGAGACCAGAGCCATTCTCGCCCAGGCGAAGTGCTCCTGCTGCCCGGTCAAGCGTTAGT
GCGCAGGGGATGCCCTCGACAACCACGACATGGGCGTCATCCGGGCCGGTGTGGCCGTCC
CCTGAACGAACGGATCCGAGGAGTACCGGCAGGCCGTCCGCAAGCTCGAGACGGCAGCGG
GCCGGGTGCACCGCAAGACCAGGCGCAAGACGGTATGGCCCAGACAGTGCGAGAGATGCG
CCTGGACGATGCGGCCACGCCGAACCACCCTCGACGAATTCCCCGGCACGATCACCCACG
GGGCCGGAAAGCTATGCGGCACATGCCAGATGCGGCAGTGGCGCGCAGACAAGGAGACCA
AGGAATCATGAGCGACAACTCCGAGAAGGTCCGCCCGAAGGTCGACCTGGAGGCGTACCG
CCACTCGATCAACATCCTGCAGTTCGTCGAACAGGAGCGCGCGAAGATCAACGCCCAGCT
CGAGGAGCTGAAGGAGGTCGAGGACGAGTCCCTCGCGATGATCCGGGAGGCCCTGGGCAA
GTCCGGCGAGATCGGCCTGCTCGACAACAAGCCGGTCGTCACCTGCCACAAGTCGTACCA
GAAGCGACTGGACCAGCAGGTGCTCAAGCAGCGGTACCCGAAGATTCACGAGGAGTGCAA
GACCGAGCTGGAGATCCGCACCCTCAAGGTGGTGGTGTGACCCGGACGCAGCCCTGCGCC
GACTATATCGAGATCGACGACCATCACATGGGCCGGATGAGTATCGAGTGCTACCGGCGC
AGGGGACACAAGAAGTCGCACAAGGGATTCCTGTGGGGGCTGTGGGACCGATGGTGAATC
CCTGCGTCGACTGCAACGAGCGGCCCCGGTACGGCAAGCTGACCCGCTGCAAGGAGTGCA
TGGAGGCACGCCGGGAGGCGAGGAGCGGACCCTGCACCATGTGCGGCGGCCCTGGATAAT
GCCGGGGCCTGTGCATCGCCTGCTACAACCGCGAGATGACCAGCGGTCGGCTCAAGCGCC
TACGGGACGAGCCGAAGGACGAGTACTGCAACCGTGGCGACGAATGCCGCAGCAAGAACA

AGCCGAGAGAGAAGCAGCGCACGTTCCGGGGCAACGTGTGCCGGAGCTGCTACACCCGCG
ACGAGTACCTGCGCATGACGCCGGAGAAGCGGGAAGCCAGACGCGAGGCCAATCGCAGGC
TCCGCGCCAGAGCCAAGGCAGATGCCGAGGCGCACAATACAATCGAGCAAGAGGAGGAGG
TGGGGAACCATGTTGAGCCAGGTGCAGCGGCAGGAGTTGCGGACCATCTGGGCCAACTTG
GACCTTGGGCCGAGGTCGAAGAACTGGGAGCCGGTGGACCGAGCAGCGCAGCAGCTGACG
GAGTTCCTCATTGACGTGCCAGTGGGTGCGGCATGAAGCGCCTCGTACCGGCCGTCGAAG
AGATCACCGCCGTCCAGTGGGGTCCTGACTGGGAGTCAATGCTTCAGAGCGCAGAACAGC
TCGGCGAGCGGGGGATGCTGTTCGACATCCGCTTCGCCACCGAGGAAGCCCGTCGGCGCG
GCGCCGACATCTTCCCCGAGCGTCACATCATGGATACCCGTTTCCGCACACACGTTGTGA
CGCACGGACTCTGGGTCGTCATCTATCCCGGCTACCGGCAGTTCCGGATCCTCTCCACCG
AGGAGCTGGACGCCCAGTACAAGGAGAAGAAGAAGTGACCAGCACCCCGCACGGCAAGCT
GATCGCCACCACCGGCGACGGCACCCACCCCAAGTTCGAGACCGAGAACTTCCCGATCGA
CGACCCGGTCTGGGTGCTCGCGTTCACCGAGGCCGGTTCGGCCGTTTCGATGTTCAAGGA
GTCGCGCTCGTACGAATGGGCGCTCTCCGCTCTCCTCCGGATCGAGGGAGTCCTCATGCG
GCAGACCGGATGGTTCGACCGCCTGGACCTCGACACACCCGACGACAAAGTCGGTGACTG
ATGTTCACCCCCGAAACACTAAAGAAGCTCATTCCCAAGCGATTCCACGAGTTCGTAGGC
CTCCTCTGCCTCGCGTGGGCGCTCTTCTGGTGAAAGGAACACCACCTCATGCGCAACTCC
ACCGCTCCTGACCGCAAGCCGAAGTTGTGCCAGTTCGCCAACTACACGCCGGGCAAGGGC
GCTGTCGGCTGCACGTTCCGCAACGAGCACCACGGCAAGCACTCGTACGAGGTCGCCGAG
ACCGTCGAGCAGGGCGACATGCGGAGCTGCGACGGGTCGGACATCTGGAAGGCCGACTTC
CATCACCACGCCTGCAGGGGTTACCAGCGTGAGCAGATCCATTCCGGGCCATACCACGCC
CACGTGGATCGGGAACGCGACCTGCGGGACGTCCAGCGGGCCGGAAGCCGCATCGCGGCG
CACTACAGCCCCGACAGCCGGGAGCTGGCCGTCTTCGCGCCTGAGACACTGCAGTACGTC
TTCGGGCTGTTCGACGAGCGGGGGATCTTCAGGACCCGCACCGTCTCGAAGATCGTTCCG
GACGGCTTCCGGCCGCTGCACTGGTCGCCGGAGAGCATGTTCGTCCTGCACACCCAGGAG
GAGCGCGAGCCGCTGACGAACGAGTAGGCGGCCGAGGAGCTGGTCTCGAAGCTGCGGGCG
GTGCCGGATCCGGTCGACGAGGACGAGGACATGCTCACCGTCGACCACGACGCCGTCCAC
CACCCGAGCGACTACCGGACGATCACCGTGACGATCAAGCACCCGAACGGGATCGAGGCG
ATCGACGTCACCCGGTGGTTCCCGTTCGCCGAGGACAACGTCCTGAAGTACCTGCTCCGC
GCGGGCCATAAGGGCGACCGGCTGGAAGACCTGCGCAAGGCCAGGCAGTACCTGGACTTC
GCGATCGAGTTCGAAGAAGAAGCACGAACCAACAAGGGATAGAACGATGTCTCACAACCA
GGCCTTCTTCAAGGAGATGCAGGGCGGCAAGGAAGCACCCCCGCTGGCCGACGTGATCCG
CGACGAGTTCGCCGACGTCCCGGACGACCAGACCATGTTCGAGTCCATCTCCGACACGCT
CACGGTCTCCGTGGAGCTGGGTATGAGCGTGGACTTCGCCGCTGCAGCAATCGCCAACGG
CCTGCTGGATCTGGGGTTCAAGAGGTGATCACGGGGGACGGCCTCGGCAAGGAGGTGATC
GAAGCCCTGGCGCGGACGGATGTGACGAAGTACGGGTTCTCCGGCCTGTCGCCCGCCCTG
CCGCTGATCGCGGGGTGAACGCCCGAGTAGGTCGAGATCCAGGACCGCCTGGCCCGCATG
ATCTTCTCGTCCGTGCTCGGCGCGCTGAGCAACCCGAAGGTGGTCGGGGGAGGGTCGATC
GTCTTCGCGAACGCGATCGGCAACACCGTGTCGCTCGTGCTGGCCGAGAACGGCTGGATC
GACGACGGAGCAAAGAACAAGATCGAGGTGAAACGGAGTGCTGGGTAACAACATCGGAGC
AGCTTTCGTGCAGCCCGCGCAGCAGATGGCCAAGGCCCTTCTAGGTATGGCGCGGAGCCT
CGACGATATAGGGAGGCAGATGCACACCGCCAATCAGCTCAAGTGGCTGGAGATGCGCAG
CAGCGAGAAGGGCTTCGAGTACAGCGACGGCGAACTTCTCACCCAGGTCGGCCGCTAGGT
CGGTACCAAGGGTGACCAGCGAGGCTGACAGCTTTCTCGACGTAGGCAGTGTGTTCGGGG
ACCAGCTCCGGCAGATCCTGATTGACGCCGTAATCGCCTGGGTGCGTGCGAGGCCGCGCT
CTCAGCAGAAGGAGATCGGGCCGTCGCAGGCCGGTGAGGTGTGTGCCCGCCGGTTGGCGT
GGTCGATGACCTCCACTCCCACCTCGAACTAGAAGGGGGATCCGCTTCCGTCGATAGTCG
GCACGTCGATGCACGGTACGATGGAAGAGGTCATGGCGCTCGCCAACGAGGCCCTCGGCC
GGGAGCGATCGCTCACGGAGTCCAGGGTCCTCGATCCGATCGCCGGTACCTGCGACCTCT
ACGACAAAGACACCCGGTCGGTGATCGACTGGAAGTTCCCCGGACACAGTGCATTCAAGA
AGGACAGCACCAAGGGACCAAGCCCGGGGTACCGCAGCCAGGTGCACACCTAAGGCCTCG
GCTACCGGAACCTGGGCAACGAGGTCGACAAGGTCGACATCATGTTCATCGCGCGCGCCG
GTCGCCTCAGCCGGTCCCACCTGTGGATCGAGGACTACGACCAGCAGCTGGCACTCGACA
CGGCCGAGCGGCTGAAGAACATCCGCAAGGTCGTCGAAAGCCTCGAGACCGACAGGTATC
CCGAACGGGCACAGCATGTTCCGGCTACACCGTCGGACTAGTGCATGTTCTGCCCGTGGT
GGAACCCGAACCCCCGAGGCAACCGTGAATGCCCCGGCACAGCGGGTGATCCGAACGGGT
TCCTGCTCCACACCGGGGGTGCCTGATGCCGGTCAGCCCCGACGACGCCTACATGGTGGT

CACGGTCAACGGCACTACCGGCCTGTACCGGTACCGGAAGGGCGGACGCCCCGACCAGTC
GCTGGAGTTCCTCGCCTCGGTGCCCGACCTCGTGGGCGTCTACCGACTTAACCCGGAGTG
ACCATGATGCTCACCTTCCTCATTCTACCCGCCCTCGCCTTCGCGGCCATCTACGTCCTG
CTGGACCGGTGATGGCAATGGGCACAGCGGAATCGATCGTACTGATCCACTGCATCACGC
TCGTCGTACTCGCAGCCATCGGCGTCTACGGCTCGCGTAAGTAGCTCCACCCCGAGCTCT
TCCCCCGACCCTGCGGGGTCACCAACCGATCAGAAAGGCTCACCAACATGAGCAGCTTCC
ACGCCAACAACTTCCTCATGGGTTCGGCCAACTCGGCGTCGTTCCTGACCCCCGGCACCA
CGGTGATGGGCGTCATCAAGCAGGAGCTGGAGGTCACGCAGCAGCGCGACTTCGACCCCG
GCGAGCCTGACTTCTGGGACAAGGACAAGACCGACTCGAAGATGCAGCTCGTCATCACCC
TGCTCACCCAGGAGCGCGACGTCAACTACCCGCACGACGATGGCGAGCGCAAGCTCTACG
TCGCCAGCCCAATCATGCGGAAGGCAATCGCGGCGGCTGTCAGTGCGGCCGGTCGCAACG
GCCTGGCCGTCGGCGGTGCACTCGCGGTGACCTACACCCAGAACGGCGTCAAGACCGGCA
AGGGCAACCAGCCGAAGGAGTACTCGGCCCAGTACGTCCCGGCGACCGGTGAGGTCAACG
TCGAGACCGGCGCGGCCGTCCCAGCGGCTGCTCCCGCTCCGGCACCGGCTCCTCCCCCGG
CACCGGCTCCCGCTCCCGCTCCGGCACCCGAGCCGGTCGTCCATCAGGCACCCCCGGTCG
CTGCTCCGGCAGCGGCGGCCGGTGGCCTCACCCCCGAGGCGCTGGCGGCACTCAACGCCC
TGTCGGGCAACCAGGGCTGATCTAGCCCACCTGAGGGGCGGGTCGGGATTCTTCGGAGTC
CTGGCCCGCCCCTTTGTTGTACCCACAGACAGGAGAGATCCATGGCAGGCAGGGTAATCC
GTCGGCTCGGCGTAGAGATCGACGAAGAGGCGAACGTCACGATCGACGGCTACGGGTGGG
CGACCGTGGACCCCGGCGAGAACGAGGGCGAAACGACGCTCTGGCTCGGCGGCGACGTCC
CGACGATCGTCATCCTCGCCCGGCAGGACCAGCTCGACGCGCCGCAGGTCGTCGCCGTCG
AGGTCTGGTCGCTCGAGCGGCTGCAGCAGATCCTGATGACCACCGCGCATCTCGCCCGAC
GCGTCGCAATCGACCCGTCCGAGCTGGACGAGGGTGACGAGTACGCATACGGCGACGAGG
ACTAGACATGCCGCACACGCTGAAGGTCACCGACCACGAAGTCGTCCGCTGGTGCCGAGT
CGAGTGCCAGCTCTGTGGATCGAAACACACTCTCCACCAATGGACATTGAAGCAGGACAT
GGGTGAGGCCGCGTTCTCGAAGGACATCTGCGTGCCCCCGCCGGAGGTGTCGACCGAGGA
GGAGGGGTCAACTTGCGTAGAAGACAGGCGGACTACCAGATCCGGAAGAACCGGCGCACC
GGGGTCTGGACCGTGCAGTGGCGCAACAACGGGCCGATCCCGCACTACTCGTTCGACGAC
GCGCGGCTGACGCTGATCAACATGCTGCGCCTCGAAATCATCTGGAACACGAATTCACGT
GGGAATAGGAGGGATTGAGATGGCGACACCGGCACATGCCCTCCGCAAGGCGATCGTACT
GCTCAAGGAGGACGGCTGGATTCAGGGAGCGATGCACACGCCCGGCGGGTACTGCTCGAT
CGGAGCGATCCGGCAAGTCGCTCTGCACGCTCTAGGCGAGGACGACGTCGAGCGCAATCA
CCAGGAGTTCATGGACGAGCTGTCCCTGCAGCGCGAGGAGACGCACCGACTCGTCCGGGG
CGTGATCCGCGACCGCTACAACCGCTCCAGCATCGTCCACTGGAACGACGACGGGCGGCG
AATGGTGTCCGAGGTGCTCGACGTCTTCGAGACCGCACTGCAGCAGGCCGACCGATGAGA
GGGTCGCTGCTGATGTCGGCCGCCGGGTCCACCACTCTGCGCCCGTCTATGATCGCGGTC
GGCTACGTCACCGCTGTCGTCGAGAACGAGGGGGTGCCGTACTTGTTCGCGATCCTGTTC
GCCCTGCTGATCGTGAGCTGGCAGGCGGAGCGGTGGTCACTGAACGGCTGCCGCGAGTAC
CAGAACAGCCGGGGCAAGGGCTGAGCGGCCGGAGCTGGAGGTCGGCGAGTCCGGCCACTT
CCTGTTCTTCCACTTCGAGGACCGCTATTGCGACGGCCTTAAGAAGGCCACCGTCTGGCT
GCAGGGGTCGATCAGTCCCTACGGAATCCCGTGCGAGTGCACACAAATCGTGGAGAGAGA
CGATGGGTGATTACACCCCACAGCACGGCACCGTCCGTGACTTCGAGGAGATCCAGCCGC
ACGGCGACGGCGCACCGGCCGAGATCGACGACCACGTGCGGATCCCTGGCAGCAACACCG
AGTGGGTCGTGACCGACGTTGTCGGCGACTTCGCCCGGCTCGTGTCCAGCGACGGCAGCC
GGACGTTCGCGTACCGGCGCACGCTCCTGGAGCCGGTCCCATGAGGCTGTCGCTCAACAT
ATTCGGCAAGGAGTTCGGGGTCGTTGTCGGGGAGTCACCGGCACCGGCCGCGAGGGTCGA
CCTCACCGAGATCTACCAGAAGTTCGAGCACATCGCCCTCGAGCTGCACGAGGGCGAGCG
GTGGGAGACCTGGCAGCGCCAGCGCAGCGCATCCGGCGGGCAGTTCGGCTTCGCGGCCAA
CTCCCGGCCGATCCAGAAATCACACACACGATGGGAACCGAGATGACACAGCCGTTCGGA
GAGTCGGTGAAGAAGACGCCGGAGCAGCGATGGTTCGACATGATCCGCAACCTGCACTTC
TGGGATGGCTTCGTCCAGGCGCGCGAGGAGAAGGACGCCCGGAGCCACGAGGAGGACTAC
GAGAGCAGCGAGCACCTGTCGCCGGTCTCCGACCCCCTCAACGCCGAGATCGTCAGTAGG
GCAGTCAAGCCCACCGGCCTGCCGGGGTTCTTCCAGCAGACCGGTGGCTACGGACCAAG
CACAAGCTGATCGTCGACATCGACTACCCGGTGCTCGCGATCGAGTCATCCACGCCGGGC
CACAGCCCCCTGTACATCGACAAAATGAGCTGACCTGGGCCTACACGCTCCGGGTGCTCG
GGGTGCTGGCCTCGGTCGGCATCGTCGAGCCGGGCTACGTGGACGCCAGCGAGCGGCTCG
GCTGCACACACCTGCGGGTGCCGTGGCTGAAGAATAAGTACCGCGCACCGCTCACGCCGT

ACGAGACGCACTGATGGCCGTGAAGCTGGCCGCCGGGGTGCGGTGGGTCGACGAGGGCGA
GGCCGGTGAACCGGCGGCCCGGCTGCTGTTCCTGAACGAGACCACCGTGAACGGCTGGGT
CGAGCACGTGCACGTAGACCTGGGGACGGACGAGGCCGAGGCGCTCGGGTGGTCGCTGAG
CAAGAATTCCATGCAACTCAAGGAGATTCGGGAGACGAGATGAACGCGCTGCCCCTAGCG
CCCTGGGAGAAATTCTGGGTGGAGCACGAGTGGTCGGAGTTCCAGCAAGAGATCGTGGGC
CTCGCCCGGCGCAAGGGGCTGCGGGTCCTGGAGATCCACGTCGCCGTCGGCGAGCGGCTG
GAGATCGTCACGGTCCCGGCCGACGGCAAGGACATCGTCTTCGAGAACGGCGAAATCACA
AGAACGACATACCAACTGCCGATCTGGGCGTAACGGAGGTGAGAGATGAAGGTCACCGGG
AAGACGGTCAAGCTGACGACCCACGCGCGACGGCGAATGGCCGAGATGGGTGTCGACGAG
CACGAGGTGCAGTTGGCGCTGAAGTACCCGGAGACCGTCGTCTGGTCCGGCAAGTACCCC
GGCTGCCAGAAGTACCGGCGCGTCCGGATCAACGTCGGTGCGAAGACCGAGGGCGACGAG
CTGGTGGGCATCACGGTCGTCTGGCCGAGCCACGAACTGTGGCAGCAGCACATCGAGAAG
ACCGGCGGCGGTCACGGACGAAAGCTGAAGCCGTGGCTGAACCACTGATGTGGAGCCTGG
CCCCGGCCCCTGCGTTCGTCGTCGAGTGGTCGGCCGAGGACGGCGAGTGGGTGGGCCTGT
GCCCGTCGCTCTTCCCCTCGCTGTCCTGGCTCGCCGAGACCCCGGCCGCCGCACTACCAG
GCATACGAGACACGGTCTGGGTCGAACTGGGCCACACATTCGAAGGAGCGCATCATGTCT
GACAGTGGGATCACCCAAGACGTCGTTACGCGGGCACCGTTCCCGCTGACAGTCGGATCC
ATCCTGGGCTTCGCCGAGAACCTGAAGGTCCGGCCTCGCGACGTGCGCGTCGACCACGTG
AAGATGGGCGCACTTAGCGCGTCATACCTGCGCCACCGCCGACCGGCCACCGAGCTGATG
CCGACGTCCGTACGGCCGAGCGTCGACCCGAGGGACGTCCACAAGCGGCACGTCAGCCTG
GTCGCCATCGAGGCGCTCCGGCTGGCCCCGAGGGTCACGACGTCCTCGGCGTCTTCACG
ACCGAGCAGGCGATGCACGGCTGGGCCGACTACGAGATCCGTGCACTGGCCGAGCATGGC
GGCACCGGCCTCTGCGTCAACCAGGGCACCCCGCGCTACTGGACCGTGGTGTTCACCGAC
CCGGTGTCACTGGAGGCGACCGGCATGGCCGCGTTCGTCGTCGCCGGAGCTACCCACGTG
GGGCAATACGACAAGGTGCTCACCGATGCCCGAGTGCCCAGCCTGCCGTGAGCCGGTCCT
GTTCGCGCTGAACGGTAACGGTAACGCGATCGTGCTCGACACGGCCCCGGACCCCGAGCG
TGGACAGGTGCTCTTCAACGATGGGCGCTCCGTCTTCGTCACCCAGCCCGAGCAGTGGGC
CGTGGTGAGGCAACACAACCTGCCGGTGTACCGGCGACACAACACGCACTGCCGCGAGTT
CTGGTCCTACAAAGTCCACCCCAGCCAGCTCGCGGAGCATACCCGAGAGGTGAATTGACA
TGCCCGAGAACAAGATCCAGGCGATCCTGGCGGCCCTGCGTGAGACCGGCATCCCGGCCG
ACGAGAACGACGTCGAAGAGTTCCTCGAGCACATGGATGACGAGGGCTTCTACGTGTCCC
GGACGGGGGAGTAGTCATGAGCGACTTCATCGAATGGCTGGACAATCTCAGCCTGTTCCA
GTGGTACGTGCTATTCGTTCTAATGGAGCTAGTATTCGGCCCCACAGACATGTTCGTCAA
GGACAAGGGGTCCGAGTACCGCGCCCTGAAGGACGACGCGCCCGGCTGGGCACCCGAGTG
GGCCATCCGGGTCGTCGCGCTGCTGTCCATAGTCCTCGTCGCCGTCGCCTGGCCGGTCGT
CACCGTCATCACGCTCTACAAGCTGACCGAGCGCGTAATCGTGTGGGGCGCAGACAAGTA
CCTGAACTGGGCGACGCGCCGGTTCCTGCGGAAGGTCGGGATCACCGACGAGGATCTCGG
CGGCAGCGTCTACGGACCCGCCCACGACGACGACTCGATCACTGTCATGTTCGACCCCGA
AGGCAATGTGGTTCAGCCTGACGGGAAGTGGAGCCAAGAGTAAGCGCAGCAAGAACCAGA
AGGAGGCGCACCTGGGCAACTCGGTGCCCGAGACGACGTCGTACGTCTTCCATGACGCCG
AGCGGCAGATCTCCAAGCTCCATGAGTTCGTGCGAGCGACGCAGCGGTTCAACCAGGACC
TGCCGCCGTTCAGCCCGCTCGAACAGCATGAGCTGGTCCCGTACGACGCCCACGAGCACG
ACCTGCTCACCCGGCGGATCTCGAACCAGCGCCGGGAGCTGCGGCACCTGAACCGCGCAT
ACCGTCGGCGCAGCGACGAGCTGGGCGCGAAGGTCACGCTGCTCAAGGACCAGCTCAAGG
TCTCGATCAAGGCGACACAGGCCCGCAACCTGAAGATCACGCTGCAGCAGGAGGAGATCG
ACCAACTGGGCGGACAGCTCGCCACTGCGACCGACGGCGGCCGACGAAAGAGAGCATTCC
GATGGGTCGGAAAGTGAAGCGGGGCCGCCAGCCGTACCTCGCGCCCGTCACGACAGGGGA
CCTGCGCCGCGACCGGGTATGGACCGAGGATCAGCTCGACGAGTTCCTCGCGAAGGTCGG
CATCCAACTCGCACCGTGGCAGCGGGATGCGTTGCTCCGGTTCGGGAATCGGCGCACGGT
CGGGGTCCACAACGGCGAGCAGAACCTGCACCACAGCGGCTGATCCGCCGGGTAGAACAC
GGTGCATCGCCTAATTAAGGAACTGGCACTCGCCCTCGGCCATGAGGTGAAGCCCACCGT
GAACGGGTTCGAGGACAGCATGGCGTTCCGGGACGAGGTGCGGCTGTGAAGCTGGTCGGG
TTCACCAGGGTCAACGGCAACGAGGTCGTCGTCAACCCGGAGCACGTCCTCGCCGTCCAC
CGCTACGGCCTGGACCACAGCGAGATCAACCTGATCAACGGGATCGACGTGCAGGTCAAG
AACCTCAGCCCCATGCAGGTCGGCCACCTCCTCGCCGAGGGGAGGCTGCCGAGAAACCCC
GTGTGACGCAGGCGAAGCACGCCTGCTGGAGTAACCCCAGGCGATGGAAGGTGCATTACC
TGGGCCGGGAGTACGTCTACTGGTCCTGGTACTGGGCCATGCGGGCGGCGAACACCCTGG

CCACCCTCGGAGGGCGCATCCCGGTCCGGGGCCTGCTCTCGGTTCAACACATTTAATGAG
TGAGGAAGTGACCATGTCGGAGAACGAGATTCGCGGATACGCCGACGCGTTCGACGAGTA
CTGGAACAAGGGCTGGAAGTCTGTCCTATGGCTGCCGAAGGGGAAGAAGTTCCCGACGTC
GTCCGTGTACACCGGCTACGACGCGGCGATCCCCAGCTCCCCCGACGTCATGGCGTGGGC
CGAGGACCATCCGGACACCTACCTGGGACTGCGGTTCCAGGCCGAGATCGTGGGCCTGGC
CGTGGACGCGTACGGCGAGAAGACCGGGGCGTCGACCATCGCGGAGGCCATGAAGCGGTA
CGGCCTGCTCCCGCCGACGGTCCGCTCCACCTCGCGGACGGACTCGACATCGGGTATCCG
CCTGTACCGCAAGCCCGCCGACATCCCACTGCACACGCAGATCAAGTTCCCCGGACTGTC
GATCGGCAACGTCGAGGTCGTCCAGGACCACCACCGGTACTGCGTGTCGTGGCCGTCCAT
TCAACCCGAGGGGCGGATGTACGAGTGGCGGGACGAGAAGGGCAACGTGGTGCCGGGCTC
ATCGATCGACGACCTGCCCGAGCTACCGCGCCCGTGGATCGAGGGACTGCGCAAGGAATC
TCACGCCGAGATCACCGCGCACCGCGATCCATTCGAGGCGCTCCGGCAGCTCCCGCAGGG
GAAGATGAGCGAGAAGGTCGGCGAGGCGCTGCGGCGCGCGAAGACCGAACTCGACTGCGC
GCCGGGGTCACGTCACGACGTCGCGAACGTCGACGTCCTGCGGCTGCTCCGTCTGGCGGA
GAAGGGGGAGCGGGGCGTCACCGAGGCGCCCGAGTCCCTGCGGTCCCAGTTCGTCGAGAT
GACGCGGGACCGGAACACCGAGCTCGAGGCCAACGCCGAGTTCGACCGGATGCTGTACGG
CGAGCGCGGGCACGACCTACTCGCCAGCACGCCATCGAATGAGAACGACTACGCGGTCCT
CGGCGTCGCCGATTACGTCCGGCAGTCCGAGACCCTGGCGAAGCAGCGCGCGCAGGCCCA
GCAGGACCCGGTCGTCGAGTGGCAGCCGCCGGCGTCGGATCCGGCCGACTCGGTCGCGGC
GTTCCTGTTCCAGGAGGAGCGGATGACCGGTCAACCGAGTCAACCTGCTGCCGGGCTGTC
GGCCGCCGACGCGTTCCTGTTCGGCATGGCACCGGGCCCGGTCGCCCACGTCGAGCAGAT
GACCGGAGCGGCGGCCGTCGCGGAGATCGAGTCCAGCGGCGAGGCCACGATGCGGAGCAA
GCTGCTCACCGTCGCGGGCCTGGCTTACGTGCCGCCGTCGGAGCCGCTGCTCGACGGGCT
GCTCTACCGGGGCACCCTCGCACAGCTCTCCGGCCCGCGGGGATCCGGCAAGACGTTCGT
CGCGCTCGGCATGGCCTGCGCGCTGGCGTCGAACCGAACCGACTGGAACGGACAGGACAT
CCCGTGGAAGCGCAACGTCAACAGCGGATCCGTCCTGTACGTCGCGGCCGAGGGGGTCTC
GGGTCTGTACGCGCGGATCTATGCGTGGTGCTCACTGCAGGGGACCGGCATCGACCCGCG
CAGCCTCCAGCTCTACGTCTACCCGGGGGCGGTACAGCTCGGTTCGTACACGCAGATGGA
GGAGCTGACCGAACTCGCTCGCTCGCTCGACTGCGTGCTACGGAAAGCTGAGCTGCCAGC
TCTACCAGCGTAGCGCGGACGTATTCCTCGGCGTGCCGTTCAATATCGCGAGCTACGCGC
TGCTGACCCATTTCTTCGCGGACCAGCTCGGACTGAAGGTCTGCGAATTCATCTGGACCG
GCGGCGACGTCCACCTGTATTCGAACCACGTCCAGCAGGCGACCGAACAACTGTCGCGCA
CGCACACGGGCTATCCGTAGCTGGCGCTGAATCACCGCGACTCGATCTTGGAGTACTCCG
CCGATGACGTCATATTCCTGGGCTACCGGCCGCACCAGGTCATCAAGGCCCCCGTTGCCG
TGTAAAGGAGGTACGGCTGTGGCCGACGACTGGACAAAAGACCCTGACGCCACGCTCGAC
TGGCATTTCGACTGGATCAACTGGCTCGAGCAGGGCGAGACGATCACCACTTCGACCTTC
GACGTGACCGCCGGAATCGCGGTCGGCTCGACGGCGAATAGGACGACGAATACGACTGTC
TGGCTTTCCGGCGGACGCCCCGGCCAGAACTACCGCGTCACGAATCGAGTCATCACATCA
GCCGGTCGCACTGACGAACGGTCCATCACTATCCGCGTCCGAGACCGGTAATTGAAGGAG
CTTCAATGGCTAACGACAACGTGCTCGGGCACCCCAGTATCACCGTGAACAAGTACAACC
CGGAGACGGTCACGGACATCACCCGCAGCCTCGGCCGCGCGCCGACGATGGAGGACTACG
CCAACATCACACCGGACGACTTCGTCACCGTGGAGGGCATTGGCCTCGAGAACGCTGGTC
TCCAGCGCCTGGCCGACCTGATCATCGGCAACGGCAGCGTGACGGCGGTCACCGCGATCA
AGGGCCTCATCGGCGTCGGCAACAGCAACACTGCCTTCAGCCCGGACCAGAGCGACCTGC
AGGGCGCGTCGAAGCTGTTCACCCCGGTGGCCAGCGCGCCGACGGCGTCCGGCGGTGTCA
TCTCGGCCTCGGCCCAGTTCGCCTCCGCCGACGCCAACTTCGCCTGGGCGGCGTGGTGCT
TCGCGATCGGCTCCGCCACGACCGTGAAGAACGCCGCTCTGGCGACCGATGGTGGTGGGT
CGGCAGTGATGCTGAACCGCAAGGTCCAGTCGCTCCGCACGAAGGCCTCCGGCGCTGACT
GGACGCTCTCCGCGACGGTCACCCTGTCCTGATCCAAAACCCAAAGGCACCTGACTTGAT
AGGCGCCTATCAAGTTGGGTGCCTGGATAGTTCGGCTTACTGACCCGCGCATGCAGAAGC
AACAAACGACCGTAAGTAGGGGTGCCTGCCATGGGCCACAAGGGAAAGCATCGCAAGCAA
ACCAACTCCAACGCAAAGCGTTTCGTAGCGACAGCAACCGGCGTCGGGGCACTCGGCCTC
GTCTCCGCTGTCGCAGGCACTCCGGCCGCGTCGGCGCACAACTGGTCCGGAGTCGCCCAC
TGCGAGTCGTCCGGAAACTGCAGCACCAACACCGGCAACGGTTTCTACGGCGGCCTGCAG
TTCACGCACTCCACCTGGGACGCGTTCAAGCCTGAAGGCGCAGCCTGGCGCGCTGATCTG
GCCACGATGGCGGATCAGATCGCGGCAGCGGAAGCAACCCTGAAGGTGCAGTGCATCGGC
GCGTGGCCGGTGTGTGGGGCGTACCTCGGCTGGCGTGGAGTCACTGCCGGCGTCGGGACC

GCGCCCTCCGCGCCGTCGTACGTCGAGCCTGCCCCGCCCCGCACCCGCCGCATCGTGG
GTGCAGCCGGTCACCGGCACGAAGTCGCAGGGCTTCCACGGCGGCCACAACGGCGTCGAC
ATCGCTGCCCCGATCGGCACCCCGATCTACGCAGCGGCGTCCGGCTCCATCGACCTCGCT
GAGTTCAACAACGACCCCGGCGGCTACGGCAACTACACCCAGCAGACCGCCGACAACGGC
GCGAAGATCCAGTACGGCCCCATCTCCGAGGTCTACGTCAGCGCAGGCGACTACGTCACC
GCTGGCACCCTGATCTCGGCGGACGGCAACGCTGGCACCTCGACCGGTGCTCACCTGCAT
CTGCGGATCAACGCCGAGGATCCGGAGGCGTACCTGATCAACCAGGGCGTCGCGCTGGAC
TGGTCCGGCCCCAGCCGCCTGCCCGTCACTCCGGCACCGGCACCTGTGTCCGCCCCTGGG
GAAGAGGTGGCGGTCGAGGACGTTCCGGTCTATCTGGCGCCGGCTCCCGAGGGAGTCGAG
ACGGTGACCGTCGTTCCGGGAGACACGCTTTCGCACATCGCTGAGGTGTTCGGGATCGAC
TCCTGGGAGACCCTCTTGAACCTGAACAAGGATGTGGTCGAGAACCCGCATCTGATCTAC
CCCGGCCAGGTACTCCCCCTGGCCTGAGAGAACGGAAGTCAGCGGCATGGGTAGCCGAAA
CAAGCTGTACTGCAGCACATCTCGGCCCTGGGCCACTGACTACCGGCGAGAGGAGGGCTG
GGCACGGCGGTTGACGAGGAAGCTCGGACGCCGCCGTGCTCAGCGTTTACTGCGCGTCGG
GCGATACGACCTGGCCTACACCCGCCAGACCAAGGATACTCAGGGATGGATGACCCACTA
GGGGGTCGACGTGCCGACTGATGTCTACACGTCGACCAGTACCTGGACCTGCCCGGCTAG
CGTTCTCTCCGTTGATGTATGGGTCTGGGGCGGCGGCGGCTCCGGCGCGGGTGCAACATC
GTTCTCCGACAACGGCGGCAAGGGATCGGGCGGTGCCTTCGCCTTCAAGACGGTCGCCGT
CACCCCCGGAAACGTCTACACCGTCACCGTCGCCACTGGCGGCACCGGACAGGGCACAAA
CGCTGGAGTGGGCGGCGGCGCATCCTGGTTCGGTAGCACTGGCACCGTCTACGCCCCCGG
CGGCAACGGCGGATCGAAGGGCCAGTCCGCCGCGGTCGGTGGGGCGGTCACCTCTGGCGC
GATCGGCACCTCGGTCTTCTACGGCGGCGCAGGGTCGGCCGGTAACCAGTCCGGCACCCG
GTCCGGCTCCGGCGGCGGCGGCGCATGCGCGAACGGCACAGGCGGCCCCGGCGGCACGCT
CTCAGGCGGCGCTGGCGGTAACGGGTCCGGCGGCCAGTTCGGCGGCAACGGCGGCGCGGG
CGTCAACACCGACGCGGACGGCAACCCCGGCGGCATACCCGGCGGCGGCGGCGCTGGTGG
TCGACGACTTAGCACGAACTCAAACGGCGGTGACGGCGGTCGCAGCGAGGTGTGGATCAC
CTACGTCACCGACATCACGATCAACCAGTCCGTCGCGTCCGGCACGACGGCGGACGCGGC
GACCGGCATCGCGCTGGCCACCCCGACCGAGACCCTGTGGTGCGACGAACTCACCCCGAG
GGTCTCGCAGTCGGCAACGACAGATACCGGCGGCGTCGGCAACGAATCCGCCAAGCCGAC
AGCGACCGTCAACCAGGCGGACACCGGCTCCGGCACTGACCTGGCCAGCCCGGTCAGCGC
GCTTATCAAGCCTGCTGACAGAGGCCAGGCCGACGATCTGGCCTCGGTCAGCATCACGGT
CGTCGGCGACACCGGACAGGGCACGGAGTCGACCAGCTATACGGCCAACACCCCGACGGC
GAACGACACCGGCTCCGGCACCGATGCCAGCACGCTGACCGCGCAGGTCACTGCAGCGAC
GGGCACCGGCACCGCCGACGACAACGGACACGCCAGCAACAGCGTGGTCGACGGCGACAC
CGGCCCAACGGCCGACGGCACCGCGACGCTGCGAACGGACTCTGGAGACGCAGCCAGCGG
GTCGGACGCGGCATCCACGACGGCGACCGTCACCCAGGGCGATACCGGATCGGCGGCCGA
CACGGTCACCGGCATCCGGCAGCTGTCGGCGGACGCCGCGCACGGCGACGATGCCAGCAC
AATGCGGATCAGCCCGGTCGGCGAAACCGGCATCGGCACCGACTCTGCGCAGATCCACGT
CACCGTCTACGGCAGTGACACCGGCAGCGGGGCAGACCTCGCGGGCGGCCGGCTGTTATC
GCCCGACGACCGCGCGACCGGTTGGAACGAGAACTCATACAAGGCCGTCAGCGGTATCCT
CGACGCGCCCCGCCTGCTCACCATCCCACCGGACAAGCGGACGGTGAAGATCGAGCGTGA
GCGGCGAACTCGCGTGGTGGACAGGGAAGTCCGGCTCGTCATCCTCGGCAAAGATCGGAC
GCCGCGCACGACCATGCCGCACCGCAACTGCCGCTCGCTCGGCGTCCAGCCAGAGAGCCG
CAAGTACATCGTGAAGCGAGAGGAGTAACCAGTGTCCGACGAGATGACCGTCGAGCAGAG
CGCGCTCGTCCGCGAGGTCCAGTCCGGCGACATGCGTCGGGCGCTGACCGCGATCCGTGG
CTACGTGGTCGACGAGCTGGACGGCACCCGGCGCCCGAAGTGCGCCATGTCCCAGCTCGA
GACCGGCGACACGCCCTCTCTCGTCCTGCGGCTGCAGAAGATCCTCGAAGAGATCGCAGC
CCTGCCGGAAGAGAAGAGGCCCGGCGAGGACGACGCACCGAAGACCAGCACCGCCCGGAT
CCGCGCCCTACGCGCCGTCGGCGACGACGAACCCATGGGCAGCAAGGCATCCCCCCGCAC
GCAAGGGGGTCCACGTCGGAGAAGCTAAGGAGGTAACCAGTGGCAGTCCTTCTCGGAGAA
CAGAATCCACGGATCGCTCATGAGCCGACATCCGTGGGTGGAGTCACATCGGCGGGCCGC
GAGGCAATCGAACTCGCCGCCGAGGCTGGCCTGACCGTCGATCCCTGGCAGCAGAACGTG
CTGCACGGCGCTCTCGCCAAGCGCGAGGACACCGTCTACAACCCATACACGGATCAGCTC
GAATACAAATGGGCCGCACGCGAAGTCGGCCTGATGATCTCCCGCCAGAACGGTTAGGGG
TCTCTCCTGGAGGCCCGCGAGCGGGCTGGCCTGTTCCTCTTCGGCGAGCGCCTGATCATC
CACTCGGCGCACCAGTTCGATACCTCCAATGAGGCCTTCGAGCGAATCCTTTTCCTGATC
GAGAACAACCCGGACTTCGAGCAAGAGGTCATGCGGGTCGACCGGTCACACGGTGAAGAG

GACATCGTGATGAAGAAGCCGAAGGGGTCCAACCTTCCACCGCAACGACTTCGGTTCCGT
ACACGGACCAAGGGCGGCGGCCGAGGCTTCACGGGTGACACCCTGATCCTCGACGAGGCC
ATGATCCTCGACGCCGACATGGTCAAGGCGCTGATGCCAACCCGGACGGCCCGACCGAAC
GTGCAGATCTGGTACACCGGCTCGGCCGGTACCCGGAAGTCGACGCAGTTCGGCCGCGTC
CGCTCCCGCGCCCTCGCTGGCGGCGACCCGCTGCTCTGGTACGTCGAGTGGTCGGCGGAT
CTGTCCACCCCGCTCTGCCCGTCCGACTGCGACGAGCACGACAAGCCCGGCGACCCGCGT
ACGTGTGCAAGGGCGAACGCCGCCTTCGGGATCCGCATCCAGGAGGAGGGCATCGAGGCG
AACTTCCGCTGAATGGACCTCGACTCGTTCGCGATCGAGCACCTCGGCGTCGGCGACAGG
CCCACCGAGGGGCAACCCTGGATCGTCATCGCCTAGCCGTACTGTAACAGCCGCTTCGAC
GCCAACTCGCAGCTCGGCAACAACTTCGTCCTCGGTGTCGACTTCGCGCCGGACCGCACC
AAGGCGACGATCCTGGCCGCCGGTCCGAACCAGCACGGCGACGTCCATCTGGAGATCACC
GGCAACGAGGACATCGGCTTCGACAACCGGGTCGGCGTCCAGTGGGTGCTCGAGCGTGTC
CTGCACATCTGGCGTACCCAGCGACCGAAGGCGGTAGCGGTCGACCCCGCCAGCGAGGCC
GGTGCGCTGATCGAGCCGCTGCAGGACGCGCAGGTCAACGTCATGACCCCGACCTCGCGC
GAGTACGGCGTCGCCTGCGGTGAGTTCCGCTCCGGGATCGTGCCGCGCAAGGGTGAGAAG
GGCTACATCACGCACATCAGCCAGGCAGCTCTCACCGAGACGATCGCGGTCTCCGACATG
CGGACCTCCGGTGACCTGTGGATGTGGAGCAAGTCGCTCTCGACGGCCGACATCACCCCG
ATCGTCGCGGCCACGCTCGCAGCCTGGGGCTCGAAGAAGATGGGCTATACGAAATCCGAA
ACCGTCGCTACCCCATGGGTATTCAGACGATGAGAGGCAGGACGGAATGAGGCTTATCGA
GCTACTGGCCGTGATCCTGGTCGCGATTGCGCTGGGCGTCACCGGCACGGTGGCCACCTT
CGGAGTCTGGACCCTGATGGCCTGGGCGGCAGCGATTCTAGTCGCGGTGTTCTCCGTGGA
GGTGACAGACTGATGGCTAAACTGTGGCACCACCTACGCGGCCGAGGTCACCCCGCCGAG
CGGCGGTTCGGACTCGACGACCTCGCCCAGTGGTACCGGCAGTACAACCTGCTCCCGTGG
GCACGGCGTCAGGGGTACAGCAACACCGAGACGAACGACAACGACTTCGAGGGCTACGTG
CGTGGGTCGTACAAGGCCAACGGCGCGGTATTCGGGGTGGCGCTGGCCCAGATGTTGGTC
TTCACCGAGGTGCGCTTCTCGTACCAGCGGTACACCGAGGACGGCCGCCCCGGCGACCTA
TACCGGCGGCCGAGCCTGAAGATCCTCGACAAGCCCTGGCCGAACGGTGCGACCGGCGAC
CTGCTGGCCCGAGCGATCCAGGACACCGACCTGGCGGGCAACCACTACGTCGTCCGCGAG
GGCAGTGGCCCGAACGCGCGCCTGCGCCGCCTGCGGCCGGACTGGGTGAGCATCATCCTC
ACCTCGCCGCCGGAGAAGGCAGTGCAGTCCGACGTCGTCGGGTACCTGTACAAGCCCGGA
GGCACCGACAACAAGGAACTCTGGAAGCTGTACCCGGTCGATGGATCGAACGGTGCCATC
GCGCACTGGACCCCGATCCCCGACCCGGAGGCGGCCTACCGTGAAAGGAGCTGGCTGACA
CCGGTTCTGCGCGAGATCATGCACGACAACGCAGCGACGAAGCACAAGCTGAAGTTCTTC
GAGAACGCAGCCACGCCGAACCTCGCGGTGTCGTTCAAGGAGACGGTGACGGCTGAGCAG
TTCAAGGAGTTCATGGAGCAGCTCGACGACAACCACGGCGGCGGAGACAACGCCTACAAG
ACGCTGCACCTCGGCGGCGGCGCGCACGTGCTCGTGATCGGCTCCAAGATGGAGGAGATC
ATCTTCATGTCCACGCAGGGTCTGTCCGAGTCGCGCATCGCTGCGGCCGGTCGGAGCCAC
CCCGCGCTCGTCGGTCTTTCCGAAGGGCTGCCAGGTAGTTCGCTGAACGAGGGCAACTAC
AAGGCGGCGCGCAACAACTTCGCCGACGCGACGATGCGGCCCCTCTGGCGCTCGCTGTGT
GCGGCGTACTCGGTGGTGGTCGAGGAGTTCGACGACTCGCGACTCTGGTACGACGCTCGC
GACGTCGAGTTCCTCCGACAGGACCAGAAAGAGCTGGCCTAGCTCACCGCGCAGCACGCG
TCCACGATCTCGAAGCTCGTGTTGCAGGGCTACACGCCGGAGTCTGTCACCGAGGCGATC
GTCAAGGCAGACCTCAGGCTGCTCAAGCACACCGTCCTCTACTCCGTCCAGCTCCAGCCC
CCCGGAACCATCGGCAAGGGCGCACTGACCGGACAGACGCCAGCTACCCAGACGGACGAC
GTCAAGAAGGTCCTGAATCAGGTCAAGAAGGACCCGAAGCCAGTGGTCGACGTCAAGAAC
CCGAAACAGCCAACAGTCGGTCAGGACAAGGCGAAATGACCGCGCACCCAAGGAAAGGAG
TGATCGAGACTGGATTTGTGTAGTCGCTCGGTAAAGTTCCGAGCCAACACGGCCGCTGGA
GACGGCGACGGCCGGACCCTGGAAGGGTACGCGGCAGTCTTCGACCAGCACACCGAGATC
AACTCGTGGGAAGGGCGCTTCACCGAACGAATCGCCAAGGGCGCGTTCAAGAAGACCCTC
CGCGAGCGCAAGCCGATCATGCAGTACGACCACGGCCGGGACTCCCGCGTCGGGCCGGTG
CCGATCGGCGTGTACGAGCACATCCGCGAGGACGAGCACGGCCTGTTCGTGTCCGGCCGC
GTGTTCGACAACCCCGTCGTCGAGCCGATCCGGCAGGCGATCGAGGGCGGTGCCGTCACC
CGCATGTCGTTCAAGTTCCGTGTCACCTGAGACCAGTGGACGGACAAGAACGGCAAGCGA
GTTCGCGGCGACGAGATCAGCAAGCTGCTCTAGGAGCCGGGCGACCGGGGTCCGCTGGCG
CGCAGGATCCGGGAGGTCTCGCTCTTCGAGGCTGGACCCGTGTCGACTCCCGCGTACGCA
GGAACCTCCGTCGGCGTCCGGATGACCGAGCAGGATCGCGAGGCTCCCGTCGCCGAGTAC
CGGCGCACCATGGCCGAGACGGAGGCCGAGCAGGACGTCGTTCGATGGCTCGAGGGTGAG

GCCCATTACGAGAACTATGTTCGGACATGGCTCTACGCTGAAACCGAGGACCGTAAGGCT
GTATCCCTCTGGCTCGCAGCCGAATCCGAATACAAGACTTCGATTGAGACCGACGCCGCC
CGGACGGGCACCTCGGCTCCCATCGAGACCCCCGAAGCAGACGCCGCCCGCACCCGGGGC
ACCTCCAATCGGGAAGACACCAACACCCCCATCCTCAAAAGGTGCATCCCCATGACCTTC
GAAGAACTGCGGGCACGTCTCGCCGAGATCGCCGTCCGCCCCTCCGACATCGGTGCCGAG
TACCGCGACGCCGAACTCTCCGAAGCCGAGCAGGCCGAGTGGGACGGCCTGGACGCCGAG
CGCGCCAAGGTCGCCAGCTCGATCGAGCGCATCGAAGCCCGCATCAAGCGAATGGGTGAG
CTGGTCGACACCGGCAGCACCGAGCGCGCCACCGACACCGGCACCCCGGCGTTCCACGCC
CGCCGCGACATGCACGACATCGACGCCGTCCGCCGCGCGGCTCGCAGCGAAGAGGACTTC
GTCGAGCGCCTCCAGGACAACGCCCGCCGGGCCACGGGGAAGATGTACTTCTCGCCGCAG
GTCTCGCGCGACGCCGCCCAGGAGCGCATCGAGCGCCTGCTCGTCAACGTCGACTCGAAG
GGTGACCTCGCCAAGCGGATCCTGGCCACCGGCACCGCGACGTACAAGCGCGTGTTCGGC
AAGGATGCTCTCTCGCAGAGCCTGCAGGATCTGACGGCCGAGGAGGCCCGCGCCCTCTCA
CTCGGTGCCGACGTCGCGTCCGCCAGCAACGGTGGCTTCGCGGTCCCCGTTCAGCTCGAC
CCGACGGTGATCCACACCAGCGCGATCGACATCAACCCGCTGCGTGGCCTGGCGCGTGTC
GTCCAGATGACCGGCAAGGAGTGGCAGGGCGTCACCTCCGCGAACGGCATCGTAGTCTCG
CGTGGCACTGAGGCCTCCACGACCGACGGCTCCAGCCAGACCCTCGCACAGCCCAAGGTC
CGTGCCAAGCGTGTCTCCGCGTTCGTCCCGTACTCTTTCGAGATCGAGCACGACTCGTCG
GCGATGCATCAGGAGATCACCCTGATGCTCGGCGAGGCGAAGGAAGCGGAGGAGGCCGGT
TCGTTCCTGACCGGCGGCCGCCAGGGCGTCAACCCGCAGGGCCTGCTCACGGGCGCCGGT
GCCGGTCAGGACGTCGCTGGCAGCATCTCGCCCGCCCTCACCTCCGGTGACATCTACGCC
CTGGAGGAGGCGCTGCCGAACCCCTACCGCGCGAACGCTCGCTTCCAGGGCGCGAAGGCG
CCCTACAACCGCATCCGCCAGAGCGACACGGCCGGTGGCGCGGACCTGCGGGAGCGGCTC
GGTGCCGGACTGCCCTCGCAGCTCATCGGCTACCCGCTGCATGACGCGAGCATCATGGAC
GGCTACCCGAAAACGGCCGCCGCCAAGCGCGTCCTGGTCCTCGGCGACTTCCGCAACTTC
CTCATCGTCGATAGCATCGGCACGAGCATCGAGCTGGTCCCGCACGTCTTCGCGACCTCG
GGTGGCGCGATCGTGCCGACCGGCCAGCGCGGCCACTTCGCCGTCTGGCGCAACAACTCC
AAGGTTCTGGTGCCGGACGCCTTCCGCGTCCTCAAGACCAAGGCATCCTGATCCACTGAC
TCCCCCGAAGTGCCGTAGCACTGTCAAAGGTGTTGTGGCCCTTCGGCCGTCGGGAGGGCG
GTAGCAATGGCACTCGGTGATCCATACATCACCAAGGACGAGCTGCGCGACTACCCGCAG
ATCCGGTCGGACGTGATCCAGTTCGACCAGAAGCTGACCGACGCCTGCTCCTCCGCCTCT
CGCGAGGTCGACCAGCACTGCGGCCGTCAGTTCAACAAGTCGGCCACGGCCACCCCCCGC
ATCTTCCAGATCGAGCGGCGGCGCAAGGTCACGGTTGACGACTTCCACACCAGCGCAGGC
CTTGTAATCGAACAGGGTGACGGGCTGGGCAACTGGGAGCCGGTCGATACCGCGTCCTGC
CTTCTGGCCCCGTACGACGCGATCGTGGACGGCCTGCCGTGGCCCTACTACCGGCTCGAG
TTCCCCGGCTGCGGCCTGGTTCCCTCGGATCGGTATCAGGCCCGGTTCCTCCGCGTGACC
TCGCAGTGGGGCTGGCCGAGCGTTCCAGCGACGGTCAAGCAGGCCGCGTTCCATATCGCC
GCGCAGACGTACCGCCTCGCCGACGCGCCCCTCGGGGTGACCGGCAACACGCTGTACGGC
GGCGTCGTCCGCGTGCAGGATCTGCCGGGCGTCGCGACGAAGCTCTGCCGGTTCGTAGTC
AATCCGATCCTGGTGGGGTGACATGACGACGCTCAGTGAGCGCCGCGACGCGCTCGCGAA
AACGATCAAAGCGGGCGTCGACAAGCCGCTGCACGCGTACGAGACGGTCGAGGAGATCGT
CAACGTGCCGTGCATCATGATCGAGCCGTTCAACGCCAACTTCGAGAGTGCGTTCCAGCC
CTGGATTCACACCTGGGAGTTCTACGTCTTCGTGCTCGCTTCTCGTTCAGCAAGTTCCGC
GACGGGCCAGAAGCTGGTCGACCAGATGGTCTCCGGCACCGGCCCGAACAGCGTGGCTCA
GATCCTGAACGAGAACTACACGCTCGGCCTGAACGGCGTCGAGGCACAGTGCTTCGGCAT
GAAGGGCTACGGCGGCTCGTTCGACTGCGGCAAGGTCGCGCACGTCGGCGCAATCCTGCA
GGTCCGAGTAACAGTCGATCCACACGCATAAGGAGGTGGCGGCAGCATGACCGCTCTCAC
AACCAAGGTTCTGTCCGACGATGGGAGCGCGCCTACACTCGCTACTCACAGCGCCTCGGT
TACCGCCCAGGTCGGCAACGGCCACAACACATTCCTGTACGTCAGCAACACCGGCGGCTC
GGCTGCCAGTGCGACGATCACCCCGCCCGGCACGACCAGCTACGGCGTTGCCCTCCCAGC
CAAGACGGTCTCCGTCCCCGGCGGCGAGGCGAAGTACATCCCGCTCCGGCGCGAGTACGC
CGACGAGTCCGGCCGGGCGACGATCGCGTTCACCGGCACCCTCGCGGACCTGAAGGTCGC
CGTCATCCGCGTCGCCTGACGGCCAGCGCGAACTTCCACAGGCTGCGCTAAGAGCAGGCC
GAGGAGGCAGCACTCCGCGCCAGCGAGACCGGCTTCTACCAGGTACTGGGCAAGAGCGCC
AGCCTCGGCGTGAAGCCCGGCAAGGTCGTCTGGATGTCGGTCGAGCACCCAGCGCGGCTG
CTGTCCTCCGGCAACGTCGAAGAGATCAACCTGCGGAGCAATACCGGTGACGAAACGGGC
CACGAAAGCCCGGACAGCACAAAGGAGTAAGGGCAATGGCAACGATCGTTCTCCGGGACT

GCTTCATCGAGGTCGATGGGGTGGACTTCTCGGATCACGTTTCGTCCGTGGAGGTTTCGC
TCAAGCAGGCGGGCGTCGCCAACACGGACTTCAGCGGCGGCGGCAAGGAGCAGGACGCCG
GTCTGAAGGATGACGAGTTCACCGTGAACTTCCAGCAGGACTTCGACGCGGCCTCGGTCG
ACGCTAAGCTCTACCCGCTGTACGACCTGGAAGACGAGTTTGTCGTGAAGGTGCGCCCCG
GCGCCGGAGCGGCCTCGGCTACCAACCCCGAATACAGCGCTACCTGCATCCTGCTCGAGT
ACCAGCCCCTCGCGGTCGAGGTCGGCGACCTCTCCGCGACCAGGGTGAAGTTCCCGACCC
AGCGCAGCGGCATCTCGCGCTCGGTCATCTGATCGGGTAGCGCCGCATGGCGATCGAACG
GTACCGGCACACCCTCTCGTTCGATGTCATCGGCGAGCGCCAGTTCCGCGAGGTCCAGAA
GGCGCTCACAGCGGCGAACCCGCAGCTCCCGCGCGACTTCAAGAACAGCGTCAACGAGGT
CGCACGCGGGCTGCGCGACGAGGCTCGGTATCAGGCTCTTCAGAAGTTCACTTCGAAGAA
GGGCCACACCGGGCTTCGTCGCAAGGCCGCCGCTGGCGCGCAGATCATCCAGATCGACAA
CGGCGTCAAGGTCATCACCTCGATGCCGGAATCCGACGAGGCGATCATCCCTCGCGGCCT
CGACGGCGTGAAGAGCTGGCGGCACCCCGTGTTCGGCAACAAGGAACGGTGGGTGCTGCA
GCGTCAAGGCTCCGACTCCTGGTTCCTCGAGACCATACGGAAGGGCCACGAGCCGCTGCG
CAACCGCCGGGTGCGCAACATCAACGACACGATCGAGAAGATCGACGACGCCAGCCACAC
CGGCTGACGCAAAGACCAATGCTGGGGCCAGGTGTTTCTGTCGCGGGTCTGCCTGGCCCC
AGCTCCCATTTCTCGCTGACCCGCGAAATCCACTGCACCACACACCAAAAGGAACCGCGA
CATCATGGCACTTCTCTCGAAGACCGACATCCTCTCCGTCGACGACCGCATGACCGTCCG
CGTCTCCGTCCCCGAGTGGGGTGGCGAGGTTCTGGTCCGCACCCTGCCCGGCCGTGAGCG
GGACGAGTTCGAGGCTTCGACCGTCAAGACGAACAAGGGCAAGCATGAGGAGAACTGCGA
GAACTTCCGCGCCCGCTTCGACGCCCTGTGCGTCGTGGACGAGAAGGGTGAGCGCCTGTT
CGCCACTGGCGCTGAGGTTTCGATGCTCGGCAGCAAGTCCGTCGCCGCCCTGCAGCGCGT
CTTCAACGAGGCGCAGAAGCTCAACGGCATGACCGAGGCTGACGTCGAGGAGCTGACCGA
ATCTTTCGAGCAGAATCCCGACGAGGCTTCTACTTCCGCCTAGCCGAGACGCTGGGGATG
ACCGTCGAGCAGATGCTGGCGGTAGTCTCCAGTACGGAGCTGGCGGAGTGGGAGGCGTAT
GAAGGCGCCTTCGGTCCGATCGGGAAGAGCTACGGAGAGCAGGCCCTCGCCAGCATCTCC
GACACGCTCGCTATCCTGCTGCGTCCGTACGGCGAGCAGTTCGACGACAACCCCGTCCCA
GTGCCCAGCAAGTACCCCCGCCCGTCGGAGTGGTACAGGCCAGAGGCCGTGGCGGACTCG
GTAGATCAGGCCGAGTTCGACAGCAACTTCCGAGAATGACCGATCACCAAAGGCCCGTCG
GCCGTTTGCCCATTGCCGCAGCCGGTCGGCGGGCTTTTTTTGTCACCAGGATGGAGGTTT
CGCATGGCAACGATGACCTCGCTCAACTTCGCGATCAGGAGTAGTTGTGACGGGACTGCG
TTGAACAAGGCGCGTGCTGACCTAATGGCTCTGCAGGCTCAGATGCGGACGATATCGTGT
GCCTCGCTGCACTTCACGGTGGACGCCGACAGCGAAGAGGCGAAAGCCAAGATCGCGGAG
CTGCGCGCGCAGGCGCGGGACATCCAGATGGGCATCGACCTGAACGACGGCCAACTCGCT
ACGGCGCAGGCCAAACTCGACATCCTGGCTCGCGATCGAGAAGTCGACGTGAGGGTGAAC
GACCATGAGATCGCGAACAAGATGGACCGGATCTCGGACTCCTTCGAGAACGCGTCGGTC
AGCGCTGTGGACTTCGGCACCTCGTCGAACCGTGCGGGCTTCATGGCCAAGGTCGGCATC
GCGGCGTTCGCCGCCGGTCTCGTCGTTCTGCCCGGACTGATCAACCTCGTCGGTGCATCG
CTGACCGGCGCGATGGGTGGCAGCATCATCGCTGTCGGCGCGCTGGCGCTTAAGGAGAAC
GAGCAGATGAAGGCGGGCTGGGAGGCCCTGTGGACCGAGATGAAGTCCACGGCCCAGTCT
GCCGCCCAGCCGATGCTGCAGCCGTTCCTGGACACCATGTCGATGGCCAAGACGCGGATC
GCGGAACTCAAGCCGGAGCTGACAGACCTCTTCGCGAGTGCAGCCCAGGCGATCCAGCCG
CTCACGTCCGGCCTGATGGATCTCGTCACTAACGCGAGGCCCGGCCTCACGCAGGCGCTC
GAGCAGTCCGGCCCGATGGTCCAAGGGTTCGCGGATGGCCGCGGTTCGCTCGGCACGCAC
GTCTCGAACTTGTTCGCCGGGATGGCAGGCGGCGCAGCGGGATTCGGTCGACTGTGGAAG
ATCACGTTCGATCAGGTCGGCCTGATGATCGAGCAGTTCGGCGTCGCTGCCGGGAAGATG
GCCGAGACGGACACCAACCCCTGGGATCGACTGCAGCCCGGCTTCAACCAGTTCATCGGT
GGCTTCCTCGACGGCCTCGTCGAATTCACGGCCGCCGTCGACAACGGGGTCGGTTACGCG
CTCTCGCTCTTCGGTCTGCTCGGCGCCGGCATGCGCGGCACGCTCGGACAGCTCGGCGAG
CTGGTGGCCGCGATCTCGAACGCCCTCATGCCCGTCCTCGACGGCACCGTGCAGGCGCTC
ACCCTCGTCATCGGCGCACTGATGTCCGGCCTCGCTCCGGCGATCAACTCTCTCGTCCCG
CTCAGCAATGCGCTGCAGCCGTTGCTCGTCATGGTCGGCCAGTCGCTGACCCAGGCGATC
AACCATCTCGCACCGCTCCTGCCCCCGAACGTGGACGCGCTCGTCTCCGGGCTCATCCCC
GCGTTCCAGGCGCTCATTCCCGCACTCGTTCCGGTGATCGAGCAGATCGCAGTAGGCCTC
ACGCCGCTGCTGGAGATGCTGCCCGTCGTGATCAACACGCTCAGACCGATCGTCGTCGGC
CTGGCCATCGCGTTCGGCCAGTTCACCCAGTGGCTGTCCCCGCTGATCCCGCACCTCATG
CAGATGTACATCGCGTAGAAGCTGATCTCGACGGCCATGGCTATCGGGCGCGGCATCATG

ATCGCGTACACGGCAGTCCGCACGGCCCTCACGGTGACGACCATCGCTCACACGGCGGCC
GTCTGGGCGAACAACATCGCGGTGAGCGCCAACCCGATCGGCCTGATCATCATCGACATC
GGCGCTCTGGTGGCAGCGATCGTATGGCTCGCGACCCAGACTCAGTTCTGCCAGACGGTC
TGGGAGGCACTGAAGGTCGCGATGGCGGATGTGTGGAAGTGGATGAAGTATGCGTGGGAT
GCCACGGTCTGATTCCTGGCCATGCGGTGGGAGCAGTTCTCCGGCACGATCGTCAACGCA
TGGAACGCCACGTGGAACCTGATCAAGGTAGCGGCACAGGCTGTCTGGGGATTCCTCACC
AACGCCTGGAACCAGTTCCTCACGGTCCTCAAGGTCACCTGGGAGGTCGTCTCCGGCGTC
ATCAAGGCGGCCTGGGACGTCTGGTGGTTCGCCATCCGCGCGAGTGTCGCGATCATCTGG
GCGTGGCTCGAAGTCGCCTGGGGTGCACTGTGGGGCACTGTCAGGCGAGTCTGGGATAGC
TTCGTAGCGCTCTTCCAGCCGCGGTGGGATGGACTCTGGAACGGCGTCAAGGTCTTCGCC
GAGGGCGTCTGGGGTGGAATCCAGATCCTCTGGAACGGACTGTGGAAGGCCGTGACGGCA
GGCTGGAACGCCTTCATGGGCTGGCTGCGTCCGATCTGGGAGTCCTTCTGGAACACCGTG
AAGCAGGTCGGTGAATCCATCTGGAACGCCGTCAAGCTCGCCTGGGAGTTCCTGTGGGAT
CAGGTCAAGCAGAAGTGGGGCTGGTGGGTCAACCTGTTCTACTCTGTCTGGCAGCCGTTC
TGGAACGGGATCAAGGCTGCAGTCGAGACCGTCTGGAACGGAATCACTCCCGCGTGGAAC
GCCTACTGGAACGGCATCAAGGCGATGTGGGAGAACTTCTCCAGCGCCATCCGTGCCGGG
TGGGAAGCCTTCTGGAACTGGGGGCGTGACTTCGCATCTGGCGTCTGGGATGCGATCACC
AGCAAGTGGGGCGAGTTCAAGCAGAAGTTCGAAGAAGTCTTCAGCACGATGGTCCACAAG
GCCCGCGAGATCTGGGACAAGATCCGAGAAGTCTTCGCGAAGCCGATCAACTTCGTCATC
CGCATCTGGAACGACCACGTCGCCGGTAAGTTCGGTCTCCCGGCACTCGACGAGATCGGC
GGGTTCGCTACCGGTGGTCGGGTCGACGGCAAGGGCACGGGAACGTCCGACGACATCCCC
GCCCGGCTCTCGCGCGGTGAGCACGTCTGGACCGCACAGGAAGTCGATGCGGCTGGCGGA
CACGGCAACGTCGAGGCCATGCGCCGCAACACGCTCAACGGTGTCGCGCACTAGGCCTCC
AGCGGTCAGGTCGAGTGGATGATCGGCCAGCAGGAGAAGTTCGCGCCTGCCCTGCAGGTC
ACTTCGGCTCAGCGGGACTTGAACGACTACCACGGCCAGGGCAAGGCGGTGGACTTCTCG
AACGGCGGCGACGCCGGTACACCGGAAGTGATGGCCTTCGCGAACTGGATCGCGGACACC
TTGGGTGCTAACACGCTCGAGCTGATCCACAGCCCGTTCGGACGGAACATCAAGGACGGC
AACAGCGTCGGCGACGGCATGGGGTTCTACGGGGCAGGCACGATGGCCCAGCACAGGAAC
CATGTCCACTGGGCAGTCGACCGCCCCCTCAACGAGGACGAGGGCGGCCAGAGCCTGCTG
GGCAAGATCTGGGGCCGCGTCCGCAAGGGCATCGCCTGGACCGTCGAGAAGTTGACCAAC
CCGATCCTCGGACCCATCCCGGATCCGTTCCTTCCCGGTGTCGGTTCGCCGTTCGCCGGA
TTCCCGAAGGCAGCGGCAACGAAGCTCCGAGACGCGTTCCTCAACAAGGTGCGCGGCGCT
GAGGGTGCATCCGGCGGCACTGCCAGTGGAGACATCGGCGGCGTCATCCCCGACGGGGAC
CGGCTCGGCATCATCAACGAGGCCCTGCGGATCACGAACAGCCCGCCTCCCTCGTCGATC
GAAGCATGGCAGCGCGGCATGAACACGCTGATCACGCGAGAGTCGGGTTGGAACGCGGGC
GCGATCAACAACTGGGACTCGAACGCAGCGGCGGGTAACGCTTCTCGCGATCTGGCCCAG
GTGATCCCGACGACTTTCGAGGCTCACAAGGCACCGGGCTACAACAACATCGACGCGCCG
GTCGACCACGTCGCGGCCTCGATCAACTACATCAAGTTTCGCTACGGCACCATCGAGAAC
GTCCCGCAGGCGAACGCCAACATGGCCCCTCAGGGCTACCGCACGGGTACCAACTCGGCT
GCGGCAGGGTGGCACCTCGTCGGCGAAGACGGACCCGAGATGGTGAACTTCCGTGGCGGC
GAGCAGGTCAAGACGTTCGACGAGCTCATCCGCGCCCTGAAGGACTCCACGTCAGGACAG
AGCATGGAGCTGGAGTCGAAGCTGACGGCCGAAATTCGCACGCTGGTGGAGAAGATCGGG
ACCGAGGTCAACACCTCCGGAGGGCGGTTCGCGCAGTCTGTCGAGCAGGCTATAGAGCGA
GTGCTTGCCACGGCCGGTATGCAGCTCAACCTCTCGATGCCGGTACCGCAGAATGCAGCC
GACGCAGCAGCGTACGCGCAGGAAGTCGCGAATCAGCTTCGCCCGCAACTGGAGATGATG
ATCGGGCAGCGTATCGGCACATGGTAATCACAAGGGGCGGGCTGGGGTTCACTACGAATC
CCGGTCTGCCCCCTTCACGTGTAGGGAGACACAATGGTTCTGTTCAACTTTCAGCCGAGC
GGCGACTGGTTGAACGACGGATGGAAGATCATCGGCGGCGTCACCGCGCTCTGGCAGGCG
CTCACCCATCTCAGCATTGCGAAGTGCATTCAGTGCCCGGCGACAACGGCAGGCGCAGCC
GTGTCCTTCCCCGTCGACACCACCAGCGTCCCCGACGGCGCGATCATCACCTCGGTGACG
GTGAAGATGCGGGTCGGACTCGGAGCTGGCGCTGCACCGTCCGGCACGGCCCCCTCGATC
ACGGTCGCGGTCGTCGCCCAGGACAACACCTAGCGCAACCTGACGCGCACCATCTACCCG
ACGAGCACCCCCACCGACTACGAGATCGCCACCTACCAGCGCGACGCGCTCGGCCTGGGG
TGGGATGTGCAGCGGCTCAACAACCTTCTGTGCCGGATCTTCTCGTACGTCGGCATCTTC
CACCTGATCCGCTGCTACCGGCTCTGGTGTGAGATCAAGCACCGGCTCCGGCCGACCGTC
GCGATCACCGCCTCGTCGGGCACCGTGACCACGCCGTCGCCGACCATCTCGTGGACGTAC
ACGCAGACCGACGGCGATCCGCAGAAGACCGTGGAGTACAAGATCTTCACGGCCGAGCAG

GTGGCCAAGGTGTCCTTCAACCCGGACACGGCGACGCCCCTCTTCCAGGACACGCTGCAG
GGCGACGTGACGAGCGTGCTGCTGCCGACGTCGTTCAACTCGAACAACTACTGGGTCTAC
ATGCGAGTCACGTCGTCATTCGGCGCGCGCTCGATGTGGGTCGGCCGTCAGTTCACGGTG
TCCGGCCAGTCGCCCGCGATCCCCGGTGTCGACGACCCGACGGACGCACTCGGCAGTGCA
GTGATCACCGTGGTGCCGGACAGCGAGTCGGGCAGCGGGTGGCTCCACATGCGGAACACC
TCGAACATGCTCAGCGCGCAGGAGGCGGTCGCGGAGTCGCCGACCGACGGCCACACGTGC
TCCGCCACCAACGCCACGATCACCCGCGCGTCCGCCGTCTCCTTCCCTGGCGGCACGTCC
TCGTGGAAGGTCACGGCGACAGCCGGGGGAGCGGCACAGGTCTCTACGGACTTCGTCGCG
ATTGAGGAGTCCCTGCCGATGACCTCGCGCGCTCAGTTCCGAGCGGCGGTCACCGGGCGC
TCCTGCCGGGTGGTGATGCGGTTCTACGACGACGGCTACGCCGAGATCGCGGGATCCGCC
CTGACCGGCAGCTCGGTGACCGACTCGACGAGCACCCGGACCGCGGCACCGGTGTACGGC
AACGCTCCGGCGAACACGGTCTACACGCGTGTCACCTTCGAGGTTCTGTCGGCGGCGGCC
AGCGCCGTGCACAACCTGGACCACCTCGGCGTCAGGTACGGCCTGAACACCCCGTGGGCC
GACGGCAGCCACGCCAGCCGCAACCTGCTCTCGGCGTACATCTCGACGGCAGAGGGCACG
CAGATCGGCGGCGAGTTCTGTACGCCGGACGCGGCTTCGACGGCGGTCACTGCGGCAGCC
ACCCGCACCGGGTCTTCTAGTTCGAACTGCTTCAAGATGACGTACGACGGCCTGTCTCCG
TCGATCGGGTTCCGAGGTGTCGGCACGGCGTTCACGTCGCCCAGGTCCGGCGCCGACTTG
AAGCTGAACAAGCCTGCGGGCACGGCGACCGGCGACCTCATGCTGGCCTTCGTCACGACG
TCGGAGTTCTGCACGATCACGCCTCCGGCTGGCTGGACCCTGGTGAACTGGGCGAAGGTC
GACGACGGCACCACGGACACCGCGATGTTCGTCCTCAAGCGCACCGCCACGGCGTCCGAA
CCGGCCTCGTGGACCGATGGCTTCCTGTCGACCAACTCGCTCCGGCGGTCGGCCGTCACG
GTGGCGTACAGCGGCGCACCGGACGTTCTGACGGACACACAGGCCGCCTCGGGTAACAAC
ACCCCGCTCGGCATGGAGACGCCGTCTCTGCACAACACGGACCCGAACGGCTGGCGCGTG
TGTGCCTTCGCGGTGTCGGACAACGCCTGCGGCGGAACTCTGACGGCGAACCGGCAGTCC
CCGTTCACGATCCCGCAGATCAGTTTCGTAGGCTCGGCGTCCCCGTGGCTCACGGCGAAC
ACCGCTGGCTCGTACACGATCAACAAGCCGTCGGGCACCCAGTCCGGTGTCGCGATGGTC
GCGACGATCGGCTACCTCGGCAACGGCGCAACAGTGAACCCGCCTGCGGGCTGGACCCTG
CGGAACCACACGACGGCGAACAACACGAGCGCCGTTCCCTACAGCCTCGCGGTGTTCTAC
CGGGTCGCTGGCGGGTCGGAGCCGGCTTACTGGTCGGGCACGATCACGGGCGGCTCGCAG
TACCGCACGACGACGACGCAGTGTCTCGCGTACCGCAACGTCGACAACGCATCCCCGTTC
ATCTCCTCGAACATCACCGGTACCCTCAACTCGAGCGCGCTGGTGACCGTGCAGGCGGCG
AACACGAACGCGCTGGCGTGGCGTGTGTCGCCCTTCGGTGGTCTATCGGAGGCGGCCAAC
AGCTTCGGCAGCACGAACGAGGTCGTCGAGAGCGTGGACAACTACGTGCAGTACTCGATC
CTGTTCGGTAGCCGCGCTTGCAACGCGGTGATGATGGCCGAATCGAACGGCGCTGTGTCG
ACGGCAACGTATGAGCGGTACGCGTACTCGAACAACTCGCTCTACGCGGGCGTCGGCTTC
ATCGGCTTCCTGCGTCCGCTCGGTACGCCTCCGACCCCGGTGGCCGACGAGACGGCACGC
ATAGCAGCCGGGCCGGTGGGTTCGGCCAACACGTGGGTCTTCGCGAGGGTCTTCGACGCG
AACGGTGTCACGCCGACGGGCAGCCAGTCGATCACCGGCATCTGGACCCCCCGATCCCGC
ACCGACAAGAACTCGATGGCTGGCTGGTTCGGCATCCTGAAGCCTGCCCAGGCGCAGAAG
GTCGGGTACGCGACGGCCACGATGAACCGCACGGTGGACGTCTCGCTGGTCAAGACCGAC
CAGATCCCGGATGCCGACTTCGTGCCGGCGACAGCATCGTTCACCGGATCGACTGCGGGC
ACGCCCTACCTGACGGTGAACTTCTACCGCGCCAACACGCTGCTCGGCAGCCAGATCGGC
GATGGCACGGCGTTCGCGACCGGCGGGTGGACCAAGTCGTCGGCGACGTTCCGCATCCCG
AGCGACACCACGCGCATGTCGGTCGGCGTCTCGGTCTCGGGTCGCAACGTCGGCAACAAC
GTCTACTGGGACCACGTCTCGCTGGCGTACGGCGTCGATGCGACCTACCGGCCGAGCACC
TCGCGTCCGGAGCACCCGGTCTGGTCGCTGCCGCAGATCCAGTACGCGGACGACGACGGC
ACCGGCTACTCGGACTGGGCGCACCTGCCCGGCCTGAAGTCCAACCCGCCTGCGTTCGAG
CCGCACTCCGGCACGGCGCTCTACACCGACCACGCGCCGATCCCGCTGACCAACAGGAAG
GACCTCGCTCGCACGGTGTCTCTCGGCCTCGGTGGCGACCTGTTCGTGTCGGAGTGGGGG
CCGGACAGCCCGGAGTTCACGTACCAGGCGGAGAATTACTGGCACAAAGACATCTCGAAC
CCGAACAACAACCTTCGGCTCAAGGTGGCCTGGGACAAGACGCAGGCGGCTAAGACGAAC
AGCGCCACAGTGTTCCAGCCTCTCGGCTCCGAACTCCCCGTGGTGCTCAGCGAGGGCTAC
ACGGGCGACACGTTCACCCTCAAGCTCACGCCGGTCAACCACGAGGAGCGCGCCGAGCTG
CGGACGATGCTCACGTCCGGCCGGACCCTGTTCCTGCAGTCGGACATCGACGACGCCTGG
TGGGTGCGCCCGGTCGGGGACCTGATCGAGGACGTGCTCCCGACCGACAAACCGCAGTCC
AATCCGCGGCGCGAGATCACCTGCCAGTTCGTGCAGGTAGCGCCAGCAGAGTGACAAGTA
GATCCAAATGGCCCGCGCGACACAACGATTCCTGGACGAGATCCGAGGCAGTCACACCGT

CTATAGCTCCGTGGACGTGATCGCTCCGGACCAGGAAACCCGGCGGCTGGTCGTGATCGA
CGGCGAGATCAACGTCGACCGGACAGCCCAGTACCGTCGCGCGGGTCGGATCGACTGCGT
AGACCTGAACGGCAGCTTCGTGCCCTTCGGCAACCAGGGCATCCTGACGCCGTTCGGCAC
CGAGATCCGGCCGTACCGGGGCGTGAAGTACGCGGACGGCACCGTGGAGGTCTACCCGCT
GGGCGTGTTCCGGCAGGCGGGTAGCTCCTTCCACGAGTCGTCGAGCCGGGGCGGCAACGC
GGGAAGCCGCATCTCGCTCCGCATGTTCGACCGATCCCGTGTGGTGGCGCGCGACAAGTT
CACGAACACGTACACGATCAACGGCGGCTCGAACATAATCACCGCGATCAAGCTGATCGT
CGGCCGGACGTTCCCCGACTTCGAGTACGACGCGGTGTCGACGACGCTCAGGACGCCCAG
CCCGAAGGTCTACACCCCGCAGGACGACCCGTGGGTGGACGCCTCGGACCTCGCGAAGTC
GATCGGCTGCGAGCTGTACTTCGACGTCAACGGCTGGGTGGTCATCGCCCCGACGACCGA
CATCGACGCGCTGCCGAACCCCGACTTCACGTACATCGAGGGGCGGGGCTGCACGACGCT
CGACCTGGCCGCCGAGTACAAGGACGAGCCGGGCTACAACGGCGTCATCGACGAGGGCGG
GGCCACGGGCGACGAGCTGCCCCCGGTGCGCGCGGAGTCGTGGGACTACGAACCCAGCTC
TCCGACGTACCGGTTCGGCCCGTACGGCGAGGTGCCGCAGTTCGTGCAGGACACCAACGT
GAAGTTCCAGGCCGACGCGCAGAAGATGGCCGATGCGCAGCTCAAGGGCTACATCGCCTT
CTCGGCCGCGCTGTCGATCAGCTCCTGGACGAACCCAGCGATGGAGGCCGGGGGCGTCAT
CCAGGTCGATCGTGACTCCATGAACATCACCCGCCTCGACACGGTCGACTCGTTCAACAT
CCGGCTCAAGAAGGACTGCGTCCAGAACCTCAAGCTCCCAACACGACGGTTGCATCGTG
ATTCGAACCTACGGGCAACCCCCGGCGACCGTCCTGACCCGTGTAAACGTTACTCGCACA
AGCACTCTCGGAGGTGAACGATGAGCGGGGTACCGACACCTGCGCCGGACACCCCCCAGG
GGGACATGACCCCGGCTGAGCAGACAGCAGCGGCCATCGCCGCCCAGGCCCTGCAGCGCC
AGCAGATCAACTCGCTGGCAGACGCGCTCACCCCGCCGACGCAGATCTCCGCGCTCGTCT
CGAAGGCGACCATCGTCACCGTCGAGTGGGGCTACCCGCCAACCGTGACAATCCAGCTCG
GCGGCGACACCACGGCCTCGATCGCGGCGGTCTGCTTCCTGGACAGCTAAGTGCCGACGG
CCGGGGACACGGTGATCGTCGTCAAGCAGGGGAGCGAGCTGTTCGTCCTCGGCCAGATGA
TGGACAACCTCGCGGCCGGGGCGATCGGCTGGCAGGCTCCGACGCTGACGACGACGTGGA
AGGCGAACCCGACGTTCATGCCGATCCTGTACCGGATGGTCTAAGACAATCGCGCGCGGA
AGATCCAGTTCCAGGGCCGCGTCTGGTGGAACGGCTTCAACACCGGCACGACACTGTTCA
CGCTCCCGGCCGACTACCGGCCCGGCGTGCAGCGCGCCTTCAATGTCACCCGAGAGAACC
TGACCGGCGGCGCGAACGCGCTGCAGCTCCAGGCGAACAAGGACGGCACGGTGACCATCT
CCGGCCACCAGCTCGAGACCGTGGGCATGACCAGCTCGGGCGGCGGCACGACCGGCACGA
CGGACACCAACCACAAGCAAAAGACGTCGGACATCGGCAACACTGGTGACCCGGTGCTCG
TCGGCTGGTGGACGGACTGGGTCGTGGGGTCGGCTGTGGGAACGAACAGCGTCACGCACA
ACCACAGCACCCCGAATCACACGCACACGATGACGAACGCGTTCTTCCCTGGATGGATCT
CGCTCGACGGCGTGGAGTTCTTCCTCTGACTAAGAGGTGCGGACGGTGCGGCGACGCAAA
GGATCTCGACGAGTTCCAGCGTCGCCGCGCCGGGTCCGAACAACGACAGTCGTACTGCAG
GCTCTGTCAGGCCGAGACGGCCGAGGAGAACAGACTGCGGGTGTCGTACGGAATCACGAC
GCGCCACTACAACGCGATGCTCGAGATGCAGGGCGGCGTCTGCGCCATCTGCTCCGAGCC
ACCGCCTCCGGGGGGAGTGCTGGCCGTCGACCACAACCACAACAGCTGTCCAGGCAAGAA
GAGGTGCGGCATGTGCCTGCGAGGCCTTCTCTGCCACGAATGCAACATGGCGCTCGGCAA
GGTCCGAGACTCCAACCAGTTGGTGCACAAAGCGAGCGGGTACCTCGACGGCGGTCACCT
CGATCTGTACCTACTGCTCGGTGAGGATCGGTAGTGCATTAGATAGTCCAGACCAATATC
AGCAAGAACAGCAGCGGGCCATAGGGCGGCCGGATCCGGCTGTTCGTCATCCACACCCAG
GAGGGCAACGGCACAGCGCGCTCGCTGGCGGGGTACCTGCAGAACCCCTCGTCCGGTGAC
AGCTACCACTACTCGATCGACAACACCGAGTGCATCGCGGTGGTCGACACGGACCGTTCC
GCGTGGGCAGTGCTCGATGCGAACGGCTACACGATCAACCTGTGCTACGCCGGTAGCCCG
GCCTCTATGTCACGTGACGAATGGATCGCCAAGTACAGCAACGCGATCGACTTCACCGGC
TGGCTGATCGCACGCGATGCGAAGCAGAACGGCTTCACGCCCTACGTCATCGGGCACCCG
GAGAGCCGCGCGGGCAAGCAGGGCACGACCGACCACTACGGCATCACCAAGGGCCTGGGG
ATCGGCGACCACACCGACGTCGGCGGCCGCGACCCGTGGGACCTGCTGCGCAAGGCGATC
GACAAGTACATGGGTAACACGGCCCCGCTGCCGCCCGCCCCGCTGCCGATCCTCAACCCG
ATCGACGAGTGCCGGAAGACGAACGACTGGCTCGGCGACGCGCTCGATGGCCAGAAGCCC
TGCCCGGACAAGAAGGGCCAGTTCCGGTACTTCAAGAACGGCGCGGTCTACTGGACCCCG
GAGACCGGCGCGCGGGCGATCCCGTCGGAGCTGCTCAAGCAGTTCGCCGACCTGAAGTGG
GAGACCGGCCCGCTCGGCGACCCGACGCACGACCGCACCATCCTCACCGGGCCGAACGGC
CGTCCGTGGGGCGTCGTGCAGGGATTCAACGGCGGCAACCTGCACCGCAAGTACGGCACC
GACGAGGCCTTCTGGACGCACGGCGAGATCGGCAACCGCTGGGCGCGTAGCGGATTCGAG

AACGGTCCGCTCGGCTGGCCGCAGTCCGACGAGCTGCCGTTCGACGGCGGAGTCGTCCAG
TACTTCGAGAACGGCCAGATCCACTGGCCCGGCAAGCTCAAGACCATCGCGCTCCTGAAC
AAGGACGGCGCGGATACACCCCTGGCTGACCAGGGTAAGTCAGAGAAGGAGATCGCAATG
ACTCTGCGAGAGAAGACCGCTCACTACCGCAAGGCCGACGAGGCTGGCATCGGCTCCGTG
CTGATGATCCTGACCTCGCTTCTCGCGCTGGGCGATGTCCTGCCCGCTGGCACGGCTGTC
GTCGTCACGACCACGATCCCCGTCCTGACCACGGCTCGCGTGTGGCTGGCGAAGAACGCC
GACCTGATCGACAACGCGGCCGACTCGATCGACGGAATGCAGCGCGACGTCCGGGCCGAC
GTGCCTACACGCGGGAAGCACGCTCGGCAGTGAGCGCCGAGTACCTGGCACTGGTCGTCA
CCATGGTCGGTCTGCTGATCGGCTCGAACGGCATCACGATGTGGCGCACCCAGCGGATCT
TCAAGACCAGCCAGTCGGAGCAGGCGAAGGCCATGGCAGTCCAGGCAATAGCGACGGCAC
ACAGCGAGCACATCGACGCGGCAGCTCAGCTTTCCCAGACAGTGCTCGGCCTGCTGGCCC
CACTGCAGTCGCAGATCAACGACTACGAGGTGAAGCTCCGCGACGCGGTGACGTACATCG
CCGACCTGCGCTCCGAGATGACCCGATCCGGGGGGACGGCACGACCGCTCCCCGAGTCCC
TGCAGGCGATTGTGACTGATCCAGCCGCCTGACGGTAGAAAGCACAAAGGCCCCCCAGCC
AAAGTCGCGCAGCGGCTCGGCTGGGGGGCCTTTTCTGCGTTC
>NewGenomeName_30
CTCCGGGCCAGTACCCACTCGTCGCATCCGTTACCAACGGCCAGAAAGTTTCCCCTGGGG
GCGTTACCTACGAACTGACCGTGGCTGACGGCCACCTGACCGCCATCACCGAAGTCGCTG
CTCCATAAGAGGTGTGTTATGCCTAAGTTCCCACAAGTAGGGCGCCTTGCGCGCTCTGCT
GCGGCTGTCCTTAGTATCTCCGCAGCAGGCTTAGGCTTTATCCAACACGAAGAAGGAACC
GTTAATCAGGCATACCTTGATTCGATTGGAAAGCCTACCATCTGCACCGGGCACACTGGC
CCCGAGGTACGCGTAGGGTTAGTCTACAGCGACGCACAATTCGCTGAGCTGCTTCACACG
GACGCCAACTCGGCTGCCGCTGCTGTTAAGCGCACGATCAAAGTCCCGCTGTACCAGTAC
GAGTTCGACGCGCTCGTCTCGTTCTGCTTCAACTTCGGGAACACGAACTGGACCACAAGC
ACTATGTTCAAGCTGATCAACCAAGGTCGGTACGACGAGGCTGGGCCGCAACTCCTTCGC
TGGCGTTATGCTGGTGGGAAGAACTGCTCCGTCCGGGCCAACAACTGCTACGGCGTTTAC
ACCCGCCGTCACAACGAGTAAGCACTATGGCAAGGACAATACTGCTCCGCGTACTGCTCC
CGCTTGCCCTTCTCGGGCTGGCTGTGGGCCTCTACGCGACGCACACGTCTCTCACTGAGG
CAAGGACAAAGCTCGATGAATAAACACTCGCTATACGCGACCTCCGGGCCTCTCAGGCTC
GCATACAGGCTGCTGTCACGGCTGTGCAGAGGCTTAGCTCGCAGACTAAAGCGCTCGTCA
GCGATGCCCTCGACAAGGAACCTGTTTACCGGGATACTCCTGTGCCTGCTGGCGTTTCTG
ACAGCCTGTGCAAGCGACTCCGCTGCAAGGATTGACCCTGACGTACCGTCCCCGCATCCA
ATCGTCTCTACGGCGACCACAGGCGGGCTGGTGAAGGGGCTGGTGGACTACGCAGACGCG
CTGGATTACTGCAACGCTTTCCGGGGATTCGGGCCAGAAGCTCAGACCCAAAAATGATGC
GCACATGCGCCCGCTCTCCAGGACATAAAGAGCGCGAGGTTCCCCCATGTGCCCTGCGCA
GAATGTCGGGCGCGCTGATTAGGGATGGCTGGTCATGGCGGTCTCTCGCTGCTACTGTCG
ATGTTGACGGGGCCAGTATGGGGCCGCTGAGCCGGGCTGACAAGCGATTTGTTACGATGG
CCGCGACAAAGGTACAGGGCACGGCAATACAGGCTATGAGGCGCTGAGAACGCGCACAGC
ACGGCATTAGGACAAGGGCGGGCAGGCAGAAGGGCACACTGTCTTCTTTTGGCTGCGCGG
GATTGTGAGAGCTGAGAGCGGTTTTCTAATTGGGCCACTATTGCGAAGGGAAACGCGATA
CCCTGCGAATGCTTAGGAGACTTGACACGAACGGTCATATCTGTCTAAGCCGGAGCAACA
GCACAGCCCAACCACACACCAGACCACGATGTCAAGACCAAACTACCAGACCGAATGACA
GACCTTGATAACAGACCGGGCACACGGTATACGCGCATGCAGGACAGCAGCTTAATGACC
ACGCGACGCACGGGAGTAACATAGTGACATCCATAATACAAGACTAGACAGACAGGAATC
ACAGTGTACTATGGGCACCACTGAGACGCCAGACAGCAACACAGCCGGACAGGATGAAAT
AAGCGGTTGACGGAGACGAGAAACACTGTACACTGCGCAACATGTTCAACGAGACGGACT
AACTGCTAAGGCAACACACTGCTAAGGCACTGGAAACTCTCTAAGCTAAGTAGCTAGCTG
GTAAGACCGACAGCCACAGAAAGTGAACAAAGCAGGTTGACAAGCGACACGCTTCACGGT
AACATCCGCAGCACAGCAACATGCTACACCGGCTTCAACGTAAAACGCTCGCTCAGCAAC
TTGAATGTGAGCTTGACACGAACGAAGCCACGCAGTAAGATTGACGGCAACAAAGCAACA
CGGAAAATTACAGCCGGGTAGTACGGGAAGCGTCTAGCATTAGCTGGTAGCGTATTGGAA
TCTAAGTAACGACGGGATCAGCGGAACTGATCGACGCTCCTAAAGGCACGGCGTCCAATC
CTTAGCTTCACGGCTGGTAGTGCAAGAGACTCGAATGCAACCGAGTACCCATGGAAATAA
CTGGGGCCGGCTTAGGCTCAGAGCTGGTCGGATACGATTACAGCAATACTGACTGTCGGG
ATGATAGTCAACTGGATCAGGTGCATAGGCGGGAGTGACTGCCGATGACTGTAGCCTGAT
AGGAACCTGCCAATACCACGGTGGTTCCTAAAGCCCTCTTATGTTCTTACTGACTGGATG
CTCTGTTGCACAGGTTACTTAGACGAGTCGGCTTTAGAAACTACCGAGGTGCAATTCATG

GCTAAGCATGCATCCATCAGTAAGGTGGAACGACAGCAGCAACGTGACTACCAGAACTTC
GGCAAGGAACTTCGCAAGCATGTCGGTGACAACAAGTCTCTGCGGTTCAAGACTACCGAT
CGTCTGTTGGCTGAGGCGGAAGGTTACTTCAAGATGGTGGCTGAGTTGTCCGCTGTTAAA
GGCGAAGGCGCAGTGACTGCAAGGCGCAAGATCCGCGAAATGCGGGACGTGCGAGAATTC
ACTGATCCTCAACCAACGAGCAAGAACGCATGGGATACTAAGCAGACCAAGGCTAAGCCG
GGAGAGTCTAGGCCGGTGCGTGGTAGTACCGCGACACATCAACGCATTATGAATGGGCAG
TCCGCCCGCAACTCACAAGGTATGGGAATCTAATCATGATTAAGCAAACTCTGATCGTCG
GCACCGCAAACATCATCAAGGCTGTTGCCTCGATCGCCAAGCGCGGCGCTAAGCTGGACG
CTGACATCCAGTTGGCCGGTGTGTCGAACCACGCTCACATCAATGAGCACAACAACGTGA
CCCTGTTTAATGATCTGTACCATTCGCTGCCGAAGGGTACCCGCAAGAATGCCTTGGCTG
AGTGGGCCATCGCTAACGGTAAGGTGAACATCAACGAAGGCCCGAACCCTAAGGACATCC
CTTTCTTGTTCGCTAAGGACAAGGTAACGAACATCGGCTTGGCGTGGCAGAAGCCTTGGT
ACGAATTCGCACCGACGCCTGACCTGATCCAAGTGTTCGACGTGCTAGCGGCCATCGCCA
AGATTCTCAAGCAAGCGGCCAAGGCGCGCAAGGATAACCATGACGTGGAAGTCAAGGGTG
AAGGCCTGCTGGAACAACTGGCTGGCATGACCACTGCGGCATAACGCCGGCACTCCCGAC
CCAAATATGGGCGCCGCAATTCCGCTGGTGCCCGAACCTCTGAGGTGCATATGCAACTCC
CAAACAACGCTATGGCCCTTGTCGCTGTGCTGGCGCTCGCTGGCTGCTCTATCAATGTCG
TAGTGGCACCGCACGCCGTGTTCGCCGTAGACAGCGCGATGGACAGCCCTGTGGACGCAT
CCGAAGGCATGAACAACGGCCACATGGGTGACAAACAATGAGCCGGGCGCTCAAGGCTGC
TGATCTGGTGCGGGTGAGCCTGCCGTCGGCGCGCAGTGCGAATGCCCGTGGGCTGCCGTT
CGGTGCCGTCGTGGAACTGGAAGGCGAGCAGGTGCTATACGGTGAGCTGCACGGCCCGCT
GTTCCGCATCAAGGCTCAAGGGAACATCCCGGCGTACCTCTTGCCAGTGTATGGTTTGAA
GCACGCCAAACAAGCAATGCGTAAGCGTGCGAGCTACGCCAACCGCCGAGGTTAATGATG
GACAACTTCACGATGCACGCCGCCGCAGCCATGCTGGTGGACGCCTTCAAGGCCGTGACT
CCTGAGTACCTGAGCAGGCTGGAGGTGCAGCCTGACATCCGTAACGACGACGACACGCAT
TGGGTGGCGCCGGAAGACTACGAGACCCTGTGCGACGACTGGGCTGTTGGTCTGCTGATC
ATCAGCAACCAGCACATCGACACGTCGAGCTATGGCGCGGCTGGCAACATCGCCTTCCGT
ACCATGCACGACCTCGGGCATCTGGTGCATGGCCTGACGTTCTCGCTGGGTCATGAAGTC
TCGAGGTCCATGGTGCAGTGTCCGGGGATCCGCAAGCACATTCACAAGACGGAGTTCAGT
GAGGTGTGCCGCGATCTGTTCCTGGCCGAAACGGTGATGATGTCGCTGTACAACGAGCGG
TATGGTGCCTTCCCTTGGGACCAGACCGAGTTTGTGATGCGCTGTTGCGCTGAGCAGTGA
CGAAGTAATGCACATCTGCGGGTGTGCATTGCTGGTTTACTCCTCCAGGCATGGTCGGAA
CTGATAGAAGCTATGACAATACAAGTGCCACGACTGGAACACCTGAGCGATGGCCTCGGC
CCTTACCGCTGGCTGCATGGTGCCCGAGGTGGGCAGCTTGACTGGGCACAGCGCGATGCT
CTGAGCATCACGGCTAATACTGGTAGCTCGCCGATGATTGGCGTTGACTACCCTTCACAC
TTCAAGTTCGGCTTTGCCAATCGTCGGCAGGCTGAGAGCTGGTGGTGTGAGAACGCACGG
CGTGAAGCGGCTGCCAAAGGCTACGCTCTAGCTGTGTACGAAGTGCCCGGCGCTGCTGTG
ATTCACGGTAACGCGCAGGTGGCATTCGGTCCAGAGGTCGCCAAGCTGTTGGCTCATATC
CCTGCATCCGAGTGGGTGACAGGCAACCTCAATATTCCCTTGGCTGCGGAGGCCGAGCAA
TGCGTACAGCAAACGGCGGGCTCGCCGCAGTCGGAACAGAACTTTGCACACGAACCACAA
CCAAAGCCGAGCGGGATGCCATCCTACTTCGTGAGTCAAGGCTCATTGCGTGGCGTAAGT
CGCTGCCAAGGAAACGTAAGCCAGATTCCCAAAGACCCGGCCCGGTTACGTGGATTCAGT
TTTGATGCTGTAGTGTTCATGGGAGACACGTTCGATCTGGACGTGGAACTCGCCCAGAAA
TCCTTCAACGAACTCAGGAGTTTGTATGGTGGAAAGACTAACTCGCTTCGATTGTGACGA
TGCAGCAATGGCCTAGGCTTGTGCCTTTGCCTGCGATGCACTGGCACACGCTGATCCTCT
GGACGTAGTGCTTGAGCCTACGCCGGAAGACCTGCTGTAACGCGAAGGCTTCGATGCCTA
CACTGGTGTGGATACCTCTCTGTCTGATGCCCACGAATCCGTCATGCTTGAGTATGGCTT
TGCTCTGTTGGAGGACGAGGACGCATAAGGGCCTCACTCACGACCCAAATAAGGACGCCG
CAATTATGGCAACGCTCAATGAATTCCGTAGCATGACTGGCCGCCAGGTAATGCACTCTC
GCCGTGGTAGCGCGGTGGGTGTAGTAGTTGGCATCGAAGCTAACTTCGTGATCGTGATCT
GGGACGGCCAGACCTTCCGCACCCGCAACAACCCTGTGTACCTGACCATTCTGCCCGAGG
ACAAGACTGTGAAAGCTGCTGACATCAACGACGTGAACAACGTGCAAAAGAACATCCTGT
CCGACCTGCTGCGCCAAGTGCGCGAGACTCCAGTAGGCCAGACCGTTGCCGGCCGTGGCA
CCGACGCCGCCGCCCGCACCATCGGCGTAGCCAACAACGTGTACAGCTTCGATGCGCTGG
CCTTGCGTGAAGCTGGCCTGATCGTTTCACAGAAGGACAATAAGGAATTCGCCTAGTGGT
TCCTGACCAGCATCGGGCACGCCAAGGCAGTCGAGCTGACCAACGTGCGCAGCAAGCCCG
AGAAGTGGCTGGTCTGGTGCCCGACTTCTGCCAAGACTCCGCAGGTTGTTCACTCCTGTG

AGCAAGAAGCGAGGGCCGTTGCTGAGAGCATGGCGCGCAACTATCCGGGCCAGTTGATCC
ACGCTGTGCAGGTACATGCGGGCTTCAAGCTCAAGGCCGAGAAGGTCGAAGTGAAGAAGA
TCGAAACCAAGCACACCATGGTGCAGGTGTAACATGGCGCGCATCACTCTGATCATGAGC
CAGCCTTCCCGTGGTGATACACTGCGGGCGCTCACGAAACAAGGCAAGCTTCCCGGCGCC
GACTTTGTTGGCCCGACGTTTGAAGATGTTAAGCTCGTCATCAAGGGCGGGTTCGCTCAT
ATCACTGGGGACAAGGTAGCGTACAGCTAACCGGCCCACGTAATCAACCGTGTCAAATTC
GAGGCTTAACCCATTCTGATCAAGAACGCCGCTTACCTGATTGGTCCGTCCAAGTACATC
GCCGACGAGGCTGCTGAGCTGCACGCTTCGGCCCGTGAGTAGGTGCTGTCCCTGCATGGC
CGCGACATCGACAAGGCGCGCAGCCAGTACAACGCCAAGGTCGCGAAGGTGCATGGCAAA
CTCAAGGGCCGCAGCGAGCGTGCCGTGGTTCTGTTCACCCGCCAGCAGGCCGCCGAGCAG
CGCCGCGTAAAGGCTGCCAAGTTGCTGGACGCGCTCAAGTGATCCGCACCGTGACCGGGC
GCATGCCAAGCGAGCCTGAGCTGCCTCGGTTGCGGGGCCGCAGCATCGACTGGATCGTCA
CTGACGACATGATCAACGTGGACTTCGCGGAGCTTGAGCGCCGCGTTGCCGCCTCTCCCT
ACTTCGCCCATCCATACGGAAGCAAACCTATGAACCGCCAGAACGTAATCGACCTGAACA
AACGCTCCGCTGTAACCGGCGACATCCTGTCCCTTCTGCGCCGTGCGCCGGGCCTCGACC
TACTGGACTACAACGAAGGTCTGGATCAAGACAAGCGCACCGTGCCAGCGGGTAGCGATG
GTCAAGAGCTGGGCCGACTGGTAAGCGATGGCTTCCAGTTCAGTGGCTTCAGTATCAACG
CCGAGCTGTTCATGACCAAGCTGCGCGGCGTCTTCTACCCGGTGCCGAGTTATAGTGGCT
AAGGTCTTCACGATATGCATGGCTGGCAACCGTGCATACATCAGCAACGAAGACCATAGC
CAGCTACGCTTTAAGCGCAAGCACGCCAAGGCTATGCGCAAGATGTTGAAGCACTGGCTT
CGATGACACTGAGTTCGTAATGCACATCTGCGGGGTGTGCATTGCGGGCAATTGTGCCCA
TCCTAAATCCCTGTTGGAGATACACCATGTCCAACGTACAAGCACTGATCACCGTAACCA
ACGGTAACGACGCCAAAGAAGACAAGCTGGTTGAGAAGGTGAACAACCAGATCGGCAAGG
CCACCGAAGCAACCGTGTCGCTGGACAAGTACCTGCAAGAGCGCGCCGCCGAAGCTGCGC
GCCTGAACATCGAAGGCGGCACCGAAGGTTCGTGCGTGTTCGGTCGTGCTGCCACTGCGC
AAGAGCTGACCGGCAAGGTCATCGCCGTGGTCGAACTGGAATGCGTCGCCAAGCTGTTGA
AAGTCATGGCCGGTGCAGGCGCAGCGTTGAAGATTTACGACGTTGCACCGAACAAGTTCA
ACCCTGTCGGCGAGCAGGTCGAGGGCACTCCAGACCCAATTCAAGGCTCCGAAGTTTCCG
ACGCTCCGGTTGGCGAAGTCGTGGACTTGCCGTTCGATGTTAACAGCATCCTCGGCGGCT
CCGACGCTCACGCCGCAGTCGCTGGCCTGTAATGCTTGACCCTCGGCTGTTTGTGCCGAC
CGATGACCTCTTGCATGCGGACTTGATACCGCATCTTGAGGCGTTGCGGACAATGGATGA
TGGTGCAGCACGGCGAGGCGGTGTTGACTTCCGGGACTTCCACGATATGGATTCCCGGTT
CAAGGCCGCAGCCGATCTGATCTGGCGTGACAGCGACAGCGTGTTCCCGATGCCGTTCCT
GTCTCCCGAGACTTGCGAGGCGTTGGTTCGTGCGTCGTCGCTGTTCTGTTACGAGAAGAA
GGCCAGCGAAGAACCAGACTTCCAAATCCCCGAGGTAGTTCTAAAGCATGAATGCTTAGG
GCGGCACGCCTCGTTGGCGGTTCTGTTTGATCAGGTGATGATTCCGGTTGCCGAGCTGCT
GTATGGCATCGAGCCGGAGGTTACGCGGAGCATCCAGCTTGCACAATATCGACCAACTGA
AACAGGCTATGGTAACTGGCATGTCGATCTGGACTCTGACTTTACTGTGGTTGTGGCCCT
TAGCAATGAGCATGCTGGAGGCGGCACAGAGGTTAAGGCACACGGCTTCTCGGAGGCTTT
CGAGGTTCCTCAGTTGCCTACCGGATCGGCTATGATCTTTCCGGGTCAGCTGTACCTTCA
CCGAGGCATGCCGGTTACTGAGGGCGTGCGCAACTTGCTTGTGTTCTGGACTGAAGGGAA
ATGAGCAATGAGTGGTTGGCCTTAGCGCAGCATTTGCCGGTGGGTGGTAACGCCAAGCAT
CCGCATTACTGTGGTGATGGCGGACCGCTGGTTGTGTTCCACAACGATACGAAATGGTCG
GCGTACTGCCACCGATGAGGGAACTTCGCGCCAGTATTCAAACCATTGCCGACGTTGCAG
GAACGCTTGGCGGCAGTTGAACGACAGCGGCTTCAAGAGGAAGCTGTTGCAACTGACTCC
CGGCCACCGATGCCAGCGGTGTATGACATGGCCGCGTGACCGCTGGAGGCACGGGTATGG
TTATTCAAGGCTGGGTTATCCCACGCCGAGGTATCGCAGAGTGGCGCCTACTATCATCCG
CCGACCCGGCGTGTGGTGTTCCCGGTATTGGAAGATGGGCGCTTGACTTTCTGGCATGCC
CGCCGCATACTCGGCACGACGGCAGCAAAGTATCTCAGCCAGCCGGGCGGGCGCGAGTGT
ACACTCCCGCTGTTCGGCGGTGGATATGTGATTGCCTTAGTCGAGGCCTTGCTGTCTGCG
TACAAGATCGGACCGGCGGGTTGCAAATCTTTGTGCCTTATGGGTGGGAAACTGCTGCCG
ATAGCCCGGGCGTGGTTGATAGTTAACCCGCAGTACACGGTAGCGATCTGGCTTGACCCT
GACGGGCCGGGCCGCAGTTCTGCAGCTGAAATCAAGAACCAACTATTGATGTTCGGGATT
GAATCCCAAGACATCCTAACGGAACGAGACCCTAAGTTCCACAGCAAGCAGGAGATTCGA
AATGTCTTTGGACATATGACTATTACAAATGATGCGAAGTCGAAAGGATTACGACAAGCT
GCACCGGGTAATGAACTCGCGTGCATTGGATCAACTGACCGCCACGATCATCACTGACTT
CGGCAGTTACTTCAAGGAGGTTCCCGAGTGTGATCTGATCCCGGTTAGCCGGGAGTTCCA

CACGTTCTTCCACACGATCTGTCACCCGCAGTTAAGCCCGGAGCAGGCGGCGGCGTACCG
CCCTGTGTTCCAGCAGATCGAGAAGCCAATCGACGACACTGCGAAGTCAATGATCATGGG
CCGGTTGTTGGAGAACAACTGGGCGCAGGACTTGCAGGACATCGCCGAGCGCTTTGCCCG
TGGTGAAGAAATCAACGTGCGCAAGCTGACGGCTAACACGCTGTCGAAGTTTGAGCAGGA
CATTGAGCGTAAGGTGAAGCTGCCTTTCGTGGCGAGGGACGAGTCGTTGTTTGACGAGGA
CGTTCGCAACGACGGGTTCAAGTGGCGCTGGACTCGCTTGGCCGAGACGATGCGGCCACT
GCGCGGCGGCGAGTTCGGCATCTTGGCGGGGCGACCTGACAAGGGCAAGACGACTGCGCT
CTCTGACCTCTGCACTTACTGGGCACGGCAGTGCAAGCTGGTCTTCCCGGACAACCCGGA
GAAGCACCAGATTCTGTGGCTCAATAACGAAGGGCCGGGCAAGCGCATCCTCAAGCGGAT
GATCCAGTCTGCGTTTGGTTGCGGTACGGCTGAGTTGATCAAGAAGCAAACGGCTGGCAC
GTTGTGGGATGAATACGAGGTAGCGATTGGCGGTGATCGGACTACGATCAAGGTCATCGA
CATCCACGGATTCAAGGCGTGCCAGGTAGAGGAAATCTTCAAGCAGCATCGACCCGGCTT
GGTGACCTTCGACATGGTGGACAACGTGAGGTTCGATGGTGAGCTTGCGAACGGTGGCAC
GAGAACAGATCAGGTGTTGGAGGCTATGTATCAATGGGCGCGCGACTGCGCTGTGCGATT
CGATTGCCCGGTACTCGCTACGTCGCAGATCAGTGCGGACGGTGACGGTGAACCGTATCC
AACTCTACCGATGCTTAAGGACAGCAAGACTGGCAAACAAGGTGCAGCCGATTTTATTAT
CACATCCGGAACGTCTAACGATCCTTCGGTGGAAGCGTTGCGTTTCATTGGGACGACCAA
GAACAAGCTACGTCTCGAAGGCCAGCCACAAAGTCCGCGTGCGCAAATGGTGATGGACGC
AACAGGAGGACGCTATGTCCCGCAATGAATACCATCCGCCGCGCTTCCGTGCAATTGAAC
AGGCCGTGTTTGAAGAGATTCGCCACGTCTGGCCAGAGGCGGGTGTGCGTGCCAACGCTG
ACATGACAAGGCCCGGATGGACTGGCATGCGTGTGCGTGTCTGCATCTTCCGGCACAACA
ACGAGGTGTTCACTGGGAGCGTGTTCCCGGAGGAACAGTTCTCGCCACCTGTCCATGTGG
TCGCCAGCACGATGCGGCGCTCGTTCACTGACGGCGTACACAAAGCGCGCACGGAATACG
AAGATGACGTACGCAATCTGCGACTATGAAACGTCGATCAAGTCCAGCTTCAAGCGGAAG
GCGAATCCCTTCCATGAGAACTGGACGGTGTGGGTAGGTGAACATCAGCACGGCAAAGAG
CCTGCGTTCATCAAGCACGATGACAAGGATCGCAGCAAGGGATGGCTGGCGCGGTTCTTT
GAACGCAACCCAGAGTGCAAAGTATTCGTAGGCTTCAACTTCAAGTTCGACGTGCTGCAC
GTGATCGCCGATGAACCGGCTGGTCTGGATGCGTGGATGGACTTCATCAACCGGGGCGGG
CAAGTGTGGGACTGTCAGCTCGCCGAGTACATTTTGTCCGGTCAAGACCCGTCGGCTCAC
ATGCTTTCGCTTGACGAGGTGTGCGTGGGTTACGGCGGGCACACGAAACCCGATGCGGTC
AAGGACTTGTGGCAGGCGGGCGTCGAGACAAGCGACATTCCCGATGACCTGATGATGGAG
TAGCTGCCGGGCGACATCGAGAACACCCGCAAGAAATTCCTCGGCCAAGTGGTCAAGGCT
CAGCAGCTCGGGCAATCCCGAAGCGTCATGCTGATCCTGGGCGCGCTGGTCTTCACGATT
GAATCCGAACGCAACGGCATGAAGGCCGACTACGAGTTGGGCTTGAAGCTGGCGGCAGAG
CTGGTAGAAGAACTGGCCGCGTTGATGCTGCGGCTTGACGAGTGCCTGCCGGGCGACCTG
CCGTTTGAATTCAACTGGGGCATTCGCATCCAGTTGTCGGCGATTTTGTTTGGTGGGGCT
GTTAACTACAAATCCCGCGAGGCGATTCTCGATGAAGACTTCAAGCAGACTTATGCACAG
AAGACTGAGTCTCACTACCTCACGTCGGACGGCAGCACTATCGGACAGGACGAATACAAC
GACGCTCTGCTCAGCGGTAAAGCTGTGCCGACACTTGTGTACTTTAGCAGCGGCAAGAAC
GATGGGGAACCGAAGACGAAACAGGTTAAGGTTAACGACCTGTCGAAACAGAAGTCCCGT
CTTGAAGACCACACGTACATCTTCCCCGGCTACACCAAGCCGAGCGACAAGTGGAAGACG
GCCACTCCGGGAGTGTACCAAACTGGCTCTGCCATCATCGAATCTCTCGGCAGCCGGGAC
ATCCCATTCCTCAAGAACTTGGCACGCCGTGCTAAACTCGACAAGGACCTAGGGACGTAC
TTCATCAAGACGAACGACAAGGGTGAGCAGACAGGCATGCGCACGCTGGTTGATCCTGAT
GGTCTGATCCACCACTTGATCAACATGACCAGCACGTTACCGGGCCGGTTCAGTTCCGGT
AACCCGAACATGCAGAACTTGCCGAAGGCAGGTGAAGGCAAGTCGAAGGCCAAGGAAATG
TTCGTGTCCCGGTTCGGGCCGGAAGGTGTGATCATTCAGTCCGACTTCTCGTCTCTGGAA
ATCTACATCCAAGCGATCCTCACGGGTTCCAAGCAGATGATCACGGACTTGCTGGCCGGT
CTTGACATGCTCGTTGCACGGGTTGCGACGACTGCCGGGATCCACTACGAGGGAGCGTAT
CGACTGTGCCACACGAAGCTGGCGGGCGGTGCGGCTGAGTTCCCTGAGTGGGTAGGCAAA
CGGACGAAGGCTAAGGTCTTCTCGTTCCAGCGTGCTTACGGCGCAGGCGCAGCCAAGATC
AGCGAATCGCCTGGCATCCCAATCGATGAGGTGTACGCACTGATCGAAGCGGAGAACACG
CGCTGCCCTGAGATTGAACCGTTCTATGCTGACCTGATGGAGAAGGTCACGAACAGCAAG
CGCGGCTGCCGCAAGGTAGTACCGCACCCGGACTTCCCGGCGAAGCAGGTTGCCTTGGGC
ACCGGCTCGTATCACACACCAGACGGTAAGCTCTACACGTACCTTGAGCAGTGCGCTCCG
AAGTTCGTGGTGGAGAAGACTGGTCAGTGGTCGTCGTTCTCGCCAACGGAAATTAAGAAC
TACATCGTGCAGGGCACCGGCGCTGAGTGGGCGAAGGCAGCATGCTGGTTGTTGGTTCGC

ATGTTCTACCCCCGCAAGAACTGGGGCGGGCTGGGCCTTCTGATCAATCAGGCACACGAC
GCCGCTTATGGTGACACGCATCCGACTGTCAAGAAGGAAGTGGCTGCTGTGATCCATGCT
TGCATGGAAGGTGCATCGCCATTCATGGCGGCGTACTTCAATTGGGACATCCCACTGCCA
GTACCAAGCGACACAATGTGGGGCGCGAGCATGGCAGAAGACAACGCCGTCGAAGGCATC
GACGAGCTGGCTCAACCGTTCCGCGAAGAAATCAAATCGCAATACATCAACCAATATCTC
TGAGAGGTTACACCATGTCCGAAGCAATCTTTGACCAAGCAGCAATCGCAGCAGCCATCG
CAGCAGCAGCGGCGCAAGAACCTGACATGAAGGAATCGCAGAAAGGCGGCGGCGGCTGGG
TTCCACCAGCAGCTGGCGGCTGCATCGTAATGCTGGTCGGCTACATCGAAACCGGCATCC
AGATCGCGCCCCCGAGCAACGTCGATCCGGTCAAGTTCCCCGGTGGCCCGGAAGAACAAG
TCGATTTGATCTTCGAAATCAGCGGCAAGGGACACGAACCGAAGGAGCTGGAGATCGGCA
CCAAGGTTCCTGAGCGCTTGACCGTAACGCGCAAGAAGTAGTTGAACGAGAAGGTGTGGT
ACTACAAAATCTTCAACACGCTGTACTATGACAAGACCGCGACGCACTTCGCCCAACTGC
TGGGCAAGCACTTCATCGCCACCATCATCCACACGCCGAACAAGAAGAACCTGCAGTCGC
CGTATGCTGGCTTCAAGTGCAAGACTGATGGCTACACCTTCAAGGCTCCGACCTACAACC
CGAACCCGATGGACATGACCGAGATTGCCATCGTACCGAAACCGGCGGTGCTGACTCTGC
TCAAGCTGTTCCTGTGGACTCACCCGTCCAAGCCGATGAGGGACTCGCTGTTCATCGAAG
GCAAGTACGACGACCGCGAGGACGAGAAGACTGGCGTGGTTACTCCGGGCCGCAGCAAGA
ACGTGGTGCAGAACAAGATTCGCGGCGCCAAGAACTTTGTAGGCTCGCCGATCTTCGACA
TCGTTAGCGGTCTGGACATCGCCGAAATCGAAGTGGCGGTGGTAGACAATACCCCGGCGC
CGGAGTCGGCTGCTGTTCCGTCTGACGACGCTGCCGCTGCTGCGCTGGCGGCGCTCTAAT
GGATGGGCTTGAAGCCGCAATAGCTGCTGCTGGCGACGAGCCTATGCCATGCTCGGTGAT
CCCGGACGTTGTGCCGGGATGCCTTGTGCAGATTGACGGCCACTACGCTGCGTACTTCAT
GGCGGGCCACGACGATACAGATAGCGGTACGGCGCCGCCTAACCTGATGCAGCGTTTAAG
CACCATCAAGGAAATGACCGGGGCGAGCGGAGTGGTTGTCCACCTGTCTGCCGACGGTTG
TACCAAGGCCGACCGCTTCCTGATTGCCACCGTGCAGCCGTATCAGGAACAGCGGGCTGG
CTCTGCCCGTCTTAAGAACTCGCGCAGCTTGCGCGAGTTCATGGAGAGCTAGAAAGGCCC
GGCGTTCACTCCGAAGATTTGGGGCGACCGCGAGGCCGATGACGGCATGGCTTACATGCA
GCAGAAGTGGGTAGCTGAAGGCAAAGGCGAGCTGATAGTGACAGCTACCCGTGACAAGGA
CATGCGACAGTATCCCGGCTGGCACATGGACTGGATGACTTACCAACTCACCTACGGTCC
CCAAGGGGCTTACGAGGTGATCGGCGCGAACGACTTGATCTATGGGCACAAGTGGCTGTG
GCTGCAAACCCTGCAAGGCGACAACGCGGATCACATCAAGGGATTGCCGTTTCACCTCAA
GCCTAACGGCAAGTGGGAATCGGTCGGCGCCGCGCGAGCGTTGGTGCGACTGGCGGGTAT
CGACTACGACGCCGATGTGTTCGATGAAGTGGTGCGGTTATACGACACGCACTACACACC
GACGGCGGGGCTGTTGCGACTGGCTGAGACGTTGCTGCTGGTATGGCTGCGGCGTGATCA
GAAGGCCACGTTCACCGACATGGCGAGCTGGTTGCAACTACGGGATGACCAGAAGGCTCG
CTTGACGGCTGCGCTGCTGGAACATCAGACACGGATGCAGGATATGAAAGCGGAGGTTAC
GCGAATTGAAGCTAACCAACAGTCAAATTAAACCAGTGCGGGACAAGATTCGTGCAGACC
AGAAAGGCTGATCTGCGCTATGCAATCTGCCTGTTATAGTTGCTGATGCTGTCCTCGATC
ATTGCCACTCTCACGGCTTTATTCGTGGTACGCTGCTTCGAAGTTGTATTAGCCTTCTTG
GAGTGCTTGAGAACAATCGCAAGCGCTTTGGGTTAGGCGACGACGCAGCATTCGCAGGCT
TCATGCAAGGCGCGCTGGCGTACATCCAGAAGCACGACACACCACAAACCCGGATGTTCC
ATCCGTCATTCAAGACCGAAGACGAGTAGCGCGTAGCGCGAAACCCCAAGGCCCGTAAGG
CACGCGCCAATGCGAGCAAGCAGAAGAAATAATGCGCGGTTCCCTGATGCGATTGTTCAG
CAAGAACAAGCACACCGAAATTCTCGCAGCCTTTGGTTGCAACGTGGGCGCGGCTGGCTA
GCACAACCGCGTGCTGGGGCCAGACAGGCCGTACAAGGTGACTCGTCAGAACGTCACATA
CTGGCGTGCGATGTTCATCGACAACAGCGACAACATGGCGAAGACCGTCCGCGAGCTGCG
CGAGAAGCGTGTGCGTCGCAAGCCCGATCCGCTGTACGCTGATCAAGTCTTCGACAGCAA
GCTCGTGTACAAGTCGATTCTGGTTATCCCGGATCAGCATGGGCCGTACCATCACCGAGA
TGCGCTGGCGTTCATGCAAGCCTTGGCCGATAAGTTCCAGCCGGAACTGACCGTCAACCT
CGGCGACGAGCTGGACTATCATGCGATGACGTTCCACGACAGTGACCCAAATCTGGACTG
CGCAGGCGTTGAACTGGAGAAGGGCAAGAAGTGGTTGCGTAAGCTGGCGAAGCTGTTCCC
GGCCCAACTGGTTGGCGATTCCAACCACGGTTCCATGGCGTTCCGTAAGACCAAGCACCA
CGGCCTGCCGGTGCAGTTGATTCGTAGCTACCGGGAACTTGTGTTCCCGAACGGCGCCGG
TGACGACTGGCACTGCGCCGAGAGCTGGCGCGTCAAGACTCCGCTGGGCGACGTGATGTT
CAAGCATCAACCAAGCGGCAGCATTTTGGTAGACGCGGCGCACAACTCCTTCAACTTGGT
GGTGGGCCACCACCACGGTAACTACTTGGACGAGTACACGGCAAGCTGCACGCACCTCTA
CTACGGGATGTACAGCGGTTGCCTGATCGACAAGGTCGCCTATGCTTTCGCCTACGGCAA

ACACTCGCTGCGCAAAACTGTTATTGGGTGCAGCGTTATCATTGATGGTCGCCCCACTTT
GGTTCCGATGGTTCTCAACAAGAAGGGCCGCTGGAACGGGAGCTTGTAATATGAGCCTTT
CTGATATGGCAACTGACTACCCTTGAGCAATCTGCCTCGGCCCGTGTAACGGACACACCG
TCACTGCCGACCCAAAAACAGTCGCCGTAAATGAGCGCGGTGCCGGGTTGAAATACGACG
GTGGCAAAGGGCGCTGGTCGCTGTTGATGGGTCGCATGTCGAAGGCGCTGGCCGGTGCGG
TTGCCGTGCAGACCTTCGGCGCCAAGAAGTACGCCGCGCAAAGTTGGGAGCAGGTGGAGA
ACAACAAGGAACGCTACCGCGACGCCTTGTATCGCCACCTAAACGCCATCGAAGCCGGTG
AGGTACGTGACCCGGAGAGCGGCCTGCCGCACTGGGATCACGTAGCCTTCAACCCACTAT
TCCTGTCCGAACTGGAGAAAGCTCAATGAGCAATCCGCACAGCGTAACTGAAATGGTGTT
CAACGGCTTTAGCGACAATCTGCAAGCAGCCATCGACACCGGCAACGAGCCGTGGGCACG
GGTGATCCTCCGTGCCGCTGTTGACAGCACCATCACCAAGGTTCACTACGCAGAACTGCG
TCAGGACTTTGAAACCGCATTCGGCGATCTGGAGGACGAATAACATGGGTATGCCTGACC
GCAAGATTCTCGTACAGAAGCCGAAAGAAACCCGCGACCTTAGCGCTAAGGCGCTGGACG
CGCTGCCGGAATTCGACTCCGGCAAGTGGGTTTTCCAGCCGAAGTTCGACGGTTCCTCGC
TGATCATCATGGTCGGCATCAACGTGGTGCGCATCTTCTCCCGTGAGGGCAAGGAATGCG
TGAGCATGCCGCCCATCGCCGAACACTTCCGCATCAACTCGGTGCCGAACATGGTTTACT
TCGGCGAGGCGTATCACCCGAACATCCTGTTCAAGGATATTTCCGGCATGTTCCGTCGGC
AGTCACCGCGGTACGAGCAGGAAGCGTGGATTTTCGATGCAGTGACCCTGCAAGAGTTCC
GCACCGGCGAGGCCAAGCGCCCGTATGCAATGCGTATGAAAGCGTTGTCGCATGTGGCGA
ACCACGAGTACGGCGTTATCCCGCCGATCACCCGTGGCAGCGTGGTCGAAGTGCAGGCCG
ACATCGACAGCATCCGCGAGTACGGCTACAAGCTGGAGCTGGACGGCTTCATGGCGAAGC
AAGCGGACGGCGAGTGGACTGCCGGTGATGACCGCGAAGGCCGCACCATCAAGCTGAAAG
AGCACAACAGCGTTGACCTTGAAGTCGTCGGCATCATTGAAGGCGAAGGCAAGTTCAAGG
ACATGGTGGGGGCCATCGAAGTCCTGTGGCGCGGCAAACATGAAGTGGTCAGCAGTGGCC
GAGTGACGAACCCCGAGCGATTGGAGTTCTTCAACGAGCCGGGCAAGCTGATCGCCAAGA
TCGTGGAAGTACATGCGTTGGGCATTACGCCTGACGGTAAACTGCGGGAACCGCGCTACC
AGCGCATCCGCGATGACAAGACAGAACCGAGCGAGTAATGAATCAGGCGGACTTAGTACC
GGAAATGGCAGACGGTGGGCGTCGGGCCGCGATAGCGAGGTTTCAAATCGCCGAGGCGGC
GAATGACGCAGCGAGCAATCCGTATGCCGCCGCGATATTCCGCCGCTTCATCAAACCTCT
GTCAGAAGGTCTGGACATTTATCTGCACAAGGCTGTGCGAGGAGTGGCAGCAAAGTCGAA
AGTGCTGCTGCGCGGTCAGGATACGCTGGCGTTGGCGTACATCACGATCCGTGGTGTGAT
CAACGCAAGCCTCTCCGAAGGGGAGGCCGTATGTGCTGGTGTGGCTATGGGCATTGGCCG
CACCGTTTACAGCGAGGTTGCACTCAGGCAATTTGAAGACCTGAACCCCGAGCTGTATTA
CACGTTGGTGCAAGACCTTCACCGCCGCATGACCAAGACCGAGCGACACAGGTTCAACGT
GATGCGATCCAGTGCCGAGAAGGACGGGACGCCCCTACCGATTTGGGAGCCTGCGTAGAA
GACGGACGTGGGCACCTTGTTGCTGGGCGCGTCTCTGGACATCGGGCTGATCGAGACATT
CGATCTAAGGTTGAAAGGGAAGACGACAAAGCACCTCCGGCTGCATCCTGACCTGCTGGC
GCTGGTCGAGCAGATCAAGGGATTCGTGGCTGGGGCGTCGCCGTTCAACCTGCCATGCGT
GGAGCCGCCGAAACCATGGTTGACACCGACCGATGGCGGCTGGCACACGATGGCGATGCG
CCGAACTCTGCCGTGCTTGGTGCGAGGGCAAGCAACAGCGACCATCGAGGACGTTGGGCC
GCGAGTGCTTAAAGCGCTGAACAAGGTGCAGAGCGTGGCGTGGGAAGTTAACGGGCGGAT
TCTGGAGGTTGCAGAGTTCGCAAGGGAGCATTTCGACGTACAGGATGTGCTGGTTAGCGA
TAAGCGCGTCGGCACGCCGGACAAGCCTCTGTTTATGCAGGCTGATCCTGTCCTCAAAAT
CGTTGACATTAACGAGTTTGAGCTTGCACAAATCACCGAGTTGCGACAGGAGTAACGGGA
ATGGCACACACAACAGAAGGTGCAGGGAGCGCATGCGGGCAGCACGAACGAAGCTATCGG
TGTGGCGAACAAGTACAAGGGGAAGCCCCTCCGGTTTGTCTACTCTGTGGACTATCGGAG
CCGATTCTATGCCTCTGGACAGGGGCCCTCCCCACAAGGTAACGACCTTTCAGAAGCGCT
AATCCACTTTGCAAGGTCTGCACCTATCAGAACTTCCGCTGGCCTATTCTGGTTCCGGGT
AGGCGGGGCGAACAAGTGGGCGGGTAACAAACTCGATAAGCAGCCGCTCGATGTCCGGGC
GCAATGGGTGATAGACAATGCCGATTTTATCTGCCGCATCGCAGAAGACCCGATCAGCCA
CCGAGAATGGACAGACGCTCATGTACCTTTCCAGTTCCTTGCGTGGTGCTTTGAGTACGC
GGCTTGGGTGCGGAATCCTGGATCGTTTCGCACTCGAATCCCACTTGGACAAGACGCCGC
TTGTAATGGTCTTCAACTCTTCTCAGCGATGCTACGAGATAGGGTGGGTGGGCGAGCAAC
GAACCTCATTCCCGACCGAGTGCAGCACGATATTTATGGACTCGTTGCCACCGCAACTGC
TGGAATCGTGCAGTCAGATATTGACGAATGCGAGATTGCCAAGCGCTGGAAGTCACATGA
AATTTCATGGTCGCTGGTCAAACGTAGTGTTATGACCCTGCCCTACAGCTCCACACGGCA
CTCCTGCCGGGACTTCATCATGAAGGAGTACATGGACAAGGGCAGCGCGCCAGAGTTCGA

CAAGCGCGAGAATGAGCCTGCTGCGCGGTGGCTCTCGTACCGGGTGTGGGATGGCATCTG
GAAGGTGGTCGTCAAGGGACGGCAGGCGATGGAGTGGTTACAGGCAGCATCGGCCATTAT
GTGCGCCGGGAACGCCCCACAAATCGAATGGAGGACCCCTGCTGGCTTCCTTGTACGTCA
GCGCTACCACGCTCGGGAAATGCTCAGGGTGAGCTGTCACAGCCTCTCAGGCAAACGTAT
CCCCATCAACGTCTAGACCTTCAAGGACGAGGGAGATCCACGGCGACACCGGAACGGCAT
CGCGCCGAACTTCGTGCATAGCTGCGACGCGGCGCACCTCACGTTCTTCACGAATGACTG
GGAGGACGAGGATGGTGGCGATCTGGCATTAGTCCACGACGACTATGGAGCCTTGGCCGA
TAAGATGCCCAAACTGCTCAGAATCCTGCGTAGGTCGTTTGTCGCCATGTACCTGTACCA
TGACCCTCTGAAAGCGCTGGCTGCGCAGGTGGAGGGGTTGCCGGAGCTTCCAGAGGCCGG
TGACTTGAACTTGGAGGACGTAACCCGGTCGGTTTACTTCTTCTGCTAATTGGGCCACTA
TAGCAACAGGAAACCTTTATGATCCAAAAAGCATTGCTACGATTCGCCCGATTCATTTTG
GGCACCAAACTCGCGGTTGTCAAGAACACGATTCGCCAGCAAGAACAAGTGATAGAGTCC
ACCACACAAGAACTGGCGGCCTCTCTATCTCGACCACCGGATGTCAAATACATGGTATCC
AGACAGGTATTCAATAAGCATGTTAAAGGCAAGCTGGAACAACCACTATTAGTTAGTACC
AGCAGCTCTGAATATGCTGCTTACTGTTTGGGTATTCAAAGAGCATGCTCTGTTATGGAG
AAGGAGTTGGTGAATGATTAAGATCAAACATTGGCTTAACGGCAAGCCGTACGATGATGT
CAGTGATGATCCCCGTGATTATTACGACAGCATGTACTGCGCTGTTAACTTCTGCCGACA
GAGCTACGTTGAAGCCGGGCAAGAGCCAATCAGCACATCCGAGGGTGTTAGTCGATTGAT
AGCCTCTGTCCTGATCATGCTGCTGGGCGAGACGTTCGTCGCGTTCTTCGATGTTGCTGA
GTCGTGGTTCGTGCGCGACAAGTACCTGAGCGAAGAACTGTTTGGCCCGCGACCGGGCAA
GACGGTTGACCTCAAAGACTTCATTGCGGCCGGCGAAGCTGTTGGCCGCAAAGAGGACTG
CCGGTATTTTGAGTTCGGAACGGGAGCCAATCCCCGGCAGCAAGCACTGGCCCGAATGTG
TCGTCAATTCGATTTCCGGCTGGCCGCAATCACACTACAGAAGGATATGTGACATGGTTT
CTGGTAAAGCACTAGGCAAGATCGGCAAGATCATCAAGAAGGTTGACCCGCTGCGCGGCG
GTGACGCCATTCTGGAATCGGTGGGCTTGCCCACGCTGACCGGCCAAGGCGACCAGAACA
TTTGGGACATGGGCCAGACTGCCGCCAAGAACGCCGCGAAGCAACAAGCCGAGCAGGCAC
GACAACAGGCCGACGCACAACATCTCGCCGCCGAGCAACAAGCGGAAGCTGTTCGCCAGC
AAACCGCAGCAGATCAACAGTCAGCGAATCAGCAGATGCAATTCCAGATGCAGCAGCAGA
ACGATCAGGCCGCACAGTCCGCTCGTAACGCCGCGCTACAAGCGAGCCGCGATTCTGGCC
AGCCAATCGGCAGCCGAGGCCGCAGCCGAGGAAAGTACGCCGAGCGTGACCACTGCCACC
TGCAGGGACAGCGCATTGACCCGCCGTAAGAACAGCGCCAACGCAGCTGACTCTGCCAAG
ATCCCGAGCATCCGCATGTGAGTCTTGGGTTTCGCACAGCCGAATCCGAATGGCGGAACC
TGCACACCAAGCGGAGCGGCTTGCTGGACAGGGCTGAGCGGCTAGCAGAAATCACCATTC
CGAGCGTGTGCCCGGATGACCAGTACGACAGTGGGCGCGATGCGCTCACTAACGGCGTGA
CGTCACTGGGCGCGCAATGTGTCTCTAACCTGACCAACAAACTTATGCTGGCGATGTTCC
CGGCATCCAGCACGTTCTTCCGGCTGGCGATGTCTGAATCGGATGCGCAAGCATTCGTGG
CCGAGAACGACCTCACGATGGACTTGCTCACGGAAGTGCTGTCGCAGGGTGAGCGCGATG
CTTTGCTGCAACTGGAAATGACCGGTGGCCGCGAAGGTCTGTATGAGGACATGACGCTCT
TGCCGATTGTCGGTAACGTGCTGATGGTCAATCGCAGCAAGGACAAGCTGGAGTTCATCC
CGCTCAAGGAGTACGTGGTACGCCGGGCGCGCAACGCTGACGTGTTGACCGTGGTCATCA
AGCAAGAGCTGGCGCACTGCGAGCTGAGCGAAGACGTTCTCGATCGCATGGACCCATGGC
GCGAAGCCGATCCCGATCTCACTCGCCAGCTCTATACGTGGTGCCGCCGCATTAAGGGCA
AGTTCCGCATCACAATGCACGTTGACCAGATTGAACTGCCGTACAGCACGTTCGGTTCGA
CCTACGCTCTGGACGACTGCCCGCTGCACCCGCTGACTTGGCGACTCCCGCTCGGCCAGC
ATTACGGTGTGGGCCGAGTCGAGGATTACTTCTCGGACTTCGGTACGCATGAAGTGGTCA
GCGAGTCCATGAGCGATGGCGCGATCATGGCTTCGCAATTCCGCTGGCTCCAGAACCCTT
CCGGTATCACCCGGCCCCAGGACTTCCAGAACAGCAAGAACGGCGACGTTATCGCCGGCG
CAGCGAACGACCTGCAACTGATCTTCGCCAACGTGGGCCAGCAACTGAACACCGTGCTGG
CAATTAGCAGCACATTCCAGCAGCGACTGGGCCAAGGCTTCCTGCTCATCTCCGCTGTGA
CGCGGCAAGCTGAGCGTGTGACCGCCGAGGAAATCCGGCTGCAAGCGATTGAACTGGAAT
CGACGTTGGGCGGGTTTTACTTCCGAGTTGCCCGCGACATCCAAATCCCTCTAGCTAATT
GGCTGATGAAGAAAGCCAGCTTTAACATCAAGGGCACGAAACTGCGCCCGGTGGTGATCA
CTGGTCTGGACGCATTTACCCGTAACGCAGATCTGGAACGCTTGATGAACTTCCTTGCGA
ACGTGACTCAGTTGGATCACATCCAGCCGCAGACTCGCATGAAGCTACAAGACTCCAACA
TCATCGCCGACATCGCGGCCGCACTTGGCGTCAGCAAGAAACGGTACGTTGCATCGGACG
ATGAAATCCAGCAACGCATGCAACAACTGCAAGCGCAACAAGCACAAGGCGGGGATGGCG
CCGCTGATGATACCACTGCACAGGAATACGCACAATGATCTTCAATCTCTTTGGCCGTCC

CTACATGGAAGAAGCTGGCGCAGAAGGCGCACCGGCTGGTGGCGCAGTATCCGCCCCGGC
AGCGGCCCCGGCGAACGCAAGCACTCCAGACCCAAAACAGGCTGCTGCACCCGCGACCGA
ACCAGCCGCCGCAGCGATCGCCTACGAGTCCACCGGCAACAGCTACGCTGACTACGCTTT
GGGCGAGATCGCCGCGGCGGGTATCGGCCCGGATCCCCCTGCCGCGCTGGCCGCGCTGGA
AGGCAACTTCACCGTGCTGAAAAACGCACTGGCTGCCAAGGGCGTGCATGGCAGCGATCA
ACTGGTGGATATGCTGACCGGAGCTGCAACCGAGGCCGCTGACGCCGAGGAAGCGCTCAA
CCAGCAAGTGACCGCCTACGTTCATGCGCTGGCCGGTTCCAAAGAGCAGTGGGACACCGT
GATGGAGTGGGGCTGCGCCAACGCTGACGAGGCCGAGAAGGAAGCGCTGAACGATCTGTT
CGGTGATCCGAAGACCCACAAGATCGCGGCGACCTACCTGCTGTCCATGTACGACCGCGC
AGGCGGTGTGCGTGAACCCGCTGCTGGTGTGATCAACCCGGATGGCGCTTCGGCCCGTGC
CGCTGGCCGTGCTGTAGAAACCCCGCTGAACCGCGTACAGTTCGCCGCCGAGGCGAAAGC
ACTGCGCGCGAAGCTGGGCGATGCCTACGTGACCTCGGCTGAGTACCAAGCACTGGGCCG
TCGCCTCGTTCGTTAATGCTAAGCGGCCCGATTCTAATTGGGCCACTATAGCAACATGGA
ACCCAACAACCTATGTCAAAAGGCTGACTCTATGTCTCTGGATAGCTTTAACGCAGTCCG
CCCCAACGCTAAACTGGGCGGCGCTGACCCGCTGGAACTGATCATAGAAGAATTCGGTGG
TATGGTCGAAGGCACTCTGCAACGTCGTTCCGTCACCGACGGCATGATCAACGTCAAGCA
GATCAACGGCACTGCGACCGTCTCCAACCACGCCATCGGCGCGACCCAACTGCAAACCAT
TCAACCGGGCAAGACCCCGAATGGTACTGCCGGGCAGTTCTCGAAGAACAGCGTGACCGT
TGACCGCACCATTCTGGCCCGCGCCATCCTGCCATTGCTGTATGTGTTCCAAACCCAGTA
CGATGCCCGTAAGGAAATCGCTCTGGAGCACGGCAAGCGCCTTGCCAAGTTCAAAGACCA
AGCGTCCCCGATCCAAGCGATCAAGACCGCCCGACTCACCGTGAACCCATACGGCAACCT
GCCGGGCCACGCTGCTGGCACCGTAGTACCCCTGGCCGCTGCCTGTGATCCTACCGACCC
AGCGAAGCTGTACCACGCCTTCGGTCTGCTGTTCGCGCAGATGATGGAGAATGACGTTGA
CCCAACCGGCGATGACGTGTGGGTATTGGTTCGTCCAACCACTATCTACGCAATGCAGGA
AGCGGAACAGGTCATCAACGGTGAGTACATCACTTCCGATGGCACCAAGATCAAGGCACA
CATCTTCACCGCGTGGGGCGTTCCAGTCGTACCAACTCCGAACCTGCCGATCTGGGTTGA
AGGTGCAACCGAGCTGCCTGACGGCGTTGCTGCTCACATGGGCGATGACTACGCTGGCGA
CTTCACCAAAGTCGTTGCTGTAGCGTTCTCGACCCGCGCCATGTTGGCCGGTTCGACCAT
CGACCTTGGATCGATGGTCTTCTTCGACGACGTGACCAAGCACTGGTACGTGGATAGCTG
GTTGGCATTCGCCTTGGCTCCAAACCGTGCCGAGTTCGCTGGCATCATCGTTACTCAGTA
ACACCACTCTGGCCCGCAACGGGTTCCCTTAATCGGGAGCCTGTTGTGGGCCATTTTCGT
AGGTACGCGCGAATGAGTTTTATCCCTGAGCTTGATGTGGAAAACCGTTGCCTTGCCACG
ATGGGTGAGCTGCCGATCAACTCGCTTGAGAGCAGCCGCAACCCAATTGTGACTAACGCA
CGGTCAGCATTCAAAGACGTACCGATTGAAGAACAGGCCGTAGGCTGGTGGTTCAACATC
GAAGTCGTCAAGCTGCAACCACAAGGAGCAATGCCCGGAGAGGGCGGCTTCTACCGACCA
CCTACCGATTGCCTCTCGCTCTCGACGGAGGATGATTACAACCCGGCATGGCTTAGCATC
CGTGACCGCAAACTGTACGACAACGGGCGTGCCGAAGATCACCTCGGTCACGATCCGATG
CAAGTCAAGATCATCCGGCTCGTTCCGTTTGACGACCTGCCGTCCAACGCTCAACGGTTA
ATCCGTGACAGCGCCGTGTTCAAGTTCCAGATGGACTACGACGGCGACGAGCTAAAGATG
CAACAGGCAGAGAATTCCTACACTGAAGCCTATGCGATCTGCATGGCTGACCACATCCGG
TCGGTCAAAGCCAACCTGCTTTATAAAGGCTCGGCTGGTTCCGCGTTGTTACGCTCTCGC
ATCCCTTACGGTCGGACTGGCCGTTGGAGGTAATATCAGCAAAGTCACAGGCTCTTACGA
GTCGTTGACCAAGGGCGTATCCGAGCAGGTCGCCCACCAGCGTTATCCCGGACAGAACTG
GGAGCAAGTCAACATGGTTTCTGACCCGGTACGCGGCCTTGCCCGTCGCCGGGGTTCCGT
GATGATGTCGGAACAGCACCGCCCGGAAATCGCGTTCGACACTCCGTCCCGCGATAACGG
CAAGTCGTCCGGCGAGCAGTCCCTGTACCTCGGCGTTAACGAGTACAGCATCATGCACCG
CAAGCGCGCAGCAGCAGGCTCGACCATGCCGATTCTGGTTGTGCTGAAGAAGACAACCGG
GAACTTCCTGCCAGTAGTCGCCCATGGCAATCTGGCGGGTTACTCGCAAAACGGATTCAA
CAGCATCACCGCAGTCGGCAATCTGGTACTGCTGGCGACCAAGGGCGTAGCATCACGGTA
CACCGCAGTCGATCAAGTCACCAACAGCCCAGCCGCTAACTCGGTGGCCGCTTGGGTGAA
GGGCGGTTCGTATTCCCGCACCTACACGCTGTCAGTTAAGCGAGCCAGCGATCAGCAGGT
TATCACGGTGCAGTACACCACGCCGACCTCGTGCTACCCGGGCATGCTGGACACCTCGGA
CAGCCCCGCAGTCATCCCCGACCCGGAGAAGCCCGGCCAGACCAAGCCAAACCCGGACTA
CCAGAAGCTGGTGCACGACCGTGTGTACGCTTGCCTGAAAGACGTGAACCAGTGGATCGG
TACAGCGGCGGCGGCGATTCAGCCAGCGGCCCTTGCTACTAAGCTCCAAGAGTTGATCAG
CCCGAGCTTCCCCGGTAGTTCCGGCCTGAACGGTTCCACCATCATCATCGACGGTGGGCA
GAGCGTGAGCTGCGATGACGGCGGTAGCGGCGAGCAGTTCATCGGCGTGGCGCGCACACT

GAAAGCTGCGGCGGATTTGACCACGGTTCACCGGTCGGGCAAGGTCGTTAAGATCAAGCC
AACGAGCGGGAACTCGTACTACGTCAAGGCCAACGTCAAGGACGGCACCACCGATGGATG
GACGCAGGTGATTTGGGATGAATGCCCCGGTGTTCTGATCACGCCAACCGCAGTCACCGC
AATCGGTGCGGTACACAACGGAACGTTCTACGTTGCACCGTCGCCAGCGGAACTCGCTGC
GCTAGCCGGTATCAACGTGCCGCCATGGGAGCCATCGTCGTCGTGCGCCCTCGACAGCCA
ATCCCTGCCAGCGTTCCTTGACAGGGAAATCAACTAAATCCGCATGTTGCAAGACCGGCT
GATCATCATCGCCGGGGCTACCGTGTTCATGTCTCGCTCTGGTGATTACTTCAACTTCTT
CCGCACGTCGGCCCTGAACACTCAGCATAACGACCCGATTGAAGTATACGCACTGGGCAG
CGAAGACGATGTGATCACGTCAGGCACTTTGCTTGCCCGCAACCTGATCTTGTTCGGGAA
GGAGTGGCAATACGCAGTGCCGGGACGTGACGCGATTACTCGGCAGAATGCGTACGTTGC
TGTGCAGTCGGGCTACCGGGACAGCAAAACCTACCCACCGGAGTCCAGCGGCAACCTGAT
CTTCTTCGGCCAGAAGCGCGAGGGCAAGCTGACGATTCAACAAATGCAGACCGGCGCTTA
CGCTGACACTCTGGATGCGTTTGAAATCACACAGCAGCTCAACAAATACCTGATCGGTGA
CCCGGTGCAGATTGAGGCGGTAACGTCACCATCCGCGCTGTTTGTTCGCACGACAGCCGA
CGCCACCGGCGTTTGGGTATACAGCTACCTCGGCTCGCAGAACAACGCGTAGGTGCTGTT
CGATAGCTGGAGCAAGTGGACGTTTAGCAAGACGCTGGGCGATATGATCGGCATCACGAC
GAGAGACGGCGACCTGTAGGTGACAACGCTTCGGCATCACCTCAATGGTTCGTGGTTCGT
TACGGATCGGTTCACGATGAACACGGATGTTCCTGACTGTCACCTCGACACCCAGCGCCG
TTACATCGCTGCCGTTTCCAAGACCATCGGCAACAACGACTATCCCGAGCTGCTGAGCGG
GGCCTACAACTCAACCGCATCCCGGTACTGGTTGCAGGATGATACCTTCGGCGAGCGATT
GGAAGCGTTGCGGGAACAGATCGGGCCACCCAACGATCCAAACCTGCTGATCGGTGCGGA
CTTCGAGAGTTACGTTGCGCTGACCAACCCGTACATCCGGGACAAGGACGGCAAGGCAAT
TCTGGACGGGCGCCTGACGCTGGGCAACATGAACGTGAACGTCTTCGAATCTTCCGCGAT
GGTCGTGAGCATCTGCGACTTGGTTAACGCTGACAGCAACGCATACGCGGAAACACTGCG
CTGGGTTCCGCGGAATGCTAACGACTGGGTACTCAACACACAGCAAGTGGCCGACACCGC
CTCGGTTGTTGTAGGCGTGTACAAAGAAGTGCGGGACTGCAAGGACAAGCTGGCTGGACG
TAGTTGGCTTCCGCTGACACTCTCGGCCATCGAATGGCAAGGGCAGTTCTACACAACACG
GAGACAGTAAATGTTCGGCATTCCCATCATGATGGCCGCTTCGGTGGTCTTGCAGAAGGA
TAAGCAGAACTCAGACATCAAGAACCTGAACCGCATCAACAAGGCGAACACCTACGAAGC
GAACCAGCAGCAACAAGGCAACGGCATTCAGGCGTCTGCGAAGGGTGCGCTTAACCGCAT
GAACCAATCCATCCAAACGCGAGTCGCGCTGACGGCCTTCGGGCAGCAGCACAACTCGCT
GGAAGCACAGCGCGCCCAACTCGGCAAGCAGATGACCACGGGCAGCTTTGCTAACCGTGT
ACACGCTGCCGAGCAGCAGGGCATGCTGGTGGCGCGGTCGAGTGCGGCGGGCGTTGGCGG
TTCTACGTCTTCCAGGCTTGAGGCAACGATGAGCGTGCGGGCCGATATGCAGCAGCAGGA
AATGCAGCAGATGTATTCCGATAAGCTGTGGGCAATCGATGACGCAGAGTAGAACAACCT
GTAAGAGCAGTATGGGATTTCCCAACAACAGAACGTGTTCATTGACGACGTGGCACCGAC
AGAGATTTATGGCCCGCCGAAGATTCCAACTAACTCCGTTGGTGACATGGCGATTGCCGG
TGCGATGGCATTCATGCAGGCTTGTAACAGCATGGGCATGTTCAGCAAGGGCGGCGCGAT
GGATGCGACTGGCGTGGGCGGTAAGCTCAAGTCGTGGTTCAGTCCACAAGGTGGCGGCAG
TTCGCCGGGCACCGGCGCTAACTCGTTCTTCCAACTCGGAGGCAGGACGTAATAGCAAGA
CAATTGGGCCAAGTAATTGGCGGCGGGCAGCAACGATATCAAGCCGCGTATGCGCCCGCA
CCGCAGAAGGATAGCACAAGCGAGCTTCTGTTCAAGATGACCCAACCCATCTTGCAGAAG
AAACTGGAAGAAGTGCAGCAGTCGAAGTTCGTGGAGGGCATGCAACGTGTGGCCACGGGA
GGGGCTTATCAGGACTTCAAGAAGGAAGACTCGGTACTGTCCAACATCTTCGGGCCATCC
GCGTCCGTAGCTGGCGCAGCCGCAATGGCTAAGGTCAAGGGCGTTGAAGACTTCAACACT
CAGTTGTATCAGGACATGCAGGATATGGCGAGCAAGTCGCCAGACGAGTTCCGCAAGCAC
GCTGTGCAGGGAATGCAGCAGCACTTGCCGGGCGATCCCGATGTCGATAACGTGTTGCAG
GCCAAGATGGTCGAAAGCCTCGGGCCGCTGACGAAGACGCAAGCCAAGGCGAACTACGCT
TGGCAGCTGCAGCAAACCACGATCAGCTTCAACGGTATGCTTCAATCTGCCGGTGACAAG
TTCCACGGCATTGCGCATCTGTTCGCACAGGGCATCGTGAGCAAGGACGACTTCGACTTG
GCGAAGGCCAATCTGCTGCAATCCAGCCTGCCTATCGCCGGGATGAATCCCCAGACGTAC
AAGAAGGCGATCATGGACAGCGCCGAGCTAGCTATGGACAAGGGCAACGCGTGGGTAGAC
CGTGTGTTCCAGCAGACCGGGATGTACGACGGGCTTGAAGCTGGTGACCAGACCAAAATG
TTCAAGGTGCGCGATGCAGCGGCCTCAAAGATCAAGAACGAGGACGGTTTTAACAAGTAC
GGCGCAGCAATCGGCGAGCTGATGGGCGGTGCGGCTGGGCGTAGTCCGGCACAGAACCAC
GAAATGGCTAACCAGATCAACCAGCGCTACATGGACGAGACAGGCAGCGAGACGGGCTTG
CTCAACATGAAGGACGTGATCAGCATGGATCGCAGCACGTACTCCCGCATGTTCAAGATG

CAGGACAAGGTGCAAGAGCTGGCGATCAAAGCGCGCCAGGACGCACAGGCCGAAGGTTAA
CTGGCACAGCAGGCAACCGAGTTGGTTCAGACCGGCAAGGGCAACTACGGTGTGGGCATG
GGCATACCGCGCAACAAGGTGGACGTAGCGTTCAACGAGGGCTTCACATCCAAGTTAGCA
ACCGGTAAGCCGGAAGACACGCTGGATATGGCAAGCCAGAACTACTCCCACGGTAACGCC
TACGTTAACCCGCAATTCCAGGGCATGCTTCTGGAGCCATTCCGCCAGATCAGTGCCGGT
AGCGTTCCGCAGCCGGAGTTCGACCGCACGGAGCAGTTCGGTGACGCATGTCAACAGCGC
CCGGAATCCGTTGGTGCGATTGATGTGTACCTCGGTAGCGAGAACGTGGTGAAGCTTCAA
CGCTACCGCGATTCGCTGGTCACGTTCCGAGGCGACATGCAACAGGCAGCCAAGGCAGGA
TGGGGCGCGCCGGTAGTGCGTGGGCCTAAGCTCAGCTCGGTGGACAACAAGCCGCTGATG
CTGGAGGCCATCCGCACTCAGATCGAAGGCAAGGGCGAAATGAACAAGCTTGATCGCTGG
TGGAAGAACGTGCCCGGCATGTCTGATGCTGAGAAAGACGTTGTGTTCAACGCAATCTCC
GGCGATGCGCAGGTCTATGTCGATAACCTCGGCATGGCTCCGCAGGCAGCAATCACTCGC
GCACTGGAAGGCTGTGGCCGTGCAACCGTTGACACCGTTGGCGCATATGCGATCCCCAAA
GAACCGAACCAGCAGCCGCTGGCGGCACTCATTGGCACCGACGATCAGACGGTGGCGCGA
GTGTTTCCGAAGTTCTTGCAGTCCATGGCTCGTAAGCAAGGCGTGGCCGTCATCTTGGCC
GGAGCCGGCAGCAGCGTGACCAGCAATAAGGTTGAAGCAACCCCATCGTCTAACGTGACG
GACCTGACCAACTGGTACGGCGGTGGCGATGCTGACGTGTCCCTGATGCGCAGCAAGGAC
GTTACGGACAAGGATGGCAAGGTTTACGGAGTCTATTCGGGCCTGCTGTTCCGGGACGAC
AAGACAGCAACCGTGAATTTCACATCCAAAGACCTGAAAGAGTTCTACGAGAACACGATC
AGGAACCGCAACGACATCACTGACAGCCTCCGCAAAGCGGGGCTTTAATAGGAGGCCGCA
TGGCTAACATTCTCGATTCAATCGAAACCAGCAAAGTAACAGTTGGCCCTGCGGCAACCT
CTGGTTCAGGCTCGCTTGCCGACGCCGGCCGCGCACAAGACTTCGGGCAGGCGAGTACGC
TCTCACCGTCTGACGCCATGGCAATCGCCAAGGGCCAGAAGCCCGCCAAACTCAATGCCT
TTGAGAAGATGTCTGCGTTCCAGCTGGCAACAGCAGGCGCCGCAGCCGCAATTGACGCGG
GCAAGTCCCGTGAGCAAGGCGCCGAAGCCGCAGCCGAGGGCTATCAAGCGGTTCGCAAAG
ACCCGTTTAAGAATTCCAGCACCAAGAACGTCGGGCACAGCCCCGCGCTGACCGATGTAG
CAGACCCAGCAACTGACGCACACGTACCGGCAATTCTGGACGAAATCATCAAGAATCAAG
CCTACAAGCAATCTGTAGGCCCGGTCGATCAAGTGGGCGCCGCAGTGCTGGAAATGACTG
GCATCGGCGCGTTGGTACGGATGCTGAGCGACAACTACAACGGTATGCGCGCCTTCAAGG
ACGACCCGAAGTTCGACAGCCTTGAGGGCCGCGAGGTTTGGAGCAAAGGCTTGAGCCAAG
AGGGCTTGGAGTGGGTGGCTGACGCCCGCAGCGCATACGAACGGCAGTACAAGCTGGCCC
GTTTGCAGGAGACACGCGACAACGACAAGAAGATCGGGGCGCGCGGCGATGGCGTCGGCA
CCGTGGTCGGGCTGACTCGTGGCGGGCTTGATTCTGCGGGCTGGGCCGCAGGTATGGGCG
TGGGTAAGGTCGCACAGTTGATCAAGGTAGGCATGGAAATGGGCGCCGTCCCGCGCGGCC
TGATAGGTGCTGGTGAGGGTGCCGCAGGTAACGTGTTGGTGGAAGCCAGTATGCAGGCCG
CTGGCGAGCATGTGACGCCGGGTGACTACTTCTACGCCGCTGGTTTCGGTGCCCTGTTCG
GGGGCGCCATCATCAGGATGACCCGCCGAGTGTCCGGTGAGCCGTCGGTACCCGAGGCTG
ATGCCGTCTCTGGCTACACCAAGGACATCGCAGAGTCCTACACCGACGGTAGCCTCAAAT
TCCACGCATGGCTTCTGGAGGAAGCTCAGAACCGGCTTCCCGAGGGTGCAACGCCCGAGC
AGATCGCCGCAGCAGCGCGTGACGTGTTCAACGAGCGCTCCAACGCCTACTGGCAGCAGG
CCACACGGGCTATGCCATAGAGCGAACGCATCCGGCCTGACGTAGACAGCACCAAGCTGT
ATGCAGACGGGGGAGACGAGCTGACAGGCACGCTGACGAAGAATCTGGAAGGGCAGTTCA
GCGACGTTGTGCGCCGGACAGCGGTGGGCGATCAGTGGGGATCCACCCCGGCGACCCTCC
CGGCCAACGCCAAGCGCCTGATCTACACCGAGAAGATGCTGCGCATGGTCGAGAACCAGC
CAACCTTCGAGCAAGCCAAGCTGGATACCTTGATTGGCAACTGGGTTGCACCGATTGCGG
ACAGCGCGATGGTGGCAAAGTTCAGCATGCCGCAATTGATCCTTGGTCGCAGTGAGCATC
CGTTCATGAAGTGGCTTGCAGCTAACCTGCAAGAGTCGCCGACGCAAGCCGGTGGCCCAC
GCACTACCGCAGCAGTCGCGCATGCGATCCGCCAACGTCGTGAAAACAGTTTCAATGCCC
GGTACAACGAGGCGTGTGGTGCATGGCGCGATCAGAACGGCGGCTCGTTGGGCAAAGACA
TCCTGTTCAAGCAGGAGCACTTCGAGGACTTCAACAAACTGGTAAGCACTGCACGGGCAA
TCCGCGCAGCCGGTATCACCGGTGACGAACATCCGAACGTTCGTGCAGCCGCCGACGTAA
TGGACGAGGGTTTCGCCCGTCTTGCAGCCGACCAGCGCACCGCCAAGACACTCGGTTCCG
AGTTGCTGCTAGATTTCAGCATCGGCTACACGCCGAGCATGCTGAACAACAAGTTCATCG
AAGAAAACCCTATGCACCGTGAGGCGATCCGTGAGGAAATCCGCAGCCAACTTCATGACG
CATGGGCAGGCATGCCGCAAGGCGCCAAGATCGCTCAAGACGTAGCGGCCACTTACATCA
ACCGCGCACGCATTGAAGGTGCAGGCGGTGCAGTAACGCCGGGGCATATCTCTGACCCGC
ATGCAGCAAGCAACCTGCGAGACGCTTTGGAGCAACACGCCCTCAGCGAAGCAGACATCG

AACGGTACATTGCCCGCGTTGGCCGTGGTGGCGCACAGCACACCAAAGGTCGCCTTGATC
TGGACTTGACCAAGCAAATCAAACTGCACGCTGGCACCGTGTTCACCCTCGGTGACGCAT
TCATCAACGACACCTCCGCGCTGTTCCGCCAGCAATCCCGCTACGTTAACGGCTACGTTA
CGCTGAGCCGCCGTGGCATCATGGGCAAGGAAGGTATCGACCAGCTCTGCGAGATGCTGG
TGCTGTCCCGCAAGGGCGCATCCGTCAAGCCCTGGGCCCACGAGCTGAACGCCTTCGACC
AAACCATGTCGGAATTCATGGGCAAGCCTTACCGCACCGCGCACCGACCGGCTGACACCT
TCCGCATCCTGACCGGCACCAGCCGACTGGGCCAAGCTGCGATCCCGCAGCTCGCCGAGA
CTGGGCAGATCGCCGCGCACTTGGGCACAGAGAGCGCAATGCGCTTTGTGAAAGCCCTCC
CACGCCTTGTGGACGGGGTACGGAAGGGCAAAACCAACGACCTCTTGCACTCGCTGGAGC
TTCCGGGCGGCGCCATCGGCGACGAACACCGCACCGATGTGCCTTGGCAGTCGTTCGATG
ACATCCAGTTGGCAGGAAAGGACGCTGCTGGTACGGTTGACCGCGCAATCCTTGGCGCTG
CACAGGCACAGTACACCCTGACCGGGCACCGGTACTTGCAGGCAGCACAGACCCGAGGCG
TAGCGGAGCAGATTCTGCACAACGTCATGCGGTATGCGACGGACGGCACCAACCATTCTG
CCCTGCGTAGCATGGGGATGAACGACAAGTTGATCGCCGCCATCAAGAAAGACCTTCGCA
ACATCGCCGAGTTCGACGACACTTGCGAACTCAAGGCATTCGACATCCGCCAGACTGACA
ACCTTGCCGCGATGCAGGAGCTTCGCCAGTTGATCGAGCGTGGTGCCAGCCATATCATCC
AGTACAACTTCATCGGCGAGCGCCAAGCGTTCGTGCATGACTCGTTCCTGCGCATCCTGA
CTCAGTTCCGTTGGTACTCCCTGACCGCATCGTCGAAACAGTGGACTCGTACACGGACTG
ACCAAGGCACCGCGAAGGCGCTGGGTATGCTGATGTGCCAGATGTCGGTGGCACTGCCGC
TGCACCGTGCCCGCGTGATGGCTGTATCGGCTCTGATGTCCGAAGACCGCGCTGCCGACT
ACCGCGAGAACAACCTACGCCCTGATATGCTGGCCCGCGCCACGCGGAACTATGCCTCTC
TGGCTGGTTCGCTTGGCCATACGGTGGATGCTGGGATGGGTGTGCTGGGCATGCAGATGT
CCGGCGTCCGCTCCGGCTCTCAGGATGTACTCGGCAACGTCCCGGCACTGGGCTACGTCT
CCAACACCGCGCAAGCGTGGAAGGATAAAGACGTGTCCGGCATTATCAAGACAATGGCCG
GCGACAACAGCGTGTTCTTGCTGCCCGCCGCACACCTCTCGCACTGGATGCAGTAAGAGT
GATCTAACCGCTAAGTGGCTCAATTCTAATTGGGCCACTATAGCAACACCAAAACCAATT
TCGGAGGCATCATGGCAGACTACTTGTCTATCAACGAGTTCCCCGGCAATGGCTCCATCA
TGCATATCGAGGTCAGCTTCGCTGGCAACAACCCCGATGTAGGCTCTGGTGCTGTTCCGT
ATTTCAAACCCGCTGACGTGAAGGCTCAGTTCTTCACTCCCGCCACTGACAGGACTCCAC
AAATCGTGGAAGTCAAGCCGCTGGGTTACGTCGGCCCAAACACCTTCATCACTCTTGAGC
CTGTGCCAACCGGCAAGATTCTTCGGGTGTACCGCGAGACCGAAGACCGTTACTCGCTGG
TCAACTTCGTAGCGTTGCAGATCGTGACGGAGCGTGACCTTGACGCACTCGCGCGGCAGA
CCGTCTTCCTCGTTGAAGTAGCGAAGGACGCCGCACAGTTCGCAATCGACACCGCCCGCG
AGGCAATCCGATACGCCACTGCTGCTACCGGCGTGGCGCGTGAAGCTCTCGCACTGGCAA
CCACGGCTGACCGCAACGCTTCGGCGGCTGTAGTCACGGCCAAAGCTGCTTCTAACGTCG
CCAACCAAGCGTTGGTCAACGCCAACAAGGCAGCACAGGACGCCGCATCGGCTGAACAGC
ACGCCACCAACGTGGAGACGCTAGCCGCTGCTGCTCAGACCAAAGCTGGCTCGGCTACCG
TTACGGCGAACCGGGCAGACGCTACGGCCAACCGGGCCGAAGGCACCGCCAAAGCAATTG
ATGGCAAAGCAACCGCTGCTGTGGCAACCGCAAACGCGGCTACCGCCACTGCCAACAGGG
CTGAGTCCACTGCAAACGCGATTGACGGCAAAGCCACACAGACCATCAACACCGCGAACT
CCGCAACCACTACCGCGAACCCAGCCAAGGCAACCGCCGAGGGTGTTGACGCGAAGGCAT
CGCAAGCATTCGCTACGGCCAACGGCGCCGCTGCTGACGTGGCAGGCGCAAACACGAAAG
CTGATCAGGAAATCCAGACGGCAAACCAAGCAAGGGCAAACGTGGCTGGCTAGGTAATCC
GCTCCGGTGATCAGATGACCGGGGTTCAGGTGGTCGAGCCGCGTGTGCAGGCTATTGCCA
AGGCTGACGGCAAGGCGATGTTTGAATGGCACGTTGCTGGGCAGACTGCGGGTATAGCAT
GGCTCACCTCTGACGGGATACTCCGATTGGGGCAGACTGGTGGTAACGGCTCAGAAGTGA
ATCAGGCCATGAACATCGGATTCAGCAGCGGGAACGGTTCTGTGAGAGGCGAATGGCGGG
CAGCTTCGATGGTATCCGTCGATGGCTACCACAAGAACGTCTACCCGCAGCCCGGTGGGG
ACATCAGCCTTCGGGCTAACGTATCCGTGTTCGGTAACATGACAGTCACCGCTGCTGTCA
ACGCCACCACCTACACTGCTACCCAAGGCAGCCTGTACCTACGGCCCGCTACGGCAGTTG
CAAACACGAACATCTGGTTCCAGAACCCGCAAGGCGGCGAGCTTGGTCTGATCTACACCG
ACCCGTGGTCTCCCATGCACTTCCGCTCAAACGGTGGCGGCGACACCTTGGCGTTCTATG
CAAACAAGACCGCACGCTTCAACGGTAACATCACTGCCCCATCTGCCGCTATCTCCGGGC
GCGTCTCTGCTGCTGAGATGCTGTCGGGCGGAACGGTGTGGTCAGGCAACGGCGCTTCGT
GGCTTGCGAACGACGGCAACGTGTACGGTCGGGTTTGGGGCGGCTACCTGTCCAACTGGG
TGAACTCCAACCTCGTCACCCGTCCCGAGTTCGGCGGTGCTGTCCGGGCTAACGTCGCAG
CATTCACCGCTGGGCAAGTCGGCACTTACGCCATGCTCCAAACTCGCGGTATGGTCAACG

GCGGGCCGGTTGCACTCGTCGGCGGTGATCAGCTCGGCTGGGCGACTTGTGCGGGCGACT
TCTCGGAAATCGTCGGGTCCGGCACTTGGCGTGTAATGGGCCGTGTGTCCACGGGTAACA
AGGTTGTTTCTACCACTATTTGGCTGAGGGTTAGCTAATGCAATTTCCGAAAACCTGTCG
GGAAGGGTTCACTGTGTACGGCGTTCGTCGTCCACGGTACGCCTCTGATTCCGGCAAGTT
CATCACCGCCGAAGTGAACTGGGGCGATAAATCCGTCCCGTACGCTATCAGCGCTCTCGA
CCCTGAACCTTGGGGCAAAGATTTGTTTGACCATTGCTTGTCGCTTGGTGTGACACCCTA
CACGGGTAAGTCCGACGCCGAGTACGCGGCAGAGGCCAATGCCGCAGCCGACCGGCAAGC
TCTGGAAGCTGAGCTGGCCGACCTGATGATTGATGTACAACTTCAACTTGCGACGCCAGA
AAAACTGGCCCGCGCCAAAGAGATCCGACTCAAACTCAAGGAGTAAAACATGCTCAGCAC
TGACGCCGCAGACGTGGTGATTCGCAATACCCCGACTGCGGAAGTCGGCGGCGCAACATT
CTACGACTCTTTTGTGTCGTTCCCAATCACTACGGTTGCGCAATGGATGGCCGGTATATG
GATTCTGATTCACGCAGGCTTCTACCTGTACGGCCGTTATCAGAGGAAATGACAACGGCA
GCTTCTAGTGATCGCCTTGGCAATCTGCACACGATGTTCACCGCTTACTGGGAGCATCGG
TTTGAAGCTCCTGAACTGGGCTTCGAAGATGAAGGCTACATCCCGCTCACGTCCAACGAC
CTTGGTGTCCTCCGGGCGTTCCTGAAAGACAACAACATCACCGCCGAACCCGGTGGCGAC
AAAGACTTGGCCCGACTGGGCGCACAGCTTGCCGCTGACCTGGAAGGCTCCGTGAACCAA
GCCGAGCTTGACTGTATCCTCAAAGACGCCGAAGGCTGGATGGGAGTTCAGAACTGATGG
AGCACAAGACGCCGCGCCACGCACAGTTGGCGCTGCTGCAACAAACCTTCCTGAGCTTCT
TGACGTTTCTGATTATCGGCATGAAGTTGTTGGGGTTTGCGACAACAGCGATCCAAAAAG
ACATCGGCCTGTACCTTGAGCATGGCCCCAAAGACCTGATGGTTCAGGCACAGCGCTCTC
AAGCCAAATCCACCATGACCGCATTGTTGGCGGTGTGGACACTGATTCAAGACCCGCGTT
CCCGTGTGCTGATTATCTCGGCTGGTGGTACGCAGGCAAACGAAATCTCGACGCTGATCG
TCCGACTCATTATGAGCATGGACATGCAGGCTTGCCTCCGACCTGACCAGCAAGCTGGCG
ACCGTGTATCCGTGGAGAAGGTCGACGTTCACAACAGCCTGAAAGGCGTGGACAAGTCGC
CCTCCGTAGTTTGCATCGGTCTTGGTGGTAACTTGCAGGGTAAGCGAGCCGTCTTGCTGA
TTGCGGACGACGTGGAATCCCACAAGAACTCACGCACCGCGACCAACCGCGAGCTTCTGC
TGCAACAAATCCGTGACTTCCCGTCCATCGCAGTTGACCGCGTGGGCGCTGGCGGCGTTA
TAGAACGTCGGGGGCGAGTGATCTCCCTCGGTACTCCGTAGACTGACGCTTCCGTGTACA
ACACACTCCCGGCAGCTGGCTTTGGCTTGCGCATCTGGCCCGGTCGTTACCCAACGGCCG
AGGAAGAACCGGCGTATGGCGAGCATCTGGCGCCGTACATCGAACAACGCGTGACCCGTG
ACCCGTCTCTGCGGGAAGGCGGCGGACCAACCGGACGTTCCGGCCAGCCAATCGATCCTG
AACTGCTGGGCGAGGCCGCACTGCTCTCCAAGGAGAACAAGCAGGGGCCAGCCTACTTCC
AGCTCCAGCACATGCTGTGTACGCCCCTGAGCGACATGGAGGGCTACATACTCGAGGCCC
AACACATCGTCGGTATGAACCTCGGCGCGCAACTGCCCATGCACTTCGTGCGTGGCATCA
GCGCTGAGCATCTGCGACAGTATCAGGTCGGCTCTCTCAAGTTCCACTGCTCGACTCCTG
TGCACATCGGCAAGGAGTTCGCTGCGCCTGCATCCCGTGTGCTTGCAATCGACCCCGCTG
GTGGCCGCAAGAACGGTGACGAAACCGGTGTGGCCGTAGTTGACCAGCTCGCTGGTAACA
TCTTCGTCCGCTCGGTAGAGGGTATCCCCGGCGGCTACTACGCCGACACGCTCAAGAAGC
TGGTGGCGTACTGCTAGCGCTGGGAGCCGCACCTGATTGTGATCGAGAAGAACATGGGCC
GTGGTGCCTTTACACAGGTTCTGCTTCCTCTGCTACGTGCTGAGGGCGTTACCTGCGCTG
TGCAATAAGTGTACAACACCGGCTAGAAAGAGCAGCGTATCGCCGACACTCTGGAGCCAA
TCGCAGCCCGTGGCGCACTCGTCTTCGACGAAAGCGTCATCCCTGACGACTGGGCTTCGA
CCCTCGGCTAGCCCGCTGACAAGCGCCAGTTGTTCACCCTGATTCACCAGTTCATCAAGC
TCACACGCGACAGAGGCGCGCTGGTTAAAGACGACCGGCTCGACGTACTCAGCATCGCCG
TAGCGCACTTCATCAACGCTCTTGCCCAAGACTCCGCACTGGTTGCGCAATCCGTGCGGG
ATCAGGAGCTGGTCAAGTTCATGCAAGACCCGCTTAGCCACAACCGCTACACCAACGCTG
CCCAAACGGGCTACGGGGAATTCGCCAATCCTACTGCAAGGAAGACAAGACGATGAAATT
CTCCACTCTGCCATCCCCCGGTCAACCCCGCGTCGGCCAGAAGCTCCGTCAGATCACTAC
CATTGCAATTTCCGCAGCCGAAATCCGCAAGGGCCAATCCGGTCTTAACTCTCCCGAGCT
GGTAGCGTTCTTCCCCGCCGCTCTGAACGCCACTCCCGGCGCTCCGGGCCAGAACCCACT
CGTCGCATCCGTTACCAACGGCCCAAAAGTTTCCACTGGCGGCGTTACCTACGAACTGAC
CGTGGCTGACGGCGAACTGGCCGCCATCACCGCAGTCGCTGCTCCATAAGAGGTGAGTTA
TGCCTACGTTCCCACAAGTAGTGCGCCTTGCGCGCTCTGCTGTGGCTGTCCTTACTATCT
CCGCAGCAGGCTTAGGCTTTATCCAACACGAAGAAGGAACCGTTAACCGGGCATACCTTG
ATTCGATTGGAGAGCCTACAATCAGCACCGGGCACACTGGCCGCGAGGTACGCTTAGGGG
TATTCTACAGCGGCGCAAAATGCGCTGAGCTGCTTCGCACGGACGCCAACTCGGCTGCCG
CTGCTTTTTAGCGCACGATCAAAGTCCCGCTGTACCCG

>NewGenomeName_31
TGGATGGCTGATGGGACCCGAACCCAAACTATAGGGGATTACAGGGGAATTGCGGGGAC
TTTTGCCTTTGATTTTGCAGGGTTTTGCGTGTTCCTTTACCCCGGCATCCCCCGCATTTG
TCCCAAGATTTGCCCCAAGCTACGGCGTGTCGCCAGGCTTCCCCGCAGGCGAATGCGTCC
CGGAGTCGCTGGGCACCGTCACGCTGCCGGGCGACACGGCGAACTGGCGCACCTCGGCAT
TGGCGCGCTGGGTTTCCCCGGGGGTGCCGAACCAGAACGCGAGGGTCTGCTTCAACTCGT
TGCACCGGTAACCGATGACCGTCTCTACCGTCAGGCTGGCGGTGGCGTCGCGCAGCAGGC
CGTCCGCCAGGCCCGATAACACGAAATAGACGATGCCCAGGGCGCCCACCAGCAGCCCGG
CGGTGATCGTCGGCCGTACCCTGTCGTTGGGCTGCTGCGCGGCCAGCTTGAGGGCGCTTT
CGCGGTCGGATGCCTCGGCAGCATACTGCGCCGCTGCGGCTTGCAGGCGGTTCTGTTCGG
CCGTCACCGCGAGTTGTTGTAGTTGCTCGCGCGAATTGGTTTCCAGTTCCTTGAGTTTCA
TGGCCGCCTGCGGGTCGGCCAGCAGCGCCGTGGACACGGCGTCCGGCGTCGAGTCGGTGC
CCAAGGCTGTAGCCACCAGGCCGCCTACGGCGGCGCCGGCCGGGGCGCCCAAGATACCGC
CCAGGATAAGGGCGGCTTTGCCGACAACGCCGGCTAGGTCTTTCCAGTCCATTTAGATGT
CCTCTGCAGCGTATTTCAGGTTTCCGGCGACCCGCCGCGCCCAGTCCTTGCCGAACGTCG
GCCAAGTGGGCAGGTTGGTGTAGAACTTCATGCGATGTGAGTTGAACAGCGGGGCCAGCA
TGGTGGGGTTCATCCCGCCCGCGGCATTGAGTGTCAGGGGGGCCGATCTGTCCGTCCTGCG
CGGTGCCCACGGCAGCTTGCAGCCACTTGGCCGCCTGGCCGGTGCTGTGGTTCACCGCAG
CGTCGAACACCTGGAAGGCCACGGCCCGAGGCAGCCTGTCGCCGCGAACCGGCGTCCAGT
AACGCGCGCGGTAGATTGCCTTGGCCGTCTCGCGTGGCATGTCGCGCATCGGCCCGGTGT
AACCGTTAGCTCGCGCTACGGCGGCGGTCACGCCCCACATAGTCTCGCCGCCGGGGTCGG
CCGGGTTGTTGGAATAACCGCCCTCATGGCCGATGAGGCGGTCGAAAGCGGTGTCGAAGT
CCATGTCAGTGGCCCCCAAGTTTCGAGGTGATGCCCGCCCAGACCGCCGCGCCCAGGGCG
ACGGCCACCAGGCCGACAAGCGCGAGGAAGCCGTGGTCGGCGGCCTTGCGCATCCGACAA
CCGAAGCACAAGTCCTCGCGGAATTCTTCGACCTGTTCCGGCACGTCCACTTCCACGCCC
AAGATGGCAAACACCTTCTTGGCGGCGCGGTCGGCGGCCTCCTGGCACGCCGGGGTATCG
CAGTGCTGGTCAGACATGGCGCTCCCTTATTCGCCGCCCAGCAGTTCGGCCTTGGTAAAG
CCGAACCGCTCGATGGTGCTCAAGTCCTCCACGTCCTGCCACACGGGGTCGATGGTCGGC
CCTTCGTAGTCGAGCGTGTCGTACCCGTCGGGGTAGGTCTGCGCGTCCTGTTTGCGCGTG
ATGCTGCCGCGCAGGTAGTCGAGAAAGTCGCCGTGGGCCGGCGTGCCGGCGAGGGCGTCC
AAGTCCTCGCGGGTGTTAATGACACGCGATGCCATATTGTTCCTCCATCTAGGTGAAAAG
GTTGTGGGTGTCGGCCCATTGCGCATGACCCGACCACGAGGCGAGAAACCGCTGCAGAGA
TTGATCATCGCCGTGCTTGATGAAATTGGCGACCTTGCGCTTCGCCCGCTTGACCGAGGA
CTTGCGCAGCAGCTTGTGGGTGGGCCATATCCGATAACCCAAGAAGTTGATGCCCCGCGA
TACTGGCGCCACCTGCCAGTGGCTGATCTTGAGGCCCACGCGCTCGCTGGCGAAGTCGCG
CAGGCGATAGAACACGGCGCGCAATTCCTCGGGGTCGTCGCCCAGCACCACGATGTCGTC
CATGTAGCGCGCCCAATGCCGCTGCTTCAATTCATCGTGCAGCAGCCGGTCAAGCGCGCC
GCCGTACACGTTGGCGAACTGTTGCGAGGTGAGGCTACCGATAGGGATGCCGACGCCCTC
GTCGGGTAGCACCACGCGCAGCAGGCGGCGGGTGGCCGCGCAATGAATTTTCTTGTCGAT
CATCGCGTGCAGTGCCGCGCGGTCGATGCTGGGGAAGAACTTGGAAAAATCCGACTTCAA
GAAGTGCGTGGCGCGGCTCCGCCGCAATTCGGCCTGCACGTGTCAGACGCCCGCGTGCGT
ACCCTTGTCAGGGCGGCAAGCGTAGGTGTACGGCAGCAGGCCTGCCTCGAAGATCGGCGC
CACGATGTTGCACAAGGCGTGCCGCACAAGGCGGTCTTTGAATTCGAGCGCCGAGATTAG
GCGGGGCTTCGGTTCGTAGACCAGGAATTCGCGGTAGGGGGCGCTCTCGTAATTGCCGGC
TTTGAGTTCAGCTTGCAAGGCCAGCAGGTTGGCGAGGTCGTACTCCTTGAATTCCAGATA
GCCCCACGTCCGGCGCTTGCCGTGCGAGGTCTTGCGGTACGCATCGAGCAGATTTTCCCA
GGTGGTGATCTGGTCGATGAGGTTGCGATGTCGTTTTCCCATTGAAGAAGTCGGCCCCGC
CTTTCCTATTTCAGTAATCGACGTTTTGCCGAACCGTGTAACGTGTTCGCCGAAGCAAGA
CAGAACCGGCTGACCACCTATTGAGGAAAGGGCAGCCCGCCAAGCCGTAGCGTTGGCAGA
GCCATGCAAGAAGGTGAGGGGCACAGACGCCGCGCGCCCCGATGTTCGCGTTCGAGTTCG
ACGGCCCGTTGTTCCAGTTCGCAGCGCGAGAACCCGAGTTCGACGTGTTGTTCCAGTTGC
CGCCGAATAGCGCAGCAGCGTTATTTCCCAGCCGGCCCTTTGCGATTCACGCGGGCAATC
CAGGAGCCGAGAATGCGCCCCACTTCGGCGATGAGCACTTGTGCTGTCTCGACCTGATGC
GGCGTCATAGCGTGCGGTTTCGGAATGCCCGCGAGAAAGCGCAACCAAAATCGCAGGATG
GCAAGCCCGGCGTCCGCTGCGTAAAGCTTGCTCACCTGATTGGACTTGCCCGCCACGATG
AATAATTCGACCTGCCCGAGCAGGCACTTCAGGAACATTTCCTGCGCAACGCCGTGCTTC
CTCGGGATGCTTTGCGCAATGGGAAACAGGTACGAAATAACCCTATCGTACCGTGCCAAA

ATGAGCATTTGGTAGTAGCACTTTGGCGCTTCCTCGATGGGTTCCATCGGTTGCCTTGGG
GGGGCCTTTCGGCCCCGCTACTCAAGAATCTGGTGGTCACAGACGCCGCGCGCCCCGAAG
AACGCGAACGAGAACGACGGCCCGCTGTACCAGAGCGCAGCGCGAGAACCCGAGAGCGAC
GTGCCGTTCCGGTCGCCGCCGAATAGCGCAGCAGCGGGCTGGGGGTACACCGATCCGCGG
CCGCTAGTGTTGGCCGTGTATTCGGATGCGCCATTCACGCCGCCGGTCTCGTTACCCCAC
GTCCACAAGCAACCGGACGCCAGCACAACGCCCCACTTGGACGTGAAGATGTTCCACGCG
CTCGTGGCGCCCGTGCCGTTTACGCCGGTGGTGGGCACGGCGGTGCCGCCGCTGGACGTA
GCCTCGGTCGTGCCGAAAGCCAGCGCCTGGAATTCGTTGTAGTTGGGCAGGCGCTTGCCG
TGGTGAGTCATGACCTCGGCGCAGTTGTACCAGGCGCCGTCGCTCTAGGCCGCGCTGCCG
TCGCCGCCGAACTTGGTAGATTTCTTCGGTGGCGCGCTGCCGTCCGCGATGGTCACGTTG
TAATTGCTGGTGCCATCGGTCAGGTGGTTGACGCCTACCAGATAGATGTCCGCCCAAAAC
GCGCCGGCCACCAGCGTCATGCAGCGCGGGTCGAGCGCAGCCGGGCGGAACTTGAAGTCC
CACAGGCTGTATTCGTTGCTCTGCGCCGTTGTGTTTCCGCCAGCCTGCGCTGCAGCGTTG
CTTCCCGGCGCATACTGGAAGCCGACCACCTTGGGCGCCTTGGTCGAGGTGTAGCAAGTG
GGCGCCGAAGAGTTGGAATCGGCGCGCACCGTGCCATCGTCGCAGACGTAGATGGCGTAG
TCGGTGCCGGCCGTCAGCGCCGGCATGGTGATGGCCGTGTCGACGGTGAACTGCACCAGC
TTGCCGTTGACCTCCACGATGGTGCCAGCCTTGATGCTGGCCGTTGCGTTGCCGGTGCGC
GTGAGGGCGACGGCCGACTTGTCGGCCTTGATGAAAGCCGTTTTCACCAGATCGTGCCGC
GCCAGGGGGAAGCCGCCAGCGGTGGCACCGTCATGCACAACGACCGTGTTCTTGTCGGTA
TCGACGGTAATCTCACGGGCGGCGCCCGTGAAGGTGGCGTGCTGGGCGGTCGTGCCGCCG
CGGAACTGGACTGCGGTACTCATACGGTTGGCTCCTAGTTATGCGATGTCGCCCCGGTCA
GCCAGGCCAATGTTGGCCCGCGCTACCGGAGGGTCGGCAAGATCGGACAGGTTGTGGGCG
GGCACCAGCGCGCCGGATACCGCGGCGAGAATGGCCACCAAGTTGTTGGCTGCTTCGCTG
ATGACCGCGATGTTCTCGGCCACGGCCGCGATGTTCTCGGCGTTCGCCCACACTGCGCCC
AGCACGCCGGCTGGCGCTGCGGGATTGCTGGCCGGCGACTGGTCGCCGTAGTCGATGACC
ATAGGTTGCCCGGTCAGGTCGTTGCCCAGCTAGGCGACGGCGGCCTCGTTTTCGGCCACG
GTGGAAACGTCGGCAGCGATGCCCGCGACGGTCTGCACGTCTGCGAGGTTATCGACAACA
GTGTTGACGTTTCCTTCGTTCGCCGCCACCGCATTGATATTGTTGGCATTATCGACTACT
GCGTTGATATTCGCCTGGTTGGCGACCGCTGCTTTGATGTTGTCGGCATTGCCGACCGCT
GCATTGATGTCCGCTTGGTTAGCAGCGGTCGCATTGATGTTGTCTGCGTAGTCGGCTACG
ACGTTGATGTTTGCCGCATTGCCTGCCGTCGCCTGCACGTCGTCGATATTGCCGGCTACC
GGGCCCACGTCGAGGATGTTGGACGCCACGGTGTTGACGTTGGCGTTGGCACCGGCCACT
TCCTTGATGCTCGCGGCGTTGGCGGCCACGGCGTCGCTATTTTCCAGGTCGCCGGCCACG
GATACCACATTGTCGATGTCGTTGGCGACAGCCAACATGCTCGACAGGTGCGTGCTGACG
GTGCTCACGTCGCCGATGTTTTCAGCAACCGTGTCCACGTTGCCGATACCGTTTGCGACA
ATGACGATGTTCCCGCCGGGCGGCGAGGTGTTGCCGATGGGGGGTACGGCAATGCCGCCA
AAGTCGTAGGACACGCCGGCCGCCCAGGTTCCGCCGAGGTAGCCCGCCACGGTATCGACG
GCGCCAACGCTGGCCGCCACCGTGTCGATGTCCGCCTCGTAGGTGGCCACGGCGTCGATG
TTGGCGAGGTGGCCGTACACGGCGGCAACCAGCGGTAGCTGTTCACCAAGCTGCACCAAA
ATCTCGCTCGATGACAGACCGCCAGAAATGGGGGCCTGCACGGTTCGGCTAAGGTTGTCG
GCGAGTTGCTGGATTTGGATAACCAGCCGGTCAAGCGCGTGCTCGATGACCTTGGGGTCG
AAGCCGCCCTGGTCGTTCAGTGTTTCGGGCTGGGTATTACGGACTTCGCTGGTCAGCGTG
ATCTTGTAACCAGTGGCATAGGTCGCCAGCGTGGTGAGTGTGCCGCCGGGATTGGAGTCC
TGGTTCTCATTCAGCGCAACGGTGTAGTCCGTACCCAGGGTCAAGGTGGATTCCACACCG
TCTGCGTCGCTCAAAATGGCGACGATTTCGCCGGTGTTGAACACCTTGAAGTCGAACGGG
AATTGTGTGGTCAGGCCATTGCAGGGATACGGCCCCGCGACCCGATCTGTGCTTTGGACG
GACATGGTAAAAACCCCTGGAGAAGCATCGCAAGGCTACGTCCCCAGGGGTGAGGCACGG
GCACCCTTAGCGGGCCTCTGGGAACCCGAACCCGATAGCCGCCGGGTTCTGCGTTTTGCC
TTCGGCCAGGGCCTGCACGCCGGTAACGGTGCGGTTGATCTGCGCGCTGGGCAGGCCGAT
CAGGTCGCCCACGACATTGACGGCAGTTTTCCGGAAAGCGTCGTCGAACTCGCCCTGGTG
CGTCTGGGTGGCGAACCGGCCCAAGGGGGCAATCATGCGCTGGCCGGCCGGCCCGGTGTA
GTCGCGGCCCATGTCGTTGGCGCCTGTCACGGTCTTGGCCGCTTCCCCGAACTCACGCAC
GACGACCATGCGGCCCAACAGGTAGTCGATATGGTTGGCGAGCAGTTTCTTGGCGAGTTT
TTCAGGGTCGTGGTCGCCCGAATCGCCAGGCGTCAGCGCATCCTTGAGGAAGAAGCCCAG
CACGGCCCGCACCACATAGGGCATGGCGTAGTCCGCCGCCAGCTTCGCGCACTTGGCCGG
AGTGTCCGCGCTCATGGTCTGCGCCACGCCCATGTTCAGGGCCGTGTTCATGAACGAATA
GAACACGGTGAAAAGCCGCTGCGCTGGGCCGCCGCGCTCAATAGCGGAAAGGTCTTTAGT

CTGCCCGCCACTCTGGGCATCTCTGACCGCCTGGTCGGCCAGGGACACGGCGCGGGTTTT
GTCGTTGCCCTCGGCGACGGCCTTTTCGTAGGCTCCCCACCACGTCGGCAGGTCCACCAT
CTGCTGGCATCGCGGCATCAGGAAATAGGCATTGCGGCCGACCCATTCCTTCGTGGCAGA
ACCATCCTGCACCTGATTGCGCAACTCGTTGAGTTCGCGGAAGCGCGTCCGCGCCCGATT
CGCCATGAACGAGGACTTGGCGTTGATCTCGCGCGTAAGACACCCCGGGGCGGTTAGGTA
CTTCATGACGCCGCGGCCTACCCACGGCGCGCCCACGCGCACGATGGACTGCGTGATGCC
CAGCGGTTGCATTAGGGCGCTCGTGACGTAGAAGCCCAGGCCCGACGCGCTGACGCCCTG
TCGCAGGCGGGACAGCGCGATGTCCACGGCAGCATCTGCGCCGCGCTCACCCTCGGCGAT
GTCGGCCGCCCAGGTCTTGAACTGCTGCTTGGGCTCGGGGCCGTAGTGCTCGCGGAAGGC
GCCGTCGATGGTCTGCGAACGCTGCAGCCGGTTGGCGTCGATCTACCACTCGTGCCACGC
TAGGTCGTGAATCACATCGTTGACGCCCGAATACAGACCGGACAGGGTATAGAGCAGCGG
GCGCCCGGACAATTCCTCGACGCGCGACTTGGTGAAGCTGCGCCGCGTAGTGGCGGTGGT
ATAGGCCCCTTGCTACTGGCGGCGCGCGCTTTCGGTGTCCGAGTGTTCTTCCGCACGCTG
GCTCGCGTCCGGGTCGTACTTAATCGGATAGTAGCCGCCGCGTAGGGTCAGGGTCTGTCC
GTCTGCCGTGGTGATGGTCAAGGGGCGCGGCTCGACCCACGCCGGCTCCTTGCCGTACAC
GCGCCGTTCTTTTGCGGCGATCTGCGGCCGGTAGCCCTCGAAGTGGTCCCGCACCGCCTG
CACCGCTTGCCATTCCTGCGCAGTCAGCGATTGCAGCACCGGCGCGATCTGCGCCGGCGT
CCACCCTTCGCCGCCCGGCAGGCGCTGCATTTTGCCCTCGTTGCCCATGTTCAGCGCGAA
GGTGAGGCGGGATTCGCGGTTCAGGCTTCGGCCGATGGTCGGGAAATGCTGCCCTTTGCC
TCCCATCTTGCCGAGGGCGAACTCCGGCGCGAGGATTTCCAACAGCTTGCCCGTGGCCTC
GCCGCGCCTCGTGGTTTCCATGTCGCCGCGCTCGTTTGCCGAACGCACGAAATATTCCCA
CATAGGGCCGCCGTCCTTGCCGCCGTCCATGACGCGCGCCCAGGTCGCTGCCTTGATATG
GGTGGCCCAATAGCGTTTGAGGCCCTGCACCGCGCGGCCGGCGTTGGTTGTGGGCGTGCG
GGTGTCGGCGCGGCGTCCCTGCGCGTGCGCCCGGATGCTGTCGGCGATTTCATCGCGCAC
GGCCTCGTAGTCGCGCTGGTGGGCGGCCGTCAGCAGATTGCGCTTCAAGCGGCCCAGGTG
ATCGAGCTGACGCACGCTATCGACAAGGCCGCGGAATTCTTCAACGGTCGTGTTCTTGTA
GGACGTGCGGTACGCCTCGTTTTCGAGTTCGGCCGGGATGTCCGGCTCGAACCCGGCCTC
GCGCTGGCTGCGTAGCCAGTCGGCCAGGGCCGTGCGATTGTCTACGGCCTTGAGGCTTTG
GCCCTTGCGCAGATCGAACCGTTCCAGCAGATTGGCGATCTGGTCGGCATAGTCCACGTC
GATGCCCTTGATGTCGCCGGCAAACTTGCCCAGGTATCGCAGGCCGCGCTCGACCTCATC
CTGGGCGGCGTAGGCCGTGCGTGCGGCCTCGTGCTGGATAAGGTGGTTACGCTTCTCGGC
CGCTGCGGTGGCGAGGTCGCCGGCATTCATCGCGCGGTCGGCATTGCGTGCCACGCGCAC
CTCGGCGTAGGTGTACTGGCCGGGTCGGATGTCGCGCACCTTGAGCCGGGACACCAAGGC
ACGCGCGTATTCACGTGCCGCTGCGGGCAGCACGCGGCGCACGATGGGTCGGCCGCGCCG
GTCAGTACCGATCTGCTGGGTCGGGCCAGCCTCAACGCGGGCCAGCGCGTTGGCCTCGGT
CGCCAGGAAGCGGGCGCGGGCAGCATTGTGAATGGCGCTATCGGCGGCGCGCTCGATGGC
TTCCGGCGTGGACAGTTCGCCGAACTGTTCCAGCATCCGCACGTCGGTAAGCGCCTAGAT
TTCCTCGCCGCGCGGGAGCGCTTCCAGTAGGCGGCGCACCAGTTCGTCACCTGACGTAAA
GCCGAACACTTCGGCCAAGGTATCGGAGTGCATCCCCTCACTGGCCGCGAGGCCCTTCTT
GCCGGTGGGCAGGTATCGCCAGATAGCGTTTTCCCCCTCGCCGTACATTTCCTTGAGGGC
CGGCAGCGACTACTTGGCGCCGTCGACGTTGGCCTACTCGGCGGCGCGGCGCCCGTTCTG
CGGCGCGCCCGGCGGCAGTTCCAGTTCACCATGCAGCAGGTAGCGGTCGGCCCGGTACAC
CGGCTGCGCATTGAATGCCTCGGAGACGTGGCGGCGCAGGTCGGCGCGGCGGGCGGCGGC
ATCCTTTGGCAGTTGCTTCATGATGCGGCCGCGTGGATTGCGCAGCCACTGCATGTCGCG
CAGGCCGCGGTCTTGCAGGTCTTGAATAGCGTCGTCGGTCGCGTCCACGCCGAGGCCCTC
ATAGGCAGCGAATTCTTCGGGCGTCATGCCAGTCTGTTCCGCCGTGGCGAACAGTGGCAT
CATGCTGCGGCCCTGTTCGGCCAGTGCGATCTGCGCGCCGGTGGCGAGCATCCGGTCGAA
CACGATGCGCACCGCGGGCGTGAGTTCGACGTTCAGGGCCTCCAAGTCGCGGTACACGTT
GAGCAGCCACGCGCGGAACCGCTGGAAAAGGCCGTGCATTTCAATGCTGGGTGCCTTGCC
CTCGAAGAGATACGGCTCGAAGCCGCGGGCGAACTGCTCGTGGTGGGCGCGCATCGCCTC
GAAGTCCAGATCGTTCCACGCGGCCAGGTCGCGCAGCCCGAACCAGTCAAGTAGCGTTTG
CGCATCACGCTTCACCTCGGTTGCGGCGTCGATGAGCTTGGCACCTGCGCGCTGCTTCTC
GGCCAGGCGCGTGGCGATGTCGAGCTGCACATCGAGCAAAAAGTGTCCGGAGTCATGCAG
GAACGTGGGCAAATCCGCATTATTAAGCAGGGAAAGGGTGAGCGTGGCGGGGTCAAAAGT
GCCGCGCGCCCGGCCCCCGCCCTGGTAGAACTTGTCTATTTTCTGATCTACACTAGCGTC
CGAGGCGTCGTCGGGGCGAGCCGTGCCATCCTCGACAAAAAGGTCGGTAGTGCCTGTCCT
CGTACCGGCCACGCCTCCTTTCTTCGGGAAGGCGGTCAGCAGCCAGTGTTTTCCTTGATC

GTCCCACGTCAAGCGCACGCCGGCCCGATGGTCGGCGGATTCCAAATTGACCCGGTACTC
GGTGCGGCTCGTGACCTTCATGGCTGACAGGATGCCCTGTAGGTCGTCCAGCACCTCGGG
GTGCCACTTCGCCAGTTTCGCCAGGCCGTAGCCGTCGCTGCGGCGTATGCCTTCTTCTCC
CCATACCAAGTCGATGTCGCCGATGTCCGGGCGATGCAGCGCGCCGATGGCCTTGCCACT
TTTCATTTCCAACAGCTTGGCAATGGCGCCCTTGGCGTCGCCCTGGAAGTCGCGCAGCAA
CGGCCCGAACGGGCCGTCCTCTTGATCGAACTGCTGACCGCCGGCCACGCTTTCCGCCGC
CAAGCGCAACGGGTATTCCTGGGACAGCGCGTCGGGCGTAGTACCCAGGCGCGCAGCCTG
CACCGCATATAAATTCCCGACCATCGACGAATAGGCGTCATTCACCTGCGGCGTGAAACG
CGCCGCCGTGTCCAATTGCGTCTTGACGGCAGCGCGCACGGCCTCGGACGATTGCTTGAA
TGCGTCGTCGCCTTGTTGCTCGCCAAGGGCGCGCTCGACTTCGGCCCGCAGTTCTTCGGC
CTGGGATTGGATCTACTGCTGCGCCTCGGCGCGGCTGAATCCTTCCGGCTCGGTCTTGAG
GTGGTCGAGCAGGCCCTGCGGGGCTTCGGTCGGCGCGATGCGCGCGGCGTAGTCCTCCAC
CGGAAGCGCGACCTGCCCGCCGGTTTGCAGGGCCTCGGGCAGTTGCGCGGCGACAGTCGG
GGACACTGCGGCCACCTGTTCGGCCAGCCCGGATTGCAAGAGCGTATTGGCGTCGATGAA
CACCTGCTGCACCGGGCCGTTCTCGGCCGCATTGGCGCCGAACTGCTCGAACGTGTCGGG
GTCGCGCGCGAGCACCTTGTCGGCTGCGGCCAATTGTTTCAAACGTGCGAGCAGTTCGGC
CTGCTGCTGGGCCGCCTGCGCACGTTGCGTGCGCTGGTCGGCCGTGGTGATGGTGCGGTC
AATGCCGCGCATGACCGCCACTTTCCCGCCCACGCCCACTAGCGTGGCGATGAGGGTTTG
CGCTGCGGCTCTGGGACGCTCGGCCATGTAGGCGGAAAACGGTTTCTCCTTGTTCTCGGG
CAGAACGGCCCATTGGTTCAGGTCTTGCAGTACGGTAGCGACCTCCTCGCCCGGCACCTC
GGTGGCGACCTGATGGGCCAGGGTCTTGAGCAGCGGCGAGCCGGCGTGCAGGTCGCCGAT
AAGGCGCGACACCGGCAGCTTTTCAGTGGCGTATTCGATGGTCGCCTGCGACACCGCAAG
GGGCAGCGCCTGCGCTGGCGTGAGGCCCTGCGAGCGGGCATCCTGATAGGACTGCCCGCC
GGTCCTCGAAGCAATGCCGCCCAGGGCCGCCGCCTGGCCGCCGGGCAGCAGTGCCATCGG
AAGCATGAGCACGTTCGCCGTGAGGGACTGTACGCCGGACGAAACACCACTGCCCAAGGT
GTCAGACTGCGGCGGACTTAGCTGCTTGGCGAGGTCTTTGGACGGCGCACCCCATTGTGC
GAACTGAACGGCGACAGGTTGCAGCGGATTTGCCGGCAGAATGGTGCCGACCAGATTGTT
GGGCCATTCAAAGACGGACTGGAACAGACCGGCCGCGCCGCGTTTGGCCTGGGCGAACCC
CGCGCCAATATCGCCAATGAGGGTGTTCTTCTGGTCGGGTGCGCTGACCAGATACTTCAC
CGCCGTGCCCAGGCTGGACAGTACCGACTCGATGCCGGTCATGTTGTCGACGTTGTCGTG
CTCAATCTTGGCCTTTTCCACGTCGGCCAGCAGCGCTGCCGTGGCCGGCGAGGTCTTGCC
CAGCGTGTCGAAGTCGATGGCGCCGATGGTGTCCTGCCGTTGCGCTTCCTTCGGCTGGTT
GAGCACGGTTTCAATTGGCAGGTTGGCGCGACGCGCCACGCGCTGCGCTTCCACGTAGGG
GTCGGGGTCAGTGTCCGATGCGAGAACGAAACCGGCCCGCGCTGCCTGCCCCTGGTCGGG
ACGCGGCAAACCGGCAACAGCGGCATCGAACGGATCGGCGGTAGTGCCCTGGGCGGTTTG
CTGGCTCAGCGTGCCGCCTACGGCGGCGTCGAATTCGTTGCTCATTTACGGGAAACCTTC
AAATTCCAGTACGCATTGAGGTTTTGCGAGTCGGACGGCGAGTCGATGCCTGCGCGCTTG
TATGCCGCCTTGATGCCGTCCTTGGTGGCGCTGTCGATGTCGCCGACCTTCAAGGTCAAG
ATCGGAACGGACGAATTCGAGAACCCGCCGCGCACAGTGGCGTTTTTCATGGACAGCGCG
TCGAGGTGCTGCTCGACCTCGGCGTCAGTGAATTTCTTGCCGGCCTCGCGCTGCGCGGCG
ATGAAATAGCCGTCGATGAATTTGCGAATGGCGCCGACGCGCTCGGCATCGCTGCTGCCG
TCCTGTGGCGTCGGGTCGACGCCCATCATGCGCAGGCGCGTGTTGAGTCCCGCCTTGATA
GCCTGGCTGTTCAAGTCGCCCGGCCCGTTGGTGCTGGGTGCCGCACCCAGACGCTTGGCG
CGCTCCTGGGAAAAATGCTTGAAGTCGGACTCAGACAGGCTGGTGCGTAGTGCGTAGAAC
TCGTTGTCGGACATGCGCGCCAACTCGTCCGGGCTGTTGGTCAGCTTTTTGTAGAGCCAC
AGTCTCGTGATGTCGTCGCCTGTGGCTATGCGCTCGGCCGCGCCCATAACGCTAGTCAGC
TTCGCGGGCGGGATGTTCGCGCGAATGGGCACCGGCAGTTCCGAAAACCGCCCGACGTTC
TCGTACAGGCCGCGCAGCGCAGTGGCGACGGCCTCGTCATCGCGTTGCTTGAGTGCGGCC
GTCTGCATGTTGAATTGGCGCTCGGCCTCCACGCGCGCAATCTTGAGCCGTTCCGGGTTG
CCGGCCAGGCGTGGGTCGTTCTGCAACTGCGCCTCGATGTCGGCCAGTGTCGGCCGCGCT
GGGCGCCCTTGGCCGGCGTTGTACGCCTGCATGTTATTAACCACGTAGTCCTGCGTTTCC
TTCGGCAGTAGCGCCAGCCAGTTGCCATCCTTGGCGGCTTTCAGCACCGATTGCAGCGCG
CCGGGGCCGGCGTTGTAGGCGACGTAGGCTTTCGGCAGGTCGCCGCCGAATCCGCGCGAT
TGCTTCTGGAAATAGGCCATGCCCAGCGCGCGGTTGTAGGCGGCATCGTTGCGGTAACGG
TTCTCATCCCACGGCAGGCCCGCCAGTTTGGCGGCCTCGGGCGCGGTGTCGTACATGACC
TGGGCGATGCCGACCGCGCCTTTGGGCGAGGTAAGCGGTTCTCCGTCCGCGCCGAACTGT
CGGCCCCCTGACTCGATGCCCACGGCGATGTTGAACGCCCGCTAGCCATCGCTCACCTGG

ATGGGAACCTTGCCGACAGCATCGGTAGCGGCAGCAAGTCCGGCGCGGCCGTCCATGTCC
TTGGTGATATGACCGCGCACCGTGAGGATGACGTCGGCGTCCATCTGCCCGGAATATTTG
TTGAGGAAGGCCGAGGCATAGGTCGGGTTGTTTTGGTCCAGCGTCGACATGTGCGCAATC
TTGTGCCCGGTGCTGTTCAGTTTGCGCGCCTGTGCCTCCTGCCACTCGGCGGATTTGCCC
AGCAATTGCGCCTGCCGGAAGGTTTCGGCCTTTATGCGATCGACAGCCGAATTGATGGCG
TCGGGGTTGTTCCAGTACAGGGCGATGTCGCGCAGCGCCGTGGACTGGATGCCCTCGGAC
GTGGAAAGCGCGGAGGTCTTGTAGTCGCTGGCCCCGTGCTGCACAGCTTGACCACGGAAC
GAGGTCAGGATGTCGTTGGAATGGAGCGCGAAAGCCCGGCGCTGGGCGTCTTTGCCTAGG
GTGCCAGAAATTTCGTCCAGTTGCTTCTTTAGGTTATCGCCGTACTCGTCGGCCAGCGGC
TTGCCTTCCGGCCGTTCCAGCGCGTTGATACCCTTGAGGTTTGCGAAGCCGGTGTCCTTG
TCATAGGTCAGGCGCAGCGCGGCTTCCTTGGCGCGGTTAAGTGCGTCGTCCACGCGCAAC
TGGTTGGCCTGCTGCACCATGCTGTCGGCGATGCGGCCCACGTCGTTGCTGAACTGGCGC
GCGCCTTGGCAGAACTCCCGGGCCTGCTGGTTCGCGGTGTCCTGGAAAGCGGGGGAATCC
GCGCCGCGGAACGTAGGCGTAGCGAAGCTGGGCGTCGCCAGGCGCGCCTGCGGCACCGTG
TTGGGCGAAACCTGCGGGGAATCATAAACGGGAACGCGCGGCATGGTTCTTACCCTTTGG
ACTGAGAATACCGATACCAGGAATCAGCCACGTCGCCGGCGCCGGACAACAGCGACGAGA
AACCCGCCCCTACCGGGCTGATGCCGGCGGCTGATCCGCTTTTCAGCAGTGCGTCGGCTT
CCATGCTTCCGGCGCCGGTGCGCAGATTGATACCTTGCACCTTGGCCTGGATGCGGGTGT
TGAGCCCTTGAAGCCCGGCCTGCAGTTGCGCCTCCATGCCCTGCGCCCGGTAGCCCCAGG
CAGTACGCGCAGCATTGGCGCGCAGCGTTTCGGTGTCGATCTACTTCACGATGTCGCTGG
AAACCTGCACCTCGGCGGCGCTGCCTTCGCCCAGGTAGATGCCGTTGGCGGCCAGCGCCG
CACGCTGGGCGCGTTTAAGTTGCCCGGCTTTCAGGGTCTGCGCTGCGATCTGCTGCTGGC
CGGCCTCAAGTGCCGACTGCGCGCTCAACTCGGCGATGCTCGCGTTGAGGTCGCCGGTCG
CCAGAATGGGGTCGGCATTGGCGTTGCCAATAGACTCGATGGTGTCCGCGTTGGTGTTGG
CGATACCCGCGTTGATGCGGGCAAGGTTCGACTGAAATTGAAGGCTGCTTTGCTGCGACC
GCGCGCCGTAAAAGCTGCCTACCAGGCCCGACCCCATGCTGGCGGCGGATGCGCCCAGGG
CGACGGCTGATGTTGCTCCTGACATGGCATTTTCTCCTATGCGCCGATGGCGATTTCCGC
CGACATCGACACGATCATCAACGGCAGAGGGTCGGCCTGCCGCACAAACAGTTGCCCGCT
GCCGCCCCATTTCGGCGACAGGACGAGCGGAATTTCCTCACTCTTGAGTGCAGGCGGTGA
GCCGTAGGGTTCGGACGTGCGCTGCTTCAGCTCGGTGAGCGCGTCCGCGTGCGGGCCGGC
GAAGATGCCGGACGACCGATGCACGCGCAACCACAGCTTGTTGATGTTCTTGACGCGCCC
TTGGCCGAAGCTGCCGTCCAGTTGCATCGCAACCCGCAGGGTTTGCAGTTCTGCGGTGAT
GGGCAGGCCGATATGCACGGTGCCGGCCTCCACGTCCAGGTCAATGGCGCCGTCAGTTAC
GACGCGCTGCGGATGCACCGCGCCATCGGCCAGGATGCTGACGGTTTTCCCTTCGAGGTC
CTCCAGCCCGGTGATATGGCTTACCGGCTCGCCCGACTACGTAAGGCCGCTATCGACGAT
GAAGGCGTCCTCCTGGGCATCGAACTGGCGCGAGGCCATGCGATCGACATACCGCACCTC
GTTGCCGCCAATGGTGCGACGCACGTCGGCAAAGAGCCGGTCCTCGTTACCCTCGGCCAC
CACCGCGCAGGACTCGAACACGCCGTCCGTGTCGTGCTGGTGCCACGCGCCGATCTGTTG
CTCGGGCACGTAGGTGAGGCCCAGCAGCTTGCCGCTGCTGCTAATGAACCACACGATAGG
CTGCGGCGCCTTCGCGTAGGCTATGTCCATAATGTCGAGATTGTCGAACAGGTGCGCCGC
GCGCAGGGACAGGTCGCCGGTGACGAAACCGTTGGCCTGCCAGTTGTAGGCCAGTTCGCG
CACGTGGCCGCCCCGCACGGCGCCGTAGATGGTCGTGTTGTTGCCCACGATCGGCTGCAC
GTCGGTGGCCCCGACGTAGGACTGCGGCCGCACGCTGATGGTGGTGGGCGTTATCGCGGG
GGAGTTCACCGACGCCACGCGCCATTCTCCCGAAGACGTGAGCAGCAGCAACTCGGTGAG
CGGCACGATATGGCGGATGGCGTTGGCCTCGCTCGCGGCCACCCGAAAAGCAACCCGATC
GTCGTCGCGCACGGGAAGCGAGTACGACATGGCCGATTCGGTGCCACTGCGCGTCATCCA
AATGTTCTGCGGCTTGTTGGTAGCGCCGGCAAAGCATGGGCGTTGCTCGAAGTAGGACAC
GGCCGCGGGGTAACCGCCGGCGGTGTTGAACACGGCGTCGTAGATCCGGGGCGTGACGGA
CAGGACGGGCGCGACATTGTCGTCCACCAGCGAGGTGCCGGTGGTCTGGCTGATATAGCC
GTACAGGCCGCCTTGCTCATTATAGACGTTGTAGCGCGACGCGACGGACGAGGCCGACCA
CGCGATGGTGTTGGCGCCGCCGTTGGTGAAGAGGTTGTTGGTGCAGGTGCCGGCCGACGA
GGGAGCGGACTCGGTTCTACCCTCGGCATCCAGGCCCGTGACCACGTAGCGGTAGGTGTA
GTCGGTGCCCTTGTTGTTCGACGTGGTGGTCACGCTCGTGGGCGTAGCCACCGGCGACGT
GAAGGCGATGGTCGCGAGTTGCCAGTTGGTCGCGCCCAGGCGGCGCAGTTCACGCGTCGC
ATAGTTCGGATGCACGAGCGTGAGCACGTCAGCCGACTGGACAAAGTGGATGTTGAACAG
GTCGGCTTCGGCGTAGGGATTGGCGATGTCGTAGGGCACGGCGCCGTCCAGCAGCGTGCC
GCCGTTGGTATGGAAGCGAAAATAACCCGCACCCAACTCGATCACCATTGTTTGCATGAC

CGAGTAGGTGAACGGGATGAGCCGCACCTTCTTGGCCGAATCCTTCACCTCGCGCACGAA
GGCGAAGCCGGCGCGGTTATCGGCCTTGCCTTGGGGCTTGACCACGAAGTTGCGGCAGAT
GGCGAGGCCACTTTGGTACTTCACGTCGTCGATTCGCCCGAACATTTCCGGGCTGATTTC
GCCGCCGCCCAATGAGCGTTGAAGGACGCGGACGTTGGACATCACCCCCTCCAACCGTTC
GGCAAGCCGGGGATATTGCCGTCCACGAAGCCGGCGCCGCGGGCGCGCATCCATTCCGGC
ATATGGGCCGGCTTGGTCCTGCGTTGGTTGGCATCGGACACCATCGCCTGCGACAGGTAT
GCCTGCATCGCGCCGACGCACCGCTTCGATGCAGCGGCGTCGACGTCGCCCTTGAGCAGT
GGCCCCGCCAGCATCGAGGCCAGGTGCCACGCCAGGGCCTGCACGAACAGCGGCGAGAAC
TAGGTCGTGTCCTTCACCAAGCTGACATAGCGCGCCACCGCGTTGACCCGATTGGTCAGA
ATGATGTCGGCGCCGGTGTTGTCGATTTCGCATCTAAACGGCTGCGGGTTGTCGATACCG
GCCTCGATGTTTGCCGCATCGTGCGGCAGTACGGCCACGATCTTGAAGGCGTCGGCGGGC
TGGGCGTAGGCGTAGCGCCACTCGGGCCGGGAAATGCACATCGCGGCAAGCTGCGCGCAT
TTGGCGGCGAAACCCCATTTGTGCAGTTCGAGCAGCGAGTCGCGCGCGAACGGATAGAAG
CGCGCGCAGTATTCCGCCTGCACGGAACCCTCGGGCGGGTTGATGCCCGCCACCGTCGCC
TCGTCGCCCAGGTAGGCCAGGGCCAGATTGCTGATGTCAACCTCGGATGCCATCGAAGCG
CCTCACGAAAAAGCGGGGGCACAAAGCCCCCGTAAGTTCGCGGGTATCACCCCACCGAAG
GAGACTCGTTACACCAAGTCGCCGGCGGACTTGTCGTCCGAACCTTGCGGGTTATTGTCG
GCGGGCTGCTTGGGGGCCTTACCGGCGCGGGGTGCGTGCTGGGCCGCCGAAGCCGGCGCG
AACTAGGACGCGTTCGCGCCGTCCGGTACGTCGAACTTGTCGCCCGGCTCGCGCAGCTAG
CCTAAATAGCCCTGTTTCAGTGCGATCACTTCCATGCTGTCACTTCGTTACGCTATGCGC
GGGCTGTCGGGATAAGCCACGTTCTGTTGGATGCCGGTCACGACCTGCGCCGAGAACTTG
CCGGCCGTCGGCGGGCCGGTGGCGACGATGTAATAGACACGGCAGTACCGGCGCAGCTTG
GTGGGCATAGGGATGACCACCTGTTTACCGGCGGCGAGGTTGGCCTTGCCGATGGCGCCG
GTGGCGGCCACGTATGCGAAGGTGGCGTTGTCCGCCGAGTCCTGCACCGAAAACGTGACG
GTCGCGGCGCCGGCGGCGTCGGCCGATTCGTCCACCGTGACGACCATCTTGCTGCGGTCA
TCCATGCCGGTGTTGGGATTGGCCTGGCCCAAGTCGATCACTTCGGTGGAGGCCGCGCTC
GCGGTGACGGGCTGGCCGTCCGAGACTTGGAGCAGCTTGTCGATAATCATGGCTGGGATT
CCTTTCTGGATTCGTGATCTGGGGCGGTCGCCCGCCCCCGGCCTGTTACACCACGCGGGC
CTCGGTCAGCAGCAGCGCATCGGTACGGCGGCCCGGGATGCCGTCGAACGCGACCACCTT
TTTGCCGGCGATTTCTTCCATCGTCAGCGTTGAGGCCGCCAACTTGTTGGTTATCTGGCG
GCGCAGGAAGCTGCGAATTATGCGCGGCATGTAGAAGGCCGGGAGGCCCATGTCCACGTT
CGGGATGGGTTCGATCGCCTGGGTCATCAGGTCGATGAGGTCGGCGACCGCGCTGGCGTT
CTTCGTCAGTTCGGACACGTCCACATTGGCGATGCGGACGACGTAACGCCAGTCGCGCAG
GGTCAGGCCGATGTCCCATTTGTAGTGGGTGCGGTAGCCCTGGTAGCGGCCGCCGGCGGC
GTTGATCAGCGTGCCCTCGCCGAGGTCACTCGATGGCAGGCCGGCCTGCGAACCCTTCGG
GGAGATGGTATGCAGGGTGTTCGGTCCCCACACCGTCAGCCAGATGGATGCGTTGTCGCT
GCCCGTCCCGCCGGCATCGATGATGGTCTGGCCGTTCTCCGCGGACAGTCTGTTGAAGCT
CGGCGTCAGGCCCATGAACTTCTCGGCGTCGATGCTGGAATCGCCGTAGAACAGCCTGGT
CGCCTGGGTCTGGTTCATGCCTTCGATGAAAGCGCGGTCTTCGGACAGGCGCCAGGCGGC
GGAATTGCCGTTGAGGTCGGCCAGGGCCTTGTCCACGTCGGCGTAGGTTTCCAGCATCCC
CATGCCGTCCTTGACCTGCACGGTGCGGGACTTCTCGGGCTGCACGCCATAGTTCAGCTT
GCGACAGGTGCCGGCGGGCAGGCCGCTGCGAACCGTGGTCTTGTGGTCGGTGAAAGCGTT
CGCCTCGATGACGGTCATGGCGTCGAGGATTTCGTTGGTTTCGTTCAGCATTTCGACGAT
CTGCGGGTCGATCTTGCCGTCCGGGGTCATGCGGGTCGCGACATCGGCCAGCGTCGGGTT
GGTGGTGGAAAGAGTAGGCATTTGGAACGCTGCTTTACGGGATCATGTTGGAGGCTGCAT
AGAGGCGCCGCGCATCGCCCTGCTTGGGCTGGTTGCCGTTGTTGCCGGCCACGAGGCGGT
CTTCACTGATTGCCAGGCCGGCCCGGTAGAACACCCGGTTGATTTCGGGGTGATTGCCCA
GGCCGGTTTTGTTCAGCAGCGTGGTGAGTTCGGGCCTGCCGAAGGCGTCGCGCGCCTTCT
TGGCCGTGGCATGGTTTTCTTGCAGCTTGTCGCCGCCGAACTCCTTGTCGGCCGTGGACG
AGGCAACCCAGGTTTCGGGCAGGCCGCCGATGTCCGCGTAGAGCTCGCCAACTTGCTCGG
CATGGCGCGCCTGCATCACAGGCAGCATCTTGTCGAGCACCAGTTGCGCCTTGTCATGGG
GCATGTGGCGTTCCTTGGCGACTTTTGAAAAGGCGCCCAGCACACCGTGGTCGAATGCCA
CGCCTTCGGGGGCCTGGAATTCGTATTGCTCGGGTGCGCCTTGCGGCTTCGCCTTGTCGT
CGCCCTCGGTCTTGGCAGCGCCCGCGGCCGGGTGTTGGCCTTGGGTGCCCTGCTCCTGGG
TCGGCTGTTGCTGCTGTTGGCCGTCGTTGCCCGCACCAGTAGCGGGTTGCTCGGCGGCCG
GTTGCGATGCGGGCTGGCCTTCAGTGGTCGTTGCGGCTTCCGTCATCAGCGTTTCGGTTG
TCATGGTTCTGTTCCTTCACCATCACGCCATAGAGTTCAGGGCAGAGCGTGTGGATTTGC

GCCAGTAGCCGCAGGCCCGCGTTCCGGTTCCATTCGGCCAAGGCCATAGCCATCGCGTTG
GTGTTGAACGAAAGCCGGTACACGCCGGCTCGTTCCAGAAACCGCCACACGATGCGGCGC
CCCCGCTTGCTGCTCATCAGCCACTTAAAGTCCGCTTCCTCGGTATCCTTCGCCAGGCGG
CGTCGCAGGTCGGTGTCGGCCTGCGCTCGTTCTTGTCCCCGAACATCGGTGGGGTCGTAA
TTGCTCATGCGCGCGCTCTATGGGCTGGACGAGGAGGCACGGGCACCCCATCAGGCCGAC
ATGCTGCTGGCGCCGTAGAGGCGCTGCGCCGCACTCGACTCATCGCGCGGCTGCACGTCC
ATGTCGGTGATCTGCAAATCGACATTGCGTGAGGCCTTTCCGTCATCGCGGGATCTGACC
GACTGCACGACGGCGCGCGCCGTGATTTCGGACACGGAACCGACTGCCGGCAACGTGTCG
ATGCCCAGCCTTTCGAGAGCTTCGGTGTTCGGGTTGATGCACAGCCCATACGGGTATTCG
GGCGTGTTGTCCGAGGCCGATTCACAGCAAAGCATCGAGCCGGCTTCCATCGGGGTCATC
TTCATGTTGGTGAGCGGCATGTCGGCCTCCTATCAGGTGTACCCGCTGAATGCGCGCGTC
ACCTCAGTGAGCGCGTTTCGCTTGCTGGTATCGACGCTGCCCAGCTTGGCCGCCGTGTCC
GCGCCCTGGTTCTACAGCGCGGCCTGATGCGCGGCCTGCTGCTGGTCTGCGTGTTGCTTG
CGGATAAGCGCCACCTGATTGCCCGGCACTATCAACTCGGGGTCGATGCCCAGCATTTCG
GCGTAAGTGTCGGCCCAGCGGTCGGCGTCGAACTTGTCGAGCACTTCGGGCTTGATGCCG
GCCACCGCGCCCAGGTTGCCAACGACACTATCAACCGAGTTCGACGCAATGGCGCGATGG
GCCGGCGCGCACATGGAAATGAACTCCACGTTCAGGTCAACGCCCTGCATTTCCTGCGCC
GGGGGCGGCAGGATGTTGGCCTCGACCATGCGCTGAAACGTCAATTCGATGAGCGGGTCG
AGGATTTCGTTGTGCATCCGTTCAAGCACCGGCCCCAGAATGAGCAGCTTTTCCTCGTTC
CGCTCGGCAACTTCGGTGGCGGTCATCTGCGGATTGGTGCCGTTGGCGAGCATGAGGAAC
AGGTCGGCGTAGAAACTCGCCTTGATGCGCTCGGGCACGTCCACGATGTCAGCCAGAAGG
TGCGACAGGTCGAGTTTGACCTTGAACGCGGTACGGATGCCACCGTTAGGCGCAGCCGCG
TCCACATAGGACAGGCCCCCCGGCACGGTGCTGATGTCCTGGTTCATGGCGGACACCGGC
TGTTGCAGCGGCGGGTTCCTCTTGTAGTCCATGGCCTGCGCCATGCGCAGTTGCTCATGT
TGTAACTGGCGCACGTCGCCCAAGGCTTCCATTGCGGGGCTGTTGCCGTAGATGTCACCG
CCGACCAAGGCCCAACGCGGGCACAGTGCGCGGAACGACCGATAGCCTGATTCGCGCAGC
GTGCGGGTTTCGTCGGCGCCTGGCTCGAAATAGACCGACTTCCACGCCATGTTGCGGTCG
TCGCGCTTGCTCGGGTCGCGGTCGGCGCGCGGCTCGATGGCGTGAATGACCGCAACCCAC
TGTTCCAGCGCGCCGCGGTCGAACAGGCTTTGCACGGTGGTGCTGCACGTGTCCTTGCCG
AATTCGCGCACCATCTGCGCGACTGTGATCTGGAATTCTCGATACAGCGTATTGACGCGG
CCCTGGTTGTCGGCCGCGATGGCGTATTCGCCGGCCGTCAGCGAATGGTGATAGACCACG
GCGTCGAAGTCGGTCAGCACGATGCTGCTGGCCGTGCCGAACGCGCCGAGTTCTTCGTAC
ATGGAGGGCAGCGCGCGATAGGTGTTCGATTTGGCCAAAATCATCAGCATCAAGTGCGTG
ACGTTCGCCAGCCACGCCTTGACGGCGGAGGACTCGTCCAGTTCGGGAATCGAGGTTGTC
AGGCGGAACCAGGGGCGGGCAGGGCTTGTCATGCCCGCCATCATCCCCGCAGCGAGCACG
CGCAGCGCGCGCGTGCCGGTGTTGTCGAGGATGTTGTTGTGCCGCTTCTCGCCGCGGTTG
CGATCTTGCACGAAGAAGCGCCGCGCGCGCGCCAGCAGGTAGTCGCTGATTTCCTTCCAG
TGCGACATCCAGCTTTCGCGCTCGGTGCGCAGTTGCCCCCAGCGCGAAAGCAGCAGTTTG
CGCTCGGTTTGCTCGGCCACGTTATTGCCCCAGCAGAGTGGACTTGACCAGGGTGAGGGC
CGATTGGTCAACGCCCTGCGGGCCGGTCAGCATGGTGCTGGACTGGCCCGACTTTGCCGC
GCTGCCGACAGCCGACAGCGCGCGCTGCGAATTGGGCTGCTTCTGGTTCGCGCGGTTTAA
CGACTGCTGCGAGTCGAGCAGTTGCTGCTGCATGATCTTGGCCTGCTGTTGGTAGGCATC
GGTCTGCTTCTGGTATTCCTGCGTCAGCCGGTCGGATTCGGCTTTCGCCTGGTCGGCCTG
CTGCTGCGCCTGCTGTTGGGCTTGTCGCTGCGCGCTGCGTTGTGCCTTTTCCTGCTTGTT
CGACATGTACTTGCTCGCCCCGACGCCAACGGCGGCGCCCACGGCGACAGCGGTAACGGC
TCCTGACATGATCTATTCCCCCGTGAAGGTGATGGTTTCGGCCCCCTGCTGGTGGGACAG
CAGTAGGTCGGCATCGTCGGTGAATTCGGCCTCGGCCTCGGCCAGCGACCGCGCCTCGAT
GGGGAACAGCATGGTCAGGTCGGTGTCGGCGTGCGCCACGAATGCCTGTTTGCGGCCGGC
ACTGGCCGGGATGACGTGGTAGCCGCGCAATTCGACGGCCTCGCCGCCAATGAACACGGT
GGCGTGCCCGCTGAAAATCAGCTCGGTGGATCCCTTGATGAGCGCGCCGGTCCGTGTGAC
GCCAGCTGGGATGCGGATGGGTCGCGCGTACACGCCGGCGTGGACCAGGTGGGCCGTGGG
AGTCACGACCTGCGGCAGCTCCGCGAGCGAGCCTTCCAGGAGGCGCACGCTGTCGATGGC
CTCGGCCGACATCGCCGGCAGGTGCGGGCGGGGGGCGACGATGGCCGTCATGCGTGCAGC
CCCCGGTAAAACGTGCGGTTGGTTTCGCGGTAGCCGGCGGCGGGCAGCATCTGCACCAGG
CGACCCTCGGTCGGGCCGCACAGGTACAGGCCGGTAGCGCCCAGGTCGCGGGCCAGTGCC
TCGGTGGCGCGCAGCAGCGCCATGCCGGCGCGACCGGGCCGATGCGCCGCGGCTACGAAA
ATGGACTCCGTGGTGCCGATCAACTGGCCTTTGAAGTGCGGCAATGGCGTGACCAGCAGG

CAGGCGAAACGCACCAGTTCCTCGCCCCGGAATACGCCAAGGAAGCGCGCCACGCCCGCG
GCTTCCATGCCGGCATAGGCGTGATGGTCAGGCGCACGCCCGGCGACGTCGGCATTTGCC
GATTCATGCGAGTAATCCGCGCACAGACTGGCGAAGGTGGGCGCGTCGGCCAATTCAGCG
AAGGCGACGTGACGGACGATTGCGGTTTCCATGCCGTGCACAGTAGCGGCGACCGCGCGG
GGCACGGGCACCCCGCTACATGCGCGCGTAGGGGTCGTAGTCCAGCCGGCTGCGCGCCTG
GCCCAGCGCCTCGACGGCTGCGCGCTTCGGCGTGTCCATGAGCGCGAGCACGTAGGCGCT
GCCAAAGTCCGGCGAGCGGCCGATCTTCTCGATGATGTCCTCGCGGCTTGCCACCTTGAG
CGTGGCGCCGGACAGTGACCACGTGGGGGCGATGAGGTTGGCGAGCAGCCGCGGGTCAGG
CGGCAGAGCGATGCCCGTGTTGTTGGTCGGGTCGAGGGCTTCGCGCATTCGCCACCACAA
CTCGCTGCGCAAGTTGAAGAAGCGCAGCCGGCCCGACTGGTCGGTGCCGCGCGCGGCCTT
GGCGACATTGACGCCGACGACCTGCTGCTTGGCCTGCGCCAGGAAGTCGTAGGGGCTAGC
GCCTACGCCGATCACGTCCAGGTGGATGACCGCGTGGTCGCGCAGCGCGGGGATGGCGAG
GCCGGCCACGGTCGGGCCGTCCGGCGTGTCTTTGCCGGGGTCGGTCAGCGGCACGTCGAA
ACACATGGCGTGCCGGCGGGACAGGATGGTGTTGTCGCGCCCGCCGCGGGCCACGTCCAC
GCCCAGGCTGTCCATCGGTGCCAGTCGGTCGGGCTGCTTCCACCGGGCCTGCGAGGCCTC
GACCCATGCTGTCGGAACAACCTGCCACGGGTCGTGCTCGATGCCCGCGTTGAAGTCGCC
GTACAGCATCTGGCTGCGCAATGGCTCGGGCAACTATTGCAGGGGGACGAGATACCCGTT
ATCGACGTAGTACGTGTTATCGGTCTCGCGCGCCGGAATGAAGGTGCGCGACTTGGGCTG
GATGATGTCCTCGGGGTTGTAGTCCGCGGGGTCGAAGTCGTTCTCGACGCGCCCACCCAC
CAGCACAAAGGGTGCCGACGACAGCGGGTTGCCGTCCGAGTCAAACCACGTGTCGCGCGA
TCCGCTGTTACGGTCGGGCAGCATCGCCACCCAGCGCAGCGCGCCCGGCGCCGTAGGATA
CAGCGGGTGCTTCTTGTCCAGCCACGGGGCGAAAAAGTCGAGAACCCACCGGCCCTCGCT
AGTAGTCGGCGGGTTGAATGTCATGAGCACGCGCGACCGCTGGCCCGGCTTGTTTGTGCG
GTTCAAGCCCATGACGAAGCGCACCTGTTGCTCGCGCTGCTCGGTCACTTCGTCGAATGC
CTTGAGGTCGTGCGGCCGGCCCTGCCACCGGCGCTCGTCGCCGGGATTGTCCAGGCCGGC
CAGTTCGCATAGCCGGCCACCGGGCAGGCGCCAGAAGCCCTTTTGCGAGTTGTAGCCGTC
CGTGACGTCCATGATTTCCGTCATGCACTGCACGAAGCCCTCGGTCTGTGCTTTGTCGCG
CCTCACTATGAGGGCGCGCTCGTCCTTGGTCAGGGTCAGGCACGCGATGAGGTCGGTTTT
GCCGCAGCCGGCAGCGTCCCCGGCGCCGATGATAACGGCTTCGGAGTTGTACGCCGCGGT
CTGCGGCCCAAGCAGTGGCCACCACGGCGGCGTGGTCGCCAGCAGCGCGTCCACCTCGGC
GAGTTCTGCCCGCGTCATGTACGTGATCAGCCGGCGGATTTCGGCAACCCTGCACATACC
AGCACCGCGGCGGGGTCGTCGTCCTCACCGGCGGCAGCACGCTGGGCGCGCTGCTGGGCG
GCGGCAACGATTGCCGCGATGCGCGCGGCGCGCTCGGTATCGTTGATCTGCACCGGGCCG
CCGCTGGGGCCGGGCAGTTCCATGCTGGTCTTGTCGCCGTACTTGGTCGGATGCCACTTT
GCCAGTAGATTCAGGCGGGTTTCGACTTGCAGCTTGGGGTGGCCCAGCATGTCGCCGCCC
TTGACCTTGTAGCCGTGGTCCGATTCCTCGGTTTCCTCGCCGGTCTGCGGCGTATGGGCG
ATTTCGAGGCTTTCCTCGGCGATGGCATCAAAGCCCGCGCGGCGGGTGCGCTCCATGCGC
TCGGCGAACTCGGGGCGGGCCTCAATCCAGTGGTACACCGTGCGCCATGCCGGCATGTGT
TCGTCTAGGCAGCTTTGGCGCAGCGGCTCGCCGTCTGCGATGTGGTCGCAGATTTCGGTG
CCGATGGCTTCGTTGTAGATCGGGGCGTTTTTCATGCGCGCAGTTTTGCGCGCGTCGCCG
AAGGCACGGGCACCCGCCGGCCTGGTCGGCTCGCGCATCGCGCGCGATGGATGCCATCAA
CGGACAGGCACGGTCTTGACGCGCACCGCGAACTGGCCGCGCCGCTCGTATGGGCAGATT
TTTGCAATCGCGCCCTTGGACTGCTCGAACTTCTCGGCCAGCACGGCGTAACTCATGCCC
TCGACCTCGTGCAGTTCCCGGATGCCCTCGACCTCTGCATCCGTCAGTTTGGCATTCGGG
AGATCCTCGCCGACTCGCAAGCCGTCATCATTCACCTCCACGGTTTCCAGTATGGCACCG
CCCTCCTTGAACGTGCTATTTTTTGCTTGCCTCGTTTGCCTCGTTTCTCATGCTTTTTTG
AGTAACTTTCTCAAGTACACGTATGAGAAACTTCCTAAAAAATGGGGTAGAAAAGAGGCA
AACGAGGCAAACCCAAGTTGCCCGGCGCTTTGAGGACGTAATAAAATGCGTATTGCAAAG
CCGGGCCACCGCCAGCGCGCCCGCTGCGATTCTATAAGGCGCAAGAAAATGCGAATAAGT
AAAAAATTGCTGATTGCCATAGGCGTGCTGTTTTTGGCAGGCTGCGCCGCCCCGAGGGAG
CGGGCGAATTGGGTAATGGTCGGCGAAAGCAAGGCTGACGGCAGTGCGACCTTGGGCAAC
GACGTACCCGCTAAATACGGCGTCACGGAAACCCCTGTCGAGTGGGACATCAACCATGCC
AACACCGAAGCCGACCGACGCTGCAAGAACTGCTGCTACAGCGGCGCGGATATGTACCGT
GAAGGACAATTCCCAATCCGGAAAACGTGTCATGCTTAGGGCGTCAGCCCCTGCTGGTCG
AAAATCTACCGGGTGCAGTACGAGTGCCTTAGCCTGCAAAGCTGACGCCGCGCGCATCAC
TCGAAGTCCCCCACGCGCCGCACGCGGATGCCTCGACGACCCTTGCCGCATATTCCTTGC
GTGCTGTCGATAGAAGGAAATCCTTTGCTGTCCAGCCGGCGCCCCAGGCTCTTGGCCGAG

GCAATGAACCGTAGTTCGTCGCGCGCCTTGGCGAAGGATTCCCACGACGCCCACAGGCGC
GCGTTGCTTTCCACGTAGGCCGGGCCGACCTCGCAGCACTCGTCCAGCCATTCGGCCAGC
AAATCCATGTCGCTCTTGTAGTCGTCGCGCGCCTGGCGCACCTCGCCCGGCGGCCGCAGG
CCCTGGCGCTGGTATGCCAGCGCGCCGCTCACGCACCACGCGAGGATGCCGGCGGCCTCG
GCCGCCAGCTTCTCGGCGCGGTCGGGGTCTTTCGTCAATTTCAAGTCCTGGTCGAAGTTG
CGCGTAAAGGGCACCGGCAGCAGGCGGCGCCAGATGGCGTGGTCGTCGCCCTTGACGATG
GGCCGGTGGTTGGTCGGCACCAAAGCTACCCAGGTGGGCGCCACCTCCACCGTGGTCTTG
GAATACAGGCCGCGCGCCGGCAGCGGGTCGCCGCCGGTCATGGACTGGATGAGGCCCTCG
CGCAACTCGCTGCCCTCGCCCGGCTCGGACACGTACACGAAGCGCGCGCCGCGCAGCCGC
AGCACGTCCTCGCGCGCGCTGACCGCGGTGGCGCCGTCCGCGCCGCTTCTCAAGAGCGTG
CCCGCGCTCGCCATCTTTGCGTGCTCGCCCAGCACGTCGCGGATGGCACCCAGCACAGTG
GACTTACCGTTGCTGCCCGACCCGGAGGGGATGGCGAGCACGTCCTCGGAGGGCTGGGCC
ATCAGGCTTTAGCCGATGAGGCGCTGGAAGAAGCCGATCATGTGGGCGTCGCCGAAGAAC
ACGTCGGCGACGGTATGCTCGACCAGCGGGCAGGTGGCCGCGGCGTCGTATTCCGTAGCG
GTGATCGTGGTCACGCGGTACGCCTGGTCGGGCGGCAGCAGCTTGCCGGTGTTCAGATCG
ACCACGCCGTTGCCCCCGCCCAGCAGGTGCGGCGCCTTGTCCAGGTCGGCCACGCCGACG
ACCACGCGCGGGTCGGACTGCGCCAGGCTCACCATGTTGCGCACCATGACCGCGCGCTGG
GACACGGCGCAGAACTTGAAGAACTCGGCGCGCTCGGGGTCGGACTCGATGGCCTTGGCC
TCGTCGGGCAGCGCGCAGATGGTTCCCTTGGCGAGGTGTTCCAGTTCGACGCCGGCGGCG
CGGCGCCAGTAGATGCCTGTCCAGGTGAACCAGCCGTCAAGTTCCGGCACGTACATGAGG
CCGTCGCCGTCGTGGCCGAGCATCCTCTCGGCATTGCCGAACTCGGTCATCTGCCGGCGC
TGCTTGTGGAACGGCACGACCTTGCGGCCCCCGGCCATCGCGGCGCGCACGTCGGCCACG
GGCAGCGTGGTGACGGTCAGTTCCTTGAACCGGGCGCGGATGAGGCCGGCCAGTTCCGCG
CGCAGTGCCAGATCGGTGCCCGGGGCCTCGCCAGCGCGGCGCGCCACGTCGTTCACAAGG
TCGATGCTGTCGGCGCACGCGAGGATGAGCGCCTTGGCATCGTCCACCGCGGTGGGCTTA
TCGGCGCGAACTGCGTCGCGCTTGCCCTGGTCGCCGATCTTGAGCAGCCAGCGCGCCGTC
GTCGGGTTCCGGCCGGAATTGCCGAAGCTGTCCCAGCACCGCGCCACGTCCTCGCTGCTG
GCGTAGTTGCTGGCCGTCGCCGACCACATGTCCCACAAAGCCAGGGCCTCGCCGCTGCCA
TCGAACTCATGATGCAGCGACATGCCGACTTTCAGCCAGGTGTCGTAGTCCTCGTTATCG
ACGTAGGCGACCAGGCGCCGCGCTTCGGCCAGGTCGATACCCACCGGCGGCTCGTAGGCC
AGGAGCGGGTCATCCTCGGGCGCCGAGGTCAGGCCCGTGCGGAACTGGCTGCGGGTCACG
CGCGCGAGGCCGCATTACTCTGCCATCGCCTCGAACACCTGCAAGGCTTCCTCGACTTGG
GCCTCGGTCATGACCGGCAGGTCGGACGCGCGCATGGCGTCCAGTCCGCCGAAGAAGTCA
GTCCACTCATTAGGCCGGCCGGTGTCGGGGTGGACGTAGTAGGTGACGAACTGCTGGCCC
TTGCCCAGCACTTCAAGCCGGTGGCGGTCGCCGGCCAAGTCCTCGAACCACGCGCCGGTG
GCCTTGCCCCAGCCCTCGGACTCGGCGCGGTAGGCCAGCAGAATCTTGGGCGCGTTGCCC
ACGCGCTCGCACGTCGCGCCCAGGTGCTCCTGGCACCAGGCGTCGAAGCGCGCGGCCAGT
TCCGCGGCGGTCGTGTCCACGTCAATGGCGACGACAGGCCGTGCCCCCTGGCCGCAGAGG
ACACCCACGCCGTGCTCGGGGTATCGCGTCAGGTCGGCCGCGCCCAGGCGCGCGGTTTGC
CAGTTGTCCAGCGCCGGGCGCTTGTGGCCGGGCTTGAAGGGGATGATGAGGTAGCCGTTG
CGCAGCAGGGCGCGGCTGTGTGTTTGAAAATCGGAACTCTAATTGTCGTCCCCCTGGTCA
GATCAAATCGGCCACGCCGTAGAACTCGGCCTCGGCTGCTGCCAGGCGCGTGCGCGAGAT
TTCGTCATACGCTGTCGCGCGCTCGATGCCGCTGAATCGTCGACCAGAAAGGACTGCAGC
GACCCCTGTTGTGCCGCTTCCTGCGAACGAGTCTGGCACCACCCCCCCGGCATGACAGG
CAGTACCAGTTCGCGCATCAGTGCCGTGGGCTTGCCACTCAGGTGCCGCTTGTCGTCGCG
GCGCACGACGGCCTGGATGCAGCCGTCGAACGTGACATCGTGGTCCAACTGCACGGCCGC
GCCCTTGGTGCCCCACACCACGTATTCGCACCCGTGCCGGAAACATCCTTTGTGCGGCGC
CCGGGCGCCACGGCCCTTGTCCCAGGCGATGAGGCCGCTCCAGAACACGCCGCCGGCCTG
CACCGCGTCGGTCATCGGCGCCAGTTGGCGCCTGTCGGTGCAGGCCATGAAGTAGCCGCC
GGGCTTGAGCACGCGCACGCACTCGGCGATCCACAAGGAACACCAAGTCAGATAGCTGCG
CTGGACGCGCGACTCGCCGGAAAACGTCGGGTAGCGCCCCTGCGAGCCGCTTTGCTTGTA
CTTCGCGTCGGGGTCTTTGGCCTTGTCGTCGCGCGCGAACCCGCCGCTGCTGTAGGGTGG
GTCGGTGATAACGGCGTCCACGCTTTCGCTCGGCATGGCGATGAGCGCGGGCAGGGCCTC
GCCCTGAATCAGTTGCCAGGGGGCGCCGGTCATCGCGTCACCTGCTGAAAGTCGTACAGG
TTGAGCACGAGGTGGCGCAGCTTCTCGACCACGCTGTATCGCGGGTCTTTGTGCCGGCCG
CTGTAGATGCGGCAGATGGTGGACTGGGATACGTCGGTGGCATCGGCGATCTGTTCTTGC
GACCAGCCGGCGCGAATGAGGGCGCAGACCAATTCGTCGGGCCGCTGGGTGTAGGCGGCG

GGAATTGTCGGTGTTGTGGCGGGCATGGCTTTCTCCATCGGAATGCGTCAACGCATTATA
TGCCTAGTCGCATAAAATGGAATAGCCTTAAATAAAACCATGCGTTAACGTATATTTCCC
CGATGACCACGCTTGCGCAAAACATCAACTACCTGTTCCGTGTCCGGGGCGAAAACGCGA
CTTCGATAGCCGAGAAAGTCGGCATATCCTAGCCGACACTGCACCGGATACAGAAGGGCA
TCAATCAGCGCCCGAGTGGGGAAACGCTACGCAAGCTGGCGGACTACTTCGGCGTGACGG
TGGACGTTCTGCTCACGCGAGACCTGTCTACGGGCTTGCCGGCCGAGGCAAACGCGCGGG
CCAACGTGCGCGAAGGCCCGAACATCAGGGGACTGGTGCCGCTGCTCACCTCGGTGCAGG
CCGGCGAGTGGTGCGAGATAGCGGAGACTATTCAGCGCGACGAAGCTAAGGTATGGTGGC
CTTGTCCGGTGAAGCACGGGCCCCGCACGTTCTGCCTGACGGTCGTGGGCGAAAGCATGA
AGAACCCCGGCGCGCGGCCCAGCTACGAGCCTGGCGACGTGATATTCGTTGACCCCGATG
TGCCAGCGAACCCTGGCGACCGCGTGGTGGCCCGGCTGGAATCCCAGGTTGCGGCGACAT
TCAAGCAGTACCTGGAAGAAGACGGCCGGAAGCTGCTCAAGGCGTTGAACCCCGACTAGC
AACCGCGCTATATCCCCATCAACTGCGACGCCACGATCTGCGGCGTGCTCATCGGAAAGT
GGGTGCCTGAATGATGATCTACTACCTGACATACCTTGCCGCCGGCGTGCTGCTGGGCCT
CGCTGTGTTGCCGTGGAAACAGCGCCTCGGTTCGCGGGAAGCCGTGCTCCTTTGGCTGCT
CGCCTGGACCTGCTTCTGGCCCTACCTGCTGCTTTACGCAGCGTGGCAGGGCCTCAAGGC
CTGCCGTGCCGCTTGGTGGGCCTACACCGGCCACAAGCACCAGTAACTGCTGCAGCACCG
CCTGACCAGCCCGCAGCGCGCGGTCTTTTTCACGCTTAAAAATATGCGCTGACGCATTGA
CATGGGCAAATTCCTTTCCCCATAATGCGCCTACGCATTTAACGAAAGGCGAAGCCCATG
AACCTGCGCACCCTCACCGACCAAGAATTCCTGCGGCACGCCCTCGCCGTGCAATACCCC
CTCACCAGTTCCGCCATAGAGGCCGAGTGCCTGCGGCGTTTCGAGGCGCTGCTCGACACG
CACGACTCCGAAGCCTACGAACTGGGCACGGAACGCGAACTCGGCGCCAGCGTCCTGTGC
GAAACCCTCGACACGCTGGACGCCCACGACATTGAACCCGGCGCGCTCGGACAACTATGC
GAGGCCATGCTGATCGGCCCCGCCAACACGGCCGCGTTGCTCAACGCCCTGGCCGCTGCC
GGCCTCGACGCCCCCCAATCCCTCAAGGCCGAACTCGACCTTGCCAAGCAGTTCGGCGCC
CTCGTTGAGGACGCGGCGGATGTTTTCTCGCGCCTGTCCGAACTCACCACCACCCCAAAC
AAGGAGTAATCACCAAGTCCCTCGAAGCAGCAATCCACGAAAACACCAGCGCGTTGCGCG
ACCTGATCGCCGCCATCAAGGCGGGCGTCCCGACAACCGCCGCCCAGGTCGCTGCGGTCG
CCGCCGAGGCCCCGGCCGCCCAGCAGGAAGCCAGGACCGAAAAAAAGCCGCGCGCCACGT
CTGCCAAGACGACGGCAGCGACTGCCCCTACCCCCCGTACTGCTGAGGTGAGGGCGGCCG
ATGCGCCCGAATCCAAGGGCGAGAACTCCGCGCCCGTGCAGGCTGGTGATGCCGGCCAGG
GTGCTGCCGCCGGCGACGCCGCTCCGACGTATCAGGACACCGACGACGCTGTGACCAAGC
TGGCCCGCGTCAAGGGACGCGACGCCGCGGTCGCTGTCCTGTGGAAGTTCGGCGCCGCGA
AGCTGCCCGACGTGAAGCCCGAGCAGTTCGCCGACGTGCTCGCCGCATGCAATCAGGCGA
TGGAGGGCTGACGCCATGACCGCCCCCATCAAGAAAACCCCCAAGAAGGCGGCGAAGTCC
GCCCTCTGCACCTTGCTGGTGCGGCGCGTCTGCCGGTCGGACTACACCAGCCGCAACGGC
TTCGCCTGGCCGCAGACGGTCGGCGCCGAGATTAGCGCGCCCGACTGGAAGGACAACAAG
AACTGCGGCCACGGCCTGCACGGCTGGCTGTTCGGCCAGGGCGACCACGGCTGCGTCGAT
TACTGGCAAGACCCGGACGCCAAGTGGTACGTGCTGGAAGTGCCCAGCGACAGCATCGTG
ATGCTGGGCGGCAAGTGCAAGTTCCCGCGAGCTGTCGTGCGCTTCATCGGGTCGCGCAGC
GACGCTGCGGGCTACATCCTCGCCCGTGAACCCAAGGCTATGAATGTCGCCGTGATCGGG
CTTGTGCGCGAAGTGGGCGATGGTGGAGTTGCGGAAGTGGGCGCCCTCGGCACGGACACC
GCAGGCTATCGCGGCACGGCCACCGCTGGCTATCGCGGCACGGCCACCGCTGGCTATCGC
GGCACGGCCACCGCTGGCTATCGCGGCACGGCCACCGCTGGCTATCGCGGCACGGCCACC
GCTGGCTATCGCGGCACGGCCACCGCTGGCTATCGCGGCACAGCCACCGCTAGCTATCGC
GGCACGGCCACCGCTGGCTATCGCGGCACGGCCACCGCTGGCTATCGCGGCACGGCCACC
GCTGGCGAATCCGGCACGGCCACCGCTGGCTATCGCGGCACGGCCACCGCTGGCAAATTC
GGCACGGCCTCCGCTGGCGAGAAAGGAGAACTCCGCATCCAGTGGTGGGAAGCCAAGGCC
GAACGCTACCGCACCGTCCTGGGCTACCTCGGCGAGGACGGTATCAAGGCCGACACCAAG
TACCGCCTCGACGACAACCACAAGTTCGTGGAGGTGTCGGAAGGACCCCGCGCAAATTCC
ATGTTGACGGTAGCTCCGCGCCCGCCGGGCACATCTTCGTGTTCGGCAGCAATCTGCGCG
GCACACACCTGGGCGGCGCAGCAGCCGCTGCACACAGAGACTAAGGCGCGGCCTGGGGCG
TTTCCGAAGGGCGTACGGCCACGCCTACGCCATCCCCACGGTCGCCGTGACCGTCGTGT
CGCGCGAGTCGCTGCCCGCCATCGGCGCGGCTGTCGAGCGTTTCATCGCCTACGCCACGG
CCCACCCGCATCTGCTTTTCTTCATTACTCGCGTCGGCTGCGGCATCGCAGGCCACCTCG
ACGCCGACATCGCGCCGATGTTCCGCGACGTCCCCGCGAACTGCTCGCTGCCCGACACGT
GGGCGGCGATTCTCGAAGATTAAAGCGCGGCGGCGCGGGTGGTACTGGCATGAGCACGCA

CGCGCAACTTTCCCCGAGCAGCGCGCACCGCTGGTCGCACTGCCCCGGCAGCGTGGCCCT
GGAAGCCGAATCCCCGGATGATTCCAGCGACTTCGCCGACGAGGGCACGGCCGCGCACGA
ACTGGCCGCCATGTCGCTGACGAACGGGCTGGACGCCAGCGCCTTCCTGGGCCGCATCAT
CGAGGTGAACGACAAGGGCTGGTAAGTCACCGCCGAAATGGCCGAGCCCGTGCAGAAGTC
CATCGACTACGTGCGCGTCTTGGGCGGTGAACTGATGGTCGAGCAGCGGTTGGGCGTCGA
GGCTGTCACTGGCGAAGCTGGCGCCAAGGGCACGTCCGACGCCGTGGTGGTGCTGCCTGA
CGAACTCATCGTTGCCGACCTGAAATATGGCCGTGGCGTGAAGGCGGACGCCGAGCGCAA
CAAGCATCTGGCGATCTACGCACGCGCCGCCCTGGACGAGTTCGGGTTCCTGGGTGACTG
CAGGCGCGTGCGCCTGGTCATCGTGCAGCCGCGCCTCGACCATATCAGCGAGTGGGACAT
GCCCATCGCCTCGCAGCGCGAAGGCGCGAGCCTGGATTCCTTCGTCGCCAACAGCAAGCC
GGCCGCTGAGCGTGCGCGGCAGTTCATCGGCAAGCCGGCCGCCGAGGTCAAGCCCGACGA
CCTGGCCCCCGGTGAGAAGCAATGTCGATTCTGCAAGGCCAAGGCCTCGTGTCCGCCGCT
GCGCGACCACGTGCTGTCCACCGTCGCCGACGACTTCGTGGACGTGTCCAGGACGGTGGC
GCCGCGGATCGAGCACGCCGCCGAGCGCACGGTAGACAACGCCATCCTCGGCAACCTGCT
GGGCGCGGTCGATCGGGTCGAGTCCTGGTGCAAGGCCATTCGCGCGATGGCCGAAGCCGA
ACTGCTGGCCGGCCATCCGGTGCCCGGCTTCAAGCTGGTGGAAGGTCGCCGTGGTGCCCG
CCGCTGCACGAACGACGCCGAGGTCGAGGCCGCGCTCAAGGCCATGCGTCTCAAGGTCGA
GCAAATGTACGACCTGTCCCTCATCAGTCCGACCACCGCCGAGAAGCTGCACAAGGCCGG
CGACAACGGCCCGTGCCAGTGGCCGAAGTTGGAGGGCTTCATCACGCAGTCCGATGGCAA
GCCCAGCGTCGCCCCCGAATCCGACACGCGCCCGGCCCTGGTCATCCAAGCCGCAGCCGA
CGACTTAGCGGACGTGAGCGGGGAAACCGTGGAGGACTTGGTGTGAGGAACCGCCCGCCT
GTTCAAACGACGCTGCCGCCCGAGGCCATGCGCCTGCTGCACCAGGCGGCACAAACGCCC
ATCACCCGAGCCGACCCGCTCGCGCGTGTGAATGCCATCGAGAAAGCCACCGAGCGGGTC
AAGCGTCAATACCCCCATTTTTTCAAGGAGTGAACACCATGAAGCTGAAACTCAACAACG
TGCGCCTCGCTTTCCCCGTTCTGTTCGAGGCCAAGACCGTCAACGGCGGGGGCAAGCCTG
CTTTCTCCGCATCGTTCCTGATCGTCCCCAGCGATGCGCAAGTCAAGGCGCTGAACCAGG
CCATCGAGCAGGTCGCCAAGGAAAAGTGGGGCGCCAAGGCCGACGCCGCGCTCAAGCAGA
TGCGCGCCCAGGATAAGGTGTGCCTGCACGATAGCGTCCTCAAGGCCAACTACGACGGCT
TCCCCGGCAACCTGCACGTGTCCGCGTACCGCGCCACGCGCCCGCTGGTCACCGACACAG
ACCGCAGCCCCCTGGCCTAGGCGGACGGCAAGCCCAACTCGGGCTGCTACGTCAACGCCA
GCATCGAACTGTGGGCGCAGGACAACAACTACGGCAAGCGGGTGAATGCGGGCCTGCGCG
GCGTGCAGTTCGTGCGCGACGGCGACGCCTTCGCGGGCGGTGGTGTCGCGAGCGAGGATG
AGTTCGACGACATCACCGAGGGCGCCGCGGCGGCCGACCTGGTGTAACCCAGCGCCCCGG
CCTTCGGGCCGGGCGCTTTGGCTGAGGCGGACCGAGACGCGTCTTGCACGCACCGGAGAT
AAGCCCCGGCGCCCCAGCCAAAGCGAACACACCCAGACAACGCCCTGACCTACTTCGACG
AATACGACTAAGACTACGACCAGGAGCCGCGCGTCGTCACCTGCAAACGCTGCGGCAAGA
CCGGCCTGCGCTGGGAAGATGACGGCGGCGAGTGGGTGCTGATGGAAGGGCACTACAAGA
TTCACCGTTGCGACATTCGCAAGGCCGCTCTCGACGACTTCGAGGCGGTGTGATCAGCAC
CCTATTCCTAGACCTAGAAACCTGCTGCCAGGTGCCTATCCAGAACGGCACGCACGCTTA
CGCCGAGAAGGCAGAGGTGTTGCTGTCTGCTTACGCGCTGGACAATGGCCCCGTGTACGT
CTGGGATGCCGCCAGCGGCGAAAAGATGCCGGACGATCTGCGGTGGGCGTTGATCGACGC
GAACGTCGCGCTCTGCGCCCACAATAGCCACTTCGACCGCAGGGTGCTGCGGCACGCCAT
GCCCGACTATCCGCTGGCGCTGCCGCGATGGCGGGACACGATGGTCAAGGCCCTGGCGCA
CTCGCTGCCCTGCGCGCTGGGGGATCTGTGCGACATCCTCAAGGTGCCGACCGACAAGGC
CAAGGACAAGGCCGGCAAGCAGCTAATCATGCTGTTCTGCAAACCGCGTCCAGCCACCAG
CAAGCTGCGCCGCGCCAAGCGCGACACGCACCCCGCCGAGTGGGCCTAATGCGTGGAGTA
TGCCGGCCTCGACATCGAAGCCATGCGCGTGATTGACCCGAAGCTGCCGGCCTGGAACTA
CCAAGGCACCGAGTTGGCGCTATGGCACCTCCACCAGACCATCAGCGACCGCGGCGTGAT
GGTCGATACCGACCTCGCCAACGCCGCCATCCGCGCCGTGGAGCATACGCAGAACGTGCT
CGCGGCGCGCACCAGTGACATAACCCTGGGCGCGGTGCAGGCGGCCACGCAGCGCGAAGC
CATGCCGCGCCACGTGCTCGAGGCCTACGGCCTCGACCTGCCAGACATGCAGCAATCGAC
GCTGGAAGGACGAATTGCCCACCCCGACCTGCCGGCCGAACTGCGCGAACTGCTGGGTAT
CTGTTTGCAGGCCAGCACCACCAGCACCAGCAAATACAAAACCCTCGCCAAGGGCGTGAG
CAGCGACGGCCGCCTGCGCGGCACGTTCCAGTTCAACGGCGCCAGCCGCACCGGACGCTG
GGCCGGCCGCCAGTTCCAACCGCAAAACCTGCCGCGGCCTGTGCTCAAGCAGGACGCCAT
CGAGCGGGGCATCGCGGCTCTGAAAGCGGACTGCGCCGACCTGCTGTTCGCCAACGTCAT
GGAACTGACCAGCAGCGCGATACGCGGCTGCATCGTGGCGCCCGGGGGCAAGAAGCTGGT

CGTCGCCGACCTTTCGAACATCGAAGGACGCATGATTGCGTGGCTCGCCGGCGAGGATTG
GAAGCTGGCCGCTTTGCGTGAATTCTACGACGGCGCCGGCCCCGACCTCTACAAGCTGGC
CTACGCCAAAGCCTTCGCGGTATCGCCTGATTCGGTCGATAAAGAACAGCGCCAGATTGG
CAAGGTCATGGAACTGATGCTGGGCTACGAGGGCGGCGTCGGTGCCTTTCTTACCGGGGC
GGCTACCTACGGCATCGACCTTGACGCGATGGCCGACGCAGCGTGGCCTTGGATCGTCGC
CGACATCATCGCCGAGGCCGAGCAGTTCCTCGCCTGGCGCACCGAGCAGCGACTCGGCGA
CTTCGGCCTCAAGCCCAAGACCTTCGTGGTCTGCGATGCGCTCAAGCGCCTGTGGCGACG
CGCGCATCCGGCGGTTTCGTCATTCTGGAAGGAACTGGAAGAAGCCGCACGCCTGGCGGT
GTTGCGGCCGGGTACGACCTACGACTGCCGCACGCTCAAGGCCCGCCGCGACGGCGCCTA
GCTGTGCATCCGCCTGCCGTCCTGCCGATTTCTCTGCTACCCCAGCCCGGAACTGGATGA
CGCCGGCAAGTTGTCCTACATGGGGGTGAACCAGTACAGCCGGAAATGGTCACGCCTCAA
GACCTACGGCGGGAAGCTGGCAGAAAACGTTACGCAAGCCGCAAGCCGCGACGTGCTCGC
GGCCAACATGCCCGCCATCGAGGCGACCGGCTACCAGATCGTGCTGTCGGTACACGACGA
AAACAGCACCGAAGCCGACGACCGCGACGAAGTCAGCGCCGCTCACCTGGCCGGCCTGAT
GGCTACCAATCCCCCCTGTGCCGCTGGCCTGCCGCTCCCCGCGGCCGGCTTTGAAGGCTA
TCGCTATCGGAAGGACTGACCCATGAAATTCATTCGACTGTATCGCCTCTACCGCGGCTA
CGGTTTCCGCCGTTCGCTGGCTTTCAAACTCGCATGGGGGCGCGGCCATGCGTGAAAGCG
ACATCGAGAAATACCTCGTCGAGCGCGTCACGAAGCTGTGCGGCGAGGTGCGCAAAGTGC
GCTGGATTGGTCGCAACGGCGCCCCCGACCGCCTGGTCATGCTGCCCCATCGTACCGTGT
GGGTAGAACTCAAGGCCCACGGCGAGAACTGACTCCCTCATCAAATCCGCGAGCATGAGC
GGAGGCGCCGCATGGGGCAGCGCGTCGCCGTGGTGGATTCGCAGGAAGGCGTGGACGAGG
TGCTGGCGTGAGTAAACGCAAACCTATTACCACCGCCAGCGAAGCAAAATGCGCGGTCAA
GGTTAGGTACGAGACCCGGCGCGTCGCTGAAATTTATGCGGCGCGGTTGGCGGGACTGCG
CGGTGTGCGGCTGTATGTCTATAAGTGTCCGCACTGCATGAAGTGGCACCACACCAAGAA
GCAGCCATGGAACAACCCCATCGACGACTTCCCGGTGGTGGCATGACCTACCGCGAGAAA
GACATCGCCTACGAAAATGGCCGCGTGTGGGTGCTCAGGACATCAGCTAGCTACACGGTT
TTCGTGTCTGGCGTGACGCATTCCACCAGCGACAGCGCCTACGAATTGAGCGATGCTGAA
TCCGGCATCGCAAAGGCACGGGCCGACTATCTGGCGCGGAAGCTGAATCCCGCCGGGTGC
TTAGTATGACGGTCCGCGACAATATGCTTCGTGTTGAAAATGCCCGCGTGCGTGTGCAAG
AACACCGTATGTGCGCGAGATTGCATGAGGATAGAACGCACTATTTCGTCGAGTACCCAC
CGGGGTTTCGATTCGGTAGTTGCCGCGTGTCGAAGGGCATGGGTGAGGCTCGGGCGCTTG
ACCTGCAGCTGACCAGGATGGAGCAGCACTGGAAGGACGAGCAGCTATGACCCGCCGCGC
CTTCGTCCCCCGTGAGTATCAGCAACCTGTCATCGATCACGTGCTCGACGTGCCGCGCGA
CGCGGTGTGGGCCGGCATGGGAATGGGCAAGACGGGTTCCGCGCTCACCGCGCTGGACAT
CCTCGAACTGGTCGAACCAGGCCCCTCGATGGTGCTGGCGCCGCTGCGCGTGGCCGCGAG
CACCTGGCCCGACGAATCGACGAAGTGGGCGCACCTGCGCAACGTCGAGGTGTCGGCAGT
GGTCGGCACGACCGAGCAGCGCCGCGCCGCGCTCAAGCGTCCGGCCAACGTGTTCACCCC
GAACTACGACAACCTGCCGTGGCTGATCGAACAACTGGGCGACCGCTGGCCCTTCCGCAA
AGTGGTGGCTGATGAATCGACGAAGCTAAAGTCGTTCCGGTTGCAAGAGGGCGGCAAGCG
CGCGCACGCGCTGGTCCGTGTCGCACATGCCAAGGTTGACCGATTCATCGAACTGACCGG
CACGCCCAGCCCAAACGGGTTGCAGGACTTGTGGGGGCAGGCTTGGTTCCTGGACAAAGG
CGAGCGCCTGGGCCGCAGTTTCGGCGCCTTCAAATCGAGGTGGTTCCAGTCCATCCAGGT
ATGCAGCGACCCCCATGCCGTGCGCCTCGAAACGTTGCCGTTTGCCCAGCAACAGATCGA
GGACAGGCTGCGCGATCTGTGCCTGTCGCTGGACGCCCGCGACTATTTCGACTTCAGCGA
ACCCATCGTCAACGTGGTGCGCGTCGAACTGCAGGCCAAGGCCCGGCGTTTATACCGCGA
CATGGAGCGCGAGGCGTTTCTCGCCCTGCAATGCGGTATTGAAATTGAAGCATTCAATGC
CGCCAGCAAGACCATAAAGTGCTTGCAACTCGCGAACGGCGCAATCTATACCGACGACAC
GGCGACCACCTGGGCCGATGTCCACGATGTCAAGCTACAGGCCCTTGAATCCATCGTCGC
CGAGGCGGCCGGTATGCCGGTGCTGGTGGCGTACCACTTCAAGTCCGACCTCGCGCGCCT
ACAGCGTGCCTTCCCCCAGGGCCGCGCGTTCGACCAGAACCCGCAGACTATCCGCGACCG
GAACGCAGGAAAAATCCCGCTGCTGTTCGCCCACCCGGCCAGCGCCGGCCCCGGGCTGAA
CCTGCAAGACGGCGGCAACATCCTGGCTTTCTTCGGCCACTGGTGGGACTTAGAACAGTA
CCAGCTAATCATCGAACGTATCGGCCCGACGCGCCAGGCGCAGGCCGACCATGACCGCCC
GGTGTTCATCTACCACATTGTCGCCACCGACACGATGGACGAATTGGTCATGGCGCGCCG
CGAGTCCAAGCGCGAGGTACAAGACCTGCTACTGGAAGCTATGAAAAGAAGAAGAACGTG
ACCACGCAAACCAACGCACTCGACCACGAGGAAGGCGGCGGCCACTACAAGGATATGCCC
ATCCAGCCTGTCAAATTCATCCACAAGAACGGCATCGGCTATTTCGAGCGCAACATCATC

AAGTACGTGAGCCGGGGGCGGAAGAAGAACGGCGTCGAGGACTTGAAAAAAGCCCGCCAC
TATATCGACCTGCTGATCGAACTTGAAGTGGGCGGCCATGAGTGAAACCGTTGTGCGCGT
GGCCGCGGCGCCGTATGTCACCATCCCTTTGGCGGCCGTCATGACAGGGCTGACAGAAAA
AGCCATCAGACGCAAGATCGAGGAAGGGAAGTGGGTCGAGGGCCGGGAATACCGGCGCTC
GCCGGACGGCGGAATTTTTATATCACTCAAGGGGTATCAGCAATGGGTCGAAAAGGCGAC
GGCGTAGAAACTCACGGCAGCGCGGTACGGCTGTCCTTCACCATCGACGGCGAGCGAACA
CGGCCGCTGCTCATGATCGACGGCAAGCCTTTGCCGCCTACCGCCGGAAACATGAAGTAC
GTGCGCCGGGTCGAACAGGAAATCCGCGAGCGAATACGCCACGGCACGTTTAGAATGGCC
GAGTATTTCCCGGCCAGTGGCGCGGGCAGCGGGCTGACGGTGAAGGGCTGGCTCGACACG
GGGCTCGACACGCAGAGCGTGGAGAATTCGACCAAGGCAGGCTACGAAAGCGCGGTCAAC
CTCTGGAACAACGCGGCAGCCCTCGTCGATAGGGAAACGAAAGCCATACAGGCCACGGTC
GGCTCGTTGTCTCTGCGCGCGCTCAAGCCCAGTCACCTGCTGACGGCGATTGCGTATCGG
CCCGATCTGACCGGCGAGACGGTGAACAACTACGTGTCGGTCATCCGCCAGGCGCTCGAC
TTGTCCGTGGCCGACAAGATCATCAAGGAAAACCCAGCCAGCGAGGTACCGCGGCAGAAA
CACCAAAAGCCCCCGCCTGACCCGTTGACCCGCGCCGAGGCTGAGGCCATCATCGCCGAC
ATGGCGAAGCACTACCCCGAGCAGGTCTATAACACGGTCGAGGCGTGGTTTTTCAGCGGC
CCGCGCACGTCCGAGGTATTCGGCCAGCGATGGCCCAACGTGGACTTGTTCAGCCCGAAA
CCGGCGATTGTCGAAGCGTTGGTGCGCGGCGAGCAGAAGGACAGCACTAAGACCAACGTG
GCCCGCGACGTGCTGGTAAACAGTCGCGCGCTGGACGCGCTCAATCCGCAGGCCAAGCAC
ACCCGGATGGTCGGCGATCACGTGTGGAACGACCCCCGGTACGGCACGCCCTGGACGGAA
GAGCCCGCCTTCCGGCGCAGCTACTGGACGCCGTGCCTCAAGCGCCTGGTGATTCGCTAC
CGCCGGCCCTACATGATGCGGCATACCTACGCCACCATGATGCTGATGGCGGGGATGACG
CCGGCATTGTGCGCGCGGCAGCTTGGGCACTCGATTGAAATGTTGCTGCGCACCTACGCG
AACTGGTTGGACGGCGCGTAGAACGATCTGGAAATGGCCCGCTTCGAGGCAACCCTAGGT
GCGGATTTGCCCCAAATTCGTCCCAAACCATAAGGCTCAAGCACGTAAGTACTTGAGCCT
TAAGGAACTTCTT
>NewGenomeName_32
TGAAGAGAGGTGAGATATTTCCAAGGGAGAACGCGCGACATTTCCGGTATGAGGCGGGAC
GGAACGGGATCACACGGAACGGCGCTTGCAAACCACTTGCGTAGGTTGAAACAGAAATAT
ATCTCTCGGGGGCAAGGCCCGCGAGGGCCGCGTCAGGCCGAAGGCAAGGCTCGCGAGGAG
ACAGTGGTCAACGGTGGAACCGCGTAGCGGTTGTCCCCGCCCTTGTGGTGGGGGCAACAC
CGTTGTCCACTGGCGGACGCGGGATCTACGACGACCCCTGCGGACCGATTCTGCGGCATT
ACATCGACGGCAGCGGAGAAGTCTGAGGGGCGGTTACCTACCTGTCAAGGCAGCCTCCTC
GGGCTCAATCGGTAGAAGTGGGGCTCGCGAACAGGTCGCGTTGGCGAGCGGCGATCTCCT
CGCGCCGGACCGCCTTGATGATCTTGTAAATCCACTGGAGTGACACGCCGAACTTCCGTG
CCAGCTCGGCATGGTTGTCGCCGCGGAATTCGTCGAAAATCTGCCCATCTCGCTGGGACA
GCCGGTACGACACACCCACCGGAAAGTAGATGTTCTGTCCGCCCCAGTGGCCGGCCATGC
GGTCGGCAATCTCGCGCCCGAGCTGGTTCGCCTGGTCGGCGCCGATGTCGGCCAGCTCGA
TCAGTGCCTGGGCGACCTGCAGAGACAAGTCGACCAGAAGCTCCGGCCCTTTGCTTTTGA
ACGTTTCGTCCTTCATACGGCCTCCGTCGTCCTGTCACGCCATTTTTTGAGGTGCTCAAT
CACACGGGATGCCTGGGCGGAACTGAGCCATTCGAGCGCGTCGACGTGCATCATGCGCTT
TACGAAAGCGCCAAGCGCTTCCTCAGACCGGTTTTGCACGACGCCCCGATCCGCCAGCTC
AAGCCAGATGCCGCGAATCAACTTCGATTGCTCGTCGTCCGCCTGACTGCGCGCGCCCTT
TTTGGAACGCACTTTAAAGCCGCAACGCTTCAGGTGGTCCAGAACCCTTTCCAGTTCTGG
AACGGTCAGGTCGGCCGCGGATTCCTTCTTTCCAATCTGCATCAGAACAGCGTGGTAGCA
GTCATCCGGCATCGCAAGATCACGCTTTGCGACGTGAATCAGGCGGATGAGCTTTTGCCG
AGCCTGAGGCCCGCGATTAGATTTCGAGTACATCGCTGTCGTCCGCTTCGAAGGAAAATT
CGTCGTATTCAACGACTCGCAGGCCGCTCGATCCATATACCGGCGGGAACCCCTCGATGC
GGCCGGCGTTAAAGGCATCCTGCGGAGAAATCTCCTCAACGGAGGCCGCGAGCAAGCGAG
CAGCAAACAGCTTCAGAAACGCCTGGAGCGGGCCATGCCGCTCGATGTCTGCATCACCCT
CAGACCAGAATCGAACCAGCTCGCAGAGCTTCTCTTCGGTCAGGATGTTGTGATCGACAT
CCACCACGATTTCGTAGCTGAAGTCGTGCGTCAGCACGTATTTTTTTATGTTCGTCATGT
CGTCCCTCGCCGTGCGATTGCTTTCGTGTCCTGGTCGATCGCCGCACGGGTCGACGCCCA
TCGCATCGGGCGTCGCCGCGCAGCTCGACGATTACTTGACGGCGTCCTGCAGCCCCTTGC
CGGCCTTGAACTTCTGGGCTTTCGACGCTGGAATCTTGATTTCTGCGCCGGTGGCGTGAT
TCCGTCCGGTACGGGCGGCCCGCGCGCCCACGCTGAATACGCCGAATCCCGTCAGCGTCA
CCATGACGCCCTTGCGCAGCGACTTCGTGATACCTTCGAGAACGGCTTCCAGCGCAAAGC

CGGAGTCGGTTTTCGTCATGCCCGTTTCTGACGCCACGTGATTGATCAGGTCCGATTTGT
TCATAAATGCTCCGTATGGTGGATACCGCGAGACGCTCGCGGCCAGCTAAAGTGCTCGAT
GTCGCAGTTACGCGTTGGCGATGTCGAGCGGAATCTGTACGTATTGGTCCGTGTCGCCGA
CGCGCTCGTAGACGCGGACGTACGACTTGCTACCGATCACCTGCAGTGCTTCGCCGATGG
CTCGCATTGCCTCCAGCCAGCGCGGATCGGTGATGTCCAATCGGCGCAGCGCGAGCACGC
GGCCTGTGTTGATCTGGCCTTCCTTGTCGGTCGCAAACGCCTCCGTGACGATCGCCTGGA
TATCGGGGCGTGCGTCCGTGGTCCAATCGCGAAGGCACTCGTCGATCATCGACTTCGCGG
CCTGCATGCGTTCATCGAACGCGATGCGGTCTTGGATGGCACGCTGGATGCGGTAGCGGC
CGTCGAACGAGTACAGGGTGACGTTGCCGTTCTTGCCGCCAACCTTCGCGCCATACTCCT
CGGCCGAAAGGTCGATGAAGGCCGAGATATCACCGAAAATTCGGGCTTTCAGCTCGACCA
GCCCCTTCGATCTCGTTTTGGCTTCGTCCGCCAGCTCGCGAACGAGGCGATCGCGCTCAC
GGTCGATCGGCTTGATCATGGTTTCCGGAATCAGGCAGCCTTTCGCGTCTTGCCAGTAAC
CATTCGGAATCTGTTTCTGTTCCATGTGGTGCTCCTCTCGATGAGGTATTGAAAGCTCGC
TAGCGGGTCCACCAGCGGCGTATTTCCTTGCGGCAGAGGCAGACGAGAAAGACGATTCCG
ATGGCGATAACTACGGCGTCGCTCATATCAATAGGCTTTGCCGCCTTCAGCGAGTCGGTT
TTCGGGCTTGTGGTCCGGACGCTGCGCATTGAAGGCCAGTTTCTCAACGATCGCGCCAGC
TTCGTCTTATTGCTTCGCGCCGGCCAGGTCGAATATCCGAATCACCGCGTCCGCGAGTTC
GACTTCGATCATCGGGCGATGGGGAAGCTTGTCGTCCATCAAACCCTTTCGATGACCTTC
CATTGCCTCGGCGATTTCGGAAACGATCAACATCAGCTTCTCTGGTACGTTGTTCGCGGC
TCGCAGATCGGTGCCGGTCTTCAGGTCCGTCCACCCCCCAGACTGAAACGATGCGCCGAA
GCAGCAATCCGTAAGTGCCTGAGCTGCGTGCAGAAGGTGTTCGTTACTCGCTTCCATGTT
GTGCTCCTTGAAAATCGGTGGGTAAGGCATGAGTCGTTGAATCAGGAACCTTCCGAGAAA
CCGACTTATCAAGCTACCCCCCATGTCACTCTGACGCTCATGAAAACGACGTAGGCAAAC
TGACGATTGCCTTGCCGCTGGACAGTGACGCCGCCCGCACGCTCGCGCAGCACGTCGGCC
GACTTCGCGAGGTACGGGCCGAGCTGGATCGTCGGCCGGCCGCCGTCGCGCGGCGAGATC
TCTTCGGCCAGCACGCGGTAGCCCAGGCCGCGCAGCGCCCGCGCGCAAGCGTGCAGGAAC
GTGAGACGCGAGACGCACTCCGCGTAGAAAACCCGCGTGCCGTCAGGCAGCAGACTTCGC
GGTGCGGGCAGGTTCATTTGCACGATGGCGCCCATGTCAGACTCCCTTGATCACGTCGGC
GGTGATGACCGGCACGCCCAGGCCCGCGGCAAGATTCATCGCGGCCGTCAGGAGGTTTCC
GACCGCGAGCGGGTACAGCAGCGACACCGTTTCGGCACGATCGCGTCGCGTGCTGGTCAT
CGTGAGCCGAGTGCGCAGCGCGTCGACGCCGCTTGCGTCGATCACGTCGCCGACCGGCTT
GTCGAGACGGCCGAACTTGAATCGGAGGTTTTCGTCCAGGCGCGGGCCGTCGAGCGGGGC
CAGCTCGAGCATTCCGCATCGCTGCACGACTTCCCGAACGTCCTGGTTGCGCTCGGACAG
CTTGACCTTCAGCTCCGGCTGGCCGATCAGGATGATGGACAGCAGCTTCTTAAATCCCAT
CTCCAACTCGAAGAAGCGCTTCAGGTGCTTGAGCGTCGCGATCGGCAGCGCGTGCGCTTC
GTCGATCACGAGGCAATGCTGGTAGCCCGCCGAGTGGCTTTCCTTCAGGGCCTTGTGGAG
CTGCGCGAAGCGGGCTTCCGGGCTGCTCTTGACCTTCTCCAGCGGGGCCACGGCGGCCAT
GATTGCTTCCGCGATGTGCGTCGCCTTCAGCGTCTTGCCTTTATGGTCGTTGTCCTCCAT
CGCCAGCACGTACGGCTTGATGACGATGATCGGATGACTCTCGCGCATCACGCGGTCTTC
GAGGTCGCGCATCAGCGTCGTCTTGCCGCCGCCCGATTCCGCGACGACGGCCAGGAGCCC
GCCATGCTTCGCAGTCTGGAACATTGCCTCGCGCACGTAGCGAATATCGGGGCTGACGAA
CATGTCTTCGTGCGACTGGATGTCATCCGCGAAAGGGTCACGGAACAGGCCGAAGTCCTT
ACGGGTGGCTGGTGCGAGAACCTGTTTGCGCAGTAACATGGATTCCTCCTTGTTGAGATC
GGTATTGCTGTTCGGCTGGGAGACCGAGTGGCCCGTCGTATCCGCCAAGACATCGGTCGG
GCCACCCACCTTTTGCACTTCATCAAAAACACCTGGGCCTGCGTCCGACACCCCTTTGTT
TTCCAGGTAGTCGAGAATTCGCTCCCGCAGGTCGAGGTCGTCGAGGCTGCGCGGCCATAC
GCCGTGATTCACGATCTGATCGACCGCCGCCTGCGACAGATTCAGGTGTTCCGCAAGTTC
GGCCTGCTTGATAGAGGCGCGTTGCAGAACGCTTTTCAGGACCAACATCATTGACCTCCT
GCCGCTGCGCGAACGAGCGGCAGCGGTTGCTGTTGACCCGCTCGCGGGCCGGTAAGCTCG
GCGACGATCGCGTCGAGCTGCTCTTGCGGTACGCCGTCCGGATAGCGCTGTTGCAGCCAG
CGGAACCGGTCCGCGCTCCAGTCGACGCCGGCAGCTTCGACCGCGGCCTTGATCTGCTTT
GCCGCCTCGATCAGCGAGAGCGGCGCGAGTTCGACGCGCGGCGCAACGAGGTCGTGTTCC
GTGCCGCGGCGCGGCAGGTACGTCGGAAGATCGGCTTTGTCGAGGTGCTTGTACGGGTCG
AGACGGCCGCCGAACGGCAGTGCCTTGCCCTAGCGAGCTGCTTCAGCGTAGGCAGGAGTC
GACGTACCTGTCACGAGCTGCTCGATCTCTCGAAGCGCATGCTGCGCTGGCGTATCGGCG
TGCCGCCGGTAGCTTTCGCCGATCACTGCGGCAGTCTCGGCATAGCCGAAATCGCCACGT
ACGATGAGCGGAACGGCGAAGTACGTCTCGCGCCCGTCCTCGCCAGTCAGGACCACCTGG

GCGGCATCGTCACTCCACGGATTGCGCGTGATCATCAGCTTCTCGCCGACCATGACGCCC
GGCACCGACGACACGTCGTATTCCTCGCCACGGAACGACATGCGCAGCTTCGGCGTGACC
TCCCGACTTTCCGGCGAGGCAACCGCCAATTCGCGGCACACATCGATCGACGGCGCCTTG
ATGAGTTGCTGGGCGGTAATGCGCATCCATGCCTCACTGCGCGTCGCACCATGCCGGCTG
TGCGTTTCAGTGGCGTTGAAGTGCATACGCCAGCGTTTCGCAAGCGCGTTCAGCTCGTCG
AGGCTGTTCACCGGCTGAAACTTGAGGCCCGCTTCGAACTTGCGCTCGATGAGGTTCCCC
GCGTTCTCAACCTGTCTGGTGGCACGGGCGTTGCCAACCTTGTGGACAATCAGCTCGATT
CCGAGCGAGCGGCACAGGTTGCGCGTCATGGACGCCGTGTTCGCCGATCCGGCGTCGAGC
ATTAGAATGCGTGGCACACCGTGCAGCAGGTCGGCCCCGACGCGCTCCTGCATAGCGTTG
ATGAGCGTCGAGCAGAGGTTCTCGCCCGATTCCGCACCCATGACGTACTCGGTGTACATC
CAGTCGCTCGCGTTGTCGGAGATTTCGTAGCTCCACACACGATCGGCCGCGATCCGGGCG
AGGTTCTTTGGCTTGTTCTTGTAGAACTCAGCGTGATCCATCACGCGCAGGCCGTTCGCA
CGGGCATCGGCTACCGGCTTCAGGTAGTACAGCACGCACAGGCTCGCGTCGATCTGCCAC
ACGTGATTCGGATGCAGGCGTGCCAGTTCAGTCACTGGGGCCGGTGCGAGAAGTTGGTCG
GGATGGACGCCATACATGCGTAGCGCGCGTTGAATCGCGCTATCGGACAACGGCCGCAGC
TCGCCGGTGGACTCGTCGAGAAATTCAGCCCGGATCATGCCATTCGCGCGCAAAATCTCC
GCCGCGTCACCGACCGTATACAGGCGCTTGCCGTTCTTACGCGTCGACTCCATGAGCGTT
GCTGAAATCAGCATCGCTTCGTCGCGCGTCAACGAGCTTTGACCGGCGTCGACACGGCGT
TTGCGTTGCGTGGTCACGGTGGCTTCCTCCAGTTTGCGGATCAGGGTGGTGAAATACAGG
CCCAGCTCGCGGCAGGCCGCGTCGTAGATCGCACCTTTTTTGCCGTGGCCGGCCGCGCGC
GCTGCCTGGGCGACAGCAACAATGCGTTCGTCCAAGACGGCACTCATCGTCGCGAGCCCC
CGAATCAGTTAGCGGCGCGTGAGAAGTCGGCATCCGCTGACCCGGTCGACGTATCACGCA
GCCATTGCGGGACGTCGTCTCCGTACGCAGCGGCCTTGACGCCGAACTCGCTTCGAAGCT
GGTTGAGCGACAACTGGATTTGCCCGAGGACGCCAGCCCTGAAGTCGTCGTGCGGCGCGC
CGTGCGTCTCGGCGTGCTGCGCCAAGGTTTCGAAGGCGGCACGCAGGTTGCCGCGAATAA
CCGACTCCGCCTCGAAGGTGATCGCGCTGGTTTCCTTGCGGATTTCCGCTCCTTCTTCGT
CCGGCGTGACCTTCTTGACGCGCGTCTTCTTCGCGGCCAGCTCGTCGATCTTCGCGTTCT
TGTCGGACAGCAGACGCGTTTGCGCGGCGGCTTGTTCGCGTGCTTCCCGAAGCCCACCAC
GCAGTTCCTTCACGCTCATGGTGGCGATATCGTCGAGCTTCAGCTCGCCGGTTTGGCCGG
TCAGCTCCAGCTCTTCGATCTGTTCGGCGTCGAGGACCAGCATTTCAAAAAGCTTGGTCT
GATTTCCGGCCGCCTCCAAAAGCGGCGTTAACGCCGCTTTTGAAAACTTGGTCGCCGACG
CCATGAACCGTTGAGCTACACGCGGCTCAATCCCCAGAACATCAAGACGCGCACCAAATT
GGCCGTGCTTATATGCCTCCTTCAGTACTCGAAGGCCACGACCAACCTCGAGGCTCGCTT
CCACACTACGACGCATGTTCGCCGCAATGTCGCGTTGAATCAGGTCCAGGTCGGTGCAAT
CGGCCGGCAACTGGTACCCGAGCTGTGTAGCGAAAGCTCGGATTGTCGAGTCTCGGTCTT
GATCAAGCACGGCGAGCTGATTCGCTGCGTCGACCATTGCGGGCATGCCCGGAACATCGG
CGTCGGTCACGGTCGTCGCGACGTTGTTGGTGCTGCTCGGCTTTCGACCTTTCGTCATGA
TTTCGCTCTCACAAATCGGTTACTTCGGCGCGGTCCAGAATCTGAACGCGCCAGGTCTCG
GCGGGCACCGCCTGGAGAGCGCGCAACGATTTCTCTGTGGACTCTTTGGCTGTTGCTACG
ACTTCGTGCTCGGCGCACTCCAGGCTGGTGATTACCACGCGGTTGAAGGAATCCCCAGGC
GGGTACACGCAAGCGGTGTACGTGGTGAAATTGGCTGACACGGTTGCTCCTTTACTGATT
CGTCAGGCGGGTTTGAATGCTGTCGAGGCGCTCGCTGGCGGCCTCGAGGCTACGGAGGAT
GGCGACGGCGTGGCGCCCCAGCTTCACGGATGGACGGATGCGGCCCGTTTCGGGAATACG
CTCCGCGAACCCCATCGCCTCAAGCGTCGCGACATACCGCGTGATGTTCGACGGCGAGAG
GTTCGTTGCCTTGGTGAGTTCGCCGGGCGTCAGGCCATGCGCGAAATGACCGAGCAGAAC
GTTGAGAACCTCCAGGACCTTCTCAGCGGACTTCGTGGTTGCGTTCGCGGTCATTCGGCT
GCTCCAAGGTCGAGTTCGGGCTGTACATGGCGCTCGACGTTGCCGCGATGCCATGCAAGC
CCTTCCATTGCTTGCTGGATCGTCGCGATCGTTTCGTCTGCATTGGCCGAGCCAGTGTAG
AAGTCGAGCAATTGCCCGACTGCGGCGTTGAGCGTGCGTTGCAAGATCTGCATGTCTTCG
GCCGTCGCGTCGCGGCCGGTCGCGATGTCGATGATCAGCCGACCGGCGCTTGCCGCCACC
CAGCGAGTGACGAAATCGGCACCGCACGCGTCCTCGAACGGCCGGATCAGAGAGATCGGC
ATACCGCCTGACTGGAACCATTTGTAGAGAGTCCAGTGATCGGCGAGCCCCATGTGTTCC
GAGATGCGTTCGATGCTGAGATTGCGCCGCTCGCGCGCATGCTCTTTACAGAGTTCAAGC
GCGTGCCGAAGGCTATGCGGCTGAATACGTTTCCAATTGCGGCGGCTCATTGGGAAGCCC
CATTCAAAACGGTTTTCGGCCGATTCCAAACAAGTTCCCATTTTGCGCCTATGCAACAAC
CTTGCGACTCGTAGAATGAAAAGCGGTTACTCCTACGGGGAAAATGTTCCATGACGGATT
CGGATTTCAGTGAGTTAGCGGCACGTGTGGACGCTGTCGGGCAAACGATGCTTCGACTTA

TCGGCCACCTGGAGGGACAGGGCTGCATCGACGGCGTTCGCTTTTCGCAGGCGCTGCGTC
GTGTCGGCGCTGCTCGTCGCCAACTGCCCGATCCGATACAGGCACGGGGCGGCGAAGTTG
TCCTGCAGATGGTGCAGCTGCTTGACGAAGCGAGGTCTCGCCGATGACCGGGCTGTCGAC
GTGGTGGCCGAAACAGTTGCAGCGCATGAGGTCTTTCACGCTGCGACGCGGTGTCGAGGC
AATGCGAGCGCTTTTGCCGGGTCGGCGCAGATTTCGCCAGCCTTGAGCCCCAGCTTGACG
GCGATTTCGTGCGCCTGGCCGCGGACACATTTTTCCGGCCGCCCAGAACCTCGAATACG
AGATTGGGAGAGAATTTGTTCGCGATCGCCCACTGGGTGATCGAGATACCTTTCGATTGA
AGCTCCGCGCGGGCTTCAGCGGCGGTACGCAGTTTCATTGAAGCTCCTGTTAAGGGGCTG
TCGGCACCGTGGAAAGTGCCGACTAGGTTGCCGTTCGTGGTGGACGGTGTGAATGAAGT
ATGGTATGCGTTTGATTACCTGTCAAGCGCCATTTGGGTAATCGTATGGATTCAATAGGC
GCGCGCCCGCGCGAGGAGCGTGAGCGGTTGGGGTATATCCAGACGGCATTAGGGGCGCTG
GCGGAGGTGACCAAGCAATCGCAGATCAAGTACGAGAAAGGCGAGCGTTCGCCGGACGCC
AGCTACCTCTCGGCAATCATGCGTGTCGGGGCGGACGTCCAGTACATCGTCGGGGCGATA
CGATCGTCGATAGCGTTGGCACCCGATGAACAAGAACTCGTTTCGCGGTACCGGTCCGCG
TCGCTTGAGGTGAAAGCCGCTGCGATTGGCGCGCTGGCGGCCGCAAGCACGACGCGCCAG
GAGCAGGTCTTGCACGGATCCGTAGGACCGGCCATAAAGGTCGAGGAAAACCTCGATCAG
CAGGGGACTAGTTTTTTCGGTAAAGGCAAGAAAAGAAAATGACCAATGACGCTAACGGGA
AACAGGAGTTCAACAGGCCGGTCGGGCAAGCCATACAGGCCGGTGAAGCAAACATCGCTG
ATCGCTCGGTGAATATCCGGTCCGGCTCAATCGGGAGCGTTGCTGGTCGCGACATCGTCA
ACGTCAACCACGGCGTCCCGTTCTCGAACAGCAATGTCGTGAACCTCCAGTTCGGCCCGA
AGGAGCCCGAAGAAGTCCAGTTCGTGACCAACCATCAGAAGGGTATGATCATGGAGCTGG
TCGGGCAGATCGCCGGCGCGACGGAAATGATGTTCTCAAGATTTCCCGGGCCGTATTGG
CTCGCGCCGGTGCCAAGCGGGTCAAATGGATTCGCAGCGATAGATACGTCGATATCGAGC
AATACCTGTCGTCCTGGCTGAACCGCGTAGCGATCAAGTCCACCGCACCAAGCGCCGCCC
AATCTACGCCCGCTCGGGACAACGTTTGCGAAGACTCACGCCCTGAGCTGACGACGCAAC
TCCAGCTCACCCAGGAGCGGCTCGCATCCACCCGAACGATGCTGAAAGTCACGATGTTCG
CGGCACTCTTGGGCGGCGTCATCCTCGGCTACTACGGCTGGTCCAGTCATCAAACAATCG
ACCAACTCCAGGCAGCATTCGGCGGCTGCCAGTATGCGGGGAAAACGTACGCGGTCGGCA
GCATCATCGACAACTCCGAAGCGCCCGATGTTGAGTGTGTTGTGACGTCGGTCGGCAAGC
CCGGTGTATGGCGCGATCTCAGCGCACGACGCAAGCACTGACGAGCCGGCCGGCGATTCA
GTCGCGCAGGCTTGAACGAGCATTAATTTCTTCCCGTAACGGGAGGCAGCAAAATGACCA
ATCCGAAGAACAATTCGAATTGCGACTCCACCAACAAGGGCGTGCCGACGCGTGTCAGCG
TCGACTACGGTGAGCGGCGCGACTACAACGATATCAACAAGGGTTTAGAAGTGGTCAACA
CGCTGCCGCCGCCTCCGCCGCTGCCGACACGGGATAACAGCAATGGGAACGACCAATCTT
GGCCTGCAGTGGCTCAACCAGCTCTTCGACATTCGCCGCTCGATCCGTTATCACAACCGC
CGGCGCGCGTTCTTCGATCGCCTCGACCAGTTGACTAACATGCTGTCCGTGATCTTCGGG
TCGACGGCGGTCTACGGGGTGCTTGAGCAGCAATACAAGGCGGTCGCGCTCGTCGCTGCG
GGGCTCGTGACGGTGCTGTCCGCGATCAACCTGGTCGTGGGTTCGTCGCAGCGCGCGCGG
GCGCATGCGGATTTCGCACGCCAGTTCATCGGCTTGGAAAAGCGCATGGCACTTTCGGTG
CCGGACGAAAGCGTGTTGCTGGCGGTGCGCAGCGAACGGCTGACGATCGAGGCCGAGGAA
CCGCCGGTCCTACACGTGCTCAACGTGATGTGCCACAACGAGCAAATGCGGGAGATGGGA
TACGCGGACGATCAGCTCGCAAAGGTCGGCTTCTGGCAGCGGATGTTCTCCCAGCTCTTC
GATTTTCAGGAGCACGCGCTGCGCTCATCGAAGCCGTAGAATTCGGCTTGCTGTCGGCGG
TGCCTGTGCCTATGCTGGATGGTTCCCGACTTGGAGTCATCCCATGAACATTCACCCCGT
CATTGCGTCATTCGAACATCAGGCCAAGATCCTCGACGTGACTGGCGACCAGGAGAACTC
CGACGATGCAATCGCGGTGCTCGCTGGTTGGATCGACCTGTCGCTCGAACGCCTTAACGA
ATTCGATGTCAGTACGCTCGTAAGGATCGGTGGGCTGTTGTATCGCGATGGTCTGAATCG
GAGGAACGCCGCGCAAGCCGACCGCTTAAGCCGATTAAAAGACCTACCCGTACATGCCGT
CCAAGATGACGGCATATCCACTACGGGAGGTCATATGCCCTTCATCAAGCGCTTCCCGCG
GCTGACGAGCTGGCTCGTCGCCGCGATCATCCTCGTTGCAGCCATCGCGCTTTTCTCGCT
GCAGCAACTGCCCGTCGCCCTCTACAAGCTGAGTCTCGTCAGCCTCGCGGCCGTCGTCGC
GTACTGGTTCGATCGCGGGCTTTTCTCGTACGCGCGGCCGGACATCTATCTCGAAACCGA
CTGGCGGCATGGACCGGACGGACTGACGCCCGAAGCCGACTATCGAGTCGCCAAGGGCGA
TGAGCAGGTGTTCGCCGCGTCCATGCTTCGCCGCGCGGTGATCGTCCTCGGCGTCGTAGT
CGGCGTCGCGCTGGGTCTCTGACCATGCGCGTCCTGATCGCCTTTGTCGTCGCGTTGCTC
GGCGCCGCCGCGCCCGTGGCCGCGCAAGTGCCCGCCGAGGCGCTGCCGTACCGCGCCGAG
CTGACGCGCAACGCACGTGCCGTCTGGGGCATGGATGCGCCAGTCTCGTCGTTCGCGGCG

CAAATCCACCAGGAGAGCCGCTGGCGCGCGGACGCCGTCAGCGTCGTTGGTTCGCGCGGG
ATGTCTCAATTCATGCCCTCGACCGTCGACTAGATCGCGGGCGCCTATCCGGCCGAGCTG
GGCGAGGAGCAGCCGTTCAACCCGTCCTGGTCGATTCGTGCGCTCGTGCGGTACGACCGG
CACCTCTGGGAGCGCATCACGGCGGCCGGTGCATGCGAGCGCATGGCGATGACGCTGTCG
GCCTATAACGGCGGCCTGGGCTGGGTCTATCGCGCTCAGCGCGTGACGGCTGCCGGCGGC
GCCGATCGGCAACGCTGGTTCGGACATGTCGAGCGGTTCAACGCCGGCCGGCACGCGGCC
GCTTTACGCGAGAACCGCGTCTACCCGCGCGTGATTCTGCGCACGTTCGAGCCGCGTTAC
GACAAGGCCGGAGTCGGGCCGGGAGCGTGCTCATGAACTTTCCTGCCCTGCTTTCGCGCG
GCCAGTGTGTCGCGGCGGCGGCCGGCGCTGCGGCGATCGGCGCACTGGCGGCTGGCGCGG
TCGCTTTCTTCGGCGGCTATCACGCCGGTGAGCTTGCCGGCGACGCGAAGGTCGCCAAGC
TCGAACGCCAAGACGCAGACGGCGCGCGCGATGCCGTCGCACAAGCGCGCATCAAGGAAC
GCGCGGAGACGCAGCGCGCGGCAGCGCTCGCCGGCGACCTGCTCGCCGAGAAAGCCCGGC
ACGCGCTCGAATCCGACGAACTGAAACGGAGAATTGCCAGTGTCACGAGCCAATACCGCC
CGGCGCCGGATGCGCCTCTTCAGGATTTGCCTCGCTGCGTGTTCACTGCCGGCTGTGTCC
GCGTGTGGAACGCCGCCGCCGGCGCCGGTGGTGTGCCCGCGACCAGTGCTGCCGCCGGAG
CTGTTGCGCCGGCCCGCGCCGATGACGCCCTTGATTCCGGGGTACGCCAGGACGACATCC
TCGCCCACCACGTCGACGCCAGCCGCCGCAGCCGCGACATCGAATCGCAACTGAACAAGC
TGATCGACTACATCGAGGGAGAAAAGCAGTGACGTTACAGGTGGAATTCTGGCAATTGGT
GTCGATGCTCGCGACGTTCATCGGCCTTCTGATCGGGGCCGGCAAGGTGTTGCTCGTGCA
GATCGAGCGCCACCAGTCCGACCGCGATCAGAACCAGGAAAAACAGATCAAGGCGGTGCT
CGAGCAGATCAGCCGGCAGGCGGACAACACGGCGCGCCTGGAGCGCGATTTCCTGCGATT
TCAGGCGGACCTACCGCTGCAGTACCTGCGCCGCGAAGACTACGTGCGCAACCAGACTGT
CATCGAAGCCAAGCTCGACGCGATCGCGCTCAGATTCGAAAACTTACAACTCCGGGGAAA
TCAATGCCGCCCATCAATCCGCTGGGAATCGACCACGCCAAGGTGCGCCGCGAAACGCTG
CGCTGGTATCTGATTCTGGCCCTGTAGAACGCGCGGCCCGAGGAAGTCGTCGAGGACGTG
ATGCAGATGACGATGCGCTCCATCTTCGCCGACATCACGGCGCTTGAGGTGCGCAAGGAA
CTGGACTACCTCGCCGATCGCGTGCTGGTGAAGCTGCGCAAGGAACCGTCCGGCCGCTGG
TGGGGCGATCTCACGCGCTACGGCGTCGACATCGCCGAGTACACGATCGATTGCGAGCCG
GGCATCGCGCGGCCGGCAAAGTACTGGAGCCAGTGACATGGGGCGCAGCAACGGCGTGCA
ACGCCTGCCGAAGCCCGTGCGCGAATGGCTCGAAAGCGCGCTCGTCGAGGGCAACTTCAC
GGGCTATCAGGAGCTGAAGCAAACGCTTCGCGACAAGGGCTACCAGATCAGCAAGTCGGC
GATCCATCGGTACGGGCAGCGTATCGAGCGCCGCTTCGCCGCGATCAAGGCGAGCACGGA
AGCCGCACGCATCCTGACCGAAGGCGCCGCGGACGATCAGGACGCCCGCTCCTAGGCTGT
GATCGCGCTCGTGCAGACCGAGATGTTCGAGTCCATCGTCAACCTGCAGGAAGCGACCGA
CGAAGACGCCGATCCAGGCGAACGTATCGCGCTGCTGTCCAAGGCCGCGAAGAACATCGC
GACGCTGGCCCGCGCGAGCGTGAACCAGACGAAATTCCGCCTGGAGGTGCAGGCACGCGC
GGAAGCGGCCGCCGCGGCCGTCGACAAGGTCGTCAAGAGCAGCGGCCTGTCCGATGACGC
GGCCGACGCGATCCGTCGTCAGATTCTCGGGATTGCAGGATGACGACCGTAGAAACCCGT
GCGGATCGTGCGCCCGCCGTCCCGCTGCCGTATCAGCAGAAATGGTGCGCCGACACGTCG
CCCGTCAAGGTCTGCGAGAAGTCGCGCCGCGTTGGCTTGTCCTGGGGCGAAGCAGCCGAT
TCAGCGTTGCTCGCAGCCAGCCAGCGCGGCATGGACGTTTGGTACGTCGGTTACAACAAA
GACATGGCGGAGGAGTTCATCCGGGACTGCGCCGACTGGGCCAAGTTCTACAGCCTCGCG
GCCGACGAGATCGAAGAAACCGACGAGGTCTTTCAGGACAAGGATGGCGACAAGTCGATC
CTCGCGTTCGTGATCCGCTTCGCGTCGGCCTTTCGCGTCACGGCACTGTCGTCGCGCCCG
TCCAACCTGCGCGGCAAGCAAGGCCGCGTGATCATCGACGAGGCAGCGTTCCACGAGCAG
CTCGGCGAGCTGCTCAAGGCCGCGATGGCGCTGCTGATGTGGGGCGGTCAGGTTCACATC
ATTTCGACGCACGACGGCGTCGACAACGCGTTCAATGAGCTGGTCACGGACGTCCGTTCC
GGCAAGAAGCCCTACAGCCTGCATCGCATCACGATCGCCGACGCAGTCCAGGACGGGCTC
TACCAGTGCATTTGCCTGCGCAAGGGCGAAGCCTGGACGGCCGAAGGTGAAGCCAAGTGG
GTCAAAGATATTCGCGCGTCGTACGGCGCGGATGCTGAGGAAGAGCTGGACTGCGTGCCG
AAGAACAGCGGCGGCGCATGGCTGTCGCGCGCGCTGAGCGAGTCCCGCATGTCGGCTGAC
ACGCCGGTGCTCCGCTGGGCCTGCAAACAGGGTTTCGAGGTGCTTCCCGACCACAGCCGC
GCAGCCGAATGCCGCGATTGGCTCGAAGTGACGCTCGGCCCGCTGCTCACGGCGCTCCCG
GTCGACGCCCGCTCGTACAACGGCGAGGACTTCGGACGGACTGGCGACCTGACGGTCCAC
GTGCCGCTGATCGAACAACAGAACCTGGTTCGCCGCGTGCCGTTCATCGTCGAGCTGCGC
AACGTGCCGTTCCGGCAGCAGAAGCAGATCGCCTTCTACCTGCTTGATCGGCTCCCGCGC
TTCACGGGCGGCGCCTTCGACGCGCGGGGCAACGGCCAATACCTCGCAGAAGTCGCGATG

CAGCGCTACGGCGCATCGCGCATCCAGCAAGTGATGCTGTCCGAGTCGTGGTATCGCGAG
CACATGCCGCCCGTGAAGGCGGCGTACGAAGACGGCACGATCGACGGCCTCCCGAAGGAT
GCCGACGTGCTCGCGGACCTGCGCGCCGTACAGGTCATCAAGGGTGTGCCGCGCATCCCG
GACGTCCGCGCGACCGGCCAGGACGACGGCAAGCGCCACGGCGACGCCGCCGGAGCGGTT
GCTCTGGCGTATTACGCGAGCCGTGAACTGAACAAGGGACCGGTGACCGCCAAGTCGCGG
CGGCGCCGCTCCAGCGTCCAAAGGACAGAGGGTTACGCATGAGCAAGGGTTTGTGGGTCA
GCCCCACCGAGTTTGTTACGTTCGGCGAGCCGGACAAGTCGCTTTCGTCGCAGATCGCGA
CGCGGGCGCGAAGCATTGATTTCTTCGCGCTCGGCATGTACCTGCCGAATCCGGACCCGG
TGCTCAAGGCACTCGGTAAGGACATCAGCGTCTACCGCGAGCTGCGGGCGGACGCGCACG
TCGGCGGGTGCGTGCGCCCCCGCAAGGCGGCCGTGAAAGCGCTCGAATGGCGCCTCGACC
GTGGCAAGGCGAAGAGTCGTGTCGCGAAGTCGATCGCCGACGTCTTCGCGGACCTCGACC
TGTCTCGGATCGTGACCGAGATGCTGGACGCCGTCCTGTACGGCTATCAGCCGATGGAGA
TTACCTGGGGCAAGGTCGGCAACTACATCGTGCCGATCGACGTCGTCGGCAAGCCGGCAG
ACTGGTTCGTGTACGACCCGGAGAACCAGCTCCGCTTTCGCAGCAAGGATCACTGGATGC
AAGGTGAAGAGCTGTCGGCACGGAAGTTCCTGGTGCCGCGCCAGGAGGCGACGTATCTGA
ACCCGTACGGCTTTCCGGACCTGTCGATGTGCTTCTGGCCGACGACGTTCAAGAAAGGCG
GCCTCAAATTCTGGGTGCAGTTCAGCGAGAAGTACGGTTCGCCGATGCTGGTCGGCAAGC
ATCCGCGCAGCGCATCCGATGGCGAAAAGAACCTGCTGCACGACTGCCTTGAGGACATGG
TGCAGGATGCTGTCGCCGTGGTCCCGGACGATTCCTGCATCGTGATTAAGGAAGGAGCCG
GCAAGACCGGTAGCGCCGACGTCTACGAGCGCCTACTGCACTTCTGTCGCGGCGCGGTAT
CGATTGCCTTGCTCGGGCAGAACCAAACCACCGAGGCGACGTCGACGCGCGCGTCCGCGC
AGGCCGGGCTTGAGGTGAAGGACGACATTCGCGACGGCGACAAGGCGGTCGTCTCCGAAG
CGATGAACATGCTGATCCGCTGGATTTGCGACCTGAACTTCGACGGCGCCGATCGACCCG
TCTTCGACATGTGGGAACAGGAACAGGTCGACGAGATCCAGGCCGGCCGCGACCAAAAGC
TGACGCAAGCCGGCGCGCGTTTCACGCCGGCATATTTCAAGCGTGCGTTCAACCTGCAGG
ATGGCGACCTGGACGAGCGGCCGCTGCCGGTGTCCGACGTTGATACGGTGGGTGGCGCAT
CCTTCGCAGAGTTCGAAGCGCCCGAGCAGGACGCGCTCGACGCCGCACTGAACACCTTGT
CCGCGCGCGACCTGAATGCCGACGCGCAGGCGCTGGTCGCGCCGCTTTTAAAGCGGATTG
CGAACGGCGCGAGTGCCGACGAGCTGCTCGGCATGCTGGCCGAGCTGTATCCGAGCCTCG
ACGCGGACGCGCTGCAGGAACGGCTTGCCCGCGCGATCTTCGTCCCGAACCTCTGGGGGC
GTCTCCATGCCTGAAGCGGTCGACCTCGGCTACTGCATGAAGCCGCCGCCGAAGAAGGCG
ATCGAGTACTTGCGCAGCAAGGGCTACGAGATCACCTGGGATTGGGAGGAGCTTTGGCAG
GACGCCCACGCGAAGGCATTTACGGTTGCCAAGGTCACGCGCCTGGACATCCTGCAGGAC
ATTCGCAACGCGGTCGAAACCGCAATCAGCGAAGGCAAGACGTTGCGATGGCTCACGAAA
GAGCTGACGCCCGTACTGCAATCCAAGGGCTCGTGGGGAAAGCAGGATCACGTCGACCCG
GATACCGGCGAAGTCAACCAGGTTCAGCTCGGCAGCCCGTGTCGCCTGCAGACCATCTAC
CGAACGAACCTGCAGACGGCCTACATGGCGGGCCGCTACGCCGAGCAAATGGCGAACGTC
GAAGATCGGCCGTACTGGATGTGCGTGGCGATCCTCGACAGCCACACGCGCCCGAGCCAT
CGGGCAATGAACGGAAAGGTGTTCCGTTACGACGATCCGTTCTGGCACTCGTTCTACCCG
CCGAACGGCTGGGGCTGCCGGTGCCGCGTGGTGGCGCTGTCGCACGACGAGATCATCGTG
CGAGGCATCAAGGTCGAGGCGGCCGGCGACCGCCTCGGCACGACGCTCAAGCTGGTCAAC
GAGAAGACCGGCGAGCTGCGCGAGGTCGCGACGTTCCGCACCGTCGACCCAGTCACGCGC
CGCGAAATCGTCGTGTCGCCGGACGTCGGCTGGAGCTACAACCCCGGTGCCGCGGCGTGG
CAGCCTGACCTGTCTCGAGACACGGTTGATCTCGGCACGATCGCCAGGGGGGAATTGCAG
TGAGCGATTTCGTGAATTTCCAGATCGACGACTCGGCGCTGCGCACGCGCTTGCTGCAGC
TCGAACAAGCCGGCCATCAGAAGGCTGGCGCGATGCGCAAGATCGCGCAGCCGCTGGTGC
TGGTCACCGAGGACAACTTCGCCGCGCAAGGCCGGCCGCGGCGGCAGGCATTGTCGGAAG
CGACGATTCACATGCGCGACGGCGGCAAGAAGGCGTACAAGAAAAACGGCGAGCTGACCG
CGGCCGCGTCGCGTCGCAAGGCCGGTCTGATGATCCTGCAGGACAGCGGGCAAATGGCCG
CGAGCGTCTCGACGGATCACGACGTCAATTCGGCCGTCATCGGCAGCAACAAGGAATACG
CCGCGATTCACCAGTTCGGCGGGCAAGCCGGTCGCGGATTGAAGGTCACGATTCCCGCCC
GTCCATGGTTGCCCGTTACGGCTGATGGCGAGCTGCAACCTGAAGCGGTCGAGCCGGTGC
TCAACACGATCCTGCGCCCCTTGATGGACGCCGCGAACCGCCGCTAACCTGTCGACGAGC
CGATCGCGCTCTGCGCGCCTCCGGACCACCCGAGGCGCGCAACGATAGCCACAGCGCCCT
GACCCTGCCCCGTAAAGTTTTATAAAGGCTTTACGGGTTTGGCTCGAACCTGCTGCGCTC
CCTGCATCACGCGTATCACGCCGGCGCAATCGTAAAGCCGATTAACAGACCCGTGCCGTT
CGTCGCCCGATGATGGGCGCCATGAACGCGAAACCAATCCACCTTTTCCGGGCAGGCACG

CAGACCGACATGAACGGTCGCGTGCTTGATTTCGCCGAAACGGACCTCGCCGCGACGGCT
GCTGCATACGATCCGAAAGTCCACGAAGCGCCGATCTTCATCGGCCATCCGCGCGACAAC
GCGCCGTCGTGGGGCTGGGTCGCTTCTCTCTCGGCGTCGGCCGGCAACCTGCAGGCCGAA
CCCATCCAGGTCGATCCCGCATTCGCCGAACTCGTCGGCGCCGGGCGCGACAAGAAGATC
AGCGCCAGCTTCTACCACCCCGATTCGCCGCACAACCCGGTGCCGGGCGTGTACTACCTC
CGTCACGTCGGGTTCCTCGGTGCGCAGCCGCCGGCCCTCAAGGGCCTGCGCGACGTCAAT
TTCAGCGACGGCAACGAAGGCGTCGTCGAGTTCAGCGACTGGGGCCAAGCACTCAATGCG
GGCCTCTGGCGCAGCCTGCGCGAATGGCTGCTGACGCAATTCGGCCGGGACACGGCTGAT
CAGGTCATCCCCGATTGGCAAATCGAGTCGATCCGCGAAGTCGCGCGACAAGACGATGCG
CCGACCAGCGCATTCGCCGAACGCGGGGCGGCAACCGTACCCACCACCACGAAGCAGGAG
AAAGACGCAGTGACCCCTGAGAAAAAGGCCGCACTGGAGGCCGAAAACACCCAGCTCAAG
CAGCAGCTCGCCGACGCGCAGGCGCGTGAGCGCAAGGCGGCGGACGACCCCCGCCACAGC
GAGCATGTGTCGTATGCCGAGCAGCTCGTCAAGGGCGGCACGCTCGACCCGAAGCACAAG
GATGCAGTCGTCGCATTTCTCGACTTCACGGCACGTGAGCCGTTCGAGTTCGGCGAAGGC
GATGCCAAGCAGCCGCTGGCGAGCTCATTCCGTTCGTTCCTCGGCGAGCTGCCGAAGGTC
GTCGAATTTGGCGAGCACGCCACGCGCGAGCGCGCCGGCGTGACGACGACGGGCGCCGAC
ACCGTCGAGTACGGCGAGAACGTCGATCCGAAGCGCGCCGAGCTGGACGGCCGCATTCGT
GCATACACGCGCGAACACAAGGTCGATTACGCGGCGGCCGCGAACGCGGTGATCCGTTAA
TAACCGGCGCAAGCCACAGATAAGGCCATTGAGCCAGGACAAATGATGGGACGTCTCTCG
TAACTGCGAATCGTCGATCCGGTGCTGACGAATCTCGCGATCGGTTACACGAACGCCGAA
TTCATCGGCCAGAGCCTGATGCCGGTCGTCGAAGTGGAAAAGGAAGGCGGCAAGATTCCG
AAGTTCGGCAGGGAATCGTTCCGCCTCTACAAGACGGAACGCGCGCTGCGCGCCCGGTCG
AATCGAATGAACCCTGAAGACCTCGGCAGCATCGACATCGTCCTCGACGAACACGATCTC
GAGTATCCGATCGACTACCGCGAAGACCAGGAATCGGCGTTCCCCCGGGAACAAGCCGCC
TTCCAGACAGCGACCGAAGCGATCCAGTTGCGCCGCGAAAAAATGGTCGCGGACCTCGCG
CAGAACCCGAACAGCTACGCCGGGGGCAACAAGAAGCAACTCAGCGCGACCGAGAAGTTC
ACCGCGGCCGGCAGCGAGCCGGTCGGCATCATCGAAGACGGCAAGGAAGCGATCCGCACG
AAGATCGGCCGCCGGCCGAACACGATGGTGATCGGTGCCTCGGCCTACAAGACGCTGAAG
GCCCACCCGCAGCTCATCGAGAAGATCAAGTACTCGATGAAGGGCATCGTGACCGTCGAC
CTGCTGAAGGAAATCTTCGAGGTCGAGAACATCGCGGTCGGCGAAGCCATCTATGCCGAC
GACAAGGACCGCTTCACGGACATCTGGGGCGCCAACATCGTGCTCGCCTACGTTCCGCTG
CAGCGCGGCGGCCAACAGCGTACGCCGTATGAGCCCTCCTACGGCTACACGCTGCGCAAG
AAAGGCAATCCGGTCGTCGACACGCGCATCGAAGACGGCAAGCCCGAACTGGTGCGCAGC
ACGGACATTCTCCGGCCGTACCTGCTCGGCGCGGACGCCGGCTACCTGATCTCGGGCATC
AACGGCTGATCGCTCGCGGCATCGTATCCCGTACTGACCACGTCTCGCCTTCGGGCGAGA
CCCACCCGAACGGAACATGACTCATGAAAACGCATCAACCGATCCTTACCACGTCCGTTA
CCGCTGCCGCCGGTCTGAACCGCTTCCAGTTCGTCGGCTTCGATGGTGGCGTGTGCGCAG
CCGGCGCGAAGGCGCTCGGCGTTGCCGAAACGACCGCCGATGTCGGCGAGCAAGCGCCGG
TCAACCTGCTCGGCGTCATCCTGGTCATGGCCGCCGCACCGGCCGACGAGCATGCCGAGG
TCGAGTCGGACGCGGCCGGCCAAGCCATTCCGAAGGCAACCGACGAATCGAACGGCTACG
CACTCGATGCGGCGACGGCGGCCGGCGACGTCATCCGCATCCTGCGGGGTATTTGACGTG
CGTTATTGCACGTTCGCCGACCTGAAGCTGGCCGTGCCGGAACGGACGCTCATTGAGCTG
ACGAACGACACGACCACCGACTACGGCGCACCGGGGCCGACGACGATCAATACCGACATC
GTCGAAAGCTCGGTGCGCCAGGCGGAGGAAATCGTCGACGCGCACTTGCGCGGCCGCTAC
CACCTGCCGCTGTCGCCGGTTCCGACGGTCATCAAGGATGTCACGGTCAATCTGGCCCGG
CACTGGATGTATGCCCGTCGACCGGAAGGCGCCGCGCTCCCGGACACCGTGTCGCAGACC
TTCAAGGGGTCGATGCACATGCTCGAAAAGATCCGCGACAACAAGCTGACGATCGGCGAC
CCGAGCGGCCCGGCGACGCCCGAGCCGGGCGAAATGAAGGTCCGCGCGCGCCGTCGGCAG
TTCGACGCGGACCTGTTGGAGCGCTTCTGATGGCGACGACGCTTGAAATGGTCGATGCCA
TCGTCGCCCGGCTGCGCGTCAAGCTGCCAGCGCTGGTGACCGAGTACTTCCCGGAGCGCC
CGGACGAATACCGGCTCAACCATGCGATCGGCGCGTTGCTGGTCAGCTATCCGGGCAGCC
AGTACGACACGACCGTCGACACCGACATGGTTGTTCAGCCGCGGCGAGTGAAGTTTGCCG
TCGCGATCGTGCTGCGCCAGCTCAACGGCCGCGGCGGTGCCATCGACGTGCTCGATCACG
TTCGCACCGCCCTGGTGGGCTTCCGCCCGCCGGACTGCAAAAAGCTCGCGGCGGTCAGCG
ACAAGTTTCTCGGCGATTCGGCCGGCCTGTGGCAGTACGTGATCGAGTTTTCGGCGGGCG
CCGTGATTGTCGAAGACGCGGAGCCGAACGACGGACCGCTGTTGACGCAAGTTACCTACG
AGGAGGAATCGTGAAATACCAGTACAGCGGACCGACCAGCGGCGTCACGCTGCAGGACGG

CGAAGACGTCCAGGAAGTGATGCTTCATACGGGCGCAGACGTCGAGCTGCGCGAAGAGCA
CGAGTACACAGCGACGCTGCTCGCGATGGGCTACCTCAAGCCGGCCGCAGCGCCGTCGAC
CAAGCCCGCGCGCCCCAGCGTGCCGGAAGACCAACCGAAGACCTCCGCGAACGCGGTCGC
CGTGAAGGGAGTCTGAGCGATGGCGGCAAACTATTTGCATGGCGACGAGACCATTGAAAG
GGAAACCGGGTCGCGGCCCGTCAAGGTCGTGAAGTCGGCGGTGATCGGCTTGATCGACAC
CGCACCGATCGGGCCGGTCAATACGCCGGTGCAGTCGCTGTCCGACGTCGACGCGGCGCA
GTTCGGCCCCCAGATGGCCGGCTTCACGATCCCGCAAGCGCTCGACGCGGTCTACGACTA
CGGCAGCGGGACGGTGATCGTGATCAACGTGCTCGACCCGGCCGTACACAAGAGCAACGC
GGCGAACGAGCCCGTCACGTTTGACGCGGCAACGGGCCGTGCAAAACTCGCGCATCCGGC
CGCGGCGAATCTCGTGCTGAAGAACGACTCGGGCGGCACGACGTACGCCGAAGGCACCGA
TTACGCTGTCGACCTGATCAACGGCGTCATCACCCGGATCAAAACCGGCACGATTCCGCC
GGGTGCGACGGCGGCCAAGGCGACCTACGACTACGCGGACCCGACGAAGGTCACGGCCGC
GGACATCATCGGCGCCGTGAACGCAGCCGGAATGCGCACGGGGATGGAGGCGCTGAAGGA
CACGTACAACCTGTACGGCTACTTCTCGAAGATTCTGATCGCGCCGGCGTACTGCACGCA
GAACTCGGTCTCGGTCGAGCTGGAGGCGACGGCCGTCCAGCTCGGGGCGATCGCGTACAT
CGATGCGCCGATCGGCACGACGCTCGCGCAGGCGCTGGCCGGGCGCGGGCCGGCGGGCAC
GATCAACTTCAACACGTCCAGCGACCGCGTACGCCTCTGCTACCCGCACGTGAAGGTCTA
CGACACCGCGACCAACGCGGAACGCCTGGAGCCGCTCTCATCGCGTGCGGCGGGCCTTAG
CGCACGTGTCGACCTGGACAAGGGCTACTGGTGGTCCAGCTCGAATCAGCAGCTCGTCGG
CGTCACGGGCGTCGAGCGGCCGCTGGCGGCGATGATCGACGATCCGCAATAGGACGTGAA
CATGCTCAACGAACAGGGCATCACGACCGTGTTCAGCTCGTACGGCTCGGGCTTGCGCCT
GTGGGGCAACCGCACGGCGGCATGGCCGACCGTGACGCACATGCGCAACTTCGAGAACGT
GCGCCGTACGGGCGACGTCATCAATGAATCGCTGCGCTACTTCAGCCAGCAGTTCGTCGA
CGCGCCGATAGACCAAGGGCTGATCGACTCGCTCGTCGAGTCGGTGAACGGGTTCGGGCG
CAAGCTGATCGGTGACGGCGCGCTGCTCGGCTTCAAGGCATGGTTCGATCCGGTCCGCAA
CCCGAAAGAGGTGCTGGCGGCCGGCCACCTGCTCATCAACTACAAGTACACGGTGCCGCC
ACCGCTCTAACGCCTGACGTACGAGACCGAGATCACCTCGGAGTACTTGCTCACCCTGAA
GGGAGGTAACTGATTATGGCGGGTGGCGTCCAAATCAACCGGATCACGAACGCCAACGTG
TATCTGAGCAACAATTCCATGCTCGGCAAGACTGAGGAAATCAAGCTGCCGGACATCCAG
GCGATCATGGCCGAGCAAAAGGCGCTCGGCATGATCGGCAAGGTCGAGCTGCCGGGCGGC
CTGGACAAGCTCGAAGGCGAGATCGAGTGGAACGCGCTCTACGCCGACGTGGCGAAGGCG
ATGGCGAACCCGTTCAAGGCCGTTCCGCTGCAATGCCGATCGAACATCGAGACGTATGGC
GCACAAGGCCGCGTGCGGGAAATCAGTCTCTTGACGTACCTGACCGTGATGTTCAAGAAG
AACCCGCCCGGTACGTACAAGCAACATGAGAACGCCGATTTCAGCTCCGCGTTCGGCGCG
ACCTAAATCAAGCAGGTCATCGACGGCGAGGAAGTGCTGGAGCTGGACTATCTGGCAAAC
ATCTTCCGCTTCGGCGGCGAAGACATGCTGGCTGACTTCCGCGCCAACACCGGCGGCTAA
CCCCACACCTGCGCAGTCGTTGTCGTCTTGGCCCGCTTCGGCGGGCCTTTTTTAATCTGC
ACTGAAAGTGCGAACGCGCGCGCGAATCGACTATGTGTGCTCTACTACCACGGAGCGCGT
CGTGAAAATCCCCCTCAAATTTCCCGTCAAACTTGCTACCGGCCAGACGCTCACGGAACT
GACGTTGCGTCGCGGCAAGCGCAAGGAAATGGCCCTTGCGGCCAAATACAGCGAAGACCC
GGGCGAACAGGAAGATTTCCTGCTCGCCATGCTGACCAACCTGACCGTCGAAGACATCGG
CGAACTCGACCTCGCCGACTCGAAGCGGCTCATGGACTCCTTTCGCAGCATGGTTGAGGT
ACGGGATACCGCCGGAAATGCGGGCGCCCGGCGATCAGCCGCCGAGCAAGGAAACGCAGG
CGCTGGACTCGGCGCTGCGACCGCTGGATGAGGTGTTGCTACTGGTGCTGAAGATTCAGC
CGTCCGAGATCGATGAACTGGATATGGACGACTACTGGCACTGGGTCGACGCCGCGGAGC
GGGAAATCAAGCGGCGCGTCGACGCGACAAAGCAATCCTGATGTCAGGCGGCCCGGCGCC
CGAGCCGGCCGCAAAGCCACACGATGGCCTTGATCGCCGCCGCGATGAGGTAGGAGCCGG
CCACGACGGCCGGCCCGAGGAAGGCGAGGCCGAAAAACGCCACGATGCCGAACACGATCG
GCACGGACCACCAGGGCAACTCGATGAACAGCCAGACGGCGAGCACGACGCCGGCAATCG
CGATCACGGTGTAGGTAATGCCTTCGGCGATGGACTGGATGTTCATGATCGGCCTCGTCA
CTTTCCGGTAAGGGTATGGCAAGCAAATTCTATATTGGCGTAAAGATCGGCGCGACGCTA
CTCGGTAGCTTCGGCGCCGCGCTTTCCGGCACGCGCACGACGCTGAATGGCCTCGGCCGC
GTCGCCGACGAGCTGCGCGCGAGGCATACCTGCCTGGGGGACGCGATGGCGCGGGCCGTT
GCGCATCCGATGCGCATCATCGCCGAGCTGCGCGGCCAGTACGACCGCCTCGGCCGCACA
ATCGATCAGGTCCAGGCGAAGCAAGCGGCCCTTGCGACCCGTCTGGCGCGCGGCGCCGCG
CTGCGTGAGCAACGCCAGGGGCTCGGCGCCGACATGCTCGGCACATACGCGACGGCGGCT
GCCACGGCCGCGCCCGTAATCGGCGCTGTTCGGCAGGCCGTCACCTTCGAAGCCGGCCTG

CGCGACATCGCGATTAGCGGCAAACTGACCCGTGATGAAGAGTTCCGGATCGGCGAGACG
ATGCGCCGTGCGGCCCTTGCCACGAGCCAGGGTCACAACTCCATTCTCGAAGGCGTCGGC
ACACTGGTCGCTGCCGGCATGGACGCGAAGGAAGCCGGTCAGAAATCGAACCTGCTCGGG
CGCGTGGCGACGGCCACGAACGCCGACATGAAAGACCTCGCCGGCATGGTGTACTCGTTC
TCCGAGACGCTCGGGATCAAGGGCGACGCGGCGCTGAAGGAGGCGTTCAACCGCGCGGCC
TACGGCGGCAAGCTCGGACGGTTCGAGCTGAAGGATATGGCCAAGGCTCTGCCGGAAATG
ACCGCGGCGTTTGCGGCCAAGGGCATCAAGGGCCAGGACGCGCTGACGCAGATCATCGCC
AGCCTCGAAGTCGGCCGCGAAGGCGCCGGCAGTGGCGACGAAGCCGTGACGAATCTGCGC
AACTGGCTGTCGCACATGAACGCGAAGGCGACCATCGACGCCTACAAGAAGGCCGGCGTC
GACTACCAGAAATCGAAGTCCAACCTTGTCGCCGGCGGCTATTCGAGCTACGAAGGCTCG
CTGCAGATCGCACAGAAGTTCATCGCTTCGCGCGGCGAGGCGTTCATGAAGCAGTGGAAA
GCGGCGGGCGCCAAGGGCGATGAGGAAGCACAGCGCAAGCTGATGGAGAGCTTCGGCCTG
AATGAGGTGTTCCAGGACATCCAGACCATCAACCACCTGCTCGCGATGCGTCAGGGCTGG
GACAAGTATCAGCAGAACAAGAAAGACATGGGTAGCGCGCAGGCGCTGAATACCATCGAC
CAGGACTACGTGCGCCGCGCGGAGCTGGCGACCGTCGCGTGGGCCCGCTTTCAGACGCAG
ATTGCGGACCTCGGGATCACGGTTGGCCGCGCGCTGCTGCCGTCGCTGACGGACTTGATG
AACACGATCACGCCGCTGATCCAGCGCACCGCGCAGTTCGCGGCCGCCCATCCCGGCCTG
ATCCGCGGCGTGGTCGGCTTCGCAACGGCCGTGATCGGCATGAAGGTCGCGACGCTCGCG
GCCGGCTGGGGCCTGAATTTCTTCGTCAAGTCGCCGCTGAACATGGTCAGCACGGCATTG
ACGACGGTCGGGGCGAAGTGGACGCTGTTTCGCGCGCTCTGGGCCGGTGGCGGCTCCCGC
CTGTCCACCGTGTTTCAGATTTTCTGCATGGGCGCGGGCGCGGCCGGCAAGTTCGCGGCC
GTGATCGGGCGTGCCGGGAGCCGGTTTATGGGATTCGGGCGCGGCGCGCTCGTCGTTGGC
CGCGCGCTGCTCCCTTTCGGGCAGGGCATGCTGATGACGTTTATCGGCCCGCTGCGGCTG
CTCGCCCAGGGCGGGATGCTGCTGGCCCGCGTCCTGGGCGGCCAGCTCGTGAACGGCCTG
ATGCTGGCCGGGCGCGCGGTGCTCTGGCTCGGGCGCGCGCTGATGCTCCATCCGATCGGG
ATTGCCTTCACCGCGATCGCGGTCGGCGCGTACCTCATCTACCGCTACTGGACGCCGATC
AAGCAGTTCTTCGGGGGCATCTGGGCTTCGATTCGTACCGCGTTCGCGGGTGTGCTCGGG
TGGTTCGGCGCTGGGCTCCCGAAAACCTTCACCGATTTCGGCCCCCACCTCATCGACAGT
CTGGTCAACGGTATTCGCAATCGCTTCACGGCTGCGAAAAACACGCTGATCGAGTTCGGC
AGCAACGTGAAAGCGTGGTTCGCGAACACGCTGGGGATCAAGTCGCCGTCCCGTGTATTC
ATGGGTTTCGGCGACAACATCGCCCAGGGCGCCGCAATCGGCATCGGGCGCTCGTCGGCG
GTCGCCGCGCGCGCGGCCGCCGGCATGGCGACGCAGGCCGCGGCCGCGGCGTCCCTGCAG
CGCATCAACGCAGCTCGGGGCGGATCGCCGGCTGGCGCATCGGTAGCCGGCTCTGGGATC
ACAGTTCACTTCTCGCCGACGATTACGGTCCAGGGCGGCTCACCGGACGGCGTCAAGGAT
CAGGTCAAGCAAGGGCTCAACCTGTCGTTGCGCGACCTGGAGCGCATGCTCAAAGACCTG
CTCGCGAAGTGCGAGCGCCGCGCGTACAGGAGCTGACGTGTTCGCAATTCTTGGAGATAT
CGAATTCGACCAGATCGGATACTTCGACGGGTTCGATGCGACCTACGGTGCCGACTACGC
CGAACACGCGCTGCTGCAGAGCAAGCCGCGGCTGCAACGCATGGGCGACAAGCTCGACGA
GATTCTGATCGCGCTGTCGTTTCACTACTGGTATTGCGATCCGGAAGCCGAGCTGGCGAA
GCTGCGCGCGGCGGTGAGCGCAAAACAGGCGATGGCGCTCGTTTTTGGCAACGGCGACTA
CAAGGGCTGGTTTGTACTGACCGAGGTTCAGTCGACCAGCAAACAAACCGACACGTCGGG
AACCGTGCTGTCCCTCGACGCACAGATCACCCTGCGCGAGTTCGTCGGAGACAAGAAAAA
CCCGTTCAAGCCGCCGGCCGTACAGCCCAAGGTGCCGCCGGCGGCCTCGCAGGCGGTCTC
CAGCGTCACATCGGCGGTGGCCACCGCGCGGGGCACGATTCAGCAGGCAGTGACCTGGGC
CAACCAGGCGCAGTCCGCGAGGCGCGTGGCCGTCGACGCGGTCAAGGTCGTCCAGAAGCT
GAAAGACGATCCGTTCGCAGCATTGAGTCGCTCGTCGAGCGTGCTGACCAACATCAAGCA
AGCCGCTGATCCACTGGCGAAGCTGTCTCCAGCGCTGGCGTCCCTGACCGACCAGATTCC
CGAGGCGGCCGGCATCCTGCGTGCCAGCAATACTGCGCTGGATGCGGTCCGATCGGCGCA
AGGCGGCTTGGCGAACGCGACGATCGGGACGATTACCGGCGCAATCGACCGGGCCGCGGG
CCAGCTCTCGACGGCGAGCATCGCGCTAAGTTCCGCGGCGCCGAGTCTGAGCAAACTGGC
CGCGAAAGTCGCGACGCGGAGGATTTGATGTTTCTGACCCACATCACAACCGAGGGCGAA
CGGTGGGATCAAATCGCCTACCGGTATTACGGCGACCCGTTTGCCTATGAGCGGATCATT
GCCGCGAACCCCAGCGTGCCGATTACGGCGGTTCTCGCGAGCGGCATTGCGCTGTCGATT
CCGGTGGTTGCGGCCGACGACGTAGCCGATGAGGAGCTTCCGCCGTGGATGCGGTGATCG
ACACCCCGACGTCGGTTGCCGACGTCCCTGAGCCCGTTTTCACGCTCGTGTACGAGCAGA
AGAACATCACCAACGACATCGCGCCCTACGTGCTGCCGGTGGCGTATACCGACTTCTTGT
CAGGCCAGTCCGCCGAGTTCGAGGTCGTGCTCGAAGACACGGATGGACGAGGGCTCGATG

CGTGGTATCCGGGCAAAGGCGACGCGCTGACGCTGAAGATCAGCTACGCCGGCGCGCCGT
TGCTCGCGTGCGACCGCTTCGAGATCGACGAGATTGGCTTCGACGAACAGCCGTCGACCG
TGAGAATCCGCGGACTCGGTACTGGCGTGAAGGCATCGGTCCGAAGCCGCAAGGCCAAAG
AGTACGAGCACACCACGCTGGCGACGATCGCGGCTCGCGCGGCGAAGCGGAACCACCTGA
CGTTCACCGGCCGCATTCGGGATACTCGCATCGACCGAGTCACGCAGTATCAGGAGCAGG
ACGTAGCGTTCCTGACGCGGCTCGCGCGCGAGTACGGCTATGCGTTCAAGATTTCGGGCA
GCAAGCTGATCTTCAGCGAACTCGCCGACCTGCGAGGATCGGACGCGGTCCAGCAGTTCA
AGCGCGCCGACTTGAAGTCGATTCGCCTGCGCGACAAGATCAAAGACGTCTACGCGCAGG
CCACGGTAGGCTACCACAGCCCGAAAACGAAGAAGCTCGTCGTCTACGGCGTGACGGGTG
ACTCCGTTGGCGTCGTCGGCCAGTCTGAAGTGGCAGCGGGCAAGCGGAAACAGTCCGGTC
AGTCGACGAGCGGCGACACGCTGCGGCTTTCCGCACGGGCCGGCTCCAAGGCAACGCTGC
AGACGAAGTCACGCGCGGCGCTTGACCGGACGAACCTCCAGCAGATTGGCGGCTCGGTCG
AGATGCGGGGTGACACGAAGCTGGCCGCCGGTGCGTCGATAGAGCTGCTCGAACTCGGCA
AACTGTCCGGCAAATACCTCATCGAGTCGGCGCGACAGCGTCTCGATCGCGGCGGCGGCT
ACCAAACCGAAGTCGCGCTGAAGCGCTCGGCGCTCGCCGTCCAGGCCGGCAAAGGCGGCG
GCACCGCGAAAAAGTCAACGAAAGGGCTGCAGGTCTACGGCGTCACCGCGAAAGGCGACG
TCGGCGTTGTTGGAACCAAGCCCGTAGCGGCGAAAGGCAAGAAAAAATGAGCGAGACCCT
CGACGAGTTCGGCACGACGATCAAGTTCGGCACGGTCAGTGCGTCGAAGCCCGGCTTCGC
GCGCGAACGGCTGACCGACCTCGGCAACATGAGGACCATGTGGCTGCCGATCGCGTACCC
GGAGACCCTGGCCGATCAGGCTTGCTGTACCTACGATAACGGCGAACAGGTCGCGGTGCT
CCTGGACGGCCGGTGCGAGGACGGTGTGATTCTCGGCGCCATCTACTCCGATGCCGATCG
TCCGCCCGTCACGGACCCGAACAAGTTCATCGTCCGCTTCAAGGATGGCGCGGTGCTCGA
GTACGATCGCGCGACCCACACGCTGACGTGCTCCGGCATGCAGACCGTGATCGTCGATTC
GAAGTCGGAAATCATGCTGCGCGCGTCCGACAAGGTGACGGTCGACGTGCCGGAGACCGA
GTTCACGCGTAACGTGACAGTCAAGGGCAAGCTGACCTACCAGGGCGGCATGGCCGGATC
GGGCGGCGATGGCGCTGCGCTGACGGGCAAAGTGAACGTCGACGGCAACGTGAAGGCGAC
TGGCACCATCATGGATGCCGGCGGCAACTCGAATCACCACTCCCACTGAGCTTTTAGCCG
GCTTTAATATCCCCCCGACTGGACGCGCGGCAACATGGCCGCATGACCCAGCTCTCCGAC
ATCACCTCCGTTCACTGGCAGCCGGCCCTTAACCGCGAAGGCGTTGTTGAGGGCGTGGAT
GACATCGATCAGGCGATTCGCCTGATTCTCGGGACGCCCCAGGGCAGCGTTCCGCATCGC
CCCGAGTTCGGCTCCAAGCTCTATCTGTATCTCGACATGCCGATCGACCGCGCCACGCCG
CATGTTGTGCGCGAGTCGGTCGACGCGATCCGCCGGTGGGAGCCGCGCTGCGAGGTCGTG
CGTGTCATTCCGTCGATCGCTGAGTCGCGTGAAACGATCCGCGTGCAGTGGCGCCTTGCT
GACGGCGTCATTAGTGAGACGGAGGAGCCGCGATGACGCTTGGTGAACCGGATTTCATCG
CCCGTGATCCAGACGCCATTACGGCCGGGATCGTGGCCGCCTACGAGGCGCGCACTGGCA
AGACGCTGTATCCGGCGCAGGTCGAACGCGTCCTGGTCGACATCATTGTCTACCGTGAAA
CCCTCGTACGCGTCGGCATCCAGGAGGCCGCGAAAGAGAACCTTGTCGCGTTCGCCCGCG
CTCCCATGATCGACTACCTTGGCGAGCTGGTCGGCGTGACGCGGCTGCCGGCGCAGCCCG
CGAAGACGACGCTGCGCTTCTCGATCTAAGAAGCGTTGCCGTCGAACCTGCTGATTGCAG
CCGGCACGCGTGTCGGGACGAGCGAGGGCGCTGTCTCGTTCGCGACCGACACCGACGTGA
CGCTCGTCGCGGGCCAGTTGTCGATCGACGTGGCCGCGACGTGCGAAACCGCAGGCTCGA
TCGGGAACGGCTGGCAGCCGGGGCAACTCGGCTCGCTCGTCGACGACCTGGGCGACGTCG
ACGTGACGGTCGCTAACACCAAGGTCAGCGCGGACGGCTACGAGGAGGAAACGGACGATC
GTCTGCGTGAGCGTATCCGGCTCGCACCACAAGCATTCGGCACGGCGGGCTCCCGGCTCG
CGTACGTGTTCCACGCCAAGAGCGCGCACCAGTCGATCGTCGACGTCGGCGTAATCGGTC
CGGAAATCGAAATGCGCGACGGCCGGCTGGTTTCCGTCAACGGCGTTCAGCCAGGCAGCG
TCCAGGTCTACCCGCTCGTCGACACGGGGCTGCCTAGCGAAGCGATTCTCCAGCTCGTAC
GCGACAACCTGACCGACGAGCGTCGGCGGCCGCTGACCGATTACGTCGACGTCCGAACGC
CGACGCCGGTCGACTACGCGATCGACGTGCGTTTGACGCTGTACAAGGATGCCGGCGCCG
ACACAACGATCGCGCAGGCACGTACGGCCGCAGAAGCCTACCGAATCGATCGTGCCGCCG
GCCTGGGGCGCGATATCGTCCCGCGCCAGCTCAGTCGCGCCGTGCAGGTTCAGGGCGTGT
ACGACGTTGACCTGCCGGGACTAGCACTGCGCGTGCTGACCGCCAGCGAATGGGCGCGGT
GTACGGGTGTCAGTGTCATCCCGATCGGGGTGGCGAATGGCTGATCTGCTGTTGCCGCCA
GCGCTGGCGAAAGACCCTCGTTTCAAGGCCTTCGCCCAGTTGGCAGAGCGCCTCGACAAC
ATCGACATGTGGCCGCTCCAGGTCTACCTGATCGATGGCGTCGACGCATCCGCACTGCCG
TTCCTTGCAGACCAGTTTTCGGTGATGGGCGAGGACGGCTGGAGTCTCGCCGAGTCGGAT
GAGGCCAAGCGTGCCTTGATCAAAGGGGCACTCGAGCTGCACCGCTACAAGGGCACGCCC

TGGGCGGTCAGAGAAGTGATTCGGTGCCTAGGCTTTGGCGGAGTTGAGCTTGTCGAGGGC
ATCGGTGGCGCCTACTACAATGGAAAAGCCGCCATGACAGCGTAATGGTGTATGGCGGC
AATGGATTGTGGGCGGCCTATCGGGTCATCCTGCTCGATCGCGCGATCACCAACGATCAA
GCGGCGTTGCTGCGCACGACGTTGGCTGCGTTCGCGCCGGCTCGATGCGTACTCGCGAGT
CTCGAATACCGTCGTGTGCCGATGCGCTACAACGGCGCCGCCCACTATGACGGACAGTAC
AACCACGGGAGCAGTTAATCGGCAAACTCGTCGAATCTTCCCAATGGGAGGAAGATGTTT
ACCAAATCGAAACCGCCGATCCGGTCGAAGGTGGCCCGGACGGGGTTTCGAACAAGCAGG
CGAAGCAGCTCGGCGGCCGCACGCGCTACCTGAAGGCGCAGGTCGAGCAGTCGCAGACCG
GGCTTGCGCAGCATATCGCCGCGCCCGACCCGCATTCGCAGTACGCGACGAAGTCGGACC
TCGCGGCAAGGCTTGCCGAACTGGTCGGCCAAGCGCCCGGCATGCTCGATACATTGAAGG
AATTGGCGGAGGCGCTCGGAAATGATCCGAATTTCGCGACGACGATTACAAATCAACTCG
GGCTGAAGGCGCCCATCAACTCGCCCGTCCTTACTGGTACTCCGAAGGCGCCGACGCAAG
TTCAGGGGGATAACTCGACGGCGCTGGCGACGACTGAATTCGTGAAGCAGTCTGGAGAGT
CGTATTCCAGCATCCAGGGTATTGCCACGACGGCAACGTTGAACAGCGGACATGTGGGGG
CGTTTATTTGGGCGTACGGCGTAGGGATCACCCTTACGCTGCCACCGGTGGCTGGCGTTA
CTAATGGTGCGACGATCACAATCGGAGCGCCGGTCACCGTGACTCTCAAGAGCAATGCAT
CGGAGAACATCAACAGCCAGTTCGGAGTAACGTCGAACACGTTCGCTCTGAATGCGGGCG
AGCAAGCGCAGTTCATCAGCAACGGTGGCGCGTGGTACTTGTCGAGCTACACGACGATTC
TTGGGGTGACGCCGCCGCAATTCGACAACACGACGAAACTCGCGACGACCGGGTTCGTGC
AGCGAGCAGTCGGTAATTACCGATCCGCCAACGGAAACGTAGGAAGCGCCACTCTCAGCA
CATCCGACGTTGGCTGCCTTATCACTTGGGACGGAAACGGCACGTTGACGCTGCCGGATG
CCACAACCGTGTCAGGCGGCTCTGCAATCCGCGTGTTCACGTATGCTGGCCCGATTGCTT
CCGTTCTAGCGGCTAACAAACCTGGGCAATTGAAAGTGCCTGCCAGCGGAGCTGACACTT
ACACATTCGCTGTTGGTGCTTTCGGGTTCTTCGAGCTGATTAACGAAAGCGGTATCAAGT
GGGACGTGGCCGGTGAGTTGTTTAGCCAGTACGGAACGACTGCCGCGCAGTTCGATAACA
GCTCGAAACTTGCGACGAAGGCGTTCATTCAAGGTGCGCTTGGGAATTTCCGGAATCAGA
TCGGGCTTAGTGCGGGAACGACGCTGAATGCCAATGCTTGGGGCAACGCGTATACGCTCT
TCGGAGGTTCGCCGTATAACGTGACCTTGCCGTTGCTTTCATCTGGCGTTAATGGTGCGG
CGATCCGTTTCTCGACCATCGGCTCGACCGCATACACGTTGGTTGGGGCTGGGTCCGATG
CGATCTTCAACGGCACTACGTCGAACACGCTCGTACTCAATCCGGGAGACACCGTCACAT
TGGTTTCGTCAGGTGTGTGGGTCATGTTTGGCGGATCGAGGTCGCTTGCTGCATCGGGCG
TGATGAGCGGTCCTGGATGGAAGACGCAGGCGCAATTCGACAACAGCACGAAGCTCGAAA
CGACGGCGTTTGTGCAGCGGGCCGTCGGCTCGGTCGTCGGATCGATGCGCAACGCCAGTA
TGTCGGTTCCGACGGTGAGCGCATCCGCGACGTTTACCGCAGATGAGATCGTTGTCGAGA
CGGTGCTCGGCGGTGCCGCGTTCCGAATCGCAAACTTCAATGCAACGATCAATCTCGGGA
CTACAGGCGCGGGCGGGATGGATACTGGTACTGCACCGGCATCCGGTTTTGTTGCTATCT
ATGCAATTTACAACCCTACGAATGGGACGACGGCTCTTCTTGCCACCAATGCGACTTCAA
TCGTTGCTCCGAACTTGTACAGCGGCGCCAACATCCCCGCCGGCTATGCGGCCAGTGCGC
TTGTATCGGTATGGTCAACCTACTCTAGTCGCCAGTTTGGCGTAGGCTTTCAGGTCGATC
GGACCGTGAACTTGACTAGCTTTTCGGTTCTGAGCACAAGTGTCCAGCAGTCTAGTTACG
CACCGTTCTCGGTGGCAAGCTACATTCCGCCGAACGCGAAGCTTGTATCGGGGTACACGA
ACTGTGGATCGACCGCCGCATCGATCATAGGTAGCGCTGTCAGTGGATCGTCAACAGGCA
TCGGATCGGCCGGCACGAATGGGCAGTCGATCCTAACCCTTGGCGCACCGTTTAGCGATG
TGCCGATGGTTACCCCGCAGACGCTGTTCTATGTTTCAACGGCAAGCGCTGTAACGATGA
CCTTCTCGCGTTTTATATCGAGTTACAAATTCTGAGGAACAGCATGGAGTTGCTAAACGT
TCAATTCAGTGACGATACTGAAACATCGATTATTTCGGTCTTCAACTGTCCACAGGACCC
GTTGGCGTGGCCCCATCAAGGACAGGTATCACTCGACGATCCTCGATACCTCGCGTATTT
CTCAAGCGTCCCGGAGGCGCTGCTGGCAGGCCTACCGGAGCCGATCGAAGGGCAATCAGC
GTAACTTCGGGGTGTTGCTGGTGAATATGTTCGCCGCGGAGCGCCCGAGATTCACACCGG
GGCGCTCGACATATCGATGGATGAGCATTTCGAGCAGGGCTGATCCAGGTCGTGCAATGG
CGCACAGTACGGCACTTCGATAGTGGCCGTTCAGCTGCAGCAGGCCCGCGTCGCCAAATA
GTGTTGAGCCGGAAAGTGCAATGAAGAAGCCGTGAAACAAATATATGCCGTACGATGCAT
CGGAAAGCGCATATGCCATACGGCTTCCCAGCACTTTGGTCGCGAACGTTGCCCTAGACG
AGTCTTCCGTTCCTGTGACTGTGAGCACGTACAACATAATGGATAGTATCGGCAACAACC
ACCATTGCCCGTGGTAAGTCGATTCCTGTTCAAAAGGGAGTGTTGCGGTCAGCGTCGCGA
TCGCTGCCTTCTGCCGGAAAGTCACGCCGAATGGAATCTCAGCGATCAGCATGCCGACCA
AGAAAAGGGAAAGTTTGAGTGGCAAGAAACCGGGCTCGGGGAACACGCCGGTGAGTGTGC

GAGCGATCGCGAATGCCGATACGCCTAATAGGATAGCAGTGGTGACGGCGTCCGCTTTCC
GCATCAGAATATAGAGGAAAGGAAAGGCAAGATAAAACTGCATTTCGAGACTCAAGCTCC
AGTCATGAAGCATGGTGGAGAACGTATAAAGCGGCAGCAGGCCAAAAGCGAACTTGACGT
GGGCTGCTAGGTTGCCTGCCGTGTAAGTGATAGTCGAAGGATCATAGATCCCACCACTCC
CCCAAAAATCCGGGTTCAATAGCTGCAGTACCTTGTAGCCCTTGAGGAACGTTGTCGAAG
TGACAACCGCGAATACGAGAGAAACGTATTACGCTGGCGCAATACGGAAGTATCGTCGGA
TATAGAAGCGTAGCCATGAAGACGGTCGTGTGATCGGCTCGACCGAATCTCGCAGTCTGG
CGTTGTAAGCCATGAGATAGCCTGAGATCATAATGAACAGATCTACTGCAAGCTTCGGAT
CAGGTATAGGGGTGAGAGTCCATCCCCCCCAGATCATGCAATCGGCGACCACTACCCAGA
TCGCGGCGTAAGCGAGCAGACCGTTCAGGTACGGAAGTCCTTCGTCATCGTCAGCTCAC
AGTAAATTGAAGGGCGCAGTTTAGCACCCGCAAGTTTTGACCGACCGCCTACTGCCGGCA
ACAGCTGCAATTTCTTTCTACGTATCGCAAGACATCCGCTCCCGAATTTATCGCGCGTCG
ACGTGCTGAAATATCGCGCCGCGCTTCA
>NewGenomeName_33
ACCGGCGCTTATACTCAGTACTCCAGCAATCTACGGCGAGCCCTGGAATGGCTATGAGTC
GCAACTACTCCTACAAAAGGTGGGTCTTTACAATTAATAACCCTACCTTTGAAGATTATG
TTCATGTTTTCGATTTCAGCACGCTCGACAATTCCAAGTTCGCAATCGTCGGAGAGGAAA
AGGGCGACAACGGCAAACCTCACCTTCAGGGATTCCTGAATCCACGAAGAAATGCGAGAG
CTGCCGCCCTTGAAGAGTCTCTGGAAGGAAGACCCTGGCTCTCTCGTGGCCGTGGATCGG
ACGAAGATAACAAAGAATATTGCTCCAAAGCGTCGACTTATCTGAGGTTTGGTAAACCAG
GCGGTAAGGGTCGATCCTCCCAGCTCTCCGATGCGACATCCGCTGTGCTCGCTGGTCTCC
CGCTGACTGACGTTGCACGGAAGTATTCGACGACTTATGTTATCTTTGGGCGTGGCCTCG
AACGTCTTCGTCCCCTGATCGTTGAAACGCAACGTGATTGGAAGATAGAAGTCATGGACT
TGATTGGTCCGCCAGGGACCGCAAAGAGCCGTTATGCGTTTGAATTTCCCGCCGAAAACA
AGTATTACAAACCACGCGGGAAGTGGGGGGACGGTTACTCGGGACATGACGTAGTCGTCA
TGGACGACTTTTATGGTTGGGTACCTTACGATGATCTGCTTCGGCTAACTGACCGTTATC
CGTTACGTGATGAGTTTAAAGGTGGCATGACCCAGTGTGTTGCTAAGACGTTGATCACAA
CTCGCAACAAAGAGCCCCGTAATTGGTATAGAAGGGAGCTCGATCTCTCCGCCTTATATC
GGCGCATTAATAAGTATCTTGTCTGAAATGTTGACAAGTATGAACCTGCCGCCGCTTGTG
CCCTCCCCTTTCCAATAAACTACTGATACAAAGTTGGCGTTTTATGTCTTTATTAAGGTG
AACAATCGAAAGGGATATCCACTTGGGCAGCTGGTTATGGTGAACACTTGAGAGGGGTAT
ACACTTAGGCAGCTGGGTAAGGTGAACACTCGTAAGTGATAAACAGTTGGGCAGCTGGTT
ATGGAGAACCCTCGAAAGGGATATAGACTTGGGCAGCTGGGTTAAAGTTAGGAAAGGACC
ACTTTTATGTCTAGAACCCGGTGAAATGACCAAACTTAATGTAAAACTTAAATTGAGCTA
CTATATCCTATATCCCCTCGTCGCCGGTCGCGTTATATGGTGTTCTGAGGCTCATGCCCA
TGCCGTAATGTACTACCTGGTCTTGACTGCAATTTATCCAGGTGAATTTTTTACGTCCTA
GGAACAATGTACTATACTTGGTGCCTTCTCCTGCCCCTTGAATTGTTGGTTTCGGTACTA
AGGACCGTCTGTGGACTCTCGATGGGTACCACGGCCGCCTGCTGCTGGTTGATACGTATG
GGTCAATGGCCCATCCTGTAGTTGATGTCCTCAGGATTTGCCCGTCCTTTTCAATGATCG
TTGAGCCTTGTGGTTTGCTTTTTTGGTAAATGTTGTAGGATGGTCCCAGGGTCACAGCAA
CCCCTTGTATTATGTAATAGTAGTAAGGGTAGTGGTAATCTGGCTAACCCCCTTGCATAT
AGCTATTTGTGTCTCCATACATTGCTCATCTCATGTTCATTCCACAGATGTTGTTTCCGT
GTGCTGGTGGGTCTGTGTATTGAGCCCCATCTTCTAGACTTAAGCTCTGCCATCTCCCCG
ATGCCGTTGGTCCAGTTCCGGATCCGAAAAATCCAAATACGGTAAGTCTGGTGTCGTTGA
ACGTCACTACTGAAAATCTTCGTATTGGTCGTCCGATACGTAGCCTTCGTCTTCTCAATC
TCCTACGTAGACCCCGCCTCCTCCTCACATCACGATATGCACGCCGATAGGAGCTGAGGC
GCATCTGCTGTGTGGGCAGTGACTTCCTGTAATGTATAAAAAGACCGCACAAGCGCCGTT
ATATTATT
>NewGenomeName_34
ACCGGCGCTTGTACTCCGTACTCCGGGCCAAGGGGATAGCAACTGCAATGGCGAAGAGCG
GCAACTACTCATACAAGAGGTGGGTCTTTACCATTAATAACCCTACCTTTGAAGACTATG
TTCATGTTCTAGAGTTTTGCACGCTCGACAATTGCAAGTTTGCTATCGTCGGCGAGGAGA
AGGGCGCGAACGGCACGCCTCACCTTCAGGGATTCCTGAACCTCCGAAGTAATGCGCGAG
CTGCCACCCTTGAGGAGTCGCTGGGAGGAAGAGCCTGGCTGTCTCGTGCCCGGGGATCTG
ACGAAGACAATGAAGAATATTGCGCCAAAGAGTCGACATACCTTCGAGTTGGTGAACCAG
TCTCCAAGGGTCGATCCTCTGATCTGGCCGAAGCGACATCCGCTGTGATGGCTGGTGTGC
CGCTAACTGAGGTGGCCCGGAAGTTCCCCACGACTTATGTTATCTTTGGGCGTGGCCTGG

AACGCCTCCGTCACCTGATCGTCGAGACGCAACGTGATTGGAAGACCGAAGTCATCGTTC
TGATTGGTCCGCCCTGCACCGGAAAGAGCCGTTATGCATTTGAATTTCCCGCCGAAAACA
AGTATTACAAACCACGCGGGAAGTGGTGGGACGGTTACTCGGGAAATGACGTAGTCGTCA
TGGACGACTTTTATGGTTGGTTGCCGTATGATGATTTGCTGAGAATTACCGACCGTTACC
CGTTGCGGGTTGAATTTAAAGGCGGTATGACTCAGTTTGTGGCTAAAACATTGATCATAA
CAAGTAACCGCGAGCCTCGTCATTGGTACAAGAGCGAGTTTGACCATTCAGCTCTGTACC
GCAGAATTAACAAGTATCTTGTGTATAATATCGACAAGTATGAACCCGCCCAGGCATGTA
CCCTCCCGTTCCCAATAAACTACTGAGACGAAATTGCCGTTAGTCGTTTATTAAGGTAAA
CACTTGGCAGGGTATTACACTTGGGCAGATCGGTTAAGGTTAGGCACTTGGCAGGGTATT
ACACTTGGGCAGCTCAGTTAAGGTTAGGCACTTGGCAGGGTATTACACTTGGGCAGCTCG
GTTAAGGTTAGGCACTTGGCAGGGTATCACACTTCGGCAGCTCGGTTAAGGTTAGAACAA
GATCTCGTTTATGCCTAGAATCCAGTGAACTGTCCAAATTTGATATAAAATGTGAACTGG
GCCTCTATGTCGTATTGTGCATTCACACCTGTTGTGTTGTCCGGCTTCCGTAGACTCATG
CCCATGCCGTAATGCACTACTTGATCTTGAGTGGAGTTGATCCAGGTGAAATTTTTGCCT
CCGAGGAAGAAGGTAGAGTGCTTCGTACCTTCACCCGCTCCTTGTATGATGGGTTTAGGT
ATGAAGTACCGTCTGTGCACTCTGGAGGGGTCCCACGTTCTCCTGCTGCTGGTTGAGCCG
TACGGGTCTATTGACCATCCCGTCGTGGATGTCTTCACTATTTGCCCGTCCTTGTATATC
ACCGTTGAGCCTTGACTCTTGCTCTTCTGGTAAATGTTGTATGCCGGTCTGAGAGTTATG
GCTACCCCCTTGATCATGTAGTAATCGTAAGGGTAGTGGTAGAACGGTGTTGAGCCGGTC
ATGTAGCTGTTCGTGTCCCCGAACATCGCCCATCTCATGTTTAAGCCGCATATATTGTTG
CCACGGGCCGGTGGGTCTGTGTATTGTGCGCCATCTTCTAAGCTTAAGCTTTGCCATTTG
CCTGCTGCAGTCGGTCCTGTGGTGGAGCCGAAAAAGCCAAATACGGTGTTCCTCGTGACC
TTATAAGTCACTACGGAAAACCGTCGTCGGGGTCTTGCGATTCGTAGCCTTCGTCTTCTG
AATCTCCTACGTAGACCCCGCCTCTTCCTCCGACCACGATATGCACGCCGATAGGTGCGG
CCGCGCATCGGCAACGTGGGCAGTGACTTCCTGTAAGATATAAAAGACCGCACAAGCGCC
GTTATATTATT
>NewGenomeName_35
ATTTTAACTTGCATGTTCTTTGAGGTTTATATCGCTAGGGTGAATAAGGCATTGGTTTCC
TTAGTCAATTAAAGGAGGATATGCGCTATGCTGGCGATACTAGACTAAAGTCTTGAGTAG
GCTAAACGTTTCGATAACGTGATGGCCCGCATAGATAATTCAATGGCTTTTGATCCCGAA
TGGAGGAGCTCAGGGAATATGTACGAGGGTGCTGTAGGACTTCAACTCCCAGTGGTTAAG
GTCTTTAAAGCACTGGATCCTAAAACAGGTCGTCGTCTTATTTTGATCGGTACGGACTGT
GGTTCCGTGGTGATCTTCGAACATAACGGTCGTGTCAAGACCGCATCCAAGTTTGCCTTG
GCGTATAACGCTGACCCTGCCTTAGACTTTATGTTGGGAGGAAGTCGGCTCTCAATCGCC
CAATTTAGTCTGATGATTACGGATTATGATATCGAAGAGAACATCGGTATCAGTCTCCGT
CGCTTGATTCAACAGGTGAACCGGAGCACGCACGATAAGAACAAGGAAGAGAAGATCATA
AAAACCCCAAAGGGAATACCCCCGTCTAAAGTACTGTCAGGGTTCTTTGGTTGGGGGGAG
TCTGCTTAACACCTGAACGAATGAAACCCAACATCCACAAGGCTGTACTTTCGCACACAT
GAGGATAGTCCATGAAAACGTTTTTCCTGTCTATTTCAACCGGCTTCACTTTCCTGGTCT
TGCAACTGGACAATGCGGCGGGTAATGAAGAAATCATGTTCAACTGCACCTTGAAGGACG
GTGGTGTGGTCAAGCTGACCCAGATTACTCAAGGTGACCTGTGGACCCTTACCATCGGGT
ACCCGGAAAAAGTGCAAGTCAGTTTTACCATGCCTGGGAATGACATGGGTCGGCAGTTCT
CATACCATCGCGATAGCAATCTCGCTACCCGTGAAGTCTTCATAACCAACCCGACGGGTA
ACTGGTTCTATACCCTCGGGGTGGATCATAACGGTAAGGCTAAAAGCGGTTATATCCAAG
TTATGAACAGCGGAACAGAAACAGCCTACGAACATTGTGTGACAGGAAGCCTTCGTGAGC
AGTTCGAAAAAGAAGGTGCCTTTGTCAACATGACGGTTGTGGATTGAACCTGTTGGGGGA
GTGAGCGTTTGCTCCCCCTGTTCCAATCTTTATTTTTTTTTCTAGAAGGGATACCGCCA
ATGGAAGCCGATGCGAAAACCCTTGGAGAAATCAAATTACTGTCTGACATCAAACTTATC
CTAAACGGCAGAATTCCCACGGATTTAGTACACAAGGTCTTTAACCACTTGAAGCATAAT
CATCAGGGTAGGTATAACCACTACTACTTGCTGGATTCCCTCAACGTCGCTAAGCGTCTA
ATAGACCACCAGGATAATCTAACATAACATGATGTTTTGCTTATCTACACTATTATCCTG
GTACTTGGGACGGGTCGTCCTATTACGAAACGTCATCCCTATGAAGTCTCTCCAGGAGTA
GGGTGGATGTTTCTTCGGTATTATGCGGACGGGATCTTTACACCTGAGGACGAACGTTTC
ATTTCACATTCCCGTCGGCCTATCAAAGCAGATACTATTCGAATGTCTTCCTACACCTTC
GTTGAGTTGGTCATTCACACCACTAAGCGCTTAACGGATGTCGTCAATCAGAAGTATCAA
AAGCTGTACGACGAGTTTGCTGTACTCACTGGATTACCCCATTGTTCTATGAAATTGAAT
AAGGCCTTTTTGGAGGAATATGGTCCTAAAGGTAATCCATGGGATGGGTTCACCACTTCA

ACTAAGGACATTTTCGCTTTAGGAATATCCAACTTCAAGAAGAAAGTCGGTCCATTCGTG
GACGAACTTAACGTTCGATGAGAGGATAAACCTATGTACAACGTTATAGCAAATGCGGTG
CTTTTGACCACATATGCTGCAGTCATGCTGCTAGTTGTTCAGCGCTATCTAGAAGCTATG
GGTATGCGTTACTCCATTAAGCGGATAGAGAACGGCCGTATTTTTAGCTTCTCGTTAGGA
GTATACGCAGGGAAGATACGGATCCTGCATTCTCCGGACTGTACAGACCCGATCTTCAGA
CGCCGTTATGATGGATGGGAAGTGACCTTTCCTGAACACTTTACGGAAGAGCAGATGGAC
CATTACATCATGCACCACGAACCCTTTGGGGAACACAATGCGTCTCATTCGAAGGATTTT
GTTGTCTTCAGAGAAGTCATGATTCGCAAGACCAATGAGGTTTCGTGCGTAGATGGGAAT
ATCTTCTGCCAAGCGTAATGAACCCATAAAGTACCCTCCCTACCCCGCAAAGGGTAGGGA
GGGTATGCAGTACACCTTTTTTGTTTAACCCATGTACAAAGGACCGGCAGTGCCGTCCAC
TTGGGCTTTCTGTTCGATCATCCGTTCGATGGTACCGGATGGCGACGAGGTGACAGCCGC
AGAACGCTGTTTGAAGCCCGGAGGGGCAGCAATGGCATCCGGGTTGTACAGCGGAAGGAG
TTTCAACATCTGACGAGCAATCTGTTTCACCGCCAGGGTGTTGAATTCCACCAAACCGTT
GAACACCATGGTGATCTGACGGATTGCGCCTTCTTCGCCCTTGTTACGTTTGATTTCAAT
CGGAACAGAGGTACGGGGCATCATACCAACCACCAAGGCAGCGTGAGCAATGTCCTTCAT
GGTCTGGGTGGGTTCGAAGTAGATGCACGAGACCGATACTTCATCGATCAGCATGTCACC
TGGATTATCCAGGATGACAATCTTGGCGTTCAGGACTTCCGAATCCATGACCAGCCATTG
TTGCCAGACCTTGAACATCTTGGTGAACACTTCACCGATGCAATCGTAACCAGTATGAGT
GACTTGACCCATCGAACGGGTAGCGCCCGACGGAATGGCGAGTACGTGACCCGTCCATTC
CATTTGGGCGAACTGAACTTCAGTCATGTCACGGAGACCTTCGAACGACTGAGAACGGTT
TTCAAACCACGCCTTACACAGCGAATGCAACGCAGTGCCACTCGGCAGTCGGGAGAAGGC
AGCAGGAATCGAGAGGACAATACACCACCCCAGTTGACGGACGTGAGGTTGTGCGCTGAC
ATACTCGTAGACGTTACCAGCCCAACCGTACATACCCCCGAATTGGGCGTTAATGACCGG
ACGGTTTTGCAGGTCCAGAGCCCGTACGAAGGGATCGGACTTCGGGAGTAGGGTCGTACT
GGACCGAGGAGGGAAGTTCGTTTCATTAGCCATCTTTCATTCCTCAGGCCGCAGTGGTGG
CCAGATCCTGTTGGTTGTAAGCGGAGAGATCGAATTCCATCATGTACTTGGCTTTGTTAA
ACCAAGCGTGGAGAATCTCACGCAGTTTCGCACGGGATCCGAGCGTTCCAGTTTGGTGGA
ACGTTTCTGCGACGATGTTGGAGAGTTCACCCCCTACTGCGTTACGGCACGCGCTCGCGA
TCTCGTCTTTCATGATGGAGGCGTAGTTATCTGCGGTGATGGTACGGTCACCACAACCGA
TGCGCCATTGATCCGCGGAGATCTTCTCCATCGCCACGCACAGGAACGGGTTAGCCAGGT
CCTTGAGGACCGAGTCGGGGTTCGGGTGAATGGTGGGGAGGCCCGGACGGTACGTTTGAA
CGTCCCAGTCCCAAGGCTTCAGGGAGATATGGCCGTTGCTGAAGTTCTCAGCCGCCACTT
CATCTTCTTCGAAGACGATGTTGGGGGAGTGCATCAGACGGAGTTAACGGTTATCCGCAT
GATCCGGTGCGTTCGGAGTGACGATCAAACGGTCGATGTTACCTGTCCACAGCGCAAAGG
CATAGGCCAGGTCGATGTTACCCGAGAAGTACCAGCCGGTCGGTTCATCGGTGACTTTCG
CTTCGATGAGGTTCACAGCTGCACGGCAGGCTGGGGTACCCCACTTCTCAGACTCGACCG
TCATGCGCAGTCGATTGGTGATCATTGCAGCCCGAGAATAGATCTCTTCGAGACTGTTCT
TCTCACCCGGTCTCCAGACACAGGCGTCTGCCACAACCAGAATGTCCTAACGGGCACCCA
GGAACTGTTCGGCTATATCTTTTACTTCTTGGGAATAACCAATATCCCAGAAGATCGATT
GACGGTTACGGGTGGTGTGTTTCATTTCCACACCATTCACGTAACTGGTCAGATCCGCCA
CCATCAGTTTGTAGGTGATGTCCCACGCCTGAAGGAGAGTGAGAGGATGCTCGGTGTTAG
TCAGCAGACCAAAGAGATCGTTGACTGTCGGCTTGGTAACGTAATCCGGTACCTTACCGT
CCTTATCCAGGAACGGCGAAATACCGCCCGAGGATTTAATAGCGCCGCTCAGGTCCCACT
TGATAACACCCGAGGTGGTGATGGCGTAGTAAGGCGCGCCGTTGTGGTGGGTGCAGGTGA
ACGGGTTCATGTGCTTGTAGTACACACCGGGAACCCCGACTTCTACCAGGGTGTCATTTT
CGGGCTGTTCCACCACATAGATCATGTGACACAGGGTGTCGATGTTGTCCTGGTAAACGA
CCAGGTCGTTTAACGGAGCCGGAACCGGGTTACCCGGGGCGTTCTTGTTACGACCGGTGA
AGGAGCCGAAACCCGTTTGCAGGCTGTACTGAACGCCGTTGAGGGACACCGGGAACAGGG
TGAACTTGGCACTTTCCTGTTGGAGGGTGGTTTTCGAATACACACGAGTCCCATCAACTC
AATCGGTGAACTGACGGAGGTCGTAAGGGAAAGTCCCCGTGGACCGAACGAATTCGGAAA
TGGAACGCCAATTCAGCGCGTCCGCACGGACACCCAGGTTAAGGCCCGACTTGTTGTATT
CGTCACGGACACCAGCCACCCCTTCGAACAGGAGGAGTACCAGGGTTTCAGGGGTATCAC
CTCTGGCGGCGATGGCGCGGACTTCGAGCGCGCCGGGTTCTTTGCCCGCTGCGGCATCAT
CGATTTTGATTTCGATGTTCAGACCGTTCGGGTAGGTTTTACCCGGGATCGGAATACGCT
CGCCGTTTTCGTTATACTTCCAACGGCCGTTGGCAACTCGTTCGTAGGCCTGACCTTCCA
CTTTCTGAACAAACGCAGAGAGCGAGGTTTTCGAGACCACTTCGTTGACCGAGAGGGGGC
GAATGCCGATGGTATTCTGGCGACCCAGGGCGAACGCCTGGATCAGGACCGACGTTGGGT

CGTAGTAGGGCGACTCCTCGTCGAAAATGTCTCCAAAGATCTTTTTGACGTCTTGGGGGG
CTACCCATTGGGTGCCTGCTTTAGACGCCAGTTTTCCTGTGGGGAAGATACCGTGGATAA
CAGGAAGGTGGAGCGGGGTGGTTGGCGGGGACGTGTCATATTCTGGAATAGAGTTGTCCC
GAATCCGACTAATGTCCACCTTTCCAGGGACGATTTTGTTGAAAACACTCATGGCAACCT
CGTGGGGGAAAAGTTTGAAGCAATCGTATGAACCCATTCTCAGGGCCGTACAGTGTAATA
AGGCACTGTCCCATAACATGTTATTTTAAGGAATATCTACCATGCTGATCAATGCCTATG
ACACCACGGTCGGTAAAATGATCCGGGCGTCTCACCACGCGGACGAAGTCATTAAAACCC
TTCATCTGACCACTAATCTGTCTCCCACTAAGAAAGACAAGGTGTACGTTATTACGCACG
CCACCTGCGTTCGATTTTCAACCATTGCTTTTCCGATTACCTTACAGACCCATACCCGCC
AAACCATTACGGTGTATGACGAACGTCCGTACCGGGATAAACAGAACCGAGTCACGAACC
AGACTGACCTGACCATTATGGGACTGGCGGCGTTTCTACAGCAGGATGTTGCCGAGTTGA
ACCTCAGCCCGGTTAAGCTGAGTCGCAATATGGTAACCAAAGGCTTTAGTGACGCGTTAA
CCCAACGGTTAACTATGCGGGGTGGTTTGGATATCGTAGAGTCGTCGACCCTGAAGGTGT
TGATTGGGTATTATGTGGTTAGTCTTCAAGAGAACCCGAACAATGACCTGGTCTTTGTGG
TTGAGAATGTCCTTCGTAATGTGTACGGGAGGGAAAAAGACTTTGTATTGGGGGTCATTG
CTGATCTCCCCCATCTGAGTACGCTGGAAGACCTCCTCAGTGAGATTCGTAAGAATCCTG
TGTTGTATAAACTCAAAGGGATGGGTCTTAAGGACTTCATTGCTGTGGTGGGTGGATTGG
CCTTTACGGGGCTGGGGAAACAGGTCTTTGAAGCTGCGTCTGAAGCACCTTGCTTGTTGA
CGGCCTTTGTCTATGGCGCAGCACGTTTCAGGGCGCTGAATAAAACACCGGTGGGTATGG
CATTGGATCCTAAATACAACAAAGGGATCCTGGATACCTTCCTTAAGAACATCGATTACA
CGTATGATCTCAACGGGTAACAGTTATGACCATGAAAGGGTTTGATACGGATAACCCATT
GCTGGCCTACGCGACAGACCACCTGTGGGGTAACCCGGAAGAGAATAACCAGTACTAGGT
GCGTACAGTACGGCTCAGTGATTACTACGGAGATGTGGCCAACTTCTCCTTTATGGGCAC
ATGGCGGAGTTTGCCTAAGCGGGATACGTTTTATTACGTCTTCTCTGTAGGGGGGTTGGA
TGCAGACTATTGGAACTCCCATACCAACTTACTGAAACGGAATCCCTTAGACCGGTGGAT
GAACCTGGCCGAGCTGTGTAATCGCCGGGGTGTTCAGCTGGATATCTACAACACCAAAGG
GTACCAGTACAGCCGGAATAAAGCTTGGGTCATGATGACTTACGATAAGCTTACGTTTAT
TGCCCTGGAGAAGTATAATACCTACCCTACGCCAACGGGTCAGGAAATGTTCTTTCGATG
CTACACCGCTTCCAGTGAGGTGGCGAAGAATGAACAAACCGCGAATGCTGCCAGTAACCC
GTACGTTTATGAAACCATGGTGTACGAGAGTCAGGCTGAACTGGCGGTCTTTAATAACCG
GTACTTAGCCTGTAAAAGCAAACCTGAGTTTACCGGGGTGACGGTGAATGGGGTATATTG
GCACAGTAATCCAGGGTCGATTCCTAACTTGGTCCTAGGGGATGTAGTGGAGTTCTTCCA
TGACACCACTGTGATCCGTACGGAGCTGTACGCGTGCAACACTCTGCAAGACTTCTATTC
CACCTTGGATAACAAACGGAAACTGATCCTGCATCCTCCTAAACGCCCAGGTGACTTTAC
CCTTCGGTATTTTGATGACAACGATTACTACCTGTTGGGTAAAGGGTCGACAGGTCTGTA
TTTCCATCGGAATGCGGTGAGTACGATTCGTCAGTTGACCCATGTGGATGTTGCCATTGC
GGATTACCAGATCCAAGCCGCCAGTAATTACCACAACGACCTCAGTGAGGTGAAGGATAG
TCGTATCCTGGTATTGGTTCTTAAAACGGATTGGGAATACTCTTGGCCGCATGATAATCA
ACGGATCCGTTACTTCTCCCGCATGACTGATGAAAACATCCTTAAAGCCATGACAGGTGA
TCGAGCAACGGTGCCGGAGTGGACCGCTCCTAATCTGGAAGCCGGGGTGGTGATGAGTTT
TACCCGGAGTCAAATGAAAGCGCTGACCGTAGGGGGGGCTCGTAAAGCGGTGGGGTACAA
TGCGGCAACTCGGGTCCTCAGTGAAACCCCCGTTAAAGCGGTATACGAGGCGGGTGGTCG
AGGGGTTGAGATCCCCAACAGTTACCGAGTCATTTGTACCGCTTGGGAACATGATGAGAA
CGGGTGCCTGTTGGAATACCACAATGTCAATAATATTCGGTACTACGCACCTCGGAATCC
GAAGTGTCAACTCGTTGAGTTCACCTGTGGTCAATACGGGGGAACATTGGACTATGTGGT
AACCAATACCGATTTACCCTTAGACCCCTTTAATGGGTTCAGGGTGTACACTGCTCACTT
CAACGTGGATCGAGGTGAAATCACCGGTGCGTTAACGGATGTCACTGGGGATAAGAAGAT
CTACTAGGTGGTTAATGAGGTCTTACTCTGGAAAGGATTGGATCCCGTTAACCAACGTGG
GGTGGTAGTCTTCAATACCCCGACCTTGACCTATGGGTTTCAACTGGATCACATTGCTCA
CAGCTTGAGCTTTGCCATTACCCTCATTTATGAACCGGGTGGATTGGCGGTGCCTCTGAT
TCCTGCTCAAATCGATATTTGGATGAACGGTCATCCATTGATTGATAATGTCGATTGGAT
CTTTGACAACGGGTACTGCTACATGATCAACAAACAGTTCATCCGACCGGGTGCTCAACA
GTTTGTGGTACGGTGCCATGACCTGTGGGAGGATAAGGAACGTCCTAAGGTAGAAACCGA
ACTCGGGTTCGTGGACGGTGGGGTGATTGGACGGTTTAAACGGTATAACCTGAGGGAAGA
CCGGGTAACACGTACCGTCATTGGAGGTCGTTTGGTGGTAACGGACGACGTGCCTACGGC
CGAGCTGTCTTCTCAAGATAACCTCTGGAATCACCTCAATGGGTTACCGTACATGGTCAA
ACATGTCTTCCAACCCATAAAGTATGTCGTACCCTATGACAACTTCCCAGGGTGTAAAGA

GAGTCGGGAGATTGATCAACGGGTGAGTGACTACTTGACGGAGTACTGCCCTAAGCCGTT
GGTGGATCCTGTGATACCGACCCTTGAGGATAAGTACCGGTTGTTCAGCCCCTTCCTGAA
TGTGGTGGTGAATGCGATTAACAACCGGTTGTGGGTGGTTGCGAATCGAGTAGACGGTGA
AGAGACGTACAGTCCTCAGATCATTGAGGAACAGGTCAGTCCGTACAAGTGGTGGTTGAA
GTATGACTCGGTGACCCGGGACTACGACATCCGCTATTTTGCCGTTATGCCCTACGCGAA
TGATGAACTGCTCTCGGTAACACCCAACCAGTTTGTGTTCATTAAGCAAGTGAACGACCT
TTACTTGAAGTCTGTCTGTGTCATTGAAGGCCACTTTGAAGTAAGTAACAGTGACTAAAG
GGGATATTCATGTTTGACAACAAACCCAGCACGAGTGCTGAGCGAGCGGTGGCGGGTACT
GATTTGGCTCCTGAGAATGACGGCCAACGGTACAGGATCCATTATCTGCCTGACATCTAT
GAACCGGACAAGCATCCGCCGGAGGACATTGTTAAGTACGTGGTTCCCCGGGAACGGGAA
ATGGTGATCGATGTTCCCAATAAACAGTTCCTGGAGGGGATTCATCTGGATTACCAGGGG
AAGACCTTGAAGTCTAACTTGGTTCCGTGGGAACCTCGGACCAGTGAGGACGGTACCACA
ACTGAACAAGACTGGATCTTCGGTCTATGAGGAGGTCCTCTGTTGGGTGAAGTCTTGCTG
AGTGTGGACTACAGCCAACGACCTAACGTAGCGCGTGTTGACAGCACCATCATGCGACCC
GGCGCAGCGTACCCTAAACTCTACCTCGGTAACGACGCCAGTGACAACGGTCGGATCATC
AGTGCCCAGTACGACAAGTCGGCGAATATGATCAGCAACAAGGTACCTACCAAGTTGGCT
GAGATCGTAGACCGTACGAACCTGGAGATTATGACCACGGGTCCTTTCTGTGTCACGGAG
AACGAGGAGGCCCTGAAAGACGGTACGCGTTGTACGTTGGTGTTCTATGACGAGGGGGGT
AACTTCATTCCCCCGGTTCAACCTCTAATGGTCCAACAGTGTGCATACATGCGGGATCAC
CAGATCGGTACCAAGTATGTCACGGAGATTGAGCTGCTCACACGGTGGTTTACCAATACA
GTTAATGCGGAGAAGATTATCATTCCGGTGAACGTATTGGTAACGGCTATTGAGTTCAGG
GCGGTGATTCATTACTCGGATGGCAATACCAGTGCGCCCATGCCGGTGAACGGTGAACGG
TTCAACCTGTATGGGTTGAATCAATGGCGCCCCACCTGGCCGGGTCAAGAGGGTGAGATC
GTACTGACCCTTAAACTGGCAGCGAATGAACAGCTTTACATTGCCAAACCCGGGAGTCCC
GATCACATCAGTCGAACATATTCGGTAGAAGCCGGTCCTGTTAAAGGCGCGTACTCGCCG
AAGATCTACACCTTCCCGCAATGGGATCCGAGTATTTCGGGGTATCGACTCCAACATTGG
CGGTTGGATCTGGATCGGAAAACGGCCATTGATGTGACCGCTCAAGTGAAGTTCAATGAG
AAGAGTGAAGTCTGGCGGCCTTCTGCCTACGGGGTTGCTCAATCGTTGATCTTTAACTTG
AACCTGAGGGATGTATCGAGTCAGTACGAGAGTGTGATCTTCCGGCAGTACACCACCATT
CTGTTGTATAAAGGCATCAATGGGCCGGGTAAACGCTTTGACGTGTCCTTCTGGCAGAAT
AAACCGTCGTACGAAGCGAAGTTCATTGTGGCTAAGAAAGCCGGAGCTGCTACCACGGTC
AATGTGGCCAGCGGGTACGGTAATCAAACGGATTGGGTGAAAGCGTTGTATTGGGGGGTC
GAACCCAGCTTCAATCGGGTCGATGAAGAGTAAGCTCCTTTACCCACTCATTTCTACCTG
GTTCATGAAGACGGGCATTCCTGGCGGTATCCGATCTCGGATTGGAATAAGAACAACGCC
ATCAACATTGAACTTCAGAAGGGTAAGACCTGTTTCCTTCGTTGGGTGGAACGCCAAACG
TCTGGTGATCAGAAGCAACTGGCCGTTCCTGGTGTAACGGTTGAATTTCCTTAAGGAGGA
AACGAAATGACCAGTACGGTTAACGTAACCGCTCATTGTACCGAAGATAAGGAAGTAGTG
GTTCGGGGTCTGACGCCTGAAGATATCTCGCAAGAGGTTATTCTTCAGAACGGTAAGAAG
ACGGAAGTGGTGGTGTTCGGCGCTAAGCACGTCATCATTCACGAACGGACTCGTTTGGAA
CCCGAAGGTTAACGGCGGTTGATCCTATATTTAAAGAGTTACCCTGGGCGGCGCCCAGGG
TTACTTGATTGTTATAGGGTTTTCGTGGAGTTGATGTATGACAGACGAACAAGACACATC
CCTTTCCTATGCTAAATGGTTGTTTAAGTACCTGAGATGCAACAACCTGCCCAAAGAACT
GATAGATGATAAACGCATCCGTATTGTTTGGGAGGCTCGCACAGACGATGAAACCCTTTT
AGCTCACTTCAATGAAGACAGCTTAAGTGGTATTAAGACTGTTCGTTTTCTCAAGGACTT
TTTCAACTACCAAGCGGGTTATGATCGGGAGACCCGGAATGCCAGTTTCCTCAGAACCAC
CGAAGTGTTCAGGCAACAGGGGATCAATAACTTTTACTTCATCCTCCAACTGAACAATCC
CCTGTTGAAAGGGGTGGAACCCTTTGATCCTACCCCCACTCCTGAGCAACAGGTGTGGGT
CCTGGAGGAGTGTCGGAGTAACTTTTGGTACTTCCTACGGGAAGTTTGCCGGTTAAAGCC
GAATCAGCCCTTCTTAGCGAACCGGGGGAATATCAGCTTTATTTGGTCGTATTTGAATCA
CATTACCATCTACAGGATCATGCCGCGGCAACAGGGTAAGCAGCAGCGGAACAGTGCTAA
GGTTCTTATTGTGCCTAAGGATACCCTGAAGACTATTCCCCCTCAGGATACTTGGAAACG
GATTGAACACCTACGGGTGGGGGATCAAGTCCTGGACCGTTCCGGGAAGCCGTGTCAGGT
GATTGGCATTCATCCCCAGGGTAAACGCCGGTTATATCGCGTCCTTACCAGTGACGGACG
AGCCACGGATGTAGGGACTGAACATCTGTGGACCTTGAAGGATTATTCCAACTGCCTCAA
TGGACGTGCACTCTGGAATGACTATTCGACCGTTGATGTCATTCACCTGTTGAAGAAGAA
GGTTAAAATTCACTTGCCGTTACCGGCTCCGGTACCCGGGAGTGAACAGGACCTACCTAT
TGACCCGTACGTTCTCGGGTTGATCTATGGCGGACAAGATCAAGACGGTAAGGTTATTAT

CCCCACGCGTACGGATGCGGTTAAACAGTACGTAGTTGATCATCTCCCTCGGGGGGTGAC
AGTTATTCAGGTAGTGGCTAACAGTTGCTTAGAACGGACTGATAACCAACCTTATTTATT
CAACAGGGTACACTGTTTACCGGATCAATACCTAGAAGCCCCTTTAAACGCCCGGAGAGA
CCTCTTACAGGCGTTTTTAAACGTTAGGGGTAAGGTTCGTAGGGGTAAGGTCTTTATAGC
CTTAAATCGCGTCCTAGGAGGTCAACTGGCGTATCTAGCCCGTAGTCTAGATGGAACCGG
TAAAGTCACCAAGAACGGAGTAGAGATTACCCTACCGGAAGAGGTGCCTCCGTTCAAGTT
CAGGGAAGAGAATGTCGTCTTTGATAATCCGTTGCTGATCGAGCGGGTGACTTTCGTAGG
GGATGACGATTGTACCTGTATTGTAGTCGATAACCGTGAACAGTTGTATTTGACCGATGA
CTTCATTGTGACCCACAACACGGTATCGGTTCAGGTGATTGACTTCTGGTTAACGTACAT
CATGGGCCGGGGGTATACGAGCCACTTGATTACCTTGAAGTCGATTAACCGAGCTCACTT
CATTGCGGCTATTAAGCAGATACGGAGTTCCATTCCCTCGTATTTGATTAACTCGACCTA
CAAGGACTAGGATGCTGGTACCAGTCTGACCTACAAGGCCTTTGGGGCAGATAACGTTAA
TACGCTGTACATTAACGTCCCTCAGATCAGTCAGGATGCAGCAGGGGATCTCGGTCGGGG
GTTACGGGTAGGGACTACCAACTACGATGTGTCCGGGTATATTCGGTTCATCGACACCAT
CATTGACGGGCGTTCCCCGTCCTCGCTAACGGAAATGGCGTTGTGCGGTGAGCAGGGGTT
GCCGTATGGGATTACGCACATTACCACCCCCAATACCACGCTCCATCCCAGCGGGGAGTT
TATGTTCAATAAACTCATGAGTGCAACGGAGTGGCGGGACAAGTTCTTTGATTGCTTCAG
TGAGAGTCACTTGAGGCAGATGTTACTCAGGGCTTCCCCTACGAAGACAACCTCTCCGTC
AGTCTCCATGGTGTACAATTACCTTCAACTGGCTAAGGACAAAGCCTGGGTGCGGGAAAC
CATTGACCTGCCGGGCTCGAGTTTGGCGAAAGCTAAGATCGACTTATTGCTCATGTGGGT
GGAAGATGGGGAGAATCGGTTGTTTGACGATGTGACCCGGGAAGCCATTAACAACATGAA
GCGGGACGTGGTTTGGAGTAAGGAGTATCTGGACTGTAACCTGTACGTGGACTTCTTTGT
GACCCAACAGGAATTGTTGGAGATGGCAAAGAGGGAGTACAATGACCATTTCCTGATTGG
TGTGGATAAGTCCTCAGCCATTCACAAGGATGCGTGCACCACTGTGATTCGCAGTATGAA
GACGGGTAAGGTCATAGGGGTGGGACGATATCCCTTAACCTTCTTGGATGATGTCACAGC
GATTGTTGTTGATTTGCTGGCCCTGATTCAGAACCGCGCCTTGATCATAGAGCGTAACTA
CGCACATCACATGATCGACAGTCTGTTGATTATGCTGCCAGCTAAGAGTATGGATCCCTT
TAAACGGGTCTTTAACCAAATCTACCAAGACACCGTCAATAACGCCAAGGAGTTTGAGGT
AGTCCAGAACACCAAATTTGCTTACCGGAATAAAGCGTTCTACCTGAAGTATAAACAATA
CTTCGGGCTAGTGACGACCAAGACCACTCGGGACGTCCTGTACGGCCTCGTCCAAGAGGC
GGTTGGGAATACCGGGTACGGCCTCTGTTGTGCGAAGCCCGCGGATGAATTGATCAACCT
GAAACTTAAAGGGGATCGAATCGACCACGATGGGAAGCAGCACGATGACTTGGTCATTGC
GTGGCTACTGAGCTACTGGTTCATTAAGCTCGGGGAGAACAAGTCCTTGTACGTGATCCC
TCCGGGGATTGCCTTAACGGACACCCGTAACCTACTGAACTCTGCTCAGAACCAAGGACG
AACGGAGTATGAGCCGTACGTTGTTCAACTCATCGATAAAGTCCGGAGTAAGGTTCACAG
CCTAACTGAAGAACTGATGAGCACCCAGGACAATATGTTAGCACTTCGTCTAGAGGTGGA
GATTCGGAAGTTAGCCAAGATGTTGCCTCCGGAACAGAATCGCATGATGACCATTGACGT
GCTGTTGGAGAACGCTAAAGTGGAACGCAACAAACGCCTACTGCAACAACGCAGAACAGC
ATAAGACCTGACCCTCCTGGAGCCCGTGTGGGGCTCCAGGAGGTATCGGGTTATTTCTGC
TTCAGAAAGGGCGGTATAACCCACTTAGTTACAAGGGTTACGCTACAGCAAGCAGTGCAT
GTTCCAGTTCTTGCAGAGTGTGACCTTTAGCCGGCTTGCCGTTGGCCAATAGGTTGAAGG
CTTCAGAGGCGGTGGTATGATCCAGATACCAACGGTAGTTCGGCGAGCTTTCGTCGAACA
TCGGCGAGCGGGAGGGTTCACCTGGTGGTGTAGGGATCCAAGGGAAGATCATGAACGCAC
GGATTGACGCTGAACCACTTTCCACCATCACACTAACCATGGTGGGCGTATGGCACGAAC
AGAGAAGCTTTTGGGTCAAGGGCAGTGCGTGCATAGTGTCTGCTCCTACGCAGTCTGCAT
GAAAAAATTCCTTGTAACTGGTTATTCGAAATAATCCTTGGACAGTACGCGCAGTACAAT
GAACAAGCACAACGCCGTACGAGTGGTTAATACCCACTGAGGGGTTTTACGTCCGGTAAT
GGCAATCACAATCTGGTCACCTAATTCCCGAATCGTCTTAACCATGTCATTCCGACTCCG
ACTCGCCCCGTAAGCACCGCGCATCTTGATCAACACGTTGTAGACTTTACTGCGTTTGAT
GTTGTTGGCGTGGAGGTAATCAAACAGGTGCAATACGACCGTTTCAGCAAACTGTTTGTA
CTCAGGGGTTTTGGGGTTGTGGTACCGCTTAGGGAACTCTTGCAGGGTATACGTCAGCAT
GTCCATCCGAACATCCTCCAGTAAACTGGCTGCATAGTTCATTAACTCTTCCTTGTAGAA
CGAGGTTTCCTCTGTCAGGATACGGCCCAGGTACTGAACGTGTTGATTCACGTCTTTCGC
TACCGACTTAATGGTCAGTTCATCTCCGAGGTTAACCTTAGAGTTCTCCAGTTTAACGAT
GTTGGTCTTGTGCTTGACATCGTGGAAGACTTTGTTAATGTCCTTAATCACCCTTCTAAG
CCTGTTCTGAATATCTCCTACCATGTAAACGATTTTCTTATCATCGTTCATCTGGGTAAA
GGCTTCGTAGGGGATACCGGTCTTAGGTTTCAGGATAAGCTCCGCCCGAGCCTCAATCAA

CGACCTCCAGCTTCCATACCGTTTAATGTCATACTTCAAGCTCAACCGGTTACAGGTCTC
CAAGACCACCTCTTTACGGGCGGTAAAGTGGTAATCGTTGTGAATGATCGAAGTGAGGCA
TTTGTAGTGGTACATGCACACCACATCTTTCATGGCTTGATGTTTAACTTTCTCCGGTAA
CTTCGAATGATGAATCCGGTACAGCAAGTAAGCAATGGTCATATTGAAAGCATCCCCTAC
GACCGCCCACTCTCTCTTGATGGCTGCCACGTTGTGGGGGTTCTCTTTGAGTTCCTCTTC
ATCCAGGTCAATGATGTCATTGAACCATTGGTTACGGTCTGCGGTGGTAAAGGTGATCTT
CTGCAACCCTAGGTACGGGCTCCCAAAGAACTCCATGTGGTTCACCACACCCATTCGGCG
GGTAATGACCGCATACACGTACTTCTGAAGATCACTCGCCCACCGAGGGGTAATCTCCAA
GTACGTCGCCAGTCGGGGAAAGATCTCCTGAATGGCATGGTTACTCTGGAATTCCACCCC
TGACAGTAAAGCTTCATTCCCTTCCACGACTCAGAGGTCACTATCAGGAGTCTTTGCCTC
TTCAAAGGGACTAAAGATATCCTGGTCCCCTACGAGGTCTTCCGTTCCGTCTAATACTAA
CATTGAAAACCCCTCTTGGTTAATTCCGACGTGGTGTTGCCCTCCGTATTTCTTCAATAC
GGGCTTGTTTATCCGACCACGTCATGCAGTTGATCCATCTGTTCTTGAAATACTCTTTGC
ATTGGGTCCACGCGTCACTGTAGGCCTGTATATCGTCCCGTATGGCGTCCAGGGGAACGC
CTGAGCGATAAACCGCCTCTTGGGTAGGTCGCCTACCGGTTTTGTAAATATACGCCTTAA
CGGCCAATTCAACGAGCTCTGCGAACTGCCGGTAATGACGAGGGTGAATACTGCTCATCC
CTTCATCGTATTCCAAGATTACTTTAGCACTCATGGAATACGTTCCCGCATTTAACCCAA
AGATCACAAAGCAGTTATTACCGGTCATATGGACATTGGTGAACGGGACTGGCATACTTC
GGTTATAACTCAATCCCCCCAGTAAGCCATCCATCATGTCATTCAGTACTCCGGACCCAC
ACATCGTGTTCTCATTCACCCGGGCTCCTAACTGACCAACTGCGGAACTCATACTCCCCA
GGGAGACTTCTGTCACACTGATGATTTTACCACCCCGAGTCGTCGCTTCCGGTATATTCA
CCTCTACACACCCATTCCCCCGATCACGAATCATGGAACCTCCCAGATCCAACAACTCCG
TCTTCCCACCCCCTACGTTACACTCCGGTAGAACAATACGGTGAATCACTTTCTCCCGTA
TACCCTGTTCCACGGTTGCCTGGTTAACCAGGTTATACCAATTCCCCGCATAGTTCGCAT
TGGGGGTTTCAAAAGCGAGCTTCAACAAGTACTCATCGATATCAGTGCCACTTACGACAC
GATTTATAGCATGGTCTACTGGATTCATTCAAGGTGACCTCGTCTAACAAACCTTAACGG
GTTTATATAAACCCGTTAACTAGGATTAGGGAATAATAACATCTTCCAGTAATTCATTAT
TCGGTTTCCATTATATGTTCCACTTACCCAATTCTCATTTTGGAGGAGGGGGTAAGATTC
TTTAGGACTGAGCGACATGAGCCTACGGCGAGTGGAGTGAAGGAGTAAAGAATCTTGGGG
GAGGAGGCCCTACCGAAGGTAGAGTCCAGAGAAGAGAAAAGAGAAAATCCGAAGGAAAAA
CCTATTACTCCGTAATAGCTTTTGCTCTTTATATAAGCCGCGGAGGGCCTTATAAGGCAA
TAAAATTTAACTTTAGTATTTATTATTATTATAGGAAATTGCTTTTTTATTTAAAAAAGG
AAAAATGAAGAGGAAATGAAAATGGGAAAAATACCTTTTAAATATTCCCTAAATTTCATT
TGCTATTAAAGAAGTACCCCTTTCAAAGTCAATTACCCCGACCGGCAGTCCCGAACAGGG
AAGCCGGCGTACAGGGTTTATAATGCAATACTGAAATAGGGTTAATGGATTTCAGTTCTA
CATTATCATTGGGTAATAAAAGAATACAGTTATTCGTATTGATGTGTTTTGATAAACCCA
GAAGAGGAAGAGTGTGTAATGACAACGATTGCTTATCGTGACGGTGTAATGGCGGCGGAT
ACTCGTGTGGTCAACGGGGCCGATATGATCAACCTCGGAGAGCAACGTAAGATCCACGAA
CCGGGGGAAGGACAGTACTGGAGTGTCAATGGTAACCATATTATCTGTTTTGGTTTGTCG
GGGAGTCTATCGCATCTGTTTGCCTTGAAAGAACTGTTGAGTGAAGGGATTACCTTTAAG
ACCAAAGCACCTGACCACCTCCTTGGGGACTCGAATATGCTCTGCGTGGGAGAACAGGGT
AATGTTTGGCTGGTTTCAGCATTTAAACAACGAGACGGGAGTATGGTGTTTACGGAGTCA
TGCGTACAACCCCCGTTTGCAATTGGATCGGGGGGTGTTTACGCCAGTTCGGTGATGGTG
GTCGGTAAAAGTGCGGTGGATGCAGTTAAAGTGGCGTGTAAGTTGGATATCTTTTCTGGC
GGTCGTATTGATACCTGGGAACTCCCCCCTATTCCAGAAACCCCCAGTAAGCGCCCTGAC
CTGGCCGCCGATAAGACGGCAAAATAATCCCGAGGGGGTAAGACCCTCAGAATGTAGGCG
GTGGAAACTAGAGTGTGTAGGTATTTTATGCCGATGACCTGCATTCACTCCATCGCCGGA
CGTGGTGGGAAGGTTAGGAGTGTTAGACACCCACCACCCCTCCACTGCCCGAGCCTATTC
TGTCAGGCTTGAGAGGGTTGCCTACAAAACGCTCCGCGCCTCCAGCTCTATGTCACTATG
ATGTAGAGCTGGAGGTCTTACTTCTTTTGTTTTTTATTTACCCATGAGGGTATATCTTAT
ACCGTTAAAGTGACCTGTTTCCACGCCATGGTTCTCTAGGTCACTGTAAGGGTACAACGT
TATTGATCCTCTTGGATGCGCTAACGGGTGGTGTTATGGTCATATCGGCGGGTGCCCACC
CGTTAGTCTTTTTTTCGATTACGCTAGATAATTTGAAACCTAAATTATTAGCAGGCTATT
GGAGACTAGTCCTTTTTAATGACTTCCCTGTAAACCAAAGAGAGCGATTCGATATGTCTC
GGATTCAATACTACCTCATGGGCGGCGCGGGTATCAACATTGGTGCGGGTTTGAAAGAAT
ATTCCCGTACGCGTGTGAACAGTGATCCGGGGATGGTAGGTTTCGATACCAGTGATAAGA
ACTTCACGGCCGCGTTCCCCATTGAGCAACTCGAAGGGGTGAACGGGAGTAGTAAGGATT

CCACCAAGCACTATGACAAACTCCTCCCCTTCGTAGCGGCTAACCTGATGAAACACCGTC
CCGGTGTTCACAACATCATCGTAGCCAGTACCGCTGGTGGTACTGGGCGTGTTATGGCGT
TGATTGCGTTGCGCCTGCTGGTCGAGTCGGGTAAAAACGTCATTCTCTGCCTGATCTCGG
AACACACTTCCCTGGTGGATAAAGCAAACGCAGTCAATGCCGTCCGTTCCTTTGCTAACC
AGTGCCAACCCAATCAGCTGAACCGTCCGGTGTGCTACATGGAGTTCCTCAATACGCCGA
ACCTGACCCGTAAGGAAGTCACCCTTCAGGCGATTGATTCACTGAATCTCCTGAGTCTCC
TCTTTGATCCCGGTAATACGGAAATCGATGAGGAAGACATTAAACGCTTCTTCAATTACT
CGGCGGGGGTAAAACCAGGCCGGCGTAGAGTAGTATTCATTTCTACGAACAGAATGGTG
CAGCGGACTATACCGGTAAGGTACCGGTCTCGGTCGCCAGCCTGTACCCGAACAACGATG
ACGTGGTTCCTCGGTTTATGGGATCGGTTTACCGGACGACGGGTATTGTCTCCGCCAACA
GTGATCGTCCGGAGGACATCAAAGAGCTGCACGTGACCCTCGATCACGGTGAAGCCCTGG
AAGAACTGGAGAAAGAGATCGAAGGGCTGGAAACGGTTCAAGCTCAAGCTGCCGTTGACT
TCTCTCAACAGAACGACATTTCGGAAGGATCGAAAGATCTGGGCTTCACGCTGTAAGACA
GACAGTGAATCGCTAAGGTAGGGAGCTCACGCTCCCTACCTTAGCCTTTATTACGCTTTG
GGGTATTTTTTATAATTAGTTTTTACTAAAATGGGCATTATTATACAGGCCTAATTAAAT
ATAGGGGTTAGAAGGTGTCTATAAGCTTCTCTGTTGATTTATCGAGGGGTTACCTATACC
GATCTACCGGTTCAGGTAAAAACGTCGTGTTAAGGAAAATAGACCAGTTTGACGTGGTAC
ACTGTTTACAGGCATTAGGCTACCACAACCTGAACGAACGCAGTCTGAATCGTCGAGCTA
TGGTCTTTATTCCGTCTCTGGAGGATGACATCACGAACTTTACGATCGGTGTACCGAGTG
ATGCACAGGTTCGTATTCGTGGGATTGGGAAGTGCTCCCAACGGTTATTGATTACATTTG
TTAATTAAGGAGACCTTGTCCCATGGTACTGTTCAATGTGAAGGAGATGGCGAATGGTAT
CCTCATGCCATCTGAATTGTCTCGGGTAGAACGGGAGACCATTATTAAGGACTTGATTGA
AACGGCCTTGAATATCTTCAATAACCCTTCTTTGTGCGACGGGATCCTGGATGACCTGTT
GGAGCATTATGAGTTTAACGTAGGGATACTGAATACCGACCGTATCGTTCTGGATCTCGT
AAAGGTCATTAAACACACCGCCCGTAAGCTGGAGTGGGACAGTCGTTTGAAGGCGAAGGT
GGAGTTCAAGCAGGTCGGGAGTGGGTATCATAAAGCACGTATTGTAATGGATTTGGATGA
AACCCTCCAAGCGTTGTCTTCGAATGTAACAGAACCGGTTCGCCTCAACGACGTTATTAG
TGACCATCCAGAAGTGGAGATGCTCAATGAAATCATTTCACTACGATCTAGCATAGCCCC
AAGGAGAGTTCCGGTACTACCAGATCGACGTCCAGTGGTTGTTAAAGGAAATCAGGAGTC
TGGTTGGGTACCTGGTTACCGGGAGCATTACCGGAAAATCGGTTACTGAACATTACGACG
AAGTAGCCTTAGGGGTCCTACAGAAAGCCCTTGCTGAGTGTTTAGTCTTTAACCGCGGCT
TTGAGTTGGAAGAGTCTCCTAACGTCTTTCCTCGTAGGCAAGCGGATAAGTATCTACTAC
CGGACGATCGAAATGAGTTGGTGATTGATGCAGTAGCTCATTTAAGCCGTTTAATAACCC
TCCCCAGGCCCTATACTGCGTTTAGTCACCCGGTGGTGTACCGGGTGCTGCCGAATGGCA
ATCTCTTAGTTGGGATCGATAAGCGGGATTTATTATGATTACGAAAACCATTCCCCTCGC
GTTGGTGAACCGTTGCTTTACGAACGCTAGCTTAAAGCCTGAGCAGACCGATTCGGTGAT
AGCGACCCTGGTACATATCTGGCGAGTGAATGATCATAACGGGGGAATGACCCGGGAGTT
GATTTTCAATGAATTGGACTTGGATGAACTGACCGCTCAAACCCTGTGGAACACTCTTCA
ACAGCCATTACTGGCGATTAGTCAGCAAATTCGTAAGTTAGCACTGGCTGGGCGATTGGT
TAGCTGGAAAGTTCTCCCCCTCACCATTATCTTGGAGATAGAAGATGAAAACCTTGTCCC
ACTCAGTACCTATACCCCCGTTGACCGATCATAAGGAATTCCTGGGGGCGTATAAAGCGT
TGTTGAACGCTTTACAGATACAAGAGAACATTCCTTTCCCGTTGTCTGCTGGTTTTACGC
ATCCGGTTGAAAAAGAGAACCAGGCGCTCACCACCCGCTTCTTTCACGATAAGAGCTTGT
TCCAACAACTCAAGTTGAACCATCCTTTACCTCCGTTACTGGAAGTGAGGGTCCACAACC
ACATGGCCACCTTTAATTTCGGTTACCCAGAGTTCGGTCAAGATGAAAAAGCGTTTCCTC
CGAGTTGATTATGAGCGGATAGCTGAATACGTGTTAGACCGTTCGTTGAAAACTGACGAC
CTGCTGGTGGATACACCGTCGAATGTCATTGACCTGTACCTCACCCACGCCTTACTTGCT
CGTCATTTAAAGCACTTGGAGGTCGCCCTCCAACCATTCAATAGGAACGGTGAATGGGCG
TTTTCCCTGGAGATTGAGGGTGAAGACGAGGATCCCCAATTGACCGAAGTGTTGTTTTCC
GATCAAGGGGAAGACATCGTCCACACCTTCCAGGTGAGCTTACGACCGCGTGCGGTGGTT
CACCTTCAGGGTAGAGTCGTGGTGTTACCCCTGATCAAAATAGAAGCGCTTACACGAGGT
CAATATGGCAGTTACTCCCAGACTCAATGACATTGTGGATTTTCAATTGGTACGTAATGG
CATCCTTGGGGATGAACGGGTGGGGGTGTTGGTCATCTCAGCCTCTATGACGTATTAAGC
GGCTAAGGCCATGGATCCTCAGATCAATGTAAAACACAGTAACCTGTTTCAATACTTCCA
GAATAAAGTGGGTCGGGTAGACGATCCTTCCAAGTATCCGTACTTCGCGGTACAACTCAG
CAATGGGCAATTTGAGGTCATTGGTGTCCCCTGGGTGAACGATGCGACCTTTAAGACTAT
CGAAGGGCGTATTCGCAGCTACACGATCACCAATTACCAGGAATCCATGGCAGGCCCTAT

TGCCAACATGCTGCGTAACCTGGGTGCGAGTTATACCGTGCATGATACCACCACGACCAA
GAATTAATTCACTACCACATCCTCTATTTAAACGGGGGATGTGGTAGAACCTTTATTTTT
TCGTCGCGTGTCTTTTTTAATAGACGTTTTGAACAGAGAGAAGGTCGTATGAATGTTCCA
GCGACTCAAGAGACTGAACCTTATCAGTCACCCTATATAGCGAAAACGTATGTAAGTAAT
TGTAATTTCCTGAAAGCGTACCACTACTTCGCGGCCCGTTACCTCTCCATTGTGTACGGG
TGGGACTATGAGAACCTGCTAGAGAATTGTGAGAAGGTCTTCGTACCGAATGAGAATGGG
TTAAAAGAAGCCAAGTTCGGGGTCTTTAAGAAAGACAAGTACGGAGATCGTGGCCCGACG
GTAATGGGAACCCGGCAGTTCTTTAACGCAGTCCAAGAGAACAACTGGCACCTCTCCCCA
TCCTTGGTCGCGTATACCAATACAGAGGAAGAACAATCTGTTAACGCGGCGGGCACCGAA
GAGTTTATTGGGTTCCGTCGGTTGTACAAAGGTAAGAAACAAGCCGCTAAATACGTCGGG
GACGAGGAAGCGGAGAAGGCGTTCCATGAAATTCAGAATGCCTTAAAGATCTTTAATAAT
TCTCAATCCGGCGGGATGTCGTCATCCGGTACCCCTTTGTTCAATAAGTCCGGTCATACG
ACAATCACCAGAACCTGCCGAGCCCTCACCAGTACGGCTAACCTGATCAATGAACGGATA
ATTACCGGTAACCGCTTATTGTTAACTTACAACAAGACGATGGAACTGTTCGTCAGTCAG
CTCCAGTTCGCCGGCCGTGATTTAATCCAATCGGTGATTGATGAGTACGGGATGACGTAC
GCCACGGTCGACCAGGTCATGGACATGGTGAGACGGTGTACCGCGTACTACTGGGATAAC
CCTGTTAAACTCAGGGCTATTGAACAGTTCTTGAGGGATCTCAGCCCCTTAGAACTGACC
ATTATCCTCTGTACCCAGGATTTGAGAGGGCTGTATACCACCAACCCAGATATGATTAAA
CGGTTCTTTAAGGAATGGTGTGATATCCCGGAGTTTCCGGAAGGGGTTAAGGAAGAGGAT
TATCCTAAACCCTTGAATGGGGATTATTACATCCTGTGTTTAACCAACCTTGGGGAAAAG
TCCAGTAAAGCCCAAATCAACTTCTTGAATGGTTATCACATGGAGGTTGAGAAACGCTGG
GGGAACTTCATTACTGCGTTCCTCAAATCTTCTATCCCGCCCACCGACATCTACAGCGTC
AAAGAGTTGGTTCGTGAAAACGTAATGACCTCTGATACAGACGCCGGTATTTACAGCATG
GATCAACTGATCCAAGAGTTCTCGACTGACCGTACGGTTTCGTTGAGGTTCAATGGCGTT
CTAACCTTCTGCATTCGCAACAGTGCGATTGACCAGCACGCTATCCTTAGTCGCGACATG
AAGGTGGCTAAGAAGTACGAGCGTCGGTTGAACATGAAGAATGAGTACGTGTATGCGTCT
TATGTCACGACATCCATGTCGAAACACTACTTCGCGTTGCAACTCATGGTGGAAGGGGTC
TTGAATAAGAAACCGAAGTTGGATTTGAAGGGTGTTCACTTACGAGGGGTTAAAATTGCG
GAATTGGTGAGGAAATTCACCGGGAAACTCATGCGGAAAATCTTGAATTCACTGTACAGC
AGTGAGAAGCTGGATGCGCCTAAGATTTTGAGGGAAGTGGCTGACATCGAGCGTCAGCTG
TTTGCCAACCTGGAAGAAGGGGGTTGGGCGTGGTTAACGATTGAAGGGGTTAAGGACCCA
TCAGCCTATACAGACCCAGAGAGTAACGAACGGTACATCAACCATTTGTTCTGGGACGAG
GTATTGGCTCCTAAGTATGGAGATACCCCCCCATTCCCATATAAAGCCTACAAGATCAAC
CTGACCTAAACCGGTAAGAAGAAACTGGAGGGTTTCTTTAACAGCATCGAGGACCCGAAT
TATAAAGCCCTGTTGATGGATCACCTTAAAGGACGAGATAAGCTGAGTTCCAGCTATATC
CCGACCGATCAGATTGATTAAATGGGAGGAATCCCTAAAGATTTTATTCCGTTTATTGAC
AAACGCCAAATCGTTAGTCATAACCTAAAATCCATTTATGCGATTCTAGAAGCGTTAGAG
CTCTTTATCTTAAATACTAAGGTAACGAGGCTGGTTTCTGATGAACATTGAGGGTGGGTT
TACGCTCAGAAACGAGGCGAATCAATAACACTGACATACCGAGAGAGGAAGCCCTAAGCC
TGCCCCTCTCGGTAGTTTTTATTTAACTTTTTCAATAAGCTTATTGGGTAAGTCAATTTC
TTGACCTCCCACAAGATGGCAGCGTGCTATTTCTTGAGATTAATACGGTTTAGCGTATTC
AACTCGTCTGCCAGTTCCAAGAACCATCCTTTGAAGTACGATTCAGGGATCCGTTGGTAG
TTATTCACCCGTTTCAAATGACTTCGTTCCACCACACTGTTAATGTCAGCCGCGGGGGAA
TTCCCGTGGTTGCAAATACTCAACTAGATGGAATACAGCTTCTTTAAAGCCGGTTCCCAA
ACCCAACTGGTTTGCGGAAACAACGCGTTCTTACCCGCCGGAACGTAGTTGAAGTAGGGA
TGTTTATAAATCGACTCAAGGGTAACGAGGAGGTGTTCAAGGTTAAAGAGTCTGCGTCCT
GTTAAGGCCTCTCCCAGGAAAGCCAGGTAGAGGTCCATTAACCGTTGTTCACTGACCGTA
TTAACCACCACCTTATCCGAGTTCATCGCACCCTTCAGTGGTTGTTGGTGAACGAAGTGG
TCGTAGAGGGTGTTAATGACCGTAAGTTGGTTATGAATTAACTGGGCGTTGGGTAAGGGG
ATGTCGGCGACAAAGGCTAGTAGACCCGTACCCCGCTCGTTGGCCATTTGCTGGTACGCC
CACGACGCAACAGCCAACTCCACCAGGTCCATGCCTATAATGGCCAAGTCATTGATCTGT
CCGTTAACCGTCTTATCCCGGTTGAGTGGGTGGGAATAACATCGTTCGGTAACCGTAGAA
CACAACGGTACCACAGGACACAGGGATACAAAGTCAATAGGAGCACTGTATTGCTTGGTA
TTCTCAATCAGACACCTGTGTTCCCGTACACTATTCCGGTAGAACCCATTGGTGATTGCT
TTACCCACATGACCCAACGAGGTAATATTAAACAACGTACATAATGAATACGCCCGGAAC
CTCGTATAACTGACCACGTAAGAGAGATCCCAATCCTTATTGATGCTTAACTGTTGAAGT
AATCCTACCAGGACGTGTTCGTTCCCGGTATGGAATGGCTGCGCCCTGACGTAGTGGAAA

AAGGGTTTACGGTTAAACGCTGTTTGCCGTTGCAGGTTACCCAAATCACCGACCCGTAAC
CGGGCATAAACCGTGTTAGACGGTTTAGGGAATGACAAGGTTAGTATGTGAGTATTTTTC
CAGTGATGAACTATTATATAGCACACCACGGTCTCCCGGTTCGTCCGGGAGTCGTTGGAG
TGCAATTTCGAAAATGGTATGAGTTTGAACAAGTACCTATAGCATTTCAGCCTAAAAAGC
CGGACTGTGACCAAGTGCTAGCATTTTTCAAACCCATATTACCAGTGTGAATCGGGACAA
TGATATTTGTGTGTGTAGGCAAACACTACGCGATTCCGTTATAGCGAAGCAAACCCTTTC
TTAAGAAAACGATGGAGTTTACCCCATGGCTGTGAATAAAGGTCGTTCGTCCGAATCCAC
CACTCCGAACTCTTCCTGGACCGATGAAGAAGCCGGCGAGAACGGGTTGAGCGCCAGCGT
ACGTCAAGACACCACCGGTCTGTCGGGCCTCTTCAGCCTGACCTCGATGACTTCGGACAA
CCGTAATATGGTGGAAGTCTCCGAAGTCGCCAAGATCATCAAGGAACGTTTCACCCGTAT
CGCGGAGGCTACCACTCCGGAAGCTCAGCGCAACATCGTTCCGAACGTGGATGAAATGAC
CGCCGGCAACTCCAGCATCCTCCCGGGCCTGGTACTGCACAAAGTCATCAACAACAAACT
CTGGGTAATGGTTGTCCTGTTCTCCAACAAGGACCTGACCATTTCCTCGGAACCGATCCG
TATCAACACCCTCAACGGTATCCAACAGCAAATCTCGGTGCCGCTGACCCCGACCAACTA
CGCCAACGAAGCGGTCATGAAGAACTTGGTGTCTTATTACACCCGTGTGGCGGAACAGCA
AGGTGTGAAAGGGGTTGAAGTAATCAACATGGTCGTGGTTGACCAGGAGATGCTGAACCA
CCCGGAAGCCGGCGATCCCAAGGACCGTCCGGCCAAGCTGGCGGGTTACCTCACCAAGCA
GTGGGAAACCGGTGTTCTGGTCCGTGCGACCATCGAGATTGCCAGCGCTGGCGTTCCGCT
GCCGTCCCCGTTCGCGGATCCGAAGCCTCCCTTCGGTAAAGACGGTTGTGCGGAAGCGCG
GATTACCGCTATCCAAAAACGTGTTACCAAGGCGTTCACCCTGAGCGCTTCGAACATGGA
AGTGGTGGCGGCTACCATCAGCGACCTCAACCGTAGTGGTGGTGTCTCTTCCCAGAACAA
CAGCAAGGAGATCGCACGCGCTACGGCTATCGTCAGCCTGGCAGGTATCACCATGCAGGC
TCACCAAGCCAACCTGGCTGCCAGCCGCGGTGCCGCTCAGCACAATGACCTGATCAATCA
GTTCCTGAACCTGACCGGTGGTGTTTACCCGCACGGCTACCGTCCGCTGCACCCGGTGAT
CACCATGGAAACCGCGTACGCCGGTGAAATGATGAACTTCAACCAGGGTCTGTTCCCGTT
CTTCTTCGGGCTGTACCTCCTGATGACGACCAACACCGACTACGTCTTCTCTGAAGCACT
GTGTAAGGTGAGCGTGGGTCAACGGGGTAACCTGAGTGCACTGGAACCGCGTATCGACAG
TCTGCTGGGTGGTGTGAACCCGCCGAACCGTCTGAAACTGAATGACAAAAACATCACGGA
TATCGATGTTGTTAACCAATGGATTCGTCAGAACGTTTCGCAACACGCGATCTTCCGTTC
GAACATCGTCCTGAATGGTCCGGATTCCGCGATCAACAACTTCCTCAGCCGGCTGAGCCA
GCAAAACCGTTGTAAGGAAGTGGGGATTGTGGTATCGGTGATCGATGCGATGACCAAGAA
CAAGTTCTCTCAGATCATCGAGCAGAACCGTTCGGCGCGCACCGGTTGGACTCCGGAAAA
ACCGGTACTGCACCGCACCCCGATCCTGGTGGTCAACGGCTTCGCTGAAGCGCCCAATGG
TAAGAAACTGAATACCTAGGAAGTGGATGAAATGCTGCTGGGCCACTACAAAGGTGCGAA
CGGTGCCCAGGCGATGATGAACTACCTGGCGGTCATGTACGGTATTACCAGCGAAGACCC
CAAAGCCCGTTGCCAGAAGCTCAACCTGGAGCTGAACCAATCGCTGTTCAACGGCGCGGT
TCACATCAACAGTTACTCGCAAGCGGCGGTCTGGGATCCGAACTTCATGGCAGCCCTGGC
GCAACCTATGGAAAGCCTGGGTAGCATGAACGCAGCGAACTCCGCGGCCAGCTTCCGTCC
GAATCAGGCTGTTTGTGTGCCGGGTGTAGGTCTGGCTACTACTGCCTTTGCAGGTGGTAG
TTCCCTGTCCAACCCGAACCTCATGAACGCCTTCCTGGGCGGTTTTGCGTTCGGCTGATA
GGAGAGTGTGCTGAAAGAGTGAAGGTTAGGGGGCTTTCGGGCTCCCTAACCTTTATTCCG
CTTTAGTCTTTTTGGAGTTTATTCGATGGAACTACCTGAACTAACTCCGAGTCTTTCGTT
GGCCATTAAACGGTTAACGGAATACAGTGAAGAAGTGCTTGATCCCCTTAAGGACTTTGT
CGGCTACGCCAGTAAGCTGGATTTTTCCTTGGAGGATGATCCTCGGTTCTCGATGCCGGC
CTATCCCGATTTTGAAGACTACGGCTACCTGCACGATACCAGCCGTCTTAAGCAAGTCTA
CCTGAATGACTTCGGTTTCAATGTGGAAGAAGACCGAGAAGCCCTCAGTCGCTTAACCCG
GATGGAGTTTGAGGGTAATTCGTTTGATACCGTTGCGCGGTGTTTGTGTAAGGACGGCAG
GGGACTCCGGGGTAACTACCTCCTGGATACCCAACGGGTGTGTCCACGGTGTGGGAGTAA
AGCAGAACTCTTCTTGGATCAAGGGGAAGACACCCGGATCTGGCTCAAATGTTCCGAAGT
GGTGAAGAAGTTCGATAATATCGGGTTCTTTACGACCTTCTTTAACAATATCAGCATAGG
AAACCCGTCCCCCAAGATCAATGTACCGCGTTACTTCATTGATCCTCTTTATCGAACCAA
GGTTAAGAAGCAACGGAATGGAACCCTCATTGCCATTAACCAGATGCTCGGTGACTTGGA
GATCCACCATGTCGACCTGAACACGCTCTATGATCACTGTGATCGGATCATGGAATACAT
TCTGGTAGGTCCAGGTGAGCGCTGGTCGAAGTACCGGGGAGGGGAAGGTGCTGAGATCCT
GGAACTGTATCATCGGTATAAACACATCGCGTTCTGTAATTACATGAAGGTACCCAGTCG
GTACTGCATGGTGCTGGAACGGGTGGGTAAAGAAGTCCGGAGTTATGAGCATCAGCCTGA
AACAGCAAAGCTCTATAACGCCATTGCGGATACCATGAAGTCCAGCAATTGTTATCAGCT

CACTGAAAAGGACTTGCAACGCAATGTGGACATTGTGGGTAAGAACCTGGTGGCGTTAGC
TGATCAATTCAGGAGTGTCAATAACCCCAAAACCGTGTTTAATATACACGGGATCAACCG
TAAGCAAGTGTGCGCAGGTCCGGTGCCATTAACGGGACGGTCGGTGATTACCCCTCAAAC
GGGTATTATTAATCACTCTGAAGTGATTATGCCGTGGAAGACGGTCGTTACCATCCTGGA
ACAGCCCATTACGAACCACCTCTATCCTTTTGCGCATACCCCCCGGAGTGCCCGTAAGAT
GATCTATAACGCGGCCTATCGGATCGTTCCAGAAATCGATGCGTTCTTACGGCGTGCGGA
GGAGAGTCGTAAACTCCTGGTTCAAATGGGGCGTAACCCCTCGATTGAGTACCTGAGTCG
GCGCACCAATTACCTGATGGTGAACCGGGATCTGGAAGATGAGACTATTAAGATCCCCAT
TACTTCAGTCGGTCCTTACAATGCTTAACAATGATTACCCTATTCCTCTACCAGGAGGAA
TTCGTGCAGATAGAAAGGATATACACAACTCCGATTAAACATCCAGAGGAAGTTGGGTTT
TATAAACATCCAACATTGCCGATTTGGTTGTCTAAAGACGACGTTCGGATCCTCACAGAA
ACAGGTTTCGTGGAACCAAGCATAGGCGGCGAATACAAGTACTACATTCGTCAGCACCTC
CAAGTGTTGAAACTAGAGACATTTCTTGAGAAGCCCAGCTCAACTATCAAGTTGTGGGGG
AATCACAGGGATGGCGACAAGCGCAATAATCACCTGGAAAACTTGGAGTGGTGTACTCCG
TCAAGGAATTTGATCCATGCGTTTAAGACCGGTCTTAGGTCGGATAAACTTCCCGGTTTA
CTCACCGATCTAGAAACAGGTGAGGTCTAATCATTTACGAGTTTAAGGGAATGCGCTACC
TACTTAGGTATCAATCCTGGTACACTCACTGTTTTCATGAAATCTGATAGAAAGTATCCG
CTCAAGTTTAAGTACTCTGTCCGTCTAATATCGGAAGAGCCTAGCAAACTGACTGCTGAC
GATATTGGTAAAGTAAGGAAGGGTGGATCCAAACCTTGCAAGATCGTCAATAAACTCACT
AGCGAAATAAAGCATCTGGCCTTTGAGAAAAGCATTCAGGAACTGTTCGGTTTATCTGAG
AGACGTTAACAGCGTGCTTTGAAAGAACGTGGATACGAAGACTGGATTTTCGAAGCAGTG
AAGACGTATGAAGAATACGTCGAGTGTTTAAAGGATGATAAGGCGAAGACTGTAAAGTCG
AACCGACATCTTTCTTTAGTCAAGGAAAAACAGTTGGCTACAGCTAAGAAGATTGTGGTT
ACTAACAATCTGACAGGCGAAATAACTGAATTCGACAATCTTAGTGACTTCTCAATGGCG
AATGGTTTCATGATAAGCGAGATCAACCGCGCTTCGAAAACCAAAGCTTCATGGAGAGAA
TTCACATTTTAATATCTGGAATAGGGTAATTGTTAAAGGCTCACCAGGCACGAAAGTCCT
GAATGAGAATCTCTTGAATTGCTGGAACTCTAACAAAAGTGAACCACCTTACGTAACCGT
AAGGGGACGAAAGTCAGAAACAACGGTTCACCGGTCCTACGCTAACCAATAACGCTAAGG
GATACTGACAAGGGGCAACCAGCAGCGAAGCCTGTTGTTATTCAACAGGAACGTTCAACG
GCCATTCGATGACCCGTAATCTGATTACGGCGAGATGTAGGGTAGCAAGCTAATGGCACC
CGAAACAGGAGACACCTAATCCCTAACCGGGAATGGTGATGCTATGGTCTGACCACGGTT
CAATAGACCGTGCAGGTGTCTGTGCACACCGGGTAATGAGTAGCGTCATTACTGGACAAG
TGGATTTTGATGGTGATTCCATGTATGCCATCTTCTTGCTTGACTTGGAGTCTAAAGCGA
AAGCCTACGGGGGATTAGGTCATCATCAGGTCTTGGATATCAACGTTACCTTCAAGATCA
GCAAGTATGCGGGACAAACCGCTACCAACCTGATGAATTTCAATACCCTCATGCTACAGT
AACCGATTGAGTAAGGAGGTGAAGTGTCGATGCGAAGTGCTAATGCCTTCAGTCACGCGA
TCTCAGGCAGTTTTACCGAAGAGTCCATAGCCAAGTACGCCGACTACGTAAACAACTCTA
CAGCGCTCCTACAGATCGCCGGAGGCTGGTTGGGGGAACAAGCCAAGAAGACCCTGGATT
GTTTTACCAGCTTCGTAAACTCCCGCGCGTGGGAAGTGGGTAAACGCCTGTTGAATAAGA
ACGACGGGGAGTACGTCGGTCGGTATTCCATTGGCTACCTGGGGAATATTGAAGCCCTCC
AAGGTGCTGAGGGATATATGCGGGACTACATCATGGCCAATCCCACCATGATGCAGCTGT
ACCTGGACGGTGAAATTGAAGGGTATGGTGGGGAGTTCAGTAAGTTCTGTTCAGGTGTTG
GGGAGGATAACCTCTTCTATCGCCGCAGTATGAATGGGTTGCTGAATTTCACCACTGTGG
ATGATAAACCGCATCTACGCCATACCCATTACCACGACAGCCTAGGTGCTAAGATCTCCT
TCAGGGAACGGTGTGATGTCCAGAAAACCTGGGCGGCCATCGATCACCATCGGGCTAAAT
CCATGTTCGACCTCACCAGTCCTGCCGGGAATAAGCTGAAGAGCGCTGAAGAAGAAACCA
CCGAGGAGTAAGGTGTCTAAACCAAGCAGGGGTGGTTCCCTGCTTGGTTTATTATGACCT
TTTATTTTGTCTTTCTATAGAGACCCTTCTTTCTTTAGGGAGTTAACAGTATGGCGCGGT
TTTGCGTAGGGACAATGGACACTAAGTTAGGGTGGGAGACTCAGTCTCCTGATACCGCTA
TTTCACGTCATGTGACCTGTTGGATTACCAGTCGAGAGAACCAAGGTAAACTGATTGGAG
GTGTGCCTAGTTTCTACATGATCTTTAAAGACTTCGCTCAATACCCCGATCAAGTGGTGG
AACAAGTCCAGGACAAATTCAAGGCGTACCTGGAGGAATTGTTCGACGAAGTCATGGTGG
GTGTAAGTCGACAAAATGTTACTGGGAGTGTCAATAATTACAGGCTCATACTGACCGCTC
GTGTCGTAGTGGACAATATGAAGTATGACTTGGCGCAAACCATTCTGATCACCGGTGAAC
TGTACAAAGTTCTGGACTTAGAGAGACTTAAACGATGACTGACAAACACAACGAGGACTT
CCGGGGTAAAGTTCCTGATCATATCCAAGAGATGCACCTGGAGAATGACCTTGGTTGGTT
GAAGGACTTGAAAGTCCACGAACTGGATGGTAAACTCCTGCTGGGTGAGCCGGGGGAAGA

ACTCCCCTTTAACGCGAGTCATGTGATTGAAGAGAAGGAATTCGTTCAGAAATGGTTGAT
TCCCTTTGCGCTGGGGAATTCCACCTGAGTGAACTACTTCTCCTTCCAGGAATGGGGCTC
CTTTACCTTTAACGGAACCCGGTCTGTGTTGGTGGTTGCCCGGGATGCTGAAACCGGTAG
ATACGAACCGACTCTCTTGGTTCCGCCGATTATTTCCAAATCCCTGACTGCGGATTACCC
CTACAAGCTCCGGATGGCCTCGCAGGCTATTTACAATATCAGTGAAGACAACCGCCAGAA
AAAGAATCTGAACGCCAGCTTGAAGGTTGCGAATGCCTTGGCGGATAGCAAAATAGGTCT
GGAAGCTATACCCACGACCTTGACGGATTTGATTACTCCGGCCTCCTTTGAGAATATCTC
GGTCGTTCCGGAAGTGGAGCGTAAGGTTTACTGGATCCGGGATGTTATCCGGAAAGGAAC
TGAAACCAATCCCGACGACCTGACTCGTGCCCGGGAGTTGTTCTTTAAAGAACACAAAGG
TGAAACCCTGACTCAGGAAGAGTTGAAATTCCTAAACCAACTGAGTTTAGGGGATTATGA
GATTGAAGACCGTCTGGCTGACGCACCGGGTGAACAGGCGGATAGCGAATCGCCAGACGA
TCCCAACATCGATCCGTTGGCGTGTTAAGGTCGTAAGGGGTACATTATTCATGCGGATTC
TTTGGACTTCCGACCACCATACGCTGCACCAGACAACCCCCACTACTCATGTACTGGGGA
ATATGTCGACGTTTTACTTTAAAGATCACGATCTGAACAATATTGATCTGTCGATCTTTG
GTGGGGACCTCACAGAACGCATGGTAGAGGCCCCTAATCCTGATTTCATTAAACTCAAGG
AATGGGCCAAGGTTTACCTGAAGGCGTGTGACAAGGCCTATACAGCCGTTCTAGTACTGG
AAGGGACCCACTCCCACGACAGGGGTCAGCCTAAACACTTGGAGACGCTGGCTCCCGAGG
GAATGGATTTCCGGTATGTGTATGCCATGTCCATTCAGTATTACCCCCAATGCAACAATC
TCAGGATTCTCTGTGTTCCTGACAAGATGGGAACCCTGACACCTGATGAGATTTGGGACA
AGACTGTCGCATTGCTCAATTAACACAAACTGAAGCAAGTGGACTTGATTGTTTTTCAGG
GTGCGTGGGAGCATCAATTGCCGGTGCAAGTACGTCACAAGGCTCACCAGTTAGCCCGAT
GGGAAACCATTGTTTAGTACTTCATTCTGTCGGGTCATATCAACGTCCCCTCTGAAGTGG
GTAAGATGCGGTGTTCGGGTTCCTTTGACCGTACAGCCCACGGGGAAGAACACCCGAAAG
GGGGGTATTTGGTTGAGCTTGACCTGGAGAAGGAAACCGCCACTTCAACCTTCCAGGAAA
TTAAGAACGCCCTTCCGTATTTAACTCTGAAAGTCAAGGAAGACATTACGACCGAACAAT
TGGTCTTGGACCTCCAGCAGTTCATCCGCCGGAAGAAACTCCCGTACCACAGCCAAATCC
GGGTCATGGGTGGTGCAGCGGATATCGTTAAACCGGTTATACGTATCTTTGAGAAAGAAT
TCCCCTACTTTGGCTTTAAAGCGGAGAATGCGAAAGCCAATGAACAGCATGTCCAAGACG
TCCTGTTCAATAACCAAACATACGACTACCCTCAGTTAACGAAAAAGAACTTGGCATCAG
CCCTGTTACCTGAATGTCAAGGTGTCTTCGAGAAGGAAGGGATCTCTGATGAAGAAGCCT
TGAAAGTCCTGGAGGAATTCCTATGAGAAGTACAGGTGCTTTGGGAATGTCCGTAGGGAC
ATCGTTGGCGTTTGAGAGTGATGCGGGTTCTCTAATACGTAATTCGGATACCATCCTGCT
GAACCTTATGACGCTAATACGGAATGCGTATGGCGCGTATGAGAACAAAGAGGAAAAGGA
TTAACTGACATCAGATCAACTGGTAGAGGCGGTTACCAGTGATCTGAAGATCCTGGCAAA
ATGGATGGAAGAGACGCGTAAAAGTAAACCGGTTGAACTGGTGGTGTGTTATCCCACCTA
TACGGGTCTTAATACCCGTTTCCGTCATGCGGATCTCAAACTCCCTACGACGAAGAATCA
AATCCGGTACGATACCCTCTCGAAAGAGGCAGCAAAGAAGCTGTATACGAAATACGAGAA
ACTGTCGTCTAAGACAGACATCGGTATGCCTGAATTAAAAGGGCGGGGTATTGTATTGAC
GCACCACATGGTGGACCTCGCGGTATCGAACAGTGTTGGACGTTTAATACTGTTGGAGTC
GTTTACGGGTAAAACCAAACCCTTTACCCAGTGGTACACTAAACTGACGGTAGGGGAGGA
TCTGTTTTACATGCCGTTTAACCGGTTCACCATCCAGATCTTTGGTGACCGTTCAACCAA
TTTCATGTCGTCCTCAACAGGGATTAAGGAGCTGGTGAAGAAACTGGCGCTGGATAATAA
GTGGACGTCTGCCACAACACTTGGACGAATCAGAAGTCATATCAACAACCTGCCACAAGG
GGTAGATCGAGCCGGTCTACAACTGATGCTGAACGGTTAGTCTCCCATCTATTCTCACTG
AAAATTCTATCTTTATTGGTTAGGAATTTCGAATTTAAGGTAACCTCTCATGAGTGACAA
TCAACATCGCAAGTTTACCCCGCGTAATAAAACCCGCCTGAATGACTGGAAACAGCCTCA
TCCAACGACCTTGGAACCTTTGCCGGGAGCAAAGTACCCGGCGCAGCGGATGTTCGAGTG
CAAGAACAATGGGGCGTTTCCCATTGTCTTCAAGTTCAACGACGGCATCTTTGAGAAAGG
TTCCAACAGTAACCACAAAGAAGTCGAACTGAACTATTCGGATCGGGGCGCGCTCTTCGA
AGCGATTATTGAGGCCTCGAATGATGCGAACTTTGGGTTCCGTCAGATCCCGATTTCGAG
GAAGCAGATGATCTTCTCGGGTGGGAGCGCGCGTCAATCGGAGAACCCGATTGTTCACGC
GACCTTTACCATCATCCGGGATAAGAACGGTGTTATCGACCTGGGCTACACCAAAGGGGT
TTACAAAGTCCCGCTGGTTTTCCGTGGTTCCAACTTCACGACGGTATACGACAAGAATGA
AGCCGGTGAACGCGATGAAGACCAGGGTCTGATGTCCCGTTGGGTAGCTCGGGCCTGGGT
GCGTTTCTACCAGGATCTAATGAATGAGATCGAGCAGGCGGCTTGGGAACCCCCGAAACC
GCGATACGGTGGGGTAAATAACAACCGGCAGCCTTCCAATGACACCAGTTCTGCCACACA
CGATGACTTCGATGACGTGGAATTCTAATTAGACCAAAAAGGGTAGGAGGGGTTGCCGCT

CCTACCCGGTCTATTGGTTTGGTTAGAATACTTTAAACCTACATTATCTTCCCGTAATCC
TGGACTATTCTGTTTGGAGTGAACGATGTTTAACATCGAGGTTAAGCGGAAATCCAAGAA
GACGATTAGAGCCATCATTGTGTCGTTGAATGAGAAGAGCCTCCGCTTTAACGGTGATGC
AACAATCAAACTTGAACGGGTAATTAAACAAAAGGACGCGAGTGATCCTAATCTCTTAGG
TTGTTTCAACAAGTATGTGGAGAACATCTTCGATACGGATAAGAAGATTGAACTCTTTAA
GTTGTATGAAAGGGCGTATCAGATTGCCGAAAGGCCTACGTATAAGGACTACCGGGTTGA
AATCGCTGAAATTAAACCGATTACCAATGCTATTCTAAACCTCATAGATGTTCCCAAGTA
CTGTAGCTTCATCCAGTATTCCGAACCCATGGTAGTTCCTCCTGACCTGAGCGTGGCCTC
CAGTAAGGGGGATTACCCGGAACAAACAACCATTACGGATTACGATTATAAAGAGTTGGT
TAAGCTGACCTTTGTGATCCGTTCGGTTTATCCGATCATCTTCTCGTTAATTGCTCGCTT
TGATGAGGCAATGGGATCAGGGTACAGTGAGCAGGTGTGTGTGGGTCTGATTAAAGATAA
CGCCCACATTGTTAACCTGTATGGGTGGAAGAAGCTTCAGACCTATGTGAACTTTGCCAT
TGGTAAACGAGGCATACCCCAGCAAGCAGACAGCATTGGGCGTGTAGAGTATTTCGTGGA
GCGGGTTCTGTATCATACCCTCTTCTCCCGGCTGTGCTGTGCGGTCATCCCCGAAACCGA
AGAAGGGAAGCACCTGGCCACCGCCATTAACGCTTCGGTACGCCATCATGGATCGATTGG
GGGTGGGTTTAAAGCCAAGGATGCCGAGGCAGATGAGGATGATAAGCGGTCCTTGTTGGA
ACGGTATCATATCAACGAAGAGATCAAGGCGGTAAATGAAACCGTAGACGCTGAGTATTT
CAGTTTAGGGTTGTATGACGAGAATGACAATGAACGGTACGTGGATCGGTTCAAGATCCC
GTGTCTGGGCCTAGGTATAAAGAATCCCCAGCTGGTGGAACAGGTCTACGACAACATCCC
CCCGAACTGGGACATGACGTTGAACAATCATGTCATAACATTTCTTCAACTGACGTTCGT
CGATGCGATCTCTCCTATGATCTTCTGGAGTTGGGACGGGCATCGACTGCGGGCAGCCAT
TGCCTTGGCTCAGGTCCGTTTGAGCGAACAAGGGTATCATTACCTTCCGTCGGTACTGGG
AGCGAGTAATAATCCCGGAGGCATGCGATCCCTTTCGGATGGTTTGCGGTTGAACACCGA
AGATAAAGAATTCCTGGTCAGTATTTGTGACATCCAAAGTCGTAATGACAAAGGACGGTC
CTTTAATGAAGCCGTGGAGGTCGCAACGGAGTTCTTGGAAACCTTTGGGTGTGGGCAATG
GAAATCGAACCTGTAATATGGGGTATTGGATGATCCTGAAGTCTATCAACGGGTAGAACG
CGCTGCGTTGTTCGACCCGGAGGTTGATTTGGAAATGAAGAATGAGTTCATGCGGTTAGT
TAGACAAGTCAATACCTGGAAGTATCATCCGTAACTAGACAGAGGAAACAAACATGTCCG
AAATGTTGGTGACATTGGCCCGAGCCATTTTTGGTATGGGGAATAGCAACCACGATCACG
TTCATTGTCATAACCAACTCAACCTGGACAGCACTGAGGTGGGTAACCTCATCATGGAAC
AGGTCAATGGAGGTGTTACCGCGGGTACCCTGAATCGCATTGCTGCCTCCAGCGGTAACC
TGTCCCAGCTTCCTCAAGGCTACGTCTCCGTCGAAGAAGGCTTCGGCGTACGGCGTGGAA
TTGGACAACACACGTTCGTCATTGAAAGCCATTCACACTTCCATTCGGAAATGTGTGTGG
TGGGGTCCCTCTATGGAGGCACTGCGTCGGAACAAGGTATCTCGCATGACACCATGTTCG
TTCAGGAACGGACTTGGTCAACCTTGACGACCAATACCCATGACAGTCAAGGCTTCCCGA
TGACCAAGCGGATGATCGACAGTTCCCATCAGTTCCTCATGGGATCGGTGGATCAGAACA
ACGACCTGAAAGCCGTACGTCCGTTGGATGTCGGGAATGAAGCCTTGGGGTTTGCCGCCA
GTGAAGCAGATGGTTCTGAGATGATGTATGCCGGGACGGTGGGGAGCGACCTCAAGCACA
ACACCTTGATGTCGAAGACCCTCAACCTCAACCCCACGCATTATTCCCGGGAGTTGCTCA
ACCTGGCCACCAATGCAGGGTTCCACTCCTCCAATGGCAGTTCGGTGGAAGTGGAACTGT
CCAACGGATTGATCGGCGAATCGATTGGAGAACTCTCCCCGCTCGAAAACCCTTTCATGC
GGGCAATGATGCAATCCACCGGTCAGTACATGTTGGGTGGGTTCCAGGGGTTCAGTATTG
GGGAAACCTATCAGGTCTTCGATAACCTGTTGGATGTCATGAACCTCAACCTGTTGAATC
CCAGTTCCTTTGCGGAAGACAACACCCTGCTCACCAGCAGTGAATATGGGTCTGCGGGTA
ACCATGAGGTGATTGCTACGGAACTGGCGATGATGTCGGTTCACTTGATGTTGCAGTGTG
GTCTCTCCCGCATACGGTTCTCCGCCAGCAATAACCTCATGGAATATAACACGAACATGG
ATGATGGTGTGGGTGTCGCTTTCGTCATCGGTGAAGCCATGTCCATGTTCAACAATGACA
TGTTCGTGGAAAACCGGGTGGAGGACTTCAAACGTCTGATTGCCCAACACTTCTTCTCGA
AATACTCGGGACCTCATGTTCATTTGAGAACCTTGATGAATATTGAAGTGGAGTGTTACA
TGTTTGGTGAGATCGTTATTACGACTTCGTTCAATGGGGAAGCCAACGAGAAAACCTTCA
CCAACGCTACCTACTACATTAATGATACCAGCAGCAGCATTGCCGGTAATGAAAACGGCC
TGTTGGAAGCCAAGAACTTCCTGACCGGTATTCGTGAATACTTCAGCTAAGAGGATAACC
GCTCATGCACGAAACCAATGAACTGAACAAACTGTACGCAGCGATGTGTTTGTCGTGGGG
CGCGCAGGTTAAGGAACAGGGACGACTCGTCTTTATGATCGATGGGGAAGAATTCCCACT
CCGTATTGATGTGATGACCCTCCACCTTCCGCTGAGCGAAGTCCTGGATGGGAACTGCGT
GGATAAGGTCTTCTTCCACCCGGCCTGTGAAAACATCGTGTCGAAAGAAACGGAAGTCCT
CAAGATCATCCGGCGGATGACCTGTATGAAACTGCTGGATACCTCCCGTCGTATTCCGAT

GGTGTTATTCACCGTTGCAGGCCACAAGCCCAAGAGTACCTGGAACCAACGTATCCACGA
CATGCTGGAGCCCCTGAAAGGTGCCAAGCGTGCGGTACGGGATGAACTCAACGCATTTTT
CAACCGTATGCACGTGGAAGTGGAAGAGAATGGGTTGGATAACCGTTTCATTCACTTTAA
GGTATCTAAAGGCGGTGGTACCAGTAAGACCACGGGTCAACGGATCTATTACAAGACCCG
TCCGGAGTTTCCGTTCTATACGGAAATCGTTCGTCGTCTGGCACGTTCGGATGGTCAATC
GGATAACCAGACCATTGAAATCAATAACCACACGGTCTCCCGAGCGGCACTTCGTCTGGC
CGCCCATATCTTCCAGAACGTCCTTCCGGCGGTAAACAGTCCCAGTGACTATGTGTTTGA
ATCGACTTCCCCGGTGGCGTCTCGTCTAGTGTCCTACATGGGGTGCTATGCGGAAATCGC
AGATCAATTGAATAAGATTCAGAATACCCTCCGATCTGATTTCGATAAAGCTGGGTTGTA
CCCGATTGATCTGAGCTGGACGGAACGCCTGGAAGAACTACCGGATATTTATCGCCAAGT
CCCAGTACTGGATTACAACAGCCACAACACCCATGATGAAGTCCAGCATCAAGCAGTGAG
TCGGAATGACATGGGTGGTATGCTGTCGGTTACCAGTAACAACCAACAGGGTGTTTCTCA
ACATCAATCCCAACCGCAGCACGTTACAGGAATGAGCGGGGACTATGAAGTATCACCTCC
GCAGATGCAAGCGGGAGACCGGTATGTCCGTACTGAGATCGATTACGTCGGTCAACGGGT
TCTCCACCATGCGGTTAACCAAACTACCAATAACCCGGTCGTCTACATCTGTAGCCGTAA
AGGGAACTTCCTGCATCGCTCTGAAACCAACATGATGCTCCAGCAAATGGGTATGTGGGG
TGGGATGGGTGGTATGATGGGGGGAACGATGCTGACCAACGGGATGGTTCAACTGCCGAA
CGGTATGATCGAGAATCCGCAGATGATGGGGGGTGTTTCGATGCCCACACCTACCGCCGC
ACCGAGTGTATACCCGGACGCCGGTATGGGGTTTGACCTGGGAGCCCCAGGTATTTTCTA
AGCAACAATAACCTTACCAGTACCTAGCGGGATATCCCGCTAGGTACTGTGTTTTCTCTA
TTGTATTCCAGTATTTTTACGCTTGGGTCGTATTACTACGTGCAACGGCCTGTGTCAAAT
GGGCCTCGTCAATTATCTTGTAACTTTGCATGCCCGCACAATCGATAAAGGGATCTTCAA
TCCCATTCAGATAGGCGCTGACCCACAGCATATGGTTTTCAACCCCTTGAACCCTCAGGA
ACCGATAGAAGTCATACCGATAGGCATACAGTTGTGCGTGAGAGATGATTTGAATCTCCT
GGGTTTGACGGAGAAGTATTTCCCGTTCGGGCCGTACCAAGACTTTGAACCGTTCAGTAA
AGAAGAAATCACGTCCTTCACTGAAGTTAACACTAGACAGTGGCATGGAGAAGATCCTTA
TCGATGCAGCTAACCATTTTTGAACCTATATTATTTCTCTGACCCGACATAAGGAATTAT
CCCAATGGAATACTTAACGACCCAACGAAGTCGATTGTATCCGAGCCTGGATACTCGTCT
GGTATTAAACAAAACCATAGGGTGTTCACGTTTCATATGGAACGCTCTACTGGGTAATCA
TAAGGAAGAATACGGTTTTCGGGTAGAGGATCCTGAAACTCATCATAAGCCGAGTTGTTC
TTTAAGTTCCTTTAATCAAAAGCTGAACGAACTTAAAGTTGAATACCCCTGGTTAAATGA
GGTCAGTTCTGTTGCTTTACAACAATCAGTTCTTCATTTTACCAAGGCTTTCAGTGTGTT
CCTCCAGGGTATTAATGGTTATCCAAAATTTAAATCTAAACGAGATCCCGGCGGATTTAA
CCTCAGTAGTGCAGCGGGAACTTTCGCCAAGGACGGCAAGATGCGAATCCCGAAGATAAA
GGGATACACCACCTGTCGACCTAAACTCGCGTTGGATTAGGAAATTAGGAACTACAGCAT
TGTCAAAGATCGGGTTGGGAATTACTTTGGCAATTGCTTATTAACTAAACCTTGTCTACC
GAATACGGCTAAAGGTCAAGGACTGGTGGGGGTTGATCTGGGGATTAAATCCTTAGCGGT
GGCGAAGCGGTCTACAGGTGCAATCTTTACCTTGGAGAATCCTCGAACCTACATGAAGTA
CCACCGTAGACGTGTACGACTTCAACGGCGATTAGGCAAGAAACAGAAAGGTAGTCAAAA
CCGTAATACAGCTAGAATTAAACTGGCCAAACTAGCACGTAAGGTCGGCAATATTCGCAA
GAACACCCTGGATACCTTCACCTCTAAACTGATTAGCGAAAACCAAGTAATCAGTATTAA
GGATTTGAATGTTAAAGGGATGCTGAAAAACAGCCGGTTGGCTAAGCATATCCAAGACTG
TGGATTTAGCACCCTCCGGAGGATGTTGGAACACAAGGCTATACGGTATGAATCACCGGT
TATTATTAGCCTGGTCCATCGATTTTACCCCAGCACCCAGTGTTGTTCAGCGTGTGGTAT
TAAACGTGAGATCAAACTGACCGTTAATGACCGGGAATGGACCTGCGCTGAATGCGGGAC
CTCTCACGTCAGAGACATCAATGCTGCAACGAATATCCTGTATGAAGGACTTCGGTTAGT
GGTCTGATAGAAAATTTACTCTTTGGAGGAACTACCGAAGCCTACTCGGGTTGTTAAGGC
TGTGGAGCATCAGTGCGGTTAAGCTGGGCACTGAAGCAGGAAGTTTGAGGTTAAAACATT
GAGATAAAGACAGTCTTATCTCATTAGTGAAAAGACTGCTGTTGACTGTGATGGAGCCGT
TATGAGTATACAAAAATTGAATCAGGACCTGAAAGGTGTAAGTAGTGAGATCCGTGCCGC
ACTGATTGCAGAGGATACGGATCTTACCCCTGACAGAGCAGCGGGTCAATTCCTCCAGGA
AGTCCGTTACAAGTATCCCTGCGGGGAGAATCAAATCCATGTTGCTTTAGTGGGTGTCAG
CAGTAACCTGATTCGCTTCCCGCACGAAGCCTCCGCGGGTCGTCTCTATATGCCGGGTAA
CATGATCCCTAAATCCGTGCCGACCAAAGGAGCGGGTGAACGGATGATGGTAAGCGGGTA
CGAATTGGAGGAACATTACGGTGGAACGGCTCGTAAGGTCGAAGCCCCTGAGAATATGCT
GGTGGAAGAGATCTTCTTCGTTCAGAGTCTGGTTCCGGGTAAGGATACGGATGACTGGAA
TTCGTTTTACGTGGTCTTTAAGAACGACGAAGAGAACGCCTACGAATTTCTGGAGCTACC

TCGGTATAACACCCATAACCTCTACGTTGGGTTCGAATATAAATACGATAAAGAGAAACT
CCGTAAGCTCCGTAAAGGTGCCACTTTCGCTAAAAGGACCGTCTTCGCTAAGAGTCCCCG
TATCAGTGAGAATGGGGAATGGTGTTTTGCAATGCCGACCTTGGTAGCCGCCTATTCGGA
CCAAAGGACTGAGGAGGATGGCGTGCTTATCACCCGTTCCTATGCGGAACGTTCGGCTTG
TATGTTCAAGCACGAGTTCAGCTATGAATGGAATGAGGATGAATGGATTCCTCTGATGCT
ATACGGAACGGACGAGAACCCCCAACCGCTCCCTGAGTCGGGTGATAACATCCGTGAGGA
TGGGTTCGTCATGGGCATCCGTCGCCGCATCACAGAGAACGCGCTGGTTTCCCTGACCAA
GAAGTCCCTACGAGAACCGGATCTGGTGTACGACAAGTTGTTCTATGCGCCGTTCGATGC
GGTTGTGATGTCAGTCACCGTTCGATCGGATCGGATGGCGAACCGGTCCAAGAACCGGGG
AACGGATTAGATCGAACAACACCACAATGCCATGCTGTACCGCTATGAGAAACGCCAGAA
TGCAATGTGGAACAATGTGATCCGGTGGTACGAAGGGAAGATCGCTGCCAACCGCGGGGA
AGACATTCCCATTACCTTTGACCTCGATACCTTCATTCGGAACGCGTATGGTAACTCTAC
CCGCAATGGGTTGGGTCGTATTAACCCGTTGGCCCGTGTGGAACGGTGGGATAAACTGAA
AGATTAGAACGTGACCATCGAACTGAAGGAACGGGTCAAAGGTCGTGTTAAATTCAAGTA
CGCGGGTTTGAACGGTGACAAAGGGGTGTGTGTACGGGTCATTGAAGACGACGAAGCTCC
CACCTATCCGGATGGGACTCGGGCGGAGTTCATTAAAGATAACGTACCGGCGTTCCGTCG
CCAGATCTTTTCGTTGCTCATGGAACTGAGTATCAACTATGTGAACATGAACGTTCAACG
GGAAGTCAGACAGCTACGGCTACAAGGGGATTATGAAGGCGCTTTCCAGGCGTTGATGAC
GTACTATGAAACCGGTTTCCCTGAATTCAGTGAATTGGTTGGTCATGCGTTGGTTACGGA
GGAGGAGAAACGTGAGCACGTCGACGATGTCTGTCGCCGGAAGATTTCCGTGCATGTGGT
CGGTAACGCGAAGTTGTATGGCGTGAACCTGATCAACGCCCTGCGTCGTCAATAGCCGTG
TAAGCCGCAGAAAGCCATTGTCGTGAACGCACTGGGAGAACGGGTGGAAACCGAGAACCC
TAACCTTATCAGCTGTCAGGACATTATGCTCCTGGATAAGTTTGGTACGGACATGAGTGC
GCAGAGTATGCCGAAGGCTAACCTCTTTGGTTTGCCAGCGAAGATGAACGAAGGCAGTAA
ATACGCCACCCCCATGAAAGACAGTAATAACAAGAATACCGGTGAAACCGAAGGCCGGTT
GCAACTCAGTCACGCGGGCGGGCAGGAAATGATCAAGCAACTGGCCCTGGCGTATTCCCC
CGAGAACCAGAATAAAGCCTCCCAACGGGTTATTCGTGCGGATGATCCGTTTGAGATTCC
TCGGTTGATTAAACCGGATGAGTACTTGGGGAGTCGGGCGTTGAAAATGGCGGTGGGAAT
GTTGTCGGATTCGGGTTATCGGCTGCGAGCTGAACTACCCAGTGATCGTCGGGAATATCC
AACCTTTAACATGGATGAAATCCCAGGGTTAGATGACGAGACCTTGAAGCAGCTTCAGTC
ATCCGAGAAACCCACTCAAGAACGTTAACCCCATGATAGACTCACGGTATGGAGAGGAAT
GTAACTCATGGTAAGGAAGATCAAACTCCCTGAATTGGCTAATTTACCAGAAGAAACTGT
TCTCCGTTGGTTAAACACCCGCCGCTGGGTGGAGGTCACGGATGACCTGGACCCAGTCGC
TCTAACGGAAACCTACGCCCTCATTGTTACCTGGCATGGAATGATCATCCACCGGCATTA
TAAGGATGTCCCCTATTCCATTAAGGAGATGGTACCCAGTAACAAGGTGGAAGGGGGTGA
AGACACCGTCTATAACGATGGAACCAACGCGACGCCTTTTAACCAGTTCTTAAAGGTCAT
TCTTCCTACGATTATTGACCCGGTGGAAGTGGACGGATTAAACGGCTTATTCATATCTG
GCAGAATAAGCTGAATAACCTGCTGGTGGTGATGAGTGAACGTTACCTTATTTCAGCCAC
CGCTGAATCCGTAGCGGAGATCATGGAAGATGAAGGGATCTCTGGAATACGGGACCGTGT
ACTGAGCAAGGAGATTTCAATTCATGAGAGTGAGCGGGAGTTTGCGGATTATGTCCGGAC
CGCTCATTCCCTTGCCAACAACACCCTCACCCTGCTGTCGCGTACGGGGGGGTGGCCAT
TAACCACGCGTATCAGAAGACGATCATCCGGGCTAAAGTCTTTGACCTGAACAACTCGAT
CATGCCGAATGCGATTACGGTTCCGTATGCGCATGGGATTACCAACCTGGCTGATATGCT
GGGTGAACGGAATGCAGCAGGTAAAGCCGGGGTTTTGTCTTCAAAGATAAAAATCCCTGG
TGGATGGAAGACCATGGGTAACATCCGGGTGGGTGATGAAGTCGTTACACCCGATGGAGG
TCCCGCTTAGGTGCTGGCAGTTCACCCACAAGGTGTAACCAAGGCTGTACGGGTGCACTT
CAAAGACGGTCGATATACTGCCGTCAGTCCAGATCACCTGTGGAAGGTTAGACGTCATCA
TTGGTGCAACGATAAAGCCATGGCTAAACTCACTCGGGAGGAAGTGGAAGAACGTATATG
GCGAGTCATCACCACCAATGAGTTGAAAGACTATATTGGTCTGTCAACTAAGGTATACGT
CCAACTGATTGAACCTGAAAGAAATACCGATAAACCCTTCAAAATTCACCCGTATGTGCT
CGGTGTCTTGTTGGGGGTTGGATGCATTAGTCAAAAGGCTGTGGATATCACCAAACCTTA
TCAGCAGCTATTCGATAAGGTCCAATCGCTATTGCCAGAACATTTGGAATGTGTTTGGCG
ACCCAACCGTAAAGGTGATGGAGAACCTAAAACGTTTGGGATCCGTTTTAAAAATCGTCG
GTCCGAACAGCACTTCAACTGGCATATCCGTGATGGCCTGAAGGAACTGGGCTTGTATGG
TATGCGGTCGTGGGGAAAAGTCATTCCTGAAGAATATCTTCACGGATCGGCTAAACAACG
TTTGGAGCCCCTCCAAGGACTGTCGGACACAGACGGAACTGTGGACAAACACAAACCGGT
TAGCTTTAGCTCTTCGTCTAAGTTACTCTCCCTCGGAGTGCAATACTTGGTCAGAAGCCT

AGGGGGGACGGCACGTCTCCAAGAACGTACTCCCCACTACACCCACAATGGAGAAAAAG
GGAAGGTCGTACGGACTATCGGGTTTATATCCGATACCCTCGACCAGAAGAACTCTTCAC
CCTAGACCACAAACGGGAACGTGCGGTCTCGCACCAGCATACGGAAACCCTGAGACTGCA
AGTAACTCATATTGAAGAAAGTCCTGATGAGGAGACACAATGCATTACGATCGATCATCC
TGATCATCTGTACATTACAGACGACTTCATTGTCACCCACAACAGCCTAACCAATAACGG
CAAGGCCCTGAAATCATCGGAATGGTGCCACCGGAAGGTCCACATCCTGTCCGCTGTGGT
GGCGGATATCGATCATTTCATGGACTGTGGAACGACCACTACAGTGCCGATCCGTATTCC
CAATATGCAAGCGGCAATGGCACCGTTGGGTAAATTCAGGGTAACCGATACAGGAGAATT
AGAACTCATTGATCAAACCACGGTGTGGTCAATTAAGCCAGGGGAGTGGGTTAATATTCA
TAGTGTGCCCGTCTGTAATCCCTACAATAGTGCTCCACCCTGCCGGGTATGCTACGGGAT
GATGGCGAGCTCTATCCCGTATAACGTGATGATGAAGAAAGGGGCTGATCTCGGGATGTG
GTGTACCACCTCGATCTGTAACCCCATCGGCCAGGGCATGCTCTCCACCAAGCATTTCAT
TAGGAATGCAACGACCCGTAAGTTCGCTCCTGCGACTAAGGATAAGAACGTTATCAATTC
CAATGGTGACGATATATTCCTGACTAAGAAACTCTGTGCACCGGGCACGGAATTGGTGCT
CAAAGCCGATATTGTAAACATCCTCTCGGATATCCGTTCCTTGGATGTTCTGGACAACCT
GTGCCTGGATAAACTCCCGTACTTCTCTGAAGTCAGATTCCGCTATAAAGTCGAAGACAT
CATGATGGGGGGTAAAACCCTCCATCAGCATGCGGCTTGTACCTCGATGTCGTCTCGGAA
TTCCCGGTTCTCGTTGGAGTTCCTGAACTATCTCCTGAGTAAAGGTTGGGCGAACGAAGG
TAAGAAACATATTACCGTTGATTTGAGTGAATGGAATACGCTCACTCCTATCTTCACACT
GCCGTATGTTCGTGAAGACTTGCACATGCACCGGGCTCGGGTAGAGAACTTCATCACGTT
CAATAAACGCAATAAGGCCTGGAGACAACAGATCGTTACACCCCGTCTCTTTGGTGAGGT
CTTCGCGGAATACTGGGGATTGATCAACCAGGAAACGAAAGGGATTAACATCATCCATCC
TGAGGTCTTGCTCTATTCCACGTTGTGTCGGGATCCCATGCGCGGGGATTATTCCCTGGC
GAATGGTTAAGGACCTAAGTATTTCGTGAACTTCCAGGAGTGTATCAAAGGCAGGGGGGG
GGGTATGCTCATGATCTACGAGAACCAGCAAAATGTGCTAAGTGATCCTAAGACATTTCG
TGTGGTAAACCGTCAGGGGAGTCCGCTGGAGTGCTTCTTTATCTTAGCGGTGAGTTGAGT
GGGTGAACCCGTTCGTTAGTGTGGACCTCTTAAGGGGGTCCTCGGAATCCCCCTTAAGAG
GTTGATTATGCGAGCAACAGCAACTATCCGCAAAGCGAATCATTACTTCAGAATCAACGG
GTACTATGAGGAATTCGCGGTTCGGGTAATCCTACCGTTTTGTAAACTGAATCTCTGCAA
GATAGCTAAGAAGCCAATACCGGGTACCCGGAAACAGGCGTGGTTTGTTCAACACGTGTT
TGCTCGGTCGAATTATGACAAGAGTGAATACCGGCTACCTATTGAAACCCTTAAAGAGTT
TGTGGAATTTGCGGGCTACCGGGGGTATAAAGAATCCCGCTAGAAGATTCAGGATGAACC
GGAGATCGAAGGCAAGGATGTTGTATTCGAATTCATTCCAGGGTTTGATTAGTTCAGGGA
AGGTCAAGGTGAATGGATTGAGTACATGCTGAGCGATGGGCACCTGAAGGTCAATAACGC
CTCCACAGGCTTTGTGAAGATGCAACCGCTGTACGCGAAGATCAAAGTCCCGGGTGGTTG
GAAGACAATGAAGGAGATGACCGTCGGTACCGAGGTCATTGCAGCGGATTGTACGGTCAA
TCAGGTGACTGGTGTTTACCCACACGGTAAACAACCCATTTACCGTTTGCACTTCGAAGA
CGGGCGTTATACCGATGCAGGACTGGATCTCCTGTGGAAAGTGTTCACAGAGGAGACTCA
GGCATGGACAGTCGTTAATACCCGTTCCGTACAGACCCTGTTGGCGAAAGAGCCGGATGG
GGTATTTATCCCTTTGTGTGAGCCTGAAGACGGTCCGGAGAAGCCCTTTGTAACGGACCA
ATTGGAAGGTTCCCGCCAACAACGGTTGGAACACCTACGACGACTCATCGATGAGAAAGG
GTATGTGCGTGGTGACGGTAGTCTGTCTTTTTCCAGTGAGGATGAGGTGGAAAGCACTAC
GGTTCAATACCTGGTTAGGAGTCTAGGGGGTATTGCCCGAAAGGTACCGTCTACAGGACT
GTACCGCCGCAGGCAATACCGGGTGTACATCAAGCACCCTCGTCCTGAGGAACTCTTTAC
CCTGACGAATAAGGGTGGCTCCCTGACATCAAAGTCGGGTAACCAGTCCCTTAAGCTCCG
GGTGAACCGCATTGAATTCATTGGGGAACATGATGCCCAGTGCATCTCTGTAGCACACCC
GGATCGTCTGTACATCACGGATGACTTCATCGTCACCCACAACACCTATATGGCCATCCA
CAGCATGGTGAAGATTGGTAAACGGACCTTAATCACCATGCAACCCCGTTACCCGATCAA
TTGGGTACGGGAACTCAACAAGATCCTTAAGCTCATTCCAGGTGATCTCTTAATCTGGGA
GAATACCCTGGGATCGTTGTATGAGTGTTTAGAAGACGGGAAGTTTGATCCTAAGGTCAT
TATTATTCCGATGTCACGTATTGAGGTCTTCCTCCCTAAAGGGAAAGAGACCCGAGACGG
GTTACACATGGATGATTTGATCAAACGGATCAATCCGGGATTAAGGATGATTAATGAAGG
GTACGAAGCGATTCATCAGATCTTCTTGTCCTTAATGTTTGGGAATATCAAGAAACTCTT
CCTGCTCGCAGCCACCTTGAAAGCAGACGATCCCTTTACGAACAAGATGTACCAGTACTT
GTTCCCTCAACGGTTACGGTTAAAGGAGGCGGAACCCGAGCATTACATCGATATAGTGGC
GTACCTGTATCGCCTCAACACCCGTCGTTACCAACTCAAGACGGAGCAGTTTGGCCCGTA
TAACGATAAGACGGTTGAAAAGAGCATCTTAAAGTCGGGTGTACTCCTGGCATTCTACTT

TAAATTGGTCAATAAGGCCTTTAAGGAGTATTACCTCAATGTGAGGCGTGAAGGAACGAA
GTGTTTGTTCTTCTTCTCCAGGGTCAGTATGTGCGATGCCATGTTGGAACTGTTCAGGAA
AGAATATCCCGGCTGGGATTTCGAAACCTTTACTGGGGACGATTCCAAGCTGAAAGATAA
GAAGGACCAGTTCTTAAAGCACGAGATCATCATTACAACTCCGAACAGTAGTGGACCAGG
TAAGCATATCGGGGGGTTGGTGACGATGATCTGCGCCCACACGGTTGCGTCGACTCAGGC
TAATAAGCAGATCATTGGACGACTCAGGGCGTTGTCGGCTAAGTTCAATAATGAGAGCGA
CCCTGCCTTTGTGTTCCTGGTGTGTTTGGACTTAGCGAAGCACGTGGAATATTTAGCCAA
ACGGGAATAGGTGTTCTTTGAGAAACAGAAGACCTTCAAGCGTATCAATTCGGAATGCTC
CCTGGACTGAGGTCCGAGGGGGTTTCCCTTTATGTCGGGGTACCGCTACTATGCCGCTAT
TCAAACGTCAGCCCATGCTGATCATTTCATGCCCTAACATCGGACAGATCAGCTGGGTAG
CCACCTAGTTGGTCGAGGACTCCCTTTACCACATCTTTGCGGATGGTTCACTCGTTGAAG
ATGATATTCTGGGAGAATGGTTAGATACCGAAACCGACAACATCCTTGTTGATGAAGAGG
GACAGGTTAAAATGTCCCCCCTACTGCACGAGCAGGAATATTGGAACCTGGTTACGGATC
CTCACGCCATGACCGAGTTTTACATGGACGAAGCCCTCGGTATTCTGGATCACCTTTTCC
AGTTAAATAAGGACACGCTCGCAGAGGTGTCAAATACGCTATCTGTTGAACGTTACTGGG
TAACCCGACATCTCCCTAAGCATAGGCTCATAAAACTCTATGGAGACCCTAAATGACCTA
TTCCTGCAACCCTCACAGCCGTACTGTTAAGTCCCAGAACATCAGAACCCCCGAAATGGA
GTTTTTGCGTCAGAAATTCATCCTGAAGATTCTGGTAACCGAACGGGTTCGATGCTTCAT
GGATGAAGTGACCTTTGATATGTCGGGTGAATTTATCCGTAAGTTCATTACAGTGCCTCC
GGAGTATACGGAACCTGAAACCATCGAACAGTACATCCAAACCGAGGTCCTGGACATCAT
GGAAGCGTTTAAGCAGTATGAGGAAAACGCAGTGTACCAGGAGGATTTAAAAGCCCTGTT
GCCGTATAAAGGCCATCGGTGGAATCTGGTGTCCTCAACAGACTCCGGAGTCCTGATTAC
AGTCGAGGCGTAGGGTGTATGAAGTCTGTTATCATTAACCTCGATCCAGATACCCGAGCG
GTGCTGTATGCGTATGTTTCCAATACACTCATGGATTTCATCCTGATAGAAGCCTTAACG
ATCGCTTTAAAGGACTATTCGTCATCCGTAGAAACGCTCCAAGACGCAATAGCGGATCCT
GAATACTACAAAAATGATCCGGATACGAACATTGAACAGTCAATTGAATTACTGCTCGAT
CTACTGAAAGAGCTAGACCGACAGGACGGAGAAGATGTTGAGTCTCCCTTCTACGACGTG
TCCAGTTATTCGGATTGGCTCAGGGATAAACTGTGTTTAGCCATTAGCTCCATAACAGAT
GCTAATAGCCCCTTGGATTTCATGCTGTTGAATGATGTCCTCACTCTTCCTGAACTCAGT
AAACAGTTCATCTTCCAAGGGAAACACCTGAATAACCGTGTTGTCATGTTGCTAACCGTA
GTCGATGACCATCTCTAGACAGTACACATATACTACCTCCCTTCCGCAAGGAAGGGAGGT
AGTAGTCGTTTATTTATTTATTCCAATGCCATCTCCAAGTCTACGAGGTTCGTACGGCTG
CCCGCTCTGTGGTGTGCCTTCATGCGCTGCAATAAAAATGCAGTAGGAATCAAGACCACC
TCAGAGCTGAGACGATGCCCTAAAGCCCCTAGACCTTCCCCACAACAGGTCTGACAATAG
TAACCGTCCTTTAGGGTACAGAACTGAGGGACTCTGGCTTGTACGGGTTTATTAACGTAA
CGATCCTGGTTTTCAGCGGTGAGCTGTATCAGGACTTTCCGATCCTGGATCCACGTTCCT
ACCCAGTATCCTTTATTGGTTGGGAGGATCGTAACGGTTTCGGTCACCGGGGTACCGCAA
TCCACGACATCCTCGCTAACGGCCGTACGGCCTATTAGACGCAACGTTTCCTTAACCCTA
GCCCCACCTTCACCGGTGGACATAGAACGGCTGTAAGAGCCCTCTACACCCGTGTTAATG
TAGTCTGCCAAGAAGTTCGGATCCCAACGCTCCGCCAGGGAGTTAGGGAGCAGTACCCAA
CCGTCTCCTGAGGCATTGGATTCAATACCGAAGGAAATGAACATCCGTTCACGCGAGTTG
TCAATAAACTTCTTTGCAATAAAGAACTTACGGCTAGGACCAGACATTACGATCTTCATG
TCCAGCGCCACACACTGATCAATAATCGAGGTAGCAACAACCGGATCATTGAGTTCGTCT
TTATGCTCTTTGAAGAGACGATCCCTGACTTTAATGACTTCAGGACTTACCGTTAGGGCA
TCTACTCCACCCGGTTTAATGAAGTGAGACCCTAACCCTTCCAGGAACTGCCAGTTATGG
GAGAACTTCAAACACTGGTCTACACTGGCCTTCCCTGCAGGTACCGCTTCCCCCGGTGGG
GGATTATACACCATGACAGTTTCCAAGATGTTCAAGATAACCGGTTTGGTGGACTCCTGG
TTAAGGTAGAGTTGGGTCTTCCCGAAGCATTCCCAGAAAAGGATAACGTTGAAGAGTAAG
AGTTCAAAGGTAGTCGTGACCGCTTTAACTTGCAACACTGGACGGAAGTCTCCCGGGGGT
ACCAGGATTTCATCCATGTAGAACAGGGGTTCATTCACCTCCCCATTTACGGTATGGGTA
GAACCCTCTACCTCGACATTGAACCGATTATCCTCCACAAAGACAGCATAGGGCTATTTC
TTGAACTGTCCAGAGGAATCATCGTCTTCAAACTGGATAGTAATAATCGATTGAATAGCC
GCCTTTTCGTTATAGGCTCGGTTATTCAGAAAGAGTTTAAGGTAGTCGAGTTTAGTCATG
GGAGTTCCACTCGCTGAATAAGGTCTTCAATACGGGTCAACGCCAGAGGGTCTTCAATGA
CCGGATAGAGGTATTGGGTCATCTTATCCTTAAGCCATTCGTTATTGATTGCAGACACCA
GGAAGAACCCAATAACCTCCTTGGCATACTGAAGGGTACTGTCTTCCGTTTGTTGAGCCA
GGAGAACTTCCAAGTTACGACTGTAGAAACTCAGGAAGCTCTTTACACGCCCTCCCAGTT

GTCCGTTCTTCTTAACGTGTTCAAAGGCCAGAGTTCCTTCAATAAGGGTAGCATTCGCCC
GAATACGCTGTTCAATAGGGGTGCGAACGTTCTCAATCTCCTCGGGGTTAAAGAGAGCGT
CCTTAATGGCTTTAAGGGTGACTTCTGAAACATCCTCAATAAAGTTTTCGTACTCTGACA
GATCGAGGTCTTCACCCAGGTAGAGTTCCATCACCAGGAACAGTCGGTTCACAGGGGGGA
TGTCATACGCCTCCAAGACGTGTTTAAGACCAATCAGGTCTTCGTAGTCCGGCAACTCCA
TAAAGAACGGCAACAACCGAGTTAAAGCAGGGAGGTTGGCTTCACCCAGGAAATCGTGAT
TGATAACGAACCCAAGCTGTTCGAGCACATCGATAATATTGGTCGTGAGGATCAAATACA
CCCGATGCTTCTTAGCCGGGATTTCCAAAGCTTCATTAATCAACAGATCAGTCAGTTGAC
TGGGCATCAAGGACTCAGGTTGACTGAGCCTAATTAAATTAAACGATTCATCCAATTCTT
TAGCCTGATTGGCGGGTATGGCGTTCCGGAGTTCTGACCAGAGACCCCCTACCATGGTGT
CAGCCAGGTACGCTCCCTCTTCCAGGGTAACAGATTCTTCACGCTCAATAATCATTGGGT
TAACTCTCGTAAAGTGCGTTTCCAAAGGGAATCAAAGCCAATGTCGACTAAACAGCAACA
GCGTCGGGTTAAGAAACTCAATAAACGCAAGCAAGTGAGTAAGCAAAAACAACGGGCTAA
GAACATCCAGGACGCTTACCTCCGACAGGACTTTGGTAAAGGCTACCAGGGGGAGGCCCT
GAGTAAGGATGCCTTGTACGATAAGGTCAGGGGTGAAAACAAGGCCTTCTCGGTACGCGA
CCTTACTAATAACCGTCAACGTCTGGCTGAGCTGGCTGAACAGATGAAGCTGACTTGAGA
TGAGGACATCCCTCAGCTCTTCAGTGGGGAAGAACTCCTCAACGCTATCAACGACATGAT
TACCGTCACCATTAAGCTCCACAGTGGGGTGATCGTGTACAACCGTCTGGTTGAAGAACG
CCGCTTTGATATCAGTGAGGATGACCGTGCCGTACTGGAGAACTACGAACGCAAAGTGGT
GGAGTTAGTGGAAGATGTCTCAGTCGTCACGACCCTGCATGAAGCAGGTCAACAAGCCGA
GGATTACTTTGAAGTCGTCCTTAACCTGGCCGATGTCATGGCAGCCCTCAGTGAGGAGTT
TGGGGAACCGGTTCTGGATCTCATTACCCAACAGGGTGATCTGATCGAAGCCTATGCCGT
TGAACACAAACCTGAAGGACTGTCTGTCTACGATTACATGTGTCAACTTCATAGTGAATG
TATTACGGTTATCCAACAGTTGTACGGTGAGAGCCGGCTGAATGACGGCTTTAACGAATT
GCACACCCTGGAGCAGGAACTCCCAGATTCCCCTGAAGAGATCCTAGGTGATAACCTCCC
CTACCCTGAGAAGGAAGAGGTTGGCGAATTCATCGCTATTTCCAACGATCCTGTCATTAA
TCCCCAGTAAGGGACTGTTTGTATGTCTGAGAATGTAACCCCGAACGAAAACCCTGAACT
GACTCCCCCTTCGGAGTTGCGCAAAGCTGTTGAACAAACGATTCCCAAAACGGAGCCTTC
CAGCCACTTTGGCTTCATGGATACCCACACGCCTCAGAATACACCCATGAAGGAGGTCCT
GGAACCGCTCAACCCCATTACCAAAGAACTGGTTGAAGACGGTCGAGCTAAGGGGTTCAC
CAGTATCTTCCTGGGGGTTGGTAAAGAAGAGAATGAGAAAGCGGCTGAACTGATTACCAA
CTGGAATGCGTGGAACACGTTTAAAGAATTGAATAAGGTAGGGGAGGTGTCCCCAACAGT
CTTTGCATCAGCTAAGCAAGACTGGGATGCCTTTGTTCAAGAGAACTACCCGGATGTGAA
TCCCTATGAGTTGGAGAAGACCGCTAGTGATCTGTTCAAGTTCATGAACAGTCTTCAGGA
GATCGTCCAACTTCGTACCCGCATGCTGCGGGAAGAGAATATTACTAACGCCTCGCGCCG
GTTTGATAAACTCACCATGACCGATATCGTTGGTAAAACCCCGTCGGTGAATAAGAAAGG
GTTGAGTATTGCAGATCGCATGATTCGCTCGACCATTCGTTCATCGGGGGGTGTATACCA
GTATAACCAACAGCTGCGTAATTCGTTCCTGAGTATTACGTGGGAACGTCCTCAGAAGCT
GGAAGTCGCCGATCTCATCAACAACATTAACCGCCGTATTCGGGGGTATGTACGACGGGT
CAGTGCGAACGCCATGGCGTTGTCTTACATCGCGGGATATATTGAGATCTGGGATTGGTT
GAAGCCCCCCTTGGTCAGTAGCAGTGTCAAAGGCCTAGCGGATTTCGATGACCTCATCAA
GGTGATCCGTATTACGGACATGCCGGTGATCTGTGGAGGTCTCTTGGCGTCGATCACGGA
TGACGGCGTCCAAATGGACCTCCGCTGCATGACCCCCAGCTGTGACTGGTATCAGTTCGA
TCTCATTGATCCGACTAAGTTGGTGCAGCGTCGTCCGTCGATTGAAACCGATGAAGAAGC
GGCCTTCTTTGGTAAAATCTTCAGTGGGCGTTAACAACACACCGTCGATGAAATCCTCAA
GATGATCGAACAGTCCACCTACGGGATGGACTATCGGCACGTGCACAACGAGGATCAATC
CATTCGTCTGAAAGTAGGACCTCCCAGTCTGTCGGATGCCTTCGCTACCTTCGACTATTT
CGTAGGACGTGTAGATAAGCAGTTGGCGGATATTAGCAACAGAGTCAACACGGAACAAGA
CCTGGAAGAGCAACGTCTCCTTCTGCTTAACAGCTTGCGTGGGGCGGAGTACATGCACTG
GGTGGAAGAGTTCGCTATTATGCCACCTCCGGGTGAGGACAGTGAACCCCAGGTCATTAA
GCGAGCCAAGGAAGACCCTAACTAGTTCAATCCAGGTCTTATGGACTCCCTGCTGACCGA
TGAGGTGATGAATAAGAACTTGGTGTTGTTCGTCTACAATAGAGCGCCTTATCTGACCCG
GACCTTTATTGGTGTCCGTAACTTCGTGTGTCCGAAGTGCGGTACGGAGTCGGATGCTCA
ACAAGAGGATCGTAAACGCGGGTATACCCCGATCGATGCCTTTATGAGTTTTTTATCCA
TTCCCAGCTGATGTTGATGAATCTGGCGATGGAGCGTCAAAAGGTAAACAACGAAGCCCT
CTCCTGATTGGCGGTAAAGCGATTCGCAATCCGGAGTTCTCGGCGGTTTACTTAAACCTC
CAGAAGCAAGATCTCAGTGGTCTCAAGGAAGAGACTATCCTGTCGAACAACCAAGCGTTG

CATGACATCAACATGGGATATGTCTCACCCCGTAATCTCCCCCCTCACCAATCGATTAAG
TTATTTGCGTCTGAAGTCATGTATTACCACCCCTTCTCGTATGAGAGTATAGGGGAGTAT
TACGGGACTTTTCGGCTGGATGAATACCTGCCCTTAAGGGATTACTTCGAATTGCGCGCT
AACAGTGCAGACACGTTGGTTAAGGCCGTTCAAAAGGGACGGGAAAAACGGTTGGTAATG
GAGAAAGAGCATCTGAAGGAAGAGGAAGAGAAACAGGCGAGGAATGGGGGTAAACGAACC
GCCTCGGAGACGGTTACTATGGATGCATTACTGGATCAATTGTCTAAACTTCGTTAAGAG
AATGGGTAACCTGGGGGCCTAGAGCCCCCAGGTTATCTTATGACATACATGGTGACACCT
CGTCTCTATATCGCTGTCTAACTGAAAAGGGCCCTCCTAATGTCCAACGATTACAAAGCA
CCTGAGGTTACTGACCTCGGTAAGAAAGAAGAAAGTCCGAAGACCCAACCTACGCCTTCC
ATCCCCAATACGGTGGCTACGGCTCAGGGTGCTAAATTCGAACCCGTACCTCTCCGTATT
GGCATTGGCCAAATGGTCAATGGCAAAGGTAATGCGGTCACGGATATCATGAAAGAACGG
ATTGTCTGTCACCTGACCTACCTGGCCGGTAAGAAAGGCTTCACCAGTGATAGTGAACGG
GAGGATGAACAGACCTCCTTTATCGAGATCATTGGTTCTACACTGACCCTTCCGTTTCCC
CAGTACGACATCGTTACTTACTACCTGTTTGCGGTTATCCGGGAGAACCGGGAAATCTTT
GCCAACGGTACGGCATTCCGCTTTACCTACAAGCTCCGTAAGGAGTACCCGATAGAGAAC
GTTAAACTCTATCACACCTACATGACCTTCCTGACGTTCATCGCGGGTAACTGCGAAGCT
CGTCATCGCCTTAACCGCATCATTGGTCTTGGAACGGCTATCCGCGATCTTCCTCGTAAG
GGCAAAGAGAACGCCACCCAGTACTTCAATAAGGTGCAAAACGTTTGATTTCCCTTTAGA
CTCTTTATCCAAGGAACGCTTCCAATGGACAGTGTAGTACTTACCGAAGAAGACGGTAAA
GGTTATGAAATCCCGGATCTCCCTCATATCGGGATTGTGTATGATTCGCTTAACGATACC
CTGGATCAAGTGGATCGGGGTGTTGCAGGTACGGAAAGCCTGACCGCCTCCCAACACTAC
CTGGTGGGTGCACTCGCTGCGAACCGACCTGGTTACTCGGTGACCGGTAACGAAGGCTTT
ATGGCCTATATCGGTGCAGGTTTTAAAGCCGCGTATGACTGCGTGGTAAAGATGTTCAAG
AGCTTCTTGGACTTCTCCTTCAAGCGTGATGTCTCTCAGAAGACCTCGGATGCTAAGAAG
GAGATTGATGACCTCCAGACAAAACTGGAGGTGGTTCAGACCGGGGGTCAGACGGAAGCG
GAAACCCAGGAAGCCCTGCACTCCATGAAGGTCCAGGTTAAGGAACAGGCACAATCTGGC
TCTAACATCGACGGGGTAGACGCAGAAGAATGGGTGGAGAAAGAGCCGAAGGCCTCCGCT
ACCCATGAGGAGAAGAAAGTCGCGGTTAAGACCCTGGCCAAAGAGCTTCCTAAGGTCAAC
ACCCGGGGCAAGAAACGCCTCCAAGGTGTCATTGAGCGGATGATGAAAACTCAGCAATAT
TTGGTGAGGGTGATTGATATCGCCGATAAAGCAGCTCGGGAAACCCTGAATGAAAACCTC
ATGCGGGTAACGGACTCTATGAAACTCCTCCGCACCAATGTTCATCAGTCTACTGAGCAA
CTGAGTCGAGTGAAAGACGTCAAAGACCTGACGGCTGTACACAACGTCATGAACGGTGTT
ATTAAAGAGATCGATACCTTTAAGAATACTGCTCAGGCCGTGCAGAGTGTTCGCGGCAAA
ATGGCTGACAAGATTAAGATCGTTGAACAGCAATTGGCCGAGGTGGCTAAAGGTGGAGGT
GACACCAAAACGACTCAAGCAGCGCTGGATGAACTCCGCAAGATCATGAACTTCCTGGTT
AAATGCGGTAAATGTATTACTAACATCATTGAAGACAATGTGAAGCTCCAGAAAGACTTT
AATGGTGTCTTCGGGCTGTAACCCTGGTAGCCCTTACTACCCCTCTCACCGTGAAGGGAG
AGGGGTAGTAAGGTCTATTATGCGGTGTCTTGCACGGGTTCTTGCGTATCGATGTTAATA
CGGGATTTACGGTTACGAGCGGTCGCTTGCATCTCCTTAAGCAGTTCCTCTCGACCTGTT
TCCACCATTAACCGTTCAAACTCATCCTTCAATTGATCGAATTGAGGGAATCTAAACAAA
TCATGGCATAATCCAATGGCGTTCGCTTCTGCACTGCTCGCAAAGTTAACCCCAATCTGC
CTGAACTTATTGCCGTCAGCGCTTTGAATCCGAAACACTTCCATTTGTGTGGTACGATTC
CCAACGTTCATTGCCAAGGTCGCGTGAGGGTACGCACCTTCAACATCGATATCGGACCAA
TCTGTACGCCCAGTGCTGTATACATCAGCGAGTCCATGGAAGAGCTGAGCGCCGACATCC
GCATTCTTCTCGGTATTGAGTAACGCAATCCAGTTTGACAACGGAGGCAGTTTATCAATA
ATGGATTGATCGCGTTTACGAGGGGTACTCCCCCCCACAAACCCATACCGGCGTGCAAAG
AAACTCAAGGTATCACTGACCAACTTGGGTTGAGAGACGTAGTTAAAATACTCCGAATAC
TTCAACAACATAGGAAGTGACAATGACAAGTCATTGGTCTTCTCATTGATCTCTTCAATA
ACGAAGTCATCCCCAATGTTGTACATGCAATACAGATACCGCCATTTCCGTTGCATGTAA
CGGTGCCACTGAGGCGTACCCGGTCGGATATGAGCACCTTCTTCGGTATACAGTTTACCC
GGTACGTTCACATGCTTCGCTGCTCCTTCTAACGAAACATCAGGAACTTTACCTTCGGAC
GGTTGACGCTTAATGCGGAAGAACGACATCGCGTGTAACCATGGCCAGGTGGCCATGGTT
CGAACAGTGGGCCATTTCTGTTGAGGTTCCAAGGGCATTTTAGAACCGTCTTCCTTGACT
TTGTGGGTTGTCCCTGAGTTCAGCTCGTAATACCGAAATTCCTTAGGAATACTGGGATCG
CAATACACATCCTCCAATCGTTACCCTTCATTCCTAAGGGCTTCCTCATTCTTGGTCATG
TCGTATTCAGCATTCCAACTGGCGACCCAGTCCGGTTGCCATTCGTGCATCTTGTTGATA
CACGCCACCACCACTAGACCTGGGGTATCAAAGAGTTGGTATTGAACCACACACAGACGC

CGTTTAAGGGGTTCACTCAACCACTTGCCTTCAGCTTTTTTGAGGTGCTGAAGAATCGTC
GCGTCATCCGGTTCTTTAAACCAACTCCGTACGGCTGCAAAATACGCCTTACGCTTCATG
GTAACCGAGGCCATCATAATGGGGTTACCGTGAGCATCCATGTCGGTTTCAACGTCGTAC
GCTGCTACGGTAAAGGGTTCCTTCTCCTGATGTTCTCCATATTTCTTAAAGAACTGGTGT
TTAATGTGCACCGGAGGGGTTTGGTCTAAGCCGAAGACGTAAGGACTTTCTTTAGCTTCC
GCTAGACTGGCTTTATAATCTGCTCGCCCATAGAGCTGTTAAATCACATTAAAGGCAATC
TGTGCCCGGGGAGCCTTGTACTCCCGACACCAGGATTCTTCAATGTAATCTTTATGCTGT
TTGTACTTCCGTTTGTTCTCTTTAACAATGTAGTACGGTTGCTTATAATCCTTAATCGAA
ATAAACGATTGACTTCGACCACCATCTTCATGAATATTGGTGATCTTTACACACGTTAAA
TCACTGCCCCTGTTGAACCTGCTGACCGAGTACTTACTGTGTTTGCAGACTTTAGCGACA
ATGGCTGAAGTATTCTCGACCATTCCTTTACCCTGCATTTTATGATAGCTTTGGACACTA
TACCAATAGGGGTATAGGTCGGTATTTACAGTAGGAGTAACCCGTTAATGTTGGACATGA
GTTTTATTACCCGAAAGGTAGCGGGTACGGAGTTCATACAGTTCGAGAATGCCACCTTTA
AAGACGAATTAACCGCTTGTTTGGATCAGTACATAACGATTACTGATAAGCGTGTGGTAT
TATCCCCTGAAAACCAAGTGGACGTACTGGGACTCATTAAGAAACACACCGGCTTTAGCA
ACATAAAGCTGGAGTTGAACGACAGTGGTAACCTGTATCTCGACGCGGGGTATTTCTCCC
CGTACCATGTTCTGAACAACACGGGTACTGATGTGTTGAGTCCAGCCTCTAAGCCTACCC
TGTATCGGTGGTTTGTGGAGAACAAGAACAAGATCTTTAAAAGAGGTGTTGATTACCGCT
CCGGTAAAGTGCCTGGTTCGTTCCAAGACGTCCTCTTGACTATTGTGATCAATGTCAACG
TGCATCAGTTCTTCCCGTTGGATAAGATCACCAAATGGAAAGTCTCCATGGGGGGCGCGC
TAGCGGGTGGTATTGCTCACGAACTGGGACATGCCTTTGGGGGGGGTGAGCTCCTGCTGA
CTCACCTGGAAGACAACCTGCTAGCCCGAGCGGGCTTGGATCAGTACCGTAAAGCCACCA
AAGCAGAAGAACGGGTGATTATCCGGAAAGACATCGCTGCTATTCTGGATGTCCCAGAAG
CCAAGTTGGAGGAACTGAAGAGCTTTGCCGCCCAAGAAGATGGAGAGAACTACCTCCTCT
ACTACACCGAACTTCTCTCTCAACGGAATAACCGTCGTCCCCTGTCAGTTGTGGTTCCTG
AGATGACCAGTGAAGTGATTGCGGATATGTACGCCATTCGTATGGGCTTCAGTAAGGAAG
TGATGGCTGGGATCAGCGCTTTGGTGGACCTTGGTGTTTTTACCATCGCGACGGAGACCT
TACTGTTCGGTGCCGTTGCAACACTCTTCTTGTTTTGGCCTACAGCCGCTATCACTATCA
TTGTTACCGGGGGAACGACGGCGCCGGGGGGTATGTTGTGGGGGGTCTTTGCCCTTAGTT
CGGCCTTCGCGTATTTCCAAGCCGGGTATTCCGGAGTCTACAATTCCGACCACCGCCGTA
TGGAAGATGCTCTGCGTCAATTGATTGCTAAACTGAAAGCCAGTAAAACTGTTTCCAGTG
AGGAACGGGTACGGTTGATTGACGACTTCACCAAAGCCCTGGAAGCGGTTAAGAAACTTA
AACCCTGGTACGACAACACTGTCCTCTACCGCTTTATTGGTTGGGTTTTCTCAGGCAGCG
ATTTCCGTAAGCAAGAAATCGAACACTTTACTCAATCCCTTAATAACCATGAGCTAGCAG
TCTTCTCCAGTCAAGTGAAAACGCTCGAAGGAGTTTATAATGTCTCATCTCGCCCATGTA
AACGATTACCGGGCTTACCTGATCAGTCAAGGGGTTAGGTGCTCGTATGCGCTGACCACG
GCCTGCCAGGAAGCCTACATCCGCGCCATTCTCAAACGCCATGCTCGTTCCCAGGACAGT
TTCACCCTGGCAGAAGACGAAGAGAATAGCATCTGGGTGCAGCTCGATAAGTGGAATTCG
GTGTATCCGATCAATGCTTCCTTCATCGTGGAATCGGTCAAACTCCGTCTGACCCGTATT
CGCGGCTGTCTGATGAAAGAAGGTAAAACACCCGGTAATGCGGAATTCGATGTGTATCTG
GATGTGAAGCGAACCTGTCCCGAGTCGGGTGACACCACGGTGGATAAACTCGATGCCACA
GACAATGATCTGTTCTGTGAGCAGGTTCATTTCCTCTACTATGAATTGGTGAAAGGCAAT
GAGTAATCCAGTGAGTTGGTATTTCCAATTGAGTGATCCGGTTGACGGCGTGCTCCCAGG
TGATGATAAACAACTCATTGACCTGGATGAGTACTACGCAGAAACCGGTCAACCCGTGGT
GGATGCGATGGTCCAAGGGAGTGAGAGCCTCGCGCTCAATCGCTTCTCTCGGGACATTGC
TATCCAACTCAAACTGAAGAATGCGTATGAAATGGATCCTTTCCCAACGGAACGTAATGC
CGTGATGGGAGCGGAAGGGTTTATCTCTAGCATTACGGAAGGGTTCAAGAAGTTCATTGA
AGGGATCATCAAGTACATCAAGATGGCCTTTAAATGGGTTCTGGAACACATTAAAGGTCT
CCTGGGCTTCCGTAAGAGCGCGCGTATTAACCAGGCGATTAATGACTCCTTTGAGAACCA
CCGGGAGGAGTTCCGTTAGACTTTTAACGGTCTGGGCTTCCCAAGTGCGAATTACTACCT
GTAGAAGTTCATTGGGGAGTTCCCCAATGCCACCGACCGCATTGGTCAACTGACGAACCT
CCGTACAAAGTTCGAAACCGATGAAGAGAGTATCGAGGCTCTTCGTAAATTCTTCCCACT
CTTCGAACAAATCATTGGTAAACTAACGGAGGCTTCTGACAAGGCAATACGTGCGCCTGA
AAACCTTAAAAGGAACATCCGTAACGAATACAACGCTACCCGGGGTCGGGCTCGCCTCCG
AGAGTGCGTAGACGGTGCGAACTCCCCTGAGGTTATCCGAGTCCAGAAAGCGGTGTTGGA
AACCCTGACCTTCGTTAAACCGGATGGTTTAGTTGAACAGATGGGTCAGTTCCTGGAAAC
CCTCTACAGGGTGAAGTTCGAAAACCAGGCGTTGAAGGAAGGGTTCCATACCGTCCGAGA

GACCCTTAAAGAGAACATTCAGGCTCAACGGGTGAAGTTGACTCGGGTGAACAGTCCGGT
GATCATGGATCTCGTTCAGCAGCTCAATGGAGCGTATCTGACGATCTCGGATAAGGCGAT
CGACATGTCTCGGATTCGTCTGAGTGAGTTGGGTAATCTGGTAGATGGAACCGATGCTGA
GAAGGTCAAGAGCATAGCGGGATACTACCAAGTCCCTGGACTCCTCAGTGATTACCAGGG
CGTCGCGGTTGCCATGCGTAACTTCAGCCAATATTGCCAATCGGTAGCCACTCAGCTGTT
GGCGGTTGAAAACCAAGTAGCAGCGATGGTGGGTTGGTATCACCGTTTTCACATGTGGTA
TTACCATGGTCTGCTGGATGACATTGAAAAGCTTCGTGAAATCAATATCCAAGCTCGCGA
GCCGGGGCACTCTCCTGTGGCGAATGCGGAGGGTTATCCGACATTGACGTTGGATTTTAT
TCCGGAAGCGGATGCAAAGACCTTCCTGGAGAATATGAGTGGGACGATGAGAATCGTGAT
TGAAAACGACATTGGTGAGCTTCGTTCCTAGTACAATGCCTTGGCACGACAAACTGGATG
GGGAAGACCCGTATGAATCGTGAAGTGTCTGAACTGGGTATTAAAGCCCTGCACGAACTG
GATTTGATCCGTAAGACCAGTGAGGACGACCTCGCTCTGCTGAAAGAAGGGATAGGACCT
GAAGCCGCTTATGTCATCATTGACCGGTACTGGTCGGGTGAACAGGCGGAAAAAATGAAG
GTTCGTGTTACGGCTCAGTTCCCTAAGGACAACGGGAATATTATCGTCAGCACGTCGTTG
GGTGAACAGGCTGGTTATATCCTGGCTCTGGGGGCTACCAAGGCCTTCTTTGCAGTGGCG
TTGCCCAGGATCGGGGCTCGACTGGAACAGGTGTTCTCGTCCATTAACCAAGTAGACCTG
AATGACCCCGTCGCCCTGGAGAAGTACAACGAACTCCGTAAGGAACCAGCCCGGTTCTTT
GAGTACTTTGCTAACGGGTTTTTCGAAGGGAAAGAAAAGGTTGCAGACGGTGAGATTACC
TGTTGGACTCAACTGCGTGATCACGCCCGTTGTGTGGTTGACGGATATTACCTCTTCCTG
GATGACTATGACTTCAATTTGAAGCAGGTTAAGGAGAACTTCTACCAGTAGTACCTCTCA
CCCTCTTCGATCAAAGAGCAATCTCTGGCCGGTATCGATTACTACACCCAACAACTTGGT
GTTCGGGTGCGGTTGGCTAAGTTGTTCTCGTGTTTCTTGGACGAGTTGATTGAGGGTAAT
CTTCCCTCTAAAGACACCGAGTAACGTAAAGGTGCTCCTACCCACTCCCTTAACGGGGAG
TTGGTAGGGGTATACGTCTTAAATGGTGGTCGTCATTCTGACCTCATGGGGTTGGAAGAT
GATATCAATGGCTTCCTGTATCGATACCAATCCATCGCTGGTTAGGGCGAGTCGTTTACG
GATGCTGAAACCAGTGAGGTCATCCAAGTTACTGATGACATCCACGGTACTGTCGCCCGC
AAACGCACTCAACTTAACACTCACCACCTCTTCACTCACATTCTCCTTTAAGGCTTCTGC
AAGGTCTAAGGTACTGACTGTTGTCTTATCAAACAACGCCATATTGCGTTGGCGAGGAGT
GCTCTCCTCCAAGGCGGCGCGGAGGTTGGGGTGACGGTAGCTATTTTCCGCTAGGTAGAA
GGTGACCGAGAATGCCAGATCATGCCGAAGGGTGGATTCATAGTTACTGTTGATCACCAC
CCGCTGATACCCCAGCTTACTCCGAGGTTGATAGAACAAGTCAGTTTGGTCAATCGCTTC
CGAGCTAAAGGCCAGCATGTCTTGGTTAATACCGTTGACAAAGAAGTCCTTCGTGTATTT
AGCAAATTGGATATCTTATACGTCTTTGCTGTACTGATAGTTCGCATCGAAGGCAATGAA
GTCCCAATAGTACTTCAGTTTACGGGGTTCCAACTCAACCGGAACACCATTCTCGTAAAC
GATATCAGTCCCCCTCCGGTACAACAACCGCATTGTACCATCCCGGTTATAGACGATGTC
ACCCGCCTTATGTTCGATCACTGGTTTCCCTTCACTGTCGAACACAATCTGTCCATTCTC
ACGTTTCACCTGGGTAGTATCATACCGTTCCGGGACATCCGCTTCGTAGTGGTTGTACTG
GGCTTCACCCACCAGGGGACGAATCCGACTGTACAGGTTACCCAACCGACGACCGAACTC
AACTTTGTAATGGGTTTCAATCACCCCCAACATCGGGATATCAAAGATGGTCTCATCAAT
CTTGTCATCGATTTGAGACTGAGTCACCTCTTTATCCCCCGCTCGGGTAAAGATAAAGGT
GACATCTGTGGTCAGTTCACTACCGGTCTTACTCTGCTGACTTCCAAACAGCCAGAAGTT
AGTAAAGTACAAAACGTCTGCCACATCCACATCAAACTGGGTGTTTAAGGTGAACTCCCA
GACCCGTTCCTTATTGGGTAACACTCCCACATGTTTACCTGCTACACTGGCCAGGCTGTT
ACTGTCCAGTGGGTTAATGGACAGTTGGACCCCTACACTGGTATTCGTCAATTCCTTATA
CGAATCCCCCGATTGAGTCACCAAGGTGATGAGATACCCGGTTTCTCGGTGTTCAATGGT
GATTTGTCCTACGCCCAGTTCCAGACCCAACGCCGCGTCATAGTCCACAAAGGTTTGATA
CCGAATGATCGGAGAGTCCAAATGATAGGTCCTGAGGACGGCTGGCTTGTTCGTGGTATC
CAGTACGTACTAGAAAGGGGTATAGACCAAGGTATTCTCCGCCACCATGTCCACCTGCTG
TTCAGGGGTCATGTTGAAATAGGCATCCCGAGTCAACTTGTTCACCAGAACACTGGTGGG
TTTATTGATGTTGAACAACACAGTGTTAGGAATAGTCACCCGTTCCCCGTCGTCCAATAC
CACCCACCTAGCTACCAGGTCTTCGACTGACGCTAAATAGCTCCCGACGTAACACGCCAT
CGGTGCGTCGAAGCCTTTGTTATCCTGAATAGGCAATTCCTTCGTTAATGCAAAGGAACA
CTTCGTCATGTAGTCAATGACCTTAACCGAACTGTACCCGCGTTTCTCCGCCGCCCCTTT
CAGGTTGTTTTCGGTAATCGGGAGGGTGCGATGTTGGCGACCGTCGATCACCCGTTGTTT
AATTTCCGTAAAGGGAATGGCATCTGTACCGCCCGAGACAATCGCATCCATCTTCCAGGC
AACACCCCACGCATTCACCAGAGGGACTGAATAGGGATCCAACTTATCAGACCCATACCG
ATAGTCCCGGTAGGCGGGTTTAATGTCCATCGGGGACACCGTGGTAAGGTCTTTGGTTAA

CGCCCCTTTGGTGGTATATGTGTAGATGTTGATAATCCCCCGGCCAATGTCAATGCCTGA
CGTGATGTACACATCTGGGATCTCATACGAGAACCGTTTCTTCACGGTCTCGAGACTGAG
GGCTAGGGTTACATTCAACGGATCAAAGACATCTTGGTAGTAGGACACCCGGCTCTCTTT
CAGTTCCCCATTCCGTTGCAACATCGCACGAACCCCATACAGGTTGTCCGTGTACTCAAT
AATCCCCCGACAGCCAGAGGCTTCGTTAGACGTGAGGTTCTCCGTGGCCTTACAGCTCAA
TTGAGCTACGGGAATCGATACCATCACATACCGACGGTCAACCACTTGGTTGAAGGTACG
GTCCAAGAGGTTCGTGGAAATAGGCATAAAGGGGTTAGGCGAGCTTTCGTCGTAAACGAT
CTTCAACCCATTCCGTTCGTTGTACTGGATCCGTATCCCATTGACGACCCAGAAGTCGTA
TCCGTGGACATTCAAAATGGGATCCTTTGGTACGAGGATCTCTTTATACACCACGGTTTT
CTTACCGACGGTTTTACGACGGTCCACAGTAATCTTCAGGAAGTTCTCAATATCCATGGT
GAGAATCATCACTGAAGTCGAGGGATTCGCAAAGAACCCAAACCGCTCTTCATCCGACAT
GTTCATGCTCAGCTCACTGACATTCCGAGCGTGCAGCAGGAACTTACGGCTGTTGACATC
CAAGAGGCGGTTAATGAAGCCATGCGACGTACCCAGGATTAAATCCATGGCCCAAACAAA
GGGGTGACTCTTACTGTTGCGTTTTACGGTCCCGTTGAACCAGTTATTCTCGACCTCATT
CACAATGAGGTTAATACCCCGAATGGGGTTGTTTGCAATACGTTCCAGTTCATCTTGACT
AATAGCCATTAGCGTTTACCCCACAATTCCAGTTCCATAGTCGCCAGATTAATCCAGGGA
TACGAGCCGTAACCCGACAAGTACCTTTCCCTGAACGGACGTTGGTAGTAATACTGATTC
CGGACCCGAGGGTGCAGGTTAGGGTTGACCCTCAACGTGGTACGGTTAAAGGCATCTGCG
ATTTGGATATTGTTAAACCGGAATCCCTGACAATCAAAGGAGATATCCAGTTCATCAAGC
CCTTGACCCCTCAGACTGTTCTGAGTACGGTCAATGGTACTGTACGCCCCCGACGGGAAG
GTCGTAGGCCACCCACCCCCACGGCAGAAGACCCATTCAATATTCCGCATGTTCTTGTTC
ATGATGAGGTGGTAAATACGGGTGGTGTAGTCCTTATAGTTCTGGAACAACGCATTGGGG
TACGGTTCCATCCCTTCATCCCCAAGGGTCACCGCTTCTATGTAGTGAACCCACACTTCG
TTCATTTACGCCACTACGTTGTGTTTAATGCCGTGATAGGTTTGTCTCAGGGTGTAGTCG
TAGTTGGCTCTCAAGACCCCATCGACCCACGCATACACTTCTTTACGGAACCCAGGCTCA
CTTTTGCCCGCCGGTAACGAGAGATCAGGAAACCCTGAACTGACTTTAAGAAAGTTCGTT
GCGGGTGGGATCCAAGGGTTCAAGGGAACCAGGGGAGTAATATCGTTCGCATTCGCCGAT
CCCCAAACCGGATCCAGTAACCCTTTTACGTACGCGTTCAAACTGTTCCGATGAGGACGG
TACCATGAGACCAATTGAGGATGAAGGATTACATTCTGATCCGACAGATTCAGCATGGGA
CGCGACATAAACGCTAAGCCTATGGTGTCGTCTGGGATGGGCGCTGACTGATTGCCAGGC
CCTAGAATACGAAATCCATGTAACAGGTTTGTCAAGGCGTTATCAAATCCAGGACCGCCA
TACTCACGGAACGCGTAATTCCACCATTCCAGGATGGTTTCCTTCGTGACTTCTCTGCTG
GGTGGCGTATTCCCAGGTGTTCTGTCCAACCAAGCGTCGTAATTATTCGGGTCTTCAGCC
ATTGTTTATTACCTTAAAGTGATCAGGGGTTTATCATGATAGGACAAGTATTGTGTCTAG
GTTCCTTCTTCTTGGACATCGCCAAGAAGCTGTATCCTGAGAGCAAGGATATCGCCTCGG
CAGCCCAAGTAACGGACCGGGCCAAACATTCATACAATGTGATTTCCACGAGCTCGGTAC
AACGTAGTGCAGCCCGCACTCTTTTGAATCCGATGGTCGCCATTGAGGGGTCCCTTATCC
ACCAGGAATACACCCCGGATCTCATGACGGTGGTGACCCTACGGGATATTGCCGCATAAC
TGACTCACCTGGCGTTGCAGAATTTCGTAGAAGTGGGGGTTAAGCTGGAGAACCTAATCG
GGAGAATCAGTCCCAACCGCGGTGGGATGATGAGTCTCTTCTCGGGACTGGAAGCGATGG
ATAACAACATCAAACCCAAAGACCCAGCTACCCCGAACCAAGTTCAGGTCAATAGCAAGA
CCTACAGTGAACTGAAGGAGTTCACCCACCTGGCGGGGGGTAAAGTGGTGAACGCCACGC
TCTACGGAGCGAATGGTAACAAGAGCGAAGTTCCCCTCACCATTCGTCAGATCCCGATTC
CGGTGGCCCATGAGAACCTGGTGACCCTCTTGTCTGCCGCTAAACCCGATGAAGGGTTGA
AGATGCGATGGATCATGACGAAGAATGGTGAGATCACCACGCCTCAGTGGTTCGTTGGCA
GTGACGACGTTAAAGCCCGTTTTAAAGCGAAAACGGGTGATACCTCCGGGTATTACAAGG
AAGCCATGAAACGGGATACCCAGAATACACTGGAAGCGATTCGTACGGGGAGCATTAGCA
TGAACTCCATGGCGAACACGGTCATCATCACTCAAGATACGGCGACTCAGCTCGAACTGG
AAATCGGTCGTAAGTTTGCGGACACGGTTAGCCGTGAGATAATCATCGAGAGTATTTCGG
CGAATACCCTCGTTATTGTGAATGATGACCGGGGTATTTTCACGTTCTACAACGATGGTC
AAACCATGCCGGAATTTTACACACGTCGAGACTTGTCCGTTAAGTCCAAGAAAGAAGGCT
CTGCGAACACGCTACAAGATCTCGTTAAACTGCTCAACGGGGGTCTCTAATGAATATCTT
TGAATACGTGAAGGGTGTTAAGACGTTCAAGAAGAACGAACTGATAACCAAGCTCACTAC
CCTCACGGTGGCCAGTGACGACATGATTTCGAACCTCGAGCGAATGGTAGCGAATAAGGT
GGATTTGAGCCATGAAATCAATCAGTGGGTGATTACCAAGAGTATTGTTCGTGAACTGGA
CAATAACGGGTACCGGGGTGTAGGCTTGGCGGGTGCTACCCGGGGATCGTGTGATACGAT
TATCAGTTTGGTCCCCGGGCTAACGAAAGGGGTGAATGGGTATAAAGACGAAGTCTGGGA

CGGTAAGCTCCTGACCTTGCGTCAAGTCAATATCCTGAACCTGATGGAACACGTGGAGTA
TTGGTTGAAATACACCAATATGGTCTACAGTGTTCTGCTGAACTTGAACAACCAGTCAGT
CACCACCCCTGAGAAGGCCTTGGATAAGGCCGACTTGAAACTGGTGAATGGGAACCTGGA
GTTCTACAAGTCGGTTACCTTGGACCCCCTGAAAGGGTCTCGTCGTCTCCTGGAGGGTCT
CGATGATTTTCCGGATGTGGAAGTCAGTGAAACCTCGTTGGCTGTTCTGGAAGCCACTGA
TCCTAAAGCCGCTCCGGATTACCTGAAGCGAGGCTTTGGGGTCCATCTGCTGAATCCGAA
GGATTGGATCGGTTTGGCGAACGTGAACCTGAACATTGCACGTATCGAGAACATGCGTCG
GGACAACCAACTCTTCGCCATGAAGATCTCGAAAGCTGTGAACATGAAGAACGGTGTCAA
CGACGCCGAAATCGATCGTCAGTTCGAGGTGCTTCAGGACGAGATCATCAAGAACGTTAA
CACCATTGAAACCATCGAGAGACGCTATGAATAGTTTTCGTCGCTCCCCCAATGTGTTTG
TGAACGCAAAGCTGACGGATGCGGAGATGACAGAGGCCTTTACCAACCTCAATCTCCTCC
GGAAGGATGCGTACAGTGATTTGGAATTGGTCATCAAAGGTCATGTGGCCATTCAAACCA
TTCTGGATGTCAGTCGGCTGGTGGGTCCCAGTTTCCGTCAGTTTATGGAAGCCAACTACG
ACAGTGGCCGTGGACCTATTGCAGGGTTGAACAAAGACATTCTTCATTACCTCAATGGGC
GTATGGGTCACCACTCGCTGATTACGTCGCTCCGTATTGAAGAGCGTAAACTCCTGAGTG
CGTCGAAAGTGCGTACAGCGACCTACCGTCAAACCCAAACCACGGGCCGTCCGCTTCCGC
TCCTGGAAGACGGCTACATCGTCCACGACTACGATCTCTATCGCCTAATGTCCGGTATCA
GTCCTCCCAATGTAGGGTGGACCTTGTTACTACTGGGCGGTGAGAATTACTACTGCTGAC
CCTTAGGTGGAGAAGAGTGCAATGAATGATGATGAAAAGACGTTGGCTGCTGAAATGGCT
GCGGTCACTGCAGTCAAATCCCAGGAGATCGTGGAAGCCGCCCGTTCCACGGCGGAACTG
TCGGAGTAGCAGAACAGCCTCGATGATCACTTCGATCGCGTGATTGAAGTCCAACGCACG
TTGAACACTCTGGAAGCCCACGAGGTGACACCGGAGTTGGCTGAACAGCTCGATAAAGAA
CTGATTGCGGCAGAAGCCCTCCCAGAGGCCGTAGAAGCGGCGGAAGCCTTGGGGTTTACC
CTTCTCCCTAAGTCATACCTTGCGACGCGTCTAGCGGGCTGAGAGACCTTCCTGAGCCAC
TTCTTTCAGAGCTTCCGGGAAGTAGTCAGTTTGATTGGAACCTTCTTCCGGGATTCGTAC
GTTCTCTTTATGGAATCCCAGGAGAGTCTGAGTAAGCAGATTGACCTGCTGGAAACTCGG
TTGTCCTCTGTCGAGAAGTTTGGCAGTCAAGGTACCTTTATCCTGGGTCACCGTCTCTTT
AACCGGTTTAAGATCAACGGTAAAGTGGATGAGAACTGGTCCGGTAACCTGACCAAGCTC
AACCATACCCTGTCGGGTCTGAGTAATAACTATTACCTCAATAGCAAGAATGCGTTGGCG
TCATCATTCAGTTACTTCGGTGGGTTTGCAGGTCTGACCCAAGTGGAAGTCGATGAACTC
CTCTGCATGATGCCGGTGAGTCTACCATCAACGCTGTTTAAGGAATGTACTTATCCGGAT
AAACGCCGTTCGGGACCTAACGTGCTGGCTAAACGGTCGGTCGAGCTGGTGGGTGGTGCG
TACTCCTACGATGTTCGCAGTACCGTTAAACCGGTGATCCTGAAGAACCCTGACGACATT
CGAGACTTTGTGATCCGGTACACTGAAATTGATCACACCGGTTTCGATAACGACGAAGAG
TATACCCTTCGGGATGTTGGGGCTGAAATGAAGGCGTTGTCGAGTGAAGACATTATGACT
GTCATCAAACTCCTGCGTCACCTCTTGAAAGACTGGGGTAAGGTCATTGAAATGGCGGAG
ACCCACCGCGTAACGGATTCGGATTACAACGACATCATTAAAGGTTTCCTGGAAGCCGAT
ATCAGTGATGAGGCGAAGACGGAGTTGTCGAAGACGTTCGCTAAAGTCGTTCGTAAGAAC
CAAACCGAGCTCTTGGGTATTCGCGCTGAGGTCTCGAACTACCTGACTCTGATTGTCAAC
GGGATGGTGTCTATCTGCAACGATTCCATTGGGCTTTACGAAGAAAGCCCGGACTAAGCG
GGGTGAGGGTATGAGCGCGGGTTATAAAAGCCAATAAGTTGATGCCCTCGAACAACACCA
GGACCTGATGCAGGCTCACTCAGAACTCCTTGAGGTGGGCAGTATCATGGGAATGGATGA
TGTCGAGGGCAGTGAGGCGTTGAAAGAAGCCGCCATGGACAGTTTGTCTTCGGTAGGTCT
GGGTTTGTTCAGTGTGAGTAAATGGGTCGGGGGTAAAGCGATTGATGGCTTTATTCATGG
TATGACGACCGCAGGTAATCAACTGAACAAGAGTTTCAATGACAACCAAGGATTTATTAA
AAATGTCCTTAAAGACGCTGGTAAGGTGGCTGACAAAGATATTCAGGTTACTGGCCAGAA
GATTGGACTGATTACCGCGGCCGGTAATCCTGACTGTATTCTCCACGACATGGAACTCCT
GGTTAAGGACCTCGAATTCGTTGAACAGCACGCCAAAGACCTGATGGGGTTCTTGGACCG
TGAATTGGTGACCTTGAGGCAGTTGAAATCGGCTAGGAAGTCCGAAGACAGTCACAAGGT
CATTGACGCGTTCAACGGGTTGGAATACCCGGTTATGAGGTTCGCTGACCGGTTGGATAA
GGCGTATAAGTCTGAGGGGCTACCGGGTGGTCGTTTCTTCCTCTTCAAACAGGAAGAAGA
CGATAAACCTGCGGTGTATTCCATTGGGGGTGAACCGCATGAAGGGGAAGGGGTGACCCT
GAACCTGTCTAAAACCGAGGTCACTGACATCCTGAATAAACTCGGGAAGGTCAATGCGTT
GCACTTGCGGGTTAAGGTATCGTATGAGAGTTATCTCGTATTCATCAAATATTGGGCAGA
AATGGTCAAAGGTGTTGATGGTAACTTGAGTCAATTGGAGCAAGTGAGTAAGACGGTAAT
AGGCGAAGGTGAGAAGCTATTGGCCGGGAACATCGGGGCTCTCGCATTTTATAGCGGCTT
CACTCCTCGTGTGGTCGGTTATACCGATAAGTACATTCATGGTGTACTTGGGGTATTCGC

TTGATTTGTTTATAAACAATCATTACCTTTCGTTAACGAAACAAAGGAAACAACTCATGA
CTATTCTCGAACGCTACACTGGCATGGAAGAGCTTCTGGAGAACGGCGCGGCCAACCTCG
AAGATGGTGTGACCGAAGCCGCTGTCGAAGAAGTTCGTGCTGACATCGCCGAGGTTGTGG
CAGAGGTAGAACAGGTCGCGGCTGCAGTCGAAGAACTGCGTGATGACCTGGAAGAGCACG
AAGAAGTCGTCGAAGAACTGGCTGAAGAAGTCGAAGGCCTGGATAGCATGCTGAGCTCGG
GTCAGTTCAGCTCCGTTGGTTTCGCTCACAGCTACAACAAGGCCCTGCGCCTGAACGCCA
AGCTGGCCGGCCAATCCTTCGAACGCCGGGGTGCGGAATCCATCTCCGACGCTGCCACCG
TCAAGTTCGCTTCGGTGACTGGCGGTGAAGCCTTCATGGACACCGTGAAGAACGCCGGTA
AGAAAGCCGCCGAGTTCATCAAGGCGATCTTCAACACCGTCATCAACTTCTTCGTGGGTC
TGTCCAGCACCGCAGCCGGTATCGACCGTCACCTCGCACAGCTCGCCGACAAACCCGGTA
AAGCCGACAAGCTGAAAGAGAAAATCAAGCTGGGCAAATGGAACATCGGTGTCGACTAGG
CCAAGAACGGCGCGGACATCCTGGACAAAGGTCCGTTCACCAAAGTCCTGAGCGATTCCC
TGCCGGCCTTCATCGACATCGGCAAGAACCTCGACGGCGTCAACGCCTCCCAGTTCAAGA
CTGCGTGCAACAGCCTGGTCGCTGACGTCAAAGCCGTTGTGAAAGAAACCGGCGGAAATG
CTACTGAGAAAGGTTCCGGTGACAAGCGTCAAGTCCTGGCTGCACGTGCCGGCTTCGGCA
TCTATCTCACCTTCGAAGAGAAGGTCGATACCGATGAAGAAGTCCTGGCTGCCGCGCGTT
CCATCAAACTGTCCTTCGGCAAGAACAAGGACGCCTCCGAGTTCGCCACAGGTGAAGCGG
CTGTCGAGGCAACCAAAGCACAACTGGAAAGCCTGCTGAAAACTGTGAGCAGTACCGTAT
CCGTTCTGAAAGACAGCAAGATCAGCCAGAAGTTCTCCAAGGCTGAACGTGTCCGTGTCG
TCGTCACCCTGAACGCCAAAGGTGGTGACCAGGACGGCAAGGCCGGCACCGACAAGGCGA
TCAAACTGTTGCGCTCGCTGTTCAGCACCGGTGCGAGCCTGACCCAGTCGGTCAACCGTC
TGCTGGCTTACCAAGCTAAGCAATCGCTGGATCTGGTTGCGGCTCACCTGTAAGGTCGAG
TGTTAGGATCTATTCCTTGAGGTACTACAGGTAGGGGGATCTCCCCCTACCAGTACCCTT
TATGTCGCAATACTGTAATTTCGATGCTATGTACTCAACTTGACTATTAGGTTGTTATCG
ATGAATACGCATCCTGTAGCGCCCCTCCGATACGTGAATTCACTGACGGTGTATGAAGAG
TATGAGGAGGATAGGGAGGGTTATAATCTAGGCAACGACGAAACGACTATGGTTGCACTC
TCCAGAGAGTACGACAAGATCAGAGGATTAGAGAGTGTCTACACCTACGTTCTCCACCGA
CATCAGAATCGGTTGACTGCTTCGACCGGTACTGAGGGGTTTATTGATTGCGTTAAACAC
GGTATCGGTAAACTCATTCAAGTGGTTAAGGATTTCTTTAAGTGGATTTGGAGTTTCTTT
GGGACCAAAGGGAAAGCCCTGAAAGACAAAGGAGATGAACTGGAGGTCGCTCTTAAAGCC
AATGGTGTACGGGAGGATGACATTTCGTATCCCAAAGCCGCCTTTTATATCTTCCCTAAA
ACGACCAAGTTACCGTCGAATGTGGCGTGGGTGGGTAGCGTCATCGGGGATATTGAGCGT
GGGATTAAGACGATTGAACGGTACAGTGCAAGTGTGGGGAACTTCTGTAAGGAATCAGGT
CGTCTGATTGACCATTCGAAATACGCAGAATTCACCACCAAACAGAAGGCCTTCTACCAA
GAGGTTAAGGGTCTGTTAGGAGTGAACTCGAAGCAGGAAGCCACCCTGGTGGTTAAAGAT
GACCTGGTCGTGGGGAAGAACACCGTGGAAGTGACGATTAACCTGACGCGGGGGCTGGGT
GTCCCGTCGGCTGATCGAGTGAAGAACGCGACCTTTAAGACTAATGTTTCGCAGGTGCGC
GAACTCCTGGTCAAGCACAATCGTCTAACAGAATCCCTCAAGGAGATGATCACTAAATTC
CATTCGTTGGAGAATGACTTTGTAACGGCGCTAATGGCAAGTTTGGACGTTAAAGAGGCG
TCTCCTGAAGCTCAGGCTGCAATTAAGGAGATGAAGGCCATGATTCGTAAAGCCATGGCG
AGTCTAAAAGTCATGGAGACGGAAGTGTATCGGAGTGCTAACGCTGTTTACGCGGTTTTG
GAAGCCGCTGTGAAGAGAACGAGCTAAAGGTCATGCATCTTAGCTTATTGAATCAATCTG
TTGTGGTGAAAGCCGCGCTGGGTGGTTACACGGACATTAACCCTCGGTTTGGGATTACCA
CGAGGATTAACGATACCGTGTTCAAATACGCCCCTCATTGAGAACTGCGTCAGAGTGTTC
TGATTGAGCTTTTGGATCTTGGAGAATTCCTGAAGGGGAATCGCCAGGACATTAGCCAGG
TGACGTATAAGGAGACCCCTGAGATCGTTGTGAGTCTTCTCCAGGAAAAGCTGGGGGTGT
TCCCAGAGGAACAGGAACCAATTGTTCAACCCAGCACCCCCCTCGTCCTCAAGGAGGATG
ATCCTCATACCACGTACTTTGAACTCTGGCGGAGTTTTGTAGGGTTACCCGGTCTCTACC
GTCACGAAGTCGACTTGGGCCTGGTTAACGACACTCTCATAATTGACGCACGTTACTCGA
AGATTTACAAGGGTAATGTCACCGTTAAGGTTAAGTAAGGGGACGATCATGAACGCAGCC
TATCGTTTAGGTTTACTGGGGTTGATCCTGGGGGAAGTGGGTAAGGACGTTCTCTCCGAG
GAGAAGACCCCCGAATTGGTTAACTACCTGGAAAAACATGGTGTTAGTCAATTAACTGAA
GAACACATCATGGGTCAGATTGTCAACACACTGAATATTATCAGTCGTGAGTTTGACTAT
TACGACGCCAGTGAGTTGAAAGTGAATACTGTACTGAACCGTTACCTGAAGGAAACCCTG
AGCAGTATTGAGAATGGAATCTATCAATTCCCTCGGGATATTTCCATGCTGATTTGTTCG
TTGGGGATTAAGGGTGGGGCGTTTACGGAGACGGAAGGAGTAGGTGAACTGATCAGCACG
GGTAAAGGGTTGGTCAATTACCTGCTGGAACAGGATGAGGGTAGTTTGAGGACTACCCCA

GCTGAGGTGTATTCTGCTACCGCTCGTACACTGTAATACATTGACAAGGTTCTGAGTTTG
GTGGACATCTTTGTAACATCTCGTCAGGGGCTGTTGGCGGGCTTGGAAGAATATGCGCCG
ATGGACTACACCTACCTCCAACCGATTGAGGTCAATCATTGGGGGGTTCCTGGGTTGGAG
TGTGCGGGAGGTGATACCTGTGAATGCGGGTGTGTGCTCGACCACCCATTGCATGTTCTG
CAGGGCTAGGAAGATTACCTGGATGGTATTCAAAGGACCGACCGTGATTATTTCCTGACC
GTTGCTGAAATGAAAGGGGTTAAACTCCGTTCCATTGAAGGGAGTGAGGGTCAAGTTTTC
GACTCCATTAAAGCGGTTGCGCGGAATGCGTACCAAGCGGCTTTCAGTGGGTGGGAACAA
CTGAAAGCCTGGTACGACTCGTCTGAGAAAGAAAAAGCTGACAACGACGCGGTAGCAAAG
ACTGGGGAGGACAATAAGAAAGCAGTCCAAACCATGCCCACTAAGAATGTCACTATCAAT
GACAAGGCGCGTGAGGGGATTAAGACCCTGGCGGAGAAGGTGGATGTCTCTGAGGCAATG
TCTCAGGTTGTGAATCGGCTTCGGACACCCGCGGACGTAGGGGGTGTGATCCATGGATTG
TTGGGTCTTCTTAAGAAAGAAGAAGTCAACGGTGAGAAACTCCGGGCTGATAAGAAAGCG
GTTGATACCACCCTGGCTCAATTGAGAAAAGGGTCACAGAAGACCAGTGGGAATGATGAA
GACAAAGAGGCAGTTAGTGCGGCTAAGGCAGCGGTGCAGGAAACGGTTAAAGCTGCTAAA
GAAGCCGTCGCGGAGGCCAAGAAGAAGATTGGTGAACACGATAAGTTGGTTAAAGGTCTT
CGTAAAGCCATTAAAGGTATTACACCCCACATCTTTATTTAAGGGGGTGGGAACACTGGG
GGGACGAACACATGAAGACACTTCATCCACGTGTCTTGGAACAGATTAAAGCACAATTGC
CCAAAGACAGCGATATACTGGGTACTCAGCCTGTTGATGAAGACGTTGGGTATGTGGTGG
TTCAGTTGAATAACGACGATACCATGCTCCCTATCCCGAAGAAGGATATTGGAGATTACA
TGGGGGTCAGTATTCCTGAACTCCCGGTGGGTGCCCTGGAGCCGTTGGCTCAGGTGTTTA
AACGGATCAGTAATGCGTTTAAACCCTTCCTCGTGGAAGGGGTGGACTACACGGTTCCAG
AAGGGAAGGTAGACCTGCCCAAGAACGCACCCTCCCCTTTCCAGATAGTCACCATGGAGA
TCGCTCCGACCAGTATCCTGTGGAAAGGACGCCTGGTCCTTAAACTGCTCAATAAAGCCC
GCTTTGGACCTTCAGGGGTTCTTAAAGCCACCGTTTTGGATTCCCCTCGTCTCCCGATGG
CGTTAAGTTCTAAAATCTTCCAGGGGAATGGGAACCTCTTTACGGAGAACCGGTCTCGGT
TGTCTGACACCTTCTGTCAGCAGGTGGTTGCTTACCTGAACATCGTTAAGATCCAGTCGG
TTACGCTGGATCGGCTGAAAGGGGGAGAACTGATCGATAACGTATCGGATTGTTTCTCTG
GCTTGGTGATCATTAAGCTTGAGGAGGGTCCTCAACTGTTTATTCGGTACCGTTCCGAAG
GGGAAGAAGATCCTCATCCATCGCCCGGTGACGTAGACCCGGAATAACCAACGTCAGGTA
CTGGGGAAGGAGAAGGGGGGAACCCCTCTCCCTCCTAGACCACATCGTGCAGGCAGTGAA
TATGCTTAAAACTTTTGTTGAATGCGAAGAAAACTACGACGCTTATTTGCGTCCTGCGAT
TTATGTTTCCATTAAGGCTGTATTGAACTTCTACGGGCTAGACTCCGCGGCCGCCATTTA
CTACAACGGTGAGAATGAGATTGCGAATAACGTAGGGAGCAATATTAGTGACGGTCCTCA
TTCAGATCGGTATTCGGATGGAATTTTCAGGAACAATATCTTTATTGTTCCTGAGGTGAA
CCGTAGTGAAGCTTGGACGAACCGTCGTCAGATGGTGGAGGGTCCCGAACTGTACAACAA
GGAACTGGGTATCCTGTTGTGCCCTACGTTTGAGAACAAACGGTTTGATGTCAAAGTCGT
CTCGATGTTCAACTCAGAGAACACCGCCCGGCAAATGAAAGACCGTATGAATCGGCTCAA
GGAGAACCAAGTCGTTGACTTCACCTTCTCCCCAACGACCCATATGGTCATTAATCCTCC
TATTCTGGAATTCCTAGAGGTGATTTACGACCTCTTGGAGAAGAACAACCCGTCGGTACC
CGACCCGTCTGAATGGTTGGGTAAGATGTCGCAATTTCCGGTGCGTGTGATCAGTAACGT
AGCGGGTAACCACAAACGGGTAGTTATCCCGTGTAAACAGAACCGGGTCGGCATTCAGTT
CTCCGAACCCTTTGTAGCGAAGACGAATAAGAGCGCGACTTATGGTCGGTGGGAAGTGGA
GTTTACGTACAGCTTCCTCTTCAATGATTTCATTGGGTGGGAGCTTGAGTTTCCCCTGAA
CATTTATCAGGATAAAATATCCACTCAGTATATTCCGAGGGTCTAACCTCAACATGAATT
ACCGGAGTTCATTAAAGCCGCTCCTGAAGTAGAAGCCGGACCGGAGATTATGTTCCCGGA
AGGGTTCAGACTCACCCCCTTCTGTTTGAAGCTGTCTAAGCATAACCCTTGGGCGTGGCC
TAACTTCGATTGGATGCAACCGGTGATCCAAGCAAGACTCAAAGTCGAGGATGAACCCAC
TCAGGTACTTTGTAACTTATTTCATATTCCTGACTTTGTTGGGTACGACAATGCTAAGGC
GTATTTAGTTCGTCGTCATGCGATTGCCTTCCAGCATTTTGAAACCCCTTTTCTGAGTCA
AGTCTTTGAAAATGACCAGTTAATTGCCCCTGAACCCCTGACCCTCGATGAGGAGGGGTC
TATAACGCTTCACAGCAAGCCTGTTTTAAACAAAACATATCGAGTAGTGGTAACCCTCGA
CTATGCGATAAGAGACTATTCTGACGCATTCTGGAGCGATTTAATCGATCATCCTGAAGA
CTGGGGTATTTTGCCTCAAATCTTTGATTGGTTCGATTTCAGTGAAGTGCCTGAACCGTG
GTACTTACACATCAACTACCTCCGGAAACACATCCATAAGCGATGGGGTCGTTGGACCGA
GCGCTTTAACCGATATGAGATGGGCTTTGGTTTGTAGGCCTACCGGAACGATTAAGAAGG
AAATGAAACCATGCCTCTGGATATTAACCCTATCGGAAGGGCTATTCCGATTGAACCTGA
AACGCCCCGGATCTACAGTGAGACGTATCGACATTCCATTGTGGATTCCGTTTGGCAACC

CGAACAGAGCCTGCTCTCTGAAGTTGAAGGACGTCCTCGTCTCTGTCAGTACTTCCGTCA
ATTCCTGAATCCAGGTGAAGAACCTAAACCCTTCTCCCCTGGAAACACCGATACGTATCC
GAGTTACGTGCATATTCACCGGTTACCCCTCAAGATCGATGGGGATGAGACGTTTAACTT
TGATCCTCAGACCGGTGGATCCACCAAGATCTTTAACTTCTGGTTTGCCACCGATGCCAA
CCCTATTCGTCATGATGTGTTTATTGTGGATATTGGGGATGGTCGAGCAGGGCTCTTTGC
GTGTACGGAACAACCGGAAATTCGGAATTTTACCGCAAATAAGGTGTACTTCGTTACCGC
GGTATTGAGGGGTATCCTCGACCAGGAGACGGATGCCATTCTCCAAGGACGGGTGGTCCA
ACACCGGGTGTATTACAAAGATTCTGTACTCCATGGAGGGGCTTCCCTGATTACTAAAGG
GGAAGAGGAGGTTGGACAGCAACTCTTCCAGTGGAATCAAACCATCGCCAGTCATATCCT
GAAACGGTTATGCTGGTACCCTGAACGCACCATCGTGTTCGATAAGAAGGGGATCAATGA
GAAGGATAATGTGTAAGACCCTTATCTGGTGAACTTCCTGTCTGCGTTCATTACCCAGGA
CATGCGACAGAACTACCCACCCATTAACCAGTTCTCGGTTCAGTACGGGGGATTGGAGTA
CAGCCGGTAGGGAACGACCAACATTTGGGAAGTGTTATTTAGGGGGGATTTGAATCTCTT
GAAGGTGTGCGACAACAAAGCCGCCTTGGTGGAAGTGACGCGGTTAATGAACAGCCGGCT
GTACGGTAACCTGGCGTCGAGTAAGATCCGGTGGTTCGTGACCACCAACCCTAAGGACTT
CATTGCGTATCGGGTTCATTTCAATATGGATGGGTATCCCATTGTTATTCCCGGGAAACA
AGAGGACATTACGTACCTCTTTAGTCAAGGCTTTTACCAAGGGAAACCCGAAGGCGCCTT
TTAGAACCTACTGTACCGAACCCTCACCACGAAAATCACGGATCATAAAGAACTCTTAGC
CTATTGCGAAGGGTACATTGATAAGGAACCGGTGGAACAACTGTACGAAGGGGCTATTCT
GCAGATGCTTCTGAAGGACGCGCGTAAATTCGGAGGTCCGCTATGAGCATGCCCTTAAGT
CGGTATAACACCATACGCACCCGACTAATCGAACTCTACAACATTCTGAATAATCGGTCC
TTCGATGTGTGGGTAACGTCCGCAAAGCTCATGAGTTTGGAAGAACTCCGGGATCGGGAA
GAATACTCCCAGGAGGGGTTCGTTGGAGATCCGAATTACTACAACGAATACCAAGTTCGA
CGGATGAGTGTTCCCAGGATCATTGATATCCTACCTAACTTGTCCAGTGAGAAGGATTTG
GGATTCAGGGAACCTCAGATTGTGATTCCGTTGATCTACGAATCCATTCAGGAGTACATT
TGCCTCTGGTGTGAACTCTTTAAGAAAGCACCGGAGTTTCCAACACCTGCCATTGATGAA
CTCCGTAGGCTGGAACTCCTGGCGTATACCCTCTTTAATTTCTACAAACGGATTAAACCG
TACCTGGATAAGAAGAAACTACGGGAACTGGGGGGTGATGTTCAAGAGACCGATAAACGG
AACCTAATGCAGTTGTCAGCGCTGTTTAGTATCAACGCCATGGGAACCCCCAAAGCTGAG
ATCAGTTTCGTCAGTCATTTGGATGAACTCTTCGGGGACGATACGTTGAACCCGGGTCTC
TCACTCAGTGAACAGCCGTATATCACGAACAAAGCTGTAGCGGATAGTTTAGCCACGCAT
GATACACCTGAGGATCTCGGTCCTTGGTTGTTCAATAAACCGACAGGATTACCGTGAGTA
TGGAATTAGCACGTCCATTAGCTAACTTAATCGCCTTAGTAAACTCGGTGAATACGCAAC
GGTCGGATAAGAAGATCCGACTGTTTGCAACGATCATTACTCCTGTGGAACAGATTGAAC
TGATTATCCCCACGGGGATGGCTCGGGTGTCGATGTATTCAGCGAACTGTTCCGATGGCT
GGCGTATTCAGGGCAACTTGCAACCCGGGGTTTACGTGAACCGAATCATGCCCTATAAGG
ACAATCTCTTTATTGAAGTGGTCGAACGTCAAGGCCTTCATCAAACCATGAGTCGGTTCT
GGTGTATCCCCCTGAGTGACCCGAACCCTGAGATGGGCGGTAACAGTGTCCATTTGGCTC
ACCTGGAAGGTAAGGACAGTATTAACCTGATAACGGTTAAGTTCCAGATCATTGAACTGG
GGTACGCTTTGCTGAAGAATGAGATGGTGTCTGACAAGCACTTAATGACCACCTTGGACG
ATGCACTCCACTACCAACTCACCAAATACGGTAAGCAACTTCAGTTGACGGGTAAGGATG
CCTTTAGAGGGGTGGACATTGAGCGTCCTATCGATAACCCTCGGGTCTTTAAGTTGATTG
ATATACCGTCTGCGGTGCCTTTAACCCAGTTAGGGATGTGGATGCAGACCCATGAGGAGT
TTGGGATCTATACCAAAGGCTTTGGGATGTATTCCCAGAAAGGCCTTTGGATGATTTACC
CGCTCTTCAAGATCGGTCGGTATGAGAACGGTAAACGGGTACTGAGTATTTATCCACTAC
CGCCTCATGCAGTCCCTACCCTCCACGCCACGTACTTTACCCAGGACCGGGGTACAACGA
TTCTGGCAACCGGTACAGGCCGTAATGTAGATGGAGCAGACATTGATCGTCAGAATGAGG
GTACGGGTAAACGGTTGATCAGTTCGGATGCGGTCATGGGTGAAGTGGGTAACTACTACG
GTAAGGGTCAGTCAGCATCGACCCGTCCTGATTCCCTCTCCGAATACCAAACCGCTAAGC
GGAGCAGTGGGGAGGAGATTATACCCTTTGAACCGACCCATACCAATAACCTCTGTAAGC
AATTATCTGTGAACTTTTACAACGATGGGTCGTTGGAAACGATTCAGTGGAATAATTCGA
ACAGGGACTTACTAGAACCAGGTATGCCTGTACGGTTCTTCTTTATGAGTGGAGATGATC
GCTTGAAGTACAAGGAGGGTACTCTGATTGGTGTTCGTACTGAACTCATGAAGGATACGG
AAAATCCAGCTATTATCTTTAGGGAACATTCTGCTTTAACCCTCTTCTTGAATGATAAGG
TGTTTGTTGCTCCTTAGGTAATTTATCACTTAATGTATTTATTGTATTGTAAACTGGCTG
ACAAGCGGGTGGGGAAAGGGGTTGATTTCCTAATAAAGAGTGGAGTAGTTACCGAGTAAC
TACGCCTTCCTTGTTCTATCTTTTGATCTTTCTCTATTCTCTTTAGATCTTTCTTTTCAT

TTATACGGAATACAGAGAGGGAAGAGAGGGAATACCTCTTATACCCCCGAAAAAAAGGGG
GGATGAGGAATAAGGAGAGAGAACCGTAGGTTTATCAGTATTCCAACACGTTCTGATTAA
TACATTCAGCAAGCAGAGCTGAGAAGATTGCTCTGCCATTACCGATTCACTACCGGAGTA
ACTTCCATGACTGGGGATTTCGACCATCTCAGTAACCGTCAACTGGCAAACTTACTGGCG
GGTTACCATTTGTACGGCACTAAGCGGACGAGCGACATACCGGGGCCTGTAATGCTGAAA
CTCATCCGAAAACGCCTCCGGGCATCTGAACCTCTACCTGATATCATTCCCTGTCTCGAC
GACGGCAAAGACGAACGTGTGCTCGGTCTGTTTGGTGAACGCATCACGGTTAAAGGAAAA
GAAAACCAATGACACATCGTGGGAATAATGAAATTAAGAATACATTTGATCTCTTACACG
TGCGTGTAGAGGATCAAACGAATGCGGGAATATTAATACGGTGATTCGAAAGGGAGGCGA
AGATTACCTTTGCTAAGGCGGATACGTTGTTTGTGATGCTGGTAAGGCGTCTAGCAGCGC
TTAGGCGAGGCGATCAGGTAGAAAGGAGTACCGTCGAGGCGGGTATGGGCAATCGGCTCT
ATTGGAAGCTTACAGAAGCGTACAAAGTACTTCAAGATAAACCCACTATTGTTCCTCCGT
TGTGAAAAGTCCCTGACGAGTTGGAAGAGGTCTTCTTAGCCTTTACGAAGACAGAACCGT
TGTATCAAGAAGGCCCCGGGTATTACGGGGCGGTGTATGCAGGTGACCGGTTACTGACCG
CACCACCGCCTGTAGACTTTGAGTCATGAAAACAGTAAATGTTTACGGGTATACGCACTC
ACTACCCCCTTACTGACCTCCCTACCTAAGCCGAGCTTAGGTAGGGGGTGTTATACCGCA
TTTGAGCTATAGGGGAACTCAAACCTATATTACCGGCGTGATTATACCCAATGGATCATT
TAGGAGATTCGGTTTATGCATTACCAAATGGTGTTTGAACAAGACCTATTGGGTAAAACC
CATACACATGACACCGGTCTGTTCATTAACGGTGAATCCAAACGGTTTTTACCACGTTGT
TGGATCATAACGAATCGGTGTTGTATGACGTGTACGACGGACACCTGGTGATTATCCGTG
AAACCAGCGAGGGGGAAGTGGAGGAATACCTAAGCGCTCGGTCGTATGATTACCAGACGT
TACCGTTGTTGGTGGTGATTGCGTGCTATAAGCACAATAAGCGGGTGGTGAAGGTGGGGC
GGAGTACCCATACCCTGATGTTCCACGACAACAAGGACGGGCGGTTTGAAGCCGTATTAG
AAGCCGGTAAAGTCACCCTCCACGATAAGGAGGGGTTAGTCTTTACCCAAGACGGGGATT
ATTACCTGTCTCTACCGGAAGAACACGTGGCGAATATCATGCTGGAACGGTTGAATCACA
GCTTCAAGTTCTTTGGCGAGAAATACAAGGTTCGGTTTAAAATAGGACGGAACTATGCGT
ATTTCCCGCTAACGGAAGTCTTTGCAGACACTGAGGTTCGGAATATACGACTGCGGTTGG
ATAACCTAACGATTACTCAACAGAGCTGGGATCGTATTCGGGATCAGCTCATGGACCTGT
TAGAAGATTACACCCAACCTACTCGAACTGTTCGTATGGATCTACCCATTAAGGGGGACT
AATGGCGTATCAGGAATATGGATCTACTTTCGTTCACGACGGTAAAGTCTACCACCTCAA
TGACTTCCTCCGTCGAAGGGAAACACTCCCCGTTAAAACGGTTCCCTTAAAGGACTTTGA
ATGGCGCCTGGCGTATGCCGTTGAAGAGGATAAGGACAGGATTAAGCGAGCGGACTATTC
CTTTCCGTTGTTGATTACGGAATGGGAAGGGGAGTGGGTGATCATTGATGGCTGGCACCG
GTTCCTTAAAGCCCATTGGGATGGGCAATCCGCCTTGAACGTTACGGTCAGTCCAACGGA
CTGGTTTAAAGGTAGAGGAAGGCCCTTTAAACTAAAGGGTTACCGTAATCGCGTTACATA
ACGTGAGATACCCCTACCCCCTAGCGCTCAGCCTAGGGGGTAGGGGGTCTATTATTCATT
TTGTCTTTACATGGCGTCCGATTCTTTCACGACATCACCATTCTCATTAAAGCGTTCTTG
CAAAGAACGTCTAAAGGTGGGTTTACCATTAATGTCATGGACAAGCCCTAACCCGGGATC
AAGATCATAGATAAAGAACCGCTTCTCAGGTGAACACCCTTCACCCAGTTGTTTACCAAT
GTAACAGGTAAAGTCATTCTTCATTGACGTCTTCGCCACATGAATGGTAATGACACCATC
CACTTCGTTGGTAATCTTCGTAGACGTTTCTGTCATTGACTTACCAGAGACTTCCCGAGC
GAAGTAGACCTCAGACTCATCATCCAGTTCTTTTAGTAGTTTCTTAGCTTCAGAGCTCAA
CTGGTGAGGTGTTCAGAAAGAAATACCCCGCGCAATCATAAATGACCGGACAGTGCGGAA
CAGTAATTGAAGTTTATCGGACTTATTCTCCCCAGGGAGTTTATCGAAATTCTGCAACGC
CAAGTAATAATACCCGTACAGAATGATCTCGTGACCTTTGAGTTCCAATTGCCGTACCCG
AGCGAACATCTTATCCCGGTTGTCTTTATGGTCTTCAATCTGGTTAATCACCAGGTACCA
CCCATTCTTCCGGAAGCAGTCCGCAATAGCGGTTACAATGTCTACGCTTTCAGTCACCTG
AAAGTCCCGGTCTTCTTTGTACTTGGTGGTTACGGCCAGCTTGTAGATCCGACGAATCAC
CAGCTCCATGGCATCCTCAGCCGATTCATACAGGATCGTTGGAATCTTAGCCTTATTCCG
CAGCATGGGTTTGTTGTAAATCCCCGCTGAAGCAATCAGGTGAGCCATACCGAGAGACTT
ACCTCGGTTCGTCATGGCTTCTAGGAGGTACATCTTACCTCAACGTAATCCTCCATCTGG
TTCCAATCCTTGAATGAGACCTTGAATACCGGTTTTAATGATCCCTTCTACACTGTTCTC
CGTCTTCGCCATCTCGATGAGATCGAGCATACTTTCCGGCGTGCCTGTATTGACGGAACT
CACCACCTCGGATTGGCGTTCCCCATACGCGTTATTCACCCGCTCTTGGATCAAATCAAT
CAATTTAGCAAAGTCTTCGGGTTTGGTATCAACGAGGTCCTTACACAGGACTTCTATCAT
AGCTTTCTTGAACTGCTTCTCGAAGTCCTCATCTTCCATACTGGAACGAATCTCTGTGAT
CTGTTTATACACCAGACGACGAGACAGTTCCTCTGTTTCCAGGTCTTCCAATCCAACATT

CACCGCATCCCTGAGACTGTCATCATTCTTGCAGAAGATCGCTACCCGCTGCATCAGGAA
CGATTTTATAAGTGGATCGTCATTGGACTGGTCCAGCAGCCAGCTGATTGAAGACCGGAT
GCCGTCCCTGACACGTCGGTCTTGTGACATCACATCGGGATTTGGGGCTGGGAGTTCTTC
CAGTAAGTCTGTCAGTTCTTCGATCAGGTTAGTGACTTTTAGTTTTTTGGCCTGATAAAT
TGCTGATAACAGTTTTACTAACACCAGCAAATCGTTCATGCTAACCCCTGAGAGTGTAAA
GTAGATGATTTTTAAATACCCTAACGGTTTACCGTTCGAGTTGTATATGGTAACCGGGGA
GTTGGTTGACACCCTGGTGGCTCAATCCATCCCGCTCTGTGGAGTGAAAGAAATTTCTGC
ATACCATGACAAGATCACCTTTGGTGAAATGGCTAACTTTGTAGCTTTGCAGTATGCGGC
GGCCAAAGCCTTTGGTGTTGAAATGCCTGACCCTATGGCGTACTTTGGTCATGGGTATAA
CGCAGGGATGGTAGCCGAAGCATTTAATGGGGTGGACAGTGATGCAATAGCCACCCTCCT
TCGTACCGCCAATCATTTCCGATCGACGGTGAATGGTCAAGTGATTCTCATTGGACTCGA
GGTAGGGGACCATAAAGCCACCGCTAGCTGGGAGAAACTTCAAGCCAAAACAGTCTCAAC
CGCCGTATTGGAAACACTTTATGCAAGGGTCGGTTATTTGGAAAGTCATTTCGGTCCCGT
ACCGAAAGCAGGTTTAGGGGAATTGACTCTGGCTGAAGTGTTCAAGCTGATCCACCTTCA
AAACCCCTGATTAAAAGGCTGCATGAATTTTATGTGCAGTTCTATTGCGCAACAAACATG
TTTGCTACTCATCTCGATCAGGATTTAAATCCATGGCAAAGCAACTACGAACCTCCATTA
ATGGAGACAACAATCTTTTCAGCGATGTCTGCAAGTCCCTCGTAGCCGGTAGTCTTTCCA
TTATCACCGGTAACGAGAGCTTCGCAGACTTCCGTAGCACTCTTCAGAGCGCGGGCGGTC
AGGAAGGTTTTCTGGTCAACCGTCTGATTGATCGTTCGCTCCCGAACGTCAGTAGTAACG
AGAGCGCTGAACTGCACACGCGCTTCAACTCGGCTACTTACGACATCGTCCAAGGTATCC
TGGAACGCAAGAGCCGTGACCTGATCCCCATGGACTGCGTTCGTATCTTCCAAGAAGCCG
GTGATAACTTCGCCAAGGTGGAAGGTGCTGAAGGCTTCTCGATGCAGAACTTCGAGGGTC
GTGAGCAAGAGATCCGTGCCTCCAGCCTGACTCTGAACGCCCAGAGCCACCTTCAAACCG
AAGGCGGTGAAGCCCTGTTCGCCACCGTCGGTGTGCGTTATCAAGACGAAGGCGCTGAGC
TGATCGTTCGTGCAGCAGGTCTGGGTACCTATGCGTACGGCAACACCCCGTGGCAATCGG
CTTATGAACTGCGTCCGATCTTCGGCCTGCTCCGCAACGGCGATATGTTCAAAGACGAAG
CCCTGGCCGTCTACCCGGTATACCCGGACGTTAACGCGAGCGAAGCCCTTGCGTTCTTCG
TGGCTGAGAGCGTCATCGCCCCGCGTCCTGTGAAATGCGCGCAAGGTGATGCCTACGGTC
GTGGTGAGCACGAAACCCAAATGTTGGCGGTTCCGGTATCGATTCCGAACCTGCTCGCCC
TGACCCAAGCGCCGGGCCAACGTCCGTGGAATTCCACCGACGAACTCGAATCCCACTCCA
TCTCCGTGTCTCTCATCGCCGCAAGCGGTAAGCTGGACGGTACTGATATTTCGTTCTTCA
ACAAAACCGGTGCGATGTCGAACAACATCTTCGCTCCAGCCAACAACGCCCAGACGAGCG
ATGAGCGCCAACTGAACCTGCAAGTCAAGCAACTGCCGGGCTTCTCGGTGAGCGACAAAG
ACGGCAAAGTGGTGGGTGAAACCATTTTCGCCAGCTTCAAGTCCGCAGGTTGCGAACCGC
TGCTGGCCTTCCGCCTGTCTGGTAGCTGCCAACGCCAGACCAACGAGTTCAACCTGCAAC
CGGGAACTGTGACCATCACCCCTGTTCGCAACATCACTACCGCTGTGGTCACCAAGATCG
GCTTCGCTGACAGCACGGTTCAAGCCCTGATGCGCTCCTTCACCGAAGGTAAACTGATCG
GTGCTATTACCACCCAGAACGTATCGAACACCAACCGCGGTAACTTCGGTTATCGGATTG
AAGTGTACGATGCCAAGAAACACCTGTCGGTGAAACGTCGTTCTCCGATCTCGGTGAAAT
GCCCGTTCTCGGCTGATGACGTCAACCAGGCCAGCCTGGACTTCGCACTGAGCCCGATGT
CGGTGGTGATTAACAACCAATGCTCCGCTCACGCTTTCCAAGCGGCTGAGCAGCACCTCG
AGTACGTCACCTCGATTACCGGTCAACCGGTGGTGGCCAACGACCAAGGTTCGGAAGTGC
TCGCGGGTCAGCACTTTGTAAGCCCGATCGCCATCAACCGTAGCTTCAAGCTGGTTAATG
TGATCTCGGCTAACAGTTCTTCGGATGTGTTCGTGTCGGTGGCATCCGCCATTACCAACG
AGATCGCTGATATCGTTGCGGCCCTGAACACCAAGTCCTTCTTGGCATCCATTGCGGAAT
ACAGCACTGGTAATACCAAGAACGCCTGGACGGTGGTCGTCCATCAGAACTTGAACCGTT
TCATCTTCCGTTCGGGTGACGCACGGACCATTGGTCCGATCACCAACCTGAAAGTGGTTG
AAACCAAGTTCGACAGCATGATCGGTAAGATCTACATCGTTCCGACCAACGATTCCAACA
ACGAAACCATTAACCCGCTGGCGCGTATTGGTGTTCTGGTATCGAAGGAGAACATCGTTG
TACAGGGTAACATCACGCGTGACAACAGCGACTTCGGTGTAGTGATGACCCTGCCGAGCT
ACAAACACTGGCCGCTCAACGTGGTCATTGGTTCTCTGGTCATCGAAGATGCTGAAGACC
TGCTGAGCGACAAAGGTCTTATCAACCAACTGGCCGTCAAACGTGTCAAGGTTGAGAACA
TCGAGCATGCGAAACCCGACCCAAACGCCAGTCCGTGAGTCCTGCGAATGACGGGCGGCT
TGAGTATCCCGAGTTGCCCTAGTAGGTACCTCACGGAAATTGCGTGTACCATTAGCCCTA
CCCACTCCCCTAACCGGGAGTGGGTAGGGTTATATTTGCAGTTATACCGTTTATTGGAAT
ATTTTGAACCTACATTATCCTTGGGTACAAGACAATAGGGTATCTGACAGCGCACCAGAT
GATCTTGACACTAACTTGAGGATCCTCACCATGAATGACGTTTCACCCTACTGGCATTTC

CGGCTGACTTATGATATCACCAATATGAGCAGCAAACAGATTATTGTCAAGGCGAAAGGA
GGGTTGAATTACCTTATTCGCAAACCACTAGAGGCTTCGTGGTATCCCGGGGCGATGATC
GTTATCAAATTCGATCGTCTGTATGTCGGTGATTTCAGTTTTGATAAGTCAGCAGCGCGG
AGTAAATTCGATCGTCTGTTGATTGATAAGATCCAAGCTCACCTGAGCTTTGTGTCGAGC
AAAGCGTCGCACGTGGACGTTCGGCAGTATCATGAGTTCGGCGTCACGATCAAGCTGAAC
AGGGATGTGGTTGAACACCTGGAAGGTGAACCGATCCACAGTGATATCCTCGGTATCACG
TTGTATAACCGGGAAGAGAATACCGGCGATCTTTATCCCCTGGATACGCCAGACTACACC
CTCAATGACCTGATGAAATGGGGAATCGGTGAGAGTGATGAGCCGGGAGGTCTTCATTAC
TACGTGTATCTGAACGATCCCCTGAGTGTTCAAAACCCGTTGTACACCAATGTCATGGTT
AACGCGGTAATGGTCCCTACGGTCGGGGAGATGAATAATGATCATGGGTTGTATGTGGGA
TTGGTCTGCGGTAATTCACAACCGAAGAAGCTATTCTACACCTTTGACGATCTCTGCGAT
GAAACCCTGGGTTTCCTGGGTATCTTTAAGACCAAAGCAGAATGTGAGATGGAGGGAAC
ACGAAACGGGCCATGACTGGCGAGAAGAAAGTGGTTGAACTGAATAAACAACTGAGTTCA
GCCAACAACGAGATTTCCAAACTGAAGGACTTGGTAGACGAGAATGTCCGTAAGAGTGTT
GACTTGGGTATCGAGCTCACCCACCTCAAAAGTGCTCATCGGTTGTAACTCATGCCAGCG
GGATATGAACGTCAACTGGAGGTGAATCGGCTGCAAATCAACAATGATGCGACCAAAGTA
CGGGGTGAAATGGACAAGATCTTGAGTAAGGCGAATCTGGAGTTCACCCGCCAGCGGGCC
AGCGCTAAAAACTGTGGAGAAGTGGCTAAGGCGGTTGGGGCTATTGCAACCGTGTTTGCT
GCGGGGTAGAAACTATGTACTTCCCGATATGGTAGGTAGGTAGGATGGACCGGCTACTCA
TTAAACACATTGAAAATTCCATGCCGAAGTTCAATAAGTTACTGGTGGATGGATTTCATC
AGAAGGAGTTTAGTAATGCGTTAGGAGTGTTCGATCATTCCATGAGGGCCATTCTGAAAA
GTGTTGAGAAACGGGGTGTCTTTTACAAGAAAACCGTACGAGCTTCACCTCGGGAGTTCA
TTGACTACTTGATCAACAGCAGTCGTAAAGTCTTCGATGTGCATAAGGAAAGCTTGTATC
CGGTTAAGATCTGGATCCAGTATAAGGACCGAGGGGGTAAGTTAACGGACTTTTACACCT
ACACGATGCTCCCGTATACGGATAAGTACGGGGATCTGTGGTTGAGGGGATCGTTGTATA
ACCCTCAGATCGTACTCGCTGAACAGGGTCTACCCGTAACGAAAGAGAACGCCTTGTTCA
TGAAAGTGCTGGATTTCAAGTTTAAGATTGGAATCGAGCATTTCAATTTCTGTCAAGTCT
TTACCGACACAGGGATTGAGACCCATCGTCAAACGGATATCAACCTGGCGGCTAAACGGT
TCTACAGTCCAACCGGATCCCGGAGGATCAAGGACAGTAAGACCCCTGTTCCGTTGTTGG
CGTGGTATATCTTTGCGGACATGGGCTTCAGTACGGCCATGGAGGAATATGCGGAGTGTG
ACTTCCGTATAGGCTCTCTGGATGCGCTGCTTAACGAATGCAAGGCAGAAGAGCGGTGGG
AAGTCTTTACCGGTCCTAAGCGTGCTTCGGATAATAAACCTTCGTTAGGTGATTATGTCA
GTTTGGACATCGGTATTGCGGTACGGAACAAGTCTAGCTGTCGTAAGGAACTGAGCGCAG
TCGGGTTACAGTACAGCTGTGCTTTATTGTTCATTGCGAACTGTTGTTCGTCGTACTTTG
ATATTGAACGGTTGGATGATCCGGACTATTGGAAGCTGATCATAGGGCGGTGTTCCGTTA
AGGCGGGTGACAGTGACGAGTACATCATGCGTTTGATGTATGAGCACTTCGATTCCATTC
GGGAGTACCTGGAGGAAGATTCGATTAAGAAGTTCGCTGCTAAATCGATCGTAGTGACCA
ATATGTTTGATCTGTTCAACTAGATCATTGCGAACCGCAGTGAGATTGTCCAGACCACCG
ACCGTGCGTCAGCGTTCAGTAAAGACTTAGCCAGTTTGGAATTCACGCTGGACAGTCTGC
TGACGTCTGCCAATAACTTCAAGCAGGATATCAAGAACAACTCGGAAATCAACCAGAAAA
AGGTGGCGAGGTTCTTGACTCACAATTTCCCTATTAAGGAGATCGACAATGCACAATACG
CCAACCTTATCCTGGAACCCACCCCTACGGATGATCCGTTCGTAGACTACAGGTTGGGCT
GCATGCCCCAACCCAAGGTCTATACCGGTGGTGGTAAGCGTAAACGGGGTGACTTCGACA
CGTCAGATAGCTCAACCTTTAGCCACGCGTCGCTTCCCTTTGTTCATAGTTTTTACGGG
TTACGAATCCTAAGCCTGATGCACGGGGATTCCTTAATCCAAGCATTTATCTGATCATTA
ATCGTACAACGGGATTAGACCCTCAACTTAAAGAACTCTACGAACTAACAGATAAACGCC
TCAAGAAAAGAGAACCATGGCCATGGCAAACATCCTCCGTCCATCCGGACAAGTCAACCG
AGGAACCGTCTCAATAGATCCTAATGCGCCCATCCCCGGGGCTTCTGGTACGACGATGGG
CCTCGGCGCCCGTCAAGGTATGTACAACCAGATGTCACCCTCCATGCAACTCCGTGGTGT
TCTCGAACACGGCCTGCAAGGGAATAGTCAGGGTGGGGGTGGGGGTTCCATCCATTGGGA
ACGCTACGGTAACTCCCACCATGAAGACGCTGCACTCCGGGAGATCCGGGAGAATGCGAA
TTCTGAAATCGACAGTAACCAACTCTTCTTGGAGAAGTTTTCCCGTGCTGGGGTGGAATT
GATTCAGCAGCTAGGAAGACGACAAGGGGCGTTCGATAACGAATAGCGGGAGACCTTAGA
AAACTTTCGTTTTGTTCCCAAAACCCCGGTTAAATGTCACATTCGGGATGAATTCGTTAA
CAACCTGAACAAGTACCCTGAAACCCTCCGCTTTATCTCAACGGCCTCTGTGCCGATGTT
TGTCCAGCGACTGATTGTCCTGGGTAAACAAGGGTCTTCGGAGTTGACGACGTCGGACTA
TATGAGATCGGCAGTCTTTGCCTATCGGTCTGTTTTAACCATGGAGATGATCTGCTGGTT

GTGCAAGAGTCCGGCAGGTCGACAGTTCACGTATAGGTTGACTCCTGAAATCCAGAAAAT
GATCGCGAACCTGGAGCATTTCAAGGATACGTTTAGCCTCGCCTGTAACACGTTAGGTTG
TACGAATCCTTATGGGTCCCTGGTGTATGAGGTGAAGACGTCAACCACGACGGAGAGCCA
TCTCAGCCACGAAGCACAAACCGCCTTCCTTCATTCTAACTTTGGAGCCAACAACCGCGT
GGAATCCCAGAACGATCCAACCACGGAAGACATCTTTGAAATGGTGAGGGAACGGGCTGA
GCTGGCCAGACGGGACAACTAGGTGTATACTCGCCCTGAAGAACCTCGGTTCCAACCCCC
AGAAGTCCAACGCTGGAATAAGGTCCGGAATGATCTTGAAAACCTGACCGTTAAGAACAA
GGATGAGTCTGATCTCACAAGTTATTTCCAATACATCGGTAAACCCAAGCATTATCTGAT
TCCGGAACCGGATTGGAAGAGGATAAAGAAGGTTTATAAACGCCACCCTGAACAGTCTCC
TCAAGAAGAGAGTATCCTCCCGGGTTGTTTCCGTATTGTGATTATTGACCTGGAAAAGGA
TAACGGCTGGTTCAGCACTGTTGTAAGGAGTCGTACACTCGATATGGTGACTGCTCTGTC
TAGCCCTGAAAAGCTGTTGCCGTTGCTGGAAGCTACGGAAGACGCTAATGACGTGGTGGT
CCTGCCGGTGGAGAAGGTGGTAGAAGATACCAAGTCCCTGGAGATCACGCTGGAGACCTG
TGAAGAATTGGAAGGGATCCCAGTGGTTACGGTGTCTGAAGGCATCGTCAGCGCGACGTC
TAAGAAGCTGATGGCGGCTGTGGTGACGACCAACACCGCCCTGAGTAAGAACATGCCAGG
GACCAACGCCACGTCGTTCTCCGCTACGATCTGGGATACCTTCACCTGTTCCGATGCTCG
GGATAAAGTCCGACTCAAGGACTATCTCCCGTTCCTGTTTAAAGACTTTAAGGAGGGCGA
TCACTCCCTGTCGTATTACCACCGGATGACAGCGGTTAAGGAGTACTTGGATGAAGGGAT
TCTGGATGAGGAGTTGGGTGAGTTCTTTCGGACTCGTCTCACCGCAACGGTGAATGCGTG
GTTCTTCAGTTCCCTGGGGTACGAATGCCTACCAAGCTCACGGAACCATCTGTCGGTAAC
CGACATCGTCGAAGACTTCGACGAGCTGGATAAGATCCTGGAAGAAGATCATCCTGAAGG
GTACCGACGGTTCAATGAACCGGGAGGGCCGGGTAACTACCTGACGGAACAAATGAAACT
GTTCGTTCCCGATGGGAAATACGATGGTGTGGAAAGTGAAGAGGGTGATCTAAGGCAAGA
AGCTCAGATGAAGTTGGAGTTACCGTTAGAACGCCGCTTCCACATCACGGTGATCAATAA
GCGCAATGGCCCGTTGCCGGATCCATCGGGAGAACCCTTGATCATTAAGCGCAGTCGATT
CCCGGAGTACTTCGATCTCATAGAGAAAGGATTCAAGGGAACGATGAAAGAGACGGATAA
CCTGGACGTTATTGATAAACTGATTCAACTCTCCGGAGGTGATCAACTCTGGTTATTCAG
TTACTCAGCCGTCGATATGAATGTCGCCACTCTTCGGGTGGTCAGTCGGAGAAGCCCCTT
GGTCCTGTTGGATCTCTTCTGAATACTTTATTTAGTCGCGATTGAATTAAAACTAGTAAC
CTACCCTAGTGGATCTCCACTAGGGTAGGTATTCCTTATTGGTTTATTTATTTAACTTAC
AGTTATTTTCAGATCTAAATAATCAGGGTGAATAGCAACAATCCTATTAATTGTTAACTG
TTATACTTCCCATTTCCTACACACGAACGAACATTCACACATGACCACTTCCAATGACAA
AGCCAAAACCACCAACAAGTCCTTCACCGGTGAGCGCAAGGAAGGTTTCGCAGCCACAAC
TGTCGCCGGGATCGTCGGAGGCGTCCTAGGTGCCGCCGCTCATTTCAATGGCACCAAAAA
CATCGGTTCATCCCTGATCGCCGGTGCGGCCGGTGTGACCGTCGGTTGGGCGGCGGGTCG
TCTGGTGTCCCCTGTTCAACACCTCGGTACGCCAGCTACCATTCTGGGTAGTGCCTGGTC
GGGTCTGCTCGGTGCGTCGATCGCCATCACCGCGGGTGTACTGGCTGGTTCGGTAATGGG
TGCTGCCCAAGAAGCCCTGCCGAGCGCCGGCGAGTAAGTTCTGCCCCAAGGAGAAGGTTA
TCCCTTCTCCTCTTCTTTTCAGTAACCCGCTCAGCCAGGAAGGTGTTTATTATGAAATCC
AGCGGCCTGCTTCTTCTGTGCGTTGGTGGTATGTTCGGCTTCTTGTTCGTGAACGAGAAC
TACGGCCAAGAGACTCAGGAAGATGAATACCAAGAAGTGATCTTGGGACATCGTATTGCT
CCTAACGGGCAAGAAATCCTGGTCTACCAACACTGAAGAACCCACCCCCCGCTTCCTTGT
CCTGTTTAAGAGGGATGAGGAGGCGGGTTCTTTACGTCTCTTTCAATTTTCACATAGGGA
AATACCATGCTCACGCCAGATGAAGCGAAGAAACGTCGGGTAAGAGTGGCTGAAATCTTT
AGTCTGATTTCGGAGGCTAATCAGCTTCTGGTTAAGGTCTCAGCCTCCGCGAATGCTAAC
AGGAGTGAGGAGATGTTTGCGTTATCCATGTCTCTGCTGGAAGAGGCGGACACCAAGGTG
AATGCGTACAAAAAGGAATTCCCCAATTGTCTCTCCTTGAAGTATAACCGTTGGATGCAA
GACGGGGTGAATTCTGAGGGTGGGGTGAGTAATAAACAAGGGCTTCCTCAACCCCCTCTC
TTGACTGAAGACAATCAGCTCTCCCCTGAGTGGGTGACCTGGTACAGTCAGGTTATGGGG
TATTCCCATTCCCATCCCTTGGAGGTGGGTGAACTTCTGTTGAATGCGTATCAGGACCTT
CCTCCCTCCGAAGTGTAAAGGATTAACGGCAAACAGTCTCCCCCCTTAGGGGAGACTTTC
TTGAGAAAGACGTATGTTTAGAATGAATAAGGGTTGTTACCCTACCGGAATAAATCCTAG
TGGAAAGTTTCTGAGTCAAGAGGAATTGTGGAAACTCCCCAATGGGACTTATGTAACCTC
CCCTTCTCACGATCATCACGTCTATCAACACGCCCGTCGTGTTGAAGTTCGAGATGGTCA
ATGCTGGCTGGTCACTGATCCCAGGTATATCTATTCGCTTGAAGACTTCTTTAAATACAA
CCTGCTCTACAGGAAAACCGCGTGAAAAAGACCTGGATGTTGATGTCGCGTAGGAGACAT
TCACAGAGCAGTATGGGATTTACCTAGCGAGTAAACTCGGCTCATTCATCGTTGATTAAA

AACCTTTTGTCATTAATCCACGTATAGGACACCGTTATGTCGAAGAAAATGCCGCCGGCC
TTCCGTAAACTGCACCTGGAAGCCTTGTACACCTACCTGAATGAAACCACGGGCGCGTGT
GTCATCCTGGTTAACACCGACGCCGGCCCTGTAGTGAAAGAAATCGGTCAAGGGAACCCC
AGGGTCTTCTTCAACATCGGAGGGTTCGCGGTGAAGTACCTTTATTTCCATGAGGAATAC
GTTCATTTCGAATGCCGTTGCCAGGGGAAGGTCTACGAGCTCTTCATCAATTATGAAGAT
ATCTTGGGTGTGCGGTATAACAGAGACTTGATTTACCTGGGTGGGGTGTGCCGAATAGTA
GGTCAGGATGAAGACGGTCGGTTGATGCTCGCCCCTGGTGAAATCGCTCCGCCAGAACAG
GATGATAAGAAAAAATCCTCCGTCCCCAAACCTCAGAACGGACACACCTCTACGTTCAAC
GTCATTGAAGGCGGTAAAGACTAACCCCTCACTATCAATACCCCCGGTAGGGATAACCTA
CCGGTGGTTAAATTATTCTGTTGAGTGTATCTAATACGTACATTGACCAGAATCATTTTG
AGTTTCATAAACACCTGAGGTAATCCAAAATGCTACCGATTAAATCACGGCTACAAGAAA
TGATCATCAACGCCCAACCCCTCCCCGGCGTTGTGGATGACATTTACAAAGGCTACGAGG
AAGTTAGGGTGTTCTTCTCCAACTTCGATCGGGTAATCACTCAACTCCCTTTCGATAAGG
ATATTCTCAATCACCTGCCCCGGGGTGAAGCCAAGACGGAGGACTTCTTCTATCTCCACA
CCATCTACCAGGAGCAGAACAAAGGCGCCATCCATGGCTTCGGAGAAGGGTATTCAGGGA
ACATTCGTTTCCGGATTAAGATCGTACGGAACACTAAAGGGGCCATTGCAGGGCTGACCG
TATGGACTCTGGGCTGGGGAATGAAAGATCTGGAACGTGATTTCAAGAACAAACACCTGT
TGCATGGGGGTGTGGATTACAAAGACGAACTCCTGGGTAAAGTGGTTCGTCATATTGCAT
TGGTCAAACTCTTCTACATCGAAGACATTTAACATCTTTCAGAAGGAGTAACCCGCCCTG
GTAACAGTTCGTAAGGAAAGCAAAGAACGGTCTCCTCTGTCGAGTCCCATCGTAGGCGAA
CGGGATTCCGTTGATTTCCATAACAACAAAGAACACCTGGAGAATGGGACATATCGAGTT
ATCGAGTACGACGGGGAAATCAATTATTGTGTGTTAGACTATCTCGTTGAATCTTGGTAA
AGCGAGGAAGAACACATGGAATTCCTCGATAATCTACACATGGACCATCTGGCCGAGAGA
ATAGGCGAGCCATACTGCGAAGACTTGGGAGAACCGTTCACCCAAGTCCTACATGACAAT
TTCAGTGACCTTTTGACTTAACAACACGAGGGGGTGGTATGAACACGTTTTACATCATTC
AGGTCGACCAACGTTCCACTTCCCACCCGGGAACAATCACTGAGTATTACGCCGAGGATC
CCTCTTCTCGTGGTTATCCCGATTTCAGCAAGTCCTGGATGGACTGTAGCACTTTCCACA
GCCTCGGAAGTGCTACTGAAGTCATTAACCGGATGCTCACTGATAAGAACACTCCCTTCG
GTGATGGTTCGGTATATCCTCCCCTCACAGTCCGGATACTGCGTCGTGATTACAACGCAC
CTGTTCCCGGTACGAAATACGAGTTCGATCTCGCCATTGTCGAGTTCTCCTACACCGATT
CCTCCGACTTGAAAGTCAAAACAGTTGATCGCTGGGGAGTCGACTTCATATGGGGTGGAG
GAGATAAACTCGCCAGGACTTTCTACAGCGTCGAAACGAATACCGATCAACCCAACATGC
CCCACTGGCCGGGTAATAATGAACTAAGTCTCTTCTACCCCAAGGCATTGGAGGGCGCTC
ACCCGTGGGTGTCCTGGTATATCCGCAGTCATGAACTTGAGTTCACTTCCGCATCAGTTA
AAACTGCTTTGGAAGAGAAAGACAAGGTCATCGGTAAAATCAGTAACCAAAGTTCCAATG
TGGAGCTTTACGACGAGATGTTTCCTAAAGCAGGCGACCGTGTTGCCGTGGGGGATCTAG
TACTCCTGGGGGATGTTCTAGCGCTTCGTAATAAGAACTCCGATCTCACCCTGGAAGAAA
TCCACCAACAAAAATTGTCCCGTCTCGTTTCGAATAAACAATAAAGACAGTCAGTCAACA
GAGGCCCTGTGAGGCCTCTGTTGGTATTTATTTCAGTAGTCAAGGAATTTGTAATGGCTA
GAAAAACCCTGTGTATGTTGATTCGCCATCACCAGTACGCGCTGGTAACCAATGACATCC
AAAAGAGCGACTTCTTTTACACCTGCCCGTTTGAATCGACCGTTGAAGGCCAACCGATTC
TCTGTGCGGTGTTCTACAAGAAACCCAACCCCGAACGCATTAAAGCTGCGTATTCGCTGA
CGTCTCTCCCCTGTCATTTGACACTTGAAGCATGGCAGAGCCTGATTGAGGATGCAGAAC
AACGCGCTATCCTCGTTACTCCCAGGCGGACCTATGATTTCCGTGGGGAGGAAATCAACG
TGTGTAATGGAGAGAATAACGGGTTTACCTTTGGAGCGGATGAATTCGTTCACCTTTTTA
GTAAGTACCTCCACGTTAACGTTCCGTTGGGCGCTCTCTACGCCGACTAGCAAATTGGTG
AAGCATTTGAAGTTATCGCTGTAAAACCCCGTAACTAGGAGGAGTGTTATGGGTACCCAA
ACGATCAGTTCGGCGAGTAACCCACAACCTTCCATTTCGTATTCGGGTGATTTATCGAAA
GAACGATCAAAGAAGTATTTGAATCGATGGCGAAGAATCTTCAAGATATCCAGCGCCTGA
AGGGTCCTAACGCAAGGGTGTGTATTACCTTAGAGATTGACGGCGAAATAGTCGATTTCA
TCCTGCATCAGGGATATAAACATCAACCCACCCTCTTAACTGCGATTATTATCTCAATCC
GTATTCACAATGTTGAAGGTATCCATCCCCAGGGAGTCATCATGATGTACAATGATGGCG
AATTGTCGATGGGAATCTACAAAGATGCTAAGTACGGCTGCGAAACGTTGGAAGGTGTGC
TTCGTGTCGCCGAAGAATACATGAGTGAAGCCGAAGAAACCCCCCGCACCCTCCATTGAT
GGAATCCTCTACCCCGCGTCCTACCTCCCTACTCCCTTAAACGGAAGTCATCATGTCTAT
TTCCTACATCATCCGGATTGGTTCCCGTCTCAATAACCAACGAGATGCCGTTACACGCTT
CTATAACGTTCATGGTGGGGCGTTTGACCATCCCCGCTATATGACCCACTGGTACGACGC

AAAAGCATTCACAACGTTCCCTGAAGCCGTTAAAGTGGTTAAACCGATCACTGATGAGGA
ACCGGGGTTGTATTCCAAAGATCCTTACCGGGCGTTCTACGACCTCTGCTCCGCAGGGTT
GGATCGCCGTATAGACCCGGATGGGTCCTACACCTTCGTGATTTACATTATCCGTTGCAA
TTCGGAGGATCCTTCGGACCCTCGAATGGAAGTGGTGGATCGTTGGGATGTGGAGTTCTA
TCGGAATAAAACCCTACCCGACGGTCAGTTCAGTTCTATCGTATACCACGCGGAGGATAA
CCCCGAGGAGAGTGACCCCGGACGCTTCTACGTCCCAGGGGACAACGAACCCAGTGTCTT
CTACTGTGACCGATTGGGTGGCGCGCATCATTGGATCCATTGGAACATCATTACCCATGA
GCTCGAATTCACCCACAAGAACATCGAATTCGCTAAGGATGAGAAAACCCGGGTAATCGA
ACAACTGCTGGAACAACAGAGGTGGGTGGCGTACTACAGTGAGAAATTTCCTCTCACTTC
CCGAGAAATCGTTGTTCACCAACCCCTGCTGTTGGGGTATGTGTTGAGTAATCGTAATGA
CAATCCTTCCCTGTCGTTGGAAGAGGTCCATTACCAAGAAATCACCTGGCGCAACACCAA
ACCGTCTGCTCATCACTGAGCATTCCGTTAATCCTTATTCCACCGATGGAGAATGAACAT
GGGAATGGTCCATTGGGCTCGATTGGTTAAAGAGTGCCTCCGAGTTCAAGCCGTCAGCGA
TGATCGCTACCTGGGGGTCTGCTCAGGATTGAGTTTGTTGTCCCACAAGTACTTCCTGGC
CAACGATCTCCCCCTCGAAGCCAACTACCCGGTGCTTACAGCCGCCTTTAAGGAACGGGC
AGACCTGGGCCCACCGGCTAAGGAATCGGTTATGAGCGCTGAACACGCCCAAGAACTGGT
TGAAACGGTGGAGGAGACCCTTCGTTACTACTTCAAGTTCTATGCGTGGGGTTGTAACCT
GAAGGCTTCCTTCTGGGCGGAGGTAAAGGGTCAGCATTAATGTTCCACATCCGTTACAAT
CTATGTCGGGCACTCTTTTTCTAGAGGAGGTAACCCTATGCCACGTGTATCACGTAAACA
AGCCAACCTTATGCGAGACGCCGCCCACAGCGCTGAGTTCGCTAACAAGGTGGGTATCGA
TCACGAGGTGGCTAAGCATTGGTACGAGGAAGACAAGAAGGTGGGGTGGTTCGACCCCAG
TTCTAATAAAGCGGAATCTTCGAAAAAGAGCCCTACCAAGTCCAGTTAGTCGTTTCAATA
ATCTTTCCCTTGTAAGGGGTGCTCTCGTAACAGTAACCCTTAGCGGATACTCGCAGTCGT
CCGTGGCCGAGAGTATAAACAGTACAGGTCTATCTATTCCTTGTGATGGGGTGAATAGGT
ATGACAATGGCGAACGGACCCCTTTCGATTCAAGTTTAACACTGGAAGAAAAACTCAATG
AATAAATATTGTGATCTTTATGACGAGTTGGAACGGTTTGTTGGGCTGGTTAAACCTGTT
GAATTTGGACTCCAACTGGATTTTAAGGACCATCAGATTATCGGGAAGTTGAGATCTGTT
GGTCTGCACTTGCCGCGTCAATCGGGTAAAACGACCCTGTTGTCCAAGTGGTGTTCGAAA
CACCCTGACGAAGTGTTCATCGTACACGGGTCTTCTCCATGGTTAGAGGCTTTCAAAAGA
CTTTCAAACCTTCCCGAACTCCCCCCTTGGGTTTCGATCTCCAAAATACGAGCCAACCAA
CCCTTGCTTACGGATCACTCGACCCTGAAGAAGATTAAATACGTGGTCGTCTATGGTTCG
TCGGTTATTTTCTCCTACTGTGGACTCAAGCGTAAAGACTGCAACCAATGGGTGGCTGAC
ACCCTTGGAACTGAAATCGTGGTGATCCATTTAGGTTAAGTTTACTCCGTGATAGCTCAG
TAGGTAGAGCAACTGTTTCGTAAACAGTAGGTCCAGGGTTCGAACCCTGGACACGGAACC
AAACATCGTTACCCCAAAGACTGGGACACACATGCTCGGCACCACCCTCCCCCAGATGCC
CTGCGCCGTCCCGCTCCGCCAGCCATCCTGTTCTCTTCTCTCTTCCAGGGTGGACCGCGG
CTTCTTGACACGGGTGACGGTGTACCTGTCCCCTACCTTTCTCAGAGGGGTGGGGAACAG
GACCTTTTCTGTATCCGCTATGTGCAGAGGATAACGTCATGTCGTTCATTATAAAGGTCA
TGCGAGAGGGCTGTGGGAATGGCACCTCCCGTGACTATTAAGGCATTATTGTGGGTCAGG
GGGATCGGTTTGCTATCTCTTGGGATCCGCATAAAGAACAGCGGGAGCTTACGGTCGAGT
TTGCTAATGGGAGAACCTCGCTCCGGGTGATTGATGGGGATCTTTACGTGGTGAATGCGG
ACGGTAAGACCATTTCCGGTACCCTCAAGAATAAGCCCTTGATCCCCGGTACTTCCCGCT
CGAACCCCCGAGACAAACTCTCTTCTAAGCTCTAACCCGTAAACACCGTGTAAACACCCT
CCTACCTCCTTTACAGGGAGGTAGGAGGATTTATTACGGTATAGCGTATTTTAAACCTAG
ATTATTGATGCGTTAAGCGACGAGGAATAAATCAATGTTTGCTGAAAAACAAATGAAAGA
ACTTAGCAGTGCTCGTAGAGAGGATGTGTGGAGTTATGACATATTTGAAATGACACTATT
CCTGATGGAAACAGACTGTGATGGTGGCGTCCCCTTCTTGAAACATATCACCATGTACAG
TTTCAACAAGATGCTCTCTGGAGAATCACTAGATCGTTATGCGATTGGACTGATGAATAA
AATGATCTACGATACTGAAGGCCATGACGCCAAGGGAACCGTAGAAAGGGTTGTGTTACA
ACGATTGACTGAAAGCGAGATCCTGGAAGCCCACGCCTCCCTCATGATTTACATCACCAA
AGCTAAGGAATTCCTGGAAGACCTCAGTCAACGCTTCCCCGATCACTTCCGGAGTGTCAG
CCGAGAAGAAACACCCAAAAAGTTCTGCTTTGCGAATAAACCGTCTCCGGAAGGGACGGT
CAGGGAACTCGCCGCTAAATACGGTAAGTCGATCAGTGAAATCCGTAAACTCAAAGCAGC
CGGTCGTCTTCATGAACTAGCACAGGGGTAATTGGTCATGTTTTGGAAATTCACGTGTTA
CAACAAGGACACTTGGGAAGCCCCCAACCAAATGACCTTGATAGAAGCGGTCGAACTCTT
TATACGGGAAAGCCAATACAGCCAGTGGGATATTCACTTCGTGGAGAATCTCCACTAAAC
TGTTGTTAAACAATCTGTTAAACAGAAGGATAGCATTATGAAACCCTTTCGAGTACCTGT

TGGACGTCTCAGTAAAGATCGACTGGAATAGCTCATCCGTCATCGTAAAGAAAAGAAACG
TGAATGTCCCTTTGGTGTGGCTCACTTCCCCAAAGGAACGATCGCGGGATTCGAGTCCCC
AACCCAGCGTTTTCTGAAAGAGGACGAACGTGAGTTTCCTGCTCAGCGGTACGCAACGTT
CGAAGCGGAAGTGGAGAGTGTAGTCCCCAACGGACCGAACAGTTGGGTGGCTGTTCTAAA
GGACCGTACGGCGATTAAAATCGACTGGCGCGCAAGATCATTAAACGGGGGGATGGTCC
TCTCCGCGCCGCGCACAATGACGTGGAGGACAGTCTGTGGAAAACCAGAGCCACCCACCC
CCTGGATCCTGACACCCTCCTTCCCATTAACAAACACCCCAAACACTACTACATCGACTC
GATCTACCATGTTATCTACGGAAAATTGGAAGAGGTTAATAAAAATAACTTCGCCACCCA
TCTGAACGATGTGGATAAGTTGATTTCGCTGATGTGCGGTGATATCATTCGTCAGTCAAC
CTCCTGAGGTGATGGTGTTTACTGCCATTATCTCATCAGTAATAAGAAGTTCCGCAAGAT
CTTCAACCGTTGTCTGCCGCGTGCGCGCACCGGCCGGCGTAAAGAAGCTGAACGTCAGGC
CAGGATCGAAGCATTCGTATAAAACAGGACGTTTGATGACCGTTATGAAGACGATTACGA
CGACCCGGGGGTGTGAAGAAGTGTTAGAAGCCCCTTAACCTGAAGACCGCCCCACTCCGT
CTTAAAAGGAATAACTCCTACGTGAAGGAGGGGATTCACGTAAATGCCGTTAAATCACAC
GTGTAGTCCCCAAGCCGTCTCTAAACGGATCTTCGGTAGAAGCCGAACGGTTTACTTAAT
GGACCGGTTAGCCGTTAAGCTACCGAGAGTGACAGATTAGATACGCCATCACTGTATTTT
GGAGAAGGTTGCGAAACATAACGAAACACCCTTTCAACGAGCCAGCAGCGGGCATGTCCT
TCAGGTCGGTGACAACGGGTCTTGCCATGTGGGGGATCACGGGACCGCCATCGCTGGAAA
AAATAGCGTCGCCACCGCTGCGAACAATGGACACGCTGTTTCTGGAGCCTATGGGACAGC
CATCGTTGAACGCTTTGGTATCGCCTCTGCCGGAAATAAAGGCAGGGATATCGCCGGAGA
TTGCGGCAGTGCCACCGCTGGAGATCATGGTATGGCCACCGCGGGAAATTACAGTACGGC
TACTGTCGGGTACTTAGGTACTGCCACCGCCGGAAAGAAAGGGAAAGCTACCGCTGGAGA
TTGGGGGACTGCTATCGTTGGACTCGATCGCGTTGCGTCTGCTGGAAAGAATGGAAAGAT
CCACATCCAGTACTTCGACAGTGTAGCCAATCGTTACCGCACAGCAGTTGGGTATATCGG
TGAGGACGGTCTCGAACCTGGCGTTCCTTACCAATTGGAGGAAAACCAGGAGTTCGTAAA
GGGAGACATTGCCATGACAAAATTCTCTACAGAGGGCCACTTGACTATACTCTTCCGAGG
ATTGGGTGTAGGTCTTATCAGCGTCCAGGTCGTCAGGCTCACTAAGGGTATAGGACATCC
CAAACCCTTTTTGCATATCGAGGGATCTGAAGACAAGGGTTACCGTTTGCTTCATAACTG
GAGTGAATGGATTGATCAGATCTTTGAGGCTAAGAAAGCTGTACTCTTCATTGAGTCCCT
CTACCCCCCTGTCACACCCGATAAAGAGACCGTGAAACTTACAGTGCCCGAGGATCAGCA
AATCCTGGTTAAAGTACGTCGAGTGAATATGCTGGCCTTGGATGCTGTTGTGTTCCACAT
TGACACCCTGGGTGGGGCGTAACAGGTATCCTATACCGAGCAACTGACCCCCGATCTCAA
TCTGTTGACTCATGTTGAGTTAGTGGTCATTTAAGGACGTCAATCATGGCTAAAAAGAAC
CTTAAGGTGTTCAAGACCCACGGGGAATTCGATACGGCGGTGATTCGTCGTGCTCGACTT
ATCCTCGACGCCAGGGGTTACCGGGGGAAGATATGTGTGAACGCACTCGGTGAAAGTTCT
GCATGGAATAAAGCAGATCGCAATGAGGACGGTCATCGTCTGATCGACGACGACACTATC
GCCGTAGTGGCTCGCTCGATGGTTTACGACACTCTCGATTACGGAGAGTAAGTTTTAACA
CGTTGACCGTATTATATACGGACAACATGTCGTTTTAATCCGCGTCTGCGGGTCCCGCCT
GAACTTAAGGGTGATAAGGTGTGGTACTCGTCGAGTATGAGCTTGGCAAAACTCATACTC
CGAACCCTATGGACGGTATAGCCGCTATCGTCAAGTCACACTGTTGAGCTATTGACCTGG
ACTTCAGTCTCCTTTTCGGTGCTGTCGGCGGGGAGTTTCTCGGACGGTGAGGCGGCTTGT
GAAGGTGCTAAACTAGCGCTGGATGAACACCTAGCAAGTTAAGGGGAGGAGGGACAGCCA
TCGGTTCTAAGCAGATGGGTTCCTCCTCCCCTCGCTCCTTTTATTTTTTTTGTCTTAATA
CCGTGTTACTCGACCGGTTTCTAAAGGATACAGACTGTACGAATATTATGTCTTATCCCC
CGACTTACCAAGAGGTTAAAGCGATGTCGAAAAACAACGATCAGAAGAACCCCTCCACTC
CTGAGGACGAGAAGGCTAAAGCGGTAACCACCCCTCCGGTGACTCCTCCCGCTCAGCCAG
CTCCCGCTACCCCTCAAACGTCTCCCAAAGCCTCGAAAGACGAGAGTGAAGAGGATAACC
AGGACTTCGACAACATTCTAATCAATGTACTGTCGCTCTAGGACAATGGGGAATTCAATA
AGGAACTCCTTATTGAGATGGTGGAAGAAGTCCGTAAGGTTACCCGTCCGGGTCTTATCC
CGGAAGCATACTACATCGTGGTCGCCGTACAGCGTTATGTGGAATTCGCCAAACGCAAGA
AGATTGACCTGAAAAACCTGTCGATCCAGGTGGGTAATCCTGAAACCTCGGTAGAACTGC
GCTCGCTGAACTTCTACAGTGCAATGGAAGGCCTGGGTCCGACCTTCTTCATGCCGTACA
TCATTTAGAGGAAAGAGTAACACTGAATAAACCCCTAACCCCTAGCCCCATTAACAGGGC
TAGGGGTTAGGGGGTATAACATTTATTTTTGATTAAGCAAAGAGATTGGTATCGGGACTG
TTTTCTTCAGGATTGGTCCCATCCTCATTGCCTTCTTCACCCTCAGGAGAGGTTTCTTCT
TCCTCCTCTCCTTCAGTACCTGCTTCAGAACCTTCCTCTTCCTCTATACCTTCCGGTTCT
TCCTCTTCCCCGGGAGGGGTATTCTCTCAGGCGTTCTGATCCCCTTCAGGGAGATTCGCC

TTATCCGCGGCTTCTTGAAGGTCTTTATGAGCCTTATCGATCTTCTTGGCGTGTGCCTTA
ATGACCTTAGAGTCCGCATCACGAGTCCCCGCAATCAGGGCTGCCAGGAACTCCCCAACA
TTCTGACGCTGACTCACAACGGCGTTCACCAAGGAAGCAAGACCACCACCCTTACCATCG
TTAAGGATGTCGTCTAATGGCATCGGGAGATTATAGCGCCTGAAGGCTTCAACCGTGAGG
ACTGACTTCACTTGCTCGTTAATCGCTGTCACGATCTTGTTATTCCCTTCTCCATCATCG
GATTCGGGAATACCCAAGAGCTCAGCAATCTGTTTCAGGTTACCCGAATGACCTGCCATA
GCGACCCACGACTCCACCAGCTGACTCACCGTATCCAAACTGTCCTTGAGCTTAGCAGTA
GGTTCAACAGAACTGGGGATCGGGAAGTGGGCTTTAAGGTTAGTCAAGAAGTCAACGAGA
ATCATTTTAATGAACTCTTCGTCAGACCCTTCCTGTTTCTCATTACTGTCCGGTGACCAG
AGTTTCTTATTCTCCGAGATCTTGGTCACCAACTTCTTCATTAAAGGCACGTTCACGTGA
GCATGCTTACGTTCAAAGTCAATAATGAAGTCTGCCAGGCATTCTTGCCAGTTAACGATT
TGGTTCAGGAGCATCTGGTGTTCAGCCAGGGCCTCAATCTGGAAGTTGTTCTGATCTTCA
CTGACATCCAACCAACTCCGAGACAGGTGGAAGTAGTTGGCGATCTTGTTCGACAGCTCC
GTATGACTATTCTGATCGACCGGACGGAAGATGTTCTTTTCCTATTGATCGAGCTGAATA
TCCGAGGCGGGGAAGTAAGCGTTCTCGCCCGCATTGACTTTAACCGTTAAGGAGTTCTCC
CTCATGGTATCCACAAACATGGGGACTGACAGTAGAGATGTTGACAGAATACTGTGCAAC
CGTGGGTTCGCTTCAAAGAACGCCGCCCTGGTCGGCGCAATCCCCGCAAAACTCTCTGCG
TCGTTCTCCTGGAGGTTAATCGTCATTTGGGTGTGGGGTGTAGCCGCCTCCAGGTTCGCC
AACGCGTCAGCGAGGTCTAAGGCCGCTAAACGGGCAATGTGCATTTTAGCCATTTGAGTC
AACGACTGACCAATCCCTAAACGGTTATAGTGCAGGGCCATATAGGTCATCGCTTCCCCA
GGCACATACAAACACCGAATCCCCTGTCTCCGGAAAATGCGTTGCAGGAAGATCTTGTTG
GCTTCCTCATCGATCTCAATACTGATGTTCTTCCCACGACCACTGAGGACTGACTGGATG
TACCGTCGAATGATATTCGATTTCGAGATCTCAATGAACTCGATCATATCGAAATCACAT
TCTTTCCCTTCTTGGATTTGACGAAGCTGTTGAACCAGGTTGTTCGTACTACCGTTTTTA
GGGCGGTTAGCAATCATTTTGGCATTGCTTCGGTTACTCTGGTAGTACTCGAAGTCATTC
GTATTCTTCAGGAAACTCCCATGCCGAGGATCTGTGAGGAGGATAAAGTCCGTTTGAGTT
CCATTACTCCCGTTGATGTGAATCGGAATCACCGCTTCACTGGGAACCTTGTAGGTAATC
CCCCGTCCATAGGGTGCAATCTTCAAGGCATCGACGGGTTTACTGAATTGAAGGATCTGA
CCCACCGGATTCCGTTGAGGGAACAAACGGTTACTCAACTCGTTAAATTCACCTTCCGTT
AAGTTAACGGTAGTGGCTTGAGGATCAGCAGGCTTCATACGTTTGCGTTTCCTACCTTCC
GCATCCGTATAGTGATGTTGATCATCTCCAGGGTTCGTACGGGCCACCAGAGCGGGTTGG
ATAATAAGGTCCAACCCTTCCGCTCCCGCTACCGCGGCAACATTCGACCGACGTTTACTT
TCCTCGAACTTCTGGAGGTACAAGACCGCTGGTTTATCGGTCAGGGTAATATCAATCAGC
TGAGCCACTTCCTTCGAGACAGAGTCTTTATCGTCTTCAAAGATATTGAATTCAACTTCA
GCATTCTGAGAAGAAGGGTTCAGGAAAAGGGATTCTAGATCACTGACGGTGTTATTTGTA
TTATGACTGGTTCTATTAACGACAAAGACACCTTTCTTCTTCACCGGGACTTTACCGTTG
ACGACTACGAACTCATGACTTAGCATCTCTTTAGCCTGGGTCATGAACTGTTCGTTACCG
GCAACATCCCCGGATTGCTCCGCACCGTTGATCAAATAATCGAGACCTGGGCGACTCAAG
TTAAACAGAACATAACTCCAGGTATTGTAGAGAATATCCGCGATCATTTCCCTCAAATCT
TTCTCGATCTTATATTCATTGGTATAGTAGCCATCCCAGATGGTGAGGAGTTCATCATGC
AATTCTGCATTTTTAATGGGTGACGGTTCGGTGTCGTAGGTGAGGATTTTGTCTTGCTTC
CCATTGGGGTAGAGTAGACACGTCTGCCAGATCAAGGTGGCTTTGTCGATATACGGCGTG
ATTAACCGCAAATCAGTAGCCGCGTTAATGTTGTTGCTAATGATATTACTCAGCTTTTCC
AGTTTGTAAGGGTTAACCGTCCGATGACCGTTGATCTCATCCCGTTCAGGGGATGAACCG
TTACGAGGCGTTGCATTACGCAGGGTCGCTGCAACCACTGGGTTTTGAGTCGTCAACGAT
TTACGCAAAGCAGCAAAATCCACCTGCGTGTTACCCTTGGACTGTCTACGAGTTGTATCG
ACCATTCTTAACCTCTTCGACCGAACTATGGCTAATATTGAATTTAATGCTTATCTGACT
GACGTGTTTCGTCTAGTCAGAACCATGGTCATCAAAATAGAAGCCATTGCCTTACGTGAT
AACAAAGTCCTGGAGGAAGCGGGTTATCCTGTCGGGTTGGATAAAAGGACCTGGCGGTAC
TACATGAACCTGAATGGGGACTACCACCCCAATGATCAGATCATGACCACTACGTCGCTT
GATACCGGTGTCGAAATTGTCTTCAATAAAGACAACTTGGTGACCCATTTAGCGACGGCC
AGGGAGTATGCCACCGGTGGCTATTGGTTCAATCGCTTGGTTGAACGGTACCCGGGACAA
GCTGAATTGATTCGCGGCATTATTGCACCGATCCCCTACGAGGAGACCATAGAGGCCGAG
GACTATAAGATCTTCAAATACAATAAGGCTTTCGTGCAGTGGACTGAGGACCAATTGATC
CCTCAACTCCAGGCTTGGATCTACAGTGAAGTGGAACAAGTCTTCCGCCACGCATACCGG
TTAACAGATGACTTGATGCTCCCTTTCGGGATTATGTTGCTGTAAGCTGATCTGTTCTAA
ACCAATTGTACCGTTCGGCATGAAGCCATTGGGACGCGGTACGCCCATGATTTCTATATT

TGGAGTCACATTGATTCCTACGGGGCGTTATCCAAGTACTAGGAAAGTTTAGATCGGTAT
CAAACCATGTGGTTGTAACGCAACATTGCGTGGATTGATAACAACCCCGGTCAGCAGTAT
ACCTTTGGGAAGTCGATGGACAACCTCCTGACCCACGCACGTATTCCCCTGGCCAAGTTT
GATATGGTGGAGAATACAGCGAATCAGTTGTAAGAATTGGTTCCCAATCCCCTGTACCGT
GGGCTCCAGCCTAACTTGCAGGAAGACTACGGTCGAGAATCCACGTTTTTCGATAGCCAA
CAAATGATCGATAAACAACAACCCTTGGCAAAAGAGAACCCTAATCAAGCGTCCATGTAC
TATCACGACGCGTTGCAGAAAGGGAAACACAGTAACCATTCTGAACTGCCCACGAAGACG
TTGGAATCCATAAAGATGGATTACACCAACCGTCACATCGATACCAAGATGTCGGTAGTC
TTTAATGAATGGATTTACTTAACCGCTATGGGGTATTTTAAAGGGCGTATTCTGGTTACC
GATCCTAAATCCGGTAAACAGGTCAGACTCCCGGATGGGGATGCCTACTACGTGTGGCGG
TACTTGGTGGACTTCGCCAGGGATCAAAAACCGGTCAACATCTGGCCGGCGTATTACCAC
AATGTATTGAAAGCGGTTCCTCCAACCCTGGAAGCCTTGATTGATATTGGGGGTCCTTCG
TTCATTACGCCGATTATGGCGTATGACATCCGTAACGTGTGGATTCCGGTGAACACCTTT
ATTGCACCGGAATACCTCATGGCGTATTCTGAAGAAGTCTACGCACTCATGTGGAAACAC
AAGAAGATGTCAAGTCAGTTCTACGACTTGAACATGCGGGCGCGGATGGAGAACACCACG
AAGTTCATGTACGAGTCTGGAATCGCTACGATCACGGTCCTGACTACCTACCAACAACTC
CCGGACCGGTATGAGTTCGACTTTACGGAATACACTCCGGAAGAAGCCCGTAACTTTGCC
TGGGAGATCTGCAAACGCAACACGGGTTGGGACAGCAACAATAACCCGTCCTTGACGATT
AAACAGTCAAACCTCATTGATATCATGATGTAGCTGTCGTCTTACACCATCCAGGTCGTT
AAGGAAATCGATGACGGTACGGACTTAACAGAGCTCCCCAGTGAGATCTTTATTGGGGAT
CCCCGGTGGATTGGGAAGGAAAATGGCGCCTTCGGGGATTACACCCATGTTCAGATGGAT
ATTCCCGCTCATTTCGATGGTGTTCGCACCGTTGAAGAGGTTCATCCCCTGGTTGAACCT
ATGATCCCTGAGGTGGCAGCGATTGCGGAAGGGTATGGGGTGGCGCGTAGCGATGATATC
TTTGAACGGGTGGATTTCAGCACCAACCTGTTGGACTACGCGGTTCGGATAATCGATGAC
TCCTATTTCAGGCTGTTACCAGCAGATCGTATGGGCCTCCCTGAAACTAATTACGGGCGT
CTGGTATTTAAAGAGCTGCATAAGGAAACCCCTGTAGGTCCAGCGGACTATGGGCGCCTG
GTGTTTAAGGAGCTGAGTCCCCTGACGGTGATTGACCCAGCTAACTACGGCAGACTGGTC
TTAAGAGGGCCGGTTAAGGCGATCACTCCTAGTGGAAGACTTCAATTTAAAGACGTTAAT
AAGGACTAAAGGAGAAGAGTCTAATGACCAACCAGTTGAGACTCATTAAGGCGTCTTTTT
GTCGTCTTCACGGGTTAGATAAGGCAACGGCCTTGAACATTGAATATCAAGGCCGTAATC
CCACCCCCCTCAATGCGATGTTCATCCTTCACGGGGACGGCATCCCTTGCACACGGTATG
AGCACGAACGCATCAGTTTGACGACCCACACCCTGAATCGTTCCCTGGACCTTGACAGCA
GTCTTAAACTCCACCGTTCTGATATTAACGCTGAGATGATCAGCGCTTGATGTAATACCC
ATACGAACGAATCCCTGCTGCCCCAGGACGTGGGTAAACTCATTATTGGAGACCGCGTGA
CAGTGATCTGTTCCACGGATTCTATGCGCTTTAAGCACTCCTTTTCGATGGCTTTTAAAT
AGGAATCACCATGGCACTTTCTGCTAAAGAACTCGAGGATCATAATCGATCTGAGGTTCC
CGCTCAGACGACCACGAATACCTCGATGGGTAATCTGATCGCGAAATGTATCGTCACTGG
TCAACCGATTACAATTCCCAAGTACACCACGCTCAATGAGCATTATGGTATTCTGGCTGA
AGAATCCCTGGGAAGTAAGAATTCCCGAAATTTCTACTTGAAGTATTTCGGGATTGGTGT
AAGGGGATCCAACTGTGATGGTAAAGACAGTCGGGGGGTCAGTCGATTGAAGGTTAACCA
ACACCAACCCATTGACATGCACTTGTTCACCGCAGTTCCCTTTATTGCACTTCCTTTGGA
CGCCCCTTTGGACGTCCTTAACCGCGAGAAGTTTCGGATGAGGACCCAGGAGGCTAGAAA
CGGTATTCCTTAGGCGTTCTATTGGTTGAAGTTGGTTAATTTCGATAACTATAATCCAAC
CGAAAACAAGATCACTCGTGATCCTGTAACCGGTCTGGAGGATGTTAAACCTTACATTCA
CCGTAAAGACGTCCTGGACAACCCACAACCGGTTGACTTTACCTGTGAGGGTAACATTCC
TGTCAGCAATGAGTACATTAACAGTTCGGCTATTTTGGACTGTTCGTTGACTCAATCTGA
TTTGCGTGAGATTTCACAAGCCTGCAAAATTTACTACGGGGATGCGGGCTACGCATCCAT
TAACGAAGTGGGTGTTGCCTATGGGATTGATACTCAATTCCGTGGGGAGATTGCTGGAGG
CGCGACTATCCAGTATACCGAGGTCATGAGTGCAGTCTTTGCTCACTACATGACCGAGCG
TGATGCTCGCAATGCGCTCGCTAACATCAAGATCCAGCTTGCGTTTGACCACGGTCCAAG
CGCGCCCATGCTCTTGCATACCAACTCAACTCAACCCCCAGCTGGATAAGGAAACCGACC
CATGTAAATCCCAGCTCTACTAAAAGGAGAAGTTCACCTTGCACTGGTTGAGAATATGAC
GGCAGGTCAGGCAATTGAGATAGCCAACGCCTCCTATCAAACCACCCTGAAACCGGAAGA
CTTCAAGGAAGCCCAAGCCATCGTTGATGGTGATAAAGTCATTCTGGACCTGCTCGACCT
TGTGAGGGGTGAGGTTGTGGTGCATTTCGTCAACAAAGCGGGTGTTACACGTAAGCCGCT
GGACGATACCACCCTGGTCAATCCCATGGCGGCGGTCGTGAATGCCGATGAGAACATCAT
CTGGGCGGGTCCACCGGAGTTTGTGGGGAGTGTCACGGAGCTGGATTGGCTGAGGTATTT

TACCGACGCGCTACCACCCATGACCCCTGATCCGATGGACTATGCCAAACGGATGGCCAC
CCTGCTGAATCAGTACAACATTGCGGGTAGTCGGTGGACCAATAACTGGAGTGCGGCCCT
TCCACGCTCTGTGACGGCATTCCACGTGGCATATCGTGGTGACGCCCCATGGGCTCCGTC
CAAGTTCATGTTGAATGATCTTGTGTCTGAGTTCATCGTGGTGATTTACCTACCCTACGC
CCACCCCGCCTGTTACCTGTGCTTTGCGTCTTAAAGCCCTCTGCTAGTGTACGGGTCTTA
GCCATAGATCCGTCCACTACCTGTATGGGGGTGAGTGTCTTCGACATCAATATTGCTAAA
GCTCGACCGTTTAAGTTGATTTACGCGAACACGATCTTTGGTAATAAGTTGTTGTTTGAC
ATTCCAGCACAATTTGATGATCGCGCGGACACTGGTATTGCGGCAAGAAGTTGCGGTATG
GCGCGCAGTTTAAGTCAACTGTTAGCTATCTTCGAACCCGACAGTGGGATCTGTGAGGTT
AACTTCCTCGGTATGTCTCCGCTTACATTTAAGCAGTTGATTCAAGTGGTCTCCTTGTTA
GGTGAAACCTTCGTTACCCAGGGTATTCATTTGTCGTATCTCCTCCCTCGTTTAGCTAAA
GCCGTGGTGGGCGCAGATTTCAGTGGGAGTAAGAAGGAGGATGTTCATAAAGGGGTGATG
GACTATCCGTGGATAGACGCCGGCGAGATTGATATGACGGTTCTGGATGAACACTCCGCT
GACAGTGTCGCTATCGGCCTCTATCGGTGTGAGCAGATCGCCCGGCATTACGGTGTCTCA
GAGAGGTTAACGTATGACTGAAACAACTCAAGACGATGACCCAGAGCTACCCGCCAATTC
TCCTCCTGTTGAAGGAGGAGTGGAACGGGAGATCTTTGGTAAATGGAAAGTATTGTTGAT
TAACCTCGGTGGTATCCTTTCCGTGTGGGTGGTGTGTTTAACGGCACTGATTAACTCGTT
TACTATTTACCTGTCAGTAACCACCAATGGTAAATGGCCCAGTGAGATGAGTATGTTCAT
CATGATGGTGGGGCCGGTGATCTGTGCATGGGGGTGGATGAAGGTTAACGGTACCTTAAA
GACTATTATGTCTCAATCGGGGTTTAGTAACAAAATGAGAACCAAGGTGGCTGAGATCAT
TGCGCCTAAGCCCAAGACAGAATAAACCCAATAGGAACCCTACCTGTAGCCTTTGCGGGC
TACAGGTAGGATTTATCACGCGTCTGTCATTTAGGTAGAGCTAAACTCTCAATTAACACA
TTGATGTTAGCAATCAACTGTGAAATGGTACGCTGGCTGGCGTATAACTTCATGTTGCTG
TCATCCAACGCCCGGGGCGTATTGATCAGCAGTACCCGGGTAACTCGATAACAGAGCTCT
CGTGCCTCCCGAGAGGGTTTCTCCAAGCAGTCATACTCCAGCAGAAAGGTCGTTAAACTA
CTTAACTGACTAGGAGGCATCTCCGCGGGTGGGTTCTTGAGTTGAGGGAACACTTGGGTC
AGCCTTTCCTGAGCTGCCAGCACTTGCTTCCGGTACGTGTTGATCCTTACTTGCTCCGCG
TGAACCTGGAGGATTGAGTTGGTCTCGGACAGTTTGGGATTGTTGCTGCATCCCACTAGG
ATCAGGGATACTAGACAGATTAACATCCACCCCATACTTTTCAAGTAGTTTTGCATAGCC
TGAGTTGTCCTCTTCAGTTCGCTGTTCCAACTGACGGGCGAGGGTTTGAGTCAAGGTGGC
TGTGGCATTGGAATTGTTTTCAATGTTGTCAGCCACCCGTTGCAACTGGTTGGTAACGCG
CTGAATCCCATTAACCGCTTCTACACTGATAGTGGGGTTACGTGGCACAATAGCCTGCCA
CGCGACGACCGCCAGAATCAATATTAGAACCACGTCTCTAATCCAGGTCATAATGGATCC
TCCTAACGGTATTGGGTTCATAACCGGTCAATCCTCTTGAATAACACTTCGAATGTAAAG
GTGGTAACCGTTGTACAACGAACTGGGTTCGAAGCGATTGGTATACCCGTGAGAAAGGTT
TCCACCGTTGGTAATACCGGTGGTCTTGTTGAGGAATTTACGTTGTATGCCTAGGTCGAT
GTCTAAAAGTCTCCTGTTGATGATTTTGCGAGTGAAGTACATGGGAATAAGGCCATTACT
GACCATTAAGGGAATAGAACGGGTTTCATGGGTGTGGTAGGTGAAGGGATACTGGTACGT
GGTGATGGGCTCCACGGACACATACAGGTTGGGATTATCCAGTACGATGGGGATGGTACT
GGGATCGGTTAACAGAGTCTTCCAGAAGCTCTCTGTACTGAAGAACCGTTTATCCACGAC
TTCACATTCTGGATCAATGATCCGACTGAGGTCGATCATAGATTTCGGTATAAAGAGGGC
GGAAAACCAATCCACCTTCTCCTTACGCAGCGTTAAGGTACGGTCACTCATTACCTGTAC
CACGTCATTGAGGTTCAACCGACCTCCAATACTCATCCAGACTGTCTTATTCCTTAGACT
GACCGGAGACCCTACGTGCAGGAAGTCGTAACTGTCGTCATCCTCAAAGTCGATATCCGA
CTCTTGGAAGGTGTAGGTCTTGAGTTTAGAGAGGGTATTGAAGTTAAGGTAGTTCACATG
AATGTTGTCATTCACATTGAAGTGCTTCCCACCGTTCAACAGGTACCCCGCCTGGTCATC
CGCCACTGCTCGTACCTAATGCCCTCCATTGATCGTCCACAACCCCCGTTCAACCAAGGC
GTTATAATCCACCGCCGTTCGATCGGTCTTGATGACCCGAATATCCGGCGCGCTTGTGAC
GTCGAGGTGCTCCTGGCGATCATTGGCGATCTTAGCGTCACCAGGATACAGGGTAAACCA
TGTGTATTGGATGTCTTGAGCCGTTACGTACCGGTACTCATTGCCGAGGAGGGTATTACG
GGTCTTAATGGGGACATTGATCTGGGTGTTTAACCACTGTTGGATCACCCCATCGAACAA
CAGGAACTGGTTATGATAGTCCGCCAGGTCAATGACCACATCCCGGCCGTACAACCCATC
TTACACCACAACCTTTAATTCACAAAACGTAGCCACCAGTTCTTTGGTGGTCATCTCACT
GATATCCACCAATTCCTCTACTCGGGGGAAGATTTGGTTGATCCCAACCGCCCTTTTATA
GATGTACATCGGGGTTCAACTCCTTAACGATTCTATGAAATCTACCCAACAGGCCCCATG
GGTACTATACTCTCTAGGGGATTAATCATAAAATGCCTTATTCATTGACTTTGGAGAAAT
TCTAATGGCAAACATCGTCTATGGTTGGAACCCCTTCCAGGAACGCGTCGATAGCATAGT

GACGAACGAAATCATTAAAACCTCTGGTAATCTCAACCGCGTCGAGTTCGTTCCCCGAGC
AGCTCCTTTCTTTGCTCACAACTTCAAACTGTACCGTCAAGGGAGTAACCAACCACTCCT
CCCGGGACAAGACTTCATCTTTGGTCATCCTTTCCAACGGTTTATTAAGACCTATCAGAA
GAACGTGTACAGTTGCGTGATTCTGCTGAAACCGGTTGAAGTGGTCCTGATGGGGGAATA
TGACACCATTGGCGGACCGTTCGTGTTGGATGATGTGGCGCACGCTACCTTGGTAGCCTA
CATCAGTAACAGCCCCCGGGAAGCCCTCTGGGAGAACCTGGTCAATGTCCCGACCGAGTT
CCCTTCGGATCCTCATGAACATCCGGCTGCCCAGGTGTATGATTTTGCTCAGATGATGGA
TTACCTGCGTGACCTGATCTTGGCGGTTACTCAAACCATCTCCGATTCCCCATCGACCTT
GAAGACCCTGTTGGAACAACACCTCGCGGCTGACTTGGCGCAAGCCCACAGGGAAGATAA
GGGAATGCTGGGTCTCGACAATGTAGAGAACTTGGGCAGCGCCACGGTCAATGATATTAC
GGGCAATTCGGACAACCTCGTCGTCACTGTAGCCGTTATGAAGGGAGCCCTCCGTCGACT
GGCTGCGGGTACGCTGAACCTTAATTAAGGAGCCGGTTATGCATTTTCCAAGCGTCCGGG
AATACAAAACCGATATTGTTCACAATAACCTGGAGAACCAAATTCGAGGTGAACCGTTTA
AAGTCGATCCAGGAATCTGGTCCCGGATTATTGTGCCTATGCACAGTCCGTTCTTTGTCG
ACAGCCGGATAGTGACGTTCCCGAACGGTCAACCCGTGGACGACTCCCAGTCCCGTATTT
ATCGCTTGATGCCTCGGTTGACGGAACTCACCGCTCAGAAAGTTGCGTGTATGATCGAAC
TGCTGGACCCCACGATTACCGAAGGGAAGTTGGAGTACGATGTGGTAGGTGAATGCAGCC
TGTTTGATACCACCCTGCTCCACATGGTGATTGCTGCCTCTGAAGATGACAGTCCGGTCT
GGTGGGAGAACATCAAGAACAAACCCGTGCTCTTCCCACCGGAGTGGCATACTCACAGCC
TACTCTTCGACATCATGGCCTTTGGTGACTTTGTTGAAGCCTTGGGGATGATCTCTGACG
CGGTTGAAACGCATGGGAAACCGTACCTTCAGATTAAGATTGACCATGCCTTTGAACTGT
TCAATCATTACCTGGGGATTTACAAGAAGTCACTGGCTGATTTCCTGGCTCGTCATAAAG
CGAGTTATAACAGCCACGGACTGACGGCCTTGCAAGTGGGATTGGAGAAAGTCGACAACT
TCCCCACCACTACCGCTTCCTGTGCCTTGGAAGGTAGACGTGACCGTCACCTCACGGTAT
CGGGACTTCAAGCCATTATTGACCACTATGGCTTTAATCCCAAGGACTTCATTGAGAACG
GTAAACTTCCCATTGCTCAATTCGGTTATACGAACTTCATTCCCCCGTCTATTGATGGAT
CCTTTGAAGGCTTGGGGGGTAAGAGAGAATGTGCAGCCATTACCATGGAGGCGGATGGCA
GCATCGTCTTTCTGGCTAACCGAATGGATGGGCGTGTTGACGGACTTAATTACTCGGTTC
TGGAAGATCCCTATACCAACCCCCGGTTAATCTATACCAGTTACCGCTATTCCCACCCTC
TGCTCTTAGCGGAGAATGCGGAATGTGATCGGATTGTCCAAGGGAGTGGACGTGAAGTCA
TTATGCTGGGGGGTATTCGTAAGAACTTGTTTTACATTGGATTGACCAATGGTACCCTGG
ATCCCACTAAACACATTCTCACACCCATTAAACTGGATGCGCTAAAGGCTCCCTGGAATG
GCAATCCGAACTTCTCCTGGAATACTTGGTTCAGAAGTTTGAGCGTGGAGTATCTGGGGA
ACTGGATCTACATCTTCCACGCCCACAACGTGGAGAATGGAGAACAAGCTGATGGTGTAA
CGGATTACCGGTATCGGTACCTGTACCGGGTTAGAACCAGTGATGTCAACGCCCAGATCC
CGGTAACCGTTTCGTTGGTCCTCCCCACCTTTAAAGACGGTGACGGTGTTCAATGGAATA
ACCCACCGTTCTTCCGGTGGTGTACCCCTCAGGGGTATCAGCGGGTGAATAACAATGATA
CCTGGGAACGGTATCACTTTACCTTTAAACAAAGTGATAACCTCGCCTTTACGGGGATTT
ACCATTCCCAGCCAACGGTGGTGTGCCCTATTCCGAACAAGCCGGGTAAATACCTGGTAC
GCTTTATGGCGGCCTGGTGGGGCCGGTACGTGGTTCCTGGACTAATAGGTACCTGGGACC
TCCCGCTAGAGATGGTGTATGAGATGGACCCTGAAACCGGTGTGATAACCCTCCTCCATC
AAACACCTCGACGGGAAATCGATTTCCTGAACATTCCGTCCTCTGGCGACCCAAAAAACC
GCCGCCTTAACCAATTGGTGTTCAACGATTCGGAACAAGGCATGGAAGTGCTGGATGATG
AGACGATTGGGGCGTCCTACGGGGCCTATCAGTCGTTCCCCCGGTCAGCCTTCTACGTTG
TACCCAGGTCGTTCAAGTACCGGTATGTCGTCATGGACCGGACGTGGATGACCGACTTAG
GGGATATTGAAAACCAGCAACTCATTGCGGAGAAAATTACCAGACCGATTGCTTCCTCGA
TCAAACCACGTTCCTTCTTGTTGGGCAATGGCGGGGATGTGTATACCGCGGGGACCAATA
CCCTACCGAGTGTTATCACCAACCAACTGTGGTACCGGCAGTCCCCTGGGAAGTTAGCGG
AGCGTCCTGAGGTCTCTAACCTCTTCTTCAACAAGGTTGTTTCTCGTCCTCTGAGTAACG
CGGTCTTCCGGGTGGAAGGTCCTCCTCAGGTGGGTGGAGCCTATGTGACTGTCCCTTCTA
ACCTGTTGGATCAGTACAACACGGACTTGGGGGAATTCACCTTCTGTATGGGAAAGCAGA
AAGCGAACCTCGACCTGTCGAAAGCAGAAGGGGAATGGCCCGCTGGTAATTTTCCTCAAG
ACATCAAGGTTATGTCAGGACATACTCAGCAGATCAATCCGGATGGTTCGATGACCATGG
TACCTACGGGGACTATTCTCTACCCATCGTCCATTACGGCTCGGTTTGTAAACCATGTCG
CGAATCCGACTGCCTGTAGAAACTCCCCCGCGATGTTTATCGGGCTGTGTGATCCCACTG
GTTTCCTGACCGATAAGTTCGGTTGGTTACCGGTGATTATGATCATCAACTACGGCGAAC
CTGGCACCACGTCTCGTCACTGGACACTCTTGGTCATCGACCCGGTTTATACCGGACCCG

TCAATGCCCGAGTGGTCAGTGATTATACCGTGCTGGATGTGGTGCACAATGTCCATCTAC
AGGGTGCTACTGGGTTGACACCTGCCAGCTGGGATGCCTTCCTGGGCGGCGGAGCTATCA
CATCGGGTGGGGGTGCGATGAGGGTAGGGTATTACCCCAAGAGCGCGACCGAGTTGGAAG
GCTTTATTGACACTGGGGTCATTGCAGCAGGTCCAGGAGACTCACTCACCTCGTTTGGGA
CATTCCGGTATACGAACCGTAGTACGAAACGGTGGACTGAATGGATACCTAGTATTACCG
GTCAAAGTGGGGGTGCTTACCACAAGACCGTTACACCGGATAACGGGGTAGCTAACCAAA
TGGCGTACAACGTTTCCACTGGGGGAGCCGCTACGATCTTTGAAGGGTCGGTGAGTAACA
CCTTGCTGGGATCGGTTTACCCCGCTACCGGTTGGTACTTGTTCTTCCAATCGGAACTCA
GGGTGGTCTTCAACGAACAGAACTACACGTTGAAGTCCATCTCCGTGGATTTGTCGGCAA
TAGCCCCTAATCCCGCTAATAAGACGTTCTATTTGTACGCAGTTCTTAAGAACGGGAATG
CGAGCTACGAAGTGACTGTGGAGAAACGAGCTGAGACACCGTTTATTATGTGGGTCGGAC
GAGCGGTTACGAATGACAGTCAGATCCTCACCATTGAACGGGTTAATGTCTTTACGATTA
ACGGGAACCGGATCTCCGAGACGAAACGGGGGAACTGCATCCCGGCGTCGTCAGGCTTGG
TGAATGCCGAAGGTCAACTCCCGTGGTTACGTTCTTCTGAAATGCTTCCGTAGACCCAAG
GTGGGAGAGGGTTAATGCCCATCCCTCCTTCTTTTCAATGTTTAGTCCATGGATCCGTTT
AAAATCGTTCAATTTGATATTCGTCGAACGCGCCCGGAGAATAAGATCACCGGGGAGAAA
ATGCTGATCAAGGATAAGACCTGTCCTTGGTTGATCCCGAATGGGTCCCCGTTCTTTGCT
GACCCTGGCCTGTTCACGTTGTATGACGAACGGGGTGCCGAAATGACGCTCAACCGGGAT
TACTGGTTGGAGGAAGCCCTTATCCCGTTCTGTGAGATCAGTGGTCGAAATGTTTGTTGT
TTCATTCGGTTAACCGAGAGTATTCGTCAGAACAACGCTTTCGTAACCGGAGACTACCAA
ACCATTGGGGCGTGGTTCGTACCGCGTAATAACCTGGACGAATGGCTGGAAAAAATGCGG
GAAGGGAAGATTCCCATTAAGTGGGAGAATGTCTTCGTTGTTCCACCCACCCTACCGCCC
GAATACCATTTGCATAAAGCTGATACCGAAATTGGTGATTGGTATGAGCTGACGTTCTTC
CTGGATACCATGGCGAACATTTACCGGAGCCGAGATCCAGAGTTGGAGGATCAATCGGAA
GTTGTGATCAATGAGGCGTTTACGAAGCTCAGGAACTTCCGTGACGCCCAGATGCGTCGT
CTTGTCGAACACGACAAGAATTACAACGCCCCTCACCTCCAAACGAAGACCAACGTTAAC
CTGGGCAATCTGGATAACTTTGCTACAGCTACTCCCGAACAGGATAAGGCGGGTACGGCT
GATATCCTCTTCAGTACCCCGCTGGGTGTTAAGGAACTGGTCAGGGGGTATACGCCGGAT
AAGTCGGCTTCCATGCGTTCAGGGATTATTGCCATTAGTTGATTAGGAGGAACGGATTAT
ATCCCTCCGGGTATTAGTGGTTCGTTTGAAGGTCGGGGGGGTTTTACCCCGGACTGTGCA
GCGGTATTGGAACTGAATGATAACCTGGTCTTTACTCGGGCTCATTACGACGGACGGACG
GAAGGGTTGTATTACACTGTGGTTCGGAATCACTTGAGCCTGAAGCCCGACGTGTCCTTT
ACCGGGTATAAATACCAGCCCCCGTCTTTAACGGCCATTGGGTTCGTACCCAACCGGGTC
TTTACAGGGAGTGGTCGGACTATCATCATGGTAACCCGTGATGGGACGAACCAGGTGTAC
GTTGCGCTGACCAATCGTACATTGGATCCTTCTGCGCATGTTTTTGTCAAATCTAACATT
GATTTGGCTATCCAAGATCTGGGGACGTCAGTTAGTCGAACGGAGTGGAGCCAAATACAC
CCGTTGGGTCATTGGGTCTACTTGTTTGTCACGGCGATGACTGAAGGGCAATGGGGTGGT
TGGGGGCATAGTCGTACACGGGCCTACAGAGCCCCTCTCAGCGCGATTAAAAATGGGCAG
CCTCTTACCTTCAGTCGGGTTAAAGTCTCACGGACGAACGTCCCAGGGGCCTTACAGAGC
AATGTTGACTACATTGAGTTCACCCCTGCTCCTCCAGACGGGGAATTCAGTACTTACGGC
CCCATTACCGTTGCGCCTTATAAGGCCAAGAAAAGGGTTAAGGGATCCCAACGACGTCCC
CTGATGTTCTTCTCTGAATCCAGTGACATAGGACCTCAATATTGTTCCATGCATTACTTC
GGGATGATGTTCTTTACTCGGAACATCAACGGGGTGGACGGTGTAACCGGGGTGTCCATC
GATTGCAGTTACGATTTCAACCCTGAAACAGCGCCCTTCGTAGTGGTTAAACAACCCCCC
GTACAGACCGTTGACCTGAGCAAGATCATCAGCAAAAATTCAGCGGCCTCTGTGCATTAT
GCAGGTATTAGTGACGTCTTCTTCAAAATCTCTGATCACTCCAGTCACCTCCTGTTGGAG
AATAAGGTGATTGTTGGGATGTCTCGTCTAGGGTTGAGGGTCTCTTTCCCGGGGTTGTTT
AGCCGTTTGCAGTTCAACAAAAACCCGAATGTAACCGCCAGTGGGATGTTGCGGGATTAC
ATGCACGCGGACAATGCGAACTTCTCGGGGATGGGGGTGTGGGAAACGCCCAGTTACCCT
ATTAAGAACGGAGGGCGAATCAGTGCAGTGGGGTATGAACTTGCCGGGGGGTATGTCTGG
GCAGACAGCGCTGGTGAACATAACCGGTCGTTCTTCTACCGGAAGGTCAGCGGTGAATAC
GCCGTCCGTCCTGGGATTACCAACAGCAACTACCCCACCTTATTGGCTCGACAGATCAGC
GCAGAGGTGTACGAGAACCGGTTAGACCGTAATACTTCACTGCTCAGTGTAACCGGGAGT
GCGGCGTTCCTGACCGCTGAAGGGATTGAAATGGGGAACAGTTGTTTCTCTGTAATGGGA
ATGAACCATTTCTACGACCTGGCCTCAGGGGTTGAATCCGATACCACATGCCCTCGGGAG
TTTACTTTTAAAGCCCCTAACGGAAATGGCATCGTGTTATCCTGCCCCAGAACCTTCAGT
CGGGTAATGGACACTGGGGATCAAACCTTTACCATGAAGGGTGCAAGCCATCGGGGTGTT

AAGCGTAACGTCATGGATAAACTGATCCAGTTGATCCCCGCTACCCTCGGGGAGAACTGG
GCGGTTGAGTTGATGGTGATCAATCCCATCAGTGGTAAAGGGTTCAGTCGGATGAACTAC
GCGCTGGCGTTGGTTCATACCCGGGACATGGAAGGGGGTCGGTATGTGGTCAAGAGTAAA
CTGTTGGCCTGTGCACCGGTGATTGGTAATCCAGTCTCGGGGGTCTACCCGATTGATGAC
CTTACGATTCTCGATCAAACCGCATTTATCACCACGGTGAACAACAGTGGGGATGGGGAT
GAGTGGAGCGACACAGAAGCGGATGTTGTGGCGTGTCCTCAGCTGTCGCTTTATGTGGAT
GAAGCGACGAATACCCTCTCCGGGCGTATTTCGTCGTTGTACAACGTCCGAGGCGAAGCC
TTGGGGTTCAACATAGCAGTCCTCACCTTCAGCCTTATTAAGACCACTCAGAAATTCTCC
AGTGTTACACCCTTTAACAACGGGGAAGGGATGTCCGCGGCGTGGTGTGTGCAGAACTAC
GCCAATCCTAACGCCGGGTTAATTACCCAACAGCAAGGCTACGCCTTTGAGGACACTGGC
GGTGCAGGGTATGTAGAGAAGATCGGCACGAACACGTATTTGATTGGTTCCTGTTACCCG
GAAGTGGGTTGGACGATTTTCTTCCAAGAAGAAGTCGAGATGATGATAAACGGTACCGTG
TACAAAATGCCCGGTGGGTCTATCGACTTTAGGAATGTGTCCTCGAATCCTCGTAACAAA
ACGTTCTACATCTATGCGACGGTTGAAGAGTCGGAAGGACGGTATATTGTCTCCGATGTT
AATCTGCGGCGGTCCAGTGAGTTGCTGCATGTCGCAACCGCCGTGACGAACGCCAACCAG
ATTGCCAGTATTACCCGGCACAAAGTCTTTACCATCTGAGAGTTCCTATTAAGTTACACA
CGGACGGGAGGGATTATTCCGGTGGGTTCTGGTTTACCGCAGGATGAAGGTACGTTTAGC
TTCTTGAAAGCCTCGGAACTTTTACCTTAAGGTAAAGGGGGAGTAAATCTCCCCCTGATC
TTTTAAATGGAGTAGCTGTATGATTATCCTTGGCGGGGAACCCCCTTTCATCGAAAACCC
TGAACCCAAGTTCGGTGGAGTGGTCTCCAAGCTCAATGAGATGACCTACAAACTGGAACC
GTGTGTTCACAGCTACAATGAGGGGACTAAAGAAAAGATCCGGTTGTTCAACGCAAACCT
GAATGATTTCATTAACCAACCCATTGTCCACATTGACGCCCACATCAATTCCCGGGGGGC
AGTCCACGGTGAAACCAAACGGACGATTGGGTTGGGCCTTAAGGACAACTACCGGACGGC
TACCCTGGCGGAACAACAGTCGCTAACGCCTGTGTTGGCGTATGTGACCCCTCACGGTGC
TCGCGCTGCGGTTGTAGCCAACAACGTCGGTTATAATGGCGCGGATTATCAACCCAATGA
CGTGCTTCAAATCTCGTCGTTCTACAACCCGGATGAAAACCCCGTAGTTCCTCCAACGGT
GGTTCAACCCATTCGTTATTTTGGGTACTCCTTGAATGTCGGGATGATGATCAACAACGA
CCGTCTGGTGTTGTCTCCGGTTACCGATCCGTCTCGTTACCAACGGTACACCGCCTTCTT
GTCTGGTTCTCTGGCGAACGCAGGGGGAACACAGATCTCGGAGATTCAGAACCTCAACGC
GTATTACACCGGACGGAATAGGAAGGCGGTAGGAGGGTTTACCAGTTCGGGCAAAGTGGC
CTTCTTTAAACCCCTGGCTGAGAAGAAGATCTACGAGTTCAAGAACACCCTCCCATTAAC
CCCCAGTAGTGATAACTTCCTGCTGTATCAGGGGTATAGCACGGCGGTCTATAAAGGACT
GGGGGTTAGTTGGGAACGAGTCGGTACGGTTATGACCCTAAAGCACTATTTCTTCAACGT
GAACTTCATGGAAACCAATCCCACTTTGGTGGAGCAGGTGACTGCGTAGTACACCGCCCG
GTTTACTCAAATCAATAAAACACCGTATACCGGACCCGCTAACGGGAGTCATTCGTCCGA
CATTAATGACTTTGTGACCCTGAGTGCAGGTAAGACCCTGGAGATGAATGGAGGACTGCA
ACCCACCACAGCCCTCATCTGGAACATTCAAGACCGGGAAATCTACCTCTTTGTCGTTAT
TCCGGTGAATGTGGTCCAAGGGGGTATTCGTCGGCCGTATAACCTTTCCTTCGAGGAATC
CAATGTCCCGGGTACCTTGGAGAATGGAGGAACGGCAAGCTTTACCCAACTGGGTAATTT
GGTCAAGGACATTCTTCAACCGAATCTGCTACCCCCTGCTGACCCTAAATGGTTGCGGTA
CGCGAACACCTGGGATTTCAATAATCCGACCTGGTATCCTGGGATCGTTCTGAAGTCAGG
GGAAGTGTTTAAAGCGGTCGCCACGAAGAACCGTCTACGGGTTAAGCGATGTACCACACC
CCATAAAGGCTTGAGGGAATGGCTGTTGGGAGAACGGGCTGAAGTGGGGATGAATGAAGT
TCGTACAGAGTTTTATCCTCCGTCACGCCATAGTCCTTTTGGGCCGGTTCCTGATCGCTT
ATTCCCCATTGAACACACCGCTGGTACCACGCGGTATCTGGCTTATTCGGTTGACAGTGA
AACTGGGTTGTATGAGTGGAGTGAGTTGACCTGGTCGAATACCTCCATTGTGGGTGTAAC
CACGGGGAATAAGTTCGTTATTGTTCCAGCGGAAGTTGGGATTATTAATAGGGAGTTGAC
CCGTATCCCTAGGGCCTTGGCGTCGTTTGATAGCCTGCTGGTGGATGGAGTCAGTATCAA
CGCCCTGTGTTTCACACCGGGTTACCAGTATACCGGGTATGCTTCAATCTCCATGGCGAA
TGGACGAGTCCAGGAGGGCGCTGAGGTCAAACTCTCCCCAACCAGCTTGATTACCCTGCA
AGCCGCCAGTGGGGGTATTCTGGCTCGTGCAGCCATTGCGAACCCTCAAGTCAACAACGC
CCTCGGGGAAAGTGTGATCAACGTCTTTGCTGTCGCCGATAACTTCGCGTTGGTGGTGAT
TACCCATGGGATGTGTTACGCAGAGGCTGCTGTGGTTCCGTATACGGTGAGTGGTGGGGT
CTTTAGGCCGAACTTCCCCTCCGCTACGGGCTTCGTTACTAAACCGGTTACCCCAGCCGG
TATGGTCGTACCGGGCACGTACCGCTATACCAATTCCGGTGATAACCCGTATATGACCTA
CGTGGATGTCCAAGTGATTGAAACCCAACGGTATGAATGGGCGGTTGTGGTCAGTCGACC
CTTTGGGGACCTCTACGGGCATATCAGTTTCAAAATAACGGATTACCCGTTTACACCCAC

CTACACCGTAGGACGGCTCAATCCAGCGAAACTGTATACGGCGTCATCACCCTTTGATAC
GGTGAACGAAGATCACCCGCCTGTCTTGATCCCTCGGAAGGGAATCTACCAGTACGACCC
TGCTAATACCGGCTACTCCACGAATCTGTTGGCGGTCGGAGGAGCGGGGGTGGTGGATCC
GTACGACGTAAATGAAAGCGGCTGGGTGAGTATTCCTGCTCGATCCAGGGTGGTTATAAG
GGGACGTGCGTATATCCTGGATACGGAATACCCCATTAAGGTCAACCCTACCGGAACGAC
GTACTGTTATTTGGTACGCCAAGGGGATGCGTTGGTAGGGTTGGGGTCTCCCCTACGCCG
AGAGGTCAATAACTCGGAAGTCCTCTTTGGGACTGCAACGAACGGGATTCTTACCATCGA
TCATTCGTACATCGTGATGGGTAGTCATGTGGTGAGTTCTAGCCGTCGGGGTACGGCTAT
TCCCACCTTTGCGGATCACGGGAACTTGGGGGTTAACACCTTCTTTACCCGACGTGACAT
CATTGATTAACCCTTAATGAATCCCCCTACCTCCCCGTTAAGGGAGGTAGGGGAGGTCCA
GTGGATTAAGCCACGGGTGGCGAGGTGGTTCCATTGCCAGGGTCTGCACCTCCGTGAATA
TGGAAGATCAGAGACACTCTCACGGTCCTCACATCGGTTTGTCCAATAACAAGACCTGTA
CTCACACTGTTACCGGTACGAGTGGTACTCCCGTTTTGGGTATAATTACCCTCTTGAGTA
TAGTTCCCTTCTTGTTCGGTATTGCCCGTATGGGTAATGTTCCCTTCCCAGTTCGTCTGA
CCAATGGCGATGTTCGCCAAATTGGCTTTCCTGCTAAGAGTGTCGGACTGAAGGTTAAAG
GTCTTCGTTAAGACATTGATTTCCTTCTCCGCATGAAAGCTAATACTGTCTTTGGTGTAA
AAGGCCATCTTCTCCTTATTGACGGCAAGGGTGGACCCTTCCTTGTTGGTATAGGTGAGC
TGATGTTTGTAATCATCGAAACTGAAGATACTCCCATCAGTTCCTCGAATATCGATGCGG
CCTTCTTTGGCATTGACTTGGATATCAAAGCCCGTCCGTTCCCCGTTGGACTGAGGGGTC
CGTAGTACCACTAACCCGTCGTGGGTACTGACCCTGGCTGTATAGAAGTTATCCAGGTTA
AAGTCCACGTCCTGAGCCAATCCAGGGTTAGCCCCCCAGCCCCACTAAACCGTCTCTAAG
CGGAAACGATCCGTATTGTCCCCAAAGATGTCCCAGTAATACTGGTTCTGACCTGTGATT
TGAAACACCCACACCTTAGCCCCTTCCCGGACATCCGGTGAGGTAAGTCGGTTCCCATTC
CCAATGGATCGCCAGACGGCTGGTACGGTGTTTGACACCAGGGTTTTAGTGGTAATCATC
TCCCCTTCTTTATTCTGGCTGGTTACTTCCTTCTGTTCTGTTTTAGGCAGGGTTCTCCCA
TCGGCGGTAGGAGAATGCGATGGAAGGTGGATCATTGCTTCGTGGCTATTGGTGTCCTTC
GTGGCGGCAACCGTCCCTACACCCAACCATTTAACCACGTTCACTAAACTCATACTCGCG
TACTCATTATCTGCACACTCACTTTACTTAAAGAATTCATACCATGTTTATATTAACTAG
ATTGATCCTAGAGAATTACGTTCCTCCGTTAAGCAGCGGAATCATACGGGTAGAATTGGA
TCTGAATCATTTGGTTAACCTGTTGATCGCTCAGAACGGTGTCGGGAAAACCAGTATCCT
CCGAGAAGCCAATCCCCTGGCTCCTGAAAATGCCAACTACCGCGGTATTGGACGTAAGTA
CGTGGAGATTAAACGGGGATTGGACAGTTTCGTTTTGGATTCTAAAACAGACTTACGCAA
CGGTCACAGTTTCAAGTTTAACGGGAAAGAGTTAAACAAGAATGGAACCGTCACGGAACA
GAAGGACTTGGTATGGATCCAATTCCGGTTAGACAGTACTATCAGCCGTATTCTGTCCGG
GCTTCAACCCAACAGTCTGTTTTTAGCGATGCCAGTGGCTCATCGTAAAGACTTCTTTAT
GTTCATCTACCCGAATGACACGAGTTACGCCTTAGGGGTCTTTAACAAGCTTAAGACAGA
ACGTCGTGAACTGTCGGCTGCAATCAAGAACCCCGTCATGCGGTATACGGAAGAGAGTGC
TAAACTCAAGCAGATCACCGAATGTGGTCCGGAAGAGCCGGAACGTCGAGTTAAGACGAT
TGAGACTGAACTGAGGGAGTCCTTGTTGGTACGAGGGGGTTTGGAGCAGGTGAAAGTAGA
CCCTGAGCTCCAAAGTAAGATTGCTACCTTCAAACGTCTGGCTGAGCAGATGACATTGAA
CCGAGTCAATGGGTTCTTTGAGACTGAGGCTGAACTCAGACAGTGAATCGAAACAGTTCA
AGCTATCTTGAGTGTACACGAAGGCCAAGCCACTGCCATTCAAATGGTTATCAGTGAGCA
CGTAGGGGTTTTGGAAGGAATGGAGGAGATCAAAGAAGATCCCTTTGTCTTCCATCATCA
AGCCGAACAACTCAAGGTGGAACTCACTGCTCTCCGGGAAGAATACAAACAACATGGGGT
GCTATTGTGTAATTACCCTTTGTTCAACGATGACGCCTATGACTTTACCGGTTTGGAATT
GGTGTACGAGGCCTTGGTGGCTCAATTGCGTCGGGTGGTGAATTCCAGTACTCCTGAGTT
AACCGGTGGGCGGTATAAAGAGTATACCGTCAAACTGGAGCAAACCCGTAATCAACTGAG
GGCCTCTAAGAATGAGCTGGACACTCTCACCCACCAGTTCAAACATTATGATGCCACGGA
GCTGGTGACTTGTCCGGAATGTACCCATGAATTCAAGGTCGGGATTACCGCGAATGAACT
GCGTGCAGCACGGGTTAAAGCGGAAGCCCTTAAGTTGAGTGTGACTAAACTTGAGCAGGA
TGTTGAAACCCTGGAAAGATTAGTCGAACAGGACAGTGAGTGGTACCAAAGCATGTTAGC
TCTACATCAGTCCTGCCGGATGAATGGTGATGTTAAGTGCCTTCCTGAAGTGATTAAAGC
ATACGACATGGGTAAGTCGGATACCAACATCCTTTTAAACGCCCTCAGGGCCTTCATGGG
GCGTTTTAACTGTAAGAGACGTATCGAGGCCCTACTGGAAGAAGAAAAGCTCTTCAACAC
GCGCATAGGGCTGTTACAGAAGGATTCGGTATCAGACATCGCTACCTACTTAAAGAGTCT
GGAACACGACCTGGAAGTGGAGAACAGTCGTATTGGTTTCTACCGTCGGCGTGTTGAACA
GCTTATAAGGAACCTAAAGACCATCCACACCTACACCCGAGACCTGGAGAGCCTCCGCTG

TCTTAAAGACGAGATCTTCAAAGGGTTGGTACAGCAAGGGTTGGTGGAACTGCGTAAACG
GGTTGATGAACGGATTCATTTGCTAACGGATGAGAAGGATGAATACCTCGCCTCCATTAT
CGAAAGCCGTTAGTTGAGTGCGGTTGTCACGTCTATTTCGGAAGACATTAATCGTTTAAA
GCGCCGTCTGAAGATTGTGGATGGGTTAATGAACGGTCTGTGTCCGAATAAGGGGTTGAT
CGGTAAGCAGATGACGGATTTCATCAAGACCTATTGTGCCAATATGAATGCGGTGACTCA
ATCAGTGTGGAACACCATCCTGTTTGTTAAACCCTGCCATAAGGACAACGGGGATTTGAA
CTGGAAGTTCCCGGTGGTTACCGGTGATGATGAGCCCTCGCCAGATGTCAGTGATTGCAG
TTTGGGTGAAGCAGGTATCATTGACTTTGCGTTCCGTCATGTGGCCATTCGTTACCACGG
TAACTTCCCTCGGACGCTGGATGGGGTGGGTACCACCTTCGATGAAATCAAACGCGGTCG
TTTCTTCAATTTCATTCACGAGTTAACCACCCAGAAGGACGCTGAACAACTCTTCATGTT
TAGCCATTTCATTACTCAATACGGAATGTTCACCAACCCGAATGTAATTGCGATGTATTA
TGAAGGGTTGAGTTTACCGGGTGAAGTCAATCAACACTGTGTTATCAATGAATTTTAAAC
CTACATTATTCATTTGCAAGGAATACCTCTAATGAACGTTCAACTCCCACGATCGGTTAC
CCTGCATGCCTATCTTCAACCGGACGGTACACTTATGCTGGGGCCGGGTCAGGCTCAAGT
AACCATTAACTACCATTCGGATCCTCAAGAATCCATTAATGCGTGCATTGTCTTTGGAGG
GGCAATGCAAGCGATTGTGACTTTTGATGAAACCGGTCAGACCCCCGTGACTTATACGCT
GTTGAATCCAGGGGGTGATTACACCGTTGAGCAGTCGGTTTTTAACATCTTCTCCTATTT
CGGAGTCAGTGCTCCGATGAACTAGGAGGCATGAGTCAGGAACAAAGCTGAAGTGATAGA
ACGGTACCGCGAACTGGTCGAAGACCTCACCCTCCCGCCGCATCAGGTGATCCTGTCGGC
GGGTGCCGCCTTGGTGTTAATGGGCATCCGAGAGGAAACAAGTGACCTCGATGTGGATGT
CCCTACCAGCGTGTTCAACTGGGCGAGTAATAAGTTTGGGGTTATCGAGAACGAAACCGT
GAATAAACGGGTTCAATACACCCCCCTGGTCGATCTCCATGAATACGACGAGAACACGGG
AGTGGTGTGTATTGAAGGGGTGTGGTTGTACAGCCCTACGGAGTTCCTGAAACAGAAACA
CTGGCTCACGAAGCTGGCTGATCGAACGGACGTTAAGACCCAACAGGATCACCGTGATAT
CCGCCAACTGGAAATTCTCACTCAATCTCAACCATTCACTGCACCCGCATTGTTGTAAGG
TGAGGTTACGGTATGTTTTACAGTCAATTGGAAGTGGTAGAAGCCGTTCAACGGTTCTCT
GAGGCTCATAACACCCCATTGGATAAGATCGCTATTGGCGGAGGTGGGGCTATCATGCAC
ATGGGGGGTCGTGAAACCACCTATGATTTGAACATTTGGGGGGAGAGTCCGTTCTTTGAA
CTCATTGCTGATACCCAGAACGCTCTCCTCAGTTCTAGGAAAGACAATGTGTTCCACGTT
GAAACGGTTATTGATTCCATGGATCAAACCGATCACTTCACCACACGTCGCCATTACTGG
GTGCGTAAACCTAACCTGTATTTCCCCACTGTTCTTGTTAAGCTGGACCAATGTCCTGAA
GTCTCCATCTTCGATGCGATGTCATTGTTGATTCAGAAACGAGGGAGCTACAGTCGCCCT
GAACGAGGAGTCCGTCGTAAAAAGGATTACAGCGACATCTGTTTCTTGAATGCGTTGCTT
AAGGAAGAACACAAAGTCAGGGCTTAACCTTAGGACAGTACCAGGGGATCACTCCCCTGG
TACGTCTTCCCCCTTATACGGTCTAGGAGATTACAATGGGTAAACACTCACCTGTTGAAC
GGATTAAATACCTCCATGAACAACTGACGTATCACGCCCATCTCTATTTCGTCGAGAACC
GGAACGAGATCTCTGATGAAGCGTATGATGTGTTGGAGCAAGAACTCAATGAGTTGTACG
ACCGATACCCTGAAATAGCGGCTAAGTTCGATTTCTATAATCGTCCCGTTCCTATTCATG
AACCCTCGGGGGAAACCATTCAACCCGTACGGTTCGAGGAACCCATGCTCAGCTTGAAGA
AAGCCTTGAGTGTGGAGGAGGCGGATACATTCCTGTTAAAGTTCCCGGATGGTACAAACT
TCTTTTACGAAGAGAATCTGGATGGGTTGGCCCTGGAGTTGGTGTATGCACAGGGACAAA
TGGTCAGTATGAGTACCCGGGGGTCTGGATTAGTCGGGGAAGATGTGACGCATTCCATAG
TTCTCTTCGATGAAGCTACACTTCCTCGTACCTTAAGGACACTCCCTGACCGTCATAAAC
CCCAGGGGGATCGATTGGTTATTCGGGGTGAAGGGTACATCAGTCTGGAACGCTTCGATT
CTCTTAATGAAACAGGGGGTAAACGGTCCAATCCCCGTAATTCCGTGAGTGGGTGGGTGC
GGTCCCTCCCTCAGAACCAACGGGAGGATATCCGAGGAACGCTGTACTTCGCGGCGTATT
ACGCCAGTGATCGATTGGGGACGAAAGCCTATTCCGAATTGAGGCAGGTTCTCTTAGGCC
TAGGGTTTGGTGTTCCTGAATCCAGGGATTGGATTGCGGTCTCAGAGAACCGGAGGGGGA
ACATTCGTCCGGAGGATGGGATTGTGATTAAAGTCGACAAGTTCAAGGAACAAGACGCCA
GTGGAAATGGGAGTAAATTTCCTCACTGGGCGATTGCTTACAAGGTCCCTGCGGAAGAGG
CGTTGGCCCAGGTTGAGTGCTGTATCTGGAATACCTCTCGGTTTGGTCGGGTAGTACCGG
TGCTCCAGTATACCCCTGTAACGGTCCAAGGGGTTATCGGTCAGTCTGCGTCGTTACATA
ACTACGGTTCGTTTATGGACTTAGGGTTATCAATCGGGAATCGGATTGTTATTACCCGTA
ACAACGATGTGATCCCTCGGTTGAACCGGGTGGAGGAGTGTGGGGAAGGAGACCTGCTAG
AAGCGACTACAAGCTGCCCTAGCTGCGATTCTCTCCTAGAGGTAGTGGTGGGTAGGGAAA
GTTCCGAACTCGTCTGTAATAACGTCGCAGAGTGCCCTGCGCAGATCGTTATGCGGTGCG
TAACGACGTTCGATAAGTTTGGACTGGATGTAGACGGTTTAGGACCCGTTAAGGTCAGTG

AATTGTTTTAGAAAGGATGGGCTAAGCAACCAGCTGATATACTTACCCTTCCGGAAGCCA
TTAAGGATCCATGCCTCACCCCGACGACTCGAAGAGGTTTGGAGCGTGTCCTTCACGAAC
CCGTTCCTTTACATCGCTTCTTAAAGGCGATGGGTATCCCGGATATTGGGATCACTTTGG
CTAAACGCCTAGCCAACGCCATCCCTGAAGGGAAGATCGAAGAATCCCTTACCAATCCTA
AGTTCCTACAAAGTGTACGAGGGTTTTCAGTGGGGATTGCTTACCGGGTCGTGAACGCGT
TCGACTCCCCGGTCTTTAAGGACAACTTCCACACCCTAAAAGGTTTGTCAACTCTCCAGG
ACACCGGAACGCAGGACGACTGCATTAAAGTCTGTATTACCGGCAACATTGGACCTTCCC
GAGGAGAACTCTGTAACCTGTTCGCGAAGGAAGGGATTGAATTCAGTGATCGGCTTACCC
AGGACTGTGCCTTCCTCATTGTGGGTGATCGTCCTGGTAAATCGAAACTGTTAATTGCAA
CCGAATCGGGTATACCTATGCTAATTGCAAAGGATTATTCGAGCATTGGCTCCCTCATTC
AGTTCATTAAAGGACACTGAGAATGACTGTTCAAACTGACACCAACGACCAACAACACAC
TACCACCGACCCGGTAGTAGCAATGAAACTCAAGAAGGCCGAGAAGGCTCTGAAACACTC
CAAGGAACTGGGTAAGAAGATGAAACACGAAAAGGAACAGTTTGTTAAGAAATCCATTAC
CCAACCCACTGAGAATAATGAGTACCGTTCCGCCCCTGAGCCTTCTAAGCTCAAGGTGGT
GGTTGACGCTAAGGAACTGGTGGCTCTCGATACCAAGCCGTACGGCGAATGGATCCGTCC
CAAAGAAGGACACGATCTTTTTGGGAATACATTCTGGTCAGTCGGTGTAACCGCGGAAAT
CGCCCTCTTCTCTAATTTCAAGACAACTTTTTGCGGAGTTGAACTCGATCTCTAGGTCGA
CTCCTATTCTGAAGTTACGCTGAAAACCCGTACTGACGTTCCTTGCCTTAACGTCAGTAA
GCGTAAGGGGAGTGCTGTGGTAGTCAACTCGGTGGTTAATCGTTGTGAGTTTGAAGGGGA
GAATTGTCTTCTGAATGTCGAGATTAACGACTGTGTGGTTAACAAGAGCAAGGTGGTTCG
TACACGATCGACCGAATTGCGACCAATCAACGAACTGATCGACTTCCCAAAAAGAGCGGA
TGTATTTAGACGAGGGGTCGATCAACGCGCCCGGTACTACGACTGCCGTTTCATCGAATC
CGAGATTAAAGATTCTCCCATCAATGGCAAGGGCTTAGTGAAAGAGTCGTATGTGGATCG
TACCCACATTACCGCTGGGAAGTTTGCCATCGTTAAATCGCATCTTACCACCACCGCCCT
CACCGCATCCATGCTGTATCTCTCGAAAGCTTAGTTTCATGGTTGTTACTTGAGCGTTTC
TGATACGCTGTTCCTTCGCAACTTCACCCTCCGGAATAAGACGCTTTACATGAGGTCACT
CTACGCGCCTAATAAACTGGGTCTTACGGTGATCGAACTTCCGTTCCATCCTCAAAATCT
TATTCTGCACCGCACCACAAAAACACAGTATGTAATCTCCATCCGCTCGGGTGGTTCGTC
CGGCCTTGAACTGGGGAGCGACGTAAGTTGCGAAGAACTCAGAGAAAAATTGGGTAAACT
CTTGATAGAAACCCCGATGGTCAATAACGAGATCTCTGAAGACGGACTCCTCTTCAAAAG
CATCTTGAATTATGTCCTGAAGTCGGTGATTTCTCGGATGAAGATCATCAAGTTGATGGA
CGATCTGATGGCTGTTAACCGTATTATCACATCAGATTTCGAGGATCCTGAGTCCGTCCC
GTATTGTTAGTACAAAACACCTTACTCCCCAGGTAATCCTGGGGAGTAAGGGTTTAATTG
CCTACTTACCTGTAAAAGGGAGGTTAACGAATGTCCAAAACAAAGAGGAATGACCGATAT
GTCATTTATACCGATGGGAGCGCGTTAGGGAATCCAGGTCCGGGCGGTTACGGTGTTTAC
GCTATATACAAAGGGGAAGAATTCTCCCTGAATAAAGGGTATTACAAAACCACTCACAAC
CGTCAAGAACTCATGATTCTGATTGCCGCACTGGAAGAGTTTGGTCCTAATATCAAAGTG
GACATCTTCACTGACTCCCAATACGTCATTAAGTGTGCTACTCAATGGTTGAGAGGATGG
ATACGGAACAACTGGATGGGGTATAACTCTGGGCAACCGGTTAAGAACCGCGATCTGTTG
GAAGTCCTCAATGAACTCTTGAAGAAGAATAAGGTCAAGTTCCATTGGGTTAAAGGTCAC
TCTGGGGATGAAGGGAATGAAAAGGCAGACCAATTAGCCAAGGATGCTGCTAATAATCCG
GAAGCCCACGATATGGAATACATGAAAGTCTTAGGGTTCTAATCAATCAACCGTGGAGAT
TAACACTATTAAAAGCTTTATTGATGTATCAGTAGTAGACGATGGGGGTAAACAGTTCCT
CTTCGGTATTACTGCGAATCAAAAGACGGGACGACCCGAAGTCGTAGGATATACCGATCC
TGATTATTTCAGAGAGGGTGCTGACCGTGTGTTTGAACAGCTTATCCGGGAAGAACAACG
AAAGGAAGCGGATATTAAACTCCAGTTAGCCTTTATTCAGGTGGTCACGGAACATCCCGG
TAAACAACTGCAACTCACGCTTTGCGACTGCGTAAATGGACCGTCAGCTGGAGAATTTTA
TTGGGTGGAGGATCGGTGTTATACGGCTGACGGAGTGTCCTTCCTATCGAATGCTCCGGT
GTGTATCTATCATAAGAACTGTCTGGATGGTACTGCGGCAGCTTGGGCGATAAGACGTGC
ACTCCCCTTGGTTGAATTGGTGGCTATGCGATATCATGATGATCCGTACGAACTCTTTGG
CCCGAGGTTGGAATATCTACAAGGGAGAATGGTCTATTGTGTTGATTTCAGTTTTAATAA
AGCAGTTGTAGAAGCGATTGCTAATTTACCGGATACAGGCTAGTTGATCTTGGATCATCA
CGTGAGTGCTCAACGGGAACTGGGTGAACTCGTCGTTGGTTATTCCGGGAAATCAACGCT
GGGTGATCGGGTTGAGATTATCATCGATATGACACGTTCAGGCGCTAAGTTGACGTACGA
CTACTTCCACCCTCAACAACCGACGCCTCCGGGTATTCTGGCGGTACAAGATCGGGATCT
GTGGAAATGGGGGTACGAAGGCAGTAAGGAATGGACCGCAGCTACCTTTGGGTATCCCCT
CACCCTTGAGAGTTTCGATAAACTGATTCATCGGGATTACCAAGACCTGGTAGAAGAAGG

TCGTACCGTCCTTCGTAAGCAGGAATTGGATGTCGAGAAACTGCTCCCGAGTGCGCGGCC
TTACACGTTGGAGGGGTACAACTGCTTGATTGTTAATGCCAGTTATTTCCACGCCAGTGA
TGTATGGGATGAACTGAAGGGTGGAGACGGTATTGACTTTGTAGTCACTTACATGGATGG
TAAGGATAGCGGGATCTTCTATCTACGGGGTAAGGACAAAGTGGACCTAGCGGAAGTGGC
TGAACGTTTTTGAGGAGGCGGCCACCCTAACGCAGCAGGGTTCACCTTCAGCTTCAACGA
TCCTCGTTTCTCCCAGTCACACCTGTTACTATATTCCGGTACTTAACTGGACACTCACAC
ACTGTACTTAATCATTGACCTGGTAGCTAAATCCGTTTTTATTGGTGACTTAGTGACTCG
CGGGGTCGACCATCGACTTATGAAGTCTATCCGATATTTGGAGAATCGGTAGGTGATTAA
AACAGTTACGGTGACCCAATGTTTACGTAACTAACAGGTATTCCATTTGATGGGGGGTGG
TTAGTATAAAGGCTATTCACCCTGTTCTCTCTATTTTTTTTTTCGGGATAAGTGGAATGA
ATAATTACGGTTATGTGGATGTGTCCTTGGTAGGACCTGAAGTAGAGGAAATCTTTGTCT
TGCTCTTGGAGTGTGGTCTTGCACCGGCTGCGGAGTATAACCTGCATTGTAACCTGATGT
ACGATGAACAGGCAGAAGAACCGCTGGTGAAACTGAATCCTGATGCGAAAGTCACCGCCT
ACGTGACAGGGATTGATGTACTGGGAGATGGATTGGTGTTTAACCTTACCAGCAAAGACC
TCGCTGATGAACACCGCAGTTTGAAAGACGGTGGATATACCCACTCCTTTGATGCCTTCT
TACCGCACATGCCTGTCACCTATGACTTCGATGAATACGACATGTTGAAAGTGAAGAAAG
CTTTCGCAACCTGGGGTGGTCGACAACTCACCTTCAGTAAAGAATCGTTCGGAACCAAAT
AAGACCCGATACTACCCCTACCCTTGACCGGGTAGGGGTAGGTTTAAGGGAGTAAAAATT
TAAGTTAAGGAATGAAACAAACCTACATTACCAGGGTGAATAGACAACGGGATTGTTTGC
GGGTAAGTGACTTTCCCTTCTAAAGACAGAAAACGGTTATAAAGATAAGCGTGACTACTG
ACTTCAATGTCGCCCTGGCGAATGTCTTGAGTGACATCGGTGAAAAGACAGACCGTGAGG
AGATCCTGTCGATCATCTACCACCACCTCTTCGACGAGACGCGTTTCAATAAATCAAAGC
GTCCGTCTGCGCTGAAGAAGATCAACGGGGCGACTCGTTCCCGGTTTAAGATGAACGACG
TTCGTCACGCCGGTAAGAAACCCGAGAAGTTGATCGTAACGAATAAACTGTCCGGTCATG
TTGACATTCCCGTCCGTGTGATGGGTGAAGTGAATGAACAGGCCTCGCAGGCAGAGACTG
TTCAGGCAACCCCCACTGAAACTGATGCAATGACCTACCTGGAGAACACCATGAAAGAAG
AAAACCAACCGCTCGCCCAACAACTCCTGGAACGCTCCCAAGGTGCCCGCGCCGCCTATG
AAGAACAGGCGGGGGCTTCCAGCTGGTCCGACAGCAATGACAGCGACGAGGTCGTCGATG
TACTGGTGGTGACCTTCAAGGCCGATAACATCCTGTCCTACACCATCGAAGCGTGGCTGA
TCAACGGCGGTCTCCCGACTCACCTGAAGACCGAACTGGAAGAGCTCGTCAAGAGTCAAG
ACCACTCGTCCCCGGCGGGTGCGCTCCGTGCCTTCCTGGACGACCACCCCGGACTCAGCC
AGGAGTTCATCAATCAGTACAGCAAGGCGGTAAAAGACATCATCGCCACCGGCGTGGAAG
GTGAAAAGGCGCGCCGTGATGCGGCTAAAGCCCAACGGCAAGCGCCGGGTACCTTCAAGG
AACGTGTCGTCTACACCCTCCGGGGCGGTCGTGAAGAAGGCTTCGGTACCGGCGCGGTGG
CGGCTGCTGCGGCGATCATCGGCGGTGGTGTCGAAATGGCATCACGTGGGAATGTGAGCA
TCGGTTCGGTTGTGGGTACGGGGCTGGGTGCGGCGGCCGGTTACTTCGCGGCTGAGAAAG
CAGAAGGGCTCATGAAGAGCGAACCGGGTCGTTCCATCGTGAGTGGTTCGATCGGTCTGA
TCCTGGGTGGGGCGGGTTCCAACCTCGGACGGACCACTCAAGGCCTTCTGGGCTACGGTC
CTCAGCAGCCCCAAGGCGAGCCCGTAGCGACGGTTTCCGTTCGGGAGCAGGTCACGGCAC
ATCCGGAACCGGTTCCTCAATCCCAGGGCATTCTGGCCACGGTAGCTTCCCTGTTCTAAG
CAATACGGAATAACGGCGAACATGATTAAACTCAGTTAATCGACTGACAGTGCTCGAATA
CGAGGGGTTATCCCCTCGTAGTTGGGTATTCGTTCTCTATTTGTTAATACACCTACATCA
TTCTTGCGTGATGAGAGTGTTTATTTATGTTAGAGGTAAGGTTGTGAACACATCCAACAC
ACCACATAAATCCCTCCAGTTCAGCCATCAGTTCTTCTACACCATGGTGTATAAGGCTGG
GTACTACGGAGCTCCACTCGAATTCAAGGATTACTCCTTGTCTTCGTGCCAATTCAATTG
CCTCTACTTAGGGTATATCCACGGGTATTCTGAGACGAGAAAGAAAGGGATTTGGAATTT
CCTTAAGAATGGCTACCTTAAAGTGCGGTATATAAGAACCCTTATTGGGGATTCCCAAGC
CCAGTTTGAGAAGGACTATGGGCATATCCCCCAAATTCGTTATCACCTTAATTCATCTGA
ATAAGCGAGGTTGATATTAATGGACGAATATGTCAACGATCCAACGCTCCCTAACTTCGA
TCAACAGATCCGAGCGCTCCAATACCTTAAAGACCATTCTACCCGTAATCCCAAGAAGAT
GATGAAGATGTGTCTGAGGATGTTGCCGGAACTCCCTGGGTTCATTAGGGACGCCTTTAT
TACCAACATTATTCTGGGGAAAGATCCTATTCACTGGCTGGAACACGCATTCGCTGAAAT
GGACCAGTATCCTGTCTTAATGAAACAGTTTATTGAGGAAGGGAACCATGGGACGATCTT
TGATAAGGGAACCCTCTATGGTTTCAGCCTGAAGACCAACCCGAAGTGGTTGAGTAACCA
AGATGGGGAATGGGACCCTGAGGAAGAGCAGGCGATTATCGATCAGGCGCTGAATGCATG
GATGGTTAAGGAGCGTCGTCGAAGCTTCCGAGGGCGTATTGTGCTCATCCAAGACTTTGG
GTTCTCCGGGATTACGTTGCTGGTCGATCACGCTTTTACGGATGAATACTGGGAGGCATG

TCAGAAACAATGGGAACTGGAGGATAAACGCCAGTTGGATCAAGAACTTAGGGCACTTCT
TTCTAAGGCAGGTTGTTGAGCATCACGGTCTGCGGAACAACATCTTCTCGCAGGGCGAGG
AATACCTCAAAGCCCGCAAATCCACCACTCAGGCTCTTGTTACCTTGAACATCAGCTATA
AGGAGCTTAAGTAATGCAATACACCCAAGAGAGTTTGGCCGAGACTCCAATGGAACAACG
TATCGAAGCGATGCTGTTCGGACTCCAAACCCTGACCTTCCAAATGAACACCTCGATCGT
CGAGTTCTACATCGGCGCGGTCAAGCTGAAAGCGCAGCCGTGTGCTCTGGTCGCTGACAT
CGTCGTCGAGCGCGATGGTGAACCGCTGGTGCATTACCGCGCCCGTTCCGAAGGCACCAC
CATTTCGATACCGAAGTCCAGTGTCAGTGTGCAGAAACTCGTCGAGTACCTCGAGAAGTT
CTCGGTCATGGTGTTCTACGCTATTCACCGTGCGTTTATCAAGGAACATTGCGATAACCG
CACTACCCTCATCGATCCCCAAGAGTAATCTTACTTAGGGGTAGGCAACTACCCCTTCCC
CGATCCTGGGTTATTCTCTGCTGGATCGGAATAATCAACGGTTAGGAGCCCATGAAACCT
AAAGTCGAACACCCCAAGGTTCCCTATACCCCCTTCTTAACCTTTCCCGAATACCTTGTT
GCTACCGATGTGAAGCTACAGGTTAAGGTGTTTAAGAAGGAATTGGAACGGATTAAGGAC
GATCCCACTCACGAACTGAATACCTTGGTCCTGTAACCCGCTTAACCACGCCCACTCTGT
AAAAGGAGTTGGGGTGGCGGGTGTTTATTTCTTTTAGTTTAAAACGTATTTATTCTCAAA
CCTACAGTATTCATATGAATGGAGCAATATGATTATTGTTTAACCCAAAGGAGGAACACT
TCATGACAGACCGTTCGTTGAAAACTGACCGAGATGATGAATGCGGCGGGGGCCCGCTCA
CGCAGTTTGTCGACTACGTTACGAACAAAGACCATATACGGAATCACGAGAAATACTTGA
TGGGTCGGTATGCTCGGGGGACGGAATCGCGACTGGAAGCGTGGATTAAATTCCAGTACG
TCTTCGGGAGACCGTTCGGTGAACGGTACGTTGGGGATCTCAAGTTCAGGGTCTATTACG
ATTTCGATACAGGGTTCTAGTTGTTTTTCGTTCCCTTTGCGTCCGGTCTCCGTCTGGAAA
GACAGGGGGATGCACCTATGGTGTTCAGCCCAGATCCCAATCAACCGGGTAATGAGAAGG
TGTTCATCAAATTCCTGGTTGAACATCAGGCTAATCGTCAGAAATAAGAACGGGTATTAC
GATGACCAAATCCCCTTGGAGTCCTGAGGAAGAAGTTCCTATGATTCTTCCTAACGGAGA
GTTCAATCCTAAAAGGAGAACGTGGTACGGTAACCAATATGGCCTGAAACCTAAGGAGGT
CAGTTATCATCAGGTTGAACGGGTTAAGATGATGCCTGAACCAGATCTGGTGACGAGGTC
AGGTAAACTGAATCCTGTTTGGATCACCTGATACCTGGTTATCAACAAGTTCACTACACC
CACCGCTCGCAATGCGGGTAAAGCGATTCTTGCTTTACGAGCTAAACAGACTAAGGAGGA
AACGGCATGAGCACACGACGTTTAATGAACCGTCCGCCCGTAAAGCCACTGGGTCTCCAG
TTTGATCGGGAAGCGTTGTGGTTGGCTCGTATCAGCTTCATGATGACGATGGAGATAGGG
ACATCTCAAGGGGATGAATTCCTAGATGCTGAACGTCGGTTTAGCAAGGATGCCATTGAA
CTCACTACTCTTTTCCTCTCGGGTAATAAGAACTGAATGTCAACTCTACGGGAAATAGCT
GAATGGGTACGTGAGCAATCCTTCAAGACCCAGGAGAGTCAACAAAATGTCCCTGTCGCA
CCAATTCAAGAAATACCCGTTGGCAACGAACTTGCGTCATACAGAAGTTCCGTGTTTCAA
TACGAAGATGTACGACTTGCCTCAAGTTAACGCTTCGTATGGGAAATCCCATGTCACACA
GTTCTTCCTCAGACGGGCGCTGTTGAATAAAACGAAGTAACGTTCTGGACTAAAGAGAAT
AAGGAGTTCTGCGTTCATGAAACACTTTATCTTTGCAATTCTGCTGGTCCCCGTGGTAGC
GTTCGGTGCGGTTAACCGTGAGAGCGCGCTCAATGGTCTTGAGACCTACACCTGTGAAGG
GTACACTGACAACGTTGTTGATACCGTTGAGGGGAGTATTGAAATCATAACGGACAATCC
GATCCAGTGGGAGAGGACCGTAGTGCTACGAACGACTCGGGGATATGTAATCAAAGCGAA
TTCACTCTTCAAGGAAGATCAAATCCTGATGGCAGAACCTTTGATAACGAAGAAGGCTGA
CTATGCCGACAATGATTTCATCATGTTGTTTAAGAAGTTCAACGATGTAGGAACCCCCTA
TTTCCTCCTCCTCGTTACTGAAGATCCCACTGCCAGCAGTGAAGATGAAGATGAGAACCC
CATTGTCCGGGTCGTCACCATAGCACCCTGTCGTGTGAGTTAACTTCCGGTGCAGAACAG
GAGGGACATCCCTCCTGTTCATCCACATTAAGATAGGACACGCCCTATGACAAATCGTTT
AGACATTCTCCCCCGGTTTCATCAGATTGAGGGGATACGGGAGACGATCATCGACTTCTC
TACCCGACTGTTCGACTACGAGTTTGCCTTGTCGGGAATACCCGCCCACCTCAAAGACCA
ATACGAAGTCCCAAACTCGGCAAACGCTTATGATGATCTCTTCGCGGATACGGATCTCTG
TATTCTGGCCTTGGATGAGGATACGCCCATCGGTATGCTGCTGTCCGCCTACGAACGCCC
CAATGCCATCGAAGTCTTGTTTGTAGAAGACAAGTACCGGAATAAGCACGTCGGTAAGGT
TCTGGTTGAACAATTCCACCGCATTAAGCTGGGTCATCCGATCTTTGGCACCGTAATGGC
GTGGAATCCCTCCGCGCGTCGCTTCTACGAACGGAATGGTTTTATCTTTAAGGAAGATAG
CCAACATGGAGAACAGGCCTTAAAGGGTGTACGTTCTCTCCTGGATTAAACCCTTACTTC
CTACTCACTTCCTAGCAACAATGTTGGAGTCTAAGATGACTACGATTGTGTTCAGTCACC
GCGTCCTCCCTCCTGTCTTCCGTTCCGTCCACGGGGTTTATGAATCCACGGCGATTAAAA
CCAAGGACGGACGACTCAAGTTGATAACGGGTTTCAGTTTCGGGCAAATGGGTGAAATCA
TTCGCGTAGGGTACATCGACCCCCAGAAACTAACTGATCCCCGGGAACTGAAAGCTTACC

ACGACCAGGTCTTGGAAGTCATTAAACAGTTCGAGAACTTACAGTTCTGGGTTAAACAAC
AGATTCGAGAGTACGAAAGAGGTGGGTATAAGAGCTTACCTGAATACGGCTCTTTCGTAC
TCCAACAAGAATACCCCATCCTGATCCACGGTAAGATTAAGGGCGCGGCGTTGAAAGTCG
AGTTATTTGGTAGAGACATCAATGAAATGGTTTTCTCGACCACGCTGAATATCACTGATC
TCGAACGTATCTTCCTCGATAAGTTGGCCGTCATCGTTGACGGTTTCCGGAATCACCTTT
GTCACGTGGATAATAACGTCACTCTGGATCAATTGTTTTAAGGAGAACGGTATGAAATGG
ATTGTTTTGATCACCACGCTCCTGGTTTCTCTGGTTGTAAATACCAACGAAGATAGCTTT
GTCTGTCACCAATACGGGTTTACCCAAGACCCTGAGCGTTTCCATTCCCTGTCCATGAGT
CCCCAGGTTAATACGTTCGACCTCACCGACGACCGAGTAATCTACCAAGGCCGTTTGTTT
ATCGGGGTGGAACCCCGAGACTACGACTTTGATGACACGTTCGTCACCTACCTGACTGCG
GAACGCAATGAGATTTTGTACTTCTACTTAGTGGGCGAGCAACCTGAAATAGGGATCAGT
CGCTTGGTAGAAGACTCAGATACGTTTTTCAGCGATAAAGCGCTGTTTACGCACTGTTCA
TTCATCCCTAAAGGAGGAAGCGACGTTGACGATGCTTCCCAACATTCATCTCGTGTTAAA
ACCGGAGGAATTAGCGGACTTATTCCCGTACGACATTGTTAAAGTAAAGGCAGACAAAGA
CACGGGGATTGTTATCGCCCACGGCGCCCTTTGCTTTTTCATTAAAGGTATCCTTCCGGG
TATTAAGACAGGGGACACTTTGTTCTTGTACAAGGACATGCTAATTCCGAAAGAACAACC
TCTTTACATTTAAGGTGACGTATGACAGTCGAGTTTATTCCCACACCTTTGATGATGCAA
CACATTACCTCAGGGTATAGTGAAATCAACCAACAGGGACGAACGGCTCTAGAGATCCAA
GAGAAATCGAGTGGTGTACTGGAGCTAATACTGTTCAGTATGCAGCGGGTGAGCGATGCC
TCGCGGACCTAGGGGTACAGGTCCAAAGACTACCGAGAGGTCTCGGTCCGAGAAGCACAG
TACCAGGCAGGCCTGGAGCTGCTCTATAAAGACCTTCTCCAGGCATTCGAAGCGAGGGTC
GAAAACACCACCTCCACGCTCAAGAGTTTCAAGGACGAAAATGGGCTATGGTAAAGGTTG
AGGTTAAACACGGACTCTCAGACTCCCAGCGAGAGGTGTTCAAAACCCAATGGCTGGATG
TGGGGGTCAGTGTGGTAACTCTGACGCGGGGAGGTGACGGCAGCCTAATAGGGTACGTGT
ATACTTTCAGTGGTAATAACCCTATTGGGGTCCAGCTTCGTTTACAGGATCCGTCACTTT
CCCTGACGCCACAACAGCTAGCCAGTTTTAAGTCTGCGATACGGAAGGGGCGGTTTGTTC
TAAAGACACGCGATAAGAAGAGGTGAATAGTGAATGTGTGCACACAGTGGAATAAACGTG
TCTTCATACGTCAACATGAGCCTCTCTTGAGTCAAGAGAAGACCAACGCCCTCATTAAGG
ATGTAACCACGGACTACCTCCAAACTGCGGTATCGATCGTGTTGAGAACTGTTCCCCATA
CCACGTTGGATGGTAAACCGAACCCTCGCTGGATTCTGGGTGTCGCTCCAGGGTTCCCGG
ATTATGATGTCGAAGCACTGATCGGGGTTAATAACGGACTGCGTAAGGAAGAAGACGACG
TCATCGATGAATGTGTCTTCCACCGGAATGAGTCGGCTAATTACCGGAATCTGGATGAAG
AACTTCAACCGTTTATTAAGCCCGCTGCACGGTTCAATGAGAATAACAAAATCGAATTCA
AGTTACTGGGCGGTTGGAAACCTTTTAACCCCGCTGTGTCGGTTTCTGTACGGAAAAAAA
TGAATGAAGACAAACACTTCACCGCCTGAGTACATCATCAGTGAGAACGGTGACTGGTTC
CATATCAGTGGGTATTGTGGGGTGTTCTCCCCGTGCTTGTGTCGGATGGGTGGGAAGTTC
CATGAGGCTAACTATGTCTCGTTAACCCCGATTAATGTCTGGCAGTGGGAAAACCTGAAA
CAGGTTCGTATTCGTAAACACCGCACCGATGAGACCAATCCCTTTGCCTTTGCCCATTCG
TTCCCCAAAGTGCGTTATGACCACCTGGTAGAAAAAGTGGGGGAGACGGTTGCGCACCTG
CTGGTTCACGAGGCTCTCTTGGCTGAAATAGACATGTCGCTGTTTCGCCACATGGATCGT
GTGTAGGGATGGGGTGGTGCTGTCCCCTTTATCCTGGTGTGTAAAGAGCGAGACCGTTAC
CCTGAGGTCCTGGAACATTGGTTCACTGTATTGGTTAACGAATCCGCGTACTTGAAAAGC
ATGGATGAGGACATGGTGCAACGATATCTCATCCCTACTGAAAGCTGTCGGGCTAAAGCC
CACTCTCACTAATTCCGCAGACCCGCTCCTATTCTTTACCTCGGAATGACAACTCAATCG
TTAATGACCTATAAGGAACCTGAATGACTACCATGAAAGTCCAACAAAAACTGGGTCTGG
CTGCTTTTGCTATCCTGTCGGACCAACTGAAAGCAGAAGGTATTCACGTGATTAATCCGG
TAGAGGGCGAGGTGGTTACCGCTGACGATGTGGTTGGTAAAGTCCTGGCTGCCTTCAACC
TGGGCAACAAGATTGAAGTGAACGCGGACGTCCCCGCTAGCAATAAAGGGGTGAATTACC
GGTTCGAACGGATTGGGGGAGCGTACTACCTGGTCCGGAAGTAGCTAACGACTCGCCCTC
CTGCCTCATTGGCAGGAGGGTGTATGTCCTTTATTTGTTATGAGGTGGTTATGTCAAGAC
AATCGTTGTTGGAATATGGAAGAGGGTGGTTATGCAACCAGAGTGGTGAGGGTAAAGAGG
AATGGACCGGGGGTCACGCGGTGTTCTACTCCATGAGTGTCAGTCCTTTAGCTTATCCCG
CCACCACCTTTTGGACGGGTCGTTTCAATATCCACATGGAGAACATCACTGTTCGCTTAC
ATTCACACGATAATGATCCTGAATTGGTGTTGTTCTTCAATAAGCTCAATCTACTGGAGC
AGGGCGTACGGGAGTATTGCAAGGCGTTCAGAAAGGTCCTCAATAACACGAAGAAACCCC
TAAAGGACCCCGTCTATAAGCGACAGTGGCTCAACCCTGAGAACATTGAGGCGCCCTTTA
CGGGGTATTATTTCTATTGCGTCGATCAGGATAAGGCAGGCCGGTTCTGCGTTGGGGATT

GCCACCGGACCATTGTGATGTGGATGGACATTCACCACAATGACGGGAGGGTGTTTAACA
AAGCGTTGTTATAAGTTCAACTCAGCTCTCTCCAATGCCTGGTGACCTATTTGAAGGAGG
CTCAGGTTAAGATCAAGAGTCTTCGTAAGCAATACAAGGTCTTAGGTTGGGATAAACCGC
AGGAGCTCCAAAGTGACACGTCCTCTGAGGACGTTCTTCCCGATCCCCAGTAGAAATTAA
TAAACCTATATTACTCAGGTGTAATGCGATTAGGTATGGGTTAACAGGAGTACGAAGATG
TCTCAAACGCTACTGGTACATGGTAAAGATGCCCTAGGAATCATTAAACAGGTCTTGAGT
GAGGTGTATGACGCCGTCACTTCGACCATGGGTCCGAATGGTCAGTTGGTGATGATCAAG
AATGGGGTCTCCACCAAGACCACCAAGGATGGGGTGACGGTAGCCCGTTCCATTCGCTTT
GCAGATGAAGCCCATGAATTGGTGAACCGGGTAATTACCGAGCCGGCTACGAAAACCGAT
GAGGAATGCGGGGATGGCACTACCACCACCATCATGCTGACCCATGCGCTCTATCACCTG
TTCAAGGACTTCCCAGGTTTCCAACACCACCGGAACATTGAAGACTTGGTAGAGCGGGTT
ATCCAACGTTTGGACTCCATGGCGATTCGGGTGGAAGTGGATGACCCTCGGTTGTATCAG
GTGGCCCTGACCTCGTCTAACCAGGATGAAAAGCTCGCCCGACTGGTTAGTGAGCTCTAT
GCGAATAACAAAGGGGGTTATCCGGATATTGAACTCAAGGAAGGGGTCAACTTCGAAGAC
CAGATCGAACAAACTACAGGGCGCAATATCCGTATGTTCTACGCGAATCCCTGGTTTGCG
AAAGGCCACGAAGGGGGTGTGACAGAATTGACGGGATTTACGGCGTTCGTTATCGATCGT
CAGATTGACAAGGAAGACAGCCAGAAGTTGATTGATACGGTGAATCACCTTGTGAAAACT
CACAAACAACACCTGGCCCTTCCCATCCTGCTGATTGCACGATGTTTCGAAGAAGCCTCT
AACTCGACCCTGATGCAATTGAATGCGGCGCACCCAACCCTGGTAGGAGACGGACGTCCT
TGGTTAATCCCGTTGAGTACGCCGGTAGGTGGTGCAGTTGGGACGTCTGAACTGCAAGAC
ATCGCGGTCATGCTGAACGCCCCTATGCTCAGTGACGTAGCGGACCTGACCAAGTTGGAT
ACCCATTCGATCAATGGCCAACACGGCCAACTGGAACAGGGGGGCAATCGGTCTAACCTG
AAGAGTACCACACCTAAGGATGAAGACCGGATCGAACAACATGCTCGGGGTATTGAGGAA
CTGTTGGAAGGGTTTAGCCTCAGCGATAAGTTCTCTGTACGCGCCCGGTACAACGAACGG
CGTATTCGGGCCCTACGGGGTAAGTTGATCACCATTTCCATAGGCGGCGAAACCTATTCT
GAAGTCAAGGAACGGGTAGACCGGTACGAGGATGCGGTGAAAGCCCTTCGTTCCGCCTTG
GAGGACGGCATCCTTCCTGGCGGTGGCGTATCCCTGGTTAAGGCGGTCTTCGGTACGATC
AAGGAAGGCCTCGAAGATAACGCCCTGAGCGCTGAGTTTGCTACGCGGTATATCAACTCA
GGCATTGCCAATGAGTTGATGCGACTGTCCACTATTCAGCATAAGCTGTTGTTCATGGAC
ACGGCGCTGTATAAGGAAAATGGGTGTTTCCATTTCAATGATGATTGGCCCAATACACCG
ACCGTCATGAATCTGGCAACTGGTGAGATTGATACCCCGGAGGGATTGGGTATTTATGAC
ACCGCGTATGCCTCTATCACTGCGCTCAAAGGCGGACGCCAGACCGCTAAGATCTTGGCC
ACTAAGAAGACACTGATGCTAGGGGAGAAGATGTCGGCGGTTAAGGTTCGGTAAGGGGTT
TGTAAAGGAGTAGGGGGAAGACCCCGACTCCTTTATTTTTGTTTTAAGGAAATCGATGGA
TGACGAAGCATTTGAAGGAGGTAGACTTCCTATGCAGTGAGTCTTGTAACTTCTTTTGAC
TTACTCCCTTTAGACCACCTTTAAGAGGATTAAGCAATGGATTCAAACCTGCCAATCCAA
ACGAACTTCCTCGACAAATTCGTCGAGTTCAACAAAGACCGCTACAAAGACCAGGCGCAA
ATCCTCGCCCTCTTTGACGGCCTGACCCTCGCGGACATCACGATCTCCGACATCACCGCC
GACGGCGCCAACCCCGAGCTGCAAAGCGCCACGGTCACCTCTGTGAGCCGCAACTTCAAA
GGTGTCGAACAAAAATGGCTCCCGGCGAACATCAACACTCCGATCGCCTTCGTGGTGGTG
AACAAAGGCGCTCCCCTGACCGGCGAAGACCAACTGGCGGAGCAGACCACCCCGGGTGCC
TACGTTTACCTGGTCGGTGAACGCGAAGAAGCGGTTCAACGCGTGGGCCTGGTGCTGGCT
TCCACCACCACCACTGAAACCCAAGCCGCCGACGCCCTGGCGCTGTTCAAGGCCAACTTC
GAATACGTCCTGGACGACGCCTATGTCGATACTGCCACCGGCAAGGTGCTCAAGGTAACC
ACCAACCCGTTCACCCACGGCGAGCTCGTGTACGAAATTGGTAAGGCTCCGATCATCGAG
ATTCCGGAAACTGACTACGGTCGTCTGGAATTCAAAGAAATCGTTCAACCCGAATCTCAA
CCGTAAGCTAGATAACTACCAAGTGGGCTAGCCCACTTGGTAGTTTACCTCTCTTCGTAC
CTCATTTCAATACCAGTGAATTTCAAATCTACATCATCAGTGTGAGCCAGGGGCAATAAA
AGTTCACGGATGAATGAATTCTCTTGCAACTGGTGCTTTAAATGTCATTAGGAATATTCA
CCATGAGTATGAGTACAGCTAAAAGGGGTAACTTTTCTACGTCCCCCGGCAAGGAAGACG
ATGATCTTCCTCTGCACGAGATCCGGGATGCCTTTAATCCCGCGTTTATGAAAACGTTTG
ACTAACTGGGCCTCGACCCACAATTGATCAAAGAGTTTATTAACGCGCTTTATCTGCTCG
ACAACATCGTTAAGACCCCCAACTTCGTATCGGGCCGAATTCTGAATAACCGAGATATCG
AAGCGTACGGCGAAGACGCGGTCTTCGCGTTCAACCGGAATATCAATATCCCGTACTACT
GCGTGCGGTCGGGTATTACCGTGTCTGAAACGGTGTACAAATTGCAGGACCAGTTTCTGG
AACGTGATTATCCCAAAACGATCCTGAGTATTCTCCGCCCCATGCGCACGCCCGGTATTG
ACGTGACCGTAGACGGCCATCCTTACAACGTAGGGATTACCTTCAAAACGCCTCATCCTT

TGTTCGGTTTGGTGAATTACCAAGATCTCCGGACTGGGATGAACACTTCAGTCACCTTCC
TACTCACTCGGGTGGGACTTTGATCCTCTACCTGCTCCACTGGGCTTAACGCTCCTGTGG
AGCGGGGTAGGGGTTATTACCGCTTTAAATTTTTCAATACACCAACCCAAAAAGGTAATA
ACCATGCCGAAGAATGAGAACAATCCTGATAAACAAGGTCTTACGTCTATCACTGATGAA
GACTTGGATAAGGCACTCAACGTACTGGACAACGCAGGGGAAGACGTAACTCCGGTGTCC
GAAGAGACCACTACGCGGCATCCGGGTACGTCGGATATCATCAACCGCTATTCACATCCC
GCCCTTCAGCGGAAGAAGGCAACCCCTGAATACATCGAGTGGAAAGCCGCTCTCAAACAG
AAGGAGGACCGATTAGTGGAATCCTTGAAGACTTCGGAGACCCGGGAACATGAAGAGAAT
GTCCTGAAAGGGAATACCCTGGGTACTAAACCGAAGACCAAGCAGGTAGAAGGACTGTTG
GAGATGGTGGTTCGTCAGAGGGCTGAACTCACCCAAGAAGACACCGTAGACGCCGCAGAA
CGCGCATTGGCAGCGCTGAAGCCGGTGCCGGAATATAACCGTAAGAAACGTATTCGACTG
TGGTGTATTGTGGTCGGGCTCTGGGTGGTTGTGGGAAGTGGTCTCTATGTGGGGTGGATG
TTCGTCCACAAACAAATGCTCACCGGTCATTACAACGGGGTCGCGGAGTGCAAAGTCCCT
TACGGGGATTTTGAAATTACCGGTAAGCGGTACCACCGCTACGCGTATCAATCGATTCTG
GGTTACCACCGCGTGGATGAGGGCACGGTCGAAGAAGTCACCGAAGTCAGACTCCCTGGG
GATAAAATCATTATCCTGGGATTCGATGCGAGTAAAGGGAACTGGTGGCGGATGACCTAT
GACCGGGGAGAATTCGGTACCGTTAAACTCAAACCTACAGATAAGTGCTGGTTCGTCGGC
GAGAAAGACCGTACCACCCTGGTAGCTTATCGCTCGTTCTGCCATTAAACGGTAACCTTA
CCGAGGGAGTCAATCCCATGCTGAATAGTTCAGACCAACCTGTGGGTCTCTTGTCCTGTC
GATTGTACATCGCTCCCAACGATGGTTCTTTCTTTACCGGTCATTTGGAAAAGTCCCACA
AGAAACGGTGTCACTTAGTACGCCACAAGCTCCAATACGCGATCCGGTATTATTCATCCG
TTAAACTGCCCGAGGGGTGGTTGGTGGGGATCAATGACCGGGATGCAAGCCGGTATTACT
TCTTCCCTACCCAGGGAAAACGGAGGAGTATTCTGGTTCCTTTACCCAGTACAGCGTACC
GAGAGATCTGGTTTAACCTGTTTAAAGTGATCTGGAGGACTGGTACCCGGGAGGCTATCT
ACCTGCACAGTGTGGCGTATCCCCATCTCATGGAGCTCTTGACCGAATCCTTAGTCCCCA
GAGTGATTTCTGTTGAAGGTGAAAGGAAAGGGGAAGGGTATATTGCGTCTCTGGAATGGA
AGCCTGAAAAACCCGGTCGGTTGATCGTAACAACGACCTTGGATAATGCTAAAAACCACA
GTGTTCCGATCCAATGGCATCCCCACGATGCGTTGGTTCGTCTCATCCGAAAGTAAGAGG
AATACATCATGAAAGAAGAAAACCAGAAGAAGACCAACAGTTTCCAAGGTGGCGATGTTT
GGGTAGTGTCTTCTAAGAAGCATCCAGGTAAATCGAAATTCCTCCCGGTAAACCTCATGG
GACCGGATACGATCGAGAACCGTGACCAACTGGTCCTCCAAGTTCATCACCGTCTGACCG
AAGGCCAGAAAGCGTTCTACCACACGCTGGAAACCTACAGGCTGTGTAAGGCCTACAGTT
CGTCACCCGATCCCCGTACGTTGGTGATCAACATCGCTCCCGGTCTTCCCCACACCAAGG
AAGAGAAAGACGCTCCTCGCCGCAGCATCGAGCTGGACGCCGGCATTAACCTCGATGCGG
TTATCAGCGAACGGGAGGGTGGGGCCTTCAAAATCGTCATCGTGGGAACCGCTACCCATA
CGTCTTATTTGGTGATCCGTGAGGACGTTGCCTTCTCGCTTATCATCGATAAGGTGTCTT
TGTCGGTTCAGGATGCGGTAGTCACCCTGTACGCCAGCAACCTCAGCAAAGGTCGAGGGC
TCTTCGAGAACCTCGCTAAACACCTTCCGGACTTCTTCTACAAGCTGTTTAAAGCGGTCT
TCCCGGAAATGGTTCGTCTGGAAGTCACGCTCACTCACATTCATGAAGACAGTGTTCGCG
TTGCGCTGTACTGGGATCCTAGGGGACCTAACAAGAATCTCATTGCCTGCAATGGGTTTG
TCTTCCCGTTGGACATTTCCCAGTTCCCGGGTAAACAAATCTCCCGGGGGTATAAACTCC
AGAAGAAAGAAACCCGTCATGTCGCTCACAAAGAAAAGACATCTGACCGGTCAGTCAAGA
CCCAGCGTCTGTTGGAAAAGTTCTCGAAATAGTCTGTGAGTACGAGGTGGGGGGTAACAC
CCCTACCTCTACCTATGCTGTAACGCGCTGTAACACCCTTTAAAGAGTTTTTAAGATGAA
AGGTACCCTACCCCATTACCAGATCAGAATTATCGCTAGAAGTGCCTTTACAGACGATTT
AAAGGAGAGACTCAAACCACTGTATGAAAACCTCTTTCACCACGATCTCCATCTCGTCAT
TGGTACTAAAACAACCCGTAAGCTGTTTAAACCCTCCGCCCGTAAAAATCCCCACCCCTC
CGTTATTGAGAAACGGTACGAAGACTTCTTCAAAGTAACCGACAAGATAGTACTCGTTAC
TCACTTGGGAGAAGCCGTGGGCTATCTGGCGGTGGTTAAGATCAAGATCACCTCACTCTT
TGTTGAAGAAGACGTCCGGAAGCTAGGCTTAGGGCGTATCATGGTGTCCAAGTTACAACG
GGGTGGACATCCTGTGAACCGGTTCCTCTACGTTCAATGCTTTACCCCAAATACAGCTGG
TATCAACTTCTACCGGAAAGTTGGGTTTAACGAAATTGACAGAGGAGAGATCTACACGGT
TTTGTTAATGGAATAAACACCGTACTAGGAGTAGCCCAAAAGGGCTACTCCTAGGGGTGT
ATTTTATTTTGGTTTCAAAAGAAGCGGTAGAGATCACGGTAACGTCCTGCTTGGTAGTAA
TCTCCTGATCCAATGGTGGAGGAATTAACACCCCCTACCAGCTAAATCCCACTGTCATCC
GTGAGCATCTCAAAGGCATACCGTTTAGTTACCGTGGACATTTGCTTGTACCAACGGTCT
TTCAGTATGTCGTAAGTATGGAACACGATGTAACCGTTTTCAACACCACTGGGAATCAGG

AACTGTTTCCACGAAGTCCCCCCAGTCCAGCTACTGGCAATAGGTGCTTCTGTTACCGTT
CTCCAGGTGTTGGTAACGGGATCGTATTCCCTGACCAATCTTGACGTACCCGCGGGGTCG
TTCCATCCCCCGAATACATAGATCTTTCCATTGAACTGTCCGATGTAATGCCCATTGAGC
GCCATCGGCATATCCGCTTTACGGGTCCAGACGTTGGTGGTGGTGTCATACTGGTGGTTG
AGGGCTGTGGGTTTATTGGTACTCCCCATGTAACCCCGCATGCAGAAGATCGTCTTGTCA
TCCAGGGTAATACATCGACCTTGTCCTCGGGCTCCATCCGGGAGGTTATTCAAGTTCGTT
GAAGACCCTGTTGAAGGATTCAATTTCCACACGCGACTCACATAGTTCCCATTATTACCG
GTAATACCCCAGAAGGAACTGTTGCAGTAACACCCGCCACTCCAGGCCCATACTGAGATA
TTCCCGGTAGTGGCCCACGTACGGGTGGTAAAGTCATAACGGGTATACGTCTGTTGTTGA
CCCCCACCGGCAAACTGACCTCTGAAGATATGAATCTTCCCTCCACCGATACCCGTACTG
AAGTTAGCAATACCGTTTAAAGGAACTTGAGTCACCCATTCCATTCGTCCCAGAATGTAG
GGGTTATCATCAGAAGGGGTAAATAGTTGACTCTCTAACATAAAGCCGCTCCTAAAGAGA
GGGAGTTAATCCGTCACTTACTGATTTCAGTTCAACACAAACAAAGGACCCGGAACGGAA
GGGAGTTCCTCTGCGACGGTGGCTGCTTCGGCTCTATCCATAAGCGACAATATCGTTTCC
AGTCGTCCCTGGATACGAGAAGTCACTTCAGTGGACAAGTCAATGGTTTCTTCGGCGGTC
AGAGAGAGCGTTACACCTTCCTTGGTACGAATAGAAGCGGAGCAGGGTTTCTCCTCACGC
AATGCTTCTTTCGCCTGGATACTCAAGGCCCAAAAATGGATTCGGTGGGTGGATTCGATC
AAGAGGGTGTGGGCCGTCTCCCCGAATCGCACTTCCATGCCTGTGTAGATCGCCGCTGCA
ATCACATTCTGGATTTCGTTCTTACGAATATCCTTAGCGGATCGGAGGCGTTCTGCGACT
TCCCCAGGGGTAAAATCCCGAGTGGTCCACTGCTGGGTCCAATACCCATCAATCTGTTTA
GGGGCAAGTTCAACCGTTACATCCCCGGCGGGTGGCTGGGTCAATTGAACGACAGCCATC
AGGAAGGGTTGTCCTTCCAAGGCACTGGCTTGTAGCTTACTGGGAATGGGCCAGTCAGGG
AAATTCTTCCGAACATCCTCAATGTAAAGAGGATATTCCCCCGTACGCGTGTCAATCAAG
GCGGTGCTTTCATACACCACAGTTACCCTATCCATACTCATTCTCCTCATTACGTGATTT
TAATCCAATACCCTTGGGAACCGCCAAAGAGGTAGAGGGCTTGGTTGTAGTTCACCCCCC
ACGCACTCGTAGCAACACCCGAGCCCAAACTTCCCAATAGCACCCACTTGTTCAACCGAG
GTTCAAAATACTGAATTGGTTGATCATTAACACTCCCCGCAATCCCGTAGAGTTCTCCAT
TGTTCCCCCGTACAAAGGCTGCACCAACTGGGATACCAGGAAGAGTCACCGTTGTCCAGG
TGTTCGTAGCCGGGTTATACTGGTACATCCCAGGGTTGTTCGCCCCCGCCACTACCCCAC
TAATAACACTCACCTTCCCTTGGTAAACCATTACCGTAAAGTCAGAACAATCTCCACTCT
CAGCGAAGGCCTTAAGGACAGTGCGGGTATTGGCAACAGGATCGTACGCGTTCAAGAAGC
CTTTATTCGTGGTGGGTGTTCCTGACTGTACCCCGCAGATGTACACCTTCCCATTGAGAT
AAACCGCTGCTCCTGTAAGCGGAGTCAGGGGCATATTAGGTAAACGGGTAACGATGTTCG
TAGTAGGATTATACCCGTACGCATCATTCGCAGCGCCTCCTAGGAAGTAAAGAACATCCT
TACCATCAGAAACCAGGATGGAGTTTTTCCGGGGGGGATTGGTAGGCCCTGTTGGACTCA
GGGTTTCAAGTACCCGGGTTCCAAAGTTAACCCGACGGAACTCTTTGGTAGCGGTCTCTA
CCCCACCGTAGTAGTACATCTGGTTTCCATGGACACATCCCCGTCCGGTATTGAATAACA
ATAAACCCGGCCAGATATCCATCGCCTCACCCTGACCTTCAATAAACGGGTACGTCCCCT
TGGCATACGGAATAGGGCGCCATCCCTTGGTAGGGACATAAGAATCACTGGCAGAGTACG
TGACATTCCTAATGGTGTACCACCCCCGATACGCTTGAGGGGTACCGGGTTCATTACCCG
TAATGTCTTTACCCCAGTTGGTAATCCCGTCAGTATTCCCACCACTGGCCGCTTGATTCA
TCCCCAGTTCTGCATCCGTGAACCGAGTGGGCCAGTTGAATCCATTGGGATCCGATACAT
GGACCTTATACAACAACCGGTCCCATTCAGACGTTGCTGTATTGCCATCACTCCCCATGG
TCAAGAGACCTACGGTGTAAATAACCCCCCGGTACCGTAGCTCATAGCTCCCTGTCCGTA
GACCGGCTTGGGTCAACAGAATACGCCGAACATTATTCCGGATCGGCATTTGAGGAATGT
AACAGGTTTCATTGTGGTTCGAAAACTTCAACCACGGTTTGGCTTTATTGATCACTTTAC
CAAAGGCAATCCCAGTGAGTTTCGCTAAATCCTCGTAACCAAACAATTCCGTTGCAGGAG
TAATCCCGTACCATCCTTGGGCAGGACTACCTGCTTTCAGTTTAGAAGGCCCCGGATTAG
GACCGTCCGGAAAGGCATTCGCGGTCGATGTAAAGAGTAACGCTTCTATCATGATGACTT
ATACCCCAAAAAGAATACCAGGGAGAGCCCGTAGTGCCCCCCTGGTAGTCATACTGTTAA
GGAACAACAGGCCCCAACGCACCAATCCATTTTGTTCCATTCCAGGTAAAGACGTAGACC
AGTTCAGATTCACTGACGGATGGGGGAGTTCCGTTATGCCACACCAAGGGTGTTGCATCC
TTACCAGCGAAGGTCACAATACCCCCTCCGCCGGTGACATTAAGCGTCAGGACCATCCCA
CGTACCGGGGCTTTAGGTCCATCAGCAATCACCATTTGCTTACCCCCCGCCGCGGTGTTA
TTGATCTGGATGTACTGATCCACCAACGGATTGATTTCCCCACTGGTCGTAAAGACCTTA
ATCGGCAAATCATAGCGGTCAAAGGTGAGATCCAACTCCTTCCATTCAGGAATATCCGTA
CCGGCACGGAAGACCCGAACGTACTGTTTACCATCCTTGGCCTGGAGGCCTTCAATACCC

TTCGGAAACTCAATAGCTTTCCATTCGGTACCGTCACCTTCTGCATTGACAGCCTGGGCG
TACTACGACCCTGGAACCGCATTCGGTACGGGACCAATAGGGAGGGGGACCCAACGGTTA
CCCAACCGGACAACCCGGCCTTCAGACTCCATGGGGGAATTGACATCACCCGCCACCAAA
CGTCCCCACAGGACCCAACCCCGATCAACGGTCTTGTAGAACACATTGAAGGTCTCAGAG
ACTGCCCAGTCACCGACTCGACCACTGAAGGCATCCGAAGGTGCGTCTACACCCGGCGGC
AACGTAAGCCACCGGCTGCCTTGCTCACCCTGAATACCGGGTTTCCCTTGAGGTCCGCGA
GGACCGTCACCCCCGGTCTTACCGGTTTGACCGCGCCATTCACCGGTTTCACGCCAGGCG
TTAGTCGACGTACTCCAGATGGCTAGGGTGTTGGACTCCGCCAGGTAATACGCATCCCCC
GCAACCCCCGCAGGAATAGCCGCTTGTAAAGCTGCCAGGTTCGCATAATCCCCTTTAATG
GGTACCGTAGGACCTTCGGGGCCTTGTACACCCCGTTCCCCGTTCCTACCCGGTTCACCC
CGGAAATCAAACCCAGGACTCCACGCGCGCTCATCATTCACGAAATACGTTAAATGATGC
TCCAGAACGAGGATAGCGTCATCTTGCTGCTAACGACCTGACGCAGGCAGTCCCGCCAAG
GTGGGTACCACATCCACCATGTTCAACCCAGCACCGGGAGCGCCATCACTCCCTGGCATG
GACACCGGACCCACGTTATCCCAACGAGTTCCGGTATTCACCCAGAGGTCTTTATTCAGG
TCAACGTAGGCTTTACCTTGAGTTTCCTCGTTAATCGGAGGAGGATCGGCATCCGCAGGT
GTCAGGACCTTAATGATGTCCAAGGACTTACCATTCAGGCCCTCCTTACCGTCCTCACCA
TCCGTCCCTTTAAAGACACCCAGATTCGCCCATGCATTCGAAATGAATCCATAGATCGCG
TTTTCATCACGAACAGCATAGACCCATTGATCTTCAGGATTCGCAGGAAGATCCGCTACT
GTGTCAACCACACCCCGAAGAACAATGTTGGTACCGTCACGACCATGGAGGCTGGCTAGG
AACTGTTCTTTACTGCCGGTGTTCCCTTTGGAAATCCAGAGTTCGTACGTGGACTGGCCA
TTGATACCATCTTCCCCTTTAAAGGGACCCAGGTCCAACCAGGTCGAACTCGCTGCACTC
CATACGTACAACCGAAGGGTGTCTCGTGTCACATACCCATCGTGGTTAGCCGGAGGGTTG
GGTAAGTTGTCCAGATCAACACGAGTCCCTACAGTCCCCAAGACAGTCAGGTTCTCACCG
GGTTCTCCTTTCATGGCCGCCCAGAAATCATCTTCCGTCTTACCTTGGTTCTCGGGTTTA
TTCAACCAGACCTGTACACTTTTCAGACCACCCGGTCCTGCATCACCCGTTTCCCCTTTA
AACCCCCCCAGCTTCCGCCACGCGCTGGTCAGGTAGATGTACAGGGTATTCTCATCCTGA
ACCGAATAAAAATCCTGTTCTACACCGGGGTTGGTGAGGTCATCTATGGTCGCTACGGTG
TCTTTAACGAGCAAATTACGACCTTCAACACCGTTTCTACCATCTTCTCCCTTGAATTGA
CCCAGAATTACCCAGTTATTCTCAATCCACATACGGACTGTATTGTCCGCACGAACCGAC
CAGGCAGCCTGTTCAACCGCGTTACTCGCGAGTTCACTGGCCAGGTCTACCGCCCCTTTC
ACCACGATACTCTTACCGTCGGTACCGTCCTTACCGTCCTTGCCCTTACACTGGCCGATA
TTAACCCAATCCGTACGGTCTTCAATGTAGATCCACAGGTGGCCCGTATCCAACGTCACC
CAGCCATGTTGATTAACCGGGTCTTTTGCATTCAGCGCATTACGGTCAGCCGCCGTTCCC
TGTACCTGAAGGTTAGCCCCGCGTTCACCCCGAATGGCTTCCTGCCACTCTGTAATGGTT
TTGTTCTCGTTACCGGGAATCTTCTTCCAGGTTTCGAACGCTTCATCTCCCTGAGCACCT
TTATCTCCTCGTTGGGAAGCCATCAATTCCTGAAGGGTCTTTTCTTCATTCCCAGGAATC
TCTTTCCACAGCTTGAAGGTACTCTTCCCGTCAGGACCTCGAATGTTCAAACTTTCCCAG
ACAAACTCACCCGCTGTTTTACCCGGAACCAAAACATGAAAATCATTCCGCGACAGGTAG
GCATCTCCGGGTTTATTCCCTTCAGGCAGTGCGAGATCGCGGTTGTCAGGCCCAACGCCC
AACAGGTTTATCCCCCGTTCCCCTTGCAAGGAACCCGAAGACGCCCATTCGTTACCGTTC
CATACCCGTAGGGCAAAGTTAACGAAGTACGCGGTACCGATCGGAAGCTCTTTGGTATCA
ATAGCTTCCAGTTCAGCTTCCGAATTGAGTTTACCTTTAAGGACAACCGCTCGAGTTGAG
AGGGATTCTAGACTCTCCTTCAACCACCGGGTACGCGAATCCAACACCCGAGCTTGGAAG
TTAGCCGCACCCTCGCCGTTGTCTCCCCCGAGAACTCGTACGTCAATCACCAAAGCCGGG
AGGTGTTCTTCCCATCGAGACTCCTCGACAATCGCTGGTAGTTCACCCTCTGTTACTGTT
TCATCGCCAATAGCCATTTAAGAACTTCCTCTGATTAAGGGGTAAAAACAAGATGGGTGT
TCAATTGACCCACCCGCACGGTGGTCGTTTGAGCGACCCCTTTCTTCAACACAATCACGA
CCCGGAGTATCAAGGTCTTAGGTGCGCTGGCCAATTCAAGGATTTGGGTATCGAAGATTC
CCTAGAGATCCCCTTGACGCGGAGGGTAATTCAGTTCAGTGTTCTGACCCGAGCGCACAG
TTTGGACTTTATCGTTCTGGTAGGTTACTTCCAGTTCCAACCCAACTTGCAAGACTGCTT
GCGCATCGGTTGGAATCGCCCAACGACCCTGAGCTACCAATTTCATCCCTACGGTCAGGT
TTGTAATAGGGAGATCCTGTTCCATTCCCAGGGTCGGTTGATCACCGGTAGGCGTGCCCG
TAAAGGACATTACCTGGTATTCACCCCCCGAAGGGTTGGTTTCCTTGGTAATAGCGACGG
TCAAACCGATCAGTCGATTACCCCCAATACCGGTATTCGAGGCGATCTCAACCGTGGTAG
TGGTAGCACCTCCCGAATCCCCAGCACTACCATCATTACCGATCAGCAAAGGGTTGGGGT
TAATGCTCCCGAAGTAGTGGGCGGTCTCCGGCGGTGTAGTGCTGAACGGGACGTAACTGG
TTGGCAAGGTAATGGTATTCTGGAACTTCTTGATCACCGCAGTGGCGACATGGTTACCGA

TGATCTCGGCTCCTTTGGGGCTTGGGTGTACACCGTCCCCCAGCATGCCCGCCAGCGGCT
CATTGACATTACCCGGCTCTACCATGTCATCCCAGGGGTTAACCACGATAACACCCCGTT
TAGGCAGCTCAGAGAGCATCCACAGGTCCACTTGGTAATGAATAGCCAATTGTTCATCAC
TGAGCCCAATAGCGGCTTGTTCTACCGGTGTTTCAGCGATCACAATGACGGTTTTGCCAT
TGGCCAGCAATGTGGCGTAAATCTCGGAGAGGTTATTGATCGTTTCATTGAACGTCTGGT
TACGCAGACGGTCATTCCCCGAATCCAAATGGATAATGACATCGAACTTATCCAAGGAAG
CCAGCAGTTGGGGAAGACGTTGGGCTTGGTGGGAGGTAGTATTCCCATGGACACCGGCGT
TCATGGCATCCGGGAAGGCTACGGCTCCCTCGGTCTTCGCACTGGTCCAGAAGGCCAGAC
CAATGTTACCCATCACGCACTGGTAAGCCCGGCTATCACCGTATACCACGATATTGCGGT
TAAACAGGAACCGGTCTTCACTTACCGCATTGACTTTGGTGGTCAGGGTATTCACTTCTT
CTTTCAAATAACGGGTACGAGATTCCAGCACACGTGCTTGCCTGTTCGCATTGCCGGTGC
CTTCTTCGGTGGCTTCAACCTTATCATCCGGCATCAGAGCCAGCAAAGGTTCAACCCATC
CCGGTTGTTCTTTAATCACTACGGGAATGTCAGCCATGAGAATAAGTCCTGTTTAATGCG
TGAGGGTGCCGTCGTAACGGAATTGTCCGTTAAAACGAGGAATGGCGTCGTAATGGCTTT
CTACGACAGCGAGAGTACCGGTAACCGTGTTGGAATCAATGATAGCCTGAGTGAACTCCC
CGTCGAGGGTAATTTGATCATCCGTCAAGTCGTAGTAGCATTTTGCTTTGATCAGGGCTT
TAATGTGTTCAGGGTTACGACTCCCTTTGCACACCAATACCGCACCTTCAATGATATCTT
CGTCTCGTTTGACTTGGTAAATACCTTCCACCCCCTGGGTTTGTAAAACGTCCCAATCAA
CCGGATCAGGGGTTTCCAGCATGGAGACCCTATTCTTCACATAATCCGCAGGGAGGTACC
GTTGGTTCTTACCGGTCAGTTTACGTTCGGAATCGACGTTGACCACCCGAGCAAATTCAC
CACCTTCTACCTCAACTTTCCTGAGGTCAGAGAGGGTAGAATTGCCCAGGGTGAGTTGTT
CTAATACACTAACAATGTTCGGTTCATCGGAATAACGTTCTTTATTAAAAGCGATGAACC
GTTCCAGCAACAGTAGATGGTTTGGAAAGTCCATAGGCGTGTCCACAGAAGTAGACAGAG
GGTTCGGTCATTAGACCATAAAATTGGTAAAGAAAATAGTACCCAAACCCATATTCTTCA
ATTACCTGGTTATTTAACTTGAGGTTCTTTTCAAACCTACATAATCAGGGTGAAGCACAG
ACAGAGACCGTTCAACGGTCAAGAAGCCTTGTCAATGAATACCCATCAGGCCAAACAGGG
GTTAGCCCAGCGTGTTTAAACCCTGCGTCAAAAAGCTCGTCGTTATGCCCGTCAGGAGAT
GTGTTCCCTAGAACCGTCTTACCGGCATCATTACCGTCAACTGAGCTACGAAGCCAGCCG
TATGGCAGATCGCCTTCAACGTCAACTGTGATTGGTGTTTACCCCCCTTCCCCCACACAC
AGGTCTGAGATGAGCAACGTTATCGATATGTTCAGCTGGCGGGACTCGAAGAATAAGCGT
TCGAGTCAGTTCTAAGTGCTTCATCGTGAAATCGAAGCACTGTACGTCATTCAGAACCGG
GCGCTTCAGGAGATCGCTTCAGCGGATACGGTCTTGGCGTATGAACAAGCTAAACTCTTC
CTGATGGATGTCAGGATTGAAATCTACACCAAGAGCATTGCGTTGAGTCAGTGTATCTAA
GGACATTTAACGTCCAGTGGTAATCAAGGCTATATTACTTGGGTGATTAGGTACACAACA
TTCATTCCTTTTTAGCGGTTTACACCATGGCTAAAAAGTCGAACTTCACTATCGATAACT
ACAACGCCGTCCTGGAAGCTGTTAAAGCCAATAACGGTTACGTGATCGACACCCACGGTG
GTAGCCGTGGACCTTATCGTGCTGCTGGTATCGCCGCACTGGTGAAGGACGAAAGGGTTA
CCCCGGTGCTGCTGTGCCATGGTTCCTACTTCTGCCCGAACTCGCTGCACAAGGAAGCCG
ATGGTTCGGTGACCCTCTCCAGCTGGTCGGTCGGTGTTACAATCTTCACCCACCAACACG
GTGGGCTGGAAGAAGCGATCCAAGCCTACAACAAGGAGCAATCCGAGTGGGCGTTGTCGG
GTGGTCAACTGATGCATGTACGCATCTTCGACTACACCGACGAAACCGCAACTGTGTTCG
GCGAGCCAGTTCATCTGGCAGGCGAAGTCGTGGGTGCGCTGGCAGCACAAGAGCCGATCG
CCAAGGTGAACATCCCTGCTCAAGAACGAGCAGTTTTGTATACGTAATCTGACTTCATTA
GGAGCAATGCCATGAGTAATGTAATGCACGTTAGCACCATCGCCGGTCGTACCGCCCTGA
CGGTGATCACACCCGACGACCTGCCGGTTACCAACTGGATGGAAAATGTGCTTGCTGTAC
AGATCCAGAAAGTGGTTGACGGAGGTAAGGGTTGGCCCAACGACGCCGGGTTCAAGGTGG
AGGTTACCTACCACACTTGGCCATAGGGTTGGGACCGGGAAGCGTACACGCACTTGTGTA
CCAACCGCGCCTAGCTGGACTCCTACATCAAGGACCAGCACGAGTGGGCCCGTTGTGAGG
ACAACATGGTGAAGATCAAGATCTACAACTGGGACCTCGGTCCGAACTTCATGTAAACTC
GTGAGTTCGGTGGTCCTGCGTTGCGGAACAGCCCTCTGAGCCGTCCTTAGTTGACGTAAG
GCTAGCGCTACCTCCCTGCCAGTGGCAGGGAGGTACTACTCTTTTTATTTTTTGCTTCAT
TTCCATTTTTATAGTTCACTCAACACAACGAGCAAAGTAATGCCTGACACTAACAACGGT
CCTGTGGAAATAAAGATGGAACGTGTGCAGTTCAAAAGAATAAAAGTATGGGGTCTTGGT
AAATTCTGGTTACTCATCAAACGCTTACTGAGTAACGACACCGATTATAAGAACACTTGA
CGAACGCATGGCTAAGTCAATGGAACTAAGGGTAATTAACTTCTAGTTCTAATCAGGTCT
ATATTACCTTTGTGTATATTAATGCGTGGTTGCCTTCCTATTTTTAAGACCGAATAAACA
CAAAGGATGTTATCTATGAGTCAAGTCAAGAAAGCGACCAAAGGTGTTCTGAAAGGTCAC

AGTCGAACGGCCGATAAGTTTGTATTATGGATGCCAGTTGAGTTACGCGATAAGGTTAGC
GAACGCGCAACGGCCAATGGGCGGAGTATGAATGCGGAATTTATCATTCTCATACAGGCT
GCGACAGGCAGTGGAACACAGGTGGAACCCAAATAAGGACTATTCCATCGGTAGGTGAC
CCTGCTCGGGTTGAAGGGCTCCCGGGTGTGGTGACTGAATTGATACCGACACCGACTGGA
TTAAAGATCCGGGCAAAAACCTGTGAGGGAGAGGATATTTATTCGTTATGGGAGATTGTT
TCATATGACTCGTTTCGAGAACTCGGATAAGGAAAAGAAACCCAGTGTTCCTTTGAAGAA
CAAAATCGGGTTTGCGGGGATAGGGCCCGACGATGAGCCCATGTCGTGGACACTCACCCT
CCCCGGTGAAAACTCGGTGGGTTACGCAGAACCCTCTCGACCTGAGAAAGAAGACCTGAT
CCCTCAACCGATCTCTTCCGATTAAAAGCGTTGTATACCCCTACCCAGCCCTTTTCGGCT
GGGTAGGGGTTAATCTCTTTATTATTGTTTACTTCACTTTGGTAACACACGACGATCTGA
CCAGTTCGGTTATGTCGATCCTCGTAGCTGTCACCGTCACGGTGATCTTATCCACCTCGA
CCACCCCACTCAGCAATCCTTTCAGCTCATCGAGTCGCTTATTGAGGGCTTCCTTATGGG
TGTCGTTACTGTAGTGGTTAATTTGACCTTCCACCTTGGCAATCTTGCTCTCCTGTGCTT
GGCGGGTCTCAAGGATGCTCACCCAACTGTTGGACTTTGCTCCTTCACAACGACCGATTT
TGAGGACAACTTCCTTAGCGGAATTGAGGTCTTTGATCGTTGTAACTTTAGGCAGGCCGA
TAAGTCCATTCACGTATCGCTGGATACCAATAACCATGCCGTCCGCTACGCCAGAATCCT
CACCTCCATCCGCCAGATTCTGGTACTTCGCTTTACGCTCATTCCAACGACCCACCGCGG
CTTCCAGTTTGGCGACCTGGCTGCGCATTAGACTGCTGTGAATGACTCTGGAATCAAAGA
CTTCCTTGATCACCTTTTTAACCTGTTCAGGTTGGTGTTCCACCGGGAGGGCTTTAATGT
CGTCAAGTTTCTTAACTACGGTCTCCTTAGCACTTTGGTCCTCGATCTTGTTGGCGATCT
GAGTGGCTTCTTGGAGAACAGGACCCACGTTGGTCGTTGTCACCTTAGGACTCGCTACGG
CGTCTATCGCCTTTTCAGCGGCCTTAGCGGATTCATCTACCTTATCCAGTTTGTCTTTAA
CGTTCTTCTTGAAGAGACCCCCGAAGATCGACTTGAACAGGTTCTTGATGTATGCCCAGG
CTTTCTTAATCCCTTCGCCAATCGACGACAGGAACCCCTCACTCCCCGCCACTTCATTAC
GGGTCAGGTAACGCGCGGTATGGATAACGGACAGAGGGTAACGCTGATTCGCAGTCAGGT
CCATTCCTTCAGTTCCCATGACCGTATCCGAAAGACCATCGAGTACTTGCTCAATATGTC
CCATTTCTGGAACACCGTCCATCAGTTCGTCTTCGTATGCAACCGTGGTGGCTTGCTGTT
CCATTATTTGCTCCTTTAGGGTTAAAAGTGTGAGAGAACACTCCGGGAGGGATACAAGGA
ATGGTTTTCAGGTTCATCCCCCATATCAAACATCTCTTGGCCGGTGATATTACCCAACAA
CTCATGGCCCTGTTCTTTACTCATAAGAATGTCCACTTGTTCCTTAGGCTATTCCATGAT
GTTAATACTGCGGGTCATAATGTTGTGTTTATCCCCGGTGTCCATATCCAAGAGTTTAAA
GTAACATGGAGCATCCTGACCGGTACGCCAGGTACGGGCTTGGACCTGTTTCACTTCATA
CTCTCGGAAAGGGGCATTCAACAGAATGGTCAGGTTCGCCATAATCAAGGGATACCCTTC
CTTCAGGGAATCAAAGATAGCAATCAACGCACGGAGAGTGGGGTCTTTCTCAAAGCGTTT
GGTATTGGTGTCTCGTTCGTTGCTGTTTTCACCGTTTACTGTCACATTCGGATACCCGAC
CTTCGTGAGGTATTCCTCGCAGAGTTTAAGGGCATCCACGTAGGAGGTAAAGATCAGAGT
CTCCTTCTCCACGTTATCGATGAGCTCAGGGAGACCTGCGTGCGCAATGGTGGCTTTAAT
AGCGTTAATACGGGCTCGTCCTAAAACGTTCCCTAACGCCTCTCCACGCAGTTTTAGACC
CAAGTACTTAACCGCGGACTTAATGTTCCTGAAATCCTTCAGGGTGGTCCCTTTAAGGCC
CTTCTCGATGTCTTCTTCCACCAGCTTACAGAACTGACTGTCTTGGTTATCCGTAAAACT
GTTGTACCCCCGGGTTGTGAACCGGTGAACAATCTGTTTGTACTTCACCAGATGCTCAAC
CGCTTTAGCATTCTCCTGGATAGACAATTCATACGACGTCATAACATCGTTGTAGAATTC
CAGGAAGCTCGGCATATGCTTCGGATAGAACGCAATCCGTTCTCCAATGCAAATCTGCAT
TTGGAGGCGAATAGCATCCAAGGTGTATTGATCCGAATTCGGGATCTTCACCTTAACCAG
TTCCATAGGAGGAGGATCGCCCAATCCCACCAGTTCAGGAATGGTGAACTTCATACGTCC
AATACGTCTACTCAGCAAATCCATTAACGAGGGACGGTTACGTCAATAGGACGCCATTAA
GAACTCCCGAGCCTTCTTATCGAAGTAGGGATCGATCATACAGGTCGTTGGGAAGATTTC
ACTCCCCTGAGCCTTTAGCGGCGTCCCTGATATCGGTAGGTGATGCCCGAACAGTTCCAT
GTCGCACCATTCGATCAACTTCCGTGTACGGGCTGCTTTAGGGTCATTGAAGTTCTGGGA
TTCATCCAGAATCATCTTCAAAGCGCCTTTATGCTTCTTACTCAGCGCAATGAGGAAGTT
CTCCAAGTATTTCCCCATACCACTGGTCATGTAATCGTAATGGACGATGTAGAACTCATA
CCCTTCTTCTACGACCTTACCGCTGATACTGGTCCAGATACGGGGTGGAACCTTAAAGTG
CTGGAGGGGTTGTTCTACCCAGACCTTATCCACAATGTTCATAGGGCAAAAGACAACCGT
CGTAGCCGTATTGATCAATTGAGACCACGCCAAGGACGTAAAGGTAGTGGGGGTCACGAT
GTAGTCATCCACCACATACAGCTTCTCGTCATTATCCTAGGCAATGCAGCAAGTCTCTTG
GTTCTCCACTTTCTCCGAGTACGCCAGCTTCAGTTGCAGGTCTTCATACTGGGAGATGTT
ATACATTGCTTGAATACGGGGATTGTCTCCAATTAACCCAGTCATCATCTTGCCGACGTC

TCGGTGCTTAAAGTCAACCTTATACAAATCGTTAACCAACACTGGCTCCGTAGCTGTTCC
TCCAATGGACCACATCAACCGACGGAAGTTCTCCATCCCAATCCGGTTCTCGTGATACGC
GGATAAGACGGATTCACTGATGAAACACCCCACGTGTTCAATCACCGATGTGACTATATT
CAAGCGATCCCGATACGGGAGCTCCAATACGTTGTCCCCTATTACAACGTCGGTATCGAC
CAGCTCCCTTGCAGCGACTACAGAAGACGTATCGCAATGGGGGTTGCATCCATTGATCTC
ACCCACTAAGGGGAAGTACACATACTCTTTACTGGCTTTATGGAGTACTTCCAACGTGGT
TAAGATTTCATTGACCCCATTCACCGTGGTTTTCCACAAATGATACGGATGAGAGACAGC
CGTTCTACCGTCTTCGAAATAGAAGCGATAAACTTCCGTGATCCCCTGGGGGTAAATACT
CTCCACACACGCGCTATCACCCCCGGGGGTCACGACGATATCCTTGACCTTTAGGTCACC
CAAGGCTATCCACCCATTTGGGAGTTTAACCCGGGTTGGGAGAGGCATGGCTTTTCCTGA
ACCCGGATTACCATCCAACAACATCCCTTTCAAGTGATAACTGTTCACAATGATGGGGTA
TTGCTGAAGAAAGTCCAACTGCTGAGGAAAGGGAACTGTGTTGAACTGCTTCAGTTTCTT
AAGATCAAAAGGTTTACCTCCCGGATTAACCGTATCTTTGATCCATGTCTCGGTTTCAAT
GAGTTCCTTCAATTCACTCAACGCTCGTCTGCGGGTACGGAGGTTCCGTATTTTGAGGAG
ATCCCCAACAATGGGGTGCGATTCTACCAGGAAGAAGTTATGCACCATAATGGTGGCCCA
GGATTCGCGTCGAATCTGATGTTTCGTTAAGAGAGAAGTGCTGTAGAACTTCTCAATGTC
TTTGGCTAAATCGCAATAGGACACACCCGTAATGTGTACGTAGTCTCCCGTTTGTTTGAT
CTTAACTGAAAACAGTCCTAACATTGAAGTCGTCCTTAAAGAGGGGGTTTAAAATGCGTC
AAGTAGAAGTAGGATCCGCGGTCTATAACAACCTGGCTCGTGTATACGACAACCTGTGGG
GTGGAGAATTGGTTGAGGTAGGCCAACGGGTAGAAGGGGAAACGGAATATCTAGATGTGC
TGACCCTCGGAGATGTCGAGATCCATTACTTCTTTACCCTCATTAAAGAAGGTAAAGAAG
AAGCCTACGTGCGGTATACCCGTGACAACGGTACCCTCACGTTCCGACTGAACGAGAAGT
GGACCCAATACCTCTTCAATGACGAAATCTTCACCATTCCAGAAGAGATCGTAGAGGGTA
TGTGGAGGGTCTCTATAGAGTTCCCCAGTAGTTGGTTACAGGTCGCGCCTAAACACCCCG
CTGATGATGTGCTTCAAGATGACCTGAAAACCACGGATAAACTGGGGATTAAGCTCTCCA
GTAAACTCCGTCATGCCGTGGATGTTATCACCAAGAAAAGCAACCATATGGTTGAATGGG
TGGGTAAACGTGACCACGACCTCTACCTCACGTTCAAAACCAAGAAAGACGACCTTTCTT
ACTTTACGGTTCGGTTTGATAAAGTCTACCCCGAAGTCCCTAAAGAGGGGATTACCCTCG
GAGAAGGGGAAACGTTGGAGATTGATAACCCCGATAACCGTCAGGAAGTTAAGGACCTTA
TTAAGACTATCGGTGAACTGGAACACCAAGTGAACCGACAGATTCAATATTACCGCAAGC
ATGCAAACCTGACTGATGCAGGTAAAGCACTCTTCGAGCAGGAAGTCCGTACCCTTCTAT
TTGCCGCCAGTCACTTCAGCAAACGCATTTATCCTTTCAACAGTGTGCATGGTATGGAAT
GGTTGGATAAGGCGGCTAACGAGATCCTGGAGCGCCTGTACGCCTACAAAGCCACCACAG
ACGTTCCCTTTGGGTTGGATGTGAACAGTTGGTTCGTGTGGAGTTATCCACTGGATGGAA
TTGAGCCGGATAAGGTCACGGATGAATCGGTTGACTTTATTGAGTGCGTACTTAATCGGT
ACACGGAGAGTTTTGTTGATATCCTGACGGGTTTCCTGATGGATTTGAACCAGACGGAAG
TCATCCGTCCGTATCACTACGTCGTGCCTGCTGACCTCAGTGACGGATACTCGATCGGCT
CGATTCATCGGGTAAAATAACGAAAGCGACTATACTCCAGGAGAGCCGTTGGGCTCTCCT
GGAGTAGTCGGTATTACGGTACACCCAATCGAGACTCTAGATCCTTAATGTATTCATCCC
TGGCCACTAAGGCCACTGCCAGATCTCGAATACGAGACAAATCATTACTGTCATCCGTAA
TCGTGTTCTTGCGGGTGGTTTCAAACACATCATGCTCATTCTTCGAGACATTCCCAATGA
CCGGTACAGTTCCCAAAGTGGATTTCCCTTCCCCTCCGAAATGGGCTTTGATGTGGGGAT
CGAAATGTAACATCGCCCAGACAAGGTTATCCTTCGTCTCAGGGGGTAACGCACCACAGT
CGGTGACAATACACATGCGTTCGTACGAATACCCATCTAACAACGGGAAACTATTCAGGT
AGGTAGTCAGCACATAGACCAGAGGACCACTCCGCGGTATTAACGACACCACCACGGCGT
TTTCACTGATCGCCTTTTCCAGTACCGCGGGGTAATCTGCTTCGCTGAGTCCAATGGGTT
TAAAGACCCGCTCGTAGATCTTAATCTTCAGTCCCTGCATTTCAGGGATCGTTCGAGTGG
CCTCTACGGTGTAGAACGTATTGGGATTCACCACGGTTGAGAAAGGTGCGATAGTTTCAA
ACCGTCCGGCTGCACCAATGGTTGGAATGATGTTTTGCTCAGCCATTCATTAATCCTCCT
CTACAATCCCGTCTTGAATCATTTGCCACCGGGTGAACATCACGTATTTCACTTGACCAA
TACGCTTGGCCACACAGACCTTATCCAAGCGAGTCAGACGGCTGACCCCGGGAGGCAGCA
AAGAGAACGGCGTAGCCACTTCAGCGGCTGCTACCAGGTCCTCCATTCCACGGATAAACG
CCATAGTAGCCTCACCCATGGATCCAAAATCAGGATCCAGGGCGGGGATCAATTGGTACT
GGGGAACAATTGAACGAATCTTCTGCATGCCTTGACGGTTGTTCTTTTTAGCTACGAACC
CAATCTGAACGGATTAATACAGCATGGGTAGGGTTTGGAAGGATTTTATGATGAAGTCTT
CTGGTATTGTTGGGACCAAGTACTGCTTGGTAATATCGATTTCAGTCTCACGATCGCCAA
TGCGGGAGAACGAGCTAGCACGGGTGGTGCGGTCTACAATCCCAATCATGTCGAAACGGG

GGATGGCAGAGAACTCCAGCGGGTTGAAGAGATCGGGGATCTTTTCTTCCCAATCTTCAC
GAGGATACTTCGAATCTTTCAAGATTTCATCTTGAATCTGTTCGTAGAGCGGATCTTCCG
CCTCGGTACCATTACCCCAGGACAGGATAGACCAGTACCCCATGTTCAACTTGCCGGTGT
TAATCAGGTCAAGTATCTGGAATTCTTCGACGTTGCGCTCTGTATAGGGCCAGACTGCAT
TCCGAGTCGCTTCATGTTCACGTTGCGCGATGACCGTCGGGGTGTCCGGTGCGAACCGCT
CAGCAATCTGCTGATAGTTCATGGTCATCAGCTTGTCCATCTCGTTTAACGGGATGGGGT
GGATAATCGCAAAATGAACACGGGGATATTGGATCAGGAAGTACGCGTCAGCCATCCACA
AGTAAAACGGCTGCTTATCACCACCCACGCCATGATAGCCACGAACAAAGCTCGGTAGCC
AGATCGTGTTATTGGTGACCATATCACCAACGTCTTCAATCACAACATTGGTGGTGAATT
GGGCTTTGAGTAGCGCAAGGGTGTTAGGACGACTGTCAGTGATTTTCCCGCTTAGAGCTT
GTTCATACAACCAGTTGGAGACACCGATTTGAGCTTCAGCAATCGCTTGCGGCATAACAA
TCTCTGTTCCATCGGCTTCGGAATAGAAATTGACCAATACGGTTGCACTGGGAATGTTCG
CAGCCAGGGTAAACTTACCCGTGTCTTTGGCATAAGTGTTGGACTTGTTCGACAGCTCGC
CAATAGGGGAGGTTGTGAGTAGACTGTAGTCGTGAAGCTTTGCTATTAATGCGAAAGAAA
GGAACGTGGTACTCATGATGGGCTCACCGTTGATGTAAGGAATACGCCCATGGTTTTAGG
TATTGCAAAGGAGATGGCGGATAGATTCATAGAATTCGTTCTTAAATTCTTCAAAGGGGA
GTCTGCTGAAGACCAACTGAGCAAAGCCCTTAAAGTTTCAGTACTTACTATTGCCCTGCT
ACTGTTTGGTTTGTTTGCATTGTTGAGTAACAACCTCAACCTGCGCATGGATCTGGCGGA
TGCTGAAATTGGTCTAGTTAAGATTAATATGCTCTGCGACCCTACTGTGGGGGAAGGGGG
ACCTTTAAAAGAGTTCGTTCGTATCAACGATGCGTTGAGTCGACAACTAGACAGTGTTAA
GAATCAAAATATCATCTTGTCGAAACGAACCATTACTGCAACGGCTGACAACAAGGTACT
TAAGGAACACCTACTTATGGTCCTTGAAGAGAACAAGATTCTACAAAATAATAACCATAT
GCGGTTGAGAAAATGTACACAATAGGGACCGGTTAAATTCCACATGGATGTGTATCCCAT
GTACACGCACCTACTAGAATGAGTTAACGTAATGGAAGCCATCACACCGTCTATCAACTG
GGTTATGTACCTCCTGGTAGGACATAACAGCAAGTTAAAGAAAAACGGCTACTTCCTGGG
TGGTCATGGGTATTCTCACGATATCCAACAAGAAGGTAAGCGTAAGCTAACCAAGGATGT
CCCGACGTCGGGGGGGTATTATACCGGTAATCCTCAGAAGACCAAGACGGCTGTAACGAT
TCGGGATATTGTGTCGTTGACCGTAGTGAATGCGGAGAGTGAGCTGGAAGCCCTCTTGAA
GGGATATTGGGAAGTCCTGAATGCCCTTACCACCGATCCCATCCTGAAGTACCTGTGTGT
CAGTACCGAACACAAAGTCTTGGAAGCCTTGAGTAAGGTGGACAAGAAGAAAGTCGAAGA
CGCTGAAGAGTTCAAACTCGGAAGACTGGTTTTAAGCGCAACGGAGCGAGCGGTGTTGTT
AGACATCCTCACCTGCTTGGAAATAGCCAGTCAAACAGACTGTCGGGTGGTCTTCGATGT
CCCAGGTGTTGCAGAAGGGGCTCAAGGGTATAAGGAAGCTGCCAAACAATGGGACCTTGC
TGAAGCGATCAGACTGTTTGGGTTTATGAAGGAAACCAACCTGGAAGTGGTTTCCCGTAA
GGAGTATGAAAACCCAGAGGTGGAATTCAACAAGATCGTCAGTGCCTCTCGGTGGTATTT
CAATACCTTGGATCCTGAAGCGTTCTATAAAGAACAACACGGGTATCGAGTGTACGGGTT
TGGTAAAGTCGAACGGGATAAAACGTACTACGGTAAGCTCACCACGGATGTGACCTACTC
CAAACTCTACACCACACGACCCCTTCGTTTACTGGATAAGTTGTTTGAATTTACGGTAGA
ACGGATTGCCAATCCCGAGGGGTATTTGTCAGCAGGGGATCCGAACCACCTTGTTACCAA
GGATGTGGCGCTGTTAATGGTTCATTTCCCAGGGGTTCCTAAGGAAAAGGAACCGATCTC
CCCTTTCACCAAACAGAACACGAAACCGGTTCTGATGGAGCTTATATCCCCGGTCTTGAT
GTCGTATCGGATCGGGGATTTGTTAATTGGGATGGATATTCTGTTTGAAGGGTTTATGAA
CAAGGATACCGAGAACAGGTATGGGTATCTGACGTTCTACATTATCACGGATTTGATTTA
CCAGAAGGAAGTCAATGGAAAAGGGGTGGAGAAGCTTAAGCTGGATCCAGCCTTTAACCA
GACTCGGGAAACGATCAGTTTAAAGGCCACTCATCCCAGCGCAATTAAACCGGTAACCAT
CAAGCTGTCCATTGGGTACGACACTCCGGATTGGAACGCTTTTAATTCGATTACGGACCC
TAAGGTTAAGGTCTGGTGTGTGACAGACACCCGGAACTAGGAAGGTTTGCGGTACGCGAC
AATTGCCGAGACGGATGAGTTTATCTATATCCATACGTCGGCGATTGCTAACCTTCGGGT
GTTAACCTTGGCGGAGTTAGGACGAAGTACTGATAAAGCGTGAACATTATACCTACTGGG
AGGTGTGTACCTCCCAGTAGGTATACGTATGGTACTATTTTGCTTTGGTTACAATCGTCT
CGGTCTGATCACGGAAGACGGCCGCCAGTTCGTTCCGGAGGGTCATCATCGCTCCGCAGA
TGTCCGTAAACTGCACAAAACGATTAACCACCAACTCCACTTCGGTCAGAATCTTCTCAT
CCACGACGAGATCATTGGTTGTGATCTTCTCAACCAGGAGTTGTCCCAGTTCATAGATCC
GAGACAATTCCTGACTGGCCATTTCAACATCCCGAGCGCCCAGTAGCTTGGCCGACACGT
TGTAGGTTTCCAGTAAGTTGTACAGTTCTTCGAGATTGACGTAAACTTCACCCAGGGTAC
ACGTCTTGCGGTGGGGGTTGGATGGGAGACCTTTAAGGAACTTTTCGGAACGGTCCAGTA
CCGTATCAAAGTTGGTAATGGTCCAACGGAAACGGGTTTCAGGACGACCGGTCTTAATCA

GTGGTTTAAGCCAATCGTTTAGTCGAATTGCTTCGGTTTACATACTACTGACCAGATAGA
CCCCATCCGTGGGTACGAATTGGGTATAAGCCATCATATTACCGAGGCCGGGTGTGTAGG
TCTCCGGGACGATGACTGGCAACCCAGCGTTCTTAACAAAGAGAATCTCTTTCCGCTGGA
TCGTTTAACGGAACTTTTCAACGTCCACAGTGATAACCGACTTTTCGAAGGCGGCCAGAC
GGCGGTCTATAGAGGCACCGTACTCAGAGAAGAAAAGACCCACACTTTCCCCTACACTGA
GTTTTTCAGCCCCCAAGACTAAGAGAAGCTGGGGATTCTTGATCCGTTCGATGAAGTCTT
TCGATGTAAAGGAATGTTCCATAACTGAAAATCTCATGAGTATAGTTTGGAGAATGGGTT
TTTACGCAGACACATCTAAAACAGGGCGTTGCCCTTTCAGCTAAAGTTCATAAAATACGA
GGAATAAGAGGAACATGAGTACGCCTCCTCTGTTTACCCAAGAGTTGGAATCGGCGGATA
CGTTAATTGTAAATCATCCGTTTTCCCCTATTTTCGATATGTTAAACACCGAGATCTTGA
TGAGTGCATCAGGGGTGGCCTATAACAACGGGGGTCTTTGTCGTAACCATGCTATCGTCG
GTGGGAACAACACCCAGAAGACCGGGATGACCGTACTGGGTATGGCACGGGTCTTGATTC
GGTTTAAAGGGTCTGTTGTCTTCTTCTTTGATATTGAGGCCACCTTCTCCGTAGACCGTC
TGGCGGAAATGATAGACCGAGAGATCGGTATTCACGGGTACTTCAACGAACAGATACTGG
GCAAGCGCTTCTTCTGCTTCTCTCGGAATGATTTGGTAAATCTCTGTGATGGCACCTTTG
TTCATGATAAGTTCAAATGGTTGAAGGGGAAGATCGCTGAACAGATTAAGGCCAAAGCCG
ATATCTTCATGAAGACCCCGTTTACGGACAGCAAAGGCAACCCCGTTAAAATCATTACCC
CTATTCTGTGCGTGGTCGACTCTATCTCTGAATTGCCCTTCCATCGGGTAGCGGAACATT
TCCAAGAAGGGGATGTTGATCATGGCGGAGAGAAGAAGACGCGCGATCTGGTGATTGGGA
ACCTCCGTCGTATCGTCTATGAAGACGCCGATATCTTGGGTGGGGTATCGGGTTGTTACC
AGATTTGGGTTGCTCAGGTTGTTGATAAGATCAACATGACTCGGCGTCCTGAGGAGAAGG
AATCTATTTTCATCCGTCCGGGTAATAAGCTGAAAGGTCCTAAGGCCATGATGCGAATTC
CGCAGATCGGTCACGACATCCTTAAAGGTTCAGTGCTTAAAGGCGGAAGTAATGGTCAAG
AGTGGTTGTACCCGAATCCCTTTGGTAAAGATGTTATTGTGACTCCTGATGCCAAAGGAA
TGCCTGACTTGATGATTTATAATAATCAACCCTGTCGGAATAAGGCGGTGATGTCAGGTA
TTAACGCGTTCTTCATTGGTTCCCAGTCCATGGGAATTCATGAAGGTCTGACCATGTACC
ATGTACTGAAGACAGCTAAGTACTTCGGCCTGTAAGGGAGTGACCGCAGTCATCACTGCA
TTCTGTACCCTGATGTGAAGGTGGGACGTACCACTGTATGGGAAAATACCATTGAAGACC
GTAAATTCCTGCGAGCACTGACCATCATTTACCACTTGTGGTTCACGCAAACCTTCTGGC
TGACACTTCCCCACAAATAGCGTCTGACTCCTCAGGCATTGTACGACGGTATTATTGCTC
AAGGACTGGAATGGAACGATATTCTGGAGAACACAGTAGACTACTGGTACACGAATCCAG
ATATTGACAAGTACACTGTTACAACGTATGAACTCATTCGCATTGCACTGGGTGAACGTA
AACCTTACTGGAAAGAGAAAGGTGCACCTAAGGCTATTATTTAAATAAAGGGTGTGAGGG
TAGCCTGAGTCTTCCCACCCCGACTTGGTAAAGGACCCCCTCATGGAAACAGCACCGTGC
CCTACCACCATTCTCATTACGGCGGACAGTCACTTTATTAATCTTTGCCTGGATCCGTTC
GACTACATCAGGCGGTTGAAAGATCGAGTTTCTGCCGTGCGGGAGGATAACCCGTTGTTG
TATACCGTCAGTGTTCGCTATGGTGTGGGCGAGATTGACAGCGAAATCCCGGTGATTGAA
GTGAATGACCGGAATAAGACCCTCTTCAGTCAAACCTTGGAGAACTGCTCCATGTTGTTT
GACGAACTCCAGGCGATGAGTACGGTCGAAAGTGATCCCTTTATTAACTCCGCCAAAGAA
GTGATTACATTGGCCAACAAAACGTTTACACATTGCAAATACCCGAGGACAGTGTAATGG
CTGGCAACCGGAAGGCATTTGAAGAACATGTGTATAAAGCCTTTGGTCCTCTGACCCAAG
GGGGTGGCAATAAGAAGATTTATGTGAAACTCTTTGCCACCATGCACGATGACGCTTTTG
AAGACTTCGTCCAGTGGTTGGAAAAAGGGAATCCGTTAGCGATTTGGGCCAGTAACTTTG
TTCCTGAAGAGAAACTGAATTTCGATAACCTGATGAGACTCTGTCAACAGTTCGGTATTG
AAGTTGAACACCAACTGGTTATTTACGACGAAGACACTGGGATCAAGTCACTGACCGCTT
ATAAGGCGATTGTAGGACGTGCTGAAGTCCGTAAACAACGTCAGATGTTGACCAAGAAGT
TCAGTGCGGCTAAGAACGACTACGAAATCGATGACCTCACCGGTGAACCCATGGGGGACA
GCCGGAGTGCGGGGATCAGCGGTCCTGAGGTTACCGTACTGCATAACCTGGGTTTGAACA
TCATGGCGAATGAACTGTAAACCGTTAAAGGCGGCGACCAAGAGGCCTTTAAGGCGTACA
AGAACGATTTGTTTACCATCGGTAAGACCACCACCAATGGGAGCCTGCGTAAAGGCTCAG
GTATCAAGGTGTTGAGCACGGTCCACCACCTCATGCGTGGTCGTCATATCGACACTAACG
TTAACCAGAAGTGATCTCTCCCCATGGATAAACTCATTCATTTGTCGAACACCATTAAAG
AAGTATGGTTGGACCTGTCAACCGGAGAAGCTCCTCCTCCCATTCTTCAGACCTTCTCGT
CGCTTATCAACCGTGCTTTGATCTGTTTTAATGAAGCCTATTCAGTCTTCATGAACCAAG
TGGGAAGTAGTAGTGATCTCTTCGACAGTTGGGTCTGTATTCAAATGCTGGTTATCACCC
GCTTGGGCGAGCATCAGGTGGCGTTCAGTCAGTTCAGTCAAATGTTCAAGGAAGTCTTTA
AGACCATTGAAGGGGGTAATGGACTGGATATTTCCGACGAGAATGAATATAAACGCTGGA

GTTTCTTGATTGCTTTAGCGTTTAGGGTGTATACCACTCATTTCATTGTACCCCCACCTA
CAGGACCTAAGGCATGAATCGGGTATTAACCGCCTACGCGGATGTGGACAGTCTCTTCGA
TTGTAGGAGGGGATTGATTCAGAAGTGGTTAACACGAGACATGGATGTTAGTGATGAAAA
CAGCTTGAATGATGAAGCCTCTGCAGCGTATTAAGAGGAACGCCGGCTTCAAGGGGACAC
CCTGTGGGAACTTCATATTGAGAAGTACTACAGGGAGAGAAGGATGGATACGTTTAACTA
TCCGTTCTTTGGGTTCACCCGACAGATATTCAGGGAACTCTATCAGAAACGGAGTTTACG
GAATTGGGGGTACGGGTATTACCCCACAGGGTTCCTGTCTAATTTCGTCAAGGTGATTAT
CGAGCATGAACAACGGACCGATAAACCTATCGAGATCAAAGGGGTGACCCTGACGATCAA
CTGTTTCCCTTATGTCATGGATACAGCCCTTAAGGAGGAGTTGATCGAGCACGTTAAGAC
TCGGTTAGGCTACCGGGTAGAGGTGAAGACCGTTGTTGACGACATTATCAATGCAACACC
CTCCTACTATCGTCAGTTCGATTATGTCTTCAAATACGACTTCCTGTTGAATGAAGACTA
TAAGTCCTTCTTTGAGAACGTCGGTAAACCGCTTATTCCTGATACGGCGTTTTTGGTTCC
AGATCTCCTGGTTCAACAAACCGAGTATTTGGAAGGGAGTATTTAAGACCGTATCTTCGC
CCAATACATTGCGCTGGCTCCTGTATTGAAATTGATTCCGATCAACAAAGGTTTCTATGA
TTACTTGACGGAATAACTCCTTTATTAACTGGCTGGGGATCTCCCCACCCAGTTATGTTC
GCTTGGTTAAATTTCCTCCCCATCGATATCCACCAGCTGAATCCCCTGCACCAGCACTTC
TTCCGTAATGGGAATCAAGGTAGGATCCAGAGGCAGGGGTTTAGAGGGATCCAGTACGAA
CTGCGAGAACTCAAAGGTGGGGAGTTTAATCCCTCCACTTTGAATACGCTCCATAGCCTC
CAGCATGTGGTTAAGGGCCAGTCGGTTGCCTTCATTCTCTTTCTTCTTAGCCCGCTCTTT
ACGGTCATCCCGTACGGTCTTCTCAATCTCCGCAATAATCTGGGTGACCCCTTCCAGCAG
ATGCGCGTTCTTCGGATTATCCAAAGACGCAGACATAGCGTATTCCTGAATGAAAGCCCG
GTTACGTTGTACTACCTAAACCAGTTCTTCCTGTTCGATATCCTCCAGATTACCAGTCAT
TGCCTTACGGAGATCTTCCAGAACCGTTTGCATTTTATCCGTATCCAAAGGACTGGCTTT
CTTCTTTTCCTTTTGTGGTGTGTCGTCAGGCGTTTCCATGATTTTTCAGACCTACATTAT
TCGTTTGAGTGGTTAGGAGTATGTGTAATTGGCACTAACTAAACGGGCTCGATTCAGGTG
GTTAAGCTGGATACCTGGTATAGGTAACCTGTTTAACCGAGAACTGGATTCCCTCACGAT
CGAGCGGCTGACTGAGATTAATCAACGGTTCTCTTAGGCTTCAGTACGGGTCGCAGGTCA
GATCCAGGATCTATACGATTGCTTAAAGCCCCTAAATACGTCGTTTGACATCGATCCGAG
GGTCTACCTACCCTTACCTATTGAACTGTCAACAAAGTCCTCTAAGGACGCTCACAGGCT
GGTAGAGACGGTACTGTCAGGTTACTCGTGGAACCCTGACGAACACTTCGTGAGTAACAT
GATCGAACACTACCCCAGTTTCCTAGACTGGTACAGTAACGGTTACAGTCTCGACGTGTA
TTACCAGGGAATGGTGAAACTCATGGAACTGTATTGTGCCTTATTCCACGTTATACCGAA
TGAGGACGATCCTGTTGAATGTCATCCTGTCGTAGAGAACTTCTTAACCAGCCGGTGGTT
TAGGCTGATGGTTATTGACCTGATCAAAATACTGACTCTGGTACTTATCCAACGTCGGGG
AGGTTAAATGGAAGAAAACAGAGAGAGACCCAAAGCCAGCCGGTTGGAGAGTATCTCCCA
CAAGTTTGTACGGGATGAAGAACTCAGAGATCCCCCCGCCCGGTAATTCCGGGCAATTCT
GAACAAAGTGGTTCCTACCCCTCACAAGTGGAACAACTACTTACGGGATTATCTGGAGTA
TATGGTGACCACCCGAGATCCCCAACGGGCTAAGACCGAACGCATTGCCCGGCAAGGTAA
CATTAAGGCAACCTATTTCCAAAGCTATACGTTAACCTTCCAGAAACTCTTGGAAGGGTT
GTCTATCCTACGGATGAAATCTTGCAGAATCATCATCGAGGTTGTTGATGAACACGGTGA
AATACTCACAGTGGAAGAAACTATTCGCATCGTAGAGAAGAAGCGTTTGGAGAAACCCAT
TGTGGATGAACCGGAAGAGTGAATGCAGGAGTAGGTGCGCTAACGCACCTAGTCCCGTTC
CCCCGTTATTCTTTATTTTTTGGAGTGCCGCTATGGGTATCCTTGACGGGTTCAATCTGT
CTTCGGGTATTAATGCCTTTAAGAAAGAAACCGAAACCGCAATGCGGGGGGTTAAAAGTG
AAACCCTAACCCCTATTGATCTCACCGCGAGTCGCCTTAAAGAACGGGTGGCTCTTAGTA
ACCCGAATGACACCACACTCACGTCAACCATCACCGCGTACCGGAGTTCGGTTGTAGAAG
CACTAGACGGTGTTATTGGAGCCCCGTCAGGCGGTTTACTCAATACGAAGGACATTACCC
GAGCCATTCGTATTGATAGCAATGGGGTCAGTTTCAGTGATGATGACTTGATTACCGCGG
CAGGGTGTCAAATGGGCGTTAACGTCTGGGGTAAAAACGGCGCGAAGCAACTCATCGCAG
ACACTCTCACCGCAGAATTCAATCGGCTCACTGGATGGAATGTGGGTCAAATTATCCAGG
TCGGTTATGGTGACAAAAGTTCAGCCTGCCGTATCAATGACAGTTGGAGAGCTCAGCTCG
GTAGTGCCACCATCGATATGCTCAGGGATTATACCGGTATTGATGAGTTTGCAGACGTCA
GTGTTCAATCGGCGTTTTACAACGCAATGCTGTACAACTCAGCGATGTACGGGATGAGTG
ACAGTTATAAAACCCAATGGGATAACTACCCGTATCTCTCTCTGATACAAGATGCCTTTA
TTGAAGCCATTCAGTACATGATTACGAATGGGGATGTTGAGAGTATCAGTAAGGTCTTTG
AACTCATTAATACCGAAGGGAAGAATACCTTACTGAGTAAATACCCGGATTTCATTGAAA
CCCTCTTTACGAAGTTCTCTTTCGACAGTCAAGTCCACAGTGAAGAGCACCCCGCCATTC

GTACGAAACGCTTAAACGTTTTAGAAACGGTAGCGGGTAAAGAGTGGTATTACCGGGAGA
CCCAATTCGGTAAAGCGTACAACCTGGGGTTGGTATCGAAGATCAGTGACGACATGGTAA
CCCTGTTCACGCCTGTTGAAGAGTTGATTCCCCTCTTAACCACTGGGGGGATCTTTCAGA
CTGAACGGGCAACGGATGTATTGAATCAATCCTTCCCGAATGCCCCTATTCGCACTAAAT
AACCCTAACGAAGACTCCCCTACCCCAATTAAGGGGTAGGGGAGTTATTACTGAATAAGT
TCGTTTTGTATTGATTAATAGAGGGTGTGATTTCCGCTTAGTAGACTCGGGGGCGTGAAA
CCCCCGAGTATACTGGCTTTCTATCTTGCAAGAGGGCGTGTGGCCAAGCGAAGCAGATCT
GCCGCTATCGAGTCTTTTATCTTAGCCGCGATGTTATCAGAACGAATACTGGTCTTAATA
TCCGTGACCACACCCGTCATTTGACAGTTCAAACGCGACCACCGCATAATGGTATCCAGG
TAGTCCATCCCTGTGATACGACTGGCAAAGTTGTTGAACGCACTGTCAGCCGACAGAATA
CGACTGACCACCGCATTCTGATTAGTAAGATCCAGGATACTAATGGACCGATCAATGGGA
AGGGTGACCAGGGGTTCCAGATCGGTAATCTGTAACCTGGCCGTCATATTCAACGGTTTA
CGATCACGCGTCCACCCCGCATGTCCTTCCCCCAGGGTCAGATCCAATTGAGTCACCAGA
CGGGTACGGATGATTCCACGAGACTTACAGAAAGCTTTCACGTAGAAGGGGGAGGTATGG
GTTCCACCGCCTGAGGTATTACCGGCCACTAAGGGCAGTAGGAGGGAAATCAATACCCAG
ATCTTAACAATCTGCTCATACGGGTGTGCGTAGTTACAAACTGACTTAATGGTGTAGGTC
TCCGTATGGAGATTGCAGGTGGATTCAGTCCAGTGGTCAGGGATCTTGTCGTAACTGTTA
TTGGCAACAGCCAGGGGGATATTTCCAATAACAGACCCTGAAAGGGCTCCCATAGCCGCG
TCTTTAACAATGTTAATCAGTCCATCGACAATGGCTAAGCCAGTTTGACCTTTAGCCACA
TCGAAGGTGAAGTCATTGGTAGTGCGAACAATGGAATTGAACTTTTCCGCCATAGGCGAT
TGCCCAGTGCTATTAGAGAAGGAATCCCGAGTAGCTCCCCCGTTCCCCTCTACGTCGAAG
GTGATCCCATCCATACCACCGGCTACAGCGGTTTTAACGAGGTCGTAAATATCTCCCATC
CAGGTACGATCGTTAGGGTTGTCTTCGTAGTAGAACTGATCCTCATTGTTTATAGCGGGA
TTAGCGGTTACTGAGGGGGTAGCGGGAGAGGGGCGTTGAGGCCGTTCGTTGGGGTTTACA
AGAGGAGCTGAAGTACCATAAGACTCACCCGTTCCCATAGGCTGCATGGTGGAGGTGAGT
TGTCCGTCAGGTCCGGTTGGGGGTTGTTCCAGTTCAAGGTCGGCAATTCCATATTGCTTA
ATACGTGCAGGGCCGATGTCTTGATAAGAGCCTCGGTTGAGGTAGGCACTGTCTTCTTCA
ACGTAGTTGGGTTCGTTATCACCCCGGCGTTTACCGACTGTGCTCATTTCCTTTTCAATA
TACTCTTGACAAGGTCTGCCAGCGGTTACTGAACGATCGAAGGTGACTTCTTGAAGGATT
TGTTTGGCTCGTTCCAATTTCTGTTGGGGGGAGTTAATTGGAGTGTCGTTGTCCATAGAG
GCCAGACGCTCCAACATTACCCGGTGTTTACGCAGCCCTTTATTGACCAACTGCATGAGG
TCAATAGTCCCATCCTGGTTGACGCAATCAGGAAAGAGGGTAGACAAACTGGATACGACT
TCACTGTTGTCGTAATCGGGTTTAAGACCGTACAGGGGATCGGTCTGTTGCTGGTTCATC
TTAGGTAGGATGGGATCGATGTACCGCAGTTTCACCATGAAGTCATTCAGTACACCGGTA
GCCACCATGGTGTACGCCCCAACCGCAGGCTTAACAGTCCAGATCTGATGCTTGGGAGAA
TCTGCTAAGAATGCCAGGAATTGGGTGCTGATACTCATCAGCTGCATAGGCCAAAAGGCA
ATAGAACCGATGGCTTGTCCCATGTAGAACGACAGACTGGGCGCCCGCCCTTTGTTCGCA
ATGATAGCCGCTGCGGGAGAGAACATGTTTGTAAGGAATGACAACAGACCAGCGAACTGA
GCCACGCCTGGGGTAATGGTTAAGAGGGTGGCGTTGTTGTCGTAGATCTCTTTATACATG
GAGCCGAATCCCCCTTCTGAGGTGACCATTAACCGACCGTAACGCGGATCGGTCGCAGGG
CTCCATTCAGGCACAGGGTTCACAAAGCGGATATCCCCTGTTGCACTACTGAAAACGTTG
AAATATTTGTCCCAATCGAGGTTGTCTAATACTTCACTCGGTGGGATACCTCGGGTTAAC
AGACGGATCGATTTGGTGATAATGTACCGGTCGCGTGTCGTCACCTGAGCCATCGTAAAT
AAATTCCTAAGGTTAGGTAAAAGAGGCGAGGAGTACATCAGTACTCCCCTATTAAGATGG
ATCAATTCAAACGGACGGTCTTATCGGTTCCCTGATTCACCTGTTTGTCTTGCATCTCCG
CCATGCGACCCAGTACCTTGTAGATCTCAGCCAGAATACTGTTCGTATCCGAACTGTTAT
CACCCAGTTTACCCAACGAGGCCTTAAGCTCAGCCACCAACGCAGAGTCATCACTACGGG
TGGTCGTAAGTGCCTCGGTACCCCGAGGGGACTGAAGAACAGGTTCAGACGGCGCAGGCG
GAACCGGTTGAGGTGCAGAGGGAGGTGGAGAACCCTCATTAGACCTCGGAGTCGAGATCG
GAACATCCGTAGCCGGGTTACCAAAGGTGGGAGTAGACGTAGGTGGAACGTTCCGTTGCA
TAGGTCCTACCACACCACCGCCCAGTCAGGTGGTATCCTGTCCTTTCACGGACTGCCCGC
CTTGCACAGCGTTAAGGAACTTGTTCGCTGCTTCAGGGGACTTAAAGGCCGCGTGGATAT
GCCCGCCCGTACCGAGTGCCGTTCGACTGCGGTATTCATTGATGACTTGGAATTCCTGAG
GGCTTAACCCTGCCCGACGAAGAATATCCGTTACAATGGCAACCGCTTGGTCACTTCCTT
GTGCACCATTCACCAGGGTGAAGTCTAACGCCAGGCCTTTAGGATGAGCGCCTTTAGACC
CCTTATTGATGTGGTAGGCGTCATTCAGTGCAGAGAAGTACCGGAAACCCGGAACTGTCT
GTTGAATGATTTTACCCAGTTCTTCCACAGCAGGATGGTGTTCACCCCCCGCAATGGCTT

CAGGGGATTTCAGGAGCAATCCACTGCTTCCCGGTGTATTGGTGTTAACGATCATCCCAC
CGTTAACACCTGACACGTATTGTCCCCCTCCGCCTCCCATGGTTCCATTCAGGGACGGCA
GACTACCACCCACAACGCCCGGAGCCGGTCTACTGACTTGAGGACGCTGGGGTTCGCTGG
GGGCTTCTTGACCGTACAAGTCGGACCCGGTTTGGCTTACTAACCCACTCCCAGCCGTTT
TATCAGCTGTGGTCAGTTCACCACTTTGTAGTTTCTTCAGGTAATTCAGATAGAGCGCAT
AGCGCTGAGGCATTTCAGGTAACGAATTACCTCCAGTCAACCCTGTTGCCGCTCTACCGA
AGTCAACGGTTTGGCTAATACTTCTCAAGAGCTTAGAGTTCCTGAAGAAGTTCACCGCAT
TGGCTGCAATGACGTTCGGATTGTTACTGGCCAGCTCTGGTCTGTTCTCCCGATCAATAC
CCAGCTCTTGGCCAACCCTCCGGTAATTAGCGCGACGCGTCAGCTGAATAAACCCACGCC
TTCGGTAACGGTACCCATCACCGGCTTCCCTGTTCCCTAAGGAAGCACCTTTACCCCCAC
CGTACACGGTATTGGCGATGGCCACTTCACCGGCTTGTACGAGCGCTCTCCCTTGTTCCA
TGCTCTTCACTTCCCTGAAGAGTTGAACCAAGCGTTCCGGACTGCTGTACTTCATGTTCT
CAGTGGTCCGGGAATAGCCTGAAGACTCGTAGTTGGTAAGGGCGAGCATTTCAGCGATTT
CACGAGGGTCCGTAAGGCCTTGTTTCAGCATCTCACGGATGATCAACTGCTCCGCCAACG
GCTTAGGCACACTCACCCCTCGATCCGTTCCTTGGTTCGTACGTACACGGGTCATATCCA
AATGACTGGTGTCCGAATCAGCCGTAATAGGCGTGAAACCAAAGGCATGCTCAGGAGTTC
TGAACCGACCGTCGCTGCTGTACGTATTCCCAGCGTCTCCGCCGAAGGTACCGTACATCC
CTCCTAGAGCAGAATCTCTCCGAGACCGGAATTGTCCTGGGTTAGAAACATTCGCTTGTT
GGTCGGTGAACCCACCCCCTACCCGGTGAGGACTGATCTTCTCAGCCCGGGTTTGGGCGT
CGGTCTTAACCGCTTCCAATTCAGGGTCTTTGAGTTTGGCTTGTACCGACGCTTCCCCCA
AGAGGTTAAACATCCTGTCAACTTTATCCGACCGTCCGCGAGAGGTTTGCCAGGCGAAGG
GTGCTGCTCTCACATTCCACACCGAGATGGTAAAGCCAAAGACGTCATTGACTTGGGTTT
GAGTCAGTTCCTTGGCGATCTCATACCGTGCGGTAGCGGAAAGGGTTCTCCAAACCACAC
CCTGTAGCCCCCGACGGTATTTCCTCATCAGGGTCATGTAGTTGGTCAGCACCGGCAGGA
ACCGATCCCTGAACCACTTGCACCAGGCATCCGCGTACCGATCATCCACCCGGAAGGCGT
GCTTGAACCGAGCGAAGAGTTCACCTATGTGGCCCTTGAACACCATGTCATCCCCTTTCG
CCATAAAGAGGGATTCACAATGACGTTCCAACTTGAGGACGGCTTCGACGCGCCAGGGGA
CATCCTTATCGTTCCCGTAGGTAGTCACCCGGATAGCGGTAAGCATATCCAAGGCTTTAC
CTTCTGGCATCAGGTCTTGGATATAGATCTGGCCGACAACCGCATTGGCGGTATACGCTT
TCCCTAACCGATCGATTTCACCTTTAACCTCCTTCAGGCGATCGGTGGCCTCCATCTTCT
CCCCGAAGTCTTTCCGCTGGGTACTCCGGTCTTCCAGCTTCCGTTCCAGTTGAACCCGTT
CCTTCTGAAGGGACTCCAGACTTTGGCCTTTAACCGTAGAGACCGCAAAGGGATCAATGG
TTGTATCTTTAGCCCCGACATACTTCTTAAGGGCATCCAAAAGGTGACCCACCCGGATGA
TCGTCATTTGTTGATCAAGGAGAGGAGTCTCTTTGTCAATCTTAGCTACGATCTGGTACG
GAGAAGGCACCAACCGACTGAGGGCACTGTGGGCTTGTTTGGCCACTTTGTACACGATTA
AGTCAGACGGATCGTCATAGTCCTTCAACGATCGCAGTTTAACCGCATCCAAACAGGCCA
GGTAGGTCAAGTAAACGGGTTTGAACCGTCCATTGAACCAGGTAAAGACTTCACCAATTT
CATTACGGTCTTGAGGATTGCCTACGAAGAGACGAAGGACTTCCTCAATAGGCGCTTGCT
TAGACAGCGACGCGCGCCCATTCCCAATCACCACATGGTCTTTGAGCAGGTTCTCAACGT
TGAGGATTTTAGCCGCCAGGGGACCGTGAGGATCTGAGACACCGTACTGAACCATCCGTA
TATGAATTTGCTTCCCTTTACCTGAACCAAACATGGAATCCCCTCCCCATTTAGAAGTCC
CCAACATCACCCCAAAGGGACTGTACATCGAAGACCCCAGGGCATCCGTAGTAGGACTCT
TTCCTTTGACCTTTTCGAAAATCCATTACGCAATGGTCTGTCCACCCAGTAAGCCCGCGG
TGGCACCCACTTGTTGGTAAAGACTATTACCGGCTTTACCCACTTTAAGGATTTGGTCGG
TGGTTCCGAGTTTCTTCGTTTCTTCGTCAGTAGCGACCTGGTACATTTAGTACGCAGACA
ATCCACCTGCGCCAATCAAACCGGCGCGTCCTGCACCCAGTTTACCCAAGTGTCCGAGTC
CACCCAGACCATTCAATAACCCACCGAGTCCACCAGCGACGCCCTTAGCGATAATCGTCA
ACAGTTTAAGTCCCATACGACCGAACTTAAACAATGCACTGAACCCGCTCCAGAGGACCT
CAGAACCCAGGAAGCTGGTCAGCCACTCGAAGCCTTTCTTAACACCCCCTACCAAACTGC
TCAGGAGACCGAAGATACCGAACTTCTTCTTCTTACCGAGTTCTTTCTCATCCTTCATAC
CGAAACCGAAGTTTTCAGCAATCGAGATGATGGCGTTTTTAACGGTGTTGTCTTTCTCTT
CCTGGAGTTTTTGCGCACAGTCGGCCAGGGAGTTAGCTCGGGTGGCATCATTGGACGACG
CTGACCGACGGAACCGATCGGTGTTGTCCCCTTCCCCCGCATTTCCTTTAGCGGCTCGAA
AGCTCTCCAGGGCGTGTTTAAGCGCTGGATTCTTTTCCATAAGGGCTTCGGTAAGGGTAA
GATCGTTTCGCTCTCCTAGGGACCCTGGAGGGCCTTCTGGGCCATCTTGGGAACCTGGTG
CCGGGGGAGTGAAGGGACTGGGGCTACCATCCGGTGAGTTATACCCCCAATGTTTCAAGA
GGAGGTAATAGATCCGATCCATGGAAGTGACCATTGGAGAGCGATCCGTACTGAACGGAT

TCTTAACCCGACCCGCCATGCCTTTCGCATACCCACCTGCGCGGTCTTTCAATTTACCGA
AGAGGTCCAACCCGAACTGTCCGGCTTGTCTAAGGCACGCGCCCAGCTTGTTGATACTGG
TCCCCATGGAGGTTTTAAGTCCACGGTCGTATTCTTCAGCCGTGATCAGGGTATTCCCTT
CCTTGTCATACACCGGGCCATCGATCTCATCCCATCCCTGGAGTTCAACAAACCCGTCTC
CACTCCGTTTGAAGTATGGACCTTTACCAAAGCGACTCCCCAGCAATACCGGTTCGTCTT
CACCTTCAATGTAAATATCCATCCGGTACAGACGGGTACGAGCTTTATCCCAAAACCCTT
CAATGCAGTTAACAGGATCCAGCCAGTTAAAGGCTTTCATAATGAGATCCCGAACCTTAT
TCAGGCCTTCAAGGAATACTTCCTTGTTGTCTTCGGTAAAGAGTTTAGCCGCTAAGCGGC
TAGCGCCAATAGCGGGTCCATTGGCCAGGTCTTTAATACTCCCAGTAATCTGATCCCACC
CCTGAATGATCTTCGCGTTGAGCATATCCAGATACTCACCCTTAAAGAGTTTCAAGGATT
CAAGGATCGGGGTATCAGACCCTTCTTCGTACAGGTCTTCCGTGTTCTCGGCTCGATGAG
CAGCCGCTCGGGCACGCAACTTGTTATATGCCATCCCTAGCGCCAAGCCACCTCCTACCA
ATGCAGCACCTTTCGGGTTGTAGATCGCCATACCCGCCAATCCACCCAGTAAGCAACCCA
GTACAAACGGTTGGTTATCCAGGAGTTTGTCTATTCCCTTATTAAACAGATCCCCAAACG
ACGTACTCTTAATCCGTTCCAGAATACTCCGTTTTTGTTGCTGGAGGGTTTCTTCAGCTT
CAGGAGTGGGTTTCTCGTAGGGTTGACGTTCCAAGATCTTGGTGAGGATATCGTTCCGGT
TCCCTCCCAGTTCTACCAGTTTACGCAGCAGGCTGTTCCCTTGAGTCACCTGGTCGTTCA
GAGCGGTGAACATCGAAGACGGATGCGTAAGACCCGAACGACTTACACCTCCCTGTTCAA
TATGTGCCCCGAGTTTATCCAAAGTTTCCCGTACACCCTGAAGGGTTTCATTCAACTGAG
CGTTCTCAGCTGCAATCATACCACCGGAGTCTCGACGCCCTCTTCCCCGTCTGCCAAAGG
AACGGGTCTTACGCACCGCTTCTTCCAGAGGGTTGTCATACACCATAGCACGGTCAGGGT
TATCAATGAAGGACTGGAGGACGTTTCGGAAGGTGTCCATATTGACTTCATCGTGGCCAT
TTTCAGACTTGATGATGCCAGCGTCTTTCATGACGTTGTAATACCCAGACGAACGCAGCA
TATCGACGCGCTCGGCAATGTCCGGCATAAACCGACCCAGGAGGTTGGATTGTTCCGTTA
ACTTGGCGGCGCGTTCTCGGGCTTGTTCTGTAGGAAGACGAGTAATAGCGCTCGCTCGGT
CCAGGGTACTGCCCTCCTTAAAGGCCGTAAAGGCTTCCGGGGTAATGTCAAAATTCCGTT
GGAACAATTCCCGGATTTCATCCGCTACCTGACGGTTTACCCCTTTGGTCTCCAAGTTCA
TGTACACGTACGGACTGAACCCTTTCCGTTTATCCGCATTCTGATCCAGTTGCATCGCCA
ATACACGGCGGGCTTCGTTGGACAATTCCTTATTGGGGTCAATGAAATCCACCAATCGAA
GGGACGTATCCGCTTGGGACATGACTTGGTCTTTATCCATTACCCGGTTAAAGGTATCCG
CTTCCTTTTGGTTATGGCTGACCAACTTACCCCGTACGTAGTCATAACTCGACGCGTTAA
GGGTATCATCCCCCGTACGGATCTTTTCAATCGACAAGTGAATGTGGCTGAGCAGTTCGG
GGATCACTTCATTCAGGGTACCGGAGGACTGTTTGTTCCAGATCTCCGGTTCGTGGGCAT
CCATTAACCCCCGACGTTTTAGGCAGTAACGACTCCCGGTGGGTTGGTACAACTCATCCA
ACAACGCCGCCATGCCTTTATTCGCCCCTTCCTTCAGCGTATTAATAACCGTCCATTCCA
CTTTAGCCAAGGGCTTTTGACCTGAACGGAGCGTACTGAGGTATTGTTCGTAGTCCCCGT
CCTCGTAAAACGAGGATCCCCAGTTGTGGTATTCCGAAAGGGTAAAGACCATTCCTTCCG
CATTTCTGAGGTTATACGACGCTATGTTACCCAAATCCGTGAGTTGCGCGTACGCGTTAT
CCGCCCACTTCCCCAGTTCCGGGAATTGTTTCTTGAACTTAGCAACGTATTCCTTTCCCT
TACGGCAATTGATCATCCGGGGGAGGTTGCTAATGAACATTTGGGCCGCAACGTTACCCA
CCATGGTCCCTACGTTGAGCGGAATGCCTTCCGACATCTCCGCCGCCATGCGCATGGCGC
CAATCAGGTCACCTGCACCTCGGATTCCGTCCCCTCGCGCATCCTTACCGAACCGCTCCT
TAATAAACTCACGTATTCCTCCAAAGGAGCCTTTAACCGTATTGAAGACCGATCCCCGAA
TGCTATTACGGCTCGCTTGACTGTGGGTGGTCTTTTCGTAATCACTCTTAGCGGAGTTCA
GGGCAATGGTCTTCAGTTCAGCAATCATCCGGTGATTACTGGCTTCCATGAACTTGTAGA
ACTTCGCGCTGGTCAAATAGGAACGGGCCAACAGGTTCACCTTGAGGGTATCATTCCTCA
ATTGAACTTTACGTTGGTAATTAACCATTTGTTCCAGCAGTTGGTTGCTCCGCCCAATAC
TCAGGTTCAGGGTATTCAACCCCCCTATGGTGCGGCCACCTACTTCGGTCATCATGGCGG
TAATACTTTCACCCACCCCAATGACCGTTTCCCGGTCTAACAGGGAGTTAGCGTCTTCAC
TCCCCAGTAGGTCTTTCACATCATTTTCGCCCACATTTTCCATGGACGGGGTGTCACTCG
CCGACGACGTATATGGCTTCCAGCTGCTGAAGTCATTTGGACTGAACCGAGTCGGCCCAC
TGGCTACACCCCTGGTGATCTTATTAGGAGCTATTTTACCTGACTTATCACCACCCCGTT
TATCCAAGTATTGGAGGTCCACGACTGCATCGTAGCTTTCACGTTTAAGTTCGTCTACGA
TCTCCCTCCGACGACGGTTAATGTCCGACGCCGTAGTAAAGGCAGTCCCCCAGGTCTGAG
GGAGGACCATCTTCAGGGTGTTCAACCGAGCATCGGTGTCCCCTACTGTCTGATTCACTA
CACCTGACAAAAACCCGGATGCTACCGAACGAGTGAACACCGGTTTCTTCCCGTCGAAAT
CGGTATCGAAATCAATATCCCCAGTAAAGGATCCTCTCCCCAATCGAAATCATCGAATG

TATTATCCGCCATTGTTAATGCACTCCCGCATAAGGAAACCGTATGAAACTGACTAACAT
TACCCTCTTAGACCCCCGGACGATTGTTCCGGGTGTCTTTAAACCTATTACGTCTACAGA
CTCATTTGAAGGGATGACCCAGAACCTCAGTGATGACGGTCTCTTCTCCCTTGACATCTT
CGGTAAACTGGGCAGTAAACAACGTGATAACACGGAAGCGTATCTCGACGTTAAACTTAC
CATCTTCAATCCCACTTACCTAAAAGCCCTTATACCGATCAAAAGTCTGTATCATTCAAT
TTTGAAAGGGTCGGAATACGCGGTATGCGATCCCGAAGTGAAGGATTTCATAAAATCGAA
CATCCTGGAAGGGGAAAAGGGTTTCGCCTTCTTCCTGAAACACTTCGATGAAACCGTCTT
CTCCCTGACCCCCTCTCATAAACGCAAACAACGGGTGGAACTGGCGAACCAATTCCGTAA
CACGGCGTTAACGGATAAGGTCCTGGTCATCCCCGCGGGTCTCAGGGATATCCAATTCAA
TCCAGATGGTTCCCACACCGAACCTGTAATCACTGAATACTACCGCGCCTTAATGTTCCG
GGTGAGAGTGATCCTCGGGAATAAGGAAGATGGCCTGAACCCCCTTTACGATACCGTTCG
GTGGGGGGTACAGAATGCTTTCTTGGAATTGGATAATTACCTCTTTGGTTTACTCCAGGG
GAAAGGGGGTGTTCTCCAGCGTCGTATGTGCACCCGAGGGGTTCACAACAGCACCCGGAA
CGTCATTACCGCCCGTAAAGTCTCCAGGACACGTTTAGACTTCGACGACGGTGTAGGTCC
TAACTCTGCGGACATGGTACTCTACCAAGCCCTGCTGAACTTCCCTTATGTCTGCATCAA
TGCGTTAACCAACGGCTACCTTTCCAACATCTTCACCCCCAGCTCTCAAACCGCTAAGCT
GGTGAATAGCAAGACCCTGGAAAACGAATACGCTGAAGTCTCCCCTGAGGTGGTTGAGAA
ATGGACCACCGCCACCGGGTTAATGAAACTCTTCAACGGGTTTGCCGATCCCTCCTTACG
GAGTAAACCCATTGTCATTGAGTGGAAATACTTAGTCCTCATTTATGATGATGGGACTGA
TGTCTGTGTTCTCCATGACATCCACGATCTCCCGCAAGGGAAAGACCGTAAATGGGTGAA
ACCTATCACCTACATGGAACTCTTCTACTTGAGTTGCCACAGTGTCATCGAAGAACAGAT
TACAGGACAAACCCGGTATCCCCTTACGGGGATTGGCTCTATCTTCCCGGCTAAGGTTAA
CCGACGTACCACCACCAATGCGAGCCGGCGGGGCATTCGTAATCCGATTGATTGGAGTGT
GCGGGAAACCTGTAAACGGTTCCCTAAACGTGAAGCCCACCCCGATTACTTCGATGCCAG
GTCCGTAGACCCCTCTAGAGAAGCTTTGCTCGACAGTGACAATGAATAGATAGTGATGCA
TGATTTAAACTCACTATACAACTCCTATTGTATGCGGAAGTGTTCGCATTCGACCATAAG
GAGTTTTAAAGTTACTAACGTGTTTTGGAAAGAAATACGTGTTGTCTACAACCAACACGG
TTTACAATGTCACCGATAAGACAGATGTTCCAGTCCATCGGATGACAGACGGGAATACGT
TCTTCATCGCACGGGGCGATGAAAACCAACTGGTTAAGATCAAACTGGGGTTAATGATCC
TCCATGGAGAAGGAAAGTTCCATCTTCCTCTCTCTGAATGCGAACAGGTGAAAGTAGGTT
TTCAGGATGTGGATGCAGGAAACTTTGCAGCGGATAACCTTTACTTGGTCTATCCTGAAG
AGGGCATCCCTTAGCCTGAACTCTCTGGGTACTACTACATCCCTGGGCTTGAACTCAACG
CAATCAACCGAGCCGGTGTGGTATACCGAACAGTTAGAAAGAATCTTTACCTACCGTATC
TGGGTGGTAACACCGTTCCTAACAGTTTTTACCCCTTTGTTGGACTGGATGTGGTCAGTG
GGGATGAACGCAAGTATGTCCACCGGCTACTGGCGGAGACCTTCGGTAAGCCGCCAAAGG
GCTACCCCAAGCTACTGGTCGACCATAATGACGGTAACAAGATGAACTTCAAGCTCTTTA
ACTTGATTTGGGTTACCGCTAAGGACAACTCCTTCAGAGCGTACAACAATCAAGAAGCGA
GAGCGGACAATAATCCCTTGGAGATTTTTGACAGGGAATTAGGTCAAGTCTTTAAGTACG
TCAGTCAAAATGAGTTTGCGAGAACCATGGAGGTGGATTCTTGGTGCATTAGTCTGGTAC
TCAAACGTCCTCGTTCAGTGTACTAGAACATGTACGGTCTTCGTCGGAAGGGAGATACAC
GTGACTTCCAAGAGATCTGGGACTATAAGCCCCCTGTGGGTAAGAAGATCCTTGCCCGGA
ATGTCTTCACCGGTGAAATCACGGTCTATAATTCATTGGCGGAGGCCTCTAGAGCAACAC
AGGTAGGGACAACAGGCATCCTGACGTCCCTGAGTAACACCAACCCGAGACCCGCGTTTA
AAGACCATCAATTCACGCGGGAGTCTGACGAGACCCCTTGGCCTGATTTGAATGAATACG
AACTGGAATGCATCCGGCGCAAGATGCACCCTGCTACAAGTGTGTATGAAATGTTTGATT
CGGTAACCCAGGAGACGTCGATTGTGCATGGCGTTGATGCAGTTACCCGAATCACCGGAG
CCAATCCCCGTACCGTTATCGTTTGCGCACGAGAAGGTAAGTTGCTCCATTCACGTTATC
GTATCAAGAAACTTACACGTTAACGAGTTTATATCCATCACTTAGTCCGGCTAGGCACGA
AAGTCCTAGACCGTATTCCTCTGAATTGCCGGGAGTTCACAAAAATCTGTTGGCTACAAC
GTAACTGGAAACAGTAAGCGTGAATGCGACCGAAAGGTAGAAAGAACAACAGACCTGACC
TACGCTAACCAATAACGCTAAGGGTCGTAAACAAGGGACAATCAGCCGCTAAGACGCTAC
CGAGTATTAAACCCTATGCGTAAAGTTCAACGACTAAGGGAGTTATGACCCTGTACACCT
ACCAATGATGGGGGTGGAAGTGGAGGAGACCTAAGGGGGAATAGACCTATTCCCTACGGT
CAAGATATAGTCTGGCCCTGTTTAATAAACAGGCAGGCACCTGAGTGTGCCGGGTGCTAG
TACGGACTGACCACCGCGGGCGATCAATTGTCGAGCCATTCTGTTGTAGCGGAAGACTGC
AAACACCAGGCCTACGAACTCTTTGGGAAACGGGACTATTACATCAGTGGTACGGGTCGG
CTTTTGTACGATCCGATCAATGAACCGATTCTCTTTATGTTTAAAGCAGCGACGAGCGGC

TTAGAGGATATTGAGGTATGAGCAATCCAATTACGTATTCCGAGTTCTTTAAGCACTATG
TGCTGCGTAAGAAACAAGACGTTGTATACCCTAAACTCCTGGGTATTGGTGAAGTGACTC
TTCCTAGGGGGAGTTTACTGCCCTTCGTACCGAATACGATTAACGAAAAGGGACCGGGTA
GTAGTGAAGCGTTTATCAGTAACTTCCCTAAAGAGGTCTTTATTGAGTTTGTAAGGGACG
GGTATAAGCCGGTGATGGGGCATGGGCGGACAGGCGCCTATGACTACCCTAAGGCGGTTA
AGGCGTACCGGGCGAGTCACTACATGTACAGCCGGGTGCGAGAGATCAGCACGGTGTACA
CCAAACAGAACGTGTTGGTCGCTAAGAACTACGGGATGATTCCTCAGGGGTTCGTTTACC
GTCCGAGTATTATCGTGAATTATGAAAAATACTACAGCATGACGCACCTGCTCATTGAGA
ACATCAATGCAGAAGCCGAGAAAGGGGTGCGTCGGCGGTATATGCGGATTGACCTGCCGT
TGAACCTGCCGTCCCTGGTTGAGCTTGAGAAAGACTACGGACGGTTGATTGATGGGTTTA
ATGATGGGCATCCTGTCCTGATGCGCAATATGTTGGAGAGTACGAAGGCTGAGAACTGCT
ATTGGCTGTTGGACATACTGGGGTTCCTGTTTGGCCAGTATTCGTGCAGTTTGTGCGGTA
AACTCACCGACCAGGCGTATAAGGATTTGCATATCCTCTTTACGTCACAAAGTAAAGTCA
TTGTAGTACATCTGGGGCTGTTGAAGGGTTGGTTAGATGAAACAAATCCTAAAGACGAGA
ACACCTACCCCTCTGCTCGGAAAGAGTTTGAAAGTCTGAAGACATTGACCCGCCATGATG
CGGTTAAACGTTTCTATTTGGCTTTGATGAATCTTTCTCGTGGAGGCGTTCCTGAGAATG
TACTCATTAAAGAGGAAACGAAAAATGTCATCCCAATTGCCGGAGAGAAAGGTACCGTTG
ACCAAGTGGTTGCGGGAACGTCACGAGAAGAAGAAGAAACATCCCATCTTCCAGAAA
CCCGAGACCATACTGGCTCGGGCACGGTTCCCCGTGCCCATCGTCCTGCTGGTTCTATCC
TTGATGTTTTCAGCCGAAGTGAAGGATCTGGCGAGGCACATAACGAAGACCAAGACACAG
CGGGAGAACGAGGTGCTGATTCAAATCTTGAAGAGTGAACATCGCCGGTAGATGATGAAC
TGCTGGAGGTGGAGAAAGTCCAAACCGAGATCAGTACCGACAAGAATGTGTTTAAGACGC
CTGAGAGTGGTGTAAAGCGGGCGTTGGAGGAGCGGGGTAAGGAAGGTACGCTGACGGTGG
CTGAACATGATTTCTTCATGCGTAAAGCGACCCGTTATCAGCACATTGAGATGGAGAACG
GTCAAACGTTCGAAAAGTGCATTCAGATCAAACCCGAAAAGGTTAAAGACCTGGGTGGAG
AGATTGAAGCTGAGTTTCTCCCCGTACTGGATGAGAGCATGTTGCGGAGTCGTGCCAAAT
CCCTCAAGATCGAATATCCTCAGAAGTTTTTACACCGGGATATTGGTCGAATGGTGTTGG
GTCTTCAGAACGCTGGCTTTGCGATGAACGACTACCGGCGTGAGAAGGTCGTCAGCGTAG
AAGGCAGTTACGAGGTCCACAGCATTCAGTTACACGAAGTCAATGGTGATCAGGTCACTG
TACATACTCGGTTGCCTACGGTGCTGAAACACGGCACCTACGTCATTGGTGGTGGTAAGC
AACACATGCAACTGCAACGTCGGGAGAAACCTTTCCGGAAGATCAGTCCTCGGGAAGTGG
TGCTGTCGAGCTAATACGACCGTAAGCTCATGGTGAGTCGTAGTCCGAAGATGGTGGATA
ACCTCTCTACGTTCATGGTGAAGCAAATCTCCCTCCAGAGTAAGCGGAAAGGGTATACGG
TCACTAAAGGCAGTACGTGGGATCGCTCCCTCAAAGCCCCCCGGATCTACAGCATTCTGG
CTCGTCAGTACAAGTACATTACTATCGACGGGTTGGTATTGGACTTCCGGTTGAAGGATC
ACCTGGAACTCCACCCTGAGTTCAAAGCCTATACCCGCCCTGATCGGTTCTTGATTGGGG
TAAAAGACGGATTACCTTTGATCATTGATGATTAGGGTAACCTGTACAAAGGGGAACAGG
AACTCGGTACTGTTGAAGACTTGCTCGGTATTGATGTTCGTAAAGCGCCTTTGACACACG
TGGTGATTAACATCAGCGGGTATTCTTTTCCGTTGGGAGTGGTCCAGTGCTTCTACTTCG
GTATTGATGAACTGTTGCGGGTGATTAAAGCCATGACGCGTTCGGTACCCGTAGTGACTC
GCCCTCAATTGACGGAGGATGAATACGCGATTCAGTTGAATGACGAATACTTGATCTTTA
ACCGTCGGGAGAAGCTCACCACGTTGATCTTCGGTGGAATGCCGAAGTTGAACAACATCA
GTAACTTCAGTCGGAGTGATTTGAATAATAACAGTATTGGGAGTGCGTTAATGGGAGACC
CTCGGGTTCGCCCATCTCAGTTCCAGGAAATGAAGAACCTCTCCGATCTCTATATCGATC
CCATTACGAAAGAGCTCTTGAAAGAGATGAAGCTGTCTGAAGACTTCCATTACTTGCTGA
TTGATGCGGCTAAGGCATTGGAAACGAATTACAGTCGACACGAGGTGGAGATTTAAGAGC
AACGCATTGTAGGGTATGAACTCTTCGCGGGTCATATCTACACGGAATTTGTAAAGTCCA
GTCGTCAATAACGGAATAAGGGGAAAGGACGTAAACACAAACTGGACTTTAATCCGGACG
CGGTTATTATTGCCATTGGTACTGACCGGTCTACGAACTAGGTGGAGGATGTGAAGCCAG
TTCACCAAGTGAAGGACCAAGAGGAAGTGACGTTTGGGGGTACGGGTGGTCGTAGTGAAA
TCACCATGGTCAAACGCGCACGGACTCAGGTGGAGAGTTACAAAGGGGTCATCAGTGAAG
CCAACATTGACAGTGGTAGAGTGGGTTATGTGACCTACACCACCTCTGATCCGGGTATGG
CTGATTTCCGAGGCAATGTTGATCCCAAGAACAAACGTACTCCTACCGGTGACGGTTCGG
TAACTATGAACCTGATGTACGGTGGAACGCATGACGATTGAGCTGTTGAGAAAACAATAG
ATCACTCAGTCCAACCAGGGAGTAAGCCCTTGTTACCATTACCTTTAATTGCGGGAACCT
ACTGAGAGCCTTATCTACCAAACCCGAGCAGGAAACCAACGGGTGGCCAACCATACCGGG
TTGGGGACGGTAATAACGATAAGGATTGGGGAACCCTACGCAGCGAAGCACCTTCGTATT

GTCGAGAGGACAAGATAGGGTGAACGTTCAACGACTACGGATGAAATATCCGGTAGAGGG
GTCAGGATGCTACTTCCATAAGTAAGGTTGCCCTGTATGCTGGAGGTGTGAATAACACTG
AGTACAGGGATGAAATATAGTCTCGTCATATAAGTGAAAACCTTAGAGCGTGGTGCTACG
CGCATCGGCGGATAGGATATCCGTGAAGAAACGGTCAAACGAGCAGTATTCACATCGACT
CAGGCCTCTCAAGCAGTGATGGCTAAGAACTACGAGATCTTTACCCTGAGAACGGGTTAC
GAGAATGTACTGGCTCACCGGACCTCTGACCTCTACAGCAAAGTAGCCGCTGAAGATGGT
AAGGTCACTAAAGTGGAGAACGATCGCTTACTCGTCCAATACAAGGACGGAACAACGGAC
TCGTATCCCTTGGGGTTGGTGATTGGGGAAGCCAGTGGGGAATATCATCCCCACAGCCGT
ACCACACATCTGAAGGTCGGAGATACCTTCCGTAAGGGAGGTGTCATCGGTTGGGATCAA
AACTGGTTCGGTCGCGATCCCTTCTGCCCAGGGCAAGTCGGGGTTAAGATCGGTAAAATG
TGCCAGATCGTTATGGCGGAAGATCAGGATTTGTACGAGGACTCTATTGCAATTTCGAAT
GAATTGGCGATGGAAGCACGTACCCCATTCATTAAACCCAGTCAGTTTGCCGAGGACGTG
TCAAACAACCTGGTAATGCGGGTTAAGGTAGGAGATTACGTCGAATAGGACACGATTTTA
TGTGATATCGAAGAGCCTCACTTGGTGGAGGGCGAGGAGCTTCCTGAGCTCGAAGCAGAC
ATCAACAAACTCGGTATCAAACAGATCCGTTCGAAGCATCATGTAAAGATCGTACACATC
GATGTCCGGTACAACTCTCCCTTAGACAAGATGTCTGACTCGTTCCGTTCGTTCATTGTT
CAGAAGGATAAGGAAACGAAAGGGAAAGGGTTGGTGGAAGGAACCGGTGTCGAGAACAAT
GCCATTTCCACGATGTTCAATCTTAATCGTCCCATGTTAGGTCCAGGTAAGGCGTTCATT
ACGTTCTACATCGAGTCGTTAGACCCGAGTACCAATGCGGATAAGTATGTCATTGGGAAC
CAGATGAAAGCCACTGTGGGTCGTATCATGGAAAGACCGCTTAAGACCGCCAGTGGGATT
GTCATTGATGTGAAGTCGAGCTTTAAAGGGATGTTTAACCGAATGGTGCTCAGTTTGAGG
AACAAGCTGGTTGCTAACGAGTATGGTTACCAAATCACTCGCCACGCCATTAAACTCTAC
AGAGGTAAATGACGATGAACTTCCGGACGAAGAACGGTTACCGAGACCTGCAAGCGTTGG
TTAAGGAACTGGGTCTTCATACCGGGCAGGTTGACGGTGTGTGGGGGAAGGGAACTTCCT
CCTCCACTGAAACCCTGCTCCGGGGGTATGCCGAGGTGGTGGGTAAGAATACCGGAGGTA
TCGGTCTCCCCATCTCCTCGGATGCCAGTGGTTATAACGTCATTACAGCGCTCCAGCGTA
ATCTGGCCTTCTTGGGTCTCTATACCCTGACTGTGGATGGTATTTGGGGGAATGGGACCT
TGAGTGGCTTGGATAAGGCATTCGAGGGGTACAAGGAGCGTTACCGCACGCCGACCTACG
ACATTGCGTGGAGCGGTAAAGTCAGTCCTGCCATTACGGCGAAAGTGAAGGATTGGTGCG
GCGTTCATGTGCCGAATCACCGAGGTCCTCATTGGCTGATGGCCTGTATGGCTTTTGAGA
CCGGCCAGACATACAGCCCGTCTATTAAGAATGCGGCGGGTTCTGAAGCGTATGGGTTGA
TTCAATTCATGAGCCCAGCCGCCAATGATTTGAATGTTCCGTTGAATGTGATCCGATCTA
TGGATCAGCTGACTCAATTGGACGTGGTCTTTAAGTACTTCGAAATGTGGATGAAACGGG
GTAAGCGTTATACCCAGGTGGAAGATTTCTATTTGACGATCTTCCATCCAGCTTCAGTAG
GTAAGAAGGCTGATGAAGTCCTCTTCCTTCAGGGGAGTAAAGCGTATCTCCAGAACAGGG
GGTTCGATGTAGATAAGGATGGTAAGATCACCCTGGGTGAAATTTCTTCTACAATGTACA
CCACGTATTACTAAGGTCTGTTGCCTGAAAATCGTCACGTTATTTCGTATTGATTCACCT
TCTTTTCCGTAGGAGAGTTTCCCTGTGAAAGCAGAACTAATCAACAATATCATTGCACTG
GCGAATACCGCGGTGTTCGATAAACAGACGATCCGTAAGGTAGGGCTGGAGTACATTGCG
CCGCTGGATCCCAGTATTCAGGCTGAGAAGATCAGCAAGGCCCTCACGAAGCGTGTTGTT
AAACAAGGAGCGAACCAATGATCAGTAAGGACAGCCTGGCTTTGGCAGAGAACATCGCCG
TCGCGCTCCCTGAAGGCACTGTAGTCCCATCCACCCCGATCCTTCAGGGGTTGAATACCC
TGAGTTATGGGGCGTTCCCGTACGAGGAGGACTTCCGGGATAAGGTGGTTAAAGTGACTT
CCGAGATCACCGAGCATTCGGCAGCGATTGAGGCGGGGAGTGACAAAGCCGCTGAGACCA
TCCGCGGTGCGTTCGAGATGGTGAAGTCCTACGGCGTCCCGATGTCGGACGCCATCCAGG
AGAAAGTGGGTCTGCTGTACAGCCGAGGTGATCTGAACTGGTCGTCGTTGCGTCATTTCA
ATGTCACGTACATCAATATCTCTGATCCGTTCTTTGACTCGGCTCTTTATCCTACTGAAG
TCAAGAACAAGGCGTTGTCGTTTGACAGTGTGGGTCTCGATGTCCTGAAACGGTTGGTAT
TTGAATTCGCAACGGAACAGGATATCCGGGATTACGTGGCAACGTCTCACCCAGAGATTA
ATGCCATCCTGGATTCCAGACTCTAAGACAACGTGTACGCGTACTACGCGATTACGATGC
TGGCCGATCTCAAATCCCTTTTCCACTACAAGGGTGAGGCGACCTTTTACTTCACGCGTG
TGAAATCGGTACGCCTTAACCTGCTGCTGAAGACCTACGTGATCTTGACGAAGATGTACA
GTCAGGAAGACCCGGCGTTCCTGGTTAAGGGGTCGTTGGAGGATTACCGGGAGTATATCA
ACCTGCTGTGGAATGGAATGACCCATTACCTCATCGCGTTGAAGAACACGGTTCAACTTT
ACCGTAACCGTAAAGCGGCTATCTACGACGCTGAAGCCTCAAAGTTGGTGGACTTTAAAC
CAGTTGATGCCCTGGACGTTACCGTTAAGGGATTGAGTGGTAATGTCGTCGTGTATTACT
GTGATGAGGTCCTCAAGACAGTAACCGGTAATGGGGGGAGTTTGAACGACTGTGTGATCG

CCTCGATCTATGGGCGTCTTACCGGTCGTCCGAATGGCTTCCTGGACCTCATCAATAACC
CTGACGTGGTTAAAGACTTGACGGCTAACTACTTCGAGGGTATTCACCGTCCACTGAACC
AAGATGCCCGTAAACATTTCATTAACAACAGTGTTGGGGCTATTGTTCAGTTCCTGAATG
AACGGGAAGAAGCTCGGGCCGCGTTGTATGAGCATTTGGATGCAGAAGGGTCTTCGGTCC
GTACCCTCATTGAAGAGAAACTGAGCGGTGACCTGGCCGTTCTCGACACCCTCTACGAGA
ACGACGTCCGCAAGAGCAGTGGCGTTGAACCTGTGAGCATGGAGATTGAGGCGCAACGGG
CGAAAGAGGTCATCTTCCAAACCAAACTCATCCCTAACTTCTTGAAGTTGTTGGGGTGTG
AACTGGCTGCGGAGATCATTGAGCTGACCTTTGTTAAGCTCGGTGAGGAAGACAACCTCA
ACAGCAAGCGTGAACGCCTCCATGGGGCGTTGATTGAACTGCTGGTGGGTGAACTGCTGG
GAGTTTAATGAATGGACGTTACTTCTCTCGAGCGAGAACGAAGTAAGATCCGTAAGGCGT
ACACCCTCCACCACGATCACAGTGTCACTGCAAATCGTCCTCTGGAAGTTCATATTCCCA
AATGTTTTACTGAACATGGGTTAGCGTTTATTGGGGATAAGGTGACCACTACGTTAGTGG
CGGGTGTTGTCATCCCGGGGGAGTGTTACTCCCCTTGGGTGGCGTTGACGGATGTCACGA
TGATCCCTATGAACGTTCGGGAGACGGGTATTGACGGCGTTCAGTACCTAATCCTGGAGT
TTGAAGAAGGGGATACCCTTATTGAGAATCTTAGGTATATCCAAGACCCCAATAAGCCCT
ACTTCTATTTCACTGAGTTTGACATCTACGCGAAGATTCCTTGGTACTTATCAGACAATG
ATGGCACCTCCTTGTTTGATAACGCTAAGAGACAAACAGGCAGTGAAGTTGGTGATACGC
CGCAACACATGCGGATCTTTAATTCCGTTATGATGCGGGATCCGGATAACTTGGACAATC
CGTATCGCAACAGTCAAGCCATGCTAGACGGTCGCCCTCCGGTATGGGTAGGGTTGAATA
CCAGCTCAATGCTAATTGACGGCACCTACTTGAAACTCACCGGAGGTTACCTCCAAGACA
ACACGGTGGCTGCTATTATCAAGCAAGACACCAAAGTAACCGATCTGGAAATGATCGTTA
AAGGGGTCCCAGATGAGTAATACTTTAACCTTTGAGAATACCCTGCTGGCAAACAGTGGT
AAACGGGGTATTCTCAAACCCATGGACAGTTCGGGGTACTACCGGATCAATGCGGGAGGG
TTCAATATCCCTAACCGTTCCGGTATTACCTACCGGGTGAATGACTACATTCGTGAGTGT
GCTCGTCCAGGCAGTGATTTTAACCGTCGGGCGAACGAAGGCCAGATGAATTGTGAAATC
GGCCAGCCACCTCAGTATGACAAGATCTTGGTCAATGGCGAAGTGGTTCGCAAGCAGATC
ACGGATCTCTTCGAATGGATTAACCGCCTTCGGACCATTGACTTTGATAATGTCTGTGAT
CATATTCGTAAAGTCCATTGGATCTTTACGGGTGGACCGAACGATCCGGTTTACAATGAC
ATTGAAGTGATTCCGTGGGCGAAGAACCCACATCATAAGCAACTGCTGCAAGAAAGTCTG
GATAATCCGGATATCAATACGGCGTTTAGTATCCGTACGGTAACCAAGCCTGAGAACTTT
GGCGATAAAACGCGCGAGGTGGACTACTGGAGCACGTATGACGCTGTCATAGAGCAGGGT
ATACTCCGTGCGTGTAAACACCTCAAAGCGGGCTTGGAGAGCCTTCTGGACGATTATTGC
GCTGAGGGTGGTGAAATGGAGTTCACCACGACCATGGAAGAGCTTTTATTTCTCTGCGAT
CAGAAGAGGCGTTCACCTGAAGTGGCGGAACGTGAAGCGGGTACGGAAAGCTTTGTTCGA
GTACAGGAAATGCTGGATGATCTCCGTAGACGAACACCTGCTCAATCCAGACCTGCGCGG
TTGTTTGTGTCCAGTTCGTTGGACGTGTTCCGGTAAGCCTGAGCCAGCATGCCTAGCCTT
GGGGTAGACCCCAAGGCTAGGTTATTAATTTTAGCTGTTTGTTTATTCTTATAGTCCGTT
GCGGCTAATCATTTAAACCAGAAAGAATTTCCTTATGTTGATTGAAAAGCTTAGTTTCCA
AACCCTGGTGCTTCATCTGCGTGAGGTGAAGGGAGGGTTTAATGTTCTCCAGGGATTGTG
GGAAACCGCCCAGAAGGAATGGGAACGTTCCAAGAATGCTGATACAGACATCCATTTCAT
CAAAAGCGTGTTTACTTCCACTCAGAAAACCGATGCGGTCCGGATCTATGTGGCGAAGCT
GGCTAAGCTGTACATGAACACCGTGGGGGTACTGCCGGGGATGTGGTCCAAACATACTCT
GACCCATGAGCATATCCTGACCAAAGCCACTGTGGACAAGATTTGCACCATGGAAGTGAA
CAGTTCCGACCAGTTGTTCGAATTGGTTGCCACCCGTGTATTGGGAGTTGATAAAACCCT
GTTGGAGAATATCCTGACCGATGAAGTCAAACCCCGGGATCGCGCCCGTATCCTGATTAA
CTACCTGGGGTACCGTGTGGATGAACCGGGTGTCCCGGCAGAGGACTACCCTGACCTCAT
CAGTAGCCTGAGTGTGTTGATCGGTAACAATGGGTTTACAGATGAGTTCTTTGAGAACCT
GTTGAAGGAAGCAGAAACCTACTTCGATAGCGGATCGGTTGACTTCCAACAAACCCCGTT
GGATGTGTTGGAGAACTTCCTGCACCAACCCTGCAAGCAATAAGGGGTTTTGCCCAAAAG
ATTTCAAGTCTATATTACTTGGGTGAGCGACATCAGGACCTACCTGACGAGGGCAGTACA
CCTCTTCCCTTGCTTTGTCTTGCTGTCGTCACACCACCACTTTTCGACAGTAAGAGGTTG
TTATGTCGATAAATAAAGAATACGGCGATTGGGTGGAAAAGGGTGTTAAGGGTCTGGTAG
ACTCGCACGACTACAACAAGGCCACCAACCACATCACGTTCGATTCGGAGAAGGTGGAAC
TGCCTAAGGGTGTTACCCAAGACAGTATTCTCGAGCACGTCAATTTCTTCAACGATATCA
GCGCGCAGGTTGAAATAGCCACCTCCCTGATCGCCCGGAGTCAATTCGAAGACAACAAGG
AACTGACCACCGTCGACGGCACCCTGAACCTCGGGGGATTGAACATCAATTCCCAGCATC
ACCTGAAACAGAAGGTGGGTGAAGATTATTTGTGGGGGCAAAGCACCACCGCAGTCGATT

ACACGCATACCGAAGAACAAGCCGTTTGGTTGGATACCCAACGTACGGCCAGCCAGGAAC
AGGCCGCCAAGCTGTCCGAGTAAGCATTCCTTCAATAAAGCTAGTGACCGTTTGTGTCAC
TAGCTTTATAGGGACTAGTTTTTGATTTAGGAAACGGACCCCTGTTGACCGTTACCTTCG
TCAGACCTCAATGGAGACAGAGAATGAAGTGGTTAAAAGCGTTGCTTGACCTTCTCTTTG
GGAGTGGGGAAGATCAGCGTGAGACGAGGGACGTGAAGTTGGAGAACCTCCGCGAAACCT
TGTTACCTCGGGTTAGATTCCCTTTGGAAAGTCACCACGAAGACAATTATAAACCCCTCT
TACTCCCAGAGTCTTCTAAAGTGATTGGAGAGAAGGAGCAGAAATGGAATGAAGGATAAC
TTCACGTCGGTAGGTAAACGAATATGAGTCATGAACGGTGGAACACGGCTCGTTTTGAAG
CCATCAATTGGGAGGACAATCAACGCGGTATCCCTATTGGTATTAAGAACAACCTGTATT
TCCGGTTTGACTATACCCGTAAGGGTGAAGGCTGGGAGGTTACCCTCTGGAATACAGAAG
GGACGATTGCCCGGATTGAAGATAGCCCGACCGGGTTCTCATTCTCCAGTTACCGAGTGG
ATCTCGTAGATGCCGAGTTGATGGCTGAGATCCATACCCAATTGAAAGATCAGATCAATA
AGGTTAAACCCACTGAACTGATCGAGGTAGGGGGGTGAAAGCATCTGAATCACACCGTCT
TTACGGAACCAGATGCGGAAGAAGGGTCTCTCGGGCCTTATGATCCAGCCGAACCGATTT
CAAGACCACGAGGGGTACCTATGCTAAGTAATCACATCATTACGTTCGAGATGGCACTGG
AAGCTGAACCTTCGGTGATTCGGACATTTGAATATAAACCCTTGAGGGTTATGATGGGAC
AGCTTGAAACCTTCATTGTCATTGAACAGGGGAAGAAGGATGTGGTGTATGCCACGATCG
AAATCCCCAGTGACGGTGATATTAGTGTGCATAATGAAGGGAGTCCGTTTAGCCGTAAAG
ACACTGAAAAAGCCCTCTCGCTGAGCTTTGAACAGATCTCAGAGAAACTCAAAGCTACAA
CACCCCCATCTTCCACTCTCAACTAAGAGGTTTATTCCATGATCATCCCTAAACCAGTCG
CTAAAGAACCGCACGAATTCAAGAAGTACGGCGAATTCACCTACGGGGTTCATTCTCCCA
GTGTTGAAGCCTTGATGCCTGTTAACGAGGCCACGATCTTGGGCCAAGAAGCCACCCTCA
AGATCCTGAACGAACTGGATAGTGCAGTCATGGGTTCTGTTCGTTCCCTGAACAACCAGT
ATGAACTCTTCCGTCAAGGGATTGCCTACGGTCTCCCGTACCGTATCAGTACCGACGGGA
ATATCGAGTTCGGAGTGTACCAACGTACCAAGAAAGCTGGGGATCAACGCCTGAGTAGCT
GCTTGTCCCTGGCCCCCGGGGGTCATATCGAGAAGGAAGACATTTCCGACTACCCGGACG
CTGATAAGTTCTACGGGGAAACTTCGGTGATCGATTGGGCTTATACCGTGGAGGCTAACC
TCCGGCGTGAATTCCTGGAGGAGATCGCCTTCTACTCATTTACCTGGCGTGATATCACCG
TAGAAGTTACTCAGAAGGCGTTTCCTATTGGGTTCTTGATGGCTAAGGGTGAACCCGGGT
ACGTTGGGAATATTCCCGTCGGTGTCATCTGTCTAGATCCAGTTGTGGAACACAACGCCA
CGTTCGATATGTCCAACGAAGTGAACAACAAAGAAATTGGGTGGTTTACGGTCAAATAGT
TGGGCGCCCAACATACCGGCGAAGACGCCCACATCAAGTCGGATGACGGTGAACTCGTTC
GCTTCGAACCCTGGTCAGGTTTGATCATCAACCGAATTGACCAGATTGTGGATATCATCA
AGGGGCAGGGTCAGTGAGAGGGGATTCCTTCTGTACCAAAACAACCAGTAAGACACTTTT
GGACACGTTGTTCCAACTGTTGGAGGTTCTTAAAGAACAGGGGAAGGATCAAGCGACCAC
AGTATACCAGACCGAAAGTCGTCACGGGTTGACCGCTGTTATCAGTCCGAATGGGGTTTG
GTTCTACCATTTCCCATCCCGGGAAACACCGGTGGCTAAACTGACCTCTACGGGTGAGTA
TGTCTCTTTGGGCATTATCAGTCTGTCCACGCGAGAACGGATCGCTTTCCACGAGGAAGA
TCTCACCGTGGTACTTAAAGAGATCATTGCACACTATCAACCGCGATAACCAGGCGCTCA
GTAGGTAGGGGTATCCCCTACCTACTGGTTTCTTTATTTATTTGGAGGAAAGAGGATGGC
TGTTATTAATTTGGCTTGGAGTGGTGGGGTGGAATCCGTGGCGGTGTCTCATAAAGCCAT
GAAAGCCGGCCATACGGTGAATCCATGCTTGGTGAATGCCACGGGCAGTGCATGCGTGAC
CTTGGTGGAATTAATGGCACTCGCTAAAGTGGAAGTTTATTTCAGAGATCACACGAATGA
CTATCCAGGGTCCATTGGAAAGACCTTCGTGATGATGGACACGAGTATGGCCGTTCCTTC
CATTTACACCATTGATAGCAACAGACAGAGTCCATTCGTTCATATTGCTAATGAATCCCT
GAATGTGGTATTGGGGATGATGCAAGCGTCTCGGAGTAACATTCACCTATTCCCCAGTAC
GTGGGTAGGGTTCATACAAGAAGATGGCTCAGAACACACATTAAATATCAATGAATGGAG
CGAAGTAGATTACCAGGTGCTGCTGAATCTTCCCGCCACACTCGGTAAATTGGCGGGCAC
GGACTTAGGTTGTCGGCCATTCATAGCCCCATTGTGGGATGTTCCTAAGCGAATCCTTTA
TAACGATCTTCCCGGGGAATTTAAAGAGCTAGTACTCCCCAATCTCATTTCACATTGGTA
TACTCTAAAAGAGAATGATGAAGTGGTCCTTACGGTCTCTAAGGAGAAGGAAGTAGAGTG
GGAAAGGGCGGGTATCCCTCTACCGACACAACGCTTTGTCTTTCCTTTGAAAGATTCCAC
CCCTGCGATGAGGTTATTGGTAGGGTTGCCCACCCCTAAAGACCTAGTGATAGAAGATGA
TCTTGGTGGAACACTGCATGAGTTCACCCAGGGTATCATCAGGAGGAAATCCGGTTACTG
GATGAACGATTATGAAGAAGTTAAGGGGGTTTATCTGGATTTGATTAATGATATCTTACG
GAGTAGAGAATAGCCTTTAACACTGGAGTATTCGTTATGGCTAACAACGACGTTAAAATC
GACCGTAAACTACTGGAATCCCTCGGTAAGGTTTACGATCATATTCCTGAAGAACGCCGT

CCGTACGACCCCAGCAGACCTGAAGGGGATGCGGGTATCCTCAAGACGTAAGAGAAACCC
AAGCGATTCAACTTTGGAATAAAAATCGGGAAATGACTATGAATTTCGCTAGTCTCTACT
CCGGAATGTTTTGTGTGGTGGAGGGGTTGGATGGAACGGGTAAGACCACTGTTGTTAATC
GCATGACGGACTGGTTAGTAGAACGTGGTGTGGATGTGGTTAAGACCCGAGAACCGGGTG
GGACCACGTACTGTAACCGGATCAGGGATTTGCTCCTGTCGCTGGATGAGGGTGGAGAAG
GTCCTTGCCAAATGACGGAACTCCTGTTGTTCAATGCCGCTCGGGTACAACACCTTGAAA
CCTTGGCTAAACCCAGCTTGCAGGCCGGGAAGTTTGTACTCTGTGATCGGTATGTGCATA
CGACCATAGCGTACCAAGGGGAAGCCCGGGAATTGGGGGTAGGGAAGATCCTGGAGTTAC
ACCGCTTGCTGACCCAAGACTTTTGGCCTCAATTTGCGTTCATGCTGGATGCACCGGTTG
AGGTGGTGTTGGACCGAGTCCATCAACGGGGTGGTATGAACCGTCTGGATTCTCAAACGG
TTGAATTCTATGAAAAGGCCCGTACAGCGTATCGTCGTATGGGGCAGACAGGCCGTGCTT
GCGCACTCATTGACGCCACTGTGAGTGCAGACGAGGTGTTCAATCAAATGACCACCTATC
TGGAACGACTGTGTAATAAATATTAGAACACATCGCTACTGCCTAGCCGTAATGGGCTAG
GCAGTAGGTTGGCGT
>NewGenomeName_36
CCGCGGCTTGTAAATGAACGAACAACAATTTTTTAGCGTAGCCGTCGAAATTATCGGCGC
GTTGGGCGGTTGGTGGATGCCCCTTATGTGGCAATTGCCTAAAGAACTTCAACAGCAAGA
TTCAAAGTTTGCCGATAACGTCGGCAATATCTAAGTTCTTGTAGCCGGGCACTACGTAAA
GCGCGACGATATGAACCGCGATATTACCGCAATATTCGCAAAGCTTGAACGCATCGAAAA
CAAACTAGACAATAAGGCCGACAAATGAAGAAGCCCGAACTTATCGAATATGGGCTCGAT
TACCGGCGCTTTTAGTCGGTTCGACTGGCCGCAGTCGGCACCGCAATTACTGGCCTTCTT
GTTGCGTGTCCCGACGCAGCGTTGGCCGCTTGGGCTATCCTTCCGCACGAATTGAAGTCG
TCGATTCCGCCGCAGTATATGCCGCTTATCGGTGTTGTTGTTTTCGCGCTTTCCATCGTT
GCCCGCCTTGTCAACCAAAAGAAATTGCACAAGGGGCCGAAAGATGAATAACGGACTTCC
TTGGGTAGCCGAAGACCGAAAGTATATCGGACTTCGCGAACACACTTCGAAGACTTCGCA
TAACACGAAACTTCTTGCCATGCTTGACAGCATGGGCGAATTTTCCAACGAGTCCCGCGA
TTGGTGGCACGACGACGAAACACCTTGGTGCGGACTGTTCGCCGGCTATCGCGTGGGCGT
TGCCGGGCTCTACGTCGTCCGCGAATGGTACAGGGCGCGGGCATGGGAAGCCCCGCAGCT
TACGAAGCTTGACCGGCCCGCCTACGGCGCGCTTGTGACCTTCACGCGAAGCGGCGGCGG
CCACGTCGGTTTTATTGTGGGCGAGGATGCGCGCAGAAATCTTATGGTTCTTGGCGGTAA
TAAGTCGAACGCCGTAAGTATCGCAGGGTTCGCAGTATCACGCGTCACAGGCTATTTCTG
GCCGTCGTTCTGGCGAAACAAGACAGCAGTTAAAAGCGTTCCTTTTGAAGAACGTTATTC
GCTGCGGCTGCTGAAGTCGAACGGCGAACTTACGACGAATGAAGGGTAAAGGGGCATCGC
ATGTTTTCCGTAATTTGGGCCAAGTTTGGCAAATGGCTTGCCGCAATCGGCGAGGGCCTT
GTCATGCTGGCAAGTATCTTTTATGCCGGTCGCAAGTCGGGCGCAACCAAGGCCGACGGC
AACGCAGCAAACGTACGGGCAAACGACGGCGAACCAATCGCAGTTCGCCAAGTTAACAAC
GCCCGCGTAGCTTCCGAACAACAGGTAAAGGCGGTTCAAAATGCGAAAGAAGTTACTTCC
GATAATTCTACTGTTGACGACCACGAAGTCACTAAGCGGCTGCGCGACGAATGCTCGCGC
AACTGAATCACAAAGCATCGCCGACGCAGTACAAACGAAGCCCGTTGTAATCGACACGGC
TTGTAAATGGGTTGCGCCCTTTTGGATTTCGAAGGCCGACGGCTTGACCGCTGGCACGGC
CCGGCAAATTCTGAACCATAACGAAGCCGTCGAACGCAATTGCGGGCCGTAGTCGCCGAC
TAAATCAGCGGACAAGTCTTAACAGAACGCGGGGCTTGCGGTCCAGTGAAGCCCACGACG
ATTAAACTAGTCCGACACTATGTCCGACGAACTGAAGGAAATCGAAAAAAAGGCCGCATA
CGCGGTCCTTCTTTTGAAAGAACGCGACCCGTTTAAGGCTGCCCTTTCGCTGTTTCCGAA
CAACACGAATCGCGGGCTATGGGTTGCGAACCATTTGCCGAACGACGACGAAGTTAATGC
CGAACAAAAGCGCCTTACCGACTAAGGCGGCGATAGGGCATTTCTTCCGGGCAATGGCGA
CCTTGCCCATGACATTAGGCCGCGTATGCAAGGTACGACGCTTCCGCACGGCTTAACCAT
TACGCCGACGCCCGAAGATTACGCGAAGCTTGCGAAGCTTTACGCCTACGGTCGCGGGTT
CATCGGAAAACCGCAAACGAACGTTAACGTAAGGACGAACGTAAACCGCGTCGTCGACAT
GCCCGTGTTCGCTAACGAAAGCGAATGGAAGCGGAACCCGCACGTCAACAGCGCGAATT
GTTAGAAAATGCACGCACTCGCCATTGTTACGAAAGGGGTGAAGCCTGTTCCGTATGAAG
TCGTATTTAAGCCCCTTCCGGGGTCTCAGACTATTGCCCTTTGGTTGATGGCAGCGCACA
CGCTGTACGAAGGCGCACGCCGCCACGGCAAGACGCTTACGCAGCTTATGCGCTTTTACC
GTAACGTCGGCAAAGGTTACGGCAGGTTGTGGCGCGGCCTTATCTTCGATTTGGAATTTG
TGCATTTGGGCCGCCTTGTTGCCGAATCGAAAAAATGGTTCGGAGATAACGGATAGCTAA
AAGACGGCGGCAAGTTTTACGAATCAACGTCTGCTTATAAATGGGTTTGGCCGACTGGCG
AAGAACTGTTGTTTGGGCACGTTAAGAAGCTGTCGGATTACGAAGGCTTGCACGCTCACG

CATACCCGTTTATCGGTTGGAACGAACTTACGAAGCATCCAAGCGGCGACCTTTACGACA
GATTTAAGTCGATCAACCGCTGTACGTTCGACCCGATTAAAGACACGCCGAAAGACCCGA
AAACCGGGCGCTATTTGACGCCGAACGGTTTACCGCTGCCGCCTGTAAAGTGCGAAGTAT
TCAGCATGACGAACCTAAGCGGCCCCGGTCACAATTCAGTACAACGTCGCTTTATCACAA
TTGCCCCGCGCGGCACCGTAGTTCGTCGAGAAATTCAGATTTACAGCCCGGCGACCGAAA
AAGAAGAAACGCTCGTAATTAGCCAAATTGCTATATTCGGTTCGTATAAGGAAAACCTAT
ACCTTCCGGCGTCGTATATCGCCGAACTGGAAAGTATCAAAGAACCGAACTTGCGGAAGT
CTTGGCGATTCGGCGATTGGGACGTTACCCCAGGCGGCGCAATTGACGACCTTTGGCAAT
CGCATATACACGTTGTACCGCGCTTCGTCATTCCGCCAAGCTGGCGAATCGACAGAACAT
ACGACGACGGGTATTCGCATCCTTTTAGTGTAGGCTGGTGGGCGGAAGCGGACGGCTCCG
ACGCGACCATAGTTCTTTCGGATGGCTCCGAATTTACGTTTTACCCGCAACCCCGTTCGC
TTATTCAGATGTTCGAATGGTACGGATGCGCGAAAGACGAAAAGGGCGAATATATACCGA
ATAAAGCTTTGAAGCTTTCGGCGTCGAATATCGCAAAAGGCATAAGCGACCGCGAAATTT
CGCTAATGGCGAACGGCTGGATTTTGTCGCAGCCTTGGCCCGGCCCTGCCGACAACCGCC
TTCGCCCAGTAATCGAGAGCGAACTAGACACGGGCCAAAAGCTTATGTCGAAGAAGGGCG
TGCGCTGTCCTGCATCGGACAAGTCGCCGGGTTCGCGCGTAATCGCGTTGCAGCTTTTCG
GCGACCGGCTAGAAGCGTCTGTTAATCGCGAAGGGCCGGGTATATACTTTATGTCGAATT
GCGTTGAAAGTAGTGATTTGCTGCCCACGCTGCCGCACGACGAAAAGAAGATAGACGACC
TAGACACGAACGCAGAGGGCCATTGTTACGATATGGTGTGTTACCAGGTATTGAAAGGCG
CGAACAAAGCGGCAGCGAAATTCAAACTTGTTATGCCAACTTAAAGGAACCGAAATCATG
CCGAACGTTACATTTATTCGCCCCGAACTTGGTAAGCTGCAGCCGCTGTTTTACCTGATT
CGCGACGCAATCGCGGGCGAACCAACGGTAAAGGGGGCGCGGACCACTTATCTTCCGATG
CCGAACGCCGAAGACCAATTGAAAGAAAATAAGGCGCGTTAGGAAGCGTACTTGAAGCGG
GCCGTTTTCTATAACGTAGCCCGTCGAACCCTGTTCGGATTGGTCGGTCAACTGTTCATG
CACGACCCGGTTGGAAAGGTGCCCGCGCTGATGAACCCGAGTGTCGCGAACGCGACGGGT
TCGGGTATCCACCTTACGCAGCTTGCAAAGCGCGGCGTATCGCTGAACCTTGCTTATTCC
CCAGCCGGGCTGTTGGTCGATTACCCGACAACCGCAGCCGAAGGCGGCGCGTCGATTGCC
GACCTTGAAGCCGGTCGCATTCGTCCGACCCTGTACGTATGTGCGCCGACTGAAATTATC
AATTGGCGAACGACCAACCAGGGCGCGGGAGAAGTTTTATCGTTGGTCGGACTGGTCGAA
ACTGGTTGCGCTGCCGATGATGGTTTTGAAATGAAAACGTCGGTGCAACTTCGCGGTTTG
CGTTTGGACGAAGAAGGTTATTACGTCCATGAAATTTCGCGCGAACCGCAGCCGACCAAA
GCCGACGCGACGAAGATTCCGAACGGCAATTACCAACAATACTTCGTTTATAAGCCGCCC
GACGCACAAAGTAAGCGCCTTACCGAAATTCCGTTGATGTTCATCGGTTCGGAAAAAAGC
GATTCGAACCCGGACAACCCGAACTTTTACGACCTTGTGTCGCTGAATATGGCGCATTTT
CGCAATTCGGCGGATTACGAAGAAAGTTGTTATATCGTTGGACAGCCGACGCCTGTTCTT
ATCGGACTAACCGAAGAATGGGTTACGAACGATCTTAAAGGTTCGGTTAACTTCGGTTCG
GTCGGCGGTATTCCGCGTCCGGTCGGAGCCGACGCGAAGTTGCTTCACGAATCCGAAAAC
ACCATGCTTAAAGAAGCAATGAATACCAAAGAACGCCAAATGGTCGCACTTGGCGCAAAG
CTTGTTGAGCAGAAGGAAGTGCACCGCACGGCAACGGAAGCCGAAATGGAAGCCGCTTCG
GAAGGTTCGACGTTTTCCAGAGCAACGAAGAACGTTTCGGCGGCCTTTGAATTGGCGTTG
AAATGGGCGGCCCGTTAGGTCGGTCAAGCCGACCGCGGCGTTAAGTTCGAACTGAATACC
GATTTCGACATTGCCCGCATGACGCCGGACGAACGCCGTTTGCTTGTTGAATAATGGCAG
AAAGGCGCAATCACCTGCGAAGAAACGCGAACCGGCTTGGGTAAAGCTGGCGTCGCAACC
GAAGACGACAGCAAGGCGATAGAAAAAATCGCCAAGGATACCGCCGGAGCAATGGCCCTT
CCGACTCCTGACAACGTCCCGGGCGATGGCGGCGGCGGCGATAATGTGGGCCTCAGTGTA
TAAGGGGCGCAATCATGGCACTATCGGACAATAAGCGATTGTACGATATTGCGACGCCCC
TCGCTGTTTACGTCGAAGACGTGAAAGTTTGGCAGTCCCGCCGATTCGGGTTCGTACTAC
GCGAAGTAAACTTGAAACTAACACGCCTTCTTGGCCGTGTATGTTATAAGACCTTTGACG
GTCTTTCGAAGGCGCAGTTAAACAAGCTTGTTTCGGAATTACGGGAATCGCAATCGAAGA
TTTACAGCGCATACACGCAAACGCTACTTGAACAGTTAAATGAGTTCATTCGGGCCGATT
TGGAAGTAAACCGGCGCGCATGGGTTACGGATTATATCGAACTTGACGGCGAAGAAAGCG
ACGGCATTATTTCGGACGAAAAAGCGATACAGTTTCTTTTAGAAGTACCCAACGCCGATG
CGAACCCTTTGTCTGGTTTGGCCGCTGTTACAGGAAGCGACGAACGCATTTGGTCGCAAG
TTACGAATACGGCAATTCCGGCAAAAGGTTTGTAGTTGCTGCCGTTTATCCAGACGTTTA
CGAATTCGGCACAATCTGGCGTCGAAAGCATGATTCGCACAGCTTGGCCGAACCGTTGGA
CTGTCGAAGAAACTTTAACAGCACTTGTTGGCGATGGTACCGCAGCGCAAGGCACGCCGT
CGCAGTTGCACCGGGTCAACGCGCAAGCCGCGTGGGTAATCCATACGGCAACCGCGCACG

TTGCGGCGTTCGTTGCGGCTGGCGTTGAGTCTGCGGCATTCGGTCGTTACGTCTGGTATT
CGGTAATCGACGGAAAGACAACGGACATTTGCATAAGTCGAAACCGGCGAATCTACCGCT
TCCGCGAAGTGCCGTAGCCGCCCGCGCATATCCGTTGCCGGTCGCACGTTGCCCAGGCAA
ATACCGATAGTGACTTAGCCGAAGAAACGTTTCATACTTGGGTTGCCCGTCAACCTGCCC
AAGTGCAAGACGACGTATTAGGCGACGACGGCGGCGAAGCTTTGCTCGATGGTCGCTTAA
AGGCGAAGGATATTCCGAAGTATGATGCGGACAGGTCGCTAACTTACGACGAATTCCGAC
TCAAGATTAAAGAAATTCTTTCACGCTGATTCGGTGAATCGGCATAATCGCAAGGAATCC
TGGAAATGGCGCTGAAAAAGAAACTTACCAAAGAAGAACACGCGAAGCTTTCGGACGCAC
TGAAAGCCGAATATATCGAAGACGGCGACGGCTTCCGCCTTGACGATGACAGTGACGAAG
ATACGGACGCTTTGAAGCGCGCCAAAGACCGCGAAGCGCAGTTGCGGAAGGACGCTGAAA
AGGAAGCGAAAGAACTTCGCGAACGGCTGGAATCTATCGAAGGCGACGACGCGCGCACGC
AAGGCGACATTGCGACGCTTGAAATGTCTTGGCAATCGAAGCTTGAAAAACAGCGCGAAG
AATACGCAGGCAAGGTCTCCAAGCATACTTCGCATACGACCAAAACGCTTGTCGATAATG
TCGCTTCGCAGCTTGCCCACAAGATTTCGAACGCCACGGCAATCATCATTCCGCATATCA
AGTCGCGGCTTATCGCAGAGTTCGAAGTCGACGCGCCGGTTACGCGCGTTTTGTAAAAGG
ACTGCAATCCGTCGGCCCTTACTATCGACGAACTGGCAAAAACATTCGTTGCAAACAAGG
ATTTTTCTGCTATTATCACGGCTAGTAACGCGTCCGGCGGTGCAGGTAAGCCTTCGCAGA
ACGGCGGCGGTGCCCCGAAATTTAACGGTCAATCCGGCAAACCCGCCGACCGTTCGAAGA
TGAATCCCGCATAACCTGCGGCGCATCTTCCAGAAGCGAAGGCAAACGGAACTAAGGACG
TTTAATCATGGCACTCCCCGACCTTGCTGTTTACTCCGAATACGCTAGTTCGGCTTTCTC
CGAAACCCTGCGCCAACAGGGCGATTTGTTCAACACTGCGACGGGCGGGTCTATCTTGCT
GCAATCGGCAGCGCACCATGGCGACTTTTCCGACGTTGCCTTTTACGCCAAGGTAACGGG
CGGCCGTGTTCGCCGTCGTAACGCCTATGGTTCCGGCACCGTCGCCGAAAAGGTCTTTAA
ACACCTTGTAGACAAGTCGCTTAAGGTCGAAGCCGGTACGTCGCCTGTTCGCCTTGACCC
CGGCCAAGTCCGTTGGATTCAGCAGAACCCGGAAGTGGCAGGCGCGGCAATGGGGCAACA
ACTCGCCGTCGCTACGATGGCGGATATCCTGAACGTTGGTCTTGGTTCGGGATATTCGGC
ACTGTCGCAGGTTTCCGACGTTGTATACGACGCGAACGCGAACACCGATGCCGCCGACAA
GCTGCCGACTTAGAAAAACCTTAACAGCGGCTAAGCCAAGTTCCGCGACCAATCATCGCA
CATTGCCGCATGGAGTATGCACAGTACGCCCATGCACAAGCTGTACGGTTCGAACTTGAC
CAACGGCGAACGCCTGTTTTCTTACGGCACCGTGAACGTTGTTCGCGACCCGTTCGGCAA
GCTTCATGTTATGACCGATTCGCCGAACCTGGTTGCCGCAGGTACGCCGAACGTTTATCA
CATTCTTGGCCTTGTGCCGGGCGGCGAGCTGTTTGGTCAAAACAACGACTTCGACGAAAG
CCAAGAACCCAAGAACGGCGACGAAAACGTTCTTCGGACTTACCAAGCCGAATGGTCGTA
CAACATCGGCGTTAAGGGCTTCGCTTGGGACAAGGCAAACGGCGGCAAGTCGCCGACCGA
TGCGTCGTTGTTCACTTAGACCAATTGGGACAAGTACGCAATTTGGCATAAAGACCTTGC
GCGCGTTGTCGTCAAGACCAACTCACCGGCCGCAGGTGGACGGGGCTCCGCTCCCGTACT
TCTGGTCCTTCACAAATTCAAAGCAGTTTCGAAGATGAAACCGGCAAAAATTCTGTTATT
CCTGGACGGCAACGCGCCGACCCCCGAAGCCTTCGCAACCGCCGCCGAACTGAACGCGCA
AGTTATGTTCAGGCAGGCCCGCGCTGTTCCCTCCGAACCGCATTACCTCGAAATCTGCGA
CGGCGTTGCGGGCAAGGTTCCGAAGCTGTACCCCGACCCTCAGCCGGCAGCGAGCCAAGC
ACTCAAAAAGTAGGCCGCCGAGCTTAAAGCCCTTACTTTGGAGGTCCGCGACGCCCCGGC
ACCGAAGGCCAGCGGCAAGGCCGCCGACAAGTCCCCAGCCGCTACGCCGGGCCAGACCCC
GGCGCGGGGCCAGAAGGCCCCGGCACCGGCGCCGCAGGGCGGCAGCGCCCCGGATTGGAA
CGCCAACCCGGCGCAGTAACGAACGGACGGCCTGTCGACTAATGTGGGCAGGCCGTTTCA
ACAACGGACTAAGGAGTCATCGCAATGCCGACCAAAAAGGTTGTTTATTTCACGGCAGGC
ATTAACGCAACGGCTGGCGAACTTGCCGACATTGCGATGCTCACCGCAGCCGCCGAACCG
CAATACGAAGTTTTGGTAAACAACGTCGCAGCAAATGCCGAATACGGAGAAACCGACCGA
ATCATTACGTCGGATTACGTCGCGGGTACTGCGCCTTCGGCTTATTCGGTAGTAGACGTT
ATCGACCACGACGCAATCCCGAAACAGGTGCTTACGGCGACGCAAGCAATCGTTAACGAC
GCCGAAGCCTTGTTCGTTCCGGTTACTGGCACTTACACGACGACGGCACCAGTTAGCGTT
GCGAACGGCGTTGTAACCGGCATCGTTCTTTTGTAAGGACGTGGAAGCATGGCAATTACA
ACCGCTGTCGAAGACGGAAGCGGCGTAACAAGCGCGAACTGTTACGTAAGCTTCGCAGCC
GCGCGCATTTACCATTCGAATCGCCGCGTCGATCTTCCGTTGGACGACGACGAATTGGCG
GCTATGCTGATTCATTCAACGGATTATTTAGAAACGCCGGCTCGCCGGTTCCAAGGCAAG
CCAACTACGACGACGCAGGCCTTGCAATGGCCGCGAACTGGCGTCTTTCTGAACGAAGAC
GAAGTACCGTCGAACGTTAATCCGAAGTTTCTTATTGCCGCGAAAGTTCAACTTACGATG
GCGATTAACGCAGGCTTCGCCCCTCAACCGAACGTTTCGCCGCAAGACTACGTTACGCGC

GAAAAGGTCGGGCCGATTGAAACGGAATACGCCGTCCCGCTGTCGGTTGGGATCATGCCC
ACCGTTACCGCAGCGAACGCGCTTCTTCCGCCGCTGTTTGGCGGATGCTCTTCGAACAAG
TTTGCGCTTCGAACAATAAGGGTTTGACGAATGGCACAATTCGATCGCGCAATTAAGACG
GCCCAACAGCTTCTCGCGAAGAACGGCGAAAAGGTAAAATGGCGCGTTATCGAAGACGTT
ACGCCAACCGGCCGGAACAAGCCTTGGGAACCCCGCCCGGATTTGCCCGAAGACAAAGAC
GTTACCATTTTCTTTTTGCCAGTTGACCGGCAGACGCGAGAAACCTTAAACTTCATTCAA
GGCACCTAAGCTCCGAAGGGTTCCGGGATGGGGCTTATGGGCAATGTTCCCTTTGCCCCG
AATTTGAAAGACGTTGTAATTCGCAACTGGGTTGAACTTCGCTTGGCTTATATCGACGTA
TATTCGCCGAACGGGCAAAAGGGACTTTATACGATCGTATTTCAAGCATGATTGAATTCG
ACCAAGTTAAGGAAGAAGTAGACGCGCTTTTTCTTGCAACTTGGAACGCAGGAAGCGCGG
CAATTGCGGGCTATGTTCCCGAAATTCGTTGACTGGGCGTGCAACATCGCGACTTGCCGT
ACGGTTCGAAGTTTTGAGTTCGGCTGTCGAAGCAAACCGTTGTTGAAGAACAAGCGACCC
TCTCAACCTGCGAAGGAGTACCGGGGCAACGAAAATACACGGCGTCGGGTGTTGTCTTCC
TACAAATCTTTTGCCCGACATCGAATACGCAAGCGTTCGAACTTGGGCAGAAATCTGCGA
AGCTTGCCCGCAATGCTTTTCGCGGGACATCAACTCCGGGTAAGGTTTGGTTTCGCTATA
GGCGAATTAACGAACTTCCGCCCGAAGAACTTTACGAACGGCTTAACGTCGTTACCGAAT
TTCAATACGACGAAATAGGGTAAGGAGTTCTTAAGATGGTGTGCGAAATTGCCAAAATCG
ACAGCACCATCACCGGACTTGCCTTCGCCCAAGAAGAATGCTTGAAGCAAATTCCGACGA
CGCCCGTTTGGGATGGACTGGAACCCAATAGCTATTCGGACTTCGGTGGCGAATTTTCGA
CCGATGCCCGCGCGCCTATCGACCCGTCGCGCCAGAACAAAAAAGGCACGATTATCGACC
TTGACGCATGGGGCGGCTTCCACGCGCACTTTACGAAAACAAATCTTGCGAGAATTCTGC
AAGGCTTCTTTTTCCCAGACGCGCGCGAACTTCCTTCGACGCAGCCGCTTAACGGCGCAT
AGGTCACGCTTACCGACGTTACCGCAATCGACAGCACTTACGCCGCAGCTTCCGGGCTTG
GCGTGTTTGGTGCCGATAAGCTGGTATATGCGACCGGCCTTGCCAAGGCGGCGAACAACG
GCCTTAAAACCGTCGTTTATGCTACCGCTGCTGTCGTCGTTGCGGCTGAAACCTTGATTG
GCGAAACCCCGCCCGTAGGCGCGAAGCTGGAATGCGTCGGTCGCCAGCTTGCCGCCGCAG
ACGCGAACAATGCCGTAACCGGCAACGTCGTTTCGCTTGTCGTTACCGCTGGCGACTTTA
CGACCATGCCCGAACTGTTCCCCGGTCGTTGCGTATTTGTCGGCGGCCATGTGACGGCAA
ACCGATTCGCTAATAATGTCGGCTATCCCCGCATTAAGTCGGTTGCAGCGAAGGCGCTTG
TTTTCGACGACGTAACTTGGCAGGCCATCAACGACACCGGAACCGGCAAGTCGATTCGTC
TTTTAGTCGGAACTGTTATCAAGAACGAAATGACCCCGGCGCTTATCAAGCGTCGTTCGT
ATCAAATCGAACGCACCTTGGGCGAAGGTTTGAACGGTACGCAGTGCGAATATCTGGAAG
GCGCAGTACCGAACGAATGTACGTTGAACGTTCCGCAGGCCGACAAGCTGAACGCCGACC
TTTCGTTTGTTGCATGCGACAACAGTTATCGCAGCGGCGACCCCGGCGACGAACAGAAAA
GCGGAACCCGCGTGCCTGCGCCCGGCGAAGATGCTTACAACACTTCTTCGGACGGTTCCC
GAATAAAGATGGCCGCTCACGACGCCGCGTCGTCGAACCCCGCCGCCCTGTTCGGCTACG
TTTCGGAAGCGAACGTTTCGATTAACAACAACGTTACGCCGAACAAGGCGGTCGGCGTGT
TGGGAGCGTTCGATACTTCGGCGGGTAACTTCGAAGTCGGAGGTTCGATTACCGCTCACT
TTACGACCGTCGCAGCGGTTAAGGCTGTTCGCGCGAACGCCGACGTTGGTTTGTCGGTAA
TCAGTGCGGCCAAGAACCCCGGCTTCGTGCTCCATATTCCGTTGCTTTGTTTGGGCGGCG
GTCGTCAGATATTCAAAAAAGACGCGCCGATTACCGTTCCGCTGGAACCCGCATGCGCAA
AAAACGCGTTCGGCTATACGATGCTGTACGAAGTGTTTTCTTATCTGCCGAATCTGGCTA
TGCCGGACTAATTGCGGTAGACTCTAAGAGCCGGGTAATACCGGCCCTTTCGTCATGCAA
TCGGAGTAAATCAAATGTCTGGACTGTTTAAGCAATTCAAAACGAATTCGACCAAGGAAG
TGGAAGGCGACGAAAGCGAATTTCTCCAAGCCCAGATCGACGACGGCAGCGTTCCGACTT
TCGTTATCTCCCGCATGGGCAAATCGAACAAGTCGTATTCAAATGCGCTTGACGCGGCGA
CCCGTCCTTATCGCCGTCAAGTCAAACTTGGCACGCTGAAAAACGCAGTCCCCGAATCGC
TGTTTATGGGCGTGTTCGTCGATACCGTATTGCGCGGCTGCAAGAACGTTAAGGGCGAAG
ACGGCAAGGAAATTGCGTATTCGAAAGACGCCGAAATTTCGCTTCTTTCCGAACTGCCGG
ACTTTTACGAACGTTTGCAGGAAGAAGCGAAGTTGGCTTCAAACTTCCGCGAAAAACCTT
TGGAAGCCGAAGCTAAAAACTAACAGAATTTTTGGCGCACCTGTTGGAACTTGGCCCACA
CGCACAGGCGATAGCAATTCAAGCGATGCGCGGGGGGCAACCGTTACCCGAACGCATCGC
TAACGCACCAGTACTTGAAGTCGGCTTGCAGTTGTATTTGCAAGCCTTCTTCGACCTAGA
TAGCGTACGGTCCGACAGCAACGGTTTAACGCCGATACCTTGGACAAGTATTGCGGCTTA
CGCAAGGGCTTTCCAGTTTGACGAAGATCAAAGCGATGACTTGTATCATTTTATGCGAAA
GCTTGATTCGGAACATTTTAAGAAACTAGCGGACAAACAGAAAGCGGCGGCATCGAATGG
CAATAAGCCGGCTAGACGTAGCCGATAGGCTGGAAAAGAAAGCGAAGGCAATAGACGAAG

CGGCCTCGCAAATCTCCGTCGATACCGCTCTGGCTATTGTGGGCGACGTGGCTTACAAGA
CGCCCGTAGATACCTCGCAAGCTTTGTCTAACTGGATTGTAACGCTAGAAAGTCCGTCGG
GTCAATACATCAAGGAGTATGTTCCGGTTTCGCAGGGTTCAACGCAACGCGCTTCGGCGG
CTGAAAGGTTGAATTCTGCGAAGCTGGTTTTACGGAACAAGAAGCCCGGGCAAGCGATTT
TTATTACGAACAAACTTACGTATAGCCGCAGGCTGAACGACGGTTATTCAGCACAAGCCC
CAGCGGGGTTCGTCGAACGCGCGGTTTTAATTGGCCGCTAAATGCGTAAGAAGTTCAAGA
TTAAGGATTAGAAGAAATGGCCGACGAAAACATAGAAATTTAGGTTCAAGACAAAGTTTA
GCCGTCCATGTCGACGAAGCTTCGCACAATCGCCAGCGAAGCCCGCAACGCCGACGCCGC
AGTTAACAACTTTCAAACTCAGCTTTCCGGAATCAGAGTCGGCGCCTAGTCGTAGCTTAT
CAGCGCATCGGCCGGCGCAACGCGCTACCTTCAACAAGGCGCACTTGCCGCGCAACAACT
GGCAGCCGAACAGTAAGGCCCCGCACGCGCCGCAGCGCAGGCCGCAGCCGCCCAAACGCG
CGACGCCACGGCTGCAACCCAAGGGGCCACGGCGCAAGCCATCATTGCCACGGCAACGCA
CCGCACGCAGACGGTCCAACAGCAGACGGCCAACGCAGCGTAGCGCCTAGCGACAGAACA
GCAGCGGACAGCCGTTCAGACGGCCAACGCGGCGGCAGCAAACGACGGGCCCGCCCTTGC
CGGCCTGCGGCTGCAACAGGCGCAAGACCGGGCCGCTAATTCCACCCGCAACGCGACTTC
GGCCCTTGGCGGATACATTCGAACCGCTGCCGTAATTCTTGGCTTAACGATTTCTTCAAA
CGCAATTCTTGCAAGCGCCGACGGATAGGTTAAGCTTCAAAACAAATCGCAGAACTTAAC
GAAGTCGCAAGAAAATGTCAACACGCTTACGAACAAACTTTTCGAACTTTCGAACCTCAC
GCGCGCGGAATTGAAGAAACGGCGCCAGCGTTTACGCGCTTCGACCGCGCTTTGGCGTT
TATGGGCAAGTCGCAAGAAGATTCGTTGCGAATGACCGAAACCATTAACAAGGCTTGGAT
TTTTTCCGGCGCAACAGCGCAAGAAGCGTCGTCGGCTTTGTTGCAGCTTTCGGAGGGCTT
CAACGCCGGTAAGCTGCCAGGCGACGAATTCCGCGCCGTTCCGGAAAATATGCCAATCGT
TTTGGACGCGGTAGCGAAGGCGCTGAAAGTTCCAATTAACCGGGTAAAGGAACTTTCGAC
CGAAGGTAAGATAACTTCTGAAGTTCTGGTTGAAGACTTTAAGCTTATACAAGACCAAGT
AGCCGCAAATTTTAACAAGACGACGCCCACAATCGGCCAAAGCTTGACGGTTCTTCGCAA
TAACGCAATTCAGTTCTTCGGGGAACTGAACAAGGCGACCGGCTTTACTGCCGGGCTTTC
GCGCGCGATTATTTGCCTTGGCGAAAACTTGAAAACGGTTGCCGTAATCGTTGCCGGACT
TGGCGTCGCGTTGCTTGTTGCCTTCGGTCCCCCGTTGGTTGGAGCGCTGGCCGGTGCGAC
GGCTCCGGTAAAATCCTTTACGCTGGCGCTTGCTTCGAACCCGCTTGACCTTATCGTCGT
CGCACTTTCGGCTGCGATTGCTTACCTTGCGCTTTTCCGCTACGAAATTAACTTAGGAAT
CGACGACTTTACGACGTTGGGCGATTTCTTCCCCGCGGCCTTTGAAGGCATCGGCCAAGC
AATCGACGACGTTAAAACTATCGCGTCGAAATTGTGGGCAGAACTGCGCGACTTCGCAAG
TGCCGCACTTGGCGAAATTAGCTCGAACGTTGACGATTCGACTTCTTCTTGGTCGGACAC
CTGTGCCGCATTCTTCCAAACCAACCGTACAGGTTGGGCAGGTGAGCTGGAAAACGCCGC
AAAAGTTCTTGACGCAATCGCCGGGCTTTTGACCGGGGCCGGAACGTTTGCAGGCCTTGC
ACTGGCCGAAGTTGTTATTTCGGTTCAAAACGGAATCGCAAACGCTGACAACGTTGTTGC
GGGTTGGAGCGAAAACGTACCGAATAAAGCCATTCAAGCCGCGAACAGGCTTCGCGCGAT
GGTTGGTAAGTCGGCTTACGAACTTGTCCAATTGCAACGCCTGGGCAGCGCAAGCCAAAG
CGAATTCTAATCTTGGAGCAAGCTTTGGGCGCAATGGCTGGAAGATGGATGTAACAGCCA
AGGCGGAGCAATGAAAAACCTGTTAAACGGCCTTTTTGACCGTACGCAGCAAATCGGCGC
GCGGCGCAGGGCCGCAGGAAGTGGGCAGCTTCACGGGGCCGGTACGTCGCAGTTTGCCGG
GCCTACCGCCGCGAACGCGGCGAAGGCTACGGAACGTCGCGCGCTGGCAATGGCAAAAAT
CAATATGCAGCTTGATAACGAATTCGCGCGTTTATTCCAGCTTCAACCGCAGCGCGAAGC
GCAAGTGAAGTTCGACCAAAACGAAGAAAGGTTGATTCTGAAGAAGATTAAGATTACTTC
GGAAGAAGCCGAAGCAATCAAGGCGAAGATTAAGGGCGTTCAAGACGCGACCGAAGTTCA
ACGCCAGTTCGACGCAATTTCCGCCGAAGCTGTCAACCCGTTGAAGGAATACAACGCTTC
GCAGGACGCGGCGAACAAGCTTCTGCAAATGGGCGCAACTACGCAGGAACAGCACGCCCG
CGAAGTAACGAAGGCGTCGGAAGCTTACTCAAATTCGCACGACCCGTTGCGGCAGTATAA
CCGCGACCTTGAACGGCAATTGCAGCTTTTGGAAATGCTGCCGAAGCAAGGCGACATCGA
ACAGCAGATTATGCAAGTTCAGAACGATTTGCTTGAAAAGGGCATCGTTCTAAACGAAAC
CGAATTGGCGCAGCTTCGAGAAAAGGTGTTGCTTATTCAGCAAGTAAACGCCGGTTCCCA
GAAACACGCGGCGTTGTTGGACGCCAGCGTAAACAAGCGCCAACAGTTTATCGACCAACT
TAAAGAGATTCAGAACTACCGCAACAATAGCGGAAGCAGTTTCAACGCAGGCGACCAAGC
CGAAGCAACTAAGAGCATGTTGCAAGGCATGAGTATTGAGACGACGGAGTTTCAAACGCA
TCTCAACGCGCAACTGGCGCAATATCAAACTTACGTCGAACAGCTTAGAATGCCGAACGA
ACAACGTGTGATTAGCGACCAAGAATACGCCGCCGCAAGTAAGCAACTTGTATTGCAGCG
GCAAAACCTGTACTTGAATTCGGCAAGCAGTTTCTTCGGAAACTTGGCCGCGTTTCAACA

GTCGAGAAATGAGAAAATGGCGGCGGTCGGAAAGGCCGCAGCAATCGTGCAAGCCATGAT
TAATTCGTATCAATCGGCAACTTCGGCTTATTCTGGGATGGCGTCGATTCCTCACGTCGG
CCCAGCGTTGGGCGCGGCTGCGGCAGCGGCTGCAATTGCCGCAGGCTTGGCGACCGTTCA
GCAAATCCTGTCGCAGAATACCGGCTTTCAAATCGGTGGTTATACCGGGTCAATGGGTGT
AAGCGAAATCGCGGTCGTCGTACACGGCCAAGAATTCGTTATGAACGCATCGGCAACCAA
CCGCATGGGCGTTGCCGAACTTCAAGCCCTGCAAACGGGCGCGCCCAGTGTTCAGCGAAA
CGACCAACAGGCAATGGCGGCTAGTGTGGGCAGGGGCGGCAATTCCGAACCCGGGCCCGC
GCCGGTTGGCAATGCACCTTTAAGCGCCGTTGTCGTACAATCGATAGAAGCGGCCTTAGC
AGGGCTTTAATCTTCGGACGGCCGGGCTTTCATTTTGGGAACCATCGAACAAAACGGCGG
CACCGTCGCAAAAATCGTCCGATTTAAATAAATGGGTTTTGCAATTGGAACATTTGCGAA
AGGCGGCGGGGACGATAGCCATTATCAGGTTTTGGCAGTTATCAAAACGCTGGCGGAAGC
TAACCGCTGGCCAACCCATCGTTACGTGTCAACCGGACTTGACCGCTAATTAATTTTGAA
AGGCGTTGGCTTGTCCGGTCTTGAAGAAATCTTTGTAGGGTTCAAGTGTTACCTAAGCGT
CGGCGGCGATTATTACAATATTAATTGTGGAACTTTCGTCGGTTACGTGAGCGGAAACAG
TTTCGAAACACAACCGGGCGCGCACTTTAGCGCAATACCGGCCCACAATTACGCGATTAC
GTATTTCATATCGGCCAACGCCCAACGAAACGTCGGATGCTACAAAGTCGGTACGCCGGT
TTATGAACATTTTTACGTCGGCAATTGGTTTCAGTATGCGCGGCCCGGTGAATATCCGTC
GCCGCTTGTTTGTGCTAGCATGTTGAACGGGTCAGAAGCAAAGCGTTTTCCAGACGTCAA
CCAGCAATTCCCATATCCGGGCGGATATTATTCGACGGCCTGTTATATGTGGGTTCGTTA
TCAGACCGGCCTTACGTCAAAAGTTTGGTCGTATCCTAACACAAATGCAAATACCGCAAG
CGGTCCGCCCCTTGCAGGGCCGCAGGGTTATAACACGCTTGTTCCCGCTGGTGATGATTA
CCAACTTGAACCTATAAACCTTTCCCAATTAGAAACAAGCGCCGGAATGGGCAACGTTTG
GGGGGAATTGGACGGCGTTTATTTTTGTTCCGGTTTCAACAATGGCCCCGAAAACGTGCT
TCAAATGGGCGGAAGTTCTGTTGAAGACCAAACAGGAATGACGTTACATCAAGCCGTAGA
CGCAATTATAGCAGTTGGCGGAACCGCTTTTGCAATGTGCCAGAACGGCAACCGTACAAC
TTGGCGCGATTTCGTCGCTTTGGAAATGAAATAATGGCCTACTATAACGGAAGTGCCTCT
CCTTTCGCGGATTTGAAAACCGCTATTGAAAATGCTTGCGTCGCTAACGGATGGACGCTT
TCAAACGGCATTCTTTCGAAGAACTGTTGTTTTTTCCAACTTGTCCCCACTACGCCCCAA
CTTACGTTGCAAGGCGAAACCGGACAAACTGGTTCAACACTGAACGCCGGCCCGACGAAC
CTTGTAAAAGTTATGTCGCCAACTGGAACGCCTATCGTTTTTCCAATCAATTACGAAATT
CACGTAATGACTTCGCCGGACGACGAAGTTGACTGCGTGTTGAACTATAATTCGGATTTC
TACCAACAACTGCCGTTCGGAAAGTCGTTGATTTCCGGCATTGGTGGAACCGGCCCATGG
TTTACCGGCAGTTACGATAGTCGCATATAGCAAGCCGGAAGCGGAAACACTTTCTATATG
ACTTCAGGTAGTGTCATGGGTTCTGCCGGAACGCGCAGTTTTTAGGCAATAGCAGGCTGA
GTTTTCTTCCACGCGCTTGGAAGCAAGAAGGGATATACTGGCAATTATTTTCATCGAGGG
ATTGACGCTGTAAATTGGTATAACCCAAGGGGTTCGAATTTGAGATACCCGCATTGAGCG
GGAATAATAGATAGTTTGCCGGATTCTTCAAACATGACAACAGTTCTTGTGCCAGTAAAA
GGCATTAAGTCAAGAAGTCACGGCGGCCTTACGATAATCGCAAACCCGCCAAGCGTTCGT
GTTTGTCGAATTGATAATTTGGAACCGGGAAGTTTTGTCGTTTACGGGGCCTATCAATGG
AAAATTTACCCATTTTACAGAAAGAACCTTGCGCAAAGGAAAGGTTCTTCAAATGCTTTA
CAAAGCGGAACTTTCGGATACGCTATAAAATACAATGGAAGTTAAAGGATGGCCGGAAGA
ATTGGCGAGTTTACGCAACAGTATATATATGGCGGAGATAACCCCGATTATTCTTCCGAA
CTTGACAGATATTCGGAAGTTGAATATTTGCAGGTCGGCTTTGCCGTAATCGGGCGCCCC
ATTTTCGGCCCTTCTGCGGATCGGTGCGTACATGCGGCGAACCGCTCGCCTATTGACGGT
AAGGAACAGGCGACCTTTCTTCGCGATTTTTATTTTCGCGTTCACGTTTCGCCGTAACTT
TTGGATTTACAAACCGATGCGTCGTCACAAACGCGCGGATACAACGTTTGGTACGCATGG
CCCGACCTTGCGGCGAATCTTGACGACATTTTGGTAAGTAATCCGGTCGGTATTGAAAGT
ACAGGCCAGGAGACGCCGTATGCAATGCCGCCACGTCAAGAACTTACTTACGAAATAACC
GCAGGCGTTTCGGGGCAGCCGAATATCCATGTTGAAGTGCAATTTGATTTTTCAAACGTC
GGCGACCCTTTACCGATTTTGACTACTGGCGTTGGTGCTGTAAAATTCGACATTGTGCCC
GAAGTTCCGTTGAACGAACCTTGGGAAGGGTTAAGCGACAATATTGTAGCAATAGACGGA
ACACAACAGCGCATAGCACTTCGCGGCGAAGTTCCGCGTATTGCAGAACGCTTGAAAGTA
ATTTTTGATTCTGTCGAAGCCCTGATCCGTTTTTACGGCGACGTTATGTCGGCAGTCGGT
CGGCTTTGGATTCCCGAATTTCAGTACGCGTCCCGAATTACTGTCGCAAGCAATAGCGGC
GAGCTTCAACTTTATCTCGACCCGACGAAGACCGACGTTCGCGATAGCGAATACGGTTTG
GTTCAAACGCCGCTTGTCGCTGTGCGTTTTGAAATTGACGTATTGACAGCCGACTGGACT
CCGCTTGGTTCGCCATTGCTGTTCGACATTCCCGAAGGGTCGTTAATCGTTCCGGGGTCA

CCTGAGCTTATTGACGACAATACAGCACTTGTGCGGTATTCCGTCGGTACCGTCGGCGAA
GTAGAAATTAACGCGAAGATGATTCGCCAGCGTTCGCAACTTACCCGCCCCGGCGCAACC
GTCCTACTTGAACAGGTCAGCGGCGTGCCAGTTTTGTCGAAGCGCCCGCTTGCGTACGAC
CTTATACGCGACGAAGTTTCATCCGGCCAAATTTCAATCGACAATCAAACCGGACTTGCC
GAAATTATCTCGCGTTGGGATTATAGCCGCATCGGCGGTCCGCGAAGCTTCAAAGTAATT
CGTATTTCGAATCCCGAAGAAATGGAATATTGGAAATCAATTCTTGCGTATGCACGCGGA
CAGGCGCGCAAGTTTAGGAGGCCCACATATCGATCCGATTTGGCCTGGGCCGTGCAACCG
TCCGACGGGGCTGCCGTCTATAGCGTAGAAGAGTCGGAGTACGCCGAAAAAATTTGGCAA
ATTATAACGCATCGCTGTCTTGAAACAGACAAAGCGTCCGGCATTCATAGAACTGAAATA
ACAGGCGCGTCGAAAGGGGAAGAAGACGTTTCTATAATTGTTTTAAATCGCCCGCTTCCG
AGCGGGACCGAATGGATGGAAGTTAAGCGAATTTAGTACTTGTTGCCGGTACGCCTTAGC
GACGATAAAGTAGAATGGAAGCATTTCGGTCTTGAAACAATCTTGAATCTTTCAATTCGA
ACGGCGGAACCCTAATGTCTGACTACGACGACAAAGAACAAAGCGTAGGCGATAACGCGG
GATTTGAACTTTACGAATTCGTCGGTTCGTATCGCAATTATTACTTGACTTCCGACGCCC
TGTGGCACGACTTCAACGGTGCGACATATAACCCCGTTCCGGGTTTGAAGCGCAGTCCGC
TGATTGTCGGAACGCACGAAGACGACAACGTAGATAAGACGGTTACGCTTCCGATTACGG
AACACATCGTTACAGACTATGCGCTTCAAACTACGCCGCCATCTTTGGTTTTGACGATTT
ACCGTCGCCAGAGCTACGCCGCAACTTGGGTTGCGGACATGAAAGTGCCGATTGCTTCAA
TTTCGGTAGACGACGAATTTGTAACGTTCAGAACGCCAAGCAAGTTCGGTTCGATGCTGT
CGGCCAATATACCGAACGTTTACGTACAGCCGCCTTGCAATAACGTTTTGTTCGACGAAT
GTTGCAAGGCAACCCGCGTTACCAATTCGCTAAATACGCAGGTTTGGGCGGTCGAAGGTC
GTAATATTTCGATTCCGTCGCTTGGCGGTATTCCCGACGGTTGGTTTATCGCCGGCGAAA
TCGCAATACCTGCGCGCAACGAACTTCGAATGATTGTCGCGCAAGCCGAGGGAATTCTAA
CGGTAAATTACGGATTCGCGCTTATTTCTGTCGGCATTTCGGTTCAGGTTACCGCAGGTT
GCGACCATTCTTACAACGAGGCCAACGGTTGCCCCAAGTTCAACAATCAACCGAACTGCG
GCGCTTGCCCGTTAGTTCCGGGCGAATCGAATAACGTATGTCAAAGCGGGGCTCATTAAC
AAATGTGGATTGCAATTGTCTACGGCGTCGCCTTCTTGCTTATGGCGGCGCTTATGCCGA
AACCGAACATCGAAAACGCACGCGCGGCGAAACTTGGCGACTTCCATGTTCCGCGTTCTA
AGTACGGCGACCCGATGCCGCTAGTGTGGGGAACAGTCCGCCAGAAATCGCCTATAACGG
CTTGGTTTGGCGACTTCGGTCCGGTGCCAATTCCTAAGAAAATGAAGACCGGGCTGTTTA
GTTCGAAGAAAGTTACGGTCGGTTATAAAAATTATCTAGGCATTGATTGCATTTTGTGCC
TTGGCCGCGGCGTTCGACTTAGTAATTTGCTGACCGGTACTTACCTTGTTTGGGAAGGCA
ACGTAACTTCGTCCGGCGATATTTACATTAACAAGCCGTACTTGTTTGGGGGCAAAGACG
AACGCCGCGGCCTGCAAGGTACGATTTCGTTTGATGACGGTCGTTTTGAGCCGCCGCAAG
ATTCTTACTTGGTTTCTAAGCTTGGCCCGAATGTTCCGGCTTACTACGGCTTGGCCTGTG
CGTTGTTCAAAGCCTTTTATATCGGAACAGGTACGAAGCCCGAACCTCTCAGCTTCGAAA
TTCAGCCCATTACATCCGGCTTGCACGCGACTTATTCAATATTGCCAAACGTGTTGCACC
TTAACCCGATGGAAATTGTTTACGACGCAATGACGCAAAAATGGGGACGCTTCGGCAACC
TTGAAAGCGAAATTGATATGTCTTCGTTTGCGGCCTGCGCGCAAATGCTGTACGATGAAG
GCTTAGGTATGTCGCTTATTGTTCGATCTGCGATTAGCGGAAAGGACTTGATGGAAGAAG
TAATGCGTGTCGCCGACGGCGTGTTGTATCAAGCCCCGGCAACGGCGAAAATTGTTGCGA
AACTTCTTCGCCAAAATTACACGGTTTCCGAACTTCTTGTTCTTGACGAATCTTCGGATA
AAGAATTTCGCAACTTGCAGAAAACAACATGGGAAAACACTTTTAACCAATGTCGCGTTA
CATTCAAGAACCGCGCGAGTAGTTACGACGACAGCGTAGCAATTACCCATGACTCCGTAA
ATATCAACTCTCCAAACCGTGTTAAGTCTACGGAAGTTTCGACGCCCGCCGTTACCCACG
CAACGGTAGCAAGTAAGATTGCGGTGCGCCAACTTACGATTTTGAACGTTACGCTTTACC
AGTGCGACTTGACCGTTAACCACAAGGCGCAGAACGCGCGCCCCGGAAGCGTGTTTGTTC
TGAATTGGTCGCCGTTCGGCATTTAGAATATGGCTATGCGTCGTACGAAAATTGATTTCG
GCGAACTTAGGTCCAACCAAATCAAGCTTTCTTGAGTTAAAGAGTGCTTTTCGTCTTCGA
CGCTTACGTTCGCACCGCCCGAAGGTTCAGGATGGCCGCCGATTGGTTCGGAACCCACAC
CGGTTACAACGCGGCTTTTTTTTACGCCGCCGGCGTGCCTTACAGGATACGACGACAGCG
AAACCAAGGCTGCGTTTGAGTCTGCCGGGCGTCTGTACCTTATCGCAGTTGCGCCGGGTT
CTACGTCCATTTCTTACGAGGCAATGACGACCCCGGACAATTTCAGGTCGTCGCCTATTC
TGTCGCTTGAAAGCGCCTCGTACAACGGCGGCGGCGTGTTGCTGAACACCTACGCTTCGA
CCATCGACAGCGAAACCCGCAACGATACACGCGGTAGCCTTATCGTAACCGGCGTGTCGC
AAGCAAGCAGCGAAGGGTTGCAGCAATACAAGATGCTCGACCAAGCGCGCGACGCTTCGG
ACTTCCGGATGATTAATAATGAACTGTTTGTCTATGTGGGCTTCGTTGACAATGGCGGCG

GTCAAATTACGTTCCCGAACGTATATCGCGGGTTTCTTGATACAACACCGGGTAATCTTG
CGGCGAACGACCGCGTTTGGTTCATTAGCGGAACAGTCGGACTGTTGCCGGAACTAGTTG
CGGGCGGAACGACGCGATACGTTAAGCTTTTGATACGACTTCGGGCGGGAATCTTGATA
TTTAGTTGGCTCCGTCGTTCAGCGGATCGTTTACCAACCGGGCCGGGTTGCCGCTGCCGC
CGCAGTACCTAACGCTAGCACAAAGCCGCACGCCAAGCCCACAAGTAGGCGCAACGCCGA
TTGCAGTTGCATGGCGCAACCGCAGCCGGGTCGATACCCTGTTGCGCGTGTATGTCGACA
CGGCGAACACGCGGGAAGCTGGCACCGAAACCCGCATTCGCTGGCGCGTAGGCGGCGGCG
GTTATACGACCGTTACGACGGCCGGAAGTTCAACAACCCTTGACGGAACGGGCAAGTACG
GTACGCTAGAAGTTATTGTAAATTCGTTTATTGCGTCGAATTCGAACTATTCAACGTACA
GCGATCCGCTAAAAATGACCCTAACGGCACCGCCACGGCCGTAATCAAAAGAACGACAGC
GCGGCGGTTTTGGCCTTGTTCAACCGCCCGCAGGCGTACAGCGTTTCGACCGCTTCGTTA
ATATAACAGTCGTAATTTATATCGTCGGGAAATTCCGTCGGCAAGTCCATTACAGGGCGT
GCGCCGTCGGTTTTCCCGACTTTGTTTCCGCTGGATACGTAAGCAATATACCCGGGTTCG
TTCTTCGGGTAATACCAACGAACAACCTTACCAATATAACGACTGTTCTTTTCGCCGCAG
CCGCGCACGTTCTTTACTGCAAGGAAGCGCCGGAAGTTGCGGCTCTCTTTAATCGTCTTT
TAGAACGGCGTTCCCTCAACAAGGAAGCAAACAAGCCCGTCGGAACAAATCAGGGTTTCG
GGCTTCTTCGAAAGAATCGAATGCAAGGCCGACCCGAGTTCGCAATAAGCGCCCTTCTTG
TTTGCGCCCAATTGTACGTCAATATAGCGCGCATCGGGGTCGCCGCCGTCTTCCTTGATT
GCGACATAACTGTTAACGTCGCGGCTGAATACGGCCTTAAACCGCGTTTGTTCGGTCTTG
TATCCTGTACATTCTTCCCAAGCCGCAATAAGCGCCCTGACTTCGTCGTGTCGCGATTTG
TGATACTTCGAAATAATGCCGTCGGTATTGCCGGAAATAACTTCGATGCCGATTTGTTCC
AAAGCTTCGATAAGCATCAGCAACACAAGTTGCCCGGTAATCGATAGTTGCAACATAAGT
TGCGTCACGTAAAGCGTCGAATACTTGTTGCCAAGTTTGCCGAAGCTTCCGATAGTGGTA
ATCTTCAAGCTGTCGGCAACGGTCTTCCATTTCTTCGCGGCTAGGCGGGCGCCCGCTTTC
TTTGCCTTGGCCGCTTCGGCTTTGGTGTGAATTCGAGTACTTACGATTTTTTCGTAAACG
GTAAAAAATGCGTCGACCAAGTGCGGCGGGCAAAGTCTTTGATTAAGGATTGTTCGCGGG
TAAAACGATCCTACGCCGTTGTCTGCCAAAATTATTTCGTCGGTCGCAGAGCGCGCCGTT
TTCTTTTCGGTTAAATGCAAACGGCCCATGCCTGGTTTATACGTCCAACCGCCTATGGTT
ACTTTCAGCTCTTATTGTTCGGGCGGCATGGTTTGCGACCCCAAGCCGTCAAGATAGAAG
CGGGCGTTTCGCACGACTTCCAGCATTTCGCGACGCTGCGTCGTTTGATAGCAAACGAAG
TCGGGCACGTCGTAAACCAAGACCGTATCGGCGGATAGTGTGGGCTACTTTGGATAATAG
CCAACGACTTATTGAAGTTCGGAGTAAATAACCGCGTCGGCTACTTGCGCGTCTGATTTC
GACCGAAGGTCTACGCCTTATTTTTCCGACATTTCGGCCCGCAATTTCAATTCGGGCGCA
AGTTCGTTCAACATAAGTTACGTATTCGAAAGGTCGTTGCAGCAATAAGGCCCCACTACA
TCGGCGTCTTCCTGCGTCAATACATGGTCTTCCGGAATGGGCAAGTCTCGCAAACGTTCG
CAATGCAAGCGCCCTTCGTACAGCTCCAACGAACCTTGAAGCGGCTCGACTTCGATAAGG
TCTATATGATTCTATCGCCCAATTTGGACACCGTCCTTCTTTTCAAACGCGAACTGCGTT
ACCTTTTCAGCACCATAGTTCGGCCCGCTATTACTGATGAAATCGGACGCTTCTTTAAGC
TTCTTGCAATCTGCGCCGCGTGCGGCTAGTTCAATCATCGGCATATCATAATTGCGGGAA
TTGAAGCCCACAATACAGAACCGCCAAAGCATGCAAAGAAGCTTCGTCGGGTTAAGATCC
TGGTCGGCGCTGCGCTCGAATGCGACGAACTTTCCGTTATCAAGCGACTTTAACGCCACG
ATAAAGAAATTTACGTAAGTTTCAACGTCGAAGACGATGACACTTCCGGCAGGCATTGCC
GTTCGTTCTTCGTCCGACATAGATTCGAGAGGCCGCAAAGCCTGCCGAATGCTTGCGGCC
ACCTTATCGACGACGCGGCTTTTCTTTGCAACAATAAAGCCTTTTTCGTTAATCATGCGG
AAGCCCCTTAAAACGGAATGTCGTCTTCGTAATTGTCCGACGGTTTATGCGTCGCTTCGC
TGCCAAGGTCAAGACCCATCAGAACGCCGGCCTGGTTTTCGCCGAAAAATACGACTTTGT
TTGCGTCCGCAGCGAAATGCCCTTTCTTTTACGCATGTTCGACGGAAAGAAGCAACTTCG
CAGAAAAGCCCATTGGTTCCGGCAAGCCTTAGATTTTGTTCTCCGAAGCTTCTTCTTTGT
GCATCTTCGCCAAAACCGCCCCCTCGTCGAAAAATACGTTCCCGTTCTGGCTGAACGACT
GAATAGCGCGAAAGCCGTTGTAAGCATCGTCGGGAACCGGCCAAGGGTTCAAGCCTTCAA
CTTCGAAAACGACTTGGCAATTTGGGTAACGTTCGCCGTAAAGTTGCGTTTTGATAAACG
CCCCGTTTTCGAAATAGAACGTTGCCGACGACGCCGAAAAGCCGTAGCCGGTAAGGGCCG
GGGCAGCCTTGGCGACAGCGACCGCCGCGCACTTGTGTAGCATCATGTCGGGGGGAAGGT
CGATAGCGTGCCACGCTTCCAGCAAGGCCGCCGCGTTGGTCGCGACGGCGCTTCCGGCTT
GCAGCAAGACGGCAGCATAGGTCGCATTCGGAGCGCTGTCTGTTGCAAGCCCTGCAACCG
CAGCGAAGGCTGCTTAAATACGGTCGACGATTGTGGCGGACTGCGCGTAGGGCGCGGAAA
TCGGCACGTCGTCGAAGCCCACACAAGGAACCAACGCCCGGACCCCGCCAGACGAAACGG

CAAGGGCGTTCACGGTAAGCTGCGCACTCGACAGTTCTTCCCCGACCTTCGACAGCCCAT
CGATAAACTGCAACGCATGCGGACACGCTACCAAGTCTTCTTCGATAGGATGCGCAACGG
TCAAAACCCCGTGGGAAGCCGCCGCCCAATTGTGGGCAATATGGCCGAACTGAACGTTCG
TCGGCCCGGCCTTTTTCTGAGCCATGGCAGCGAACTTCAACGCCGCCAAAAACGACGCCG
CAGGGCTTGGCACTTTGGCTTTGGTCTTTCCGGCTGCGGCTTGCATTCGCGCCCTTGGCT
TGCCCTTTGTCGGTTCTGCGGATGGCGCTTCGTCTTCGGGCGGAAGCGGAATATTACCGT
CGATTTTCATTTTCGGCCCCTTGTTCGTCAAGTTTTGGAATTGCCGCTTGATTATAGTTC
GCCAAGTCTAGCGTTTCTATTAGCGCGTGTTGCAACCAATTGCGACACGACAACGGGATA
TTATCCGTCGTTACGCCGTACTCGGCAAGTCCAACGATTGAACGTTGCAACAGTAATTCG
CGGTGCATTTCTACTTTGGAATCCGGTGAAATGAGGCCAGTTCGCGCAACTTCAAAGGCG
ATTCTCGCCGCCGCCAAAGGGCTTTCGACTTCCAAGGCGTCTTCGGCAGTTGCGCCGCAT
TCCGCGACTTGTTCGTTTTTCCATTCGTCAAAGCTTTTATAATGTTGCTTGATTTCACCA
TTCAGCGGAAAGAACTACGGGATACTTTTTGTTCACCCCTACGCGGACCCGGCGCGGCGT
GCGCAGTTCTGAAACACGCTGCGAAGCTTGATAACTTGTCGGCGGCGGTTCTTCGGCGTG
CCGCGGCCGCCACCAATCGCGCGAACGTTACGCATCAAGCCCCGGATGTTCAAGCATTAT
AAATTCGTTGAAGATTTGGAAGCCGCAAAAGTAACTAACTTTAATCATCCGCTGTTTCGT
TAAATTTTCGTTTGCGTCGCGCTTTTCGTGCAAATTGTAAAGTACGTTTTGAACGTCGAA
ATATTCGACTATCGGCGCGTCGCTTCTCAGCGGTTCCAGAGTTCCACCAGTCGCCAACAA
CTTCGTTTCTAAAATAAATTCGGTGCCGCAGTTGATACACTGCCGGGCCGCAGCGTAATT
ATATACGCCTCATATATCGCAAATACGAACGGGCGCGTCGCCGCCGCCTCTGACCGGTCT
GTTCGGAATTGGCGGGTCATTGATTGGGCCTAGCCTTCGAACGTTTACGGCGAACTCCGC
GTACAGACAATTTTGTTTGCCAGTATCCGGCGACGGTCAGGTTCCGCGTCCCAACTTTTG
TACGTGTTTGCCCGGCGATAGCCTCGGGTTCAAATCGCCGATCAAGCCAATGGGCAGATG
GTCGAAACCCCTTGTAAGTTTCTGCCCCGATACCAAGCCGCGAAGTTCGCCAGCTTTGAA
AGCGCGCAAACGGTCGGTATTGGTCATCGAAGGCAACTTGGAATTAACCGCCAGAACTTC
CAAGCCGTAAGACCGAATTCCGTTTGCGGGGTGTTCGGTATTATCAATACGTGTTGCGAA
GATAAGCCAAGTCGAAGAGTCGCAAGCTATATCGGTCGTTTCCCGAACAGCGTTAAAAAC
AACTTCGTCGGTGTCTACGGCGGCCTCTAGTTGCTTGCTGCTGAATTGGCCAGCCGTTAC
GCCTACTTACGACAAGTCGATTTAGGTTCGCGTCGGCCTTGCGCTGAGCGCCTTGAAGTA
GCCTTCGGCGATAAGTTGGTTAAACGATTCAACGCCGGTTATATCGTAACAAATGACGGT
AAAAATTCCGTTGTCGGTAATTAGCCCGATTTTCATTCGATAGGGCGTTGCCGTGAAGCC
GATAACTTTAAGATGCGGGTTAATCTTGCGAAGTTCGGCGATAATATATTGGTAATGCGA
ATCTTCCTTGTCGCTTAACAAATGGCATTCGTCGATTAACAGCTACTCGCGCCAACCGAA
TTGGCGCAGATGCACCGGGCGACCGTCGTCTTCTTCAAGCCCGGGTTTAATCGCAGGCGC
TACGGACTGAACGCCGCCAAATACGATTGGCATAATCATATCGCGGGAGTTCAAGCCTGC
CGAATAAATGCCCATCGGCGCGATAGGCTAAATCTACATAAGTTTTTCGGCGTTCTGTTC
GATTAGTTCTTTAACATGCGTAAGCCTCATTATTCGTTGATTCGGCCAGTAACCGAAAAT
TCGGCGAACAAAATTTGCAATTACGACCGACTTTCCGGATTCAGTCGGCATAGCGACCAT
GGGGTTTCCAACCCCGCCGCGCTGGAAATAATCGAATATTGAATATTCCGCTTCGTCTTG
ATACTAACGCGCAACATAAATGTTAGACTTCCCTCGCGACGGCGGTAATTGGTGAATAAG
AATCGCAACCCGCAGCGATGAAATCTCCCGGAACTACGCCGTTGTGAACTTCGCAAAACC
ATTCGCCGTTTTCGACAGGGTTTGCGGCCTTGCAGCTTCGGCAGTTCTTTTCCGGATTCG
CGCCCTTGTGGCAAATCTCCTTCGCCGCAGAATATCCGCACTTGTGAAAAGTCGTATTGT
CCGACAAGCGCGGCGGCGCTTTCTGCGACAGAATGATTTCTTCGGCCTTTTCCATGATTT
GTTGGCCTAGCCGATGGTCTAGCTTTACATCTTCGCAATGCAAGTCGTCCTCGTTCTTGT
TAATGTTTAGGTAAAGACCATAACGAAAATGATATTCGTTGCCACAGGTCGAAGTTTGGG
CGAAATGTTGCGGCTTGCATACTGCCATTCCTTTCCACATTAAGTCCCAAACGCCCTTGA
CTGTTCTGTTGCTCTTGAATTCCAGCAAAACGGGTTCGTCGTTACCGGCGCGGTCGGGAA
GCCAGGCGATACCGTCAAGCGACCCGCCGAAGTGCCCCATTACGCCGGAGATGCGGTATA
GTCGGAATTCCAAGCCGTCGGCTTTCCCGCGTGGGTCTTGTTGGGCGTAATACGGATTTT
CGTTCGTATTAAGGGCGCAAGCCAACGCCAGTTCTTCGCCTTCCTTCAATACGCCGAAAC
TGTCGCTTTCGGCTTGGCAAAAGAATCCGTCGCGTTTTTCGTAACAACATTTAACGGAGA
TGCCTTCCAGGCATTCGATAAAGCGGGCTTCCTCACGGAGCCCACGATTAAACAAGCGTT
GCTTGCGACCGTCCCTCTTCTCCAGAAAACAGCAACGGAAGATATACCAAAGCTACCGCT
TTAATTCGTCGCCTATCAACGACGCGCTGCGGTTCAGAAGGTGCCCGCCGTCATAGGAGC
GAACGCAATATTCGTCTATATCTTCTTTTATGCGCTTTGCAAGGGCAGTTGCAACGTCGG
GCGCGTCAAGGTCTACACTTAGTTTTCCTGCGTCGGATTTCGTCGGCTGCGCGCCGTTCA

GCGGTAATTGTGCGTTCTTCGCGTCTTTGCTGTTTTGCATCGTTCTTCACCTTGCGCCGG
ATAAGGGCTTCGTAATTGTTCGCTAGATAATCTGCCGAAAATGTAGCAACGTCTTCAAGT
TGGTCTTTCGACAGCTAAGATATATGGCAATCCGACGGGTCGATTCATAATTCGTCCGCA
AGCCAATTGTAAGCCTTCGCGCGTGACATTAGGCCAGTTTGCCCAAGGCGGTCAAATTCG
TAATGGGCTTTTGGTCGAAGCTGGCGCGTTGTTCGGTCGGCCATACGACCCAACGGAATA
AACGTGCGGGGATGGCAACCAACGGCGGCGCGACAATCATGGCAATAATAACCGTGCGGC
CAATTACCATAAGTTCGCCCGTAAAACTTATCGTTCTTAGTTTGTTCAATGTTAACGGAA
CAACAAGTATCGCATTGTTCGGGCATCGGCAATGCGTCCTTTTTTTTCGACATAATTTCG
CATCCGCCTTAAAAAGAAACGCCGGGGCCGGGTAAGACCCCGGCGAACCTACGCCGCTAC
GGCGTTACGCCTTAACGCTGGCCCCAAGGTGCCGCGCCAGCCGGTGCGCCGCCCTGCGCG
GGCTGGCCGCCCTGTTGCCCGGCGCCGCCCTGCGCGGGCTGCTGTTGGGCGGGCTGTTGG
GCAGGCTGGCCGCCCCATGCTGCGCCCTTCGGCTGTTGGGCCCGTTCCTGCGCGGGCTGT
TGCCGGCCTCCCCAAGCGGCACCGCCTGCGGGCTGCTGCGCGGGTTGCTGCTACGGTTGA
CCGCCCCAAGCCCCGCCGTTCTGGCCCTGCGGCTGCTGCGGCTGCTGTCCGAAGCCTGCG
ACGCCCTGCGGCTGCTGGTGCTGCGGCTGCGCTGCCGGTGCGCCTGCGCTAGCCTTGCCC
GGTTCGTCGCCGTTCCTATCAAAGACCTTTTTAACTTCGGTATACTGCGGGTCGTTTTTC
TGCGGCCCGACTTCGATAAGAAACGGCCGGTTGTGAAATTGCGACGAATCGGTAACTTGG
AACACGCTGATTACGTGGCAGATTGCCGAAAGCTGCCGATGCGCGATTTCGACGGTCTGT
TGGTTGGAATGTTACAGGCTCAGGCTATAAGCGCCGGTCGTGACATGCTCCGGGCCGTCG
ATAATCCGCAAGGTCAGCTGCAGATAACCGCCGTCGTTCGCCTTGTTGGCCTTGACTTCG
GCAGATTCGACGATTGCCGGATGCTTGCCAATCGGCAGGCGTCCGACGCCTTGGGTAGGG
TCGAACTGTTGGGCGTTGAACGCTTGGATAAGTTGCATGATGTTTCACCTTTTCGAAAAG
TCTTCGCCAATGGTTACGGCGGCGAAGTAAACCGTTTTATTGCATCGCTTTCGCGAACAA
TGCGGTAAGGTCGCGCTGTTCATGTCCGTGAAGATTCCCAAAACGGTCGCGCGCGAAAAC
ATCGGGAATTTCCTTCGTTCGCAGCGCCCGAACCGGCTTCGGCATTCCGCGTACGCTTGC
TTCACCCAAGTGCCTAACGTTATCGAACAAATACGGAACTTTAACGTTAAGGTCTTTGCC
CGGAACGAACGGGTGTTTTTGCATGATCGGTTCGTAAGTAACTTCGCCGTTCTGCAAAAT
CGTTTGCCGTCGGTTCTCAATAGGGGCTTGTTTCGCAATCATTACGATCTGTATTTCCGG
CATGTAATACAAGTCGTTCGCGATGTTCATTACACGTTCGGACATATTGCCGTAAGCCTT
CATACCGTGTTTGACCTTGCCGAATTCGTCGGTCAAGATAATTTTGGCGATGTTCGAAAT
ACTGTCAATACCCAACGTATCGAAATACGCAGCTTCGCGCGATTTAATCAACCATTCGAA
GAATTCGACCGTAAGCGCGGGCGTATACGCTCCCCATGCGGGAACGTTCGAACCGCGCAT
GGACAGCATACCGGGTTCGGTCAAAAGCAAAACCGGTCGCGGCGCAGTATTGATAAGTGG
CGTTTTGCCGGAACCGGGCGCTCAGAATACGACGGACTTTACGCCGGCGCGGCGGGCCAT
TTGCCACGCCGGTTTTAATTGCGACATTTGCACAACTTGCACCTTCGGGAATGACCCCGG
CGCTTGTGGGCAACCGGGGCGGGGTTGTTACTTCGACTTCGGCTCGACAAGTTCAAGCGC
GTGCGTGGCCTCCTTCGACGTTACAACTTCGTGGAAACGCTTACGGAACTTGTCGTGAAG
GTTCTTGAACTCGGTAACGGAAAGTTCGGGCTTCCATTTTACCTAACGTTCGGCGATAAA
TGCGCCTTCGGGGCCGGTCTTTTCGATTTTCTGCAAAGCCTCTTCGACGGCTTCTTGGCC
GCCAGCGAAGCTGCGGTTAATCTTGAAAACGGCTTTCAGTTTGTAGCCTACGCCAAGTTC
GACGTTTTCGGTTCCTGCGCGCAAAGCTTCGGGGTTGAAGTTGAAAGCGTTTTTCAGAAT
TTCGGGGCGCGATGCGGATTCGGCTTCTTTCGCTGCGGCAAGCTCCTTCGTTGCTTCCTG
CCATTGCCGAATCAAAGCGTCGCGGTTAGCGTTTTCGTTGAAAGACATTGTTGTTCCTCC
TTCGGTTGGGCCGCCGCACAATTGCGCCGGTATGTACGAACTCTACGACGGCCCTTCGTT
GATGTCCAGCCCCTTGTTAAAGGATTTTTCGTTCTTCGGACAAGTCGCGATTGTTGGCGT
CGAATTCGGTAAAGGCGTTCGGGAAGCGACGACGAAGCTTCGCGATGTTGACCAACTGCA
CTTCGCCGAAGTTCGAACCAACGGCGTGCACCAAAAGCGCATCGTACCAAAAGCCGTCGG
CGTCTGCTTCAAACGCGTTAATAGCGGGGAAAGCTTCGCCTACGGCTGCGGCGCTTAGGT
CTTCCAACAGTTCGTCCGCTTCGGCCACTTTGCCGATAATTGCATGAATGACGTTAACGG
CTGCGGCGTCGTGTTCGAGATGGTCGTAAATCCAAGCGGGAAGCTTCGCCAGCGTTGCCG
CGTTAGTTTCCGGGGCCGGAACGCCCGTGTCGCGACCGAAGAACATGGCTTTCTTTATTG
CGTCAAGTTTGTTAGGGGCTTCGACGACATCGGCGATGGTTTCGCGGAAGTATGCAAGCG
GCACGCGGTCGCCGTAATACTTGTTGGACGCCGTAACGTGGGCTTCTTCGATATAGTCGA
AGGGGTCTGCGGGTTCGTTCATACGCACACCTTTTAGAAGGGCCGGGCACCATGCGCCCG
CGTTCCAGCACTGTAACCGACCGCGCCTTTCCTGTCAACAACAATTGTAAGCAAGCAAGA
AGCCGGGTATGCCGCCCACAATTGCTAGAAGGTGGCGTTATGGACATTGAAGAACTTACG
ATGCGGGTAAAGAACGCAAGCGTCGAGCGGTCGCTGTGGGCACTTGCCGCGCAATGCAAC

GTTTCGCACGAACTTATAAGAAAGCTTTTGCTTGCGAATTCGGCGCCGAACATAACGGCG
ACGACTTATAACAAGATTGACGAAGGGTTGGCAAAGAATAGACTTTAAGAACATTCTGCA
CGAAATGCGGATTTGCGCGCAATGGGTGGTTTGGCGATAAGAAGACACGGAGTGAAAGAA
ACCGACGAAGGTTCCGTATTCAGCTAAAACCGGGCACCTGGCCGGCGTAATCGACCCGAA
CACTTGGGCCGGGTTCGACGAATGCTTTAACGCAATGTCGTCCGGTTGGTATGCCGGAAT
CTGTATCGTATTGACTTAAAACGACCCGTATTCTTTTATCGACCTTGACGACACAAAAGG
CGACCAAACGGCGCTTGACCGCCAAATAAAGATTTTTAACGAATTCAACAGCTACGCCGA
ACGGTCGCCTTCCGGTTCGGGGTTGCATATCATCGTTAAGGGCGCGATTCCGTCGGGCCA
CCGTCGTTCGTTTATCGAAGTTTATTCGTCGCTTCGTTAAATGACAATGACGGGCGGTAT
TTACCGTAACGCGCCGGTTAACGATTGCAACGAAATATGAAACCTTCTTTGGGGCCAAAT
GGGGCAAGGTTCGGTCGCCGTCGCGCATTATGCCAGACTTGCCCAAGCCAAGGAAACCGA
CGAACAGGTTTATAACCGCGCAGTCGCCGCAGGCACTAGCGACAAGTTCGCAGAATCGTA
CGCGGGCAAATGGGAAGGTATGTACGCTTCGCACTCCGGAGCCGACTTTGCATTGGTCGA
TATTATCGCGTATTATACCCAGATCCGAGCGCAGATTTCGCGAATGTATCGCAGGTCGGG
CTTGGGGCACCGTGACAAAGCAAAACGCGACGATTACGTTTCGTACATGCTGAACAAGTG
TTTCGACCGCAGGTTGCCGCCCGATGACGTTGACGGATTGCGCAACAAGTTAGCCGAAGC
AATAGAAAAGAAAGAAGCCGCCGACAGGGCCGGCGCGTTGTCCCAGAACAGCGAAGCGAC
GCCGCATCCGAAAGCCCCGGCCCCGAACCTTAACGAAGCTTCGAAGGTATACAGCGTCCC
GCCCGGATGAGTCGGAGAAATCGCGCAATACATTTAAGCACAGGGGCCGCGCCCGGTTCC
TGAAATCGCGGTGGGCGGTCCCCTTGGCGTGGTCGCCGGTATTGTGGGCAGGGCCTACAA
CATTTACGGCACTGGCCTTAATCAATACGTTCAATTGCTCGCACCGACCGGAACAGGTAA
AGAAGCCATCGCAAGGGGCATTGATAAGCTTATGGCGCAGGTCATCCGAACCGTTCCCGC
CGCGTCGGACTTCCTCGGCCCCGGCGAAATTGCTTCGTCGCAAGCGATTATTATGTATAT
GTGGCGCGTGCGAAAGTCGTTCGTTTCGGTGGTTGGCGAATTTGGCATTTATCTTCAACG
AATGGCATGCGTCAACGCACCGCCGCACCTTATCGGGCTTCGTCGCTTCCTGTTGGATGC
TTACAACAAGTCGGGCGAAGGCAAGGTACTTCGACCTTCGATTTATTCAGACAAGGACAA
GAACACCGCCTCAGTAATGGCCCCGTCCTTTACTTTGCTTGGCGAATCGACGCCCGAAAA
GTTTTACGAAGGTTTACACGAAGGTTTGATTTCCGAAGGTCTGTCGCCGCGTTTTACGAT
GATTGAATAGAACGGCGACCGCCCGGCGTTGAATCCGGGGCACCTGTCGGCCCAACCTTC
TTACGGACTTGTAGACAGGCGTTCGACCTTGTGCGCCCATGCGCTTATGTTGAATTGGCA
ACATAAGGCGATTCACGTTCAAACCGACGCAACCGCCCGGGAACTGTTCCAGCAATTCGA
CGCGCACTGCGACGCGAACATTAACACAAGCGACCGCGAAGTTCGACGGCACCTTTGGAA
CCGGGCGCAAGTAAAGGCGTTGAAGTTGGCCGGAATTATAGCCGTTGGCCTCAACCCTTA
CGACCCGACCATTACCGCCGACGTTGCGTGTTGGGCGATCAACCTTGTCGTCGCCGACGT
TCGAAACTTGCTTGCCCGCTGAGATGCTGGCGAAATTGGCATAGACAACGACGAATCGAA
ACAGCGTGCGAAGGTTATTGCAGCCGTTTAAGATTTCATCGTTTCACCTTGGCCGGACGT
TGCGAAGTACGCAGGCGAACGAATGTCCAACCTTCATTCGAACCGAATTGTTCCGTACAG
CTACGTTCAACGCATCCTTGCCGCTGTTGCCGTCTTTTGTAAAGACCGCATCGTCGCAAC
CGGGGCAATCAAGCGCTCCTTGAAGACCCTTTGCGAGCGCGTCGATCTGCATGAAGTAAG
CCGGGCCACACTGGCGAAGGATTACGGAACAAGGGCCGTTGCATGCTTGGTCGCGCATCC
GGGCGTTTTCGGCCGGTAACGCGCGCCCGCCAGTGTGGGCAGAAGGGGCCGCATTCGGGC
GGCTTTTTCGTGCCTGTCAAAAAAGTTCGTCGTACCTGTTGACGTGGAATCGAAGGTATC
GAATATTTGAACAATCGAAGCAAGCAACGAAGGACTAACGAACATCAACAACTATGAAAC
TTTTTTAGAAGAATTCCAACGCATCGGCGAAATTCGCCAAGGTAACGCAGTTCTTACCAA
AGACCGCGAAGGCTTCCGTTTCCGCAACGCGCGAACTCTGTTTGCGAAGTTCCGTCGCCA
CTTGTCAGCCAACGGCAGCGCCGAACGTGTCGAAGCGTATTGGAACGGCATTGTCGCCAA
CATGCGGGCCGCTCACGCAGCTTAACCCCGTAGGCCAGCCCCGGCCCTTGGCACGCGCTC
GGGGCCGCACAGCCTGTCCACAATCAAGGGAAGCCGCAGCCATCAACCTAGCCCCCGCAG
ACATTCCAATAGACACGTTCCGCAAGCCCGGCTCGTCGGCATGGGCTCGCAGAGTCGAAA
CCTGCGTTCGCATTACCCATATTCCGACCGGCGTTTTTGTCGAACGCTGCGACCTTCGTT
CGGCGCACGCTAACAAGTCCGCCGCAATGGCAGACCTTCGAAAGAAGCTTGACGAAATGA
CAAAGGCGATGCCCCGCATTTATTGGACGTATTACCCGAAATACCGTCGCGGGTATTGGC
GCGTTTGCGCAATGCCGAAGCGTTGGAAGCCGGAAGACGGGCCAGCAGGGGACAAGGCGC
AAGAATTCGCCCGTCGCTTGAATGATGCGATACCTTCGAAGATTGAACAGGAACTCGCTC
GACTGCCGGTTGTTGATACGGTTAAACATTTGAAAGACCTTCCGCCCGGTTCGTACATTG
TTTCGGGCGACGCTGCGGCAGAATTGGCCCGACAGTTTGCAAAGCGTTTCGATGAATTCT
TAATTGTTTCGAAGGTTCTTCGCGTATAATAGCCGACGAAACGTATGATGGCCCGTCATA

ACGCTTAAACCCTTGATACATAAGGCTTTTAACGGATTTATAGCAAGTATAACGTTAAAC
GCCGTTCCACCTGACCCGGCGGGACTCTTTATTAAAAGTCACTATTGATGAAAAGCTATA
TTATAGCTATGATATTGTATTATCTTATTGTATTTCCTTTATTTTCTAGGACTTAGGGAC
TATACAGACCCTTAAACACGTATAACGTTTCTAAATTGATTAAATGCTGGCCACTTGATA
AATTGTTGCCTATGCTTCGGCTATTGATTAAGGGGCTTGCCATGACGAAACCGACGACGT
AGTAAGGCCGACCGACGGGCGTTATTCCGAAGCGTGCGCCCTGCCGCCAATTCGGGCGCG
AAGTTGCGGCTAATATGAAGGTTTGCCCGTAATCCTTGAAGTTGACCGGGCTTGTATCTA
CTGCGAAGGAGTTAAAGAAAGGACCGACGAAACAAAGAAGGTTCAAAATCTACCGTCGTC
GCCGAAGGCCCTTGCGCATGTCGAAGTTTCACCGGACAGTCAACCGGAACGAACGCCCGA
CGAAAATGACGCGAAGTATCCGTTTCGTTCGTTGCAAGTTGGGGAATCGTTTGCGATACC
GATTGCAGACCTGAACCCCGGAACGTTGGCGTCGCTGCGTTCGTCGGCCAGATCTTACGG
CAAGAGGCTTAACCGGAAGTAGCGCGTATTCGTTCACGACGAATATAAGTGCGACGAAAT
GGCCCGGCTGAAATGAACAACGACGCGCGGCCGATTGTGGGCAAACTGGTTAACGTATTC
GGATCGTCGTATCTTGGCCTTCGGTCGCAGTGTCCGAAAGCATTTGTCATACGACCAACA
GCGACAGGCCATCGGGCCGTCGTAATCATTGAAGAAAGGGCAAGGCATGTACGAAATCGA
AACCGGCCATAAAATGCCGAAGCAACGTCGCAAGGCTTGAAGCGAACCGACGGCGGTTCA
ACAGGCGTTCAACGTCTACGCCGACGCATAAAAGGCCGTTTATGGCGTGCGCCCGCTGTC
GTTCACGTATGACCGCGAAAGCGGCTTTATTCGCGTCGAAAGCAGCGGCGGCGTAAGCGG
CGCAAGGCTGCGCGAAATGACGAAACAGCTTCGTTACCGAAAAGTCTAAAAAAAAATTAC
TTCAAAACGTTGACGTACTGTCGAAGCCATCGTCCCATATACCCATACCAACGAACACAA
AGAACCGCCGAAATGCAAATTCTTCTCCAAACCAACCGGAACGCGGGAACCTTTCAAGCT
GGATTCGGACCCAATATGCTTTTAAGTGGCTGGTGCCAAGGGGTTTTGGCTATCAGCGAA
TGGGGTTCGTACTATTTGAACGAAGACGTTTCGCGCCATAGGCAAGCATTTCTTTTCAAC
ATGAACGACATTTAATAAACATGCGCGTCGGGCGCAACCCGGCATTTCAAAGGGGTTATA
ACAATGTGCGTCGTCTGCGAGAAGAAACAGGCCGTCGAAGTTTCCGGGCCGAACAAAGAA
GACCGCGCCAACATCCTTGGCTTGGTCGAAAAGCTGGCCGAAGCCCTGGGCGATGTTCTG
CAAGTCGCGCGTGAAGTACACGAAGCCAAGCCGGAAGCGTTCCAGCCGGCACACGTCGCG
AGCTTTCAGAACGCCGCCGACTTGCTTAACGACCGGGACGAAATGCCCGACGCCCTGTTG
GCCCTGCTGTTGGCCGCCCTTGGGCGCGGCGTCAAGGTCGAAACAATCCGCGTCGAACTG
CGCGAAGGCGTAACGCCAGAACAGGCCGTCGCGCGTGCAATGGCCGAAAGCGAAGGCAGG
ACCGGAACGAAACACTAACCAAAGCGCCCCGGCTGTTGTATAGTCCGGGCCTCTTTCTAT
CATGGGGAACGAAAGAACATGGACGGCGAAGTAGACCAAACAGCCGACCGAATAGAAAAC
GAAATAAGCTTTACGGTCGGCGAATTATGGCGAAAGTCCGCAGCGATACCCGCACGCCGT
CCCGGCGAATGTGATTTTTGCGGCGAAGACTTCGCGCGCGTTGTCGAAGTTACCGACCCG
CGAACGGGCGAAGTCGTCGAAAGCTGCTGTCGCTGTCGTGACCGTAGGGGCATCGCATGA
CGCCCCCATCCCGTGCCAAGTCTGGCACCGAACACGCGCACAAAACGGCGCTTTTTGTCT
ATGCTGCTGTCGCAGACTAGCACGGCTTCGACGTTGCCGACGAATAGTCGCAGACCGGCA
AGTTACCGAAGCGCGACCCTGATGCACCGCCGAAGGTGCCCGCCCTTGAATGATTCCACG
CCATACCGAACGGCGGCAGTGGCGGCGACGACGACAAGTCCCGCACGATACGCGGCGCGC
AGTTGAAGGCCGAATGCGTGCGGCAGGGCGTAGACGATACGGACTTGCCTTGGCCTGTTT
ACGGACGGTGCCCCGCAGATTCGCTGATGATGATGATGAAATGGGCCGGTCTTTATATCA
AAATGAAGAAGCCGACCGAACGTCCGAAGTCGGAAACCGCAAAAGGCGGCGTATCGGACG
CACAACGAGGTTTCCGCGAATACGCATAGCGCGTCGCAAATAGTTGGGCCTTCTGCTATG
ATTGGGAACAAGCGGCGTCGTATCTTCGTTGTTATTTCGAATGGGGTACGCAATGAAACT
ATATGTTGACGATATGACCCGGCAAGACCCGGAAAAGGCCGCGCTTTGTATGCGCGCATT
AATCAAACTTGACGGTAAGAAGGTCGAACGCTGCGTAACAGCGGACGAAGAAGGGCGTTA
CGTCGTCGCATATATTTCGCAAGAGGACCCGCGATTTAAGGCCGAAGTAATTAAGCAAGG
TCGCGACTGTTGGCCGGTCGAAACGTTGTACGCCGTTGTTAGCATCGGTGACCCGGACGC
GAAGTCGTGAATCGTCCTCGTTGTGAGGTGACCTGCGCCCGGTTCAAAGCTGCGGCGCTG
GCCCTTGTGGGCTGGCCCCTTGACCGCATCGCCGGGCATTTGTCCGTTTGCTATGGCGAA
CGGTCTTACGTCGAAGGTGGCAAGCTGTATCGCGCGTCGAAGTTGCCGCCGTATCAACCG
CACTTGATAAGTGACGAACAATGAAAAAGACCGACGAAGAATTGATAGACAAAGCCCGCA
ACATTGCGCGTTCGAAACGCTGTTTCGTCGTAAGGAAGCCAGCTTATTTGCTGCTGTACC
GCGAATGCACGCCGCGTAACGTATGCGTGGGCAAACGGTCGTCGGCTTCGGCGTTGCTAC
AACCTGCGAAAAAGGCTTGACGCAGCGTCGAAGCTTTCTTACACTTCTTCGTAAGTTGAA
CGAAGCAAGGAGAAACGACCACGCCCGCATATAATGTCACAACCGAATTCGAAATGACCG
ACGGTCGCGTTCTGTCTTGCGAATATGGCGTATCGTATACGCCCGCTTACATTAGAGGCC

CGCCCGAAGATTGTTACCCGGGAGAAAGCGACGCGGGCGAACCGACGTACTTTATCGACG
GCGAAGAAGTCGATTATAAAGACCTTCCAAAAGGCTTGGACGCAATCGCCGACAAGCTTT
ATTACGCGGAGCCGGGCGAATATTCGTATTCGCAATCGGACGTTGACACCGAACCGGATT
ACGAACCGGATTACGATTACTGTTAACGAAGGAGTAACGAAAATGTCTTGGCTTGTCGGA
TGGGGCGTACTTTCCGTCGGCGTTGTTGTCGGCTTCTTTTTTGCGGCCTTGCTGCGGGCC
AATGGGCGGGTCGTGCCGGTTCCTTCGGCTACCGTTACCGTCGTGTCGCCGCAGTAACGT
GCCCGCATGGGCTACGCGCTGGCAAGAGCTGGCGACTTGCTGGACAGCGGCGACCTTGAT
TACGTGTCGGGCGGCATTATGGTGCAAGGCGTAGGGCACGGCGTCGTTATTATCAAAGGC
GATTTGTCGTTGCGGGTGGCCAAAGAAGAGTAAGGACGAAGCGCGCGAAATGTCCGAAGC
CGTAAAGGCCCACCGGGCGAAGATTGACGGTTGCGCCCGCCATCGCGTCGAAATAGCCGA
CCCGCCGTAAACGTTCGGGCAAAAGTTCACTTGTTCGAACTGCGGCGGTACAATGGACGC
CGTACAGGCGTTCCGATACTGTCAAGTCTTCAAGGCCGCAGTCGGCGACCCGAACGAAGT
TATACCGGGTTACGAATAACGCTTGACAAGCGGCGAAGGTTCAACGAAAGTCAGACGTAA
CGAAGAGACCCTGTTAAGGAGTTGCCGAAATGACCGTTCTAATTTTGCTTCTTTGCACCG
TTGCCGCTTTGTTCATTCTTCGCATGTTCGTTCTTGCGATATTGGATTGGTTCGAACCAC
GCGAATACGCCGAACGTATGCGCCAAGCTGAACAGCGCGCGGCGCTGGAAAAGGCAAGGC
GGGCCAAGTGTCGATGATCTACGTACCGCGCGAAGCGCTGTCCAAATGGGACGCGCGAAT
TATTGAACTTGCAGAACACGTCGCCGGATGGTCGAAAGGGCCGCGTAAGCGCATCGGCGC
GGCTATCGCGAGCCCGAATAGGTCTATTGCGTCGCTTGGGTACAACGGCACGCCGCGCGG
CTTCGACGACGCCGTTTTTTTGACCATCACACGCGACGAACGACACGCGGTCGTAATTCA
TGCCGAAGACAACGCAATTCGACAGGCTGCGCCGGGCGAGGACTTGTCCGCGTGTACGCT
GTACGTTTCGCCGCTGTTTCCTTGCGCCATTTGTGCCGACCGCATCGTTCGGGCCGGTAT
CGGTCGCGTTGTCGCTCATTGCGGGCATATATCGCCAGACTGGCCCGCGTGTGACGACGA
AGCCGAACGCATCTTTAACTAAGCGGGCGTCGAATGCCTGTTGACAATGGATTAACGAAC
AGGATACCGCTAGACCATTCGCAAATCGAAAGCTTGTTGTTCTATCAAGACCCGACGACG
CAAAACCATATCGTCGCACCGAAGGCCGACCGGTTCAGCCAAGCCGGAAGCTTCGAAGCA
TTCGGGCCGCGTGTTGCCGTTGACCCGGCGTTGTATAACTTGCTTGCAGCAGCGCAGACA
ATGTACCAAGCCCTTTCGCTGCAATACGCTTACCTTCAAGCTCAAATCGAAGTTATCGAA
CGCTTGCCATAGGCGGGAGAACTTGATAAGCTGTTGTCGTCGTTAATCAAAGCGCAGAAC
TCTTGCCTAATGGCCCAACGTATAGCGCAAGTCGGTTACGAAAATGTCGCAAATTCACTT
GACAAAGGTTCGAAGCGGTCTTAAGCTTGAACCATCGAAACGAACAAAGGTGCCCAAAAT
GAACCGTAAGCTCCTTGTTATCGCTGTAATCGTCGCCTTCGTCGCGTTGCTTGGTATGGC
CGGGCAAACCGACTTCGAAAACGAAGTCCGCGCGCAGCTCTTGTATTGTGACATCGTAGC
GAACGGCGTTTGGCCGCATTACGACAAAATTTACGATTCTGAATGCACGCCCGAACGCAT
CGTAGAAATCTAAGAAATTTTGCGTTGACCGCTTGACACGAAGAACGGTCGGCGTAAAAA
CAACCCGTCGCAACAACGCGACATAACCTAAACGTTAAGGAGTTACACAAAATGGCTACC
AAGAAATCGAGCGCGAAGAAGAACACCGCCGCCCCGGGTATCGGCCTTGCCGAAGTTGTT
GCAGCCGGGCCGAATGGCCTGTATACTTCGCCTGCCGTAGATGGCCCGCTTATCGTAGCC
GGTATGTTGGAAATCAACCCGGCAGTTACCAACGAAGCGGGCGAAATCGCAACCCGCGCA
ACCCAAAAAGGAACCGACGAAATGAACCAGAAGACTGAAACCGGCATCGCCGCAGCTTCC
GCCCCCGTCGCTACTTCCGTCGCCATCGAAGACAACGTACCCATGCCGACCGGCAGCGGA
CGCGGTCGCGGCGGCAACTTCTATCCGTTCGACGCGCTAGAAGTCGGCCAAAGCTTCTTC
GTTCCGAACAGCGAAGACAAGCCGAACCCCGCGAAGTCGCTGGCTTCGACCGTTTCCAGC
GCAGCCGCCCGTTTGGCCGTGCATTCCGAAGAAGGCGGCACCATGACCAACAAGAAGGGC
GAAACCGTGCCCGTTGTGGTCGAATCCCGCAAGTTCGTTGTTCGATCGGTCGAAGGGGGC
GCGCGCGACTGCCGTACCAAGTAAGCAACGGCGCGGGCTATGGCCCCCCCTACGACTTCG
CTTCAACGGAAACCCCGGACATTGCGCCGGCGTTTTCTTTTGCCTTTCTTGTCAATTTGT
GCTAATCCGTGCTAATCTTCGGCCGTATCGATTTCGGGG
>NewGenomeName_37
TGTTTGACTAACGATCTCTTCACTTACTATGCCTCCAAATCAACGGAGGCTTTTCTACAT
GCAACAACACTCAACCGCTTCGGACTTCTAGCGCAAACCATTAATCGACGCAATTAAAGC
TCTTGTCGAAACTGCTGGTATGCCTGTGCTTGCTCAATCTCCTAACCTCCGAATCTGGGA
ACAGTGCAACAATCGCGTAAAACTGCTGGAAGAAATTCTAAATCATTACAATAACGGTAT
TCGCCGTCATGATAACGGTGACTTCTTTCTCCACCCTGAATGCCAACTCGCCACTACAAT
TGCGTATCGTTCTCACAAAAAGGGTTATGACCCTCGCTTGAAACTATACCCTGAGTCATT
CACTCTGGACGATATTTGTACTGGCAAACCCATTCCTCAGACTGCACCTTCTGAGCTTCA
ACTTACTGGTGAAGTCTGCGAGGATAACCGCCTTACTGTAGTCTCCATAAACGAAGAGTT

AGACGAATGGTACAACGTGCTCGGACAACGTGATATTAATAACACTATTGAGGAGAAACC
TCTCGGAAACGCAAAAATGGACTTAATGAATGAAACACCTGTCTATAAACACTGGTATGA
ACTGGTAACAAAACGACCCGTTAAATCAATCAGAGAGGACTACAACTATGCTAACGCTAA
GGGTACTAAAGCTGAGTGTTTGCATATCCTTGAGGAGTCCACCATGCAATCCCGCCTTCG
CATTATCGTCCAGAGATTGATCAATGCTATGCGTACTGCCCCCACTGAAGGCTGGTATGA
TGTATTCGATACTCTCACCTTAGCTGATGATCGTACCAAGTACTTCTAAGCTAACCCCAA
TGCTCGTCGTGACTATTATCGTGATATTGGCCGTATGGTTCTGACTCCCGAGGGTCGGTC
GGTCCATGATTCATATTCGGACTGCTACCAGTACTTTTATATGCCTGAGTATGGCACCGA
GCATGGCCCATTACACTTTCACGCAGAGCATCTTATGCGCACACTTCCTTTGGGTTCTCT
CGACTCTATCTTTAGTGATTTGGTGTGTACTAATCGGCAAATAAATCGCTTGCAAAACAC
ATCGCCTTATGGTTACTCTATGGCCATCGCAGTCCAGTATTCCCCGGATGCTCTCTATCG
CGCCGGATGGCTCAGGCCTGTCGATCCGAACGAAGAACCGCTAAAATCCACCTCGTACAT
GGCTGTAGGCTTCTACGTTGCTAAATACGTTTATAAAAAATCTGATGTTGACATAGCCGC
AAAAGGCTTAGGTACTCGAAAATGTAACGCTACATTAAAAACGCAGATGGCTCTTTGAAC
ACAGAAACTATTTCGAATTCGAATGACCCGCAACTTCTGGATGAAACTACTCCCAATGAC
GACGCTGACCGCGGAGTGTTTAATCCAACTTACCCAAGTGGGTTACTAAGTGACCCCGTT
GAAGAACATCTTGAAGCAGAACGGCAATAATGACCTACGATTGAAGCTGGCAGTGCAATC
AGTAGCCGACGTGTTGGAGGCGCAACCTGTGACGACTAATCTGCTCAAATTCATGCGCAA
TTTGACTCGAACAATCGGAGCGTCCATCCTACAGAGTTTTATCGCTTCAATGACGCAGAA
ATTAGCAAATACGGATATTTCTGGTGTGACTAAAAACTACGATACTAAAGCAGGAATGGC
TGTTGCTGACTTCCGACTCAAATCAAAGTGGACTGCTGGTGGAAAATGAAGAGCTACAAT
CCCAACTTAAAGCACTCGAGGGCGTCTTCCTGCGCAACCTATCGTCATCATCTCAACGTG
GTAGCAAAGACTGACGCGCTTGATGAAGAAATATGGCTCGATATGATCGGCGCTCTTCTT
AAGGACTGGTTCCGCTATGAACAACATTTCGTACATGGCAAAGAATCAATGCTTGACATT
CTAAATGAACGTGGCTTATTATCCACCTTGCCAAATGACTGAACTTACAAAAGAATCAAA
AATGACAGCAAATGAGGATGCAGTAGCCTTTCAAACGGTTATCATCTCCATCTAACTTAT
TCAGGCATCTTCTGTTCTTGACCTGACTGAGGACGATTTCGAATTGTTAACTCCTGAACG
TGTATGGATTGCAACTGACCGCTCCCGTGCTCGCCGCACTATTGAGGCTTGCGGCTATGG
ACCTCTGGACTTTGTGGGATACCCTCGCTTTCCTACTCCTATTGAGGTTACTGCTGCCGT
CATTTCTTATTATGGTTATGCCATTAACATTCAAACGGCATGGCTTATTATGGAAGGCGC
TCAACTCACCGAAAACAGCATTAATGGTGTCGCTGCCCCGGTTAAAGCCGGTTCACTCTT
CGCGTTTAACTTGCGTGTACGCGCCGGAAACACCGAGCTTATTACCCAAGCAGAAAACAA
TGCGCGTGAACAACTGCGCGCTCAAGGAGTGATGGAAGTCTAAAAAATCTGTTCGCCTCT
CTGGTGGCAAATCTAAAGGTGCTTGCCTCTGGTATGTTCGCGGCACACAATTCTAATCTC
TAAGGGGCTTCGGCTCCTCTATTTAATTATACAATATGTCGATCGTACAAACTTCCGCTG
AACGTATTACGCATGACCTCTCACGTCTGGTATTCGAAGGTGCTAGAATCGGAGGACTTA
AAACTATCTCCTGCACTCCGGTTGTCGCTGGTGATTCTAACTATACTGAAATGGTAGGCG
CTATTCGTCTTGCTCCTCTCCGTCGTGGTCGGGCTGTCGACTCTCGAGTCGACGTCTTTA
CTTTGTGTATCCCTCAACGTCATGTCTATGGTCAACAAGGGATTGACTATAGGAAGATTG
GCGTTAACGCTACTCCTCTGGCTCCAGTAACTTGATCCTCAGGCTTTGATTCTGGTGCCT
ATCTTGGCACTATCCCGTCAACTGATTTGAGGGTTCCTAAATCCCATCATCAATCTTATC
TTAATATTTAGAAAAACTACTTTAAGCCGCCGTGGTCTAATGACCCCACCTATGCTAACC
CATCTAATAAGCACGCTGAGGATTACAAATGGGGCGTTCGTGTCGCTAACTTAAAATCTA
TCTGGACCGCGCCCCTGCCTCCTGCTTCATTGACTAGGCAGGATATGACTACTGGCGCTA
CCACTATCGATATTATTGGCTTACAAGCTGCCCATGCCAATCTCCACACTGAACAGGAAC
GTGACTATTTCATGAGTCGCTACCGCGATATTTTGAAAGACTTTGGCGGTCACACGTCCA
ATTACGGTGATAACCGTCCTCTGCTCCTGATGCGCTGTGAATCCTGGGCCTCCGGCTATG
ACGTCGACGGTACTGACCAAACTTCATTGGGTCAGTTCTCTGGTCGCGTCCAACAAACCT
TTATTCATAAAGTGCCTCGCTTCTTTGTTCCTGAGCATGGCGTACTTTTGTCTTTATCTG
TTACTCGCTTCCCCCCTACTCACGAAAGTGAAATGCACGATCTCGTGGGTAAGGAATCTC
TGACTTATACTGACATTGCCTGTGACCCTGCTCGTATGGCAAACCTGCCTCCGCGTGAAA
TTGAGATATAAGACCTCTGTCACTCTGCTGTTACTGGTAAATTCAAAATTGCCGAAGGTC
GATGGTATCGTACTCAACCTGACCGCGTCGCCTTCCCTTATAACTCTCATGACAGCTTCT
CTTACTATTCAGAACTGACGTCAACGGAGATTCTCGAACGTGTACTCGTTCAAACCGATA
ACTATGAGGAAATCTTCCAGTCTATGCAACTTGCACACTGGAACATGCGAATTAAATTTA
ACTCTACTGTATAACGTCATATGCCTACCACTTGTGATGGCATCATGACTTAATACAACA
AAACGTTGTACGGGTACAAATCTTCATGTGTGACTGTTCAAGGGTGAACAAGGGAAGCGC

file:///E|/Curro/Simulación/paper/submission/Additional%20file%204.txt[27/06/2014 19:27:32]

GTTAGCTAATTTACTTAAGGAACTTCTCATGTTTCAAAATTATCTCTCTAAACACAATTC
TCCGTTAACTTCTCAGTCTATTACTGCTATAAAAACACCTGCGGCTGCTGGTCCGACTAT
TTCAACACCGGAGCTTAGCAGCACTACTATTTTCGCTGGCTTAATTCTGACTGCTGCCAC
TACTCATTCTGGTGTCGCCCATGCCGTTCCTATTGACGAAACCAATCTTACTGACAATCA
GGTCTTATCGGTCGCCGCTTCTTTATCTGGCGTACCGTCTGATGGTATTGCTATTGGTAT
TCGCTTTGAGGTCGCTGGCGGTACCGTCCCGACAGCTCTCCCTGCTCTTGACTCTGCTTA
TCCGGTGGGATTCTTCACTTCTGGCGCTGCTCTGTCATGTGAAGATGCCGTATCTATTCC
GACGCAGCCTGTTACTGCTGGTAATGCTGTATATGCTGGTATTCTTATCAGGGCCAAATC
TTGGTCCGCTGGCGTCGTGTCCGGCCTCTTATCTGTTAATCAGGTAAACCGTGAAACTAC
TGTACTCTAACCGCTTAAATCAGGTCTAAGCTATGTTCGGGGCAATCGCTGGAGGTATTG
CCTCCTCTCTTGCTGGTGGACTACAGAACAAAGCATTCGGTGGAGTTTCTTCCGCTCCCC
AATCTGGCGTACAAGGTAATGTCCTCGCTACTGACAATAATGTAATGGGTGCTAATGATG
CTGGTATCAAATCCCCTATTCAGGGTTCTAATCCTTCCATTGGTCAACGAGCTGCTCCCG
TACCTATATCGGGCCTTCTTTCCGATACTGGCAAAGACGCCCTATCTCCAATCACTGGTG
CTTGCGTTGATAAGCTTATGGAAAATACCGGAATGTCTAAAGCTGTTGCTGCTAAAGGTA
TGGATATGAAAGATTATCTTGCTTCTGCGTTCCCCGAACTCAACCCGTGGGAACGTTCTG
GCGCTCGTGCTTCTTCTGCTTGAATGGTTGATGCTGGATTCCAGAACCAAAAGGAATTAA
CTCGAATGCAGCATGACAATCAAAATGAAATCGCTAAGATGCAGCACGATACTCAAAAGT
ATATCGCGGGTGTTCAGTCTGTTACTTCTCGGCAACATACTAAGGACTCTGTGTATGCTC
AAAACGAAATGCACCAATACAACCATAAAGAATCTATGTCACGCGTTGGCGCGATTCTCG
AGAACACAAGCCTCACCAAACAACCACAAAATTCTGACATTATGCGGCAAATGCTTCCTC
AGGCTCAAACCTCAGGTCAACACTTTACAAATGACCAAATCCAAGATCTTATTCGAAGGG
TCGGCGCTGATTTTCATGCTGTTAGTGATAACACTGATCGAACAAATGTAGAAACGGACC
GCTCTAAGCAGGAGGTCCAAAACTCTCGTTACGCTTCGTCTCAATTCGGTAAATGCGCCA
AGGATGCATCAAATGTCATGAGTGACGCATCTTCTGGAATTCTTGATTATTTCGTGGCG
TTGATAATGCTGTCGAGGATACTTGGAACAATTATTTCAAAGATGGCAAGTCGGATGGGA
TAATCTCAAACCAACGTACCAAAAAA
>NewGenomeName_38
ACCTTGACATAACCATAACTACATATCTATCATGCCTCCAAATCATCGGAGGCTTTCTTA
CATGCAACAAGCTAGTGACTTCTACAACAGACCACATATCGACGCTCTCAAACCGCTCGT
TGAAACCGCTGGTATGTCTGTGCTTTCTCAATCTCCAAACCTCCGTATCTGGAAACAGGT
AAATTACCGTATCAAACTATTGGAAGAAATCCTTGCTCATTACACCAATGGTATCCGCCG
TGATGACAACGGCGACTTCTGGATGAATCCCAATTCTCAACTCGCAACCACAATTGCCTA
TCGTGCCCATCAAAAAGGACACAACCCAAAACTTATTCAATACCCTGATACATTCACTCT
GGATGACATCATCCGTAAAAAGCCATTACCACAAAACGCGCCTGACGAATTAAAACTCTC
CGATGAAATCGGGGAAGACTATCGCCTTACAATCATCTCTCTAATCGAAGAAATGCAAGA
AATCTACGACGTGCTCGGACAACTTGATATTAATAACACTATAGACCACAAACCTATCGG
TAACGCCCACTGGAATCTACTCTATGAAAAACCTGTATATAAACACTGGTATGAACTGGT
ATCAAAACGACCCCTTAAATCAATCAGAGACGACTACAACTATGCAAAGGCTAAGTGTAT
TAAGGATGAATGCTCGAAAATCCTTGAGGAATCCACCATGAAATCACGTAGAGGCTTTAC
CGTACAAAGACTAATGAATGCTATGCGCACTGCCCACACTGAGGGCTGGTACGTTGTATT
CGATAATCTTACTCTCGCCGATGACCGCCTTAAGGATTTCTATGAGAATCCCAATGCTCT
CCCTCACTATTTCCGTGATATTGGTCGTATGGTTCTCACTGCCGAGGGTCGTTCTGTCCA
TGATTCATCTTCGGACTGCTATCAGTATTTTTGTGTGCCTGAGTATGGCACCCAGCACGG
CCGTTTACATTTTCACGCAGTGCACCTTATGCGCACACTTCCTCTGGGTTCTATCGACCC
TAACTTTGGCAAGCTGGTGCGTACTAATCGGCAAATAAATACCTTGCAAAATACATGGCC
CTATGGTTACTCTATGCCCATCGCGGTCCGGTACTCCCAAGACGCATTTTCGCGCGCTGG
CTGGCTCTGGCCCGTTGATACAAAGGGCGAACCTCTTAAAGCTACCTCGTATATGGCTGT
CGGCGTCTACGTCGCTAAATACGTTAACAAAAAATCAGATATTGACATGGCCTCTAAAGG
TCTAGGGAATAAAGAATGGAACAATTCACTCAAAACCAAAATCAACCTGCTGCTCAAGAA
AATTTTTCGCATCAGGATGTCTCGCAACTTCGGAATGAAGCTGCACACAATGAGTCACCT
CTCAGCGGAAACTCTAATCCAACTGACCCAAGTGGGCTACGACGTGACCCCGTTCAACAA
CATCTTGAAGCAGAACGCCAAGAAAGAGCTACGATTGAGGCTGGCAAAGAAATCTGTCGC
CGACGTTTTGGAGGCGCAACCTGTGACGACCAATCTGCTCAAATTCATGCGCAATTTGAC
CCGTCAAATCGGAGTGTCCAACCTGCAGAGTTTTATCGCTTCAATGACAACGAAATTAAC
AAGTATGGATATTTCTGATGAAACCAAAAACTACGTTGATACTGCAGGAATTACTGCTAC
TCACTTACGAATTAAATCGAAGTGGACTGCTGGTGGAAAATGAAGAAATTCAATCTGAAC

TTAAAAAATTCGAGAAGCTCTTACTTCGCCACCTTTCGCCACCACATGAACGTTCTGGCA
AAGACTGACGCTCTCGACGAGGAAAAATACCTAAATATGCTAGGTGCTCTCCTCAAGGAC
TGGTTCCGTTACGAAGAACATTTCGTACATGGTAAACAATCAATGCTTGACTTATTAAAT
GAACGTGGACTATTATCCACATCAACAACCAACCCAAAAGGAAATACAAAATGAACTCTA
ACGAATCTGCTGTTGCCTTCGCTACTGCTCTCGCATCTATCAAGCTCATTCAGGCTTCAT
CTGTGCTGGACCTCACCGAAGACGACTTCGATTTCTTAACCCGTGACCGTGTATGGATTG
CCACTGACCGCTCCCGTGCTCGCCGCGCTATTGAGGCCTGCGTATATGGTACATTGGACT
TTGTCGGATACCCTCGCTTTCCTGCTCCTGTTGAGTTTATTGCTGCCGTCATTGCTTATT
ATGTTCATCCCGTTAACATCCAGACAGCATATCTTATTATGGAAGGCGCCGAGTTCACCG
AAAATATTATCAATGGTATTGACCAGCCAGTTAAGGCCGCTGAACTATTCGCTTTTACCC
TGCGTGTACGCGCCGGAAACACTGACCATGTTACACATGCAGAAACTAACGTACGTGAAC
AATTACGTGCTCAAGGAGTAATGTAATGGCTAAAGGCTCTCGTCGGCGTTCGCCGTCCCG
CTTTAAAGGTGGTCGCCTCTGGTATGTTGGCGGCACACAATTCTAATCGTCTAGGGGCTT
CGGCCCCTCTATTTAAGGATATAAAATGTCTAACGTTCAAACGTCCGCTGACCGCATTCC
TAACGACCTTTCTCACCTCGTCTTCGAGGCTGGTAAAATCGGCCGTTTAAAAACTATCTC
CTGGACTCCAGTTGTCGCTGGCGACTCATTTGAAACTGAGATGGTAGGCGCTATTCGCCT
TTCTCCACTCCGTCGCGGCCTTGCCGTCGACTCTCGCGTGGATATCTTCTCTTTCTATAT
TCGGCATCGTCATATCTATGGTCAAGAATTCATTGAATTCATGAAAGCTGGCGTTAATGC
CACTCCTCTCGCACCTGTTACTACTGCCTCTGGCTGGGATGCTTCTGCCTATCTTGGCAC
TATTCCATCCTCAAACCTCAAGGTTCCTAAATTCTTAAATCAAGGATACCTTAATATTTA
CAACAACTACTTTAAGCCACCGTGGGATGCTGACCTTACATACGCTAACCCATCTAACAT
GCCTGAACAGGATTATAAATGGGGCGTTCGTGTCGCTAACTTAAAATCTATCTGGACCGC
CCCCTTACCTCCTAACTCTAAAACATCAGAGACTATGACCACTGGCGCTACGTCTATTGA
TATTATGGGCCTGCAAGCTGCTTATGCTAAATTGCACACCCAACAAGAACGTGACTACTT
CATGACTCGCTATCGCGATATTATGAAAGACTTCGGAGGTCACACCTCCTATGACGGTGA
TAACCGTCCTCTGCTGCTTATGCGCTCTGAATTTTGGGCCTCTGGCTATGACGTCGACGG
TACTGACCAATCCTCTTTAGGTCAGTTCTCCGGTCGCGTCCAACAAACATTCAATCACAA
AGTACCTCGCTTCTATGTACCTGAACACGGCGTCATTATGACTCTCGCCGTTACTCGCTT
CCCTCCTACTCATGAAATGGAGATGCACTATCTTGTCGGTAAAGAATCTTTAACCTATAC
CGACCTCGCCTGCGACCCTGCTCTTATGGCTAACCTGCCTCCGCGCGAAGTATCCCTTAA
GTAATTCTTCCACTCCTCGCCGGACTCCGCTAAGTTCAAAATGGCCGAGGGCCAATGGTA
TCGCACGCAGCCTGACCGCGTCGCATTCCCTTATAACGCACTTGACGGCTTTCCTTTCTA
CTCTGCTCTGCCGTCTACTGACTTAAAACAAAGTGTCCTAGTTAACACTGATAACTACGA
CGAAATCTTTCAGTCTATGCAACTCGCACACTGGAATATGCAAACTAAATTTAATACTAC
CGTCTATAGACATATGCCTACGACTCGTGATTCAATCATGACCTCATAAAGCCTATAACG
TGACAGGAGGAAAAATTTTAATTTTTCGCCCCCTGTCAGGTTGAACAAGCGCAAGCGCGT
TAGCTGCCTAACTTAAAAGGAAATTCAAATGTTTCAGAAATTCATCAAAAAACATAACGC
TCCGTTAACTTCTCAGCCCGCTATCTCTAAGGCTGTTACTCCATCTATGACCGCCGCCCC
TCTTGTCACTACACCTCTCGGCGTGTCTTCTACTCAATTATTCTTGGAACTTACAACCAC
TGCCGCCGCTGGCGGCTTCGCTTACGCTATCCGCGTAGACAATTCTAACCCTACTGAAAA
TCAGGTCTTCTCCGTCTGTGCTCACATCACATCTTACGTGGACCCTAAATATATTGCGTG
CCTCGTTCGCTTTGAGGTCACTGCTGGTACCACTCCTACCGCTATGCCTCCTGTCTATGA
TGCTTACCCTATCGCTGGTTACTATGACGCTGGCTCTTATACTGTTGAAGACTGCGCTAC
TATTCCTACTCACGCTGTCTCTTCTGGCAACGATGTATATGTCGGCCTTATGCTTTTCTC
GCGCATGTGGCCCGTTGCAAAACTATCCGGTATCGCTTCCCTTAATCAGGTAAACAAGGA
AGTAACCGTTCTCCAACCTCTTAAATAAGGTATTCTCTATGTTTGGTGCAATCGCTGGCG
GTATCGCCTCATCTCTTGCTGGCAGACTCATCAACAAAGCCTTTGGGGGCGGTCAATCCG
CCTCCTCTACTGGCGTTCAAGGTGATGTCCTCGCTTCTGACAATAACGTAATCGGTGCTA
ATGATGCTGGTATCAAATCCGCTATTCAAGGCTCTAATCCACCCAATGGCCAACAAGCTG
CTCCCAATGCTATTTCGGGCGTTCTCGCTGACACTGGCAAAGACGCCCTATCTTCAATCA
CTGGCGCTGGCGTTAATAAACTTATGGAAAAAATCGGACTATCTAAAACTGCTGCTGATA
AAGGCAAGGACACAAAGGATTATCTTGCTTCTGCATTCCCCGACCTCAACCCGTGGGAAC
GTGCTGGCGCTGGTGCTTCGTCTGCTGGCATGGTTGACGCTGGCTTCCAAAACCAAAAAG
AATTAACCCGCATGCAGCTTGACAATCAAAAAGAAATCGCTAAGATGCAAAACGACACAC
AAAAGGATATCGCTGGTATCCAATCTGTCACATCCCGGCAAAATACTAAGGATGCTGTCT
ATGCTCAAAACGAGATGCTCGAGTATAACCAAAAAGAATCTATGTCACGCGTTGGCTCTA
TTCTCGAAAACACAAGCCTCACCAAACAACAACAAACTTCTGAAATTATGCGGCAGATGC

TTACTCAAGCTCAAACCGCAGGTCAACATTTTACAAATGACCAAATCAAAGAACTTACAC
GAAAAGTTGGTGCTGATATTGATTCTGTCCGCGCTAACACTGACCGTACACATGTAGAAA
CTCAACGCTCAAAACAGGAAGTTCAAAACTCTCGTTATGCTGCCTCACAAGTCGGTAAAA
CCGCTAAGGATGTCTCTAATGCAGTTCCTGATACAGCTAGTTCTATTGTTGATTATTTTC
GTGGTATTGATAACAAAGTGGCAGACGCTTATAACAACTATTTCAAAGATGGTAAATCCG
ACGGAATCGGGTCGAACATCAAATAA
>NewGenomeName_39
TTGTTGACATTCCCTAAACCCTTATGAGACTCTTGCTTCAACGAATGAAGTAGGAGTCTT
TATTATGAAACCCGCTTACCGCTTGACTCATCCGATTCTCGGCTCTTGGTTGTTTAAGTG
CCCCGTTTATATCCGTTATGAGCACCTTCATCACGGTCGTCTTATCAAGGTTCGCGCTGT
TCGTGATTTGTCCCCGCGTCAGCAGCTTATTGCTAACCCTAAAAACTGCTTTGACGTTGT
TAATGCTGAGATTGACGTTACTTATTGTTCTGTCACTTATTTTAATTATGAGGCTGAGGA
ATATACCGAGTGTGATTCAGAAACTATGGATAATATCCTTGCTTATATTAACTCTCCTCA
TTCCCTTCGCTCTGAGGAGGATTCATCCGTTGATGTTGACGATATGGCGTGGCTTTATGA
AATTGCGGAGTAAGTTATGGAAACTGTTGTCGGAAAATTGATTGCTTTGGCTGTTGTTGC
TCTTATGCTGTATGGAATTGCCCGCTTAATTCGGAGGTTCCCGTGAAGTATTTTAGTCTT
GTGGATGAACATTATGCTTATTGCCCTGATACGAGGATTTTGCACATTACTCGTAGCCGT
GTTTCTGACCCTCGTTATGAGCGTCGCTTTCTTACTGATGAGGAACATTTTGCACTTTTG
GATTGGTTGAAAAATCGCCCAACTTATATTAATTCTTGACAGGCCTTTAAAATACCCTCT
ATATTGCCCTCATAGTTTCACTACGAGGGCTTTTTTGCATGGCTTCTGAAATCTTCTCTG
ATGTTGTTAAGTCTGTTTGGTCGCACGCTACGGCTAATCACGCTCATCTTCGTATGGCTG
TTCAGGTTGATAATCGTAGTCGCCTTCTTTCTGATTTGGTTTACCAGTTGGAAAACCGCC
TTTCCGAAGAGACTCATTTGGATGATGAGACTTATGCCCTTTATGAATCTCAGACTGGCC
TTTTGGAAGATCATATGGCACTGGTTCGGAAGTGTGCAGCCCAACTTGATAATAGTAACA
CTATAGACCACCGTACCCCTTTGGATGCCGAATGTGTTTTCCTGTTTGAAATGATTTGCC
CAGGCGTTCGCTACGGTAAAACGTTGAATTCTCTTTGGACTAAGTATGTTGCTCAATGGC
CTGAGAAGTTAATTCGTCAAGAGTTAGACATGGTTAAGCCTTTATCCTTTTAGGATGAGG
TTGCTAAGTACATGGAGAAGAGGCAGGAAAAAACACGCAAAAATCGCATGACTCAAAAAG
TAATTAATGAGATGCGTATTGCTCACCAAAAGGGCTGGTTTTTCGTATTTGATACGTTGA
CACTTGCTGATGATCGTTTGCAGGCTTTTAATGAAAACCCCAATGCTCTTCGTGATTCTT
TCAGGACTGTTGGTCGAGCTGTTCTGCGCGCTGAAGGTCGTTCGGTTAAGGATTCCTACA
ACGATTGCTATCGCTATCTTTGTGTGCCGGAGTTTGGAGGTCTGCACGGTCGTCTTCATT
GGCATGTTGTGCCTATGGTGCGGACTCTTCCTTTGGGAAGTTACGACCCTAATTTCGGTC
GCAAGGTACGTAATTATCGCCAGATTAACTCGTTCAGTGGCATGTGGCCTTATGGCTTCA
CGCAACCTATAGCTGTACGCTATCAACATGATGCTTATTCTCGCAAAGGTTGGTTGTGGC
CTGTTGACAAATCAGGTAAAGCAATGCAGAGTAAGCCCTATCAGGCGGTCGCATGGTATG
TGACCAAATATGTAGCTAAGCAATCTGACCAGCGTCAGAAAGCTATTACTGAGAGACAAA
AAAAATGCAAGAATCCACTTATGGCAATCTGTCTGAAAAAGGAATTCAGTGTTCGCAACA
GCCGGAAACTCGGAATGGAGTTACCGTCGATGGCGCATCTCAGCAACAAAGTGCTTCTGG
AGCTGAGCCGAATCAGCTTCGATTCCAGCCCTCTGTATCAGATAGTGAAAGAGAACGCCA
AAAAGCAATTGACCTTGAACATCGGCGCGCTGCCTTTGCACGTCATTTTGGATGTGCGCC
CGGAAGTGAGAAGTATGTTGAAAAATATCCGTCGTTTGATGAAAAAGACACCCGAGTTCA
ATTGGCAGAGTTCTATCGCTTCAATGACGGTCACCTTAAAAAATGGGGATATTTCTGATG
AAGCACGTCAATACATTAATGATGCAGGAATTACGCCTTTTGACTTGCGAGCTAAAGCGA
CTCAAACTTTCGGCGGTAAGTGACCCGGATTTTTCTCAGGAGTAGATTCATGCTGAGCTC
GACTCGTTACTACGCAAGTTATCGCGCTACTTTGACTAAGCAGCTGATGTTTTTGACTAA
AGCTGACTTTACCAATGATGATGAAAAATGGCTTAATGCCTTTGGTAATTTGCTCCGTCA
GTGGTTTCAGATTGAGGATTGGAAAGGGAATCATAAAAAATTGCTTGACGATTTAAAAAA
ACGTGATTACATTTGAGCTGTTTCAAGGTTTCTTACCCATTAAATGTTAGGAGAATGTTA
TGTCTAATCTTGTTGCTACTGATGTTAACTTTGCTACTTCTGTTGCTGCTCTGAAGATGC
TTCAGGCTTCCGCTGTTCTGGATATTACCGATGAGGATTTTGATTTCCTGACTGGTGATA
AAATTTGGATTGCTACTCACCGCAACCGTGCGCGTCGCTGTGTTGAGGCTTGTGGTTATG
GAACACTGGACTTTGTGGGATACCCTCGCTTTCCTGCTCCTGTTGAGTTTATTGCTGCCG
TCATTGCTTATTATGTTCATCCCGTCAACATTCAGACGGCCTGCCTTATCATGGAAGGCG
CTGAGTTTACGGAAAACATTGTTAATGGCGTCGAGCGTCCGGTTAAAGCGGCTGAGCTCT
TCGCGTACACCTTGCGTGTTAAGGCCGGATTCAAAGAAACGGTTCTGAGCGCTGAAGAGA
ATGCTCGTCAGAAACTTCGTGCAAGTGGAGTAATGTAATATGAAGAAAGCTCGTCGTTCC

CCTTCTCGTAAGAAAGGCGCTCGTCTCTGGTATGTAGGCGGTTCTCAATTTTAATTAACG
GGGCTTCGGCCCCTATAAGAATTCTTAAATGTCAAACGTGCAAACTTCCGCTGAACGTAT
TCCTCACGACCTTTCTCATCTGGTTTTTGATGCAGGCAAAATTGGTCGTTTAAAGGTTGT
TTCTTGGACTCCTGTTGTTGCAGGTGATTCATTTGAGCTTGATATGATTGGCGCTATTCG
CCTTTCCCCGCTTCGTCGTGGCCTTGCTGTTGACTCTCGTGTTGACCCGTTCACGTTTTA
TGTCCCTCATCGTCATGTATATGGTGAGCAGTGGATTGATTTTATGAAGCAAGGTGTTAA
TGCTGCTCCTTTGGCCCCTGTTCAGTGCTCTCGCGGTTGGGATAGTGCTCAGTATCTTGC
TACTATTCCGTCTAGTAACTTAAAGGTTCCTAAGTTTCTTCATCCAGGTTACCTGAACAT
TTACAATAATTACTTTAAAGCTCCGTGGATGGATGACCTGACTTATGCCAACCCGTCTAA
TATGGAGAGTGAAGATGTTCGTTTTGGTGTTCGTGCATGTAACTTGAAAACTATTTGGAC
TGCTCCGCTTCCTCCGGATACTGAAACCTCTCATTCTATGGAGACTGAAACCAACTCCAT
TGACATTATGGGTTTGCAGGCCGCTTATGCTCAATTACATACTGAGCAGGAGCGTGATTA
CTTCATGACTCGTTATCGTGATATTGTTAACGAATTTGGAGGTAGTACTTCTTATGATGC
GGATAATCGTCCTTTGCTTGTTATGCATTCTGAGTTCTGGGCCTCAGGCTATGATGTTGA
CGGCACCGACCAAAGCTCTCTCGGTCAGTTCTCCGGTCTTGGCCAGCAGACGTTTAAGCA
TTCAGTACCGCGATTCTTCTGCCCTGAGCATGGCACTATGTTCACACTCGCATTGGTCCG
CTTCCCGCCGACTCATGAAATGGAGATGCATTACTTAGTTGGTAAAGAAGATTTGACTTA
TACTGATTTGGCTTGTGACCCTGCTTTGATGGCAAACTTGCCTCCTCGTGAGGTTAATAT
GTCTCAGTTCTTCCATTCTGGAAGTGCTAGTGCTAAGTTTAAGATCGCTGAAGGTAAGTG
GTATCGCATGCAGCCTGATCGCGTTGCATTGCCGTATAACTATCTTGATGGTTTTCCGTT
CTATAGCGCGATTCCGTCTGATGATATTAAGCAGCGCGTTCTGGTTAATACTGATAACTA
CGATGAGGTTTTCCAGTCTATGCAGCTTGCGCACTGGAATATGCAGACTAAGTTTAATTG
CAGCATTTATCGTAATATGCCGACCACTCGCGACTCTATTATGACCTCTTGATTATGTGG
GGCTTCGGCCCCCTTTAAGGATATTGATTATGTTTCAAGTTTATGTATCTAAACATGTTG
CTCCGTTCACCTCTGTGAATCGTGCCTTTTCTATTGCCCCTGCCGCCAATCCCGAAGTTG
TTAACTCTGGTGGGATTGTTGCAGGTCGTTCTACTTTTCGCATTGCTTCTTCTATTGCTT
CTGCTTCCAATGGTAGTGGTTTTGCTTTTGTTCAGATGATGGATCCGAATTCTCCTGATT
CACAGCAGGTTTTCTCTGTTGCTGCTTCGTTGTCCTTTAGTGTTGATTCGAATTATATTC
CTTGTGTTGTTCGTTTTGAGAGCACTGTTGCTCAGCCTACCAGCTTTGATATTGCAACCG
CTGATTTTATTCCGGTTGAGGCTTCTGCCCTTTCTCCTCGTGCGCTTAGCGTTCGCGATT
GTGTTACCGTTGATGTTAAGCCTCGTACTGAAGGCAATAATGTTTTTGTTGGTGTGGTTT
TCTTCTCTGCTGCCGCTAATGCTGGTAATGTCACTGGTGTTGTTTCGATGGCTCAGGTTG
ACCACGAAGTTACAGCTTTGCAGCCGCTTAAATAAAAGGCTGCCGCACTCCCGGTTAGAT
GCCTGCCTAGTGTAGGGCAGACCGAGCCGTACGGAGATACCCGATAAACTAGGAACGTGG
AAGGCGCAAGCCGCCTTCCCTCCTGCTAAGCCCCAAAAGGAGTTACAGCGATGTTAGGTG
CCGTTGTAGGTGGTATAGCTTCAGCCTTAGCTGGTGGAGCCGCTTCTAAGCTGTTTGGTG
GTAAACCTGCAGCTCAAGCCGCTACCGAGTCCTCTGGCCTTTCTAATGGTCAAGGCGTTA
TTGGTATGGATCAAGATGCTGGTATTCAGTCAGCTATTCAGGGCTCTAATGTTCCCAATG
GTCAGATTCCTGCGCCCACGCAGACTGGTGGCGTTATGTCTGATGCTAAAAATATGATTA
AGAATGCTGGTAAAGATTTGCTTGATGGCGTTATGACCGCTGGAAGTGATCGCATTAAGC
AGGCATTGGTTGATAAGTTGAGTGGTAACGATGCAAAGAAAAAAGGACAAGCAACCCGTG
ATTACTGGGCTGCGGCCTTCCCTGAGCTGAATGCATGGGAACGTGCTGGAGCTGGTGCTT
CCGGTGCCGGATTGGAGTCTTCCGGCCAGAATCAGCAAAAGGGGTTGATGCGTATGCAGC
TGGATACTCAGAAGGATATTGCTAAGATGCAAATGCATAATAATTTGCAGATTGCTGGTA
TTCAGTCCGCTACTTCTCGCGAGAATACGAAGGATTCAGTTTATGCTCAGAATGAGATGC
TTAAATTTAAGCAACAGGAGTCACAGGCACGCGTTGAGTCTATCCTCGCCAACACTGACC
TTACGGTTAAACAGGCTGCCCATGAAATTATGAGAATGGCCCTTACTCGTGCTCAGGAGA
CTGGTCCGCATTTAACTAATAGTCAGATTATGGCATTGGAGAAAAAAGTTTATGCAGAAA
TCGGAAAAATTCATCAAGATACTCAAAACGCTCGTTATGGTAGCTCTCAAGTTACTGCTG
CGGCGAAAGATGTTACGAATATGATTACCGATGCCACTTCTGGTGCTGCCGATTGGGTCT
CTCAACAGTGGAATTCTTTCTTTAAGGATGGCAAGTCTAATGGTATTCCCCTCAATACCA
GAAAATAA
>NewGenomeName_40
CGATTCCCGGTGGTTACTATTCCATCACCAGTTTAGCCTTAACACAGAATGTCAAGATGG
ACGAATCGCTCGACTTCGCCCGCCATTCGTTGAAGGCGGGGGGCCGGAGGCCCACCGGTT
GGCCCCATGCAACGAGTGGCGCACCTAAAGGGGGGCACGTCATCCGTACAGGGGGGTACG
TCATGCTTACAGGGGGGTACGTCTCAAAGGGGCGTCCCCGTACCGGGAGTTGCGTCACGC

GTACAGGGGGGTACGTCACTGCCAATCACAAGCCGCCACGTTGCGAAAGTGACGTTTCCA
AAATGGGAGGCGCCAGCCTCTCTAAATATTTAGCGCTCTTACCGGTCGGCAGTAGCTATA
CGCATGGCGATTCGGGTGGATGCTCGGGAACGGCCGACAATCGGCCGCTGGGGCCAGTGA
ATTGGCGCTTAGCGGAGAGGGGCAAACTGGACCCAGCTGAGCCGGGCAGGGGCAAGTAAT
TTCAAAAGAACGCTCTCCGAGAAGATTCTCCACTAGGACCATCGACGGTGTTCAGCGCAC
CAACAAGATCACGGGCGATGAAAACCCCTCAAGGCAGCGACATCCGGATTGGTATCGCTG
GAATTACAATCACTCTATCGCTGTGTCGCTACACGAGTGCTCCCGCTCCCGCGCTAAGAT
ATGCAACTGCGGACAATTCCGAAAGTACTGGTTTCAAGAATGTGCCGGACTTGGGGATCG
ATCAACCCAGGCCTCCCTCGCAGAGGCGATCGTGCGACCCCTCCGAGGAAAGGGTACGCG
GGGTAAAAGACACCTTGAAAACCACTACTCCCAGACAACCCCGGACCGCAAAAAGGCGTA
AAAGATTGTAAGATGGCCAGACGAGCTCGCAGACCGAGAGGTAGAATTTACTCCTTCGGA
AGAAGACGGTGGCACCACCTCAAGCGACTTCGACGAAGATATAAATTTCGAAATCGGAGG
AGTCAGCGGTTTCGTAGACGAGGTTTTGGGAAGGCCTTTCACAACCCCCGCCCCGGTACG
GATAATGCAACGCTGCCGAACCCCCAATCTACTATGACTATCCGCTTCCAAGGGCTCTTC
TGTCTTACGGAAGGACTCATTCTGCTTAAAAACAGCACAGCTGGGGGCTATGCAGACCAC
ATGTACCGGGCGAGTGTCGCCAAGATATTCGAGAACATGAAAGCGTTCCTGCTAACCCCA
ATGAACCTGACATACGGGAGCAAGATCGGAGGCCCAATCACCGGTGAGTTGATTACGGAC
GGGTCTAGATATCAAGCCGCGGACAATTGGACTAATTGCTGGCTGCCGCTAGATAATAAC
GAGTCCTCCGCTACACCATCGGCATTGTGGAGACGGGCCTTAATGAGATTGCCGCCTACG
GACTCTTGACGGTTCTTTAATCACTCCAAGCAGACGACACTGCAAGACATGGGTCGCCGG
TTTGGGGGCTGGCGCCTGTTCCGACACATTAAAACCCAGTTTCACCTCCTTGCCACTAAA
AATGGGGGATCCTTCAGCCCTGTGGCGAGTTTTCTCTTCCAGGGAGAGTAACTCCCGCGT
AGAGATAATGTTATTTACAGCAGCGATCACCCGAACCGGGGGCAAGAAGGCAGACAACCG
ATGACGGGGGGCATTGCTTATGCGACCGGGAAAATGAGACCCGACGTGCAGCAGTACCCT
GCTATGCCCCCAGACCCCCTGATCATCACCGCTACTACAGCGCAGGGCACGCAAGTTGGC
TCCCTGAATAACACGCAAGCTTTGTGGTCATGTGTCGCAGATTTGCGCTTTGCTATACTC
ACAGTACACGGTGCACAATCGTCTTTTCCTCCAGCGCAACGTTCATTTTCTAGACTGTCC
TTCAACCCCCGTAAGGCGAGAGGAGCCGGGGTACCCGAGGGCAAGAGATGGCACATGCTG
GTGCCGCTCGGCACGGAGGCCAGCGCCGACAGCTTCATGTCAACACGCGCATCAGAGCTG
GACACTAATTTCGTTACGTTCTAAGTAGGGCCAGGCACAAATAAGTAGCAACAGTACAAG
TCCAGCACAGCTCTCTACGCGCTATCGGAGCCGCTAATGATCATCGAAGCATGGGCAATA
CTACGCGTCCAGTCTTTCTGGGAGCTGGGTGACAGGGAGAGGCCATACCCATGGGAATTC
AACTAGGCGACGAGCACCAGGTACTGGGAGAGGCAGGCCTGAAGAGTGGGGGGGGCAAAA
GCCCCCCCCCCTTAAACCCCCCCCTGGGTGCGAATCCCCCCCAGGATCCCCTTTTATATT
GCACACAATAAACGCAGAAAATAGATTTATCGCACTCTA
>NewGenomeName_41
CTCTACCTACTATACTTGCAAATTATCGGCATCAAGTTTTGCCATTAAGAGCATATAATT
TGTTATTTAATGAGTATTTTCGTGACGAGAATGTCCAGGAGTCTTTACCTGTTTGGACAG
GAGATGCTGACCCTAAGGCTGATCCGACTACTGGAGAAGAATATCAAGAGGATGATGCAG
TTCCTTATGTATATAAGTTAATACGTTGCAATTAGCGATATGATTAGTTTACTTCTGCTC
TTGCGGGTTTGCAGAAAGGTCCTTCTATTGGAATATGTATTACAGGTGGAGATTCGGGAC
GTCTTCCGGTTCATGGTTTAGCGATTAGATCTTATTTGGGTGATTCTTCTGAGGATCAGT
GTAGTTTGGGCGTTTCTTATGTAAACGGGTCATAGAGATGGGTTACTGCAGACGGTCGTT
TGGCTTCTGCAATGGGTAGTGTTCCTGTTGGTACTACTGGTAATTTTCCTATTGATAATG
TTGTGCCGCCATCTTATTTTGGTACGACTGTTGCCCAAACTGGTAGGCCATCCGCTTCTT
CGTCTCCGCCTTTTGTCACGCGTTATTTTCCTATTTATGTTGATTTAGCGGCTTCATCTT
CCGTTACGATTAATTCGCTTCGTAATCCGATTACTTCGCAACAGTGGTTTCAGAAGAGTG
CTCGTTTTGGAAGTATATAGGTTGAATCTGTTCAAGGTCATTTTGGCGTTCATCTTGGTG
AGTATCGTGCTCAGCGACCTATTTAGTTAGGTGAATCTGAGCCTTATGTTCATGTTAATC
CTGTAGTACAGAACTCATCTACAGAGTCCGCTTCTCCTCAAGGAAATCTATCTGCTTATG
CATTATCTTCAGATACGAAACATTTTTTTACGAAGTCTTTTGTTGAGCATGGTTATGTTA
TAGCTCTTCGTGCCCCTACGGCGGATATAACTTATCAGCGACGTTTAGTGCGTCAGTGGT
CAAGATTTAGTTGTTCTGATTATTATTGGCCTACTTTTGCTGAATTGGGAGCGCAGCCTG
TTTATAATAAAGAGTTTGATTGCCAATGAGATACTGGTATGGATCCTTGTGGTCCTGAAG
TTAATGATGTGCCTTTTGGTTATCAAGTGCGTTATGCGGAGTATCGTTATATGCCTTCGA
TGGTTACTGGATTATTTAGATCTAACGCTACAGGTACTGTAGATTCTCGGCATTTGTCTC
AGAATTTTGTGAATTTACCAACTTTGTCTGAGACTTTTCTACACGATAATACGCCGAGAG

ATAGATCGTTAGCAGTTCCTGATTAGACTGATTTTATTGGTGACTTCTACTTTAATTATC
GTTGTATTAGGCCTATGCCTTTGTATTCTGTTCTAGGTTTAAGAAGGATTTCAGATCCCA
ACGAGAGTAACCCAGTGAATTCGAGAAGAGCAGCGAAAGAAAATAGTAGTCAATGTAACG
TAGTGGAATTGAACAATACTGTTCTGAAGCGAAAAGTATGCATTGGGCCACGTAAATTAC
ATAATTTTAGAGGCATTAAGAGTTTCACGGAGAATGTTGTTAGATTCAAAGGAAATTATG
TGAATTCCGCCGGAAGTGTTAGAGGAGATGATCTTCAAGGTTTTTATTCCACTCAGTCTA
TTTCCAGTGGTAAGCTGGCAAGCCTTCTTAATAATTTGCCTGTGCCTTCTCTAGGTAATG
GTTTAATTATTGGGATGTTTGGCGGTCTTGCTTCATGCCTTCTTTCTTATAGAGCTGTTA
AAAAGAAAAATAAGGTTATGCAGGATATTACTAAAAGACAAATGGCTCTTCAGGAGCGAA
TGTGCAGCTCGGCTGTTCGTCGGCATGAGAAGGACTTAAAGAAGGCAGGTTGGAATGCGC
TTTTAGCATGTGGGGGATCTACTTCTACTCCTCAAGCTGCTTTTTATTCTCCTGTTAATC
ATATGGAGTGAGGACTTAATTCTGCGATATCAGTTGCGGCTAAATTTTTTGAATATCAGG
GTTTAGCTTATGCTGATCTTCAGGGTCGTTTGAATTCTGCTATGAGCGTGGTTCAGTTGG
CTTATGCTGTTCAGAATTATAAAAGGAACTATGGACAGTTTGGTGATGTTGCATATTGGT
CTGATCGACATGCTGGCGAGTGGTTGCCTGCTAGGCTTTTCTATTTGTTAAGAAAGTATC
CCTTTGGAATAGCGGTTTCAGCTGCAAATTCTGGTTATGCTGTTCCTAAGGGTGCTAAAG
GTGTTAATTTGATGGTTTCGAATTTGAATAGAACGGCTTTTCAGCGTAATAATTCTAGAT
ACTATGTTTCGATGGGATGGAGAAGTTAATCAAGTTTAGAACGATTTATGATGAGGAGCG
TCCTGCTCCTGATTTGGAGTGTAAGGATGAACGTCTATGTATAGCTTATCAATGTACTGA
GACGTCTCTAGCAAAATTGGTTAAGTTCGAGAATCAGAATCCTTCTTATTTACATGCATT
TGCTCTTGATTCTACTCGTCAACCGGAATATGGAGAGGGGCCAACTCGTTTGGATTATCA
AGACCCTTTAGAGATTGTAGCTCGTAGTGAAGAGGCTTTTTATTCTTTACCTGCGAATAT
TCGAGGTAATCTTTCGAATCCAATGGAGTTTTTGTCATGATTGGATGACCCTGCTAATTA
TGATGAAGTTGAGAAGGTAGGTTTATTGGATCCTGAGAAAGGTCAGATAAGAGAATCTAA
GTGACAAAAAGATCAAAAAGATGAGGTTTCTACTGAGGATAAATAGCGAGCGCTATATCC
ACTTGCCTATATAGCCCGAGCGACACAAAATATATAAAATCTTAATAGAAGGATGTAATT
CGATGGTTGGTAGAAGACGTTTGAGTAGAGGAATAAGTAGAAGAATTTGTAGAAGAGCAG
AAGCTAGAGTTGGAAGAAGGCGTAGCTCATTTAGTGGTGGTTGTAGATTGTAAGGCAAAA
AAAATGGAGTATCTTTATAATGAGCTACTGCTTAGACTCATATCTAATATCCGTTTATAT
CCGTTCAACACAAAGGAAATCAGATTATATGTGTACTGATCCTATTATACCTATAGTTCA
ATATAAAGTTCCAGTGAAATCTTCGTTAGGTGTTGTGGATTGGTCTACATTTAGGTCTAA
GTTTAAGTCTAATCGGTTTTTTTTCGAGAAGAATGTTGTTCGTCGTGCGGTAAGTAATGA
AGATGAAGCTTTTAGATTTACTGAGCAAGTGAAGACAGTTAGTTATTTATCTACTTATGA
CCTCGATGGTTATCACCAGGTGAAGCAGTTTTCTTTTCCTCATCTTTGTAGGAAATGTTC
TGAGTGTTTCTAGTAGCGTTCTAAGGATTTAGCGGTTCAAGCTCCTATGACAGCGCGTTT
TCATGAGGAGAATTCAGTTTTGATTCATTCCTATGATAATGATCATTTAGGCGATCATCT
TTTAAGTTATGATCATATTCGAGTTGTTCAGAAGCGTCTGAGTCGTTATGTGGATTATCA
CTATAGCAAAAAGATTAAGTTTTTGACTGTAGGAGAATATGCTGATAAGAAAGATCGTCT
GCAATGGTATATGATTGTTTCTGCTTGGAAGCCGTAATCTGACAAACAATTAGTGCCTCA
TTTAGGATGAAGGTATCGAACGGATGTTCGGTGTCGTTCTCGAAAGCTTAAGGAACTATG
GAAATTCGGTTAAGTTGACGTAGATGAAGCTACACATGGTAATATTTTTAATGTAGCTCA
TTATGTGCAGAAAAAGATTGTGGTTGGATGTGACTTAGATTCTTATAAGTCTTGTTCTAG
GAGTGAGAAGACGACAACTCCTCAAGCTTTAGCTTTTGCTTCTTTTTTTTCATATTTAAG
GCAATTTCTTACGACTCAGAGTATAGTTTTAAATTGTTTCAAATACTGATTTCCGCGTTA
TTTTAAGGGTTTATTGAGGATGTTGGTTTCTGCGGATTCGGAGTTTGATCCTGAGTATTA
TAATGGTTTTAGGAAAAGGTTACTTAGTGTATGTTGTTATTCGTTGGCAAATAAATATTT
TACATATTTAGAATGCTTAGTTGAAGTTTTGCGAGTTTTGAATATTCATGATTTATACCA
GGGTGCGCTTAGGTGTATGGATCAATCTTTTCCTATGCCGCAAGCTGGTGATCATGATGG
AGACTATAATACTAGTTAGGAGATCTGATGCATAAGTTTTGTTATTCAATTTATGATCGA
AAGGCTCGGCCTTAGGGAGATTTGATCTCTTTGCCTTCAGGTGAGAAAGAGGCTGCTATT
CGATGTTTTATAGATGTTGTGATGGATTCAGATTCTAAGAATATTTTGCATCGATATCCT
GAGGATTTTGATTATTGCTTTATTGGTCATTTTGATAAGGATAAAGGACGCCTTTATCCT
GTTGATGCTGGGATAGGTACGATTATTAATGCAGGAGAGTTTTTTTTAGATTCTGAGTAT
AGACAAGAAGAGGTTTAATATGCCTAAAGGCCGAAAGCTTCCGTCGATTGTGAATAATCG
TTACTCAGTGGTACCGACAGCCACGATTAGGCGTTCTTGCTTTGATCGGTCTCACGGTTA
TAAGAGCACGTTTGATTTGAATTATTTAGTTCCATTTTTTGGTGATGAAGTTCTTCCAGG
AGATACTTTTTCTCTATCGGATACGCATCTTAGTCGTTTGACTATGTTAGTGCAACCGAT

CATGGATAATATTCAGTCAACTACCCAGTTTTTCTGTGTTCCCAATCGTTTGCTTTGTGA
TTATGCGGAATTGTTTATTACAGTAGGTGATGAGCCTGTAGCATGGACAAGCACTAATCC
TGCTAATGAGTATTTTGTGCCTCAAGTAACTTCTCCGGACGGAGGTTACGCAGGGAATTG
TATTTAGTACTATTCTG
>NewGenomeName_42
AAAACATGTACTACCCCACCCCTTTTACAAAAATTCCCCCAATGGAAATTTGAAAAGGAA
ATGAAAGAGAAAATCCATTTCCAACACTAGCCATTCTAAATATCAATCATCTATTATCTT
CTAAAATCCTTTCATCTGTTTCTATTATCATTATATCACAATTTAGAAAAATGTCAAGAA
ATAAAATCACTAAATAAATAAAAAATAATTTCAAAAATCTCTTGACAATCATTTTAGTAA
ATGCTATAATGGTTATAGTTAGAAATGAGATAAAAATATTACTAAAAATAAATTAAAAAT
AATTTCAAAAAACACTTGACTAGCATTTTGAGAAATGCTATAATAGTATCAGTTAAAACA
AAGGAGAAAGAAAACATGAAAATGAAAGCCTTATATGTAAATCACAACGATGAAACTTTT
GCAGAACTTACACACGGGCAAGCGCTAGATGTAATCTTTCATGCTGATAACGCTGAAATG
TATGGCGACCATGTAATCCAGATTGTAGACCTAGGAAATGAGATTAAACTTGAATTGTAT
AGAAATAATGAATACATTCCATTTGCTGGTTACTATGAACGAGTGAAAACACTGTCTGAA
CTATACACAAATATTTCGGACTGGTTTGAAGAAGTTGAGTAAAGGAGATAATAAAAATGG
AATTAAGACAATTATCAGTAAACAACATTGAAAATCTTACTAAAAAGATTATTGACCTTA
AAACGCTAACGTTTAAGCCAGATGAATTTATAGAAAGCTCTATGGTATGTAGCGAGGAAC
TTATAACCAGCACACTTAAAGTTATGTATGTGTTTAGTGTTAAGTGGGATTTAAAAGATT
AATTTTACATAGATTTTTTCTATAAAATTTTAAATAATAGAAACGATGAATTACACATTA
AACGATTAAAATTTATTTGCTATTGTTGGTTAAATACAGAAAAATAATTTACTTTCTTAT
TAAGTTTATCACTTGCTTGCTATTTGAAAATGTGGTAAACTTAAATAAGAAAATAAATAA
TATTTATTTTCTTAAATGTAAAAAGCAAATTTTAAAGAAAGGAGTAAAAGCACTAGACAG
ATTTACCTATCAAATGTAGCTTTAACGCTACGCAGGTCACTTTCAATCTTTACAAAAATG
AAGATGGGAATGTGACTATCACTACTGAACAAGTGACGATAAACCAATGTCGCCAGCTTC
CTTACATTGAACGTTATCTGAAAGAGCGTTTCAAGGGCTAACTCACTATTGAGGTAGTAG
ATTATGAATATAAAAGTTATTCGGCTTCTATCCCTTTCGCTACCGCCTTAGAATACGCAG
AGGAACAACAAGCGGAAGGGGTGTAATTAAATGGCTTTAACACCAAAACAAAGGAAGGTA
AGACGGGACTATTTAACCAGAAAGAAAAGCACACTACAACAGCAGGGCGCCTCTAATGCT
GAAATAAAAGCTATTATGGGCGGTCGGTGGAATTTTGCAGGAATGAGTGATAAGGAGCTA
GAACGTGCCTATGATGGGATTAAAAGCAAAGGTCGTACTCAAGTATTTGGCAATCATGTT
TACACTAGTGACTATGTAAAAAAAGCTAAGGCATGGTACGGGGATAAGTTTTCTGTAGAA
AAGCTGACTCAAGGTTTTCGCTCTTCTGAACGATCAGAGTTGAACCGCTTTCATTCAGTT
AAGGAAGTCAAAGAATACCGCTCAGAACGTGACAGAGAAGCCAAAGAACGCTCTATACAA
GCGGTGGAAGAGATGCACTATAATACAAGAGACGCAGGCAACAAGTCGCAATAAAAAGCC
ATTAAGTCTATGATTTCACGCATAAGGCGAATGAGTGCCAGCAACTTTTGTGCATTTCTA
ACAGGCGGACGGTCTGACAAGGTTTCATTTGATAATGTCATGGTTTTTGTAGACACGGAC
GGTAAGGAAACGGCTTTTGAATTTCAGGACAGTCTAGCCCGTGAAATCCTTGATAATGTA
GATAAGTTTTCCAAGCAATTTGTTTCTGATATGAGAAGACGAAAGAAGCGAGGGAAGAAG
TGACTTGCTACTATGCAGGCGACTTTGAAACAACTACAAACGAGGAAGACACAGAGGTAT
GGCTATCTTGCTTTGCTAAAGTTATTGACAATGACAAGTTAGACACATTCAAGGTAAAGA
CTAGCCTAGAGGATTTTCTAAAATCGCTCTATCTTGACCTATACAAGACTTATACGGAGA
CGGGAGAAAATGAATTTATCATATTCTTTCATAATCCCAAGTTTGACGGCTCTTTCTTGT
TATCTTTCTTTCTAAATAATGATATAGAATGTACTTACTTTATTAACGATATGGGGGTAT
GGTATTCTATTACGCTAGAATTTCCAGACTTTACGCTACCTTTTAGAGATTCTTTAAAAA
TCCTTAATTTCTCAATCGCTACGATGGCTGGACTGTTTAAAATGCCAATTGCTAAAGGGA
CTACACCTTTCCTAAAACATAAGCCAGAAGTGATTAAGCCAGAATGGATTGACTACATTC
ATGTATACGTTGCAATACTAGCCCGTGGTATCTTTGCTATGTACTATGAAGAAAATTTTA
CAAAGTACACATCAGCAAGTGAAGCGCTGACCGAGTTTAAACGGATTTTCAGGAAGTCTA
AACGAAAATTCAGAGCCTTTTTCCCGATCTTAGATGTAAAGGTGGACGACTTTTGCCGTA
AGCATATCGTGGGGGCTGGACGTTTGCCAACCCTAAAACACAAGGGGCGAACCTTGAACC
AGTTGATAGATATTTATGACATTAACAGTATGTATCCTGCTACTATGCTACAGAACGCTT
TGCCAATCGGAATACCGAAACGATACAAAGGCAAGCCCAAAGAGATAAAGGAAGACCACT
ATTATATCTACCACATTAAAGCCGATTTTGACTTAAAACGTGGCTACCTCCCAACTATCC
AGATTAAGAAAAAACTGGACGGTTTAAGAATCGGAGTCCGCACTAGCGACTATGTGCCAA
CATCGAAGAACGAGGTCATAGATTTAGATTTGACTAATTTTGACCTTGATTTATTCTTAA
AGCACTATGACGCAACTATCATGTATGTTGAAACACTTGAATTTCAGACAGAATCGGACT

TGTTTGATGATTATATCACAACTTACAGATACAAGAAAGATAACGCACAAAGCCCAGCAG
AAAAACAAAAGACTAAGATTATGCTAAATAGCTTATACGGGAAGTTTGGCGCTAAAATCA
TATCTGTTAAGAAACTAGCCTATCTGGACGATAAAGGTATATTACGCTTTAAAAATGACG
ATGAAGATGAAGTACAGCCCGTTTATGCACCCGTGGCGCTTTTTGTAACATCTATTGCCC
GTCACTTTATCATCTCAAACGCACAAGAAAACTATGATAATTTCTTATATGCTGATACAG
ACAGCTTGCACTTGCTCCATTCTGACAGCCTTGTCCTTGACATTGACCCGTCAGAGTTCG
GCAAGTGGGCGCATGAGGGGAGAGCCGTTAAGGCAAAATATTTACGCTCTAAACTGTATA
TAGAGGAACTGATAAAAGAAGACGGGACAACGCACCTAGACGTTTAGGGCGCAGGTATGA
CCCCAGAAATCAAGGAAAAAATCACTATTGAAAACTTTGTTATCGGGGCAACCTTTGAGG
GTAAGCGTGCAAGTAAGCACATTAAAGAGGGTACGCTGATTTATGAAACAACCTTTAAAA
TCAGAGAAACAGACTATCTTGTTTGATGGCCTTATACTCTCGGTATACCGCTCATTTTTT
AAAAATTTATTACATAAACAACAGGTTGAAGATAAAAAAGGGTACTACTACCAGAAATCA
AACAACGCACGTAAAAATACTATTTTTCTAAAGTCCTATCTAAAAGCACATTATGCCTAT
GAAAATTTTGACTATATCATGGAATTATACAAATTTGTTTCACAAGGATTTGATAAAATT
TCAATCAATGCTTTTTACAATCTTTGCAACTATTTAGAAGAAAATAAAATCTATTATCTG
TCTTCTAACGCTCTTTACGATGGTTACGAGAAATCCAAAAATCGTCAAAACGATTTAGAA
AATCACAATACAATCATCACACCATTAAGATTTTTAAAATCATCAAACGGAGATAAACAA
AATGTCTAAACAAACACAAAAACATGAAAACTTTGATACAGTTGTGGCTCAGGCTACAAT
CACAGCAACGTCTAACAAATCAGACGGCAAGAATAAGCAAAAGAAAGCGACTAAAGCCGT
TTACCTTGTCCCAGCGACAGAAGAAGACACTAAAAAGCTCGTAGACTTTGGCATGACTTT
ATACACACCAGACACGGAGAAGGACCCAGACGCAAAACCTTACTTTAATGTTAAGGCAAC
CGAAAATGTGAAAATTTTCACAAGTGAAACGGACTTTGAAGAGGTCAACTTCGGGGTATC
TTACGAAGACGTAAACCCAGAGACAGGGGAAATCACAGTCAAGAAGACCCCGAACTACAA
AACAGAAGAACCCGTACACGTTGCGATTATGTTTGTTGAGGGTGGCGACAATGGAAACGA
CTTTTTCCGTGTTAATGCTCTGATGATGGCTGACACGTTGACCCTTGAAGAAGTGCAACC
CGTGAACCCGTTCGCTGGATTGTTTGGAAAATAAGAAAGAGCCTTCCCAATCGGGAAAGC
TCCAATTATAAAGCGTTTTTCATGGCTTGAGAAGTCAGTTGGTTACAATGACTCGCACCA
GCAAGCACCCCTTGAGGTGTAACCATCTTGCCAGCACTAGACAGGCGTTGAAAAGCCTTA
CAGGTTTATCATATCATACTTGCTTTATTTTGTCAAGTATGATATACTTTTGTTAAAAAT
TGAAAGGAGAGGGCTATGACCTCACAGGAATGCCTCGCAGTGCTAGACGCTGCAATGGCA
AAAGTCGGAAATGATGTAGAAATTGAGAGCCTAACGGCTGACTTGATTGAAATTAAGGCT
TTTGTCGGAGAAATTGACACAGTTGTCTCAGTCTTGAATGAAGACGTTGAGCGCCCAAAT
CTTAAAAACGGTAACTTACGTTCGGCTAATAACGAACTTTACCGCCGTTTAGGGCAACAA
GATGAAATCATGAAACAAGCACAAGAAGACATGAGCGTAGTATCAGCAATCAATGCTGTT
ATTTAAAGAAAGGAAGAAAAGAAGATGAAACCATTTTCACAAAGCATTAACTGGTATCCT
AACAAGGCACTAGACGCACTCAAGGACTAACCAGAAACAGTCGCAGAAGTTACTCCGCCA
GCAGTTATGCCTGCCGACACACCAGCGCAGGAAGTGCCAAACTACCCAGCGCAAGCCCAG
CCAGCGAACTTGAGGGCGTAGAAATGAACATCGACCACGAAAACATTGTAGAAGAGGGAG
AAGAATAGAATGGCTAATAAAATTACCACTTTCTAATCAGGACAAACTGGTAAACAAATT
TCAAACATTGACCTATTGAACTCTATCCGCACCCGTGCAAGTGCTGACTATCAGGCAGAT
ATTACAGTTCTTGAGGGCGCACGCATTAACCAAGCAACAGTTCCATATGAGGATTTTCAA
AAGCACCCTAATGAGTTTTTCACAGCCTTGGTCAATCGTATCGGGTCAACCGTTATCAAG
GCACTTACTTATGTAAATCCGCTTGCTATTTTCAAGTCTGAAACCTTTGAGTTTGGGGAC
ACATTCCAAGAAATCTATGTACACCCAGCGGAAAAGAAAACCTATGACGCTAAGTCAGAC
GTAAGCCCTTTAAAATTCGCTGATACAGAAATCGAAGCCTTTTATCACACTTTGAACAAT
GAAAACTACAATGAGCGTACCTTTGAACGTGCTTGGATTCAGAAAGCCTTTGTTTCTGAC
ATGGCTTTTGACGAGTTTGTGGACAAAATGTTTACATCATTGCTATCATCTGACACGCTG
GACGAGTACCAAGCAGTTAGGGTGTACTTGAGAAATCACTTGCGGAAGTCTCTTATACAG
ACCTTGAAAGGTAACGATAAGACAATTGCCGTGGCAGGTACGAAGATTGACGAAACAAAA
CAGGACTTTGTCGTAGACTTTAACCAGTCTCTAATTAACTTGTCTAAAGGTTACAAAATT
CCAAGCCGTACAACGTTTAACAACCCTGTAGGCGTGCCAAACATGACGGCGATTGAAGAC
CAATATCTGGTTATCTCAGCAGAATTTTCTACACATCTTGACATGTTACTTGCTAACGCC
TTTAACATGGATAAAGCAAGCGTACTCGCTCGCACAATCGTAGTAGATGATTTGGAAAAA
TTCACGGGAGAGGACGCAAACAATGGACGCAAGCCAGTTGCTTTCCTTATTTCTGCTAAG
TCTATCATTAACAAAGATAAACTGGTACACATGGAAGCAATCCGCAACCCTCGCAACATG
ACCTACAACTATTTCTACCATCACCACTACATGACCAGCCTTTGACTTTTTGAAAACATT
CATTTCTGGTATGTTTAGGAAGCCTAAAGGCTGACCAAGGGCGGCCAATAGCCCGCCTAT

TTTATTAAGTGAAAGGGGACTAAATGAGTTACAAGAATTACAAGCGACATCTTGGTAAGA
TTGAGGTAAACAAAGAAACCGTAGAGCGTAACCGTCTAGCCTTTTTTGAGTTTTATTTCA
ATTATTTCTATAATATCGTGGTAAACTATTTCACTTGGGAGGGTTTGCCTAATGACTTTG
ACGAGTTGGTTATAGAAAAGAAGCTGATAGAAAATGGGCATGTAGCTTTTTTTCATGATG
ACACTTTTGGCTATATCGCACAAGGTGGACCACGAGGGGAAGGCTTAAACCCTTATGACC
AGCCTTTGACCTATCAACCCGTCAACGCTAGCAGTATGAACTATTTTAAACAAATGGAAA
TCGCTAATACTGAAAATGATTTTAGAGTTATTGAAGACTTACACAAGGATAATCCAGACA
AGATTAAAAGACCTTGTATTGTGATTCCTAACAATAATTTCTATGAGCCGTTTATAGGCT
ATTTAGAGTTATTTTGCGAAAAGTTGGCAGATATTGAGCTTACGATTCAATTAAATAGAA
ATGCACAAATTACACCGTATTTCATCTTTGCGGATAATACAAACGTGCTATGTGTGAAAA
ACATCTTTAATGAGATTGCCAATTTTGAACCCGTTGTGTATCTCAACAAGCAGATAGACC
AAGACGGACAAGACAGCTTTAAACAGTTGTCAGACTATATCCAAGTATTCCGAACGGACG
CACCGTTTTTACTTGACAAGTTGCACGATGAGAAATTACGAGTGATGAACCAGTTGCTGA
CTTTCATTGGGATAAATAACAACCCATCAAATAAAAAAGAGCGTCTGGTAGTCTCAGAAG
CTATTTCTAATAATGGGGTTATGTCAGCGAATAGTGAGGTAGGCTGGAAGTCCAGAAGAA
AAATCGTTGAGCTCATCAATAAATGTTACGGGCTAAAAATCAGCGTAAAACCAGAGGAAA
CGATTCAGCAGTTTAACCTTGACAAAATGGCGCTAGACTTGGCAGAAAAGGAGGGGACAA
TCATTGACCCAGAATAACACAACAGCAACGATTGCCACCTTTTTAAAATCCAGATATAGA
AATCCCGTGACGGGACGGCTGGACGGCTTGGCAGTAGATGAAAACGGCGAGTTTCTGCAT
TACAACACGATTATAGACCAGACCAATAACGAGTTATTTAAGGACATGCAGCTAGTCAAT
GGAGTTTCAGACAATTTCAAGAAAGAGTTTTGCAAACACTTTTACAACAGGGAGATAGAA
CTTGAGACTTTCGCACGTTGCCAGATAGCCCTTGAGGAAGTTCTAAACAATGAGTGTTGC
AATCTAATTAAATATCTCGCTGAAATCAGAAACAAGGCTATCAAGGACTTAAATCAGTCT
ATGAATATTGACACGGTTGGAAACCAGAAAGCAGACGGGCAAGCCTTGCAAATCGCAAAC
ACCACACCCCAAGAGCGGAAAGAAATCGTCTTTACTGAGCGCTACGGCGTGATAGAGTAC
GCTGACAACTTGGTAGAAAACCACCAGAAAGACAACGCTGACACAAAAAGCAACGTCTCA
AGGTGGAGCGGTTCAAGCCTTGCCGAACACTTCCAACATAACGCAGAATTAAAGGATATA
CAGTTTCAGATATATAATATATGTGATAAGCTGTCTTTACAAGTCTTTTAGAAAGGTGTA
TAGATGAAAGATTTATCAAGCGCTAAAATCCTAAAAGATGATAGTATGTTAGAAGAAATC
ATGCTTTTCAGCTTTCAAGACTTTGCTTATAGTGATGATGGATTATATTATATCCAGTCA
AATAGCAGACGGTTAGGCGACTAGTCTAAATTGTGGATAAAATTAAAGCCTATCTGTTAT
CATTATGAAAGCATTGAAAATGAAACTTTCTGGACTATCCGCAAAAGCTACAAGCCGTTA
CAATCCATTAAGGCGTTGTTATCCATTCGCTTTAAGATTGTGGGAGCGTACTATAGCTTT
GAAAAACTGACCAGCAAAAGCAAGCTGAAAGGCTTTGGCAGAGTGATAGACGATAACAAC
TATTTTTCACGCATACCCCCTGTAAATGAGGTGGTACATTGGGATACCGGGGTTATCGTA
ACACCTAACTATCAAATGAACATCACAGGACTAAAAGAAACCCGAGTAGAGGTTGACGGT
CAGCAACTCTTTGAAGATTGGTCTACCTTTAAAATCAATGTTACCAACGATAGAAAGGGA
GTACCTCGCACAGTTATGACAGCAGAAAAAGGACATGAAAACCTATGATAATTATCAACT
TGTCCGAGACAACGGACACGCAACAAATCGAAGTCACGGGACACGGAGACGATATAGACC
AGTCTTGCGCCCGTGTATCAACCGTTTGCGATTGTATTTACTTATTCTTAAAATCTAACA
TAGATGATTATGTTAAAAAAGATGGTTATACATTGCTACGGATTTTTAAAAAACTTACTG
CGGTACAAACTTTAAAAGCTATTTTAAGTTACATCGTAACGCTTGAGCAACTTTATAAAA
ATTCAATTAAGGTTATAAATAAAGAAAAAGAGGTAGAAACAAATGGCGAAGACAACTACA
CTTGTACGAGGTATTCACTCATGGATTAAGTTCCAAAAACACCAAGGAGTGGAGAGCCTT
TCTCTTGAGGGTAAGCAGGCGCTTTCTGACTTGTCACAGGATAAAAACGGGGACACAACC
CTTATTTTAAATGCAGATGTTGATAAATTAAATACTATTCATTAAGCTATACCTTTTATT
TCTGTTTCAGAAAAATTTTCAGGAGTTGACCCTAATCAAATGAAAACAGCAACCGTCAGC
CAAGATTTAACAGAATTCCCTCTAAATAATGGGGAACTGGTTAAATTTAACAAAGAAAAA
GATGGTATTTCTGTAAATGATGAAGAATTGAAAGCAGAAATTTCTCAACTTATTGAAGCT
AAATTGACAGGGTTTCCAGTTGTATTAAATTATGAACATATTTTACTTGATAATTTTTCA
AAAATTGAAACATCTATCAATATTTCCACTTATCAACCGTTTAATGATTTTGTGGAAGTA
ACTTTGAGCAATCCAGCTACACATGAACAAGACACTGTTATTATTTCAACAAATGAGTTA
TACCATGGAAAAATGATTTTTAATAGATTTGTTTGCAGTGTTATAGTTGAAAAAGATGAT
AGACATATTATTGATATAACTGTAAATTCTCATAAAGAAGCTGACAGAATCCATGTTACC
ATAAAATGGTTTACACAATACAGAGGACAAAAAATCCTTGACGTAAATGAAATTGGTCAA
GACGCACACTTTGAGGGATATGAAAACCCTAAAGTAGAATTGACTTATAGTCCTATTCCA
CCAGCTGTAAGCACACCAACCGAAACACCAAGCCCAACCCCTGAAACAACTGGAAACACA

CAGCAACCAATTGCAGAAGAATCTCAATAAAATAAGAAAGGATTTTAAAACATGAATCCG
GAAGAATTTAAAGATGAATTTTTTAGGGCTTACCGTGGACGCTATTAGTCTTACTGGGTA
GAACGTTGGGGGCTTATCCCCTCAATCCCTACCAGCTTTGAAAACGCCAATTCTATCTAC
GAGCTGTTGGCGTGGCTACAGCGTGCCTTTAAGCAGTTGCTTGACGACTTTGTGGCGTTG
GAAAGCTAACTTGAAGACTATAAGAACGCTTTAACTGAACTCATAGAGCAACTTATACCT
TTGCTTATCCTTAGATACATGAAGAGCAAGGAAGCGGACGACTGGTTTAATAAAAAAGCG
GCCATCTACTATAACAAGCTTATCAAGCCTTATATTGACGCAGAAATAGCTAAAGTCAAT
AAGAAAATCGCTGACCTTGAAAAGAAAGTAGATGATGACGTTAAGCGACTAGATGGACGG
ATTGACGCTTTAAACGATAAGCTAGAAAAAGAAATCAAGAAACTTGTCGACAGAATCACA
CAGGAAGTCGCTAAGTTAAACGAGCGTATAACAGCAGAAAACAACGCACTCAAGGAGACA
ATCGAAGCGCTAGAAAATCTAACGCGGGCTTGCAAAATGCTTTGCGTAAAATCATTGAAA
ACCTTGAGGGGTCAGGCGCATGGACTGGTGGGCTTACTGGTCGCTTTAACCAAGGGCGCA
ACATCGCAACAGGTAATATTAACTTGTTTGGTGGTACACCAGACGGTTCAAGCTTTATCC
GAACCAACAGCGGAAGCACAGAAAACGACCTCGCAGGAGGTATCTAATGCCTTTAGAAAC
ACGCTTTTTAACCTCTACTACAGCCAACGTAGAAAACTTTGGTACAGGTGTTGCCCCTTG
GACGGAAGCCTATGCTAATGCTTGGCAGTTCTCAGGGGACACAGACTACGGCTACATGAC
CAACGGGAACCCGACCTATCTACAGTACGGGCAAAATGACCCGTCTGTATGGGCGTCCAT
GCGTTTCTGGGGCGAATCCGTTGAAATCCTAGAAGAGACAAAAAACGATGTTAATTCTAT
CACGGCTAAAATCAGAGTTAAAGTCCTCTTTTGGTGGTCAAAACGGGTCAGCTGAAACGC
TGGGTATCGGGTAGAATATGATATTAAAATCTATGGGCGCACCGTTTGGACGTTTAGCGG
ATATACAACCGATGAAGTCATTAAAAATGATGAAGTTGCCCAAGACTTTCCCGTAACCAT
ACCAGCCGAAGAAAGTTCAGCAGCCAATGCCTTAAATATAAATGTATCTTATCCAGACGG
GCAATATTCAGACAATTCTTTCTATGTTGGTGTGTACCGATATAATATCAACAAGAAAAA
GCCTAAAACATTAAAGCCGTGGGCAATCCGTAAGAGCGGAATCTTTAAGACCTTAAACCG
TCCCTCTGGAATCTTCCAACAGCGCAAAAGCAGTTGGCAAGACATCAGGGAACAGCCAGC
AAACGCAGTCGGGCAAGCAGTCACAGCACCCCACAGCATGAGAAAGTCGGGTCAATGGAT
GGGACAAGGACAGATAGGACAAGAATAAGGGAGGGTTTCAGCCCACCTAATTTTAAAGGA
GAAGACATGCAAGAATCAACCAAGATATGGCTTTATGCCAAAAGCCCTTTTAAAAACGAT
TATGCCAATGTGATTAACTTTGAGACAAGGGAAAGTGTGGAAGACTTTTTCACAAAGAAG
AATCCGCATATAGAAATTGTGTACGAGTATGACAAGTTTCAATATACCCAAAGAAACGGC
TCAATCGTAGTTTCTGGACGGGTAGAGAATTATGAAAATGTGACTTATGTGCGCTTTATC
AACAACGGAAGAACCTACTATGCCTTTGTTTTTGACGTTCTCCATATCAATGAAGATGCT
ACACCCATTATTTACGAGGTGGACGTTTGGAACACCTACCAGCACGAACTAAAGGCGCTT
AATGTGATTGGTCAAGTGGAACAGCAAACCGTACCTAATGTGTTGTCGGCGCTGAAAGAC
AGTCAGCAAGGTTTTTCAGTTGGGGCGAAATACGCTACACGGGCTGGAGAAGTTGGAATC
GATACGGAGTGGCTTGTAGTAGTCGCAAAACCTACGATTATAATGACCACCCAGGCAAAC
CGCCCCGTCAATATGAGTTATTCAGGTATGCAAAAAACCTTTAAATACTTTTTTATTCCT
GTAAATTTGAAAAGCGGAGCGTCCAGCCGCTTATTTTTCAGGGCAAAAAGTATGATAGCT
TTTACCTTGAGAACCTGTATAAGCACCTTTTCGGCTTGAATCAAGACGGCAGTAGCACCG
TAAACCAGATTGTGAACATGTATTTAAGCCGTGACATCGGGGTAAAATTCATAGAAACAA
CCGAGGGCGACAAGACCTCTATAGACATCTTGTCTAATATCACGGGAAGCGTTGCAGAGA
TTGGCAGAAAGAACAGCCGAAACTATCGGACATCTGGAAGTTCATCAGACGGAGGCAGTG
GAAGCACCAACGAAAGCGGAGGCATTTCAACCGAGGAAAGCCGTGTTAGACTGGTTACTA
GAATCATTAAAAAGCTAGTACCAGACGCAACAGCGGAGAGTATCGCTGGAATTATCGGGA
ACTTTTCAGCAGAAAGCAACGTCACAGCCAAGAAATACGAGGCAGACTATGCTACTGGGT
ACGAGTACGAGAAAATGGAATCAGAGCCAACAGCCGAGAACCTTATGGGAAGCTGGGGCG
CTTTTGCGAGTCTCTACTCTATCAGCTTAAGTGAAGCTGGATATAGAGGGTCAGACGGCA
ATCACTGGATAGGTATTGGGATAGGACAGTGGCCGGTACCGAGGGCGGAGGAGCTTTTGA
ACTTTGCAAGAAGTGAAGGGAAATCATTATGGGATTTTAACCTACAATTTCAATTTATGA
ACCAAGAGAGCCGAGCCGATACGTTTAGACGGGTAGCCAGTTCCAGAGCAAGCGCCAGGA
ACAATCCAAGGGACTGTATGAACAACTGGGAGGGGGTAGCCTATAAAGAAGCGGAACGCA
TAGAGCACTAACGCCTGGTTATCTAAGATACAAGACGAACTACAGAAAGGGTAAACAATG
GCAGAAGCAACAGAAACGCTAAAAGCACTAAATGAAATCAAGTCACGGGTAGGAACAACC
GTGGGAAGTGGGCAATGTGACGGCTAGTCGGCTTGTATTCTCAACTCTTGGGCGGATGCG
ACATCGGGGGAGGTATCAACACCCCCAACCCCAACGGCAACGGCAGACAAGCCAGTGGAA
GCGATACACAACGGGGTATGAGTGCCAGCAACATTGGGGGCGACTATGTCTGGGAAGCGC
TGGGCTGGAAAGTCCGATTTGACCCGTCTTGGGCTGATTTACGGGTAGGGTGCATTGTCT

GCTATATCCCAAGCGGTAGCAATATCTGGGGGCATACGTCTGTTATTTCAGCGGTCAACG
GCTCAAGCTATGCCGTTATAGAGCAAAATTATGCTTGGAATGGCTACACCACAGAACGCA
CGGGTATAGATACGATTGTCAATATTGAAAGTATTATCTACCCTCCCGAAGTTGTCGCAG
GTGGCGATATTGGAGAGATAACAGGTAACACAGGAGATAGACAGTTAGGAAACGGGGACT
ACTCAAAAACCGCCTTTGACGTTGAAGCCTTACTAATTGAGCTGGACGTATTTTTTGACT
ATCGCCCTAATGTATATGAAATCCCGCACCTCCTCAAGATAGCTTATGACCAGATACAAG
AGGGGCTACGCTCATACATGGGTAAAGACGACCTAGAAATAGAAGTACAACTATTAAATA
GTGAGTTTACGGAGATAGAGCTTTATGATATTTACGGTAACAGTTATGTGTATCAACCGC
AGTATTTACCGAGAACCATAGACGAAGCACACGAGTATAATGTTATTGTAAGCAGATGCC
TTGGGGATAGCAATCAGGTTCATATCAATTTTCTTGAGTATAACAACGCTAACTATGTAA
GCTATGCTGATAAGAATATTCTGGATAGATTGGAAAGTGGCGACTGGGCGGAACACAATC
CAGAGCATTTTAAATACGGTTTGAATGACGTGACAGGGAAAAGCGTGGCTATTTTGAATG
ACGCAGAAGCCAGCTATATCCAGAGCCATAAGAATCAAATGGAACATACACAGCTTACGT
TTAAAGAGAATCGGGACATGCTGAAACAGAGCGTAGACCTATCTGATAAACAAGTCGCTA
CAGCGAACTCACAAGCCAGCTATAACGCACAATTTGCCGTAGATAGCGCCAATATCAACC
AATGGACGGAGGGCGCTAGTGGTATCTTAAACGTAGCTGGAAATCTCTTAACAGGGAACT
TTGGGGGCGCACTTGGTGGGCTTGCGTCTGGCGTTATGAAAGTCTTTAACGCTAACCGTG
ATTATAATGATAAAGTGGTGCAACAAGGTTTCACAAGCGAAAACAACGCTTTGAAATCTC
AGTCAATCGCACTTGCAAACATCAAATCTAAGATAGCACTTGACCAGTCTATCAGAGCTT
ACAACGCAACGATGGCAGACCTACAGAACCAGCCTATCAGCGTGCAACAAATCGGGAATG
ACCTTGCTTTCCAGAGTGGGAAAAAACTGACAGACGTTTATTGGAAAGTCTCCCTAGCTC
GAAAAGAAATCATGGGACGGGCAAACGAGTACATAAAATGCTATGGGGTGCTTGTCAACT
GGTTCACTAATGACGCTCTAAGTGGAATGAGGTCAAGAAAACGTTTTAATTATATCAAGA
TGATTAACGTTAACCTTGGAACACTAAGAGCCAACCAGTCGCACATGAACGCTATTCAGG
CTATTTTTCAGTCAGGTGTCAGAATATGCAATTATTCAGCCAATAAAGAAGACGGCATTT
TGTTTGATAATCAGAAATACAACCCGAATTTTTAAAAGTATGATATAATGAAATAGAAAG
GAGTGATTTTCTATGGAAGCAACTGAAAAATGGTACAATCCGCAGAAAATGCTATCTTAT
AACCAGTATTTAAACTTTGTTATGGGTGGTCGTGGAATTGGTAAGACCTTTGCACTCAAG
AAATATCTGTTTAAAAGGTTCATTGAGAAAGGGGAACACTTCATCTACCTAAGGCGGAAC
AAGTCAGAGCTTGACCGTATCGACAAGGACAAGTTTTTTACTACCGAGTTATTAAAGCAA
GTTTTCACTAACTTTGAAGTGATTGACAGCGACGCCAGCAAAATCCAAACTCAGATTATT
TTCAAAGCCGACAACATGGAGGAAGAGGAAAATATACTTGTTTTATCTTCTACTAAAATT
ATTCTTAACGGGAAAATCGTTTGCTATCTCAAGAGCCTTTGTACATGGGTAGACTTGAAA
GGGTCAGAGTATGATGATGTAATGAGTATTCTATACGATGAAGTATTGATAGACGTTACC
AGTAAAAAGAGGTATCTCGATAACGAGGTGGAGGCGTTACTAAATTTCATCTTCTCAGTA
TTCCGAAGACGGGACGGGTGTCATGCTTATCTACTATCAAATGCAAGTAATTTCAACAAC
CCTTATTTTGGCTTTCTCAAGTTCTATGACGACAACGGCAAGCGCTTTTACAATCTGAAA
CAATATGCAGCCTTGATAGAGATCCCCCCTCATTCAGCCTTTCAAACAGAGGAAGAAAAA
GAGAGCGGATTCTTTAAGCTATTGAGTAAGTCAAGTATTTATGAAAGCGTTGCTAATAAT
GAATTTCAGAGTAAGAACGACAAGAATATAGCAAAAATTAAAGGCTCGAAGTCTAGGCTA
TATAGTTTCTATTGTGACGGTACTTTCTTAACGGGGTACTATATCGACTATATGGTATAT
AGTGCTAAAGAGTTTGACAAAAATTTGACCGCTTATTGTTTAGAAGTGGAATAGGTGGAA
GACGGGTTTGTGTACTTGAATAAAGCCAGCGCACTAGGTAAAACTTTACGGAGTTTGTAA
TTAAAAAATACGTTAATTTATGAAGATTTAGAAACTAAAAACAAATTTATAGAGGTTATC
AATCATGTTATATAATATTATGCTAGAAGTTGCTAAATGCGACTATATCACACTTCTTTT
TGCATTGATTCTGTCTGACTTTATCACAGGCTCTTTAAAGCCTTGGAAGTGGAAAGTGAC
CGATAGCTGGACAGGGTTAAAAGGAGTTATCAAACATACCCTAACATTTATTTTTTACTA
GACTGTAGCGGTATTCTTAACATATATTCACGCTATGGCAGTCCGTCAGATTTTGCTTAT
TATCATTAACTTCTACTATGCACTTTCAATTATGGAAAGTCTTGCTGTTATGGGTGTCTT
TATCCCTAAATTTATGACGGATAGAGTACAGGAAGAATTGCAGAAATACACCGCACAACT
AGACGCAGGAAAAGACCTGCTAGAAGAATTTAAAGGAGAAAAGAAATAATGGTTAAAAAG
AATGATTTATTTGTAGATGTTTCAAGTCACAACGGTTACGATATAACAGGTATCTTGGAG
CAAATGGGAACAACTAACACCATCATTAAAATTTCTGAAAGTACGACCTATTTAAACCCT
TGCTTGTCTGCTCAAGTGGAGCAGTCAAACCCTATTGGCTTTTATCACTTCGCACGCTTT
GGCGGAGACGTAGCAGAAGCCGAAAGAGAAGCGCAGTTTTTCCTTGACAACGTGCATATG
CAAGTTAAATACCTTGTATCGGACTACGAGGACCACCCAAGCGGATACGCACAAGCGAAC
ACTAACGCATGCTTAGGCTGTATGCAGATGATTGCTGACGCTGGATATAAACCTATTTAT

TATAGTTATAAACCGTTTACACATGATAATGTGGACTATCAGCAAATCCTTGCACAGATC
CCTAATTCTCTATGGATTGCAGGCTATGGCTTAAACGATGGTACAGCTAACTTTGAATAC
TTCCCAAGCATTGACGGATAAGATGGTGGCAGTGTTCTAGTAACGCGTTTGACAAGAAT
ATTGTACTGTTAGACGATGAAGAAGACGACAAGCCAAAGACCGCTGGAAAGTGGAAACAA
GACAGCAAGGGGTGGTGGTTCAGACGAAACAATGGCAGATTCCGTTATAATAAATGGGAA
AAAATCGGTGGTGTGTGGTACTACTTCGATAGTAAAGGATATTCCTTAACGAGCGAATGG
CTCAAAGATAATGAAAAATGGTACTACCTCAAGGACAACGGCGCATTGGGGACTGGCTGG
GTGCTAGTCGGGTCAGAGTGGTATTATATGGACGATTCAGGCGCTATGGTTACTGGTTGG
GTCAAGTATAACAATAACTGGTACTATATGACAAATGAACGTGGTAACATGGTTTCTAAT
GAATTTATTAAGTCTGGAAAAGGTTGGTATTTCATGCACACAAACGGAGAGCTTGCAGAC
AATCCAAGTTTCACGAAAGAACCAGACGGGCTTATAACCGTAGCATAAAAAAGAAAAGCT
AGAAGGATTTTCCTACTAGCTGTTTTTATAGTCTGCTATAATTTTATAGGCATCTTCGTC
TGGATTATCCAGAGCGATGGAACAGATTGCCGACAGGACGCTGTTCATCTGATTGTATTT
CTGTAAGTAGTGATTTTCTAGCTGTTTGTAATTGCTGATATGCTTTTCATATCCAGCCAG
AGAAAAGGAGTGGTGTAGGTTTATGAGTTGCTGGGTCAGTCATGCCTTGTCTAGCCCGTC
AGGTATGCTTTCATCATTTCTGAAAGCGTTTCTAAATAGCTTGTAAATGAATGTATAACC
AGTTCATCAAGCGTTACCATTCCCCGAACGCTGGAAGCGTTAGGCTTGCAATCTTTTGAC
CGTGTAACGTTAAACTGTTTTCAAATTGTAAGGCTATTTCTTGCTGTTCTAAGCGTGTTT
TGTCTTCCATAGTATTAACCCCCTACGTTTTCAAGATAAGCGACTAGACGCAAAATAGAA
GCCGTGTTACTATCGTTTTCTCGCTTTAACTTCGTGATGGTGCTTTGTTGTTGCTCAAGT
TGTTTTTGTTGCTTGAAAATGGTATAGGTCAAGAGTGACATAATCACTAGAAAAACCAAG
ATGGCAAGGTTACCAGAAGCGAACCACCAAAACCAGAAACGACCTTTTTTGTTAAATTTG
TTATATGAATTTTTCCTTTTCTTAATACCTCAATTTGAATAATTCTCTTTCTACTGATAA
ATAAGTAACCTCTAATATACCTTTGTAAGCTGTAGACCCAGTCTGGTTGGTAATGTTGTT
CTACATCATATAAGTTATAAGTAGTGGCTCAAATGCCCTCTATATCGGCCATTATAGGCT
TTATACGACTAATAGCATAATCTAAGGTAATGATAGGAAAGCCATAAAAATAATATTGCT
TGACGACCTTTCTAACCGGTTCTATTTAAGTTGGAGAAATCTTGTTTAAATCGTTTATCT
AGTAACCGTCCCTTCCTTGTACATAGCGCTGAAACGCTGTTTTTAAACAATAATGTGAAT
ATTTTGAGTATTTCAAGTTTTTCACTTCCTCAAAGATAATAAGCGTGTTTAAGCGCTCAT
TTTTAAAATAGTTCTCATGGACTTCTTTATATCCATGATTTCTTTATACCTCTTTTTCTG
TTACCAGTTTATCGTCTGGAAACTTATCAATACATTGTATTTTAAGAGGAGTTACCCCCT
CAAGAATAATCTGAAACTGTGCTTTAATGTTTAGCCTCCTATTTGTACAAATACACGTTT
TCGTTTCCACAATATCCAACATATTCTGAACCAAAATAAGATAGGAAACTTTCAAAATTT
TCAACGCTCCAAGGCTTCTTTTGAACCCTCTACATAAGAGTATAACCCTTTTTCTGCTAG
GTTTGCTTTCCCTACAAAAATACAGGTGTTGCTATCTGTAAAACCTTGCCTTTTAGCAAG
TTTCACTAATTTATCTTTCATGTTCATGCCTGCTTGCATAGTCATCGCATACTGGTTAAT
GATAATCATTTTGTTATACCTATTTATCTTTCTGATACCATTATAGCATTTCTCAAAATG
CTTGTCAAGTGTTTTTAGAAATTATTTTTAATTTATTTTTAGAAATGTTTTTATCTCATT
TCTAACTATATACATTGTATCATTTTATAAAATGATTGTCAAGAGATTTTTGAATTTATT
TTTTATTTATTTAGTGATTTCATTTCTTGACATTTTTCTAAGTTGTGTTATAATGGTAAT
AGAAACAGATGAAAGTATTTTAGAAGATGATAGATGATTGATTTTTAGAATGGCTAGTGT
TGGAAAGGGATTTTCTCTTTCATTTCATTTTCAAATTTCCATTGGGGGAATTTTTGAAAA
AGGGGTTGGGGAGTACATGCTTT
>NewGenomeName_43
ATACAGCTTTATCTGTTTTTTAAGATACTTACTACTTTTCTTAGTGGAAACTATTAGTGG
CTGTGAGGTCAAAAATGAAATTAAACCTTAGGATAGGGGTTATTTCTAATTGAGCTGGTA
TAGGCCCTTTTCTTTTTTGTGTCAGGTTAACAATAACATCTTTTAAGACCATCCTTAATA
GCTCGTTCTTTTTCTCTGAGCTTGATTCAATGTGATACTGGTCCATTATTTTATTTATGT
TGTTTCTCGCTGTATTACTGTCTATTTCTGATTGTGTATCTTGTAATCAATTGAGCTCAT
TCTTTGCATTTTGGAGCTCTTTAAATTCTTCATCTAATGCAGCTTTTCTCTTCAAAAAAA
GTTCATCACTGTAAATGCCACTTTCATATTTATCGAAGATGAAGTTCTCTTTGTTTTTTA
GCTCTTTCTCTTTTTGTGAAAGGTGTTCAGACATTTGCTTTTTTGTTTTCATGTTACTGT
TGTCGTCTTCGTACTTTGAAAGCATCGAATTGATGTGTTTTGTTAAAGTACTATCATTTA
AGTCATTTAAGGAAGAGAGGTACTCCAGAATTGCATTTTCAACATCGTTATATCTCACAT
ACGTACACTTATTTTTCTTACATGTGAGCGATTTAACGTGATAAACGCTTTCAGTTCCAT
CTTTGTTTTTTCGTATGCGTTTAGATTCGTATTTAGATAAAGGCTCACCACATTTTGAGC
AAGTAAAAACACCGGCCAGTTCACTTAATTCAAATTCATCTTTATTTGGCAAAAGGGGCA

CTTTGTTTGCGATTTTTAATTGTGATTGTTGAAATTGTTCTTTCTCAATTATAGGAGCAT
GCGCATCTTGTACTACAATTTGTTCTTTTTCAGGGCTAATTGTTCTGTTTCCGTCTTTTG
TTTTTTCTCTAACCTTATATTTAACAGTTCCTATATAAACTTCATTTTGGAGAATTGCTT
TGATTGTGTATTGATTCCAACGTTTTTTCGCTGACGGTTTAGGGATTTGTAAATTTGTTA
GATGTGAAGCAACTGCTGTATACCTATAATCTTTTCCATTCAACCCATTTACAAAGATGT
TAAAAATTAATTGAACTACTTTTGCTGCATCCTCAACTGGATCTAGTTTAGGCGTCTTTT
TGTTTAATTGGTATCCATAAGGTGCCAGTCCAGATATCCATTTTCCCTGTGCTGCATAAG
TGTATTTAGCGCCTGTCATACGCTCCCTAGTCATTTCAAATTCTTCTCTAGCCATAAATA
ACTCAAAACGTATCTGGCGCATGTCAACAGGGTTTCTAGGGTCATAAACCTTATATGGTG
TAATTATGATCAATCGTTTGCTTTGAAGGAGGTTAACAATTTGTCCTGCATCGCTATAAC
TCCCACGACTAAGTCTTGTTATTTCTTTAACAGCAATTGCCTGATATTTGCCTTCCTCAA
GATCTCTAAGACATTCTTTGAAAACAGGTCGACCATCAATGCTTTCTCCTGATCCAATCT
CCATTTTTAATTCATAAGGGATCTCAATGGCCGTAAGTATTTTATTCATAAGTTCTTTTT
GTTCTGTTAGTGTGTCTTCACCTGTTCTTTTTTTTCGTTCCATCTCCTGACGAGAACGTC
TAAGATACCCTAAAATATTTGTGATGTTGTAAGAATTAACAATGTTTTTTAACTCCAAAA
ATTTAGCACCTCTTTATTTGTGTTTAATGTCAAAACAGAAACTTAATTAAGAATATTTTA
TCTGACATTGAATACGAAAACAATACAATAATTTGAAGGAATTGAATTGATTGAAATTTT
ATGAAAAACCTTCTTGAACATGGGTTATAATTAGTTCATCTAAATTCATTGTAAATACTT
TATAAATCCAGGAGGGCTTTATGAATATTCGCTTTTCAATGTTAGTTTGTGTATCTATTA
TTTTCTTCACTGGTGGGTGTGCTGAAAGTAGTGCGAATTCAAATGATGGTTCTAAAAATA
AAAATGAGTCTAAAGAGGAATCCTCCGAAGAAGGTGTAAAGGAAAATGATAATAAACTCG
CGGATACTCCAAATATGGATGATTGTATTGAAGTGTACGGAAAAGAAGAGTGTGAACAGA
TCACTGAGTACTATAAAAGTGAAGAAGGGCAAAAGGAGTTGGATGCAAGCGAAACAAATA
AATATTCTGCAAAGTCTCAAACAAGTACTACACATAAAGATATCTATGAAAAAGTATCCT
GGCTTGTTAAAGAAATGTTCGGAGTACCAGAAAGAACAATTCCAGCTGATTATGAAAAAA
GTTTGGTAGAAAAAAGGGATGATGGTTTATATAACATTCAATCTTCTTATGAAATAAAAG
GAACTGGAAACCAAGCGGATACTTACTATGAGTTTGAAATGTTGATGGATAATAAATACA
ACTTGATAGACGCATATTTTCCCGGCAGCACAGGAAGATATTCACGCCCTATGGTGTATG
ACAAACTTAAAAACATGGAAATACCAGAAGTAAAGAAAGTATCTCCAGAGGAAGAGAAAA
GGGAAGAGAAAAAACGTGAAGAAACAATGAAGAGTATTTATGGGGAAGAGCATATAAAGG
ACAATAAATAAGTAGCAGTAAAAACAAGTGTACGTTAAGTAGTTTAAATAAAATTCACAT
TTTATTTAAACCTGAAAGCTGCTTGAATACATCACAAATATAAAGTATCCTGTATATGTA
TCATAGTTGGTGTATCAACCACTATAGGCCAGAGAGTTCCCTCACATAATCTCTGGCTTC
TTTTATTAACCTTACATTATTATTTATGGAGGATAGTATGGACAATCGAAAAATGCTTCT
TAGACAGAAGTTGAAAATAAACAAACAAAAAAGCCTCCGAGTAACATTGATGTCAACACT
GCCTCGTGATATGGCAACTACTATTGAGGATTGTAGTCTAATCACATCTCCTGAATTAGA
AAGAATCCTAGATAAAGTTCAGAAAAAATGCAACTTTGAGTTACATAAAAATGATTTTGC
AATTAAATACAGTGAGCATAGAAAAGAATACAGTTGGGAATATGAAGTGATCAATCATGT
GCAACAAACTAAGCTTCCTGATGAACAAGTTTACTTATATTTAGGGATAGAGGATAGCCC
AATTTTTCTGATTAACGGTAACTGGATAATAGATAACTTTAATTTTCTGTGGGAACAAAT
AAATAACAACGATTTATGGATTATTGACTTTCATTTTAAATATGGGCTTCTAGTGTCTCG
ATATGGCGGATATTTAGATCATGATCCTAACCCAAATGAAATTATTTATGCAGTCACTGA
ATGGGGGATACAAAATTGATTTATACAACAGAGAGTTGCAAAGGGCTTTGGATCTGCATT
TTTGGTGGATTTGTAAAGTGATTTACTTAGTGAAGGAAATTAAAGAATGAAGGGGCGAGC
CGCATTGTATAAAGCATAAGTTAATTCGTGGATTACGTGGTATGATAATCTCAAAGACGA
AGGGGGATAAGCATGTTTAAAGAAGTAGACTATTGCACTTGTGAAGTGTCCAAAGTTTAT
ACCGAACAAAATGATATGGGACATTGGGATGTATGTGATATCTGCAAGAAAGTCATTGAA
GATACTTTTGAGTACTTTAATTACAATTCAGATGATTATAATGGATGAAATGATTTGACA
AAATTTTTGAGTCAACGTGACACTCTGTTTTGCTTTACATCTGCTAATATGATAATAAGG
TAATAAATAGCCAGGCGTATTGCCGAAGGGGAGAGCGCTTATTTTTATAACGAGTTAAGC
CCTCCCATTGTTAAGCGAGGATCAAAAGAGTAGGAGGCCTTCTCAGTGAAAAGGCTAAAC
AGGTGGACACTAAAAATAAGCTTGAAAAATTTCCTTCCAAGCTCAATGATTTAACTGTCA
ATTTTAAACCTAAAGAACCCGATAGTTTTAAAAAGTGTTACTAAACTCATTTGGTAGACC
AAGCTATTTTTTTATATTTTTACTATGATTATTAATAAACCATTTCTCAGCAAAATCCAC
TGCTTCTGTAAGTGAAAAAAGGCGGCAATTAGCAGAACCCACTTTGCCAGCTTTCATATT
ATGTTCTGCTTCAAACGCTTGACCGACTTCTTGAAATAGTTCTTCTCTAAATTCAAGTTC
TTTATATTCTTTCCATACTCTTTTACCTTCTACTATTATAGGTGCACCCCTGTTTATAAT

TTTTTGATAAGGAATTCTGTACTCAGCAAGATGGAAGCAGGTACTACTATCAAGATCAGC
ACCTAATAATAATACATATGATTCCCGTATATAAAGTTTCCCCAGATGTGATTGTTCCCC
CAGTCCGAATTCTAAAGGATGTTGATTAAGGATTTTGTTTTTATGTTTTCCCCAAGCTAC
AAAGGAGTAGTTTGGGTGATTACTTCTTTTAACTTCTGGATAAGACCTGAATAACTCTTC
TATTTGCCCCATCCCTCTAGTAGTGGGGGTATAGTTAGAATTATAGGCTGGCATACTTTC
TCTAATAATATCCCCCCACTCCACTGGTACGGGAGGGTTTCCCCATTCTTTTGGGTCAGA
TAATTCCACGGATTGAGAAGGCATCACGATGGAACCTTCTTCAGTAACAACATCTATTAA
TGCTCGAATCACAGCAACAGCTCCACCATTTACCCATCCTATAGAAGATAAAGAGGAATG
TACAAGTACGGTCAGCCCTTTCTTTAATCCGAAAGCTTTCAAATCTTCTGTTATAGACTG
TTTTGTTCTTGGAAAAGTTGTCCTTTCAACTATTTTTTTCATTTAGTTCATCTCCTATCT
ATAACCTCTTATTAGATACTCTGTAAGTTTGCAACAACTAAATTAGCAAGACCCCTTGAT
GAAAGGTGTTGTTTTTTTATTCATCTTCTTAAACTAACTGACCCGTTAATTTAAGTGGAA
GTTATTACAATCTCTTTATATTTAGTTTAACATAAATAGCCAAGTAAACTTAGAAATCCA
TTTTAAACTACATAAAATATAAGAAACTCCGTTATCCATAATATTAAATCAACCTTTATC
TTTAACTTAGCCATATAAAAAAAGAAAAGCCCAAAAACGGGCTTTTTATTTTGTTTGCTC
TCTTAGTATAGCTACTATTTTATCAAGAGAATGTTCATCAATATTTCTAATGGTACGCTT
GCTACTCATTCCAAGCTTACCTTTGAATTTTAAATGGAGGATACCCAATTGCATCGTGGC
CATTCCCAAGGGATTTGGAGCAATATCGAAGTCAACTTCTTTGATACTTTTATATTCAAC
AACTTCAGTATCTTCACCCCCAAAAAAACCGCCTTTCATAGTGACAAATAGAAGTTTCTT
TTCAGTGATAACCATAAAACCAAATTTAGTAAGCTTTGGATCTGCAGAAGTCATATGTAA
AATCTTTTCCTTTTTCCCAACTTCCTTTGTTAAAGCTTTAATTGCATACTCATAAAACTT
TCTTTCTGATTTGGGGAATTCTTGAATGATGGACTCAAGATCTTTTAAAAGTATTGGATA
ATCTATGATTCCATACTTAGAGTTTAATTTATGAAATTTTTGTCCCATCACTTATCTCCT
TTCATCGTTCACAAGCATAGTTGTCATGATCCCTGTCCATTTTAGATTGGTAAGCAGGGT
GGGAGCTAGGCACTCAATTCGGATATTTCTTTCTTAACTCTGTGCAATTTTTAAATGTTT
CTGTTCCTCCAGTTGTCTCTGAAGAAGCTTCTTTTTCAGTTGTAGTCGAAGTTTCACTTG
TTGCCTTTGGAGTTGTAGGCTGTGTTGCTGCCGGTTTACATGTTGTTGCTTTTTTAACTG
CAGTGGTTTTCTCTTTCACACAGCCATTCAATCCTCGGTCAGTCACATATCCATTCTTGC
TCCAGATTGAAAGCTTCTCTGATTTTGCTTCTTGCTCGTCTTTCTTAAATTGATCTATGT
ATTTCGTATTTGGCTCATATACATACGCTACTCTGGCCAATCCTTCTTTTAGCAATGTTT
CCTGAACAGATTTGCCATCCACATAAACATAAGCTAATAGTCTTCCGTATTTATCTCTGC
GATCACCTTTGTCAAATTCAAGCTGCAGCTTACCACTGTTGACCAATTCTTTAGTTCGTT
TTGACGCATCCTCACCATATGGTTGAACACAAGAATTTGGTTTCTTTGTTTCAGGTGTGT
CTATGAGCAAGTAGCGTACTGTGTCTACATTTCCGTTGTAGGTAACTTTAATTGTGTCAC
CATCAACAGCTCTATCTAGAGTAACATCGACCAATTCCTTTTTGTCTTTTTCCTGAGACT
TGTCAGCAGTTTTTTCTTTAGTAGATTCCTTTTGTTCTGTTTTCTTTTCCACATCCGTAG
ACGCTTGTGGTGTTTCTTTTTCTGTGCTTACTTTTTCAGAATCATATGAGCTGCAGGCTG
GTAACGATAAGCTCAAAGTGAACGCTGCAAGACCTAATAATAATTTCTTCAACTTCAGTT
CCCCGTTTAGAATGTTTGTTCTCTATTTATATCGCTAAACCATCAATAATTATAATCCTG
TATCCAATTTTTGTGAATTAAATTATTCCTTAAATTTTGTCTTGTGATGAAATAAAACTG
GCGACTGTCATTGTAATTAGTTTAGCTGCATTTTCATTTTCGATCTGCTCTACAAAATGA
TTCCCGTCTTACTTTTTTATCGAATAAAAAATTGATAAAATAAAATAATTTGGCAGGTTT
TTTGTTATATTAAATATATAAACTCTAGATGATATGAAAGGGCGGGTCAAGAAATGAAAC
ATGATAGTCTTTTAGATGCAACATCCCAATATGGACCACAGGATCTTTTATTAGGAAGAG
ATGGGCTAATATCTACTGGATATTGGGCAAAAACTCAAGCTAATATTCTTACTGGAACGG
GTTTGCCTGTGAGCAATGATGAGATGAAAACTTATTTGAACTTACCGAAAAACGTTGAAA
AACCTCAAGATTTTCAGAAATTATATGATGTTTATAACGCATATAACAAGTTGTGTGACT
GGTGGTTGGAGAAATTATTAAGCTATGTTAATAAAATGGCTAATGATATATACAATGTTG
GGTCTACCACTTCGGAATTGATAAACGAAGCATTACAACCAGGTTTAAGGGCAAGTACAA
CTGCAAGCAATGATCAAGATCGACAAGATGCTAATCAAGATTTTCATGAGGTCTGTCTTA
TACTTATGAGAGAGCTCCATGAAAATCAACAATCTATGAAAGAGGTTCAAAGTCTACTAA
ATTCCTTTCTTCAAGGAGGTAACAACTTGATAGGTGTTACACAATTGAACAATTCTTTTG
AAGAAGTAATGACTTATTTAGATTCACAATACAATGATGTGAGCGAGATACATGATTTAT
TGAATATACTCATGCAATTTAAAAAATTGTTGGGTGAAAGTCTTGAAGAGCATGAGATAA
ATGAGAAAGTAAAATTCAGCTCTGAGTTAGGCCCTTTAATCGGGTATATAGTATCCGAAA
TGTTAGAGTATTCTGATGTTCAACTGTTTAAACAACGAATTGATAATTTTCAGAATTTCA
ACGGTGTAGATGCACAAGTTGCATTAGTTGTAAAAGTTTTAAGTCTGTTTCACGGCATCA

ATATAGACTTGCATAATGTAGTATACCAGGCAGAAAGGTCTCTGGAGTTTATAGAACAAA
CTGAAGGATACTGGCATTCTATAGCAAATCAATTTTATTCACTCGCACAAGGGTTTGAGA
ATGAGGATATCAATAAGCTTTCTATGGATCTTGATAATGCTGCGGCCACTTGGGAAGCTG
TTGCTAATAAGGCAAAAGAGTTTGTAACAAATTCATACCAAGGATAAAAAATGGGTAATC
TGAAAAAGTGTAAAAAGAGGGTTAAAAATAACCATCCTCCATTGCTTTAGTCAGTAGGGA
AAAGTCAGCACCATAGTCTTCTTCATAAACCCATTCATAGCCTATTTGACCCCAATTTGG
GACTTGATCAGGTCACAATTTTCCGAAACTAGCTTCAACTTCTTTGAATTTCTTTTTTAC
TTTACGATAACCTTGTGTAAACAATATTTTTGCTCTAAGAGCTTCAAATGAATAACCATA
GCTAATATTGGCTCCTTGTCTTAATCGAACAATTCAAGGATTCAGTATAGGCGTTTGTAA
TGGGAGAATTGAAATAGTTGAATATCTCTTCTTCCCAATTATTCATTGACTTAATCAGGT
TTTTAAAATAAGTCATTAACTCTTTAGGAACCTTTGAAAGCCAGTTTTGAATCATATTTG
TTAACATTATTACTTTTACCAATATGAATAGAAAGAAATCCAAAAAGAGATGCCGAAACA
TCACTTTGATGTCAATATGGGGTGATTTATTTGTCTAACTTTGTTCATAAGACGGTAAAT
GGCTTGAAAAGTCTTTTAGATGAAAAAGGAGCCATTCTATTGTTTTGTTCAGAAGGTGAT
ATCATGGAGTTCCTTGTTACATTTTCACCGGACAAAGCAACCCTGAATGACATTCAAAGC
TTTGAGGCTAAACACCAGCTTTCCCTTCCAGAGGATTATCAGAAGTTCATCAGACTACAT
AACGGTGCGAAAATTTTTGAGATTCTTTCTGATGGGGAGAATATAGGTGGAGGACTTCAG
CTATTTAGCCTTGAAGAGATTGAAGAGGAACTAAAATATGAGGATTTGTTTGAAGGTATC
AATGGTATCCCAATTGCCTACTTATTGGAAGAATGCCATTTAATGATCGATTAAGATAAA
ATTAATCAAGGTGATCCTAATTATCTCTATAAATTTGAAAGTGGTTTAGAATATAATCCA
TTAAATTTAAACTTTGAAATATTTTTAGACCGGTATATATTGGCTAATGGGGAACCTTTT
TGGGATTGGCGCTACTATACCGCAGAAAATTATTATAGAACAAGATAGTGTGATGTATAA
GACATTCAGTTCTGGCTGTCTTTATCTGTTATTCAAGCAAATGATTGTGGTAAAATTTAC
ATAGAGTAGGCTTGAAAGGAGAATGAAATGAAGGTGTTTGAAGCTGATTCATTGCTCTCT
GATGCGGATAAGAGAACCAAAGAGTATAAGGAACTAAGGTCGCAAATGGTGAAGCTCAAA
AAGGCGTTTAAAGCTGTAGCTGACCTAGACGACAGCAAGTTTTCAGGTAAGAGTGCAGAC
AACATTAAAGCATTTTATCATGACCATGTCGGCGTTACAGATCAATGGATTGATCTTATC
GATATGAAAATTGTTTTCTTAAGCAGCATTGCTGCAAAACTTGAAGACGCTAAGATGTCT
GATGCATACATAGAAGAATCCTTCTTAGAACATGACCTTGTAAATGCATACACAAAATCA
AAATCCATTATGTCTGAACGGAAAAAAGCAATGAAAGACATTTTAAACGACATCAATGAT
ATTCTTCCACTTGAAATATTCTCAACAGAAGACTTGAAAGACAAACTTTCCTCTGCAGAT
GACAAACGTGAAAAAACAATCGATAAGATAAACAAGCTTGATGAGGATTTAAGAACAGAG
CACGCTGAGACTGAACAAAATGAACAATTCATCCAGCAAGATTTTAAAAAGCTGCAGGAA
TCAACAGGCAAAGGGAAGAACGCCACACCGATTCACTATAGCGCCAAAGCGTATAGAGAA
AGTGACATACATAAGAAAAAAGGCCATATTGAGCAGCATTCAGAATCTTATTTAACCGTG
AAGAAAGAAGAAGCAAAGGAACGTGAAATCAAGGAATTAAAGAAAAAACTTAATGACGGT
GTGTCTGATCCTGATGAATATTTAGTGATTGCTTAGAAAGTATGGTACGAGTATCTGGAG
CCAGCTCAAGTACAGCTTGCAGTGCAAATTGAACAAGCAAAGCAGCTAGAAGGAGCAGGA
GAAATCACCTGGGACATCGTAAAAGGTGTAGGGGTAGGCTTATATGACGTTGGCAAAGAC
ACTGTAACCGGCTTATGGGACTTCATCACCGATCCAGGAGAAACATTGTCCGCGCTTGGC
AATGCAGTTATTCATCCAGTTAAAACATATGATGCAATATCTGCTGCAATTAAAGAGTCA
TATCAAAAGGATATGGTGAATGGAGATGCCTATTCAAGGTCAAGATGGGTAACGTATGCA
ATTGGCTCAGTGGCTGCAGCTGCTATCGGAACAAAAGGTGCAGGAGCCATTAACAAAGCT
GATGCAGCAGGCAAGGTAATAAATAAAGCTAGTCAAGCAGGGAAGAAAATTAAGGCCGTC
AAGATTCCAGATTTGCTGCCTTATAACCCTAAGTACTATCTTGCTATGGCAGGGGACGTG
CCATACAATGTAGTGGATGGAGAAAACTTAAAAAATCAGCTAATGAGTTTTGCTAAAGGG
TCAGATAAAGAAGTAAAACCTTTCGATGTCGTTGATTATAGACCAAGTAACAGTCCTTTA
GAGAATCATCACGGTGTAATGGACGTATGGGCTAAACACAATGTTCCGAATTATGTCCGT
AGAGGGAGTAACACACCAACAGTAGCTTTAACTAAGGAACAACACAACGCTACCAAGAAA
GTATATAGAGAATGGTTGTTCGAAAAAACAGGGAAGAAAGTTGGCGGCAAAGTAAACTGG
AAAGAAGTATCCCCCAGAGAAATACAAGAACTTACTGAGAAGATGTTTGATGCTGCTAAT
GTTCCTAAAAAAGCTAGACAGCGATATTATAACGCATTTAATCAATACAACTTTAGAAAG
TAGGTGCGGATTTGAGCATTGACATGTTAATCAAGAAGATTGCTTCTACATCAGACTGTC
GGCTGTTTGAAGCTGATGGTCTCCCAGTAATAGACGAGAAGCATTAACTACCAAAAGATA
TTAGTGAATTTTATGAACAATGTGGTGGAGCAGTTTTATATGAAAATGCGGATTATCCAA
TATACATAGTACGACCAGCTGAGTTTGAATTAGCTAATCCCATTATTGTTGGGGAGCTCT
GTGAAGAAGACATTTCATCCGAGTGGTATATTGTTTGCACGGATGGAAAAGGAGAATACC

TAACAATTGACTTAAACGATCAGCGAAAAGGAAAATGCTATGACAGTTTTTTTGATCGAC
ATGGAATAGTCGGCGAAACACAGGTGATAGCAAGTTCACTTACAGATTTAATACAGAGAC
TGCTTGAAAATAAGGGTAAACATTGGTGTTGGTTAAGGGATGATAATGTATCACTAGGCG
ATGCATATGATGGCATAGAAATTGAATAATCTATTAAACAAAAGTATTGCAATTGTTCAT
TGCTAAAGATGTGCAATGCGGAGGGTATTAAAATGAGCTTCACTAAGATCGAACAAAAGT
TGAAAGAGTTTACGATTGATGCTGGCAGATATCCTAGCATTAATACAAATGAACAACTCA
AAGAGCTTGAAATGTACATAGGCAATCAATAACCTTCAGATTATAAGGACTTTTTGAAGA
AATATGGAGGATGTTATTTAGAAAGTAAAAAAACAACAGATGAGATTGAGTATGACGTTT
GCTATAAACCCTTAGAGAAACATCCATGGATGGGCAAGGGCGATGATACACAATTGTTAG
AAGGTTTTTATGGTTTAGCGAATGATCATAACAGTCTTCAGAAAGCAATCGATACATATT
CTGATCGCTTTCCTAGAAATATTATTCCTATCGCAAGCTCTGCAGGCGGGAATGAAATAT
GCATGGATATCGATAATGGAAAGATATTGTTTTGGGATCATGAATTAAGTCATCCCGACA
AAGACTTTTTCTTAATTGCCAATGCGTTTGAGGAGTTTGTCTTCAGTTTAGTTGATGAAC
CAATTGAAGCTGATAAAGAAGATGATGGAATTCTGTATATTGAATTAGATGATGACTTAC
TAAGCAGTTAAGAAATCGTATGGAAATAACTGGAGGGAAAGTTAAAATGGCAGTTACTTA
TTGGACAGGGGAGAATCTTACACTGCGAGCAATTCAACCGGAAGATATCGTTATATTTGA
TTTGTTAGATGATGAAATTCTGCGAAATATGGACTCGCTACATTTTCCTCGTTCAGCTAA
TAACATGAGAGAATGGGTTGAAGAACAATTAGAAAAAGATGAATTCCGTTTTATTGCAGT
TGAAAGTGATAATAATATAGTAGGTATGATCCAAACCTTTGACTGTGATAGGAAAAATGG
TACCTTCGGTTATTATTTGGCTGTATTCGAGCCATATAGGGGCAAAGGATTTGCCAAAGA
AATGATCTTGATGGTTCTTCGCTTTTTCTTCTTGGAACTTGCTTATCAAAAAGTAAATAC
AACTGTTTATTCTTTCAATAATCCATCCATACGATTACATGAAAAATTAGGTTTCGTGAA
AGAGGGACAACTAAGAAAGATCATTTTCACTAAAGGTGCCTATTATGATGGTATTTGCTT
TGGGATGACAAGAGAAGAATTTGAACTGAATCATGGATAATATCACTTGTGCATCGATAA
AGTACTGGGTTCCAATAAGATAATGGTTTTATTCACCAAGCAATAGTATTGCAAATACCA
TTTTTATATGGTTATAATTAGGGTAATGATACAATTATATAATTATTTTTTGCATTTTGT
TTAATTAAATGCATAAAATAAAAAGACCAGGGTGTTGGCAGCACCCTGGCTCTGTACAAA
AGCTGCCCATCAAAGGGCTTGCTCGTTGTCAAATCTGCGTAGGCCTAACCCTTCAGGTGT
CCAAACTCAAGGGAAGGTCTATTTTTTCTTTTGGCTTGACGTTACAATCATCATTATATT
GAAGATCAACAAGACGGTATTTAATAACAATCCGCCAAAATTGATCATTATCATTAATGA
TTCGTAAACAGTCATTTTCCCACCCCTTTCATAGGGGATGAGCAAGACTCCCTTGAGTGA
GCAAACTTTATGTACAGGAAATATTATACTTTACATAAATGTCCTTGTCGTTAAAAATTC
ACAATAAATTGACAATATGAAAAGAACTTGCTTAATGCAGGTTCTTTTTTTATTTCAAAT
ATTAAACAATTCCCTTCAAATGACGATATATAACATGTAAACTTGTACCAAGGAGGATGT
AGGAAATGAAGAAGAGAATTACATATTCACTGCTTGCTCTTCTAGCAGTTGTTGCTTTCG
CTTTCACTGATTCATCAAAAGCAAAAGCGGCAGAAGCATTACCACTCTTCTATCTACAAC
TTACAGGGATTACATCAGACGGAAACGATTTTGCTTGGGATAACCTCACTTCTAGTCAAA
CCTAGGCGCCAAACGTTTTGAAGGGGAATAAACTATATGTGAAAGCTCGTTTTATGGGTT
ATACCAAGTTAACAGTTATCACAGGCAAAGTCGGTAAGAATCTGTTGTATAACGGTACAG
CTAAAATGTTTAAATCTGATGCTATCCTTGGTCAAAATAAGGTGGTAATAGGCTGGGATA
GATATTTTGAGATTCCCATGGTCGCACTTCAAGATAACTCTATACAGATTAAAGCATTGA
GCTCAGGCACCACTTTTGTTTACAGTCAAAAAATTGATTTTGAAAGAGAGTGAACCAGGT
GATTAGTACTTCTAATTATGATGTAACTAAAGATCCAAAATTCTTAGCTTTTTGTAGCTT
AGCAACAGAAGGGTATGGACACAACAATACTCAGCAATCTTCAATAGGTTTTAACGAGTT
TTGCGAAAAACTTGAGAAAAATGCGATCGTTAAACCCGAGAATAAAAAAAGCACGCTTGA
TTTGAATAAGTCTTTAGAAGACTGGAAATTGAGATTTACTGCAGGCGGAAACCTTTCTAC
AATGGATTCGCAGGTATTAGATGTTATCAAAAAAGCTTATAACTTAGGCATGGTTAATAA
AGATAATATGCTATTACGAAATGAAGCAATTAATGCTTATAGAAATTATATTTAAATAAT
GAAAACAATGCCCAGCTTAAATAAAGAGCTGGGCATTTTACTTATTGGGGTTATTTGCTT
TCAAATACTCTCGATGTTCTTTCTCCAATTTATTAATATCATCTTTTAATATATAGATCT
TATATTTTTCCTTTGCTTCTCTAGACAGATTATCTTTTAAATACTCACGGAGCTTTGCTT
CACTTTTGGTTTGATCCATGCCAATTGTAATTGCCGGATCTGTTTGACCAATTTGCACAC
TGGATATACCAAATTCATCCCGAATATAATCAATTACTTCCAAAGGGGACTTTTTATTTG
CTTCGATTAAAGTAATACCTGACATAATCAAAGCAGGAACTAAGACCAAAAATCCGATTA
GCCTCTTCTTCATTAAGCATTACACTCTCCTTCATGGAAAAGAATCCCTCCTTTATTAAT
ACTACGCAAATCATCATTGACCTTTATATTTTATTACCAACATTTACATCGAACTTTTGT
TCTGGTTTATCTTGAATGAAGAACGTTTGTTCTGTTAATATTAGATCAATAAAGGAGTGG

GGCGATATGCTTAGAGATCGAGGAACAATCAAATGGACATCAATGATGCTTCCAGAACAT
TTGACACAGCTTAAACAAGATTTGATTGATGTATCAAAAATTGAAAAGCCATCCTTAGAT
GATCAACAAATTGAAGAGATGGATATTCTCGTCTCTGAGGCACTTGAATTTAACAAAGAG
TTGCAATTTAAACTTTTTCACAATGGATTCGTTGAAAATGTCACCGGCAGCGTCCATTAC
ATAAATTTTGAACAACAAAAGCTTCACGTAAAAGACCAGAATGACAATACAGCTTATATC
AACATGAATAACATCATAGGAGTTACATATAATGATTGATTACTCACAATTACCACGTAA
GAATATACTTTGTGTCGATATGAAGTCCTTTTATGCTTCTGTATCGGCTGTAACAATGGG
GCTGAACCCTTTAACATGCTATCTTCCTGTTGTAGGGAATACGGAGAGACAGGGAAGTGT
ATTATTAGCTGCATCTCCTGCACTTAAAAAAGATTTTGGAATCAAAACAGGATCGAGACT
ATTTGAGATACCTGAAGATCCAAGGATACACATTGTAAATCCACAAATGAAGCTTTTCAT
CAGAGTTTCAACTGAAATTACAAAGCTGTTTTACAGATTTGTTCCTGAGAAATGTGTCCA
TACGTATTCAATTGATGAATCTTTTTTAGATGCAGGAAAAGAAGATCCTGAAGAAATGGC
CAAAGCAATCCAAAGAAGCATGTGGAGAGAATTTGGTTTGATGTGCACAGTTGGTATTGG
AGACAATATGCTACTCAGTAAGCTTGCACTTGACCTGGAGAGTAAGAAAACAAATAGTGG
CATTGCACTTTGGAGATATGAAGATGTACCAAATAAACGCTGGAAGGTTCGCCCTTTGTC
TAAAATGTGGGGGATAGGAGGGAGGATGGAAAGAAACCTAAATCGGATGGGAATATCGAC
TATAGGTCAGTTAGCCAAATTTCCTTTAGACCTGCTTGAAAAGAAGTTCGGAATAATGGG
AAACCAGTTGTACTACCATGCTCACGGAATTGATTTATCAGAAATAGGTGCTCCGTTGAT
GCAAGGTCAGATTAGTTTCGGTAAGAGTCTGATTTTACTGAGGGATTACACAAGGAGTGA
AGAGATTAAGGCGGTTCTTCTGGAGATTTGTGAAGAAGTCGCAAGAAGGGCACGTACACA
TAAAAAAGTTGGTCGAACAATCAGTCTGGGAATTGGGTACAGTAAGGATGAGCTTGGTGG
TGGTTTTCATCGTTCCAAAACAATTGATCTTCCTACAGGTATCACGATGGATATTTATAG
ATGCTGCTTAATGCTTTTTAATAAGTTTTACTCGGGTAAGACTGTGAGAAGTGTCTCAGT
CACGTTATCGAATATTGAGGATGATGTTAATCAGCAGCTGAGTTTATTTGAAGTGGATAA
TGAAAAGAGAAGGAAACCCGGTTTTGTAATGGATGGGATTAGAAGTAAATACGGCTCTAA
AGCGATTCTGAGAGCAGTTTCTTATACACCAGCAGGAACTGCACTTCAACGAGCTGGATT
AACAGGTGTGCATAAGAGTTAAGATAAATTTAAACTTATATAACACATCGCTTAAAGTTT
TTTTGTTTTAAAAACTTAAAAAACATGGTAAAATTATATAAAAACATAAGAAAGAGTGAT
TATATGGAATATGTAGTTGTGATAATCATTTTATTAGCACTTTTCTTTATTTTTACTGTT
TTCCTAAACACACGTTATAGTTTTGATGAAAAATGCTTAGTCTTAAAATTTGGTTTATCT
AAAACAGAAATTCCAATTAATCAAATAGTTAGTATTAAAGGATCAGACAAGTGTGGAGTT
GCAGATAATATCGATTATAAAATTGGTATGCCATATGCTCAACCTGATAGAATTGTTATT
GAAACTACAAATAAGCGTTTTCTAGTTTTTTTAAATGGAGCTCAACAATTTATTCAAAAG
TATAAAAGGGTTAGTGTTTGAACATAAAAAAGTACCTTCTTACAATAGAAGGTACTTTTT
TGTATCTATAATTATTAAAAATTTACCTAAATTTTTATCATTATTAATTCAAAATAAATC
CATAATAGTCAATTTTATTTAGTGTATTACAACCAATTCTGTTTATTGATAGGTAAGAAA
GTTTTTTTTCTATGATTTATGAACAAGTTTCCTTATAATTTTCAAAAAAAAATAAAAAAT
ATGGTTGAATTTAGAATTATCTTCCTTTATATTAAAAAATGTAATCCGGATTGCAAACAA
ATGGGGAGGTTTTACAAATGGAAAAGCTATTTAAAGAAGTTAAACTAGAGGAACTCGAAA
ACCAAAAAGGTAGTGGATTAGGAAAAGCTCAGTGTGCTACGTTGTGGCCACAATGTGCTA
GTGGCGGTACAATTGGTTGTGGTGGCGGAGCTGTTGCTTGTCAAAACTATCGTCAATTCT
GCAGATAAAACATTTGTAGAGGGAATATTTTAAATATTCCCTCATATTTAAAGCGGGGAT
TGAAATTGAGTAAGAAAAAGAAATATGTTCTTACTAAACAGTTTAATAGTCATGATTGTG
GACTAGCTTGTATCTCGACAATTTTAAAGTTTCATAACCTTAACTATGGAATTGATTTCT
TACTAGACCTAATTGGGGATAAGGAAGGCTATAGTTTTAGAGACTTAATTGTTATTTTTA
AGAAGATGCGGATAAAAACTAGGCCACTTGAATGGCAGGAAAATAAGACATTCGAAGCCC
TAAAACAAATAAAGCTCCCTTGTATAGCTTTGTTTGAAGGGGAGGAATATGGACATTACA
TAACAATATACGAAATTAGAAATAACTATTTACTTGCTAGTGATCCTGATAAAGACAAAA
TAACTAAAATAATAAAAGAGGATTTTGAAAGTAAATTCACAAACTTTATATTAGAAATTG
ACAAAGAGTCAATTCCTGAAAAAGAAAAAGATCAAAAACAACATTCTTACGGTTTTAAGG
ACATACTTTTTAGAAATAAATTGATCGTTTTTGTGATTTTATTGACTTCCTTGTTCGTTG
TGGGTCTTGCTGTAGCTGGGTCGTTTTATATAAAGTTTCTAGTTGACCTAATTATCCCAA
GAAGCTTAAGAGAATCTTTAATCACAATCACTTTAATATTCATAAGTATGGTCTTAATAA
GGTGCATCTTCGATTTTGTAAGATCATATTTGATAATAAAATTGTCTTACAAAGTTGATA
AAGAGATGTCAAATGTTTATTTTCATAAAGTAACAAAATTACCTATTAATTTTTTTGAAA
ACAGAGAAGATGGAGAGGTAATTTCTCGATTCAATGATGGTATATATATTAAAGACTTTT
ATAGTGCTAACTTTGTTACTGCAATAATTGATATAATTTTAATACTGGGATTTGGAGTTA

TTTTATATAGAACAAATAACATTCTTTTCTTAACAATTATTCTCCCGATTTTGCTACTTT
CATGTCTAGCGATTTTGTTGTTTGATCATCTTAAAAAGAAAAATCAAAAACTGGTGGAGG
ATAAGGCTAAATCTACCTCTCTTTTAATTAATTTTCTGAAGAATATGACAACTGTTTATT
CTTTAAATAAAACCTCGTTTTTTTTAGAAAAATTTCATCTTACAAATGATAAACAATTAA
ATTCAACCTTTAGTGTAGCAAAGGCAGTTATTAGCAATGAAAAACTGAAAGGATTAATTA
AAAACTCTTTTACCATAATTATTCTATGGGTTGGGACAAGACAAGTTCTAAATGATTCAA
TGAGTTTGGGTACACTGCTATTTATAAACACATTAGCAGCCTTTTTGCTAAGTTAACTAG
ATCGTATATTGAGTATTCAATCAGATCTTCAGAAGGCACATGTTGCTTCCATAAGATTTT
TTGACGTAGTAAACTATCCAGTTCAGCAAGATAGCAATGAGAATTTAACTGAACTTGATT
TTATTCAGAATATCAAAACAGTTAATCTTAATATTGGGGCAGACCCAATGCGTTATATAG
TTGAAGATATTAATTTAATATTAGACAGAAAGGATAAAGTCCTTATTATTGGAGAAAGTG
GTACTGGAAAAAGTACGCTTGCAAAAAGTTTGTCTAAAGTGTATAAAGTACCCGATAAGT
CAATTTATTTAAATGGATTAGATATTAATCGATACGATCATTTATCAATAAGAAAAAGGA
TTGTGTATATTGACGAAAATCCTTTTCTTTATAAGGGAACCATTAAAGAGAATCTTTGCA
TGGGAGAGATTTTTGCTCAGAACGAAATTGAAAACGCGTGTATAATGTCCCAATGCCATG
AATTCATTTGTACCTTGGACACGCAGTCCAGTTATAAATTGTCTGAAAACGTTTCCTATC
TATCTACAGTACAAAAACAACGGTTAGCATTAGCAAGAGCAATTTTACATCAACCACAAG
TATTAATTTTAGATGAGTCATTATCTAACAGTGATCCGGATAACACAAAATTGATTTATG
AAACCTTTCACAGGCTGGATTGTTTAATTATTCTTATAACTCATAATGACCCAAGTAACT
TCAAATACAATAAAAAATTAGTATTCAGAAACAATTGGATTATAGAGTCGAGCTACTCGG
AAAATAAGGAGTATTCTATATGAAAAAGTGGATTGTTTTATTTCTTGTTTTAATAGCAGC
AGCCATTAGTATTTTCGTTTATGTTTCTACAGGTAGCGAAAAACCTTTTTATAATGATAT
AAATTTAACTCAATATCAAAAAGAAGTAGACTCTAAAAAACCTAAATTTATTTATGTTTA
TGAGACAAGTTGTCCTCCTTGTCAAGAAATAAAACCTGAGTTAAATGAAGTAATTAAGAA
AGAAAAGTTAAAAGTACAGGCTTTAAATATTGAAGAAAAGGAAAATTATGACACTGAATT
TTTAGATAAATATAATTTGAATAAAACTCCAACGATTCTCTATTACAAAGATGGCAAAGA
AAAAGATCGGTTAGAGGGCTATAGAAGTGCAAGCCAAATAGAAAAGTTCTTTGATAAAAA
TGGTGATAGACAATGAAACTGAGTGATATTTATTTGGAATTAAAGAAAGGCTATGCCGAT
TCTTTATTGTATTCAGATTTCCCATTGTTGGTTAATATAATGGAATATGAAAAAGTTATT
GATGTGATGTCAATTCAATCTTTGGTTGCAGGTTATGAAAAATCAGATACTCCTACAATA
ACATGCGGTATTATAGTTTATTACGAAAGCAAGAGAATTAAAAAGTGTTTAAATAGTGTT
AAAGATGATTTTAACGAGATTATTGTTCTAGATTCATACCCCACTGATGATACCGTTGAT
ATTATTTAATGTGATTTTCCTGATGTTGAAATTAAATATGAACAGTAGAAGAATGATTTT
TCCTATGCTAGAAATAAAATTATAGAGTATGCTACTTCCGAATGGATTTATTTTATTGAT
GCAGATAATTTATACTCTAAAGAAAACAAAGGGAAAATAGCTAAAGTAGCTAGAGTTTTA
GAGTTTTTATCTATTGATTGTGTAGTTAGTCCATATATAGAAGAATATACTGGACATCTA
TATTCTGATACACGAAGAATGTTTCGGCTCAATGGTAAAGTTAAATTTCATGGGAAAGTG
TATGAAGAACCTATGAATTATAATCATAGTCTACCTTTTAATTTCATTGTGAACCTTAAG
GTTTACCATAATGGATATAATCCTTCAGAGAATAATATAAAATCAAAAACACGAAGGAAT
ATAAATCTCACAAAAGAAATGTTAAGATTGGAGCCCGAAAACCCAAAATGGTTGTTCTTT
TTCGGCAGAGAACTAAATTTACTTGATAAAGATGAAGAAGCAATTGATTATCTGAAAAAA
TCAATAAACAACTATAAAAAATTTAATGATCAAAGACATTTTATAGATGCTTTAGTGCTA
TTATGTACTTTATTATTGCAGAGAAATAATTATGTTGACTTAACTTTATATTTGGATATA
TTGGAAACTGAATATCCAAGATGTGTTGATGTTGATTACTTTAGATCTGCAATTTTGTTA
GTAGATATGCAAAATAAACTTACTTCTTTAAGCAATATGATTGATGAAGCTCTTACAGAC
GAGAGATACAGTGCTATAAATACAACAAAAGATCACTTTAAAAGAAGTTTAATAAGCCTT
AATATTCAACTCGAAAATTGGGAAAGAGTAAAAGACATATCAGGGGAAATTAAAAATGAT
AATATGAAAAAAGAAATTAAACAATATCTTGCCAACTCACTCCACAATATTGAACACGTC
CTGAAAGGAATTGAAGTATGAATACAAGATATGTAAAATCATTTTTTTTATTACTGTTTT
TTCTCTCTTTCTTTGGCACAATGGCTAGTTTATTCTACAGTGAGTTCAGGCATTTCAAAC
CATGTGTTCTATGTTTGTATCAAAGAATAGTTCTATATCCTATACCTATTATCTTACTAA
TAGGCTTATTAAAAAAAGATCTTAATTCGATATTTTATGTTGTTTTCCTTTCATAAATTG
GATTGATTATTGCGTTTTATCATTATATTATCCAACTTACACAAAGCAAAAGTGTCGTAT
GTGAATTTGGAACCAACAGCTGCGCAAAAATTGAAGTAGAGTATCTAGGCTTTATTACAT
TACCCTTAATGAGTTCAGTATGTTTTGCATTGAGATTTGGTATAGGACTGAAATTAATTA
TCAAAAGCGAGAAATTAAAACAAAATCAAAATGTATATAATTGAATAAGAAGTAACCCGC
CTTGTAAGAGGGAGGGTTACACAGGTAATCCGAAAAAAGCTATTTAAACAAGTTCTCTTT

TTTTAGAACTTGTTTTTGTTTTTTTCCTTTTGCAGTTATGTAATTGTTTTTGAACCATGC
TGCGGTAGTAGTACTGGTTGTGAAGACGATAGTGAAAATTGTATATAAACAATCATATAA
ATGATTTATGTCCTCTTCGTTAATGATGAATGCTGCTTTACCCAGCATCACCATTATTTG
ATTGAGTAAAGCTATAGCGAGCAAAAGAGTCCTAACAATTGTGCCTTTATCAATATTCTC
AAACATTGAATTCCTCCTACTTAAAATTTTGTTCGATTTTATCGAGCTTTTCAATTACAA
CATCGTACTTTTCACTGAATATACCCAGTACTTCGTTTTGAGAATCGATTTGATTACAAA
GTTTCGACTCTCTTTCCTTACTAGTTTTCATTACGTAGAAAAGTAGCCAACAAAATAAAA
CAGCAAATGGCCCCTGGGTACTTAAATATTGTGCTATATCCATTTCCATAAAATGTTCAC
CACCTTATTACCATAGAAAAGGATATTTACTGAAACATATTGATCAATAAACATCCTCCG
AGATAATTGAATATAATTAGATTGTTGATCCAAACTGTCCCGAGATATATCCACGTTTAC
CTTTATAAATAACTTCCCAGTAGCCATTGGAATTGTTTTTACCTTTAACTGAACCAGAAA
TTGAAATAGTGCTTCCAAGCTTAACTGTGCCAATATTTTTAGAACTATTTCGATCGGGCT
TGTCCATTACGATTGCAGCGCTTGATACACCGACAATTTTGATTTTTCCTACAGATTTAA
TAGATGAGGAACTTGAAGAAGTTGATGTTGTTTTTGGGGCAGAGGAAGAGGTTTTAACTG
ATCCGGTAACATCAACATATTTGTCAGAAGCAGTGATGTAATATGTAGTGCCTTTTGAGT
TCTTAACTTTGTATTGATAGGCAGATCCAACTTTAACTTTCTCTACAACTGTAGGAAATC
CGATGCCTTTATTTACTGTGCCGACAACATCTTTATCTTCCCAAGATGGTTTTGAATAGA
AGCGAAGTCCGTCAACTTTTGATTTTGGTGAACCACTTACTGCTGATGAAGAGGAATGGG
AGGAGGTTGTTGGTTTAGTTGGAGAAGCTTTTGAAACATATTTGCCTGACATTTTTGCCT
TTACTCGATTTTTAAAGTCTACAAATTTTTGGCTGTTAGATACCCATGGTGCAGGGCAAT
TCTTATGGGTGATGTCATAATGACGAACAATATCATCAATAGGATCTAAACCAAACTTAT
TACATAATTCAACGAACACATCCTCAGTTTTTTCAACTGTATCTGAATGGAATGAACCGT
CTTTTTCAAGGCACATTTCAACTCCAATAGAAAGAAAGCTAGCGTTAGGTTTCAGCGCAG
CAACTCCTCGATAAGGATTTCCGGCACTATCTCTTTGCTGAATATCATTTGCATGGTAAG
CTACTTCAATTAATCGAATGATACAAATTGCTTCAGCCTTATCTACGAAAATGTGAGCTG
ATTCATAAATTTGTGCATTACTGAAGTATCTTCGATGATTGTCTGCACCTGCACCCGGAT
TGGCTGTATAGTGTAGGACGCATTTTTTCACACCCTGTAATTTCAAACCTGGTCTAGTAT
ACTTATTGACTGGAATGTAGCTATTAGTGAAAACTGACATAAAAACATCTCCTTAATTTC
TAATTAAAAAGAGTCTCGAATGGACCATTTTCATAGCGATTTCGAAACTCGTTGAATTGA
ATCTCATTATTGTTGGATGTTCCATAAATTTCATGGAAAAGATTGTGGATTTTCTTAGTT
AGAGGGATTCCGAGTCCATAATTTAAATGAAGTCTTACACATTCATTTTTAATTTCCTCA
AGCTCATTGACTGTAAAGTCGCCAATTATAGAATTCATAACTTAAATTGCTCAGTGCTTC
TTTAACAATCTCGCTAAAATTCTTTTTATAGTGGTGCACATGAAGGTCTTTTGTAGAGTT
AGTTATTGCACATTTAAATGAATATTTCTCTAATGAGGGCTTCGTCCAAACTTCATAAAC
AGCTTCTCCTAAAGTTTTATGTAACGAAGAAATACCGCCTTTCCATCTAGAGCTATTTTC
GCCTGTGAAAAGAAGTAAATAACACTTTCTGCAGTTTGCTAAACCTCTTACCATGTTATG
AAGTGGAGCGTATTGAACTCCAAAGTCTATATGTTCCTTGCGTAGAAAAGGGAGATTTTC
CTTATCGTGATGGTATGTATCGAATAAAGGGATATACGCACGATCAGAAAAAGATGCTTG
TGCACATTCAACAGAGTATTTTTTAGCACCTTTACATTGAAAACACTCTTAACCTTTAAA
GAAGTTGCAGGGAAGGTGCTCCCATATATGTCCTTCAGGGCACTTAGCTTTTAGTTTAGA
ATGACAGTTTTTATAGGTGCTTAAAATCTAATAGCCTTTAAGGGCATATCTACTTTTCAC
AGTACTTATGTTAAGCCTTTTTCTAAGGGAGCCTTCAATTATCCTGCATTCTGGACATCG
AGTACCTTTTTTAAAGTGTTCGTAGTTTACTTGCCATGTGTGTTTATGTCGACATCTGAC
TTTCACCTTTGTTTTCGAATTTACCATGTCTCCAGAAATGTGAATGTAGTTATTCTGAGC
TTATAAATCAATAAATTTCGTTTGAGCTTCTTCACGTTTTTTTAAATGATAATCTGATAT
CTTTCTTGCCATCTATCCACCTAATAATTAAATTTGAGCACAAATAAAAGAGAAGGGGAC
TAAACCCAATCTCTCTTTATCTCACGTATGCTCTTGTTATCTCTGTTTTTCAAGAATGTA
TTTTGAATAAAATCTATATTTTATCTAGAACGTAACCACCACCTTTATTGTTCCTTTATT
CTTAATCCAAGTTCAAGCTAGGTATATTACCTACGTTACCCGACACTCGTCTTCTATCTA
TATCTGATGGAGTTCTAATGAAAGCTGTTCCTTCAACATCACTATAAACTGAGTTTCCAG
TTACAATTAAATCGTTTGTTCCAGTGTCTTCTTGAATAAAGTAAAGGTGATAATCAGAGT
GGCATTGCTTGAAAACATTATCAGAGAAAACAGCTTCTCTTACAGATCCCCAAATTGCGC
GATCAATATTATTAGTGATAATGTTTCCTGAACAAATTGCATTTTGAGAGGTTTTGTACC
GGACGAAGGTTTTTAAGGATTTTGCGAGGTTATTCTTAGTGAAGATAATTGCAGCATTAC
CCACCCAAAGGGCAGTACCGCTAAATGTGATCTCAGAGTCAATTAGTTTAATAATTTCTC
CTTCAGCAACGTTGATGCACTCATTGGCTATATTGGAAATATACATTCCTTCAATCGTTA
TAGACTTGCCGATCCCGTGAATACTCACTCCTCGGTCTGTAGCATTAACCAAGCTGCAGT

CTTTCATTTTAAATGATTTTATATTTCCACCGGCGAACAATCGGCAGCTACTCTTATTTA
CACTGCTGTTGCCATCAATTTCAATGCCTTCTATATAATTGTGATCTGTGATTCGCAACA
ATTCATCAAGTGAGTGATTTGGATTTCGAAGGAGTTTAGTGTAGGATCGTCTTTCACCAA
CAATTGAGAAATTTGAAGGGAGTTCAATTTTTTCACTAGCGCTAGTTGTATTCTGCTTAC
CGATTAAATATGTACCTTTCGAAAAATGTAGTTTCCCTCCACCTTTTTCAGATAAATCAG
TAAGGGCTTTTTTAAACTTGGCTGTATTATCGATTAAGCCATCTGGTGAGGAGCCTAAAT
CCAATACATTCCAAGTAGGCTTGGATTCAATAAACTCTACACGTCTGTTTAGACTTGTTG
CCAAACCTCTTCCTTCTTGAACTTCAAAAAATCTGCTATCTGTATCGTGATAAGGATATG
AAATTGTTAAGGTATCGACCACTGCACGTTCAATGCCATCACTAATGTTATTAATATATT
TTGCTTCAACAGCAGTTTGAGAGCTTCCACCACCATCTAAAAGAAATGCATTAATACCGC
CTTCAGATAAAAATAAAGATGGCATATTATAAGCTGTAATACCACTGGATCGAAGTGAGC
GGCCATCTACAGTAATGAGAACTAGTGTACCATCAGATCTTTCCCCAATGGCTTTTCGAG
GATGCTTAACATTTAAATGTGCCCATGTAGTCATTTGAGCCGTTCTTGGCTTAGAATCTT
TAACTAGCCAAATGCCAAAAGCAAAACAGTTTCGTGCGCCTTTAGAAATTAGGATGTCGG
CATCAACTTCTCTGTTTCCGTAAACCTTTATAGTTCCATCATCAAAAAATACACAAGCCT
CAGCGCCTGTGTATCCAGCAGCGTCATAATCTTTGTAGAGAACTCCGTCTTTAATTTGAT
GCCCCATGACTTCACCATTTGATCGCCAACCACAAGCGTTTATTGCAACAACAGATCTTT
TTCGTTTTGCCATACTTAAAACTGTTTCACGATTTGTTGTACCAAAATAAGAAGATGTGG
GATCTATGGATTTTTCAAAGTCATATGCAAACGTTTTTTGCACCATACTTTTTTTAGCTT
CGGTTGTTTTAGGAGTAACCTTTGTAACATAGTACTCAATACCAAGTGTGGCATCCTGTT
TAAAAGAACTTGTTACTGTAAAATATCTGTCATCAGTTAATACCTTGTTTAAAATTTCTG
TTGTGTTCGTTTCTACTTCATTAAACTTCTGGGTAATCTTAGGAGCGGTGTATTCTTCTC
CTTCTGAAGATAGTAGTTCAACAGGCATCGGAACATATTTATCTAAATTTTGATCATATC
TATAAAAACCAGCCATAACTTTCCTCCAATAAAAAAGATCCCGGAGGGGATCTTATTCAG
TTTTGTACCATACTTGTCCTGAATCTGGTGCTGTATCTGAAACAACAACTCGTTTTTTCT
CAGGTGAAAAAGAGGCATCCTTATACCAGATATAATTTGCATTAAATGGTTCTGTTGCGC
TTAGGAGTATATTAAAACCTTCATATACTTCGGAAGTTCCAGTATCTACCCACTCAAATC
CATCCCATCGATAAACGATTTTTGTTTCTTTAACAGGCACCGTCCAACCAATCAGTGGGG
TTGGGTAATAGGTGAAAATATCTGAATAGGTATAGACAGATGGTTTATAAATTTTTCTTG
TGTTTTCTACAACTTCTTCATAATTTGATGTAGCCTGTCTACACCAGGCTGTCACTTCTT
GGCATCGTTTTGTAACTCGTTCACATTCAGCTATACGTTCATTCATTCGAATTATTGTAT
CTTCAGCTTAATCGATATGTCCTTGAAGAGTTTCAATAACCATATTCCCTTGTCGTTTTA
TCCAAATTCGAGAGGCAGGGAAGAAGGAAGCTCCTTCACCACTATAATTAAAGGTTAGCG
ATTTTCCTTCATTTGATGCATTAAAAAAGACAACTCCCATAAGGTAATCAACCTTAAAAT
AGTTGTCGTCAAGTTCTCCATCTTCAATCTCTCGCCATTCCTTGTTGTCCCCAGTAACTC
CAACACGAAATTCTCTGTTCGGAATCTCAGTTAGGAGTGCTCTTCCATTAAATATTGTCA
ATGTTTCATTGTAAGTTAGATAGGGGTCTTCAACAGAACCGATTCCCTTTTTACTTAATA
TTGGATCATTATATAATTTAGCAAAATCAGCCAGTTTAGTTCACCTCCGTTAATTTTGTT
GGTAGGCTTCCCAGATATCTTTAACGTGTAATTTATTTCCTGTGCAGTTACTATCTGAGC
CTGTTACAAATAGATTGTTTCCCAAAGCACCGTATCCTAAATCTCCTCCAATAAGGGAAA
GTCCAGTTGAAGTAACTTGATAGGCATAGCCCCCACTTGGCTTTCAATTACTAGTTGAC
TGTCTTCAGGTGAAATCGGAGTTATTTTGACTAAATCAGGAGAAAGAGTAAGGGGGATTT
GCTTACTGAGAGTGCCGTCTCCTGTATAAATACCTTTTGCAAACTTGGGAGGAATGGGGA
TGTTTTCAGCTAATGCATAATCAGATGCTGCTCGTCCACCTAGCATCTCTGCATTACCGT
CTATTGAACCACTAATGATCCCTTATTCATTAAACACTGGTATAGAGTTTGGAGAAGTCG
TTGTTGAAGCTGTAAAACCATTCAAGGAGTCTGCTGAACCTGCAGATGACACAACCCATT
CTTCTCCGTTAAATAGTTCTTGTTTATTGTTCTTTGGATTGATCCAGATTGTCCCTTTTT
CCGGATCTTCTGGCTTAGCTTCCGTGGAGATTGTATAGAGGCCATTAACTCTTCCACTAA
GACTTCCTTTAACTTCAACCGAATTAGAGGGGAGGTAGGGGTTCTCTGAAGGAAATTGGG
GTTGAATAATGTCGGAAAGTATACGTCCGCTGCTATCAATAGACGCTTCAACAGTCCGGT
AGCCTTGACTTCCAAACGTATAATACTTATTTGAAACCTGGCCTGTAAGGGTTGCGATTC
TCTTATCATAGCTAACCGATAGATACTGTTCAGATGACATCTTTGATCCAAATATATAGG
TGTCCGAAGATGTATCTGAGTAACAGTGGACAAGGGATCCGTCAATATTGTACTTATTCT
CTTTAGAATTTGGGAACTGCCATTCAATGGAGATATCAACCGATCCATTATCGTTGACAA
CATGTGTTATTGCAGTTCCGTTATTAAGAATAGTTGGGTTTGCCACAGGAGATGAGATAC
GATCATTACGTGCATTGTAATTAACAAGAAGTGTATCATAATCAATTTTTTTCTTATTAT
ACTCTGTGGATGCTTTCTTAGTTGTATACAAAGTATTAGCAAATTCCTGTTCCAATGACC

TGGCTCTTTTACCGTTTGAGAGTGTAACCTGTATACTCTGTTGATCAAAATCGAAAGTCA
TCCCTGAAAGGGTTGCTTTGACATCGGTTCTGAAATTCTTATTAACTACCCTGATTATAT
CTCCGAGATATATTCTATCCCAAAATGCCTTTTCGCTATTAACATTAAATAGGTTTACGA
TGTTCGTTCTGATATCAACAGGGCGAGCGTTTCTATTGCTCAGTTCCTCTAACCCTTTTT
CGTAAAGCTCTGTCTCATCAAAAATATTATCATTAGTCCAATCATCTTCAAATATAAAAT
AAGACAGTTGTTTCTTAAGCTCCTCACCAAGAAACTTGTCCATCGATAAACGGTCTTTTA
GTTTTTCTATTTCCTGAGAAATGTTTGTGATTTGCGAGTTTATTCTGGATATTTCAGCTT
TTTTGGCGATAACTTCTAATAGTTTTGCATCTCTTTGTTTAATAAGATCTTTTGTGTCGT
CACCGGCTTTTTTCGCTACGGTTATCTTATCTAAAATTTGCTGTAGCTCAATGTTTTCAA
GAGTGAAGAGTTTATTTTCTTGAGTCGTTTTTTGGGTTTCTAAATCCCTTTTTTGGTTTA
AAAGGAGGTGGAAAGAAGAGCCTTCTTTATTGACAAGTTCATTATAATCCAAAATCGCAT
GACATAAATCATCATCCATGTAATCACAGTGTGTTATTACATTTCTGTTTTTATCACGTT
TAAAAGGATATAAAAAATAAGAAAAATCGTCCAAGTAACTCTGTCCAGTAGGGTTTACCG
AATTAATCACAATTCCGTCTTTACCTGTGATGGTTGAACGTGTTACGACTTCATCAATAT
CAATGGTATCTTCCATATCAATCATAAATCGTTCAGGGGAGAATTGGACACCTTTGTACT
TGGATACTGTGTCTTTCTTATAAAAAGAGACAGTGTTCTTTACAGTGTCGAAGACTGGGA
CTGCCTCAAAAGTTTCCCATATCGTTTTTAAAAACTCATATCTTGTTGAAGTGACATCAA
ATGATCGTCTTTTTTCATTAAATGAAGGATCAATATAACCGATATTGAGTCTTGTGCCTT
TAAGACAATCAGTGGCCACTTCCTGTAGATTCTTTGAAACTCCTTGATACGACCTTATTT
TGTTTTTATGAAGGATGTACGGTAGTCCTTGACATCTAACTTGTATTGTTTGCTCCTCAT
TTTCTGATTTTGTTAAACCGGTTATCACAAACCATTCAACTCTGTTTAAGGATTCTGCCT
TGATAAGATACCACTCACGCATTAGTTTTGCCACATGGTTTGGCTTCATCAAAAAATTAT
ACCTGGCTTTGAGTGGAATATTATAAGATAGATCATTTAAATCAGCAAAGCTTAGATTAA
GAGATACATTTGAGAAATCCGCAATATTAGCGATTTTCTTTTTATTTGGTTTTGCTAAAG
AAAGGAGGATTTCGCCTGGTTTAAACTTATTTAACAAAATACATCCTCCTATAAATATTT
GTATCGGTATTTTAATCGAATATAGCACTCACGATTTATTTTAATTCTGTTTTTACCTCT
TGTTAAAATCACATACTCTTCACTTACTTGGTCATAACATTCATAACCCGAAATACTTGA
AGTGATCAATTTTTTATTGGAATCAACATTGATAACTTCATGATGTTTTATTCCCGTAAA
CTTCAGAGGTGTTCTATATAAAGTTAAGTTCTCAACAGTTATGTCACCGTCGCCTATCTT
CTTAAATTCAAGAGATAACGGAATGTCCACATCTCCTATGTTTTCAATTTCAATATTCTT
AACACCTGATGTGACATCAAACCAATGAGTTGTTGTTTCTTGTCCATATTTATAGGGGAA
TACCTTCATTGTGAGCTTCACATATCCATCGTTAGAACAGTTATGAACTAAATCTGTAGC
GTTTATTGGCAAGGCGAAATAAATTATGGCTAAATTTCTACTAAAGGAGAGAGGTTTATA
ATCATCTACATTTAACCAACGAGCAACTCTACGAACATTTTTTTCATCTAGATGATTTTC
TATGTAAAAATTCAATGGGATCTCAAATGGCTCAAGTTTGACATTGTCTACATATGGTTC
TGAAGATAGCCTAGTGTATGTTTCATTTACTGTTCTATTTGCAATAAACGACTCCTTTAA
AAGTCCTCCTTCTGTATTTACATTTACGGCTCCTAATTCATAAGAAGGGATATTATTGAA
CATAAAGTATTGACTTTCTCTAATCATGAGTTTCCACCTCAAGTTAAGTTAAATAAAGAG
TCTGCTTTTGAAGACTCTTACATGCTACCATTTAGTTTTACAACTTCGTTTTTAATGCTT
TCAAACATCGATCTCGCTCCCGATTCTCCTCCTATTAGCTTATCAATGTGGAAAGTATTC
GTTTAATTAATTGTTTTATCTCCAGAAGAGGCAATTGTTTTTGAGGGGATCTCAATCTTC
TTTAAATTTGGAATTAAACTGTTTGTTAACATTGATTGATTAACGTTAGGAACTAATGAA
GGAATAGAAGTAACTCCTTTTTTAATAAGGTCTGCTAATTTTACTCCTTGGCCCCATTTT
GGGGAATCGTCCACAGCGGTATCACGAACAGCTTTTACCGTATCAAGGATGTTGGATGTG
TCAGTTTTATTTAGGATCAGTTCTTTGTCATGTAGGAATGCAAGTTTACCAGCACCTAAT
CCTGTTCCAGTGTACCCTCCAGAAGCGAAAGAGGATACTTTTTTACCTGTCGTGTTGCCT
TTGACAGCAGTATTCAGTGCATTAGATGCTTCTTTGAGTTTATCAATCAGGTTGTTTGAA
ATACTTTTTCCAATGGACTCCATATTGGTATTAATAAACTTAGAAAACTCATTAAGCTGC
TTAGCGATATCGGTGATTTTTCCATTCATAATCTTATCCTCAAGCTTTTTAAAGGCTCGT
TCATCATTTACAAGATTATCGTGTTTATTGTTGATTGACTCCTCATCTTTTTCGAGCTGA
TCTTGGAGCGCTTCTTTCCGTTTGTTACTTTCGCGATCCTTTAGAAAATCATCAAGGTCT
AACTGCTCTTTTTGAAGCTGTTCAGTTAGTTCTTTGACTTTTGACTTTCCGAATTCAGAA
TCATCAAGAGAGTATTGATTAATTTGGTCAGTCAACTTTTGAATACTGTCCTGTCTTTCT
TTTAATTCTTTTTGAAATTTAGCCTCGTCATCAGTCTTGTCTATCTCAGCGATCAAGTCT
TGAGTCGCTTTCTGATGCGCTTCAAACTCAATATCACGCATTTTTTCGTACATCTCGTTG
TAGATGGATACAACTTCATCAGCCAATGATTTATAGATCTCCTTGATCGACTTCTTGGTG
TTATAAAGCTCAAGGTTAAAATCTTTCTGTTTATCTTTCCAGCTTTGCATTTCTTCAGTG

ATCTGTTCCTGGATGTCAGGAAAACCTTTCGCAGCTTTCTTTTGTTCTTCAAGCTGCTTA
ATGTATTTCTTCGCTTCCTTTTGTTGTTGTTGAATGAGCTTTATTTGCTTGCTATAGTAT
TTAACCTTGTCTTCATCTTCTTCAGTCATTGAAATTTTGTTATGGACATATTTAATTTTC
CCTTGGGTTTTAGAAGATGACTTTTCAATTGACTTAAGTGTCTCATCAACTTTAGATTGA
ACAAGTTGTTTCTGTAGCTCACGAACCTGGTCTTGAACAGAAATTAAATCTAGCTTGGCC
TGTTTAAGCTCTTCTTGAAGCTGTGCACGTTGAGCGGAGTTCAATGCTTTATTTGTTTTA
ATTTCTTTTTGAATCCAATTAACTTTTTGTTGTTGGATTTTAGCTTGCTCTGCCACAGCT
TTTTTCTGATCAGAGGTGTATTTTCGGCATTCCTTGCTGTCAGAAGTGTATCTGTTAGCC
ATTGACTCATCTTTTGCTATCCGAACATCAAAATCTCCAATTCTTTTATCAAACTCATCG
AGTTTAGATTGAACTAGTTCATACTGAAGTTCTTGAATCTGATCACTGACTGAACTGATA
TCTCCTTGGAGGGAGCGAAGATCAGATTTAGCCTGAGCTATACCTTGTTGTCGTTCTGCC
TCAGCTTGTGACGCATCTGAAATAGAAGTTCCAATACCTTGCATGTACTTTTCAGGGTCG
ATTGTTTTTCCATTTTGTTCGATCTGTAAATGAAGGTGGTTCCCAGTCGAGTTTCCTGTA
CTACCAACTTTACCAATAGTTTGACCGGCTTTAACTGATTGACCTGCTTTTACAGAAGGA
GTGTTAAGCATGTGCATGTACTTGGCAACTGATCCATCATCCTGTTTAATAACAACCCAG
TTACCTGCAGTTTTACTGTAGCCATCAATTGGGACTTTACCACTTTGAAGAGATTTAATT
GCTGTACCTGCTTTTGCAGCAAAATCAGTTCCTTTGTGTGGGGAGGAGCGGAGACCAGAT
TCCTGTTGTCCATATTTGGAGCTTACCCTAAAAGCGCTATTATTTGTATAATAGCTGGCG
ATTGAAGAAGTGGCAGATGAGAGCAATTTGCTATAGTTGGCCATAATCTTCTTGACGTAA
TTCTGTGTTTCTTTAAAAGGAGGGATACCACCATATTTAATTACGTTACCAGGCCCAGCA
TTATATGCAGCCAATGCTTTTTCAACATTACCGCCACATTTTTCAAGTTGTTGGGCGAGG
TACTTTGTTCCACCCATAACACTTTGATAAGGATCGTAAGCGTGATTTACTCCTAAGCTT
TTTGCTGTTGCTGGCATCAGTTGCATTAATCCCATGGCACCTACACCAGATCGTGCTTTA
GCATTAAACCCTGATTCTCGCTGAAATACAGCTGCAATGAGGGCAGGGTCAACATTGTAT
TTACTAGCTGCTGAATTTATGTAGCTTGAATACTTGCCTGAATATGATCCACCAGTTGAG
GAGGGGGTTCCACCAGAAGAAGTTGTAGAGGTTACAATACCGTATTGAGTAATGTTACCG
GATTTGATTTGATCTTGAAGCAGCTTATCTTGTTCCTGCATAAGCTTTTTCTTTTACTGA
AGTGCTTTAATTTCTTTCTTGATTGCATCTCGAAATTTCTGAGAGTATTTAGGATAATCA
TTGACCTGCTTGTTGTACTTGTCAATCTCAGCATTAACTTTTTCTAATGCTTCCTTGTAT
TTATCAACAACATACATGGAAGTCTTGGTTTCTTCATTGGCTTTTTCTTGTTGATCTGTC
CAATTCTCCAATGATGTTCCAGCTTCAACAAGTGCCTGTTTATTCATTTCTTGTGTACTG
GAGGCTTCTTCAGAGGCAGAAATATAAGATTCTAAAGCTGATTTAACGCTTTTCATTGCT
TCAATTTGACTATTCGAGTATCCACCAGGTTGAAGCATTTTCTCTTCAAGCTTTTTAAGT
TCTTTTTTTGCATCTGCAACATTATTAATTGACTTAACTTCTAGGTCTGACAGTTCGGCC
TCAGACATATCAAGCTTTCGTTCTTTTCGTAGCTTTTTCAGGCTGTCAATCGGTAAGGTA
TCAGCGTTTAAAGTTTTGATAGCGTTGTCAACTTCTGTTTTCATCAATTTATTGCTGTAG
GTAACAATGTCGTTATAAGCATCAAGTTTAACTTTTCTTTGTTTGATAACTTCATCACGG
TTAATTTTCACCACGCCATTTTCGATGCTAATAGCCTGGGCAAGTTCCTTATCTTTTTGA
ATAAGGGTATTAGCTTCATTTGCAGAAATACTTTTACCTTCAGCCATTTTTTCCAGAAGA
TCATTGAGAGGAGCTCCTTGATCTTTGGTGTTATTAAAAATATCCCCATTTAAGACATCT
TGAACGGAATCGAACTGCATAGCATCTGACATTTCTTTAATAGCTGCCTTAATATCATCA
ACACTATTTGCATCCAGAGCTTCTTTTAGCTTCTTACCAAAATCTTCTGCCTCGTAACCT
GCTTCTGCCAGCGTCTCACCTAAATCACCAACTTCAGGTTTGACGGAAGATAAGCTCTTA
TCCCCATCTTTTATGTTCTTCTGTGCTTTGTCGAAGCTCATTTTAAAAACATCAATAGAA
GAATCGGATTTGGAGTATGTTTCCAAGAGACTTTGAAGATCTTTTTTTGCGTTATCGAAA
GCTGTTTCATCGCCTGGATGTAAAGCTTTTTGCATTTTTTCTTGAAGCTTTCCTAAAGAA
GAGGAGAATTTTTCTAATTCTTCAGGATCTAAATCTTCTTTTAAGTTAAGTTTGTTGACA
ACATCACTAATACTCGTATTTAAAGTATTACTGATATCAATTGAACTATAAGCATTTGCA
ATTGAAAGGACGCTATCTTTAACTTTAGCATTTCCACTCCCAATGTCAGATTGAGCTTTG
AGCATACTTTGTTTAGCTTTCTCAGCTACAACCTTATAGTCATCGTCATCTGCAATGAGA
TCCCATTCAGGTCTACCTTTATCGTTGTAGTCAGCTATTTGTTTGTACTGCTTTAATTCA
TCCTTAGACTTTTTAATTTCCTTAGAAGCGTCTTCGAATGTTATCTTTGCGCTGTCTCTT
GTTTCTTGTTTCTTGAAAGCCAAATCCTCTTTAGTATTCTCACTCGCTTTTTCAAGCTCT
TTATTTCTCTTAAGAATTGCATTTCCTTGAGAATCATAGCCTTTAACTAATGCAGGCAAA
GTTTGTGCTAATTGCTGAGTGACTTGAATGTATTCTTGCTCTTCATCTGAAGTTAAAGAT
CTTGACTCTTTAACTTTTTGAAGCTCTTTATATTGCTGTATTAGTTTATCAGTGGAGTCT
TTATTGGTCGTAATTGCTTCGACATTGGTTTGCTGGCTCTGCTCAAAATCATCTTTAGCT

TTTTTAGCTTCTGCAAAAGAAGAAATTAATGATTCTAGCGGCCATCCCAAAGCAGCAAAT
GCACGGCCAACTAAAGTTGAAACAAGCAACCCTCGAAGAGCAGTTTTTAGAACTCTTGAG
GCGATTGCTGCACGAGTCATACCAGCTTCTAGCCCAGCAGTCGCTAAAGTTTCTTGCCCC
ATTGCACGTGTGCCCAAAATTAGGCTGCTGGCTAATGTGCGGGTATTCTTACTGAGCAAA
AGGGTTGCAGTGCTTACTGCAGCTAAAAGGGGAGGTCGGAACCCAACTGATTTGATTACT
CCTGTAGAAGCGTTAAGCAAAGAACCAGCGGCTTGAGTAAATTCAATTAATCCGTCGCTA
ATAAAAGCATCAGAAGCTGCAATAGCAAATTCAGTGAAGTTATTTTGAAGCTTATTTACC
CTAGCTTGTAGACTATCTGCATACTTTTGCTGCTCACTCCAAGCACTTCCTGTTGAGTTA
GCCGCAGTTTTAGCCGCATTCTGAGCAATAGAGAAGTTGTTCATCATTGCATTAAAACGG
GATAATTGATAAATACCAGCTACTCCAATTGACGTATTTTGTTTCTGAGCATCAGAAAGC
GTATCCCACTTACCAGCAACTTCACTAATTAAATCACTTGCTGATTTAGCTTCAACACCA
GCTGTTTTGACTGAGATACCAATCTGTTCTAACCCTTTAATTGAGCTTTGATTATTCCCA
ATCCGCGCGAAAATTGTCTTTAAGCAGTTCCCGACGATATTCCCTGATTCACGTGTTGTA
CTAGCAATTGCAGTTGTATAACCAATAAGATAATTTAGCTCTACCCCGAATGTAGAAGCA
GTTGAACCAGATTTACGGATAGAATTGGCCAGACCTAGAGTTGTAACAGCATAGTTATTA
TCAACCTCATTTAATTTATCTGCAATTGATATTGAATCATTTGCTGCAATATTAAAGTTG
GGCATTGCTGCCGTTAGAGTGTTAACTGTATCATCCGGAGTTAAATCAGAGACATTTTGA
AGGACTTGGGCAGTTTTCGTTAACGTGGAGAGCTCACTTTCATCGAAACTCATTCTCCCA
AAATCGCCTGTCATTTGAATAATATCTGTGATTGTATTTGAAAGTGTATCACCTAAGTCA
ATAGATTCTTGGAGAAGTTCATTATATTTATAATCCGGCTCATTCATAACACGGCGAATA
TTTGTCATGAGAGTATCAATTTCTATTGCCTGGGATACCATTTCTTTAAGTCCAGAGATA
GCTCCGTAGAATAAAGAACCGGAGATTAAATAGGTGGACATGCTTTTGAAGGTTTGGGTT
AGTTCTGCTCCAAAAGAAGAGGCTTGATTAGCAGCTGTTTGAGCGTTGGAGGCTAATTCT
CTACATTGCATATTCAATCTTTGAATTTGTGATCTGATATTATTGCTTCCAGTGCTTACA
TTAAGACTATATACCGCATTCAAATAATCTTGAACAGCTTGTCTATTACTAGATCCCATA
CAACTGCCATACCGTGTATTTAGGTTTTGAACATTTACTTGTGCCTGTCGCTGAAATAAT
TCAATGGTTTTCTTTAATTCATTGTTCTTCGCAACTGCCGCAGATTTATCATCTAACATC
CTTATCCTATTTTGCAGTGCTTCAATTTGTTGAGCGGATTGAGCTGTGTTTATTTTTCGG
CCAAGAGATGAGATGGACGTATCAGTAACAATACCCTGCTGTCTAAGCTTCTCTAAATCT
TGCTTAAGCTGCTCAATTGCTCTCCTTTGTTGGTCATAATTAGTTGTAGTTTTTGAGGAG
GTCGAATTAGTTTTAGGATCAGTTGTATAAACAATATCATCGAACCCGTGGCGGTTTTTC
TGCACTACGTTTGTTGGCTGTCCTTGCAGATTTCTCTGCACAGTCTTTTTTTGCACCTAT
CCTAGTTTCTCAGTGGCCTGTGTTAGCTTGTTAACCTCTTGAGTTTCTTGCTTTAATGCT
GTATTACGATTGTTGATTATTTTTGTTTCACGTTGAAGTATCTCACCATTTTGCTTATAC
TGCTGGGTGAACTTTTCAACTGATCCGTCAGCATTCTTAATTACTGTTGAGGTTTCTTTA
GCTGTTTGATTATAGGATTTTAGGTGTTTCTGATATGTGTCGATAGCAGAGGAGAATTCT
TGCAGAGCTTTTAAGGTTGTAGAATCAATGTTTGTATTGAGCTTGAGGGAGTTCAGTTTC
TTTTCCAGGGATTTAATTTGCTGATTTAACTGTTCGACAGTTTTGGATGAGGTATCAGCT
GGCGGGGTTAGTATAATTCTGAGGTTTTGACTCAAGTAAGAATCACTTCCTTTCAGTAGG
GGAGAGGACAAAGAAAAAAGCCACTCAGTTATTGAGCCACTTTGTTTGCTTTCTTAATAA
TTACATCCATAACACCATGCCAATATTCTGCATTCGTAAGTGCTTGTTGAATGACATTGC
TATCTTTTTTATACCCCTGAATATTATACCCTTCAACTTCTTGTCTGGGGAGAAACTCGA
TAAAATGGATAGGCTTACCGAGGTCTTCAACAGTAATCTCTAGCGTGACAGATTTGACCA
TTTCATTTTGTATTGAAGAGGCAGACAACCCTCCAATTGCAGCTACAATTCCTCCAGCTA
ACAGGCCACCAGCTACGATTCCTGATTTTGATACTTTAGAAATTATTTGATTGTCTAAAG
CAATTTCGGATTCTATAACATTGGAAAAAGGGATTGTGTATGCTTCAATATGTTCATCTT
TATTAAGCTGATATACTTTCACAAGGGAATCAGATTCCTTAGATGCAATTGTAGTTTTGT
AAGTATTAAAGAAATTGTCTGGATTAAAACCACCGTCGAAGTGCTTAAGTGTTTCCGCAT
TTTCTTTTAGTTTATCTATATTTTTTTGGGCTTTAATTTTATAGGGCTCAAGTGCTAATA
GGGCTAAACCTACAATTATTAAGCAAATTCCCATGCCTCTCCAGCCGTCCATTAGCAAAA
AGAAAAGACAGAATAAAAAAATAGCCACCATAAGCCATAAACTTATACCCCATTCAACCAC
CCCCGGTATCATTCTCCTACATTATACCGTTGTTTCCAGTCATGGAGAACTGTATAAGTG
GAAAGTGAAATCATTAGTTCTTTTCTGATTTTATCTGAATGAGCAGTTCTTTGAGATAAT
CTGCAGTTGTAGCATTTTCTTTGTCTTTTAAAATATTACGAAGTGTCCATAAGCAAATTA
TGATTGTGATTAATACGTAAATTAATGCATTCAATCGGTTTCCCTCAGTAATTTTTGATA
TATTCACACCAAATAATACTTGAAATCCTATAAACGACGTTCCCAAAATCAAACTTCCAA
TCCCCCAAGTTTTAGATGGATCATATTGCGATTTTATTCCTTCCGTCGTTGCAATGGCAG

ATGATAATTCAGACGGGCTTAAAGATTTTATTATTTGGACTTCCCCTGCATGTGTATGAT
AATCTTGTCCATTAATAGGAAATTCATGCTTGATAAACTTAATTAATTCTTTAGGATTCA
TTTCAGCCAGATTGTGCTTTAATACTTTTTTATTGAACATTTTCATCACCTCTCCATATT
ATCGACAAATACGCAAAGGATTTTAATGGAAATTAGGTTTCTGAGAGTACGTTTCTCTAA
TTTTCAACATGAATGAATAGTCCTTTTTAAGATTTATACACAGTCATTCTCCAAGGAAAA
ATGACTCAATTTTTCGATTTTCTATGTAAATCAAAAAAGAATCATGTTAGAGTAGGTGTG
CTGAAATAATTCAAAAAATCATTTTGCAATATGATTAAAGTAATATTGCCAGAGAGTTCG
GGTTGGACAAAATTGTAAGTATTTAATACACTGGGGCTGCAATCAAATATGCACCGGGAA
ACAATTGCTAAACGGGGGAGTTTAAACGAACAACAAGAAAAATATCTTTGATATCGTAAT
GTACATTATTTTCGGTGTGTTAAGTCTTTTTCTAGTTGCAAAAACTGATTATGGCACTGG
AGTTTTAGTGTTTGTTGCAATTTTATACCCCGCTGTAATTGCTTATACAATTAAGCAAGT
ATTTAGTAATTCAGATTCTTAAGGTGTAACACAAGGAGGATTTCAATTGAGAAAGAAAAG
AGTTATTACTTGTGTTATGGCTGCATCATTGACTTTAGGCTCACTTTTACCTGCAGGTTA
AGCTACTGCAAAGGAGGACTCTAAGCCAACCCCTTCTTACGAAGAAGTAGCTCTACATTA
CAAAATGAAAAGTGAAAAGATTTCATCGAACGGGAAATTAGTTGAAATTGAGTATGTGAG
TGGAAATGAAACTCACAAAGTTCAGATGAACGGAAATGATCATACTGTAAAAGTCGATGG
CATAGAACAAAAAGGCTTAAACTTTGAGTATGATGAAAATGCTGCTAACAGAACAAATTA
TGAAAACAATAATTTGAAATCAAATGAATTCACAACACAGGCTGCAAAACCTAAAAAGGG
ATACCATTATGTGGGGACTTTATCTGGGCATACAAAAGCAGCTAAAAATGCGTTATCAGT
TACAATGTCATTAGACGGTATTGTCCCTGGCTTGGGATGGGGGAGTAAGGCTGCCACTAT
TTTATTCTCTTATTGGGCAAACGAACAAATCCCTGATGCGTATTATAAATATGATTTATA
CGAAAAAGGAGCAATGACTGATAGTTGGTATCAGTATGCTACAGTTCAATTTTTTGAAGA
TAAAGCCCATAAAAAGAAAATGGGCAAACCGTGGACTAGTACTCCTGCAAAAGAAGATTT
AACTAAGAGCTAATTCTATAATCACTTTGTCTTTATCGGGGGACAAAGTGATTCTTTGTT
TTATACATAAGCAAACTCCCTCAAACGAGGAATCTTGATTCTGAATAAAAGAGGGATTTT
ATGCTGAATTGAATAGAATTTCTCTGATGGTTAACACTAAGCTCAAATTATCAATTGGAG
GAGATATCGTGAAAGTAATCCATGGAATCAGAGTATATGAAAAAGGAGAAAAAGTCTTTT
TTGAAACTGAAATGCCATCCATTCCAGAAAACATGTATTCTAAATTCGGGTGGAAAATTA
TCGAAATAGATGGTAAGAACTATTGTGCCCCGATGGAGGAGGAAGAATATATACATATTG
TTGCAAAATATTTAGGGATTTCACCGAGTGAAGTTGATTTAAACTTAATTCATTGTGGAA
CGATGGGGGACAATGGTTGTTTTGGAGATTGTACCGGAAATAGATTTTGTAAAAGGTGGA
GTACTGGAGATTCAACAGGATGTATTTGTGGTGCTTAAAACTAAACAAATATTTGATTTT
TTTCTTCTTCAAAATCCCGTAAATCATAATGTTTGATTGTTGTTGAGACATCTTCATGAT
GAGCTACATATTTGCTTATCAATTCAATTTTGACTTTCTTAACTTCTAGGAGATAAGTGA
TGCACGAAGCTTTAAAGAGGTGGGGGTTTATACGTCGACCAAGGATGTCTGATAACACAT
CAGAGCAAAAATAATCAGCCCAAGACTCTGACATCCGTTTAGGCTCTCCGCCATACTGAG
TAGTGAAGAGGTATTCATGATCATAGCCACGTTTTTCATGCCATAAGCGAAGATACTCCA
GTGCTTCTCTGTTAATCATGTACTCAAGAGGTTTTCCTTCACCTTTACCTTTTCCAAATA
CCTTATGACTCATCACGTATGACTGACCCTCTGGAACAGGGTAATCTAATATCTCTGTTT
TTAACTGTATGAGTTCTGCTCTTCGCCCGCCAACATTAAAGGCAGTAGCAAGCCATGCCA
TTCCCAAATAATTTTCGTCCTCTTTTAGGGCGTCCATCATCAGTTTATAATCATCATATG
TAACTTTAACTTTTTCATAGGTTGTGGTTTTAGGAATAGCTGGAAGCCCGCGGGTGTAAT
TTCTGAATTTCTCATAATTATGATCTTCTTCTGCAACAACATTTTCGATATAATTATTTA
ACGATGATACACCGGCTTTTTTTAAAGCAATCCCACTTGAAGACATCCCACGATTCTTTA
AGAAACTTTGATACCTAATGAAATCACGCTTTGAGATCTTATAAAGGTTTTTCCCGTTAA
GAGAGGTATGTACCCACCAAAAGAATTGGCGAACGGAGGAGTAATACTGCTCTCGTGTTT
TATCTCTAAATGAATGAGCATCTAAAAACTCTCGAACCAAGTTTCTGTGCTCTTCATCTA
CTTGCTCCCACATTGATTCCGTGATCTCGGGGAGTCTTATTGCTCGAGATCTGAGCATAT
TCTTTTCAATTGGTTTGGTCATTAATTCACCACCATTAGTCTGTTTTAATACCATCTGCG
TTTAAATCTTTTTTTAGCGCTGCGGTTAATCGTCCATCTCTTAAGGTCTCAGAGGTATTC
TTCATAAAAGGACGAGGGTTACCGTATCCATATCCATATGAATCAGGGTATGTATACCCC
TGCCCGGTTTCAATAACTGTAGCAATGTCTTTTCCATCATCTTCTCTAATATTGTCCAAT
GACACCCCGTTGCTTTCGTTGGTGGTTATAAAGGCATCTTTAACTTGTCCTGTGCGTTCA
TAAAGTAGGGGATCATATGCACCGTAAACATCTTCAACAATATGCTCTTGTCCTGTTCTT
ACCATAATTTGTTTCACATTATAGGAAGACTTATGAATTGATTGAATTGCAGCTTGTTCA
ACCATAGCTTTGATATCTTTAATTGTTTTCGCCACTAACTTCACCAGAACCAGTCTCTAT
TTCTTCTACCTTTTCTAGAATAAGCTTGCTGATTTCCTGTTGGCTCATAGTTAACAAGTC

TTCAGTGATTTTTTCTAAAGCTTTTGTTGCATCCTTAAGCTTCTTCATACTTTCTTTTGG
AAAACTGTTAATAATCATAGGGAAGAATTCGGAATCTACAAGCTTAAGGTACCATTTAAC
TTTATTTTTGATGTCATTCGGAATCCCCAAATCTGAAAATTCTTTGACAATTGAAAAGAA
TACCCTTTGAACAGTGTTGATTTTACTAAAATCAATACCTTTATCTTCAGCTTTTTGTTG
ATCAGAGATCATTTCAGTTAGCATTTTTGCTAACCGAGAAGGAGAAAAGTATCGGTCGAT
GAATACATGTACATCATCGGTTAATTGGACTTTTTCTTTCTTGTCATACTTATTGACACT
TTCCTCAATTAAACTTAAATTCAGTTTTTTTGATGCCATTTAATATCCTCCTTAATATCC
TCGTTTTGAGCAAACAATGATAACGTGTTTATTCTGTAGATAAGGTAACTGAAGTATAGG
CTTCCCCAACAATTTCTTGATATTGTTCAGGAGTAATTAAGTTATTGTCTACTCCTTCTT
TGAGTTCTTCCTTAGTTACATCATCGTAATAGTAAGCTTCTTTAACCTGATCAAGAGTTG
CCCAGTTTTTGTTTAGGGCTATAACCCAAAAATTGCTCATGCTGTTGTCCCAACTTTCAA
GTTTTTAACTTCATTTTGTAAATTTAAGATTATGCGATTTTGTTTTAAAATTAAAATTCT
GGCTTCAGCTAGTGATTGTCCAAGGAATTCCACATCAGAAGGTTCTGCAGGGGGCGGTGA
GAGGCTGTCTATATATTCTTGAGAGGCTGTTTCTATCCAAACTTGTTTTGATAAATCAAA
TCGTGGAAGGTAATAGGAGGGAGAGTCAGGTAATGGAACAGAAGTGCAATTATCGGGAAA
AATATAATCTCCCTTTTCATCGATTTCAGTTACAACTAGGGGTTCAACGAACATAAAATT
TTCGTCATATTTATAGCCTTGTATAAAGACTTACCTCCTAAGTCAACGGAGCAACTACAT
CTATGTAGTAACCCGTTATTGCAGCACTGTTGTCAGCTGATACCCCACTTAGCTGAAGGT
CACCATTAGAGTTTACAAATAATTTACTTCTGCCGAAAGTTCCACTCACTTCAACAGACT
TAACAGCACCTTGTGTTGGAGAAAATTCAGTTGGAATAGAAGCAAATACTACATCTCGGT
TTGTTACAACGTGCCCACTAAGCTGGAAAGTCCCAGCTACTTTACGATATTGAAGTGGCC
TTGAGCCTGTCTTTGCGCCATTTTTAGATGTAACGCTTAACCAAGGCACGTTATCAATTT
CACTGCTACTTATCAATCGTTCCCAACTTGACCGTGCTCCGTATATCAATGATTTTCTGT
AAGTCTTATTATCATTAGTCAGAACAATAGCTTGACCATAAAGTTTATTAGCACTACATG
TCCAAAATCCCCGCCCGTTACTTGGAGGAGCTTCTAAGGCAGCCTTATCATATCCGAAAT
ATGTTAAAGTAGAAGAATTCAAAAGTTCAGAATGGAAGTCGCTTGTTATAGAAACTAGTG
GTTGACCATCATCGCTTGATATTTTAGTTAAGTGAGCAGAATTCCTTTTACTTCTTTCAG
TGCTAGTGATATGACGCTCATTATCTTTTATGTGAGCATCAAATTCTAATTTTGTCGCTT
GTTTATCATTCGTAAGGTTTCCTAACCCGACCTGAATTGCAGTTACTTTATGGGGACTGT
TTGTGTTCTGCGAATGTGCGTCAGTATACTTTTTAGAATTGCTTTCCGCGGCATTCCAAG
TAGTTCTCTCTGGAGCTGTAATATGAATAATTGTATCATGAGCATGTTGATCAAATTCAG
TTTTAGTTGCTTGTTTTTCATTCAGGACATTTGCTAAACCAACTTGGCTTTTTATTACTG
AATGGGGATTGAGAGTATCGTTTAGATGTTCGTCAAATTCCTCTTTAGTTGCTTGGACAT
TATTTAAAACATTGCCCAAACCAACCTGCTCTTTGGTGACTAAATGAGGATTAGACTTAT
CATCAGTATGTTTCTTGAGCTCATTCTGTTGATATTCCATAAACAAATTTGTTTTTTCTG
TTAATGCTTGTGCATGCTCAAGCATATGTATTTCAGCTCTGTCATTGGATCCTCTTAATG
AATTGATCTGAATATTGTTTTGTTTGAGTTGACCGTAGTATTTACTGCTGCTCATCTGAA
CTCACCACAATCCCTTTAACAGTTACATCACCAGTAACAGAAACTACTACGAACATAACT
TTAAACAATCCAGCAATGTCGAATTCCCAATACTCATTAGTATTTAAAGTACTGCTCCCA
AGTTGGAAGTCTGTTTTATTGGTGCCACTAAGTGCAGTTTTTTCGCCATTTTCATCAACT
GCATAAAAATTTATTTCTCGTGAAGTTGAGGAACCAGAGATCTTAACAGTAAGTTCCCGA
AAATGACCCACTACAAATTCTTCACCTTCAGATGGGGTAGTTGCGGCTTCGTGAAATGTA
AATGAAGTTTTGTCTGGAATCGTGATTACGACATTTTCAGTCGTTTCTGTCAACAACTCC
ACCTCCTTAATTTGAATAAGAACTGTCTTTTATCTAGAAAAACAAGAGGGGAGGACTTAT
TCAAAAAGCCCATCCTCTTGCAGCTTATTCCACATACCTTCCCAAGCTGGGGTAGAGGAA
GGGGATGACTGAAGCGAGCTTAAAGGTTCGTACTCATTGAAGTCGCCTTCAAAGCGTTGC
TTGAAATCATCAACACTGATATAGGATCCATCTGATAGTAATAGGAATTTTTGTTCCATA
CCATCAAATTGTTTGAACAGACCACATACTTCATCAAATGTCATTATTCCATCCCCCAAC
CAACGATTTTTAACCCAGTTAGATCAACTGTACCGGCTGCTTTAACTGTCAGATGTATTT
TTGGAGCACTGAAAGTTAATTGTTTTTGTAAAGGGTAATTCTGAACAAAAACAATGCTGT
TGAATACAACAGAATTATCATTACCTGATGTAGTTGCAGAATAAAATGGGATGTTTTTCG
GTACGGAATAAATACTTGCTAAAACCTTGCTGTATGAGCTTTCTGTTTGAGTTGTGTACA
TAGCAACACCGTAAGTTCTGAAAACACCAAGTGAATCGATTAGATCAACTGTATAAGACT
TAGTTGCACCTGTAGCTATTGTATCAGCATCAGTAACAACAAATTCAGAATAGGATTTTT
GAACTTGGGTTTTAATTGCTGCAGTGTTTTTAACCAAAACAGGGACAGAAGCCGTATTAG
TTATATTTACATCAAGTGCAGTGTTGGCCACATTAACATTAAGAGGCTGGTCATTATTGA
GACCAACCTCCAAGGGGACAGTCGAACGTTCAATTGATACAGCTGTTATGGGATTACCTG

TCCCATCATCTTTTGCAGAGGTGTATTTATCACCGTCCTGGTTTAAAAAAGCAATAATAA
ACGCCTCCTTAATTATTCAGTTGTTCGGCCAAGATCTGAGCTTTGAGATGAGCCAGTTCC
TTCATCACTAGTGACAACGCCCTTTGTCCCATCAGCTTTACGTTTTACACGAGCAAACTT
ACGGATTTTTCCGTTATTATCAGCAAGGGCTCGATAGCCGATTTCCGGAGTGTATGCATT
TCCTTCTTCAAACGACATATCTGCTTCACCAGAGAAGTTTACTTTAGAGAGCTGAATATA
AAGGTCACTGTAGATTTTTGAAGTCTTAGGATCGTATTCAATTGTGTGAATTTCAAAGTA
GTAATTCTCAGAGAATTTTTCACCGTTAATTTCAACAGTCTCGGCTTCAACTTCAATTTG
ATAATGAACTGCTAAAGCTTCACCCTTGGTTGCAAAGGTATCAGGCACAGTAACTGTTTT
AGATGCAGCGTCAACTTCAATTTTATCTCCATCTGCGTTAGTTAAAGCAACTTTAGACAA
TGGGAGATATGAAAGGATAACCGTACCAGTATCACTAACTGTTAAGCTTTCAGCTTCCCA
CACAGAAATTGTTTCGTTTTCAAATTTTACACCATGCTGCATTGCCATGAAATCAAGATC
AAAGAAAGCATTTAGGACATTACCCGATACTACTTTACTTGAATTAATAACATCCAGGTC
TCGGTTACCCCATCCGCCTTTTAAAATGTCTTGTTGAACTTGTTGGGAAAACTGAGTCAT
TTGTGTAACACCTGAAACAATAACCTTATTATCGCTCAGACCTTTAGCTGTAATTTTTCC
AACTTCATGGATTACAGTTTGTTATGCCAAATTGTTTCCTCCTAAATAAAAATAAAATGC
GACTAAAGTTAAGTAGCCTCCAAATAATTGCTCGATATTTTTAGCATCTTTAGTGCTCAG
GTGATAAGACTCTTCCTTGTAAAGATTAATGTGACTACTCCAATCACTTACTTTGACATC
AGGAGAGACGGTAGCAAAAAGTGTGGTTGTGTTATAATTCATAACTTCAGCCATCCTGTA
GAACGACAAAGATAGTTGATACATCGTCATTTCAGATATTTCTTCATACGTGTATCCGTT
AAATGCAGCAACACAGCTTACTATATCCTTTAAATCACTTTGAGAATCTTGTTGCTTGAG
TGATTTACTTATATCATGGAACTCCTGAAGTTCATCATTGTCAACGACCTTGTCTTCAGT
GATACAATGCATGTCTAGTATTAGTTTACGGAGACGGGGAAAATTATGTTTTCCAATCAA
CGATAGGGAATCCTTGTTTATCAATACTTTTGAAAAAACTTTGAAATAAGCCTCGTGAAA
ATCAGGGAGTACCGAATGTACAATTTCATAAAGATCTCTCTTTTTCATTTCAATAATAAA
TGGGTCAAGCGAGCCATCCTCATTAAACCTTGAATATTCTTTAATAAGACTTTTCTTATT
CATCCTCAACATGCTTAGTTCTTTGGTGTAGAGAGGATAATCTATTAAGCGGATGAAATC
AATCTCTCCTATTTCAGTCTGGATCGGTTTTCCTAAGAAAAAGAAATCCTTTAACTCATT
CATTTACTTGCACCAAAAGTAAGGATCATCTTGGAGCCCAAATATCCGTCAGGGGGATTA
CTAATAAGCAAACGTTTATAGTCTACTGTTACACCAAAACCAGCAATATTTTTATTGAAA
AATAAATTTGAAACCCTGTCGATGATTTTCAAACTTCGATACTCAGATATCTCAAATGTA
TTAATGTGTGTGTATACATCGATCATCAATTCGTGATTTTACAACATAAAGCTTTTGTTT
GTCGGCTTAGGCAATGCATTCCCTAAATAAATACACATCCTACATAATGGTGAATCCGAT
AGATCATCTGTTTTAGGTGCTCGCTTGAATATGGTATTGATTATAGCGGGAGAGTCATTT
GTGGAGTCATAATAATTTTCGAGTGATTGAACATCTGGATGAGAAGGGGAGAGAGGATCA
GTTTTGTAATACAAGAGACGATTCAGTTCAACATCATCCATAACTAATCTGAAGACTTTT
GTCATCTGTTCAACAGTCATGCTCATGTCTTTTCACCAACTTTCTTTTTGGCAACCAATT
TTATTGTTCCGCGGTCTCCATAAACTTTAGAATAGTCAATATCATCGACTTGATAATCTT
CGCCAAAGAAGGTGAGGGTAAGTCCGATTTTCAATTTTTCATGAACAAGATAAGGAATTG
TTATGTTTGCTTGACCATCAGGAAGATTTACCGCCAATTCAGTGCCATTTATTGAAGTTG
TCCGTTCGAATATGCAGGGGACTTCAGTTGTTTCGCCAGGTACTTTTTCATAAATTGGCT
TACCAGTGATTTCGTTAATTTTGCCAGTATCGATTAGGTTGTCTTCTGGAGTAAGAAAAA
ATGATGTTCCACATATTTTTATCTCAGCCTTTTTGTAAATCTGATTACTGAGAGGGTGGG
AGGTGATGAGCCATGTTTCATCCTTAAACTTAACAACTCCGCCACGATAAATGTCAGCAA
TTTCACCTAATATATATCTTGCAGAACCATCGCCACTAGAGTACTTAGAAGTGGTCATTA
CTGTTTTTTCGATTCCATCAATTGTGACTTTTTCAGATTCAAACCCCTTAAGGCCTTGTT
GGAAAATAAGCTTACCATCATGAAGAAGTATATTATTAATGTTAACCTTGTGGTAATTAC
TGTAGTCCTTCATTCGAATCACCGTCTGTGACTAAGTTATCCCTAAGTCTGCTTATTTCA
CCACTTACTGTAAAAATAGTGTCCCTGATTTCTGTGAGTTCAGGAGCACTTAGCAGCTTT
TTATTAAGGTATTTCTCCAATTTGAAGAGGACTGAATTGCTTCTTTTACACAGTTTATTG
CAAACAACATCTAAGTCCTCAAGCTTTATTTGTCCATCTTTATGTGCCGATTTAACTTTA
ATTGTACTCATAGATAATCCACCGCAGCGTTCAATATCAAGCGCTCAATTTCGTCTTTCT
GCTCCATTACACTATGTTTAAGTGAATTGAGTTGCGTACCAAAGTTCTTGATCCCCACGT
CATTCGTGGAGGGCTGCCACGTATTCTCAAACAAGGTCTTTTTATTTAACAAAAACATGT
ATCTCAAGAAGTGTGCAAGAATAAGAAGATCATCCTCAGATAATTCTCGATTAACAGTTT
CAGTTGAATTATCAGCTTTTAAATTGTCTCTAAGCCGGTTATTGAAATGAAGGATTGCAT
TTCGAATTAATTTATAAATGTCCTCTTCTTGTTGAGGAACATCAAAATCGGACGTCTCGC
AGTTGTTTAAAAAGGTTTCCCATATTTGATCATAAGATGTCATAAAAATCCCTCCTCTTA

GTCAAAGAGAAGGGAAGGATCAGCGACTCCCAACCAATTAGCCAAAACTTTTAATTTGCC
TGCAGCAATGTCATCACTAAATTCTGAAGCAACGTCAATAATAAATTGCTTCTCAGAATC
CACTGTGATTTTATCGAGCTTATTTTTTAGTTGAGCAATATTTCCCGATTTAATCATCTT
TTCAATTTCTTCTTTGGTATGAATGTTATTTTCATAAGCCTCCGACTCAACAATAGAAGC
TTTAATCTCTTTGGTTGTCTCGTTATCTTCAACAATAACTAATTCACCTTTATCGAAACA
AACGCTGTTCATTGTGAGCCATTCAACAACTTCTTGTGGACCTTCTTTAATATCAGACTT
CCCATTTTTACTACCAGACCAAGTGTATTGTTTATGTCCACCATCGCCGGTATACCCAAC
AAAATAAGACGTACTTCTGTATCGTGCTAACTTAATTTTCTCAGTCAAATTTACATCCTC
CTAAAAAATCCTCATATTTTGATTTAGATAGATACTGCAGCTTGTTCTTCAATAATTCCG
ATTGCTTCTCCAAAAAGAAGGTTCACAGAAGCATATTGAACAATTTTCATTTTGATTCTT
TCATCTTCGATATCTTGTTCTGTCAATTGACGCAGTCCGCCATACTCAACAACAGAATAT
GGTTTTTGAGAAACGCCGCCGGCAAACATATAGCCTTTGTTGATAGGAAGTTCGACTTTA
CTATTTGTATCATCTGTAAATGGGTTAGTGAGGTTAACAGCAGTTGTTCTTCCGATAGTT
GTTAGATTTAGAGCAGTAAGGAGCTCTCCTTTTACTTCTTCTGTTAAGAAGTTCTTAAAC
GTAGAGTCTGTTCCTTGTTGGAATACGAAGTAGCCAATGAGAAGAGTGTCAGCGAGGAAG
ATTGGTTTTCCACCATAACGTTGAAGCACGGAAGCGACTTTGTTGTATTGTTGAAGAGTA
AGGTTTGAGCCTGTTTGAACATTTTTTGCAGGGATTTTACCAGCTGTAATTGCGCTTGCA
GTTAATTGATGAATTTTATCGAGGTACAAACGGACTTTAGCATCTGCGATATCATTCACC
AATTTATTGAAGTAAACAATTGAATCTGTTACGAGATCTAGAGGTTAATAATAGAAACCT
GTTGACATAGTTTTCGGAACAACAGGAACTGTTTCTTGTCCTTCAACACGAACTAAATCA
ACACCAGAGCCTGTAGCAGACCAAATGACTTATGCTTTATTCTTTTTTGGAATTTTAATT
TCTTTAATATTGCCTCGTTTTTCTTGTTGGACATTGGCCAGAAGGGAGAGGAGCTGTGTT
ACTCTTGGTTTCGCAATTTCATCAGCTTGTTCAACGACAAGGTTATTAAATTGATGGAGC
ATGGAAGGGTCAGGTGTAGTACCGCCATCACCAAACACTTTCTTAATGTAAGTTTGGATA
TCAGCTTTGCCTGTACCGTCCATTTTGTTATTTAATACACGGCTAAACAAGCCTTTGATT
TTCACAGTATCTAATTTCATTTAAAATACTCCTTTTATTGAAATTTATTTGTTATGTATT
AGGCCTCGATTACTTCAAGGCGAACAAGTTTTTGTCCCATTGTGTAAACAAGGTCATCTT
CATTATGTACAACTAGGAACTTAGCAGAAGCATCAGCATAATCCTCATGAGGTGAAGTAG
GATCACTTAGAATATACTTTTGAGTAGCAATATCAAAGTGAGCCACTTGACCTTGTTTAA
CCTCGGTCACTCCTGTATTAAATGAAAATGCAGAAACATCAAAACGAGTATAAGCTGGCT
CAAGGATAACAATTCGAGCATGTTCTCCTACACCGTTATAAAAATCTCTCATTGATACAC
CAAGGTAGCGTGCTTCAGGAGATGCAATTAAATATGCTTTTTTTGTTTCATCTGATAGTT
TCGAAGCTGTACGGTTCCCCTCAGCATCAAAACCAAGTTCAACAAGCATAAAGTTATTAA
TGTCTGAACCAGTCACTTTTGCACCGTGAGCAACTGTTTTGATTTTTAAAGAATTCAAGT
TTCCAGTAGTATGATTACCTACTTCAGTAAGGGCTTTTTGTAATCTTGTAGCCATTTTTT
ATTCCTCCAAGTTTAAAATTTATTGTGAAAAGCGTGATTCAAAAGAGTCATCGTTAGGGA
TTAAATTTTCCCGTTTACTTGAAAATTCTCTAATGGTTGTATTTGTTTCAGTAGGAACAC
TAACTAAATCAACTAACATTGTGTTAAGTTGTAATACAGCTTTTTCTCCTTCTTCATCTT
GTTTAACCGAAGCATGGATAAGGTTTTGTACTTCTTCTGTACTGAATTTTTCCTCAGCAT
TTAAAGCTTCAAATTTCGCCTTATAGAATTCATTCTTCTCACTTAGTTTTTGCTCTAATA
AAGTTTTCTCGTGTTTTTCTTTATAAGGCTTCAATTGTTCCACTTCAGAATTTAATTGCA
CAAGCTTTTCACTGGCAGTGTTGAATTGCTGTTATATTCCTACCTTATCCTTATTGATTT
GATTTACTTGTTTCGTTAAATCTTTGATTTGTTCATCTTTCTGGTTAAGTTGTGATTGAA
TAGGCTCAGGAACAACTTCCTCCCAGTTGCGCGTCATGAATACCTCAGTTTTAATGTCAA
AATCAATTGAAACAGTATCGCCTGTTCTTGTGTAATTGAACTTAAAGTATTTGTCATAGC
TATTTTCATCTGACCAGCTATACACGTTTACGATGAAATACGTATCATATACATCTGCAA
TAAATGAATCTGATTCTTTATCCAGGGTTGGATCTAATTGGCTATATAGAAGCGTCCGCA
CGTCAGAGTGAGACAATTCACACCCTTTTCTAAACTTATTCATTTTTTCACCTTCCTCAT
TATTTTGTCTTGTAGCTGCTTGAGCCACTAGTCTTTCAAATCGTTGAAGTTCATTAAAGC
TTAGAAGTTTGGAAGAATCATAGGTGGGGAGGACAATGTCATGCTCTCCACGTTTCCCTG
AATTTAAAATTGCATGACCTTCGAAATAGATGGGGGACTGTAGGTGTTCGATACCATCTT
GTACAGTGTAATTTGAATAAAGGGTCTTACAACTTGTATTAATGTTGATGCCTCTACCAT
ACCATTCAAGTAAAAGTTCACATGCATCTTTAAAACGAGAGCTCCAAAGTATTGCATCTG
CTGGCAATACTTCTTTTTTTCCATCTGGGGTTTCAATTTCAGTTATGTATCCCTCGGATG
TAAAGACACCGATTGGGACAGTATCTCTTTTTACTTCAAGTTCTCCGTGCTTATCTGTGT
CTAGATAAGCTTCATGAGTTCCCAATGCATCTGTAGAAGTATTTAATTCATCAACCTCGT
AATATTTTGCAGCAATCGGTTTATTAATAATTGTTGAAGCAGCTTCAAGAGCAACATCCT

TACAAATTACTGTATTATTGTGAGCGACATCGAAATCAAAAATGACAAAGCTACATGACA
ACTTTGTTGGGTCATCTGATGTTTTAATCTCATTCAATTGAAGTTGCCATAGTTTTTTCT
TTTGCTTCTTTGTCAAATAATTTCACCTCCCTTCAAAAGTGAAGTTTTAAATAGGATTGT
CATTACCGTTGGATGTAGCTGACTTCACTGTGTTGTCGTTGTTTTTATTACCTTCATTTG
GGCGGCCAACTTCATTCCCAGTGAAGGTATATGAAGTTTGATAAGGTCTAATTTTTTCTT
GGAGTTTCAGCTCTTCAGTTTCATATAGAGTTTGTTGAAGGTAACTTACCCAAGACACTC
TTGCCAAATTGTCAACTACGTGCTTAATAGACCAGCCCTTATCGTTTAATTTAATTAAGA
TGTCCATTTTTTCTTTAAGAGTGAGCGGCTTGTCTTTATCGTAATTCATATAGTAATTGT
CTTTTTGGGCAGCAGGCAAAACAAGGTTGAATAACTTTTGATAACCCTCTTGTTCAATAC
CCTCCATTAAAACGCCAATACGCTTATAAGATGTATCCAAGTTTAATGACGAAGTTGCGT
AGTTACCGCCATCACCATTTAAAAGAGGGCCGGATAATCCGTAAGCAGATTGAATGTCGC
TATTGATATGATCAAATTTTGCTCCATCTAATCCATCTGCTTTAACATCTGGAAAATTGA
TATCAGCAAAGTCAGGGATTGATACTACAGTCACTCCATCTTTTTGGTTTTTTTCCAAAG
CCGTTTTAACGCCACCATGAATTTTTTGTTTAACTGCTTTAGGCAGCTTCATGTTTGTGT
ATTCGCCATTACCTTTATCTGTTCCAATCGTTAGAACGGCATCAGCGTTAATAATTTTAT
TTGCAATCGATCTTTCAACATCTTTAAGTTTCTTTTTATGGAGAACGTCATATAGACCTG
GTGTAACCCAAGATGTTCCCAATTCTTGATTTCTTTTTAGCGTCCCTGTGCGTAGTGGAA
AAGTTCTTTCTTGTGGCAATTCCTTAAACCGATATTTCTCTCGATCTTTCATGAAGTTTT
CATAATCGCTTTGTTTAATATAAGGGGAGAGGCTTTTTAACAATTCGTTTCTTTGATCAT
CTTTATACTTAGTGAACAGCTCCATGTCAACTACACATACCCAGTCTGCGTTTCTTCTAA
AGGAGGGGAATACATATTTAATTTCATCAAAAATAAACGGATACGGGCTCTTAGCATCCC
CAATCCATATACCAACAAGAGTACCAGCTGTAGCTACCTGTTTTAACAGATCCCTAGTAA
GTCTTTTGTGCGTAACCTTATGTAAGGATTTATTCAAAAGGGAAATATGCTTATCTGAAG
ACTTAACTTTATTAAAGGAGTCAATTTTATAGTTGAGGGTAGGGAGGGCTTCAATCAATT
CAAACAATTGGTGAATCTCAGCAGTCGAAATGTAAAAATACTGAGCAAGATCTTCAATTT
CTTCTTGAAATTCATCGGGGTTAGAAAAGTAATTCTTTAATTGTTCGGCCTCAATTTCCG
TAACAATGCCATTGGAAAACATATTTGAGATAAAGCCGGAAGCAAATGTAGAAGTATAAG
TTGAATAGTCATTCAGAATTTGCTTATACTCTTCAGACTCAATGTCAACTTTATTTAAGG
TTACCATGTTTCACCTCATTTCTTTTAAAAATAGACTAATTCATCTTCAACGTCATATTC
AGTTTGTTTGTTTAACTGTCTTTCTAATACAGTAGCAATATCGTTGCCATAAGCCACTGA
GCTATATCTGTCTTTTCGTTTACTCTTTGGCTCTTTAAGCTTAACTTGTCCGTTATCGCT
ATATTCAGCTTCTAAGTTGATCATTTCATTTATTAGAAGGGTAATCTGAGCATAGCTTGA
AAGGAATTTTCCTTTAACTTCAGGAGATAGCCCTTCATAGCCTTTAAATCTTTTCAGGTA
TTCTTTTCCTTCATTTCGTTGATAGGAATTTTGATTTTTCCTCGTTTGAATCCATCTTT
TAGTAATACCGCAATTTCGCTGATCAATTGTGCATTACCTTTAATGCTATAAATTACTTT
TTCAGCATTTTGATAAGTACAACGCGCAGCCATTCTTTCATCGTTAATACATGAAAATGG
CTCATATTCTTTTGCACGTTCTTTATCATAAAAAGGCTGGCATAATGCATCATACACACC
AAGTCCGATACTTTGAGTATCTAATACAATATAATCGCAATCATAATCTTCGTATATTTG
TCTAATTCTTGTTGCTTGAGTTCCTGTGTGACCTCCTACGATGCTCTCCACGTATACAAT
ATGACGATCATGTCCATTGGAGTTTGGTATCAGTCTGAATACAGTGTAAACACTGGCATC
ATTATCCTTACCGGCCATTCCAGCGATATCATTGCTTACCAGTCTAATTTCTCCTGGTTT
CTTGCCTTTATATTTGAAGTTGGAATCCTTGATTAGACTGTAATAGTCAGGAGGGAAGAG
GGGAGAAGCAAGCTTGCGGTTCTTTTCAATGTCTTCGGATTTAAAATAGGCTTTTTCAGA
TTCACCAAACCAGAGAGCTTCCATTTCCATAGACCAACCGATAGGATCAAAGTCTTCTTC
GGCCATTTCATCTCTAAACTGATCTTTATCCAAAAGACCTTCTTTAATTGCGATCTGATA
AGGGAGACCACACACAAAATATTTTGATCTTTTCATCATGGCATTGTAGTAAGTTATGAA
TCTGTTAAATGACCAATGAACCTTGTACCAGCAGGAAGACAAGTAAAATTCTTTGTTTCG
TTCTTTTAAGTGAGCGTATTCTTCTTTTTCGAGATATTTAGGAGAACGGGGAGCTGTCAG
GAATTTTCGAAGTACTTTACTGATGATTTCAAAATCAACCATTCTGAATTCATCTACAAT
AAGGAGGTTTGCACGCTTGGAGCGGGCGCCATCATTTGAGGCTACGATTTTAATCCAGCT
GCCATTATGAAACTCCACTTTAGCATCATTTGTTGAGGTTTTCAAATCTTCAATTTCTCG
CCTTAAATTAGGTGATTCTTTGCGTAAGTCATCAATTTTCTCTATGACTTCACGAGCTTG
TCTCTTTGTGCCCGATGCGAAAACTATTTTAGTACCTGGGTAGAGTATGGCTTGCACACA
GCAGTATACAGAGATTAGCCAAGTTTTACCCTGCCCACGACTGGCCAGGTACATAAAATA
ATGTTTATGTACCATCATGTAAATTAAAATACATTGAAACAATTTAAGTGTAATACCCAA
GTATTCTTTTACAAAACGATGTGGGTTTGCTCTATAGAAAGAAGTCCATGCGCCGATACC
ATCCATTAATCTTTCAGAATTTGATTTCTTTTTAAAGTTGTTTCCCTTTTTAAAAATATT

GATTCCTCGACTGTGCTTGTTGCGATCTGTTGTAAAATTTTATGTGAGGTCATTTTCTC
GATCCTCTTCTTCTGCAACAGGTTCTTCAACAGTATGCTTATTCATTTCTTCGCAATATT
CCTCTGAATATTGATTTTTCAAACCAAGCATTCGAGACAGATGCCCAAGGAAAAATACCT
TAATGTATTTCCCGATTTTATCAGGATCTTTCCATCTAGGCTGTGGCTCAGGAATAGGTC
TCTGATTCTCATATTTTTTGATCAGAGTGCCAAAAGACTCTTGTTCATCTCCACTTGCTC
CAGTTTCTTGAACGGGTTTTAAATTACTTGACCCAAGTAAATCTTGAAGAGTTATTTGCT
GCTGATTGACTTTTTCACCATTTTCACGCCGTTTGCGTATATCTAGCCGAGTCAAACATA
TTTTATTTATAAGAAGTTCCATTCCCTTAGAGTCGCATTCATACCTATTCGTGAAGTCAA
TGTACTCATTCTGAAGCCAAATAAAATCTTCAACATCAAGACCACGACCCCAGAATTGCA
TTAATTCATCTATGTCTTCCTTAGATATATCTTCTGTACTTTTAGCTAGTAACAATTGTG
TTGTATATTCTTGTTCTTCTTCAAAGTCAAACTCACTGTCTGCCCAAGTTTTTGACTTGT
AATCTTTCATTCGGATATTTTTCATATAAGTACCTAACACATTTCCAGCTGATTTTTTCT
TGGACTCTGATTCATTAACAGCGGAAACCCACAAGTCGTAAACAAAAGGACGGTCAATCA
TTCTTAAGATGTTTTCAACATTCTTTAAGTTATCTTTGTCTTTTTGGAATTCATTCTGGA
GACAGCTCTTACATACTGACAGTTTGCCTGTTGCAGAATTGATAGGAGATTCCGATATAT
AATATTGATTGGCATTTTGTTCTTTGCCGCAGCATGAGCATTTTATCTTTTCAGCAGACA
TATTTCACCTCCCTAAAGTTTATGTAAACGCATTTTACGAAACGCCCAGCAACAGAGCGG
AAGGGGAGTACCGCAATCTTCGCTGGGCGTTCTAAAAAGGTGCTTAACTATAGGTCAAAT
CACCACTGGGAGACATTTCGATATCTTGTGTTTTTAACTTTTCTATCACCATAGCCATTA
ACCCTTTACCAATTCCACATGTATCAATCAACAGTTTGTCAGGGCGTTTTTCTTTTAAAA
TCTGAACCAATAGGTCTGCTTGATATCCGAATGTCTGAGAAGCTATTCTTCTTTTAATAT
TTTCAAATTTGGAGTCTTTAACATTAACAACTAAAACTGTTGTAAAATCTGTCCGGGGAT
CAACGATTAACAACCTATTTCCTTTTGGAGAATCTACGCCTATTTTATAATCAGCAATGT
TTTCGCTTTCAACATTGCTACCTAAGGTGTAAAAATTAACTTTTGATTTTGATGCAAGTC
CCATTAGTTTATGTAATTTACTAATAACAGTAGGTATCTCTTTATCACATTTGTATCCAA
ACTGCTTGAGTTGGGGAAGTGTTTCTAAGTGCTTACTCAGATATATAAAGTCTTCATTCG
TCATTTTCTTCTTCATCCTCAATCGTTATTTTGTATTAATTTAATAAACCTTTTTGCAGT
CAACCATTAATGCGCCTCCAATCTAAATAAAATTAAAGTTTTATCAAAAAAAGATTAATA
GTTTAACTTTAGGTTTAAAAATCCACTATTGGATGTTGACATCTGATAAAAAAGTGGATT
ACACTATTTATGTATTACGATATCTAAAGGGGAGATCACGTTATGGAAAACAAAGTTACA
TTATCAGATCTTGTACGTAAAAATATGCCAGTGATTTTAAAGGGTTCAAAACACCATCTT
ACAGAAAAAGAGCTGCTTGAAGAACTAGCAAAAAAGATACCTGAACTTGTTAAGAATGGT
GAATATAGAAATGGAGTTCTTCGAGGTGTATTAAATAAATTATCTACACAGCCAGTTGAT
CGATTAGGATTTTCACGTGAGGGTAATCGAGTTAAGTATTATTTCATTACAGATAAAAAA
ACTGAGCTTCAAAATGTTATTAGAAATCTCATCAGTGAGAATAAAGAAAAGGAATTATTG
ACAGTTGATTACTTGAATGATTCGCCTGAAACAATTGACTATAGAAAAGATGTATCTACT
CACGTTAAAGACTTAGAGGGATTTACAAGATAAACATAATTAAAGAAGGCCTAATAGGAG
CCTTCTTTAATTTAATATGTAATCGTATTGAATAATCCGACCTTTACCGGACTCATAAAT
TGAAAGGTTTGCTCCAGCTTTGGCACCTGTCATTAAGCTGTCACTGTATTCATCGGAACC
CATTACAGAAGGGAGTTGTATAACTTGGATATTATGAGTGGCTGCTTCACCTACAGTGAG
CATGTTTCCGTGGTGGAAGTGGGAGATGTACATGTAATCGTCGACTTTTCGTTTCATCTG
TGAGATGTCACGAATGGCATTCTTTTTGTTTTTAATCTGGTGTCCGTGACATGCGACAAT
TTCAAGTTCAAGTAATTTAAAATCCACAATTCCTTCGTCGTATAAAGGAACTCCAATTCG
CTCATTGTCTTTGAGTACGTCATGGATATAAGTAGCTATAATACGCTCTACATCTTCTTT
AGGCATTTCCGAGCGATTTGTATTATGTAATCTTAGTTCAGTATGATTTGCAGAAGGGAT
ATGTATGTACTTAATTTTGACGTGCTTAGAAAGTTCTAAAAGCCATTCAGCTTTGTACCT
AGAGTATTTTATCACGTGGTCAATAAACCCGTACTGAAGGGCAGTTAATTGCGATACGCG
CAATGCCATACCTTCGACACTGTCAGCACCATTTAACACGACCAGCTCATCTAAATTTTT
TTTTTGAATGTCCTCAACAGTTTCAGAAAGAATCTGATTCATACGCTGCAAATAACTCTG
TTGGTTGTATTCATTGTTGTTGCTTTTAAATTGCTTCCCGAAGTGTTCATCTCCAAACCC
TAGAACAGCAGCTCTTTTTCTTTCGTTCTTATTTAATGGATAGAAGGAAGGGGGAGGGAG
AGTACCTACTTTCTCAATTGCTTCAGTTACAGTTTCATATAGCAGCTCTGTTCGGCCTTT
GACGCGTGTGTTTTTATGTATATCATGCTTTACGGCTTGAAGCTTTTTTCTCTCTTCCAT
GATTTCAACCTTTTTCGGTTCCAATTCAAGGAGAGAATTATTTAATTCATCTGACCTTTC
TCGTTGGTATTCAACTCCTTCAATAAAGTTGTTGAACCATTTTCTATAAGCTGATTCACC
TTTAGATTCACCAGTTTCCTTGTTGATTAACTCTATGATTTCTTCCCAGTTTAGATTATA
AATGTCTTTATTAGAGCAGATTCTTGTTTTCCATTCTTTAAGGTTTTCATCTGAGTGACG

CTTAGTTTGAAAAGGATCGATCATTCAGTTAGTCATCTCCTTACTCAACGCCTTTAACAG
GAAGTTCATTTTCTTCTTTTACAGTAATTGAAACATTTTTTCCATTAAACTCTGAAAGAA
TTTCTTTGAAATCATATGTGTATTCAGCTTCTTTAGTTTGTTCAGTAACTTCCATTACGT
CCATATCAAAAAAGCCTTTAACATTAATTTGATGTACTTTTTTGCTTGCCATTTAAATTC
CTCCAATAATTTTCTATTTACGTACAATAAGCTCCTTCGGAAGCCCGATGATCCGAAGCA
TCGGTAACTTCCGAGAAGGGGATATAAAGGAGATGAAAGATAAGTCAGATAGGCGTTGGG
GAAACGCCCGAAGGAGATTATTGATAACTTGCGAACCGGAAGGCTCGTAAACACATTCCG
GCTGATCAGTAAAACGCAAGCACAAAAAGGCCTTCTAACGATTGAGAAGACCTTGTTCTG
ATTACATTCTAACCCCGTTTCACCTACGTCATTAGCTCACGTTGGGACGCAAGCCTTCAG
ATCGACAGGGATTACGCTGATTTCGGGTTCAGCGACTGAAACCTTTGAAGTTGACAAACT
ATAAGTAAGTCGAAATATCAAAAGATGCCAAAATAACGTCTTCAGGAACTCCTTGTACAT
CATGGTACATGAGCATATAAACCGGTAATCGCTGTGCGAAAACTGATTCTCCTTCTGCAC
ATGGCAGATGAGCTAATTCATTTAAGAACTGTGTAAAATCTCCGAATTCACAGCGAAGCC
TTGATTAGTAGCGGCAATGGAAGGAATCCCAAAGCTGGACGTATCGCGGGATCTTTAGTG
TCCCCCACGCGGGATTAATCGGCTACTCTCGATCAGACAATGAAATACTAACTCTAAACC
TCGACACATTATGGTGGCGAAACACCAATAATGAGAAGGAAAGACCCATTATAAGCCTTC
TCAATGGCGTTTTCAGTCTCTCTTGTCAGGTTAAACGCCTTATTGAGAAACAAAGCGTCT
CAAACTCTCGCCATTTATTTTGCACAGTTTTCTCTGACCCGTGTAAGGAGGTATGTGCAT
GGGAAAGCTAAGTTTCTTGACGGAAAAGTAGCGTATATGTTACTAAGTGAAAGGAAAAGG
CAAGACCGAAAATAATCCTTTTCTGTGAAATGACAGCGGTGATCAAGCGCCCAAAACCAT
ATCAATTATTTTACGAGGGTATGTAATTTCAAAGCCTCGTTTACCCGGAAAAATTTTGTT
GACCGCATAATAAGCATCGAATCTTACAAGGGCATTATTTAAAGGGAAGGGAGACGCATC
TACATTCCCTAAGTGTAAGGCGAAGCAGTACACCGCAATTAATATTCAGGGATACATGTC
ACCCATAGTAAGACTGTGATGGGATTCACCCTACATAAAAAACTTGAGTAGCAAAGGTCT
TACCGACATGTTTAGCCCGATAATAGCCATTTATCTGTCTCCTGAAGATACAGACATCCG
AGTTAAACTCGCACCCTCATAAATGACTATTCTCCGACTAACTGCAATGACCAGCCGCAG
TTTTGTGTCAGAATGATACAGAAAAGAAGGGAGTAGATATTGTTTAATCGAGAAAAGCTA
GTTCACACTGGAGAGGAATCTCCACTAATATTCATTTGGATTAGTCTTTACCTTCATAAA
TGAACATTAGTCGAGAACTGATATCCCAAATATCAGTAATCAACTAATTATTGACGGCAC
CCCCATGCCATAAATAAATAACTATAATTAATTAACCCGGAGTTGAAGATGAAAAGCGAA
AAGAGGGGAGGTCGCCAATTTCCTCCATAACTTCCAACTCTGCCACTTTTAGGAGCATAA
GCTCTTTGTGACAAAGCGTGCAGGTAGCCTTTCCTGTCGTCCTCATTAACTGATTATTTA
GCTTTGACTGTGTCTTTAAGTGCTTTTGCTGGCTTAAATGTAGGTGCTTTTGTAGGCGGA
ATATCAATTTCCTCTCCTGTCTGGGGATTTCTCCCTTTACTAGCTGCGCGTTCACGAACT
TCAACAGGTCCGAAACCAGGAATACTTACTTTTTCTCCACTTTTTAATGCCTCTGAAATA
GTGTCAAAGACTTTTTCGACACTGGGAGCTCCTTCTTTTTTAGATACCCCTTGTTTTTCT
GCCCCTTTAGCGATTAGTTCTGTTTTATTCATAATCAATTTCCTCCTAAGTATTATTAAA
TTTATTCTAAATGAACAATAAAGATTGTCTTTTATAAAGACGGGGGCAACGTACTAGCCC
TTTTATGTGGAAATTAGCTTTCTGTCTCCCTTATGACGATTATCTCCAAATGTGCTCTCA
ACCCTGTAGCACCAAGGGATAAAGACACTTTTTTGTTGAAGTTTTTTTCGGTAAAATCGC
TGTACACCTTGGGGGAGTAAGGCTCAAGGCACTTTCTAACGCTGGTTAATTCTCTTTTTG
TTATATTTTTTGTATTTGTCACGTTCTTTTTTCTCTCCACAAGACTGACAGTACTTCGCT
TTGTTAGATGTTGGCTTAAAAGCTTTTCCACAAATACAGCATCCTTTAAAGGTCTTTAAA
TTATACTTAATGTTCTCCAGTACAATATCGCCAAAGCACTCCCACAGTGTCGATTTGTAT
TTACTTTTTTTCTTATATAGATGTTTTACCAATACATCTGTAATAAATCCGTCATCATTA
TGTATTTCCAATAGTTTTTGTTTGATAATCTTATAGACATAAAGTTTTTGTCCTGGTTTA
GCTTCTTCATCATTCATGAGCCATTCTTTATTTCGATCAAGACGTTTATATTCATTTATT
ACGGCTTCATTAAGTTTAATCTCTTTGTTTTTGAGTAAGAAGCGGTAATCAAATTTTCCT
GCAACTGCAGCAAAGTTGATTCGGTCAGAAGGGATAATGGAGTCTAACTTATTTACTGTA
CTTAAATTAATCGATTCTACGCTATGTTATTCTTTATCCTTTGCATTGATGAAGAAGTGG
GGCACTTTATTATTTATGTAATCTTTGATTTTCTCATCAGCGTGATCAGGGCGGGTGGGC
ATGAATAAGGTTTTTGCAAAATCGATAGTAAAGTTATTCTCCATGCATAACCATTTGATC
ACGTCCAGGTTTATATTGTCACTGTTCCATATCTTAGTGATGTTGTTACTGTACTCCCCG
ATATTGATTCCATAAGCAAGAGTTAGTGCTTCATAGATGTTTCTGCTATTAAGCTCTTGT
TTCTGGGCTACAGGCATTTCATAATACAAAGGAACGATGTCCGCCATATTACGCTTCGCA
ATATTGACGATTAGCTCATCAGAAATAATAAAGGCCTTATCCCCTTCATTGTCAAACTGC
AGCAGCTTGGATATCGGATCATGAGTGCTGGTATAAACACCTGGGGTAATGAACCACTTT

TCATATTCCTCATTTTTCTGGTTCCACCTAACACCATGCTCTCTGAATAGGTGAGGGGAG
CGGAGGATATCAATATACCCTTCATCATATAAAGAACAATGGACATCGCTTCCTGAAAGT
AGTCCTTTTGGACTCTCAATTCCAAGAAACAATCTTTCACAAAAAGCATATAGGTCAGGA
CATAAATTTGTGTACCGGGCGTCACTGACAAGTAATTTTCCTGATTCGGCGTCCTTAATC
TTACTCTTCTTCTCATTCTTAATGATTTCTTTGGTGTGATCATCATTTAGCAGCTCAGGA
TATATTAGTAGAGCTTCTTGAAGGCTTGTCTTATGTTTATTTTTCTCGGTAGCCCCTAAA
ACTTTCATCATTGTTTCTTTATCAGTGACTAATCGAGTAATCTCACTAACAGTCTTTGAG
CTGATTTGTTTCAATTCCTCATCTGTGATATCCGTGAGTGTTTGTAGCATCTGGTAAGTC
AGTTTTCCCTCAACAGATGGATCTTCTTCATTTAATTTAGCTCCTAAACTTCCATACTTT
TTAAATTTATAGCGATAACCATCCCAAGAATCATAGTACCTCCACATCTTAAACTGGCTT
TTCGTAAAAATTATTTGGATATCATCATTAATAATGTGCCATTCTTTACCGTAGACGTCT
TTAACTATAAATGAACTGTGTTTTTCAGCAAACTTTCTAAAGTCAAATGGAACAAGTAGC
CCCTTAACCCAAGGTAATCTGCCCATAAAGCTTTTCTGACTCAAACTAGGGAGCGTCATT
CCACAACCATCTGTATGTTCTATAGGAATATCCATAATCATACGTGTGATTTCATATGTA
TCCCGGTCAATATAATCAACTAGGCTGGAAACATTTGTTTCTAAGTCATTTACGACAATT
GCTTTATCAATATCAATTTCCCATGAACTGCTGGCGCTACTCGATAAGGACATGTAGCTA
TTCCATTTGTTTATGCTGCTTCCACCTTGAGCATTGATATGCTCCACACTAAGACCACAA
GTTAAAGCATTTTGATACTTATCTAAGGTGCTTTGCTTGATAAAACAAGATTTTTTTGTT
CTTATTTGACCGGCACTGCTAGTGAAATAAACGTATTTCTCATTGTTATGTATGAATCCT
TTATCAATAATGTCCCTTAAAATTTGAAAGTGATAAGTTTGGACAACCATGATCTCTTCA
GAGAGAGAGTTTTCTTTGATTCCCAGCGTTCGAGTTAAGACTGATTCAAATAATGTAATC
ACATTATTATCTTTCAGAGAATCTGTTCTAAGTGTTCTTATTTGATTATGCTCGTTAAAG
GCGTTATAAAGCTTTTCTTTTAAGGAAATAATCCTTTGCGTTAAGTACTTCTTATGTTTT
TTATCAACATGTTCAAGTTTTCTGAGATGATCTCTGTACCTATATGATGTTAAAGTCTTG
TTATGTAACTTGTTTTCTTGATCATTATAAAAAGCAGAAGTGTCAATACTGTAAATATGT
ACTTGTTTATTTAGGCCCTCTTTTTTTCCTTTCAATAAATTCTCCCCTTATTACTTTTAT
GCATATTTTTTGATTTAATAATCGCTTTTTAGAAACTTATGTACAAGATTTTTATATGTA
TAGCTCTTTTTATCGATTGTTCCAAATAGTTTATAGTCCTCATAAAGAGTGTTTTCTTCT
TGTGAAGCAGTTCCTTCAATAATAGCAGTCTCTAAAAATCCAATTAAATTACTAAACATG
AACGTTACTTTCTCTGAATCCTTTTGCATTATCTCACCTCCTCACTTATGGAGTTATTAT
ATACATATTATTTGTATTTGTCTATTGTTTTTTCGCTAGTGTGTTAATAGCTCCAGGTTA
AGAGGTGAATTCTGAAGCATTTAGAGAAGGGGGGATTATCGTAATCTAAAAACATGCTTA
TCGTAAAAAATATTAAAGAGTTTAAAAAGCCAGTAAACATAAGGAGATTTGCTGTATCGA
ATGGTGATTAGGGGATAAACGATCGTAAAACATAAGTGAGAGTAGGGGAAAGTGGTTGCT
TTATCTAAATATTGAAGAAATCGAGTAAATTTGAAGAGGGGAAAATGTTGATATTTAAGG
CTTTTACGATAGCGATTACGATGTGAAAAAGGGTGATTTTGAGGTGGAATTCGGAAAATG
AAAATTTGGATAGGGTGTGGAAATGGAAGTGCTATGGGTACATCTGTTCCCTTAATTTTA
CCATTAGATGTAAATATACCCCCATATATTGGTATTGTAATGCGTGTATAAGGAACATTA
TACGTGCATTTAATGACTTGGGCAGGTCTGCACCAATGCTATCATAATTCCAGGTCAAAC
ACAAGAATTAATTTTTTAAAAATAACTTGTTTTTCAATTTAAAAATGAATAACCATTCAT
TAAAAATAATCGTATTTATAACGCATCCGAGGGAATCGAAGACATAATTTATTCTTATCC
ACTTACATTACTATAGCATATCTTCATATATAACCCTTGTATTTACTGACCATCTCAGCC
TTGAAAGGTAGTCGGCAACTTGTCTTTAACTAAGGTTCTCTTTTTCAAATCAACATCTCC
TTCTGTAAACAGTTTGTTCCTACACTGTTAACAATTAAAGAAACCTTTAGTTTCTCCCGA
TATATTTTATGAAACTTTTTGCTTCTTTTTGTTTACGAAACTATTTGCTTATATAATAAT
AGTCAAAGTGAGGAACCTACTATGTTTGGCGAACGTTTAAAAAAATGCCGTACCTCCAAA
GGATATAGCCAGCAACGGATGGCGGATTTCTTAGGAATTACGAGACAGAGATATGGGAAA
TATGAAATTGGTAAGGCTGAACCTGATTTAAAAACCCTTACTAAATTAAGCAATATATTA
GGAGTATCTACTGACTTTCTTCTAAAAGGCACACATGCACAATTTGATCTCGATGAGATA
TTAAATGATCCAGAGACTTTAATTGCTGGATATAATGGAAAGATCTCGGAAGAGCAAGCT
AAAGAGTTACAGTATTATCTTTTAAAAAAAGAGTTCGAAGAACATTAGTAGCTATTCTTA
ATTCTTCTGGTAAAACACTTGTTTAATAAAAAGAAAACAAACTGTTTAACACCATTAAGT
TAAATGAAAGAAAAGAAAAAATAACTGGAGAAAAAAGGGGAAATGAAATTGTTTAAAAAA
TTGGGGATTTTACTATTAATCACTTCACTGATATTGCTCGCGGCTTGCAAAAATAGTGAA
GAGTCTTCTTCATCATCAGAAGACACAAACAATGCAACTGATACCAACACATCAGAAAGC
CAAGACATCTCAGTTAGTGGTCCTGAAAAAGTTGGGGATGTTTATGAAATTGATGGTGGT
ACAGCTAATGTAATGGCCATCAGCAATAAAGAAACCACTGTGAAAACTGGCCCGATCCAA

TTTACTGTAAAAAAAGTAATTGCCGCTGTTGCCAACGAACAACTACCTTTTATTGATGTT
CAAATTGAATCAGAAAATACATCTGATGAAGTAGTTCGTTTCAGACCAAGTCTAGCACAA
TTAGCAACAAGCGCTAGGGTACAGATCGATGAGCCATCATTGTTGGAAAGTGACAGGCTT
CTCGACGAGTACGTAGGAAAAGTACATGATAGTAGATCCATCATATATGTATTTGATAAC
GAAGACGATCTAAAAGATTTAGAATCAATTCGGCTGAGAATCTCTTCTCCATTTAATGAG
GACTTAAAGAATTTAGGGGACAAGCTAGACTTAAAAATTAACTTAGAGCATTAAAAACAA
GCCCTATTTTGGGGCTTTTCTTTAGAAAATAAAGTCGAACATAAGTTTTTATTTTGTTGG
TAGGAGGCTTTACCTTGTATCATATTTCACTTCTTGAGGAAAAAAGACGAAAAATTTACA
CCGGATCCCATCATACATTCCACCTAATCTCACTCATACATTAACACGCTCTCAGAGCCA
ATATAACACCTCTCTAAGCATCCTTAATCAATTCACAATCCATTTGTATTCTGGTTCATT
CAAACAGCACACAAGGCATCTTAATACCATCCATCAACACACAGATAACTTACCACAAAT
ATAAAAAATATGTATATAAAAATCATGAATTATGTTATAATAGAGTCATAGGAAAGGAGG
TGTACATAGTGCTTGAGAAAATAGGTATCGTAGTTGCTTTCCTCATATCTTTAACGGTTC
TTACAATCAACAGTCTAACAATAGTTGAGAAGGTAAGAAACCTAAAGAATGGGACAAGCA
TGAAGAAAAAGCGTATACACAAGCGGCTCCGACCAAAGAGACAACGCCAACGTATACGCC
GATGAGAGCTAAGCTAAAGGGGAATGAACTTCTCCTTTAGCTACTACCCATATTATAACA
TGATCAATCACATTGCAAACATGAATCGATTCTCATTATGGTTTACCCATATCACATTCA
TTGGCTTATTCTTAATGTTTCAACTCATCAAAGATTGCTTCAGCAGCGAAGGACAGGCGC
TAATCAACACAATTTTTGTTGTCACATGTATCATTGCCATATTGTTATGGATCATCTATT
GTGTATTCCTTAAACTAAGAAACAAGTCACACTAATCATATAGGCCTCATGGTATAATTA
GATCATTGAGGTGAGATGATTGGAGAAAAAACGCCTGGATGCTATTCAGCAGCTAACAAA
GGAATTGGAAATGCTCGAGAAAGATATTGACTCCATCAAAGAAGCAACTGTCAGAATTGA
GAAAGACCTTTTAGAGTACAGAGAAGAGATAAGCAAAGTAAAACAAGATGATTCAGTATT
AATCATGCAGCAACACAAGGATAATTAAATATGCGTTATAAATAAGGGAGCGGTAAGGCA
ATTATCGTTGCTTATTTTTCGATACAAAATTAAAATATAAGGAGGATATATGGAAAGAGT
TAAAGTTATCGATTCTATTATGGGATCTGGTAAAACAACTTACATTATTAAGATGATGAA
TGAGGCACCAAAGAATGAGCATTTTATCTTTATCACACCATATCTTGATGAGGTAACAAG
AATAAAGAGGGCATGTACGAATAGAAAGTTTTACGAACCAAAAATACATAGTGAGGAAGG
GGAAGCGCTCTATAAACTGGATTCATTACATAAACATTTAGCAGATAACAATGACATTGT
AACAACTCATGCTTTATTCAGTATGGCAAATTAAACAACTAAAGAATTGATTTACTCAGG
CAATTATACTCTTATCCTGGATGAGACTATGGAAGTGGTTAAGAAAATAAATATATCCAA
GGATGATCTGGATATGCTGTTCCAAAATGAATGGATTAAGAACAATAATGGCACTACCAT
TTGGAATGATGAACAAGAACGAAATCTTAATAGGGAATATAAAGGAGAGTTCCAAACACT
TAAACATTTGGCCAAGAGTAAAAACTTAATTTTGCATAATGAGTCTGTATTGTTTTGGCA
GTTTCCTGCAGACATCTTTGCCCAATTTAAACAGGTCTATAATCTTACATATTTGTTTGA
TGCTCAGATTCAGAAATATTATTATGACATTAACGGAATAGAATATGAACTTTATGCAGT
AGTCAAAGACAATGACAATTATAAATTAATGCAGCACAGTAGGCAATTTGATAAAGCAAA
GAAGGATGTCCTAAGACGTAAAATTAAAATATATGAAGGTGACTTGAATAAAATTGGTGA
TGATTATTATGCATTATCAAAAAACTGGTTTGAAAAACGTTCAGTGTTACATAAACGGCT
TAAGAATAATATTCTGAATTACTACCAGAATATACTTAAATCAAACTCTAAGGGTAATCT
TGGGACAACTTTTAAATCACATAAAAGTAAACTAAGTGGGAAAGGTTATACGAAAGGCTT
TTTAGCCTGTAACATAAAAGCAACGAACGAATACAGTCACAAAAGATCTTTGGTTTATTC
AATAAACAGATTCGTTAATCCTGCAATTGATGATTATTTTAGATCCAAGGGAATAATCAT
AAACGAGGACAATTTTGCTTTATCTGAGATGATTCAATGGATTTGGAGATCAGCTATAAG
AAATGGACAGGATAGTAATATATATGTCCCATCATCACGAATGAGAAAGCTGTTAATGGA
TTGGTTAGAGAACCAGAGATAAGAATAGCGTATGAAAAAAAAAGCAATAAATCGTTTTTA
AAACCCTTGGTACATAAGGGTTTTAAAGGGGTCTCTCGTAGAAGAAAAGAAGTAAATAAA
TTAAATAATAAAATAAGAGAATGGGGGTGCTGCTAATTGTTCAAAACGGTTCGTTCGTCA
CCTGAACAATTTTTGCTCACACCACCCCCAAACCCCCTCATGAGCAATTAATATTAACTC
TATATCAAATACAAAAAATATGTATTTAATTATTGATTATTAAAGATTATCAAGTTATAA
TATAAATAACAAATACAGATAATATGTATAAAAACAGGAGGTAGTTAAAATGAATGCGCA
ACTTTTTAATCTGGAGTCTAGACTTGATGATTTGGAAAATGAAATTAATACACAGTACTG
TGAGTTAGATACTAATCTTGATGCTCTTAAATCCAATCGTATTGAGCTGGAATCACAGCT
TGAAAAATTTGAGTCTAGTCTTACAAATAGCTTACAAGGAAGCATTTCAAATAACTGCCG
AAATGACCTCCTTAATCTGGGATACACGCTTTCACAAGTTGATTGCATGTCTGACGAAGA
AGTTTACGCTGCACTCGATAAAATTGATGAAGAAATTCCTAATACTGATCAAGATTATTC
TACGGGATGTGAAGATGTTGAAAAACAAATCATTGAGATGAAGAGCGACTATTTCATTGA

TCGAAAAGAAAGAGGGTCAGGGAATTTCGATGAGGCATGGGAAGGAGAAATCCTTGATTT
AGAATTTGAGTACACGGTTCTTTGTTTAGAAAAGTGGTTAGAACCACTCAATTATATTAT
TACCTGGGAGGGATAGGAATTAGCTTTAACAAGTGATTATACCGTTTTAAATGAGTCACA
ATAGTTCAAAGCAGCGATTCTTTTCTTGAATAAAGAATGTTTAGCTTGAGATATATATCA
AAATAAAATGCATATCTTAAATAGAATATGGATAGTGAAAGCTGTACAGAACAAAATTGG
AGATGGTCATGATTAATCTTCAAGAAGACAGGTTCATTAAATAATCGCATAGAATTAGGG
ACAGAGAATTAAAAGAAAAGAAGCAGGTTTGGAACTCTATGGGGAATGACCTCCTTAGTG
AAGGAATTGCAAAAGTAATGAAGAACTTAAATAATAGACTTTAAAATTTGAGAAAATAAT
TAGGTGATATATTTGGAATATATAGCTCTTGAATCCTCCTTTTTAGAGTACGATATTAAT
GAACCGATTAAAATCTATACCGGCCACTTTACACTTGAAGTTGCTGATGATTTTTTTGAA
ATCTTGGGAGAAGTTAAAATTGCATTTTTACCAAAAGCTAGACTATTATTTGAAGGAGCC
ATTTCAGGCAATCTTAGCAAGCTTTTCGAATTTGAAAAGGCAATGAAAAGTAATAATATG
ATGATTAATGTACCTGGATTTATGAAATCTGAATTTCTTATTTCAGGTATAACAGACGGA
AGTAAAGGGAACAAAGTATCAGGGATACTAAAACGATCTATTTTAACCTCAGCTGAAACT
AAAGTGAATAGAATGGAATTCACGGTGGTTAACTTCGTGAATGATTTAGGTAGAAGGATT
GTGCATGGAAGGTTTAAATTTTCAGGGAGAACAAAATTAAAATATAAAGATTGGGAAATA
ATTTTGGATAAGCAATATGACTATTCAAACAAAAAAATCTTTGATAGATTAAAAAATTCG
GGTGGATACTTAATAACACATGTAGGATATTTAAAAAGAGTGGATGATAAATTATTTGAT
ACTAAAGAAGTAGAACCACTTATTTCAGGGCTTTATTGGTTATTATCCTTTTCAGCAGGT
AGACATGTTGCTATCCCTACATTGGAAGGATACCACAACGAGGAGGTAATATGGAGTAAA
TACCAAGTTCCATTAATAGATGGCTGGACTAATAATATTACTTGGTTTCCAAAACAAAAA
TCTCCATCCTTAGAACATTTATTACCATAGGTGATTGAGAAGCAAGAAGATACTTTTTGG
AATAAAGTTTTATGGGAAGTGTTGAGTTGGTACTCCCAAGCACATTCATCAAGTATAGTA
GAAAACAAGGTGGTATCTGTTCAGGTTGCATTGGAAACTTTGGCGTGGGTGTATCTTATT
GTACACAGAAAATCCAATATTAGTAAATCGAAATATAAGTATATGAATGCAGCAGAGAAA
TTTAGAGAAATATTATCTAGATTTTCAATTGACTTATCAATACCAAAACTCTTTATTGAC
ATTAAGGATAATTATGACGATGGCCCACACCTTTTTACTGTATTTAGGAATAAGATAGTC
CATCCTACAAGGGAAGTTGATTTTGACAATCCGATAGATAAGCTCCATGTACTTTATCTT
GGTGTTTGGTATCTTGAATTACTAACACTTGGAATATTGGGATATGAAGGAAGTTATGTA
AACCGTTTAAAGTTACCTATAATAGAAGGAGTATATGAATTTGTCCCTTGGAAAACAAGA
GATAATTAAAAATGTCTTTATAAGGATTTACAGTCAGTTGGCGATAAAAATTTCTCAGAG
TTGCCATACAAAAAACATTTGCTTTTGACTAAGTATCTATGTTGTAAGAAGTTAAAAAGG
TTTAAACGGTTTTCGATTTAGATCACGAATAACAACGATAAAACGAACTTAGATTTATGC
ATTATTGACCAAGTCTTATCTGGGTCTTTTTTATGTATCAGTTGATTACTATAAGGTATC
AATAAAAGACAAATGAAAAGAGTGGAATTATGCTTAGATCAAATTTAAAGTCCATAATAG
ACGAAAGAAAGATCAGTATCCGGAAGCTATCTAGAGATATTGATCATGAGTATCCAACTG
TCAGAAAGCTTTATAATGACGAAATGGAGCGGTATCCAAGAGATCTGTTAGATAAAGTCT
GTACATACCTAAACATCGAGCTGCAGGAATTGCTGATATTCGAAAAAAGCCATAACCATA
TCGATCACTCACGATGAAAATGGCATACTAAAGTTACAAGCATAGGAAGGAATTACAACT
TTTCCAGGTCTACATAAAATCGTTCTTATATGGAAAAGGTGAACAATTAATAAGGAGAAC
ATAAATGGATTTGTATAGAAAAGCTAAAAGGCTAAAAAAAGAATTATCGAAACATGAGGA
AATTAATGATTCTTTGAAAGAGCTTGCATTTGATATTTTTAAAGAGTTAACAGTAAAACC
TGAATTTATAGACAAAAACACCAATGATGTTATTGGGGAGGAAAAAATTAACACCAGCAC
CTTTACAAATAAAGAAAGGTTAGACATTTTAATTGAATGGACTATTGAAATAGATCCAAG
ATTAAGTTTTATTATTGATAAATGCGATATTCCAATAGAAAGAACGGGTGGGGATGATTT
AGATGCTTTTATGGAGAATGTTTATACAGAATATATAGAAGAATTGCATAAGCTACCCAA
TAGAAAATCATTCGGTTCATCAAAAATCACAAAGATAGAATCTTTTGCGCAACAAATTAC
TAAAACAATTGAACTTTACTTAGATGGTTATGTGCATTTAGCATATGCAGAATTTGATAA
AGGAATGAATATCTTTTCTGAAGAGGTTAACATTGAAGAGCTGCTCTTATATAACATTGA
TAGTATTCGTATTCCTTGTAAATTCTTTTGAATGAGAACCAGTAGCACAGAGGTTTTTTC
TAAAGATGATATGTTTCATATACCATTTGAGAAGAGAGGAATAATACGCACAAATCGTTT
TAGTATACCAGGATTTCCATGTTTTTACGTTGGAAGTTCATCACTTGTTTGTTGGGAAGA
ACTAGGAAGGCCTGACCTAAATACTACCTACACTTCGGTTTTTCACCTTGAGGATGCAGA
TATAAAAATTCTGGATTTAAATGTTTCACCTACGGAACTGGCAGATAATCTAAAGAAATT
TTTTGAAATAACATTTAGAAGAAAAATATATAACTTTAATGCATACTTTATGACAAGGAA
TTTAATATCTGCATGCCTAATTAGAGTAAAAAAACAAAAAGACATTTTTAAACCAGAATA
TATCATTCCTCAATTTTTGCTTGAATGTGTTAAACAGACAGAGTCTTCGGAATACTGGGG

AATCTGTTATTTGTCTTCTAAGATTAACAGACAAACAATTGAAAATTTCAAGCTATATAA
GAATTATGCTATTCCTGTTAGGCAAAGAAAAGACAGTGGACATTGTTCGTTATTGGGCGA
GACATTCCAAATATCTGACCCCGTAGCTTGGGAAACGTTCCAAAGTCATTAGGACTCACC
AGAATCGCTTCCTATCAAAACTCCATTATTTCCAGAAACCGAAGGGACTCATCATGATAA
CCATAAAACAGAGTTGGGGAGACTAGAGGTCTTTTTAATTAGATATAGATCAGTTGTTAA
AGACCAACTGTAACTGAAATCTAAAAACTACGATTTTATAGGGAAATGTGAAACAAGCGG
AGGTGCTGATTTGAGCCTCAATCAAAAAGCGTTAAACAGTTATGTTTATACTCTGGCGTT
TTCAAGTTTGTCATTTGGTCTAATTTTTGGTCTGTATCTGTTTGTTTACTCTGGATTGAT
GGCAATAGCATTAGTCCCAATTGCCATCATTGCATTTTACGCTTTAATCACTTACTTGGT
GTTTGCTGCCCCTCTGCAAGTGTGGCTAAGAAGAAGGCGCAGAAAATTCAGTTTGATCAA
TTTTTTAATTTATATTGCAGTGGCCTTTTCAGCTGTGTTTTTGTTCTGGTTTGTTGATTA
TCCGCCAAATGCTCTTACCATGTTCAGAAGCTTTGAGTATTACATTATGAGTATTGTAGC
TGCGTTTATTTATTGGTTTTGGGATTCGATATTCCTACGAAATTAACATATTAAATTATG
AATTTCACACCGTTTACAACAAGTAAGAAGGCTGCTGATCCAAACAGTAAATATTCGGAC
AGTTTTTGTTTGCTTAATATCGCTTCAACTCCTACTAATGATAAAAGGGCGGCAACAAAT
AATTGCACAACAATCAACAATAATTCATTTTGATTGAACAATGAGTATACGCACAAGACT
AATACAATGCAAGAAACCGAAATTTTTGCAACTTTTAGCAAGGTATCACTCCTTATGCTA
ACCTTAACACTATTACGCGTCTTAATCCGAGAGCATAAAGAATCAAGATAAAAGAAGTGT
TTTAAACAAAAAGAGCATTACACTAAATGCTCTTTTTCATGCCACACCTTCTGCAGTCAC
GTAAAAATATTCCACTCTTCACAGAGCTTTTAAACAAAGTGTAATCACAATTGTCGCAGC
GGCCGTATTTTACATCAGGATGTTCTTTGTAATCATATACGATTGAAGTGTCATATCCGT
TTGTTTCGTATTTCTCTTCCACAAGATCACCTACATAAATTATTCAACATACTCAAGAAT
ACCAAATTTCGTTCGATATAGGGAGGGAACAAATGATTGGATTAGCTTATTTTTTAATTA
TTTGGCTTGGAGTTGGATTATTGACTGGCATTAAGTTTATTTTTGTTGATCAGGTCTATG
ATGAAGAGTTTAAAGAACGCATGGATAAAGAAACAGCAGCGGGCATGGAAAGGACTTTGG
CCAGCCTGTTTTTCAAAAATAAGCTTAATGTGATTGCTTTTTTTATGTTAATTGGTTTAC
TGCCATTGGCAATGAGGATTACAAAATAATTTAAAAGAGGTTGATTTATGCCTTTGATTT
ATTATTTTTATGTACTGCAGTTTGAAAACAAGGAATACTTTAAAGCATTTAAATTAGGTG
AGAGTGGTTATTTGACATCTAGCGACCTCCATGAAGCTTCTAAAATACATAATATGTTAG
AAGTCCTCGAGGTAGCAAGTGAGCTTAAAACGAAGTGTAATGTGCAATGTGAGGTAAGGG
AAATTCAAGTCGTAAAGCGTTAGGAGTGTTCAGGTGTATTGGATAGAGTGGATTGAGAAT
GGAGAAAAGAAAAACATTGTTGCAGAAGGTTGGATTGAATGGGCTGCTATACTTGAAGAC
TTGTATCAGAAGCGGTTCGAGTATGTTGAATGGAAGCGGCTATAACATCTTCACTATTCT
ATAAATATGAGGTGGTTGATTATATGAGTGACCAAAATGAAAAAAGTCCTTCATGGGTAG
GTGACATTATTAAGCTGAGCCCTAAATATTTATTGGGACTAGCAGTTTTTTCTGGAATTG
GGCTTTGGCTAGGGAATGTTGGATTAGGAAAAACACTTGGTATAAAAGATGCTATAAATA
GCTATAAACTTTACCTAGGGCTTGTTTTCTTAGCATCAACCAGTTTCATTTTATCTCGCT
TTATTTGGTGGATAAGTTTGGGGATAAAAAATAAAATTGACCAAAAATACGGTTATAAAC
TGCAAAAAGAAAGACTCCGAAATCTTAATAGGAGGGAAAAACAAATATTAAGCCCCTACA
TCTTTGACGATGTTCGGTCTGTGGAATTAAGTCTAACTGATGGGACAGCTCAGGAATTAG
AACATTTAAAGATTATTTATAGAAGTTCTAATATTAGTAATCGTGGTTCTTATTTTGCAT
ATAACATACAGCCTTGGGCGAGGGGATATTTAACTAAAAACAAGCATTTATTACATGAGT
CAATAGATCACATCTTATAAGACATACAGAAAGGAAATATTCAAATTTGAATAATATAGG
AGAAATAATTTCTAATTTTGAAGGTATCATTGGGGCTTTATTGGGAGTTATACTCACTTT
AATTCTTACTCACATATTAAAGCACTTTGGGCAAATTAAATTTTATATAGTTGACTTTGC
AATATATTTTAAAACCGATAATGATGGTTGGGGAACTAATGTTATGCCATCTAAAGATGA
AGCAAAGCAAATAGAAATACACTCCCAGATTGAAATATACAATGGCGCTGAGATCCCCAA
GGTTCTACGGGAATTTAAATTTTGCTTTTACAAAAACACAAATTTAATTGTTTCTGTTAC
CCCTGATGATAAAGCAACAACTGAAGAGTTTGCTGAGTTTGGCTATTACAGGGATAAGTT
ACTTAATATTAATGTGCCTTCTAAGCAAATCATCGCTATTAATATAATAAAATTTTTAAA
CGAAAAGGAAACTAAACAAGTGAAAAAATGCAATAGGGTTTATCTTGAGGCAAAAGATCA
TAACGGGAAAATGTACAAAGTATTCTTAGGGGAGTTTTAAAATGAAAAACACATTTTTAT
ATTTTAGATGGGAAGACCTCCATGGTCAAATAGGTGTTGATTCCTTTAATTTATTGAGAG
CAAGTTATTCTAATTTGAGTGAGCAGCAATTAGTAGAGTTAATTAAAGAGTTGATATCTA
TAGAGCGGGAGGATATCGCTGCAAAATTTGATATACACCTATCTGAGAACGCCCCAGTTT
TCGATGAACGGCAACATGTGGTTTATAAAGGCGTTGCCGGAGATATGAACTACAAAGACA
TGTTGCTTTCTTTGGTAACGGCATTAGGTTTGACTAATACACTTGACCATGTGCAGAATA

TCCTGTCCTTAGCTAAATGCTTAAGAAGTGTTGATAGAGAAATTTTCGCTAGGTTTGCAA
AGGATATTGCGGATGAGGTGTATTATAGCCTTAAGTAGGACCTTTTGCTTTAGAAGAACA
CAATGAGGTTTAGTCCTCATTGTGTCTAGTTAAAATAGGAATTTTAAAGAGATTACAAAT
ACAAAAAATATGTATAAAATAATTGACTCGTAATGTGATCTATTGTATTATTAAGTTAAC
CAATAAAGACAACAAAAAGAAATGAGGGGAAATAATGGAGTTAATAAGGATAGCTATGAA
GAAAGACTTGGATAATGACAACTCTTTAATGAATAAATGGGCAACAGTAGCTGGCCTTAA
AAACCCCAATCCTCTTTATGACTTCTAAAACCATGATGGGAAAACATTTAATGAATTTTC
TTCAATAGTCAACATTGTTAAGAGTCAGTATCCAGGCCGTGAATATGAATTAATGAAAGA
TTACTGTTTAAACCTAGTTGTTAAGACAAAGGCAGCAAGAAGTCCATTGGAGTATGCTGA
TGCAAATATGTTTTTTGAAATAGAAGATGTTTTAATAGATTCAATGATTTCTTGCAGCAA
TATGAAAAGTAAAGAATATGGAAAAGTGTATAAAATACATAGAGAACTGTCTAACAGTGT
TATTACTGAATTTGAGGCAGTGAAAAGACTCGGCAAATTAAATATAAAAACACCTGAAAT
GAATTCTTTCTCAAGACTCTTGCTGCTTTATCATTATTTAAGCACTGGTAACGTTTCTCC
GATGGCCCAACTTATAAAACAAATTGACCTAAGTGAGATTTCTGAGAACATGTACATTAG
AAATACATATCAAACAAGAGTTCATGTTCTAATGTCTAATATAAAGTTAAATGAAAATTC
ATTAGAGGATTGCAGAGAGTACTCTAAAAAGGCATTGGAAAGTACAAATATCCTGAGATT
TCAGGTTTTCAGCTACTTAACTATTGGCAACTCTCTATTATTTTCGAATTATGAATTGGC
TCAAGAAAACTTTTTAAAAGGGCTAAGCATTTCTGTTCAAAATGAAAATTACAACCTGAT
TTTCCAGCAGGCTTTGTGCTTCTTAAATAATGTATGGCGCAAAGAAAATAAGTGGATTAA
TTTTGAATCTGATTCAATTATGGATTTGTAGGAGCAAGCTCATTGTTTTATCAACTTTAA
CGAAAAATCCAAAGCAAAAGAAGTTTTGGATTAACTAGATCTTTTAGTTCACAACGATTA
TGAGCTTGCAAAGCATTATTATTTGAAAGCAAGACTCGAACAAAATAAAGCATGTTTCTA
TTCTTCAATCGAGTATTTTAAAAAGTCTAAAGACAAATTCCTTATTAGGCTGCCACTGTT
AGAACTGCAAGAGATGGGTGAAAATCAAAAACTTTTAGAATTACTTTTACGTTAAAGGAG
GTGAGACAGTGAAAAAACTTATTATGGCTTTAGTTATCTTGGGCGCACTAGGCACTTCTT
ACATAAGTGCAGATTCTTCAATCCAACAAGCTTCAGGTGATTATGAGGTTGCTGGAATGC
CACGTGGAGCATAAAATCCATTGACACATAAAGTTATTAGTATTATTATTTATTTAATTA
AATTAAACAGAGAAAAGGAAGACGTTTGGCTCTTTTGAGCTAAGCGTCTTTTGTAGTGTT
AAGGCCATCACTTAAATATTAGGTTCTAATAACATCTAGTGATCAACTTCAAATACATAC
ACCCTAATTTAATGAAATACATTACAAAGCAGCACATTCGCAAAAAAATTGCGTAGAATG
TGCTATTTGTCGTTAAAAAAATCTTTACTTCCCTTTTGTAATGCATAACAATTGCTATAG
ATGCAAAAGATAAAAAATATGTATATAGGAGGTTGGTGGTATGTCCGCAATTAGTTACTT
AAAAAACAGTATGACAATGCTTAAAACCATTTACCAAAAGAAAGTTGAAAGCTTAGTTAA
AAATGATTTGTTTTTTCATGAAAAAAGCATTGAAAAGTCAAAAATAATGAAGAATGAAAA
TGTTCGAAGACAACTAACTAAAGGATACATGAAATTGCTAAGCGAATACAAGGAGGATTA
ATGATGCATGTTGTAGAACTTAGGTCTACAAATCATAAAGATATTGATGCCGATTTTGTA
TTAAATGCTAAACAAACTTACATAGAGAGTGTACTAAACATTAGGAAAATGATTGTTAAT
GCAAAAACTGAAGATGATCTACATGGTGCAAAAATAGAGATAGCAGCATTATTAAAAGAT
CTAAATAGAGTATTATTAGGTGGAGATTGATTAGAAGGAAGCATTGAAAATAATCCGCAT
TTTAGATCCCTAATACATTTTGTGAAGAATTTAAAACGACACATTGCAATTGAATTTGAA
GAGTTTATTTATCAACCGTAACATATACGCACTGCAGTCTATAGTATACATAAAATTAGG
AGGTTGACATGTCAGAGCGAATAAAACAGCTAATGGTCAAACGTGGCATCACAATAGAGG
AATTGTCGAGGGAGACAATGGTTGATATGCAGACATTAAACAAAATCATTGAAATGCCAG
ATGAATCAGATGCTACAACCATAAACCTTATCGCTCTGGTGTTGAATGTCTCTATTGATG
AGTTATTAGATGAGAAAGGAGGCGAAGATAATGCAAAATAAAATTAAACAATTAAACAAT
TATGCGGTTTACGATGATATCGAGGGCATTTTAATCAATAAAGATATAAGAAGTAGCTCA
GGGAATTCTAACTATATGATGCCTTCATCAAGTAGAAGGGTGTCGAACACCAGAAAGAAT
TATGAAGGGGATATTAAGCAGTTCTTTAGTGTGATTAAAGGTAAAGATGTCAAAAGTTTA
GTTCCCGATGATTTAGTTGTAAGTAAAAGCGAATTAAGCAACTATGTGAAGTATCTTCAG
GAAAAGGGATTAGTTAATAAGAGTATTAACAGAAAAATGACCGCCTTGAAGATGCTCTAT
ACATATTTGGAGCATGATTATAAGGACTATATTGACTTGTCGGTGTTTAATACTGTCGAA
AGGCTTAAAACAGTAATTAAAAACTGGGATAAAACAACGCAGACAGAAGCCGAAAGAATC
GCTCAGGATATGTATATAAATGAAAGACAGAAACCTTTAATGAAAAAGCTGTTTGTTAAA
TTCGCCATCAGAACTTCTTTTCGTGTAAGTGCGATTTTGCGAGTAAGATGGAAAGACATA
CAGCTTGATGAAGGTACAGGCCATTATATAGTAACAGTTATTGAAAAAGGATCTCAGGTT
GTGTCTACAGGCATTAACCAGGTATTTTATTAGGAATTGTTGCAGCTGAAGGAAGAGGAT
GACAGCGAAACTGAATTGGTTTTTCAGGGGCTTTCGGAACAATCTTTACGACACTCCTAA

AAAAGGTCGAAAAAAAGGTTAGTAATACCTCCTGAAAGAAAATTAGACTTACACTCATTT
AAGGGTGTAGGAATTGACTATGTTTATGAGAATTCTGGTCACGGTTTACTTGCAGCAAAA
GAACAAGGAAATGATAAAAACACATTAACAACAGAGAGATATATGAGCAGAAAGATTAAC
ATAGCGAACTCTGCTGGTGTAACAATGGATGAAAAAATCGATTTAAATCCACTATATGAA
GCAACCCAAGAGGATTTTATTAGTTTTTTTGAAAACGCTGATCTTGTTACATTAAAAAAG
TTTATAAAGCATGTAAATGAGCGATAATCATTTCTTCGGGATATTACTCAGTTCAAATGA
TAATTCTTCTTGATTTACCAGATTGCCTTTGGTAACTTTTAATTTAGCTACATGACATGA
GGTGAAAGGCTATATGACAGTGATCTTTGATCAGTCTGCAAATCAGAAACTGCTTTCAGA
AATGAAAGATGCTATCTCGAAAAATAAACACATAAGATCTTTTATTAACGATATTCAATT
AGAGATGGCTAAAAATAAAATTACTCCAGGGACAACACAAAAATTAATTTATGATATAGA
AAATCCAGAAGTCGAAATTTCTAAAAAACATATGTACTTTTTAGCCAACTCCCTATACTC
AGCCCTTGAAAGTGAAAGGTTTCATCCACGAAATTACTTCACAGAAACGGATATGAGAGA
AATTGAAACGTTATGGGAAGGATCTGTGGAGGAAGATATAAAATTTCCGTATACATTCAA
ACAAGTTGTAAAGTATTCGGATGATAATTATTTCTTCCCCATCACTGCTAAAGAGTTGTT
TATGCTATTTGAAAATAAGTTATTGCACTATAATCCTAGTGCTTAAAGCACGAACAAAAC
CAAAAAACTAGAGGGCTCAGATATTGAGATACCTGTACCGCAGCTCAATAAACAATCGGT
TGAAGAAATAAAGGAACTGTTATTAGATGGGAAATTAATTACATCAGTTTTTACGTTCAA
TGCACGTGTTGGAAGCGCAAGTTGTGGCGAAGAATTAAAATATGATGACGACACTATGTC
TCTTACAGTGACTGAAGACACCATTTTAGACGTTTTAGACGGGTATCGCCGGCTAAGAGG
CATTACTATGGCTATAAGACAACATCCTGAGTTAGATCATTTGTTTGAAGAAACCTTTAG
AGTGGACATCTATAACTACACTCAAAAAAGGGCAAGAGAGCATTTTGGGCAACAAAACAC
AATAAACCCAGTTAAAAAATCTAAAGTGGCTGAGATGAGTCAAAATGTTTATTCAAATAA
AATTGTTAAGTTTATTCAGGACAATAGCATAATTCGAGATTATATAAAGACAAATGGAGA
CTGGATAAATCAGAATCAAAACTTACTTATAACTTTTTCTGACTTCAAAAGGGCAATTGA
AAGAAGCTATTCTAAAAAAGATTTTTCTACTCAAGCAGACATCTTCAAAACTGGAAGATA
CCTTACATCTTTCTTTGATGCTTTAGCTACACAAGATGTAGATGAGTTCTTAGGTGATAT
AGCAAAAGAACGGAAGAGAAGTTTTGTAAACAACGATTTGTTCATTAATGGTTATGTGGG
ATTAGCTAAGAAATTGCAATTAGATGGTGTAAGCCTAGACGAGTTGGAAAGTAAGATTAC
TGATGTTTTAGGCTCTATAGATTTTAGTAAGAAAAATAAGTTGTGGGATGAATTAGGTGT
AGTAGACAAGAATGGAAATGATAAATCACCACAAAAGATGTGGAATTTCTTCAATAATTT
AAAAATAGACGAGTAATTACTTAGGAGTTGTTTGTTGCAATGTTCAATAGTGAGATTAAG
GACAAATATTTAGATACCTTATCTGAGGGTATGGTTATGCAGATGAGGCCTATTTTTGCA
AAAGCAGAGATTACTGAGACTTTATATAATAAAGACATATATGATTTCACATCAATGCAA
ATTTTAGAACTTATACGATCATTCGATCAAACCACTATCGGTAGTGTTCGAAGAACCTTA
GCATTATTGTCATCGTATGTTGATTGGGCAATTTCATATAAGTTAAGTAAAGGATTAACA
AATTTAGCAAGAACTATTTCTGAAGAAGAGCTTTATGAATGTCTCGGGGACAAAAAATTA
TATATTACTTATAGTGAATTAGAGGAAATGGAAAATAAATTAGTTAATTATAAATCTAAG
GCTGTGTTAAGACTACTTTTTGAAGAAGTTTCTGGTTTAGCTCATTCTGAATTGCTAAGT
TTAACGAAGAAGCAAGTTGAGGATGCAACGCTTAACGGTAACGTTTTAACCCTCTATGAT
TCAAAGCACGGTGAACGAAAACTAAAAGTTAGTAGTGAATGTCTTGTTATTGCCTTAAAT
GCAGCTCTGGAAACTAAATATAAATTAAAGAATGGGAAGGCAAAAGGCCAAACAAAAGAA
GTCTTTTTAGTTGAAAATGATTATGTAGTTAAAACAAAAAGAACGTCCAACAAAGGAGAC
GTCCAAGCAAGTAAATTTGTCATAACTAATCTAATTACTGATATATCTGAGTTCTTTAAA
ATTAACTTTTTAACACCAAATACGATTGTTAGATCTGGTCATTTGTATAGAGCATATCAG
CTTTATAACGAAAAAGGGGTTATTGATAACTCTGTAAGGTATCAGATTATAGATGACTTT
AATTTAATGGTAAAATCCAAATATCGAGCATTTTATTCAATGCAAGATTATATTAATGAG
GAAGAAGTTAATAAATATTACGCAGAAGAACTAGGTCTTAAAGAGACGACCATTTAATTA
TGAAATCCATTTGACTATTTTGGTTAAATGGATTTTTCTTTTATCTCAAAAAATTGAGAA
AAAATTCGCATTTCGACAAAAATAGACAAAATAATCACTTTAAATACATGTTTAAATGTA
GTAAGATGGTCATGTAAACCATATGGTTTACTAAGGAGGAGACCAAGACCAAAGTCTTAG
TCGTTTCAATGCCTTTCAACCTTGAATTCATAGAGGTCTTCTACATTACAATCTAAAGCA
GCAGCAATTCTTTTTGCAGTTCGAATAGACATATCTACTTTAACTAATCGATTATAGTCG
CTCAGCTGCTGTTTCTTAATCCCAGTTATCTCTGAAAGTTCATTGATTGTAATGTCCTTC
TTTTTACATAATTCAGGTATCAAGCATTGCCCGATCTCAACTTTAATCATAATCGGACAT
CCTCCTGCGTTCTGTTATGGAAAGACATAATACATATCATACCATACGAAGCCGATAAAT
AACCACTAGCATAACACCTACTTTCTATGTATCATGATTATACAAAACAGAACGCACGTT
CCCTTAACACCTCAAGGGTTTATCACAGAAAATTGAACATAGGAGAATGAGGATGGCAGG

TTATTTAAACAATATTGCACTGAATCTGGAGATTGTTCTCAAAAACAAAGCAGATAGTCC
AGAAGTCTCTGAAACATTAGTAACCAGGATTTGTGAAAATTTACTTTTATCTAAAGAAGT
CTCGTTTCTAAAAGCTGACGGATCAGTTGAAAATTTTAAATTAAGTGATATGGAATATGA
AATAACAAATACAGAAGAATTGCCTGAGTAAAAATAAAAAAAAGAAAGACTCCATTAAAC
ATAATGTAATCAGCAGCAAGAGGAGAATGAAGGTATTGTTCTGAATATTTTTGTTGATCA
AGATAATTACAAAGAGGTTAGTCTGAAACTTACAAAAAAATTGCTGACTTCAGAACATTA
TCAATTCCTACTTTGTTTCAAGGGAGAGAAATTAGATATTACAATTTCAGTTACACCACT
AAGCCTCGTTAAGCTTAGGGATGACATCAATGAATTGATCTTTATGTTCTCAGATTAAAA
TTAGATTCGGTCACATAAATTGGCCGAATCTAATTGACAGATATTAAATGATGGGTTATC
GTTTATTCAAATACAAATAATATGTATATAGGAGATGGTAAAGAGAGCTATGTTGCTTGA
TGAAAAGCTCGATAAATTAATGAAAACGATTCTGCGATTAAAAGCATACAAAGAAGAGGA
AAATTTACGAAGAGTCATCGGAGAATTTCATTCAATAATTGATTATGCTTACGAGGGGAT
GTATATAGCTGAAGATATGTTAAGAGAAGAAGAAAATAAGGGCAAAGAAGTAAGTACATA
TTGAATTGTGTAATTTTCACTAAGGTAACAAAATTAAAATTAGTGGATAAAATGTAAGTT
TCATCGAGATTCGAATTGGAGGGGAATATCACTTGGAATTATCATTAGATGAATTGAAGC
TCTGTCTTAAACCATTAGTGTTTTTCGGTGAGTTAAACCTTGAAATCAGTGATTATGAAG
AAGGTAAGAAAATTTAAGTGCTGGATCATTATGAAGGGTCTTTAATTAATTTAGCAGACC
AAACGATCAACGAAAATTATGTGTGTACTACATGTAATTGTACTTTATATACCAATGAAA
ATAATGAAGTATGTTTCATAGAGCATCCGTATCGTGCAATCACACCTGTAAATAAAGATC
AAGTGATTCATTTAACTAAGCTAATTGGAGCAATCATAATTACGGACGAGGAGGATCCAA
TTGAATGAACACAGCATACAGAGTTTGGGACGGCGAGCAGATGCATTATTGGGATGATGA
AGGTTTGAGCCTTATCATCAAAAGCAACGGCGATTGGACTTTGAATCGTTTATACACAGA
TGTTTTGGTTCCGGTTGTAGACAGCACGAATAGAAACGCGGCTCTCATGTGGGGAGCAAA
GGTAAGAGGCAAATTTATTTATGATAGGTCTATAGTAAAAATAACGAGTGATGATAAAGA
ATCATCAGATGTTTGCGAAGTTAAGTTTTCAGATGGCGTTTTCCAACTAGATGTATCAAA
AATTTCCGCTGAATATGACGTGACTGCCGTCGGGTGGGTGGAATATGCAACTATTGAAGG
AATCGGAGACGTTTATCAAAATCCTGAGTTATTGGAGGGTGTGAAATGACAATCAATTTG
AAAGTAAAACAAGAGAAGCGCAAAGGGTTATCATTTAATGATATCCAAGATGGATACTTC
ATTCTAAGAAATGACGATGTTTGGATTGTGAAAATGGCTGTTACAAATAGAAATAAGATA
CATTTAATCGATTTAGAGACGTTCCATGTTAAAACTGTATCAACAAAGAACGACTTGAAA
AGTTTGTTTGAAGATTGGAGCCGAATTAAGAGATTAAGTCCGAAACAGGTGAATTTAAAT
ATCGGGTTCCAATGGAAAGGGTAAAGGAGGGCGCGGAGTGACAAGTGAAATGCAATTACA
GGCACAGATCGACGTTATCGAAAAAGAAAATAAGGAATTGCGTCGCAGGAATGAGGAATT
AGGGCAGACGGTCGAATGTCAGAACACACAGATCGTCACTCAAAATTGGAGACTGTTGTT
TTTCGCTTCATCCTGGATCGTTTATGGAATTGTTAGTGCTATAAAACACCTTTGGGGATA
AGGAGGGCGTTGAGTGATGGAGGAAATAGATAACAACGAATTGAAGTTCCGAATGATTAC
GCCGGATGGAGAATCATTTCTGGTTAATTCTGCACCTATTGAAGGCTGGCAAAATGATAG
TTGGAAGACGTTTGATGAACAGAAACGGGTTTTCGATGCAGAGATTAAAAAAGCATTGGA
ACACTGTAACGTACCGTTGGAGGAAACAATTGAACGGGCTGAATTAGTCGTAGATAAGGA
CGACAACACTGTTCTGACCATAGACAAACTACCGGTTCTAATCTTATATAAACCTGAATT
TTTATATTCGAAAGGAAAAGCAGTTCAAAGATACAAACGACTTTATGAGGAGGACGTGGA
ATGAGGGAATTTCATTTGCACAAATATCCCGTGACATCACTTGAAGGGAATGAGTATGCC
GTTAGCATTTATAACGATAGACACTCAAAAGGTTTTGTCAAAGTATCTTTATATAAAAAG
GTGCGCGGCTTTTTCAGGAATGAAAAATTCAAGTGTCTTACAAGAGAAGGAGACTTCGCT
CCGAGTTATTTCGAAGAAAAGTGGGATTACGATTACATACAGATGGCAATCAATGAAGTT
ATTAACTATGAAAACTCCATAAAGGAGCAAATTAGTCATGAAAATAAACAAAAGGCTGCA
ATAGAAAAAATTTGAAGCATGGAGTGGCCAGGAGGTGTAACTGTTATTTGAGGTTAATGTG
AAAACAAACCAAAGAGAAAAATTCATCTGAAATGGTATCCCATATGATGAACTTGATACA
CAAATCATTCATTTAATTGATATTTTAAATTTCAGAATTGGATTAAAAACACGCCACTGT
TGTTTTGGGCATAAACCATACGAAGAAATTCAAGTGATGTTTGAAAACGAAGTGAACATA
AAAGAAGATCAGATTCTAGAATTAGCAGAACTAGCGGGTAGGGAATGGAAAGGCCTACAG
TTAACCTTTAGCAAATGGGCAAGATTTTCCCCATTGATGTTTAAGTGGTCATTGGTGTTA
TCGAAGAGATTCAGAAACCCAGAAGATCCAAATAAATACCGTTATCTAAGATCAGTTGAA
GAATTCTTTGAGAGCTATGCTGCAAAGAAGTGATAAAAGATGCATTTTAAATTAAATGAG
ACGGAGGTATTTAATGGAAAGTTATCTAACAGTAATTGAACAGAATGGACAGCTTTTGGT
TGACAGCCGTGAAGTGGCAGAGATGGTAGGTAAGAGACATACAAATTTACTTAGAAGTAT
TGATGGATATGTAGCGATCTTACTAAACGCAAAATTGCGTTCAGTGGAATTTTTCTTAGA

AAGTACATATAAAGATGCAACTGGTCGCTCACTGAAGCATTTCCATTTAACCCGAAAAGG
ATGCGATATGGTTGCTAATAAGATGACTGGTGCTAAAGGGGTATTCTTCACAGCTCAATA
TGTTTCTAAATTTGAAGAAATGGAGAAGGCGTTAAAAGCAAGACCGTCCCTAATTGATAC
ATATCTAGATATCAATGAAGATGAACGTGCCATTGCTTATTTCACTGAAAGAAAAGCAAA
ACGAGAATTGCAGGAGCAATTAACATTGGCTGAACCACAAGTGGAGAAATATGATCGCTT
TCTTAATACAGATGGATTAATGAAAATAGGACAAGTGGCAAAAGCAATTGGCATAAAAGG
AATGGGCCAAAACAATTTATTTCGCTTTTTGAGGGAAAATAAAGTTCTTATTGATGGAAC
AAACAAATACGCCCCATATCAAAAATATGTAGAAAGAGGATTCTTTCAAGTGAAGACCCA
GGAGACAAGTGTTGGAATTAAAACAATTACTCTTGTTACTCCTAAAGGTGCTGATTTTAT
TGTTGATCTTCTAAAGAAAAATGGGCACAAAAGAGAAATAGCATCATAGATTTGAAAACT
TTAGATAAAAGTAAAATTTTAAAGAGAATCGGAGGATTGAAGATGGAGAATAATGTTTTA
TACGGAGTTTACAGTACTAGATCACGAAAGTTTTGCTTTGGAATTGAGGAGCCTTCAAAA
ACAAAAGCAAGAAAAGAGCTATTCAATCTAATTGGTACAGATGCATACAAATGGCGTTTT
GAAATAAGAAAAATTAAAAGAAAGTAGATGGAAAAATGAAATTTGCACCAATGGATAAAG
TTAAATTTAAAACAGCTAGTCATCTCAACAAACCTCGAACTTTAAAAAAAAGAGTTCCTG
AATTAGACGATCCTTTACTCGGCGAATGCTGGGAATTTGAAGAAGATGGTTTGAGACAAT
TTGATTGGGAGGAGAACTACGAATTTGTCGCTAGACCCAAACATTTTAATTGGGATTAAA
AACAGAGTCTAATTGAAGTTTAATGAGAATCGGGGGAATAAAAATGGAGAAAATGAACCT
GCAAAAAGAAATAACAATATTTGATTTAAATAAAATCAAACCTGGCACAAAAGTTCAAGT
AACTTGGTATAAAGGAACAGAGATGGAGTATACACATAATGGCGAAGTTATCATTAATAA
TGGGGAAAAGTTTTATTATAATTACGTTGATAAAGAAGGATATGTAGGTCACTGTCATGT
AAACGCACTTGATTTGAAAAACTATCCTTACAGTCTAATTGTTGAGATTAAATCAAAATA
AAATTGAAGTTTTAATCAAATACAAATGAATAATCAATCCAGAAATAAGGGAAATTAACT
AATAAAATTGTGAGGAATTTTCAATGAAACGATTTATTTGAGTTTTATCCTTTTTAAGTA
TTATTGTTGCCTATCCCATTCAAACAAACGCCAGTCCAATGCCTTGCAGTGTAATACTGG
AGCCGGTAGATAAAAATCTTAAAAATGCTAAAGGTGTAGCAATAATCTATAAGGTGCAAT
TGAACCCACCGAGTGCTGCCAGAACCAATATAAGTATTCTTGCTGTCCATCTTCCTGCAC
CTTCCTCTTTTGGAAATTACGACAGTTACGAAGGATTTGCTACTAAACCAGGTGAGATTA
GTTGGCGGTTTAAACTTTATCCTACTCCTGAGGAAGAGAGTCCTAGTTGGGCGGGAAGAA
TTGACACAATTTCGGCCGAAATGAAGAATGTTAAAGTGCAAGTGCGTCTGTCAAATTCTA
GCACACAAAAGTTGGGGCCAAGTATATTAACGAAAAATTTCGAGTCCTGTTATTGAATGT
CTTGGGGCAACAGTGTAAAACAAACTTGGAATGGATTGATGTAAATGATTAAATCGATTA
TTTTACCTGAAGAAAACACAAAAATAACTGTAGGTAAACCGATAAATGATGAATCAAATA
CAAAGGTAATTGCTATTTATGACTATAGAGAAGAACCAGAAGAAGCTTTCTGGGTACACT
TATCAAAGGGGAATGATCTATTTGTAGATAACCATGAAGTCATTGTTGAGTATGAATAGG
TCTGTTAATAATGGTTGAATAAAAAGACTGTTTTAAAGAAAGCGTGAGAACCGATAGTCA
ATGTCAAAAAACTTAGTGATCCAGAATATATTAAGCAGATGGAAATTGAGAGAAATGAAC
GTGAGCGGATATATCAAGAAAAGGAGTCTATAAGATAGAAAACTGTGTGCTTCACTGGTC
ATAGGCCGAACAAATTGGGTGGATACGATATGAAAAATCCAACAATGCTTAAACTCAAAG
ATAAGCTGCTTGCAGTAATTGAAGAATTAATCACTAAGGAAAACAAGTATAGGTTTATAA
CTGGAGGAGCGTTAGGAACTGATCAGGCTGCTTGTTGGTGTGTGCATATGCGAAAGAACT
AACACCCTCATATCAAAAACATAATTTCCACACCTTTCAAAGAACAAGATAAAGTTTGGT
CTGCAGACCAAAAAATAGGGTACAAACGTATGCTTAATGTAGCAGATGAGATTATTAATG
TTGAAGAATTAGATAAATATAAAGTTAGTGGAGAGGAACCAGGAGAGTTTTCGCCAGCTC
AGATGCAAAAGCGAAATGAATACATGATTGATCACAGTGAGGCAATAGTAGCTGTTTATG
ATGGAAGTAAAAGTGGTACAAGAAACTGCTTAAATTACGCCAAGAAAACTTACTTAGGAC
ATCAGCTATGGAGACTTCATCCTGTTTTTAATTTTGAATTAGACATTACGTATTTTGTTG
GATGATAAGGAGAGGTGAACTTTTCTGAAACTAATAGACGAAAGAACATATGAAGAACGA
TTGTACATATATTCTGATGATATTGAAGCCAGAAAGCATTTCGTAAGTGAATTAAAACCA
AAAGGGTGGATGGTGGACAATTGCTGGGTTGGGATTGATCTAAATCAAATAAAACAGTTT
TATCGATTCAAGAGGCAATTAACTGATGATTATGTGTTTAACAGATAGACTAAAGAGCAG
GGGGCAATTAATATAGATGAAAAATTAAGTGATAGTAAAAAGCATAAAATAATACACGGA
TATTATCACAAGAATTGTATAAGTTGTAAAAGCCGCTTACCGGCAACTGAAGAGAATTTT
TACTCTGTGAAGAAAAATAAGGATGGACTTCATTCATACTGTAAAGCTTGTGTATTAAAA
AAGGCTAAAGAAAGCATGTTGAAGAATTATGACAAACAGCTTGAACGAATGAGGGAAAGA
AATCTTCTTCCAGGGATGAAAGAGGCCAAAAAGAAATACAACTCAAGTTTGAGAAAGAAA
AAAACACAACAAATCTGGCAAGAAAAGAACAAGCGAAAATTAAAAAACAACCGTTTACAA

AGAGATGCTCACAAGAAACATAACATTACAGCTGTGCAGTGGCAAAAGTGCAAAGACTAT
TTTAACAACAAGTGTTCCTATTGCGGGCTGAAAATTGAAGATCATAAGATATTATTCAAA
GGAAGCTATATACAAAGTGATTTTCATAAAGAGCATGTATATCACAAAGGGGCTAATGAT
ATAAGCAACTGCATACCTGCTTGTAAATCATGCAATAGCTCAAAGCACGACTTTGCATTT
GAAGAGTGGTACAACTCATCAAACAAAAACTTTTCATCCGAAAGATTACTAAAGATCAAA
GAGTGGCTAAATCGTTTTAAAGAGGAACGACAAGATTCAGAATGGAGGACAAAAGGATAG
TCTGTAACAAATACATAATTGATACTGTAATGATACTTGCACAAGTAAATAGAGGGGAAT
TTGAAAACATTGAGCGCAAAAAATCTCCTAAAAATCAAAGAGGGGGGAACACCCCCTCGA
TAAATCAATAATTTGCTTGGTACTTCCCTACATACCATCCATCTTTATATTCAATGCCTT
TTAAGTACCAGTTTCTACCGTTTTCGTCTTTCTCAATATTTTTAAAATCATTAATATTGT
GATGGTAAATATACTTTGTATGCAATTCTTTCACACTTATTTCGTCTTGAACAATGTTAT
TCTCTTTAACCCTAGCAGATGGGCTAGTCGCGAATGCAGGAGAAGCAGAAACGAGTAATC
CAAGAGATAAAACAGAACCAGTCAATACTTTTCTTAATTTCATTTGAAGCATCTCCATTT
CCTTTTAATGTATTGCAATTACATTATACGGTTTTTAAAGAAGGCAATATAGCGAAATTA
CTCCCATACATATGGAAATTGTGTGTCGGTTAATGTCGTGATAGATGAGAAGGCAAATGG
AGAGATGGATTATAGGATGGAATCATAAACAACAGTAGAATAAAAATAAAATAAGTGGAG
GTTAAGTTGGTGATAAAGAGGAATCTGCTTAGCAACCACGTTGATGAGATTATCGGAGAA
TATTACGCTGCTAAAGGATATTCAGTCCATAGCATTGACCGCCAGGAAAATGGACAACTA
ATCGTTGTTACGGAGCGAGTAGCAGAGGAGAAGGAAAATGCAAAAGTTGATATAGCATTT
GATTTTGTACATAGAAGACCACATAAGAAGAAGTATTTAGCATAAAAAAGGCAAAGAGGA
ATTAACACTCTTTACCGTTAGGTTTCTTTTTTAGACGATCACAGATAAATTTCATTACAC
TGATAGCTATTGCAACAGATCCAATTATTAATGTGATTTGGAGGATGAATATTACTCCGG
AATTTATATTCACATTGTCAAATAAGTCACTCGACAAGGAAAGAAGCTTATGAATACCCA
AGGCAAGTACGAAAAAGGTGAGGCATATTACGACAAGGGATGCTTTGGAATAATCTTTAG
TGTGGTCAAGATTATAAATTGCTATTGATACAAATAATAAACCCGTAGCGATCTTGTGGG
ATAGCGAATTATTCGCCGAAAATGATTGCACTACTATGCTTAAGAAAAATCCAGTAATAC
CGATTGTTTTACTCAAGGAATCACCTACTCTTATGTAAGTTAAGTATGAGTTTACCAATT
AAAGGGGATTTTCTCAAGATGATACACATTAAAGTTAAATTAAAATCGTGATTTAAAGGA
GCGGAGATAATGAACACTTATGTAGTCGATGACGGCAAGTATAAATGTATTTATGCGGGA
ACTGAGAAAGCAGCTGCTTTTAATAATGAATTTGAAAACGGAACAAGAGTAAGGGGTTGG
TTTGAAGGCCATCACATAAAAACGTTTGAGAAAGAACAAAGAGAAGTCTCTGGAGTAGGA
GAATGGATCGTTAAGTATCATGCTGCTACTGAATTGCAAAAGGAAGTAAATCGTTTGGAA
AAGACCTATTTCAAGAAGAAAGAGTTGCTAGACACTATTAGACAAGCTGATGGGGTCGGA
AGGGAAATCAATGATAAAAAGAAATCAATACTCAATTACAGTGGATGTTAATGGAGGAGA
ATATAATCAGGAGTTTGTTATTTTAGCGGATTCGCTGAGTAAAGTTGGGCGAGATAAGAT
AATTGCTGATGGTGTGACAATTCAATTTAATGACCAGATTCTTGAATTGAATAGTAATAA
AGAAGATTCGAATGATTATTATAAGGGTCAAACAATAACTGTTAAAGAAGATTGCCTAAA
TAACTTCTGGAAAGGCGGAACCGTTACTGTTGAAGATGTAGATATACATAGAGGCATATT
GATAGACGGTGTCGTTTACTTAGAAAAACAAGTGGTAGACAAGTATTTTTCCAAAGAAGA
GGAATGACATGTACAGGAAGATTTTCGATATGGTTAACGGTAGTGAAGAGCAATCAAAAA
TAACTGATTGGCTATATAAATTAATGATTGAGGACGAACATTCACTTTACAAAATCGACA
TTAGAGTTTCGAAAAGGAGGATTTCTGAACAAGGAGATATTTATAGAGAGGAGGGAGAAT
ACATAGAGAACGCAGATCTTCAAACTTTACCAATCGGGACAAAATCCTTTGTGCAAAACG
GTTGTTGGAAGGGTGAAATTGTTTCTAATGAAAAGAAACATTTATATATGCCACAGATTG
GAGAGATGAGGGAAATCAAAGTTGGATCAACTGGGTTGAATATAGAGATTGAATGAAAGA
TAGTTTTTAACTAAACATATTAGGATGGATCGTTATCGAAAAAGCACACGTATGGATCAC
GATCATAAAGTATTAATTTCTATGCCGGCGTTTTTCTTCGAAGTGATCAGGATAATTCTG
TTTAGATTTCGATTTGAACACAAAATAAAGTCCTGCAATTAAGATGTAGTCCATGATTTT
GTCAAATGACCAATTCACGAAACAATCTGAGATTAATTCCATTTATTTCACCTTCGAGAC
AAAAATGTCCAGTCTCTTTTATATGGCAAGCGTATGACAACCTGCTTGCAGTAATAAAGG
TGAAATGAATCTTGGAAATTTAAAAAGGTTTAAAGAAAAATAGCATTTAAAATAAAATAT
TTATTTTAATGGAAGGAGGTAACCGTAGTGCCAAATCAAGAGCAAATAAAACGCATGAAC
GATATTAATGATCTGATAAAGCTTATTGCAAGCATTGATCATCGTACATTCTACCGCAAG
TCAAAGGATCGTATAGCGTATTTCAGGTTTAAAAAAAAACTGTTCTTCGTTGATGATTAC
ACGGTTGATGACGTTTATCCGTATGAAATGGGATACGGGAGACCAAACGGCTTTTCGCAT
GGAGGCAACAAGTGGCAACTGGTGAACAGTTTTAGAAAGTTCATCATAACAGGGAAATGC
GGCGAGCTGAGGGATTATAAAGAAATATGGGCTTACAGCTATGAAGGATGTATGAAAATC

CGTCAGAAGGCAAAAGAAATAGGGTTTATAGAGACTGTTGATTATCCTTATAGCTTCGAT
GAATGGATGAATACTAATTGATCCATGTACTAAATTTTCATTGAATTTTAAAAGGCTAGG
CTCTAAGAAGTTATGTAACGGAGAGAATACACTGTCCAGGATTCAAAGGAAGGTATGCGG
AGTTTAACGTGCTTAGTCTTAAATCTGTGCTTAACGTTACATAAGATCGGTTCGATAAAT
ACATATTCATCAGATTCAGACTTAATTTGTTTCATAGAGTGGACCTTAGTTCGTTCTGCA
TGTGGCATAAATTCCATAAATCCAGCTGAAGTTCCATCAGGATAAGACAGCAGGAATTTT
ATATCCTTTTTGGTGTAGCCAGTTATGAGAACCTCAGTGTAATCGTAATAAATCACTTTA
AGCCAATTATGCGAACGTTTATTGATCTCATAAGGGGAGTAAGCTTTCTTGATTACGATT
CCCTCTAAATGCTTTTCTTTGGCCAGGTTGAAGTAAGCAAGACCGTTACCTTGAGTTCCC
TCTATAACAAGGACATTAGGATGATCCAGTTCAAGTGAATTCAGAACTGTCTTACGCTCA
GTGAGTGGCTTAGCTGCAATTGATTGGCCGTCTTTGTAGATAACATCAAAGACACAGTAA
ACAATATTATGAGCTTATTTTTTGGACATAAAACGTTCCATTACAGCCTGAAGGTCGGGA
GCACCACCTGAGGCAGCTACAATAACTTCACCATCGAGCACTGTTCCATCTGGAATATCT
AAGTCCATGAGTTCTGGGAACTTACTTGATACTTCGTTGTTGTGACGAGTGTATAGCTTA
ATCGGATTATCAAATTTAGAAAGGATGAGTCTGATTCCATCAAATTTGAGCTCAGTGATA
TAGTTCTCGTCGTTAAAAGGCTCTTTTCTTGAATGCAAGAGCATTGGCGATACAAACAAA
ATATCACCTCCTACTTAGAACATATTAGCTAAGCGAAGGTGATATATAAAGCGATATGGC
AGTGGTACTTAATGGGATTCAATGAGCTCAGGTGAATTGTTTTTAGGTGAATTCACTAAA
GATGAAACCTGGTATGCTTCCATGTCATCAGCATCATATGGCTGCAGTAAACTTTGTAGA
TAATCAGGATCAGTATTTTTAGGGTTGAGTCATTCCTTTTCGTTCTCATCAGTAAGGATA
ACCGGCATCCGATCATGGATGTCTACCATAAGCTCATTAGGCTTTGTTGTAATGATTGTG
CAAGTGTATAAAGGATTGCCTTCTGGTGTGTTCCACTTTTCGTATAAGCCTGCAAAAGCG
AAGAGATTGGACGATTTAAGTTTAATCCGCATAGGAATCTTAGTCTTTGGATCAAGGCGT
TTCCATTCATAAAAACTGTCAGCCGGTATGATACAACGTTTGCTTACGAGCGGCTTTCGA
AAGCTGGGTTCCTCGGACAATGTTTCAGCACGGGCATTAATCATTTTATAGCCGATCTTT
TCGTCTTTAGTCCATGGAGGGATCAGACCCCATCTAAGTTTGCCCAGACGGTTATTGGAT
CCATCGTTGATGATTGTCAGGATGTTTTGTGAAGGAGCGACATTATAGCTAGGGTGATAT
TCACCTTCAGGTAAGAATTGATCTATATTGAATTGCTCAATGATGTCATCAAAGTCAGAA
AATAAAGTGAACCTGCCACACATGTTCATCATCCTTTAGGGTTTTTGAATATTGTACAGG
CTTGATACACGAAAATCAAAAAGGAGGAATGTGATGCCGCAGCAAACAGTTGAGGTTAAA
GACGTTGATGTGTTGATTAGGGGAATATGGAGAAAGAAAAAGTTCACCGATATTCAAAAG
GGGCAAACCTTTAAGATTGAGGAGAACGGAAGAACAAAGAAATACATAGCGAGAACAGAT
CCTTATTGGGATGACATGTTTGAAACTGACATTATTGATTTGTTGGATAAAAATAAAATT
AGAAGAAATAAGTAAACAGAAATCTAGATAAAAACGATATTTTAACCAGAATCCAGGGAG
GGAATTGTAATGAACTTATATGAAATTATGTTAGAGCACTTTGCACCCAGGGGAAGTGAG
CGGGGTATCTTTACATATTTGCTTGCACAATCTGATGAAGAGGTATATGAATGGTTGAAA
ACTGATCCGAGCTTGTCTGACGGCAGGGCAGTCTATACCCCATATCAAGACAACGAAGCA
AACGGTAAAACATAGGCATTATATCATCAGAGCTTTGATATTGTTGGTCATGAAAAATAT
AAAGATCGAATGATTCGATTAAAAGGTGAGTTGAATGATGAAGGTGAACTAACCGATCTT
TATTACGGAATGACCTTAGTCGGGTGGAGTATGGTGAAATCGGATATTCCATCGGAACAA
ATTGAATTGTTAAAAGATACTGGCATCAGTATTGAGTCTGCATAAAAGTAATTTTTTATT
CGGAGAAGGGGGTGGTCCTAATGTGATTGCTTATAAGATGAAAGTAAAAAATAAAAATAA
TCGTATTTAAGGAGAGTTGTTGATTGTTAAAGTTTAAAAACAGAATAGTAGAAAAAAGAG
TTAAGGGGGCAGAGAAGGCAAGCTTTGGCGGGCCAGTCCTTCTCTACCGCACACTTCGCA
AGGTTACGAAGCTATATAAGTATATCACAGACAAGAAGTCTTTTCCAATCCTAGTTACTT
CATTGTTTGCATTGTTTAATCCCAATTCAGTTCTAGCTGCAGATAAGTATAGTAATTTTG
AAGAGTTGAAAGAAAATGAGTCACCTTTGAGCTATAACATTCTAACTACCGATGTTGACA
GGAGAGTGCTTATTCTGGCACCCCATGGTGGAGGTATTCCAGGAGGGACTAGTGCGCTTG
CAAGAGAATTAAGTAAGTCTTACTCTGCCTATCTATTTGAAGGTTTAAGGATTCCAGGAG
CTTCTGAGTTACATATTACAAGTACGAATTTCGATGAACCTCAAGCTTTAGATTTATTGA
GTAAGCATGATTTAACAATCTCAATTCACGGGTATGCATCAAGTAAGAAACACACTTTAG
TTGGTGGGACAGACAGAGAAAAAGCAGCAAAGATCACATCATTACTTACCGATGCAGGCT
TTTCTGCAGAATTACTGTCTGAAGATTCCCGTTTAGCAGGCACAAATGAGCAGAACATTG
CTAATAAAAATAGCACTGGTATGAGTATTCAACTTGAAATAAGCACTGAACAACGAAGAG
AAATGTTTAATACTTTTACACTTGCTGGAGGCAATGGAACGCAGAATCAAGTTTTCTATG
ACTATATTGCTGTTCTAACTAAATTCACTAATGAAAATGTGTATTGTATGGCTGGTGTAG
CACCATGACATTGTGGAATAAATCCATACTATTCTATGTTGACTTTGATCAGTGGGGAAT

AAATGCTTTTAACTCGAATCCAATTGAAACAACTAAAGGATTTAATGAAGCTCTTATGTA
TGCATCAGAGAACAACTTTCCTATTGTTGAAGTTCCAAAAGGGAATTTTATTATTGATTC
AGTAAATACATTAAAGCAACGAAATCCTGAAATTGGTGGGGGAATTAAAATCCCATCAAA
TATGGAGCTCCTTTTGGATCCAGAAGCAGTGTTTCAAGTTAACCCTAATGGGTATCAGGG
CTCTTCTTGTTTTTATATTGGGCTTGCAGAGAACGTAATAATTAGAGGAGGTCGTATTAT
AGGTGACCGGTATCAACATGATTATTCTCTAATTGATACCGATAGAAAAACACATGAATG
GGGATTTGGAATACATGTTCGTGGAAGCAAAAATGTTTTGATTGAAAATGTACAAATCTC
AGATTGTATTGGAGACAACATTTGGATTGCAGCTCACGGAATGATGAATTTCCCAGGGAT
GGTTTATACGCCTTCCAAAAGTGTTACCGTAAGAAAATGCGAACTAAAAAGAGGGAGAAG
GAACAATTTAGCTACTAACGGTTGAGAAAGATTATTGGTTGAAGACTGTGATATAGAGGA
AGCTGGAGGAAATACAATTGGCCCTCAACTAGGTATTGATTTAGAGGGCTACGGAGAAAA
CGGAAGAAAGTATGATCATCCTTATGAGTTAACGATATCGGATTGCAGCTTTAGAAAAAA
CGGTCGTGGTTCGGTTACTGCTCATACAAGCGGTAAAGTTTCCATCAAAGATAACGACTG
TGACAATGTTATTTCATATGGCTACAGTACAGATGTGAGTATTAAGGGTAACAAGATAAT
AAATGAAGGGGGGTCTAAAGAGTACGGAATAGACTCTGTAGGTGGTTCGAGCACTGAGAC
TGGCAACAGAATTCAAATAACTGATAACAATATTCAAGGATTTAAAATAGGCATGATGAT
TAGAGGGAAAGGGGTATCGATTGATAATAATACCGTAAAGAACGCTTCAAATTGTGCAAT
AGCAACACATATGGCCGAAGATGTTTCCATTTCAAACAACAGAATACAGGACAGCGATTG
TATTCAGATCCAGGTGAGAAATTCATCAGATATTAAAGTAAGGAATAATAAAGGGAAAGG
TACAACTTCAGCATATGCAATTAAAGTGATGGATTCGAATGACGTTAAATTCTTAAATAA
TACGTTCTCCAATCTTTACGGAGGTCTATATTGTGAGAGATCTCAGGCAGTCAGAATTAA
GCTAAACGATTTCTTATTGCGTGGAAAAGGTTACGGGATATATTGGGATAAAGATTCAGA
AGTCTTCCTAACAAGGAATGAGATATTTGAACCAAGAAATGTTGCAATTATGGGGGCAGC
TGATATGTACAATTTCAGAATAAGTGATAACCAGGTCTATAATTGCAAAGCAATCATTGC
GATCCACTTAATAGGCGGTTCTGAGCATATGGTTAGAGGAAATGAACTTATGTTTAACAG
TGATTCAGATCAAGGCTATGGAATATATTTAAACGGGACAAAAAAGGTTCGATTAATAAG
GAATGATGTTCAAGGTATTGGTGCAAGAGTGCTTTCGCATCCATTTGCAACGTTTAATGC
TTCCAGTACAACTTTAATACATAATAAATACGACAGCGGCACAACTAGATTAGCCTTAGA
TGATACAGTAATAGATTATAAATAAAAAATTCTTAACGAGAACACTTGTTCCCTTTATGG
GTTTTGGTATATAATTTAGTAGTACCAAAAATTTCAAAACAGGGGATAAGAGTATGAGTG
AGCAGAAGCTTAGAATCGGAGAAATACGGTATGAGGTTTTCGATGATTATGATCCATTAA
CAGAGGACTATTTAGGAAAGAACCTAGCAAATGCTTATTTTATTGCTCAAATTAAAAAGT
AAAATGTAGTTCAAGTAGGCAATAGGATAATTAATCACTCAAGCGATGTTAAAATTGGTT
TTGATGCATTCGGGGAAGAATCTAAAGAGAAAATCAAAGAGTATCTTCTGGCTGCCTTTA
GTCTCATAGCAGAAGGGGCTAAAGACGATGGTCTAGTAGAATATTTATAAGGCAATGCTG
GGCTAGTCTCTAAGATTAGCCTAGTTATCATACGAATAAAGGGGTTAACTGAATGTCAAA
CATCATTTCTAAAGAGCAAGATGATGCAATTAAATACTTTAGAAACAAATTAAATTTATC
AGACAAAGACTTATACATACCGTTGATTAATTTTGAACTACTTAGAGACAAGAACGCGCT
ATATGCGAACATTCTTTATAAGCTGTATAAAAATGATCCCTCTTTGTTTATTAGAGCTTT
AAAGGAAGGTTATGTGGTTAATCAGCCAATTGCATTTGATGAGGCCATTGTGCGATTGTT
TAATGGAGAAGAACTGGCTATTGTACATAAAACAACAGGCAGAAGATACAATGTAAATGT
GAAAATGAAGCAGCTGCCTGATGGCTTTTCATTGCAAACAATGGATATGTGCTTGTGGAG
TGAGCTTGTTTGAAAATAGCAAACTCTTGACCTATATAATGAAAATATGATAGAATATAG
AAGAAATAGATAATTGAGGAGATGTTTTTGTGGAGTTTAAAGTTGGACAAGACGTATCAG
AAATTTGGAATATCCATGGATCAATTCTTCCGGAAGTTTTAATGTATATGTTCCCTCGTT
CAGACGAATCTTATGATTGGGAATTTGTAAATGACAACGGAAGACATATCTTTACTGCAT
GGAGAAAAAGTGAACCTATTCCTACATTAGAGGAAATTGAAAAAGCAGCAATTGAGCTTG
AAGAGAAGAAAAATGCACCTAAGCCAAAAACTCTCGAAGAAAGAGTTGCTGATTTAGAAA
AACAAGTAGCTTACTTAACGAGTAAGGTTGAAGGAACAAACTTACAAGAGTCCCTTATTG
GGGCTTTTAAATTTAAATAAAATGATAATTTTATTCGAATTAAAAAGAGCGCAAATGAAA
TACAAAAATAAAAAATATAAAAGTTTATAAAACATGTAGACAAAGATAAAAAATATGTAT
ATAATAAAACCGAGTTAAAGGGAAGGAGGCGAATTACTTGGAGGTACTTGGGATAACAGA
GAAAGCGTTAAACTATTACAGAGAAAACGTTAAAGACAACAAGACCATTACTCCTGATCA
GGCACTGTTAAAGATGATAAGGAATGTCCTACTTGTCAAAGAAACTCATCCTGAGCGAGT
GAAAAAACGTTTATTTTGCACAGAGTACGCTTACGGAAATATGATAATCAAAGTTAACAG
AAAAAAGCAGGTATTTGAAATTGTAAATAAATCAGGATGCTTTTCTGACCAAAATGGTTG
GAAGTTCCCACAAAGAAGGTACATAGAACTTAGTAAAGAACTTGGAATCAAAGACTGCAA

GTTTAGAAAAATTACATATTCAAAGAAAAATCACAATAAACAAAAATAAAAATAGAGGAT
GATTAAAAATATGACAGAAAACAAAACGGTATTACGTGAAGCATCCAACGTTGTAACAAT
TGAAGGAACTTTAGCTGAAGTACGACACACTGAATGGAAAAATGGAAATGGACTAAATAT
TGAATTAGACATTGAGGTTGCACCTAACGAAGTACATACAGTTAAAGGCTTTTCCAAGTA
TAAGAAAGATGATGGTACTGATAATGCAGTTGCAAAGGGGTACCAGACTATTGTAAGCGA
GTATAAGTCAATCGCAAAACACGGTAGAGATGAAGCTGACAAAGTAAGAATTACTCAAGG
TAAAATTAGCTTAAATGAGTATTATTCACAGGGGATTTTTAAATCATATCCACAATTAAT
GGCTAACTTTGTGAACAGACGGGATGGTAATGAAGAATTCAATCCCAGAGCAGAATTTGA
TGTTGAGGTGTTTGTAAAGAACGTAACCGAAGAAAAAGTAAAAGGGGAAGAAACAGGTAG
AGTTAATTTAAATGGCTATATTCCTGTGTACAACGGAAAAGTAATTCCTTTTACATTTGT
AGTCACAAAAGAAGGCTCACAATATGTTGAAAATAATTATGAAAAGGGAACTACCGTTAA
CGTTTTTGGGAAGTTCATTAATTATAAAGAACAAAAAGTAACAACCAAAGTAGCGGCATT
TGGAGAAGATAAGAAAGAAATTACTTCAATTACAAGAAGAGAGTATCTAATTACAGGTGG
TATTGATCCTTATGATGAGGATAGTAAGAATGCTTTTAATGCAGATGCAATTAAAAAAGC
GTTGACTGAAAGAGAGATTTGTCTAGACGAGCTTAAGAAAGAAGGAAATAAAGAAAACAA
TAAAAAGTCGGGGTTTGGTGGAAGTGCTCCTAATAACAAGCCTTCAAAGCCGGTTGAAAT
TTCAGATGATGATCTCCCTTTCTAAAATAAAATAGATGATTAATCTAATACATAACTGGG
GTGAGCTCCGACTCACCTAACAAATTCAAAATAAAAGGAGAACTGAAATGGCAATCGATA
TTTTCAACCCACAAGTTTCTGTACTAGCAAAAGGATTAGAAGGAAAAGTTATCACAATCT
ACGGTTCTAACAACTTACGTAAAACTAAGCAAAGCACTCGGATGAAGAAACCGTTATATC
TGCCATTCGAAAAAGGATTAAATGCTATTGCCGGTGTCCTATTCATGCCTATCAATAGTT
GGGCTGATTTTATAAAGGTAAACAAGCAGTTAACCAAAAATGCTGAAAAGGCCAAAGAGA
TGTATCACACAATCATTGTTGATGAAGTAGACGCTTTTGCTAAATATGCGACTAGATATA
TCTGCGAGCAATATGATGTAGAACGGATTAAAGATGGTAATGATGGGTTTGGCCTTTGGA
AAGAGTATGAAACTGAAGTATGGGAAGAAATCAATAAATTAATTGGCGTAGGATTTACGG
TTATCTTTATTGCTCATGCTGCTGAAGACAAAAAAGGCAAAGTATATCCGAAGGGGGATA
AACGAGTTTTAGCTCTAGTAATTGATAACAGCGATATTGTTCTCTATCTAAGTTCTAATG
GTGTTGATGAAGATAGAAAAGTAATCAACTCAAGTGCTTGGTTAGCTGAAACTGAAGGGC
ATTTTGCTCGTAGCCGATTTGATTACATTGACACGTATCTTCCTGAATTTACTGCAGAAA
ACTTAGAAAAAGCAATTATTGAGGCAGTCGAAAGACAGGAGCAAGCAGAAGGAATTGTTG
CTGTTACATACGAAGAGCAAAAACAAAACAACGCTTCAGAAGAACTTGATTTCAACTCAT
TAATGGATCAAATTAAAGAAATTGGCATGAAGCTTAATGAAGAAGGCCGTTTAGAAGAAG
TTAATGAGATTACAGAGAAGCATTTAGGTAAGGGTGTAAAAGTTACTGAGTGCAGCCGTA
AGCAAGTAGGTGTCATGTCTGTAATTCTAGATGATCTAAAAGACCTTCTAGCAGAATAAA
AGAGGAGAGATTATTCTCTCCTCCTTATTAGGAGTGATTATTTGGGGAGACAAGTTAAAT
GTCCTTATTGCGAGACCAAGTTAGACAAAGACTCAGCCATTCCTTATAAAAAAAGATATT
ACCATGAAAAGTGTTTTAATACCTGGAAGCAAGAATCAGATCACAGAAAAGAGTTAATTC
AAAACATATGCAATTTATATGGTCTTACATCTCCAACTGGCATGATGTTGAAACAAATTA
AAGAGTTTCAAGAGGAATATGGGTATAACCTTAAAGGCATTGAACTAGCGCTTAGGTACT
TTTATGAAACTTTGGATAATCAACCAGGAGAAGGTGATGGCATTGGAATCGTTCCATTTG
TTTATGATGAAGCAAAGCGGCATTACATTAGACAAAAAGCCATCCGAAAATCAGCTGAAG
ATCCAAAGAATCATAAAAGAGAAGAAATTACTTTGGTCATAAAAAAAGGAATGAGAAAGA
AAAGTGGGCTAGTTGATATCTCAATGCTATAGGAAGGAGAGTACATTTGCTACAAGACAA
ACAGGCAATAATTCAAGTTTTAGGGAGCATTTTAAAAGATCCCACAATCTTGTCAGAGAG
CAATAAATATAAGATAACTACGGATGATTTTCCTTCAAGGTTTCATTCAATATTATTTTT
TGCCATGAGCAACCTATTTCATCAAGGAACTGAAGTATTGAATGACGTTGAAATAGATGG
ATATCTAAAGGATTACGATATTCAATATAAAATTTTCCATGATAATGAGGGTCTTGAATA
TATAGAAAAAAATACAAGGGTTGGCAGTTGTCGAAAACTTTGATTACCACTATAAAAGATT
AAAAAAGTTTAGGCTGCTTAGAGAAATGGATGGCTTGGGATTCGATATTAAGGAAATATA
TGATGAATCTCTGATTGACCCAAAAGAACAAGAAAAGATGCAAGAACAGTTTGATAAGAA
GTCGATTGATGAAATTTGGACAGCTTATGAAATGAAGATTGTAGACATAAAAGAAAAATT
CAGAACTTCATCTGTAAGTGTGGGAATCCAGGGTGGAGAAGGTATTAATGAATTATTGGA
TTCATTCGAAGAGTCGCCGGATATTGGAGTTCCATGAAATAGCGAAATGCTTACTTCAAT
TTTTCGGGGATCACGGAAGAAAAAGTTCTATCTGCGCTCAAGTATTACAGGTGGCGGCAA
AACAAGAAACATGGTCGCTGACGCTTGTCGCTTAAGTGCAACTGAACTTTATGATCCTAA
AAAGAAGGAATGGGTATCAAACCCATGGAGCGAAAGTTCTACAGTTATTTCAACAGAAAT
GATGGCCGAAGAATTGCAAAGTTTAGCACTTGCCTACATAAGTAGTCTAGAAGAGAAAAA

AATCCTTAGAAACACAATCAATGAAAAAGAAAAACAACTTGTTCGTAAGGCTGCTAAGGT
TCTTCAAGAGTCTAACATATGGTTCGAACATCTGCCTAATTTTAATATTCAGGAGATTGA
AAGTACAATGGAAAAGAACGTCATTAAAACCAATGTAGAGTACGTTTATTTCGATTATAT
TCACTAATCAGTAACCATTTTTTCAGAGATGAGCAAAATAAGCGGTGTCAACTTGAGGGA
AGACCAAAGTCTTTTACTTATGTCAGATAAGTTAAAAGGCGTATGTAACAAATACGATGT
CTATATGATGAGTGCAACTCAGCTAAATGGTGATTGGAAAGAAGCATGGTTAAAAGGACA
AGTAATAGATGCTTCTTACTTGAGAGGAAGTAAGGCCATCGCAGATAAAACAGATACTGC
AATGATAATTCTTCCTTTAAGTAAAAAAGAGAAGGATCCAATTGATCCAATTTTAAAAGA
AGGATTTTATCCAGAGCCAACTTTTGTTACACATGTATTTAAAAATAGGGGAAATGAGTA
TGACAAAGTTAAAGTCTTCTCCCATATCAATATGGGCAACATGCGGATAAAGGACTGTTT
CACAACAAATCTTGACAATGAACTAATCAGAGTTGAGAAATTGAATATAAAAGCAGGATG
AAGGGTGTAGCGCCCTTTGAAGTATGATAAAGACAGAGTAAAAGAAAGCCTAACCATTGA
GGATATACATAAGATTTTAAAAGATTTAGGTAGAGAGAACAATCTCTGGGATCAACAAGG
AAATCCAATTTACAGAACCGTTTGTCACAATGCTTCTGGTGGGAGCTATAAGCTGTATTA
CTATCATGAAGCAAAACAGTTTCACTGTTATACAGAGTGTGGGGATAATTTTGATGTATT
CGAACTTGTTATAAGAGCAAAAAGCCAAAAAGGAAGTAATATCTCTTTTAATCAGGCTAT
CGAATATGTTGCCAAAATAGCAGGTAGAACATTTGGATCTGGGAATAGAGAGACATACAT
AAACAATGATTTGATTGATGACTGGGAATGGATGGGGAAGTTCAAAAAGAAGAAAAAAAT
ACATATTGAGCTCCCCAGCTTTAATGAAACGGTTCTAGATGTATTTGTGCCTTATCCTCA
CCAATTGTGGCTAGGTGAGGGAATAAGTCACAAGACATTAAAAGGGTTTGAGATTGGGTA
TTATTTTAGACCTCATACAGAATGGGTTACCATTCCTCATCGAGATTTAAATAATAGGTT
GATTGGTATACGTAAGCACTCAATGATTAAAGAAGAAGTTGATGCAGGCTATAAATATAT
GCCTTTAAAAGTAGGCAATATCTTATATAACCATCAAACAACGATGAATCTGTATGGATT
ACATAAAACAAAAAATTCGATAGAAGGGTTCAAGAAAGTATTGATTTCGAATCAGAAAA
ATCAGTATTAAAATGCCAAGACTTTTACGGTGAATCAAACTTCACCTGTGCTGTTTGTTC
AAGTAATATTTCTGATTTTCACCGTGACATTTTATTGTATCTTGGTGTTGAAGAAGTTTT
TATTGCTCTTGATAAATATCGACCACCAAAAGAACATGAAACAGAGGAGAAATATCAAGA
AAAACTGGTTGAATACCAGAAGAAAATTCTAAAGCTCGCAGCAAAATTTACTCCTTATGT
TCGTGTATATGTTTTGTGGGAATATGAAGGCTTACTGGATTATAAGGACAGCCCAGCGGA
CAAGGGAAAGGAAACTCTAGAGGAGCTAATCAGAAGGAAAATTGAGATTGGCACAGATGA
AGGGGGAATTTAATGGAGTATAGACTAATTGGCGACAATGATTATAATTTCGATCCGTTA
GCTACTATCTTAAAAAATAGAGGCATAGAATATCCAAAGTTGTTTGTTAATGTTGATCAA
AGTTCAGTTATGCATTATTCAAAGCTTAATAATATTGATAAAGCTGCAGATTGTCTTATC
AGGCATTTAAATAATAAAAATAAATTGTTTGTTCAGGTAGACAGTGATGTTGATGGATAC
ACATCCAGCTCAATCATTATAAATTACATAAATAAGATTTGTCCGAAAGCTAATATACAC
TACAGAATTCAAGATGGGAAAGAGCATGGGATTTTTATTGATACAATTCCTGATGATGTT
GACTTAGTCATAATCCCAGATGCAGGATCAAGTCAATTTGAGGAACATGAGGCTCTTAAT
AAGAGAGGCACAGAAATAATTGTTATTGATCACCATGAATGTGAACGAGCGTCTGAACAT
GCGATCGTAGTAAATAATCAACTTTCGCCTAATTATTCGAATAAAACTCTGACAGGTGCA
GGAATGGCCTATAAATTTTGCCAGGCAATTGATGAAAAGCTAAATAAAAATGAAGCCGAA
CAATTCTTAGAGCTTGTATCTATTGGTAACATTGCTGAGTCGGCTGATTCAAGAAACCTT
GAGACCAGGTATTTTATGAATGAAGGCTTGAAGAAAATAAAACATCCATTATTAAAGAAG
CTGTTTAAGAAGCAAGAGTTTTCAACCAAGGGTGACAAGAGCATACAGAATACACAGTTC
TTTATTAACCCTTTAATTAACGCTGCCATTACGGTTGGAAGCAGTGAAAAAAAAGATCAA
ATGATGAGAGCATTCCTCCTTTCTAAAGAAAAGGTTCCCTACAAAAAACGTGGCAAAGT
GAAACTGAGCTTGTATCAATACATGAAGACACAGTTAGGATTCTAGGAAATCTAAAAGCA
TAGCAGAAACGGATTGTAGATGCAGCTGTAGTGGAAATTAAAAATTGAATAGAAGAGAAA
AGTTTAACAGCGAATAAAGTACTCATTGTTTACATTGAAGGAATTCTAGATAAAAGCCTA
ACTGGACTGGTGGCCAATCAGCTTGCAGAAGAATATAAAAAGCCGGTCTTGTTAGGCAGA
AACGATCCCGAAAAAGGTAAAGATATCTTGAGTGGCTCTATACGAGGATATGATAAAGGG
TTTATAAAGGATTTTAAAAAAGAGCTTATAGATACTGGATTATTTGAGTTTGTTGGAGGT
CACCCAAATGCTGCGGGCTTTTCAATTAAACGACAGAACTTAATCCTGGTGAACAAAGTG
CTGAATGCAAAATTTAAAGACATAAATATCGAAGAAGATATTCAGAATGTTGATTTTGAG
ATCCCAGCAAAACGATTGACAAAAGAATTTATCCTTCAACTTGATGGTTCCAAAGACTAT
TGGGGTTACAAAGTTGAAGAACCCTTAATAGCTATAACGGATCTTGAAATTGAAGTCGAA
CAAATTGAGCATTTAGGGAAAATGAATAAGACAACTGTTAAATTTAAGCATGGCGATATT
GAATACATAAGGTTTAAAAGTGATGAGAATTACTTTAATCAGCTTACTGCATCAAATGGA

ACGTTAGTCATTAATGTAATTGGCAAAGCAAAGGCAAATGAATTCAAAGGCAAGAAAACA
CCTCAAATCGAGATTTATGAATTGGAGGTGGTTCGCACAAAACAAAAAGAACTTGTGTTT
TAAGGGGGAGAAAAGTTGATAGGATGTCACTGTCACACAGATAGGAGCAACATAAGGCTT
CTAGACTCTACAAACTCAGTTAAAGAACTGCTCAAAACTGCAGTAAAGATGGAATATAAG
GGATTGGCCATAACAGACCATGAAGTCCTCTCAGCACATTTAGATGCTATTCGAACAGTT
AGAGAAATGAAATAGAAGGGGGATATGCCAGAAGATTTTAAACTGATATTGGGCAATGAA
GCGTACCTAGTCGATTCTTTAGAGGAAGTTCGAGATAACTATAAATCGGGAGTGACAAAA
TTTCCGCATTTCTTGATGTTGGCAATGGATCCAAAAGGACATGAGCAGTTAAGAATACTG
TCTTCTCAAGCCTGGGAAAACTCATTTTATACAGGAACAATGGAAAGAGTGCCGACAGTA
AAAAAGGATGTAGAGGAGCTATTAAGTAAAGATCCAGGCCACATCATTGCTACCACAGTT
TGCTTGGGGTCTGAGGTAAAAATTCACCTGTTAAAGATAAAGGCTTTTGAAGAAACTGGT
GATTCTCAGTCAATTAAGCAGCACAAACTAAAGATTCATGAGTTTATAACTTGGTGTATA
GAGGTTTTTGGGAAGGATAAGTTTTTTATCGAGCTTCAACCTGCACTGAGTGAAGAACAG
ATTTACTGTAACAAGAAGCTGATAGATATAGCCAACGGGTATGACTTGCAAATGATTGTT
ACAACAGATGCTCACTATCTAAGACCAGAGGATAGAGCAATGCATCAAGCCTTTTTAAAC
GCTAAGGATGGAGAAAGAGAAGTTGATTCCTTTTATGAAGGCTTTTTCGTTCAAAACGTT
GATGAAATTCACGAGAGAATGGACTACATTGATAAAGAAGTCATCGATCAGGCCATAAAA
AATACAATGCTCATTGGTGAGATGATTGAAGAATATACTATAGAGCACGAACCAATTATC
CCTAAGATGGAGCTTCCTAACTTTAAATTAAGACATTTATTTAAACCAGCATGTGATCAA
TATGAATATATAAAAAAGATGTCTGAATCAGCAGATGAACAAGATAGATATCTCCTTAAG
TTAATTGAGGTCGGATTTGAAGAGAAATTAAGAACAAGCGAACTAACGAGAGAAGCTTTT
CATAAAATATTGAACAGGATTAACGTTGAACTGGGTGAACTTTGGGAAATCAGCCAAAAG
CTGAATCAGTCTATGTCTTCTTATTACTTAACAGTTAGAGAAATCATTAATATTATTTGG
GATGATCAGTGTGGAGGAAATAGTTTAGTTGGGGCAGCCAGGGGAAGTGCTGCAGGTTAC
TTAGTTAATTATCTACTCGACAATACTCAAATTAATCCAATGCAATATGATTTACCACAT
TGGAGACATATACATAAATCGAGACCTGACATACCAGATATCGATATTGATACTGAAGGA
TCAAAAAGACAAAAAATTCTTAAGGCACTAAGAGAAAGGTTTGGAGACAAGCGTGTTCTT
CAAATTGCTACTTTTGGAACTGAGGGTTCAAAATCAGCGCTTCAGACAGTATGTAGAGGC
TTAGGAATCGATAATGATATATCCCAGTATTTAAGTGGAATGATTCCATTTGAAAGAGGA
TCTAACTGGCCTTTGAAGCATTGTTTTTATGGTGACAAAGAAACTGGCAGGAAGCCAATT
AAAGAGTTTATTAGGGAGGTTGAACAATACCCGAATCTTAAAGAAACAGCTTTAAAAATT
GAAGGGTTAACTAATAAGCGCTCTTCTCATGCAGCCGGAGTGATTATCTTTAACGATGAA
TATACAAAGTCGAATGCAATGCTGAAAACTCCTAAAGGAGCTTATATTACACAGTTTAAT
ATGGGTGACAGTGAAGCCATGGGCTCAGTAAAGTTTGACCTTCTTACAATTGAAGCTTTA
GATAAGATTAGAGTAACATTAGACCAATTAATCGAGAATAATGAAATTGAATGGCAAGGA
AGCTTGAAGGAAACATTCAACAAATACATCCATCCAGACGTAATTGAGTATGAAGATGAT
AAGCTATGGGAAATCGCTGGTAATGGGGAAATCATGGATTTATTCCAGTTCTCAACAGAA
GTCGGTCATCAATCAGTTGTCAAAGTAAAACCTAAGAACTTACTTGAAGCAGCAGTAACC
AATTCTTTAATGAGGCTTATGTCAGACGGTGAAGAACAGCCTGAAGATACATATGTTAAA
TACAAAAATAATCTTAATAAATGGTATGAAGAAATGACACGGTACGGTCTAAGCGAAAAG
GAAATCAGAGTAATGGAAAGGCACCTTAAGGACATTTATGGTGTTGCTGATACTCAAGAA
GTGGTTATGCAGATGGTTATGGATAAAGATATAGCTAACTTTGACATTAAAGAATCAAAT
TATCTTAGGAAATCCATAGCAAAAAAGAAAGAAGATGTACTAAAAGAAGTGGAAGAATTG
TTTTTTAAAAAAGGAAAGGAGATTGGCACTTCTGATAACCTGTTGAATTATGTCTGGAAT
GTTCAATTTAAAAGACAGTTTTGCTACAGTTTTAGTTTACTTCATACCTTGGCATATTCA
ATTATTGCAGTACAGGAATTGAACTTAAACTATCGATATAATCCTTTATACTGGAATACT
GCTGGTTTAACGGTAAACAGCGGAGGAATAGATACCGAGGATACAAAAGACAACAAAAAG
ACAGCTGCTACATACTACGGAAAAGTTGCTTCAGCCATCGGAAACATTAGACAAAGAGGG
ATTAAAATAGACCTCCCAGATATAAATAAGGCTGATTTTGGTTTTAGGACTGATATTAAC
AACAATTCAATTTTATTTGGACTTAAAGGAATGAGTCGAATTGGAGACGATGTTATTCAT
CATTTAGTTCTAAATAGACCATATAGTGACTTTAACGACTTTATTGAAAGAATCTTTAAG
AGCGGCATTATTAAGAAAGGACAAGTAATCCAATTAATAAAAGGAGGCTGCTTTGATTCT
TTTGGAAACAGGCAAGAAATCATGAAGGCCTTTATTAGCTTAATATCAGAACCAAAAAGT
AAGCATACGTTGTCTAATTTAAAAATGCTAATTGAAAACAACATTGTTCCTTCAGAATTT
GCACAAGAAGTGAGATTCTTTCGTTTTAAAGATTACATCAGCAAAAAGGTGTACAAAACA
TTAAAGTCGCCAAAAGATAAACTTTTTTTATTGGACGATGTATCAGCTTCGTTTTATAAC
CAGCAATTCCGTGAGGATAGTGTAGTTGACATGTTAAACGGGCAGCTTGTCATTTCTGAA

AAAGCTTTTAAAAAAGAATGTGATAACAAGATGTCTAATATAAAGTCCTGGATAACAACT
GAAGAACCACTCAAGAAATTGAATGATTGTTTATTAATAAAAGAGTGGAAAAATACGCC
GATGGGTCATTACGAAAGTGGAAATGGATTCATTGAGCTATTATTATAATGACCATGAG
CTTTCTGGTGTAAACTTTGCCAAGTATGATATTGCTGACTTTTATAAACTGCCAGCAGAG
CCGGTCAAAGGTAAACCTTATCAATGGAGAGGGAAAACTCTCTATGAATATGAGACTACA
CGGATTATAGGCACTGTTTTAGATAGGGATAAAAACAAACATGCAATTACTCTTTTAACA
CCTACAGGGGTGGTTACAGTTAAACAGTGGTCGGGCAGGTTTAGTCATTACAATAAACAG
ATTTCTAGATCCATTGGTGGCGGAAAGAAAGAGGTAATCGAGACATCTTGGTATACCAGA
GGAACATTGCTCATGTTTACTGGTTTCAGAAGAGGTAACAATTTTATTCCGAAGGTCTAT
AAAGATAGCATATATAATCACACAGTCTGCAGAATAGATTTTGTTGATAATGAAGGAAGT
ATGAGTTTGACAACTAAAAGGGCAGAAATATAAATCTTAACTGATATAGGAGAGATAAAA
ATTTTCAAAAAACTTATAGACAAACATAAAAAATATGTATATCATAGAATTAACAAAATG
GCACTTTATGCTACAATTGGTTTGTTAGGAGTAGGGCTCGTATATAGCGCTAAAAACTTG
TATACACATCAAGATAATCAAGTCTCAATAAAAAAGTCATTTTATTTAAATAAAAAAGAG
GTGAGGCAAAAACTTATTCACGAAATTGACGTTCCAAGAATCCTTCCTAGGCTAAATAGT
GAGGAAGAAAAGCAGGCTGAAAGTAGAAAAAAGGATCTTAATGCGACAATTACATATCTA
ACAGAAGAAAATAAAAAAGCAGCAAAACATACAAAAACAAAAAAAGTGCAAAAAACCAAT
ACAAAGAGAAATCTTGATAAAGCAGTTTCAAAAGGTACAAACGCAAAGGCAGTGAAGAGT
CATGACGTTGTTGCTACTGCTTATAGTGCGTTTTGCTCGACAGGGTGCACAGGGAAAACA
AGAACCTGCTACGATGTATCAAACACATCCTATTACAATGGAAAAAGAATAATTGCTGTT
GATCCAGAAGTAATTCCTTTGTATTCATTAGTGCAAGTTTCATATGAAGGAAATAGCTTT
CAAGAATATGCAATAGATACAGGAGGGGACATCAAAAACAATCGTATTGATATTCTAATG
GACAGTGAGCAAGAAGCAAATGCATTTGGTCGTAAAAATGTAAGAGTAAGCTGGTAAATC
GATCCAAATCACTGAAAGGTATAATCATTTCAACAATTCGTTCGATGCAATACATAACAT
GAAATTTTTCCCAAGGAAAACTATTACATAGAGAGTGAAGAGGATGTTCATTTTGGATAA
AGAGGCTAAGGTAAAATCAACTGGAGAGCGGGGGATTATTGAAGCGATCTATCCTGAAAC
AGAAACAGTAGAACTCTGTTATTATGATGGAACTTATGATGAAAGACGTTTTGATGATGT
TGTTATGGCAACAAGCAGTTAAAGTGCATGACAAAAATAAAAAATAGGGTGAGATAATTG
AAGTGTATTCAAATTGAAATGTCATTCACAGACGAATATGGACAGGTGACCCGATTAAAT
AAGACTTATAAACCGTCGATTATTGAAGAACATAAAGGGGAAATCCCTAGATTGTTGCTA
GATGATTTTAAGAGGTTCTTGTTGTCCCTTGGGTTTAATGAAAAACAGGTTTCGAGAATA
GTAACAGAAGATTAAGAGGTTTTTTATTGAGGGGAGGTGGTATTAAATTGCCTAAATTCT
GGAGTTATCCTGTTGGACTAGCTGTAGAAATTAACAATAATGCACGATATGGATGCGCAC
ATCATGTGGGGCGAAAAGGAAAGATTATCGAGCATTTACATTCAGCTACATATGACTATG
CAGTTAGCGATGAAACAGGTGACATTACTTACTTTAAAGAACATGAATTAACACCACTAA
AGGGAGGTTTAGCTTATGTTTTAAAAAGGTCAAAAGGTAATTGTTGATTTTACAGATGAG
ATTGGAGCTGTTTCGAAAGTTGATTATCGATACAACCAGGTAGAAGTAAAGTATCCTGAT
GGCACTTACCAGGTTGTTGGATTTCATAAAGTTAGAAAGGTGGAGGATTAATGACATTTA
TTATCTTGGAGGGGCCTGATTGCTGCTTTAAATTAACAATTGCAGCAAAGCTAACTAAAG
TACTGAAGTATCCAATTATCAAAGGTTCAACCTTTGAGTTGGCCAAAAGCGGAAATGAGA
AGTTATTTGAACACTTCAATAAACTAGCTGATGAAGACAACGTGATTGTTGACAGGTTTG
TTTATTCTAACTTGGTATATGCAAAGAAATTCAAAGATTACTCGCTCCTTATAGAGCAAC
AGCTTAGATTTATTGAGGATAGAATTAAAGCAAAAGCGAAGGTTGTATACTTACACGCTG
ATCCAAGCGTTATTACGAAAAGGTTGAGGGTACGTGGAGATGACTATATAGAGGGAAAAG
ACATTGATTCAATTTTAGAGTTGTACAGAGAAGTTATGAGCAATGCGGGATTACATAAAT
ATTCATGGGATACTGGACAATGGTCAAGTGACGAAATTGCTAAAGACATAATCTTTTTAG
TGGAACAGGGGAATTTATGAAAAAAGTAATTGCGATTGACATGGATCAGGTCTTAGCTGA
TTTACTAAGTGATTGGGTAGCTTACATTAACACATACGATGATCCTTTTCTCAAAGAGAA
AGATATCCTATGTTGGGATATAAAAAAATATACAAATACCAACAATAATGTTTATAGACA
TTTAGATTACGATTTGTTCAGAAATCTAAATGTTATAGAAGGGAGCCAAAGAGTGACAAA
AGAGCTGATGAAGAAATATGAAGTGTAGGTTGTGACTACAGCAACAAACCATCCAGATTC
CCTTAAAGCAAAGCTTGAATGGCTTACAGAATATTTTCCATTTATTCCTCATATCAATGT
TGTGCTTTGTGGCAATAAAAACATAATTAAAGCAGACATCATGATCGATGATGGAATACA
TAACTTAGTATCATTTGAAGGAATGAAGATACTATTTGATGCTCCCCATAACAGGAATGA
AAATAGATTTATTCGTGTTATGAATTGGGAAGAGATTGAACGAAAGTTACTTTAAAATAT
CTCATTTATCAAGAAGGGAGAAAACAGTGGAACGTTCAATTAAAAGACTGTCAGCTTCTT
GTGAGTCGACAATTCATCACAAGGTATATCAAATTATGAACGAAGCAAAAACAGAGTTTG

AAAAAGTGCTAAAGAAATTAAAATAAACGATCAATTATATTTAGAGTAAAAATAAAATAT
ATGGAGGTTGTTTGTTGAGTAAACTACGTGTAATGAGCCTTTTTAGTGGAATTGGTGCAT
TTGAAGCTGCATTAAGAAACCTAGGCGTTGATTATGAACTGATTGGCTTTAGTGAAATAG
ATAAATATGCAATCAAATCTTATTGTGCAATTCATAATGTAAGTGAAACATTGAATGTTG
GAGACATAAGTAAAGCTAAGAAAGATAATATTCCATATTTTGATTTGTTTACAAGTGGAT
TTCCTTGTCCCACTTTTTCAGTAGCCGGCGGCCGTGATGGTATGGAATATAAGTGTAGCA
ATTGTTCGCACGAGCATTTAATTACTTATGAGGATTATAAGAAGGGAGTCATATGCCCGA
AGTGTGAAGCTGTTTCTAAGGCTAAAGATGAACGGGGAACGCTCTTTTTTGAAACAGCTT
TGTTAGCAGAAGAGAAGAAGCCGAAGTTTGTGATATTAGAAAATGTAAAAGGGCTAATTA
ATAGTGGCAATGGACAAGTGTTAAGAATAATTAGTGAGACTATGAACAATATAGGCTATA
GGATTGACCTAGAGCTACTTAATTCAAAGTTTATCAATGTTCCAAAGAATCGGGAACGGG
TATACATAATTGGCATTAGAGAAGATTTGGTTGAAAATGAACAATGGGTTGTAGGTCTGA
AGCTAAACGATGTTCTGAGTAAAGGTAAAAAGAGATTGCAAGAAAGAAATATAAAGAGTT
TTAATTTTAAATGGCCTTTACAAGACGCTGTCACAAAGAGGTTGAGAGAAATTCTTGAGG
ATTTAGTTGATGAGAAGTATTACTTAAATGAAGAAAAGACGAAGAAACTTGTTGAGCAAC
TAGGAACCGCACCGCTACAAAAACAAGAAGTAAGGGAGCCGTTAATGGTGGGACACGTGG
ATTCAAAAGGTCACGATGCCATCAAAAGAGTTTACTCGCCTGAAGGACTTTCACCGACAT
TAACTACAATGGGCGGCGGTCACAGAGAACCTAAAATCGCAGAAAAGCAGAAAGAAGTCA
GGGCAGTGTTAACGCCAGAAAGAGAAGAGAAAAGACAGAATGGAAGGCGTTTTAAAGAAA
ACGGAGAGCCGGCTTTCACGGTTAATACAATTGATCGACATGGTGTAGCAATCGGAGAAT
ACCCAAAATATAAAATTAGAAAGCTCTCTCCATTAGAATGTTGGAGACACCACGCATTTG
ATGACGAAGATTTTGAAAAAGCTTTTGCAGCAGGAATAAGTAACTCACAATTATACAAGC
AAGCCGGTAATTCAATTACAGTTAGTGTGCTTGAGTCTATATTTCAGGAATTAATACATA
CATATGTTAATAAAGAATCTGAATAAAATTTGTCTTTTAAACCAATATAAAATAAGAGGA
GTGGATCGATTGGATAGTTACCCAGAGTCCCTAAAAACAGAGACAGAGGAGATTAAAGAG
CGTGTTAGGAATGGAAATATCAAAGAAGACAAGATTAAAGAAATTGCAGAAACGACAGTT
GAGTTTTTGAAATCAGAGGAGAAAAGACATAAATACTTTTCTGAAGTTGCTGCAGCTATG
GCTGATAACTTAAGTGAGTTTTTCAAGTCGTATTTAAAAGGAGAGTGAATATGTTAACTG
ATCAAGAAAAAATTGACTTGGTAAACGCTCTTGATTTTGTAGTTATTGAACCACATACAC
AAAGCATTTACGTACATAACGAAGAAAAGACTAATGGAGTATTAGCTAAGGTTTTGCACA
CTATTTCAGTAGATGAAAATATTGAGAGCTTTAAGAAAGGTAGTCTAATTGATATCTTTC
CAGCAGCAATGCAAGAAGCCGGTGCGGAAGGATTTAAAGATGGCCAGTTTGTGATTATGC
CAAAGAAATTTTATGTTGATCAATGTTAGGCGATGAGTAAGGAAACCGAGCGGTTAACTA
ACCTAAACAATCTACACAATATTAAACCAAATACATATCAAGGCTTGATTCATTAAATTG
TTTCTAGAAAAAATTAAAGGATAAAGGGATGTTTATTGAGAAAGTATTATGTAAGTTGTA
AAGACAGCAAAGGTGAAAATGCGTCTCTAGTTATTGAGGCGCTATCACCTGAACTTGCAA
AAGAACAAGCATACGAAGTTTATGAGGTAGGGGATATTTATAATGTAAGCCTGGGCGAAG
GAAAGTCGAAGAATTGCCTAGACCGAAAATATTCCCCATACATAAAAAATGACAATGGAA
AAGCAATAACCATATTTTCATAGGGAGAGGGTATCATTAAGGATATTGTTAGGGATCTTG
TAATTGATGATATAGATGCTGCTGAAGGAGTATTGGATAAGCTAAATGTTTATCTAGAAA
ACACTTTAAGGCCTAAAGAAATGCGTGTTTGGGAGCGGTGTGATAAAGATATGATGTCTG
TTTTTGCAAAGCTGAAAAATAGTAAACAAATTATTTAAAAACCGATAGACAAAAACAAAT
AATATGTATATAATAAATTTACGTTGAGGCGAAGGAGAGTGTTAATGAATCATATATGTG
ACATCTGTAAGGAGTACATAAGCGGAAAAACAATTTGTCTTAGGTTCAGCGATGAAAAAA
CTTATGTAGACTTCAACTGCTGTGAAAGTTGTGCAAAGGGTTATTCCGATCACGTGAAAA
ATGACTGCAGCAATTTAAGTGTTAAGAAGACAGTAGAACATTTAGGATTAAATATCAAAT
ACAAAATAAGAGGATAAAATATTCCTTTTATACAAATGTAAAAATAAAAAACCCCATAAG
AAGTTTTATGGCGTCTATCGAACAGGGAGGTGCTTCAAAGGCTCTGGATTTACTCTGAGA
TTAGTATAGGAATGTAGCACCAACAATGATCAATAGGATGAATAACACCACGATCAAGAC
GAAGCTGCTTCGGTATCCGCCGCCAGAGTATCCACCAGAATATCCGCAGTAGAATCCCAT
ATTGCCACCTCCTTTAGTAAGGATAGTATATGGTATGGCAAAGGTTAAAAATTCGACATA
GATTTCAGTCTAAAAAAGAAAAAATGAAGACTTTGTCTAAATTTAAGAGTTGAATAAGGA
GAGTTGATGAATGAACATGTTTGTAATTGCTGCAAAGAGTGAAGGGAAATATCTCTATGG
TTCCCACCCACATATTTACTCAAATCTTAAACAAGCAGAAAATGCATTGAAGACAATGAG
AAAAAACGGAAAGTTAACTGAAAGAGACAAGGTATATGGACTAGACGGTCTGATGTTGGT
GGATTTATAAAAAGGAAAGATATAGATGGGGAAATATGATCAAAGGAAATACTAATGATT
CCCCGGGCAAGCGCAGTATACGACAAATTAAAACAAGCAGATAGAAATATTAGGGGAGAT

GAGAGTATGCAAAACTTAGATGCACCGATTTACGAAGTTAAACAGGAAAGTGACTGGTAT
AAATCTGAGAAGAAACGCAAAGAGGATATAAACTCTTTCTTTGATAAATTCGGAGAAAAG
TATGGAGTCAAGGAAGGCTTCTCGTTTTATCACTCTGAGTACTTTGGAGTCTACGAAGGA
ACAGAAGCGTATGAAATATTTAAAAATGACATCGTTAAAAATCATGTCGACGGATTCTAT
GCTTTCAAAAAACGATCTAAATAATTTAAAGAAATAAAAGCTATGATTGAGCAAATTGAA
GAAGTGTATCCATTTAGGTCTCATGACGAGCTGGGGTTAAACAACATGACTGGTCCTCAA
TGGATAGGTGTCAGGTGGTTCTTTGGAGTTAAAACTGAACAGCTTGTTAATGGAGACAGT
GTGGTTGCTACTGACTATAAAGATTATCTAAAAATTGTAATGGATCATTGAGATTAAAAT
TCAATTTTTAAAGAGACTTATAAGGAGGTGAATAAGTGGGACGCCATAAAGCAACGATTG
AAGGATTGGTTATGAAAGAAAGGTATTACTCACACCGTGCCCCAGGCACAGAGAGGTGGA
TTACTCAGCCAGTGTGAAAGGTAACTCGTACTGAACCAATCTTCGAGGGCTATATAGATA
TTGAGCCTATAGAAATTGGAGGCAAAGTTTATATCCCTGGACTTAATGAATATGTCATAG
TAACTGACGGGCAGCGAAACATACATAACAAGTGGACATATCAAACAGACAGAGTTATTA
AGACAATTATAGATGAAAAAAGCTTGAAGGAATGCGAAGAACACAATACTAGGAAAGCAA
AAAACAATGATACTCAAAATCAAAGACAGATAAAAACTTCCTGGTTGCAAAGGCTAACTA
AAAAAGACTATAGGAGAGATATTTATTGAAGAAGGATACAAAAGATATTTGGAGCGGATT
CTCTATGGGGTCAGGTTCGTTAATTGTAGTTGGATTACTTCTTTTTGTTGAAGCGTTGAC
TATGTCACTAATTGTTTATTACGGATTGAATCATGTGATAAATCCTTTGCTTATTGATAC
ATACAATATTCAAAATGTCCATGTCACTTTACCTCATGCATTTGTTATTGGTGTTTTACT
CAATGTATTTGTCAAAGGTGTACAACGGTCAGATCAGGAAAAAGATGAGAACATTTTCAA
GAAAGCCGGTAAGTCTTTATTTCATTCAACTTTCGCATTGATTGTTCTATAGGTTAGCAC
ATTGTTTATCTAAGGAGGCACTCAAGATGAGTGATGATAAGGCTGCAAAGATTATTTTCA
AAAACAAATCAACTGGTAAAATTGTCGGCGAAATGAAAGCTACATCTTTAGCTGCTGACA
CCATTTTGCAAAACGACAATAAGTCTAAATGAGAGTTTTATTTTAAGCCGAAATTTTCAA
AAGGAGAGTTAGATATGATCTTAAAAGAAATTGCCGTATATGAAGAAACACTGAATAAAC
ATTTACCAAATGAAACAAGAATGGTTCTTCATTACAAAAATGTAGAGGCACCTGAAAACG
AAGATAAACTCACAGTAAGGATTAGTAATGATTTATTGGTTTATAAATGGGTTGAACTAG
GCGGTGTATCTAAGGGCACTTATAAAATGAAAATGACTAGTCTAGAAGATGAAACAAGCG
AAGTTAATGTGAAAATCGAAAGAATTCAAATGAACTCAGACAGAGATTTGGCGGTAGAAA
TAGAATTTAATGCGGTCAATCCATTCCAGAGCGTTGTGGGTGTACCTGGTATTGATTCGT
TCTCCATAAAAGGATGAGTTTAAAGGGATTAGTGGATTCTAAATCTAAACAATAAATATT
AAGAGGAGCATTGTGCTATGGGAATTAAGGCAGCAGTTTTTGAAATAAAAGCAACTTGTT
ACGCTCATGAAGGATTTATTGACGAAAGTCCATCTGTTCAAGGAGCAGCGTTAGAACAAC
TAGGCAAGGATATGGTAGATCATTTGCTCGAAAACGGAGTTGATGATGTGAAAATTAAAG
GCGATTACGTTGAGGAATTGGAAGTTGAAAAACCCATTATGAAGTATTTTGAAGTGTTTG
ACCCCTATTATGCATTGATTAAGGCTTATACAAAAGAAAAGGCAATGGAACTGTATACAG
ATACTGTTACAGATGATGATGATGGAGTGTTAAGTGATGAAATGACAGAAGTTGGGCAAG
TTTATGCGGCGATACAACATGGAAGGGCGCCAGGAGAAGATAAAGAGCACATGCCGTTTA
AACAGGTTCTTGAGGAGATTTCTAATGACGAAGAGATGGTATTGCTTATTGATGGCAGTT
TACTTTAAAGAATAAAAATAAATTAAGAGGATGATGAAGATGAAAATGAAATATGGACTT
TATTGTATGGGATCCCTTGTTAATACTTATGATGATGCGATTGAGGCTCATAATGATGCA
GTATAAGCTCAAGAAGAAAGCGGAGTACCGCATGAAGTAAGAGAAATTCCATAAAAATAA
GAGGAGGATATTATATGATTCAAGGGTTTATAAAGATCAGAAACTTCATCTTCTAGAAG
ATCCTATACAGCAGTACACCCTCATGAAAGTTGAAGAAAATGCAGTATGTGTATACCGGT
GGATCGATGATTATAGACACAAGATTGAAAGATTCACAGATGCTGAAGAGACTAAAAAAC
TCCTTGGAGAAGGGTGGCCGAAACAATAGAAGACACAATGAAATTTATACAGAAACAATT
GGAGGACGAATAGATGTCGCAAAGTAATTATAGGCCGTCAGTTCCTAGATGGGTTGGCGA
TATACTAGAGCTAGACAAGAAAAGAAGACAAAATCAGTACAGAGGCTCACTAACGTCAGG
TCAAGAGAAGAAGGACTGGGACGAGTGGAAGCGTAGATATTCAAGAAAATTAAAGTACGC
AAGATTAAACGGATGGACGATCGAAGAAGAGTGACAATTCCTAAGGAGGACGAATAGGTA
CTGCTTGAATCTAGATAAAATCACAGCTTTAATCAAAATACAGAAAATAAGGAGATGTAA
AATGGGGGCAGCTAGACGTATTGATCCAACTCAACCATATGTAAAAACGAAGAACATTAT
TAACTTTACAGTAGCCACTGAGAACACTCATAATCATTTAGCTGATGGGCAATCTTTCCC
TGTATTAAAAGGAGATATTATTGCAACTGACCAACAAGGAAATCAATTTGTTGAATTAGA
AAAGAATCTAGATGATTACGTTCCCGTTAAGAAAAGTTCCTTAGATGAAAGTATGGCACA
GGGCTACATGGAAATGGGGGACATTAATCATGAGATATCAGAAGCATTCAATTACGCTGA
AAGTGAAGCTGCAACAGTGACTACAAAATTAATTACAGGGGCTTATAACGGTTAGTGATC

ATTACATATGAGAGTAAAACAGGAAATGTAAAAAGGCTTGTCAAAGCGCTGCAACAAGAG
TTTGACGTTGAGGCAATTGAAATCACTGATGATACGATCATCAATCAAGGATTCATACAT
ATTACATATACGATAGGCTTTGGGGAAGTACCTGAAAGGACTTTGAGTTTTATCAATAAG
AATAAAAATAAAATTAGAGGAGTTGCTGTTAGTGGTAACAAGGTTTGGGGTGATAACTAT
GGTTTAGCTGGAGACAAGCTTTCAGCTAAGTTCCACACACCATTGTTATTAAAGTTTGAA
CTTAGTGGAACGAAACAAGATCTACAGAAAATCATTCAGGAGGTACAACTTATTGACAAA
CACAATACCAAATTGGATCAAGCTCAATAATGAAATCATGATCCAGAAAGATGGGAAGTA
TCAATTTGAAAAGGATAAGGAGGCCGTACATAGTGACTTCGTTGATTACATAAATCAAAA
CACAGTCTTTTTCCATGATCTGAAAGACAAGCTGGACTATCTGATTAAAAATGATTATTA
CGAGGAAGAATTCTTAAGCAAATATACATTCGAACAGATTAAATCAATCTATAAGATTGC
TTACAGTTACAAATTCAGATTCCCTTCTTTTATGAGTGCCTTTAAGTTCTACAATGACTA
CGCATTGAAGACAAACGATAAAACAAAGATCCTGGAAAGGTATGTGGATCGTGTCTCAAT
TGTGGCTTTATATTGCGCTGATGGCGATTACGAGAAGGCTGTTGAGGAAGTACATACTAT
GATGAAACAAGAGTATCAGCCAGCAACACCTACTTTCCTTAATGCTGGACGTAAGCGAAG
AGGTGAAATGGTGAGCTGCTTCTTACTTGAAGTAGGCGACAATTTGAATGATATTTCACG
TGATATTGATATTTCCATGCAGCTTTCTAAGCTAGGTGGAGGAGTAGCATTAAATCTAAA
CAAACTAAGAGCCAAAGGTGAGGCGATTAAAGACGTAGAGAATGCGACTAAAGGTGTCGT
AGGTGTTATGAAACTTCTAGATAATGCCTTCAGATATGCCGACCAAATGGGTTGATTTGG
CCCCTTTCATCAGCAATGGTGATCGAAAACCTCTTTAATGCATGGGAACTCCTACAGGGA
CAATCATGAGCGAAGCAAGACTAAGTCTTGAACGTGCAACGACTAGCCGAAAGGCGTAGG
CTGCAAGCTATTGGCAGTCGAGACAGGAGGCACCCTTAGAGGGTGAAGATATAGTCTAAC
CTTCATGTTAACATGAAGCAGCCATATGGCGGGACGTGCTTAGCGAACACGTTTGAATGG
TCTCCAAAGACAAGGATCAGGAGCAGTTTATCTAAGTGTATTCCATCCAGACAATACAGA
CTTCCTGGATACCAAAAAAATAAGTGCTGATGAAGATGTCCGAGTTAAACCACTGGCTAT
TGGCGTAGTTGTTCCAGACAAATTTATTGAATTGGCAAGGGAAGACAAGGATTATTACAT
GTTCTATCCGCATTCAGTATACAAGGAGTATGGGCAGTATCTAGATGAGCTAGACATTAA
TGAAATGTATGATGAGCTTGTTGAAAATCCAAGAGTTAGAAAAGCAAAGGGAAATGCTCG
AAAGTTGTTGAAGCAATTGGCTATTCTAAGAAACGAATCTGGCTATCCGTATATTATGTT
CGCTGACAATGTAAATAAAGTGCATCCAAATGAACATATTTCAAAAGTGAAGTTTTCAAA
TTTGTGCGTTACTGGAGAAACACTTCTTTTAGCTGAGAATGGATATGAAAAAGCAGCCGA
TCTTTATAAAAAGCGGAATGATCTTAAAGTGGTCATTGATAATAGAACAAAAGATTTTGC
AGTTGGTAGCAAAGGTACCACAATTGTAGATGCAATTCCAATGCAACTGACTAAAAAAGA
TGCGGAAATATTTAAAGTCAAAACGAAGCAAGGATATGAAATTAGGGCATCTGAGTGGCA
CAAATGCTATGTGAAAAGAGATGGTGTAATTCAAAAGCTCCAATTGAATCAACTGAAAAC
AGGCGATAAATTATTAGTCCAATCAGCTGAAGGAGCATACGGGAAAATTCATGAACCCGA
TCTTGCATACATTATGGGCATTATAGCTGGTGATGGAACCATTACTGTAAAAACTGCCAA
GATTTATTTATACGATAATAAAAAAGTTCTTGAGCAGAAAGTTACTGATGCAGTTCACCG
GATTATTCAAAAACACAAGGTTGTTCGTGCTTATAAGCACAATACATCTCTTCTCCCAAC
ATTTAATATGGCCAATCCAGAAAAACAAGATTTGCTCTACATGAATAGTACTGTCTTATT
TGACATCTTGAAGAAGTTCGGTATGAACAAAGAAACAAAAACAAGAGTCCCAGAATTCAG
TTTTCAAGCTAATAAGGAAACGGAAGCAGCTTATTTATCGGGTTTATTCCAGACTGATGG
GTGTGTAAATGCAAATCATAAAGCCAAAGCATTAACTATTGAGTTAACATCCATCCACTA
TGAGAGCCTTCAGGACGTTCAGAAGCTGTTACTGAACATGGGTGTTTATACAACGATTTA
TTCTAACAACAAGCGCTCACAAGAACTCCTTCCTGATGGAAAAGGTGGTTCTAAGCTTTA
CAATGCCAAACCAGCACACAAAATCAGTATTTAAGATAGAAACTCAAGAGAGTTGTTTAT
GAGTATTGTTGAAATGAAAGAATACGATGTTTATAAATTCAATTTGTTAACTGAGACATT
GCAACCCAAGTCAAGAAAGCCAAAGCATGATTTTACAGCTGAAATTATTAGTATTGAGGA
AGATGGTGTTGAGGATGTTTATGATACAGCACAAGAAGACTATCACTCTCTGATTTTCAA
TGGAATTGTTACTGGTAACTGTTCAGAGGTACTTCAATCGTCACAAGTATCAGTTTATAC
GGATTACGATAAAGAGGATGAAATTGGCTTAGCTATCTCATGTAATCTTGGCTCAATGAA
CATTGTAAATGTAATGAGTAATCAATCAATTGCTTCAACAGTCAGAATAGCAATTGACTC
ACTGACAACTGTCACAAGGAAAACAAACATTGTAAATGGTCCAGCAGTTGCGAGAGCAAA
TACATTAATGAGATCATTTGGTCTAAGGCAGATGAACCTCCATGGATTTCTAGCTTAAAA
TAATATTGCTTATGACAGGGAAGAAGCTAAGGATTTTGCAAATACATACTTTATGATGGT
TAACTTCTACTCACTGCAGCGTTCTATCGAAATTGCACGGGAAACAGGGGAGACATACTA
CAAGTTTGATGGTTCGACTTATAAATCCGGCGAGTATTTCGAAAAGTACGGAACAAATGA
TTATAGCCCTCAGTATTAAAAGGTTAAAAAGCTATTTGGAGATCAACATATTCCTAATAT

TCAAGATTGGATGAAACTTAAAGACGATGTAATGAAATATGGTCCTTATCATTCCGATAG
ACAAGCTATTGCGCCTACGGGAAGCATCTCATATGTTCAATCATCTACAGCAGGTGTAAT
GCCAATTATGGAGAGAATTGAGGAACATACATACGGAAACAGTAAGACATATTATCCGAT
GCCAGGTTTATCGGCTCAGAATTGGTGCTTCTATAAGGAAGCATACGACATGGATATGTT
TAAGGTAGTTGATCTTAATGCAACAATTCAGCAGAACGTCGACCAAGGGATTTCATTTAC
GTTGTTCTTAAAGGATACGATGACGACGAGAGACCTAAATAGAATAGACCTCTACGCACA
TCATCGTGGAATTAAAACATTATATTATGCCAGAACCAAAGATACGACCCAAGAGGGATG
CTTGTCCTGTGTAGTTTAATAGGTACGTTTACGTTTTGGTTCAAAATTAACAGGATCTCC
CGGACGTCGTTTGCACTTAATTTTGTTAAATTTTCTCATAAGAGCATTACAAAGTAAACC
TAATATAAGCTTCAAAATATATTCGATTAAACATGATAAAAGCCTATCACACAACCAGCC
AGTTGAAATGACTGTACTCTCTAACAAAGATATAAGGTTGTCTAAAAAATTCACTGTCTT
TCATTCCTTTCGAATGTTTGACAGTAATATAGGAGTCTGTCAATTCAGCTTTTAAATTCA
CTGCATTTTTTTCGGAAAAAAATATTTCAATTGAAAGGACATGATTAATTGACAAAAATT
TATGACGCAGCAAACTGGTCAAAGCATGAAGACGATTTTACCCAAATGTTCTATAACCAA
AACGTGAAACAGTTCTGGCTTCCGGAAGAGATTGCTTTAAACGGCGATCTCCTCACATGG
AAGTACCTCGGAAAAAATGAGCAGGACACTTATATGTAGGTACTGGCCGGACTTACGCTT
CTTGATATAGAGAAGGGGAATACGGGGATGCCGATCGTGGCTGAACACGTTGATGGACAT
CAAAGAAAGGCAGTTTTAAATTTCATGGCCATGATGGAAAATGCTGTTCATGCAAAGTCT
TACAGCAACATCTTTCTAACATTGGCTCCAACCGAGCAGATCAATGAAGTCTTTGAATGG
GCGAAAAACAATAGGTTTCTTCAAAAGAAAGCTAGAACAATTGTTTCAGTCTATAAAACA
ATCAAGAAAAACGATGAAATTTCTTTATTTAAAGGAATGGTTGCATCTGTGTTCCTGGAG
AGTTTTCTTTTCTACTCAGGGTTTTATTACCCACTTTATTTTTATGGACAAGGAAAGTTA
ATGCAAAGTGGAGAGATCATTAACCTGATTATTTAATAGTCCCTTTCGTCGGCAACGGCA
AATGTGAACCTCTCTCATTGCTGGAAAATCCTTATTTGGACAATCAGCAGCGAAGCTATG
CGAACCCAAAGGAGGTGAAAATGATAAGGAAAGAAGTCGAAGAAGCACCTTGGTGGATAA
CGGAAACGGGAATTATCATATCAAAAAAATTAAAGAAACCAAGAACGACATTTATTACTC
CACATGGCTATGAAATGATAGGATACACGCATCCGAAAAAAGGAACACAGAACTATTTAG
TACATAGGTTAGTCGCAAAATATTTTATTTATGATATACCAAAAGTAATGTTGGTAAACC
ACATAGATGGAAATAAACTTAACAACCACGTTCGGAACTTAGAAATAGTTACACCTAAAG
AAAATACTCTACATGCAATGAAAATTGGATTAATGTCAGGACAACCTGGAGAAAGTAATT
CAATGTCAAAGCTCACTAATATGGAGGCAACAAATTTAATCTATGATTTGATTGCTGGAA
TGAACAATGTTGAAGCTGGTGAAAAATACAGCCTTCATCCTCGTTAAGTTAGTCTAATTC
GTCATAAAAGAAGATGGAACACTTTATGGGATCGCATAGAACGTTCAACGACTATCGCAT
AGGCGGCGAAATTCCGCAAAACGAGTCGGGCGCAAGCTATTGGGGTGGGTGAGAAACCCT
TAAATCGAAACGGGAGGCATCCTACAGGGATGATGATATAGTCTGCTCCTTACGGTAACG
TAAGGCGGTTGCGACAGAGCAACGAACCGAAAGTAGCGAACTCGGTTGAACTAAGGGTAT
TAGAGACGAAGCGATACATGGCGTATATGTAGGATTGTTAGCTCAAGAGATTTATAAGAA
GCAAACGCCACAGAAGCAAAAAGAACTGTACGCATGGGCTTTAAACTTACTGCAGGAACT
TTACGAAAATGAATTGGAGTATACAGAAGATGTCTATGATCAGGTTGGATTAGCTCCAGA
TGTAAAGAAATTCATCAGATACAACGCAAATAAAGCTTTAATCAATCTGGGCTTTGATCA
TTGGTTTGAAGAAGAGGATGTTAATCCAATTGTTATCAATGGTTTGAACACTAAGACTAA
ATCCCATGACTGCTTTTCAACTAAAGGGAACGGATACAAAAAAGCAACAGTGGAGCCTTT
AAAGGATTCAGATTTCATTTTTACCGAGAAAGGATGTATTCATTGAGATTAATTGAATTA
GAGCAGCCTAATTGCAATCCATGTAAAATGGTGTCCAATTACTTAGAACAAGTAAATATT
CAATTTGAGACTGTTGACGTTACACAGGAGCCAGAAGTAGCAGCAAGATTTGGTGTCATG
GGAGTACCGGTAACCATTCTGCTGAGTGATCAGGGAGAAGAACTAAACCGAAGTGTTGGT
TTTAAGCCTAATGAACTTGATGAGTTACTAAAGGAATTACGATAAAAGGGGAATTTTAAA
CAAACTTGAATTAAAAGGAGCTAATACATAATGCAAATTAAAATCAAATACGTAGATGAA
ACATAAACAAGAATTAACAAACTGGAGCAAGGTGATTGGATTGATCTTTGTGCAGCTGAA
GATGTAGCGATCAAAAAAGATGAATTTAAACTTGTTCCATTGGGAGTGGCCATGGAATTG
CCTGAAGGGTACGAAGCACATGTCGTTCCTCGTTCGAGTACGTATAAGAAATTTGGTGTG
ATTCAAACAAGCTCAATGGGTGTTATCGATGAGTCTTATAAGGGTGACAACGATTTTTGG
TTCTTTCCTGCTTATGCGTTACGTGACACAAAGATTAAGAAAGGGGATCGAATCTGCCAG
TTCAGAATTATGAAGAAGATGCCGGCAGTTGACTTGATCGAGGTAGACAGTCTAGGCAAC
GGTGACCGAGGTGGGCACGGATCAACTGGAACGAAGTAATTGGATTTTGTATTTGAAATA
TAGATTATTTTAAAAGTCATCTTGAAAAACATTTTTAATCATATTACCTTTTTCTAGGAA
AGGGGATTAAAATATGAAAATAACTATTGAAGAATTACCTGAATTAAGGATTGCTTATTT

CCGAAACTTTGGAGAGTATGGTGGTAAGCAAAACAAAGAACTAATGGAATCTTTTAAAAA
GTGGGCACAGTTGAATGGTGTATTTCATAACTCTGTAATTTTAGGAATTCCACAAGATGA
CCCAAGTATTACTCCTAAAGAGGAATGCCGTTATGATGTTGGTGTTGTATTAAATGAAGA
TTTTAATGTGCTGCAACCAGCTGAAGTTGGTAAGTTACCTGGAGGGAAATACGCTGTTTT
CTTGCTTGACCATACAAAAGAGGCAGTAAGTGAATTTTGGGGCAATATTTCTCTGAAAT
AGAAAAAAATAGTCTAACTATGAGAGGAGAACCAATTATAGAGAGATATACTTCACAAAT
GATTGATAATCACTTATGTGAAGTATTAGTTCCTATCCAATAAAGAAGGTGGGTAATTTC
CCACCTCCTATATATGTATAAGTTAAATGCTCTGGATTAACTCGTGTATTTTATCAAGAT
TAATGGTAATGTTGCATATTGCAATTACGAAGTATAGAGTCCCTTGGTATCGTTCTTTGT
TAACAAAACAGTTAATTCCAAAACCAATCAAAAATGCAATTATGATTAGCTGAACGAACA
CAAATAATTGTGAATTAGGACTGAAGAAAGAATAAACCAATAATAACAATAGAATCAGTA
AGCTAACGATTTTTAATGTTTTTCGCAAATCAACACTCCTTTAAATACATTGTTTTTAAT
TTACCATAAAAGATTTTTGTTGGAAATATTTCCGAATAAAAGAATACTTTTATCTAATAT
TTCAGGAGGAAGAGATTATTAACAGCAAAGAAAGAGATTCACAAGCGAAATATGACGACA
TGCTTTATAGGAACGGTCTTTGCTTTGGGTTTCTTCAGTTACAAGGACTTGAAGATGAAT
TTATTTAACATATGAGACAAGTTGCTGAGTACGAGAAAGACCTGAGATACAAGAAGGCAG
CAGCTAACTTCATGAAGATGCATGACCAAACCTGGGTGGAGGATTAATTGTTAAAGGATA
AAAATAAAATAATCAAGAGTGTTGAAAACATCAACAAACTTGAAGAAGGGTTGTCGCTAT
TTGAATAAGGTGACGAAGAATATTTAAGTGTATTTGTGAAAATTCAGGGGCTATATGATG
AAACCTCAGATACTGCTTTAGAGTGTTTTAAAGAGTTGGCTACAAAAATTAGGAAAATTG
GTCAGAAACGGATTATAAAAGGGATTGATCGGTTGCCACAGACAATTAAAGAAAACATTG
CTGATCAAGTGAATGATTTTAAAGGGGGAGCTATTTGAATAAAAGCAAATATTAACAGTA
TAGAGTTTGAGGGTGAGTAAAAGCCAACCCAACACAAATATAAAATTAGGAGATGTTTAC
TATAACATATGTAACTTTATTTCTATCAGCTTATCTAATTGTATTAAACATTAATGAGGT
CAGGTTGATTAGTCGAGGGGAAAGCAATACGTATATAAAAGTAAAGAGTGTGCTTGATAA
TTCAACTCCAGAAGATGCGAAACGAAATAAGAATTTCATTTACCTGTTCACTTTGCTTAA
GGGGAGTTCTTTAATCATCCCCTTGGCATATAACGGTTTAATTATGCATGAAAATATCCT
GATGCTCTTTTGGATAGCATTTTAAGTTATATACACAGTCCTAAGCATGTTTAAGGTTTT
AGACGTGTTGGAGGCCGAAACAGTGAGCCATAGTAGGTATATTTATTTGGCTTATGTCTT
TGGGAACCTTATCTTTGCTTTGAGAATTATTTTTCATATAATGTGAAATTGTGTTTAGCT
GCACTCATTATACGGCTCAAGCCCTTTTAGCATTTGTCAGGGCTAAGTAAATGGATTTAA
GAATGAAAATAAAATAACTATAGACAAATACAAATAATATGTATAATATCATAATAGAAG
ATGATGAAATGACAGTTTTTTTATCTAATATACAAAAACAAAAAATATACATTTTTTAAA
ACTAATATTTTATTCAAACTTTAAGGAGGTGATTCATTGGAAGTAGGAGACACAATACAT
AACACCAACGAACAGATAACAGCCTTAGAGAAAAAGAAATACCAAATTGAAACAACTTTA
CTTGAAAAACAAAGGGATCTGTTAAAACTCGAAACACAGCAAAACAAAGCTAAGCTTGAA
TTGTTATTCGAGCTAAGTGAAGTCTTAACCCAATTGGAAGGTGAAGAGTGGGTAAGTGCC
ACTATTGCTTTACGAATTATTAAAAGAAATAAAAGAAAGTACTTGGATCTCTTTGATCTT
AATGATGACAAAGCATACGTAAATAAGGATAAATTTAAATTTCTTCATGATGAATTCTTT
GGGCTGAAACAACAATTAAATGATATCTGAGGAGTTGTCAGTTGACGGTAGCGAAATTGG
AGGTGACAAATATATGTGTACATGTGAAGAGGGTAGTGGCAAATGTTCTTATTGTGACGG
AGCGTTGGATATATTCTAAATGAAGAGAACGAAATTTAGAGAGTGGCTAATCAAAGGAGG
CAATAAAATGGTACAGACATTACACAGTGCAAGTCGAGGAGCTTATTAATAGTAGTTAGT
GACATAAAAAGAGGTGGAAACAATACCATTAGTTCTGTATGGATTTAGAGAAGACTTAAA
AAAGAGAGGGGAATTATCCCCCTCATAAATTAATGAAATTGACCGCCCATGTTTTGTTGA
GCTAAGCGAACTAAACGTTTAGTGATTTCTCCACCAACTGAACCGTTTGCACGAGATGTA
GTCTCAGCGCCTAATTGAACACCAAACTCAGAAGCTATTTCAAGTTTCATTTGTTCAATA
GCTGAAGCTGCTTGAGGAATTAGTAAATCATTATTGTTGTTTGATCTTGATCTACTTTGT
TGAGCCATCTTATTCATCTCCTAAAGTGTAATGAAAACAATCTTGTTTGATTCATATTAT
GGAGAGATTATTTTAATTTATACACGCCAAAGAAATAAATTTTTCATTAAGAAAGGTGGA
GACAAAATGAAAATTGATTATGTTTCAGATCTCCATATAAATCATTGGATTCCTTGGAAT
GTAAATCAGATTAAGTGGGAGAAGCGAACAAGAGAAATTGTAAATAGGTTAATCTCAAAC
GGTAATGGGGACGTACTGGTCATTGCCGGCGGTTTTACTGAATGGAATCAACAAACTTTA
TGGGTTTTAGATGAGGCAGCAAAGCAATACGAGAAGGTTTACTTTACATATGGTAATCAT
GATCTTTATGTACTTAGCAAGAGCCAAAAAAGGAAATACTCAGACTCGTTAGGCAGGTTG
AATGATCTAATTCAAAAAGCTGCTGACATGAAAAATGTAACACCATTAATTAAGACAACT
GAAACATACAAAGGAAAAGTCTTTGCAGGAGATGTAATGTGGTACCTTCCAAAAGGAATC

GAAGGATGGGACTTCTGTAAAGGAGTATCTAACGATTCTAATTACATTTGGCTTAATGGT
TATAACAAAGTAGATGGTGTAAGAGCGATGTGGAAAGAATCAATGGATTGGTATGAAACG
CTTGAAAATACACAAGTTGATGAATTTGGCTCACATGTTCCACCTGTTCACAATCCTTAT
TCTCCATTTGAGCCTAATACATGCTACATGGTTGATGTCCCGTTCATTAACGCAAAACAC
TGGGTTTGTGGACATGATCATTTACAAGCTGAGTTTTACAAAGATGGAACGAGCTTTCAC
ATGAATTGTATCGGATACCCGTATGACTATGATAATTATCCGAGTGTCAATGTGATACCT
GGTGAAGAAGTAGATTCATATAAAACGTTTGAGCTTAAAACATTCGAGATTTAGATTTAA
TTAAAATTCTAATTTTATACAGAAGGGAAGGGGAGACATGGAAAATGAAGTTGTGTTTTT
TTGTAGAAAATGCAATCATCACTTGTTTGCTAAAAATCCTATGATAAACACATTAAAGGT
TATATCTGAAATTGATTGCCCTAATTGTGGAGAAGAGGGTTATCATAATTGGATTCTTGC
GCATATAGGTGATTCAGAGAAAGAAAAGGAGAACTTCAATTGGAAGTAGATAAAATGTTG
AATTTATTAAGAAAGAGGTGTAGCTATGATGGACAGTAGAGAACAAATTGATTGGACTTG
TAATGAATGCAATTTTAGTTGGATAGGTGACAATTCAGATTTTTCATGTCCTTCTTGTGA
TGAAATCGATATAAAGCCAAAAAATAAGATACTTGATTGATTAAGACGTTGATTTCATTT
AGTGGGATAAACTCAATGAAAGAGATTGAATTTGAAGAAGTTAAAAAATTTGTTGAAGTA
CTGTCTCGGCGACTTAGATCAACCTAGGTGATAAAAATTCATATCAGTTATGCAGAATGG
TTAGTTGCAGAAGTCGAACGCTTAAGGGGGTTAGTCAAGTGAGTAATAGTTTAGATGTAC
AAGGAGGGATTGAATGGAGAACGTAATGTCCTGGTTTAATAAAGACTTTGAAATTAAATC
AGACAATAACAATGATAAAACTTTATTGTGATTATTTGATCTTATGAAGGAAAGTCTCAT
GACTTATTTCAATATTGAAAACACTTCTGATATTCATGAGTTTATAAAAATCGCAGTAGC
AAAGAATGATGTTGATTATCCCTTCCTTGAATGGATAAAGGAAAAAGGTATTCCAAGATT
TAAGAATATTGATTTTAATAAGCTACCCAATGACGATCGCTTTCTTGCCATGATTGAGTT
TGATGAATTTGTTCTGCAAAATGAAATGGATTTTCAAGATCCTGAAGAGGTTCGAGGGTG
TATTATTTCAACAGTTAATAGCCTGAAGGAATATATTAATATCTGCCACAATAAAACATG
ATTGGAGGAAAGATACCTGATTGTTACAGCCTGGATATTGTTAATTGTGTTTGGTTTATT
CGCTTTATCAGATTTTAACTTAACTGAGAACGAGACAAAGCATATTAAGTTTTTCATTTT
AATGAAATTTGTTTCTGTCTTTATAGCTGCTATTGCAGCAGGAGTGATTTCGGGAGGATT
ATTTCAATGATTGAAATTTTTAAAGATACAGGAGCTACCCATGATTTAGTTTATCATGCT
AAGATTAACACGTTTGTTTGGGATGTGGAATTTGACATTGTTTTATCAGACAGTAAGGAA
TTAAATAAATGTTATTTTGTGAAATGCTTTAATCCGTATCGAATAAATGGAAAGTGTGAT
TTTGCGGTTAGTTCAATAGATATATTTTCCGAAGGTAAACGTCTCTTAATTGAAAATGAA
TTCAATTTTAAAATTACAAAGGCAGTCCACGTGGCAACATCAAAAGACGTAACTGAAATA
GTTTTGCTTTTAAGTGAGAGAATCAGTAGTCCATTCCCAATTGTAAAAGAAGTTGTTTAT
CTAGATTAAGGTAAATAGGAACAGGATACAATCGGTCTTTTATTTAGAATAAAAATAAAA
TTAGTGGAGGTATTATGTGTAATCGAAATGTTATTACAATTCCATATGAAGAGGATATGT
CAAAGTATTCAATACTACACCAGGTAGGGGGGGGAATTGAGTATTTTCAAAAAGAGTATT
CACAATATCCAATGTTCGAATTTGATAGTGAAGAGGATTACAACGAATATAAATGCTTAA
TAATGCACCTTAAAAAGAATAAAAAAATCTCTAGTTTCTCTTTTTAAAGAGTATGGTAT
TGCAAACAACAGTTTAATATGCAAAAAGTAATGAACTAAATATTGCACATATGGGAAAGA
GGTGAGAAAAAATTAACGAGGCTTATCATTCAACTCAAACACTGTTAAACAAGATGGATAG
ACAAATGAAGACAGTGAAGGAGGCCATTGAAGAGAAAGATTTGGAACGAGCACATCGTAA
CTTAATTAACTTGGCTGACAATAATGAGGAATTAATGCAGGAAATAAGGTGGGTCAAAAA
GGGAACCATATTATAAGTTATTTGGTTAATTGTCTGCGCTCTTTAAAGTTCCATACATAA
AGCTCTTCTATAGAGCAACCTATGGCATCAGCAAATGTCATTGCTGTTCCAATATTCATA
TTTGATTTAAAACCACCTACATAATCATTAATACGTTGTTTTGATAATCCAGTACAATCA
ACAAGATCTTCAATTGAAACGCTATATTCATGCATGAGCTCATTGAGTCTTGAACCAATT
GGTTTCCATTGTTTTTTCACTATGCACACCTCTTTCAGCAACGATTTTAGCACATACATA
ATTTACCTTCAATATAGAATAGGAGAGTGATTGATTGGGAGCAAACAATCAAGGGAAAGT
TTTTGAAGCGAACATTGAAAAATCTGCAGCAGATCAAAAGTTATTCTTTTACCGGATTAA
AGATGTTAACCCAATGTTCTTGAAAAGAGGGTCATCAGTATCAAAAAATAAATATGATTG
CTTTCTGTTCTTTAAGGGGTACTTGTTCCCCTTTGAGCTTAAGTCGACAAAAAGCAAGTC
CATTTCCTTCAGCGAGAAAATCATAAAGCCGCAGCAGATTAAATATTTAAGGGAAGCAAC
ACAATACCCAAACATAATCCCTGGTTTTCTGTTTCAATTTAGAGAGCCGGAAAACAAAGT
TTATTTCGTACATATTAATGATTTCCTTACATATAAGAACATAGCTGAAAAACAGTTGAA
ACATACATATAAGAATCAAGTAGACAAAGCCAGTATCCCGATTGCGATTTGTGAAGAAAT
CGGTACTGAAGTGCGCTCGATGAAAAAGAAAGTGAATTATACATATTATTTAAACAAGCT
GTGCGGTGAACTAATAAAGAAAGAACAGTCAAGAGACAAACCATTACATACATATAATAC

TCCTGTGAAAACGGGGGTGTAGCCTTGGAACCTTACCAACCTTATGAGGAATTAAAGAAA
AAAACAATAAAGGTAATCCAGAAAGAAAATTACAGTATTCGATATATAACTCAGGTTGAA
GCAAGTAATGACTTAGATGAGTTTTACAAACAATTTGATCAACATTTATTAGAAGCTGCT
CTGGAAAGGAAGGCAGAGGAAGTTTCTTTTTTTCGTTAGATATGTAGTAAGTAACTTAAT
ATACAGCTTTATCTGT
>NewGenomeName_44
TTAGTATTACCCGCCGCCTGGGGCACCGGGGCACCGCAGCTATTGGCTGCCTTGCGGAGG
TGCCCGCCCCTAGGGAGGCGTAAATGGCGCCGTTAGGCGCCCGTTAATCTCCGGAGGATC
ACACTCGTCCGGGAACCATCCCGTCCAAGGAGGGCTATGGCTGTCGCCGTTGGTGTTTCA
CTCTTAACAACCCTACAGACGGCGAAATCGAATTCGTCCGTTCTCTCGGGCCTGACGAAT
TCTACTATGCCATCGTTGGACGGGAAAAGGGTGAGCAAGGCACTCCCCATTTGCAAGGCT
ACTTTCCTTTTAAAAATAAGAAGCGACTGAGCGCGCTTAAGAAAATGCTGCCGCGAGGTC
ATTTTGAGGGCGCTAAAGGGAGCGACGCGGATAACGAGAAGTATTGCAGTAAAGAGGGGG
ACGTCATACTTACCCTGGGCATTGTGGGGAGAGACGGTCACCGCGCTTTCGATGGAGCTG
TTGCTGCCGTGATGTCCGGACCCAAAATGAAGGAAGTCGCGCGAGAGTTCTCAGATATCT
ACGTCAGCCATGGGCGGGGCTTGCATAGCCTCTCGCTATTGGTTGGTTCCCGCCCGCGTG
ATTTTAAGACTGAGGTTGACATCATTTACGGGCCACCGGGGTGTGGCAAGAGGCGATGGG
CCAATGAGCAGCCTGGGACCAAATATTATAAAATGCCCGGTGAATGGTGGGATGGATATG
ATGGGGAAGATGTCGTCATCTTGGACGACTTTTGTCGGTGGCTTCCTTATTGCGAGATGC
TCCGCCTCTGCGACCGTTACCCACATAAAGTGCCAGTTAAGGGCGCTTTTGTGGAGTTTA
CGAGCAAGAGGATCATTATCACGAGCAATAAGGCCCTGGAGACCTGGTACAAGGAGGACT
GTGACCCGAAGCCACTGTTCCGGAGATTCACTCGTGTTTGGTGGTACAACATTGACAAGT
TGGAACAAGTCCGGCCAGATTTCCTCGCCCGCCCCATCAATTTTTGATAGTCTCGGTGAT
GTTTAATAAAGTTAGGCGTCGGTCCGAAGGCCCGATGCCGCAGGGGGGGACCCCCTGCCG
GAGGGTTCGCAGGGCCGTCAGGCCCGAGAACCCGACCAGCCCGGAGGGCCTAGTCTGTAT
CGGGGGGGGGGCCCCGGGGGGTCCCCCCGACAGGACGAACACGGTAGCGACGAAGGCGCC
AATAAACACTCAAAAAGGTATTTGCTGCCTGAGTCTTTATTAAGTACTGGGATTGTTAGG
GGCAAACTGACGGAATTGAACATATATAGTGAGCTTGGTTACATAAGTGATCGTTTGTTC
TGGGTGAGGGAAGCTGAAGCCAATGCCGTAGTGCCTGACTTTCGCTCCTGCTGCGTTGGG
TCCTCCTTGTAGTGGGATCCAGCCGGTTCTGGCGATGTTTAGCCACAATGCTGCAGACTG
GTTCGCTGTGGTGAGGTCGTTTATCGTTATTTGTGGTTTTGGTTTGAGGAGACGTTTGAA
TCCCCTGCTAACATACCATTTTCTGGCACCGTCGAAAGGTGCCAGTGGGTCTTGTGTTTG
GTCTGCAACAGTTTTAAATTTACTTATCCTGGAGTCTTGGATTACGGCCGTGTGGCCGAA
TCCGTCTCCTTGTATGGTGTAGTGTCCCCATGTGGGCCTCATTTCCATTTTAGCTAGCTT
AATCCGGTAGTTTTCGAAATTCAGTGTTTGTGGGTTTGGTGTGTTTGTTATAAAGTCTGA
CAATGCATAGGTTACAAAGTAGGAGCTAAAAATTAGGTTGCCAGTACTGGTGGTTTGTTT
TTGAATTTGGAATTTGAATTGGCGTGTGAGTCTGAGAGTGTAAACCCTATTGGTTGAGAA
ACGGCGTCTGCGGAAGTGTCTACGTCGCCGGCGGTATCGCCTGATGTGACGTCTGCGGTA
GTATGGGCGGGCATACCGTCGTCTAACCTGAAATATAGCGCATGCGTAGTTAGAGGTGCC
CCATAGGCGGCGG
>NewGenomeName_45
ACAAAACTGACAAATATTGTCAACGCTCCTATGCGTGGGGGTATACGTTTCTAAGGTGTG
TACTGCTTTATGGACGCATCCTACTCATGGTCCGCTAAAATGCGGTCAATGTATAGAGTG
CCGTCTTGCGTATTCGAGAGAGGGGGCTATCAGGATAACCCACGAGCAAATGATGCACGA
GAGATCTTGTATGCTCAACCTTACATATGATGATGATCATTTACCTTTGCACGGCTAGCT
GGTTAAAGCTGACATGCAAAAGTTTTTTAAACGTCTTCGTAAGGCAGTATACAAATTTCG
CTATGTTGCTTCTGGTGAGTATGGAGACGCTAGTAGGCGTCCCCATTTTCACATTGCACT
GTTTGGTCAAGACTTTGAATTTGATCGGCAGCAATTCGGCAGCTCTAATGGAGATAAAAC
TTACACAAGTCAAACTGTTTTTAGATACTGGCCACAGGGTAATCACCTAATTGGTACTCT
TAATTTTGAAAGCGCAGCGAATATCGCAAGATATATACTTAAGAAAATTAAGGCTTCTGA
AAAGGTCTCACCTTTGCCGTTACACGTAGATAAAGAGGATGGGGAAATAATATTTCCTAA
TCCTGAGTTCTTAACAATGTCGAAGGGCATTAGTACAGGGTGGTTTAAAGATTACTTCAT
GTCGGATGTTTCTCCGTCTGCAAGTGTTGTAACCATCCAAGGCACTAGGGCACCAGTCCC
TAGGTACTATAAAACTTTATTAAAGGAGTTGGGTCATGATTTGAGTTTAGATATGCAGTT
TCGCTCTTCAGCGCGAGCTGATTTGGAAGTAGAGCATAAACGATACGAAAAATCTTCCTG
TTCGAAAGATCGCAAGACAAAATGTCAGCGAATCTAGAGTTCAATTATCAAAACGTACAA
TTTAAAGGTCAAAAATGTTGCAATATACAGTTTCTGTTAAGGATAGGGCTGCTGAAGTTT

TCAAACGCCCGTTTATTGTTCCACATCGTAATGTGGCTGTTCGTGATTTTACTGATGAGA
TTAATCGTGCTAGTGCCGATAATCCATTGAATAAACATCCTGATGATTTTGATTTGTATT
TGTTGGGACAATTTGACGATACCGTCGGTTCCTTCATTCGTGAAGGTGCCCCAATTGTTC
TCGTCCGTGGTATGGACGTTGTTCAAAGCTCTGTTTGACCCTTGCACCCCTTAGGGGGTA
CTTTTTTATTTTTAGGAGTAATTATGTTTTCTAATAAATCTGCCAGTGCTCATGATTTCG
CAATGGTTCCTAGAGCGGATATTCCGCGTTCTAAGTTTTCTATGCAGAAAACGCTTAAAA
CTACTTTTGATAGTGGTTTTTTAGTTCCTTTTATGTGTGAGGAGCTTTTACCCGGTGATA
CGTTCAACACTAAAGTTACTATGTTCGGTCGTCTCGCGACCCCCATTTTCCCAGTCATGG
ATAATCTCCATTTGGACTCGTTCTTTTTCTTTGTTCCTAATCGTCTCATATGGTCAAATT
GGGTTGAATTTAGGGGGCAGCAGGATAATCCTGCCGATTCTATTTCCTACTCTATACCTC
AACAAGTTTCCCCAGCTGGGGGTTAAGCTGTTGAGGCTTTGCAAGACTATTTTGGCTTGC
CAACTCTCGGGCAAGTTGGCTCTGGTCTTACTGTTTCACATTCTGCGTTACCTACCAGAG
CTTACAACTTGATTTATAACCAATGGTACCGAGATCAGAATTTACAAAATTCTGTCGTAG
TTGATCCTGGTGATGGTCCTGATACATCACCTAGTACTAAGTACACTTTATTACGTCGTG
GTAAACGACACGATTATTTTACTTCTGCTCTACCTTGGCCTCAAAAAGGTGGTAATGCTG
TTACTATCCCTTTGGGTACTAGAGCTCCTGTATATGGTGTTGGTGTAGCTACAGCAACTT
CTGGTAATGGTTCTACCGCTGGTATTAAAGATCCAGCCGGTCTTAATCAAACATGGACTA
ATGCTACAACAGCAGCCCCATATATTAAAATGACAACACCTGTAGGTGCATCAGCATTGC
CCGATATTTATGCATATTTATCTGCAGCTACAGCTGCTACTATTAATCAGCTTCGTCAAT
CTTTTCAGATTCAAAAGTTGCTTGAGCGTGACGCCCGTGGCGGTACTCGGTATACCGAGA
TTATTCGCTCACATTTTGGCGTTGCTTCACCTGATGCACGCCTTCAACGCCCTGAATATT
TGGGCGGTGCTTCTACTAATATTAATATTTCACCTATTGCTCAAAATAGTGCTACTGGTT
TATCTGGTTCTACTACCCCTATCGGCAATGTTGCCGCTTTTGGTACTTTTTTAGGGAAAG
AACATGGATTTACACAATCCTTCGTCGAGCACGGTTATGTTATTGGTCTTGTTTCTGTTC
GTGCTGACCTTACTTATCAGCAGGGTCTTAGAAGACACTGGTCTCGTAGTACTCGCTATG
ATTATTATTTTCCTGCTTTCGCTACTCTTGGTGAGCAGTCTGTTTTAAATAAGGAAATTT
ATGTTACTGGTAATACAACTCAAGATAACTCGGTTTTTGTTTATCAAGAACGTTGGGCCG
AGTATCGTTATAACCCCTCGGAGATTACCGGCTTATTTAAGTCTACTTCTTCGGGTACTA
TTGACCCATGGCATTATGCGCAAAAATTCACATCTTTGCCTACATTGAATAGAACTTTTA
TTCAGGATAATCCGCCTTTAGCACGCAACCTTGCTGTTGGTGCTTCTCCTACTGGATAGC
AGTTTCTTCTCGATTCTTTTTTTACTACTACGGCTGCTCGTCCTATGCCTATGTACTCTG
TACCCGGCTTGATCGATCATTTCTAATATGCTCGATTGGCTTACTTCACCTATTGCTTCT
ACTGTTTCTGGTATTGCGGGTTTTCTTGGTCAACAAGATACTAATGCTGCAAACCAAGCA
AACGCACAAGCACAAATGGATTTTCAAGAAAGAATGTCCAATTCTGCTTATCAACGACAA
GTTAAGGATATGGAAGCTTCTGGTTTAAATCCTATGCTTGCTTATATCAAAGGTGGGGGC
GCAAGTTCCCCAGCTGGTTCTATGGCTACTTATCAAAACCCAGCTGCAGCTGGTGTTTCT
TCTGCTTTAGGGTCTGCACAAGTTTCACAAACTCATGCTAGTACAAGGCAGACTTCTGCA
CAAACAGATTATCTTACCGGTCCTCAAACTGAGATGACTAATCAACAGATTAACAATCTA
AAAACTGATAACGTAAAGACTACTCAAATTATTCGTTATCTTAATGAAGAATTTCAAAAC
TTGATGAAACAAGGTTTGAATTTAACTGAAGTTGGTAATCAATTACGTGAAAGCGTTAGT
TTGATGAAGTCTCAAATTACTAATTTTGGAGCTTTAACTACTTCTACACAGTTCCAAGCT
AAGATTAATGATTTTAATCAGCAGTTGTTAAAAATGGATGTAGATGCTGCTAAACCACCT
GGTAATTTTGGTCGTGAATATAACCAATATAGACCTCTAATTGAATTTCTCAAATCGTTT
GTTAAAGCCAGATAATGGCTAGATTTTTTTTAAAGGAAATGAAAATGAAGACTGTTTTTT
GGCGTTCTCCATATAACTATGATAGGGATCTAGCCAGCGATAAAGCTGGCCTTTCTTGTA
ATGATCCTTCTTTAACGCAACAGCAGTTTAAAGAAGAGTCTGATAAAAATACGATTGTTG
ATCGTTTTATGAAGTCTGGAGTTTTGCCTAATCCAATTAATATGCCTCAATATATTGATT
ATGAAGGCGTTTTTGACTTTCAATCTGCTATGAACACTATCCGTCAAGCAGATGAGAATT
TTATGCGTATGGACGCTAAAGTTCGTGCTAGATTTAATAATAGTCCCCAAGAGTTCCTCG
AGTTTTTCGGTAATCCCGAAAATCAAGAAGAGGCGATTCGCTTGGTGTTGGCTACGCCTC
AAGCCGTTGTAGAAACGAAAGTTTCTGCTGCGGAACCGACGTCTAAGTCGGAATAATGGT
ACAGTTCGCTACTTGATGTAACTGTACCTAGTGACACCAACTTCTAGGGAGAATGAAATG
AAACCTTTGCACAGATCTTTTGTAAATAAAAACAGTTCTGCTAAACAATTTAAATCTAAT
GTTGGCAAA
>NewGenomeName_46
TAGACTGAAAGCAAATCGCACTACGTCAGCAAATTAGCAAAAGGCACCGCAACGTGGGGG
CTGGAGTCTCTAAATGCGCACAAGGCACCGAACATGCCTTCTTATTACCCATTAAGCGCT

TATCAATGCGCAGACGGACACATTATCTTCCACGAGAGTAGACGACATGGCACAGTAAAG
TCATTATCTTTACCCTGCAGCCAATGCGTTGGCTGCAGATTAGCACGCTCACGCCAGTGG
GCTATTAGATGTATGCATGAGGCCCAAATGCGTACACAAAACTGTTTTATAACTCTCACA
TACAATGATGACAATCTCCCAAGCGATAAATCTTTATGCGACCGACCCTATCAACTCTTT
ATTCAAAGATCTCGAAAACGGTACTTTGGACTCAAAATACGTTATTACATGGTTTGTGTG
TATGGCGAAAATTCCGACCGACCTCACTTCCACGCCTGCCTCTTCGGAATCGATTTCGAT
GATGAGAAGCTATGGAAAAGGACTTCCGCTAATTCTATGTTATATACATCCAGGGACCTT
AAAATACTCTCGCCATTTGGTTATTCCTCCGTTGGAAACGTTACTTTCGAATCGGCGGCC
TATGTCGCGCGATACATTATGAAAAAGATGACAGGGAAAAACGCAGAGGATCATTACACA
GAAATAAACTCCGAAACAGGGGAAATACTTACACATAAACCCGAGTTTATGAAAATGGCT
CTTAAGCCTGGTACAGCTTACTAATGGTATAAAAAATACTCTTCCGATGTCTATCCCAAC
GATTACGTGGTAACTCGTGGAAATAAAGTCAAACCTCCAAAATATTATGACAGAAATTCC
AAAATTGACAATCCATATGAGTTTGACGAACTACTTTACCTCCGAGAAAAGTCTACTAAA
CTACATTTTGAAGACAATACGCGTGAACGCTTGCTTGTAAATGAGCAAGTTACAAAGGCA
AAACTTCAAAAACTAAAACGTAACCTCACTTAAGGACAATACCTCATGAAAGTAGTACTA
TGTTCTGTAAGAGACAGAGCAGCTGATGCGTTCGGTCGACCAATGTTTGTACCCTCTGTT
GGTGTCGCAATTAGGAGATTTAGCGTCGAAGTCTACCGCCAGTCTGATGATAATCAGCTA
CATAATCACCCCGATGATTTTGATTTATATGAAATAGGCGAGTTTGACGACAACTCAGGT
ACTTTTGCTTTACATGTCCAACCAAAACTGTTATCTTTAGGAAAACAGGTCAAAATACCT
AAGGAATGGTTTTAAATAAGCCGACTCAAAGGGTGTATGTTTGGGTCGGAACGATAAGGA
GCTTAAAAACATGCATCGTAATCAATCGGTAGATGTCCATCAATTTACTATGATACCAAA
GGCCGATATACCTTGGTCTTCGTTTGATAGCCAGTCTACACACAAAACAACTTTCGATGC
TGGCTATCTCGTACCCGTGTACGTGGATGAGATGCTCCCAGGCGATACATTTCGCTTGAA
TATGACTGCGTTTGCTCGATTAGCAACGCCACTATATCCAATTATGGATAATATGCATTT
AGATTCAATCTTTTTATTTGTACCCAATCGATTGATTTGGGACTATTGGCAGAAATTTAT
GGGTCAATAAACGAATCCAGGGGATTCAATTAGTTACGTTGTACCCCAACAGTTAAGCCC
AACTGGTGGTTACGCTATAGGATCACTACAAGATTACATGTGACTACCGACTGTTGGACA
AGTCGGAAATTCAAGCACTGTGTCTCATTGTGCTTTCTGGCCACGTGCTTATAACTTCAT
ATATAACGAATGGTTTAGAGATGAAAATCGACAAAATACTGTTGTTGTTGATACTGATGA
TGGCCCTGATACTGTTACTGCTTACGTATCCCGTCGGCGTGGAAAACGCAAAGATTATTT
TACGTCCGCACTTCCATGGCCTCAAAAAGGTGATGCCGTTACACTTCCATCAGGAACTTC
TGCACCTGTTAAGTTCCAAAATATTAGTGGTAGTGGTACTGCATTAACTGGTAGTGTGAT
TGAGTTTTCAGGTCGTGATAACGATACTCCCGCAATATTTTCAGCACGAAACTACGGAAG
TAGTACAGGAAATGCAAATCTACCTATCGCTAATACCGCAAACCTGTATGCTGATTTAAG
CGCAGCAACAACTGCAACTATTAATCAATTACCACAATGTTGCCAGATTCAAAAACTGTT
AGAAAGGGATGCGTGTGGTGGTACACGTTATACTGAAATTATTCGCTAACACTTTGGTGT
TATTTCTCCTGATGCTCGCTTACAGCGTCCTGAGTACATCGGGGGTGGATCGACCAACAT
CAATCATAATCCGAGCGCTCAAATATCAGGTACGAGCGCTAGTGGAACTAATCCCCCTTT
GGGTACACTTGCTTCTATGGGTACTGCCTTGGCTCATAACAATGGCTTGACTTACTCGTC
TACTGAACATGGCGTAATAATCGGCATGGTCTCAGTTCGTGCAGACTTGCCTCATCAGCA
AGGTCTGCAACGTATGTGGTCTAGATCAACACAATACGATTTTTACTTCCGAGCTTTTTC
CGCTTTAGGTGAGCAAGCTGTTCTTAATAAGGAAATCTATGTTACAGGTGGGTCTGGGGA
CAATGATGTATTTGGCTATCAAGAACGCTAGGCAGAATACAGATACTCTCCTAGCCGCAT
TTCAAGTTTATCCAGTTCTACTGCTGCTGGAACTATTGATGCCTGGCATTTTGCCCAAAA
GTTTCCAAGTGTACCAACGTTAAAGACTACGTTTATACAAGACACACCTCCGGTTAGCCG
AGTAGTGGCAGTTGGAGCATCTGCTAACGGACAACAATTTATTATTGATAGCTTTTTTGA
TGTTAAAAAAGCACGACCAATGCCAATGTACAGCGTACCTGGCTTAATCGATCATTTCTA
ATAGGATTGATGCCCGAGCTGCGTTTGGTGGTCAGTTGGGCTTTTGAGGCCAACAGAAAA
CCAACCAAAAAAATTGGAATATAGCTCAGGCAGCTAATCAAGCTGGTGCTGAGCAAGCGC
AACGTCAAAGGGATTTTCACGAACGTAGGGGCAAAACTCAATATCAAAATGCTATAGAGG
ATATCCAAAAAGATGGGATAAGCCCCATGCTTGCATATTCACAAGGTGTAGCGGGTACCA
AATCGGGAGCGATAGGGCAGGTGTCGACGGCAAAAGTAGGTAATGCTATTGGATCTGCAT
TGCAAGGTTATCAGACTATGGCAATGACTCATGCGGATTTAGACTTAAAAGACGCAACTA
CAAAAGGTACAACCGGTCAAACTATAGAAACAGAAGCCGATAAGATAAAAACGCCTCAGG
ATATAGGATACACGTTAGAAAATAATAAGTTAAACCAGCAACAACAAAAAAACTTGACCG
AAGCTTTAGCTAAACTTACGGCTGAATTAGCTAATGTGAAGCCAAGTACTGCGCAAACAT
CTGCGCCAACAAAAAACATAAAAGAATTACAAACCCGATCCGCTGACCCTTTTGGTATC

GTGATATTAAAAAAGGCGTTAAAGGGATTCATTAAGGTTTTAAAAATAACCCTTCATAGG
TTGTTCCTCATTACACAGCTGAAATTATATATAAGTCAGGTAAAGACTGGGTAAAACATA
AATATCGACAATAGATAGGCAAATAATGAGTAAAAAAACTGTGCTTTTACGTACACCAGA
CAACTACGACAAAGATGCTGCGTCAAATGAGTCATGGTTGCATTGTGGGGATGCCTCCCT
GGCTCAGCAGCATTTTAAAGACGAATGCGATATTATTAATATCCTTCGTCAATTCGACAC
GACGGGCCAATTGCCCGGACAGCCATTAACGTCTCGTAATGGCGACTTTAGCGGAATTGG
TGACTACCATACCGCATTGTACCGCGTTATCGCGGCTCAAGATGAATTTGACCGCTTGCC
GGCTCAAATCGGAGCTATGTTCGATAACGAACCACCAAATTTAATAGAATTATTAGAAAA
TTCTGACAATCGACCCGAGACTGAAGAGCTCGGATTAGTTGAAAAAGCCGTGGAACTGGG
GCAAAGCACAGTTACTCCACTTGATGTAACTGTGCTAGGAGACACCAAAACCCCAAATGC
TCATTAAAAGGAATCCTAAAAAATGATGTATAGCAAACCAGTAAATAAACGTCGTTCCGC
TCGTTCCTT
>NewGenomeName_47
ATTGCACACCTAATTTCGAAAATGGCATGTGATACACCTTTTTATGTCAATAACCCCCGC
TACCCTATCTACAGCAACTACCGGCAAGTTCAGGTTCCTTGTGGAAAGTGTTCAGCATGT
TTGTCCAGACGCACTAGAGTCTGGACATTTCGTTTAAAAACTCACGCAAAAAATGCTATA
TCTTCTTACTTCATTACTCTTACTTACGATACCGGATATGTACCTATATCAAGTAGGGGT
TTCCTTACACTCCATAAACGAGACGTTCAACTCTATTTTAAAAGGCTTAGGAAACTTCAT
TCAAAAGATCACGAACCCCTAAAATATTATTTAGCAGGAGAATTTGGAAGCAAAACATTT
AGACCCCATTATCATATCATACTATTTAATGCCAACATCGACCTAATTCATAAAGCATGG
GACAAAGGAGAAGTCCACCTAGGGGAATTAACAGAGCCTTCGGCCGCATACACTGCAAAA
TTTATAAACATAGGAAAAGTTATACCAGTGCACAAAAATGATGATAGACTGCCAGAATTT
AGTTTAATGAGTAAGAAACTAGGACTTAACTATTTATCAGAAAAAATAATTCGATATCAT
AGAAGCGATATTGAAAGAAATTTCATAACATTGGAAGACGGAAAGAAAATAAGCTTACCT
AGGTACTTTAGAGCAAAAATTTGGACAGAGCGAGAAAGAAGAATACAAGCAGATAAACTA
GCACAAAAATTCAAAGAGTTAGAGCAACAAAAAGAAACAGAGTACTACACAAAAAATCAA
ATATTGGAAGGATATGTACAACTTAAAGCAAGTGGAAAAGCCCATAGAATTATTACACAC
GAAAAACGAGCCCGAGAAGGGCGCAATAAAAGTTAGATCAAGCTTCAGTTATCAAGACAA
AACAGAGGAGCAGGAGGAGAAATCATCAATGGAACCAAGCCAAACAGTTACAGACATGAC
TTTGTCATTATCAGAACTAGTACAACGATACACCAGAGGACAATCAGTGGCAACCTTTAC
ACCCGTTTATTACGGAGAAGAGGAAGAATTCGCAGACGCTAGCAGAATGGATCCTATTGA
GCGCATAGAGTATGCTCGATTCATTAGAGAAAAATACTCAGAAACCAGACACTCCCTAGC
CGAGCAGCAAGCTGGCGACGAATGAGCAGCCGAGCCGCAAATGAGCGATGGGCAAGAAAA
AATTGAACAAACAACCACCCCACATGTATAATAACAAAAGAGTAAAAAAAGGGAAGTTTA
ACGGTACTACCGCTAAACTTCCCGATACGTGGCGCTATGGCAAAAAAACAAAAGCGCAAT
TGCAAAAGGCCTTCGAAGAGGAGGCCAAACACTAATAATCCTTGATATATTAGTGCTAAT
TGACACCAAGTAAAAACGAGAGCCTGCGAGAGTAAAAACGAAGATAGAAAATTAAACCCA
AAAAACAAAAAAGTTAATGGATCCAGTAACTACAGCCGCAGCAATCACAGCCGGCGTCAG
TGCGCTATCGGGCGGAGCCCAAGCATACGCCACAGGAAAACAAAACAAAAAATCTCGTGC
ATTCTCCAGGGAGATGTACGAAAAAACAAAAGCAGACAATTTGATGTTTTGCTATATGCA
AAACCAGTATAATTCACCAGAGCATCAAATGGCAAGATTAAAATCTGCAGGATTAAACCC
AAGTATGGTATACGACAAAGGCGGGGCTATTCAATCTGCTGGAAATATACCAACACCAGA
TGTTCAAGGAGGACAATTCAGAACTCCCGACTTTGCGCCAATATCAGGAGCTGTACAAGG
ATATTTTGATACCAAAATAAAACAGGCTCAGTACGATAACTTAATGGCTGCAAATACATC
CATTCAACGAGAAGCTATTTTAAAAGCTGCACAAACATTGGCAGCAACAGAAAGTACAAA
AGGTCAATCTATAGCCTATGCATTGGCACAAACAAACTTTCAATATTCAGTAGAAGGAGC
GAGATTAGCAAACGAGCAAAACAGAGCAAACACACAATATACACTTTCTGAAAATGAAAG
AAAGTCAGCCTTACAGGCACCTTCTTTAGCAGCAGCAGTAGAAAATGTATGAAGAATCAA
AGCGGAAACTGCTAATACAAATGCTGCAAGAGGAAAAATAAACCAAACAATTAAAGACCT
CCAACAAGATTAAAGATCAAAAGAATTTGAAGAAGATTTAGCAAAACAAGGGATATACAA
AAATGACCCTTTATGGTCAAGGGCATTAGCTCAATTTATTGAAGGTCTCACTGGAGGTAT
GTCTATGAAAGATTTAGGAACAAACACAGGAGGATTGTTTAACCAATCAGGATTTAAAAA
TGCAACTAAATCAACAATGTCATGGTGGATTCCCAAATAAAACATTACAAAAAAACGTCT
GAAGAAATAAAAAAAGATGTAAATGATTTGATAGATCAAATAAATAAATTAGTTTTAGAA
AATGAATCAAATGATGCGGTAGCATTAAGCAGATTAGATTCTGTATGTTCTTTACTACAA
ACTACCCTTATTCATATTAATAACTTAAACAGCAAAACCCAATGCGCTACAGAAGAGGCG
GAAGATCCCGCAGAAAAGGCTACGGCCGCAGAAAAAACAACACTTACTGAGTTAAAAGAG

GAGGCATAAGACTATAATGGCAAATGCAAACTTATTTAACTCGGTTCAGCTACCTAAAGT
AGCTAGCAATGTATTCGACCTTTGACATGATGTGATAATGTCGTTGAAAATGGGTGGTCT
TTACCCAACATGTGTAATGGAATGCGTACCAGGTGACAAAGTAAAAATAGGCACTGAAAC
GATGCTTCGATTTGCACCACTTATCGCACCAGTTATGCACAAAGTAAATGTTACAACTCA
TTACTTCTTTGTGCCAAACAGAATATTATGGCCAAACTGGGAACAATGTATTACAGGAAA
CTTAGATGTACAAGCACCATGGTTTAGACTATTTCCTTCACCAGCTTCAGGAGGATTTCC
TGTTAAATCTTTAGCGGATTATTTAGGATATCCAACTTTGTTAACGCCAAACGCTATACC
ATATCCTGAGCCTGGAGCTCAAGTAGGAAGCCCTTACCCTGTAGCAGCTTACAACAAAAT
TTATAATGAATACTACAGAGACCAAAGTCTTCAATCTCCAGTTTTAGATTCATTAACAGA
TGGAGAAAATGATCCATTTAGGAACCTAGCAGCAGGACCTGTTAAAAAAAGAGCGTGGCA
GCATGACTATTTTACATCATGTTTACTATGGGCACAAAAAGGTGACGCTGTAACTATTCC
AATAGGAGATGTAACTATCAACTATAATGCAGCTGCTGGAGGAACTGTTATGCGACAGGT
AGATAGCACACCATATATAAACCAAACAGATCTTAATTATTCAGATGGTGGAGGACAACC
TAGAGCAAGAGGAGTAACAGGGACACGTTACAATATTGATAACTCCAGCCAACTATCAGG
AACTGTAGAAGCAGCAGATATTAACTCACTTAGAAGAGCATTTAGACTTCAAGAATGGTT
AGAAAGAAATCCTAGAGGAGGAACAAGATACATTGAAATCATACTTGCTCACTTCGGTGT
AAAGTCTTCAGATGCTAGACTTCAACGTCCAGAATACTTAGGTGGTTCAAAAGGTAAAAT
GGTAATTAGCGAGGTATTATCTACCGCAGAAACAACACTCCCAGTAGGTAACATGGCAGG
ACATGGTATATCCGTATCAGGAGGTAAAGAATTCAAGTACAATGTTGAAGAACACGGGTG
GATTATGGGAATAATTTCTGTAACACCAGAACCAGCATATCAGCAAGGAATTCATAGATC
ACTCAGCAAATTAGATAGACTAGATTATTTCTGGTCAACCTTTGCAAATATTGGAGAACA
AGAAGTATTAGGGAAAGAAATTTATGCAAATGGTATAAACATTGGAGAAACATTCGGTTA
TGTTCCCCGGTATGCAGAATATAAATTCTTAAAGAGAAGAGTAGCAGGAGAAATGCGAAC
TTCACATGATTACTGGCACTTAGGAAGAAAATTCAGTGCAGCACCAAACTTAAATGGAGC
ATTTATTGAATGCGACCCTAGTACGCGAATCTTCGCAGTTGAAGACGCAGAAGTTGACAA
CATCTACGGACATATCTTTAACAAAATTAAGGCGATTAGAAAAATGCCGAAAT
>NewGenomeName_48
GTGTGCACATCTCTATGGTCACTTCCCACCCATGGCCCTACGAAGTGTGGTAAGTGTATA
GAGTGCCGTTTGGCTTATTCGAGAGAATGGGCTATTCGTATAACTCACGAGCAACAGATG
CACAAGGTGTCTTGTATGCTGAACCTCACGTATAACGATGATTGGTTACCTGAACATGGT
CAACTTTTTAAAGATGACCTACAACGGTTTTTCAAGCGGTTGCGTAAGGCTGGTTTTAAG
TTTCGTTATGTTGCCTCAGGTGAGTATGGTGATCTTTCTAGAAGGCCTCATTTTCATATT
GCGTTATTTGGTTTGGATTTTGCTCATGACCGTTTACGGTTTGGCAGTTCTAATGGTGAT
TTTACTTTTACTTCTGCAACCGTCTCTAAGTTCTGGCCAAGGGGTAATCATTTGATTGGT
TCCCTTCATTTTGAATCCGCTGCATACATTGCCCGAGATATCTTGAAAAAGATTAAGGGC
TTCCAAAATCCTGAACCGTTTTACATTGATGATTCTACTGGTGAAGTTGTTTTGCCTAAT
CCCTAATTTCTGGTTATGTCTAAAGGCATTGGCCGCTCTTGGTTTAGGGATTTCTTCATG
TCGGATGTACTTCCGCATGCTTCTGTTATTACTCCCCAGTGCTCTAAAGCCCCAGTTCCA
CGTTTTTACAAAACTTTGTTAAAGGAGGTTGGAACCGACCTTGCACTGGATATGCTGTAT
AGGTTTTCGGTTCGTGCCGAATTAGACCTCGAGCGTAAAGCTTATGAGGACCTTCCGGTC
CGTAAGCGTTCGCGTTCTTTTGTTAGCTCTACTAGAGCGTCTCTTTAAAAACGTATAATT
TAAAGGATTTGTCATGATTTTATTTGTTGTTTCTGTTAAAGACCGCGCTGCCGATGTTTT
TAACCGTCCCTTTTTTGTTCCTCATCGTAATGTTGCTATTCGTGTGTTCACTGATGAAGT
GAATCGTGCATCTGGTGATAACCAGTCGAATAAGCATCCCGATGATTTTGATTTGTATTT
GTTGGGCGAATTTGATGATGCACGTGGCGCATTTGTAAATAATGAGCCTCTGGTTCATGT
TTGCGCAAAAGACGTTTTGCAGTCGTCATGACCCTTGTGGACCTTCGGGGCCACTTTTTT
TAATTTCATTTTGGAGTTTTTATGTTTCACAATAAATCGGTTGATGCACATAGTTTTGCA
ATGGTCCCTCGTGCTGACATCCCCCGTTCTCGAGTTTCTCTGCAGAAAACTCTTAAGACT
AGTTTTCATAGTGGTCTTCTAGTTCCTATTATGTGCGAAGAGGTTTTACCCGGAGACAGA
TTTAATGTTAATGTCACTATGTTCGGCCATTTGGCTACACCCATTTTTCCGGTTATGGAT
AATCTCCATTTGGACTCATTCTTTTTATTTGTTCCTAATCGTTTGGTTTGGACGAATTGG
GTTAAGTTTGTGGGGGAGCAGGATAACCCTGCCGATTCTATTTCTTACACTATCCCTCAA
CAAGTATCCCCAGCTGGTGGATACGCTATCGGGGCCTCACAGGACTACCTTGGTTTACCG
ACTGTGGGTCAAGTTACCGCTGGTGCTACGGTTTCACATTCGGCGTTACCTACCCGAGCC
TATAATTTGATTTATAACCAATGCTTTAGGGAAGACAATTTACAGAATTCTCGTGTTGTT
GATAAAGGTGATGGCCCTGATGCCACACCTTCTACTGTTTATGCTTTGCAAAGACGCGGT
AAGCGTCATGATTATTTTACTTCTTCTTTACCATGGCCACAGCAAGTTGGTACTCCTGTT

TCTCTTCCTTTAGGTACTTCTGCGCCTGTTTATGGTACTGGAAAGACTCTTGGTCTTTTC
GATGTTGTTACTAATTTAGGTTTGCATGCTGATACTACTAATACATTGCGTAATGCTTCT
TCTTCTTACAATACCAATATTGGTTCTATTCCTACTGGTGTAGGGGGTGTTATTAGTAAG
AATCTTGGTGGTGTTGCTTCTGGTGTTTCTGGTTTATATGCTGATTTGTCGGCTGCTACG
TCTGCGACTATTAATCAGCTTCGTCAATCTTTTCAGATTCAAAAGTTGCTTGAACGTGAT
GCTCGGGGTGGTACTCGTGACACTGAGATTTTGCGTTCTCATTTCGGCGTTACTTCGCCG
GATGCTCGCTTGCAGCGTCCTGAGTATTTAGGTGGTGGCTCTACTCTTATTAATATTTCT
CCTATTGCCCAAACCACTGGTACTGGTATTTCTGGCCAGACTACTCCTCAGGGTAATTTA
GCTGCTATGGGTGTTTATCATGCACACAACCATGGGTTTACCCAGTCGTTTGTTGAGCAT
GGTTATGTTATTGGTGTTATTGCTGTTGGCGCTGATTTGACTTATCAGCAAGGTTTACGG
CGTCATTGGAGCCGTTCTACTCGTTATGATTATTATTTTCCTGCTTTTGCGATGCTTGGT
GAGCAAGCTATTTTAAATAAGGAGATTTATGTTACTGGTGGTTCGTATGATTCGAATGTT
TTTGGTTATCAGGAGCGTTGGGCTGAATATCGCTACAATCCTTCTGAGATTACTGGTCTT
TTTCGTTCTACTGCCGCGGGTACTATTGACCCATGACATTATGCGCAGAAATTTACTGCG
CTCCCTACTTTGAATTCAACTTTTACTGAAGATACACAGCCTCTTGCTCGAAATTTGGCG
GTTGGTTCTGCTGCTAATGCTCAGCAGTGTTTGCTTGAGGCGTTTTTTAATATTAACGCT
GCTCGTCCTCTTCCTACGTACTCTGTACCTGGGCTTATCGATCATTATTAAGCCTCTATC
ATTCGCTTCGGGGAATGATTGAGGGAAAAGTTTTTATGTCTTTTTTTGAAACTCCTAAGA
GTGGTTTTGATTCTGTTAGTTCAGTTGTTAATGCCATTCCTTCTAATATTATTTCTGCTG
GTGCTTCTCTTCTTGGTTTTAAGGGTCCAGCTAATACCAATGCTGCTAATGTTGCATTGG
GTAATCGCCAGATGGATTTTCAAGAGCGTATGTCTAATACTGCTTCACAACGGCAAGTTG
AGGATATGAAGGCTGCTGGCCTTAATCCTATGCTTGCTTATATGAAAGGTGGTGGTGCTT
CTACGCCTTCTGGTGCTATGCCTCAGGTTCAGAACCCTTATGCTGCCTGTGCTACAACTG
GTTATCAGTCTGCCCAAACCCAAGTTGCTAATAAGCAAGTTGGTAAGGTTGGCGCTGAGA
TTGAGAATGTGGATGCTGATACTATTAAGAAGCGTGCTGATACTCTTTTATCTCTAGCTA
ATAAGTAATTGTCTTTTGCTTTTGCTGATGAGAAGCGTTCTTCTATTAATCTTCAGTCTT
TTCAAGCTAAGAAGATTGCTGTAGAGACTAAGAATATTCCTATAGAGGGTGATCGGTTAG
TTGCTGTTATTAAACAGCTTGTATAGTCTGTTAAGCATATTGAAGCGCAGACGTTGACTG
AAGAGCAGCGTAAGCAGGAAATTTTCGCTCTTGCTTTGAAGACTTTGAATGAATCCGTTT
AGGTGGCTGCTGATTTAAAAGCTATTAACCAAGCTGACAATTTGGGTAAGGAGTTTGGAC
AGTTCAAACCTTTGGTTGATAATGTTATGAACATTTTTCGTATGTTGAGACGTTAAGGAG
TTTTTATGTTATTTGCTTCCGCGTATGATAATTTTGATGCTCGTTGTGACGACTTTGGTT
TGAAGTGTTTAGACGATGCTTTGAGTCAGCAGCAATTTCGCGATGAGTCTGATATCAACA
ATATTGTTGCTCGTTTTATGAAGACTGGTCATTAACCTGATCCAGTCTCTATGCCCCAGT
ATGTTGATTATGAGGGCGTCTTTGATTTTCAGTCTGCTATGAATGTAGTTCGACAGGCTG
ATGAGAAGTTTATGCGGATGGACGCAAAAGTTCGTGCTAGATTCCATAATTCCCCTCAGG
AATTTTTGGAGTTTTTCGCTGATCCAGCGAATTCTGAGAAAGCGGTTCGCCTTGGTTTGG
CTTTGCCAATACCTAAGGAGACTCCTCCGGTCGATGCAGCTCTTGCTGCTTAATCGTTTT
TGGGCACAGTTCGCTACTTGATGTATCGGTGCCAATTGTCACCTTTTTGTCTTTCTGTTC
TACTGGAGTAACTATGAAGCCTTTGCACCGTCACAATGCCAATAAGCCCTCAAGCGCTTC
TTCTTTTAAACGTAACATATATACGACTAAGTTGATTAATATCACTGCTGGCCCGATGCG
CGGTGGTATACGCCTCTAAGGTTCT
>NewGenomeName_49
ACCGGCCACTTGGAGCCACGGAGCCCCTTGCAGCTATGCCTCCGCAAGAGGGGGAAGCAG
CGGCTAAGAGGCTGTGCTTCACGCTGAATAACTACACAGATGAAGAGGTGTCCGCCGTGA
AGGCGTGGAACGAGAGTGAGTACCATTACGCTGTCGTTGGACGCGACAAGCGGGAGAATG
GTACGCAGCATTTGCAGGGCTACATTCATCTAAAGAAGAAGGCTCGGCTAAGCACTCTGA
AAAAAATACTTAGCCGTGCCCATTGGGAGAAAGCTCGCGGTTCGGACAGCGACAACGAGG
CGTACTGTACGAAAGATGGGGACGTGATACTTACCCTTGGCATGCCGGTGGAAGGGAACC
GTAGCGATATCCCCGGAGCTGTTGCCGCCGTGAAAGCCGGAAGTCGAATGGTGGATATCG
CGCGAGAGTTCAGTGAAGTCTACGTCAAGTATGGGCGTGGCTTACGTGAACTGGCGCTTC
TGATTGGAAAGAAACGGCGAGATTTCAAAAGCGAATTTATCGTTGTCACCGGTCCGTCGG
GCGTGGGCAAGTGCCGTTATGCTAATTAGTATCCAGGCACTAAGTTTTATAAGATGAAAG
GGGACTGGTGGGACGGCTACTCTAACGAGGATGTTGTCGTTATTGACGGCTTATATGGAT
GGATACCTTTTTGTGAGTTACTGCGTCTCACTGATAGGTATCCACATAAGGTTCCCGTGA
AAGGATCCTATGTGGAGTTTAAGTCGAAGGTCGTCACAGTGACCAGCAATACGCACACCG
ACTCGTGGTACAATCAAGAGAAGTGCTATCTACCCGCGTTGTATCGGCGGATAAATAAGT

GGCTGACGTGGAATGCCATCCGGTTCGAGGATGCACCGGATTGTATGAAGAACTACCCTA
TAAACTATTGATTACGCCCCCTCCCTCATAGTGCCTTTCGAGGAAAGAGCAATCGCGGCC
AGCCAAGGCGGCCGCGTTCACGGGTACTTCGCGAGCGCACCGGCGCGAGCGAAGTTCCCT
AGTGAAGTTCCCCGAGGAAAACACTTGAGATGAGGCGATTTATAATTTTTACACTTAATA
AATAAAGACAGGAACCAGTATTGTATGTCTGTACTTTATTTAGGGTTTGCGAGGTGGGTT
AGGTTCATTTGTCTGAACTCAACATATATCGTCACTTTTGCATGGAATAGTAAAGCGTCG
TGGGGCTGGGGCCAACTGATGCCGAGGCCGTAGTGCTTTACTCTAGAGCCCCAGCTGGGT
AAGTTTTGGTCTCCTTTCTTCATCATAGGTATCTATGCGGATCTGGGGTTGGTTAGCCAT
TGCGCTGCAGTAACGTTTCCCGCGTCGGTGTCCGGAATGGTAATTTGGGGTCGGGATCGG
AAAAGACGCTTGAATCCTTTGCGGAGGTCCCGCTGTCTGGCGCCGTCCCAGTTTGCTAGC
GGGTCTGTCTCCAGGACTGGCTTCTTGTAGAATTCTTCAAGTTGGTTGTCTTGAATGGGC
ACACTGTGCCCTATGCCATGCCATGATCCCCAGTTCTCGTTTAGTGGACGCATTTCGAAT
TTTGCCAGCCGGATCCTGTAGTCTTCGAATGGTAGCCTCCAATTAATTGTTCCCCAGTCG
AGGAAGTCGTCCAGCATTATGATGAGGCCGTCGGTAGCCCATTTCAGGGTAGTGTCACCT
GAGCCGGCGCGTTCGTGTCTGTACAGGCGGAGAAACTTGAATCGGTATACTCTGTTAGTT
CTGTGACCACGCCTATTGGTGAGTCTGCGTCTGCGCCAGTAACGTCGTCGCCATCGCCGT
CTAATCGCCTGTGTCTGTCGGCGTCTCCTAGCAACCTGTATGAAAGAGCCAATCACGAGG
TGTCACGTAACCGTGGCTCCAAGTGGCAGTTGAGTGTT
>NewGenomeName_50
ACCGGCGGGCCGGCGCCATGGGGCCATCTGGCCCCTGGCCAAGCATGCGCAGTAGTGGTG
CATGCTCGATGGCCGCGCAGCGGCCTTTATTTGCGGAGCATGCGCTGAAGGCAGCGCAGC
GGCCGCTATCTGCGAAACATCACGGCAGCACGGCGAGACAGCGGAGCCCGCCGCTGGTGC
TTTACTCTGAATAACTACACTCCCGAGGAAGAGGAGACCGCTCGCAATCTGATTCAAGAT
GCAGATAAGGACGCGTTTGCGATAATCGGCAAAGAAGTTGGCGAAAGCGGTACTCCTCAT
CTGCAAGGCTTCATGCATTTTAAGCAAAAGCAGCGGCATACCGCTCTAAAGAAACTTATT
CCTCGCGCGCATTTTGAGAAAGCTCGCGGCAGTGATCAGCAGAATGCTGATTATTGTGGT
AAAGACGGCGAAATACTTACCATGATCGGTACTCCGAGTGATACGAATTCGAGTGATCTT
GCGGGAGCTGTTGCCGCTGTGTAACGCGGAAGTCAAATGAGTGAAATCGCGCGAGAGTTC
AGTGAAGTCTACGTCAAGTATGGGCGGGGCCTCCGTGATCTCCAGTTGCTGATTGGTTGC
CCGCCCCGCGATTTCAAAACAGAAGTCATCGTTCTGATTGGCCCAGCTGGCTGTGGCAAG
TCAAAATTGGCCAATGAGATGGAAGGGTCTAAGTTCTACGAGATGAAAGGTGGTTGGTGG
GATGGTTATGACAATCAAAATATTGTCATAATCGATGACTTCTACGGTTGGCTCCCGTAC
TGTGAGTGTCTACCTCTGTGTGACCGTTACCCTCACCGTGTGCTGGTGAAAGGTGACTAC
GTGGAATTCACCAGCAAGAAGATTGTGTTTACCAGCAATCGCCATGTGGATGGGTGGTGG
ACAGGTGAAATTGAGAAGAGTGCCTTCTACCGACGGATGAATGTGTACAAGTTTTATGAG
ACTGGCGAATTCAAGGACATGCCCGGACACATGCTCCCGCACCCAATCAATTATTCGGCG
CGAGATGAAAACACCCCCCTCGGGATGCCAGTCGTTTTCATCTCGCGCCCCCGAGTCGCG
GAGCTCGCGAAGCGAACGGAGCGCGAGTGGCGCGCGAGATTGTCGACGACCGACGCGCGT
AGCGCGGAGGGAGGAGACCACTTGCTTAGAATTTAATATGCTATCTTTATTAAAGAGATC
CAACAACATTCCTGTCTATGTCTTTTTCATTGTCAGTAATTGTTAAGCAGTCCCCGAACC
TTGACAGGTCTTCTTCAGATTTCTTCAACATTATATGAGCGAACTGCCTGAATGTTACAT
ATATAGTTATTTCTGCATTATATCTCAGTTGCAGCCCAGGTGAAGGCCTAGAGAATGCCA
GCCCGTAGTGCACTACTCTTGTACCGTCCCTCTGGGGCCCTGCTGGCCTTTTTAAAGGTA
ACCAGCCTTATCTACTGCTATTCAGCCACATTGATGCTGAAAAGTTAGAGGCTGTCATAT
CTGATATGGTTAGTTGAGGCTTTGGGCGTACCAGCCTCTTAAACCCTCTGACCAATTTCC
ACGCCCTCGCCCCGTCCCAGTTATCCAGTGGGTCTTGCGTTTGATCCTGTAATTTTATAT
CTTTAACTCCGGCGTCCAGAACTGGGACTGTGTACCGCCAGCCCCTTGCAATTTTTGTAG
TGACACCTTCAGGCCTCAGCACCACTTTTGCTAAATTAATCCTGTAATCCTCGAACGGCC
ATTCTGCCGGTATTCCCTCTTGACATATAAAGTCATTCAGGTTGAAGGTGAATAAATCTG
AGGCCCACTTTTGTGGGTCAGTCTTGGATGGGAAGTCCACTGTGAATTGCCGTCTAAATC
TGAACAAGTATATTCTGTTCAGTCTGTGCGCTCTTCGGTTCCTGGGCAACCTGGGACGTC
TGCGCAGTCGTCTGTGAAACCGCCTTCTCCGGAAACCGCGAGTAACACGCCTCCTTCTGG
TTGCTGAGCGACGTACCTAAAAAAAGAATGGCCCCACGGCCCGCCGTATAGTATT
>NewGenomeName_51
CTAGTAGTACATACAAAGTGTTTCCCCCCTACGACCTCCTTATGCTCACCCCAATGCGAG
CACCCATATGGTGACTTTAGGTTTACTTTGGTTATACTTGAAGTGTACTTGAACTGTACT
TTAGGCGTACTTAATCCTTAACCTTATGTGTGACATTGGCCTTACTACTAGATGTAGTGC

TTACGGGGGACTTTAGGGGACTATATGAAGGCCTCTTACTGTCTCTATCTGTAAGTCAAC
CTCTGAGTATCTCCTTATGTCACCACCTTAAGTCATCTCGTTACGTCACCACCTTATGTC
AACACCTTCAGTCCTCTCCTTATGTTACCACCTTAAGTCAGCCTCTATCCCTATGTTAAC
TGTCAGTCGGTGACTTCAGTCTATAGACATTATGTTACTACTCTTAGAGGGACTACTTAG
AGGGTACGTAGAGACTACTTAGGTACTACTTAGAGACTACTTAGGGACTACTTATAGGCT
GCTTAGGGACTACCTATAGGCTACCTAGAGACTCCTTATGGGCTACCTAGAGACTACTTA
TAGGCTATCTAGCAAGACCCTTACACTTAATACGATTCCCTATTGGGAAGGTGTCTATCA
CTATGCCTTTCCTTATAGGTATAAATTAGAGTGCTAACTTAAAGAACAATCAAAGAACAC
CTAAAGAACACGTAACGAGGACTTTTTCTTACTTTAAGTATTGACTCTAAGATTAGATAC
TATATTATTCATCTCAAGCCAAAACAGTGAGTCGCCCTCTGATACCACATCAGGTGTCAA
GGTGACAATCACTAGGTTGACATGCAATAACACTAAGACGTACAATGATTCTCACAGATT
CACATAGATTCACCGGTCTTTAACAACATCGATAGTAACACACTTGAGAATGACAAATAG
TGATTAACTCAAGGTGACTCGAAGAGTGAAGCCTTTATGTTAAGCACTAACTTACTAACT
AATGAGTTCATCACAGTATGAAGAATTACTCTGGCGGGTACTCCAGATATAAGTCAATCT
CTAGGTACTTTGGGACACATCATTTGACAGGTCTAACGCCACTAGGTGTAGTGACTGCTG
CTCTCTTACTCATCGCAATGCGTAAGTGAAACAAGTAGGGCATTAACTACTATCGCGGTA
AGCAGCTCGCTAAGTCTCTAATTGTTCCTGGCAGCCCTATTAAAGGGTCTGAGGATACAC
AAGGCTAAGCTCTAGCACTTGAAACAAGGCCTGGTCTCTACAACTAACCTTCTACTATCT
TAAATAGGACACACTCTAATGAACTACACCGATATTCACGCACGTCTGGCAATCATCAAG
TATGTCCCTATGAGTGAACTGGACAAGCGCCAGCCTCTTCTGGTTGAACTAGCTGCTAAC
ATTGTGAACGGTAACACTAATACCGGTAACGATACAGGTGGGTCTAAGGGTCTTGTGAAT
CAGGACTGGTGGCATATCTTTAGGTAACTCATGGGTGATGCAGGGTTCAACCAGTTAGGT
AATGGTCACTTCAGTGCAGCTTACTCAGGCTAAATTTTACCCGGTCGAGCCATTAATGTT
GGCTTGAAGAAAGAGTACTCAGGACCTTCATACACTGCGTTCTGTAGGATGCACCAAGGT
CGAGCTGGTATCCCTAACATCTATCACGTAGCACGTCACGCAGGCCGCTACACAGTTGTA
CTTGACCATTTGAGTGGGTCTAAGCGAGACACTAATGAGACTCACGCTAAGTATCCGTAT
GTTGCAAGAGCACTCATTGAATATAACGATATCAATGTCGAAGCTCGTGTACATTTTGTT
GGCACTGAGTTTATCGAGGCGTGCAAGATGTTTCGTGAGTTCTACTACGGGATTGCGGCT
TTAGATATGCACACTGGGAACAGTATGTTCGACAAGAACGATGTGGCGCACTTCTCTGAC
CCTGTTAGCACATCTCATGACCGTGAACCTGAGGATAGCTTCCCGTTGGACCTCGATGCA
ATGATTGCTGAGGTCGAGGCGATAGCTAACGGGCGTATCATCGAACGTTGTCGGAACCGT
AAGGCAAAGCGTGAGCCTAATGGTACATTCCGTGCTGAACATAAAGCGGCAATGAAGCGC
CGCAAGTGTGACCGTAAGTTATCTGAATGTAGAGCTGCACGGGTAGGTAAGTGTGAAACT
CAGCAGGACCACTAACATTTAATGTCCCGCCAAAAGGAACACAAACTAATCGACCGAAAC
GAACGTCGTGTTGGGTTTCACGTGGGTCCTGTATGGCATGACTTCGGGTTTCTCAATGGG
AACTCAACGATAGACAAAGTTAACCCAGTGTACGACTTTATGTTGGAAGTACCTGACGGA
TTAGCTATTCAGGCAGGAGGTTCACTATTGATAGATAAAATACTAGACGCACACCTGATG
GGATGATACGGTGTATCTTCAAGGTCGGTACTAACTACTGGGCGTTATAGATAATCTTCC
TTAACAAACTGCGGCAACAATTACTAACTTACTCAACGCACCAAAGAACGACTACTCTGA
TATCGCTAATGCCATCCAGCCGTACAACATCCTGGCTGCCCATTACGGTGCCCAGCTCGC
AGCTACACGGCTCAAGTTGGAACATGTAGCGCACACTGAGGAGGAGAAGCGTTCCCTTAA
GGCAATGGAGCGTCAAATCAAACCAGGTGAGTTCGGTGATAACACGGTAGCTAGACCGCT
CCTCTCATCCCTTCATCCAAAGTTTATCGAAGCGTGGAACACTTCGTTTATTGATGTAGA
AGCAAAGCGTGGTAATCGTCCAGTGGCCTACATTCTGTAACAGAAGGTTACACATGAAGC
TGCTGCGTTCATCACTCTGAAAGTGACACTCGCATGCTTGACGAAAGAAATGTTCACCAA
CCTGCTGTCGGTAGGCACTAAGAATGACCGCAGAATTGAGGACGAGTTACGCTTTGGGCG
CATCTGTGACGAAGTAGCGACGCACTTTAAGAACCACGTACCGGAAGCACTGAATAAGCG
AGTCGGTATCGTCTACAAAAAAGCATACATGCAAGCTGTAGAGGGCAAGTTGCTCGATGC
CGGTCAACTGCAAACCAAGTGGACTAGTTGGACACCTCAAGAATTAATTCACACTGGTGT
ACGTATGTTAGTGTTAATCATTGAGTGTACTGGTCTCGTTGAGTTGCACCGTCCGTTTGC
TGGCAACATTGAGAAACACGGAGAGTACATCCAATTGACCGAAATAAATGTTGACCTACT
GTCTAAACGTGCTGGTGTCCTGGCTGCAATTGCTCCAATGTAACAACCTAGTGTAGTCCC
TCCTAAGCCTTGAACTTCTCCCGTGGGTGGTGGCTTTTGGGCTGCTGGTCGTAAGCCTCT
CTCTATGCTCCGCACTGCTTCCAAGAAGGCGTTAGAGCGTTACAACGGTGTGTATATGCC
TGTAGTCTACAAAGCAGTGCACATTGCACTGCTTACTCCTTCGAAGATTAACAAAGAGGT
TCTGGCAGTCGTCTACGAGATTGTGAAATAGAAGCATTGCCTGGTTGCTGACGCACTTCC
TATTGAACATGGTGAAGTACCAGTGAACCCTGTAGACATCGATACAAACGAAGCGTCTCT

CAAAGGATGGAAGAAAGCAGCACCTGCAATGTATCGTAAAGAGAAAGCTCGTGTGTCTCG
TCGTATGAGCATGGAGATCATGTCAGGTAAGGCGAATAAGTTCGCACAATTTAAAGGTAT
CTGGTTCCCTATGAACATGGACTGGCGTGGTCGTTTATACGCAGTACCAAAGTTTAACCC
ACAAGGTAACGACATGACTAAAGGTCTGCCGACATTAGCTCAAGGTAAAGCAATTGGTGT
GGAAGAGTTCTACTGGTTGAAGATTCATTGTACTAATACTGCTGGTGTTGTCAAGTTGGA
CTTCGGAGAACGCATCAAGTTCATTGTTGACAACCATAAGAACATCATGAGCGTTGCAGC
TGACCCTATTTGTAACAGTTGATGGACTGAACAATACTCACCTTCCTGCTTCCCCGCGTT
CTCCTATGAGTATGCAGGTGTGCAACATTACGGGATGAACGAGAAATGCCCTCTGGCTCT
GGCGTTCGATGGTAGCTGCTCTGGTATTAAACACTTCCCTGCAATGCTGCGTGACGAAAT
GGGTGGACGTGCTGTTAACCTACTGCCAAGCAAAGAAGTACAAGACATCTATCGTATCAT
AGCTGAGCGTGTGAACGAGGTTCTTCATCAAGATGTAATCAACGTTGCTCACATGGAAGT
AGAAACAATGACTAATAAGGACACTGGAGAACTCACCGAGAAGCTCAAGCTATGGACTAA
AGAACTGGCTGGTCAATGGTTGGGCTGGGGTGTAACACGTAATGTAACTAAGCGTTCCGT
AATGACCTTGGCATATGTTTCCAAAGAGTATGGCTTCCGTGACAAAGTGTTAGAGGATAC
CAATGAGCCAGCTAACGACGTTGGCAAGGGCTTGATGTTCACTCGACCTAACCAATCAGC
TGGCTACATCGCTAAACTGATTTGGAATGCAGTAACAGGCACAGTGGTTGCAGCAGTTGA
GGCGGTTCACTGGTTGCCGTCCGCAGCTAAGCTTCAGGCTGCTGAAGTTAACGACAAGAT
GACTAGAGAGATTTTTCGTAACCGTTCCGCTGTAGCCTGGGTAACTCCAGATGGCTTCCC
TGTGTGGCAGGAGTATCGCAAGGCAGTACAGACCCGCTTGTACTTAATGTTCTTAGGTCA
GATTCGTCTACGACCTACAGTGAACACCATTAAGGATAGCGGAATTGAAGCGCGTAAGCA
GGAGTCAGGCATCCCACCTATCTTTGTCCACTCAATGGATGGAAGTCACCTTCGAATGAC
TGTATTTCGCGCCTATGAGATCTACGGTGTGGAGTCCTTCGCACTGATTCATGACCCATT
TGTTACTATCCCGGCCGTTGCTGGTAACCTGATTCAGCCTGTACGTGTAACAATGGTGAA
CACCAACGACGAAAACGTTTTGCTTGCATACGTCTATGACCAGTTAGCTGATCAATTACA
TGAATCAAAGCTTGATAAGATGCCTGAGATGCCAGCTAAAGGGTCTCTAGATATCCAAGA
AATCCTCAAGTCAGACTTTGCGTTCGCCGAGCGGAATTTAATTCGACTCCCTATTGGTAG
ACCCAAGGGTTTCCCTACTTTCAGATACACTTTAAGATATACTCTAAGAGGCACTTTAAT
TTGACTTTAAGATGAAACAACCTCATGTGTACCAACTTTGAACCAAACACTAAGCATATC
GACGCAATCAACGAACATGGTACTGAATGGCAGGAACGTAAAGACCACTTCAAGAAACGC
CACAATACACAGCGTGGTAACTCTGAGAAACGAAACTGGAAGGATGCACTCTAATGAGTA
GCTTATATAGTGGGAACCTAAACGCCTTCAAGGCTGCTCCTGACAGAGTCTCTAAGATGG
TCTTAGTGGTCGCAAGTGAGGACTGGTATGAGTCACATGTAAGTCTGAACTGAAAGTGGC
TACGCATAGTGGACAAGGAAGGTAAGTGGATTCACTCCATCACTTGTATTCATCAAGACG
AAGACGTTCTATACAGTATGGAAGCCGCGTTGTTGAACCGAATGTATGACCAGTTGAAAG
ACTGGAAGTAATACGACTCACTATTGCGAAAGAGTGAAACTCAAAGTCATCTAAAAGGGG
GCCGTTGAGATTAACTTTGTGATTAACTCTTGATTAAACCTTAACCAACACAAACACATA
TACATCTATAGGACTGCCACAATGCTAATTTCATTCAAGACTTCACCACATAAAGCTGTT
TTATTCGTAGAGACTTCCGTTAAGAAAGCTCTCAACAAAGCTGGCTACCTTATCCGCGCT
GTAAAGTATGATGGTATTCGTGGTAAAATTGAAGTTGACAGTACGGCTGACGGCATGTGG
CTCTCCCGTGTCTGTAAGACCATCCCTGCCCTTGAGCACATCAATCGGTTCGACTCTCGC
TGGCAGCGTCTTCTCAGAGATGACTCCTGTATCTTCCCTGATGGTGTCATGCTTGATGGT
GAACTGATGGTCAAAGACGTAGACATGAACACAGGGTCTGGCCTCCTGCTTACCAGATGG
AGTAACCCTAAGAATTTCAGTACCAACGGTAACGCTTTGGCATCTGACTTCGCTATGAAG
TCTGCGATGGTTGCCTACATCTTGTCCTCTGATAAGCTTAAGGTTCTTCTGTATGGTGTC
ATGCCGTATGGCGCTCTGGTTTCTGGTGAGACGTACAAAGTGATGAACCTTCTGATGCGT
GAGCTCATTAAGGCAATGCTTCCGTTAATGACCGAGTACTTCCCTGAAATCAAATGGAAC
ATCTCTGATACCTATGAACTCTACTACTATGCGGAACTGACAGCACTCTATAGGAAAGCT
CGAGCCGATGGTCAGGAAGGCCTAGTAGTGAAAGACCCTCTGGCATTCTATCAGCGTGGT
AAGAAGGCTGGCTTCTGGAAGCCAAAGCCTAAGTGTGAAGCTGATGGTATAATTCATTCC
GTCAATTGGGGAACACCGGGTCTCTCTAATGAAGGCTTGGTGATTGGCATTCATGTTCTC
TTAGAGACTGGTCGCCATGTCGCAGCTAACAATATCTCACAGAGTCTTATGGAAGAGCTC
ACAGCTAACGCTAAAGAACACAGTGAGGACTATTACAATAGATGGGCCTGTCACGTAGCC
TACATGGAGGAGACGAGAGATGGATCATATCGAAATCCCTCATTCATTAGGATCAGTGGT
CTAGAGTCACACCAAAGGGTCTAGTGGTGAATCCCTCAATGGTCAGTCCTTAACAAGGGG
CTAGCCTTTAGCGTTTCTAATTAGATTTACTATGGAGGAATAACAACATGCTATTCCTAA
TTGTTGCTATCATCGCTCCCACACTTGCCTTATTTATCGTTGACGACAACTGCTGGCCTG
ATTGCTAATACGACTCACATATGGATGACACACGTATGCTACGATTAAACTTCAATAGAT

CTAATGGCACCAACTCGGTTCGTGAGTCTAGCCGCTCTCTGGCCAATGATACCGAGAAAC
ATGAAGTCATTCCAAAAGTGCTTGGAGCTACGATTGAGTTAGCACCTCGTGTTCACGCTG
TGATTACTACTGGCTTATCTAAAGAAGCAACAATTGGCTCCCGGACTTTCATTCCGGTTC
TCTTCACCCGTTTCCCTAAAGTCCGTCTGGTCATAGAGTGTATCATAGTGGTTTGCAAAT
GTATGGTTGCACTGCATGTATAGAGAATGACCACCGCCACCCACATACCTGTGAGCCTGT
AGCTAAAACACAAGATTTAGATTGTGTGCGCGCTCAGAGCCACTATATGGTGTTTGATGG
CATCGATGCGATCGAAGTGATTGCCCGATCGATGACCCGAGAACAGTTCAAAGGGTACTG
CTTGGGGAAATTCCTTAAGTAATGTCAAAGTGCTGGTAAGAAATCCGAGCGGGCATTCTT
AGAGAAGGACATTGCAAAAGCTGGGTTCTATGGAGAGCTGTACTAGAAACATAAAGGTAA
ATGTTATGCTTAAACCTAGTGAATGGCGTGTGAGAAAATTCAACTAAACTGGCAATGTCG
ACTAGCTCGAAATGTACAACCTGTGGAAAGGGAGAGGACAATGAGCATTTTAAACAAGGG
GTCACTGGTTAATGGTGACATGAAGTTCTTAGCTACTGTAGAAGGCAAAGGCCGATCATT
CGAAGTATGTGTGTTCGATGCGCCCCTTGCAGAGCCCATCGAATTAGCTGAGTGGCAATA
TGTCCCAGCTGGTTTCGCAGTGACCCGCATCCCTCCAGACACAAAAGTTTAATTGGACTC
ACAATTGGGCGACGCATAGTTTCCCACTTTGAACATTAATAAACTTAAGGGGAAACTTCA
TGGCTTTCACTAAACGTAAAGTATTCACTACTGCTCGTTGTATCGCTGAACAGTACTGCT
ACCTGTCTAAGCCTGACTTTGGTAATGGTGAATTCAAGAACGAACGTGGTGTCTATAAAG
TATCACTAACTGTATCCAACGAACACCCTCACGGTCAGAAAATGCTTGTTGAGATTGGCG
GGGCCCATGAGACGCCTTATGCTGCAAGTATGGAAGAGTACGCAGCTAACCCGCCGAAAG
TGGTTAGGGGTAAGAAACCGCTGAACCCATATGTTGGCGACATGCCTTTCCTGGTTAACG
AAGATGGAACCAAAAAGTTCACCTTAAAGTTCTATGGTTCATACGCTAACAGTACGACTG
GAGATAATAGACCAATCAACATGTCTATCATGGACTCCAAAGGTAAACGCATTCGTGGTG
AGCGTCTAGCTATCTCTGGTGGCTCCGAGCCCAAGTTTTAATACAATCTGTTCCCTTATG
GCCGGTCTGCTGTGGCAGGTGCCTCCGTTAAGCAACAACTGGATTCCATTACGCTGATAA
AGCTGGTTGAGTTCGGTGGCAGGGGTGAGGATGACTGGGCTGATGAAAACGAAGAAGACG
GTTCTGTTGCTGTTGAAGCACAGACCCGTAAGCCTCAGAACGGCTCTGGCTGGTATGAAG
AACCAGAACAGGACGATGAAGATGATAATGAGACCTGCGACTTTTAATATACGATAGGGA
GGGCACATGGCAGGTTATGGTGCTAAAGGAAATAACAAGGTGGGTATGTACCGTAGTGGA
CTCGAAGAGAAGAACATGAAACTCTTAGGGTACAAGGGTGTTATAGCTGAGTACGAAAGG
TGGAGCGTTCCTTATGTGGTTCCCGCAAGTAATCACCAGTATCCACCAGACATTTTATTG
CCTAACGGTGTACTTGTGGAGCCGAAAGGCTTAGGGGAGCGTGACGCCCGCAAGACACAC
CTGCTGATTCGAGAACAGTACCCCGACTTGGATATACGCCTGGTCGTCTCACGCAGTCGC
ACCAAGACTTAAAAGGGGCCTCCTAGAAGTTATGCGGAGTTCTGTGAGAAACATGGCCTC
TTATTTGCTGACAAGCTGATTCCAGTAGCGTGGCTGAAGGAAGCATCCCGGCATGTAACA
TTAGATAAGCTGAGGACCAAGATGGAGAAGAATAAATGAGTACGGTACAATTTTAACCAC
GCAGTTTTACAGAAGCAATCTTCGTACACTGCAGTGCAACACACGCAACTATGTACGTCG
GTGTCCGAGAAATCTGTCAGTGGCATAAAGAACATGTTCGGTTAGCAATCGGCTATCATT
TTGTGATTCGCCGTGATTGGACCATTGAGGAACGTCGCCCAGTAGAAGTCAATGGCTCTC
ACGTTAATAGCTGGAACAGTAAGTCAGTTGGCACGTGTGTTGTTGGTGGTATTGAAGATA
AGTGCCACTTTGAGGCAAACTTTACGCCTGCTCAGATGGTCTCTCATAAAGAGAAACTGG
CAGACCTGATGAACGTGTATCCAGACGCTGAGGTGAAAGTTCAACATGATGTTACACCTA
GAGCGTGTCCGTCATTCAACTTGAGCCACTGGCAGAAGACTGGAGAACTAGTGACAAGCG
ATTGGGGCAAATTAATAGAACTCACTTGGGGAGAGCCGAAAGGTTTCTCCCTTTATTCGC
TAAAAATGATTTAGGAGTATACACAATGTCTAACCCTAAAGAGCATGTGTTCGTCGATTT
AATCTGTGTACTTGTCATTCGTGGAGCCTGCCTGCGTCCGTTCCTATAGGAGGTATCTAA
TAGATATGGAACAACAGCAAGCTAGCGTGTTCCTGTACTACCTGCCGTGTGAAAACTGCG
GGTCGTCAGATGGTGACTCAATGTACTCCGATGGACACAGGCACTACTCCGTGTGTGACC
ACTTTGTCCCTCCCTCGGATGAGACTCGTAACCAAGAATCAACCAAGCGTCGTCGCTCAT
GTAACACTGGAGGTGACAAAATGTCTAACCTTTTAAATTTTGTGGATTCAGAGGGTCGCT
ATGCGAGCCTCAAAGCTCGTGGCCTTATGGATGCTATCTGTCATCAGTATGGATATTGGG
TAGCGAAGGTCAACGGCGAGATGCTTCAGGTCGCAAACTACTAAGACTCTGACGGAAACC
TTGAGGGTCAGAAGGTGCGCGACGAGAATAAAGAGTTATCTGCAAAGGGGAAACTCAAAG
CTGACCTATTGTTCTGTACGCAGTTATGCAATTGTGGCAAGCAGATTGTAGTTACAGAAG
GTGAGATTGATTGCTTGACTGTAGCTCAACTTCAAGCACAGAAGTATCCCGTTGTGAGTC
TCCCTATGGGTGCTCGAGCCGAAAAGAAAACATGCTCTGACAACTATGAACACTTTGACC
AGGTCCACGAGATTAACCTTATGTTCCATATGGATGTTCTCGGTCGTAAGGCGATCGTAA
AGTGTGCTCCTGTCCTACCTAGTGGTAAAGTCCGAGTCGCTGCACTGCCTCTTAAGGATG

CCAGTGAGTGTTTACTCAATGGACAAGCCAGAGCAGTAACCGACCAGTTTTGGAATGCAC
AACCATGGGTGCCTGATGGTGTAGTCTGTGCTCTGTCTCGTTAATACCGAGTGCTTGAAG
CTATGGTCAAAGAGGTAACAAGGGGTCTTCTATTCACGGGTGAAACGAAACTAAATGATA
TGACACTCGATGCTCGTGGTGGTGAAGTCACCATGATCACTTCAGGTTCCGGTATGGGTA
AGACCACGTTCGTTCGTCAAGAGATGCTCCAGTGGGCCAAGGGCGCTGCGAAGGTAGGCT
TGGCCATGCTTGAAGAAGCCGTTGAGGAAACTGTTCAGGACTTAATGGGCTTGAACAATC
ATGTCCATCTACGTCAAGACAAAGAATTCAAGATGAAAATCTTCGAGGATGGACGCTTCG
ATGAATGGTGTGGTGAACTGTTTAACACAGAAATATTCCACCTGTATGATTCCTTTGCGG
AGTCAAAGGAAGAGTGTGTCTTTGCGAAGCTGGCCTACATGGTCGATGGCTCAGACTGTA
ACGTCATCCTGTAAGACCATATTTCTATTGAGCTATCTTGTATGGAGGAAAACTCTGAGG
CACGTAAGACGATTGACCGCTTGCTGACCAAGCTGAAAGCCTTTTCGAAGACTAAAGGAG
TGGTCGTTGTGGTAATCTGTCAGCTCAAGAATCCTGAGAACGGGAAAGCACATGAAGAAG
GTCGTCCAGTAAGCATTACTGACCTAAGTGGCAGCGCTTCTCCACGCTAACTATCAGATA
GTATCATTGCTCTCGAACGCACCCATCAAGGGGATTATCCAAACCTGGTTCAACTTAGGG
TGCTTAAGGGTCGCTGTACTTGTGATACTCGTATGGGTGGGCATATGGCCTACATAAATG
AAAGAGGATGGCTTGAACCGAGTGTCTCACCTGAAGATGTGGCAAGTACAGATAGCGGAT
CGGGACCAGAAGAACACGGTCAGGACTTCTGATGGTAAACCTATACAACTCACTAATGGG
GCAGACAACCTGTTCCCACTCATCAATCCACTTAAAAAGAAGCACAATGTTTAACTTCAT
CATCACTCCCGGTAAGCTGGTAGTGAAGCTCTAGCTCTTTGAAGCTAAACGTATTAACAA
CAAGGCGCGTAAAGATCCTGAGCGGGCACAGCGCTTGGCTAAACATGCACGTAGGATGTC
TGAAGCTGCAAGTGCTGGTGTAATGCCTGCTGCTAAGATTACCGCTAAAGCATCCAACCT
CGACAAATTCTTCCTGTAAGGAGTAGGATATCATGGCCGTTAAATTCCCTGGCATTACCA
TTCGTCTCTCGGTCACCGTTGACCAATATGCTCGTCGGGTTCACGTCAAGGTTCGTAACG
GAAAAGCCACTCTGGTCTACCGTTGGAAAGACCACAAGTCTACCAAGGCGCACACACAAC
GGGTACCTTTTGTTGATCTGCAGGTGGGTCGTCTATCATCTTCCGTTGCGGTAGCTGCAA
CTGTGGCTGTAGCTGGGGACTATGTACGTCAGGCTATGCTAGGCACCACAATTGAAGCTG
AAGGCAACATGCTTGTTGAGAAGTCTTAGGCGTAAGAAGAGTTAAACTAAAGGTCATCTC
GGGGGGGCCTTCTTGATTAACTTTTGTAAGAAAACTATTCAACTAGCTAATGGCGTAATA
GCACTGTTACTATCCCACTTAGAAGCAAATGGGTTGCTGCACACCGTTAGTCAATTTCAC
TGTGCGGTGACTTACGACTAAAAAACCTACCAGTACATCCGTTATCGACCTGCTCACTTT
GGGGCATACATGGATGCTCTCAAAGATGAGGTGGCTCGCGGTGGTCTCATTGTGTTTCAT
AATGGCCCGAAGTATGACGTCCCAGTAATCTAGTTGCTCGCCAAGAAAATGTTAGGACGT
GATGTCGAGTTCCCTAAAAAGAACGTCTTGCATACGCTCGTGCTGTCCCGCTTCGCTTAC
TCAAACATCAAGGACACCGATGCTCGGCTGTGTCGTGCATGCAAGCTACCACGGAAACGC
TTTCGTTCCCACGGTCTGGAGGCTTCGGGTTATCGCTTTGGTGAAGTGAAGGGTGAGTAC
AAGGACGAATTCAAGGCCTCACTGGTTGAGGCTGGTGAAGGCTACGTGGTTGGTCTGGAA
TGGTTGATGTTCAACGAGGACATGCTGGAATATCATGTTCAAGACGTTGTCGAAACCAAA
GCTCTCTTCGAGAAACTTGATAGTAACGCATTCAATCTCCCTGAGCTGGAACCTTATGGA
AGCACTGAGGCAGCACGTTTCTGGAAGGGTAGCATGGAGGCTGACAAGCTGGAACACGAT
GGTTCATGGTTGGTAGCTAAGATGGAACGTGGTGGTTTCCCTATTGACGTCAAGTGCTTA
GAGAATCTTTATGCTGACCTTGCTGGTCGCCGTGGTGAATTTCTGGTCGAGTTGACATCC
ACTTGCGGCTCACGGTATCAGGTTAAGGGTGGAGTTGAAGCATTCCGTCACCCAAGGACT
GGTAAGCCTCTCACTAAGTAATCTTACGTGATATTTCCGAAACAGGGAGGTATCTTTAAA
AACCTATAGAACAAGAAGCAACATAAAGGTCTGGAGCCTTGTGAACTGGCCAATCGTGTT
TACGTAGAGGGCGCTCCATATACGTCTGTAGAGCACGTCGTGTTTCACCCTTCTAGTCGT
GACTACATCACTCGTGTAGTTACTAAACCGGGCTGGGTGTCCCTTGAGCTCACTCGGAGT
GGTGCACCAAAGGTAGACGATGAGGTCTTAGAGCACGTTAAGGTAGACGACCCTGGTGCA
AAACGTTGCATCAAGTTGTCTAAAGACGCCCTAATGATTCAGAAGCGTATCGGTCAGGTT
GCTGAAGGTGATAACAGTTGGTTCCGAACGATGGGAGAGGATGGTCGTATTCTCGGTAGC
GTTAACCCGAACGGTGCAGTAACTGGACGTGGAACCCATAGTTTCCCTAACGTGGCACAG
GTGCCTTCTATCCGGGGTGCTTATTGTGAACCTTGCCATGCTGCTTTCGGTTCAGAGCAT
AACAAAAAAGAGGATAAGCCAGACCTTTGGATTCAAGTAGGTGTGGATGCGTCAGGCCTT
GAGCTACGTTGCTTGGGTCACTTCATGTTTCGAAATGACGAAGGTGAGAACGTTGAGACT
ATCGTTACTGGTGATCCACATACAACGAGCCAAATTGCCCCTGGGTTACCTACAAGCGAA
AACGCTAAGACGTTAAACACAATGGGCGTCTATAAACCTATCTCATTCGATGGGCATCCC
TAAGGGACAATATCCAGCTAAATGATATTCTGTCGCTTTTAACTAAGGAGGCTCCATTTG
TATTCACTGAACCATAACTAACCTCAATCCTCACAGACCGCGCCTCCCAGGAAGTACCCT

CAGGGGCACTTTCAGGATAAGAAGTTTAAGGGTTGCAATTTGGTATTTAACCCCAAGTCT
CCAGTTGAGTTATACGGCTCCGATAGGTGTAAGGCTAGGGGAATCCAGAAGGCATATCTT
CTGCGCAGCTACGACATAACTCTTGATGACTACGCTGAGAAGATGGACTGCCAGAAAGGT
CTATGCACGATATGTCGCACCGAGGGTTTTGCTATGAACCCAAACGTGCACAGGATGAAA
CTTGTTGTTGAACACTGTCGCACCAGAGGTAAGTTTAGAGGTTTATTGTGTCACAACTCC
AATCGTGCATTAGGGCTTCTTAAAGAAAACGTGCATTCTCTACAGGCTGCTATTAAATAT
CTAAATGTGGAACGACTCCCACGGAAGGGCGTAGGGCCAAGTGGCTCGAAACGGTAAGGC
GCTGAGATGCAAGATATAGTTTGCTCTGCATGGCAACATGTAGCAGTTCATAAGAGAACG
GGCACGGAGTAACGACCCCTGATGAACACTGAGGTATCTACCGATTTTTACATGGTGCCG
GTACTGCAAAGATTGGCCAGATTCTTGGAGGTACTTCTGAGGATGGTAAGAGAGTCATCA
AGAACTTCCTTGAGTAAACAACGGCGATCGCTGCTTTACGTGAAGCAATCACAGGGGCAC
CCCTCAAGGACTCGAAGTGGGTTGGTGGCGAACAAATGCTAACTTGGAAACGTCGTTGGT
TTCCTGGCTTGGATGGTCGTAAGGCACACGGCCGTAGTTCTCACGCTGCGTTGAGCACCT
TGTTGCAGTCTGATGGTGCTCTGCGTTGTAAACTGTGGATTGTCAAGACAGAAGAGACGC
GCCTTGAGACGGGCCACAAGCACGGCTGGATGGTGGCTTCTGCCTGATGGGATGGATAC
ACGATGAGCAACAGCTTGCTTGCCGTACTGAAGAGATAGCCTAAGTTGTAATCGAAGTGT
CTCAACAGGCAATGCGCAGGGTTGGAGACCACTTTAACTTCCGGTGCCAACTGGATACTG
AAGGAAAGATGAGACCGAATTGGCCTGCAAGTAACTAATTAACAGGCGAAATATATCATG
GCTATTACTAAACGTTTTAAAGTACCATTCGAAGTAACCGCTGTAATCAGCTCTGCGATG
GAGAAACAATGTACTGATGGATTGATTCAGTTGTCTAAGAAGGCTGCTGGTGGTGAAAAT
CATAGTCCCCTTGACCGCGAGTTTCTGGTTCCAGCATTGACCCAGAGTGTCGAGGGTGCT
ATCGCCTCTTCTTCGAAACACGGTATGCGCGGGGTTATTCGCACAGAGTTTGCGGAAGCA
GGACTGAAAAACTCTCCAGTAACTACTCGTGAGGTGAAGTATTGGGTGTAAATATACGTG
TACTGGCGGCTATCAAAAACTGCCGTAAGACCTTTCAGTCAAACTTTGTGCATAAAGATT
CGTCCCTAGTAGCTGAGGCTGCAAGCCGTGGTCATATCCCTTGACTATCAATCGATGGGC
GTAAAGCTGCTGCTTGGGAAGTAACAGGTGCTGGTGTCCGTTTCCTGATTGAGATTGGAG
GTTGCGTATGGATTTGCTTACTGACAACGTACAGGTGACTCGTGAAGAGTTGAACGACAG
GCAGATGTACATTCAGTCCCGTGAGAAAGTCCAAGCTTTCCTGGATGCTCTGAGAGCCTG
TGGTGTGGACATTTGTGATGGCGACCTAGATACTTTCGAAATGGGATATAGGGATGATGG
CTTATGACTGCTATTACACTGAAGGACTTCTATGAGTTACGCCAAGGTAGACCCGATAAG
GGAGTCTAGGTGATGGATGGCGACCGCCTGGTATTCCAAGCGATGGCAGCAAGTGGAGTC
GAAACCCAATGGGATTATGACACTTGGAGTTTAGGGTGTGACCACGGGAAAGCGCGGAAC
GTCATGGTTGAGTGCGTTAGGTGCTATGAGAGCCGTAAGAAGGCTTGGAGTGAYGCACCT
ATCGTGATGGCCTGAACTTACTATGTGAACTGGCGTAAGGAATTAGTTGACCCAACCTAT
AAGGAGAACCGTAAGGCGTCGCGTAAGAGTTTAGGCTCCCGGTCGTTCGTCGAGCATGTT
CAGGCCTTTGAGGCCTGGACGTCTATCCTTGAGCCTCGCTTAGAAGGTGATGACGTAATG
GGACTCAATGGGTCTGGTGCTGAACACTTTGGGTTCAAGAATGCTGTACTGGTCTCCTGT
AATAAGGACTTTAAGACTATCCCTAATTGTGACTTCTTGTGGTGCATTACTGGTAACATG
TTGAGTCAAGAACAGACTACCGCTGACTATTGGCACATCTATCAGTCCATTAAAGGTGAC
GTGACTGATGGGTATGGTGGCCTTCTGGGCTGGGGTGAGACAACTGTTGACTGGTTAGAG
AACCCGTATGCCTATGAGCAGGTTGAGAAAACCTTTAAGTCTGGTCCTCGTACGGGACAC
ACAGTTATTGAATGGAAGAAGGTGGACATGTGAGACAGATCCTTGTGGGACGCTATTGTG
ACCCATTGTGCCAAAGCTGGGATGACTGAAGAGGAAATCATAAAGGAATGTCAGATGGCT
CGCATCCTTCGTGTCGAAGAGTACAACTTTATCGACAAGGAGATGTCCCTACGGTCTCCT
GAACAGTTACATTCGTGAGGGCCACAGTTATAGCTTAGAGTGTGTAACTCTAGGCATTCT
TCTCTATGTTAAATTAATACGATTCACGATTGGAGAGGGGTGCCTATATGATACCTACTT
TAGGTTCTAACTTCAAGGAGATCGGTAATTAAGTTAACGCCTATCAAGAAGTACATTGAG
AACCCACAAGATATTCCTAAAGTTCCTCTGAGCGTCTTGGAGCTTCTTCAAGTTCAATAC
AAAGCTGAATATGCTATTCAGTCTGGCCGTATCGACCGATTGAAACAGGCTGGGTGGTCT
GAAAGCTACATCGCTGGGTTCCTCGCGGGTCTCAACTATGCCTCTCAATCCTTTGACGAT
ATGGAGGCAACCCGTAAGGAGCAGGCGGATTCCTAATAACAGGAGGTAAGCTAATATGTT
TTTCACCAAAGATTAAAAGGCCAAAGGTAGATACCAATCAGGTACGTGCAATCGACCCTG
CGCCACTAACAGAGACATCAAAAGGTATTCTGTTTGGTGGTCACGACACGGTTGCACCGA
AAGATGGCCCAAGTCATGCAATAACTACAGGTGGTAGGAAGTCCCTCAAAATGAAACTTG
AAGACAGGCCATCCCAGGCACTTAGGAGTGACAATTCGCAGAGACGGACCGGGTCCAAAG
CGTCTACCCGCAAGTCCTTATTCAACGACAACAAGTAGTCAGGAGGTCAATCATGGGTTT
GGGCCAGAGCTTAGACAAGGGTTTCAAGAAAGTACCTAAGACACTCGAAGGTATTATTAA

AGGTGCTCTAGGAGCTGCTGCTAGTATGCTGGGTGGTGGTGTGCCAGATGTCGCACAAGC
TGCACAGATTGTTGAGCCACCAAGCCAAGGAGAGGTGAACACTGATGACAGTTCTCAGAC
TGAAAGTGGCAAGAAGAAAACACGTGCTAGCGGTAGGAAAGCACTGAGTGTCGCTCGCAT
TTCTGGAGGTGGCCTAAATATCTAACACGGAGGCGCTAAATGGCAAGCTCACAGAAACGC
GAAGGCTGCGCTGAGAATGGTGCCAAGTCGTTGGCTGATGCATTGAAGAACGACCGGAAC
TCCTATGAGACCCGTGCGGAGAACTGTGCTAAGTACACCATTGCCTCGTCGTTCCCGAAG
GACTCCGATAACGACTATAATGGCTATACAACTCCGTCTCAAGCGGCGGGCGCTTGTGGT
CTGAACAAGTTGGCATCTAAGCTCGAGCTTGCTCTGTTGCCTATGCAAACTTCGATGAAG
CTGACCATGTCTGAGTTCGAAGCGAAAAAATTAGTAGGACTACCAGCTGAACTGGTGAAG
GTAGAAGAAGGTCTATCTATCGTCGAGCGATTATTGATGAACTACATCCAGTCAAACTCT
TACCGTGTTACACTCTTTGAGACCCTCAAGCAGTAGATCTTAGCGGGTTACGCTCTGCTG
CACATTCCTACACCGGCATGTTCTTACAACCACATGAAGTTATACCGTCTGTCTTATGAT
GTTCCCCAACGAGACGCATTCGGCACGGTACTACACATTGTGACCTTGGATTAGACTGCC
TAAGCAGCACTTACCGGATTCGTAAGGAACTCTATGGACTCTAGTCAGGAGCATAAGGGT
GACGAAATGATTGATGGGTACACTCAAATATGTCTCGATGAGGAATCTGGAGAGTACCTG
AAGTACGAAGAGATTAATGGCGTGGTAGTCCAAGGCACAGATGCTTCTTATCCGGTGTAC
GTTTGTCCGTATATCCCGGTTCGCACCGCGCGCATTGATGGTGAATCATATGGTCCTTCG
TACTCTGAGGAATAGTTGGGTGACTTACGTTCACTTCAGAACCAACAGGAAGCAATCGTC
AAGATGTCTATGATTAGTTCAAAGGTCATCGGCTTGGTTAACCCAGCTGGTATCACACAG
GTACGTCGCTTAACGAAAGCACAGACGGGTCACTTTGTTTCTAGTCGTCCAGAAGACATC
TCGTTCCTTCAGTTCGAAAAGGCTGCTGAATTCTCTGTCGCGATAGCTGTGAGTGAACTA
ATTCGAGCCCGCTTGTCCTACGCCTTTATGTTGAACTCTGCGGTACAGCGTACTGATAAG
CGTGTGAAAGCTGAAGAGGTTCGCTATGTTGCAGCTAAACTGGACGATACCCTTGGTGGT
GTCTATTCTATTCTCCCACAGGAACTTACGGTAGCTATGTTTCGTGTACTACTGAAGGAA
TTTCAAGAAACCAACCAGATACCTGAGTTACCTAAAGAGGACGTTGAGCCAAGTACCAGT
ACCGGGATGGTAGCATCGGGGCGCGGTCAAGACCTCTACAAGCTGGAACGTTGTATTGCC
ACGTGCTCAGCTCTTGCTCCTATTCACGGAGACCCGGCCATCAACATTGCGAACATTAAG
CTGCGCATTGCGAACCCTATTAGTATCGACAGTTCTGGCATCGTTAACACACCTGAAGAG
AAGCCACAGGAAATGTCTGAGGCCGCACAAGTTACTGCTATGCAGAATGCTGCTGGGTCT
GCTGGTGAGGGTTCTGGTACAATGGCTCCTGCAAGTCCTGAGAAAATGAAAGCGGCTGAT
GCACAGGCTGGCATGGCACCTTATTAATACAACTCACTAATGAGAGAAAGACTATGTCCT
CCCTATGATTTATACATTTTATTATCAAAGGAGATAGAATATGTCCGGTGACTCTAATGC
TGATGTTTACGCAGCGTTCGGTGTGGACTCTGAGCTTGTCGGAGGGTCCACTCCCACTGA
CCACGAACAGGCCATGCTTGAACTCGATGTTGCTGCCCGTGATGGCGAGGATGAAATCGA
GATATTAAGCACTGATGTCCATTACGGTAATCCAGACCCGTTTGGTGAAGAAGACAATAA
TCGTACTGAGATTCGTATATCCACTGATGGTGAACCTGAAGAGACTGGAGAGGGTGTGAT
TGATTACTATGAGAGCACTGAAGAAGAAGCCTCAAAAGAGTTTGCACGTGTGGGTGACGT
TCCATAAGAACTGAGTGAAGCCTCAAGTCAACTAGTAGGGCATGAAGCTGGCTTCGAAGA
GATGTTCAATCAGGCTGCTGAACGTGGACTGTCTGAAGAAATTATCGTCCGTATCCAACA
GGAGTATGGTGAAGATGGTCTGTCCGAAGAGTCCTATGAAGAACTGGCTCAGGATGGGTA
TTCTAAGTCCTTCGTTGTGTCCTACATTCGTGTACAGGGAGAACTGGTCTAACAGTACGT
TCAGTGTGTTATGGAGTATGCTGGTAGTGAAGCACAGTTCCAAGCAATCTACAATCACCT
CGAAGTGTCCAATCCGGATGCCGCCGAGTCTCCGGTTAGTGCTCTGGAAACCCGTGAACT
TGCGACCGTTAACGCTATCGTGTATCTCGCTGGTGCAGGCTATGCGAAAACTTTCGTTAA
AGGTGCTGCCCGTAGTATTTCTAAACGTGCCGTTCCTGCTGGACCTCTACGAGCCAAACA
GTCTGGCTTTGAGTCACAGGCTGAGGTGTTTGCTGCAATGTCTTACCCTCGATACCGCTC
GGACTGTATGTTCCGTGCTGAAGTTCAGCGTAAGATGATGTACAGTAACTTCTAAAAATA
ATACGACTAACTATTGGAAGAGACGTCACGGTGTCCCACTTTAAGTGATACACAATGAGA
ACCAACTCGGGTCAAGTAGTACCTCACACATTAAAACTTTATAAACCAACAGTAGGAGAA
TAAACATGGCTAACACGAATCGTGGTCAACAAATGGGTAAAGACCAAGGTAATGCCATGA
GAGCTGGGGACAAACTGGCTCAGTTCCTGAAAGTCTTTGGTGGCGAAGTTCTGACTGCGT
TTACCCGTACCTCCGTAACTATGAACAAACACCTTGTTCGCTCTATCCAATCTCGTAAGT
CTGCTCAGTTCCCTCTGCCGCGTCGTATAAAAGCTGCTTACCTATAACAAGGTGAGAACC
TCGATGACAAACGTGAAGACATCAAGCACACTGAGAAGGCCATTAACATTTATGTAGTAC
TTACTGCTGACGTTCTGATTTAAGACACCGTAGACGCTATGAACCACTATGACGTTCGCT
CTGAGTACACTGCACAGATGGGTGAATCTCCTGCAATGGATGCTGGTGGTGCAGTACTGG
CGGAAATGGCTAAGTAATGTAACCTTCCAACTGCTAACAATGATAACATTGGTGGTCTTG

GCAAGGCTCACGTACTTGCAGTTGGCGACCACGCTACCCTGCAAGGTGACCAAGTTAAGC
TAGGTCAGGCGATTATTGCACAGTTGACTCTGGCTCGTGCCAAACTGACTGGCTGCTATG
TACCATCTAGTGACCGTGTGTTCTACACTACTCCTGATAACTTCTCTTCGATTCTGGCAG
CTCTGATGCCTAAGGCTGCAACCTATCAGGCTCTGATCGACCCATCTACTGTTTCTATCC
GCGACGTAATGGGCTTTGAAGTAATCGAAGTTCCTCATCTTACTGCGGGTGGTTCTGGTG
ATAACCGCGCTTAAGAAGGTGTAGCACCTACTAATCAGAAACACTCATTCCCTGATACTG
CTTCTGGTGATACTTATGTTGCTTTGGATAACGTGGTTGGCCTGTACAATCACCGCTCTG
CCGTTGGTAATCTTAAGTTTAAAGATATGGCTCTGGAACGAGTACGTCGTGCCAACTTCC
AAGCAGACCAGATTATCGCTGAGTGTGCAATGAGAAACTGTGGTCTACGCCCTGTCGCAT
GTGGTGCGCCGGTGTTCAAAAAGGATTAACTTCTCGCAAACTCATAGGGGCCTCTGAGTC
GCTTACTGAAGTTGAGCAAGATCTAACCCCCCATCAGAAAGCTGCCCGTACCCGTGCGGC
AAACAAGGCTCGTAAAGATGCTGAGTTAGCTGCTATTAAGTATCCATTATGAGAAACCCC
TTGGGTGCCTTCGCGGGTGCTTGAGGGCTTTTTTCGCTACAATCCGGATACACTTATGCG
TTCCTATGAAACTACCCTAGAGAAAGCCGAAGAGTTGGCTGCTGTCTACGACATCATGGT
CCCTATTGGTGAACCCCCAGTATCCACTCTGGAAGGTGAGACTAATGCGGATTTGGATAA
CGCACGTCGAGTTCTCAATAAGACTTACCGACAGATTCACTCGAAAAGATGGCACTTTAA
CATTGCGGGAGGACAACAGTTGCCACCTGATGTGTTCAACGGTTTGATTCGTTACATCTC
AGACTACCTGTCTGTACTCTGGGAAGGTGGAGCAACTGCCTACGTTAACCGTGGTGAATA
TCTCTTTGATCGGACGACCGGAACTGATATCTTTGAGGTCCCAGTTACTGTGACCATCAT
CAAACTCCGTGAGTTCTATGGGATGCCTAAATGCTACCGCTCATGGATTGCCCCCTAGGC
AGCCCCTCAGTTTAACAACAGGTTCTTTGGTGCACATGAGATTGACGCAGTGCAAGCTGA
AAAGGAACAAGCTGCCAAGATGCAGTTTCACGACTATGATCTGAACTTCAGTAAGTGCAA
CATGTTAGATGGTGATGCATTCACAGATGGTCCACTATATCCTTAACGATAACATTTTAT
AGGAGGTGTCTAATGGCTCTTATTTCACAATCCGTCTAGAACCTGAAGGGCGGTACCAGT
CAACAGCCGAACCTCTTAAGGTTCGCCGAACAGGGTTCCAAACAGATTAATGGTTGGTCC
TCGGAGATTGAGGGTCTTCAGAAGCGTTCACATTTTATATTCATTAAGACCATTGGAGAC
CAGAATGCCCTTGGTGCCAAACCTCTAGATCACCTCATCAACCGTGATAGTGCCGAACAG
TATAACGTAGTGTTTCCGGGAGAGGGTGTTCGTGTGTGCGATCTAAACACTAAAGAGTAC
GATGTAAAGGGTGGGTAGTCCTACGTTAAGGTAGTGTACACTCGAGATGACTTAAGGATC
ATCTCTGTGCCTGACTACACATTTATCGTTAATCGCAATATGGTTGTGCGCCCAGAGACT
AACCCTTTGTATACCCGTAAGGAGAATGGAGTCCGCTTGATTAACAGCCGCGGGTTTCTG
TTTGGTTTTACGTTGGCATTCTCTATTAACAACACAAAGATTGATTTCGTGATTGCCCAT
GGGGACGGACCTGAACATTCTAATCATAGTGACCCACAGTGGCTCGTTAAGAAACTTGCT
GGTCTCGCGCGTTTGAATGTTGCCTTTAAGGGCTGGAGGTTCACCGAAGGGCCTGCGTAC
AGCCCTGTGGTTGCTCCAAGTAACAGTCAGTGTAACTCTCTGTCCGCTGAAGATGGCTAC
GCCGACCAGGTGATGAACGCAGTGATGCACACCGTCCTGTCATTCAGTACGTTGCCCGTG
GAAGCCCCGAATGGATAAACAGTGGAAATAGTTGGGCACACCTCTATGACAACCGATATG
TTCTTCGTTCAGTACGACAATATGAAGAAGGTATGGAAAGAGGTTTCTGGTTGGGGTGTA
CAGGAGGGACTTAACGGAGGCAAGATATCTCATGCTCTGGTCCGACAGGCTGATGGGTCA
TTCCAGATGCAAGTCCTACCGTGGACACAACGTTCCTGTGGTAACCTGGACACTAACCCT
ACGCCGCCTATTGGTGCACAGAAGATTAACGATGTATTCTTCTTCCGGAACCGCATAGGG
TCCCTCGCTGGTGAGAACATTGTGATGTCCCGTACCTCTAAGTATTTTTCATTGTCCCCT
GCATCAGTAGCTAACCTGTCCGACGAAGACCCTATCGAAGTGGTTGTGTCTCATAACCGA
ATCTCTATCCAGAAGTACGCCGTACCGTTCTCTGAGGAGTTGCTCCCATGTTCAGACCAA
GCACAGTTCGTGTTGTCGGCTCATGGTATCCTCTCACCTAAGTCAGTGGAGTTGAACCAC
ACGACCGAGTTCAATGTGTCAGGCCAAGTGAGTCCTTCTGGCGTTGGGCGCGGTGGTTAC
TCTGCGTCACCTCGTGCTTCCTATACGTGACTTAACCGTTATTATGCGGTGCAGGAGTTT
ATCTCTGTGAAGTCTGCTGAGGATATGAGTGCTCACGTTCCTAACTACATTCCGGACGAT
GTTTTCTCTATACGAGGCTCCAGCACTGAGAACTTCATCTCTGTGTTGTCTTCAAATCCT
CCAAGCAGAAGCTTCCTCTAGAAGTTCCTCTACATGAACGAAGAGATAGCACAACCGTCG
TGGTCACATTGGGGACTAGGAAGTAACGTTACCGTACTGGCCTGTGACTCTATTGGTTCA
ACGATGTACCTTGTGTAGCGCAATCAGTGAAACACTTGGATGTGTCGTCGTCACCTTACG
AAGAACAGCATCGACTTCCCTGATGAGCCGTATCGTCTGTACATTGACAATACGGAGCAG
TATGTGATTCCTTAAGGTTCATTCAATGATGGCACTTACGCTACCACTGTGTAACCTGTA
GATGCCTATGGGATGAGATTTTGTTCCGGTAAGTTCTACATTGTGGCCTCTGAGGGACTG
GTGTCATGGTTCGAGCCTCCTCGTGCTGGTTGCCCTAATGGTGTACCTATGTCAACAATG
AATGGGAACCGTGAGGGTGAGAAAATCTACGTTGGGTCGGCTATCAGCTTCCGCTATGTC

TTCTCTAAGTTCCTCATTAAGAAGACCGCTGATGATGGCTCTATAGCTACTGAGGACATT
GATCGTTTACAGTTACGGCGAGTGTGGGTGAACGAAGAGGACTCTGGTGCATTCATAGTG
GAAGTGGAGGCCACCTCTCGACAGTTCAGCTATGATATGGCAGGTGCACGCTTGGGTTCC
AATGTGTTACGTGCTGGTGGACTTAATGTTGGTACCGGTCAGTTCCGCTTCCCGGTGACT
GCTAAGGCACAGTTCAATGAAGTACGTATTATCTCTGAACAAACCACACCACTAAACGTT
ATCGGTTGTGGTTGGGAGGGTAACTACCTTCGTCGTTCTTGTGGTATCTCATTGAAGCAC
ACCGTCTAACTCAAACAGTGAACAATACGACTCACTATTGGGGAACGGTGTTTATACTCT
AGGAGAGAAACTCTATGATTATCCTACCTACAACAGAACGTGACTTTGTGTTATTCAACC
CATCAGAAGATGACATTGCTGAGGCCAAATCCTACAGCATTGAATCTTCATTTCCACCCG
CCTCTGAGTGTGTGACAATGAGTTTACCTGGGGTACCTTTAGCTATTGGAAGCAGCTGTG
GTGACCAATTGTGGTTCGTAACGTCTGACGAAGTGTGGAGACTATCCAGACCTGCTAAAC
GGGAGTTCCTCAAGCTCATCCTAGAGTACCGCGATACGATGCTAATATAGTCTCCAGTAA
TCTGGTACTCTGCCGGGATAGGCAACAAGTCCCACATTAGGTTCTCGATCGCTATCGGTG
CGGTATTCCATAATGAGTCTACTGGTGACAGTAATCAATTCCAACTATTCACAATAGGAT
GCAAAATTATGTACTGGGTGGCGGCTATCCCTCTCGCTATGCAAGGTACTTCCATTATTA
TGGTAGGTATGAAAGGTGAACTAGCAAAGGCTGCACAGATTGACCAAGGTCGTCCACAAA
GTTGGCAGGCGCTGAAAGAGATGAATAATACCGATTCTAATCTTAAGTTAGAATCCCGTG
ACCTCATCGACTCAACTGTCCAAGAGATGCCCCAAGCCAATATGAACCGTGTTAGAAATA
TGGGGTTAATCCGTGCGGCTGTCCGTGAAGGATTGCTTGAAGGAAACCCTATGGACCGCG
TACAACGCGTGACCGAGGGTGACTTCCAGCGAGAGTCTCAAGGTTTGTCCGAGAACAACC
AACGGGACTATTTTGTCGTCTTAGGGAACCGTTTAGCTAACCAGGAGAACACTGTTGGTC
AGATTAAAGAAATCAACAGGTCCGGGCTAAAACTAAAAGGTCGATTAGAGCAAATGATCG
ACGCGTTAGGTATGGGTATGGGTCAACTGGCTGGTATCGCAACTTCTGGTGGACTGTTCG
ATGGCAAGTCGACTGAGAAGGGTACCACTAAGGTTAAGACCCCGGGCGTTAAATGAGCAA
ACCAAGATGGTAAATAAAGGAGGTACAGTTTATGAGTAAGTTAGCGCAAGCATTAGGTAA
CATGAACCCTCCGTCTACTAGTCTTCTGCGTGGCACTCGTCGCATGGAGGTAAAAGGTGC
AACAGTCTATGAAGACCCTAAATATGCGGCGAAGTCTAAGCTGCTTGGCACTGTAGGTAA
ACTGTCTGAGATGGGTGCGGATGTATAAATGCAGAATGACCAGAGCGAGAAGGCCAATGC
AGACGAACGCTATCACGAGATTATTCATAAGCTGACTTCTGAGCAACGCAGACAGGCCAT
GAGGAACGGAACCCTGTTATATCAGTATGACCAGTACGGAATGCGAGCACTCAAGCTTAA
GACTGGACGTAACGCTGCGGATCTAGTCGATGACGAAGTGGCACAGAAGGTGCAGAACGG
TGAGTTCCGCACTCGGCAAGAGTTGGAAGAGTTCCGTAACAGTCGACTGCAAGAAGCATC
TATGAGCTATGCGGAACAGTTTGGTATTAATGAGACTGATGACTTCAAGCAGAAGGGTTT
CAACTCCGACATCACTCAACATAACATTGCCCTCGATGGTGCTCACGATAACTCCCTGAT
TGACCACGCTCAGAAAGGTGCTGGCATGAACAGTCGCGTCGAGTTAAACTCTGTGTCGCA
CGACCCGGATAACCTACGATTTCCGTATGCTGGTGAGTTCTTTGAGAACTACGTCACTGC
CGGATTGACGACTAGGAGTTTCCCTAGTGATAACCAAGCGTTGCAAATGATTTGCCAAGG
TCTGTCTGACGTGGGTAACCGTGAAGGTGGCGGGCAGTTCCTGCAACAGATTGAGAAGCG
TAAGGTGAAACTCCACGGAAAAGAGACAACCTACAAGGAACTGATGGGCGCCGAACAGTG
GAACAATCTGATGTATAAGACTCAAGATAATGAGTTCCAGTTGAACGCTAAGACGACCTA
GGTGTTCCAAATTATTTTGGACTTTGTGTTGAACCAGGAGAACATAAGCACTCGTTGGAA
AGCGCTTCAATCTATGAAGGCCGAACCGGATACATTGCAACCGGGCGAATAGATGACACC
TGAAGGTCAAGCGTTAATCAATGCACAGACACAGATGCAGGACGGCATGAATCGGGAGAC
CTCTGAGTTCGCCAAGCAAATGGACAAACAACTGAATTCGATGAACAAAACGAACGTCAT
GGATACACCGTTCCATAAGAGACTCAATGGTCAGTACGTGTCTACTGCGCATGTCTAGAT
GCCGACCAAACATAACACAGGCGAGTTCACTCATAGTGATATGGTTATCTATGCGAACAA
GAAGCTGGCCGATATTAATCATATGAATATTCCAGAGGCACACAAAGGCCGAATCAAGCT
GATCTACCTCGAGGCTGACTCCGAGACGTGTGCTTTCCGTACTGCGGCTGGTGAACTTAT
TGGTGACGCTGAGAAGGAATGGACGTTCACTGTGATTAACGGGAAGATGCCAGAAGATGG
TGTTGTGGCACTTAACGCCCTTCGACGTGTTCGTAATGCTGACCCCGACCTGTTCGCTGC
ATTGTATCCTGATAAGGCCGAGATGTTCCTCACTATGGATATGATGGCCAACCAAGCGAG
TGACCCACAAATCTTAATGCATGCAGCTAAGGATCGGCAGTGTCTAACCAAAGTGATGCA
GTATGAGGATGATAAGTATTGGGCTTCTCTAATGGACAACTCGCAGTCCCCTGAGATTAA
GTACATGCCACCACTCCGCCAGAACGATGCCCGTAAGATTTCTGACTCTGTTAAATACCG
CACTAGTAACCCTGATATGGCAATGCAACAGGTCGCTTGGTATCGCATAGTTCACACTAC
GACTCTTACAGATGATTATGCTGACGGGGATACAATTGATGACTTAACGAGAAACTCTCT
GCGTGTCACGGTTGATCCGGAATCTTGGAAACAAGGTAAGGACATAATAGACACAGCCGC

TAAGAAACTCGCTGTGACTAACCCTTGGGTGACTCGGATGCAGGTCACTGTGTTTGAACG
TGGTGATTCTATCTACTTGATGACCACGACCGGACAGGAAAACGTTCGGGACGATAAGTA
GTTGCTCTCTAAGATGTGCAAAGAGACTCAAGCGAAGCTGGATGAGGAAGCCCGTAATAA
GGCACACCAAGATTCCAACAAGCGAACTCTACATACACGGGCTCTGAACCGCAAGGGTGA
ACGTGATGATAAGAAGACTAAACGCTCTGACAGCATGTATGATAGCGTGAGTGGCAAAGG
TTTTCTGGATACACTTACTGGTAAAGACTAATAGAGCACGATAGGAGGTTCCAATCGGAT
AAGTATAACCCGAACGAACCGCATGAATATGATGCGTTACTTCAAGAGGGTGCTGACGCA
CATGGAGTCACCTTTGGCCTTCTCCCAAAGGTCGGTTGGGTAGAGTCCCGCTTTAAGCCC
ACGGCTCAATCACCAACAGGTCCAAGAGATGTCATGCAGTTCGCTAAGGCATCAGGTCAG
GCTTATAGTCTGCAGAAGGATGAAGACTTCACTGACCCTGCTAAGTCAATTGATGCAGGT
GCTCGTTACCTTTCAGACTTCGGTAAGAAGTACAACGGGGATGGATTTAAAGCTGCCTTA
GTGTACATCCAAGGTGAAGGACGTAATTGTAAGCCTCGGTTAGATGGCTACGTTTCGGGC
AACTTTGCGAGCATTGGGGACGAAGTCCGTAACTACCTGCGTTCACTGCAGGATGTTGCT
AAGTCTCCTAAGAGTGGAGACCTTGAGACATTCGGCGGTATCACCCCAACGTTTAAAGGC
ATTCGGCTGGGAGCTGATATGAGCGGTATCGGGAAGAAAGGAAGTGTGACAACTGGGCTC
CCTGAGTCCCACGACATGTCCTTTAGCGGTAAAGAACAGAACGCTCTCAATTAGCCGTTC
GGAAAAGATTATTGGGAAGCCAAAGGGAAAACTCTTGACGAAGCCTACAAGCGTTCAACC
TTCATTGGATTGGGTCATGCTACTGAAACAGAACTCACCAACTCAACCTTGGGTGTTGCC
TTCCGTGCTGGTAAGCATGAGGATGGTTTCGATGTTCTTACTGACGTACTTCAACCGACA
CGGTTTAATAGCCACATTTGGACGCCTGAAGAACCGGTGAAGATTAGGACTGAAGTGAAG
AACCCTGGGTACATGAACGTTGTACTTGTGGGTTCCGCTGAGAACGTTGAGGAACTAATC
AAGATGGCTAACGACAACTATGAGACTGACGCTAAGGCTGCAGACGCGGGCCTAGGTGCC
AAACTGTCCGGTGGTCTTATTGGTGCTGGTATTGACCCTCAGACCTATGTTCCCATTGCT
GGGACTACCGCTATGGGGTTCAAGTTGGTGAACCAAGCGTTAATGGTAGGTGCTGGAGCT
GGTGCACTTAATGTTGCCTCTGAAGGCCTACGTACTTCGATTGCTGGAGGTGAGGCCCAC
TATGCTGAAGCTGCCTTAGCTGGGATGCTAATTGCGGGTGGACTGACTGCTGTGCCAGAT
GGTGTAGCTGGTGGTCTCCGTAAGTCTGGTGCTGAATATATTGAGAACCCGTTTGCTGCT
GCACAGATGCGCTTTGAGGCCCGTGAGACTACACGCTACCCTGGTGGATATGCTGCAAGC
CGTATGCCTCCTAGTGAAGACCGAGTGTTCTCACGGCACAATGCCGTTGAGTATGCAGCA
CTGGAAACCGAACCTGGTGCTGTAGTACTGAAGTATGGTTCCATTATTAGTCATACCAAT
CTAGAGAACCCTTTGACAGCCATAGAGTTTGCCGAGGTGGACCCCGAAGGTGCTGCTTGG
GGATTGCCGATGCGCGGCCCGAGTGTAATAGGTTTGTGAACACCACGTTCCGACCAAGCT
GAAATCCGGGGTCTCGCCAAGCACTTAGTGCGCTAACCTTCAGGTATGCAGTCTGGCTCC
GGCGGTAAGTTAGGGGCTACGGCTTCCGATATCAAAGAGCGACTTCACAGTACAAACCAG
ATAACCTACAATGACCTCTATGGTTCAAGGAAGGATGCAATGGCTGACCCTCAGTGGTGT
GTGGGTGCGTCCAAGAGTGGTGCTCAAGTTGCTCGTCAAGAAATCTTTCGTCGCGCAGCT
GTGGCTATCGAGCCTCCTGATCTTCGGGCTAACTTGACCAAGGCAGAGCGTTAGCTGATG
GACAGCGTGAAGGAACATTTTGACCTCAAGCGTGAGATGATGGAGAACCCGTCGATGTTC
CGTCACAAGGCCAACTCTATATTCCCTATCAGTCGCCACAAAGGGACTTACGTTCCTCAT
GTGTACTCGAGGGAAGCCAAGCGGTAGTACTCTCACGCTCTGGGGGGTCCCGATGGTCTA
CAGGAAGGATTTGCTGCAAGCTGGATGACACCCTATCGCTCACGACCTGATGTGAAACCG
CGTGTTGATGAAAACTTAGCGGAAACCTTAGGTATTGACGTTAAGCCTGTTACTGAAGAG
ATGGTCATGAAACACGCTAGTGGCAAGGCATATGTTATCGCCAAGGCTGTTCGGTTCAAC
TCTTCCAGTGTCGTTGATGATAACATTGAGGGTATTGTTGGAATCGAGAACAACTCATTC
CTAGGGGCCCGTCACCTGTTAGATTCAGACATGCCTGTAACACTCCCTAACGGACACCAT
TTCTCCCTGAATGACTTACGTGACTTCGATATGAAAAGCGTCACGCCAGCCTATGACCGC
CGTGTTGATGGTCATATTGCTCTCATGGGTGCTAGAGGGAATACTACTGCTGAACTCGTA
GCTGAAAACAGGATGATCGATAAGTAGTGTGAAGGTAATGGCACAGAAAGGGGTGAGGTA
GGAGCACTGAAAGATACCATTAAGATTCTCAATGGCCGCGCTCGGCGTAATACGGATACT
TTGGGAGACAGTATGGTTCGCGCTCCGTCCGATATGTCCTTCTTTACGAAGCACGCATAC
CTGGGCTTGCACAACGTAACTGAAATCTCTGGTATCTTGGTAAAGAGTAACATTCGTGCG
TTGCTCCATGGAGTCCCAGCACTTTGGGACTTAGCGTTCCGTAACAAGACTGTCTCTGGT
TGTGAACTCAATAAACTCAACTCAATGGTGTTCGGCAGAGAGCTCGACCAGTTAATTCGA
CCGTCCCGTCAGGACATCATTCAGCGTCTGCGAGAGTCCACCGAAACACCAGATGTAAAC
CCTATGGTTGCTGGTTCTATCAAGCATACGACTCAAGAGTTAGCTGCCCGCCCACCTCTT
ACAAAATTCCTTAATGGGACGTCAAACTACCTTCTGGAGATGGGTCGACAGGTTGTGATG
GGGGATGTGGTAACTCATGCTGTTACAGGCAAAGGTGGCAACAAGTGGAATAAGGGTGAC

ATGCTGAAGTCTGCGAGTATCTCTAAGGAACAGTGGGGAGGCATTCAGTCTCTCATTAGG
GAGAACGTAACTCGTGGCGAAGACGGTAAGTTTACCTTCAAGGATAAGCGTAAGCTGGCT
AGTGATCCTCGTGCCATGGACTTATGGCGTTTAGCTGATAAGGTTGCAGACGAAACGATG
CTCCGACCTTATAAGGTTTCTTTACAGTATTCCCATGCGTTTGGTGCCACCGTTAAGTTC
GTCCTGCAGTTTAAGCCGTTTGTCATCAAGTCGATGAACCCTCAGTTGATCCGCTCTGGT
AACGAAGCCTTAAAGAACCAACGCGCTATGGACATGGCGTTGACCTATGCTATCTCTGGT
GGTATTGCTGGCTCCTACTATGTGGCACAGGCTCATTTGAAAGCTGCTTGCCTACCTAAA
GAGCAACAGAAAGACTACTTGAAGAAACCCCTCCACCCTAAGATGATTGCCTACTCCGAG
GTCTCCAGAACTTTACACTTTGGGGCTCCGCTGACTATTGCTAACTTCGGTATGTGAGCT
GCAGGTTATGACCAAGGTCCTATGGTCCGCTCAAGTATTCTACCTATTGGTGAGGACAAA
AGTGAGAACAATCAGGCTGTATCTTCTGGTGACATGAGTGATTCCATTATGGGCGCTATT
GGTGAACAGGTGCCTCCTCTCGGAATGGCTGGTTCGGCTTTTCCTGCTAGTCGGAATGCT
TATGGCGTCCTTACAGCTCCGAACAAACTGACCGAGCTTGAGATGATGACTGGCTAAATG
AATGCTCACCGAGACTTGATACCCAACGACCCAATCTCTCAACAGATGCTCATAAAGTTC
TATGAGGTCAACGGTGTGTATATTAAGTCTAACAAGAAGTAAACAATACGACTCACTATT
GCGGAGGCTCTCTTAGCTGCCCCTTTCTATTTACCGTTTATACCTCACCAAAAGGAGGAT
ACAGATGGCTAACAAAAATTACACTGTACGTCCTTAACCGCTGAACGGTTCCGTCAACTT
CACGATTACCTTTGAGTATCTACCACCTAAGTTCGTCCTAGTGACACTTAGAGGGAAATA
CCGAAGGGAGTTGGTTCTTAATCAGGAGTACCGGTTTACTGATAAAACCCAAATCAGAAC
CGGCAGTACATGGACTGCTGCGGTCGGCTATGAGATGATTGAGATTCGTAGATTCACTTC
TGCAACCGACCGTCTGGTTGACTTTGCTGATGGCTCAATCCTTCGCGCATTTGACCTTAA
CATGTCACTGATTCAGACACTACACTTAGCTGAGGAGGCACGTCACCTTACTGCGGATAC
TATTGGGGTCAACTATGATGACAACCTACATGTCCGTGGTCGTAAGTTTCTAAACCTAGC
GTTTGCTACCAGTAACTATGATGTTGTCCCTCTGAAGCAGATTACTGACCGTGAGTCCTC
TGGGTGAAACGCTCTGACCAAAGCTAGTGAACATGCTGACCGTTCTAACAAGGAAGCCAA
TCGCTCACGGGACCAACCTGAGCGGGCTAAACAAGAAGCTCACCGCCCGACCCATAAGGG
AGGTGTCTCTGCGACACAAGCAGTCGAGGCCAATAGGCAAGCTGACCGTTCCAACTCTGA
GGCCAACCGTTCTAAAGGTTAAGCTGATGGGATGACCGCCAGTGTGGAAGCTGCTAAGGC
CTATGCTGAGAGAGCCAGTATGGAAGCGAATCGTTCCCGTGCTGAGGCTAATCGTGCTGC
AGATCAAGTTACTAAGGCCGCTGCAGAGGTTTCCAAAGCTGCTGCACATGTGTCTATTGC
TAAGATTCAGGGCGACCCTTCCAGTACTGTGGCTAATCGTGCGAAAAGTGCAGCAGACCG
TGCTAAGACTGAGGCCCATAAGTTCGGCAATATCAATGAGTTCGCTGGAACTCTCGAGAA
GGAGGAGGGAGTAACACCAACTTTCAAACCGGGGCTTAAACTTCGTTCTGGTGACGTCTA
TGCCGATAGTGGTATCTTCACCTAGAAAACACAGGGTGGCGACTGCAGCCAGTGGACTGG
ATCTCTGACACCAGATGTACAACATGATATAGAAGGAGTAGAGAACGTCGCTTATCTCCT
TGAGCGAGCTACAACTCTGAGTAAAGAGCAATTATATGCTAAGTGACTATATAATAAGGG
TGTTGACGAAAAATTCTTTATCAGGCGGTATCCTCGAGATGCACATTTTGACTGACGCGC
CGAATGTGCTCTTATCTGCTCTTCTGGTAGGAGTATCCCAGCGGATGGTAACTTATTCAT
CTTGAATTATGCGGCGAATCTTGATACATGGGTTAATATGCGCCTAGGTGCGCACGCCTA
CAGCAAGGGTGAGGTTAACATGATGGTCGATGGACCACTCACAACACACCAAAGTGATGC
TCGTTGTGCACGCAAGAGCTATGGTTAGACTGTGGTATGGCAACGCTCTGCAGGTGGCGG
TGTTTCAGTAAGCCTCTCACAGGCTGTCCGCTGGAGAACTATCTGGATACTAGTTAATAA
TGGCACGTGTTGTGTTCAGATTGGAGCTGCTGCTACTTAGTTCGTGGTGGTTATGGGTGG
TTGGTTGAAGTTCACAATTTCCATCAACGGTAGATCTTTCCGTAACGACCAATATCGAAA
TACAGTACCTGAGCAAATCTTAGTTCTCAACTTATAAGGACAATACAAAAGGACCATGGA
TGGTCCTTACCAATGAGGAGGACACAATGTTGTCTTTAGACTTTAATAATGAAGTTGTGA
AGGCTGCGCGGATTGCAGGGACAGGTGTGGCCGATGGTGCTTCCCGTCTGTTTTGCGGAC
TGTCGTTTACCGAGTGGTTCCACTTGGCCGCAATTGCCTACAGAGTGGCTGAAATCTGTG
GCACGGTAGTCGCTGTGATTATCAAGTGGAAGAAGAAGGGTAGAGATGTCTAACGAAAAG
TCTTTAATACAATGCCTTGAGATGCTGGACACTGAGGTGGCATAACGTATGCTGAAAGAC
CTTCAGGATGGTGAGAAGCGCACACCTCTACTGTATTACGCAATCGGTAAACTGTTAGAG
CGCCATAAGGCCCAAGTCTCCAAGTTACAGCCAGGCCATAACATCCTTGGTGGAATGGCA
GCTGGACTCGAAGAGTACCAGAACCTAGTGGGTGCTCATGGTCTCGCTGAAGACGAGAAG
TACACGCTGAACTTATCGAACAAGGCAAGGCCACAACATCTAGTGGTCTGTCTGATATTT
CCACACAACTCACGGTGACGCTACGAGAAACGTGAAAATACGGGAGGGTAGTTATGCTCA
AGAATTTAAAGAGGTACGCCTTAATCGTTGCGTTTGCCTTCGGGATTACCCTTGCGTATC
ACCTCTGCTATGACAGAGCTAACACTAAATGAGAACAGCAGGAGAACAATGAGTACGTCA

AAAAATCTGAGGGGACTGCGGATAAACGGGCCGCTATCAATGAAGTCTCACAGCAATACC
AAGAAGAGCTTGCGGCGCTGGAGGGCACCACTGATAGGTTCATTAATGATATGGGTCACT
ACGGTAGGCGGCGGAGTCACAAACGAGCAGACACCTCACGCGAACTACAAGCGCACGTTG
GATGCCTCGTTGATGCTCGAGCCCAACTTGACATAGAGTTTAGATGACGTCTTATCGGAG
TAACTCAAAGAGGGGATGCGAGGATTAAGTCGCTCAAAAATACAATCAAATAAATGCAAC
ATAAGGAGAAAGACAATAGGTAACGTTAGAATCAGAACCAAATCCACTCTCAACGGACGT
GCACCGGATAGTCTGGACGTGTGGGATCAGAACAGTGCGGGCCTTCTAGTGGGTCGTATT
GAACTTCCTGAGTTCTCTATGGGAACAGTAGAAGGATACACAAAGTTAGAATCTCAGCTG
AAACTGTCTAATGTAGCTATGTAGGTGTACCCCAACTTCACTGCATTCAACGCTGCGAGG
AGCGGAAACATTGAGTCTAAAGTTGGCAGTGGATATATGACTGTGTTACTTAATAGAGCT
AAAGGTGACACATGGGGTCTAGCACAGATTGATGTACATAAAGAATGCGATGACTCTACT
CGACACCCTGAGCCAAATCCGTCTCCTGAGATGGACGTCGCGGCTATCACTAAGTACTTT
ACGCCCTATGTGGATAACCCTGAGTGCCTCCTCGAGTGGAGCTAGATAACCGCTATAGGT
GGTTCAGAACGTAACCGTCGTCTGCCGCACGAGAGGTTTGGTGTTGCTCTGGTTAACTCT
ATACAGCATATGTGCTCACAAACCCTCAAGATTCTTCAGGATGCGCTAGGTAGCACAGCA
CCCACCGAGTAACACTAGGAAGCCGCACTGTCCAATCAAGCAAACAAGAATCCGCACATA
GTGGCGCAACTTAAGGGTGACTTCGTAGCTTTCTCGTTCGTCATATGGACGGCTCTGAAC
TTACCTAAACCAAGCAAGAGCCAGATTGATATGGCACGTACACCCACTGATGGTGACCAC
AAGAAGTTTATCCTTCAGGCGTTCCGTGGTATCGGCAAGTCCTTTATCACTTGGGCGTTC
GTTGCGTGGGTATAATGGAGGGACCCTCAACTTTATGTTCTGATTGTGTCCGACTCTAAG
GAACATGCTGATGCTAACTCCATCTTCATCGAGCACATCATTGACCTGCTACCGTTCCTC
TCTGAGCTGAAACCTCGACTGGGACAGCGTGACTCTGTGATTAGCTTCGATGGTGGCTTA
GCTAATCCAGACTATTCTCCCTCTGTGAAGTCTGTCGGTATTACTGGTCGGTTAACTGGT
AGCCGTGCTGATATGATCATTGCGGATTACGTTGAGGTCCCTGGTAACAGTTTTACAAGT
GCTGCTCGTGAGGAACCGTGGACGCTGTTAACTGAGTTTGCTGCATTACTCAAGCCATTA
CCAACCTCTCCTGTTATCTACCGAGGGACGCCTCAGACCGAGATGACTGTCAACAAGGAA
CTTGAGGATATCAAAGGGTACAGCACAGTAATCTGGCCCGCACATTAGTCACGCAATGAT
GCTAAATCTCTCTATTAGGGGGGCCGTTTGGGTCCTATGCTTAAGGCTGAATATGATGAA
GGCTTTGAGTTACTCCGTGGACAACCTACTGACCATCTTCGATGCTATAAGGTCGATCTT
CGTGAACGTGAACTCGAATCTGGCAAAGGTGGTTACAGTTTTCCTTTCATGCTTAACCCT
AACCTTAGTGATGCCGAGAAGTACCCGTTACGTCTCCGCCGCGCTATCGTGTGCGCTGTA
GACCCTGAACGTGCCCCCCTGCCAAACTTGTGGTTGCCGAACCGTCAGAACAGCAATGAG
GAGCTTCCTAACTTTGGCCTGAAAGGTGATGACGTCCATAGCTTCCACACGTGCTCCTCT
CGAACCACTGATTACCAGTCTAAGATTCTTGTCATTGACTCCTGTGGTCGTGGTAAGGAT
GAGCCCGCGTTTGCTGTCCTCTATTCCCTGAATGGCTACATCTACTTGATGGAAGTCAGT
GGAGTCCGTGGAGGCTCCGATGAAGCTACGCTAGAGAAGCTCACTAAGAAGGCCAAGCAG
TGTGAGATTCAGACAGTAGTCCAAGAGTCCAACTTTGGCGACGGTATGTTCGGTAAGATC
ATCTCTCCTGGTCTCCATAAGTACCACAAGGCTCCACTCGAAGAGATACGAGCTAAGGGC
ATGAAGACGATGAGGATCTGCGATACCATTGAACCATTGATGGGCTCCCATAAGCTAATC
ATTAGTGATGAAGTGATCCGAGAAGACTATCTGACTTCTCGTGTCCTAGACGGGAAACAA
GATATTCGCTACAGTGCGTTCTATCAGATGACCCGTATGACTCGTGAGCGAGGCGCTGTA
GCACACGATGACCGACTTGATCATATCGCATTGGGTATTAAATGGCTACGCGAGGGGATG
ATTGTAGATAGCAAGATAGGCGAAGAAGAGATGACCCTTCAGTTCCAGGAGGCACACATG
GAGAAACAGACGATTGGTGATGACCAGATTCATAGCCTTAATGTTGGTGGCGTGGACATC
TATTATCTGGACGATGCAGGAGGCTCTAGCTTCATCGACTGGTAACAGCTACCCAACAAG
AAGCAGGTGTGAGTAATGAGAGTGGCAATTGATAGTATAACAGGTCGCCTCACCTTCCTA
GGGTGTAAGACTAAAACTGGCTACGGTAGGATTCGTGTTAATGGCGTACGATGGATGGCC
CATAGATACGCCCGGCACGTCCACCTGAAGCGTCCCTCAAATGAAGGATGTGTGGTGACG
CATTTGTGTGCTAACCCTTCGTGTGTGAACACTGAGCACTTCTCAGAGGGAACACATAAA
GTAATCATAGAGGGCTGTAGGTCGAAAGGTAGTATGGCCCATAGTGATTCTTTAGGTGTT
TCTCCCGGTAGGCACCAAAGTCACATCGATTCGATTGAAAAGGCAGTTAATAGACGACTG
TAGGCTGTACTTGATGATAATGGTACAGCTTCTGAGGTTGCTAAGAGACTTGGCGTAAAC
CCCCAAAGGGTCAAGAAGGGTCGGTCTGGTAGACTAACTATGCACATAAAGTGACTTAAT
ATTCACAATGGGCACCCTTTGGAGTGATGTGTAAAAACCTAATGAAATCAAGGGTGTCCC
TAATACAATTCACTATTGGGAAGGGCCCCCTATGACAAGTTAAAGACACTTAAAGATTGC
ATATGCATAGAGCATACTTAGTGACTAGTATTAGTTACTACTCTTAGTGACTACCCTTAG
TGACTACTTTTGGTGACTACCCTTAATGACTACTCTTATTGACAACTCTTAGTGACTACT

CTTAGAGACCCGTGCAGTGAGGGGTGATAGTAATAGTAATTACAACCCCACTATCAGTGT
AACGATAAAGCACACCAAGAATGACGCTGTATACGCTTACTATACGTGCACGAGGAAATG
GCAACGCATCAAACTACTTGTAAATTTCTCGTTGTACTTGCTGGTGCCATTGGTAGCACA
TCTGGTTCTGAACACATCGGTGAACTGGAAACTCTGGTTTGTTCTATACTCACTTGTGTT
CATTAGACTACTCAATGACCCTTAGTGTGCTCGTAGTAATTATGGTCGATATACATCTTC
CCATATAATCACTGCAAGAAGTCAACTCTAATAGTAACCCTTATGTGCCCTATATGAGCC
GACGCGGTGTCTTAGAGTCTATTAACGTCTATTAGTACCTCTTAGTACGTCTTAGCGTCC
CTATGGGCCTCTAGTGTGCGCTCAGGACCACCTTATGATAGACACTCCAAGTGGTGGACC
TCATGAAACTCTTGGTATCCCCCCATTAAGAAGCCTACAGAACAATCCGATTGAGTATCT
AATAGTACATACAAAGTGTTTCCCCACTATGGCCTCATTATGCTCACCCCAAAGAGAGCA
CCCCTATGGTGACTTTAGGTTTACTTTAGGTGTAATAGAGGTGTACTTGAGGTGTACTTT
AGGTGTATTTAATGCCTAACCTTATGTGTGAGATTGGGTTTACTACTAGATGTAGTGCTT
AGGGGGGACTTTAGGGGACTATATGTAGGCCTATTTCTGTCTCT
>NewGenomeName_52
CGATTTTGTGATTGAAATTGATTTGATTTGATAAGATATGAGGAGTGATTGAGTTGAAAT
AATCACAACTTGGCGATCTCGTGAAAAATCTAGTGCAAATCGATCCTCCCTGGCTAGAGA
TTGTATCTTTTCGTTCTCGACCGTTCACGAAACCATCCGGCGTAGTTCTCCCGCGTTGTT
TCCAGTTTGCGACACAAAAAGAACAAAATTATACACCATATAATAATAATTAACACAATA
GCTTAGTTATATATATTAGTTTAGGTGATCTTTTATGTATAGCGTGTTCCTTAGGGTATG
TGGTAGGGTGTGTTTGATTGATATTCCTGTCTGTATATGATTTAGCCAATTGGGATTGCT
GTTGGCATTTGGCTTCAAAAATGTATACACGTATTTAAACCTGCACAAAATCGTGAACAC
CTGAAAAGCGACGGAACAAGGATTTGTTCTCAGAAAAATCATCAACTCGAAATTTTTTTT
TGTGTGCACTTGGGTATGGAACGTTAGCATACATAAAACTACTTGACTCGAACAAACGGC
GGCGATAATACGCCTCCACCAATTAACCAAACAGGAGGATACCCAATCATCGCAGCTACC
ATAGAGTTTCGCAACAGCTTGCGGATGCTGGCTCATAAGCTGACAGACGGAGAGCCATAG
GAATTCGTAATCACTGACGCAACCATTACCGATAAACTGGTTGAAGAAGACCGGAAGAAG
ATTAACGCTTCGTATTTGCGCACGATAATCAATCGTGTTACAGAGGTGAAGGAGATCGGA
ACGGTTTCAATTACTCGCACTGAGCCGGTCGAGGAAGCGCCAGGTTTTAAGGTGGAAATC
AACCGCGAGGCAAAGCTGCAGGTATTCACCAAATGAGAGATTCCGGCACTGGAAGAGCGG
ATCGGTGAGCGAATCGCCAAACGGTGCGTGAACACAATGCCAAATATCACGTATCTGGAA
GGTGAACAACTCCACGGCGCGGCAATCGGGATTAAGCGTTATCAGGATCTGATTAAGTCA
TGTGCAACGGAGGTATAAGCCATGAAATACGAACGCAAAGAGAAGATCGCAAAGGTCATA
GTTGATGAGTTTGGTCGCGTCGAACTCACACAGGACCAATCGGAGGAAATCCAGGAGGCA
CACAGACACATGATTTTACCACGCGTGATTGTCAGATCTGGAGGCAGGCAATTTAAATAC
GTGGTTGCTTCTCCAGCGCTTAAAACGCCTGTAAAAGCATAGCTTAATAGGGGCTAAGAA
TTCGGAGTCGTCAAGGAGCCCGTAGATTGAAAGGATTCGCCTTTGACGCCGCGATGTGGT
GTATTATTATTGAAAGTCATGGCGTGGCGCTGGTTGTGTTTGGTTTTATTTTATTGGGGG
TTTTTGATGAATAAGAATCTTTTAGATCATGCCCTGGTGTTCTTTATCGCTGCTCCCGGA
TTTTCGCTGTTGTCAATTCCATTTTCAATCCTAGCTTTTGCCGCGTCAATGCTGAATTTA
CAGATTACATCCTGATTGGTCAACACCACTCTTTGTTAAAACTGAATGCGGGAGGTTTGA
CATAATTACCCCATTGACACGAAATGAGGAATATAAAATGTTTGGACTGAAACGCGTAAC
AGTTGAGATCCTTAATGATGGCTCTTATAACGGCATGTAACACTTATCCTTCCAGGTCCG
GATTGAGAATTGCAATGATTGTGGGATTGAAGGTGGAATGGTTGACGTTCAAATTGATGC
ACTTATTGCGGCTGGCTATGACCAAGAGAAAGGCCAATATGCCAATATACTGCATGGCCA
TGAAAGCGACACACATTTCCCGTTCACATATGGCGAATGCCGGATTGTTGATTGAGGTTC
TAAATTGAGCAGTTGGCACCAGGTGAACGTAATGCGATTCCACCGACACAGAACAAAGAT
AATGCTAAATAACACCAATTGCTAAAACTAAATCAGGGGGATTTGTCATAGTGTCCCTAT
TTTAGTTAAGCAAATGAGAATCAATAATCTTAAGCATCTAAAAGAGAAGCTTCGCGATCA
CATTTAAATACTATCAGCCTGACAACGAAGATACCGACGCCACTAAAACGCGCGGAGAGT
GGAATGATGGTGGATGAACAGGATTTAAACTTGAGTATTTCGTTAATATGCACAACAACT
AAACTCCAGATAACATCCCTGGCAAAGGTGGGTATTACATAATGGCGGCTTTTGGCCAAG
AAGGAAAACGCGAGGATAACGATCTGTTCGGCGCGTTGAACTATTTAAATATCAAGCCTG
GGCAGGTCAATTCAAACGGTGTTCTATTCCCTGCTTCCGTAGATGCTAAAGATTACGATT
ACATTTGCAATGTTGAATATATGCCAGTCGCCGCCACCCTGTTAGGTCTTAAGCGTGAGA
AGGTCTAATGGCACGAATAGTAATCTAAACCGCGCCGCGTTGAAGTACATGACGTTGTTA
TTAGCTTCCTTTCCGGTTATATTGGTGGCTACAAGAGCCGTAATAATTACGGCGCTGACA
TCAACATTAGGAGCGGTACTAGCGTAAATATCAACAACATCACGAATTGCTAAAACAGAA

TTTTGGTCTTGCTGTATAGTAAGCTCATACCAAACAACGAAAAGGATAGAAAAATGGATT
GATCACGGCACGCGGTTTATGGCTTACACGTCAAAGGCGGTAGAGGTTCAACATATGCGC
ACCTGCTTCATGTTAACGAGCGCGGCCAGGTTGATGTCGCAATGCATGTTGACCAACTGG
TTCAAGGCAGTGAGTCCGACAGATTTAGACCTGATTTCGTTGAATATGATCTCGCGTACA
GCCACGACCAATGGCTAAATGGATATAAGTTTACAATGCTGGATAGCCATGTTATTGACA
CATGCAAGTCAGCAAAACGAGACTATAAGCGCATGGTTCGCAAAGTTTAGAAGGAGAGAG
CGCAATAGCTCAGAATGTTAAGCGGTGATGTGAGCTGATTGGTACAAAGCCCGCATGATG
CAACAAGAGAAGATAGAAAGATGTCAAAAGACTCAAATAAGCAGTTTCTGAAATACGGCT
CCATGATGGTGCTCGGTACCGTTTTGGTGGCTGGGATCGCGTATAGGATGGTTGACTGTG
CGGGGTAATGGATGTTTATGATCAACTAGTGGGGGAGGGACTACGCTAAGCCCCATGTTG
CGACAAAACAGCGTTTCGTAATTTTAGACCTTGACGGCACATTGTCGAATGGTGAGCACT
GCTAGCACCTGTTGCCGTAAGTGGATTTGCATCTTGCTGAAAGCTGGAGTGAGTTTCATC
ACGCCGCCACGGATGATAATCCAGTCCAGGATACGATAGCCGGTAGCAATGCCATGTTTG
ATGCCGGATACGTTGTGATCATCCTAACTGATCGGATTGATGAAGTTGAGGCAGAAACGC
GAGCATGGTTAGCTGAAATCGTAGTTTGTCTCGATTGGCTGCTTAGGCGCCGCGCGGATG
ACAACCGGGAGAATTCCGTGATCAAAGACGAAGTCTTTCGGACTTTCGGACTTGATCGCA
TTTTCGCATGTTAGGCTTACTCACCAAAGGTTATTTCATCCTTGCGCGGCCTGGGCTTTA
CTCTTTACCAGGTGATTGTTGACGGCGAGCCGTGACACGATCACTGAATATCTGACGGCG
TAAAGAAATTGAGCACTGACAGCAAAGTTTATTGAAGATGGGCCTGGCGATAATGTGCTG
ATTTACATAAACGGTTAATCAGTGTGTATTGATTGCAAATATTGGGATGATACAAATTTG
CTGTTTCGCGGAATCTTTGACGAGAAAGGAGCGTTGGAGTTAGCGGAGCACATCAAGCCG
ATTTCGGAAGAATTACCAGAATAGAGGGTGTATTATGTTGACTTTTATCTTCATCGCTAT
TGTTTTAATTCTGTATGTTTACGGCTCTTTCCTGATGCCAACGCTACAAAAAGAGGCTGA
CGCTGATGATAAGGATAGTTGGAAGCCGATTGTTTTGTGGCCTGGCTATATGATCGTGCC
GATTTGCGAACTAATCTTCGATGGTGAGTTCAAGTGGTAAATAGCGCTTTTTGCTAAAGC
TTGCTTAGGTACATTTGTTATAGTGTGCCTTGTGGAGACAAGCAAGGAGAATGACAAATG
AAACACTACAGGTGCGGCCGTCTCATTTCTCGTGACGCTGGGATTAATATTATTGTTGCA
TATTATGACAAGATGGAATTTTTTGAATCCATTTACACAAGGGGCGTTTTTAATTCTATG
GTTGATGCTGGATTCATGAAAGAGTGCGATCCACCAAGTGGGAAGCCGTATTATAAGAAT
GTTGAACTCTGGCATAACGCCGTATAGGTGACAATTCTGTTAATTGGCCCTATGGTGATT
ATTTTCTTATGATGCTTGACAAGCGTAAAGAGACGGAAAAAAAGATCAGCTATCTTCGTG
TTCTTGCCGATAGAATGCAAAATGGCAAAGTAGGAGTCAGTGATGCTACTATTGAAGCGC
GTAATATTCATTGGATCGTTGAAGAAGAAGCATTAACACCATAAACTGTTAAGGCGGTAA
ATAATGGCAAAAATTAACATCCTTAATGCGCCCCCTGGGGCTGGGAAAGACAGTCTCGGC
TAGATGGTTTCTTTCTATTCGCCGGGTGTTCAATGCCGTATCAATAGCTTTAAAGAGCCA
ATGTGTGAAGTGGCATTGTCTACCCTGGGTAAAGAGAACTATCAGCGATTCATGTTGGCT
TATAATGGCGGAGGGTAAAACGAAAGGCCGCAAACCTTACTGATGAGTATGTCCCCGCGT
GAGTTCATGATCTGGATTAGCGAGGATGTAATCAAACCTCGATTCGGCAAGGATTATTTC
GGCGTAAGATTCAACTAGACAATAAATGAGTGTGATGCTTCGGTTGTTTGCATTGATGGT
GGTTTTCCATATAAAATTATCGCTCTAATCAATGCGGGCAATAAGGTGTTGCTATGCCGC
TTGCATCGCGACCGGTTCACGTTTGGAGGTAATAGCCGCAACTACAGTCGAATTATCCGC
TATTACCATAAAAACGGATATAGGGAGCACGTTTTCTAATTGACTGAGAACATGCCAATG
GGAACCGTAGACACGATTATCAGCATGTTCTTTAAATAGCACGAATTGCTAAAAATTGCG
CAAGGCCATTTGATATAGTGGTCTTATTGAAGAGAAGCAACCAATCAGTCGAATAATCAA
TGCAAATTAAATGAACATAATCAAACACCATGAATCTGACACTTGGCGCGATTTACCATA
GGGAAGAAGGTGTAAACGGGTTTACCCCAACTGTTACGCATGAATACGGCAGTCAATTCT
TCTTAAACGCTAAAGACGGTTTATCTGAAGATGTTGGCTCTTTCGTGCTTGCAGCGTTTG
ACAGCATGGAGGAATAAAATGTTCTGGATTCATTCGCCGCAAGGCTTGCTTTAGGTGACT
GAGGTTGAATTAGAAGATTTACGAGACGGCGAGACGGTAAACATCATTGCGAAGGATTCT
AAAGGCAAGACGCTTAAAAAGTATGTTTTTAACAATCCTGTTAAGTTTGAAAAACATGAT
TAAGATGTTTTTAATATTTATCCTGCTTGTCATAATTGGCCATTTTTTGATTGCCGCTGA
TTGTGTTCAATTTGGTGGCTCTGTGTTGGGCATGACTGGTTATTACGTTGTGTATAAAGG
GAATGCATTAGATTAATGGAAAAATGCAGGTACGCGCATTCGTAAAGATATCTAACAGAT
GGATTTATTCAGCTAAAACGCCTTCCACGTTTAGCGTGTACGGTTAAAAGACTGCTCTCG
CATGGGGTCGCATCATTGCAGACAGCTATGGATCCGAGATGCTGGAGTATTACGCAAACA
TGATTGAGAAGACCCTAAGTGTAGGGGGCCAGGCTGAATATAAGTGTTGCGTAGAAGAAG
AAATTTCTTGTTGGTAAGGGGAATTAACATGATTGCTAAAAATAAACTTCACAACCTTTT

AACTACTGGTACTGATTATCAGGTACTGAATAGCACAAAAGACCGTTATCTTATATACTG
TGATACTGGTGATCCCATGTGGTTGCGTAAGGATTTTTCTAAATCGAACACCTCAACAA
TGCAAAGCGGATCTCGGATGAAGCCTGCGCACTAGCAGAGCGTGCACGAATTTGCGAATT
CGTAGCAGGGCAGGTTAGAAATTCTTGATTGCCTGATGGCGATCTCTTCAAATCAATGCG
TGGTGCAATGACGGTTGGAATTCTAGATCGCGATATTGAACAATCAGCTTACTATGTAAT
TGAGAATGCGTAATTAAATGCGTTGAGTAACTCAAAGCATAACGAATACGTCAAAGTATT
AGAGCAACAGCTTGAAGTGCTGCAAGTGGCGGTAGGTGAAAATAAAATGACTCATACAAA
GGTTGTAATAGCGTTGGCTGATGCTTTGCTTGGCGTTTACCCATGAATGAAGTGCCAAAA
GCACTTTTTGTTAAGGCTTCTGCGAGGTCATTTGTTAAGGTTTCGTCTTTGAAGCGAAAG
ATGGAGATTTACAAACTGTCTGTGTACACTGGAAAGTTGTTTGTAAGAGAAAACCTTATT
TACGCCGGCACTTATGAGATAATCATTGATGAAGTTCGCGTAGTTCCGGCTGATTAAGAT
AACTAGAAATATTGCGGAGTAAAAGCCGATAAATGGGATAAGGCCGCTTTCTTTCTTGTT
GTTATTAGCCGCGTCAAGTTTTATGCAATGGAGGAGGCCGGATGGTCACAATTAGCCTAT
CAGAGGAACAATCGAAAACATTATTGGGTGGCCTTGGCTGTGGAATGGGTAGCGGTAGCG
AGGCGGTTGTTAACATCAAAGAGGAAGTAGCAAGAGAGCTTTCTACAAGGCTTGAGCATC
AACTGATGCCAGCCTCAACAAGCGGGGCGTAGTTCGCTGTGTGGAGGCATGGAAACGGCT
TTAATGAAATAGATGTTGAGAAGTGGAAGCGAAAAAACGAGGTGTTAGCATGGTTTACAT
TCGCTCTTACGGAGTTGGCTAGTTTGGTAACAAACAGCTCCGAAAAATTCACCACAGGCT
CGATGAAGGAATTTCACAGCAACGCGTTTCAGGTGGTGGGGTTCTGGCGTTTGAGTCAGT
GGAAAACTAAGAAAGTGAGTGGATGGATCGCTAGCAGGTAAAGGATCTGATTGTTGGTGA
GCTGTCATGCCTGATCGACGAGCTCACAACGGACTTGCCAGGGTCGCGGATAACCTATGA
GCCAGGTTCAACGCTACGGGACTGCTGGAAGGTAAAAACGCTAAGCAACAGCCGTGATAT
TCTGGACGGCAAATGTAAGGAAAATTGATAGCTTTTAAAGCGCCTCAATCGCATTCTAAG
AGGCGCATTTTTTGGGAAGGTGATTCTAGGCTTGAATACATAAAACCGTCGCAGTGGTTC
GCACAACACCTAGAAGAAGCGTTAGAGCGCGGCGACACTTACCCAGCGTTGCACTTCTTT
GATATGTTTCAGCTTTAGAAATCAAGCGGACCGTAAAATGTTAGGACTTAATGAGGCTGA
AGATAACATTGTTAAACGGGCTGGAAAAGAGTACGAGAAGGATCTGAAAGTTGAAGTAAC
AAGCAACAACATATATGACCATATTGCGGCTGGAATTATCGCACAACATAACACGACACT
TTTTACCCTCATCACTCGAACAATGTTTGTATGGCTTGCTGGCTATGTTCCTGGTCGATG
GGGTAAGCCTGTCCAGCATGAATAATAAGCACTTGCATCACTGAAGGTTAACGGCTTGTT
GTCTGGCTGGTAGATTAAACGGCGTGTACATAAGAGCCTCTATAGAGCAAAGCGCAGCGC
GTTAGCCGTAAAGTCAAATCGGATGGGATTGCCTTGCAAATGACCGTCGAGCGAGCCGTA
TCCCGCAATCACTCAGGAGATTAGTTGATGAAATCCACATTGCTTTTCAAAAGAAGAGTT
TTACACCCTCGTAACGAACTTCCTGTTATTCGCGTGATGGTTTCAGAAGAAGCTTTCGCT
ATTGATGTTCAAACGAATGGTGTCATAATCTCAAACAAAGAAGATACAAAGGCGTTCTCA
TTTCCCAAACACGACATCATCCAGATTATCGAAGAGGAAGTAAATATTGCAGAATAAAGC
GAGTGCAATGGTAACTACCACCATTGAACGTGACCGCAAGAAAACGGGATACAGAAAGCG
TTCTGCATAAGGCGAGGCGGTTTGAGACAGGATCGGTGCTTTTACGGGAAAATTGCAAGA
GGATACGCCGGATGTTCTTCAAAGTTAATTTAGCTATCAGGCGCATGGGTCGCAATTGCG
GATCATGCAAGCTGCAATTTGAAAAGGTTGTTAGAGCATGCATAGCCAAAATGCCTGTAA
GATTTGCTAATGAATATTCTGGAGCAGATTAATAAACGAACGAATATTCAATCAATTATG
CCACGGCTATATAATACTGATGTTTATCGCTGGTTTTGGTTTTTGTGCGATGTTTTGTTT
TGTTGTTTACCGTCTCTGTTCGTGGTGTGCTCGTCGAAGAATTAAAGAAAACGATCTTGT
GATCGCGTCTTACATAAAGAAAAGCGATGTTGGGGAAATTACACGAAATTACGAAGAGAT
TGCTGATGAACTTGCATTCAGTCACAAGTTTGGAAAGCCTGGAAGCATTAAGTATATTGA
TTGATTCAACCCAAAAAGATTACCCGCCTTGTGCGGGTTTGTTTGTCTCTGTAATTTAGC
TATTATCACGCCTCAACAGGAGGATTTTATTAGAGCTAAAAAAACAGCAGTGCCAGCGGC
GGAAAGTCTAAACTTTAAAACGTTATGAAATAGGCGAGTATCCGAACTGGTAGGTAGTGA
GATTGAAAAACGCGCAACGCTAACGACGGAACAAGTCATGACCCTGATTGTTCATTATTT
TGAGTGGGCGGTGCTAAACAGCATTAAAGCGGGCGAAACCGCATCATTTCAGGGTTGAGT
GTATCAGCATACGATTCACAAGCCGCGTATTATTACATGGGAAGGGCTAAAACTGTTTTG
CGGTTTTTCCGGCACAGCACTAGCAAAGGGGAAACTTAAACCAGGCTATGATGGTATTAT
TGGGTTGACTGAATCGGGAATAAAAGAACAAAAATACCAGTCACCTGCAAACGGAATGTT
TTACAGCACCATGATCGCAAAAGGGCTAAGCATTGATAAAGGCCACACGATCAATAATAG
CACCAACCGAAAAGAGCAAGATAATACAGAACAAGCCATGAAAAACGCAGCCACAGGCGT
CCTAAGCAAGTTTTAAAGGTGAATGCTATGTTGATTTGGAAAGTTTTCACAAAGCTTGAA
AAGCTGGCTATTCAGGAATTAGGCACACACGATTTTGATGATTTCATTAAAAGTTGGTTC

ACGATCCAGCAGGGCGAAAAAAGGAATCCTAACTGGCATCACCTTTGTATTGCAAGGGCA
ATAGATGAAATTATTGAAGGTGTGCGCAAAGATACCATTTTTAAGCTAACGCCAGGCTCA
CGCAAAACTGAATTGCGTTCGATCTACTTTCCTCCTTAGTCATATTTGAAATGGAACAAC
GTGCGAAATCGTATTACTTCTTTCGCTGATACACATGTTGAACGTAACAGCGAGCGTGTG
CGAGATCGAGTTAACTCGCGAGAATGGCAAGAGTTTCCCCCCGCAAAAGCTGGTACAAGC
ATGGATGACGAATTCCAGAACCTTAATGAGGCTGGCAAAGGTCGCCTGGTAACGATTAGT
AAATCGATTGGAGGTCAGATTACTCGTAGCCTTGGCGGTTACATCACGCCTGGCGTTTAT
TCCGGTTGTGTGACGCTTGATGATCCAGAATAGCAGGATGAGATGTTTTTGGAGCTTAAG
AGTAAGCGCGGTCAACTGATCGCAAAGAACACCAGTCGATCACGTCGCGCACAGTCAGAA
ACGTCGATTATCATTATTCAGCAGCGTCTAAACGCGCAGGAAATGACCTGGTTTCTGAAG
AATGATGGAATGGGTATTGCATTGCTTCAGATATCAATTCCGTCGAGGGTTACGGTGCAA
TACGGGATATCGCTTCCTGCTTGCTTACAGTCTCATTTTGAAGAAGATGTGCTTCCGTCT
GAATCCCTCGTTATTGACGGCGCGAAGTATTAGTCTTTTTGGCCTAGCAAGGAGATCATT
CATGATCTTTAAACGCTACGCGATGCGGATTTTTACAGGTTCCTTTGTGCTTATCAGCAA
GAGCCAACTGCGCTTGGGGGTCCCGCGTTTAACGTTGTATCGCTCTAGTATTAGGAAACC
AGAGATAAATCAACTATGCCAAAACCGGATCGCTTTGATTATACGTTTATTGCAGCTGCT
ACTGGTCACAAAGAAGGTGAGCTAACCGACTACAGCGTTCCATGTTATTGGGGCATGTCC
AAAGTCCGTATAAATTTTTTCGACGTTGTGCGCGGAAGATGTGAAGCGCCAATGTTAGAC
ACGCAGCTAAAAGTGTTTGTTCATCAGTGCTGGAACCGAAACAAAGAATGCGGAAACTTA
GGTTACATTAACCTGGAACATAAAGTCAGCGGTACACGTTTAGTCCAGATTTTCCGCAAA
GCATATACGAGAGATATTAGTCCAGTGCAACGACGTAAAGACTGAGTAAGGCGCTGCATG
GACGCAGAGCCAGTGATCAAGAATGGTTATGTTGCATTGCCGGAATCGCATCAGAGGCTC
GCTGAATTTATGGCGGAGGCGGCGGCATTCTCTTGTGATGATAGCCATCCTCATGATGAT
ATTATTGATAACCTTTTTGATGCGGTTAATATTTGGCTTACCCTTGCAGACAATGAGGTT
GATAGAATGAAGCGTTTACATGGAATAGCGAAGCGTTGATGTATAAAATCAAAGACTGGA
ATCTTCCGGCCTTTTCATTATGGGGGAATTTATGAGCGACAAGGTAAAGGCCATTACTAA
GGAAGAGGGCTACGACAAAATTTACAGATCTAATGATGGAACGTATTCGCCAAAGGCTTT
TTAAATGCAAAGATCGGCATTTAAAGCGTTATCGCAATTTTACGAAGAAGATGGAATGAC
GCGGCGCATTGTCGATGTTATTCGCCAAGAAATGGTAACACTTGGTTTTAAGGTTGAGGG
CGTGACAAATGAAAAGTCGTTTAAATCTTGTTGGGATGAATTGCGAATACATGCTGAAAT
TATTAATGCGCTTTCATGGTCGCGTTTAATTGGCGGTTCCGCAATGCTGACTATTGTTTC
TGATAACAAATTGCTGAAAGCAACTGTAAAGTCTGGTGCACAACTTTAAGATCTTCGCGT
ATAGCATCGATATCAGATCGCGATGCATGCACGTAAGACTAATGCGCTGAGCGTTCGTTA
CGGTGAGACAAAACTGTATAAGACCGCACCAGGCGGAGATATACCAGAATTCTTTGATCA
TTTCTCGCGTATTTGCATCATTGATGGTTAGCGCGTATCGAGTGAGCAACCCAGTCAGAA
TCATGATTTGAGCGCAACTATTTTAAACTAGCGTTTAATTGAGGCAATGGTGGATTAGAA
CCACTTCCACGTAATAGCAACGCAATTGCTACGCCGAAGACTACAAGAAGTTTTGAAAGC
GCGTGATCTGGCTATAATGTGCGACGATGAAGTAAGACGCTATGCGGCGCGTTTGCGTTT
GGCTCAGGTTGTGGACAAAAGCGGAGTTGGTAATGCAATTGGTATTGATGCCACCGATGG
GGAATATGAGGTTCTCAATTCTGAGGTTTCCGGGGTTCCAGAGTTCTTACAGGAAAGGAC
TTATCGAATTGTCGCACTAACGGGCATTCATGACATCACCCTCAAAACTGAGCACACTGG
CGGCGTTTGAGCGAGTGAAAAGACGGCGCTAGTGAAGGTGTATACGTTGATTGATCGCAA
GCGCGTAAGGTATTACGAGCCGATTCTTGAATACCTTTTGCCGTTCATGATCTCAGAAAC
AGAATGGTCTATAGAATGCGAACCGTTAAGCGTTCAGAGTGATAAGGACAAAGCTGGAAT
CATGGCGAAGGACGATGAATCTGTTGTTAAATTGAAAGCTGAACACGCAATTACTCTCAA
AGACACCTGAGACACTTAGCGATCCATTAGTCCAGATCTGTAAATTATGGACAATGACAA
CATTGAACTGCCGGAGCCGGAAGATTTAGATCCCGGGTCAGGGCATGAGGGAGGATTGAA
TAAATGCTGTTTTCCGATATGCCAGAGAGCGACGTTGAAAAGGCGCTTGATGATGTGGCG
ATTCATATCACTGAAAACAATGCTGTTGCAGAGGCGGCAATTGCCGGCGTTAGGTTGCTG
GATTGCGGGTTCGCCGTTAGTACATACAAATACCACACTCGTCAATTTATCAAACTGGCA
AAGCAAACAGGAGGCGCAGATAATCGATCCGTTATCATTCTGATTGGTGGCGATCCCGGT
TACATGAATTCACGTTGGTTCAATTCAAAATGCATAACCTGGCGAACACTTATTGAGGCG
TCCGCAAGGAAATTTGCGGATAATGTGCCTGATTATTGAGATGTGCAACAGCTTTTAAAT
GAGTTATGGGACCAAGCATAAACAAGAGCGCAGACCCGAAAGCGCCTAAGAAATTATAAG
GCGTGGGCTAAAAACAGGGCGACTGTTTTTGTTGGTTTGTGGTATTCTGTGTTAATATCT
CAACGCGTGGTTGATACCGGAGTTACCCATTACATTTGTCAAACTTAGGATGATAAGAGG
GTTAGACCTGACTATGCTAAATTGGACTGGAAACGGAACTCATTGGCAATGTAGCACATA

TTCCTATGGCATGAATATAATTGTCGTTGTAGGGCGCTACCGGTTAGGGAGACAGAGGAA
GAGGTTTATTCATGAAGAATGTACAACGTTTTGATAGCGTCAGAGTCAACGCGCATTTTG
ATGCACACGGCTTTTTAGTGGACCGGCTTGTTGTGACGACAATTGGTTTGCTGATTTATC
AAACACCAGTAGGCGAACGTAGTGAAGTTAGGCGAGCAAGTGAATTGTTTAAAGCTGATA
GCCTGCCTTGTTACGGAGGCAAGCCGATCACAGTAGGTCACGTCACCCTAACGCCTGAAA
ATGCGCGTGACGTTGTAGTTGGTACTTGTTAAGGCGCTGGCATTCCAAATGGGATCGGAG
GTGAATCACCTTTGAACATTTATGCAAATGATGCAATTGAAAGCGCCAAGAGAAAACGAT
CGGCTGAAATGAGCGTTGGTTATACGTAGATTGATATTGATAAGCCTGGTTATGGATCTA
ATGAAAATGGTGAATTCATCTTTGAAGAAGATCTAAAAGAAGATGAACAGCCGCCTGAAG
GATAGGTAAAATTTGATGCGCTTCAGACAAGCAATAGTGTTAACCATATCGCGTTGGAGT
CTTAGGGTCCCGCTGGCATGGCGACAGTTAAGCGTCATAGCGAGCAAGAATTCCCGCATG
ATATCGACTTTAAATTAAATAAAGGGGACGACGTTATGACCAGTAAAATTTAACTTGATA
GTGCTGTTAAACTTGACGTACGAAAAGAAGTTGCTACTTATATTGAAACCGGGAAAGCAG
ACGCTAGAGCGGCAAAAACTAAAGCCGATATCCAGGAAACAGCGCGCGATGCTCTAAATC
CGAGGTTTGACGGCGTTGCGGCGCAGATTGAAGAAGCAGTGCAAAAAACAAACACACACG
CTGACAAATATGCCGCACTAGTAGCTACCGCTTAAGATATAGGCGTATAATGCGACGGCC
TAGCAGCCAAAGAAATTCAGATTGCATATGTGAAAGAGGTCATGGGTACTGACATTAGGG
CAAACGCTGATGCATATATTGATACCGCTTTTGATATCGCAGGAGAATCTGATATAATGG
CTGCCCATCGCATCGCAGTAGCAGGTGATGCAGCTAAAAACAATCACGATGCAGCGGGAT
CCACTCTTGATCCTTCCGCACGTCTGAATAAAGCTAACCAAGAAGAAAAGATTATGGCTC
AGATTTCAGCAAGTTACCATGCTGCTCCGGCTCGCGCCTTGCCTGGTCAAGTGTCTGACA
CCTCCGCGTATAACATCGTTGGCTCTTGTGTTCGTGATGGTGATGGCGGATCAACAATTT
ATGTTGGCGTTGGTGTTCAACATGGCGGAGTTAGCGCAAGCGGTGAGAAATTAATCAAGG
CTATCGGGTAGTCTGGTAATACTTATGGTGTCGCTCTTCGATCCAACTTCCAGACCACGT
CTAAAGATGGTCGCATGGTTTATGAGGCTGGAAGCTGCATCAACGTAACGACTAAAGGGA
CCGGTTGGAGGGTTTCTGTAGAATCAGAAGTGCCAGCATTCAAAACACCCATCAAAACTG
ATAATTCAGAACAGGTGGATTGAACGTCTGGCTCAATTGAAACTACTTGGATCGCAACTG
GAGACATTACGAAATTGTAAGGTCTAAAACTCGTTGAGGTCCCACTGGTATAATAATAAG
GCCGCTTTATTCCGACCTTTTTTATATGGTGTTTTTATGTCTGATTACGGTTCTATAATA
GGTATAGTTGAAAATGGGTGCTCGGTAGATTCAAGCCTTTATAAGATAGACGGCTCCCCG
TTTATGGATGTGTCAGCCGTGTTTGGTAAGGCCGTATGTGTTGATCGCATAGCTTGCGGT
TACAAAATAATCCGCCACAATCTTAATTAAAAATCAAAGCATTACGGAATCGTTACGCGT
GATTATATGGATTTTTGAAAAGAATCTTACGACGCCGGAGATCCGGTTTACGTAAGAACT
CAAGGTCGAATTGCGGTAATCACTCAGGACGAACAAGATATTTCATGGTATAAAGATCCT
GTTTTGGTTAAAGACAATGGCTGTAAATCAACAAGTGGATTTCCTACCAGTTGGTTTTTT
TCTGATGTATACGTTAGATATTCACACGGATATATCTCTCTTTCAGTTCAGTTGTCGCGT
GCTAGCGAAAAAAACACTTGAGCGTTTATCAAAAAAATGCGGTATTACAGCCCTTATTAGG
ACGGCTTTTCTATGCGGGGGAATATTATGTCTAAAGAAATGAAATATAATGAGTTTGAAG
CTAACGTGATGTACAACCAGATGCAGTTTCGCGGCGCTAAAAACGACGCATCTGAGACGG
GTATTCGGACGTCTCAAGAGCTTTTTAAACTTAAGGCTCAGCTCTCTGAGAAAGAATAAC
CGCCAGGTTCCGAGACGCTTATATTCCCTGTGAGGTTGGAACTTTATGATAAAGATAAAA
CCTTTGAATATCATGCCTTAGACAAAGTAGGACTCGCAAAAATCATCTCTGATTCCACTG
TTGGTCTGTCAATTCTTGACGCGCTGATGACTTCGTAATTAGGTAAAGTTTTCCCCCTCG
GCAATGCGTTCCTTATTTCCATTGACGAAAGCAAAGCAGGTCAACGCACCGGCAAGAGCC
TATCAACCCGGAAGGCCAACGTCGCACAGACTGTGCGTGATCAGCTTGTATGTCACCTGG
TTTTCTAAGGCTCTAAACCTCATAAAATTTTCAGCGTGCCTGAGCTTCCAAACCAGACAA
CTTTCAACACCGCAGGTTGGAACTACGCCGCAGGTCCTGGCAACAAGGCGGAAACCGCAC
ACGATGAACTGGAGCAAGCTATTGAAAAAATCGTAACGCTGATTAACGACCAGACTCGCG
GTAATATTATCCTGATTCCGCAGTCAATGCGTAAAGTTCTGATGGTTCGGATGTCAGAAT
CCACCATGAGCTATCTTGATTATGTTAAGGAGCAGAGTGGGGGTGACACTTTCGAATCCA
TGTCCGAGCTTGTGGATATCGACGGTTCAGGCACTAAAGCGGCGCCGGTTTACGAAAAAG
AACCGATAAACATTAGCATTGTGATTCCAGAAGCGTTTAACATGCTGCCCACTCAACCTA
AAGACCTGCATTTCAAATTGCCTTGCACCTTTCAATGAACTTGCCTGGCGATTTATCGTT
CGTTGTCGCTTGTTCTGATTAAAGGTTTTGTAGTACGCTGATACTTCAATTAATATAGGG
AGCCTTTAAAGGTTCCGTTTTTAATCTCTGGAGAAAAGCTAATGGCTAAAGAAAAAGAAG
TTACAGTGCTAAACACTGCCGTTGAACTGATTATTATTGATCGGGATGATGTTATGACAG
ATAACGAAATTACAGTTTCGGAAAACGTGTTAGAACGGCACCGTCCAAAATCGCTGCATG

CCCAGGGCAAGTTAACCGTAAAAGAGAACAGAGAGCTAAGCGATAAGATCATTGGAGCGG
CCAACGAAAACTGCAAGCCGAATCCATCCGATGGCAAAAGGAAGGCACAGCTTGAGAAAG
GCGGCGAGGATTAATTAGAAAGGGCGCTCATGTAGCGCCTTTTTTATTATGGAGAATACA
ACATGAATGATGTCGAGATCCTAGAGCAAATTTATAAAATCGCGACAGCATTCAAGAAAG
TAGATCCAGAATTGATTCATGCATGGATTGAACAGGCTACAGCTTCCGATTGCCAAAAGG
ATTTGCAAGACATATATTTCCGCGCTGGTGCTCTTGACACTTTACCCCTTATGACTCCTG
ACTGAGCTCTGAAAGGCTAAAGTTAGAGCGTAGAAAGCGTCTCGCACCGTATAGAGTCAT
TTGCCCTGACTTGTAAGATTATGCAAGCGTTCAGCAAAGTTAGCCACCACACAAGGGGTA
ACTCGTTGCGCCAAACTCCGTGCAGTAAAATGTACGAAGTCCTCAACAAGAAAAAAGACG
TAGGATTTGGCCTTACACCTGCACTTCATCGGAGGTGCTCTCGATGAATTATGAACAAAT
CAGTTCTATGGAATCGGCTGGAACTAATTTTTGTATAGTTGGAACGGGTGAATTTGATTG
CATTACTTGACTTGGAAGCGTTGACATTGTTGGCGGTATTCAGGATGAAAAACCCGAAAT
AAAGGTGAAGATTAAAGGTCTTGTGAGAGCACCACGGACGCGAGAAGTTGACGGAGAGCT
AATTAGAGGTACCGACTAGTTAGGCGTTTTTAATGCTGAGGTAGAGCGTAAAAACGTATA
TCAGATTGTTAAAGACGGCGAGCGTTATATTATGGTTGAAACAAGACCAACCAGAACAAC
ATGCATTACCGTTTCTTACCGTCCAGTAATGGGGAGGGTTGCGGTTCATGGCTAATTATA
CGATGCGCTAATTTCATTGAAATGTTGACACATGGCTCAAGTAAGTAGCAAGGGGATTGA
ATGACGTGATTCAGCTTTTTGGTGAAAAGGTTTACGGCGCATTAGTTGATATGGCGCCAA
TGGACACAGGACGATTAAAAGCCAATATTCAGATCACACCAAACACGCCGCCTCTTCTCG
CGCTCACTCAATATGATCCTGATGGGGGAAAAATAAAGGCGAAAGATCGCAGGACGATTT
AAGCTTAATTGCATGGCGGCGGCGCTATTAAATCAGTATACTGTACAAATATGCAGATTT
ACCCTAATGCTTTTGAATATGAACATTCGAAACATTTGCCAGCGTGTGTATTTGGTATCG
TTGAGATCCGGTTGGGCTCTTACTTGACGGAAGCGATAAGGGAGGATAGAAAGAACAATG
CAGTATGGGTTATCTGTCGCGGCAAGAATGGCGCTGTATCAAGAATATGAAAACGAATAT
ATGATTGATTATGAAAACGTTGAAGTTCCGCCACCAAAAGGCGGAGGCATTTGGCTGAAA
TATAACTACACAGAAGCAGATACAATAATACATGATCTTAAACGGAAATGCATTAGCTAT
ATTGGAATGGTTCAGATCGGAATTGAATTCCCACCAGGATGAGGAATTGATACGGCTCGC
AAGCTGGCAAAAAATCTTGCTGATTTCATTGAAGGTCGTAAAATGCTTTCGAATGGCTAT
GTTTCAGCAGGTCCAAAACGGCATCAAGTTCAGAAATCAGAATCAAGCTGGTTTTAGCCA
TCGCGTTTCTATCTACGTTTTGAAGGTTAACAAAGGGGGCTATTATGCACTTGCCTATCG
TAGCACAGATTTTTATTGAGTAAGCTCGCGCACAGTCGGTAAGCCCGACCAACGTAAGTA
CTGCCACCAAACCTGAATTCACGCTTGAAAGCGGCGGGTATGCATTCAATAAAGGTGATT
ACATTATGGTTACCGCATCAAGTTGGGGTAAATTGATTGGTCGCGTTTGACGGGTGTCGG
ATGTTAAATCAACAAAGGTTACTGTTGACGGTATCGTCGCAACCGATACTGACGTTTTTC
ACGACCGCTCTGTAACTGTGAGATTTGCAAAAATCAACGGTTGGACAGAGATTCCATGCA
TACAGGACATGGGGCAGGACGGCGACGAGCGGCAGTATTACAATTACCAATGCTTAAACG
GTGACCAGGAGCCGCAGTTACCAACCTATAAAAGTGCGGTATCACTGACTACCACATTTT
CGCATGTATACGATAACCCGATTTATCCGCTCGTGCGTAACGCTGACGAATCCGGCGCCG
TTAAAGGATTTTGCATGTATGTTCCAACAGCGTAAGAAATGCGCCTTTGGGCTGGTGTGC
TTTCCTTCAACGAGATCCCACAAACGGCGGTAGCCGAAATGGAAACCGTTTCTTTGTCGG
TATGCGTGAAATGTCGCTTTACTTTCATCCCTGCTAACCATGAATAATATGAAGGGGCGC
ATTGCGTCCCGTATTTATCTGATGTAATATCTCATGACTGTTTAACCACTCAGGAGATAT
TACAATGGCTGAAATAAAATTGACTCTTCCCCCGCTTCCAGATTTCAAACTTCGAGTCAA
ATTTGTATTGCGGAATGGTAAGTTGCAGAAAATTGTCTTACCGTTTAAGCATAAAAATGC
CAGCGAGATTCAAGAGCTTTACCAGAAAGAAGCTATTAAAGATCCTGAATTCCTCATGAA
TGTTGCCGTTGGTTCGGACCTGGAATAAGAATTCACCGAAGAAAACGCTCAAATGTTAGT
TGATTATCATCCTGGCTCGGGGCTGAATCTTATGGGATGTTATTTAAGTCCGCTGGCAAG
GCGACGCGTAAAAGACTAAATCGGGTGTTTTATCTCTATTACCTGAAACCGCCAACAGCT
GCAGAATTGCAAGCCGTAGGATTAACGCGCGCAGACTATAAAGGAGAAGCTCCGCCAGAG
GTCATCTTTGGTGAAAGTCTCATGCAATCATGGGATATATTTTGCGCGATGTGTACACAG
TGGCGTTGTGCTCGCGATGGTGCTTGCCATTTTGATTATAATGATTTGCCTATGTTATTC
AGAATCTATAAACTAGACGATGAAGAACAGGCCTTAAGTGACTTGCGCATAATGGAACCA
AAAGCACTGGGAATGATGCAAGCAAATAACATATAAAGCGCCTACGGGCGAGTTTTTTAT
GAGGGTCAAATATTTGGCTGAACTATTTGCAGGTTTAACACTTGACGTTAACGTCTAGCA
TTTACACAGGGCTGTGAAGTCATTGCATGACTTGTTAAAAATCAAACGACGAAGGAAAGA
AAGGCGTTGATAGCTTTGTTGATGAGGGAGAAATTGCAATGCAAAGAGCAAAACAATTAG
CTGAAGAATTGGCGATACAGAGCCGTGAACTAAAAAGCATTCAGGACACTGTGGCTCCGA

CAGCGGCTCATAAGCGCAAGCTAATCCAGGCCTCAAGTGATCTTGATAAGCTATGGCAAC
AGGCAACTGTTACCGATGAAACTTTTTTCCAGATGGGGAGTATGCAGGAAACTCGGATCA
TTAAGCTGGACCGTACTACTAAGGCGTTAACGGAAGAAGGCCTCGCAGAACTTCAGGAAT
CAAAAGTCAAAGAGAACGCAGCTTTAGCCGGTAAAACTTTCCTTCCCGATCTGGACACGC
AAACCCCCGCTCTTGGAACAACATAAGCCGGATTGCTGCAAATGAAAGCGGCGCACCTTG
GCGTCAGTGCTCAGGCGGCGTTATTTCTCGCACAGTTTAAAGCGCAAGTAAAACCGATGA
AGTTAGCCTGAATGACCGCGGGGCAATATTACCAGGCCATGCGAATGCTTCCCGCTCATA
TCCCTTACGTAGTAGCGTCGCTGGCTTCCGGTATGCCTGTTTGGTTGGTTGCAATCCAGC
AGGGTGGACAGATTAAAGATTCTTTCGGCAGTGTTGCTAATACGTTTAAAGCGCTGATGT
CGTTCCTGAATGCGACAAACGTTGGCCTTGTCGCATGAGCCGATTCAATGGGTGCTCTTG
TTAAGGCTGGCTATGATGCGTATAAATCGCAACGTGACTTGCCGGAGGCGTTGGTGGTGA
CTTGTGGATATCCTGGCACAAGCGGCGCACTGTTCGATAGGCTGGCGCGGGACATTAATT
ACAGCACTGATGCGTCTATAGGAAACATTCGATACACCTCTACAGAGCTGGCAAAATCCG
GTAAGTTCATGATTAATCAGATTAAATCAATCACGGAGACTACCGCTCAGTGGACAACCG
TAACTGGTGAATCAAGCGACAAGATAACCCATTCCTTTACTAAGAGTGCGCGCGATCGGG
TTAAAGGTCTTGCAGAGCTGAACGAGCAGTTCAACTTTTTAAGCGAAGGGCAGTCAATTT
TTATTGCAGGCCTGGAGTAAACGACAGGCAAAACGGAAGCGGTAATGGCGGCGAACAAGT
TATTTGCAGATGTTATGGATCTGAGATTAGCTAAACTGGCATAATCCGCAAGGTCATTAG
AAAAGATGTGGGCGAACATTAATATATTCGAGTCAGATGGATGGGGTTCGGCTGGGGATC
ATACGATGGCGGCGCTTAACCTGATTATTGACGTTGTAGCCGGAACCAGTGAGTAGGTTC
GTTACTTGCTTAATCAGGGTGCTATCCTCATTGGTGAGTTTATCGTGTCTGCAACTAAGC
CTATGCAAAAAATACCTGGTCTTGATGGAGTTTGTGATTCAGTAATTGCACAGCAGCAAC
AGATTATTTATAATTCAATAGAGCAAAACAAGGAGTTATTAAAGTCTATTGCCGAACGTC
ACGATAGTGTCAGAAAAGGCGATCAAGGTCACATTGACATGATGAAAACGCGTGCGGCAG
ATGACCAGCAATATCCTGCTAAAACAAAGGAGAATATCAGGAAGGAAGCCGAGGAATTAG
CCGAAGGCGATAAAAAGAAAAATGCTGAAAAAGTTTTAGTATCGTCTGGCGCTGAGCTGG
AAGAACAATACCACCGTGATATCCCGCCTTAAGAAAGTCAATTTAGAGTTTTGAGAGAGC
ATAAGACGATCACTGATACCATAAGCCAGCAACGTAGATCTTTATGGGCAGAACAAGCTA
AAATTCAGACTTTGCATGTAGCCTCAACAAAGCGGCAACTGACTGATGAAGAAAAAAGCA
TTTTAGACAACCAAGATAAGATCCTGGCGACGGCTGAACAAATAGCCCTTTTAGGCGATC
AAATTGTTGAACAAATGCGCCTTAATCAGTTGCAAGATAGCGACATCAAGTTTATCCGGC
AGCAGAAGGGAGCAATGGAAGCACAAGCAAAAACCAGAGGGTTAAGCGCGCGGGTAGCAG
TAAGAGAGGCGGCACGTGCACCGGTTGACTCTGAATATCTCGCTAAGGGAGGACAGAAAG
AAGATCCGCAGCCTAAAGCCATGACGGATGCCTTAGACAACCGATATAAAGAGGAAGATG
CCAAACGAGATGATTGGTCGTCTGGCGCTATGGATCCCTTTGTGGACTATGCCGAGGAAG
CAATGAACATGTATGATAATATCGCTAACATAGCCAGCCAGGCGCTCAACGGCCTAGCGC
AGAAACTGGCTGACTTTTTGACTATTGGATAAGCTAACTTAGAAGACTTTGGAAAATCGA
TCACTAGCCTAATAGTGCAGATGATCTCAAAGATGATTATCTTCATTAGCATTTTCGGTA
TGATGTGCGGCCCAACCAGGGCGCGTAGGAGCCTTATAAGCGGTGCTAGTTTTGCTGGGG
GCGGTTATACTGGCGAGGGTGGCAAATATGATCGGGGTGGCTTTGTTCATACAGGTGACT
TTGTTTCCCCCAAAGGGGCAACGCAAAGGATCGGAGCTAAAAACCTGTATCGCATGATGC
GTGGTCATGCTAACGGCGGTCCAGTAGGAAGCCTGACCACCGGCGGCGCTAGGATCAGTC
GCGGCGCATCACAATTTGCGTTTGGTGATATCAACGTTAGCATTGACAACGGTCAATATC
CAAAAGGGATGAAAACTTATATTAAGATGATTTTCACCGATATGATTCAAGGTTCATGCT
GGCAAGACGAAGAGGTTTATTACTTTGTTCATGGCAGGGCTTAATGTTATATGAATTTAC
TTGCTCTATGTAGATTCAGAACGGCGGCGGTGTAATGACAACCACCAATAACGACAGGGA
TGTGGTTTTTGGGAACGCTTATCGCCAAAAGGCAACTTCTGGATTTAACACGGAGCAGAG
GGAGTTGTCTAATGTTTACGCTGGCACCGATTATAAAAAATTGAATGCCTTCATGACGGA
TCACGGATTAATGCCTTTCTTGTGGAGGATACCGGATGGTGATTTAGGTTTGTTTACGGT
TAAAGTCGGAACGGTAGGGCTAAGGCCAATAACCACCACCGTTCAGGAAGTCAAAGCTAC
ATTTACCGCAGAATGTCCCTCCATGCGATAAATCAAAGCCGCTATCGAGCGGCTTTTTTA
TTGTTTCTATAATGGTGCTTTATCCGAGGGCATTGAATATGGCTGAAAAACAGATCAAAA
AGACTTTCGTCAATTGTCTGCCATCGCTTTTCCCTGGTAAAATAATTCCGCTTGTAGTGG
TGGTCGGCACAAACTGTGGTGCGCAGGTTTACCGCTTTCATTCTGGCAATATCGCGTACA
CTCCAGAGGAATTAATGCAAGGGCTTGATACTGGAATATTCCCTCCGAAAGATCTTAAAT
TTCGTGGCTAGGTTTACGGGGCGCGGCCTTTACGGAGTAAGGGTTTTGGCTTTACAAGTG
ACGGAACGGCAGCGAAACCACAATTAGCGCTTTCAAATTTGGATAGCCGATTATGTGCAG

TAATTAGATCATTTATGGGAGTGATGCAAATCAAACTGACAGTTTGAGTTACATCAGGTG
ATTTAATTAATGAAGAATTAAACGTGGAGGACGGCGAATATAGAAAGTTTGTTTATTATA
ATGTGCGTCCAAATTTTGCTAACCAGACAGTTGGACGGTTTGAATTAACATCACCTTATG
GAACGGATGATATTATGATCCCCCCACGACTCACGCAAAGCATTTGTTATTGAGCGCAAA
GGGGATGGTATAGAAGCGGGAAAGGTTGCGGATATAACGGATCGGCAATGTTCTATAATG
ACGATAATCCGGTAGGATATACAAGCAAGGATTTTTGCGCCGGAACAGTGACAGCCTGGA
AATTGCGCTTTGGCGAACTAAAAGAATTACATTTCGGCGGTAGCGCGGTTACATCAGTAT
TGAGTAAAAAATAATGATTAACTTTAGAATTAAAGTTGAACTTATGCGCCATGCCCATGA
CGTTTACCCTAATGAATGTGGCGGCCTTGTAACCCAAAACCCACGCGAACGGAACTATCT
CCGGATCGACAATGTACGCCAGATGCCGGAAAAACATTCTGGAATGGACGCTGAACAATA
TGCGGACGTTGAGGACGGCGCCTCTGATATCCTAGCAGAACCTCACAGCCACACAGGGGT
AGGGGGAACAACAATCCCTATCGCACCCTATTTGTGGATGTGTGATGAAGCTTGTCTTTC
TTGGGTTATCTTTTCGGTAGCAGAAGCAAACGTCAGAATCATAAAGCCACAATCGCGGCC
TTTGATTGGTTGCCCTTGGTCGCTTGGCGCGGCTGATTGTGGGGGACTTCTTTTGGCCAG
GCATAAAGAGCAGGGCGTGATCCTGAATGATTTCACAAAGCCTTATCAATGGTGGAAACA
TGAGTTAGGTGAAAACCTATATCAAGAGAATTATTGAAAAGAGGGCTTTGTTGAAACGGG
CGAACAACCAAAACACGGAGACATTAACATAATGCAGCTTCAACTGCCAGTATGGCACCA
TGCCGGAATTTATTAAGACAACAATGAACTATTGCATCATCCATTCGGCAAGTGGTCGCG
GGTTGATTGAAATTCTGGATCGTATCAGGAACACTCACAAATGCAACGCAGACATAAGGA
TCTTAAAAATGATTAATGTGATTTACGTAATAAAATCTGGCTCATTGGGTCGCCGCTTTT
GTCTTTTCCTTACTTTTGCTATTGATAGTTGGCCTGAATGCGCAAGGGCATTATAAAGCG
AGGTTGAAGGATTATAAGGATTGATGAAAAGTGAAATCGGATAAAAGATAAAATTTGCAA
TTTTCGTTGATGATAAAAATCTTTGCCTGAATACTGTGAGAGACTGGCGTTGCACTCGTA
AGGTTAGAATCGTACGAGTTCCAACCGTATTAAAATCTGGAGGCCTATTTCAAGTTGTGT
TAGGAGCTGTAATTATAGTAGCTGCATTCTATACTGGTAGCGTTTCAATTGGTGCCTTGC
GGGCTCTTTCTTCATCAGCTCTTATGATGGGCGGGGCTATAATTCATGGCGGAGTAATGC
AGATGAGTAGCCCACGACTGGGAGCCCTAAGAATGGAGAGTTAAAGCGCGGAGAATCAAC
CATCATCTGCGTTTGGTGGTGCTGTTAATACTACGGCGGCCGGTTATCCGGAATGTTTAC
CTTATGGATATAGAACGGTAGGCGGCGCTCTATTCAGAGATGGCTCATACGCAGATGATA
AACTTTAATAATATAAACCCGCCGTTCCGCGGGTTTTTTGTTACATGTACAACGGCAGCA
CGTTTAAATAGCACAACAGGTTGACAGCTATGATCAAAGCAATGATAACTGGCAGTGAGT
GCGGTTCTGCAAAGCCTCATACACCTGTTGAAATCGAAGGTAACCTAATTTCTATCAACA
GGAGCAGAATTCTTTTAGCCGTTTGTGCTGGTGAAGTTGATCCAAAGTTTTCATTGAAAG
ATTAATATTTTGATGACGTTCCGGTTATGAATTAGGATTGTTCACTAAACTTTCAAAATT
TTGAGGCAGAATTCAGACCAGGGACGCAAACGCAGGATTATATTCAGGGCTTTACTGATA
CAGCCAGCAAAGTTTCTGCTGCCCGCGCTCTGACAGCCGCAGCGCCGTATATTATGTCTG
TTATCCATAAGATCCTTTCTGCGATCCGTATTATAATTTTAATGTCTCGCGGGGTTAGTC
AGGAAGATAACGGAAATCTAACAGGTGTACGCGTTGAATATGCGGTTGATATTGGTGTTG
TTGGTGCTGATTAGAAAGAGGTTTTGCATGCTGTAACTGAAGGTAAAACAATGAGCGGTT
ACGACAGAAGGCGAAGAGTTGATTTACCTGCTTTTAATGAGTGTGTCTGATTGCGCGCCC
GACGCCTTACAGAAAGCACGTCAGCAAGGGTAACGGATCTGATTATTATGCAAAGCTCTG
CGGAACGTGTAGACGCTAAATTTCCTTACCCACTGACTAGTTTAGACTACGTTGAATATG
ATAGAGAATTATTCCCTAATGCGTTACCGAATAACCGCATCAAAAAGAAATGGAACATCA
TAAATGTTACGTCGAATTACGAACCGATTAGCCGCTTCTATTCAGGGGTATGGGATGGAC
TCTGGAAAAGTGCCTGGTCTAATAATCCGGCTTTTGTACTATATGATTTAACAACCAATC
CGCGTTATGGACTCGATCAAATAGAGTCCGGCGCGGCGCTTGATAATTGGAGCATTTACG
CATATGCGCAACATTGCGATCAGATGGATCCAGACGGTAAAGGCTGAACCGAGCCGCAAT
ATCTTGGGGACGTTGTACTCCAGAGCTAAGTAGAAGCGTATCAGCTAGAACGTGATATTT
GATCCATCTTCCGTAGCATAAGCCTTTCGAATGGTGAGAGCGTTGCAATCGTGATCGATG
AGCCTAGCGATGCGTCATACAACTTTACTAATGACAACGGGGTTAACGGTGAATTCACTA
ACACGTTTGCCAAGGAAAAAAGCATGTACACGGAATGCAATGTGAATTTCGCCGACGAAC
TAAACATGTATCAACAGGATCTTGAGGGAGTTTTCGATACAGAGGCCGCGTTACGCTTTG
GCTATCACCGAACATCTATTACTCCGACTGGAGGCACACGACCTAGAGAAGCCAACCGCC
GTGGACTCTGGATTATGAGAACCAACGTCAAGGGCACAACGGTAAACTTTGCTACAGGTC
TGGAAGGTATGATCCCAACCGTAGGTCATGTGGTCGTTGCATCTGATAACTTGTGGTCCA
GGCCTCTAACGCTGAATCTATCATAGCGCTTGCTGGAGTTTCGCGGGTTTAAAGTGTTCA
CGCCGTTTGAGCTAGACGCAAGAGCAGGTGATCGCATTCTGGTAAATAAGCCGGATGGTA

ACCCAGTTGGTCGAATCATTGCCCCTTTGAGTGATAAGGGTTAAACGCTAACGCTAAACA
TGACTTTTGGTTTTGACGTTCACCCGGACACCCTTTTTGCAATTGAGCGGACCGATACTG
CACAGCAACGCTGTGTTGAGACCGGAATCACTAAGGGCGCTGGTGATGAAGAATTTTCCG
ACAACTTTACAGCTGTTGATTACGATCCGAACAAATGCGATGTAATTGATTTTGGCGTAA
GCATTGATGACAGTCCGACTTGAATCGCCCAGCCTGACATTTTGCTAGCACTGCAAAACG
TCTAGATCGAATCTTATTCGCGTGTTGTCCCAGGCGCGGGCGTCGAAACAATGCATGTTT
CAGGGGATAAGGTCGAATATGCAAGCATTTATTAAATGCAGTGGAGAAAAGACCATGGTA
CTTGGAACAGCACGCCGCGAACAGCCTGCAACGAAACGGAAGTTGAAGGAAATTATGCTG
CTCACTATCACGTAAGGCTTAGATCTGTTGCAGCGAATGGTTCAGCGTATGGATGGTCTG
CGATCGTAAATGCAGGATTAACCCGAAACGAGGGAGAGCCGGAAGAGCCCATTAACCTTA
CAACGTCGGACAATGAAGTTTTCGGAATAGTCGTAAAGTAGTGTATGCCAGAAGAAAGCG
GAGACAGGGCCTTAATAGAGTTGCAGCATGCGCCAAACGGTTCTGATGATCATCCCATTG
TTGATGAAGGAACGCTCTTAACGCTGGTACCGTTCCCGCAACATGAGTATTGGCATTCAA
TACTGCCAGCGGATCTTGTTGTTTGTTACAAGGCAAGGGCATTTGAGAAAATCGTTAACG
TTTCGGATTGGACTGATTTTGTGCGCGGCATGGCTTCTGTCGATACGAGCATTAACTCGG
ATCAGGTTAAGGTCTATATTGAACATTCTGATGGCTATAAGTGTTTGATGGAAAACGCGA
TAAAGACCATTGATAAGATACACAGCACAACGGGACCAGTGATTGAAAACGCAATAGCTC
ATGATCGAGGTGTTCGACGTATGACAGTTGAAAACCGAAAGCGTAAAGCGAAGTTCTTGC
AATCGTTGCAACTCATTGCAGACGAATGAGAAGCCAGGGTAACCCAGGTCACTCAAACGA
GTGCGCAATTTGCCAAGAAATTAAGGGCTCAAAATAGCGGGGTGAGAGGAGTGATTGCTA
ACAGCATTGCGACCATTAGCCAAAGGATTGTTCAGCTTACAGATACTTTTGAGAGTGAAA
TTGATGGGTTTAACCAGGATATCAGAGCGTATATAACTGATGTTAACCCGGCAATCACTA
ATGAAGCGGATGAGCAAGCGTCAGCGGATAGGGCGTTATCAACACATACTGGAGTTACGC
AATTAGCAGAGAATCAGAAACTTGATCCTTGTGTTAATGCAGATAGCGTTGGTGCCATGT
ACGGCGTTCAGTTTGTAATCAGGTATAACGGGCAGGAATATAGCGCAGGTTTGGCGCTAT
CTCTTGTCGCTGATGGTGGCGAAGTTAAATCACAATTCCTGTTTGATGCAGGACGAATCG
CGACCATCTACAATGCTCAAAGTTCAGCCTTTACAGTGCCCTTTCTTGTTGAAAATAATC
AGGTGTCTATCAATAGTTTGCTTGTTAAAAATGGTCCTATCGGTAACGCACAGATTGATA
CTTGCATCAACTATAACAACAGGCAGAGCGGGTTTGGAGGATGGGCGATTAACAAAGAGT
GTTATGCAGACTTTAACGAGATAACGGTAAGGCGTACTGTTTTCGTAAATGCCGGTTCGA
TCACTGGTAACGTCTTCGCTACTGATGGTTGGTTCACGGGTACTGTTGACGCAGAGAAAA
TAGAGGTTTAGGTTGCAAAGGCTGTCGTCGTTCCGCTTAATGGATGGGTTCTATTTCCAG
CGGTGAACTACAACCCACGTCTTGTGATCCCTTATGTTGGGATTCTGGGGTACAAATATC
CAGGTGGTACATCGGGCAGAGGTACTGTTTGGGTTGATTCAACATATGGCGGGCACCTTG
CTAACGAGCAAGCATCTGTAATGCGTGGCTGATCAAGAGGTTATGTACTGTCGCCAGAAC
TTAACGCAACCACGCTCTCATATGGGGGAGATCTAAACCACGCAAACGCAGTACCGGTCT
TTACAGTTCTGCTATTCAAATCATAAAAGTAGGAAACTCAATGCCCTGGCTTAACGGTCG
GGGCATGTAGTTTTCTTTTCTTCCACTATGTTCACAACCGAATGAGGCGTAAAATTGAAA
ACGTCTTAATAACAAAAAGAACATTTTCAATATCTATATAATACATACACTTAGAGTTAA
TTATTAATATATATATCTATATGTATCCTTATTGTTTCCATTGTTCTATATGGTTTGTGG
TGTTTGTGTGTTTTCTATTCGTTGATGGTTATATATCACATGGTATCTATATATATATTG
TATGCAGTGTCAATGCGTGAAAAATGGCATACAAAAACAACAATTGGTAGAATGATAAAC
TTACGATGTGATTTTGTTTTCAATGCTAGTTGTAAACGTATTCAAATCAAAAAGAATACC
TAAATCTTCTTGACTACATGAAACAAAGACATATAATGCAGCCATACCAACGAGAGGATG
ACAAAATGAGCGATTTTAAAGTTTACTCTTATGACCCGTTAACCAATTAATGATATCACT
ATCATTAAACTTGGGCGACGGACTACGTTAGCGGTTCAAGCCTGGTAGAAATCATTGCCA
GTTGTTCGGCAGCAGGGAAATACAGACCACGCGAGGAAATCAAAGCGCCTCTTTTCGGTA
CGTAATCGTATACAATCATTAAAAGAAGTGTATTGTTAGCTAAAGAGTACGGACGACCGC
CAGCGTCGGATGATTACGACAAATTAATCACAGGCCAAACGGCATTGGCCAGCAAGTTAC
AATCTTTTGGCCTGACTGGTACGAGCGGTAAAAGTTATCCAGACCTGATAATAAAGATGG
TTGATTGTGGTGACGATTTTAAGGTACATTGGCTTATTGAAATGATCGCAGAGAGCCAGG
CGCGAGCGGATAACGTTGAATTGTTTCCGGCTAAATCTTACTACGCTTGCGTTATCATGC
GCCGCGTAGTGGAAGACATCACGGAGCACAACGCTTGCATGAATAGCCCAACCGGACAAC
GCGAGCTAGCAATTTTCGGCATTATCAAAGGCGTGAAAGTAAAAGTAAGGATTGCCCAAG
TTGCTATTTCCAAAGATGTGCTTGCAACAGTGATTTCCGGCTATGATGGTGATTGTAATC
CGGGATATGAATCCACGCGATTTGATGAAGCGTTTTTGATCACGGATGATATAACGACGT
GTCGCGTTAACCCGTCAGACTTTGCCAGGCTGGCTTTCAATCATGGCTACTCCAAGAAAA

TGCCATTGCCGCATGACCTATTCAAGAAAGTGTATCAAGCAGAAACAGCCCCCGTAGACG
TCTGTTTGCTTCCGGAGGATAAAACGGAGCCTTCTTTACGGGTTGCATACCCGATGACGG
ATGAACAATTGAAGTTCGGACGGCTTCAAAACATCAGTGAGCGAAACCAGTTTGAAGAAT
GCCAGGCAAATGACAGTTGGCCTTCTTACACAAACGGAGAGCCTGAAAATGATCTTCAAG
GACCTAATTGGGATCGTCGTCAATACAAAGGTTTGTTATAAATTGCTCTATATGCTAAAA
AAGCCATTCGATGCGTGGTATACTCGACGCATCAACCAACGAGAGGGAGAAAATAAAAAT
GGAACAGCAACTCAATCAACAAGAAATTCAAGATCTGCGCGATCACGTAGGCGGATTTGT
AAACGCAGTACGAATTATGCAACGCCACCGCGCACGCGTAATTGATCCAATCGGATCACC
GGAAACAATTGAAAATGTCCGACAGGGTGCATATTCGATTCATAGCGTCATGTTAATGGG
TCTGCAAATGGAAAATAATAAAGACCATCGCAAGATGCTTCATCAGGCAAGTGAAATCAT
GATCCACCATTTGATTGATTATAACAATGAGAACGAAGAAGAGCATTATTTAACACTGCG
GCGCAAGCGGCCATTATTAAACCGGAGTCAATGAAATGAAATTTTCCGAGCAAAACGCAA
AGGTACTTAAGGCATTGTTTGGGGCGCTTCGATTATTCACCAAAGTAAAAAAGGGTATGC
AAAACGCGCACCTGAAAAATAAATATGTAACCCTTGAGAGTGTTCTTGCTGCTATCTTGC
CTGGCTTTAATGATAAGGATTTATTCCTTACTCAAGATCAGAAAGTGTGCGAAGATTCGA
AATCAAGCACGGATTTAACTCGCTTCATTCACCTTGAATCAAATGAATGGGTTGAATAGA
GTTTCACGTAACCAACGCAGAAATTACAACCACAAGGCGGCGGTTCAACGATCAGCTATG
CCCGACGTTATGCTGTTTGGACGGCACTTGGCCTGGCAACGGCTGACGATGACGCAAACG
ATGCCACCAAAAACGCGCAAGATTGGAATAAAGATCTTGATGCTTGCGATAACCTTCCAT
ACTTGCAAGAGACGTTTAAAACTCCTTAGAAGCAATCTGACGCCGCCAACCGCCGAAACA
TGAAAGACCAATACGACAAGCGGAAAGCTAATATGGAAATCGGAAAGGCTCGCGGCTTTA
ATTCGCCTGAACAAGCCGCGAATGTTGCTAAGGATGAAAAGCTTGATAAGGAACCGCCAG
ACTAAGTAAAATCTTAGTCTATCACTGTATTTGAATAATAATTTGCGGGGCGTACTGCCC
CTATAAGGGGAAATAATGCACGTTAGCACTGGTGAAATTGGCAAAGACCCTTTTGGTCGG
CGAGGGCAGAACGGCACACTTTTTATTGTTGAGCCTTCAGAATCACAGAAAGATCGATAG
GGTAACAGCCAATACACAAATTACAAATTATTCGTTAATGCAAAAAGCGAGGGAATGAAA
AATTGGTATCGTAAAGCATTCGAGCTGTGTAAAATGATTTCTGGGACGGGCGACCAGTGG
CTCATTGCTAGCAACGAATATAACGGGCAGATTTATAAGACTCTAACAAGTCCCGGATTT
GCGAAGTTGCATTTTAGCAAGCGCGGTGGAAGTCAAGCACCACAACAGCAACGACCACCT
CAGCACCAGCCACAACCGAAACAACAAGCATCACAAAACAACGAGCATCCAATGGACATG
TCCGACGACATTCCATTCTAAAAATAAAGGGGCTTGCGCCCCTTTTCCACCGTCTTCGAA
ACCCGAAACAGCAATTATCTTAACTGAGATCCGGCTTGCAAGTTCAAGCTGTTGTGCGTT
AAGCGTTCCTGTATTCATGATAGACAACTGTGCACACATAGGCCCAAGAGCGGTAAAATA
AGTAAAGGTTGATTGCATCATATCCAGTTGTTGATGACCATCATTCACAATCGCTTGTGC
GGCTTATTCACCCAATAGAAATTGGTTCATTGCAAAGAATTGGTAAATCAAAAGAGGAGG
CGTTGCGCTTCCCTGTGTATTATGGTAGCTCTGGAATCGTTTCGGCATTACCTCCAGCCA
ATCCGACAACCATTTTTTTAATTGTGCAAAACCAGCTTTTAAACTTGAAATTTGACTTT
GTAACGCTTCATTTTTACCCAGCAACTCTTGAACAGCAGCAATCAGATCAGAAATAACTG
CTCGATAATTAACGTTCACGATTTCTAATTCATCACCGTTATCATCTGTTGCAGTGCCGC
CAGTGAAAACGTAAGTGGCGTTTATTTCAGCCATTTGTTGCGCAAGGAATCCACGCTGCA
AAACATCTTTTCTCTTATAATAAAACTCCGTAACATAAATCTGTTAGACGCGATACCATG
CGCAAGGCTTAGGATCTTCAAATCTATACTTAATGCGAACTTATTATCCTTGCATAGCGT
ATCGTCCCTCTGCGGCATAGAATCCATTATCGTTTCTCATTGAATACCATATGACGTTAC
AGCCGAATGTGTTTAAAGAACAAACATATTGATACGCCTGCCCAGGAGTACCAACTAAAT
AATTTTGAATTCCGAATCATGAGCTATTAATATAGGTAAGAAGGTTTGAGCCATCGCTAG
ATCGCCGTGATTTCTCCTTTATTAGAGCAGCAGTACTAGCGCGGCCTTGTGCGTAATAAG
CTTTAGCCCATACGGCATTAAAAGTAACATTCTTATCAATTCCAACCCCTAAATATCGTC
TAGCTTGAGCGACATTATCTGCACCACTGCCACCGGATACCATTGGTAGATCATTTAGTA
CTCCAGCGGAGCTATCAAAAACCCCACAATCACCAGCGCTGTAAACGAATAAATAAGAGT
CTTTAACAGGTGCCCAAAGTGACGTTAGTTCTCACAGTCTGTCAACCGGTTCAACAAGCA
GGTGTTCATGAGCTTTTGCAACAGTTGTTGCAACAGTAGCCCCGTGATCAATACCTAGAG
CCGACCAACCTTTTAGATCGCGATTGAACATACCCCATGTGCCATCATGCCTTACAGATA
TCCCGTTTAAACCATCTGGAGAAAGCATTGACATAAGAACAGAATCCTGAATAATTCTAT
TAAGTTGCAGATTTATTTTTGCTTGCGTAACGTTTTCAGCACCAGTTCCGCGAGCGCAAA
TAGAAAGTTCCGCTGTGCTGTTGTTGTCATTACGTGCAACTCGCCAGTCTCCGTTGTTGG
AGATCCGCAATATATAAGTCCCTGCAGTATTTCGTAAACAGTTGTATGCGGCAGTATCTG
GATGTTGTACGGAATTAACAGCCTCAATTTTCAAAGCTTCCCTTGCAAGGTCTTTGTTTG

CATGATCATTAAGGTTTTGATGCTTATGCAGCAGAGTTTCTGGTTGCGCCTGTTGCGCCC
ATTTGCGAGCTTCATCACACGCGTCCACCGCTTTATTTCAAATACGGTTGTCTCAGTAA
CGGCAGAATCCTTAATCTCTTGTGTTTGGTTTTTTATTGCGTCCGTGTCTGCCCTAATCT
AATTGGTTTCAGCGACAGCGGCGTCTTTAATCCGTTGCGATTCATTAACAGCGGTATCTT
TATTCTGTTGCGTTTCAGTAACAGCGTCTTCCTTTATTGTTTGCGTGTGGTTTTGTAATT
GTTCAGTCCCGGCTCAGTTTGCATCAGTATTTTGCGAGGAAGCCTCAGCGCGTGCAACAA
AATCCTGTATTGTTTATAAATCAAATTTCTTGAAAAATTCAAACGCATCCGCAATAACAG
TTTTTTTACTTTAGTAAGAACCCAGCGGGTCCGGTACCCCTTGCGCTATTCCATCGATAG
TTAACGAGTCAGTTAACAGGATCACATAATTTCCGTTTTATACTGGATCGAAGTCCGTCT
GGATCGCTTTCGTTTCCGTTTCGCTTTCAATATCGCTATTCGCAGCCAATTTACGAGGCG
ATGTGAGAAATACGATTGTTGCACCAGTACTGATAAGAGAATGAGGATCACGCCAGTTTG
TGTCAGTACCAGTAATAACGTCGTGCGCGTCCATTGTAGCCGTGCCAGTTCGATAGAAAG
CCATAATTATACCTCATTTTATTGATTGAAATAGTTTATTGATTACGCGATATTGCTCTG
TTACATGCAATACAAACACCGCCTTAGGGGGGTTCGTCAAATGCAACGAGAAGTTAATGA
AACAGTTACATCGAAACGTGTTACACGCAGGATTAATATTATAACAAGACACATACCTTG
TAAAGTCTCCGCTCTATTCCCTTTTCCAATTTACCGTTAACTGGTAACATCACGTCTAAT
TCGCGATCATCGGCAGCAATCAAAGTGTCGCGACCATTAACCTGCTAGACAACCCGCGTA
TTACGCTCTCCTTTGTATCTGTATGCAACAATTTCAAAATTTGAAGGTGAGAAACACGCC
CATACTTCTTGCCTCGTCGCCAGGTGTATACGTTGCGCATCAATCCAGACATAGCAAAGG
TAAATTGTTCTGTCAGTCTTCCCAATTATCGCAACGCTTCCTCGCGTGTAAGCTTTCGCG
TAAAAACTGCTATTACCTTCCGCGTTGACGAAACGAATATCGCACATCTCATCATCCTAT
TCATCACGATGCACCAGGTGGCTAGGAATGGCGTGTATCTGCTCGCATTTTCCATTGTTA
TGAGTTTTTACGCCAATTTGATACGATTCGACTTCTAATGAATAAAACACCTCGAAAACG
CTTAGAGTAGTTATTCCCATGCGTTCAGCTTTATCCATCACCTCAACACAATTTTCATGC
GGTGCTTGCGATCCCAAATTGTAACCAGAATCACGTTTTGCCGCGTTGTAAACCATGTTG
GCAAGCTCAACAGGAACCAAGTTTAACCATTCGCCTAAAATGTCGATGTCTTCACTGTAA
TATTCACTACGAATGCGCATGAATAATCCGATCACTGAATCATTACCGCATTGGCTCCAA
ATGTTGTCGAGGTCGCCTTTCTCGTCAATTATGTCAGTCCAACAAAAGCGATCTTTACCG
CCGCAATAAGGGCAAGGCTGATGTTTGCCGTTAAAGGTTATTTGAGGAATCTCGCAAAAT
TTCATTAATAACGCTTGCCACAGGACCCGCATCTTGGGGATAACTTCTTCTTTTTGATAA
TGAATTTCTCTACCTCCGAATAAAAAGCCTGGCACGATTATAAACCATGCCAGGCCGGAA
TTTGTAGCATGCAGTGCTATCGAAGGAAATCACAACGCGTTTCATCTGATTGGCTTCCGT
TGCTTTGCGCCGTTCTTCCTTCGCCCTTTCGCGTTCAGCTTTACTCCGCGCTTTCTCCTT
CTCGCGTTCTTCTGGACGCATAGAACGCAACGGATCGTAAGGCCGCACTAATCCGATTGC
TTCCGCTACCGCTTTGGTTCCAAATCACATCAGCTTTCGGTTGCTGCACCGATTCTCTAA
AGGCTTGCCGTTATTATCAAATTCGATATCTGGACGGCAAGATGAAGCGCGGAAGCCGTT
AAAACCTTTGCGACGGTCCTCTTTCTGGCTTAGTTCAGCGCCTTTAGTTAAAATCATTCG
CTACTATTTGCATCCCCATACTGTTTTAAGGTTAACGCCAATAGCACGCGGCAGGGCAGC
GCGACCGCCATAAGCGTTGACTACTTCATCAAGCCGCGCCTTTAATCCTGCTCTGACTGC
GTACTCTAATACAACATAACCGCGAACAGGTGATCGACGTTTACGATCTTTACGACGTGT
TACGCCGTTGTTACCTTTGATTGGTCTTTTATCGATTTCGCTAGTTGAGGAAGAAATTTT
GAATTCGCACATATTTTCACCTATAGCACTTTTTGCTAAAAAACTTGCTTGAATTTGTGT
ATGATATACCGCACATGGCATTTAGTCAAAAGGATGAAAAAGGATTCCTACCATTGCAAC
GCAGATTGCAGATCTGGGAGAGGGTAGAATCAAGGCAATCCAGAAGCGTTGCACGGTTGG
TGAGTTTGTCCCGTATAAATACCAATGCTTTGTATACGCTGAAATTGCAGAGCGCTTGAG
TAAGTATATTAATCCGTTTTTCGTCAAAGCGGCCGTGTCGGCAGGTAAAACCAACAGCTT
TGCTATGGTTGCGGCTCTGTGCACGAAAATGGGATTAAAATCGTTAATTCTGGCGCGTCT
ATCTGAGATTGTTGAAGAGGATCCAAAAGAGATTAGCGCCTTTGTTGTTCCGAATTCCGT
TTATTGCTCTGGCCTGAATACCAAACACGCTTATTTCCCGACTGTTGCTGAATCGGAAGG
TACTGTAAATGGCGGATTGCATAATGCGTTAGATGATTACGTCGCATAGGCTTTAGGTAT
AGATGAGTGTCACCAAGTCAACGGGAGTGAGGTTCTTAAAGCCGAGGGAAACGGCGAAAG
CATAGAGCAGATGATGATGCCGAAAGGTTAACCAGATGAAGAGAAACCGTTGCTGATTGG
TTGAGGCCGCGAAAAATACACCATTATCATTGCAGAAATGCGCCGCGGTTGTCTTGAGAT
TTACGGTCATCAGTTGCGTATATGTGGAATGAACCGATCGGAATATCGCGGAATTGAGCC
GATACTTGTCGAAGATAAAAGAATTCCTTGGTTTTGGCGGGAGCAGAGCACAAACATTGA
TACTGAGTACCTGGTAAAATTTCGGTCAGTTGATCGCACTTATTTTGGCAATCTGGGTGA
TTTGGGTGACGACTTATCAAAATTTACGCCCGTGCAAGAATTTGGGGTGGATGATTATGA

CCGGGAGCAATTAAAGGCCATGAGTTACAAGATTCACCAATCAGGAACAATGACGCAGAA
ACTATTGCAGGTGGATCACGAGGTGATGAAATACCGCTTGTGGACCCTTCTCACTTCCGC
TGACGATTGGCACTGCAAAGTGGCGGCGGCGGCGATTCCGTCTCGCACGAAAAATTACAT
TCTTACCGGAAAAACTCGAGAGAGTCAGCGGCGATTATGGCTTGATGATTCCTATGAATG
GCGAGTGAAATAGATTTTCCTGGTTCAGGCGCTAACAACTAGCGTTGACGTTCCGCCGTG
GGATACCTCTGTAATTTTGCGCAAGATCGGAAGTCTTACTCTATTAACTCAGCTCTTGGG
TCGTGGTATGCGTCAATGACAGACTTACCATAAAGTAGTTTTGGGATTGAACAAAACACA
TCATCTTGGTTTGGAATTATCCGGCGCGATGACTGAGATGGGTAATTTGTATTTCGATCC
AATGCATGAGCAAGCGCAATTCCAGTTGCGTAAATCGCAGGATATAGATCCGAAATGTTG
ACCGATTGGCCGAACGGAAATTGCATTCGTTGCGGGGGGTTGCCTTAACGAGGATGAAAA
CGGAAATCGTCTTGAGCACTTTGGGACGTTCCAGGAATGTGAGGATCAGATCGATCAACG
TCCAAAGACAGTTATTATAACCGGTTGCGGCGGAAAAAGTGACGTTGCGGAGCGTGTATG
CCGATGCTGTAATGATCAGTTACGAGATCCAATTGATGGCTTATCCGGCAAGGGGTACTG
TAAAAACGATTGGTGCGACGTGTTAGATTTTAATGTGACGCTGACAAAAAACCAGGCTGG
CGTTAGGTTCTGAGACACCTTGCTAGATAGTGGCGGCGTAGAATTCAGAGCTTACGCAAA
ATATTTCCCTAAATAAGACCACAAGATATGCGAAACGCTTTGGACAAAAGCGGTTTGAAC
ACACGTTGTAGATCGACAGTCTGCGAACTATATCATCACTTGCAGCCACGCAATTAACAA
CATGAGTTATGCGACGCAAATATGGCCACCAAATCACGTAACGCAACGCAAGAACACAAA
GAAAGAAGATTTAACAACATTGAAGGAATTTGAATAGGGTTATTGATTCAGGTGAGTATC
TGGAGTATTACGACAAGTCCGATCCAGACGATAGAAAGGATGAATCCCACCAGGTCGATT
CTTATTCATGGCTAACATATGAGGTGCCCATGGGGCTTGTTTGTCACACTAAAAACGAGG
GGAAGAAAACCATCGGCACAGCCTTAAAGGATCAGCAAACGGGTGTGAAGAAGGGAGTTA
GCGACATTCTAATCCGGACTGGTTTTTTGGGCTGCAAGTATTCATTTATAGCCGGTGACC
TTAAACGCGCCAACGAGAAAGGCACGAAGATCAGAGACGAACAACAGAAATTCTTGCGAC
GTGTCAGAGAGTGCGGAGGTTTTGCCTCCGTATGCTATGGAGTAGAGCTAGTAAAATTGG
CTATCGCATAAGCTACAAAATAGCGCGTTTTGTTAAAACAGGCGCCACGAAATGCGCCAT
GATGCACCCATCAGCACGAAACGGAGAATTAACCAAGAAATAGATGTTATGAGTTGGAGC
TTTATCACTAGCTCTTGTCGGTTGCATGGATAAACCAAAATCATGTACTTGCGGAACCGA
AGCGTTTGAGGTTACAAGCCTCCCTATGAAAGTTGGGCAACGCAAAAGCACAGGCGATGT
AATGGATTCTATCAGCGTAAATCAATATAAGTTGTGTACGCCTATTGGAGCGGCTTTGTA
CACCATTAATGAAATCACAATTGATATTATAGTTAACTTTTATGAGGACACTTTAACTTG
AGAGGTAAAATATAATGGCTAAAGATATTCAGGACAAAACCTCAATTGACCCTTTCGCAC
AAGAAATGTATTTTCATGAGCGGCAACACGGCACATATTCAGATATTGAGTTGCGTTCAG
GTGGCCATTATCAGCCAGTTATAGAAAACCCAATGTTCAAGGTTAGCAGCAACCCTCATG
CAGGAAGGAAAACGCCCGACGTTTTGCGTGACCTGTGGGAAACTACAGACGAAATGGTTG
AATACCTTGCGGAACGTTACGGCAGATTTCATCTTGACACAGCAGCAACGGAAGATACTT
AAAAATGTGATAAATTTTGCAGCAAAGAAACGAACTGCTTAAAGAGCTCGTGGGGTATCC
AAAAGCACGTTTGGTTAAATCCACCATAGAGCCCCCCAGCTCTGCTTATCAAAAAAGCAA
TTGAGCAGTTGGAAGACGGAGCCCAGATTGATATCTTACTTTCGCCTGAAAATTCAACCA
CATGGTTTAGGGACCCACAAAAACACGCGGCCGAAATTATCTGGATTCCTGGTGAAACCT
GGAAGGATGAAGCAGGTCGCGAATACTCCCGAACTGGTCGAGTAGCGTTTATTTCTGGTA
TGGCAGGTGAACCAGTTAGCGGTAACAATAAAGGTAGTGTAATTTTTATGATGCGCCAAT
TGGAAGAAGGTGACCAACAGCTAACGCACTATGTGAAGAGTTGCGACATTTGCCATTCCG
TAAAAAATAAACGAGCCAAAGCGCGCAGTATTTAAAATGAGGATTCGATTAGCCGATCAC
AAGAAATGGGGTTTGTTTGTTGAGCTGACACTTCGTTTAATTGATATGCAGGGTTATAGC
GCTGATAAAATGGAGCTGATAAACTGACTGGATATGACATTCGACGAAATTAAAGTTGCT
CCTTTGTAAATGTGGGAAAGAGAAATTGAGCACGACCTGATACAAGACTAACGCAGAGAG
GGTGCATGATTTTTTAATGTGAAATAGCCCTTTTTGTTAAGACCCGGTACGGCGGGCTTT
GTATTATGCTATTAATGGAAAACAAGAGGTTTTCAACGATGAAAATTGTAAACGTCGAAT
ACTTCAAAGATGGGCAATGGCTGACTAAAGAATCATTATTTATGCTGGACCGCAAAAATC
CACAGGTCATTTCGCCAATGCTTTGGAATGACGCAATGGAGTTACTTAAAAAGGCTTTCG
GTTATGAAAGTGACAAAAGGTAATAAGGCAACTAGGGAGTTAGAGAAAAAATGGGGTCCT
GATGTAGGGATCTGCAAAATAGCGCGATATTTCGACATCAAAGACGTATGCGTTATTGCC
AGTAAAGAAATGGCCTATGTGGAAGAACAGCCGCGCAAGGTTCACCGCGGTCCGGCAATT
GCGTCAACAATCGACTAGAAAGAAGTTATTAACAACACTCAAGACCAAAAAATATATTTC
AAATGAGATACATTTGCGTTATTACTTTTCCTCCCGTTATAGCTTGGGTTATGGTTCATA
TGTGAGGTATGGGGTTGTCATGAACTATCACGATTTATTCTCATATGAAAATGGAGCGCT

TACAAGAAATGTTGCTAAATCTAAAAGATGTAGGATTGTCAATCCTGTTGGATTGATCAA
AGGAAGTCGTTACTAGCAAACAACTGTCTCAGGTAACAATGTTGTTGTTGATCGTATAAT
ATGGGAACTGCAAAACGGGAAAATTCCAAATGGAAGGGACATAGATCAGATAAATCACGT
TAGAGCCGATAACGGGATTGAAAATCAAAAAATTGTTGATCACAAAGATAATCATAAAAA
CGTATCAATGCAATCAAATAATATTACTGCGGTTTGTGCGGTCAGGTTTATAGTAAGATG
GAATAAGTTCAGAGCATCAATAAAGGTTTACTGGGTAACTATATCTTTGGGATACTTAAC
CAGCATTGATGATGCTAACAATGCCGGAAAGAGAGCAAATGATAGATTTGATTTTCACAA
AAACCACGGAATTTAATAAATGACTTCTAAAAAAATAACGGATGAGCAATTGATTGCAGA
GCGCGAAGCGGGTTTAAAGTTGCGAGAGATTGCTTAATATAACGGAAAGATCTTACGCCA
GATTGAGCACCGTCTCATCTAATTGGCTAAGCTTGGCGAACTCGCAACTATCGGATCACC
TGGGTTTGCGGTAATAGGTGAATCGCACCTTATACGTGATCAGGATGAGGTGATAATTAC
TCGGACCAAAAGGCAGAAAGATAAAGAGCAATTAGAAGCAATTCCGCAAGCTGCTGTAGA
AGCATTTTCGGAAGAAGTGCGGCGCATTGAGCCGTAACCGGATACAATGACTGTTTACTT
GCAAAAGCTGGCGCTATATCCGATCTTTCTTATTCAGATTGGCGCACTGGCTCATAAGCT
TGAAAGCGGTGAGAAGTAAGATACGGTAACGGCGGAGAGCGTCATGTATGCGTTTATCGA
TTAAGCAGGTGATAAAGCGCCTAATACCCACAATGCTGTGTTGCTGATTGGTGGTGATAT
GATTCAGGGCGACGGTCTGGTGGCTGTAACACCTGCAGGCGGTCACTTTGTGGATCAAGA
TAGCCGTTACTTAAAACTTGATTATGTTCCTATCCGCGCCACGCGTCGAGTAGTAAGCAG
ATAGCTGGCAAAACATAAAGAGGTTTAATTGCAGATCATTGGAGGTAATCACGACCAAGC
AGGAATGATATGGCTGCGTGCTGCAACGGAGCCGACGTATGAGAATGAGCCGCGCGTAAA
GCATGATGTTTCTCTGCATGTCGTGCATCACACGCAATGTGGAAAACCGTTCCTGGCTTA
TCATCAGGGTTAAACTAGATGCAAGGCAGAAACGTATTTACTGATGTATGCAGCGGATTG
ACGGGAAGATCTCGGAAACTCCAATTCAATGTATGCGCATGAAGGGCATTGTCATCAATG
GACTGTTCCGGAAAAGATCCTTGGCGTTGTTGAGGTGCATACCACTATGGGGGCTAAATA
TACTTATCCCACGCGTGACGGTTGGTGTTCACACCGACGAGCGGCGGTAATTAGCTATAA
TAAGGAATATGGCGAGATAGGCCGCCTTGTGTTCTATCCAGACATGATTGCATAATCAAA
TTCTCACCTAAAAAGGCTAGCGAACTGCCAGCTTTATTTTGTTTTTGTCGTTTACGCAAT
AAGACTATTTTTGACATTTCTAATTTAATTAAACTGAGGATCAGATCCTGACGGACATTC
TTTTAATGGCGGAAAACTCTTTCGGAGGTGCAACCGCAGTTGGAAATTTTACAGGTGAGT
TTTTGCTTGCACTTGCAGCGTTGATGTTGTTCGCTATTTACGGCTGTTGGGGCGCATGGC
TGAAATCGGGTGATAGTAAATCGATCGGAGAGGCTCGAGATTCAGGTGATTTATAAACGA
CGCTTAACCCAGGGCAGAAAGAGAGGGAGTAATGACAAGCACGAAAGGTATAGCCGCCAC
CGTCACAGGGGCGGCTTTAATGTTTACATCGCCTTTGATTGAAGAAATCGAAGGCGTAAA
GTATAGGCCTTACAAGGCTATTGCCGGTATCTGGACTGTCTGCCCCTGCACCACAGGAAA
TCATGTGATTCTGGGAAAGAAATATACCAGGTGAGAGTGTGCTGCGGTATTAGCAAAGCC
TATGAAAGTAGGGGCTGACAGTGTTGATATAGCTTTTAAGTCTGATATTCCTATGTGTAT
GCGAGCGGCTTTGTACTTATTCACATTCAACGCTGGATAAGGGGCATCTAGGAAATCACC
CAGGTTGAAGAACATCAATAAGGGTGTTTTGTATGGTGGTTGTGGCGAGCGTGGGAGCTG
GACATATTACCGGGGCCCGAAAACGGGCAAAAAGGATAAATCATAGGGATTGAAGAATCG
CCGCGCTGTTGAATATAACTATTGTTTAATGGATATGAATAAATGAAAAGAAAATAACTG
GCAAGTATGCTGTGCAATGGACTTTCTGGCTGTTCAGCTTCAAGCGTGTTACCTTGTGTG
ATTGGTACTAAGCCTTATATAACGGCACAGGCTGGAGCGGAGAATGTGAAACAGACTGTT
GGTGCAACCGCAAAGCAGCATACCTCAAGCAAGTAGGAAACCACACTCAAAGAAACGGGA
GTTGGTCAACTTGACACATCCAATATGAAGCAGGTGCGTATCTCAAGCATCAAGGGTGAT
AACATTAAGGCGGAAAGCTTAGAAATTCGTAATAATGACGGCCTGGACGTCCCGGCAATC
GAAATGGGTTTATTGATGTTCATTACTGATATGCCGGCAGGTTGAACGCTAACACGTAAT
TAGGCGGCATAAGCCGCCTGTCCTTTGACGCAAATTTGGTAACATCAAGTAGCAATTCAC
AATTATGATGTGTAAGTTTATGCTCTTGCTCCAAAAATGCTCGAATATCACTGTAAAGCA
ACAACATTAACGCATAGACAAAATTATCATGCGGAGTTTGCATCGCTGAACTATCTAACA
AAATATGATCAATCAAATGCTGGCTTGCGGAATAATTCTACATCGCTCCTTTTCCCAAAG
TTCATCATCTCCCACGCATAAAGATTGGAATCACCTTCCAGGGTATGAAACTCAACATCA
AACGGCCTCTTCATCTTGTTTTTGCATCAGTACAGGGCTTGACCGCCGTCAATGTATGCA
TAACCCATCACCCGCGCTATAAACTCTTTGCTTGAGCTTGAGGCGAAATAGCGCGGCGTT
ACCCCCGCATTTTAGGTGAACCTTACACATTCACAGTGACGGAATAAAAATCTTGCTAAC
TCCTGGCGGTTGAATTTCTAGCGAGCCTCGCAAACTCGATACAGATCTAATAAGAACGTG
ATTACCCCATTAACGCAGGATTTACATACAGAAATTTACCAACGCTGCAAACATAATTCT
TTTCTTCAAGCATAGGCAATAATTGTTCCTTAACTCGTTTCATCACGCCTGATTGACCTG

TAAACGGCTTAAACTTACGCCCACTTTCATAGATAGCCCTTGCATTGATAACGCCTTTAT
TTTGACGTGAGATCTTAATTATGCCTTCAATTAACTTGATCATTTCTACATCATCGCCAG
CGAGACCGGAAGCATTCCCGGAGTTGATATATGTTGTGCGTAATTCGCTGACCATCACCA
AAGCCTCTTGCATTGTCTCCACTTAAATTCCTCGGGACTTTTGCGACGTACCGTTTGGAT
TAAACCAATGGCGGATCGTGTGCATTACTGACGAAATGCGAATCACTGGCTTATCCATTT
GAACCAAAGTACCACGCAACATTAGGTGTGAATATTTCCCGCCGTCTGCTCAATATGGCT
CTAATTTCTGGCGAGCAATATTAAGAACGCGCATTGGGGACCGTGACGGCTTCAATACAA
CCTTATGCTCAGTCATGATGTTATGAATCAACCGCAAATACTGCGCCTTTAATTCTTTAT
TAACTGACGTGTAATCAAGATCACCATATTGATCAATAGAGACACCCTCGCCTAACATTG
TTTTTTCACGTACTAGCAAGAAAAGTTCACTAACAACGATAACCCCCGCCCCTGCATCTA
CGATCCCTTTTATCATTTCATACTGGACAATGACGCGCGGGCATCCAAAGGCAACTAAAC
TCACAATGTTTTCAGAGTTCGCGCGAGCTAATGAAACGTGTCCGGCGTCCCAAGCTTTTA
GAACTAACTGGTTGTTAGTCTTGGGCTCACTATTAGCGAATGTCAATCCGAGTTAGCTGT
TGATGCTGGGTGCTTCAGCGGAAATGACGGCAAAGTTACCCTCTCAATTGTAGATCCTGC
CTAACCCTTTCGGCGTGGTGTCTGATACCTGAAAAAAGATATCGCACATTTTTTCTAACT
TTTCCTCTAATTCCTCCTGTTCCTCAAACAACGCCGCTAACTCACTGCAGAAATGTTCAG
CTTTCAATTCTTTTTCAACGCCGTTAAATTTAGCTGCAATCTTCTTTCGCTCTTTCTTGC
GTTGTTCGTTTATGAGATCTACTTCACAGCCCATGGGCGCAATAGCAAGGCTGTTGCTTG
CTGATTGACCGGTTGCCGGAGGTTGACGGGTTACAGCATAACGCGCAGTTGGTTGTTCAG
TGCCATGGTACTCAACCCGGAAACGTCCAACCATAGCGGCGGAGACGCAACCAATGAAAT
GCATGTAGGCCGATGATTTAGGAAATTGAACAGATTGCGCAATGCTACGTGACCGTTTAC
CCACAACATCAAGATCAGTCCCCAAAGAGATCACAGGATAGCGGTCATTAGCGCCGTTGA
TATCCTTAACGGGTTCCCAAAAGCTGGAAGATTGGCGGTATCCGTTAGACTGTATGGTAA
CGCGGAGCTGCGATGTACTCTGCGATTATGCAATGGCTATAAGTTGTTAACGCGTCGATT
TGTTGGTGTCAAAATCAAACATCTAAAAATCTCCTTCTTGTGGCCGCTATTTATAACGGC
AACATTCTACGACCTTTTAGCAATTCGTACTATGTGATTGGCTCAACCCTGCGCAATAAC
TTTCCGTTTATAATGCTATGACATTCCAGAGTTTTAGGATCCAATGCGAGCGAAAGATGG
TACGTATAACCTATCAGCAACTTACCAGGCTATTCCCTCACCTTGTAATACTTATCGCCA
GGTTTAAACATTGCCGAGTCGCTGTAAAGGACTTTATACCGGAATTCGCCCATCGTTGTT
GGGTAGAGCCTTGTATATTCAACGCCTTGAAACATTTGTAAAATCTTTCAAACAGATAAG
CGCCACCGGCGGCACAGTAGCCGACTTTCTCGCGATACAGCGTGCATCGGTCGCCGTTTT
CGTCGAACACATGTCCATGAACACCACCTAACATTCGGGCTTGTTCAATTAGGTAACCCT
TATCAGCATTGAAAGTTTTCTTGATCTGCATGGAATGGTTTTCCCATGTGCATTTAATAG
TTTAGGTCCTAACATTTCGGAATGTTAATCCGGGTAAGTTCACGTGAATCGAGTTCCCAT
CGAACCTATCGAAGGTATCATCAGTTTCGTCTACCCGCGCTTTGTATAGGCAGTTATCAG
ATCCGATTAGTTTGATGGATATGAATTTAGCCATTTGGTACATCTCCTTTTCTTTTCGTT
GAAAGCGTCATGCCGGATACGTGATCCGGCGTTTTAACAAAAAGTCCTATCTTTGATTTG
CCATGCTTACAACAAGCGCAAAGCCTCGCGGAGTGAGTCCGCGGATCAGCTTTGTACGCT
TAGACTTACCTCCTAGCTTTTGCCGGCATGGATTGGTGTTCCCTGTTGGCAATACTGCGT
TTGTATCCGGCTTCTCATAACCCTTATCCGTCCAAAGACAAGTTTCTTTGGGGTACGCAT
CGCCAGCAGGAATGGTGTCACGAAACTGAGGATATTAATCACCTTCCTGCAACAGACCAC
CAAACGCGAAAGGATGCAATATCAAAGGGGGCTGGCGCCAGATCGATGACAGGAAGCTAA
CAGCATGCTCGATCACGTATGGCACATTGAACCAATCCGCAACTTCAGCCGCAATCTTCC
ATGTTGAAACTGGCTTATCCGGGAGTAGCGGATCACGTACACGCGTTGCGGAAAGGTGAC
GCGAATCGTTAACATCTAAACCAGTGCAGGGAGGAGACGCGATCACGCAATCAGGCTTCC
CGGAAACCTTATTCCGACCCTTAAAGCTGAAATCTGAATCAATCCAAACGTTCAAAAACT
CAAGGCTGGAATGACTTACGCGCACTGAATGGTAATCACCATGATCAGCATCATCATAAT
TGAAACAGATTACTTTGTGACCACATTCAGCCCAAGGCAACGCATCAAAACCGGCACCGC
TAAAAAGAGAAAAAACTTACATACTTAAATCCTTTGTGTATGGCATTGTGAGGCGTTTTG
TTTGTGGTGTCGCAACTGTATAGGCTACGGCCTGAAATCGCGCCATAATTTGAAACCGGG
CGCAATTTTTTGACTTACAGGATGGGCATTTTGTTTTGTACCCGCTTCCGCCGTGTTTTG
GACGGTAAATTTTTGCAATAAGTGTCATTTTAAGAACTCCGTAGAAGGGGAAACAACGCT
TACAAGATGGATCGAATTTACAACCGCAATCATTCATTATTCCGTTAGGATCAGAAAACA
TTACGCTTTTTTCTTGATCCATATGCATACCGCCTAATGCATCATCAAGCGTCACTTCAA
GATAGGCAACTTTAAGCGCGCATTCCGTGTTGAGTCCACCCACGTCCTCTTCATGGAGGC
GAGCAAATAAAGCATGTTGAATTATATCGGCTCCACTTCTTAACAAAGAAGATCATTTGG
TCAATCAACGCGTAAGCACCGCCTTCAGTCATGCGGCGAATTTTTCCCGCTGGGTCTTCG

CCAACAGCGTAACCAGATTTTTCGCGGGTAATCGATAACTGATCGAATCCAGAAGCGCGA
TTCATATACAAATCTACTTTCCCGTGAGTGCTGAAAATGTTCATGATGGATTCAGTTTTC
AGTTGCATGTTTGATGCTGCATTTTGTTTATGTGAGCTTATGATACCGATGAGAGTGAAA
TCCTTTTTATCAATTCGTGCTCTTTACGGCGAAAAACGGATATCCTGTTATATCTTCAGT
TCTATAACGTGCAGTCCGAATCCAACAGCCGCTTTAGCTCATAATTCAACCGTTTTTATC
ACGAATGTTCACGCGGTAAATTCTTCCGGTTTTTATGTTTTTCAAGTATGTCATAATCTA
ATCTAATGCTATTCTTTGAACTCTTGAACATATGCGCATTTGCGCGGCGACTCCTGGACT
TCTAGGATTCCACAGTCACCATAAACCAGAAAGCAATATTGGTAAAGGTTGCCGCCTACG
TTTTCCTCTGCGATCTGAATTCCGGTACACCAACCGCCACCAAACCTACGAACGAAATGA
TAAGCGGAACACCTGCAATTAATTTGCATACGCTTTCTTATTGCCGTGGTTTTCCTGAGA
ACCCCTGTTTATTTTTTCTCTCTTAATACACATGCCATCTGATTCAGATGAATCAAAAAG
ACCAATATGGATTTCTTTCGCGCCTACTTTTAATATTGGTCTGCATTATTTAGCTACGGA
ATGCCACTACACACCGTTAAACCCTGATTTAGTTGATTTGCAATATGATTGATTTTTCCC
GTATTCATTTGCACTAACAAAGCGTAGATTTTCGATCCTGTTATCAGTCCTATCGTGGTT
TATATGATCAATTTTCATTCCATCTGGAATTTTGCCATGATGCATTTCCCAAACGATTCG
AGCCGCAAGATAACTTTGATCATTTAGAGACATCTGTATGTAGCCATCATTTCTTAAGCT
TCCGGCAACCTTTCCAGTCCATTCATTATTTCTTGCAGAGTCCCTATATGATCTTATGAT
TGTGCGCGGCTTTCTGATAAGACTTCCGCAATCATATATAGAAAAGTCGTGCCGATCCAT
TGCAAATTCTTCGGTTAGTTTTGATGAATGGATCAAGCCAAAACAAACCGGATCGCTTTT
TACAAAAAGTGCGATTTAGTCAGATTCCATTACGATTACTTGTTCCTAACGCGATCGCAC
AAGCTTGATATCTGCAAACGTAGTGGGACGTCGCTTCGTCAATTCCTCATAGGTATCAGT
TGCAATGCAAGCGCTATCACATTCATAGCACAGAACGCGTTTACTATATGCGCAACTAAT
TGCAACCGCTCGATATGTATTCTTGATCATTTCATCTCCTCGATTGAATAAACATGATCA
ACGACTGGCGCGTGTACGCGATTTTTATCTTCAGTAACGCAAAGGGAAAGAGGCAAGCGG
CGATTGTCTGTAATATGCAAGTGATTTTGTTTATCCATCAAAATTCCAGATCCGCGATCT
TCATCTAATCGGCGAACTCTCAACATACCATCGCCCCGCATATTTATTTTTGTTTTCTTG
ACCACAAATTTTTGACCGTTCATTTATTTATCCACCACAGTTTTCAATACGGTATTAAAT
GCCAGTAGTTTACATTTCTTTCCAGTCTCAAACACTGCGATATTCCAGGTATTTCCGCTG
AGATATTCAACCATCGCATCAATATCGTCTCTGATTAGTGTGCCCTTCTTGAATAAGCCA
TTTTCATGCCTCGAATCGCGAACAGTAGCCACACTTTCGAATTGCGTTTGTTGTCCAGAG
TTCATCTTCGATTCTCCTGATTGGTTGATGTGTGTATTTTGCCTGAATAAGATCAAGCAC
GTCTAACAATAAGTGCTATTTCAAAACATAAAAGCCCCGGCGCTAACAGTACTATCTTTC
ATTATATGCCGCGCCCCCAGGCCACAAGCTTTGCTGTGAAATTCTTGCGTCGTCACCGCA
TCACCTTGCATTGTTTGAAGCGCAATAATTAGCACGTGCATCATTTTTCGTATCCTTAAT
TCTATGCAAGATTAATGTGAATTGATGCACTTCCGAGAATTTCAGCGGTTCCAATCTCAA
CCATGTATACCTATTCATGAGGGCTAAATGATCTGTATTCGTGGAATAGGTTAGCGATGC
GCATAACCGGAAGTTCACTAGGTTTTGGGTTAGGCACTGCATATTCATTGCCAGTGCGCT
TAATGCGTATGAGAATCATTTTATACTCACGCGGTCCAAAAGATAGAAATTAGTGAGTTT
GTACGACTCCGGCCATAATTGCGAATAACAACAGATGAGTTTTCGCCTTGCCGATGCCCT
AAAGTGATCGGCATTTCAGAACCATCACCTAATTCATCCCCAAACTTTTTGGACTTTTCT
CGCCTCGCGCAAATGCAACCAAATTTACCAGGAGTGATATTGCAGTATATGTTAGCAAGG
GGAAGCTTAACCCCTTCTAAGTCTACCCTTTGCGTCAAACTGCGGAGGCTAATCGTGGAA
AACATAAATCTCCATTCCAAATTCTCCGAGTACTTGAGAATTGTAAGATCCTGCGCGATC
AGCTAACAAAAACGCCTGGCACAGTGTTCCATGCGAGCGGGATACTGTCTTGCTGTTTTC
GTGTCACCAGGTAATCAAGGTAGTCATGGCTAAATCCTCAGTTGATTTCAATGTAGTAAT
CACAGCACTTCGCTGTTGAATGGTTCTAGCATAAAATGCTATTCCTGCGATAGACCGACT
TGGCTAGAACGATGAGGCCCATGAACACATCATTTTTTGTATCGAACCCATTCACTCTCC
TGTTACGTGATCGTTATATGATCAGTTGATTATTGTAGTGTT
>NewGenomeName_53
CCCGCCTACCGGGAAAACCGGCGCGCCCTCCTGCCGCTGCCTCGGCGTGCCTACGGCAGT
CTACGCGTGGGACATATCGCGTGCGCAATGCGTGCGTAGCGAATTTCCTCCTTCGCACTT
GCATCGTGAGGTTAGAGCGTACGGCGGGTCAAAATGTTCTAGATAGGATCGTCCTTCTAG
GGATTAGGAATGTCGTAGGCGACCAGGTGGATCCCTGGGGCCTCCCCTCATCCCTTCTCT
GATAGGGGTTTGGCTCCTGGGTTACCAGTGATGACTTGACGCCTCCCTAGGGCTGGCCGA
CCCCGTTTGCCCTGCCCCGCCTCGACGCTTTACCCGCGTCCGGCCATGGATGCCCTGAGA
GCGTCCGGCTCTCTTCCCGTCTGTCCGTCGTCGCCTGTGAGCTCCAGTTACCGCAGGGTC
TCGGGGGCTGCGTCTGTCCCCGCCGCCCTCCCTTCTCTCATACTAGTGCGCCAATTCTTG

TCAATATGGCTACCACTACGTTAGATACTGAATGGTGACAGAAGGGGCAACAATAGGTCT
GTCGAGTCCGCAGACATCTTGAGGGCGTTAGGCATACGCACTAGCTAAGGCAAAGCGATG
TGGAGGTTCTGGCCTAATGACTAGGCGAGGCGGGGAACGGGACTCGCCAACTAGTACTCG
TCCATCAAGGCACGGTAAAGGGTAGCCAAGCCTGACACGTTTAACGTATGCTATAAAACT
CGAGGCGATACTCAAGATAGACGCACGTAACCGGGTGCAGGCTGAGAGTTAACGTGCTAA
TTTGTCGTCCCTTGGCTCTAGACGTTCCGCCGGTGCTAGGCCTTGGTCAGGGAAAGCCCC
TGGTCGAAGGTAGCCGGCATCGCTACGCGCAAGTTGCGATGGTGAGGAGACACAGAGTAC
GATCTGCTGAGATTACGACGGTCGACACCCTCACAGCTATTTAACTATTCGGGTCTCCCG
TCCAATGCCATTTAATCAACGTGAAGGATATACGTCTTCCAGGCTAAGCTAACATAGTGC
TTGTCAAGAAAGTCTAAAGCACGTACGATAAAGTCCAAGCTAGCATGTCAACGCTGGACC
CCTTTCCCTTTCGTATTGCAGGGGTGACCGCCGTCTTCAGTAATTCCGGTATCGTATGGT
AAACCAACCTCCGTGGGGACGGTTACACAGCCAAACTAACCACCGAACGCCTAATTCGAA
CGACGGTCATCAAGCAGGCCCCAAACGGTGACGCGAAGACAGGGGCCGGGCTCTGGCCAA
CGAATGAGTAGTAAGCTTACGTAGCTCTACGACTGATTGCTGTTCACATGTACACTGAGC
GGAGAGTTATCTTACCCGATTACGCACCTCGAACTTGCATTGGCCCGACGAGATTACACC
CTTCGCACCGACGGGGTTAGTGATCCTTGCCGTAAATGAGGAAAGTGATAGTCCCGAGTG
CGTACCCTAACCTTGTTGGCGAACCTTGGCCACTGCAGGCTGATCCTACATTGAATCTAG
GCAGTCGGGGAGTAATGCACTGCCCCGGATGATGGAGAAGCAAGCACAAGCTCGAATGGG
AGGCTCAGCCATGCAAGAGAGTATTCCCCTCTTAGATCCCAAGCTGAAACCTTAAGCGCA
CCGTCAGCAGCGCCGAACCGCAACCCTACGGCCTAACATGAGCGATATGCAGACGCGAGC
CGAACACGACCTGCGCTGGCGATTGCAACTCGTTCCCAACTCTAACGATGCTGGCAGGTT
GAATGAGCGGGCAGTGGCGTTGGATGATGAGCGCCAGCGTAACTACGTGTGCAAAAATCG
TCATTGCCGGAAACTGACCGGATGCAGGAATTCGCAATCAGGATAGCCGACTGTCGGCGT
CATCCGCCTTCAAGGGACACGGTATGCCATTGTGCCTTAACGCGTAGCCCGGCCCACGAC
CTCTATAAGGCTATGCCCAGCGTATACCACTTCGCAATAACGCCAAATTTCACCTGGGTA
CTGTTGCCTTATTCCCACAAAACCGTGCGGCTCGTTGGGCAGTAGGACACCCACCTGGCG
TACTCAATACTACGTGCTGAATGCTATGAACCATTATGATGAGATAAACAAGCGCGCTGT
CCAAGCCGTGTAACCGCTCGCCTTGGGAAGGCGCTTGTACCATGGTGCTCACGGAGTAGT
CCGTGTTCCGGACTAGTCGCTGAAGCTGGTGCCCAAAGGCCCCCTCTGAAAGGCTTTGGC
CGGTTGATTCTAGAGCTTCGGCCAAACCACTGTGAGCGCAGTATAGTCCTACGCCAGGAT
CTTCCCGCGTGTATAAATCAAGGAAGGCAGCAGCGACCGACACCGCGCGGCAGCCTGGGA
AAGGAACAAGTGTATGCGAGTCGGCACCCTGGCCGTGGGGGTTGATTTCCTCTGTCAGTT
AGCGTTGCTGCTGCACCATTCCGCCGAAGAGGAAGCGAAGGGCCTTGACCTAAAGGCTGC
CGACTCCCTGTAGAGATGCAAGGACTAGTTGGGGTCTGACACCATTCAGGGTTAAAACCG
ACGTAGCCCGTACCTTCCGAAAATTGTCGATAGGGCAAGTGTTGACGGTGAACGAACAGA
ATATGCGAAAGAGCTTCAAGCGCAACGCCGAGAATGATTTTTTGGGATGTTGGCTGTACG
TAGGCCTAGGCGGGCTAGTGCATTTATGCTAGCAATTGTCGTAAGCTCGTCCCTCCTGCT
CAGCGCCCCGCAGCCTCGCTAGCGGAAGGTAACGAACTACCACCGGGACGACGACCGCCG
CTTAACGGGTGCACTCGGGGCTCGAGAAGCGGGAAGACGAGGTGCGTCTGCGGGCTGTAA
TCGGGAACATCCGGTACGGCGCACGCTTGGCTACCGTATGCTGTCGCGTGTGACGAAAGG
GAGGGAACGGAACCGGGTAGTTCTACGGCCACAGTGGCGCACTTATCACACGACGCAATC
CCAAAAGCTAACGTTCTCAGCGCATGTCCCGCAAGGTGTCCAACTATCCAAAGAGCGTAA
AACATTTTCCACGCCAAGATCGAGCTTAAGCAACACGCCGTTAGCAAGGAGCCGATGGAG
ATCATGTGCTGGGCGATTTGCTTCGCTTTGGTGGCACGCGATATCTGCCCTGACGCGATC
GCCGCGTTGAGTCGTACCGTTTAGGAATACCTTGATAGGTTTGGTGCCCCGCCAGTAAGT
TTCGTGTACAACGCGTTGCAGCGTATCCGCTCCTGTCCTGAGCATTTGCGTGTATACCAT
TCGGTGGCCGAGGGCGGTAAAGCGTTCTGCTAATTCTGGCGCGAGTACTCACGAAGCTGG
GACCAGCATGTACGTAGACGTTCAAGAAGTATGCCACAAGGGCATCTAAGCCTGCCAGAA
CTGGGCGGGCGAGGAATGCGAGCATATCATGTGCCACGCCTTATGGCGTGGATTTACTGT
GCACCTCAGCAGCTCCGACAATAGCTAGCCTGCCAGGGTAGCTATTGGGGCCTACGTGCC
TAGCACCCTCTCCTGGGCGAACTCCATCGTTGGGGCACTGGGCAGGTAGCGTCGGCGGAG
CCTTCCCACGACTGCCATTCGTGCAGGCATACTAATGGAACTGCACCGGGACGGTCACGA
CGGGGGGACGCCTTAAGTCCTGGACTCGCATCCCGAGACCCTTTTTGGTAACGAGACAAC
GAGGGACTAGGAACTTATAAGAATTCTCGTAGCTGACAAGGAGGCTACGATCAAGCGCTA
ATAGGGTATTCCGGGTAGTAAGCAAGTTTCCATATATACCGTAGATCAGCAATCCGATGA
CCCTCAGACGTTACACGCCCAGCGGGAGTGCCTCAAGTATCACTTTAGACCCGACGTCAA
AATGGAGGACGGGCAGACCAGCTTGTACGGCAATGCCAGGGCCAACCCATGGTTCCGGGC

CCTCAACCGCTTGGCGACGTTCGCGTTGTACCGAGCGACTAACTACGAGGAGGAATTGCC
GCTGGAGGTGACCAAGTTCAGAGAGCTTGTTCCAATCTTTAAAGAGATGGGAGCCCCCAT
GAGGCTGTTGGAAGACAGGTATGGTCTAATTTGGTCCGACGCTCTTTGTCAGAGACAGTT
ATTCTCGCCGTCCGTGTGGCTCCCGAACGTACCGCTGGTTTTTGTCGGAACTTTCCCCGT
AGCACCGGACTGGATAGTAAACGGTGCGGTGGTGGTGCGAAGCGTGATGAGACATGAGAA
GAACGACCTCTCACCGGGGTCTTCGGCATCTATCTGTTCCTGTTCTCGCTAATGTGGAGG
CTCTCCACATAGCCGCAAAGCGGGTTCCGTACAGATGTGCGTCTACTACGTCAACGTGTT
GTTAGCGTTTTTCGTACATTTGGACTCAACCGGAGGCGGGTCGAACTGCAATGGGGGGTA
AAACTACGACTACCCGTCAAGGACTACCTCTCTTAGTGAACCAGTCTGAAGCGTAGGCCA
TCGCTTACGTACCATGCCCGGGCCCGGTAACGCGCATAATGCAGTACCCTACTTCCTCGA
GCAAAACGATGCGGTCGTCCGGGCACAGCGCTTCCAGTCCTGCTAGCCTCTGTCTGTACA
GGAATCTGGGGCACTGGTAGAGGATTTAGTTGACGCGGTTTGACTTGATGCACCGAGCTC
TGTAGCTCCGGGCTCTGCCCCGTCTGGGGAAATTTTTCGCAATGCCGGGGAGCGGTTGAT
CTATCCGGGCACATACCAAGCCTGAGCGCCGGTCCACGCCCGACTAAAACGAGGAGTGGA
TTTTTAGCTAGTCCGCTACCTGAGGCTCTCTCAACCCGGTCATTAGAAGCACCCCGAAGG
AGACCGAACGTTTAGAGTGGTAGTGTCTATCCGATAACAACGCAGCGATGTAATGAGTCG
GTATATAGGTACAACCTAATCGAGTTGCCACCGTGTGGTGAGTGGCGCCACGCTATATCT
AGTTGCTCCTAGCGGTGTCGCAGCCGGACCACCTGTGCAACCCAGTGCACGACGCGGTGT
CCGGAGGGTCCTTTTATAAGACTGGGCTGCGTATAGTTTACGAAGACCCCCTATCCGACT
TGGGCATGGTTGCTATAGCTCGAGGTTGTCATCACGCATTCGAGCGGACTCGTGTGTCGG
TGCGTTATACTGTCTCCCGCGTTCTTACGACCAGAGAGCGAGACGATGACCTCGACGCTT
ACGGTTGACGGTATAGTACTCGTGGCTTATAAATCAGGCGGTTCCATGAGCGAGACGGAC
TGTCTCCTGGAACGTTCCAGACTGGCGGAGGGCCTCACACGCACGGTGCGCTTCAAGTGC
CACGGCAGTTAGGCATGGGAGGCCGACAAGGTGAGCCAGTATCTCGAGTCCGAGGCACTG
GAGCGATGGCTGTAGCAGGGGGGTATCAACTGCGGTAGCTGCCAAGATGGTCATGCCTGG
CCTTTTGTGTGGTACAGAGAATAGACTAGTCCGTCTTGGCTAAACCTTGGGCGTAGTGTG
TGATCGTCGGGCCTGAACCTGACGACCTCTGCGGGGCAAGCGATCGCCCCAGCGCGAGGG
AAGGTATCGCGGCCAGAGACCACCCGAACATCCGCGAAGTGAGGCAGCGAATGGATCCGG
ACCAGGTTACCGTCGAGGCAGTGTGGTTCTTGAGTACCCAACCGCATGATGGCCGCACCA
GTACCCGCTAAGTGAATGAGAAAGGAACTGGCGCGCCATCGTTAAAACTGGCCCGTGTAC
ACAAGGAGATGCCGTGATACGTGAACCGCGTCAAACAGACGAACACCCCACCCCAGACGG
CGCTCTCGGAACGGATGGTCTAGAAACTGTAATGATCGCGTACGCTTATGTTTCTGAAGT
AGTGGCCCCGGATGTCGCAACGCGCTGTGATCTGCCACGCAGTATACGCTACATCACCGT
TGCATGGGATCTCCGATACCCCTCCAACATAGCCAGCGGCTCTGTGGTAAAAGTCGACGG
GTATGGAAGTATAGCAATTTCTGTGGGCGACGTCATGAACGTGGCCCCTAGCCTGACCAA
GGCAATCGCTTTCGAGCTGTGCGCCGCCCTCAGATCTGAAATAGAGCAACTGTAGGGGCT
AAAGCAGGCCGTAAGAGCAACCGCGGGAACACAGCCAACCGCCGCGTCGATTTCTGCCCT
GACAGAGCTTTCCGTGAGAACCCCGATGTCAGTTCCCTCCTGCCCTCGCTGCTACGGCGA
ATTGGTGTGGTGAAAGCGTAGGAGTACTCCCTGAGTTTAAAGTCGGGCGTATTAGTGGCC
AGAGGTAGACTGTATCACCTGTCAACGGTCGCGTATACTCGCAGAGTTGAAGAGTCGTTG
TGCAAGGCTACCCGCATGTGGAGTTAAAGCTTATCCGGTAAGATGACCGCGGTCCCAACG
TCGGCCGGAACCGCTCCGCTGCGTAATAGGTAGGAGTTAGCCTGGCGATTTAGGATGAAC
TCGGTGAGGAGTAGCAGACAACAGTTTTTGCATAGAGTCGGGCTAAGGATCTCGCGCCCT
CACCTACTACCCCCCGGCTCTACCGCGAGGGAGCGCCACCATCAGCAACCATATCCAATA
AGTTCACGACCAAGCGCGTTCCGTCCATCCCAAGGCAGCCGAGCTGTTAGCTAAACCCAC
TACCCTACGCATGTAGGTGGTGTCTTTGGAGCGCCTCCAGAGGATCGCCTAGAGGGATGC
CGGGTCCTTCGCCAGTGGACGCCAGGACGCCAAGATGGTCAGCAGCGGCGCGGTAGTGGG
CAAGGCTAAGAAGGAAGCCGAGCGGCAGCGACGCGTCCTCGTTGAGGCCTGCTACGAAAG
CGCTTCGGACAGTATCAAATCTAGGCATATCGCGGACGTGCGGCGTCCGCAGCCTGGAAA
CATTCCTGAGGTCGCTGATCATCCTAGCGAGTAGCATCGGGTCTTAGTGCAGGAGGATGG
CCGCATTACCCGCCCCCTTGGCGTCTACGAGAAACAGCGACGCTCGGTGCTCAATGTCGG
CAACGACGAGGTTGGACCATGGAAGTTGTTAAGAACGTGAACGGCCCTGCCCGCTGGGCT
GTCCACGGTAAGGCGGATGCGGATGACATGGACCCGCGCATGACCTTTCAGCAGGCGCAA
CAGGAAACCGAGGAGCTGGCTCGCGTGCGCTAGTACGTGCACCGGTACCCCGGAGAATAC
TAGGGCTCGTGCAACGAGCTTGAGGAGGCGAATCACCCGATCTCATGGCTAGATGGAACC
TGGAGAAACCAACCCTACACATGCCAGCTCCTCGAGGCCAGGTAAGTGAACCAGGATGAT
TTCGTGAAAGAGCTGGAAAGGATCAGGACGTTGACGGTGTGCCGCTTCAGTACCACAGG

AAGCCAATCACTACTTTGCACCTGATAGCCTACGTTCTGGCCGGTGCTGTAGCATGAGTA
GACTGGAAGCGATCCCAGCAAAACGGTTAGTTTGAAGTAGAGCTGTGAGCATTAACTCGG
GCTGTAAGTAACGTTGAGTAGGCAAGACAGTCTAAGCCATATGTTCCGTGAAGTGATTCA
CGTTCGTAGATTCCGCGTAAAGCTTTCCCACCTCTATCGGGCCCCTGATTTACCAGGGGC
CCTCGATGGTTCCAACGCCCTGCACGCGTAGTCCGAGGCACTGTGGCCTCAGGATGCAAA
ATGGCAAAGAAGTCATAGAGATAGACATGAGCCAGCATTATGTGAAGCCCAAGCGTAATT
CCTGGCACTACGCACAGAGCTGCTGGGGGCACGCTGATAGCTGTGAAATCTGGACCACCC
CGCGCGTAGTAACCCCTCTAGTCAACAGAGAGTGGTAACTCCTAGAGGCAGTAGCTATTG
CGCCGACTGCTGAAGTGACCCAGACATGACCTCCGCCCACGCGCCGGTAGGGCGGGGCTG
CATTATGCGTCATCTCACAATGTCCGACCGGTCGGACCTGCAGGAGGCAGTGGAGTGGGA
TTTAGCGAATTGTGCCGATGAGATTGTCTTCTTCGCGCCGATTTATCAGATTCTAGAGAG
CACCATCACTTGTCAAAGGGCAGTAAGCTTTGCCCTCTCGACACGACCCCTCAGATAGGC
CGGTGCGAGCCGGCGCTGGTTGAGGCAAGGGAAGGAATACTTGAGTATGTACCAATGGGC
AATTCTGACGGCGAGTACATCAGTACCCTAAAGGCCAAGACAGCTCTATACGCCCACAGC
CAAAGGCAATCGGTACGGCCAACCGGGAGAACGAGTTTTTGCAAACGGCGGAGCCTCTGG
CCCGCCGGCAACCCGCGCGCACTTAGCTCCGGGAGGTGAAACGTTAAAGCTGCGCAATCG
GCAAAGCCATCGAGTGTTGCTGGGGGAACAGCCACCGCTGTCGCGAGGGCCGGCAGGTGA
AGAGGAAACCAGTTCTCGTGGGGGCAGACGTCCAGCCCCAGGAACCCCTTAGACCACGGA
CCGACGATCCTAAACGCACAGTAAGCGAATCGCTGCGCCGCGGCGTTGACTGAAATCAGA
TTGTCAATGGCATCAACCTGTTTGCTGTACTGGCAAAGGCCCCCGTTTAGATTAACCGCC
GCGAGGGTAAGTTGATACACGCCATTGCGGAGGGGTTGTTGGGGCTCTCTAAAGGCCCAG
TCCAGCCGAAGTAGTGGTCGGACTGACACCAAGGTCAGCGCCCGACTGTGTTCCCTCCTG
ATCAGTACGATCAACGTACGAGGCAGTGTGTGATCATTGAGGACTACCTCTCAGCGATCT
AGATGAGACGGGCGTAAAAAGAGCCGGAAACTAATTCGAATGCGGTGCTCGGGAGTCGAG
TGCCTAAACGCCTGGCGGCCGCTCACCTCGAAGCAGATGGGGTGCGCTACGCCTCCGACG
GAGATCAATCCAGGGTTGACGCGTGGCCAGACGCCCCCCCCCGCTTACGTGGCCGCCGCA
AGGCAGTGCTAATCTGCAAGGCTCCACTATGTTTAGACCTTAAGGATATGAAGACGCCAC
TGGTAGAGGAGTTGAATCATGGGTCGCCCCCCAAGGGCGGAGGAATACGGGGCTATCGAT
CGTCCCCACGCTGCCGGGGTGAAGGCGCAGCGCGCGTACCGACTGTACAACGGTCCTGCG
GGCTTTTGGCACCGGATTGGGACGTTGCGCGTCATGTAAAGATAACATTGCGGCTGGACG
TACATCCGGGATGAGAAATAAGCAACCGTCACGAACCGATCCACATTTTCCGGTCCTGGA
TCAGAGATAATGGTAACGAGAGACAGTCGGGGCAATACCGGAAGCTTACCTAGGCGGTCC
GCACGTAACGTGTGCCCTAGGAGCAATATGCCCGGGTGGAGCGTCCGCTGACTTCTGGGG
GCGATGCCTTGCTGGTCCTATCTGGCGACGGTCAGGAGTATGGTCTCGGATATTATGACC
CTTAAAGCGCTGTGCTAGAGGTAGACGGAACGAACGCTGATATTAACGGTTCCCGTCGGG
AAGGTTGGCCCCGAGGCGCAATTCCCATAGGACTGGGATCCGGTATCCCACCACGGGTAC
CCTGTTAGAGAGTACCCGTACGTCGGTGCAATCGGCTGCCAGGTGAATCACCTGTGCGTT
CATAGCCGGGTGCTGGTCGTGCCGGAACGCACCGCATTGCTGAAGCTGCGCCAGAACGTT
GACAAGATTCCCATTTCGAGAGTCAAGAACAATCTGCGCGGGCGGGAAGTCTCCATTAGT
GCCGGTGCTACGCTCGCCCAGCATAAGACCGGGGCAGCTGTTGATGTAATGTTCGACCCC
TGTCGCTGTGTGCATGAGATGGAGCTCGGTTTGGCCCAGAATGCCACCTCCGAGTGGTTA
AGTCCCAGCATCGACCACAAACCGAAACTGGCGGAGAACGACTCCGCCAGACTGCTCCAG
CTTCATGTCTTGAAGGCGCATCTCTACGGCCTCCTGGGTGGTGCCTCAGTGATTTTGACG
GGGCGCCCAGAGAATGGTAAGATGAGCCTCATGGTACCCACCCTTGCGCGCTTTGCGGAA
TAGACAGACCGATACTCCGTCACGGAGCGAGCTATCCTTTGGCTGAACACCGAGGGTCTG
TGTCAGCGATTCATGCCACCGATATACGGGGCGGCCATAGGCGCTGGCACGGACTTGTTA
GTGGGCTTGTGCGACGCCGTCGCGCTGCGTGATGAAAACTCCTTCTAGGTTCGCAGTACT
GATCGTGTCCGTTTACCCGTTATGCATTTTGCGTGCTCGGATTAAATCGAGCAAGGCATC
GCATCAATCAGGCCCGCTATGGTCGTCTTGGATCAGACGTCGTCCTTCCGGCCGGGTCAA
CGGGCCAGGAGTATGTGCAAGGTCGATGAGGTGGAGGAGAAGTCGCCCGCCGGCCATGCT
CTGTCGCAGCGCCACGACTTGGGGCGCCAAGGGACTGTACACATCAGCGTCGTGGCTGTG
ATCATGCAGTAGCCGGCGTACTCGGGCCTCAAGGTCAGCAATACCGGTAGCAAGGGCGCG
CACGATCTCATGGTGACCATCGGTTCGCTGGTTGAGCCCGCAATGCCGCTGCTTCGTGGT
TTCAGCACGCTGAAACCGAGGGTCCAGATGGCCAATGTACCAAGCCATATTATCTCGGAA
TTATTGTTGGGTGCAGAGAGATGTGACTTCGACCACGGTTCGTAAACAGTGGAGAGTAAG
CGTCACAAACCTTGCGTAGTATGCCGGGCACGGTCTTTTCTTATTACAAGCGGGGCACAA
GTGATACCACGCTTCATTCATCTTATCCGACGCCAGCCCCCCCTATATCGCACAGCTGGA

CGAGCGCATGGAAATGCACATCAACAATGTTCTGTACGACCTGGGTAGCATACGTGTATG
GATCAAGACATTGGCCTCAAAAGCACTCATATAGAGACAGACGAGGTGGAGGTCGCATTT
CATGACGCATGACTCCTTCCACAGCGGTCTCATAGTGACACCCGGTTACTGGAACTGCTC
TTCCATTCCGAAGTCGCGCGCCCCTCTGAGCACATCATCATGCACGATCCTGTCTTCCAA
ACGAAAGATCATGGAACCTTTACGTAGGCCAGGTGCCTCGCCCTTAATTTGCCTCACGTC
GAAATGCATCGAACTGGACAACTTGTTGGTCATGAATGAGACCCACCTGGGCACGTACCC
CCAACTAATACCAGCGGGACGGTGGATCTCGTACAATGTTCCTGAGTAGCCGGCTTGGAA
GGAGTAAGTGTGCTTGCCTTTCCACAGCGGACCGTCGAACCTTATGCTGTGCTGGAACGC
GACGCGAAACCTACGTTGGTATCTCTCTACAGGAATGGCAAAGGTAGTTAACGTCACTCC
AAAAGCGGAGCGGGTCGGCAACCTCCGGGCACACGGCTATTGAATACCCGAACACGTGTG
CTCGATACTGGGGCATCCCCGTACCACAGATGAAGCCTAGTACCCTTAAATTATGCATTG
GTCCTAAGATCCGTCCCCTCTGGCATTCTACCGCGCACCAGTCGGAGGACGGGCTCCAGG
AGGCACACTACAGGCGTATAGACGCAAGCACGCTCCATGAATGTTAGAATGTAGCTGGGT
ACATCGCACCGATCCGCCGGCCCTGACAAGGGGATGTGCAGTAGACGTCACGCGAGGGCA
TTAAATGCAGGAACTTGAGTGGAATTGCGGTTCACGTTTCTCGAACCCAGTTCTCCGCAT
TTACACCACCGCCGAGAGATCATGGCGGGGTGCTCCAGATAAGCGACAGGTCGTATGAGG
AGATGGCCATACTACTACGCCTGCTTACGCGGCTGCACGGCGAGGACCTGAAAAATGTGC
AGGTCCTGATGTTCGACCTGGGAATACTAAGGGAGTATTAAGGCGGGGCCAAGGCTGCCT
TTAGGAATGTGCTGCCCTTAGACGAACGTCGTCCCCACAGTGGTTTCTAGGTGCGGCCCC
CATTCCTTCCGATGATCGAATGCCGGAGGCCCCTGTCCATCTAAATAAAGTCTTCGCTCG
ACTACACACTCTCTATTATCAGGGAGCTGTCCCAGCGTGTCCGGACCGTCGAGTGCGGAG
GCCTGATCCTCATGGACGCCCTCCGCCTACTTCGCTACGGCAAGATCTCCGGGTGCTGGA
AGGTGAATCCTGCTTTCCGCGCCCCGTTGGCGATTATGATTGAGACGGATAGTATTGTCA
TCGACGCCATGTGGGCAGGCCCCGTTGAGTCGTCCCCCGATAAGATCGTCAGTTGACCGG
TCCGTATGGCACACGGGGATGAAGTAAACGCACGCGGGTCGACGCATCAACACAGGGCCT
GGGACAACCTTAGTTATCCCCCAAGCGCGTATGAGGGCGGCCTATCCCAAACTCCCTATT
ATTGCCGCGCCCGTATCGTCCCCTATATGGAGGGAACTAAGGTTGGCAGTAAGCGCCTCC
CAACGTTCGAGCGGTTTTGTGAGAGCGACTGGGCAATTGGATTGACAGCGACACATCGAC
TTCTATGTTGCGGCCTGGCCACAGGCCAAAGGGCGGGCTGGGACTAGGGCGTGTCCCGTA
TAAGTCCCGACCCTGACAAGCAAGGAACGTCCGGCTGGATGGTGGGCAGTTCGGATCCGG
TTCCCTCGGTGTGGGCGAGGTGTGGTTAATCTAATAGTGGCCTAAAGGAAGGTATGGGCA
ACCGATGCTGCGTTAGATACCAACCCCTGATCCTGGCGGCTAGCGTCCCCGCCCCTGCCT
GAGAGCCACGTGGACATGCCCGTTGGATGTGTCCGCGCCCACGGACACATCCGTAGCCGG
GTACTGCATCCGCACATCAGGTCCCAGGGGAACGAACCCAGCTAAGATTGGATCATACTC
GATGGGGTAGTGCCCCTCGTATCACCCATCGGTGTCCACGAGGTGTACTGGATCGTGACG
CGCTACCGCAACGACTTCACGAGGTCCCTCAAACGTAGTGGCCGGGTGTATTGTACCCAT
TTCGCCGAGTACCTCCGGTCTAACGCCCCCCAAACGTACCCCAGGTTTGACGAGAAAGCG
TCGAAGTTCGGTGGTATTCCCAAAGTCGATGCAGTCAAGGAAGACTGCAAGGAGTGGCAC
CACATGAGGAAGATCGAATCGGAGCTCCGCGCCGAGTACTCCGCCTTTCCCAACGGGAAG
GTTCAGCACACTCGTCGCATCTTGCGCAGGCTGATGACGGTGCTCGAGGAGAGCGTTCTG
TGGGGGAAGTTGGTGGAGCGCTGCGCGGAGTCGCTGGCTTCCGATTCCTGCGAGCTCTAC
CGCTGGTACCTGAACACCGAGGGTGCAGTGGCCAAATGGACGACTCAGGAACCTGAGCTG
CACGAGCTGCGAGTCTCCGTACGGCAGCCGCGGACCGCTCTGCCTACTCAATTGCACTAC
CACAGTCGGAGTGACTACTATCGCAGCGCCTGACTGTTAAGCGGTGCGTTGAAATGCCGG
ACCCGCATACCTCGCACCCATGCTGGTGGACACTTACTGTACGAGAAATATGACTTTGCC
ATGCTCGGTGACTCACTCACCGCTTTGCTCTACCCCAACGGACCTGTCGAGGCGGCCGTC
ACACACTTGACCCGGGAACACAAGAACCCCGATGGCTAGAAGGCCGGCCAGAACAAGACC
CAGCTGAAGGGTCATAAGGTGGAATATGATCACGCACAGATGAAGTGTTTTGACCCGGTG
TTTGTGTCTCCCGACACCAAGTGGACGCAGCAACTGCCTTCAGACGTCTTGGAGGAGTAC
GCGTTAGCCGGCACGGATTCTCGCTGGACCAGGCTATGGAACGGCGTCTGCCTCCGGTGC
GACGTGAAGTCCGCGGCGTATAACACAAGCTCGGAGGTGCCGGAGGTACTGGCCTAACCC
ACCATCTAACCTAACGATAAGCTTGAACCCACGGTGAGCCTGGCGAAGGCTCACGCCACG
CAGTACCGCTCAGCGGCGTTCGACAAGGAGTGCAAGGCAATAAAGGTCATTGGGATGGAA
CAGGACGTCCAACCGGACGGGAACGACCACAACAGATTGAAGCTTCACGCAACGGACTGA
GATTGCCTGACTCTCTCGTACCGGCGCCTCCAGAAAATTCCTCGAGCCGGCAAACTTAGG
GTTATGGTGAGTGTCGAGTCTCTCTTCTGCAATGAGGGGCGGATAATGGCAGTGGACTAT
TCAGTCCTGTAGATAGTGAACTTGGCTGCGCTCAGCGCCGACTACAACTTGGTTATGTAC

CCCGGCCACGGCTCATACTCTCAATGCCTGCGTCTGGCCGCAAGGACCTTTGAACCCGAC
AATCAGGTGCTGCACAAAGCAGTCGAGAAGAAGCCAGATCCGGAGCATAAACGGTATAAG
TAGACGCGGACGTAAAAGCAGCCAAGAGGATCCACTGTCTGGGAAGGGCCCGGTGCCGCG
GGGAACGCAAGTGCCTCGGATTTATCTGTCGTGACCGCCGAAGGTTTCTACGAAACGGAC
ACGTGCCTATTCCCGCTTAGCATCCTGTTCCGTAGGCTAATGCTCGCTAAGGTCGAGCAT
AGGGACGGCATGACTAAGGGCCGCCAAAGAGATCAGCACGACGCCAGTGGGGCGCGACGG
TTCTTCAGCGGTGGCTCCCAGTAGGCGCCAGCTAGTGCACGTTACAGCTGCCGGTAGTGG
AGACTTTAGAACAAGGAGGTTTGCCACCGGGACCTCGAGTGAATGGACGGTCTGATCGCT
AATTCCTGGTACCAGATCGAGGCTTTCTCACTCATGGCGGTAGCGGCAGTACGAGTACTA
AGTTGGGTCTGTTTCGGGAAATCCCTATTGAACTCTGAGTTGCCGCAAGGGCGCGTGTGC
TTCATCAACAAAACCCGGAATCGGCTTTAGCGCGACGTAGACAAATTGCGCGAACGGGTG
GTGGCCTTCCAGGTTAGGGGCAGCATGCAAGAATAGTAAACGTAGAAGACCGCTAGCTTG
CTCACAAAAAGTGCGCATTTCGCGTTCCCTACACTCGCTCAGGGAGGGCTCAATAGGGCA
CCCAGGGAGCACATGCACTGGGCAACTCCAATATCCACACCAATCACTGGCCCTAACGGT
TGATTGGCTGTACGCAGTACCCCAGGACGCTCGCTACTCTTGGGCCAGACAGACATGTAA
TAGGGATTGCGCAGGCTTGGGGTACCGACATGACCCAGGCATCGGTGGTCCTCTCCGTCA
CGGTGGCCAGGCTTACTCAGGCGTACTTGGCATCCACGTACCCACTTAGTTCGTGCCCAT
GGTATGGGTCTAGGCCGCCGGCACCGCGCAACAGCAGAGCGAGGTGGCGACGAGACATGG
CCGCTGCTGCCAAACCCAGGTGACTCCACCTGCCTACCCCGATCCCCTGGGGGACGCCAT
GGTAGGGCGACTCTACTTGTCCAACCTGAACGTGGTCGAGAGCTTTGACGCGTCCCCTGT
ACCGGATATAGTATAATGCAGAAACTCCCTGAACAACTGGCCCAGGCCAACGTGGGCGCC
TAACCCACGTCTCTAGAACTGTGCCAACGCTTCAAGTGAAGGGCCGGTGGTAAGGACTAC
CCCGGAGCCAACGTCGTCGACTAACTCGTGCGGTACGTCTTGATCGGTATGCACGCATAG
GGAACTCAAGGAACGTCCGCGGACCCTGCCCTTCATTACCAATGGGGCTGCACCATTGTC
GGTGGGGGCAATCAGACCGTATACCGCACGCCCGGCCTCTGGTTGACCTATCATATGATG
ATATGCAACAACGCGCATCCGGCCACTAGCATCATCTCCGGCAATATGACCTGTGTGGGG
CCGGCCAAGCCCTTCATGTGAATGCTCCGCGACGCCGCCCTCGTGAAGATCGTGCACAAC
GTCGTGCAGGAAGAGCCCAGGTAGAATCGCAGACTGATGATCGTAATCGTGAGCGAGCCG
CCCCTCGTCAGGCCCAGCAAGAACGAGTACAAGACTCCCCGCCTTCGCGACTACCCGTTC
GCCCTCCTCCTGCGACCACATCCGACCAAGCAAGCCTCGGACGACATCCACATGGAGGGC
ACAAACGAGACCACGAGCCCGTGCAACAACTGCCTCAATGATAGGAACCCGCTGGCTATA
GTCTTGCCTCGATCGGCCCTCGAGCAGTGCCTGTGCCGCACGAGGAACCTGCCCTCGCCA
GAGCAGCTCGACGCTTCTCCCCTTGCCACGCCCCAGTTATCCCAAACGCCGCCCCCGCCT
CCCATCCCGCCAACCCCGGCTATCTCCGCTACCAAAACGTACATGGCGCCTGCTGCGCCG
CCGGTCCAGGTAGTGGCCCAGGTGGCCGCTGTGCCACCGGTGACGGTAGCGGGAGTCGTC
CTGGACTTGCCCTATAAGGTAGGTATCCAGGCAGGGGCGGGGATACCGCTGTATTGGCGC
TTATCCGAGTCGGCGTGGTAACCGCGCCCGGTGACAATTTCGACATGACGGTGCCTAATC
GACTACCGCTGTTGGACGGCGTTGGGGTGCCTTACCGCTTTAGCGCGAGTGTGAAGACTC
ATAACACTGATGATCTAGGGGCTGACAAGGTGACGCTGGCCAACCAGTGAGGCTCTGGTC
GCGAGGATTCCCGCAAAAACCTCGCTCGCCAGGGGGGTCCGCATAACCTGGAGTGACTGT
ATGCGACGCGGAAGCCCTACTAGTGCACTCGCAAAGCTACGCCTAAGTCGGCGCTCTTGG
GGCCGCAAGGGTGGCTCCTGGCTAAGGAGACGCTCATCGATGACGGAATCCCCGAGAAGA
TCGTGGTCCGCCTGAAACGATACAGGTAAGACGGCGAGCCCCGGGTCCTGGACGGGGTGC
CCTCCGGCGTTCGGTCCGTCAGGGCGGCTGAATACAAGGACCTCCGGTCGACACCAGCCC
CCGTCCATAACGGGGAGAAGGGAAGGATCGATGTCCTCAACTAGGGCGAACGCATTGTCT
GCATCAACACCGGGTGCACGTGATCCCTCTCGCTCCGAGCCACGGGGCATAGGGGGGGGT
TCATCACGGCGTAGGGTTTCAACGGCGAGACTGTAGATCACGTCCCAGGCCTGGCTCGCT
GTCGGCTGAAGAGCGTCGTAGTCGCCGGTATGCGGGAGGTGCTAGGTGGTGTAGTGACCG
AGAACGAGGGGGCGAACTTAATCTTTGGGGTCTAAGCTAGCATTGGCCAGGATACGCTGG
CCGAAACCCATGTACTGTTACTGCACCGCCCCGACCAGGAGTGCGCGTCCCGCTACATCT
GTGAACTGGCCCTCAACCCAGACCTGCAGTAGTCGTTGGACTGGATGAACGGTAAACACC
CACGAGTCGACAACCAGAAGCTTGAGGAGCGTGATGCGTTAGCAGAGTAAGACGAAGCTC
AGAGGCGTGTGGTTCACCCATCTACCCAAAGAGCTGGGACGCCTCTGTCAGTCCTGTTTT
ATAATGAGTGAGTTGAGCATTGCACGCGTAGCGGTGGTTGATAACCACCACGATACCGGC
GGACTCCGGTGCGACATCTATCGGTCGTGTAATGCGGCGGGAGGCCTGGTAGGCAAGGCA
GTGGGTAGGTGGGGTACCAGGAGGACTAGGTATGGGGCCATCACGGCGTTCCTGGAGAAT
GGGTTCCAGGACCTGAAAAGCCACGGGCCTGAGTTAATGTATCCAGCCCACGAGCCACTC

GATCTAGACGCTGAGGGAAAGGGGGTAAAGGGCAGTCATGAAGCACCAGTGCGCGTGGAT
TCCTCCGAGGGCCGCAAGAACCCTGGTCAGGATCCCTGCCGAGTATCTGCCTCGTTACAG
GAATTAGTTACCTGGAAGCGGCCCTACCGGATGCATTGTTGGTTGGCGACGGTAACACGC
CACACGGTGCACATAACCTCAGCTATAGAGGACGAGGGGTGGTAAGTCAGGAACTCTTAC
AACAATTTGCACGACAGGTTTGACAGAAGTCTCGCACTAGCAACTGTGAAACTACGGACG
GGCTGACCGGAGGTGCCACGAAAACTGCGGGCTAGGGGACCCGCCCCTTGGAAGACGGCC
CATGTATTTTGAGCAACGACAGCGCGACCCGTCCCAAAGTCGGGCCCGTCAGGGGACTAC
GGAAGCAGACCCAAGGTCTTAACCAGTTAAGGCAGGAATCGAACATACGCAGCTAGCGGG
CGCACTGTTTGTATGTTGCTATCGTGATCGACTCGGAGCTCGACTGGGGCTAGAGCATAG
GGGAGGATGGGAAGCCTGTAGCTGCGATCTTGGAGCGGCTATCGGGAGGAAAAGGTGGAG
TGGAACAAAACGGTACCTGATTCGACCGGCTGAGGAATTAAGCCCGTCTCCTGATGGATT
GTTATAATCCAGCTCGCCCGCATCGGGTAATGGGAACAATGGTGATCGCGAAGCGCCGGT
ACGCGTTCACGAGAAAAAAGCTGGAGTGGTGGACCGGCAAACAGTTGACCGCAAAGAAGC
GCGAACATTTGCAGTGCCCCAGTAGGTCATGGTGGAACCACTACTTGGCCGGCAAACGCG
AATCCTGGGAGGAGATGCAGCACTCCATAATTGAAGACTGCATCTCGGTGCGAGGGTTGG
AGTTTAGCTAGCACCCCGGGTACCGTTGTTATAGGCACGTGCGTGTCCTCAACGATGAGC
AGGGGATCGGCTTTCCCAAATCCTCCTCGCACGCCCTGACGGTCGTAGAGACGACTGTCC
CCTGGTCCTGTTAGCACGAGTTTGGGCATTGCAACGGAGCAGGCGGTTGCAACGTTAGCC
GCAAAACCTTACGCTCGAAGCAGATGCGTTAATCTCACCCATGCAAGTGAACTCCCCAGT
CAATACGAGCGCTGTTTCTACGCAATTCCCCACAAACTAGTCCGAGCGTTCGGTTATCAG
TACTGTAGGGAGGAGGGTCCTGCATCCGCGGCTAAGGACGGTCGAGCTATAGAACCGCAT
CTTGTGGGCGGAGAAAGGAGCGCCGCCGCTTACATAGCGGACACCAGCAGGTCTGGCTGG
ATCGCCCTTCTACGCTCCGCCAGTGGTGGTGGGCTCGCCTTGCGGGCCGTGCGTCGCCTA
CTGAGTATCGCTGTGCGTCCTATCCGTGAAGTTGGCAAGCGGAACATCCCACATTTCAAC
CGGCAAGAACTGAACGTTAGTACGTAATATAAGGCTGCCCTGGAACGCCGGATCGCCCAA
AACCGCGACGGACTGGCTCACGATGGTGAGCACGGCTAGTCGCTCTTGGAACACGTTGCC
ACGCCTTAACTGATCCTCAGGGTACTGGATAATCCGAGGGCGAGCACTGATGAGTGAGTG
CATGTGCTGCGTGGGAGGCACTGGGAGCTGGAAACCAGGGCCACGGAGATACGTGAATAC
AGCATTACGGGACAACAACGAGCTGGGCCCGTAACACACCAGGCGCCTAGATCATCCCTT
TACGCTCGTGGCTGAGGCGTTCGGGGAAAACAACATTGGCCGGCAGTCCCACAAGCGCTA
AGGCCCCGCGACCAAGGGCCCGCGCGAAAGCCAGGTCCAGCGTGCGAACCGCCACTTCGC
TCAGGCTAAAATCCACCAGCGTCGCGAAGTTCAAAACGGCCCGCGAGGCGGCAGGGTCTA
GGAATGCCCTTCTGTACGTCACTTGACCGCCCGCACGTACTACCAATTGCGGGAAGCAAT
TGATCTTGTTGTGCACTGCAAGACACGCGGCCCAAGGAGCCTTCTGTGTGCCTGGCCCTG
CCGGGGGCGGACAGAGACGCACGCCGGTGTGAGCGGCGGGGTCGTACTGGCCCACATCAT
GCGGCACACCGCCGCGTCAAGAGCCACCCTAAGCTGCATCCCCCACGTCCTCGGGCGAGC
CATCCTGCGCGAGGCATTTGTGTAGGATGGATACCGTACGACGGCACGATACTTCGACCT
GACTTGGTTGTATGTGCGTAGAGCGGGCACGTCCAGGAAAGGGCGCACCCCGATTACGAG
TGGTCCTTCCGTAAACAAAGTCCGGGTAGGTCAGTTAAGCCGGAACATAAGGACCAGTGG
CTACTTCCACGTCGGCGAGCACGGCCGCGAGGCGACGATGGATACTGCGCGGGGGGAGAT
ACGCGAACTAGGTCCGGGTGTCAATTCGGATGCGAATGGCGTCGTCACGGTTGAGGAGCG
GAAGGTCGTGGCTTACGTGTCGTCTTAGTTTAGGGGAGACTCGCCCATGGTATTCACTGT
GCCGACCGCATGGGACGGGAGCTCAGGCGGGGGGCCCTGCACTGGGGCTATTCAGCTCTA
CTTACCACTGAGCCGTTTGACCTGCCGTACTCGCGCGCCGTTTCGACCGGAATTACGGGA
CAACCCGTGGAGATGCACCGATGTGATCGCCTGTGCGAACTAGCTTCAGGCCGCGGCTTT
CAGAATAAACTCCCGCACGCGGAAGCTTATCAAGGTGCTGTGACGCAGCGGCCGCGGTGT
GATGGGTATGCGTAGGCGGGAAGCGCCTCCCTAGATGGCTTCCGCATTCCAAGACACCAG
GGACATGGACTCGTATCCCTAAATGTCCCTGAAGCGGTTTACGCGTTGAGGGCGTCCGTA
GCCCGTCTCGCCGATGGACAAGAAGGAAGGCCACCAAGCTGACCAGCCCGTAGGCAAGGC
CCCCCATGTCGCCGCCCGCCCTTCACCTCGAAAATGGGTGCCGACGTTCACCTACAGCCA
TGGCGGGGACGAGTAGCGTACGGTTTTGAACCGGCCAGAGGACCAGACTTATAGGGAGCT
GTTGCACCTGGACGAGATCCGCCCGAGGGATTAAAGACGCTTCTATCGGACCAAGGTGCG
TGTACCGTATATTACCCGCTATCACATGGCCCGCTTCGATCAATGATCAACCTTGGTAGT
TGCGCGCAACTCTGATTTGTACGTCGCCCTGTAAGCACCGCCAGACAAGTGCATCGCCGA
AGCCTACTCAAAGCTCGGGTCATTCGCTGCTTAGTGGCACTTGCGCGTGGACAAGCCTTC
CCGATAGCGATCCACTTACGGACGTCGCCTCCGGATCCACAAAGAAGCGACCGGCTCTGA
CATCCTGTCCTTATCCTCGAGGCTGCACGACCCGATGGGTGGCGAGTAAGTTTCCCTCAA

TCAGTGCGTTGGCGACTCCAAGCCGCACTCCTACCCCTAGATGGCCGAGGAGGAGGACGC
CCGGATCGACCTCGACACAGAAACCCCGGCCTCTAAGGAGCGGCACCTGGCCCAGCTGTG
GCCAAAGAGGGACTGTCCCTTCGCCCTGGCTAACGAGCCCGTCATCACCTACGTATTTGG
AGAGAAGTCGCCGGGTGTACAGGAGGGCACTTCCGACTCCGTGGGAGAGCAAGAGGCGAT
CGTACCCGGTTGCGTTAGTCTCTTGCAGCCATAATACCACTCGCGCTATGTGCTGGTCTC
GGCTATCGAGCATGCTAAGCCGGGCGGCCCCGCACCAATTGGTGGCGTGCGGGAGCAGGA
ACGCAGCGCCACTGACTTCTCACCGATGTTGAGTTTGATGCCGACTGGACAACTCGGCGA
CCACGATGACGGCGTCGGCCCGAAGCAACAGGTTAAGAACGGGCCGTTGGGTGTCACGGA
GATCGTGGTGGTAGAGGCGCGCTACTTTACGCGACCCCATAGGCTGCGTGCTGCCGCGGA
AGACAACTTCGTACACGTCCTAGGCGCACCGCGCCTAACCTACACGGCCCGACTGAAAGA
TGACAATGGGAATGCAGTGGTGGCGATCACCGAGAAGTTTTGGACACACACCTCTAGCGC
CGAGGAAGTGCACACTTCTATCCGGCTAGGGTTCGTGCCGCTGTACTCGGATATCGATTC
TAGGCCTGTGTGTCTAGGGGCGCCGGTGCAAGAGAGTGTAGGACCACGGGCGAAAAGGGT
CCCGGACATGACCGTGTTCCGCAGACCGCCATTTAGGTTCTTTGTACTGTCGCACTATAA
TGACCTGCATGACATCGTCCCCAAATAAAGCGAGGCAGAGGTTGAGTGCCCATCGGGCCA
GCTCGTGTTGTTGGTCAGGACCTAGGAATCCCAGCCAACCCGCCCCGGTTTGGACGGGAA
CACAGTCATATACACGACAGGTGAGGGTAGTGTGATCGCTGCCTTTATAACCACTTGCTC
AACGCCGGTATCTCTTCACCTTGCCCACTTTGAGATACCACTGGCGGCAGACAGCTTCAC
GCCCTAACGGACCCTGCTACATGGAGGAGTCGCAGGGAGTGTATTACGATCATCATTGTA
CACCACTTCAGGTTGTTAAGGCTCGTGCTGTGGAGGGGGAGTGCTCACTGGTGCGCCTGG
TGTCCTTGGTAACCATGGGTCGAATGGCAACCGGGATCGCTGTAGTCGCCGCGGACGATG
TCCTGCAGATCGCCGACCTCCTCTCGGTGTAGTGGCCGTACATCCTGCCCCAGTTCACGA
ACGCGGGGGTGGCCCCACCGTCCGTTCGCCCCGAAAAGAATCGTGCCCGGCAACCGAACA
TCACGCCGAGATCCCTTTCTCCCAGTAATGATGCGGGGTAATAACAGACAAGCTCCAGGA
GGTTATATGGCTAAACCGATTATAAAGGAAGTGGGTAGTGTCCTCAGGGGCACCATTGTC
GCTGGGCATAAGAGCGGTGGCGGCGAGGTTGGCGGCGGTGAGAAGGATTCCTCGAAACAA
GCACTGGCAGCGTTGCTGAAGAAGCAGCGCCCTGTAGCCCAGAGGCTACAGCTGCTCCTG
AGCGAAGATAACATACCAACGATTGACACAGGTTGCAATGGCTAGGCGCAGAATGAAGCC
GCCGCTAAGGAGCGTGGTGCACGTTGCGTAGTTAACGTACGTTCCCTTGCCATTAACATC
GGACACCAATCTGACAGCAAACCCCTGCAATGTGGGCGGTAATCGTAGATCCGACTGCGA
GACAGAAGGCCCAGGGCTTCAAACAGTTGTCCATAGCCTTTCTGTTGGTGCCCACTATCG
AGAACACGCGCGAGTCGGAATTACTATAGTACGTCTTGCCCGCTGCCGGCGCCTTCCTCA
TAAGCAACTTGCCGCCGAAGGTGGCGGTTAGACTCTTTCCACGTGGCTGTGTTAGCTCGC
CGATCGAGCTAGACGAGACGCGACAAGAACTCGCCGCCACTAACGTTACCGAACAGTCCG
AACTGCACCGTCGCACTGCCGACCTGGAGCTACTCGTTGCTCCGCTCCACTTCTTATACG
ATGGCCTGGCGTTGCTGCACCGCCACCTCATGCTTTTGATAGACACCGGCATCGCCCGCG
AATAGGGCGACCCTGTCCTAGTTAAGGATCCCTGCTGGCCTATGCAGACGTACACGGTGC
GGAGCAGCTCTTACTGCCACACCGCCCTGAATGTAGTCCGCCAACAGATGGTGCACCCTG
AACTGACTACCGCGACCCATGCAGACGTCCAGGACCAGCTCATTGCCAAACGCAACTCGG
ACAATACCGCCTAGTAACGGTTGATCGGGGCGCTCACTACCCTGAGCGGGAGGTGCATCC
CAGCATGGGACGAGCAAGAAGGTTAGTGAGTCGGCCCGGGCTCTAGCTAATTTGATGACG
TATGTTCGACTGTTCCCGATGGTTGGATCGCACCCCACGTCGAGCAATTCGCTGCTGGCC
ACGTAGAGCAGTACAGTGGCCAATCCTCTCGTCTGCTCTTCTTCAGCGTGAACCTTGGCC
TGGGCAAGTTCGAGGAGGGGAGTATGGTCAGCCTCTGGGACGTTGCAAATCGCGGCGCGG
TGGACGTCTATGGTGACATTAAGACCGGTGGCTTCGTACCGGGCCATGTGGATTCAGTTG
CGAAGTAAGAGCAAAGCGATTTCAAACCTAACGGCCAGGCCTTCGCCAGTTTCGAGAGGT
TCGTGATGCGCTAGACGCGAGGTGACATGTACACCGAGTCAGTACGACACACTGAACTCG
TGAGGGGAGAGGTGTTTCGAACTGTGGCTCCGACGTCACCACACCTCCTGGGCGTCGCGA
ACAATCGTCCGGCTGAAACGTTCGCGTAGCCAGGGGCGTACCTGAGCACGCACGGGGCAT
CGCGAAGTTACGGCGGGATGCTTCTCGGGATGCCCCCGAGCGTGTACCGTCTGAGCATTA
GCAGCGGAATCCTGGCGCTGTCCAGGACCATGGAGTCCACCAATATCCTGAGCGTAAGAC
TGTAGACCGGAGATAGCGACCTGACGCTGGACCAACTCACTAACCTATTCGCTCTTGAGA
AGGTCGTAATGGGTATGTTAGCCTACATCCTTGTTGGCTTCTCTATGTTGTCTTAAGACA
CACCCGCTATGGCTGCAGAGGCCGAGCAGGAATCTCCCTTTACATAACAACCACTGGCAG
TAGTTCGCGGGGCGCTGGTCGCCCAGAAATTAGTAGGAGTCCTAACATCATGTGTGGAGA
GGCGCAAGCTGTGGTATCGGTGCCGGCCCCCACGGTGAATCGACGCCTACCTCGCCGCC
TGCTCCTGTAGCTGGCCCGCTAGCTTACGTATTTGCTTCTTCCGACGCTGCTCGTTAGGG

TTCAAACCAGCACGCTGAGTCCACGGCAGCCACATCCGAGCGTGGTCGCCTCCTCGCACA
GACTGGGTAGGACGTGTTCTGCGACCTGGCTGCAACCCTAAGTATCGGCATGGTAAATCT
CATCATCCATAGTAAGCACCTGGAAATCTCACGGGCTTTTTCTAAGCCTATCGGGGAGGG
[truncated: 499,457 more chars]
